# Supplementary material for: Patterns of compensatory mutations in rpoA/B/C genes of multidrug resistant M. tuberculosis in Uganda
Source: PLoS One. 2025 Dec 4;20(12):e0328957. doi: 10.1371/journal.pone.0328957 (PMC12677784; doi:10.1371/journal.pone.0328957)
Supplement: S2 File — (ZIP) [file pone.0328957.s002.zip › Variants A_S1_L001_001.bam.html]

 

Calling SNPs/INDELs (computing variant list in .vcf format) from A\_S1\_L001\_001.bam

*by SAMtools/BCFtools:*

Howto

Important aspects

This takes up to one hour!!! **Please wait ...**

Variants A\_S1\_L001\_001.bam

|  |  |
| --- | --- |
| Variants |  |

|  |  |
| --- | --- |
| |  | | --- | | *by GATK* | |

|  |  |  |
| --- | --- | --- |
| |  | | --- | | A\_S1\_L001\_001.bam | | | computed 2016-10-27 using PhyResSE v1.0 (Ref. NC\_000962.3) | |

|  |  |
| --- | --- |
| 1071  variants called Export in VCF format |  |

|  |  |  |  |  |  |  |  |  |  |  |  |  |  |  |  |  |  |  |  |  |  |  |  |  |  |  |  |  |  |  |  |  |  |  |  |  |  |  |  |  |  |  |  |  |  |  |  |  |  |  |  |  |  |  |  |  |  |  |  |  |  |  |  |  |  |  |  |  |  |  |  |  |  |  |  |  |  |  |  |  |  |  |  |  |  |  |  |  |  |  |  |  |  |  |  |  |  |  |  |  |  |  |  |  |  |  |  |  |  |  |  |  |  |  |  |  |  |  |  |  |  |  |  |  |  |  |  |  |  |  |  |  |  |  |  |  |  |  |  |  |  |  |  |  |  |  |  |  |  |  |  |  |  |  |  |  |  |  |  |  |  |  |  |  |  |  |  |  |  |  |  |  |  |  |  |  |  |  |  |  |  |  |  |  |  |  |  |  |  |  |  |  |  |  |  |  |  |  |  |  |  |  |  |  |  |  |  |  |  |  |  |  |  |  |  |  |  |  |  |  |  |  |  |  |  |  |  |  |  |  |  |  |  |  |  |  |  |  |  |  |  |  |  |  |  |  |  |  |  |  |  |  |  |  |  |  |  |  |  |  |  |  |  |  |  |  |  |  |  |  |  |  |  |  |  |  |  |  |  |  |  |  |  |  |  |  |  |  |  |  |  |  |  |  |  |  |  |  |  |  |  |  |  |  |  |  |  |  |  |  |  |  |  |  |  |  |  |  |  |  |  |  |  |  |  |  |  |  |  |  |  |  |  |  |  |  |  |  |  |  |  |  |  |  |  |  |  |  |  |  |  |  |  |  |  |  |  |  |  |  |  |  |  |  |  |  |  |  |  |  |  |  |  |  |  |  |  |  |  |  |  |  |  |  |  |  |  |  |  |  |  |  |  |  |  |  |  |  |  |  |  |  |  |  |  |  |  |  |  |  |  |  |  |  |  |  |  |  |  |  |  |  |  |  |  |  |  |  |  |  |  |  |  |  |  |  |  |  |  |  |  |  |  |  |  |  |  |  |  |  |  |  |  |  |  |  |  |  |  |  |  |  |  |  |  |  |  |  |  |  |  |  |  |  |  |  |  |  |  |  |  |  |  |  |  |  |  |  |  |  |  |  |  |  |  |  |  |  |  |  |  |  |  |  |  |  |  |  |  |  |  |  |  |  |  |  |  |  |  |  |  |  |  |  |  |  |  |  |  |  |  |  |  |  |  |  |  |  |  |  |  |  |  |  |  |  |  |  |  |  |  |  |  |  |  |  |  |  |  |  |  |  |  |  |  |  |  |  |  |  |  |  |  |  |  |  |  |  |  |  |  |  |  |  |  |  |  |  |  |  |  |  |  |  |  |  |  |  |  |  |  |  |  |  |  |  |  |  |  |  |  |  |  |  |  |  |  |  |  |  |  |  |  |  |  |  |  |  |  |  |  |  |  |  |  |  |  |  |  |  |  |  |  |  |  |  |  |  |  |  |  |  |  |  |  |  |  |  |  |  |  |  |  |  |  |  |  |  |  |  |  |  |  |  |  |  |  |  |  |  |  |  |  |  |  |  |  |  |  |  |  |  |  |  |  |  |  |  |  |  |  |  |  |  |  |  |  |  |  |  |  |  |  |  |  |  |  |  |  |  |  |  |  |  |  |  |  |  |  |  |  |  |  |  |  |  |  |  |  |  |  |  |  |  |  |  |  |  |  |  |  |  |  |  |  |  |  |  |  |  |  |  |  |  |  |  |  |  |  |  |  |  |  |  |  |  |  |  |  |  |  |  |  |  |  |  |  |  |  |  |  |  |  |  |  |  |  |  |  |  |  |  |  |  |  |  |  |  |  |  |  |  |  |  |  |  |  |  |  |  |  |  |  |  |  |  |  |  |  |  |  |  |  |  |  |  |  |  |  |  |  |  |  |  |  |  |  |  |  |  |  |  |  |  |  |  |  |  |  |  |  |  |  |  |  |  |  |  |  |  |  |  |  |  |  |  |  |  |  |  |  |  |  |  |  |  |  |  |  |  |  |  |  |  |  |  |  |  |  |  |  |  |  |  |  |  |  |  |  |  |  |  |  |  |  |  |  |  |  |  |  |  |  |  |  |  |  |  |  |  |  |  |  |  |  |  |  |  |  |  |  |  |  |  |  |  |  |  |  |  |  |  |  |  |  |  |  |  |  |  |  |  |  |  |  |  |  |  |  |  |  |  |  |  |  |  |  |  |  |  |  |  |  |  |  |  |  |  |  |  |  |  |  |  |  |  |  |  |  |  |  |  |  |  |  |  |  |  |  |  |  |  |  |  |  |  |  |  |  |  |  |  |  |  |  |  |  |  |  |  |  |  |  |  |  |  |  |  |  |  |  |  |  |  |  |  |  |  |  |  |  |  |  |  |  |  |  |  |  |  |  |  |  |  |  |  |  |  |  |  |  |  |  |  |  |  |  |  |  |  |  |  |  |  |  |  |  |  |  |  |  |  |  |  |  |  |  |  |  |  |  |  |  |  |  |  |  |  |  |  |  |  |  |  |  |  |  |  |  |  |  |  |  |  |  |  |  |  |  |  |  |  |  |  |  |  |  |  |  |  |  |  |  |  |  |  |  |  |  |  |  |  |  |  |  |  |  |  |  |  |  |  |  |  |  |  |  |  |  |  |  |  |  |  |  |  |  |  |  |  |  |  |  |  |  |  |  |  |  |  |  |  |  |  |  |  |  |  |  |  |  |  |  |  |  |  |  |  |  |  |  |  |  |  |  |  |  |  |  |  |  |  |  |  |  |  |  |  |  |  |  |  |  |  |  |  |  |  |  |  |  |  |  |  |  |  |  |  |  |  |  |  |  |  |  |  |  |  |  |  |  |  |  |  |  |  |  |  |  |  |  |  |  |  |  |  |  |  |  |  |  |  |  |  |  |  |  |  |  |  |  |  |  |  |  |  |  |  |  |  |  |  |  |  |  |  |  |  |  |  |  |  |  |  |  |  |  |  |  |  |  |  |  |  |  |  |  |  |  |  |  |  |  |  |  |  |  |  |  |  |  |  |  |  |  |  |  |  |  |  |  |  |  |  |  |  |  |  |  |  |  |  |  |  |  |  |  |  |  |  |  |  |  |  |  |  |  |  |  |  |  |  |  |  |  |  |  |  |  |  |  |  |  |  |  |  |  |  |  |  |  |  |  |  |  |  |  |  |  |  |  |  |  |  |  |  |  |  |  |  |  |  |  |  |  |  |  |  |  |  |  |  |  |  |  |  |  |  |  |  |  |  |  |  |  |  |  |  |  |  |  |  |  |  |  |  |  |  |  |  |  |  |  |  |  |  |  |  |  |  |  |  |  |  |  |  |  |  |  |  |  |  |  |  |  |  |  |  |  |  |  |  |  |  |  |  |  |  |  |  |  |  |  |  |  |  |  |  |  |  |  |  |  |  |  |  |  |  |  |  |  |  |  |  |  |  |  |  |  |  |  |  |  |  |  |  |  |  |  |  |  |  |  |  |  |  |  |  |  |  |  |  |  |  |  |  |  |  |  |  |  |  |  |  |  |  |  |  |  |  |  |  |  |  |  |  |  |  |  |  |  |  |  |  |  |  |  |  |  |  |  |  |  |  |  |  |  |  |  |  |  |  |  |  |  |  |  |  |  |  |  |  |  |  |  |  |  |  |  |  |  |  |  |  |  |  |  |  |  |  |  |  |  |  |  |  |  |  |  |  |  |  |  |  |  |  |  |  |  |  |  |  |  |  |  |  |  |  |  |  |  |  |  |  |  |  |  |  |  |  |  |  |  |  |  |  |  |  |  |  |  |  |  |  |  |  |  |  |  |  |  |  |  |  |  |  |  |  |  |  |  |  |  |  |  |  |  |  |  |  |  |  |  |  |  |  |  |  |  |  |  |  |  |  |  |  |  |  |  |  |  |  |  |  |  |  |  |  |  |  |  |  |  |  |  |  |  |  |  |  |  |  |  |  |  |  |  |  |  |  |  |  |  |  |  |  |  |  |  |  |  |  |  |  |  |  |  |  |  |  |  |  |  |  |  |  |  |  |  |  |  |  |  |  |  |  |  |  |  |  |  |  |  |  |  |  |  |  |  |  |  |  |  |  |  |  |  |  |  |  |  |  |  |  |  |  |  |  |  |  |  |  |  |  |  |  |  |  |  |  |  |  |  |  |  |  |  |  |  |  |  |  |  |  |  |  |  |  |  |  |  |  |  |  |  |  |  |  |  |  |  |  |  |  |  |  |  |  |  |  |  |  |  |  |  |  |  |  |  |  |  |  |  |  |  |  |  |  |  |  |  |  |  |  |  |  |  |  |  |  |  |  |  |  |  |  |  |  |  |  |  |  |  |  |  |  |  |  |  |  |  |  |  |  |  |  |  |  |  |  |  |  |  |  |  |  |  |  |  |  |  |  |  |  |  |  |  |  |  |  |  |  |  |  |  |  |  |  |  |  |  |  |  |  |  |  |  |  |  |  |  |  |  |  |  |  |  |  |  |  |  |  |  |  |  |  |  |  |  |  |  |  |  |  |  |  |  |  |  |  |  |  |  |  |  |  |  |  |  |  |  |  |  |  |  |  |  |  |  |  |  |  |  |  |  |  |  |  |  |  |  |  |  |  |  |  |  |  |  |  |  |  |  |  |  |  |  |  |  |  |  |  |  |  |  |  |  |  |  |  |  |  |  |  |  |  |  |  |  |  |  |  |  |  |  |  |  |  |  |  |  |  |  |  |  |  |  |  |  |  |  |  |  |  |  |  |  |  |  |  |  |  |  |  |  |  |  |  |  |  |  |  |  |  |  |  |  |  |  |  |  |  |  |  |  |  |  |  |  |  |  |  |  |  |  |  |  |  |  |  |  |  |  |  |  |  |  |  |  |  |  |  |  |  |  |  |  |  |  |  |  |  |  |  |  |  |  |  |  |  |  |  |  |  |  |  |  |  |  |  |  |  |  |  |  |  |  |  |  |  |  |  |  |  |  |  |  |  |  |  |  |  |  |  |  |  |  |  |  |  |  |  |  |  |  |  |  |  |  |  |  |  |  |  |  |  |  |  |  |  |  |  |  |  |  |  |  |  |  |  |  |  |  |  |  |  |  |  |  |  |  |  |  |  |  |  |  |  |  |  |  |  |  |  |  |  |  |  |  |  |  |  |  |  |  |  |  |  |  |  |  |  |  |  |  |  |  |  |  |  |  |  |  |  |  |  |  |  |  |  |  |  |  |  |  |  |  |  |  |  |  |  |  |  |  |  |  |  |  |  |  |  |  |  |  |  |  |  |  |  |  |  |  |  |  |  |  |  |  |  |  |  |  |  |  |  |  |  |  |  |  |  |  |  |  |  |  |  |  |  |  |  |  |  |  |  |  |  |  |  |  |  |  |  |  |  |  |  |  |  |  |  |  |  |  |  |  |  |  |  |  |  |  |  |  |  |  |  |  |  |  |  |  |  |  |  |  |  |  |  |  |  |  |  |  |  |  |  |  |  |  |  |  |  |  |  |  |  |  |  |  |  |  |  |  |  |  |  |  |  |  |  |  |  |  |  |  |  |  |  |  |  |  |  |  |  |  |  |  |  |  |  |  |  |  |  |  |  |  |  |  |  |  |  |  |  |  |  |  |  |  |  |  |  |  |  |  |  |  |  |  |  |  |  |  |  |  |  |  |  |  |  |  |  |  |  |  |  |  |  |  |  |  |  |  |  |  |  |  |  |  |  |  |  |  |  |  |  |  |  |  |  |  |  |  |  |  |  |  |  |  |  |  |  |  |  |  |  |  |  |  |  |  |  |  |  |  |  |  |  |  |  |  |  |  |  |  |  |  |  |  |  |  |  |  |  |  |  |  |  |  |  |  |  |  |  |  |  |  |  |  |  |  |  |  |  |  |  |  |  |  |  |  |  |  |  |  |  |  |  |  |  |  |  |  |  |  |  |  |  |  |  |  |  |  |  |  |  |  |  |  |  |  |  |  |  |  |  |  |  |  |  |  |  |  |  |  |  |  |  |  |  |  |  |  |  |  |  |  |  |  |  |  |  |  |  |  |  |  |  |  |  |  |  |  |  |  |  |  |  |  |  |  |  |  |  |  |  |  |  |  |  |  |  |  |  |  |  |  |  |  |  |  |  |  |  |  |  |  |  |  |  |  |  |  |  |  |  |  |  |  |  |  |  |  |  |  |  |  |  |  |  |  |  |  |  |  |  |  |  |  |  |  |  |  |  |  |  |  |  |  |  |  |  |  |  |  |  |  |  |  |  |  |  |  |  |  |  |  |  |  |  |  |  |  |  |  |  |  |  |  |  |  |  |  |  |  |  |  |  |  |  |  |  |  |  |  |  |  |  |  |  |  |  |  |  |  |  |  |  |  |  |  |  |  |  |  |  |  |  |  |  |  |  |  |  |  |  |  |  |  |  |  |  |  |  |  |  |  |  |  |  |  |  |  |  |  |  |  |  |  |  |  |  |  |  |  |  |  |  |  |  |  |  |  |  |  |  |  |  |  |  |  |  |  |  |  |  |  |  |  |  |  |  |  |  |  |  |  |  |  |  |  |  |  |  |  |  |  |  |  |  |  |  |  |  |  |  |  |  |  |  |  |  |  |  |  |  |  |  |  |  |  |  |  |  |  |  |  |  |  |  |  |  |  |  |  |  |  |  |  |  |  |  |  |  |  |  |  |  |  |  |  |  |  |  |  |  |  |  |  |  |  |  |  |  |  |  |  |  |  |  |  |  |  |  |  |  |  |  |  |  |  |  |  |  |  |  |  |  |  |  |  |  |  |  |  |  |  |  |  |  |  |  |  |  |  |  |  |  |  |  |  |  |  |  |  |  |  |  |  |  |  |  |  |  |  |  |  |  |  |  |  |  |  |  |  |  |  |  |  |  |  |  |  |  |  |  |  |  |  |  |  |  |  |  |  |  |  |  |  |  |  |  |  |  |  |  |  |  |  |  |  |  |  |  |  |  |  |  |  |  |  |  |  |  |  |  |  |  |  |  |  |  |  |  |  |  |  |  |  |  |  |  |  |  |  |  |  |  |  |  |  |  |  |  |  |  |  |  |  |  |  |  |  |  |  |  |  |  |  |  |  |  |  |  |  |  |  |  |  |  |  |  |  |  |  |  |  |  |  |  |  |  |  |  |  |  |  |  |  |  |  |  |  |  |  |  |  |  |  |  |  |  |  |  |  |  |  |  |  |  |  |  |  |  |  |  |  |  |  |  |  |  |  |  |  |  |  |  |  |  |  |  |  |  |  |  |  |  |  |  |  |  |  |  |  |  |  |  |  |  |  |  |  |  |  |  |  |  |  |  |  |  |  |  |  |  |  |  |  |  |  |  |  |  |  |  |  |  |  |  |  |  |  |  |  |  |  |  |  |  |  |  |  |  |  |  |  |  |  |  |  |  |  |  |  |  |  |  |  |  |  |  |  |  |  |  |  |  |  |  |  |  |  |  |  |  |  |  |  |  |  |  |  |  |  |  |  |  |  |  |  |  |  |  |  |  |  |  |  |  |  |  |  |  |  |  |  |  |  |  |  |  |  |  |  |  |  |  |  |  |  |  |  |  |  |  |  |  |  |  |  |  |  |  |  |  |  |  |  |  |  |  |  |  |  |  |  |  |  |  |  |  |  |  |  |  |  |  |  |  |  |  |  |  |  |  |  |  |  |  |  |  |  |  |  |  |  |  |  |  |  |  |  |  |  |  |  |  |  |  |  |  |  |  |  |  |  |  |  |  |  |  |  |  |  |  |  |  |  |  |  |  |  |  |  |  |  |  |  |  |  |  |  |  |  |  |  |  |  |  |  |  |  |  |  |  |  |  |  |  |  |  |  |  |  |  |  |  |  |  |  |  |  |  |  |  |  |  |  |  |  |  |  |  |  |  |  |  |  |  |  |  |  |  |  |  |  |  |  |  |  |  |  |  |  |  |  |  |  |  |  |  |  |  |  |  |  |  |  |  |  |  |  |  |  |  |  |  |  |  |  |  |  |  |  |  |  |  |  |  |  |  |  |  |  |  |  |  |  |  |  |  |  |  |  |  |  |  |  |  |  |  |  |  |  |  |  |  |  |  |  |  |  |  |  |  |  |  |  |  |  |  |  |  |  |  |  |  |  |  |  |  |  |  |  |  |  |  |  |  |  |  |  |  |  |  |  |  |  |  |  |  |  |  |  |  |  |  |  |  |  |  |  |  |  |  |  |  |  |  |  |  |  |  |  |  |  |  |  |  |  |  |  |  |  |  |  |  |  |  |  |  |  |  |  |  |  |  |  |  |  |  |  |  |  |  |  |  |  |  |  |  |  |  |  |  |  |  |  |  |  |  |  |  |  |  |  |  |  |  |  |  |  |  |  |  |  |  |  |  |  |  |  |  |  |  |  |  |  |  |  |  |  |  |  |  |  |  |  |  |  |  |  |  |  |  |  |  |  |  |  |  |  |  |  |  |  |  |  |  |  |  |  |  |  |  |  |  |  |  |  |  |  |  |  |  |  |  |  |  |  |  |  |  |  |  |  |  |  |  |  |  |  |  |  |  |  |  |  |  |  |  |  |  |  |  |  |  |  |  |  |  |  |  |  |  |  |  |  |  |  |  |  |  |  |  |  |  |  |  |  |  |  |  |  |  |  |  |  |  |  |  |  |  |  |  |  |  |  |  |  |  |  |  |  |  |  |  |  |  |  |  |  |  |  |  |  |  |  |  |  |  |  |  |  |  |  |  |  |  |  |  |  |  |  |  |  |  |  |  |  |  |  |  |  |  |  |  |  |  |  |  |  |  |  |  |  |  |  |  |  |  |  |  |  |  |  |  |  |  |  |  |  |  |  |  |  |  |  |  |  |  |  |  |  |  |  |  |  |  |  |  |  |  |  |  |  |  |  |  |  |  |  |  |  |  |  |  |  |  |  |  |  |  |  |  |  |  |  |  |  |  |  |  |  |  |  |  |  |  |  |  |  |  |  |  |  |  |  |  |  |  |  |  |  |  |  |  |  |  |  |  |  |  |  |  |  |  |  |  |  |  |  |  |  |  |  |  |  |  |  |  |  |  |  |  |  |  |  |  |  |  |  |  |  |  |  |  |  |  |  |  |  |  |  |  |  |  |  |  |  |  |  |  |  |  |  |  |  |  |  |  |  |  |  |  |  |  |  |  |  |  |  |  |  |  |  |  |  |  |  |  |  |  |  |  |  |  |  |  |  |  |  |  |  |  |  |  |  |  |  |  |  |  |  |  |  |  |  |  |  |  |  |  |  |  |  |  |  |  |  |  |  |  |  |  |  |  |  |  |  |  |  |  |  |  |  |  |  |  |  |  |  |  |  |  |  |  |  |  |  |  |  |  |  |  |  |  |  |  |  |  |  |  |  |  |  |  |  |  |  |  |  |  |  |  |  |  |  |  |  |  |  |  |  |  |  |  |  |  |  |  |  |  |  |  |  |  |  |  |  |  |  |  |  |  |  |  |  |  |  |  |  |  |  |  |  |  |  |  |  |  |  |  |  |  |  |  |  |  |  |  |  |  |  |  |  |  |  |  |  |  |  |  |  |  |  |  |  |  |  |  |  |  |  |  |  |  |  |  |  |  |  |  |  |  |  |  |  |  |  |  |  |  |  |  |  |  |  |  |  |  |  |  |  |  |  |  |  |  |  |  |  |  |  |  |  |  |  |  |  |  |  |  |  |  |  |  |  |  |  |  |  |  |  |  |  |  |  |  |  |  |  |  |  |  |  |  |  |  |  |  |  |  |  |  |  |  |  |  |  |  |  |  |  |  |  |  |  |  |  |  |  |  |  |  |  |  |  |  |  |  |  |  |  |  |  |  |  |  |  |  |  |  |  |  |  |  |  |  |  |  |  |  |  |  |  |  |  |  |  |  |  |  |  |  |  |  |  |  |  |  |  |  |  |  |  |  |  |  |  |  |  |  |  |  |  |  |  |  |  |  |  |  |  |  |  |  |  |  |  |  |  |  |  |  |  |  |  |  |  |  |  |  |  |  |  |  |  |  |  |  |  |  |  |  |  |  |  |  |  |  |  |  |  |  |  |  |  |  |  |  |  |  |  |  |  |  |  |  |  |  |  |  |  |  |  |  |  |  |  |  |  |  |  |  |  |  |  |  |  |  |  |  |  |  |  |  |  |  |  |  |  |  |  |  |  |  |  |  |  |  |  |  |  |  |  |  |  |  |  |  |  |  |  |  |  |  |  |  |  |  |  |  |  |  |  |  |  |  |  |  |  |  |  |  |  |  |  |  |  |  |  |  |  |  |  |  |  |  |  |  |  |  |  |  |  |  |  |  |  |  |  |  |  |  |  |  |  |  |  |  |  |  |  |  |  |  |  |  |  |  |  |  |  |  |  |  |  |  |  |  |  |  |  |  |  |  |  |  |  |  |  |  |  |  |  |  |  |  |  |  |  |  |  |  |  |  |  |  |  |  |  |  |  |  |  |  |  |  |  |  |  |  |  |  |  |  |  |  |  |  |  |  |  |  |  |  |  |  |  |  |  |  |  |  |  |  |  |  |  |  |  |  |  |  |  |  |  |  |  |  |  |  |  |  |  |  |  |  |  |  |  |  |  |  |  |  |  |  |  |  |  |  |  |  |  |  |  |  |  |  |  |  |  |  |  |  |  |  |  |  |  |  |  |  |  |  |  |  |  |  |  |  |  |  |  |  |  |  |  |  |  |  |  |  |  |  |  |  |  |  |  |  |  |  |  |  |  |  |  |  |  |  |  |  |  |  |  |  |  |  |  |  |  |  |  |  |  |  |  |  |  |  |  |  |  |  |  |  |  |  |  |  |  |  |  |  |  |  |  |  |  |  |  |  |  |  |  |  |  |  |  |  |  |  |  |  |  |  |  |  |  |  |  |  |  |  |  |  |  |  |  |  |  |  |  |  |  |  |  |  |  |  |  |  |  |  |  |  |  |  |  |  |  |  |  |  |  |  |  |  |  |  |  |  |  |  |  |  |  |  |  |  |  |  |  |  |  |  |  |  |  |  |  |  |  |  |  |  |  |  |  |  |  |  |  |  |  |  |  |  |  |  |  |  |  |  |  |  |  |  |  |  |  |  |  |  |  |  |  |  |  |  |  |  |  |  |  |  |  |  |  |  |  |  |  |  |  |  |  |  |  |  |  |  |  |  |  |  |  |  |  |  |  |  |  |  |  |  |  |  |  |  |  |  |  |  |  |  |  |  |  |  |  |  |  |  |  |  |  |  |  |  |  |  |  |  |  |  |  |  |  |  |  |  |  |  |  |  |  |  |  |  |  |  |  |  |  |  |  |  |  |  |  |  |  |  |  |  |  |  |  |  |  |  |  |  |  |  |  |  |  |  |  |  |  |  |  |  |  |  |  |  |  |  |  |  |  |  |  |  |  |  |  |  |  |  |  |  |  |  |  |  |  |  |  |  |  |  |  |  |  |  |  |  |  |  |  |  |  |  |  |  |  |  |  |  |  |  |  |  |  |  |  |  |  |  |  |  |  |  |  |  |  |  |  |  |  |  |  |  |  |  |  |  |  |  |  |  |  |  |  |  |  |  |  |  |  |  |  |  |  |  |  |  |  |  |  |  |  |  |  |  |  |  |  |  |  |  |  |  |  |  |  |  |  |  |  |  |  |  |  |  |  |  |  |  |  |  |  |  |  |  |  |  |  |  |  |  |  |  |  |  |  |  |  |  |  |  |  |  |  |  |  |  |  |  |  |  |  |  |  |  |  |  |  |  |  |  |  |  |  |  |  |  |  |  |  |  |  |  |  |  |  |  |  |  |  |  |  |  |  |  |  |  |  |  |  |  |  |  |  |  |  |  |  |  |  |  |  |  |  |  |  |  |  |  |  |  |  |  |  |  |  |  |  |  |  |  |  |  |  |  |  |  |  |  |  |  |  |  |  |  |  |  |  |  |  |  |  |  |  |  |  |  |  |  |  |  |  |  |  |  |  |  |  |  |  |  |  |  |  |  |  |  |  |  |  |  |  |  |  |  |  |  |  |  |  |  |  |  |  |  |  |  |  |  |  |  |  |  |  |  |  |  |  |  |  |  |  |  |  |  |  |  |  |  |  |  |  |  |  |  |  |  |  |  |  |  |  |  |  |  |  |  |  |  |  |  |  |  |  |  |  |  |  |  |  |  |  |  |  |  |  |  |  |  |  |  |  |  |  |  |  |  |  |  |  |  |  |  |  |  |  |  |  |  |  |  |  |  |  |  |  |  |  |  |  |  |  |  |  |  |  |  |  |  |  |  |  |  |  |  |  |  |  |  |  |  |  |  |  |  |  |  |  |  |  |  |  |  |  |  |  |  |  |  |  |  |  |  |  |  |  |  |  |  |  |  |  |  |  |  |  |  |  |  |  |  |  |  |  |  |  |  |  |  |  |  |  |  |  |  |  |  |  |  |  |  |  |  |  |  |  |  |  |  |  |  |  |  |  |  |  |  |  |  |  |  |  |  |  |  |  |  |  |  |  |  |  |  |  |  |  |  |  |  |  |  |  |  |  |  |  |  |  |  |  |  |  |  |  |  |  |  |  |  |  |  |  |  |  |  |  |  |  |  |  |  |  |  |  |  |  |  |  |  |  |  |  |  |  |  |  |  |  |  |  |  |  |  |  |  |  |  |  |  |  |  |  |  |  |  |  |  |  |  |  |  |  |  |  |  |  |  |  |  |  |  |  |  |  |  |  |  |  |  |  |  |  |  |  |  |  |  |  |  |  |  |  |  |  |  |  |  |  |  |  |  |  |  |  |  |  |  |  |  |  |  |  |  |  |  |  |  |  |  |  |  |  |  |  |  |  |  |  |  |  |  |  |  |  |  |  |  |  |  |  |  |  |  |  |  |  |  |  |  |  |  |  |  |  |  |  |  |  |  |  |  |  |  |  |  |  |  |  |  |  |  |  |  |  |  |  |  |  |  |  |  |  |  |  |  |  |  |  |  |  |  |  |  |  |  |  |  |  |  |  |  |  |  |  |  |  |  |  |  |  |  |  |  |  |  |  |  |  |  |  |  |  |  |  |  |  |  |  |  |  |  |  |  |  |  |  |  |  |  |  |  |  |  |  |  |  |  |  |  |  |  |  |  |  |  |  |  |  |  |  |  |  |  |  |  |  |  |  |  |  |  |  |  |  |  |  |  |  |  |  |  |  |  |  |  |  |  |  |  |  |  |  |  |  |  |  |  |  |  |  |  |  |  |  |  |  |  |  |  |  |  |  |  |  |  |  |  |  |  |  |  |  |  |  |  |  |  |  |  |  |  |  |  |  |  |  |  |  |  |  |  |  |  |  |  |  |  |  |  |  |  |  |  |  |  |  |  |  |  |  |  |  |  |  |  |  |  |  |  |  |  |  |  |  |  |  |  |  |  |  |  |  |  |  |  |  |  |  |  |  |  |  |  |  |  |  |  |  |  |  |  |  |  |  |  |  |  |  |  |  |  |  |  |  |  |  |  |  |  |  |  |  |  |  |  |  |  |  |  |  |  |  |  |  |  |  |  |  |  |  |  |  |  |  |  |  |  |  |  |  |  |  |  |  |  |  |  |  |  |  |  |  |  |  |  |  |  |  |  |  |  |  |  |  |  |  |  |  |  |  |  |  |  |  |  |  |  |  |  |  |  |  |  |  |  |  |  |  |  |  |  |  |  |  |  |  |  |  |  |  |  |  |  |  |  |  |  |  |  |  |  |  |  |  |  |  |  |  |  |  |  |  |  |  |  |  |  |  |  |  |  |  |  |  |  |  |  |  |  |  |  |  |  |  |  |  |  |  |  |  |  |  |  |  |  |  |  |  |  |  |  |  |  |  |  |  |  |  |  |  |  |  |  |  |  |  |  |  |  |  |  |  |  |  |  |  |  |  |  |  |  |  |  |  |  |  |  |  |  |  |  |  |  |  |  |  |  |  |  |  |  |  |  |  |  |  |  |  |  |  |  |  |  |  |  |  |  |  |  |  |  |  |  |  |  |  |  |  |  |  |  |  |  |  |  |  |  |  |  |  |  |  |  |  |  |  |  |  |  |  |  |  |  |  |  |  |  |  |  |  |  |  |  |  |  |  |  |  |  |  |  |  |  |  |  |  |  |  |  |  |  |  |  |  |  |  |  |  |  |  |  |  |  |  |  |  |  |  |  |  |  |  |  |  |  |  |  |  |  |  |  |  |  |  |  |  |  |  |  |  |  |  |  |  |  |  |  |  |  |  |  |  |  |  |  |  |  |  |  |  |  |  |  |  |  |  |  |  |  |  |  |  |  |  |  |  |  |  |  |  |  |  |  |  |  |  |  |  |  |  |  |  |  |  |  |  |  |  |  |  |  |  |  |  |  |  |  |  |  |  |  |  |  |  |  |  |  |  |  |  |  |  |  |  |  |  |  |  |  |  |  |  |  |  |  |  |  |  |  |  |  |  |  |  |  |  |  |  |  |  |  |  |  |  |  |  |  |  |  |  |  |  |  |  |  |  |  |  |  |  |  |  |  |  |  |  |  |  |  |  |  |  |  |  |  |  |  |  |  |  |  |  |  |  |  |  |  |  |  |  |  |  |  |  |  |  |  |  |  |  |  |  |  |  |  |  |  |  |  |  |  |  |  |  |  |  |  |  |  |  |  |  |  |  |  |  |  |  |  |  |  |  |  |  |  |  |  |  |  |  |  |  |  |  |  |  |  |  |  |  |  |  |  |  |  |  |  |  |  |  |  |  |  |  |  |  |  |  |  |  |  |  |  |  |  |  |  |  |  |  |  |  |  |  |  |  |  |  |  |  |  |  |  |  |  |  |  |  |  |  |  |  |  |  |  |  |  |  |  |  |  |  |  |  |  |  |  |  |  |  |  |  |  |  |  |  |  |  |  |  |  |  |  |  |  |  |  |  |  |  |  |  |  |  |  |  |  |  |  |  |  |  |  |  |  |  |  |  |  |  |  |  |  |  |  |  |  |  |  |  |  |  |  |  |  |  |  |  |  |  |  |  |  |  |  |  |  |  |  |  |  |  |  |  |  |  |  |  |  |  |  |  |  |  |  |  |  |  |  |  |  |  |  |  |  |  |  |  |  |  |  |  |  |  |  |  |  |  |  |  |  |  |  |  |  |  |  |  |  |  |  |  |  |  |  |  |  |  |  |  |  |  |  |  |  |  |  |  |  |  |  |  |  |  |  |  |  |  |  |  |  |  |  |  |  |  |  |  |  |  |  |  |  |  |  |  |  |  |  |  |  |  |  |  |  |  |  |  |  |  |  |  |  |  |  |  |  |  |  |  |  |  |  |  |  |  |  |  |  |  |  |  |  |  |  |  |  |  |  |  |  |  |  |  |  |  |  |  |  |  |  |  |  |  |  |  |  |  |  |  |  |  |  |  |  |  |  |  |  |  |  |  |  |  |  |  |  |  |  |  |  |  |  |  |  |  |  |  |  |  |  |  |  |  |  |  |  |  |  |  |  |  |  |  |  |  |  |  |  |  |  |  |  |  |  |  |  |  |  |  |  |  |  |  |  |  |  |  |  |  |  |  |  |  |  |  |  |  |  |  |  |  |  |  |  |  |  |  |  |  |  |  |  |  |  |  |  |  |  |  |  |  |  |  |  |  |  |  |  |  |  |  |  |  |  |  |  |  |  |  |  |  |  |  |  |  |  |  |  |  |  |  |  |  |  |  |  |  |  |  |  |  |  |  |  |  |  |  |  |  |  |  |  |  |  |  |  |  |  |  |  |  |  |  |  |  |  |  |  |  |  |  |  |  |  |  |  |  |  |  |  |  |  |  |  |  |  |  |  |  |  |  |  |  |  |  |  |  |  |  |  |  |  |  |  |  |  |  |  |  |  |  |  |  |  |  |  |  |  |  |  |  |  |  |  |  |  |  |  |  |  |  |  |  |  |  |  |  |  |  |  |  |  |  |  |  |  |  |  |  |  |  |  |  |  |  |  |  |  |  |  |  |  |  |  |  |  |  |  |  |  |  |  |  |  |  |  |  |  |  |  |  |  |  |  |  |  |  |  |  |  |  |  |  |  |  |  |  |  |  |  |  |  |  |  |  |  |  |  |  |  |  |  |  |  |  |  |  |  |  |  |  |  |  |  |  |  |  |  |  |  |  |  |  |  |  |  |  |  |  |  |  |  |  |  |  |  |  |  |  |  |  |  |  |  |  |  |  |  |  |  |  |  |  |  |  |  |  |  |  |  |  |  |  |  |  |  |  |  |  |  |  |  |  |  |  |  |  |  |  |  |  |  |  |  |  |  |  |  |  |  |  |  |  |  |  |  |  |  |  |  |  |  |  |  |  |  |  |  |  |  |  |  |  |  |  |  |  |  |  |  |  |  |  |  |  |  |  |  |  |  |  |  |  |  |  |  |  |  |  |  |  |  |  |  |  |  |  |  |  |  |  |  |  |  |  |  |  |  |  |  |  |  |  |  |  |  |  |  |  |  |  |  |  |  |  |  |  |  |  |  |  |  |  |  |  |  |  |  |  |  |  |  |  |  |  |  |  |  |  |  |  |  |  |  |  |  |  |  |  |  |  |  |  |  |  |  |  |  |  |  |  |  |  |  |  |  |  |  |  |  |  |  |  |  |  |  |  |  |  |  |  |  |  |  |  |  |  |  |  |  |  |  |  |  |  |  |  |  |  |  |  |  |  |  |  |  |  |  |  |  |  |  |  |  |  |  |  |  |  |  |  |  |  |  |  |  |  |  |  |  |  |  |  |  |  |  |  |  |  |  |  |  |  |  |  |  |  |  |  |  |  |  |  |  |  |  |  |  |  |  |  |  |  |  |  |  |  |  |  |  |  |  |  |  |  |  |  |  |  |  |  |  |  |  |  |  |  |  |  |  |  |  |  |  |  |  |  |  |  |  |  |  |  |  |  |  |  |  |  |  |  |  |  |  |  |  |  |  |  |  |  |  |  |  |  |  |  |  |  |  |  |  |  |  |  |  |  |  |  |  |  |  |  |  |  |  |  |  |  |  |  |  |  |  |  |  |  |  |  |  |  |  |  |  |  |  |  |  |  |  |  |  |  |  |  |  |  |  |  |  |  |  |  |  |  |  |  |  |  |  |  |  |  |  |  |  |  |  |  |  |  |  |  |  |  |  |  |  |  |  |  |  |  |  |  |  |  |  |  |  |  |  |  |  |  |  |  |  |  |  |  |  |  |  |  |  |  |  |  |  |  |  |  |  |  |  |  |  |  |  |  |  |  |  |  |  |  |  |  |  |  |  |  |  |  |  |  |  |  |  |  |  |  |  |  |  |  |  |  |  |  |  |  |  |  |  |  |  |  |  |  |  |  |  |  |  |  |  |  |  |  |  |  |  |  |  |  |  |  |  |  |  |  |  |  |  |  |  |  |  |  |  |  |  |  |  |  |  |  |  |  |  |  |  |  |  |  |  |  |  |  |  |  |  |  |  |  |  |  |  |  |  |  |  |  |  |  |  |  |  |  |  |  |  |  |  |  |  |  |  |  |  |  |  |  |  |  |  |  |  |  |  |  |  |  |  |  |  |  |  |  |  |  |  |  |  |  |  |  |  |  |  |  |  |  |  |  |  |  |  |  |  |  |  |  |  |  |  |  |  |  |  |  |  |  |  |  |  |  |  |  |  |  |  |  |  |  |  |  |  |  |  |  |  |  |  |  |  |  |  |  |  |  |  |  |  |  |  |  |  |  |  |  |  |  |  |  |  |  |  |  |  |  |  |  |  |  |  |  |  |  |  |  |  |  |  |  |  |  |  |  |  |  |  |  |  |  |  |  |  |  |  |  |  |  |  |  |  |  |  |  |  |  |  |  |  |  |  |  |  |  |  |  |  |  |  |  |  |  |  |  |  |  |  |  |  |  |  |  |  |  |  |  |  |  |  |  |  |  |  |  |  |  |  |  |  |  |  |  |  |  |  |  |  |  |  |  |  |  |  |  |  |  |  |  |  |  |  |  |  |  |  |  |  |  |  |  |  |  |  |  |  |  |  |  |  |  |  |  |  |  |  |  |  |  |  |  |  |  |  |  |  |  |  |  |  |  |  |  |  |  |  |  |  |  |  |  |  |  |  |  |  |  |  |  |  |  |  |  |  |  |  |  |  |  |  |  |  |  |  |  |  |  |  |  |  |  |  |  |  |  |  |  |  |  |  |  |  |  |  |  |  |  |  |  |  |  |  |  |  |  |  |  |  |  |  |  |  |  |  |  |  |  |  |  |  |  |  |  |  |  |  |  |  |  |  |  |  |  |  |  |  |  |  |  |  |  |  |  |  |  |  |  |  |  |  |  |  |  |  |  |  |  |  |  |  |  |  |  |  |  |  |  |  |  |  |  |  |  |  |  |  |  |  |  |  |  |  |  |  |  |  |  |  |  |  |  |  |  |  |  |  |  |  |  |  |  |  |  |  |  |  |  |  |  |  |  |  |  |  |  |  |  |  |  |  |  |  |  |  |  |  |  |  |  |  |  |  |  |  |  |  |  |  |  |  |  |  |  |  |  |  |  |  |  |  |  |  |  |  |  |  |  |  |  |  |  |  |  |  |  |  |  |  |  |  |  |  |  |  |  |  |  |  |  |  |  |  |  |  |  |  |  |  |  |  |  |  |  |  |  |  |  |  |  |  |  |  |  |  |  |  |  |  |  |  |  |  |  |  |  |  |  |  |  |  |  |  |  |  |  |  |  |  |  |  |  |  |  |  |  |  |  |  |  |  |  |  |  |  |  |  |  |  |  |  |  |  |  |  |  |  |  |  |  |  |  |  |  |  |  |  |  |  |  |  |  |  |  |  |  |  |  |  |  |  |  |  |  |  |  |  |  |  |  |  |  |  |  |  |  |  |  |  |  |  |  |  |  |  |  |  |  |  |  |  |  |  |  |  |  |  |  |  |  |  |  |  |  |  |  |  |  |  |  |  |  |  |  |  |  |  |  |  |  |  |  |  |  |  |  |  |  |  |  |  |  |  |  |  |  |  |  |  |  |  |  |  |  |  |  |  |  |  |  |  |  |  |  |  |  |  |  |  |  |  |  |  |  |  |  |  |  |  |  |  |  |  |  |  |  |  |  |  |  |  |  |  |  |  |  |  |  |  |  |  |  |  |  |  |  |  |  |  |  |  |  |  |  |  |  |  |  |  |  |  |  |  |  |  |  |  |  |  |  |  |  |  |  |  |  |  |  |  |  |  |  |  |  |  |  |  |  |  |  |  |  |  |  |  |  |  |  |  |  |  |  |  |  |  |  |  |  |  |  |  |  |  |  |  |  |  |  |  |  |  |  |  |  |  |  |  |  |  |  |  |  |  |  |  |  |  |  |  |  |  |  |  |  |  |  |  |  |  |  |  |  |  |  |  |  |  |  |  |  |  |  |  |  |  |  |  |  |  |  |  |  |  |  |  |  |  |  |  |  |  |  |  |  |  |  |  |  |  |  |  |  |  |  |  |  |  |  |  |  |  |  |  |  |  |  |  |  |  |  |  |  |  |  |  |  |  |  |  |  |  |  |  |  |  |  |  |  |  |  |  |  |  |  |  |  |  |  |  |  |  |  |  |  |  |  |  |  |  |  |  |  |  |  |  |  |  |  |  |  |  |  |  |  |  |  |  |  |  |  |  |  |  |  |  |  |  |  |  |  |  |  |  |  |  |  |  |  |  |  |  |  |  |  |  |  |  |  |  |  |  |  |  |  |  |  |  |  |  |  |  |  |  |  |  |  |  |  |  |  |  |  |  |  |  |  |  |  |  |  |  |  |  |  |  |  |  |  |  |  |  |  |  |  |  |  |  |  |  |  |  |  |  |  |  |  |  |  |  |  |  |  |  |  |  |  |  |  |  |  |  |  |  |  |  |  |  |  |  |  |  |  |  |  |  |  |  |  |  |  |  |  |  |  |  |  |  |  |  |  |  |  |  |  |  |  |  |  |  |  |  |  |  |  |  |  |  |  |  |  |  |  |  |  |  |  |  |  |  |  |  |  |  |  |  |  |  |  |  |  |  |  |  |  |  |  |  |  |  |  |  |  |  |  |  |  |  |  |  |  |  |  |  |  |  |  |  |  |  |  |  |  |  |  |  |  |  |  |  |  |  |  |  |  |  |  |  |  |  |  |  |  |  |  |  |  |  |  |  |  |  |  |  |  |  |  |  |  |  |  |  |  |  |  |  |  |  |  |  |  |  |  |  |  |  |  |  |  |  |  |  |  |  |  |  |  |  |  |  |  |  |  |  |  |  |  |  |  |  |  |  |  |  |  |  |  |  |  |  |  |  |  |  |  |  |  |  |  |  |  |  |  |  |  |  |  |  |  |  |  |  |  |  |  |  |  |  |  |  |  |  |  |  |  |  |  |  |  |  |  |  |  |  |  |  |  |  |  |  |  |  |  |  |  |  |  |  |  |  |  |  |  |  |  |  |  |  |  |  |  |  |  |  |  |  |  |  |  |  |  |  |  |  |  |  |  |  |  |  |  |  |  |  |  |  |  |  |  |  |  |  |  |  |  |  |  |  |  |  |  |  |  |  |  |  |  |  |  |  |  |  |  |  |  |  |  |  |  |  |  |  |  |  |  |  |  |  |  |  |  |  |  |  |  |  |  |  |  |  |  |  |  |  |  |  |  |  |  |  |  |  |  |  |  |  |  |  |  |  |  |  |  |  |  |  |  |  |  |  |  |  |  |  |  |  |  |  |  |  |  |  |  |  |  |  |  |  |  |  |  |  |  |  |  |  |  |  |  |  |  |  |  |  |  |  |  |  |  |  |  |  |  |  |  |  |  |  |  |  |  |  |  |  |  |  |  |  |  |  |  |  |  |  |  |  |  |  |  |  |  |  |  |  |  |  |  |  |  |  |  |  |  |  |  |  |  |  |  |  |  |  |  |  |  |  |  |  |  |  |  |  |  |  |  |  |  |  |  |  |  |  |  |  |  |  |  |  |  |  |  |  |  |  |  |  |  |  |  |  |  |  |  |  |  |  |  |  |  |  |  |  |  |  |  |  |  |  |  |  |  |  |  |  |  |  |  |  |  |  |  |  |  |  |  |  |  |  |  |  |  |  |  |  |  |  |  |  |  |  |  |  |  |  |  |  |  |  |  |  |  |  |  |  |  |  |  |  |  |  |  |  |  |  |  |  |  |  |  |  |  |  |  |  |  |  |  |  |  |  |  |  |  |  |  |  |  |  |  |  |  |  |  |  |  |  |  |  |  |  |  |  |  |  |  |  |  |  |  |  |  |  |  |  |  |  |  |  |  |  |  |  |  |  |  |  |  |  |  |  |  |  |  |  |  |  |  |  |  |  |  |  |  |  |  |  |  |  |  |  |  |  |  |  |  |  |  |  |  |  |  |  |  |  |  |  |  |  |  |  |  |  |  |  |  |  |  |  |  |  |  |  |  |  |  |  |  |  |  |  |  |  |  |  |  |  |  |  |  |  |  |  |  |  |  |  |  |  |  |  |  |  |  |  |  |  |  |  |  |  |  |  |  |  |  |  |  |  |  |  |  |  |  |  |  |  |  |  |  |  |  |  |  |  |  |  |  |  |  |  |  |  |  |  |  |  |  |  |  |  |  |  |  |  |  |  |  |  |  |  |  |  |  |  |  |  |  |  |  |  |  |  |  |  |  |  |  |  |  |  |  |  |  |  |  |  |  |  |  |  |  |  |  |  |  |  |  |  |  |  |  |  |  |  |  |  |  |  |  |  |  |  |  |  |  |  |  |  |  |  |  |  |  |  |
| --- | --- | --- | --- | --- | --- | --- | --- | --- | --- | --- | --- | --- | --- | --- | --- | --- | --- | --- | --- | --- | --- | --- | --- | --- | --- | --- | --- | --- | --- | --- | --- | --- | --- | --- | --- | --- | --- | --- | --- | --- | --- | --- | --- | --- | --- | --- | --- | --- | --- | --- | --- | --- | --- | --- | --- | --- | --- | --- | --- | --- | --- | --- | --- | --- | --- | --- | --- | --- | --- | --- | --- | --- | --- | --- | --- | --- | --- | --- | --- | --- | --- | --- | --- | --- | --- | --- | --- | --- | --- | --- | --- | --- | --- | --- | --- | --- | --- | --- | --- | --- | --- | --- | --- | --- | --- | --- | --- | --- | --- | --- | --- | --- | --- | --- | --- | --- | --- | --- | --- | --- | --- | --- | --- | --- | --- | --- | --- | --- | --- | --- | --- | --- | --- | --- | --- | --- | --- | --- | --- | --- | --- | --- | --- | --- | --- | --- | --- | --- | --- | --- | --- | --- | --- | --- | --- | --- | --- | --- | --- | --- | --- | --- | --- | --- | --- | --- | --- | --- | --- | --- | --- | --- | --- | --- | --- | --- | --- | --- | --- | --- | --- | --- | --- | --- | --- | --- | --- | --- | --- | --- | --- | --- | --- | --- | --- | --- | --- | --- | --- | --- | --- | --- | --- | --- | --- | --- | --- | --- | --- | --- | --- | --- | --- | --- | --- | --- | --- | --- | --- | --- | --- | --- | --- | --- | --- | --- | --- | --- | --- | --- | --- | --- | --- | --- | --- | --- | --- | --- | --- | --- | --- | --- | --- | --- | --- | --- | --- | --- | --- | --- | --- | --- | --- | --- | --- | --- | --- | --- | --- | --- | --- | --- | --- | --- | --- | --- | --- | --- | --- | --- | --- | --- | --- | --- | --- | --- | --- | --- | --- | --- | --- | --- | --- | --- | --- | --- | --- | --- | --- | --- | --- | --- | --- | --- | --- | --- | --- | --- | --- | --- | --- | --- | --- | --- | --- | --- | --- | --- | --- | --- | --- | --- | --- | --- | --- | --- | --- | --- | --- | --- | --- | --- | --- | --- | --- | --- | --- | --- | --- | --- | --- | --- | --- | --- | --- | --- | --- | --- | --- | --- | --- | --- | --- | --- | --- | --- | --- | --- | --- | --- | --- | --- | --- | --- | --- | --- | --- | --- | --- | --- | --- | --- | --- | --- | --- | --- | --- | --- | --- | --- | --- | --- | --- | --- | --- | --- | --- | --- | --- | --- | --- | --- | --- | --- | --- | --- | --- | --- | --- | --- | --- | --- | --- | --- | --- | --- | --- | --- | --- | --- | --- | --- | --- | --- | --- | --- | --- | --- | --- | --- | --- | --- | --- | --- | --- | --- | --- | --- | --- | --- | --- | --- | --- | --- | --- | --- | --- | --- | --- | --- | --- | --- | --- | --- | --- | --- | --- | --- | --- | --- | --- | --- | --- | --- | --- | --- | --- | --- | --- | --- | --- | --- | --- | --- | --- | --- | --- | --- | --- | --- | --- | --- | --- | --- | --- | --- | --- | --- | --- | --- | --- | --- | --- | --- | --- | --- | --- | --- | --- | --- | --- | --- | --- | --- | --- | --- | --- | --- | --- | --- | --- | --- | --- | --- | --- | --- | --- | --- | --- | --- | --- | --- | --- | --- | --- | --- | --- | --- | --- | --- | --- | --- | --- | --- | --- | --- | --- | --- | --- | --- | --- | --- | --- | --- | --- | --- | --- | --- | --- | --- | --- | --- | --- | --- | --- | --- | --- | --- | --- | --- | --- | --- | --- | --- | --- | --- | --- | --- | --- | --- | --- | --- | --- | --- | --- | --- | --- | --- | --- | --- | --- | --- | --- | --- | --- | --- | --- | --- | --- | --- | --- | --- | --- | --- | --- | --- | --- | --- | --- | --- | --- | --- | --- | --- | --- | --- | --- | --- | --- | --- | --- | --- | --- | --- | --- | --- | --- | --- | --- | --- | --- | --- | --- | --- | --- | --- | --- | --- | --- | --- | --- | --- | --- | --- | --- | --- | --- | --- | --- | --- | --- | --- | --- | --- | --- | --- | --- | --- | --- | --- | --- | --- | --- | --- | --- | --- | --- | --- | --- | --- | --- | --- | --- | --- | --- | --- | --- | --- | --- | --- | --- | --- | --- | --- | --- | --- | --- | --- | --- | --- | --- | --- | --- | --- | --- | --- | --- | --- | --- | --- | --- | --- | --- | --- | --- | --- | --- | --- | --- | --- | --- | --- | --- | --- | --- | --- | --- | --- | --- | --- | --- | --- | --- | --- | --- | --- | --- | --- | --- | --- | --- | --- | --- | --- | --- | --- | --- | --- | --- | --- | --- | --- | --- | --- | --- | --- | --- | --- | --- | --- | --- | --- | --- | --- | --- | --- | --- | --- | --- | --- | --- | --- | --- | --- | --- | --- | --- | --- | --- | --- | --- | --- | --- | --- | --- | --- | --- | --- | --- | --- | --- | --- | --- | --- | --- | --- | --- | --- | --- | --- | --- | --- | --- | --- | --- | --- | --- | --- | --- | --- | --- | --- | --- | --- | --- | --- | --- | --- | --- | --- | --- | --- | --- | --- | --- | --- | --- | --- | --- | --- | --- | --- | --- | --- | --- | --- | --- | --- | --- | --- | --- | --- | --- | --- | --- | --- | --- | --- | --- | --- | --- | --- | --- | --- | --- | --- | --- | --- | --- | --- | --- | --- | --- | --- | --- | --- | --- | --- | --- | --- | --- | --- | --- | --- | --- | --- | --- | --- | --- | --- | --- | --- | --- | --- | --- | --- | --- | --- | --- | --- | --- | --- | --- | --- | --- | --- | --- | --- | --- | --- | --- | --- | --- | --- | --- | --- | --- | --- | --- | --- | --- | --- | --- | --- | --- | --- | --- | --- | --- | --- | --- | --- | --- | --- | --- | --- | --- | --- | --- | --- | --- | --- | --- | --- | --- | --- | --- | --- | --- | --- | --- | --- | --- | --- | --- | --- | --- | --- | --- | --- | --- | --- | --- | --- | --- | --- | --- | --- | --- | --- | --- | --- | --- | --- | --- | --- | --- | --- | --- | --- | --- | --- | --- | --- | --- | --- | --- | --- | --- | --- | --- | --- | --- | --- | --- | --- | --- | --- | --- | --- | --- | --- | --- | --- | --- | --- | --- | --- | --- | --- | --- | --- | --- | --- | --- | --- | --- | --- | --- | --- | --- | --- | --- | --- | --- | --- | --- | --- | --- | --- | --- | --- | --- | --- | --- | --- | --- | --- | --- | --- | --- | --- | --- | --- | --- | --- | --- | --- | --- | --- | --- | --- | --- | --- | --- | --- | --- | --- | --- | --- | --- | --- | --- | --- | --- | --- | --- | --- | --- | --- | --- | --- | --- | --- | --- | --- | --- | --- | --- | --- | --- | --- | --- | --- | --- | --- | --- | --- | --- | --- | --- | --- | --- | --- | --- | --- | --- | --- | --- | --- | --- | --- | --- | --- | --- | --- | --- | --- | --- | --- | --- | --- | --- | --- | --- | --- | --- | --- | --- | --- | --- | --- | --- | --- | --- | --- | --- | --- | --- | --- | --- | --- | --- | --- | --- | --- | --- | --- | --- | --- | --- | --- | --- | --- | --- | --- | --- | --- | --- | --- | --- | --- | --- | --- | --- | --- | --- | --- | --- | --- | --- | --- | --- | --- | --- | --- | --- | --- | --- | --- | --- | --- | --- | --- | --- | --- | --- | --- | --- | --- | --- | --- | --- | --- | --- | --- | --- | --- | --- | --- | --- | --- | --- | --- | --- | --- | --- | --- | --- | --- | --- | --- | --- | --- | --- | --- | --- | --- | --- | --- | --- | --- | --- | --- | --- | --- | --- | --- | --- | --- | --- | --- | --- | --- | --- | --- | --- | --- | --- | --- | --- | --- | --- | --- | --- | --- | --- | --- | --- | --- | --- | --- | --- | --- | --- | --- | --- | --- | --- | --- | --- | --- | --- | --- | --- | --- | --- | --- | --- | --- | --- | --- | --- | --- | --- | --- | --- | --- | --- | --- | --- | --- | --- | --- | --- | --- | --- | --- | --- | --- | --- | --- | --- | --- | --- | --- | --- | --- | --- | --- | --- | --- | --- | --- | --- | --- | --- | --- | --- | --- | --- | --- | --- | --- | --- | --- | --- | --- | --- | --- | --- | --- | --- | --- | --- | --- | --- | --- | --- | --- | --- | --- | --- | --- | --- | --- | --- | --- | --- | --- | --- | --- | --- | --- | --- | --- | --- | --- | --- | --- | --- | --- | --- | --- | --- | --- | --- | --- | --- | --- | --- | --- | --- | --- | --- | --- | --- | --- | --- | --- | --- | --- | --- | --- | --- | --- | --- | --- | --- | --- | --- | --- | --- | --- | --- | --- | --- | --- | --- | --- | --- | --- | --- | --- | --- | --- | --- | --- | --- | --- | --- | --- | --- | --- | --- | --- | --- | --- | --- | --- | --- | --- | --- | --- | --- | --- | --- | --- | --- | --- | --- | --- | --- | --- | --- | --- | --- | --- | --- | --- | --- | --- | --- | --- | --- | --- | --- | --- | --- | --- | --- | --- | --- | --- | --- | --- | --- | --- | --- | --- | --- | --- | --- | --- | --- | --- | --- | --- | --- | --- | --- | --- | --- | --- | --- | --- | --- | --- | --- | --- | --- | --- | --- | --- | --- | --- | --- | --- | --- | --- | --- | --- | --- | --- | --- | --- | --- | --- | --- | --- | --- | --- | --- | --- | --- | --- | --- | --- | --- | --- | --- | --- | --- | --- | --- | --- | --- | --- | --- | --- | --- | --- | --- | --- | --- | --- | --- | --- | --- | --- | --- | --- | --- | --- | --- | --- | --- | --- | --- | --- | --- | --- | --- | --- | --- | --- | --- | --- | --- | --- | --- | --- | --- | --- | --- | --- | --- | --- | --- | --- | --- | --- | --- | --- | --- | --- | --- | --- | --- | --- | --- | --- | --- | --- | --- | --- | --- | --- | --- | --- | --- | --- | --- | --- | --- | --- | --- | --- | --- | --- | --- | --- | --- | --- | --- | --- | --- | --- | --- | --- | --- | --- | --- | --- | --- | --- | --- | --- | --- | --- | --- | --- | --- | --- | --- | --- | --- | --- | --- | --- | --- | --- | --- | --- | --- | --- | --- | --- | --- | --- | --- | --- | --- | --- | --- | --- | --- | --- | --- | --- | --- | --- | --- | --- | --- | --- | --- | --- | --- | --- | --- | --- | --- | --- | --- | --- | --- | --- | --- | --- | --- | --- | --- | --- | --- | --- | --- | --- | --- | --- | --- | --- | --- | --- | --- | --- | --- | --- | --- | --- | --- | --- | --- | --- | --- | --- | --- | --- | --- | --- | --- | --- | --- | --- | --- | --- | --- | --- | --- | --- | --- | --- | --- | --- | --- | --- | --- | --- | --- | --- | --- | --- | --- | --- | --- | --- | --- | --- | --- | --- | --- | --- | --- | --- | --- | --- | --- | --- | --- | --- | --- | --- | --- | --- | --- | --- | --- | --- | --- | --- | --- | --- | --- | --- | --- | --- | --- | --- | --- | --- | --- | --- | --- | --- | --- | --- | --- | --- | --- | --- | --- | --- | --- | --- | --- | --- | --- | --- | --- | --- | --- | --- | --- | --- | --- | --- | --- | --- | --- | --- | --- | --- | --- | --- | --- | --- | --- | --- | --- | --- | --- | --- | --- | --- | --- | --- | --- | --- | --- | --- | --- | --- | --- | --- | --- | --- | --- | --- | --- | --- | --- | --- | --- | --- | --- | --- | --- | --- | --- | --- | --- | --- | --- | --- | --- | --- | --- | --- | --- | --- | --- | --- | --- | --- | --- | --- | --- | --- | --- | --- | --- | --- | --- | --- | --- | --- | --- | --- | --- | --- | --- | --- | --- | --- | --- | --- | --- | --- | --- | --- | --- | --- | --- | --- | --- | --- | --- | --- | --- | --- | --- | --- | --- | --- | --- | --- | --- | --- | --- | --- | --- | --- | --- | --- | --- | --- | --- | --- | --- | --- | --- | --- | --- | --- | --- | --- | --- | --- | --- | --- | --- | --- | --- | --- | --- | --- | --- | --- | --- | --- | --- | --- | --- | --- | --- | --- | --- | --- | --- | --- | --- | --- | --- | --- | --- | --- | --- | --- | --- | --- | --- | --- | --- | --- | --- | --- | --- | --- | --- | --- | --- | --- | --- | --- | --- | --- | --- | --- | --- | --- | --- | --- | --- | --- | --- | --- | --- | --- | --- | --- | --- | --- | --- | --- | --- | --- | --- | --- | --- | --- | --- | --- | --- | --- | --- | --- | --- | --- | --- | --- | --- | --- | --- | --- | --- | --- | --- | --- | --- | --- | --- | --- | --- | --- | --- | --- | --- | --- | --- | --- | --- | --- | --- | --- | --- | --- | --- | --- | --- | --- | --- | --- | --- | --- | --- | --- | --- | --- | --- | --- | --- | --- | --- | --- | --- | --- | --- | --- | --- | --- | --- | --- | --- | --- | --- | --- | --- | --- | --- | --- | --- | --- | --- | --- | --- | --- | --- | --- | --- | --- | --- | --- | --- | --- | --- | --- | --- | --- | --- | --- | --- | --- | --- | --- | --- | --- | --- | --- | --- | --- | --- | --- | --- | --- | --- | --- | --- | --- | --- | --- | --- | --- | --- | --- | --- | --- | --- | --- | --- | --- | --- | --- | --- | --- | --- | --- | --- | --- | --- | --- | --- | --- | --- | --- | --- | --- | --- | --- | --- | --- | --- | --- | --- | --- | --- | --- | --- | --- | --- | --- | --- | --- | --- | --- | --- | --- | --- | --- | --- | --- | --- | --- | --- | --- | --- | --- | --- | --- | --- | --- | --- | --- | --- | --- | --- | --- | --- | --- | --- | --- | --- | --- | --- | --- | --- | --- | --- | --- | --- | --- | --- | --- | --- | --- | --- | --- | --- | --- | --- | --- | --- | --- | --- | --- | --- | --- | --- | --- | --- | --- | --- | --- | --- | --- | --- | --- | --- | --- | --- | --- | --- | --- | --- | --- | --- | --- | --- | --- | --- | --- | --- | --- | --- | --- | --- | --- | --- | --- | --- | --- | --- | --- | --- | --- | --- | --- | --- | --- | --- | --- | --- | --- | --- | --- | --- | --- | --- | --- | --- | --- | --- | --- | --- | --- | --- | --- | --- | --- | --- | --- | --- | --- | --- | --- | --- | --- | --- | --- | --- | --- | --- | --- | --- | --- | --- | --- | --- | --- | --- | --- | --- | --- | --- | --- | --- | --- | --- | --- | --- | --- | --- | --- | --- | --- | --- | --- | --- | --- | --- | --- | --- | --- | --- | --- | --- | --- | --- | --- | --- | --- | --- | --- | --- | --- | --- | --- | --- | --- | --- | --- | --- | --- | --- | --- | --- | --- | --- | --- | --- | --- | --- | --- | --- | --- | --- | --- | --- | --- | --- | --- | --- | --- | --- | --- | --- | --- | --- | --- | --- | --- | --- | --- | --- | --- | --- | --- | --- | --- | --- | --- | --- | --- | --- | --- | --- | --- | --- | --- | --- | --- | --- | --- | --- | --- | --- | --- | --- | --- | --- | --- | --- | --- | --- | --- | --- | --- | --- | --- | --- | --- | --- | --- | --- | --- | --- | --- | --- | --- | --- | --- | --- | --- | --- | --- | --- | --- | --- | --- | --- | --- | --- | --- | --- | --- | --- | --- | --- | --- | --- | --- | --- | --- | --- | --- | --- | --- | --- | --- | --- | --- | --- | --- | --- | --- | --- | --- | --- | --- | --- | --- | --- | --- | --- | --- | --- | --- | --- | --- | --- | --- | --- | --- | --- | --- | --- | --- | --- | --- | --- | --- | --- | --- | --- | --- | --- | --- | --- | --- | --- | --- | --- | --- | --- | --- | --- | --- | --- | --- | --- | --- | --- | --- | --- | --- | --- | --- | --- | --- | --- | --- | --- | --- | --- | --- | --- | --- | --- | --- | --- | --- | --- | --- | --- | --- | --- | --- | --- | --- | --- | --- | --- | --- | --- | --- | --- | --- | --- | --- | --- | --- | --- | --- | --- | --- | --- | --- | --- | --- | --- | --- | --- | --- | --- | --- | --- | --- | --- | --- | --- | --- | --- | --- | --- | --- | --- | --- | --- | --- | --- | --- | --- | --- | --- | --- | --- | --- | --- | --- | --- | --- | --- | --- | --- | --- | --- | --- | --- | --- | --- | --- | --- | --- | --- | --- | --- | --- | --- | --- | --- | --- | --- | --- | --- | --- | --- | --- | --- | --- | --- | --- | --- | --- | --- | --- | --- | --- | --- | --- | --- | --- | --- | --- | --- | --- | --- | --- | --- | --- | --- | --- | --- | --- | --- | --- | --- | --- | --- | --- | --- | --- | --- | --- | --- | --- | --- | --- | --- | --- | --- | --- | --- | --- | --- | --- | --- | --- | --- | --- | --- | --- | --- | --- | --- | --- | --- | --- | --- | --- | --- | --- | --- | --- | --- | --- | --- | --- | --- | --- | --- | --- | --- | --- | --- | --- | --- | --- | --- | --- | --- | --- | --- | --- | --- | --- | --- | --- | --- | --- | --- | --- | --- | --- | --- | --- | --- | --- | --- | --- | --- | --- | --- | --- | --- | --- | --- | --- | --- | --- | --- | --- | --- | --- | --- | --- | --- | --- | --- | --- | --- | --- | --- | --- | --- | --- | --- | --- | --- | --- | --- | --- | --- | --- | --- | --- | --- | --- | --- | --- | --- | --- | --- | --- | --- | --- | --- | --- | --- | --- | --- | --- | --- | --- | --- | --- | --- | --- | --- | --- | --- | --- | --- | --- | --- | --- | --- | --- | --- | --- | --- | --- | --- | --- | --- | --- | --- | --- | --- | --- | --- | --- | --- | --- | --- | --- | --- | --- | --- | --- | --- | --- | --- | --- | --- | --- | --- | --- | --- | --- | --- | --- | --- | --- | --- | --- | --- | --- | --- | --- | --- | --- | --- | --- | --- | --- | --- | --- | --- | --- | --- | --- | --- | --- | --- | --- | --- | --- | --- | --- | --- | --- | --- | --- | --- | --- | --- | --- | --- | --- | --- | --- | --- | --- | --- | --- | --- | --- | --- | --- | --- | --- | --- | --- | --- | --- | --- | --- | --- | --- | --- | --- | --- | --- | --- | --- | --- | --- | --- | --- | --- | --- | --- | --- | --- | --- | --- | --- | --- | --- | --- | --- | --- | --- | --- | --- | --- | --- | --- | --- | --- | --- | --- | --- | --- | --- | --- | --- | --- | --- | --- | --- | --- | --- | --- | --- | --- | --- | --- | --- | --- | --- | --- | --- | --- | --- | --- | --- | --- | --- | --- | --- | --- | --- | --- | --- | --- | --- | --- | --- | --- | --- | --- | --- | --- | --- | --- | --- | --- | --- | --- | --- | --- | --- | --- | --- | --- | --- | --- | --- | --- | --- | --- | --- | --- | --- | --- | --- | --- | --- | --- | --- | --- | --- | --- | --- | --- | --- | --- | --- | --- | --- | --- | --- | --- | --- | --- | --- | --- | --- | --- | --- | --- | --- | --- | --- | --- | --- | --- | --- | --- | --- | --- | --- | --- | --- | --- | --- | --- | --- | --- | --- | --- | --- | --- | --- | --- | --- | --- | --- | --- | --- | --- | --- | --- | --- | --- | --- | --- | --- | --- | --- | --- | --- | --- | --- | --- | --- | --- | --- | --- | --- | --- | --- | --- | --- | --- | --- | --- | --- | --- | --- | --- | --- | --- | --- | --- | --- | --- | --- | --- | --- | --- | --- | --- | --- | --- | --- | --- | --- | --- | --- | --- | --- | --- | --- | --- | --- | --- | --- | --- | --- | --- | --- | --- | --- | --- | --- | --- | --- | --- | --- | --- | --- | --- | --- | --- | --- | --- | --- | --- | --- | --- | --- | --- | --- | --- | --- | --- | --- | --- | --- | --- | --- | --- | --- | --- | --- | --- | --- | --- | --- | --- | --- | --- | --- | --- | --- | --- | --- | --- | --- | --- | --- | --- | --- | --- | --- | --- | --- | --- | --- | --- | --- | --- | --- | --- | --- | --- | --- | --- | --- | --- | --- | --- | --- | --- | --- | --- | --- | --- | --- | --- | --- | --- | --- | --- | --- | --- | --- | --- | --- | --- | --- | --- | --- | --- | --- | --- | --- | --- | --- | --- | --- | --- | --- | --- | --- | --- | --- | --- | --- | --- | --- | --- | --- | --- | --- | --- | --- | --- | --- | --- | --- | --- | --- | --- | --- | --- | --- | --- | --- | --- | --- | --- | --- | --- | --- | --- | --- | --- | --- | --- | --- | --- | --- | --- | --- | --- | --- | --- | --- | --- | --- | --- | --- | --- | --- | --- | --- | --- | --- | --- | --- | --- | --- | --- | --- | --- | --- | --- | --- | --- | --- | --- | --- | --- | --- | --- | --- | --- | --- | --- | --- | --- | --- | --- | --- | --- | --- | --- | --- | --- | --- | --- | --- | --- | --- | --- | --- | --- | --- | --- | --- | --- | --- | --- | --- | --- | --- | --- | --- | --- | --- | --- | --- | --- | --- | --- | --- | --- | --- | --- | --- | --- | --- | --- | --- | --- | --- | --- | --- | --- | --- | --- | --- | --- | --- | --- | --- | --- | --- | --- | --- | --- | --- | --- | --- | --- | --- | --- | --- | --- | --- | --- | --- | --- | --- | --- | --- | --- | --- | --- | --- | --- | --- | --- | --- | --- | --- | --- | --- | --- | --- | --- | --- | --- | --- | --- | --- | --- | --- | --- | --- | --- | --- | --- | --- | --- | --- | --- | --- | --- | --- | --- | --- | --- | --- | --- | --- | --- | --- | --- | --- | --- | --- | --- | --- | --- | --- | --- | --- | --- | --- | --- | --- | --- | --- | --- | --- | --- | --- | --- | --- | --- | --- | --- | --- | --- | --- | --- | --- | --- | --- | --- | --- | --- | --- | --- | --- | --- | --- | --- | --- | --- | --- | --- | --- | --- | --- | --- | --- | --- | --- | --- | --- | --- | --- | --- | --- | --- | --- | --- | --- | --- | --- | --- | --- | --- | --- | --- | --- | --- | --- | --- | --- | --- | --- | --- | --- | --- | --- | --- | --- | --- | --- | --- | --- | --- | --- | --- | --- | --- | --- | --- | --- | --- | --- | --- | --- | --- | --- | --- | --- | --- | --- | --- | --- | --- | --- | --- | --- | --- | --- | --- | --- | --- | --- | --- | --- | --- | --- | --- | --- | --- | --- | --- | --- | --- | --- | --- | --- | --- | --- | --- | --- | --- | --- | --- | --- | --- | --- | --- | --- | --- | --- | --- | --- | --- | --- | --- | --- | --- | --- | --- | --- | --- | --- | --- | --- | --- | --- | --- | --- | --- | --- | --- | --- | --- | --- | --- | --- | --- | --- | --- | --- | --- | --- | --- | --- | --- | --- | --- | --- | --- | --- | --- | --- | --- | --- | --- | --- | --- | --- | --- | --- | --- | --- | --- | --- | --- | --- | --- | --- | --- | --- | --- | --- | --- | --- | --- | --- | --- | --- | --- | --- | --- | --- | --- | --- | --- | --- | --- | --- | --- | --- | --- | --- | --- | --- | --- | --- | --- | --- | --- | --- | --- | --- | --- | --- | --- | --- | --- | --- | --- | --- | --- | --- | --- | --- | --- | --- | --- | --- | --- | --- | --- | --- | --- | --- | --- | --- | --- | --- | --- | --- | --- | --- | --- | --- | --- | --- | --- | --- | --- | --- | --- | --- | --- | --- | --- | --- | --- | --- | --- | --- | --- | --- | --- | --- | --- | --- | --- | --- | --- | --- | --- | --- | --- | --- | --- | --- | --- | --- | --- | --- | --- | --- | --- | --- | --- | --- | --- | --- | --- | --- | --- | --- | --- | --- | --- | --- | --- | --- | --- | --- | --- | --- | --- | --- | --- | --- | --- | --- | --- | --- | --- | --- | --- | --- | --- | --- | --- | --- | --- | --- | --- | --- | --- | --- | --- | --- | --- | --- | --- | --- | --- | --- | --- | --- | --- | --- | --- | --- | --- | --- | --- | --- | --- | --- | --- | --- | --- | --- | --- | --- | --- | --- | --- | --- | --- | --- | --- | --- | --- | --- | --- | --- | --- | --- | --- | --- | --- | --- | --- | --- | --- | --- | --- | --- | --- | --- | --- | --- | --- | --- | --- | --- | --- | --- | --- | --- | --- | --- | --- | --- | --- | --- | --- | --- | --- | --- | --- | --- | --- | --- | --- | --- | --- | --- | --- | --- | --- | --- | --- | --- | --- | --- | --- | --- | --- | --- | --- | --- | --- | --- | --- | --- | --- | --- | --- | --- | --- | --- | --- | --- | --- | --- | --- | --- | --- | --- | --- | --- | --- | --- | --- | --- | --- | --- | --- | --- | --- | --- | --- | --- | --- | --- | --- | --- | --- | --- | --- | --- | --- | --- | --- | --- | --- | --- | --- | --- | --- | --- | --- | --- | --- | --- | --- | --- | --- | --- | --- | --- | --- | --- | --- | --- | --- | --- | --- | --- | --- | --- | --- | --- | --- | --- | --- | --- | --- | --- | --- | --- | --- | --- | --- | --- | --- | --- | --- | --- | --- | --- | --- | --- | --- | --- | --- | --- | --- | --- | --- | --- | --- | --- | --- | --- | --- | --- | --- | --- | --- | --- | --- | --- | --- | --- | --- | --- | --- | --- | --- | --- | --- | --- | --- | --- | --- | --- | --- | --- | --- | --- | --- | --- | --- | --- | --- | --- | --- | --- | --- | --- | --- | --- | --- | --- | --- | --- | --- | --- | --- | --- | --- | --- | --- | --- | --- | --- | --- | --- | --- | --- | --- | --- | --- | --- | --- | --- | --- | --- | --- | --- | --- | --- | --- | --- | --- | --- | --- | --- | --- | --- | --- | --- | --- | --- | --- | --- | --- | --- | --- | --- | --- | --- | --- | --- | --- | --- | --- | --- | --- | --- | --- | --- | --- | --- | --- | --- | --- | --- | --- | --- | --- | --- | --- | --- | --- | --- | --- | --- | --- | --- | --- | --- | --- | --- | --- | --- | --- | --- | --- | --- | --- | --- | --- | --- | --- | --- | --- | --- | --- | --- | --- | --- | --- | --- | --- | --- | --- | --- | --- | --- | --- | --- | --- | --- | --- | --- | --- | --- | --- | --- | --- | --- | --- | --- | --- | --- | --- | --- | --- | --- | --- | --- | --- | --- | --- | --- | --- | --- | --- | --- | --- | --- | --- | --- | --- | --- | --- | --- | --- | --- | --- | --- | --- | --- | --- | --- | --- | --- | --- | --- | --- | --- | --- | --- | --- | --- | --- | --- | --- | --- | --- | --- | --- | --- | --- | --- | --- | --- | --- | --- | --- | --- | --- | --- | --- | --- | --- | --- | --- | --- | --- | --- | --- | --- | --- | --- | --- | --- | --- | --- | --- | --- | --- | --- | --- | --- | --- | --- | --- | --- | --- | --- | --- | --- | --- | --- | --- | --- | --- | --- | --- | --- | --- | --- | --- | --- | --- | --- | --- | --- | --- | --- | --- | --- | --- | --- | --- | --- | --- | --- | --- | --- | --- | --- | --- | --- | --- | --- | --- | --- | --- | --- | --- | --- | --- | --- | --- | --- | --- | --- | --- | --- | --- | --- | --- | --- | --- | --- | --- | --- | --- | --- | --- | --- | --- | --- | --- | --- | --- | --- | --- | --- | --- | --- | --- | --- | --- | --- | --- | --- | --- | --- | --- | --- | --- | --- | --- | --- | --- | --- | --- | --- | --- | --- | --- | --- | --- | --- | --- | --- | --- | --- | --- | --- | --- | --- | --- | --- | --- | --- | --- | --- | --- | --- | --- | --- | --- | --- | --- | --- | --- | --- | --- | --- | --- | --- | --- | --- | --- | --- | --- | --- | --- | --- | --- | --- | --- | --- | --- | --- | --- | --- | --- | --- | --- | --- | --- | --- | --- | --- | --- | --- | --- | --- | --- | --- | --- | --- | --- | --- | --- | --- | --- | --- | --- | --- | --- | --- | --- | --- | --- | --- | --- | --- | --- | --- | --- | --- | --- | --- | --- | --- | --- | --- | --- | --- | --- | --- | --- | --- | --- | --- | --- | --- | --- | --- | --- | --- | --- | --- | --- | --- | --- | --- | --- | --- | --- | --- | --- | --- | --- | --- | --- | --- | --- | --- | --- | --- | --- | --- | --- | --- | --- | --- | --- | --- | --- | --- | --- | --- | --- | --- | --- | --- | --- | --- | --- | --- | --- | --- | --- | --- | --- | --- | --- | --- | --- | --- | --- | --- | --- | --- | --- | --- | --- | --- | --- | --- | --- | --- | --- | --- | --- | --- | --- | --- | --- | --- | --- | --- | --- | --- | --- | --- | --- | --- | --- | --- | --- | --- | --- | --- | --- | --- | --- | --- | --- | --- | --- | --- | --- | --- | --- | --- | --- | --- | --- | --- | --- | --- | --- | --- | --- | --- | --- | --- | --- | --- | --- | --- | --- | --- | --- | --- | --- | --- | --- | --- | --- | --- | --- | --- | --- | --- | --- | --- | --- | --- | --- | --- | --- | --- | --- | --- | --- | --- | --- | --- | --- | --- | --- | --- | --- | --- | --- | --- | --- | --- | --- | --- | --- | --- | --- | --- | --- | --- | --- | --- | --- | --- | --- | --- | --- | --- | --- | --- | --- | --- | --- | --- | --- | --- | --- | --- | --- | --- | --- | --- | --- | --- | --- | --- | --- | --- | --- | --- | --- | --- | --- | --- | --- | --- | --- | --- | --- | --- | --- | --- | --- | --- | --- | --- | --- | --- | --- | --- | --- | --- | --- | --- | --- | --- | --- | --- | --- | --- | --- | --- | --- | --- | --- | --- | --- | --- | --- | --- | --- | --- | --- | --- | --- | --- | --- | --- | --- | --- | --- | --- | --- | --- | --- | --- | --- | --- | --- | --- | --- | --- | --- | --- | --- | --- | --- | --- | --- | --- | --- | --- | --- | --- | --- | --- | --- | --- | --- | --- | --- | --- | --- | --- | --- | --- | --- | --- | --- | --- | --- | --- | --- | --- | --- | --- | --- | --- | --- | --- | --- | --- | --- | --- | --- | --- | --- | --- | --- | --- | --- | --- | --- | --- | --- | --- | --- | --- | --- | --- | --- | --- | --- | --- | --- | --- | --- | --- | --- | --- | --- | --- | --- | --- | --- | --- | --- | --- | --- | --- | --- | --- | --- | --- | --- | --- | --- | --- | --- | --- | --- | --- | --- | --- | --- | --- | --- | --- | --- | --- | --- | --- | --- | --- | --- | --- | --- | --- | --- | --- | --- | --- | --- | --- | --- | --- | --- | --- | --- | --- | --- | --- | --- | --- | --- | --- | --- | --- | --- | --- | --- | --- | --- | --- | --- | --- | --- | --- | --- | --- | --- | --- | --- | --- | --- | --- | --- | --- | --- | --- | --- | --- | --- | --- | --- | --- | --- | --- | --- | --- | --- | --- | --- | --- | --- | --- | --- | --- | --- | --- | --- | --- | --- | --- | --- | --- | --- | --- | --- | --- | --- | --- | --- | --- | --- | --- | --- | --- | --- | --- | --- | --- | --- | --- | --- | --- | --- | --- | --- | --- | --- | --- | --- | --- | --- | --- | --- | --- | --- | --- | --- | --- | --- | --- | --- | --- | --- | --- | --- | --- | --- | --- | --- | --- | --- | --- | --- | --- | --- | --- | --- | --- | --- | --- | --- | --- | --- | --- | --- | --- | --- | --- | --- | --- | --- | --- | --- | --- | --- | --- | --- | --- | --- | --- | --- | --- | --- | --- | --- | --- | --- | --- | --- | --- | --- | --- | --- | --- | --- | --- | --- | --- | --- | --- | --- | --- | --- | --- | --- | --- | --- | --- | --- | --- | --- | --- | --- | --- | --- | --- | --- | --- | --- | --- | --- | --- | --- | --- | --- | --- | --- | --- | --- | --- | --- | --- | --- | --- | --- | --- | --- | --- | --- | --- | --- | --- | --- | --- | --- | --- | --- | --- | --- | --- | --- | --- | --- | --- | --- | --- | --- | --- | --- | --- | --- | --- | --- | --- | --- | --- | --- | --- | --- | --- | --- | --- | --- | --- | --- | --- | --- | --- | --- | --- | --- | --- | --- | --- | --- | --- | --- | --- | --- | --- | --- | --- | --- | --- | --- | --- | --- | --- | --- | --- | --- | --- | --- | --- | --- | --- | --- | --- | --- | --- | --- | --- | --- | --- | --- | --- | --- | --- | --- | --- | --- | --- | --- | --- | --- | --- | --- | --- | --- | --- | --- | --- | --- | --- | --- | --- | --- | --- | --- | --- | --- | --- | --- | --- | --- | --- | --- | --- | --- | --- | --- | --- | --- | --- | --- | --- | --- | --- | --- | --- | --- | --- | --- | --- | --- | --- | --- | --- | --- | --- | --- | --- | --- | --- | --- | --- | --- | --- | --- | --- | --- | --- | --- | --- | --- | --- | --- | --- | --- | --- | --- | --- | --- | --- | --- | --- | --- | --- | --- | --- | --- | --- | --- | --- | --- | --- | --- | --- | --- | --- | --- | --- | --- | --- | --- | --- | --- | --- | --- | --- | --- | --- | --- | --- | --- | --- | --- | --- | --- | --- | --- | --- | --- | --- | --- | --- | --- | --- | --- | --- | --- | --- | --- | --- | --- | --- | --- | --- | --- | --- | --- | --- | --- | --- | --- | --- | --- | --- | --- | --- | --- | --- | --- | --- | --- | --- | --- | --- | --- | --- | --- | --- | --- | --- | --- | --- | --- | --- | --- | --- | --- | --- | --- | --- | --- | --- | --- | --- | --- | --- | --- | --- | --- | --- | --- | --- | --- | --- | --- | --- | --- | --- | --- | --- | --- | --- | --- | --- | --- | --- | --- | --- | --- | --- | --- | --- | --- | --- | --- | --- | --- | --- | --- | --- | --- | --- | --- | --- | --- | --- | --- | --- | --- | --- | --- | --- | --- | --- | --- | --- | --- | --- | --- | --- | --- | --- | --- | --- | --- | --- | --- | --- | --- | --- | --- | --- | --- | --- | --- | --- | --- | --- | --- | --- | --- | --- | --- | --- | --- | --- | --- | --- | --- | --- | --- | --- | --- | --- | --- | --- | --- | --- | --- | --- | --- | --- | --- | --- | --- | --- | --- | --- | --- | --- | --- | --- | --- | --- | --- | --- | --- | --- | --- | --- | --- | --- | --- | --- | --- | --- | --- | --- | --- | --- | --- | --- | --- | --- | --- | --- | --- | --- | --- | --- | --- | --- | --- | --- | --- | --- | --- | --- | --- | --- | --- | --- | --- | --- | --- | --- | --- | --- | --- | --- | --- | --- | --- | --- | --- | --- | --- | --- | --- | --- | --- | --- | --- | --- | --- | --- | --- | --- | --- | --- | --- | --- | --- | --- | --- | --- | --- | --- | --- | --- | --- | --- | --- | --- | --- | --- | --- | --- | --- | --- | --- | --- | --- | --- | --- | --- | --- | --- | --- | --- | --- | --- | --- | --- | --- | --- | --- | --- | --- | --- | --- | --- | --- | --- | --- | --- | --- | --- | --- | --- | --- | --- | --- | --- | --- | --- | --- | --- | --- | --- | --- | --- | --- | --- | --- | --- | --- | --- | --- | --- | --- | --- | --- | --- | --- | --- | --- | --- | --- | --- | --- | --- | --- | --- | --- | --- | --- | --- | --- | --- | --- | --- | --- | --- | --- | --- | --- | --- | --- | --- | --- | --- | --- | --- | --- | --- | --- | --- | --- | --- | --- | --- | --- | --- | --- | --- | --- | --- | --- | --- | --- | --- | --- | --- | --- | --- | --- | --- | --- | --- | --- | --- | --- | --- | --- | --- | --- | --- | --- | --- | --- | --- | --- | --- | --- | --- | --- | --- | --- | --- | --- | --- | --- | --- | --- | --- | --- | --- | --- | --- | --- | --- | --- | --- | --- | --- | --- | --- | --- | --- | --- | --- | --- | --- | --- | --- | --- | --- | --- | --- | --- | --- | --- | --- | --- | --- | --- | --- | --- | --- | --- | --- | --- | --- | --- | --- | --- | --- | --- | --- | --- | --- | --- | --- | --- | --- | --- | --- | --- | --- | --- | --- | --- | --- | --- | --- | --- | --- | --- | --- | --- | --- | --- | --- | --- | --- | --- | --- | --- | --- | --- | --- | --- | --- | --- | --- | --- | --- | --- | --- | --- | --- | --- | --- | --- | --- | --- | --- | --- | --- | --- | --- | --- | --- | --- | --- | --- | --- | --- | --- | --- | --- | --- | --- | --- | --- | --- | --- | --- | --- | --- | --- | --- | --- | --- | --- | --- | --- | --- | --- | --- | --- | --- | --- | --- | --- | --- | --- | --- | --- | --- | --- | --- | --- | --- | --- | --- | --- | --- | --- | --- | --- | --- | --- | --- | --- | --- | --- | --- | --- | --- | --- | --- | --- | --- | --- | --- | --- | --- | --- | --- | --- | --- | --- | --- | --- | --- | --- | --- | --- | --- | --- | --- | --- | --- | --- | --- | --- | --- | --- | --- | --- | --- | --- | --- | --- | --- | --- | --- | --- | --- | --- | --- | --- | --- | --- | --- | --- | --- | --- | --- | --- | --- | --- | --- | --- | --- | --- | --- | --- | --- | --- | --- | --- | --- | --- | --- | --- | --- | --- | --- | --- | --- | --- | --- | --- | --- | --- | --- | --- | --- | --- | --- | --- | --- | --- | --- | --- | --- | --- | --- | --- | --- | --- | --- | --- | --- | --- | --- | --- | --- | --- | --- | --- | --- | --- | --- | --- | --- | --- | --- | --- | --- | --- | --- | --- | --- | --- | --- | --- | --- | --- | --- | --- | --- | --- | --- | --- | --- | --- | --- | --- | --- | --- | --- | --- | --- | --- | --- | --- | --- | --- | --- | --- | --- | --- | --- | --- | --- | --- | --- | --- | --- | --- | --- | --- | --- | --- | --- | --- | --- | --- | --- | --- | --- | --- | --- | --- | --- | --- | --- | --- | --- | --- | --- | --- | --- | --- | --- | --- | --- | --- | --- | --- | --- | --- | --- | --- | --- | --- | --- | --- | --- | --- | --- | --- | --- | --- | --- | --- | --- | --- | --- | --- | --- | --- | --- | --- | --- | --- | --- | --- | --- | --- | --- | --- | --- | --- | --- | --- | --- | --- | --- | --- | --- | --- | --- | --- | --- | --- | --- | --- | --- | --- | --- | --- | --- | --- | --- | --- | --- | --- | --- | --- | --- | --- | --- | --- | --- | --- | --- | --- | --- | --- | --- | --- | --- | --- | --- | --- | --- | --- | --- | --- | --- | --- | --- | --- | --- | --- | --- | --- | --- | --- | --- | --- | --- | --- | --- | --- | --- | --- | --- | --- | --- | --- | --- | --- | --- | --- | --- | --- | --- | --- | --- | --- | --- | --- | --- | --- | --- | --- | --- | --- | --- | --- | --- | --- | --- | --- | --- | --- | --- | --- | --- | --- | --- | --- | --- | --- | --- | --- | --- | --- | --- | --- | --- | --- | --- | --- | --- | --- | --- | --- | --- | --- | --- | --- | --- | --- | --- | --- | --- | --- | --- | --- | --- | --- | --- | --- | --- | --- | --- | --- | --- | --- | --- | --- | --- | --- | --- | --- | --- | --- | --- | --- | --- | --- | --- | --- | --- | --- | --- | --- | --- | --- | --- | --- | --- | --- | --- | --- | --- | --- | --- | --- | --- | --- | --- | --- | --- | --- | --- | --- | --- | --- | --- | --- | --- | --- | --- | --- | --- | --- | --- | --- | --- | --- | --- | --- | --- | --- | --- | --- | --- | --- | --- | --- | --- | --- | --- | --- | --- | --- | --- | --- | --- | --- | --- | --- | --- | --- | --- | --- | --- | --- | --- | --- | --- | --- | --- | --- | --- | --- | --- | --- | --- | --- | --- | --- | --- | --- | --- | --- | --- | --- | --- | --- | --- | --- | --- | --- | --- | --- | --- | --- | --- | --- | --- | --- | --- | --- | --- | --- | --- | --- | --- | --- | --- | --- | --- | --- | --- | --- | --- | --- | --- | --- | --- | --- | --- | --- | --- | --- | --- | --- | --- | --- | --- | --- | --- | --- | --- | --- | --- | --- | --- | --- | --- | --- | --- | --- | --- | --- | --- | --- | --- | --- | --- | --- | --- | --- | --- | --- | --- | --- | --- | --- | --- | --- | --- | --- | --- | --- | --- | --- | --- | --- | --- | --- | --- | --- | --- | --- | --- | --- | --- | --- | --- | --- | --- | --- | --- | --- | --- | --- | --- | --- | --- | --- | --- | --- | --- | --- | --- | --- | --- | --- | --- | --- | --- | --- | --- | --- | --- | --- | --- | --- | --- | --- | --- | --- | --- | --- | --- | --- | --- | --- | --- | --- | --- | --- | --- | --- | --- | --- | --- | --- | --- | --- | --- | --- | --- | --- | --- | --- | --- | --- | --- | --- | --- | --- | --- | --- | --- | --- | --- | --- | --- | --- | --- | --- | --- | --- | --- | --- | --- | --- | --- | --- | --- | --- | --- | --- | --- | --- | --- | --- | --- | --- | --- | --- | --- | --- | --- | --- | --- | --- | --- | --- | --- | --- | --- | --- | --- | --- | --- | --- | --- | --- | --- | --- | --- | --- | --- | --- | --- | --- | --- | --- | --- | --- | --- | --- | --- | --- | --- | --- | --- | --- | --- | --- | --- | --- | --- | --- | --- | --- | --- | --- | --- | --- | --- | --- | --- | --- | --- | --- | --- | --- | --- | --- | --- | --- | --- | --- | --- | --- | --- | --- | --- | --- | --- | --- | --- | --- | --- | --- | --- | --- | --- | --- | --- | --- | --- | --- | --- | --- | --- | --- | --- | --- | --- | --- | --- | --- | --- | --- | --- | --- | --- | --- | --- | --- | --- | --- | --- | --- | --- | --- | --- | --- | --- | --- | --- | --- | --- | --- | --- | --- | --- | --- | --- | --- | --- | --- | --- | --- | --- | --- | --- | --- | --- | --- | --- | --- | --- | --- | --- | --- | --- | --- | --- | --- | --- | --- | --- | --- | --- | --- | --- | --- | --- | --- | --- | --- | --- | --- | --- | --- | --- | --- | --- | --- | --- | --- | --- | --- | --- | --- | --- | --- | --- | --- | --- | --- | --- | --- | --- | --- | --- | --- | --- | --- | --- | --- | --- | --- | --- | --- | --- | --- | --- | --- | --- | --- | --- | --- | --- | --- | --- | --- | --- | --- | --- | --- | --- | --- | --- | --- | --- | --- | --- | --- | --- | --- | --- | --- | --- | --- | --- | --- | --- | --- | --- | --- | --- | --- | --- | --- | --- | --- | --- | --- | --- | --- | --- | --- | --- | --- | --- | --- | --- | --- | --- | --- | --- | --- | --- | --- | --- | --- | --- | --- | --- | --- | --- | --- | --- | --- | --- | --- | --- | --- | --- | --- | --- | --- | --- | --- | --- | --- | --- | --- | --- | --- | --- | --- | --- | --- | --- | --- | --- | --- | --- | --- | --- | --- | --- | --- | --- | --- | --- | --- | --- | --- | --- | --- | --- | --- | --- | --- | --- | --- | --- | --- | --- | --- | --- | --- | --- | --- | --- | --- | --- | --- | --- | --- | --- | --- | --- | --- | --- | --- | --- | --- | --- | --- | --- | --- | --- | --- | --- | --- | --- | --- | --- | --- | --- | --- | --- | --- | --- | --- | --- | --- | --- | --- | --- | --- | --- | --- | --- | --- | --- | --- | --- | --- | --- | --- | --- | --- | --- | --- | --- | --- | --- | --- | --- | --- | --- | --- | --- | --- | --- | --- | --- | --- | --- | --- | --- | --- | --- | --- | --- | --- | --- | --- | --- | --- | --- | --- | --- | --- | --- | --- | --- | --- | --- | --- | --- | --- | --- | --- | --- | --- | --- | --- | --- | --- | --- | --- | --- | --- | --- | --- | --- | --- | --- | --- | --- | --- | --- | --- | --- | --- | --- | --- | --- | --- | --- | --- | --- | --- | --- | --- | --- | --- | --- | --- | --- | --- | --- | --- | --- | --- | --- | --- | --- | --- | --- | --- | --- | --- | --- | --- | --- | --- | --- | --- | --- | --- | --- | --- | --- | --- | --- | --- | --- | --- | --- | --- | --- | --- | --- | --- | --- | --- | --- | --- | --- | --- | --- | --- | --- | --- | --- | --- | --- | --- | --- | --- | --- | --- | --- | --- | --- | --- | --- | --- | --- | --- | --- | --- | --- | --- | --- | --- | --- | --- | --- | --- | --- | --- | --- | --- | --- | --- | --- | --- | --- | --- | --- | --- | --- | --- | --- | --- | --- | --- | --- | --- | --- | --- | --- | --- | --- | --- | --- | --- | --- | --- | --- | --- | --- | --- | --- | --- | --- | --- | --- | --- | --- | --- | --- | --- | --- | --- | --- | --- | --- | --- | --- | --- | --- | --- | --- | --- | --- | --- | --- | --- | --- | --- | --- | --- | --- | --- | --- | --- | --- | --- | --- | --- | --- | --- | --- | --- | --- | --- | --- | --- | --- | --- | --- | --- | --- | --- | --- | --- | --- | --- | --- | --- | --- | --- | --- | --- | --- | --- | --- | --- | --- | --- | --- | --- | --- | --- | --- | --- | --- | --- | --- | --- | --- | --- | --- | --- | --- | --- | --- | --- | --- | --- | --- | --- | --- | --- | --- | --- | --- | --- | --- | --- | --- | --- | --- | --- | --- | --- | --- | --- | --- | --- | --- | --- | --- | --- | --- | --- | --- | --- | --- | --- | --- | --- | --- | --- | --- | --- | --- | --- | --- | --- | --- | --- | --- | --- | --- | --- | --- | --- | --- | --- | --- | --- | --- | --- | --- | --- | --- | --- | --- | --- | --- | --- | --- | --- | --- | --- | --- | --- | --- | --- | --- | --- | --- | --- | --- | --- | --- | --- | --- | --- | --- | --- | --- | --- | --- | --- | --- | --- | --- | --- | --- | --- | --- | --- | --- | --- | --- | --- | --- | --- | --- | --- | --- | --- | --- | --- | --- | --- | --- | --- | --- | --- | --- | --- | --- | --- | --- | --- | --- | --- | --- | --- | --- | --- | --- | --- | --- | --- | --- | --- | --- | --- | --- | --- | --- | --- | --- | --- | --- | --- | --- | --- | --- | --- | --- | --- | --- | --- | --- | --- | --- | --- | --- | --- | --- | --- | --- | --- | --- | --- | --- | --- | --- | --- | --- | --- | --- | --- | --- | --- | --- | --- | --- | --- | --- | --- | --- | --- | --- | --- | --- | --- | --- | --- | --- | --- | --- | --- | --- | --- | --- | --- | --- | --- | --- | --- | --- | --- | --- | --- | --- | --- | --- | --- | --- | --- | --- | --- | --- | --- | --- | --- | --- | --- | --- | --- | --- | --- | --- | --- | --- | --- | --- | --- | --- | --- | --- | --- | --- | --- | --- | --- | --- | --- | --- | --- | --- | --- | --- | --- | --- | --- | --- | --- | --- | --- | --- | --- | --- | --- | --- | --- | --- | --- | --- | --- | --- | --- | --- | --- | --- | --- | --- | --- | --- | --- | --- | --- | --- | --- | --- | --- | --- | --- | --- | --- | --- | --- | --- | --- | --- | --- | --- | --- | --- | --- | --- | --- | --- | --- | --- | --- | --- | --- | --- | --- | --- | --- | --- | --- | --- | --- | --- | --- | --- | --- | --- | --- | --- | --- | --- | --- | --- | --- | --- | --- | --- | --- | --- | --- | --- | --- | --- | --- | --- | --- | --- | --- | --- | --- | --- | --- | --- | --- | --- | --- | --- | --- | --- | --- | --- | --- | --- | --- | --- | --- | --- | --- | --- | --- | --- | --- | --- | --- | --- | --- | --- | --- | --- | --- | --- | --- | --- | --- | --- | --- | --- | --- | --- | --- | --- | --- | --- | --- | --- | --- | --- | --- | --- | --- | --- | --- | --- | --- | --- | --- | --- | --- | --- | --- | --- | --- | --- | --- | --- | --- | --- | --- | --- | --- | --- | --- | --- | --- | --- | --- | --- | --- | --- | --- | --- | --- | --- | --- | --- | --- | --- | --- | --- | --- | --- | --- | --- | --- | --- | --- | --- | --- | --- | --- | --- | --- | --- | --- | --- | --- | --- | --- | --- | --- | --- | --- | --- | --- | --- | --- | --- | --- | --- | --- | --- | --- | --- | --- | --- | --- | --- | --- | --- | --- | --- | --- | --- | --- | --- | --- | --- | --- | --- | --- | --- | --- | --- | --- | --- | --- | --- | --- | --- | --- | --- | --- | --- | --- | --- | --- | --- | --- | --- | --- | --- | --- | --- | --- | --- | --- | --- | --- | --- | --- | --- | --- | --- | --- | --- | --- | --- | --- | --- | --- | --- | --- | --- | --- | --- | --- | --- | --- | --- | --- | --- | --- | --- | --- | --- | --- | --- | --- | --- | --- | --- | --- | --- | --- | --- | --- | --- | --- | --- | --- | --- | --- | --- | --- | --- | --- | --- | --- | --- | --- | --- | --- | --- | --- | --- | --- | --- | --- | --- | --- | --- | --- | --- | --- | --- | --- | --- | --- | --- | --- | --- | --- | --- | --- | --- | --- | --- | --- | --- | --- | --- | --- | --- | --- | --- | --- | --- | --- | --- | --- | --- | --- | --- | --- | --- | --- | --- | --- | --- | --- | --- | --- | --- | --- | --- | --- | --- | --- | --- | --- | --- | --- | --- | --- | --- | --- | --- | --- | --- | --- | --- | --- | --- | --- | --- | --- | --- | --- | --- | --- | --- | --- | --- | --- | --- | --- | --- | --- | --- | --- | --- | --- | --- | --- | --- | --- | --- | --- | --- | --- | --- | --- | --- | --- | --- | --- | --- | --- | --- | --- | --- | --- | --- | --- | --- | --- | --- | --- | --- | --- | --- | --- | --- | --- | --- | --- | --- | --- | --- | --- | --- | --- | --- | --- | --- | --- | --- | --- | --- | --- | --- | --- | --- | --- | --- | --- | --- | --- | --- | --- | --- | --- | --- | --- | --- | --- | --- | --- | --- | --- | --- | --- | --- | --- | --- | --- | --- | --- | --- | --- | --- | --- | --- | --- | --- | --- | --- | --- | --- | --- | --- | --- | --- | --- | --- | --- | --- | --- | --- | --- | --- | --- | --- | --- | --- | --- | --- | --- | --- | --- | --- | --- | --- | --- | --- | --- | --- | --- | --- | --- | --- | --- | --- | --- | --- | --- | --- | --- | --- | --- | --- | --- | --- | --- | --- | --- | --- | --- | --- | --- | --- | --- | --- | --- | --- | --- | --- | --- | --- | --- | --- | --- | --- | --- | --- | --- | --- | --- | --- | --- | --- | --- | --- | --- | --- | --- | --- | --- | --- | --- | --- | --- | --- | --- | --- | --- | --- | --- | --- | --- | --- | --- | --- | --- | --- | --- | --- | --- | --- | --- | --- | --- | --- | --- | --- | --- | --- | --- | --- | --- | --- | --- | --- | --- | --- | --- | --- | --- | --- | --- | --- | --- | --- | --- | --- | --- | --- | --- | --- | --- | --- | --- | --- | --- | --- | --- | --- | --- | --- | --- | --- | --- | --- | --- | --- | --- | --- | --- | --- | --- | --- | --- | --- | --- | --- | --- | --- | --- | --- | --- | --- | --- | --- | --- | --- | --- | --- | --- | --- | --- | --- | --- | --- | --- | --- | --- | --- | --- | --- | --- | --- | --- | --- | --- | --- | --- | --- | --- | --- | --- | --- | --- | --- | --- | --- | --- | --- | --- | --- | --- | --- | --- | --- | --- | --- | --- | --- | --- | --- | --- | --- | --- | --- | --- | --- | --- | --- | --- | --- | --- | --- | --- | --- | --- | --- | --- | --- | --- | --- | --- | --- | --- | --- | --- | --- | --- | --- | --- | --- | --- | --- | --- | --- | --- | --- | --- | --- | --- | --- | --- | --- | --- | --- | --- | --- | --- | --- | --- | --- | --- | --- | --- | --- | --- | --- | --- | --- | --- | --- | --- | --- | --- | --- | --- | --- | --- | --- | --- | --- | --- | --- | --- | --- | --- | --- | --- | --- | --- | --- | --- | --- | --- | --- | --- | --- | --- | --- | --- | --- | --- | --- | --- | --- | --- | --- | --- | --- | --- | --- | --- | --- | --- | --- | --- | --- | --- | --- | --- | --- | --- | --- | --- | --- | --- | --- | --- | --- | --- | --- | --- | --- | --- | --- | --- | --- | --- | --- | --- | --- | --- | --- | --- | --- | --- | --- | --- | --- | --- | --- | --- | --- | --- | --- | --- | --- | --- | --- | --- | --- | --- | --- | --- | --- | --- | --- | --- | --- | --- | --- | --- | --- | --- | --- | --- | --- | --- | --- | --- | --- | --- | --- | --- | --- | --- | --- | --- | --- | --- | --- | --- | --- | --- | --- | --- | --- | --- | --- | --- | --- | --- | --- | --- | --- | --- | --- | --- | --- | --- | --- | --- | --- | --- | --- | --- | --- | --- | --- | --- | --- | --- | --- | --- | --- | --- | --- | --- | --- | --- | --- | --- | --- | --- | --- | --- | --- | --- | --- | --- | --- | --- | --- | --- | --- | --- | --- | --- | --- | --- | --- | --- | --- | --- | --- | --- | --- | --- | --- | --- | --- | --- | --- | --- | --- | --- | --- | --- | --- | --- | --- | --- | --- | --- | --- | --- | --- | --- | --- | --- | --- | --- | --- | --- | --- | --- | --- | --- | --- | --- | --- | --- | --- | --- | --- | --- | --- | --- | --- | --- | --- | --- | --- | --- | --- | --- | --- | --- | --- | --- | --- | --- | --- | --- | --- | --- | --- | --- | --- | --- | --- | --- | --- | --- | --- | --- | --- | --- | --- | --- | --- | --- | --- | --- | --- | --- | --- | --- | --- | --- | --- | --- | --- | --- | --- | --- | --- | --- | --- | --- | --- | --- | --- | --- | --- | --- | --- | --- | --- | --- | --- | --- | --- | --- | --- | --- | --- | --- | --- | --- | --- | --- | --- | --- | --- | --- | --- | --- | --- | --- | --- | --- | --- | --- | --- | --- | --- | --- | --- | --- | --- | --- | --- | --- | --- | --- | --- | --- | --- | --- | --- | --- | --- | --- | --- | --- | --- | --- | --- | --- | --- | --- | --- | --- | --- | --- | --- | --- | --- | --- | --- | --- | --- | --- | --- | --- | --- | --- | --- | --- | --- | --- | --- | --- | --- | --- | --- | --- | --- | --- | --- | --- | --- | --- | --- | --- | --- | --- | --- | --- | --- | --- | --- | --- | --- | --- | --- | --- | --- | --- | --- | --- | --- | --- | --- | --- | --- | --- | --- | --- | --- | --- | --- | --- | --- | --- | --- | --- | --- | --- | --- | --- | --- | --- | --- | --- | --- | --- | --- | --- | --- | --- | --- | --- | --- | --- | --- | --- | --- | --- | --- | --- | --- | --- | --- | --- | --- | --- | --- | --- | --- | --- | --- | --- | --- | --- | --- | --- | --- | --- | --- | --- | --- | --- | --- | --- | --- | --- | --- | --- | --- | --- | --- | --- | --- | --- | --- | --- | --- | --- | --- | --- | --- | --- | --- | --- | --- | --- | --- | --- | --- | --- | --- | --- | --- | --- | --- | --- | --- | --- | --- | --- | --- | --- | --- | --- | --- | --- | --- | --- | --- | --- | --- | --- | --- | --- | --- | --- | --- | --- | --- | --- | --- | --- | --- | --- | --- | --- | --- | --- | --- | --- | --- | --- | --- | --- | --- | --- | --- | --- | --- | --- | --- | --- | --- | --- | --- | --- | --- | --- | --- | --- | --- | --- | --- | --- | --- | --- | --- | --- | --- | --- | --- | --- | --- | --- | --- | --- | --- | --- | --- | --- | --- | --- | --- | --- | --- | --- | --- | --- | --- | --- | --- | --- | --- | --- | --- | --- | --- | --- | --- | --- | --- | --- | --- | --- | --- | --- | --- | --- | --- | --- | --- | --- | --- | --- | --- | --- | --- | --- | --- | --- | --- | --- | --- | --- | --- | --- | --- | --- | --- | --- | --- | --- | --- | --- | --- | --- | --- | --- | --- | --- | --- | --- | --- | --- | --- | --- | --- | --- | --- | --- | --- | --- | --- | --- | --- | --- | --- | --- | --- | --- | --- | --- | --- | --- | --- | --- | --- | --- | --- | --- | --- | --- | --- | --- | --- | --- | --- | --- | --- | --- | --- | --- | --- | --- | --- | --- | --- | --- | --- | --- | --- | --- | --- | --- | --- | --- | --- | --- | --- | --- | --- | --- | --- | --- | --- | --- | --- | --- | --- | --- | --- | --- | --- | --- | --- | --- | --- | --- | --- | --- | --- | --- | --- | --- | --- | --- | --- | --- | --- | --- | --- | --- | --- | --- | --- | --- | --- | --- | --- | --- | --- | --- | --- | --- | --- | --- | --- | --- | --- | --- | --- | --- | --- | --- | --- | --- | --- | --- | --- | --- | --- | --- | --- | --- | --- | --- | --- | --- | --- | --- | --- | --- | --- | --- | --- | --- | --- | --- | --- | --- | --- | --- | --- | --- | --- | --- | --- | --- | --- | --- | --- | --- | --- | --- | --- | --- | --- | --- | --- | --- | --- | --- | --- | --- | --- | --- | --- | --- | --- | --- | --- | --- | --- | --- | --- | --- | --- | --- | --- | --- | --- | --- | --- | --- | --- | --- | --- | --- | --- | --- | --- | --- | --- | --- | --- | --- | --- | --- | --- | --- | --- | --- | --- | --- | --- | --- | --- | --- | --- | --- | --- | --- | --- | --- | --- | --- | --- | --- | --- | --- | --- | --- | --- | --- | --- | --- | --- | --- | --- | --- | --- | --- | --- | --- | --- | --- | --- | --- | --- | --- | --- | --- | --- | --- | --- | --- | --- | --- | --- | --- | --- | --- | --- | --- | --- | --- | --- | --- | --- | --- | --- | --- | --- | --- | --- | --- | --- | --- | --- | --- | --- | --- | --- | --- | --- | --- | --- | --- | --- | --- | --- | --- | --- | --- | --- | --- | --- | --- | --- | --- | --- | --- | --- | --- | --- | --- | --- | --- | --- | --- | --- | --- | --- | --- | --- | --- | --- | --- | --- | --- | --- | --- | --- | --- | --- | --- | --- | --- | --- | --- | --- | --- | --- | --- | --- | --- | --- | --- | --- | --- | --- | --- | --- | --- | --- | --- | --- | --- | --- | --- | --- | --- | --- | --- | --- | --- | --- | --- | --- | --- | --- | --- | --- | --- | --- | --- | --- | --- | --- | --- | --- | --- | --- | --- | --- | --- | --- | --- | --- | --- | --- | --- | --- | --- | --- | --- | --- | --- | --- | --- | --- | --- | --- | --- | --- | --- | --- | --- | --- | --- | --- | --- | --- | --- | --- | --- | --- | --- | --- | --- | --- | --- | --- | --- | --- | --- | --- | --- | --- | --- | --- | --- | --- | --- | --- | --- | --- | --- | --- | --- | --- | --- | --- | --- | --- | --- | --- | --- | --- | --- | --- | --- | --- | --- | --- | --- | --- | --- | --- | --- | --- | --- | --- | --- | --- | --- | --- | --- | --- | --- | --- | --- | --- | --- | --- | --- | --- | --- | --- | --- | --- | --- | --- | --- | --- | --- | --- | --- | --- | --- | --- | --- | --- | --- | --- | --- | --- | --- | --- | --- | --- | --- | --- | --- | --- | --- | --- | --- | --- | --- | --- | --- | --- | --- | --- | --- | --- | --- | --- | --- | --- | --- | --- | --- | --- | --- | --- | --- | --- | --- | --- | --- | --- | --- | --- | --- | --- | --- | --- | --- | --- | --- | --- | --- | --- | --- | --- | --- | --- | --- | --- | --- | --- | --- | --- | --- | --- | --- | --- | --- | --- | --- | --- | --- | --- | --- | --- | --- | --- | --- | --- | --- | --- | --- | --- | --- | --- | --- | --- | --- | --- | --- | --- | --- | --- | --- | --- | --- | --- | --- | --- | --- | --- | --- | --- | --- | --- | --- | --- | --- | --- | --- | --- | --- | --- | --- | --- | --- | --- | --- | --- | --- | --- | --- | --- | --- | --- | --- | --- | --- | --- | --- | --- | --- | --- | --- | --- | --- | --- | --- | --- | --- | --- | --- | --- | --- | --- | --- | --- | --- | --- | --- | --- | --- | --- | --- | --- | --- | --- | --- | --- | --- | --- | --- | --- | --- | --- | --- | --- | --- | --- | --- | --- | --- | --- | --- | --- | --- | --- | --- | --- | --- | --- | --- | --- | --- | --- | --- | --- | --- | --- | --- | --- | --- | --- | --- | --- | --- | --- | --- | --- | --- | --- | --- | --- | --- | --- | --- | --- | --- | --- | --- | --- | --- | --- | --- | --- | --- | --- | --- | --- | --- | --- | --- | --- | --- | --- | --- | --- | --- | --- | --- | --- | --- | --- | --- | --- | --- | --- | --- | --- | --- | --- | --- | --- | --- | --- | --- | --- | --- | --- | --- | --- | --- | --- | --- | --- | --- | --- | --- | --- | --- | --- | --- | --- | --- | --- | --- | --- | --- | --- | --- | --- | --- | --- | --- | --- | --- | --- | --- | --- | --- | --- | --- | --- | --- | --- | --- | --- | --- | --- | --- | --- | --- | --- | --- | --- | --- | --- | --- | --- | --- | --- | --- | --- | --- | --- | --- | --- | --- | --- | --- | --- | --- | --- | --- | --- | --- | --- | --- | --- | --- | --- | --- | --- | --- | --- | --- | --- | --- | --- | --- | --- | --- | --- | --- | --- | --- | --- | --- | --- | --- | --- | --- | --- | --- | --- | --- | --- | --- | --- | --- | --- | --- | --- | --- | --- | --- | --- | --- | --- | --- | --- | --- | --- | --- | --- | --- | --- | --- | --- | --- | --- | --- | --- | --- | --- | --- | --- | --- | --- | --- | --- | --- | --- | --- | --- | --- | --- | --- | --- | --- | --- | --- | --- | --- | --- | --- | --- | --- | --- | --- | --- | --- | --- | --- | --- | --- | --- | --- | --- | --- | --- | --- | --- | --- | --- | --- | --- | --- | --- | --- | --- | --- | --- | --- | --- | --- | --- | --- | --- | --- | --- | --- | --- | --- | --- | --- | --- | --- | --- | --- | --- | --- | --- | --- | --- | --- | --- | --- | --- | --- | --- | --- | --- | --- | --- | --- | --- | --- | --- | --- | --- | --- | --- | --- | --- | --- | --- | --- | --- | --- | --- | --- | --- | --- | --- | --- | --- | --- | --- | --- | --- | --- | --- | --- | --- | --- | --- | --- | --- | --- | --- | --- | --- | --- | --- | --- | --- | --- | --- | --- | --- | --- | --- | --- | --- | --- | --- | --- | --- | --- | --- | --- | --- | --- | --- | --- | --- | --- | --- | --- | --- | --- | --- | --- | --- | --- | --- | --- | --- | --- | --- | --- | --- | --- | --- | --- | --- | --- | --- | --- | --- | --- | --- | --- | --- | --- | --- | --- | --- | --- | --- | --- | --- | --- | --- | --- | --- | --- | --- | --- | --- | --- | --- | --- | --- | --- | --- | --- | --- | --- | --- | --- | --- | --- | --- | --- | --- | --- | --- | --- | --- | --- | --- | --- | --- | --- | --- | --- | --- | --- | --- | --- | --- | --- | --- | --- | --- | --- | --- | --- | --- | --- | --- | --- | --- | --- | --- | --- | --- | --- | --- | --- | --- | --- | --- | --- | --- | --- | --- | --- | --- | --- | --- | --- | --- | --- | --- | --- | --- | --- | --- | --- | --- | --- | --- | --- | --- | --- | --- | --- | --- | --- | --- | --- | --- | --- | --- | --- | --- | --- | --- | --- | --- | --- | --- | --- | --- | --- | --- | --- | --- | --- | --- | --- | --- | --- | --- | --- | --- | --- | --- | --- | --- | --- | --- | --- | --- | --- | --- | --- | --- | --- | --- | --- | --- | --- | --- | --- | --- | --- | --- | --- | --- | --- | --- | --- | --- | --- | --- | --- | --- | --- | --- | --- | --- | --- | --- | --- | --- | --- | --- | --- | --- | --- | --- | --- | --- | --- | --- | --- | --- | --- | --- | --- | --- | --- | --- | --- |
| |  |  |  |  |  |  |  |  |  | | --- | --- | --- | --- | --- | --- | --- | --- | --- | | **Position** | **Reference** | **Sample** | **Quality** | **Type** | **Region** | **AA Exchange** | **PAM1** | **Known Variant** | | 1977 | A | G | 980.77 | SNP | intergenic |  |  | - | | 4013 | T | C | 1134.77 | SNP | Rv0003 (recF 3280:4437 DNA replication and repair protein RecF (single-strand DNA binding protein) CDS) | Ile245Thr | 11 | - | | 7362 | G | C | 761.77 | SNP | Rv0006 (gyrA 7302:9818 DNA gyrase (subunit A) GyrA (DNA topoisomerase (ATP-hydrolysing)) (DNA topoisomerase II) (type II DNA topoisomerase) CDS) | Glu21Gln | 27 | - | | 7539 | A | G | 1081.77 | SNP | Rv0006 (gyrA 7302:9818 DNA gyrase (subunit A) GyrA (DNA topoisomerase (ATP-hydrolysing)) (DNA topoisomerase II) (type II DNA topoisomerase) CDS) | Thr80Ala | 32 | genotype | | 7585 | G | C | 1110.77 | SNP | Rv0006 (gyrA 7302:9818 DNA gyrase (subunit A) GyrA (DNA topoisomerase (ATP-hydrolysing)) (DNA topoisomerase II) (type II DNA topoisomerase) CDS) | Ser95Thr | 32 | genotype | | 9304 | G | A | 894.77 | SNP | Rv0006 (gyrA 7302:9818 DNA gyrase (subunit A) GyrA (DNA topoisomerase (ATP-hydrolysing)) (DNA topoisomerase II) (type II DNA topoisomerase) CDS) | Gly668Asp | 6 | - | | 11879 | A | G | 765.77 | SNP | Rv0008c (- 12311:11874 Possible membrane protein CDS) | Ser145Pro | 12 | - | | 14467 | G | C | 1189.77 | SNP | Rv0012 (- 14089:14877 Probable conserved membrane protein CDS) | Ala127Pro | 13 | - | | 14785 | T | C | 1355.77 | SNP | Rv0012 (- 14089:14877 Probable conserved membrane protein CDS) | Cys233Arg | 1 | - | | 18091 | G | A | 905.77 | SNP | Rv0015c (pknA 18762:17467 Transmembrane serine/threonine-protein kinase A PknA (protein kinase A) (STPK A) CDS) | silent (Thr224) | 9871 | - | | 21795 | G | A | 30.74 | SNP | Rv0018c (pstP 23181:21637 Phosphoserine/threonine phosphatase PstP CDS) | Pro463Ser | 17 | - | | 22334 | C | T | 804.77 | SNP | Rv0018c (pstP 23181:21637 Phosphoserine/threonine phosphatase PstP CDS) | Arg283His | 8 | - | | 26959 | C | G | 582.77 | SNP | intergenic |  |  | - | | 32075 | T | C | 1257.77 | SNP | Rv0029 (- 32057:33154 hypothetical protein CDS) | Trp7Arg | 8 | - | | 32405 | A | G | 549.77 | SNP | Rv0029 (- 32057:33154 hypothetical protein CDS) | Thr117Ala | 32 | - | | 34044 | T | C | 1031.77 | SNP | intergenic |  |  | - | | 34226 | A | G | 1172.77 | SNP | intergenic |  |  | - | | 37031 | C | G | 1019.77 | SNP | Rv0034 (- 36867:37262 hypothetical protein CDS) | silent (Ala55) | 9867 | - | | 42967 | G | C | 1224.77 | SNP | Rv0040c (mtc28 43365:42433 Secreted proline rich protein Mtc28 (proline rich 28 kDa antigen) CDS) | silent (Pro133) | 9926 | - | | 45672 | A | C | 1116.77 | SNP | Rv0041 (leuS 43562:46471 Probable leucyl-tRNA synthetase LeuS (leucine--tRNA ligase) (LEURS) CDS) | Asp704Ala | 10 | - | | 45673 | T | C | 1157.77 | SNP | Rv0041 (leuS 43562:46471 Probable leucyl-tRNA synthetase LeuS (leucine--tRNA ligase) (LEURS) CDS) | silent (Asp704) | 9859 | - | | 47909 | G | A | 900.77 | SNP | Rv0043c (- 48100:47366 Probable transcriptional regulatory protein (probably GntR-family) CDS) | silent (Arg64) | 9913 | - | | 54304 | C | T | 1258.77 | SNP | Rv0050 (ponA1 53663:55699 Probable bifunctional penicillin-binding protein 1A/1B PonA1 (murein polymerase) (PBP1): penicillin-insensitive transglycosylase (peptidoglycan TGASE) + penicillin-sensitive transpeptidase (DD-transpeptidase) CDS) | silent (Leu214) | 9947 | - | | 55553 | C | T | 51.77 | SNP | Rv0050 (ponA1 53663:55699 Probable bifunctional penicillin-binding protein 1A/1B PonA1 (murein polymerase) (PBP1): penicillin-insensitive transglycosylase (peptidoglycan TGASE) + penicillin-sensitive transpeptidase (DD-transpeptidase) CDS) | Pro631Ser | 17 | - | | 55553 | C | CCGT | 994.73 | INS | Rv0050 (ponA1 53663:55699 Probable bifunctional penicillin-binding protein 1A/1B PonA1 (murein polymerase) (PBP1): penicillin-insensitive transglycosylase (peptidoglycan TGASE) + penicillin-sensitive transpeptidase (DD-transpeptidase) CDS) |  |  | - | | 55683 | C | T | 173.90 | SNP | Rv0050 (ponA1 53663:55699 Probable bifunctional penicillin-binding protein 1A/1B PonA1 (murein polymerase) (PBP1): penicillin-insensitive transglycosylase (peptidoglycan TGASE) + penicillin-sensitive transpeptidase (DD-transpeptidase) CDS) | Pro674Leu | 3 | - | | 56334 | G | C | 851.77 | SNP | Rv0051 (- 55696:57378 Probable conserved transmembrane protein CDS) | silent (Ser213) | 9840 | - | | 62049 | A | G | 847.77 | SNP | Rv0058 (dnaB 60396:63020 Probable replicative DNA helicase DnaB CDS) | Arg552Gly | 1 | - | | 69989 | G | A | 1121.77 | SNP | Rv0064 (- 68620:71559 Probable conserved transmembrane protein CDS) | Gly457Asp | 6 | - | | 70816 | A | G | 1160.77 | SNP | Rv0064 (- 68620:71559 Probable conserved transmembrane protein CDS) | Asn733Asp | 42 | - | | 71190 | T | G | 623.77 | SNP | Rv0064 (- 68620:71559 Probable conserved transmembrane protein CDS) | Ser857Arg | 6 | - | | 71336 | G | C | 90.28 | SNP | Rv0064 (- 68620:71559 Probable conserved transmembrane protein CDS) | Arg906Pro | 5 | - | | 71453 | T | TC | 187.80 | INS | Rv0064 (- 68620:71559 Probable conserved transmembrane protein CDS) |  |  | - | | 71584 | C | CCGAGCGCTGTTCTGGCGCT AATCTGACGCTAGAATAG | 10507.73 | INS | intergenic |  |  | - | | 75940 | G | C | 1206.77 | SNP | Rv0068 (- 75301:76212 Probable oxidoreductase CDS) | Val(s)214Leu | 3 | - | | 79162 | G | A | 1150.77 | SNP | intergenic |  |  | - | | 80616 | C | G | 1449.77 | SNP | intergenic |  |  | - | | 87893 | C | T | 1429.77 | SNP | Rv0078B (- 88004:87798 hypothetical protein CDS) | Val38Ile | 33 | - | | 92199 | T | G | 977.77 | SNP | Rv0083 (- 90400:92322 Probable oxidoreductase CDS) | silent (Thr600) | 9871 | - | | 103962 | C | T | 78.28 | SNP | Rv0094c (- 104663:103710 hypothetical protein CDS) | silent (Pro234) | 9926 | - | | 104712 | C | T | 602.77 | SNP | intergenic |  |  | - | | 104962 | G | A | 565.77 | SNP | Rv0095c (- 105215:104805 hypothetical protein CDS) | Ala85Val(s) | 9867 | - | | 105021 | G | A | 580.77 | SNP | Rv0095c (- 105215:104805 hypothetical protein CDS) | silent (Ser65) | 9840 | - | | 105031 | T | G | 641.77 | SNP | Rv0095c (- 105215:104805 hypothetical protein CDS) | Asp62Ala | 10 | - | | 105045 | G | C | 640.77 | SNP | Rv0095c (- 105215:104805 hypothetical protein CDS) | Asp57Glu | 56 | - | | 105060 | G | A | 475.77 | SNP | Rv0095c (- 105215:104805 hypothetical protein CDS) | silent (Asp52) | 9859 | - | | 105063 | G | A | 464.77 | SNP | Rv0095c (- 105215:104805 hypothetical protein CDS) | silent (Phe51) | 9946 | - | | 116000 | T | G | 981.77 | SNP | Rv0101 (nrp 110001:117539 Probable peptide synthetase Nrp (peptide synthase) CDS) | Val2000Val(s) | 18 | - | | 117403 | C | T | 1063.77 | SNP | Rv0101 (nrp 110001:117539 Probable peptide synthetase Nrp (peptide synthase) CDS) | Ala2468Val(s) | 9867 | - | | 122109 | A | G | 1181.77 | SNP | Rv0103c (ctpB 122173:119915 Probable cation-transporter P-type ATPase B CtpB CDS) | Leu(s)22Ser | 28 | - | | 122794 | T | G | 1503.77 | SNP | Rv0104 (- 122317:123831 hypothetical protein CDS) | Phe160Val | 1 | - | | 125830 | G | GA | 2108.73 | INS | Rv0107c (ctpI 130541:125643 Probable cation-transporter ATPase I CtpI CDS) |  |  | - | | 129716 | G | A | 997.77 | SNP | Rv0107c (ctpI 130541:125643 Probable cation-transporter ATPase I CtpI CDS) | Arg276Cys | 1 | - | | 130449 | C | A | 909.77 | SNP | Rv0107c (ctpI 130541:125643 Probable cation-transporter ATPase I CtpI CDS) | silent (Ala31) | 9867 | - | | 131174 | T | TG | 1258.73 | INS | intergenic |  |  | - | | 132417 | C | G | 186.84 | SNP | Rv0109 (PE\_PGRS1 131382:132872 PE-PGRS family protein PE\_PGRS1 CDS) | Arg346Gly | 1 | - | | 133839 | C | T | 1024.77 | SNP | intergenic |  |  | - | | 138788 | C | T | 871.77 | SNP | Rv0115 (hddA 138513:139673 Possible D-alpha-D-heptose-7-phosphate kinase HddA CDS) | silent (Phe92) | 9946 | - | | 146087 | T | C | 1270.77 | SNP | Rv0120c (fusA2 147771:145627 Probable elongation factor G FusA2 (EF-G) CDS) | Asn562Ser | 34 | - | | 154283 | T | C | 1099.77 | SNP | Rv0127 (mak 154232:155599 Maltokinase Mak CDS) | Ser18Pro | 12 | - | | 172815 | C | T | 1039.77 | SNP | Rv0146 (- 172211:173143 Possible S-adenosylmethionine-dependent methyltransferase CDS) | Thr202Ile | 7 | - | | 177067 | G | C | 913.77 | SNP | intergenic |  |  | - | | 177857 | G | A | 589.77 | SNP | Rv0151c (PE1 179309:177543 PE family protein PE1 CDS) | Leu485Leu(s) | 4 | - | | 188800 | T | C | 584.77 | SNP | Rv0159c (PE3 188839:187433 PE family protein PE3 CDS) | Thr14Ala | 32 | - | | 194681 | G | C | 839.77 | SNP | Rv0165c (mce1R 194815:194144 Probable transcriptional regulatory protein Mce1R (probably GntR-family) CDS) | silent (Leu45) | 9947 | - | | 196642 | C | T | 763.77 | SNP | Rv0166 (fadD5 194993:196657 Probable fatty-acid-CoA ligase FadD5 (fatty-acid-CoA synthetase) (fatty-acid-CoA synthase) CDS) | silent (Asn550) | 9822 | - | | 206339 | T | C | 979.77 | SNP | Rv0174 (mce1F 205231:206778 Mce-family protein Mce1F CDS) | Leu370Pro | 2 | - | | 218204 | C | T | 843.77 | SNP | Rv0186 (bglS 216269:218344 Probable beta-glucosidase BglS (gentiobiase) (cellobiase) (beta-D-glucoside glucohydrolase) CDS) | Arg646STOP | 2 | - | | 219120 | A | G | 1039.77 | SNP | Rv0187 (- 218705:219367 Probable O-methyltransferase CDS) | Asp139Gly | 11 | - | | 223942 | T | C | 393.77 | SNP | Rv0192 (- 223564:224664 hypothetical protein CDS) | Ser127Pro | 12 | - | | 225323 | T | C | 1041.77 | SNP | Rv0193c (- 226571:224724 Hypothetical protein CDS) | Lys417Glu | 4 | - | | 227098 | T | C | 1114.77 | SNP | Rv0194 (- 226878:230462 Probable transmembrane multidrug efflux pump CDS) | Met(s)74Thr | 22 | - | | 231114 | C | G | 1080.77 | SNP | Rv0195 (- 230899:231534 Possible two component transcriptional regulatory protein (probably LuxR-family) CDS) | silent (Ala72) | 9867 | - | | 232110 | C | T | 745.77 | SNP | Rv0196 (- 231647:232231 Possible transcriptional regulatory protein CDS) | Thr155Met(s) | 32 | - | | 232172 | C | G | 622.77 | SNP | Rv0196 (- 231647:232231 Possible transcriptional regulatory protein CDS) | Leu176Val(s) | 4 | - | | 234477 | T | G | 673.77 | SNP | Rv0197 (- 232231:234519 Possible oxidoreductase CDS) | Tyr749STOP | 2 | - | | 234496 | C | CGT | 1304.73 | INS | Rv0197 (- 232231:234519 Possible oxidoreductase CDS) |  |  | - | | 237709 | C | T | 897.77 | SNP | Rv0200 (- 237206:237895 Possible conserved transmembrane protein CDS) | silent (Ala168) | 9867 | - | | 256640 | T | G | 1325.77 | SNP | Rv0214 (fadD4 256064:257677 Probable fatty-acid-CoA ligase FadD4 (fatty-acid-CoA synthetase) (fatty-acid-CoA synthase) CDS) | Ser193Ala | 35 | - | | 257835 | C | T | 1161.77 | SNP | Rv0215c (fadE3 258856:257783 Probable acyl-CoA dehydrogenase FadE3 CDS) | Arg341His | 8 | - | | 257982 | T | G | 907.77 | SNP | Rv0215c (fadE3 258856:257783 Probable acyl-CoA dehydrogenase FadE3 CDS) | Asp292Ala | 10 | - | | 261869 | T | C | 793.77 | SNP | Rv0218 (- 260924:262252 Probable conserved transmembrane protein CDS) | Cys316Arg | 1 | - | | 265244 | C | T | 1097.77 | SNP | Rv0221 (- 264067:265476 Possible triacylglycerol synthase (diacylglycerol acyltransferase) CDS) | Ala393Val(s) | 9867 | - | | 265554 | A | C | 806.77 | SNP | Rv0222 (echA1 265507:266295 Probable enoyl-CoA hydratase EchA1 (enoyl hydrase) (unsaturated acyl-CoA hydratase) (crotonase) CDS) | silent (Val16) | 9901 | - | | 278681 | C | G | 883.77 | SNP | Rv0233 (nrdB 278585:279529 Ribonucleoside-diphosphate reductase (beta chain) NrdB (ribonucleotide reductase small chain) CDS) | His33Asp | 4 | - | | 281628 | C | A | 872.77 | SNP | Rv0235c (- 282614:281166 Probable conserved transmembrane protein CDS) | Leu(s)329Phe | 1 | - | | 285772 | A | C | 721.77 | SNP | Rv0236c (aftD 286851:282649 Possible arabinofuranosyltransferase AftD CDS) | silent (Pro360) | 9926 | - | | 285871 | A | G | 482.77 | SNP | Rv0236c (aftD 286851:282649 Possible arabinofuranosyltransferase AftD CDS) | silent (Val327) | 9901 | - | | 310973 | G | A | 996.77 | SNP | Rv0259c (- 311517:310774 hypothetical protein CDS) | Ala182Val(s) | 9867 | - | | 311613 | G | T | 1002.77 | SNP | Rv0260c (- 312659:311514 Possible transcriptional regulatory protein CDS) | silent (Val349) | 9901 | - | | 312060 | T | G | 999.77 | SNP | Rv0260c (- 312659:311514 Possible transcriptional regulatory protein CDS) | silent (Arg200) | 9913 | - | | 312944 | C | T | 1131.77 | SNP | Rv0261c (narK3 314168:312759 Probable integral membrane nitrite extrusion protein NarK3 (nitrite facilitator) CDS) | Val409Ile | 33 | - | | 313670 | T | G | 1137.77 | SNP | Rv0261c (narK3 314168:312759 Probable integral membrane nitrite extrusion protein NarK3 (nitrite facilitator) CDS) | Asn167His | 18 | - | | 325038 | G | A | 1098.77 | SNP | Rv0270 (fadD2 324567:326249 Probable fatty-acid-CoA ligase FadD2 (fatty-acid-CoA synthetase) (fatty-acid-CoA synthase) CDS) | Gly158Ser | 16 | - | | 325039 | G | A | 1033.77 | SNP | Rv0270 (fadD2 324567:326249 Probable fatty-acid-CoA ligase FadD2 (fatty-acid-CoA synthetase) (fatty-acid-CoA synthase) CDS) | Gly158Asp | 6 | - | | 332357 | A | G | 1464.77 | SNP | Rv0276 (- 331748:332668 hypothetical protein CDS) | Ile204Val | 57 | - | | 333637 | A | G | 48.77 | SNP | Rv0278c (PE\_PGRS3 336310:333437 PE-PGRS family protein PE\_PGRS3 CDS) | Trp892Arg | 8 | - | | 333640 | G | A | 55.77 | SNP | Rv0278c (PE\_PGRS3 336310:333437 PE-PGRS family protein PE\_PGRS3 CDS) | Arg891Trp | 2 | - | | 333641 | C | T | 56.77 | SNP | Rv0278c (PE\_PGRS3 336310:333437 PE-PGRS family protein PE\_PGRS3 CDS) | silent (Gln890) | 9876 | - | | 333892 | G | C | 279.78 | SNP | Rv0278c (PE\_PGRS3 336310:333437 PE-PGRS family protein PE\_PGRS3 CDS) | Arg807Gly | 1 | - | | 335597 | C | G | 62.74 | SNP | Rv0278c (PE\_PGRS3 336310:333437 PE-PGRS family protein PE\_PGRS3 CDS) | silent (Gly238) | 9935 | - | | 335606 | T | C | 62.74 | SNP | Rv0278c (PE\_PGRS3 336310:333437 PE-PGRS family protein PE\_PGRS3 CDS) | silent (Gly235) | 9935 | - | | 336773 | A | G | 119.03 | SNP | Rv0279c (PE\_PGRS4 339073:336560 PE-PGRS family protein PE\_PGRS4 CDS) | silent (Gly767) | 9935 | - | | 337820 | G | A | 179.84 | SNP | Rv0279c (PE\_PGRS4 339073:336560 PE-PGRS family protein PE\_PGRS4 CDS) | silent (Gly418) | 9935 | - | | 337959 | A | C | 51.74 | SNP | Rv0279c (PE\_PGRS4 339073:336560 PE-PGRS family protein PE\_PGRS4 CDS) | Ile372Ser | 2 | - | | 338020 | A | C | 54.74 | SNP | Rv0279c (PE\_PGRS4 339073:336560 PE-PGRS family protein PE\_PGRS4 CDS) | Cys352Gly | 1 | - | | 338100 | T | C | 90.28 | SNP | Rv0279c (PE\_PGRS4 339073:336560 PE-PGRS family protein PE\_PGRS4 CDS) | Asn325Ser | 34 | - | | 338453 | A | G | 77.28 | SNP | Rv0279c (PE\_PGRS4 339073:336560 PE-PGRS family protein PE\_PGRS4 CDS) | silent (Ala207) | 9867 | - | | 346275 | C | G | 1449.77 | SNP | Rv0284 (eccC3 345635:349627 ESX conserved component EccC3 ESX-3 type VII secretion system protein Possible membrane protein CDS) | Pro214Arg | 4 | - | | 352580 | G | A | 1204.77 | SNP | Rv0289 (espG3 352149:353036 ESX-3 secretion-associated protein EspG3 CDS) | silent (Leu144) | 9947 | - | | 356528 | A | G | 911.77 | SNP | Rv0292 (eccE3 355880:356875 ESX conserved component EccE3 ESX-3 type VII secretion system protein Probable transmembrane protein CDS) | Asn217Asp | 42 | - | | 359488 | G | A | 1002.77 | SNP | Rv0295c (- 359748:358945 hypothetical protein CDS) | silent (Arg87) | 9913 | - | | 364563 | A | C | 580.77 | SNP | intergenic |  |  | - | | 373282 | TA | T | 1233.73 | DEL | Rv0305c (PPE6 375711:372820 PPE family protein PPE6 CDS) |  |  | - | | 384380 | A | C | 1040.77 | SNP | Rv0315 (- 383602:384486 Possible beta-1,3-glucanase precursor CDS) | Lys260Thr | 8 | - | | 386432 | C | G | 1489.77 | SNP | Rv0318c (- 387099:386305 Probable conserved integral membrane protein CDS) | Gly223Ala | 21 | - | | 390828 | T | C | 1199.77 | SNP | Rv0323c (- 391251:390580 hypothetical protein CDS) | Ser142Gly | 21 | - | | 403980 | G | A | 1534.77 | SNP | Rv0338c (- 405841:403193 Probable iron-sulfur-binding reductase CDS) | Ala621Val | 13 | - | | 404326 | T | C | 1591.77 | SNP | Rv0338c (- 405841:403193 Probable iron-sulfur-binding reductase CDS) | Arg506Gly | 1 | - | | 411582 | G | A | 1277.77 | SNP | Rv0342 (iniA 410838:412760 Isoniazid inductible gene protein IniA CDS) | Ala249Thr | 22 | - | | 412017 | C | G | 846.77 | SNP | Rv0342 (iniA 410838:412760 Isoniazid inductible gene protein IniA CDS) | Gln394Glu | 35 | - | | 414486 | C | T | 916.77 | SNP | Rv0344c (lpqJ 414941:414381 Probable lipoprotein LpqJ CDS) | silent (Glu152) | 9865 | - | | 420008 | A | G | 1120.77 | SNP | Rv0350 (dnaK 419835:421712 Probable chaperone protein DnaK (heat shock protein 70) (heat shock 70 kDa protein) (HSP70) CDS) | silent (Ala58) | 9867 | - | | 424320 | T | TC | 1674.73 | INS | Rv0354c (PPE7 424694:424269 PPE family protein PPE7 CDS) |  |  | - | | 427310 | TTGCCGAGGTTTGCAC | T | 2699.73 | DEL | Rv0355c (PPE8 434679:424777 PPE family protein PPE8 CDS) |  |  | - | | 428563 | G | A | 661.77 | SNP | Rv0355c (PPE8 434679:424777 PPE family protein PPE8 CDS) | silent (Asn2039) | 9822 | - | | 435708 | G | A | 1387.77 | SNP | Rv0357c (purA 436769:435471 Probable adenylosuccinate synthetase PurA (imp--aspartate ligase) (ADSS) (ampsase) CDS) | silent (Thr354) | 9871 | - | | 444667 | T | C | 853.77 | SNP | Rv0366c (- 444815:444222 hypothetical protein CDS) | His50Arg | 10 | - | | 450234 | C | T | 937.77 | SNP | Rv0373c (- 451803:449404 Probable carbon monoxyde dehydrogenase (large chain) CDS) | Ala524Thr | 22 | - | | 454295 | T | C | 995.77 | SNP | Rv0376c (- 454372:453230 hypothetical protein CDS) | silent (Pro26) | 9926 | - | | 457452 | T | G | 827.77 | SNP | Rv0381c (- 457823:456915 Hypothetical protein CDS) | silent (Thr124) | 9871 | - | | 459399 | A | C | 1309.77 | SNP | intergenic |  |  | - | | 467497 | C | CG | 1430.73 | INS | Rv0388c (PPE9 468001:467459 PPE family protein PPE9 CDS) |  |  | - | | 467508 | C | CG | 1465.73 | INS | Rv0388c (PPE9 468001:467459 PPE family protein PPE9 CDS) |  |  | - | | 467516 | G | C | 856.77 | SNP | Rv0388c (PPE9 468001:467459 PPE family protein PPE9 CDS) | silent (Ser162) | 9840 | - | | 467526 | C | G | 834.77 | SNP | Rv0388c (PPE9 468001:467459 PPE family protein PPE9 CDS) | Gly159Ala | 21 | - | | 467546 | G | C | 789.77 | SNP | Rv0388c (PPE9 468001:467459 PPE family protein PPE9 CDS) | Asp152Glu | 56 | - | | 467557 | A | C | 888.77 | SNP | Rv0388c (PPE9 468001:467459 PPE family protein PPE9 CDS) | Leu(s)149Val(s) | 9867 | - | | 467564 | A | C | 985.77 | SNP | Rv0388c (PPE9 468001:467459 PPE family protein PPE9 CDS) | His146Gln | 23 | - | | 467585 | G | C | 1154.77 | SNP | Rv0388c (PPE9 468001:467459 PPE family protein PPE9 CDS) | His139Gln | 23 | - | | 467590 | T | C | 1211.77 | SNP | Rv0388c (PPE9 468001:467459 PPE family protein PPE9 CDS) | Thr138Ala | 32 | - | | 467621 | T | G | 993.77 | SNP | Rv0388c (PPE9 468001:467459 PPE family protein PPE9 CDS) | silent (Gly127) | 9935 | - | | 467638 | G | T | 1056.77 | SNP | Rv0388c (PPE9 468001:467459 PPE family protein PPE9 CDS) | Gln122Lys | 12 | - | | 473192 | G | A | 1106.77 | SNP | Rv0393 (- 472781:474106 Conserved 13E12 repeat family protein CDS) | Ala138Thr | 22 | - | | 475178 | T | C | 1154.77 | SNP | Rv0395 (- 474940:475344 Hypothetical protein CDS) | Val80Ala | 18 | - | | 482666 | C | T | 1951.77 | SNP | Rv0402c (mmpL1 483231:480355 Probable conserved transmembrane transport protein MmpL1 CDS) | Gly189Asp | 6 | - | | 483874 | C | T | 638.77 | SNP | intergenic |  |  | - | | 488796 | G | A | 1217.77 | SNP | Rv0405 (pks6 485731:489939 Probable membrane bound polyketide synthase Pks6 CDS) | Val(s)1022Val | 13 | - | | 489935 | G | C | 847.77 | SNP | Rv0405 (pks6 485731:489939 Probable membrane bound polyketide synthase Pks6 CDS); Rv0406c (- 490705:489887 Beta lactamase like protein CDS) | Arg1402Pro; silent (Thr257) | 5; 9871 | - | | 493934 | T | C | 1522.77 | SNP | Rv0409 (ackA 493851:495008 Probable acetate kinase AckA (acetokinase) CDS) | silent (Arg28) | 9913 | - | | 502589 | C | G | 1367.77 | SNP | Rv0417 (thiG 502366:503124 Probable thiamin biosynthesis protein ThiG (thiazole biosynthesis protein) CDS) | Ser75Cys | 5 | - | | 503354 | G | C | 1907.77 | SNP | intergenic |  |  | - | | 513257 | T | C | 1070.77 | SNP | Rv0425c (ctpH 515321:510702 Possible metal cation transporting P-type ATPase CtpH CDS) | Met(s)689Val(s) | 9867 | - | | 524095 | A | G | 1050.77 | SNP | Rv0435c (- 524533:522347 Putative conserved ATPase CDS) | Ser147Pro | 12 | - | | 541201 | A | G | 917.77 | SNP | Rv0450c (mmpL4 541491:538588 Probable conserved transmembrane transport protein MmpL4 CDS) | silent (Leu97) | 9947 | - | | 545925 | G | C | 67.77 | SNP | Rv0456c (echA2 546803:545889 enoyl-CoA hydratase EchA2 (enoyl hydrase) (unsaturated acyl-CoA hydratase) (crotonase) CDS) | Asp293Glu | 56 | - | | 551525 | A | C | 869.77 | SNP | Rv0459 (- 551198:551689 hypothetical protein CDS) | silent (Arg110) | 9913 | - | | 572346 | G | T | 982.77 | SNP | Rv0483 (lprQ 571710:573065 Probable conserved lipoprotein LprQ CDS) | Ala213Ser | 28 | - | | 573262 | A | G | 582.77 | SNP | Rv0484c (- 573801:573046 Probable short-chain type oxidoreductase CDS) | silent (Gly180) | 9935 | - | | 577507 | T | C | 630.77 | SNP | intergenic |  |  | - | | 578032 | C | T | 1481.77 | SNP | Rv0488 (- 577664:578269 Probable conserved integral membrane protein CDS) | silent (Ala123) | 9867 | - | | 580772 | T | A | 285.78 | SNP | intergenic |  |  | - | | 580773 | GGGGGCACCACCCGCTTGCG GGGGA | G | 2965.73 | DEL | intergenic |  |  | - | | 582010 | G | C | 689.77 | SNP | Rv0492c (- 583378:581489 Probable oxidoreductase GMC-type CDS) | Pro457Ala | 22 | - | | 587974 | G | A | 568.28 | SNP | Rv0497 (- 587377:588309 Probable conserved transmembrane protein CDS) | Ala200Thr | 22 | - | | 590436 | T | C | 853.77 | SNP | Rv0500 (proC 590083:590970 Probable pyrroline-5-carboxylate reductase ProC (P5CR) (P5C reductase) CDS) | silent (Ala118) | 9867 | - | | 591628 | T | C | 1095.77 | SNP | intergenic |  |  | - | | 595232 | C | T | 667.77 | SNP | Rv0504c (- 595302:594802 hypothetical protein CDS) | Gly24Glu | 4 | - | | 597816 | A | G | 1035.77 | SNP | Rv0507 (mmpL2 597199:600105 Probable conserved transmembrane transport protein MmpL2 CDS) | silent (Ala206) | 9867 | - | | 598475 | G | A | 815.77 | SNP | Rv0507 (mmpL2 597199:600105 Probable conserved transmembrane transport protein MmpL2 CDS) | Arg426His | 8 | - | | 610120 | T | G | 1124.77 | SNP | intergenic |  |  | - | | 623472 | A | G | 62.74 | SNP | Rv0532 (PE\_PGRS6 622793:624577 PE-PGRS family protein PE\_PGRS6 CDS) | Asp227Gly | 11 | - | | 623508 | C | G | 106.28 | SNP | Rv0532 (PE\_PGRS6 622793:624577 PE-PGRS family protein PE\_PGRS6 CDS) | Ala239Gly | 21 | - | | 628113 | C | T | 933.77 | SNP | Rv0536 (galE3 627248:628288 Probable UDP-glucose 4-epimerase GalE3 (galactowaldenase) (UDP-galactose 4-epimerase) (uridine diphosphate galactose 4-epimerase) (uridine diphospho-galactose 4-epimerase) CDS) | Ala289Val(s) | 9867 | - | | 630722 | G | C | 462.77 | SNP | Rv0538 (- 630040:631686 Possible conserved membrane protein CDS) | Arg228Pro | 5 | - | | 637319 | G | A | 1100.77 | SNP | Rv0545c (pitA 637463:636210 Probable low-affinity inorganic phosphate transporter integral membrane protein PitA CDS) | Pro49Ser | 17 | - | | 648002 | T | G | 1873.77 | SNP | Rv0556 (- 647959:648474 Probable conserved transmembrane protein CDS) | Leu15Arg | 1 | - | | 663410 | A | C | 91.77 | SNP | intergenic |  |  | - | | 663418 | A | C | 107.77 | SNP | intergenic |  |  | - | | 663419 | G | A | 118.77 | SNP | intergenic |  |  | - | | 663420 | C | A | 109.77 | SNP | intergenic |  |  | - | | 663429 | T | G | 125.77 | SNP | intergenic |  |  | - | | 665293 | A | G | 1416.77 | SNP | Rv0572c (- 665383:665042 Hypothetical protein CDS) | Phe31Leu | 13 | - | | 669398 | T | C | 647.77 | SNP | Rv0575c (- 669745:668579 Possible oxidoreductase CDS) | silent (Gln116) | 9876 | - | | 672097 | C | T | 140.03 | SNP | Rv0578c (PE\_PGRS7 675916:671996 PE-PGRS family protein PE\_PGRS7 CDS) | Gly1274Ser | 16 | - | | 672491 | C | G | 143.90 | SNP | Rv0578c (PE\_PGRS7 675916:671996 PE-PGRS family protein PE\_PGRS7 CDS) | silent (Gly1142) | 9935 | - | | 673238 | A | G | 241.80 | SNP | Rv0578c (PE\_PGRS7 675916:671996 PE-PGRS family protein PE\_PGRS7 CDS) | silent (His893) | 9912 | - | | 685461 | C | G | 1233.77 | SNP | Rv0587 (yrbE2A 685129:685926 Conserved hypothetical integral membrane protein YrbE2A CDS) | silent (Ala111) | 9867 | - | | 685608 | T | C | 1693.77 | SNP | Rv0587 (yrbE2A 685129:685926 Conserved hypothetical integral membrane protein YrbE2A CDS) | silent (Leu160) | 9947 | - | | 685869 | G | A | 1002.77 | SNP | Rv0587 (yrbE2A 685129:685926 Conserved hypothetical integral membrane protein YrbE2A CDS) | silent (Leu247) | 9947 | - | | 686972 | T | C | 974.77 | SNP | Rv0589 (mce2A 686821:688035 Mce-family protein Mce2A CDS) | Phe51Ser | 3 | - | | 690465 | T | G | 317.78 | SNP | Rv0591 (mce2C 689059:690504 Mce-family protein Mce2C CDS) | silent (Leu469) | 9947 | - | | 698968 | G | A | 605.77 | SNP | Rv0601c (- 698994:698524 Two component sensor kinase [first part] CDS) | silent (Gly9) | 9935 | - | | 706709 | C | T | 474.78 | SNP | Rv0612 (- 706324:706929 hypothetical protein CDS) | Ala129Val | 13 | - | | 721498 | G | A | 983.77 | SNP | Rv0629c (recD 721732:720005 Probable exonuclease V (alpha chain) RecD (exodeoxyribonuclease V alpha chain) (exodeoxyribonuclease V polypeptide) CDS) | Leu79Leu(s) | 4 | - | | 731487 | G | T | 1222.77 | SNP | intergenic |  |  | - | | 754186 | A | G | 1007.77 | SNP | Rv0658c (- 754409:753693 Probable conserved integral membrane protein CDS) | Leu75Pro | 2 | - | | 761155 | C | T | 1276.77 | SNP | Rv0667 (rpoB 759807:763325 DNA-directed RNA polymerase (beta chain) RpoB (transcriptase beta chain) (RNA polymerase beta subunit) CDS) | Ser450Leu(s) | 35 | resistance | | 764916 | T | C | 800.77 | SNP | Rv0668 (rpoC 763370:767320 DNA-directed RNA polymerase (beta' chain) RpoC (transcriptase beta' chain) (RNA polymerase beta' subunit) CDS) | Leu516Pro | 2 | - | | 767939 | G | A | 910.77 | SNP | Rv0669c (- 769597:767684 Possible hydrolase CDS) | silent (Arg553) | 9913 | - | | 775639 | T | C | 679.77 | SNP | Rv0676c (mmpL5 778480:775586 Probable conserved transmembrane transport protein MmpL5 CDS) | Ile948Val | 57 | - | | 780433 | A | C | 1017.77 | SNP | intergenic |  |  | - | | 781395 | T | C | 1180.77 | SNP | intergenic (Rv0682-165nt) |  |  | - | | 818798 | G | T | 1430.77 | SNP | Rv0726c (- 819640:818537 Possible S-adenosylmethionine-dependent methyltransferase CDS) | silent (Ile281) | 9872 | - | | 820246 | C | A | 594.77 | SNP | Rv0727c (fucA 820499:819843 Possible L-fuculose phosphate aldolase FucA (L-fuculose-1-phosphate aldolase) CDS) | Cys85Phe | 0 | - | | 836538 | A | G | 98.28 | SNP | Rv0746 (PE\_PGRS9 835701:838052 PE-PGRS family protein PE\_PGRS9 CDS) | Asn280Asp | 42 | - | | 836658 | A | G | 106.28 | SNP | Rv0746 (PE\_PGRS9 835701:838052 PE-PGRS family protein PE\_PGRS9 CDS) | Thr320Ala | 32 | - | | 837033 | A | G | 189.90 | SNP | Rv0746 (PE\_PGRS9 835701:838052 PE-PGRS family protein PE\_PGRS9 CDS) | Thr445Ala | 32 | - | | 839194 | A | G | 49.74 | SNP | Rv0747 (PE\_PGRS10 838451:840856 PE-PGRS family protein PE\_PGRS10 CDS) | silent (Thr248) | 9871 | - | | 839334 | A | G | 154.90 | SNP | Rv0747 (PE\_PGRS10 838451:840856 PE-PGRS family protein PE\_PGRS10 CDS) | Lys295Arg | 19 | - | | 839348 | A | G | 144.03 | SNP | Rv0747 (PE\_PGRS10 838451:840856 PE-PGRS family protein PE\_PGRS10 CDS) | Ser300Gly | 21 | - | | 839515 | G | A | 79.77 | SNP | Rv0747 (PE\_PGRS10 838451:840856 PE-PGRS family protein PE\_PGRS10 CDS) | silent (Ala355) | 9867 | - | | 839516 | A | G | 87.77 | SNP | Rv0747 (PE\_PGRS10 838451:840856 PE-PGRS family protein PE\_PGRS10 CDS) | Thr356Ala | 32 | - | | 839519 | C | G | 90.77 | SNP | Rv0747 (PE\_PGRS10 838451:840856 PE-PGRS family protein PE\_PGRS10 CDS) | Leu357Val(s) | 4 | - | | 839520 | T | C | 87.77 | SNP | Rv0747 (PE\_PGRS10 838451:840856 PE-PGRS family protein PE\_PGRS10 CDS) | Leu357Pro | 2 | - | | 839534 | A | C | 58.77 | SNP | Rv0747 (PE\_PGRS10 838451:840856 PE-PGRS family protein PE\_PGRS10 CDS) | Ile362Leu | 22 | - | | 839545 | C | CGG | 327.82 | INS | Rv0747 (PE\_PGRS10 838451:840856 PE-PGRS family protein PE\_PGRS10 CDS) |  |  | - | | 840496 | C | G | 48.74 | SNP | Rv0747 (PE\_PGRS10 838451:840856 PE-PGRS family protein PE\_PGRS10 CDS) | silent (Gly682) | 9935 | - | | 841764 | G | C | 1380.77 | SNP | Rv0749A (- 841874:841737 hypothetical protein CDS) | silent (Thr37) | 9871 | - | | 844283 | C | T | 597.77 | SNP | Rv0752c (fadE9 844414:843242 Probable acyl-CoA dehydrogenase FadE9 CDS) | silent (Ala44) | 9867 | - | | 846256 | C | A | 507.77 | SNP | Rv0754 (PE\_PGRS11 846159:847913 PE-PGRS family protein PE\_PGRS11 CDS) | Ala33Asp | 6 | - | | 852910 | C | T | 755.77 | SNP | Rv0758 (phoR 852396:853853 Possible two component system response sensor kinase membrane associated PhoR CDS) | Pro172Leu | 3 | - | | 854252 | GCC | G | 1573.73 | DEL | intergenic |  |  | - | | 857696 | A | G | 1253.77 | SNP | Rv0764c (cyp51 858037:856682 Cytochrome P450 51 Cyp51 (CYPL1) (P450-L1A1) (sterol 14-alpha demethylase) (lanosterol 14-alpha demethylase) (P450-14DM) CDS) | silent (Ala114) | 9867 | - | | 859769 | A | G | 1758.77 | SNP | Rv0766c (cyp123 860072:858864 Probable cytochrome P450 123 Cyp123 CDS) | Ser102Pro | 12 | - | | 869679 | C | T | 735.77 | SNP | Rv0776c (- 869763:868984 hypothetical protein CDS) | Gly29Arg | 0 | - | | 874835 | C | CCG | 3119.73 | INS | Rv0781 (ptrBa 874233:874943 Probable protease II PtrBa [first part] (oligopeptidase B) CDS); Rv0782 (ptrBb 874732:876390 Probable protease II PtrBb [second part] (oligopeptidase B) CDS) |  |  | - | | 880562 | G | T | 1216.77 | SNP | Rv0785 (- 879340:881040 hypothetical protein CDS) | Cys408Phe | 0 | - | | 882257 | T | C | 1753.77 | SNP | Rv0787 (- 881459:882418 hypothetical protein CDS) | Tyr267His | 4 | - | | 888774 | G | A | 691.77 | SNP | intergenic |  |  | - | | 893733 | T | G | 914.77 | SNP | Rv0800 (pepC 893318:894619 Probable aminopeptidase PepC CDS) | Leu139Arg | 1 | - | | 900221 | T | C | 1282.77 | SNP | Rv0806c (cpsY 901330:899732 Possible UDP-glucose-4-epimerase CpsY (galactowaldenase) (UDP-galactose-4-epimerase) (uridine diphosphate galactose-4-epimerase) (uridine diphospho-galactose-4-epimerase) CDS) | Val370Val(s) | 18 | - | | 903550 | T | C | 408.77 | SNP | Rv0808 (purF 902111:903694 Amidophosphoribosyltransferase PurF (glutamine phosphoribosylpyrophosphate amidotransferase) (ATASE) (gpatase) CDS) | silent (Ala480) | 9867 | - | | 903913 | T | C | 858.77 | SNP | Rv0809 (purM 903725:904819 Probable phosphoribosylformylglycinamidine CYCLO-ligase PurM (AIRS) (phosphoribosyl-aminoimidazole synthetase) (air synthase) CDS) | silent (Gly63) | 9935 | - | | 905636 | A | C | 1013.77 | SNP | Rv0811c (- 906340:905234 hypothetical protein CDS) | silent (Pro235) | 9926 | - | | 906857 | A | G | 1063.77 | SNP | Rv0812 (- 906423:907292 Probable amino acid aminotransferase CDS) | Ile145Met(s) | 6 | - | | 919384 | T | C | 1430.77 | SNP | Rv0825c (- 919553:918912 hypothetical protein CDS) | Tyr57Cys | 3 | - | | 921813 | C | G | 876.77 | SNP | Rv0829 (- 921575:921865 Possible transposase (fragment) CDS) | Ala80Gly | 21 | - | | 927496 | TAACGGCGGC | T | 877.75 | DEL | Rv0833 (PE\_PGRS13 925361:927610 PE-PGRS family protein PE\_PGRS13 CDS) |  |  | - | | 944941 | A | G | 847.77 | SNP | Rv0848 (cysK2 944938:946056 Possible cysteine synthase a CysK2 (O-acetylserine sulfhydrylase) (O-acetylserine (thiol)-lyase) (CSASE) CDS) | Arg2Gly | 1 | - | | 945214 | G | A | 1322.77 | SNP | Rv0848 (cysK2 944938:946056 Possible cysteine synthase a CysK2 (O-acetylserine sulfhydrylase) (O-acetylserine (thiol)-lyase) (CSASE) CDS) | Gly93Ser | 16 | - | | 946647 | A | C | 810.77 | SNP | Rv0849 (- 946056:947315 Probable conserved integral membrane transport protein CDS) | Thr198Pro | 4 | - | | 949535 | T | C | 1274.77 | SNP | Rv0853c (pdc 951118:949436 Probable pyruvate or indole-3-pyruvate decarboxylase Pdc CDS) | silent (Ala528) | 9867 | - | | 955304 | A | G | 1456.77 | SNP | Rv0859 (fadA 955077:956288 Possible acyl-CoA thiolase FadA CDS) | silent (Ala76) | 9867 | - | | 955524 | A | G | 851.77 | SNP | Rv0859 (fadA 955077:956288 Possible acyl-CoA thiolase FadA CDS) | Ser150Gly | 21 | - | | 955983 | C | T | 831.77 | SNP | Rv0859 (fadA 955077:956288 Possible acyl-CoA thiolase FadA CDS) | Pro303Ser | 17 | - | | 956644 | C | T | 866.77 | SNP | Rv0860 (fadB 956293:958455 Probable fatty oxidation protein FadB CDS) | Leu118Leu(s) | 4 | - | | 968426 | A | AGCCGGGTTG | 2217.73 | INS | Rv0872c (PE\_PGRS15 970244:968424 PE-PGRS family protein PE\_PGRS15 CDS) |  |  | - | | 979314 | C | T | 979.77 | SNP | Rv0880 (- 978934:979365 Possible transcriptional regulatory protein (possibly MarR-family) CDS) | silent (Ile127) | 9872 | - | | 979704 | G | C | 1475.77 | SNP | Rv0881 (- 979362:980228 Possible rRNA methyltransferase (rRNA methylase) CDS) | Gly115Arg | 0 | - | | 980475 | T | C | 743.77 | SNP | Rv0882 (- 980225:980509 Probable transmembrane protein CDS) | Leu(s)84Ser | 28 | - | | 986463 | G | C | 1576.77 | SNP | intergenic |  |  | - | | 990001 | G | C | 1091.77 | SNP | Rv0890c (- 992596:989948 Probable transcriptional regulatory protein (probably LuxR-family) CDS) | Pro866Ala | 22 | - | | 990626 | T | A | 815.77 | SNP | Rv0890c (- 992596:989948 Probable transcriptional regulatory protein (probably LuxR-family) CDS) | Leu657Phe | 6 | - | | 992307 | C | T | 1228.77 | SNP | Rv0890c (- 992596:989948 Probable transcriptional regulatory protein (probably LuxR-family) CDS) | Cys97Tyr | 3 | - | | 993346 | A | C | 1191.77 | SNP | Rv0891c (- 993455:992598 Possible transcriptional regulatory protein CDS) | Val37Gly | 5 | - | | 1010204 | C | CG | 1961.73 | INS | Rv0907 (- 1010136:1011734 hypothetical protein CDS) |  |  | - | | 1020044 | C | T | 665.77 | SNP | intergenic |  |  | - | | 1025106 | T | C | 1469.77 | SNP | Rv0919 (- 1024684:1025184 GCN5-related N-acetyltransferase CDS) | silent (Phe141) | 9946 | - | | 1037012 | T | C | 893.77 | SNP | Rv0930 (pstA1 1036999:1037925 Probable phosphate-transport integral membrane ABC transporter PstA1 CDS) | Met(s)5Thr | 22 | - | | 1037911 | C | T | 1329.77 | SNP | Rv0930 (pstA1 1036999:1037925 Probable phosphate-transport integral membrane ABC transporter PstA1 CDS) | Arg305STOP | 2 | - | | 1044557 | A | AACGCC | 2668.73 | INS | Rv0936 (pstA2 1044317:1045222 Phosphate-transport integral membrane ABC transporter PstA2 CDS) |  |  | - | | 1047165 | T | C | 803.77 | SNP | Rv0938 (ligD 1046136:1048415 ATP dependent DNA ligase LigD (ATP dependent polydeoxyribonucleotide synthase) (thermostable DNA ligase) (ATP dependent polynucleotide ligase) (sealase) (DNA repair enzyme) (DNA joinase) CDS) | Cys344Arg | 1 | - | | 1056916 | T | G | 975.77 | SNP | intergenic |  |  | - | | 1068151 | T | C | 1269.77 | SNP | Rv0956 (purN 1067561:1068208 Probable 5'-phosphoribosylglycinamide formyltransferase PurN (GART) (gar transformylase) (5'-phosphoribosylglycinamide transformylase) CDS) | silent (His197) | 9912 | - | | 1068432 | A | G | 1185.77 | SNP | Rv0957 (purH 1068205:1069776 Probable bifunctional purine biosynthesis protein PurH: phosphoribosylaminoimidazolecarboxamide formyltransferase (AICAR transformylase) (5'-phosphoribosyl-5-aminoimidazole-4-carboxamide formyltransferase) + inosinemonophosphate cyclohydrolase (imp cyclohydrolase) (inosinicase) (imp synthetase) (ATIC) CDS) | silent (Pro76) | 9926 | - | | 1070702 | T | C | 748.77 | SNP | Rv0958 (- 1069883:1071262 Possible magnesium chelatase CDS) | Ser274Pro | 12 | - | | 1074558 | G | A | 984.77 | SNP | Rv0962c (lprP 1075114:1074440 Possible lipoprotein LprP CDS) | Pro186Leu | 3 | - | | 1075279 | T | C | 1363.77 | SNP | intergenic |  |  | - | | 1076309 | G | T | 1016.77 | SNP | Rv0964c (- 1076678:1076196 Hypothetical protein CDS) | Pro124Thr | 5 | - | | 1077312 | A | G | 589.77 | SNP | Rv0966c (- 1077835:1077233 hypothetical protein CDS) | Val(s)175Ala | 9867 | - | | 1079927 | C | A | 883.77 | SNP | Rv0969 (ctpV 1078743:1081055 Probable metal cation transporter P-type ATPase CtpV CDS) | silent (Thr395) | 9871 | - | | 1081681 | T | C | 972.77 | SNP | Rv0970 (- 1081052:1081684 Probable conserved integral membrane protein CDS) | silent (Val210) | 9901 | - | | 1087050 | C | T | 705.77 | SNP | Rv0974c (accD2 1087345:1085756 Probable acetyl-/propionyl-CoA carboxylase (beta subunit) AccD2 CDS) | Cys99Tyr | 3 | - | | 1087193 | G | C | 1139.77 | SNP | Rv0974c (accD2 1087345:1085756 Probable acetyl-/propionyl-CoA carboxylase (beta subunit) AccD2 CDS) | Asn51Lys | 25 | - | | 1093406 | A | G | 1019.77 | SNP | Rv0978c (PE\_PGRS17 1094356:1093361 PE-PGRS family protein PE\_PGRS17 CDS) | silent (Val317) | 9901 | - | | 1093928 | G | A | 52.74 | SNP | Rv0978c (PE\_PGRS17 1094356:1093361 PE-PGRS family protein PE\_PGRS17 CDS) | silent (Asn143) | 9822 | - | | 1094538 | T | G | 682.77 | SNP | intergenic |  |  | - | | 1096633 | T | G | 1213.77 | SNP | intergenic |  |  | - | | 1098698 | C | G | 455.77 | SNP | Rv0982 (mprB 1097508:1099022 Two component sensor kinase MprB CDS) | silent (Gly397) | 9935 | - | | 1100234 | T | C | 1383.77 | SNP | Rv0983 (pepD 1099066:1100460 Probable serine protease PepD (serine proteinase) (MTB32B) CDS) | Leu390Pro | 2 | - | | 1105345 | C | T | 919.77 | SNP | Rv0988 (- 1105116:1106276 Possible conserved exported protein CDS) | Thr77Ile | 7 | - | | 1106422 | T | C | 1530.77 | SNP | Rv0989c (grcC2 1107382:1106405 Probable polyprenyl-diphosphate synthase GrcC2 (polyprenyl pyrophosphate synthetase) CDS) | Ile321Val | 57 | - | | 1106485 | C | T | 1178.77 | SNP | Rv0989c (grcC2 1107382:1106405 Probable polyprenyl-diphosphate synthase GrcC2 (polyprenyl pyrophosphate synthetase) CDS) | Glu300Lys | 7 | - | | 1109975 | A | G | 1308.77 | SNP | Rv0993 (galU 1109272:1110192 UTP--glucose-1-phosphate uridylyltransferase GalU (UDP-glucose pyrophosphorylase) (UDPGP) (alpha-D-glucosyl-1-phosphate uridylyltransferase) (uridine diphosphoglucose pyrophosphorylase) CDS) | Gln235Arg | 10 | - | | 1120281 | C | T | 659.77 | SNP | Rv1003 (- 1120022:1120879 hypothetical protein CDS) | Ala87Val | 13 | - | | 1126889 | G | C | 801.77 | SNP | Rv1007c (metS 1127003:1125444 Methionyl-tRNA synthetase MetS (MetRS) (methionine--tRNA ligase) CDS) | Arg39Gly | 1 | - | | 1127648 | C | A | 1623.77 | SNP | Rv1008 (tatD 1127089:1127883 Probable deoxyribonuclease TatD (YJJV protein) CDS) | Thr187Asn | 9 | - | | 1149551 | C | T | 1011.77 | SNP | Rv1028c (kdpD 1151686:1149104 Probable sensor protein KdpD CDS) | silent (Glu712) | 9865 | - | | 1150585 | G | A | 1060.77 | SNP | Rv1028c (kdpD 1151686:1149104 Probable sensor protein KdpD CDS) | Pro368Ser | 17 | - | | 1163134 | T | C | 1166.77 | SNP | Rv1040c (PE8 1163376:1162549 PE family protein PE8 CDS) | silent (Gly81) | 9935 | - | | 1165521 | T | TA | 1364.73 | INS | intergenic |  |  | - | | 1168715 | C | CT | 1267.73 | INS | Rv1046c (- 1169228:1168704 Hypothetical protein CDS) |  |  | - | | 1169307 | C | T | 1231.77 | SNP | intergenic |  |  | - | | 1170404 | C | A | 46.74 | SNP | Rv1047 (- 1169423:1170670 Probable transposase CDS) | Gln328Lys | 12 | - | | 1178116 | T | C | 1854.77 | SNP | Rv1056 (- 1177628:1178392 hypothetical protein CDS) | silent (Thr163) | 9871 | - | | 1184605 | C | A | 1352.77 | SNP | Rv1061 (- 1184015:1184878 hypothetical protein CDS) | Asp197Glu | 56 | - | | 1186775 | C | T | 448.53 | SNP | Rv1063c (- 1186823:1185741 hypothetical protein CDS) | Val17Ile | 33 | - | | 1191990 | T | G | 518.77 | SNP | Rv1068c (PE\_PGRS20 1192148:1190757 PE-PGRS family protein PE\_PGRS20 CDS) | silent (Ala53) | 9867 | - | | 1200418 | A | G | 867.77 | SNP | intergenic |  |  | - | | 1208148 | G | A | 1103.77 | SNP | Rv1084 (- 1207636:1209657 hypothetical protein CDS) | silent (Leu171) | 9947 | - | | 1220680 | T | C | 980.77 | SNP | Rv1093 (glyA1 1220574:1221854 Serine hydroxymethyltransferase 1 GlyA1 CDS) | Val36Ala | 18 | - | | 1224367 | T | C | 961.77 | SNP | intergenic |  |  | - | | 1248978 | T | C | 1026.77 | SNP | Rv1125 (- 1248082:1249326 hypothetical protein CDS) | silent (Ala299) | 9867 | - | | 1249918 | T | G | 1268.77 | SNP | Rv1126c (- 1249935:1249330 hypothetical protein CDS) | silent (Val6) | 9901 | - | | 1263633 | C | T | 577.77 | SNP | Rv1135c (PPE16 1264128:1262272 PPE family protein PPE16 CDS) | Ala166Thr | 22 | - | | 1280683 | G | GCGAAGT | 3118.73 | INS | Rv1153c (omt 1280846:1279998 Probable O-methyltransferase Omt CDS) |  |  | - | | 1281118 | T | C | 1421.77 | SNP | Rv1154c (- 1281484:1280843 Hypothetical protein CDS) | Thr123Ala | 32 | - | | 1284154 | G | T | 846.77 | SNP | Rv1157c (- 1284171:1283056 Conserved ala-, pro-rich protein CDS) | Asn6Lys | 25 | - | | 1292102 | A | G | 765.77 | SNP | Rv1162 (narH 1291065:1292741 Probable respiratory nitrate reductase (beta chain) NarH CDS) | silent (Pro346) | 9926 | - | | 1302563 | AG | A | 1013.73 | DEL | Rv1172c (PE12 1302681:1301755 PE family protein PE12 CDS) |  |  | - | | 1305141 | C | T | 1502.77 | SNP | Rv1173 (fbiC 1302931:1305501 Probable F420 biosynthesis protein FbiC CDS) | silent (Thr737) | 9871 | - | | 1311016 | C | T | 302.78 | SNP | Rv1179c (- 1313299:1310480 hypothetical protein CDS) | Ala762Thr | 22 | - | | 1313337 | A | AG | 1034.73 | INS | intergenic |  |  | - | | 1313338 | A | C | 513.77 | SNP | intergenic |  |  | - | | 1315191 | A | C | 950.77 | SNP | Rv1180 (pks3 1313725:1315191 Probable polyketide beta-ketoacyl synthase Pks3 CDS) | STOP489Tyr | 1 | - | | 1315884 | G | A | 727.77 | SNP | Rv1181 (pks4 1315234:1319982 Probable polyketide beta-ketoacyl synthase Pks4 CDS) | silent (Ala217) | 9867 | - | | 1327890 | G | A | 913.77 | SNP | Rv1186c (- 1329305:1327689 hypothetical protein CDS) | silent (Asp472) | 9859 | - | | 1328222 | T | C | 1061.77 | SNP | Rv1186c (- 1329305:1327689 hypothetical protein CDS) | Asn362Asp | 42 | - | | 1328687 | G | C | 1168.77 | SNP | Rv1186c (- 1329305:1327689 hypothetical protein CDS) | Pro207Ala | 22 | - | | 1332774 | A | C | 612.77 | SNP | Rv1189 (sigI 1332092:1332964 Possible alternative RNA polymerase sigma factor SigI CDS) | His228Pro | 5 | - | | 1341102 | C | T | 1092.77 | SNP | Rv1198 (esxL 1341006:1341290 Putative ESAT-6 like protein EsxL (ESAT-6 like protein 4) CDS) | Arg33Cys | 1 | - | | 1341103 | G | C | 1127.77 | SNP | Rv1198 (esxL 1341006:1341290 Putative ESAT-6 like protein EsxL (ESAT-6 like protein 4) CDS) | Arg33Pro | 5 | - | | 1341254 | G | A | 1318.77 | SNP | Rv1198 (esxL 1341006:1341290 Putative ESAT-6 like protein EsxL (ESAT-6 like protein 4) CDS) | silent (Ala83) | 9867 | - | | 1341319 | A | G | 1403.77 | SNP | intergenic |  |  | - | | 1341624 | G | T | 65.28 | SNP | Rv1199c (- 1342605:1341358 Possible transposase CDS) | Gln328Lys | 12 | - | | 1354590 | C | T | 1203.77 | SNP | Rv1212c (glgA 1355661:1354498 Putative glycosyl transferase GlgA CDS) | Gly358Arg | 0 | - | | 1357977 | C | T | 451.77 | SNP | Rv1215c (- 1359444:1357759 hypothetical protein CDS) | Glu490Lys | 7 | - | | 1365837 | C | CG | 1074.73 | INS | intergenic |  |  | - | | 1368349 | G | C | 809.77 | SNP | Rv1225c (- 1368721:1367891 hypothetical protein CDS) | Pro125Ala | 22 | - | | 1374065 | T | C | 632.77 | SNP | Rv1230c (- 1374197:1372962 Possible membrane protein CDS) | Ser45Gly | 21 | - | | 1375724 | A | C | 845.77 | SNP | Rv1232c (- 1376168:1374861 hypothetical protein CDS) | Cys149Gly | 1 | - | | 1382628 | T | C | 1093.77 | SNP | Rv1239c (corA 1383042:1381942 Possible magnesium and cobalt transport transmembrane protein CorA CDS) | Lys139Glu | 4 | - | | 1391868 | G | A | 1645.77 | SNP | Rv1248c (- 1393052:1389357 Multifunctional alpha-ketoglutarate metabolic enzyme CDS) | silent (Asp395) | 9859 | - | | 1393626 | A | G | 714.77 | SNP | Rv1249c (- 1393982:1393194 Possible membrane protein CDS) | silent (Leu119) | 9947 | - | | 1396922 | T | C | 1431.77 | SNP | Rv1251c (- 1399240:1395821 hypothetical protein CDS) | silent (Thr773) | 9871 | - | | 1411210 | T | G | 922.77 | SNP | Rv1263 (amiB2 1410431:1411819 Probable amidase AmiB2 (aminohydrolase) CDS) | Val260Val(s) | 18 | - | | 1413148 | C | T | 1010.77 | SNP | intergenic |  |  | - | | 1414021 | C | T | 626.77 | SNP | Rv1266c (pknH 1415840:1413960 Probable transmembrane serine/threonine-protein kinase H PknH (protein kinase H) (STPK H) CDS) | Arg607Gln | 9 | - | | 1416410 | A | C | 1115.77 | SNP | Rv1267c (embR 1417347:1416181 Probable transcriptional regulatory protein EmbR CDS) | Leu313Arg | 1 | genotype | | 1430158 | G | A | 1340.77 | SNP | Rv1279 (- 1430062:1431648 Probable dehydrogenase FAD flavoprotein GMC oxidoreductase CDS) | Ala33Thr | 22 | - | | 1433114 | G | A | 1511.77 | SNP | Rv1280c (oppA 1433440:1431665 Probable periplasmic oligopeptide-binding lipoprotein OppA CDS) | silent (Gly109) | 9935 | - | | 1440469 | C | G | 1259.77 | SNP | Rv1286 (cysN 1438907:1440751 Probable bifunctional enzyme CysN/CysC: sulfate adenyltransferase (subunit 1) + adenylylsulfate kinase CDS) | silent (Pro521) | 9926 | - | | 1445781 | A | G | 912.77 | SNP | Rv1291c (- 1445834:1445499 Conserved hypothetical secreted protein CDS) | silent (Ala18) | 9867 | - | | 1445922 | A | G | 1063.77 | SNP | intergenic |  |  | - | | 1457144 | C | T | 931.77 | SNP | Rv1300 (hemK 1456565:1457542 Probable HemK protein homolog HemK CDS) | Arg194Cys | 1 | - | | 1465155 | C | T | 1666.77 | SNP | Rv1309 (atpG 1464884:1465801 Probable ATP synthase gamma chain AtpG CDS) | Ala91Val | 13 | - | | 1468208 | A | C | 561.77 | SNP | Rv1313c (- 1469505:1468171 Possible transposase CDS) | Leu433Arg | 1 | - | | 1469314 | G | T | 106.03 | SNP | Rv1313c (- 1469505:1468171 Possible transposase CDS) | silent (Gly64) | 9935 | - | | 1469316 | C | T | 109.03 | SNP | Rv1313c (- 1469505:1468171 Possible transposase CDS) | Gly64Ser | 16 | - | | 1469317 | C | G | 126.03 | SNP | Rv1313c (- 1469505:1468171 Possible transposase CDS) | silent (Ala63) | 9867 | - | | 1471659 | C | T | 864.77 | SNP | intergenic |  |  | - | | 1480945 | C | G | 625.77 | SNP | Rv1319c (- 1482501:1480894 Possible adenylate cyclase (ATP pyrophosphate-lyase) (adenylyl cyclase) CDS) | silent (Thr519) | 9871 | - | | 1480948 | C | T | 603.77 | SNP | Rv1319c (- 1482501:1480894 Possible adenylate cyclase (ATP pyrophosphate-lyase) (adenylyl cyclase) CDS) | silent (Glu518) | 9865 | - | | 1480972 | T | C | 712.77 | SNP | Rv1319c (- 1482501:1480894 Possible adenylate cyclase (ATP pyrophosphate-lyase) (adenylyl cyclase) CDS) | silent (Glu510) | 9865 | - | | 1481185 | A | C | 2004.77 | SNP | Rv1319c (- 1482501:1480894 Possible adenylate cyclase (ATP pyrophosphate-lyase) (adenylyl cyclase) CDS) | Asp439Glu | 56 | - | | 1481321 | A | G | 909.77 | SNP | Rv1319c (- 1482501:1480894 Possible adenylate cyclase (ATP pyrophosphate-lyase) (adenylyl cyclase) CDS) | Val394Ala | 18 | - | | 1481468 | G | T | 1061.77 | SNP | Rv1319c (- 1482501:1480894 Possible adenylate cyclase (ATP pyrophosphate-lyase) (adenylyl cyclase) CDS) | Ala345Asp | 6 | - | | 1482627 | T | C | 1150.77 | SNP | Rv1320c (- 1484217:1482514 Possible adenylate cyclase (ATP pyrophosphate-lyase) (adenylyl cyclase) CDS) | Thr531Ala | 32 | - | | 1483652 | A | G | 1140.77 | SNP | Rv1320c (- 1484217:1482514 Possible adenylate cyclase (ATP pyrophosphate-lyase) (adenylyl cyclase) CDS) | Leu189Pro | 2 | - | | 1484708 | A | C | 1420.77 | SNP | Rv1321 (- 1484279:1484959 hypothetical protein CDS) | Ser144Arg | 6 | - | | 1488433 | A | G | 55.77 | SNP | Rv1325c (PE\_PGRS24 1489965:1488154 PE-PGRS family protein PE\_PGRS24 CDS) | silent (Asp511) | 9859 | - | | 1488434 | T | G | 32.77 | SNP | Rv1325c (PE\_PGRS24 1489965:1488154 PE-PGRS family protein PE\_PGRS24 CDS) | Asp511Ala | 10 | - | | 1488435 | C | A | 32.77 | SNP | Rv1325c (PE\_PGRS24 1489965:1488154 PE-PGRS family protein PE\_PGRS24 CDS) | Asp511Tyr | 0 | - | | 1499274 | C | G | 414.77 | SNP | Rv1330c (pncB1 1500559:1499213 Nicotinic acid phosphoribosyltransferase PncB1 CDS) | Gly429Ala | 21 | - | | 1501468 | G | C | 937.77 | SNP | Rv1332 (- 1500926:1501582 Probable transcriptional regulatory protein CDS) | silent (Pro181) | 9926 | genotype | | 1502795 | T | C | 1135.77 | SNP | Rv1334 (mec 1502641:1503081 Possible hydrolase CDS) | Met(s)52Thr | 22 | - | | 1522435 | G | C | 1389.77 | SNP | Rv1355c (moeY 1524032:1521885 Possible molybdopterin biosynthesis protein MoeY CDS) | Thr533Ser | 38 | - | | 1526819 | C | A | 1008.77 | SNP | Rv1358 (- 1526612:1530091 Probable transcriptional regulatory protein CDS) | silent (Arg70) | 9913 | - | | 1536251 | G | T | 940.77 | SNP | Rv1364c (- 1537644:1535683 Possible sigma factor regulatory protein CDS) | Ala465Glu | 10 | - | | 1537710 | AAC | A | 2385.73 | DEL | intergenic |  |  | - | | 1537771 | G | C | 1565.77 | SNP | intergenic |  |  | - | | 1544349 | T | G | 1263.77 | SNP | Rv1371 (- 1543359:1544828 Probable conserved membrane protein CDS) | Trp331Gly | 0 | - | | 1547125 | T | C | 1664.77 | SNP | Rv1374c (- 1547530:1547072 Hypothetical protein CDS) | Thr136Ala | 32 | - | | 1552547 | G | A | 957.77 | SNP | Rv1378c (- 1552655:1551228 hypothetical protein CDS) | Arg37Trp | 2 | - | | 1570566 | C | A | 606.77 | SNP | Rv1394c (cyp132 1570969:1569584 Probable cytochrome P450 132 Cyp132 CDS) | Arg135Leu | 1 | - | | 1573605 | C | T | 529.77 | SNP | Rv1396c (PE\_PGRS25 1573857:1572127 PE-PGRS family protein PE\_PGRS25 CDS) | Gly85Ser | 16 | - | | 1588899 | G | T | 1123.77 | SNP | Rv1412 (ribC 1588567:1589172 Probable riboflavin synthase alpha chain RibC (RibE) CDS) | silent (Ala111) | 9867 | - | | 1597405 | G | A | 532.77 | SNP | Rv1422 (- 1596881:1597909 hypothetical protein CDS) | silent (Pro175) | 9926 | - | | 1605170 | C | T | 862.77 | SNP | Rv1429 (- 1604878:1606146 hypothetical protein CDS) | Ala98Val(s) | 9867 | - | | 1609840 | A | G | 906.77 | SNP | Rv1431 (- 1608083:1609852 hypothetical protein CDS) | silent (Pro586) | 9926 | - | | 1612624 | T | TATCGGTACCGGTGCGCCAG GG | 4821.73 | INS | Rv1435c (- 1612950:1612342 Probable conserved proline, glycine, valine-rich secreted protein CDS) |  |  | - | | 1613035 | T | C | 948.77 | SNP | intergenic |  |  | - | | 1620135 | A | G | 968.77 | SNP | Rv1442 (bisC 1619791:1622091 Probable biotin sulfoxide reductase BisC (BDS reductase) (BSO reductase) CDS) | silent (Gly115) | 9935 | - | | 1630148 | A | C | 1202.77 | SNP | Rv1449c (tkt 1630199:1628097 Transketolase Tkt (TK) CDS) | Tyr18Asp | 0 | - | | 1633344 | A | G | 302.78 | SNP | Rv1450c (PE\_PGRS27 1634627:1630638 PE-PGRS family protein PE\_PGRS27 CDS) | silent (Asn428) | 9822 | - | | 1636991 | T | C | 99.03 | SNP | Rv1452c (PE\_PGRS28 1638229:1636004 PE-PGRS family protein PE\_PGRS28 CDS) | silent (Gly413) | 9935 | - | | 1636996 | G | C | 108.03 | SNP | Rv1452c (PE\_PGRS28 1638229:1636004 PE-PGRS family protein PE\_PGRS28 CDS) | Arg412Gly | 1 | - | | 1637015 | A | G | 69.80 | SNP | Rv1452c (PE\_PGRS28 1638229:1636004 PE-PGRS family protein PE\_PGRS28 CDS) | silent (Ala405) | 9867 | - | | 1637018 | G | C | 37.79 | SNP | Rv1452c (PE\_PGRS28 1638229:1636004 PE-PGRS family protein PE\_PGRS28 CDS) | silent (Gly404) | 9935 | - | | 1637035 | C | T | 66.77 | SNP | Rv1452c (PE\_PGRS28 1638229:1636004 PE-PGRS family protein PE\_PGRS28 CDS) | Ala399Thr | 22 | - | | 1638211 | T | C | 51.74 | SNP | Rv1452c (PE\_PGRS28 1638229:1636004 PE-PGRS family protein PE\_PGRS28 CDS) | Thr7Ala | 32 | - | | 1638212 | T | C | 49.74 | SNP | Rv1452c (PE\_PGRS28 1638229:1636004 PE-PGRS family protein PE\_PGRS28 CDS) | Val6Val(s) | 18 | - | | 1638235 | T | A | 46.74 | SNP | intergenic |  |  | - | | 1638238 | A | T | 62.28 | SNP | intergenic |  |  | - | | 1639594 | C | A | 1282.77 | SNP | Rv1453 (- 1638381:1639646 Possible transcriptional activator protein CDS) | Pro405Gln | 6 | - | | 1639961 | C | T | 688.77 | SNP | Rv1454c (qor 1640660:1639674 Probable quinone reductase Qor (NADPH:quinone reductase) (zeta-crystallin homolog protein) CDS) | Val234Ile | 33 | - | | 1650072 | A | G | 731.77 | SNP | Rv1462 (- 1649526:1650719 hypothetical protein CDS) | Asn183Asp | 42 | - | | 1655829 | G | GC | 225.77 | INS | Rv1468c (PE\_PGRS29 1656721:1655609 PE-PGRS family protein PE\_PGRS29 CDS) |  |  | - | | 1672598 | A | G | 738.77 | SNP | Rv1482c (- 1673299:1672457 hypothetical protein CDS) | silent (Ser234) | 9840 | - | | 1676290 | C | A | 1156.77 | SNP | Rv1486c (- 1676883:1676017 hypothetical protein CDS) | Lys198Asn | 13 | - | | 1689349 | C | T | 598.77 | SNP | Rv1498c (- 1689920:1689303 Probable methyltransferase CDS) | Arg191His | 8 | - | | 1692141 | A | C | 1323.77 | SNP | Rv1501 (- 1691890:1692711 hypothetical protein CDS) | silent (Ile84) | 9872 | - | | 1693561 | A | G | 1929.77 | SNP | Rv1502 (- 1692924:1693823 Hypothetical protein CDS) | Tyr213Cys | 3 | - | | 1696464 | C | G | 1486.77 | SNP | intergenic |  |  | - | | 1698911 | G | A | 964.77 | SNP | Rv1508c (- 1699894:1698095 Probable membrane protein CDS) | silent (Gly328) | 9935 | - | | 1706034 | A | G | 650.96 | SNP | Rv1514c (- 1706595:1705807 hypothetical protein CDS) | Cys188Arg | 1 | - | | 1706119 | T | C | 984.77 | SNP | Rv1514c (- 1706595:1705807 hypothetical protein CDS) | silent (Ser159) | 9840 | - | | 1706262 | T | C | 1210.77 | SNP | Rv1514c (- 1706595:1705807 hypothetical protein CDS) | Lys112Glu | 4 | - | | 1726541 | G | A | 1037.77 | SNP | Rv1527c (pks5 1728409:1722083 Probable polyketide synthase Pks5 CDS) | silent (Tyr623) | 9945 | - | | 1728837 | A | G | 836.77 | SNP | intergenic |  |  | - | | 1736577 | A | G | 1452.77 | SNP | Rv1536 (ileS 1736519:1739644 Isoleucyl-tRNA synthetase IleS CDS) | Glu20Gly | 7 | - | | 1736992 | G | C | 1698.77 | SNP | Rv1536 (ileS 1736519:1739644 Isoleucyl-tRNA synthetase IleS CDS) | silent (Leu158) | 9947 | - | | 1751042 | C | T | 1034.77 | SNP | Rv1547 (dnaE1 1747694:1751248 Probable DNA polymerase III (alpha chain) DnaE1 (DNA nucleotidyltransferase) CDS) | Pro1117Ser | 17 | - | | 1752561 | T | C | 290.78 | SNP | Rv1548c (PPE21 1753333:1751297 PPE family protein PPE21 CDS) | Asp258Gly | 11 | - | | 1753519 | G | GC | 1662.73 | INS | Rv1549 (fadD11.1 1753510:1754037 Possible fatty-acid-CoA ligase FadD11.1 (fatty-acid-CoA synthetase) (fatty-acid-CoA synthase) CDS) |  |  | - | | 1757105 | A | C | 1263.77 | SNP | Rv1551 (plsB1 1755445:1757310 Possible acyltransferase PlsB1 CDS) | Gln554Pro | 8 | - | | 1757399 | T | C | 969.77 | SNP | intergenic |  |  | - | | 1759252 | G | T | 1224.77 | SNP | Rv1552 (frdA 1757681:1759432 Probable fumarate reductase [flavoprotein subunit] FrdA (fumarate dehydrogenase) (fumaric hydrogenase) CDS) | silent (Ser524) | 9840 | genotype | | 1760292 | A | G | 1137.77 | SNP | Rv1554 (frdC 1760175:1760555 Probable fumarate reductase [membrane anchor subunit] FrdC (fumarate dehydrogenase) (fumaric hydrogenase) CDS) | Met(s)40Val(s) | 9867 | - | | 1763855 | A | G | 1322.77 | SNP | Rv1559 (ilvA 1763428:1764717 Probable threonine dehydratase IlvA CDS) | Asp143Gly | 11 | - | | 1778430 | T | C | 855.77 | SNP | Rv1570 (bioD 1777859:1778539 Dethiobiotin synthetase BioD CDS) | Met(s)191Thr | 22 | - | | 1789446 | C | T | 38.74 | SNP | Rv1588c (- 1789836:1789168 Partial REP13E12 repeat protein CDS) | Val131Ile | 33 | - | | 1789564 | C | T | 34.74 | SNP | Rv1588c (- 1789836:1789168 Partial REP13E12 repeat protein CDS) | silent (Arg91) | 9913 | - | | 1789650 | C | T | 220.80 | SNP | Rv1588c (- 1789836:1789168 Partial REP13E12 repeat protein CDS) | Ala63Thr | 22 | - | | 1789654 | A | G | 237.80 | SNP | Rv1588c (- 1789836:1789168 Partial REP13E12 repeat protein CDS) | silent (Leu61) | 9947 | - | | 1789671 | C | T | 256.78 | SNP | Rv1588c (- 1789836:1789168 Partial REP13E12 repeat protein CDS) | Ala56Thr | 22 | - | | 1789675 | A | C | 263.80 | SNP | Rv1588c (- 1789836:1789168 Partial REP13E12 repeat protein CDS) | silent (Gly54) | 9935 | - | | 1789678 | C | G | 258.78 | SNP | Rv1588c (- 1789836:1789168 Partial REP13E12 repeat protein CDS) | Val(s)53Val | 13 | - | | 1789742 | G | C | 51.77 | SNP | Rv1588c (- 1789836:1789168 Partial REP13E12 repeat protein CDS) | Thr32Ser | 38 | - | | 1789746 | A | G | 46.77 | SNP | Rv1588c (- 1789836:1789168 Partial REP13E12 repeat protein CDS) | Leu(s)31Leu | 3 | - | | 1789766 | T | G | 744.77 | SNP | Rv1588c (- 1789836:1789168 Partial REP13E12 repeat protein CDS) | Asp24Ala | 10 | - | | 1798355 | G | A | 997.77 | SNP | Rv1597 (- 1798294:1799052 Hypothetical protein CDS) | Gly21Asp | 6 | - | | 1803265 | G | A | 1302.77 | SNP | Rv1602 (hisH 1802664:1803284 Probable amidotransferase HisH CDS) | Ser201Asn | 20 | - | | 1804409 | C | A | 1184.77 | SNP | Rv1604 (impA 1804039:1804851 Probable inositol-monophosphatase ImpA (imp) CDS) | Pro124Gln | 6 | - | | 1817976 | A | T | 1226.77 | SNP | Rv1618 (tesB1 1817615:1818517 Probable acyl-CoA thioesterase II TesB1 CDS) | His121Leu | 4 | - | | 1827998 | C | T | 1215.77 | SNP | intergenic |  |  | - | | 1832350 | G | A | 731.77 | SNP | Rv1629 (polA 1830665:1833379 Probable DNA polymerase I PolA CDS) | silent (Gln562) | 9876 | - | | 1836286 | G | C | 804.77 | SNP | intergenic |  |  | - | | 1847919 | C | G | 960.77 | SNP | Rv1639c (- 1848458:1846989 hypothetical protein CDS) | silent (Thr180) | 9871 | - | | 1854300 | T | C | 835.77 | SNP | Rv1644 (tsnR 1853606:1854388 Possible 23S rRNA methyltransferase TsnR CDS) | Leu232Pro | 2 | - | | 1856777 | G | C | 1194.77 | SNP | Rv1647 (- 1856774:1857724 Adenylate cyclase (ATP pyrophosphate-lyase) (adenylyl cyclase) CDS) | Ala2Pro | 13 | - | | 1864698 | C | T | 324.77 | SNP | Rv1651c (PE\_PGRS30 1865382:1862347 PE-PGRS family protein PE\_PGRS30 CDS) | Ala229Thr | 22 | - | | 1870129 | G | A | 1146.77 | SNP | Rv1656 (argF 1869922:1870845 Probable ornithine carbamoyltransferase, anabolic ArgF CDS) | Val70Ile | 33 | - | | 1879671 | T | C | 60.77 | SNP | Rv1661 (pks7 1875304:1881684 Probable polyketide synthase Pks7 CDS) | silent (Gly1456) | 9935 | - | | 1885772 | G | A | 1004.77 | SNP | Rv1662 (pks8 1881704:1886512 Probable polyketide synthase Pks8 CDS) | Ala1357Thr | 22 | - | | 1894300 | G | GGTCTTGCCGC | 4603.73 | INS | Rv1668c (- 1895342:1894224 Probable first part of macrolide-transport ATP-binding protein ABC transporter CDS) |  |  | - | | 1901493 | T | C | 756.77 | SNP | Rv1676 (- 1901047:1901751 hypothetical protein CDS) | silent (Ser149) | 9840 | - | | 1907296 | G | C | 960.77 | SNP | Rv1682 (- 1906403:1907320 Probable coiled-coil structural protein CDS) | silent (Ala298) | 9867 | - | | 1907384 | G | A | 1191.77 | SNP | intergenic |  |  | - | | 1917972 | A | G | 949.77 | SNP | Rv1694 (tlyA 1917940:1918746 2'-O-methyltransferase TlyA CDS) | silent (Leu11) | 9947 | - | | 1931179 | C | A | 910.77 | SNP | Rv1704c (cycA 1931456:1929786 Probable D-serine/alanine/glycine transporter protein CycA CDS) | Arg93Leu | 1 | - | | 1933988 | G | A | 1659.77 | SNP | intergenic |  |  | - | | 1937115 | G | A | 1432.77 | SNP | Rv1708 (- 1936360:1937316 Putative initiation inhibitor protein CDS) | silent (Arg252) | 9913 | - | | 1944402 | T | C | 1243.77 | SNP | Rv1716 (- 1943576:1944406 hypothetical protein CDS) | Val276Ala | 18 | - | | 1950767 | T | C | 933.77 | SNP | Rv1724c (- 1951051:1950632 Hypothetical protein CDS) | silent (Lys95) | 9926 | - | | 1960284 | C | A | 775.77 | SNP | Rv1733c (- 1960487:1959855 Probable conserved transmembrane protein CDS) | Gln68His | 20 | - | | 1963566 | C | G | 1345.77 | SNP | Rv1736c (narX 1964186:1962228 Probable nitrate reductase NarX CDS) | Met(s)207Ile | 2 | - | | 1967237 | C | A | 1299.77 | SNP | Rv1739c (- 1967637:1965955 Probable sulphate-transport transmembrane protein ABC transporter CDS) | Arg134Leu | 1 | - | | 1970691 | G | A | 589.77 | SNP | Rv1743 (pknE 1969004:1970704 Probable transmembrane serine/threonine-protein kinase E PknE (protein kinase E) (STPK E) CDS) | Arg563Lys | 37 | - | | 1978166 | G | T | 1189.77 | SNP | Rv1750c (fadD1 1979567:1977969 Possible fatty-acid-CoA ligase FadD1 (fatty-acid-CoA synthetase) (fatty-acid-CoA synthase) CDS) | Gln468Lys | 12 | - | | 1982961 | GC | G | 1036.73 | DEL | Rv1753c (PPE24 1984775:1981614 PPE family protein PPE24 CDS) |  |  | - | | 1983273 | G | A | 563.77 | SNP | Rv1753c (PPE24 1984775:1981614 PPE family protein PPE24 CDS) | silent (Gly501) | 9935 | - | | 1983276 | G | C | 604.77 | SNP | Rv1753c (PPE24 1984775:1981614 PPE family protein PPE24 CDS) | Val500Val(s) | 18 | - | | 1987394 | C | T | 927.77 | SNP | intergenic |  |  | - | | 1993808 | A | T | 1021.77 | SNP | Rv1760 (- 1993153:1994661 Possible triacylglycerol synthase (diacylglycerol acyltransferase) CDS) | Glu219Val(s) | 17 | - | | 1994939 | G | A | 1315.77 | SNP | Rv1761c (- 1995054:1994671 Possible exported protein CDS) | Thr39Ile | 7 | - | | 2022868 | T | C | 644.77 | SNP | Rv1783 (eccC5 2019257:2023432 ESX conserved component EccC5 ESX-5 type VII secretion system protein CDS) | silent (Ser1204) | 9840 | - | | 2023442 | A | C | 1111.77 | SNP | intergenic |  |  | - | | 2033748 | G | C | 1104.77 | SNP | Rv1795 (eccD5 2032240:2033751 ESX conserved component EccD5 ESX-5 type VII secretion system protein Probable membrane protein CDS); Rv1796 (mycP5 2033729:2035486 Probable proline rich membrane-anchored mycosin MycP5 (serine protease) (subtilisin-like protease) (subtilase-like) (mycosin-5) CDS) | silent (Arg503); Gly7Ala | 9913; 21 | - | | 2039011 | G | C | 162.90 | SNP | intergenic |  |  | - | | 2045310 | A | G | 309.78 | SNP | Rv1803c (PE\_PGRS32 2046842:2044923 PE-PGRS family protein PE\_PGRS32 CDS) | silent (Ile511) | 9872 | - | | 2046073 | A | G | 90.28 | SNP | Rv1803c (PE\_PGRS32 2046842:2044923 PE-PGRS family protein PE\_PGRS32 CDS) | Val257Ala | 18 | - | | 2049065 | T | C | 877.77 | SNP | intergenic |  |  | - | | 2049097 | G | C | 986.77 | SNP | intergenic |  |  | - | | 2051746 | T | C | 847.77 | SNP | Rv1809 (PPE33 2051282:2052688 PPE family protein PPE33 CDS) | silent (Ala155) | 9867 | - | | 2052035 | G | T | 1542.77 | SNP | Rv1809 (PPE33 2051282:2052688 PPE family protein PPE33 CDS) | Val(s)252Leu(s) | 9867 | - | | 2055271 | A | G | 1162.77 | SNP | Rv1812c (- 2055359:2054157 Probable dehydrogenase CDS) | Leu30Pro | 2 | - | | 2057774 | A | T | 978.77 | SNP | Rv1815 (- 2057528:2058193 hypothetical protein CDS) | Ile83Phe | 8 | - | | 2058447 | G | A | 1013.77 | SNP | Rv1816 (- 2058256:2058960 Possible transcriptional regulatory protein CDS) | Leu(s)64Leu | 3 | - | | 2074806 | A | G | 742.77 | SNP | intergenic |  |  | - | | 2079730 | G | A | 898.77 | SNP | Rv1833c (- 2079789:2078929 Possible haloalkane dehalogenase CDS) | silent (Asp20) | 9859 | - | | 2094911 | ACAGCGT | A | 4459.73 | DEL | Rv1844c (gnd1 2095188:2093731 Probable 6-phosphogluconate dehydrogenase Gnd1 CDS) |  |  | - | | 2096186 | A | G | 1147.77 | SNP | Rv1846c (blaI 2096599:2096183 Transcriptional repressor BlaI CDS) | silent (Thr138) | 9871 | - | | 2101786 | C | T | 928.77 | SNP | Rv1854c (ndh 2103042:2101651 Probable NADH dehydrogenase Ndh CDS) | Val(s)419Val | 13 | - | | 2109523 | C | CG | 1805.73 | INS | intergenic |  |  | - | | 2116903 | C | T | 1094.77 | SNP | Rv1867 (- 2115764:2117248 hypothetical protein CDS) | silent (Gly380) | 9935 | - | | 2128870 | A | G | 1059.77 | SNP | Rv1878 (glnA3 2128022:2129374 Probable glutamine synthetase GlnA3 (glutamine synthase) (GS-I) CDS) | silent (Leu283) | 9947 | - | | 2133468 | T | TTCGCATGCCGTCACC | 2529.73 | INS | Rv1883c (- 2133692:2133231 hypothetical protein CDS) |  |  | - | | 2135870 | T | C | 1307.77 | SNP | intergenic |  |  | - | | 2143328 | G | C | 1442.77 | SNP | Rv1895 (- 2142521:2143675 Possible dehydrogenase CDS) | Val(s)270Leu | 3 | - | | 2147022 | A | C | 1333.77 | SNP | Rv1900c (lipJ 2147633:2146245 Probable lignin peroxidase LipJ CDS) | Ile204Met(s) | 6 | - | | 2149855 | C | CA | 2126.73 | INS | Rv1902c (nanT 2150274:2149006 Probable sialic acid-transport integral membrane protein NanT CDS) |  |  | - | | 2155168 | C | T | 712.77 | SNP | Rv1908c (katG 2156111:2153889 Catalase-peroxidase-peroxynitritase T KatG CDS) | Ser315Asn | 20 | resistance | | 2163375 | T | C | 621.77 | SNP | Rv1917c (PPE34 2167311:2162932 PPE family protein PPE34 CDS) | Asn1313Asp | 42 | - | | 2163412 | A | G | 682.77 | SNP | Rv1917c (PPE34 2167311:2162932 PPE family protein PPE34 CDS) | silent (Val1300) | 9901 | - | | 2163415 | C | A | 688.77 | SNP | Rv1917c (PPE34 2167311:2162932 PPE family protein PPE34 CDS) | silent (Pro1299) | 9926 | - | | 2163417 | G | C | 744.77 | SNP | Rv1917c (PPE34 2167311:2162932 PPE family protein PPE34 CDS) | Pro1299Ala | 22 | - | | 2163419 | C | T | 702.77 | SNP | Rv1917c (PPE34 2167311:2162932 PPE family protein PPE34 CDS) | Ser1298Asn | 20 | - | | 2163421 | C | G | 631.77 | SNP | Rv1917c (PPE34 2167311:2162932 PPE family protein PPE34 CDS) | silent (Thr1297) | 9871 | - | | 2163444 | T | C | 114.77 | SNP | Rv1917c (PPE34 2167311:2162932 PPE family protein PPE34 CDS) | Asn1290Asp | 42 | - | | 2163790 | A | C | 579.77 | SNP | Rv1917c (PPE34 2167311:2162932 PPE family protein PPE34 CDS) | silent (Pro1174) | 9926 | - | | 2165286 | A | C | 750.77 | SNP | Rv1917c (PPE34 2167311:2162932 PPE family protein PPE34 CDS) | Ser676Ala | 35 | - | | 2165503 | T | A | 802.77 | SNP | Rv1917c (PPE34 2167311:2162932 PPE family protein PPE34 CDS) | silent (Ala603) | 9867 | - | | 2165928 | G | T | 1436.77 | SNP | Rv1917c (PPE34 2167311:2162932 PPE family protein PPE34 CDS) | Pro462Thr | 5 | - | | 2184781 | G | T | 861.77 | SNP | Rv1933c (fadE18 2184957:2183866 Probable acyl-CoA dehydrogenase FadE18 CDS) | silent (Gly59) | 9935 | - | | 2195922 | T | C | 1100.77 | SNP | Rv1944c (- 2195934:2195344 hypothetical protein CDS) | Thr5Ala | 32 | - | | 2195923 | A | C | 1112.77 | SNP | Rv1944c (- 2195934:2195344 hypothetical protein CDS) | Asp4Glu | 56 | - | | 2196879 | T | A | 2230.77 | SNP | Rv1945 (- 2195989:2197353 hypothetical protein CDS) | silent (Leu297) | 9947 | - | | 2196882 | A | G | 2348.77 | SNP | Rv1945 (- 2195989:2197353 hypothetical protein CDS) | silent (Lys298) | 9926 | - | | 2196964 | A | C | 60.77 | SNP | Rv1945 (- 2195989:2197353 hypothetical protein CDS) | Asn326His | 18 | - | | 2196969 | G | C | 97.77 | SNP | Rv1945 (- 2195989:2197353 hypothetical protein CDS) | silent (Ala327) | 9867 | - | | 2196970 | C | A | 93.77 | SNP | Rv1945 (- 2195989:2197353 hypothetical protein CDS) | His328Asn | 21 | - | | 2198421 | C | CG | 1679.73 | INS | Rv1947 (- 2198024:2198425 Hypothetical protein CDS) |  |  | - | | 2207591 | T | TC | 1523.73 | INS | intergenic |  |  | - | | 2211826 | A | G | 767.77 | SNP | Rv1968 (mce3C 2211626:2212858 Mce-family protein Mce3C CDS) | silent (Lys67) | 9926 | - | | 2216443 | C | A | 721.77 | SNP | Rv1971 (mce3F 2215257:2216570 Mce-family protein Mce3F CDS) | Ala396Glu | 10 | - | | 2220512 | T | G | 1075.77 | SNP | Rv1977 (- 2219754:2220800 hypothetical protein CDS) | silent (Ser253) | 9840 | - | | 2223293 | T | C | 1134.77 | SNP | intergenic |  |  | - | | 2228967 | A | G | 828.77 | SNP | intergenic |  |  | - | | 2251999 | A | G | 1560.77 | SNP | intergenic |  |  | - | | 2258433 | C | G | 1100.77 | SNP | Rv2010 (vapC15 2258273:2258671 Toxin VapC15 CDS) | Ala54Gly | 21 | - | | 2260151 | A | G | 323.77 | SNP | intergenic |  |  | - | | 2260154 | C | T | 318.77 | SNP | intergenic |  |  | - | | 2260171 | T | C | 634.77 | SNP | intergenic |  |  | - | | 2260174 | C | T | 605.77 | SNP | intergenic |  |  | - | | 2260196 | C | CA | 942.73 | INS | intergenic |  |  | - | | 2260199 | C | T | 570.77 | SNP | intergenic |  |  | - | | 2260212 | G | T | 541.77 | SNP | intergenic |  |  | - | | 2260214 | G | C | 527.77 | SNP | intergenic |  |  | - | | 2260220 | C | T | 412.77 | SNP | intergenic |  |  | - | | 2260222 | C | G | 474.77 | SNP | intergenic |  |  | - | | 2260231 | T | C | 496.77 | SNP | intergenic |  |  | - | | 2260525 | C | T | 902.77 | SNP | intergenic |  |  | - | | 2264782 | C | A | 990.77 | SNP | Rv2017 (- 2263998:2265038 Transcriptional regulatory protein CDS) | Ala262Glu | 10 | - | | 2265059 | T | G | 1194.77 | SNP | intergenic |  |  | - | | 2266487 | G | C | 924.77 | SNP | Rv2020c (- 2266720:2266421 hypothetical protein CDS) | silent (Leu78) | 9947 | - | | 2266504 | T | TA | 1544.73 | INS | Rv2020c (- 2266720:2266421 hypothetical protein CDS) |  |  | - | | 2266508 | A | T | 645.77 | SNP | Rv2020c (- 2266720:2266421 hypothetical protein CDS) | Asp71Glu | 56 | - | | 2266511 | GT | G | 1216.73 | DEL | Rv2020c (- 2266720:2266421 hypothetical protein CDS) |  |  | - | | 2266517 | T | C | 685.77 | SNP | Rv2020c (- 2266720:2266421 hypothetical protein CDS) | silent (Glu68) | 9865 | - | | 2266550 | G | T | 625.77 | SNP | Rv2020c (- 2266720:2266421 hypothetical protein CDS) | silent (Gly57) | 9935 | - | | 2266553 | C | G | 735.77 | SNP | Rv2020c (- 2266720:2266421 hypothetical protein CDS) | silent (Ser56) | 9840 | - | | 2266583 | C | G | 586.77 | SNP | Rv2020c (- 2266720:2266421 hypothetical protein CDS) | Glu46Asp | 53 | - | | 2266598 | G | C | 632.77 | SNP | Rv2020c (- 2266720:2266421 hypothetical protein CDS) | silent (Leu41) | 9947 | - | | 2266604 | C | G | 619.77 | SNP | Rv2020c (- 2266720:2266421 hypothetical protein CDS) | silent (Ser39) | 9840 | - | | 2266613 | G | GC | 967.73 | INS | Rv2020c (- 2266720:2266421 hypothetical protein CDS) |  |  | - | | 2266624 | G | T | 591.77 | SNP | Rv2020c (- 2266720:2266421 hypothetical protein CDS) | Leu33Ile | 9 | - | | 2267983 | C | T | 1054.77 | SNP | Rv2023c (- 2268108:2267749 Hypothetical protein CDS) | silent (Ala42) | 9867 | - | | 2269780 | T | C | 643.77 | SNP | Rv2024c (- 2270240:2268693 hypothetical protein CDS) | Asp154Gly | 11 | - | | 2270102 | A | G | 1145.77 | SNP | Rv2024c (- 2270240:2268693 hypothetical protein CDS) | Trp47Arg | 8 | - | | 2273627 | C | T | 616.77 | SNP | Rv2027c (dosT 2274508:2272787 Two component sensor histidine kinase DosT CDS) | silent (Gly294) | 9935 | - | | 2278641 | A | G | 731.77 | SNP | Rv2031c (hspX 2278932:2278498 Heat shock protein HspX (alpha-crystallin homolog) (14 kDa antigen) (HSP16.3) CDS) | Phe98Leu | 13 | - | | 2282787 | C | T | 1202.77 | SNP | Rv2037c (- 2283721:2282747 Conserved transmembrane protein CDS) | Cys312Tyr | 3 | - | | 2283964 | C | A | 1106.77 | SNP | Rv2038c (- 2284796:2283723 Probable sugar-transport ATP-binding protein ABC transporter CDS) | Arg278Leu | 1 | - | | 2285251 | C | A | 722.77 | SNP | Rv2039c (- 2285641:2284799 Probable sugar-transport integral membrane protein ABC transporter CDS) | Val131Phe | 0 | - | | 2287121 | A | G | 940.77 | SNP | Rv2041c (- 2287846:2286527 Probable sugar-binding lipoprotein CDS) | silent (Asp242) | 9859 | - | | 2288860 | C | A | 1340.77 | SNP | Rv2043c (pncA 2289241:2288681 Pyrazinamidase/nicotinamidase PncA (PZase) CDS) | Val128Phe | 0 | - | | 2294876 | G | A | 1015.77 | SNP | Rv2048c (pks12 2306986:2294531 Polyketide synthase Pks12 CDS) | silent (Arg4037) | 9913 | - | | 2294894 | G | C | 958.77 | SNP | Rv2048c (pks12 2306986:2294531 Polyketide synthase Pks12 CDS) | silent (Leu4031) | 9947 | - | | 2294896 | G | A | 948.77 | SNP | Rv2048c (pks12 2306986:2294531 Polyketide synthase Pks12 CDS) | Leu4031Phe | 6 | - | | 2294903 | G | A | 999.77 | SNP | Rv2048c (pks12 2306986:2294531 Polyketide synthase Pks12 CDS) | silent (Phe4028) | 9946 | - | | 2296042 | G | C | 1139.77 | SNP | Rv2048c (pks12 2306986:2294531 Polyketide synthase Pks12 CDS) | Pro3649Ala | 22 | - | | 2300237 | A | G | 671.77 | SNP | Rv2048c (pks12 2306986:2294531 Polyketide synthase Pks12 CDS) | silent (Ala2250) | 9867 | - | | 2300546 | A | T | 605.77 | SNP | Rv2048c (pks12 2306986:2294531 Polyketide synthase Pks12 CDS) | His2147Gln | 23 | - | | 2300552 | T | G | 554.77 | SNP | Rv2048c (pks12 2306986:2294531 Polyketide synthase Pks12 CDS) | silent (Pro2145) | 9926 | - | | 2300555 | A | G | 622.77 | SNP | Rv2048c (pks12 2306986:2294531 Polyketide synthase Pks12 CDS) | silent (Asp2144) | 9859 | - | | 2310543 | G | A | 946.77 | SNP | Rv2051c (ppm1 2310755:2308131 Polyprenol-monophosphomannose synthase Ppm1 CDS) | silent (Gly71) | 9935 | - | | 2329533 | A | G | 971.77 | SNP | Rv2072c (cobL 2330146:2328974 Precorrin-6Y C(5,15)-methyltransferase (decarboxylating) CobL CDS) | Leu205Pro | 2 | - | | 2334007 | A | G | 1392.77 | SNP | Rv2077c (- 2334294:2333323 Possible conserved transmembrane protein CDS) | silent (Ala96) | 9867 | - | | 2335494 | A | G | 1061.77 | SNP | Rv2079 (- 2335355:2337325 hypothetical protein CDS) | Tyr47Cys | 3 | - | | 2340621 | C | G | 1708.77 | SNP | Rv2082 (- 2338709:2340874 hypothetical protein CDS) | Pro638Arg | 4 | - | | 2341636 | C | G | 924.77 | SNP | Rv2083 (- 2340871:2341815 hypothetical protein CDS) | Leu256Val(s) | 4 | - | | 2345037 | C | A | 951.77 | SNP | Rv2088 (pknJ 2344411:2346180 Transmembrane serine/threonine-protein kinase J PknJ (protein kinase J) (STPK J) CDS) | silent (Leu209) | 9947 | - | | 2345542 | G | T | 791.77 | SNP | Rv2088 (pknJ 2344411:2346180 Transmembrane serine/threonine-protein kinase J PknJ (protein kinase J) (STPK J) CDS) | Ala378Ser | 28 | - | | 2346672 | T | C | 1238.77 | SNP | Rv2089c (pepE 2347324:2346197 Dipeptidase PepE CDS) | Asp218Gly | 11 | - | | 2352043 | C | G | 875.77 | SNP | Rv2092c (helY 2352054:2349334 ATP-dependent DNA helicase HelY CDS) | silent (Leu4) | 9947 | - | | 2355511 | G | A | 991.77 | SNP | Rv2097c (pafA 2356677:2355319 Proteasome accessory factor a PafA CDS) | silent (Val389) | 9901 | - | | 2357268 | TGCC | T | 394.75 | DEL | intergenic |  |  | - | | 2358104 | G | C | 625.77 | SNP | intergenic |  |  | - | | 2361604 | C | G | 764.77 | SNP | Rv2101 (helZ 2360240:2363281 Probable helicase HelZ CDS) | Val455Val(s) | 18 | - | | 2362041 | C | A | 693.77 | SNP | Rv2101 (helZ 2360240:2363281 Probable helicase HelZ CDS) | Pro601Gln | 6 | - | | 2368564 | TA | T | 1891.73 | DEL | intergenic |  |  | - | | 2369971 | A | G | 762.77 | SNP | Rv2110c (prcB 2370601:2369726 Proteasome beta subunit PrcB; assembles with alpha subunit PrcA CDS) | Tyr211His | 4 | - | | 2374245 | C | T | 1437.77 | SNP | Rv2114 (- 2373834:2374457 hypothetical protein CDS) | Gln138STOP | 8 | - | | 2384735 | C | T | 950.77 | SNP | Rv2124c (metH 2386067:2382489 5-methyltetrahydrofolate--homocystein methyltransferase MetH (methionine synthase, vitamin-B12 dependent isozyme) (ms) CDS) | Asp445Asn | 36 | - | | 2386389 | G | A | 887.77 | SNP | Rv2125 (- 2386293:2387171 hypothetical protein CDS) | Gly33Ser | 16 | - | | 2387733 | T | C | 190.84 | SNP | Rv2126c (PE\_PGRS37 2387972:2387202 PE-PGRS family protein PE\_PGRS37 CDS) | silent (Glu80) | 9865 | - | | 2400467 | G | A | 1325.77 | SNP | Rv2141c (- 2401722:2400376 hypothetical protein CDS) | Thr419Ile | 7 | - | | 2409982 | C | T | 735.77 | SNP | Rv2151c (ftsQ 2410641:2409697 Possible cell division protein FtsQ CDS) | silent (Pro220) | 9926 | - | | 2415656 | G | C | 964.77 | SNP | Rv2155c (murD 2416394:2414934 Probable UDP-N-acetylmuramoylalanine-D-glutamate ligase MurD CDS) | Arg247Gly | 1 | - | | 2421842 | C | A | 866.77 | SNP | Rv2160A (- 2422278:2421643 hypothetical protein CDS); Rv2160c (- 2422003:2421662 hypothetical protein CDS) | Gly146Val(s); silent (Arg54) | 21; 9913 | - | | 2424925 | A | G | 692.77 | SNP | intergenic |  |  | - | | 2436673 | G | A | 901.77 | SNP | Rv2174 (mptA 2435909:2437459 Alpha(1->6)mannosyltransferase Possible conserved integral membrane protein CDS) | silent (Glu255) | 9865 | - | | 2439401 | A | G | 138.90 | SNP | Rv2177c (- 2439947:2439282 Possible transposase CDS) | Tyr183His | 4 | - | | 2439458 | C | T | 77.28 | SNP | Rv2177c (- 2439947:2439282 Possible transposase CDS) | Ala164Thr | 22 | - | | 2439460 | A | G | 113.03 | SNP | Rv2177c (- 2439947:2439282 Possible transposase CDS) | Ile163Thr | 11 | - | | 2440953 | G | T | 1300.77 | SNP | Rv2178c (aroG 2441720:2440332 3-deoxy-D-arabino-heptulosonate 7-phosphate synthase AroG (DAHP synthetase, phenylalanine-repressible) CDS) | silent (Arg256) | 9913 | - | | 2447282 | T | G | 1052.77 | SNP | Rv2185c (TB16.3 2447500:2447066 Conserved protein TB16.3 CDS) | Glu73Asp | 53 | - | | 2453645 | A | C | 906.77 | SNP | intergenic |  |  | - | | 2454835 | C | T | 790.77 | SNP | Rv2191 (- 2453819:2455756 hypothetical protein CDS) | silent (Leu339) | 9947 | - | | 2465997 | T | G | 790.77 | SNP | Rv2201 (asnB 2464997:2466955 Probable asparagine synthetase AsnB CDS) | Ile334Ser | 2 | - | | 2494263 | G | A | 777.77 | SNP | Rv2223c (- 2495399:2493837 Probable exported protease CDS) | silent (Tyr379) | 9945 | - | | 2499726 | G | A | 1204.77 | SNP | Rv2226 (- 2498832:2500373 hypothetical protein CDS) | Asp299Asn | 36 | - | | 2509140 | G | C | 849.77 | SNP | Rv2236c (cobD 2509375:2508434 Probable cobalamin biosynthesis transmembrane protein CobD CDS) | Ser79Cys | 5 | - | | 2509722 | A | G | 1025.77 | SNP | Rv2237 (- 2509489:2510256 hypothetical protein CDS) | silent (Pro78) | 9926 | - | | 2516538 | C | T | 1060.77 | SNP | Rv2242 (- 2515304:2516548 hypothetical protein CDS) | Thr412Met(s) | 32 | - | | 2521342 | T | C | 846.77 | SNP | Rv2247 (accD6 2520743:2522164 Acetyl/propionyl-CoA carboxylase (beta subunit) AccD6 CDS) | silent (Asp200) | 9859 | - | | 2523205 | G | GCGC | 2382.73 | INS | intergenic |  |  | - | | 2525722 | CG | C | 1349.73 | DEL | Rv2250A (- 2525402:2525821 Possible flavoprotein CDS); Rv2251 (- 2525565:2526992 Possible flavoprotein CDS) |  |  | - | | 2529680 | A | G | 846.77 | SNP | Rv2256c (- 2529874:2529341 hypothetical protein CDS) | silent (Thr65) | 9871 | - | | 2531742 | A | G | 1069.77 | SNP | Rv2258c (- 2531897:2530836 Possible transcriptional regulatory protein CDS) | silent (Ala52) | 9867 | - | | 2534562 | GGA | G | 1336.73 | DEL | Rv2262c (- 2535552:2534470 hypothetical protein CDS) |  |  | - | | 2549596 | T | G | 1422.77 | SNP | Rv2277c (- 2550029:2549124 Possible glycerolphosphodiesterase CDS) | Asp145Ala | 10 | - | | 2551572 | A | C | 1036.77 | SNP | Rv2280 (- 2551560:2552939 Probable dehydrogenase CDS) | Thr5Pro | 4 | - | | 2562752 | G | A | 1331.77 | SNP | Rv2290 (lppO 2562599:2563114 Probable conserved lipoprotein LppO CDS) | Val52Ile | 33 | - | | 2565052 | T | G | 1715.77 | SNP | intergenic |  |  | - | | 2586127 | A | G | 1027.77 | SNP | Rv2314c (- 2587290:2585917 hypothetical protein CDS) | silent (Gly388) | 9935 | - | | 2594763 | C | T | 597.77 | SNP | Rv2322c (rocD1 2595364:2594699 Probable ornithine aminotransferase (N-terminus part) RocD1 (ornithine--oxo-acid aminotransferase) CDS) | Gly201Asp | 6 | - | | 2598400 | A | G | 703.77 | SNP | Rv2326c (- 2599947:2597854 Possible transmembrane ATP-binding protein ABC transporter CDS) | silent (Asn516) | 9822 | - | | 2604156 | TCAGTGCCAA | T | 2649.73 | DEL | Rv2330c (lppP 2604222:2603695 Probable lipoprotein LppP CDS) |  |  | - | | 2612632 | C | A | 389.77 | SNP | Rv2337c (- 2612987:2611869 Hypothetical protein CDS) | Gly119Val | 3 | - | | 2630158 | C | G | 307.77 | SNP | Rv2350c (plcB 2630319:2628781 Membrane-associated phospholipase C 2 PlcB CDS) | silent (Arg54) | 9913 | - | | 2630161 | A | G | 275.77 | SNP | Rv2350c (plcB 2630319:2628781 Membrane-associated phospholipase C 2 PlcB CDS) | silent (Asn53) | 9822 | - | | 2630173 | C | G | 142.77 | SNP | Rv2350c (plcB 2630319:2628781 Membrane-associated phospholipase C 2 PlcB CDS) | Leu(s)49Phe | 1 | - | | 2630176 | C | G | 138.77 | SNP | Rv2350c (plcB 2630319:2628781 Membrane-associated phospholipase C 2 PlcB CDS) | Leu(s)48Phe | 1 | - | | 2630182 | G | A | 131.77 | SNP | Rv2350c (plcB 2630319:2628781 Membrane-associated phospholipase C 2 PlcB CDS) | silent (Ile46) | 9872 | - | | 2630184 | T | A | 127.77 | SNP | Rv2350c (plcB 2630319:2628781 Membrane-associated phospholipase C 2 PlcB CDS) | Ile46Phe | 8 | - | | 2630188 | C | T | 142.77 | SNP | Rv2350c (plcB 2630319:2628781 Membrane-associated phospholipase C 2 PlcB CDS) | silent (Glu44) | 9865 | - | | 2630206 | T | G | 84.77 | SNP | Rv2350c (plcB 2630319:2628781 Membrane-associated phospholipase C 2 PlcB CDS) | silent (Gly38) | 9935 | - | | 2630211 | G | A | 70.77 | SNP | Rv2350c (plcB 2630319:2628781 Membrane-associated phospholipase C 2 PlcB CDS) | Pro37Ser | 17 | - | | 2630215 | A | G | 52.77 | SNP | Rv2350c (plcB 2630319:2628781 Membrane-associated phospholipase C 2 PlcB CDS) | silent (Pro35) | 9926 | - | | 2631556 | C | G | 358.77 | SNP | Rv2351c (plcA 2632075:2630537 Membrane-associated phospholipase C 1 PlcA (MTP40 antigen) CDS) | Gly174Arg | 0 | - | | 2631565 | T | C | 647.77 | SNP | Rv2351c (plcA 2632075:2630537 Membrane-associated phospholipase C 1 PlcA (MTP40 antigen) CDS) | Ile171Val | 57 | - | | 2631574 | T | C | 665.77 | SNP | Rv2351c (plcA 2632075:2630537 Membrane-associated phospholipase C 1 PlcA (MTP40 antigen) CDS) | Thr168Ala | 32 | - | | 2631583 | G | A | 731.77 | SNP | Rv2351c (plcA 2632075:2630537 Membrane-associated phospholipase C 1 PlcA (MTP40 antigen) CDS) | Leu165Leu(s) | 4 | - | | 2631599 | G | A | 729.77 | SNP | Rv2351c (plcA 2632075:2630537 Membrane-associated phospholipase C 1 PlcA (MTP40 antigen) CDS) | silent (Ile159) | 9872 | - | | 2631620 | A | G | 714.77 | SNP | Rv2351c (plcA 2632075:2630537 Membrane-associated phospholipase C 1 PlcA (MTP40 antigen) CDS) | silent (Gly152) | 9935 | - | | 2631962 | T | G | 88.77 | SNP | Rv2351c (plcA 2632075:2630537 Membrane-associated phospholipase C 1 PlcA (MTP40 antigen) CDS) | silent (Gly38) | 9935 | - | | 2631967 | G | A | 37.77 | SNP | Rv2351c (plcA 2632075:2630537 Membrane-associated phospholipase C 1 PlcA (MTP40 antigen) CDS) | Pro37Ser | 17 | - | | 2631968 | A | G | 43.77 | SNP | Rv2351c (plcA 2632075:2630537 Membrane-associated phospholipase C 1 PlcA (MTP40 antigen) CDS) | silent (Cys36) | 9973 | - | | 2631971 | A | G | 144.77 | SNP | Rv2351c (plcA 2632075:2630537 Membrane-associated phospholipase C 1 PlcA (MTP40 antigen) CDS) | silent (Pro35) | 9926 | - | | 2631977 | G | C | 149.77 | SNP | Rv2351c (plcA 2632075:2630537 Membrane-associated phospholipase C 1 PlcA (MTP40 antigen) CDS) | silent (Ala33) | 9867 | - | | 2639364 | C | A | 90.77 | SNP | Rv2356c (PPE40 2639535:2637688 PPE family protein PPE40 CDS) | Gly58Cys | 0 | - | | 2656225 | A | G | 1119.77 | SNP | Rv2377c (mbtH 2656430:2656215 Putative conserved protein MbtH CDS) | Val69Ala | 18 | - | | 2660319 | C | G | 1115.77 | SNP | Rv2379c (mbtF 2662085:2657700 Peptide synthetase MbtF (peptide synthase) CDS) | Glu589Asp | 53 | - | | 2676886 | G | A | 921.77 | SNP | Rv2384 (mbtA 2675936:2677633 Bifunctional enzyme MbtA: salicyl-AMP ligase (SAL-AMP ligase) + salicyl-S-ArCP synthetase CDS) | silent (Pro317) | 9926 | - | | 2680658 | T | G | 1313.77 | SNP | intergenic |  |  | - | | 2695378 | C | G | 1059.77 | SNP | Rv2398c (cysW 2695799:2694981 Probable sulfate-transport integral membrane protein ABC transporter CysW CDS) | Gly141Ala | 21 | - | | 2701259 | A | C | 596.77 | SNP | Rv2403c (lppR 2701290:2700535 Probable conserved lipoprotein LppR CDS) | Leu11Arg | 1 | - | | 2704884 | A | ACAGCGACCATATCGCCGAG CT | 5834.73 | INS | Rv2407 (- 2704697:2705518 hypothetical protein CDS) |  |  | - | | 2705659 | C | T | 1053.77 | SNP | intergenic |  |  | - | | 2713795 | C | T | 1320.77 | SNP | intergenic |  |  | - | | 2718852 | T | G | 1050.77 | SNP | intergenic |  |  | - | | 2721013 | A | G | 402.77 | SNP | Rv2424c (- 2721777:2720776 Probable transposase CDS) | silent (Arg255) | 9913 | - | | 2723277 | G | A | 1202.77 | SNP | Rv2425c (- 2723308:2721866 hypothetical protein CDS) | Pro11Leu | 3 | - | | 2734074 | T | C | 447.77 | SNP | Rv2436 (rbsK 2733230:2734144 Ribokinase RbsK CDS) | Val282Ala | 18 | - | | 2751804 | C | T | 724.78 | SNP | Rv2450c (rpfE 2752180:2751662 Probable resuscitation-promoting factor RpfE CDS) | Arg126Gln | 9 | - | | 2752698 | C | A | 1652.77 | SNP | intergenic |  |  | - | | 2760152 | A | G | 1035.77 | SNP | Rv2458 (mmuM 2759779:2760687 Probable homocysteine S-methyltransferase MmuM (S-methylmethionine:homocysteine methyltransferase) (cysteine methyltransferase) CDS) | Tyr125Cys | 3 | - | | 2779136 | T | C | 1063.77 | SNP | Rv2476c (gdh 2782262:2777388 Probable NAD-dependent glutamate dehydrogenase Gdh (NAD-Gdh) (NAD-dependent glutamic dehydrogenase) CDS) | Ser1043Gly | 21 | - | | 2786952 | A | G | 1107.77 | SNP | Rv2482c (plsB2 2789283:2786914 Probable glycerol-3-phosphate acyltransferase PlsB2 (GPAT) CDS) | Cys778Arg | 1 | - | | 2794793 | G | A | 919.77 | SNP | Rv2486 (echA14 2794350:2795120 Probable enoyl-CoA hydratase EchA14 (enoyl hydrase) (unsaturated acyl-CoA hydratase) (crotonase) CDS) | Met(s)148Ile | 2 | - | | 2795160 | C | T | 727.77 | SNP | intergenic |  |  | - | | 2807237 | G | A | 918.77 | SNP | Rv2491 (- 2806665:2807288 hypothetical protein CDS) | Val(s)191Val | 13 | - | | 2809621 | T | C | 1144.77 | SNP | Rv2495c (bkdC 2809939:2808758 Probable branched-chain keto acid dehydrogenase E2 component BkdC CDS) | Thr107Ala | 32 | - | | 2816034 | G | A | 1076.77 | SNP | Rv2501c (accA1 2816880:2814916 Probable acetyl-/propionyl-coenzyme A carboxylase alpha chain (alpha subunit) AccA1: biotin carboxylase + biotin carboxyl carrier protein (BCCP) CDS) | Pro283Ser | 17 | - | | 2816296 | A | C | 1058.77 | SNP | Rv2501c (accA1 2816880:2814916 Probable acetyl-/propionyl-coenzyme A carboxylase alpha chain (alpha subunit) AccA1: biotin carboxylase + biotin carboxyl carrier protein (BCCP) CDS) | Asp195Glu | 56 | - | | 2818837 | A | G | 1339.77 | SNP | Rv2503c (scoB 2819127:2818471 Probable succinyl-CoA:3-ketoacid-coenzyme A transferase (beta subunit) ScoB (3-oxo-acid:CoA transferase) (OXCT B) (succinyl CoA:3-oxoacid CoA-transferase) CDS) | silent (Gly97) | 9935 | - | | 2821077 | CGG | C | 2380.73 | DEL | Rv2505c (fadD35 2821596:2819953 Probable fatty-acid-CoA ligase FadD35 (fatty-acid-CoA synthetase) (fatty-acid-CoA synthase) CDS) |  |  | - | | 2821342 | C | T | 693.77 | SNP | Rv2505c (fadD35 2821596:2819953 Probable fatty-acid-CoA ligase FadD35 (fatty-acid-CoA synthetase) (fatty-acid-CoA synthase) CDS) | silent (Ala85) | 9867 | - | | 2827984 | G | T | 1022.77 | SNP | intergenic |  |  | - | | 2828019 | T | C | 1133.77 | SNP | intergenic |  |  | - | | 2828517 | A | G | 1181.77 | SNP | intergenic |  |  | - | | 2829779 | T | C | 228.80 | SNP | Rv2512c (- 2829803:2828556 Transposase for insertion sequence element IS1081 CDS) | Thr9Ala | 32 | - | | 2830525 | C | A | 1222.77 | SNP | Rv2513 (- 2830161:2830583 Hypothetical protein CDS) | Thr122Lys | 11 | - | | 2830879 | A | C | 1563.77 | SNP | Rv2514c (- 2831338:2830877 hypothetical protein CDS) | STOP154Gly | 21 | - | | 2831382 | T | G | 1396.77 | SNP | Rv2515c (- 2832591:2831344 hypothetical protein CDS) | Lys404Gln | 6 | - | | 2832071 | G | C | 102.77 | SNP | Rv2515c (- 2832591:2831344 hypothetical protein CDS) | Ala174Gly | 21 | - | | 2836257 | G | A | 773.88 | SNP | Rv2519 (PE26 2835785:2837263 PE family protein PE26 CDS) | Gly158Asp | 6 | - | | 2855259 | A | G | 1035.77 | SNP | Rv2531c (- 2857781:2854938 Probable amino acid decarboxylase CDS) | silent (Ala841) | 9867 | - | | 2865760 | A | G | 1376.77 | SNP | Rv2542 (- 2865130:2866341 hypothetical protein CDS) | Thr211Ala | 32 | - | | 2865882 | T | C | 1210.77 | SNP | Rv2542 (- 2865130:2866341 hypothetical protein CDS) | silent (Val251) | 9901 | - | | 2866552 | C | A | 1217.77 | SNP | Rv2543 (lppA 2866468:2867127 Probable conserved lipoprotein LppA CDS) | Leu29Met(s) | 4 | - | | 2881597 | AG | A | 1239.73 | DEL | Rv2561 (- 2881409:2881702 hypothetical protein CDS) |  |  | - | | 2882101 | G | C | 843.77 | SNP | Rv2562 (- 2881758:2882147 hypothetical protein CDS) | Arg115Pro | 5 | - | | 2888201 | T | C | 732.77 | SNP | Rv2566 (- 2886373:2889795 Long conserved protein CDS) | Leu610Pro | 2 | - | | 2889633 | T | C | 768.77 | SNP | Rv2566 (- 2886373:2889795 Long conserved protein CDS) | silent (Ala1087) | 9867 | - | | 2891267 | C | T | 998.77 | SNP | Rv2567 (- 2889795:2892449 Conserved hypothetical alanine and leucine rich protein CDS) | silent (Gly491) | 9935 | - | | 2891728 | A | G | 671.77 | SNP | Rv2567 (- 2889795:2892449 Conserved hypothetical alanine and leucine rich protein CDS) | Gln645Arg | 10 | - | | 2894208 | G | A | 1127.77 | SNP | Rv2569c (- 2894408:2893464 hypothetical protein CDS) | silent (Ser67) | 9840 | - | | 2897375 | T | G | 546.77 | SNP | Rv2572c (aspS 2897803:2896013 Probable aspartyl-tRNA synthetase AspS (aspartate--tRNA ligase) (ASPRS) (aspartic acid translase) CDS) | silent (Arg143) | 9913 | - | | 2899538 | A | C | 1077.77 | SNP | Rv2575 (- 2899339:2900220 Possible conserved membrane glycine rich protein CDS) | Asp67Ala | 10 | - | | 2910461 | G | T | 742.77 | SNP | Rv2584c (apt 2910900:2910229 Adenine phosphoribosyltransferase Apt (APRT) (AMP diphosphorylase) (AMP pyrophosphorylase) (transphosphoribosidase) CDS) | Ala147Glu | 10 | - | | 2911293 | C | G | 1058.77 | SNP | Rv2585c (- 2912677:2911004 Possible conserved lipoprotein CDS) | Cys462Ser | 11 | - | | 2912294 | T | G | 904.77 | SNP | Rv2585c (- 2912677:2911004 Possible conserved lipoprotein CDS) | silent (Ala128) | 9867 | - | | 2923391 | T | C | 655.77 | SNP | Rv2592c (ruvB 2924233:2923199 Probable holliday junction DNA helicase RuvB CDS) | silent (Pro281) | 9926 | - | | 2927939 | T | C | 1224.77 | SNP | intergenic |  |  | - | | 2939373 | G | C | 466.77 | SNP | Rv2611c (- 2939962:2939012 Probable acyltransferase CDS) | Ser197Cys | 5 | - | | 2939657 | T | C | 572.77 | SNP | Rv2611c (- 2939962:2939012 Probable acyltransferase CDS) | Ile102Met(s) | 6 | - | | 2944833 | G | C | 174.84 | SNP | Rv2615c (PE\_PGRS45 2944985:2943600 PE-PGRS family protein PE\_PGRS45 CDS) | silent (Ala51) | 9867 | - | | 2944857 | T | G | 263.78 | SNP | Rv2615c (PE\_PGRS45 2944985:2943600 PE-PGRS family protein PE\_PGRS45 CDS) | Gln43His | 20 | - | | 2954439 | T | C | 972.77 | SNP | Rv2627c (- 2954748:2953507 hypothetical protein CDS) | Arg104Gly | 1 | - | | 2965900 | C | T | 1318.77 | SNP | intergenic |  |  | - | | 2974933 | A | G | 985.77 | SNP | Rv2650c (- 2975234:2973795 Possible PhiRv2 prophage protein CDS) | Ile101Thr | 11 | - | | 2982955 | C | T | 958.77 | SNP | Rv2665 (- 2982699:2982980 Hypothetical arginine rich protein CDS) | Pro86Leu | 3 | - | | 2984740 | A | G | 693.77 | SNP | Rv2668 (- 2984733:2985254 Possible exported alanine and valine rich protein CDS) | His3Arg | 10 | - | | 2994499 | G | A | 692.77 | SNP | Rv2678c (hemE 2995062:2993989 Probable uroporphyrinogen decarboxylase HemE (uroporphyrinogen III decarboxylase) (URO-D) (UPD) CDS) | silent (Leu188) | 9947 | - | | 2996876 | A | C | 1045.77 | SNP | Rv2681 (- 2996739:2998055 Conserved hypothetical alanine rich protein CDS) | silent (Leu46) | 9947 | - | | 3005185 | G | T | 1240.77 | SNP | Rv2688c (- 3005650:3004745 Antibiotic-transport ATP-binding protein ABC transporter CDS) | Pro156Thr | 5 | - | | 3006361 | CG | C | 1193.74 | DEL | Rv2689c (- 3007062:3005845 Conserved alanine and valine and glycine rich protein CDS) |  |  | - | | 3009692 | A | G | 1316.77 | SNP | Rv2691 (ceoB 3009344:3010027 TRK system potassium uptake protein CeoB CDS) | Thr117Ala | 32 | - | | 3012688 | GC | G | 1029.73 | DEL | intergenic |  |  | - | | 3015966 | G | A | 773.77 | SNP | Rv2701c (suhB 3016735:3015863 Inositol-1-monophosphatase SuhB CDS) | Ala257Val(s) | 9867 | - | | 3017465 | T | C | 887.77 | SNP | Rv2702 (ppgK 3016858:3017655 Polyphosphate glucokinase PpgK (polyphosphate-glucose phosphotransferase) CDS) | Ile203Thr | 11 | - | | 3020515 | G | C | 1114.77 | SNP | intergenic |  |  | - | | 3041871 | G | T | 1155.77 | SNP | Rv2729c (- 3042475:3041570 Probable conserved integral membrane alanine valine and leucine rich protein CDS) | Ala202Glu | 10 | - | | 3054081 | A | G | 1023.77 | SNP | Rv2741 (PE\_PGRS47 3053914:3055491 PE-PGRS family protein PE\_PGRS47 CDS) | silent (Gly56) | 9935 | - | | 3054321 | A | G | 331.78 | SNP | Rv2741 (PE\_PGRS47 3053914:3055491 PE-PGRS family protein PE\_PGRS47 CDS) | silent (Gly136) | 9935 | - | | 3065954 | T | C | 1434.77 | SNP | Rv2752c (- 3066191:3064515 hypothetical protein CDS) | Thr80Ala | 32 | - | | 3069778 | G | T | 1525.77 | SNP | Rv2756c (hsdM 3070083:3068461 Possible type I restriction/modification system DNA methylase HsdM (M protein) (DNA methyltransferase) CDS) | Phe102Leu | 13 | - | | 3080795 | A | G | 1493.77 | SNP | Rv2771c (- 3081033:3080581 hypothetical protein CDS) | Leu80Pro | 2 | - | | 3100153 | G | GA | 2313.73 | INS | Rv2790c (ltp1 3100169:3098964 Probable lipid-transfer protein Ltp1 CDS) |  |  | - | | 3103682 | T | C | 936.77 | SNP | Rv2794c (pptT 3103940:3103257 Phosphopantetheinyl transferase PptT (CoA:APO-[ACP]pantetheinephosphotransferase) (CoA:APO-[acyl-carrier protein]pantetheinephosphotransferase) CDS) | Met(s)87Val(s) | 9867 | - | | 3112675 | G | A | 819.77 | SNP | Rv2804c (- 3113094:3112465 Hypothetical protein CDS) | silent (Asp140) | 9859 | - | | 3118000 | A | G | 647.77 | SNP | Rv2812 (- 3116818:3118227 Probable transposase CDS) | Arg395Gly | 1 | - | | 3121878 | A | ACTCG | 1115.73 | INS | intergenic |  |  | - | | 3131469 | T | TTGTCGGCGA | 2141.73 | INS | Rv2823c (- 3131773:3129344 hypothetical protein CDS) |  |  | - | | 3133536 | T | C | 1307.77 | SNP | Rv2825c (- 3133539:3132892 hypothetical protein CDS) | Lys2Glu | 4 | - | | 3137058 | G | A | 1213.77 | SNP | Rv2830c (vapB22 3137224:3137009 Possible antitoxin VapB22 CDS) | Ala56Val(s) | 9867 | - | | 3143100 | A | G | 1166.77 | SNP | Rv2836c (dinF 3143628:3142309 Possible DNA-damage-inducible protein F DinF CDS) | Cys177Arg | 1 | - | | 3162805 | C | G | 304.78 | SNP | Rv2853 (PE\_PGRS48 3162268:3164115 PE-PGRS family protein PE\_PGRS48 CDS) | Arg180Gly | 1 | - | | 3165937 | G | A | 915.77 | SNP | Rv2855 (mtr 3165205:3166584 NADPH-dependent mycothiol reductase Mtr CDS) | Val245Ile | 33 | - | | 3170460 | C | T | 954.77 | SNP | Rv2858c (aldC 3170723:3169356 Probable aldehyde dehydrogenase AldC CDS) | Val(s)88Val | 13 | - | | 3175335 | C | T | 1498.77 | SNP | Rv2863 (vapC23 3174992:3175372 Possible toxin VapC23 CDS) | Thr115Met(s) | 32 | - | | 3177884 | C | A | 1139.77 | SNP | Rv2866 (relG 3177822:3178085 Toxin RelG CDS) | silent (Arg21) | 9913 | - | | 3183561 | G | C | 488.77 | SNP | Rv2872 (vapC43 3183382:3183825 Possible toxin VapC43 Contains PIN domain CDS) | silent (Pro60) | 9926 | - | | 3186860 | T | G | 935.77 | SNP | Rv2874 (dipZ 3184847:3186934 Possible integral membrane C-type cytochrome biogenesis protein DipZ CDS) | Tyr672Asp | 0 | - | | 3186911 | GT | G | 1177.73 | DEL | Rv2874 (dipZ 3184847:3186934 Possible integral membrane C-type cytochrome biogenesis protein DipZ CDS) |  |  | - | | 3190145 | TC | T | 1487.73 | DEL | Rv2880c (- 3190678:3189851 hypothetical protein CDS) |  |  | - | | 3226181 | A | C | 953.77 | SNP | Rv2916c (ffh 3226285:3224708 Probable signal recognition particle protein Ffh (fifty-four homolog) (SRP protein) CDS) | silent (Arg35) | 9913 | - | | 3228143 | G | T | 966.77 | SNP | Rv2917 (- 3226363:3228243 Conserved hypothetical alanine and arginine rich protein CDS) | Arg594Leu | 1 | - | | 3232759 | G | A | 310.77 | SNP | intergenic |  |  | - | | 3247316 | C | G | 1442.77 | SNP | Rv2931 (ppsA 3245445:3251075 Phenolpthiocerol synthesis type-I polyketide synthase PpsA CDS) | Asp624Glu | 56 | - | | 3247851 | G | A | 878.77 | SNP | Rv2931 (ppsA 3245445:3251075 Phenolpthiocerol synthesis type-I polyketide synthase PpsA CDS) | Ala803Thr | 22 | - | | 3247853 | C | T | 836.77 | SNP | Rv2931 (ppsA 3245445:3251075 Phenolpthiocerol synthesis type-I polyketide synthase PpsA CDS) | silent (Ala803) | 9867 | - | | 3247856 | G | C | 921.77 | SNP | Rv2931 (ppsA 3245445:3251075 Phenolpthiocerol synthesis type-I polyketide synthase PpsA CDS) | silent (Arg804) | 9913 | - | | 3247864 | C | CTAGG | 1870.73 | INS | Rv2931 (ppsA 3245445:3251075 Phenolpthiocerol synthesis type-I polyketide synthase PpsA CDS) |  |  | - | | 3247865 | GCAAA | G | 1959.73 | DEL | Rv2931 (ppsA 3245445:3251075 Phenolpthiocerol synthesis type-I polyketide synthase PpsA CDS) |  |  | - | | 3247874 | G | A | 749.77 | SNP | Rv2931 (ppsA 3245445:3251075 Phenolpthiocerol synthesis type-I polyketide synthase PpsA CDS) | silent (Arg810) | 9913 | - | | 3247877 | T | C | 842.77 | SNP | Rv2931 (ppsA 3245445:3251075 Phenolpthiocerol synthesis type-I polyketide synthase PpsA CDS) | silent (Phe811) | 9946 | - | | 3247883 | T | C | 1145.77 | SNP | Rv2931 (ppsA 3245445:3251075 Phenolpthiocerol synthesis type-I polyketide synthase PpsA CDS) | silent (Ser813) | 9840 | - | | 3248028 | G | A | 871.77 | SNP | Rv2931 (ppsA 3245445:3251075 Phenolpthiocerol synthesis type-I polyketide synthase PpsA CDS) | Val862Ile | 33 | - | | 3248074 | G | A | 781.78 | SNP | Rv2931 (ppsA 3245445:3251075 Phenolpthiocerol synthesis type-I polyketide synthase PpsA CDS) | Arg877His | 8 | - | | 3248075 | C | T | 830.78 | SNP | Rv2931 (ppsA 3245445:3251075 Phenolpthiocerol synthesis type-I polyketide synthase PpsA CDS) | silent (Arg877) | 9913 | - | | 3253488 | G | A | 51.77 | SNP | Rv2932 (ppsB 3251072:3255688 Phenolpthiocerol synthesis type-I polyketide synthase PpsB CDS) | Arg806His | 8 | - | | 3253489 | C | T | 32.77 | SNP | Rv2932 (ppsB 3251072:3255688 Phenolpthiocerol synthesis type-I polyketide synthase PpsB CDS) | silent (Arg806) | 9913 | - | | 3256494 | A | G | 1243.77 | SNP | Rv2933 (ppsC 3255685:3262251 Phenolpthiocerol synthesis type-I polyketide synthase PpsC CDS) | silent (Gly270) | 9935 | - | | 3269581 | A | G | 1177.77 | SNP | Rv2935 (ppsE 3267737:3272203 Phenolpthiocerol synthesis type-I polyketide synthase PpsE CDS) | silent (Ala615) | 9867 | - | | 3270784 | A | G | 1236.77 | SNP | Rv2935 (ppsE 3267737:3272203 Phenolpthiocerol synthesis type-I polyketide synthase PpsE CDS) | silent (Gln1016) | 9876 | - | | 3296843 | A | G | 970.77 | SNP | Rv2947c (pks15 3297840:3296350 Probable polyketide synthase Pks15 CDS) | Val(s)333Ala | 9867 | - | | 3300196 | G | A | 1043.77 | SNP | Rv2949c (- 3300570:3299971 Chorismate pyruvate lyase CDS) | silent (Phe125) | 9946 | - | | 3304753 | G | T | 1176.77 | SNP | Rv2952 (- 3304441:3305253 Possible methyltransferase (methylase) CDS) | Ala105Ser | 28 | - | | 3308606 | G | A | 1220.77 | SNP | intergenic |  |  | - | | 3317702 | C | T | 994.77 | SNP | intergenic |  |  | - | | 3332626 | C | T | 1433.77 | SNP | Rv2976c (ung 3332754:3332071 Probable uracil-DNA glycosylase Ung (UDG) CDS) | silent (Leu43) | 9947 | - | | 3336587 | T | A | 361.77 | SNP | intergenic |  |  | - | | 3336646 | T | A | 84.96 | SNP | intergenic |  |  | - | | 3336825 | T | C | 823.77 | SNP | Rv2981c (ddlA 3337917:3336796 Probable D-alanine--D-alanine ligase DdlA (D-alanylalanine synthetase) (D-ala-D-ala ligase) CDS) | Thr365Ala | 32 | - | | 3338603 | G | C | 849.77 | SNP | Rv2982c (gpdA2 3338999:3337995 Probable glycerol-3-phosphate dehydrogenase [NAD(P)+] GpdA2 (NAD(P)H-dependent glycerol-3-phosphate dehydrogenase) CDS) | Pro133Ala | 22 | - | | 3358235 | A | T | 1000.77 | SNP | Rv2999 (lppY 3357602:3358567 Probable conserved lipoprotein LppY CDS) | Met(s)212Leu(s) | 9867 | - | | 3363338 | A | G | 878.77 | SNP | intergenic |  |  | - | | 3367765 | G | A | 801.77 | SNP | Rv3009c (gatB 3368793:3367264 Probable glutamyl-tRNA(GLN) amidotransferase (subunit B) GatB (Glu-ADT subunit B) CDS) | silent (Gly343) | 9935 | - | | 3371719 | G | A | 719.77 | SNP | Rv3012c (gatC 3371730:3371431 Probable glutamyl-tRNA(GLN) amidotransferase (subunit C) GatC (Glu-ADT subunit C) CDS) | silent (Ile4) | 9872 | - | | 3379742 | T | C | 49.74 | SNP | intergenic |  |  | - | | 3379751 | A | C | 51.74 | SNP | intergenic |  |  | - | | 3379757 | A | C | 48.74 | SNP | intergenic |  |  | - | | 3379763 | G | A | 76.28 | SNP | intergenic |  |  | - | | 3379784 | C | A | 159.90 | SNP | intergenic |  |  | - | | 3379788 | C | G | 208.84 | SNP | intergenic |  |  | - | | 3381641 | G | T | 99.03 | SNP | Rv3023c (- 3382622:3381375 Probable transposase CDS) | Gln328Lys | 12 | - | | 3382598 | C | T | 528.77 | SNP | Rv3023c (- 3382622:3381375 Probable transposase CDS) | Ala9Thr | 22 | - | | 3382738 | G | A | 1503.77 | SNP | intergenic |  |  | - | | 3392342 | G | A | 1293.77 | SNP | Rv3032 (- 3391534:3392778 Alpha (1->4) glucosyltransferase CDS) | Arg270Gln | 9 | - | | 3394302 | A | C | 1361.77 | SNP | Rv3034c (- 3394921:3394019 Possible transferase CDS) | Leu207Arg | 1 | - | | 3402816 | C | T | 1124.77 | SNP | Rv3042c (serB2 3403162:3401933 Probable phosphoserine phosphatase SerB2 (PSP) (O-phosphoserine phosphohydrolase) (pspase) CDS) | Gly116Glu | 4 | - | | 3415180 | ACACCTAGGGGGTGG | A | 2248.73 | DEL | intergenic |  |  | - | | 3423426 | G | A | 1103.77 | SNP | Rv3061c (fadE22 3425427:3423262 Probable acyl-CoA dehydrogenase FadE22 CDS) | silent (Leu668) | 9947 | - | | 3425854 | C | T | 924.77 | SNP | Rv3062 (ligB 3425584:3427107 Probable ATP-dependent DNA ligase LigB (polydeoxyribonucleotide synthase [ATP]) (polynucleotide ligase [ATP]) (sealase) (DNA repair protein) (DNA joinase) CDS) | Pro91Ser | 17 | - | | 3428917 | C | A | 1038.77 | SNP | Rv3063 (cstA 3427243:3429519 Probable carbon starvation protein A homolog CstA CDS) | Arg559Ser | 11 | - | | 3440464 | T | G | 1485.77 | SNP | Rv3077 (- 3439541:3441352 Possible hydrolase CDS) | silent (Arg308) | 9913 | - | | 3440468 | G | C | 1390.77 | SNP | Rv3077 (- 3439541:3441352 Possible hydrolase CDS) | Gly310Arg | 0 | - | | 3449852 | T | C | 1098.77 | SNP | Rv3083 (- 3448504:3449991 Probable monooxygenase (hydroxylase) CDS) | Ile450Thr | 11 | - | | 3456666 | A | G | 767.77 | SNP | Rv3089 (fadD13 3455761:3457272 Probable chain-fatty-acid-CoA ligase FadD13 (fatty-acyl-CoA synthetase) CDS) | silent (Ala302) | 9867 | - | | 3462135 | G | C | 936.77 | SNP | Rv3093c (- 3462764:3461760 Hypothetical oxidoreductase CDS) | Cys210Trp | 0 | - | | 3466426 | G | A | 1018.77 | SNP | Rv3097c (lipY 3467091:3465778 PE-PGRS family protein, triacylglycerol lipase LipY (esterase/lipase) (triglyceride lipase) (tributyrase) CDS) | silent (Val222) | 9901 | genotype | | 3473996 | G | GA | 1697.73 | INS | intergenic |  |  | - | | 3477917 | C | T | 752.77 | SNP | Rv3109 (moaA1 3477649:3478728 Probable molybdenum cofactor biosynthesis protein A MoaA1 CDS) | Pro90Leu | 3 | - | | 3480435 | A | G | 1051.77 | SNP | Rv3113 (- 3480074:3480742 Possible phosphatase CDS) | His121Arg | 10 | - | | 3481475 | G | A | 488.77 | SNP | Rv3115 (- 3481451:3482698 Probable transposase CDS) | Ala9Thr | 22 | - | | 3482432 | C | A | 100.03 | SNP | Rv3115 (- 3481451:3482698 Probable transposase CDS) | Gln328Lys | 12 | - | | 3486328 | G | A | 1628.77 | SNP | intergenic |  |  | - | | 3486977 | A | G | 1242.77 | SNP | Rv3121 (cyp141 3486509:3487711 Probable cytochrome P450 141 Cyp141 CDS) | Lys157Glu | 4 | - | | 3490749 | C | T | 857.77 | SNP | Rv3125c (PPE49 3491651:3490476 PPE family protein PPE49 CDS) | Leu(s)301Leu | 3 | - | | 3503895 | C | T | 1059.77 | SNP | Rv3137 (- 3503393:3504175 Probable monophosphatase CDS) | Pro168Leu | 3 | - | | 3505027 | G | A | 1117.77 | SNP | Rv3138 (pflA 3504195:3505283 Probable pyruvate formate lyase activating protein PflA (formate acetyltransferase activating enzyme) ([pyruvate formate-lyase] activating enzyme) CDS) | Arg278His | 8 | - | | 3515582 | C | T | 1100.77 | SNP | Rv3150 (nuoF 3515412:3516749 Probable NADH dehydrogenase I (chain F) NuoF (NADH-ubiquinone oxidoreductase chain F) CDS) | silent (Ser57) | 9840 | - | | 3518167 | A | G | 774.77 | SNP | Rv3151 (nuoG 3516746:3519166 Probable NADH dehydrogenase I (chain G) NuoG (NADH-ubiquinone oxidoreductase chain G) CDS) | Ile474Met(s) | 6 | - | | 3518555 | A | G | 510.77 | SNP | Rv3151 (nuoG 3516746:3519166 Probable NADH dehydrogenase I (chain G) NuoG (NADH-ubiquinone oxidoreductase chain G) CDS) | Thr604Ala | 32 | - | | 3556275 | A | G | 1182.77 | SNP | Rv3190c (- 3556687:3555422 Hypothetical protein CDS) | Leu138Pro | 2 | - | | 3569029 | T | C | 961.77 | SNP | intergenic |  |  | - | | 3580636 | CT | C | 2412.73 | DEL | intergenic |  |  | - | | 3581414 | A | G | 1224.77 | SNP | Rv3204 (- 3581315:3581620 Possible DNA-methyltransferase (modification methylase) CDS) | Thr34Ala | 32 | - | | 3590686 | G | GC | 1193.73 | INS | intergenic |  |  | - | | 3591063 | T | C | 875.77 | SNP | Rv3213c (- 3591492:3590692 Possible SOJ/para-related protein CDS) | Lys144Glu | 4 | - | | 3594394 | G | C | 246.78 | SNP | intergenic |  |  | - | | 3594395 | G | A | 193.80 | SNP | intergenic |  |  | - | | 3594398 | T | C | 260.78 | SNP | intergenic |  |  | - | | 3594400 | A | G | 234.80 | SNP | intergenic |  |  | - | | 3604821 | G | C | 513.77 | SNP | Rv3228 (- 3604726:3605718 hypothetical protein CDS) | silent (Ala32) | 9867 | - | | 3614982 | T | C | 1508.77 | SNP | Rv3239c (- 3617603:3614457 Probable conserved transmembrane transport protein CDS) | silent (Leu874) | 9947 | - | | 3622040 | G | T | 973.77 | SNP | Rv3242c (- 3622211:3621570 hypothetical protein CDS) | Arg58Ser | 11 | - | | 3622441 | A | C | 872.77 | SNP | Rv3243c (- 3623091:3622249 hypothetical protein CDS) | Val217Val(s) | 18 | - | | 3640047 | G | A | 886.77 | SNP | Rv3260c (whiB2 3640141:3639872 Probable transcriptional regulatory protein WhiB-like WhiB2 CDS) | Pro32Leu | 3 | - | | 3644061 | C | T | 1140.77 | SNP | Rv3263 (- 3643177:3644838 Probable DNA methylase (modification methylase) (methyltransferase) CDS) | silent (Ile295) | 9872 | - | | 3687908 | T | C | 1166.77 | SNP | Rv3302c (glpD2 3689442:3687685 Probable glycerol-3-phosphate dehydrogenase GlpD2 CDS) | Tyr512Cys | 3 | - | | 3689523 | G | T | 765.77 | SNP | Rv3303c (lpdA 3690938:3689457 NAD(P)H quinone reductase LpdA CDS) | Cys472STOP | 3 | - | | 3691061 | A | C | 81.77 | SNP | intergenic |  |  | - | | 3696179 | T | C | 995.77 | SNP | Rv3308 (pmmB 3694864:3696468 Probable phosphomannomutase PmmB (phosphomannose mutase) CDS) | Val(s)439Ala | 9867 | - | | 3697152 | T | C | 1198.77 | SNP | intergenic |  |  | - | | 3699253 | C | G | 1148.77 | SNP | Rv3311 (- 3698121:3699383 hypothetical protein CDS) | Pro378Arg | 4 | - | | 3704596 | G | C | 969.77 | SNP | Rv3317 (sdhD 3704437:3704871 Probable succinate dehydrogenase (hydrophobic membrane anchor subunit) SdhD (succinic dehydrogenase) (fumarate reductase) (fumarate dehydrogenase) (fumaric hydrogenase) CDS) | Val(s)54Leu | 3 | - | | 3711910 | G | A | 121.03 | SNP | Rv3327 (- 3711749:3713461 Probable transposase fusion protein CDS) | Trp54STOP | 0 | - | | 3714211 | G | T | 1199.77 | SNP | Rv3328c (sigJ 3714332:3713394 Probable alternative RNA polymerase sigma factor (fragment) SigJ CDS) | Pro41Gln | 6 | - | | 3718357 | C | T | 1360.77 | SNP | Rv3331 (sugI 3717090:3718598 Probable sugar-transport integral membrane protein SugI CDS) | Pro423Leu | 3 | - | | 3721806 | G | C | 1523.77 | SNP | Rv3335c (- 3722600:3721731 Probable conserved integral membrane protein CDS) | silent (Gly265) | 9935 | - | | 3730385 | C | G | 492.77 | SNP | Rv3343c (PPE54 3736935:3729364 PPE family protein PPE54 CDS) | Arg2184Pro | 5 | - | | 3730386 | G | T | 538.77 | SNP | Rv3343c (PPE54 3736935:3729364 PPE family protein PPE54 CDS) | silent (Arg2184) | 9913 | - | | 3730466 | A | G | 1298.77 | SNP | Rv3343c (PPE54 3736935:3729364 PPE family protein PPE54 CDS) | Ile2157Thr | 11 | - | | 3730582 | G | A | 38.77 | SNP | Rv3343c (PPE54 3736935:3729364 PPE family protein PPE54 CDS) | silent (Asn2118) | 9822 | - | | 3730624 | C | T | 336.74 | SNP | Rv3343c (PPE54 3736935:3729364 PPE family protein PPE54 CDS) | silent (Ser2104) | 9840 | - | | 3730741 | G | A | 248.78 | SNP | Rv3343c (PPE54 3736935:3729364 PPE family protein PPE54 CDS) | silent (Gly2065) | 9935 | - | | 3732517 | A | G | 231.77 | SNP | Rv3343c (PPE54 3736935:3729364 PPE family protein PPE54 CDS) | silent (Ile1473) | 9872 | - | | 3732525 | A | T | 263.77 | SNP | Rv3343c (PPE54 3736935:3729364 PPE family protein PPE54 CDS) | Phe1471Ile | 7 | - | | 3732553 | A | G | 298.22 | SNP | Rv3343c (PPE54 3736935:3729364 PPE family protein PPE54 CDS) | silent (Ile1461) | 9872 | - | | 3732624 | A | G | 365.77 | SNP | Rv3343c (PPE54 3736935:3729364 PPE family protein PPE54 CDS) | Leu(s)1438Leu | 3 | - | | 3735508 | G | A | 1374.77 | SNP | Rv3343c (PPE54 3736935:3729364 PPE family protein PPE54 CDS) | silent (Leu476) | 9947 | - | | 3735907 | C | T | 142.03 | SNP | Rv3343c (PPE54 3736935:3729364 PPE family protein PPE54 CDS) | silent (Ser343) | 9840 | - | | 3735931 | G | A | 135.03 | SNP | Rv3343c (PPE54 3736935:3729364 PPE family protein PPE54 CDS) | silent (Ser335) | 9840 | - | | 3735967 | G | A | 41.74 | SNP | Rv3343c (PPE54 3736935:3729364 PPE family protein PPE54 CDS) | silent (Ser323) | 9840 | - | | 3736628 | T | G | 719.77 | SNP | Rv3343c (PPE54 3736935:3729364 PPE family protein PPE54 CDS) | Glu103Ala | 17 | - | | 3737661 | TG | T | 478.73 | DEL | intergenic |  |  | - | | 3738516 | C | CTGCCGCCGCTGCCGCCGT | 2044.73 | INS | Rv3345c (PE\_PGRS50 3742774:3738158 PE-PGRS family protein PE\_PGRS50 CDS) |  |  | - | | 3746409 | A | G | 374.77 | SNP | Rv3347c (PPE55 3753184:3743711 PPE family protein PPE55 CDS) | Leu2259Pro | 2 | - | | 3747403 | C | A | 590.84 | SNP | Rv3347c (PPE55 3753184:3743711 PPE family protein PPE55 CDS) | Gly1928Cys | 0 | - | | 3750226 | CGCCGGTGTTGGCGTT | C | 5000.74 | DEL | Rv3347c (PPE55 3753184:3743711 PPE family protein PPE55 CDS) |  |  | - | | 3752207 | A | G | 1014.77 | SNP | Rv3347c (PPE55 3753184:3743711 PPE family protein PPE55 CDS) | silent (Ile326) | 9872 | - | | 3752654 | A | T | 342.78 | SNP | Rv3347c (PPE55 3753184:3743711 PPE family protein PPE55 CDS) | silent (Gly177) | 9935 | - | | 3752662 | A | G | 298.78 | SNP | Rv3347c (PPE55 3753184:3743711 PPE family protein PPE55 CDS) | Leu(s)175Leu | 3 | - | | 3752761 | A | G | 128.03 | SNP | Rv3347c (PPE55 3753184:3743711 PPE family protein PPE55 CDS) | Leu(s)142Leu | 3 | - | | 3752778 | A | G | 37.74 | SNP | Rv3347c (PPE55 3753184:3743711 PPE family protein PPE55 CDS) | Val136Ala | 18 | - | | 3752934 | A | T | 114.03 | SNP | Rv3347c (PPE55 3753184:3743711 PPE family protein PPE55 CDS) | Val(s)84Glu | 10 | - | | 3753116 | C | T | 552.77 | SNP | Rv3347c (PPE55 3753184:3743711 PPE family protein PPE55 CDS) | silent (Pro23) | 9926 | - | | 3753164 | T | G | 796.77 | SNP | Rv3347c (PPE55 3753184:3743711 PPE family protein PPE55 CDS) | silent (Pro7) | 9926 | - | | 3766777 | A | G | 271.77 | SNP | Rv3350c (PPE56 3767102:3755952 PPE family protein PPE56 CDS) | Leu109Pro | 2 | - | | 3766778 | G | C | 285.77 | SNP | Rv3350c (PPE56 3767102:3755952 PPE family protein PPE56 CDS) | Leu109Val(s) | 4 | - | | 3766815 | G | C | 151.77 | SNP | Rv3350c (PPE56 3767102:3755952 PPE family protein PPE56 CDS) | Val96Val(s) | 18 | - | | 3766816 | A | G | 136.77 | SNP | Rv3350c (PPE56 3767102:3755952 PPE family protein PPE56 CDS) | Val96Ala | 18 | - | | 3766819 | C | G | 122.77 | SNP | Rv3350c (PPE56 3767102:3755952 PPE family protein PPE56 CDS) | Gly95Ala | 21 | - | | 3766858 | G | T | 106.77 | SNP | Rv3350c (PPE56 3767102:3755952 PPE family protein PPE56 CDS) | Ala82Glu | 10 | - | | 3766859 | C | G | 117.77 | SNP | Rv3350c (PPE56 3767102:3755952 PPE family protein PPE56 CDS) | Ala82Pro | 13 | - | | 3766860 | G | C | 122.77 | SNP | Rv3350c (PPE56 3767102:3755952 PPE family protein PPE56 CDS) | silent (Ala81) | 9867 | - | | 3766893 | C | G | 49.74 | SNP | Rv3350c (PPE56 3767102:3755952 PPE family protein PPE56 CDS) | silent (Ala70) | 9867 | - | | 3779671 | C | CGGCAACGGT | 871.74 | INS | Rv3367 (PE\_PGRS51 3778568:3780334 PE-PGRS family protein PE\_PGRS51 CDS) |  |  | - | | 3792796 | G | A | 1236.77 | SNP | Rv3378c (- 3793248:3792358 Diterpene synthase CDS) | silent (Asn151) | 9822 | - | | 3798095 | A | C | 1482.77 | SNP | Rv3383c (idsB 3798489:3797437 Possible polyprenyl synthetase IdsB (polyprenyl transferase) (polyprenyl diphosphate synthase) CDS) | Val132Gly | 5 | - | | 3817117 | C | A | 718.77 | SNP | Rv3399 (- 3816129:3817175 Possible S-adenosylmethionine-dependent methyltransferase CDS) | Ala330Glu | 10 | - | | 3820407 | A | G | 70.77 | SNP | intergenic |  |  | - | | 3820545 | A | G | 32.77 | SNP | intergenic |  |  | - | | 3823159 | A | T | 956.77 | SNP | Rv3403c (- 3823863:3822262 Hypothetical protein CDS) | silent (Val235) | 9901 | - | | 3826684 | C | T | 958.77 | SNP | Rv3408 (vapC47 3826548:3826958 Possible toxin VapC47 Contains PIN domain CDS) | Ser46Leu(s) | 35 | - | | 3829770 | T | C | 1107.77 | SNP | Rv3410c (guaB3 3829910:3828783 Probable inosine-5'-monophosphate dehydrogenase GuaB3 (imp dehydrogenase) (inosinic acid dehydrogenase) (inosinate dehydrogenase) (imp oxidoreductase) (inosine-5'-monophosphate oxidoreductase) (IMPDH) (IMPD) CDS) | silent (Pro47) | 9926 | - | | 3838871 | A | G | 816.77 | SNP | Rv3420c (rimI 3839062:3838586 Ribosomal-protein-alanine acetyltransferase RimI (acetylating enzyme for N-terminal of ribosomal protein S18) CDS) | silent (Ala64) | 9867 | - | | 3841654 | T | G | 48.77 | SNP | intergenic |  |  | - | | 3841662 | T | C | 470.77 | SNP | intergenic |  |  | - | | 3841663 | C | T | 66.77 | SNP | intergenic |  |  | - | | 3841851 | T | C | 1346.77 | SNP | Rv3424c (- 3842076:3841714 Hypothetical protein CDS) | Asn76Asp | 42 | - | | 3841925 | G | A | 235.77 | SNP | Rv3424c (- 3842076:3841714 Hypothetical protein CDS) | Ala51Val(s) | 9867 | - | | 3841926 | C | G | 158.77 | SNP | Rv3424c (- 3842076:3841714 Hypothetical protein CDS) | Ala51Pro | 13 | - | | 3841927 | T | C | 208.77 | SNP | Rv3424c (- 3842076:3841714 Hypothetical protein CDS) | Leu50Leu(s) | 4 | - | | 3841931 | G | A | 199.77 | SNP | Rv3424c (- 3842076:3841714 Hypothetical protein CDS) | Ala49Val | 13 | - | | 3841934 | T | G | 205.77 | SNP | Rv3424c (- 3842076:3841714 Hypothetical protein CDS) | Asp48Ala | 10 | - | | 3841940 | C | A | 185.77 | SNP | Rv3424c (- 3842076:3841714 Hypothetical protein CDS) | Gly46Val(s) | 21 | - | | 3841960 | A | T | 253.77 | SNP | Rv3424c (- 3842076:3841714 Hypothetical protein CDS) | silent (Ala39) | 9867 | - | | 3841971 | T | G | 303.77 | SNP | Rv3424c (- 3842076:3841714 Hypothetical protein CDS) | Lys36Gln | 6 | - | | 3842147 | G | C | 34.77 | SNP | intergenic |  |  | - | | 3842148 | C | G | 36.77 | SNP | intergenic |  |  | - | | 3842164 | G | C | 41.77 | SNP | intergenic |  |  | - | | 3842186 | T | A | 52.77 | SNP | intergenic |  |  | - | | 3842204 | G | A | 57.77 | SNP | intergenic |  |  | - | | 3842211 | T | C | 62.77 | SNP | intergenic |  |  | - | | 3842227 | A | C | 77.77 | SNP | intergenic |  |  | - | | 3842228 | A | G | 102.77 | SNP | intergenic |  |  | - | | 3842620 | A | G | 1106.77 | SNP | Rv3425 (PPE57 3842239:3842769 PPE family protein PPE57 CDS) | Thr128Ala | 32 | - | | 3842625 | A | G | 1099.77 | SNP | Rv3425 (PPE57 3842239:3842769 PPE family protein PPE57 CDS) | silent (Pro129) | 9926 | - | | 3843001 | G | A | 53.77 | SNP | intergenic |  |  | - | | 3843354 | A | G | 83.77 | SNP | Rv3426 (PPE58 3843036:3843734 PPE family protein PPE58 CDS) | Thr107Ala | 32 | - | | 3843356 | T | C | 109.77 | SNP | Rv3426 (PPE58 3843036:3843734 PPE family protein PPE58 CDS) | silent (Thr107) | 9871 | - | | 3843361 | C | A | 99.77 | SNP | Rv3426 (PPE58 3843036:3843734 PPE family protein PPE58 CDS) | Ala109Asp | 6 | - | | 3843362 | C | A | 72.77 | SNP | Rv3426 (PPE58 3843036:3843734 PPE family protein PPE58 CDS) | silent (Ala109) | 9867 | - | | 3843363 | A | G | 105.77 | SNP | Rv3426 (PPE58 3843036:3843734 PPE family protein PPE58 CDS) | Asn110Asp | 42 | - | | 3843407 | CG | C | 1151.73 | DEL | Rv3426 (PPE58 3843036:3843734 PPE family protein PPE58 CDS) |  |  | - | | 3843696 | T | A | 324.77 | SNP | Rv3426 (PPE58 3843036:3843734 PPE family protein PPE58 CDS) | Leu(s)221Met(s) | 9867 | - | | 3843704 | G | C | 380.77 | SNP | Rv3426 (PPE58 3843036:3843734 PPE family protein PPE58 CDS) | silent (Thr223) | 9871 | - | | 3843714 | T | C | 472.77 | SNP | Rv3426 (PPE58 3843036:3843734 PPE family protein PPE58 CDS) | Cys227Arg | 1 | - | | 3843749 | G | T | 327.77 | SNP | intergenic |  |  | - | | 3843751 | G | T | 311.77 | SNP | intergenic |  |  | - | | 3843752 | A | G | 374.77 | SNP | intergenic |  |  | - | | 3843753 | G | A | 311.77 | SNP | intergenic |  |  | - | | 3843760 | T | C | 432.77 | SNP | intergenic |  |  | - | | 3844756 | GC | G | 2195.73 | DEL | Rv3428c (- 3845970:3844738 Possible transposase CDS) |  |  | - | | 3844992 | T | A | 1369.77 | SNP | Rv3428c (- 3845970:3844738 Possible transposase CDS) | Ser327Cys | 5 | - | | 3846580 | T | A | 105.77 | SNP | intergenic |  |  | - | | 3846582 | T | G | 140.77 | SNP | intergenic |  |  | - | | 3846591 | C | T | 123.77 | SNP | intergenic |  |  | - | | 3846605 | G | A | 463.77 | SNP | intergenic |  |  | - | | 3846607 | A | C | 531.77 | SNP | intergenic |  |  | - | | 3846622 | G | T | 436.77 | SNP | intergenic |  |  | - | | 3846687 | A | G | 834.77 | SNP | intergenic |  |  | - | | 3846704 | A | G | 759.77 | SNP | intergenic |  |  | - | | 3846707 | A | C | 539.77 | SNP | intergenic |  |  | - | | 3846716 | C | T | 326.77 | SNP | intergenic |  |  | - | | 3846718 | C | CATGGCTAG | 1743.73 | INS | intergenic |  |  | - | | 3846727 | C | T | 319.77 | SNP | intergenic |  |  | - | | 3846728 | A | G | 340.81 | SNP | intergenic |  |  | - | | 3846729 | CGGAT | C | 1061.73 | DEL | intergenic |  |  | - | | 3846741 | G | T | 378.77 | SNP | intergenic |  |  | - | | 3846743 | C | G | 383.77 | SNP | intergenic |  |  | - | | 3846764 | C | G | 756.77 | SNP | intergenic |  |  | - | | 3846773 | T | TG | 1221.73 | INS | intergenic |  |  | - | | 3846774 | T | G | 637.77 | SNP | intergenic |  |  | - | | 3846777 | C | A | 616.77 | SNP | intergenic |  |  | - | | 3846779 | T | G | 637.77 | SNP | intergenic |  |  | - | | 3858011 | G | C | 861.77 | SNP | Rv3438 (- 3857397:3858239 hypothetical protein CDS) | silent (Leu205) | 9947 | - | | 3859893 | C | T | 1229.77 | SNP | Rv3440c (- 3859976:3859665 Hypothetical protein CDS) | silent (Glu28) | 9865 | - | | 3862472 | GA | G | 1172.73 | DEL | intergenic |  |  | - | | 3864995 | T | C | 624.77 | SNP | Rv3447c (eccC4 3868238:3864528 ESX conserved component EccC4 ESX-4 type VII secretion system protein Probable membrane protein CDS) | Ser1082Gly | 21 | - | | 3868520 | T | C | 877.77 | SNP | Rv3448 (eccD4 3868352:3869755 ESX conserved component EccD4 ESX-4 type VII secretion system protein Probable integral membrane protein CDS) | Ser57Pro | 12 | - | | 3877421 | A | G | 1153.77 | SNP | Rv3456c (rplQ 3877432:3876890 50S ribosomal protein L17 RplQ CDS) | silent (Pro4) | 9926 | - | | 3879331 | G | A | 866.77 | SNP | Rv3459c (rpsK 3879692:3879273 30S ribosomal protein S11 RpsK CDS) | Ser121Leu(s) | 35 | - | | 3883173 | T | G | 1151.77 | SNP | Rv3465 (rmlC 3882834:3883442 dTDP-4-dehydrorhamnose 3,5-epimerase RmlC (dTDP-4-keto-6-deoxyglucose 3,5-epimerase) (dTDP-L-rhamnose synthetase) (thymidine diphospho-4-keto-rhamnose 3,5-epimerase) CDS) | Ser114Ala | 35 | - | | 3884748 | G | A | 67.28 | SNP | Rv3467 (- 3883964:3884917 hypothetical protein CDS) | Gly262Asp | 6 | - | | 3885886 | T | C | 1360.77 | SNP | Rv3468c (- 3886069:3884975 Possible dTDP-glucose 4,6-dehydratase CDS) | Ile62Val | 57 | - | | 3892530 | G | A | 1592.77 | SNP | Rv3476c (kgtP 3893720:3892371 Probable dicarboxylic acid transport integral membrane protein KgtP (dicarboxylate transporter) CDS) | silent (Ile397) | 9872 | - | | 3892671 | A | G | 1844.77 | SNP | Rv3476c (kgtP 3893720:3892371 Probable dicarboxylic acid transport integral membrane protein KgtP (dicarboxylate transporter) CDS) | silent (Val350) | 9901 | - | | 3895269 | G | C | 898.77 | SNP | Rv3478 (PPE60 3894426:3895607 PE family protein PPE60 CDS) | Glu282Gln | 27 | - | | 3895281 | T | C | 873.77 | SNP | Rv3478 (PPE60 3894426:3895607 PE family protein PPE60 CDS) | Trp286Arg | 8 | - | | 3895282 | G | A | 826.77 | SNP | Rv3478 (PPE60 3894426:3895607 PE family protein PPE60 CDS) | Trp286STOP | 0 | - | | 3896340 | T | G | 880.77 | SNP | Rv3479 (- 3895820:3898885 Possible transmembrane protein CDS) | Leu174Arg | 1 | - | | 3898408 | A | G | 1021.77 | SNP | Rv3479 (- 3895820:3898885 Possible transmembrane protein CDS) | silent (Ala863) | 9867 | - | | 3898637 | G | A | 1461.77 | SNP | Rv3479 (- 3895820:3898885 Possible transmembrane protein CDS) | Val940Ile | 33 | - | | 3899644 | G | C | 94.77 | SNP | Rv3480c (- 3900402:3898909 Possible triacylglycerol synthase (diacylglycerol acyltransferase) CDS) | Val253Val(s) | 18 | - | | 3923611 | A | G | 1203.77 | SNP | Rv3504 (fadE26 3922471:3923673 Probable acyl-CoA dehydrogenase FadE26 CDS) | Asn381Asp | 42 | - | | 3929262 | TGGCAACGGC | T | 527.87 | DEL | Rv3507 (PE\_PGRS53 3926569:3930714 PE-PGRS family protein PE\_PGRS53 CDS) |  |  | - | | 3930300 | TGGCAACGGC | T | 601.73 | DEL | Rv3507 (PE\_PGRS53 3926569:3930714 PE-PGRS family protein PE\_PGRS53 CDS) |  |  | - | | 3934542 | T | G | 37.77 | SNP | Rv3508 (PE\_PGRS54 3931005:3936710 PE-PGRS family protein PE\_PGRS54 CDS) | Ser1180Ala | 35 | - | | 3934699 | G | A | 101.03 | SNP | Rv3508 (PE\_PGRS54 3931005:3936710 PE-PGRS family protein PE\_PGRS54 CDS) | Ser1232Asn | 20 | - | | 3934733 | G | C | 76.88 | SNP | Rv3508 (PE\_PGRS54 3931005:3936710 PE-PGRS family protein PE\_PGRS54 CDS) | silent (Gly1243) | 9935 | - | | 3934734 | G | A | 54.79 | SNP | Rv3508 (PE\_PGRS54 3931005:3936710 PE-PGRS family protein PE\_PGRS54 CDS) | Ala1244Thr | 22 | - | | 3942640 | T | C | 183.90 | SNP | intergenic |  |  | - | | 3944582 | CGG | C | 296.80 | DEL | intergenic |  |  | - | | 3946929 | C | T | 54.74 | SNP | Rv3514 (PE\_PGRS57 3945794:3950263 PE-PGRS family protein PE\_PGRS57 CDS) | Ala379Val | 13 | - | | 3952800 | G | A | 1513.77 | SNP | Rv3516 (echA19 3952544:3953335 Possible enoyl-CoA hydratase EchA19 (enoyl hydrase) (unsaturated acyl-CoA hydratase) (crotonase) CDS) | Gly86Asp | 6 | - | | 3954664 | C | A | 759.77 | SNP | Rv3518c (cyp142 3955521:3954325 Probable cytochrome P450 monooxygenase 142 Cyp142 CDS) | silent (Ala286) | 9867 | - | | 3958403 | A | G | 1022.77 | SNP | Rv3521 (- 3957521:3958432 hypothetical protein CDS) | Asn295Asp | 42 | - | | 3958797 | C | T | 1481.77 | SNP | Rv3522 (ltp4 3958448:3959512 Possible lipid transfer protein or keto acyl-CoA thiolase Ltp4 CDS) | Ala117Val | 13 | - | | 3959418 | C | T | 836.77 | SNP | Rv3522 (ltp4 3958448:3959512 Possible lipid transfer protein or keto acyl-CoA thiolase Ltp4 CDS) | Thr324Ile | 7 | - | | 3983080 | A | C | 1138.77 | SNP | Rv3543c (fadE29 3983140:3981977 Probable acyl-CoA dehydrogenase FadE29 CDS) | Ser21Ala | 35 | - | | 4005607 | T | C | 981.77 | SNP | Rv3564 (fadE33 4005247:4006203 Probable acyl-CoA dehydrogenase FadE33 CDS) | Leu(s)121Leu | 3 | - | | 4009653 | C | T | 1536.77 | SNP | Rv3568c (hsaC 4010199:4009297 3,4-DHSA dioxygenase CDS) | Ala183Thr | 22 | - | | 4013927 | G | A | 1602.77 | SNP | Rv3572 (- 4013511:4014041 hypothetical protein CDS) | silent (Ser139) | 9840 | - | | 4018414 | C | CA | 2041.73 | INS | Rv3576 (lppH 4018358:4019071 Possible conserved lipoprotein LppH CDS) |  |  | - | | 4018415 | G | A | 1124.77 | SNP | Rv3576 (lppH 4018358:4019071 Possible conserved lipoprotein LppH CDS) | Gly20Arg | 0 | - | | 4018802 | CAA | C | 1926.73 | DEL | Rv3576 (lppH 4018358:4019071 Possible conserved lipoprotein LppH CDS) |  |  | - | | 4024273 | T | C | 1059.77 | SNP | Rv3581c (ispF 4024347:4023868 Probable 2C-methyl-D-erythritol 2,4-cyclodiphosphate synthase IspF (MECPS) CDS) | Val25Val(s) | 18 | - | | 4026899 | G | A | 902.77 | SNP | Rv3585 (radA 4026444:4027886 DNA repair protein RadA (DNA repair protein SMS) CDS) | silent (Gln152) | 9876 | - | | 4034827 | C | T | 700.77 | SNP | Rv3593 (lpqF 4034352:4035710 Probable conserved lipoprotein LpqF CDS) | Ala159Val(s) | 9867 | - | | 4055801 | G | A | 1615.77 | SNP | Rv3616c (espA 4056375:4055197 ESX-1 secretion-associated protein A, EspA CDS) | Thr192Ile | 7 | - | | 4059904 | A | G | 931.77 | SNP | intergenic |  |  | - | | 4060100 | G | A | 549.77 | SNP | Rv3619c (esxV 4060268:4059984 Putative ESAT-6 like protein EsxV (ESAT-6 like protein 1) CDS) | Leu57Leu(s) | 4 | - | | 4060201 | G | A | 377.77 | SNP | Rv3619c (esxV 4060268:4059984 Putative ESAT-6 like protein EsxV (ESAT-6 like protein 1) CDS) | Ser23Leu(s) | 35 | - | | 4060210 | T | A | 368.77 | SNP | Rv3619c (esxV 4060268:4059984 Putative ESAT-6 like protein EsxV (ESAT-6 like protein 1) CDS) | Gln20Leu | 6 | - | | 4060230 | G | A | 464.77 | SNP | Rv3619c (esxV 4060268:4059984 Putative ESAT-6 like protein EsxV (ESAT-6 like protein 1) CDS) | silent (His13) | 9912 | - | | 4069292 | G | A | 800.77 | SNP | Rv3630 (- 4069175:4070470 Probable conserved integral membrane protein CDS) | Ala40Thr | 22 | - | | 4088885 | G | A | 732.89 | SNP | Rv3649 (- 4088781:4091096 Probable helicase CDS) | silent (Arg35) | 9913 | - | | 4091590 | C | A | 1028.77 | SNP | intergenic |  |  | - | | 4095001 | CG | C | 2195.73 | DEL | Rv3655c (- 4095300:4094923 hypothetical protein CDS) |  |  | - | | 4095050 | G | A | 1427.77 | SNP | Rv3655c (- 4095300:4094923 hypothetical protein CDS) | Pro84Leu | 3 | - | | 4099397 | C | T | 888.77 | SNP | intergenic |  |  | - | | 4100975 | T | C | 869.77 | SNP | intergenic |  |  | - | | 4107490 | C | T | 1433.77 | SNP | intergenic |  |  | - | | 4109796 | G | A | 752.77 | SNP | Rv3668c (- 4110481:4109783 Possible protease CDS) | Pro229Leu | 3 | - | | 4111303 | G | C | 1281.77 | SNP | Rv3669 (- 4110827:4111345 Probable conserved transmembrane protein CDS) | Val(s)159Val | 13 | - | | 4117361 | AC | A | 1565.73 | DEL | Rv3677c (- 4118052:4117258 Possible hydrolase CDS) |  |  | - | | 4120926 | A | G | 124.77 | SNP | Rv3680 (- 4119795:4120955 Probable anion transporter ATPase CDS) | Asn378Asp | 42 | - | | 4120983 | A | G | 633.77 | SNP | intergenic |  |  | - | | 4121032 | C | T | 91.77 | SNP | intergenic |  |  | - | | 4137732 | G | A | 1026.77 | SNP | Rv3695 (- 4137206:4138138 Possible conserved membrane protein CDS) | Gly176Glu | 4 | - | | 4139670 | C | T | 761.77 | SNP | Rv3696c (glpK 4139755:4138202 Probable glycerol kinase GlpK (ATP:glycerol 3-phosphotransferase) (glycerokinase) (GK) CDS) | Cys29Tyr | 3 | - | | 4148377 | C | T | 1361.77 | SNP | Rv3705c (- 4148962:4148318 hypothetical protein CDS) | Gly196Ser | 16 | - | | 4148669 | C | T | 1310.77 | SNP | Rv3705c (- 4148962:4148318 hypothetical protein CDS) | silent (Thr98) | 9871 | - | | 4155050 | G | A | 1117.77 | SNP | Rv3710 (leuA 4153740:4155674 2-isopropylmalate synthase LeuA (alpha-isopropylmalate synthase) (alpha-IPM synthetase) (IPMS) CDS) | Val(s)437Val | 13 | - | | 4156099 | C | A | 1363.77 | SNP | Rv3711c (dnaQ 4156729:4155740 Probable DNA polymerase III (epsilon subunit) DnaQ CDS) | Val(s)211Leu(s) | 9867 | - | | 4156683 | C | G | 1045.77 | SNP | Rv3711c (dnaQ 4156729:4155740 Probable DNA polymerase III (epsilon subunit) DnaQ CDS) | Trp16Ser | 5 | - | | 4159830 | C | G | 1065.77 | SNP | intergenic |  |  | - | | 4160407 | A | G | 1179.77 | SNP | Rv3715c (recR 4160500:4159889 Probable recombination protein RecR CDS) | Leu(s)32Leu | 3 | - | | 4162073 | C | T | 886.77 | SNP | Rv3718c (- 4162258:4161815 hypothetical protein CDS) | silent (Gln62) | 9876 | - | | 4162339 | A | G | 1658.77 | SNP | Rv3719 (- 4162306:4163718 hypothetical protein CDS) | Thr12Ala | 32 | - | | 4170984 | C | T | 1090.77 | SNP | Rv3725 (- 4170214:4171143 Possible oxidoreductase CDS) | silent (Cys257) | 9973 | - | | 4179179 | A | G | 942.77 | SNP | Rv3729 (- 4178285:4180615 Possible transferase CDS) | Ile299Val | 57 | - | | 4182695 | G | A | 972.77 | SNP | Rv3731 (ligC 4181758:4182834 Possible ATP-dependent DNA ligase LigC (polydeoxyribonucleotide synthase [ATP]) (polynucleotide ligase [ATP]) (sealase) (DNA repair protein) (DNA joinase) CDS) | Arg313His | 8 | - | | 4186230 | G | A | 1076.77 | SNP | Rv3735 (- 4186089:4186577 hypothetical protein CDS) | Ala48Thr | 22 | - | | 4187485 | T | C | 1442.77 | SNP | Rv3736 (- 4186634:4187695 Transcriptional regulatory protein (probably AraC/XylS-family) CDS) | silent (Ala284) | 9867 | - | | 4187817 | A | G | 1057.77 | SNP | Rv3737 (- 4187699:4189288 Probable conserved transmembrane protein CDS) | Asp40Gly | 11 | - | | 4189841 | T | C | 768.77 | SNP | Rv3738c (PPE66 4190232:4189285 PPE family protein PPE66 CDS) | Tyr131Cys | 3 | - | | 4198611 | CG | C | 1419.73 | DEL | intergenic |  |  | - | | 4204441 | A | G | 844.77 | SNP | Rv3759c (proX 4205373:4204426 Possible osmoprotectant (glycine betaine/carnitine/choline/L-proline) binding lipoprotein ProX CDS) | silent (His311) | 9912 | - | | 4210274 | A | G | 1030.77 | SNP | Rv3764c (tcrY 4211009:4209582 Possible two component sensor kinase TcrY CDS) | Cys246Arg | 1 | - | | 4212196 | A | T | 1722.77 | SNP | intergenic |  |  | - | | 4214751 | C | A | 784.77 | SNP | Rv3769 (- 4214615:4214887 Hypothetical protein CDS) | Thr46Asn | 9 | - | | 4215467 | G | A | 1492.77 | SNP | Rv3770c (- 4215775:4215200 Hypothetical leucine rich protein CDS) | silent (Gly103) | 9935 | - | | 4221490 | C | G | 1229.77 | SNP | Rv3776 (- 4221089:4222648 hypothetical protein CDS) | silent (Leu134) | 9947 | - | | 4222073 | A | G | 859.77 | SNP | Rv3776 (- 4221089:4222648 hypothetical protein CDS) | Met(s)329Val(s) | 9867 | - | | 4222882 | A | G | 1027.77 | SNP | Rv3777 (- 4222694:4223680 Probable oxidoreductase CDS) | silent (Leu63) | 9947 | - | | 4239250 | G | A | 887.77 | SNP | Rv3792 (aftA 4237932:4239863 Arabinofuranosyltransferase AftA CDS) | Gly440Asp | 6 | - | | 4239891 | T | C | 1599.77 | SNP | Rv3793 (embC 4239863:4243147 Integral membrane indolylacetylinositol arabinosyltransferase EmbC (arabinosylindolylacetylinositol synthase) CDS) | Ile10Thr | 11 | - | | 4242643 | C | T | 536.77 | SNP | Rv3793 (embC 4239863:4243147 Integral membrane indolylacetylinositol arabinosyltransferase EmbC (arabinosylindolylacetylinositol synthase) CDS) | silent (Arg927) | 9913 | genotype | | 4243222 | C | A | 1259.77 | SNP | intergenic (Rv3794-11nt) |  |  | - | | 4245055 | C | A | 859.77 | SNP | Rv3794 (embA 4243233:4246517 Integral membrane indolylacetylinositol arabinosyltransferase EmbA (arabinosylindolylacetylinositol synthase) CDS) | Thr608Asn | 9 | genotype | | 4247429 | A | G | 1322.77 | SNP | Rv3795 (embB 4246514:4249810 Integral membrane indolylacetylinositol arabinosyltransferase EmbB (arabinosylindolylacetylinositol synthase) CDS) | Met(s)306Val(s) | 9867 | resistance | | 4250742 | G | A | 1106.77 | SNP | Rv3796 (- 4249878:4251005 hypothetical protein CDS) | Gly289Ser | 16 | - | | 4252891 | G | A | 995.77 | SNP | intergenic |  |  | - | | 4254290 | T | G | 680.77 | SNP | Rv3798 (- 4252993:4254327 Probable transposase CDS) | Leu433Arg | 1 | - | | 4255922 | A | G | 1755.77 | SNP | Rv3799c (accD4 4255948:4254380 Probable propionyl-CoA carboxylase beta chain 4 AccD4 (pccase) (propanoyl-CoA:carbon dioxide ligase) CDS) | silent (His9) | 9912 | - | | 4257220 | A | G | 1072.77 | SNP | Rv3800c (pks13 4261146:4255945 Polyketide synthase Pks13 CDS) | silent (Arg1309) | 9913 | - | | 4257849 | G | A | 1073.77 | SNP | Rv3800c (pks13 4261146:4255945 Polyketide synthase Pks13 CDS) | Arg1100Trp | 2 | - | | 4260268 | G | C | 583.77 | SNP | Rv3800c (pks13 4261146:4255945 Polyketide synthase Pks13 CDS) | silent (Ala293) | 9867 | genotype | | 4287195 | A | G | 828.77 | SNP | Rv3822 (- 4286721:4287935 hypothetical protein CDS) | Thr159Ala | 32 | - | | 4301570 | T | C | 1242.77 | SNP | Rv3827c (- 4302789:4301563 Possible transposase CDS) | Asp407Gly | 11 | - | | 4302036 | T | C | 971.77 | SNP | Rv3827c (- 4302789:4301563 Possible transposase CDS) | Thr252Ala | 32 | - | | 4306155 | C | T | 953.77 | SNP | Rv3831 (- 4305757:4306239 Hypothetical protein CDS) | silent (Ser133) | 9840 | - | | 4309213 | C | T | 693.77 | SNP | Rv3835 (- 4309047:4310396 hypothetical protein CDS) | Ala56Val | 13 | - | | 4311528 | G | A | 747.77 | SNP | Rv3837c (- 4311707:4311009 Probable phosphoglycerate mutase (phosphoglyceromutase) (phosphoglycerate phosphomutase) CDS) | silent (Ala60) | 9867 | - | | 4315691 | C | T | 802.77 | SNP | Rv3843c (- 4316596:4315568 Probable conserved transmembrane protein CDS) | silent (Glu302) | 9865 | - | | 4338595 | GC | G | 2991.73 | DEL | intergenic |  |  | - | | 4338732 | G | A | 867.77 | SNP | intergenic |  |  | - | | 4349187 | G | A | 1335.77 | SNP | Rv3871 (eccCb1 4348827:4350602 ESX conserved component EccCb1 ESX-1 type VII secretion system protein CDS) | Gly121Arg | 0 | - | | 4351039 | G | T | 1408.77 | SNP | Rv3872 (PE35 4350745:4351044 PE family-related protein PE35 CDS) | Glu99STOP | 17 | - | | 4356110 | G | C | 821.77 | SNP | Rv3877 (eccD1 4355007:4356542 ESX conserved component EccD1 ESX-1 type VII secretion system protein Probable transmembrane protein CDS) | silent (Leu368) | 9947 | - | | 4359997 | C | T | 839.77 | SNP | intergenic |  |  | - | | 4366272 | G | C | 1266.77 | SNP | Rv3884c (eccA2 4366838:4364979 ESX conserved component EccA2 ESX-2 type VII secretion system protein Probable CbxX/CfqX family protein CDS) | silent (Ala189) | 9867 | - | | 4367911 | A | G | 305.78 | SNP | Rv3885c (eccE2 4368521:4366908 ESX conserved component EccE2 ESX-2 type VII secretion system protein Possible membrane protein CDS) | Ile204Thr | 11 | - | | 4375620 | T | TA | 2078.73 | INS | Rv3892c (PPE69 4375683:4374484 PPE family protein PPE69 CDS) |  |  | - | | 4375628 | G | T | 1175.77 | SNP | Rv3892c (PPE69 4375683:4374484 PPE family protein PPE69 CDS) | Thr19Lys | 11 | - | | 4379680 | C | G | 955.77 | SNP | Rv3894c (eccC2 4380452:4376262 ESX conserved component EccC2 ESX-2 type VII secretion system protein Possible membrane protein CDS) | Arg258Pro | 5 | - | | 4382054 | T | C | 1383.77 | SNP | Rv3896c (- 4382851:4381943 hypothetical protein CDS) | silent (Ala266) | 9867 | - | | 4382275 | G | T | 993.77 | SNP | Rv3896c (- 4382851:4381943 hypothetical protein CDS) | Gln193Lys | 12 | - | | 4383144 | C | CCGGGG | 2657.73 | INS | Rv3897c (- 4383640:4383008 hypothetical protein CDS) |  |  | - | | 4384057 | G | A | 560.77 | SNP | intergenic |  |  | - | | 4400660 | AC | A | 1960.73 | DEL | Rv3911 (sigM 4400186:4400854 Possible alternative RNA polymerase sigma factor SigM CDS) |  |  | - | | 4408087 | CG | C | 2153.73 | DEL | Rv3919c (gid 4408202:4407528 Probable glucose-inhibited division protein B Gid CDS) |  |  | - | |  | | export |

elog
