# Supplementary material for: Patterns of compensatory mutations in rpoA/B/C genes of multidrug resistant M. tuberculosis in Uganda
Source: PLoS One. 2025 Dec 4;20(12):e0328957. doi: 10.1371/journal.pone.0328957 (PMC12677784; doi:10.1371/journal.pone.0328957)
Supplement: S2 File — (ZIP) [file pone.0328957.s002.zip › Variants C_S3_L001_001.bam.html]

 

Calling SNPs/INDELs (computing variant list in .vcf format) from C\_S3\_L001\_001.bam

*by SAMtools/BCFtools:*

Howto

Important aspects

This takes up to one hour!!! **Please wait ...**

Variants C\_S3\_L001\_001.bam

|  |  |
| --- | --- |
| Variants |  |

|  |  |
| --- | --- |
| |  | | --- | | *by GATK* | |

|  |  |  |
| --- | --- | --- |
| |  | | --- | | C\_S3\_L001\_001.bam | | | computed 2016-10-27 using PhyResSE v1.0 (Ref. NC\_000962.3) | |

|  |  |
| --- | --- |
| 1208  variants called Export in VCF format |  |

|  |  |  |  |  |  |  |  |  |  |  |  |  |  |  |  |  |  |  |  |  |  |  |  |  |  |  |  |  |  |  |  |  |  |  |  |  |  |  |  |  |  |  |  |  |  |  |  |  |  |  |  |  |  |  |  |  |  |  |  |  |  |  |  |  |  |  |  |  |  |  |  |  |  |  |  |  |  |  |  |  |  |  |  |  |  |  |  |  |  |  |  |  |  |  |  |  |  |  |  |  |  |  |  |  |  |  |  |  |  |  |  |  |  |  |  |  |  |  |  |  |  |  |  |  |  |  |  |  |  |  |  |  |  |  |  |  |  |  |  |  |  |  |  |  |  |  |  |  |  |  |  |  |  |  |  |  |  |  |  |  |  |  |  |  |  |  |  |  |  |  |  |  |  |  |  |  |  |  |  |  |  |  |  |  |  |  |  |  |  |  |  |  |  |  |  |  |  |  |  |  |  |  |  |  |  |  |  |  |  |  |  |  |  |  |  |  |  |  |  |  |  |  |  |  |  |  |  |  |  |  |  |  |  |  |  |  |  |  |  |  |  |  |  |  |  |  |  |  |  |  |  |  |  |  |  |  |  |  |  |  |  |  |  |  |  |  |  |  |  |  |  |  |  |  |  |  |  |  |  |  |  |  |  |  |  |  |  |  |  |  |  |  |  |  |  |  |  |  |  |  |  |  |  |  |  |  |  |  |  |  |  |  |  |  |  |  |  |  |  |  |  |  |  |  |  |  |  |  |  |  |  |  |  |  |  |  |  |  |  |  |  |  |  |  |  |  |  |  |  |  |  |  |  |  |  |  |  |  |  |  |  |  |  |  |  |  |  |  |  |  |  |  |  |  |  |  |  |  |  |  |  |  |  |  |  |  |  |  |  |  |  |  |  |  |  |  |  |  |  |  |  |  |  |  |  |  |  |  |  |  |  |  |  |  |  |  |  |  |  |  |  |  |  |  |  |  |  |  |  |  |  |  |  |  |  |  |  |  |  |  |  |  |  |  |  |  |  |  |  |  |  |  |  |  |  |  |  |  |  |  |  |  |  |  |  |  |  |  |  |  |  |  |  |  |  |  |  |  |  |  |  |  |  |  |  |  |  |  |  |  |  |  |  |  |  |  |  |  |  |  |  |  |  |  |  |  |  |  |  |  |  |  |  |  |  |  |  |  |  |  |  |  |  |  |  |  |  |  |  |  |  |  |  |  |  |  |  |  |  |  |  |  |  |  |  |  |  |  |  |  |  |  |  |  |  |  |  |  |  |  |  |  |  |  |  |  |  |  |  |  |  |  |  |  |  |  |  |  |  |  |  |  |  |  |  |  |  |  |  |  |  |  |  |  |  |  |  |  |  |  |  |  |  |  |  |  |  |  |  |  |  |  |  |  |  |  |  |  |  |  |  |  |  |  |  |  |  |  |  |  |  |  |  |  |  |  |  |  |  |  |  |  |  |  |  |  |  |  |  |  |  |  |  |  |  |  |  |  |  |  |  |  |  |  |  |  |  |  |  |  |  |  |  |  |  |  |  |  |  |  |  |  |  |  |  |  |  |  |  |  |  |  |  |  |  |  |  |  |  |  |  |  |  |  |  |  |  |  |  |  |  |  |  |  |  |  |  |  |  |  |  |  |  |  |  |  |  |  |  |  |  |  |  |  |  |  |  |  |  |  |  |  |  |  |  |  |  |  |  |  |  |  |  |  |  |  |  |  |  |  |  |  |  |  |  |  |  |  |  |  |  |  |  |  |  |  |  |  |  |  |  |  |  |  |  |  |  |  |  |  |  |  |  |  |  |  |  |  |  |  |  |  |  |  |  |  |  |  |  |  |  |  |  |  |  |  |  |  |  |  |  |  |  |  |  |  |  |  |  |  |  |  |  |  |  |  |  |  |  |  |  |  |  |  |  |  |  |  |  |  |  |  |  |  |  |  |  |  |  |  |  |  |  |  |  |  |  |  |  |  |  |  |  |  |  |  |  |  |  |  |  |  |  |  |  |  |  |  |  |  |  |  |  |  |  |  |  |  |  |  |  |  |  |  |  |  |  |  |  |  |  |  |  |  |  |  |  |  |  |  |  |  |  |  |  |  |  |  |  |  |  |  |  |  |  |  |  |  |  |  |  |  |  |  |  |  |  |  |  |  |  |  |  |  |  |  |  |  |  |  |  |  |  |  |  |  |  |  |  |  |  |  |  |  |  |  |  |  |  |  |  |  |  |  |  |  |  |  |  |  |  |  |  |  |  |  |  |  |  |  |  |  |  |  |  |  |  |  |  |  |  |  |  |  |  |  |  |  |  |  |  |  |  |  |  |  |  |  |  |  |  |  |  |  |  |  |  |  |  |  |  |  |  |  |  |  |  |  |  |  |  |  |  |  |  |  |  |  |  |  |  |  |  |  |  |  |  |  |  |  |  |  |  |  |  |  |  |  |  |  |  |  |  |  |  |  |  |  |  |  |  |  |  |  |  |  |  |  |  |  |  |  |  |  |  |  |  |  |  |  |  |  |  |  |  |  |  |  |  |  |  |  |  |  |  |  |  |  |  |  |  |  |  |  |  |  |  |  |  |  |  |  |  |  |  |  |  |  |  |  |  |  |  |  |  |  |  |  |  |  |  |  |  |  |  |  |  |  |  |  |  |  |  |  |  |  |  |  |  |  |  |  |  |  |  |  |  |  |  |  |  |  |  |  |  |  |  |  |  |  |  |  |  |  |  |  |  |  |  |  |  |  |  |  |  |  |  |  |  |  |  |  |  |  |  |  |  |  |  |  |  |  |  |  |  |  |  |  |  |  |  |  |  |  |  |  |  |  |  |  |  |  |  |  |  |  |  |  |  |  |  |  |  |  |  |  |  |  |  |  |  |  |  |  |  |  |  |  |  |  |  |  |  |  |  |  |  |  |  |  |  |  |  |  |  |  |  |  |  |  |  |  |  |  |  |  |  |  |  |  |  |  |  |  |  |  |  |  |  |  |  |  |  |  |  |  |  |  |  |  |  |  |  |  |  |  |  |  |  |  |  |  |  |  |  |  |  |  |  |  |  |  |  |  |  |  |  |  |  |  |  |  |  |  |  |  |  |  |  |  |  |  |  |  |  |  |  |  |  |  |  |  |  |  |  |  |  |  |  |  |  |  |  |  |  |  |  |  |  |  |  |  |  |  |  |  |  |  |  |  |  |  |  |  |  |  |  |  |  |  |  |  |  |  |  |  |  |  |  |  |  |  |  |  |  |  |  |  |  |  |  |  |  |  |  |  |  |  |  |  |  |  |  |  |  |  |  |  |  |  |  |  |  |  |  |  |  |  |  |  |  |  |  |  |  |  |  |  |  |  |  |  |  |  |  |  |  |  |  |  |  |  |  |  |  |  |  |  |  |  |  |  |  |  |  |  |  |  |  |  |  |  |  |  |  |  |  |  |  |  |  |  |  |  |  |  |  |  |  |  |  |  |  |  |  |  |  |  |  |  |  |  |  |  |  |  |  |  |  |  |  |  |  |  |  |  |  |  |  |  |  |  |  |  |  |  |  |  |  |  |  |  |  |  |  |  |  |  |  |  |  |  |  |  |  |  |  |  |  |  |  |  |  |  |  |  |  |  |  |  |  |  |  |  |  |  |  |  |  |  |  |  |  |  |  |  |  |  |  |  |  |  |  |  |  |  |  |  |  |  |  |  |  |  |  |  |  |  |  |  |  |  |  |  |  |  |  |  |  |  |  |  |  |  |  |  |  |  |  |  |  |  |  |  |  |  |  |  |  |  |  |  |  |  |  |  |  |  |  |  |  |  |  |  |  |  |  |  |  |  |  |  |  |  |  |  |  |  |  |  |  |  |  |  |  |  |  |  |  |  |  |  |  |  |  |  |  |  |  |  |  |  |  |  |  |  |  |  |  |  |  |  |  |  |  |  |  |  |  |  |  |  |  |  |  |  |  |  |  |  |  |  |  |  |  |  |  |  |  |  |  |  |  |  |  |  |  |  |  |  |  |  |  |  |  |  |  |  |  |  |  |  |  |  |  |  |  |  |  |  |  |  |  |  |  |  |  |  |  |  |  |  |  |  |  |  |  |  |  |  |  |  |  |  |  |  |  |  |  |  |  |  |  |  |  |  |  |  |  |  |  |  |  |  |  |  |  |  |  |  |  |  |  |  |  |  |  |  |  |  |  |  |  |  |  |  |  |  |  |  |  |  |  |  |  |  |  |  |  |  |  |  |  |  |  |  |  |  |  |  |  |  |  |  |  |  |  |  |  |  |  |  |  |  |  |  |  |  |  |  |  |  |  |  |  |  |  |  |  |  |  |  |  |  |  |  |  |  |  |  |  |  |  |  |  |  |  |  |  |  |  |  |  |  |  |  |  |  |  |  |  |  |  |  |  |  |  |  |  |  |  |  |  |  |  |  |  |  |  |  |  |  |  |  |  |  |  |  |  |  |  |  |  |  |  |  |  |  |  |  |  |  |  |  |  |  |  |  |  |  |  |  |  |  |  |  |  |  |  |  |  |  |  |  |  |  |  |  |  |  |  |  |  |  |  |  |  |  |  |  |  |  |  |  |  |  |  |  |  |  |  |  |  |  |  |  |  |  |  |  |  |  |  |  |  |  |  |  |  |  |  |  |  |  |  |  |  |  |  |  |  |  |  |  |  |  |  |  |  |  |  |  |  |  |  |  |  |  |  |  |  |  |  |  |  |  |  |  |  |  |  |  |  |  |  |  |  |  |  |  |  |  |  |  |  |  |  |  |  |  |  |  |  |  |  |  |  |  |  |  |  |  |  |  |  |  |  |  |  |  |  |  |  |  |  |  |  |  |  |  |  |  |  |  |  |  |  |  |  |  |  |  |  |  |  |  |  |  |  |  |  |  |  |  |  |  |  |  |  |  |  |  |  |  |  |  |  |  |  |  |  |  |  |  |  |  |  |  |  |  |  |  |  |  |  |  |  |  |  |  |  |  |  |  |  |  |  |  |  |  |  |  |  |  |  |  |  |  |  |  |  |  |  |  |  |  |  |  |  |  |  |  |  |  |  |  |  |  |  |  |  |  |  |  |  |  |  |  |  |  |  |  |  |  |  |  |  |  |  |  |  |  |  |  |  |  |  |  |  |  |  |  |  |  |  |  |  |  |  |  |  |  |  |  |  |  |  |  |  |  |  |  |  |  |  |  |  |  |  |  |  |  |  |  |  |  |  |  |  |  |  |  |  |  |  |  |  |  |  |  |  |  |  |  |  |  |  |  |  |  |  |  |  |  |  |  |  |  |  |  |  |  |  |  |  |  |  |  |  |  |  |  |  |  |  |  |  |  |  |  |  |  |  |  |  |  |  |  |  |  |  |  |  |  |  |  |  |  |  |  |  |  |  |  |  |  |  |  |  |  |  |  |  |  |  |  |  |  |  |  |  |  |  |  |  |  |  |  |  |  |  |  |  |  |  |  |  |  |  |  |  |  |  |  |  |  |  |  |  |  |  |  |  |  |  |  |  |  |  |  |  |  |  |  |  |  |  |  |  |  |  |  |  |  |  |  |  |  |  |  |  |  |  |  |  |  |  |  |  |  |  |  |  |  |  |  |  |  |  |  |  |  |  |  |  |  |  |  |  |  |  |  |  |  |  |  |  |  |  |  |  |  |  |  |  |  |  |  |  |  |  |  |  |  |  |  |  |  |  |  |  |  |  |  |  |  |  |  |  |  |  |  |  |  |  |  |  |  |  |  |  |  |  |  |  |  |  |  |  |  |  |  |  |  |  |  |  |  |  |  |  |  |  |  |  |  |  |  |  |  |  |  |  |  |  |  |  |  |  |  |  |  |  |  |  |  |  |  |  |  |  |  |  |  |  |  |  |  |  |  |  |  |  |  |  |  |  |  |  |  |  |  |  |  |  |  |  |  |  |  |  |  |  |  |  |  |  |  |  |  |  |  |  |  |  |  |  |  |  |  |  |  |  |  |  |  |  |  |  |  |  |  |  |  |  |  |  |  |  |  |  |  |  |  |  |  |  |  |  |  |  |  |  |  |  |  |  |  |  |  |  |  |  |  |  |  |  |  |  |  |  |  |  |  |  |  |  |  |  |  |  |  |  |  |  |  |  |  |  |  |  |  |  |  |  |  |  |  |  |  |  |  |  |  |  |  |  |  |  |  |  |  |  |  |  |  |  |  |  |  |  |  |  |  |  |  |  |  |  |  |  |  |  |  |  |  |  |  |  |  |  |  |  |  |  |  |  |  |  |  |  |  |  |  |  |  |  |  |  |  |  |  |  |  |  |  |  |  |  |  |  |  |  |  |  |  |  |  |  |  |  |  |  |  |  |  |  |  |  |  |  |  |  |  |  |  |  |  |  |  |  |  |  |  |  |  |  |  |  |  |  |  |  |  |  |  |  |  |  |  |  |  |  |  |  |  |  |  |  |  |  |  |  |  |  |  |  |  |  |  |  |  |  |  |  |  |  |  |  |  |  |  |  |  |  |  |  |  |  |  |  |  |  |  |  |  |  |  |  |  |  |  |  |  |  |  |  |  |  |  |  |  |  |  |  |  |  |  |  |  |  |  |  |  |  |  |  |  |  |  |  |  |  |  |  |  |  |  |  |  |  |  |  |  |  |  |  |  |  |  |  |  |  |  |  |  |  |  |  |  |  |  |  |  |  |  |  |  |  |  |  |  |  |  |  |  |  |  |  |  |  |  |  |  |  |  |  |  |  |  |  |  |  |  |  |  |  |  |  |  |  |  |  |  |  |  |  |  |  |  |  |  |  |  |  |  |  |  |  |  |  |  |  |  |  |  |  |  |  |  |  |  |  |  |  |  |  |  |  |  |  |  |  |  |  |  |  |  |  |  |  |  |  |  |  |  |  |  |  |  |  |  |  |  |  |  |  |  |  |  |  |  |  |  |  |  |  |  |  |  |  |  |  |  |  |  |  |  |  |  |  |  |  |  |  |  |  |  |  |  |  |  |  |  |  |  |  |  |  |  |  |  |  |  |  |  |  |  |  |  |  |  |  |  |  |  |  |  |  |  |  |  |  |  |  |  |  |  |  |  |  |  |  |  |  |  |  |  |  |  |  |  |  |  |  |  |  |  |  |  |  |  |  |  |  |  |  |  |  |  |  |  |  |  |  |  |  |  |  |  |  |  |  |  |  |  |  |  |  |  |  |  |  |  |  |  |  |  |  |  |  |  |  |  |  |  |  |  |  |  |  |  |  |  |  |  |  |  |  |  |  |  |  |  |  |  |  |  |  |  |  |  |  |  |  |  |  |  |  |  |  |  |  |  |  |  |  |  |  |  |  |  |  |  |  |  |  |  |  |  |  |  |  |  |  |  |  |  |  |  |  |  |  |  |  |  |  |  |  |  |  |  |  |  |  |  |  |  |  |  |  |  |  |  |  |  |  |  |  |  |  |  |  |  |  |  |  |  |  |  |  |  |  |  |  |  |  |  |  |  |  |  |  |  |  |  |  |  |  |  |  |  |  |  |  |  |  |  |  |  |  |  |  |  |  |  |  |  |  |  |  |  |  |  |  |  |  |  |  |  |  |  |  |  |  |  |  |  |  |  |  |  |  |  |  |  |  |  |  |  |  |  |  |  |  |  |  |  |  |  |  |  |  |  |  |  |  |  |  |  |  |  |  |  |  |  |  |  |  |  |  |  |  |  |  |  |  |  |  |  |  |  |  |  |  |  |  |  |  |  |  |  |  |  |  |  |  |  |  |  |  |  |  |  |  |  |  |  |  |  |  |  |  |  |  |  |  |  |  |  |  |  |  |  |  |  |  |  |  |  |  |  |  |  |  |  |  |  |  |  |  |  |  |  |  |  |  |  |  |  |  |  |  |  |  |  |  |  |  |  |  |  |  |  |  |  |  |  |  |  |  |  |  |  |  |  |  |  |  |  |  |  |  |  |  |  |  |  |  |  |  |  |  |  |  |  |  |  |  |  |  |  |  |  |  |  |  |  |  |  |  |  |  |  |  |  |  |  |  |  |  |  |  |  |  |  |  |  |  |  |  |  |  |  |  |  |  |  |  |  |  |  |  |  |  |  |  |  |  |  |  |  |  |  |  |  |  |  |  |  |  |  |  |  |  |  |  |  |  |  |  |  |  |  |  |  |  |  |  |  |  |  |  |  |  |  |  |  |  |  |  |  |  |  |  |  |  |  |  |  |  |  |  |  |  |  |  |  |  |  |  |  |  |  |  |  |  |  |  |  |  |  |  |  |  |  |  |  |  |  |  |  |  |  |  |  |  |  |  |  |  |  |  |  |  |  |  |  |  |  |  |  |  |  |  |  |  |  |  |  |  |  |  |  |  |  |  |  |  |  |  |  |  |  |  |  |  |  |  |  |  |  |  |  |  |  |  |  |  |  |  |  |  |  |  |  |  |  |  |  |  |  |  |  |  |  |  |  |  |  |  |  |  |  |  |  |  |  |  |  |  |  |  |  |  |  |  |  |  |  |  |  |  |  |  |  |  |  |  |  |  |  |  |  |  |  |  |  |  |  |  |  |  |  |  |  |  |  |  |  |  |  |  |  |  |  |  |  |  |  |  |  |  |  |  |  |  |  |  |  |  |  |  |  |  |  |  |  |  |  |  |  |  |  |  |  |  |  |  |  |  |  |  |  |  |  |  |  |  |  |  |  |  |  |  |  |  |  |  |  |  |  |  |  |  |  |  |  |  |  |  |  |  |  |  |  |  |  |  |  |  |  |  |  |  |  |  |  |  |  |  |  |  |  |  |  |  |  |  |  |  |  |  |  |  |  |  |  |  |  |  |  |  |  |  |  |  |  |  |  |  |  |  |  |  |  |  |  |  |  |  |  |  |  |  |  |  |  |  |  |  |  |  |  |  |  |  |  |  |  |  |  |  |  |  |  |  |  |  |  |  |  |  |  |  |  |  |  |  |  |  |  |  |  |  |  |  |  |  |  |  |  |  |  |  |  |  |  |  |  |  |  |  |  |  |  |  |  |  |  |  |  |  |  |  |  |  |  |  |  |  |  |  |  |  |  |  |  |  |  |  |  |  |  |  |  |  |  |  |  |  |  |  |  |  |  |  |  |  |  |  |  |  |  |  |  |  |  |  |  |  |  |  |  |  |  |  |  |  |  |  |  |  |  |  |  |  |  |  |  |  |  |  |  |  |  |  |  |  |  |  |  |  |  |  |  |  |  |  |  |  |  |  |  |  |  |  |  |  |  |  |  |  |  |  |  |  |  |  |  |  |  |  |  |  |  |  |  |  |  |  |  |  |  |  |  |  |  |  |  |  |  |  |  |  |  |  |  |  |  |  |  |  |  |  |  |  |  |  |  |  |  |  |  |  |  |  |  |  |  |  |  |  |  |  |  |  |  |  |  |  |  |  |  |  |  |  |  |  |  |  |  |  |  |  |  |  |  |  |  |  |  |  |  |  |  |  |  |  |  |  |  |  |  |  |  |  |  |  |  |  |  |  |  |  |  |  |  |  |  |  |  |  |  |  |  |  |  |  |  |  |  |  |  |  |  |  |  |  |  |  |  |  |  |  |  |  |  |  |  |  |  |  |  |  |  |  |  |  |  |  |  |  |  |  |  |  |  |  |  |  |  |  |  |  |  |  |  |  |  |  |  |  |  |  |  |  |  |  |  |  |  |  |  |  |  |  |  |  |  |  |  |  |  |  |  |  |  |  |  |  |  |  |  |  |  |  |  |  |  |  |  |  |  |  |  |  |  |  |  |  |  |  |  |  |  |  |  |  |  |  |  |  |  |  |  |  |  |  |  |  |  |  |  |  |  |  |  |  |  |  |  |  |  |  |  |  |  |  |  |  |  |  |  |  |  |  |  |  |  |  |  |  |  |  |  |  |  |  |  |  |  |  |  |  |  |  |  |  |  |  |  |  |  |  |  |  |  |  |  |  |  |  |  |  |  |  |  |  |  |  |  |  |  |  |  |  |  |  |  |  |  |  |  |  |  |  |  |  |  |  |  |  |  |  |  |  |  |  |  |  |  |  |  |  |  |  |  |  |  |  |  |  |  |  |  |  |  |  |  |  |  |  |  |  |  |  |  |  |  |  |  |  |  |  |  |  |  |  |  |  |  |  |  |  |  |  |  |  |  |  |  |  |  |  |  |  |  |  |  |  |  |  |  |  |  |  |  |  |  |  |  |  |  |  |  |  |  |  |  |  |  |  |  |  |  |  |  |  |  |  |  |  |  |  |  |  |  |  |  |  |  |  |  |  |  |  |  |  |  |  |  |  |  |  |  |  |  |  |  |  |  |  |  |  |  |  |  |  |  |  |  |  |  |  |  |  |  |  |  |  |  |  |  |  |  |  |  |  |  |  |  |  |  |  |  |  |  |  |  |  |  |  |  |  |  |  |  |  |  |  |  |  |  |  |  |  |  |  |  |  |  |  |  |  |  |  |  |  |  |  |  |  |  |  |  |  |  |  |  |  |  |  |  |  |  |  |  |  |  |  |  |  |  |  |  |  |  |  |  |  |  |  |  |  |  |  |  |  |  |  |  |  |  |  |  |  |  |  |  |  |  |  |  |  |  |  |  |  |  |  |  |  |  |  |  |  |  |  |  |  |  |  |  |  |  |  |  |  |  |  |  |  |  |  |  |  |  |  |  |  |  |  |  |  |  |  |  |  |  |  |  |  |  |  |  |  |  |  |  |  |  |  |  |  |  |  |  |  |  |  |  |  |  |  |  |  |  |  |  |  |  |  |  |  |  |  |  |  |  |  |  |  |  |  |  |  |  |  |  |  |  |  |  |  |  |  |  |  |  |  |  |  |  |  |  |  |  |  |  |  |  |  |  |  |  |  |  |  |  |  |  |  |  |  |  |  |  |  |  |  |  |  |  |  |  |  |  |  |  |  |  |  |  |  |  |  |  |  |  |  |  |  |  |  |  |  |  |  |  |  |  |  |  |  |  |  |  |  |  |  |  |  |  |  |  |  |  |  |  |  |  |  |  |  |  |  |  |  |  |  |  |  |  |  |  |  |  |  |  |  |  |  |  |  |  |  |  |  |  |  |  |  |  |  |  |  |  |  |  |  |  |  |  |  |  |  |  |  |  |  |  |  |  |  |  |  |  |  |  |  |  |  |  |  |  |  |  |  |  |  |  |  |  |  |  |  |  |  |  |  |  |  |  |  |  |  |  |  |  |  |  |  |  |  |  |  |  |  |  |  |  |  |  |  |  |  |  |  |  |  |  |  |  |  |  |  |  |  |  |  |  |  |  |  |  |  |  |  |  |  |  |  |  |  |  |  |  |  |  |  |  |  |  |  |  |  |  |  |  |  |  |  |  |  |  |  |  |  |  |  |  |  |  |  |  |  |  |  |  |  |  |  |  |  |  |  |  |  |  |  |  |  |  |  |  |  |  |  |  |  |  |  |  |  |  |  |  |  |  |  |  |  |  |  |  |  |  |  |  |  |  |  |  |  |  |  |  |  |  |  |  |  |  |  |  |  |  |  |  |  |  |  |  |  |  |  |  |  |  |  |  |  |  |  |  |  |  |  |  |  |  |  |  |  |  |  |  |  |  |  |  |  |  |  |  |  |  |  |  |  |  |  |  |  |  |  |  |  |  |  |  |  |  |  |  |  |  |  |  |  |  |  |  |  |  |  |  |  |  |  |  |  |  |  |  |  |  |  |  |  |  |  |  |  |  |  |  |  |  |  |  |  |  |  |  |  |  |  |  |  |  |  |  |  |  |  |  |  |  |  |  |  |  |  |  |  |  |  |  |  |  |  |  |  |  |  |  |  |  |  |  |  |  |  |  |  |  |  |  |  |  |  |  |  |  |  |  |  |  |  |  |  |  |  |  |  |  |  |  |  |  |  |  |  |  |  |  |  |  |  |  |  |  |  |  |  |  |  |  |  |  |  |  |  |  |  |  |  |  |  |  |  |  |  |  |  |  |  |  |  |  |  |  |  |  |  |  |  |  |  |  |  |  |  |  |  |  |  |  |  |  |  |  |  |  |  |  |  |  |  |  |  |  |  |  |  |  |  |  |  |  |  |  |  |  |  |  |  |  |  |  |  |  |  |  |  |  |  |  |  |  |  |  |  |  |  |  |  |  |  |  |  |  |  |  |  |  |  |  |  |  |  |  |  |  |  |  |  |  |  |  |  |  |  |  |  |  |  |  |  |  |  |  |  |  |  |  |  |  |  |  |  |  |  |  |  |  |  |  |  |  |  |  |  |  |  |  |  |  |  |  |  |  |  |  |  |  |  |  |  |  |  |  |  |  |  |  |  |  |  |  |  |  |  |  |  |  |  |  |  |  |  |  |  |  |  |  |  |  |  |  |  |  |  |  |  |  |  |  |  |  |  |  |  |  |  |  |  |  |  |  |  |  |  |  |  |  |  |  |  |  |  |  |  |  |  |  |  |  |  |  |  |  |  |  |  |  |  |  |  |  |  |  |  |  |  |  |  |  |  |  |  |  |  |  |  |  |  |  |  |  |  |  |  |  |  |  |  |  |  |  |  |  |  |  |  |  |  |  |  |  |  |  |  |  |  |  |  |  |  |  |  |  |  |  |  |  |  |  |  |  |  |  |  |  |  |  |  |  |  |  |  |  |  |  |  |  |  |  |  |  |  |  |  |  |  |  |  |  |  |  |  |  |  |  |  |  |  |  |  |  |  |  |  |  |  |  |  |  |  |  |  |  |  |  |  |  |  |  |  |  |  |  |  |  |  |  |  |  |  |  |  |  |  |  |  |  |  |  |  |  |  |  |  |  |  |  |  |  |  |  |  |  |  |  |  |  |  |  |  |  |  |  |  |  |  |  |  |  |  |  |  |  |  |  |  |  |  |  |  |  |  |  |  |  |  |  |  |  |  |  |  |  |  |  |  |  |  |  |  |  |  |  |  |  |  |  |  |  |  |  |  |  |  |  |  |  |  |  |  |  |  |  |  |  |  |  |  |  |  |  |  |  |  |  |  |  |  |  |  |  |  |  |  |  |  |  |  |  |  |  |  |  |  |  |  |  |  |  |  |  |  |  |  |  |  |  |  |  |  |  |  |  |  |  |  |  |  |  |  |  |  |  |  |  |  |  |  |  |  |  |  |  |  |  |  |  |  |  |  |  |  |  |  |  |  |  |  |  |  |  |  |  |  |  |  |  |  |  |  |  |  |  |  |  |  |  |  |  |  |  |  |  |  |  |  |  |  |  |  |  |  |  |  |  |  |  |  |  |  |  |  |  |  |  |  |  |  |  |  |  |  |  |  |  |  |  |  |  |  |  |  |  |  |  |  |  |  |  |  |  |  |  |  |  |  |  |  |  |  |  |  |  |  |  |  |  |  |  |  |  |  |  |  |  |  |  |  |  |  |  |  |  |  |  |  |  |  |  |  |  |  |  |  |  |  |  |  |  |  |  |  |  |  |  |  |  |  |  |  |  |  |  |  |  |  |  |  |  |  |  |  |  |  |  |  |  |  |  |  |  |  |  |  |  |  |  |  |  |  |  |  |  |  |  |  |  |  |  |  |  |  |  |  |  |  |  |  |  |  |  |  |  |  |  |  |  |  |  |  |  |  |  |  |  |  |  |  |  |  |  |  |  |  |  |  |  |  |  |  |  |  |  |  |  |  |  |  |  |  |  |  |  |  |  |  |  |  |  |  |  |  |  |  |  |  |  |  |  |  |  |  |  |  |  |  |  |  |  |  |  |  |  |  |  |  |  |  |  |  |  |  |  |  |  |  |  |  |  |  |  |  |  |  |  |  |  |  |  |  |  |  |  |  |  |  |  |  |  |  |  |  |  |  |  |  |  |  |  |  |  |  |  |  |  |  |  |  |  |  |  |  |  |  |  |  |  |  |  |  |  |  |  |  |  |  |  |  |  |  |  |  |  |  |  |  |  |  |  |  |  |  |  |  |  |  |  |  |  |  |  |  |  |  |  |  |  |  |  |  |  |  |  |  |  |  |  |  |  |  |  |  |  |  |  |  |  |  |  |  |  |  |  |  |  |  |  |  |  |  |  |  |  |  |  |  |  |  |  |  |  |  |  |  |  |  |  |  |  |  |  |  |  |  |  |  |  |  |  |  |  |  |  |  |  |  |  |  |  |  |  |  |  |  |  |  |  |  |  |  |  |  |  |  |  |  |  |  |  |  |  |  |  |  |  |  |  |  |  |  |  |  |  |  |  |  |  |  |  |  |  |  |  |  |  |  |  |  |  |  |  |  |  |  |  |  |  |  |  |  |  |  |  |  |  |  |  |  |  |  |  |  |  |  |  |  |  |  |  |  |  |  |  |  |  |  |  |  |  |  |  |  |  |  |  |  |  |  |  |  |  |  |  |  |  |  |  |  |  |  |  |  |  |  |  |  |  |  |  |  |  |  |  |  |  |  |  |  |  |  |  |  |  |  |  |  |  |  |  |  |  |  |  |  |  |  |  |  |  |  |  |  |  |  |  |  |  |  |  |  |  |  |  |  |  |  |  |  |  |  |  |  |  |  |  |  |  |  |  |  |  |  |  |  |  |  |  |  |  |  |  |  |  |  |  |  |  |  |  |  |  |  |  |  |  |  |  |  |  |  |  |  |  |  |  |  |  |  |  |  |  |  |  |  |  |  |  |  |  |  |  |  |  |  |  |  |  |  |  |  |  |  |  |  |  |  |  |  |  |  |  |  |  |  |  |  |  |  |  |  |  |  |  |  |  |  |  |  |  |  |  |  |  |  |  |  |  |  |  |  |  |  |  |  |  |  |  |  |  |  |  |  |  |  |  |  |  |  |  |  |  |  |  |  |  |  |  |  |  |  |  |  |  |  |  |  |  |  |  |  |  |  |  |  |  |  |  |  |  |  |  |  |  |  |  |  |  |  |  |  |  |  |  |  |  |  |  |  |  |  |  |  |  |  |  |  |  |  |  |  |  |  |  |  |  |  |  |  |  |  |  |  |  |  |  |  |  |  |  |  |  |  |  |  |  |  |  |  |  |  |  |  |  |  |  |  |  |  |  |  |  |  |  |  |  |  |  |  |  |  |  |  |  |  |  |  |  |  |  |  |  |  |  |  |  |  |  |  |  |  |  |  |  |  |  |  |  |  |  |  |  |  |  |  |  |  |  |  |  |  |  |  |  |  |  |  |  |  |  |  |  |  |  |  |  |  |  |  |  |  |  |  |  |  |  |  |  |  |  |  |  |  |  |  |  |  |  |  |  |  |  |  |  |  |  |  |  |  |  |  |  |  |  |  |  |  |  |  |  |  |  |  |  |  |  |  |  |  |  |  |  |  |  |  |  |  |  |  |  |  |  |  |  |  |  |  |  |  |  |  |  |  |  |  |  |  |  |  |  |  |  |  |  |  |  |  |  |  |  |  |  |  |  |  |  |  |  |  |  |  |  |  |  |  |  |  |  |  |  |  |  |  |  |  |  |  |  |  |  |  |  |  |  |  |  |  |  |  |  |  |  |  |  |  |  |  |  |  |  |  |  |  |  |  |  |  |  |  |  |  |  |  |  |  |  |  |  |  |  |  |  |  |  |  |  |  |  |  |  |  |  |  |  |  |  |  |  |  |  |  |  |  |  |  |  |  |  |  |  |  |  |  |  |  |  |  |  |  |  |  |  |  |  |  |  |  |  |  |  |  |  |  |  |  |  |  |  |  |  |  |  |  |  |  |  |  |  |  |  |  |  |  |  |  |  |  |  |  |  |  |  |  |  |  |  |  |  |  |  |  |  |  |  |  |  |  |  |  |  |  |  |  |  |  |  |  |  |  |  |  |  |  |  |  |  |  |  |  |  |  |  |  |  |  |  |  |  |  |  |  |  |  |  |  |  |  |  |  |  |  |  |  |  |  |  |  |  |  |  |  |  |  |  |  |  |  |  |  |  |  |  |  |  |  |  |  |  |  |  |  |  |  |  |  |  |  |  |  |  |  |  |  |  |  |  |  |  |  |  |  |  |  |  |  |  |  |  |  |  |  |  |  |  |  |  |  |  |  |  |  |  |  |  |  |  |  |  |  |  |  |  |  |  |  |  |  |  |  |  |  |  |  |  |  |  |  |  |  |  |  |  |  |  |  |  |  |  |  |  |  |  |  |  |  |  |  |  |  |  |  |  |  |  |  |  |  |  |  |  |  |  |  |  |  |  |  |  |  |  |  |  |  |  |  |  |  |  |  |  |  |  |  |  |  |  |  |  |  |  |  |  |  |  |  |  |  |  |  |  |  |  |  |  |  |  |  |  |  |  |  |  |  |  |  |  |  |  |  |  |  |  |  |  |  |  |  |  |  |  |  |  |  |  |  |  |  |  |  |  |  |  |  |  |  |  |  |  |  |  |  |  |  |  |  |  |  |  |  |  |  |  |  |  |  |  |  |  |  |  |  |  |  |  |  |  |  |  |  |  |  |  |  |  |  |  |  |  |  |  |  |  |  |  |  |  |  |  |  |  |  |  |  |  |  |  |  |  |  |  |  |  |  |  |  |  |  |  |  |  |  |  |  |  |  |  |  |  |  |  |  |  |  |  |  |  |  |  |  |  |  |  |  |  |  |  |  |  |  |  |  |  |  |  |  |  |  |  |  |  |  |  |  |  |  |  |  |  |  |  |  |  |  |  |  |  |  |  |  |  |  |  |  |  |  |  |  |  |  |  |  |  |  |  |  |  |  |  |  |  |  |  |  |  |  |  |  |  |  |  |  |  |  |  |  |  |  |  |  |  |  |  |  |  |  |  |  |  |  |  |  |  |  |  |  |  |  |  |  |  |  |  |  |  |  |  |  |  |  |  |  |  |  |  |  |  |  |  |  |  |  |  |  |  |  |  |  |  |  |  |  |  |  |  |  |  |  |  |  |  |  |  |  |  |  |  |  |  |  |  |  |  |  |  |  |  |  |  |  |  |  |  |  |  |  |  |  |  |  |  |  |  |  |  |  |  |  |  |  |  |  |  |  |  |  |  |  |  |  |  |  |  |  |  |  |  |  |  |  |  |  |  |  |  |  |  |  |  |  |  |  |  |  |  |  |  |  |  |  |  |  |  |  |  |  |  |  |  |  |  |  |  |  |  |  |  |  |  |  |  |  |  |  |  |  |  |  |  |  |  |  |  |  |  |  |  |  |  |  |  |  |  |  |  |  |  |  |  |  |  |  |  |  |  |  |  |  |  |  |  |  |  |  |  |  |  |  |  |  |  |  |  |  |  |  |  |  |  |  |  |  |  |  |  |  |  |  |  |  |  |  |  |  |  |  |  |  |  |  |  |  |  |  |  |  |  |  |  |  |  |  |  |  |  |  |  |  |  |  |  |  |  |  |  |  |  |  |  |  |  |  |  |  |  |  |  |  |  |  |  |  |  |  |  |  |  |  |  |  |  |  |  |  |  |  |  |  |  |  |  |  |  |  |  |  |  |  |  |  |  |  |  |  |  |  |  |  |  |  |  |  |  |  |  |  |  |  |  |  |  |  |  |  |  |  |  |  |  |  |  |  |  |  |  |  |  |  |  |  |  |  |  |  |  |  |  |  |  |  |  |  |  |  |  |  |  |  |  |  |  |  |  |  |  |  |  |  |  |  |  |  |  |  |  |  |  |  |  |  |  |  |  |  |  |  |  |  |  |  |  |  |  |  |  |  |  |  |  |  |  |  |  |  |  |  |  |  |  |  |  |  |  |  |  |  |  |  |  |  |  |  |  |  |  |  |  |  |  |  |  |  |  |  |  |  |  |  |  |  |  |  |  |  |  |  |  |  |  |  |  |  |  |  |  |  |  |  |  |  |  |  |  |  |  |  |  |  |  |  |  |  |  |  |  |  |  |  |  |  |  |  |  |  |  |  |  |  |  |  |  |  |  |  |  |  |  |  |  |  |  |  |  |  |  |  |  |  |  |  |  |  |  |  |  |  |  |  |  |  |  |  |  |  |  |  |  |  |  |  |  |  |  |  |  |  |  |  |  |  |  |  |  |  |  |  |  |  |  |  |  |  |  |  |  |  |  |  |  |  |  |  |  |  |  |  |  |  |  |  |  |  |  |  |  |  |  |  |  |  |  |  |  |  |  |  |  |  |  |  |  |  |  |  |  |  |  |  |  |  |  |  |  |  |  |  |  |  |  |  |  |  |  |  |  |  |  |  |  |  |  |  |  |  |  |  |  |  |  |  |  |  |  |  |  |  |  |  |  |  |  |  |  |  |  |  |  |  |  |  |  |  |  |  |  |  |  |  |  |  |  |  |  |  |  |  |  |  |  |  |  |  |  |  |  |  |  |  |  |  |  |  |  |  |  |  |  |  |  |  |  |  |  |  |  |  |  |  |  |  |  |  |  |  |  |  |  |  |  |  |  |  |  |  |  |  |  |  |  |  |  |  |  |  |  |  |  |  |  |  |  |  |  |  |  |  |  |  |  |  |  |  |  |  |  |  |  |  |  |  |  |  |  |  |  |  |  |  |  |  |  |  |  |  |  |  |  |  |  |  |  |  |  |  |  |  |  |  |  |  |  |  |  |  |  |  |  |  |  |  |  |  |  |  |  |  |  |  |  |  |  |  |  |  |  |  |  |  |  |  |  |  |  |  |  |  |  |  |  |  |  |  |  |  |  |  |  |  |  |  |  |  |  |  |  |  |  |  |  |  |  |  |  |  |  |  |  |  |  |  |  |  |  |  |  |  |  |  |  |  |  |  |  |  |  |  |  |  |  |  |  |  |  |  |  |  |  |  |  |  |  |  |  |  |  |  |  |  |  |  |  |  |  |  |  |  |  |  |  |  |  |  |  |  |  |  |  |  |  |  |  |  |  |  |  |  |  |  |  |  |  |  |  |  |  |  |  |  |  |  |  |  |  |  |  |  |  |  |  |  |  |  |  |  |  |  |  |  |  |  |  |  |  |  |  |  |  |  |  |  |  |  |  |  |  |  |  |  |  |  |  |  |  |  |  |  |  |  |  |  |  |  |  |  |  |  |  |  |  |  |  |  |  |  |  |  |  |  |  |  |  |  |  |  |  |  |  |  |  |  |  |  |  |  |  |  |  |  |  |  |  |  |  |  |  |  |  |  |  |  |  |  |  |  |  |  |  |  |  |  |  |  |  |  |  |  |  |  |  |  |  |  |  |  |  |  |  |  |  |  |  |  |  |  |  |  |  |  |  |  |  |  |  |  |  |  |  |  |  |  |  |  |  |  |  |  |  |  |  |  |  |  |  |  |  |  |  |  |  |  |  |  |  |  |  |  |  |  |  |  |  |  |  |  |  |  |  |  |  |  |  |  |  |  |  |  |  |  |  |  |  |  |  |  |  |  |  |  |  |  |  |  |  |  |  |  |  |  |  |  |  |  |  |  |  |  |  |  |  |  |  |  |  |  |  |  |  |  |  |  |  |  |  |  |  |  |  |  |  |  |  |  |  |  |  |  |  |  |  |  |  |  |  |  |  |  |  |  |  |  |  |  |  |  |  |  |  |  |  |  |  |  |  |  |  |  |  |  |  |  |  |  |  |  |  |  |  |  |  |  |  |  |  |  |  |  |  |  |  |  |  |  |  |  |  |  |  |  |  |  |  |  |  |  |  |  |  |  |  |  |  |  |  |  |  |  |  |  |  |  |  |  |  |  |  |  |  |  |  |  |  |  |  |  |  |  |  |  |  |  |  |  |  |  |  |  |  |  |  |  |  |  |  |  |  |  |  |  |  |  |  |  |  |  |  |  |  |  |  |  |  |  |  |  |  |  |  |  |  |  |  |  |  |  |  |  |  |  |  |  |  |  |  |  |  |  |  |  |  |  |  |  |  |  |  |  |  |  |  |  |  |  |  |  |  |  |  |  |  |  |  |  |  |  |  |  |  |  |  |  |  |  |  |  |  |  |  |  |  |  |  |  |  |  |  |  |  |  |  |  |  |  |  |  |  |  |  |  |  |  |  |  |  |  |  |  |  |  |  |  |  |  |  |  |  |  |  |  |  |  |  |  |  |  |  |  |  |  |  |  |  |  |  |  |  |  |  |  |  |  |  |  |  |  |  |  |  |  |  |  |  |  |  |  |  |  |  |  |  |  |  |  |  |  |  |  |  |  |  |  |  |  |  |  |  |  |  |  |  |  |  |  |  |  |  |  |  |  |  |  |  |  |  |  |  |  |  |  |  |  |  |  |  |  |  |  |  |  |  |  |  |  |  |  |  |  |  |  |  |  |  |  |  |  |  |  |  |  |  |  |  |  |  |  |  |  |  |  |  |  |  |  |  |  |  |  |  |  |  |  |  |  |  |  |  |  |  |  |  |  |  |  |  |  |  |  |  |  |  |  |  |  |  |  |  |  |  |  |  |  |  |  |  |  |  |  |  |  |  |  |  |  |  |  |  |  |  |  |  |  |  |  |  |  |  |  |  |  |  |  |  |  |  |  |  |  |  |  |  |  |  |  |  |  |  |  |  |  |  |  |  |  |  |  |  |  |  |  |  |  |  |  |  |  |  |  |  |  |  |  |  |  |  |  |  |  |  |  |  |  |  |  |  |  |  |  |  |  |  |  |  |  |  |  |  |  |  |  |  |  |  |  |  |  |  |  |  |  |  |  |  |  |  |  |  |  |  |  |  |  |  |  |  |  |  |  |  |  |  |  |  |  |  |  |  |  |  |  |  |  |  |  |  |  |  |  |  |  |  |  |  |  |  |  |  |  |  |  |  |  |  |  |  |  |  |  |  |  |  |  |  |  |  |  |  |  |  |  |  |  |  |  |  |  |  |  |  |  |  |  |  |  |  |  |  |  |  |  |  |  |  |  |  |  |  |  |  |  |  |  |  |  |  |  |  |  |  |  |  |  |  |  |  |  |  |  |  |  |  |  |  |  |  |  |  |  |  |  |  |  |  |  |  |  |  |  |  |  |  |  |  |  |  |  |  |  |  |  |  |  |  |  |  |  |  |  |  |  |  |  |  |  |  |  |  |  |  |  |  |  |  |  |  |  |  |  |  |  |  |  |  |  |  |  |  |  |  |  |  |  |  |  |  |  |  |  |  |  |  |  |  |  |  |  |  |  |  |  |  |  |  |  |  |  |  |  |  |  |  |  |  |  |  |  |  |  |  |  |  |  |  |  |  |  |  |  |  |  |  |  |  |  |  |  |  |  |  |  |  |  |  |  |  |  |  |  |  |  |  |  |  |  |  |  |  |  |  |  |  |  |  |  |  |  |  |  |  |  |  |  |  |  |  |  |  |  |  |  |  |  |  |  |  |  |  |  |  |  |  |  |  |  |  |  |  |  |  |  |  |  |  |  |  |  |  |  |  |  |  |  |  |  |  |  |  |  |  |  |  |  |  |  |  |  |  |  |  |  |  |  |  |  |  |  |  |  |  |  |  |  |  |  |  |  |  |  |  |  |  |  |  |  |  |  |  |  |  |  |  |  |  |  |  |  |  |  |  |  |  |  |  |  |  |  |  |  |  |  |  |  |  |  |  |  |  |  |  |  |  |  |  |  |  |  |  |  |  |  |  |  |  |  |  |  |  |  |  |  |  |  |  |  |  |  |  |  |  |  |  |  |  |  |  |  |  |  |  |  |  |  |  |  |  |  |  |  |  |  |  |  |  |  |  |  |  |  |  |  |  |  |  |  |  |  |  |  |  |  |  |  |  |  |  |  |  |  |  |  |  |  |  |  |  |  |  |  |  |  |  |  |  |  |  |  |  |  |  |  |  |  |  |  |  |  |  |  |  |  |  |  |  |  |  |  |  |  |  |  |  |  |  |  |  |  |  |  |  |  |  |  |  |  |  |  |  |  |  |  |  |  |  |  |  |  |  |  |  |  |  |  |  |  |  |  |  |  |  |  |  |  |  |  |  |  |  |  |  |  |  |  |  |  |  |  |  |  |  |  |  |  |  |  |  |  |  |  |  |  |  |  |  |  |  |  |  |  |  |  |  |  |  |  |  |  |  |  |  |  |  |  |  |  |  |  |  |  |  |  |  |  |  |  |  |  |  |  |  |  |  |  |  |  |  |  |  |  |  |  |  |  |  |  |  |  |  |  |  |  |  |  |  |  |  |  |  |  |  |  |  |  |  |  |  |  |  |  |  |  |  |  |  |  |  |  |  |  |  |  |  |  |  |  |  |  |  |  |  |  |  |  |  |  |  |  |  |  |  |  |  |  |  |  |  |  |  |  |  |  |  |  |  |  |  |  |  |  |  |  |  |  |  |  |  |  |  |  |  |  |  |  |  |  |  |  |  |  |  |  |  |  |  |  |  |  |  |  |  |  |  |  |  |  |  |  |  |  |  |  |  |  |  |  |  |  |  |  |  |  |  |  |  |  |  |  |  |  |  |  |  |  |  |  |  |  |  |  |  |  |  |  |  |  |  |  |  |  |  |  |  |  |  |  |  |  |  |  |  |  |  |  |  |  |  |  |  |  |  |  |  |  |  |  |  |  |  |  |  |  |  |  |  |  |  |  |  |  |  |  |  |  |  |  |  |  |  |  |  |  |  |  |  |  |  |  |  |  |  |  |  |  |  |  |  |  |  |  |  |  |  |  |  |  |  |  |  |  |  |  |  |  |  |  |  |  |  |  |  |  |  |  |  |  |  |  |  |  |  |  |  |  |  |  |  |  |  |  |  |  |  |  |  |  |  |  |  |  |  |  |  |  |  |  |  |  |  |  |  |  |  |  |  |  |  |  |  |  |  |  |  |  |  |  |  |  |  |  |  |  |  |  |  |  |  |  |  |  |  |  |  |  |  |  |  |  |  |  |  |  |  |  |  |  |  |  |  |  |  |  |  |  |  |  |  |  |  |  |  |  |  |  |  |  |  |  |  |  |  |  |  |  |  |  |  |  |  |  |  |  |  |  |  |  |  |  |  |  |  |  |  |  |  |  |  |  |  |  |  |  |  |  |  |  |  |  |  |  |  |  |  |  |  |  |  |  |  |  |  |  |  |  |  |  |  |  |  |  |  |  |  |  |  |  |  |  |  |  |  |  |  |  |  |  |  |  |  |  |  |  |  |  |  |  |  |  |  |  |  |  |  |  |  |  |  |  |  |  |  |  |  |  |  |  |  |  |  |  |  |  |  |  |  |  |  |  |  |  |  |  |  |  |  |  |  |  |  |  |  |  |  |  |  |  |  |  |  |  |  |  |  |  |  |  |  |  |  |  |  |  |  |  |  |  |  |  |  |  |  |  |  |  |  |  |  |  |  |  |  |  |  |  |  |  |  |  |  |  |  |  |  |  |  |  |  |  |  |  |  |  |  |  |  |  |  |  |  |  |  |  |  |  |  |  |  |  |  |  |  |  |  |  |  |  |  |  |  |  |  |  |  |  |  |  |  |  |  |  |  |  |  |  |  |  |  |  |  |  |  |  |  |  |  |  |  |  |  |  |  |  |  |  |  |  |  |  |  |  |  |  |  |  |  |  |  |  |  |  |  |  |  |  |  |  |  |  |  |  |  |  |  |  |  |  |  |  |  |  |  |  |  |  |  |  |  |  |  |  |  |  |  |  |  |  |  |  |  |  |  |  |  |  |  |  |  |  |  |  |  |  |  |  |  |  |  |  |  |  |  |  |  |  |  |  |  |  |  |  |  |  |  |  |  |  |  |  |  |  |  |  |  |  |  |  |  |  |  |  |  |  |  |  |  |  |  |  |  |  |  |  |  |  |  |  |  |  |  |  |  |  |  |  |  |  |  |  |  |  |  |  |  |  |  |  |  |  |  |  |  |  |  |  |  |  |  |  |  |  |  |  |  |  |  |  |  |  |  |  |  |  |  |  |  |  |  |  |  |  |  |  |  |  |  |  |  |  |  |  |  |  |  |  |  |  |  |  |  |  |  |  |  |  |  |  |  |  |  |  |  |  |  |  |  |  |  |  |  |  |  |  |  |  |  |  |  |  |  |  |  |  |  |  |  |  |  |  |  |  |  |  |  |  |  |  |  |  |  |  |  |  |  |  |  |  |  |  |  |  |  |  |  |  |  |  |  |  |  |  |  |  |  |  |  |  |  |  |  |  |  |  |  |  |  |  |  |  |  |  |  |  |  |  |  |  |  |  |  |  |  |  |  |  |  |  |  |  |  |  |  |  |  |  |  |  |  |  |  |  |  |  |  |  |  |  |  |  |  |  |  |  |  |  |  |  |  |  |  |  |  |  |  |  |  |  |  |  |  |  |  |  |  |  |  |  |  |  |  |  |  |  |  |  |  |  |  |  |  |  |  |  |  |  |  |  |  |  |  |  |  |  |  |  |  |  |  |  |  |  |  |  |  |  |  |  |  |  |  |  |  |  |  |  |  |  |  |  |  |  |  |  |  |  |  |  |  |  |  |  |  |  |  |  |  |  |  |  |  |  |  |  |  |  |  |  |  |  |  |  |  |  |  |  |  |  |  |  |  |  |  |  |  |  |  |  |  |  |  |  |  |  |  |  |  |  |  |  |  |  |  |  |  |  |  |  |  |  |  |  |  |  |  |  |  |  |  |  |  |  |  |  |  |  |  |  |  |  |  |  |  |  |  |  |  |  |  |  |
| --- | --- | --- | --- | --- | --- | --- | --- | --- | --- | --- | --- | --- | --- | --- | --- | --- | --- | --- | --- | --- | --- | --- | --- | --- | --- | --- | --- | --- | --- | --- | --- | --- | --- | --- | --- | --- | --- | --- | --- | --- | --- | --- | --- | --- | --- | --- | --- | --- | --- | --- | --- | --- | --- | --- | --- | --- | --- | --- | --- | --- | --- | --- | --- | --- | --- | --- | --- | --- | --- | --- | --- | --- | --- | --- | --- | --- | --- | --- | --- | --- | --- | --- | --- | --- | --- | --- | --- | --- | --- | --- | --- | --- | --- | --- | --- | --- | --- | --- | --- | --- | --- | --- | --- | --- | --- | --- | --- | --- | --- | --- | --- | --- | --- | --- | --- | --- | --- | --- | --- | --- | --- | --- | --- | --- | --- | --- | --- | --- | --- | --- | --- | --- | --- | --- | --- | --- | --- | --- | --- | --- | --- | --- | --- | --- | --- | --- | --- | --- | --- | --- | --- | --- | --- | --- | --- | --- | --- | --- | --- | --- | --- | --- | --- | --- | --- | --- | --- | --- | --- | --- | --- | --- | --- | --- | --- | --- | --- | --- | --- | --- | --- | --- | --- | --- | --- | --- | --- | --- | --- | --- | --- | --- | --- | --- | --- | --- | --- | --- | --- | --- | --- | --- | --- | --- | --- | --- | --- | --- | --- | --- | --- | --- | --- | --- | --- | --- | --- | --- | --- | --- | --- | --- | --- | --- | --- | --- | --- | --- | --- | --- | --- | --- | --- | --- | --- | --- | --- | --- | --- | --- | --- | --- | --- | --- | --- | --- | --- | --- | --- | --- | --- | --- | --- | --- | --- | --- | --- | --- | --- | --- | --- | --- | --- | --- | --- | --- | --- | --- | --- | --- | --- | --- | --- | --- | --- | --- | --- | --- | --- | --- | --- | --- | --- | --- | --- | --- | --- | --- | --- | --- | --- | --- | --- | --- | --- | --- | --- | --- | --- | --- | --- | --- | --- | --- | --- | --- | --- | --- | --- | --- | --- | --- | --- | --- | --- | --- | --- | --- | --- | --- | --- | --- | --- | --- | --- | --- | --- | --- | --- | --- | --- | --- | --- | --- | --- | --- | --- | --- | --- | --- | --- | --- | --- | --- | --- | --- | --- | --- | --- | --- | --- | --- | --- | --- | --- | --- | --- | --- | --- | --- | --- | --- | --- | --- | --- | --- | --- | --- | --- | --- | --- | --- | --- | --- | --- | --- | --- | --- | --- | --- | --- | --- | --- | --- | --- | --- | --- | --- | --- | --- | --- | --- | --- | --- | --- | --- | --- | --- | --- | --- | --- | --- | --- | --- | --- | --- | --- | --- | --- | --- | --- | --- | --- | --- | --- | --- | --- | --- | --- | --- | --- | --- | --- | --- | --- | --- | --- | --- | --- | --- | --- | --- | --- | --- | --- | --- | --- | --- | --- | --- | --- | --- | --- | --- | --- | --- | --- | --- | --- | --- | --- | --- | --- | --- | --- | --- | --- | --- | --- | --- | --- | --- | --- | --- | --- | --- | --- | --- | --- | --- | --- | --- | --- | --- | --- | --- | --- | --- | --- | --- | --- | --- | --- | --- | --- | --- | --- | --- | --- | --- | --- | --- | --- | --- | --- | --- | --- | --- | --- | --- | --- | --- | --- | --- | --- | --- | --- | --- | --- | --- | --- | --- | --- | --- | --- | --- | --- | --- | --- | --- | --- | --- | --- | --- | --- | --- | --- | --- | --- | --- | --- | --- | --- | --- | --- | --- | --- | --- | --- | --- | --- | --- | --- | --- | --- | --- | --- | --- | --- | --- | --- | --- | --- | --- | --- | --- | --- | --- | --- | --- | --- | --- | --- | --- | --- | --- | --- | --- | --- | --- | --- | --- | --- | --- | --- | --- | --- | --- | --- | --- | --- | --- | --- | --- | --- | --- | --- | --- | --- | --- | --- | --- | --- | --- | --- | --- | --- | --- | --- | --- | --- | --- | --- | --- | --- | --- | --- | --- | --- | --- | --- | --- | --- | --- | --- | --- | --- | --- | --- | --- | --- | --- | --- | --- | --- | --- | --- | --- | --- | --- | --- | --- | --- | --- | --- | --- | --- | --- | --- | --- | --- | --- | --- | --- | --- | --- | --- | --- | --- | --- | --- | --- | --- | --- | --- | --- | --- | --- | --- | --- | --- | --- | --- | --- | --- | --- | --- | --- | --- | --- | --- | --- | --- | --- | --- | --- | --- | --- | --- | --- | --- | --- | --- | --- | --- | --- | --- | --- | --- | --- | --- | --- | --- | --- | --- | --- | --- | --- | --- | --- | --- | --- | --- | --- | --- | --- | --- | --- | --- | --- | --- | --- | --- | --- | --- | --- | --- | --- | --- | --- | --- | --- | --- | --- | --- | --- | --- | --- | --- | --- | --- | --- | --- | --- | --- | --- | --- | --- | --- | --- | --- | --- | --- | --- | --- | --- | --- | --- | --- | --- | --- | --- | --- | --- | --- | --- | --- | --- | --- | --- | --- | --- | --- | --- | --- | --- | --- | --- | --- | --- | --- | --- | --- | --- | --- | --- | --- | --- | --- | --- | --- | --- | --- | --- | --- | --- | --- | --- | --- | --- | --- | --- | --- | --- | --- | --- | --- | --- | --- | --- | --- | --- | --- | --- | --- | --- | --- | --- | --- | --- | --- | --- | --- | --- | --- | --- | --- | --- | --- | --- | --- | --- | --- | --- | --- | --- | --- | --- | --- | --- | --- | --- | --- | --- | --- | --- | --- | --- | --- | --- | --- | --- | --- | --- | --- | --- | --- | --- | --- | --- | --- | --- | --- | --- | --- | --- | --- | --- | --- | --- | --- | --- | --- | --- | --- | --- | --- | --- | --- | --- | --- | --- | --- | --- | --- | --- | --- | --- | --- | --- | --- | --- | --- | --- | --- | --- | --- | --- | --- | --- | --- | --- | --- | --- | --- | --- | --- | --- | --- | --- | --- | --- | --- | --- | --- | --- | --- | --- | --- | --- | --- | --- | --- | --- | --- | --- | --- | --- | --- | --- | --- | --- | --- | --- | --- | --- | --- | --- | --- | --- | --- | --- | --- | --- | --- | --- | --- | --- | --- | --- | --- | --- | --- | --- | --- | --- | --- | --- | --- | --- | --- | --- | --- | --- | --- | --- | --- | --- | --- | --- | --- | --- | --- | --- | --- | --- | --- | --- | --- | --- | --- | --- | --- | --- | --- | --- | --- | --- | --- | --- | --- | --- | --- | --- | --- | --- | --- | --- | --- | --- | --- | --- | --- | --- | --- | --- | --- | --- | --- | --- | --- | --- | --- | --- | --- | --- | --- | --- | --- | --- | --- | --- | --- | --- | --- | --- | --- | --- | --- | --- | --- | --- | --- | --- | --- | --- | --- | --- | --- | --- | --- | --- | --- | --- | --- | --- | --- | --- | --- | --- | --- | --- | --- | --- | --- | --- | --- | --- | --- | --- | --- | --- | --- | --- | --- | --- | --- | --- | --- | --- | --- | --- | --- | --- | --- | --- | --- | --- | --- | --- | --- | --- | --- | --- | --- | --- | --- | --- | --- | --- | --- | --- | --- | --- | --- | --- | --- | --- | --- | --- | --- | --- | --- | --- | --- | --- | --- | --- | --- | --- | --- | --- | --- | --- | --- | --- | --- | --- | --- | --- | --- | --- | --- | --- | --- | --- | --- | --- | --- | --- | --- | --- | --- | --- | --- | --- | --- | --- | --- | --- | --- | --- | --- | --- | --- | --- | --- | --- | --- | --- | --- | --- | --- | --- | --- | --- | --- | --- | --- | --- | --- | --- | --- | --- | --- | --- | --- | --- | --- | --- | --- | --- | --- | --- | --- | --- | --- | --- | --- | --- | --- | --- | --- | --- | --- | --- | --- | --- | --- | --- | --- | --- | --- | --- | --- | --- | --- | --- | --- | --- | --- | --- | --- | --- | --- | --- | --- | --- | --- | --- | --- | --- | --- | --- | --- | --- | --- | --- | --- | --- | --- | --- | --- | --- | --- | --- | --- | --- | --- | --- | --- | --- | --- | --- | --- | --- | --- | --- | --- | --- | --- | --- | --- | --- | --- | --- | --- | --- | --- | --- | --- | --- | --- | --- | --- | --- | --- | --- | --- | --- | --- | --- | --- | --- | --- | --- | --- | --- | --- | --- | --- | --- | --- | --- | --- | --- | --- | --- | --- | --- | --- | --- | --- | --- | --- | --- | --- | --- | --- | --- | --- | --- | --- | --- | --- | --- | --- | --- | --- | --- | --- | --- | --- | --- | --- | --- | --- | --- | --- | --- | --- | --- | --- | --- | --- | --- | --- | --- | --- | --- | --- | --- | --- | --- | --- | --- | --- | --- | --- | --- | --- | --- | --- | --- | --- | --- | --- | --- | --- | --- | --- | --- | --- | --- | --- | --- | --- | --- | --- | --- | --- | --- | --- | --- | --- | --- | --- | --- | --- | --- | --- | --- | --- | --- | --- | --- | --- | --- | --- | --- | --- | --- | --- | --- | --- | --- | --- | --- | --- | --- | --- | --- | --- | --- | --- | --- | --- | --- | --- | --- | --- | --- | --- | --- | --- | --- | --- | --- | --- | --- | --- | --- | --- | --- | --- | --- | --- | --- | --- | --- | --- | --- | --- | --- | --- | --- | --- | --- | --- | --- | --- | --- | --- | --- | --- | --- | --- | --- | --- | --- | --- | --- | --- | --- | --- | --- | --- | --- | --- | --- | --- | --- | --- | --- | --- | --- | --- | --- | --- | --- | --- | --- | --- | --- | --- | --- | --- | --- | --- | --- | --- | --- | --- | --- | --- | --- | --- | --- | --- | --- | --- | --- | --- | --- | --- | --- | --- | --- | --- | --- | --- | --- | --- | --- | --- | --- | --- | --- | --- | --- | --- | --- | --- | --- | --- | --- | --- | --- | --- | --- | --- | --- | --- | --- | --- | --- | --- | --- | --- | --- | --- | --- | --- | --- | --- | --- | --- | --- | --- | --- | --- | --- | --- | --- | --- | --- | --- | --- | --- | --- | --- | --- | --- | --- | --- | --- | --- | --- | --- | --- | --- | --- | --- | --- | --- | --- | --- | --- | --- | --- | --- | --- | --- | --- | --- | --- | --- | --- | --- | --- | --- | --- | --- | --- | --- | --- | --- | --- | --- | --- | --- | --- | --- | --- | --- | --- | --- | --- | --- | --- | --- | --- | --- | --- | --- | --- | --- | --- | --- | --- | --- | --- | --- | --- | --- | --- | --- | --- | --- | --- | --- | --- | --- | --- | --- | --- | --- | --- | --- | --- | --- | --- | --- | --- | --- | --- | --- | --- | --- | --- | --- | --- | --- | --- | --- | --- | --- | --- | --- | --- | --- | --- | --- | --- | --- | --- | --- | --- | --- | --- | --- | --- | --- | --- | --- | --- | --- | --- | --- | --- | --- | --- | --- | --- | --- | --- | --- | --- | --- | --- | --- | --- | --- | --- | --- | --- | --- | --- | --- | --- | --- | --- | --- | --- | --- | --- | --- | --- | --- | --- | --- | --- | --- | --- | --- | --- | --- | --- | --- | --- | --- | --- | --- | --- | --- | --- | --- | --- | --- | --- | --- | --- | --- | --- | --- | --- | --- | --- | --- | --- | --- | --- | --- | --- | --- | --- | --- | --- | --- | --- | --- | --- | --- | --- | --- | --- | --- | --- | --- | --- | --- | --- | --- | --- | --- | --- | --- | --- | --- | --- | --- | --- | --- | --- | --- | --- | --- | --- | --- | --- | --- | --- | --- | --- | --- | --- | --- | --- | --- | --- | --- | --- | --- | --- | --- | --- | --- | --- | --- | --- | --- | --- | --- | --- | --- | --- | --- | --- | --- | --- | --- | --- | --- | --- | --- | --- | --- | --- | --- | --- | --- | --- | --- | --- | --- | --- | --- | --- | --- | --- | --- | --- | --- | --- | --- | --- | --- | --- | --- | --- | --- | --- | --- | --- | --- | --- | --- | --- | --- | --- | --- | --- | --- | --- | --- | --- | --- | --- | --- | --- | --- | --- | --- | --- | --- | --- | --- | --- | --- | --- | --- | --- | --- | --- | --- | --- | --- | --- | --- | --- | --- | --- | --- | --- | --- | --- | --- | --- | --- | --- | --- | --- | --- | --- | --- | --- | --- | --- | --- | --- | --- | --- | --- | --- | --- | --- | --- | --- | --- | --- | --- | --- | --- | --- | --- | --- | --- | --- | --- | --- | --- | --- | --- | --- | --- | --- | --- | --- | --- | --- | --- | --- | --- | --- | --- | --- | --- | --- | --- | --- | --- | --- | --- | --- | --- | --- | --- | --- | --- | --- | --- | --- | --- | --- | --- | --- | --- | --- | --- | --- | --- | --- | --- | --- | --- | --- | --- | --- | --- | --- | --- | --- | --- | --- | --- | --- | --- | --- | --- | --- | --- | --- | --- | --- | --- | --- | --- | --- | --- | --- | --- | --- | --- | --- | --- | --- | --- | --- | --- | --- | --- | --- | --- | --- | --- | --- | --- | --- | --- | --- | --- | --- | --- | --- | --- | --- | --- | --- | --- | --- | --- | --- | --- | --- | --- | --- | --- | --- | --- | --- | --- | --- | --- | --- | --- | --- | --- | --- | --- | --- | --- | --- | --- | --- | --- | --- | --- | --- | --- | --- | --- | --- | --- | --- | --- | --- | --- | --- | --- | --- | --- | --- | --- | --- | --- | --- | --- | --- | --- | --- | --- | --- | --- | --- | --- | --- | --- | --- | --- | --- | --- | --- | --- | --- | --- | --- | --- | --- | --- | --- | --- | --- | --- | --- | --- | --- | --- | --- | --- | --- | --- | --- | --- | --- | --- | --- | --- | --- | --- | --- | --- | --- | --- | --- | --- | --- | --- | --- | --- | --- | --- | --- | --- | --- | --- | --- | --- | --- | --- | --- | --- | --- | --- | --- | --- | --- | --- | --- | --- | --- | --- | --- | --- | --- | --- | --- | --- | --- | --- | --- | --- | --- | --- | --- | --- | --- | --- | --- | --- | --- | --- | --- | --- | --- | --- | --- | --- | --- | --- | --- | --- | --- | --- | --- | --- | --- | --- | --- | --- | --- | --- | --- | --- | --- | --- | --- | --- | --- | --- | --- | --- | --- | --- | --- | --- | --- | --- | --- | --- | --- | --- | --- | --- | --- | --- | --- | --- | --- | --- | --- | --- | --- | --- | --- | --- | --- | --- | --- | --- | --- | --- | --- | --- | --- | --- | --- | --- | --- | --- | --- | --- | --- | --- | --- | --- | --- | --- | --- | --- | --- | --- | --- | --- | --- | --- | --- | --- | --- | --- | --- | --- | --- | --- | --- | --- | --- | --- | --- | --- | --- | --- | --- | --- | --- | --- | --- | --- | --- | --- | --- | --- | --- | --- | --- | --- | --- | --- | --- | --- | --- | --- | --- | --- | --- | --- | --- | --- | --- | --- | --- | --- | --- | --- | --- | --- | --- | --- | --- | --- | --- | --- | --- | --- | --- | --- | --- | --- | --- | --- | --- | --- | --- | --- | --- | --- | --- | --- | --- | --- | --- | --- | --- | --- | --- | --- | --- | --- | --- | --- | --- | --- | --- | --- | --- | --- | --- | --- | --- | --- | --- | --- | --- | --- | --- | --- | --- | --- | --- | --- | --- | --- | --- | --- | --- | --- | --- | --- | --- | --- | --- | --- | --- | --- | --- | --- | --- | --- | --- | --- | --- | --- | --- | --- | --- | --- | --- | --- | --- | --- | --- | --- | --- | --- | --- | --- | --- | --- | --- | --- | --- | --- | --- | --- | --- | --- | --- | --- | --- | --- | --- | --- | --- | --- | --- | --- | --- | --- | --- | --- | --- | --- | --- | --- | --- | --- | --- | --- | --- | --- | --- | --- | --- | --- | --- | --- | --- | --- | --- | --- | --- | --- | --- | --- | --- | --- | --- | --- | --- | --- | --- | --- | --- | --- | --- | --- | --- | --- | --- | --- | --- | --- | --- | --- | --- | --- | --- | --- | --- | --- | --- | --- | --- | --- | --- | --- | --- | --- | --- | --- | --- | --- | --- | --- | --- | --- | --- | --- | --- | --- | --- | --- | --- | --- | --- | --- | --- | --- | --- | --- | --- | --- | --- | --- | --- | --- | --- | --- | --- | --- | --- | --- | --- | --- | --- | --- | --- | --- | --- | --- | --- | --- | --- | --- | --- | --- | --- | --- | --- | --- | --- | --- | --- | --- | --- | --- | --- | --- | --- | --- | --- | --- | --- | --- | --- | --- | --- | --- | --- | --- | --- | --- | --- | --- | --- | --- | --- | --- | --- | --- | --- | --- | --- | --- | --- | --- | --- | --- | --- | --- | --- | --- | --- | --- | --- | --- | --- | --- | --- | --- | --- | --- | --- | --- | --- | --- | --- | --- | --- | --- | --- | --- | --- | --- | --- | --- | --- | --- | --- | --- | --- | --- | --- | --- | --- | --- | --- | --- | --- | --- | --- | --- | --- | --- | --- | --- | --- | --- | --- | --- | --- | --- | --- | --- | --- | --- | --- | --- | --- | --- | --- | --- | --- | --- | --- | --- | --- | --- | --- | --- | --- | --- | --- | --- | --- | --- | --- | --- | --- | --- | --- | --- | --- | --- | --- | --- | --- | --- | --- | --- | --- | --- | --- | --- | --- | --- | --- | --- | --- | --- | --- | --- | --- | --- | --- | --- | --- | --- | --- | --- | --- | --- | --- | --- | --- | --- | --- | --- | --- | --- | --- | --- | --- | --- | --- | --- | --- | --- | --- | --- | --- | --- | --- | --- | --- | --- | --- | --- | --- | --- | --- | --- | --- | --- | --- | --- | --- | --- | --- | --- | --- | --- | --- | --- | --- | --- | --- | --- | --- | --- | --- | --- | --- | --- | --- | --- | --- | --- | --- | --- | --- | --- | --- | --- | --- | --- | --- | --- | --- | --- | --- | --- | --- | --- | --- | --- | --- | --- | --- | --- | --- | --- | --- | --- | --- | --- | --- | --- | --- | --- | --- | --- | --- | --- | --- | --- | --- | --- | --- | --- | --- | --- | --- | --- | --- | --- | --- | --- | --- | --- | --- | --- | --- | --- | --- | --- | --- | --- | --- | --- | --- | --- | --- | --- | --- | --- | --- | --- | --- | --- | --- | --- | --- | --- | --- | --- | --- | --- | --- | --- | --- | --- | --- | --- | --- | --- | --- | --- | --- | --- | --- | --- | --- | --- | --- | --- | --- | --- | --- | --- | --- | --- | --- | --- | --- | --- | --- | --- | --- | --- | --- | --- | --- | --- | --- | --- | --- | --- | --- | --- | --- | --- | --- | --- | --- | --- | --- | --- | --- | --- | --- | --- | --- | --- | --- | --- | --- | --- | --- | --- | --- | --- | --- | --- | --- | --- | --- | --- | --- | --- | --- | --- | --- | --- | --- | --- | --- | --- | --- | --- | --- | --- | --- | --- | --- | --- | --- | --- | --- | --- | --- | --- | --- | --- | --- | --- | --- | --- | --- | --- | --- | --- | --- | --- | --- | --- | --- | --- | --- | --- | --- | --- | --- | --- | --- | --- | --- | --- | --- | --- | --- | --- | --- | --- | --- | --- | --- | --- | --- | --- | --- | --- | --- | --- | --- | --- | --- | --- | --- | --- | --- | --- | --- | --- | --- | --- | --- | --- | --- | --- | --- | --- | --- | --- | --- | --- | --- | --- | --- | --- | --- | --- | --- | --- | --- | --- | --- | --- | --- | --- | --- | --- | --- | --- | --- | --- | --- | --- | --- | --- | --- | --- | --- | --- | --- | --- | --- | --- | --- | --- | --- | --- | --- | --- | --- | --- | --- | --- | --- | --- | --- | --- | --- | --- | --- | --- | --- | --- | --- | --- | --- | --- | --- | --- | --- | --- | --- | --- | --- | --- | --- | --- | --- | --- | --- | --- | --- | --- | --- | --- | --- | --- | --- | --- | --- | --- | --- | --- | --- | --- | --- | --- | --- | --- | --- | --- | --- | --- | --- | --- | --- | --- | --- | --- | --- | --- | --- | --- | --- | --- | --- | --- | --- | --- | --- | --- | --- | --- | --- | --- | --- | --- | --- | --- | --- | --- | --- | --- | --- | --- | --- | --- | --- | --- | --- | --- | --- | --- | --- | --- | --- | --- | --- | --- | --- | --- | --- | --- | --- | --- | --- | --- | --- | --- | --- | --- | --- | --- | --- | --- | --- | --- | --- | --- | --- | --- | --- | --- | --- | --- | --- | --- | --- | --- | --- | --- | --- | --- | --- | --- | --- | --- | --- | --- | --- | --- | --- | --- | --- | --- | --- | --- | --- | --- | --- | --- | --- | --- | --- | --- | --- | --- | --- | --- | --- | --- | --- | --- | --- | --- | --- | --- | --- | --- | --- | --- | --- | --- | --- | --- | --- | --- | --- | --- | --- | --- | --- | --- | --- | --- | --- | --- | --- | --- | --- | --- | --- | --- | --- | --- | --- | --- | --- | --- | --- | --- | --- | --- | --- | --- | --- | --- | --- | --- | --- | --- | --- | --- | --- | --- | --- | --- | --- | --- | --- | --- | --- | --- | --- | --- | --- | --- | --- | --- | --- | --- | --- | --- | --- | --- | --- | --- | --- | --- | --- | --- | --- | --- | --- | --- | --- | --- | --- | --- | --- | --- | --- | --- | --- | --- | --- | --- | --- | --- | --- | --- | --- | --- | --- | --- | --- | --- | --- | --- | --- | --- | --- | --- | --- | --- | --- | --- | --- | --- | --- | --- | --- | --- | --- | --- | --- | --- | --- | --- | --- | --- | --- | --- | --- | --- | --- | --- | --- | --- | --- | --- | --- | --- | --- | --- | --- | --- | --- | --- | --- | --- | --- | --- | --- | --- | --- | --- | --- | --- | --- | --- | --- | --- | --- | --- | --- | --- | --- | --- | --- | --- | --- | --- | --- | --- | --- | --- | --- | --- | --- | --- | --- | --- | --- | --- | --- | --- | --- | --- | --- | --- | --- | --- | --- | --- | --- | --- | --- | --- | --- | --- | --- | --- | --- | --- | --- | --- | --- | --- | --- | --- | --- | --- | --- | --- | --- | --- | --- | --- | --- | --- | --- | --- | --- | --- | --- | --- | --- | --- | --- | --- | --- | --- | --- | --- | --- | --- | --- | --- | --- | --- | --- | --- | --- | --- | --- | --- | --- | --- | --- | --- | --- | --- | --- | --- | --- | --- | --- | --- | --- | --- | --- | --- | --- | --- | --- | --- | --- | --- | --- | --- | --- | --- | --- | --- | --- | --- | --- | --- | --- | --- | --- | --- | --- | --- | --- | --- | --- | --- | --- | --- | --- | --- | --- | --- | --- | --- | --- | --- | --- | --- | --- | --- | --- | --- | --- | --- | --- | --- | --- | --- | --- | --- | --- | --- | --- | --- | --- | --- | --- | --- | --- | --- | --- | --- | --- | --- | --- | --- | --- | --- | --- | --- | --- | --- | --- | --- | --- | --- | --- | --- | --- | --- | --- | --- | --- | --- | --- | --- | --- | --- | --- | --- | --- | --- | --- | --- | --- | --- | --- | --- | --- | --- | --- | --- | --- | --- | --- | --- | --- | --- | --- | --- | --- | --- | --- | --- | --- | --- | --- | --- | --- | --- | --- | --- | --- | --- | --- | --- | --- | --- | --- | --- | --- | --- | --- | --- | --- | --- | --- | --- | --- | --- | --- | --- | --- | --- | --- | --- | --- | --- | --- | --- | --- | --- | --- | --- | --- | --- | --- | --- | --- | --- | --- | --- | --- | --- | --- | --- | --- | --- | --- | --- | --- | --- | --- | --- | --- | --- | --- | --- | --- | --- | --- | --- | --- | --- | --- | --- | --- | --- | --- | --- | --- | --- | --- | --- | --- | --- | --- | --- | --- | --- | --- | --- | --- | --- | --- | --- | --- | --- | --- | --- | --- | --- | --- | --- | --- | --- | --- | --- | --- | --- | --- | --- | --- | --- | --- | --- | --- | --- | --- | --- | --- | --- | --- | --- | --- | --- | --- | --- | --- | --- | --- | --- | --- | --- | --- | --- | --- | --- | --- | --- | --- | --- | --- | --- | --- | --- | --- | --- | --- | --- | --- | --- | --- | --- | --- | --- | --- | --- | --- | --- | --- | --- | --- | --- | --- | --- | --- | --- | --- | --- | --- | --- | --- | --- | --- | --- | --- | --- | --- | --- | --- | --- | --- | --- | --- | --- | --- | --- | --- | --- | --- | --- | --- | --- | --- | --- | --- | --- | --- | --- | --- | --- | --- | --- | --- | --- | --- | --- | --- | --- | --- | --- | --- | --- | --- | --- | --- | --- | --- | --- | --- | --- | --- | --- | --- | --- | --- | --- | --- | --- | --- | --- | --- | --- | --- | --- | --- | --- | --- | --- | --- | --- | --- | --- | --- | --- | --- | --- | --- | --- | --- | --- | --- | --- | --- | --- | --- | --- | --- | --- | --- | --- | --- | --- | --- | --- | --- | --- | --- | --- | --- | --- | --- | --- | --- | --- | --- | --- | --- | --- | --- | --- | --- | --- | --- | --- | --- | --- | --- | --- | --- | --- | --- | --- | --- | --- | --- | --- | --- | --- | --- | --- | --- | --- | --- | --- | --- | --- | --- | --- | --- | --- | --- | --- | --- | --- | --- | --- | --- | --- | --- | --- | --- | --- | --- | --- | --- | --- | --- | --- | --- | --- | --- | --- | --- | --- | --- | --- | --- | --- | --- | --- | --- | --- | --- | --- | --- | --- | --- | --- | --- | --- | --- | --- | --- | --- | --- | --- | --- | --- | --- | --- | --- | --- | --- | --- | --- | --- | --- | --- | --- | --- | --- | --- | --- | --- | --- | --- | --- | --- | --- | --- | --- | --- | --- | --- | --- | --- | --- | --- | --- | --- | --- | --- | --- | --- | --- | --- | --- | --- | --- | --- | --- | --- | --- | --- | --- | --- | --- | --- | --- | --- | --- | --- | --- | --- | --- | --- | --- | --- | --- | --- | --- | --- | --- | --- | --- | --- | --- | --- | --- | --- | --- | --- | --- | --- | --- | --- | --- | --- | --- | --- | --- | --- | --- | --- | --- | --- | --- | --- | --- | --- | --- | --- | --- | --- | --- | --- | --- | --- | --- | --- | --- | --- | --- | --- | --- | --- | --- | --- | --- | --- | --- | --- | --- | --- | --- | --- | --- | --- | --- | --- | --- | --- | --- | --- | --- | --- | --- | --- | --- | --- | --- | --- | --- | --- | --- | --- | --- | --- | --- | --- | --- | --- | --- | --- | --- | --- | --- | --- | --- | --- | --- | --- | --- | --- | --- | --- | --- | --- | --- | --- | --- | --- | --- | --- | --- | --- | --- | --- | --- | --- | --- | --- | --- | --- | --- | --- | --- | --- | --- | --- | --- | --- | --- | --- | --- | --- | --- | --- | --- | --- | --- | --- | --- | --- | --- | --- | --- | --- | --- | --- | --- | --- | --- | --- | --- | --- | --- | --- | --- | --- | --- | --- | --- | --- | --- | --- | --- | --- | --- | --- | --- | --- | --- | --- | --- | --- | --- | --- | --- | --- | --- | --- | --- | --- | --- | --- | --- | --- | --- | --- | --- | --- | --- | --- | --- | --- | --- | --- | --- | --- | --- | --- | --- | --- | --- | --- | --- | --- | --- | --- | --- | --- | --- | --- | --- | --- | --- | --- | --- | --- | --- | --- | --- | --- | --- | --- | --- | --- | --- | --- | --- | --- | --- | --- | --- | --- | --- | --- | --- | --- | --- | --- | --- | --- | --- | --- | --- | --- | --- | --- | --- | --- | --- | --- | --- | --- | --- | --- | --- | --- | --- | --- | --- | --- | --- | --- | --- | --- | --- | --- | --- | --- | --- | --- | --- | --- | --- | --- | --- | --- | --- | --- | --- | --- | --- | --- | --- | --- | --- | --- | --- | --- | --- | --- | --- | --- | --- | --- | --- | --- | --- | --- | --- | --- | --- | --- | --- | --- | --- | --- | --- | --- | --- | --- | --- | --- | --- | --- | --- | --- | --- | --- | --- | --- | --- | --- | --- | --- | --- | --- | --- | --- | --- | --- | --- | --- | --- | --- | --- | --- | --- | --- | --- | --- | --- | --- | --- | --- | --- | --- | --- | --- | --- | --- | --- | --- | --- | --- | --- | --- | --- | --- | --- | --- | --- | --- | --- | --- | --- | --- | --- | --- | --- | --- | --- | --- | --- | --- | --- | --- | --- | --- | --- | --- | --- | --- | --- | --- | --- | --- | --- | --- | --- | --- | --- | --- | --- | --- | --- | --- | --- | --- | --- | --- | --- | --- | --- | --- | --- | --- | --- | --- | --- | --- | --- | --- | --- | --- | --- | --- | --- | --- | --- | --- | --- | --- | --- | --- | --- | --- | --- | --- | --- | --- | --- | --- | --- | --- | --- | --- | --- | --- | --- | --- | --- | --- | --- | --- | --- | --- | --- | --- | --- | --- | --- | --- | --- | --- | --- | --- | --- | --- | --- | --- | --- | --- | --- | --- | --- | --- | --- | --- | --- | --- | --- | --- | --- | --- | --- | --- | --- | --- | --- | --- | --- | --- | --- | --- | --- | --- | --- | --- | --- | --- | --- | --- | --- | --- | --- | --- | --- | --- | --- | --- | --- | --- | --- | --- | --- | --- | --- | --- | --- | --- | --- | --- | --- | --- | --- | --- | --- | --- | --- | --- | --- | --- | --- | --- | --- | --- | --- | --- | --- | --- | --- | --- | --- | --- | --- | --- | --- | --- | --- | --- | --- | --- | --- | --- | --- | --- | --- | --- | --- | --- | --- | --- | --- | --- | --- | --- | --- | --- | --- | --- | --- | --- | --- | --- | --- | --- | --- | --- | --- | --- | --- | --- | --- | --- | --- | --- | --- | --- | --- | --- | --- | --- | --- | --- | --- | --- | --- | --- | --- | --- | --- | --- | --- | --- | --- | --- | --- | --- | --- | --- | --- | --- | --- | --- | --- | --- | --- | --- | --- | --- | --- | --- | --- | --- | --- | --- | --- | --- | --- | --- | --- | --- | --- | --- | --- | --- | --- | --- | --- | --- | --- | --- | --- | --- | --- | --- | --- | --- | --- | --- | --- | --- | --- | --- | --- | --- | --- | --- | --- | --- | --- | --- | --- | --- | --- | --- | --- | --- | --- | --- | --- | --- | --- | --- | --- | --- | --- | --- | --- | --- | --- | --- | --- | --- | --- | --- | --- | --- | --- | --- | --- | --- | --- | --- | --- | --- | --- | --- | --- | --- | --- | --- | --- | --- | --- | --- | --- | --- | --- | --- | --- | --- | --- | --- | --- | --- | --- | --- | --- | --- | --- | --- | --- | --- | --- | --- | --- | --- | --- | --- | --- | --- | --- | --- | --- | --- | --- | --- | --- | --- | --- | --- | --- | --- | --- | --- | --- | --- | --- | --- | --- | --- | --- | --- | --- | --- | --- | --- | --- | --- | --- | --- | --- | --- | --- | --- | --- | --- | --- | --- | --- | --- | --- | --- | --- | --- | --- | --- | --- | --- | --- | --- | --- | --- | --- | --- | --- | --- | --- | --- | --- | --- | --- | --- | --- | --- | --- | --- | --- | --- | --- | --- | --- | --- | --- | --- | --- | --- | --- | --- | --- | --- | --- | --- | --- | --- | --- | --- | --- | --- | --- | --- | --- | --- | --- | --- | --- | --- | --- | --- | --- | --- | --- | --- | --- | --- | --- | --- | --- | --- | --- | --- | --- | --- | --- | --- | --- | --- | --- | --- | --- | --- | --- | --- | --- | --- | --- | --- | --- | --- | --- | --- | --- | --- | --- | --- | --- | --- | --- | --- | --- | --- | --- | --- | --- | --- | --- | --- | --- | --- | --- | --- | --- | --- | --- | --- | --- | --- | --- | --- | --- | --- | --- | --- | --- | --- | --- | --- | --- | --- | --- | --- | --- | --- | --- | --- | --- | --- | --- | --- | --- | --- | --- | --- | --- | --- | --- | --- | --- | --- | --- | --- | --- | --- | --- | --- | --- | --- | --- | --- | --- | --- | --- | --- | --- | --- | --- | --- | --- | --- | --- | --- | --- | --- | --- | --- | --- | --- | --- | --- | --- | --- | --- | --- | --- | --- | --- | --- | --- | --- | --- | --- | --- | --- | --- | --- | --- | --- | --- | --- | --- | --- | --- | --- | --- | --- | --- | --- | --- | --- | --- | --- | --- | --- | --- | --- | --- | --- | --- | --- | --- | --- | --- | --- | --- | --- | --- | --- | --- | --- | --- | --- | --- | --- | --- | --- | --- | --- | --- | --- | --- | --- | --- | --- | --- | --- | --- | --- | --- | --- | --- | --- | --- | --- | --- | --- | --- | --- | --- | --- | --- | --- | --- | --- | --- | --- | --- | --- | --- | --- | --- | --- | --- | --- | --- | --- | --- | --- | --- | --- | --- | --- | --- | --- | --- | --- | --- | --- | --- | --- | --- | --- | --- | --- | --- | --- | --- | --- | --- | --- | --- | --- | --- | --- | --- | --- | --- | --- | --- | --- | --- | --- | --- | --- | --- | --- | --- | --- | --- | --- | --- | --- | --- | --- | --- | --- | --- | --- | --- | --- | --- | --- | --- | --- | --- | --- | --- | --- | --- | --- | --- | --- | --- | --- | --- | --- | --- | --- | --- | --- | --- | --- | --- | --- | --- | --- | --- | --- | --- | --- | --- | --- | --- | --- | --- | --- | --- | --- | --- | --- | --- | --- | --- | --- | --- | --- | --- | --- | --- | --- | --- | --- | --- | --- | --- | --- | --- | --- | --- | --- | --- | --- | --- | --- | --- | --- | --- | --- | --- | --- | --- | --- | --- | --- | --- | --- | --- | --- | --- | --- | --- | --- | --- | --- | --- | --- | --- | --- | --- | --- | --- | --- | --- | --- | --- | --- | --- | --- | --- | --- | --- | --- | --- | --- | --- | --- | --- | --- | --- | --- | --- | --- | --- | --- | --- | --- | --- | --- | --- | --- | --- | --- | --- | --- | --- | --- | --- | --- | --- | --- | --- | --- | --- | --- | --- | --- | --- | --- | --- | --- | --- | --- | --- | --- | --- | --- | --- | --- | --- | --- | --- | --- | --- | --- | --- | --- | --- | --- | --- | --- | --- | --- | --- | --- | --- | --- | --- | --- | --- | --- | --- | --- | --- | --- | --- | --- | --- | --- | --- | --- | --- | --- | --- | --- | --- | --- | --- | --- | --- | --- | --- | --- | --- | --- | --- | --- | --- | --- | --- | --- | --- | --- | --- | --- | --- | --- | --- | --- | --- | --- | --- | --- | --- | --- | --- | --- | --- | --- | --- | --- | --- | --- | --- | --- | --- | --- | --- | --- | --- | --- | --- | --- | --- | --- | --- | --- | --- | --- | --- | --- | --- | --- | --- | --- | --- | --- | --- | --- | --- | --- | --- | --- | --- | --- | --- | --- | --- | --- | --- | --- | --- | --- | --- | --- | --- | --- | --- | --- | --- | --- | --- | --- | --- | --- | --- | --- | --- | --- | --- | --- | --- | --- | --- | --- | --- | --- | --- | --- | --- | --- | --- | --- | --- | --- | --- | --- | --- | --- | --- | --- | --- | --- | --- | --- | --- | --- | --- | --- | --- | --- | --- | --- | --- | --- | --- | --- | --- | --- | --- | --- | --- | --- | --- | --- | --- | --- | --- | --- | --- | --- | --- | --- | --- | --- | --- | --- | --- | --- | --- | --- | --- | --- | --- | --- | --- | --- | --- | --- | --- | --- | --- | --- | --- | --- | --- | --- | --- | --- | --- | --- | --- | --- | --- | --- | --- | --- | --- | --- | --- | --- | --- | --- | --- | --- | --- | --- | --- | --- | --- | --- | --- | --- | --- | --- | --- | --- | --- | --- | --- | --- | --- | --- | --- | --- | --- | --- | --- | --- | --- | --- | --- | --- | --- | --- | --- | --- | --- | --- | --- | --- | --- | --- | --- | --- | --- | --- | --- | --- | --- | --- | --- | --- | --- | --- | --- | --- | --- | --- | --- | --- | --- | --- | --- | --- | --- | --- | --- | --- | --- | --- | --- | --- | --- | --- | --- | --- | --- | --- | --- | --- | --- | --- | --- | --- | --- | --- | --- | --- | --- | --- | --- | --- | --- | --- | --- | --- | --- | --- | --- | --- | --- | --- | --- | --- | --- | --- | --- | --- | --- | --- | --- | --- | --- | --- | --- | --- | --- | --- | --- | --- | --- | --- | --- | --- | --- | --- | --- | --- | --- | --- | --- | --- | --- | --- | --- | --- | --- | --- | --- | --- | --- | --- | --- | --- | --- | --- | --- | --- | --- | --- | --- | --- | --- | --- | --- | --- | --- | --- | --- | --- | --- | --- | --- | --- | --- | --- | --- | --- | --- | --- | --- | --- | --- | --- | --- | --- | --- | --- | --- | --- | --- | --- | --- | --- | --- | --- | --- | --- | --- | --- | --- | --- | --- | --- | --- | --- | --- | --- | --- | --- | --- | --- | --- | --- | --- | --- | --- | --- | --- | --- | --- | --- | --- | --- | --- | --- | --- | --- | --- | --- | --- | --- | --- | --- | --- | --- | --- | --- | --- | --- | --- | --- | --- | --- | --- | --- | --- | --- | --- | --- | --- | --- | --- | --- | --- | --- | --- | --- | --- | --- | --- | --- | --- | --- | --- | --- | --- | --- | --- | --- | --- | --- | --- | --- | --- | --- | --- | --- | --- | --- | --- | --- | --- | --- | --- | --- | --- | --- | --- | --- | --- | --- | --- | --- | --- | --- | --- | --- | --- | --- | --- | --- | --- | --- | --- | --- | --- | --- | --- | --- | --- | --- | --- | --- | --- | --- | --- | --- | --- | --- | --- | --- | --- | --- | --- | --- | --- | --- | --- | --- | --- | --- | --- | --- | --- | --- | --- | --- | --- | --- | --- | --- | --- | --- | --- | --- | --- | --- | --- | --- | --- | --- | --- | --- | --- | --- | --- | --- | --- | --- | --- | --- | --- | --- | --- | --- | --- | --- | --- | --- | --- | --- | --- | --- | --- | --- | --- | --- | --- | --- | --- | --- | --- | --- | --- | --- | --- | --- | --- | --- | --- | --- | --- | --- | --- | --- | --- | --- | --- | --- | --- | --- | --- | --- | --- | --- | --- | --- | --- | --- | --- | --- | --- | --- | --- | --- | --- | --- | --- | --- | --- | --- | --- | --- | --- | --- | --- | --- | --- | --- | --- | --- | --- | --- | --- | --- | --- | --- | --- | --- | --- | --- | --- | --- | --- | --- | --- | --- | --- | --- | --- | --- | --- | --- | --- | --- | --- | --- | --- | --- | --- | --- | --- | --- | --- | --- | --- | --- | --- | --- | --- | --- | --- | --- | --- | --- | --- | --- | --- | --- | --- | --- | --- | --- | --- | --- | --- | --- | --- | --- | --- | --- | --- | --- | --- | --- | --- | --- | --- | --- | --- | --- | --- | --- | --- | --- | --- | --- | --- | --- | --- | --- | --- | --- | --- | --- | --- | --- | --- | --- | --- | --- | --- | --- | --- | --- | --- | --- | --- | --- | --- | --- | --- | --- | --- | --- | --- | --- | --- | --- | --- | --- | --- | --- | --- | --- | --- | --- | --- | --- | --- | --- | --- | --- | --- | --- | --- | --- | --- | --- | --- | --- | --- | --- | --- | --- | --- | --- | --- | --- | --- | --- | --- | --- | --- | --- | --- | --- | --- | --- | --- | --- | --- | --- | --- | --- | --- | --- | --- | --- | --- | --- | --- | --- | --- | --- | --- | --- | --- | --- | --- | --- | --- | --- | --- | --- | --- | --- | --- | --- | --- | --- | --- | --- | --- | --- | --- | --- | --- | --- | --- | --- | --- | --- | --- | --- | --- | --- | --- | --- | --- | --- | --- | --- | --- | --- | --- | --- | --- | --- | --- | --- | --- | --- | --- | --- | --- | --- | --- | --- | --- | --- | --- | --- | --- | --- | --- | --- | --- | --- | --- | --- | --- | --- | --- | --- | --- | --- | --- | --- | --- | --- | --- | --- | --- | --- | --- | --- | --- | --- | --- | --- | --- | --- | --- | --- | --- | --- | --- | --- | --- | --- | --- | --- | --- | --- | --- | --- | --- | --- | --- | --- | --- | --- | --- | --- | --- | --- | --- | --- | --- | --- | --- | --- | --- | --- | --- | --- | --- | --- | --- | --- | --- | --- | --- | --- | --- | --- | --- | --- | --- | --- | --- | --- | --- | --- | --- | --- | --- | --- | --- | --- | --- | --- | --- | --- | --- | --- | --- | --- | --- | --- | --- | --- | --- | --- | --- | --- | --- | --- | --- | --- | --- | --- | --- | --- | --- | --- | --- | --- | --- | --- | --- | --- | --- | --- | --- | --- | --- | --- | --- | --- | --- | --- | --- | --- | --- | --- | --- | --- | --- | --- | --- | --- | --- | --- | --- | --- | --- | --- | --- | --- | --- | --- | --- | --- | --- | --- | --- | --- | --- | --- | --- | --- | --- | --- | --- | --- | --- | --- | --- | --- | --- | --- | --- | --- | --- | --- | --- | --- | --- | --- | --- | --- | --- | --- | --- | --- | --- | --- | --- | --- | --- | --- | --- | --- | --- | --- | --- | --- | --- | --- | --- | --- | --- | --- | --- | --- | --- | --- | --- | --- | --- | --- | --- | --- | --- | --- | --- | --- | --- | --- | --- | --- | --- | --- | --- | --- | --- | --- | --- | --- | --- | --- | --- | --- | --- | --- | --- | --- | --- | --- | --- | --- | --- | --- | --- | --- | --- | --- | --- | --- | --- | --- | --- | --- | --- | --- | --- | --- | --- | --- | --- | --- | --- | --- | --- | --- | --- | --- | --- | --- | --- | --- | --- | --- | --- | --- | --- | --- | --- | --- | --- | --- | --- | --- | --- | --- | --- | --- | --- | --- | --- | --- | --- | --- | --- | --- | --- | --- | --- | --- | --- | --- | --- | --- | --- | --- | --- | --- | --- | --- | --- | --- | --- | --- | --- | --- | --- | --- | --- | --- | --- | --- | --- | --- | --- | --- | --- | --- | --- | --- | --- | --- | --- | --- | --- | --- | --- | --- | --- | --- | --- | --- | --- | --- | --- | --- | --- | --- | --- | --- | --- | --- | --- | --- | --- | --- | --- | --- | --- | --- | --- | --- | --- | --- | --- | --- | --- | --- | --- | --- | --- | --- | --- | --- | --- | --- | --- | --- | --- | --- | --- | --- | --- | --- | --- | --- | --- | --- | --- | --- | --- | --- | --- | --- | --- | --- | --- | --- | --- | --- | --- | --- | --- | --- | --- | --- | --- | --- | --- | --- | --- | --- | --- | --- | --- | --- | --- | --- | --- | --- | --- | --- | --- | --- | --- | --- | --- | --- | --- | --- | --- | --- | --- | --- | --- | --- | --- | --- | --- | --- | --- | --- | --- | --- | --- | --- | --- | --- | --- | --- | --- | --- | --- | --- | --- | --- | --- | --- | --- | --- | --- | --- | --- | --- | --- | --- | --- | --- | --- | --- | --- | --- | --- | --- | --- | --- | --- | --- | --- | --- | --- | --- | --- | --- | --- | --- | --- | --- | --- | --- | --- | --- | --- | --- | --- | --- | --- | --- | --- | --- | --- | --- | --- | --- | --- | --- | --- | --- | --- | --- | --- | --- | --- | --- | --- | --- | --- | --- | --- | --- | --- | --- | --- | --- | --- | --- | --- | --- | --- | --- | --- | --- | --- | --- | --- | --- | --- | --- | --- | --- | --- | --- | --- | --- | --- | --- | --- | --- | --- | --- | --- | --- | --- | --- | --- | --- | --- | --- | --- | --- | --- | --- | --- | --- | --- | --- | --- | --- | --- | --- | --- | --- | --- | --- | --- | --- | --- | --- | --- | --- | --- | --- | --- | --- | --- | --- | --- | --- | --- | --- | --- | --- | --- | --- | --- | --- | --- | --- | --- | --- | --- | --- | --- | --- | --- | --- | --- | --- | --- | --- | --- | --- | --- | --- | --- | --- | --- | --- | --- | --- | --- | --- | --- | --- | --- | --- | --- | --- | --- | --- | --- | --- | --- | --- | --- | --- | --- | --- | --- | --- | --- | --- | --- | --- | --- | --- | --- | --- | --- | --- | --- | --- | --- | --- | --- | --- | --- | --- | --- | --- | --- | --- | --- | --- | --- | --- | --- | --- | --- | --- | --- | --- | --- | --- | --- | --- | --- | --- | --- | --- | --- | --- | --- | --- | --- | --- | --- | --- | --- | --- | --- | --- | --- | --- | --- | --- | --- | --- | --- | --- | --- | --- | --- | --- | --- | --- | --- | --- | --- | --- | --- | --- | --- | --- | --- | --- | --- | --- | --- | --- | --- | --- | --- | --- | --- | --- | --- | --- | --- | --- | --- | --- | --- | --- | --- | --- | --- | --- | --- | --- | --- | --- | --- | --- | --- | --- | --- | --- | --- | --- | --- | --- | --- | --- | --- | --- | --- | --- | --- | --- | --- | --- | --- | --- | --- | --- | --- | --- | --- | --- | --- | --- | --- | --- | --- | --- | --- | --- | --- | --- | --- | --- | --- | --- | --- | --- | --- | --- | --- | --- | --- | --- | --- | --- | --- | --- | --- | --- | --- | --- | --- | --- | --- | --- | --- | --- | --- | --- | --- | --- | --- | --- | --- | --- | --- | --- | --- | --- | --- | --- | --- | --- | --- | --- | --- | --- | --- | --- | --- | --- | --- | --- | --- | --- | --- | --- | --- | --- | --- | --- | --- | --- | --- | --- | --- | --- | --- | --- | --- | --- | --- | --- | --- | --- | --- | --- | --- | --- | --- | --- | --- | --- | --- | --- | --- | --- | --- | --- | --- | --- | --- | --- | --- | --- | --- | --- | --- | --- | --- | --- | --- | --- | --- | --- | --- | --- | --- | --- | --- | --- | --- | --- | --- | --- | --- | --- | --- | --- | --- | --- | --- | --- | --- | --- | --- | --- | --- | --- | --- | --- | --- | --- | --- | --- | --- | --- | --- | --- | --- | --- | --- | --- | --- | --- | --- | --- | --- | --- | --- | --- | --- | --- | --- | --- | --- | --- | --- | --- | --- | --- | --- | --- | --- | --- | --- | --- | --- | --- | --- | --- | --- | --- | --- | --- | --- | --- | --- | --- | --- | --- | --- | --- | --- | --- | --- | --- | --- | --- | --- | --- | --- | --- | --- | --- | --- | --- | --- | --- | --- | --- | --- | --- | --- | --- | --- | --- | --- | --- | --- | --- | --- | --- | --- | --- | --- | --- | --- | --- | --- | --- | --- | --- | --- | --- | --- | --- | --- | --- | --- | --- | --- | --- | --- | --- | --- | --- | --- | --- | --- | --- | --- | --- | --- | --- | --- | --- | --- | --- | --- | --- | --- | --- | --- | --- | --- | --- | --- | --- | --- | --- | --- | --- | --- | --- | --- | --- | --- | --- | --- | --- | --- | --- | --- | --- | --- | --- | --- | --- | --- | --- | --- | --- | --- | --- | --- | --- | --- | --- | --- | --- | --- | --- | --- | --- | --- | --- | --- | --- | --- | --- | --- | --- | --- | --- | --- | --- | --- | --- | --- | --- | --- | --- | --- | --- | --- | --- | --- | --- | --- | --- | --- | --- | --- | --- | --- | --- | --- | --- | --- | --- | --- | --- | --- | --- | --- | --- | --- | --- | --- | --- | --- | --- | --- | --- | --- | --- | --- | --- | --- | --- | --- | --- | --- | --- | --- | --- | --- | --- | --- | --- | --- | --- | --- | --- | --- | --- | --- | --- | --- | --- | --- | --- | --- | --- | --- | --- | --- | --- | --- | --- | --- | --- | --- | --- | --- | --- | --- | --- | --- | --- | --- | --- | --- | --- | --- | --- | --- | --- | --- | --- | --- | --- | --- | --- | --- | --- | --- | --- | --- | --- | --- | --- | --- | --- | --- | --- | --- | --- | --- | --- | --- | --- | --- | --- | --- | --- | --- | --- | --- | --- | --- | --- | --- | --- | --- | --- | --- | --- | --- | --- | --- | --- | --- | --- | --- | --- | --- | --- | --- | --- | --- | --- | --- | --- | --- | --- | --- | --- | --- | --- | --- | --- | --- | --- | --- | --- | --- | --- | --- | --- | --- | --- | --- | --- | --- | --- | --- | --- | --- | --- | --- | --- | --- | --- | --- | --- | --- | --- | --- | --- | --- | --- | --- | --- | --- | --- | --- | --- | --- | --- | --- | --- | --- | --- | --- | --- | --- | --- | --- | --- | --- | --- | --- | --- | --- | --- | --- | --- | --- | --- | --- | --- | --- | --- | --- | --- | --- | --- | --- | --- | --- | --- | --- | --- | --- | --- | --- | --- | --- | --- | --- | --- | --- | --- | --- | --- | --- | --- | --- | --- | --- | --- | --- | --- | --- | --- | --- | --- | --- | --- | --- | --- | --- | --- | --- | --- | --- | --- | --- | --- | --- | --- | --- | --- | --- | --- | --- | --- | --- | --- | --- | --- | --- | --- | --- | --- | --- | --- | --- | --- | --- | --- | --- | --- | --- | --- | --- | --- | --- | --- | --- | --- | --- | --- | --- | --- | --- | --- | --- | --- | --- | --- | --- | --- | --- | --- | --- | --- | --- | --- | --- | --- | --- | --- | --- | --- | --- | --- | --- | --- | --- | --- | --- | --- | --- | --- | --- | --- | --- | --- | --- | --- | --- | --- | --- | --- | --- | --- | --- | --- | --- | --- | --- | --- | --- | --- | --- | --- | --- | --- | --- | --- | --- | --- | --- | --- | --- | --- | --- | --- | --- | --- | --- | --- | --- | --- | --- | --- | --- | --- | --- | --- | --- | --- | --- | --- | --- | --- | --- | --- | --- | --- | --- | --- | --- | --- | --- | --- | --- | --- | --- | --- | --- | --- | --- | --- | --- | --- | --- | --- | --- | --- | --- | --- | --- | --- | --- | --- | --- | --- | --- | --- | --- | --- | --- | --- | --- | --- | --- | --- | --- | --- | --- | --- | --- | --- | --- | --- | --- | --- | --- | --- | --- | --- | --- | --- | --- | --- | --- | --- | --- | --- | --- | --- | --- | --- | --- | --- | --- | --- | --- | --- | --- | --- | --- | --- | --- | --- | --- | --- | --- | --- | --- | --- | --- | --- | --- | --- | --- | --- | --- | --- | --- | --- | --- | --- | --- | --- | --- | --- | --- | --- | --- | --- | --- | --- | --- | --- | --- | --- | --- | --- | --- | --- | --- | --- | --- | --- | --- | --- | --- | --- | --- | --- | --- | --- | --- | --- | --- | --- | --- | --- | --- | --- | --- | --- | --- | --- | --- | --- | --- | --- | --- | --- | --- | --- | --- | --- | --- | --- | --- | --- | --- | --- | --- | --- | --- | --- | --- | --- | --- | --- | --- | --- | --- | --- | --- | --- | --- | --- | --- | --- | --- | --- | --- | --- | --- | --- | --- | --- | --- | --- | --- | --- | --- | --- | --- | --- | --- | --- | --- | --- | --- | --- | --- | --- | --- | --- | --- | --- | --- | --- | --- | --- | --- | --- | --- | --- | --- | --- | --- | --- | --- | --- | --- | --- | --- | --- | --- | --- | --- | --- | --- | --- | --- | --- | --- | --- | --- | --- | --- | --- | --- | --- | --- | --- | --- | --- | --- | --- | --- | --- | --- | --- | --- | --- | --- | --- | --- | --- | --- | --- | --- | --- | --- | --- | --- | --- | --- | --- | --- | --- | --- | --- | --- | --- | --- | --- | --- | --- | --- | --- | --- | --- | --- | --- | --- | --- | --- | --- | --- | --- | --- | --- | --- | --- | --- | --- | --- | --- | --- | --- | --- | --- | --- | --- | --- | --- | --- | --- | --- | --- | --- | --- | --- | --- | --- | --- | --- | --- | --- | --- | --- | --- | --- | --- | --- | --- | --- | --- | --- | --- | --- | --- | --- | --- | --- | --- | --- | --- | --- | --- | --- | --- | --- | --- | --- | --- | --- | --- | --- | --- | --- | --- | --- | --- | --- | --- | --- | --- | --- | --- | --- | --- | --- | --- | --- | --- | --- | --- | --- | --- | --- | --- | --- | --- | --- | --- | --- | --- | --- | --- | --- | --- | --- | --- | --- | --- | --- | --- | --- | --- | --- | --- | --- | --- | --- | --- | --- | --- | --- | --- | --- | --- | --- | --- | --- | --- | --- | --- | --- | --- | --- | --- | --- | --- | --- | --- | --- | --- | --- | --- | --- | --- | --- | --- | --- | --- | --- | --- | --- | --- | --- | --- | --- | --- | --- | --- | --- | --- | --- | --- | --- | --- | --- | --- | --- | --- | --- | --- | --- | --- | --- | --- | --- | --- | --- | --- | --- | --- | --- | --- | --- | --- | --- | --- | --- | --- | --- | --- | --- | --- | --- | --- | --- | --- | --- | --- | --- | --- | --- | --- | --- | --- | --- | --- | --- | --- | --- | --- | --- | --- | --- | --- | --- | --- | --- | --- | --- | --- | --- | --- | --- | --- | --- | --- | --- | --- | --- | --- | --- | --- | --- | --- | --- | --- | --- | --- | --- | --- | --- | --- | --- | --- | --- | --- | --- | --- | --- | --- | --- | --- | --- | --- | --- | --- | --- | --- | --- | --- | --- | --- | --- | --- | --- | --- | --- | --- | --- | --- | --- | --- | --- | --- | --- | --- | --- | --- | --- | --- | --- | --- | --- | --- | --- | --- | --- | --- | --- | --- | --- | --- | --- | --- | --- | --- | --- | --- | --- | --- | --- | --- | --- | --- | --- | --- | --- | --- | --- | --- | --- | --- | --- | --- | --- | --- | --- | --- | --- | --- | --- | --- | --- | --- | --- | --- | --- | --- | --- | --- | --- | --- | --- | --- | --- | --- | --- | --- | --- | --- | --- | --- | --- | --- | --- | --- | --- | --- | --- | --- | --- | --- | --- | --- | --- | --- | --- | --- | --- | --- | --- | --- | --- | --- | --- | --- | --- | --- | --- | --- | --- | --- | --- | --- | --- | --- | --- | --- | --- | --- | --- | --- | --- | --- | --- | --- | --- | --- | --- | --- | --- | --- | --- | --- | --- | --- | --- | --- | --- | --- | --- | --- | --- | --- | --- | --- | --- | --- | --- | --- | --- | --- | --- | --- | --- | --- | --- | --- | --- | --- | --- | --- | --- | --- | --- | --- | --- | --- | --- | --- | --- | --- | --- | --- | --- | --- | --- | --- | --- | --- | --- | --- | --- | --- | --- | --- | --- | --- | --- | --- | --- | --- | --- | --- | --- | --- | --- | --- | --- | --- | --- | --- | --- | --- | --- | --- | --- | --- | --- | --- | --- | --- | --- | --- | --- | --- | --- | --- | --- | --- | --- | --- | --- | --- | --- | --- | --- | --- | --- | --- | --- | --- | --- | --- | --- | --- | --- | --- | --- | --- | --- | --- | --- | --- | --- | --- | --- | --- | --- | --- | --- | --- | --- | --- | --- | --- | --- | --- | --- | --- | --- | --- | --- | --- | --- | --- | --- | --- | --- | --- | --- | --- | --- | --- | --- | --- | --- | --- | --- | --- | --- | --- | --- | --- | --- | --- | --- | --- | --- | --- | --- | --- | --- | --- | --- | --- | --- | --- | --- | --- | --- | --- | --- | --- | --- | --- | --- | --- | --- | --- | --- | --- | --- | --- | --- | --- | --- | --- | --- | --- | --- | --- | --- | --- | --- | --- | --- | --- | --- | --- | --- | --- | --- | --- | --- | --- | --- | --- | --- | --- | --- | --- | --- | --- | --- | --- | --- | --- | --- | --- | --- | --- | --- | --- | --- | --- | --- | --- | --- | --- | --- | --- | --- | --- | --- | --- | --- | --- | --- | --- | --- | --- | --- | --- | --- | --- | --- | --- | --- | --- | --- | --- | --- | --- | --- | --- | --- | --- | --- | --- | --- | --- | --- | --- | --- | --- | --- | --- | --- | --- | --- | --- | --- | --- | --- | --- | --- | --- | --- | --- | --- | --- | --- | --- | --- | --- | --- | --- | --- | --- | --- | --- | --- | --- | --- | --- | --- | --- | --- | --- | --- | --- | --- | --- | --- | --- | --- | --- | --- | --- | --- | --- | --- | --- | --- | --- | --- | --- | --- | --- | --- | --- | --- | --- | --- | --- | --- | --- | --- | --- | --- | --- | --- | --- | --- | --- | --- | --- | --- | --- | --- | --- | --- | --- | --- | --- | --- | --- | --- | --- | --- | --- | --- | --- | --- | --- | --- | --- | --- | --- | --- | --- | --- | --- | --- | --- | --- | --- | --- | --- | --- | --- | --- | --- | --- | --- | --- | --- | --- | --- | --- | --- | --- | --- | --- | --- | --- | --- | --- | --- | --- | --- | --- | --- | --- | --- | --- | --- | --- | --- | --- | --- | --- | --- | --- | --- | --- | --- | --- | --- | --- | --- | --- | --- | --- | --- | --- | --- | --- | --- | --- | --- | --- | --- | --- | --- | --- | --- | --- | --- | --- | --- | --- | --- | --- | --- | --- | --- | --- | --- | --- | --- | --- | --- | --- | --- | --- | --- | --- | --- | --- | --- | --- | --- | --- | --- | --- | --- | --- | --- | --- | --- | --- | --- | --- | --- | --- | --- | --- | --- | --- | --- | --- | --- | --- | --- | --- | --- | --- | --- | --- | --- | --- | --- | --- | --- | --- | --- | --- | --- | --- | --- | --- | --- | --- | --- | --- | --- | --- | --- | --- | --- | --- | --- | --- | --- | --- | --- | --- | --- | --- | --- | --- | --- | --- | --- | --- | --- | --- | --- | --- | --- | --- | --- | --- | --- | --- | --- | --- | --- | --- | --- | --- | --- | --- | --- | --- | --- | --- | --- | --- | --- | --- | --- | --- | --- | --- | --- | --- | --- | --- | --- | --- | --- | --- | --- | --- | --- | --- | --- | --- | --- | --- | --- | --- | --- | --- | --- | --- | --- | --- | --- | --- | --- | --- | --- | --- | --- | --- | --- | --- | --- | --- | --- | --- | --- | --- | --- | --- | --- | --- | --- | --- | --- | --- | --- | --- | --- | --- | --- | --- | --- | --- | --- | --- | --- | --- | --- | --- | --- | --- | --- | --- | --- | --- | --- | --- | --- | --- | --- | --- | --- | --- | --- | --- | --- | --- | --- | --- | --- | --- | --- | --- | --- | --- | --- | --- | --- | --- | --- | --- | --- | --- | --- | --- | --- | --- | --- | --- | --- | --- | --- | --- | --- | --- | --- | --- | --- | --- | --- | --- | --- | --- | --- | --- | --- | --- | --- | --- | --- | --- | --- | --- | --- | --- | --- | --- | --- | --- | --- | --- | --- | --- | --- | --- | --- | --- | --- | --- | --- | --- | --- | --- | --- | --- | --- | --- | --- | --- | --- | --- | --- | --- | --- | --- | --- | --- | --- | --- | --- | --- | --- | --- | --- | --- | --- | --- | --- | --- | --- | --- | --- | --- | --- | --- | --- | --- | --- | --- | --- | --- | --- | --- | --- | --- | --- | --- | --- | --- | --- | --- | --- | --- | --- | --- | --- | --- | --- | --- | --- | --- | --- | --- | --- | --- | --- | --- | --- | --- | --- | --- | --- | --- | --- | --- | --- | --- | --- | --- | --- | --- | --- | --- | --- | --- | --- | --- | --- | --- | --- | --- | --- | --- | --- | --- | --- | --- | --- | --- | --- | --- | --- | --- | --- | --- | --- | --- | --- | --- | --- | --- | --- | --- | --- | --- | --- | --- | --- | --- | --- | --- | --- | --- | --- | --- | --- | --- | --- | --- | --- | --- | --- | --- | --- | --- | --- | --- | --- | --- | --- | --- | --- | --- | --- | --- | --- | --- | --- | --- | --- | --- | --- | --- | --- | --- | --- | --- | --- | --- | --- | --- | --- | --- | --- | --- | --- | --- | --- | --- | --- | --- | --- | --- | --- | --- | --- | --- | --- | --- | --- | --- | --- | --- | --- | --- | --- | --- | --- | --- | --- | --- | --- | --- | --- | --- | --- | --- | --- | --- | --- | --- | --- | --- | --- | --- | --- | --- | --- | --- | --- | --- | --- | --- | --- | --- | --- | --- | --- | --- | --- | --- | --- | --- | --- | --- | --- | --- | --- | --- | --- | --- | --- | --- | --- | --- | --- | --- | --- | --- | --- | --- | --- | --- | --- | --- | --- | --- | --- | --- | --- | --- | --- | --- | --- | --- | --- | --- | --- | --- | --- | --- | --- | --- | --- | --- | --- | --- | --- | --- | --- | --- | --- | --- | --- | --- | --- | --- | --- | --- | --- | --- | --- | --- | --- | --- | --- | --- | --- | --- | --- | --- | --- | --- | --- | --- | --- | --- | --- | --- | --- | --- | --- | --- | --- | --- | --- | --- | --- | --- | --- | --- | --- | --- | --- | --- | --- | --- | --- | --- | --- | --- | --- | --- | --- | --- | --- | --- | --- | --- | --- | --- | --- | --- | --- | --- | --- | --- | --- | --- | --- | --- | --- | --- | --- | --- | --- | --- | --- | --- | --- | --- | --- | --- | --- | --- | --- | --- | --- | --- | --- | --- | --- | --- | --- | --- | --- | --- | --- | --- | --- | --- | --- | --- | --- | --- | --- | --- | --- | --- | --- | --- | --- | --- | --- | --- | --- | --- | --- | --- | --- | --- | --- | --- | --- | --- | --- | --- | --- | --- | --- | --- | --- | --- | --- | --- | --- | --- | --- | --- | --- | --- | --- | --- | --- | --- | --- | --- | --- | --- | --- | --- | --- | --- | --- | --- | --- | --- | --- | --- | --- | --- | --- | --- | --- | --- | --- | --- | --- | --- | --- | --- | --- | --- | --- | --- | --- | --- | --- | --- | --- | --- | --- | --- | --- | --- | --- | --- | --- | --- | --- | --- | --- | --- | --- | --- | --- | --- | --- | --- | --- | --- | --- | --- | --- | --- | --- | --- | --- | --- | --- | --- | --- | --- | --- | --- | --- | --- | --- | --- | --- | --- | --- | --- | --- | --- | --- | --- | --- | --- | --- | --- | --- | --- | --- | --- | --- | --- | --- | --- | --- | --- | --- | --- | --- | --- | --- | --- | --- | --- | --- | --- | --- | --- | --- | --- | --- | --- | --- | --- | --- | --- | --- | --- | --- | --- | --- | --- | --- | --- | --- | --- | --- | --- | --- | --- | --- | --- | --- | --- | --- | --- | --- | --- | --- | --- | --- | --- | --- | --- | --- | --- | --- | --- | --- | --- | --- | --- | --- | --- | --- | --- | --- | --- | --- | --- | --- | --- | --- | --- | --- | --- | --- | --- | --- | --- | --- | --- | --- | --- | --- | --- | --- | --- | --- | --- | --- | --- | --- | --- | --- | --- | --- | --- | --- | --- | --- | --- | --- | --- | --- | --- | --- | --- | --- | --- | --- | --- | --- | --- | --- | --- | --- | --- | --- | --- | --- | --- | --- | --- | --- | --- | --- | --- | --- | --- | --- | --- | --- | --- | --- | --- | --- | --- | --- | --- | --- | --- | --- | --- | --- | --- | --- | --- | --- | --- | --- | --- | --- | --- | --- | --- | --- | --- | --- | --- | --- | --- | --- | --- | --- | --- | --- | --- | --- | --- | --- | --- | --- | --- | --- | --- | --- | --- | --- | --- | --- | --- | --- | --- | --- | --- | --- | --- | --- | --- | --- | --- | --- | --- | --- | --- | --- | --- | --- | --- | --- | --- | --- | --- | --- | --- | --- | --- | --- | --- | --- | --- | --- | --- | --- | --- | --- | --- | --- | --- | --- | --- | --- | --- | --- | --- | --- | --- | --- | --- | --- | --- | --- | --- | --- | --- | --- | --- | --- | --- | --- | --- | --- | --- | --- | --- | --- | --- | --- | --- | --- | --- | --- | --- | --- | --- | --- | --- | --- | --- | --- | --- | --- | --- | --- | --- | --- | --- | --- | --- | --- | --- | --- | --- | --- | --- | --- | --- | --- | --- | --- | --- | --- | --- | --- | --- | --- | --- | --- | --- | --- | --- | --- | --- | --- | --- | --- | --- | --- | --- | --- | --- | --- | --- | --- | --- | --- | --- | --- | --- | --- | --- | --- | --- | --- | --- | --- | --- | --- | --- | --- | --- | --- | --- | --- | --- | --- | --- | --- | --- | --- | --- | --- | --- | --- | --- | --- | --- | --- | --- | --- | --- | --- | --- | --- | --- | --- | --- | --- | --- | --- | --- | --- | --- | --- | --- | --- | --- | --- | --- | --- | --- | --- | --- | --- | --- | --- | --- | --- | --- | --- | --- | --- | --- | --- | --- | --- | --- | --- | --- | --- | --- | --- | --- | --- | --- | --- | --- | --- | --- | --- | --- | --- | --- | --- | --- | --- | --- | --- | --- | --- | --- | --- | --- | --- | --- | --- | --- | --- | --- | --- | --- | --- | --- | --- | --- | --- | --- | --- | --- | --- | --- | --- | --- | --- | --- | --- | --- | --- | --- | --- | --- | --- | --- | --- | --- | --- | --- | --- | --- | --- | --- | --- | --- | --- | --- | --- | --- | --- | --- | --- | --- | --- | --- | --- | --- | --- | --- | --- | --- | --- | --- | --- | --- | --- | --- | --- | --- | --- | --- | --- | --- | --- | --- | --- | --- | --- | --- | --- | --- | --- | --- | --- | --- | --- | --- | --- | --- | --- | --- | --- | --- | --- | --- | --- | --- | --- | --- | --- | --- | --- | --- | --- | --- | --- | --- | --- | --- | --- | --- | --- | --- | --- | --- | --- | --- | --- | --- | --- | --- | --- | --- | --- | --- | --- | --- | --- | --- | --- | --- | --- | --- | --- | --- | --- | --- | --- | --- | --- | --- | --- | --- | --- | --- | --- | --- | --- | --- | --- | --- | --- | --- | --- | --- | --- | --- | --- | --- | --- | --- | --- | --- | --- | --- | --- | --- | --- | --- | --- | --- | --- | --- | --- | --- | --- | --- | --- | --- | --- | --- | --- | --- | --- | --- | --- | --- | --- | --- | --- | --- | --- | --- | --- | --- | --- | --- | --- | --- | --- | --- | --- | --- | --- | --- | --- | --- | --- | --- | --- | --- | --- | --- | --- | --- | --- | --- | --- | --- | --- | --- | --- | --- | --- | --- | --- | --- | --- | --- | --- | --- | --- | --- | --- | --- | --- | --- | --- | --- | --- | --- | --- | --- | --- | --- | --- | --- | --- | --- | --- | --- | --- | --- | --- | --- | --- | --- | --- | --- | --- | --- | --- | --- | --- | --- | --- | --- | --- | --- | --- | --- | --- | --- | --- | --- | --- | --- | --- | --- | --- | --- | --- | --- | --- | --- | --- | --- | --- | --- | --- | --- | --- | --- | --- | --- | --- | --- | --- | --- | --- | --- | --- | --- | --- | --- | --- | --- | --- | --- | --- | --- | --- | --- | --- | --- | --- | --- | --- | --- | --- | --- | --- | --- | --- | --- | --- | --- | --- | --- | --- | --- | --- | --- | --- | --- | --- | --- | --- | --- | --- | --- | --- | --- | --- | --- | --- | --- | --- | --- | --- | --- | --- | --- | --- | --- | --- | --- | --- | --- | --- | --- | --- | --- | --- | --- | --- | --- | --- | --- | --- | --- | --- | --- | --- | --- | --- | --- | --- | --- | --- | --- | --- | --- | --- | --- | --- | --- | --- | --- | --- | --- | --- | --- | --- | --- | --- | --- | --- | --- | --- | --- | --- | --- | --- | --- | --- | --- | --- | --- | --- | --- | --- | --- | --- | --- | --- | --- | --- | --- | --- | --- | --- | --- | --- | --- | --- | --- | --- | --- | --- | --- | --- | --- | --- | --- | --- | --- | --- | --- | --- | --- | --- | --- | --- | --- | --- | --- | --- | --- | --- | --- | --- | --- | --- | --- | --- | --- | --- | --- | --- | --- | --- | --- | --- | --- | --- | --- | --- | --- | --- | --- | --- | --- | --- | --- | --- | --- | --- | --- | --- | --- | --- | --- | --- | --- | --- | --- | --- | --- | --- | --- | --- | --- | --- | --- | --- | --- | --- | --- | --- | --- | --- | --- | --- | --- | --- | --- | --- | --- | --- | --- | --- | --- | --- | --- | --- | --- | --- | --- | --- | --- | --- | --- | --- | --- | --- | --- | --- | --- | --- | --- | --- | --- | --- | --- | --- | --- | --- | --- | --- | --- | --- | --- | --- | --- | --- | --- | --- | --- | --- | --- | --- | --- | --- | --- | --- | --- | --- | --- | --- | --- | --- | --- | --- | --- | --- | --- | --- | --- | --- | --- | --- | --- | --- | --- | --- | --- | --- | --- | --- | --- | --- | --- | --- | --- | --- | --- | --- | --- | --- | --- | --- | --- | --- | --- | --- | --- | --- | --- | --- | --- | --- | --- | --- | --- | --- | --- | --- | --- | --- | --- | --- | --- | --- | --- | --- | --- | --- | --- | --- | --- | --- | --- | --- | --- | --- | --- | --- | --- | --- | --- | --- | --- | --- | --- | --- | --- | --- | --- | --- | --- | --- | --- | --- | --- | --- | --- | --- | --- | --- | --- | --- | --- | --- | --- | --- | --- | --- | --- | --- | --- | --- | --- | --- | --- | --- | --- | --- | --- | --- | --- | --- | --- | --- | --- | --- | --- | --- | --- | --- | --- | --- | --- | --- | --- | --- | --- | --- | --- | --- | --- | --- | --- | --- | --- | --- | --- | --- | --- | --- | --- | --- | --- | --- | --- | --- | --- | --- | --- | --- | --- | --- | --- | --- | --- | --- | --- | --- | --- | --- | --- | --- | --- | --- | --- | --- | --- | --- | --- | --- | --- | --- | --- | --- | --- | --- | --- | --- | --- | --- | --- | --- | --- | --- | --- | --- | --- | --- | --- | --- | --- | --- | --- | --- | --- | --- | --- | --- | --- | --- | --- | --- | --- | --- | --- | --- | --- | --- | --- | --- | --- | --- | --- | --- | --- | --- | --- | --- | --- | --- | --- | --- | --- | --- | --- | --- | --- | --- | --- | --- | --- | --- | --- | --- | --- | --- | --- | --- | --- | --- | --- | --- | --- | --- | --- | --- | --- | --- | --- | --- | --- | --- | --- | --- | --- | --- | --- | --- | --- | --- | --- | --- | --- | --- | --- | --- | --- | --- | --- | --- | --- | --- | --- | --- | --- | --- | --- | --- | --- | --- | --- | --- | --- | --- | --- | --- | --- | --- | --- | --- | --- | --- | --- | --- | --- | --- | --- | --- | --- | --- | --- | --- | --- | --- | --- | --- | --- | --- | --- | --- | --- | --- | --- | --- | --- | --- | --- | --- | --- | --- | --- | --- | --- | --- | --- | --- | --- | --- | --- | --- | --- | --- | --- | --- | --- | --- | --- | --- | --- | --- | --- | --- | --- | --- | --- | --- | --- | --- | --- | --- | --- | --- | --- | --- | --- | --- | --- | --- | --- | --- | --- | --- | --- | --- | --- | --- | --- | --- | --- | --- | --- | --- | --- | --- | --- | --- | --- | --- | --- | --- | --- | --- | --- | --- | --- | --- | --- | --- | --- | --- | --- | --- | --- | --- | --- | --- | --- | --- | --- | --- | --- | --- | --- | --- | --- | --- | --- | --- | --- | --- | --- | --- | --- | --- | --- | --- | --- | --- | --- | --- | --- | --- | --- | --- | --- | --- | --- | --- | --- | --- | --- | --- | --- | --- | --- | --- | --- | --- | --- | --- | --- | --- | --- | --- | --- | --- | --- | --- | --- | --- | --- | --- | --- | --- | --- | --- | --- | --- | --- | --- | --- | --- | --- | --- | --- | --- | --- | --- | --- | --- | --- | --- | --- | --- | --- | --- | --- | --- | --- | --- | --- | --- | --- | --- | --- | --- | --- | --- | --- | --- | --- | --- | --- | --- | --- | --- | --- | --- | --- | --- | --- | --- | --- | --- | --- | --- | --- | --- | --- | --- | --- | --- | --- | --- | --- | --- | --- | --- | --- | --- | --- | --- | --- | --- | --- | --- | --- | --- | --- | --- | --- | --- | --- | --- | --- | --- | --- | --- | --- | --- | --- | --- | --- | --- | --- | --- | --- | --- | --- | --- | --- | --- | --- | --- | --- | --- | --- | --- | --- | --- | --- | --- | --- | --- | --- | --- | --- | --- | --- | --- | --- | --- | --- | --- | --- | --- | --- | --- | --- | --- | --- | --- | --- | --- |
| |  |  |  |  |  |  |  |  |  | | --- | --- | --- | --- | --- | --- | --- | --- | --- | | **Position** | **Reference** | **Sample** | **Quality** | **Type** | **Region** | **AA Exchange** | **PAM1** | **Known Variant** | | 1977 | A | G | 374.77 | SNP | intergenic |  |  | - | | 2540 | G | A | 173.90 | SNP | Rv0002 (dnaN) | Met(s)163Ile | 2 | - | | 4013 | T | C | 463.77 | SNP | Rv0003 (recF) | Ile245Thr | 11 | - | | 7362 | G | C | 501.77 | SNP | Rv0006 (gyrA) | Glu21Gln | 27 | - | | 7539 | A | G | 300.78 | SNP | Rv0006 (gyrA) | Thr80Ala | 32 | genotype | | 7585 | G | C | 469.77 | SNP | Rv0006 (gyrA) | Ser95Thr | 32 | genotype | | 9304 | G | A | 418.77 | SNP | Rv0006 (gyrA) | Gly668Asp | 6 | - | | 11879 | A | G | 303.78 | SNP | Rv0008c | Ser145Pro | 12 | - | | 14785 | T | C | 219.84 | SNP | Rv0012 | Cys233Arg | 1 | - | | 18091 | G | A | 333.78 | SNP | Rv0015c (pknA) | silent (Thr224) | 9871 | - | | 21795 | G | A | 62.74 | SNP | Rv0018c (pstP) | Pro463Ser | 17 | - | | 22334 | C | T | 171.90 | SNP | Rv0018c (pstP) | Arg283His | 8 | - | | 26959 | C | G | 223.84 | SNP | intergenic |  |  | - | | 32075 | T | C | 270.80 | SNP | Rv0029 | Trp7Arg | 8 | - | | 32405 | A | G | 219.77 | SNP | Rv0029 | Thr117Ala | 32 | - | | 34044 | T | C | 323.78 | SNP | intergenic |  |  | - | | 34226 | A | G | 416.77 | SNP | intergenic |  |  | - | | 37031 | C | G | 253.80 | SNP | Rv0034 | silent (Ala55) | 9867 | - | | 42967 | G | C | 585.77 | SNP | Rv0040c (mtc28) | silent (Pro133) | 9926 | - | | 52733 | C | T | 311.78 | SNP | intergenic |  |  | - | | 54304 | C | T | 190.31 | SNP | Rv0050 (ponA1) | silent (Leu214) | 9947 | - | | 55553 | C | T | 186.90 | SNP | Rv0050 (ponA1) | Pro631Ser | 17 | - | | 56334 | G | C | 455.77 | SNP | Rv0051 | silent (Ser213) | 9840 | - | | 62049 | A | G | 296.78 | SNP | Rv0058 (dnaB) | Arg552Gly | 1 | - | | 69989 | G | A | 432.77 | SNP | Rv0064 | Gly457Asp | 6 | - | | 70816 | A | G | 363.77 | SNP | Rv0064 | Asn733Asp | 42 | - | | 71336 | G | C | 84.28 | SNP | Rv0064 | Arg906Pro | 5 | - | | 75940 | G | C | 232.84 | SNP | Rv0068 | Val(s)214Leu | 3 | - | | 78705 | G | A | 108.77 | SNP | Rv0070c (glyA2) | silent (Tyr64) | 9945 | - | | 79162 | G | A | 381.53 | SNP | intergenic |  |  | - | | 80616 | C | G | 221.84 | SNP | intergenic |  |  | - | | 92199 | T | G | 375.77 | SNP | Rv0083 | silent (Thr600) | 9871 | - | | 104712 | C | T | 171.90 | SNP | intergenic |  |  | - | | 104962 | G | A | 377.77 | SNP | Rv0095c | Ala85Val(s) | 9867 | - | | 105021 | G | A | 280.78 | SNP | Rv0095c | silent (Ser65) | 9840 | - | | 105031 | T | G | 226.84 | SNP | Rv0095c | Asp62Ala | 10 | - | | 105045 | G | C | 350.78 | SNP | Rv0095c | Asp57Glu | 56 | - | | 105060 | G | A | 308.78 | SNP | Rv0095c | silent (Asp52) | 9859 | - | | 105063 | G | A | 317.78 | SNP | Rv0095c | silent (Phe51) | 9946 | - | | 116000 | T | G | 335.78 | SNP | Rv0101 (nrp) | Val2000Val(s) | 18 | - | | 117403 | C | T | 360.78 | SNP | Rv0101 (nrp) | Ala2468Val(s) | 9867 | - | | 122109 | A | G | 489.77 | SNP | Rv0103c (ctpB) | Leu(s)22Ser | 28 | - | | 122794 | T | G | 204.94 | SNP | Rv0104 | Phe160Val | 1 | - | | 125830 | G | GA | 830.73 | INS | Rv0107c (ctpI) |  |  | - | | 130449 | C | A | 393.77 | SNP | Rv0107c (ctpI) | silent (Ala31) | 9867 | - | | 131174 | T | TG | 888.73 | INS | intergenic |  |  | - | | 133839 | C | T | 528.77 | SNP | intergenic |  |  | - | | 136950 | C | T | 467.77 | SNP | Rv0112 (gca) | Ala221Val(s) | 9867 | - | | 138788 | C | T | 414.77 | SNP | Rv0115 (hddA) | silent (Phe92) | 9946 | - | | 146087 | T | C | 399.77 | SNP | Rv0120c (fusA2) | Asn562Ser | 34 | - | | 154283 | T | C | 344.78 | SNP | Rv0127 (mak) | Ser18Pro | 12 | - | | 177857 | G | A | 248.80 | SNP | Rv0151c (PE1) | Leu485Leu(s) | 4 | - | | 188800 | T | C | 384.77 | SNP | Rv0159c (PE3) | Thr14Ala | 32 | - | | 194681 | G | C | 314.78 | SNP | Rv0165c (mce1R) | silent (Leu45) | 9947 | - | | 196642 | C | T | 62.74 | SNP | Rv0166 (fadD5) | silent (Asn550) | 9822 | - | | 206339 | T | C | 526.77 | SNP | Rv0174 (mce1F) | Leu370Pro | 2 | - | | 218204 | C | T | 443.77 | SNP | Rv0186 (bglS) | Arg646STOP | 2 | - | | 219120 | A | G | 284.80 | SNP | Rv0187 | Asp139Gly | 11 | - | | 222486 | C | T | 232.80 | SNP | Rv0191 | silent (Ala66) | 9867 | - | | 223942 | T | C | 104.03 | SNP | Rv0192 | Ser127Pro | 12 | - | | 225323 | T | C | 457.77 | SNP | Rv0193c | Lys417Glu | 4 | - | | 227098 | T | C | 455.77 | SNP | Rv0194 | Met(s)74Thr | 22 | - | | 229433 | C | T | 301.78 | SNP | Rv0194 | silent (Tyr852) | 9945 | - | | 231114 | C | G | 259.80 | SNP | Rv0195 | silent (Ala72) | 9867 | - | | 232110 | C | T | 140.03 | SNP | Rv0196 | Thr155Met(s) | 32 | - | | 234477 | T | G | 99.28 | SNP | Rv0197 | Tyr749STOP | 2 | - | | 234496 | C | CGT | 258.87 | INS | Rv0197 |  |  | - | | 237709 | C | T | 280.78 | SNP | Rv0200 | silent (Ala168) | 9867 | - | | 256640 | T | G | 562.77 | SNP | Rv0214 (fadD4) | Ser193Ala | 35 | - | | 257982 | T | G | 297.78 | SNP | Rv0215c (fadE3) | Asp292Ala | 10 | - | | 261869 | T | C | 308.78 | SNP | Rv0218 | Cys316Arg | 1 | - | | 265244 | C | T | 151.03 | SNP | Rv0221 | Ala393Val(s) | 9867 | - | | 265554 | A | C | 449.77 | SNP | Rv0222 (echA1) | silent (Val16) | 9901 | - | | 278681 | C | G | 404.77 | SNP | Rv0233 (nrdB) | His33Asp | 4 | - | | 281628 | C | A | 340.78 | SNP | Rv0235c | Leu(s)329Phe | 1 | - | | 285772 | A | C | 162.90 | SNP | Rv0236c (aftD) | silent (Pro360) | 9926 | - | | 285871 | A | G | 92.28 | SNP | Rv0236c (aftD) | silent (Val327) | 9901 | - | | 310973 | G | A | 412.77 | SNP | Rv0259c | Ala182Val(s) | 9867 | - | | 311613 | G | T | 416.77 | SNP | Rv0260c | silent (Val349) | 9901 | - | | 312060 | T | G | 336.78 | SNP | Rv0260c | silent (Arg200) | 9913 | - | | 312944 | C | T | 364.78 | SNP | Rv0261c (narK3) | Val409Ile | 33 | - | | 325038 | G | A | 269.80 | SNP | Rv0270 (fadD2) | Gly158Ser | 16 | - | | 325039 | G | A | 269.80 | SNP | Rv0270 (fadD2) | Gly158Asp | 6 | - | | 332357 | A | G | 276.80 | SNP | Rv0276 | Ile204Val | 57 | - | | 338100 | T | C | 55.74 | SNP | Rv0279c (PE\_PGRS4) | Asn325Ser | 34 | - | | 346275 | C | G | 322.78 | SNP | Rv0284 (eccC3) | Pro214Arg | 4 | - | | 356528 | A | G | 277.80 | SNP | Rv0292 (eccE3) | Asn217Asp | 42 | - | | 364563 | A | C | 266.78 | SNP | intergenic |  |  | - | | 373282 | TA | T | 540.73 | DEL | Rv0305c (PPE6) |  |  | - | | 384380 | A | C | 329.78 | SNP | Rv0315 | Lys260Thr | 8 | - | | 386432 | C | G | 306.78 | SNP | Rv0318c | Gly223Ala | 21 | - | | 390828 | T | C | 143.03 | SNP | Rv0323c | Ser142Gly | 21 | - | | 391111 | G | A | 482.77 | SNP | Rv0323c | silent (Thr47) | 9871 | - | | 401763 | T | C | 373.78 | SNP | intergenic |  |  | - | | 403980 | G | A | 557.77 | SNP | Rv0338c | Ala621Val | 13 | - | | 404326 | T | C | 307.78 | SNP | Rv0338c | Arg506Gly | 1 | - | | 412017 | C | G | 373.77 | SNP | Rv0342 (iniA) | Gln394Glu | 35 | - | | 414486 | C | T | 450.77 | SNP | Rv0344c (lpqJ) | silent (Glu152) | 9865 | - | | 420008 | A | G | 360.78 | SNP | Rv0350 (dnaK) | silent (Ala58) | 9867 | - | | 424320 | T | TC | 262.77 | INS | Rv0354c (PPE7) |  |  | - | | 428563 | G | A | 126.03 | SNP | Rv0355c (PPE8) | silent (Asn2039) | 9822 | - | | 435708 | G | A | 396.77 | SNP | Rv0357c (purA) | silent (Thr354) | 9871 | - | | 454295 | T | C | 232.84 | SNP | Rv0376c | silent (Pro26) | 9926 | - | | 457171 | A | G | 142.03 | SNP | Rv0381c | Val218Ala | 18 | - | | 457452 | T | G | 99.28 | SNP | Rv0381c | silent (Thr124) | 9871 | - | | 459399 | A | C | 62.74 | SNP | intergenic |  |  | - | | 467497 | C | CG | 264.77 | INS | Rv0388c (PPE9) |  |  | - | | 467508 | C | CG | 287.77 | INS | Rv0388c (PPE9) |  |  | - | | 467516 | G | C | 173.90 | SNP | Rv0388c (PPE9) | silent (Ser162) | 9840 | - | | 467526 | C | G | 220.84 | SNP | Rv0388c (PPE9) | Gly159Ala | 21 | - | | 467546 | G | C | 188.90 | SNP | Rv0388c (PPE9) | Asp152Glu | 56 | - | | 467557 | A | C | 187.90 | SNP | Rv0388c (PPE9) | Leu(s)149Val(s) | 9867 | - | | 467564 | A | C | 255.80 | SNP | Rv0388c (PPE9) | His146Gln | 23 | - | | 467585 | G | C | 280.78 | SNP | Rv0388c (PPE9) | His139Gln | 23 | - | | 467590 | T | C | 254.80 | SNP | Rv0388c (PPE9) | Thr138Ala | 32 | - | | 467621 | T | G | 271.80 | SNP | Rv0388c (PPE9) | silent (Gly127) | 9935 | - | | 467638 | G | T | 253.80 | SNP | Rv0388c (PPE9) | Gln122Lys | 12 | - | | 473800 | G | C | 229.84 | SNP | Rv0393 | silent (Thr340) | 9871 | - | | 475178 | T | C | 331.78 | SNP | Rv0395 | Val80Ala | 18 | - | | 483874 | C | T | 239.84 | SNP | intergenic |  |  | - | | 488796 | G | A | 348.78 | SNP | Rv0405 (pks6) | Val(s)1022Val | 13 | - | | 489935 | G | C | 368.77 | SNP | Rv0405 (pks6); Rv0406c | Arg1402Pro; silent (Thr257) | 5; 9871 | - | | 493934 | T | C | 329.78 | SNP | Rv0409 (ackA) | silent (Arg28) | 9913 | - | | 499919 | C | G | 240.84 | SNP | Rv0413 (mutT3) | silent (Gly69) | 9935 | - | | 502589 | C | G | 120.03 | SNP | Rv0417 (thiG) | Ser75Cys | 5 | - | | 503354 | G | C | 750.77 | SNP | intergenic |  |  | - | | 513257 | T | C | 243.80 | SNP | Rv0425c (ctpH) | Met(s)689Val(s) | 9867 | - | | 524095 | A | G | 233.84 | SNP | Rv0435c | Ser147Pro | 12 | - | | 533729 | C | T | 544.77 | SNP | Rv0444c (rskA) | Val(s)21Met(s) | 9867 | - | | 541201 | A | G | 142.03 | SNP | Rv0450c (mmpL4) | silent (Leu97) | 9947 | - | | 551525 | A | C | 351.78 | SNP | Rv0459 | silent (Arg110) | 9913 | - | | 573262 | A | G | 270.80 | SNP | Rv0484c | silent (Gly180) | 9935 | - | | 578032 | C | T | 272.78 | SNP | Rv0488 | silent (Ala123) | 9867 | - | | 580772 | T | A | 142.03 | SNP | intergenic |  |  | - | | 580773 | GGGGGCACCACCCGCTTGCG GGGGA | G | 1914.75 | DEL | intergenic |  |  | - | | 587974 | G | A | 284.80 | SNP | Rv0497 | Ala200Thr | 22 | - | | 590436 | T | C | 494.77 | SNP | Rv0500 (proC) | silent (Ala118) | 9867 | - | | 591628 | T | C | 277.80 | SNP | intergenic |  |  | - | | 595232 | C | T | 95.03 | SNP | Rv0504c | Gly24Glu | 4 | - | | 597816 | A | G | 270.80 | SNP | Rv0507 (mmpL2) | silent (Ala206) | 9867 | - | | 598475 | G | A | 395.77 | SNP | Rv0507 (mmpL2) | Arg426His | 8 | - | | 610120 | T | G | 524.77 | SNP | intergenic |  |  | - | | 623508 | C | G | 62.74 | SNP | Rv0532 (PE\_PGRS6) | Ala239Gly | 21 | - | | 628113 | C | T | 298.78 | SNP | Rv0536 (galE3) | Ala289Val(s) | 9867 | - | | 630722 | G | C | 277.80 | SNP | Rv0538 | Arg228Pro | 5 | - | | 637319 | G | A | 232.96 | SNP | Rv0545c (pitA) | Pro49Ser | 17 | - | | 648002 | T | G | 406.77 | SNP | Rv0556 | Leu15Arg | 1 | - | | 661306 | CT | C | 341.75 | DEL | Rv0570 (nrdZ) |  |  | - | | 665293 | A | G | 397.77 | SNP | Rv0572c | Phe31Leu | 13 | - | | 669398 | T | C | 181.90 | SNP | Rv0575c | silent (Gln116) | 9876 | - | | 673238 | A | G | 100.28 | SNP | Rv0578c (PE\_PGRS7) | silent (His893) | 9912 | - | | 680206 | T | C | 34.77 | SNP | Rv0584 | silent (Thr326) | 9871 | - | | 685461 | C | G | 122.79 | SNP | Rv0587 (yrbE2A) | silent (Ala111) | 9867 | - | | 685608 | T | C | 229.84 | SNP | Rv0587 (yrbE2A) | silent (Leu160) | 9947 | - | | 685869 | G | A | 373.78 | SNP | Rv0587 (yrbE2A) | silent (Leu247) | 9947 | - | | 686972 | T | C | 620.77 | SNP | Rv0589 (mce2A) | Phe51Ser | 3 | - | | 690465 | T | G | 225.84 | SNP | Rv0591 (mce2C) | silent (Leu469) | 9947 | - | | 698968 | G | A | 206.84 | SNP | Rv0601c | silent (Gly9) | 9935 | - | | 712271 | A | G | 158.10 | SNP | intergenic |  |  | - | | 721498 | G | A | 240.84 | SNP | Rv0629c (recD) | Leu79Leu(s) | 4 | - | | 754186 | A | G | 415.77 | SNP | Rv0658c | Leu75Pro | 2 | - | | 759199 | G | T | 398.10 | SNP | Rv0666 | Val(s)22Leu(s) | 9867 | - | | 761110 | A | T | 284.78 | SNP | Rv0667 (rpoB) | Asp435Val | 1 | resistance | | 762968 | G | A | 146.77 | SNP | Rv0667 (rpoB) | silent (Lys1054) | 9926 | - | | 763486 | T | G | 41.77 | SNP | Rv0668 (rpoC) | silent (Leu39) | 9947 | - | | 763550 | T | C | 66.77 | SNP | Rv0668 (rpoC) | Tyr61His | 4 | - | | 763570 | G | C | 38.77 | SNP | Rv0668 (rpoC) | silent (Arg67) | 9913 | - | | 763573 | G | T | 38.77 | SNP | Rv0668 (rpoC) | Val(s)68Val | 13 | - | | 763589 | A | G | 48.77 | SNP | Rv0668 (rpoC) | Ile74Val | 57 | - | | 764428 | G | C | 30.77 | SNP | Rv0668 (rpoC) | silent (Arg353) | 9913 | - | | 764434 | A | G | 30.77 | SNP | Rv0668 (rpoC) | silent (Lys355) | 9926 | - | | 764435 | A | C | 30.77 | SNP | Rv0668 (rpoC) | silent (Arg356) | 9913 | - | | 764441 | A | C | 30.77 | SNP | Rv0668 (rpoC) | Ile358Leu | 22 | - | | 764452 | T | C | 30.77 | SNP | Rv0668 (rpoC) | silent (Gly361) | 9935 | - | | 764461 | A | C | 30.77 | SNP | Rv0668 (rpoC) | Glu364Asp | 53 | - | | 764470 | C | T | 30.77 | SNP | Rv0668 (rpoC) | silent (Val367) | 9901 | - | | 764471 | A | C | 30.77 | SNP | Rv0668 (rpoC) | Asn368His | 18 | - | | 764473 | C | G | 30.77 | SNP | Rv0668 (rpoC) | Asn368Lys | 25 | - | | 764498 | T | G | 30.77 | SNP | Rv0668 (rpoC) | Ser377Ala | 35 | - | | 764503 | G | C | 33.77 | SNP | Rv0668 (rpoC) | Val(s)378Val | 13 | - | | 764548 | G | A | 30.77 | SNP | Rv0668 (rpoC) | silent (Gly393) | 9935 | - | | 775639 | T | C | 343.78 | SNP | Rv0676c (mmpL5) | Ile948Val | 57 | - | | 781395 | T | C | 135.03 | SNP | intergenic (Rv0682-165nt) |  |  | - | | 789688 | CG | C | 736.73 | DEL | intergenic |  |  | - | | 818798 | G | T | 561.77 | SNP | Rv0726c | silent (Ile281) | 9872 | - | | 837033 | A | G | 99.28 | SNP | Rv0746 (PE\_PGRS9) | Thr445Ala | 32 | - | | 839471 | T | C | 66.77 | SNP | Rv0747 (PE\_PGRS10) | Leu(s)341Leu | 3 | - | | 839472 | T | A | 57.77 | SNP | Rv0747 (PE\_PGRS10) | Leu(s)341STOP | 9867 | - | | 839515 | G | A | 110.77 | SNP | Rv0747 (PE\_PGRS10) | silent (Ala355) | 9867 | - | | 839516 | A | G | 127.77 | SNP | Rv0747 (PE\_PGRS10) | Thr356Ala | 32 | - | | 839519 | C | G | 127.77 | SNP | Rv0747 (PE\_PGRS10) | Leu357Val(s) | 4 | - | | 839520 | T | C | 127.77 | SNP | Rv0747 (PE\_PGRS10) | Leu357Pro | 2 | - | | 839534 | A | C | 127.77 | SNP | Rv0747 (PE\_PGRS10) | Ile362Leu | 22 | - | | 841764 | G | C | 257.80 | SNP | Rv0749A | silent (Thr37) | 9871 | - | | 842107 | C | T | 442.77 | SNP | Rv0750 | silent (Ala25) | 9867 | - | | 846256 | C | A | 198.84 | SNP | Rv0754 (PE\_PGRS11) | Ala33Asp | 6 | - | | 852910 | C | T | 368.77 | SNP | Rv0758 (phoR) | Pro172Leu | 3 | - | | 854252 | GCC | G | 1009.73 | DEL | intergenic |  |  | - | | 857696 | A | G | 358.78 | SNP | Rv0764c (cyp51) | silent (Ala114) | 9867 | - | | 859769 | A | G | 205.84 | SNP | Rv0766c (cyp123) | Ser102Pro | 12 | - | | 869679 | C | T | 196.84 | SNP | Rv0776c | Gly29Arg | 0 | - | | 874835 | C | CCG | 1357.73 | INS | Rv0781 (ptrBa); Rv0782 (ptrBb) |  |  | - | | 880562 | G | T | 239.80 | SNP | Rv0785 | Cys408Phe | 0 | - | | 882257 | T | C | 357.78 | SNP | Rv0787 | Tyr267His | 4 | - | | 887391 | T | C | 354.78 | SNP | Rv0794c | Met(s)416Val(s) | 9867 | - | | 888774 | G | A | 44.77 | SNP | intergenic |  |  | - | | 888992 | A | C | 265.80 | SNP | intergenic |  |  | - | | 893733 | T | G | 489.77 | SNP | Rv0800 (pepC) | Leu139Arg | 1 | - | | 900221 | T | C | 223.84 | SNP | Rv0806c (cpsY) | Val370Val(s) | 18 | - | | 901806 | GTCCGGA | G | 1216.73 | DEL | Rv0807 |  |  | - | | 902282 | T | C | 36.77 | SNP | Rv0808 (purF) | Leu(s)58Leu | 3 | - | | 903550 | T | C | 229.84 | SNP | Rv0808 (purF) | silent (Ala480) | 9867 | - | | 903913 | T | C | 240.84 | SNP | Rv0809 (purM) | silent (Gly63) | 9935 | - | | 906857 | A | G | 240.84 | SNP | Rv0812 | Ile145Met(s) | 6 | - | | 918995 | A | G | 475.77 | SNP | Rv0825c | Leu(s)187Leu | 3 | - | | 919384 | T | C | 472.77 | SNP | Rv0825c | Tyr57Cys | 3 | - | | 921813 | C | G | 100.28 | SNP | Rv0829 | Ala80Gly | 21 | - | | 928633 | G | T | 93.28 | SNP | Rv0834c (PE\_PGRS14) | Thr618Lys | 11 | - | | 928634 | T | A | 92.28 | SNP | Rv0834c (PE\_PGRS14) | Thr618Ser | 38 | - | | 944941 | A | G | 513.77 | SNP | Rv0848 (cysK2) | Arg2Gly | 1 | - | | 945214 | G | A | 340.78 | SNP | Rv0848 (cysK2) | Gly93Ser | 16 | - | | 946075 | T | G | 555.77 | SNP | Rv0849 | Phe7Cys | 0 | - | | 949535 | T | C | 342.89 | SNP | Rv0853c (pdc) | silent (Ala528) | 9867 | - | | 955524 | A | G | 350.78 | SNP | Rv0859 (fadA) | Ser150Gly | 21 | - | | 955983 | C | T | 255.80 | SNP | Rv0859 (fadA) | Pro303Ser | 17 | - | | 956644 | C | T | 94.28 | SNP | Rv0860 (fadB) | Leu118Leu(s) | 4 | - | | 976897 | TGG | T | 584.73 | DEL | Rv0878c (PPE13) |  |  | - | | 979314 | C | T | 233.80 | SNP | Rv0880 | silent (Ile127) | 9872 | - | | 979704 | G | C | 360.78 | SNP | Rv0881 | Gly115Arg | 0 | - | | 986463 | G | C | 440.77 | SNP | intergenic |  |  | - | | 990001 | G | C | 378.77 | SNP | Rv0890c | Pro866Ala | 22 | - | | 990626 | T | A | 545.77 | SNP | Rv0890c | Leu657Phe | 6 | - | | 993346 | A | C | 587.77 | SNP | Rv0891c | Val37Gly | 5 | - | | 996469 | A | T | 590.77 | SNP | intergenic |  |  | - | | 1010204 | C | CG | 518.73 | INS | Rv0907 |  |  | - | | 1020044 | C | T | 416.77 | SNP | intergenic |  |  | - | | 1025106 | T | C | 476.77 | SNP | Rv0919 | silent (Phe141) | 9946 | - | | 1028999 | A | T | 361.78 | SNP | Rv0922 | Ser439Cys | 5 | - | | 1037012 | T | C | 229.84 | SNP | Rv0930 (pstA1) | Met(s)5Thr | 22 | - | | 1037911 | C | T | 199.84 | SNP | Rv0930 (pstA1) | Arg305STOP | 2 | - | | 1047165 | T | C | 87.81 | SNP | Rv0938 (ligD) | Cys344Arg | 1 | - | | 1056916 | T | G | 128.03 | SNP | intergenic |  |  | - | | 1068151 | T | C | 374.77 | SNP | Rv0956 (purN) | silent (His197) | 9912 | - | | 1068432 | A | G | 313.78 | SNP | Rv0957 (purH) | silent (Pro76) | 9926 | - | | 1070702 | T | C | 263.80 | SNP | Rv0958 | Ser274Pro | 12 | - | | 1074558 | G | A | 629.77 | SNP | Rv0962c (lprP) | Pro186Leu | 3 | - | | 1075279 | T | C | 508.77 | SNP | intergenic |  |  | - | | 1076309 | G | T | 370.77 | SNP | Rv0964c | Pro124Thr | 5 | - | | 1077312 | A | G | 315.78 | SNP | Rv0966c | Val(s)175Ala | 9867 | - | | 1079927 | C | A | 185.90 | SNP | Rv0969 (ctpV) | silent (Thr395) | 9871 | - | | 1081681 | T | C | 308.78 | SNP | Rv0970 | silent (Val210) | 9901 | - | | 1087193 | G | C | 326.78 | SNP | Rv0974c (accD2) | Asn51Lys | 25 | - | | 1093406 | A | G | 195.90 | SNP | Rv0978c (PE\_PGRS17) | silent (Val317) | 9901 | - | | 1098698 | C | G | 389.77 | SNP | Rv0982 (mprB) | silent (Gly397) | 9935 | - | | 1100234 | T | C | 284.80 | SNP | Rv0983 (pepD) | Leu390Pro | 2 | - | | 1106422 | T | C | 816.77 | SNP | Rv0989c (grcC2) | Ile321Val | 57 | - | | 1109975 | A | G | 487.77 | SNP | Rv0993 (galU) | Gln235Arg | 10 | - | | 1126889 | G | C | 476.77 | SNP | Rv1007c (metS) | Arg39Gly | 1 | - | | 1127648 | C | A | 95.41 | SNP | Rv1008 (tatD) | Thr187Asn | 9 | - | | 1149551 | C | T | 448.77 | SNP | Rv1028c (kdpD) | silent (Glu712) | 9865 | - | | 1150585 | G | A | 320.78 | SNP | Rv1028c (kdpD) | Pro368Ser | 17 | - | | 1163134 | T | C | 396.77 | SNP | Rv1040c (PE8) | silent (Gly81) | 9935 | - | | 1168715 | C | CT | 1119.73 | INS | Rv1046c |  |  | - | | 1169307 | C | T | 280.78 | SNP | intergenic |  |  | - | | 1178116 | T | C | 355.77 | SNP | Rv1056 | silent (Thr163) | 9871 | - | | 1184605 | C | A | 553.77 | SNP | Rv1061 | Asp197Glu | 56 | - | | 1186775 | C | T | 96.28 | SNP | Rv1063c | Val17Ile | 33 | - | | 1200418 | A | G | 359.78 | SNP | intergenic |  |  | - | | 1215136 | G | A | 65.28 | SNP | intergenic |  |  | - | | 1220680 | T | C | 454.77 | SNP | Rv1093 (glyA1) | Val36Ala | 18 | - | | 1224367 | T | C | 195.90 | SNP | intergenic |  |  | - | | 1244380 | C | A | 155.84 | SNP | Rv1121 (zwf1) | Ala225Asp | 6 | - | | 1244644 | T | C | 158.90 | SNP | Rv1121 (zwf1) | Ile313Thr | 11 | - | | 1248978 | T | C | 384.77 | SNP | Rv1125 | silent (Ala299) | 9867 | - | | 1259418 | C | T | 275.78 | SNP | Rv1133c (metE) | silent (Gln643) | 9876 | - | | 1263633 | C | T | 307.78 | SNP | Rv1135c (PPE16) | Ala166Thr | 22 | - | | 1269703 | G | A | 285.78 | SNP | Rv1142c (echA10) | Pro86Ser | 17 | - | | 1280683 | G | GCGAAGT | 575.77 | INS | Rv1153c (omt) |  |  | - | | 1281118 | T | C | 415.77 | SNP | Rv1154c | Thr123Ala | 32 | - | | 1292102 | A | G | 332.78 | SNP | Rv1162 (narH) | silent (Pro346) | 9926 | - | | 1302177 | C | T | 317.78 | SNP | Rv1172c (PE12) | Gly169Ser | 16 | - | | 1313337 | A | AG | 312.75 | INS | intergenic |  |  | - | | 1313338 | A | C | 100.28 | SNP | intergenic |  |  | - | | 1315191 | A | C | 329.78 | SNP | Rv1180 (pks3) | STOP489Tyr | 1 | - | | 1315884 | G | A | 425.77 | SNP | Rv1181 (pks4) | silent (Ala217) | 9867 | - | | 1327890 | G | A | 539.77 | SNP | Rv1186c | silent (Asp472) | 9859 | - | | 1328222 | T | C | 248.80 | SNP | Rv1186c | Asn362Asp | 42 | - | | 1328687 | G | C | 264.80 | SNP | Rv1186c | Pro207Ala | 22 | - | | 1341102 | C | T | 153.90 | SNP | Rv1198 (esxL) | Arg33Cys | 1 | - | | 1341103 | G | C | 160.90 | SNP | Rv1198 (esxL) | Arg33Pro | 5 | - | | 1357977 | C | T | 308.78 | SNP | Rv1215c | Glu490Lys | 7 | - | | 1358794 | G | A | 273.80 | SNP | Rv1215c | silent (Pro217) | 9926 | - | | 1365837 | C | CG | 298.75 | INS | intergenic |  |  | - | | 1374065 | T | C | 263.80 | SNP | Rv1230c | Ser45Gly | 21 | - | | 1375724 | A | C | 314.78 | SNP | Rv1232c | Cys149Gly | 1 | - | | 1380911 | T | C | 101.77 | SNP | Rv1238 (sugC) | silent (Arg76) | 9913 | - | | 1380935 | G | C | 102.77 | SNP | Rv1238 (sugC) | silent (Ser84) | 9840 | - | | 1382628 | T | C | 368.78 | SNP | Rv1239c (corA) | Lys139Glu | 4 | - | | 1391868 | G | A | 410.77 | SNP | Rv1248c | silent (Asp395) | 9859 | - | | 1393626 | A | G | 400.77 | SNP | Rv1249c | silent (Leu119) | 9947 | - | | 1396922 | T | C | 359.78 | SNP | Rv1251c | silent (Thr773) | 9871 | - | | 1411210 | T | G | 523.77 | SNP | Rv1263 (amiB2) | Val260Val(s) | 18 | - | | 1413148 | C | T | 406.77 | SNP | intergenic |  |  | - | | 1414021 | C | T | 473.77 | SNP | Rv1266c (pknH) | Arg607Gln | 9 | - | | 1416222 | A | G | 59.77 | SNP | Rv1267c (embR) | Phe376Leu | 13 | - | | 1416232 | A | G | 91.77 | SNP | Rv1267c (embR) | silent (Cys372) | 9973 | - | | 1416234 | A | C | 66.77 | SNP | Rv1267c (embR) | Cys372Gly | 1 | - | | 1416410 | A | C | 523.77 | SNP | Rv1267c (embR) | Leu313Arg | 1 | genotype | | 1430158 | G | A | 275.80 | SNP | Rv1279 | Ala33Thr | 22 | - | | 1433114 | G | A | 570.77 | SNP | Rv1280c (oppA) | silent (Gly109) | 9935 | - | | 1440469 | C | G | 463.77 | SNP | Rv1286 (cysN) | silent (Pro521) | 9926 | - | | 1445781 | A | G | 442.77 | SNP | Rv1291c | silent (Ala18) | 9867 | - | | 1445922 | A | G | 694.77 | SNP | intergenic |  |  | - | | 1457144 | C | T | 148.90 | SNP | Rv1300 (hemK) | Arg194Cys | 1 | - | | 1463706 | C | T | 54.77 | SNP | Rv1308 (atpA) | Thr160Ile | 7 | - | | 1463722 | C | A | 44.77 | SNP | Rv1308 (atpA) | silent (Gly165) | 9935 | - | | 1463729 | C | G | 63.77 | SNP | Rv1308 (atpA) | Gln168Glu | 35 | - | | 1463750 | A | C | 44.77 | SNP | Rv1308 (atpA) | Lys175Gln | 6 | - | | 1465155 | C | T | 443.77 | SNP | Rv1309 (atpG) | Ala91Val | 13 | - | | 1468208 | A | C | 138.03 | SNP | Rv1313c | Leu433Arg | 1 | - | | 1471659 | C | T | 183.90 | SNP | intergenic |  |  | - | | 1471878 | C | T | 101.77 | SNP | Rvnr01 | rRNA | rRNA | - | | 1471896 | T | C | 445.77 | SNP | Rvnr01 | rRNA | rRNA | - | | 1471900 | C | T | 407.77 | SNP | Rvnr01 | rRNA | rRNA | - | | 1472100 | T | C | 107.77 | SNP | Rvnr01 | rRNA | rRNA | - | | 1472101 | G | A | 88.77 | SNP | Rvnr01 | rRNA | rRNA | - | | 1472106 | G | A | 302.77 | SNP | Rvnr01 | rRNA | rRNA | - | | 1472127 | C | T | 1530.77 | SNP | Rvnr01 | rRNA | rRNA | - | | 1472129 | G | C | 1592.77 | SNP | Rvnr01 | rRNA | rRNA | - | | 1472137 | G | A | 1756.77 | SNP | Rvnr01 | rRNA | rRNA | - | | 1472150 | T | A | 1684.77 | SNP | Rvnr01 | rRNA | rRNA | - | | 1472151 | C | T | 1695.77 | SNP | Rvnr01 | rRNA | rRNA | - | | 1472172 | T | C | 1710.77 | SNP | Rvnr01 | rRNA | rRNA | - | | 1472203 | G | A | 1525.77 | SNP | Rvnr01 | rRNA | rRNA | - | | 1472210 | A | C | 1128.77 | SNP | Rvnr01 | rRNA | rRNA | - | | 1472213 | G | C | 1134.77 | SNP | Rvnr01 | rRNA | rRNA | - | | 1472215 | A | G | 1135.77 | SNP | Rvnr01 | rRNA | rRNA | - | | 1472225 | C | A | 1099.77 | SNP | Rvnr01 | rRNA | rRNA | - | | 1472234 | T | C | 808.77 | SNP | Rvnr01 | rRNA | rRNA | - | | 1472236 | C | G | 809.77 | SNP | Rvnr01 | rRNA | rRNA | - | | 1472240 | G | A | 773.77 | SNP | Rvnr01 | rRNA | rRNA | - | | 1472338 | A | C | 43.77 | SNP | Rvnr01 | rRNA | rRNA | - | | 1472344 | C | T | 541.77 | SNP | Rvnr01 | rRNA | rRNA | - | | 1472379 | T | G | 522.77 | SNP | Rvnr01 | rRNA | rRNA | - | | 1472382 | G | A | 444.77 | SNP | Rvnr01 | rRNA | rRNA | - | | 1472389 | G | A | 36.77 | SNP | Rvnr01 | rRNA | rRNA | - | | 1472400 | C | T | 394.77 | SNP | Rvnr01 | rRNA | rRNA | - | | 1472412 | A | C | 56.77 | SNP | Rvnr01 | rRNA | rRNA | - | | 1472415 | T | G | 60.77 | SNP | Rvnr01 | rRNA | rRNA | - | | 1472422 | T | C | 58.77 | SNP | Rvnr01 | rRNA | rRNA | - | | 1472530 | G | A | 255.77 | SNP | Rvnr01 | rRNA | rRNA | - | | 1472537 | C | T | 147.77 | SNP | Rvnr01 | rRNA | rRNA | - | | 1472541 | T | G | 252.77 | SNP | Rvnr01 | rRNA | rRNA | - | | 1472544 | C | G | 197.77 | SNP | Rvnr01 | rRNA | rRNA | - | | 1472545 | A | T | 261.77 | SNP | Rvnr01 | rRNA | rRNA | - | | 1472557 | G | A | 428.77 | SNP | Rvnr01 | rRNA | rRNA | - | | 1472571 | G | C | 433.77 | SNP | Rvnr01 | rRNA | rRNA | - | | 1472573 | C | T | 444.77 | SNP | Rvnr01 | rRNA | rRNA | - | | 1472579 | G | A | 343.77 | SNP | Rvnr01 | rRNA | rRNA | - | | 1472581 | A | T | 417.77 | SNP | Rvnr01 | rRNA | rRNA | - | | 1472598 | A | C | 517.77 | SNP | Rvnr01 | rRNA | rRNA | - | | 1472616 | G | A | 821.77 | SNP | Rvnr01 | rRNA | rRNA | - | | 1472655 | G | A | 487.77 | SNP | Rvnr01 | rRNA | rRNA | - | | 1472658 | G | A | 494.77 | SNP | Rvnr01 | rRNA | rRNA | - | | 1472661 | A | G | 548.77 | SNP | Rvnr01 | rRNA | rRNA | - | | 1472669 | T | TCGG | 905.73 | INS | Rvnr01 |  |  | - | | 1472676 | CCTTCCTTGG | C | 1772.73 | DEL | Rvnr01 |  |  | - | | 1472688 | T | TACCCG | 1026.73 | INS | Rvnr01 |  |  | - | | 1472690 | C | G | 355.77 | SNP | Rvnr01 | rRNA | rRNA | - | | 1472697 | T | A | 370.77 | SNP | Rvnr01 | rRNA | rRNA | - | | 1472700 | C | T | 340.77 | SNP | Rvnr01 | rRNA | rRNA | - | | 1472708 | T | A | 654.77 | SNP | Rvnr01 | rRNA | rRNA | - | | 1472713 | T | C | 1184.77 | SNP | Rvnr01 | rRNA | rRNA | - | | 1472716 | C | T | 1122.77 | SNP | Rvnr01 | rRNA | rRNA | - | | 1472733 | G | A,C | 592.29 | MUL | Rvnr01 | rRNA | rRNA | - | | 1472734 | C | T | 840.77 | SNP | Rvnr01 | rRNA | rRNA | - | | 1472741 | G | A | 889.77 | SNP | Rvnr01 | rRNA | rRNA | - | | 1472742 | C | G,T | 732.29 | MUL | Rvnr01 | rRNA | rRNA | - | | 1472744 | A | G | 1477.77 | SNP | Rvnr01 | rRNA | rRNA | - | | 1472750 | C | A | 79.77 | SNP | Rvnr01 | rRNA | rRNA | resistance | | 1472767 | G | A | 1739.77 | SNP | Rvnr01 | rRNA | rRNA | - | | 1472779 | G | A | 870.77 | SNP | Rvnr01 | rRNA | rRNA | - | | 1472781 | C | T | 1632.77 | SNP | Rvnr01 | rRNA | rRNA | - | | 1472786 | C | T | 891.77 | SNP | Rvnr01 | rRNA | rRNA | - | | 1472793 | A | T | 1575.77 | SNP | Rvnr01 | rRNA | rRNA | - | | 1472803 | T | A | 1459.77 | SNP | Rvnr01 | rRNA | rRNA | - | | 1472895 | C | T | 1229.77 | SNP | Rvnr01 | rRNA | rRNA | - | | 1472953 | G | A | 200.77 | SNP | Rvnr01 | rRNA | rRNA | - | | 1472954 | T | G | 198.77 | SNP | Rvnr01 | rRNA | rRNA | - | | 1472957 | C | T | 237.77 | SNP | Rvnr01 | rRNA | rRNA | - | | 1472958 | A | T | 206.77 | SNP | Rvnr01 | rRNA | rRNA | - | | 1472959 | T | A | 205.77 | SNP | Rvnr01 | rRNA | rRNA | - | | 1472970 | C | T | 190.77 | SNP | Rvnr01 | rRNA | rRNA | - | | 1472971 | GT | G | 360.73 | DEL | Rvnr01 |  |  | - | | 1472974 | A | G | 242.77 | SNP | Rvnr01 | rRNA | rRNA | - | | 1472975 | T | A | 214.77 | SNP | Rvnr01 | rRNA | rRNA | - | | 1472977 | G | A | 209.77 | SNP | Rvnr01 | rRNA | rRNA | - | | 1472982 | G | C | 244.77 | SNP | Rvnr01 | rRNA | rRNA | - | | 1472987 | G | T | 192.77 | SNP | Rvnr01 | rRNA | rRNA | - | | 1472988 | T | G | 167.77 | SNP | Rvnr01 | rRNA | rRNA | - | | 1472992 | A | C | 221.77 | SNP | Rvnr01 | rRNA | rRNA | - | | 1473002 | G | T | 219.77 | SNP | Rvnr01 | rRNA | rRNA | - | | 1473004 | T | A | 237.77 | SNP | Rvnr01 | rRNA | rRNA | - | | 1473008 | C | A | 195.77 | SNP | Rvnr01 | rRNA | rRNA | - | | 1473009 | T | C | 250.77 | SNP | Rvnr01 | rRNA | rRNA | - | | 1473035 | G | A | 1022.77 | SNP | Rvnr01 | rRNA | rRNA | - | | 1473053 | T | A | 748.77 | SNP | Rvnr01 | rRNA | rRNA | - | | 1473056 | A | T | 633.77 | SNP | Rvnr01 | rRNA | rRNA | - | | 1473062 | T | A | 631.77 | SNP | Rvnr01 | rRNA | rRNA | - | | 1473068 | A | G | 586.77 | SNP | Rvnr01 | rRNA | rRNA | - | | 1473070 | G | A | 492.77 | SNP | Rvnr01 | rRNA | rRNA | - | | 1473088 | A | G | 155.77 | SNP | Rvnr01 | rRNA | rRNA | - | | 1473093 | C | A | 81.77 | SNP | Rvnr01 | rRNA | rRNA | - | | 1473094 | T | A | 44.77 | SNP | Rvnr01 | rRNA | rRNA | - (C would be genotype) | | 1473099 | T | A | 40.77 | SNP | Rvnr01 | rRNA | rRNA | - | | 1473122 | T | A | 35.77 | SNP | Rvnr01 | rRNA | rRNA | - | | 1473127 | G | A | 40.77 | SNP | Rvnr01 | rRNA | rRNA | - | | 1473145 | C | T | 55.77 | SNP | Rvnr01 | rRNA | rRNA | - | | 1473147 | G | C | 49.77 | SNP | Rvnr01 | rRNA | rRNA | - | | 1473148 | G | A | 40.77 | SNP | Rvnr01 | rRNA | rRNA | - | | 1473150 | T | G | 82.77 | SNP | Rvnr01 | rRNA | rRNA | - | | 1473161 | A | C | 120.77 | SNP | Rvnr01 | rRNA | rRNA | - | | 1473163 | C | T | 100.77 | SNP | Rvnr01 | rRNA | rRNA | - | | 1473164 | C | G | 130.77 | SNP | Rvnr01 | rRNA | rRNA | - | | 1473166 | G | A | 166.77 | SNP | Rvnr01 | rRNA | rRNA | - | | 1473177 | G | A | 655.77 | SNP | Rvnr01 | rRNA | rRNA | - | | 1473179 | C | T | 646.77 | SNP | Rvnr01 | rRNA | rRNA | - | | 1473192 | A | G | 825.77 | SNP | Rvnr01 | rRNA | rRNA | - | | 1473199 | CA | C | 1610.73 | DEL | Rvnr01 |  |  | - | | 1473202 | C | T | 877.77 | SNP | Rvnr01 | rRNA | rRNA | - | | 1473205 | T | C | 722.77 | SNP | Rvnr01 | rRNA | rRNA | - | | 1473226 | C | T | 1419.77 | SNP | Rvnr01 | rRNA | rRNA | - | | 1473352 | C | T | 1265.78 | SNP | Rvnr01 | rRNA | rRNA | - | | 1473384 | A | G | 210.77 | SNP | intergenic |  |  | - | | 1474330 | A | T | 97.77 | SNP | Rvnr02 | rRNA | rRNA | - | | 1474565 | T | A | 92.77 | SNP | Rvnr02 | rRNA | rRNA | - | | 1474570 | G | T | 66.77 | SNP | Rvnr02 | rRNA | rRNA | - | | 1474571 | G | T | 95.77 | SNP | Rvnr02 | rRNA | rRNA | - | | 1474578 | A | G | 117.77 | SNP | Rvnr02 | rRNA | rRNA | - | | 1474584 | C | G | 230.77 | SNP | Rvnr02 | rRNA | rRNA | - | | 1474586 | T | A | 247.77 | SNP | Rvnr02 | rRNA | rRNA | - | | 1474779 | G | A | 429.77 | SNP | Rvnr02 | rRNA | rRNA | - | | 1474780 | C | T | 432.77 | SNP | Rvnr02 | rRNA | rRNA | - | | 1474782 | G | A | 421.77 | SNP | Rvnr02 | rRNA | rRNA | - | | 1474790 | C | T | 471.77 | SNP | Rvnr02 | rRNA | rRNA | - | | 1474798 | C | T | 534.77 | SNP | Rvnr02 | rRNA | rRNA | - | | 1474804 | C | T | 578.77 | SNP | Rvnr02 | rRNA | rRNA | - | | 1474823 | C | G | 877.77 | SNP | Rvnr02 | rRNA | rRNA | - | | 1474827 | C | T | 889.77 | SNP | Rvnr02 | rRNA | rRNA | - | | 1474830 | A | G | 942.77 | SNP | Rvnr02 | rRNA | rRNA | - | | 1474831 | A | C | 907.77 | SNP | Rvnr02 | rRNA | rRNA | - | | 1474844 | G | T | 1261.77 | SNP | Rvnr02 | rRNA | rRNA | - | | 1474866 | C | A | 1251.77 | SNP | Rvnr02 | rRNA | rRNA | - | | 1474869 | G | C | 1140.77 | SNP | Rvnr02 | rRNA | rRNA | - | | 1474896 | A | G | 1033.77 | SNP | Rvnr02 | rRNA | rRNA | - | | 1474902 | T | C | 860.77 | SNP | Rvnr02 | rRNA | rRNA | - | | 1474904 | G | C | 847.77 | SNP | Rvnr02 | rRNA | rRNA | - | | 1474913 | T | A | 860.77 | SNP | Rvnr02 | rRNA | rRNA | - | | 1474920 | G | A | 711.77 | SNP | Rvnr02 | rRNA | rRNA | - | | 1474931 | G | A | 68.77 | SNP | Rvnr02 | rRNA | rRNA | - | | 1474938 | G | A | 74.77 | SNP | Rvnr02 | rRNA | rRNA | - | | 1475122 | C | T | 348.77 | SNP | Rvnr02 | rRNA | rRNA | - | | 1475129 | G | T | 474.77 | SNP | Rvnr02 | rRNA | rRNA | - | | 1475154 | C | A | 810.77 | SNP | Rvnr02 | rRNA | rRNA | - | | 1475167 | T | A | 718.77 | SNP | Rvnr02 | rRNA | rRNA | - | | 1475175 | G | A | 626.77 | SNP | Rvnr02 | rRNA | rRNA | - | | 1475182 | T | A | 618.77 | SNP | Rvnr02 | rRNA | rRNA | - | | 1475188 | C | T | 584.77 | SNP | Rvnr02 | rRNA | rRNA | - | | 1475574 | C | A | 298.77 | SNP | Rvnr02 | rRNA | rRNA | - | | 1475672 | C | G | 216.77 | SNP | Rvnr02 | rRNA | rRNA | - | | 1475673 | T | C | 275.77 | SNP | Rvnr02 | rRNA | rRNA | - | | 1475687 | C | T | 305.77 | SNP | Rvnr02 | rRNA | rRNA | - | | 1475688 | G | A | 312.77 | SNP | Rvnr02 | rRNA | rRNA | - | | 1475696 | T | C | 351.77 | SNP | Rvnr02 | rRNA | rRNA | - | | 1475703 | A | G | 344.77 | SNP | Rvnr02 | rRNA | rRNA | - | | 1475707 | T | A | 322.77 | SNP | Rvnr02 | rRNA | rRNA | - | | 1475713 | C | T | 398.77 | SNP | Rvnr02 | rRNA | rRNA | - | | 1475716 | A | G | 611.77 | SNP | Rvnr02 | rRNA | rRNA | - | | 1475803 | T | C | 447.77 | SNP | Rvnr02 | rRNA | rRNA | - | | 1475804 | G | C | 542.77 | SNP | Rvnr02 | rRNA | rRNA | - | | 1475816 | C | G | 602.77 | SNP | Rvnr02 | rRNA | rRNA | - | | 1475817 | A | G | 756.77 | SNP | Rvnr02 | rRNA | rRNA | - | | 1475843 | G | A | 567.77 | SNP | Rvnr02 | rRNA | rRNA | - | | 1475849 | G | A | 670.77 | SNP | Rvnr02 | rRNA | rRNA | - | | 1475853 | C | T | 595.77 | SNP | Rvnr02 | rRNA | rRNA | - | | 1475858 | T | C | 948.77 | SNP | Rvnr02 | rRNA | rRNA | - | | 1475866 | T | A | 97.77 | SNP | Rvnr02 | rRNA | rRNA | - | | 1476225 | T | G | 282.77 | SNP | Rvnr02 | rRNA | rRNA | - | | 1476229 | C | T | 307.77 | SNP | Rvnr02 | rRNA | rRNA | - | | 1476251 | T | C | 996.77 | SNP | Rvnr02 | rRNA | rRNA | - | | 1476252 | T | G | 553.77 | SNP | Rvnr02 | rRNA | rRNA | - | | 1476260 | A | G | 967.77 | SNP | Rvnr02 | rRNA | rRNA | - | | 1476268 | A | T | 231.77 | SNP | Rvnr02 | rRNA | rRNA | - | | 1476275 | T | A | 189.77 | SNP | Rvnr02 | rRNA | rRNA | - | | 1476279 | G | A | 135.77 | SNP | Rvnr02 | rRNA | rRNA | - | | 1476280 | A | C | 757.77 | SNP | Rvnr02 | rRNA | rRNA | - | | 1476293 | C | T | 379.77 | SNP | Rvnr02 | rRNA | rRNA | - | | 1476294 | A | G | 296.77 | SNP | Rvnr02 | rRNA | rRNA | - | | 1476295 | C | G | 307.77 | SNP | Rvnr02 | rRNA | rRNA | - | | 1476296 | C | T | 270.77 | SNP | Rvnr02 | rRNA | rRNA | - | | 1476297 | C | T | 103.77 | SNP | Rvnr02 | rRNA | rRNA | - | | 1476301 | A | C | 74.77 | SNP | Rvnr02 | rRNA | rRNA | - | | 1476302 | G | A | 280.77 | SNP | Rvnr02 | rRNA | rRNA | - | | 1476311 | G | C | 325.77 | SNP | Rvnr02 | rRNA | rRNA | - | | 1476312 | T | C | 304.77 | SNP | Rvnr02 | rRNA | rRNA | - | | 1476313 | G | A | 305.77 | SNP | Rvnr02 | rRNA | rRNA | - | | 1476332 | G | C | 814.77 | SNP | Rvnr02 | rRNA | rRNA | - | | 1476338 | C | T | 499.77 | SNP | Rvnr02 | rRNA | rRNA | - | | 1476353 | G | T | 1058.77 | SNP | Rvnr02 | rRNA | rRNA | - | | 1476358 | T | C | 1070.77 | SNP | Rvnr02 | rRNA | rRNA | - | | 1476372 | T | C | 1154.77 | SNP | Rvnr02 | rRNA | rRNA | - | | 1476382 | A | G | 1209.77 | SNP | Rvnr02 | rRNA | rRNA | - | | 1476383 | T | A | 1096.77 | SNP | Rvnr02 | rRNA | rRNA | - | | 1476408 | G | A | 1547.77 | SNP | Rvnr02 | rRNA | rRNA | - | | 1476425 | G | A | 1219.77 | SNP | Rvnr02 | rRNA | rRNA | - | | 1476428 | C | T | 1411.77 | SNP | Rvnr02 | rRNA | rRNA | - | | 1476466 | C | T | 1265.77 | SNP | Rvnr02 | rRNA | rRNA | - | | 1476481 | T | C | 1461.77 | SNP | Rvnr02 | rRNA | rRNA | - | | 1476506 | T | C | 1157.77 | SNP | Rvnr02 | rRNA | rRNA | - | | 1476512 | C | T | 369.77 | SNP | Rvnr02 | rRNA | rRNA | - | | 1476514 | C | T | 439.77 | SNP | Rvnr02 | rRNA | rRNA | - | | 1476519 | C | G | 463.77 | SNP | Rvnr02 | rRNA | rRNA | - | | 1476524 | C | A | 415.77 | SNP | Rvnr02 | rRNA | rRNA | - | | 1476525 | A | G | 406.77 | SNP | Rvnr02 | rRNA | rRNA | - | | 1476530 | C | T | 425.77 | SNP | Rvnr02 | rRNA | rRNA | - | | 1476536 | G | A | 398.77 | SNP | Rvnr02 | rRNA | rRNA | - | | 1476537 | A | G | 432.77 | SNP | Rvnr02 | rRNA | rRNA | - | | 1476538 | A | G | 433.77 | SNP | Rvnr02 | rRNA | rRNA | - | | 1476540 | C | G | 489.77 | SNP | Rvnr02 | rRNA | rRNA | - | | 1476547 | C | T | 1132.77 | SNP | Rvnr02 | rRNA | rRNA | - | | 1476567 | C | T | 1319.77 | SNP | Rvnr02 | rRNA | rRNA | - | | 1476573 | A | T | 1248.77 | SNP | Rvnr02 | rRNA | rRNA | - | | 1476577 | T | G | 1018.77 | SNP | Rvnr02 | rRNA | rRNA | - | | 1476584 | C | T | 1260.77 | SNP | Rvnr02 | rRNA | rRNA | - | | 1476595 | C | T | 1033.77 | SNP | Rvnr02 | rRNA | rRNA | - | | 1476607 | C | T | 1021.77 | SNP | Rvnr02 | rRNA | rRNA | - | | 1476608 | C | G | 1025.77 | SNP | Rvnr02 | rRNA | rRNA | - | | 1476621 | C | A | 836.77 | SNP | Rvnr02 | rRNA | rRNA | - | | 1476624 | T | A | 817.77 | SNP | Rvnr02 | rRNA | rRNA | - | | 1476628 | T | A | 843.77 | SNP | Rvnr02 | rRNA | rRNA | - | | 1476629 | C | A | 838.77 | SNP | Rvnr02 | rRNA | rRNA | - | | 1476630 | A | G | 886.77 | SNP | Rvnr02 | rRNA | rRNA | - | | 1476637 | C | G | 982.77 | SNP | Rvnr02 | rRNA | rRNA | - | | 1480174 | C | G | 48.77 | SNP | Rv1318c | silent (Leu217) | 9947 | - | | 1480945 | C | G | 49.77 | SNP | Rv1319c | silent (Thr519) | 9871 | - | | 1480948 | C | T | 59.77 | SNP | Rv1319c | silent (Glu518) | 9865 | - | | 1481185 | A | C | 108.77 | SNP | Rv1319c | Asp439Glu | 56 | - | | 1481321 | A | G | 966.77 | SNP | Rv1319c | Val394Ala | 18 | - | | 1481468 | G | T | 223.77 | SNP | Rv1319c | Ala345Asp | 6 | - | | 1482627 | T | C | 440.77 | SNP | Rv1320c | Thr531Ala | 32 | - | | 1483652 | A | G | 357.78 | SNP | Rv1320c | Leu189Pro | 2 | - | | 1484708 | A | C | 433.77 | SNP | Rv1321 | Ser144Arg | 6 | - | | 1488433 | A | G | 38.78 | SNP | Rv1325c (PE\_PGRS24) | silent (Asp511) | 9859 | - | | 1488434 | T | G | 51.78 | SNP | Rv1325c (PE\_PGRS24) | Asp511Ala | 10 | - | | 1488435 | C | A | 51.77 | SNP | Rv1325c (PE\_PGRS24) | Asp511Tyr | 0 | - | | 1499274 | C | G | 383.77 | SNP | Rv1330c (pncB1) | Gly429Ala | 21 | - | | 1501468 | G | C | 299.78 | SNP | Rv1332 | silent (Pro181) | 9926 | genotype | | 1502795 | T | C | 231.84 | SNP | Rv1334 (mec) | Met(s)52Thr | 22 | - | | 1532827 | A | T | 195.90 | SNP | Rv1361c (PPE19) | Phe269Leu | 13 | - | | 1536251 | G | T | 202.84 | SNP | Rv1364c | Ala465Glu | 10 | - | | 1537710 | AAC | A | 594.73 | DEL | intergenic |  |  | - | | 1537771 | G | C | 118.03 | SNP | intergenic |  |  | - | | 1544349 | T | G | 585.77 | SNP | Rv1371 | Trp331Gly | 0 | - | | 1547125 | T | C | 347.78 | SNP | Rv1374c | Thr136Ala | 32 | - | | 1552547 | G | A | 230.84 | SNP | Rv1378c | Arg37Trp | 2 | - | | 1570566 | C | A | 127.03 | SNP | Rv1394c (cyp132) | Arg135Leu | 1 | - | | 1570998 | GC | G | 676.73 | DEL | intergenic |  |  | - | | 1588899 | G | T | 303.78 | SNP | Rv1412 (ribC) | silent (Ala111) | 9867 | - | | 1588919 | G | T | 318.78 | SNP | Rv1412 (ribC) | Trp118Leu(s) | 0 | - | | 1597405 | G | A | 197.84 | SNP | Rv1422 | silent (Pro175) | 9926 | - | | 1597437 | C | T | 218.84 | SNP | Rv1422 | Ala186Val | 13 | - | | 1609840 | A | G | 308.78 | SNP | Rv1431 | silent (Pro586) | 9926 | - | | 1613035 | T | C | 314.78 | SNP | intergenic |  |  | - | | 1620135 | A | G | 232.84 | SNP | Rv1442 (bisC) | silent (Gly115) | 9935 | - | | 1630148 | A | C | 391.77 | SNP | Rv1449c (tkt) | Tyr18Asp | 0 | - | | 1634633 | A | T | 54.77 | SNP | intergenic |  |  | - | | 1634636 | T | A | 54.77 | SNP | intergenic |  |  | - | | 1639594 | C | A | 418.77 | SNP | Rv1453 | Pro405Gln | 6 | - | | 1647687 | G | A | 51.77 | SNP | Rv1461 | silent (Gln233) | 9876 | - | | 1650072 | A | G | 400.77 | SNP | Rv1462 | Asn183Asp | 42 | - | | 1663761 | A | C | 52.77 | SNP | Rv1475c (acn) | Ile762Met(s) | 6 | - | | 1663767 | C | G | 56.77 | SNP | Rv1475c (acn) | Val(s)760Val | 13 | - | | 1663788 | A | G | 66.77 | SNP | Rv1475c (acn) | silent (Ser753) | 9840 | - | | 1663804 | T | A | 30.77 | SNP | Rv1475c (acn) | Tyr748Phe | 28 | - | | 1674465 | C | T | 44.78 | SNP | Rv1484 (inhA) | silent (Leu88) | 9947 | - | | 1675496 | C | T | 257.80 | SNP | Rv1485 (hemZ) | silent (Thr160) | 9871 | - | | 1676290 | C | A | 311.78 | SNP | Rv1486c | Lys198Asn | 13 | - | | 1676769 | C | T | 349.78 | SNP | Rv1486c | Ala39Thr | 22 | - | | 1677293 | A | C | 475.77 | SNP | Rv1487 | Asp118Ala | 10 | - | | 1689349 | C | T | 252.78 | SNP | Rv1498c | Arg191His | 8 | - | | 1692141 | A | C | 151.03 | SNP | Rv1501 | silent (Ile84) | 9872 | - | | 1693561 | A | G | 323.78 | SNP | Rv1502 | Tyr213Cys | 3 | - | | 1696464 | C | G | 255.80 | SNP | intergenic |  |  | - | | 1698911 | G | A | 230.80 | SNP | Rv1508c | silent (Gly328) | 9935 | - | | 1706034 | A | G | 281.78 | SNP | Rv1514c | Cys188Arg | 1 | - | | 1706119 | T | C | 314.78 | SNP | Rv1514c | silent (Ser159) | 9840 | - | | 1706262 | T | C | 515.77 | SNP | Rv1514c | Lys112Glu | 4 | - | | 1711117 | C | T | 303.78 | SNP | Rv1520 | silent (Val30) | 9901 | - | | 1726541 | G | A | 250.80 | SNP | Rv1527c (pks5) | silent (Tyr623) | 9945 | - | | 1727059 | G | T | 348.78 | SNP | Rv1527c (pks5) | Pro451Thr | 5 | - | | 1728837 | A | G | 290.78 | SNP | intergenic |  |  | - | | 1736577 | A | G | 504.77 | SNP | Rv1536 (ileS) | Glu20Gly | 7 | - | | 1736992 | G | C | 534.77 | SNP | Rv1536 (ileS) | silent (Leu158) | 9947 | - | | 1747151 | C | G | 186.90 | SNP | intergenic |  |  | - | | 1751042 | C | T | 231.84 | SNP | Rv1547 (dnaE1) | Pro1117Ser | 17 | - | | 1752561 | T | C | 135.03 | SNP | Rv1548c (PPE21) | Asp258Gly | 11 | - | | 1753519 | G | GC | 209.80 | INS | Rv1549 (fadD11.1) |  |  | - | | 1757105 | A | C | 442.77 | SNP | Rv1551 (plsB1) | Gln554Pro | 8 | - | | 1759252 | G | T | 346.78 | SNP | Rv1552 (frdA) | silent (Ser524) | 9840 | genotype | | 1760292 | A | G | 364.78 | SNP | Rv1554 (frdC) | Met(s)40Val(s) | 9867 | - | | 1763855 | A | G | 322.78 | SNP | Rv1559 (ilvA) | Asp143Gly | 11 | - | | 1778430 | T | C | 257.80 | SNP | Rv1570 (bioD) | Met(s)191Thr | 22 | - | | 1779370 | G | C | 429.77 | SNP | Rv1573 | silent (Thr19) | 9871 | - | | 1780048 | A | G | 187.77 | SNP | Rv1574 | His40Arg | 10 | - | | 1780586 | C | CG | 377.74 | INS | Rv1575 |  |  | - | | 1781577 | G | A | 265.80 | SNP | Rv1576c | Thr163Ile | 7 | - | | 1788613 | C | T | 326.78 | SNP | Rv1587c | Gly184Asp | 6 | - | | 1789650 | C | T | 150.77 | SNP | Rv1588c | Ala63Thr | 22 | - | | 1789654 | A | G | 220.77 | SNP | Rv1588c | silent (Leu61) | 9947 | - | | 1789671 | C | T | 245.77 | SNP | Rv1588c | Ala56Thr | 22 | - | | 1789675 | A | C | 301.77 | SNP | Rv1588c | silent (Gly54) | 9935 | - | | 1789678 | C | G | 219.77 | SNP | Rv1588c | Val(s)53Val | 13 | - | | 1789766 | T | G | 693.77 | SNP | Rv1588c | Asp24Ala | 10 | - | | 1798355 | G | A | 427.77 | SNP | Rv1597 | Gly21Asp | 6 | - | | 1803265 | G | A | 486.77 | SNP | Rv1602 (hisH) | Ser201Asn | 20 | - | | 1804409 | C | A | 451.77 | SNP | Rv1604 (impA) | Pro124Gln | 6 | - | | 1817976 | A | T | 371.77 | SNP | Rv1618 (tesB1) | His121Leu | 4 | - | | 1832350 | G | A | 197.84 | SNP | Rv1629 (polA) | silent (Gln562) | 9876 | - | | 1836286 | G | C | 189.90 | SNP | intergenic |  |  | - | | 1846446 | A | G | 46.77 | SNP | Rv1638 (uvrA) | silent (Glu902) | 9865 | - | | 1846455 | G | C | 40.77 | SNP | Rv1638 (uvrA) | silent (Leu905) | 9947 | - | | 1847919 | C | G | 440.77 | SNP | Rv1639c | silent (Thr180) | 9871 | - | | 1851974 | C | T | 492.77 | SNP | Rv1640c (lysX) | Ser21Asn | 20 | - | | 1854300 | T | C | 263.80 | SNP | Rv1644 (tsnR) | Leu232Pro | 2 | - | | 1856777 | G | C | 570.77 | SNP | Rv1647 | Ala2Pro | 13 | - | | 1864698 | C | T | 92.28 | SNP | Rv1651c (PE\_PGRS30) | Ala229Thr | 22 | - | | 1885772 | G | A | 318.78 | SNP | Rv1662 (pks8) | Ala1357Thr | 22 | - | | 1886337 | C | T | 184.90 | SNP | Rv1662 (pks8) | Thr1545Ile | 7 | - | | 1894300 | G | GGTCTTGCCGC | 905.37 | INS | Rv1668c |  |  | - | | 1901493 | T | C | 494.77 | SNP | Rv1676 | silent (Ser149) | 9840 | - | | 1907296 | G | C | 451.77 | SNP | Rv1682 | silent (Ala298) | 9867 | - | | 1917972 | A | G | 175.90 | SNP | Rv1694 (tlyA) | silent (Leu11) | 9947 | - | | 1931179 | C | A | 378.77 | SNP | Rv1704c (cycA) | Arg93Leu | 1 | - | | 1933988 | G | A | 407.77 | SNP | intergenic |  |  | - | | 1944402 | T | C | 186.90 | SNP | Rv1716 | Val276Ala | 18 | - | | 1950767 | T | C | 598.77 | SNP | Rv1724c | silent (Lys95) | 9926 | - | | 1957946 | G | A | 629.77 | SNP | Rv1731 (gabD2) | Met(s)90Ile | 2 | - | | 1960284 | C | A | 160.90 | SNP | Rv1733c | Gln68His | 20 | - | | 1967237 | C | A | 392.77 | SNP | Rv1739c | Arg134Leu | 1 | - | | 1976798 | C | T | 263.80 | SNP | Rv1748 | Pro67Ser | 17 | - | | 1978166 | G | T | 207.84 | SNP | Rv1750c (fadD1) | Gln468Lys | 12 | - | | 1982961 | GC | G | 317.75 | DEL | Rv1753c (PPE24) |  |  | - | | 1983213 | T | C | 180.77 | SNP | Rv1753c (PPE24) | silent (Pro521) | 9926 | - | | 1987394 | C | T | 480.77 | SNP | intergenic |  |  | - | | 1992323 | G | GC | 160.87 | INS | Rv1759c (wag22) |  |  | - | | 1992974 | T | C | 500.77 | SNP | intergenic |  |  | - | | 1993808 | A | T | 175.90 | SNP | Rv1760 | Glu219Val(s) | 17 | - | | 1994939 | G | A | 431.77 | SNP | Rv1761c | Thr39Ile | 7 | - | | 2000298 | A | G | 442.77 | SNP | Rv1767 | Val75Val(s) | 18 | - | | 2004674 | T | A | 30.77 | SNP | Rv1770 | Val266Asp | 1 | - | | 2033748 | G | C | 442.77 | SNP | Rv1795 (eccD5); Rv1796 (mycP5) | silent (Arg503); Gly7Ala | 9913; 21 | - | | 2041215 | C | T | 267.80 | SNP | Rv1800 (PPE28) | Thr588Met(s) | 32 | - | | 2045310 | A | G | 61.74 | SNP | Rv1803c (PE\_PGRS32) | silent (Ile511) | 9872 | - | | 2046073 | A | G | 49.74 | SNP | Rv1803c (PE\_PGRS32) | Val257Ala | 18 | - | | 2049065 | T | C | 493.77 | SNP | intergenic |  |  | - | | 2049097 | G | C | 403.77 | SNP | intergenic |  |  | - | | 2051519 | T | G | 309.78 | SNP | Rv1809 (PPE33) | Ser80Ala | 35 | - | | 2051746 | T | C | 406.77 | SNP | Rv1809 (PPE33) | silent (Ala155) | 9867 | - | | 2052035 | G | T | 477.77 | SNP | Rv1809 (PPE33) | Val(s)252Leu(s) | 9867 | - | | 2055271 | A | G | 263.80 | SNP | Rv1812c | Leu30Pro | 2 | - | | 2057774 | A | T | 126.03 | SNP | Rv1815 | Ile83Phe | 8 | - | | 2058447 | G | A | 260.77 | SNP | Rv1816 | Leu(s)64Leu | 3 | - | | 2060845 | C | T | 510.77 | SNP | Rv1817 | silent (Ala417) | 9867 | - | | 2074806 | A | G | 270.80 | SNP | intergenic |  |  | - | | 2085550 | C | T | 349.78 | SNP | Rv1837c (glcB) | Val(s)478Met(s) | 9867 | - | | 2094911 | ACAGCGT | A | 1360.73 | DEL | Rv1844c (gnd1) |  |  | - | | 2096186 | A | G | 382.77 | SNP | Rv1846c (blaI) | silent (Thr138) | 9871 | - | | 2109523 | C | CG | 540.73 | INS | intergenic |  |  | - | | 2111907 | A | C | 186.90 | SNP | Rv1864c | Val68Gly | 5 | - | | 2113914 | G | A | 173.90 | SNP | Rv1866 | Ala259Thr | 22 | - | | 2116903 | C | T | 224.80 | SNP | Rv1867 | silent (Gly380) | 9935 | - | | 2128870 | A | G | 195.90 | SNP | Rv1878 (glnA3) | silent (Leu283) | 9947 | - | | 2133468 | T | TTCGCATGCCGTCACC | 732.73 | INS | Rv1883c |  |  | - | | 2135870 | T | C | 257.80 | SNP | intergenic |  |  | - | | 2143328 | G | C | 404.77 | SNP | Rv1895 | Val(s)270Leu | 3 | - | | 2147022 | A | C | 465.77 | SNP | Rv1900c (lipJ) | Ile204Met(s) | 6 | - | | 2149855 | C | CA | 1061.73 | INS | Rv1902c (nanT) |  |  | - | | 2154798 | C | T | 256.80 | SNP | Rv1908c (katG) | Trp438STOP | 0 | - | | 2163375 | T | C | 278.77 | SNP | Rv1917c (PPE34) | Asn1313Asp | 42 | - | | 2163412 | A | G | 168.77 | SNP | Rv1917c (PPE34) | silent (Val1300) | 9901 | - | | 2163415 | C | A | 174.77 | SNP | Rv1917c (PPE34) | silent (Pro1299) | 9926 | - | | 2163417 | G | C | 140.77 | SNP | Rv1917c (PPE34) | Pro1299Ala | 22 | - | | 2163419 | C | T | 135.77 | SNP | Rv1917c (PPE34) | Ser1298Asn | 20 | - | | 2163421 | C | G | 147.77 | SNP | Rv1917c (PPE34) | silent (Thr1297) | 9871 | - | | 2163790 | A | C | 62.74 | SNP | Rv1917c (PPE34) | silent (Pro1174) | 9926 | - | | 2165286 | A | C | 54.74 | SNP | Rv1917c (PPE34) | Ser676Ala | 35 | - | | 2165503 | T | A | 114.82 | SNP | Rv1917c (PPE34) | silent (Ala603) | 9867 | - | | 2165928 | G | T | 317.78 | SNP | Rv1917c (PPE34) | Pro462Thr | 5 | - | | 2184781 | G | T | 271.80 | SNP | Rv1933c (fadE18) | silent (Gly59) | 9935 | - | | 2196879 | T | A | 401.77 | SNP | Rv1945 | silent (Leu297) | 9947 | - | | 2196882 | A | G | 425.77 | SNP | Rv1945 | silent (Lys298) | 9926 | - | | 2202917 | C | T | 168.90 | SNP | Rv1957 | His112Tyr | 4 | - | | 2207591 | T | TC | 555.73 | INS | intergenic |  |  | - | | 2209403 | C | T | 300.78 | SNP | Rv1966 (mce3A) | Ala26Val | 13 | - | | 2211826 | A | G | 99.28 | SNP | Rv1968 (mce3C) | silent (Lys67) | 9926 | - | | 2216443 | C | A | 230.80 | SNP | Rv1971 (mce3F) | Ala396Glu | 10 | - | | 2220512 | T | G | 367.78 | SNP | Rv1977 | silent (Ser253) | 9840 | - | | 2223293 | T | C | 693.77 | SNP | intergenic |  |  | - | | 2228967 | A | G | 489.77 | SNP | intergenic |  |  | - | | 2247625 | A | C | 516.77 | SNP | intergenic |  |  | - | | 2251999 | A | G | 424.77 | SNP | intergenic |  |  | - | | 2260151 | A | G | 81.77 | SNP | intergenic |  |  | - | | 2260154 | C | T | 58.77 | SNP | intergenic |  |  | - | | 2260171 | T | C | 194.77 | SNP | intergenic |  |  | - | | 2260174 | C | T | 190.77 | SNP | intergenic |  |  | - | | 2260196 | C | CA | 331.73 | INS | intergenic |  |  | - | | 2260199 | C | T | 181.77 | SNP | intergenic |  |  | - | | 2260212 | G | T | 174.77 | SNP | intergenic |  |  | - | | 2260214 | G | C | 64.77 | SNP | intergenic |  |  | - | | 2260220 | C | T | 39.77 | SNP | intergenic |  |  | - | | 2260222 | C | G | 68.77 | SNP | intergenic |  |  | - | | 2260231 | T | C | 79.77 | SNP | intergenic |  |  | - | | 2260525 | C | T | 185.90 | SNP | intergenic |  |  | - | | 2264782 | C | A | 214.84 | SNP | Rv2017 | Ala262Glu | 10 | - | | 2265059 | T | G | 367.77 | SNP | intergenic |  |  | - | | 2266487 | G | C | 628.77 | SNP | Rv2020c | silent (Leu78) | 9947 | - | | 2266504 | T | TA | 1363.73 | INS | Rv2020c |  |  | - | | 2266508 | A | T | 514.77 | SNP | Rv2020c | Asp71Glu | 56 | - | | 2266511 | GT | G | 1094.73 | DEL | Rv2020c |  |  | - | | 2266517 | T | C | 563.77 | SNP | Rv2020c | silent (Glu68) | 9865 | - | | 2266550 | G | T | 566.77 | SNP | Rv2020c | silent (Gly57) | 9935 | - | | 2266553 | C | G | 575.77 | SNP | Rv2020c | silent (Ser56) | 9840 | - | | 2266583 | C | G | 495.77 | SNP | Rv2020c | Glu46Asp | 53 | - | | 2266598 | G | C | 473.77 | SNP | Rv2020c | silent (Leu41) | 9947 | - | | 2266604 | C | G | 357.77 | SNP | Rv2020c | silent (Ser39) | 9840 | - | | 2266613 | G | GC | 703.73 | INS | Rv2020c |  |  | - | | 2266624 | G | T | 327.77 | SNP | Rv2020c | Leu33Ile | 9 | - | | 2269780 | T | C | 55.74 | SNP | Rv2024c | Asp154Gly | 11 | - | | 2270102 | A | G | 527.77 | SNP | Rv2024c | Trp47Arg | 8 | - | | 2273627 | C | T | 229.84 | SNP | Rv2027c (dosT) | silent (Gly294) | 9935 | - | | 2282787 | C | T | 175.90 | SNP | Rv2037c | Cys312Tyr | 3 | - | | 2284329 | T | C | 77.77 | SNP | Rv2038c | Val156Val(s) | 18 | - | | 2285251 | C | A | 389.77 | SNP | Rv2039c | Val131Phe | 0 | - | | 2287121 | A | G | 374.78 | SNP | Rv2041c | silent (Asp242) | 9859 | - | | 2288817 | G | C | 78.77 | SNP | Rv2043c (pncA) | Thr142Arg | 1 | - (T would be resistance) | | 2288941 | C | A | 144.77 | SNP | Rv2043c (pncA) | Gly101STOP | 21 | - | | 2294876 | G | A | 238.96 | SNP | Rv2048c (pks12) | silent (Arg4037) | 9913 | - | | 2294894 | G | C | 289.78 | SNP | Rv2048c (pks12) | silent (Leu4031) | 9947 | - | | 2294896 | G | A | 281.78 | SNP | Rv2048c (pks12) | Leu4031Phe | 6 | - | | 2294903 | G | A | 283.78 | SNP | Rv2048c (pks12) | silent (Phe4028) | 9946 | - | | 2296042 | G | C | 133.03 | SNP | Rv2048c (pks12) | Pro3649Ala | 22 | - | | 2300237 | A | G | 218.84 | SNP | Rv2048c (pks12) | silent (Ala2250) | 9867 | - | | 2300546 | A | T | 62.74 | SNP | Rv2048c (pks12) | His2147Gln | 23 | - | | 2300552 | T | G | 54.74 | SNP | Rv2048c (pks12) | silent (Pro2145) | 9926 | - | | 2300555 | A | G | 53.74 | SNP | Rv2048c (pks12) | silent (Asp2144) | 9859 | - | | 2310543 | G | A | 231.84 | SNP | Rv2051c (ppm1) | silent (Gly71) | 9935 | - | | 2329533 | A | G | 378.77 | SNP | Rv2072c (cobL) | Leu205Pro | 2 | - | | 2334007 | A | G | 233.84 | SNP | Rv2077c | silent (Ala96) | 9867 | - | | 2335494 | A | G | 318.78 | SNP | Rv2079 | Tyr47Cys | 3 | - | | 2340621 | C | G | 533.77 | SNP | Rv2082 | Pro638Arg | 4 | - | | 2341636 | C | G | 367.77 | SNP | Rv2083 | Leu256Val(s) | 4 | - | | 2345037 | C | A | 106.28 | SNP | Rv2088 (pknJ) | silent (Leu209) | 9947 | - | | 2346672 | T | C | 347.78 | SNP | Rv2089c (pepE) | Asp218Gly | 11 | - | | 2355289 | G | T | 450.77 | SNP | Rv2096c (pafB) | silent (Arg8) | 9913 | - | | 2355511 | G | A | 206.84 | SNP | Rv2097c (pafA) | silent (Val389) | 9901 | - | | 2358104 | G | C | 437.77 | SNP | intergenic |  |  | - | | 2361604 | C | G | 323.78 | SNP | Rv2101 (helZ) | Val455Val(s) | 18 | - | | 2362041 | C | A | 195.90 | SNP | Rv2101 (helZ) | Pro601Gln | 6 | - | | 2364786 | A | C | 433.77 | SNP | intergenic |  |  | - | | 2368564 | TA | T | 569.73 | DEL | intergenic |  |  | - | | 2369971 | A | G | 172.78 | SNP | Rv2110c (prcB) | Tyr211His | 4 | - | | 2374245 | C | T | 231.84 | SNP | Rv2114 | Gln138STOP | 8 | - | | 2382196 | G | A | 280.78 | SNP | Rv2123 (PPE37) | Ala376Thr | 22 | - | | 2386389 | G | A | 216.84 | SNP | Rv2125 | Gly33Ser | 16 | - | | 2400467 | G | A | 446.77 | SNP | Rv2141c | Thr419Ile | 7 | - | | 2415656 | G | C | 226.84 | SNP | Rv2155c (murD) | Arg247Gly | 1 | - | | 2418817 | G | A | 257.78 | SNP | Rv2157c (murF) | Ser63Leu(s) | 35 | - | | 2424925 | A | G | 180.90 | SNP | intergenic |  |  | - | | 2439519 | G | A | 410.77 | SNP | Rv2177c | silent (Arg143) | 9913 | - | | 2440953 | G | T | 304.78 | SNP | Rv2178c (aroG) | silent (Arg256) | 9913 | - | | 2447282 | T | G | 191.78 | SNP | Rv2185c (TB16.3) | Glu73Asp | 53 | - | | 2453645 | A | C | 339.78 | SNP | intergenic |  |  | - | | 2465997 | T | G | 404.77 | SNP | Rv2201 (asnB) | Ile334Ser | 2 | - | | 2494263 | G | A | 52.74 | SNP | Rv2223c | silent (Tyr379) | 9945 | - | | 2496985 | G | C | 100.28 | SNP | Rv2224c (caeA) | silent (Arg13) | 9913 | - | | 2499726 | G | A | 262.78 | SNP | Rv2226 | Asp299Asn | 36 | - | | 2509140 | G | C | 106.28 | SNP | Rv2236c (cobD) | Ser79Cys | 5 | - | | 2509722 | A | G | 352.78 | SNP | Rv2237 | silent (Pro78) | 9926 | - | | 2521342 | T | C | 421.77 | SNP | Rv2247 (accD6) | silent (Asp200) | 9859 | - | | 2525722 | CG | C | 199.80 | DEL | Rv2250A; Rv2251 |  |  | - | | 2528170 | A | G | 583.77 | SNP | Rv2253 | Asn63Asp | 42 | - | | 2529680 | A | G | 480.77 | SNP | Rv2256c | silent (Thr65) | 9871 | - | | 2531742 | A | G | 467.77 | SNP | Rv2258c | silent (Ala52) | 9867 | - | | 2534562 | GGA | G | 449.74 | DEL | Rv2262c |  |  | - | | 2551572 | A | C | 359.78 | SNP | Rv2280 | Thr5Pro | 4 | - | | 2562752 | G | A | 85.56 | SNP | Rv2290 (lppO) | Val52Ile | 33 | - | | 2586127 | A | G | 217.84 | SNP | Rv2314c | silent (Gly388) | 9935 | - | | 2598400 | A | G | 329.78 | SNP | Rv2326c | silent (Asn516) | 9822 | - | | 2612632 | C | A | 169.90 | SNP | Rv2337c | Gly119Val | 3 | - | | 2614193 | C | T | 185.90 | SNP | intergenic |  |  | - | | 2624272 | G | A | 341.77 | SNP | Rv2345 | Gly151Glu | 4 | - | | 2630158 | C | G | 102.77 | SNP | Rv2350c (plcB) | silent (Arg54) | 9913 | - | | 2630161 | A | G | 86.77 | SNP | Rv2350c (plcB) | silent (Asn53) | 9822 | - | | 2630173 | C | G | 81.77 | SNP | Rv2350c (plcB) | Leu(s)49Phe | 1 | - | | 2630176 | C | G | 81.77 | SNP | Rv2350c (plcB) | Leu(s)48Phe | 1 | - | | 2630182 | G | A | 79.77 | SNP | Rv2350c (plcB) | silent (Ile46) | 9872 | - | | 2630184 | T | A | 77.77 | SNP | Rv2350c (plcB) | Ile46Phe | 8 | - | | 2630188 | C | T | 74.77 | SNP | Rv2350c (plcB) | silent (Glu44) | 9865 | - | | 2630206 | T | G | 35.77 | SNP | Rv2350c (plcB) | silent (Gly38) | 9935 | - | | 2630211 | G | A | 30.77 | SNP | Rv2350c (plcB) | Pro37Ser | 17 | - | | 2630215 | A | G | 43.77 | SNP | Rv2350c (plcB) | silent (Pro35) | 9926 | - | | 2631556 | C | G | 93.77 | SNP | Rv2351c (plcA) | Gly174Arg | 0 | - | | 2631565 | T | C | 87.77 | SNP | Rv2351c (plcA) | Ile171Val | 57 | - | | 2631574 | T | C | 93.77 | SNP | Rv2351c (plcA) | Thr168Ala | 32 | - | | 2631583 | G | A | 83.77 | SNP | Rv2351c (plcA) | Leu165Leu(s) | 4 | - | | 2631599 | G | A | 155.77 | SNP | Rv2351c (plcA) | silent (Ile159) | 9872 | - | | 2631620 | A | G | 90.77 | SNP | Rv2351c (plcA) | silent (Gly152) | 9935 | - | | 2631914 | C | G | 36.77 | SNP | Rv2351c (plcA) | silent (Arg54) | 9913 | - | | 2631962 | T | G | 33.77 | SNP | Rv2351c (plcA) | silent (Gly38) | 9935 | - | | 2631967 | G | A | 44.77 | SNP | Rv2351c (plcA) | Pro37Ser | 17 | - | | 2631968 | A | G | 53.77 | SNP | Rv2351c (plcA) | silent (Cys36) | 9973 | - | | 2631971 | A | G | 84.77 | SNP | Rv2351c (plcA) | silent (Pro35) | 9926 | - | | 2631977 | G | C | 88.77 | SNP | Rv2351c (plcA) | silent (Ala33) | 9867 | - | | 2646016 | C | T | 361.77 | SNP | Rv2364c (era) | Asp220Asn | 36 | - | | 2656225 | A | G | 233.84 | SNP | Rv2377c (mbtH) | Val69Ala | 18 | - | | 2660319 | C | G | 285.78 | SNP | Rv2379c (mbtF) | Glu589Asp | 53 | - | | 2667672 | G | A | 318.78 | SNP | Rv2381c (mbtD) | silent (Arg866) | 9913 | - | | 2680658 | T | G | 346.78 | SNP | intergenic |  |  | - | | 2695378 | C | G | 628.77 | SNP | Rv2398c (cysW) | Gly141Ala | 21 | - | | 2701413 | G | C | 149.77 | SNP | Rv2404c (lepA) | silent (Arg612) | 9913 | - | | 2701416 | T | C | 153.77 | SNP | Rv2404c (lepA) | silent (Lys611) | 9926 | - | | 2701419 | G | C | 128.77 | SNP | Rv2404c (lepA) | silent (Arg610) | 9913 | - | | 2701422 | C | G | 155.77 | SNP | Rv2404c (lepA) | silent (Thr609) | 9871 | - | | 2701423 | G | C | 129.77 | SNP | Rv2404c (lepA) | Thr609Arg | 1 | - | | 2701545 | C | G | 123.77 | SNP | Rv2404c (lepA) | silent (Leu568) | 9947 | - | | 2704884 | A | ACAGCGACCATATCGCCGAG CT | 2806.73 | INS | Rv2407 |  |  | - | | 2713795 | C | T | 294.78 | SNP | intergenic |  |  | - | | 2718852 | T | G | 363.78 | SNP | intergenic |  |  | - | | 2726145 | G | A | 246.80 | SNP | intergenic (Rv2428-48nt) |  |  | resistance | | 2734074 | T | C | 82.28 | SNP | Rv2436 (rbsK) | Val282Ala | 18 | - | | 2743962 | G | A | 212.89 | SNP | Rv2444c (rne) | silent (Thr341) | 9871 | - | | 2751804 | C | T | 36.74 | SNP | Rv2450c (rpfE) | Arg126Gln | 9 | - | | 2752698 | C | A | 438.77 | SNP | intergenic |  |  | - | | 2760152 | A | G | 216.84 | SNP | Rv2458 (mmuM) | Tyr125Cys | 3 | - | | 2779136 | T | C | 150.03 | SNP | Rv2476c (gdh) | Ser1043Gly | 21 | - | | 2786952 | A | G | 278.80 | SNP | Rv2482c (plsB2) | Cys778Arg | 1 | - | | 2794793 | G | A | 326.78 | SNP | Rv2486 (echA14) | Met(s)148Ile | 2 | - | | 2795160 | C | T | 409.77 | SNP | intergenic |  |  | - | | 2807237 | G | A | 577.77 | SNP | Rv2491 | Val(s)191Val | 13 | - | | 2809621 | T | C | 314.78 | SNP | Rv2495c (bkdC) | Thr107Ala | 32 | - | | 2816034 | G | A | 238.80 | SNP | Rv2501c (accA1) | Pro283Ser | 17 | - | | 2816296 | A | C | 133.03 | SNP | Rv2501c (accA1) | Asp195Glu | 56 | - | | 2818837 | A | G | 233.84 | SNP | Rv2503c (scoB) | silent (Gly97) | 9935 | - | | 2821077 | CGG | C | 882.73 | DEL | Rv2505c (fadD35) |  |  | - | | 2821342 | C | T | 195.90 | SNP | Rv2505c (fadD35) | silent (Ala85) | 9867 | - | | 2825336 | G | T | 281.78 | SNP | Rv2509 | Arg220Leu | 1 | - | | 2825882 | G | A | 218.85 | SNP | Rv2510c | Pro403Leu | 3 | - | | 2827984 | G | T | 195.90 | SNP | intergenic |  |  | - | | 2828019 | T | C | 144.03 | SNP | intergenic |  |  | - | | 2828517 | A | G | 404.77 | SNP | intergenic |  |  | - | | 2829779 | T | C | 55.74 | SNP | Rv2512c | Thr9Ala | 32 | - | | 2829823 | G | A | 35.74 | SNP | intergenic |  |  | - | | 2830525 | C | A | 275.80 | SNP | Rv2513 | Thr122Lys | 11 | - | | 2835520 | G | T | 64.77 | SNP | Rvnt30 | tRNA | tRNA | - | | 2835527 | C | T | 114.77 | SNP | Rvnt30 | tRNA | tRNA | - | | 2836257 | G | A | 298.78 | SNP | Rv2519 (PE26) | Gly158Asp | 6 | - | | 2855259 | A | G | 111.03 | SNP | Rv2531c | silent (Ala841) | 9867 | - | | 2865760 | A | G | 400.77 | SNP | Rv2542 | Thr211Ala | 32 | - | | 2881597 | AG | A | 539.73 | DEL | Rv2561 |  |  | - | | 2881818 | C | T | 699.77 | SNP | Rv2562 | His21Tyr | 4 | - | | 2888201 | T | C | 392.77 | SNP | Rv2566 | Leu610Pro | 2 | - | | 2888973 | C | T | 300.78 | SNP | Rv2566 | silent (Leu867) | 9947 | - | | 2889633 | T | C | 184.84 | SNP | Rv2566 | silent (Ala1087) | 9867 | - | | 2891267 | C | T | 163.90 | SNP | Rv2567 | silent (Gly491) | 9935 | - | | 2891728 | A | G | 162.90 | SNP | Rv2567 | Gln645Arg | 10 | - | | 2894208 | G | A | 171.90 | SNP | Rv2569c | silent (Ser67) | 9840 | - | | 2897375 | T | G | 307.78 | SNP | Rv2572c (aspS) | silent (Arg143) | 9913 | - | | 2899538 | A | C | 406.77 | SNP | Rv2575 | Asp67Ala | 10 | - | | 2910461 | G | T | 257.80 | SNP | Rv2584c (apt) | Ala147Glu | 10 | - | | 2911293 | C | G | 241.80 | SNP | Rv2585c | Cys462Ser | 11 | - | | 2912294 | T | G | 346.78 | SNP | Rv2585c | silent (Ala128) | 9867 | - | | 2922936 | TGGCGGTGAC | T | 544.87 | DEL | Rv2591 (PE\_PGRS44) |  |  | - | | 2923391 | T | C | 178.90 | SNP | Rv2592c (ruvB) | silent (Pro281) | 9926 | - | | 2927939 | T | C | 221.84 | SNP | intergenic |  |  | - | | 2928696 | G | T | 130.03 | SNP | Rv2601 (speE) | silent (Ala103) | 9867 | - | | 2939373 | G | C | 62.28 | SNP | Rv2611c | Ser197Cys | 5 | - | | 2939657 | T | C | 127.03 | SNP | Rv2611c | Ile102Met(s) | 6 | - | | 2945167 | G | T | 80.77 | SNP | intergenic |  |  | - | | 2954439 | T | C | 329.78 | SNP | Rv2627c | Arg104Gly | 1 | - | | 2965900 | C | T | 176.84 | SNP | intergenic |  |  | - | | 2969775 | C | A | 228.77 | SNP | Rvnt32 | tRNA | tRNA | - | | 2969781 | A | G | 229.77 | SNP | Rvnt32 | tRNA | tRNA | - | | 2969793 | T | C | 300.77 | SNP | Rvnt32 | tRNA | tRNA | - | | 2969819 | A | T | 322.77 | SNP | Rvnt32 | tRNA | tRNA | - | | 2974933 | A | G | 100.28 | SNP | Rv2650c | Ile101Thr | 11 | - | | 2981660 | G | T | 33.77 | SNP | Rv2662 | Arg60Leu | 1 | - | | 2982955 | C | T | 290.78 | SNP | Rv2665 | Pro86Leu | 3 | - | | 2990655 | C | T | 33.77 | SNP | intergenic |  |  | - | | 2996876 | A | C | 248.80 | SNP | Rv2681 | silent (Leu46) | 9947 | - | | 3005185 | G | T | 535.77 | SNP | Rv2688c | Pro156Thr | 5 | - | | 3006361 | CG | C | 852.73 | DEL | Rv2689c |  |  | - | | 3009692 | A | G | 592.77 | SNP | Rv2691 (ceoB) | Thr117Ala | 32 | - | | 3015966 | G | A | 108.03 | SNP | Rv2701c (suhB) | Ala257Val(s) | 9867 | - | | 3017465 | T | C | 425.77 | SNP | Rv2702 (ppgK) | Ile203Thr | 11 | - | | 3020515 | G | C | 322.78 | SNP | intergenic |  |  | - | | 3041871 | G | T | 509.77 | SNP | Rv2729c | Ala202Glu | 10 | - | | 3054081 | A | G | 299.78 | SNP | Rv2741 (PE\_PGRS47) | silent (Gly56) | 9935 | - | | 3054321 | A | G | 41.74 | SNP | Rv2741 (PE\_PGRS47) | silent (Gly136) | 9935 | - | | 3062515 | T | C | 558.77 | SNP | Rv2749 | Val4Ala | 18 | - | | 3069778 | G | T | 527.77 | SNP | Rv2756c (hsdM) | Phe102Leu | 13 | - | | 3080795 | A | G | 863.77 | SNP | Rv2771c | Leu80Pro | 2 | - | | 3100153 | G | GA | 649.80 | INS | Rv2790c (ltp1) |  |  | - | | 3103682 | T | C | 472.77 | SNP | Rv2794c (pptT) | Met(s)87Val(s) | 9867 | - | | 3112675 | G | A | 337.78 | SNP | Rv2804c | silent (Asp140) | 9859 | - | | 3118000 | A | G | 231.84 | SNP | Rv2812 | Arg395Gly | 1 | - | | 3121782 | G | A | 51.74 | SNP | Rv2815c | Arg16Trp | 2 | - | | 3129500 | G | A | 497.77 | SNP | Rv2823c | silent (Phe758) | 9946 | - | | 3131469 | T | TTGTCGGCGA | 1283.73 | INS | Rv2823c |  |  | - | | 3133536 | T | C | 725.77 | SNP | Rv2825c | Lys2Glu | 4 | - | | 3137058 | G | A | 184.90 | SNP | Rv2830c (vapB22) | Ala56Val(s) | 9867 | - | | 3143100 | A | G | 88.28 | SNP | Rv2836c (dinF) | Cys177Arg | 1 | - | | 3170460 | C | T | 236.80 | SNP | Rv2858c (aldC) | Val(s)88Val | 13 | - | | 3175335 | C | T | 533.77 | SNP | Rv2863 (vapC23) | Thr115Met(s) | 32 | - | | 3177884 | C | A | 406.77 | SNP | Rv2866 (relG) | silent (Arg21) | 9913 | - | | 3183561 | G | C | 40.77 | SNP | Rv2872 (vapC43) | silent (Pro60) | 9926 | - | | 3186860 | T | G | 142.03 | SNP | Rv2874 (dipZ) | Tyr672Asp | 0 | - | | 3190145 | TC | T | 225.84 | DEL | Rv2880c |  |  | - | | 3199932 | C | A | 95.77 | SNP | Rv2890c (rpsB) | silent (Gly17) | 9935 | - | | 3226181 | A | C | 320.78 | SNP | Rv2916c (ffh) | silent (Arg35) | 9913 | - | | 3228143 | G | T | 164.90 | SNP | Rv2917 | Arg594Leu | 1 | - | | 3232815 | A | G | 321.78 | SNP | intergenic |  |  | - | | 3247316 | C | G | 240.84 | SNP | Rv2931 (ppsA) | Asp624Glu | 56 | - | | 3247851 | G | A | 86.28 | SNP | Rv2931 (ppsA) | Ala803Thr | 22 | - | | 3247853 | C | T | 70.28 | SNP | Rv2931 (ppsA) | silent (Ala803) | 9867 | - | | 3247856 | G | C | 91.28 | SNP | Rv2931 (ppsA) | silent (Arg804) | 9913 | - | | 3247874 | G | A | 109.03 | SNP | Rv2931 (ppsA) | silent (Arg810) | 9913 | - | | 3247877 | T | C | 128.03 | SNP | Rv2931 (ppsA) | silent (Phe811) | 9946 | - | | 3247883 | T | C | 335.78 | SNP | Rv2931 (ppsA) | silent (Ser813) | 9840 | - | | 3248074 | G | A | 340.78 | SNP | Rv2931 (ppsA) | Arg877His | 8 | - | | 3248075 | C | T | 348.78 | SNP | Rv2931 (ppsA) | silent (Arg877) | 9913 | - | | 3256494 | A | G | 310.78 | SNP | Rv2933 (ppsC) | silent (Gly270) | 9935 | - | | 3269581 | A | G | 260.80 | SNP | Rv2935 (ppsE) | silent (Ala615) | 9867 | - | | 3270784 | A | G | 224.84 | SNP | Rv2935 (ppsE) | silent (Gln1016) | 9876 | - | | 3296843 | A | G | 171.90 | SNP | Rv2947c (pks15) | Val(s)333Ala | 9867 | - | | 3300196 | G | A | 203.80 | SNP | Rv2949c | silent (Phe125) | 9946 | - | | 3304753 | G | T | 340.78 | SNP | Rv2952 | Ala105Ser | 28 | - | | 3308606 | G | A | 227.84 | SNP | intergenic |  |  | - | | 3317702 | C | T | 291.10 | SNP | intergenic |  |  | - | | 3330620 | G | A | 130.03 | SNP | Rv2974c | Arg248Cys | 1 | - | | 3332626 | C | T | 408.77 | SNP | Rv2976c (ung) | silent (Leu43) | 9947 | - | | 3336825 | T | C | 360.77 | SNP | Rv2981c (ddlA) | Thr365Ala | 32 | - | | 3338603 | G | C | 245.80 | SNP | Rv2982c (gpdA2) | Pro133Ala | 22 | - | | 3358235 | A | T | 356.77 | SNP | Rv2999 (lppY) | Met(s)212Leu(s) | 9867 | - | | 3359870 | A | G | 38.77 | SNP | Rv3001c (ilvC) | silent (Gly239) | 9935 | - | | 3359884 | A | G | 37.77 | SNP | Rv3001c (ilvC) | Leu(s)235Leu | 3 | - | | 3360007 | T | C | 37.77 | SNP | Rv3001c (ilvC) | Thr194Ala | 32 | - | | 3363338 | A | G | 338.78 | SNP | intergenic |  |  | - | | 3367765 | G | A | 327.77 | SNP | Rv3009c (gatB) | silent (Gly343) | 9935 | - | | 3371719 | G | A | 454.77 | SNP | Rv3012c (gatC) | silent (Ile4) | 9872 | - | | 3402816 | C | T | 612.77 | SNP | Rv3042c (serB2) | Gly116Glu | 4 | - | | 3415180 | ACACCTAGGGGGTGG | A | 1111.77 | DEL | intergenic |  |  | - | | 3418923 | C | G | 284.80 | SNP | Rv3058c | Ala152Pro | 13 | - | | 3423426 | G | A | 517.77 | SNP | Rv3061c (fadE22) | silent (Leu668) | 9947 | - | | 3425854 | C | T | 421.77 | SNP | Rv3062 (ligB) | Pro91Ser | 17 | - | | 3428917 | C | A | 524.77 | SNP | Rv3063 (cstA) | Arg559Ser | 11 | - | | 3440464 | T | G | 353.78 | SNP | Rv3077 | silent (Arg308) | 9913 | - | | 3440468 | G | C | 357.78 | SNP | Rv3077 | Gly310Arg | 0 | - | | 3456666 | A | G | 351.78 | SNP | Rv3089 (fadD13) | silent (Ala302) | 9867 | - | | 3462135 | G | C | 353.77 | SNP | Rv3093c | Cys210Trp | 0 | - | | 3466426 | G | A | 398.77 | SNP | Rv3097c (lipY) | silent (Val222) | 9901 | genotype | | 3473996 | G | GA | 822.73 | INS | intergenic |  |  | - | | 3477660 | T | C | 581.77 | SNP | Rv3109 (moaA1) | silent (Pro4) | 9926 | - | | 3477917 | C | T | 371.78 | SNP | Rv3109 (moaA1) | Pro90Leu | 3 | - | | 3480435 | A | G | 383.77 | SNP | Rv3113 | His121Arg | 10 | - | | 3481475 | G | A | 128.03 | SNP | Rv3115 | Ala9Thr | 22 | - | | 3486977 | A | G | 764.77 | SNP | Rv3121 (cyp141) | Lys157Glu | 4 | - | | 3490749 | C | T | 225.84 | SNP | Rv3125c (PPE49) | Leu(s)301Leu | 3 | - | | 3501829 | C | A | 283.80 | SNP | Rv3136 (PPE51) | silent (Ser12) | 9840 | - | | 3503895 | C | T | 107.03 | SNP | Rv3137 | Pro168Leu | 3 | - | | 3505027 | G | A | 33.77 | SNP | Rv3138 (pflA) | Arg278His | 8 | - | | 3515582 | C | T | 261.74 | SNP | Rv3150 (nuoF) | silent (Ser57) | 9840 | - | | 3518167 | A | G | 175.78 | SNP | Rv3151 (nuoG) | Ile474Met(s) | 6 | - | | 3518555 | A | G | 130.03 | SNP | Rv3151 (nuoG) | Thr604Ala | 32 | - | | 3556275 | A | G | 501.77 | SNP | Rv3190c | Leu138Pro | 2 | - | | 3569029 | T | C | 195.90 | SNP | intergenic |  |  | - | | 3580636 | CT | C | 688.73 | DEL | intergenic |  |  | - | | 3581414 | A | G | 252.80 | SNP | Rv3204 | Thr34Ala | 32 | - | | 3590686 | G | GC | 209.87 | INS | intergenic |  |  | - | | 3591063 | T | C | 208.84 | SNP | Rv3213c | Lys144Glu | 4 | - | | 3594400 | A | G | 44.74 | SNP | intergenic |  |  | - | | 3604821 | G | C | 96.28 | SNP | Rv3228 | silent (Ala32) | 9867 | - | | 3614982 | T | C | 354.78 | SNP | Rv3239c | silent (Leu874) | 9947 | - | | 3619569 | G | C | 45.77 | SNP | Rv3240c (secA1) | silent (Thr321) | 9871 | - | | 3622441 | A | C | 164.90 | SNP | Rv3243c | Val217Val(s) | 18 | - | | 3644061 | C | T | 355.18 | SNP | Rv3263 | silent (Ile295) | 9872 | - | | 3652367 | C | T | 33.77 | SNP | Rv3270 (ctpC) | silent (Asp614) | 9859 | - | | 3662367 | C | T | 497.77 | SNP | Rv3280 (accD5) | silent (Tyr102) | 9945 | - | | 3669811 | C | A | 293.78 | SNP | Rv3288c (usfY) | Trp63Cys | 0 | - | | 3687908 | T | C | 161.90 | SNP | Rv3302c (glpD2) | Tyr512Cys | 3 | - | | 3689523 | G | T | 308.78 | SNP | Rv3303c (lpdA) | Cys472STOP | 3 | - | | 3696179 | T | C | 183.84 | SNP | Rv3308 (pmmB) | Val(s)439Ala | 9867 | - | | 3697152 | T | C | 581.77 | SNP | intergenic |  |  | - | | 3699253 | C | G | 141.03 | SNP | Rv3311 | Pro378Arg | 4 | - | | 3704596 | G | C | 629.77 | SNP | Rv3317 (sdhD) | Val(s)54Leu | 3 | - | | 3710395 | C | T | 41.74 | SNP | intergenic |  |  | - | | 3710396 | A | G | 46.74 | SNP | intergenic |  |  | - | | 3710397 | T | A | 39.74 | SNP | intergenic |  |  | - | | 3714211 | G | T | 504.77 | SNP | Rv3328c (sigJ) | Pro41Gln | 6 | - | | 3718357 | C | T | 267.10 | SNP | Rv3331 (sugI) | Pro423Leu | 3 | - | | 3721806 | G | C | 652.77 | SNP | Rv3335c | silent (Gly265) | 9935 | - | | 3730385 | C | G | 187.77 | SNP | Rv3343c (PPE54) | Arg2184Pro | 5 | - | | 3730386 | G | T | 186.77 | SNP | Rv3343c (PPE54) | silent (Arg2184) | 9913 | - | | 3730466 | A | G | 407.77 | SNP | Rv3343c (PPE54) | Ile2157Thr | 11 | - | | 3730624 | C | T | 47.74 | SNP | Rv3343c (PPE54) | silent (Ser2104) | 9840 | - | | 3732624 | A | G | 151.03 | SNP | Rv3343c (PPE54) | Leu(s)1438Leu | 3 | - | | 3735508 | G | A | 387.77 | SNP | Rv3343c (PPE54) | silent (Leu476) | 9947 | - | | 3736628 | T | G | 357.31 | SNP | Rv3343c (PPE54) | Glu103Ala | 17 | - | | 3737661 | TG | T | 317.75 | DEL | intergenic |  |  | - | | 3739772 | GTTGCCGGCGTCACCGCCGT TGAGGCCGGAGCCGCCGTTG CCGCCGTTGCCGCCGGCCGC GCCGCTCCCGTTGCCGGCGG TGCCGCCC | G | 183 | DEL | Rv3345c (PE\_PGRS50) |  |  | - | | 3744261 | C | T | 445.77 | SNP | Rv3347c (PPE55) | Gly2975Glu | 4 | - | | 3746409 | A | G | 55.74 | SNP | Rv3347c (PPE55) | Leu2259Pro | 2 | - | | 3747403 | C | A | 145.77 | SNP | Rv3347c (PPE55) | Gly1928Cys | 0 | - | | 3752207 | A | G | 269.80 | SNP | Rv3347c (PPE55) | silent (Ile326) | 9872 | - | | 3752654 | A | T | 95.28 | SNP | Rv3347c (PPE55) | silent (Gly177) | 9935 | - | | 3752662 | A | G | 98.28 | SNP | Rv3347c (PPE55) | Leu(s)175Leu | 3 | - | | 3752761 | A | G | 45.74 | SNP | Rv3347c (PPE55) | Leu(s)142Leu | 3 | - | | 3752778 | A | G | 48.74 | SNP | Rv3347c (PPE55) | Val136Ala | 18 | - | | 3753116 | C | T | 61.74 | SNP | Rv3347c (PPE55) | silent (Pro23) | 9926 | - | | 3753164 | T | G | 55.74 | SNP | Rv3347c (PPE55) | silent (Pro7) | 9926 | - | | 3766777 | A | G | 65.77 | SNP | Rv3350c (PPE56) | Leu109Pro | 2 | - | | 3766778 | G | C | 55.77 | SNP | Rv3350c (PPE56) | Leu109Val(s) | 4 | - | | 3766815 | G | C | 72.77 | SNP | Rv3350c (PPE56) | Val96Val(s) | 18 | - | | 3766816 | A | G | 72.77 | SNP | Rv3350c (PPE56) | Val96Ala | 18 | - | | 3766819 | C | G | 74.77 | SNP | Rv3350c (PPE56) | Gly95Ala | 21 | - | | 3766858 | G | T | 51.74 | SNP | Rv3350c (PPE56) | Ala82Glu | 10 | - | | 3766859 | C | G | 55.74 | SNP | Rv3350c (PPE56) | Ala82Pro | 13 | - | | 3766860 | G | C | 55.74 | SNP | Rv3350c (PPE56) | silent (Ala81) | 9867 | - | | 3774199 | T | TC | 467.73 | INS | Rv3363c |  |  | - | | 3792796 | G | A | 161.79 | SNP | Rv3378c | silent (Asn151) | 9822 | - | | 3796694 | A | C | 438.77 | SNP | Rv3382c (lytB1) | silent (Arg248) | 9913 | - | | 3798095 | A | C | 219.84 | SNP | Rv3383c (idsB) | Val132Gly | 5 | - | | 3800460 | G | A | 211.84 | SNP | Rv3386 | silent (Glu123) | 9865 | - | | 3817117 | C | A | 205.84 | SNP | Rv3399 | Ala330Glu | 10 | - | | 3820545 | A | G | 33.77 | SNP | intergenic |  |  | - | | 3823159 | A | T | 168.90 | SNP | Rv3403c | silent (Val235) | 9901 | - | | 3826684 | C | T | 273.80 | SNP | Rv3408 (vapC47) | Ser46Leu(s) | 35 | - | | 3829770 | T | C | 349.78 | SNP | Rv3410c (guaB3) | silent (Pro47) | 9926 | - | | 3838871 | A | G | 170.90 | SNP | Rv3420c (rimI) | silent (Ala64) | 9867 | - | | 3841654 | T | G | 48.77 | SNP | intergenic |  |  | - | | 3841662 | T | C | 43.77 | SNP | intergenic |  |  | - | | 3841663 | C | T | 54.77 | SNP | intergenic |  |  | - | | 3842211 | T | C | 34.77 | SNP | intergenic |  |  | - | | 3842228 | A | G | 30.77 | SNP | intergenic |  |  | - | | 3842452 | C | A | 98.77 | SNP | Rv3425 (PPE57) | Gln72Lys | 12 | - | | 3842620 | A | G | 218.84 | SNP | Rv3425 (PPE57) | Thr128Ala | 32 | - | | 3842625 | A | G | 224.84 | SNP | Rv3425 (PPE57) | silent (Pro129) | 9926 | - | | 3843001 | G | A | 131.77 | SNP | intergenic |  |  | - | | 3843024 | A | C | 118.77 | SNP | intergenic |  |  | - | | 3843025 | A | G | 92.77 | SNP | intergenic |  |  | - | | 3843032 | G | A | 98.77 | SNP | intergenic |  |  | - | | 3843354 | A | G | 177.77 | SNP | Rv3426 (PPE58) | Thr107Ala | 32 | - | | 3843356 | T | C | 180.77 | SNP | Rv3426 (PPE58) | silent (Thr107) | 9871 | - | | 3843361 | C | A | 136.77 | SNP | Rv3426 (PPE58) | Ala109Asp | 6 | - | | 3843362 | C | A | 172.77 | SNP | Rv3426 (PPE58) | silent (Ala109) | 9867 | - | | 3843363 | A | G | 180.77 | SNP | Rv3426 (PPE58) | Asn110Asp | 42 | - | | 3843407 | CG | C | 436.73 | DEL | Rv3426 (PPE58) |  |  | - | | 3843531 | A | G | 57.77 | SNP | Rv3426 (PPE58) | Met(s)166Val(s) | 9867 | - | | 3843696 | T | A | 118.77 | SNP | Rv3426 (PPE58) | Leu(s)221Met(s) | 9867 | - | | 3843704 | G | C | 131.77 | SNP | Rv3426 (PPE58) | silent (Thr223) | 9871 | - | | 3843714 | T | C | 217.77 | SNP | Rv3426 (PPE58) | Cys227Arg | 1 | - | | 3843749 | G | T | 114.77 | SNP | intergenic |  |  | - | | 3843751 | G | T | 126.77 | SNP | intergenic |  |  | - | | 3843752 | A | G | 133.77 | SNP | intergenic |  |  | - | | 3843753 | G | A | 124.77 | SNP | intergenic |  |  | - | | 3843760 | T | C | 124.77 | SNP | intergenic |  |  | - | | 3844756 | GC | G | 631.73 | DEL | Rv3428c |  |  | - | | 3844992 | T | A | 439.77 | SNP | Rv3428c | Ser327Cys | 5 | - | | 3846580 | T | A | 102.77 | SNP | intergenic |  |  | - | | 3846582 | T | G | 140.77 | SNP | intergenic |  |  | - | | 3846591 | C | T | 160.77 | SNP | intergenic |  |  | - | | 3846605 | G | A | 206.77 | SNP | intergenic |  |  | - | | 3846607 | A | C | 175.77 | SNP | intergenic |  |  | - | | 3846622 | G | T | 160.77 | SNP | intergenic |  |  | - | | 3846687 | A | G | 112.77 | SNP | intergenic |  |  | - | | 3846697 | C | CG | 285.73 | INS | intergenic |  |  | - | | 3846707 | A | C | 64.77 | SNP | intergenic |  |  | - | | 3846764 | C | G | 117.77 | SNP | intergenic |  |  | - | | 3846774 | T | G | 69.77 | SNP | intergenic |  |  | - | | 3846777 | C | A | 66.77 | SNP | intergenic |  |  | - | | 3846779 | T | G | 59.77 | SNP | intergenic |  |  | - | | 3847215 | T | C | 99.28 | SNP | Rv3429 (PPE59) | silent (Gly17) | 9935 | - | | 3859893 | C | T | 62.74 | SNP | Rv3440c | silent (Glu28) | 9865 | - | | 3862472 | GA | G | 583.73 | DEL | intergenic |  |  | - | | 3864995 | T | C | 543.77 | SNP | Rv3447c (eccC4) | Ser1082Gly | 21 | - | | 3868527 | TG | T | 357.74 | DEL | Rv3448 (eccD4) |  |  | - | | 3869796 | C | G | 237.22 | SNP | Rv3449 (mycP4) | silent (Leu15) | 9947 | - | | 3876530 | G | A | 274.80 | SNP | Rv3455c (truA) | Ala98Val(s) | 9867 | - | | 3877421 | A | G | 224.84 | SNP | Rv3456c (rplQ) | silent (Pro4) | 9926 | - | | 3879331 | G | A | 172.90 | SNP | Rv3459c (rpsK) | Ser121Leu(s) | 35 | - | | 3883595 | A | C | 97.28 | SNP | Rv3466 | Asp24Ala | 10 | - | | 3885886 | T | C | 62.74 | SNP | Rv3468c | Ile62Val | 57 | - | | 3892671 | A | G | 653.77 | SNP | Rv3476c (kgtP) | silent (Val350) | 9901 | - | | 3895269 | G | C | 99.28 | SNP | Rv3478 (PPE60) | Glu282Gln | 27 | - | | 3895281 | T | C | 99.28 | SNP | Rv3478 (PPE60) | Trp286Arg | 8 | - | | 3895282 | G | A | 106.28 | SNP | Rv3478 (PPE60) | Trp286STOP | 0 | - | | 3896340 | T | G | 338.78 | SNP | Rv3479 | Leu174Arg | 1 | - | | 3898408 | A | G | 195.90 | SNP | Rv3479 | silent (Ala863) | 9867 | - | | 3898637 | G | A | 701.77 | SNP | Rv3479 | Val940Ile | 33 | - | | 3899644 | G | C | 77.77 | SNP | Rv3480c | Val253Val(s) | 18 | - | | 3909627 | C | T | 419.77 | SNP | Rv3490 (otsA) | silent (Gly464) | 9935 | - | | 3933131 | C | G | 109.03 | SNP | Rv3508 (PE\_PGRS54) | silent (Gly709) | 9935 | - | | 3934542 | T | G | 48.77 | SNP | Rv3508 (PE\_PGRS54) | Ser1180Ala | 35 | - | | 3940802 | A | G | 106.28 | SNP | Rv3511 (PE\_PGRS55) | Asn396Asp | 42 | - | | 3942640 | T | C | 79.28 | SNP | intergenic |  |  | - | | 3952800 | G | A | 289.78 | SNP | Rv3516 (echA19) | Gly86Asp | 6 | - | | 3958403 | A | G | 278.80 | SNP | Rv3521 | Asn295Asp | 42 | - | | 3958797 | C | T | 262.80 | SNP | Rv3522 (ltp4) | Ala117Val | 13 | - | | 3959418 | C | T | 374.77 | SNP | Rv3522 (ltp4) | Thr324Ile | 7 | - | | 3967432 | G | A | 106.28 | SNP | Rv3530c | Ala130Val(s) | 9867 | - | | 3969423 | GGCC | G | 744.73 | DEL | Rv3532 (PPE61) |  |  | - | | 4005607 | T | C | 315.78 | SNP | Rv3564 (fadE33) | Leu(s)121Leu | 3 | - | | 4013927 | G | A | 488.77 | SNP | Rv3572 | silent (Ser139) | 9840 | - | | 4018414 | C | CA | 612.73 | INS | Rv3576 (lppH) |  |  | - | | 4018415 | G | A | 273.80 | SNP | Rv3576 (lppH) | Gly20Arg | 0 | - | | 4018802 | CAA | C | 265.80 | DEL | Rv3576 (lppH) |  |  | - | | 4024273 | T | C | 195.90 | SNP | Rv3581c (ispF) | Val25Val(s) | 18 | - | | 4026899 | G | A | 462.77 | SNP | Rv3585 (radA) | silent (Gln152) | 9876 | - | | 4034477 | G | A | 180.94 | SNP | Rv3593 (lpqF) | silent (Ala42) | 9867 | - | | 4034827 | C | T | 95.28 | SNP | Rv3593 (lpqF) | Ala159Val(s) | 9867 | - | | 4037794 | GC | G | 435.73 | DEL | Rv3595c (PE\_PGRS59) |  |  | - | | 4039514 | G | A | 74.77 | SNP | Rv3596c (clpC1) | silent (Asp397) | 9859 | - | | 4039517 | G | C | 76.77 | SNP | Rv3596c (clpC1) | silent (Pro396) | 9926 | - | | 4039533 | T | G | 33.77 | SNP | Rv3596c (clpC1) | Asn391Thr | 13 | - | | 4048038 | G | GGCGACC | 700.74 | INS | Rv3605c |  |  | - | | 4055801 | G | A | 385.77 | SNP | Rv3616c (espA) | Thr192Ile | 7 | - | | 4059904 | A | G | 313.78 | SNP | intergenic |  |  | - | | 4060100 | G | A | 164.90 | SNP | Rv3619c (esxV) | Leu57Leu(s) | 4 | - | | 4067387 | G | A | 393.77 | SNP | intergenic |  |  | - | | 4069292 | G | A | 185.90 | SNP | Rv3630 | Ala40Thr | 22 | - | | 4086547 | G | A | 195.90 | SNP | Rv3646c (topA) | silent (Pro237) | 9926 | - | | 4091590 | C | A | 479.77 | SNP | intergenic |  |  | - | | 4095001 | CG | C | 346.74 | DEL | Rv3655c |  |  | - | | 4100975 | T | C | 342.78 | SNP | intergenic |  |  | - | | 4109796 | G | A | 284.80 | SNP | Rv3668c | Pro229Leu | 3 | - | | 4111303 | G | C | 275.80 | SNP | Rv3669 | Val(s)159Val | 13 | - | | 4117361 | AC | A | 1068.73 | DEL | Rv3677c |  |  | - | | 4120983 | A | G | 182.77 | SNP | intergenic |  |  | - | | 4132664 | G | A | 397.77 | SNP | Rv3691 | silent (Thr49) | 9871 | - | | 4139670 | C | T | 280.78 | SNP | Rv3696c (glpK) | Cys29Tyr | 3 | - | | 4148377 | C | T | 607.77 | SNP | Rv3705c | Gly196Ser | 16 | - | | 4148669 | C | T | 412.77 | SNP | Rv3705c | silent (Thr98) | 9871 | - | | 4152338 | C | T | 355.78 | SNP | Rv3709c (ask) | silent (Glu382) | 9865 | - | | 4155050 | G | A | 596.77 | SNP | Rv3710 (leuA) | Val(s)437Val | 13 | - | | 4156099 | C | A | 264.80 | SNP | Rv3711c (dnaQ) | Val(s)211Leu(s) | 9867 | - | | 4159830 | C | G | 440.77 | SNP | intergenic |  |  | - | | 4160407 | A | G | 508.77 | SNP | Rv3715c (recR) | Leu(s)32Leu | 3 | - | | 4162073 | C | T | 218.74 | SNP | Rv3718c | silent (Gln62) | 9876 | - | | 4162339 | A | G | 443.77 | SNP | Rv3719 | Thr12Ala | 32 | - | | 4179179 | A | G | 484.77 | SNP | Rv3729 | Ile299Val | 57 | - | | 4182695 | G | A | 395.77 | SNP | Rv3731 (ligC) | Arg313His | 8 | - | | 4187485 | T | C | 384.77 | SNP | Rv3736 | silent (Ala284) | 9867 | - | | 4187817 | A | G | 179.84 | SNP | Rv3737 | Asp40Gly | 11 | - | | 4189841 | T | C | 242.80 | SNP | Rv3738c (PPE66) | Tyr131Cys | 3 | - | | 4198611 | CG | C | 1030.73 | DEL | intergenic |  |  | - | | 4204441 | A | G | 471.77 | SNP | Rv3759c (proX) | silent (His311) | 9912 | - | | 4210274 | A | G | 322.78 | SNP | Rv3764c (tcrY) | Cys246Arg | 1 | - | | 4212196 | A | T | 536.77 | SNP | intergenic |  |  | - | | 4214751 | C | A | 304.78 | SNP | Rv3769 | Thr46Asn | 9 | - | | 4215467 | G | A | 185.90 | SNP | Rv3770c | silent (Gly103) | 9935 | - | | 4221490 | C | G | 269.80 | SNP | Rv3776 | silent (Leu134) | 9947 | - | | 4222073 | A | G | 55.74 | SNP | Rv3776 | Met(s)329Val(s) | 9867 | - | | 4222882 | A | G | 455.77 | SNP | Rv3777 | silent (Leu63) | 9947 | - | | 4231405 | A | C | 162.90 | SNP | Rv3785 | Asp29Ala | 10 | - | | 4242643 | C | T | 216.84 | SNP | Rv3793 (embC) | silent (Arg927) | 9913 | genotype | | 4243709 | C | T | 84.28 | SNP | Rv3794 (embA) | silent (Ala159) | 9867 | - | | 4245055 | C | A | 274.78 | SNP | Rv3794 (embA) | Thr608Asn | 9 | genotype | | 4247429 | A | G | 361.78 | SNP | Rv3795 (embB) | Met(s)306Val(s) | 9867 | resistance | | 4250742 | G | A | 294.78 | SNP | Rv3796 | Gly289Ser | 16 | - | | 4255922 | A | G | 400.77 | SNP | Rv3799c (accD4) | silent (His9) | 9912 | - | | 4257220 | A | G | 309.78 | SNP | Rv3800c (pks13) | silent (Arg1309) | 9913 | - | | 4257849 | G | A | 126.03 | SNP | Rv3800c (pks13) | Arg1100Trp | 2 | - | | 4260268 | G | C | 188.90 | SNP | Rv3800c (pks13) | silent (Ala293) | 9867 | genotype | | 4287195 | A | G | 215.78 | SNP | Rv3822 | Thr159Ala | 32 | - | | 4302036 | T | C | 220.84 | SNP | Rv3827c | Thr252Ala | 32 | - | | 4306155 | C | T | 489.77 | SNP | Rv3831 | silent (Ser133) | 9840 | - | | 4311528 | G | A | 265.78 | SNP | Rv3837c | silent (Ala60) | 9867 | - | | 4312542 | G | A | 397.77 | SNP | Rv3838c (pheA) | Ala43Val(s) | 9867 | - | | 4315145 | T | C | 332.78 | SNP | Rv3842c (glpQ1) | Lys140Glu | 4 | - | | 4315691 | C | T | 400.77 | SNP | Rv3843c | silent (Glu302) | 9865 | - | | 4335216 | G | A | 566.77 | SNP | Rv3859c (gltB) | Pro289Leu | 3 | - | | 4338595 | GC | G | 816.73 | DEL | intergenic |  |  | - | | 4338635 | T | G | 186.90 | SNP | intergenic |  |  | - | | 4338732 | G | A | 380.77 | SNP | intergenic |  |  | - | | 4341452 | CG | C | 515.73 | DEL | Rv3864 (espE) |  |  | - | | 4341636 | G | A | 197.84 | SNP | Rv3865 (espF) | Gly24Asp | 6 | - | | 4344495 | T | G | 55.77 | SNP | Rv3868 (eccA1) | silent (Leu394) | 9947 | - | | 4351039 | G | T | 303.78 | SNP | Rv3872 (PE35) | Glu99STOP | 17 | - | | 4356110 | G | C | 178.90 | SNP | Rv3877 (eccD1) | silent (Leu368) | 9947 | - | | 4362955 | C | T | 351.78 | SNP | Rv3882c (eccE1) | Val156Ile | 33 | - | | 4366272 | G | C | 316.78 | SNP | Rv3884c (eccA2) | silent (Ala189) | 9867 | - | | 4367911 | A | G | 265.80 | SNP | Rv3885c (eccE2) | Ile204Thr | 11 | - | | 4372010 | A | G | 607.77 | SNP | Rv3888c | Ser233Pro | 12 | - | | 4375628 | G | T | 273.80 | SNP | Rv3892c (PPE69) | Thr19Lys | 11 | - | | 4379680 | C | G | 635.77 | SNP | Rv3894c (eccC2) | Arg258Pro | 5 | - | | 4382054 | T | C | 362.78 | SNP | Rv3896c | silent (Ala266) | 9867 | - | | 4382275 | G | T | 218.84 | SNP | Rv3896c | Gln193Lys | 12 | - | | 4383144 | C | CCGGGG | 1487.73 | INS | Rv3897c |  |  | - | | 4400660 | AC | A | 600.73 | DEL | Rv3911 (sigM) |  |  | - | |  | | export |

elog
