# Supplementary material for: Patterns of compensatory mutations in rpoA/B/C genes of multidrug resistant M. tuberculosis in Uganda
Source: PLoS One. 2025 Dec 4;20(12):e0328957. doi: 10.1371/journal.pone.0328957 (PMC12677784; doi:10.1371/journal.pone.0328957)
Supplement: S2 File — (ZIP) [file pone.0328957.s002.zip › Variants D_S4_L001_001.bam.html]

 

Calling SNPs/INDELs (computing variant list in .vcf format) from D\_S4\_L001\_001.bam

*by SAMtools/BCFtools:*

Howto

Important aspects

This takes up to one hour!!! **Please wait ...**

Variants D\_S4\_L001\_001.bam

|  |  |
| --- | --- |
| Variants |  |

|  |  |
| --- | --- |
| |  | | --- | | *by GATK* | |

|  |  |  |
| --- | --- | --- |
| |  | | --- | | D\_S4\_L001\_001.bam | | | computed 2016-10-27 using PhyResSE v1.0 (Ref. NC\_000962.3) | |

|  |  |
| --- | --- |
| 1348  variants called Export in VCF format |  |

|  |  |  |  |  |  |  |  |  |  |  |  |  |  |  |  |  |  |  |  |  |  |  |  |  |  |  |  |  |  |  |  |  |  |  |  |  |  |  |  |  |  |  |  |  |  |  |  |  |  |  |  |  |  |  |  |  |  |  |  |  |  |  |  |  |  |  |  |  |  |  |  |  |  |  |  |  |  |  |  |  |  |  |  |  |  |  |  |  |  |  |  |  |  |  |  |  |  |  |  |  |  |  |  |  |  |  |  |  |  |  |  |  |  |  |  |  |  |  |  |  |  |  |  |  |  |  |  |  |  |  |  |  |  |  |  |  |  |  |  |  |  |  |  |  |  |  |  |  |  |  |  |  |  |  |  |  |  |  |  |  |  |  |  |  |  |  |  |  |  |  |  |  |  |  |  |  |  |  |  |  |  |  |  |  |  |  |  |  |  |  |  |  |  |  |  |  |  |  |  |  |  |  |  |  |  |  |  |  |  |  |  |  |  |  |  |  |  |  |  |  |  |  |  |  |  |  |  |  |  |  |  |  |  |  |  |  |  |  |  |  |  |  |  |  |  |  |  |  |  |  |  |  |  |  |  |  |  |  |  |  |  |  |  |  |  |  |  |  |  |  |  |  |  |  |  |  |  |  |  |  |  |  |  |  |  |  |  |  |  |  |  |  |  |  |  |  |  |  |  |  |  |  |  |  |  |  |  |  |  |  |  |  |  |  |  |  |  |  |  |  |  |  |  |  |  |  |  |  |  |  |  |  |  |  |  |  |  |  |  |  |  |  |  |  |  |  |  |  |  |  |  |  |  |  |  |  |  |  |  |  |  |  |  |  |  |  |  |  |  |  |  |  |  |  |  |  |  |  |  |  |  |  |  |  |  |  |  |  |  |  |  |  |  |  |  |  |  |  |  |  |  |  |  |  |  |  |  |  |  |  |  |  |  |  |  |  |  |  |  |  |  |  |  |  |  |  |  |  |  |  |  |  |  |  |  |  |  |  |  |  |  |  |  |  |  |  |  |  |  |  |  |  |  |  |  |  |  |  |  |  |  |  |  |  |  |  |  |  |  |  |  |  |  |  |  |  |  |  |  |  |  |  |  |  |  |  |  |  |  |  |  |  |  |  |  |  |  |  |  |  |  |  |  |  |  |  |  |  |  |  |  |  |  |  |  |  |  |  |  |  |  |  |  |  |  |  |  |  |  |  |  |  |  |  |  |  |  |  |  |  |  |  |  |  |  |  |  |  |  |  |  |  |  |  |  |  |  |  |  |  |  |  |  |  |  |  |  |  |  |  |  |  |  |  |  |  |  |  |  |  |  |  |  |  |  |  |  |  |  |  |  |  |  |  |  |  |  |  |  |  |  |  |  |  |  |  |  |  |  |  |  |  |  |  |  |  |  |  |  |  |  |  |  |  |  |  |  |  |  |  |  |  |  |  |  |  |  |  |  |  |  |  |  |  |  |  |  |  |  |  |  |  |  |  |  |  |  |  |  |  |  |  |  |  |  |  |  |  |  |  |  |  |  |  |  |  |  |  |  |  |  |  |  |  |  |  |  |  |  |  |  |  |  |  |  |  |  |  |  |  |  |  |  |  |  |  |  |  |  |  |  |  |  |  |  |  |  |  |  |  |  |  |  |  |  |  |  |  |  |  |  |  |  |  |  |  |  |  |  |  |  |  |  |  |  |  |  |  |  |  |  |  |  |  |  |  |  |  |  |  |  |  |  |  |  |  |  |  |  |  |  |  |  |  |  |  |  |  |  |  |  |  |  |  |  |  |  |  |  |  |  |  |  |  |  |  |  |  |  |  |  |  |  |  |  |  |  |  |  |  |  |  |  |  |  |  |  |  |  |  |  |  |  |  |  |  |  |  |  |  |  |  |  |  |  |  |  |  |  |  |  |  |  |  |  |  |  |  |  |  |  |  |  |  |  |  |  |  |  |  |  |  |  |  |  |  |  |  |  |  |  |  |  |  |  |  |  |  |  |  |  |  |  |  |  |  |  |  |  |  |  |  |  |  |  |  |  |  |  |  |  |  |  |  |  |  |  |  |  |  |  |  |  |  |  |  |  |  |  |  |  |  |  |  |  |  |  |  |  |  |  |  |  |  |  |  |  |  |  |  |  |  |  |  |  |  |  |  |  |  |  |  |  |  |  |  |  |  |  |  |  |  |  |  |  |  |  |  |  |  |  |  |  |  |  |  |  |  |  |  |  |  |  |  |  |  |  |  |  |  |  |  |  |  |  |  |  |  |  |  |  |  |  |  |  |  |  |  |  |  |  |  |  |  |  |  |  |  |  |  |  |  |  |  |  |  |  |  |  |  |  |  |  |  |  |  |  |  |  |  |  |  |  |  |  |  |  |  |  |  |  |  |  |  |  |  |  |  |  |  |  |  |  |  |  |  |  |  |  |  |  |  |  |  |  |  |  |  |  |  |  |  |  |  |  |  |  |  |  |  |  |  |  |  |  |  |  |  |  |  |  |  |  |  |  |  |  |  |  |  |  |  |  |  |  |  |  |  |  |  |  |  |  |  |  |  |  |  |  |  |  |  |  |  |  |  |  |  |  |  |  |  |  |  |  |  |  |  |  |  |  |  |  |  |  |  |  |  |  |  |  |  |  |  |  |  |  |  |  |  |  |  |  |  |  |  |  |  |  |  |  |  |  |  |  |  |  |  |  |  |  |  |  |  |  |  |  |  |  |  |  |  |  |  |  |  |  |  |  |  |  |  |  |  |  |  |  |  |  |  |  |  |  |  |  |  |  |  |  |  |  |  |  |  |  |  |  |  |  |  |  |  |  |  |  |  |  |  |  |  |  |  |  |  |  |  |  |  |  |  |  |  |  |  |  |  |  |  |  |  |  |  |  |  |  |  |  |  |  |  |  |  |  |  |  |  |  |  |  |  |  |  |  |  |  |  |  |  |  |  |  |  |  |  |  |  |  |  |  |  |  |  |  |  |  |  |  |  |  |  |  |  |  |  |  |  |  |  |  |  |  |  |  |  |  |  |  |  |  |  |  |  |  |  |  |  |  |  |  |  |  |  |  |  |  |  |  |  |  |  |  |  |  |  |  |  |  |  |  |  |  |  |  |  |  |  |  |  |  |  |  |  |  |  |  |  |  |  |  |  |  |  |  |  |  |  |  |  |  |  |  |  |  |  |  |  |  |  |  |  |  |  |  |  |  |  |  |  |  |  |  |  |  |  |  |  |  |  |  |  |  |  |  |  |  |  |  |  |  |  |  |  |  |  |  |  |  |  |  |  |  |  |  |  |  |  |  |  |  |  |  |  |  |  |  |  |  |  |  |  |  |  |  |  |  |  |  |  |  |  |  |  |  |  |  |  |  |  |  |  |  |  |  |  |  |  |  |  |  |  |  |  |  |  |  |  |  |  |  |  |  |  |  |  |  |  |  |  |  |  |  |  |  |  |  |  |  |  |  |  |  |  |  |  |  |  |  |  |  |  |  |  |  |  |  |  |  |  |  |  |  |  |  |  |  |  |  |  |  |  |  |  |  |  |  |  |  |  |  |  |  |  |  |  |  |  |  |  |  |  |  |  |  |  |  |  |  |  |  |  |  |  |  |  |  |  |  |  |  |  |  |  |  |  |  |  |  |  |  |  |  |  |  |  |  |  |  |  |  |  |  |  |  |  |  |  |  |  |  |  |  |  |  |  |  |  |  |  |  |  |  |  |  |  |  |  |  |  |  |  |  |  |  |  |  |  |  |  |  |  |  |  |  |  |  |  |  |  |  |  |  |  |  |  |  |  |  |  |  |  |  |  |  |  |  |  |  |  |  |  |  |  |  |  |  |  |  |  |  |  |  |  |  |  |  |  |  |  |  |  |  |  |  |  |  |  |  |  |  |  |  |  |  |  |  |  |  |  |  |  |  |  |  |  |  |  |  |  |  |  |  |  |  |  |  |  |  |  |  |  |  |  |  |  |  |  |  |  |  |  |  |  |  |  |  |  |  |  |  |  |  |  |  |  |  |  |  |  |  |  |  |  |  |  |  |  |  |  |  |  |  |  |  |  |  |  |  |  |  |  |  |  |  |  |  |  |  |  |  |  |  |  |  |  |  |  |  |  |  |  |  |  |  |  |  |  |  |  |  |  |  |  |  |  |  |  |  |  |  |  |  |  |  |  |  |  |  |  |  |  |  |  |  |  |  |  |  |  |  |  |  |  |  |  |  |  |  |  |  |  |  |  |  |  |  |  |  |  |  |  |  |  |  |  |  |  |  |  |  |  |  |  |  |  |  |  |  |  |  |  |  |  |  |  |  |  |  |  |  |  |  |  |  |  |  |  |  |  |  |  |  |  |  |  |  |  |  |  |  |  |  |  |  |  |  |  |  |  |  |  |  |  |  |  |  |  |  |  |  |  |  |  |  |  |  |  |  |  |  |  |  |  |  |  |  |  |  |  |  |  |  |  |  |  |  |  |  |  |  |  |  |  |  |  |  |  |  |  |  |  |  |  |  |  |  |  |  |  |  |  |  |  |  |  |  |  |  |  |  |  |  |  |  |  |  |  |  |  |  |  |  |  |  |  |  |  |  |  |  |  |  |  |  |  |  |  |  |  |  |  |  |  |  |  |  |  |  |  |  |  |  |  |  |  |  |  |  |  |  |  |  |  |  |  |  |  |  |  |  |  |  |  |  |  |  |  |  |  |  |  |  |  |  |  |  |  |  |  |  |  |  |  |  |  |  |  |  |  |  |  |  |  |  |  |  |  |  |  |  |  |  |  |  |  |  |  |  |  |  |  |  |  |  |  |  |  |  |  |  |  |  |  |  |  |  |  |  |  |  |  |  |  |  |  |  |  |  |  |  |  |  |  |  |  |  |  |  |  |  |  |  |  |  |  |  |  |  |  |  |  |  |  |  |  |  |  |  |  |  |  |  |  |  |  |  |  |  |  |  |  |  |  |  |  |  |  |  |  |  |  |  |  |  |  |  |  |  |  |  |  |  |  |  |  |  |  |  |  |  |  |  |  |  |  |  |  |  |  |  |  |  |  |  |  |  |  |  |  |  |  |  |  |  |  |  |  |  |  |  |  |  |  |  |  |  |  |  |  |  |  |  |  |  |  |  |  |  |  |  |  |  |  |  |  |  |  |  |  |  |  |  |  |  |  |  |  |  |  |  |  |  |  |  |  |  |  |  |  |  |  |  |  |  |  |  |  |  |  |  |  |  |  |  |  |  |  |  |  |  |  |  |  |  |  |  |  |  |  |  |  |  |  |  |  |  |  |  |  |  |  |  |  |  |  |  |  |  |  |  |  |  |  |  |  |  |  |  |  |  |  |  |  |  |  |  |  |  |  |  |  |  |  |  |  |  |  |  |  |  |  |  |  |  |  |  |  |  |  |  |  |  |  |  |  |  |  |  |  |  |  |  |  |  |  |  |  |  |  |  |  |  |  |  |  |  |  |  |  |  |  |  |  |  |  |  |  |  |  |  |  |  |  |  |  |  |  |  |  |  |  |  |  |  |  |  |  |  |  |  |  |  |  |  |  |  |  |  |  |  |  |  |  |  |  |  |  |  |  |  |  |  |  |  |  |  |  |  |  |  |  |  |  |  |  |  |  |  |  |  |  |  |  |  |  |  |  |  |  |  |  |  |  |  |  |  |  |  |  |  |  |  |  |  |  |  |  |  |  |  |  |  |  |  |  |  |  |  |  |  |  |  |  |  |  |  |  |  |  |  |  |  |  |  |  |  |  |  |  |  |  |  |  |  |  |  |  |  |  |  |  |  |  |  |  |  |  |  |  |  |  |  |  |  |  |  |  |  |  |  |  |  |  |  |  |  |  |  |  |  |  |  |  |  |  |  |  |  |  |  |  |  |  |  |  |  |  |  |  |  |  |  |  |  |  |  |  |  |  |  |  |  |  |  |  |  |  |  |  |  |  |  |  |  |  |  |  |  |  |  |  |  |  |  |  |  |  |  |  |  |  |  |  |  |  |  |  |  |  |  |  |  |  |  |  |  |  |  |  |  |  |  |  |  |  |  |  |  |  |  |  |  |  |  |  |  |  |  |  |  |  |  |  |  |  |  |  |  |  |  |  |  |  |  |  |  |  |  |  |  |  |  |  |  |  |  |  |  |  |  |  |  |  |  |  |  |  |  |  |  |  |  |  |  |  |  |  |  |  |  |  |  |  |  |  |  |  |  |  |  |  |  |  |  |  |  |  |  |  |  |  |  |  |  |  |  |  |  |  |  |  |  |  |  |  |  |  |  |  |  |  |  |  |  |  |  |  |  |  |  |  |  |  |  |  |  |  |  |  |  |  |  |  |  |  |  |  |  |  |  |  |  |  |  |  |  |  |  |  |  |  |  |  |  |  |  |  |  |  |  |  |  |  |  |  |  |  |  |  |  |  |  |  |  |  |  |  |  |  |  |  |  |  |  |  |  |  |  |  |  |  |  |  |  |  |  |  |  |  |  |  |  |  |  |  |  |  |  |  |  |  |  |  |  |  |  |  |  |  |  |  |  |  |  |  |  |  |  |  |  |  |  |  |  |  |  |  |  |  |  |  |  |  |  |  |  |  |  |  |  |  |  |  |  |  |  |  |  |  |  |  |  |  |  |  |  |  |  |  |  |  |  |  |  |  |  |  |  |  |  |  |  |  |  |  |  |  |  |  |  |  |  |  |  |  |  |  |  |  |  |  |  |  |  |  |  |  |  |  |  |  |  |  |  |  |  |  |  |  |  |  |  |  |  |  |  |  |  |  |  |  |  |  |  |  |  |  |  |  |  |  |  |  |  |  |  |  |  |  |  |  |  |  |  |  |  |  |  |  |  |  |  |  |  |  |  |  |  |  |  |  |  |  |  |  |  |  |  |  |  |  |  |  |  |  |  |  |  |  |  |  |  |  |  |  |  |  |  |  |  |  |  |  |  |  |  |  |  |  |  |  |  |  |  |  |  |  |  |  |  |  |  |  |  |  |  |  |  |  |  |  |  |  |  |  |  |  |  |  |  |  |  |  |  |  |  |  |  |  |  |  |  |  |  |  |  |  |  |  |  |  |  |  |  |  |  |  |  |  |  |  |  |  |  |  |  |  |  |  |  |  |  |  |  |  |  |  |  |  |  |  |  |  |  |  |  |  |  |  |  |  |  |  |  |  |  |  |  |  |  |  |  |  |  |  |  |  |  |  |  |  |  |  |  |  |  |  |  |  |  |  |  |  |  |  |  |  |  |  |  |  |  |  |  |  |  |  |  |  |  |  |  |  |  |  |  |  |  |  |  |  |  |  |  |  |  |  |  |  |  |  |  |  |  |  |  |  |  |  |  |  |  |  |  |  |  |  |  |  |  |  |  |  |  |  |  |  |  |  |  |  |  |  |  |  |  |  |  |  |  |  |  |  |  |  |  |  |  |  |  |  |  |  |  |  |  |  |  |  |  |  |  |  |  |  |  |  |  |  |  |  |  |  |  |  |  |  |  |  |  |  |  |  |  |  |  |  |  |  |  |  |  |  |  |  |  |  |  |  |  |  |  |  |  |  |  |  |  |  |  |  |  |  |  |  |  |  |  |  |  |  |  |  |  |  |  |  |  |  |  |  |  |  |  |  |  |  |  |  |  |  |  |  |  |  |  |  |  |  |  |  |  |  |  |  |  |  |  |  |  |  |  |  |  |  |  |  |  |  |  |  |  |  |  |  |  |  |  |  |  |  |  |  |  |  |  |  |  |  |  |  |  |  |  |  |  |  |  |  |  |  |  |  |  |  |  |  |  |  |  |  |  |  |  |  |  |  |  |  |  |  |  |  |  |  |  |  |  |  |  |  |  |  |  |  |  |  |  |  |  |  |  |  |  |  |  |  |  |  |  |  |  |  |  |  |  |  |  |  |  |  |  |  |  |  |  |  |  |  |  |  |  |  |  |  |  |  |  |  |  |  |  |  |  |  |  |  |  |  |  |  |  |  |  |  |  |  |  |  |  |  |  |  |  |  |  |  |  |  |  |  |  |  |  |  |  |  |  |  |  |  |  |  |  |  |  |  |  |  |  |  |  |  |  |  |  |  |  |  |  |  |  |  |  |  |  |  |  |  |  |  |  |  |  |  |  |  |  |  |  |  |  |  |  |  |  |  |  |  |  |  |  |  |  |  |  |  |  |  |  |  |  |  |  |  |  |  |  |  |  |  |  |  |  |  |  |  |  |  |  |  |  |  |  |  |  |  |  |  |  |  |  |  |  |  |  |  |  |  |  |  |  |  |  |  |  |  |  |  |  |  |  |  |  |  |  |  |  |  |  |  |  |  |  |  |  |  |  |  |  |  |  |  |  |  |  |  |  |  |  |  |  |  |  |  |  |  |  |  |  |  |  |  |  |  |  |  |  |  |  |  |  |  |  |  |  |  |  |  |  |  |  |  |  |  |  |  |  |  |  |  |  |  |  |  |  |  |  |  |  |  |  |  |  |  |  |  |  |  |  |  |  |  |  |  |  |  |  |  |  |  |  |  |  |  |  |  |  |  |  |  |  |  |  |  |  |  |  |  |  |  |  |  |  |  |  |  |  |  |  |  |  |  |  |  |  |  |  |  |  |  |  |  |  |  |  |  |  |  |  |  |  |  |  |  |  |  |  |  |  |  |  |  |  |  |  |  |  |  |  |  |  |  |  |  |  |  |  |  |  |  |  |  |  |  |  |  |  |  |  |  |  |  |  |  |  |  |  |  |  |  |  |  |  |  |  |  |  |  |  |  |  |  |  |  |  |  |  |  |  |  |  |  |  |  |  |  |  |  |  |  |  |  |  |  |  |  |  |  |  |  |  |  |  |  |  |  |  |  |  |  |  |  |  |  |  |  |  |  |  |  |  |  |  |  |  |  |  |  |  |  |  |  |  |  |  |  |  |  |  |  |  |  |  |  |  |  |  |  |  |  |  |  |  |  |  |  |  |  |  |  |  |  |  |  |  |  |  |  |  |  |  |  |  |  |  |  |  |  |  |  |  |  |  |  |  |  |  |  |  |  |  |  |  |  |  |  |  |  |  |  |  |  |  |  |  |  |  |  |  |  |  |  |  |  |  |  |  |  |  |  |  |  |  |  |  |  |  |  |  |  |  |  |  |  |  |  |  |  |  |  |  |  |  |  |  |  |  |  |  |  |  |  |  |  |  |  |  |  |  |  |  |  |  |  |  |  |  |  |  |  |  |  |  |  |  |  |  |  |  |  |  |  |  |  |  |  |  |  |  |  |  |  |  |  |  |  |  |  |  |  |  |  |  |  |  |  |  |  |  |  |  |  |  |  |  |  |  |  |  |  |  |  |  |  |  |  |  |  |  |  |  |  |  |  |  |  |  |  |  |  |  |  |  |  |  |  |  |  |  |  |  |  |  |  |  |  |  |  |  |  |  |  |  |  |  |  |  |  |  |  |  |  |  |  |  |  |  |  |  |  |  |  |  |  |  |  |  |  |  |  |  |  |  |  |  |  |  |  |  |  |  |  |  |  |  |  |  |  |  |  |  |  |  |  |  |  |  |  |  |  |  |  |  |  |  |  |  |  |  |  |  |  |  |  |  |  |  |  |  |  |  |  |  |  |  |  |  |  |  |  |  |  |  |  |  |  |  |  |  |  |  |  |  |  |  |  |  |  |  |  |  |  |  |  |  |  |  |  |  |  |  |  |  |  |  |  |  |  |  |  |  |  |  |  |  |  |  |  |  |  |  |  |  |  |  |  |  |  |  |  |  |  |  |  |  |  |  |  |  |  |  |  |  |  |  |  |  |  |  |  |  |  |  |  |  |  |  |  |  |  |  |  |  |  |  |  |  |  |  |  |  |  |  |  |  |  |  |  |  |  |  |  |  |  |  |  |  |  |  |  |  |  |  |  |  |  |  |  |  |  |  |  |  |  |  |  |  |  |  |  |  |  |  |  |  |  |  |  |  |  |  |  |  |  |  |  |  |  |  |  |  |  |  |  |  |  |  |  |  |  |  |  |  |  |  |  |  |  |  |  |  |  |  |  |  |  |  |  |  |  |  |  |  |  |  |  |  |  |  |  |  |  |  |  |  |  |  |  |  |  |  |  |  |  |  |  |  |  |  |  |  |  |  |  |  |  |  |  |  |  |  |  |  |  |  |  |  |  |  |  |  |  |  |  |  |  |  |  |  |  |  |  |  |  |  |  |  |  |  |  |  |  |  |  |  |  |  |  |  |  |  |  |  |  |  |  |  |  |  |  |  |  |  |  |  |  |  |  |  |  |  |  |  |  |  |  |  |  |  |  |  |  |  |  |  |  |  |  |  |  |  |  |  |  |  |  |  |  |  |  |  |  |  |  |  |  |  |  |  |  |  |  |  |  |  |  |  |  |  |  |  |  |  |  |  |  |  |  |  |  |  |  |  |  |  |  |  |  |  |  |  |  |  |  |  |  |  |  |  |  |  |  |  |  |  |  |  |  |  |  |  |  |  |  |  |  |  |  |  |  |  |  |  |  |  |  |  |  |  |  |  |  |  |  |  |  |  |  |  |  |  |  |  |  |  |  |  |  |  |  |  |  |  |  |  |  |  |  |  |  |  |  |  |  |  |  |  |  |  |  |  |  |  |  |  |  |  |  |  |  |  |  |  |  |  |  |  |  |  |  |  |  |  |  |  |  |  |  |  |  |  |  |  |  |  |  |  |  |  |  |  |  |  |  |  |  |  |  |  |  |  |  |  |  |  |  |  |  |  |  |  |  |  |  |  |  |  |  |  |  |  |  |  |  |  |  |  |  |  |  |  |  |  |  |  |  |  |  |  |  |  |  |  |  |  |  |  |  |  |  |  |  |  |  |  |  |  |  |  |  |  |  |  |  |  |  |  |  |  |  |  |  |  |  |  |  |  |  |  |  |  |  |  |  |  |  |  |  |  |  |  |  |  |  |  |  |  |  |  |  |  |  |  |  |  |  |  |  |  |  |  |  |  |  |  |  |  |  |  |  |  |  |  |  |  |  |  |  |  |  |  |  |  |  |  |  |  |  |  |  |  |  |  |  |  |  |  |  |  |  |  |  |  |  |  |  |  |  |  |  |  |  |  |  |  |  |  |  |  |  |  |  |  |  |  |  |  |  |  |  |  |  |  |  |  |  |  |  |  |  |  |  |  |  |  |  |  |  |  |  |  |  |  |  |  |  |  |  |  |  |  |  |  |  |  |  |  |  |  |  |  |  |  |  |  |  |  |  |  |  |  |  |  |  |  |  |  |  |  |  |  |  |  |  |  |  |  |  |  |  |  |  |  |  |  |  |  |  |  |  |  |  |  |  |  |  |  |  |  |  |  |  |  |  |  |  |  |  |  |  |  |  |  |  |  |  |  |  |  |  |  |  |  |  |  |  |  |  |  |  |  |  |  |  |  |  |  |  |  |  |  |  |  |  |  |  |  |  |  |  |  |  |  |  |  |  |  |  |  |  |  |  |  |  |  |  |  |  |  |  |  |  |  |  |  |  |  |  |  |  |  |  |  |  |  |  |  |  |  |  |  |  |  |  |  |  |  |  |  |  |  |  |  |  |  |  |  |  |  |  |  |  |  |  |  |  |  |  |  |  |  |  |  |  |  |  |  |  |  |  |  |  |  |  |  |  |  |  |  |  |  |  |  |  |  |  |  |  |  |  |  |  |  |  |  |  |  |  |  |  |  |  |  |  |  |  |  |  |  |  |  |  |  |  |  |  |  |  |  |  |  |  |  |  |  |  |  |  |  |  |  |  |  |  |  |  |  |  |  |  |  |  |  |  |  |  |  |  |  |  |  |  |  |  |  |  |  |  |  |  |  |  |  |  |  |  |  |  |  |  |  |  |  |  |  |  |  |  |  |  |  |  |  |  |  |  |  |  |  |  |  |  |  |  |  |  |  |  |  |  |  |  |  |  |  |  |  |  |  |  |  |  |  |  |  |  |  |  |  |  |  |  |  |  |  |  |  |  |  |  |  |  |  |  |  |  |  |  |  |  |  |  |  |  |  |  |  |  |  |  |  |  |  |  |  |  |  |  |  |  |  |  |  |  |  |  |  |  |  |  |  |  |  |  |  |  |  |  |  |  |  |  |  |  |  |  |  |  |  |  |  |  |  |  |  |  |  |  |  |  |  |  |  |  |  |  |  |  |  |  |  |  |  |  |  |  |  |  |  |  |  |  |  |  |  |  |  |  |  |  |  |  |  |  |  |  |  |  |  |  |  |  |  |  |  |  |  |  |  |  |  |  |  |  |  |  |  |  |  |  |  |  |  |  |  |  |  |  |  |  |  |  |  |  |  |  |  |  |  |  |  |  |  |  |  |  |  |  |  |  |  |  |  |  |  |  |  |  |  |  |  |  |  |  |  |  |  |  |  |  |  |  |  |  |  |  |  |  |  |  |  |  |  |  |  |  |  |  |  |  |  |  |  |  |  |  |  |  |  |  |  |  |  |  |  |  |  |  |  |  |  |  |  |  |  |  |  |  |  |  |  |  |  |  |  |  |  |  |  |  |  |  |  |  |  |  |  |  |  |  |  |  |  |  |  |  |  |  |  |  |  |  |  |  |  |  |  |  |  |  |  |  |  |  |  |  |  |  |  |  |  |  |  |  |  |  |  |  |  |  |  |  |  |  |  |  |  |  |  |  |  |  |  |  |  |  |  |  |  |  |  |  |  |  |  |  |  |  |  |  |  |  |  |  |  |  |  |  |  |  |  |  |  |  |  |  |  |  |  |  |  |  |  |  |  |  |  |  |  |  |  |  |  |  |  |  |  |  |  |  |  |  |  |  |  |  |  |  |  |  |  |  |  |  |  |  |  |  |  |  |  |  |  |  |  |  |  |  |  |  |  |  |  |  |  |  |  |  |  |  |  |  |  |  |  |  |  |  |  |  |  |  |  |  |  |  |  |  |  |  |  |  |  |  |  |  |  |  |  |  |  |  |  |  |  |  |  |  |  |  |  |  |  |  |  |  |  |  |  |  |  |  |  |  |  |  |  |  |  |  |  |  |  |  |  |  |  |  |  |  |  |  |  |  |  |  |  |  |  |  |  |  |  |  |  |  |  |  |  |  |  |  |  |  |  |  |  |  |  |  |  |  |  |  |  |  |  |  |  |  |  |  |  |  |  |  |  |  |  |  |  |  |  |  |  |  |  |  |  |  |  |  |  |  |  |  |  |  |  |  |  |  |  |  |  |  |  |  |  |  |  |  |  |  |  |  |  |  |  |  |  |  |  |  |  |  |  |  |  |  |  |  |  |  |  |  |  |  |  |  |  |  |  |  |  |  |  |  |  |  |  |  |  |  |  |  |  |  |  |  |  |  |  |  |  |  |  |  |  |  |  |  |  |  |  |  |  |  |  |  |  |  |  |  |  |  |  |  |  |  |  |  |  |  |  |  |  |  |  |  |  |  |  |  |  |  |  |  |  |  |  |  |  |  |  |  |  |  |  |  |  |  |  |  |  |  |  |  |  |  |  |  |  |  |  |  |  |  |  |  |  |  |  |  |  |  |  |  |  |  |  |  |  |  |  |  |  |  |  |  |  |  |  |  |  |  |  |  |  |  |  |  |  |  |  |  |  |  |  |  |  |  |  |  |  |  |  |  |  |  |  |  |  |  |  |  |  |  |  |  |  |  |  |  |  |  |  |  |  |  |  |  |  |  |  |  |  |  |  |  |  |  |  |  |  |  |  |  |  |  |  |  |  |  |  |  |  |  |  |  |  |  |  |  |  |  |  |  |  |  |  |  |  |  |  |  |  |  |  |  |  |  |  |  |  |  |  |  |  |  |  |  |  |  |  |  |  |  |  |  |  |  |  |  |  |  |  |  |  |  |  |  |  |  |  |  |  |  |  |  |  |  |  |  |  |  |  |  |  |  |  |  |  |  |  |  |  |  |  |  |  |  |  |  |  |  |  |  |  |  |  |  |  |  |  |  |  |  |  |  |  |  |  |  |  |  |  |  |  |  |  |  |  |  |  |  |  |  |  |  |  |  |  |  |  |  |  |  |  |  |  |  |  |  |  |  |  |  |  |  |  |  |  |  |  |  |  |  |  |  |  |  |  |  |  |  |  |  |  |  |  |  |  |  |  |  |  |  |  |  |  |  |  |  |  |  |  |  |  |  |  |  |  |  |  |  |  |  |  |  |  |  |  |  |  |  |  |  |  |  |  |  |  |  |  |  |  |  |  |  |  |  |  |  |  |  |  |  |  |  |  |  |  |  |  |  |  |  |  |  |  |  |  |  |  |  |  |  |  |  |  |  |  |  |  |  |  |  |  |  |  |  |  |  |  |  |  |  |  |  |  |  |  |  |  |  |  |  |  |  |  |  |  |  |  |  |  |  |  |  |  |  |  |  |  |  |  |  |  |  |  |  |  |  |  |  |  |  |  |  |  |  |  |  |  |  |  |  |  |  |  |  |  |  |  |  |  |  |  |  |  |  |  |  |  |  |  |  |  |  |  |  |  |  |  |  |  |  |  |  |  |  |  |  |  |  |  |  |  |  |  |  |  |  |  |  |  |  |  |  |  |  |  |  |  |  |  |  |  |  |  |  |  |  |  |  |  |  |  |  |  |  |  |  |  |  |  |  |  |  |  |  |  |  |  |  |  |  |  |  |  |  |  |  |  |  |  |  |  |  |  |  |  |  |  |  |  |  |  |  |  |  |  |  |  |  |  |  |  |  |  |  |  |  |  |  |  |  |  |  |  |  |  |  |  |  |  |  |  |  |  |  |  |  |  |  |  |  |  |  |  |  |  |  |  |  |  |  |  |  |  |  |  |  |  |  |  |  |  |  |  |  |  |  |  |  |  |  |  |  |  |  |  |  |  |  |  |  |  |  |  |  |  |  |  |  |  |  |  |  |  |  |  |  |  |  |  |  |  |  |  |  |  |  |  |  |  |  |  |  |  |  |  |  |  |  |  |  |  |  |  |  |  |  |  |  |  |  |  |  |  |  |  |  |  |  |  |  |  |  |  |  |  |  |  |  |  |  |  |  |  |  |  |  |  |  |  |  |  |  |  |  |  |  |  |  |  |  |  |  |  |  |  |  |  |  |  |  |  |  |  |  |  |  |  |  |  |  |  |  |  |  |  |  |  |  |  |  |  |  |  |  |  |  |  |  |  |  |  |  |  |  |  |  |  |  |  |  |  |  |  |  |  |  |  |  |  |  |  |  |  |  |  |  |  |  |  |  |  |  |  |  |  |  |  |  |  |  |  |  |  |  |  |  |  |  |  |  |  |  |  |  |  |  |  |  |  |  |  |  |  |  |  |  |  |  |  |  |  |  |  |  |  |  |  |  |  |  |  |  |  |  |  |  |  |  |  |  |  |  |  |  |  |  |  |  |  |  |  |  |  |  |  |  |  |  |  |  |  |  |  |  |  |  |  |  |  |  |  |  |  |  |  |  |  |  |  |  |  |  |  |  |  |  |  |  |  |  |  |  |  |  |  |  |  |  |  |  |  |  |  |  |  |  |  |  |  |  |  |  |  |  |  |  |  |  |  |  |  |  |  |  |  |  |  |  |  |  |  |  |  |  |  |  |  |  |  |  |  |  |  |  |  |  |  |  |  |  |  |  |  |  |  |  |  |  |  |  |  |  |  |  |  |  |  |  |  |  |  |  |  |  |  |  |  |  |  |  |  |  |  |  |  |  |  |  |  |  |  |  |  |  |  |  |  |  |  |  |  |  |  |  |  |  |  |  |  |  |  |  |  |  |  |  |  |  |  |  |  |  |  |  |  |  |  |  |  |  |  |  |  |  |  |  |  |  |  |  |  |  |  |  |  |  |  |  |  |  |  |  |  |  |  |  |  |  |  |  |  |  |  |  |  |  |  |  |  |  |  |  |  |  |  |  |  |  |  |  |  |  |  |  |  |  |  |  |  |  |  |  |  |  |  |  |  |  |  |  |  |  |  |  |  |  |  |  |  |  |  |  |  |  |  |  |  |  |  |  |  |  |  |  |  |  |  |  |  |  |  |  |  |  |  |  |  |  |  |  |  |  |  |  |  |  |  |  |  |  |  |  |  |  |  |  |  |  |  |  |  |  |  |  |  |  |  |  |  |  |  |  |  |  |  |  |  |  |  |  |  |  |  |  |  |  |  |  |  |  |  |  |  |  |  |  |  |  |  |  |  |  |  |  |  |  |  |  |  |  |  |  |  |  |  |  |  |  |  |  |  |  |  |  |  |  |  |  |  |  |  |  |  |  |  |  |  |  |  |  |  |  |  |  |  |  |  |  |  |  |  |  |  |  |  |  |  |  |  |  |  |  |  |  |  |  |  |  |  |  |  |  |  |  |  |  |  |  |  |  |  |  |  |  |  |  |  |  |  |  |  |  |  |  |  |  |  |  |  |  |  |  |  |  |  |  |  |  |  |  |  |  |  |  |  |  |  |  |  |  |  |  |  |  |  |  |  |  |  |  |  |  |  |  |  |  |  |  |  |  |  |  |  |  |  |  |  |  |  |  |  |  |  |  |  |  |  |  |  |  |  |  |  |  |  |  |  |  |  |  |  |  |  |  |  |  |  |  |  |  |  |  |  |  |  |  |  |  |  |  |  |  |  |  |  |  |  |  |  |  |  |  |  |  |  |  |  |  |  |  |  |  |  |  |  |  |  |  |  |  |  |  |  |  |  |  |  |  |  |  |  |  |  |  |  |  |  |  |  |  |  |  |  |  |  |  |  |  |  |  |  |  |  |  |  |  |  |  |  |  |  |  |  |  |  |  |  |  |  |  |  |  |  |  |  |  |  |  |  |  |  |  |  |  |  |  |  |  |  |  |  |  |  |  |  |  |  |  |  |  |  |  |  |  |  |  |  |  |  |  |  |  |  |  |  |  |  |  |  |  |  |  |  |  |  |  |  |  |  |  |  |  |  |  |  |  |  |  |  |  |  |  |  |  |  |  |  |  |  |  |  |  |  |  |  |  |  |  |  |  |  |  |  |  |  |  |  |  |  |  |  |  |  |  |  |  |  |  |  |  |  |  |  |  |  |  |  |  |  |  |  |  |  |  |  |  |  |  |  |  |  |  |  |  |  |  |  |  |  |  |  |  |  |  |  |  |  |  |  |  |  |  |  |  |  |  |  |  |  |  |  |  |  |  |  |  |  |  |  |  |  |  |  |  |  |  |  |  |  |  |  |  |  |  |  |  |  |  |  |  |  |  |  |  |  |  |  |  |  |  |  |  |  |  |  |  |  |  |  |  |  |  |  |  |  |  |  |  |  |  |  |  |  |  |  |  |  |  |  |  |  |  |  |  |  |  |  |  |  |  |  |  |  |  |  |  |  |  |  |  |  |  |  |  |  |  |  |  |  |  |  |  |  |  |  |  |  |  |  |  |  |  |  |  |  |  |  |  |  |  |  |  |  |  |  |  |  |  |  |  |  |  |  |  |  |  |  |  |  |  |  |  |  |  |  |  |  |  |  |  |  |  |  |  |  |  |  |  |  |  |  |  |  |  |  |  |  |  |  |  |  |  |  |  |  |  |  |  |  |  |  |  |  |  |  |  |  |  |  |  |  |  |  |  |  |  |  |  |  |  |  |  |  |  |  |  |  |  |  |  |  |  |  |  |  |  |  |  |  |  |  |  |  |  |  |  |  |  |  |  |  |  |  |  |  |  |  |  |  |  |  |  |  |  |  |  |  |  |  |  |  |  |  |  |  |  |  |  |  |  |  |  |  |  |  |  |  |  |  |  |  |  |  |  |  |  |  |  |  |  |  |  |  |  |  |  |  |  |  |  |  |  |  |  |  |  |  |  |  |  |  |  |  |  |  |  |  |  |  |  |  |  |  |  |  |  |  |  |  |  |  |  |  |  |  |  |  |  |  |  |  |  |  |  |  |  |  |  |  |  |  |  |  |  |  |  |  |  |  |  |  |  |  |  |  |  |  |  |  |  |  |  |  |  |  |  |  |  |  |  |  |  |  |  |  |  |  |  |  |  |  |  |  |  |  |  |  |  |  |  |  |  |  |  |  |  |  |  |  |  |  |  |  |  |  |  |  |  |  |  |  |  |  |  |  |  |  |  |  |  |  |  |  |  |  |  |  |  |  |  |  |  |  |  |  |  |  |  |  |  |  |  |  |  |  |  |  |  |  |  |  |  |  |  |  |  |  |  |  |  |  |  |  |  |  |  |  |  |  |  |  |  |  |  |  |  |  |  |  |  |  |  |  |  |  |  |  |  |  |  |  |  |  |  |  |  |  |  |  |  |  |  |  |  |  |  |  |  |  |  |  |  |  |  |  |  |  |  |  |  |  |  |  |  |  |  |  |  |  |  |  |  |  |  |  |  |  |  |  |  |  |  |  |  |  |  |  |  |  |  |  |  |  |  |  |  |  |  |  |  |  |  |  |  |  |  |  |  |  |  |  |  |  |  |  |  |  |  |  |  |  |  |  |  |  |  |  |  |  |  |  |  |  |  |  |  |  |  |  |  |  |  |  |  |  |  |  |  |  |  |  |  |  |  |  |  |  |  |  |  |  |  |  |  |  |  |  |  |  |  |  |  |  |  |  |  |  |  |  |  |  |  |  |  |  |  |  |  |  |  |  |  |  |  |  |  |  |  |  |  |  |  |  |  |  |  |  |  |  |  |  |  |  |  |  |  |  |  |  |  |  |  |  |  |  |  |  |  |  |  |  |  |  |  |  |  |  |  |  |  |  |  |  |  |  |  |  |  |  |  |  |  |  |  |  |  |  |  |  |  |  |  |  |  |  |  |  |  |  |  |  |  |  |  |  |  |  |  |  |  |  |  |  |  |  |  |  |  |  |  |  |  |  |  |  |  |  |  |  |  |  |  |  |  |  |  |  |  |  |  |  |  |  |  |  |  |  |  |  |  |  |  |  |  |  |  |  |  |  |  |  |  |  |  |  |  |  |  |  |  |  |  |  |  |  |  |  |  |  |  |  |  |  |  |  |  |  |  |  |  |  |  |  |  |  |  |  |  |  |  |  |  |  |  |  |  |  |  |  |  |  |  |  |  |  |  |  |  |  |  |  |  |  |  |  |  |  |  |  |  |  |  |  |  |  |  |  |  |  |  |  |  |  |  |  |  |  |  |  |  |  |  |  |  |  |  |  |  |  |  |  |  |  |  |  |  |  |  |  |  |  |  |  |  |  |  |  |  |  |  |  |  |  |  |  |  |  |  |  |  |  |  |  |  |  |  |  |  |  |  |  |  |  |  |  |  |  |  |  |  |  |  |  |  |  |  |  |  |  |  |  |  |  |  |  |  |  |  |  |  |  |  |  |  |  |  |  |  |  |  |  |  |  |  |  |  |  |  |  |  |  |  |  |  |  |  |  |  |  |  |  |  |  |  |  |  |  |  |  |  |  |  |  |  |  |  |  |  |  |  |  |  |  |  |  |  |  |  |  |  |  |  |  |  |  |  |  |  |  |  |  |  |  |  |  |  |  |  |  |  |  |  |  |  |  |  |  |  |  |  |  |  |  |  |  |  |  |  |  |  |  |  |  |  |  |  |  |  |  |  |  |  |  |  |  |  |  |  |  |  |  |  |  |  |  |  |  |  |  |  |  |  |  |  |  |  |  |  |  |  |  |  |  |  |  |  |  |  |  |  |  |  |  |  |  |  |  |  |  |  |  |  |  |  |  |  |  |  |  |  |  |  |  |  |  |  |  |  |  |  |  |  |  |  |  |  |  |  |  |  |  |  |  |  |  |  |  |  |  |  |  |  |  |  |  |  |  |  |  |  |  |  |  |  |  |  |  |  |  |  |  |  |  |  |  |  |  |  |  |  |  |  |  |  |  |  |  |  |  |  |  |  |  |  |  |  |  |  |  |  |  |  |  |  |  |  |  |  |  |  |  |  |  |  |  |  |  |  |  |  |  |  |  |  |  |  |  |  |  |  |  |  |  |  |  |  |  |  |  |  |  |  |  |  |  |  |  |  |  |  |  |  |  |  |  |  |  |  |  |  |  |  |  |  |  |  |  |  |  |  |  |  |  |  |  |  |  |  |  |  |  |  |  |  |  |  |  |  |  |  |  |  |  |  |  |  |  |  |  |  |  |  |  |  |  |  |  |  |  |  |  |  |  |  |  |  |  |  |  |  |  |  |  |  |  |  |  |  |  |  |  |  |  |  |  |  |  |  |  |  |  |  |  |  |  |  |  |  |  |  |  |  |  |  |  |  |  |  |  |  |  |  |  |  |  |  |  |  |  |  |  |  |  |  |  |  |  |  |  |  |  |  |  |  |  |  |  |  |  |  |  |  |  |  |  |  |  |  |  |  |  |  |  |  |  |  |  |  |  |  |  |  |  |  |  |  |  |  |  |  |  |  |  |  |  |  |  |  |  |  |  |  |  |  |  |  |  |  |  |  |  |  |  |  |  |  |  |  |  |  |  |  |  |  |  |  |  |  |  |  |  |  |  |  |  |  |  |  |  |  |  |  |  |  |  |  |  |  |  |  |  |  |  |  |  |  |  |  |  |  |  |  |  |  |  |  |  |  |  |  |  |  |  |  |  |  |  |  |  |  |  |  |  |  |  |  |  |  |  |  |  |  |  |  |  |  |  |  |  |  |  |  |  |  |  |  |  |  |  |  |  |  |  |  |  |  |  |  |  |  |  |  |  |  |  |  |  |  |  |  |  |  |  |  |  |  |  |  |  |  |  |  |  |  |  |  |  |  |  |  |  |  |  |  |  |  |  |  |  |  |  |  |  |  |  |  |  |  |  |  |  |  |  |  |  |  |  |  |  |  |  |  |  |  |  |  |  |  |  |  |  |  |  |  |  |  |  |  |  |  |  |  |  |  |  |  |  |  |  |  |  |  |  |  |  |  |  |  |  |  |  |  |  |  |  |  |  |  |  |  |  |  |  |  |  |  |  |  |  |  |  |  |  |  |  |  |  |  |  |  |  |  |  |  |  |  |  |  |  |  |  |  |  |  |  |  |  |  |  |  |  |  |  |  |  |  |  |  |  |  |  |  |  |  |  |  |  |  |  |  |  |  |  |  |  |  |  |  |  |  |  |  |  |  |  |  |  |  |  |  |  |  |  |  |  |  |  |  |  |  |  |  |  |  |  |  |  |  |  |  |  |  |  |  |  |  |  |  |  |  |  |  |  |  |  |  |  |  |  |  |  |  |  |  |  |  |  |  |  |  |  |  |  |  |  |  |  |  |  |  |  |  |  |  |  |  |  |  |  |  |  |  |  |  |  |  |  |  |  |  |  |  |  |  |  |  |  |  |  |  |  |  |  |  |  |  |  |  |  |  |  |  |  |  |  |  |  |  |  |  |  |  |  |  |  |  |  |  |  |  |  |  |  |  |  |  |  |  |  |  |  |  |  |  |  |  |  |  |  |  |  |  |  |  |  |  |  |  |  |  |  |  |  |  |  |  |  |  |  |  |  |  |  |  |  |  |  |  |  |  |  |  |  |  |  |  |  |  |  |  |  |  |  |  |  |  |  |  |  |  |  |  |  |  |  |  |  |  |  |  |  |  |  |  |  |  |  |  |  |  |  |  |  |  |  |  |  |  |  |  |  |  |  |  |  |  |  |  |  |  |  |  |  |  |  |  |  |  |  |  |  |  |  |  |  |  |  |  |  |  |  |  |  |  |  |  |  |  |  |  |  |  |  |  |  |  |  |  |  |  |  |  |  |  |  |  |  |  |  |  |  |  |  |  |  |  |  |  |  |  |  |  |  |  |  |  |  |  |  |  |  |  |  |  |  |  |  |  |  |  |  |  |  |  |  |  |  |  |  |  |  |  |  |  |  |  |  |  |  |  |  |  |  |  |  |  |  |  |  |  |  |  |  |  |  |  |  |  |  |  |  |  |  |  |  |  |  |  |  |  |  |  |  |  |  |  |  |  |  |  |  |  |  |  |  |  |  |  |  |  |  |  |  |  |  |  |  |  |  |  |  |  |  |  |  |  |  |  |  |  |  |  |  |  |  |  |  |  |  |  |  |  |  |  |  |  |  |  |  |  |  |  |  |  |  |  |  |  |  |  |  |  |  |  |  |  |  |  |  |  |  |  |  |  |  |  |  |  |  |  |  |  |  |  |  |  |  |  |  |  |  |  |  |  |  |  |  |  |  |  |  |  |  |  |  |  |  |  |  |  |  |  |  |  |  |  |  |  |  |  |  |  |  |  |  |  |  |  |  |  |  |  |  |  |  |  |  |  |  |  |  |  |  |  |  |  |  |  |  |  |  |  |  |  |  |  |  |  |  |  |  |  |  |  |  |  |  |  |  |  |  |  |  |  |  |  |  |  |  |  |  |  |  |  |  |  |  |  |  |  |  |  |  |  |  |  |  |  |  |  |  |  |  |  |  |  |  |  |  |  |  |  |  |  |  |  |  |  |  |  |  |  |  |  |  |  |  |  |  |  |  |  |  |  |  |  |  |  |  |  |  |  |  |  |  |  |  |  |  |  |  |  |  |  |  |  |  |  |  |  |  |  |  |  |  |  |  |  |  |  |  |  |  |  |  |  |  |  |  |  |  |  |  |  |  |  |  |  |  |  |  |  |  |  |  |  |  |  |  |  |  |  |  |  |  |  |  |  |  |  |  |  |  |  |  |  |  |  |  |  |  |  |  |  |  |  |  |  |  |  |  |  |  |  |  |  |  |  |  |  |  |  |  |  |  |  |  |  |  |  |  |  |  |  |  |  |  |  |  |  |  |  |  |  |  |  |  |  |  |  |  |  |  |  |  |  |  |  |  |  |  |  |  |  |  |  |  |  |  |  |  |  |  |  |  |  |  |  |  |  |  |  |  |  |  |  |  |  |  |  |  |  |  |  |  |  |  |  |  |  |  |  |  |  |  |  |  |  |  |  |  |  |  |  |  |  |  |  |  |  |  |  |  |  |  |  |  |  |  |  |  |  |  |  |  |  |  |  |  |  |  |  |  |  |  |  |  |  |  |  |  |  |  |  |  |  |  |  |  |  |  |  |  |  |  |  |  |  |  |  |  |  |  |  |  |  |  |  |  |  |  |  |  |  |  |  |  |  |  |  |  |  |  |  |  |  |  |  |  |  |  |  |  |  |  |  |  |  |  |  |  |  |  |  |  |  |  |  |  |  |  |  |  |  |  |  |  |  |  |  |  |  |  |  |  |  |  |  |  |  |  |  |  |  |  |  |  |  |  |  |  |  |  |  |  |  |  |  |  |  |  |  |  |  |  |  |  |  |  |  |  |  |  |  |  |  |  |  |  |  |  |  |  |  |  |  |  |  |  |  |  |  |  |  |  |  |  |  |  |  |  |  |  |  |  |  |  |  |  |  |  |  |  |  |  |  |  |  |  |  |  |  |  |  |  |  |  |  |  |  |  |  |  |  |  |  |  |  |  |  |  |  |  |  |  |  |  |  |  |  |  |  |  |  |  |  |  |  |  |  |  |  |  |  |  |  |  |  |  |  |  |  |  |  |  |  |  |  |  |  |  |  |  |  |  |  |  |  |  |  |  |  |  |  |  |  |  |  |  |  |  |  |  |  |  |  |  |  |  |  |  |  |  |  |  |  |  |  |  |  |  |  |  |  |  |  |  |  |  |  |  |  |  |  |  |  |  |  |  |  |  |  |  |  |  |  |  |  |  |  |  |  |  |  |  |  |  |  |  |  |  |  |  |  |  |  |  |  |  |  |  |  |  |  |  |  |  |  |  |  |  |  |  |  |  |  |  |  |  |  |  |  |  |  |  |  |  |  |  |  |  |  |  |  |  |  |  |  |  |  |  |  |  |  |  |  |  |  |  |  |  |  |  |  |  |  |  |  |  |  |  |  |  |  |  |  |  |  |  |  |  |  |  |  |  |  |  |  |  |  |  |  |  |  |  |  |  |  |  |  |  |  |  |  |  |  |  |  |  |  |  |  |  |  |  |  |  |  |  |  |  |  |  |  |  |  |  |  |  |  |  |  |  |  |  |  |  |  |  |  |  |  |  |  |  |  |  |  |  |  |  |  |  |  |  |  |  |  |  |  |  |  |  |  |  |  |  |  |  |  |  |  |  |  |  |  |  |  |  |  |  |  |  |  |  |  |  |  |  |  |  |  |  |  |  |  |  |  |  |  |  |  |  |  |  |  |  |  |  |  |  |  |  |  |  |  |  |  |  |  |  |  |  |  |  |  |  |  |  |  |  |  |  |  |  |  |  |  |  |  |  |  |  |  |  |  |  |  |  |  |  |  |  |  |  |  |  |  |  |  |  |  |  |  |  |  |  |  |  |  |  |  |  |  |  |  |  |  |  |  |  |  |  |  |  |  |  |  |  |  |  |  |  |  |  |  |  |  |  |  |  |  |  |  |  |  |  |  |  |  |  |  |  |  |  |  |  |  |  |  |  |  |  |  |  |  |  |  |  |  |  |  |  |  |  |  |  |  |  |  |  |  |  |  |  |  |  |  |  |  |  |  |  |  |  |  |  |  |  |  |  |  |  |  |  |  |  |  |  |  |  |  |  |  |  |  |  |  |  |  |  |  |  |  |  |  |  |  |  |  |  |  |  |  |  |  |  |  |  |  |  |  |  |  |  |  |  |  |  |  |  |  |  |  |  |  |  |  |  |  |  |  |  |  |  |  |  |  |  |  |  |  |  |  |  |  |  |  |  |  |  |  |  |  |  |  |  |  |  |  |  |  |  |  |  |  |  |  |  |  |  |  |  |  |  |  |  |  |  |  |  |  |  |  |  |  |  |  |  |  |  |  |  |  |  |  |  |  |  |  |  |  |  |  |  |  |  |  |  |  |  |  |  |  |  |  |  |  |  |  |  |  |  |  |  |  |  |  |  |  |  |  |  |  |  |  |  |  |  |  |  |  |  |  |  |  |  |  |  |  |  |  |  |  |  |  |  |  |  |  |  |  |  |  |  |  |  |  |  |  |  |  |  |  |  |  |  |  |  |  |  |  |  |  |  |  |  |  |  |  |  |  |  |  |  |  |  |  |  |  |  |  |  |  |  |  |  |  |  |  |  |  |  |  |  |  |  |  |  |  |  |  |  |  |  |  |  |  |  |  |  |  |  |  |  |  |  |  |  |  |  |  |  |  |  |  |  |  |  |  |  |  |  |  |  |  |  |  |  |  |  |  |  |  |  |  |  |  |  |  |  |  |  |  |  |  |  |  |  |  |  |  |  |  |  |  |  |  |  |  |  |  |  |  |  |  |  |  |  |  |  |  |  |  |  |  |  |  |  |  |  |  |  |  |  |  |  |  |  |  |  |  |  |  |  |  |  |  |  |  |  |  |  |  |  |  |  |  |  |  |  |  |  |  |  |  |  |  |  |  |  |  |  |  |  |  |  |  |  |  |  |  |  |  |  |  |  |  |  |  |  |  |  |  |  |  |  |  |  |  |  |  |  |  |  |  |  |  |  |  |  |  |  |  |  |  |  |  |  |  |  |  |  |  |  |  |  |  |  |  |  |  |  |  |  |  |  |  |  |  |  |  |  |  |  |  |  |  |  |  |  |  |  |  |  |  |  |  |  |  |  |  |  |  |  |  |  |  |  |  |  |  |  |  |  |  |  |  |  |  |  |  |  |  |  |  |  |  |  |  |  |  |  |  |  |  |  |  |  |  |  |  |  |  |  |  |  |  |  |  |  |  |  |  |  |  |  |  |  |  |  |  |  |  |  |  |  |  |  |  |  |  |  |  |  |  |  |  |  |  |  |  |  |  |  |  |  |  |  |  |  |  |  |  |  |  |  |  |  |  |  |  |  |  |  |  |  |  |  |  |  |  |  |  |  |  |  |  |  |  |  |  |  |  |  |  |  |  |  |  |  |  |  |  |  |  |  |  |  |  |  |  |  |  |  |  |  |  |  |  |  |  |  |  |  |  |  |  |  |  |  |  |  |  |  |  |  |  |  |  |  |  |  |  |  |  |  |  |  |  |  |  |  |  |  |  |  |  |  |  |  |  |  |  |  |  |  |  |  |  |  |  |  |  |  |  |  |  |  |  |  |  |  |  |  |  |  |  |  |  |  |  |  |  |  |  |  |  |  |  |  |  |  |  |  |  |  |  |  |  |  |  |  |  |  |  |  |  |  |  |  |  |  |  |  |  |  |  |  |  |  |  |  |  |  |  |  |  |  |  |  |  |  |  |  |  |  |  |  |  |  |  |  |  |  |  |  |  |  |  |  |  |  |  |  |  |  |  |  |  |  |  |  |  |  |  |  |  |  |  |  |  |  |  |  |  |  |  |  |  |  |  |  |  |  |  |  |  |  |  |  |  |  |  |  |  |  |  |  |  |  |  |  |  |  |  |  |  |  |  |  |  |  |  |  |  |  |  |  |  |  |  |  |  |  |  |  |  |  |  |  |  |  |  |  |  |  |  |  |  |  |  |  |  |  |  |  |  |  |  |  |  |  |  |  |  |  |  |  |  |  |  |  |  |  |  |  |  |  |  |  |  |  |  |  |  |  |  |  |  |  |  |  |  |  |  |  |  |  |  |  |  |  |  |  |  |  |  |  |  |  |  |  |  |  |  |  |  |  |  |  |  |  |  |  |  |  |  |  |  |  |  |  |  |  |  |  |  |  |  |  |  |  |  |  |  |  |  |  |  |  |  |  |  |  |  |  |  |  |  |  |  |  |  |  |  |  |  |  |  |  |  |  |  |  |  |  |  |  |  |  |  |  |  |  |  |  |  |  |  |  |  |  |  |  |  |  |  |  |  |  |  |  |  |  |  |  |  |  |  |  |  |  |  |  |  |  |  |  |  |  |  |  |  |  |  |  |  |  |  |  |  |  |  |  |  |  |  |  |  |  |  |  |  |  |  |  |  |  |  |  |  |  |  |  |  |  |  |  |  |  |  |  |  |  |  |  |  |  |  |  |  |  |  |  |  |  |  |  |  |  |  |  |  |  |  |  |  |  |  |  |  |  |  |  |  |  |  |  |  |  |  |  |  |  |  |  |  |  |  |  |  |  |  |  |  |  |  |  |  |  |  |  |  |  |  |  |  |  |  |  |  |  |  |  |  |  |  |  |  |  |  |  |  |  |  |  |  |  |  |  |  |  |  |  |  |  |  |  |  |  |  |  |  |  |  |  |  |  |  |  |  |  |  |  |  |  |  |  |  |  |  |  |  |  |  |  |  |  |  |  |  |  |  |  |  |  |  |  |  |  |  |  |  |  |  |  |  |  |  |  |  |  |  |  |  |  |  |  |  |  |  |  |  |  |  |  |  |  |  |  |  |  |  |  |  |  |  |  |  |  |  |  |  |  |  |  |  |  |  |  |  |  |  |  |  |  |  |  |  |  |  |  |  |  |  |  |  |  |  |  |  |  |  |  |  |  |  |  |  |  |  |  |  |  |
| --- | --- | --- | --- | --- | --- | --- | --- | --- | --- | --- | --- | --- | --- | --- | --- | --- | --- | --- | --- | --- | --- | --- | --- | --- | --- | --- | --- | --- | --- | --- | --- | --- | --- | --- | --- | --- | --- | --- | --- | --- | --- | --- | --- | --- | --- | --- | --- | --- | --- | --- | --- | --- | --- | --- | --- | --- | --- | --- | --- | --- | --- | --- | --- | --- | --- | --- | --- | --- | --- | --- | --- | --- | --- | --- | --- | --- | --- | --- | --- | --- | --- | --- | --- | --- | --- | --- | --- | --- | --- | --- | --- | --- | --- | --- | --- | --- | --- | --- | --- | --- | --- | --- | --- | --- | --- | --- | --- | --- | --- | --- | --- | --- | --- | --- | --- | --- | --- | --- | --- | --- | --- | --- | --- | --- | --- | --- | --- | --- | --- | --- | --- | --- | --- | --- | --- | --- | --- | --- | --- | --- | --- | --- | --- | --- | --- | --- | --- | --- | --- | --- | --- | --- | --- | --- | --- | --- | --- | --- | --- | --- | --- | --- | --- | --- | --- | --- | --- | --- | --- | --- | --- | --- | --- | --- | --- | --- | --- | --- | --- | --- | --- | --- | --- | --- | --- | --- | --- | --- | --- | --- | --- | --- | --- | --- | --- | --- | --- | --- | --- | --- | --- | --- | --- | --- | --- | --- | --- | --- | --- | --- | --- | --- | --- | --- | --- | --- | --- | --- | --- | --- | --- | --- | --- | --- | --- | --- | --- | --- | --- | --- | --- | --- | --- | --- | --- | --- | --- | --- | --- | --- | --- | --- | --- | --- | --- | --- | --- | --- | --- | --- | --- | --- | --- | --- | --- | --- | --- | --- | --- | --- | --- | --- | --- | --- | --- | --- | --- | --- | --- | --- | --- | --- | --- | --- | --- | --- | --- | --- | --- | --- | --- | --- | --- | --- | --- | --- | --- | --- | --- | --- | --- | --- | --- | --- | --- | --- | --- | --- | --- | --- | --- | --- | --- | --- | --- | --- | --- | --- | --- | --- | --- | --- | --- | --- | --- | --- | --- | --- | --- | --- | --- | --- | --- | --- | --- | --- | --- | --- | --- | --- | --- | --- | --- | --- | --- | --- | --- | --- | --- | --- | --- | --- | --- | --- | --- | --- | --- | --- | --- | --- | --- | --- | --- | --- | --- | --- | --- | --- | --- | --- | --- | --- | --- | --- | --- | --- | --- | --- | --- | --- | --- | --- | --- | --- | --- | --- | --- | --- | --- | --- | --- | --- | --- | --- | --- | --- | --- | --- | --- | --- | --- | --- | --- | --- | --- | --- | --- | --- | --- | --- | --- | --- | --- | --- | --- | --- | --- | --- | --- | --- | --- | --- | --- | --- | --- | --- | --- | --- | --- | --- | --- | --- | --- | --- | --- | --- | --- | --- | --- | --- | --- | --- | --- | --- | --- | --- | --- | --- | --- | --- | --- | --- | --- | --- | --- | --- | --- | --- | --- | --- | --- | --- | --- | --- | --- | --- | --- | --- | --- | --- | --- | --- | --- | --- | --- | --- | --- | --- | --- | --- | --- | --- | --- | --- | --- | --- | --- | --- | --- | --- | --- | --- | --- | --- | --- | --- | --- | --- | --- | --- | --- | --- | --- | --- | --- | --- | --- | --- | --- | --- | --- | --- | --- | --- | --- | --- | --- | --- | --- | --- | --- | --- | --- | --- | --- | --- | --- | --- | --- | --- | --- | --- | --- | --- | --- | --- | --- | --- | --- | --- | --- | --- | --- | --- | --- | --- | --- | --- | --- | --- | --- | --- | --- | --- | --- | --- | --- | --- | --- | --- | --- | --- | --- | --- | --- | --- | --- | --- | --- | --- | --- | --- | --- | --- | --- | --- | --- | --- | --- | --- | --- | --- | --- | --- | --- | --- | --- | --- | --- | --- | --- | --- | --- | --- | --- | --- | --- | --- | --- | --- | --- | --- | --- | --- | --- | --- | --- | --- | --- | --- | --- | --- | --- | --- | --- | --- | --- | --- | --- | --- | --- | --- | --- | --- | --- | --- | --- | --- | --- | --- | --- | --- | --- | --- | --- | --- | --- | --- | --- | --- | --- | --- | --- | --- | --- | --- | --- | --- | --- | --- | --- | --- | --- | --- | --- | --- | --- | --- | --- | --- | --- | --- | --- | --- | --- | --- | --- | --- | --- | --- | --- | --- | --- | --- | --- | --- | --- | --- | --- | --- | --- | --- | --- | --- | --- | --- | --- | --- | --- | --- | --- | --- | --- | --- | --- | --- | --- | --- | --- | --- | --- | --- | --- | --- | --- | --- | --- | --- | --- | --- | --- | --- | --- | --- | --- | --- | --- | --- | --- | --- | --- | --- | --- | --- | --- | --- | --- | --- | --- | --- | --- | --- | --- | --- | --- | --- | --- | --- | --- | --- | --- | --- | --- | --- | --- | --- | --- | --- | --- | --- | --- | --- | --- | --- | --- | --- | --- | --- | --- | --- | --- | --- | --- | --- | --- | --- | --- | --- | --- | --- | --- | --- | --- | --- | --- | --- | --- | --- | --- | --- | --- | --- | --- | --- | --- | --- | --- | --- | --- | --- | --- | --- | --- | --- | --- | --- | --- | --- | --- | --- | --- | --- | --- | --- | --- | --- | --- | --- | --- | --- | --- | --- | --- | --- | --- | --- | --- | --- | --- | --- | --- | --- | --- | --- | --- | --- | --- | --- | --- | --- | --- | --- | --- | --- | --- | --- | --- | --- | --- | --- | --- | --- | --- | --- | --- | --- | --- | --- | --- | --- | --- | --- | --- | --- | --- | --- | --- | --- | --- | --- | --- | --- | --- | --- | --- | --- | --- | --- | --- | --- | --- | --- | --- | --- | --- | --- | --- | --- | --- | --- | --- | --- | --- | --- | --- | --- | --- | --- | --- | --- | --- | --- | --- | --- | --- | --- | --- | --- | --- | --- | --- | --- | --- | --- | --- | --- | --- | --- | --- | --- | --- | --- | --- | --- | --- | --- | --- | --- | --- | --- | --- | --- | --- | --- | --- | --- | --- | --- | --- | --- | --- | --- | --- | --- | --- | --- | --- | --- | --- | --- | --- | --- | --- | --- | --- | --- | --- | --- | --- | --- | --- | --- | --- | --- | --- | --- | --- | --- | --- | --- | --- | --- | --- | --- | --- | --- | --- | --- | --- | --- | --- | --- | --- | --- | --- | --- | --- | --- | --- | --- | --- | --- | --- | --- | --- | --- | --- | --- | --- | --- | --- | --- | --- | --- | --- | --- | --- | --- | --- | --- | --- | --- | --- | --- | --- | --- | --- | --- | --- | --- | --- | --- | --- | --- | --- | --- | --- | --- | --- | --- | --- | --- | --- | --- | --- | --- | --- | --- | --- | --- | --- | --- | --- | --- | --- | --- | --- | --- | --- | --- | --- | --- | --- | --- | --- | --- | --- | --- | --- | --- | --- | --- | --- | --- | --- | --- | --- | --- | --- | --- | --- | --- | --- | --- | --- | --- | --- | --- | --- | --- | --- | --- | --- | --- | --- | --- | --- | --- | --- | --- | --- | --- | --- | --- | --- | --- | --- | --- | --- | --- | --- | --- | --- | --- | --- | --- | --- | --- | --- | --- | --- | --- | --- | --- | --- | --- | --- | --- | --- | --- | --- | --- | --- | --- | --- | --- | --- | --- | --- | --- | --- | --- | --- | --- | --- | --- | --- | --- | --- | --- | --- | --- | --- | --- | --- | --- | --- | --- | --- | --- | --- | --- | --- | --- | --- | --- | --- | --- | --- | --- | --- | --- | --- | --- | --- | --- | --- | --- | --- | --- | --- | --- | --- | --- | --- | --- | --- | --- | --- | --- | --- | --- | --- | --- | --- | --- | --- | --- | --- | --- | --- | --- | --- | --- | --- | --- | --- | --- | --- | --- | --- | --- | --- | --- | --- | --- | --- | --- | --- | --- | --- | --- | --- | --- | --- | --- | --- | --- | --- | --- | --- | --- | --- | --- | --- | --- | --- | --- | --- | --- | --- | --- | --- | --- | --- | --- | --- | --- | --- | --- | --- | --- | --- | --- | --- | --- | --- | --- | --- | --- | --- | --- | --- | --- | --- | --- | --- | --- | --- | --- | --- | --- | --- | --- | --- | --- | --- | --- | --- | --- | --- | --- | --- | --- | --- | --- | --- | --- | --- | --- | --- | --- | --- | --- | --- | --- | --- | --- | --- | --- | --- | --- | --- | --- | --- | --- | --- | --- | --- | --- | --- | --- | --- | --- | --- | --- | --- | --- | --- | --- | --- | --- | --- | --- | --- | --- | --- | --- | --- | --- | --- | --- | --- | --- | --- | --- | --- | --- | --- | --- | --- | --- | --- | --- | --- | --- | --- | --- | --- | --- | --- | --- | --- | --- | --- | --- | --- | --- | --- | --- | --- | --- | --- | --- | --- | --- | --- | --- | --- | --- | --- | --- | --- | --- | --- | --- | --- | --- | --- | --- | --- | --- | --- | --- | --- | --- | --- | --- | --- | --- | --- | --- | --- | --- | --- | --- | --- | --- | --- | --- | --- | --- | --- | --- | --- | --- | --- | --- | --- | --- | --- | --- | --- | --- | --- | --- | --- | --- | --- | --- | --- | --- | --- | --- | --- | --- | --- | --- | --- | --- | --- | --- | --- | --- | --- | --- | --- | --- | --- | --- | --- | --- | --- | --- | --- | --- | --- | --- | --- | --- | --- | --- | --- | --- | --- | --- | --- | --- | --- | --- | --- | --- | --- | --- | --- | --- | --- | --- | --- | --- | --- | --- | --- | --- | --- | --- | --- | --- | --- | --- | --- | --- | --- | --- | --- | --- | --- | --- | --- | --- | --- | --- | --- | --- | --- | --- | --- | --- | --- | --- | --- | --- | --- | --- | --- | --- | --- | --- | --- | --- | --- | --- | --- | --- | --- | --- | --- | --- | --- | --- | --- | --- | --- | --- | --- | --- | --- | --- | --- | --- | --- | --- | --- | --- | --- | --- | --- | --- | --- | --- | --- | --- | --- | --- | --- | --- | --- | --- | --- | --- | --- | --- | --- | --- | --- | --- | --- | --- | --- | --- | --- | --- | --- | --- | --- | --- | --- | --- | --- | --- | --- | --- | --- | --- | --- | --- | --- | --- | --- | --- | --- | --- | --- | --- | --- | --- | --- | --- | --- | --- | --- | --- | --- | --- | --- | --- | --- | --- | --- | --- | --- | --- | --- | --- | --- | --- | --- | --- | --- | --- | --- | --- | --- | --- | --- | --- | --- | --- | --- | --- | --- | --- | --- | --- | --- | --- | --- | --- | --- | --- | --- | --- | --- | --- | --- | --- | --- | --- | --- | --- | --- | --- | --- | --- | --- | --- | --- | --- | --- | --- | --- | --- | --- | --- | --- | --- | --- | --- | --- | --- | --- | --- | --- | --- | --- | --- | --- | --- | --- | --- | --- | --- | --- | --- | --- | --- | --- | --- | --- | --- | --- | --- | --- | --- | --- | --- | --- | --- | --- | --- | --- | --- | --- | --- | --- | --- | --- | --- | --- | --- | --- | --- | --- | --- | --- | --- | --- | --- | --- | --- | --- | --- | --- | --- | --- | --- | --- | --- | --- | --- | --- | --- | --- | --- | --- | --- | --- | --- | --- | --- | --- | --- | --- | --- | --- | --- | --- | --- | --- | --- | --- | --- | --- | --- | --- | --- | --- | --- | --- | --- | --- | --- | --- | --- | --- | --- | --- | --- | --- | --- | --- | --- | --- | --- | --- | --- | --- | --- | --- | --- | --- | --- | --- | --- | --- | --- | --- | --- | --- | --- | --- | --- | --- | --- | --- | --- | --- | --- | --- | --- | --- | --- | --- | --- | --- | --- | --- | --- | --- | --- | --- | --- | --- | --- | --- | --- | --- | --- | --- | --- | --- | --- | --- | --- | --- | --- | --- | --- | --- | --- | --- | --- | --- | --- | --- | --- | --- | --- | --- | --- | --- | --- | --- | --- | --- | --- | --- | --- | --- | --- | --- | --- | --- | --- | --- | --- | --- | --- | --- | --- | --- | --- | --- | --- | --- | --- | --- | --- | --- | --- | --- | --- | --- | --- | --- | --- | --- | --- | --- | --- | --- | --- | --- | --- | --- | --- | --- | --- | --- | --- | --- | --- | --- | --- | --- | --- | --- | --- | --- | --- | --- | --- | --- | --- | --- | --- | --- | --- | --- | --- | --- | --- | --- | --- | --- | --- | --- | --- | --- | --- | --- | --- | --- | --- | --- | --- | --- | --- | --- | --- | --- | --- | --- | --- | --- | --- | --- | --- | --- | --- | --- | --- | --- | --- | --- | --- | --- | --- | --- | --- | --- | --- | --- | --- | --- | --- | --- | --- | --- | --- | --- | --- | --- | --- | --- | --- | --- | --- | --- | --- | --- | --- | --- | --- | --- | --- | --- | --- | --- | --- | --- | --- | --- | --- | --- | --- | --- | --- | --- | --- | --- | --- | --- | --- | --- | --- | --- | --- | --- | --- | --- | --- | --- | --- | --- | --- | --- | --- | --- | --- | --- | --- | --- | --- | --- | --- | --- | --- | --- | --- | --- | --- | --- | --- | --- | --- | --- | --- | --- | --- | --- | --- | --- | --- | --- | --- | --- | --- | --- | --- | --- | --- | --- | --- | --- | --- | --- | --- | --- | --- | --- | --- | --- | --- | --- | --- | --- | --- | --- | --- | --- | --- | --- | --- | --- | --- | --- | --- | --- | --- | --- | --- | --- | --- | --- | --- | --- | --- | --- | --- | --- | --- | --- | --- | --- | --- | --- | --- | --- | --- | --- | --- | --- | --- | --- | --- | --- | --- | --- | --- | --- | --- | --- | --- | --- | --- | --- | --- | --- | --- | --- | --- | --- | --- | --- | --- | --- | --- | --- | --- | --- | --- | --- | --- | --- | --- | --- | --- | --- | --- | --- | --- | --- | --- | --- | --- | --- | --- | --- | --- | --- | --- | --- | --- | --- | --- | --- | --- | --- | --- | --- | --- | --- | --- | --- | --- | --- | --- | --- | --- | --- | --- | --- | --- | --- | --- | --- | --- | --- | --- | --- | --- | --- | --- | --- | --- | --- | --- | --- | --- | --- | --- | --- | --- | --- | --- | --- | --- | --- | --- | --- | --- | --- | --- | --- | --- | --- | --- | --- | --- | --- | --- | --- | --- | --- | --- | --- | --- | --- | --- | --- | --- | --- | --- | --- | --- | --- | --- | --- | --- | --- | --- | --- | --- | --- | --- | --- | --- | --- | --- | --- | --- | --- | --- | --- | --- | --- | --- | --- | --- | --- | --- | --- | --- | --- | --- | --- | --- | --- | --- | --- | --- | --- | --- | --- | --- | --- | --- | --- | --- | --- | --- | --- | --- | --- | --- | --- | --- | --- | --- | --- | --- | --- | --- | --- | --- | --- | --- | --- | --- | --- | --- | --- | --- | --- | --- | --- | --- | --- | --- | --- | --- | --- | --- | --- | --- | --- | --- | --- | --- | --- | --- | --- | --- | --- | --- | --- | --- | --- | --- | --- | --- | --- | --- | --- | --- | --- | --- | --- | --- | --- | --- | --- | --- | --- | --- | --- | --- | --- | --- | --- | --- | --- | --- | --- | --- | --- | --- | --- | --- | --- | --- | --- | --- | --- | --- | --- | --- | --- | --- | --- | --- | --- | --- | --- | --- | --- | --- | --- | --- | --- | --- | --- | --- | --- | --- | --- | --- | --- | --- | --- | --- | --- | --- | --- | --- | --- | --- | --- | --- | --- | --- | --- | --- | --- | --- | --- | --- | --- | --- | --- | --- | --- | --- | --- | --- | --- | --- | --- | --- | --- | --- | --- | --- | --- | --- | --- | --- | --- | --- | --- | --- | --- | --- | --- | --- | --- | --- | --- | --- | --- | --- | --- | --- | --- | --- | --- | --- | --- | --- | --- | --- | --- | --- | --- | --- | --- | --- | --- | --- | --- | --- | --- | --- | --- | --- | --- | --- | --- | --- | --- | --- | --- | --- | --- | --- | --- | --- | --- | --- | --- | --- | --- | --- | --- | --- | --- | --- | --- | --- | --- | --- | --- | --- | --- | --- | --- | --- | --- | --- | --- | --- | --- | --- | --- | --- | --- | --- | --- | --- | --- | --- | --- | --- | --- | --- | --- | --- | --- | --- | --- | --- | --- | --- | --- | --- | --- | --- | --- | --- | --- | --- | --- | --- | --- | --- | --- | --- | --- | --- | --- | --- | --- | --- | --- | --- | --- | --- | --- | --- | --- | --- | --- | --- | --- | --- | --- | --- | --- | --- | --- | --- | --- | --- | --- | --- | --- | --- | --- | --- | --- | --- | --- | --- | --- | --- | --- | --- | --- | --- | --- | --- | --- | --- | --- | --- | --- | --- | --- | --- | --- | --- | --- | --- | --- | --- | --- | --- | --- | --- | --- | --- | --- | --- | --- | --- | --- | --- | --- | --- | --- | --- | --- | --- | --- | --- | --- | --- | --- | --- | --- | --- | --- | --- | --- | --- | --- | --- | --- | --- | --- | --- | --- | --- | --- | --- | --- | --- | --- | --- | --- | --- | --- | --- | --- | --- | --- | --- | --- | --- | --- | --- | --- | --- | --- | --- | --- | --- | --- | --- | --- | --- | --- | --- | --- | --- | --- | --- | --- | --- | --- | --- | --- | --- | --- | --- | --- | --- | --- | --- | --- | --- | --- | --- | --- | --- | --- | --- | --- | --- | --- | --- | --- | --- | --- | --- | --- | --- | --- | --- | --- | --- | --- | --- | --- | --- | --- | --- | --- | --- | --- | --- | --- | --- | --- | --- | --- | --- | --- | --- | --- | --- | --- | --- | --- | --- | --- | --- | --- | --- | --- | --- | --- | --- | --- | --- | --- | --- | --- | --- | --- | --- | --- | --- | --- | --- | --- | --- | --- | --- | --- | --- | --- | --- | --- | --- | --- | --- | --- | --- | --- | --- | --- | --- | --- | --- | --- | --- | --- | --- | --- | --- | --- | --- | --- | --- | --- | --- | --- | --- | --- | --- | --- | --- | --- | --- | --- | --- | --- | --- | --- | --- | --- | --- | --- | --- | --- | --- | --- | --- | --- | --- | --- | --- | --- | --- | --- | --- | --- | --- | --- | --- | --- | --- | --- | --- | --- | --- | --- | --- | --- | --- | --- | --- | --- | --- | --- | --- | --- | --- | --- | --- | --- | --- | --- | --- | --- | --- | --- | --- | --- | --- | --- | --- | --- | --- | --- | --- | --- | --- | --- | --- | --- | --- | --- | --- | --- | --- | --- | --- | --- | --- | --- | --- | --- | --- | --- | --- | --- | --- | --- | --- | --- | --- | --- | --- | --- | --- | --- | --- | --- | --- | --- | --- | --- | --- | --- | --- | --- | --- | --- | --- | --- | --- | --- | --- | --- | --- | --- | --- | --- | --- | --- | --- | --- | --- | --- | --- | --- | --- | --- | --- | --- | --- | --- | --- | --- | --- | --- | --- | --- | --- | --- | --- | --- | --- | --- | --- | --- | --- | --- | --- | --- | --- | --- | --- | --- | --- | --- | --- | --- | --- | --- | --- | --- | --- | --- | --- | --- | --- | --- | --- | --- | --- | --- | --- | --- | --- | --- | --- | --- | --- | --- | --- | --- | --- | --- | --- | --- | --- | --- | --- | --- | --- | --- | --- | --- | --- | --- | --- | --- | --- | --- | --- | --- | --- | --- | --- | --- | --- | --- | --- | --- | --- | --- | --- | --- | --- | --- | --- | --- | --- | --- | --- | --- | --- | --- | --- | --- | --- | --- | --- | --- | --- | --- | --- | --- | --- | --- | --- | --- | --- | --- | --- | --- | --- | --- | --- | --- | --- | --- | --- | --- | --- | --- | --- | --- | --- | --- | --- | --- | --- | --- | --- | --- | --- | --- | --- | --- | --- | --- | --- | --- | --- | --- | --- | --- | --- | --- | --- | --- | --- | --- | --- | --- | --- | --- | --- | --- | --- | --- | --- | --- | --- | --- | --- | --- | --- | --- | --- | --- | --- | --- | --- | --- | --- | --- | --- | --- | --- | --- | --- | --- | --- | --- | --- | --- | --- | --- | --- | --- | --- | --- | --- | --- | --- | --- | --- | --- | --- | --- | --- | --- | --- | --- | --- | --- | --- | --- | --- | --- | --- | --- | --- | --- | --- | --- | --- | --- | --- | --- | --- | --- | --- | --- | --- | --- | --- | --- | --- | --- | --- | --- | --- | --- | --- | --- | --- | --- | --- | --- | --- | --- | --- | --- | --- | --- | --- | --- | --- | --- | --- | --- | --- | --- | --- | --- | --- | --- | --- | --- | --- | --- | --- | --- | --- | --- | --- | --- | --- | --- | --- | --- | --- | --- | --- | --- | --- | --- | --- | --- | --- | --- | --- | --- | --- | --- | --- | --- | --- | --- | --- | --- | --- | --- | --- | --- | --- | --- | --- | --- | --- | --- | --- | --- | --- | --- | --- | --- | --- | --- | --- | --- | --- | --- | --- | --- | --- | --- | --- | --- | --- | --- | --- | --- | --- | --- | --- | --- | --- | --- | --- | --- | --- | --- | --- | --- | --- | --- | --- | --- | --- | --- | --- | --- | --- | --- | --- | --- | --- | --- | --- | --- | --- | --- | --- | --- | --- | --- | --- | --- | --- | --- | --- | --- | --- | --- | --- | --- | --- | --- | --- | --- | --- | --- | --- | --- | --- | --- | --- | --- | --- | --- | --- | --- | --- | --- | --- | --- | --- | --- | --- | --- | --- | --- | --- | --- | --- | --- | --- | --- | --- | --- | --- | --- | --- | --- | --- | --- | --- | --- | --- | --- | --- | --- | --- | --- | --- | --- | --- | --- | --- | --- | --- | --- | --- | --- | --- | --- | --- | --- | --- | --- | --- | --- | --- | --- | --- | --- | --- | --- | --- | --- | --- | --- | --- | --- | --- | --- | --- | --- | --- | --- | --- | --- | --- | --- | --- | --- | --- | --- | --- | --- | --- | --- | --- | --- | --- | --- | --- | --- | --- | --- | --- | --- | --- | --- | --- | --- | --- | --- | --- | --- | --- | --- | --- | --- | --- | --- | --- | --- | --- | --- | --- | --- | --- | --- | --- | --- | --- | --- | --- | --- | --- | --- | --- | --- | --- | --- | --- | --- | --- | --- | --- | --- | --- | --- | --- | --- | --- | --- | --- | --- | --- | --- | --- | --- | --- | --- | --- | --- | --- | --- | --- | --- | --- | --- | --- | --- | --- | --- | --- | --- | --- | --- | --- | --- | --- | --- | --- | --- | --- | --- | --- | --- | --- | --- | --- | --- | --- | --- | --- | --- | --- | --- | --- | --- | --- | --- | --- | --- | --- | --- | --- | --- | --- | --- | --- | --- | --- | --- | --- | --- | --- | --- | --- | --- | --- | --- | --- | --- | --- | --- | --- | --- | --- | --- | --- | --- | --- | --- | --- | --- | --- | --- | --- | --- | --- | --- | --- | --- | --- | --- | --- | --- | --- | --- | --- | --- | --- | --- | --- | --- | --- | --- | --- | --- | --- | --- | --- | --- | --- | --- | --- | --- | --- | --- | --- | --- | --- | --- | --- | --- | --- | --- | --- | --- | --- | --- | --- | --- | --- | --- | --- | --- | --- | --- | --- | --- | --- | --- | --- | --- | --- | --- | --- | --- | --- | --- | --- | --- | --- | --- | --- | --- | --- | --- | --- | --- | --- | --- | --- | --- | --- | --- | --- | --- | --- | --- | --- | --- | --- | --- | --- | --- | --- | --- | --- | --- | --- | --- | --- | --- | --- | --- | --- | --- | --- | --- | --- | --- | --- | --- | --- | --- | --- | --- | --- | --- | --- | --- | --- | --- | --- | --- | --- | --- | --- | --- | --- | --- | --- | --- | --- | --- | --- | --- | --- | --- | --- | --- | --- | --- | --- | --- | --- | --- | --- | --- | --- | --- | --- | --- | --- | --- | --- | --- | --- | --- | --- | --- | --- | --- | --- | --- | --- | --- | --- | --- | --- | --- | --- | --- | --- | --- | --- | --- | --- | --- | --- | --- | --- | --- | --- | --- | --- | --- | --- | --- | --- | --- | --- | --- | --- | --- | --- | --- | --- | --- | --- | --- | --- | --- | --- | --- | --- | --- | --- | --- | --- | --- | --- | --- | --- | --- | --- | --- | --- | --- | --- | --- | --- | --- | --- | --- | --- | --- | --- | --- | --- | --- | --- | --- | --- | --- | --- | --- | --- | --- | --- | --- | --- | --- | --- | --- | --- | --- | --- | --- | --- | --- | --- | --- | --- | --- | --- | --- | --- | --- | --- | --- | --- | --- | --- | --- | --- | --- | --- | --- | --- | --- | --- | --- | --- | --- | --- | --- | --- | --- | --- | --- | --- | --- | --- | --- | --- | --- | --- | --- | --- | --- | --- | --- | --- | --- | --- | --- | --- | --- | --- | --- | --- | --- | --- | --- | --- | --- | --- | --- | --- | --- | --- | --- | --- | --- | --- | --- | --- | --- | --- | --- | --- | --- | --- | --- | --- | --- | --- | --- | --- | --- | --- | --- | --- | --- | --- | --- | --- | --- | --- | --- | --- | --- | --- | --- | --- | --- | --- | --- | --- | --- | --- | --- | --- | --- | --- | --- | --- | --- | --- | --- | --- | --- | --- | --- | --- | --- | --- | --- | --- | --- | --- | --- | --- | --- | --- | --- | --- | --- | --- | --- | --- | --- | --- | --- | --- | --- | --- | --- | --- | --- | --- | --- | --- | --- | --- | --- | --- | --- | --- | --- | --- | --- | --- | --- | --- | --- | --- | --- | --- | --- | --- | --- | --- | --- | --- | --- | --- | --- | --- | --- | --- | --- | --- | --- | --- | --- | --- | --- | --- | --- | --- | --- | --- | --- | --- | --- | --- | --- | --- | --- | --- | --- | --- | --- | --- | --- | --- | --- | --- | --- | --- | --- | --- | --- | --- | --- | --- | --- | --- | --- | --- | --- | --- | --- | --- | --- | --- | --- | --- | --- | --- | --- | --- | --- | --- | --- | --- | --- | --- | --- | --- | --- | --- | --- | --- | --- | --- | --- | --- | --- | --- | --- | --- | --- | --- | --- | --- | --- | --- | --- | --- | --- | --- | --- | --- | --- | --- | --- | --- | --- | --- | --- | --- | --- | --- | --- | --- | --- | --- | --- | --- | --- | --- | --- | --- | --- | --- | --- | --- | --- | --- | --- | --- | --- | --- | --- | --- | --- | --- | --- | --- | --- | --- | --- | --- | --- | --- | --- | --- | --- | --- | --- | --- | --- | --- | --- | --- | --- | --- | --- | --- | --- | --- | --- | --- | --- | --- | --- | --- | --- | --- | --- | --- | --- | --- | --- | --- | --- | --- | --- | --- | --- | --- | --- | --- | --- | --- | --- | --- | --- | --- | --- | --- | --- | --- | --- | --- | --- | --- | --- | --- | --- | --- | --- | --- | --- | --- | --- | --- | --- | --- | --- | --- | --- | --- | --- | --- | --- | --- | --- | --- | --- | --- | --- | --- | --- | --- | --- | --- | --- | --- | --- | --- | --- | --- | --- | --- | --- | --- | --- | --- | --- | --- | --- | --- | --- | --- | --- | --- | --- | --- | --- | --- | --- | --- | --- | --- | --- | --- | --- | --- | --- | --- | --- | --- | --- | --- | --- | --- | --- | --- | --- | --- | --- | --- | --- | --- | --- | --- | --- | --- | --- | --- | --- | --- | --- | --- | --- | --- | --- | --- | --- | --- | --- | --- | --- | --- | --- | --- | --- | --- | --- | --- | --- | --- | --- | --- | --- | --- | --- | --- | --- | --- | --- | --- | --- | --- | --- | --- | --- | --- | --- | --- | --- | --- | --- | --- | --- | --- | --- | --- | --- | --- | --- | --- | --- | --- | --- | --- | --- | --- | --- | --- | --- | --- | --- | --- | --- | --- | --- | --- | --- | --- | --- | --- | --- | --- | --- | --- | --- | --- | --- | --- | --- | --- | --- | --- | --- | --- | --- | --- | --- | --- | --- | --- | --- | --- | --- | --- | --- | --- | --- | --- | --- | --- | --- | --- | --- | --- | --- | --- | --- | --- | --- | --- | --- | --- | --- | --- | --- | --- | --- | --- | --- | --- | --- | --- | --- | --- | --- | --- | --- | --- | --- | --- | --- | --- | --- | --- | --- | --- | --- | --- | --- | --- | --- | --- | --- | --- | --- | --- | --- | --- | --- | --- | --- | --- | --- | --- | --- | --- | --- | --- | --- | --- | --- | --- | --- | --- | --- | --- | --- | --- | --- | --- | --- | --- | --- | --- | --- | --- | --- | --- | --- | --- | --- | --- | --- | --- | --- | --- | --- | --- | --- | --- | --- | --- | --- | --- | --- | --- | --- | --- | --- | --- | --- | --- | --- | --- | --- | --- | --- | --- | --- | --- | --- | --- | --- | --- | --- | --- | --- | --- | --- | --- | --- | --- | --- | --- | --- | --- | --- | --- | --- | --- | --- | --- | --- | --- | --- | --- | --- | --- | --- | --- | --- | --- | --- | --- | --- | --- | --- | --- | --- | --- | --- | --- | --- | --- | --- | --- | --- | --- | --- | --- | --- | --- | --- | --- | --- | --- | --- | --- | --- | --- | --- | --- | --- | --- | --- | --- | --- | --- | --- | --- | --- | --- | --- | --- | --- | --- | --- | --- | --- | --- | --- | --- | --- | --- | --- | --- | --- | --- | --- | --- | --- | --- | --- | --- | --- | --- | --- | --- | --- | --- | --- | --- | --- | --- | --- | --- | --- | --- | --- | --- | --- | --- | --- | --- | --- | --- | --- | --- | --- | --- | --- | --- | --- | --- | --- | --- | --- | --- | --- | --- | --- | --- | --- | --- | --- | --- | --- | --- | --- | --- | --- | --- | --- | --- | --- | --- | --- | --- | --- | --- | --- | --- | --- | --- | --- | --- | --- | --- | --- | --- | --- | --- | --- | --- | --- | --- | --- | --- | --- | --- | --- | --- | --- | --- | --- | --- | --- | --- | --- | --- | --- | --- | --- | --- | --- | --- | --- | --- | --- | --- | --- | --- | --- | --- | --- | --- | --- | --- | --- | --- | --- | --- | --- | --- | --- | --- | --- | --- | --- | --- | --- | --- | --- | --- | --- | --- | --- | --- | --- | --- | --- | --- | --- | --- | --- | --- | --- | --- | --- | --- | --- | --- | --- | --- | --- | --- | --- | --- | --- | --- | --- | --- | --- | --- | --- | --- | --- | --- | --- | --- | --- | --- | --- | --- | --- | --- | --- | --- | --- | --- | --- | --- | --- | --- | --- | --- | --- | --- | --- | --- | --- | --- | --- | --- | --- | --- | --- | --- | --- | --- | --- | --- | --- | --- | --- | --- | --- | --- | --- | --- | --- | --- | --- | --- | --- | --- | --- | --- | --- | --- | --- | --- | --- | --- | --- | --- | --- | --- | --- | --- | --- | --- | --- | --- | --- | --- | --- | --- | --- | --- | --- | --- | --- | --- | --- | --- | --- | --- | --- | --- | --- | --- | --- | --- | --- | --- | --- | --- | --- | --- | --- | --- | --- | --- | --- | --- | --- | --- | --- | --- | --- | --- | --- | --- | --- | --- | --- | --- | --- | --- | --- | --- | --- | --- | --- | --- | --- | --- | --- | --- | --- | --- | --- | --- | --- | --- | --- | --- | --- | --- | --- | --- | --- | --- | --- | --- | --- | --- | --- | --- | --- | --- | --- | --- | --- | --- | --- | --- | --- | --- | --- | --- | --- | --- | --- | --- | --- | --- | --- | --- | --- | --- | --- | --- | --- | --- | --- | --- | --- | --- | --- | --- | --- | --- | --- | --- | --- | --- | --- | --- | --- | --- | --- | --- | --- | --- | --- | --- | --- | --- | --- | --- | --- | --- | --- | --- | --- | --- | --- | --- | --- | --- | --- | --- | --- | --- | --- | --- | --- | --- | --- | --- | --- | --- | --- | --- | --- | --- | --- | --- | --- | --- | --- | --- | --- | --- | --- | --- | --- | --- | --- | --- | --- | --- | --- | --- | --- | --- | --- | --- | --- | --- | --- | --- | --- | --- | --- | --- | --- | --- | --- | --- | --- | --- | --- | --- | --- | --- | --- | --- | --- | --- | --- | --- | --- | --- | --- | --- | --- | --- | --- | --- | --- | --- | --- | --- | --- | --- | --- | --- | --- | --- | --- | --- | --- | --- | --- | --- | --- | --- | --- | --- | --- | --- | --- | --- | --- | --- | --- | --- | --- | --- | --- | --- | --- | --- | --- | --- | --- | --- | --- | --- | --- | --- | --- | --- | --- | --- | --- | --- | --- | --- | --- | --- | --- | --- | --- | --- | --- | --- | --- | --- | --- | --- | --- | --- | --- | --- | --- | --- | --- | --- | --- | --- | --- | --- | --- | --- | --- | --- | --- | --- | --- | --- | --- | --- | --- | --- | --- | --- | --- | --- | --- | --- | --- | --- | --- | --- | --- | --- | --- | --- | --- | --- | --- | --- | --- | --- | --- | --- | --- | --- | --- | --- | --- | --- | --- | --- | --- | --- | --- | --- | --- | --- | --- | --- | --- | --- | --- | --- | --- | --- | --- | --- | --- | --- | --- | --- | --- | --- | --- | --- | --- | --- | --- | --- | --- | --- | --- | --- | --- | --- | --- | --- | --- | --- | --- | --- | --- | --- | --- | --- | --- | --- | --- | --- | --- | --- | --- | --- | --- | --- | --- | --- | --- | --- | --- | --- | --- | --- | --- | --- | --- | --- | --- | --- | --- | --- | --- | --- | --- | --- | --- | --- | --- | --- | --- | --- | --- | --- | --- | --- | --- | --- | --- | --- | --- | --- | --- | --- | --- | --- | --- | --- | --- | --- | --- | --- | --- | --- | --- | --- | --- | --- | --- | --- | --- | --- | --- | --- | --- | --- | --- | --- | --- | --- | --- | --- | --- | --- | --- | --- | --- | --- | --- | --- | --- | --- | --- | --- | --- | --- | --- | --- | --- | --- | --- | --- | --- | --- | --- | --- | --- | --- | --- | --- | --- | --- | --- | --- | --- | --- | --- | --- | --- | --- | --- | --- | --- | --- | --- | --- | --- | --- | --- | --- | --- | --- | --- | --- | --- | --- | --- | --- | --- | --- | --- | --- | --- | --- | --- | --- | --- | --- | --- | --- | --- | --- | --- | --- | --- | --- | --- | --- | --- | --- | --- | --- | --- | --- | --- | --- | --- | --- | --- | --- | --- | --- | --- | --- | --- | --- | --- | --- | --- | --- | --- | --- | --- | --- | --- | --- | --- | --- | --- | --- | --- | --- | --- | --- | --- | --- | --- | --- | --- | --- | --- | --- | --- | --- | --- | --- | --- | --- | --- | --- | --- | --- | --- | --- | --- | --- | --- | --- | --- | --- | --- | --- | --- | --- | --- | --- | --- | --- | --- | --- | --- | --- | --- | --- | --- | --- | --- | --- | --- | --- | --- | --- | --- | --- | --- | --- | --- | --- | --- | --- | --- | --- | --- | --- | --- | --- | --- | --- | --- | --- | --- | --- | --- | --- | --- | --- | --- | --- | --- | --- | --- | --- | --- | --- | --- | --- | --- | --- | --- | --- | --- | --- | --- | --- | --- | --- | --- | --- | --- | --- | --- | --- | --- | --- | --- | --- | --- | --- | --- | --- | --- | --- | --- | --- | --- | --- | --- | --- | --- | --- | --- | --- | --- | --- | --- | --- | --- | --- | --- | --- | --- | --- | --- | --- | --- | --- | --- | --- | --- | --- | --- | --- | --- | --- | --- | --- | --- | --- | --- | --- | --- | --- | --- | --- | --- | --- | --- | --- | --- | --- | --- | --- | --- | --- | --- | --- | --- | --- | --- | --- | --- | --- | --- | --- | --- | --- | --- | --- | --- | --- | --- | --- | --- | --- | --- | --- | --- | --- | --- | --- | --- | --- | --- | --- | --- | --- | --- | --- | --- | --- | --- | --- | --- | --- | --- | --- | --- | --- | --- | --- | --- | --- | --- | --- | --- | --- | --- | --- | --- | --- | --- | --- | --- | --- | --- | --- | --- | --- | --- | --- | --- | --- | --- | --- | --- | --- | --- | --- | --- | --- | --- | --- | --- | --- | --- | --- | --- | --- | --- | --- | --- | --- | --- | --- | --- | --- | --- | --- | --- | --- | --- | --- | --- | --- | --- | --- | --- | --- | --- | --- | --- | --- | --- | --- | --- | --- | --- | --- | --- | --- | --- | --- | --- | --- | --- | --- | --- | --- | --- | --- | --- | --- | --- | --- | --- | --- | --- | --- | --- | --- | --- | --- | --- | --- | --- | --- | --- | --- | --- | --- | --- | --- | --- | --- | --- | --- | --- | --- | --- | --- | --- | --- | --- | --- | --- | --- | --- | --- | --- | --- | --- | --- | --- | --- | --- | --- | --- | --- | --- | --- | --- | --- | --- | --- | --- | --- | --- | --- | --- | --- | --- | --- | --- | --- | --- | --- | --- | --- | --- | --- | --- | --- | --- | --- | --- | --- | --- | --- | --- | --- | --- | --- | --- | --- | --- | --- | --- | --- | --- | --- | --- | --- | --- | --- | --- | --- | --- | --- | --- | --- | --- | --- | --- | --- | --- | --- | --- | --- | --- | --- | --- | --- | --- | --- | --- | --- | --- | --- | --- | --- | --- | --- | --- | --- | --- | --- | --- | --- | --- | --- | --- | --- | --- | --- | --- | --- | --- | --- | --- | --- | --- | --- | --- | --- | --- | --- | --- | --- | --- | --- | --- | --- | --- | --- | --- | --- | --- | --- | --- | --- | --- | --- | --- | --- | --- | --- | --- | --- | --- | --- | --- | --- | --- | --- | --- | --- | --- | --- | --- | --- | --- | --- | --- | --- | --- | --- | --- | --- | --- | --- | --- | --- | --- | --- | --- | --- | --- | --- | --- | --- | --- | --- | --- | --- | --- | --- | --- | --- | --- | --- | --- | --- | --- | --- | --- | --- | --- | --- | --- | --- | --- | --- | --- | --- | --- | --- | --- | --- | --- | --- | --- | --- | --- | --- | --- | --- | --- | --- | --- | --- | --- | --- | --- | --- | --- | --- | --- | --- | --- | --- | --- | --- | --- | --- | --- | --- | --- | --- | --- | --- | --- | --- | --- | --- | --- | --- | --- | --- | --- | --- | --- | --- | --- | --- | --- | --- | --- | --- | --- | --- | --- | --- | --- | --- | --- | --- | --- | --- | --- | --- | --- | --- | --- | --- | --- | --- | --- | --- | --- | --- | --- | --- | --- | --- | --- | --- | --- | --- | --- | --- | --- | --- | --- | --- | --- | --- | --- | --- | --- | --- | --- | --- | --- | --- | --- | --- | --- | --- | --- | --- | --- | --- | --- | --- | --- | --- | --- | --- | --- | --- | --- | --- | --- | --- | --- | --- | --- | --- | --- | --- | --- | --- | --- | --- | --- | --- | --- | --- | --- | --- | --- | --- | --- | --- | --- | --- | --- | --- | --- | --- | --- | --- | --- | --- | --- | --- | --- | --- | --- | --- | --- | --- | --- | --- | --- | --- | --- | --- | --- | --- | --- | --- | --- | --- | --- | --- | --- | --- | --- | --- | --- | --- | --- | --- | --- | --- | --- | --- | --- | --- | --- | --- | --- | --- | --- | --- | --- | --- | --- | --- | --- | --- | --- | --- | --- | --- | --- | --- | --- | --- | --- | --- | --- | --- | --- | --- | --- | --- | --- | --- | --- | --- | --- | --- | --- | --- | --- | --- | --- | --- | --- | --- | --- | --- | --- | --- | --- | --- | --- | --- | --- | --- | --- | --- | --- | --- | --- | --- | --- | --- | --- | --- | --- | --- | --- | --- | --- | --- | --- | --- | --- | --- | --- | --- | --- | --- | --- | --- | --- | --- | --- | --- | --- | --- | --- | --- | --- | --- | --- | --- | --- | --- | --- | --- | --- | --- | --- | --- | --- | --- | --- | --- | --- | --- | --- | --- | --- | --- | --- | --- | --- | --- | --- | --- | --- | --- | --- | --- | --- | --- | --- | --- | --- | --- | --- | --- | --- | --- | --- | --- | --- | --- | --- | --- | --- | --- | --- | --- | --- | --- | --- | --- | --- | --- | --- | --- | --- | --- | --- | --- | --- | --- | --- | --- | --- | --- | --- | --- | --- | --- | --- | --- | --- | --- | --- | --- | --- | --- | --- | --- | --- | --- | --- | --- | --- | --- | --- | --- | --- | --- | --- | --- | --- | --- | --- | --- | --- | --- | --- | --- | --- | --- | --- | --- | --- | --- | --- | --- | --- | --- | --- | --- | --- | --- | --- | --- | --- | --- | --- | --- | --- | --- | --- | --- | --- | --- | --- | --- | --- | --- | --- | --- | --- | --- | --- | --- | --- | --- | --- | --- | --- | --- | --- | --- | --- | --- | --- | --- | --- | --- | --- | --- | --- | --- | --- | --- | --- | --- | --- | --- | --- | --- | --- | --- | --- | --- | --- | --- | --- | --- | --- | --- | --- | --- | --- | --- | --- | --- | --- | --- | --- | --- | --- | --- | --- | --- | --- | --- | --- | --- | --- | --- | --- | --- | --- | --- | --- | --- | --- | --- | --- | --- | --- | --- | --- | --- | --- | --- | --- | --- | --- | --- | --- | --- | --- | --- | --- | --- | --- | --- | --- | --- | --- | --- | --- | --- | --- | --- | --- | --- | --- | --- | --- | --- | --- | --- | --- | --- | --- | --- | --- | --- | --- | --- | --- | --- | --- | --- | --- | --- | --- | --- | --- | --- | --- | --- | --- | --- | --- | --- | --- | --- | --- | --- | --- | --- | --- | --- | --- | --- | --- | --- | --- | --- | --- | --- | --- | --- | --- | --- | --- | --- | --- | --- | --- | --- | --- | --- | --- | --- | --- | --- | --- | --- | --- | --- | --- | --- | --- | --- | --- | --- | --- | --- | --- | --- | --- | --- | --- | --- | --- | --- | --- | --- | --- | --- | --- | --- | --- | --- | --- | --- | --- | --- | --- | --- | --- | --- | --- | --- | --- | --- | --- | --- | --- | --- | --- | --- | --- | --- | --- | --- | --- | --- | --- | --- | --- | --- | --- | --- | --- | --- | --- | --- | --- | --- | --- | --- | --- | --- | --- | --- | --- | --- | --- | --- | --- | --- | --- | --- | --- | --- | --- | --- | --- | --- | --- | --- | --- | --- | --- | --- | --- | --- | --- | --- | --- | --- | --- | --- | --- | --- | --- | --- | --- | --- | --- | --- | --- | --- | --- | --- | --- | --- | --- | --- | --- | --- | --- | --- | --- | --- | --- | --- | --- | --- | --- | --- | --- | --- | --- | --- | --- | --- | --- | --- | --- | --- | --- | --- | --- | --- | --- | --- | --- | --- | --- | --- | --- | --- | --- | --- | --- | --- | --- | --- | --- | --- | --- | --- | --- | --- | --- | --- | --- | --- | --- | --- | --- | --- | --- | --- | --- | --- | --- | --- | --- | --- | --- | --- | --- | --- | --- | --- | --- | --- | --- | --- | --- | --- | --- | --- | --- | --- | --- | --- | --- | --- | --- | --- | --- | --- | --- | --- | --- | --- | --- | --- | --- | --- | --- | --- | --- | --- | --- | --- | --- | --- | --- | --- | --- | --- | --- | --- | --- | --- | --- | --- | --- | --- | --- | --- | --- | --- | --- | --- | --- | --- | --- | --- | --- | --- | --- | --- | --- | --- | --- | --- | --- | --- | --- | --- | --- | --- | --- | --- | --- | --- | --- | --- | --- | --- | --- | --- | --- | --- | --- | --- | --- | --- | --- | --- | --- | --- | --- | --- | --- | --- | --- | --- | --- | --- | --- | --- | --- | --- | --- | --- | --- | --- | --- | --- | --- | --- | --- | --- | --- | --- | --- | --- | --- | --- | --- | --- | --- | --- | --- | --- | --- | --- | --- | --- | --- | --- | --- | --- | --- | --- | --- | --- | --- | --- | --- | --- | --- | --- | --- | --- | --- | --- | --- | --- | --- | --- | --- | --- | --- | --- | --- | --- | --- | --- | --- | --- | --- | --- | --- | --- | --- | --- | --- | --- | --- | --- | --- | --- | --- | --- | --- | --- | --- | --- | --- | --- | --- | --- | --- | --- | --- | --- | --- | --- | --- | --- | --- | --- | --- | --- | --- | --- | --- | --- | --- | --- | --- | --- | --- | --- | --- | --- | --- | --- | --- | --- | --- | --- | --- | --- | --- | --- | --- | --- | --- | --- | --- | --- | --- | --- | --- | --- | --- | --- | --- | --- | --- | --- | --- | --- | --- | --- | --- | --- | --- | --- | --- | --- | --- | --- | --- | --- | --- | --- | --- | --- | --- | --- | --- | --- | --- | --- | --- | --- | --- | --- | --- | --- | --- | --- | --- | --- | --- | --- | --- | --- | --- | --- | --- | --- | --- | --- | --- | --- | --- | --- | --- | --- | --- | --- | --- | --- | --- | --- | --- | --- | --- | --- | --- | --- | --- | --- | --- | --- | --- | --- | --- | --- | --- | --- | --- | --- | --- | --- | --- | --- | --- | --- | --- | --- | --- | --- | --- | --- | --- | --- | --- | --- | --- | --- | --- | --- | --- | --- | --- | --- | --- | --- | --- | --- | --- | --- | --- | --- | --- | --- | --- | --- | --- | --- | --- | --- | --- | --- | --- | --- | --- | --- | --- | --- | --- | --- | --- | --- | --- | --- | --- | --- | --- | --- | --- | --- | --- | --- | --- | --- | --- | --- | --- | --- | --- | --- | --- | --- | --- | --- | --- | --- | --- | --- | --- | --- | --- | --- | --- | --- | --- | --- | --- | --- | --- | --- | --- | --- | --- | --- | --- | --- | --- | --- | --- | --- | --- | --- | --- | --- | --- | --- | --- | --- | --- | --- | --- | --- | --- | --- | --- | --- | --- | --- | --- | --- | --- | --- | --- | --- | --- | --- | --- | --- | --- | --- | --- | --- | --- | --- | --- | --- | --- | --- | --- | --- | --- | --- | --- | --- | --- | --- | --- | --- | --- | --- | --- | --- | --- | --- | --- | --- | --- | --- | --- | --- | --- | --- | --- | --- | --- | --- | --- | --- | --- | --- | --- | --- | --- | --- | --- | --- | --- | --- | --- | --- | --- | --- | --- | --- | --- | --- | --- | --- | --- | --- | --- | --- | --- | --- | --- | --- | --- | --- | --- | --- | --- | --- | --- | --- | --- | --- | --- | --- | --- | --- | --- | --- | --- | --- | --- | --- | --- | --- | --- | --- | --- | --- | --- | --- | --- | --- | --- | --- | --- | --- | --- | --- | --- | --- | --- | --- | --- | --- | --- | --- | --- | --- | --- | --- | --- | --- | --- | --- | --- | --- | --- | --- | --- | --- | --- | --- | --- | --- | --- | --- | --- | --- | --- | --- | --- | --- | --- | --- | --- | --- | --- | --- | --- | --- | --- | --- | --- | --- | --- | --- | --- | --- | --- | --- | --- | --- | --- | --- | --- | --- | --- | --- | --- | --- | --- | --- | --- | --- | --- | --- | --- | --- | --- | --- | --- | --- | --- | --- | --- | --- | --- | --- | --- | --- | --- | --- | --- | --- | --- | --- | --- | --- | --- | --- | --- | --- | --- | --- | --- | --- | --- | --- | --- | --- | --- | --- | --- | --- | --- | --- | --- | --- | --- | --- | --- | --- | --- | --- | --- | --- | --- | --- | --- | --- | --- | --- | --- | --- | --- | --- | --- | --- | --- | --- | --- | --- | --- | --- | --- | --- | --- | --- | --- | --- | --- | --- | --- | --- | --- | --- | --- | --- | --- | --- | --- | --- | --- | --- | --- | --- | --- | --- | --- | --- | --- | --- | --- | --- | --- | --- | --- | --- | --- | --- | --- | --- | --- | --- | --- | --- | --- | --- | --- | --- | --- | --- | --- | --- | --- | --- | --- | --- | --- | --- | --- | --- | --- | --- | --- | --- | --- | --- | --- | --- | --- | --- | --- | --- | --- | --- | --- | --- | --- | --- | --- | --- | --- | --- | --- | --- | --- | --- | --- | --- | --- | --- | --- | --- | --- | --- | --- | --- | --- | --- | --- | --- | --- | --- | --- | --- | --- | --- | --- | --- | --- | --- | --- | --- | --- | --- | --- | --- | --- | --- | --- | --- | --- | --- | --- | --- | --- | --- | --- | --- | --- | --- | --- | --- | --- | --- | --- | --- | --- | --- | --- | --- | --- | --- | --- | --- | --- | --- | --- | --- | --- | --- | --- | --- | --- | --- | --- | --- | --- | --- | --- | --- | --- | --- | --- | --- | --- | --- | --- | --- | --- | --- | --- | --- | --- | --- | --- | --- | --- | --- | --- | --- | --- | --- | --- | --- | --- | --- | --- | --- | --- | --- | --- | --- | --- | --- | --- | --- | --- | --- | --- | --- | --- | --- | --- | --- | --- | --- | --- | --- | --- | --- | --- | --- | --- | --- | --- | --- | --- | --- | --- | --- | --- | --- | --- | --- | --- | --- | --- | --- | --- | --- | --- | --- | --- | --- | --- | --- | --- | --- | --- | --- | --- | --- | --- | --- | --- | --- | --- | --- | --- | --- | --- | --- | --- | --- | --- | --- | --- | --- | --- | --- | --- | --- | --- | --- | --- | --- | --- | --- | --- | --- | --- | --- | --- | --- | --- | --- | --- | --- | --- | --- | --- | --- | --- | --- | --- | --- | --- | --- | --- | --- | --- | --- | --- | --- | --- | --- | --- | --- | --- | --- | --- | --- | --- | --- | --- | --- | --- | --- | --- | --- | --- | --- | --- | --- | --- | --- | --- | --- | --- | --- | --- | --- | --- | --- | --- | --- | --- | --- | --- | --- | --- | --- | --- | --- | --- | --- | --- | --- | --- | --- | --- | --- | --- | --- | --- | --- | --- | --- | --- | --- | --- | --- | --- | --- | --- | --- | --- | --- | --- | --- | --- | --- | --- | --- | --- | --- | --- | --- | --- | --- | --- | --- | --- | --- | --- | --- | --- | --- | --- | --- | --- | --- | --- | --- | --- | --- | --- | --- | --- | --- | --- | --- | --- | --- | --- | --- | --- | --- | --- | --- | --- | --- | --- | --- | --- | --- | --- | --- | --- | --- | --- | --- | --- | --- | --- | --- | --- | --- | --- | --- | --- | --- | --- | --- | --- | --- | --- | --- | --- | --- | --- | --- | --- | --- | --- | --- | --- | --- | --- | --- | --- | --- | --- | --- | --- | --- | --- | --- | --- | --- | --- | --- | --- | --- | --- | --- | --- | --- | --- | --- | --- | --- | --- | --- | --- | --- | --- | --- | --- | --- | --- | --- | --- | --- | --- | --- | --- | --- | --- | --- | --- | --- | --- | --- | --- | --- | --- | --- | --- | --- | --- | --- | --- | --- | --- | --- | --- | --- | --- | --- | --- | --- | --- | --- | --- | --- | --- | --- | --- | --- | --- | --- | --- | --- | --- | --- | --- | --- | --- | --- | --- | --- | --- | --- | --- | --- | --- | --- | --- | --- | --- | --- | --- | --- | --- | --- | --- | --- | --- | --- | --- | --- | --- | --- | --- | --- | --- | --- | --- | --- | --- | --- | --- | --- | --- | --- | --- | --- | --- | --- | --- | --- | --- | --- | --- | --- | --- | --- | --- | --- | --- | --- | --- | --- | --- | --- | --- | --- | --- | --- | --- | --- | --- | --- | --- | --- | --- | --- | --- | --- | --- | --- | --- | --- | --- | --- | --- | --- | --- | --- | --- | --- | --- | --- | --- | --- | --- | --- | --- | --- | --- | --- | --- | --- | --- | --- | --- | --- | --- | --- | --- | --- | --- | --- | --- | --- | --- | --- | --- | --- | --- | --- | --- | --- | --- | --- | --- | --- | --- | --- | --- | --- | --- | --- | --- | --- | --- | --- | --- | --- | --- | --- | --- | --- | --- | --- | --- | --- | --- | --- | --- | --- | --- | --- | --- | --- | --- | --- | --- | --- | --- | --- | --- | --- | --- | --- | --- | --- | --- | --- | --- | --- | --- | --- | --- | --- | --- | --- | --- | --- | --- | --- | --- | --- | --- | --- | --- | --- | --- | --- | --- | --- | --- | --- | --- | --- | --- | --- | --- | --- | --- | --- | --- | --- | --- | --- | --- | --- | --- | --- | --- | --- | --- | --- | --- | --- | --- | --- | --- | --- | --- | --- | --- | --- | --- | --- | --- | --- | --- | --- | --- | --- | --- | --- | --- | --- | --- | --- | --- | --- | --- | --- | --- | --- | --- | --- | --- | --- | --- | --- | --- | --- | --- | --- | --- | --- | --- | --- | --- | --- | --- | --- | --- | --- | --- | --- | --- | --- | --- | --- | --- | --- | --- | --- | --- | --- | --- | --- | --- | --- | --- | --- | --- | --- | --- | --- | --- | --- | --- | --- | --- | --- | --- | --- | --- | --- | --- | --- | --- | --- | --- | --- | --- | --- | --- | --- | --- | --- | --- | --- | --- | --- | --- | --- | --- | --- | --- | --- | --- | --- | --- | --- | --- | --- | --- | --- | --- | --- | --- | --- | --- | --- | --- | --- | --- | --- | --- | --- | --- | --- | --- | --- | --- | --- | --- | --- | --- | --- | --- | --- | --- | --- | --- | --- | --- | --- | --- | --- | --- | --- | --- | --- | --- | --- | --- | --- | --- | --- | --- | --- | --- | --- | --- | --- | --- | --- | --- | --- | --- | --- | --- | --- | --- | --- | --- | --- | --- | --- | --- | --- | --- | --- | --- | --- | --- | --- | --- | --- | --- | --- | --- | --- | --- | --- | --- | --- | --- | --- | --- | --- | --- | --- | --- | --- | --- | --- | --- | --- | --- | --- | --- | --- | --- | --- | --- | --- | --- | --- | --- | --- | --- | --- | --- | --- | --- | --- | --- | --- | --- | --- | --- | --- | --- | --- | --- | --- | --- | --- | --- | --- | --- | --- | --- | --- | --- | --- | --- | --- | --- | --- | --- | --- | --- | --- | --- | --- | --- | --- | --- | --- | --- | --- | --- | --- | --- | --- | --- | --- | --- | --- | --- | --- | --- | --- | --- | --- | --- | --- | --- | --- | --- | --- | --- | --- | --- | --- | --- | --- | --- | --- | --- | --- | --- | --- | --- | --- | --- | --- | --- | --- | --- | --- | --- | --- | --- | --- | --- | --- | --- | --- | --- | --- | --- | --- | --- | --- | --- | --- | --- | --- | --- | --- | --- | --- | --- | --- | --- | --- | --- | --- | --- | --- | --- | --- | --- | --- | --- | --- | --- | --- | --- | --- | --- | --- | --- | --- | --- | --- | --- | --- | --- | --- | --- | --- | --- | --- | --- | --- | --- | --- | --- | --- | --- | --- | --- | --- | --- | --- | --- | --- | --- | --- | --- | --- | --- | --- | --- | --- | --- | --- | --- | --- | --- | --- | --- | --- | --- | --- | --- | --- | --- | --- | --- | --- | --- | --- | --- | --- | --- | --- | --- | --- | --- | --- | --- | --- | --- | --- | --- | --- | --- | --- | --- | --- | --- | --- | --- | --- | --- | --- | --- | --- | --- | --- | --- | --- | --- | --- | --- | --- | --- | --- | --- | --- | --- | --- | --- | --- | --- | --- | --- | --- | --- | --- | --- | --- | --- | --- | --- | --- | --- | --- | --- | --- | --- | --- | --- | --- | --- | --- | --- | --- | --- | --- | --- | --- | --- | --- | --- | --- | --- | --- | --- | --- | --- | --- | --- | --- | --- | --- | --- | --- | --- | --- | --- | --- | --- | --- | --- | --- | --- | --- | --- | --- | --- | --- | --- | --- | --- | --- | --- | --- | --- | --- | --- | --- | --- | --- | --- | --- | --- | --- | --- | --- | --- | --- | --- | --- | --- | --- | --- | --- | --- | --- | --- | --- | --- | --- | --- | --- | --- | --- | --- | --- | --- | --- | --- | --- | --- | --- | --- | --- | --- | --- | --- | --- | --- | --- | --- | --- | --- | --- | --- | --- | --- | --- | --- | --- | --- | --- | --- | --- | --- | --- | --- | --- | --- | --- | --- | --- | --- | --- | --- | --- | --- | --- | --- | --- | --- | --- | --- | --- | --- | --- | --- | --- | --- | --- | --- | --- | --- | --- | --- | --- | --- | --- | --- | --- | --- | --- | --- | --- | --- | --- | --- | --- | --- | --- | --- | --- | --- | --- | --- | --- | --- | --- | --- | --- | --- | --- | --- | --- | --- | --- | --- | --- | --- | --- | --- | --- | --- | --- | --- | --- | --- | --- | --- | --- | --- | --- | --- | --- | --- | --- | --- | --- | --- | --- | --- | --- | --- | --- | --- | --- | --- | --- | --- | --- | --- | --- | --- | --- | --- | --- | --- | --- | --- | --- | --- | --- | --- | --- | --- | --- | --- | --- | --- | --- | --- | --- | --- | --- | --- | --- | --- | --- | --- | --- | --- | --- | --- | --- | --- | --- | --- | --- | --- | --- | --- | --- | --- | --- | --- | --- | --- | --- | --- | --- | --- | --- | --- | --- | --- | --- | --- | --- | --- | --- | --- | --- | --- | --- | --- | --- | --- | --- | --- | --- | --- | --- | --- | --- | --- | --- | --- | --- | --- | --- | --- | --- | --- | --- | --- | --- | --- | --- | --- | --- | --- | --- | --- | --- | --- | --- | --- | --- | --- | --- | --- | --- | --- | --- | --- | --- | --- | --- | --- | --- | --- | --- | --- | --- | --- | --- | --- | --- | --- | --- | --- | --- | --- | --- | --- | --- | --- | --- | --- | --- | --- | --- | --- | --- | --- | --- | --- | --- | --- | --- | --- | --- | --- | --- | --- | --- | --- | --- | --- | --- | --- | --- | --- | --- | --- | --- | --- | --- | --- | --- | --- | --- | --- | --- | --- | --- | --- | --- | --- | --- | --- | --- | --- | --- | --- | --- | --- | --- | --- | --- | --- | --- | --- | --- | --- | --- | --- | --- | --- | --- | --- | --- | --- | --- | --- | --- | --- | --- | --- | --- | --- | --- | --- | --- | --- | --- | --- | --- | --- | --- | --- | --- | --- | --- | --- | --- | --- | --- | --- | --- | --- | --- | --- | --- | --- | --- | --- | --- | --- | --- | --- | --- | --- | --- | --- | --- | --- | --- | --- | --- | --- | --- | --- | --- | --- | --- | --- | --- | --- | --- | --- | --- | --- | --- | --- | --- | --- | --- | --- | --- | --- | --- | --- | --- | --- | --- | --- | --- | --- | --- | --- | --- | --- | --- | --- | --- | --- | --- | --- | --- | --- | --- | --- | --- | --- | --- | --- | --- | --- | --- | --- | --- | --- | --- | --- | --- | --- | --- | --- | --- | --- | --- | --- | --- | --- | --- | --- | --- | --- | --- | --- | --- | --- | --- | --- | --- | --- | --- | --- | --- | --- | --- | --- | --- | --- | --- | --- | --- | --- | --- | --- | --- | --- | --- | --- | --- | --- | --- | --- | --- | --- | --- | --- | --- | --- | --- | --- | --- | --- | --- | --- | --- | --- | --- | --- | --- | --- | --- | --- | --- | --- | --- | --- | --- | --- | --- | --- | --- | --- | --- | --- | --- | --- | --- | --- | --- | --- | --- | --- | --- | --- | --- | --- | --- | --- | --- | --- | --- | --- | --- | --- | --- | --- | --- | --- | --- | --- | --- | --- | --- | --- | --- | --- | --- | --- | --- | --- | --- | --- | --- | --- | --- | --- | --- | --- | --- | --- | --- | --- | --- | --- | --- | --- | --- | --- | --- | --- | --- | --- | --- | --- | --- | --- | --- | --- | --- | --- | --- | --- | --- | --- | --- | --- | --- | --- | --- | --- | --- | --- | --- | --- | --- | --- | --- | --- | --- | --- | --- | --- | --- | --- | --- | --- | --- | --- | --- | --- | --- | --- | --- | --- | --- | --- | --- | --- | --- | --- | --- | --- | --- | --- | --- | --- | --- | --- | --- | --- | --- | --- | --- | --- | --- | --- | --- | --- | --- | --- | --- | --- | --- | --- | --- | --- | --- | --- | --- | --- | --- | --- | --- | --- | --- | --- | --- | --- | --- | --- | --- | --- | --- | --- | --- | --- | --- | --- | --- | --- | --- | --- | --- | --- | --- | --- | --- | --- | --- | --- | --- | --- | --- | --- | --- | --- | --- | --- | --- | --- | --- | --- | --- | --- | --- | --- | --- | --- | --- | --- | --- | --- | --- | --- | --- | --- | --- | --- | --- | --- | --- | --- | --- | --- | --- | --- | --- | --- | --- | --- | --- | --- | --- | --- | --- | --- | --- | --- | --- | --- | --- | --- | --- | --- | --- | --- | --- | --- | --- | --- | --- | --- | --- | --- | --- | --- | --- | --- | --- | --- | --- | --- | --- | --- | --- | --- | --- | --- | --- | --- | --- | --- | --- | --- | --- | --- | --- | --- | --- | --- | --- | --- | --- | --- | --- | --- | --- | --- | --- | --- | --- | --- | --- | --- | --- | --- | --- | --- | --- | --- | --- | --- | --- | --- | --- | --- | --- | --- | --- | --- | --- | --- | --- | --- | --- | --- | --- | --- | --- | --- | --- | --- | --- | --- | --- | --- | --- | --- | --- | --- | --- | --- | --- | --- | --- | --- | --- | --- | --- | --- | --- | --- | --- | --- | --- | --- | --- | --- | --- | --- | --- | --- | --- | --- | --- | --- | --- | --- | --- | --- | --- | --- | --- | --- | --- | --- | --- | --- | --- | --- | --- | --- | --- | --- | --- | --- | --- | --- | --- | --- | --- | --- | --- | --- | --- | --- | --- | --- | --- | --- | --- | --- | --- | --- | --- | --- | --- | --- | --- | --- | --- | --- | --- | --- | --- | --- | --- | --- | --- | --- | --- | --- | --- | --- | --- | --- | --- | --- | --- | --- | --- | --- | --- | --- | --- | --- | --- | --- | --- | --- | --- | --- | --- | --- | --- | --- | --- | --- | --- | --- | --- | --- | --- | --- | --- | --- | --- | --- | --- | --- | --- | --- | --- | --- | --- | --- | --- | --- | --- | --- | --- | --- | --- | --- | --- | --- | --- | --- | --- | --- | --- | --- | --- | --- | --- | --- | --- | --- | --- | --- | --- | --- | --- | --- | --- | --- | --- | --- | --- | --- | --- | --- | --- | --- | --- | --- | --- | --- | --- | --- | --- | --- | --- | --- | --- | --- | --- | --- | --- | --- | --- | --- | --- | --- | --- | --- | --- | --- | --- | --- | --- | --- | --- | --- | --- | --- | --- | --- | --- | --- | --- | --- | --- | --- | --- | --- | --- | --- | --- | --- | --- | --- | --- | --- | --- | --- | --- | --- | --- | --- | --- | --- | --- | --- | --- | --- | --- | --- | --- | --- | --- | --- | --- | --- | --- | --- | --- | --- | --- | --- | --- | --- | --- | --- | --- | --- | --- | --- | --- | --- | --- | --- | --- | --- | --- | --- | --- | --- | --- | --- | --- | --- | --- | --- | --- | --- | --- | --- | --- | --- | --- | --- | --- | --- | --- | --- | --- | --- | --- | --- | --- | --- | --- | --- | --- | --- | --- | --- | --- | --- | --- | --- | --- | --- | --- | --- | --- | --- | --- | --- | --- | --- | --- | --- | --- | --- | --- | --- | --- | --- | --- | --- | --- | --- | --- | --- | --- | --- | --- | --- | --- | --- | --- | --- | --- | --- | --- | --- | --- | --- | --- | --- | --- | --- | --- | --- | --- | --- | --- | --- | --- | --- | --- | --- | --- | --- | --- | --- | --- | --- | --- | --- | --- | --- | --- | --- | --- | --- | --- | --- | --- | --- | --- | --- | --- | --- | --- | --- | --- | --- | --- | --- | --- | --- | --- | --- | --- | --- | --- | --- | --- | --- | --- | --- | --- | --- | --- | --- | --- | --- | --- | --- | --- | --- | --- | --- | --- | --- | --- | --- | --- | --- | --- | --- | --- | --- | --- | --- | --- | --- | --- | --- | --- | --- | --- | --- | --- | --- | --- | --- | --- | --- | --- | --- | --- | --- | --- | --- | --- | --- | --- | --- | --- | --- | --- | --- | --- | --- | --- | --- | --- | --- | --- | --- | --- | --- | --- | --- | --- | --- | --- | --- | --- | --- | --- | --- | --- | --- | --- | --- | --- | --- | --- | --- | --- | --- | --- | --- | --- | --- | --- | --- | --- | --- | --- | --- | --- | --- | --- | --- | --- | --- | --- | --- | --- | --- | --- | --- | --- | --- | --- | --- | --- | --- | --- | --- | --- | --- | --- | --- | --- | --- | --- | --- | --- | --- | --- | --- | --- | --- | --- | --- | --- | --- | --- | --- | --- | --- | --- | --- | --- | --- | --- | --- | --- | --- | --- | --- | --- | --- | --- | --- | --- | --- | --- | --- | --- | --- | --- | --- | --- | --- | --- | --- | --- | --- | --- | --- | --- | --- | --- | --- | --- | --- | --- | --- | --- | --- | --- | --- | --- | --- | --- | --- | --- | --- | --- | --- | --- | --- | --- | --- | --- | --- | --- | --- | --- | --- | --- | --- | --- | --- | --- | --- | --- | --- | --- | --- | --- | --- | --- | --- | --- | --- | --- | --- | --- | --- | --- | --- | --- | --- | --- | --- | --- | --- | --- | --- | --- | --- | --- | --- | --- | --- | --- | --- | --- | --- | --- | --- | --- | --- | --- | --- | --- | --- | --- | --- | --- | --- | --- | --- | --- | --- | --- | --- | --- | --- | --- | --- | --- | --- | --- | --- | --- | --- | --- | --- | --- | --- | --- | --- | --- | --- | --- | --- | --- | --- | --- | --- | --- | --- | --- | --- | --- | --- | --- | --- | --- | --- | --- | --- | --- | --- | --- | --- | --- | --- | --- | --- | --- | --- | --- | --- | --- | --- | --- | --- | --- | --- | --- | --- | --- | --- | --- | --- | --- | --- | --- | --- | --- | --- | --- | --- | --- | --- | --- | --- | --- | --- | --- | --- | --- | --- | --- | --- | --- | --- | --- | --- | --- | --- | --- | --- | --- | --- | --- | --- | --- | --- | --- | --- | --- | --- | --- | --- | --- | --- | --- | --- | --- | --- | --- | --- | --- | --- | --- | --- | --- | --- | --- | --- | --- | --- | --- | --- | --- | --- | --- | --- | --- | --- | --- | --- | --- | --- | --- | --- | --- | --- | --- | --- | --- | --- | --- | --- | --- | --- | --- | --- | --- | --- | --- | --- | --- | --- | --- | --- | --- | --- | --- | --- | --- | --- | --- | --- | --- | --- | --- | --- | --- | --- | --- | --- | --- | --- | --- | --- | --- | --- | --- | --- | --- | --- | --- | --- | --- | --- | --- | --- | --- | --- | --- | --- | --- | --- | --- | --- | --- | --- | --- | --- | --- | --- | --- | --- | --- | --- | --- | --- | --- | --- | --- | --- | --- | --- | --- | --- | --- | --- | --- | --- | --- | --- | --- | --- | --- | --- | --- | --- | --- | --- | --- | --- | --- | --- | --- | --- | --- | --- | --- | --- | --- | --- | --- | --- | --- | --- | --- | --- | --- | --- | --- | --- | --- | --- | --- | --- | --- | --- | --- | --- | --- | --- | --- | --- | --- | --- | --- | --- | --- | --- | --- | --- | --- | --- | --- | --- | --- | --- | --- | --- | --- | --- | --- | --- | --- | --- | --- | --- | --- | --- | --- | --- | --- | --- | --- | --- | --- | --- | --- | --- | --- | --- | --- | --- | --- | --- | --- | --- | --- | --- | --- | --- | --- | --- | --- | --- | --- | --- | --- | --- | --- | --- | --- | --- | --- | --- | --- | --- | --- | --- | --- | --- | --- | --- | --- | --- | --- | --- | --- | --- | --- | --- | --- | --- | --- | --- | --- | --- | --- | --- | --- | --- | --- | --- | --- | --- | --- | --- | --- | --- | --- | --- | --- | --- | --- | --- | --- | --- | --- | --- | --- | --- | --- | --- | --- | --- | --- | --- | --- | --- | --- | --- | --- | --- | --- | --- | --- | --- | --- | --- | --- | --- | --- | --- | --- | --- | --- | --- | --- | --- | --- | --- | --- | --- | --- | --- | --- | --- | --- | --- | --- | --- | --- | --- | --- | --- | --- | --- | --- | --- | --- | --- | --- | --- | --- | --- | --- | --- | --- | --- | --- | --- | --- | --- | --- | --- | --- | --- | --- | --- | --- | --- | --- | --- | --- | --- | --- | --- | --- | --- | --- | --- | --- | --- | --- | --- | --- | --- | --- | --- | --- | --- | --- | --- | --- | --- | --- | --- | --- | --- | --- | --- | --- | --- | --- | --- | --- | --- | --- | --- | --- | --- | --- | --- | --- | --- | --- | --- | --- | --- | --- | --- | --- | --- | --- | --- | --- | --- | --- | --- | --- | --- | --- | --- | --- | --- | --- | --- | --- | --- | --- | --- | --- | --- | --- | --- | --- | --- | --- | --- | --- | --- | --- | --- | --- | --- | --- | --- | --- | --- | --- | --- | --- | --- | --- | --- | --- | --- | --- | --- | --- | --- | --- | --- | --- | --- | --- | --- | --- | --- | --- | --- | --- | --- | --- | --- | --- | --- | --- | --- | --- | --- | --- | --- | --- | --- | --- | --- | --- | --- | --- | --- | --- | --- | --- | --- | --- | --- | --- | --- | --- | --- | --- | --- | --- | --- | --- | --- | --- | --- | --- | --- | --- | --- | --- | --- | --- | --- | --- | --- | --- | --- | --- | --- | --- | --- | --- | --- | --- | --- | --- | --- | --- | --- | --- | --- | --- | --- | --- | --- | --- | --- | --- | --- | --- | --- | --- | --- | --- | --- | --- | --- | --- | --- | --- | --- | --- | --- | --- | --- | --- | --- | --- | --- | --- | --- | --- | --- | --- | --- | --- | --- | --- | --- | --- | --- | --- | --- | --- | --- | --- | --- | --- | --- | --- | --- | --- | --- | --- | --- | --- | --- | --- | --- | --- | --- | --- | --- | --- | --- | --- | --- | --- | --- | --- | --- | --- | --- | --- | --- | --- | --- | --- | --- | --- | --- | --- | --- | --- | --- | --- | --- | --- | --- | --- | --- | --- | --- | --- | --- | --- | --- | --- | --- | --- | --- | --- | --- | --- | --- | --- | --- | --- | --- | --- | --- | --- | --- | --- | --- | --- | --- | --- | --- | --- | --- | --- | --- | --- | --- | --- | --- | --- | --- | --- | --- | --- | --- | --- | --- | --- | --- | --- | --- | --- | --- | --- | --- | --- | --- | --- | --- | --- | --- | --- | --- | --- | --- | --- | --- | --- | --- | --- | --- | --- | --- | --- | --- | --- | --- | --- | --- | --- | --- | --- | --- | --- | --- | --- | --- | --- | --- | --- | --- | --- | --- | --- | --- | --- | --- | --- | --- | --- | --- | --- | --- | --- | --- | --- | --- | --- | --- | --- | --- | --- | --- | --- | --- | --- | --- | --- | --- | --- | --- | --- | --- | --- | --- | --- | --- | --- | --- | --- | --- | --- | --- | --- | --- | --- | --- | --- | --- | --- | --- | --- | --- | --- | --- | --- | --- | --- | --- | --- | --- | --- | --- | --- | --- | --- | --- | --- | --- | --- | --- | --- | --- | --- | --- | --- | --- | --- | --- | --- | --- | --- | --- | --- | --- | --- | --- | --- | --- | --- | --- | --- | --- | --- | --- | --- | --- | --- | --- | --- | --- | --- | --- | --- | --- | --- | --- | --- | --- | --- | --- | --- | --- | --- | --- | --- | --- | --- | --- | --- | --- | --- | --- | --- | --- | --- | --- | --- | --- | --- | --- | --- | --- | --- | --- | --- | --- | --- | --- | --- | --- | --- | --- | --- | --- | --- | --- | --- | --- | --- | --- | --- | --- | --- | --- | --- | --- | --- | --- | --- | --- | --- | --- | --- | --- | --- | --- | --- | --- | --- | --- | --- | --- | --- | --- | --- | --- | --- | --- | --- | --- | --- | --- | --- | --- | --- | --- | --- | --- | --- | --- | --- | --- | --- | --- | --- | --- | --- | --- | --- | --- | --- | --- | --- | --- | --- | --- | --- | --- | --- | --- | --- | --- | --- | --- | --- | --- | --- | --- | --- | --- | --- | --- | --- | --- | --- | --- | --- | --- | --- | --- | --- | --- | --- | --- | --- | --- | --- | --- | --- | --- | --- | --- | --- | --- | --- | --- | --- | --- | --- | --- | --- | --- | --- | --- | --- | --- | --- | --- | --- | --- | --- | --- | --- | --- | --- | --- | --- | --- | --- | --- | --- | --- | --- | --- | --- | --- | --- | --- | --- | --- | --- | --- | --- | --- | --- | --- | --- | --- | --- | --- | --- | --- | --- | --- | --- | --- | --- | --- | --- | --- | --- | --- | --- | --- | --- | --- | --- | --- | --- | --- | --- | --- | --- | --- | --- | --- | --- | --- | --- | --- | --- | --- | --- | --- | --- | --- | --- | --- | --- | --- | --- | --- | --- | --- | --- | --- | --- | --- | --- | --- | --- | --- | --- | --- | --- | --- | --- | --- | --- | --- | --- | --- | --- | --- | --- | --- | --- | --- | --- | --- | --- | --- | --- | --- | --- | --- | --- | --- | --- | --- | --- | --- | --- | --- | --- | --- | --- | --- | --- | --- | --- | --- | --- | --- | --- | --- | --- | --- | --- | --- | --- | --- | --- | --- | --- | --- | --- | --- | --- | --- | --- | --- | --- | --- | --- | --- | --- | --- | --- | --- | --- | --- | --- | --- | --- | --- | --- | --- | --- | --- | --- | --- | --- | --- | --- | --- | --- | --- | --- | --- | --- | --- | --- | --- | --- | --- | --- | --- | --- | --- | --- | --- | --- | --- | --- | --- | --- | --- | --- | --- | --- | --- | --- | --- | --- | --- | --- | --- | --- | --- | --- | --- | --- | --- | --- | --- | --- | --- | --- | --- | --- | --- | --- | --- | --- | --- | --- | --- | --- | --- | --- | --- | --- | --- | --- | --- | --- | --- | --- | --- | --- | --- | --- | --- | --- | --- | --- | --- | --- | --- | --- | --- | --- | --- | --- | --- | --- | --- | --- | --- | --- | --- | --- | --- | --- | --- | --- | --- | --- | --- | --- | --- | --- | --- | --- | --- | --- | --- | --- | --- | --- | --- | --- | --- | --- | --- | --- | --- | --- | --- | --- | --- | --- | --- | --- | --- | --- | --- | --- | --- | --- | --- | --- | --- | --- | --- | --- | --- | --- | --- | --- | --- | --- | --- | --- | --- | --- | --- | --- | --- | --- | --- | --- | --- | --- | --- | --- | --- | --- | --- | --- | --- | --- | --- | --- | --- | --- | --- | --- | --- | --- | --- | --- | --- | --- | --- | --- | --- | --- | --- | --- | --- | --- | --- | --- | --- | --- | --- | --- | --- | --- | --- | --- | --- | --- | --- | --- | --- | --- | --- | --- | --- | --- | --- | --- | --- | --- | --- | --- | --- | --- | --- | --- | --- | --- | --- | --- | --- | --- | --- | --- | --- | --- | --- | --- | --- | --- | --- | --- | --- | --- | --- | --- | --- | --- | --- | --- | --- | --- | --- | --- | --- | --- | --- | --- | --- | --- | --- | --- | --- | --- | --- | --- | --- | --- | --- | --- | --- | --- | --- | --- | --- | --- | --- | --- | --- | --- | --- | --- | --- | --- | --- | --- | --- | --- | --- | --- | --- | --- | --- | --- | --- | --- | --- | --- | --- | --- | --- | --- | --- | --- | --- | --- | --- | --- | --- | --- | --- | --- | --- | --- | --- | --- | --- | --- | --- | --- | --- | --- | --- | --- | --- | --- | --- | --- | --- | --- | --- | --- | --- | --- | --- | --- | --- | --- | --- | --- | --- | --- | --- | --- | --- | --- | --- | --- | --- | --- | --- | --- | --- | --- | --- | --- | --- | --- | --- | --- | --- | --- | --- | --- | --- | --- | --- | --- | --- | --- | --- | --- | --- | --- | --- | --- | --- | --- | --- | --- | --- | --- | --- | --- | --- | --- | --- | --- | --- | --- | --- | --- | --- | --- | --- | --- | --- | --- | --- | --- | --- | --- | --- | --- | --- | --- | --- | --- | --- | --- | --- | --- | --- | --- | --- | --- | --- | --- | --- | --- | --- | --- | --- | --- | --- | --- | --- | --- | --- | --- | --- | --- | --- | --- | --- | --- | --- | --- | --- | --- | --- | --- | --- | --- | --- | --- | --- | --- | --- | --- | --- | --- | --- | --- | --- | --- | --- | --- | --- | --- | --- | --- | --- | --- | --- | --- | --- | --- | --- | --- | --- | --- | --- | --- | --- | --- | --- | --- | --- | --- | --- | --- | --- | --- | --- | --- | --- | --- | --- | --- | --- | --- | --- | --- | --- | --- | --- | --- | --- | --- | --- | --- | --- | --- | --- | --- | --- | --- | --- | --- | --- | --- | --- | --- | --- | --- | --- | --- | --- | --- | --- | --- | --- | --- | --- | --- | --- | --- | --- | --- | --- | --- | --- | --- | --- | --- | --- | --- | --- | --- | --- | --- | --- | --- | --- | --- | --- | --- | --- | --- | --- | --- | --- | --- | --- | --- | --- | --- | --- | --- | --- | --- | --- | --- | --- | --- | --- | --- | --- | --- | --- | --- | --- | --- | --- | --- | --- | --- | --- | --- | --- | --- | --- | --- | --- | --- | --- | --- | --- | --- | --- | --- | --- | --- | --- | --- | --- | --- | --- | --- | --- | --- | --- | --- | --- | --- | --- | --- | --- | --- | --- | --- | --- | --- | --- | --- | --- | --- | --- | --- | --- | --- | --- | --- | --- | --- | --- | --- | --- | --- | --- | --- | --- | --- | --- | --- | --- | --- | --- | --- | --- | --- | --- | --- | --- | --- | --- | --- | --- | --- | --- | --- | --- | --- | --- | --- | --- | --- | --- | --- | --- | --- | --- | --- | --- | --- | --- | --- | --- | --- | --- | --- | --- | --- | --- | --- | --- | --- | --- | --- | --- | --- | --- | --- | --- | --- | --- | --- | --- | --- | --- | --- | --- | --- | --- | --- | --- | --- | --- | --- | --- | --- | --- | --- | --- | --- | --- | --- | --- | --- | --- | --- | --- | --- | --- | --- | --- | --- | --- | --- | --- | --- | --- | --- | --- | --- | --- | --- | --- | --- | --- | --- | --- | --- | --- | --- | --- | --- | --- | --- | --- | --- | --- | --- | --- | --- | --- | --- | --- | --- | --- | --- | --- | --- | --- | --- | --- | --- | --- | --- | --- | --- | --- | --- | --- | --- | --- | --- | --- | --- | --- | --- | --- | --- | --- | --- | --- | --- | --- | --- | --- | --- | --- | --- | --- | --- | --- | --- | --- | --- | --- | --- | --- | --- | --- | --- | --- | --- | --- | --- | --- | --- | --- | --- | --- | --- | --- | --- | --- | --- | --- | --- | --- | --- | --- | --- | --- | --- | --- | --- | --- | --- | --- | --- | --- | --- | --- | --- | --- | --- | --- | --- | --- | --- | --- | --- | --- | --- | --- | --- | --- | --- | --- | --- | --- | --- | --- | --- | --- | --- | --- | --- | --- | --- | --- | --- | --- | --- | --- | --- | --- | --- | --- | --- | --- | --- | --- | --- | --- | --- | --- | --- | --- | --- | --- | --- | --- | --- | --- | --- | --- | --- | --- | --- | --- | --- | --- | --- | --- | --- | --- | --- | --- | --- | --- | --- | --- | --- | --- | --- | --- | --- | --- | --- | --- | --- | --- | --- | --- | --- | --- | --- | --- | --- | --- | --- | --- | --- | --- | --- | --- | --- | --- | --- | --- | --- | --- | --- | --- | --- | --- | --- | --- | --- | --- | --- | --- | --- | --- | --- | --- | --- | --- | --- | --- | --- | --- | --- | --- | --- | --- | --- | --- | --- | --- | --- | --- | --- | --- | --- | --- | --- | --- | --- | --- | --- | --- | --- | --- | --- | --- | --- | --- | --- | --- | --- | --- | --- | --- | --- | --- | --- | --- | --- | --- | --- | --- | --- | --- | --- | --- | --- | --- | --- | --- | --- | --- | --- | --- | --- | --- | --- | --- | --- | --- | --- | --- | --- | --- | --- | --- | --- | --- | --- | --- | --- | --- | --- | --- | --- | --- | --- | --- | --- | --- | --- | --- | --- | --- | --- | --- | --- | --- | --- | --- | --- | --- | --- | --- | --- | --- | --- | --- | --- | --- | --- | --- | --- | --- | --- | --- | --- | --- | --- | --- | --- | --- | --- | --- | --- | --- | --- | --- | --- | --- | --- | --- | --- | --- | --- | --- | --- | --- | --- | --- | --- | --- | --- | --- | --- | --- | --- | --- | --- | --- | --- | --- | --- | --- | --- | --- | --- | --- | --- | --- | --- | --- | --- | --- | --- | --- | --- | --- | --- | --- | --- | --- |
| |  |  |  |  |  |  |  |  |  | | --- | --- | --- | --- | --- | --- | --- | --- | --- | | **Position** | **Reference** | **Sample** | **Quality** | **Type** | **Region** | **AA Exchange** | **PAM1** | **Known Variant** | | 1977 | A | G | 999.77 | SNP | intergenic |  |  | - | | 4013 | T | C | 1731.77 | SNP | Rv0003 (recF) | Ile245Thr | 11 | - | | 7362 | G | C | 939.77 | SNP | Rv0006 (gyrA) | Glu21Gln | 27 | - | | 7585 | G | C | 1532.77 | SNP | Rv0006 (gyrA) | Ser95Thr | 32 | genotype | | 9304 | G | A | 1221.77 | SNP | Rv0006 (gyrA) | Gly668Asp | 6 | - | | 11370 | C | T | 1475.77 | SNP | intergenic |  |  | - | | 11879 | A | G | 1027.77 | SNP | Rv0008c | Ser145Pro | 12 | - | | 14202 | G | C | 791.77 | SNP | Rv0012 | silent (Leu38) | 9947 | - | | 14785 | T | C | 1845.77 | SNP | Rv0012 | Cys233Arg | 1 | - | | 21795 | G | A | 213.84 | SNP | Rv0018c (pstP) | Pro463Ser | 17 | - | | 22453 | G | A | 1346.77 | SNP | Rv0018c (pstP) | silent (Tyr243) | 9945 | - | | 24698 | GCCGCGTTGCTCGGGGTAA | G | 4361.73 | DEL | Rv0020c (fhaA) |  |  | - | | 26959 | C | G | 855.77 | SNP | intergenic |  |  | - | | 29482 | CA | C | 1632.73 | DEL | Rv0025 |  |  | - | | 30519 | C | T | 1078.77 | SNP | Rv0026 | silent (Gly266) | 9935 | - | | 30688 | T | G | 988.77 | SNP | Rv0026 | Ser323Ala | 35 | - | | 30943 | C | T | 819.77 | SNP | Rv0026 | Pro408Ser | 17 | - | | 31077 | C | T | 1740.77 | SNP | intergenic |  |  | - | | 32349 | CCCGCGCGTCGGCGATGCGT CGCGTCGAGTCGGCGATG | C | 14038.73 | DEL | Rv0029 |  |  | - | | 34044 | T | C | 1309.77 | SNP | intergenic |  |  | - | | 37031 | C | G | 1074.77 | SNP | Rv0034 | silent (Ala55) | 9867 | - | | 42967 | G | C | 1197.77 | SNP | Rv0040c (mtc28) | silent (Pro133) | 9926 | - | | 50557 | T | C | 1149.77 | SNP | Rv0046c (ino1) | Arg190Gly | 1 | - | | 51750 | C | T | 1863.77 | SNP | intergenic |  |  | - | | 51949 | A | G | 1547.77 | SNP | Rv0048c | Val250Ala | 18 | - | | 51954 | T | A | 1472.77 | SNP | Rv0048c | Glu248Asp | 53 | - | | 53785 | C | G | 1296.77 | SNP | Rv0050 (ponA1) | Ile41Met(s) | 6 | - | | 54394 | A | G | 1164.77 | SNP | Rv0050 (ponA1) | silent (Ala244) | 9867 | - | | 55553 | C | CCGCCGT | 1405.73 | INS | Rv0050 (ponA1) |  |  | - | | 57393 | A | T | 730.77 | SNP | intergenic |  |  | - | | 62049 | A | G | 797.77 | SNP | Rv0058 (dnaB) | Arg552Gly | 1 | - | | 62657 | G | A | 1112.77 | SNP | Rv0058 (dnaB) | silent (Pro754) | 9926 | genotype | | 66249 | T | G | 580.77 | SNP | Rv0062 (celA1) | Leu233Arg | 1 | - | | 66285 | C | G | 613.77 | SNP | Rv0062 (celA1) | Ala245Gly | 21 | - | | 69871 | C | T | 1624.77 | SNP | Rv0064 | Leu418Phe | 6 | - | | 69989 | G | A | 1440.77 | SNP | Rv0064 | Gly457Asp | 6 | - | | 70816 | A | G | 786.77 | SNP | Rv0064 | Asn733Asp | 42 | - | | 71336 | G | C | 210.84 | SNP | Rv0064 | Arg906Pro | 5 | - | | 71584 | C | CCGAGCGCTGTTCTGGCGCT AATCTGACGCTAGAATAG | 12026.73 | INS | intergenic |  |  | - | | 74390 | G | A | 1348.77 | SNP | Rv0066c (icd2) | Ala41Val | 13 | - | | 75264 | C | T | 1425.77 | SNP | intergenic |  |  | - | | 75940 | G | C | 985.77 | SNP | Rv0068 | Val(s)214Leu | 3 | - | | 79504 | T | TCGGTGGACCCGGTGGACC | 3805.73 | INS | Rv0071 |  |  | - | | 80616 | C | G | 1330.77 | SNP | intergenic |  |  | - | | 83982 | A | G | 1135.77 | SNP | Rv0074 | silent (STOP412) | 9867 | - | | 92199 | T | G | 1662.77 | SNP | Rv0083 | silent (Thr600) | 9871 | - | | 95591 | G | T | 1795.77 | SNP | Rv0087 (hycE) | Glu60STOP | 17 | - | | 97388 | G | T | 1263.77 | SNP | Rv0088 | Trp154Cys | 0 | - | | 103836 | G | T | 219.80 | SNP | Rv0094c | Asn276Lys | 25 | - | | 104712 | C | T | 405.77 | SNP | intergenic |  |  | - | | 104838 | T | G | 772.77 | SNP | Rv0095c | Glu126Asp | 53 | - | | 104962 | G | A | 951.77 | SNP | Rv0095c | Ala85Val(s) | 9867 | - | | 105045 | G | C | 994.77 | SNP | Rv0095c | Asp57Glu | 56 | - | | 105060 | G | A | 966.77 | SNP | Rv0095c | silent (Asp52) | 9859 | - | | 105063 | G | A | 954.77 | SNP | Rv0095c | silent (Phe51) | 9946 | - | | 112247 | A | G | 873.77 | SNP | Rv0101 (nrp) | silent (Ala749) | 9867 | - | | 116000 | T | G | 1472.77 | SNP | Rv0101 (nrp) | Val2000Val(s) | 18 | - | | 122109 | A | G | 1720.77 | SNP | Rv0103c (ctpB) | Leu(s)22Ser | 28 | - | | 125830 | G | GA | 1848.73 | INS | Rv0107c (ctpI) |  |  | - | | 131174 | T | TG | 1569.73 | INS | intergenic |  |  | - | | 132417 | C | G | 47.74 | SNP | Rv0109 (PE\_PGRS1) | Arg346Gly | 1 | - | | 133839 | C | T | 1037.77 | SNP | intergenic |  |  | - | | 137850 | GT | G | 1908.73 | DEL | Rv0113 (gmhA) |  |  | - | | 144390 | G | A | 869.77 | SNP | Rv0119 (fadD7) | silent (Ala114) | 9867 | - | | 146087 | T | C | 1513.77 | SNP | Rv0120c (fusA2) | Asn562Ser | 34 | - | | 147985 | C | T | 1408.77 | SNP | Rv0121c | Ala120Thr | 22 | - | | 150898 | C | G | 31.77 | SNP | Rv0124 (PE\_PGRS2) | Pro456Ala | 22 | - | | 154283 | T | C | 1379.77 | SNP | Rv0127 (mak) | Ser18Pro | 12 | - | | 155293 | G | A | 615.77 | SNP | Rv0127 (mak) | silent (Pro354) | 9926 | - | | 163573 | A | C | 1201.77 | SNP | Rv0136 (cyp138) | silent (Arg70) | 9913 | - | | 174684 | C | A | 861.77 | SNP | Rv0147 | silent (Arg483) | 9913 | - | | 177596 | T | C | 1355.77 | SNP | Rv0151c (PE1) | Ile572Val | 57 | - | | 177857 | G | A | 882.77 | SNP | Rv0151c (PE1) | Leu485Leu(s) | 4 | - | | 178946 | C | T | 1541.77 | SNP | Rv0151c (PE1) | Ala122Thr | 22 | - | | 187738 | G | A | 1779.77 | SNP | Rv0159c (PE3) | Arg368Trp | 2 | - | | 188800 | T | C | 870.77 | SNP | Rv0159c (PE3) | Thr14Ala | 32 | - | | 194681 | G | C | 835.77 | SNP | Rv0165c (mce1R) | silent (Leu45) | 9947 | - | | 196642 | C | T | 1694.77 | SNP | Rv0166 (fadD5) | silent (Asn550) | 9822 | - | | 200332 | C | A | 978.77 | SNP | Rv0170 (mce1B) | Phe146Leu | 13 | - | | 206339 | T | C | 1197.77 | SNP | Rv0174 (mce1F) | Leu370Pro | 2 | - | | 216586 | A | G | 657.77 | SNP | Rv0186 (bglS) | silent (Gln106) | 9876 | - | | 220050 | C | T | 1388.77 | SNP | Rv0189c (ilvD) | silent (Leu558) | 9947 | - | | 223942 | T | C | 553.77 | SNP | Rv0192 | Ser127Pro | 12 | - | | 225323 | T | C | 1513.77 | SNP | Rv0193c | Lys417Glu | 4 | - | | 227098 | T | C | 2041.77 | SNP | Rv0194 | Met(s)74Thr | 22 | - | | 231114 | C | G | 1253.77 | SNP | Rv0195 | silent (Ala72) | 9867 | - | | 232574 | G | T | 698.77 | SNP | Rv0197 | Gly115Val | 3 | - | | 234477 | T | G | 1173.77 | SNP | Rv0197 | Tyr749STOP | 2 | - | | 234496 | C | CGT | 2700.73 | INS | Rv0197 |  |  | - | | 251468 | G | T | 1049.77 | SNP | Rv0210 | Arg450Leu | 1 | - | | 261869 | T | C | 652.77 | SNP | Rv0218 | Cys316Arg | 1 | - | | 265554 | A | C | 1281.77 | SNP | Rv0222 (echA1) | silent (Val16) | 9901 | - | | 265968 | C | G | 889.77 | SNP | Rv0222 (echA1) | silent (Arg154) | 9913 | - | | 278681 | C | G | 999.77 | SNP | Rv0233 (nrdB) | His33Asp | 4 | - | | 283614 | T | C | 753.77 | SNP | Rv0236c (aftD) | Ser1080Gly | 21 | - | | 284623 | G | A | 863.77 | SNP | Rv0236c (aftD) | silent (Thr743) | 9871 | - | | 285772 | A | C | 744.77 | SNP | Rv0236c (aftD) | silent (Pro360) | 9926 | - | | 285871 | A | G | 807.77 | SNP | Rv0236c (aftD) | silent (Val327) | 9901 | - | | 293628 | A | AC | 810.73 | INS | intergenic |  |  | - | | 302200 | C | T | 1303.77 | SNP | Rv0251c (hsp) | silent (Gln151) | 9876 | - | | 304679 | G | T | 1241.77 | SNP | Rv0252 (nirB) | Gly605Val | 3 | - | | 304923 | A | G | 1146.77 | SNP | Rv0252 (nirB) | silent (Lys686) | 9926 | - | | 310973 | G | A | 1388.77 | SNP | Rv0259c | Ala182Val(s) | 9867 | - | | 311613 | G | T | 1017.77 | SNP | Rv0260c | silent (Val349) | 9901 | - | | 320038 | G | A | 1126.77 | SNP | Rv0266c (oplA) | Pro373Ser | 17 | - | | 328641 | G | T | 1383.77 | SNP | Rv0272c | silent (Thr356) | 9871 | - | | 333786 | G | C | 40.77 | SNP | Rv0278c (PE\_PGRS3) | Ala842Gly | 21 | - | | 333789 | G | T | 39.77 | SNP | Rv0278c (PE\_PGRS3) | Ser841Tyr | 1 | - | | 333892 | G | C | 319.77 | SNP | Rv0278c (PE\_PGRS3) | Arg807Gly | 1 | - | | 335810 | CCCGCCGGCGCCGCCGTTG | C | 1737.74 | DEL | Rv0278c (PE\_PGRS3) |  |  | - | | 335971 | A | G | 147.77 | SNP | Rv0278c (PE\_PGRS3) | Leu(s)114Leu | 3 | - | | 336005 | G | A | 321.77 | SNP | Rv0278c (PE\_PGRS3) | silent (Ile102) | 9872 | - | | 336047 | C | G | 271.77 | SNP | Rv0278c (PE\_PGRS3) | silent (Ala88) | 9867 | - | | 336050 | A | G | 268.77 | SNP | Rv0278c (PE\_PGRS3) | silent (Tyr87) | 9945 | - | | 336053 | G | C | 246.77 | SNP | Rv0278c (PE\_PGRS3) | silent (Ala86) | 9867 | - | | 336074 | T | C | 379.77 | SNP | Rv0278c (PE\_PGRS3) | silent (Ala79) | 9867 | - | | 336081 | A | G | 360.77 | SNP | Rv0278c (PE\_PGRS3) | Val(s)77Ala | 9867 | - | | 336082 | C | T | 348.77 | SNP | Rv0278c (PE\_PGRS3) | Val(s)77Met(s) | 9867 | - | | 336191 | C | T | 1098.77 | SNP | Rv0278c (PE\_PGRS3) | Met(s)40Ile | 2 | - | | 336380 | A | T | 156.77 | SNP | intergenic |  |  | - | | 336400 | C | G | 130.77 | SNP | intergenic |  |  | - | | 336403 | C | G | 107.77 | SNP | intergenic |  |  | - | | 336405 | A | G | 128.77 | SNP | intergenic |  |  | - | | 336504 | G | T | 488.77 | SNP | intergenic |  |  | - | | 336535 | T | G | 318.77 | SNP | intergenic |  |  | - | | 336537 | T | G | 282.77 | SNP | intergenic |  |  | - | | 336540 | G | T | 260.77 | SNP | intergenic |  |  | - | | 336546 | T | G | 264.77 | SNP | intergenic |  |  | - | | 336557 | C | CT | 368.73 | INS | intergenic |  |  | - | | 336560 | T | C | 157.77 | SNP | Rv0279c (PE\_PGRS4) | silent (STOP838) | 9867 | - | | 336562 | A | ATGG | 564.73 | INS | Rv0279c (PE\_PGRS4) |  |  | - | | 336590 | G | C | 266.77 | SNP | Rv0279c (PE\_PGRS4) | Ile828Met(s) | 6 | - | | 336592 | T | G | 268.77 | SNP | Rv0279c (PE\_PGRS4) | Ile828Leu | 22 | - | | 336611 | G | C | 364.77 | SNP | Rv0279c (PE\_PGRS4) | silent (Ala821) | 9867 | - | | 336617 | G | C | 289.77 | SNP | Rv0279c (PE\_PGRS4) | silent (Pro819) | 9926 | - | | 336620 | T | C | 260.77 | SNP | Rv0279c (PE\_PGRS4) | silent (Thr818) | 9871 | - | | 336691 | T | C | 84.28 | SNP | Rv0279c (PE\_PGRS4) | Ser795Gly | 21 | - | | 336698 | C | G | 49.74 | SNP | Rv0279c (PE\_PGRS4) | silent (Gly792) | 9935 | - | | 336701 | A | G | 82.28 | SNP | Rv0279c (PE\_PGRS4) | silent (Gly791) | 9935 | - | | 336707 | G | A | 60.28 | SNP | Rv0279c (PE\_PGRS4) | silent (Asp789) | 9859 | - | | 336708 | T | C | 74.28 | SNP | Rv0279c (PE\_PGRS4) | Asp789Gly | 11 | - | | 336710 | A | G | 81.28 | SNP | Rv0279c (PE\_PGRS4) | silent (Ala788) | 9867 | - | | 336728 | G | A | 252.77 | SNP | Rv0279c (PE\_PGRS4) | silent (Gly782) | 9935 | - | | 338020 | A | C | 139.03 | SNP | Rv0279c (PE\_PGRS4) | Cys352Gly | 1 | - | | 338100 | T | C | 182.90 | SNP | Rv0279c (PE\_PGRS4) | Asn325Ser | 34 | - | | 338453 | A | G | 105.03 | SNP | Rv0279c (PE\_PGRS4) | silent (Ala207) | 9867 | - | | 338618 | C | G | 201.84 | SNP | Rv0279c (PE\_PGRS4) | silent (Gly152) | 9935 | - | | 338789 | G | C | 459.77 | SNP | Rv0279c (PE\_PGRS4) | silent (Thr95) | 9871 | - | | 338790 | G | A | 452.77 | SNP | Rv0279c (PE\_PGRS4) | Thr95Ile | 7 | - | | 338791 | T | C | 497.77 | SNP | Rv0279c (PE\_PGRS4) | Thr95Ala | 32 | - | | 338792 | G | C | 514.77 | SNP | Rv0279c (PE\_PGRS4) | silent (Ala94) | 9867 | - | | 338810 | G | C | 410.77 | SNP | Rv0279c (PE\_PGRS4) | silent (Ala88) | 9867 | - | | 338813 | G | A | 431.77 | SNP | Rv0279c (PE\_PGRS4) | silent (Tyr87) | 9945 | - | | 338816 | C | G | 358.77 | SNP | Rv0279c (PE\_PGRS4) | silent (Ala86) | 9867 | - | | 338844 | A | G | 795.77 | SNP | Rv0279c (PE\_PGRS4) | Val(s)77Ala | 9867 | - | | 338845 | C | T | 732.77 | SNP | Rv0279c (PE\_PGRS4) | Val(s)77Met(s) | 9867 | - | | 338903 | G | C | 431.77 | SNP | Rv0279c (PE\_PGRS4) | silent (Ala57) | 9867 | - | | 340372 | T | C | 463.77 | SNP | Rv0280 (PPE3) | Ser337Pro | 12 | - | | 340953 | G | T | 797.77 | SNP | Rv0280 (PPE3) | silent (Ala530) | 9867 | - | | 346275 | C | G | 830.77 | SNP | Rv0284 (eccC3) | Pro214Arg | 4 | - | | 356528 | A | G | 1186.77 | SNP | Rv0292 (eccE3) | Asn217Asp | 42 | - | | 361415 | G | A | 1215.77 | SNP | Rv0297 (PE\_PGRS5) | Ala28Thr | 22 | - | | 361994 | A | T | 53.74 | SNP | Rv0297 (PE\_PGRS5) | Asn221Tyr | 3 | - | | 361995 | A | T | 53.74 | SNP | Rv0297 (PE\_PGRS5) | Asn221Ile | 3 | - | | 362667 | C | T | 113.03 | SNP | Rv0297 (PE\_PGRS5) | Ala445Val(s) | 9867 | - | | 372913 | A | C | 1575.77 | SNP | Rv0305c (PPE6) | silent (Gly933) | 9935 | - | | 373282 | TA | T | 1516.73 | DEL | Rv0305c (PPE6) |  |  | - | | 376774 | T | C | 1230.77 | SNP | Rv0307c | silent (Ala94) | 9867 | - | | 382984 | G | A | 1873.77 | SNP | Rv0314c | silent (Ser186) | 9840 | - | | 384380 | A | C | 2027.77 | SNP | Rv0315 | Lys260Thr | 8 | - | | 386432 | C | G | 1786.77 | SNP | Rv0318c | Gly223Ala | 21 | - | | 388725 | C | T | 1486.77 | SNP | Rv0321 (dcd) | silent (Tyr48) | 9945 | - | | 390828 | T | C | 872.77 | SNP | Rv0323c | Ser142Gly | 21 | - | | 391853 | A | G | 1243.77 | SNP | Rv0324 | Thr168Ala | 32 | - | | 395784 | G | A | 1328.77 | SNP | Rv0330c | Leu102Phe | 6 | - | | 397275 | G | C | 728.77 | SNP | Rv0331 | Ala359Pro | 13 | - | | 403980 | G | A | 2033.77 | SNP | Rv0338c | Ala621Val | 13 | - | | 404326 | T | C | 1275.77 | SNP | Rv0338c | Arg506Gly | 1 | - | | 405750 | T | G | 1589.77 | SNP | Rv0338c | Tyr31Ser | 2 | - | | 414486 | C | T | 852.77 | SNP | Rv0344c (lpqJ) | silent (Glu152) | 9865 | - | | 420008 | A | G | 1645.77 | SNP | Rv0350 (dnaK) | silent (Ala58) | 9867 | - | | 424320 | T | TC | 1408.73 | INS | Rv0354c (PPE7) |  |  | - | | 427310 | TTGCCGAGGTTTGCAC | T | 3153.73 | DEL | Rv0355c (PPE8) |  |  | - | | 429171 | C | T | 1384.77 | SNP | Rv0355c (PPE8) | Ala1837Thr | 22 | - | | 433539 | T | C | 960.77 | SNP | Rv0355c (PPE8) | Met(s)381Val(s) | 9867 | - | | 454295 | T | C | 1520.77 | SNP | Rv0376c | silent (Pro26) | 9926 | - | | 457452 | T | G | 866.77 | SNP | Rv0381c | silent (Thr124) | 9871 | - | | 459399 | A | C | 909.77 | SNP | intergenic |  |  | - | | 463974 | T | C | 637.77 | SNP | Rv0386 | silent (His188) | 9912 | - | | 467497 | C | CG | 575.73 | INS | Rv0388c (PPE9) |  |  | - | | 467508 | C | CG | 776.73 | INS | Rv0388c (PPE9) |  |  | - | | 467516 | G | C | 494.77 | SNP | Rv0388c (PPE9) | silent (Ser162) | 9840 | - | | 467526 | C | G | 562.77 | SNP | Rv0388c (PPE9) | Gly159Ala | 21 | - | | 467546 | G | C | 637.77 | SNP | Rv0388c (PPE9) | Asp152Glu | 56 | - | | 467557 | A | C | 546.77 | SNP | Rv0388c (PPE9) | Leu(s)149Val(s) | 9867 | - | | 467564 | A | C | 569.77 | SNP | Rv0388c (PPE9) | His146Gln | 23 | - | | 467585 | G | C | 830.77 | SNP | Rv0388c (PPE9) | His139Gln | 23 | - | | 467590 | T | C | 842.77 | SNP | Rv0388c (PPE9) | Thr138Ala | 32 | - | | 467621 | T | G | 1007.77 | SNP | Rv0388c (PPE9) | silent (Gly127) | 9935 | - | | 467638 | G | T | 941.77 | SNP | Rv0388c (PPE9) | Gln122Lys | 12 | - | | 475178 | T | C | 904.77 | SNP | Rv0395 | Val80Ala | 18 | - | | 483935 | T | G | 1608.77 | SNP | intergenic |  |  | - | | 489073 | C | G | 1366.77 | SNP | Rv0405 (pks6) | Leu1115Val | 11 | - | | 489935 | G | C | 1572.77 | SNP | Rv0405 (pks6); Rv0406c | Arg1402Pro; silent (Thr257) | 5; 9871 | - | | 498557 | C | A | 1189.77 | SNP | Rv0412c | Asp355Tyr | 0 | - | | 501615 | C | G | 1089.77 | SNP | Rv0415 (thiO) | silent (Leu156) | 9947 | - | | 502589 | C | G | 1190.77 | SNP | Rv0417 (thiG) | Ser75Cys | 5 | - | | 503354 | G | C | 1674.77 | SNP | intergenic |  |  | - | | 505536 | T | G | 47.77 | SNP | Rv0419 (lpqM) | Cys151Gly | 1 | - | | 513257 | T | C | 1014.77 | SNP | Rv0425c (ctpH) | Met(s)689Val(s) | 9867 | - | | 514245 | C | T | 1076.77 | SNP | Rv0425c (ctpH) | Val(s)359Val | 13 | genotype | | 514684 | C | T | 777.77 | SNP | Rv0425c (ctpH) | Arg213His | 8 | - | | 524891 | C | A | 1258.77 | SNP | Rv0436c (pssA) | Gly167Val | 3 | - | | 541201 | A | G | 2256.77 | SNP | Rv0450c (mmpL4) | silent (Leu97) | 9947 | - | | 544461 | C | T | 216.78 | SNP | Rv0453 (PPE11) | Arg430Cys | 1 | - | | 546914 | G | A | 939.77 | SNP | intergenic |  |  | - | | 551525 | A | C | 1020.77 | SNP | Rv0459 | silent (Arg110) | 9913 | - | | 565655 | A | G | 1393.77 | SNP | intergenic |  |  | - | | 573262 | A | G | 997.77 | SNP | Rv0484c | silent (Gly180) | 9935 | - | | 580772 | T | A | 309.77 | SNP | intergenic |  |  | - | | 580773 | GGGGGCACCACCCGCTTGCG GGGGA | G | 2958.73 | DEL | intergenic |  |  | - | | 590436 | T | C | 1272.77 | SNP | Rv0500 (proC) | silent (Ala118) | 9867 | - | | 595321 | T | G | 621.77 | SNP | intergenic |  |  | - | | 595399 | AT | A | 945.73 | DEL | intergenic |  |  | - | | 597816 | A | G | 1857.77 | SNP | Rv0507 (mmpL2) | silent (Ala206) | 9867 | - | | 598475 | G | A | 1980.77 | SNP | Rv0507 (mmpL2) | Arg426His | 8 | - | | 599165 | A | C | 2235.77 | SNP | Rv0507 (mmpL2) | Glu656Ala | 17 | - | | 610120 | T | G | 1602.77 | SNP | intergenic |  |  | - | | 623472 | A | G | 146.03 | SNP | Rv0532 (PE\_PGRS6) | Asp227Gly | 11 | - | | 623508 | C | G | 151.03 | SNP | Rv0532 (PE\_PGRS6) | Ala239Gly | 21 | - | | 628895 | G | A | 922.77 | SNP | Rv0537c | silent (Phe279) | 9946 | - | | 630722 | G | C | 974.77 | SNP | Rv0538 | Arg228Pro | 5 | - | | 631689 | A | G | 420.77 | SNP | intergenic |  |  | - | | 637319 | G | A | 1287.77 | SNP | Rv0545c (pitA) | Pro49Ser | 17 | - | | 648002 | T | G | 1843.77 | SNP | Rv0556 | Leu15Arg | 1 | - | | 649436 | CCGGA | C | 2774.73 | DEL | Rv0557 (mgtA) |  |  | - | | 657269 | A | G | 1138.77 | SNP | Rv0565c | Ser68Pro | 12 | - | | 663410 | A | C | 190.77 | SNP | intergenic |  |  | - | | 663418 | A | C | 153.77 | SNP | intergenic |  |  | - | | 663419 | G | A | 173.77 | SNP | intergenic |  |  | - | | 663420 | C | A | 178.77 | SNP | intergenic |  |  | - | | 663429 | T | G | 173.77 | SNP | intergenic |  |  | - | | 665293 | A | G | 1681.77 | SNP | Rv0572c | Phe31Leu | 13 | - | | 669398 | T | C | 1261.77 | SNP | Rv0575c | silent (Gln116) | 9876 | - | | 673238 | A | G | 158.90 | SNP | Rv0578c (PE\_PGRS7) | silent (His893) | 9912 | - | | 685461 | C | G | 910.77 | SNP | Rv0587 (yrbE2A) | silent (Ala111) | 9867 | - | | 685608 | T | C | 1373.77 | SNP | Rv0587 (yrbE2A) | silent (Leu160) | 9947 | - | | 686655 | C | T | 1338.77 | SNP | Rv0588 (yrbE2B) | Thr243Ile | 7 | - | | 686972 | T | C | 1036.77 | SNP | Rv0589 (mce2A) | Phe51Ser | 3 | - | | 690465 | T | G | 564.77 | SNP | Rv0591 (mce2C) | silent (Leu469) | 9947 | - | | 698828 | G | A | 771.77 | SNP | Rv0601c | Ala56Val(s) | 9867 | - | | 698968 | G | A | 1029.77 | SNP | Rv0601c | silent (Gly9) | 9935 | - | | 712693 | A | G | 1035.77 | SNP | intergenic |  |  | - | | 721410 | T | C | 1442.77 | SNP | Rv0629c (recD) | Asp108Gly | 11 | - | | 726816 | G | C | 763.77 | SNP | Rv0631c (recC) | Phe497Leu(s) | 2 | - | | 728556 | GC | G | 1556.73 | DEL | intergenic |  |  | - | | 746045 | G | C | 489.77 | SNP | Rv0648 | silent (Leu1109) | 9947 | - | | 754186 | A | G | 1135.77 | SNP | Rv0658c | Leu75Pro | 2 | - | | 761109 | G | T | 1651.77 | SNP | Rv0667 (rpoB) | Asp435Tyr | 0 | resistance | | 765150 | G | A | 1743.77 | SNP | Rv0668 (rpoC) | Gly594Glu | 4 | genotype | | 767414 | G | A | 1199.77 | SNP | intergenic |  |  | - | | 768579 | C | G | 1301.77 | SNP | Rv0669c | Gly340Ala | 21 | - | | 775639 | T | C | 1224.77 | SNP | Rv0676c (mmpL5) | Ile948Val | 57 | - | | 781395 | T | C | 1436.77 | SNP | intergenic (Rv0682-165nt) |  |  | - | | 782246 | G | A | 1673.77 | SNP | Rv0683 (rpsG) | Val105Ile | 33 | - | | 788259 | G | T | 1343.77 | SNP | Rv0688 | Gly107Val | 3 | - | | 794480 | CG | C | 2351.73 | DEL | Rv0694 (lldD1) |  |  | - | | 800219 | T | C | 1554.77 | SNP | intergenic |  |  | - | | 820483 | G | T | 577.77 | SNP | Rv0727c (fucA) | Ala6Asp | 6 | - | | 828176 | G | C | 1536.77 | SNP | Rv0736 (rslA) | Asp13His | 3 | - | | 836538 | A | G | 61.74 | SNP | Rv0746 (PE\_PGRS9) | Asn280Asp | 42 | - | | 839334 | A | G | 50.74 | SNP | Rv0747 (PE\_PGRS10) | Lys295Arg | 19 | - | | 839348 | A | G | 55.74 | SNP | Rv0747 (PE\_PGRS10) | Ser300Gly | 21 | - | | 839496 | G | A | 204.77 | SNP | Rv0747 (PE\_PGRS10) | Gly349Asp | 6 | - | | 839515 | G | A | 208.77 | SNP | Rv0747 (PE\_PGRS10) | silent (Ala355) | 9867 | - | | 839516 | A | G | 207.77 | SNP | Rv0747 (PE\_PGRS10) | Thr356Ala | 32 | - | | 839519 | C | G | 178.77 | SNP | Rv0747 (PE\_PGRS10) | Leu357Val(s) | 4 | - | | 839520 | T | C | 178.77 | SNP | Rv0747 (PE\_PGRS10) | Leu357Pro | 2 | - | | 839534 | A | C | 180.77 | SNP | Rv0747 (PE\_PGRS10) | Ile362Leu | 22 | - | | 841764 | G | C | 1479.77 | SNP | Rv0749A | silent (Thr37) | 9871 | - | | 841924 | C | T | 1321.77 | SNP | intergenic |  |  | - | | 850591 | C | T | 1361.77 | SNP | intergenic |  |  | - | | 851133 | T | G | 704.77 | SNP | Rv0756c | Thr112Pro | 4 | - | | 852910 | C | T | 850.77 | SNP | Rv0758 (phoR) | Pro172Leu | 3 | - | | 854252 | GC | G | 1526.73 | DEL | intergenic |  |  | - | | 857696 | A | G | 1748.77 | SNP | Rv0764c (cyp51) | silent (Ala114) | 9867 | - | | 864184 | G | A | 981.77 | SNP | Rv0771 | silent (Glu15) | 9865 | - | | 865737 | C | T | 1437.77 | SNP | Rv0772 (purD) | silent (Ala384) | 9867 | - | | 868488 | G | A | 797.77 | SNP | Rv0775 | Glu28Lys | 7 | - | | 869440 | C | T | 1266.77 | SNP | Rv0776c | silent (Leu108) | 9947 | - | | 872108 | C | G | 864.77 | SNP | Rv0778 (cyp126) | silent (Ala226) | 9867 | - | | 874835 | C | CCG | 4258.73 | INS | Rv0781 (ptrBa); Rv0782 (ptrBb) |  |  | - | | 877224 | C | A | 2096.77 | SNP | Rv0783c (emrB) | Gly406Val | 3 | - | | 880562 | G | T | 1101.77 | SNP | Rv0785 | Cys408Phe | 0 | - | | 880935 | C | T | 1083.77 | SNP | Rv0785 | silent (Gly532) | 9935 | - | | 882257 | T | C | 1507.77 | SNP | Rv0787 | Tyr267His | 4 | - | | 888774 | G | A | 609.77 | SNP | intergenic |  |  | - | | 893733 | T | G | 1401.77 | SNP | Rv0800 (pepC) | Leu139Arg | 1 | - | | 896356 | C | T | 975.77 | SNP | Rv0803 (purL) | silent (Thr179) | 9871 | - | | 897209 | G | C | 1351.77 | SNP | Rv0803 (purL) | Glu464Gln | 27 | - | | 900221 | T | C | 1306.77 | SNP | Rv0806c (cpsY) | Val370Val(s) | 18 | - | | 902413 | C | T | 639.77 | SNP | Rv0808 (purF) | silent (Val101) | 9901 | - | | 903550 | T | C | 1313.77 | SNP | Rv0808 (purF) | silent (Ala480) | 9867 | - | | 903913 | T | C | 1054.77 | SNP | Rv0809 (purM) | silent (Gly63) | 9935 | - | | 906857 | A | G | 1042.77 | SNP | Rv0812 | Ile145Met(s) | 6 | - | | 918316 | T | C | 1099.77 | SNP | Rv0824c (desA1) | silent (Gln145) | 9876 | - | | 919574 | T | C | 1764.77 | SNP | intergenic |  |  | - | | 920747 | G | A | 1072.77 | SNP | Rv0827c (kmtR) | silent (Val129) | 9901 | - | | 921813 | C | G | 1550.77 | SNP | Rv0829 | Ala80Gly | 21 | - | | 923065 | T | A | 1557.77 | SNP | Rv0831c | silent (Gly215) | 9935 | - | | 927110 | A | G | 62.74 | SNP | Rv0833 (PE\_PGRS13) | Ser584Gly | 21 | - | | 928075 | T | C | 445.77 | SNP | Rv0834c (PE\_PGRS14) | Asn804Ser | 34 | - | | 928076 | T | C | 441.77 | SNP | Rv0834c (PE\_PGRS14) | Asn804Asp | 42 | - | | 928158 | CTCGCCGCCG | C | 1314.73 | DEL | Rv0834c (PE\_PGRS14) |  |  | - | | 929440 | T | G | 265.78 | SNP | Rv0834c (PE\_PGRS14) | Asp349Ala | 10 | - | | 931417 | C | T | 1564.77 | SNP | Rv0835 (lpqQ) | silent (Pro155) | 9926 | - | | 945214 | G | A | 1861.77 | SNP | Rv0848 (cysK2) | Gly93Ser | 16 | - | | 949535 | T | C | 1041.77 | SNP | Rv0853c (pdc) | silent (Ala528) | 9867 | - | | 950230 | C | T | 947.77 | SNP | Rv0853c (pdc) | Asp297Asn | 36 | - | | 955524 | A | G | 1426.77 | SNP | Rv0859 (fadA) | Ser150Gly | 21 | - | | 957117 | T | C | 1026.77 | SNP | Rv0860 (fadB) | silent (Asp275) | 9859 | - | | 968426 | A | AGCCGGGTTG | 1167.73 | INS | Rv0872c (PE\_PGRS15) |  |  | - | | 968775 | G | A | 33.77 | SNP | Rv0872c (PE\_PGRS15) | silent (Val490) | 9901 | - | | 969602 | C | G | 61.74 | SNP | Rv0872c (PE\_PGRS15) | Gly215Arg | 0 | - | | 976897 | T | G | 1182.77 | SNP | Rv0878c (PPE13) | Gln436Pro | 8 | - | | 978373 | T | C | 826.77 | SNP | intergenic |  |  | - | | 979704 | G | C | 1297.77 | SNP | Rv0881 | Gly115Arg | 0 | - | | 979859 | T | C | 1331.77 | SNP | Rv0881 | silent (Ala166) | 9867 | - | | 983485 | T | G | 1284.77 | SNP | Rv0885 | Ser242Ala | 35 | - | | 986463 | G | C | 1813.77 | SNP | intergenic |  |  | - | | 990001 | G | C | 1168.77 | SNP | Rv0890c | Pro866Ala | 22 | - | | 993346 | A | C | 2234.77 | SNP | Rv0891c | Val37Gly | 5 | - | | 1010204 | C | CG | 1907.73 | INS | Rv0907 |  |  | - | | 1025106 | T | C | 1661.77 | SNP | Rv0919 | silent (Phe141) | 9946 | - | | 1028944 | C | T | 1362.77 | SNP | Rv0922 | silent (Tyr420) | 9945 | - | | 1037012 | T | C | 820.77 | SNP | Rv0930 (pstA1) | Met(s)5Thr | 22 | - | | 1037911 | C | T | 1262.77 | SNP | Rv0930 (pstA1) | Arg305STOP | 2 | - | | 1047165 | T | C | 808.77 | SNP | Rv0938 (ligD) | Cys344Arg | 1 | - | | 1049508 | C | T | 710.77 | SNP | Rv0939 | Ala366Val(s) | 9867 | - | | 1068151 | T | C | 1469.77 | SNP | Rv0956 (purN) | silent (His197) | 9912 | - | | 1068432 | A | G | 1247.77 | SNP | Rv0957 (purH) | silent (Pro76) | 9926 | - | | 1070702 | T | C | 1042.77 | SNP | Rv0958 | Ser274Pro | 12 | - | | 1074558 | G | A | 1578.77 | SNP | Rv0962c (lprP) | Pro186Leu | 3 | - | | 1075279 | T | C | 1886.77 | SNP | intergenic |  |  | - | | 1076309 | G | T | 855.77 | SNP | Rv0964c | Pro124Thr | 5 | - | | 1077312 | A | G | 1088.77 | SNP | Rv0966c | Val(s)175Ala | 9867 | - | | 1079927 | C | A | 776.77 | SNP | Rv0969 (ctpV) | silent (Thr395) | 9871 | - | | 1081681 | T | C | 1300.77 | SNP | Rv0970 | silent (Val210) | 9901 | - | | 1087193 | G | C | 869.77 | SNP | Rv0974c (accD2) | Asn51Lys | 25 | - | | 1090292 | C | G | 866.77 | SNP | intergenic |  |  | - | | 1092340 | C | CG | 1193.73 | INS | Rv0977 (PE\_PGRS16) |  |  | - | | 1093406 | A | G | 805.77 | SNP | Rv0978c (PE\_PGRS17) | silent (Val317) | 9901 | - | | 1093928 | G | A | 66.28 | SNP | Rv0978c (PE\_PGRS17) | silent (Asn143) | 9822 | - | | 1095850 | A | T | 42.74 | SNP | Rv0980c (PE\_PGRS18) | Val201Asp | 1 | - | | 1095851 | C | T | 100.03 | SNP | Rv0980c (PE\_PGRS18) | Val201Ile | 33 | - | | 1095852 | A | C | 124.03 | SNP | Rv0980c (PE\_PGRS18) | silent (Gly200) | 9935 | - | | 1095855 | A | G | 120.03 | SNP | Rv0980c (PE\_PGRS18) | silent (Gly199) | 9935 | - | | 1096470 | G | C | 45.74 | SNP | intergenic |  |  | - | | 1096508 | C | G | 55.74 | SNP | intergenic |  |  | - | | 1096510 | T | C | 49.74 | SNP | intergenic |  |  | - | | 1096633 | T | G | 1421.77 | SNP | intergenic |  |  | - | | 1100234 | T | C | 956.77 | SNP | Rv0983 (pepD) | Leu390Pro | 2 | - | | 1102788 | G | T | 1304.77 | SNP | Rv0987 | Val83Phe | 0 | - | | 1106422 | T | C | 2305.77 | SNP | Rv0989c (grcC2) | Ile321Val | 57 | - | | 1106877 | T | C | 1172.77 | SNP | Rv0989c (grcC2) | Tyr169Cys | 3 | - | | 1109975 | A | G | 1526.77 | SNP | Rv0993 (galU) | Gln235Arg | 10 | - | | 1126889 | G | C | 1888.77 | SNP | Rv1007c (metS) | Arg39Gly | 1 | - | | 1127648 | C | A | 1803.77 | SNP | Rv1008 (tatD) | Thr187Asn | 9 | - | | 1148255 | G | A | 2029.77 | SNP | intergenic |  |  | - | | 1149551 | C | T | 1302.77 | SNP | Rv1028c (kdpD) | silent (Glu712) | 9865 | - | | 1150585 | G | A | 1190.77 | SNP | Rv1028c (kdpD) | Pro368Ser | 17 | - | | 1155067 | C | CG | 1663.73 | INS | Rv1030 (kdpB) |  |  | - | | 1159854 | C | T | 809.77 | SNP | Rv1035c | Ala70Thr | 22 | - | | 1163134 | T | C | 1534.77 | SNP | Rv1040c (PE8) | silent (Gly81) | 9935 | - | | 1164336 | G | A | 1338.77 | SNP | intergenic |  |  | - | | 1164361 | G | A | 1468.77 | SNP | intergenic |  |  | - | | 1164571 | A | G | 1393.77 | SNP | intergenic |  |  | - | | 1165521 | T | TA | 2524.73 | INS | intergenic |  |  | - | | 1168715 | C | CT | 1831.73 | INS | Rv1046c |  |  | - | | 1170404 | C | A | 45.74 | SNP | Rv1047 | Gln328Lys | 12 | - | | 1174515 | C | T | 940.77 | SNP | Rv1051c | silent (Ala62) | 9867 | - | | 1178116 | T | C | 2179.77 | SNP | Rv1056 | silent (Thr163) | 9871 | - | | 1190093 | A | C | 1033.77 | SNP | Rv1067c (PE\_PGRS19) | Leu(s)111Trp | 0 | - | | 1191741 | G | A | 345.77 | SNP | Rv1068c (PE\_PGRS20) | silent (Tyr136) | 9945 | - | | 1200418 | A | G | 1192.77 | SNP | intergenic |  |  | - | | 1220680 | T | C | 1136.77 | SNP | Rv1093 (glyA1) | Val36Ala | 18 | - | | 1224367 | T | C | 961.77 | SNP | intergenic |  |  | - | | 1231587 | C | T | 1405.77 | SNP | Rv1104 | Pro96Leu | 3 | - | | 1237562 | G | C | 812.77 | SNP | Rv1111c | Arg211Gly | 1 | - | | 1244655 | G | T | 910.77 | SNP | Rv1121 (zwf1) | Ala317Ser | 28 | - | | 1248978 | T | C | 940.77 | SNP | Rv1125 | silent (Ala299) | 9867 | - | | 1251199 | C | T | 1281.77 | SNP | Rv1127c (ppdK) | Gly69Glu | 4 | - | | 1252164 | T | C | 1630.77 | SNP | Rv1128c | Glu270Gly | 7 | - | | 1256806 | C | T | 927.77 | SNP | Rv1131 (prpC) | silent (Asp225) | 9859 | - | | 1275957 | T | C | 1160.77 | SNP | intergenic |  |  | - | | 1276321 | T | G | 1591.77 | SNP | Rv1148c | Gln476His | 20 | - | | 1276322 | T | G | 1604.77 | SNP | Rv1148c | Gln476Pro | 8 | - | | 1276360 | T | G | 1538.77 | SNP | Rv1148c | silent (Ile463) | 9872 | - | | 1276363 | T | G | 1504.77 | SNP | Rv1148c | silent (Arg462) | 9913 | - | | 1276366 | C | T | 1498.77 | SNP | Rv1148c | silent (Pro461) | 9926 | - | | 1276931 | T | G | 988.77 | SNP | Rv1148c | Asp273Ala | 10 | - | | 1277869 | G | GT | 2422.73 | INS | intergenic |  |  | - | | 1281118 | T | C | 1685.77 | SNP | Rv1154c | Thr123Ala | 32 | - | | 1292102 | A | G | 1420.77 | SNP | Rv1162 (narH) | silent (Pro346) | 9926 | - | | 1292464 | C | T | 1412.77 | SNP | Rv1162 (narH) | Ala467Val(s) | 9867 | - | | 1302161 | C | G | 1019.77 | SNP | Rv1172c (PE12) | Gly174Ala | 21 | - | | 1306259 | A | G | 1309.77 | SNP | Rv1175c (fadH) | silent (Ala656) | 9867 | - | | 1307598 | C | G | 812.77 | SNP | Rv1175c (fadH) | Cys210Ser | 11 | - | | 1312176 | C | T | 1529.77 | SNP | Rv1179c | Arg375Gln | 9 | - | | 1313337 | A | AG | 1469.73 | INS | intergenic |  |  | - | | 1313338 | A | C | 796.77 | SNP | intergenic |  |  | - | | 1315191 | A | C | 1193.77 | SNP | Rv1180 (pks3) | STOP489Tyr | 1 | - | | 1315884 | G | A | 1221.77 | SNP | Rv1181 (pks4) | silent (Ala217) | 9867 | - | | 1327890 | G | A | 1329.77 | SNP | Rv1186c | silent (Asp472) | 9859 | - | | 1328687 | G | C | 907.77 | SNP | Rv1186c | Pro207Ala | 22 | - | | 1339432 | G | C | 417.77 | SNP | Rv1196 (PPE18) | silent (Ala28) | 9867 | - | | 1339511 | G | A | 73.28 | SNP | Rv1196 (PPE18) | Val(s)55Met(s) | 9867 | - | | 1339512 | T | C | 40.74 | SNP | Rv1196 (PPE18) | Val(s)55Ala | 9867 | - | | 1339516 | G | A | 38.74 | SNP | Rv1196 (PPE18) | silent (Gly56) | 9935 | - | | 1339837 | G | C | 67.28 | SNP | Rv1196 (PPE18) | silent (Thr163) | 9871 | - | | 1339839 | C | A | 64.28 | SNP | Rv1196 (PPE18) | Ala164Glu | 10 | - | | 1339841 | A | G | 51.74 | SNP | Rv1196 (PPE18) | Thr165Ala | 32 | - | | 1339861 | G | C | 68.28 | SNP | Rv1196 (PPE18) | Glu171Asp | 53 | - | | 1339864 | G | C | 67.28 | SNP | Rv1196 (PPE18) | silent (Ala172) | 9867 | - | | 1339867 | G | A | 62.28 | SNP | Rv1196 (PPE18) | silent (Pro173) | 9926 | - | | 1339868 | G | C | 68.28 | SNP | Rv1196 (PPE18) | Glu174Gln | 27 | - | | 1339869 | A | T | 62.28 | SNP | Rv1196 (PPE18) | Glu174Val(s) | 17 | - | | 1339873 | G | C | 67.28 | SNP | Rv1196 (PPE18) | Met(s)175Ile | 2 | - | | 1339878 | G | A | 64.28 | SNP | Rv1196 (PPE18) | Ser177Asn | 20 | - | | 1339880 | G | C | 67.28 | SNP | Rv1196 (PPE18) | Ala178Pro | 13 | - | | 1339882 | G | C | 67.28 | SNP | Rv1196 (PPE18) | silent (Ala178) | 9867 | - | | 1339885 | T | C | 68.28 | SNP | Rv1196 (PPE18) | silent (Gly179) | 9935 | - | | 1339894 | C | T | 442.77 | SNP | Rv1196 (PPE18) | silent (Leu182) | 9947 | - | | 1340667 | A | G | 1079.77 | SNP | Rv1197 (esxK) | silent (Ser3) | 9840 | - | | 1340830 | G | A | 78.77 | SNP | Rv1197 (esxK) | Ala58Thr | 22 | - | | 1340916 | G | A | 58.77 | SNP | Rv1197 (esxK) | silent (Glu86) | 9865 | - | | 1341023 | A | G | 80.28 | SNP | Rv1198 (esxL) | silent (Gln6) | 9876 | - | | 1341083 | C | G | 48.74 | SNP | Rv1198 (esxL) | silent (Ala26) | 9867 | - | | 1341099 | A | G | 78.28 | SNP | Rv1198 (esxL) | Ile32Val | 57 | - | | 1341114 | A | G | 142.90 | SNP | Rv1198 (esxL) | Thr37Ala | 32 | - | | 1341120 | A | G | 181.84 | SNP | Rv1198 (esxL) | Ser39Gly | 21 | - | | 1341148 | C | T | 261.28 | SNP | Rv1198 (esxL) | Ala48Val(s) | 9867 | - | | 1341152 | C | T | 246.90 | SNP | Rv1198 (esxL) | silent (Ala49) | 9867 | - | | 1341624 | G | T | 138.90 | SNP | Rv1199c | Gln328Lys | 12 | - | | 1342581 | T | C | 445.77 | SNP | Rv1199c | Thr9Ala | 32 | - | | 1360209 | T | C | 1758.77 | SNP | Rv1217c | silent (Ala531) | 9867 | - | | 1362006 | T | C | 1451.77 | SNP | Rv1218c | Gln243Arg | 10 | - | | 1365410 | G | A | 612.77 | SNP | Rv1222 (rseA) | Ala23Thr | 22 | - | | 1365837 | C | CG | 1278.73 | INS | intergenic |  |  | - | | 1368961 | G | A | 387.77 | SNP | Rv1226c | silent (Leu445) | 9947 | - | | 1372703 | C | A | 965.77 | SNP | Rv1229c (mrp) | Val(s)83Leu(s) | 9867 | - | | 1374065 | T | C | 977.77 | SNP | Rv1230c | Ser45Gly | 21 | - | | 1375724 | A | C | 1219.77 | SNP | Rv1232c | Cys149Gly | 1 | - | | 1382628 | T | C | 1709.77 | SNP | Rv1239c (corA) | Lys139Glu | 4 | - | | 1393626 | A | G | 1016.77 | SNP | Rv1249c | silent (Leu119) | 9947 | - | | 1393994 | C | T | 1396.77 | SNP | intergenic |  |  | - | | 1396510 | G | A | 1404.77 | SNP | Rv1251c | His911Tyr | 4 | - | | 1396922 | T | C | 1730.77 | SNP | Rv1251c | silent (Thr773) | 9871 | - | | 1411210 | T | G | 1127.77 | SNP | Rv1263 (amiB2) | Val260Val(s) | 18 | - | | 1413148 | C | T | 1442.77 | SNP | intergenic |  |  | - | | 1414021 | C | T | 962.77 | SNP | Rv1266c (pknH) | Arg607Gln | 9 | - | | 1416222 | A | G | 58.77 | SNP | Rv1267c (embR) | Phe376Leu | 13 | - | | 1416232 | A | G | 39.77 | SNP | Rv1267c (embR) | silent (Cys372) | 9973 | - | | 1416234 | A | C | 65.77 | SNP | Rv1267c (embR) | Cys372Gly | 1 | - | | 1426079 | C | T | 1304.77 | SNP | intergenic |  |  | - | | 1440469 | C | G | 1882.77 | SNP | Rv1286 (cysN) | silent (Pro521) | 9926 | - | | 1444202 | C | G | 1139.77 | SNP | Rv1290c | Leu(s)282Phe | 1 | - | | 1445781 | A | G | 1122.77 | SNP | Rv1291c | silent (Ala18) | 9867 | - | | 1457144 | C | T | 684.77 | SNP | Rv1300 (hemK) | Arg194Cys | 1 | - | | 1461915 | C | G | 1494.77 | SNP | Rv1307 (atpH) | Leu25Val | 11 | - | | 1468208 | A | C | 1285.77 | SNP | Rv1313c | Leu433Arg | 1 | - | | 1471539 | G | A | 2044.77 | SNP | Rv1315 (murA) | Val(s)407Met(s) | 9867 | - | | 1471659 | C | T | 1773.77 | SNP | intergenic |  |  | - | | 1480174 | C | G | 234.77 | SNP | Rv1318c | silent (Leu217) | 9947 | - | | 1480176 | G | T | 144.77 | SNP | Rv1318c | Leu217Met(s) | 4 | - | | 1480184 | G | A | 144.77 | SNP | Rv1318c | Pro214Leu | 3 | - | | 1480186 | C | T | 102.77 | SNP | Rv1318c | silent (Ala213) | 9867 | - | | 1480189 | C | G | 129.77 | SNP | Rv1318c | Leu(s)212Phe | 1 | - | | 1480195 | G | T | 137.77 | SNP | Rv1318c | silent (Arg210) | 9913 | - | | 1480197 | G | C | 108.77 | SNP | Rv1318c | Arg210Gly | 1 | - | | 1480198 | T | C | 125.77 | SNP | Rv1318c | silent (Pro209) | 9926 | - | | 1480204 | C | T | 104.77 | SNP | Rv1318c | silent (Pro207) | 9926 | - | | 1480205 | G | A | 112.77 | SNP | Rv1318c | Pro207Leu | 3 | - | | 1480219 | C | T | 203.77 | SNP | Rv1318c | silent (Ala202) | 9867 | - | | 1480231 | G | C | 103.77 | SNP | Rv1318c | Phe198Leu(s) | 2 | - | | 1480233 | A | C | 79.77 | SNP | Rv1318c | Phe198Val | 1 | - | | 1480242 | A | G | 108.77 | SNP | Rv1318c | Leu(s)195Leu | 3 | - | | 1480243 | T | A | 79.77 | SNP | Rv1318c | silent (Ala194) | 9867 | - | | 1480945 | C | G | 978.77 | SNP | Rv1319c | silent (Thr519) | 9871 | - | | 1480948 | C | T | 852.77 | SNP | Rv1319c | silent (Glu518) | 9865 | - | | 1480972 | T | C | 1010.77 | SNP | Rv1319c | silent (Glu510) | 9865 | - | | 1481185 | A | C | 831.77 | SNP | Rv1319c | Asp439Glu | 56 | - | | 1482627 | T | C | 1462.77 | SNP | Rv1320c | Thr531Ala | 32 | - | | 1483894 | G | GT | 1824.73 | INS | Rv1320c |  |  | - | | 1484708 | A | C | 1774.77 | SNP | Rv1321 | Ser144Arg | 6 | - | | 1487084 | C | T | 743.77 | SNP | intergenic |  |  | - | | 1495326 | A | G | 1594.77 | SNP | Rv1328 (glgP) | Lys255Glu | 4 | - | | 1499274 | C | G | 636.77 | SNP | Rv1330c (pncB1) | Gly429Ala | 21 | - | | 1519431 | C | T | 1244.77 | SNP | Rv1353c | Val(s)185Val | 13 | - | | 1526819 | C | A | 1292.77 | SNP | Rv1358 | silent (Arg70) | 9913 | - | | 1532777 | T | C | 39.77 | SNP | Rv1361c (PPE19) | Gln286Arg | 10 | - | | 1533059 | A | G | 174.77 | SNP | Rv1361c (PPE19) | Ile192Thr | 11 | - | | 1533060 | T | A | 183.77 | SNP | Rv1361c (PPE19) | Ile192Phe | 8 | - | | 1533077 | A | G | 298.77 | SNP | Rv1361c (PPE19) | Val186Ala | 18 | - | | 1533159 | C | T | 1228.77 | SNP | Rv1361c (PPE19) | Ala159Thr | 22 | - | | 1533162 | T | C | 1255.77 | SNP | Rv1361c (PPE19) | Thr158Ala | 32 | - | | 1533163 | G | C | 1295.77 | SNP | Rv1361c (PPE19) | silent (Ala157) | 9867 | - | | 1533208 | C | G | 1285.77 | SNP | Rv1361c (PPE19) | silent (Gly142) | 9935 | - | | 1533583 | G | A | 1534.88 | SNP | Rv1361c (PPE19) | silent (Tyr17) | 9945 | - | | 1536251 | G | T | 1433.77 | SNP | Rv1364c | Ala465Glu | 10 | - | | 1538659 | T | G | 1731.77 | SNP | Rv1366 | silent (Arg90) | 9913 | - | | 1539200 | G | T | 1250.77 | SNP | Rv1366; Rv1366A | Gly271STOP; silent (Gly7) | 21; 9935 | - | | 1546541 | A | C | 1715.77 | SNP | Rv1373 | Gln177Pro | 8 | - | | 1547125 | T | C | 1657.77 | SNP | Rv1374c | Thr136Ala | 32 | - | | 1550020 | C | G | 720.77 | SNP | Rv1376 | His291Gln | 23 | - | | 1552547 | G | A | 798.77 | SNP | Rv1378c | Arg37Trp | 2 | - | | 1553633 | T | C | 780.77 | SNP | Rv1380 (pyrB) | silent (Gly134) | 9935 | - | | 1553801 | G | A | 1201.77 | SNP | Rv1380 (pyrB) | Val(s)190Val | 13 | - | | 1563717 | C | T | 1244.77 | SNP | Rv1388 (mihF) | silent (Val8) | 9901 | - | | 1570566 | C | A | 1205.77 | SNP | Rv1394c (cyp132) | Arg135Leu | 1 | - | | 1573660 | T | G | 955.77 | SNP | Rv1396c (PE\_PGRS25) | Arg66Ser | 11 | - | | 1580680 | C | T | 989.77 | SNP | Rv1404 | silent (Ala30) | 9867 | - | | 1588899 | G | T | 833.77 | SNP | Rv1412 (ribC) | silent (Ala111) | 9867 | - | | 1589383 | A | G | 1345.77 | SNP | intergenic |  |  | - | | 1591152 | C | T | 1626.77 | SNP | Rv1415 (ribA2) | silent (Asp252) | 9859 | - | | 1605149 | C | T | 1505.77 | SNP | Rv1429 | Pro91Leu | 3 | - | | 1609840 | A | G | 1077.77 | SNP | Rv1431 | silent (Pro586) | 9926 | - | | 1612278 | G | GCGTCGA | 3538.73 | INS | Rv1434 |  |  | - | | 1612624 | T | TATCGGTACCGGTGCGCCAG GG | 3814.70 | INS | Rv1435c |  |  | - | | 1613035 | T | C | 1099.77 | SNP | intergenic |  |  | - | | 1617735 | G | A | 1091.77 | SNP | intergenic |  |  | - | | 1618615 | CCGCCGCCGGTGCCGCCGGC GCCGCCGTCGCCGCCGG | C | 1919.80 | DEL | Rv1441c (PE\_PGRS26) |  |  | - | | 1618711 | C | G | 46.77 | SNP | Rv1441c (PE\_PGRS26) | Gly325Ala | 21 | - | | 1618999 | C | T | 217.80 | SNP | Rv1441c (PE\_PGRS26) | Gly229Asp | 6 | - | | 1624791 | C | G | 867.77 | SNP | Rv1446c (opcA) | Arg192Pro | 5 | - | | 1627351 | T | C | 1028.77 | SNP | Rv1448c (tal) | Thr244Ala | 32 | - | | 1630148 | A | C | 1794.77 | SNP | Rv1449c (tkt) | Tyr18Asp | 0 | - | | 1632737 | C | T | 112.03 | SNP | Rv1450c (PE\_PGRS27) | Gly631Ser | 16 | - | | 1636826 | C | A | 43.74 | SNP | Rv1452c (PE\_PGRS28) | silent (Gly468) | 9935 | - | | 1636980 | G | T | 42.78 | SNP | Rv1452c (PE\_PGRS28) | Pro417His | 3 | - | | 1636981 | G | T | 42.90 | SNP | Rv1452c (PE\_PGRS28) | Pro417Thr | 5 | - | | 1636983 | C | G | 45.79 | SNP | Rv1452c (PE\_PGRS28) | Gly416Ala | 21 | - | | 1639594 | C | A | 1205.77 | SNP | Rv1453 | Pro405Gln | 6 | - | | 1643654 | G | T | 1315.77 | SNP | Rv1458c | Leu203Ile | 9 | - | | 1644362 | C | T | 84.77 | SNP | intergenic |  |  | - | | 1645334 | C | T | 1174.77 | SNP | Rv1459c | Val269Ile | 33 | - | | 1645802 | T | C | 1378.77 | SNP | Rv1459c | Lys113Glu | 4 | - | | 1646431 | G | C | 714.77 | SNP | Rv1460 | Glu82Asp | 53 | - | | 1650072 | A | G | 912.77 | SNP | Rv1462 | Asn183Asp | 42 | - | | 1669358 | C | A | 818.77 | SNP | Rv1479 (moxR1) | His26Asn | 21 | - | | 1676290 | C | A | 1228.77 | SNP | Rv1486c | Lys198Asn | 13 | - | | 1677388 | G | A | 1445.77 | SNP | intergenic |  |  | - | | 1680631 | G | C | 964.77 | SNP | intergenic |  |  | - | | 1689349 | C | T | 907.77 | SNP | Rv1498c | Arg191His | 8 | - | | 1689571 | C | G | 1960.77 | SNP | Rv1498c | Cys117Ser | 11 | - | | 1692141 | A | C | 1818.77 | SNP | Rv1501 | silent (Ile84) | 9872 | - | | 1693561 | A | G | 2467.77 | SNP | Rv1502 | Tyr213Cys | 3 | - | | 1698911 | G | A | 1859.77 | SNP | Rv1508c | silent (Gly328) | 9935 | - | | 1704554 | C | T | 824.77 | SNP | Rv1512 (epiA) | silent (Ile154) | 9872 | - | | 1706119 | T | C | 1501.77 | SNP | Rv1514c | silent (Ser159) | 9840 | - | | 1709432 | C | T | 1276.77 | SNP | Rv1517 | Leu188Phe | 6 | - | | 1711645 | T | C | 1210.77 | SNP | Rv1520 | silent (Gly206) | 9935 | - | | 1711692 | G | A | 1129.77 | SNP | Rv1520 | Arg222Gln | 9 | - | | 1713192 | A | G | 1505.77 | SNP | Rv1521 (fadD25) | Val297Val(s) | 18 | - | | 1724722 | T | C | 891.77 | SNP | Rv1527c (pks5) | Thr1230Ala | 32 | - | | 1728622 | C | G | 1759.77 | SNP | intergenic |  |  | - | | 1728837 | A | G | 1552.77 | SNP | intergenic |  |  | - | | 1752561 | T | C | 820.77 | SNP | Rv1548c (PPE21) | Asp258Gly | 11 | - | | 1753519 | G | GC | 2265.73 | INS | Rv1549 (fadD11.1) |  |  | - | | 1759252 | G | T | 1313.77 | SNP | Rv1552 (frdA) | silent (Ser524) | 9840 | genotype | | 1760292 | A | G | 1512.77 | SNP | Rv1554 (frdC) | Met(s)40Val(s) | 9867 | - | | 1769469 | C | A | 1243.77 | SNP | Rv1564c (treX) | silent (Ala711) | 9867 | - | | 1772742 | G | A | 969.77 | SNP | Rv1565c | Ala363Val(s) | 9867 | - | | 1778430 | T | C | 494.77 | SNP | Rv1570 (bioD) | Met(s)191Thr | 22 | - | | 1789516 | A | G | 182.77 | SNP | Rv1588c | silent (Gly107) | 9935 | - | | 1789564 | C | T | 228.78 | SNP | Rv1588c | silent (Arg91) | 9913 | - | | 1789565 | C | A | 348.77 | SNP | Rv1588c | Arg91Leu | 1 | - | | 1789650 | C | T | 358.77 | SNP | Rv1588c | Ala63Thr | 22 | - | | 1789654 | A | G | 434.77 | SNP | Rv1588c | silent (Leu61) | 9947 | - | | 1789671 | C | T | 446.77 | SNP | Rv1588c | Ala56Thr | 22 | - | | 1789675 | A | C | 430.77 | SNP | Rv1588c | silent (Gly54) | 9935 | - | | 1789678 | C | G | 373.77 | SNP | Rv1588c | Val(s)53Val | 13 | - | | 1792777 | T | C | 804.77 | SNP | Rv1592c | Ile322Val | 57 | - | | 1792778 | T | C | 815.77 | SNP | Rv1592c | silent (Glu321) | 9865 | - | | 1798355 | G | A | 755.77 | SNP | Rv1597 | Gly21Asp | 6 | - | | 1803265 | G | A | 1617.77 | SNP | Rv1602 (hisH) | Ser201Asn | 20 | - | | 1804409 | C | A | 1374.77 | SNP | Rv1604 (impA) | Pro124Gln | 6 | - | | 1817976 | A | T | 1547.77 | SNP | Rv1618 (tesB1) | His121Leu | 4 | - | | 1833684 | G | C | 1602.77 | SNP | Rv1630 (rpsA) | Arg48Pro | 5 | - | | 1834130 | G | A | 1366.77 | SNP | Rv1630 (rpsA) | Glu197Lys | 7 | - | | 1836286 | G | C | 674.77 | SNP | intergenic |  |  | - | | 1847919 | C | G | 1060.77 | SNP | Rv1639c | silent (Thr180) | 9871 | - | | 1849119 | G | C | 1839.77 | SNP | Rv1640c (lysX) | Arg973Gly | 1 | - | | 1854300 | T | C | 1515.77 | SNP | Rv1644 (tsnR) | Leu232Pro | 2 | - | | 1856777 | G | C | 1360.77 | SNP | Rv1647 | Ala2Pro | 13 | - | | 1864811 | T | C | 517.77 | SNP | Rv1651c (PE\_PGRS30) | Asn191Ser | 34 | - | | 1868578 | C | T | 970.77 | SNP | Rv1654 (argB) | Ser246Leu(s) | 35 | - | | 1872906 | G | T | 716.77 | SNP | Rv1659 (argH) | Ala90Ser | 28 | - | | 1875207 | C | T | 1244.77 | SNP | Rv1660 (pks10) | Leu350Leu(s) | 4 | - | | 1875544 | T | G | 37.77 | SNP | Rv1661 (pks7) | Leu(s)81Val(s) | 9867 | - | | 1884697 | G | A | 990.77 | SNP | Rv1662 (pks8) | silent (Gly998) | 9935 | - | | 1885772 | G | A | 1228.77 | SNP | Rv1662 (pks8) | Ala1357Thr | 22 | - | | 1885840 | G | A | 946.77 | SNP | Rv1662 (pks8) | silent (Glu1379) | 9865 | - | | 1894300 | G | GGTCTTGCCGC | 2604.73 | INS | Rv1668c |  |  | - | | 1894422 | A | G | 1147.77 | SNP | Rv1668c | silent (Asp307) | 9859 | - | | 1896387 | T | C | 1621.77 | SNP | Rv1670 | Trp90Arg | 8 | - | | 1901493 | T | C | 1080.77 | SNP | Rv1676 | silent (Ser149) | 9840 | - | | 1907296 | G | C | 1691.77 | SNP | Rv1682 | silent (Ala298) | 9867 | - | | 1916137 | A | G | 980.77 | SNP | Rv1691 | silent (Leu63) | 9947 | - | | 1917972 | A | G | 870.77 | SNP | Rv1694 (tlyA) | silent (Leu11) | 9947 | - | | 1931179 | C | A | 1094.77 | SNP | Rv1704c (cycA) | Arg93Leu | 1 | - | | 1931564 | G | A | 360.77 | SNP | Rv1705c (PPE22) | Thr364Met(s) | 32 | - | | 1933988 | G | A | 2745.77 | SNP | intergenic |  |  | - | | 1940258 | G | A | 942.77 | SNP | Rv1712 (cmk) | silent (Glu220) | 9865 | - | | 1944107 | A | G | 964.77 | SNP | Rv1716 | Ser178Gly | 21 | - | | 1944402 | T | C | 784.77 | SNP | Rv1716 | Val276Ala | 18 | - | | 1950767 | T | C | 1646.77 | SNP | Rv1724c | silent (Lys95) | 9926 | - | | 1951626 | GC | G | 1158.73 | DEL | Rv1725c |  |  | - | | 1952601 | C | T | 591.77 | SNP | Rv1726 | silent (His250) | 9912 | - | | 1956524 | G | A | 1677.77 | SNP | Rv1730c | Thr241Met(s) | 32 | - | | 1960284 | C | A | 1429.77 | SNP | Rv1733c | Gln68His | 20 | - | | 1960391 | G | A | 1548.77 | SNP | Rv1733c | Pro33Ser | 17 | - | | 1962071 | AGG | A | 2129.73 | DEL | intergenic |  |  | - | | 1964719 | C | T | 1355.77 | SNP | Rv1737c (narK2) | Val(s)218Met(s) | 9867 | - | | 1967237 | C | A | 1175.77 | SNP | Rv1739c | Arg134Leu | 1 | - | | 1983135 | T | G | 45.77 | SNP | Rv1753c (PPE24) | silent (Pro547) | 9926 | - | | 1983195 | A | G | 186.84 | SNP | Rv1753c (PPE24) | silent (Gly527) | 9935 | - | | 1983198 | C | G | 199.84 | SNP | Rv1753c (PPE24) | Val(s)526Val | 13 | - | | 1983313 | T | G | 851.77 | SNP | Rv1753c (PPE24) | Asn488Thr | 13 | - | | 1992323 | GC | G | 697.73 | DEL | Rv1759c (wag22) |  |  | - | | 1993808 | A | T | 1135.77 | SNP | Rv1760 | Glu219Val(s) | 17 | - | | 2007502 | G | A | 665.77 | SNP | Rv1773c | His89Tyr | 4 | - | | 2008870 | C | G | 1373.77 | SNP | Rv1774 | Leu347Val | 11 | - | | 2014185 | C | T | 1084.77 | SNP | Rv1779c | Ala99Thr | 22 | - | | 2020144 | G | A | 1292.77 | SNP | Rv1783 (eccC5) | silent (Glu296) | 9865 | - | | 2022868 | T | C | 767.77 | SNP | Rv1783 (eccC5) | silent (Ser1204) | 9840 | - | | 2026148 | C | T | 1023.77 | SNP | Rv1787 (PPE25) | Ala283Val(s) | 9867 | - | | 2030634 | G | C | 105.03 | SNP | intergenic |  |  | - | | 2030720 | C | T | 51.74 | SNP | Rv1793 (esxN) | silent (Asp9) | 9859 | - | | 2030855 | T | C | 461.77 | SNP | Rv1793 (esxN) | silent (Ile54) | 9872 | - | | 2030862 | T | C | 591.77 | SNP | Rv1793 (esxN) | Leu(s)57Leu | 3 | - | | 2030942 | G | A | 1108.77 | SNP | Rv1793 (esxN) | silent (Ala83) | 9867 | - | | 2037698 | G | A | 1454.77 | SNP | Rv1798 (eccA5) | silent (Thr333) | 9871 | - | | 2039901 | C | T | 1109.77 | SNP | Rv1800 (PPE28) | Ala150Val | 13 | - | | 2045310 | A | G | 369.77 | SNP | Rv1803c (PE\_PGRS32) | silent (Ile511) | 9872 | - | | 2049065 | T | C | 1186.77 | SNP | intergenic |  |  | - | | 2049097 | G | C | 1124.77 | SNP | intergenic |  |  | - | | 2051746 | T | C | 718.77 | SNP | Rv1809 (PPE33) | silent (Ala155) | 9867 | - | | 2052035 | G | T | 1274.77 | SNP | Rv1809 (PPE33) | Val(s)252Leu(s) | 9867 | - | | 2054805 | G | A | 1111.77 | SNP | Rv1812c | silent (Pro185) | 9926 | - | | 2055271 | A | G | 894.77 | SNP | Rv1812c | Leu30Pro | 2 | - | | 2056450 | T | C | 1831.77 | SNP | intergenic |  |  | - | | 2057141 | G | A | 1983.77 | SNP | Rv1814 (erg3) | silent (Pro207) | 9926 | - | | 2057774 | A | T | 933.77 | SNP | Rv1815 | Ile83Phe | 8 | - | | 2065383 | C | T | 649.77 | SNP | Rv1820 (ilvG) | silent (Ala195) | 9867 | - | | 2074514 | G | C | 236.80 | SNP | intergenic |  |  | - | | 2074754 | C | T | 1278.77 | SNP | intergenic |  |  | - | | 2081449 | T | TA | 2777.73 | INS | Rv1835c |  |  | - | | 2087935 | A | G | 869.77 | SNP | intergenic |  |  | - | | 2090921 | C | T | 673.77 | SNP | Rv1842c | Gly389Ser | 16 | - | | 2094911 | ACAGCGT | A | 2882.73 | DEL | Rv1844c (gnd1) |  |  | - | | 2096186 | A | G | 1088.77 | SNP | Rv1846c (blaI) | silent (Thr138) | 9871 | - | | 2108141 | T | C | 621.77 | SNP | Rv1860 (apa) | Phe136Leu | 13 | - | | 2109523 | C | CG | 2603.73 | INS | intergenic |  |  | - | | 2115210 | A | C | 958.77 | SNP | Rv1866 | Asn691His | 18 | - | | 2116903 | C | T | 1073.77 | SNP | Rv1867 | silent (Gly380) | 9935 | - | | 2123169 | T | G | 2187.77 | SNP | intergenic |  |  | - | | 2128870 | A | G | 678.77 | SNP | Rv1878 (glnA3) | silent (Leu283) | 9947 | - | | 2132152 | T | C | 1708.77 | SNP | Rv1881c (lppE) | Ile60Val | 57 | - | | 2133468 | T | TTCGCATGCCGTCACC | 3060.73 | INS | Rv1883c |  |  | - | | 2134325 | C | T | 1501.77 | SNP | Rv1885c | Arg183Gln | 9 | - | | 2135870 | T | C | 1068.77 | SNP | intergenic |  |  | - | | 2137521 | A | ACTCCGATCAC | 7069.73 | INS | Rv1888c |  |  | - | | 2143328 | G | C | 1568.77 | SNP | Rv1895 | Val(s)270Leu | 3 | - | | 2147022 | A | C | 1592.77 | SNP | Rv1900c (lipJ) | Ile204Met(s) | 6 | - | | 2152952 | A | C | 1071.77 | SNP | intergenic |  |  | - | | 2155168 | C | G | 1061.77 | SNP | Rv1908c (katG) | Ser315Thr | 32 | resistance | | 2157856 | G | A | 388.77 | SNP | Rv1911c (lppC) | silent (Val44) | 9901 | - | | 2158582 | G | A | 1718.77 | SNP | Rv1912c (fadB5) | silent (Gly170) | 9935 | - | | 2160998 | G | A | 1214.77 | SNP | Rv1915 (aceAa) | Gly179Asp | 6 | - | | 2161343 | G | GT | 1293.73 | INS | Rv1915 (aceAa) |  |  | - | | 2162803 | T | C | 689.77 | SNP | intergenic |  |  | - | | 2163375 | T | C | 1378.77 | SNP | Rv1917c (PPE34) | Asn1313Asp | 42 | - | | 2163412 | A | G | 1106.77 | SNP | Rv1917c (PPE34) | silent (Val1300) | 9901 | - | | 2163415 | C | A | 959.77 | SNP | Rv1917c (PPE34) | silent (Pro1299) | 9926 | - | | 2163417 | G | C | 1158.77 | SNP | Rv1917c (PPE34) | Pro1299Ala | 22 | - | | 2163419 | C | T | 917.77 | SNP | Rv1917c (PPE34) | Ser1298Asn | 20 | - | | 2163421 | C | G | 858.77 | SNP | Rv1917c (PPE34) | silent (Thr1297) | 9871 | - | | 2163444 | T | C | 72.77 | SNP | Rv1917c (PPE34) | Asn1290Asp | 42 | - | | 2163790 | A | C | 1276.77 | SNP | Rv1917c (PPE34) | silent (Pro1174) | 9926 | - | | 2165286 | A | C | 1254.77 | SNP | Rv1917c (PPE34) | Ser676Ala | 35 | - | | 2165428 | T | A | 164.77 | SNP | Rv1917c (PPE34) | silent (Ala628) | 9867 | - | | 2165503 | T | A | 1212.77 | SNP | Rv1917c (PPE34) | silent (Ala603) | 9867 | - | | 2175062 | C | T | 929.77 | SNP | Rv1922 | silent (Ala332) | 9867 | - | | 2180796 | CCGCCTTGGCCTTGTTTGAC CAT | C | 8258.73 | DEL | Rv1928c |  |  | - | | 2187274 | C | A | 2028.77 | SNP | intergenic |  |  | - | | 2192250 | G | T | 83.77 | SNP | Rv1939 | Val53Phe | 0 | - | | 2202833 | G | A | 957.77 | SNP | Rv1957 | Asp84Asn | 36 | - | | 2203754 | C | T | 1556.77 | SNP | Rv1959c (parE1) | Arg75Gln | 9 | - | | 2204260 | A | G | 787.77 | SNP | Rv1961 | Ser17Gly | 21 | - | | 2207591 | T | TC | 2818.73 | INS | intergenic |  |  | - | | 2211826 | A | G | 999.77 | SNP | Rv1968 (mce3C) | silent (Lys67) | 9926 | - | | 2216443 | C | A | 1083.77 | SNP | Rv1971 (mce3F) | Ala396Glu | 10 | - | | 2220512 | T | G | 1140.77 | SNP | Rv1977 | silent (Ser253) | 9840 | - | | 2223293 | T | C | 1490.77 | SNP | intergenic |  |  | - | | 2225456 | A | T | 1146.77 | SNP | Rv1982c (vapC36) | Leu126His | 1 | - | | 2225882 | G | A | 821.77 | SNP | Rv1982A (vapB36) | Arg74Cys | 1 | - | | 2228967 | A | G | 1225.77 | SNP | intergenic |  |  | - | | 2233751 | G | C | 1278.77 | SNP | intergenic |  |  | - | | 2239349 | G | A | 1018.77 | SNP | Rv1996 | Ala116Thr | 22 | - | | 2247677 | A | C | 1554.77 | SNP | Rv2002 (fabG3) | silent (Ile6) | 9872 | - | | 2248179 | A | G | 1263.77 | SNP | Rv2002 (fabG3) | Ser174Gly | 21 | - | | 2251999 | A | G | 1232.77 | SNP | intergenic |  |  | - | | 2253453 | T | C | 1664.77 | SNP | Rv2006 (otsB1) | silent (Arg484) | 9913 | - | | 2260151 | A | G | 462.77 | SNP | intergenic |  |  | - | | 2260154 | C | T | 479.77 | SNP | intergenic |  |  | - | | 2260171 | T | C | 816.77 | SNP | intergenic |  |  | - | | 2260174 | C | T | 772.77 | SNP | intergenic |  |  | - | | 2260196 | C | CA | 822.73 | INS | intergenic |  |  | - | | 2260199 | C | T | 517.77 | SNP | intergenic |  |  | - | | 2260212 | G | T | 383.77 | SNP | intergenic |  |  | - | | 2260214 | G | C | 379.77 | SNP | intergenic |  |  | - | | 2260220 | C | T | 315.77 | SNP | intergenic |  |  | - | | 2260222 | C | G | 320.77 | SNP | intergenic |  |  | - | | 2260231 | T | C | 354.77 | SNP | intergenic |  |  | - | | 2260525 | C | T | 1009.77 | SNP | intergenic |  |  | - | | 2263760 | C | T | 746.77 | SNP | Rv2016 | Pro112Leu | 3 | - | | 2264782 | C | A | 1677.77 | SNP | Rv2017 | Ala262Glu | 10 | - | | 2265059 | T | G | 1238.77 | SNP | intergenic |  |  | - | | 2266487 | G | C | 1272.77 | SNP | Rv2020c | silent (Leu78) | 9947 | - | | 2266504 | T | TA | 1911.73 | INS | Rv2020c |  |  | - | | 2266508 | A | T | 1049.77 | SNP | Rv2020c | Asp71Glu | 56 | - | | 2266511 | GT | G | 1756.73 | DEL | Rv2020c |  |  | - | | 2266517 | T | C | 1038.77 | SNP | Rv2020c | silent (Glu68) | 9865 | - | | 2266550 | G | T | 1150.77 | SNP | Rv2020c | silent (Gly57) | 9935 | - | | 2266553 | C | G | 1278.77 | SNP | Rv2020c | silent (Ser56) | 9840 | - | | 2266583 | C | G | 1300.77 | SNP | Rv2020c | Glu46Asp | 53 | - | | 2266598 | G | C | 1291.77 | SNP | Rv2020c | silent (Leu41) | 9947 | - | | 2266604 | C | G | 1172.77 | SNP | Rv2020c | silent (Ser39) | 9840 | - | | 2266613 | G | GC | 2174.73 | INS | Rv2020c |  |  | - | | 2266624 | G | T | 1169.77 | SNP | Rv2020c | Leu33Ile | 9 | - | | 2269780 | T | C | 917.77 | SNP | Rv2024c | Asp154Gly | 11 | - | | 2270102 | A | G | 1168.77 | SNP | Rv2024c | Trp47Arg | 8 | - | | 2282787 | C | T | 1004.77 | SNP | Rv2037c | Cys312Tyr | 3 | - | | 2285251 | C | A | 1528.77 | SNP | Rv2039c | Val131Phe | 0 | - | | 2287121 | A | G | 1118.77 | SNP | Rv2041c | silent (Asp242) | 9859 | - | | 2295692 | C | T | 95.03 | SNP | Rv2048c (pks12) | Val(s)3765Val | 13 | - | | 2296042 | G | C | 986.77 | SNP | Rv2048c (pks12) | Pro3649Ala | 22 | - | | 2300237 | A | G | 557.77 | SNP | Rv2048c (pks12) | silent (Ala2250) | 9867 | - | | 2300546 | A | T | 1057.77 | SNP | Rv2048c (pks12) | His2147Gln | 23 | - | | 2300552 | T | G | 917.77 | SNP | Rv2048c (pks12) | silent (Pro2145) | 9926 | - | | 2300555 | A | G | 946.77 | SNP | Rv2048c (pks12) | silent (Asp2144) | 9859 | - | | 2301965 | G | A | 979.78 | SNP | Rv2048c (pks12) | silent (Pro1674) | 9926 | - | | 2307701 | G | A | 1612.77 | SNP | intergenic |  |  | - | | 2311099 | C | G | 425.77 | SNP | Rv2052c | silent (Pro473) | 9926 | - | | 2325009 | C | T | 1709.77 | SNP | Rv2067c | Glu288Lys | 7 | - | | 2329533 | A | G | 1062.77 | SNP | Rv2072c (cobL) | Leu205Pro | 2 | - | | 2330324 | G | A | 875.77 | SNP | Rv2073c | Arg214Cys | 1 | - | | 2334007 | A | G | 1883.77 | SNP | Rv2077c | silent (Ala96) | 9867 | - | | 2335075 | A | G | 1462.77 | SNP | Rv2078 | Glu6Gly | 7 | - | | 2335494 | A | G | 999.77 | SNP | Rv2079 | Tyr47Cys | 3 | - | | 2338768 | G | T | 1175.77 | SNP | Rv2082 | silent (Pro20) | 9926 | - | | 2338773 | G | A | 1152.77 | SNP | Rv2082 | Arg22Gln | 9 | - | | 2338866 | T | G | 1210.77 | SNP | Rv2082 | Leu53Arg | 1 | - | | 2338912 | A | C | 1402.77 | SNP | Rv2082 | silent (Arg68) | 9913 | - | | 2338961 | G | A | 1410.77 | SNP | Rv2082 | Val85Ile | 33 | - | | 2338990 | G | C | 1323.77 | SNP | Rv2082 | silent (Ala94) | 9867 | - | | 2338994 | G | A | 1205.77 | SNP | Rv2082 | Ala96Thr | 22 | - | | 2339605 | A | G | 111.03 | SNP | Rv2082 | silent (Pro299) | 9926 | - | | 2339608 | AGCAGCG | A | 418.87 | DEL | Rv2082 |  |  | - | | 2340621 | C | G | 1058.77 | SNP | Rv2082 | Pro638Arg | 4 | - | | 2341636 | C | G | 353.77 | SNP | Rv2083 | Leu256Val(s) | 4 | - | | 2345037 | C | A | 1163.77 | SNP | Rv2088 (pknJ) | silent (Leu209) | 9947 | - | | 2348446 | C | G | 1068.77 | SNP | Rv2090 | Phe358Leu(s) | 2 | - | | 2357268 | TGCC | T | 279.80 | DEL | intergenic |  |  | - | | 2358029 | T | TG | 1334.73 | INS | intergenic |  |  | - | | 2361604 | C | G | 639.77 | SNP | Rv2101 (helZ) | Val455Val(s) | 18 | - | | 2362041 | C | A | 1037.77 | SNP | Rv2101 (helZ) | Pro601Gln | 6 | - | | 2368564 | TA | T | 2493.73 | DEL | intergenic |  |  | - | | 2369326 | C | G | 1386.77 | SNP | Rv2109c (prcA) | Arg135Pro | 5 | - | | 2370533 | A | C | 1436.77 | SNP | Rv2110c (prcB) | silent (Ser23) | 9840 | - | | 2372550 | G | C | 40.77 | SNP | Rv2112c (dop) | Pro7Arg | 4 | - | | 2386389 | G | A | 813.77 | SNP | Rv2125 | Gly33Ser | 16 | - | | 2387733 | T | C | 128.03 | SNP | Rv2126c (PE\_PGRS37) | silent (Glu80) | 9865 | - | | 2396883 | G | A | 972.77 | SNP | intergenic |  |  | - | | 2401825 | T | C | 554.77 | SNP | intergenic |  |  | - | | 2415656 | G | C | 965.77 | SNP | Rv2155c (murD) | Arg247Gly | 1 | - | | 2416156 | G | A | 1055.77 | SNP | Rv2155c (murD) | Thr80Ile | 7 | - | | 2424008 | G | T | 62.74 | SNP | Rv2162c (PE\_PGRS38) | silent (Ala277) | 9867 | - | | 2424925 | A | G | 937.77 | SNP | intergenic |  |  | - | | 2426630 | C | T | 682.77 | SNP | Rv2163c (pbpB) | Arg153His | 8 | - | | 2438955 | G | A | 1146.77 | SNP | Rv2176 (pknL) | Gly339Arg | 0 | - | | 2439204 | A | G | 258.77 | SNP | intergenic |  |  | - | | 2440926 | G | T | 1824.77 | SNP | Rv2178c (aroG) | Asp265Glu | 56 | - | | 2441590 | T | C | 974.77 | SNP | Rv2178c (aroG) | Gln44Arg | 10 | - | | 2449284 | C | T | 1515.77 | SNP | Rv2187 (fadD15) | silent (Tyr375) | 9945 | - | | 2454856 | C | T | 1062.77 | SNP | Rv2191 | silent (Arg346) | 9913 | - | | 2460628 | C | A | 1792.77 | SNP | Rv2196 (qcrB) | silent (Ala317) | 9867 | - | | 2462871 | G | A | 802.77 | SNP | Rv2198c (mmpS3) | silent (Ala59) | 9867 | - | | 2475207 | G | A | 586.77 | SNP | Rv2210c (ilvE) | Ser255Leu(s) | 35 | - | | 2487490 | AG | A | 1655.73 | DEL | intergenic |  |  | - | | 2499726 | G | A | 1040.77 | SNP | Rv2226 | Asp299Asn | 36 | - | | 2506888 | T | G | 1339.77 | SNP | Rv2232 (ptkA) | Val204Gly | 5 | - | | 2509140 | G | C | 717.77 | SNP | Rv2236c (cobD) | Ser79Cys | 5 | - | | 2509722 | A | G | 1436.77 | SNP | Rv2237 | silent (Pro78) | 9926 | - | | 2512237 | T | TC | 1001.74 | INS | Rv2240c |  |  | - | | 2516567 | G | C | 1765.77 | SNP | intergenic |  |  | - | | 2521342 | T | C | 935.77 | SNP | Rv2247 (accD6) | silent (Asp200) | 9859 | - | | 2523205 | G | GCGC | 1803.73 | INS | intergenic |  |  | - | | 2523709 | G | A | 930.77 | SNP | Rv2249c (glpD1) | silent (Arg361) | 9913 | - | | 2525722 | CG | C | 1048.73 | DEL | Rv2250A; Rv2251 |  |  | - | | 2526026 | G | A | 911.77 | SNP | Rv2251 | silent (Ala154) | 9867 | - | | 2526974 | T | C | 1544.77 | SNP | Rv2251 | silent (Pro470) | 9926 | - | | 2527120 | C | T | 889.77 | SNP | Rv2252 | silent (Asp44) | 9859 | - | | 2529680 | A | G | 1184.77 | SNP | Rv2256c | silent (Thr65) | 9871 | - | | 2531742 | A | G | 1511.77 | SNP | Rv2258c | silent (Ala52) | 9867 | - | | 2532017 | G | C | 207.77 | SNP | intergenic |  |  | - | | 2534562 | GGA | G | 1572.73 | DEL | Rv2262c |  |  | - | | 2537780 | G | A | 1135.77 | SNP | Rv2264c | Arg191Trp | 2 | - | | 2540326 | T | A | 1204.77 | SNP | Rv2266 (cyp124) | Tyr75Asn | 4 | - | | 2557095 | G | T | 1581.77 | SNP | Rv2284 (lipM) | silent (Leu317) | 9947 | - | | 2559686 | C | T | 1732.77 | SNP | intergenic |  |  | - | | 2573756 | C | A | 1393.77 | SNP | intergenic |  |  | - | | 2574022 | C | T | 1137.77 | SNP | Rv2302 | silent (Arg70) | 9913 | - | | 2577461 | C | G | 835.77 | SNP | Rv2306A | silent (Ala118) | 9867 | - | | 2578626 | A | G | 1202.77 | SNP | Rv2307c | Met(s)24Thr | 22 | - | | 2580954 | G | C | 1652.77 | SNP | Rv2308 | Arg179Pro | 5 | - | | 2582348 | G | T | 879.77 | SNP | intergenic |  |  | - | | 2585809 | G | A | 687.77 | SNP | Rv2313c | Ala33Val(s) | 9867 | - | | 2586127 | A | G | 971.77 | SNP | Rv2314c | silent (Gly388) | 9935 | - | | 2589216 | G | C | 1719.77 | SNP | Rv2316 (uspA) | Val(s)127Leu | 3 | - | | 2598400 | A | G | 1685.77 | SNP | Rv2326c | silent (Asn516) | 9822 | - | | 2603797 | G | A | 1955.77 | SNP | Rv2330c (lppP) | silent (Ile142) | 9872 | - | | 2608117 | C | A | 1241.77 | SNP | Rv2333c (stp) | Asp69Tyr | 0 | - | | 2610702 | T | C | 1387.77 | SNP | intergenic |  |  | - | | 2611891 | T | A | 1351.77 | SNP | Rv2337c | Asp366Val | 1 | - | | 2612632 | C | A | 720.77 | SNP | Rv2337c | Gly119Val | 3 | - | | 2617673 | G | A | 1267.77 | SNP | Rv2340c (PE\_PGRS39) | silent (His412) | 9912 | - | | 2623232 | C | G | 947.77 | SNP | Rv2344c (dgt) | Gly174Ala | 21 | - | | 2626004 | G | A | 1062.77 | SNP | Rv2346c (esxO) | Leu57Leu(s) | 4 | - | | 2626011 | G | A | 1194.77 | SNP | Rv2346c (esxO) | silent (Ile54) | 9872 | - | | 2626018 | T | C | 34.77 | SNP | Rv2346c (esxO) | Glu52Gly | 7 | - | | 2626101 | C | A | 285.77 | SNP | Rv2346c (esxO) | silent (Leu24) | 9947 | - | | 2626105 | A | G | 429.77 | SNP | Rv2346c (esxO) | Leu(s)23Ser | 28 | - | | 2626108 | C | G | 393.77 | SNP | Rv2346c (esxO) | Gly22Ala | 21 | - | | 2626110 | G | C | 444.77 | SNP | Rv2346c (esxO) | silent (Ala21) | 9867 | - | | 2626262 | C | T | 225.77 | SNP | Rv2347c (esxP) | silent (Glu86) | 9865 | - | | 2630158 | C | G | 422.77 | SNP | Rv2350c (plcB) | silent (Arg54) | 9913 | - | | 2630161 | A | G | 432.77 | SNP | Rv2350c (plcB) | silent (Asn53) | 9822 | - | | 2630173 | C | G | 270.77 | SNP | Rv2350c (plcB) | Leu(s)49Phe | 1 | - | | 2630176 | C | G | 263.77 | SNP | Rv2350c (plcB) | Leu(s)48Phe | 1 | - | | 2630182 | G | A | 212.77 | SNP | Rv2350c (plcB) | silent (Ile46) | 9872 | - | | 2630184 | T | A | 208.77 | SNP | Rv2350c (plcB) | Ile46Phe | 8 | - | | 2630188 | C | T | 217.77 | SNP | Rv2350c (plcB) | silent (Glu44) | 9865 | - | | 2630206 | T | G | 145.77 | SNP | Rv2350c (plcB) | silent (Gly38) | 9935 | - | | 2630211 | G | A | 139.77 | SNP | Rv2350c (plcB) | Pro37Ser | 17 | - | | 2630215 | A | G | 132.77 | SNP | Rv2350c (plcB) | silent (Pro35) | 9926 | - | | 2630224 | T | G | 92.77 | SNP | Rv2350c (plcB) | silent (Gly32) | 9935 | - | | 2631556 | C | G | 79.77 | SNP | Rv2351c (plcA) | Gly174Arg | 0 | - | | 2631565 | T | C | 104.77 | SNP | Rv2351c (plcA) | Ile171Val | 57 | - | | 2631574 | T | C | 84.77 | SNP | Rv2351c (plcA) | Thr168Ala | 32 | - | | 2631583 | G | A | 134.77 | SNP | Rv2351c (plcA) | Leu165Leu(s) | 4 | - | | 2631599 | G | A | 161.77 | SNP | Rv2351c (plcA) | silent (Ile159) | 9872 | - | | 2631620 | A | G | 216.77 | SNP | Rv2351c (plcA) | silent (Gly152) | 9935 | - | | 2633009 | GCATACCCGCCCCAGCACCC CC | G | 5785.73 | DEL | Rv2352c (PPE38) |  |  | - | | 2637541 | C | T | 2481.77 | SNP | intergenic |  |  | - | | 2638997 | G | A | 530.77 | SNP | Rv2356c (PPE40) | Ser180Leu(s) | 35 | - | | 2642383 | C | T | 1245.77 | SNP | Rv2360c | Ala66Thr | 22 | - | | 2645270 | C | T | 1317.77 | SNP | Rv2363 (amiA2) | silent (Gly317) | 9935 | - | | 2652174 | C | A | 1023.77 | SNP | Rv2372c | Val218Phe | 0 | - | | 2655691 | T | C | 1658.77 | SNP | Rv2376c (cfp2) | Gln142Arg | 10 | - | | 2656225 | A | G | 1722.77 | SNP | Rv2377c (mbtH) | Val69Ala | 18 | - | | 2660091 | T | G | 1389.77 | SNP | Rv2379c (mbtF) | silent (Ala665) | 9867 | - | | 2660319 | C | G | 1224.77 | SNP | Rv2379c (mbtF) | Glu589Asp | 53 | - | | 2671061 | C | A | 1091.77 | SNP | Rv2382c (mbtC) | silent (Ala181) | 9867 | - | | 2676498 | A | C | 797.77 | SNP | Rv2384 (mbtA) | Gln188Pro | 8 | - | | 2680658 | T | G | 2218.77 | SNP | intergenic |  |  | - | | 2695378 | C | G | 1565.77 | SNP | Rv2398c (cysW) | Gly141Ala | 21 | - | | 2700239 | G | A | 1169.77 | SNP | Rv2402 | Val(s)571Met(s) | 9867 | - | | 2703915 | CGCGGGCCGCCCAGCGGCCC GCTGAGGAGCCGGGCAGTCA GCCCCGCCCGGCGACGAT | C | 3848.73 | DEL | intergenic |  |  | - | | 2704884 | A | ACAGCGACCATATCGCCGAG CT | 32729.73 | INS | Rv2407 |  |  | - | | 2713795 | C | T | 1179.77 | SNP | intergenic |  |  | - | | 2718852 | T | G | 1465.77 | SNP | intergenic |  |  | - | | 2720660 | C | CCATTTCGGCA | 3902.14 | INS | intergenic |  |  | - | | 2720665 | ACCGGTCGGAT | A | 3022.73 | DEL | intergenic |  |  | - | | 2720676 | T | A | 717.77 | SNP | intergenic |  |  | - | | 2721013 | A | G | 750.77 | SNP | Rv2424c | silent (Arg255) | 9913 | - | | 2721562 | C | G | 1138.77 | SNP | Rv2424c | silent (Ala72) | 9867 | - | | 2730752 | T | C | 1366.77 | SNP | Rv2435c | silent (Ser666) | 9840 | - | | 2734074 | T | C | 118.03 | SNP | Rv2436 (rbsK) | Val282Ala | 18 | - | | 2737572 | C | A | 772.77 | SNP | Rv2439c (proB) | Ala226Ser | 28 | - | | 2739174 | T | G | 825.77 | SNP | Rv2440c (obg) | silent (Gly171) | 9935 | - | | 2751804 | C | T | 738.77 | SNP | Rv2450c (rpfE) | Arg126Gln | 9 | - | | 2752698 | C | A | 2317.77 | SNP | intergenic |  |  | - | | 2752854 | G | A | 2280.77 | SNP | Rv2452c | silent (Asp47) | 9859 | - | | 2759082 | T | G | 1373.77 | SNP | Rv2457c (clpX) | Lys136Thr | 8 | - | | 2760152 | A | G | 1097.77 | SNP | Rv2458 (mmuM) | Tyr125Cys | 3 | - | | 2760241 | G | A | 1092.77 | SNP | Rv2458 (mmuM) | Glu155Lys | 7 | - | | 2760985 | G | T | 1696.77 | SNP | Rv2459 | silent (Leu44) | 9947 | - | | 2772619 | G | C | 1566.77 | SNP | Rv2469c | silent (Ala139) | 9867 | - | | 2773931 | T | C | 1433.77 | SNP | Rv2471 (aglA) | Met(s)123Thr | 22 | - | | 2779136 | T | C | 900.77 | SNP | Rv2476c (gdh) | Ser1043Gly | 21 | - | | 2786952 | A | G | 1380.77 | SNP | Rv2482c (plsB2) | Cys778Arg | 1 | - | | 2788745 | C | T | 962.77 | SNP | Rv2482c (plsB2) | Arg180Gln | 9 | - | | 2808506 | G | C | 1271.77 | SNP | Rv2494 (vapC38) | Arg66Pro | 5 | - | | 2809621 | T | C | 1250.77 | SNP | Rv2495c (bkdC) | Thr107Ala | 32 | - | | 2818837 | A | G | 1225.77 | SNP | Rv2503c (scoB) | silent (Gly97) | 9935 | - | | 2821342 | C | T | 876.77 | SNP | Rv2505c (fadD35) | silent (Ala85) | 9867 | - | | 2827984 | G | T | 1204.77 | SNP | intergenic |  |  | - | | 2828019 | T | C | 1487.77 | SNP | intergenic |  |  | - | | 2828822 | G | T | 253.78 | SNP | Rv2512c | Gln328Lys | 12 | - | | 2829779 | T | C | 319.78 | SNP | Rv2512c | Thr9Ala | 32 | - | | 2830525 | C | A | 1407.77 | SNP | Rv2513 | Thr122Lys | 11 | - | | 2841238 | C | A | 1090.77 | SNP | Rv2524c (fas) | Ala2699Ser | 28 | - | | 2843168 | C | T | 630.77 | SNP | Rv2524c (fas) | silent (Glu2055) | 9865 | - | | 2855259 | A | G | 961.77 | SNP | Rv2531c | silent (Ala841) | 9867 | - | | 2862052 | C | T | 795.77 | SNP | Rv2538c (aroB) | Ala209Thr | 22 | - | | 2865760 | A | G | 1541.77 | SNP | Rv2542 | Thr211Ala | 32 | - | | 2865882 | T | C | 1682.77 | SNP | Rv2542 | silent (Val251) | 9901 | - | | 2866503 | G | A | 30.77 | SNP | Rv2543 (lppA) | silent (Pro12) | 9926 | - | | 2866551 | C | G | 322.77 | SNP | Rv2543 (lppA) | silent (Ala28) | 9867 | - | | 2866569 | C | A | 408.77 | SNP | Rv2543 (lppA) | silent (Thr34) | 9871 | - | | 2866578 | C | A | 473.77 | SNP | Rv2543 (lppA) | His37Gln | 23 | - | | 2866580 | A | G | 583.77 | SNP | Rv2543 (lppA) | Asn38Ser | 34 | - | | 2866607 | G | A | 573.77 | SNP | Rv2543 (lppA) | Gly47Asp | 6 | - | | 2866647 | G | A | 435.77 | SNP | Rv2543 (lppA) | silent (Lys60) | 9926 | - | | 2866671 | G | A | 471.77 | SNP | Rv2543 (lppA) | silent (Glu68) | 9865 | - | | 2866677 | A | C | 428.77 | SNP | Rv2543 (lppA) | silent (Leu70) | 9947 | - | | 2866863 | C | G | 593.77 | SNP | Rv2543 (lppA) | silent (Ala132) | 9867 | - | | 2866876 | A | G | 525.77 | SNP | Rv2543 (lppA) | Ile137Val | 57 | - | | 2866880 | C | T | 509.77 | SNP | Rv2543 (lppA) | Ala138Val | 13 | - | | 2866882 | G | A | 588.77 | SNP | Rv2543 (lppA) | Ala139Thr | 22 | - | | 2867207 | C | G | 781.77 | SNP | Rv2544 (lppB) | silent (Ala28) | 9867 | - | | 2867230 | G | A | 157.77 | SNP | Rv2544 (lppB) | Gly36Asp | 6 | - | | 2867231 | C | T | 152.77 | SNP | Rv2544 (lppB) | silent (Gly36) | 9935 | - | | 2867236 | A | G | 135.77 | SNP | Rv2544 (lppB) | Asn38Ser | 34 | - | | 2867240 | C | T | 113.77 | SNP | Rv2544 (lppB) | silent (Pro39) | 9926 | - | | 2867245 | A | C | 162.77 | SNP | Rv2544 (lppB) | Lys41Thr | 8 | - | | 2867251 | C | G | 123.77 | SNP | Rv2544 (lppB) | Pro43Arg | 4 | - | | 2867254 | A | G | 84.77 | SNP | Rv2544 (lppB) | His44Arg | 10 | - | | 2867263 | G | A | 187.77 | SNP | Rv2544 (lppB) | Gly47Asp | 6 | - | | 2867298 | C | A | 67.77 | SNP | Rv2544 (lppB) | His59Asn | 21 | - | | 2867347 | A | G | 49.77 | SNP | Rv2544 (lppB) | Gln75Arg | 10 | - | | 2871977 | C | G | 769.77 | SNP | Rv2552c (aroE) | silent (Ser13) | 9840 | - | | 2880702 | G | C | 1434.77 | SNP | Rv2560 | Val210Leu | 15 | - | | 2881597 | AG | A | 1057.73 | DEL | Rv2561 |  |  | - | | 2885734 | G | A | 698.77 | SNP | Rv2565 | Gly375Asp | 6 | - | | 2888201 | T | C | 534.77 | SNP | Rv2566 | Leu610Pro | 2 | - | | 2889633 | T | C | 925.77 | SNP | Rv2566 | silent (Ala1087) | 9867 | - | | 2891267 | C | T | 1377.77 | SNP | Rv2567 | silent (Gly491) | 9935 | - | | 2891728 | A | G | 1324.77 | SNP | Rv2567 | Gln645Arg | 10 | - | | 2894208 | G | A | 1115.77 | SNP | Rv2569c | silent (Ser67) | 9840 | - | | 2906978 | C | T | 660.77 | SNP | Rv2582 (ppiB) | silent (Leu55) | 9947 | - | | 2910461 | G | T | 774.77 | SNP | Rv2584c (apt) | Ala147Glu | 10 | - | | 2911293 | C | G | 1409.77 | SNP | Rv2585c | Cys462Ser | 11 | - | | 2912294 | T | G | 1219.77 | SNP | Rv2585c | silent (Ala128) | 9867 | - | | 2913078 | C | G | 1048.77 | SNP | Rv2586c (secF) | Val(s)312Leu | 3 | - | | 2917173 | G | A | 868.77 | SNP | Rv2589 (gabT) | Gly272Arg | 0 | - | | 2923391 | T | C | 686.77 | SNP | Rv2592c (ruvB) | silent (Pro281) | 9926 | - | | 2927939 | T | C | 1713.77 | SNP | intergenic |  |  | - | | 2935682 | C | A | 865.77 | SNP | Rv2608 (PPE42) | silent (Arg213) | 9913 | - | | 2939373 | G | C | 1125.77 | SNP | Rv2611c | Ser197Cys | 5 | - | | 2939657 | T | C | 546.77 | SNP | Rv2611c | Ile102Met(s) | 6 | - | | 2946570 | G | A | 1209.77 | SNP | Rv2618 | Gly46Asp | 6 | - | | 2953367 | T | C | 1066.77 | SNP | intergenic |  |  | - | | 2954439 | T | C | 1446.77 | SNP | Rv2627c | Arg104Gly | 1 | - | | 2958749 | G | A | 1734.77 | SNP | Rv2631 | Gly393Asp | 6 | - | | 2961922 | C | T | 90.28 | SNP | Rv2634c (PE\_PGRS46) | Asp174Asn | 36 | - | | 2974933 | A | G | 292.78 | SNP | Rv2650c | Ile101Thr | 11 | - | | 2983613 | G | A | 46.74 | SNP | Rv2666 | silent (Gly181) | 9935 | - | | 2984740 | A | G | 636.77 | SNP | Rv2668 | His3Arg | 10 | - | | 3001395 | T | C | 2092.77 | SNP | Rv2684 (arsA) | Val(s)261Ala | 9867 | - | | 3005185 | G | T | 1330.77 | SNP | Rv2688c | Pro156Thr | 5 | - | | 3005618 | C | T | 1495.77 | SNP | Rv2688c | silent (Ala11) | 9867 | - | | 3009692 | A | G | 1334.77 | SNP | Rv2691 (ceoB) | Thr117Ala | 32 | - | | 3015140 | C | T | 1820.77 | SNP | intergenic |  |  | - | | 3017276 | A | G | 1504.77 | SNP | Rv2702 (ppgK) | Asn140Ser | 34 | - | | 3017465 | T | C | 1678.77 | SNP | Rv2702 (ppgK) | Ile203Thr | 11 | - | | 3037377 | T | C | 528.77 | SNP | intergenic |  |  | - | | 3041871 | G | T | 1407.77 | SNP | Rv2729c | Ala202Glu | 10 | - | | 3054081 | A | G | 1503.77 | SNP | Rv2741 (PE\_PGRS47) | silent (Gly56) | 9935 | - | | 3054321 | A | G | 286.78 | SNP | Rv2741 (PE\_PGRS47) | silent (Gly136) | 9935 | - | | 3057137 | C | T | 1215.77 | SNP | Rv2743c | silent (Lys32) | 9926 | - | | 3067901 | G | A | 1231.77 | SNP | Rv2754c (thyX) | silent (Thr15) | 9871 | - | | 3069167 | A | G | 1183.77 | SNP | Rv2756c (hsdM) | Leu306Pro | 2 | - | | 3078178 | C | T | 1592.77 | SNP | Rv2769c (PE27) | Val(s)270Met(s) | 9867 | - | | 3080795 | A | G | 1547.77 | SNP | Rv2771c | Leu80Pro | 2 | - | | 3086208 | G | T | 1105.77 | SNP | intergenic |  |  | - | | 3086788 | T | C | 1561.77 | SNP | intergenic |  |  | - | | 3089776 | G | A | 939.77 | SNP | Rv2782c (pepR) | Arg196Trp | 2 | - | | 3098497 | A | C | 1118.77 | SNP | Rv2789c (fadE21) | Ser148Ala | 35 | - | | 3099091 | TCCG | T | 3449.73 | DEL | Rv2790c (ltp1) |  |  | - | | 3100151 | A | ACGACC | 2483.73 | INS | Rv2790c (ltp1) |  |  | - | | 3103682 | T | C | 882.77 | SNP | Rv2794c (pptT) | Met(s)87Val(s) | 9867 | - | | 3105748 | C | T | 754.77 | SNP | Rv2797c | Met(s)520Ile | 2 | - | | 3113491 | A | AG | 2230.73 | INS | intergenic |  |  | - | | 3113872 | A | T | 890.77 | SNP | Rv2807 | Glu72Val(s) | 17 | - | | 3114597 | C | A | 1306.77 | SNP | Rv2807 | Leu314Met(s) | 4 | - | | 3118000 | A | G | 628.77 | SNP | Rv2812 | Arg395Gly | 1 | - | | 3119345 | C | T | 954.77 | SNP | intergenic |  |  | - | | 3122201 | A | AG | 208.80 | INS | intergenic |  |  | - | | 3122202 | C | T | 128.90 | SNP | intergenic |  |  | - | | 3122206 | CTGCAGCT | C | 521.80 | DEL | intergenic |  |  | - | | 3122217 | C | G | 165.84 | SNP | intergenic |  |  | - | | 3122221 | GT | G | 151.80 | DEL | intergenic |  |  | - | | 3122224 | C | CGGCGCGATTG | 619.80 | INS | intergenic |  |  | - | | 3122225 | A | T | 126.90 | SNP | intergenic |  |  | - | | 3122228 | C | CCG | 292.80 | INS | intergenic |  |  | - | | 3129359 | C | T | 2278.77 | SNP | Rv2823c | silent (Lys805) | 9926 | - | | 3131469 | T | TTGTCGGCGA | 2926.73 | INS | Rv2823c |  |  | - | | 3133536 | T | C | 1851.77 | SNP | Rv2825c | Lys2Glu | 4 | - | | 3135912 | G | C | 534.77 | SNP | Rv2828c | Thr141Arg | 1 | - | | 3137058 | G | A | 1473.77 | SNP | Rv2830c (vapB22) | Ala56Val(s) | 9867 | - | | 3157349 | C | T | 981.77 | SNP | Rv2848c (cobB) | Gly58Asp | 6 | - | | 3159998 | CAA | C | 3104.73 | DEL | Rv2850c |  |  | - | | 3162805 | C | G | 248.80 | SNP | Rv2853 (PE\_PGRS48) | Arg180Gly | 1 | - | | 3170663 | T | C | 867.77 | SNP | Rv2858c (aldC) | Thr21Ala | 32 | - | | 3177884 | C | A | 1511.77 | SNP | Rv2866 (relG) | silent (Arg21) | 9913 | - | | 3183561 | G | C | 149.77 | SNP | Rv2872 (vapC43) | silent (Pro60) | 9926 | - | | 3186860 | T | G | 1064.77 | SNP | Rv2874 (dipZ) | Tyr672Asp | 0 | - | | 3189433 | C | T | 1446.77 | SNP | intergenic |  |  | - | | 3190145 | TC | T | 1902.73 | DEL | Rv2880c |  |  | - | | 3207290 | C | A | 571.77 | SNP | Rv2897c | Gly218Val | 3 | - | | 3219790 | G | A | 1089.77 | SNP | Rv2912c | silent (Ile24) | 9872 | - | | 3226181 | A | C | 870.77 | SNP | Rv2916c (ffh) | silent (Arg35) | 9913 | - | | 3227488 | A | G | 964.77 | SNP | Rv2917 | Ser376Gly | 21 | - | | 3228143 | G | T | 434.77 | SNP | Rv2917 | Arg594Leu | 1 | - | | 3231091 | C | A | 1652.77 | SNP | Rv2920c (amt) | Val(s)472Val | 13 | - | | 3232703 | G | A | 130.77 | SNP | intergenic |  |  | - | | 3232759 | G | A | 420.77 | SNP | intergenic |  |  | - | | 3247316 | C | G | 961.77 | SNP | Rv2931 (ppsA) | Asp624Glu | 56 | - | | 3247851 | G | A | 875.77 | SNP | Rv2931 (ppsA) | Ala803Thr | 22 | - | | 3247853 | C | T | 843.77 | SNP | Rv2931 (ppsA) | silent (Ala803) | 9867 | - | | 3247856 | G | C | 916.77 | SNP | Rv2931 (ppsA) | silent (Arg804) | 9913 | - | | 3247864 | C | CTAGG | 1781.73 | INS | Rv2931 (ppsA) |  |  | - | | 3247865 | GCAAA | G | 1827.73 | DEL | Rv2931 (ppsA) |  |  | - | | 3247874 | G | A | 840.77 | SNP | Rv2931 (ppsA) | silent (Arg810) | 9913 | - | | 3247877 | T | C | 900.77 | SNP | Rv2931 (ppsA) | silent (Phe811) | 9946 | - | | 3247883 | T | C | 1044.77 | SNP | Rv2931 (ppsA) | silent (Ser813) | 9840 | - | | 3248074 | G | A | 1125.77 | SNP | Rv2931 (ppsA) | Arg877His | 8 | - | | 3248075 | C | T | 1114.77 | SNP | Rv2931 (ppsA) | silent (Arg877) | 9913 | - | | 3249411 | G | A | 1375.77 | SNP | Rv2931 (ppsA) | Gly1323Ser | 16 | - | | 3251956 | C | G | 1411.77 | SNP | Rv2932 (ppsB) | silent (Arg295) | 9913 | - | | 3256494 | A | G | 1355.77 | SNP | Rv2933 (ppsC) | silent (Gly270) | 9935 | - | | 3258274 | A | C | 669.77 | SNP | Rv2933 (ppsC) | Ser864Arg | 6 | - | | 3266288 | C | G | 899.77 | SNP | Rv2934 (ppsD) | silent (Ser1347) | 9840 | - | | 3269581 | A | G | 1487.77 | SNP | Rv2935 (ppsE) | silent (Ala615) | 9867 | - | | 3270784 | A | G | 1070.77 | SNP | Rv2935 (ppsE) | silent (Gln1016) | 9876 | - | | 3289923 | C | A | 1403.77 | SNP | Rv2943A; Rv2944 | silent (Ala73); Pro45His | 9867; 3 | - | | 3296843 | A | G | 366.77 | SNP | Rv2947c (pks15) | Val(s)333Ala | 9867 | - | | 3303669 | C | A | 1270.77 | SNP | Rv2951c | Ala194Ser | 28 | - | | 3308489 | C | T | 1266.77 | SNP | Rv2955c | silent (Pro19) | 9926 | - | | 3308606 | G | A | 1698.77 | SNP | intergenic |  |  | - | | 3336646 | T | A | 95.77 | SNP | intergenic |  |  | - | | 3336679 | TA | T | 307.73 | DEL | intergenic |  |  | - | | 3336825 | T | C | 805.77 | SNP | Rv2981c (ddlA) | Thr365Ala | 32 | - | | 3338603 | G | C | 1174.77 | SNP | Rv2982c (gpdA2) | Pro133Ala | 22 | - | | 3352244 | A | G | 1473.77 | SNP | Rv2994 | Thr326Ala | 32 | - | | 3352932 | C | G | 1232.77 | SNP | Rv2995c (leuB) | silent (Thr179) | 9871 | - | | 3355161 | C | T | 753.77 | SNP | Rv2997 | silent (Arg21) | 9913 | - | | 3358235 | A | T | 1464.77 | SNP | Rv2999 (lppY) | Met(s)212Leu(s) | 9867 | - | | 3363338 | A | G | 1169.77 | SNP | intergenic |  |  | - | | 3363866 | G | A | 1595.77 | SNP | Rv3005c | Leu223Leu(s) | 4 | - | | 3367765 | G | A | 1066.77 | SNP | Rv3009c (gatB) | silent (Gly343) | 9935 | - | | 3381641 | G | T | 140.84 | SNP | Rv3023c | Gln328Lys | 12 | - | | 3401871 | A | G | 613.80 | SNP | Rv3041c | silent (Ala16) | 9867 | - | | 3402816 | C | T | 1112.77 | SNP | Rv3042c (serB2) | Gly116Glu | 4 | - | | 3404376 | C | G | 1321.77 | SNP | Rv3043c (ctaD) | silent (Thr182) | 9871 | - | | 3415180 | ACACCTAGGGGGTGG | A | 6295.73 | DEL | intergenic |  |  | - | | 3420388 | C | T | 1048.77 | SNP | Rv3059 (cyp136) | silent (Phe299) | 9946 | - | | 3425854 | C | T | 1146.77 | SNP | Rv3062 (ligB) | Pro91Ser | 17 | - | | 3428374 | C | G | 2070.77 | SNP | Rv3063 (cstA) | His378Asp | 4 | - | | 3428917 | C | A | 1594.77 | SNP | Rv3063 (cstA) | Arg559Ser | 11 | - | | 3435765 | G | T | 1273.77 | SNP | intergenic |  |  | - | | 3440464 | T | G | 1155.77 | SNP | Rv3077 | silent (Arg308) | 9913 | - | | 3440468 | G | C | 1142.77 | SNP | Rv3077 | Gly310Arg | 0 | - | | 3440895 | G | A | 1400.77 | SNP | Rv3077 | Arg452His | 8 | - | | 3442631 | G | T | 1562.77 | SNP | intergenic |  |  | - | | 3450725 | T | C | 1210.77 | SNP | Rv3084 (lipR) | silent (Val243) | 9901 | - | | 3455220 | G | A | 453.77 | SNP | Rv3088 (tgs4) | Arg294Gln | 9 | - | | 3455686 | G | C | 2032.77 | SNP | Rv3088 (tgs4) | silent (Leu449) | 9947 | - | | 3456666 | A | G | 1049.77 | SNP | Rv3089 (fadD13) | silent (Ala302) | 9867 | - | | 3458659 | G | A | 1580.77 | SNP | Rv3090 | Arg150His | 8 | - | | 3462135 | G | C | 1139.77 | SNP | Rv3093c | Cys210Trp | 0 | - | | 3462145 | A | AGGCGC | 2337.73 | INS | Rv3093c |  |  | - | | 3465812 | G | A | 1156.77 | SNP | Rv3097c (lipY) | Pro427Leu | 3 | - | | 3473996 | G | GA | 2187.73 | INS | intergenic |  |  | - | | 3480474 | G | A | 1415.77 | SNP | Rv3113 | Gly134Glu | 4 | - | | 3480722 | G | A | 1413.77 | SNP | Rv3113 | Ala217Thr | 22 | - | | 3482432 | C | A | 272.78 | SNP | Rv3115 | Gln328Lys | 12 | - | | 3486977 | A | G | 2245.77 | SNP | Rv3121 (cyp141) | Lys157Glu | 4 | - | | 3503895 | C | T | 674.77 | SNP | Rv3137 | Pro168Leu | 3 | - | | 3505027 | G | A | 360.77 | SNP | Rv3138 (pflA) | Arg278His | 8 | - | | 3517548 | A | G | 1213.77 | SNP | Rv3151 (nuoG) | Glu268Gly | 7 | - | | 3518167 | A | G | 1109.77 | SNP | Rv3151 (nuoG) | Ile474Met(s) | 6 | - | | 3518555 | A | G | 628.77 | SNP | Rv3151 (nuoG) | Thr604Ala | 32 | - | | 3528084 | A | G | 67.77 | SNP | Rv3159c (PPE53) | silent (Gly360) | 9935 | - | | 3528099 | A | C | 142.77 | SNP | Rv3159c (PPE53) | silent (Gly355) | 9935 | - | | 3528102 | C | A | 108.77 | SNP | Rv3159c (PPE53) | silent (Ser354) | 9840 | - | | 3528117 | T | C | 170.77 | SNP | Rv3159c (PPE53) | silent (Leu349) | 9947 | - | | 3528119 | G | T | 158.77 | SNP | Rv3159c (PPE53) | Leu349Ile | 9 | - | | 3528120 | A | G | 179.77 | SNP | Rv3159c (PPE53) | silent (Asn348) | 9822 | - | | 3528129 | G | T | 292.77 | SNP | Rv3159c (PPE53) | silent (Gly345) | 9935 | - | | 3528140 | G | A | 236.77 | SNP | Rv3159c (PPE53) | Leu342Leu(s) | 4 | - | | 3528144 | G | A | 241.77 | SNP | Rv3159c (PPE53) | silent (Gly340) | 9935 | - | | 3528158 | A | G | 417.77 | SNP | Rv3159c (PPE53) | Leu(s)336Leu | 3 | - | | 3528159 | G | A | 414.77 | SNP | Rv3159c (PPE53) | silent (Asn335) | 9822 | - | | 3528165 | G | A | 405.77 | SNP | Rv3159c (PPE53) | silent (Gly333) | 9935 | - | | 3528192 | G | A | 319.77 | SNP | Rv3159c (PPE53) | silent (Asn324) | 9822 | - | | 3528198 | A | G | 344.77 | SNP | Rv3159c (PPE53) | silent (Asn322) | 9822 | - | | 3529067 | G | C | 1823.77 | SNP | Rv3159c (PPE53) | Arg33Gly | 1 | - | | 3544710 | T | C | 1077.77 | SNP | Rv3176c (mesT) | silent (Pro197) | 9926 | - | | 3556275 | A | G | 1278.77 | SNP | Rv3190c | Leu138Pro | 2 | - | | 3556607 | G | T | 1363.77 | SNP | Rv3190c | Ser27Arg | 6 | - | | 3567600 | G | A | 1227.77 | SNP | Rv3197 | Val(s)193Met(s) | 9867 | - | | 3580636 | CT | C | 1552.73 | DEL | intergenic |  |  | - | | 3581414 | A | G | 1153.77 | SNP | Rv3204 | Thr34Ala | 32 | - | | 3583295 | C | T | 1425.77 | SNP | Rv3206c (moeB1) | Gly139Asp | 6 | - | | 3590686 | G | GC | 1811.73 | INS | intergenic |  |  | - | | 3591063 | T | C | 782.77 | SNP | Rv3213c | Lys144Glu | 4 | - | | 3595036 | C | T | 837.77 | SNP | Rv3218 | Pro190Leu | 3 | - | | 3597737 | C | T | 1591.77 | SNP | Rv3221c (TB7.3) | Val(s)10Val | 13 | - | | 3603355 | G | A | 879.77 | SNP | intergenic |  |  | - | | 3604821 | G | C | 326.77 | SNP | Rv3228 | silent (Ala32) | 9867 | - | | 3614982 | T | C | 2065.77 | SNP | Rv3239c | silent (Leu874) | 9947 | - | | 3621044 | T | C | 853.77 | SNP | Rv3241c | Ile71Val | 57 | - | | 3621423 | A | G | 1084.77 | SNP | intergenic |  |  | - | | 3622441 | A | C | 1016.77 | SNP | Rv3243c | Val217Val(s) | 18 | - | | 3625065 | T | G | 798.77 | SNP | Rv3245c (mtrB) | Met(s)517Leu | 3 | - | | 3629928 | T | C | 1276.77 | SNP | Rv3249c | Thr154Ala | 32 | - | | 3649543 | G | A | 1319.77 | SNP | Rv3268 | Asp42Asn | 36 | - | | 3663889 | C | A | 67.77 | SNP | Rv3281 (accE5) | Asn67Lys | 25 | - | | 3678813 | T | A | 1135.77 | SNP | Rv3296 (lhr) | Ile680Asn | 3 | - | | 3683037 | C | T | 1704.77 | SNP | intergenic |  |  | - | | 3689523 | G | T | 733.77 | SNP | Rv3303c (lpdA) | Cys472STOP | 3 | - | | 3691061 | A | C | 91.77 | SNP | intergenic |  |  | - | | 3692357 | G | C | 894.77 | SNP | Rv3305c (amiA1) | Ala151Gly | 21 | - | | 3704596 | G | C | 804.77 | SNP | Rv3317 (sdhD) | Val(s)54Leu | 3 | - | | 3709949 | G | A | 1056.77 | SNP | Rv3324c (moaC3) | silent (Leu100) | 9947 | - | | 3714108 | G | A | 794.77 | SNP | Rv3328c (sigJ) | silent (Thr75) | 9871 | - | | 3714211 | G | T | 1343.77 | SNP | Rv3328c (sigJ) | Pro41Gln | 6 | - | | 3714757 | A | C | 1025.77 | SNP | Rv3329 | Gln122His | 20 | - | | 3717562 | C | CGGT | 1805.73 | INS | Rv3331 (sugI) |  |  | - | | 3718357 | C | T | 1093.77 | SNP | Rv3331 (sugI) | Pro423Leu | 3 | - | | 3721806 | G | C | 881.77 | SNP | Rv3335c | silent (Gly265) | 9935 | - | | 3727746 | G | A | 722.77 | SNP | Rv3341 (metA) | Gly87Ser | 16 | - | | 3730390 | G | C | 951.77 | SNP | Rv3343c (PPE54) | Phe2182Leu(s) | 2 | - | | 3730394 | G | A | 900.77 | SNP | Rv3343c (PPE54) | Ala2181Val | 13 | - | | 3730582 | G | A | 128.03 | SNP | Rv3343c (PPE54) | silent (Asn2118) | 9822 | - | | 3730648 | A | G | 82.28 | SNP | Rv3343c (PPE54) | silent (Ser2096) | 9840 | - | | 3735813 | C | G | 432.77 | SNP | Rv3343c (PPE54) | Glu375Gln | 27 | - | | 3736628 | T | G | 1079.77 | SNP | Rv3343c (PPE54) | Glu103Ala | 17 | - | | 3738044 | G | T | 252.78 | SNP | intergenic |  |  | - | | 3744452 | A | C | 1085.77 | SNP | Rv3347c (PPE55) | silent (Pro2911) | 9926 | - | | 3746409 | A | G | 526.77 | SNP | Rv3347c (PPE55) | Leu2259Pro | 2 | - | | 3752207 | A | G | 884.77 | SNP | Rv3347c (PPE55) | silent (Ile326) | 9872 | - | | 3752809 | G | A | 377.77 | SNP | Rv3347c (PPE55) | Leu126Leu(s) | 4 | - | | 3752813 | G | A | 348.77 | SNP | Rv3347c (PPE55) | silent (Asn124) | 9822 | - | | 3752821 | T | C | 388.77 | SNP | Rv3347c (PPE55) | Met(s)122Val(s) | 9867 | - | | 3753116 | C | T | 281.90 | SNP | Rv3347c (PPE55) | silent (Pro23) | 9926 | - | | 3753164 | T | G | 558.77 | SNP | Rv3347c (PPE55) | silent (Pro7) | 9926 | - | | 3766860 | G | C | 42.74 | SNP | Rv3350c (PPE56) | silent (Ala81) | 9867 | - | | 3783058 | G | A | 1496.77 | SNP | Rv3370c (dnaE2) | silent (Ser561) | 9840 | - | | 3786162 | G | T | 1032.77 | SNP | Rv3371 | Asp411Tyr | 0 | - | | 3791438 | T | C | 2853.77 | SNP | Rv3377c | Lys306Glu | 4 | - | | 3798095 | A | C | 1993.77 | SNP | Rv3383c (idsB) | Val132Gly | 5 | - | | 3798451 | C | G | 1794.77 | SNP | Rv3383c (idsB) | silent (Gly13) | 9935 | - | | 3805526 | T | G | 1398.77 | SNP | Rv3390 (lpqD) | Val(s)221Gly | 21 | - | | 3812821 | A | C | 838.77 | SNP | Rv3396c (guaA) | Leu(s)420Val(s) | 9867 | - | | 3815477 | G | T | 1499.77 | SNP | Rv3398c (idsA1) | silent (Ala210) | 9867 | - | | 3817117 | C | A | 1311.77 | SNP | Rv3399 | Ala330Glu | 10 | - | | 3820407 | A | G | 71.77 | SNP | intergenic |  |  | - | | 3820545 | A | G | 102.77 | SNP | intergenic |  |  | - | | 3823159 | A | T | 1269.77 | SNP | Rv3403c | silent (Val235) | 9901 | - | | 3826684 | C | T | 1175.77 | SNP | Rv3408 (vapC47) | Ser46Leu(s) | 35 | - | | 3829770 | T | C | 1073.77 | SNP | Rv3410c (guaB3) | silent (Pro47) | 9926 | - | | 3838871 | A | G | 1285.77 | SNP | Rv3420c (rimI) | silent (Ala64) | 9867 | - | | 3854174 | G | A | 1550.77 | SNP | Rv3435c | Ala207Val(s) | 9867 | - | | 3859893 | C | T | 1144.77 | SNP | Rv3440c | silent (Glu28) | 9865 | - | | 3862472 | GA | G | 1553.73 | DEL | intergenic |  |  | - | | 3863814 | C | A | 795.77 | SNP | Rv3446c | Gly240Cys | 0 | - | | 3864995 | T | C | 742.77 | SNP | Rv3447c (eccC4) | Ser1082Gly | 21 | - | | 3866350 | C | T | 741.77 | SNP | Rv3447c (eccC4) | Gly630Glu | 4 | - | | 3872726 | C | T | 884.77 | SNP | Rv3451 (cut3) | Pro37Leu | 3 | - | | 3873242 | G | A | 1481.77 | SNP | Rv3451 (cut3) | Gly209Asp | 6 | - | | 3874722 | AT | A | 2651.73 | DEL | Rv3453 |  |  | - | | 3876910 | C | T | 1004.77 | SNP | Rv3456c (rplQ) | Ala175Thr | 22 | - | | 3877421 | A | G | 1759.77 | SNP | Rv3456c (rplQ) | silent (Pro4) | 9926 | - | | 3883626 | A | G | 1221.77 | SNP | Rv3466 | silent (Pro34) | 9926 | - | | 3883796 | G | T | 214.78 | SNP | Rv3466 | Arg91Leu | 1 | - | | 3883797 | G | A | 135.90 | SNP | Rv3466 | silent (Arg91) | 9913 | - | | 3884791 | C | A | 305.78 | SNP | Rv3467 | Asn276Lys | 25 | - | | 3884906 | A | G | 887.77 | SNP | Rv3467 | Lys315Glu | 4 | - | | 3885886 | T | C | 1900.77 | SNP | Rv3468c | Ile62Val | 57 | - | | 3887921 | A | G | 989.77 | SNP | Rv3470c (ilvB2) | silent (Phe294) | 9946 | - | | 3890310 | G | A | 754.77 | SNP | Rv3473c (bpoA) | Arg142Trp | 2 | - | | 3892671 | A | G | 2473.77 | SNP | Rv3476c (kgtP) | silent (Val350) | 9901 | - | | 3895269 | G | C | 919.77 | SNP | Rv3478 (PPE60) | Glu282Gln | 27 | - | | 3895399 | C | A | 340.77 | SNP | Rv3478 (PPE60) | Pro325Gln | 6 | - | | 3895400 | A | G | 303.78 | SNP | Rv3478 (PPE60) | silent (Pro325) | 9926 | - | | 3895403 | A | C | 308.77 | SNP | Rv3478 (PPE60) | silent (Ala326) | 9867 | - | | 3896340 | T | G | 1337.77 | SNP | Rv3479 | Leu174Arg | 1 | - | | 3898408 | A | G | 1165.77 | SNP | Rv3479 | silent (Ala863) | 9867 | - | | 3899124 | G | C | 1792.77 | SNP | Rv3480c | Leu427Val(s) | 4 | - | | 3922380 | G | A | 2019.77 | SNP | intergenic |  |  | - | | 3925733 | T | G | 1263.77 | SNP | Rv3506 (fadD17) | Phe282Val | 1 | - | | 3927202 | G | A | 267.78 | SNP | Rv3507 (PE\_PGRS53) | Gly212Ser | 16 | - | | 3929089 | GACGGCGGCA | G | 1106.74 | DEL | Rv3507 (PE\_PGRS53) |  |  | - | | 3932559 | A | G | 31.77 | SNP | Rv3508 (PE\_PGRS54) | Thr519Ala | 32 | - | | 3932564 | T | C | 31.77 | SNP | Rv3508 (PE\_PGRS54) | silent (Asp520) | 9859 | - | | 3932600 | T | C | 31.77 | SNP | Rv3508 (PE\_PGRS54) | silent (Phe532) | 9946 | - | | 3932601 | T | G | 31.77 | SNP | Rv3508 (PE\_PGRS54) | Ser533Ala | 35 | - | | 3932609 | A | C | 32.77 | SNP | Rv3508 (PE\_PGRS54) | silent (Gly535) | 9935 | - | | 3934542 | T | G | 77.28 | SNP | Rv3508 (PE\_PGRS54) | Ser1180Ala | 35 | - | | 3934699 | G | A | 65.28 | SNP | Rv3508 (PE\_PGRS54) | Ser1232Asn | 20 | - | | 3934733 | G | C | 53.77 | SNP | Rv3508 (PE\_PGRS54) | silent (Gly1243) | 9935 | - | | 3934734 | G | A | 48.77 | SNP | Rv3508 (PE\_PGRS54) | Ala1244Thr | 22 | - | | 3940802 | A | G | 237.80 | SNP | Rv3511 (PE\_PGRS55) | Asn396Asp | 42 | - | | 3941497 | G | C | 33.77 | SNP | Rv3511 (PE\_PGRS55) | silent (Gly627) | 9935 | - | | 3941499 | C | A | 30.94 | SNP | Rv3511 (PE\_PGRS55) | Ala628Asp | 6 | - | | 3941834 | G | C | 56.28 | SNP | intergenic |  |  | - | | 3942481 | C | G | 99.03 | SNP | intergenic |  |  | - | | 3942640 | T | C | 299.78 | SNP | intergenic |  |  | - | | 3943019 | C | G | 83.28 | SNP | intergenic |  |  | - | | 3943079 | C | A | 74.28 | SNP | intergenic |  |  | - | | 3952800 | G | A | 1259.77 | SNP | Rv3516 (echA19) | Gly86Asp | 6 | - | | 3958403 | A | G | 1254.77 | SNP | Rv3521 | Asn295Asp | 42 | - | | 3959418 | C | T | 1355.77 | SNP | Rv3522 (ltp4) | Thr324Ile | 7 | - | | 3969763 | C | T | 670.77 | SNP | Rv3532 (PPE61) | Gln141STOP | 8 | - | | 3982149 | T | C | 578.77 | SNP | Rv3543c (fadE29) | Glu331Gly | 7 | - | | 3985547 | G | T | 1341.77 | SNP | intergenic |  |  | - | | 4002899 | C | T | 1105.77 | SNP | Rv3561 (fadD3) | silent (Ala421) | 9867 | - | | 4003130 | G | A | 1024.77 | SNP | Rv3561 (fadD3) | Val(s)498Val | 13 | - | | 4005607 | T | C | 1119.77 | SNP | Rv3564 (fadE33) | Leu(s)121Leu | 3 | - | | 4024273 | T | C | 1491.77 | SNP | Rv3581c (ispF) | Val25Val(s) | 18 | - | | 4026899 | G | A | 1280.77 | SNP | Rv3585 (radA) | silent (Gln152) | 9876 | - | | 4031626 | G | A | 111.03 | SNP | Rv3590c (PE\_PGRS58) | silent (Gly511) | 9935 | - | | 4034827 | C | T | 1551.77 | SNP | Rv3593 (lpqF) | Ala159Val(s) | 9867 | - | | 4037283 | T | G | 214.84 | SNP | Rv3595c (PE\_PGRS59) | silent (Gly256) | 9935 | - | | 4047967 | A | C | 766.77 | SNP | Rv3605c | Leu72Arg | 1 | - | | 4051716 | C | T | 919.77 | SNP | Rv3610c (ftsH) | Val390Ile | 33 | - | | 4053050 | A | G | 638.78 | SNP | Rv3611 | Asn34Ser | 34 | - | | 4055801 | G | A | 1105.77 | SNP | Rv3616c (espA) | Thr192Ile | 7 | - | | 4056124 | G | A | 1754.77 | SNP | Rv3616c (espA) | silent (Leu84) | 9947 | - | | 4059904 | A | G | 1276.77 | SNP | intergenic |  |  | - | | 4060100 | G | A | 584.77 | SNP | Rv3619c (esxV) | Leu57Leu(s) | 4 | - | | 4060201 | G | A | 498.77 | SNP | Rv3619c (esxV) | Ser23Leu(s) | 35 | - | | 4060210 | T | A | 549.77 | SNP | Rv3619c (esxV) | Gln20Leu | 6 | - | | 4060230 | G | A | 562.77 | SNP | Rv3619c (esxV) | silent (His13) | 9912 | - | | 4063682 | G | T | 1315.77 | SNP | Rv3624c (hpt) | Leu75Met(s) | 4 | - | | 4069292 | G | A | 621.77 | SNP | Rv3630 | Ala40Thr | 22 | - | | 4086802 | G | A | 1489.77 | SNP | Rv3646c (topA) | silent (His152) | 9912 | - | | 4091760 | C | A | 1970.77 | SNP | intergenic |  |  | - | | 4094346 | T | TCGGCGCCGGCGGCGCCGG | 2744.73 | INS | Rv3653 (PE\_PGRS61) |  |  | - | | 4095001 | CG | C | 2516.73 | DEL | Rv3655c |  |  | - | | 4098261 | G | A | 302.78 | SNP | Rv3660c | silent (Ala296) | 9867 | - | | 4100971 | C | T | 778.77 | SNP | intergenic |  |  | - | | 4100975 | T | C | 808.77 | SNP | intergenic |  |  | - | | 4111303 | G | C | 1155.77 | SNP | Rv3669 | Val(s)159Val | 13 | - | | 4115012 | G | A | 970.77 | SNP | Rv3673c | Thr49Met(s) | 32 | - | | 4117167 | GGC | G | 2010.73 | DEL | intergenic |  |  | - | | 4120926 | A | G | 295.77 | SNP | Rv3680 | Asn378Asp | 42 | - | | 4120983 | A | G | 770.77 | SNP | intergenic |  |  | - | | 4121109 | A | C | 1586.77 | SNP | intergenic |  |  | - | | 4146314 | G | T | 722.77 | SNP | Rv3703c | Pro193Gln | 6 | - | | 4150647 | C | G | 984.77 | SNP | Rv3707c | Gly132Arg | 0 | - | | 4151855 | A | G | 849.77 | SNP | Rv3708c (asd) | silent (Pro121) | 9926 | - | | 4154051 | G | A | 1725.77 | SNP | Rv3710 (leuA) | silent (Arg104) | 9913 | - | | 4156099 | C | A | 1089.77 | SNP | Rv3711c (dnaQ) | Val(s)211Leu(s) | 9867 | - | | 4159195 | T | C | 1661.77 | SNP | Rv3714c | silent (Pro209) | 9926 | - | | 4162339 | A | G | 2282.77 | SNP | Rv3719 | Thr12Ala | 32 | - | | 4182695 | G | A | 1664.77 | SNP | Rv3731 (ligC) | Arg313His | 8 | - | | 4187485 | T | C | 1478.77 | SNP | Rv3736 | silent (Ala284) | 9867 | - | | 4187817 | A | G | 1155.77 | SNP | Rv3737 | Asp40Gly | 11 | - | | 4192588 | G | A | 865.77 | SNP | Rv3741c | Ala89Val(s) | 9867 | - | | 4197138 | C | CT | 1575.73 | INS | intergenic |  |  | - | | 4198611 | CG | C | 2251.73 | DEL | intergenic |  |  | - | | 4204441 | A | G | 1578.77 | SNP | Rv3759c (proX) | silent (His311) | 9912 | - | | 4205120 | A | G | 1279.77 | SNP | Rv3759c (proX) | Leu85Pro | 2 | - | | 4205550 | G | A | 1207.77 | SNP | Rv3760 | Val(s)5Met(s) | 9867 | - | | 4207911 | G | A | 2308.77 | SNP | Rv3762c | silent (Ala322) | 9867 | - | | 4209518 | T | A | 1540.77 | SNP | Rv3763 (lpqH) | Cys158Ser | 11 | - | | 4210274 | A | G | 1199.77 | SNP | Rv3764c (tcrY) | Cys246Arg | 1 | - | | 4212840 | T | C | 1934.77 | SNP | Rv3766 | Val183Ala | 18 | - | | 4221490 | C | G | 1397.77 | SNP | Rv3776 | silent (Leu134) | 9947 | - | | 4222073 | A | G | 469.77 | SNP | Rv3776 | Met(s)329Val(s) | 9867 | - | | 4222882 | A | G | 1401.77 | SNP | Rv3777 | silent (Leu63) | 9947 | - | | 4223172 | T | C | 724.77 | SNP | Rv3777 | Val160Ala | 18 | - | | 4229087 | C | T | 1288.77 | SNP | Rv3782 (glfT1) | silent (Asn247) | 9822 | genotype | | 4231228 | G | GT | 2350.73 | INS | Rv3784 |  |  | - | | 4232863 | G | C | 772.77 | SNP | Rv3786c | Asp245Glu | 56 | - | | 4242643 | C | T | 1130.77 | SNP | Rv3793 (embC) | silent (Arg927) | 9913 | genotype | | 4242803 | G | C | 970.77 | SNP | Rv3793 (embC) | Val(s)981Leu | 3 | genotype | | 4243622 | G | A | 1421.77 | SNP | Rv3794 (embA) | silent (Thr130) | 9871 | - | | 4247729 | G | A | 962.77 | SNP | Rv3795 (embB) | Gly406Ser | 16 | resistance | | 4249408 | G | A | 943.77 | SNP | Rv3795 (embB) | silent (Pro965) | 9926 | - | | 4252878 | T | A | 801.79 | SNP | intergenic |  |  | - | | 4252879 | C | CCCACG | 489.52 | INS | intergenic |  |  | - | | 4252881 | T | TTTTTG | 977.73 | INS | intergenic |  |  | - | | 4255922 | A | G | 1299.77 | SNP | Rv3799c (accD4) | silent (His9) | 9912 | - | | 4257220 | A | G | 781.77 | SNP | Rv3800c (pks13) | silent (Arg1309) | 9913 | - | | 4269297 | G | C | 1302.77 | SNP | Rv3806c (ubiA) | Ile179Met(s) | 6 | - | | 4270835 | T | C | 1440.77 | SNP | Rv3808c (glfT2) | Gln482Arg | 10 | - | | 4275386 | G | C | 1598.77 | SNP | Rv3811 | Ala197Pro | 13 | - | | 4287109 | G | A | 2429.77 | SNP | Rv3822 | Ser130Asn | 20 | - | | 4292305 | C | T | 1488.77 | SNP | Rv3824c (papA1) | Met(s)290Ile | 2 | - | | 4296015 | G | A | 2048.77 | SNP | Rv3825c (pks2) | silent (Asp1197) | 9859 | - | | 4302036 | T | C | 899.77 | SNP | Rv3827c | Thr252Ala | 32 | - | | 4302161 | T | C | 1215.77 | SNP | Rv3827c | Gln210Arg | 10 | - | | 4306155 | C | T | 1363.77 | SNP | Rv3831 | silent (Ser133) | 9840 | - | | 4306472 | G | T | 1117.77 | SNP | Rv3832c | Leu114Met(s) | 4 | - | | 4306767 | G | A | 1074.77 | SNP | Rv3832c | silent (Gly15) | 9935 | - | | 4306929 | G | T | 1692.77 | SNP | Rv3833 | silent (Arg21) | 9913 | - | | 4307179 | G | A | 1519.77 | SNP | Rv3833 | Val105Ile | 33 | - | | 4314645 | A | G | 1116.77 | SNP | Rv3841 (bfrB) | silent (Leu156) | 9947 | - | | 4319986 | C | T | 817.77 | SNP | intergenic |  |  | - | | 4323355 | G | C | 2033.77 | SNP | intergenic |  |  | - | | 4338595 | GC | G | 3937.73 | DEL | intergenic |  |  | - | | 4338732 | G | A | 2167.77 | SNP | intergenic |  |  | - | | 4340006 | A | C | 1229.77 | SNP | Rv3863 | silent (Ala386) | 9867 | - | | 4351039 | G | T | 1080.77 | SNP | Rv3872 (PE35) | Glu99STOP | 17 | - | | 4356110 | G | C | 1285.77 | SNP | Rv3877 (eccD1) | silent (Leu368) | 9947 | - | | 4357123 | C | A | 1439.77 | SNP | Rv3878 (espJ) | Thr144Lys | 11 | - | | 4357597 | C | G | 1096.77 | SNP | Rv3879c (espK) | Cys729Ser | 11 | - | | 4359135 | TGGGGTTCCCGGGGTGATC | T | 4913.73 | DEL | Rv3879c (espK) |  |  | - | | 4359165 | G | C | 830.77 | SNP | Rv3879c (espK) | silent (Thr206) | 9871 | - | | 4359447 | A | G | 1955.77 | SNP | Rv3879c (espK) | silent (Asn112) | 9822 | - | | 4366195 | T | C | 540.77 | SNP | Rv3884c (eccA2) | Glu215Gly | 7 | - | | 4366272 | G | C | 867.77 | SNP | Rv3884c (eccA2) | silent (Ala189) | 9867 | - | | 4369672 | GC | G | 223.84 | DEL | Rv3886c (mycP2) |  |  | - | | 4372217 | G | A | 1619.77 | SNP | Rv3888c | His164Tyr | 4 | - | | 4374264 | G | C | 1636.77 | SNP | Rv3891c (esxD) | Gln37Glu | 35 | - | | 4375480 | C | T | 983.77 | SNP | Rv3892c (PPE69) | silent (Ala68) | 9867 | - | | 4375628 | G | T | 1309.77 | SNP | Rv3892c (PPE69) | Thr19Lys | 11 | - | | 4377909 | G | A | 1609.77 | SNP | Rv3894c (eccC2) | silent (Tyr848) | 9945 | - | | 4379680 | C | G | 1472.77 | SNP | Rv3894c (eccC2) | Arg258Pro | 5 | - | | 4382054 | T | C | 1801.77 | SNP | Rv3896c | silent (Ala266) | 9867 | - | | 4382275 | G | T | 1442.77 | SNP | Rv3896c | Gln193Lys | 12 | - | | 4383144 | C | CCGGGG | 2076.73 | INS | Rv3897c |  |  | - | | 4384417 | C | T | 799.77 | SNP | Rv3899c | silent (Leu321) | 9947 | - | | 4385111 | G | A | 524.77 | SNP | Rv3899c | Ala90Val | 13 | - | | 4386228 | T | C | 1790.77 | SNP | Rv3900c | silent (Leu27) | 9947 | - | | 4386709 | C | T | 1490.77 | SNP | Rv3901c | Ala36Thr | 22 | - | | 4387074 | G | A | 1866.77 | SNP | intergenic |  |  | - | | 4393178 | A | G | 1239.77 | SNP | intergenic |  |  | - | | 4400246 | G | A | 1406.77 | SNP | Rv3911 (sigM) | Asp21Asn | 36 | - | | 4400660 | AC | A | 1349.73 | DEL | Rv3911 (sigM) |  |  | - | | 4407977 | C | T | 1209.77 | SNP | Rv3919c (gid) | Gly76Ser | 16 | - | |  | | export |

elog
