# Supplementary material for: Patterns of compensatory mutations in rpoA/B/C genes of multidrug resistant M. tuberculosis in Uganda
Source: PLoS One. 2025 Dec 4;20(12):e0328957. doi: 10.1371/journal.pone.0328957 (PMC12677784; doi:10.1371/journal.pone.0328957)
Supplement: S2 File — (ZIP) [file pone.0328957.s002.zip › Variants G_S7_L001_001.bam.html]

 

Calling SNPs/INDELs (computing variant list in .vcf format) from G\_S7\_L001\_001.bam

*by SAMtools/BCFtools:*

Howto

Important aspects

This takes up to one hour!!! **Please wait ...**

Variants G\_S7\_L001\_001.bam

|  |  |
| --- | --- |
| Variants |  |

|  |  |
| --- | --- |
| |  | | --- | | *by GATK* | |

|  |  |  |
| --- | --- | --- |
| |  | | --- | | G\_S7\_L001\_001.bam | | | computed 2016-10-27 using PhyResSE v1.0 (Ref. NC\_000962.3) | |

|  |  |
| --- | --- |
| 1834  variants called Export in VCF format |  |

|  |  |  |  |  |  |  |  |  |  |  |  |  |  |  |  |  |  |  |  |  |  |  |  |  |  |  |  |  |  |  |  |  |  |  |  |  |  |  |  |  |  |  |  |  |  |  |  |  |  |  |  |  |  |  |  |  |  |  |  |  |  |  |  |  |  |  |  |  |  |  |  |  |  |  |  |  |  |  |  |  |  |  |  |  |  |  |  |  |  |  |  |  |  |  |  |  |  |  |  |  |  |  |  |  |  |  |  |  |  |  |  |  |  |  |  |  |  |  |  |  |  |  |  |  |  |  |  |  |  |  |  |  |  |  |  |  |  |  |  |  |  |  |  |  |  |  |  |  |  |  |  |  |  |  |  |  |  |  |  |  |  |  |  |  |  |  |  |  |  |  |  |  |  |  |  |  |  |  |  |  |  |  |  |  |  |  |  |  |  |  |  |  |  |  |  |  |  |  |  |  |  |  |  |  |  |  |  |  |  |  |  |  |  |  |  |  |  |  |  |  |  |  |  |  |  |  |  |  |  |  |  |  |  |  |  |  |  |  |  |  |  |  |  |  |  |  |  |  |  |  |  |  |  |  |  |  |  |  |  |  |  |  |  |  |  |  |  |  |  |  |  |  |  |  |  |  |  |  |  |  |  |  |  |  |  |  |  |  |  |  |  |  |  |  |  |  |  |  |  |  |  |  |  |  |  |  |  |  |  |  |  |  |  |  |  |  |  |  |  |  |  |  |  |  |  |  |  |  |  |  |  |  |  |  |  |  |  |  |  |  |  |  |  |  |  |  |  |  |  |  |  |  |  |  |  |  |  |  |  |  |  |  |  |  |  |  |  |  |  |  |  |  |  |  |  |  |  |  |  |  |  |  |  |  |  |  |  |  |  |  |  |  |  |  |  |  |  |  |  |  |  |  |  |  |  |  |  |  |  |  |  |  |  |  |  |  |  |  |  |  |  |  |  |  |  |  |  |  |  |  |  |  |  |  |  |  |  |  |  |  |  |  |  |  |  |  |  |  |  |  |  |  |  |  |  |  |  |  |  |  |  |  |  |  |  |  |  |  |  |  |  |  |  |  |  |  |  |  |  |  |  |  |  |  |  |  |  |  |  |  |  |  |  |  |  |  |  |  |  |  |  |  |  |  |  |  |  |  |  |  |  |  |  |  |  |  |  |  |  |  |  |  |  |  |  |  |  |  |  |  |  |  |  |  |  |  |  |  |  |  |  |  |  |  |  |  |  |  |  |  |  |  |  |  |  |  |  |  |  |  |  |  |  |  |  |  |  |  |  |  |  |  |  |  |  |  |  |  |  |  |  |  |  |  |  |  |  |  |  |  |  |  |  |  |  |  |  |  |  |  |  |  |  |  |  |  |  |  |  |  |  |  |  |  |  |  |  |  |  |  |  |  |  |  |  |  |  |  |  |  |  |  |  |  |  |  |  |  |  |  |  |  |  |  |  |  |  |  |  |  |  |  |  |  |  |  |  |  |  |  |  |  |  |  |  |  |  |  |  |  |  |  |  |  |  |  |  |  |  |  |  |  |  |  |  |  |  |  |  |  |  |  |  |  |  |  |  |  |  |  |  |  |  |  |  |  |  |  |  |  |  |  |  |  |  |  |  |  |  |  |  |  |  |  |  |  |  |  |  |  |  |  |  |  |  |  |  |  |  |  |  |  |  |  |  |  |  |  |  |  |  |  |  |  |  |  |  |  |  |  |  |  |  |  |  |  |  |  |  |  |  |  |  |  |  |  |  |  |  |  |  |  |  |  |  |  |  |  |  |  |  |  |  |  |  |  |  |  |  |  |  |  |  |  |  |  |  |  |  |  |  |  |  |  |  |  |  |  |  |  |  |  |  |  |  |  |  |  |  |  |  |  |  |  |  |  |  |  |  |  |  |  |  |  |  |  |  |  |  |  |  |  |  |  |  |  |  |  |  |  |  |  |  |  |  |  |  |  |  |  |  |  |  |  |  |  |  |  |  |  |  |  |  |  |  |  |  |  |  |  |  |  |  |  |  |  |  |  |  |  |  |  |  |  |  |  |  |  |  |  |  |  |  |  |  |  |  |  |  |  |  |  |  |  |  |  |  |  |  |  |  |  |  |  |  |  |  |  |  |  |  |  |  |  |  |  |  |  |  |  |  |  |  |  |  |  |  |  |  |  |  |  |  |  |  |  |  |  |  |  |  |  |  |  |  |  |  |  |  |  |  |  |  |  |  |  |  |  |  |  |  |  |  |  |  |  |  |  |  |  |  |  |  |  |  |  |  |  |  |  |  |  |  |  |  |  |  |  |  |  |  |  |  |  |  |  |  |  |  |  |  |  |  |  |  |  |  |  |  |  |  |  |  |  |  |  |  |  |  |  |  |  |  |  |  |  |  |  |  |  |  |  |  |  |  |  |  |  |  |  |  |  |  |  |  |  |  |  |  |  |  |  |  |  |  |  |  |  |  |  |  |  |  |  |  |  |  |  |  |  |  |  |  |  |  |  |  |  |  |  |  |  |  |  |  |  |  |  |  |  |  |  |  |  |  |  |  |  |  |  |  |  |  |  |  |  |  |  |  |  |  |  |  |  |  |  |  |  |  |  |  |  |  |  |  |  |  |  |  |  |  |  |  |  |  |  |  |  |  |  |  |  |  |  |  |  |  |  |  |  |  |  |  |  |  |  |  |  |  |  |  |  |  |  |  |  |  |  |  |  |  |  |  |  |  |  |  |  |  |  |  |  |  |  |  |  |  |  |  |  |  |  |  |  |  |  |  |  |  |  |  |  |  |  |  |  |  |  |  |  |  |  |  |  |  |  |  |  |  |  |  |  |  |  |  |  |  |  |  |  |  |  |  |  |  |  |  |  |  |  |  |  |  |  |  |  |  |  |  |  |  |  |  |  |  |  |  |  |  |  |  |  |  |  |  |  |  |  |  |  |  |  |  |  |  |  |  |  |  |  |  |  |  |  |  |  |  |  |  |  |  |  |  |  |  |  |  |  |  |  |  |  |  |  |  |  |  |  |  |  |  |  |  |  |  |  |  |  |  |  |  |  |  |  |  |  |  |  |  |  |  |  |  |  |  |  |  |  |  |  |  |  |  |  |  |  |  |  |  |  |  |  |  |  |  |  |  |  |  |  |  |  |  |  |  |  |  |  |  |  |  |  |  |  |  |  |  |  |  |  |  |  |  |  |  |  |  |  |  |  |  |  |  |  |  |  |  |  |  |  |  |  |  |  |  |  |  |  |  |  |  |  |  |  |  |  |  |  |  |  |  |  |  |  |  |  |  |  |  |  |  |  |  |  |  |  |  |  |  |  |  |  |  |  |  |  |  |  |  |  |  |  |  |  |  |  |  |  |  |  |  |  |  |  |  |  |  |  |  |  |  |  |  |  |  |  |  |  |  |  |  |  |  |  |  |  |  |  |  |  |  |  |  |  |  |  |  |  |  |  |  |  |  |  |  |  |  |  |  |  |  |  |  |  |  |  |  |  |  |  |  |  |  |  |  |  |  |  |  |  |  |  |  |  |  |  |  |  |  |  |  |  |  |  |  |  |  |  |  |  |  |  |  |  |  |  |  |  |  |  |  |  |  |  |  |  |  |  |  |  |  |  |  |  |  |  |  |  |  |  |  |  |  |  |  |  |  |  |  |  |  |  |  |  |  |  |  |  |  |  |  |  |  |  |  |  |  |  |  |  |  |  |  |  |  |  |  |  |  |  |  |  |  |  |  |  |  |  |  |  |  |  |  |  |  |  |  |  |  |  |  |  |  |  |  |  |  |  |  |  |  |  |  |  |  |  |  |  |  |  |  |  |  |  |  |  |  |  |  |  |  |  |  |  |  |  |  |  |  |  |  |  |  |  |  |  |  |  |  |  |  |  |  |  |  |  |  |  |  |  |  |  |  |  |  |  |  |  |  |  |  |  |  |  |  |  |  |  |  |  |  |  |  |  |  |  |  |  |  |  |  |  |  |  |  |  |  |  |  |  |  |  |  |  |  |  |  |  |  |  |  |  |  |  |  |  |  |  |  |  |  |  |  |  |  |  |  |  |  |  |  |  |  |  |  |  |  |  |  |  |  |  |  |  |  |  |  |  |  |  |  |  |  |  |  |  |  |  |  |  |  |  |  |  |  |  |  |  |  |  |  |  |  |  |  |  |  |  |  |  |  |  |  |  |  |  |  |  |  |  |  |  |  |  |  |  |  |  |  |  |  |  |  |  |  |  |  |  |  |  |  |  |  |  |  |  |  |  |  |  |  |  |  |  |  |  |  |  |  |  |  |  |  |  |  |  |  |  |  |  |  |  |  |  |  |  |  |  |  |  |  |  |  |  |  |  |  |  |  |  |  |  |  |  |  |  |  |  |  |  |  |  |  |  |  |  |  |  |  |  |  |  |  |  |  |  |  |  |  |  |  |  |  |  |  |  |  |  |  |  |  |  |  |  |  |  |  |  |  |  |  |  |  |  |  |  |  |  |  |  |  |  |  |  |  |  |  |  |  |  |  |  |  |  |  |  |  |  |  |  |  |  |  |  |  |  |  |  |  |  |  |  |  |  |  |  |  |  |  |  |  |  |  |  |  |  |  |  |  |  |  |  |  |  |  |  |  |  |  |  |  |  |  |  |  |  |  |  |  |  |  |  |  |  |  |  |  |  |  |  |  |  |  |  |  |  |  |  |  |  |  |  |  |  |  |  |  |  |  |  |  |  |  |  |  |  |  |  |  |  |  |  |  |  |  |  |  |  |  |  |  |  |  |  |  |  |  |  |  |  |  |  |  |  |  |  |  |  |  |  |  |  |  |  |  |  |  |  |  |  |  |  |  |  |  |  |  |  |  |  |  |  |  |  |  |  |  |  |  |  |  |  |  |  |  |  |  |  |  |  |  |  |  |  |  |  |  |  |  |  |  |  |  |  |  |  |  |  |  |  |  |  |  |  |  |  |  |  |  |  |  |  |  |  |  |  |  |  |  |  |  |  |  |  |  |  |  |  |  |  |  |  |  |  |  |  |  |  |  |  |  |  |  |  |  |  |  |  |  |  |  |  |  |  |  |  |  |  |  |  |  |  |  |  |  |  |  |  |  |  |  |  |  |  |  |  |  |  |  |  |  |  |  |  |  |  |  |  |  |  |  |  |  |  |  |  |  |  |  |  |  |  |  |  |  |  |  |  |  |  |  |  |  |  |  |  |  |  |  |  |  |  |  |  |  |  |  |  |  |  |  |  |  |  |  |  |  |  |  |  |  |  |  |  |  |  |  |  |  |  |  |  |  |  |  |  |  |  |  |  |  |  |  |  |  |  |  |  |  |  |  |  |  |  |  |  |  |  |  |  |  |  |  |  |  |  |  |  |  |  |  |  |  |  |  |  |  |  |  |  |  |  |  |  |  |  |  |  |  |  |  |  |  |  |  |  |  |  |  |  |  |  |  |  |  |  |  |  |  |  |  |  |  |  |  |  |  |  |  |  |  |  |  |  |  |  |  |  |  |  |  |  |  |  |  |  |  |  |  |  |  |  |  |  |  |  |  |  |  |  |  |  |  |  |  |  |  |  |  |  |  |  |  |  |  |  |  |  |  |  |  |  |  |  |  |  |  |  |  |  |  |  |  |  |  |  |  |  |  |  |  |  |  |  |  |  |  |  |  |  |  |  |  |  |  |  |  |  |  |  |  |  |  |  |  |  |  |  |  |  |  |  |  |  |  |  |  |  |  |  |  |  |  |  |  |  |  |  |  |  |  |  |  |  |  |  |  |  |  |  |  |  |  |  |  |  |  |  |  |  |  |  |  |  |  |  |  |  |  |  |  |  |  |  |  |  |  |  |  |  |  |  |  |  |  |  |  |  |  |  |  |  |  |  |  |  |  |  |  |  |  |  |  |  |  |  |  |  |  |  |  |  |  |  |  |  |  |  |  |  |  |  |  |  |  |  |  |  |  |  |  |  |  |  |  |  |  |  |  |  |  |  |  |  |  |  |  |  |  |  |  |  |  |  |  |  |  |  |  |  |  |  |  |  |  |  |  |  |  |  |  |  |  |  |  |  |  |  |  |  |  |  |  |  |  |  |  |  |  |  |  |  |  |  |  |  |  |  |  |  |  |  |  |  |  |  |  |  |  |  |  |  |  |  |  |  |  |  |  |  |  |  |  |  |  |  |  |  |  |  |  |  |  |  |  |  |  |  |  |  |  |  |  |  |  |  |  |  |  |  |  |  |  |  |  |  |  |  |  |  |  |  |  |  |  |  |  |  |  |  |  |  |  |  |  |  |  |  |  |  |  |  |  |  |  |  |  |  |  |  |  |  |  |  |  |  |  |  |  |  |  |  |  |  |  |  |  |  |  |  |  |  |  |  |  |  |  |  |  |  |  |  |  |  |  |  |  |  |  |  |  |  |  |  |  |  |  |  |  |  |  |  |  |  |  |  |  |  |  |  |  |  |  |  |  |  |  |  |  |  |  |  |  |  |  |  |  |  |  |  |  |  |  |  |  |  |  |  |  |  |  |  |  |  |  |  |  |  |  |  |  |  |  |  |  |  |  |  |  |  |  |  |  |  |  |  |  |  |  |  |  |  |  |  |  |  |  |  |  |  |  |  |  |  |  |  |  |  |  |  |  |  |  |  |  |  |  |  |  |  |  |  |  |  |  |  |  |  |  |  |  |  |  |  |  |  |  |  |  |  |  |  |  |  |  |  |  |  |  |  |  |  |  |  |  |  |  |  |  |  |  |  |  |  |  |  |  |  |  |  |  |  |  |  |  |  |  |  |  |  |  |  |  |  |  |  |  |  |  |  |  |  |  |  |  |  |  |  |  |  |  |  |  |  |  |  |  |  |  |  |  |  |  |  |  |  |  |  |  |  |  |  |  |  |  |  |  |  |  |  |  |  |  |  |  |  |  |  |  |  |  |  |  |  |  |  |  |  |  |  |  |  |  |  |  |  |  |  |  |  |  |  |  |  |  |  |  |  |  |  |  |  |  |  |  |  |  |  |  |  |  |  |  |  |  |  |  |  |  |  |  |  |  |  |  |  |  |  |  |  |  |  |  |  |  |  |  |  |  |  |  |  |  |  |  |  |  |  |  |  |  |  |  |  |  |  |  |  |  |  |  |  |  |  |  |  |  |  |  |  |  |  |  |  |  |  |  |  |  |  |  |  |  |  |  |  |  |  |  |  |  |  |  |  |  |  |  |  |  |  |  |  |  |  |  |  |  |  |  |  |  |  |  |  |  |  |  |  |  |  |  |  |  |  |  |  |  |  |  |  |  |  |  |  |  |  |  |  |  |  |  |  |  |  |  |  |  |  |  |  |  |  |  |  |  |  |  |  |  |  |  |  |  |  |  |  |  |  |  |  |  |  |  |  |  |  |  |  |  |  |  |  |  |  |  |  |  |  |  |  |  |  |  |  |  |  |  |  |  |  |  |  |  |  |  |  |  |  |  |  |  |  |  |  |  |  |  |  |  |  |  |  |  |  |  |  |  |  |  |  |  |  |  |  |  |  |  |  |  |  |  |  |  |  |  |  |  |  |  |  |  |  |  |  |  |  |  |  |  |  |  |  |  |  |  |  |  |  |  |  |  |  |  |  |  |  |  |  |  |  |  |  |  |  |  |  |  |  |  |  |  |  |  |  |  |  |  |  |  |  |  |  |  |  |  |  |  |  |  |  |  |  |  |  |  |  |  |  |  |  |  |  |  |  |  |  |  |  |  |  |  |  |  |  |  |  |  |  |  |  |  |  |  |  |  |  |  |  |  |  |  |  |  |  |  |  |  |  |  |  |  |  |  |  |  |  |  |  |  |  |  |  |  |  |  |  |  |  |  |  |  |  |  |  |  |  |  |  |  |  |  |  |  |  |  |  |  |  |  |  |  |  |  |  |  |  |  |  |  |  |  |  |  |  |  |  |  |  |  |  |  |  |  |  |  |  |  |  |  |  |  |  |  |  |  |  |  |  |  |  |  |  |  |  |  |  |  |  |  |  |  |  |  |  |  |  |  |  |  |  |  |  |  |  |  |  |  |  |  |  |  |  |  |  |  |  |  |  |  |  |  |  |  |  |  |  |  |  |  |  |  |  |  |  |  |  |  |  |  |  |  |  |  |  |  |  |  |  |  |  |  |  |  |  |  |  |  |  |  |  |  |  |  |  |  |  |  |  |  |  |  |  |  |  |  |  |  |  |  |  |  |  |  |  |  |  |  |  |  |  |  |  |  |  |  |  |  |  |  |  |  |  |  |  |  |  |  |  |  |  |  |  |  |  |  |  |  |  |  |  |  |  |  |  |  |  |  |  |  |  |  |  |  |  |  |  |  |  |  |  |  |  |  |  |  |  |  |  |  |  |  |  |  |  |  |  |  |  |  |  |  |  |  |  |  |  |  |  |  |  |  |  |  |  |  |  |  |  |  |  |  |  |  |  |  |  |  |  |  |  |  |  |  |  |  |  |  |  |  |  |  |  |  |  |  |  |  |  |  |  |  |  |  |  |  |  |  |  |  |  |  |  |  |  |  |  |  |  |  |  |  |  |  |  |  |  |  |  |  |  |  |  |  |  |  |  |  |  |  |  |  |  |  |  |  |  |  |  |  |  |  |  |  |  |  |  |  |  |  |  |  |  |  |  |  |  |  |  |  |  |  |  |  |  |  |  |  |  |  |  |  |  |  |  |  |  |  |  |  |  |  |  |  |  |  |  |  |  |  |  |  |  |  |  |  |  |  |  |  |  |  |  |  |  |  |  |  |  |  |  |  |  |  |  |  |  |  |  |  |  |  |  |  |  |  |  |  |  |  |  |  |  |  |  |  |  |  |  |  |  |  |  |  |  |  |  |  |  |  |  |  |  |  |  |  |  |  |  |  |  |  |  |  |  |  |  |  |  |  |  |  |  |  |  |  |  |  |  |  |  |  |  |  |  |  |  |  |  |  |  |  |  |  |  |  |  |  |  |  |  |  |  |  |  |  |  |  |  |  |  |  |  |  |  |  |  |  |  |  |  |  |  |  |  |  |  |  |  |  |  |  |  |  |  |  |  |  |  |  |  |  |  |  |  |  |  |  |  |  |  |  |  |  |  |  |  |  |  |  |  |  |  |  |  |  |  |  |  |  |  |  |  |  |  |  |  |  |  |  |  |  |  |  |  |  |  |  |  |  |  |  |  |  |  |  |  |  |  |  |  |  |  |  |  |  |  |  |  |  |  |  |  |  |  |  |  |  |  |  |  |  |  |  |  |  |  |  |  |  |  |  |  |  |  |  |  |  |  |  |  |  |  |  |  |  |  |  |  |  |  |  |  |  |  |  |  |  |  |  |  |  |  |  |  |  |  |  |  |  |  |  |  |  |  |  |  |  |  |  |  |  |  |  |  |  |  |  |  |  |  |  |  |  |  |  |  |  |  |  |  |  |  |  |  |  |  |  |  |  |  |  |  |  |  |  |  |  |  |  |  |  |  |  |  |  |  |  |  |  |  |  |  |  |  |  |  |  |  |  |  |  |  |  |  |  |  |  |  |  |  |  |  |  |  |  |  |  |  |  |  |  |  |  |  |  |  |  |  |  |  |  |  |  |  |  |  |  |  |  |  |  |  |  |  |  |  |  |  |  |  |  |  |  |  |  |  |  |  |  |  |  |  |  |  |  |  |  |  |  |  |  |  |  |  |  |  |  |  |  |  |  |  |  |  |  |  |  |  |  |  |  |  |  |  |  |  |  |  |  |  |  |  |  |  |  |  |  |  |  |  |  |  |  |  |  |  |  |  |  |  |  |  |  |  |  |  |  |  |  |  |  |  |  |  |  |  |  |  |  |  |  |  |  |  |  |  |  |  |  |  |  |  |  |  |  |  |  |  |  |  |  |  |  |  |  |  |  |  |  |  |  |  |  |  |  |  |  |  |  |  |  |  |  |  |  |  |  |  |  |  |  |  |  |  |  |  |  |  |  |  |  |  |  |  |  |  |  |  |  |  |  |  |  |  |  |  |  |  |  |  |  |  |  |  |  |  |  |  |  |  |  |  |  |  |  |  |  |  |  |  |  |  |  |  |  |  |  |  |  |  |  |  |  |  |  |  |  |  |  |  |  |  |  |  |  |  |  |  |  |  |  |  |  |  |  |  |  |  |  |  |  |  |  |  |  |  |  |  |  |  |  |  |  |  |  |  |  |  |  |  |  |  |  |  |  |  |  |  |  |  |  |  |  |  |  |  |  |  |  |  |  |  |  |  |  |  |  |  |  |  |  |  |  |  |  |  |  |  |  |  |  |  |  |  |  |  |  |  |  |  |  |  |  |  |  |  |  |  |  |  |  |  |  |  |  |  |  |  |  |  |  |  |  |  |  |  |  |  |  |  |  |  |  |  |  |  |  |  |  |  |  |  |  |  |  |  |  |  |  |  |  |  |  |  |  |  |  |  |  |  |  |  |  |  |  |  |  |  |  |  |  |  |  |  |  |  |  |  |  |  |  |  |  |  |  |  |  |  |  |  |  |  |  |  |  |  |  |  |  |  |  |  |  |  |  |  |  |  |  |  |  |  |  |  |  |  |  |  |  |  |  |  |  |  |  |  |  |  |  |  |  |  |  |  |  |  |  |  |  |  |  |  |  |  |  |  |  |  |  |  |  |  |  |  |  |  |  |  |  |  |  |  |  |  |  |  |  |  |  |  |  |  |  |  |  |  |  |  |  |  |  |  |  |  |  |  |  |  |  |  |  |  |  |  |  |  |  |  |  |  |  |  |  |  |  |  |  |  |  |  |  |  |  |  |  |  |  |  |  |  |  |  |  |  |  |  |  |  |  |  |  |  |  |  |  |  |  |  |  |  |  |  |  |  |  |  |  |  |  |  |  |  |  |  |  |  |  |  |  |  |  |  |  |  |  |  |  |  |  |  |  |  |  |  |  |  |  |  |  |  |  |  |  |  |  |  |  |  |  |  |  |  |  |  |  |  |  |  |  |  |  |  |  |  |  |  |  |  |  |  |  |  |  |  |  |  |  |  |  |  |  |  |  |  |  |  |  |  |  |  |  |  |  |  |  |  |  |  |  |  |  |  |  |  |  |  |  |  |  |  |  |  |  |  |  |  |  |  |  |  |  |  |  |  |  |  |  |  |  |  |  |  |  |  |  |  |  |  |  |  |  |  |  |  |  |  |  |  |  |  |  |  |  |  |  |  |  |  |  |  |  |  |  |  |  |  |  |  |  |  |  |  |  |  |  |  |  |  |  |  |  |  |  |  |  |  |  |  |  |  |  |  |  |  |  |  |  |  |  |  |  |  |  |  |  |  |  |  |  |  |  |  |  |  |  |  |  |  |  |  |  |  |  |  |  |  |  |  |  |  |  |  |  |  |  |  |  |  |  |  |  |  |  |  |  |  |  |  |  |  |  |  |  |  |  |  |  |  |  |  |  |  |  |  |  |  |  |  |  |  |  |  |  |  |  |  |  |  |  |  |  |  |  |  |  |  |  |  |  |  |  |  |  |  |  |  |  |  |  |  |  |  |  |  |  |  |  |  |  |  |  |  |  |  |  |  |  |  |  |  |  |  |  |  |  |  |  |  |  |  |  |  |  |  |  |  |  |  |  |  |  |  |  |  |  |  |  |  |  |  |  |  |  |  |  |  |  |  |  |  |  |  |  |  |  |  |  |  |  |  |  |  |  |  |  |  |  |  |  |  |  |  |  |  |  |  |  |  |  |  |  |  |  |  |  |  |  |  |  |  |  |  |  |  |  |  |  |  |  |  |  |  |  |  |  |  |  |  |  |  |  |  |  |  |  |  |  |  |  |  |  |  |  |  |  |  |  |  |  |  |  |  |  |  |  |  |  |  |  |  |  |  |  |  |  |  |  |  |  |  |  |  |  |  |  |  |  |  |  |  |  |  |  |  |  |  |  |  |  |  |  |  |  |  |  |  |  |  |  |  |  |  |  |  |  |  |  |  |  |  |  |  |  |  |  |  |  |  |  |  |  |  |  |  |  |  |  |  |  |  |  |  |  |  |  |  |  |  |  |  |  |  |  |  |  |  |  |  |  |  |  |  |  |  |  |  |  |  |  |  |  |  |  |  |  |  |  |  |  |  |  |  |  |  |  |  |  |  |  |  |  |  |  |  |  |  |  |  |  |  |  |  |  |  |  |  |  |  |  |  |  |  |  |  |  |  |  |  |  |  |  |  |  |  |  |  |  |  |  |  |  |  |  |  |  |  |  |  |  |  |  |  |  |  |  |  |  |  |  |  |  |  |  |  |  |  |  |  |  |  |  |  |  |  |  |  |  |  |  |  |  |  |  |  |  |  |  |  |  |  |  |  |  |  |  |  |  |  |  |  |  |  |  |  |  |  |  |  |  |  |  |  |  |  |  |  |  |  |  |  |  |  |  |  |  |  |  |  |  |  |  |  |  |  |  |  |  |  |  |  |  |  |  |  |  |  |  |  |  |  |  |  |  |  |  |  |  |  |  |  |  |  |  |  |  |  |  |  |  |  |  |  |  |  |  |  |  |  |  |  |  |  |  |  |  |  |  |  |  |  |  |  |  |  |  |  |  |  |  |  |  |  |  |  |  |  |  |  |  |  |  |  |  |  |  |  |  |  |  |  |  |  |  |  |  |  |  |  |  |  |  |  |  |  |  |  |  |  |  |  |  |  |  |  |  |  |  |  |  |  |  |  |  |  |  |  |  |  |  |  |  |  |  |  |  |  |  |  |  |  |  |  |  |  |  |  |  |  |  |  |  |  |  |  |  |  |  |  |  |  |  |  |  |  |  |  |  |  |  |  |  |  |  |  |  |  |  |  |  |  |  |  |  |  |  |  |  |  |  |  |  |  |  |  |  |  |  |  |  |  |  |  |  |  |  |  |  |  |  |  |  |  |  |  |  |  |  |  |  |  |  |  |  |  |  |  |  |  |  |  |  |  |  |  |  |  |  |  |  |  |  |  |  |  |  |  |  |  |  |  |  |  |  |  |  |  |  |  |  |  |  |  |  |  |  |  |  |  |  |  |  |  |  |  |  |  |  |  |  |  |  |  |  |  |  |  |  |  |  |  |  |  |  |  |  |  |  |  |  |  |  |  |  |  |  |  |  |  |  |  |  |  |  |  |  |  |  |  |  |  |  |  |  |  |  |  |  |  |  |  |  |  |  |  |  |  |  |  |  |  |  |  |  |  |  |  |  |  |  |  |  |  |  |  |  |  |  |  |  |  |  |  |  |  |  |  |  |  |  |  |  |  |  |  |  |  |  |  |  |  |  |  |  |  |  |  |  |  |  |  |  |  |  |  |  |  |  |  |  |  |  |  |  |  |  |  |  |  |  |  |  |  |  |  |  |  |  |  |  |  |  |  |  |  |  |  |  |  |  |  |  |  |  |  |  |  |  |  |  |  |  |  |  |  |  |  |  |  |  |  |  |  |  |  |  |  |  |  |  |  |  |  |  |  |  |  |  |  |  |  |  |  |  |  |  |  |  |  |  |  |  |  |  |  |  |  |  |  |  |  |  |  |  |  |  |  |  |  |  |  |  |  |  |  |  |  |  |  |  |  |  |  |  |  |  |  |  |  |  |  |  |  |  |  |  |  |  |  |  |  |  |  |  |  |  |  |  |  |  |  |  |  |  |  |  |  |  |  |  |  |  |  |  |  |  |  |  |  |  |  |  |  |  |  |  |  |  |  |  |  |  |  |  |  |  |  |  |  |  |  |  |  |  |  |  |  |  |  |  |  |  |  |  |  |  |  |  |  |  |  |  |  |  |  |  |  |  |  |  |  |  |  |  |  |  |  |  |  |  |  |  |  |  |  |  |  |  |  |  |  |  |  |  |  |  |  |  |  |  |  |  |  |  |  |  |  |  |  |  |  |  |  |  |  |  |  |  |  |  |  |  |  |  |  |  |  |  |  |  |  |  |  |  |  |  |  |  |  |  |  |  |  |  |  |  |  |  |  |  |  |  |  |  |  |  |  |  |  |  |  |  |  |  |  |  |  |  |  |  |  |  |  |  |  |  |  |  |  |  |  |  |  |  |  |  |  |  |  |  |  |  |  |  |  |  |  |  |  |  |  |  |  |  |  |  |  |  |  |  |  |  |  |  |  |  |  |  |  |  |  |  |  |  |  |  |  |  |  |  |  |  |  |  |  |  |  |  |  |  |  |  |  |  |  |  |  |  |  |  |  |  |  |  |  |  |  |  |  |  |  |  |  |  |  |  |  |  |  |  |  |  |  |  |  |  |  |  |  |  |  |  |  |  |  |  |  |  |  |  |  |  |  |  |  |  |  |  |  |  |  |  |  |  |  |  |  |  |  |  |  |  |  |  |  |  |  |  |  |  |  |  |  |  |  |  |  |  |  |  |  |  |  |  |  |  |  |  |  |  |  |  |  |  |  |  |  |  |  |  |  |  |  |  |  |  |  |  |  |  |  |  |  |  |  |  |  |  |  |  |  |  |  |  |  |  |  |  |  |  |  |  |  |  |  |  |  |  |  |  |  |  |  |  |  |  |  |  |  |  |  |  |  |  |  |  |  |  |  |  |  |  |  |  |  |  |  |  |  |  |  |  |  |  |  |  |  |  |  |  |  |  |  |  |  |  |  |  |  |  |  |  |  |  |  |  |  |  |  |  |  |  |  |  |  |  |  |  |  |  |  |  |  |  |  |  |  |  |  |  |  |  |  |  |  |  |  |  |  |  |  |  |  |  |  |  |  |  |  |  |  |  |  |  |  |  |  |  |  |  |  |  |  |  |  |  |  |  |  |  |  |  |  |  |  |  |  |  |  |  |  |  |  |  |  |  |  |  |  |  |  |  |  |  |  |  |  |  |  |  |  |  |  |  |  |  |  |  |  |  |  |  |  |  |  |  |  |  |  |  |  |  |  |  |  |  |  |  |  |  |  |  |  |  |  |  |  |  |  |  |  |  |  |  |  |  |  |  |  |  |  |  |  |  |  |  |  |  |  |  |  |  |  |  |  |  |  |  |  |  |  |  |  |  |  |  |  |  |  |  |  |  |  |  |  |  |  |  |  |  |  |  |  |  |  |  |  |  |  |  |  |  |  |  |  |  |  |  |  |  |  |  |  |  |  |  |  |  |  |  |  |  |  |  |  |  |  |  |  |  |  |  |  |  |  |  |  |  |  |  |  |  |  |  |  |  |  |  |  |  |  |  |  |  |  |  |  |  |  |  |  |  |  |  |  |  |  |  |  |  |  |  |  |  |  |  |  |  |  |  |  |  |  |  |  |  |  |  |  |  |  |  |  |  |  |  |  |  |  |  |  |  |  |  |  |  |  |  |  |  |  |  |  |  |  |  |  |  |  |  |  |  |  |  |  |  |  |  |  |  |  |  |  |  |  |  |  |  |  |  |  |  |  |  |  |  |  |  |  |  |  |  |  |  |  |  |  |  |  |  |  |  |  |  |  |  |  |  |  |  |  |  |  |  |  |  |  |  |  |  |  |  |  |  |  |  |  |  |  |  |  |  |  |  |  |  |  |  |  |  |  |  |  |  |  |  |  |  |  |  |  |  |  |  |  |  |  |  |  |  |  |  |  |  |  |  |  |  |  |  |  |  |  |  |  |  |  |  |  |  |  |  |  |  |  |  |  |  |  |  |  |  |  |  |  |  |  |  |  |  |  |  |  |  |  |  |  |  |  |  |  |  |  |  |  |  |  |  |  |  |  |  |  |  |  |  |  |  |  |  |  |  |  |  |  |  |  |  |  |  |  |  |  |  |  |  |  |  |  |  |  |  |  |  |  |  |  |  |  |  |  |  |  |  |  |  |  |  |  |  |  |  |  |  |  |  |  |  |  |  |  |  |  |  |  |  |  |  |  |  |  |  |  |  |  |  |  |  |  |  |  |  |  |  |  |  |  |  |  |  |  |  |  |  |  |  |  |  |  |  |  |  |  |  |  |  |  |  |  |  |  |  |  |  |  |  |  |  |  |  |  |  |  |  |  |  |  |  |  |  |  |  |  |  |  |  |  |  |  |  |  |  |  |  |  |  |  |  |  |  |  |  |  |  |  |  |  |  |  |  |  |  |  |  |  |  |  |  |  |  |  |  |  |  |  |  |  |  |  |  |  |  |  |  |  |  |  |  |  |  |  |  |  |  |  |  |  |  |  |  |  |  |  |  |  |  |  |  |  |  |  |  |  |  |  |  |  |  |  |  |  |  |  |  |  |  |  |  |  |  |  |  |  |  |  |  |  |  |  |  |  |  |  |  |  |  |  |  |  |  |  |  |  |  |  |  |  |  |  |  |  |  |  |  |  |  |  |  |  |  |  |  |  |  |  |  |  |  |  |  |  |  |  |  |  |  |  |  |  |  |  |  |  |  |  |  |  |  |  |  |  |  |  |  |  |  |  |  |  |  |  |  |  |  |  |  |  |  |  |  |  |  |  |  |  |  |  |  |  |  |  |  |  |  |  |  |  |  |  |  |  |  |  |  |  |  |  |  |  |  |  |  |  |  |  |  |  |  |  |  |  |  |  |  |  |  |  |  |  |  |  |  |  |  |  |  |  |  |  |  |  |  |  |  |  |  |  |  |  |  |  |  |  |  |  |  |  |  |  |  |  |  |  |  |  |  |  |  |  |  |  |  |  |  |  |  |  |  |  |  |  |  |  |  |  |  |  |  |  |  |  |  |  |  |  |  |  |  |  |  |  |  |  |  |  |  |  |  |  |  |  |  |  |  |  |  |  |  |  |  |  |  |  |  |  |  |  |  |  |  |  |  |  |  |  |  |  |  |  |  |  |  |  |  |  |  |  |  |  |  |  |  |  |  |  |  |  |  |  |  |  |  |  |  |  |  |  |  |  |  |  |  |  |  |  |  |  |  |  |  |  |  |  |  |  |  |  |  |  |  |  |  |  |  |  |  |  |  |  |  |  |  |  |  |  |  |  |  |  |  |  |  |  |  |  |  |  |  |  |  |  |  |  |  |  |  |  |  |  |  |  |  |  |  |  |  |  |  |  |  |  |  |  |  |  |  |  |  |  |  |  |  |  |  |  |  |  |  |  |  |  |  |  |  |  |  |  |  |  |  |  |  |  |  |  |  |  |  |  |  |  |  |  |  |  |  |  |  |  |  |  |  |  |  |  |  |  |  |  |  |  |  |  |  |  |  |  |  |  |  |  |  |  |  |  |  |  |  |  |  |  |  |  |  |  |  |  |  |  |  |  |  |  |  |  |  |  |  |  |  |  |  |  |  |  |  |  |  |  |  |  |  |  |  |  |  |  |  |  |  |  |  |  |  |  |  |  |  |  |  |  |  |  |  |  |  |  |  |  |  |  |  |  |  |  |  |  |  |  |  |  |  |  |  |  |  |  |  |  |  |  |  |  |  |  |  |  |  |  |  |  |  |  |  |  |  |  |  |  |  |  |  |  |  |  |  |  |  |  |  |  |  |  |  |  |  |  |  |  |  |  |  |  |  |  |  |  |  |  |  |  |  |  |  |  |  |  |  |  |  |  |  |  |  |  |  |  |  |  |  |  |  |  |  |  |  |  |  |  |  |  |  |  |  |  |  |  |  |  |  |  |  |  |  |  |  |  |  |  |  |  |  |  |  |  |  |  |  |  |  |  |  |  |  |  |  |  |  |  |  |  |  |  |  |  |  |  |  |  |  |  |  |  |  |  |  |  |  |  |  |  |  |  |  |  |  |  |  |  |  |  |  |  |  |  |  |  |  |  |  |  |  |  |  |  |  |  |  |  |  |  |  |  |  |  |  |  |  |  |  |  |  |  |  |  |  |  |  |  |  |  |  |  |  |  |  |  |  |  |  |  |  |  |  |  |  |  |  |  |  |  |  |  |  |  |  |  |  |  |  |  |  |  |  |  |  |  |  |  |  |  |  |  |  |  |  |  |  |  |  |  |  |  |  |  |  |  |  |  |  |  |  |  |  |  |  |  |  |  |  |  |  |  |  |  |  |  |  |  |  |  |  |  |  |  |  |  |  |  |  |  |  |  |  |  |  |  |  |  |  |  |  |  |  |  |  |  |  |  |  |  |  |  |  |  |  |  |  |  |  |  |  |  |  |  |  |  |  |  |  |  |  |  |  |  |  |  |  |  |  |  |  |  |  |  |  |  |  |  |  |  |  |  |  |  |  |  |  |  |  |  |  |  |  |  |  |  |  |  |  |  |  |  |  |  |  |  |  |  |  |  |  |  |  |  |  |  |  |  |  |  |  |  |  |  |  |  |  |  |  |  |  |  |  |  |  |  |  |  |  |  |  |  |  |  |  |  |  |  |  |  |  |  |  |  |  |  |  |  |  |  |  |  |  |  |  |  |  |  |  |  |  |  |  |  |  |  |  |  |  |  |  |  |  |  |  |  |  |  |  |  |  |  |  |  |  |  |  |  |  |  |  |  |  |  |  |  |  |  |  |  |  |  |  |  |  |  |  |  |  |  |  |  |  |  |  |  |  |  |  |  |  |  |  |  |  |  |  |  |  |  |  |  |  |  |  |  |  |  |  |  |  |  |  |  |  |  |  |  |  |  |  |  |  |  |  |  |  |  |  |  |  |  |  |  |  |  |  |  |  |  |  |  |  |  |  |  |  |  |  |  |  |  |  |  |  |  |  |  |  |  |  |  |  |  |  |  |  |  |  |  |  |  |  |  |  |  |  |  |  |  |  |  |  |  |  |  |  |  |  |  |  |  |  |  |  |  |  |  |  |  |  |  |  |  |  |  |  |  |  |  |  |  |  |  |  |  |  |  |  |  |  |  |  |  |  |  |  |  |  |  |  |  |  |  |  |  |  |  |  |  |  |  |  |  |  |  |  |  |  |  |  |  |  |  |  |  |  |  |  |  |  |  |  |  |  |  |  |  |  |  |  |  |  |  |  |  |  |  |  |  |  |  |  |  |  |  |  |  |  |  |  |  |  |  |  |  |  |  |  |  |  |  |  |  |  |  |  |  |  |  |  |  |  |  |  |  |  |  |  |  |  |  |  |  |  |  |  |  |  |  |  |  |  |  |  |  |  |  |  |  |  |  |  |  |  |  |  |  |  |  |  |  |  |  |  |  |  |  |  |  |  |  |  |  |  |  |  |  |  |  |  |  |  |  |  |  |  |  |  |  |  |  |  |  |  |  |  |  |  |  |  |  |  |  |  |  |  |  |  |  |  |  |  |  |  |  |  |  |  |  |  |  |  |  |  |  |  |  |  |  |  |  |  |  |  |  |  |  |  |  |  |  |  |  |  |  |  |  |  |  |  |  |  |  |  |  |  |  |  |  |  |  |  |  |  |  |  |  |  |  |  |  |  |  |  |  |  |  |  |  |  |  |  |  |  |  |  |  |  |  |  |  |  |  |  |  |  |  |  |  |  |  |  |  |  |  |  |  |  |  |  |  |  |  |  |  |  |  |  |  |  |  |  |  |  |  |  |  |  |  |  |  |  |  |  |  |  |  |  |  |  |  |  |  |  |  |  |  |  |  |  |  |  |  |  |  |  |  |  |  |  |  |  |  |  |  |  |  |  |  |  |  |  |  |  |  |  |  |  |  |  |  |  |  |  |  |  |  |  |  |  |  |  |  |  |  |  |  |  |  |  |  |  |  |  |  |  |  |  |  |  |  |  |  |  |  |  |  |  |  |  |  |  |  |  |  |  |  |  |  |  |  |  |  |  |  |  |  |  |  |  |  |  |  |  |  |  |  |  |  |  |  |  |  |  |  |  |  |  |  |  |  |  |  |  |  |  |  |  |  |  |  |  |  |  |  |  |  |  |  |  |  |  |  |  |  |  |  |  |  |  |  |  |  |  |  |  |  |  |  |  |  |  |  |  |  |  |  |  |  |  |  |  |  |  |  |  |  |  |  |  |  |  |  |  |  |  |  |  |  |  |  |  |  |  |  |  |  |  |  |  |  |  |  |  |  |  |  |  |  |  |  |  |  |  |  |  |  |  |  |  |  |  |  |  |  |  |  |  |  |  |  |  |  |  |  |  |  |  |  |  |  |  |  |  |  |  |  |  |  |  |  |  |  |  |  |  |  |  |  |  |  |  |  |  |  |  |  |  |  |  |  |  |  |  |  |  |  |  |  |  |  |  |  |  |  |  |  |  |  |  |  |  |  |  |  |  |  |  |  |  |  |  |  |  |  |  |  |  |  |  |  |  |  |  |  |  |  |  |  |  |  |  |  |  |  |  |  |  |  |  |  |  |  |  |  |  |  |  |  |  |  |  |  |  |  |  |  |  |  |  |  |  |  |  |  |  |  |  |  |  |  |  |  |  |  |  |  |  |  |  |  |  |  |  |  |  |  |  |  |  |  |  |  |  |  |  |  |  |  |  |  |  |  |  |  |  |  |  |  |  |  |  |  |  |  |  |  |  |  |  |  |  |  |  |  |  |  |  |  |  |  |  |  |  |  |  |  |  |  |  |  |  |  |  |  |  |  |  |  |  |  |  |  |  |  |  |  |  |  |  |  |  |  |  |  |  |  |  |  |  |  |  |  |  |  |  |  |  |  |  |  |  |  |  |  |  |  |  |  |  |  |  |  |  |  |  |  |  |  |  |  |  |  |  |  |  |  |  |  |  |  |  |  |  |  |  |  |  |  |  |  |  |  |  |  |  |  |  |  |  |  |  |  |  |  |  |  |  |  |  |  |  |  |  |  |  |  |  |  |  |  |  |  |  |  |  |  |  |  |  |  |  |  |  |  |  |  |  |  |  |  |  |  |  |  |  |  |  |  |  |  |  |  |  |  |  |  |  |  |  |  |  |  |  |  |  |  |  |  |  |  |  |  |  |  |  |  |  |  |  |  |  |  |  |  |  |  |  |  |  |  |  |  |  |  |  |  |  |  |  |  |  |  |  |  |  |  |  |  |  |  |  |  |  |  |  |  |  |  |  |  |  |  |  |  |  |  |  |  |  |  |  |  |  |  |  |  |  |  |  |  |  |  |  |  |  |  |  |  |  |  |  |  |  |  |  |  |  |  |  |  |  |  |  |  |  |  |  |  |  |  |  |  |  |  |  |  |  |  |  |  |  |  |  |  |  |  |  |  |  |  |  |  |  |  |  |  |  |  |  |  |  |  |  |  |  |  |  |  |  |  |  |  |  |  |  |  |  |  |  |  |  |  |  |  |  |  |  |  |  |  |  |  |  |  |  |  |  |  |  |  |  |  |  |  |  |  |  |  |  |  |  |  |  |  |  |  |  |  |  |  |  |  |  |  |  |  |  |  |  |  |  |  |  |  |  |  |  |  |  |  |  |  |  |  |  |  |  |  |  |  |  |  |  |  |  |  |  |  |  |  |  |  |  |  |  |  |  |  |  |  |  |  |  |  |  |  |  |  |  |  |  |  |  |  |  |  |  |  |  |  |  |  |  |  |  |  |  |  |  |  |  |  |  |  |  |  |  |  |  |  |  |  |  |  |  |  |  |  |  |  |  |  |  |  |  |  |  |  |  |  |  |  |  |  |  |  |  |  |  |  |  |  |  |  |  |  |  |  |  |  |  |  |  |  |  |  |  |  |  |  |  |  |  |  |  |  |  |  |  |  |  |  |  |  |  |  |  |  |  |  |  |  |  |  |  |  |  |  |  |  |  |  |  |  |  |  |  |  |  |  |  |  |  |  |  |  |  |  |  |  |  |  |  |  |  |  |  |  |  |  |  |  |  |  |  |  |  |  |  |  |  |  |  |  |  |  |  |  |  |  |  |  |  |  |  |  |  |  |  |  |  |  |  |  |  |  |  |  |  |  |  |  |  |  |  |  |  |  |  |  |  |  |  |  |  |  |  |  |  |  |  |  |  |  |  |  |  |  |  |  |  |  |  |  |  |  |  |  |  |  |  |  |  |  |  |  |  |  |  |  |  |  |  |  |  |  |  |  |  |  |  |  |  |  |  |  |  |  |  |  |  |  |  |  |  |  |  |  |  |  |  |  |  |  |  |  |  |  |  |  |  |  |  |  |  |  |  |  |  |  |  |  |  |  |  |  |  |  |  |  |  |  |  |  |  |  |  |  |  |  |  |  |  |  |  |  |  |  |  |  |  |  |  |  |  |  |  |  |  |  |  |  |  |  |  |  |  |  |  |  |  |  |  |  |  |  |  |  |  |  |  |  |  |  |  |  |  |  |  |  |  |  |  |  |  |  |  |  |  |  |  |  |  |  |  |  |  |  |  |  |  |  |  |  |  |  |  |  |  |  |  |  |  |  |  |  |  |  |  |  |  |  |  |  |  |  |  |  |  |  |  |  |  |  |  |  |  |  |  |  |  |  |  |  |  |  |  |  |  |  |  |  |  |  |  |  |  |  |  |  |  |  |  |  |  |  |  |  |  |  |  |  |  |  |  |  |  |  |  |  |  |  |  |  |  |  |  |  |  |  |  |  |  |  |  |  |  |  |  |  |  |  |  |  |  |  |  |  |  |  |  |  |  |  |  |  |  |  |  |  |  |  |  |  |  |  |  |  |  |  |  |  |  |  |  |  |  |  |  |  |  |  |  |  |  |  |  |  |  |  |  |  |  |  |  |  |  |  |  |  |  |  |  |  |  |  |  |  |  |  |  |  |  |  |  |  |  |  |  |  |  |  |  |  |  |  |  |  |  |  |  |  |  |  |  |  |  |  |  |  |  |  |  |  |  |  |  |  |  |  |  |  |  |  |  |  |  |  |  |  |  |  |  |  |  |  |  |  |  |  |  |  |  |  |  |  |  |  |  |  |  |  |  |  |  |  |  |  |  |  |  |  |  |  |  |  |  |  |  |  |  |  |  |  |  |  |  |  |  |  |  |  |  |  |  |  |  |  |  |  |  |  |  |  |  |  |  |  |  |  |  |  |  |  |  |  |  |  |  |  |  |  |  |  |  |  |  |  |  |  |  |  |  |  |  |  |  |  |  |  |  |  |  |  |  |  |  |  |  |  |  |  |  |  |  |  |  |  |  |  |  |  |  |  |  |  |  |  |  |  |  |  |  |  |  |  |  |  |  |  |  |  |  |  |  |  |  |  |  |  |  |  |  |  |  |  |  |  |  |  |  |  |  |  |  |  |  |  |  |  |  |  |  |  |  |  |  |  |  |  |  |  |  |  |  |  |  |  |  |  |  |  |  |  |  |  |  |  |  |  |  |  |  |  |  |  |  |  |  |  |  |  |  |  |  |  |  |  |  |  |  |  |  |  |  |  |  |  |  |  |  |  |  |  |  |  |  |  |  |  |  |  |  |  |  |  |  |  |  |  |  |  |  |  |  |  |  |  |  |  |  |  |  |  |  |  |  |  |  |  |  |  |  |  |  |  |  |  |  |  |  |  |  |  |  |  |  |  |  |  |  |  |  |  |  |  |  |  |  |  |  |  |  |  |  |  |  |  |  |  |  |  |  |  |  |  |  |  |  |  |  |  |  |  |  |  |  |  |  |  |  |  |  |  |  |  |  |  |  |  |  |  |  |  |  |  |  |  |  |  |  |  |  |  |  |  |  |  |  |  |  |  |  |  |  |  |  |  |  |  |  |  |  |  |  |  |  |  |  |  |  |  |  |  |  |  |  |  |  |  |  |  |  |  |  |  |  |  |  |  |  |  |  |  |  |  |  |  |  |  |  |  |  |  |  |  |  |  |  |  |  |  |  |  |  |  |  |  |  |  |  |  |  |  |  |  |  |  |  |  |  |  |  |  |  |  |  |  |  |  |  |  |  |  |  |  |  |  |  |  |  |  |  |  |  |  |  |  |  |  |  |  |  |  |  |  |  |  |  |  |  |  |  |  |  |  |  |  |  |  |  |  |  |  |  |  |  |  |  |  |  |  |  |  |  |  |  |  |  |  |  |  |  |  |  |  |  |  |  |  |  |  |  |  |  |  |  |  |  |  |  |  |  |  |  |  |  |  |  |  |  |  |  |  |  |  |  |  |  |  |  |  |  |  |  |  |  |  |  |  |  |  |  |  |  |  |  |  |  |  |  |  |  |  |  |  |  |  |  |  |  |  |  |  |  |  |  |  |  |  |  |  |  |  |  |  |  |  |  |  |  |  |  |  |  |  |  |  |  |  |  |  |  |  |  |  |  |  |  |  |  |  |  |  |  |  |  |  |  |  |  |  |  |  |  |  |  |  |  |  |  |  |  |  |  |  |  |  |  |  |  |  |  |  |  |  |  |  |  |  |  |  |  |  |  |  |  |  |  |  |  |  |  |  |  |  |  |  |  |  |  |  |  |  |  |  |  |  |  |  |  |  |  |  |  |  |  |  |  |  |  |  |  |  |  |  |  |  |  |  |  |  |  |  |  |  |  |  |  |  |  |  |  |  |  |  |  |  |  |  |  |  |  |  |  |  |  |  |  |  |  |  |  |  |  |  |  |  |  |  |  |  |  |  |  |  |  |  |  |  |  |  |  |  |  |  |  |  |  |  |  |  |  |  |  |  |  |  |  |  |  |  |  |  |  |  |  |  |  |  |  |  |  |  |  |  |  |  |  |  |  |  |  |  |  |  |  |  |  |  |  |  |  |  |  |  |  |  |  |  |  |  |  |  |  |  |  |  |  |  |  |  |  |  |  |  |  |  |  |  |  |  |  |  |  |  |  |  |  |  |  |  |  |  |  |  |  |  |  |  |  |  |  |  |  |  |  |  |  |  |  |  |  |  |  |  |  |  |  |  |  |  |  |  |  |  |  |  |  |  |  |  |  |  |  |  |  |  |  |  |  |  |  |  |  |  |  |  |  |  |  |  |  |  |  |  |  |  |  |  |  |  |  |  |  |  |  |  |  |  |  |  |  |  |  |  |  |  |  |  |  |  |  |  |  |  |  |  |  |  |  |  |  |  |  |  |  |  |  |  |  |  |  |  |  |  |  |  |  |  |  |  |  |  |  |  |  |  |  |  |  |  |  |  |  |  |  |  |  |  |  |  |  |  |  |  |  |  |  |  |  |  |  |  |  |  |  |  |  |  |  |  |  |  |  |  |  |  |  |  |  |  |  |  |  |  |  |  |  |  |  |  |  |  |  |  |  |  |  |  |  |  |  |  |  |  |  |  |  |  |  |  |  |  |  |  |  |  |  |  |  |  |  |  |  |  |  |  |  |  |  |  |  |  |  |  |  |  |  |  |  |  |  |  |  |  |  |  |  |  |  |  |  |  |  |  |  |  |  |  |  |  |  |  |  |  |  |  |  |  |  |  |  |  |  |  |  |  |  |  |  |  |  |  |  |  |  |  |  |  |  |  |  |  |  |  |  |  |  |  |  |  |  |  |  |  |  |  |  |  |  |  |  |  |  |  |  |  |  |  |  |  |  |  |  |  |  |  |  |  |  |  |  |  |  |  |  |  |  |  |  |  |  |  |  |  |  |  |  |  |  |  |  |  |  |  |  |  |  |  |  |  |  |  |  |  |  |  |  |  |  |  |  |  |  |  |  |  |  |  |  |  |  |  |  |  |  |  |  |  |  |  |  |  |  |  |  |  |  |  |  |  |  |  |  |  |  |  |  |  |  |  |  |  |  |  |  |  |  |  |  |  |  |  |  |  |  |  |  |  |  |  |  |  |  |  |  |  |  |  |  |  |  |  |  |  |  |  |  |  |  |  |  |  |  |  |  |  |  |  |  |  |  |  |  |  |  |  |  |  |  |  |  |  |  |  |  |  |  |  |  |  |  |  |  |  |  |  |  |  |  |  |  |  |  |  |  |  |  |  |  |  |  |  |  |  |  |  |  |  |  |  |  |  |  |  |  |  |  |  |  |  |  |  |  |  |  |  |  |  |  |  |  |  |  |  |  |  |  |  |  |  |  |  |  |  |  |  |  |  |  |  |  |  |  |  |  |  |  |  |  |  |  |  |  |  |  |  |  |  |  |  |  |  |  |  |  |  |  |  |  |  |  |  |  |  |  |  |  |  |  |  |  |  |  |  |  |  |  |  |  |  |  |  |  |  |  |  |  |  |  |  |  |  |  |  |  |  |  |  |  |  |  |  |  |  |  |  |  |  |  |  |  |  |  |  |  |  |  |  |  |  |  |  |  |  |  |  |  |  |  |  |  |  |  |  |  |  |  |  |  |  |  |  |  |  |  |  |  |  |  |  |  |  |  |  |  |  |  |  |  |  |  |  |  |  |  |  |  |  |  |  |  |  |  |  |  |  |  |  |  |  |  |  |  |  |  |  |  |  |  |  |  |  |  |  |  |  |  |  |  |  |  |  |  |  |  |  |  |  |  |  |  |  |  |  |  |  |  |  |  |  |  |  |  |  |  |  |  |  |  |  |  |  |  |  |  |  |  |  |  |  |  |  |  |  |  |  |  |  |  |  |  |  |  |  |  |  |  |  |  |  |  |  |  |  |  |  |  |  |  |  |  |  |  |  |  |  |  |  |  |  |  |  |  |  |  |  |  |  |  |  |  |  |  |  |  |  |  |  |  |  |  |  |  |  |  |  |  |  |  |  |  |  |  |  |  |  |  |  |  |  |  |  |  |  |  |  |  |  |  |  |  |  |  |  |  |  |  |  |  |  |  |  |  |  |  |  |  |  |  |  |  |  |  |  |  |  |  |  |  |  |  |  |  |  |  |  |  |  |  |  |  |  |  |  |  |  |  |  |  |  |  |  |  |  |  |  |  |  |  |  |  |  |  |  |  |  |  |  |  |  |  |  |  |  |  |  |  |  |  |  |  |  |  |  |  |  |  |  |  |  |  |  |  |  |  |  |  |  |  |  |  |  |  |  |  |  |  |  |  |  |  |  |  |  |  |  |  |  |  |  |  |  |  |  |  |  |  |  |  |  |  |  |  |  |  |  |  |  |  |  |  |  |  |  |  |  |  |  |  |  |  |  |  |  |  |  |  |  |  |  |  |  |  |  |  |  |  |  |  |  |  |  |  |  |  |  |  |  |  |  |  |  |  |  |  |  |  |  |  |  |  |  |  |  |  |  |  |  |  |  |  |  |  |  |  |  |  |  |  |  |  |  |  |  |  |  |  |  |  |  |  |  |  |  |  |  |  |  |  |  |  |  |  |  |  |  |  |  |  |  |  |  |  |  |  |  |  |  |  |  |  |  |  |  |  |  |  |  |  |  |  |  |  |  |  |  |  |  |  |  |  |  |  |  |  |  |  |  |  |  |  |  |  |  |  |  |  |  |  |  |  |  |  |  |  |  |  |  |  |  |  |  |  |  |  |  |  |  |  |  |  |  |  |  |  |  |  |  |  |  |  |  |  |  |  |  |  |  |  |  |  |  |  |  |  |  |  |  |  |  |  |  |  |  |  |  |  |  |  |  |  |  |  |  |  |  |  |  |  |  |  |  |  |  |  |  |  |  |  |  |  |  |  |  |  |  |  |  |  |  |  |  |  |  |  |  |  |  |  |  |  |  |  |  |  |  |  |  |  |  |  |  |  |  |  |  |  |  |  |  |  |  |  |  |  |  |  |  |  |  |  |  |  |  |  |  |  |  |  |  |  |  |  |  |  |  |  |  |  |  |  |  |  |  |  |  |  |  |  |  |  |  |  |  |  |  |  |  |  |  |  |  |  |  |  |  |  |  |  |  |  |  |  |  |  |  |  |  |  |  |  |  |  |  |  |  |  |  |  |  |  |  |  |  |  |  |  |  |  |  |  |  |  |  |  |  |  |  |  |  |  |  |  |  |  |  |  |  |  |  |  |  |  |  |  |  |  |  |  |  |  |  |  |  |  |  |  |  |  |  |  |  |  |  |  |  |  |  |  |  |  |  |  |  |  |  |  |  |  |  |  |  |  |  |  |  |  |  |  |  |  |  |  |  |  |  |  |  |  |  |  |  |  |  |  |  |  |  |  |  |  |  |  |  |  |  |  |  |  |  |  |  |  |  |  |  |  |  |  |  |  |  |  |  |  |  |  |  |  |  |  |  |  |  |  |  |  |  |  |  |  |  |  |  |  |  |  |  |  |  |  |  |  |  |  |  |  |  |  |  |  |  |  |  |  |  |  |  |  |  |  |  |  |  |  |  |  |  |  |  |  |  |  |  |  |  |  |  |  |  |  |  |  |  |  |  |  |  |  |  |  |  |  |  |  |  |  |  |  |  |  |  |  |  |  |  |  |  |  |  |  |  |  |  |  |  |  |  |  |  |  |  |  |  |  |  |  |  |  |  |  |  |  |  |  |  |  |  |  |  |  |  |  |  |  |  |  |  |  |  |  |  |  |  |  |  |  |  |  |  |  |  |  |  |  |  |  |  |  |  |  |  |  |  |  |  |  |  |  |  |  |  |  |  |  |  |  |  |  |  |  |  |  |  |  |  |  |  |  |  |  |  |  |  |  |  |  |  |  |  |  |  |  |  |  |  |  |  |  |  |  |  |  |  |  |  |  |  |  |  |  |  |  |  |  |  |  |  |  |  |  |  |  |  |  |  |  |  |  |  |  |  |  |  |  |  |  |  |  |  |  |  |  |  |  |  |  |  |  |  |  |  |  |  |  |  |  |  |  |  |  |  |  |  |  |  |  |  |  |  |  |  |  |  |  |  |  |  |  |  |  |  |  |  |  |  |  |  |  |  |  |  |  |  |  |  |  |  |  |  |  |  |  |  |  |  |  |  |  |  |  |  |  |  |  |  |  |  |  |  |  |  |  |  |  |  |  |  |  |  |  |  |  |  |  |  |  |  |  |  |  |  |  |  |  |  |  |  |  |  |  |  |  |  |  |  |  |  |  |  |  |  |  |  |  |  |  |  |  |  |  |  |  |  |  |  |  |  |  |  |  |  |  |  |  |  |  |  |  |  |  |  |  |  |  |  |  |  |  |  |  |  |  |  |  |  |  |  |  |  |  |  |  |  |  |  |  |  |  |  |  |  |  |  |  |  |  |  |  |  |  |  |  |  |  |  |  |  |  |  |  |  |  |  |  |  |  |  |  |  |  |  |  |  |  |  |  |  |  |  |  |  |  |  |  |  |  |  |  |  |  |  |  |  |  |  |  |  |  |  |  |  |  |  |  |  |  |  |  |  |  |  |  |  |  |  |  |  |  |  |  |  |  |  |  |  |  |  |  |  |  |  |  |  |  |  |  |  |  |  |  |  |  |  |  |  |  |  |  |  |  |  |  |  |  |  |  |  |  |  |  |  |  |  |  |  |  |  |  |  |  |  |  |  |  |  |  |  |  |  |  |  |  |  |  |  |  |  |  |  |  |  |  |  |  |  |  |  |  |  |  |  |  |  |  |  |  |  |  |  |  |  |  |  |  |  |  |  |  |  |  |  |  |  |  |  |  |  |  |  |  |  |  |  |  |  |  |  |  |  |  |  |  |  |  |  |  |  |  |  |  |  |  |  |  |  |  |  |  |  |  |  |  |  |  |  |  |  |  |  |  |  |  |  |  |  |  |  |  |  |  |  |  |  |  |  |  |  |  |  |  |  |  |  |  |  |  |  |  |  |  |  |  |  |  |  |  |  |  |  |  |  |  |  |  |  |  |  |  |  |  |  |  |  |  |  |  |  |  |  |  |  |  |  |  |  |  |  |  |  |  |  |  |  |  |  |  |  |  |  |  |  |  |  |  |  |  |  |  |  |  |  |  |  |  |  |  |  |  |  |  |  |  |  |  |  |  |  |  |  |  |  |  |  |  |  |  |  |  |  |  |  |  |  |  |  |  |  |  |  |  |  |  |  |  |  |  |  |  |  |  |  |  |  |  |  |  |  |  |  |  |  |  |  |  |  |  |  |  |  |  |  |  |  |  |  |  |  |  |  |  |  |  |  |  |  |  |  |  |  |  |  |  |  |  |  |  |  |  |  |  |  |  |  |  |  |  |  |  |  |  |  |  |  |  |  |  |  |  |  |  |  |  |  |  |  |  |  |  |  |  |  |  |  |  |  |  |  |  |  |  |  |  |  |  |  |  |  |  |  |  |  |  |  |  |  |  |  |  |  |  |  |  |  |  |  |  |  |  |  |  |  |  |  |  |  |  |  |  |  |  |  |  |  |  |  |  |  |  |  |  |  |  |  |  |  |  |  |  |  |  |  |  |  |  |  |  |  |  |  |  |  |  |  |  |  |  |  |  |  |  |  |  |  |  |  |  |  |  |  |  |  |  |  |  |  |  |  |  |  |  |  |  |  |  |  |  |  |  |  |  |  |  |  |  |  |  |  |  |  |  |  |  |  |  |  |  |  |  |  |  |  |  |  |  |  |  |  |  |  |  |  |  |  |  |  |  |  |  |  |  |  |  |  |  |  |  |  |  |  |  |  |  |  |  |  |  |  |  |  |  |  |  |  |  |  |  |  |  |  |  |  |  |  |  |  |  |  |  |  |  |  |  |  |  |  |  |  |  |  |  |  |  |  |  |  |  |  |  |  |  |  |  |  |  |  |  |  |  |  |  |  |  |  |  |  |  |  |  |  |  |  |  |  |  |  |  |  |  |  |  |  |  |  |  |  |  |  |  |  |  |  |  |  |  |  |  |  |  |  |  |  |  |  |  |  |  |  |  |  |  |  |  |  |  |  |  |  |  |  |  |  |  |  |  |  |  |  |  |  |  |  |  |  |  |  |  |  |  |  |  |  |  |  |  |  |  |  |  |  |  |  |  |  |  |  |  |  |  |  |  |  |  |  |  |  |  |  |  |  |  |  |  |  |  |  |  |  |  |  |  |  |  |  |  |  |  |  |  |  |  |  |  |  |  |  |  |  |  |  |  |  |  |  |  |  |  |  |  |  |  |  |  |  |  |  |  |  |  |  |  |  |  |  |  |  |  |  |  |  |  |  |  |  |  |  |  |  |  |  |  |  |  |  |  |  |  |  |  |  |  |  |  |  |  |  |  |  |  |  |  |  |  |  |  |  |  |  |  |  |  |  |  |  |  |  |  |  |  |  |  |  |  |  |  |  |  |  |  |  |  |  |  |  |  |  |  |  |  |  |  |  |  |  |  |  |  |  |  |  |  |  |  |  |  |  |  |  |  |  |  |  |  |  |  |  |  |  |  |  |  |  |  |  |  |  |  |  |  |  |  |  |  |  |  |  |  |  |  |  |  |  |  |  |  |  |  |  |  |  |  |  |  |  |  |  |  |  |  |  |  |  |  |  |  |  |  |  |  |  |  |  |  |  |  |  |  |  |  |  |  |  |  |  |  |  |  |  |  |  |  |  |  |  |  |  |  |  |  |  |  |  |  |  |  |  |  |  |  |  |  |  |  |  |  |  |  |  |  |  |  |  |  |  |  |  |  |  |  |  |  |  |  |  |  |  |  |  |  |  |  |  |  |  |  |  |  |  |  |  |  |  |  |  |  |  |  |  |  |  |  |  |  |  |  |  |  |  |  |  |  |  |  |  |  |  |  |  |  |  |  |  |  |  |  |  |  |  |  |  |  |  |  |  |  |  |  |  |  |  |  |  |  |  |  |  |  |  |  |  |  |  |  |  |  |  |  |  |  |  |  |  |  |  |  |  |  |  |  |  |  |  |  |  |  |  |  |  |  |  |  |  |  |  |  |  |  |  |  |  |  |  |  |  |  |  |  |  |  |  |  |  |  |  |  |  |  |  |  |  |  |  |  |  |  |  |  |  |  |  |  |  |  |  |  |  |  |  |  |  |  |  |  |  |  |  |  |  |  |  |  |  |  |  |  |  |  |  |  |  |  |  |  |  |  |  |  |  |  |  |  |  |  |  |  |  |  |  |  |  |  |  |  |  |  |  |  |  |  |  |  |  |  |  |  |  |  |  |  |  |  |  |  |  |  |  |  |  |  |  |  |  |  |  |  |  |  |  |  |  |  |  |  |  |  |  |  |  |  |  |  |  |  |  |  |  |  |  |  |  |  |  |  |  |  |  |  |  |  |  |  |  |  |  |  |  |  |  |  |  |  |  |  |  |  |  |  |  |  |  |  |  |  |  |  |  |  |  |  |  |  |  |  |  |  |  |  |  |  |  |  |  |  |  |  |  |  |  |  |  |  |  |  |  |  |  |  |  |  |  |  |  |  |  |  |  |  |  |  |  |  |  |  |  |  |  |  |  |  |  |  |  |  |  |  |  |  |  |  |  |  |  |  |  |  |  |  |  |  |  |  |  |  |  |  |  |  |  |  |  |  |  |  |  |  |  |  |  |  |  |  |  |  |  |  |  |  |  |  |  |  |  |  |  |  |  |  |  |  |  |  |  |  |  |  |  |  |  |  |  |  |  |  |  |  |  |  |  |  |  |  |  |  |  |  |  |  |  |  |  |  |  |  |  |  |  |  |  |  |  |  |  |  |  |  |  |  |  |  |  |  |  |  |  |  |  |  |  |  |  |  |  |  |  |  |  |  |  |  |  |  |  |  |  |  |  |  |  |  |  |  |  |  |  |  |  |  |  |  |  |  |  |  |  |  |  |  |  |  |  |  |  |  |  |  |  |  |  |  |  |  |  |  |  |  |  |  |  |  |  |  |  |  |  |  |  |  |  |  |  |  |  |  |  |  |  |  |  |  |  |  |  |  |  |  |  |  |  |  |  |  |  |  |  |  |  |  |  |  |  |  |  |  |  |  |  |  |  |  |  |  |  |  |  |  |  |  |  |  |  |  |  |  |  |  |  |  |  |  |  |  |  |  |  |  |  |  |  |  |  |  |  |  |  |  |  |  |  |  |  |  |  |  |  |  |  |  |  |  |  |  |  |  |  |  |  |  |  |  |  |  |  |  |  |  |  |  |  |  |  |  |  |  |  |  |  |  |  |  |  |  |  |  |  |  |  |  |  |  |  |  |  |  |  |  |  |  |  |  |  |  |  |  |  |  |  |  |  |  |  |  |  |  |  |  |  |  |  |  |  |  |  |  |  |  |  |  |  |  |  |  |  |  |  |  |  |  |  |  |  |  |  |  |  |  |  |  |  |  |  |  |  |  |  |  |  |  |  |  |  |  |  |  |  |  |  |  |  |  |  |  |  |  |  |  |  |  |  |  |  |  |  |  |  |  |  |  |  |  |  |  |  |  |  |  |  |  |  |  |  |  |  |  |  |  |  |  |  |  |  |  |  |  |  |  |  |  |  |  |  |  |  |  |  |  |  |  |  |  |  |  |  |  |  |  |  |  |  |  |  |  |  |  |  |  |  |  |  |  |  |  |  |  |  |  |  |  |  |  |  |  |  |  |  |  |  |  |  |  |  |  |  |  |  |  |  |  |  |  |  |  |  |  |  |  |  |  |  |  |  |  |  |  |  |  |  |  |  |  |  |  |  |  |  |  |  |  |  |  |  |  |  |  |  |  |  |  |  |  |  |  |  |  |  |  |  |  |  |  |  |  |  |  |  |  |  |  |  |  |  |  |  |  |  |  |  |  |  |  |  |  |  |  |  |  |  |  |  |  |  |  |  |  |  |  |  |  |  |  |  |  |  |  |  |  |  |  |  |  |  |  |  |  |  |  |  |  |  |  |  |  |  |  |  |  |  |  |  |  |  |  |  |  |  |  |  |  |  |  |  |  |  |  |  |  |  |  |  |  |  |  |  |  |  |  |  |  |  |  |  |  |  |  |  |  |  |  |  |  |  |  |  |  |  |  |  |  |  |  |  |  |  |  |  |  |  |  |  |  |  |  |  |  |  |  |  |  |  |  |  |  |  |  |  |  |  |  |  |  |  |  |  |  |  |  |  |  |  |  |  |  |  |  |  |  |  |  |  |  |  |  |  |  |  |  |  |  |  |  |  |  |  |  |  |  |  |  |  |  |  |  |  |  |  |  |  |  |  |  |  |  |  |  |  |  |  |  |  |  |  |  |  |  |  |  |  |  |  |  |  |  |  |  |  |  |  |  |  |  |  |  |  |  |  |  |  |  |  |  |  |  |  |  |  |  |  |  |  |  |  |  |  |  |  |  |  |  |  |  |  |  |  |  |  |  |  |  |  |  |  |  |  |  |  |  |  |  |  |  |  |  |  |  |  |  |  |  |  |  |  |  |  |  |  |  |  |  |  |  |  |  |  |  |  |  |  |  |  |  |  |  |  |  |  |  |  |  |  |  |  |  |  |  |  |  |  |  |  |  |  |  |  |  |  |  |  |  |  |  |  |  |  |  |  |  |  |  |  |  |  |  |  |  |  |  |  |  |  |  |  |  |  |  |  |  |  |  |  |  |  |  |  |  |  |  |  |  |  |  |  |  |  |  |  |  |  |  |  |  |  |  |  |  |  |  |  |  |  |  |  |  |  |  |  |  |  |  |  |  |  |  |  |  |  |  |  |  |  |  |  |  |  |  |  |  |  |  |  |  |  |  |  |  |  |  |  |  |  |  |  |  |  |  |  |  |  |  |  |  |  |  |  |  |  |  |  |  |  |  |  |  |  |  |  |  |  |  |  |  |  |  |  |  |  |  |  |  |  |  |  |  |  |  |  |  |  |  |  |  |  |  |  |  |  |  |  |  |  |  |  |  |  |  |  |  |  |  |  |  |  |  |  |  |  |  |  |  |  |  |  |  |  |  |  |  |  |  |  |  |  |  |  |  |  |  |  |  |  |  |  |  |  |  |  |  |  |  |  |  |  |  |  |  |  |  |  |  |  |  |  |  |  |  |  |  |  |  |  |  |  |  |  |  |  |  |  |  |  |  |  |  |  |  |  |  |  |  |  |  |  |  |  |  |  |  |  |  |  |  |  |  |  |  |  |  |  |  |  |  |  |  |  |  |  |  |  |  |  |  |  |  |  |  |  |  |  |  |  |  |  |  |  |  |  |  |  |  |  |  |  |  |  |  |  |  |  |  |  |  |  |  |  |  |  |  |  |  |  |  |  |  |  |  |  |  |  |  |  |  |  |  |  |  |  |  |  |  |  |  |  |  |  |  |  |  |  |  |  |  |  |  |  |  |  |  |  |  |  |  |  |  |  |  |  |  |  |  |  |  |  |  |  |  |  |  |  |  |  |  |  |  |  |  |  |  |  |  |  |  |  |  |  |  |  |  |  |  |  |  |  |  |  |  |  |  |  |  |  |  |  |  |  |  |  |  |  |  |  |  |  |  |  |  |  |  |  |  |  |  |  |  |  |  |  |  |  |  |  |  |  |  |  |  |  |  |  |  |  |  |  |  |  |  |  |  |  |  |  |  |  |  |  |  |  |  |  |  |  |  |  |  |  |  |  |  |  |  |  |  |  |  |  |  |  |  |  |  |  |  |  |  |  |  |  |  |  |  |  |  |  |  |  |  |  |  |  |  |  |  |  |  |  |  |  |  |  |  |  |  |  |  |  |  |  |  |  |  |  |  |  |  |  |  |  |  |  |  |  |  |  |  |  |  |  |  |  |  |  |  |  |  |  |  |  |  |  |  |  |  |  |  |  |  |  |  |  |  |  |  |  |  |  |  |  |  |  |  |  |  |  |  |  |  |  |  |  |  |  |  |  |  |  |  |  |  |  |  |  |  |  |  |  |  |  |  |  |  |  |  |  |  |  |  |  |  |  |  |  |  |  |  |  |  |  |  |  |  |  |  |  |  |  |  |  |  |  |  |  |  |  |  |  |  |  |  |  |  |  |  |  |  |  |  |  |  |  |  |  |  |  |  |  |  |  |  |  |  |  |  |  |  |  |  |  |  |  |  |  |  |  |  |  |  |  |  |  |  |  |  |  |  |  |  |  |  |  |  |  |  |  |  |  |  |  |  |  |  |  |  |  |  |  |  |  |  |  |  |  |  |  |  |  |  |  |  |  |  |  |  |  |  |  |  |  |  |  |  |  |  |  |  |  |  |  |  |  |  |  |  |  |  |  |  |  |  |  |  |  |  |  |  |  |  |  |  |  |  |  |  |  |  |  |  |  |  |  |  |  |  |  |  |  |  |  |  |  |  |  |  |  |  |  |  |  |  |  |  |  |  |  |  |  |  |  |  |  |  |  |  |  |  |  |  |  |  |  |  |  |  |  |  |  |  |  |  |  |  |  |  |  |  |  |  |  |  |  |  |  |  |  |  |  |  |  |  |  |  |  |  |  |  |  |  |  |  |  |  |  |  |  |  |  |  |  |  |  |  |  |  |  |  |  |  |  |  |  |  |  |  |  |  |  |  |  |  |  |  |  |  |  |  |  |  |  |  |  |  |  |  |  |  |  |  |  |  |  |  |  |  |  |  |  |  |  |  |  |  |  |  |  |  |  |  |  |  |  |  |  |  |  |  |  |  |  |  |  |  |  |  |  |  |  |  |  |  |  |  |  |  |  |  |  |  |  |  |  |  |  |  |  |  |  |  |  |  |  |  |  |  |  |  |  |  |  |  |  |  |  |  |  |  |  |  |  |  |  |  |  |  |  |  |  |  |  |  |  |  |  |  |  |  |  |  |  |  |  |  |  |  |  |  |  |  |  |  |  |  |  |  |  |  |  |  |  |  |  |  |  |  |  |  |  |  |  |  |  |  |  |  |  |  |  |  |  |  |  |  |  |  |  |  |  |  |  |  |  |  |  |  |  |  |  |  |  |  |  |  |  |  |  |  |  |  |  |  |  |  |  |  |  |  |  |  |  |  |  |  |  |  |  |  |  |  |  |  |  |  |  |  |  |  |  |  |  |  |  |  |  |  |  |  |  |  |  |  |  |  |  |  |  |  |  |  |  |  |  |  |  |  |  |  |  |  |  |  |  |  |  |  |  |  |  |  |  |  |  |  |  |  |  |  |  |  |  |  |  |  |  |  |  |  |  |  |  |  |  |  |  |  |  |  |  |  |  |  |  |  |  |  |  |  |  |  |  |  |  |  |  |  |  |  |  |  |  |  |  |  |  |  |  |  |  |  |  |  |  |  |  |  |  |  |  |  |  |  |  |  |  |  |  |  |  |  |  |  |  |  |  |  |  |  |  |  |  |  |  |  |  |  |  |  |  |  |  |  |  |  |  |  |  |  |  |  |  |  |  |  |  |  |  |  |  |  |  |  |  |  |  |  |  |  |  |  |  |  |  |  |  |  |  |  |  |  |  |  |  |  |  |  |  |  |  |  |  |  |  |  |  |  |  |  |  |  |  |  |  |  |  |  |  |  |  |  |  |  |  |  |  |  |  |  |  |  |  |  |  |  |  |  |  |  |  |  |  |  |  |  |  |  |  |  |  |  |  |  |  |  |  |  |  |  |  |  |  |  |  |  |  |  |  |  |  |  |  |  |  |  |  |  |  |  |  |  |  |  |  |  |  |  |  |  |  |  |  |  |  |  |  |  |  |  |  |  |  |  |  |  |  |  |  |  |  |  |
| --- | --- | --- | --- | --- | --- | --- | --- | --- | --- | --- | --- | --- | --- | --- | --- | --- | --- | --- | --- | --- | --- | --- | --- | --- | --- | --- | --- | --- | --- | --- | --- | --- | --- | --- | --- | --- | --- | --- | --- | --- | --- | --- | --- | --- | --- | --- | --- | --- | --- | --- | --- | --- | --- | --- | --- | --- | --- | --- | --- | --- | --- | --- | --- | --- | --- | --- | --- | --- | --- | --- | --- | --- | --- | --- | --- | --- | --- | --- | --- | --- | --- | --- | --- | --- | --- | --- | --- | --- | --- | --- | --- | --- | --- | --- | --- | --- | --- | --- | --- | --- | --- | --- | --- | --- | --- | --- | --- | --- | --- | --- | --- | --- | --- | --- | --- | --- | --- | --- | --- | --- | --- | --- | --- | --- | --- | --- | --- | --- | --- | --- | --- | --- | --- | --- | --- | --- | --- | --- | --- | --- | --- | --- | --- | --- | --- | --- | --- | --- | --- | --- | --- | --- | --- | --- | --- | --- | --- | --- | --- | --- | --- | --- | --- | --- | --- | --- | --- | --- | --- | --- | --- | --- | --- | --- | --- | --- | --- | --- | --- | --- | --- | --- | --- | --- | --- | --- | --- | --- | --- | --- | --- | --- | --- | --- | --- | --- | --- | --- | --- | --- | --- | --- | --- | --- | --- | --- | --- | --- | --- | --- | --- | --- | --- | --- | --- | --- | --- | --- | --- | --- | --- | --- | --- | --- | --- | --- | --- | --- | --- | --- | --- | --- | --- | --- | --- | --- | --- | --- | --- | --- | --- | --- | --- | --- | --- | --- | --- | --- | --- | --- | --- | --- | --- | --- | --- | --- | --- | --- | --- | --- | --- | --- | --- | --- | --- | --- | --- | --- | --- | --- | --- | --- | --- | --- | --- | --- | --- | --- | --- | --- | --- | --- | --- | --- | --- | --- | --- | --- | --- | --- | --- | --- | --- | --- | --- | --- | --- | --- | --- | --- | --- | --- | --- | --- | --- | --- | --- | --- | --- | --- | --- | --- | --- | --- | --- | --- | --- | --- | --- | --- | --- | --- | --- | --- | --- | --- | --- | --- | --- | --- | --- | --- | --- | --- | --- | --- | --- | --- | --- | --- | --- | --- | --- | --- | --- | --- | --- | --- | --- | --- | --- | --- | --- | --- | --- | --- | --- | --- | --- | --- | --- | --- | --- | --- | --- | --- | --- | --- | --- | --- | --- | --- | --- | --- | --- | --- | --- | --- | --- | --- | --- | --- | --- | --- | --- | --- | --- | --- | --- | --- | --- | --- | --- | --- | --- | --- | --- | --- | --- | --- | --- | --- | --- | --- | --- | --- | --- | --- | --- | --- | --- | --- | --- | --- | --- | --- | --- | --- | --- | --- | --- | --- | --- | --- | --- | --- | --- | --- | --- | --- | --- | --- | --- | --- | --- | --- | --- | --- | --- | --- | --- | --- | --- | --- | --- | --- | --- | --- | --- | --- | --- | --- | --- | --- | --- | --- | --- | --- | --- | --- | --- | --- | --- | --- | --- | --- | --- | --- | --- | --- | --- | --- | --- | --- | --- | --- | --- | --- | --- | --- | --- | --- | --- | --- | --- | --- | --- | --- | --- | --- | --- | --- | --- | --- | --- | --- | --- | --- | --- | --- | --- | --- | --- | --- | --- | --- | --- | --- | --- | --- | --- | --- | --- | --- | --- | --- | --- | --- | --- | --- | --- | --- | --- | --- | --- | --- | --- | --- | --- | --- | --- | --- | --- | --- | --- | --- | --- | --- | --- | --- | --- | --- | --- | --- | --- | --- | --- | --- | --- | --- | --- | --- | --- | --- | --- | --- | --- | --- | --- | --- | --- | --- | --- | --- | --- | --- | --- | --- | --- | --- | --- | --- | --- | --- | --- | --- | --- | --- | --- | --- | --- | --- | --- | --- | --- | --- | --- | --- | --- | --- | --- | --- | --- | --- | --- | --- | --- | --- | --- | --- | --- | --- | --- | --- | --- | --- | --- | --- | --- | --- | --- | --- | --- | --- | --- | --- | --- | --- | --- | --- | --- | --- | --- | --- | --- | --- | --- | --- | --- | --- | --- | --- | --- | --- | --- | --- | --- | --- | --- | --- | --- | --- | --- | --- | --- | --- | --- | --- | --- | --- | --- | --- | --- | --- | --- | --- | --- | --- | --- | --- | --- | --- | --- | --- | --- | --- | --- | --- | --- | --- | --- | --- | --- | --- | --- | --- | --- | --- | --- | --- | --- | --- | --- | --- | --- | --- | --- | --- | --- | --- | --- | --- | --- | --- | --- | --- | --- | --- | --- | --- | --- | --- | --- | --- | --- | --- | --- | --- | --- | --- | --- | --- | --- | --- | --- | --- | --- | --- | --- | --- | --- | --- | --- | --- | --- | --- | --- | --- | --- | --- | --- | --- | --- | --- | --- | --- | --- | --- | --- | --- | --- | --- | --- | --- | --- | --- | --- | --- | --- | --- | --- | --- | --- | --- | --- | --- | --- | --- | --- | --- | --- | --- | --- | --- | --- | --- | --- | --- | --- | --- | --- | --- | --- | --- | --- | --- | --- | --- | --- | --- | --- | --- | --- | --- | --- | --- | --- | --- | --- | --- | --- | --- | --- | --- | --- | --- | --- | --- | --- | --- | --- | --- | --- | --- | --- | --- | --- | --- | --- | --- | --- | --- | --- | --- | --- | --- | --- | --- | --- | --- | --- | --- | --- | --- | --- | --- | --- | --- | --- | --- | --- | --- | --- | --- | --- | --- | --- | --- | --- | --- | --- | --- | --- | --- | --- | --- | --- | --- | --- | --- | --- | --- | --- | --- | --- | --- | --- | --- | --- | --- | --- | --- | --- | --- | --- | --- | --- | --- | --- | --- | --- | --- | --- | --- | --- | --- | --- | --- | --- | --- | --- | --- | --- | --- | --- | --- | --- | --- | --- | --- | --- | --- | --- | --- | --- | --- | --- | --- | --- | --- | --- | --- | --- | --- | --- | --- | --- | --- | --- | --- | --- | --- | --- | --- | --- | --- | --- | --- | --- | --- | --- | --- | --- | --- | --- | --- | --- | --- | --- | --- | --- | --- | --- | --- | --- | --- | --- | --- | --- | --- | --- | --- | --- | --- | --- | --- | --- | --- | --- | --- | --- | --- | --- | --- | --- | --- | --- | --- | --- | --- | --- | --- | --- | --- | --- | --- | --- | --- | --- | --- | --- | --- | --- | --- | --- | --- | --- | --- | --- | --- | --- | --- | --- | --- | --- | --- | --- | --- | --- | --- | --- | --- | --- | --- | --- | --- | --- | --- | --- | --- | --- | --- | --- | --- | --- | --- | --- | --- | --- | --- | --- | --- | --- | --- | --- | --- | --- | --- | --- | --- | --- | --- | --- | --- | --- | --- | --- | --- | --- | --- | --- | --- | --- | --- | --- | --- | --- | --- | --- | --- | --- | --- | --- | --- | --- | --- | --- | --- | --- | --- | --- | --- | --- | --- | --- | --- | --- | --- | --- | --- | --- | --- | --- | --- | --- | --- | --- | --- | --- | --- | --- | --- | --- | --- | --- | --- | --- | --- | --- | --- | --- | --- | --- | --- | --- | --- | --- | --- | --- | --- | --- | --- | --- | --- | --- | --- | --- | --- | --- | --- | --- | --- | --- | --- | --- | --- | --- | --- | --- | --- | --- | --- | --- | --- | --- | --- | --- | --- | --- | --- | --- | --- | --- | --- | --- | --- | --- | --- | --- | --- | --- | --- | --- | --- | --- | --- | --- | --- | --- | --- | --- | --- | --- | --- | --- | --- | --- | --- | --- | --- | --- | --- | --- | --- | --- | --- | --- | --- | --- | --- | --- | --- | --- | --- | --- | --- | --- | --- | --- | --- | --- | --- | --- | --- | --- | --- | --- | --- | --- | --- | --- | --- | --- | --- | --- | --- | --- | --- | --- | --- | --- | --- | --- | --- | --- | --- | --- | --- | --- | --- | --- | --- | --- | --- | --- | --- | --- | --- | --- | --- | --- | --- | --- | --- | --- | --- | --- | --- | --- | --- | --- | --- | --- | --- | --- | --- | --- | --- | --- | --- | --- | --- | --- | --- | --- | --- | --- | --- | --- | --- | --- | --- | --- | --- | --- | --- | --- | --- | --- | --- | --- | --- | --- | --- | --- | --- | --- | --- | --- | --- | --- | --- | --- | --- | --- | --- | --- | --- | --- | --- | --- | --- | --- | --- | --- | --- | --- | --- | --- | --- | --- | --- | --- | --- | --- | --- | --- | --- | --- | --- | --- | --- | --- | --- | --- | --- | --- | --- | --- | --- | --- | --- | --- | --- | --- | --- | --- | --- | --- | --- | --- | --- | --- | --- | --- | --- | --- | --- | --- | --- | --- | --- | --- | --- | --- | --- | --- | --- | --- | --- | --- | --- | --- | --- | --- | --- | --- | --- | --- | --- | --- | --- | --- | --- | --- | --- | --- | --- | --- | --- | --- | --- | --- | --- | --- | --- | --- | --- | --- | --- | --- | --- | --- | --- | --- | --- | --- | --- | --- | --- | --- | --- | --- | --- | --- | --- | --- | --- | --- | --- | --- | --- | --- | --- | --- | --- | --- | --- | --- | --- | --- | --- | --- | --- | --- | --- | --- | --- | --- | --- | --- | --- | --- | --- | --- | --- | --- | --- | --- | --- | --- | --- | --- | --- | --- | --- | --- | --- | --- | --- | --- | --- | --- | --- | --- | --- | --- | --- | --- | --- | --- | --- | --- | --- | --- | --- | --- | --- | --- | --- | --- | --- | --- | --- | --- | --- | --- | --- | --- | --- | --- | --- | --- | --- | --- | --- | --- | --- | --- | --- | --- | --- | --- | --- | --- | --- | --- | --- | --- | --- | --- | --- | --- | --- | --- | --- | --- | --- | --- | --- | --- | --- | --- | --- | --- | --- | --- | --- | --- | --- | --- | --- | --- | --- | --- | --- | --- | --- | --- | --- | --- | --- | --- | --- | --- | --- | --- | --- | --- | --- | --- | --- | --- | --- | --- | --- | --- | --- | --- | --- | --- | --- | --- | --- | --- | --- | --- | --- | --- | --- | --- | --- | --- | --- | --- | --- | --- | --- | --- | --- | --- | --- | --- | --- | --- | --- | --- | --- | --- | --- | --- | --- | --- | --- | --- | --- | --- | --- | --- | --- | --- | --- | --- | --- | --- | --- | --- | --- | --- | --- | --- | --- | --- | --- | --- | --- | --- | --- | --- | --- | --- | --- | --- | --- | --- | --- | --- | --- | --- | --- | --- | --- | --- | --- | --- | --- | --- | --- | --- | --- | --- | --- | --- | --- | --- | --- | --- | --- | --- | --- | --- | --- | --- | --- | --- | --- | --- | --- | --- | --- | --- | --- | --- | --- | --- | --- | --- | --- | --- | --- | --- | --- | --- | --- | --- | --- | --- | --- | --- | --- | --- | --- | --- | --- | --- | --- | --- | --- | --- | --- | --- | --- | --- | --- | --- | --- | --- | --- | --- | --- | --- | --- | --- | --- | --- | --- | --- | --- | --- | --- | --- | --- | --- | --- | --- | --- | --- | --- | --- | --- | --- | --- | --- | --- | --- | --- | --- | --- | --- | --- | --- | --- | --- | --- | --- | --- | --- | --- | --- | --- | --- | --- | --- | --- | --- | --- | --- | --- | --- | --- | --- | --- | --- | --- | --- | --- | --- | --- | --- | --- | --- | --- | --- | --- | --- | --- | --- | --- | --- | --- | --- | --- | --- | --- | --- | --- | --- | --- | --- | --- | --- | --- | --- | --- | --- | --- | --- | --- | --- | --- | --- | --- | --- | --- | --- | --- | --- | --- | --- | --- | --- | --- | --- | --- | --- | --- | --- | --- | --- | --- | --- | --- | --- | --- | --- | --- | --- | --- | --- | --- | --- | --- | --- | --- | --- | --- | --- | --- | --- | --- | --- | --- | --- | --- | --- | --- | --- | --- | --- | --- | --- | --- | --- | --- | --- | --- | --- | --- | --- | --- | --- | --- | --- | --- | --- | --- | --- | --- | --- | --- | --- | --- | --- | --- | --- | --- | --- | --- | --- | --- | --- | --- | --- | --- | --- | --- | --- | --- | --- | --- | --- | --- | --- | --- | --- | --- | --- | --- | --- | --- | --- | --- | --- | --- | --- | --- | --- | --- | --- | --- | --- | --- | --- | --- | --- | --- | --- | --- | --- | --- | --- | --- | --- | --- | --- | --- | --- | --- | --- | --- | --- | --- | --- | --- | --- | --- | --- | --- | --- | --- | --- | --- | --- | --- | --- | --- | --- | --- | --- | --- | --- | --- | --- | --- | --- | --- | --- | --- | --- | --- | --- | --- | --- | --- | --- | --- | --- | --- | --- | --- | --- | --- | --- | --- | --- | --- | --- | --- | --- | --- | --- | --- | --- | --- | --- | --- | --- | --- | --- | --- | --- | --- | --- | --- | --- | --- | --- | --- | --- | --- | --- | --- | --- | --- | --- | --- | --- | --- | --- | --- | --- | --- | --- | --- | --- | --- | --- | --- | --- | --- | --- | --- | --- | --- | --- | --- | --- | --- | --- | --- | --- | --- | --- | --- | --- | --- | --- | --- | --- | --- | --- | --- | --- | --- | --- | --- | --- | --- | --- | --- | --- | --- | --- | --- | --- | --- | --- | --- | --- | --- | --- | --- | --- | --- | --- | --- | --- | --- | --- | --- | --- | --- | --- | --- | --- | --- | --- | --- | --- | --- | --- | --- | --- | --- | --- | --- | --- | --- | --- | --- | --- | --- | --- | --- | --- | --- | --- | --- | --- | --- | --- | --- | --- | --- | --- | --- | --- | --- | --- | --- | --- | --- | --- | --- | --- | --- | --- | --- | --- | --- | --- | --- | --- | --- | --- | --- | --- | --- | --- | --- | --- | --- | --- | --- | --- | --- | --- | --- | --- | --- | --- | --- | --- | --- | --- | --- | --- | --- | --- | --- | --- | --- | --- | --- | --- | --- | --- | --- | --- | --- | --- | --- | --- | --- | --- | --- | --- | --- | --- | --- | --- | --- | --- | --- | --- | --- | --- | --- | --- | --- | --- | --- | --- | --- | --- | --- | --- | --- | --- | --- | --- | --- | --- | --- | --- | --- | --- | --- | --- | --- | --- | --- | --- | --- | --- | --- | --- | --- | --- | --- | --- | --- | --- | --- | --- | --- | --- | --- | --- | --- | --- | --- | --- | --- | --- | --- | --- | --- | --- | --- | --- | --- | --- | --- | --- | --- | --- | --- | --- | --- | --- | --- | --- | --- | --- | --- | --- | --- | --- | --- | --- | --- | --- | --- | --- | --- | --- | --- | --- | --- | --- | --- | --- | --- | --- | --- | --- | --- | --- | --- | --- | --- | --- | --- | --- | --- | --- | --- | --- | --- | --- | --- | --- | --- | --- | --- | --- | --- | --- | --- | --- | --- | --- | --- | --- | --- | --- | --- | --- | --- | --- | --- | --- | --- | --- | --- | --- | --- | --- | --- | --- | --- | --- | --- | --- | --- | --- | --- | --- | --- | --- | --- | --- | --- | --- | --- | --- | --- | --- | --- | --- | --- | --- | --- | --- | --- | --- | --- | --- | --- | --- | --- | --- | --- | --- | --- | --- | --- | --- | --- | --- | --- | --- | --- | --- | --- | --- | --- | --- | --- | --- | --- | --- | --- | --- | --- | --- | --- | --- | --- | --- | --- | --- | --- | --- | --- | --- | --- | --- | --- | --- | --- | --- | --- | --- | --- | --- | --- | --- | --- | --- | --- | --- | --- | --- | --- | --- | --- | --- | --- | --- | --- | --- | --- | --- | --- | --- | --- | --- | --- | --- | --- | --- | --- | --- | --- | --- | --- | --- | --- | --- | --- | --- | --- | --- | --- | --- | --- | --- | --- | --- | --- | --- | --- | --- | --- | --- | --- | --- | --- | --- | --- | --- | --- | --- | --- | --- | --- | --- | --- | --- | --- | --- | --- | --- | --- | --- | --- | --- | --- | --- | --- | --- | --- | --- | --- | --- | --- | --- | --- | --- | --- | --- | --- | --- | --- | --- | --- | --- | --- | --- | --- | --- | --- | --- | --- | --- | --- | --- | --- | --- | --- | --- | --- | --- | --- | --- | --- | --- | --- | --- | --- | --- | --- | --- | --- | --- | --- | --- | --- | --- | --- | --- | --- | --- | --- | --- | --- | --- | --- | --- | --- | --- | --- | --- | --- | --- | --- | --- | --- | --- | --- | --- | --- | --- | --- | --- | --- | --- | --- | --- | --- | --- | --- | --- | --- | --- | --- | --- | --- | --- | --- | --- | --- | --- | --- | --- | --- | --- | --- | --- | --- | --- | --- | --- | --- | --- | --- | --- | --- | --- | --- | --- | --- | --- | --- | --- | --- | --- | --- | --- | --- | --- | --- | --- | --- | --- | --- | --- | --- | --- | --- | --- | --- | --- | --- | --- | --- | --- | --- | --- | --- | --- | --- | --- | --- | --- | --- | --- | --- | --- | --- | --- | --- | --- | --- | --- | --- | --- | --- | --- | --- | --- | --- | --- | --- | --- | --- | --- | --- | --- | --- | --- | --- | --- | --- | --- | --- | --- | --- | --- | --- | --- | --- | --- | --- | --- | --- | --- | --- | --- | --- | --- | --- | --- | --- | --- | --- | --- | --- | --- | --- | --- | --- | --- | --- | --- | --- | --- | --- | --- | --- | --- | --- | --- | --- | --- | --- | --- | --- | --- | --- | --- | --- | --- | --- | --- | --- | --- | --- | --- | --- | --- | --- | --- | --- | --- | --- | --- | --- | --- | --- | --- | --- | --- | --- | --- | --- | --- | --- | --- | --- | --- | --- | --- | --- | --- | --- | --- | --- | --- | --- | --- | --- | --- | --- | --- | --- | --- | --- | --- | --- | --- | --- | --- | --- | --- | --- | --- | --- | --- | --- | --- | --- | --- | --- | --- | --- | --- | --- | --- | --- | --- | --- | --- | --- | --- | --- | --- | --- | --- | --- | --- | --- | --- | --- | --- | --- | --- | --- | --- | --- | --- | --- | --- | --- | --- | --- | --- | --- | --- | --- | --- | --- | --- | --- | --- | --- | --- | --- | --- | --- | --- | --- | --- | --- | --- | --- | --- | --- | --- | --- | --- | --- | --- | --- | --- | --- | --- | --- | --- | --- | --- | --- | --- | --- | --- | --- | --- | --- | --- | --- | --- | --- | --- | --- | --- | --- | --- | --- | --- | --- | --- | --- | --- | --- | --- | --- | --- | --- | --- | --- | --- | --- | --- | --- | --- | --- | --- | --- | --- | --- | --- | --- | --- | --- | --- | --- | --- | --- | --- | --- | --- | --- | --- | --- | --- | --- | --- | --- | --- | --- | --- | --- | --- | --- | --- | --- | --- | --- | --- | --- | --- | --- | --- | --- | --- | --- | --- | --- | --- | --- | --- | --- | --- | --- | --- | --- | --- | --- | --- | --- | --- | --- | --- | --- | --- | --- | --- | --- | --- | --- | --- | --- | --- | --- | --- | --- | --- | --- | --- | --- | --- | --- | --- | --- | --- | --- | --- | --- | --- | --- | --- | --- | --- | --- | --- | --- | --- | --- | --- | --- | --- | --- | --- | --- | --- | --- | --- | --- | --- | --- | --- | --- | --- | --- | --- | --- | --- | --- | --- | --- | --- | --- | --- | --- | --- | --- | --- | --- | --- | --- | --- | --- | --- | --- | --- | --- | --- | --- | --- | --- | --- | --- | --- | --- | --- | --- | --- | --- | --- | --- | --- | --- | --- | --- | --- | --- | --- | --- | --- | --- | --- | --- | --- | --- | --- | --- | --- | --- | --- | --- | --- | --- | --- | --- | --- | --- | --- | --- | --- | --- | --- | --- | --- | --- | --- | --- | --- | --- | --- | --- | --- | --- | --- | --- | --- | --- | --- | --- | --- | --- | --- | --- | --- | --- | --- | --- | --- | --- | --- | --- | --- | --- | --- | --- | --- | --- | --- | --- | --- | --- | --- | --- | --- | --- | --- | --- | --- | --- | --- | --- | --- | --- | --- | --- | --- | --- | --- | --- | --- | --- | --- | --- | --- | --- | --- | --- | --- | --- | --- | --- | --- | --- | --- | --- | --- | --- | --- | --- | --- | --- | --- | --- | --- | --- | --- | --- | --- | --- | --- | --- | --- | --- | --- | --- | --- | --- | --- | --- | --- | --- | --- | --- | --- | --- | --- | --- | --- | --- | --- | --- | --- | --- | --- | --- | --- | --- | --- | --- | --- | --- | --- | --- | --- | --- | --- | --- | --- | --- | --- | --- | --- | --- | --- | --- | --- | --- | --- | --- | --- | --- | --- | --- | --- | --- | --- | --- | --- | --- | --- | --- | --- | --- | --- | --- | --- | --- | --- | --- | --- | --- | --- | --- | --- | --- | --- | --- | --- | --- | --- | --- | --- | --- | --- | --- | --- | --- | --- | --- | --- | --- | --- | --- | --- | --- | --- | --- | --- | --- | --- | --- | --- | --- | --- | --- | --- | --- | --- | --- | --- | --- | --- | --- | --- | --- | --- | --- | --- | --- | --- | --- | --- | --- | --- | --- | --- | --- | --- | --- | --- | --- | --- | --- | --- | --- | --- | --- | --- | --- | --- | --- | --- | --- | --- | --- | --- | --- | --- | --- | --- | --- | --- | --- | --- | --- | --- | --- | --- | --- | --- | --- | --- | --- | --- | --- | --- | --- | --- | --- | --- | --- | --- | --- | --- | --- | --- | --- | --- | --- | --- | --- | --- | --- | --- | --- | --- | --- | --- | --- | --- | --- | --- | --- | --- | --- | --- | --- | --- | --- | --- | --- | --- | --- | --- | --- | --- | --- | --- | --- | --- | --- | --- | --- | --- | --- | --- | --- | --- | --- | --- | --- | --- | --- | --- | --- | --- | --- | --- | --- | --- | --- | --- | --- | --- | --- | --- | --- | --- | --- | --- | --- | --- | --- | --- | --- | --- | --- | --- | --- | --- | --- | --- | --- | --- | --- | --- | --- | --- | --- | --- | --- | --- | --- | --- | --- | --- | --- | --- | --- | --- | --- | --- | --- | --- | --- | --- | --- | --- | --- | --- | --- | --- | --- | --- | --- | --- | --- | --- | --- | --- | --- | --- | --- | --- | --- | --- | --- | --- | --- | --- | --- | --- | --- | --- | --- | --- | --- | --- | --- | --- | --- | --- | --- | --- | --- | --- | --- | --- | --- | --- | --- | --- | --- | --- | --- | --- | --- | --- | --- | --- | --- | --- | --- | --- | --- | --- | --- | --- | --- | --- | --- | --- | --- | --- | --- | --- | --- | --- | --- | --- | --- | --- | --- | --- | --- | --- | --- | --- | --- | --- | --- | --- | --- | --- | --- | --- | --- | --- | --- | --- | --- | --- | --- | --- | --- | --- | --- | --- | --- | --- | --- | --- | --- | --- | --- | --- | --- | --- | --- | --- | --- | --- | --- | --- | --- | --- | --- | --- | --- | --- | --- | --- | --- | --- | --- | --- | --- | --- | --- | --- | --- | --- | --- | --- | --- | --- | --- | --- | --- | --- | --- | --- | --- | --- | --- | --- | --- | --- | --- | --- | --- | --- | --- | --- | --- | --- | --- | --- | --- | --- | --- | --- | --- | --- | --- | --- | --- | --- | --- | --- | --- | --- | --- | --- | --- | --- | --- | --- | --- | --- | --- | --- | --- | --- | --- | --- | --- | --- | --- | --- | --- | --- | --- | --- | --- | --- | --- | --- | --- | --- | --- | --- | --- | --- | --- | --- | --- | --- | --- | --- | --- | --- | --- | --- | --- | --- | --- | --- | --- | --- | --- | --- | --- | --- | --- | --- | --- | --- | --- | --- | --- | --- | --- | --- | --- | --- | --- | --- | --- | --- | --- | --- | --- | --- | --- | --- | --- | --- | --- | --- | --- | --- | --- | --- | --- | --- | --- | --- | --- | --- | --- | --- | --- | --- | --- | --- | --- | --- | --- | --- | --- | --- | --- | --- | --- | --- | --- | --- | --- | --- | --- | --- | --- | --- | --- | --- | --- | --- | --- | --- | --- | --- | --- | --- | --- | --- | --- | --- | --- | --- | --- | --- | --- | --- | --- | --- | --- | --- | --- | --- | --- | --- | --- | --- | --- | --- | --- | --- | --- | --- | --- | --- | --- | --- | --- | --- | --- | --- | --- | --- | --- | --- | --- | --- | --- | --- | --- | --- | --- | --- | --- | --- | --- | --- | --- | --- | --- | --- | --- | --- | --- | --- | --- | --- | --- | --- | --- | --- | --- | --- | --- | --- | --- | --- | --- | --- | --- | --- | --- | --- | --- | --- | --- | --- | --- | --- | --- | --- | --- | --- | --- | --- | --- | --- | --- | --- | --- | --- | --- | --- | --- | --- | --- | --- | --- | --- | --- | --- | --- | --- | --- | --- | --- | --- | --- | --- | --- | --- | --- | --- | --- | --- | --- | --- | --- | --- | --- | --- | --- | --- | --- | --- | --- | --- | --- | --- | --- | --- | --- | --- | --- | --- | --- | --- | --- | --- | --- | --- | --- | --- | --- | --- | --- | --- | --- | --- | --- | --- | --- | --- | --- | --- | --- | --- | --- | --- | --- | --- | --- | --- | --- | --- | --- | --- | --- | --- | --- | --- | --- | --- | --- | --- | --- | --- | --- | --- | --- | --- | --- | --- | --- | --- | --- | --- | --- | --- | --- | --- | --- | --- | --- | --- | --- | --- | --- | --- | --- | --- | --- | --- | --- | --- | --- | --- | --- | --- | --- | --- | --- | --- | --- | --- | --- | --- | --- | --- | --- | --- | --- | --- | --- | --- | --- | --- | --- | --- | --- | --- | --- | --- | --- | --- | --- | --- | --- | --- | --- | --- | --- | --- | --- | --- | --- | --- | --- | --- | --- | --- | --- | --- | --- | --- | --- | --- | --- | --- | --- | --- | --- | --- | --- | --- | --- | --- | --- | --- | --- | --- | --- | --- | --- | --- | --- | --- | --- | --- | --- | --- | --- | --- | --- | --- | --- | --- | --- | --- | --- | --- | --- | --- | --- | --- | --- | --- | --- | --- | --- | --- | --- | --- | --- | --- | --- | --- | --- | --- | --- | --- | --- | --- | --- | --- | --- | --- | --- | --- | --- | --- | --- | --- | --- | --- | --- | --- | --- | --- | --- | --- | --- | --- | --- | --- | --- | --- | --- | --- | --- | --- | --- | --- | --- | --- | --- | --- | --- | --- | --- | --- | --- | --- | --- | --- | --- | --- | --- | --- | --- | --- | --- | --- | --- | --- | --- | --- | --- | --- | --- | --- | --- | --- | --- | --- | --- | --- | --- | --- | --- | --- | --- | --- | --- | --- | --- | --- | --- | --- | --- | --- | --- | --- | --- | --- | --- | --- | --- | --- | --- | --- | --- | --- | --- | --- | --- | --- | --- | --- | --- | --- | --- | --- | --- | --- | --- | --- | --- | --- | --- | --- | --- | --- | --- | --- | --- | --- | --- | --- | --- | --- | --- | --- | --- | --- | --- | --- | --- | --- | --- | --- | --- | --- | --- | --- | --- | --- | --- | --- | --- | --- | --- | --- | --- | --- | --- | --- | --- | --- | --- | --- | --- | --- | --- | --- | --- | --- | --- | --- | --- | --- | --- | --- | --- | --- | --- | --- | --- | --- | --- | --- | --- | --- | --- | --- | --- | --- | --- | --- | --- | --- | --- | --- | --- | --- | --- | --- | --- | --- | --- | --- | --- | --- | --- | --- | --- | --- | --- | --- | --- | --- | --- | --- | --- | --- | --- | --- | --- | --- | --- | --- | --- | --- | --- | --- | --- | --- | --- | --- | --- | --- | --- | --- | --- | --- | --- | --- | --- | --- | --- | --- | --- | --- | --- | --- | --- | --- | --- | --- | --- | --- | --- | --- | --- | --- | --- | --- | --- | --- | --- | --- | --- | --- | --- | --- | --- | --- | --- | --- | --- | --- | --- | --- | --- | --- | --- | --- | --- | --- | --- | --- | --- | --- | --- | --- | --- | --- | --- | --- | --- | --- | --- | --- | --- | --- | --- | --- | --- | --- | --- | --- | --- | --- | --- | --- | --- | --- | --- | --- | --- | --- | --- | --- | --- | --- | --- | --- | --- | --- | --- | --- | --- | --- | --- | --- | --- | --- | --- | --- | --- | --- | --- | --- | --- | --- | --- | --- | --- | --- | --- | --- | --- | --- | --- | --- | --- | --- | --- | --- | --- | --- | --- | --- | --- | --- | --- | --- | --- | --- | --- | --- | --- | --- | --- | --- | --- | --- | --- | --- | --- | --- | --- | --- | --- | --- | --- | --- | --- | --- | --- | --- | --- | --- | --- | --- | --- | --- | --- | --- | --- | --- | --- | --- | --- | --- | --- | --- | --- | --- | --- | --- | --- | --- | --- | --- | --- | --- | --- | --- | --- | --- | --- | --- | --- | --- | --- | --- | --- | --- | --- | --- | --- | --- | --- | --- | --- | --- | --- | --- | --- | --- | --- | --- | --- | --- | --- | --- | --- | --- | --- | --- | --- | --- | --- | --- | --- | --- | --- | --- | --- | --- | --- | --- | --- | --- | --- | --- | --- | --- | --- | --- | --- | --- | --- | --- | --- | --- | --- | --- | --- | --- | --- | --- | --- | --- | --- | --- | --- | --- | --- | --- | --- | --- | --- | --- | --- | --- | --- | --- | --- | --- | --- | --- | --- | --- | --- | --- | --- | --- | --- | --- | --- | --- | --- | --- | --- | --- | --- | --- | --- | --- | --- | --- | --- | --- | --- | --- | --- | --- | --- | --- | --- | --- | --- | --- | --- | --- | --- | --- | --- | --- | --- | --- | --- | --- | --- | --- | --- | --- | --- | --- | --- | --- | --- | --- | --- | --- | --- | --- | --- | --- | --- | --- | --- | --- | --- | --- | --- | --- | --- | --- | --- | --- | --- | --- | --- | --- | --- | --- | --- | --- | --- | --- | --- | --- | --- | --- | --- | --- | --- | --- | --- | --- | --- | --- | --- | --- | --- | --- | --- | --- | --- | --- | --- | --- | --- | --- | --- | --- | --- | --- | --- | --- | --- | --- | --- | --- | --- | --- | --- | --- | --- | --- | --- | --- | --- | --- | --- | --- | --- | --- | --- | --- | --- | --- | --- | --- | --- | --- | --- | --- | --- | --- | --- | --- | --- | --- | --- | --- | --- | --- | --- | --- | --- | --- | --- | --- | --- | --- | --- | --- | --- | --- | --- | --- | --- | --- | --- | --- | --- | --- | --- | --- | --- | --- | --- | --- | --- | --- | --- | --- | --- | --- | --- | --- | --- | --- | --- | --- | --- | --- | --- | --- | --- | --- | --- | --- | --- | --- | --- | --- | --- | --- | --- | --- | --- | --- | --- | --- | --- | --- | --- | --- | --- | --- | --- | --- | --- | --- | --- | --- | --- | --- | --- | --- | --- | --- | --- | --- | --- | --- | --- | --- | --- | --- | --- | --- | --- | --- | --- | --- | --- | --- | --- | --- | --- | --- | --- | --- | --- | --- | --- | --- | --- | --- | --- | --- | --- | --- | --- | --- | --- | --- | --- | --- | --- | --- | --- | --- | --- | --- | --- | --- | --- | --- | --- | --- | --- | --- | --- | --- | --- | --- | --- | --- | --- | --- | --- | --- | --- | --- | --- | --- | --- | --- | --- | --- | --- | --- | --- | --- | --- | --- | --- | --- | --- | --- | --- | --- | --- | --- | --- | --- | --- | --- | --- | --- | --- | --- | --- | --- | --- | --- | --- | --- | --- | --- | --- | --- | --- | --- | --- | --- | --- | --- | --- | --- | --- | --- | --- | --- | --- | --- | --- | --- | --- | --- | --- | --- | --- | --- | --- | --- | --- | --- | --- | --- | --- | --- | --- | --- | --- | --- | --- | --- | --- | --- | --- | --- | --- | --- | --- | --- | --- | --- | --- | --- | --- | --- | --- | --- | --- | --- | --- | --- | --- | --- | --- | --- | --- | --- | --- | --- | --- | --- | --- | --- | --- | --- | --- | --- | --- | --- | --- | --- | --- | --- | --- | --- | --- | --- | --- | --- | --- | --- | --- | --- | --- | --- | --- | --- | --- | --- | --- | --- | --- | --- | --- | --- | --- | --- | --- | --- | --- | --- | --- | --- | --- | --- | --- | --- | --- | --- | --- | --- | --- | --- | --- | --- | --- | --- | --- | --- | --- | --- | --- | --- | --- | --- | --- | --- | --- | --- | --- | --- | --- | --- | --- | --- | --- | --- | --- | --- | --- | --- | --- | --- | --- | --- | --- | --- | --- | --- | --- | --- | --- | --- | --- | --- | --- | --- | --- | --- | --- | --- | --- | --- | --- | --- | --- | --- | --- | --- | --- | --- | --- | --- | --- | --- | --- | --- | --- | --- | --- | --- | --- | --- | --- | --- | --- | --- | --- | --- | --- | --- | --- | --- | --- | --- | --- | --- | --- | --- | --- | --- | --- | --- | --- | --- | --- | --- | --- | --- | --- | --- | --- | --- | --- | --- | --- | --- | --- | --- | --- | --- | --- | --- | --- | --- | --- | --- | --- | --- | --- | --- | --- | --- | --- | --- | --- | --- | --- | --- | --- | --- | --- | --- | --- | --- | --- | --- | --- | --- | --- | --- | --- | --- | --- | --- | --- | --- | --- | --- | --- | --- | --- | --- | --- | --- | --- | --- | --- | --- | --- | --- | --- | --- | --- | --- | --- | --- | --- | --- | --- | --- | --- | --- | --- | --- | --- | --- | --- | --- | --- | --- | --- | --- | --- | --- | --- | --- | --- | --- | --- | --- | --- | --- | --- | --- | --- | --- | --- | --- | --- | --- | --- | --- | --- | --- | --- | --- | --- | --- | --- | --- | --- | --- | --- | --- | --- | --- | --- | --- | --- | --- | --- | --- | --- | --- | --- | --- | --- | --- | --- | --- | --- | --- | --- | --- | --- | --- | --- | --- | --- | --- | --- | --- | --- | --- | --- | --- | --- | --- | --- | --- | --- | --- | --- | --- | --- | --- | --- | --- | --- | --- | --- | --- | --- | --- | --- | --- | --- | --- | --- | --- | --- | --- | --- | --- | --- | --- | --- | --- | --- | --- | --- | --- | --- | --- | --- | --- | --- | --- | --- | --- | --- | --- | --- | --- | --- | --- | --- | --- | --- | --- | --- | --- | --- | --- | --- | --- | --- | --- | --- | --- | --- | --- | --- | --- | --- | --- | --- | --- | --- | --- | --- | --- | --- | --- | --- | --- | --- | --- | --- | --- | --- | --- | --- | --- | --- | --- | --- | --- | --- | --- | --- | --- | --- | --- | --- | --- | --- | --- | --- | --- | --- | --- | --- | --- | --- | --- | --- | --- | --- | --- | --- | --- | --- | --- | --- | --- | --- | --- | --- | --- | --- | --- | --- | --- | --- | --- | --- | --- | --- | --- | --- | --- | --- | --- | --- | --- | --- | --- | --- | --- | --- | --- | --- | --- | --- | --- | --- | --- | --- | --- | --- | --- | --- | --- | --- | --- | --- | --- | --- | --- | --- | --- | --- | --- | --- | --- | --- | --- | --- | --- | --- | --- | --- | --- | --- | --- | --- | --- | --- | --- | --- | --- | --- | --- | --- | --- | --- | --- | --- | --- | --- | --- | --- | --- | --- | --- | --- | --- | --- | --- | --- | --- | --- | --- | --- | --- | --- | --- | --- | --- | --- | --- | --- | --- | --- | --- | --- | --- | --- | --- | --- | --- | --- | --- | --- | --- | --- | --- | --- | --- | --- | --- | --- | --- | --- | --- | --- | --- | --- | --- | --- | --- | --- | --- | --- | --- | --- | --- | --- | --- | --- | --- | --- | --- | --- | --- | --- | --- | --- | --- | --- | --- | --- | --- | --- | --- | --- | --- | --- | --- | --- | --- | --- | --- | --- | --- | --- | --- | --- | --- | --- | --- | --- | --- | --- | --- | --- | --- | --- | --- | --- | --- | --- | --- | --- | --- | --- | --- | --- | --- | --- | --- | --- | --- | --- | --- | --- | --- | --- | --- | --- | --- | --- | --- | --- | --- | --- | --- | --- | --- | --- | --- | --- | --- | --- | --- | --- | --- | --- | --- | --- | --- | --- | --- | --- | --- | --- | --- | --- | --- | --- | --- | --- | --- | --- | --- | --- | --- | --- | --- | --- | --- | --- | --- | --- | --- | --- | --- | --- | --- | --- | --- | --- | --- | --- | --- | --- | --- | --- | --- | --- | --- | --- | --- | --- | --- | --- | --- | --- | --- | --- | --- | --- | --- | --- | --- | --- | --- | --- | --- | --- | --- | --- | --- | --- | --- | --- | --- | --- | --- | --- | --- | --- | --- | --- | --- | --- | --- | --- | --- | --- | --- | --- | --- | --- | --- | --- | --- | --- | --- | --- | --- | --- | --- | --- | --- | --- | --- | --- | --- | --- | --- | --- | --- | --- | --- | --- | --- | --- | --- | --- | --- | --- | --- | --- | --- | --- | --- | --- | --- | --- | --- | --- | --- | --- | --- | --- | --- | --- | --- | --- | --- | --- | --- | --- | --- | --- | --- | --- | --- | --- | --- | --- | --- | --- | --- | --- | --- | --- | --- | --- | --- | --- | --- | --- | --- | --- | --- | --- | --- | --- | --- | --- | --- | --- | --- | --- | --- | --- | --- | --- | --- | --- | --- | --- | --- | --- | --- | --- | --- | --- | --- | --- | --- | --- | --- | --- | --- | --- | --- | --- | --- | --- | --- | --- | --- | --- | --- | --- | --- | --- | --- | --- | --- | --- | --- | --- | --- | --- | --- | --- | --- | --- | --- | --- | --- | --- | --- | --- | --- | --- | --- | --- | --- | --- | --- | --- | --- | --- | --- | --- | --- | --- | --- | --- | --- | --- | --- | --- | --- | --- | --- | --- | --- | --- | --- | --- | --- | --- | --- | --- | --- | --- | --- | --- | --- | --- | --- | --- | --- | --- | --- | --- | --- | --- | --- | --- | --- | --- | --- | --- | --- | --- | --- | --- | --- | --- | --- | --- | --- | --- | --- | --- | --- | --- | --- | --- | --- | --- | --- | --- | --- | --- | --- | --- | --- | --- | --- | --- | --- | --- | --- | --- | --- | --- | --- | --- | --- | --- | --- | --- | --- | --- | --- | --- | --- | --- | --- | --- | --- | --- | --- | --- | --- | --- | --- | --- | --- | --- | --- | --- | --- | --- | --- | --- | --- | --- | --- | --- | --- | --- | --- | --- | --- | --- | --- | --- | --- | --- | --- | --- | --- | --- | --- | --- | --- | --- | --- | --- | --- | --- | --- | --- | --- | --- | --- | --- | --- | --- | --- | --- | --- | --- | --- | --- | --- | --- | --- | --- | --- | --- | --- | --- | --- | --- | --- | --- | --- | --- | --- | --- | --- | --- | --- | --- | --- | --- | --- | --- | --- | --- | --- | --- | --- | --- | --- | --- | --- | --- | --- | --- | --- | --- | --- | --- | --- | --- | --- | --- | --- | --- | --- | --- | --- | --- | --- | --- | --- | --- | --- | --- | --- | --- | --- | --- | --- | --- | --- | --- | --- | --- | --- | --- | --- | --- | --- | --- | --- | --- | --- | --- | --- | --- | --- | --- | --- | --- | --- | --- | --- | --- | --- | --- | --- | --- | --- | --- | --- | --- | --- | --- | --- | --- | --- | --- | --- | --- | --- | --- | --- | --- | --- | --- | --- | --- | --- | --- | --- | --- | --- | --- | --- | --- | --- | --- | --- | --- | --- | --- | --- | --- | --- | --- | --- | --- | --- | --- | --- | --- | --- | --- | --- | --- | --- | --- | --- | --- | --- | --- | --- | --- | --- | --- | --- | --- | --- | --- | --- | --- | --- | --- | --- | --- | --- | --- | --- | --- | --- | --- | --- | --- | --- | --- | --- | --- | --- | --- | --- | --- | --- | --- | --- | --- | --- | --- | --- | --- | --- | --- | --- | --- | --- | --- | --- | --- | --- | --- | --- | --- | --- | --- | --- | --- | --- | --- | --- | --- | --- | --- | --- | --- | --- | --- | --- | --- | --- | --- | --- | --- | --- | --- | --- | --- | --- | --- | --- | --- | --- | --- | --- | --- | --- | --- | --- | --- | --- | --- | --- | --- | --- | --- | --- | --- | --- | --- | --- | --- | --- | --- | --- | --- | --- | --- | --- | --- | --- | --- | --- | --- | --- | --- | --- | --- | --- | --- | --- | --- | --- | --- | --- | --- | --- | --- | --- | --- | --- | --- | --- | --- | --- | --- | --- | --- | --- | --- | --- | --- | --- | --- | --- | --- | --- | --- | --- | --- | --- | --- | --- | --- | --- | --- | --- | --- | --- | --- | --- | --- | --- | --- | --- | --- | --- | --- | --- | --- | --- | --- | --- | --- | --- | --- | --- | --- | --- | --- | --- | --- | --- | --- | --- | --- | --- | --- | --- | --- | --- | --- | --- | --- | --- | --- | --- | --- | --- | --- | --- | --- | --- | --- | --- | --- | --- | --- | --- | --- | --- | --- | --- | --- | --- | --- | --- | --- | --- | --- | --- | --- | --- | --- | --- | --- | --- | --- | --- | --- | --- | --- | --- | --- | --- | --- | --- | --- | --- | --- | --- | --- | --- | --- | --- | --- | --- | --- | --- | --- | --- | --- | --- | --- | --- | --- | --- | --- | --- | --- | --- | --- | --- | --- | --- | --- | --- | --- | --- | --- | --- | --- | --- | --- | --- | --- | --- | --- | --- | --- | --- | --- | --- | --- | --- | --- | --- | --- | --- | --- | --- | --- | --- | --- | --- | --- | --- | --- | --- | --- | --- | --- | --- | --- | --- | --- | --- | --- | --- | --- | --- | --- | --- | --- | --- | --- | --- | --- | --- | --- | --- | --- | --- | --- | --- | --- | --- | --- | --- | --- | --- | --- | --- | --- | --- | --- | --- | --- | --- | --- | --- | --- | --- | --- | --- | --- | --- | --- | --- | --- | --- | --- | --- | --- | --- | --- | --- | --- | --- | --- | --- | --- | --- | --- | --- | --- | --- | --- | --- | --- | --- | --- | --- | --- | --- | --- | --- | --- | --- | --- | --- | --- | --- | --- | --- | --- | --- | --- | --- | --- | --- | --- | --- | --- | --- | --- | --- | --- | --- | --- | --- | --- | --- | --- | --- | --- | --- | --- | --- | --- | --- | --- | --- | --- | --- | --- | --- | --- | --- | --- | --- | --- | --- | --- | --- | --- | --- | --- | --- | --- | --- | --- | --- | --- | --- | --- | --- | --- | --- | --- | --- | --- | --- | --- | --- | --- | --- | --- | --- | --- | --- | --- | --- | --- | --- | --- | --- | --- | --- | --- | --- | --- | --- | --- | --- | --- | --- | --- | --- | --- | --- | --- | --- | --- | --- | --- | --- | --- | --- | --- | --- | --- | --- | --- | --- | --- | --- | --- | --- | --- | --- | --- | --- | --- | --- | --- | --- | --- | --- | --- | --- | --- | --- | --- | --- | --- | --- | --- | --- | --- | --- | --- | --- | --- | --- | --- | --- | --- | --- | --- | --- | --- | --- | --- | --- | --- | --- | --- | --- | --- | --- | --- | --- | --- | --- | --- | --- | --- | --- | --- | --- | --- | --- | --- | --- | --- | --- | --- | --- | --- | --- | --- | --- | --- | --- | --- | --- | --- | --- | --- | --- | --- | --- | --- | --- | --- | --- | --- | --- | --- | --- | --- | --- | --- | --- | --- | --- | --- | --- | --- | --- | --- | --- | --- | --- | --- | --- | --- | --- | --- | --- | --- | --- | --- | --- | --- | --- | --- | --- | --- | --- | --- | --- | --- | --- | --- | --- | --- | --- | --- | --- | --- | --- | --- | --- | --- | --- | --- | --- | --- | --- | --- | --- | --- | --- | --- | --- | --- | --- | --- | --- | --- | --- | --- | --- | --- | --- | --- | --- | --- | --- | --- | --- | --- | --- | --- | --- | --- | --- | --- | --- | --- | --- | --- | --- | --- | --- | --- | --- | --- | --- | --- | --- | --- | --- | --- | --- | --- | --- | --- | --- | --- | --- | --- | --- | --- | --- | --- | --- | --- | --- | --- | --- | --- | --- | --- | --- | --- | --- | --- | --- | --- | --- | --- | --- | --- | --- | --- | --- | --- | --- | --- | --- | --- | --- | --- | --- | --- | --- | --- | --- | --- | --- | --- | --- | --- | --- | --- | --- | --- | --- | --- | --- | --- | --- | --- | --- | --- | --- | --- | --- | --- | --- | --- | --- | --- | --- | --- | --- | --- | --- | --- | --- | --- | --- | --- | --- | --- | --- | --- | --- | --- | --- | --- | --- | --- | --- | --- | --- | --- | --- | --- | --- | --- | --- | --- | --- | --- | --- | --- | --- | --- | --- | --- | --- | --- | --- | --- | --- | --- | --- | --- | --- | --- | --- | --- | --- | --- | --- | --- | --- | --- | --- | --- | --- | --- | --- | --- | --- | --- | --- | --- | --- | --- | --- | --- | --- | --- | --- | --- | --- | --- | --- | --- | --- | --- | --- | --- | --- | --- | --- | --- | --- | --- | --- | --- | --- | --- | --- | --- | --- | --- | --- | --- | --- | --- | --- | --- | --- | --- | --- | --- | --- | --- | --- | --- | --- | --- | --- | --- | --- | --- | --- | --- | --- | --- | --- | --- | --- | --- | --- | --- | --- | --- | --- | --- | --- | --- | --- | --- | --- | --- | --- | --- | --- | --- | --- | --- | --- | --- | --- | --- | --- | --- | --- | --- | --- | --- | --- | --- | --- | --- | --- | --- | --- | --- | --- | --- | --- | --- | --- | --- | --- | --- | --- | --- | --- | --- | --- | --- | --- | --- | --- | --- | --- | --- | --- | --- | --- | --- | --- | --- | --- | --- | --- | --- | --- | --- | --- | --- | --- | --- | --- | --- | --- | --- | --- | --- | --- | --- | --- | --- | --- | --- | --- | --- | --- | --- | --- | --- | --- | --- | --- | --- | --- | --- | --- | --- | --- | --- | --- | --- | --- | --- | --- | --- | --- | --- | --- | --- | --- | --- | --- | --- | --- | --- | --- | --- | --- | --- | --- | --- | --- | --- | --- | --- | --- | --- | --- | --- | --- | --- | --- | --- | --- | --- | --- | --- | --- | --- | --- | --- | --- | --- | --- | --- | --- | --- | --- | --- | --- | --- | --- | --- | --- | --- | --- | --- | --- | --- | --- | --- | --- | --- | --- | --- | --- | --- | --- | --- | --- | --- | --- | --- | --- | --- | --- | --- | --- | --- | --- | --- | --- | --- | --- | --- | --- | --- | --- | --- | --- | --- | --- | --- | --- | --- | --- | --- | --- | --- | --- | --- | --- | --- | --- | --- | --- | --- | --- | --- | --- | --- | --- | --- | --- | --- | --- | --- | --- | --- | --- | --- | --- | --- | --- | --- | --- | --- | --- | --- | --- | --- | --- | --- | --- | --- | --- | --- | --- | --- | --- | --- | --- | --- | --- | --- | --- | --- | --- | --- | --- | --- | --- | --- | --- | --- | --- | --- | --- | --- | --- | --- | --- | --- | --- | --- | --- | --- | --- | --- | --- | --- | --- | --- | --- | --- | --- | --- | --- | --- | --- | --- | --- | --- | --- | --- | --- | --- | --- | --- | --- | --- | --- | --- | --- | --- | --- | --- | --- | --- | --- | --- | --- | --- | --- | --- | --- | --- | --- | --- | --- | --- | --- | --- | --- | --- | --- | --- | --- | --- | --- | --- | --- | --- | --- | --- | --- | --- | --- | --- | --- | --- | --- | --- | --- | --- | --- | --- | --- | --- | --- | --- | --- | --- | --- | --- | --- | --- | --- | --- | --- | --- | --- | --- | --- | --- | --- | --- | --- | --- | --- | --- | --- | --- | --- | --- | --- | --- | --- | --- | --- | --- | --- | --- | --- | --- | --- | --- | --- | --- | --- | --- | --- | --- | --- | --- | --- | --- | --- | --- | --- | --- | --- | --- | --- | --- | --- | --- | --- | --- | --- | --- | --- | --- | --- | --- | --- | --- | --- | --- | --- | --- | --- | --- | --- | --- | --- | --- | --- | --- | --- | --- | --- | --- | --- | --- | --- | --- | --- | --- | --- | --- | --- | --- | --- | --- | --- | --- | --- | --- | --- | --- | --- | --- | --- | --- | --- | --- | --- | --- | --- | --- | --- | --- | --- | --- | --- | --- | --- | --- | --- | --- | --- | --- | --- | --- | --- | --- | --- | --- | --- | --- | --- | --- | --- | --- | --- | --- | --- | --- | --- | --- | --- | --- | --- | --- | --- | --- | --- | --- | --- | --- | --- | --- | --- | --- | --- | --- | --- | --- | --- | --- | --- | --- | --- | --- | --- | --- | --- | --- | --- | --- | --- | --- | --- | --- | --- | --- | --- | --- | --- | --- | --- | --- | --- | --- | --- | --- | --- | --- | --- | --- | --- | --- | --- | --- | --- | --- | --- | --- | --- | --- | --- | --- | --- | --- | --- | --- | --- | --- | --- | --- | --- | --- | --- | --- | --- | --- | --- | --- | --- | --- | --- | --- | --- | --- | --- | --- | --- | --- | --- | --- | --- | --- | --- | --- | --- | --- | --- | --- | --- | --- | --- | --- | --- | --- | --- | --- | --- | --- | --- | --- | --- | --- | --- | --- | --- | --- | --- | --- | --- | --- | --- | --- | --- | --- | --- | --- | --- | --- | --- | --- | --- | --- | --- | --- | --- | --- | --- | --- | --- | --- | --- | --- | --- | --- | --- | --- | --- | --- | --- | --- | --- | --- | --- | --- | --- | --- | --- | --- | --- | --- | --- | --- | --- | --- | --- | --- | --- | --- | --- | --- | --- | --- | --- | --- | --- | --- | --- | --- | --- | --- | --- | --- | --- | --- | --- | --- | --- | --- | --- | --- | --- | --- | --- | --- | --- | --- | --- | --- | --- | --- | --- | --- | --- | --- | --- | --- | --- | --- | --- | --- | --- | --- | --- | --- | --- | --- | --- | --- | --- | --- | --- | --- | --- | --- | --- | --- | --- | --- | --- | --- | --- | --- | --- | --- | --- | --- | --- | --- | --- | --- | --- | --- | --- | --- | --- | --- | --- | --- | --- | --- | --- | --- | --- | --- | --- | --- | --- | --- | --- | --- | --- | --- | --- | --- | --- | --- | --- | --- | --- | --- | --- | --- | --- | --- | --- | --- | --- | --- | --- | --- | --- | --- | --- | --- | --- | --- | --- | --- | --- | --- | --- | --- | --- | --- | --- | --- | --- | --- | --- | --- | --- | --- | --- | --- | --- | --- | --- | --- | --- | --- | --- | --- | --- | --- | --- | --- | --- | --- | --- | --- | --- | --- | --- | --- | --- | --- | --- | --- | --- | --- | --- | --- | --- | --- | --- | --- | --- | --- | --- | --- | --- | --- | --- | --- | --- | --- | --- | --- | --- | --- | --- | --- | --- | --- | --- | --- | --- | --- | --- | --- | --- | --- | --- | --- | --- | --- | --- | --- | --- | --- | --- | --- | --- | --- | --- | --- | --- | --- | --- | --- | --- | --- | --- | --- | --- | --- | --- | --- | --- | --- | --- | --- | --- | --- | --- | --- | --- | --- | --- | --- | --- | --- | --- | --- | --- | --- | --- | --- | --- | --- | --- | --- | --- | --- | --- | --- | --- | --- | --- | --- | --- | --- | --- | --- | --- | --- | --- | --- | --- | --- | --- | --- | --- | --- | --- | --- | --- | --- | --- | --- | --- | --- | --- | --- | --- | --- | --- | --- | --- | --- | --- | --- | --- | --- | --- | --- | --- | --- | --- | --- | --- | --- | --- | --- | --- | --- | --- | --- | --- | --- | --- | --- | --- | --- | --- | --- | --- | --- | --- | --- | --- | --- | --- | --- | --- | --- | --- | --- | --- | --- | --- | --- | --- | --- | --- | --- | --- | --- | --- | --- | --- | --- | --- | --- | --- | --- | --- | --- | --- | --- | --- | --- | --- | --- | --- | --- | --- | --- | --- | --- | --- | --- | --- | --- | --- | --- | --- | --- | --- | --- | --- | --- | --- | --- | --- | --- | --- | --- | --- | --- | --- | --- | --- | --- | --- | --- | --- | --- | --- | --- | --- | --- | --- | --- | --- | --- | --- | --- | --- | --- | --- | --- | --- | --- | --- | --- | --- | --- | --- | --- | --- | --- | --- | --- | --- | --- | --- | --- | --- | --- | --- | --- | --- | --- | --- | --- | --- | --- | --- | --- | --- | --- | --- | --- | --- | --- | --- | --- | --- | --- | --- | --- | --- | --- | --- | --- | --- | --- | --- | --- | --- | --- | --- | --- | --- | --- | --- | --- | --- | --- | --- | --- | --- | --- | --- | --- | --- | --- | --- | --- | --- | --- | --- | --- | --- | --- | --- | --- | --- | --- | --- | --- | --- | --- | --- | --- | --- | --- | --- | --- | --- | --- | --- | --- | --- | --- | --- | --- | --- | --- | --- | --- | --- | --- | --- | --- | --- | --- | --- | --- | --- | --- | --- | --- | --- | --- | --- | --- | --- | --- | --- | --- | --- | --- | --- | --- | --- | --- | --- | --- | --- | --- | --- | --- | --- | --- | --- | --- | --- | --- | --- | --- | --- | --- | --- | --- | --- | --- | --- | --- | --- | --- | --- | --- | --- | --- | --- | --- | --- | --- | --- | --- | --- | --- | --- | --- | --- | --- | --- | --- | --- | --- | --- | --- | --- | --- | --- | --- | --- | --- | --- | --- | --- | --- | --- | --- | --- | --- | --- | --- | --- | --- | --- | --- | --- | --- | --- | --- | --- | --- | --- | --- | --- | --- | --- | --- | --- | --- | --- | --- | --- | --- | --- | --- | --- | --- | --- | --- | --- | --- | --- | --- | --- | --- | --- | --- | --- | --- | --- | --- | --- | --- | --- | --- | --- | --- | --- | --- | --- | --- | --- | --- | --- | --- | --- | --- | --- | --- | --- | --- | --- | --- | --- | --- | --- | --- | --- | --- | --- | --- | --- | --- | --- | --- | --- | --- | --- | --- | --- | --- | --- | --- | --- | --- | --- | --- | --- | --- | --- | --- | --- | --- | --- | --- | --- | --- | --- | --- | --- | --- | --- | --- | --- | --- | --- | --- | --- | --- | --- | --- | --- | --- | --- | --- | --- | --- | --- | --- | --- | --- | --- | --- | --- | --- | --- | --- | --- | --- | --- | --- | --- | --- | --- | --- | --- | --- | --- | --- | --- | --- | --- | --- | --- | --- | --- | --- | --- | --- | --- | --- | --- | --- | --- | --- | --- | --- | --- | --- | --- | --- | --- | --- | --- | --- | --- | --- | --- | --- | --- | --- | --- | --- | --- | --- | --- | --- | --- | --- | --- | --- | --- | --- | --- | --- | --- | --- | --- | --- | --- | --- | --- | --- | --- | --- | --- | --- | --- | --- | --- | --- | --- | --- | --- | --- | --- | --- | --- | --- | --- | --- | --- | --- | --- | --- | --- | --- | --- | --- | --- | --- | --- | --- | --- | --- | --- | --- | --- | --- | --- | --- | --- | --- | --- | --- | --- | --- | --- | --- | --- | --- | --- | --- | --- | --- | --- | --- | --- | --- | --- | --- | --- | --- | --- | --- | --- | --- | --- | --- | --- | --- | --- | --- | --- | --- | --- | --- | --- | --- | --- | --- | --- | --- | --- | --- | --- | --- | --- | --- | --- | --- | --- | --- | --- | --- | --- | --- | --- | --- | --- | --- | --- | --- | --- | --- | --- | --- | --- | --- | --- | --- | --- | --- | --- | --- | --- | --- | --- | --- | --- | --- | --- | --- | --- | --- | --- | --- | --- | --- | --- | --- | --- | --- | --- | --- | --- | --- | --- | --- | --- | --- | --- | --- | --- | --- | --- | --- | --- | --- | --- | --- | --- | --- | --- | --- | --- | --- | --- | --- | --- | --- | --- | --- | --- | --- | --- | --- | --- | --- | --- | --- | --- | --- | --- | --- | --- | --- | --- | --- | --- | --- | --- | --- | --- | --- | --- | --- | --- | --- | --- | --- | --- | --- | --- | --- | --- | --- | --- | --- | --- | --- | --- | --- | --- | --- | --- | --- | --- | --- | --- | --- | --- | --- | --- | --- | --- | --- | --- | --- | --- | --- | --- | --- | --- | --- | --- | --- | --- | --- | --- | --- | --- | --- | --- | --- | --- | --- | --- | --- | --- | --- | --- | --- | --- | --- | --- | --- | --- | --- | --- | --- | --- | --- | --- | --- | --- | --- | --- | --- | --- | --- | --- | --- | --- | --- | --- | --- | --- | --- | --- | --- | --- | --- | --- | --- | --- | --- | --- | --- | --- | --- | --- | --- | --- | --- | --- | --- | --- | --- | --- | --- | --- | --- | --- | --- | --- | --- | --- | --- | --- | --- | --- | --- | --- | --- | --- | --- | --- | --- | --- | --- | --- | --- | --- | --- | --- | --- | --- | --- | --- | --- | --- | --- | --- | --- | --- | --- | --- | --- | --- | --- | --- | --- | --- | --- | --- | --- | --- | --- | --- | --- | --- | --- | --- | --- | --- | --- | --- | --- | --- | --- | --- | --- | --- | --- | --- | --- | --- | --- | --- | --- | --- | --- | --- | --- | --- | --- | --- | --- | --- | --- | --- | --- | --- | --- | --- | --- | --- | --- | --- | --- | --- | --- | --- | --- | --- | --- | --- | --- | --- | --- | --- | --- | --- | --- | --- | --- | --- | --- | --- | --- | --- | --- | --- | --- | --- | --- | --- | --- | --- | --- | --- | --- | --- | --- | --- | --- | --- | --- | --- | --- | --- | --- | --- | --- | --- | --- | --- | --- | --- | --- | --- | --- | --- | --- | --- | --- | --- | --- | --- | --- | --- | --- | --- | --- | --- | --- | --- | --- | --- | --- | --- | --- | --- | --- | --- | --- | --- | --- | --- | --- | --- | --- | --- | --- | --- | --- | --- | --- | --- | --- | --- | --- | --- | --- | --- | --- | --- | --- | --- | --- | --- | --- | --- | --- | --- | --- | --- | --- | --- | --- | --- | --- | --- | --- | --- | --- | --- | --- | --- | --- | --- | --- | --- | --- | --- | --- | --- | --- | --- | --- | --- | --- | --- | --- | --- | --- | --- | --- | --- | --- | --- | --- | --- | --- | --- | --- | --- | --- | --- | --- | --- | --- | --- | --- | --- | --- | --- | --- | --- | --- | --- | --- | --- | --- | --- | --- | --- | --- | --- | --- | --- | --- | --- | --- | --- | --- | --- | --- | --- | --- | --- | --- | --- | --- | --- | --- | --- | --- | --- | --- | --- | --- | --- | --- | --- | --- | --- | --- | --- | --- | --- | --- | --- | --- | --- | --- | --- | --- | --- | --- | --- | --- | --- | --- | --- | --- | --- | --- | --- | --- | --- | --- | --- | --- | --- | --- | --- | --- | --- | --- | --- | --- | --- | --- | --- | --- | --- | --- | --- | --- | --- | --- | --- | --- | --- | --- | --- | --- | --- | --- | --- | --- | --- | --- | --- | --- | --- | --- | --- | --- | --- | --- | --- | --- | --- | --- | --- | --- | --- | --- | --- | --- | --- | --- | --- | --- | --- | --- | --- | --- | --- | --- | --- | --- | --- | --- | --- | --- | --- | --- | --- | --- | --- | --- | --- | --- | --- | --- | --- | --- | --- | --- | --- | --- | --- | --- | --- | --- | --- | --- | --- | --- | --- | --- | --- | --- | --- | --- | --- | --- | --- | --- | --- | --- | --- | --- | --- | --- | --- | --- | --- | --- | --- | --- | --- | --- | --- | --- | --- | --- | --- | --- | --- | --- | --- | --- | --- | --- | --- | --- | --- | --- | --- | --- | --- | --- | --- | --- | --- | --- | --- | --- | --- | --- | --- | --- | --- | --- | --- | --- | --- | --- | --- | --- | --- | --- | --- | --- | --- | --- | --- | --- | --- | --- | --- | --- | --- | --- | --- | --- | --- | --- | --- | --- | --- | --- | --- | --- | --- | --- | --- | --- | --- | --- | --- | --- | --- | --- | --- | --- | --- | --- | --- | --- | --- | --- | --- | --- | --- | --- | --- | --- | --- | --- | --- | --- | --- | --- | --- | --- | --- | --- | --- | --- | --- | --- | --- | --- | --- | --- | --- | --- | --- | --- | --- | --- | --- | --- | --- | --- | --- | --- | --- | --- | --- | --- | --- | --- | --- | --- | --- | --- | --- | --- | --- | --- | --- | --- | --- | --- | --- | --- | --- | --- | --- | --- | --- | --- | --- | --- | --- | --- | --- | --- | --- | --- | --- | --- | --- | --- | --- | --- | --- | --- | --- | --- | --- | --- | --- | --- | --- | --- | --- | --- | --- | --- | --- | --- | --- | --- | --- | --- | --- | --- | --- | --- | --- | --- | --- | --- | --- | --- | --- | --- | --- | --- | --- | --- | --- | --- | --- | --- | --- | --- | --- | --- | --- | --- | --- | --- | --- | --- | --- | --- | --- | --- | --- | --- | --- | --- | --- | --- | --- | --- | --- | --- | --- | --- | --- | --- | --- | --- | --- | --- | --- | --- | --- | --- | --- | --- | --- | --- | --- | --- | --- | --- | --- | --- | --- | --- | --- | --- | --- | --- | --- | --- | --- | --- | --- | --- | --- | --- | --- | --- | --- | --- | --- | --- | --- | --- | --- | --- | --- | --- | --- | --- | --- | --- | --- | --- | --- | --- | --- | --- | --- | --- | --- | --- | --- | --- | --- | --- | --- | --- | --- | --- | --- | --- | --- | --- | --- | --- | --- | --- | --- | --- | --- | --- | --- | --- | --- | --- | --- | --- | --- | --- | --- | --- | --- | --- | --- | --- | --- | --- | --- | --- | --- | --- | --- | --- | --- | --- | --- | --- | --- | --- | --- | --- | --- | --- | --- | --- | --- | --- | --- | --- | --- | --- | --- | --- | --- | --- | --- | --- | --- | --- | --- | --- | --- | --- | --- | --- | --- | --- | --- | --- | --- | --- | --- | --- | --- | --- | --- | --- | --- | --- | --- | --- | --- | --- | --- | --- | --- | --- | --- | --- | --- | --- | --- | --- | --- | --- | --- | --- | --- | --- | --- | --- | --- | --- | --- | --- | --- | --- | --- | --- | --- | --- | --- | --- | --- | --- | --- | --- | --- | --- | --- | --- | --- | --- | --- | --- | --- | --- | --- | --- | --- | --- | --- | --- | --- | --- | --- | --- | --- | --- | --- | --- | --- | --- | --- | --- | --- | --- | --- | --- | --- | --- | --- | --- | --- | --- | --- | --- | --- | --- | --- | --- | --- | --- | --- | --- | --- | --- | --- | --- | --- | --- | --- | --- | --- | --- | --- | --- | --- | --- | --- | --- | --- | --- | --- | --- | --- | --- | --- | --- | --- | --- | --- | --- | --- | --- | --- | --- | --- | --- | --- | --- | --- | --- | --- | --- | --- | --- | --- | --- | --- | --- | --- | --- | --- | --- | --- | --- | --- | --- | --- | --- | --- | --- | --- | --- | --- | --- | --- | --- | --- | --- | --- | --- | --- | --- | --- | --- | --- | --- | --- | --- | --- | --- | --- | --- | --- | --- | --- | --- | --- | --- | --- | --- | --- | --- | --- | --- | --- | --- | --- | --- | --- | --- | --- | --- | --- | --- | --- | --- | --- | --- | --- | --- | --- | --- | --- | --- | --- | --- | --- | --- | --- | --- | --- | --- | --- | --- | --- | --- | --- | --- | --- | --- | --- | --- | --- | --- | --- | --- | --- | --- | --- | --- | --- | --- | --- | --- | --- | --- | --- | --- | --- | --- | --- | --- | --- | --- | --- | --- | --- | --- | --- | --- | --- | --- | --- | --- | --- | --- | --- | --- | --- | --- | --- | --- | --- | --- | --- | --- | --- | --- | --- | --- | --- | --- | --- | --- | --- | --- | --- | --- | --- | --- | --- | --- | --- | --- | --- | --- | --- | --- | --- | --- | --- | --- | --- | --- | --- | --- | --- | --- | --- | --- | --- | --- | --- | --- | --- | --- | --- | --- | --- | --- | --- | --- | --- | --- | --- | --- | --- | --- | --- | --- | --- | --- | --- | --- | --- | --- | --- | --- | --- | --- | --- | --- | --- | --- | --- | --- | --- | --- | --- | --- | --- | --- | --- | --- | --- | --- | --- | --- | --- | --- | --- | --- | --- | --- | --- | --- | --- | --- | --- | --- | --- | --- | --- | --- | --- | --- | --- | --- | --- | --- | --- | --- | --- | --- | --- | --- | --- | --- | --- | --- | --- | --- | --- | --- | --- | --- | --- | --- | --- | --- | --- | --- | --- | --- | --- | --- | --- | --- | --- | --- | --- | --- | --- | --- | --- | --- | --- | --- | --- | --- | --- | --- | --- | --- | --- | --- | --- | --- | --- | --- | --- | --- | --- | --- | --- | --- | --- | --- | --- | --- | --- | --- | --- | --- | --- | --- | --- | --- | --- | --- | --- | --- | --- | --- | --- | --- | --- | --- | --- | --- | --- | --- | --- | --- | --- | --- | --- | --- | --- | --- | --- | --- | --- | --- | --- | --- | --- | --- | --- | --- | --- | --- | --- | --- | --- | --- | --- | --- | --- | --- | --- | --- | --- | --- | --- | --- | --- | --- | --- | --- | --- | --- | --- | --- | --- | --- | --- | --- | --- | --- | --- | --- | --- | --- | --- | --- | --- | --- | --- | --- | --- | --- | --- | --- | --- | --- | --- | --- | --- | --- | --- | --- | --- | --- | --- | --- | --- | --- | --- | --- | --- | --- | --- | --- | --- | --- | --- | --- | --- | --- | --- | --- | --- | --- | --- | --- | --- | --- | --- | --- | --- | --- | --- | --- | --- | --- | --- | --- | --- | --- | --- | --- | --- | --- | --- | --- | --- | --- | --- | --- | --- | --- | --- | --- | --- | --- | --- | --- | --- | --- | --- | --- | --- | --- | --- | --- | --- | --- | --- | --- | --- | --- | --- | --- | --- | --- | --- | --- | --- | --- | --- | --- | --- | --- | --- | --- | --- | --- | --- | --- | --- | --- | --- | --- | --- | --- | --- | --- | --- | --- | --- | --- | --- | --- | --- | --- | --- | --- | --- | --- | --- | --- | --- | --- | --- | --- | --- | --- | --- | --- | --- | --- | --- | --- | --- | --- | --- | --- | --- | --- | --- | --- | --- | --- | --- | --- | --- | --- | --- | --- | --- | --- | --- | --- | --- | --- | --- | --- | --- | --- | --- | --- | --- | --- | --- | --- | --- | --- | --- | --- | --- | --- | --- | --- | --- | --- | --- | --- | --- | --- | --- | --- | --- | --- | --- | --- | --- | --- | --- | --- | --- | --- | --- | --- | --- | --- | --- | --- | --- | --- | --- | --- | --- | --- | --- | --- | --- | --- | --- | --- | --- | --- | --- | --- | --- | --- | --- | --- | --- | --- | --- | --- | --- | --- | --- | --- | --- | --- | --- | --- | --- | --- | --- | --- | --- | --- | --- | --- | --- | --- | --- | --- | --- | --- | --- | --- | --- | --- | --- | --- | --- | --- | --- | --- | --- | --- | --- | --- | --- | --- | --- | --- | --- | --- | --- | --- | --- | --- | --- | --- | --- | --- | --- | --- | --- | --- | --- | --- | --- | --- | --- | --- | --- | --- | --- | --- | --- | --- | --- | --- | --- | --- | --- | --- | --- | --- | --- | --- | --- | --- | --- | --- | --- | --- | --- | --- | --- | --- | --- | --- | --- | --- | --- | --- | --- | --- | --- | --- | --- | --- | --- | --- | --- | --- | --- | --- | --- | --- | --- | --- | --- | --- | --- | --- | --- | --- | --- | --- | --- | --- | --- | --- | --- | --- | --- | --- | --- | --- | --- | --- | --- | --- | --- | --- | --- | --- | --- | --- | --- | --- | --- | --- | --- | --- | --- | --- | --- | --- | --- | --- | --- | --- | --- | --- | --- | --- | --- | --- | --- | --- | --- | --- | --- | --- | --- | --- | --- | --- | --- | --- | --- | --- | --- | --- | --- | --- | --- | --- | --- | --- | --- | --- | --- | --- | --- | --- | --- | --- | --- | --- | --- | --- | --- | --- | --- | --- | --- | --- | --- | --- | --- | --- | --- | --- | --- | --- | --- | --- | --- | --- | --- | --- | --- | --- | --- | --- | --- | --- | --- | --- | --- | --- | --- | --- | --- | --- | --- | --- | --- | --- | --- | --- | --- | --- | --- | --- | --- | --- | --- | --- | --- | --- | --- | --- | --- | --- | --- | --- | --- | --- | --- | --- | --- | --- | --- | --- | --- | --- | --- | --- | --- | --- | --- | --- | --- | --- | --- | --- | --- | --- | --- | --- | --- | --- | --- | --- | --- | --- | --- | --- | --- | --- | --- | --- | --- | --- | --- | --- | --- | --- | --- | --- | --- | --- | --- | --- | --- | --- | --- | --- | --- | --- | --- | --- | --- | --- | --- | --- | --- | --- | --- | --- | --- | --- | --- | --- | --- | --- | --- | --- | --- | --- | --- | --- | --- | --- | --- | --- | --- | --- | --- | --- | --- | --- | --- | --- | --- | --- | --- | --- | --- | --- | --- | --- | --- | --- | --- | --- | --- | --- | --- | --- | --- | --- | --- | --- | --- | --- | --- | --- | --- | --- | --- | --- | --- | --- | --- | --- | --- | --- | --- | --- | --- | --- | --- | --- | --- | --- | --- | --- | --- | --- | --- | --- | --- | --- | --- | --- | --- | --- | --- | --- | --- | --- | --- | --- | --- | --- | --- | --- | --- | --- | --- | --- | --- | --- | --- | --- | --- | --- | --- | --- | --- | --- | --- | --- | --- | --- | --- | --- | --- | --- | --- | --- | --- | --- | --- | --- | --- | --- | --- | --- | --- | --- | --- | --- | --- | --- | --- | --- | --- | --- | --- | --- | --- | --- | --- | --- | --- | --- | --- | --- | --- | --- | --- | --- | --- | --- | --- | --- | --- | --- | --- | --- | --- | --- | --- | --- | --- | --- | --- | --- | --- | --- | --- | --- | --- | --- | --- | --- | --- | --- | --- | --- | --- | --- | --- | --- | --- | --- | --- | --- | --- | --- | --- | --- | --- | --- | --- | --- | --- | --- | --- | --- | --- | --- | --- | --- | --- | --- | --- | --- | --- | --- | --- | --- | --- | --- | --- | --- | --- | --- | --- | --- | --- | --- | --- | --- | --- | --- | --- | --- | --- | --- | --- | --- | --- | --- | --- | --- | --- | --- | --- | --- | --- | --- | --- | --- | --- | --- | --- | --- | --- | --- | --- | --- | --- | --- | --- | --- | --- | --- | --- | --- | --- | --- | --- | --- | --- | --- | --- | --- | --- | --- | --- | --- | --- | --- | --- | --- | --- | --- | --- | --- | --- | --- | --- | --- | --- | --- | --- | --- | --- | --- | --- | --- | --- | --- | --- | --- | --- | --- | --- | --- | --- | --- | --- | --- | --- | --- | --- | --- | --- | --- | --- | --- | --- | --- | --- | --- | --- | --- | --- | --- | --- | --- | --- | --- | --- | --- | --- | --- | --- | --- | --- | --- | --- | --- | --- | --- | --- | --- | --- | --- | --- | --- | --- | --- | --- | --- | --- | --- | --- | --- | --- | --- | --- | --- | --- | --- | --- | --- | --- | --- | --- | --- | --- | --- | --- | --- | --- | --- | --- | --- | --- | --- | --- | --- | --- | --- | --- | --- | --- | --- | --- | --- | --- | --- | --- | --- | --- | --- | --- | --- | --- | --- | --- | --- | --- | --- | --- | --- | --- | --- | --- | --- | --- | --- | --- | --- | --- | --- | --- | --- | --- | --- | --- | --- | --- | --- | --- | --- | --- | --- | --- | --- | --- | --- | --- | --- | --- | --- | --- | --- | --- | --- | --- | --- | --- | --- | --- | --- | --- | --- | --- | --- | --- | --- | --- | --- | --- | --- | --- | --- | --- | --- | --- | --- | --- | --- | --- | --- | --- | --- | --- | --- | --- | --- | --- | --- | --- | --- | --- | --- | --- | --- | --- | --- | --- | --- | --- | --- | --- | --- | --- | --- | --- | --- | --- | --- | --- | --- | --- | --- | --- | --- | --- | --- | --- | --- | --- | --- | --- | --- | --- | --- | --- | --- | --- | --- | --- | --- | --- | --- | --- | --- | --- | --- | --- | --- | --- | --- | --- | --- | --- | --- | --- | --- | --- | --- | --- | --- | --- | --- | --- | --- | --- | --- | --- | --- | --- | --- | --- | --- | --- | --- | --- | --- | --- | --- | --- | --- | --- | --- | --- | --- | --- | --- | --- | --- | --- | --- | --- | --- | --- | --- | --- | --- | --- | --- | --- | --- | --- | --- | --- | --- | --- | --- | --- | --- | --- | --- | --- | --- | --- | --- | --- | --- | --- | --- | --- | --- | --- | --- | --- | --- | --- | --- | --- | --- | --- | --- | --- | --- | --- | --- | --- | --- | --- | --- | --- | --- | --- | --- | --- | --- | --- | --- | --- | --- | --- | --- | --- | --- | --- | --- | --- | --- | --- | --- | --- | --- | --- | --- | --- | --- | --- | --- | --- | --- | --- | --- | --- | --- | --- | --- | --- | --- | --- | --- | --- | --- | --- | --- | --- | --- | --- | --- | --- | --- | --- | --- | --- | --- | --- | --- | --- | --- | --- | --- | --- | --- | --- | --- | --- | --- | --- | --- | --- | --- | --- | --- | --- | --- | --- | --- | --- | --- | --- | --- | --- | --- | --- | --- | --- | --- | --- | --- | --- | --- | --- | --- | --- | --- | --- | --- | --- | --- | --- | --- | --- | --- | --- | --- | --- | --- | --- | --- | --- | --- | --- | --- | --- | --- | --- | --- | --- | --- | --- | --- | --- | --- | --- | --- | --- | --- | --- | --- | --- | --- | --- | --- | --- | --- | --- | --- | --- | --- | --- | --- | --- | --- | --- | --- | --- | --- | --- | --- | --- | --- | --- | --- | --- | --- | --- | --- | --- | --- | --- | --- | --- | --- | --- | --- | --- | --- | --- | --- | --- | --- | --- | --- | --- | --- | --- | --- | --- | --- | --- | --- | --- | --- | --- | --- | --- | --- | --- | --- | --- | --- | --- | --- | --- | --- | --- | --- | --- | --- | --- | --- | --- | --- | --- | --- | --- | --- | --- | --- | --- | --- | --- | --- | --- | --- | --- | --- | --- | --- | --- | --- | --- | --- | --- | --- | --- | --- | --- | --- | --- | --- | --- | --- | --- | --- | --- | --- | --- | --- | --- | --- | --- | --- | --- | --- | --- | --- | --- | --- | --- | --- | --- | --- | --- | --- | --- | --- | --- | --- | --- | --- | --- | --- | --- | --- | --- | --- | --- | --- | --- | --- | --- | --- | --- | --- | --- | --- | --- | --- | --- | --- | --- | --- | --- | --- | --- | --- | --- | --- | --- | --- | --- | --- | --- | --- | --- | --- | --- | --- | --- | --- | --- | --- | --- | --- | --- | --- | --- | --- | --- | --- | --- | --- | --- | --- | --- | --- | --- | --- | --- | --- | --- | --- | --- | --- | --- | --- | --- | --- | --- | --- | --- | --- | --- | --- | --- | --- | --- | --- | --- | --- | --- | --- | --- | --- | --- | --- | --- | --- | --- | --- | --- | --- | --- | --- | --- | --- | --- | --- | --- | --- | --- | --- | --- | --- | --- | --- | --- | --- | --- | --- | --- | --- | --- | --- | --- | --- | --- | --- | --- | --- | --- | --- | --- | --- | --- | --- | --- | --- | --- | --- | --- | --- | --- | --- | --- | --- | --- | --- | --- | --- | --- | --- | --- | --- | --- | --- | --- | --- | --- | --- | --- | --- | --- | --- | --- | --- | --- | --- | --- | --- | --- | --- | --- | --- | --- | --- | --- | --- | --- | --- | --- | --- | --- | --- | --- | --- | --- | --- | --- | --- | --- | --- | --- | --- | --- | --- | --- | --- | --- | --- | --- | --- | --- | --- | --- | --- | --- | --- | --- | --- | --- | --- | --- | --- | --- | --- | --- | --- | --- | --- | --- | --- | --- | --- | --- | --- | --- | --- | --- | --- | --- | --- | --- | --- | --- | --- | --- | --- | --- | --- | --- | --- | --- | --- | --- | --- | --- | --- | --- | --- | --- | --- | --- | --- | --- | --- | --- | --- | --- | --- | --- | --- | --- | --- | --- | --- | --- | --- | --- | --- | --- | --- | --- | --- | --- | --- | --- | --- | --- | --- | --- | --- | --- | --- | --- | --- | --- | --- | --- | --- | --- | --- | --- | --- | --- | --- | --- | --- | --- | --- | --- | --- | --- | --- | --- | --- | --- | --- | --- | --- | --- | --- | --- | --- | --- | --- | --- | --- | --- | --- | --- | --- | --- | --- | --- | --- | --- | --- | --- | --- | --- | --- | --- | --- | --- | --- | --- | --- | --- | --- | --- | --- | --- | --- | --- | --- | --- | --- | --- | --- | --- | --- | --- | --- | --- | --- | --- | --- | --- | --- | --- | --- | --- | --- | --- | --- | --- | --- | --- | --- | --- | --- | --- | --- | --- | --- | --- | --- | --- | --- | --- | --- | --- | --- | --- | --- | --- | --- | --- | --- | --- | --- | --- | --- | --- | --- | --- | --- | --- | --- | --- | --- | --- | --- | --- | --- | --- | --- | --- | --- | --- | --- | --- | --- | --- | --- | --- | --- | --- | --- | --- | --- | --- | --- | --- | --- | --- | --- | --- | --- | --- | --- | --- | --- | --- | --- | --- | --- | --- | --- | --- | --- | --- | --- | --- | --- | --- | --- | --- | --- | --- | --- | --- | --- | --- | --- | --- | --- | --- | --- | --- | --- | --- | --- | --- | --- | --- | --- | --- | --- | --- | --- | --- | --- | --- | --- | --- | --- | --- | --- | --- | --- | --- | --- | --- | --- | --- | --- | --- | --- | --- | --- | --- | --- | --- | --- | --- | --- | --- | --- | --- | --- | --- | --- | --- | --- | --- | --- | --- | --- | --- | --- | --- | --- | --- | --- | --- | --- | --- | --- | --- | --- | --- | --- | --- | --- | --- | --- | --- | --- | --- | --- | --- | --- | --- | --- | --- | --- | --- | --- | --- | --- | --- | --- | --- | --- | --- | --- | --- | --- | --- | --- | --- | --- | --- | --- | --- | --- | --- | --- | --- | --- | --- | --- | --- | --- | --- | --- | --- | --- | --- | --- | --- | --- | --- | --- | --- | --- | --- | --- | --- | --- | --- | --- | --- | --- | --- | --- | --- | --- | --- | --- | --- | --- | --- | --- | --- | --- | --- | --- | --- | --- | --- | --- | --- | --- | --- | --- | --- | --- | --- | --- | --- | --- | --- | --- | --- | --- | --- | --- | --- | --- | --- | --- | --- | --- | --- | --- | --- | --- | --- | --- | --- | --- | --- | --- | --- | --- | --- | --- | --- | --- | --- | --- | --- | --- | --- | --- | --- | --- | --- | --- | --- | --- | --- | --- | --- | --- | --- | --- | --- | --- | --- | --- | --- | --- | --- | --- | --- | --- | --- | --- | --- | --- | --- | --- | --- | --- | --- | --- | --- | --- | --- | --- | --- | --- | --- | --- | --- | --- | --- | --- | --- | --- | --- | --- | --- | --- | --- | --- | --- | --- | --- | --- | --- | --- | --- | --- | --- | --- | --- | --- | --- | --- | --- | --- | --- | --- | --- | --- | --- | --- | --- | --- | --- | --- | --- | --- | --- | --- | --- | --- | --- | --- | --- | --- | --- | --- | --- | --- | --- | --- | --- | --- | --- | --- | --- | --- | --- | --- | --- | --- | --- | --- | --- | --- | --- | --- | --- | --- | --- | --- | --- | --- | --- | --- | --- | --- | --- | --- | --- | --- | --- | --- | --- | --- | --- | --- | --- | --- | --- | --- | --- | --- | --- | --- | --- | --- | --- | --- | --- | --- | --- | --- | --- | --- | --- | --- | --- | --- | --- | --- | --- | --- | --- | --- | --- | --- | --- | --- | --- | --- | --- | --- | --- | --- | --- | --- | --- | --- | --- | --- | --- | --- | --- | --- | --- | --- | --- | --- | --- | --- | --- | --- | --- | --- | --- | --- | --- | --- | --- | --- | --- | --- | --- | --- | --- | --- | --- | --- | --- | --- | --- | --- | --- | --- | --- | --- | --- | --- | --- | --- | --- | --- | --- | --- | --- | --- | --- | --- | --- | --- | --- | --- | --- | --- | --- | --- | --- | --- | --- | --- | --- | --- | --- | --- | --- | --- | --- | --- | --- | --- | --- | --- | --- | --- | --- | --- | --- | --- | --- | --- | --- | --- | --- | --- | --- | --- | --- | --- | --- | --- | --- | --- | --- | --- | --- | --- | --- | --- | --- | --- | --- | --- | --- | --- | --- | --- | --- | --- | --- | --- | --- | --- | --- | --- | --- | --- | --- | --- | --- | --- | --- | --- | --- | --- | --- | --- | --- | --- | --- | --- | --- | --- | --- | --- | --- | --- | --- | --- | --- | --- | --- | --- | --- | --- | --- | --- | --- | --- | --- | --- | --- | --- | --- | --- | --- | --- | --- | --- | --- | --- | --- | --- | --- | --- | --- | --- | --- | --- | --- | --- | --- | --- | --- | --- | --- | --- | --- | --- | --- | --- | --- | --- | --- | --- | --- | --- | --- | --- | --- | --- | --- | --- | --- | --- | --- | --- | --- | --- | --- | --- | --- | --- | --- | --- | --- | --- | --- | --- | --- | --- | --- | --- | --- | --- | --- | --- | --- | --- | --- | --- | --- | --- | --- | --- | --- | --- | --- | --- | --- | --- | --- | --- | --- | --- | --- | --- | --- | --- | --- | --- | --- | --- | --- | --- | --- | --- | --- | --- | --- | --- | --- | --- | --- | --- | --- | --- | --- | --- | --- | --- | --- | --- | --- | --- | --- | --- | --- | --- | --- | --- | --- | --- | --- | --- | --- | --- | --- | --- | --- | --- | --- | --- | --- | --- | --- | --- | --- | --- | --- | --- | --- | --- | --- | --- | --- | --- | --- | --- | --- | --- | --- | --- | --- | --- | --- | --- | --- | --- | --- | --- | --- | --- | --- | --- | --- | --- | --- | --- | --- | --- | --- | --- | --- | --- | --- | --- | --- | --- | --- | --- | --- | --- | --- | --- | --- | --- | --- | --- | --- | --- | --- | --- | --- | --- | --- | --- | --- | --- | --- | --- | --- | --- | --- | --- | --- | --- | --- | --- | --- | --- | --- | --- | --- | --- | --- | --- | --- | --- | --- | --- | --- | --- | --- | --- | --- | --- | --- | --- | --- | --- | --- | --- | --- | --- | --- | --- | --- | --- | --- | --- | --- | --- | --- | --- | --- | --- | --- | --- | --- | --- | --- | --- | --- | --- | --- | --- | --- | --- | --- | --- | --- | --- | --- | --- | --- | --- | --- | --- | --- | --- | --- | --- | --- | --- | --- | --- | --- | --- | --- | --- | --- | --- | --- | --- | --- | --- | --- | --- | --- | --- | --- | --- | --- | --- | --- | --- | --- | --- | --- | --- | --- | --- | --- | --- | --- | --- | --- | --- | --- | --- | --- | --- | --- | --- | --- | --- | --- | --- | --- | --- | --- | --- | --- | --- | --- | --- | --- | --- | --- | --- | --- | --- | --- | --- | --- | --- | --- | --- | --- | --- | --- | --- | --- | --- | --- | --- | --- | --- | --- | --- | --- | --- | --- | --- | --- | --- | --- | --- | --- | --- | --- | --- | --- | --- | --- | --- | --- | --- | --- | --- | --- | --- | --- | --- | --- | --- | --- | --- | --- | --- | --- | --- | --- | --- | --- | --- | --- | --- | --- | --- | --- | --- | --- | --- | --- | --- | --- | --- | --- | --- | --- | --- | --- | --- | --- | --- | --- | --- | --- | --- | --- | --- | --- | --- | --- | --- | --- | --- | --- | --- | --- | --- | --- | --- | --- | --- | --- | --- | --- | --- | --- | --- | --- | --- | --- | --- | --- | --- | --- | --- | --- | --- | --- | --- | --- | --- | --- | --- | --- | --- | --- | --- | --- | --- | --- | --- | --- | --- | --- | --- | --- | --- | --- | --- | --- | --- | --- | --- | --- | --- | --- | --- | --- | --- | --- | --- | --- | --- | --- | --- | --- | --- | --- | --- | --- | --- | --- | --- | --- | --- | --- | --- | --- | --- | --- | --- | --- | --- | --- | --- | --- | --- | --- | --- | --- | --- | --- | --- | --- | --- | --- | --- | --- | --- | --- | --- | --- | --- | --- | --- | --- | --- | --- | --- | --- | --- | --- | --- | --- | --- | --- | --- | --- | --- | --- | --- | --- | --- | --- | --- | --- | --- | --- | --- | --- | --- | --- | --- | --- | --- | --- | --- | --- | --- | --- | --- | --- | --- | --- | --- | --- | --- | --- | --- | --- | --- | --- | --- | --- | --- | --- | --- | --- | --- | --- | --- | --- | --- | --- | --- | --- | --- | --- | --- | --- | --- | --- | --- | --- | --- | --- | --- | --- | --- | --- | --- | --- | --- | --- | --- | --- | --- | --- | --- | --- | --- | --- | --- | --- | --- | --- | --- | --- | --- | --- | --- | --- | --- | --- | --- | --- | --- | --- | --- | --- | --- | --- | --- | --- | --- | --- | --- | --- | --- | --- | --- | --- | --- | --- | --- | --- | --- | --- | --- | --- | --- | --- | --- | --- | --- | --- | --- | --- | --- | --- | --- | --- | --- | --- | --- | --- | --- | --- | --- | --- | --- | --- | --- | --- | --- | --- | --- | --- | --- | --- | --- | --- | --- | --- | --- | --- | --- | --- | --- | --- | --- | --- | --- | --- | --- | --- | --- | --- | --- | --- | --- | --- | --- | --- | --- | --- | --- | --- | --- | --- | --- | --- | --- | --- | --- | --- | --- | --- | --- | --- | --- | --- | --- | --- | --- | --- | --- | --- | --- | --- | --- | --- | --- | --- | --- | --- | --- | --- | --- | --- | --- | --- | --- | --- | --- | --- | --- | --- | --- | --- | --- | --- | --- | --- | --- | --- | --- | --- | --- | --- | --- | --- | --- | --- | --- | --- | --- | --- | --- | --- | --- | --- | --- | --- | --- | --- | --- | --- | --- | --- | --- | --- | --- | --- | --- | --- | --- | --- | --- | --- | --- | --- | --- | --- | --- | --- | --- | --- | --- | --- | --- | --- | --- | --- | --- | --- | --- | --- | --- | --- | --- | --- | --- | --- | --- | --- | --- | --- | --- | --- | --- | --- | --- | --- | --- | --- | --- | --- | --- | --- | --- | --- | --- | --- | --- | --- | --- | --- | --- | --- | --- | --- | --- | --- | --- | --- | --- | --- | --- | --- | --- | --- | --- | --- | --- | --- | --- | --- | --- | --- | --- | --- | --- | --- | --- | --- | --- | --- | --- | --- | --- | --- | --- | --- | --- | --- | --- | --- | --- | --- | --- | --- | --- | --- | --- | --- | --- | --- | --- | --- | --- | --- | --- | --- | --- | --- | --- | --- | --- | --- | --- | --- | --- | --- | --- | --- | --- | --- | --- | --- | --- | --- | --- | --- | --- | --- | --- | --- | --- | --- | --- | --- | --- | --- | --- | --- | --- | --- | --- | --- | --- | --- | --- | --- | --- | --- | --- | --- | --- | --- | --- | --- | --- | --- | --- | --- | --- | --- | --- | --- | --- | --- | --- | --- | --- | --- | --- | --- | --- | --- | --- | --- | --- | --- | --- | --- | --- | --- | --- | --- | --- | --- | --- | --- | --- | --- | --- | --- | --- | --- | --- | --- | --- | --- | --- | --- | --- | --- | --- | --- | --- | --- | --- | --- | --- | --- | --- | --- | --- | --- | --- | --- | --- | --- | --- | --- | --- | --- | --- | --- | --- | --- | --- | --- | --- | --- | --- | --- | --- | --- | --- | --- | --- | --- | --- | --- | --- | --- | --- | --- | --- | --- | --- | --- | --- | --- | --- | --- | --- | --- | --- | --- | --- | --- | --- | --- | --- | --- | --- | --- | --- | --- | --- | --- | --- | --- | --- | --- | --- | --- | --- | --- | --- | --- | --- | --- | --- | --- | --- | --- | --- | --- | --- | --- | --- | --- | --- | --- | --- | --- | --- | --- | --- | --- | --- | --- | --- | --- | --- | --- | --- | --- | --- | --- | --- | --- | --- | --- | --- | --- | --- | --- | --- | --- | --- | --- | --- | --- | --- | --- | --- | --- | --- | --- | --- | --- | --- | --- | --- | --- | --- | --- | --- | --- | --- | --- | --- | --- | --- | --- | --- | --- | --- | --- | --- | --- | --- | --- | --- | --- | --- | --- | --- | --- | --- | --- | --- | --- | --- | --- | --- | --- | --- | --- | --- | --- | --- | --- | --- | --- | --- | --- | --- | --- | --- | --- | --- | --- | --- | --- | --- | --- | --- | --- | --- | --- | --- | --- | --- | --- | --- | --- | --- | --- | --- | --- | --- | --- | --- | --- | --- | --- | --- | --- | --- | --- | --- | --- | --- | --- | --- | --- | --- | --- | --- | --- | --- | --- | --- | --- | --- | --- | --- | --- | --- | --- | --- | --- | --- | --- | --- | --- | --- | --- | --- | --- | --- | --- | --- | --- | --- | --- | --- | --- | --- | --- | --- | --- | --- | --- | --- | --- | --- | --- | --- | --- | --- | --- | --- | --- | --- | --- | --- | --- | --- | --- | --- | --- | --- | --- | --- | --- | --- | --- | --- | --- | --- | --- | --- | --- | --- | --- | --- | --- | --- | --- | --- | --- | --- | --- | --- | --- | --- | --- | --- | --- | --- | --- | --- | --- | --- | --- | --- | --- | --- | --- | --- | --- | --- | --- | --- | --- | --- | --- | --- | --- | --- | --- | --- | --- | --- | --- | --- | --- | --- | --- | --- | --- | --- | --- | --- | --- | --- | --- | --- | --- | --- | --- | --- | --- | --- | --- | --- | --- | --- | --- | --- | --- | --- | --- | --- | --- | --- | --- | --- | --- | --- | --- | --- | --- | --- | --- | --- | --- | --- | --- | --- | --- | --- | --- | --- | --- | --- | --- | --- | --- | --- | --- | --- | --- | --- | --- | --- | --- | --- | --- | --- | --- | --- | --- | --- | --- | --- | --- | --- | --- | --- | --- | --- | --- | --- | --- | --- | --- | --- | --- | --- | --- | --- | --- | --- | --- | --- | --- | --- | --- | --- | --- | --- | --- | --- | --- | --- | --- | --- | --- | --- | --- | --- | --- | --- | --- | --- | --- | --- | --- | --- | --- | --- | --- | --- | --- | --- | --- | --- | --- | --- | --- | --- | --- | --- | --- | --- | --- | --- | --- | --- | --- | --- | --- | --- | --- | --- | --- | --- | --- | --- | --- | --- | --- | --- | --- | --- | --- | --- | --- | --- | --- | --- | --- | --- | --- | --- | --- | --- | --- | --- | --- | --- | --- | --- | --- | --- | --- | --- | --- | --- | --- | --- | --- | --- | --- | --- | --- | --- | --- | --- | --- | --- | --- | --- | --- | --- | --- | --- | --- | --- | --- | --- | --- | --- | --- | --- | --- | --- | --- | --- | --- | --- | --- | --- | --- | --- | --- | --- | --- | --- | --- | --- | --- | --- | --- | --- | --- | --- | --- | --- | --- | --- | --- | --- | --- | --- | --- | --- | --- | --- | --- | --- | --- | --- | --- | --- | --- | --- | --- | --- | --- | --- | --- | --- | --- | --- | --- | --- | --- | --- | --- | --- | --- | --- | --- | --- | --- | --- | --- | --- | --- | --- | --- | --- | --- | --- | --- | --- | --- | --- | --- | --- | --- | --- | --- | --- | --- | --- | --- | --- | --- | --- | --- | --- | --- | --- | --- | --- | --- | --- | --- | --- | --- | --- | --- | --- | --- | --- | --- | --- | --- | --- | --- | --- | --- | --- | --- | --- | --- | --- | --- | --- | --- | --- | --- | --- | --- | --- | --- | --- | --- | --- | --- | --- | --- | --- | --- | --- | --- | --- | --- | --- | --- | --- | --- | --- | --- | --- | --- | --- | --- | --- | --- | --- | --- | --- | --- | --- | --- | --- | --- | --- | --- | --- | --- | --- | --- | --- | --- | --- | --- | --- | --- | --- | --- | --- | --- | --- | --- | --- | --- | --- | --- | --- | --- | --- | --- | --- | --- | --- | --- | --- | --- | --- | --- | --- | --- | --- | --- | --- | --- | --- | --- | --- | --- | --- | --- | --- | --- | --- | --- | --- | --- | --- | --- | --- | --- | --- | --- | --- | --- | --- | --- | --- | --- | --- | --- | --- | --- | --- | --- | --- | --- | --- | --- | --- | --- | --- | --- | --- | --- | --- | --- | --- | --- | --- | --- | --- | --- | --- | --- | --- | --- | --- | --- | --- | --- | --- | --- | --- | --- | --- | --- | --- | --- | --- | --- | --- | --- | --- | --- | --- | --- | --- | --- | --- | --- | --- | --- | --- | --- | --- | --- | --- | --- | --- | --- | --- | --- | --- | --- | --- | --- | --- | --- | --- | --- | --- | --- | --- | --- | --- | --- | --- | --- | --- | --- | --- | --- | --- | --- | --- | --- | --- | --- | --- | --- | --- | --- | --- | --- | --- | --- | --- | --- | --- | --- | --- | --- | --- | --- | --- | --- | --- | --- | --- | --- | --- | --- | --- | --- | --- | --- | --- | --- | --- | --- | --- | --- | --- | --- | --- | --- | --- | --- | --- | --- | --- | --- | --- | --- | --- | --- | --- | --- | --- | --- | --- | --- | --- | --- | --- | --- | --- | --- | --- | --- | --- | --- | --- | --- | --- | --- | --- | --- | --- | --- | --- | --- | --- | --- | --- | --- | --- | --- | --- | --- | --- | --- | --- | --- | --- | --- | --- | --- | --- | --- | --- | --- | --- | --- | --- | --- | --- | --- | --- | --- | --- | --- | --- | --- | --- | --- | --- | --- | --- | --- | --- | --- | --- | --- | --- | --- | --- | --- | --- | --- | --- | --- | --- | --- | --- | --- | --- | --- | --- | --- | --- | --- | --- | --- | --- | --- | --- | --- | --- | --- | --- | --- | --- | --- | --- | --- | --- | --- | --- | --- | --- | --- | --- | --- | --- | --- | --- | --- | --- | --- | --- | --- | --- | --- | --- | --- | --- | --- | --- | --- | --- | --- | --- | --- | --- | --- | --- | --- | --- | --- | --- | --- | --- | --- | --- | --- | --- | --- | --- | --- | --- | --- | --- | --- | --- | --- | --- | --- | --- | --- | --- | --- | --- | --- | --- | --- | --- | --- | --- | --- | --- | --- | --- | --- | --- | --- | --- | --- | --- | --- | --- | --- | --- | --- | --- | --- | --- | --- | --- | --- | --- | --- | --- | --- | --- | --- | --- | --- | --- | --- | --- | --- | --- | --- | --- | --- | --- | --- | --- | --- | --- | --- | --- | --- | --- | --- | --- | --- | --- | --- | --- | --- | --- | --- | --- | --- | --- | --- | --- | --- | --- | --- | --- | --- | --- | --- | --- | --- | --- | --- | --- | --- | --- | --- | --- | --- | --- | --- | --- | --- | --- | --- | --- | --- | --- | --- | --- | --- | --- | --- | --- | --- | --- | --- | --- | --- | --- | --- | --- | --- | --- | --- | --- | --- | --- | --- | --- | --- | --- | --- | --- | --- | --- | --- | --- | --- | --- | --- | --- | --- | --- | --- | --- | --- | --- | --- | --- | --- | --- | --- | --- | --- | --- | --- | --- | --- | --- | --- | --- | --- | --- | --- | --- | --- | --- | --- | --- | --- | --- | --- | --- | --- | --- | --- | --- | --- | --- | --- | --- | --- | --- | --- | --- | --- | --- | --- | --- | --- | --- | --- | --- | --- | --- | --- | --- | --- | --- | --- | --- | --- | --- | --- | --- | --- | --- | --- | --- | --- | --- | --- | --- | --- | --- | --- | --- | --- | --- | --- | --- | --- | --- | --- | --- | --- | --- | --- | --- | --- | --- | --- | --- | --- | --- | --- | --- | --- | --- | --- | --- | --- | --- | --- | --- | --- | --- | --- | --- | --- | --- | --- | --- | --- | --- | --- | --- | --- | --- | --- | --- | --- | --- | --- | --- | --- | --- | --- | --- | --- | --- | --- | --- | --- | --- | --- | --- | --- | --- | --- | --- | --- | --- | --- | --- | --- | --- | --- | --- | --- | --- | --- | --- | --- | --- | --- | --- | --- | --- | --- | --- | --- | --- | --- | --- | --- | --- | --- | --- | --- | --- | --- | --- | --- | --- | --- | --- | --- | --- | --- | --- | --- | --- | --- | --- | --- | --- | --- | --- | --- | --- | --- | --- | --- | --- | --- | --- | --- | --- | --- | --- | --- | --- | --- | --- | --- | --- | --- | --- | --- | --- | --- | --- | --- | --- | --- | --- | --- | --- | --- | --- | --- | --- | --- | --- | --- | --- | --- | --- | --- | --- | --- | --- | --- | --- | --- | --- | --- | --- | --- | --- | --- | --- | --- | --- | --- | --- | --- | --- | --- | --- | --- | --- | --- | --- | --- | --- | --- | --- | --- | --- | --- | --- | --- | --- | --- | --- | --- | --- | --- | --- | --- | --- | --- | --- | --- | --- | --- | --- | --- | --- | --- | --- | --- | --- | --- | --- | --- | --- | --- | --- | --- | --- | --- | --- | --- | --- | --- | --- | --- | --- | --- | --- | --- | --- | --- | --- | --- | --- | --- | --- | --- | --- | --- | --- | --- | --- | --- | --- | --- | --- | --- | --- | --- | --- | --- | --- | --- | --- | --- | --- | --- | --- | --- | --- | --- | --- | --- | --- | --- | --- | --- | --- | --- | --- | --- | --- | --- | --- | --- | --- | --- | --- | --- | --- | --- | --- | --- | --- | --- | --- | --- | --- | --- | --- | --- | --- | --- | --- | --- | --- | --- | --- | --- | --- | --- | --- | --- | --- | --- | --- | --- | --- | --- | --- | --- | --- | --- | --- | --- | --- | --- | --- | --- | --- | --- | --- | --- | --- | --- | --- | --- | --- | --- | --- | --- | --- | --- | --- | --- | --- | --- | --- | --- | --- | --- | --- | --- | --- | --- | --- | --- | --- | --- | --- | --- | --- | --- | --- | --- | --- | --- | --- | --- | --- | --- | --- | --- | --- | --- | --- | --- | --- | --- | --- | --- | --- | --- | --- | --- | --- | --- | --- | --- | --- | --- | --- | --- | --- | --- | --- | --- | --- | --- | --- | --- | --- | --- | --- | --- | --- | --- | --- | --- | --- | --- | --- | --- | --- | --- | --- | --- | --- | --- | --- | --- | --- | --- | --- | --- | --- | --- | --- | --- | --- | --- | --- | --- | --- | --- | --- | --- | --- | --- | --- | --- | --- | --- | --- | --- | --- | --- | --- | --- | --- | --- | --- | --- | --- | --- | --- | --- | --- | --- | --- | --- | --- | --- | --- | --- | --- | --- | --- | --- | --- | --- | --- | --- | --- | --- | --- | --- | --- | --- | --- | --- | --- | --- | --- | --- | --- | --- | --- | --- | --- | --- | --- | --- | --- | --- | --- | --- | --- | --- | --- | --- | --- | --- | --- | --- | --- | --- | --- | --- | --- | --- | --- | --- | --- | --- | --- | --- | --- | --- | --- | --- | --- | --- | --- | --- | --- | --- | --- | --- | --- | --- | --- | --- | --- | --- | --- | --- | --- | --- | --- | --- | --- | --- | --- | --- | --- | --- | --- | --- | --- | --- | --- | --- | --- | --- | --- | --- | --- | --- | --- | --- | --- | --- | --- | --- | --- | --- | --- | --- | --- | --- | --- | --- | --- | --- | --- | --- | --- | --- | --- | --- | --- | --- | --- | --- | --- | --- | --- | --- | --- | --- | --- | --- | --- | --- | --- | --- | --- | --- | --- | --- | --- | --- | --- | --- | --- | --- | --- | --- | --- | --- | --- | --- | --- | --- | --- | --- | --- | --- | --- | --- | --- | --- | --- | --- | --- | --- | --- | --- | --- | --- | --- | --- | --- | --- | --- | --- | --- | --- | --- | --- | --- | --- | --- | --- | --- | --- | --- | --- | --- | --- | --- | --- | --- | --- | --- | --- | --- | --- | --- | --- | --- | --- | --- | --- | --- | --- | --- | --- | --- | --- | --- | --- | --- | --- | --- | --- | --- | --- | --- | --- | --- | --- | --- | --- | --- | --- | --- | --- | --- | --- | --- | --- | --- | --- | --- | --- | --- | --- | --- | --- | --- | --- | --- | --- | --- | --- | --- | --- | --- | --- | --- | --- | --- | --- | --- | --- | --- | --- | --- | --- | --- | --- | --- | --- | --- | --- | --- | --- | --- | --- | --- | --- | --- | --- | --- | --- | --- | --- | --- | --- | --- | --- | --- | --- | --- | --- | --- | --- | --- | --- | --- | --- | --- | --- | --- | --- | --- | --- | --- | --- | --- | --- | --- | --- | --- | --- | --- | --- | --- | --- | --- | --- | --- | --- | --- | --- | --- | --- | --- | --- | --- | --- | --- | --- | --- | --- | --- | --- | --- | --- | --- | --- | --- | --- | --- | --- | --- | --- | --- | --- | --- | --- | --- | --- | --- | --- | --- | --- | --- | --- | --- | --- | --- | --- | --- | --- | --- | --- | --- | --- | --- | --- | --- | --- | --- | --- | --- | --- | --- | --- | --- | --- | --- | --- | --- | --- | --- | --- | --- | --- | --- | --- | --- | --- | --- | --- | --- | --- | --- | --- | --- | --- | --- | --- | --- | --- | --- | --- | --- | --- | --- | --- | --- | --- | --- | --- | --- | --- | --- | --- | --- | --- | --- | --- | --- | --- | --- | --- | --- | --- | --- | --- | --- | --- | --- | --- | --- | --- | --- | --- | --- | --- | --- | --- | --- | --- | --- | --- | --- | --- | --- | --- | --- | --- | --- | --- | --- | --- | --- | --- | --- | --- | --- | --- | --- | --- | --- | --- | --- | --- | --- | --- | --- | --- | --- | --- | --- | --- | --- | --- | --- | --- | --- | --- | --- | --- | --- | --- | --- | --- | --- | --- | --- | --- | --- | --- | --- | --- | --- | --- | --- | --- | --- | --- | --- | --- | --- | --- | --- | --- | --- | --- | --- | --- | --- | --- | --- | --- | --- | --- | --- | --- | --- | --- | --- | --- | --- | --- | --- | --- | --- | --- | --- | --- | --- | --- | --- | --- | --- | --- | --- | --- | --- | --- | --- | --- | --- | --- | --- | --- | --- | --- | --- | --- | --- | --- | --- | --- | --- | --- | --- | --- | --- | --- | --- | --- | --- | --- | --- | --- | --- | --- | --- | --- | --- | --- | --- | --- | --- | --- | --- | --- | --- | --- | --- | --- | --- | --- | --- | --- | --- | --- | --- | --- | --- | --- | --- | --- | --- | --- | --- | --- | --- | --- | --- | --- | --- | --- | --- | --- | --- | --- | --- | --- | --- | --- | --- | --- | --- | --- | --- | --- | --- | --- | --- | --- | --- | --- | --- | --- | --- | --- | --- | --- | --- | --- | --- | --- | --- | --- | --- | --- | --- | --- | --- | --- | --- | --- | --- | --- | --- | --- | --- | --- | --- | --- | --- | --- | --- | --- | --- | --- | --- | --- | --- | --- | --- | --- | --- | --- | --- | --- | --- | --- | --- | --- | --- | --- | --- | --- | --- | --- | --- | --- | --- | --- | --- | --- | --- | --- | --- | --- | --- | --- | --- | --- | --- | --- | --- | --- | --- | --- | --- | --- | --- | --- | --- | --- | --- | --- | --- | --- | --- | --- | --- | --- | --- | --- | --- | --- | --- | --- | --- | --- | --- | --- | --- | --- | --- | --- | --- | --- | --- | --- | --- | --- | --- | --- | --- | --- | --- | --- | --- | --- | --- | --- | --- | --- | --- | --- | --- | --- | --- | --- | --- | --- | --- | --- | --- | --- | --- | --- | --- | --- | --- | --- | --- | --- | --- | --- | --- | --- | --- | --- | --- | --- | --- | --- | --- | --- | --- | --- | --- | --- | --- | --- | --- | --- | --- | --- | --- | --- | --- | --- | --- | --- | --- | --- | --- | --- | --- | --- | --- | --- | --- | --- | --- | --- | --- | --- | --- | --- | --- | --- | --- | --- | --- | --- | --- | --- | --- | --- | --- | --- | --- | --- | --- | --- | --- | --- | --- | --- | --- | --- | --- | --- | --- | --- | --- | --- | --- | --- | --- | --- | --- | --- | --- | --- | --- | --- | --- | --- | --- | --- | --- | --- | --- | --- | --- | --- | --- | --- | --- | --- | --- | --- | --- | --- | --- | --- | --- | --- | --- | --- | --- | --- | --- | --- | --- | --- | --- | --- | --- | --- | --- | --- | --- | --- | --- | --- | --- | --- | --- | --- | --- | --- | --- | --- | --- | --- | --- | --- | --- | --- | --- | --- | --- | --- | --- | --- | --- | --- | --- | --- | --- | --- | --- | --- | --- | --- | --- | --- | --- | --- | --- | --- | --- | --- | --- | --- | --- | --- | --- | --- | --- | --- | --- | --- | --- | --- | --- | --- | --- | --- | --- | --- | --- | --- | --- | --- | --- | --- | --- | --- | --- | --- | --- | --- | --- | --- | --- | --- | --- | --- | --- | --- | --- | --- | --- | --- | --- | --- | --- | --- | --- | --- | --- | --- | --- | --- | --- | --- | --- | --- | --- | --- | --- | --- | --- | --- | --- | --- | --- | --- | --- | --- | --- | --- | --- | --- | --- | --- | --- | --- | --- | --- | --- | --- | --- | --- | --- | --- | --- | --- | --- | --- | --- | --- | --- | --- | --- | --- | --- | --- | --- | --- | --- | --- | --- | --- | --- | --- | --- | --- | --- | --- | --- | --- | --- | --- | --- | --- | --- | --- | --- | --- | --- | --- | --- | --- | --- | --- | --- | --- | --- | --- | --- | --- | --- | --- | --- | --- | --- | --- | --- | --- | --- | --- | --- | --- | --- | --- | --- | --- | --- | --- | --- | --- | --- | --- | --- | --- | --- | --- | --- | --- | --- | --- | --- | --- | --- | --- | --- | --- | --- | --- | --- | --- | --- | --- | --- | --- | --- | --- | --- | --- | --- | --- | --- | --- | --- | --- | --- | --- | --- | --- | --- | --- | --- | --- | --- | --- | --- | --- | --- | --- | --- | --- | --- | --- | --- | --- | --- | --- | --- | --- | --- | --- | --- | --- | --- | --- | --- | --- | --- | --- | --- | --- | --- | --- | --- | --- | --- | --- | --- | --- | --- | --- | --- | --- | --- | --- | --- | --- | --- | --- | --- | --- | --- | --- | --- | --- | --- | --- | --- | --- | --- | --- | --- | --- | --- | --- | --- | --- | --- | --- | --- | --- | --- | --- | --- | --- | --- | --- | --- | --- | --- | --- | --- | --- | --- | --- | --- | --- | --- | --- | --- | --- | --- | --- | --- | --- | --- | --- | --- | --- | --- | --- | --- | --- | --- | --- | --- | --- | --- | --- | --- | --- | --- | --- | --- | --- | --- | --- | --- | --- | --- | --- | --- | --- | --- | --- | --- | --- | --- | --- | --- | --- | --- | --- | --- | --- | --- | --- | --- | --- | --- | --- | --- | --- | --- | --- | --- | --- | --- | --- | --- | --- | --- | --- | --- | --- | --- | --- | --- | --- | --- | --- | --- | --- | --- | --- | --- | --- | --- | --- | --- | --- | --- | --- | --- | --- | --- | --- | --- | --- | --- | --- | --- | --- | --- | --- | --- | --- | --- | --- | --- | --- | --- | --- | --- | --- | --- | --- | --- | --- | --- | --- | --- | --- | --- | --- | --- | --- | --- | --- | --- | --- | --- | --- | --- | --- | --- | --- | --- | --- | --- | --- | --- | --- | --- | --- | --- | --- | --- | --- | --- | --- | --- | --- | --- | --- | --- | --- | --- | --- | --- | --- | --- | --- | --- | --- | --- | --- | --- | --- | --- | --- | --- | --- | --- | --- | --- | --- | --- | --- | --- | --- | --- | --- | --- | --- | --- | --- | --- | --- | --- | --- | --- | --- | --- | --- | --- | --- | --- | --- | --- | --- | --- | --- | --- | --- | --- | --- | --- | --- | --- | --- | --- | --- | --- | --- | --- | --- | --- | --- | --- | --- | --- | --- | --- | --- | --- | --- | --- | --- | --- | --- | --- | --- | --- | --- | --- | --- | --- | --- | --- | --- | --- | --- | --- | --- | --- | --- | --- | --- | --- | --- | --- | --- | --- | --- | --- | --- | --- | --- | --- | --- | --- | --- | --- | --- | --- | --- | --- | --- | --- | --- | --- | --- | --- | --- | --- | --- | --- | --- | --- | --- | --- | --- | --- | --- | --- | --- | --- | --- | --- | --- | --- | --- | --- | --- | --- | --- | --- | --- | --- | --- | --- | --- | --- | --- | --- | --- | --- | --- | --- | --- | --- | --- | --- | --- | --- | --- | --- | --- | --- | --- | --- | --- | --- | --- | --- | --- | --- | --- | --- | --- | --- | --- | --- | --- | --- | --- | --- | --- | --- | --- | --- | --- | --- | --- | --- | --- | --- | --- | --- | --- | --- | --- | --- | --- | --- | --- | --- | --- | --- | --- | --- | --- | --- | --- | --- | --- | --- | --- | --- | --- | --- | --- | --- | --- | --- | --- | --- | --- | --- | --- | --- | --- | --- | --- | --- | --- | --- | --- | --- | --- | --- | --- | --- | --- | --- | --- | --- | --- | --- | --- | --- | --- | --- | --- | --- | --- | --- | --- | --- | --- | --- | --- | --- | --- | --- | --- | --- | --- | --- | --- | --- | --- | --- | --- | --- | --- | --- | --- | --- | --- | --- | --- | --- | --- | --- | --- | --- | --- | --- | --- | --- | --- | --- | --- | --- | --- | --- | --- | --- | --- | --- | --- | --- | --- | --- | --- | --- | --- | --- | --- | --- | --- | --- | --- |
| |  |  |  |  |  |  |  |  |  | | --- | --- | --- | --- | --- | --- | --- | --- | --- | | **Position** | **Reference** | **Sample** | **Quality** | **Type** | **Region** | **AA Exchange** | **PAM1** | **Known Variant** | | 358 | G | C | 152.77 | SNP | Rv0001 (dnaA) | Ala120Pro | 13 | - | | 1849 | C | A | 1148.77 | SNP | intergenic |  |  | - | | 1977 | A | G | 784.77 | SNP | intergenic |  |  | - | | 4013 | T | C | 943.77 | SNP | Rv0003 (recF) | Ile245Thr | 11 | - | | 7362 | G | C | 874.77 | SNP | Rv0006 (gyrA) | Glu21Gln | 27 | - | | 7585 | G | C | 895.77 | SNP | Rv0006 (gyrA) | Ser95Thr | 32 | genotype | | 9304 | G | A | 753.77 | SNP | Rv0006 (gyrA) | Gly668Asp | 6 | - | | 11820 | C | G | 1353.77 | SNP | intergenic |  |  | - | | 11879 | A | G | 803.77 | SNP | Rv0008c | Ser145Pro | 12 | - | | 14785 | T | C | 918.77 | SNP | Rv0012 | Cys233Arg | 1 | - | | 14861 | G | T | 980.77 | SNP | Rv0012 | Gly258Val | 3 | - | | 15117 | C | G | 1568.77 | SNP | Rv0013 (trpG) | Ile68Met(s) | 6 | - | | 16119 | C | A | 1022.77 | SNP | Rv0014c (pknB) | Arg451Leu | 1 | - | | 17654 | T | G | 788.77 | SNP | Rv0015c (pknA) | Gln370Pro | 8 | - | | 17657 | T | C | 794.77 | SNP | Rv0015c (pknA) | Gln369Arg | 10 | - | | 21795 | G | A | 106.28 | SNP | Rv0018c (pstP) | Pro463Ser | 17 | - | | 24698 | GCCGCGTTGCTCGGGGTAA | G | 3131.73 | DEL | Rv0020c (fhaA) |  |  | - | | 25610 | G | C | 791.77 | SNP | intergenic |  |  | - | | 26959 | C | G | 876.77 | SNP | intergenic |  |  | - | | 28366 | AT | A | 1126.73 | DEL | Rv0024 |  |  | - | | 34044 | T | C | 1023.77 | SNP | intergenic |  |  | - | | 35608 | C | T | 689.77 | SNP | Rv0032 (bioF2) | silent (Ala438) | 9867 | - | | 36008 | G | C | 992.77 | SNP | Rv0032 (bioF2) | Asp572His | 3 | - | | 37031 | C | G | 1124.77 | SNP | Rv0034 | silent (Ala55) | 9867 | - | | 37305 | C | G | 878.77 | SNP | Rv0035 (fadD34) | Ser16Trp | 1 | - | | 37334 | A | T | 774.77 | SNP | Rv0035 (fadD34) | Thr26Ser | 38 | - | | 39022 | A | G | 604.77 | SNP | intergenic |  |  | - | | 39030 | C | T | 1241.77 | SNP | intergenic |  |  | - | | 39158 | C | G | 890.77 | SNP | Rv0036c | silent (Arg224) | 9913 | - | | 42281 | C | A | 775.77 | SNP | Rv0039c | Cys24Phe | 0 | - | | 42967 | G | C | 918.77 | SNP | Rv0040c (mtc28) | silent (Pro133) | 9926 | - | | 44768 | A | G | 853.77 | SNP | Rv0041 (leuS) | Arg403Gly | 1 | - | | 48503 | T | C | 682.77 | SNP | Rv0044c | silent (Arg175) | 9913 | - | | 49360 | C | T | 1115.77 | SNP | Rv0045c | Val194Ile | 33 | - | | 49690 | GCC | G | 1375.73 | DEL | Rv0045c |  |  | - | | 50557 | T | C | 1126.77 | SNP | Rv0046c (ino1) | Arg190Gly | 1 | - | | 51949 | A | G | 803.77 | SNP | Rv0048c | Val250Ala | 18 | - | | 54394 | A | G | 949.77 | SNP | Rv0050 (ponA1) | silent (Ala244) | 9867 | - | | 55540 | G | GCCGCCGCCT | 2027.73 | INS | Rv0050 (ponA1) |  |  | - | | 55553 | C | T | 350.94 | SNP | Rv0050 (ponA1) | Pro631Ser | 17 | - | | 62049 | A | G | 728.77 | SNP | Rv0058 (dnaB) | Arg552Gly | 1 | - | | 63146 | G | T | 1183.77 | SNP | intergenic |  |  | - | | 65150 | C | T | 973.77 | SNP | Rv0061c | Trp67STOP | 0 | - | | 68337 | T | TC | 1912.73 | INS | Rv0063 |  |  | - | | 69989 | G | A | 1153.77 | SNP | Rv0064 | Gly457Asp | 6 | - | | 70267 | G | T | 1047.77 | SNP | Rv0064 | Val550Phe | 0 | - | | 70816 | A | G | 690.77 | SNP | Rv0064 | Asn733Asp | 42 | - | | 71336 | G | C | 258.80 | SNP | Rv0064 | Arg906Pro | 5 | - | | 71584 | C | CCGAGCGCTGTTCTGGCGCT AATCTGACGCTAGAATAG | 10416.73 | INS | intergenic |  |  | - | | 75233 | C | A | 833.77 | SNP | intergenic |  |  | - | | 75940 | G | C | 1284.77 | SNP | Rv0068 | Val(s)214Leu | 3 | - | | 79480 | T | C | 1025.77 | SNP | intergenic |  |  | - | | 87468 | C | T | 917.77 | SNP | Rv0078A | Glu112Lys | 7 | - | | 92199 | T | G | 628.77 | SNP | Rv0083 | silent (Thr600) | 9871 | - | | 94388 | G | A | 1288.77 | SNP | Rv0086 (hycQ) | silent (Gly146) | 9935 | - | | 96894 | C | T | 954.77 | SNP | intergenic |  |  | - | | 97350 | A | C | 726.77 | SNP | Rv0088 | silent (Arg142) | 9913 | - | | 97357 | C | A | 712.77 | SNP | Rv0088 | Thr144Asn | 9 | - | | 99162 | TCGGTGTGCG | T | 3188.80 | DEL | Rv0090 |  |  | - | | 101539 | C | T | 805.77 | SNP | Rv0092 (ctpA) | silent (Gly319) | 9935 | - | | 103743 | GAGGAAT | G | 1733.73 | DEL | Rv0094c |  |  | - | | 103752 | T | TAGCAGCTTC | 2149.73 | INS | Rv0094c |  |  | - | | 103756 | G | T | 533.77 | SNP | Rv0094c | Pro303His | 3 | - | | 105007 | C | G | 205.78 | SNP | Rv0095c | Ser70Thr | 32 | - | | 105011 | G | A | 207.80 | SNP | Rv0095c | Leu69Leu(s) | 4 | - | | 105021 | G | A | 373.77 | SNP | Rv0095c | silent (Ser65) | 9840 | - | | 105045 | G | C | 495.77 | SNP | Rv0095c | Asp57Glu | 56 | - | | 105139 | C | A | 979.77 | SNP | Rv0095c | Gly26Val(s) | 21 | - | | 105736 | T | C | 982.77 | SNP | Rv0096 (PPE1) | Val138Ala | 18 | - | | 116000 | T | G | 1013.77 | SNP | Rv0101 (nrp) | Val2000Val(s) | 18 | - | | 122109 | A | G | 750.77 | SNP | Rv0103c (ctpB) | Leu(s)22Ser | 28 | - | | 123454 | C | T | 1124.77 | SNP | Rv0104 | Gln380STOP | 8 | - | | 123520 | T | C | 1235.77 | SNP | Rv0104 | Tyr402His | 4 | - | | 125830 | G | GA | 1077.73 | INS | Rv0107c (ctpI) |  |  | - | | 131174 | T | TG | 934.73 | INS | intergenic |  |  | - | | 132417 | C | G | 85.28 | SNP | Rv0109 (PE\_PGRS1) | Arg346Gly | 1 | - | | 132687 | G | T | 150.90 | SNP | Rv0109 (PE\_PGRS1) | Asp436Tyr | 0 | - | | 133839 | C | T | 1074.77 | SNP | intergenic |  |  | - | | 135329 | C | T | 834.77 | SNP | Rv0111 | silent (Pro460) | 9926 | - | | 139297 | G | T | 891.77 | SNP | Rv0115 (hddA) | Gly262Val | 3 | - | | 141457 | G | C | 1167.77 | SNP | Rv0117 (oxyS) | silent (Arg86) | 9913 | - | | 141623 | G | A | 1138.77 | SNP | Rv0117 (oxyS) | Asp142Asn | 36 | - | | 143207 | T | C | 658.77 | SNP | Rv0118c (oxcA) | Ser224Gly | 21 | - | | 146087 | T | C | 684.77 | SNP | Rv0120c (fusA2) | Asn562Ser | 34 | - | | 146236 | C | G | 1135.77 | SNP | Rv0120c (fusA2) | silent (Ala512) | 9867 | - | | 147262 | G | T | 719.77 | SNP | Rv0120c (fusA2) | Asp170Glu | 56 | - | | 150496 | A | G | 451.77 | SNP | Rv0124 (PE\_PGRS2) | Ile322Val | 57 | - | | 154191 | A | G | 1057.77 | SNP | intergenic |  |  | - | | 154283 | T | C | 1234.77 | SNP | Rv0127 (mak) | Ser18Pro | 12 | - | | 162151 | GT | G | 1160.73 | DEL | Rv0134 (ephF) |  |  | - | | 163705 | C | T | 527.77 | SNP | Rv0136 (cyp138) | Pro114Ser | 17 | - | | 163706 | C | T | 620.77 | SNP | Rv0136 (cyp138) | Pro114Leu | 3 | - | | 177857 | G | A | 632.77 | SNP | Rv0151c (PE1) | Leu485Leu(s) | 4 | - | | 182824 | G | A | 608.77 | SNP | Rv0154c (fadE2) | silent (Ile125) | 9872 | - | | 183091 | C | G | 1121.77 | SNP | Rv0154c (fadE2) | silent (Pro36) | 9926 | - | | 188800 | T | C | 383.77 | SNP | Rv0159c (PE3) | Thr14Ala | 32 | - | | 190816 | A | C | 919.77 | SNP | Rv0161 | silent (Ser70) | 9840 | - | | 194305 | C | CGG | 2607.73 | INS | Rv0165c (mce1R) |  |  | - | | 194681 | G | C | 503.77 | SNP | Rv0165c (mce1R) | silent (Leu45) | 9947 | - | | 195682 | C | G | 959.77 | SNP | Rv0166 (fadD5) | Val230Val(s) | 18 | - | | 196642 | C | T | 751.77 | SNP | Rv0166 (fadD5) | silent (Asn550) | 9822 | - | | 199470 | T | G | 655.77 | SNP | Rv0169 (mce1A) | Ser313Ala | 35 | - | | 203038 | T | C | 938.77 | SNP | Rv0172 (mce1D) | Ile188Thr | 11 | - | | 204094 | G | T | 829.77 | SNP | Rv0173 (lprK) | Val(s)10Val | 13 | - | | 206339 | T | C | 799.77 | SNP | Rv0174 (mce1F) | Leu370Pro | 2 | - | | 206481 | C | G | 287.74 | SNP | Rv0174 (mce1F) | silent (Pro417) | 9926 | - | | 206484 | G | T | 226.41 | SNP | Rv0174 (mce1F) | silent (Gly418) | 9935 | - | | 213147 | C | A | 869.03 | SNP | Rv0182c (sigG) | Asp332Tyr | 0 | - | | 213281 | C | T | 882.77 | SNP | Rv0182c (sigG) | Gly287Asp | 6 | - | | 215977 | C | T | 673.77 | SNP | Rv0185 | Ala88Val(s) | 9867 | - | | 217201 | T | C | 778.77 | SNP | Rv0186 (bglS) | silent (Asn311) | 9822 | - | | 222925 | G | A | 763.77 | SNP | Rv0191 | Ala213Thr | 22 | - | | 223942 | T | C | 329.78 | SNP | Rv0192 | Ser127Pro | 12 | - | | 225323 | T | C | 1445.77 | SNP | Rv0193c | Lys417Glu | 4 | - | | 227098 | T | C | 1220.77 | SNP | Rv0194 | Met(s)74Thr | 22 | - | | 230170 | C | T | 1230.77 | SNP | Rv0194 | Pro1098Leu | 3 | - | | 230576 | G | GT | 1763.73 | INS | intergenic |  |  | - | | 231114 | C | G | 896.77 | SNP | Rv0195 | silent (Ala72) | 9867 | - | | 234477 | T | G | 573.77 | SNP | Rv0197 | Tyr749STOP | 2 | - | | 234496 | C | CGT | 1053.73 | INS | Rv0197 |  |  | - | | 242977 | C | T | 781.77 | SNP | Rv0204c | Ala80Thr | 22 | - | | 249522 | T | C | 565.77 | SNP | Rv0209 | Val(s)162Ala | 9867 | - | | 251575 | G | A | 968.77 | SNP | Rv0210 | Ala486Thr | 22 | - | | 261869 | T | C | 618.77 | SNP | Rv0218 | Cys316Arg | 1 | - | | 262268 | A | T | 784.77 | SNP | Rv0219 | silent (Ala5) | 9867 | - | | 264129 | G | A | 1575.77 | SNP | Rv0221 | Met(s)21Ile | 2 | - | | 265554 | A | C | 631.77 | SNP | Rv0222 (echA1) | silent (Val16) | 9901 | - | | 267751 | G | C | 695.82 | SNP | Rv0223c | Ala5Gly | 21 | - | | 270430 | G | C | 1210.77 | SNP | Rv0226c | Pro379Ala | 22 | - | | 278021 | T | C | 592.77 | SNP | Rv0232 | silent (Arg41) | 9913 | - | | 278681 | C | G | 934.77 | SNP | Rv0233 (nrdB) | His33Asp | 4 | - | | 282892 | C | T | 264.80 | SNP | Rv0236c (aftD) | silent (Thr1320) | 9871 | - | | 283610 | G | A | 837.77 | SNP | Rv0236c (aftD) | Ala1081Val | 13 | - | | 283614 | T | C | 919.77 | SNP | Rv0236c (aftD) | Ser1080Gly | 21 | - | | 285772 | A | C | 280.78 | SNP | Rv0236c (aftD) | silent (Pro360) | 9926 | - | | 285871 | A | G | 382.77 | SNP | Rv0236c (aftD) | silent (Val327) | 9901 | - | | 288260 | C | T | 1537.77 | SNP | Rv0237 (lpqI) | Leu359Leu(s) | 4 | - | | 292679 | A | G | 347.77 | SNP | Rv0243 (fadA2) | Glu170Gly | 7 | - | | 293628 | A | AC | 807.73 | INS | intergenic |  |  | - | | 295556 | G | T | 658.77 | SNP | Rv0244c (fadE5) | silent (Gly26) | 9935 | - | | 296312 | C | T | 728.77 | SNP | Rv0245 | Ser103Phe | 2 | - | | 304923 | A | G | 1002.77 | SNP | Rv0252 (nirB) | silent (Lys686) | 9926 | - | | 305188 | G | T | 959.77 | SNP | Rv0252 (nirB) | Val(s)775Leu(s) | 9867 | - | | 308520 | G | T | 263.80 | SNP | Rv0256c (PPE2) | Ala343Asp | 6 | - | | 310973 | G | A | 964.77 | SNP | Rv0259c | Ala182Val(s) | 9867 | - | | 311613 | G | T | 833.77 | SNP | Rv0260c | silent (Val349) | 9901 | - | | 312686 | C | T | 701.77 | SNP | intergenic |  |  | - | | 325505 | T | C | 1388.77 | SNP | Rv0270 (fadD2) | silent (Val313) | 9901 | - | | 333637 | A | G | 48.77 | SNP | Rv0278c (PE\_PGRS3) | Trp892Arg | 8 | - | | 333640 | G | A | 43.77 | SNP | Rv0278c (PE\_PGRS3) | Arg891Trp | 2 | - | | 333641 | C | T | 47.77 | SNP | Rv0278c (PE\_PGRS3) | silent (Gln890) | 9876 | - | | 333892 | G | C | 136.03 | SNP | Rv0278c (PE\_PGRS3) | Arg807Gly | 1 | - | | 334641 | G | C | 93.77 | SNP | Rv0278c (PE\_PGRS3) | Ala557Gly | 21 | - | | 335971 | A | G | 75.28 | SNP | Rv0278c (PE\_PGRS3) | Leu(s)114Leu | 3 | - | | 336005 | G | A | 131.03 | SNP | Rv0278c (PE\_PGRS3) | silent (Ile102) | 9872 | - | | 336050 | A | G | 131.03 | SNP | Rv0278c (PE\_PGRS3) | silent (Tyr87) | 9945 | - | | 336053 | G | C | 131.03 | SNP | Rv0278c (PE\_PGRS3) | silent (Ala86) | 9867 | - | | 336074 | T | C | 131.03 | SNP | Rv0278c (PE\_PGRS3) | silent (Ala79) | 9867 | - | | 336081 | A | G | 122.03 | SNP | Rv0278c (PE\_PGRS3) | Val(s)77Ala | 9867 | - | | 336082 | C | T | 111.03 | SNP | Rv0278c (PE\_PGRS3) | Val(s)77Met(s) | 9867 | - | | 336113 | G | A | 41.74 | SNP | Rv0278c (PE\_PGRS3) | silent (Ser66) | 9840 | - | | 336380 | A | T | 177.90 | SNP | intergenic |  |  | - | | 336400 | C | G | 219.84 | SNP | intergenic |  |  | - | | 336403 | C | G | 185.90 | SNP | intergenic |  |  | - | | 336405 | A | G | 183.90 | SNP | intergenic |  |  | - | | 336504 | G | T | 339.77 | SNP | intergenic |  |  | - | | 336535 | T | G | 162.90 | SNP | intergenic |  |  | - | | 336537 | T | G | 144.90 | SNP | intergenic |  |  | - | | 336540 | G | T | 75.28 | SNP | intergenic |  |  | - | | 336546 | T | G | 146.90 | SNP | intergenic |  |  | - | | 336557 | C | CT | 212.80 | INS | intergenic |  |  | - | | 336560 | T | C | 158.90 | SNP | Rv0279c (PE\_PGRS4) | silent (STOP838) | 9867 | - | | 336562 | A | ATGG | 346.80 | INS | Rv0279c (PE\_PGRS4) |  |  | - | | 336590 | G | C | 118.03 | SNP | Rv0279c (PE\_PGRS4) | Ile828Met(s) | 6 | - | | 336592 | T | G | 136.90 | SNP | Rv0279c (PE\_PGRS4) | Ile828Leu | 22 | - | | 336611 | G | C | 117.03 | SNP | Rv0279c (PE\_PGRS4) | silent (Ala821) | 9867 | - | | 336617 | G | C | 76.28 | SNP | Rv0279c (PE\_PGRS4) | silent (Pro819) | 9926 | - | | 336620 | T | C | 43.74 | SNP | Rv0279c (PE\_PGRS4) | silent (Thr818) | 9871 | - | | 338618 | C | G | 41.74 | SNP | Rv0279c (PE\_PGRS4) | silent (Gly152) | 9935 | - | | 338789 | G | C | 52.77 | SNP | Rv0279c (PE\_PGRS4) | silent (Thr95) | 9871 | - | | 338810 | G | C | 39.77 | SNP | Rv0279c (PE\_PGRS4) | silent (Ala88) | 9867 | - | | 338844 | A | G | 735.77 | SNP | Rv0279c (PE\_PGRS4) | Val(s)77Ala | 9867 | - | | 338845 | C | T | 652.77 | SNP | Rv0279c (PE\_PGRS4) | Val(s)77Met(s) | 9867 | - | | 338876 | G | A | 203.77 | SNP | Rv0279c (PE\_PGRS4) | silent (Ser66) | 9840 | - | | 338903 | G | C | 407.77 | SNP | Rv0279c (PE\_PGRS4) | silent (Ala57) | 9867 | - | | 338960 | T | C | 367.77 | SNP | Rv0279c (PE\_PGRS4) | silent (Ala38) | 9867 | - | | 338963 | T | C | 378.77 | SNP | Rv0279c (PE\_PGRS4) | silent (Thr37) | 9871 | - | | 340132 | G | A | 618.77 | SNP | Rv0280 (PPE3) | Glu257Lys | 7 | - | | 340372 | T | C | 467.77 | SNP | Rv0280 (PPE3) | Ser337Pro | 12 | - | | 342146 | A | C | 1138.77 | SNP | Rv0282 (eccA3) | Glu6Ala | 17 | - | | 346275 | C | G | 1252.77 | SNP | Rv0284 (eccC3) | Pro214Arg | 4 | - | | 353309 | G | A | 603.77 | SNP | Rv0290 (eccD3) | Ser76Asn | 20 | - | | 353365 | G | A | 647.77 | SNP | Rv0290 (eccD3) | Ala95Thr | 22 | - | | 356528 | A | G | 692.77 | SNP | Rv0292 (eccE3) | Asn217Asp | 42 | - | | 362081 | GC | G | 244.77 | DEL | Rv0297 (PE\_PGRS5) |  |  | - | | 363464 | G | A | 741.77 | SNP | Rv0298 | silent (Arg71) | 9913 | - | | 364854 | C | G | 1100.77 | SNP | Rv0302 | His84Asp | 4 | - | | 366880 | T | C | 359.90 | SNP | Rv0304c (PPE5) | Asn1962Ser | 34 | - | | 368087 | AGCTGCCGGTGTTGAT | A | 3033.73 | DEL | Rv0304c (PPE5) |  |  | - | | 372913 | A | C | 979.77 | SNP | Rv0305c (PPE6) | silent (Gly933) | 9935 | - | | 373282 | TA | T | 1740.73 | DEL | Rv0305c (PPE6) |  |  | - | | 374353 | A | G | 828.77 | SNP | Rv0305c (PPE6) | silent (Arg453) | 9913 | - | | 376774 | T | C | 1019.77 | SNP | Rv0307c | silent (Ala94) | 9867 | - | | 377014 | C | T | 965.77 | SNP | Rv0307c | silent (Leu14) | 9947 | - | | 379528 | G | T | 912.77 | SNP | Rv0311 | Glu119Asp | 53 | - | | 383716 | C | T | 612.77 | SNP | Rv0315 | Pro39Ser | 17 | - | | 384380 | A | C | 769.77 | SNP | Rv0315 | Lys260Thr | 8 | - | | 386432 | C | G | 1032.77 | SNP | Rv0318c | Gly223Ala | 21 | - | | 390828 | T | C | 959.77 | SNP | Rv0323c | Ser142Gly | 21 | - | | 391853 | A | G | 797.77 | SNP | Rv0324 | Thr168Ala | 32 | - | | 392261 | T | C | 575.77 | SNP | Rv0325 | STOP75Gln | 3 | - | | 396199 | T | C | 633.77 | SNP | intergenic |  |  | - | | 396771 | C | G | 1161.77 | SNP | Rv0331 | Pro191Ala | 22 | - | | 401318 | A | G | 49.74 | SNP | Rv0336 | Tyr376Cys | 3 | - | | 403980 | G | A | 849.77 | SNP | Rv0338c | Ala621Val | 13 | - | | 404326 | T | C | 799.77 | SNP | Rv0338c | Arg506Gly | 1 | - | | 414486 | C | T | 1587.77 | SNP | Rv0344c (lpqJ) | silent (Glu152) | 9865 | - | | 420008 | A | G | 1096.77 | SNP | Rv0350 (dnaK) | silent (Ala58) | 9867 | - | | 424320 | T | TC | 1251.73 | INS | Rv0354c (PPE7) |  |  | - | | 424981 | G | A | 619.77 | SNP | Rv0355c (PPE8) | silent (Pro3233) | 9926 | - | | 427310 | TTGCCGAGGTTTGCAC | T | 1524.75 | DEL | Rv0355c (PPE8) |  |  | - | | 428698 | C | T | 478.77 | SNP | Rv0355c (PPE8) | silent (Ala1994) | 9867 | - | | 432536 | C | T | 952.77 | SNP | Rv0355c (PPE8) | Gly715Asp | 6 | - | | 441823 | C | T | 873.77 | SNP | Rv0363c (fba) | silent (Glu159) | 9865 | - | | 445780 | C | T | 754.77 | SNP | Rv0368c | Arg249His | 8 | - | | 449633 | G | A | 1313.77 | SNP | Rv0373c | Thr724Ile | 7 | - | | 450525 | T | G | 1096.77 | SNP | Rv0373c | Thr427Pro | 4 | - | | 454295 | T | C | 915.77 | SNP | Rv0376c | silent (Pro26) | 9926 | - | | 454333 | T | G | 681.77 | SNP | Rv0376c | Thr14Pro | 4 | - | | 456594 | T | C | 896.77 | SNP | Rv0380c | Met(s)76Val(s) | 9867 | - | | 457452 | T | G | 803.77 | SNP | Rv0381c | silent (Thr124) | 9871 | - | | 458893 | C | G | 1012.77 | SNP | Rv0383c | silent (Arg141) | 9913 | - | | 459399 | A | C | 592.77 | SNP | intergenic |  |  | - | | 460413 | C | T | 734.77 | SNP | Rv0384c (clpB) | silent (Lys530) | 9926 | - | | 465300 | C | T | 578.77 | SNP | Rv0386 | silent (Phe630) | 9946 | - | | 467497 | C | CG | 702.73 | INS | Rv0388c (PPE9) |  |  | - | | 467508 | C | CG | 819.73 | INS | Rv0388c (PPE9) |  |  | - | | 467516 | G | C | 432.77 | SNP | Rv0388c (PPE9) | silent (Ser162) | 9840 | - | | 467526 | C | G | 486.77 | SNP | Rv0388c (PPE9) | Gly159Ala | 21 | - | | 467546 | G | C | 498.77 | SNP | Rv0388c (PPE9) | Asp152Glu | 56 | - | | 467557 | A | C | 539.77 | SNP | Rv0388c (PPE9) | Leu(s)149Val(s) | 9867 | - | | 467564 | A | C | 545.77 | SNP | Rv0388c (PPE9) | His146Gln | 23 | - | | 467585 | G | C | 658.77 | SNP | Rv0388c (PPE9) | His139Gln | 23 | - | | 467590 | T | C | 658.77 | SNP | Rv0388c (PPE9) | Thr138Ala | 32 | - | | 467621 | T | G | 606.77 | SNP | Rv0388c (PPE9) | silent (Gly127) | 9935 | - | | 467638 | G | T | 607.77 | SNP | Rv0388c (PPE9) | Gln122Lys | 12 | - | | 475178 | T | C | 826.77 | SNP | Rv0395 | Val80Ala | 18 | - | | 477234 | T | G | 360.77 | SNP | Rv0398c | Glu29Asp | 53 | - | | 483935 | T | G | 985.77 | SNP | intergenic |  |  | - | | 484596 | C | T | 889.77 | SNP | Rv0404 (fadD30) | Pro207Leu | 3 | - | | 485725 | G | A | 1112.77 | SNP | Rv0404 (fadD30) | silent (Thr583) | 9871 | - | | 485810 | CA | C | 1303.74 | DEL | Rv0405 (pks6) |  |  | - | | 489741 | G | A | 938.77 | SNP | Rv0405 (pks6) | Val(s)1337Val | 13 | - | | 489935 | G | C | 1301.77 | SNP | Rv0405 (pks6); Rv0406c | Arg1402Pro; silent (Thr257) | 5; 9871 | - | | 491742 | T | C | 765.77 | SNP | Rv0407 (fgd1) | silent (Phe320) | 9946 | genotype | | 492150 | G | C | 505.77 | SNP | Rv0408 (pta) | Gly122Ala | 21 | - | | 497491 | G | A | 1080.77 | SNP | Rv0411c (glnH) | silent (Asp270) | 9859 | genotype | | 498531 | A | G | 887.77 | SNP | Rv0412c | silent (Ala363) | 9867 | - | | 498557 | C | A | 817.77 | SNP | Rv0412c | Asp355Tyr | 0 | - | | 502589 | C | G | 850.77 | SNP | Rv0417 (thiG) | Ser75Cys | 5 | - | | 503354 | G | C | 1303.77 | SNP | intergenic |  |  | - | | 505974 | G | A | 586.77 | SNP | Rv0419 (lpqM) | Ala297Thr | 22 | - | | 507028 | GC | G | 1179.73 | DEL | intergenic |  |  | - | | 513257 | T | C | 587.77 | SNP | Rv0425c (ctpH) | Met(s)689Val(s) | 9867 | - | | 513789 | C | T | 739.77 | SNP | Rv0425c (ctpH) | Val(s)511Val | 13 | - | | 517358 | T | C | 783.77 | SNP | Rv0428c | Asp149Gly | 11 | - | | 518987 | C | T | 498.77 | SNP | Rv0430 | silent (Arg85) | 9913 | - | | 524891 | C | A | 1278.77 | SNP | Rv0436c (pssA) | Gly167Val | 3 | - | | 533547 | C | G | 735.77 | SNP | Rv0444c (rskA) | Glu81Asp | 53 | - | | 541201 | A | G | 1064.77 | SNP | Rv0450c (mmpL4) | silent (Leu97) | 9947 | - | | 542014 | C | G | 790.77 | SNP | intergenic |  |  | - | | 542514 | C | G | 842.77 | SNP | Rv0452 | His125Asp | 4 | - | | 546357 | A | G | 963.77 | SNP | Rv0456c (echA2) | silent (Thr149) | 9871 | - | | 549363 | C | G | 873.77 | SNP | Rv0457c | Arg82Pro | 5 | - | | 549777 | C | T | 633.77 | SNP | Rv0458 | Arg35Cys | 1 | - | | 551525 | A | C | 784.77 | SNP | Rv0459 | silent (Arg110) | 9913 | - | | 555991 | A | G | 565.77 | SNP | Rv0465c | Cys106Arg | 1 | - | | 561776 | C | T | 863.77 | SNP | intergenic |  |  | - | | 561777 | C | T | 867.77 | SNP | intergenic |  |  | - | | 565655 | A | G | 1146.77 | SNP | intergenic |  |  | - | | 572591 | C | T | 1011.77 | SNP | Rv0483 (lprQ) | silent (Asp294) | 9859 | - | | 573262 | A | G | 498.77 | SNP | Rv0484c | silent (Gly180) | 9935 | - | | 575907 | C | T | 898.77 | SNP | Rv0486 (mshA) | Ala187Val | 13 | genotype | | 580772 | T | A | 296.78 | SNP | intergenic |  |  | - | | 580773 | GGGGGCACCACCCGCTTGCG GGGGA | G | 3228.29 | DEL | intergenic |  |  | - | | 583171 | A | C | 533.77 | SNP | Rv0492c | Ser70Ala | 35 | - | | 584171 | T | C | 812.77 | SNP | Rv0493c | Ser174Gly | 21 | - | | 590436 | T | C | 688.77 | SNP | Rv0500 (proC) | silent (Ala118) | 9867 | - | | 597816 | A | G | 977.77 | SNP | Rv0507 (mmpL2) | silent (Ala206) | 9867 | - | | 598475 | G | A | 1327.77 | SNP | Rv0507 (mmpL2) | Arg426His | 8 | - | | 599868 | A | G | 1176.77 | SNP | Rv0507 (mmpL2) | silent (Arg890) | 9913 | - | | 605150 | G | A | 824.77 | SNP | Rv0512 (hemB) | silent (Arg183) | 9913 | - | | 610120 | T | G | 718.77 | SNP | intergenic |  |  | - | | 611048 | T | G | 760.77 | SNP | intergenic |  |  | - | | 620625 | A | G | 776.77 | SNP | Rv0529 (ccsA) | Ile245Met(s) | 6 | - | | 621598 | C | T | 856.77 | SNP | Rv0530 | Pro231Leu | 3 | - | | 623472 | A | G | 96.28 | SNP | Rv0532 (PE\_PGRS6) | Asp227Gly | 11 | - | | 623508 | C | G | 91.28 | SNP | Rv0532 (PE\_PGRS6) | Ala239Gly | 21 | - | | 627485 | G | A | 278.77 | SNP | Rv0536 (galE3) | Val80Ile | 33 | - | | 628864 | T | C | 650.77 | SNP | Rv0537c | Thr290Ala | 32 | - | | 630722 | G | C | 780.77 | SNP | Rv0538 | Arg228Pro | 5 | - | | 637319 | G | A | 1014.77 | SNP | Rv0545c (pitA) | Pro49Ser | 17 | - | | 640954 | A | G | 1028.77 | SNP | intergenic |  |  | - | | 648002 | T | G | 950.77 | SNP | Rv0556 | Leu15Arg | 1 | - | | 648856 | T | C | 1397.77 | SNP | Rv0557 (mgtA) | silent (Gly107) | 9935 | genotype | | 655559 | G | A | 765.77 | SNP | Rv0564c (gpdA1) | Pro131Ser | 17 | - | | 655986 | T | G | 1418.77 | SNP | intergenic |  |  | - | | 657081 | C | T | 1323.77 | SNP | Rv0565c | Val(s)130Val | 13 | - | | 657142 | C | T | 1568.77 | SNP | Rv0565c | Arg110His | 8 | - | | 659341 | T | C | 606.77 | SNP | intergenic |  |  | - | | 662028 | G | A | 1317.77 | SNP | Rv0570 (nrdZ) | Gly245Glu | 4 | - | | 662911 | T | C | 848.77 | SNP | Rv0570 (nrdZ) | silent (Ala539) | 9867 | - | | 663410 | A | C | 64.77 | SNP | intergenic |  |  | - | | 663418 | A | C | 62.77 | SNP | intergenic |  |  | - | | 663419 | G | A | 60.77 | SNP | intergenic |  |  | - | | 663420 | C | A | 65.77 | SNP | intergenic |  |  | - | | 663429 | T | G | 78.77 | SNP | intergenic |  |  | - | | 665293 | A | G | 924.77 | SNP | Rv0572c | Phe31Leu | 13 | - | | 669033 | T | A | 746.77 | SNP | Rv0575c | Asp238Val | 1 | - | | 669398 | T | C | 819.77 | SNP | Rv0575c | silent (Gln116) | 9876 | - | | 670545 | G | A | 659.77 | SNP | Rv0576 | Arg233His | 8 | - | | 672491 | C | G | 76.28 | SNP | Rv0578c (PE\_PGRS7) | silent (Gly1142) | 9935 | - | | 673238 | A | G | 82.28 | SNP | Rv0578c (PE\_PGRS7) | silent (His893) | 9912 | - | | 683829 | C | T | 530.77 | SNP | Rv0585c | silent (Ala148) | 9867 | - | | 684355 | TG | T | 1091.73 | DEL | intergenic |  |  | - | | 685461 | C | G | 769.77 | SNP | Rv0587 (yrbE2A) | silent (Ala111) | 9867 | - | | 685608 | T | C | 1041.77 | SNP | Rv0587 (yrbE2A) | silent (Leu160) | 9947 | - | | 686264 | G | A | 921.77 | SNP | Rv0588 (yrbE2B) | Ala113Thr | 22 | - | | 686972 | T | C | 1663.77 | SNP | Rv0589 (mce2A) | Phe51Ser | 3 | - | | 688792 | T | TG | 1488.73 | INS | Rv0590 (mce2B) |  |  | - | | 690450 | A | C | 262.78 | SNP | Rv0591 (mce2C) | silent (Ala464) | 9867 | - | | 690465 | T | G | 289.78 | SNP | Rv0591 (mce2C) | silent (Leu469) | 9947 | - | | 694531 | A | G | 616.77 | SNP | Rv0594 (mce2F) | Asn432Ser | 34 | - | | 696917 | G | T | 660.77 | SNP | intergenic |  |  | - | | 697365 | G | A | 1223.77 | SNP | Rv0598c (vapC27) | Thr68Ile | 7 | - | | 698968 | G | A | 407.77 | SNP | Rv0601c | silent (Gly9) | 9935 | - | | 699840 | A | C | 977.77 | SNP | intergenic |  |  | - | | 705048 | C | T | 839.77 | SNP | Rv0610c | Glu288Lys | 7 | - | | 707334 | T | C | 306.78 | SNP | Rv0613c | Thr728Ala | 32 | - | | 712693 | A | G | 777.77 | SNP | intergenic |  |  | - | | 713310 | T | C | 964.77 | SNP | Rv0620 (galK) | Cys199Arg | 1 | - | | 721373 | T | G | 870.77 | SNP | Rv0629c (recD) | Glu120Asp | 53 | - | | 725190 | G | A | 93.28 | SNP | Rv0631c (recC) | silent (Phe1039) | 9946 | - | | 725365 | A | G | 277.80 | SNP | Rv0631c (recC) | Phe981Ser | 3 | - | | 732110 | T | G | 848.77 | SNP | Rv0635 (hadA) | Cys61Gly | 1 | - | | 738522 | T | G | 956.77 | SNP | Rv0644c (mmaA2) | Glu213Asp | 53 | - | | 754186 | A | G | 915.77 | SNP | Rv0658c | Leu75Pro | 2 | - | | 757139 | C | A | 1436.77 | SNP | Rv0663 (atsD) | Arg335Ser | 11 | - | | 757182 | A | G | 1510.77 | SNP | Rv0663 (atsD) | Asp349Gly | 11 | - | | 761155 | C | T | 1120.77 | SNP | Rv0667 (rpoB) | Ser450Leu(s) | 35 | resistance | | 763031 | T | C | 1405.77 | SNP | Rv0667 (rpoB) | silent (Ala1075) | 9867 | genotype | | 764931 | C | A | 1379.77 | SNP | Rv0668 (rpoC) | Ala521Asp | 6 | - | | 767414 | G | A | 884.77 | SNP | intergenic |  |  | - | | 773497 | C | A | 838.77 | SNP | Rv0673 (echA4) | Phe125Leu | 13 | - | | 774065 | T | C | 815.77 | SNP | Rv0674 | Met(s)1Thr | 22 | - | | 775639 | T | C | 1008.77 | SNP | Rv0676c (mmpL5) | Ile948Val | 57 | - | | 776100 | G | A | 1207.77 | SNP | Rv0676c (mmpL5) | Thr794Ile | 7 | - | | 776182 | C | T | 1294.77 | SNP | Rv0676c (mmpL5) | Asp767Asn | 36 | - | | 779615 | G | C | 1021.77 | SNP | Rv0679c | Asn142Lys | 25 | - | | 781395 | T | C | 1298.77 | SNP | intergenic (Rv0682-165nt) |  |  | - | | 781687 | A | G | 1730.77 | SNP | Rv0682 (rpsL) | Lys43Arg | 19 | resistance | | 782148 | C | A | 886.77 | SNP | Rv0683 (rpsG) | Ala72Asp | 6 | - | | 782634 | A | G | 1060.77 | SNP | Rv0684 (fusA1) | silent (Ala50) | 9867 | - | | 791249 | C | T | 1023.77 | SNP | Rv0691c | Ala140Thr | 22 | - | | 797736 | C | T | 1210.77 | SNP | Rv0697 | silent (Leu268) | 9947 | genotype | | 798355 | G | C | 1132.77 | SNP | Rv0697 | Ala475Pro | 13 | - | | 799666 | A | G | 694.77 | SNP | Rv0699 | Asp13Gly | 11 | - | | 807445 | G | T | 699.77 | SNP | Rv0711 (atsA) | Val371Phe | 0 | - | | 809762 | C | A | 604.77 | SNP | intergenic |  |  | - | | 809840 | G | GC | 1267.73 | INS | intergenic |  |  | - | | 811753 | C | T | 1011.77 | SNP | Rv0715 (rplX) | silent (His4) | 9912 | - | | 820483 | G | T | 455.77 | SNP | Rv0727c (fucA) | Ala6Asp | 6 | - | | 820752 | C | T | 632.77 | SNP | Rv0728c (serA2) | Arg242His | 8 | - | | 830868 | G | GGC | 1194.73 | INS | Rv0739 |  |  | - | | 835611 | C | T | 645.77 | SNP | Rv0745 | Thr153Met(s) | 32 | - | | 836658 | A | G | 55.74 | SNP | Rv0746 (PE\_PGRS9) | Thr320Ala | 32 | - | | 837033 | A | G | 100.28 | SNP | Rv0746 (PE\_PGRS9) | Thr445Ala | 32 | - | | 839194 | A | G | 99.28 | SNP | Rv0747 (PE\_PGRS10) | silent (Thr248) | 9871 | - | | 839334 | A | G | 121.03 | SNP | Rv0747 (PE\_PGRS10) | Lys295Arg | 19 | - | | 839348 | A | G | 109.03 | SNP | Rv0747 (PE\_PGRS10) | Ser300Gly | 21 | - | | 839515 | G | A | 109.77 | SNP | Rv0747 (PE\_PGRS10) | silent (Ala355) | 9867 | - | | 839516 | A | G | 91.77 | SNP | Rv0747 (PE\_PGRS10) | Thr356Ala | 32 | - | | 839519 | C | G | 124.77 | SNP | Rv0747 (PE\_PGRS10) | Leu357Val(s) | 4 | - | | 839520 | T | C | 123.77 | SNP | Rv0747 (PE\_PGRS10) | Leu357Pro | 2 | - | | 839534 | A | C | 124.77 | SNP | Rv0747 (PE\_PGRS10) | Ile362Leu | 22 | - | | 840794 | A | G | 319.78 | SNP | Rv0747 (PE\_PGRS10) | Ser782Gly | 21 | - | | 840795 | G | C | 334.78 | SNP | Rv0747 (PE\_PGRS10) | Ser782Thr | 32 | - | | 841764 | G | C | 740.77 | SNP | Rv0749A | silent (Thr37) | 9871 | - | | 842030 | C | T | 944.77 | SNP | intergenic |  |  | - | | 847995 | T | C | 809.77 | SNP | intergenic |  |  | - | | 852910 | C | T | 638.77 | SNP | Rv0758 (phoR) | Pro172Leu | 3 | - | | 857696 | A | G | 1141.77 | SNP | Rv0764c (cyp51) | silent (Ala114) | 9867 | - | | 861216 | T | G | 783.77 | SNP | Rv0768 (aldA) | Leu102Arg | 1 | - | | 866051 | G | A | 654.77 | SNP | Rv0773c (ggtA) | Arg447Cys | 1 | - | | 874835 | C | CCG | 1966.73 | INS | Rv0781 (ptrBa); Rv0782 (ptrBb) |  |  | - | | 876141 | G | T | 699.77 | SNP | Rv0782 (ptrBb) | silent (Thr470) | 9871 | - | | 880562 | G | T | 976.77 | SNP | Rv0785 | Cys408Phe | 0 | - | | 882257 | T | C | 1162.77 | SNP | Rv0787 | Tyr267His | 4 | - | | 888992 | AACCTCAACACCCCCTCAAG TGAAGGAGG | A | 8288.73 | DEL | intergenic |  |  | - | | 892416 | C | T | 682.77 | SNP | Rv0799c | Val(s)286Val | 13 | - | | 893733 | T | G | 761.77 | SNP | Rv0800 (pepC) | Leu139Arg | 1 | - | | 900221 | T | C | 974.77 | SNP | Rv0806c (cpsY) | Val370Val(s) | 18 | - | | 903537 | G | T | 785.77 | SNP | Rv0808 (purF) | Arg476Leu | 1 | - | | 903550 | T | C | 846.77 | SNP | Rv0808 (purF) | silent (Ala480) | 9867 | - | | 903913 | T | C | 898.77 | SNP | Rv0809 (purM) | silent (Gly63) | 9935 | - | | 906857 | A | G | 1084.77 | SNP | Rv0812 | Ile145Met(s) | 6 | - | | 908186 | T | C | 948.77 | SNP | Rv0814c (sseC2) | Thr100Ala | 32 | - | | 909166 | C | T | 266.78 | SNP | Rv0815c (cysA2) | silent (Leu51) | 9947 | - | | 911130 | C | T | 1055.77 | SNP | Rv0818 | silent (Asp53) | 9859 | - | | 916685 | CGGA | C | 1603.73 | DEL | Rv0823c |  |  | - | | 916690 | C | A | 621.77 | SNP | Rv0823c | silent (Ala319) | 9867 | - | | 919351 | A | C | 1285.77 | SNP | Rv0825c | Phe68Cys | 0 | - | | 921813 | C | G | 1426.77 | SNP | Rv0829 | Ala80Gly | 21 | - | | 931123 | T | C | 1312.77 | SNP | Rv0835 (lpqQ) | silent (Tyr57) | 9945 | genotype | | 932280 | T | C | 1338.77 | SNP | Rv0836c | STOP218Trp | 0 | - | | 934230 | C | G | 1006.77 | SNP | intergenic |  |  | - | | 934611 | G | T | 892.77 | SNP | intergenic |  |  | - | | 940602 | C | G | 1004.77 | SNP | Rv0844c (narL) | Gly169Arg | 0 | - | | 941845 | C | A | 1245.77 | SNP | Rv0845 | Ala219Glu | 10 | - | | 945214 | G | A | 819.77 | SNP | Rv0848 (cysK2) | Gly93Ser | 16 | - | | 949535 | T | C | 853.77 | SNP | Rv0853c (pdc) | silent (Ala528) | 9867 | - | | 951702 | C | T | 571.77 | SNP | Rv0855 (far) | Ala24Val | 13 | - | | 954101 | C | T | 876.77 | SNP | Rv0858c (dapC) | Ala274Thr | 22 | - | | 954468 | C | G | 697.56 | SNP | Rv0858c (dapC) | silent (Ala151) | 9867 | - | | 955074 | G | T | 1156.77 | SNP | intergenic |  |  | - | | 955524 | A | G | 900.77 | SNP | Rv0859 (fadA) | Ser150Gly | 21 | - | | 957117 | T | C | 1284.77 | SNP | Rv0860 (fadB) | silent (Asp275) | 9859 | - | | 960367 | A | G | 571.77 | SNP | Rv0862c | Leu(s)749Ser | 28 | - | | 968426 | A | AGCCGGGTTG | 1699.73 | INS | Rv0872c (PE\_PGRS15) |  |  | - | | 972980 | C | T | 929.77 | SNP | Rv0874c | Gly243Ser | 16 | - | | 979704 | G | C | 896.77 | SNP | Rv0881 | Gly115Arg | 0 | - | | 986463 | G | C | 1160.77 | SNP | intergenic |  |  | - | | 987585 | A | AG | 1159.73 | INS | Rv0888 |  |  | - | | 990001 | G | C | 1178.77 | SNP | Rv0890c | Pro866Ala | 22 | - | | 991896 | T | C | 595.77 | SNP | Rv0890c | Glu234Gly | 7 | - | | 993346 | A | C | 1572.77 | SNP | Rv0891c | Val37Gly | 5 | - | | 993352 | G | A | 1535.77 | SNP | Rv0891c | Ala35Val | 13 | - | | 996205 | T | C | 926.77 | SNP | Rv0893c | Lys31Glu | 4 | - | | 1010204 | C | CG | 1057.73 | INS | Rv0907 |  |  | - | | 1011511 | A | C | 764.77 | SNP | Rv0907 | Tyr459Ser | 2 | - | | 1022003 | A | C | 1452.77 | SNP | intergenic |  |  | - | | 1024346 | A | G | 705.77 | SNP | Rv0918 | Ser46Gly | 21 | - | | 1025106 | T | C | 1102.77 | SNP | Rv0919 | silent (Phe141) | 9946 | - | | 1025901 | T | G | 1138.77 | SNP | Rv0920c | Ser306Arg | 6 | - | | 1028217 | G | A | 1052.77 | SNP | Rv0922 | Gly178Asp | 6 | - | | 1034208 | CGCT | C | 1735.73 | DEL | Rv0927c |  |  | - | | 1034758 | C | T | 1089.77 | SNP | intergenic |  |  | - | | 1037012 | T | C | 717.77 | SNP | Rv0930 (pstA1) | Met(s)5Thr | 22 | - | | 1037911 | C | T | 800.77 | SNP | Rv0930 (pstA1) | Arg305STOP | 2 | - | | 1038807 | C | A | 1049.77 | SNP | Rv0931c (pknD) | Val(s)370Leu(s) | 9867 | - | | 1040251 | C | A | 1082.77 | SNP | Rv0932c (pstS2) | silent (Pro266) | 9926 | - | | 1047165 | T | C | 797.77 | SNP | Rv0938 (ligD) | Cys344Arg | 1 | - | | 1054784 | C | G | 602.77 | SNP | Rv0945 | Arg180Gly | 1 | - | | 1068151 | T | C | 1120.77 | SNP | Rv0956 (purN) | silent (His197) | 9912 | - | | 1068432 | A | G | 655.77 | SNP | Rv0957 (purH) | silent (Pro76) | 9926 | - | | 1070702 | T | C | 650.77 | SNP | Rv0958 | Ser274Pro | 12 | - | | 1071966 | A | G | 773.77 | SNP | Rv0959 | Asn238Asp | 42 | - | | 1074558 | G | A | 914.77 | SNP | Rv0962c (lprP) | Pro186Leu | 3 | - | | 1075279 | T | C | 1165.77 | SNP | intergenic |  |  | - | | 1076309 | G | T | 1181.77 | SNP | Rv0964c | Pro124Thr | 5 | - | | 1076689 | A | C | 901.77 | SNP | intergenic |  |  | - | | 1076880 | C | T | 1086.77 | SNP | Rv0965c | silent (Ala106) | 9867 | - | | 1077312 | A | G | 571.77 | SNP | Rv0966c | Val(s)175Ala | 9867 | - | | 1079927 | C | A | 546.77 | SNP | Rv0969 (ctpV) | silent (Thr395) | 9871 | - | | 1080192 | G | A | 548.77 | SNP | Rv0969 (ctpV) | Asp484Asn | 36 | - | | 1081681 | T | C | 859.77 | SNP | Rv0970 | silent (Val210) | 9901 | - | | 1087193 | G | C | 859.77 | SNP | Rv0974c (accD2) | Asn51Lys | 25 | - | | 1090188 | A | AG | 2079.73 | INS | intergenic |  |  | - | | 1093406 | A | G | 736.77 | SNP | Rv0978c (PE\_PGRS17) | silent (Val317) | 9901 | - | | 1094110 | C | T | 51.77 | SNP | Rv0978c (PE\_PGRS17) | Gly83Ser | 16 | - | | 1094228 | T | G | 134.77 | SNP | Rv0978c (PE\_PGRS17) | Gln43His | 20 | - | | 1094375 | C | G | 260.77 | SNP | intergenic |  |  | - | | 1095644 | C | T | 129.84 | SNP | Rv0980c (PE\_PGRS18) | Ala270Thr | 22 | - | | 1095678 | A | G | 126.03 | SNP | Rv0980c (PE\_PGRS18) | silent (Gly258) | 9935 | - | | 1096205 | T | C | 49.77 | SNP | Rv0980c (PE\_PGRS18) | Ser83Gly | 21 | - | | 1096299 | C | G | 47.74 | SNP | Rv0980c (PE\_PGRS18) | silent (Ala51) | 9867 | - | | 1096508 | C | G | 243.80 | SNP | intergenic |  |  | - | | 1096510 | T | C | 245.80 | SNP | intergenic |  |  | - | | 1096567 | A | G | 441.77 | SNP | intergenic |  |  | - | | 1096633 | T | G | 635.77 | SNP | intergenic |  |  | - | | 1097220 | C | T | 867.77 | SNP | Rv0981 (mprA) | silent (Ser133) | 9840 | - | | 1097442 | C | T | 1359.77 | SNP | Rv0981 (mprA) | silent (Asp207) | 9859 | - | | 1098523 | T | A | 875.77 | SNP | Rv0982 (mprB) | Leu339His | 1 | - | | 1099058 | G | A | 981.77 | SNP | intergenic |  |  | - | | 1100234 | T | C | 558.77 | SNP | Rv0983 (pepD) | Leu390Pro | 2 | - | | 1102117 | G | A | 547.77 | SNP | Rv0986 | Val(s)105Val | 13 | - | | 1102468 | C | A | 1006.77 | SNP | Rv0986 | silent (Gly222) | 9935 | - | | 1102646 | G | A | 1193.77 | SNP | Rv0987 | silent (Ala35) | 9867 | - | | 1103656 | C | T | 531.77 | SNP | Rv0987 | Ala372Val | 13 | - | | 1104690 | T | G | 978.77 | SNP | Rv0987 | Phe717Val | 1 | - | | 1105686 | C | G | 1662.77 | SNP | Rv0988 | Leu191Val(s) | 4 | - | | 1105687 | T | C | 1773.77 | SNP | Rv0988 | Leu191Pro | 2 | - | | 1106422 | T | C | 1641.77 | SNP | Rv0989c (grcC2) | Ile321Val | 57 | - | | 1107940 | A | C | 675.77 | SNP | Rv0990c | Ser54Ala | 35 | - | | 1108521 | G | A | 885.77 | SNP | intergenic |  |  | - | | 1109975 | A | G | 1448.77 | SNP | Rv0993 (galU) | Gln235Arg | 10 | - | | 1111678 | G | A | 631.77 | SNP | Rv0995 (rimJ) | Gly23Ser | 16 | - | | 1126889 | G | C | 591.77 | SNP | Rv1007c (metS) | Arg39Gly | 1 | - | | 1127493 | T | C | 554.77 | SNP | Rv1008 (tatD) | silent (Asn135) | 9822 | - | | 1127648 | C | A | 1373.77 | SNP | Rv1008 (tatD) | Thr187Asn | 9 | - | | 1144585 | A | G | 786.77 | SNP | Rv1023 (eno) | Arg8Gly | 1 | - | | 1145848 | A | C | 745.77 | SNP | Rv1023 (eno) | Lys429Gln | 6 | - | | 1148259 | A | G | 1100.77 | SNP | intergenic |  |  | - | | 1148930 | C | T | 1010.77 | SNP | Rv1027c (kdpE) | Gly60Ser | 16 | - | | 1149551 | C | T | 696.77 | SNP | Rv1028c (kdpD) | silent (Glu712) | 9865 | - | | 1150585 | G | A | 879.77 | SNP | Rv1028c (kdpD) | Pro368Ser | 17 | - | | 1151054 | C | G | 781.77 | SNP | Rv1028c (kdpD) | silent (Ser211) | 9840 | - | | 1160770 | T | A | 328.77 | SNP | Rv1037c (esxI) | Gln20Leu | 6 | - | | 1163134 | T | C | 1306.77 | SNP | Rv1040c (PE8) | silent (Gly81) | 9935 | - | | 1164336 | G | A | 799.77 | SNP | intergenic |  |  | - | | 1164619 | C | T | 801.77 | SNP | Rv1041c | Val273Ile | 33 | - | | 1165521 | T | TA | 1733.73 | INS | intergenic |  |  | - | | 1168009 | GC | G | 1002.73 | DEL | Rv1045 |  |  | - | | 1168715 | C | CT | 1796.73 | INS | Rv1046c |  |  | - | | 1168776 | T | G | 893.77 | SNP | Rv1046c | Arg151Ser | 11 | - | | 1172085 | C | A | 729.77 | SNP | Rv1048c | Glu23Asp | 53 | - | | 1173750 | G | T | 566.77 | SNP | Rv1050 | silent (Ala290) | 9867 | - | | 1175343 | C | T | 824.77 | SNP | intergenic |  |  | - | | 1177157 | G | A | 1107.77 | SNP | Rv1054 | Gly77Asp | 6 | - | | 1178116 | T | C | 1413.77 | SNP | Rv1056 | silent (Thr163) | 9871 | - | | 1181405 | C | G | 713.77 | SNP | Rv1058 (fadD14) | Ala241Gly | 21 | - | | 1190093 | A | C | 417.77 | SNP | Rv1067c (PE\_PGRS19) | Leu(s)111Trp | 0 | - | | 1191741 | G | A | 127.03 | SNP | Rv1068c (PE\_PGRS20) | silent (Tyr136) | 9945 | - | | 1194351 | A | G | 794.77 | SNP | Rv1070c (echA8) | silent (His231) | 9912 | - | | 1197157 | T | G | 617.77 | SNP | intergenic |  |  | - | | 1200418 | A | G | 1079.77 | SNP | intergenic |  |  | - | | 1201581 | A | C | 1110.77 | SNP | Rv1076 (lipU) | Gln272Pro | 8 | - | | 1202643 | G | T | 690.77 | SNP | Rv1077 (cbs) | silent (Ala309) | 9867 | - | | 1211369 | A | C | 1037.77 | SNP | Rv1086 | Ser259Arg | 6 | - | | 1212432 | C | A | 62.74 | SNP | Rv1087 (PE\_PGRS21) | silent (Gly291) | 9935 | - | | 1215104 | AT | A | 1808.73 | DEL | intergenic |  |  | - | | 1215742 | G | A | 989.77 | SNP | Rv1090 (celA2b) | silent (Gln48) | 9876 | - | | 1215743 | C | A | 909.77 | SNP | Rv1090 (celA2b) | Gln49Lys | 12 | - | | 1218658 | G | C | 168.90 | SNP | Rv1091 (PE\_PGRS22) | silent (Gly730) | 9935 | - | | 1218896 | G | A | 95.03 | SNP | Rv1091 (PE\_PGRS22) | Gly810Ser | 16 | - | | 1220680 | T | C | 1170.77 | SNP | Rv1093 (glyA1) | Val36Ala | 18 | - | | 1222636 | G | C | 1022.77 | SNP | Rv1094 (desA2) | silent (Gly226) | 9935 | - | | 1224367 | T | C | 693.77 | SNP | intergenic |  |  | - | | 1227830 | G | T | 1305.77 | SNP | Rv1099c (glpX) | silent (Thr285) | 9871 | - | | 1230778 | G | A | 1298.77 | SNP | Rv1102c (mazF3) | Thr65Ile | 7 | - | | 1232778 | GCACGA | G | 4880.73 | DEL | Rv1105 |  |  | - | | 1241386 | C | T | 1831.77 | SNP | Rv1116A | Gly2Asp | 6 | - | | 1248382 | A | G | 876.77 | SNP | Rv1125 | Ser101Gly | 21 | - | | 1248936 | G | C | 722.77 | SNP | Rv1125 | silent (Pro285) | 9926 | - | | 1248978 | T | C | 786.77 | SNP | Rv1125 | silent (Ala299) | 9867 | - | | 1250340 | A | G | 1150.77 | SNP | Rv1127c (ppdK) | silent (Ala355) | 9867 | - | | 1251199 | C | T | 1061.77 | SNP | Rv1127c (ppdK) | Gly69Glu | 4 | - | | 1252053 | AG | A | 1141.73 | DEL | Rv1128c |  |  | - | | 1252164 | T | C | 578.77 | SNP | Rv1128c | Glu270Gly | 7 | - | | 1254562 | A | G | 1043.77 | SNP | Rv1130 (prpD) | Asp3Gly | 11 | - | | 1265070 | G | T | 725.77 | SNP | intergenic |  |  | - | | 1273250 | G | GA | 1265.73 | INS | Rv1145 (mmpL13a) |  |  | - | | 1275957 | T | C | 842.77 | SNP | intergenic |  |  | - | | 1276588 | C | G | 669.77 | SNP | Rv1148c | silent (Ala387) | 9867 | - | | 1276842 | G | A | 43.74 | SNP | Rv1148c | Leu303Leu(s) | 4 | - | | 1276843 | G | C | 48.74 | SNP | Rv1148c | silent (Gly302) | 9935 | - | | 1276846 | C | G | 49.74 | SNP | Rv1148c | silent (Arg301) | 9913 | - | | 1276853 | G | C | 42.74 | SNP | Rv1148c | Ala299Gly | 21 | - | | 1276885 | C | T | 37.74 | SNP | Rv1148c | silent (Gln288) | 9876 | - | | 1277869 | G | GT | 1864.73 | INS | intergenic |  |  | - | | 1281118 | T | C | 1129.77 | SNP | Rv1154c | Thr123Ala | 32 | - | | 1281771 | T | C | 707.77 | SNP | Rv1155 | Ser115Pro | 12 | - | | 1285001 | A | C | 817.77 | SNP | Rv1159 (pimE) | Thr4Pro | 4 | - | | 1286766 | G | C | 1386.77 | SNP | Rv1160 (mutT2) | Gly58Arg | 0 | - | | 1288698 | G | A | 1354.77 | SNP | Rv1161 (narG) | silent (Gly457) | 9935 | - | | 1292102 | A | G | 920.77 | SNP | Rv1162 (narH) | silent (Pro346) | 9926 | - | | 1292679 | AACGGCGCAGCGGC | A | 2159.73 | DEL | Rv1162 (narH) |  |  | - | | 1292693 | G | T | 402.77 | SNP | Rv1162 (narH) | silent (Ala543) | 9867 | - | | 1292698 | TCTTCCCG | T | 1303.73 | DEL | Rv1162 (narH) |  |  | - | | 1292709 | C | G | 398.77 | SNP | Rv1162 (narH) | Pro549Ala | 22 | - | | 1292712 | C | CCGGGAAG | 1286.73 | INS | Rv1162 (narH) |  |  | - | | 1292718 | A | C | 413.77 | SNP | Rv1162 (narH) | Ser552Arg | 6 | - | | 1292719 | GC | G | 635.73 | DEL | Rv1162 (narH) |  |  | - | | 1293333 | C | G | 462.77 | SNP | Rv1163 (narJ) | Pro179Arg | 4 | - | | 1299305 | G | A | 910.77 | SNP | Rv1168c (PPE17) | Pro167Leu | 3 | - | | 1306259 | A | G | 1077.77 | SNP | Rv1175c (fadH) | silent (Ala656) | 9867 | - | | 1307598 | C | G | 833.77 | SNP | Rv1175c (fadH) | Cys210Ser | 11 | - | | 1315191 | A | C | 1018.77 | SNP | Rv1180 (pks3) | STOP489Tyr | 1 | - | | 1315884 | G | A | 1049.77 | SNP | Rv1181 (pks4) | silent (Ala217) | 9867 | - | | 1317655 | C | T | 721.77 | SNP | Rv1181 (pks4) | Leu808Leu(s) | 4 | - | | 1322741 | A | G | 1005.77 | SNP | Rv1183 (mmpL10) | Thr408Ala | 32 | - | | 1327890 | G | A | 1204.77 | SNP | Rv1186c | silent (Asp472) | 9859 | - | | 1328687 | G | C | 987.77 | SNP | Rv1186c | Pro207Ala | 22 | - | | 1329234 | G | A | 519.77 | SNP | Rv1186c | silent (Asp24) | 9859 | - | | 1333669 | T | G | 799.77 | SNP | Rv1190 | silent (Gly230) | 9935 | - | | 1336164 | C | T | 902.77 | SNP | Rv1193 (fadD36) | Pro124Leu | 3 | - | | 1339662 | C | A | 344.77 | SNP | Rv1196 (PPE18) | Thr105Lys | 11 | - | | 1340208 | G | A | 633.77 | SNP | Rv1196 (PPE18) | Arg287Gln | 9 | - | | 1340652 | AG | A | 1894.73 | DEL | intergenic |  |  | - | | 1340657 | A | G | 1081.77 | SNP | intergenic |  |  | - | | 1340667 | A | G | 1149.77 | SNP | Rv1197 (esxK) | silent (Ser3) | 9840 | - | | 1342581 | T | C | 263.80 | SNP | Rv1199c | Thr9Ala | 32 | - | | 1349733 | G | C | 1028.77 | SNP | Rv1206 (fadD6) | Glu134Asp | 53 | - | | 1351172 | A | G | 1433.77 | SNP | intergenic |  |  | - | | 1357308 | T | G | 407.77 | SNP | Rv1214c (PE14) | silent (Ala106) | 9867 | - | | 1360209 | T | C | 835.77 | SNP | Rv1217c | silent (Ala531) | 9867 | - | | 1361190 | G | A | 640.77 | SNP | Rv1217c | silent (Ser204) | 9840 | - | | 1361285 | C | T | 1194.77 | SNP | Rv1217c | Ala173Thr | 22 | - | | 1362006 | T | C | 1326.77 | SNP | Rv1218c | Gln243Arg | 10 | - | | 1365837 | C | CGG | 1380.73 | INS | intergenic |  |  | - | | 1367484 | T | G | 913.77 | SNP | Rv1224 (tatB) | Trp8Gly | 0 | - | | 1368322 | C | CG | 728.73 | INS | Rv1225c |  |  | - | | 1370852 | T | G | 708.77 | SNP | intergenic |  |  | - | | 1374065 | T | C | 689.77 | SNP | Rv1230c | Ser45Gly | 21 | - | | 1374201 | A | G | 710.77 | SNP | intergenic |  |  | - | | 1375044 | C | G | 821.77 | SNP | Rv1232c | silent (Ala375) | 9867 | - | | 1375724 | A | C | 621.77 | SNP | Rv1232c | Cys149Gly | 1 | - | | 1382628 | T | C | 894.77 | SNP | Rv1239c (corA) | Lys139Glu | 4 | - | | 1390763 | C | T | 1218.77 | SNP | Rv1248c | Val(s)764Met(s) | 9867 | - | | 1393626 | A | G | 803.77 | SNP | Rv1249c | silent (Leu119) | 9947 | - | | 1396922 | T | C | 1057.77 | SNP | Rv1251c | silent (Thr773) | 9871 | - | | 1406760 | T | TG | 2175.73 | INS | Rv1258c |  |  | - | | 1411210 | T | G | 675.77 | SNP | Rv1263 (amiB2) | Val260Val(s) | 18 | - | | 1413129 | CG | C | 1233.73 | DEL | intergenic |  |  | - | | 1413148 | C | T | 947.77 | SNP | intergenic |  |  | - | | 1414021 | C | T | 664.77 | SNP | Rv1266c (pknH) | Arg607Gln | 9 | - | | 1416222 | A | G | 172.77 | SNP | Rv1267c (embR) | Phe376Leu | 13 | - | | 1416232 | A | G | 178.77 | SNP | Rv1267c (embR) | silent (Cys372) | 9973 | - | | 1416234 | A | C | 176.77 | SNP | Rv1267c (embR) | Cys372Gly | 1 | - | | 1418863 | C | CGGGAGCCA | 1493.73 | INS | Rv1269c |  |  | - | | 1440469 | C | G | 912.77 | SNP | Rv1286 (cysN) | silent (Pro521) | 9926 | - | | 1443428 | G | A | 123.77 | SNP | intergenic |  |  | - | | 1445781 | A | G | 903.77 | SNP | Rv1291c | silent (Ala18) | 9867 | - | | 1457144 | C | T | 598.79 | SNP | Rv1300 (hemK) | Arg194Cys | 1 | - | | 1468208 | A | C | 651.77 | SNP | Rv1313c | Leu433Arg | 1 | - | | 1468492 | C | A | 111.03 | SNP | Rv1313c | silent (Leu338) | 9947 | - | | 1471659 | C | T | 985.77 | SNP | intergenic |  |  | - | | 1477596 | C | T | 1052.77 | SNP | Rv1316c (ogt) | silent (Gly12) | 9935 | - | | 1477818 | G | A | 790.77 | SNP | Rv1317c (alkA) | Pro434Leu | 3 | - | | 1479085 | T | C | 871.77 | SNP | Rv1317c (alkA) | Ile12Val | 57 | - | | 1480174 | C | G | 134.77 | SNP | Rv1318c | silent (Leu217) | 9947 | - | | 1480176 | G | T | 156.77 | SNP | Rv1318c | Leu217Met(s) | 4 | - | | 1480184 | G | A | 137.77 | SNP | Rv1318c | Pro214Leu | 3 | - | | 1480186 | C | T | 111.77 | SNP | Rv1318c | silent (Ala213) | 9867 | - | | 1480189 | C | G | 135.77 | SNP | Rv1318c | Leu(s)212Phe | 1 | - | | 1480195 | G | T | 106.77 | SNP | Rv1318c | silent (Arg210) | 9913 | - | | 1480197 | G | C | 55.77 | SNP | Rv1318c | Arg210Gly | 1 | - | | 1480198 | T | C | 88.77 | SNP | Rv1318c | silent (Pro209) | 9926 | - | | 1480204 | C | T | 62.77 | SNP | Rv1318c | silent (Pro207) | 9926 | - | | 1480205 | G | A | 77.77 | SNP | Rv1318c | Pro207Leu | 3 | - | | 1480219 | C | T | 97.77 | SNP | Rv1318c | silent (Ala202) | 9867 | - | | 1480231 | G | C | 83.77 | SNP | Rv1318c | Phe198Leu(s) | 2 | - | | 1480233 | A | C | 73.77 | SNP | Rv1318c | Phe198Val | 1 | - | | 1480242 | A | G | 119.77 | SNP | Rv1318c | Leu(s)195Leu | 3 | - | | 1480243 | T | A | 109.77 | SNP | Rv1318c | silent (Ala194) | 9867 | - | | 1480945 | C | G | 382.77 | SNP | Rv1319c | silent (Thr519) | 9871 | - | | 1480948 | C | T | 334.77 | SNP | Rv1319c | silent (Glu518) | 9865 | - | | 1480972 | T | C | 527.77 | SNP | Rv1319c | silent (Glu510) | 9865 | - | | 1481337 | G | A | 344.77 | SNP | Rv1319c | Arg389Trp | 2 | - | | 1482627 | T | C | 1004.77 | SNP | Rv1320c | Thr531Ala | 32 | - | | 1484708 | A | C | 1004.77 | SNP | Rv1321 | Ser144Arg | 6 | - | | 1486630 | A | C | 987.77 | SNP | Rv1323 (fadA4) | Asn257His | 18 | - | | 1490905 | A | G | 1204.77 | SNP | Rv1326c (glgB) | Ser470Pro | 12 | - | | 1498951 | G | T | 594.77 | SNP | Rv1329c (dinG) | Thr80Lys | 11 | - | | 1499274 | C | G | 568.77 | SNP | Rv1330c (pncB1) | Gly429Ala | 21 | - | | 1501700 | G | A | 758.77 | SNP | Rv1333 | Val(s)34Val | 13 | - | | 1526311 | G | T | 1073.77 | SNP | intergenic |  |  | - | | 1526819 | C | A | 861.77 | SNP | Rv1358 | silent (Arg70) | 9913 | - | | 1544255 | C | T | 1720.77 | SNP | Rv1371 | silent (Arg299) | 9913 | - | | 1546465 | G | GC | 1391.73 | INS | Rv1373 |  |  | - | | 1546703 | C | T | 693.77 | SNP | Rv1373 | Pro231Leu | 3 | - | | 1547125 | T | C | 1174.77 | SNP | Rv1374c | Thr136Ala | 32 | - | | 1552547 | G | A | 803.77 | SNP | Rv1378c | Arg37Trp | 2 | - | | 1554418 | G | A | 1097.77 | SNP | Rv1381 (pyrC) | silent (Ala77) | 9867 | - | | 1556787 | T | C | 669.77 | SNP | Rv1383 (carA) | Ser273Pro | 12 | - | | 1561939 | G | C | 1029.77 | SNP | Rv1387 (PPE20) | Glu57Asp | 53 | - | | 1563717 | C | T | 966.77 | SNP | Rv1388 (mihF) | silent (Val8) | 9901 | - | | 1570566 | C | A | 874.77 | SNP | Rv1394c (cyp132) | Arg135Leu | 1 | - | | 1573660 | T | G | 757.77 | SNP | Rv1396c (PE\_PGRS25) | Arg66Ser | 11 | - | | 1576481 | T | G | 828.77 | SNP | Rv1400c (lipI) | Thr106Pro | 4 | - | | 1576527 | G | T | 615.77 | SNP | Rv1400c (lipI) | Phe90Leu | 13 | - | | 1577241 | G | A | 365.77 | SNP | Rv1401 | silent (Pro104) | 9926 | - | | 1587031 | C | A | 1082.77 | SNP | Rv1410c | Ala246Ser | 28 | - | | 1588899 | G | T | 444.77 | SNP | Rv1412 (ribC) | silent (Ala111) | 9867 | - | | 1593331 | G | A | 822.77 | SNP | intergenic |  |  | - | | 1594906 | G | A | 651.77 | SNP | Rv1420 (uvrC) | Val289Ile | 33 | - | | 1595342 | T | C | 798.77 | SNP | Rv1420 (uvrC) | Val(s)434Ala | 9867 | - | | 1606673 | G | T | 1178.77 | SNP | Rv1430 (PE16) | silent (Ala96) | 9867 | - | | 1608276 | A | C | 876.77 | SNP | Rv1431 | Asn65Thr | 13 | - | | 1609840 | A | G | 939.77 | SNP | Rv1431 | silent (Pro586) | 9926 | - | | 1612624 | T | TATCGGTACCGGTGCGCCAG GG | 2574.73 | INS | Rv1435c |  |  | - | | 1613035 | T | C | 1227.77 | SNP | intergenic |  |  | - | | 1616061 | C | T | 839.77 | SNP | Rv1438 (tpi) | silent (Ser166) | 9840 | - | | 1617302 | G | T | 1236.77 | SNP | Rv1439c | Leu29Ile | 9 | - | | 1620181 | C | G | 837.77 | SNP | Rv1442 (bisC) | Leu131Val | 11 | - | | 1624791 | C | G | 638.77 | SNP | Rv1446c (opcA) | Arg192Pro | 5 | - | | 1625332 | C | CGGT | 2504.73 | INS | Rv1446c (opcA) |  |  | - | | 1627351 | T | C | 916.77 | SNP | Rv1448c (tal) | Thr244Ala | 32 | - | | 1630148 | A | C | 1241.77 | SNP | Rv1449c (tkt) | Tyr18Asp | 0 | - | | 1630784 | GC | G | 315.74 | DEL | Rv1450c (PE\_PGRS27) |  |  | - | | 1634609 | C | T | 96.03 | SNP | Rv1450c (PE\_PGRS27) | Ala7Thr | 22 | - | | 1634610 | C | T | 85.03 | SNP | Rv1450c (PE\_PGRS27) | Val(s)6Val | 13 | - | | 1636143 | G | T | 38.77 | SNP | Rv1452c (PE\_PGRS28) | Ala696Asp | 6 | - | | 1636826 | C | A | 86.28 | SNP | Rv1452c (PE\_PGRS28) | silent (Gly468) | 9935 | - | | 1638235 | T | A | 318.77 | SNP | intergenic |  |  | - | | 1638238 | A | T | 365.77 | SNP | intergenic |  |  | - | | 1638364 | C | T | 734.77 | SNP | intergenic |  |  | - | | 1639594 | C | A | 1147.77 | SNP | Rv1453 | Pro405Gln | 6 | - | | 1643864 | T | C | 697.77 | SNP | Rv1458c | Thr133Ala | 32 | - | | 1644362 | C | T | 58.77 | SNP | intergenic |  |  | - | | 1645802 | T | C | 1480.77 | SNP | Rv1459c | Lys113Glu | 4 | - | | 1647436 | G | A | 1151.77 | SNP | Rv1461 | Val150Ile | 33 | - | | 1650072 | A | G | 675.77 | SNP | Rv1462 | Asn183Asp | 42 | - | | 1651308 | A | G | 398.77 | SNP | Rv1463 | Glu198Gly | 7 | - | | 1651706 | C | T | 882.77 | SNP | Rv1464 (csd) | silent (Gly63) | 9935 | - | | 1659502 | G | GA | 1308.73 | INS | Rv1471 (trxB1) |  |  | - | | 1669158 | T | C | 710.77 | SNP | intergenic |  |  | - | | 1670803 | C | G | 417.82 | SNP | Rv1480 | Leu131Val | 11 | - | | 1675193 | C | G | 907.77 | SNP | Rv1485 (hemZ) | silent (Arg59) | 9913 | - | | 1676290 | C | A | 1162.77 | SNP | Rv1486c | Lys198Asn | 13 | - | | 1678706 | A | C | 897.77 | SNP | Rv1489 | Lys52Thr | 8 | - | | 1688064 | A | G | 757.77 | SNP | Rv1497 (lipL) | Met(s)42Val(s) | 9867 | - | | 1688300 | T | C | 636.77 | SNP | Rv1497 (lipL) | silent (Phe120) | 9946 | - | | 1689349 | C | T | 813.77 | SNP | Rv1498c | Arg191His | 8 | - | | 1692141 | A | C | 1046.77 | SNP | Rv1501 | silent (Ile84) | 9872 | - | | 1693561 | A | G | 1289.77 | SNP | Rv1502 | Tyr213Cys | 3 | - | | 1695037 | G | A | 1176.77 | SNP | Rv1504c | silent (Phe36) | 9946 | - | | 1695796 | C | T | 927.77 | SNP | Rv1505c | Ala51Thr | 22 | - | | 1696671 | CA | C | 2505.73 | DEL | intergenic |  |  | - | | 1697896 | C | T | 718.80 | SNP | intergenic |  |  | - | | 1698911 | G | A | 1180.77 | SNP | Rv1508c | silent (Gly328) | 9935 | - | | 1699849 | G | A | 1197.77 | SNP | Rv1508c | Pro16Ser | 17 | - | | 1701215 | C | T | 1156.77 | SNP | intergenic |  |  | - | | 1706119 | T | C | 925.77 | SNP | Rv1514c | silent (Ser159) | 9840 | - | | 1706685 | C | A | 828.77 | SNP | Rv1515c | Gly281Val | 3 | - | | 1709432 | C | T | 1331.77 | SNP | Rv1517 | Leu188Phe | 6 | - | | 1711670 | C | T | 1038.77 | SNP | Rv1520 | Arg215Cys | 1 | - | | 1713192 | A | G | 1381.77 | SNP | Rv1521 (fadD25) | Val297Val(s) | 18 | - | | 1716472 | A | G | 909.77 | SNP | Rv1522c (mmpL12) | Ser381Pro | 12 | - | | 1722200 | C | G | 721.77 | SNP | Rv1527c (pks5) | Val(s)2070Val | 13 | - | | 1722228 | A | C | 605.77 | SNP | Rv1527c (pks5) | Leu2061Arg | 1 | - | | 1728837 | A | G | 983.77 | SNP | intergenic |  |  | - | | 1733134 | C | T | 1377.77 | SNP | Rv1532c | silent (Gln139) | 9876 | - | | 1733325 | C | G | 934.77 | SNP | Rv1532c | Ala76Pro | 13 | - | | 1741352 | C | T | 1042.77 | SNP | Rv1538c (ansA) | Gly281Ser | 16 | - | | 1752561 | T | C | 515.77 | SNP | Rv1548c (PPE21) | Asp258Gly | 11 | - | | 1753519 | G | GC | 1886.73 | INS | Rv1549 (fadD11.1) |  |  | - | | 1756358 | CG | C | 1984.73 | DEL | Rv1551 (plsB1) |  |  | - | | 1759252 | G | T | 978.77 | SNP | Rv1552 (frdA) | silent (Ser524) | 9840 | genotype | | 1760292 | A | G | 1031.77 | SNP | Rv1554 (frdC) | Met(s)40Val(s) | 9867 | - | | 1760688 | C | G | 888.77 | SNP | Rv1555 (frdD) | Pro46Arg | 4 | - | | 1774659 | C | T | 1283.77 | SNP | intergenic |  |  | - | | 1776680 | C | T | 827.77 | SNP | Rv1568 (bioA) | Ala430Val | 13 | - | | 1778430 | T | C | 509.77 | SNP | Rv1570 (bioD) | Met(s)191Thr | 22 | - | | 1789446 | C | T | 103.03 | SNP | Rv1588c | Val131Ile | 33 | - | | 1789516 | A | G | 86.77 | SNP | Rv1588c | silent (Gly107) | 9935 | - | | 1789564 | C | T | 610.77 | SNP | Rv1588c | silent (Arg91) | 9913 | - | | 1789565 | C | A | 568.77 | SNP | Rv1588c | Arg91Leu | 1 | - | | 1789650 | C | T | 858.77 | SNP | Rv1588c | Ala63Thr | 22 | - | | 1789654 | A | G | 831.77 | SNP | Rv1588c | silent (Leu61) | 9947 | - | | 1789671 | C | T | 79.77 | SNP | Rv1588c | Ala56Thr | 22 | - | | 1789675 | A | C | 175.77 | SNP | Rv1588c | silent (Gly54) | 9935 | - | | 1789678 | C | G | 198.77 | SNP | Rv1588c | Val(s)53Val | 13 | - | | 1789795 | A | G | 244.77 | SNP | Rv1588c | silent (Asp14) | 9859 | - | | 1789798 | A | G | 256.77 | SNP | Rv1588c | silent (Phe13) | 9946 | - | | 1792777 | T | C | 1141.77 | SNP | Rv1592c | Ile322Val | 57 | - | | 1792778 | T | C | 1134.77 | SNP | Rv1592c | silent (Glu321) | 9865 | - | | 1798355 | G | A | 1120.77 | SNP | Rv1597 | Gly21Asp | 6 | - | | 1803265 | G | A | 1433.77 | SNP | Rv1602 (hisH) | Ser201Asn | 20 | - | | 1804409 | C | A | 637.77 | SNP | Rv1604 (impA) | Pro124Gln | 6 | - | | 1811375 | G | T | 1044.77 | SNP | Rv1612 (trpB) | silent (Gly83) | 9935 | - | | 1817976 | A | T | 837.77 | SNP | Rv1618 (tesB1) | His121Leu | 4 | - | | 1831220 | A | C | 919.77 | SNP | Rv1629 (polA) | Thr186Pro | 4 | - | | 1831226 | A | G | 1080.77 | SNP | Rv1629 (polA) | Arg188Gly | 1 | - | | 1831288 | C | T | 860.77 | SNP | Rv1629 (polA) | silent (Pro208) | 9926 | - | | 1834177 | A | C | 1299.77 | SNP | Rv1630 (rpsA) | silent (Arg212) | 9913 | genotype | | 1836286 | G | C | 663.77 | SNP | intergenic |  |  | - | | 1839759 | G | C | 1026.77 | SNP | Rv1634 | Gly198Arg | 0 | - | | 1843620 | T | C | 1136.77 | SNP | Rv1637c | Asn25Asp | 42 | - | | 1843786 | C | T | 948.77 | SNP | Rv1638 (uvrA) | Arg16Cys | 1 | - | | 1847919 | C | G | 935.77 | SNP | Rv1639c | silent (Thr180) | 9871 | - | | 1849051 | C | T | 1002.77 | SNP | Rv1640c (lysX) | silent (Pro995) | 9926 | - | | 1849609 | T | C | 828.77 | SNP | Rv1640c (lysX) | silent (Arg809) | 9913 | - | | 1849934 | A | G | 1655.77 | SNP | Rv1640c (lysX) | Ile701Thr | 11 | - | | 1854300 | T | C | 1244.77 | SNP | Rv1644 (tsnR) | Leu232Pro | 2 | - | | 1855907 | G | T | 637.77 | SNP | Rv1646 (PE17) | silent (Ser48) | 9840 | - | | 1856777 | G | C | 918.77 | SNP | Rv1647 | Ala2Pro | 13 | - | | 1859559 | C | A | 1130.77 | SNP | Rv1649 (pheS) | Ala276Asp | 6 | - | | 1877744 | A | C | 658.77 | SNP | Rv1661 (pks7) | Glu814Ala | 17 | - | | 1885385 | T | G | 982.77 | SNP | Rv1662 (pks8) | Leu(s)1228Val(s) | 9867 | - | | 1885772 | G | A | 732.77 | SNP | Rv1662 (pks8) | Ala1357Thr | 22 | - | | 1886155 | G | T | 412.77 | SNP | Rv1662 (pks8) | silent (Leu1484) | 9947 | - | | 1888373 | T | C | 1129.77 | SNP | Rv1664 (pks9) | silent (Ala116) | 9867 | - | | 1892017 | T | C | 1060.77 | SNP | Rv1665 (pks11) | silent (His264) | 9912 | - | | 1894300 | G | GGTCTTGCCGC | 2715.73 | INS | Rv1668c |  |  | - | | 1894422 | A | G | 967.77 | SNP | Rv1668c | silent (Asp307) | 9859 | - | | 1897608 | C | T | 856.77 | SNP | Rv1672c | Leu(s)200Leu | 3 | - | | 1901493 | T | C | 1094.77 | SNP | Rv1676 | silent (Ser149) | 9840 | - | | 1902337 | A | C | 954.77 | SNP | intergenic |  |  | - | | 1906078 | A | G | 289.78 | SNP | Rv1681 (moeX) | Thr277Ala | 32 | - | | 1907296 | G | C | 1026.77 | SNP | Rv1682 | silent (Ala298) | 9867 | - | | 1916137 | A | G | 630.77 | SNP | Rv1691 | silent (Leu63) | 9947 | - | | 1917972 | A | G | 758.77 | SNP | Rv1694 (tlyA) | silent (Leu11) | 9947 | - | | 1924008 | G | A | 815.77 | SNP | Rv1699 (pyrG) | silent (Pro60) | 9926 | - | | 1931179 | C | A | 691.77 | SNP | Rv1704c (cycA) | Arg93Leu | 1 | - | | 1931718 | G | C | 489.77 | SNP | Rv1705c (PPE22) | Leu313Val(s) | 4 | - | | 1933988 | G | A | 1179.77 | SNP | intergenic |  |  | - | | 1944107 | A | G | 1077.77 | SNP | Rv1716 | Ser178Gly | 21 | - | | 1944402 | T | C | 672.77 | SNP | Rv1716 | Val276Ala | 18 | - | | 1945100 | T | C | 784.77 | SNP | Rv1718 | Leu(s)98Leu | 3 | - | | 1950767 | T | C | 1530.77 | SNP | Rv1724c | silent (Lys95) | 9926 | - | | 1955760 | CGAA | C | 2583.73 | DEL | Rv1730c |  |  | - | | 1955913 | CCCGGAAGTCGATGACCGTT | C | 4356.73 | DEL | Rv1730c |  |  | - | | 1955933 | G | T | 477.77 | SNP | Rv1730c | Thr438Lys | 11 | - | | 1955941 | G | C | 491.77 | SNP | Rv1730c | Asp435Glu | 56 | - | | 1960284 | C | A | 731.77 | SNP | Rv1733c | Gln68His | 20 | - | | 1961735 | A | G | 944.77 | SNP | Rv1735c | silent (Ala18) | 9867 | - | | 1967026 | C | T | 676.77 | SNP | Rv1739c | silent (Ala204) | 9867 | - | | 1967237 | C | A | 769.77 | SNP | Rv1739c | Arg134Leu | 1 | - | | 1967543 | C | A | 841.77 | SNP | Rv1739c | Gly32Val | 3 | - | | 1971725 | G | C | 569.77 | SNP | Rv1745c (idi) | silent (Arg89) | 9913 | - | | 1980652 | G | T | 438.77 | SNP | Rv1751 | silent (Pro344) | 9926 | - | | 1983195 | A | G | 151.90 | SNP | Rv1753c (PPE24) | silent (Gly527) | 9935 | - | | 1983198 | C | G | 190.84 | SNP | Rv1753c (PPE24) | Val(s)526Val | 13 | - | | 1983235 | T | G | 319.94 | SNP | Rv1753c (PPE24) | Asn514Thr | 13 | - | | 1983239 | A | G | 364.77 | SNP | Rv1753c (PPE24) | Leu(s)513Leu | 3 | - | | 1983291 | C | T | 747.77 | SNP | Rv1753c (PPE24) | silent (Pro495) | 9926 | - | | 1983313 | T | G | 839.77 | SNP | Rv1753c (PPE24) | Asn488Thr | 13 | - | | 1984076 | GGTTGGCGTTGCCGATGTTG GCAAGGCCGTT | G | 5744.73 | DEL | Rv1753c (PPE24) |  |  | - | | 2003105 | C | T | 783.77 | SNP | Rv1769 | silent (Phe160) | 9946 | - | | 2003827 | A | C | 443.96 | SNP | Rv1769 | Asp401Ala | 10 | - | | 2009290 | GC | G | 947.73 | DEL | Rv1775 |  |  | - | | 2010614 | G | A | 564.77 | SNP | intergenic |  |  | - | | 2022868 | T | C | 529.77 | SNP | Rv1783 (eccC5) | silent (Ser1204) | 9840 | - | | 2023628 | C | G | 937.77 | SNP | Rv1785c (cyp143) | Gly334Ala | 21 | - | | 2033418 | C | T | 1480.77 | SNP | Rv1795 (eccD5) | silent (Ala393) | 9867 | - | | 2045310 | A | G | 207.84 | SNP | Rv1803c (PE\_PGRS32) | silent (Ile511) | 9872 | - | | 2049065 | T | C | 865.77 | SNP | intergenic |  |  | - | | 2049097 | G | C | 772.77 | SNP | intergenic |  |  | - | | 2049663 | T | G | 984.77 | SNP | intergenic |  |  | - | | 2050822 | G | C | 600.77 | SNP | Rv1808 (PPE32) | Gly301Ala | 21 | - | | 2051746 | T | C | 718.77 | SNP | Rv1809 (PPE33) | silent (Ala155) | 9867 | - | | 2052035 | G | T | 1059.77 | SNP | Rv1809 (PPE33) | Val(s)252Leu(s) | 9867 | - | | 2055271 | A | G | 1041.77 | SNP | Rv1812c | Leu30Pro | 2 | - | | 2057774 | A | T | 778.77 | SNP | Rv1815 | Ile83Phe | 8 | - | | 2061958 | A | G | 30.74 | SNP | Rv1818c (PE\_PGRS33) | silent (Ala239) | 9867 | - | | 2067684 | G | A | 1589.77 | SNP | Rv1821 (secA2) | Val(s)410Met(s) | 9867 | - | | 2069771 | C | A | 1090.77 | SNP | Rv1823 | Gln24Lys | 12 | - | | 2072313 | C | A | 534.77 | SNP | Rv1826 (gcvH) | Thr121Lys | 11 | - | | 2074570 | G | C | 39.77 | SNP | intergenic |  |  | - | | 2074754 | C | T | 990.77 | SNP | intergenic |  |  | - | | 2078246 | C | G | 1263.77 | SNP | Rv1832 (gcvB) | silent (Gly790) | 9935 | - | | 2078312 | C | T | 1055.77 | SNP | Rv1832 (gcvB) | silent (Asp812) | 9859 | - | | 2082053 | A | G | 690.77 | SNP | Rv1835c | Cys179Arg | 1 | - | | 2086672 | C | T | 643.77 | SNP | Rv1837c (glcB) | Gly104Ser | 16 | - | | 2093991 | C | T | 1136.77 | SNP | Rv1844c (gnd1) | Ala400Thr | 22 | - | | 2094911 | ACAGCGT | A | 2525.73 | DEL | Rv1844c (gnd1) |  |  | - | | 2096186 | A | G | 838.77 | SNP | Rv1846c (blaI) | silent (Thr138) | 9871 | - | | 2097270 | C | T | 722.77 | SNP | Rv1847 | Arg132Trp | 2 | - | | 2108141 | T | C | 627.77 | SNP | Rv1860 (apa) | Phe136Leu | 13 | - | | 2108890 | A | C | 792.77 | SNP | intergenic |  |  | - | | 2109523 | C | CG | 1787.73 | INS | intergenic |  |  | - | | 2112832 | A | C | 857.77 | SNP | Rv1865c | silent (Ala45) | 9867 | - | | 2116903 | C | T | 708.77 | SNP | Rv1867 | silent (Gly380) | 9935 | - | | 2122395 | C | T | 600.77 | SNP | Rv1872c (lldD2) | Val(s)253Met(s) | 9867 | - | | 2122976 | C | G | 1122.77 | SNP | Rv1872c (lldD2) | Gly59Ala | 21 | - | | 2123169 | T | G | 1428.77 | SNP | intergenic |  |  | - | | 2128870 | A | G | 676.77 | SNP | Rv1878 (glnA3) | silent (Leu283) | 9947 | - | | 2133468 | T | TTCGCATGCCGTCACC | 2074.73 | INS | Rv1883c |  |  | - | | 2135154 | G | T | 759.77 | SNP | Rv1886c (fbpB) | silent (Pro238) | 9926 | - | | 2135870 | T | C | 849.77 | SNP | intergenic |  |  | - | | 2137521 | A | ACTCCGATCAC | 5047.73 | INS | Rv1888c |  |  | - | | 2138436 | A | G | 904.77 | SNP | intergenic |  |  | - | | 2138453 | C | T | 762.77 | SNP | Rv1888A | silent (Gly55) | 9935 | - | | 2138730 | G | T | 737.78 | SNP | Rv1889c | Asp96Glu | 56 | - | | 2142266 | C | A | 677.77 | SNP | intergenic |  |  | - | | 2143328 | G | C | 906.77 | SNP | Rv1895 | Val(s)270Leu | 3 | - | | 2147022 | A | C | 1416.77 | SNP | Rv1900c (lipJ) | Ile204Met(s) | 6 | - | | 2151222 | T | G | 641.77 | SNP | Rv1904 | Leu(s)90Trp | 0 | - | | 2153725 | CTCATCCCCGTCTCG | C | 4746.73 | DEL | Rv1907c |  |  | - | | 2154724 | C | A | 900.77 | SNP | Rv1908c (katG) | Arg463Leu | 1 | genotype | | 2155168 | C | G | 979.77 | SNP | Rv1908c (katG) | Ser315Thr | 32 | resistance | | 2158109 | T | C | 658.77 | SNP | Rv1912c (fadB5) | Asp328Gly | 11 | - | | 2160998 | G | A | 880.77 | SNP | Rv1915 (aceAa) | Gly179Asp | 6 | - | | 2161343 | G | GT | 1261.73 | INS | Rv1915 (aceAa) |  |  | - | | 2163375 | T | C | 226.77 | SNP | Rv1917c (PPE34) | Asn1313Asp | 42 | - | | 2163412 | A | G | 1130.77 | SNP | Rv1917c (PPE34) | silent (Val1300) | 9901 | - | | 2163415 | C | A | 1035.77 | SNP | Rv1917c (PPE34) | silent (Pro1299) | 9926 | - | | 2163417 | G | C | 1078.77 | SNP | Rv1917c (PPE34) | Pro1299Ala | 22 | - | | 2163419 | C | T | 909.77 | SNP | Rv1917c (PPE34) | Ser1298Asn | 20 | - | | 2163421 | C | G | 786.77 | SNP | Rv1917c (PPE34) | silent (Thr1297) | 9871 | - | | 2163444 | T | C | 36.77 | SNP | Rv1917c (PPE34) | Asn1290Asp | 42 | - | | 2163493 | A | G | 80.77 | SNP | Rv1917c (PPE34) | silent (Ala1273) | 9867 | - | | 2163494 | G | C | 85.77 | SNP | Rv1917c (PPE34) | Ala1273Gly | 21 | - | | 2163496 | A | C | 60.77 | SNP | Rv1917c (PPE34) | silent (Gly1272) | 9935 | - | | 2163504 | G | A | 93.77 | SNP | Rv1917c (PPE34) | Leu1270Leu(s) | 4 | - | | 2163510 | T | C | 113.77 | SNP | Rv1917c (PPE34) | Ile1268Val | 57 | - | | 2163517 | A | C | 95.77 | SNP | Rv1917c (PPE34) | silent (Gly1265) | 9935 | - | | 2163520 | G | A | 37.77 | SNP | Rv1917c (PPE34) | silent (Phe1264) | 9946 | - | | 2163790 | A | C | 614.77 | SNP | Rv1917c (PPE34) | silent (Pro1174) | 9926 | - | | 2165286 | A | C | 784.77 | SNP | Rv1917c (PPE34) | Ser676Ala | 35 | - | | 2165503 | T | A | 658.77 | SNP | Rv1917c (PPE34) | silent (Ala603) | 9867 | - | | 2165938 | C | T | 281.77 | SNP | Rv1917c (PPE34) | silent (Pro458) | 9926 | - | | 2167926 | A | G | 765.77 | SNP | Rv1918c (PPE35) | Leu(s)896Ser | 28 | - | | 2172380 | A | C | 1062.77 | SNP | Rv1920 | Glu253Ala | 17 | - | | 2173860 | A | C | 681.77 | SNP | intergenic |  |  | - | | 2177366 | C | T | 994.77 | SNP | Rv1925 (fadD31) | Arg94Cys | 1 | - | | 2179825 | G | C | 1152.77 | SNP | Rv1927 | Glu51Asp | 53 | - | | 2185674 | A | G | 742.77 | SNP | Rv1934c (fadE17) | Ile172Thr | 11 | - | | 2187587 | G | C | 2035.77 | SNP | Rv1936 | Gln68His | 20 | - | | 2191498 | G | T | 1090.77 | SNP | Rv1938 (ephB) | Gly158Trp | 0 | - | | 2192250 | G | T | 41.77 | SNP | Rv1939 | Val53Phe | 0 | - | | 2196715 | G | C | 227.77 | SNP | Rv1945 | Val243Leu | 15 | - | | 2199052 | C | G | 890.77 | SNP | Rv1948c | Gly5Arg | 0 | - | | 2202500 | C | T | 677.77 | SNP | Rv1956 (higA) | silent (His121) | 9912 | - | | 2207591 | T | TC | 2347.73 | INS | intergenic |  |  | - | | 2209465 | G | A | 1094.77 | SNP | Rv1966 (mce3A) | Ala47Thr | 22 | - | | 2211826 | A | G | 391.77 | SNP | Rv1968 (mce3C) | silent (Lys67) | 9926 | - | | 2216443 | C | A | 978.77 | SNP | Rv1971 (mce3F) | Ala396Glu | 10 | - | | 2216963 | C | G | 1141.77 | SNP | Rv1972 | His124Gln | 23 | - | | 2220482 | CA | C | 1722.73 | DEL | Rv1977 |  |  | - | | 2220512 | T | G | 916.77 | SNP | Rv1977 | silent (Ser253) | 9840 | - | | 2221584 | G | C | 1294.77 | SNP | Rv1978 | Ser226Thr | 32 | - | | 2223293 | T | C | 873.77 | SNP | intergenic |  |  | - | | 2225365 | T | C | 1075.77 | SNP | intergenic |  |  | - | | 2228967 | A | G | 414.77 | SNP | intergenic |  |  | - | | 2229801 | C | G | 986.77 | SNP | Rv1985c | silent (Pro34) | 9926 | - | | 2230045 | G | A | 1310.77 | SNP | Rv1986 | Cys12Tyr | 3 | - | | 2235087 | G | A | 864.77 | SNP | Rv1992c (ctpG) | silent (Phe740) | 9946 | - | | 2241031 | AG | A | 1265.73 | DEL | Rv1997 (ctpF) |  |  | - | | 2244421 | G | T | 537.77 | SNP | Rv1999c | Leu240Met(s) | 4 | - | | 2245864 | C | A | 900.77 | SNP | Rv2000 | Pro219Gln | 6 | - | | 2247677 | A | C | 1399.77 | SNP | Rv2002 (fabG3) | silent (Ile6) | 9872 | - | | 2251313 | T | C | 1226.77 | SNP | Rv2005c | Ile191Val | 57 | - | | 2251605 | G | A | 601.77 | SNP | Rv2005c | silent (Ser93) | 9840 | - | | 2251999 | A | G | 1237.77 | SNP | intergenic |  |  | - | | 2255482 | G | A | 1297.77 | SNP | Rv2006 (otsB1) | Gly1161Ser | 16 | - | | 2260100 | C | T | 1210.77 | SNP | intergenic |  |  | - | | 2260525 | C | T | 514.77 | SNP | intergenic |  |  | - | | 2264782 | C | A | 940.77 | SNP | Rv2017 | Ala262Glu | 10 | - | | 2265059 | T | G | 957.77 | SNP | intergenic |  |  | - | | 2266487 | G | C | 878.77 | SNP | Rv2020c | silent (Leu78) | 9947 | - | | 2266504 | T | TA | 1356.73 | INS | Rv2020c |  |  | - | | 2266508 | A | T | 631.77 | SNP | Rv2020c | Asp71Glu | 56 | - | | 2266511 | GT | G | 1166.73 | DEL | Rv2020c |  |  | - | | 2266517 | T | C | 738.77 | SNP | Rv2020c | silent (Glu68) | 9865 | - | | 2266550 | G | T | 812.77 | SNP | Rv2020c | silent (Gly57) | 9935 | - | | 2266553 | C | G | 853.77 | SNP | Rv2020c | silent (Ser56) | 9840 | - | | 2266583 | C | G | 614.77 | SNP | Rv2020c | Glu46Asp | 53 | - | | 2266598 | G | C | 645.77 | SNP | Rv2020c | silent (Leu41) | 9947 | - | | 2266604 | C | G | 624.77 | SNP | Rv2020c | silent (Ser39) | 9840 | - | | 2266613 | G | GC | 1056.73 | INS | Rv2020c |  |  | - | | 2266624 | G | T | 596.77 | SNP | Rv2020c | Leu33Ile | 9 | - | | 2267015 | T | C | 1354.77 | SNP | Rv2021c | silent (Ala32) | 9867 | - | | 2268433 | C | A | 539.10 | SNP | intergenic |  |  | - | | 2268627 | G | C | 1690.77 | SNP | intergenic |  |  | - | | 2269545 | G | A | 822.77 | SNP | Rv2024c | silent (Ser232) | 9840 | - | | 2269780 | T | C | 742.77 | SNP | Rv2024c | Asp154Gly | 11 | - | | 2270102 | A | G | 1117.77 | SNP | Rv2024c | Trp47Arg | 8 | - | | 2273733 | TC | T | 1504.73 | DEL | Rv2027c (dosT) |  |  | - | | 2282787 | C | T | 535.77 | SNP | Rv2037c | Cys312Tyr | 3 | - | | 2283191 | G | C | 1083.77 | SNP | Rv2037c | silent (Ala177) | 9867 | - | | 2285251 | C | A | 1161.77 | SNP | Rv2039c | Val131Phe | 0 | - | | 2287121 | A | G | 618.77 | SNP | Rv2041c | silent (Asp242) | 9859 | - | | 2288085 | G | C | 954.77 | SNP | Rv2042c | silent (Ala199) | 9867 | - | | 2288704 | C | A | 1129.77 | SNP | Rv2043c (pncA) | Val180Phe | 0 | resistance | | 2294007 | T | C | 834.77 | SNP | Rv2047c | Thr174Ala | 32 | - | | 2295685 | C | A | 745.77 | SNP | Rv2048c (pks12) | Val(s)3768Leu(s) | 9867 | - | | 2296042 | G | C | 1148.77 | SNP | Rv2048c (pks12) | Pro3649Ala | 22 | - | | 2296181 | A | G | 467.77 | SNP | Rv2048c (pks12) | silent (Gly3602) | 9935 | - | | 2298194 | G | T | 716.77 | SNP | Rv2048c (pks12) | silent (Ala2931) | 9867 | - | | 2300237 | A | G | 341.77 | SNP | Rv2048c (pks12) | silent (Ala2250) | 9867 | - | | 2300546 | A | T | 751.77 | SNP | Rv2048c (pks12) | His2147Gln | 23 | - | | 2300552 | T | G | 660.77 | SNP | Rv2048c (pks12) | silent (Pro2145) | 9926 | - | | 2300555 | A | G | 676.77 | SNP | Rv2048c (pks12) | silent (Asp2144) | 9859 | - | | 2302033 | G | A | 539.77 | SNP | Rv2048c (pks12) | Arg1652Cys | 1 | - | | 2311099 | C | G | 394.77 | SNP | Rv2052c | silent (Pro473) | 9926 | - | | 2314689 | C | A | 807.77 | SNP | Rv2057c (rpmG1) | Arg46Leu | 1 | - | | 2315386 | C | A | 578.77 | SNP | Rv2059 | His71Gln | 23 | - | | 2316123 | C | A | 473.77 | SNP | Rv2059 | Thr317Lys | 11 | - | | 2318725 | C | T | 469.77 | SNP | Rv2062c (cobN) | Glu677Lys | 7 | - | | 2320320 | C | T | 671.77 | SNP | Rv2062c (cobN) | Arg145His | 8 | - | | 2321975 | T | C | 474.77 | SNP | Rv2064 (cobG) | silent (Asp175) | 9859 | - | | 2325895 | C | G | 676.77 | SNP | Rv2068c (blaC) | Val(s)305Val | 13 | - | | 2328543 | T | C | 512.77 | SNP | Rv2071c (cobM) | Ile145Met(s) | 6 | - | | 2329533 | A | G | 562.77 | SNP | Rv2072c (cobL) | Leu205Pro | 2 | - | | 2331620 | A | C | 684.77 | SNP | Rv2075c | silent (Gly420) | 9935 | - | | 2331789 | G | T | 962.77 | SNP | Rv2075c | Pro364Gln | 6 | - | | 2334007 | A | G | 1082.77 | SNP | Rv2077c | silent (Ala96) | 9867 | - | | 2335075 | A | G | 828.77 | SNP | Rv2078 | Glu6Gly | 7 | - | | 2335494 | A | G | 888.77 | SNP | Rv2079 | Tyr47Cys | 3 | - | | 2337179 | C | T | 1087.77 | SNP | Rv2079 | Gln609STOP | 8 | - | | 2338810 | T | C | 819.77 | SNP | Rv2082 | silent (Arg34) | 9913 | - | | 2338811 | A | G | 775.77 | SNP | Rv2082 | Lys35Glu | 4 | - | | 2339255 | A | G | 989.77 | SNP | Rv2082 | Thr183Ala | 32 | - | | 2339835 | C | T | 300.78 | SNP | Rv2082 | Pro376Leu | 3 | - | | 2340621 | C | G | 888.77 | SNP | Rv2082 | Pro638Arg | 4 | - | | 2341636 | C | G | 393.77 | SNP | Rv2083 | Leu256Val(s) | 4 | - | | 2342649 | A | AGGCGTACACAC | 6065.73 | INS | Rv2084 |  |  | - | | 2344246 | G | C | 915.77 | SNP | intergenic |  |  | - | | 2344499 | T | G | 1107.77 | SNP | Rv2088 (pknJ) | Leu30Arg | 1 | - | | 2345037 | C | A | 464.77 | SNP | Rv2088 (pknJ) | silent (Leu209) | 9947 | - | | 2346318 | G | A | 797.77 | SNP | Rv2089c (pepE) | Pro336Leu | 3 | - | | 2348446 | C | G | 546.77 | SNP | Rv2090 | Phe358Leu(s) | 2 | - | | 2354791 | A | G | 1269.77 | SNP | Rv2096c (pafB) | Ser174Pro | 12 | - | | 2358029 | T | TG | 999.73 | INS | intergenic |  |  | - | | 2358525 | A | C | 649.77 | SNP | Rv2100 | Asp46Ala | 10 | - | | 2361604 | C | G | 458.77 | SNP | Rv2101 (helZ) | Val455Val(s) | 18 | - | | 2362041 | C | A | 1023.77 | SNP | Rv2101 (helZ) | Pro601Gln | 6 | - | | 2364475 | T | C | 712.77 | SNP | Rv2103c (vapC37) | Thr16Ala | 32 | - | | 2368564 | TA | T | 1868.73 | DEL | intergenic |  |  | - | | 2369186 | G | C | 896.77 | SNP | Rv2109c (prcA) | Arg182Gly | 1 | - | | 2369326 | C | G | 1101.77 | SNP | Rv2109c (prcA) | Arg135Pro | 5 | - | | 2381572 | AC | A | 1403.73 | DEL | Rv2123 (PPE37) |  |  | - | | 2386389 | G | A | 701.77 | SNP | Rv2125 | Gly33Ser | 16 | - | | 2387733 | T | C | 217.84 | SNP | Rv2126c (PE\_PGRS37) | silent (Glu80) | 9865 | - | | 2388641 | G | A | 792.77 | SNP | Rv2127 (ansP1) | Gly9Asp | 6 | - | | 2399734 | G | A | 623.77 | SNP | Rv2139 (pyrD) | Gly339Ser | 16 | - | | 2401825 | T | C | 197.77 | SNP | intergenic |  |  | - | | 2406842 | AC | A | 2239.73 | DEL | Rv2147c; Rv2148c |  |  | - | | 2411496 | G | A | 1158.77 | SNP | Rv2152c (murC) | silent (Phe209) | 9946 | - | | 2413246 | C | A | 551.77 | SNP | Rv2153c (murG) | Val(s)36Leu(s) | 9867 | - | | 2415656 | G | C | 564.77 | SNP | Rv2155c (murD) | Arg247Gly | 1 | - | | 2421816 | A | G | 617.77 | SNP | Rv2160A; Rv2160c | Cys155Arg; Val(s)63Ala | 1; 9867 | - | | 2421975 | G | GGGAA | 1799.73 | INS | Rv2160A; Rv2160c |  |  | - | | 2424925 | A | G | 456.77 | SNP | intergenic |  |  | - | | 2425471 | T | C | 1210.77 | SNP | Rv2163c (pbpB) | silent (Arg539) | 9913 | - | | 2439401 | A | G | 99.77 | SNP | Rv2177c | Tyr183His | 4 | - | | 2440926 | G | T | 1345.77 | SNP | Rv2178c (aroG) | Asp265Glu | 56 | - | | 2442468 | C | T | 1123.77 | SNP | Rv2180c | Trp249STOP | 0 | - | | 2448458 | C | T | 735.77 | SNP | Rv2187 (fadD15) | Thr100Ile | 7 | - | | 2451700 | A | C | 719.77 | SNP | Rv2189c | silent (Ala107) | 9867 | - | | 2462871 | G | A | 525.77 | SNP | Rv2198c (mmpS3) | silent (Ala59) | 9867 | - | | 2470591 | A | C | 913.77 | SNP | intergenic |  |  | - | | 2491597 | G | T | 737.77 | SNP | Rv2221c (glnE) | silent (Arg253) | 9913 | - | | 2499726 | G | A | 1013.77 | SNP | Rv2226 | Asp299Asn | 36 | - | | 2501114 | GC | G | 1150.73 | DEL | Rv2227 |  |  | - | | 2505085 | G | A | 788.77 | SNP | Rv2231c (cobC) | silent (Ala205) | 9867 | genotype | | 2509140 | G | C | 509.77 | SNP | Rv2236c (cobD) | Ser79Cys | 5 | - | | 2509722 | A | G | 789.77 | SNP | Rv2237 | silent (Pro78) | 9926 | - | | 2510350 | C | G | 983.77 | SNP | intergenic |  |  | - | | 2516567 | G | C | 1375.77 | SNP | intergenic |  |  | - | | 2521342 | T | C | 940.77 | SNP | Rv2247 (accD6) | silent (Asp200) | 9859 | - | | 2521428 | A | G | 974.77 | SNP | Rv2247 (accD6) | Asp229Gly | 11 | - | | 2522955 | T | G | 768.77 | SNP | Rv2248 | Val199Gly | 5 | - | | 2523205 | G | GCGC | 954.73 | INS | intergenic |  |  | - | | 2525722 | CG | C | 810.73 | DEL | Rv2250A; Rv2251 |  |  | - | | 2526974 | T | C | 835.77 | SNP | Rv2251 | silent (Pro470) | 9926 | - | | 2529680 | A | G | 668.77 | SNP | Rv2256c | silent (Thr65) | 9871 | - | | 2531742 | A | G | 667.77 | SNP | Rv2258c | silent (Ala52) | 9867 | - | | 2532017 | G | C | 209.77 | SNP | intergenic |  |  | - | | 2533377 | T | C | 819.77 | SNP | Rv2260 | silent (Asp16) | 9859 | - | | 2534562 | GGA | G | 1420.73 | DEL | Rv2262c |  |  | - | | 2536599 | C | T | 455.77 | SNP | Rv2264c | silent (Gly584) | 9935 | - | | 2536628 | G | GA | 532.73 | INS | Rv2264c |  |  | - | | 2536892 | T | C | 230.18 | SNP | Rv2264c | Thr487Ala | 32 | - | | 2543395 | T | C | 1198.77 | SNP | Rv2268c (cyp128) | silent (Glu294) | 9865 | - | | 2546684 | C | A | 781.77 | SNP | Rv2274c (mazF8) | Gly41Val | 3 | - | | 2548700 | C | T | 1189.77 | SNP | Rv2276 (cyp121) | His318Tyr | 4 | - | | 2551576 | C | G | 1349.77 | SNP | Rv2280 | Ala6Gly | 21 | - | | 2551577 | T | C | 1373.77 | SNP | Rv2280 | silent (Ala6) | 9867 | - | | 2562644 | G | T | 1241.77 | SNP | Rv2290 (lppO) | Ala16Ser | 28 | - | | 2564368 | G | GC | 1144.73 | INS | Rv2293c |  |  | - | | 2566766 | C | CG | 638.73 | INS | intergenic |  |  | - | | 2566768 | A | C | 360.77 | SNP | intergenic |  |  | - | | 2573756 | C | A | 817.77 | SNP | intergenic |  |  | - | | 2578626 | A | G | 979.77 | SNP | Rv2307c | Met(s)24Thr | 22 | - | | 2581052 | G | A | 968.77 | SNP | Rv2308 | Asp212Asn | 36 | - | | 2586076 | C | G | 666.77 | SNP | Rv2314c | silent (Arg405) | 9913 | - | | 2586127 | A | G | 816.77 | SNP | Rv2314c | silent (Gly388) | 9935 | - | | 2589036 | G | C | 1330.77 | SNP | Rv2316 (uspA) | Asp67His | 3 | - | | 2589216 | G | C | 1493.77 | SNP | Rv2316 (uspA) | Val(s)127Leu | 3 | - | | 2592310 | G | A | 281.77 | SNP | Rv2319c | silent (Ile139) | 9872 | - | | 2598400 | A | G | 1166.77 | SNP | Rv2326c | silent (Asn516) | 9822 | - | | 2600260 | A | G | 808.77 | SNP | Rv2327 | silent (Arg91) | 9913 | - | | 2601576 | T | G | 1229.77 | SNP | Rv2328 (PE23) | Ser282Arg | 6 | - | | 2601760 | G | A | 1045.77 | SNP | Rv2328 (PE23) | Ala344Thr | 22 | - | | 2608117 | C | A | 1256.77 | SNP | Rv2333c (stp) | Asp69Tyr | 0 | - | | 2610381 | C | T | 931.77 | SNP | Rv2335 (cysE) | Pro217Leu | 3 | - | | 2612256 | G | T | 741.77 | SNP | Rv2337c | silent (Leu244) | 9947 | - | | 2612632 | C | A | 735.77 | SNP | Rv2337c | Gly119Val | 3 | - | | 2614909 | GC | G | 1391.73 | DEL | Rv2339 (mmpL9) |  |  | - | | 2619271 | T | C | 747.77 | SNP | intergenic |  |  | - | | 2625924 | T | C | 703.77 | SNP | Rv2346c (esxO) | silent (Ala83) | 9867 | - | | 2626004 | G | A | 685.77 | SNP | Rv2346c (esxO) | Leu57Leu(s) | 4 | - | | 2626011 | G | A | 677.77 | SNP | Rv2346c (esxO) | silent (Ile54) | 9872 | - | | 2626018 | T | C | 671.77 | SNP | Rv2346c (esxO) | Glu52Gly | 7 | - | | 2626513 | T | A | 464.78 | SNP | Rv2347c (esxP) | Thr3Ser | 38 | - | | 2626514 | T | G | 466.77 | SNP | Rv2347c (esxP) | silent (Ala2) | 9867 | - | | 2627618 | C | A | 677.77 | SNP | Rv2349c (plcC) | Gly361Cys | 0 | - | | 2627946 | A | G | 605.77 | SNP | Rv2349c (plcC) | silent (Arg251) | 9913 | - | | 2630740 | T | C | 954.77 | SNP | Rv2351c (plcA) | Thr446Ala | 32 | - | | 2631371 | C | T | 555.77 | SNP | Rv2351c (plcA) | Val(s)235Val | 13 | - | | 2632341 | C | CA | 1097.73 | INS | intergenic |  |  | - | | 2634282 | T | G | 579.77 | SNP | intergenic |  |  | - | | 2637162 | C | T | 1003.77 | SNP | intergenic |  |  | - | | 2637541 | C | T | 767.77 | SNP | intergenic |  |  | - | | 2638997 | G | A | 361.77 | SNP | Rv2356c (PPE40) | Ser180Leu(s) | 35 | - | | 2642383 | C | T | 748.77 | SNP | Rv2360c | Ala66Thr | 22 | - | | 2647905 | G | A | 757.77 | SNP | Rv2366c | Arg155Cys | 1 | - | | 2656225 | A | G | 1145.77 | SNP | Rv2377c (mbtH) | Val69Ala | 18 | - | | 2660319 | C | G | 823.77 | SNP | Rv2379c (mbtF) | Glu589Asp | 53 | - | | 2663795 | G | A | 504.77 | SNP | Rv2380c (mbtE) | silent (Ala1107) | 9867 | - | | 2673818 | C | G | 800.77 | SNP | Rv2383c (mbtB) | Val(s)674Leu | 3 | - | | 2680658 | T | G | 935.77 | SNP | intergenic |  |  | - | | 2695378 | C | G | 954.77 | SNP | Rv2398c (cysW) | Gly141Ala | 21 | - | | 2697489 | C | T | 939.77 | SNP | Rv2400c (subI) | Ala76Thr | 22 | - | | 2703902 | C | CG | 948.73 | INS | intergenic |  |  | - | | 2704884 | A | ACAGCGACCATATCGCCGAG CT | 1330.73 | INS | Rv2407 |  |  | - | | 2706663 | G | T | 1742.77 | SNP | Rv2408 (PE24); Rv2409c | Gly216Val; Thr224Asn | 3; 9 | - | | 2711722 | T | G | 634.77 | SNP | Rv2414c | silent (Pro385) | 9926 | - | | 2713795 | C | T | 937.77 | SNP | intergenic |  |  | - | | 2714526 | GGT | G | 1808.73 | DEL | Rv2416c (eis) |  |  | - | | 2718852 | T | G | 1077.77 | SNP | intergenic |  |  | - | | 2720952 | T | C | 299.78 | SNP | Rv2424c | Thr276Ala | 32 | - | | 2720954 | G | A | 285.78 | SNP | Rv2424c | Thr275Ile | 7 | - | | 2721562 | C | G | 1234.77 | SNP | Rv2424c | silent (Ala72) | 9867 | - | | 2723506 | T | C | 753.77 | SNP | Rv2426c | Leu226Leu(s) | 4 | - | | 2724180 | TCACGATCGGGTCTCCTCTA G | T | 4554.73 | DEL | Rv2426c |  |  | - | | 2729058 | A | C | 641.77 | SNP | Rv2433c | Leu26Arg | 1 | - | | 2734074 | T | C | 114.03 | SNP | Rv2436 (rbsK) | Val282Ala | 18 | - | | 2734481 | TA | T | 1824.73 | DEL | Rv2437 |  |  | - | | 2737572 | C | A | 753.77 | SNP | Rv2439c (proB) | Ala226Ser | 28 | - | | 2738274 | A | C | 888.77 | SNP | Rv2440c (obg) | Ser471Arg | 6 | - | | 2739546 | G | C | 685.77 | SNP | Rv2440c (obg) | Val47Val(s) | 18 | - | | 2740693 | T | C | 680.77 | SNP | intergenic |  |  | - | | 2741209 | G | A | 1527.77 | SNP | Rv2443 (dctA) | silent (Leu167) | 9947 | - | | 2751804 | C | T | 540.77 | SNP | Rv2450c (rpfE) | Arg126Gln | 9 | - | | 2752122 | G | C | 303.90 | SNP | Rv2450c (rpfE) | Thr20Arg | 1 | genotype | | 2752698 | C | A | 1568.77 | SNP | intergenic |  |  | - | | 2760152 | A | G | 769.77 | SNP | Rv2458 (mmuM) | Tyr125Cys | 3 | - | | 2772945 | A | AG | 1343.73 | INS | Rv2469c |  |  | - | | 2775361 | C | T | 1011.77 | SNP | Rv2472 | silent (Arg30) | 9913 | - | | 2779136 | T | C | 984.77 | SNP | Rv2476c (gdh) | Ser1043Gly | 21 | - | | 2786952 | A | G | 1102.77 | SNP | Rv2482c (plsB2) | Cys778Arg | 1 | - | | 2789798 | G | T | 1703.77 | SNP | Rv2483c (plsC) | silent (Arg409) | 9913 | - | | 2791098 | C | T | 485.77 | SNP | Rv2484c | Gly466Asp | 6 | - | | 2793810 | C | A | 1239.77 | SNP | Rv2485c (lipQ) | Arg60Leu | 1 | - | | 2794071 | G | A | 790.77 | SNP | intergenic |  |  | - | | 2800087 | G | A | 754.77 | SNP | Rv2488c | Thr265Ile | 7 | - | | 2807486 | C | A | 1262.77 | SNP | Rv2492 | Ala70Asp | 6 | - | | 2808452 | T | C | 585.77 | SNP | Rv2494 (vapC38) | Val(s)48Ala | 9867 | - | | 2809621 | T | C | 816.77 | SNP | Rv2495c (bkdC) | Thr107Ala | 32 | - | | 2809633 | A | C | 829.77 | SNP | Rv2495c (bkdC) | Tyr103Asp | 0 | - | | 2817502 | A | C | 824.77 | SNP | Rv2502c (accD1) | Phe325Val | 1 | - | | 2818837 | A | G | 590.77 | SNP | Rv2503c (scoB) | silent (Gly97) | 9935 | - | | 2821342 | C | T | 785.77 | SNP | Rv2505c (fadD35) | silent (Ala85) | 9867 | - | | 2825466 | G | A | 693.77 | SNP | Rv2509 | silent (Lys263) | 9926 | - | | 2825581 | T | G | 555.77 | SNP | Rv2510c | silent (Ile503) | 9872 | - | | 2827984 | G | T | 1061.77 | SNP | intergenic |  |  | - | | 2828019 | T | C | 922.77 | SNP | intergenic |  |  | - | | 2829779 | T | C | 302.78 | SNP | Rv2512c | Thr9Ala | 32 | - | | 2830525 | C | A | 996.77 | SNP | Rv2513 | Thr122Lys | 11 | - | | 2833329 | G | A | 1353.77 | SNP | Rv2516c | Ala62Val | 13 | - | | 2835894 | C | T | 1076.77 | SNP | Rv2519 (PE26) | Thr37Ile | 7 | - | | 2835984 | T | C | 1029.77 | SNP | Rv2519 (PE26) | Val(s)67Ala | 9867 | - | | 2841022 | A | G | 701.77 | SNP | Rv2524c (fas) | Cys2771Arg | 1 | - | | 2846569 | G | A | 706.77 | SNP | Rv2524c (fas) | Arg922Cys | 1 | - | | 2847281 | A | G | 813.77 | SNP | Rv2524c (fas) | silent (Asp684) | 9859 | - | | 2850631 | T | C | 1432.77 | SNP | intergenic |  |  | - | | 2851161 | AAACAT | A | 2612.73 | DEL | Rv2526 (vapB17) |  |  | - | | 2851167 | T | C | 805.77 | SNP | Rv2526 (vapB17) | Leu(s)26Ser | 28 | - | | 2855259 | A | G | 887.77 | SNP | Rv2531c | silent (Ala841) | 9867 | - | | 2865760 | A | G | 1444.77 | SNP | Rv2542 | Thr211Ala | 32 | - | | 2865882 | T | C | 1464.77 | SNP | Rv2542 | silent (Val251) | 9901 | - | | 2866551 | C | G | 208.77 | SNP | Rv2543 (lppA) | silent (Ala28) | 9867 | - | | 2866569 | C | A | 215.77 | SNP | Rv2543 (lppA) | silent (Thr34) | 9871 | - | | 2866578 | C | A | 288.77 | SNP | Rv2543 (lppA) | His37Gln | 23 | - | | 2866580 | A | G | 305.77 | SNP | Rv2543 (lppA) | Asn38Ser | 34 | - | | 2866607 | G | A | 1768.77 | SNP | Rv2543 (lppA) | Gly47Asp | 6 | - | | 2866647 | G | A | 1410.77 | SNP | Rv2543 (lppA) | silent (Lys60) | 9926 | - | | 2866671 | G | A | 1160.77 | SNP | Rv2543 (lppA) | silent (Glu68) | 9865 | - | | 2866677 | A | C | 1047.77 | SNP | Rv2543 (lppA) | silent (Leu70) | 9947 | - | | 2866863 | C | G | 673.77 | SNP | Rv2543 (lppA) | silent (Ala132) | 9867 | - | | 2866876 | A | G | 833.77 | SNP | Rv2543 (lppA) | Ile137Val | 57 | - | | 2866880 | C | T | 817.77 | SNP | Rv2543 (lppA) | Ala138Val | 13 | - | | 2866882 | G | A | 869.77 | SNP | Rv2543 (lppA) | Ala139Thr | 22 | - | | 2867207 | C | G | 626.77 | SNP | Rv2544 (lppB) | silent (Ala28) | 9867 | - | | 2867230 | G | A | 302.78 | SNP | Rv2544 (lppB) | Gly36Asp | 6 | - | | 2867231 | C | T | 299.78 | SNP | Rv2544 (lppB) | silent (Gly36) | 9935 | - | | 2867236 | A | G | 313.78 | SNP | Rv2544 (lppB) | Asn38Ser | 34 | - | | 2867240 | C | T | 280.78 | SNP | Rv2544 (lppB) | silent (Pro39) | 9926 | - | | 2867245 | A | C | 311.78 | SNP | Rv2544 (lppB) | Lys41Thr | 8 | - | | 2867251 | C | G | 296.78 | SNP | Rv2544 (lppB) | Pro43Arg | 4 | - | | 2867254 | A | G | 266.78 | SNP | Rv2544 (lppB) | His44Arg | 10 | - | | 2867263 | G | A | 295.78 | SNP | Rv2544 (lppB) | Gly47Asp | 6 | - | | 2867298 | C | A | 449.77 | SNP | Rv2544 (lppB) | His59Asn | 21 | - | | 2867347 | A | G | 394.77 | SNP | Rv2544 (lppB) | Gln75Arg | 10 | - | | 2867458 | G | C | 42.74 | SNP | Rv2544 (lppB) | Ser112Thr | 32 | - | | 2867459 | T | C | 43.74 | SNP | Rv2544 (lppB) | silent (Ser112) | 9840 | - | | 2867522 | A | G | 181.84 | SNP | Rv2544 (lppB) | silent (Glu133) | 9865 | - | | 2867532 | A | G | 187.80 | SNP | Rv2544 (lppB) | Ile137Val | 57 | - | | 2867536 | C | T | 136.90 | SNP | Rv2544 (lppB) | Ala138Val | 13 | - | | 2867538 | G | A | 199.80 | SNP | Rv2544 (lppB) | Ala139Thr | 22 | - | | 2867552 | A | G | 405.77 | SNP | Rv2544 (lppB) | silent (Arg143) | 9913 | - | | 2867575 | T | C | 560.77 | SNP | Rv2544 (lppB) | Val(s)151Ala | 9867 | - | | 2867594 | G | A | 611.77 | SNP | Rv2544 (lppB) | silent (Leu157) | 9947 | - | | 2867756 | T | C | 652.77 | SNP | Rv2544 (lppB) | silent (Ile211) | 9872 | - | | 2867880 | TA | T | 1725.73 | DEL | Rv2545 (vapB18) |  |  | - | | 2880702 | G | C | 1042.77 | SNP | Rv2560 | Val210Leu | 15 | - | | 2881597 | AG | A | 506.73 | DEL | Rv2561 |  |  | - | | 2881974 | G | A | 1090.77 | SNP | Rv2562 | Ala73Thr | 22 | - | | 2884068 | A | C | 942.77 | SNP | Rv2564 (glnQ) | Met(s)243Leu | 3 | - | | 2886570 | A | G | 673.77 | SNP | Rv2566 | silent (Glu66) | 9865 | - | | 2888201 | T | C | 727.77 | SNP | Rv2566 | Leu610Pro | 2 | - | | 2889633 | T | C | 548.77 | SNP | Rv2566 | silent (Ala1087) | 9867 | - | | 2891267 | C | T | 782.77 | SNP | Rv2567 | silent (Gly491) | 9935 | - | | 2891728 | A | G | 1292.77 | SNP | Rv2567 | Gln645Arg | 10 | - | | 2894208 | G | A | 626.77 | SNP | Rv2569c | silent (Ser67) | 9840 | - | | 2898522 | T | C | 685.77 | SNP | Rv2573 | silent (Tyr160) | 9945 | - | | 2903439 | G | A | 817.77 | SNP | Rv2578c | silent (Ser31) | 9840 | - | | 2906068 | C | A | 613.77 | SNP | Rv2580c (hisS) | Ala9Ser | 28 | - | | 2910461 | G | T | 422.77 | SNP | Rv2584c (apt) | Ala147Glu | 10 | - | | 2911293 | C | G | 815.77 | SNP | Rv2585c | Cys462Ser | 11 | - | | 2912294 | T | G | 1159.77 | SNP | Rv2585c | silent (Ala128) | 9867 | - | | 2919381 | T | C | 845.77 | SNP | Rv2590 (fadD9) | Val(s)504Ala | 9867 | - | | 2923391 | T | C | 408.77 | SNP | Rv2592c (ruvB) | silent (Pro281) | 9926 | - | | 2925962 | T | C | 987.77 | SNP | Rv2596 (vapC40) | Cys77Arg | 1 | - | | 2927939 | T | C | 1071.77 | SNP | intergenic |  |  | - | | 2939373 | G | C | 663.77 | SNP | Rv2611c | Ser197Cys | 5 | - | | 2939657 | T | C | 225.80 | SNP | Rv2611c | Ile102Met(s) | 6 | - | | 2944769 | G | C | 144.90 | SNP | Rv2615c (PE\_PGRS45) | Gln73Glu | 35 | - | | 2944773 | A | G | 129.90 | SNP | Rv2615c (PE\_PGRS45) | silent (Tyr71) | 9945 | - | | 2945004 | C | G | 68.77 | SNP | intergenic |  |  | - | | 2948230 | G | A | 1048.77 | SNP | Rv2621c | Ala110Val | 13 | - | | 2954439 | T | C | 834.77 | SNP | Rv2627c | Arg104Gly | 1 | - | | 2955957 | A | C | 956.77 | SNP | Rv2629 | Asp64Ala | 10 | genotype | | 2969197 | A | G | 863.77 | SNP | intergenic |  |  | - | | 2974933 | A | G | 527.77 | SNP | Rv2650c | Ile101Thr | 11 | - | | 2975227 | T | G | 749.77 | SNP | Rv2650c | Asn3Thr | 13 | - | | 2975428 | A | G | 964.77 | SNP | Rv2651c | silent (Gly116) | 9935 | - | | 2980861 | A | G | 1182.77 | SNP | intergenic |  |  | - | | 2983095 | A | G | 242.80 | SNP | Rv2666 | Thr9Ala | 32 | - | | 2983613 | G | A | 111.03 | SNP | Rv2666 | silent (Gly181) | 9935 | - | | 2983873 | A | G | 787.77 | SNP | Rv2666 | silent (STOP268) | 9867 | - | | 2984740 | A | G | 492.77 | SNP | Rv2668 | His3Arg | 10 | - | | 2988630 | C | G | 617.77 | SNP | Rv2672 | His317Asp | 4 | - | | 2994187 | T | C | 468.77 | SNP | Rv2678c (hemE) | Leu292Leu(s) | 4 | - | | 3003115 | C | G | 879.77 | SNP | Rv2685 (arsB1) | Ala378Gly | 21 | - | | 3005185 | G | T | 859.77 | SNP | Rv2688c | Pro156Thr | 5 | - | | 3007172 | CGCCGCTCCTCCCCATCGCT TTGCTCTGCATCGTCGCCGG CGCGGGTCAATCGAAGAT | C | 2080.73 | DEL | intergenic |  |  | - | | 3009692 | A | G | 961.77 | SNP | Rv2691 (ceoB) | Thr117Ala | 32 | - | | 3009834 | A | G | 975.77 | SNP | Rv2691 (ceoB) | Asp164Gly | 11 | - | | 3010420 | A | G | 825.77 | SNP | Rv2692 (ceoC) | Ile133Val | 57 | - | | 3010993 | C | T | 792.77 | SNP | Rv2693c | Gly126Arg | 0 | - | | 3017465 | T | C | 1613.77 | SNP | Rv2702 (ppgK) | Ile203Thr | 11 | - | | 3021892 | GTTCGACGAC | G | 3157.19 | DEL | Rv2709 |  |  | - | | 3024021 | C | A | 535.77 | SNP | Rv2711 (ideR) | silent (Arg153) | 9913 | - | | 3024343 | G | A | 1074.77 | SNP | Rv2712c | Thr329Ile | 7 | - | | 3024556 | C | T | 839.77 | SNP | Rv2712c | Trp258STOP | 0 | - | | 3027798 | T | C | 696.77 | SNP | Rv2714 | Val(s)245Ala | 9867 | - | | 3031168 | A | G | 438.77 | SNP | Rv2719c | Tyr124His | 4 | - | | 3033189 | G | A | 886.77 | SNP | Rv2721c | silent (Pro477) | 9926 | - | | 3036826 | C | T | 1394.77 | SNP | Rv2724c (fadE20) | Val(s)156Met(s) | 9867 | - | | 3037377 | T | C | 355.77 | SNP | intergenic |  |  | - | | 3041871 | G | T | 611.77 | SNP | Rv2729c | Ala202Glu | 10 | - | | 3046886 | T | C | 1455.77 | SNP | Rv2734 | silent (Arg22) | 9913 | - | | 3048912 | C | G | 544.77 | SNP | Rv2736c (recX) | Val59Leu | 15 | - | | 3054081 | A | G | 1039.77 | SNP | Rv2741 (PE\_PGRS47) | silent (Gly56) | 9935 | - | | 3054321 | A | G | 423.77 | SNP | Rv2741 (PE\_PGRS47) | silent (Gly136) | 9935 | - | | 3065711 | C | T | 958.77 | SNP | Rv2752c | Gly161Ser | 16 | - | | 3068497 | T | C | 1662.77 | SNP | Rv2756c (hsdM) | silent (Glu529) | 9865 | - | | 3069167 | A | G | 714.77 | SNP | Rv2756c (hsdM) | Leu306Pro | 2 | - | | 3078178 | C | T | 843.77 | SNP | Rv2769c (PE27) | Val(s)270Met(s) | 9867 | - | | 3079877 | A | G | 736.77 | SNP | Rv2770c (PPE44) | Phe194Ser | 3 | - | | 3080795 | A | G | 977.77 | SNP | Rv2771c | Leu80Pro | 2 | - | | 3086788 | T | C | 958.77 | SNP | intergenic |  |  | - | | 3103682 | T | C | 1056.77 | SNP | Rv2794c (pptT) | Met(s)87Val(s) | 9867 | - | | 3104189 | A | G | 1489.77 | SNP | Rv2795c | silent (Cys241) | 9973 | - | | 3110140 | G | T | 827.77 | SNP | intergenic |  |  | - | | 3111476 | C | T | 744.77 | SNP | Rv2802c | silent (Ser116) | 9840 | - | | 3112877 | G | A | 912.77 | SNP | Rv2804c; Rv2805 | Ala73Val; Gly4Asp | 13; 6 | - | | 3113872 | A | T | 672.77 | SNP | Rv2807 | Glu72Val(s) | 17 | - | | 3115384 | G | C | 1522.77 | SNP | intergenic |  |  | - | | 3118000 | A | G | 767.77 | SNP | Rv2812 | Arg395Gly | 1 | - | | 3127931 | T | A | 32.04 | SNP | Rv2820c | Lys114Asn | 13 | - | | 3131469 | T | TTGTCGGCGA | 3425.73 | INS | Rv2823c |  |  | - | | 3133054 | C | G | 619.77 | SNP | Rv2825c | silent (Ser162) | 9840 | - | | 3133055 | G | C | 551.77 | SNP | Rv2825c | Ser162Trp | 1 | - | | 3133536 | T | C | 1396.77 | SNP | Rv2825c | Lys2Glu | 4 | - | | 3135606 | A | G | 1018.77 | SNP | intergenic |  |  | - | | 3135912 | G | C | 656.77 | SNP | Rv2828c | Thr141Arg | 1 | - | | 3135950 | C | G | 518.77 | SNP | Rv2828c | silent (Ser128) | 9840 | - | | 3135951 | G | C | 498.77 | SNP | Rv2828c | Ser128Trp | 1 | - | | 3137058 | G | A | 889.77 | SNP | Rv2830c (vapB22) | Ala56Val(s) | 9867 | - | | 3137736 | G | T | 369.77 | SNP | Rv2831 (echA16) | Asp156Tyr | 0 | - | | 3144623 | C | T | 732.77 | SNP | Rv2837c; Rv2838c (rbfA) | Ser8Asn; silent (Glu183) | 20; 9865 | - | | 3156201 | C | CG | 1282.73 | INS | Rv2848c (cobB) |  |  | - | | 3165636 | G | A | 777.77 | SNP | Rv2855 (mtr) | silent (Ser144) | 9840 | - | | 3174496 | A | G | 924.77 | SNP | Rv2862c | Cys50Arg | 1 | - | | 3174692 | AC | A | 1011.73 | DEL | intergenic |  |  | - | | 3177452 | C | A | 1132.77 | SNP | intergenic |  |  | - | | 3177884 | C | A | 933.77 | SNP | Rv2866 (relG) | silent (Arg21) | 9913 | - | | 3180988 | C | A | 588.77 | SNP | Rv2869c (rip) | Val259Phe | 0 | - | | 3186860 | T | G | 399.77 | SNP | Rv2874 (dipZ) | Tyr672Asp | 0 | - | | 3189242 | A | G | 983.77 | SNP | Rv2878c (mpt53) | silent (Ala52) | 9867 | - | | 3190145 | TC | T | 1337.73 | DEL | Rv2880c |  |  | - | | 3193202 | G | A | 1362.77 | SNP | intergenic |  |  | - | | 3194241 | C | CG | 1204.73 | INS | Rv2885c |  |  | - | | 3200282 | C | T | 446.77 | SNP | Rv2891 | Ala6Val(s) | 9867 | - | | 3209156 | G | A | 918.77 | SNP | Rv2899c (fdhD) | Ala84Val | 13 | - | | 3214869 | A | G | 616.77 | SNP | Rv2905 (lppW) | Gln81Arg | 10 | - | | 3215622 | CG | C | 231.80 | DEL | intergenic |  |  | - | | 3218997 | T | A | 745.77 | SNP | Rv2911 (dacB2) | Leu220Gln | 3 | - | | 3226181 | A | C | 994.77 | SNP | Rv2916c (ffh) | silent (Arg35) | 9913 | - | | 3228143 | G | T | 437.77 | SNP | Rv2917 | Arg594Leu | 1 | - | | 3232703 | G | A | 501.77 | SNP | intergenic |  |  | - | | 3232759 | G | A | 1057.77 | SNP | intergenic |  |  | - | | 3238119 | CGTG | C | 2141.73 | DEL | Rv2923c |  |  | - | | 3247316 | C | G | 765.77 | SNP | Rv2931 (ppsA) | Asp624Glu | 56 | - | | 3247851 | G | A | 667.77 | SNP | Rv2931 (ppsA) | Ala803Thr | 22 | - | | 3247853 | C | T | 568.77 | SNP | Rv2931 (ppsA) | silent (Ala803) | 9867 | - | | 3247856 | G | C | 626.77 | SNP | Rv2931 (ppsA) | silent (Arg804) | 9913 | - | | 3247864 | C | CTAGG | 1458.73 | INS | Rv2931 (ppsA) |  |  | - | | 3247865 | GCAAA | G | 1491.73 | DEL | Rv2931 (ppsA) |  |  | - | | 3247874 | G | A | 621.77 | SNP | Rv2931 (ppsA) | silent (Arg810) | 9913 | - | | 3247877 | T | C | 654.77 | SNP | Rv2931 (ppsA) | silent (Phe811) | 9946 | - | | 3247883 | T | C | 708.77 | SNP | Rv2931 (ppsA) | silent (Ser813) | 9840 | - | | 3248074 | G | A | 937.77 | SNP | Rv2931 (ppsA) | Arg877His | 8 | - | | 3248075 | C | T | 923.77 | SNP | Rv2931 (ppsA) | silent (Arg877) | 9913 | - | | 3249025 | T | G | 1187.77 | SNP | Rv2931 (ppsA) | Leu1194Arg | 1 | - | | 3256494 | A | G | 901.77 | SNP | Rv2933 (ppsC) | silent (Gly270) | 9935 | - | | 3266030 | A | G | 323.78 | SNP | Rv2934 (ppsD) | silent (Ser1261) | 9840 | - | | 3269581 | A | G | 1030.77 | SNP | Rv2935 (ppsE) | silent (Ala615) | 9867 | - | | 3270784 | A | G | 783.77 | SNP | Rv2935 (ppsE) | silent (Gln1016) | 9876 | - | | 3276703 | T | G | 779.77 | SNP | Rv2940c (mas) | Thr2005Pro | 4 | - | | 3284640 | A | G | 819.77 | SNP | Rv2941 (fadD28) | Thr436Ala | 32 | - | | 3284855 | C | T | 1614.77 | SNP | Rv2941 (fadD28) | silent (Ile507) | 9872 | - | | 3296371 | G | GCCGCGGC | 2414.27 | INS | Rv2947c (pks15) |  |  | - (CGGCGCCG would be genotype) | | 3296843 | A | G | 503.77 | SNP | Rv2947c (pks15) | Val(s)333Ala | 9867 | - | | 3300005 | C | A | 1222.77 | SNP | Rv2949c | Trp189Leu(s) | 0 | - | | 3304966 | G | A | 844.77 | SNP | Rv2952 | Gly176Arg | 0 | - | | 3308606 | G | A | 1761.77 | SNP | intergenic |  |  | - | | 3310626 | T | C | 600.77 | SNP | intergenic |  |  | - | | 3314412 | A | G | 1544.77 | SNP | Rv2962c | silent (Ala237) | 9867 | - | | 3316955 | A | C | 1097.77 | SNP | Rv2964 (purU) | Met(s)143Leu | 3 | - | | 3326554 | A | C | 1048.77 | SNP | Rv2971 | Asn152His | 18 | genotype | | 3331361 | ACG | A | 865.73 | DEL | Rv2974c; Rv2975c |  |  | - | | 3335708 | G | C | 881.77 | SNP | Rv2979c | Pro14Arg | 4 | - | | 3336679 | TA | T | 370.73 | DEL | intergenic |  |  | - | | 3336825 | T | C | 736.77 | SNP | Rv2981c (ddlA) | Thr365Ala | 32 | - | | 3338603 | G | C | 752.77 | SNP | Rv2982c (gpdA2) | Pro133Ala | 22 | - | | 3352932 | C | G | 872.77 | SNP | Rv2995c (leuB) | silent (Thr179) | 9871 | - | | 3358235 | A | T | 985.77 | SNP | Rv2999 (lppY) | Met(s)212Leu(s) | 9867 | - | | 3363338 | A | G | 947.77 | SNP | intergenic |  |  | - | | 3367765 | G | A | 707.77 | SNP | Rv3009c (gatB) | silent (Gly343) | 9935 | - | | 3375165 | A | C | 742.77 | SNP | Rv3015c | Phe167Cys | 0 | - | | 3379708 | G | C | 85.28 | SNP | intergenic |  |  | - | | 3379712 | G | C | 85.28 | SNP | intergenic |  |  | - | | 3379718 | T | C | 85.28 | SNP | intergenic |  |  | - | | 3379726 | C | A | 44.74 | SNP | intergenic |  |  | - | | 3379730 | G | C | 45.74 | SNP | intergenic |  |  | - | | 3379732 | C | T | 43.74 | SNP | intergenic |  |  | - | | 3379735 | A | C | 48.74 | SNP | intergenic |  |  | - | | 3379736 | C | A | 42.74 | SNP | intergenic |  |  | - | | 3379742 | T | C | 189.84 | SNP | intergenic |  |  | - | | 3379751 | A | C | 287.78 | SNP | intergenic |  |  | - | | 3379757 | A | C | 280.78 | SNP | intergenic |  |  | - | | 3379763 | G | A | 288.78 | SNP | intergenic |  |  | - | | 3379784 | C | A | 307.78 | SNP | intergenic |  |  | - | | 3379788 | C | G | 382.77 | SNP | intergenic |  |  | - | | 3380534 | A | G | 92.28 | SNP | intergenic |  |  | - | | 3401871 | A | G | 996.77 | SNP | Rv3041c | silent (Ala16) | 9867 | - | | 3402816 | C | T | 992.77 | SNP | Rv3042c (serB2) | Gly116Glu | 4 | - | | 3404376 | C | G | 1227.77 | SNP | Rv3043c (ctaD) | silent (Thr182) | 9871 | - | | 3408150 | T | C | 1041.77 | SNP | Rv3047c | Thr53Ala | 32 | - | | 3412611 | T | C | 1568.77 | SNP | Rv3051c (nrdE) | Gln519Arg | 10 | - | | 3413574 | G | C | 1027.77 | SNP | Rv3051c (nrdE) | Ala198Gly | 21 | - | | 3415180 | ACACCTAGGGGGTGG | A | 2182.84 | DEL | intergenic |  |  | - | | 3420825 | A | G | 1038.77 | SNP | Rv3059 (cyp136) | Asp445Gly | 11 | - | | 3425854 | C | T | 821.77 | SNP | Rv3062 (ligB) | Pro91Ser | 17 | - | | 3428917 | C | A | 1191.77 | SNP | Rv3063 (cstA) | Arg559Ser | 11 | - | | 3440464 | T | G | 664.77 | SNP | Rv3077 | silent (Arg308) | 9913 | - | | 3440468 | G | C | 682.77 | SNP | Rv3077 | Gly310Arg | 0 | - | | 3446699 | C | G | 1022.77 | SNP | Rv3081 | Phe220Leu(s) | 2 | - | | 3447293 | T | G | 1106.77 | SNP | intergenic |  |  | - | | 3450725 | T | C | 655.77 | SNP | Rv3084 (lipR) | silent (Val243) | 9901 | - | | 3454263 | C | G | 1148.77 | SNP | Rv3087 | Leu447Val | 11 | - | | 3455686 | G | C | 940.77 | SNP | Rv3088 (tgs4) | silent (Leu449) | 9947 | - | | 3456666 | A | G | 751.77 | SNP | Rv3089 (fadD13) | silent (Ala302) | 9867 | - | | 3462135 | G | C | 582.77 | SNP | Rv3093c | Cys210Trp | 0 | - | | 3462822 | G | T | 624.77 | SNP | Rv3094c | Ala357Glu | 10 | - | | 3466919 | C | G | 360.77 | SNP | Rv3097c (lipY) | Gly58Ala | 21 | - | | 3467465 | C | G | 1823.77 | SNP | Rv3098c | silent (Ala66) | 9867 | - | | 3473996 | G | GA | 1671.73 | INS | intergenic |  |  | - | | 3477942 | A | G | 1024.77 | SNP | Rv3109 (moaA1) | silent (Thr98) | 9871 | - | | 3480474 | G | A | 1321.77 | SNP | Rv3113 | Gly134Glu | 4 | - | | 3480789 | T | C | 936.77 | SNP | Rv3114 | Ser11Pro | 12 | - | | 3486977 | A | G | 1293.77 | SNP | Rv3121 (cyp141) | Lys157Glu | 4 | - | | 3487108 | C | T | 1086.77 | SNP | Rv3121 (cyp141) | silent (Ala200) | 9867 | - | | 3498198 | C | T | 1103.77 | SNP | Rv3132c (devS) | silent (Glu356) | 9865 | - | | 3500149 | G | A | 1359.77 | SNP | Rv3134c | Leu201Leu(s) | 4 | - | | 3500243 | G | C | 936.77 | SNP | Rv3134c | silent (Ala169) | 9867 | - | | 3502160 | A | T | 364.77 | SNP | Rv3136 (PPE51) | Thr123Ser | 38 | - | | 3502166 | T | C | 385.77 | SNP | Rv3136 (PPE51) | Phe125Leu | 13 | - | | 3502169 | T | C | 394.77 | SNP | Rv3136 (PPE51) | Phe126Leu | 13 | - | | 3502171 | C | G | 400.77 | SNP | Rv3136 (PPE51) | Phe126Leu(s) | 2 | - | | 3502183 | T | C | 514.77 | SNP | Rv3136 (PPE51) | silent (Thr130) | 9871 | - | | 3502186 | G | A | 507.77 | SNP | Rv3136 (PPE51) | silent (Ala131) | 9867 | - | | 3502195 | G | A | 553.77 | SNP | Rv3136 (PPE51) | silent (Ala134) | 9867 | - | | 3502200 | C | T | 556.77 | SNP | Rv3136 (PPE51) | Thr136Ile | 7 | - | | 3502228 | C | A | 438.77 | SNP | Rv3136 (PPE51) | silent (Ala145) | 9867 | - | | 3502231 | G | A | 398.77 | SNP | Rv3136 (PPE51) | silent (Gln146) | 9876 | - | | 3502237 | C | G | 429.77 | SNP | Rv3136 (PPE51) | silent (Ala148) | 9867 | - | | 3502252 | T | C | 321.77 | SNP | Rv3136 (PPE51) | silent (Gly153) | 9935 | - | | 3502262 | G | A | 274.77 | SNP | Rv3136 (PPE51) | Ala157Thr | 22 | - | | 3503895 | C | T | 1003.77 | SNP | Rv3137 | Pro168Leu | 3 | - | | 3505027 | G | A | 472.77 | SNP | Rv3138 (pflA) | Arg278His | 8 | - | | 3508970 | A | G | 691.77 | SNP | Rv3141 (fadB4) | silent (Glu292) | 9865 | - | | 3509626 | C | A | 1524.77 | SNP | intergenic |  |  | - | | 3510120 | T | G | 736.77 | SNP | Rv3144c (PPE52) | Lys400Gln | 6 | - | | 3518167 | A | G | 689.77 | SNP | Rv3151 (nuoG) | Ile474Met(s) | 6 | - | | 3518555 | A | G | 579.77 | SNP | Rv3151 (nuoG) | Thr604Ala | 32 | - | | 3528064 | T | C | 105.77 | SNP | Rv3159c (PPE53) | Asn367Ser | 34 | - | | 3528065 | T | C | 77.77 | SNP | Rv3159c (PPE53) | Asn367Asp | 42 | - | | 3528066 | G | A | 62.77 | SNP | Rv3159c (PPE53) | silent (Asn366) | 9822 | - | | 3528072 | A | G | 90.77 | SNP | Rv3159c (PPE53) | silent (Ser364) | 9840 | - | | 3528084 | A | G | 143.77 | SNP | Rv3159c (PPE53) | silent (Gly360) | 9935 | - | | 3528087 | G | A | 98.77 | SNP | Rv3159c (PPE53) | silent (Ile359) | 9872 | - | | 3528099 | A | C | 198.77 | SNP | Rv3159c (PPE53) | silent (Gly355) | 9935 | - | | 3528102 | C | A | 146.77 | SNP | Rv3159c (PPE53) | silent (Ser354) | 9840 | - | | 3528117 | T | C | 233.77 | SNP | Rv3159c (PPE53) | silent (Leu349) | 9947 | - | | 3528119 | G | T | 195.77 | SNP | Rv3159c (PPE53) | Leu349Ile | 9 | - | | 3528120 | A | G | 186.77 | SNP | Rv3159c (PPE53) | silent (Asn348) | 9822 | - | | 3528129 | G | T | 221.77 | SNP | Rv3159c (PPE53) | silent (Gly345) | 9935 | - | | 3528140 | G | A | 282.77 | SNP | Rv3159c (PPE53) | Leu342Leu(s) | 4 | - | | 3528144 | G | A | 312.77 | SNP | Rv3159c (PPE53) | silent (Gly340) | 9935 | - | | 3528158 | A | G | 282.77 | SNP | Rv3159c (PPE53) | Leu(s)336Leu | 3 | - | | 3528159 | G | A | 273.77 | SNP | Rv3159c (PPE53) | silent (Asn335) | 9822 | - | | 3528165 | G | A | 230.77 | SNP | Rv3159c (PPE53) | silent (Gly333) | 9935 | - | | 3528192 | G | A | 132.77 | SNP | Rv3159c (PPE53) | silent (Asn324) | 9822 | - | | 3528198 | A | G | 157.77 | SNP | Rv3159c (PPE53) | silent (Asn322) | 9822 | - | | 3528971 | TCGC | T | 1024.73 | DEL | Rv3159c (PPE53) |  |  | - | | 3529067 | G | C | 1828.77 | SNP | Rv3159c (PPE53) | Arg33Gly | 1 | - | | 3530955 | C | G | 507.77 | SNP | Rv3161c | Val62Leu | 15 | - | | 3531603 | CCGTGGAGAGCCCA | C | 4212.73 | DEL | Rv3162c |  |  | - | | 3540882 | T | C | 1087.77 | SNP | Rv3172c | silent (STOP161) | 9867 | - | | 3542049 | G | A | 1423.77 | SNP | intergenic |  |  | - | | 3542219 | G | A | 1424.77 | SNP | Rv3174 | Gly28Ser | 16 | - | | 3550347 | C | T | 942.77 | SNP | intergenic |  |  | - | | 3550789 | C | G | 489.77 | SNP | Rv3183 | silent (Ala25) | 9867 | - | | 3556275 | A | G | 1238.77 | SNP | Rv3190c | Leu138Pro | 2 | - | | 3570896 | G | T | 679.77 | SNP | Rv3198c (uvrD2) | silent (Arg106) | 9913 | - | | 3571828 | G | C | 490.77 | SNP | Rv3199c (nudC) | Pro239Arg | 4 | - | | 3576231 | T | C | 922.77 | SNP | Rv3201c | Gln269Arg | 10 | - | | 3577497 | C | T | 453.77 | SNP | Rv3202c | Glu902Lys | 7 | - | | 3578952 | G | C | 854.77 | SNP | Rv3202c | Pro417Ala | 22 | - | | 3580636 | CT | C | 852.73 | DEL | intergenic |  |  | - | | 3581414 | A | G | 875.77 | SNP | Rv3204 | Thr34Ala | 32 | - | | 3587446 | G | A | 543.77 | SNP | Rv3210c | Leu32Leu(s) | 4 | - | | 3590686 | G | GC | 936.73 | INS | intergenic |  |  | - | | 3591063 | T | C | 778.77 | SNP | Rv3213c | Lys144Glu | 4 | - | | 3597249 | C | G | 1431.77 | SNP | Rv3220c | Gly96Arg | 0 | - | | 3600576 | C | T | 981.77 | SNP | Rv3224 | silent (Cys242) | 9973 | - | | 3604821 | G | C | 612.77 | SNP | Rv3228 | silent (Ala32) | 9867 | - | | 3610391 | A | AC | 2076.73 | INS | Rv3234c (tgs3) |  |  | - | | 3612813 | T | C | 553.77 | SNP | Rv3236c | Thr102Ala | 32 | - | | 3614982 | T | C | 1367.77 | SNP | Rv3239c | silent (Leu874) | 9947 | - | | 3617931 | G | A | 594.77 | SNP | Rv3240c (secA1) | silent (Ser867) | 9840 | - | | 3621423 | A | G | 705.77 | SNP | intergenic |  |  | - | | 3622441 | A | C | 706.77 | SNP | Rv3243c | Val217Val(s) | 18 | - | | 3625065 | T | G | 511.77 | SNP | Rv3245c (mtrB) | Met(s)517Leu | 3 | - | | 3626562 | G | A | 967.77 | SNP | Rv3245c (mtrB) | Pro18Ser | 17 | - | | 3628520 | G | A | 1158.77 | SNP | Rv3248c (sahH) | silent (Ser376) | 9840 | - | | 3638093 | G | A | 1125.77 | SNP | Rv3257c (pmmA) | Ser206Leu(s) | 35 | - | | 3643985 | A | C | 1454.77 | SNP | Rv3263 | Glu270Ala | 17 | - | | 3653988 | G | A | 632.77 | SNP | Rv3272 | Val(s)181Met(s) | 9867 | - | | 3663790 | A | C | 43.74 | SNP | Rv3281 (accE5) | silent (Ala34) | 9867 | - | | 3664457 | C | A | 757.77 | SNP | Rv3282 | Ala80Asp | 6 | - | | 3665753 | G | A | 797.77 | SNP | Rv3283 (sseA) | Glu276Lys | 7 | - | | 3670040 | C | T | 677.77 | SNP | Rv3289c | silent (Ala124) | 9867 | - | | 3674157 | T | A | 366.77 | SNP | Rv3293 (pcd) | Cys186Ser | 11 | - | | 3675504 | G | T | 1725.77 | SNP | Rv3294c | silent (Ile164) | 9872 | - | | 3678249 | A | C | 714.77 | SNP | Rv3296 (lhr) | Lys492Thr | 8 | - | | 3680932 | G | A | 440.77 | SNP | Rv3296 (lhr) | silent (Pro1386) | 9926 | - | | 3681548 | A | C | 952.77 | SNP | Rv3297 (nei) | silent (Arg77) | 9913 | - | | 3684649 | G | A | 2187.77 | SNP | Rv3299c (atsB) | Arg439Trp | 2 | - | | 3689523 | G | T | 774.77 | SNP | Rv3303c (lpdA) | Cys472STOP | 3 | - | | 3690016 | A | G | 800.77 | SNP | Rv3303c (lpdA) | Leu(s)308Ser | 28 | - | | 3693681 | A | C | 718.77 | SNP | Rv3306c (amiB1) | silent (Ala103) | 9867 | - | | 3704596 | G | C | 1020.77 | SNP | Rv3317 (sdhD) | Val(s)54Leu | 3 | - | | 3704770 | A | G | 690.77 | SNP | Rv3317 (sdhD) | Thr112Ala | 32 | - | | 3711910 | G | A | 402.77 | SNP | Rv3327 | Trp54STOP | 0 | - | | 3712372 | A | G | 126.90 | SNP | Rv3327 | silent (Leu208) | 9947 | - | | 3714211 | G | T | 533.77 | SNP | Rv3328c (sigJ) | Pro41Gln | 6 | - | | 3714757 | A | C | 990.77 | SNP | Rv3329 | Gln122His | 20 | - | | 3718316 | G | A | 732.77 | SNP | Rv3331 (sugI) | silent (Ala409) | 9867 | - | | 3718357 | C | T | 888.77 | SNP | Rv3331 (sugI) | Pro423Leu | 3 | - | | 3721174 | T | G | 1123.77 | SNP | intergenic |  |  | - | | 3721806 | G | C | 1273.77 | SNP | Rv3335c | silent (Gly265) | 9935 | - | | 3730466 | A | G | 400.77 | SNP | Rv3343c (PPE54) | Ile2157Thr | 11 | - | | 3730519 | C | G | 338.77 | SNP | Rv3343c (PPE54) | silent (Thr2139) | 9871 | - | | 3730642 | G | A | 39.74 | SNP | Rv3343c (PPE54) | silent (Asn2098) | 9822 | - | | 3730993 | A | G | 533.77 | SNP | Rv3343c (PPE54) | silent (Gly1981) | 9935 | - | | 3732247 | C | G | 67.77 | SNP | Rv3343c (PPE54) | silent (Thr1563) | 9871 | - | | 3732310 | G | A | 296.77 | SNP | Rv3343c (PPE54) | silent (Asn1542) | 9822 | - | | 3732344 | G | A | 1607.77 | SNP | Rv3343c (PPE54) | Ala1531Val | 13 | - | | 3732517 | A | G | 350.77 | SNP | Rv3343c (PPE54) | silent (Ile1473) | 9872 | - | | 3732525 | A | T | 297.77 | SNP | Rv3343c (PPE54) | Phe1471Ile | 7 | - | | 3732553 | A | G | 249.77 | SNP | Rv3343c (PPE54) | silent (Ile1461) | 9872 | - | | 3732624 | A | G | 356.53 | SNP | Rv3343c (PPE54) | Leu(s)1438Leu | 3 | - | | 3735802 | C | G | 1092.77 | SNP | Rv3343c (PPE54) | silent (Thr378) | 9871 | - | | 3736024 | A | G | 382.77 | SNP | Rv3343c (PPE54) | silent (Gly304) | 9935 | - | | 3736628 | T | G | 702.77 | SNP | Rv3343c (PPE54) | Glu103Ala | 17 | - | | 3736828 | G | T | 682.77 | SNP | Rv3343c (PPE54) | silent (Ala36) | 9867 | - | | 3737707 | C | G | 125.03 | SNP | intergenic |  |  | - | | 3738416 | TG | T | 284.75 | DEL | Rv3345c (PE\_PGRS50) |  |  | - | | 3741572 | G | A | 198.80 | SNP | Rv3345c (PE\_PGRS50) | silent (Gly401) | 9935 | - | | 3743656 | G | A | 914.77 | SNP | intergenic |  |  | - | | 3743899 | G | A | 306.77 | SNP | Rv3347c (PPE55) | Leu3096Leu(s) | 4 | - | | 3746409 | A | G | 435.77 | SNP | Rv3347c (PPE55) | Leu2259Pro | 2 | - | | 3750177 | A | T | 968.77 | SNP | Rv3347c (PPE55) | Phe1003Tyr | 21 | - | | 3750178 | A | C | 988.77 | SNP | Rv3347c (PPE55) | Phe1003Val | 1 | - | | 3750185 | C | G | 879.77 | SNP | Rv3347c (PPE55) | silent (Ser1000) | 9840 | - | | 3750187 | A | T | 853.77 | SNP | Rv3347c (PPE55) | Ser1000Thr | 32 | - | | 3750188 | C | G | 941.77 | SNP | Rv3347c (PPE55) | Met(s)999Ile | 2 | - | | 3750193 | G | A | 887.77 | SNP | Rv3347c (PPE55) | Leu998Phe | 6 | - | | 3750205 | C | T | 888.77 | SNP | Rv3347c (PPE55) | Asp994Asn | 36 | - | | 3750209 | A | G | 912.77 | SNP | Rv3347c (PPE55) | silent (Asn992) | 9822 | - | | 3750210 | T | G | 883.77 | SNP | Rv3347c (PPE55) | Asn992Thr | 13 | - | | 3750407 | G | C | 643.77 | SNP | Rv3347c (PPE55) | silent (Gly926) | 9935 | - | | 3750417 | A | T | 751.77 | SNP | Rv3347c (PPE55) | Phe923Tyr | 21 | - | | 3750421 | T | C | 760.77 | SNP | Rv3347c (PPE55) | Ser922Gly | 21 | - | | 3752207 | A | G | 816.77 | SNP | Rv3347c (PPE55) | silent (Ile326) | 9872 | - | | 3753116 | C | T | 573.77 | SNP | Rv3347c (PPE55) | silent (Pro23) | 9926 | - | | 3753164 | T | G | 892.77 | SNP | Rv3347c (PPE55) | silent (Pro7) | 9926 | - | | 3754508 | G | C | 899.77 | SNP | Rv3349c | Leu176Val | 11 | - | | 3761016 | GC | G | 179.80 | DEL | Rv3350c (PPE56) |  |  | - | | 3761191 | A | G | 301.28 | SNP | Rv3350c (PPE56) | Val1971Ala | 18 | - | | 3762013 | A | C | 912.77 | SNP | Rv3350c (PPE56) | Leu1697Arg | 1 | - | | 3770743 | T | C | 922.77 | SNP | intergenic |  |  | - | | 3775409 | T | C | 422.77 | SNP | Rv3365c | Gln698Arg | 10 | - | | 3775441 | A | C | 493.77 | SNP | Rv3365c | Ser687Arg | 6 | - | | 3778011 | G | A | 1164.77 | SNP | Rv3366 (spoU) | Arg92Gln | 9 | - | | 3778012 | G | T | 1122.77 | SNP | Rv3366 (spoU) | silent (Arg92) | 9913 | - | | 3778396 | C | T | 694.77 | SNP | intergenic |  |  | - | | 3790961 | A | C | 1269.77 | SNP | Rv3377c | Cys465Gly | 1 | - | | 3794867 | C | CCA | 1620.73 | INS | Rv3379c (dxs2) |  |  | - | | 3798095 | A | C | 1685.77 | SNP | Rv3383c (idsB) | Val132Gly | 5 | - | | 3798596 | A | G | 1443.77 | SNP | intergenic |  |  | - | | 3804299 | G | A | 303.53 | SNP | Rv3389c (htdY) | Pro165Ser | 17 | - | | 3811672 | G | T | 970.77 | SNP | intergenic |  |  | - | | 3811675 | A | G | 1013.77 | SNP | intergenic |  |  | - | | 3815477 | G | T | 655.77 | SNP | Rv3398c (idsA1) | silent (Ala210) | 9867 | - | | 3817117 | C | A | 947.77 | SNP | Rv3399 | Ala330Glu | 10 | - | | 3820407 | A | G | 462.77 | SNP | intergenic |  |  | - | | 3820429 | G | A | 77.77 | SNP | intergenic |  |  | - | | 3820545 | A | G | 290.77 | SNP | intergenic |  |  | - | | 3821845 | G | T | 695.77 | SNP | Rv3402c | Ala16Glu | 10 | - | | 3823159 | A | T | 787.77 | SNP | Rv3403c | silent (Val235) | 9901 | - | | 3823659 | A | G | 612.77 | SNP | Rv3403c | Ser69Pro | 12 | - | | 3826501 | C | T | 1079.77 | SNP | Rv3407 (vapB47) | Arg84Cys | 1 | - | | 3826684 | C | T | 675.82 | SNP | Rv3408 (vapC47) | Ser46Leu(s) | 35 | - | | 3829770 | T | C | 484.77 | SNP | Rv3410c (guaB3) | silent (Pro47) | 9926 | - | | 3830349 | C | T | 522.77 | SNP | Rv3411c (guaB2) | Ala391Thr | 22 | - | | 3830695 | A | G | 487.77 | SNP | Rv3411c (guaB2) | silent (Ala275) | 9867 | - | | 3834486 | T | C | 946.77 | SNP | Rv3415c | silent (Gln12) | 9876 | - | | 3838871 | A | G | 611.77 | SNP | Rv3420c (rimI) | silent (Ala64) | 9867 | - | | 3841652 | T | A | 44.77 | SNP | intergenic |  |  | - | | 3841654 | T | G | 118.77 | SNP | intergenic |  |  | - | | 3841662 | T | C | 145.77 | SNP | intergenic |  |  | - | | 3841663 | C | T | 100.77 | SNP | intergenic |  |  | - | | 3841790 | A | G | 2163.77 | SNP | Rv3424c | Val(s)96Ala | 9867 | - | | 3842317 | T | G | 58.77 | SNP | Rv3425 (PPE57) | Ser27Ala | 35 | - | | 3842452 | C | A | 38.77 | SNP | Rv3425 (PPE57) | Gln72Lys | 12 | - | | 3842463 | TA | T | 1561.73 | DEL | Rv3425 (PPE57) |  |  | - | | 3842620 | A | G | 884.77 | SNP | Rv3425 (PPE57) | Thr128Ala | 32 | - | | 3842625 | A | G | 874.77 | SNP | Rv3425 (PPE57) | silent (Pro129) | 9926 | - | | 3843025 | A | G | 58.77 | SNP | intergenic |  |  | - | | 3843407 | CG | C | 1549.73 | DEL | Rv3426 (PPE58) |  |  | - | | 3843696 | T | A | 220.77 | SNP | Rv3426 (PPE58) | Leu(s)221Met(s) | 9867 | - | | 3843704 | G | C | 203.77 | SNP | Rv3426 (PPE58) | silent (Thr223) | 9871 | - | | 3843714 | T | C | 252.77 | SNP | Rv3426 (PPE58) | Cys227Arg | 1 | - | | 3843749 | G | T | 200.77 | SNP | intergenic |  |  | - | | 3843751 | G | T | 232.77 | SNP | intergenic |  |  | - | | 3843752 | A | G | 189.77 | SNP | intergenic |  |  | - | | 3843753 | G | A | 189.77 | SNP | intergenic |  |  | - | | 3843760 | T | C | 271.77 | SNP | intergenic |  |  | - | | 3844733 | A | C | 698.77 | SNP | intergenic |  |  | - | | 3844756 | GC | G | 1299.73 | DEL | Rv3428c |  |  | - | | 3844992 | T | A | 1483.77 | SNP | Rv3428c | Ser327Cys | 5 | - | | 3845695 | C | T | 1295.77 | SNP | Rv3428c | silent (Arg92) | 9913 | - | | 3845986 | C | T | 625.77 | SNP | intergenic |  |  | - | | 3846605 | G | A | 694.77 | SNP | intergenic |  |  | - | | 3846607 | A | C | 717.77 | SNP | intergenic |  |  | - | | 3846622 | G | T | 608.77 | SNP | intergenic |  |  | - | | 3846687 | A | G | 810.77 | SNP | intergenic |  |  | - | | 3846704 | A | G | 694.77 | SNP | intergenic |  |  | - | | 3846707 | A | C | 665.77 | SNP | intergenic |  |  | - | | 3846716 | C | T | 97.77 | SNP | intergenic |  |  | - | | 3846727 | C | T | 109.77 | SNP | intergenic |  |  | - | | 3846728 | A | G | 167.77 | SNP | intergenic |  |  | - | | 3846741 | G | T | 162.77 | SNP | intergenic |  |  | - | | 3846743 | C | G | 224.77 | SNP | intergenic |  |  | - | | 3846764 | C | G | 461.77 | SNP | intergenic |  |  | - | | 3846773 | T | TG | 808.73 | INS | intergenic |  |  | - | | 3846774 | T | G | 396.77 | SNP | intergenic |  |  | - | | 3846777 | C | A | 417.77 | SNP | intergenic |  |  | - | | 3846779 | T | G | 420.77 | SNP | intergenic |  |  | - | | 3846791 | A | G | 778.77 | SNP | intergenic |  |  | - | | 3846843 | CAAA | C | 444.73 | DEL | intergenic |  |  | - | | 3846851 | G | A | 210.77 | SNP | intergenic |  |  | - | | 3846852 | C | G | 246.77 | SNP | intergenic |  |  | - | | 3846853 | T | C | 242.56 | SNP | intergenic |  |  | - | | 3846857 | G | A | 212.77 | SNP | intergenic |  |  | - | | 3846860 | T | G | 242.77 | SNP | intergenic |  |  | - | | 3846866 | C | A | 215.18 | SNP | intergenic |  |  | - | | 3846881 | AT | A | 257.73 | DEL | intergenic |  |  | - | | 3846886 | A | T | 230.28 | SNP | intergenic |  |  | - | | 3846897 | T | G | 250.78 | SNP | intergenic |  |  | - | | 3847022 | T | C | 41.77 | SNP | intergenic |  |  | - | | 3847052 | G | A | 30.77 | SNP | intergenic |  |  | - | | 3847073 | G | C | 59.77 | SNP | intergenic |  |  | - | | 3847099 | G | A | 40.77 | SNP | intergenic |  |  | - | | 3847215 | T | C | 1006.77 | SNP | Rv3429 (PPE59) | silent (Gly17) | 9935 | - | | 3847221 | T | C | 963.77 | SNP | Rv3429 (PPE59) | silent (Gly19) | 9935 | - | | 3847235 | C | T | 964.77 | SNP | Rv3429 (PPE59) | Ser24Phe | 2 | - | | 3847237 | G | T | 1004.77 | SNP | Rv3429 (PPE59) | Ala25Ser | 28 | - | | 3847238 | C | T | 1010.77 | SNP | Rv3429 (PPE59) | Ala25Val | 13 | - | | 3851887 | A | C | 545.77 | SNP | Rv3433c | Ser443Ala | 35 | - | | 3851888 | T | C | 618.77 | SNP | Rv3433c | silent (Ala442) | 9867 | - | | 3853284 | A | G | 1483.77 | SNP | Rv3434c | silent (Gly215) | 9935 | - | | 3859893 | C | T | 755.77 | SNP | Rv3440c | silent (Glu28) | 9865 | - | | 3862472 | GA | G | 1141.73 | DEL | intergenic |  |  | - | | 3863138 | G | A | 1428.77 | SNP | Rv3445c (esxU) | Pro43Ser | 17 | - | | 3863681 | C | G | 701.77 | SNP | Rv3446c | Arg284Pro | 5 | - | | 3864995 | T | C | 701.77 | SNP | Rv3447c (eccC4) | Ser1082Gly | 21 | - | | 3870010 | A | G | 647.77 | SNP | Rv3449 (mycP4) | Thr87Ala | 32 | - | | 3871246 | T | C | 520.77 | SNP | Rv3450c (eccB4) | silent (Gly417) | 9935 | - | | 3874722 | AT | A | 1981.73 | DEL | Rv3453 |  |  | - | | 3877421 | A | G | 1185.77 | SNP | Rv3456c (rplQ) | silent (Pro4) | 9926 | - | | 3883178 | A | C | 1143.77 | SNP | Rv3465 (rmlC) | Glu115Asp | 53 | - | | 3884906 | A | G | 768.77 | SNP | Rv3467 | Lys315Glu | 4 | - | | 3885886 | T | C | 898.77 | SNP | Rv3468c | Ile62Val | 57 | - | | 3892671 | A | G | 1262.77 | SNP | Rv3476c (kgtP) | silent (Val350) | 9901 | - | | 3893480 | G | A | 1059.77 | SNP | Rv3476c (kgtP) | Leu81Phe | 6 | - | | 3894732 | A | G | 523.77 | SNP | Rv3478 (PPE60) | Arg103Gly | 1 | - | | 3895585 | C | T | 476.77 | SNP | Rv3478 (PPE60) | Pro387Leu | 3 | - | | 3895727 | C | A | 963.77 | SNP | intergenic |  |  | - | | 3896340 | T | G | 598.77 | SNP | Rv3479 | Leu174Arg | 1 | - | | 3898408 | A | G | 1326.77 | SNP | Rv3479 | silent (Ala863) | 9867 | - | | 3899672 | C | T | 740.77 | SNP | Rv3480c | Arg244His | 8 | - | | 3901784 | G | A | 817.77 | SNP | Rv3482c | Thr108Ile | 7 | - | | 3902318 | A | AT | 1204.73 | INS | Rv3483c |  |  | - | | 3906311 | G | A | 774.77 | SNP | Rv3487c (lipF) | Arg233Cys | 1 | - | | 3908465 | A | G | 835.77 | SNP | Rv3490 (otsA) | Glu77Gly | 7 | - | | 3909235 | G | C | 1157.77 | SNP | Rv3490 (otsA) | Val(s)334Leu | 3 | - | | 3911939 | C | A | 505.77 | SNP | Rv3494c (mce4F) | silent (Pro477) | 9926 | - | | 3916386 | G | T | 726.77 | SNP | Rv3497c (mce4C) | Arg191Ser | 11 | - | | 3926653 | A | C | 713.77 | SNP | Rv3507 (PE\_PGRS53) | Asn29His | 18 | - | | 3934542 | T | G | 47.74 | SNP | Rv3508 (PE\_PGRS54) | Ser1180Ala | 35 | - | | 3934699 | G | A | 33.74 | SNP | Rv3508 (PE\_PGRS54) | Ser1232Asn | 20 | - | | 3934733 | G | C | 69.28 | SNP | Rv3508 (PE\_PGRS54) | silent (Gly1243) | 9935 | - | | 3934734 | G | A | 59.28 | SNP | Rv3508 (PE\_PGRS54) | Ala1244Thr | 22 | - | | 3940802 | A | G | 100.03 | SNP | Rv3511 (PE\_PGRS55) | Asn396Asp | 42 | - | | 3941276 | ACCGGCGGGG | A | 782.77 | DEL | Rv3511 (PE\_PGRS55) |  |  | - | | 3941834 | G | C | 92.85 | SNP | intergenic |  |  | - | | 3941836 | C | A | 139.10 | SNP | intergenic |  |  | - | | 3942481 | C | G | 45.74 | SNP | intergenic |  |  | - | | 3942640 | T | C | 122.03 | SNP | intergenic |  |  | - | | 3943744 | CCGGCAACGG | C | 535.87 | DEL | intergenic |  |  | - | | 3952308 | C | A | 1198.77 | SNP | Rv3515c (fadD19) | Asp55Tyr | 0 | - | | 3952800 | G | A | 795.77 | SNP | Rv3516 (echA19) | Gly86Asp | 6 | - | | 3958403 | A | G | 1066.77 | SNP | Rv3521 | Asn295Asp | 42 | - | | 3959418 | C | T | 701.77 | SNP | Rv3522 (ltp4) | Thr324Ile | 7 | - | | 3959730 | T | C | 877.77 | SNP | Rv3523 (ltp3) | Phe68Leu | 13 | - | | 3962187 | G | A | 1180.77 | SNP | Rv3525c | silent (Asp46) | 9859 | - | | 3964518 | G | A | 1084.77 | SNP | Rv3528c | silent (Pro225) | 9926 | - | | 3968006 | A | C | 976.77 | SNP | Rv3531c | Phe313Leu(s) | 2 | - | | 3970594 | C | G | 1263.77 | SNP | intergenic |  |  | - | | 3976595 | G | T | 913.77 | SNP | Rv3537 (kstD) | Arg409Ser | 11 | - | | 3979990 | C | G | 1389.77 | SNP | Rv3540c (ltp2) | Val(s)224Leu | 3 | - | | 3981983 | G | A | 378.77 | SNP | Rv3543c (fadE29) | silent (Pro386) | 9926 | - | | 3984321 | G | A | 873.77 | SNP | Rv3545c (cyp125) | silent (His375) | 9912 | - | | 3987370 | CG | C | 1122.73 | DEL | intergenic |  |  | - | | 3993058 | G | A | 1005.77 | SNP | Rv3554 (fdxB) | Gly125Glu | 4 | - | | 4001622 | T | C | 1213.77 | SNP | intergenic |  |  | - | | 4005114 | G | C | 944.77 | SNP | Rv3563 (fadE32) | Trp275Ser | 5 | - | | 4005607 | T | C | 412.77 | SNP | Rv3564 (fadE33) | Leu(s)121Leu | 3 | - | | 4008747 | A | G | 671.77 | SNP | Rv3567c (hsaB) | Ile179Thr | 11 | - | | 4022388 | G | A | 686.77 | SNP | Rv3579c | silent (Pro2) | 9926 | - | | 4024273 | T | C | 839.77 | SNP | Rv3581c (ispF) | Val25Val(s) | 18 | - | | 4026899 | G | A | 1020.77 | SNP | Rv3585 (radA) | silent (Gln152) | 9876 | - | | 4028752 | A | G | 1108.77 | SNP | Rv3586 | Thr288Ala | 32 | - | | 4032218 | G | A | 185.90 | SNP | Rv3590c (PE\_PGRS58) | Ala314Val | 13 | - | | 4032625 | G | T | 70.28 | SNP | Rv3590c (PE\_PGRS58) | silent (Gly178) | 9935 | - | | 4033577 | A | G | 1036.77 | SNP | Rv3591c | Phe156Leu | 13 | - | | 4034827 | C | T | 937.77 | SNP | Rv3593 (lpqF) | Ala159Val(s) | 9867 | - | | 4037283 | T | G | 125.03 | SNP | Rv3595c (PE\_PGRS59) | silent (Gly256) | 9935 | - | | 4041581 | C | T | 1126.77 | SNP | Rv3598c (lysS) | Ala454Thr | 22 | - | | 4050811 | G | A | 513.77 | SNP | Rv3610c (ftsH) | silent (Tyr691) | 9945 | - | | 4053050 | A | G | 306.78 | SNP | Rv3611 | Asn34Ser | 34 | - | | 4055801 | G | A | 1059.77 | SNP | Rv3616c (espA) | Thr192Ile | 7 | - | | 4056416 | C | A | 1594.77 | SNP | intergenic |  |  | - | | 4056685 | G | A | 1333.77 | SNP | intergenic |  |  | - | | 4056693 | G | T | 1591.77 | SNP | intergenic |  |  | - | | 4059143 | A | C | 759.77 | SNP | Rv3618 | Glu149Ala | 17 | - | | 4059904 | A | G | 590.77 | SNP | intergenic |  |  | - | | 4060100 | G | A | 415.77 | SNP | Rv3619c (esxV) | Leu57Leu(s) | 4 | - | | 4060201 | G | A | 207.80 | SNP | Rv3619c (esxV) | Ser23Leu(s) | 35 | - | | 4060210 | T | A | 174.84 | SNP | Rv3619c (esxV) | Gln20Leu | 6 | - | | 4060230 | G | A | 213.80 | SNP | Rv3619c (esxV) | silent (His13) | 9912 | - | | 4060588 | T | C | 716.77 | SNP | Rv3620c (esxW) | Thr2Ala | 32 | - | | 4069292 | G | A | 744.77 | SNP | Rv3630 | Ala40Thr | 22 | - | | 4070225 | G | A | 528.77 | SNP | Rv3630 | Ala351Thr | 22 | - | | 4088287 | A | T | 45.77 | SNP | intergenic |  |  | - | | 4088288 | A | T | 48.77 | SNP | intergenic |  |  | - | | 4088337 | G | A | 947.77 | SNP | Rv3648c (cspA) | silent (Arg65) | 9913 | - | | 4089058 | T | C | 711.77 | SNP | Rv3649 | Leu93Pro | 2 | - | | 4095001 | CG | C | 1551.73 | DEL | Rv3655c |  |  | - | | 4095295 | T | C | 937.77 | SNP | Rv3655c | silent (Glu2) | 9865 | - | | 4100975 | T | C | 824.77 | SNP | intergenic |  |  | - | | 4107074 | T | C | 762.77 | SNP | Rv3666c (dppA) | Gln4Arg | 10 | - | | 4111303 | G | C | 849.77 | SNP | Rv3669 | Val(s)159Val | 13 | - | | 4112429 | T | C | 1037.77 | SNP | Rv3671c | Ile363Val | 57 | - | | 4115890 | G | C | 1484.77 | SNP | Rv3674c (nth) | Pro2Arg | 4 | - | | 4116610 | G | A | 1911.77 | SNP | Rv3676 (crp) | Asp45Asn | 36 | - | | 4120390 | G | C | 1030.77 | SNP | Rv3680 | Gly199Ala | 21 | - | | 4120926 | A | G | 431.77 | SNP | Rv3680 | Asn378Asp | 42 | - | | 4120983 | A | G | 1037.77 | SNP | intergenic |  |  | - | | 4122287 | G | T | 505.77 | SNP | Rv3682 (ponA2) | silent (Ala124) | 9867 | - | | 4123770 | G | A | 757.77 | SNP | Rv3682 (ponA2) | Ala619Thr | 22 | - | | 4124349 | GCC | G | 1352.73 | DEL | intergenic |  |  | - | | 4133316 | A | T | 723.77 | SNP | Rv3691 | Thr267Ser | 38 | - | | 4137829 | C | T | 801.77 | SNP | Rv3695 | silent (Ala208) | 9867 | - | | 4139183 | A | AC | 501.73 | INS | Rv3696c (glpK) |  |  | - | | 4145737 | A | G | 562.77 | SNP | Rv3703c | silent (Tyr385) | 9945 | - | | 4151855 | A | G | 759.77 | SNP | Rv3708c (asd) | silent (Pro121) | 9926 | - | | 4156099 | C | A | 780.77 | SNP | Rv3711c (dnaQ) | Val(s)211Leu(s) | 9867 | - | | 4156503 | C | T | 840.77 | SNP | Rv3711c (dnaQ) | Gly76Asp | 6 | - | | 4158493 | C | T | 820.77 | SNP | Rv3713 (cobQ2) | silent (Ile89) | 9872 | - | | 4159195 | T | C | 919.77 | SNP | Rv3714c | silent (Pro209) | 9926 | - | | 4160371 | C | A | 1479.77 | SNP | Rv3715c (recR) | Gly44Cys | 0 | - | | 4161798 | C | T | 575.77 | SNP | intergenic |  |  | - | | 4162339 | A | G | 1668.77 | SNP | Rv3719 | Thr12Ala | 32 | - | | 4166441 | G | A | 854.77 | SNP | Rv3721c (dnaZX) | silent (His97) | 9912 | - | | 4167656 | A | G | 1111.77 | SNP | Rv3722c | Met(s)158Thr | 22 | - | | 4169852 | G | T | 949.77 | SNP | Rv3724B (cut5b) | Ala83Ser | 28 | - | | 4170739 | G | C | 1001.77 | SNP | Rv3725 | Val176Leu | 15 | - | | 4170964 | G | GA | 1544.73 | INS | Rv3725 |  |  | - | | 4174131 | G | C | 1567.77 | SNP | Rv3727 | Gly393Arg | 0 | - | | 4179089 | C | T | 692.77 | SNP | Rv3729 | Pro269Ser | 17 | - | | 4179832 | G | C | 1037.77 | SNP | Rv3729 | Gln516His | 20 | - | | 4182387 | G | C | 833.77 | SNP | Rv3731 (ligC) | Val(s)210Val | 13 | - | | 4182695 | G | A | 859.77 | SNP | Rv3731 (ligC) | Arg313His | 8 | - | | 4186678 | G | A | 655.77 | SNP | Rv3736 | silent (Leu15) | 9947 | - | | 4187485 | T | C | 830.77 | SNP | Rv3736 | silent (Ala284) | 9867 | - | | 4187817 | A | G | 930.77 | SNP | Rv3737 | Asp40Gly | 11 | - | | 4189210 | G | T | 489.77 | SNP | Rv3737 | silent (Pro504) | 9926 | - | | 4194501 | G | A | 1007.77 | SNP | Rv3743c (ctpJ) | silent (Phe291) | 9946 | - | | 4197138 | C | CT | 878.73 | INS | intergenic |  |  | - | | 4198611 | CG | C | 1510.73 | DEL | intergenic |  |  | - | | 4204168 | T | C | 895.77 | SNP | Rv3758c (proV) | Asn84Asp | 42 | - | | 4204441 | A | G | 1394.77 | SNP | Rv3759c (proX) | silent (His311) | 9912 | - | | 4205120 | A | G | 1161.77 | SNP | Rv3759c (proX) | Leu85Pro | 2 | - | | 4205325 | A | G | 1142.77 | SNP | Rv3759c (proX) | Trp17Arg | 8 | - | | 4210274 | A | G | 1010.77 | SNP | Rv3764c (tcrY) | Cys246Arg | 1 | - | | 4212832 | GACC | G | 2134.73 | DEL | Rv3766 |  |  | - | | 4214988 | G | A | 1045.77 | SNP | intergenic |  |  | - | | 4215233 | GGCC | G | 1856.73 | DEL | Rv3770c |  |  | - | | 4215484 | G | C | 834.77 | SNP | Rv3770c | Pro98Ala | 22 | - | | 4217557 | A | G | 849.77 | SNP | Rv3772 (hisC2) | Thr142Ala | 32 | - | | 4218416 | C | A | 538.89 | SNP | Rv3773c | Gly137Val(s) | 21 | - | | 4218941 | G | A | 1006.77 | SNP | Rv3774 (echA21) | silent (Gly31) | 9935 | - | | 4221070 | AC | A | 1561.73 | DEL | intergenic |  |  | - | | 4221423 | C | T | 713.77 | SNP | Rv3776 | Ser112Leu(s) | 35 | - | | 4221490 | C | G | 269.78 | SNP | Rv3776 | silent (Leu134) | 9947 | - | | 4222073 | A | G | 158.90 | SNP | Rv3776 | Met(s)329Val(s) | 9867 | - | | 4222882 | A | G | 962.77 | SNP | Rv3777 | silent (Leu63) | 9947 | - | | 4223172 | T | C | 641.77 | SNP | Rv3777 | Val160Ala | 18 | - | | 4229167 | T | C | 707.77 | SNP | Rv3782 (glfT1) | Val(s)274Ala | 9867 | - | | 4231859 | CGCGGTCTGTGCGCCGGGCG CACGCACCGAATTCGAGCTA TTAGCGGCCGCGGCAAGGGA CGCATTCGGCCTGGACGTCC ACCCAGCGGT | C | 553.73 | DEL | Rv3785 |  |  | - | | 4234960 | A | C | 342.77 | SNP | Rv3788 | Ile61Leu | 22 | - | | 4236237 | C | T | 914.77 | SNP | Rv3790 (dprE1) | silent (Thr153) | 9871 | - | | 4242643 | C | T | 839.77 | SNP | Rv3793 (embC) | silent (Arg927) | 9913 | genotype | | 4243460 | C | T | 868.77 | SNP | Rv3794 (embA) | silent (Cys76) | 9973 | genotype | | 4247429 | A | T | 1277.77 | SNP | Rv3795 (embB) | Met(s)306Leu(s) | 9867 | - (C would be resistance) | | 4251297 | G | C | 812.77 | SNP | Rv3797 (fadE35) | silent (Gly71) | 9935 | - | | 4252066 | T | C | 743.77 | SNP | Rv3797 (fadE35) | Ser328Pro | 12 | - | | 4254290 | T | G | 482.77 | SNP | Rv3798 | Leu433Arg | 1 | - | | 4254431 | G | A | 728.77 | SNP | Rv3799c (accD4) | silent (Asp506) | 9859 | - | | 4255922 | A | G | 1287.77 | SNP | Rv3799c (accD4) | silent (His9) | 9912 | - | | 4256210 | G | T | 946.77 | SNP | Rv3800c (pks13) | Ala1646Asp | 6 | - | | 4257220 | A | G | 706.77 | SNP | Rv3800c (pks13) | silent (Arg1309) | 9913 | - | | 4258106 | G | A | 1173.77 | SNP | Rv3800c (pks13) | Thr1014Met(s) | 32 | - | | 4262388 | C | T | 715.77 | SNP | Rv3801c (fadD32) | Gly227Ser | 16 | - | | 4264381 | G | GC | 1949.73 | INS | intergenic |  |  | - | | 4267647 | T | C | 805.77 | SNP | Rv3805c (aftB) | Asp397Gly | 11 | - | | 4279750 | G | C | 1390.77 | SNP | Rv3814c | Pro89Arg | 4 | - | | 4280708 | G | A | 654.77 | SNP | Rv3815c | silent (Val27) | 9901 | - | | 4284429 | G | A | 1255.77 | SNP | Rv3820c (papA2) | Pro466Leu | 3 | - | | 4287164 | A | G | 1086.77 | SNP | Rv3822 | silent (Gly148) | 9935 | - | | 4290135 | C | T | 1151.77 | SNP | Rv3823c (mmpL8) | silent (Leu465) | 9947 | - | | 4296015 | G | A | 1360.77 | SNP | Rv3825c (pks2) | silent (Asp1197) | 9859 | - | | 4301075 | G | C | 641.77 | SNP | Rv3826 (fadD23) | Glu422Gln | 27 | - | | 4302036 | T | C | 577.77 | SNP | Rv3827c | Thr252Ala | 32 | - | | 4303498 | T | TG | 2053.73 | INS | Rv3829c |  |  | - | | 4305063 | G | GA | 1302.73 | INS | Rv3830c |  |  | - | | 4306155 | C | T | 754.77 | SNP | Rv3831 | silent (Ser133) | 9840 | - | | 4307179 | G | A | 890.77 | SNP | Rv3833 | Val105Ile | 33 | - | | 4308395 | G | A | 1571.77 | SNP | Rv3834c (serS) | Leu174Leu(s) | 4 | - | | 4309927 | T | G | 339.31 | SNP | Rv3835 | Leu294Arg | 1 | - | | 4311664 | C | T | 638.77 | SNP | Rv3837c | Gly15Asp | 6 | - | | 4313128 | C | T | 1108.77 | SNP | Rv3839 | Pro122Ser | 17 | - | | 4314645 | A | G | 576.77 | SNP | Rv3841 (bfrB) | silent (Leu156) | 9947 | - | | 4314800 | G | A | 761.77 | SNP | Rv3842c (glpQ1) | His255Tyr | 4 | - | | 4319985 | G | C | 449.77 | SNP | intergenic |  |  | - | | 4322039 | AC | A | 768.73 | DEL | Rv3847 |  |  | - | | 4329782 | G | A | 1304.77 | SNP | intergenic |  |  | - | | 4338595 | GC | G | 2093.73 | DEL | intergenic |  |  | - | | 4338732 | G | A | 1335.77 | SNP | intergenic |  |  | - | | 4340028 | G | C | 685.77 | SNP | intergenic |  |  | - | | 4350305 | G | A | 647.77 | SNP | Rv3871 (eccCb1) | silent (Pro493) | 9926 | - | | 4351039 | G | T | 957.77 | SNP | Rv3872 (PE35) | Glu99STOP | 17 | - | | 4356110 | G | C | 791.77 | SNP | Rv3877 (eccD1) | silent (Leu368) | 9947 | - | | 4357597 | C | G | 501.77 | SNP | Rv3879c (espK) | Cys729Ser | 11 | - | | 4357804 | T | G | 661.77 | SNP | Rv3879c (espK) | Glu660Ala | 17 | - | | 4359135 | T | TGGGGTTCCCGGGGTGATCG GGGTTCCCGGCGTGATC | 2773.73 | INS | Rv3879c (espK) |  |  | - | | 4359165 | G | C | 401.77 | SNP | Rv3879c (espK) | silent (Thr206) | 9871 | - | | 4359653 | C | T | 859.77 | SNP | Rv3879c (espK) | Asp44Asn | 36 | - | | 4359828 | G | A | 984.77 | SNP | intergenic |  |  | - | | 4362568 | T | G | 660.77 | SNP | Rv3882c (eccE1) | silent (Arg285) | 9913 | - | | 4366195 | T | C | 849.77 | SNP | Rv3884c (eccA2) | Glu215Gly | 7 | - | | 4366272 | G | C | 703.77 | SNP | Rv3884c (eccA2) | silent (Ala189) | 9867 | - | | 4368084 | G | C | 579.77 | SNP | Rv3885c (eccE2) | Val146Val(s) | 18 | - | | 4372353 | G | C | 1025.77 | SNP | Rv3888c | silent (Arg118) | 9913 | - | | 4375628 | G | T | 747.77 | SNP | Rv3892c (PPE69) | Thr19Lys | 11 | - | | 4378504 | T | C | 828.77 | SNP | Rv3894c (eccC2) | Asp650Gly | 11 | - | | 4379044 | CG | C | 702.73 | DEL | Rv3894c (eccC2) |  |  | - | | 4379680 | C | G | 795.77 | SNP | Rv3894c (eccC2) | Arg258Pro | 5 | - | | 4382054 | T | C | 983.77 | SNP | Rv3896c | silent (Ala266) | 9867 | - | | 4382275 | G | T | 886.77 | SNP | Rv3896c | Gln193Lys | 12 | - | | 4383144 | C | CCGGGG | 1928.73 | INS | Rv3897c |  |  | - | | 4383655 | A | G | 355.77 | SNP | Rv3898c | STOP111Gln | 3 | - | | 4384007 | C | G | 780.77 | SNP | intergenic |  |  | - | | 4386228 | T | C | 1046.77 | SNP | Rv3900c | silent (Leu27) | 9947 | - | | 4388124 | T | C | 1033.77 | SNP | Rv3903c | Glu770Gly | 7 | - | | 4393178 | A | G | 830.77 | SNP | intergenic |  |  | - | | 4393590 | C | G | 652.77 | SNP | Rv3908 (mutT4) | Arg48Gly | 1 | - | | 4397736 | C | T | 931.77 | SNP | Rv3910 | silent (Ile380) | 9872 | - | | 4400660 | AC | A | 811.73 | DEL | Rv3911 (sigM) |  |  | - | | 4407588 | T | C | 1305.77 | SNP | Rv3919c (gid) | silent (Ala205) | 9867 | genotype | | 4407927 | T | G | 1233.77 | SNP | Rv3919c (gid) | Glu92Asp | 53 | genotype | | 4408923 | C | T | 1215.77 | SNP | intergenic |  |  | - | |  | | export |

elog
