# Supplementary material for: Patterns of compensatory mutations in rpoA/B/C genes of multidrug resistant M. tuberculosis in Uganda
Source: PLoS One. 2025 Dec 4;20(12):e0328957. doi: 10.1371/journal.pone.0328957 (PMC12677784; doi:10.1371/journal.pone.0328957)
Supplement: S2 File — (ZIP) [file pone.0328957.s002.zip › Variants J_S10_L001_001.bam.html]

 

Calling SNPs/INDELs (computing variant list in .vcf format) from J\_S10\_L001\_001.bam

*by SAMtools/BCFtools:*

Howto

Important aspects

This takes up to one hour!!! **Please wait ...**

Variants J\_S10\_L001\_001.bam

|  |  |
| --- | --- |
| Variants |  |

|  |  |
| --- | --- |
| |  | | --- | | *by GATK* | |

|  |  |  |
| --- | --- | --- |
| |  | | --- | | J\_S10\_L001\_001.bam | | | computed 2016-10-27 using PhyResSE v1.0 (Ref. NC\_000962.3) | |

|  |  |
| --- | --- |
| 1968  variants called Export in VCF format |  |

|  |  |  |  |  |  |  |  |  |  |  |  |  |  |  |  |  |  |  |  |  |  |  |  |  |  |  |  |  |  |  |  |  |  |  |  |  |  |  |  |  |  |  |  |  |  |  |  |  |  |  |  |  |  |  |  |  |  |  |  |  |  |  |  |  |  |  |  |  |  |  |  |  |  |  |  |  |  |  |  |  |  |  |  |  |  |  |  |  |  |  |  |  |  |  |  |  |  |  |  |  |  |  |  |  |  |  |  |  |  |  |  |  |  |  |  |  |  |  |  |  |  |  |  |  |  |  |  |  |  |  |  |  |  |  |  |  |  |  |  |  |  |  |  |  |  |  |  |  |  |  |  |  |  |  |  |  |  |  |  |  |  |  |  |  |  |  |  |  |  |  |  |  |  |  |  |  |  |  |  |  |  |  |  |  |  |  |  |  |  |  |  |  |  |  |  |  |  |  |  |  |  |  |  |  |  |  |  |  |  |  |  |  |  |  |  |  |  |  |  |  |  |  |  |  |  |  |  |  |  |  |  |  |  |  |  |  |  |  |  |  |  |  |  |  |  |  |  |  |  |  |  |  |  |  |  |  |  |  |  |  |  |  |  |  |  |  |  |  |  |  |  |  |  |  |  |  |  |  |  |  |  |  |  |  |  |  |  |  |  |  |  |  |  |  |  |  |  |  |  |  |  |  |  |  |  |  |  |  |  |  |  |  |  |  |  |  |  |  |  |  |  |  |  |  |  |  |  |  |  |  |  |  |  |  |  |  |  |  |  |  |  |  |  |  |  |  |  |  |  |  |  |  |  |  |  |  |  |  |  |  |  |  |  |  |  |  |  |  |  |  |  |  |  |  |  |  |  |  |  |  |  |  |  |  |  |  |  |  |  |  |  |  |  |  |  |  |  |  |  |  |  |  |  |  |  |  |  |  |  |  |  |  |  |  |  |  |  |  |  |  |  |  |  |  |  |  |  |  |  |  |  |  |  |  |  |  |  |  |  |  |  |  |  |  |  |  |  |  |  |  |  |  |  |  |  |  |  |  |  |  |  |  |  |  |  |  |  |  |  |  |  |  |  |  |  |  |  |  |  |  |  |  |  |  |  |  |  |  |  |  |  |  |  |  |  |  |  |  |  |  |  |  |  |  |  |  |  |  |  |  |  |  |  |  |  |  |  |  |  |  |  |  |  |  |  |  |  |  |  |  |  |  |  |  |  |  |  |  |  |  |  |  |  |  |  |  |  |  |  |  |  |  |  |  |  |  |  |  |  |  |  |  |  |  |  |  |  |  |  |  |  |  |  |  |  |  |  |  |  |  |  |  |  |  |  |  |  |  |  |  |  |  |  |  |  |  |  |  |  |  |  |  |  |  |  |  |  |  |  |  |  |  |  |  |  |  |  |  |  |  |  |  |  |  |  |  |  |  |  |  |  |  |  |  |  |  |  |  |  |  |  |  |  |  |  |  |  |  |  |  |  |  |  |  |  |  |  |  |  |  |  |  |  |  |  |  |  |  |  |  |  |  |  |  |  |  |  |  |  |  |  |  |  |  |  |  |  |  |  |  |  |  |  |  |  |  |  |  |  |  |  |  |  |  |  |  |  |  |  |  |  |  |  |  |  |  |  |  |  |  |  |  |  |  |  |  |  |  |  |  |  |  |  |  |  |  |  |  |  |  |  |  |  |  |  |  |  |  |  |  |  |  |  |  |  |  |  |  |  |  |  |  |  |  |  |  |  |  |  |  |  |  |  |  |  |  |  |  |  |  |  |  |  |  |  |  |  |  |  |  |  |  |  |  |  |  |  |  |  |  |  |  |  |  |  |  |  |  |  |  |  |  |  |  |  |  |  |  |  |  |  |  |  |  |  |  |  |  |  |  |  |  |  |  |  |  |  |  |  |  |  |  |  |  |  |  |  |  |  |  |  |  |  |  |  |  |  |  |  |  |  |  |  |  |  |  |  |  |  |  |  |  |  |  |  |  |  |  |  |  |  |  |  |  |  |  |  |  |  |  |  |  |  |  |  |  |  |  |  |  |  |  |  |  |  |  |  |  |  |  |  |  |  |  |  |  |  |  |  |  |  |  |  |  |  |  |  |  |  |  |  |  |  |  |  |  |  |  |  |  |  |  |  |  |  |  |  |  |  |  |  |  |  |  |  |  |  |  |  |  |  |  |  |  |  |  |  |  |  |  |  |  |  |  |  |  |  |  |  |  |  |  |  |  |  |  |  |  |  |  |  |  |  |  |  |  |  |  |  |  |  |  |  |  |  |  |  |  |  |  |  |  |  |  |  |  |  |  |  |  |  |  |  |  |  |  |  |  |  |  |  |  |  |  |  |  |  |  |  |  |  |  |  |  |  |  |  |  |  |  |  |  |  |  |  |  |  |  |  |  |  |  |  |  |  |  |  |  |  |  |  |  |  |  |  |  |  |  |  |  |  |  |  |  |  |  |  |  |  |  |  |  |  |  |  |  |  |  |  |  |  |  |  |  |  |  |  |  |  |  |  |  |  |  |  |  |  |  |  |  |  |  |  |  |  |  |  |  |  |  |  |  |  |  |  |  |  |  |  |  |  |  |  |  |  |  |  |  |  |  |  |  |  |  |  |  |  |  |  |  |  |  |  |  |  |  |  |  |  |  |  |  |  |  |  |  |  |  |  |  |  |  |  |  |  |  |  |  |  |  |  |  |  |  |  |  |  |  |  |  |  |  |  |  |  |  |  |  |  |  |  |  |  |  |  |  |  |  |  |  |  |  |  |  |  |  |  |  |  |  |  |  |  |  |  |  |  |  |  |  |  |  |  |  |  |  |  |  |  |  |  |  |  |  |  |  |  |  |  |  |  |  |  |  |  |  |  |  |  |  |  |  |  |  |  |  |  |  |  |  |  |  |  |  |  |  |  |  |  |  |  |  |  |  |  |  |  |  |  |  |  |  |  |  |  |  |  |  |  |  |  |  |  |  |  |  |  |  |  |  |  |  |  |  |  |  |  |  |  |  |  |  |  |  |  |  |  |  |  |  |  |  |  |  |  |  |  |  |  |  |  |  |  |  |  |  |  |  |  |  |  |  |  |  |  |  |  |  |  |  |  |  |  |  |  |  |  |  |  |  |  |  |  |  |  |  |  |  |  |  |  |  |  |  |  |  |  |  |  |  |  |  |  |  |  |  |  |  |  |  |  |  |  |  |  |  |  |  |  |  |  |  |  |  |  |  |  |  |  |  |  |  |  |  |  |  |  |  |  |  |  |  |  |  |  |  |  |  |  |  |  |  |  |  |  |  |  |  |  |  |  |  |  |  |  |  |  |  |  |  |  |  |  |  |  |  |  |  |  |  |  |  |  |  |  |  |  |  |  |  |  |  |  |  |  |  |  |  |  |  |  |  |  |  |  |  |  |  |  |  |  |  |  |  |  |  |  |  |  |  |  |  |  |  |  |  |  |  |  |  |  |  |  |  |  |  |  |  |  |  |  |  |  |  |  |  |  |  |  |  |  |  |  |  |  |  |  |  |  |  |  |  |  |  |  |  |  |  |  |  |  |  |  |  |  |  |  |  |  |  |  |  |  |  |  |  |  |  |  |  |  |  |  |  |  |  |  |  |  |  |  |  |  |  |  |  |  |  |  |  |  |  |  |  |  |  |  |  |  |  |  |  |  |  |  |  |  |  |  |  |  |  |  |  |  |  |  |  |  |  |  |  |  |  |  |  |  |  |  |  |  |  |  |  |  |  |  |  |  |  |  |  |  |  |  |  |  |  |  |  |  |  |  |  |  |  |  |  |  |  |  |  |  |  |  |  |  |  |  |  |  |  |  |  |  |  |  |  |  |  |  |  |  |  |  |  |  |  |  |  |  |  |  |  |  |  |  |  |  |  |  |  |  |  |  |  |  |  |  |  |  |  |  |  |  |  |  |  |  |  |  |  |  |  |  |  |  |  |  |  |  |  |  |  |  |  |  |  |  |  |  |  |  |  |  |  |  |  |  |  |  |  |  |  |  |  |  |  |  |  |  |  |  |  |  |  |  |  |  |  |  |  |  |  |  |  |  |  |  |  |  |  |  |  |  |  |  |  |  |  |  |  |  |  |  |  |  |  |  |  |  |  |  |  |  |  |  |  |  |  |  |  |  |  |  |  |  |  |  |  |  |  |  |  |  |  |  |  |  |  |  |  |  |  |  |  |  |  |  |  |  |  |  |  |  |  |  |  |  |  |  |  |  |  |  |  |  |  |  |  |  |  |  |  |  |  |  |  |  |  |  |  |  |  |  |  |  |  |  |  |  |  |  |  |  |  |  |  |  |  |  |  |  |  |  |  |  |  |  |  |  |  |  |  |  |  |  |  |  |  |  |  |  |  |  |  |  |  |  |  |  |  |  |  |  |  |  |  |  |  |  |  |  |  |  |  |  |  |  |  |  |  |  |  |  |  |  |  |  |  |  |  |  |  |  |  |  |  |  |  |  |  |  |  |  |  |  |  |  |  |  |  |  |  |  |  |  |  |  |  |  |  |  |  |  |  |  |  |  |  |  |  |  |  |  |  |  |  |  |  |  |  |  |  |  |  |  |  |  |  |  |  |  |  |  |  |  |  |  |  |  |  |  |  |  |  |  |  |  |  |  |  |  |  |  |  |  |  |  |  |  |  |  |  |  |  |  |  |  |  |  |  |  |  |  |  |  |  |  |  |  |  |  |  |  |  |  |  |  |  |  |  |  |  |  |  |  |  |  |  |  |  |  |  |  |  |  |  |  |  |  |  |  |  |  |  |  |  |  |  |  |  |  |  |  |  |  |  |  |  |  |  |  |  |  |  |  |  |  |  |  |  |  |  |  |  |  |  |  |  |  |  |  |  |  |  |  |  |  |  |  |  |  |  |  |  |  |  |  |  |  |  |  |  |  |  |  |  |  |  |  |  |  |  |  |  |  |  |  |  |  |  |  |  |  |  |  |  |  |  |  |  |  |  |  |  |  |  |  |  |  |  |  |  |  |  |  |  |  |  |  |  |  |  |  |  |  |  |  |  |  |  |  |  |  |  |  |  |  |  |  |  |  |  |  |  |  |  |  |  |  |  |  |  |  |  |  |  |  |  |  |  |  |  |  |  |  |  |  |  |  |  |  |  |  |  |  |  |  |  |  |  |  |  |  |  |  |  |  |  |  |  |  |  |  |  |  |  |  |  |  |  |  |  |  |  |  |  |  |  |  |  |  |  |  |  |  |  |  |  |  |  |  |  |  |  |  |  |  |  |  |  |  |  |  |  |  |  |  |  |  |  |  |  |  |  |  |  |  |  |  |  |  |  |  |  |  |  |  |  |  |  |  |  |  |  |  |  |  |  |  |  |  |  |  |  |  |  |  |  |  |  |  |  |  |  |  |  |  |  |  |  |  |  |  |  |  |  |  |  |  |  |  |  |  |  |  |  |  |  |  |  |  |  |  |  |  |  |  |  |  |  |  |  |  |  |  |  |  |  |  |  |  |  |  |  |  |  |  |  |  |  |  |  |  |  |  |  |  |  |  |  |  |  |  |  |  |  |  |  |  |  |  |  |  |  |  |  |  |  |  |  |  |  |  |  |  |  |  |  |  |  |  |  |  |  |  |  |  |  |  |  |  |  |  |  |  |  |  |  |  |  |  |  |  |  |  |  |  |  |  |  |  |  |  |  |  |  |  |  |  |  |  |  |  |  |  |  |  |  |  |  |  |  |  |  |  |  |  |  |  |  |  |  |  |  |  |  |  |  |  |  |  |  |  |  |  |  |  |  |  |  |  |  |  |  |  |  |  |  |  |  |  |  |  |  |  |  |  |  |  |  |  |  |  |  |  |  |  |  |  |  |  |  |  |  |  |  |  |  |  |  |  |  |  |  |  |  |  |  |  |  |  |  |  |  |  |  |  |  |  |  |  |  |  |  |  |  |  |  |  |  |  |  |  |  |  |  |  |  |  |  |  |  |  |  |  |  |  |  |  |  |  |  |  |  |  |  |  |  |  |  |  |  |  |  |  |  |  |  |  |  |  |  |  |  |  |  |  |  |  |  |  |  |  |  |  |  |  |  |  |  |  |  |  |  |  |  |  |  |  |  |  |  |  |  |  |  |  |  |  |  |  |  |  |  |  |  |  |  |  |  |  |  |  |  |  |  |  |  |  |  |  |  |  |  |  |  |  |  |  |  |  |  |  |  |  |  |  |  |  |  |  |  |  |  |  |  |  |  |  |  |  |  |  |  |  |  |  |  |  |  |  |  |  |  |  |  |  |  |  |  |  |  |  |  |  |  |  |  |  |  |  |  |  |  |  |  |  |  |  |  |  |  |  |  |  |  |  |  |  |  |  |  |  |  |  |  |  |  |  |  |  |  |  |  |  |  |  |  |  |  |  |  |  |  |  |  |  |  |  |  |  |  |  |  |  |  |  |  |  |  |  |  |  |  |  |  |  |  |  |  |  |  |  |  |  |  |  |  |  |  |  |  |  |  |  |  |  |  |  |  |  |  |  |  |  |  |  |  |  |  |  |  |  |  |  |  |  |  |  |  |  |  |  |  |  |  |  |  |  |  |  |  |  |  |  |  |  |  |  |  |  |  |  |  |  |  |  |  |  |  |  |  |  |  |  |  |  |  |  |  |  |  |  |  |  |  |  |  |  |  |  |  |  |  |  |  |  |  |  |  |  |  |  |  |  |  |  |  |  |  |  |  |  |  |  |  |  |  |  |  |  |  |  |  |  |  |  |  |  |  |  |  |  |  |  |  |  |  |  |  |  |  |  |  |  |  |  |  |  |  |  |  |  |  |  |  |  |  |  |  |  |  |  |  |  |  |  |  |  |  |  |  |  |  |  |  |  |  |  |  |  |  |  |  |  |  |  |  |  |  |  |  |  |  |  |  |  |  |  |  |  |  |  |  |  |  |  |  |  |  |  |  |  |  |  |  |  |  |  |  |  |  |  |  |  |  |  |  |  |  |  |  |  |  |  |  |  |  |  |  |  |  |  |  |  |  |  |  |  |  |  |  |  |  |  |  |  |  |  |  |  |  |  |  |  |  |  |  |  |  |  |  |  |  |  |  |  |  |  |  |  |  |  |  |  |  |  |  |  |  |  |  |  |  |  |  |  |  |  |  |  |  |  |  |  |  |  |  |  |  |  |  |  |  |  |  |  |  |  |  |  |  |  |  |  |  |  |  |  |  |  |  |  |  |  |  |  |  |  |  |  |  |  |  |  |  |  |  |  |  |  |  |  |  |  |  |  |  |  |  |  |  |  |  |  |  |  |  |  |  |  |  |  |  |  |  |  |  |  |  |  |  |  |  |  |  |  |  |  |  |  |  |  |  |  |  |  |  |  |  |  |  |  |  |  |  |  |  |  |  |  |  |  |  |  |  |  |  |  |  |  |  |  |  |  |  |  |  |  |  |  |  |  |  |  |  |  |  |  |  |  |  |  |  |  |  |  |  |  |  |  |  |  |  |  |  |  |  |  |  |  |  |  |  |  |  |  |  |  |  |  |  |  |  |  |  |  |  |  |  |  |  |  |  |  |  |  |  |  |  |  |  |  |  |  |  |  |  |  |  |  |  |  |  |  |  |  |  |  |  |  |  |  |  |  |  |  |  |  |  |  |  |  |  |  |  |  |  |  |  |  |  |  |  |  |  |  |  |  |  |  |  |  |  |  |  |  |  |  |  |  |  |  |  |  |  |  |  |  |  |  |  |  |  |  |  |  |  |  |  |  |  |  |  |  |  |  |  |  |  |  |  |  |  |  |  |  |  |  |  |  |  |  |  |  |  |  |  |  |  |  |  |  |  |  |  |  |  |  |  |  |  |  |  |  |  |  |  |  |  |  |  |  |  |  |  |  |  |  |  |  |  |  |  |  |  |  |  |  |  |  |  |  |  |  |  |  |  |  |  |  |  |  |  |  |  |  |  |  |  |  |  |  |  |  |  |  |  |  |  |  |  |  |  |  |  |  |  |  |  |  |  |  |  |  |  |  |  |  |  |  |  |  |  |  |  |  |  |  |  |  |  |  |  |  |  |  |  |  |  |  |  |  |  |  |  |  |  |  |  |  |  |  |  |  |  |  |  |  |  |  |  |  |  |  |  |  |  |  |  |  |  |  |  |  |  |  |  |  |  |  |  |  |  |  |  |  |  |  |  |  |  |  |  |  |  |  |  |  |  |  |  |  |  |  |  |  |  |  |  |  |  |  |  |  |  |  |  |  |  |  |  |  |  |  |  |  |  |  |  |  |  |  |  |  |  |  |  |  |  |  |  |  |  |  |  |  |  |  |  |  |  |  |  |  |  |  |  |  |  |  |  |  |  |  |  |  |  |  |  |  |  |  |  |  |  |  |  |  |  |  |  |  |  |  |  |  |  |  |  |  |  |  |  |  |  |  |  |  |  |  |  |  |  |  |  |  |  |  |  |  |  |  |  |  |  |  |  |  |  |  |  |  |  |  |  |  |  |  |  |  |  |  |  |  |  |  |  |  |  |  |  |  |  |  |  |  |  |  |  |  |  |  |  |  |  |  |  |  |  |  |  |  |  |  |  |  |  |  |  |  |  |  |  |  |  |  |  |  |  |  |  |  |  |  |  |  |  |  |  |  |  |  |  |  |  |  |  |  |  |  |  |  |  |  |  |  |  |  |  |  |  |  |  |  |  |  |  |  |  |  |  |  |  |  |  |  |  |  |  |  |  |  |  |  |  |  |  |  |  |  |  |  |  |  |  |  |  |  |  |  |  |  |  |  |  |  |  |  |  |  |  |  |  |  |  |  |  |  |  |  |  |  |  |  |  |  |  |  |  |  |  |  |  |  |  |  |  |  |  |  |  |  |  |  |  |  |  |  |  |  |  |  |  |  |  |  |  |  |  |  |  |  |  |  |  |  |  |  |  |  |  |  |  |  |  |  |  |  |  |  |  |  |  |  |  |  |  |  |  |  |  |  |  |  |  |  |  |  |  |  |  |  |  |  |  |  |  |  |  |  |  |  |  |  |  |  |  |  |  |  |  |  |  |  |  |  |  |  |  |  |  |  |  |  |  |  |  |  |  |  |  |  |  |  |  |  |  |  |  |  |  |  |  |  |  |  |  |  |  |  |  |  |  |  |  |  |  |  |  |  |  |  |  |  |  |  |  |  |  |  |  |  |  |  |  |  |  |  |  |  |  |  |  |  |  |  |  |  |  |  |  |  |  |  |  |  |  |  |  |  |  |  |  |  |  |  |  |  |  |  |  |  |  |  |  |  |  |  |  |  |  |  |  |  |  |  |  |  |  |  |  |  |  |  |  |  |  |  |  |  |  |  |  |  |  |  |  |  |  |  |  |  |  |  |  |  |  |  |  |  |  |  |  |  |  |  |  |  |  |  |  |  |  |  |  |  |  |  |  |  |  |  |  |  |  |  |  |  |  |  |  |  |  |  |  |  |  |  |  |  |  |  |  |  |  |  |  |  |  |  |  |  |  |  |  |  |  |  |  |  |  |  |  |  |  |  |  |  |  |  |  |  |  |  |  |  |  |  |  |  |  |  |  |  |  |  |  |  |  |  |  |  |  |  |  |  |  |  |  |  |  |  |  |  |  |  |  |  |  |  |  |  |  |  |  |  |  |  |  |  |  |  |  |  |  |  |  |  |  |  |  |  |  |  |  |  |  |  |  |  |  |  |  |  |  |  |  |  |  |  |  |  |  |  |  |  |  |  |  |  |  |  |  |  |  |  |  |  |  |  |  |  |  |  |  |  |  |  |  |  |  |  |  |  |  |  |  |  |  |  |  |  |  |  |  |  |  |  |  |  |  |  |  |  |  |  |  |  |  |  |  |  |  |  |  |  |  |  |  |  |  |  |  |  |  |  |  |  |  |  |  |  |  |  |  |  |  |  |  |  |  |  |  |  |  |  |  |  |  |  |  |  |  |  |  |  |  |  |  |  |  |  |  |  |  |  |  |  |  |  |  |  |  |  |  |  |  |  |  |  |  |  |  |  |  |  |  |  |  |  |  |  |  |  |  |  |  |  |  |  |  |  |  |  |  |  |  |  |  |  |  |  |  |  |  |  |  |  |  |  |  |  |  |  |  |  |  |  |  |  |  |  |  |  |  |  |  |  |  |  |  |  |  |  |  |  |  |  |  |  |  |  |  |  |  |  |  |  |  |  |  |  |  |  |  |  |  |  |  |  |  |  |  |  |  |  |  |  |  |  |  |  |  |  |  |  |  |  |  |  |  |  |  |  |  |  |  |  |  |  |  |  |  |  |  |  |  |  |  |  |  |  |  |  |  |  |  |  |  |  |  |  |  |  |  |  |  |  |  |  |  |  |  |  |  |  |  |  |  |  |  |  |  |  |  |  |  |  |  |  |  |  |  |  |  |  |  |  |  |  |  |  |  |  |  |  |  |  |  |  |  |  |  |  |  |  |  |  |  |  |  |  |  |  |  |  |  |  |  |  |  |  |  |  |  |  |  |  |  |  |  |  |  |  |  |  |  |  |  |  |  |  |  |  |  |  |  |  |  |  |  |  |  |  |  |  |  |  |  |  |  |  |  |  |  |  |  |  |  |  |  |  |  |  |  |  |  |  |  |  |  |  |  |  |  |  |  |  |  |  |  |  |  |  |  |  |  |  |  |  |  |  |  |  |  |  |  |  |  |  |  |  |  |  |  |  |  |  |  |  |  |  |  |  |  |  |  |  |  |  |  |  |  |  |  |  |  |  |  |  |  |  |  |  |  |  |  |  |  |  |  |  |  |  |  |  |  |  |  |  |  |  |  |  |  |  |  |  |  |  |  |  |  |  |  |  |  |  |  |  |  |  |  |  |  |  |  |  |  |  |  |  |  |  |  |  |  |  |  |  |  |  |  |  |  |  |  |  |  |  |  |  |  |  |  |  |  |  |  |  |  |  |  |  |  |  |  |  |  |  |  |  |  |  |  |  |  |  |  |  |  |  |  |  |  |  |  |  |  |  |  |  |  |  |  |  |  |  |  |  |  |  |  |  |  |  |  |  |  |  |  |  |  |  |  |  |  |  |  |  |  |  |  |  |  |  |  |  |  |  |  |  |  |  |  |  |  |  |  |  |  |  |  |  |  |  |  |  |  |  |  |  |  |  |  |  |  |  |  |  |  |  |  |  |  |  |  |  |  |  |  |  |  |  |  |  |  |  |  |  |  |  |  |  |  |  |  |  |  |  |  |  |  |  |  |  |  |  |  |  |  |  |  |  |  |  |  |  |  |  |  |  |  |  |  |  |  |  |  |  |  |  |  |  |  |  |  |  |  |  |  |  |  |  |  |  |  |  |  |  |  |  |  |  |  |  |  |  |  |  |  |  |  |  |  |  |  |  |  |  |  |  |  |  |  |  |  |  |  |  |  |  |  |  |  |  |  |  |  |  |  |  |  |  |  |  |  |  |  |  |  |  |  |  |  |  |  |  |  |  |  |  |  |  |  |  |  |  |  |  |  |  |  |  |  |  |  |  |  |  |  |  |  |  |  |  |  |  |  |  |  |  |  |  |  |  |  |  |  |  |  |  |  |  |  |  |  |  |  |  |  |  |  |  |  |  |  |  |  |  |  |  |  |  |  |  |  |  |  |  |  |  |  |  |  |  |  |  |  |  |  |  |  |  |  |  |  |  |  |  |  |  |  |  |  |  |  |  |  |  |  |  |  |  |  |  |  |  |  |  |  |  |  |  |  |  |  |  |  |  |  |  |  |  |  |  |  |  |  |  |  |  |  |  |  |  |  |  |  |  |  |  |  |  |  |  |  |  |  |  |  |  |  |  |  |  |  |  |  |  |  |  |  |  |  |  |  |  |  |  |  |  |  |  |  |  |  |  |  |  |  |  |  |  |  |  |  |  |  |  |  |  |  |  |  |  |  |  |  |  |  |  |  |  |  |  |  |  |  |  |  |  |  |  |  |  |  |  |  |  |  |  |  |  |  |  |  |  |  |  |  |  |  |  |  |  |  |  |  |  |  |  |  |  |  |  |  |  |  |  |  |  |  |  |  |  |  |  |  |  |  |  |  |  |  |  |  |  |  |  |  |  |  |  |  |  |  |  |  |  |  |  |  |  |  |  |  |  |  |  |  |  |  |  |  |  |  |  |  |  |  |  |  |  |  |  |  |  |  |  |  |  |  |  |  |  |  |  |  |  |  |  |  |  |  |  |  |  |  |  |  |  |  |  |  |  |  |  |  |  |  |  |  |  |  |  |  |  |  |  |  |  |  |  |  |  |  |  |  |  |  |  |  |  |  |  |  |  |  |  |  |  |  |  |  |  |  |  |  |  |  |  |  |  |  |  |  |  |  |  |  |  |  |  |  |  |  |  |  |  |  |  |  |  |  |  |  |  |  |  |  |  |  |  |  |  |  |  |  |  |  |  |  |  |  |  |  |  |  |  |  |  |  |  |  |  |  |  |  |  |  |  |  |  |  |  |  |  |  |  |  |  |  |  |  |  |  |  |  |  |  |  |  |  |  |  |  |  |  |  |  |  |  |  |  |  |  |  |  |  |  |  |  |  |  |  |  |  |  |  |  |  |  |  |  |  |  |  |  |  |  |  |  |  |  |  |  |  |  |  |  |  |  |  |  |  |  |  |  |  |  |  |  |  |  |  |  |  |  |  |  |  |  |  |  |  |  |  |  |  |  |  |  |  |  |  |  |  |  |  |  |  |  |  |  |  |  |  |  |  |  |  |  |  |  |  |  |  |  |  |  |  |  |  |  |  |  |  |  |  |  |  |  |  |  |  |  |  |  |  |  |  |  |  |  |  |  |  |  |  |  |  |  |  |  |  |  |  |  |  |  |  |  |  |  |  |  |  |  |  |  |  |  |  |  |  |  |  |  |  |  |  |  |  |  |  |  |  |  |  |  |  |  |  |  |  |  |  |  |  |  |  |  |  |  |  |  |  |  |  |  |  |  |  |  |  |  |  |  |  |  |  |  |  |  |  |  |  |  |  |  |  |  |  |  |  |  |  |  |  |  |  |  |  |  |  |  |  |  |  |  |  |  |  |  |  |  |  |  |  |  |  |  |  |  |  |  |  |  |  |  |  |  |  |  |  |  |  |  |  |  |  |  |  |  |  |  |  |  |  |  |  |  |  |  |  |  |  |  |  |  |  |  |  |  |  |  |  |  |  |  |  |  |  |  |  |  |  |  |  |  |  |  |  |  |  |  |  |  |  |  |  |  |  |  |  |  |  |  |  |  |  |  |  |  |  |  |  |  |  |  |  |  |  |  |  |  |  |  |  |  |  |  |  |  |  |  |  |  |  |  |  |  |  |  |  |  |  |  |  |  |  |  |  |  |  |  |  |  |  |  |  |  |  |  |  |  |  |  |  |  |  |  |  |  |  |  |  |  |  |  |  |  |  |  |  |  |  |  |  |  |  |  |  |  |  |  |  |  |  |  |  |  |  |  |  |  |  |  |  |  |  |  |  |  |  |  |  |  |  |  |  |  |  |  |  |  |  |  |  |  |  |  |  |  |  |  |  |  |  |  |  |  |  |  |  |  |  |  |  |  |  |  |  |  |  |  |  |  |  |  |  |  |  |  |  |  |  |  |  |  |  |  |  |  |  |  |  |  |  |  |  |  |  |  |  |  |  |  |  |  |  |  |  |  |  |  |  |  |  |  |  |  |  |  |  |  |  |  |  |  |  |  |  |  |  |  |  |  |  |  |  |  |  |  |  |  |  |  |  |  |  |  |  |  |  |  |  |  |  |  |  |  |  |  |  |  |  |  |  |  |  |  |  |  |  |  |  |  |  |  |  |  |  |  |  |  |  |  |  |  |  |  |  |  |  |  |  |  |  |  |  |  |  |  |  |  |  |  |  |  |  |  |  |  |  |  |  |  |  |  |  |  |  |  |  |  |  |  |  |  |  |  |  |  |  |  |  |  |  |  |  |  |  |  |  |  |  |  |  |  |  |  |  |  |  |  |  |  |  |  |  |  |  |  |  |  |  |  |  |  |  |  |  |  |  |  |  |  |  |  |  |  |  |  |  |  |  |  |  |  |  |  |  |  |  |  |  |  |  |  |  |  |  |  |  |  |  |  |  |  |  |  |  |  |  |  |  |  |  |  |  |  |  |  |  |  |  |  |  |  |  |  |  |  |  |  |  |  |  |  |  |  |  |  |  |  |  |  |  |  |  |  |  |  |  |  |  |  |  |  |  |  |  |  |  |  |  |  |  |  |  |  |  |  |  |  |  |  |  |  |  |  |  |  |  |  |  |  |  |  |  |  |  |  |  |  |  |  |  |  |  |  |  |  |  |  |  |  |  |  |  |  |  |  |  |  |  |  |  |  |  |  |  |  |  |  |  |  |  |  |  |  |  |  |  |  |  |  |  |  |  |  |  |  |  |  |  |  |  |  |  |  |  |  |  |  |  |  |  |  |  |  |  |  |  |  |  |  |  |  |  |  |  |  |  |  |  |  |  |  |  |  |  |  |  |  |  |  |  |  |  |  |  |  |  |  |  |  |  |  |  |  |  |  |  |  |  |  |  |  |  |  |  |  |  |  |  |  |  |  |  |  |  |  |  |  |  |  |  |  |  |  |  |  |  |  |  |  |  |  |  |  |  |  |  |  |  |  |  |  |  |  |  |  |  |  |  |  |  |  |  |  |  |  |  |  |  |  |  |  |  |  |  |  |  |  |  |  |  |  |  |  |  |  |  |  |  |  |  |  |  |  |  |  |  |  |  |  |  |  |  |  |  |  |  |  |  |  |  |  |  |  |  |  |  |  |  |  |  |  |  |  |  |  |  |  |  |  |  |  |  |  |  |  |  |  |  |  |  |  |  |  |  |  |  |  |  |  |  |  |  |  |  |  |  |  |  |  |  |  |  |  |  |  |  |  |  |  |  |  |  |  |  |  |  |  |  |  |  |  |  |  |  |  |  |  |  |  |  |  |  |  |  |  |  |  |  |  |  |  |  |  |  |  |  |  |  |  |  |  |  |  |  |  |  |  |  |  |  |  |  |  |  |  |  |  |  |  |  |  |  |  |  |  |  |  |  |  |  |  |  |  |  |  |  |  |  |  |  |  |  |  |  |  |  |  |  |  |  |  |  |  |  |  |  |  |  |  |  |  |  |  |  |  |  |  |  |  |  |  |  |  |  |  |  |  |  |  |  |  |  |  |  |  |  |  |  |  |  |  |  |  |  |  |  |  |  |  |  |  |  |  |  |  |  |  |  |  |  |  |  |  |  |  |  |  |  |  |  |  |  |  |  |  |  |  |  |  |  |  |  |  |  |  |  |  |  |  |  |  |  |  |  |  |  |  |  |  |  |  |  |  |  |  |  |  |  |  |  |  |  |  |  |  |  |  |  |  |  |  |  |  |  |  |  |  |  |  |  |  |  |  |  |  |  |  |  |  |  |  |  |  |  |  |  |  |  |  |  |  |  |  |  |  |  |  |  |  |  |  |  |  |  |  |  |  |  |  |  |  |  |  |  |  |  |  |  |  |  |  |  |  |  |  |  |  |  |  |  |  |  |  |  |  |  |  |  |  |  |  |  |  |  |  |  |  |  |  |  |  |  |  |  |  |  |  |  |  |  |  |  |  |  |  |  |  |  |  |  |  |  |  |  |  |  |  |  |  |  |  |  |  |  |  |  |  |  |  |  |  |  |  |  |  |  |  |  |  |  |  |  |  |  |  |  |  |  |  |  |  |  |  |  |  |  |  |  |  |  |  |  |  |  |  |  |  |  |  |  |  |  |  |  |  |  |  |  |  |  |  |  |  |  |  |  |  |  |  |  |  |  |  |  |  |  |  |  |  |  |  |  |  |  |  |  |  |  |  |  |  |  |  |  |  |  |  |  |  |  |  |  |  |  |  |  |  |  |  |  |  |  |  |  |  |  |  |  |  |  |  |  |  |  |  |  |  |  |  |  |  |  |  |  |  |  |  |  |  |  |  |  |  |  |  |  |  |  |  |  |  |  |  |  |  |  |  |  |  |  |  |  |  |  |  |  |  |  |  |  |  |  |  |  |  |  |  |  |  |  |  |  |  |  |  |  |  |  |  |  |  |  |  |  |  |  |  |  |  |  |  |  |  |  |  |  |  |  |  |  |  |  |  |  |  |  |  |  |  |  |  |  |  |  |  |  |  |  |  |  |  |  |  |  |  |  |  |  |  |  |  |  |  |  |  |  |  |  |  |  |  |  |  |  |  |  |  |  |  |  |  |  |  |  |  |  |  |  |  |  |  |  |  |  |  |  |  |  |  |  |  |  |  |  |  |  |  |  |  |  |  |  |  |  |  |  |  |  |  |  |  |  |  |  |  |  |  |  |  |  |  |  |  |  |  |  |  |  |  |  |  |  |  |  |  |  |  |  |  |  |  |  |  |  |  |  |  |  |  |  |  |  |  |  |  |  |  |  |  |  |  |  |  |  |  |  |  |  |  |  |  |  |  |  |  |  |  |  |  |  |  |  |  |  |  |  |  |  |  |  |  |  |  |  |  |  |  |  |  |  |  |  |  |  |  |  |  |  |  |  |  |  |  |  |  |  |  |  |  |  |  |  |  |  |  |  |  |  |  |  |  |  |  |  |  |  |  |  |  |  |  |  |  |  |  |  |  |  |  |  |  |  |  |  |  |  |  |  |  |  |  |  |  |  |  |  |  |  |  |  |  |  |  |  |  |  |  |  |  |  |  |  |  |  |  |  |  |  |  |  |  |  |  |  |  |  |  |  |  |  |  |  |  |  |  |  |  |  |  |  |  |  |  |  |  |  |  |  |  |  |  |  |  |  |  |  |  |  |  |  |  |  |  |  |  |  |  |  |  |  |  |  |  |  |  |  |  |  |  |  |  |  |  |  |  |  |  |  |  |  |  |  |  |  |  |  |  |  |  |  |  |  |  |  |  |  |  |  |  |  |  |  |  |  |  |  |  |  |  |  |  |  |  |  |  |  |  |  |  |  |  |  |  |  |  |  |  |  |  |  |  |  |  |  |  |  |  |  |  |  |  |  |  |  |  |  |  |  |  |  |  |  |  |  |  |  |  |  |  |  |  |  |  |  |  |  |  |  |  |  |  |  |  |  |  |  |  |  |  |  |  |  |  |  |  |  |  |  |  |  |  |  |  |  |  |  |  |  |  |  |  |  |  |  |  |  |  |  |  |  |  |  |  |  |  |  |  |  |  |  |  |  |  |  |  |  |  |  |  |  |  |  |  |  |  |  |  |  |  |  |  |  |  |  |  |  |  |  |  |  |  |  |  |  |  |  |  |  |  |  |  |  |  |  |  |  |  |  |  |  |  |  |  |  |  |  |  |  |  |  |  |  |  |  |  |  |  |  |  |  |  |  |  |  |  |  |  |  |  |  |  |  |  |  |  |  |  |  |  |  |  |  |  |  |  |  |  |  |  |  |  |  |  |  |  |  |  |  |  |  |  |  |  |  |  |  |  |  |  |  |  |  |  |  |  |  |  |  |  |  |  |  |  |  |  |  |  |  |  |  |  |  |  |  |  |  |  |  |  |  |  |  |  |  |  |  |  |  |  |  |  |  |  |  |  |  |  |  |  |  |  |  |  |  |  |  |  |  |  |  |  |  |  |  |  |  |  |  |  |  |  |  |  |  |  |  |  |  |  |  |  |  |  |  |  |  |  |  |  |  |  |  |  |  |  |  |  |  |  |  |  |  |  |  |  |  |  |  |  |  |  |  |  |  |  |  |  |  |  |  |  |  |  |  |  |  |  |  |  |  |  |  |  |  |  |  |  |  |  |  |  |  |  |  |  |  |  |  |  |  |  |  |  |  |  |  |  |  |  |  |  |  |  |  |  |  |  |  |  |  |  |  |  |  |  |  |  |  |  |  |  |  |  |  |  |  |  |  |  |  |  |  |  |  |  |  |  |  |  |  |  |  |  |  |  |  |  |  |  |  |  |  |  |  |  |  |  |  |  |  |  |  |  |  |  |  |  |  |  |  |  |  |  |  |  |  |  |  |  |  |  |  |  |  |  |  |  |  |  |  |  |  |  |  |  |  |  |  |  |  |  |  |  |  |  |  |  |  |  |  |  |  |  |  |  |  |  |  |  |  |  |  |  |  |  |  |  |  |  |  |  |  |  |  |  |  |  |  |  |  |  |  |  |  |  |  |  |  |  |  |  |  |  |  |  |  |  |  |  |  |  |  |  |  |  |  |  |  |  |  |  |  |  |  |  |  |  |  |  |  |  |  |  |  |  |  |  |  |  |  |  |  |  |  |  |  |  |  |  |  |  |  |  |  |  |  |  |  |  |  |  |  |  |  |  |  |  |  |  |  |  |  |  |  |  |  |  |  |  |  |  |  |  |  |  |  |  |  |  |  |  |  |  |  |  |  |  |  |  |  |  |  |  |  |  |  |  |  |  |  |  |  |  |  |  |  |  |  |  |  |  |  |  |  |  |  |  |  |  |  |  |  |  |  |  |  |  |  |  |  |  |  |  |  |  |  |  |  |  |  |  |  |  |  |  |  |  |  |  |  |  |  |  |  |  |  |  |  |  |  |  |  |  |  |  |  |  |  |  |  |  |  |  |  |  |  |  |  |  |  |  |  |  |  |  |  |  |  |  |  |  |  |  |  |  |  |  |  |  |  |  |  |  |  |  |  |  |  |  |  |  |  |  |  |  |  |  |  |  |  |  |  |  |  |  |  |  |  |  |  |  |  |  |  |  |  |  |  |  |  |  |  |  |  |  |  |  |  |  |  |  |  |  |  |  |  |  |  |  |  |  |  |  |  |  |  |  |  |  |  |  |  |  |  |  |  |  |  |  |  |  |  |  |  |  |  |  |  |  |  |  |  |  |  |  |  |  |  |  |  |  |  |  |  |  |  |  |  |  |  |  |  |  |  |  |  |  |  |  |  |  |  |  |  |  |  |  |  |  |  |  |  |  |  |  |  |  |  |  |  |  |  |  |  |  |  |  |  |  |  |  |  |  |  |  |  |  |  |  |  |  |  |  |  |  |  |  |  |  |  |  |  |  |  |  |  |  |  |  |  |  |  |  |  |  |  |  |  |  |  |  |  |  |  |  |  |  |  |  |  |  |  |  |  |  |  |  |  |  |  |  |  |  |  |  |  |  |  |  |  |  |  |  |  |  |  |  |  |  |  |  |  |  |  |  |  |  |  |  |  |  |  |  |  |  |  |  |  |  |  |  |  |  |  |  |  |  |  |  |  |  |  |  |  |  |  |  |  |  |  |  |  |  |  |  |  |  |  |  |  |  |  |  |  |  |  |  |  |  |  |  |  |  |  |  |  |  |  |  |  |  |  |  |  |  |  |  |  |  |  |  |  |  |  |  |  |  |  |  |  |  |  |  |  |  |  |  |  |  |  |  |  |  |  |  |  |  |  |  |  |  |  |  |  |  |  |  |  |  |  |  |  |  |  |  |  |  |  |  |  |  |  |  |  |  |  |  |  |  |  |  |  |  |  |  |  |  |  |  |  |  |  |  |  |  |  |  |  |  |  |  |  |  |  |  |  |  |  |  |  |  |  |  |  |  |  |  |  |  |  |  |  |  |  |  |  |  |  |  |  |  |  |  |  |  |  |  |  |  |  |  |  |  |  |  |  |  |  |  |  |  |  |  |  |  |  |  |  |  |  |  |  |  |  |  |  |  |  |  |  |  |  |  |  |  |  |  |  |  |  |  |  |  |  |  |  |  |  |  |  |  |  |  |  |  |  |  |  |  |  |  |  |  |  |  |  |  |  |  |  |  |  |  |  |  |  |  |  |  |  |  |  |  |  |  |  |  |  |  |  |  |  |  |  |  |  |  |  |  |  |  |  |  |  |  |  |  |  |  |  |  |  |  |  |  |  |  |  |  |  |  |  |  |  |  |  |  |  |  |  |  |  |  |  |  |  |  |  |  |  |  |  |  |  |  |  |  |  |  |  |  |  |  |  |  |  |  |  |  |  |  |  |  |  |  |  |  |  |  |  |  |  |  |  |  |  |  |  |  |  |  |  |  |  |  |  |  |  |  |  |  |  |  |  |  |  |  |  |  |  |  |  |  |  |  |  |  |  |  |  |  |  |  |  |  |  |  |  |  |  |  |  |  |  |  |  |  |  |  |  |  |  |  |  |  |  |  |  |  |  |  |  |  |  |  |  |  |  |  |  |  |  |  |  |  |  |  |  |  |  |  |  |  |  |  |  |  |  |  |  |  |  |  |  |  |  |  |  |  |  |  |  |  |  |  |  |  |  |  |  |  |  |  |  |  |  |  |  |  |  |  |  |  |  |  |  |  |  |  |  |  |  |  |  |  |  |  |  |  |  |  |  |  |  |  |  |  |  |  |  |  |  |  |  |  |  |  |  |  |  |  |  |  |  |  |  |  |  |  |  |  |  |  |  |  |  |  |  |  |  |  |  |  |  |  |  |  |  |  |  |  |  |  |  |  |  |  |  |  |  |  |  |  |  |  |  |  |  |  |  |  |  |  |  |  |  |  |  |  |  |  |  |  |  |  |  |  |  |  |  |  |  |  |  |  |  |  |  |  |  |  |  |  |  |  |  |  |  |  |  |  |  |  |  |  |  |  |  |  |  |  |  |  |  |  |  |  |  |  |  |  |  |  |  |  |  |  |  |  |  |  |  |  |  |  |  |  |  |  |  |  |  |  |  |  |  |  |  |  |  |  |  |  |  |  |  |  |  |  |  |  |  |  |  |  |  |  |  |  |  |  |  |  |  |  |  |  |  |  |  |  |  |  |  |  |  |  |  |  |  |  |  |  |  |  |  |  |  |  |  |  |  |  |  |  |  |  |  |  |  |  |  |  |  |  |  |  |  |  |  |  |  |  |  |  |  |  |  |  |  |  |  |  |  |  |  |  |  |  |  |  |  |  |  |  |  |  |  |  |  |  |  |  |  |  |  |  |  |  |  |  |  |  |  |  |  |  |  |  |  |  |  |  |  |  |  |  |  |  |  |  |  |  |  |  |  |  |  |  |  |  |  |  |  |  |  |  |  |  |  |  |  |  |  |  |  |  |  |  |  |  |  |  |  |  |  |  |  |  |  |  |  |  |  |  |  |  |  |  |  |  |  |  |  |  |  |  |  |  |  |  |  |  |  |  |  |  |  |  |  |  |  |  |  |  |  |  |  |  |  |  |  |  |  |  |  |  |  |  |  |  |  |  |  |  |  |  |  |  |  |  |  |  |  |  |  |  |  |  |  |  |  |  |  |  |  |  |  |  |  |  |  |  |  |  |  |  |  |  |  |  |  |  |  |  |  |  |  |  |  |  |  |  |  |  |  |  |  |  |  |  |  |  |  |  |  |  |  |  |  |  |  |  |  |  |  |  |  |  |  |  |  |  |  |  |  |  |  |  |  |  |  |  |  |  |  |  |  |  |  |  |  |  |  |  |  |  |  |  |  |  |  |  |  |  |  |  |  |  |  |  |  |  |  |  |  |  |  |  |  |  |  |  |  |  |  |  |  |  |  |  |  |  |  |  |  |  |  |  |  |  |  |  |  |  |  |  |  |  |  |  |  |  |  |  |  |  |  |  |  |  |  |  |  |  |  |  |  |  |  |  |  |  |  |  |  |  |  |  |  |  |  |  |  |  |  |  |  |  |  |  |  |  |  |  |  |  |  |  |  |  |  |  |  |  |  |  |  |  |  |  |  |  |  |  |  |  |  |  |  |  |  |  |  |  |  |  |  |  |  |  |  |  |  |  |  |  |  |  |  |  |  |  |  |  |  |  |  |  |  |  |  |  |  |  |  |  |  |  |  |  |  |  |  |  |  |  |  |  |  |  |  |  |  |  |  |  |  |  |  |  |  |  |  |  |  |  |  |  |  |  |  |  |  |  |  |  |  |  |  |  |  |  |  |  |  |  |  |  |  |  |  |  |  |  |  |  |  |  |  |  |  |  |  |  |  |  |  |  |  |  |  |  |  |  |  |  |  |  |  |  |  |  |  |  |  |  |  |  |  |  |  |  |  |  |  |  |  |  |  |  |  |  |  |  |  |  |  |  |  |  |  |  |  |  |  |  |  |  |  |  |  |  |  |  |  |  |  |  |  |  |  |  |  |  |  |  |  |  |  |  |  |  |  |  |  |  |  |  |  |  |  |  |  |  |  |  |  |  |  |  |  |  |  |  |  |  |  |  |  |  |  |  |  |  |  |  |  |  |  |  |  |  |  |  |  |  |  |  |  |  |  |  |  |  |  |  |  |  |  |  |  |  |  |  |  |  |  |  |  |  |  |  |  |  |  |  |  |  |  |  |  |  |  |  |  |  |  |  |  |  |  |  |  |  |  |  |  |  |  |  |  |  |  |  |  |  |  |  |  |  |  |  |  |  |  |  |  |  |  |  |  |  |  |  |  |  |  |  |  |  |  |  |  |  |  |  |  |  |  |  |  |  |  |  |  |  |  |  |  |  |  |  |  |  |  |  |  |  |  |  |  |  |  |  |  |  |  |  |  |  |  |  |  |  |  |  |  |  |  |  |  |  |  |  |  |  |  |  |  |  |  |  |  |  |  |  |  |  |  |  |  |  |  |  |  |  |  |  |  |  |  |  |  |  |  |  |  |  |  |  |  |  |  |  |  |  |  |  |  |  |  |  |  |  |  |  |  |  |  |  |  |  |  |  |  |  |  |  |  |  |  |  |  |  |  |  |  |  |  |  |  |  |  |  |  |  |  |  |  |  |  |  |  |  |  |  |  |  |  |  |  |  |  |  |  |  |  |  |  |  |  |  |  |  |  |  |  |  |  |  |  |  |  |  |  |  |  |  |  |  |  |  |  |  |  |  |  |  |  |  |  |  |  |  |  |  |  |  |  |  |  |  |  |  |  |  |  |  |  |  |  |  |  |  |  |  |  |  |  |  |  |  |  |  |  |  |  |  |  |  |  |  |  |  |  |  |  |  |  |  |  |  |  |  |  |  |  |  |  |  |  |  |  |  |  |  |  |  |  |  |  |  |  |  |  |  |  |  |  |  |  |  |  |  |  |  |  |  |  |  |  |  |  |  |  |  |  |  |  |  |  |  |  |  |  |  |  |  |  |  |  |  |  |  |  |  |  |  |  |  |  |  |  |  |  |  |  |  |  |  |  |  |  |  |  |  |  |  |  |  |  |  |  |  |  |  |  |  |  |  |  |  |  |  |  |  |  |  |  |  |  |  |  |  |  |  |  |  |  |  |  |  |  |  |  |  |  |  |  |  |  |  |  |  |  |  |  |  |  |  |  |  |  |  |  |  |  |  |  |  |  |  |  |  |  |  |  |  |  |  |  |  |  |  |  |  |  |  |  |  |  |  |  |  |  |  |  |  |  |  |  |  |  |  |  |  |  |  |  |  |  |  |  |  |  |  |  |  |  |  |  |  |  |  |  |  |  |  |  |  |  |  |  |  |  |  |  |  |  |  |  |  |  |  |  |  |  |  |  |  |  |  |  |  |  |  |  |  |  |  |  |  |  |  |  |  |  |  |  |  |  |  |  |  |  |  |  |  |  |  |  |  |  |  |  |  |  |  |  |  |  |  |  |  |  |  |  |  |  |  |  |  |  |  |  |  |  |  |  |  |  |  |  |  |  |  |  |  |  |  |  |  |  |  |  |  |  |  |  |  |  |  |  |  |  |  |  |  |  |  |  |  |  |  |  |  |  |  |  |  |  |  |  |  |  |  |  |  |  |  |  |  |  |  |  |  |  |  |  |  |  |  |  |  |  |  |  |  |  |  |  |  |  |  |  |  |  |  |  |  |  |  |  |  |  |  |  |  |  |  |  |  |  |  |  |  |  |  |  |  |  |  |  |  |  |  |  |  |  |  |  |  |  |  |  |  |  |  |  |  |  |  |  |  |  |  |  |  |  |  |  |  |  |  |  |  |  |  |  |  |  |  |  |  |  |  |  |  |  |  |  |  |  |  |  |  |  |  |  |  |  |  |  |  |  |  |  |  |  |  |  |  |  |  |  |  |  |  |  |  |  |  |  |  |  |  |  |  |  |  |  |  |  |  |  |  |  |  |  |  |  |  |  |  |  |  |  |  |  |  |  |  |  |  |  |  |  |  |  |  |  |  |  |  |  |  |  |  |  |  |  |  |  |  |  |  |  |  |  |  |  |  |  |  |  |  |  |  |  |  |  |  |  |  |  |  |  |  |  |  |  |  |  |  |  |  |  |  |  |  |  |  |  |  |  |  |  |  |  |  |  |  |  |  |  |  |  |  |  |  |  |  |  |  |  |  |  |  |  |  |  |  |  |  |  |  |  |  |  |  |  |  |  |  |  |  |  |  |  |  |  |  |  |  |  |  |  |  |  |  |  |  |  |  |  |  |  |  |  |  |  |  |  |  |  |  |  |  |  |  |  |  |  |  |  |  |  |  |  |  |  |  |  |  |  |  |  |  |  |  |  |  |  |  |  |  |  |  |  |  |  |  |  |  |  |  |  |  |  |  |  |  |  |  |  |  |  |  |  |  |  |  |  |  |  |  |  |  |  |  |  |  |  |  |  |  |  |  |  |  |  |  |  |  |  |  |  |  |  |  |  |  |  |  |  |  |  |  |  |  |  |  |  |  |  |  |  |  |  |  |  |  |  |  |  |  |  |  |  |  |  |  |  |  |  |  |  |  |  |  |  |  |  |  |  |  |  |  |  |  |  |  |  |  |  |  |  |  |  |  |  |  |  |  |  |  |  |  |  |  |  |  |  |  |  |  |  |  |  |  |  |  |  |  |  |  |  |  |  |  |  |  |  |  |  |  |  |  |  |  |  |  |  |  |  |  |  |  |  |  |  |  |  |  |  |  |  |  |  |  |  |  |  |  |  |  |  |  |  |  |  |  |  |  |  |  |  |  |  |  |  |  |  |  |  |  |  |  |  |  |  |  |  |  |  |  |  |  |  |  |  |  |  |  |  |  |  |  |  |  |  |  |  |  |  |  |  |  |  |  |  |  |  |  |  |  |  |  |  |  |  |  |  |  |  |  |  |  |  |  |  |  |  |  |  |  |  |  |  |  |  |  |  |  |  |  |  |  |  |  |  |  |  |  |  |  |  |  |  |  |  |  |  |  |  |  |  |  |  |  |  |  |  |  |  |  |  |  |  |  |  |  |  |  |  |  |  |  |  |  |  |  |  |  |  |  |  |  |  |  |  |  |  |  |  |  |  |  |  |  |  |  |  |  |  |  |  |  |  |  |  |  |  |  |  |  |  |  |  |  |  |  |  |  |  |  |  |  |  |  |  |  |  |  |  |  |  |  |  |  |  |  |  |  |  |  |  |  |  |  |  |  |  |  |  |  |  |  |  |  |  |  |  |  |  |  |  |  |  |  |  |  |  |  |  |  |  |  |  |  |  |  |  |  |  |  |  |  |  |  |  |  |  |  |  |  |  |  |  |  |  |  |  |  |  |  |  |  |  |  |  |  |  |  |  |  |  |  |  |  |  |  |  |  |  |  |  |  |  |  |  |  |  |  |  |  |  |  |  |  |  |  |  |  |  |  |  |  |  |  |  |  |  |  |  |  |  |  |  |  |  |  |  |  |  |  |  |  |  |  |  |  |  |  |  |  |  |  |  |  |  |  |  |  |  |  |  |  |  |  |  |  |  |  |  |  |  |  |  |  |  |  |  |  |  |  |  |  |  |  |  |  |  |  |  |  |  |  |  |  |  |  |  |  |  |  |  |  |  |  |  |  |  |  |  |  |  |  |  |  |  |  |  |  |  |  |  |  |  |  |  |  |  |  |  |  |  |  |  |  |  |  |  |  |  |  |  |  |  |  |  |  |  |  |  |  |  |  |  |  |  |  |  |  |  |  |  |  |  |  |  |  |  |  |  |  |  |  |  |  |  |  |  |  |  |  |  |  |  |  |  |  |  |  |  |  |  |  |  |  |  |  |  |  |  |  |  |  |  |  |  |  |  |  |  |  |  |  |  |  |  |  |  |  |  |  |  |  |  |  |  |  |  |  |  |  |  |  |  |  |  |  |  |  |  |  |  |  |  |  |  |  |  |  |  |  |  |  |  |  |  |  |  |  |  |  |  |  |  |  |  |  |  |  |  |  |  |  |  |  |  |  |  |  |  |  |  |  |  |  |  |  |  |  |  |  |  |  |  |  |  |  |  |  |  |  |  |  |  |  |  |  |  |  |  |  |  |  |  |  |  |  |  |  |  |  |  |  |  |  |  |  |  |  |  |  |  |  |  |  |  |  |  |  |  |  |  |  |  |  |  |  |  |  |  |  |  |  |  |  |  |  |  |  |  |  |  |  |  |  |  |  |  |  |  |  |  |  |  |  |  |  |  |  |  |  |  |  |  |  |  |  |  |  |  |  |  |  |  |  |  |  |  |  |  |  |  |  |  |  |  |  |  |  |  |  |  |  |  |  |  |  |  |  |  |  |  |  |  |  |  |  |  |  |  |  |  |  |  |  |  |  |  |  |  |  |  |  |  |  |  |  |  |  |  |  |  |  |  |  |  |  |  |  |  |  |  |  |  |  |  |  |  |  |  |  |  |  |  |  |  |  |  |  |  |  |  |  |  |  |  |  |  |  |  |  |  |  |  |  |  |  |  |  |  |  |  |  |  |  |  |  |  |  |  |  |  |  |  |  |  |  |  |  |  |  |  |  |  |  |  |  |  |  |  |  |  |  |  |  |  |  |  |  |  |  |  |  |  |  |  |  |  |  |  |  |  |  |  |  |  |  |  |  |  |  |  |  |  |  |  |  |  |  |  |  |  |  |  |  |  |  |  |  |  |  |  |  |  |  |  |  |  |  |  |  |  |  |  |  |  |  |  |  |  |  |  |  |  |  |  |  |  |  |  |  |  |  |  |  |  |  |  |  |  |  |  |  |  |  |  |  |  |  |  |  |  |  |  |  |  |  |  |  |  |  |  |  |  |  |  |  |  |  |  |  |  |  |  |  |  |  |  |  |  |  |  |  |  |  |  |  |  |  |  |  |  |  |  |  |  |  |  |  |  |  |  |  |  |  |  |  |  |  |  |  |  |  |  |  |  |  |  |  |  |  |  |  |  |  |  |  |  |  |  |  |  |  |  |  |  |  |  |  |  |  |  |  |  |  |  |  |  |  |  |  |  |  |  |  |  |  |  |  |  |  |  |  |  |  |  |  |  |  |  |  |  |  |  |  |  |  |  |  |  |  |  |  |  |  |  |  |  |  |  |  |  |  |  |  |  |  |  |  |  |  |  |  |  |  |  |  |  |  |  |  |  |  |  |  |  |  |  |  |  |  |  |  |  |  |  |  |  |  |  |  |  |  |  |  |  |  |  |  |  |  |  |  |  |  |  |  |  |  |  |  |  |  |  |  |  |  |  |  |  |  |  |  |  |  |  |  |  |  |  |  |  |  |  |  |  |  |  |  |  |  |  |  |  |  |  |  |  |  |  |  |  |  |  |  |  |  |  |  |  |  |  |  |  |  |  |  |  |  |  |  |  |  |  |  |  |  |  |  |  |  |  |  |  |  |  |  |  |  |  |  |  |  |  |  |  |  |  |  |  |  |  |  |  |  |  |  |  |  |  |  |  |  |  |  |  |  |  |  |  |  |  |  |  |  |  |  |  |  |  |  |  |  |  |  |  |  |  |  |  |  |  |  |  |  |  |  |  |  |  |  |  |  |  |  |  |  |  |  |  |  |  |  |  |  |  |  |  |  |  |  |  |  |  |  |  |  |  |  |  |  |  |  |  |  |  |  |  |  |  |  |  |  |  |  |  |  |  |  |  |  |  |  |  |  |  |  |  |  |  |  |  |  |  |  |  |  |  |  |  |  |  |  |  |  |  |  |  |  |  |  |  |  |  |  |  |  |  |  |  |  |  |  |  |  |  |  |  |  |  |  |  |  |  |  |  |  |  |  |  |  |  |  |  |  |  |  |  |  |  |  |  |  |  |  |  |  |  |  |  |  |  |  |  |  |  |  |  |  |  |  |  |  |  |  |  |  |  |  |  |  |  |  |  |  |  |  |  |  |  |  |  |  |  |  |  |  |  |  |  |  |  |  |  |  |  |  |  |  |  |  |  |  |  |  |  |  |  |  |  |  |  |  |  |  |  |  |  |  |  |  |  |  |  |  |  |  |  |  |  |  |  |  |  |  |  |  |  |  |  |  |  |  |  |  |  |  |  |  |  |  |  |  |  |  |  |  |  |  |  |  |  |  |  |  |  |  |  |  |  |  |  |  |  |  |  |  |  |  |  |  |  |  |  |  |  |  |  |  |  |  |  |  |  |  |  |  |  |  |  |  |  |  |  |  |  |  |  |  |  |  |  |  |  |  |  |  |  |  |  |  |  |  |  |  |  |  |  |  |  |  |  |  |  |  |  |  |  |  |  |  |  |  |  |  |  |  |  |  |  |  |  |  |  |  |  |  |  |  |  |  |  |  |  |  |  |  |  |  |  |  |  |  |  |  |  |  |  |  |  |  |  |  |  |  |  |  |  |  |  |  |  |  |  |  |  |  |  |  |  |  |  |  |  |  |  |  |  |  |  |  |  |  |  |  |  |  |  |  |  |  |  |  |  |  |  |  |  |  |  |  |  |  |  |  |  |  |  |  |  |  |  |  |  |  |  |  |  |  |  |  |  |  |  |  |  |  |  |  |  |  |  |  |  |  |  |  |  |  |  |  |  |  |  |  |  |  |  |  |  |  |  |  |  |  |  |  |  |  |  |  |  |  |  |  |  |  |  |  |  |  |  |  |  |  |  |  |  |  |  |  |  |  |  |  |  |  |  |  |  |  |  |  |  |  |  |  |  |  |  |  |  |  |  |  |  |  |  |  |  |  |  |  |  |  |  |  |  |  |  |  |  |  |  |  |  |  |  |  |  |  |  |  |  |  |  |  |  |  |  |  |  |  |  |  |  |  |  |  |  |  |  |  |  |  |  |  |  |  |  |  |  |  |  |  |  |  |  |  |  |  |  |  |  |  |  |  |  |  |  |  |  |  |  |  |  |  |  |  |  |  |  |  |  |  |  |  |  |  |  |  |  |  |  |  |  |  |  |  |  |  |  |  |  |  |  |  |  |  |  |  |  |  |  |  |  |  |  |  |  |  |  |  |  |  |  |  |  |  |  |  |  |  |  |  |  |  |  |  |  |  |  |  |  |  |  |  |  |  |  |  |  |  |  |  |  |  |  |  |  |  |  |  |  |  |  |  |  |  |  |  |  |  |  |  |  |  |  |  |  |  |  |  |  |  |  |  |  |  |  |  |  |  |  |  |  |  |  |  |  |  |  |  |  |  |  |  |  |  |  |  |  |  |  |  |  |  |  |  |  |  |  |  |  |  |  |  |  |  |  |  |  |  |  |  |  |  |  |  |  |  |  |  |  |  |  |  |  |  |  |  |  |  |  |  |  |  |  |  |  |  |  |  |  |  |  |  |  |  |  |  |  |  |  |  |  |  |  |  |  |  |  |  |  |  |  |  |  |  |  |  |  |  |  |  |  |  |  |  |  |  |  |  |  |  |  |  |  |  |  |  |  |  |  |  |  |  |  |  |  |  |  |  |  |  |  |  |  |  |  |  |  |  |  |  |  |  |  |  |  |  |  |  |  |  |  |  |  |  |  |  |  |  |  |  |  |  |  |  |  |  |  |  |  |  |  |  |  |  |  |  |  |  |  |  |  |  |  |  |  |  |  |  |  |  |  |  |  |  |  |  |  |  |  |  |  |  |  |  |  |  |  |  |  |  |  |  |  |  |  |  |  |  |  |  |  |  |  |  |  |  |  |  |  |  |  |  |  |  |  |  |  |  |  |  |  |  |  |  |  |  |  |  |  |  |  |  |  |  |  |  |  |  |  |  |  |  |  |  |  |  |  |  |  |  |  |  |  |  |  |  |  |  |  |  |  |  |  |  |  |  |  |  |  |  |  |  |  |  |  |  |  |  |  |  |  |  |  |  |  |  |  |  |  |  |  |  |  |  |  |  |  |  |  |  |  |  |  |  |  |  |  |  |  |  |  |  |  |  |  |  |  |  |  |  |  |  |  |  |  |  |  |  |  |  |  |  |  |  |  |  |  |  |  |  |  |  |  |  |  |  |  |  |  |  |  |  |  |  |  |  |  |  |  |  |  |  |  |  |  |  |  |  |  |  |  |  |  |  |  |  |  |  |  |  |  |  |  |  |  |  |  |  |  |  |  |  |  |  |  |  |  |  |  |  |  |  |  |  |  |  |  |  |  |  |  |  |  |  |  |  |  |  |  |  |  |  |  |  |  |  |  |  |  |  |  |  |  |  |  |  |  |  |  |  |  |  |  |  |  |  |  |  |  |  |  |  |  |  |  |  |  |  |  |  |  |  |  |  |  |  |  |  |  |  |  |  |  |  |  |  |  |  |  |  |  |  |  |  |  |  |  |  |  |  |  |  |  |  |  |  |  |  |  |  |  |  |  |  |  |  |  |  |  |  |  |  |  |  |  |  |  |  |  |  |  |  |  |  |  |  |  |  |  |  |  |  |  |  |  |  |  |  |  |  |  |  |  |  |  |  |  |  |  |  |  |  |  |  |  |  |  |  |  |  |  |  |  |  |  |  |  |  |  |  |  |  |  |  |  |  |  |  |  |  |  |  |  |  |  |  |  |  |  |  |  |  |  |  |  |  |  |  |  |  |  |  |  |  |  |  |  |  |  |  |  |  |  |  |  |  |  |  |  |  |  |  |  |  |  |  |  |  |  |  |  |  |  |  |  |  |  |  |  |  |  |  |  |  |  |  |  |  |  |  |  |  |  |  |  |  |  |  |  |  |  |  |  |  |  |  |  |  |  |  |  |  |  |  |  |  |  |  |  |  |  |  |  |  |  |  |  |  |  |  |  |  |  |  |  |  |  |  |  |  |  |  |  |  |  |  |  |  |  |  |  |  |  |  |  |  |  |  |  |  |  |  |  |  |  |  |  |  |  |  |  |  |  |  |  |  |  |  |  |  |  |  |  |  |  |  |  |  |  |  |  |  |  |  |  |  |  |  |  |  |  |  |  |  |  |  |  |  |  |  |  |  |  |  |  |  |  |  |  |  |  |  |  |  |  |  |  |  |  |  |  |  |  |  |  |  |  |  |  |  |  |  |  |  |  |  |  |  |  |  |  |  |  |  |  |  |  |  |  |  |  |  |  |  |  |  |  |  |  |  |  |  |  |  |  |  |  |  |  |  |  |  |  |  |  |  |  |  |  |  |  |  |  |  |  |  |  |  |  |  |  |  |  |  |  |  |  |  |  |  |  |  |  |  |  |  |  |  |  |  |  |  |  |  |  |  |  |  |  |  |  |  |  |  |  |  |  |  |  |  |  |  |  |  |  |  |  |  |  |  |  |  |  |  |  |  |  |  |  |  |  |  |  |  |  |  |  |  |  |  |  |  |  |  |  |  |  |  |  |  |  |  |  |  |  |  |  |  |  |  |  |  |  |  |  |  |  |  |  |  |  |  |  |  |  |  |  |  |  |  |  |  |  |  |  |  |  |  |  |  |  |  |  |  |  |  |  |  |  |  |  |  |  |  |  |  |  |  |  |  |  |  |  |  |  |  |  |  |  |  |  |  |  |  |  |  |  |  |  |  |  |  |  |  |  |  |  |  |  |  |  |  |  |  |  |  |  |  |  |  |  |  |  |  |  |  |  |  |  |  |  |  |  |  |  |  |  |  |  |  |  |  |  |  |  |  |  |  |  |  |  |  |  |  |  |  |  |  |  |  |  |  |  |  |  |  |  |  |  |  |  |  |  |  |  |  |  |  |  |  |  |  |  |  |  |  |  |  |  |  |  |  |  |  |  |  |  |  |  |  |  |  |  |  |  |  |  |  |  |  |  |  |  |  |  |  |  |  |  |  |  |  |  |  |  |  |  |  |  |  |  |  |  |  |  |  |  |  |  |  |  |  |  |  |  |  |  |  |  |  |  |  |  |  |  |  |  |  |  |  |  |  |  |  |  |  |  |  |  |  |  |  |  |  |  |  |  |  |  |  |  |  |  |  |  |  |  |  |  |  |  |  |  |  |  |  |  |  |  |  |  |  |  |  |  |  |  |  |  |  |  |  |  |  |  |  |  |  |  |  |  |  |  |  |  |  |  |  |  |  |  |  |  |  |  |  |  |  |  |  |  |  |  |  |  |  |  |  |  |  |  |  |  |  |  |  |  |  |  |  |  |  |  |  |  |  |  |  |  |  |  |  |  |  |  |  |  |  |  |  |  |  |  |  |  |  |  |  |  |  |  |  |  |  |  |  |  |  |  |  |  |  |  |  |  |  |  |  |  |  |  |  |  |  |  |  |  |  |  |  |  |  |  |  |  |  |  |  |  |  |  |  |  |  |  |  |  |  |  |  |  |  |  |  |  |  |  |  |  |  |  |  |  |  |  |  |  |  |  |  |  |  |  |  |  |  |  |  |  |  |  |  |  |  |  |  |  |  |  |  |  |  |  |  |  |  |  |  |  |  |  |  |  |  |  |  |  |  |  |  |  |  |  |  |  |  |  |  |  |  |  |  |  |  |  |  |  |  |  |  |  |  |  |  |  |  |  |  |  |  |  |  |  |  |  |  |  |  |  |  |  |  |  |  |  |  |  |  |  |  |  |  |  |  |  |  |  |  |  |  |  |  |  |  |  |  |  |  |  |  |  |  |  |  |  |  |  |  |  |  |  |  |  |  |  |  |  |  |  |  |  |  |  |  |  |  |  |  |  |  |  |  |  |  |  |  |  |  |  |  |  |  |  |  |  |  |  |  |  |  |  |  |  |  |  |  |  |  |  |  |  |  |  |  |  |  |  |  |  |  |  |  |  |  |  |  |  |  |  |  |  |  |  |  |  |  |  |  |  |  |  |  |  |  |  |  |  |  |  |  |  |  |  |  |  |  |  |  |  |  |  |  |  |  |  |  |  |  |  |  |  |  |  |  |  |  |  |  |  |  |  |  |  |  |  |  |  |  |  |  |  |  |  |  |  |  |  |  |  |  |  |  |  |  |  |  |  |  |  |  |  |  |  |  |  |  |  |  |  |  |  |  |  |  |  |  |  |  |  |  |  |  |  |  |  |  |  |  |  |  |  |  |  |  |  |  |  |  |  |  |  |  |  |  |  |  |  |  |  |  |  |  |  |  |  |  |  |  |  |  |  |  |  |  |  |  |  |  |  |  |  |  |  |  |  |  |  |  |  |  |  |  |  |  |  |  |  |  |  |  |  |  |  |  |  |  |  |  |  |  |  |  |  |  |  |  |  |  |  |  |  |  |  |  |  |  |  |  |  |  |  |  |  |  |  |  |  |  |  |  |  |  |  |  |  |  |  |  |  |  |  |  |  |  |  |  |  |  |  |  |  |  |  |  |  |  |  |  |  |  |  |  |  |  |  |  |  |  |  |  |  |  |  |  |  |  |  |  |  |  |  |  |  |  |  |  |  |  |  |  |  |  |  |  |  |  |  |  |  |  |  |  |  |  |  |  |  |  |  |  |  |  |  |  |  |  |  |  |  |  |  |  |  |  |  |  |  |  |  |  |  |  |  |  |  |  |  |  |  |  |  |  |  |  |  |  |  |  |  |  |  |  |  |  |  |  |  |  |  |  |  |  |  |  |  |  |  |  |  |  |  |  |  |  |  |  |  |  |  |  |  |  |  |  |  |  |  |  |  |  |  |  |  |  |  |  |  |  |  |  |  |  |  |  |  |  |  |  |  |  |  |  |  |  |  |  |  |  |  |  |  |  |  |  |  |  |  |  |  |  |  |  |  |  |  |  |  |  |  |  |  |  |  |  |  |  |  |  |  |  |  |  |  |  |  |  |  |  |  |  |  |  |  |  |  |  |  |  |  |  |  |  |  |  |  |  |  |  |  |  |  |  |  |  |  |  |  |  |  |  |  |  |  |  |  |  |  |  |  |  |  |  |  |  |  |  |  |  |  |  |  |  |  |  |  |  |  |  |  |  |  |  |  |  |  |  |  |  |  |  |  |  |  |  |  |  |  |  |  |  |  |  |  |  |  |  |  |  |  |  |  |  |  |  |  |  |  |  |  |  |  |  |  |  |  |  |  |  |  |  |  |  |  |  |  |  |  |  |  |  |  |  |  |  |  |  |  |  |  |  |  |  |  |  |  |  |  |  |  |  |  |  |  |  |  |  |  |  |  |  |  |  |  |  |  |  |  |  |  |  |  |  |  |  |  |  |  |  |  |  |  |  |  |  |  |  |  |  |  |  |  |  |  |  |  |  |  |  |  |  |  |  |  |  |  |  |  |  |  |  |  |  |  |  |  |  |  |  |  |  |  |  |  |  |  |  |  |  |  |  |  |  |  |  |  |  |  |  |  |  |  |  |  |  |  |  |  |  |  |  |  |  |  |  |  |  |  |  |  |  |  |  |  |  |  |  |  |  |  |  |  |  |  |  |  |  |  |  |  |  |  |  |  |  |  |  |  |  |  |  |  |  |  |  |  |  |  |  |  |  |  |  |  |  |  |  |  |  |  |  |  |  |  |  |  |  |  |  |  |  |  |  |  |  |  |  |  |  |  |  |  |  |  |  |  |  |  |  |  |  |  |  |  |  |  |  |  |  |  |  |  |  |  |  |  |  |  |  |  |  |  |  |  |  |  |  |  |  |  |  |  |  |  |  |  |  |  |  |  |  |  |  |  |  |  |  |  |  |  |  |  |  |  |  |  |  |  |  |  |  |  |  |  |  |  |  |  |  |  |  |  |  |  |  |  |  |  |  |  |  |  |  |  |  |  |  |  |  |  |  |  |  |  |  |  |  |  |  |  |  |  |  |  |  |  |  |  |  |  |  |  |  |  |  |  |  |  |  |  |  |  |  |  |  |  |  |  |  |  |  |  |  |  |  |  |  |  |  |  |  |  |  |  |  |  |  |  |  |  |  |  |  |  |  |  |  |  |  |  |  |  |  |  |  |  |  |  |  |  |  |  |  |  |  |  |  |  |  |  |  |  |  |  |  |  |  |  |  |  |  |  |  |  |  |  |  |  |  |  |  |  |  |  |  |  |  |  |  |  |  |  |  |  |  |  |  |  |  |  |  |  |  |  |  |  |  |  |  |  |  |  |  |  |  |  |  |  |  |  |  |  |  |  |  |  |  |  |  |  |  |  |  |  |  |  |  |  |  |  |  |  |  |  |  |  |  |  |  |  |  |  |  |  |  |  |  |  |  |  |  |  |  |  |  |  |  |  |  |  |  |  |  |  |  |  |  |  |  |  |  |  |  |  |  |  |  |  |  |  |  |  |  |  |  |  |  |  |  |  |  |  |  |  |  |  |  |  |  |  |  |  |  |  |  |  |  |  |  |  |  |  |  |  |  |  |  |  |  |  |  |  |  |  |  |  |  |  |  |  |  |  |  |  |  |  |  |  |  |  |  |  |  |  |  |  |  |  |  |  |  |  |  |  |  |  |  |  |  |  |  |  |  |  |  |  |  |  |  |  |  |  |  |  |  |  |  |  |  |  |  |  |  |  |  |  |  |  |  |  |  |  |  |  |  |  |  |  |  |  |  |  |  |  |  |  |  |  |  |  |  |  |  |  |  |  |  |  |  |  |  |  |  |  |  |  |  |  |  |  |  |  |  |  |  |  |  |  |  |  |  |  |  |  |  |  |  |  |  |  |  |  |  |  |  |  |  |  |  |  |  |  |  |  |  |  |  |  |  |  |  |  |  |  |  |  |  |  |  |  |  |  |  |  |  |  |  |  |  |  |  |  |  |  |  |  |  |  |  |  |  |  |  |  |  |  |  |  |  |  |  |  |  |  |  |  |  |  |  |  |  |  |  |  |  |  |  |  |  |  |  |  |  |  |  |  |  |  |  |  |  |  |  |  |  |  |  |  |  |  |  |  |  |  |  |  |  |  |  |  |  |  |  |  |  |  |  |  |  |  |  |  |  |  |  |  |  |  |  |  |  |  |  |  |  |  |  |  |  |  |  |  |  |  |  |  |  |  |  |  |  |  |  |  |  |  |  |  |  |  |  |  |  |  |  |  |  |  |  |  |  |  |  |  |  |  |  |  |  |  |  |  |  |  |  |  |  |  |  |  |  |  |  |  |  |  |  |  |  |  |  |  |  |  |  |  |  |  |  |  |  |  |  |  |  |  |  |  |  |  |  |  |  |  |  |  |  |  |  |  |  |  |  |  |  |  |  |  |  |  |  |  |  |  |  |  |  |  |  |  |  |  |  |  |  |  |  |  |  |  |  |  |  |  |  |  |  |  |  |  |  |  |  |  |  |  |  |  |  |  |  |  |  |  |  |  |  |  |  |  |  |  |  |  |  |  |  |  |  |  |  |  |  |  |  |  |  |  |  |  |  |  |  |  |  |  |  |  |  |  |  |  |  |  |  |  |  |  |  |  |  |  |  |  |  |  |  |  |  |  |  |  |  |  |  |  |  |  |  |  |  |  |  |  |  |  |  |  |  |  |  |  |  |  |  |  |  |  |  |  |  |  |  |  |  |  |  |  |  |  |  |  |  |  |  |  |  |  |  |  |  |  |  |  |  |  |  |  |  |  |  |  |  |  |  |  |  |  |  |  |  |  |  |  |  |  |  |  |  |  |  |  |  |  |  |  |  |  |  |  |  |  |  |  |  |  |  |  |  |  |  |  |  |  |  |  |  |  |  |  |  |  |  |  |  |  |  |  |  |  |  |  |  |  |  |  |  |  |  |  |  |  |  |  |  |  |  |  |  |  |  |  |  |  |  |  |  |  |  |  |  |  |  |  |  |  |  |  |  |  |  |  |  |  |  |  |  |  |  |  |  |  |  |  |  |  |  |  |  |  |  |  |  |  |  |  |  |  |  |  |  |  |  |  |  |  |  |  |  |  |  |  |  |  |  |  |  |  |  |  |  |  |  |  |  |  |  |  |  |  |  |  |  |  |  |  |  |  |  |  |  |  |  |  |  |  |  |  |  |  |  |  |  |  |  |  |  |  |  |  |  |  |  |  |  |  |  |  |  |  |  |  |  |  |  |  |  |  |  |  |  |  |  |  |  |  |  |  |  |  |  |  |  |  |  |  |  |  |  |  |  |  |  |  |  |  |  |  |  |  |  |  |  |  |  |  |  |  |  |  |  |  |  |  |  |  |  |  |  |  |  |  |  |  |  |  |  |  |  |  |  |  |  |  |  |  |  |  |  |  |  |  |  |  |  |  |  |  |  |  |  |  |  |  |  |  |  |  |  |  |  |  |  |  |  |  |  |  |  |  |  |  |  |  |  |  |  |  |  |  |  |  |  |  |  |  |  |  |  |  |  |  |  |  |  |  |  |  |  |  |  |  |  |  |  |  |  |  |  |  |  |  |  |  |  |  |  |  |  |  |  |  |  |  |  |  |  |  |  |  |  |  |  |  |  |  |  |  |  |  |  |  |  |  |  |  |  |  |  |  |  |  |  |  |  |  |  |  |  |  |  |  |  |  |  |  |  |  |  |  |  |  |  |  |  |  |  |  |  |  |  |  |  |  |  |  |  |  |  |  |  |  |  |  |  |  |  |  |  |  |  |  |  |  |  |  |  |  |  |  |  |  |  |  |  |  |  |  |  |  |  |  |  |  |  |  |  |  |  |  |  |  |  |  |  |  |  |  |  |  |  |  |  |  |  |  |  |  |  |  |  |  |  |  |  |  |  |  |  |  |  |  |  |  |  |  |  |  |  |  |  |  |  |  |  |  |  |  |  |  |  |  |  |  |  |  |  |  |  |  |  |  |  |  |  |  |  |  |  |  |  |  |  |  |  |  |  |  |  |  |  |  |  |  |  |  |  |  |  |  |  |  |  |  |  |  |  |  |  |  |  |  |  |  |  |  |  |  |  |  |  |  |  |  |  |  |  |  |  |  |  |  |  |  |  |  |  |  |  |  |  |  |  |  |  |  |  |  |  |  |  |  |  |  |  |  |  |  |  |  |  |  |  |  |  |  |  |  |  |  |  |  |  |  |  |  |  |  |  |  |  |  |  |  |  |  |  |  |  |  |  |  |  |  |  |  |  |  |  |  |  |  |  |  |  |  |  |  |  |  |  |  |  |  |  |  |  |  |  |  |  |  |  |  |  |  |  |  |  |  |  |  |  |  |  |  |  |  |  |  |  |  |  |  |  |  |  |  |  |  |  |  |  |  |  |  |  |  |  |  |  |  |  |  |  |  |  |  |  |  |  |  |  |  |  |  |  |  |  |  |  |  |  |  |  |  |  |  |  |  |  |  |  |  |  |  |  |  |  |  |  |  |  |  |  |  |  |  |  |  |  |  |  |  |  |  |  |  |  |  |  |  |  |  |  |  |  |  |  |  |  |  |  |  |  |  |  |  |  |  |  |  |  |  |  |  |  |  |  |  |  |  |  |  |  |  |  |  |  |  |  |  |  |  |  |  |  |  |  |  |  |  |  |  |  |  |  |  |  |  |  |  |  |  |  |  |  |  |  |  |  |  |  |  |  |  |  |  |  |  |  |  |  |  |  |  |  |  |  |  |  |  |  |  |  |  |  |  |  |  |  |  |  |  |  |  |  |  |  |  |  |  |  |  |  |  |  |  |  |  |  |  |  |  |  |  |  |  |  |  |  |  |  |  |  |  |  |  |  |  |  |  |  |  |  |  |  |  |  |  |  |  |  |  |  |  |  |  |  |  |  |  |  |  |  |  |  |  |  |  |  |  |  |  |  |  |  |  |  |  |  |  |  |  |  |  |  |  |  |  |  |  |  |  |  |  |  |  |  |  |  |  |  |  |  |  |  |  |  |  |  |  |  |  |  |  |  |  |  |  |  |  |  |  |  |  |  |  |  |  |  |  |  |  |  |  |  |  |  |  |  |  |  |  |  |  |  |  |  |  |  |  |  |  |  |  |  |  |  |  |  |  |  |  |  |  |  |  |  |  |  |  |  |  |  |  |  |  |  |  |  |  |  |  |  |  |  |  |  |  |  |  |  |  |  |  |  |  |  |  |  |  |  |  |  |  |  |  |  |  |  |  |  |  |  |  |  |  |  |  |  |  |  |  |  |  |  |  |  |  |  |  |  |  |  |  |  |  |  |  |  |  |  |  |  |  |  |  |  |  |  |  |  |  |  |  |  |  |  |  |  |  |  |  |  |  |  |  |  |  |  |  |  |  |  |  |  |  |  |  |  |  |  |  |  |  |  |  |  |  |  |  |  |  |  |  |  |  |  |  |  |  |  |  |  |  |  |  |  |  |  |  |  |  |  |  |  |  |  |  |  |  |  |  |  |  |  |  |  |  |  |  |  |  |  |  |  |  |  |  |  |  |  |  |  |  |  |  |  |  |  |  |  |  |  |  |  |  |  |  |  |  |  |  |  |  |  |  |  |  |  |  |  |  |  |  |  |  |  |  |  |  |  |  |  |  |  |  |  |  |  |  |  |  |  |  |  |  |  |  |  |  |  |  |  |  |  |  |  |  |  |  |  |  |  |  |  |  |  |  |  |  |  |  |  |  |  |  |  |  |  |  |  |  |  |  |  |  |  |  |  |  |  |  |  |  |  |  |  |  |  |  |  |  |  |  |  |  |  |  |  |  |  |  |  |  |  |  |  |  |  |  |  |  |  |  |  |  |  |  |  |  |  |  |  |  |  |  |  |  |  |  |  |  |  |  |  |  |  |  |  |  |  |  |  |  |  |  |  |  |  |  |  |  |  |  |  |  |  |  |  |  |  |  |  |  |  |  |  |  |  |  |  |  |  |  |  |  |  |  |  |  |  |  |  |  |  |  |  |  |  |  |  |  |  |  |  |  |  |  |  |  |  |  |  |  |  |  |  |  |  |  |  |  |  |  |  |  |  |  |  |  |  |  |  |  |  |  |  |  |  |  |  |  |  |  |  |  |  |  |  |  |  |  |  |  |  |  |  |  |
| --- | --- | --- | --- | --- | --- | --- | --- | --- | --- | --- | --- | --- | --- | --- | --- | --- | --- | --- | --- | --- | --- | --- | --- | --- | --- | --- | --- | --- | --- | --- | --- | --- | --- | --- | --- | --- | --- | --- | --- | --- | --- | --- | --- | --- | --- | --- | --- | --- | --- | --- | --- | --- | --- | --- | --- | --- | --- | --- | --- | --- | --- | --- | --- | --- | --- | --- | --- | --- | --- | --- | --- | --- | --- | --- | --- | --- | --- | --- | --- | --- | --- | --- | --- | --- | --- | --- | --- | --- | --- | --- | --- | --- | --- | --- | --- | --- | --- | --- | --- | --- | --- | --- | --- | --- | --- | --- | --- | --- | --- | --- | --- | --- | --- | --- | --- | --- | --- | --- | --- | --- | --- | --- | --- | --- | --- | --- | --- | --- | --- | --- | --- | --- | --- | --- | --- | --- | --- | --- | --- | --- | --- | --- | --- | --- | --- | --- | --- | --- | --- | --- | --- | --- | --- | --- | --- | --- | --- | --- | --- | --- | --- | --- | --- | --- | --- | --- | --- | --- | --- | --- | --- | --- | --- | --- | --- | --- | --- | --- | --- | --- | --- | --- | --- | --- | --- | --- | --- | --- | --- | --- | --- | --- | --- | --- | --- | --- | --- | --- | --- | --- | --- | --- | --- | --- | --- | --- | --- | --- | --- | --- | --- | --- | --- | --- | --- | --- | --- | --- | --- | --- | --- | --- | --- | --- | --- | --- | --- | --- | --- | --- | --- | --- | --- | --- | --- | --- | --- | --- | --- | --- | --- | --- | --- | --- | --- | --- | --- | --- | --- | --- | --- | --- | --- | --- | --- | --- | --- | --- | --- | --- | --- | --- | --- | --- | --- | --- | --- | --- | --- | --- | --- | --- | --- | --- | --- | --- | --- | --- | --- | --- | --- | --- | --- | --- | --- | --- | --- | --- | --- | --- | --- | --- | --- | --- | --- | --- | --- | --- | --- | --- | --- | --- | --- | --- | --- | --- | --- | --- | --- | --- | --- | --- | --- | --- | --- | --- | --- | --- | --- | --- | --- | --- | --- | --- | --- | --- | --- | --- | --- | --- | --- | --- | --- | --- | --- | --- | --- | --- | --- | --- | --- | --- | --- | --- | --- | --- | --- | --- | --- | --- | --- | --- | --- | --- | --- | --- | --- | --- | --- | --- | --- | --- | --- | --- | --- | --- | --- | --- | --- | --- | --- | --- | --- | --- | --- | --- | --- | --- | --- | --- | --- | --- | --- | --- | --- | --- | --- | --- | --- | --- | --- | --- | --- | --- | --- | --- | --- | --- | --- | --- | --- | --- | --- | --- | --- | --- | --- | --- | --- | --- | --- | --- | --- | --- | --- | --- | --- | --- | --- | --- | --- | --- | --- | --- | --- | --- | --- | --- | --- | --- | --- | --- | --- | --- | --- | --- | --- | --- | --- | --- | --- | --- | --- | --- | --- | --- | --- | --- | --- | --- | --- | --- | --- | --- | --- | --- | --- | --- | --- | --- | --- | --- | --- | --- | --- | --- | --- | --- | --- | --- | --- | --- | --- | --- | --- | --- | --- | --- | --- | --- | --- | --- | --- | --- | --- | --- | --- | --- | --- | --- | --- | --- | --- | --- | --- | --- | --- | --- | --- | --- | --- | --- | --- | --- | --- | --- | --- | --- | --- | --- | --- | --- | --- | --- | --- | --- | --- | --- | --- | --- | --- | --- | --- | --- | --- | --- | --- | --- | --- | --- | --- | --- | --- | --- | --- | --- | --- | --- | --- | --- | --- | --- | --- | --- | --- | --- | --- | --- | --- | --- | --- | --- | --- | --- | --- | --- | --- | --- | --- | --- | --- | --- | --- | --- | --- | --- | --- | --- | --- | --- | --- | --- | --- | --- | --- | --- | --- | --- | --- | --- | --- | --- | --- | --- | --- | --- | --- | --- | --- | --- | --- | --- | --- | --- | --- | --- | --- | --- | --- | --- | --- | --- | --- | --- | --- | --- | --- | --- | --- | --- | --- | --- | --- | --- | --- | --- | --- | --- | --- | --- | --- | --- | --- | --- | --- | --- | --- | --- | --- | --- | --- | --- | --- | --- | --- | --- | --- | --- | --- | --- | --- | --- | --- | --- | --- | --- | --- | --- | --- | --- | --- | --- | --- | --- | --- | --- | --- | --- | --- | --- | --- | --- | --- | --- | --- | --- | --- | --- | --- | --- | --- | --- | --- | --- | --- | --- | --- | --- | --- | --- | --- | --- | --- | --- | --- | --- | --- | --- | --- | --- | --- | --- | --- | --- | --- | --- | --- | --- | --- | --- | --- | --- | --- | --- | --- | --- | --- | --- | --- | --- | --- | --- | --- | --- | --- | --- | --- | --- | --- | --- | --- | --- | --- | --- | --- | --- | --- | --- | --- | --- | --- | --- | --- | --- | --- | --- | --- | --- | --- | --- | --- | --- | --- | --- | --- | --- | --- | --- | --- | --- | --- | --- | --- | --- | --- | --- | --- | --- | --- | --- | --- | --- | --- | --- | --- | --- | --- | --- | --- | --- | --- | --- | --- | --- | --- | --- | --- | --- | --- | --- | --- | --- | --- | --- | --- | --- | --- | --- | --- | --- | --- | --- | --- | --- | --- | --- | --- | --- | --- | --- | --- | --- | --- | --- | --- | --- | --- | --- | --- | --- | --- | --- | --- | --- | --- | --- | --- | --- | --- | --- | --- | --- | --- | --- | --- | --- | --- | --- | --- | --- | --- | --- | --- | --- | --- | --- | --- | --- | --- | --- | --- | --- | --- | --- | --- | --- | --- | --- | --- | --- | --- | --- | --- | --- | --- | --- | --- | --- | --- | --- | --- | --- | --- | --- | --- | --- | --- | --- | --- | --- | --- | --- | --- | --- | --- | --- | --- | --- | --- | --- | --- | --- | --- | --- | --- | --- | --- | --- | --- | --- | --- | --- | --- | --- | --- | --- | --- | --- | --- | --- | --- | --- | --- | --- | --- | --- | --- | --- | --- | --- | --- | --- | --- | --- | --- | --- | --- | --- | --- | --- | --- | --- | --- | --- | --- | --- | --- | --- | --- | --- | --- | --- | --- | --- | --- | --- | --- | --- | --- | --- | --- | --- | --- | --- | --- | --- | --- | --- | --- | --- | --- | --- | --- | --- | --- | --- | --- | --- | --- | --- | --- | --- | --- | --- | --- | --- | --- | --- | --- | --- | --- | --- | --- | --- | --- | --- | --- | --- | --- | --- | --- | --- | --- | --- | --- | --- | --- | --- | --- | --- | --- | --- | --- | --- | --- | --- | --- | --- | --- | --- | --- | --- | --- | --- | --- | --- | --- | --- | --- | --- | --- | --- | --- | --- | --- | --- | --- | --- | --- | --- | --- | --- | --- | --- | --- | --- | --- | --- | --- | --- | --- | --- | --- | --- | --- | --- | --- | --- | --- | --- | --- | --- | --- | --- | --- | --- | --- | --- | --- | --- | --- | --- | --- | --- | --- | --- | --- | --- | --- | --- | --- | --- | --- | --- | --- | --- | --- | --- | --- | --- | --- | --- | --- | --- | --- | --- | --- | --- | --- | --- | --- | --- | --- | --- | --- | --- | --- | --- | --- | --- | --- | --- | --- | --- | --- | --- | --- | --- | --- | --- | --- | --- | --- | --- | --- | --- | --- | --- | --- | --- | --- | --- | --- | --- | --- | --- | --- | --- | --- | --- | --- | --- | --- | --- | --- | --- | --- | --- | --- | --- | --- | --- | --- | --- | --- | --- | --- | --- | --- | --- | --- | --- | --- | --- | --- | --- | --- | --- | --- | --- | --- | --- | --- | --- | --- | --- | --- | --- | --- | --- | --- | --- | --- | --- | --- | --- | --- | --- | --- | --- | --- | --- | --- | --- | --- | --- | --- | --- | --- | --- | --- | --- | --- | --- | --- | --- | --- | --- | --- | --- | --- | --- | --- | --- | --- | --- | --- | --- | --- | --- | --- | --- | --- | --- | --- | --- | --- | --- | --- | --- | --- | --- | --- | --- | --- | --- | --- | --- | --- | --- | --- | --- | --- | --- | --- | --- | --- | --- | --- | --- | --- | --- | --- | --- | --- | --- | --- | --- | --- | --- | --- | --- | --- | --- | --- | --- | --- | --- | --- | --- | --- | --- | --- | --- | --- | --- | --- | --- | --- | --- | --- | --- | --- | --- | --- | --- | --- | --- | --- | --- | --- | --- | --- | --- | --- | --- | --- | --- | --- | --- | --- | --- | --- | --- | --- | --- | --- | --- | --- | --- | --- | --- | --- | --- | --- | --- | --- | --- | --- | --- | --- | --- | --- | --- | --- | --- | --- | --- | --- | --- | --- | --- | --- | --- | --- | --- | --- | --- | --- | --- | --- | --- | --- | --- | --- | --- | --- | --- | --- | --- | --- | --- | --- | --- | --- | --- | --- | --- | --- | --- | --- | --- | --- | --- | --- | --- | --- | --- | --- | --- | --- | --- | --- | --- | --- | --- | --- | --- | --- | --- | --- | --- | --- | --- | --- | --- | --- | --- | --- | --- | --- | --- | --- | --- | --- | --- | --- | --- | --- | --- | --- | --- | --- | --- | --- | --- | --- | --- | --- | --- | --- | --- | --- | --- | --- | --- | --- | --- | --- | --- | --- | --- | --- | --- | --- | --- | --- | --- | --- | --- | --- | --- | --- | --- | --- | --- | --- | --- | --- | --- | --- | --- | --- | --- | --- | --- | --- | --- | --- | --- | --- | --- | --- | --- | --- | --- | --- | --- | --- | --- | --- | --- | --- | --- | --- | --- | --- | --- | --- | --- | --- | --- | --- | --- | --- | --- | --- | --- | --- | --- | --- | --- | --- | --- | --- | --- | --- | --- | --- | --- | --- | --- | --- | --- | --- | --- | --- | --- | --- | --- | --- | --- | --- | --- | --- | --- | --- | --- | --- | --- | --- | --- | --- | --- | --- | --- | --- | --- | --- | --- | --- | --- | --- | --- | --- | --- | --- | --- | --- | --- | --- | --- | --- | --- | --- | --- | --- | --- | --- | --- | --- | --- | --- | --- | --- | --- | --- | --- | --- | --- | --- | --- | --- | --- | --- | --- | --- | --- | --- | --- | --- | --- | --- | --- | --- | --- | --- | --- | --- | --- | --- | --- | --- | --- | --- | --- | --- | --- | --- | --- | --- | --- | --- | --- | --- | --- | --- | --- | --- | --- | --- | --- | --- | --- | --- | --- | --- | --- | --- | --- | --- | --- | --- | --- | --- | --- | --- | --- | --- | --- | --- | --- | --- | --- | --- | --- | --- | --- | --- | --- | --- | --- | --- | --- | --- | --- | --- | --- | --- | --- | --- | --- | --- | --- | --- | --- | --- | --- | --- | --- | --- | --- | --- | --- | --- | --- | --- | --- | --- | --- | --- | --- | --- | --- | --- | --- | --- | --- | --- | --- | --- | --- | --- | --- | --- | --- | --- | --- | --- | --- | --- | --- | --- | --- | --- | --- | --- | --- | --- | --- | --- | --- | --- | --- | --- | --- | --- | --- | --- | --- | --- | --- | --- | --- | --- | --- | --- | --- | --- | --- | --- | --- | --- | --- | --- | --- | --- | --- | --- | --- | --- | --- | --- | --- | --- | --- | --- | --- | --- | --- | --- | --- | --- | --- | --- | --- | --- | --- | --- | --- | --- | --- | --- | --- | --- | --- | --- | --- | --- | --- | --- | --- | --- | --- | --- | --- | --- | --- | --- | --- | --- | --- | --- | --- | --- | --- | --- | --- | --- | --- | --- | --- | --- | --- | --- | --- | --- | --- | --- | --- | --- | --- | --- | --- | --- | --- | --- | --- | --- | --- | --- | --- | --- | --- | --- | --- | --- | --- | --- | --- | --- | --- | --- | --- | --- | --- | --- | --- | --- | --- | --- | --- | --- | --- | --- | --- | --- | --- | --- | --- | --- | --- | --- | --- | --- | --- | --- | --- | --- | --- | --- | --- | --- | --- | --- | --- | --- | --- | --- | --- | --- | --- | --- | --- | --- | --- | --- | --- | --- | --- | --- | --- | --- | --- | --- | --- | --- | --- | --- | --- | --- | --- | --- | --- | --- | --- | --- | --- | --- | --- | --- | --- | --- | --- | --- | --- | --- | --- | --- | --- | --- | --- | --- | --- | --- | --- | --- | --- | --- | --- | --- | --- | --- | --- | --- | --- | --- | --- | --- | --- | --- | --- | --- | --- | --- | --- | --- | --- | --- | --- | --- | --- | --- | --- | --- | --- | --- | --- | --- | --- | --- | --- | --- | --- | --- | --- | --- | --- | --- | --- | --- | --- | --- | --- | --- | --- | --- | --- | --- | --- | --- | --- | --- | --- | --- | --- | --- | --- | --- | --- | --- | --- | --- | --- | --- | --- | --- | --- | --- | --- | --- | --- | --- | --- | --- | --- | --- | --- | --- | --- | --- | --- | --- | --- | --- | --- | --- | --- | --- | --- | --- | --- | --- | --- | --- | --- | --- | --- | --- | --- | --- | --- | --- | --- | --- | --- | --- | --- | --- | --- | --- | --- | --- | --- | --- | --- | --- | --- | --- | --- | --- | --- | --- | --- | --- | --- | --- | --- | --- | --- | --- | --- | --- | --- | --- | --- | --- | --- | --- | --- | --- | --- | --- | --- | --- | --- | --- | --- | --- | --- | --- | --- | --- | --- | --- | --- | --- | --- | --- | --- | --- | --- | --- | --- | --- | --- | --- | --- | --- | --- | --- | --- | --- | --- | --- | --- | --- | --- | --- | --- | --- | --- | --- | --- | --- | --- | --- | --- | --- | --- | --- | --- | --- | --- | --- | --- | --- | --- | --- | --- | --- | --- | --- | --- | --- | --- | --- | --- | --- | --- | --- | --- | --- | --- | --- | --- | --- | --- | --- | --- | --- | --- | --- | --- | --- | --- | --- | --- | --- | --- | --- | --- | --- | --- | --- | --- | --- | --- | --- | --- | --- | --- | --- | --- | --- | --- | --- | --- | --- | --- | --- | --- | --- | --- | --- | --- | --- | --- | --- | --- | --- | --- | --- | --- | --- | --- | --- | --- | --- | --- | --- | --- | --- | --- | --- | --- | --- | --- | --- | --- | --- | --- | --- | --- | --- | --- | --- | --- | --- | --- | --- | --- | --- | --- | --- | --- | --- | --- | --- | --- | --- | --- | --- | --- | --- | --- | --- | --- | --- | --- | --- | --- | --- | --- | --- | --- | --- | --- | --- | --- | --- | --- | --- | --- | --- | --- | --- | --- | --- | --- | --- | --- | --- | --- | --- | --- | --- | --- | --- | --- | --- | --- | --- | --- | --- | --- | --- | --- | --- | --- | --- | --- | --- | --- | --- | --- | --- | --- | --- | --- | --- | --- | --- | --- | --- | --- | --- | --- | --- | --- | --- | --- | --- | --- | --- | --- | --- | --- | --- | --- | --- | --- | --- | --- | --- | --- | --- | --- | --- | --- | --- | --- | --- | --- | --- | --- | --- | --- | --- | --- | --- | --- | --- | --- | --- | --- | --- | --- | --- | --- | --- | --- | --- | --- | --- | --- | --- | --- | --- | --- | --- | --- | --- | --- | --- | --- | --- | --- | --- | --- | --- | --- | --- | --- | --- | --- | --- | --- | --- | --- | --- | --- | --- | --- | --- | --- | --- | --- | --- | --- | --- | --- | --- | --- | --- | --- | --- | --- | --- | --- | --- | --- | --- | --- | --- | --- | --- | --- | --- | --- | --- | --- | --- | --- | --- | --- | --- | --- | --- | --- | --- | --- | --- | --- | --- | --- | --- | --- | --- | --- | --- | --- | --- | --- | --- | --- | --- | --- | --- | --- | --- | --- | --- | --- | --- | --- | --- | --- | --- | --- | --- | --- | --- | --- | --- | --- | --- | --- | --- | --- | --- | --- | --- | --- | --- | --- | --- | --- | --- | --- | --- | --- | --- | --- | --- | --- | --- | --- | --- | --- | --- | --- | --- | --- | --- | --- | --- | --- | --- | --- | --- | --- | --- | --- | --- | --- | --- | --- | --- | --- | --- | --- | --- | --- | --- | --- | --- | --- | --- | --- | --- | --- | --- | --- | --- | --- | --- | --- | --- | --- | --- | --- | --- | --- | --- | --- | --- | --- | --- | --- | --- | --- | --- | --- | --- | --- | --- | --- | --- | --- | --- | --- | --- | --- | --- | --- | --- | --- | --- | --- | --- | --- | --- | --- | --- | --- | --- | --- | --- | --- | --- | --- | --- | --- | --- | --- | --- | --- | --- | --- | --- | --- | --- | --- | --- | --- | --- | --- | --- | --- | --- | --- | --- | --- | --- | --- | --- | --- | --- | --- | --- | --- | --- | --- | --- | --- | --- | --- | --- | --- | --- | --- | --- | --- | --- | --- | --- | --- | --- | --- | --- | --- | --- | --- | --- | --- | --- | --- | --- | --- | --- | --- | --- | --- | --- | --- | --- | --- | --- | --- | --- | --- | --- | --- | --- | --- | --- | --- | --- | --- | --- | --- | --- | --- | --- | --- | --- | --- | --- | --- | --- | --- | --- | --- | --- | --- | --- | --- | --- | --- | --- | --- | --- | --- | --- | --- | --- | --- | --- | --- | --- | --- | --- | --- | --- | --- | --- | --- | --- | --- | --- | --- | --- | --- | --- | --- | --- | --- | --- | --- | --- | --- | --- | --- | --- | --- | --- | --- | --- | --- | --- | --- | --- | --- | --- | --- | --- | --- | --- | --- | --- | --- | --- | --- | --- | --- | --- | --- | --- | --- | --- | --- | --- | --- | --- | --- | --- | --- | --- | --- | --- | --- | --- | --- | --- | --- | --- | --- | --- | --- | --- | --- | --- | --- | --- | --- | --- | --- | --- | --- | --- | --- | --- | --- | --- | --- | --- | --- | --- | --- | --- | --- | --- | --- | --- | --- | --- | --- | --- | --- | --- | --- | --- | --- | --- | --- | --- | --- | --- | --- | --- | --- | --- | --- | --- | --- | --- | --- | --- | --- | --- | --- | --- | --- | --- | --- | --- | --- | --- | --- | --- | --- | --- | --- | --- | --- | --- | --- | --- | --- | --- | --- | --- | --- | --- | --- | --- | --- | --- | --- | --- | --- | --- | --- | --- | --- | --- | --- | --- | --- | --- | --- | --- | --- | --- | --- | --- | --- | --- | --- | --- | --- | --- | --- | --- | --- | --- | --- | --- | --- | --- | --- | --- | --- | --- | --- | --- | --- | --- | --- | --- | --- | --- | --- | --- | --- | --- | --- | --- | --- | --- | --- | --- | --- | --- | --- | --- | --- | --- | --- | --- | --- | --- | --- | --- | --- | --- | --- | --- | --- | --- | --- | --- | --- | --- | --- | --- | --- | --- | --- | --- | --- | --- | --- | --- | --- | --- | --- | --- | --- | --- | --- | --- | --- | --- | --- | --- | --- | --- | --- | --- | --- | --- | --- | --- | --- | --- | --- | --- | --- | --- | --- | --- | --- | --- | --- | --- | --- | --- | --- | --- | --- | --- | --- | --- | --- | --- | --- | --- | --- | --- | --- | --- | --- | --- | --- | --- | --- | --- | --- | --- | --- | --- | --- | --- | --- | --- | --- | --- | --- | --- | --- | --- | --- | --- | --- | --- | --- | --- | --- | --- | --- | --- | --- | --- | --- | --- | --- | --- | --- | --- | --- | --- | --- | --- | --- | --- | --- | --- | --- | --- | --- | --- | --- | --- | --- | --- | --- | --- | --- | --- | --- | --- | --- | --- | --- | --- | --- | --- | --- | --- | --- | --- | --- | --- | --- | --- | --- | --- | --- | --- | --- | --- | --- | --- | --- | --- | --- | --- | --- | --- | --- | --- | --- | --- | --- | --- | --- | --- | --- | --- | --- | --- | --- | --- | --- | --- | --- | --- | --- | --- | --- | --- | --- | --- | --- | --- | --- | --- | --- | --- | --- | --- | --- | --- | --- | --- | --- | --- | --- | --- | --- | --- | --- | --- | --- | --- | --- | --- | --- | --- | --- | --- | --- | --- | --- | --- | --- | --- | --- | --- | --- | --- | --- | --- | --- | --- | --- | --- | --- | --- | --- | --- | --- | --- | --- | --- | --- | --- | --- | --- | --- | --- | --- | --- | --- | --- | --- | --- | --- | --- | --- | --- | --- | --- | --- | --- | --- | --- | --- | --- | --- | --- | --- | --- | --- | --- | --- | --- | --- | --- | --- | --- | --- | --- | --- | --- | --- | --- | --- | --- | --- | --- | --- | --- | --- | --- | --- | --- | --- | --- | --- | --- | --- | --- | --- | --- | --- | --- | --- | --- | --- | --- | --- | --- | --- | --- | --- | --- | --- | --- | --- | --- | --- | --- | --- | --- | --- | --- | --- | --- | --- | --- | --- | --- | --- | --- | --- | --- | --- | --- | --- | --- | --- | --- | --- | --- | --- | --- | --- | --- | --- | --- | --- | --- | --- | --- | --- | --- | --- | --- | --- | --- | --- | --- | --- | --- | --- | --- | --- | --- | --- | --- | --- | --- | --- | --- | --- | --- | --- | --- | --- | --- | --- | --- | --- | --- | --- | --- | --- | --- | --- | --- | --- | --- | --- | --- | --- | --- | --- | --- | --- | --- | --- | --- | --- | --- | --- | --- | --- | --- | --- | --- | --- | --- | --- | --- | --- | --- | --- | --- | --- | --- | --- | --- | --- | --- | --- | --- | --- | --- | --- | --- | --- | --- | --- | --- | --- | --- | --- | --- | --- | --- | --- | --- | --- | --- | --- | --- | --- | --- | --- | --- | --- | --- | --- | --- | --- | --- | --- | --- | --- | --- | --- | --- | --- | --- | --- | --- | --- | --- | --- | --- | --- | --- | --- | --- | --- | --- | --- | --- | --- | --- | --- | --- | --- | --- | --- | --- | --- | --- | --- | --- | --- | --- | --- | --- | --- | --- | --- | --- | --- | --- | --- | --- | --- | --- | --- | --- | --- | --- | --- | --- | --- | --- | --- | --- | --- | --- | --- | --- | --- | --- | --- | --- | --- | --- | --- | --- | --- | --- | --- | --- | --- | --- | --- | --- | --- | --- | --- | --- | --- | --- | --- | --- | --- | --- | --- | --- | --- | --- | --- | --- | --- | --- | --- | --- | --- | --- | --- | --- | --- | --- | --- | --- | --- | --- | --- | --- | --- | --- | --- | --- | --- | --- | --- | --- | --- | --- | --- | --- | --- | --- | --- | --- | --- | --- | --- | --- | --- | --- | --- | --- | --- | --- | --- | --- | --- | --- | --- | --- | --- | --- | --- | --- | --- | --- | --- | --- | --- | --- | --- | --- | --- | --- | --- | --- | --- | --- | --- | --- | --- | --- | --- | --- | --- | --- | --- | --- | --- | --- | --- | --- | --- | --- | --- | --- | --- | --- | --- | --- | --- | --- | --- | --- | --- | --- | --- | --- | --- | --- | --- | --- | --- | --- | --- | --- | --- | --- | --- | --- | --- | --- | --- | --- | --- | --- | --- | --- | --- | --- | --- | --- | --- | --- | --- | --- | --- | --- | --- | --- | --- | --- | --- | --- | --- | --- | --- | --- | --- | --- | --- | --- | --- | --- | --- | --- | --- | --- | --- | --- | --- | --- | --- | --- | --- | --- | --- | --- | --- | --- | --- | --- | --- | --- | --- | --- | --- | --- | --- | --- | --- | --- | --- | --- | --- | --- | --- | --- | --- | --- | --- | --- | --- | --- | --- | --- | --- | --- | --- | --- | --- | --- | --- | --- | --- | --- | --- | --- | --- | --- | --- | --- | --- | --- | --- | --- | --- | --- | --- | --- | --- | --- | --- | --- | --- | --- | --- | --- | --- | --- | --- | --- | --- | --- | --- | --- | --- | --- | --- | --- | --- | --- | --- | --- | --- | --- | --- | --- | --- | --- | --- | --- | --- | --- | --- | --- | --- | --- | --- | --- | --- | --- | --- | --- | --- | --- | --- | --- | --- | --- | --- | --- | --- | --- | --- | --- | --- | --- | --- | --- | --- | --- | --- | --- | --- | --- | --- | --- | --- | --- | --- | --- | --- | --- | --- | --- | --- | --- | --- | --- | --- | --- | --- | --- | --- | --- | --- | --- | --- | --- | --- | --- | --- | --- | --- | --- | --- | --- | --- | --- | --- | --- | --- | --- | --- | --- | --- | --- | --- | --- | --- | --- | --- | --- | --- | --- | --- | --- | --- | --- | --- | --- | --- | --- | --- | --- | --- | --- | --- | --- | --- | --- | --- | --- | --- | --- | --- | --- | --- | --- | --- | --- | --- | --- | --- | --- | --- | --- | --- | --- | --- | --- | --- | --- | --- | --- | --- | --- | --- | --- | --- | --- | --- | --- | --- | --- | --- | --- | --- | --- | --- | --- | --- | --- | --- | --- | --- | --- | --- | --- | --- | --- | --- | --- | --- | --- | --- | --- | --- | --- | --- | --- | --- | --- | --- | --- | --- | --- | --- | --- | --- | --- | --- | --- | --- | --- | --- | --- | --- | --- | --- | --- | --- | --- | --- | --- | --- | --- | --- | --- | --- | --- | --- | --- | --- | --- | --- | --- | --- | --- | --- | --- | --- | --- | --- | --- | --- | --- | --- | --- | --- | --- | --- | --- | --- | --- | --- | --- | --- | --- | --- | --- | --- | --- | --- | --- | --- | --- | --- | --- | --- | --- | --- | --- | --- | --- | --- | --- | --- | --- | --- | --- | --- | --- | --- | --- | --- | --- | --- | --- | --- | --- | --- | --- | --- | --- | --- | --- | --- | --- | --- | --- | --- | --- | --- | --- | --- | --- | --- | --- | --- | --- | --- | --- | --- | --- | --- | --- | --- | --- | --- | --- | --- | --- | --- | --- | --- | --- | --- | --- | --- | --- | --- | --- | --- | --- | --- | --- | --- | --- | --- | --- | --- | --- | --- | --- | --- | --- | --- | --- | --- | --- | --- | --- | --- | --- | --- | --- | --- | --- | --- | --- | --- | --- | --- | --- | --- | --- | --- | --- | --- | --- | --- | --- | --- | --- | --- | --- | --- | --- | --- | --- | --- | --- | --- | --- | --- | --- | --- | --- | --- | --- | --- | --- | --- | --- | --- | --- | --- | --- | --- | --- | --- | --- | --- | --- | --- | --- | --- | --- | --- | --- | --- | --- | --- | --- | --- | --- | --- | --- | --- | --- | --- | --- | --- | --- | --- | --- | --- | --- | --- | --- | --- | --- | --- | --- | --- | --- | --- | --- | --- | --- | --- | --- | --- | --- | --- | --- | --- | --- | --- | --- | --- | --- | --- | --- | --- | --- | --- | --- | --- | --- | --- | --- | --- | --- | --- | --- | --- | --- | --- | --- | --- | --- | --- | --- | --- | --- | --- | --- | --- | --- | --- | --- | --- | --- | --- | --- | --- | --- | --- | --- | --- | --- | --- | --- | --- | --- | --- | --- | --- | --- | --- | --- | --- | --- | --- | --- | --- | --- | --- | --- | --- | --- | --- | --- | --- | --- | --- | --- | --- | --- | --- | --- | --- | --- | --- | --- | --- | --- | --- | --- | --- | --- | --- | --- | --- | --- | --- | --- | --- | --- | --- | --- | --- | --- | --- | --- | --- | --- | --- | --- | --- | --- | --- | --- | --- | --- | --- | --- | --- | --- | --- | --- | --- | --- | --- | --- | --- | --- | --- | --- | --- | --- | --- | --- | --- | --- | --- | --- | --- | --- | --- | --- | --- | --- | --- | --- | --- | --- | --- | --- | --- | --- | --- | --- | --- | --- | --- | --- | --- | --- | --- | --- | --- | --- | --- | --- | --- | --- | --- | --- | --- | --- | --- | --- | --- | --- | --- | --- | --- | --- | --- | --- | --- | --- | --- | --- | --- | --- | --- | --- | --- | --- | --- | --- | --- | --- | --- | --- | --- | --- | --- | --- | --- | --- | --- | --- | --- | --- | --- | --- | --- | --- | --- | --- | --- | --- | --- | --- | --- | --- | --- | --- | --- | --- | --- | --- | --- | --- | --- | --- | --- | --- | --- | --- | --- | --- | --- | --- | --- | --- | --- | --- | --- | --- | --- | --- | --- | --- | --- | --- | --- | --- | --- | --- | --- | --- | --- | --- | --- | --- | --- | --- | --- | --- | --- | --- | --- | --- | --- | --- | --- | --- | --- | --- | --- | --- | --- | --- | --- | --- | --- | --- | --- | --- | --- | --- | --- | --- | --- | --- | --- | --- | --- | --- | --- | --- | --- | --- | --- | --- | --- | --- | --- | --- | --- | --- | --- | --- | --- | --- | --- | --- | --- | --- | --- | --- | --- | --- | --- | --- | --- | --- | --- | --- | --- | --- | --- | --- | --- | --- | --- | --- | --- | --- | --- | --- | --- | --- | --- | --- | --- | --- | --- | --- | --- | --- | --- | --- | --- | --- | --- | --- | --- | --- | --- | --- | --- | --- | --- | --- | --- | --- | --- | --- | --- | --- | --- | --- | --- | --- | --- | --- | --- | --- | --- | --- | --- | --- | --- | --- | --- | --- | --- | --- | --- | --- | --- | --- | --- | --- | --- | --- | --- | --- | --- | --- | --- | --- | --- | --- | --- | --- | --- | --- | --- | --- | --- | --- | --- | --- | --- | --- | --- | --- | --- | --- | --- | --- | --- | --- | --- | --- | --- | --- | --- | --- | --- | --- | --- | --- | --- | --- | --- | --- | --- | --- | --- | --- | --- | --- | --- | --- | --- | --- | --- | --- | --- | --- | --- | --- | --- | --- | --- | --- | --- | --- | --- | --- | --- | --- | --- | --- | --- | --- | --- | --- | --- | --- | --- | --- | --- | --- | --- | --- | --- | --- | --- | --- | --- | --- | --- | --- | --- | --- | --- | --- | --- | --- | --- | --- | --- | --- | --- | --- | --- | --- | --- | --- | --- | --- | --- | --- | --- | --- | --- | --- | --- | --- | --- | --- | --- | --- | --- | --- | --- | --- | --- | --- | --- | --- | --- | --- | --- | --- | --- | --- | --- | --- | --- | --- | --- | --- | --- | --- | --- | --- | --- | --- | --- | --- | --- | --- | --- | --- | --- | --- | --- | --- | --- | --- | --- | --- | --- | --- | --- | --- | --- | --- | --- | --- | --- | --- | --- | --- | --- | --- | --- | --- | --- | --- | --- | --- | --- | --- | --- | --- | --- | --- | --- | --- | --- | --- | --- | --- | --- | --- | --- | --- | --- | --- | --- | --- | --- | --- | --- | --- | --- | --- | --- | --- | --- | --- | --- | --- | --- | --- | --- | --- | --- | --- | --- | --- | --- | --- | --- | --- | --- | --- | --- | --- | --- | --- | --- | --- | --- | --- | --- | --- | --- | --- | --- | --- | --- | --- | --- | --- | --- | --- | --- | --- | --- | --- | --- | --- | --- | --- | --- | --- | --- | --- | --- | --- | --- | --- | --- | --- | --- | --- | --- | --- | --- | --- | --- | --- | --- | --- | --- | --- | --- | --- | --- | --- | --- | --- | --- | --- | --- | --- | --- | --- | --- | --- | --- | --- | --- | --- | --- | --- | --- | --- | --- | --- | --- | --- | --- | --- | --- | --- | --- | --- | --- | --- | --- | --- | --- | --- | --- | --- | --- | --- | --- | --- | --- | --- | --- | --- | --- | --- | --- | --- | --- | --- | --- | --- | --- | --- | --- | --- | --- | --- | --- | --- | --- | --- | --- | --- | --- | --- | --- | --- | --- | --- | --- | --- | --- | --- | --- | --- | --- | --- | --- | --- | --- | --- | --- | --- | --- | --- | --- | --- | --- | --- | --- | --- | --- | --- | --- | --- | --- | --- | --- | --- | --- | --- | --- | --- | --- | --- | --- | --- | --- | --- | --- | --- | --- | --- | --- | --- | --- | --- | --- | --- | --- | --- | --- | --- | --- | --- | --- | --- | --- | --- | --- | --- | --- | --- | --- | --- | --- | --- | --- | --- | --- | --- | --- | --- | --- | --- | --- | --- | --- | --- | --- | --- | --- | --- | --- | --- | --- | --- | --- | --- | --- | --- | --- | --- | --- | --- | --- | --- | --- | --- | --- | --- | --- | --- | --- | --- | --- | --- | --- | --- | --- | --- | --- | --- | --- | --- | --- | --- | --- | --- | --- | --- | --- | --- | --- | --- | --- | --- | --- | --- | --- | --- | --- | --- | --- | --- | --- | --- | --- | --- | --- | --- | --- | --- | --- | --- | --- | --- | --- | --- | --- | --- | --- | --- | --- | --- | --- | --- | --- | --- | --- | --- | --- | --- | --- | --- | --- | --- | --- | --- | --- | --- | --- | --- | --- | --- | --- | --- | --- | --- | --- | --- | --- | --- | --- | --- | --- | --- | --- | --- | --- | --- | --- | --- | --- | --- | --- | --- | --- | --- | --- | --- | --- | --- | --- | --- | --- | --- | --- | --- | --- | --- | --- | --- | --- | --- | --- | --- | --- | --- | --- | --- | --- | --- | --- | --- | --- | --- | --- | --- | --- | --- | --- | --- | --- | --- | --- | --- | --- | --- | --- | --- | --- | --- | --- | --- | --- | --- | --- | --- | --- | --- | --- | --- | --- | --- | --- | --- | --- | --- | --- | --- | --- | --- | --- | --- | --- | --- | --- | --- | --- | --- | --- | --- | --- | --- | --- | --- | --- | --- | --- | --- | --- | --- | --- | --- | --- | --- | --- | --- | --- | --- | --- | --- | --- | --- | --- | --- | --- | --- | --- | --- | --- | --- | --- | --- | --- | --- | --- | --- | --- | --- | --- | --- | --- | --- | --- | --- | --- | --- | --- | --- | --- | --- | --- | --- | --- | --- | --- | --- | --- | --- | --- | --- | --- | --- | --- | --- | --- | --- | --- | --- | --- | --- | --- | --- | --- | --- | --- | --- | --- | --- | --- | --- | --- | --- | --- | --- | --- | --- | --- | --- | --- | --- | --- | --- | --- | --- | --- | --- | --- | --- | --- | --- | --- | --- | --- | --- | --- | --- | --- | --- | --- | --- | --- | --- | --- | --- | --- | --- | --- | --- | --- | --- | --- | --- | --- | --- | --- | --- | --- | --- | --- | --- | --- | --- | --- | --- | --- | --- | --- | --- | --- | --- | --- | --- | --- | --- | --- | --- | --- | --- | --- | --- | --- | --- | --- | --- | --- | --- | --- | --- | --- | --- | --- | --- | --- | --- | --- | --- | --- | --- | --- | --- | --- | --- | --- | --- | --- | --- | --- | --- | --- | --- | --- | --- | --- | --- | --- | --- | --- | --- | --- | --- | --- | --- | --- | --- | --- | --- | --- | --- | --- | --- | --- | --- | --- | --- | --- | --- | --- | --- | --- | --- | --- | --- | --- | --- | --- | --- | --- | --- | --- | --- | --- | --- | --- | --- | --- | --- | --- | --- | --- | --- | --- | --- | --- | --- | --- | --- | --- | --- | --- | --- | --- | --- | --- | --- | --- | --- | --- | --- | --- | --- | --- | --- | --- | --- | --- | --- | --- | --- | --- | --- | --- | --- | --- | --- | --- | --- | --- | --- | --- | --- | --- | --- | --- | --- | --- | --- | --- | --- | --- | --- | --- | --- | --- | --- | --- | --- | --- | --- | --- | --- | --- | --- | --- | --- | --- | --- | --- | --- | --- | --- | --- | --- | --- | --- | --- | --- | --- | --- | --- | --- | --- | --- | --- | --- | --- | --- | --- | --- | --- | --- | --- | --- | --- | --- | --- | --- | --- | --- | --- | --- | --- | --- | --- | --- | --- | --- | --- | --- | --- | --- | --- | --- | --- | --- | --- | --- | --- | --- | --- | --- | --- | --- | --- | --- | --- | --- | --- | --- | --- | --- | --- | --- | --- | --- | --- | --- | --- | --- | --- | --- | --- | --- | --- | --- | --- | --- | --- | --- | --- | --- | --- | --- | --- | --- | --- | --- | --- | --- | --- | --- | --- | --- | --- | --- | --- | --- | --- | --- | --- | --- | --- | --- | --- | --- | --- | --- | --- | --- | --- | --- | --- | --- | --- | --- | --- | --- | --- | --- | --- | --- | --- | --- | --- | --- | --- | --- | --- | --- | --- | --- | --- | --- | --- | --- | --- | --- | --- | --- | --- | --- | --- | --- | --- | --- | --- | --- | --- | --- | --- | --- | --- | --- | --- | --- | --- | --- | --- | --- | --- | --- | --- | --- | --- | --- | --- | --- | --- | --- | --- | --- | --- | --- | --- | --- | --- | --- | --- | --- | --- | --- | --- | --- | --- | --- | --- | --- | --- | --- | --- | --- | --- | --- | --- | --- | --- | --- | --- | --- | --- | --- | --- | --- | --- | --- | --- | --- | --- | --- | --- | --- | --- | --- | --- | --- | --- | --- | --- | --- | --- | --- | --- | --- | --- | --- | --- | --- | --- | --- | --- | --- | --- | --- | --- | --- | --- | --- | --- | --- | --- | --- | --- | --- | --- | --- | --- | --- | --- | --- | --- | --- | --- | --- | --- | --- | --- | --- | --- | --- | --- | --- | --- | --- | --- | --- | --- | --- | --- | --- | --- | --- | --- | --- | --- | --- | --- | --- | --- | --- | --- | --- | --- | --- | --- | --- | --- | --- | --- | --- | --- | --- | --- | --- | --- | --- | --- | --- | --- | --- | --- | --- | --- | --- | --- | --- | --- | --- | --- | --- | --- | --- | --- | --- | --- | --- | --- | --- | --- | --- | --- | --- | --- | --- | --- | --- | --- | --- | --- | --- | --- | --- | --- | --- | --- | --- | --- | --- | --- | --- | --- | --- | --- | --- | --- | --- | --- | --- | --- | --- | --- | --- | --- | --- | --- | --- | --- | --- | --- | --- | --- | --- | --- | --- | --- | --- | --- | --- | --- | --- | --- | --- | --- | --- | --- | --- | --- | --- | --- | --- | --- | --- | --- | --- | --- | --- | --- | --- | --- | --- | --- | --- | --- | --- | --- | --- | --- | --- | --- | --- | --- | --- | --- | --- | --- | --- | --- | --- | --- | --- | --- | --- | --- | --- | --- | --- | --- | --- | --- | --- | --- | --- | --- | --- | --- | --- | --- | --- | --- | --- | --- | --- | --- | --- | --- | --- | --- | --- | --- | --- | --- | --- | --- | --- | --- | --- | --- | --- | --- | --- | --- | --- | --- | --- | --- | --- | --- | --- | --- | --- | --- | --- | --- | --- | --- | --- | --- | --- | --- | --- | --- | --- | --- | --- | --- | --- | --- | --- | --- | --- | --- | --- | --- | --- | --- | --- | --- | --- | --- | --- | --- | --- | --- | --- | --- | --- | --- | --- | --- | --- | --- | --- | --- | --- | --- | --- | --- | --- | --- | --- | --- | --- | --- | --- | --- | --- | --- | --- | --- | --- | --- | --- | --- | --- | --- | --- | --- | --- | --- | --- | --- | --- | --- | --- | --- | --- | --- | --- | --- | --- | --- | --- | --- | --- | --- | --- | --- | --- | --- | --- | --- | --- | --- | --- | --- | --- | --- | --- | --- | --- | --- | --- | --- | --- | --- | --- | --- | --- | --- | --- | --- | --- | --- | --- | --- | --- | --- | --- | --- | --- | --- | --- | --- | --- | --- | --- | --- | --- | --- | --- | --- | --- | --- | --- | --- | --- | --- | --- | --- | --- | --- | --- | --- | --- | --- | --- | --- | --- | --- | --- | --- | --- | --- | --- | --- | --- | --- | --- | --- | --- | --- | --- | --- | --- | --- | --- | --- | --- | --- | --- | --- | --- | --- | --- | --- | --- | --- | --- | --- | --- | --- | --- | --- | --- | --- | --- | --- | --- | --- | --- | --- | --- | --- | --- | --- | --- | --- | --- | --- | --- | --- | --- | --- | --- | --- | --- | --- | --- | --- | --- | --- | --- | --- | --- | --- | --- | --- | --- | --- | --- | --- | --- | --- | --- | --- | --- | --- | --- | --- | --- | --- | --- | --- | --- | --- | --- | --- | --- | --- | --- | --- | --- | --- | --- | --- | --- | --- | --- | --- | --- | --- | --- | --- | --- | --- | --- | --- | --- | --- | --- | --- | --- | --- | --- | --- | --- | --- | --- | --- | --- | --- | --- | --- | --- | --- | --- | --- | --- | --- | --- | --- | --- | --- | --- | --- | --- | --- | --- | --- | --- | --- | --- | --- | --- | --- | --- | --- | --- | --- | --- | --- | --- | --- | --- | --- | --- | --- | --- | --- | --- | --- | --- | --- | --- | --- | --- | --- | --- | --- | --- | --- | --- | --- | --- | --- | --- | --- | --- | --- | --- | --- | --- | --- | --- | --- | --- | --- | --- | --- | --- | --- | --- | --- | --- | --- | --- | --- | --- | --- | --- | --- | --- | --- | --- | --- | --- | --- | --- | --- | --- | --- | --- | --- | --- | --- | --- | --- | --- | --- | --- | --- | --- | --- | --- | --- | --- | --- | --- | --- | --- | --- | --- | --- | --- | --- | --- | --- | --- | --- | --- | --- | --- | --- | --- | --- | --- | --- | --- | --- | --- | --- | --- | --- | --- | --- | --- | --- | --- | --- | --- | --- | --- | --- | --- | --- | --- | --- | --- | --- | --- | --- | --- | --- | --- | --- | --- | --- | --- | --- | --- | --- | --- | --- | --- | --- | --- | --- | --- | --- | --- | --- | --- | --- | --- | --- | --- | --- | --- | --- | --- | --- | --- | --- | --- | --- | --- | --- | --- | --- | --- | --- | --- | --- | --- | --- | --- | --- | --- | --- | --- | --- | --- | --- | --- | --- | --- | --- | --- | --- | --- | --- | --- | --- | --- | --- | --- | --- | --- | --- | --- | --- | --- | --- | --- | --- | --- | --- | --- | --- | --- | --- | --- | --- | --- | --- | --- | --- | --- | --- | --- | --- | --- | --- | --- | --- | --- | --- | --- | --- | --- | --- | --- | --- | --- | --- | --- | --- | --- | --- | --- | --- | --- | --- | --- | --- | --- | --- | --- | --- | --- | --- | --- | --- | --- | --- | --- | --- | --- | --- | --- | --- | --- | --- | --- | --- | --- | --- | --- | --- | --- | --- | --- | --- | --- | --- | --- | --- | --- | --- | --- | --- | --- | --- | --- | --- | --- | --- | --- | --- | --- | --- | --- | --- | --- | --- | --- | --- | --- | --- | --- | --- | --- | --- | --- | --- | --- | --- | --- | --- | --- | --- | --- | --- | --- | --- | --- | --- | --- | --- | --- | --- | --- | --- | --- | --- | --- | --- | --- | --- | --- | --- | --- | --- | --- | --- | --- | --- | --- | --- | --- | --- | --- | --- | --- | --- | --- | --- | --- | --- | --- | --- | --- | --- | --- | --- | --- | --- | --- | --- | --- | --- | --- | --- | --- | --- | --- | --- | --- | --- | --- | --- | --- | --- | --- | --- | --- | --- | --- | --- | --- | --- | --- | --- | --- | --- | --- | --- | --- | --- | --- | --- | --- | --- | --- | --- | --- | --- | --- | --- | --- | --- | --- | --- | --- | --- | --- | --- | --- | --- | --- | --- | --- | --- | --- | --- | --- | --- | --- | --- | --- | --- | --- | --- | --- | --- | --- | --- | --- | --- | --- | --- | --- | --- | --- | --- | --- | --- | --- | --- | --- | --- | --- | --- | --- | --- | --- | --- | --- | --- | --- | --- | --- | --- | --- | --- | --- | --- | --- | --- | --- | --- | --- | --- | --- | --- | --- | --- | --- | --- | --- | --- | --- | --- | --- | --- | --- | --- | --- | --- | --- | --- | --- | --- | --- | --- | --- | --- | --- | --- | --- | --- | --- | --- | --- | --- | --- | --- | --- | --- | --- | --- | --- | --- | --- | --- | --- | --- | --- | --- | --- | --- | --- | --- | --- | --- | --- | --- | --- | --- | --- | --- | --- | --- | --- | --- | --- | --- | --- | --- | --- | --- | --- | --- | --- | --- | --- | --- | --- | --- | --- | --- | --- | --- | --- | --- | --- | --- | --- | --- | --- | --- | --- | --- | --- | --- | --- | --- | --- | --- | --- | --- | --- | --- | --- | --- | --- | --- | --- | --- | --- | --- | --- | --- | --- | --- | --- | --- | --- | --- | --- | --- | --- | --- | --- | --- | --- | --- | --- | --- | --- | --- | --- | --- | --- | --- | --- | --- | --- | --- | --- | --- | --- | --- | --- | --- | --- | --- | --- | --- | --- | --- | --- | --- | --- | --- | --- | --- | --- | --- | --- | --- | --- | --- | --- | --- | --- | --- | --- | --- | --- | --- | --- | --- | --- | --- | --- | --- | --- | --- | --- | --- | --- | --- | --- | --- | --- | --- | --- | --- | --- | --- | --- | --- | --- | --- | --- | --- | --- | --- | --- | --- | --- | --- | --- | --- | --- | --- | --- | --- | --- | --- | --- | --- | --- | --- | --- | --- | --- | --- | --- | --- | --- | --- | --- | --- | --- | --- | --- | --- | --- | --- | --- | --- | --- | --- | --- | --- | --- | --- | --- | --- | --- | --- | --- | --- | --- | --- | --- | --- | --- | --- | --- | --- | --- | --- | --- | --- | --- | --- | --- | --- | --- | --- | --- | --- | --- | --- | --- | --- | --- | --- | --- | --- | --- | --- | --- | --- | --- | --- | --- | --- | --- | --- | --- | --- | --- | --- | --- | --- | --- | --- | --- | --- | --- | --- | --- | --- | --- | --- | --- | --- | --- | --- | --- | --- | --- | --- | --- | --- | --- | --- | --- | --- | --- | --- | --- | --- | --- | --- | --- | --- | --- | --- | --- | --- | --- | --- | --- | --- | --- | --- | --- | --- | --- | --- | --- | --- | --- | --- | --- | --- | --- | --- | --- | --- | --- | --- | --- | --- | --- | --- | --- | --- | --- | --- | --- | --- | --- | --- | --- | --- | --- | --- | --- | --- | --- | --- | --- | --- | --- | --- | --- | --- | --- | --- | --- | --- | --- | --- | --- | --- | --- | --- | --- | --- | --- | --- | --- | --- | --- | --- | --- | --- | --- | --- | --- | --- | --- | --- | --- | --- | --- | --- | --- | --- | --- | --- | --- | --- | --- | --- | --- | --- | --- | --- | --- | --- | --- | --- | --- | --- | --- | --- | --- | --- | --- | --- | --- | --- | --- | --- | --- | --- | --- | --- | --- | --- | --- | --- | --- | --- | --- | --- | --- | --- | --- | --- | --- | --- | --- | --- | --- | --- | --- | --- | --- | --- | --- | --- | --- | --- | --- | --- | --- | --- | --- | --- | --- | --- | --- | --- | --- | --- | --- | --- | --- | --- | --- | --- | --- | --- | --- | --- | --- | --- | --- | --- | --- | --- | --- | --- | --- | --- | --- | --- | --- | --- | --- | --- | --- | --- | --- | --- | --- | --- | --- | --- | --- | --- | --- | --- | --- | --- | --- | --- | --- | --- | --- | --- | --- | --- | --- | --- | --- | --- | --- | --- | --- | --- | --- | --- | --- | --- | --- | --- | --- | --- | --- | --- | --- | --- | --- | --- | --- | --- | --- | --- | --- | --- | --- | --- | --- | --- | --- | --- | --- | --- | --- | --- | --- | --- | --- | --- | --- | --- | --- | --- | --- | --- | --- | --- | --- | --- | --- | --- | --- | --- | --- | --- | --- | --- | --- | --- | --- | --- | --- | --- | --- | --- | --- | --- | --- | --- | --- | --- | --- | --- | --- | --- | --- | --- | --- | --- | --- | --- | --- | --- | --- | --- | --- | --- | --- | --- | --- | --- | --- | --- | --- | --- | --- | --- | --- | --- | --- | --- | --- | --- | --- | --- | --- | --- | --- | --- | --- | --- | --- | --- | --- | --- | --- | --- | --- | --- | --- | --- | --- | --- | --- | --- | --- | --- | --- | --- | --- | --- | --- | --- | --- | --- | --- | --- | --- | --- | --- | --- | --- | --- | --- | --- | --- | --- | --- | --- | --- | --- | --- | --- | --- | --- | --- | --- | --- | --- | --- | --- | --- | --- | --- | --- | --- | --- | --- | --- | --- | --- | --- | --- | --- | --- | --- | --- | --- | --- | --- | --- | --- | --- | --- | --- | --- | --- | --- | --- | --- | --- | --- | --- | --- | --- | --- | --- | --- | --- | --- | --- | --- | --- | --- | --- | --- | --- | --- | --- | --- | --- | --- | --- | --- | --- | --- | --- | --- | --- | --- | --- | --- | --- | --- | --- | --- | --- | --- | --- | --- | --- | --- | --- | --- | --- | --- | --- | --- | --- | --- | --- | --- | --- | --- | --- | --- | --- | --- | --- | --- | --- | --- | --- | --- | --- | --- | --- | --- | --- | --- | --- | --- | --- | --- | --- | --- | --- | --- | --- | --- | --- | --- | --- | --- | --- | --- | --- | --- | --- | --- | --- | --- | --- | --- | --- | --- | --- | --- | --- | --- | --- | --- | --- | --- | --- | --- | --- | --- | --- | --- | --- | --- | --- | --- | --- | --- | --- | --- | --- | --- | --- | --- | --- | --- | --- | --- | --- | --- | --- | --- | --- | --- | --- | --- | --- | --- | --- | --- | --- | --- | --- | --- | --- | --- | --- | --- | --- | --- | --- | --- | --- | --- | --- | --- | --- | --- | --- | --- | --- | --- | --- | --- | --- | --- | --- | --- | --- | --- | --- | --- | --- | --- | --- | --- | --- | --- | --- | --- | --- | --- | --- | --- | --- | --- | --- | --- | --- | --- | --- | --- | --- | --- | --- | --- | --- | --- | --- | --- | --- | --- | --- | --- | --- | --- | --- | --- | --- | --- | --- | --- | --- | --- | --- | --- | --- | --- | --- | --- | --- | --- | --- | --- | --- | --- | --- | --- | --- | --- | --- | --- | --- | --- | --- | --- | --- | --- | --- | --- | --- | --- | --- | --- | --- | --- | --- | --- | --- | --- | --- | --- | --- | --- | --- | --- | --- | --- | --- | --- | --- | --- | --- | --- | --- | --- | --- | --- | --- | --- | --- | --- | --- | --- | --- | --- | --- | --- | --- | --- | --- | --- | --- | --- | --- | --- | --- | --- | --- | --- | --- | --- | --- | --- | --- | --- | --- | --- | --- | --- | --- | --- | --- | --- | --- | --- | --- | --- | --- | --- | --- | --- | --- | --- | --- | --- | --- | --- | --- | --- | --- | --- | --- | --- | --- | --- | --- | --- | --- | --- | --- | --- | --- | --- | --- | --- | --- | --- | --- | --- | --- | --- | --- | --- | --- | --- | --- | --- | --- | --- | --- | --- | --- | --- | --- | --- | --- | --- | --- | --- | --- | --- | --- | --- | --- | --- | --- | --- | --- | --- | --- | --- | --- | --- | --- | --- | --- | --- | --- | --- | --- | --- | --- | --- | --- | --- | --- | --- | --- | --- | --- | --- | --- | --- | --- | --- | --- | --- | --- | --- | --- | --- | --- | --- | --- | --- | --- | --- | --- | --- | --- | --- | --- | --- | --- | --- | --- | --- | --- | --- | --- | --- | --- | --- | --- | --- | --- | --- | --- | --- | --- | --- | --- | --- | --- | --- | --- | --- | --- | --- | --- | --- | --- | --- | --- | --- | --- | --- | --- | --- | --- | --- | --- | --- | --- | --- | --- | --- | --- | --- | --- | --- | --- | --- | --- | --- | --- | --- | --- | --- | --- | --- | --- | --- | --- | --- | --- | --- | --- | --- | --- | --- | --- | --- | --- | --- | --- | --- | --- | --- | --- | --- | --- | --- | --- | --- | --- | --- | --- | --- | --- | --- | --- | --- | --- | --- | --- | --- | --- | --- | --- | --- | --- | --- | --- | --- | --- | --- | --- | --- | --- | --- | --- | --- | --- | --- | --- | --- | --- | --- | --- | --- | --- | --- | --- | --- | --- | --- | --- | --- | --- | --- | --- | --- | --- | --- | --- | --- | --- | --- | --- | --- | --- | --- | --- | --- | --- | --- | --- | --- | --- | --- | --- | --- | --- | --- | --- | --- | --- | --- | --- | --- | --- | --- | --- | --- | --- | --- | --- | --- | --- | --- | --- | --- | --- | --- | --- | --- | --- | --- | --- | --- | --- | --- | --- | --- | --- | --- | --- | --- | --- | --- | --- | --- | --- | --- | --- | --- | --- | --- | --- | --- | --- | --- | --- | --- | --- | --- | --- | --- | --- | --- | --- | --- | --- | --- | --- | --- | --- | --- | --- | --- | --- | --- | --- | --- | --- | --- | --- | --- | --- | --- | --- | --- | --- | --- | --- | --- | --- | --- | --- | --- | --- | --- | --- | --- | --- | --- | --- | --- | --- | --- | --- | --- | --- | --- | --- | --- | --- | --- | --- | --- | --- | --- | --- | --- | --- | --- | --- | --- | --- | --- | --- | --- | --- | --- | --- | --- | --- | --- | --- | --- | --- | --- | --- | --- | --- | --- | --- | --- | --- | --- | --- | --- | --- | --- | --- | --- | --- | --- | --- | --- | --- | --- | --- | --- | --- | --- | --- | --- | --- | --- | --- | --- | --- | --- | --- | --- | --- | --- | --- | --- | --- | --- | --- | --- | --- | --- | --- | --- | --- | --- | --- | --- | --- | --- | --- | --- | --- | --- | --- | --- | --- | --- | --- | --- | --- | --- | --- | --- | --- | --- | --- | --- | --- | --- | --- | --- | --- | --- | --- | --- | --- | --- | --- | --- | --- | --- | --- | --- | --- | --- | --- | --- | --- | --- | --- | --- | --- | --- | --- | --- | --- | --- | --- | --- | --- | --- | --- | --- | --- | --- | --- | --- | --- | --- | --- | --- | --- | --- | --- | --- | --- | --- | --- | --- | --- | --- | --- | --- | --- | --- | --- | --- | --- | --- | --- | --- | --- | --- | --- | --- | --- | --- | --- | --- | --- | --- | --- | --- | --- | --- | --- | --- | --- | --- | --- | --- | --- | --- | --- | --- | --- | --- | --- | --- | --- | --- | --- | --- | --- | --- | --- | --- | --- | --- | --- | --- | --- | --- | --- | --- | --- | --- | --- | --- | --- | --- | --- | --- | --- | --- | --- | --- | --- | --- | --- | --- | --- | --- | --- | --- | --- | --- | --- | --- | --- | --- | --- | --- | --- | --- | --- | --- | --- | --- | --- | --- | --- | --- | --- | --- | --- | --- | --- | --- | --- | --- | --- | --- | --- | --- | --- | --- | --- | --- | --- | --- | --- | --- | --- | --- | --- | --- | --- | --- | --- | --- | --- | --- | --- | --- | --- | --- | --- | --- | --- | --- | --- | --- | --- | --- | --- | --- | --- | --- | --- | --- | --- | --- | --- | --- | --- | --- | --- | --- | --- | --- | --- | --- | --- | --- | --- | --- | --- | --- | --- | --- | --- | --- | --- | --- | --- | --- | --- | --- | --- | --- | --- | --- | --- | --- | --- | --- | --- | --- | --- | --- | --- | --- | --- | --- | --- | --- | --- | --- | --- | --- | --- | --- | --- | --- | --- | --- | --- | --- | --- | --- | --- | --- | --- | --- | --- | --- | --- | --- | --- | --- | --- | --- | --- | --- | --- | --- | --- | --- | --- | --- | --- | --- | --- | --- | --- | --- | --- | --- | --- | --- | --- | --- | --- | --- | --- | --- | --- | --- | --- | --- | --- | --- | --- | --- | --- | --- | --- | --- | --- | --- | --- | --- | --- | --- | --- | --- | --- | --- | --- | --- | --- | --- | --- | --- | --- | --- | --- | --- | --- | --- | --- | --- | --- | --- | --- | --- | --- | --- | --- | --- | --- | --- | --- | --- | --- | --- | --- | --- | --- | --- | --- | --- | --- | --- | --- | --- | --- | --- | --- | --- | --- | --- | --- | --- | --- | --- | --- | --- | --- | --- | --- | --- | --- | --- | --- | --- | --- | --- | --- | --- | --- | --- | --- | --- | --- | --- | --- | --- | --- | --- | --- | --- | --- | --- | --- | --- | --- | --- | --- | --- | --- | --- | --- | --- | --- | --- | --- | --- | --- | --- | --- | --- | --- | --- | --- | --- | --- | --- | --- | --- | --- | --- | --- | --- | --- | --- | --- | --- | --- | --- | --- | --- | --- | --- | --- | --- | --- | --- | --- | --- | --- | --- | --- | --- | --- | --- | --- | --- | --- | --- | --- | --- | --- | --- | --- | --- | --- | --- | --- | --- | --- | --- | --- | --- | --- | --- | --- | --- | --- | --- | --- | --- | --- | --- | --- | --- | --- | --- | --- | --- | --- | --- | --- | --- | --- | --- | --- | --- | --- | --- | --- | --- | --- | --- | --- | --- | --- | --- | --- | --- | --- | --- | --- | --- | --- | --- | --- | --- | --- | --- | --- | --- | --- | --- | --- | --- | --- | --- | --- | --- | --- | --- | --- | --- | --- | --- | --- | --- | --- | --- | --- | --- | --- | --- | --- | --- | --- | --- | --- | --- | --- | --- | --- | --- | --- | --- | --- | --- | --- | --- | --- | --- | --- | --- | --- | --- | --- | --- | --- | --- | --- | --- | --- | --- | --- | --- | --- | --- | --- | --- | --- | --- | --- | --- | --- | --- | --- | --- | --- | --- | --- | --- | --- | --- | --- | --- | --- | --- | --- | --- | --- | --- | --- | --- | --- | --- | --- | --- | --- | --- | --- | --- | --- | --- | --- | --- | --- | --- | --- | --- | --- | --- | --- | --- | --- | --- | --- | --- | --- | --- | --- | --- | --- | --- | --- | --- | --- | --- | --- | --- | --- | --- | --- | --- | --- | --- | --- | --- | --- | --- | --- | --- | --- | --- | --- | --- | --- | --- | --- | --- | --- | --- | --- | --- | --- | --- | --- | --- | --- | --- | --- | --- | --- | --- | --- | --- | --- | --- | --- | --- | --- | --- | --- | --- | --- | --- | --- | --- | --- | --- | --- | --- | --- | --- | --- | --- | --- | --- | --- | --- | --- | --- | --- | --- | --- | --- | --- | --- | --- | --- | --- | --- | --- | --- | --- | --- | --- | --- | --- | --- | --- | --- | --- | --- | --- | --- | --- | --- | --- | --- | --- | --- | --- | --- | --- | --- | --- | --- | --- | --- | --- | --- | --- | --- | --- | --- | --- | --- | --- | --- | --- | --- | --- | --- | --- | --- | --- | --- | --- | --- | --- | --- | --- | --- | --- | --- | --- | --- | --- | --- | --- | --- | --- | --- | --- | --- | --- | --- | --- | --- | --- | --- | --- | --- | --- | --- | --- | --- | --- | --- | --- | --- | --- | --- | --- | --- | --- | --- | --- | --- | --- | --- | --- | --- | --- | --- | --- | --- | --- | --- | --- | --- | --- | --- | --- | --- | --- | --- | --- | --- | --- | --- | --- | --- | --- | --- | --- | --- | --- | --- | --- | --- | --- | --- | --- | --- | --- | --- | --- | --- | --- | --- | --- | --- | --- | --- | --- | --- | --- | --- | --- | --- | --- | --- | --- | --- | --- | --- | --- | --- | --- | --- | --- | --- | --- | --- | --- | --- | --- | --- | --- | --- | --- | --- | --- | --- | --- | --- | --- | --- | --- | --- | --- | --- | --- | --- | --- | --- | --- | --- | --- | --- | --- | --- | --- | --- | --- | --- | --- | --- | --- | --- | --- | --- | --- | --- | --- | --- | --- | --- | --- | --- | --- | --- | --- | --- | --- | --- | --- | --- | --- | --- | --- | --- | --- | --- | --- | --- | --- | --- | --- | --- | --- | --- | --- | --- | --- | --- | --- | --- | --- | --- | --- | --- | --- | --- | --- | --- | --- | --- | --- | --- | --- | --- | --- | --- | --- | --- | --- | --- | --- | --- | --- | --- | --- | --- | --- | --- | --- | --- | --- | --- | --- | --- | --- | --- | --- | --- | --- | --- | --- | --- | --- | --- | --- | --- | --- | --- | --- | --- | --- | --- | --- | --- | --- | --- | --- | --- | --- | --- | --- | --- | --- | --- | --- | --- | --- | --- | --- | --- | --- | --- | --- | --- | --- | --- | --- | --- | --- | --- | --- | --- | --- | --- | --- | --- | --- | --- | --- | --- | --- | --- | --- | --- | --- | --- | --- | --- | --- | --- | --- | --- | --- | --- | --- | --- | --- | --- | --- | --- | --- | --- | --- | --- | --- | --- | --- | --- | --- | --- | --- | --- | --- | --- | --- | --- | --- | --- | --- | --- | --- | --- | --- | --- | --- | --- | --- | --- | --- | --- | --- | --- | --- | --- | --- | --- | --- | --- | --- | --- | --- | --- | --- | --- | --- | --- | --- | --- | --- | --- | --- | --- | --- | --- | --- | --- | --- | --- | --- | --- | --- | --- | --- | --- | --- | --- | --- | --- | --- | --- | --- | --- | --- | --- | --- | --- | --- | --- | --- | --- | --- | --- | --- | --- | --- | --- | --- | --- | --- | --- | --- | --- | --- | --- | --- | --- | --- | --- | --- | --- | --- | --- | --- | --- | --- | --- | --- | --- | --- | --- | --- | --- | --- | --- | --- | --- | --- | --- | --- | --- | --- | --- | --- | --- | --- | --- | --- | --- | --- | --- | --- | --- | --- | --- | --- | --- | --- | --- | --- | --- | --- | --- | --- | --- | --- | --- | --- | --- | --- | --- | --- | --- | --- | --- | --- | --- | --- | --- | --- | --- | --- | --- | --- | --- | --- | --- | --- | --- | --- | --- | --- | --- | --- | --- | --- | --- | --- | --- | --- | --- | --- | --- | --- | --- | --- | --- | --- | --- | --- | --- | --- | --- | --- | --- | --- | --- | --- | --- | --- | --- | --- | --- | --- | --- | --- | --- | --- | --- | --- | --- | --- | --- | --- | --- | --- | --- | --- | --- | --- | --- | --- | --- | --- | --- | --- | --- | --- | --- | --- | --- | --- | --- | --- | --- | --- | --- | --- | --- | --- | --- | --- | --- | --- | --- | --- | --- | --- | --- | --- | --- | --- | --- | --- | --- | --- | --- | --- | --- | --- | --- | --- | --- | --- | --- | --- | --- | --- | --- | --- | --- | --- | --- | --- | --- | --- | --- | --- | --- | --- | --- | --- | --- | --- | --- | --- | --- | --- | --- | --- | --- | --- | --- | --- | --- | --- | --- | --- | --- | --- | --- | --- | --- | --- | --- | --- | --- | --- | --- | --- | --- | --- | --- | --- | --- | --- | --- | --- | --- | --- | --- | --- | --- | --- | --- | --- | --- | --- | --- | --- | --- | --- | --- | --- | --- | --- | --- | --- | --- | --- | --- | --- | --- | --- | --- | --- | --- | --- | --- | --- | --- | --- | --- | --- | --- | --- | --- | --- | --- | --- | --- | --- | --- | --- | --- | --- | --- | --- | --- | --- | --- | --- | --- | --- | --- | --- | --- | --- | --- | --- | --- | --- | --- | --- | --- | --- | --- | --- | --- | --- | --- | --- | --- | --- | --- | --- | --- | --- | --- | --- | --- | --- | --- | --- | --- | --- | --- | --- | --- | --- | --- | --- | --- | --- | --- | --- | --- | --- | --- | --- | --- | --- | --- | --- | --- | --- | --- | --- | --- | --- | --- | --- | --- | --- | --- | --- | --- | --- | --- | --- | --- | --- | --- | --- | --- | --- | --- | --- | --- | --- | --- | --- | --- | --- | --- | --- | --- | --- | --- | --- | --- | --- | --- | --- | --- | --- | --- | --- | --- | --- | --- | --- | --- | --- | --- | --- | --- | --- | --- | --- | --- | --- | --- | --- | --- | --- | --- | --- | --- | --- | --- | --- | --- | --- | --- | --- | --- | --- | --- | --- | --- | --- | --- | --- | --- | --- | --- | --- | --- | --- | --- | --- | --- | --- | --- | --- | --- | --- | --- | --- | --- | --- | --- | --- | --- | --- | --- | --- | --- | --- | --- | --- | --- | --- | --- | --- | --- | --- | --- | --- | --- | --- | --- | --- | --- | --- | --- | --- | --- | --- | --- | --- | --- | --- | --- | --- | --- | --- | --- | --- | --- | --- | --- | --- | --- | --- | --- | --- | --- | --- | --- | --- | --- | --- | --- | --- | --- | --- | --- | --- | --- | --- | --- | --- | --- | --- | --- | --- | --- | --- | --- | --- | --- | --- | --- | --- | --- | --- | --- | --- | --- | --- | --- | --- | --- | --- | --- | --- | --- | --- | --- | --- | --- | --- | --- | --- | --- | --- | --- | --- | --- | --- | --- | --- | --- | --- | --- | --- | --- | --- | --- | --- | --- | --- | --- | --- | --- | --- | --- | --- | --- | --- | --- | --- | --- | --- | --- | --- | --- | --- | --- | --- | --- | --- | --- | --- | --- | --- | --- | --- | --- | --- | --- | --- | --- | --- | --- | --- | --- | --- | --- | --- | --- | --- | --- | --- | --- | --- | --- | --- | --- | --- | --- | --- | --- | --- | --- | --- | --- | --- | --- | --- | --- | --- | --- | --- | --- | --- | --- | --- | --- | --- | --- | --- | --- | --- | --- | --- | --- | --- | --- | --- | --- | --- | --- | --- | --- | --- | --- | --- | --- | --- | --- | --- | --- | --- | --- | --- | --- | --- | --- | --- | --- | --- | --- | --- | --- | --- | --- | --- | --- | --- | --- | --- | --- | --- | --- | --- | --- | --- | --- | --- | --- | --- | --- | --- | --- | --- | --- | --- | --- | --- | --- | --- | --- | --- | --- | --- | --- | --- | --- | --- | --- | --- | --- | --- | --- | --- | --- | --- | --- | --- | --- | --- | --- | --- | --- | --- | --- | --- | --- | --- | --- | --- | --- | --- | --- | --- | --- | --- | --- | --- | --- | --- | --- | --- | --- | --- | --- | --- | --- | --- | --- | --- | --- | --- | --- | --- | --- | --- | --- | --- | --- | --- | --- | --- | --- | --- | --- | --- | --- | --- | --- | --- | --- | --- | --- | --- | --- | --- | --- | --- | --- | --- | --- | --- | --- | --- | --- | --- | --- | --- | --- | --- | --- | --- | --- | --- | --- | --- | --- | --- | --- | --- | --- | --- | --- | --- | --- | --- | --- | --- | --- | --- | --- | --- | --- | --- | --- | --- | --- | --- | --- | --- | --- | --- | --- | --- | --- | --- | --- | --- | --- | --- | --- | --- | --- | --- | --- | --- | --- | --- | --- | --- | --- | --- | --- | --- | --- | --- | --- | --- | --- | --- | --- | --- | --- | --- | --- | --- | --- | --- | --- | --- | --- | --- | --- | --- | --- | --- | --- | --- | --- | --- | --- | --- | --- | --- | --- | --- | --- | --- | --- | --- | --- | --- | --- | --- | --- | --- | --- | --- | --- | --- | --- | --- | --- | --- | --- | --- | --- | --- | --- | --- | --- | --- | --- | --- | --- | --- | --- | --- | --- | --- | --- | --- | --- | --- | --- | --- | --- | --- | --- | --- | --- | --- | --- | --- | --- | --- | --- | --- | --- | --- | --- | --- | --- | --- | --- | --- | --- | --- | --- | --- | --- | --- | --- | --- | --- | --- | --- | --- | --- | --- | --- | --- | --- | --- | --- | --- | --- | --- | --- | --- | --- | --- | --- | --- | --- | --- | --- | --- | --- | --- | --- | --- | --- | --- | --- | --- | --- | --- | --- | --- | --- | --- | --- | --- | --- | --- | --- | --- | --- | --- | --- | --- | --- | --- | --- | --- | --- | --- | --- | --- | --- | --- | --- | --- | --- | --- | --- | --- | --- | --- | --- | --- | --- | --- | --- | --- | --- | --- | --- | --- | --- | --- | --- | --- | --- | --- | --- | --- | --- | --- | --- | --- | --- | --- | --- | --- | --- | --- | --- | --- | --- | --- | --- | --- | --- | --- | --- | --- | --- | --- | --- | --- | --- | --- | --- | --- | --- | --- | --- | --- | --- | --- | --- | --- | --- | --- | --- | --- | --- | --- | --- | --- | --- | --- | --- | --- | --- | --- | --- | --- | --- | --- | --- | --- | --- | --- | --- | --- | --- | --- | --- | --- | --- | --- | --- | --- | --- | --- | --- | --- | --- | --- | --- | --- | --- | --- | --- | --- | --- | --- | --- | --- | --- | --- | --- | --- | --- | --- | --- | --- | --- | --- | --- | --- | --- | --- | --- | --- | --- | --- | --- | --- | --- | --- | --- | --- | --- | --- | --- | --- | --- | --- | --- | --- | --- | --- | --- | --- | --- | --- | --- | --- | --- | --- | --- | --- | --- | --- | --- | --- | --- | --- | --- | --- | --- | --- | --- | --- | --- | --- | --- | --- | --- | --- | --- | --- | --- | --- | --- | --- | --- | --- | --- | --- | --- | --- | --- | --- | --- | --- | --- | --- | --- | --- | --- | --- | --- | --- | --- | --- | --- | --- | --- | --- | --- | --- | --- | --- | --- | --- | --- | --- | --- | --- | --- | --- | --- | --- | --- | --- | --- | --- | --- | --- | --- | --- | --- | --- | --- | --- | --- | --- | --- | --- | --- | --- | --- | --- | --- | --- | --- | --- | --- | --- | --- | --- | --- | --- | --- | --- | --- | --- | --- | --- | --- | --- | --- | --- | --- | --- | --- | --- | --- | --- | --- | --- | --- | --- | --- | --- | --- | --- | --- | --- | --- | --- | --- | --- | --- | --- | --- | --- | --- | --- | --- | --- | --- | --- | --- | --- | --- | --- | --- | --- | --- | --- | --- | --- | --- | --- | --- | --- | --- | --- | --- | --- | --- | --- | --- | --- | --- | --- | --- | --- | --- | --- | --- | --- | --- | --- | --- | --- | --- | --- | --- | --- | --- | --- | --- | --- | --- | --- | --- | --- | --- | --- | --- | --- | --- | --- | --- | --- | --- | --- | --- | --- | --- | --- | --- | --- | --- | --- | --- | --- | --- | --- | --- | --- | --- | --- | --- | --- | --- | --- | --- | --- | --- | --- | --- | --- | --- | --- | --- | --- | --- | --- | --- | --- | --- | --- | --- | --- | --- | --- | --- | --- | --- | --- | --- | --- | --- | --- | --- | --- | --- | --- | --- | --- | --- | --- | --- | --- | --- | --- | --- | --- | --- | --- | --- | --- | --- | --- | --- | --- | --- | --- | --- | --- | --- | --- | --- | --- | --- | --- | --- | --- | --- | --- | --- | --- | --- | --- | --- | --- | --- | --- | --- | --- | --- | --- | --- | --- | --- | --- | --- | --- | --- | --- | --- | --- | --- | --- | --- | --- | --- | --- | --- | --- | --- | --- | --- | --- | --- | --- | --- | --- | --- | --- | --- | --- | --- | --- | --- | --- | --- | --- | --- | --- | --- | --- | --- | --- | --- | --- | --- | --- | --- | --- | --- | --- | --- | --- | --- | --- | --- | --- | --- | --- | --- | --- | --- | --- | --- | --- | --- | --- | --- | --- | --- | --- | --- | --- | --- | --- | --- | --- | --- | --- | --- | --- | --- | --- | --- | --- | --- | --- | --- | --- | --- | --- | --- | --- | --- | --- | --- | --- | --- | --- | --- | --- | --- | --- | --- | --- | --- | --- | --- | --- | --- | --- | --- | --- | --- | --- | --- | --- | --- | --- | --- | --- | --- | --- | --- | --- | --- | --- | --- | --- | --- | --- | --- | --- | --- | --- | --- | --- | --- | --- | --- | --- | --- | --- | --- | --- | --- | --- | --- | --- | --- | --- | --- | --- | --- | --- | --- | --- | --- | --- | --- | --- | --- | --- | --- | --- | --- | --- | --- | --- | --- | --- | --- | --- | --- | --- | --- | --- | --- | --- | --- | --- | --- | --- | --- | --- | --- | --- | --- | --- | --- | --- | --- | --- | --- | --- | --- | --- | --- | --- | --- | --- | --- | --- | --- | --- | --- | --- | --- | --- | --- | --- | --- | --- | --- | --- | --- | --- | --- | --- | --- | --- | --- | --- | --- | --- | --- | --- | --- | --- | --- | --- | --- | --- | --- | --- | --- | --- | --- | --- | --- | --- | --- | --- | --- | --- | --- | --- | --- | --- | --- | --- | --- | --- | --- | --- | --- | --- | --- | --- | --- | --- | --- | --- | --- | --- | --- | --- | --- | --- | --- | --- | --- | --- | --- | --- | --- | --- | --- | --- | --- | --- | --- | --- | --- | --- | --- | --- | --- | --- | --- | --- | --- | --- | --- | --- | --- | --- | --- | --- | --- | --- | --- | --- | --- | --- | --- | --- | --- | --- | --- | --- | --- | --- | --- | --- | --- | --- | --- | --- | --- | --- | --- | --- | --- | --- | --- | --- | --- | --- | --- | --- | --- | --- | --- | --- | --- | --- | --- | --- | --- | --- | --- | --- | --- | --- | --- | --- | --- | --- | --- | --- | --- | --- | --- | --- | --- | --- | --- | --- | --- | --- | --- | --- | --- | --- | --- | --- | --- | --- | --- | --- | --- | --- | --- | --- | --- | --- | --- | --- | --- | --- | --- | --- | --- | --- | --- | --- | --- | --- | --- | --- | --- | --- | --- | --- | --- | --- | --- | --- | --- | --- | --- | --- | --- | --- | --- | --- | --- | --- | --- | --- | --- | --- | --- | --- | --- | --- | --- | --- | --- | --- | --- | --- | --- | --- | --- | --- | --- | --- | --- | --- | --- | --- | --- | --- | --- | --- | --- | --- | --- | --- | --- | --- | --- | --- | --- | --- | --- | --- | --- | --- | --- | --- | --- | --- | --- | --- | --- | --- | --- | --- | --- | --- | --- | --- | --- | --- | --- | --- | --- | --- | --- | --- | --- | --- | --- | --- | --- | --- | --- | --- | --- | --- | --- | --- | --- | --- | --- | --- | --- | --- | --- | --- | --- | --- | --- | --- | --- | --- | --- | --- | --- | --- | --- | --- | --- | --- | --- | --- | --- | --- | --- | --- | --- | --- | --- | --- | --- | --- | --- | --- | --- | --- | --- | --- | --- | --- | --- | --- | --- | --- | --- | --- | --- | --- | --- | --- | --- | --- | --- | --- | --- | --- | --- | --- | --- | --- | --- | --- | --- | --- | --- | --- | --- | --- | --- | --- | --- | --- | --- | --- | --- | --- | --- | --- | --- | --- | --- | --- | --- | --- | --- | --- | --- | --- | --- | --- | --- | --- | --- | --- | --- | --- | --- | --- | --- | --- | --- | --- | --- | --- | --- | --- | --- | --- | --- | --- | --- | --- | --- | --- | --- | --- | --- | --- | --- | --- | --- | --- | --- | --- | --- | --- | --- | --- | --- | --- | --- | --- | --- | --- | --- | --- | --- | --- | --- | --- | --- | --- | --- | --- | --- | --- | --- | --- | --- | --- | --- | --- | --- | --- | --- | --- | --- | --- | --- | --- | --- | --- | --- | --- | --- | --- | --- | --- | --- | --- | --- | --- | --- | --- | --- | --- | --- | --- | --- | --- | --- | --- | --- | --- | --- | --- | --- | --- | --- | --- | --- | --- | --- | --- | --- | --- | --- | --- | --- | --- | --- | --- | --- | --- | --- | --- | --- | --- | --- | --- | --- | --- | --- | --- | --- | --- | --- | --- | --- | --- | --- | --- | --- | --- | --- | --- | --- | --- | --- | --- | --- | --- | --- | --- | --- | --- | --- | --- | --- | --- | --- | --- | --- | --- | --- | --- | --- | --- | --- | --- | --- | --- | --- | --- | --- | --- | --- | --- | --- | --- | --- | --- | --- | --- | --- | --- | --- | --- | --- | --- | --- | --- | --- | --- | --- | --- | --- | --- | --- | --- | --- | --- | --- | --- | --- | --- | --- | --- | --- | --- | --- | --- | --- | --- | --- | --- | --- | --- | --- | --- | --- | --- | --- | --- | --- | --- | --- | --- | --- | --- | --- | --- | --- | --- | --- | --- | --- | --- | --- | --- | --- | --- | --- | --- | --- | --- | --- | --- | --- | --- | --- | --- | --- | --- | --- | --- | --- | --- | --- | --- | --- | --- | --- | --- | --- | --- | --- | --- | --- | --- | --- | --- | --- | --- | --- | --- | --- | --- | --- | --- | --- | --- | --- | --- | --- | --- | --- | --- | --- | --- | --- | --- | --- | --- | --- | --- | --- | --- | --- | --- | --- | --- | --- | --- | --- | --- | --- | --- | --- | --- | --- | --- | --- | --- | --- | --- | --- | --- | --- | --- | --- | --- | --- | --- | --- | --- | --- | --- | --- | --- | --- | --- | --- | --- | --- | --- | --- | --- | --- | --- | --- | --- | --- | --- | --- | --- | --- | --- | --- | --- | --- | --- | --- | --- | --- | --- | --- | --- | --- | --- | --- | --- | --- | --- | --- | --- | --- | --- | --- | --- | --- | --- | --- | --- | --- | --- | --- | --- | --- | --- | --- | --- | --- | --- | --- | --- | --- | --- | --- | --- | --- | --- | --- | --- | --- | --- | --- | --- | --- | --- | --- | --- | --- | --- | --- | --- | --- | --- | --- | --- | --- | --- | --- | --- | --- | --- | --- | --- | --- | --- | --- | --- | --- | --- | --- | --- | --- | --- | --- | --- | --- | --- | --- | --- | --- | --- | --- | --- | --- | --- | --- | --- | --- | --- | --- | --- | --- | --- | --- | --- | --- | --- | --- | --- | --- | --- | --- | --- | --- | --- | --- | --- | --- | --- | --- | --- | --- | --- | --- | --- | --- | --- | --- | --- | --- | --- | --- | --- | --- | --- | --- | --- | --- | --- | --- | --- | --- | --- | --- | --- | --- | --- | --- | --- | --- | --- | --- | --- | --- | --- | --- | --- | --- | --- | --- | --- | --- | --- | --- | --- | --- | --- | --- | --- | --- | --- | --- | --- | --- | --- | --- | --- | --- | --- | --- | --- | --- | --- | --- | --- | --- | --- | --- | --- | --- | --- | --- | --- | --- | --- | --- | --- | --- | --- | --- | --- | --- | --- | --- | --- | --- | --- | --- | --- | --- | --- | --- | --- | --- | --- | --- | --- | --- | --- | --- | --- | --- | --- | --- | --- | --- | --- | --- | --- | --- | --- | --- | --- | --- | --- | --- | --- | --- | --- | --- | --- | --- | --- | --- | --- | --- | --- | --- | --- | --- | --- | --- | --- | --- | --- | --- | --- | --- | --- | --- | --- | --- | --- | --- | --- | --- | --- | --- | --- | --- | --- | --- | --- | --- | --- | --- | --- | --- | --- | --- | --- | --- | --- | --- | --- | --- | --- | --- | --- | --- | --- | --- | --- | --- | --- | --- | --- | --- | --- | --- | --- | --- | --- | --- | --- | --- | --- | --- | --- | --- | --- | --- | --- | --- | --- | --- | --- | --- | --- | --- | --- | --- | --- | --- | --- | --- | --- | --- | --- | --- | --- | --- | --- | --- | --- | --- | --- | --- | --- | --- | --- | --- | --- | --- | --- | --- | --- | --- | --- | --- | --- | --- | --- | --- | --- | --- | --- | --- | --- | --- | --- | --- | --- | --- | --- | --- | --- | --- | --- | --- | --- | --- | --- | --- | --- | --- | --- | --- | --- | --- | --- | --- | --- | --- | --- | --- | --- | --- | --- | --- | --- | --- | --- | --- | --- | --- | --- | --- | --- | --- | --- | --- | --- | --- | --- | --- | --- | --- | --- | --- | --- | --- | --- | --- | --- | --- | --- | --- | --- | --- | --- | --- | --- | --- | --- | --- | --- | --- | --- | --- | --- | --- | --- | --- | --- | --- | --- | --- | --- | --- | --- | --- | --- | --- | --- | --- | --- | --- | --- | --- | --- | --- | --- | --- | --- | --- | --- | --- | --- | --- | --- | --- | --- | --- | --- | --- | --- | --- | --- | --- | --- | --- | --- | --- | --- | --- | --- | --- | --- | --- | --- | --- | --- | --- | --- | --- | --- | --- | --- | --- | --- | --- | --- | --- | --- | --- | --- | --- | --- | --- | --- | --- | --- | --- | --- | --- | --- | --- | --- | --- | --- | --- | --- | --- | --- | --- | --- | --- | --- | --- | --- | --- | --- | --- | --- | --- | --- | --- | --- | --- | --- | --- | --- | --- | --- | --- | --- | --- | --- | --- | --- | --- | --- | --- | --- | --- | --- | --- | --- | --- | --- | --- | --- | --- | --- | --- | --- | --- | --- | --- | --- | --- | --- | --- | --- | --- | --- | --- | --- | --- | --- | --- | --- | --- | --- | --- | --- | --- | --- | --- | --- | --- | --- | --- | --- | --- | --- | --- | --- | --- | --- | --- | --- | --- | --- | --- | --- | --- | --- | --- | --- | --- | --- | --- | --- | --- | --- | --- | --- | --- | --- | --- | --- | --- | --- | --- | --- | --- | --- | --- | --- | --- | --- | --- | --- | --- | --- | --- | --- | --- | --- | --- | --- | --- | --- | --- | --- | --- | --- | --- | --- | --- | --- | --- | --- | --- | --- | --- | --- | --- | --- | --- | --- | --- | --- | --- | --- | --- | --- | --- | --- | --- | --- | --- | --- | --- | --- | --- | --- | --- | --- | --- | --- | --- | --- | --- | --- | --- | --- | --- | --- | --- | --- | --- | --- | --- | --- | --- | --- | --- | --- | --- | --- | --- | --- | --- | --- | --- | --- | --- | --- | --- | --- | --- | --- | --- | --- | --- | --- | --- | --- | --- | --- | --- | --- | --- | --- | --- | --- | --- | --- | --- | --- | --- | --- | --- | --- | --- | --- | --- | --- | --- | --- | --- | --- | --- | --- | --- | --- | --- | --- | --- | --- | --- | --- | --- | --- | --- | --- | --- | --- | --- | --- | --- | --- | --- | --- | --- | --- | --- | --- | --- | --- | --- | --- | --- | --- | --- | --- | --- | --- | --- | --- | --- | --- | --- | --- | --- | --- | --- | --- | --- | --- | --- | --- | --- | --- | --- | --- | --- | --- | --- | --- | --- | --- | --- | --- | --- | --- | --- | --- | --- | --- | --- | --- | --- | --- | --- | --- | --- | --- | --- | --- | --- | --- | --- | --- | --- | --- | --- | --- | --- | --- | --- | --- | --- | --- | --- | --- | --- | --- | --- | --- | --- | --- | --- | --- | --- | --- | --- | --- | --- | --- | --- | --- | --- | --- | --- | --- | --- | --- | --- | --- | --- | --- | --- | --- | --- | --- | --- | --- | --- | --- | --- | --- | --- | --- | --- | --- | --- | --- | --- | --- | --- | --- | --- | --- | --- | --- | --- | --- | --- | --- | --- | --- | --- | --- | --- | --- | --- | --- | --- | --- | --- | --- | --- | --- | --- | --- | --- | --- | --- | --- | --- | --- | --- | --- | --- | --- | --- | --- | --- | --- | --- | --- | --- | --- | --- | --- | --- | --- | --- | --- | --- | --- | --- | --- | --- | --- | --- | --- | --- | --- | --- | --- | --- | --- | --- | --- | --- | --- | --- | --- | --- | --- | --- | --- | --- | --- | --- | --- | --- | --- | --- | --- | --- | --- | --- | --- | --- | --- | --- | --- | --- | --- | --- | --- | --- | --- | --- | --- | --- | --- | --- | --- | --- | --- | --- | --- | --- | --- | --- | --- | --- | --- | --- | --- | --- | --- | --- | --- | --- | --- | --- | --- | --- | --- | --- | --- | --- | --- | --- | --- | --- | --- | --- | --- | --- | --- | --- | --- | --- | --- | --- | --- | --- | --- | --- | --- | --- | --- | --- | --- | --- | --- | --- | --- | --- | --- | --- | --- | --- | --- | --- | --- | --- | --- | --- | --- | --- | --- | --- | --- | --- | --- | --- | --- | --- | --- | --- | --- | --- | --- | --- | --- | --- | --- | --- | --- | --- | --- | --- | --- | --- | --- | --- | --- | --- | --- | --- | --- | --- | --- | --- | --- | --- | --- | --- | --- | --- | --- | --- | --- | --- | --- | --- | --- | --- | --- | --- | --- | --- | --- | --- | --- | --- | --- | --- | --- | --- | --- | --- | --- | --- | --- | --- | --- | --- | --- | --- | --- | --- | --- | --- | --- | --- | --- | --- | --- | --- | --- | --- | --- | --- | --- | --- | --- | --- | --- | --- | --- | --- | --- | --- | --- | --- | --- | --- | --- | --- | --- | --- | --- | --- | --- | --- | --- | --- | --- | --- | --- | --- | --- | --- | --- | --- | --- | --- | --- | --- | --- | --- | --- | --- | --- | --- | --- | --- | --- | --- | --- | --- | --- | --- | --- | --- | --- | --- | --- | --- | --- | --- | --- | --- | --- | --- | --- | --- | --- | --- | --- | --- | --- | --- | --- | --- | --- | --- | --- | --- | --- | --- | --- | --- | --- | --- | --- | --- | --- | --- | --- | --- | --- | --- | --- | --- | --- | --- | --- | --- | --- | --- | --- | --- | --- | --- | --- | --- | --- | --- | --- | --- | --- | --- | --- | --- | --- | --- | --- | --- | --- | --- | --- | --- | --- | --- | --- | --- | --- | --- | --- | --- | --- | --- | --- | --- | --- | --- | --- | --- | --- | --- | --- | --- | --- | --- | --- | --- | --- | --- | --- | --- | --- | --- | --- | --- | --- | --- | --- | --- | --- | --- | --- | --- | --- | --- | --- | --- | --- | --- | --- | --- | --- | --- | --- | --- | --- | --- | --- | --- | --- | --- | --- | --- | --- | --- | --- | --- | --- | --- | --- | --- | --- | --- | --- | --- | --- | --- | --- | --- | --- | --- | --- | --- | --- | --- | --- | --- | --- | --- | --- | --- | --- | --- | --- | --- | --- | --- | --- | --- | --- | --- | --- | --- | --- | --- | --- | --- | --- | --- | --- | --- | --- | --- | --- | --- | --- | --- | --- | --- | --- | --- | --- | --- | --- | --- | --- | --- | --- | --- | --- | --- | --- | --- | --- | --- | --- | --- | --- | --- | --- | --- | --- | --- | --- | --- | --- | --- | --- | --- | --- | --- | --- | --- | --- | --- | --- | --- | --- | --- | --- | --- | --- | --- | --- | --- | --- | --- | --- | --- | --- | --- | --- | --- | --- | --- | --- | --- | --- | --- | --- | --- | --- | --- | --- | --- | --- | --- | --- | --- | --- | --- | --- | --- | --- | --- | --- | --- | --- | --- | --- | --- | --- | --- | --- | --- | --- | --- | --- | --- | --- | --- | --- | --- | --- | --- | --- | --- | --- | --- | --- | --- | --- | --- | --- | --- | --- | --- | --- | --- | --- | --- | --- | --- | --- | --- | --- | --- | --- | --- | --- | --- | --- | --- | --- | --- | --- | --- | --- | --- | --- | --- | --- | --- | --- | --- | --- | --- | --- | --- | --- | --- | --- | --- | --- | --- | --- | --- | --- | --- | --- | --- | --- | --- | --- | --- | --- | --- | --- | --- | --- | --- | --- | --- | --- | --- | --- | --- | --- | --- | --- | --- | --- | --- | --- | --- | --- | --- | --- | --- | --- | --- | --- | --- | --- | --- | --- | --- | --- | --- | --- | --- | --- | --- | --- | --- | --- | --- | --- | --- | --- | --- | --- | --- | --- | --- | --- | --- | --- | --- | --- | --- | --- | --- | --- | --- | --- | --- | --- | --- | --- | --- | --- | --- | --- | --- | --- | --- | --- | --- | --- | --- | --- | --- | --- | --- | --- | --- | --- | --- | --- | --- | --- | --- | --- | --- | --- | --- | --- | --- | --- | --- | --- | --- | --- | --- | --- | --- | --- | --- | --- | --- | --- | --- | --- | --- | --- | --- | --- | --- | --- | --- | --- | --- | --- | --- | --- | --- | --- | --- | --- | --- | --- | --- | --- | --- | --- | --- | --- | --- | --- | --- | --- | --- | --- | --- | --- | --- | --- | --- | --- | --- | --- | --- | --- | --- | --- | --- | --- | --- | --- | --- | --- | --- | --- | --- | --- | --- | --- | --- | --- | --- | --- | --- | --- | --- | --- | --- | --- | --- | --- | --- | --- | --- | --- | --- | --- | --- | --- | --- | --- | --- | --- | --- | --- | --- | --- | --- | --- | --- | --- | --- | --- | --- | --- | --- | --- | --- | --- | --- | --- | --- | --- | --- | --- | --- | --- | --- | --- | --- | --- | --- | --- | --- | --- | --- | --- | --- | --- | --- | --- | --- | --- | --- | --- | --- | --- | --- | --- | --- | --- | --- | --- | --- | --- | --- | --- | --- | --- | --- | --- | --- | --- | --- | --- | --- | --- | --- | --- | --- | --- | --- | --- | --- | --- | --- | --- | --- | --- | --- | --- | --- | --- | --- | --- | --- | --- | --- | --- | --- | --- | --- | --- | --- | --- | --- | --- | --- | --- | --- | --- | --- | --- | --- | --- | --- | --- | --- | --- | --- | --- | --- | --- | --- | --- | --- | --- | --- | --- | --- | --- | --- | --- | --- | --- | --- | --- | --- | --- | --- | --- | --- | --- | --- | --- | --- | --- | --- | --- | --- | --- | --- | --- | --- | --- | --- | --- | --- | --- | --- | --- | --- | --- | --- | --- | --- | --- | --- | --- | --- | --- | --- | --- | --- | --- | --- | --- | --- | --- | --- | --- | --- | --- | --- | --- | --- | --- | --- | --- | --- | --- | --- | --- | --- | --- | --- | --- | --- | --- | --- | --- | --- | --- | --- | --- | --- | --- | --- | --- | --- | --- | --- | --- | --- | --- | --- | --- | --- | --- | --- | --- | --- | --- | --- | --- | --- | --- | --- | --- | --- | --- | --- | --- | --- | --- | --- | --- | --- | --- | --- | --- | --- | --- | --- | --- | --- | --- | --- | --- | --- | --- | --- | --- | --- | --- | --- | --- | --- | --- | --- | --- | --- | --- | --- | --- | --- | --- | --- | --- | --- | --- | --- | --- | --- | --- | --- | --- | --- | --- | --- | --- | --- | --- | --- | --- | --- | --- | --- | --- | --- | --- | --- | --- | --- | --- | --- | --- | --- | --- | --- | --- | --- | --- | --- | --- | --- | --- | --- | --- | --- | --- | --- | --- | --- | --- | --- | --- | --- | --- | --- | --- | --- | --- | --- | --- | --- | --- | --- | --- | --- | --- | --- | --- | --- | --- | --- | --- | --- | --- | --- | --- | --- | --- | --- | --- | --- | --- | --- | --- | --- | --- | --- | --- | --- | --- | --- | --- | --- | --- | --- | --- | --- | --- | --- | --- | --- | --- | --- | --- | --- | --- | --- | --- | --- | --- | --- | --- | --- | --- | --- | --- | --- | --- | --- | --- | --- | --- | --- | --- | --- | --- | --- | --- | --- | --- | --- | --- | --- | --- | --- | --- | --- | --- | --- | --- | --- | --- | --- | --- | --- | --- | --- | --- | --- | --- | --- | --- | --- | --- | --- | --- | --- | --- | --- | --- | --- | --- | --- | --- | --- | --- | --- | --- | --- | --- | --- | --- | --- | --- | --- | --- | --- | --- | --- | --- | --- | --- | --- | --- | --- | --- | --- | --- | --- | --- | --- | --- | --- | --- | --- | --- | --- | --- | --- | --- | --- | --- | --- | --- | --- | --- | --- | --- | --- | --- | --- | --- | --- | --- | --- | --- | --- | --- | --- | --- | --- | --- | --- | --- | --- | --- | --- | --- | --- | --- | --- | --- | --- | --- | --- | --- | --- | --- | --- | --- | --- | --- | --- | --- | --- | --- | --- | --- | --- | --- | --- | --- | --- | --- | --- | --- | --- | --- | --- | --- | --- | --- | --- | --- | --- | --- | --- | --- | --- | --- | --- | --- | --- | --- | --- | --- | --- | --- | --- | --- | --- | --- | --- | --- | --- | --- | --- | --- | --- | --- | --- | --- | --- | --- | --- | --- | --- | --- | --- | --- | --- | --- | --- | --- | --- | --- | --- | --- | --- | --- | --- | --- | --- | --- | --- | --- | --- | --- | --- | --- | --- | --- | --- | --- | --- | --- | --- | --- | --- | --- | --- | --- | --- | --- | --- | --- | --- | --- | --- | --- | --- | --- | --- | --- | --- | --- | --- | --- | --- | --- | --- | --- | --- | --- | --- | --- | --- | --- | --- | --- | --- | --- | --- | --- | --- | --- | --- | --- | --- | --- | --- | --- | --- | --- | --- | --- | --- | --- | --- | --- | --- | --- | --- | --- | --- | --- | --- | --- | --- | --- | --- | --- | --- | --- | --- | --- | --- | --- | --- | --- | --- | --- | --- | --- | --- | --- | --- | --- | --- | --- | --- | --- | --- | --- | --- | --- | --- | --- | --- | --- | --- | --- | --- | --- | --- | --- | --- | --- | --- | --- | --- | --- | --- | --- | --- | --- | --- | --- | --- | --- | --- | --- | --- | --- | --- | --- | --- | --- | --- | --- | --- | --- | --- | --- | --- | --- | --- | --- | --- | --- | --- | --- | --- | --- | --- | --- | --- | --- | --- | --- | --- | --- | --- | --- | --- | --- | --- | --- | --- | --- | --- | --- | --- | --- | --- | --- | --- | --- | --- | --- | --- | --- | --- | --- | --- | --- | --- | --- | --- | --- | --- | --- | --- | --- | --- | --- | --- | --- | --- | --- | --- | --- | --- | --- | --- | --- | --- | --- | --- | --- | --- | --- | --- | --- | --- | --- | --- | --- | --- | --- | --- | --- | --- | --- | --- | --- | --- | --- | --- | --- | --- | --- | --- | --- | --- | --- | --- | --- | --- | --- | --- | --- | --- | --- | --- | --- | --- | --- | --- | --- | --- | --- | --- | --- | --- | --- | --- | --- | --- | --- | --- | --- | --- | --- | --- | --- | --- | --- | --- | --- | --- | --- | --- | --- | --- | --- | --- | --- | --- | --- | --- | --- | --- | --- | --- | --- | --- | --- | --- | --- | --- | --- | --- | --- | --- | --- | --- | --- | --- | --- | --- | --- | --- | --- | --- | --- | --- | --- | --- | --- | --- | --- | --- | --- | --- | --- | --- | --- | --- | --- | --- | --- | --- | --- | --- | --- | --- | --- | --- | --- | --- | --- | --- | --- | --- | --- | --- | --- | --- | --- | --- | --- | --- | --- | --- | --- | --- | --- | --- | --- | --- | --- | --- | --- | --- | --- | --- | --- | --- | --- | --- | --- | --- | --- | --- | --- | --- | --- | --- | --- | --- | --- | --- | --- | --- | --- | --- | --- | --- | --- | --- | --- | --- | --- | --- | --- | --- | --- | --- | --- | --- | --- | --- | --- | --- | --- | --- | --- | --- | --- | --- | --- | --- | --- | --- | --- | --- | --- | --- | --- | --- | --- | --- | --- | --- | --- | --- | --- | --- | --- | --- | --- | --- | --- | --- | --- | --- | --- | --- | --- | --- | --- | --- | --- | --- | --- | --- | --- | --- | --- | --- | --- | --- | --- | --- | --- | --- | --- | --- | --- | --- | --- | --- | --- | --- | --- | --- | --- | --- | --- | --- | --- | --- | --- | --- | --- | --- | --- | --- | --- | --- | --- | --- | --- | --- | --- | --- | --- | --- | --- | --- | --- | --- | --- | --- | --- | --- | --- | --- | --- | --- | --- | --- | --- | --- | --- | --- | --- | --- | --- | --- | --- | --- | --- | --- | --- | --- | --- | --- | --- | --- | --- | --- | --- | --- | --- | --- | --- | --- | --- | --- | --- | --- | --- | --- | --- | --- | --- | --- | --- | --- | --- | --- | --- | --- | --- | --- | --- | --- | --- | --- | --- | --- | --- | --- | --- | --- | --- | --- | --- | --- | --- | --- | --- | --- | --- | --- | --- | --- | --- | --- | --- | --- | --- | --- | --- | --- | --- | --- | --- | --- | --- | --- | --- | --- | --- | --- | --- | --- | --- | --- | --- | --- | --- | --- | --- | --- | --- | --- | --- | --- | --- | --- | --- | --- | --- | --- | --- | --- | --- | --- | --- | --- | --- | --- | --- | --- | --- | --- | --- | --- | --- | --- | --- | --- | --- | --- | --- | --- | --- | --- | --- | --- | --- | --- | --- | --- | --- | --- | --- | --- | --- | --- | --- | --- | --- | --- | --- | --- | --- | --- | --- | --- | --- | --- | --- | --- | --- | --- | --- | --- | --- | --- | --- | --- | --- | --- | --- | --- | --- | --- | --- | --- | --- | --- | --- | --- | --- | --- | --- | --- | --- | --- | --- | --- | --- | --- | --- | --- | --- | --- | --- | --- | --- | --- | --- | --- | --- | --- | --- | --- | --- | --- | --- | --- | --- | --- | --- | --- | --- | --- | --- | --- | --- | --- | --- | --- | --- | --- | --- | --- | --- | --- | --- | --- | --- | --- | --- | --- | --- | --- | --- | --- | --- | --- | --- | --- | --- | --- | --- | --- | --- | --- | --- | --- | --- | --- | --- | --- | --- | --- | --- | --- | --- | --- | --- | --- | --- | --- | --- | --- | --- | --- | --- | --- | --- | --- | --- | --- | --- | --- | --- | --- | --- | --- | --- | --- | --- | --- | --- | --- | --- | --- | --- | --- | --- | --- | --- | --- | --- | --- | --- | --- | --- | --- | --- | --- | --- | --- | --- | --- | --- | --- | --- | --- | --- | --- | --- | --- | --- | --- | --- | --- | --- | --- | --- | --- | --- | --- | --- | --- | --- | --- | --- | --- | --- | --- | --- | --- | --- | --- | --- | --- | --- | --- | --- | --- | --- | --- | --- | --- | --- | --- | --- | --- | --- | --- | --- | --- | --- | --- | --- | --- | --- | --- | --- | --- | --- | --- | --- | --- | --- | --- | --- | --- | --- | --- | --- | --- | --- | --- | --- | --- | --- | --- | --- | --- | --- | --- | --- | --- | --- | --- | --- | --- | --- | --- | --- | --- | --- | --- | --- | --- | --- | --- | --- | --- | --- | --- | --- | --- | --- | --- | --- | --- | --- | --- | --- | --- | --- | --- | --- | --- | --- | --- | --- | --- | --- | --- | --- | --- | --- | --- | --- | --- | --- | --- | --- | --- | --- | --- | --- | --- | --- | --- | --- | --- | --- | --- | --- | --- | --- | --- | --- | --- | --- | --- | --- | --- | --- | --- | --- | --- | --- | --- | --- | --- | --- | --- | --- | --- | --- | --- | --- | --- | --- | --- | --- | --- | --- | --- | --- | --- | --- | --- | --- | --- | --- | --- | --- | --- | --- | --- | --- | --- | --- | --- | --- | --- | --- | --- | --- | --- | --- | --- | --- | --- | --- | --- | --- | --- | --- | --- | --- | --- | --- | --- | --- | --- | --- | --- | --- | --- | --- | --- | --- | --- | --- | --- | --- | --- | --- | --- | --- | --- | --- | --- | --- | --- | --- | --- | --- | --- | --- | --- | --- | --- | --- | --- | --- | --- | --- | --- | --- | --- | --- | --- | --- | --- | --- | --- | --- | --- | --- | --- | --- | --- | --- | --- | --- | --- | --- | --- | --- | --- | --- | --- | --- | --- | --- | --- | --- | --- | --- | --- | --- | --- | --- | --- | --- | --- | --- | --- | --- | --- | --- | --- | --- | --- | --- | --- | --- | --- | --- | --- | --- | --- | --- | --- | --- | --- | --- | --- | --- | --- | --- | --- | --- | --- | --- | --- | --- | --- | --- | --- | --- | --- | --- | --- | --- | --- | --- | --- | --- | --- | --- | --- | --- | --- | --- | --- | --- | --- | --- | --- | --- | --- | --- | --- | --- | --- | --- | --- | --- | --- | --- | --- | --- | --- | --- | --- | --- | --- | --- | --- | --- | --- | --- | --- | --- | --- | --- | --- | --- | --- | --- | --- | --- | --- | --- | --- | --- | --- | --- | --- | --- | --- | --- | --- | --- | --- | --- | --- | --- | --- | --- | --- | --- | --- | --- | --- | --- | --- | --- | --- | --- | --- | --- | --- | --- | --- | --- | --- | --- | --- | --- | --- | --- | --- | --- | --- | --- | --- | --- | --- | --- | --- | --- | --- | --- | --- | --- | --- | --- | --- | --- | --- | --- | --- | --- | --- | --- | --- | --- | --- | --- | --- | --- | --- | --- | --- | --- | --- | --- | --- | --- | --- | --- | --- | --- | --- | --- | --- | --- | --- | --- | --- | --- | --- | --- | --- | --- | --- | --- | --- | --- | --- | --- | --- | --- | --- | --- | --- | --- | --- | --- | --- | --- | --- | --- | --- | --- | --- | --- | --- | --- | --- | --- | --- | --- | --- | --- | --- | --- | --- | --- | --- | --- | --- | --- | --- | --- | --- | --- | --- | --- | --- | --- | --- | --- | --- | --- | --- | --- | --- | --- | --- | --- | --- | --- | --- | --- | --- | --- | --- | --- | --- | --- | --- | --- | --- | --- | --- | --- | --- | --- | --- | --- | --- | --- | --- | --- | --- | --- | --- | --- | --- | --- | --- | --- | --- | --- | --- | --- | --- | --- | --- | --- | --- | --- | --- | --- | --- | --- | --- | --- | --- | --- | --- | --- | --- | --- | --- | --- | --- | --- | --- | --- | --- | --- | --- | --- | --- | --- | --- | --- | --- | --- | --- | --- | --- | --- | --- | --- | --- | --- | --- | --- | --- | --- | --- | --- | --- | --- | --- | --- | --- | --- | --- | --- | --- | --- | --- | --- | --- | --- | --- | --- | --- | --- | --- | --- | --- | --- | --- | --- | --- | --- | --- | --- | --- | --- | --- | --- | --- | --- | --- | --- | --- | --- | --- | --- | --- | --- | --- | --- | --- | --- | --- | --- | --- | --- | --- | --- | --- | --- | --- | --- | --- | --- | --- | --- | --- | --- | --- | --- | --- | --- | --- | --- | --- | --- | --- | --- | --- | --- | --- | --- | --- | --- | --- | --- | --- | --- | --- | --- | --- | --- | --- | --- | --- | --- | --- | --- | --- | --- | --- | --- | --- | --- | --- | --- | --- | --- | --- | --- | --- | --- | --- | --- | --- | --- | --- | --- | --- | --- | --- | --- | --- | --- | --- | --- | --- | --- | --- | --- | --- | --- | --- | --- | --- | --- | --- | --- | --- | --- | --- | --- | --- | --- | --- | --- | --- | --- | --- | --- | --- | --- | --- | --- | --- | --- | --- | --- | --- | --- | --- | --- | --- | --- | --- | --- | --- | --- | --- | --- | --- | --- | --- | --- | --- | --- | --- | --- | --- | --- | --- | --- | --- | --- | --- | --- | --- | --- | --- | --- | --- | --- | --- | --- | --- | --- | --- | --- | --- | --- | --- | --- | --- | --- | --- | --- | --- | --- | --- | --- | --- | --- | --- | --- | --- | --- | --- | --- | --- | --- | --- | --- | --- | --- | --- | --- | --- | --- | --- | --- | --- | --- | --- | --- | --- | --- | --- | --- | --- | --- | --- | --- | --- | --- | --- | --- | --- | --- | --- | --- | --- | --- | --- | --- | --- | --- | --- | --- | --- | --- | --- | --- | --- | --- | --- | --- | --- | --- | --- | --- | --- | --- | --- | --- | --- | --- | --- | --- | --- | --- | --- | --- | --- | --- | --- | --- | --- | --- | --- | --- | --- | --- | --- | --- | --- | --- | --- | --- | --- | --- | --- | --- | --- | --- | --- | --- | --- | --- | --- | --- | --- | --- | --- | --- | --- | --- | --- | --- | --- | --- | --- | --- | --- | --- | --- | --- | --- | --- | --- | --- | --- | --- | --- | --- | --- | --- | --- | --- | --- | --- | --- | --- | --- | --- | --- | --- | --- | --- | --- | --- | --- | --- | --- | --- | --- | --- | --- | --- | --- | --- | --- | --- | --- | --- | --- | --- | --- | --- | --- | --- | --- | --- | --- | --- | --- | --- | --- | --- | --- | --- | --- | --- | --- | --- | --- | --- | --- | --- | --- | --- | --- | --- | --- | --- | --- | --- | --- | --- | --- | --- | --- | --- | --- | --- | --- | --- | --- | --- | --- | --- | --- | --- | --- | --- | --- | --- | --- | --- | --- | --- | --- | --- | --- | --- | --- | --- | --- | --- | --- | --- | --- | --- | --- | --- | --- | --- | --- | --- | --- | --- | --- | --- | --- | --- | --- | --- | --- | --- | --- | --- | --- | --- | --- | --- | --- | --- | --- | --- | --- | --- | --- | --- | --- | --- | --- | --- | --- | --- | --- | --- | --- | --- | --- | --- | --- | --- | --- | --- | --- | --- | --- | --- | --- | --- | --- | --- | --- | --- | --- | --- | --- | --- | --- | --- | --- | --- | --- | --- | --- | --- | --- | --- | --- | --- | --- | --- | --- | --- | --- | --- | --- | --- | --- | --- | --- | --- | --- | --- | --- | --- | --- | --- | --- | --- | --- | --- | --- | --- | --- | --- | --- | --- | --- | --- | --- | --- | --- | --- | --- | --- | --- | --- | --- | --- | --- | --- | --- | --- | --- | --- | --- | --- | --- | --- | --- | --- | --- | --- | --- | --- | --- | --- | --- | --- | --- | --- | --- | --- | --- | --- | --- | --- | --- | --- | --- | --- | --- | --- | --- | --- | --- | --- | --- | --- | --- | --- | --- | --- | --- | --- | --- | --- | --- | --- | --- | --- | --- | --- | --- | --- | --- | --- | --- | --- | --- | --- | --- | --- | --- | --- | --- | --- | --- | --- | --- | --- | --- | --- | --- | --- | --- | --- | --- | --- | --- | --- | --- | --- | --- | --- | --- | --- | --- | --- | --- | --- | --- | --- | --- | --- | --- | --- | --- | --- | --- | --- | --- | --- | --- | --- | --- | --- | --- | --- | --- | --- | --- | --- | --- | --- | --- | --- | --- | --- | --- | --- | --- | --- | --- | --- | --- | --- | --- | --- | --- | --- | --- | --- | --- | --- | --- | --- | --- | --- | --- | --- | --- | --- | --- | --- | --- | --- | --- | --- | --- | --- | --- | --- | --- | --- | --- | --- | --- | --- | --- | --- | --- | --- | --- | --- | --- | --- | --- | --- | --- | --- | --- | --- | --- | --- | --- | --- | --- | --- | --- | --- | --- | --- | --- | --- | --- | --- | --- | --- | --- | --- | --- | --- | --- | --- | --- | --- | --- | --- | --- | --- | --- | --- | --- | --- | --- | --- | --- | --- | --- | --- | --- | --- | --- | --- | --- | --- | --- | --- | --- | --- | --- | --- | --- | --- | --- | --- | --- | --- | --- | --- | --- | --- | --- | --- | --- | --- | --- | --- | --- | --- | --- | --- | --- | --- | --- | --- | --- | --- | --- | --- | --- | --- | --- | --- | --- | --- | --- | --- | --- | --- | --- | --- | --- | --- | --- | --- | --- | --- | --- | --- | --- | --- | --- | --- | --- | --- | --- | --- | --- | --- | --- | --- | --- | --- | --- | --- | --- | --- | --- | --- | --- | --- | --- | --- | --- | --- | --- | --- | --- | --- | --- | --- | --- | --- | --- | --- | --- | --- | --- | --- | --- | --- | --- | --- | --- | --- | --- | --- | --- | --- | --- | --- | --- | --- | --- | --- | --- | --- | --- | --- | --- | --- | --- | --- | --- | --- | --- | --- | --- | --- | --- | --- | --- | --- | --- | --- | --- | --- | --- | --- | --- | --- | --- | --- | --- | --- | --- | --- | --- | --- | --- | --- | --- | --- | --- | --- | --- | --- | --- | --- | --- | --- | --- | --- | --- | --- | --- | --- | --- | --- | --- | --- | --- | --- | --- | --- | --- | --- | --- | --- | --- | --- | --- | --- | --- | --- | --- | --- | --- | --- | --- | --- | --- | --- | --- | --- | --- | --- | --- | --- | --- | --- | --- | --- | --- | --- | --- | --- | --- | --- | --- | --- | --- | --- | --- | --- | --- | --- | --- | --- | --- | --- | --- | --- | --- | --- | --- | --- | --- | --- | --- | --- | --- | --- | --- | --- | --- | --- | --- | --- | --- | --- | --- | --- | --- | --- | --- | --- | --- | --- | --- | --- | --- | --- | --- | --- | --- | --- | --- | --- | --- | --- | --- | --- | --- | --- | --- | --- | --- | --- | --- | --- | --- | --- | --- | --- | --- | --- | --- | --- | --- | --- | --- | --- | --- | --- | --- | --- | --- | --- | --- | --- | --- | --- | --- | --- | --- | --- | --- | --- | --- | --- | --- | --- | --- | --- | --- | --- | --- | --- | --- | --- | --- | --- | --- | --- | --- | --- | --- | --- | --- | --- | --- | --- | --- | --- | --- | --- | --- | --- | --- | --- | --- | --- | --- | --- | --- | --- | --- | --- | --- | --- | --- | --- | --- | --- | --- | --- | --- | --- | --- | --- | --- | --- | --- | --- | --- | --- | --- | --- | --- | --- | --- | --- | --- | --- | --- | --- | --- | --- | --- | --- | --- | --- | --- | --- | --- | --- | --- | --- | --- | --- | --- | --- | --- | --- | --- | --- | --- | --- | --- | --- | --- | --- | --- | --- | --- | --- | --- | --- | --- | --- | --- | --- | --- | --- | --- | --- | --- | --- | --- | --- | --- | --- | --- | --- | --- | --- | --- | --- | --- | --- | --- | --- | --- | --- | --- | --- | --- | --- | --- | --- | --- | --- | --- | --- | --- | --- | --- | --- | --- | --- | --- | --- | --- | --- | --- | --- | --- | --- | --- | --- | --- | --- | --- | --- | --- | --- | --- | --- | --- | --- | --- | --- | --- | --- | --- | --- | --- | --- | --- | --- | --- | --- | --- | --- | --- | --- | --- | --- | --- | --- | --- | --- | --- | --- | --- | --- | --- | --- | --- | --- | --- | --- | --- | --- | --- | --- | --- | --- | --- | --- | --- | --- | --- | --- | --- | --- | --- | --- | --- | --- | --- | --- | --- | --- | --- | --- | --- | --- | --- | --- | --- | --- | --- | --- | --- | --- | --- | --- | --- | --- | --- | --- | --- | --- | --- | --- | --- | --- | --- | --- | --- | --- | --- | --- | --- | --- | --- | --- | --- | --- | --- | --- | --- | --- | --- | --- | --- | --- | --- | --- | --- | --- | --- | --- | --- | --- | --- | --- | --- | --- | --- | --- | --- | --- | --- | --- | --- | --- | --- | --- | --- | --- | --- | --- | --- | --- | --- | --- | --- | --- | --- | --- | --- | --- | --- | --- | --- | --- | --- | --- | --- | --- | --- | --- | --- | --- | --- | --- | --- | --- | --- | --- | --- | --- | --- | --- | --- | --- | --- | --- | --- | --- | --- | --- | --- | --- | --- | --- | --- | --- | --- | --- | --- | --- | --- | --- | --- | --- | --- | --- | --- | --- | --- | --- | --- | --- | --- | --- | --- | --- | --- | --- | --- | --- | --- | --- | --- | --- | --- | --- | --- | --- | --- | --- | --- | --- | --- | --- | --- | --- | --- | --- | --- | --- | --- | --- | --- | --- | --- | --- | --- | --- | --- | --- | --- | --- | --- | --- | --- | --- | --- | --- | --- | --- | --- | --- | --- | --- | --- | --- | --- | --- | --- | --- | --- | --- | --- | --- | --- | --- | --- | --- | --- | --- | --- | --- | --- | --- | --- | --- | --- | --- | --- | --- | --- | --- | --- | --- | --- | --- | --- | --- | --- | --- | --- | --- | --- | --- | --- | --- | --- | --- | --- | --- | --- | --- | --- | --- | --- | --- | --- | --- | --- | --- | --- | --- | --- | --- | --- | --- | --- | --- | --- | --- | --- | --- | --- | --- | --- | --- | --- | --- | --- | --- | --- | --- | --- | --- | --- | --- | --- | --- | --- | --- | --- | --- | --- | --- | --- | --- | --- | --- | --- | --- | --- | --- | --- | --- | --- | --- | --- | --- | --- | --- | --- | --- | --- | --- | --- | --- | --- | --- | --- | --- | --- | --- | --- | --- | --- | --- | --- | --- | --- | --- | --- | --- | --- | --- | --- | --- | --- | --- | --- | --- | --- | --- | --- | --- | --- | --- | --- | --- | --- | --- | --- | --- | --- | --- | --- | --- | --- | --- | --- | --- | --- | --- | --- | --- | --- | --- | --- | --- | --- | --- | --- | --- | --- | --- | --- | --- | --- | --- | --- | --- | --- | --- | --- | --- | --- | --- | --- | --- | --- | --- | --- | --- | --- | --- | --- | --- | --- | --- | --- | --- | --- | --- | --- | --- | --- | --- | --- | --- | --- | --- | --- | --- | --- | --- | --- | --- | --- | --- | --- | --- | --- | --- | --- | --- | --- | --- | --- | --- | --- | --- | --- | --- | --- | --- | --- | --- | --- | --- | --- | --- | --- | --- | --- | --- | --- | --- | --- | --- | --- | --- | --- | --- | --- | --- | --- | --- | --- | --- | --- | --- | --- | --- | --- | --- | --- | --- | --- | --- | --- | --- | --- | --- | --- | --- | --- | --- | --- | --- | --- | --- | --- | --- | --- | --- | --- | --- | --- | --- | --- | --- | --- | --- | --- | --- | --- | --- | --- | --- | --- | --- | --- | --- | --- | --- | --- | --- | --- | --- | --- | --- | --- | --- | --- | --- | --- | --- | --- | --- | --- | --- | --- | --- | --- | --- | --- | --- | --- | --- | --- | --- | --- | --- | --- | --- | --- | --- | --- | --- | --- | --- | --- | --- | --- | --- | --- | --- | --- | --- | --- | --- | --- | --- | --- | --- | --- | --- | --- | --- | --- | --- | --- | --- | --- | --- | --- | --- | --- | --- | --- | --- | --- | --- | --- | --- | --- | --- | --- | --- | --- | --- | --- | --- | --- | --- | --- | --- | --- | --- | --- | --- | --- | --- | --- | --- | --- | --- | --- | --- | --- | --- | --- | --- | --- | --- | --- | --- | --- | --- | --- | --- | --- | --- | --- | --- | --- | --- | --- | --- | --- | --- | --- | --- | --- | --- | --- | --- | --- | --- | --- | --- | --- | --- | --- | --- | --- | --- | --- | --- | --- | --- | --- | --- | --- | --- | --- | --- | --- | --- | --- | --- | --- | --- | --- | --- | --- | --- | --- | --- | --- | --- | --- | --- | --- | --- | --- | --- | --- | --- | --- | --- | --- | --- | --- | --- | --- | --- | --- | --- | --- | --- | --- | --- | --- | --- | --- | --- | --- | --- | --- | --- | --- | --- | --- | --- | --- | --- | --- | --- | --- | --- | --- | --- | --- | --- | --- | --- | --- | --- | --- | --- | --- | --- | --- | --- | --- | --- | --- | --- | --- | --- | --- | --- | --- | --- | --- | --- | --- | --- | --- | --- | --- | --- | --- | --- | --- | --- | --- | --- | --- | --- | --- | --- | --- | --- | --- | --- | --- | --- | --- | --- | --- | --- | --- | --- | --- | --- | --- | --- | --- | --- | --- | --- | --- | --- | --- | --- | --- | --- | --- | --- | --- | --- | --- | --- | --- | --- | --- | --- | --- | --- | --- | --- | --- | --- | --- | --- | --- | --- | --- | --- | --- | --- | --- | --- | --- | --- | --- | --- | --- | --- | --- | --- | --- | --- | --- | --- | --- | --- | --- | --- | --- | --- | --- | --- | --- | --- | --- | --- | --- | --- | --- | --- | --- | --- | --- | --- | --- | --- | --- | --- | --- | --- | --- | --- | --- | --- | --- | --- | --- | --- | --- | --- | --- | --- | --- | --- | --- | --- | --- | --- | --- | --- | --- | --- | --- | --- | --- | --- | --- | --- | --- | --- | --- | --- | --- | --- | --- | --- | --- | --- | --- | --- | --- | --- | --- | --- | --- | --- | --- | --- | --- | --- | --- | --- | --- | --- | --- | --- | --- | --- | --- | --- | --- | --- | --- | --- | --- | --- | --- | --- | --- | --- | --- | --- | --- | --- | --- | --- | --- | --- | --- | --- | --- | --- | --- | --- | --- | --- | --- | --- | --- | --- | --- | --- | --- | --- | --- | --- | --- | --- | --- | --- | --- | --- | --- | --- | --- | --- | --- | --- | --- | --- | --- | --- | --- | --- | --- | --- | --- | --- | --- | --- | --- | --- | --- | --- | --- | --- | --- | --- | --- | --- | --- | --- | --- | --- | --- | --- | --- | --- | --- | --- | --- | --- | --- | --- | --- | --- | --- | --- | --- | --- | --- | --- | --- | --- | --- | --- | --- | --- | --- | --- | --- | --- | --- | --- | --- | --- | --- | --- | --- | --- | --- | --- | --- | --- | --- | --- | --- | --- | --- | --- | --- | --- | --- | --- | --- | --- | --- | --- | --- | --- | --- | --- | --- | --- | --- | --- | --- | --- | --- | --- | --- | --- | --- | --- | --- | --- | --- | --- | --- | --- | --- | --- | --- | --- | --- | --- | --- | --- | --- | --- | --- | --- | --- | --- | --- | --- | --- | --- | --- | --- | --- | --- | --- | --- | --- | --- | --- | --- | --- | --- | --- | --- | --- | --- | --- | --- | --- | --- | --- | --- | --- | --- | --- | --- | --- | --- | --- | --- | --- | --- | --- | --- | --- | --- | --- | --- | --- | --- | --- | --- | --- | --- | --- | --- | --- | --- | --- | --- | --- | --- | --- | --- | --- | --- | --- | --- | --- | --- | --- | --- | --- | --- | --- | --- | --- | --- | --- | --- | --- | --- | --- | --- | --- | --- | --- | --- | --- | --- | --- | --- | --- | --- | --- | --- | --- | --- | --- | --- | --- | --- | --- | --- | --- | --- | --- | --- | --- | --- | --- | --- | --- | --- | --- | --- | --- | --- | --- | --- | --- | --- | --- | --- | --- | --- | --- | --- | --- | --- | --- | --- | --- | --- | --- | --- | --- | --- | --- | --- | --- | --- | --- | --- | --- | --- | --- | --- | --- | --- | --- | --- | --- | --- | --- | --- | --- | --- | --- | --- | --- | --- | --- | --- | --- | --- | --- | --- | --- | --- | --- | --- | --- | --- | --- | --- | --- | --- | --- | --- | --- | --- | --- | --- | --- | --- | --- | --- | --- | --- | --- | --- | --- | --- | --- | --- | --- | --- | --- | --- | --- | --- | --- | --- | --- | --- | --- | --- | --- | --- | --- | --- | --- | --- | --- | --- | --- | --- | --- | --- | --- | --- | --- | --- | --- | --- | --- | --- | --- | --- | --- | --- | --- | --- | --- | --- | --- | --- | --- | --- | --- | --- | --- | --- | --- | --- | --- | --- | --- | --- | --- | --- | --- | --- | --- | --- | --- | --- | --- | --- | --- | --- | --- | --- | --- | --- | --- | --- | --- | --- | --- | --- | --- | --- | --- | --- | --- | --- | --- | --- | --- | --- | --- | --- | --- | --- | --- | --- | --- | --- | --- | --- | --- | --- | --- | --- | --- | --- | --- | --- | --- | --- | --- | --- | --- | --- | --- | --- | --- | --- | --- | --- | --- | --- | --- | --- | --- | --- | --- | --- | --- | --- | --- | --- | --- | --- | --- | --- | --- | --- | --- | --- | --- | --- | --- | --- | --- | --- | --- |
| |  |  |  |  |  |  |  |  |  | | --- | --- | --- | --- | --- | --- | --- | --- | --- | | **Position** | **Reference** | **Sample** | **Quality** | **Type** | **Region** | **AA Exchange** | **PAM1** | **Known Variant** | | 1849 | C | A | 3343.77 | SNP | intergenic |  |  | - | | 1977 | A | G | 2264.77 | SNP | intergenic |  |  | - | | 2632 | C | T | 1625.77 | SNP | Rv0002 (dnaN) | Ser194Leu(s) | 35 | - | | 3446 | C | T | 5275.77 | SNP | Rv0003 (recF) | Ala56Val | 13 | - | | 4013 | T | C | 4145.77 | SNP | Rv0003 (recF) | Ile245Thr | 11 | - | | 5078 | C | CG | 4918.73 | INS | intergenic (Rv0005-162nt) |  |  | - | | 7362 | G | C | 2621.77 | SNP | Rv0006 (gyrA) | Glu21Gln | 27 | - | | 7585 | G | C | 3073.77 | SNP | Rv0006 (gyrA) | Ser95Thr | 32 | genotype | | 8414 | C | T | 3002.77 | SNP | Rv0006 (gyrA) | silent (Asp371) | 9859 | - | | 9304 | G | A | 2116.77 | SNP | Rv0006 (gyrA) | Gly668Asp | 6 | - | | 11879 | A | G | 2125.77 | SNP | Rv0008c | Ser145Pro | 12 | - | | 12204 | G | A | 1411.77 | SNP | Rv0008c | silent (Leu36) | 9947 | - | | 14785 | T | C | 3230.05 | SNP | Rv0012 | Cys233Arg | 1 | - | | 15117 | C | G | 3671.77 | SNP | Rv0013 (trpG) | Ile68Met(s) | 6 | - | | 21795 | G | A | 493.77 | SNP | Rv0018c (pstP) | Pro463Ser | 17 | - | | 24007 | G | T | 2936.77 | SNP | Rv0020c (fhaA) | silent (Arg480) | 9913 | - | | 24698 | GCCGCGTTGCTCGGGGTAA | G | 7590.73 | DEL | Rv0020c (fhaA) |  |  | - | | 26747 | GCC | G | 2682.73 | DEL | Rv0021c |  |  | - | | 26752 | C | T | 1430.77 | SNP | Rv0021c | Gly44Arg | 0 | - | | 26957 | C | G | 1718.77 | SNP | intergenic |  |  | - | | 26959 | C | G | 1751.77 | SNP | intergenic |  |  | - | | 27487 | G | A | 2584.77 | SNP | intergenic |  |  | - | | 32103 | C | T | 1793.77 | SNP | Rv0029 | Ala16Val(s) | 9867 | - | | 34044 | T | C | 2739.77 | SNP | intergenic |  |  | - | | 34568 | TC | T | 3656.73 | DEL | Rv0032 (bioF2) |  |  | - | | 37031 | C | G | 1308.77 | SNP | Rv0034 | silent (Ala55) | 9867 | - | | 39017 | G | C | 72.77 | SNP | intergenic |  |  | - | | 39030 | C | T | 1385.77 | SNP | intergenic |  |  | - | | 42281 | C | A | 1161.77 | SNP | Rv0039c | Cys24Phe | 0 | - | | 42967 | G | C | 1824.77 | SNP | Rv0040c (mtc28) | silent (Pro133) | 9926 | - | | 48197 | C | CGG | 4299.73 | INS | intergenic |  |  | - | | 49360 | C | T | 1971.77 | SNP | Rv0045c | Val194Ile | 33 | - | | 49690 | GCC | G | 4511.73 | DEL | Rv0045c |  |  | - | | 50557 | T | C | 1234.77 | SNP | Rv0046c (ino1) | Arg190Gly | 1 | - | | 51949 | A | G | 2391.77 | SNP | Rv0048c | Val250Ala | 18 | - | | 53422 | G | A | 1060.77 | SNP | intergenic |  |  | - | | 54394 | A | G | 2104.77 | SNP | Rv0050 (ponA1) | silent (Ala244) | 9867 | - | | 54842 | G | T | 3854.77 | SNP | Rv0050 (ponA1) | Ala394Ser | 28 | - | | 55543 | G | GCCGCCT | 4837.73 | INS | Rv0050 (ponA1) |  |  | - | | 55553 | C | T | 1399.77 | SNP | Rv0050 (ponA1) | Pro631Ser | 17 | - | | 56001 | G | A | 2508.77 | SNP | Rv0051 | silent (Gln102) | 9876 | - | | 62049 | A | G | 2345.77 | SNP | Rv0058 (dnaB) | Arg552Gly | 1 | - | | 65083 | G | A | 1433.77 | SNP | Rv0061c | Pro90Ser | 17 | - | | 66632 | C | T | 1526.77 | SNP | Rv0062 (celA1) | Pro361Ser | 17 | - | | 67012 | C | T | 1995.77 | SNP | Rv0063 | silent (Thr30) | 9871 | - | | 69984 | C | A | 3367.77 | SNP | Rv0064 | silent (Ala455) | 9867 | - | | 69989 | G | A | 3276.77 | SNP | Rv0064 | Gly457Asp | 6 | - | | 70267 | G | T | 2841.77 | SNP | Rv0064 | Val550Phe | 0 | - | | 70816 | A | G | 1845.77 | SNP | Rv0064 | Asn733Asp | 42 | - | | 71336 | G | C | 338.78 | SNP | Rv0064 | Arg906Pro | 5 | - | | 71584 | C | CCGAGCGCTGTTCTGGCGCT AATCTGACGCTAGAATAG | 22979.73 | INS | intergenic |  |  | - | | 72549 | C | T | 2198.77 | SNP | Rv0066c (icd2) | Gly655Ser | 16 | - | | 75233 | C | A | 1586.77 | SNP | intergenic |  |  | - | | 75940 | G | C | 2263.77 | SNP | Rv0068 | Val(s)214Leu | 3 | - | | 80616 | C | G | 2220.77 | SNP | intergenic |  |  | - | | 85985 | T | C | 1272.77 | SNP | Rv0077c | Asn161Ser | 34 | - | | 88176 | A | G | 2960.77 | SNP | intergenic |  |  | - | | 92199 | T | G | 1678.77 | SNP | Rv0083 | silent (Thr600) | 9871 | - | | 103930 | G | C | 300.78 | SNP | Rv0094c | Ala245Gly | 21 | - | | 104712 | C | T | 1350.77 | SNP | intergenic |  |  | - | | 104824 | C | A | 1799.77 | SNP | Rv0095c | Ser131Ile | 1 | - | | 105736 | T | C | 1860.77 | SNP | Rv0096 (PPE1) | Val138Ala | 18 | - | | 116000 | T | G | 2479.77 | SNP | Rv0101 (nrp) | Val2000Val(s) | 18 | - | | 117389 | C | T | 1758.77 | SNP | Rv0101 (nrp) | silent (Thr2463) | 9871 | - | | 122109 | A | G | 2815.77 | SNP | Rv0103c (ctpB) | Leu(s)22Ser | 28 | - | | 123198 | T | C | 1625.77 | SNP | Rv0104 | silent (Pro294) | 9926 | - | | 123520 | T | C | 2706.77 | SNP | Rv0104 | Tyr402His | 4 | - | | 123745 | G | A | 3038.77 | SNP | Rv0104 | Gly477Arg | 0 | - | | 125830 | G | GA | 3437.73 | INS | Rv0107c (ctpI) |  |  | - | | 129576 | A | C | 1792.77 | SNP | Rv0107c (ctpI) | silent (Ala322) | 9867 | - | | 131174 | T | TG | 3681.73 | INS | intergenic |  |  | - | | 132417 | C | G | 276.78 | SNP | Rv0109 (PE\_PGRS1) | Arg346Gly | 1 | - | | 133839 | C | T | 3017.77 | SNP | intergenic |  |  | - | | 143207 | T | C | 1985.77 | SNP | Rv0118c (oxcA) | Ser224Gly | 21 | - | | 144670 | C | A | 2027.77 | SNP | Rv0119 (fadD7) | silent (Arg208) | 9913 | - | | 144865 | C | T | 2157.77 | SNP | Rv0119 (fadD7) | Leu273Leu(s) | 4 | - | | 146087 | T | C | 2255.77 | SNP | Rv0120c (fusA2) | Asn562Ser | 34 | - | | 154283 | T | C | 2117.77 | SNP | Rv0127 (mak) | Ser18Pro | 12 | - | | 157129 | C | T | 2169.77 | SNP | Rv0129c (fbpC) | Gly158Ser | 16 | genotype | | 169202 | C | G | 1352.77 | SNP | Rv0143c | silent (Leu327) | 9947 | - | | 170671 | G | A | 1848.77 | SNP | Rv0144 | Ala130Thr | 22 | - | | 172492 | C | G | 2406.77 | SNP | Rv0146 | Tyr94STOP | 2 | - | | 176534 | TG | T | 1934.73 | DEL | Rv0149 |  |  | - | | 177857 | G | A | 1469.77 | SNP | Rv0151c (PE1) | Leu485Leu(s) | 4 | - | | 178205 | C | G | 3014.77 | SNP | Rv0151c (PE1) | Gly369Arg | 0 | - | | 178453 | C | G | 3931.77 | SNP | Rv0151c (PE1) | Gly286Ala | 21 | - | | 181090 | C | T | 2143.77 | SNP | intergenic |  |  | - | | 188317 | A | G | 2531.77 | SNP | Rv0159c (PE3) | Ser175Pro | 12 | - | | 188800 | T | C | 1712.77 | SNP | Rv0159c (PE3) | Thr14Ala | 32 | - | | 189850 | A | G | 2022.77 | SNP | Rv0160c (PE4) | Phe197Ser | 3 | - | | 190428 | C | G | 2215.77 | SNP | Rv0160c (PE4) | silent (Leu4) | 9947 | - | | 194305 | C | CGG | 3923.73 | INS | Rv0165c (mce1R) |  |  | - | | 194681 | G | C | 1578.77 | SNP | Rv0165c (mce1R) | silent (Leu45) | 9947 | - | | 196642 | C | T | 2311.77 | SNP | Rv0166 (fadD5) | silent (Asn550) | 9822 | - | | 196841 | G | A | 1834.77 | SNP | intergenic |  |  | - | | 198401 | G | T | 3560.77 | SNP | Rv0168 (yrbE1B) | Gly248Cys | 0 | - | | 199470 | T | G | 2052.77 | SNP | Rv0169 (mce1A) | Ser313Ala | 35 | - | | 201949 | G | A | 930.77 | SNP | Rv0171 (mce1C) | Gly340Arg | 0 | - | | 206339 | T | C | 1603.77 | SNP | Rv0174 (mce1F) | Leu370Pro | 2 | - | | 206481 | C | G | 1054.77 | SNP | Rv0174 (mce1F) | silent (Pro417) | 9926 | - | | 206484 | G | T | 927.77 | SNP | Rv0174 (mce1F) | silent (Gly418) | 9935 | - | | 207079 | G | C | 1682.77 | SNP | Rv0175 | Arg89Pro | 5 | - | | 210624 | C | T | 2655.77 | SNP | Rv0179c (lprO) | silent (Leu63) | 9947 | - | | 217201 | T | C | 1571.77 | SNP | Rv0186 (bglS) | silent (Asn311) | 9822 | - | | 218599 | T | C | 2335.77 | SNP | intergenic |  |  | - | | 223942 | T | C | 679.77 | SNP | Rv0192 | Ser127Pro | 12 | - | | 224338 | C | T | 2097.77 | SNP | Rv0192 | Pro259Ser | 17 | - | | 225274 | T | C | 1719.77 | SNP | Rv0193c | Tyr433Cys | 3 | - | | 225323 | T | C | 2145.77 | SNP | Rv0193c | Lys417Glu | 4 | - | | 227098 | T | C | 2667.77 | SNP | Rv0194 | Met(s)74Thr | 22 | - | | 230576 | G | GT | 4129.73 | INS | intergenic |  |  | - | | 230920 | TC | T | 2548.73 | DEL | Rv0195 |  |  | - | | 231114 | C | G | 1845.77 | SNP | Rv0195 | silent (Ala72) | 9867 | - | | 234477 | T | G | 1286.77 | SNP | Rv0197 | Tyr749STOP | 2 | - | | 234496 | C | CGT | 3648.73 | INS | Rv0197 |  |  | - | | 243923 | CG | C | 2989.73 | DEL | Rv0205 |  |  | - | | 244857 | G | C | 1098.77 | SNP | Rv0206c (mmpL3) | Ala821Gly | 21 | - | | 249522 | T | C | 1489.77 | SNP | Rv0209 | Val(s)162Ala | 9867 | - | | 251575 | G | A | 2190.77 | SNP | Rv0210 | Ala486Thr | 22 | - | | 255373 | T | C | 2469.77 | SNP | Rv0213c | Asp193Gly | 11 | - | | 257071 | C | T | 2991.77 | SNP | Rv0214 (fadD4) | silent (Tyr336) | 9945 | - | | 261869 | T | C | 1526.77 | SNP | Rv0218 | Cys316Arg | 1 | - | | 262268 | A | T | 2306.77 | SNP | Rv0219 | silent (Ala5) | 9867 | - | | 264992 | T | C | 1083.77 | SNP | Rv0221 | Leu309Pro | 2 | - | | 265554 | A | C | 1638.77 | SNP | Rv0222 (echA1) | silent (Val16) | 9901 | - | | 266405 | C | T | 2277.77 | SNP | Rv0223c | Gly454Ser | 16 | - | | 274463 | C | A | 1919.77 | SNP | Rv0229c | Arg175Leu | 1 | - | | 276539 | G | C | 3326.77 | SNP | Rv0231 (fadE4) | Gly161Ala | 21 | - | | 278681 | C | G | 2230.77 | SNP | Rv0233 (nrdB) | His33Asp | 4 | - | | 278755 | C | T | 1672.77 | SNP | Rv0233 (nrdB) | silent (Ala57) | 9867 | - | | 279515 | GCGGCCTCGGCCTAGGCCTG GCGAGCAGACGCAAAATCGC CCAATTTCGTGCCGAATTGG GCGATTTTGCGTCTGC | G | 7606.73 | DEL | Rv0233 (nrdB) |  |  | - | | 283503 | G | A | 1228.77 | SNP | Rv0236c (aftD) | His1117Tyr | 4 | - | | 283614 | T | C | 1367.77 | SNP | Rv0236c (aftD) | Ser1080Gly | 21 | - | | 285772 | A | C | 1561.77 | SNP | Rv0236c (aftD) | silent (Pro360) | 9926 | - | | 285871 | A | G | 1295.77 | SNP | Rv0236c (aftD) | silent (Val327) | 9901 | - | | 287141 | GC | G | 2354.73 | DEL | intergenic |  |  | - | | 289253 | C | T | 2513.77 | SNP | Rv0239 (vapB24) | silent (Asp50) | 9859 | - | | 293628 | A | AC | 2209.73 | INS | intergenic |  |  | - | | 294514 | T | C | 2897.77 | SNP | Rv0244c (fadE5) | Thr374Ala | 32 | - | | 296717 | C | G | 3808.77 | SNP | intergenic |  |  | - | | 301341 | C | A | 3519.77 | SNP | Rv0249c | silent (Pro105) | 9926 | - | | 304923 | A | G | 2682.77 | SNP | Rv0252 (nirB) | silent (Lys686) | 9926 | - | | 308661 | A | G | 1549.77 | SNP | Rv0256c (PPE2) | Leu296Pro | 2 | - | | 310973 | G | A | 2667.77 | SNP | Rv0259c | Ala182Val(s) | 9867 | - | | 311613 | G | T | 1973.77 | SNP | Rv0260c | silent (Val349) | 9901 | - | | 324812 | C | T | 2467.77 | SNP | Rv0270 (fadD2) | silent (Asp82) | 9859 | - | | 325505 | T | C | 2270.77 | SNP | Rv0270 (fadD2) | silent (Val313) | 9901 | - | | 325766 | G | A | 2126.77 | SNP | Rv0270 (fadD2) | silent (Lys400) | 9926 | - | | 328569 | G | C | 2441.77 | SNP | intergenic |  |  | - | | 333685 | C | T | 554.98 | SNP | Rv0278c (PE\_PGRS3) | Gly876Ser | 16 | - | | 333892 | G | C | 400.77 | SNP | Rv0278c (PE\_PGRS3) | Arg807Gly | 1 | - | | 335261 | C | G | 30.77 | SNP | Rv0278c (PE\_PGRS3) | silent (Ala350) | 9867 | - | | 335810 | CCCGCCGGCGCCGCCGTTG | C | 1536.74 | DEL | Rv0278c (PE\_PGRS3) |  |  | - | | 335971 | A | G | 216.77 | SNP | Rv0278c (PE\_PGRS3) | Leu(s)114Leu | 3 | - | | 336005 | G | A | 204.77 | SNP | Rv0278c (PE\_PGRS3) | silent (Ile102) | 9872 | - | | 336050 | A | G | 212.77 | SNP | Rv0278c (PE\_PGRS3) | silent (Tyr87) | 9945 | - | | 336053 | G | C | 179.77 | SNP | Rv0278c (PE\_PGRS3) | silent (Ala86) | 9867 | - | | 336074 | T | C | 118.77 | SNP | Rv0278c (PE\_PGRS3) | silent (Ala79) | 9867 | - | | 336081 | A | G | 70.77 | SNP | Rv0278c (PE\_PGRS3) | Val(s)77Ala | 9867 | - | | 336348 | C | T | 1989.77 | SNP | intergenic |  |  | - | | 336380 | A | T | 119.77 | SNP | intergenic |  |  | - | | 336400 | C | G | 79.77 | SNP | intergenic |  |  | - | | 336403 | C | G | 114.77 | SNP | intergenic |  |  | - | | 336405 | A | G | 31.77 | SNP | intergenic |  |  | - | | 336504 | G | T | 618.77 | SNP | intergenic |  |  | - | | 336535 | T | G | 407.77 | SNP | intergenic |  |  | - | | 336537 | T | G | 361.77 | SNP | intergenic |  |  | - | | 336540 | G | T | 220.77 | SNP | intergenic |  |  | - | | 336546 | T | G | 368.77 | SNP | intergenic |  |  | - | | 336557 | C | CT | 566.73 | INS | intergenic |  |  | - | | 336560 | T | C | 208.77 | SNP | Rv0279c (PE\_PGRS4) | silent (STOP838) | 9867 | - | | 336562 | A | ATGG | 730.73 | INS | Rv0279c (PE\_PGRS4) |  |  | - | | 336590 | G | C | 283.77 | SNP | Rv0279c (PE\_PGRS4) | Ile828Met(s) | 6 | - | | 336592 | T | G | 326.77 | SNP | Rv0279c (PE\_PGRS4) | Ile828Leu | 22 | - | | 336611 | G | C | 316.77 | SNP | Rv0279c (PE\_PGRS4) | silent (Ala821) | 9867 | - | | 336617 | G | C | 254.77 | SNP | Rv0279c (PE\_PGRS4) | silent (Pro819) | 9926 | - | | 336620 | T | C | 181.77 | SNP | Rv0279c (PE\_PGRS4) | silent (Thr818) | 9871 | - | | 336728 | G | A | 107.77 | SNP | Rv0279c (PE\_PGRS4) | silent (Gly782) | 9935 | - | | 337820 | G | A | 178.94 | SNP | Rv0279c (PE\_PGRS4) | silent (Gly418) | 9935 | - | | 337959 | A | C | 122.03 | SNP | Rv0279c (PE\_PGRS4) | Ile372Ser | 2 | - | | 338100 | T | C | 303.78 | SNP | Rv0279c (PE\_PGRS4) | Asn325Ser | 34 | - | | 338453 | A | G | 474.77 | SNP | Rv0279c (PE\_PGRS4) | silent (Ala207) | 9867 | - | | 338565 | C | G | 197.77 | SNP | Rv0279c (PE\_PGRS4) | Gly170Ala | 21 | - | | 338648 | T | G | 320.78 | SNP | Rv0279c (PE\_PGRS4) | silent (Gly142) | 9935 | - | | 338669 | T | C | 491.31 | SNP | Rv0279c (PE\_PGRS4) | Leu135Leu(s) | 4 | - | | 338682 | T | G | 390.22 | SNP | Rv0279c (PE\_PGRS4) | Asp131Ala | 10 | - | | 338683 | C | G | 432.10 | SNP | Rv0279c (PE\_PGRS4) | Asp131His | 3 | - | | 338685 | C | G | 436.53 | SNP | Rv0279c (PE\_PGRS4) | Gly130Ala | 21 | - | | 338690 | A | G | 463.28 | SNP | Rv0279c (PE\_PGRS4) | silent (Asn128) | 9822 | - | | 338692 | T | C | 441.41 | SNP | Rv0279c (PE\_PGRS4) | Asn128Asp | 42 | - | | 338844 | A | G | 200.77 | SNP | Rv0279c (PE\_PGRS4) | Val(s)77Ala | 9867 | - | | 338845 | C | T | 184.77 | SNP | Rv0279c (PE\_PGRS4) | Val(s)77Met(s) | 9867 | - | | 338876 | G | A | 473.77 | SNP | Rv0279c (PE\_PGRS4) | silent (Ser66) | 9840 | - | | 338903 | G | C | 817.77 | SNP | Rv0279c (PE\_PGRS4) | silent (Ala57) | 9867 | - | | 338960 | T | C | 842.77 | SNP | Rv0279c (PE\_PGRS4) | silent (Ala38) | 9867 | - | | 338963 | T | C | 886.77 | SNP | Rv0279c (PE\_PGRS4) | silent (Thr37) | 9871 | - | | 339230 | G | C | 1743.77 | SNP | intergenic |  |  | - | | 340372 | T | C | 678.77 | SNP | Rv0280 (PPE3) | Ser337Pro | 12 | - | | 342146 | A | C | 2522.77 | SNP | Rv0282 (eccA3) | Glu6Ala | 17 | - | | 342873 | C | T | 2609.77 | SNP | Rv0282 (eccA3) | silent (Val248) | 9901 | - | | 346275 | C | G | 2077.77 | SNP | Rv0284 (eccC3) | Pro214Arg | 4 | - | | 350088 | C | A | 2531.77 | SNP | Rv0286 (PPE4) | Leu52Met(s) | 4 | - | | 353197 | C | T | 1471.77 | SNP | Rv0290 (eccD3) | Arg39Cys | 1 | - | | 356528 | A | G | 2033.77 | SNP | Rv0292 (eccE3) | Asn217Asp | 42 | - | | 358473 | G | C | 1350.77 | SNP | Rv0294 (tam) | Trp101Cys | 0 | - | | 363467 | G | A | 2677.77 | SNP | Rv0298 | silent (Ala72) | 9867 | - | | 363563 | G | A | 2529.77 | SNP | Rv0299 | Ala30Thr | 22 | - | | 365152 | C | A | 1608.77 | SNP | Rv0302 | Pro183Gln | 6 | - | | 367718 | G | T | 2163.77 | SNP | Rv0304c (PPE5) | Leu1683Ile | 9 | - | | 368077 | C | T | 2071.77 | SNP | Rv0304c (PPE5) | Gly1563Asp | 6 | - | | 368087 | AGCTGCCGGTGTTGAT | A | 9986.73 | DEL | Rv0304c (PPE5) |  |  | - | | 369886 | C | G | 1721.77 | SNP | Rv0304c (PPE5) | Gly960Ala | 21 | - | | 372913 | A | C | 2512.77 | SNP | Rv0305c (PPE6) | silent (Gly933) | 9935 | - | | 373282 | TA | T | 3176.71 | DEL | Rv0305c (PPE6) |  |  | - | | 376774 | T | C | 1961.77 | SNP | Rv0307c | silent (Ala94) | 9867 | - | | 384380 | A | C | 1873.77 | SNP | Rv0315 | Lys260Thr | 8 | - | | 386432 | C | G | 2448.77 | SNP | Rv0318c | Gly223Ala | 21 | - | | 390828 | T | C | 2792.77 | SNP | Rv0323c | Ser142Gly | 21 | - | | 391853 | A | G | 1618.77 | SNP | Rv0324 | Thr168Ala | 32 | - | | 392261 | T | C | 1543.77 | SNP | Rv0325 | STOP75Gln | 3 | - | | 394006 | G | A | 2732.77 | SNP | Rv0327c (cyp135A1) | Arg14Trp | 2 | - | | 396199 | T | C | 1206.77 | SNP | intergenic |  |  | - | | 402836 | G | A | 2165.77 | SNP | Rv0337c (aspC) | silent (Tyr109) | 9945 | - | | 403980 | G | A | 2817.77 | SNP | Rv0338c | Ala621Val | 13 | - | | 404326 | T | C | 2343.77 | SNP | Rv0338c | Arg506Gly | 1 | - | | 406251 | T | C | 1166.77 | SNP | Rv0339c | Asp733Gly | 11 | - | | 414486 | C | T | 2892.77 | SNP | Rv0344c (lpqJ) | silent (Glu152) | 9865 | - | | 417404 | G | T | 1473.77 | SNP | Rv0347 | Arg34Leu | 1 | - | | 420008 | A | G | 3314.77 | SNP | Rv0350 (dnaK) | silent (Ala58) | 9867 | - | | 422678 | G | A | 1001.77 | SNP | Rv0352 (dnaJ1) | Arg76His | 8 | - | | 424320 | T | TC | 2893.73 | INS | Rv0354c (PPE7) |  |  | - | | 427310 | TTGCCGAGGTTTGCAC | T | 7614.73 | DEL | Rv0355c (PPE8) |  |  | - | | 428698 | C | T | 1670.77 | SNP | Rv0355c (PPE8) | silent (Ala1994) | 9867 | - | | 428921 | G | A | 2148.77 | SNP | Rv0355c (PPE8) | Ser1920Phe | 2 | - | | 432459 | C | T | 2532.77 | SNP | Rv0355c (PPE8) | Asp741Asn | 36 | - | | 438470 | C | T | 1735.77 | SNP | Rv0360c | silent (Lys90) | 9926 | - | | 440365 | G | A | 1951.77 | SNP | Rv0362 (mgtE) | silent (Ser165) | 9840 | - | | 440878 | C | T | 1542.77 | SNP | Rv0362 (mgtE) | silent (Thr336) | 9871 | - | | 445780 | C | T | 1564.77 | SNP | Rv0368c | Arg249His | 8 | - | | 445882 | G | A | 928.77 | SNP | Rv0368c | Ser215Leu(s) | 35 | - | | 447642 | G | C | 2223.77 | SNP | Rv0370c | silent (Leu134) | 9947 | - | | 454295 | T | C | 2423.77 | SNP | Rv0376c | silent (Pro26) | 9926 | - | | 454333 | T | G | 1880.77 | SNP | Rv0376c | Thr14Pro | 4 | - | | 455024 | G | A | 2446.77 | SNP | Rv0377 | Val202Ile | 33 | - | | 455623 | CT | C | 2440.73 | DEL | intergenic |  |  | - | | 457452 | T | G | 1286.77 | SNP | Rv0381c | silent (Thr124) | 9871 | - | | 459399 | A | C | 1903.77 | SNP | intergenic |  |  | - | | 466808 | G | C | 723.77 | SNP | Rv0387c | Ala200Gly | 21 | - | | 467433 | C | T | 1016.77 | SNP | intergenic |  |  | - | | 467497 | C | CG | 1612.73 | INS | Rv0388c (PPE9) |  |  | - | | 467508 | C | CG | 1712.73 | INS | Rv0388c (PPE9) |  |  | - | | 467516 | G | C | 1052.77 | SNP | Rv0388c (PPE9) | silent (Ser162) | 9840 | - | | 467526 | C | G | 1144.77 | SNP | Rv0388c (PPE9) | Gly159Ala | 21 | - | | 467546 | G | C | 1139.77 | SNP | Rv0388c (PPE9) | Asp152Glu | 56 | - | | 467557 | A | C | 1076.77 | SNP | Rv0388c (PPE9) | Leu(s)149Val(s) | 9867 | - | | 467564 | A | C | 1091.77 | SNP | Rv0388c (PPE9) | His146Gln | 23 | - | | 467585 | G | C | 1338.77 | SNP | Rv0388c (PPE9) | His139Gln | 23 | - | | 467590 | T | C | 1390.77 | SNP | Rv0388c (PPE9) | Thr138Ala | 32 | - | | 467621 | T | G | 1334.77 | SNP | Rv0388c (PPE9) | silent (Gly127) | 9935 | - | | 467638 | G | T | 1236.77 | SNP | Rv0388c (PPE9) | Gln122Lys | 12 | - | | 473310 | A | G | 1504.77 | SNP | Rv0393 | Gln177Arg | 10 | - | | 475178 | T | C | 1176.77 | SNP | Rv0395 | Val80Ala | 18 | - | | 475915 | A | C | 981.77 | SNP | Rv0397 | Ser34Arg | 6 | - | | 476612 | G | A | 1990.77 | SNP | Rv0397A | Val(s)73Val | 13 | - | | 481526 | AG | A | 5573.73 | DEL | Rv0402c (mmpL1) |  |  | - | | 483935 | T | G | 3886.77 | SNP | intergenic |  |  | - | | 484504 | C | G | 4681.77 | SNP | Rv0404 (fadD30) | silent (Ala176) | 9867 | - | | 485561 | A | C | 3713.77 | SNP | Rv0404 (fadD30) | Ile529Leu | 22 | - | | 485730 | C | T | 3288.77 | SNP | Rv0404 (fadD30) | Pro585Leu | 3 | - | | 485810 | CA | C | 4600.73 | DEL | Rv0405 (pks6) |  |  | - | | 489935 | G | C | 2728.77 | SNP | Rv0405 (pks6); Rv0406c | Arg1402Pro; silent (Thr257) | 5; 9871 | - | | 491742 | T | C | 2045.77 | SNP | Rv0407 (fgd1) | silent (Phe320) | 9946 | genotype | | 492150 | G | C | 2123.77 | SNP | Rv0408 (pta) | Gly122Ala | 21 | - | | 498531 | A | G | 2081.77 | SNP | Rv0412c | silent (Ala363) | 9867 | - | | 498557 | C | A | 2066.77 | SNP | Rv0412c | Asp355Tyr | 0 | - | | 502589 | C | G | 2078.77 | SNP | Rv0417 (thiG) | Ser75Cys | 5 | - | | 503354 | G | C | 2764.77 | SNP | intergenic |  |  | - | | 505974 | G | A | 789.77 | SNP | Rv0419 (lpqM) | Ala297Thr | 22 | - | | 513257 | T | C | 1447.77 | SNP | Rv0425c (ctpH) | Met(s)689Val(s) | 9867 | - | | 517358 | T | C | 2051.77 | SNP | Rv0428c | Asp149Gly | 11 | - | | 517389 | C | A | 2188.77 | SNP | Rv0428c | Val139Phe | 0 | - | | 523654 | C | T | 1453.77 | SNP | Rv0435c | Ala294Thr | 22 | - | | 524891 | C | A | 2057.77 | SNP | Rv0436c (pssA) | Gly167Val | 3 | - | | 531775 | C | G | 1461.77 | SNP | Rv0442c (PPE10) | Trp147Ser | 5 | - | | 534664 | G | T | 3160.77 | SNP | Rv0446c | Phe184Leu | 13 | - | | 541201 | A | G | 2259.77 | SNP | Rv0450c (mmpL4) | silent (Leu97) | 9947 | - | | 546357 | A | G | 2040.77 | SNP | Rv0456c (echA2) | silent (Thr149) | 9871 | - | | 548326 | T | C | 2388.77 | SNP | Rv0457c | Thr428Ala | 32 | - | | 551525 | A | C | 2056.77 | SNP | Rv0459 | silent (Arg110) | 9913 | - | | 555991 | A | G | 2637.77 | SNP | Rv0465c | Cys106Arg | 1 | - | | 560540 | ACTTTTC | A | 8080.73 | DEL | Rv0469 (umaA) |  |  | - | | 562062 | GGCCA | G | 3111.73 | DEL | Rv0470A |  |  | - | | 565655 | A | G | 2311.77 | SNP | intergenic |  |  | - | | 572591 | C | T | 2196.77 | SNP | Rv0483 (lprQ) | silent (Asp294) | 9859 | - | | 573262 | A | G | 1863.77 | SNP | Rv0484c | silent (Gly180) | 9935 | - | | 579284 | T | C | 1828.77 | SNP | intergenic |  |  | - | | 580772 | T | A | 649.77 | SNP | intergenic |  |  | - | | 580773 | GGGGGCACCACCCGCTTGCG GGGGA | G | 5662.73 | DEL | intergenic |  |  | - | | 584171 | T | C | 1854.77 | SNP | Rv0493c | Ser174Gly | 21 | - | | 586477 | C | G | 1448.77 | SNP | Rv0496 | Ser28Arg | 6 | - | | 590436 | T | C | 1479.77 | SNP | Rv0500 (proC) | silent (Ala118) | 9867 | - | | 597816 | A | G | 2538.77 | SNP | Rv0507 (mmpL2) | silent (Ala206) | 9867 | - | | 598475 | G | A | 3672.77 | SNP | Rv0507 (mmpL2) | Arg426His | 8 | - | | 599868 | A | G | 2859.77 | SNP | Rv0507 (mmpL2) | silent (Arg890) | 9913 | - | | 610105 | T | G | 1588.77 | SNP | intergenic |  |  | - | | 610120 | T | G | 1501.77 | SNP | intergenic |  |  | - | | 611977 | C | T | 1543.77 | SNP | Rv0519c | Gly33Asp | 6 | - | | 623163 | C | T | 409.77 | SNP | Rv0532 (PE\_PGRS6) | Ala124Val | 13 | - | | 623472 | A | G | 106.28 | SNP | Rv0532 (PE\_PGRS6) | Asp227Gly | 11 | - | | 623508 | C | G | 106.28 | SNP | Rv0532 (PE\_PGRS6) | Ala239Gly | 21 | - | | 624395 | C | G | 175.90 | SNP | Rv0532 (PE\_PGRS6) | Pro535Ala | 22 | - | | 629433 | C | T | 2731.77 | SNP | Rv0537c | Gly100Asp | 6 | - | | 629714 | G | A | 1823.77 | SNP | Rv0537c | silent (Asp6) | 9859 | - | | 630722 | G | C | 1234.77 | SNP | Rv0538 | Arg228Pro | 5 | - | | 633562 | C | T | 1881.77 | SNP | Rv0541c | Val(s)281Val | 13 | - | | 635139 | G | C | 1812.77 | SNP | Rv0542c (menE) | silent (Gly122) | 9935 | - | | 637319 | G | A | 1737.77 | SNP | Rv0545c (pitA) | Pro49Ser | 17 | - | | 647627 | G | A | 2961.77 | SNP | Rv0555 (menD) | Val(s)444Met(s) | 9867 | - | | 648002 | T | G | 2860.77 | SNP | Rv0556 | Leu15Arg | 1 | - | | 648856 | T | C | 3023.77 | SNP | Rv0557 (mgtA) | silent (Gly107) | 9935 | genotype | | 652950 | T | C | 2022.77 | SNP | Rv0562 (grcC1) | silent (Arg60) | 9913 | - | | 655986 | T | G | 2180.77 | SNP | intergenic |  |  | - | | 657081 | C | T | 2781.77 | SNP | Rv0565c | Val(s)130Val | 13 | - | | 659341 | T | C | 1930.77 | SNP | intergenic |  |  | - | | 662911 | T | C | 1786.77 | SNP | Rv0570 (nrdZ) | silent (Ala539) | 9867 | - | | 663410 | A | C | 172.77 | SNP | intergenic |  |  | - | | 663418 | A | C | 181.77 | SNP | intergenic |  |  | - | | 663419 | G | A | 169.77 | SNP | intergenic |  |  | - | | 663420 | C | A | 143.77 | SNP | intergenic |  |  | - | | 663429 | T | G | 221.77 | SNP | intergenic |  |  | - | | 665293 | A | G | 2392.77 | SNP | Rv0572c | Phe31Leu | 13 | - | | 668262 | A | G | 1783.77 | SNP | Rv0574c | Tyr45His | 4 | - | | 669398 | T | C | 1981.77 | SNP | Rv0575c | silent (Gln116) | 9876 | - | | 670545 | G | A | 1563.77 | SNP | Rv0576 | Arg233His | 8 | - | | 672491 | C | G | 431.77 | SNP | Rv0578c (PE\_PGRS7) | silent (Gly1142) | 9935 | - | | 673238 | A | G | 478.77 | SNP | Rv0578c (PE\_PGRS7) | silent (His893) | 9912 | - | | 674702 | A | T | 507.77 | SNP | Rv0578c (PE\_PGRS7) | silent (Gly405) | 9935 | - | | 675740 | C | T | 1692.77 | SNP | Rv0578c (PE\_PGRS7) | silent (Gln59) | 9876 | - | | 684376 | T | C | 2675.77 | SNP | intergenic |  |  | - | | 685461 | C | G | 2077.77 | SNP | Rv0587 (yrbE2A) | silent (Ala111) | 9867 | - | | 685608 | T | C | 3558.77 | SNP | Rv0587 (yrbE2A) | silent (Leu160) | 9947 | - | | 686123 | C | A | 2023.77 | SNP | Rv0588 (yrbE2B) | Leu66Met(s) | 4 | - | | 686263 | G | A | 1453.77 | SNP | Rv0588 (yrbE2B) | silent (Glu112) | 9865 | - | | 686972 | T | C | 3149.77 | SNP | Rv0589 (mce2A) | Phe51Ser | 3 | - | | 688792 | T | TG | 3263.73 | INS | Rv0590 (mce2B) |  |  | - | | 690450 | A | C | 700.77 | SNP | Rv0591 (mce2C) | silent (Ala464) | 9867 | - | | 690465 | T | G | 946.77 | SNP | Rv0591 (mce2C) | silent (Leu469) | 9947 | - | | 691887 | G | GC | 2505.73 | INS | Rv0592 (mce2D) |  |  | - | | 698968 | G | A | 1629.77 | SNP | Rv0601c | silent (Gly9) | 9935 | - | | 708056 | T | C | 1326.77 | SNP | Rv0613c | His487Arg | 10 | - | | 709481 | G | T | 1656.77 | SNP | Rv0613c; Rv0614 | Ala12Glu; silent (Ser42) | 10; 9840 | - | | 712693 | A | G | 1996.77 | SNP | intergenic |  |  | - | | 713310 | T | C | 2318.77 | SNP | Rv0620 (galK) | Cys199Arg | 1 | - | | 725190 | G | A | 244.80 | SNP | Rv0631c (recC) | silent (Phe1039) | 9946 | - | | 729685 | C | T | 2467.77 | SNP | Rv0633c | Arg161Gln | 9 | - | | 740357 | C | T | 1809.77 | SNP | Rv0646c (lipG) | silent (Ala261) | 9867 | - | | 746463 | CGGTCGACGA | C | 3756.73 | DEL | Rv0649 (fabD2) |  |  | - | | 750027 | C | CA | 3849.73 | INS | Rv0654 |  |  | - | | 750033 | C | G | 2799.77 | SNP | Rv0654 | Pro12Ala | 22 | - | | 753174 | C | A | 747.77 | SNP | Rv0656c (vapC6) | Trp65Leu(s) | 0 | - | | 754186 | A | G | 2029.77 | SNP | Rv0658c | Leu75Pro | 2 | - | | 757182 | A | G | 2931.77 | SNP | Rv0663 (atsD) | Asp349Gly | 11 | - | | 759746 | C | T | 2175.77 | SNP | intergenic (Rv0667-61nt) |  |  | - | | 761140 | A | T | 2204.77 | SNP | Rv0667 (rpoB) | His445Leu | 4 | resistance | | 762434 | T | G | 1917.77 | SNP | Rv0667 (rpoB) | silent (Gly876) | 9935 | genotype | | 763031 | T | C | 2189.77 | SNP | Rv0667 (rpoB) | silent (Ala1075) | 9867 | genotype | | 767339 | A | G | 2107.77 | SNP | intergenic |  |  | - | | 767414 | G | A | 2015.77 | SNP | intergenic |  |  | - | | 771094 | C | A | 1957.77 | SNP | Rv0671 (lpqP) | silent (Gly171) | 9935 | - | | 775639 | T | C | 1778.77 | SNP | Rv0676c (mmpL5) | Ile948Val | 57 | - | | 776100 | G | A | 2216.77 | SNP | Rv0676c (mmpL5) | Thr794Ile | 7 | - | | 781395 | T | C | 2794.77 | SNP | intergenic (Rv0682-165nt) |  |  | - | | 788615 | G | C | 3260.77 | SNP | Rv0688 | Gly226Arg | 0 | - | | 798779 | T | C | 2584.77 | SNP | intergenic |  |  | - | | 798934 | C | A | 2379.77 | SNP | Rv0698 | silent (Arg34) | 9913 | - | | 799288 | G | T | 2132.77 | SNP | Rv0698 | silent (Pro152) | 9926 | - | | 807405 | C | T | 2405.77 | SNP | Rv0711 (atsA) | silent (Ser357) | 9840 | - | | 820483 | G | T | 1165.77 | SNP | Rv0727c (fucA) | Ala6Asp | 6 | - | | 820734 | A | G | 900.77 | SNP | Rv0728c (serA2) | Val248Ala | 18 | - | | 820752 | C | T | 1027.77 | SNP | Rv0728c (serA2) | Arg242His | 8 | - | | 829165 | G | T | 1681.77 | SNP | intergenic |  |  | - | | 830868 | G | GGC | 2834.73 | INS | Rv0739 |  |  | - | | 834857 | C | T | 1622.77 | SNP | Rv0744c | Met(s)30Ile | 2 | - | | 836426 | A | C | 41.74 | SNP | Rv0746 (PE\_PGRS9) | silent (Leu242) | 9947 | - | | 836454 | A | G | 55.74 | SNP | Rv0746 (PE\_PGRS9) | Thr252Ala | 32 | - | | 836538 | A | G | 143.03 | SNP | Rv0746 (PE\_PGRS9) | Asn280Asp | 42 | - | | 837033 | A | G | 357.78 | SNP | Rv0746 (PE\_PGRS9) | Thr445Ala | 32 | - | | 839194 | A | G | 98.28 | SNP | Rv0747 (PE\_PGRS10) | silent (Thr248) | 9871 | - | | 839269 | A | G | 66.77 | SNP | Rv0747 (PE\_PGRS10) | silent (Gly273) | 9935 | - | | 839279 | G | A | 140.77 | SNP | Rv0747 (PE\_PGRS10) | Asp277Asn | 36 | - | | 839284 | C | G | 67.77 | SNP | Rv0747 (PE\_PGRS10) | silent (Ala278) | 9867 | - | | 839309 | T | G | 93.77 | SNP | Rv0747 (PE\_PGRS10) | Ser287Ala | 35 | - | | 839334 | A | G | 293.78 | SNP | Rv0747 (PE\_PGRS10) | Lys295Arg | 19 | - | | 839348 | A | G | 243.80 | SNP | Rv0747 (PE\_PGRS10) | Ser300Gly | 21 | - | | 839515 | G | A | 193.77 | SNP | Rv0747 (PE\_PGRS10) | silent (Ala355) | 9867 | - | | 839516 | A | G | 204.77 | SNP | Rv0747 (PE\_PGRS10) | Thr356Ala | 32 | - | | 839519 | C | G | 159.77 | SNP | Rv0747 (PE\_PGRS10) | Leu357Val(s) | 4 | - | | 839520 | T | C | 164.77 | SNP | Rv0747 (PE\_PGRS10) | Leu357Pro | 2 | - | | 839534 | A | C | 111.77 | SNP | Rv0747 (PE\_PGRS10) | Ile362Leu | 22 | - | | 839794 | C | T | 37.77 | SNP | Rv0747 (PE\_PGRS10) | silent (Gly448) | 9935 | - | | 839799 | C | A | 40.77 | SNP | Rv0747 (PE\_PGRS10) | Ala450Asp | 6 | - | | 839806 | G | C | 108.77 | SNP | Rv0747 (PE\_PGRS10) | silent (Gly452) | 9935 | - | | 839821 | G | C | 111.77 | SNP | Rv0747 (PE\_PGRS10) | Leu(s)457Phe | 1 | - | | 840496 | C | G | 150.79 | SNP | Rv0747 (PE\_PGRS10) | silent (Gly682) | 9935 | - | | 840847 | C | T | 1056.77 | SNP | Rv0747 (PE\_PGRS10) | silent (Gly799) | 9935 | - | | 841289 | GAA | G | 3570.73 | DEL | Rv0749 (vapC31) |  |  | - | | 841764 | G | C | 3579.77 | SNP | Rv0749A | silent (Thr37) | 9871 | - | | 842153 | G | A | 1332.77 | SNP | Rv0750 | Glu41Lys | 7 | - | | 847995 | T | C | 2343.77 | SNP | intergenic |  |  | - | | 852427 | T | C | 1231.77 | SNP | Rv0758 (phoR) | Leu11Pro | 2 | - | | 852910 | C | T | 1711.77 | SNP | Rv0758 (phoR) | Pro172Leu | 3 | - | | 854252 | GC | G | 3010.73 | DEL | intergenic |  |  | - | | 856308 | C | T | 2901.77 | SNP | Rv0762c | Val55Ile | 33 | - | | 857643 | C | G | 2696.77 | SNP | Rv0764c (cyp51) | Gly132Ala | 21 | - | | 857696 | A | G | 2721.77 | SNP | Rv0764c (cyp51) | silent (Ala114) | 9867 | - | | 858464 | A | G | 2262.77 | SNP | Rv0765c | Val(s)134Ala | 9867 | - | | 872193 | GCGGAAACCACC | G | 6658.06 | DEL | Rv0778 (cyp126) |  |  | - | | 874835 | C | CCG | 6911.73 | INS | Rv0781 (ptrBa); Rv0782 (ptrBb) |  |  | - | | 876141 | G | T | 2652.77 | SNP | Rv0782 (ptrBb) | silent (Thr470) | 9871 | - | | 880562 | G | T | 2182.77 | SNP | Rv0785 | Cys408Phe | 0 | - | | 882257 | T | C | 2954.77 | SNP | Rv0787 | Tyr267His | 4 | - | | 888774 | G | A | 688.77 | SNP | intergenic |  |  | - | | 893733 | T | G | 1665.77 | SNP | Rv0800 (pepC) | Leu139Arg | 1 | - | | 894060 | A | G | 83.77 | SNP | Rv0800 (pepC) | Asp248Gly | 11 | - | | 896979 | T | C | 1783.77 | SNP | Rv0803 (purL) | Val387Ala | 18 | - | | 900221 | T | C | 2125.77 | SNP | Rv0806c (cpsY) | Val370Val(s) | 18 | - | | 903537 | G | T | 2492.77 | SNP | Rv0808 (purF) | Arg476Leu | 1 | - | | 903550 | T | C | 1815.77 | SNP | Rv0808 (purF) | silent (Ala480) | 9867 | - | | 903913 | T | C | 2007.77 | SNP | Rv0809 (purM) | silent (Gly63) | 9935 | - | | 906742 | T | C | 2555.77 | SNP | Rv0812 | Val107Ala | 18 | - | | 906857 | A | G | 2294.77 | SNP | Rv0812 | Ile145Met(s) | 6 | - | | 913918 | C | G | 1590.77 | SNP | Rv0821c (phoY2) | silent (Gly94) | 9935 | - | | 919551 | C | T | 2894.77 | SNP | Rv0825c | Val(s)1Val | 13 | - | | 921813 | C | G | 2485.77 | SNP | Rv0829 | Ala80Gly | 21 | - | | 921909 | C | T | 3480.77 | SNP | intergenic |  |  | - | | 925453 | C | T | 142.03 | SNP | Rv0833 (PE\_PGRS13) | silent (Ala31) | 9867 | - | | 927385 | A | G | 46.74 | SNP | Rv0833 (PE\_PGRS13) | silent (Gly675) | 9935 | - | | 931123 | T | C | 2714.77 | SNP | Rv0835 (lpqQ) | silent (Tyr57) | 9945 | genotype | | 931240 | G | T | 2860.77 | SNP | Rv0835 (lpqQ) | Trp96Cys | 0 | - | | 932280 | T | C | 1794.77 | SNP | Rv0836c | STOP218Trp | 0 | - | | 934230 | C | G | 2133.77 | SNP | intergenic |  |  | - | | 934611 | G | T | 1537.77 | SNP | intergenic |  |  | - | | 936620 | CCCA | C | 4150.73 | DEL | Rv0840c (pip) |  |  | - | | 937640 | C | CG | 3862.73 | INS | Rv0841 |  |  | - | | 941845 | C | A | 2016.77 | SNP | Rv0845 | Ala219Glu | 10 | - | | 942616 | C | A | 1858.77 | SNP | intergenic |  |  | - | | 945214 | G | A | 1907.77 | SNP | Rv0848 (cysK2) | Gly93Ser | 16 | - | | 945238 | G | T | 2052.77 | SNP | Rv0848 (cysK2) | Ala101Ser | 28 | - | | 949535 | T | C | 1912.77 | SNP | Rv0853c (pdc) | silent (Ala528) | 9867 | - | | 950116 | C | G | 1862.77 | SNP | Rv0853c (pdc) | Ala335Pro | 13 | - | | 955524 | A | G | 2290.77 | SNP | Rv0859 (fadA) | Ser150Gly | 21 | - | | 957117 | T | C | 1628.77 | SNP | Rv0860 (fadB) | silent (Asp275) | 9859 | - | | 957306 | C | T | 1935.77 | SNP | Rv0860 (fadB) | silent (Ala338) | 9867 | - | | 958362 | C | G | 1090.77 | SNP | Rv0860 (fadB) | silent (Ala690) | 9867 | - | | 960367 | A | G | 1833.77 | SNP | Rv0862c | Leu(s)749Ser | 28 | - | | 964969 | T | C | 137.03 | SNP | Rv0867c (rpfA) | silent (Ala189) | 9867 | - | | 968426 | A | AGCCGGGTTG | 3953.73 | INS | Rv0872c (PE\_PGRS15) |  |  | - | | 969762 | C | G | 88.28 | SNP | Rv0872c (PE\_PGRS15) | silent (Gly161) | 9935 | - | | 976897 | T | TGGG,TGGGG | 537.19 | MUL | Rv0878c (PPE13) |  |  | - | | 979704 | G | C | 2161.77 | SNP | Rv0881 | Gly115Arg | 0 | - | | 986463 | G | C | 3531.77 | SNP | intergenic |  |  | - | | 990001 | G | C | 2872.77 | SNP | Rv0890c | Pro866Ala | 22 | - | | 990850 | C | A | 2501.77 | SNP | Rv0890c | Ala583Ser | 28 | - | | 991939 | G | A | 1846.77 | SNP | Rv0890c | Arg220Cys | 1 | - | | 993346 | A | C | 3760.77 | SNP | Rv0891c | Val37Gly | 5 | - | | 996263 | G | A | 4934.77 | SNP | Rv0893c | silent (Thr11) | 9871 | - | | 1002172 | G | A | 1420.77 | SNP | Rv0897c | Arg82Trp | 2 | - | | 1007198 | A | G | 727.77 | SNP | Rv0904c (accD3) | Leu328Pro | 2 | - | | 1008460 | C | T | 2058.77 | SNP | Rv0905 (echA6) | Ser85Phe | 2 | - | | 1009500 | C | T | 2009.77 | SNP | Rv0906 | Pro186Leu | 3 | - | | 1010204 | C | CG | 4024.73 | INS | Rv0907 |  |  | - | | 1012815 | C | G | 999.77 | SNP | Rv0908 (ctpE) | Ala362Gly | 21 | - | | 1023911 | C | G | 2404.77 | SNP | intergenic |  |  | - | | 1024346 | A | G | 1447.77 | SNP | Rv0918 | Ser46Gly | 21 | - | | 1025106 | T | C | 3910.77 | SNP | Rv0919 | silent (Phe141) | 9946 | - | | 1026482 | G | A | 1873.77 | SNP | Rv0920c | Pro112Leu | 3 | - | | 1029586 | G | A | 1666.77 | SNP | Rv0923c | Pro331Leu | 3 | - | | 1034381 | G | A | 1355.77 | SNP | Rv0927c | Ala84Val | 13 | - | | 1037012 | T | C | 1545.77 | SNP | Rv0930 (pstA1) | Met(s)5Thr | 22 | - | | 1037911 | C | T | 2864.77 | SNP | Rv0930 (pstA1) | Arg305STOP | 2 | - | | 1040600 | G | A | 2023.77 | SNP | Rv0932c (pstS2) | Pro150Leu | 3 | - | | 1047165 | T | C | 1570.77 | SNP | Rv0938 (ligD) | Cys344Arg | 1 | - | | 1048225 | C | T | 2548.77 | SNP | Rv0938 (ligD) | Pro697Leu | 3 | - | | 1054784 | C | G | 2076.77 | SNP | Rv0945 | Arg180Gly | 1 | - | | 1068151 | T | C | 2588.77 | SNP | Rv0956 (purN) | silent (His197) | 9912 | - | | 1068432 | A | G | 1568.77 | SNP | Rv0957 (purH) | silent (Pro76) | 9926 | - | | 1070702 | T | C | 1734.77 | SNP | Rv0958 | Ser274Pro | 12 | - | | 1071349 | G | C | 1292.77 | SNP | Rv0959 | Gly32Ala | 21 | - | | 1072867 | C | T | 2756.77 | SNP | Rv0959 | Ala538Val | 13 | - | | 1074558 | G | A | 1704.77 | SNP | Rv0962c (lprP) | Pro186Leu | 3 | - | | 1075279 | T | C | 3040.77 | SNP | intergenic |  |  | - | | 1076309 | G | T | 1872.77 | SNP | Rv0964c | Pro124Thr | 5 | - | | 1077312 | A | G | 1781.77 | SNP | Rv0966c | Val(s)175Ala | 9867 | - | | 1079927 | C | A | 1619.77 | SNP | Rv0969 (ctpV) | silent (Thr395) | 9871 | - | | 1080192 | G | A | 1396.77 | SNP | Rv0969 (ctpV) | Asp484Asn | 36 | - | | 1081681 | T | C | 2189.77 | SNP | Rv0970 | silent (Val210) | 9901 | - | | 1084911 | G | A | 1756.77 | SNP | Rv0973c (accA2) | silent (Tyr280) | 9945 | genotype | | 1087193 | G | C | 2476.77 | SNP | Rv0974c (accD2) | Asn51Lys | 25 | - | | 1090188 | A | AG | 4228.73 | INS | intergenic |  |  | - | | 1093406 | A | G | 1592.77 | SNP | Rv0978c (PE\_PGRS17) | silent (Val317) | 9901 | - | | 1093928 | G | A | 31.74 | SNP | Rv0978c (PE\_PGRS17) | silent (Asn143) | 9822 | - | | 1095490 | A | G | 2707.77 | SNP | Rv0980c (PE\_PGRS18) | Val321Ala | 18 | - | | 1095644 | C | T | 290.53 | SNP | Rv0980c (PE\_PGRS18) | Ala270Thr | 22 | - | | 1095678 | A | G | 341.77 | SNP | Rv0980c (PE\_PGRS18) | silent (Gly258) | 9935 | - | | 1096633 | T | G | 2088.77 | SNP | intergenic |  |  | - | | 1097220 | C | T | 1754.77 | SNP | Rv0981 (mprA) | silent (Ser133) | 9840 | - | | 1098523 | T | A | 1753.77 | SNP | Rv0982 (mprB) | Leu339His | 1 | - | | 1099058 | G | A | 1583.77 | SNP | intergenic |  |  | - | | 1100234 | T | C | 1741.77 | SNP | Rv0983 (pepD) | Leu390Pro | 2 | - | | 1104690 | T | G | 3918.77 | SNP | Rv0987 | Phe717Val | 1 | - | | 1106099 | C | T | 3059.77 | SNP | Rv0988 | silent (Ile328) | 9872 | - | | 1106422 | T | C | 5541.77 | SNP | Rv0989c (grcC2) | Ile321Val | 57 | - | | 1107940 | A | C | 1337.77 | SNP | Rv0990c | Ser54Ala | 35 | - | | 1109975 | A | G | 2525.77 | SNP | Rv0993 (galU) | Gln235Arg | 10 | - | | 1110721 | T | C | 1595.77 | SNP | Rv0994 (moeA1) | silent (Arg151) | 9913 | - | | 1111852 | G | T | 2378.77 | SNP | Rv0995 (rimJ) | Asp81Tyr | 0 | - | | 1113733 | C | A | 2374.77 | SNP | intergenic |  |  | - | | 1117578 | T | C | 1968.77 | SNP | Rv1001 (arcA) | Ser132Pro | 12 | - | | 1119984 | G | A | 1499.77 | SNP | intergenic |  |  | - | | 1121663 | GGGGATGCCGATGCCC | G | 1976.73 | DEL | Rv1004c |  |  | - | | 1126889 | G | C | 1733.77 | SNP | Rv1007c (metS) | Arg39Gly | 1 | - | | 1127648 | C | A | 2468.77 | SNP | Rv1008 (tatD) | Thr187Asn | 9 | - | | 1139089 | G | C | 2138.77 | SNP | Rv1020 (mfd) | silent (Thr41) | 9871 | - | | 1144409 | T | G | 1684.77 | SNP | Rv1022 (lpqU) | Ile225Ser | 2 | - | | 1144585 | A | G | 2199.77 | SNP | Rv1023 (eno) | Arg8Gly | 1 | - | | 1148259 | A | G | 2294.77 | SNP | intergenic |  |  | - | | 1149551 | C | T | 1784.77 | SNP | Rv1028c (kdpD) | silent (Glu712) | 9865 | - | | 1150585 | G | A | 1469.77 | SNP | Rv1028c (kdpD) | Pro368Ser | 17 | - | | 1151054 | C | G | 2254.77 | SNP | Rv1028c (kdpD) | silent (Ser211) | 9840 | - | | 1152805 | T | A | 2552.77 | SNP | Rv1029 (kdpA) | Leu265Gln | 3 | - | | 1152863 | A | G | 2320.77 | SNP | Rv1029 (kdpA) | silent (Gln284) | 9876 | - | | 1153388 | C | T | 2489.77 | SNP | Rv1029 (kdpA) | silent (Asn459) | 9822 | - | | 1157010 | A | G | 2315.77 | SNP | Rv1032c (trcS) | Tyr316His | 4 | - | | 1160270 | A | G | 2421.77 | SNP | Rv1036c | Val(s)55Ala | 9867 | - | | 1160660 | G | A | 1265.77 | SNP | Rv1037c (esxI) | Leu57Leu(s) | 4 | - | | 1160738 | T | C | 1064.77 | SNP | Rv1037c (esxI) | Ile31Val | 57 | - | | 1160761 | G | A | 981.77 | SNP | Rv1037c (esxI) | Ser23Leu(s) | 35 | - | | 1160770 | T | A | 879.77 | SNP | Rv1037c (esxI) | Gln20Leu | 6 | - | | 1160790 | G | A | 935.77 | SNP | Rv1037c (esxI) | silent (His13) | 9912 | - | | 1163134 | T | C | 3074.77 | SNP | Rv1040c (PE8) | silent (Gly81) | 9935 | - | | 1164336 | G | A | 1854.77 | SNP | intergenic |  |  | - | | 1164571 | A | G | 1550.77 | SNP | intergenic |  |  | - | | 1165521 | T | TA | 2795.73 | INS | intergenic |  |  | - | | 1168715 | C | CT | 2913.73 | INS | Rv1046c |  |  | - | | 1173750 | G | T | 1733.77 | SNP | Rv1050 | silent (Ala290) | 9867 | - | | 1177184 | C | A | 2980.77 | SNP | Rv1054 | Ala86Glu | 10 | - | | 1178116 | T | C | 3169.77 | SNP | Rv1056 | silent (Thr163) | 9871 | - | | 1188985 | G | A | 298.28 | SNP | Rv1067c (PE\_PGRS19) | silent (Gly480) | 9935 | - | | 1189196 | CCGCCGT | C | 1025.73 | DEL | Rv1067c (PE\_PGRS19) |  |  | - | | 1189606 | A | G | 133.77 | SNP | Rv1067c (PE\_PGRS19) | silent (Gly273) | 9935 | - | | 1189613 | G | A | 103.77 | SNP | Rv1067c (PE\_PGRS19) | Ala271Val | 13 | - | | 1190093 | A | C | 862.77 | SNP | Rv1067c (PE\_PGRS19) | Leu(s)111Trp | 0 | - | | 1191462 | A | C | 32.74 | SNP | Rv1068c (PE\_PGRS20) | silent (Gly229) | 9935 | - | | 1191497 | T | A | 47.28 | SNP | Rv1068c (PE\_PGRS20) | Thr218Ser | 38 | - | | 1191741 | G | A | 654.77 | SNP | Rv1068c (PE\_PGRS20) | silent (Tyr136) | 9945 | - | | 1191817 | A | C | 934.77 | SNP | Rv1068c (PE\_PGRS20) | Leu(s)111Trp | 0 | - | | 1193463 | T | A | 2069.77 | SNP | Rv1069c | Thr271Ser | 38 | - | | 1200418 | A | G | 2352.77 | SNP | intergenic |  |  | - | | 1211369 | A | C | 2591.77 | SNP | Rv1086 | Ser259Arg | 6 | - | | 1212825 | T | C | 85.28 | SNP | Rv1087 (PE\_PGRS21) | silent (Gly422) | 9935 | - | | 1212827 | T | G | 86.28 | SNP | Rv1087 (PE\_PGRS21) | Val423Gly | 5 | - | | 1212834 | T | G | 47.74 | SNP | Rv1087 (PE\_PGRS21) | silent (Gly425) | 9935 | - | | 1212837 | T | C | 62.28 | SNP | Rv1087 (PE\_PGRS21) | silent (Ala426) | 9867 | - | | 1212971 | T | C | 247.80 | SNP | Rv1087 (PE\_PGRS21) | Val471Ala | 18 | - | | 1215104 | AT | A | 4551.73 | DEL | intergenic |  |  | - | | 1220680 | T | C | 2163.77 | SNP | Rv1093 (glyA1) | Val36Ala | 18 | - | | 1224367 | T | C | 977.77 | SNP | intergenic |  |  | - | | 1227830 | G | T | 2450.77 | SNP | Rv1099c (glpX) | silent (Thr285) | 9871 | - | | 1230778 | G | A | 2864.77 | SNP | Rv1102c (mazF3) | Thr65Ile | 7 | - | | 1236433 | A | C | 2622.77 | SNP | Rv1110 (lytB2) | Glu83Asp | 53 | - | | 1240744 | C | A | 2156.77 | SNP | Rv1115 | Asn186Lys | 25 | - | | 1241572 | A | G | 3928.77 | SNP | intergenic |  |  | - | | 1247391 | A | C | 2064.77 | SNP | Rv1124 (ephC) | Ser89Arg | 6 | - | | 1248382 | A | G | 1638.77 | SNP | Rv1125 | Ser101Gly | 21 | - | | 1248936 | G | C | 2952.77 | SNP | Rv1125 | silent (Pro285) | 9926 | - | | 1248978 | T | C | 2713.77 | SNP | Rv1125 | silent (Ala299) | 9867 | - | | 1250340 | A | G | 2836.77 | SNP | Rv1127c (ppdK) | silent (Ala355) | 9867 | - | | 1251199 | C | T | 2344.77 | SNP | Rv1127c (ppdK) | Gly69Glu | 4 | - | | 1251384 | G | A | 2835.77 | SNP | Rv1127c (ppdK) | silent (Ala7) | 9867 | - | | 1252164 | T | C | 2104.77 | SNP | Rv1128c | Glu270Gly | 7 | - | | 1254562 | A | G | 2189.77 | SNP | Rv1130 (prpD) | Asp3Gly | 11 | - | | 1258044 | G | GA | 5763.73 | INS | Rv1132 |  |  | - | | 1260342 | C | G | 1455.77 | SNP | Rv1133c (metE) | silent (Ser335) | 9840 | - | | 1262859 | C | T | 1410.77 | SNP | Rv1135c (PPE16) | Gly424Ser | 16 | - | | 1265828 | G | A | 1957.77 | SNP | Rv1138c | Leu221Leu(s) | 4 | - | | 1269028 | G | C | 2594.77 | SNP | intergenic |  |  | - | | 1271187 | C | G | 1759.77 | SNP | Rv1144 | Thr11Ser | 38 | - | | 1273250 | G | GA | 4802.73 | INS | Rv1145 (mmpL13a) |  |  | - | | 1275957 | T | C | 2505.77 | SNP | intergenic |  |  | - | | 1276432 | C | T | 2019.77 | SNP | Rv1148c | silent (Lys439) | 9926 | - | | 1276588 | C | G | 1773.77 | SNP | Rv1148c | silent (Ala387) | 9867 | - | | 1277869 | G | GT | 3730.73 | INS | intergenic |  |  | - | | 1281118 | T | C | 2013.77 | SNP | Rv1154c | Thr123Ala | 32 | - | | 1281771 | T | C | 1340.77 | SNP | Rv1155 | Ser115Pro | 12 | - | | 1281984 | G | A | 1429.77 | SNP | intergenic |  |  | - | | 1287372 | G | C | 2772.77 | SNP | Rv1161 (narG) | silent (Leu15) | 9947 | - | | 1292102 | A | G | 1697.77 | SNP | Rv1162 (narH) | silent (Pro346) | 9926 | - | | 1306259 | A | G | 1847.77 | SNP | Rv1175c (fadH) | silent (Ala656) | 9867 | - | | 1307598 | C | G | 1487.77 | SNP | Rv1175c (fadH) | Cys210Ser | 11 | - | | 1308317 | T | C | 2601.77 | SNP | Rv1176c | His159Arg | 10 | - | | 1313337 | A | AG | 2306.73 | INS | intergenic |  |  | - | | 1313338 | A | C | 1280.77 | SNP | intergenic |  |  | - | | 1315191 | A | C | 2123.77 | SNP | Rv1180 (pks3) | STOP489Tyr | 1 | - | | 1315884 | G | A | 2393.77 | SNP | Rv1181 (pks4) | silent (Ala217) | 9867 | - | | 1325650 | G | A | 2858.77 | SNP | intergenic |  |  | - | | 1327890 | G | A | 2859.77 | SNP | Rv1186c | silent (Asp472) | 9859 | - | | 1328687 | G | C | 1773.77 | SNP | Rv1186c | Pro207Ala | 22 | - | | 1334340 | G | A | 809.77 | SNP | Rv1191 | Arg137His | 8 | - | | 1339774 | C | G | 1428.77 | SNP | Rv1196 (PPE18) | silent (Gly142) | 9935 | - | | 1339823 | A | G | 1472.77 | SNP | Rv1196 (PPE18) | Thr159Ala | 32 | - | | 1339894 | C | T | 1450.77 | SNP | Rv1196 (PPE18) | silent (Leu182) | 9947 | - | | 1339905 | C | T | 1428.77 | SNP | Rv1196 (PPE18) | Ala186Val | 13 | - | | 1340500 | G | T | 1574.77 | SNP | Rv1196 (PPE18) | Met(s)384Ile | 2 | - | | 1340652 | AG | A | 2297.73 | DEL | intergenic |  |  | - | | 1340657 | A | G | 1415.77 | SNP | intergenic |  |  | - | | 1340667 | A | G | 1332.77 | SNP | Rv1197 (esxK) | silent (Ser3) | 9840 | - | | 1340781 | C | G | 51.77 | SNP | Rv1197 (esxK) | silent (Ser41) | 9840 | - | | 1340784 | T | C | 1358.77 | SNP | Rv1197 (esxK) | silent (Gly42) | 9935 | - | | 1341295 | A | G | 2338.77 | SNP | intergenic |  |  | - | | 1345016 | C | T | 1585.77 | SNP | Rv1201c (dapD) | Ala52Thr | 22 | - | | 1351172 | A | G | 2461.77 | SNP | intergenic |  |  | - | | 1360209 | T | C | 2209.77 | SNP | Rv1217c | silent (Ala531) | 9867 | - | | 1361190 | G | A | 1322.77 | SNP | Rv1217c | silent (Ser204) | 9840 | - | | 1362006 | T | C | 3542.77 | SNP | Rv1218c | Gln243Arg | 10 | - | | 1365837 | C | CGG | 2960.73 | INS | intergenic |  |  | - | | 1367484 | T | G | 2258.77 | SNP | Rv1224 (tatB) | Trp8Gly | 0 | - | | 1371470 | G | A | 2705.77 | SNP | Rv1228 (lpqX) | Arg184His | 8 | - | | 1374065 | T | C | 1024.77 | SNP | Rv1230c | Ser45Gly | 21 | - | | 1375724 | A | C | 2134.77 | SNP | Rv1232c | Cys149Gly | 1 | - | | 1377568 | A | G | 1452.77 | SNP | Rv1235 (lpqY) | Val15Val(s) | 18 | - | | 1378725 | T | C | 1896.77 | SNP | Rv1235 (lpqY) | Phe401Ser | 3 | - | | 1382628 | T | C | 2636.77 | SNP | Rv1239c (corA) | Lys139Glu | 4 | - | | 1386010 | GC | G | 196.77 | DEL | Rv1243c (PE\_PGRS23) |  |  | - | | 1390763 | C | T | 2681.77 | SNP | Rv1248c | Val(s)764Met(s) | 9867 | - | | 1393626 | A | G | 2184.77 | SNP | Rv1249c | silent (Leu119) | 9947 | - | | 1396618 | C | A | 1672.77 | SNP | Rv1251c | Glu875STOP | 17 | - | | 1396922 | T | C | 1931.77 | SNP | Rv1251c | silent (Thr773) | 9871 | - | | 1401033 | C | T | 2880.77 | SNP | Rv1253 (deaD) | Ser355Leu(s) | 35 | - | | 1411210 | T | G | 1598.77 | SNP | Rv1263 (amiB2) | Val260Val(s) | 18 | - | | 1413148 | C | T | 2030.77 | SNP | intergenic |  |  | - | | 1414021 | C | T | 2254.77 | SNP | Rv1266c (pknH) | Arg607Gln | 9 | - | | 1416222 | A | G | 343.77 | SNP | Rv1267c (embR) | Phe376Leu | 13 | - | | 1416232 | A | G | 352.77 | SNP | Rv1267c (embR) | silent (Cys372) | 9973 | - | | 1416234 | A | C | 378.77 | SNP | Rv1267c (embR) | Cys372Gly | 1 | - | | 1422666 | C | T | 1639.77 | SNP | Rv1273c | Gly462Glu | 4 | - | | 1422667 | C | T | 1567.77 | SNP | Rv1273c | Gly462Arg | 0 | - | | 1423435 | C | G | 2107.77 | SNP | Rv1273c | Val(s)206Leu | 3 | - | | 1430132 | G | A | 2818.77 | SNP | Rv1279 | Ser24Asn | 20 | - | | 1440090 | C | T | 2227.77 | SNP | Rv1286 (cysN) | Thr395Ile | 7 | - | | 1440469 | C | G | 2805.77 | SNP | Rv1286 (cysN) | silent (Pro521) | 9926 | - | | 1441545 | G | A | 275.77 | SNP | Rv1288 | silent (Ala66) | 9867 | - | | 1441551 | G | T | 86.77 | SNP | Rv1288 | silent (Ala68) | 9867 | - | | 1441552 | T | G | 63.77 | SNP | Rv1288 | Leu(s)69Val(s) | 9867 | - | | 1441553 | T | C | 59.77 | SNP | Rv1288 | Leu(s)69Ser | 28 | - | | 1441576 | T | C | 283.78 | SNP | Rv1288 | Leu(s)77Leu | 3 | - | | 1441579 | A | T | 285.78 | SNP | Rv1288 | Asn78Tyr | 3 | - | | 1441582 | T | C | 301.78 | SNP | Rv1288 | Trp79Arg | 8 | - | | 1443428 | G | A | 114.77 | SNP | intergenic |  |  | - | | 1445781 | A | G | 2581.77 | SNP | Rv1291c | silent (Ala18) | 9867 | - | | 1450316 | C | T | 1979.77 | SNP | Rv1294 (thrA) | silent (Ala314) | 9867 | - | | 1457144 | C | T | 1484.77 | SNP | Rv1300 (hemK) | Arg194Cys | 1 | - | | 1468208 | A | C | 1626.77 | SNP | Rv1313c | Leu433Arg | 1 | - | | 1471659 | C | T | 3015.77 | SNP | intergenic |  |  | - | | 1478357 | G | T | 1681.77 | SNP | Rv1317c (alkA) | silent (Pro254) | 9926 | - | | 1479085 | T | C | 1661.77 | SNP | Rv1317c (alkA) | Ile12Val | 57 | - | | 1480174 | C | G | 69.77 | SNP | Rv1318c | silent (Leu217) | 9947 | - | | 1480945 | C | G | 3430.77 | SNP | Rv1319c | silent (Thr519) | 9871 | - | | 1480948 | C | T | 3223.52 | SNP | Rv1319c | silent (Glu518) | 9865 | - | | 1480972 | T | C | 1528.77 | SNP | Rv1319c | silent (Glu510) | 9865 | - | | 1481185 | A | C | 2886.77 | SNP | Rv1319c | Asp439Glu | 56 | - | | 1481563 | C | T | 3138.77 | SNP | Rv1319c | silent (Glu313) | 9865 | - | | 1482627 | T | C | 1811.77 | SNP | Rv1320c | Thr531Ala | 32 | - | | 1484708 | A | C | 2869.77 | SNP | Rv1321 | Ser144Arg | 6 | - | | 1488428 | C | T | 189.77 | SNP | Rv1325c (PE\_PGRS24) | Gly513Asp | 6 | - | | 1488433 | A | G | 112.77 | SNP | Rv1325c (PE\_PGRS24) | silent (Asp511) | 9859 | - | | 1488434 | T | G | 112.77 | SNP | Rv1325c (PE\_PGRS24) | Asp511Ala | 10 | - | | 1488435 | C | A | 58.77 | SNP | Rv1325c (PE\_PGRS24) | Asp511Tyr | 0 | - | | 1489142 | C | T | 313.78 | SNP | Rv1325c (PE\_PGRS24) | Gly275Asp | 6 | - | | 1490905 | A | G | 3448.77 | SNP | Rv1326c (glgB) | Ser470Pro | 12 | - | | 1495836 | G | A | 2169.77 | SNP | Rv1328 (glgP) | Val(s)425Met(s) | 9867 | - | | 1498951 | G | T | 1491.77 | SNP | Rv1329c (dinG) | Thr80Lys | 11 | - | | 1499212 | CTCAGGCCG | C | 6619.73 | DEL | intergenic |  |  | - | | 1499221 | G | A | 1464.77 | SNP | Rv1330c (pncB1) | Pro447Ser | 17 | - | | 1499274 | C | G | 1358.77 | SNP | Rv1330c (pncB1) | Gly429Ala | 21 | - | | 1501700 | G | A | 1225.77 | SNP | Rv1333 | Val(s)34Val | 13 | - | | 1513189 | C | T | 1541.77 | SNP | Rv1348 (irtA) | Ala48Val | 13 | - | | 1523575 | A | G | 4108.77 | SNP | Rv1355c (moeY) | Val153Ala | 18 | - | | 1526819 | C | A | 3008.77 | SNP | Rv1358 | silent (Arg70) | 9913 | - | | 1533241 | G | C | 1940.77 | SNP | Rv1361c (PPE19) | silent (Thr131) | 9871 | - | | 1533583 | G | A | 2125.77 | SNP | Rv1361c (PPE19) | silent (Tyr17) | 9945 | - | | 1533624 | A | C | 2726.77 | SNP | Rv1361c (PPE19) | Phe4Val | 1 | - | | 1534551 | C | T | 2115.77 | SNP | Rv1362c | silent (Glu20) | 9865 | - | | 1536251 | G | T | 2312.77 | SNP | Rv1364c | Ala465Glu | 10 | - | | 1540141 | T | C | 1876.77 | SNP | Rv1367c | Ile169Val | 57 | - | | 1540484 | C | G | 2509.77 | SNP | Rv1367c | silent (Leu54) | 9947 | - | | 1544255 | C | T | 4484.77 | SNP | Rv1371 | silent (Arg299) | 9913 | - | | 1546703 | C | T | 2530.77 | SNP | Rv1373 | Pro231Leu | 3 | - | | 1547125 | T | C | 2658.77 | SNP | Rv1374c | Thr136Ala | 32 | - | | 1552547 | G | A | 1417.77 | SNP | Rv1378c | Arg37Trp | 2 | - | | 1554216 | C | T | 2448.77 | SNP | Rv1381 (pyrC) | Pro10Leu | 3 | - | | 1555532 | T | C | 856.77 | SNP | Rv1382 | Leu19Pro | 2 | - | | 1556430 | T | G | 1560.77 | SNP | Rv1383 (carA) | Ser154Ala | 35 | - | | 1563717 | C | T | 1668.77 | SNP | Rv1388 (mihF) | silent (Val8) | 9901 | - | | 1568631 | A | C | 2261.77 | SNP | Rv1393c | silent (Thr319) | 9871 | - | | 1570566 | C | A | 2365.77 | SNP | Rv1394c (cyp132) | Arg135Leu | 1 | - | | 1572204 | C | T | 168.90 | SNP | Rv1396c (PE\_PGRS25) | Gly552Ser | 16 | - | | 1573660 | T | G | 1531.77 | SNP | Rv1396c (PE\_PGRS25) | Arg66Ser | 11 | - | | 1588899 | G | T | 1470.77 | SNP | Rv1412 (ribC) | silent (Ala111) | 9867 | - | | 1591545 | G | T | 2184.77 | SNP | Rv1415 (ribA2) | silent (Pro383) | 9926 | - | | 1592322 | C | T | 2419.77 | SNP | Rv1417 | Ala58Val | 13 | - | | 1593331 | G | A | 1722.77 | SNP | intergenic |  |  | - | | 1595342 | T | C | 1628.77 | SNP | Rv1420 (uvrC) | Val(s)434Ala | 9867 | - | | 1599557 | C | G | 2686.77 | SNP | Rv1424c | Arg33Thr | 2 | - | | 1608276 | A | C | 3298.77 | SNP | Rv1431 | Asn65Thr | 13 | - | | 1609840 | A | G | 1399.77 | SNP | Rv1431 | silent (Pro586) | 9926 | - | | 1612624 | T | TATCGGTACCGGTGCGCCAG GG | 6427.73 | INS | Rv1435c |  |  | - | | 1613035 | T | C | 2310.77 | SNP | intergenic |  |  | - | | 1614143 | G | A | 2096.77 | SNP | Rv1436 (gap) | silent (Leu279) | 9947 | - | | 1618978 | T | C | 337.77 | SNP | Rv1441c (PE\_PGRS26) | Asp236Gly | 11 | - | | 1624791 | C | G | 1394.77 | SNP | Rv1446c (opcA) | Arg192Pro | 5 | - | | 1627351 | T | C | 1554.77 | SNP | Rv1448c (tal) | Thr244Ala | 32 | - | | 1630148 | A | C | 2627.77 | SNP | Rv1449c (tkt) | Tyr18Asp | 0 | - | | 1634609 | C | T | 504.77 | SNP | Rv1450c (PE\_PGRS27) | Ala7Thr | 22 | - | | 1634610 | C | T | 481.77 | SNP | Rv1450c (PE\_PGRS27) | Val(s)6Val | 13 | - | | 1634633 | A | T | 622.77 | SNP | intergenic |  |  | - | | 1634636 | T | A | 593.77 | SNP | intergenic |  |  | - | | 1636826 | C | A | 272.78 | SNP | Rv1452c (PE\_PGRS28) | silent (Gly468) | 9935 | - | | 1636918 | C | T | 124.90 | SNP | Rv1452c (PE\_PGRS28) | Ala438Thr | 22 | - | | 1636927 | C | T | 105.77 | SNP | Rv1452c (PE\_PGRS28) | Asp435Asn | 36 | - | | 1636928 | A | G | 131.03 | SNP | Rv1452c (PE\_PGRS28) | silent (Gly434) | 9935 | - | | 1636934 | A | G | 128.10 | SNP | Rv1452c (PE\_PGRS28) | silent (His432) | 9912 | - | | 1636936 | G | T | 117.77 | SNP | Rv1452c (PE\_PGRS28) | His432Asn | 21 | - | | 1636944 | G | T | 115.90 | SNP | Rv1452c (PE\_PGRS28) | Ala429Asp | 6 | - | | 1636945 | C | G | 124.77 | SNP | Rv1452c (PE\_PGRS28) | Ala429Pro | 13 | - | | 1636946 | C | A | 113.77 | SNP | Rv1452c (PE\_PGRS28) | silent (Gly428) | 9935 | - | | 1636980 | G | T | 71.78 | SNP | Rv1452c (PE\_PGRS28) | Pro417His | 3 | - | | 1636981 | G | T | 91.77 | SNP | Rv1452c (PE\_PGRS28) | Pro417Thr | 5 | - | | 1636983 | C | G | 55.77 | SNP | Rv1452c (PE\_PGRS28) | Gly416Ala | 21 | - | | 1638182 | C | T | 1651.77 | SNP | Rv1452c (PE\_PGRS28) | silent (Ser16) | 9840 | - | | 1638183 | G | A | 1629.77 | SNP | Rv1452c (PE\_PGRS28) | Ser16Leu(s) | 35 | - | | 1638188 | C | T | 1624.77 | SNP | Rv1452c (PE\_PGRS28) | silent (Ala14) | 9867 | - | | 1638191 | C | G | 1708.77 | SNP | Rv1452c (PE\_PGRS28) | silent (Ala13) | 9867 | - | | 1638194 | G | C | 1655.77 | SNP | Rv1452c (PE\_PGRS28) | silent (Ala12) | 9867 | - | | 1639226 | C | T | 1837.77 | SNP | Rv1453 | silent (Phe282) | 9946 | - | | 1639594 | C | A | 1542.77 | SNP | Rv1453 | Pro405Gln | 6 | - | | 1645802 | T | C | 2790.77 | SNP | Rv1459c | Lys113Glu | 4 | - | | 1650072 | A | G | 2547.77 | SNP | Rv1462 | Asn183Asp | 42 | - | | 1650406 | C | A | 1826.77 | SNP | Rv1462 | Thr294Asn | 9 | - | | 1653374 | G | C | 1380.77 | SNP | Rv1466 | silent (Gly48) | 9935 | - | | 1658900 | C | T | 1989.77 | SNP | Rv1469 (ctpD) | silent (Asn646) | 9822 | - | | 1676290 | C | A | 2771.77 | SNP | Rv1486c | Lys198Asn | 13 | - | | 1676880 | A | G | 1998.77 | SNP | Rv1486c | Trp2Arg | 8 | - | | 1681147 | G | A | 2984.77 | SNP | intergenic |  |  | - | | 1688300 | T | C | 1701.77 | SNP | Rv1497 (lipL) | silent (Phe120) | 9946 | - | | 1689349 | C | T | 2310.77 | SNP | Rv1498c | Arg191His | 8 | - | | 1692141 | A | C | 4332.77 | SNP | Rv1501 | silent (Ile84) | 9872 | - | | 1692960 | T | C | 2783.77 | SNP | Rv1502 | Ser13Pro | 12 | - | | 1693561 | A | G | 5431.77 | SNP | Rv1502 | Tyr213Cys | 3 | - | | 1698911 | G | A | 3047.77 | SNP | Rv1508c | silent (Gly328) | 9935 | - | | 1699849 | G | A | 2747.77 | SNP | Rv1508c | Pro16Ser | 17 | - | | 1706119 | T | C | 3455.77 | SNP | Rv1514c | silent (Ser159) | 9840 | - | | 1708792 | T | C | 2249.77 | SNP | intergenic |  |  | - | | 1709432 | C | T | 3128.77 | SNP | Rv1517 | Leu188Phe | 6 | - | | 1711556 | G | C | 33.74 | SNP | Rv1520 | Asp177His | 3 | - | | 1713192 | A | G | 2790.77 | SNP | Rv1521 (fadD25) | Val297Val(s) | 18 | - | | 1713923 | A | G | 4214.77 | SNP | Rv1521 (fadD25) | His541Arg | 10 | - | | 1716472 | A | G | 2170.77 | SNP | Rv1522c (mmpL12) | Ser381Pro | 12 | - | | 1728161 | G | T | 3484.77 | SNP | Rv1527c (pks5) | silent (Gly83) | 9935 | - | | 1728837 | A | G | 2839.77 | SNP | intergenic |  |  | - | | 1735739 | T | C | 2620.77 | SNP | intergenic |  |  | - | | 1748439 | C | T | 3409.77 | SNP | Rv1547 (dnaE1) | Thr249Ile | 7 | - | | 1752561 | T | C | 1412.77 | SNP | Rv1548c (PPE21) | Asp258Gly | 11 | - | | 1753519 | G | GC | 4074.73 | INS | Rv1549 (fadD11.1) |  |  | - | | 1759252 | G | T | 2043.77 | SNP | Rv1552 (frdA) | silent (Ser524) | 9840 | genotype | | 1760292 | A | G | 1938.77 | SNP | Rv1554 (frdC) | Met(s)40Val(s) | 9867 | - | | 1760407 | T | G | 2228.77 | SNP | Rv1554 (frdC) | Leu78Arg | 1 | - | | 1761765 | G | A | 2269.77 | SNP | Rv1557 (mmpL6) | Gly8Ser | 16 | - | | 1763482 | G | A | 3190.85 | SNP | Rv1559 (ilvA) | Gly19Arg | 0 | - | | 1764225 | C | T | 1751.77 | SNP | Rv1559 (ilvA) | silent (Ala266) | 9867 | - | | 1778430 | T | C | 1422.77 | SNP | Rv1570 (bioD) | Met(s)191Thr | 22 | - | | 1779370 | G | C | 1644.77 | SNP | Rv1573 | silent (Thr19) | 9871 | - | | 1780586 | C | CG | 2730.73 | INS | Rv1575 |  |  | - | | 1781251 | C | T | 1110.77 | SNP | Rv1576c | Glu272Lys | 7 | - | | 1781811 | T | C | 1665.77 | SNP | Rv1576c | Asp85Gly | 11 | - | | 1784237 | GA | G | 2739.73 | DEL | Rv1581c |  |  | - | | 1788613 | C | T | 2082.77 | SNP | Rv1587c | Gly184Asp | 6 | - | | 1788627 | C | G | 1883.77 | SNP | Rv1587c | silent (Leu179) | 9947 | - | | 1788630 | C | G | 1734.77 | SNP | Rv1587c | Glu178Asp | 53 | - | | 1789650 | C | T | 269.78 | SNP | Rv1588c | Ala63Thr | 22 | - | | 1789654 | A | G | 377.77 | SNP | Rv1588c | silent (Leu61) | 9947 | - | | 1789671 | C | T | 548.77 | SNP | Rv1588c | Ala56Thr | 22 | - | | 1789675 | A | C | 573.77 | SNP | Rv1588c | silent (Gly54) | 9935 | - | | 1789678 | C | G | 523.77 | SNP | Rv1588c | Val(s)53Val | 13 | - | | 1789735 | C | T | 1190.77 | SNP | Rv1588c | silent (Pro34) | 9926 | - | | 1789933 | G | A | 1864.77 | SNP | intergenic |  |  | - | | 1792777 | T | C | 1855.77 | SNP | Rv1592c | Ile322Val | 57 | - | | 1792778 | T | C | 2026.77 | SNP | Rv1592c | silent (Glu321) | 9865 | - | | 1798355 | G | A | 1970.77 | SNP | Rv1597 | Gly21Asp | 6 | - | | 1802047 | C | G | 2875.77 | SNP | Rv1601 (hisB) | Gln5Glu | 35 | - | | 1803265 | G | A | 2340.77 | SNP | Rv1602 (hisH) | Ser201Asn | 20 | - | | 1803290 | A | C | 2439.77 | SNP | intergenic |  |  | - | | 1804409 | C | A | 2274.77 | SNP | Rv1604 (impA) | Pro124Gln | 6 | - | | 1811375 | G | T | 2601.77 | SNP | Rv1612 (trpB) | silent (Gly83) | 9935 | - | | 1812448 | C | T | 2273.77 | SNP | Rv1613 (trpA) | silent (Tyr30) | 9945 | - | | 1817976 | A | T | 2752.77 | SNP | Rv1618 (tesB1) | His121Leu | 4 | - | | 1818286 | C | T | 3558.77 | SNP | Rv1618 (tesB1) | silent (Gly224) | 9935 | - | | 1827468 | C | A | 2166.77 | SNP | Rv1625c (cya) | Val(s)160Leu(s) | 9867 | - | | 1832509 | C | G | 1848.77 | SNP | Rv1629 (polA) | silent (Thr615) | 9871 | - | | 1833025 | C | T | 1368.77 | SNP | Rv1629 (polA) | silent (Asp787) | 9859 | - | | 1836286 | G | C | 1224.77 | SNP | intergenic |  |  | - | | 1836417 | C | A | 1660.77 | SNP | Rv1632c | silent (Gly138) | 9935 | - | | 1839759 | G | C | 2227.77 | SNP | Rv1634 | Gly198Arg | 0 | - | | 1846020 | C | G | 2861.77 | SNP | Rv1638 (uvrA) | silent (Gly760) | 9935 | - | | 1847919 | C | G | 2742.77 | SNP | Rv1639c | silent (Thr180) | 9871 | - | | 1849609 | T | C | 1959.77 | SNP | Rv1640c (lysX) | silent (Arg809) | 9913 | - | | 1852877 | A | C | 1102.77 | SNP | Rv1641 (infC) | STOP202Ser | 28 | - | | 1854300 | T | C | 2014.77 | SNP | Rv1644 (tsnR) | Leu232Pro | 2 | - | | 1856617 | C | T | 3140.77 | SNP | Rv1646 (PE17) | Thr285Ile | 7 | - | | 1856777 | G | C | 2404.77 | SNP | Rv1647 | Ala2Pro | 13 | - | | 1859559 | C | A | 2796.77 | SNP | Rv1649 (pheS) | Ala276Asp | 6 | - | | 1862954 | GC | G | 2203.73 | DEL | Rv1651c (PE\_PGRS30) |  |  | - | | 1863584 | G | T | 763.77 | SNP | Rv1651c (PE\_PGRS30) | Thr600Asn | 9 | - | | 1864924 | A | ACCGCCGGCCCCGCCGTCGG GT | 1520.77 | INS | Rv1651c (PE\_PGRS30) |  |  | - | | 1873700 | G | A | 1708.77 | SNP | Rv1659 (argH) | silent (Leu354) | 9947 | - | | 1877326 | G | C | 1596.77 | SNP | Rv1661 (pks7) | Val(s)675Leu | 3 | - | | 1882348 | G | T | 1539.77 | SNP | Rv1662 (pks8) | Gln215His | 20 | - | | 1884110 | C | CGCCCCGTGCGATTTGCCGA GGG | 9112.73 | INS | Rv1662 (pks8) |  |  | - | | 1885772 | G | A | 1794.77 | SNP | Rv1662 (pks8) | Ala1357Thr | 22 | - | | 1894300 | G | GGTCTTGCCGC | 7121.73 | INS | Rv1668c |  |  | - | | 1894422 | A | G | 2271.77 | SNP | Rv1668c | silent (Asp307) | 9859 | - | | 1901493 | T | C | 2027.77 | SNP | Rv1676 | silent (Ser149) | 9840 | - | | 1907296 | G | C | 2419.77 | SNP | Rv1682 | silent (Ala298) | 9867 | - | | 1908598 | G | A | 1721.77 | SNP | Rv1683 | silent (Arg335) | 9913 | - | | 1915963 | G | A | 1792.77 | SNP | Rv1691 | silent (Arg5) | 9913 | - | | 1916137 | A | G | 1384.77 | SNP | Rv1691 | silent (Leu63) | 9947 | - | | 1917972 | A | G | 1142.77 | SNP | Rv1694 (tlyA) | silent (Leu11) | 9947 | - | | 1923908 | C | T | 2059.77 | SNP | Rv1699 (pyrG) | Thr27Ile | 7 | - | | 1925136 | G | A | 2369.77 | SNP | Rv1699 (pyrG) | Val(s)436Val | 13 | - | | 1926029 | T | C | 2567.77 | SNP | Rv1700 | Tyr150His | 4 | - | | 1927329 | C | A | 2818.77 | SNP | Rv1702c | Arg416Leu | 1 | - | | 1931179 | C | A | 1133.77 | SNP | Rv1704c (cycA) | Arg93Leu | 1 | - | | 1931718 | G | C | 1399.77 | SNP | Rv1705c (PPE22) | Leu313Val(s) | 4 | - | | 1933988 | G | A | 3626.77 | SNP | intergenic |  |  | - | | 1941617 | G | C | 1154.77 | SNP | Rv1713 (engA) | Asp444His | 3 | - | | 1944107 | A | G | 1480.77 | SNP | Rv1716 | Ser178Gly | 21 | - | | 1944402 | T | C | 1857.77 | SNP | Rv1716 | Val276Ala | 18 | - | | 1946999 | T | G | 2414.77 | SNP | intergenic |  |  | - | | 1950767 | T | C | 3607.77 | SNP | Rv1724c | silent (Lys95) | 9926 | - | | 1952743 | G | A | 1632.77 | SNP | Rv1726 | Val(s)298Met(s) | 9867 | - | | 1960284 | C | A | 1899.77 | SNP | Rv1733c | Gln68His | 20 | - | | 1967237 | C | A | 1721.77 | SNP | Rv1739c | Arg134Leu | 1 | - | | 1971725 | G | C | 748.77 | SNP | Rv1745c (idi) | silent (Arg89) | 9913 | - | | 1972901 | C | T | 1183.77 | SNP | Rv1746 (pknF) | Ala255Val(s) | 9867 | - | | 1977646 | G | A | 3095.77 | SNP | Rv1749c | silent (Ala80) | 9867 | - | | 1983135 | T | G | 83.77 | SNP | Rv1753c (PPE24) | silent (Pro547) | 9926 | - | | 1983313 | T | G | 1174.77 | SNP | Rv1753c (PPE24) | Asn488Thr | 13 | - | | 1986985 | C | G | 2266.77 | SNP | intergenic |  |  | - | | 1989057 | AGAGAGGACTTCATCGATGC G | A | 16641.73 | DEL | Rv1758 (cut1) |  |  | - | | 1989237 | T | C | 2301.77 | SNP | Rv1758 (cut1) | Phe66Leu | 13 | - | | 1991894 | G | A | 235.78 | SNP | Rv1759c (wag22) | silent (Gly228) | 9935 | - | | 1992323 | G | GCCCC | 633.73 | INS | Rv1759c (wag22) |  |  | - | | 1992751 | T | A | 2332.77 | SNP | intergenic |  |  | - | | 1993561 | T | C | 3395.77 | SNP | Rv1760 | Cys137Arg | 1 | - | | 1993808 | A | T | 2083.77 | SNP | Rv1760 | Glu219Val(s) | 17 | - | | 1995528 | A | G | 2317.77 | SNP | Rv1762c | silent (His105) | 9912 | - | | 2001811 | G | C | 945.77 | SNP | Rv1768 (PE\_PGRS31) | Gly400Arg | 0 | - | | 2003252 | C | T | 1932.77 | SNP | Rv1769 | silent (Ala209) | 9867 | - | | 2003827 | A | C | 2390.77 | SNP | Rv1769 | Asp401Ala | 10 | - | | 2010614 | G | A | 1854.77 | SNP | intergenic |  |  | - | | 2010880 | T | G | 3281.77 | SNP | Rv1777 (cyp144) | silent (Gly75) | 9935 | - | | 2011568 | G | C | 2848.77 | SNP | Rv1777 (cyp144) | Glu305Gln | 27 | - | | 2017026 | G | A | 1793.77 | SNP | Rv1781c (malQ) | Gln151STOP | 8 | - | | 2022868 | T | C | 1845.77 | SNP | Rv1783 (eccC5) | silent (Ser1204) | 9840 | - | | 2023628 | C | G | 1730.77 | SNP | Rv1785c (cyp143) | Gly334Ala | 21 | - | | 2025977 | C | G | 1766.77 | SNP | Rv1787 (PPE25) | Thr226Arg | 1 | - | | 2034676 | C | T | 3315.77 | SNP | Rv1796 (mycP5) | silent (Ile316) | 9872 | - | | 2035040 | G | A | 1862.77 | SNP | Rv1796 (mycP5) | Val438Ile | 33 | - | | 2043519 | G | A | 2162.77 | SNP | Rv1802 (PPE30) | Gly46Ser | 16 | - | | 2045310 | A | G | 1147.77 | SNP | Rv1803c (PE\_PGRS32) | silent (Ile511) | 9872 | - | | 2049065 | T | C | 1904.77 | SNP | intergenic |  |  | - | | 2049097 | G | C | 1841.77 | SNP | intergenic |  |  | - | | 2050822 | G | C | 1465.77 | SNP | Rv1808 (PPE32) | Gly301Ala | 21 | - | | 2051345 | G | A | 1502.77 | SNP | Rv1809 (PPE33) | Gly22Ser | 16 | - | | 2051746 | T | C | 1617.77 | SNP | Rv1809 (PPE33) | silent (Ala155) | 9867 | - | | 2052035 | G | T | 2875.77 | SNP | Rv1809 (PPE33) | Val(s)252Leu(s) | 9867 | - | | 2055271 | A | G | 1480.77 | SNP | Rv1812c | Leu30Pro | 2 | - | | 2055746 | C | T | 1987.77 | SNP | Rv1813c | Ala123Thr | 22 | - | | 2056184 | C | T | 2968.77 | SNP | intergenic |  |  | - | | 2057774 | A | T | 1812.77 | SNP | Rv1815 | Ile83Phe | 8 | - | | 2058949 | GC | G | 2609.73 | DEL | Rv1816 |  |  | - | | 2059654 | C | T | 1923.77 | SNP | Rv1817 | silent (Asp20) | 9859 | - | | 2061433 | T | TCCGCCGGCG | 743.82 | INS | Rv1818c (PE\_PGRS33) |  |  | - | | 2061958 | A | G | 156.90 | SNP | Rv1818c (PE\_PGRS33) | silent (Ala239) | 9867 | - | | 2062440 | C | T | 583.77 | SNP | Rv1818c (PE\_PGRS33) | Ala79Thr | 22 | - | | 2074754 | C | T | 1698.77 | SNP | intergenic |  |  | - | | 2084697 | G | T | 1676.77 | SNP | intergenic |  |  | - | | 2087652 | C | T | 2142.77 | SNP | Rv1838c (vapC13); Rv1839c (vapB13) | Val(s)1Met(s); silent (Arg87) | 9867; 9913 | - | | 2094911 | ACAGCGT | A | 5975.73 | DEL | Rv1844c (gnd1) |  |  | - | | 2096186 | A | G | 1962.77 | SNP | Rv1846c (blaI) | silent (Thr138) | 9871 | - | | 2097480 | G | A | 2328.77 | SNP | Rv1848 (ureA) | Glu45Lys | 7 | - | | 2108141 | T | C | 1170.77 | SNP | Rv1860 (apa) | Phe136Leu | 13 | - | | 2108890 | A | C | 1837.77 | SNP | intergenic |  |  | - | | 2109523 | C | CG | 2504.73 | INS | intergenic |  |  | - | | 2110074 | C | T | 1589.77 | SNP | Rv1862 (adhA) | silent (Thr177) | 9871 | - | | 2116903 | C | T | 1984.77 | SNP | Rv1867 | silent (Gly380) | 9935 | - | | 2118846 | C | T | 1764.77 | SNP | Rv1868 | silent (Gly500) | 9935 | - | | 2118936 | G | A | 1601.77 | SNP | Rv1868 | silent (Pro530) | 9926 | - | | 2122976 | C | G | 1819.77 | SNP | Rv1872c (lldD2) | Gly59Ala | 21 | - | | 2123169 | T | G | 3050.77 | SNP | intergenic |  |  | - | | 2128870 | A | G | 1624.77 | SNP | Rv1878 (glnA3) | silent (Leu283) | 9947 | - | | 2133468 | T | TTCGCATGCCGTCACC | 1908.73 | INS | Rv1883c |  |  | - | | 2135870 | T | C | 2275.77 | SNP | intergenic |  |  | - | | 2137521 | A | ACTCCGATCAC | 11833.73 | INS | Rv1888c |  |  | - | | 2138453 | C | T | 2621.77 | SNP | Rv1888A | silent (Gly55) | 9935 | - | | 2142250 | C | T | 1833.77 | SNP | intergenic |  |  | - | | 2143327 | CG | C | 4672.73 | DEL | Rv1895 |  |  | - | | 2147022 | A | C | 3208.76 | SNP | Rv1900c (lipJ) | Ile204Met(s) | 6 | - | | 2151780 | G | T | 1585.77 | SNP | Rv1905c (aao) | Gln206Lys | 12 | - | | 2153184 | T | G | 2422.77 | SNP | intergenic |  |  | - | | 2154724 | C | A | 1620.77 | SNP | Rv1908c (katG) | Arg463Leu | 1 | genotype | | 2155168 | C | G | 1576.77 | SNP | Rv1908c (katG) | Ser315Thr | 32 | resistance | | 2158109 | T | C | 2091.77 | SNP | Rv1912c (fadB5) | Asp328Gly | 11 | - | | 2158905 | C | A | 2238.77 | SNP | Rv1912c (fadB5) | Gly63STOP | 21 | - | | 2160998 | G | A | 2094.77 | SNP | Rv1915 (aceAa) | Gly179Asp | 6 | - | | 2161343 | G | GT | 1566.73 | INS | Rv1915 (aceAa) |  |  | - | | 2163375 | T | C | 1139.77 | SNP | Rv1917c (PPE34) | Asn1313Asp | 42 | - | | 2163400 | A | G | 347.77 | SNP | Rv1917c (PPE34) | silent (Gly1304) | 9935 | - | | 2163403 | A | G | 245.77 | SNP | Rv1917c (PPE34) | silent (Pro1303) | 9926 | - | | 2163412 | A | G | 453.77 | SNP | Rv1917c (PPE34) | silent (Val1300) | 9901 | - | | 2163415 | C | A | 654.77 | SNP | Rv1917c (PPE34) | silent (Pro1299) | 9926 | - | | 2163417 | G | C | 547.77 | SNP | Rv1917c (PPE34) | Pro1299Ala | 22 | - | | 2163419 | C | T | 314.77 | SNP | Rv1917c (PPE34) | Ser1298Asn | 20 | - | | 2163421 | C | G | 384.77 | SNP | Rv1917c (PPE34) | silent (Thr1297) | 9871 | - | | 2163444 | T | C | 498.77 | SNP | Rv1917c (PPE34) | Asn1290Asp | 42 | - | | 2163790 | A | C | 1660.77 | SNP | Rv1917c (PPE34) | silent (Pro1174) | 9926 | - | | 2164095 | T | C | 3979.77 | SNP | Rv1917c (PPE34) | Asn1073Asp | 42 | - | | 2165286 | A | C | 2010.77 | SNP | Rv1917c (PPE34) | Ser676Ala | 35 | - | | 2165503 | T | A | 1742.77 | SNP | Rv1917c (PPE34) | silent (Ala603) | 9867 | - | | 2165938 | C | T | 774.77 | SNP | Rv1917c (PPE34) | silent (Pro458) | 9926 | - | | 2167310 | A | G | 2389.77 | SNP | Rv1917c (PPE34) | Met(s)1Thr | 22 | - | | 2167926 | A | G | 1801.77 | SNP | Rv1918c (PPE35) | Leu(s)896Ser | 28 | - | | 2170461 | C | T | 1655.77 | SNP | Rv1918c (PPE35) | Gly51Glu | 4 | - | | 2172526 | A | C | 2370.77 | SNP | Rv1921c (lppF) | STOP424Gly | 21 | - | | 2173860 | A | C | 1349.77 | SNP | intergenic |  |  | - | | 2187587 | G | C | 2468.77 | SNP | Rv1936 | Gln68His | 20 | - | | 2195888 | G | A | 1954.77 | SNP | Rv1944c | Pro16Leu | 3 | - | | 2199052 | C | G | 2379.77 | SNP | Rv1948c | Gly5Arg | 0 | - | | 2205371 | A | C | 2094.77 | SNP | Rv1962A (vapB35) | Val(s)60Gly | 21 | - | | 2207591 | T | TC | 5366.73 | INS | intergenic |  |  | - | | 2209465 | G | A | 1519.77 | SNP | Rv1966 (mce3A) | Ala47Thr | 22 | - | | 2211826 | A | G | 975.77 | SNP | Rv1968 (mce3C) | silent (Lys67) | 9926 | - | | 2216345 | C | A | 1931.77 | SNP | Rv1971 (mce3F) | silent (Pro363) | 9926 | - | | 2216443 | C | A | 1844.77 | SNP | Rv1971 (mce3F) | Ala396Glu | 10 | - | | 2220512 | T | G | 2172.77 | SNP | Rv1977 | silent (Ser253) | 9840 | - | | 2220940 | CGCCACGAT | C | 9073.73 | DEL | Rv1978 |  |  | - | | 2221584 | G | C | 2721.77 | SNP | Rv1978 | Ser226Thr | 32 | - | | 2223293 | T | C | 2977.77 | SNP | intergenic |  |  | - | | 2228967 | A | G | 1426.77 | SNP | intergenic |  |  | - | | 2229801 | C | G | 2515.77 | SNP | Rv1985c | silent (Pro34) | 9926 | - | | 2233136 | G | C | 2184.77 | SNP | Rv1989c | Pro55Arg | 4 | - | | 2235169 | GACAATCCGACGTTTTGCAC CATGATCTGCCGCGAGCGCC GGGCATGGTCTAGGGCTTGG GGCA | G | 8632.73 | DEL | Rv1992c (ctpG) |  |  | - | | 2244421 | G | T | 2308.77 | SNP | Rv1999c | Leu240Met(s) | 4 | - | | 2245916 | T | G | 2283.77 | SNP | Rv2000 | silent (Ala236) | 9867 | - | | 2247677 | A | C | 3888.77 | SNP | Rv2002 (fabG3) | silent (Ile6) | 9872 | - | | 2251999 | A | G | 2250.77 | SNP | intergenic |  |  | - | | 2260100 | C | T | 2997.77 | SNP | intergenic |  |  | - | | 2260151 | A | G | 758.77 | SNP | intergenic |  |  | - | | 2260154 | C | T | 651.77 | SNP | intergenic |  |  | - | | 2260171 | T | C | 1155.77 | SNP | intergenic |  |  | - | | 2260174 | C | T | 983.77 | SNP | intergenic |  |  | - | | 2260196 | C | CA | 1582.73 | INS | intergenic |  |  | - | | 2260199 | C | T | 917.77 | SNP | intergenic |  |  | - | | 2260212 | G | T | 780.77 | SNP | intergenic |  |  | - | | 2260214 | G | C | 771.77 | SNP | intergenic |  |  | - | | 2260220 | C | T | 712.77 | SNP | intergenic |  |  | - | | 2260222 | C | G | 732.77 | SNP | intergenic |  |  | - | | 2260231 | T | C | 756.77 | SNP | intergenic |  |  | - | | 2260525 | C | T | 2038.77 | SNP | intergenic |  |  | - | | 2264782 | C | A | 1860.77 | SNP | Rv2017 | Ala262Glu | 10 | - | | 2265059 | T | G | 1843.77 | SNP | intergenic |  |  | - | | 2266487 | G | C | 2680.77 | SNP | Rv2020c | silent (Leu78) | 9947 | - | | 2266504 | T | TA | 4809.73 | INS | Rv2020c |  |  | - | | 2266508 | A | T | 2275.77 | SNP | Rv2020c | Asp71Glu | 56 | - | | 2266511 | GT | G | 4256.73 | DEL | Rv2020c |  |  | - | | 2266517 | T | C | 2473.77 | SNP | Rv2020c | silent (Glu68) | 9865 | - | | 2266550 | G | T | 2652.77 | SNP | Rv2020c | silent (Gly57) | 9935 | - | | 2266553 | C | G | 2612.77 | SNP | Rv2020c | silent (Ser56) | 9840 | - | | 2266583 | C | G | 2546.77 | SNP | Rv2020c | Glu46Asp | 53 | - | | 2266598 | G | C | 2614.77 | SNP | Rv2020c | silent (Leu41) | 9947 | - | | 2266604 | C | G | 2462.77 | SNP | Rv2020c | silent (Ser39) | 9840 | - | | 2266613 | G | GC | 4279.73 | INS | Rv2020c |  |  | - | | 2266624 | G | T | 2696.77 | SNP | Rv2020c | Leu33Ile | 9 | - | | 2269780 | T | C | 1505.77 | SNP | Rv2024c | Asp154Gly | 11 | - | | 2270102 | A | G | 2603.77 | SNP | Rv2024c | Trp47Arg | 8 | - | | 2275764 | G | A | 1763.77 | SNP | Rv2029c (pfkB) | Leu221Phe | 6 | - | | 2282787 | C | T | 1445.77 | SNP | Rv2037c | Cys312Tyr | 3 | - | | 2284456 | T | G | 2854.77 | SNP | Rv2038c | Glu114Ala | 17 | - | | 2285251 | C | A | 3200.06 | SNP | Rv2039c | Val131Phe | 0 | - | | 2285558 | G | A | 2572.77 | SNP | Rv2039c | silent (Cys28) | 9973 | - | | 2287121 | A | G | 2808.77 | SNP | Rv2041c | silent (Asp242) | 9859 | - | | 2289047 | G | A | 3078.77 | SNP | Rv2043c (pncA) | silent (Ser65) | 9840 | genotype | | 2289365 | CG | C | 3842.73 | DEL | Rv2044c |  |  | - | | 2294290 | A | C | 2584.77 | SNP | Rv2047c | Asn79Lys | 25 | - | | 2296042 | G | C | 1572.77 | SNP | Rv2048c (pks12) | Pro3649Ala | 22 | - | | 2296181 | A | G | 667.77 | SNP | Rv2048c (pks12) | silent (Gly3602) | 9935 | - | | 2296876 | C | G | 771.77 | SNP | Rv2048c (pks12) | Gly3371Arg | 0 | - | | 2300205 | A | AC | 1015.73 | INS | Rv2048c (pks12) |  |  | - | | 2300206 | T | C | 495.77 | SNP | Rv2048c (pks12) | Ile2261Val | 57 | - | | 2300209 | CG | C | 716.73 | DEL | Rv2048c (pks12) |  |  | - | | 2300237 | A | G | 959.77 | SNP | Rv2048c (pks12) | silent (Ala2250) | 9867 | - | | 2300546 | A | T | 1477.77 | SNP | Rv2048c (pks12) | His2147Gln | 23 | - | | 2300552 | T | G | 1547.77 | SNP | Rv2048c (pks12) | silent (Pro2145) | 9926 | - | | 2300555 | A | G | 1556.77 | SNP | Rv2048c (pks12) | silent (Asp2144) | 9859 | - | | 2301782 | T | C | 136.98 | SNP | Rv2048c (pks12) | Val1735Val(s) | 18 | - | | 2306472 | T | C | 1597.77 | SNP | Rv2048c (pks12) | Tyr172Cys | 3 | - | | 2307586 | CGTCG | C | 3796.73 | DEL | intergenic |  |  | - | | 2309203 | G | A | 1554.77 | SNP | Rv2051c (ppm1) | Ala518Val | 13 | - | | 2311099 | C | G | 1131.77 | SNP | Rv2052c | silent (Pro473) | 9926 | - | | 2319244 | C | T | 1606.77 | SNP | Rv2062c (cobN) | Asp504Asn | 36 | - | | 2323108 | TGC | T | 2726.73 | DEL | Rv2065 (cobH) |  |  | - | | 2325320 | T | C | 2679.77 | SNP | Rv2067c | Asp184Gly | 11 | - | | 2328543 | T | C | 1199.77 | SNP | Rv2071c (cobM) | Ile145Met(s) | 6 | - | | 2329533 | A | G | 1495.77 | SNP | Rv2072c (cobL) | Leu205Pro | 2 | - | | 2331620 | A | C | 1583.77 | SNP | Rv2075c | silent (Gly420) | 9935 | - | | 2331789 | G | T | 1562.77 | SNP | Rv2075c | Pro364Gln | 6 | - | | 2333215 | C | T | 1849.77 | SNP | Rv2076c | Cys25Tyr | 3 | - | | 2334007 | A | G | 2475.77 | SNP | Rv2077c | silent (Ala96) | 9867 | - | | 2335075 | A | G | 2902.77 | SNP | Rv2078 | Glu6Gly | 7 | - | | 2335080 | T | C | 2937.77 | SNP | Rv2078 | Leu(s)8Leu | 3 | - | | 2335494 | A | G | 1886.77 | SNP | Rv2079 | Tyr47Cys | 3 | - | | 2338136 | G | A | 438.77 | SNP | Rv2081c | Pro124Ser | 17 | - | | 2338142 | AG | A | 690.73 | DEL | Rv2081c |  |  | - | | 2338181 | G | T | 541.77 | SNP | Rv2081c | Arg109Ser | 11 | - | | 2338194 | AC | A | 304.73 | DEL | Rv2081c |  |  | - | | 2338275 | G | C | 577.77 | SNP | Rv2081c | silent (Thr77) | 9871 | - | | 2338416 | C | G | 1274.77 | SNP | Rv2081c | silent (Ala30) | 9867 | - | | 2338422 | C | T | 1035.77 | SNP | Rv2081c | Trp28STOP | 0 | - | | 2338425 | A | G | 1073.77 | SNP | Rv2081c | silent (Phe27) | 9946 | - | | 2338428 | G | A | 1036.77 | SNP | Rv2081c | silent (Asn26) | 9822 | - | | 2338431 | A | G | 1098.77 | SNP | Rv2081c | silent (His25) | 9912 | - | | 2338434 | C | T | 1011.77 | SNP | Rv2081c | silent (Ser24) | 9840 | - | | 2338457 | G | A | 994.77 | SNP | Rv2081c | Arg17Cys | 1 | - | | 2338677 | T | C | 774.77 | SNP | intergenic |  |  | - | | 2338679 | C | T | 696.77 | SNP | intergenic |  |  | - | | 2338692 | C | G | 1202.77 | SNP | intergenic |  |  | - | | 2338701 | C | T | 1007.77 | SNP | intergenic |  |  | - | | 2338702 | A | G | 1091.77 | SNP | intergenic |  |  | - | | 2338704 | C | G | 1084.77 | SNP | intergenic |  |  | - | | 2338707 | C | T | 1059.77 | SNP | intergenic |  |  | - | | 2338810 | T | C | 1574.77 | SNP | Rv2082 | silent (Arg34) | 9913 | - | | 2338811 | A | G | 1576.77 | SNP | Rv2082 | Lys35Glu | 4 | - | | 2338866 | T | G | 1781.77 | SNP | Rv2082 | Leu53Arg | 1 | - | | 2338912 | A | C | 1810.77 | SNP | Rv2082 | silent (Arg68) | 9913 | - | | 2338961 | G | A | 2075.77 | SNP | Rv2082 | Val85Ile | 33 | - | | 2338990 | G | C | 4012.77 | SNP | Rv2082 | silent (Ala94) | 9867 | - | | 2338994 | G | A | 3935.77 | SNP | Rv2082 | Ala96Thr | 22 | - | | 2339240 | G | A | 2246.77 | SNP | Rv2082 | Gly178Ser | 16 | - | | 2339255 | A | G | 2580.77 | SNP | Rv2082 | Thr183Ala | 32 | - | | 2340621 | C | G | 5589.77 | SNP | Rv2082 | Pro638Arg | 4 | - | | 2341636 | C | G | 922.77 | SNP | Rv2083 | Leu256Val(s) | 4 | - | | 2342649 | A | AGGCGTACACAC | 9335.73 | INS | Rv2084 |  |  | - | | 2344246 | G | C | 2297.77 | SNP | intergenic |  |  | - | | 2345037 | C | A | 1329.77 | SNP | Rv2088 (pknJ) | silent (Leu209) | 9947 | - | | 2345085 | A | C | 1272.77 | SNP | Rv2088 (pknJ) | silent (Ala225) | 9867 | - | | 2346929 | C | T | 2400.77 | SNP | Rv2089c (pepE) | silent (Leu132) | 9947 | - | | 2348446 | C | G | 1193.77 | SNP | Rv2090 | Phe358Leu(s) | 2 | - | | 2348708 | G | A | 2669.77 | SNP | Rv2091c | silent (Val195) | 9901 | - | | 2353385 | G | C | 2202.77 | SNP | Rv2095c (pafC) | Gln311Glu | 35 | - | | 2357268 | TGCC | T | 531.73 | DEL | intergenic |  |  | - | | 2358029 | T | TG | 2185.73 | INS | intergenic |  |  | - | | 2361604 | C | G | 1079.77 | SNP | Rv2101 (helZ) | Val455Val(s) | 18 | - | | 2362041 | C | A | 2145.77 | SNP | Rv2101 (helZ) | Pro601Gln | 6 | - | | 2367060 | TA | T | 6259.73 | DEL | intergenic |  |  | - | | 2368564 | TA | T | 6740.73 | DEL | intergenic |  |  | - | | 2369186 | G | C | 2508.77 | SNP | Rv2109c (prcA) | Arg182Gly | 1 | - | | 2369326 | C | G | 3386.77 | SNP | Rv2109c (prcA) | Arg135Pro | 5 | - | | 2372550 | G | C | 175.77 | SNP | Rv2112c (dop) | Pro7Arg | 4 | - | | 2381100 | G | A | 1582.77 | SNP | Rv2123 (PPE37) | silent (Pro10) | 9926 | - | | 2382286 | GC | G | 1934.73 | DEL | Rv2123 (PPE37) |  |  | - | | 2382289 | G | T | 1203.77 | SNP | Rv2123 (PPE37) | Asp407Tyr | 0 | - | | 2386389 | G | A | 1248.77 | SNP | Rv2125 | Gly33Ser | 16 | - | | 2387733 | T | C | 359.77 | SNP | Rv2126c (PE\_PGRS37) | silent (Glu80) | 9865 | - | | 2387742 | G | A | 323.77 | SNP | Rv2126c (PE\_PGRS37) | silent (Phe77) | 9946 | - | | 2388641 | G | A | 2101.77 | SNP | Rv2127 (ansP1) | Gly9Asp | 6 | - | | 2391747 | A | C | 1641.77 | SNP | Rv2130c (mshC) | Leu238Arg | 1 | - | | 2402765 | G | A | 2391.77 | SNP | intergenic |  |  | - | | 2413246 | C | A | 1147.77 | SNP | Rv2153c (murG) | Val(s)36Leu(s) | 9867 | - | | 2415656 | G | C | 1360.77 | SNP | Rv2155c (murD) | Arg247Gly | 1 | - | | 2418560 | C | T | 1398.77 | SNP | Rv2157c (murF) | Ala149Thr | 22 | - | | 2421816 | A | G | 760.77 | SNP | Rv2160A; Rv2160c | Cys155Arg; Val(s)63Ala | 1; 9867 | - | | 2421975 | G | GGGAA | 2295.73 | INS | Rv2160A; Rv2160c |  |  | - | | 2423447 | G | A | 331.77 | SNP | Rv2162c (PE\_PGRS38) | silent (Arg464) | 9913 | - | | 2423819 | C | G | 62.74 | SNP | Rv2162c (PE\_PGRS38) | silent (Gly340) | 9935 | - | | 2423822 | A | G | 55.74 | SNP | Rv2162c (PE\_PGRS38) | silent (Gly339) | 9935 | - | | 2424114 | C | A | 157.90 | SNP | Rv2162c (PE\_PGRS38) | Arg242Leu | 1 | - | | 2424151 | C | A | 53.74 | SNP | Rv2162c (PE\_PGRS38) | Ala230Ser | 28 | - | | 2424925 | A | G | 1266.77 | SNP | intergenic |  |  | - | | 2425471 | T | C | 3208.76 | SNP | Rv2163c (pbpB) | silent (Arg539) | 9913 | - | | 2434749 | C | T | 1718.77 | SNP | intergenic |  |  | - | | 2435696 | C | A | 2127.77 | SNP | Rv2173 (idsA2) | silent (Arg284) | 9913 | - | | 2439519 | G | A | 558.77 | SNP | Rv2177c | silent (Arg143) | 9913 | - | | 2440926 | G | T | 3930.77 | SNP | Rv2178c (aroG) | Asp265Glu | 56 | - | | 2440935 | G | C | 3875.77 | SNP | Rv2178c (aroG) | silent (Ser262) | 9840 | - | | 2448458 | C | T | 2171.77 | SNP | Rv2187 (fadD15) | Thr100Ile | 7 | - | | 2449826 | C | G | 1579.77 | SNP | Rv2187 (fadD15) | Ser556Trp | 1 | - | | 2452452 | G | A | 1223.77 | SNP | Rv2190c | Thr274Met(s) | 32 | - | | 2462871 | G | A | 1551.77 | SNP | Rv2198c (mmpS3) | silent (Ala59) | 9867 | - | | 2467098 | A | C | 1541.77 | SNP | Rv2202c (adoK) | silent (Ala310) | 9867 | - | | 2470591 | A | C | 1744.77 | SNP | intergenic |  |  | - | | 2472029 | T | G | 1164.77 | SNP | Rv2207 (cobT) | Trp207Gly | 0 | - | | 2477984 | G | A | 1873.77 | SNP | Rv2212 | silent (Ser265) | 9840 | - | | 2478619 | G | A | 628.77 | SNP | Rv2213 (pepB) | silent (Leu94) | 9947 | - | | 2480809 | G | C | 1218.77 | SNP | Rv2214c (ephD) | Ala298Gly | 21 | - | | 2494430 | C | G | 2131.77 | SNP | Rv2223c | Gly324Arg | 0 | - | | 2499726 | G | A | 2254.77 | SNP | Rv2226 | Asp299Asn | 36 | - | | 2504177 | C | T | 1077.77 | SNP | Rv2230c | silent (Glu144) | 9865 | - | | 2506183 | G | A | 1745.77 | SNP | intergenic |  |  | - | | 2509140 | G | C | 1146.77 | SNP | Rv2236c (cobD) | Ser79Cys | 5 | - | | 2509722 | A | G | 2107.77 | SNP | Rv2237 | silent (Pro78) | 9926 | - | | 2516271 | T | C | 1495.77 | SNP | Rv2242 | Met(s)323Thr | 22 | - | | 2516567 | G | C | 2445.77 | SNP | intergenic |  |  | - | | 2521342 | T | C | 1913.77 | SNP | Rv2247 (accD6) | silent (Asp200) | 9859 | - | | 2523205 | G | GCGC | 3016.73 | INS | intergenic |  |  | - | | 2525722 | CG | C | 2742.73 | DEL | Rv2250A; Rv2251 |  |  | - | | 2526974 | T | C | 2277.77 | SNP | Rv2251 | silent (Pro470) | 9926 | - | | 2529680 | A | G | 1970.77 | SNP | Rv2256c | silent (Thr65) | 9871 | - | | 2531742 | A | G | 2003.77 | SNP | Rv2258c | silent (Ala52) | 9867 | - | | 2531964 | C | G | 1628.89 | SNP | intergenic |  |  | - | | 2533377 | T | C | 2052.77 | SNP | Rv2260 | silent (Asp16) | 9859 | - | | 2534562 | GGA | G | 2806.73 | DEL | Rv2262c |  |  | - | | 2536312 | C | T | 1459.77 | SNP | Rv2263 | silent (Arg224) | 9913 | - | | 2536625 | C | CG | 891.73 | INS | Rv2264c |  |  | - | | 2540554 | T | C | 2194.77 | SNP | Rv2266 (cyp124) | Ser151Pro | 12 | - | | 2541477 | C | G | 1822.77 | SNP | intergenic |  |  | - | | 2541779 | G | A | 2788.77 | SNP | Rv2267c | silent (Tyr344) | 9945 | - | | 2542543 | G | A | 3878.77 | SNP | Rv2267c | Arg90Cys | 1 | - | | 2562644 | G | T | 2492.77 | SNP | Rv2290 (lppO) | Ala16Ser | 28 | - | | 2564368 | G | GC | 2397.73 | INS | Rv2293c |  |  | - | | 2566063 | C | A | 2054.77 | SNP | Rv2294 | Ala246Glu | 10 | - | | 2566766 | C | CG | 2769.73 | INS | intergenic |  |  | - | | 2566768 | A | C | 1761.77 | SNP | intergenic |  |  | - | | 2566783 | G | A | 1806.77 | SNP | Rv2295 | silent (Ser4) | 9840 | - | | 2573756 | C | A | 2810.77 | SNP | intergenic |  |  | - | | 2578626 | A | G | 2252.77 | SNP | Rv2307c | Met(s)24Thr | 22 | - | | 2586127 | A | G | 1234.77 | SNP | Rv2314c | silent (Gly388) | 9935 | - | | 2587852 | C | T | 2262.77 | SNP | Rv2315c | Gly318Asp | 6 | - | | 2589216 | G | C | 2740.77 | SNP | Rv2316 (uspA) | Val(s)127Leu | 3 | - | | 2591172 | G | A | 2000.77 | SNP | Rv2318 (uspC) | Ala219Thr | 22 | - | | 2598400 | A | G | 2311.77 | SNP | Rv2326c | silent (Asn516) | 9822 | - | | 2600260 | A | G | 2378.77 | SNP | Rv2327 | silent (Arg91) | 9913 | - | | 2601576 | T | G | 1469.77 | SNP | Rv2328 (PE23) | Ser282Arg | 6 | - | | 2604156 | TCAGTGCCAA | T | 6645.73 | DEL | Rv2330c (lppP) |  |  | - | | 2608117 | C | A | 2341.77 | SNP | Rv2333c (stp) | Asp69Tyr | 0 | - | | 2612632 | C | A | 2153.77 | SNP | Rv2337c | Gly119Val | 3 | - | | 2615413 | A | G | 3630.77 | SNP | Rv2339 (mmpL9) | Thr241Ala | 32 | - | | 2619271 | T | C | 1270.77 | SNP | intergenic |  |  | - | | 2624986 | T | G | 1395.77 | SNP | Rv2345 | Val(s)389Gly | 21 | - | | 2625924 | T | C | 1630.77 | SNP | Rv2346c (esxO) | silent (Ala83) | 9867 | - | | 2626095 | C | G | 1555.77 | SNP | Rv2346c (esxO) | silent (Ala26) | 9867 | - | | 2626513 | T | A | 929.77 | SNP | Rv2347c (esxP) | Thr3Ser | 38 | - | | 2626514 | T | G | 1062.77 | SNP | Rv2347c (esxP) | silent (Ala2) | 9867 | - | | 2626600 | G | A | 876.77 | SNP | intergenic |  |  | - | | 2627946 | A | G | 2714.77 | SNP | Rv2349c (plcC) | silent (Arg251) | 9913 | - | | 2637541 | C | T | 2327.77 | SNP | intergenic |  |  | - | | 2638690 | ATTTCCGCTGCCGAAG | A | 2721.73 | DEL | Rv2356c (PPE40) |  |  | - | | 2642383 | C | T | 2031.77 | SNP | Rv2360c | Ala66Thr | 22 | - | | 2652254 | G | C | 2097.77 | SNP | Rv2372c | Ala191Gly | 21 | - | | 2653691 | G | A | 1355.77 | SNP | Rv2373c (dnaJ2) | silent (Phe99) | 9946 | - | | 2656225 | A | G | 2615.77 | SNP | Rv2377c (mbtH) | Val69Ala | 18 | - | | 2656235 | C | T | 2546.77 | SNP | Rv2377c (mbtH) | Asp66Asn | 36 | - | | 2656635 | G | A | 2118.77 | SNP | Rv2378c (mbtG) | Pro357Ser | 17 | - | | 2660319 | C | G | 1964.77 | SNP | Rv2379c (mbtF) | Glu589Asp | 53 | - | | 2661039 | G | A | 2750.77 | SNP | Rv2379c (mbtF) | silent (Ile349) | 9872 | - | | 2680658 | T | G | 2533.77 | SNP | intergenic |  |  | - | | 2688700 | C | T | 1137.77 | SNP | Rv2394 (ggtB) | Pro231Ser | 17 | - | | 2690160 | A | G | 2319.77 | SNP | Rv2395 | Asn30Ser | 34 | - | | 2693194 | CG | C | 295.74 | DEL | Rv2396 (PE\_PGRS41) |  |  | - | | 2695378 | C | G | 2294.77 | SNP | Rv2398c (cysW) | Gly141Ala | 21 | - | | 2697489 | C | T | 1671.77 | SNP | Rv2400c (subI) | Ala76Thr | 22 | - | | 2700222 | T | C | 2698.77 | SNP | Rv2402 | Leu565Pro | 2 | - | | 2704884 | A | ACAGCGACCATATCGCCGAG CT | 1521.73 | INS | Rv2407 |  |  | - | | 2712328 | C | T | 1729.77 | SNP | Rv2414c | silent (Pro183) | 9926 | - | | 2713795 | C | T | 1557.77 | SNP | intergenic |  |  | - | | 2718852 | T | G | 2108.77 | SNP | intergenic |  |  | - | | 2720069 | G | A | 2600.77 | SNP | Rv2423 | Gly158Glu | 4 | - | | 2720444 | C | T | 2174.77 | SNP | Rv2423 | Ser283Phe | 2 | - | | 2720895 | G | A | 191.80 | SNP | Rv2424c | His295Tyr | 4 | - | | 2720952 | T | C | 191.80 | SNP | Rv2424c | Thr276Ala | 32 | - | | 2720954 | G | A | 194.80 | SNP | Rv2424c | Thr275Ile | 7 | - | | 2721562 | C | G | 2062.77 | SNP | Rv2424c | silent (Ala72) | 9867 | - | | 2721746 | C | G | 1475.77 | SNP | Rv2424c | Gly11Ala | 21 | - | | 2723506 | T | C | 2300.77 | SNP | Rv2426c | Leu226Leu(s) | 4 | - | | 2724180 | TCACGATCGGGTCTCCTCTA G | T | 6589.73 | DEL | Rv2426c |  |  | - | | 2726105 | G | A | 2802.77 | SNP | intergenic (Rv2428-88nt) |  |  | genotype | | 2729058 | A | C | 2720.77 | SNP | Rv2433c | Leu26Arg | 1 | - | | 2734074 | T | C | 624.77 | SNP | Rv2436 (rbsK) | Val282Ala | 18 | - | | 2737572 | C | A | 1244.77 | SNP | Rv2439c (proB) | Ala226Ser | 28 | - | | 2738221 | G | A | 1472.77 | SNP | Rv2439c (proB) | silent (Ile9) | 9872 | - | | 2740693 | T | C | 1405.77 | SNP | intergenic |  |  | - | | 2744388 | G | A | 2018.77 | SNP | Rv2444c (rne) | silent (Ala199) | 9867 | - | | 2751804 | C | T | 1055.77 | SNP | Rv2450c (rpfE) | Arg126Gln | 9 | - | | 2752698 | C | A | 3866.77 | SNP | intergenic |  |  | - | | 2753869 | A | G | 2682.77 | SNP | Rv2454c | Val(s)293Ala | 9867 | - | | 2758009 | C | A | 1544.77 | SNP | Rv2456c | Val(s)62Leu(s) | 9867 | - | | 2760152 | A | G | 1561.77 | SNP | Rv2458 (mmuM) | Tyr125Cys | 3 | - | | 2771383 | A | G | 1664.77 | SNP | Rv2467 (pepN) | Ser800Gly | 21 | - | | 2779136 | T | C | 1801.77 | SNP | Rv2476c (gdh) | Ser1043Gly | 21 | - | | 2782498 | G | A | 3567.77 | SNP | Rv2477c | silent (Asp515) | 9859 | - | | 2786952 | A | G | 2097.77 | SNP | Rv2482c (plsB2) | Cys778Arg | 1 | - | | 2787921 | G | GT | 3852.73 | INS | Rv2482c (plsB2) |  |  | - | | 2791098 | C | T | 1043.77 | SNP | Rv2484c | Gly466Asp | 6 | - | | 2795957 | ACCCGCCGGCGCCGCCGTTG CCGCCCGCGCCGCCGGT | A | 1344 | DEL | Rv2487c (PE\_PGRS42) |  |  | - | | 2797726 | T | C | 2410.77 | SNP | Rv2488c | Tyr1052Cys | 3 | - | | 2798595 | C | T | 919.77 | SNP | Rv2488c | silent (Ala762) | 9867 | - | | 2800282 | A | C | 1443.77 | SNP | Rv2488c | Leu200Arg | 1 | - | | 2802221 | C | G | 38.77 | SNP | Rv2490c (PE\_PGRS43) | Gly1339Ala | 21 | - | | 2803138 | GCCACCGGCA | G | 1058.73 | DEL | Rv2490c (PE\_PGRS43) |  |  | - | | 2804296 | G | C | 355.77 | SNP | Rv2490c (PE\_PGRS43) | silent (Ala647) | 9867 | - | | 2804481 | C | G | 326.78 | SNP | Rv2490c (PE\_PGRS43) | Gly586Arg | 0 | - | | 2807486 | C | A | 4337.77 | SNP | Rv2492 | Ala70Asp | 6 | - | | 2809621 | T | C | 1789.77 | SNP | Rv2495c (bkdC) | Thr107Ala | 32 | - | | 2817747 | G | A | 1859.77 | SNP | Rv2502c (accD1) | Ala243Val | 13 | - | | 2818837 | A | G | 1898.77 | SNP | Rv2503c (scoB) | silent (Gly97) | 9935 | - | | 2819919 | G | C | 1628.77 | SNP | intergenic |  |  | - | | 2821342 | C | T | 1259.77 | SNP | Rv2505c (fadD35) | silent (Ala85) | 9867 | - | | 2825431 | G | A | 2301.77 | SNP | Rv2509 | Ala252Thr | 22 | - | | 2825466 | G | A | 2128.77 | SNP | Rv2509 | silent (Lys263) | 9926 | - | | 2827984 | G | T | 1970.77 | SNP | intergenic |  |  | - | | 2828019 | T | C | 2138.77 | SNP | intergenic |  |  | - | | 2829779 | T | C | 987.77 | SNP | Rv2512c | Thr9Ala | 32 | - | | 2830525 | C | A | 2784.77 | SNP | Rv2513 | Thr122Lys | 11 | - | | 2836773 | C | T | 2722.77 | SNP | Rv2519 (PE26) | Ser330Leu(s) | 35 | - | | 2840999 | C | G | 1122.77 | SNP | Rv2524c (fas) | silent (Ser2778) | 9840 | - | | 2841022 | A | G | 1111.77 | SNP | Rv2524c (fas) | Cys2771Arg | 1 | - | | 2847281 | A | G | 2187.77 | SNP | Rv2524c (fas) | silent (Asp684) | 9859 | - | | 2855259 | A | G | 1967.77 | SNP | Rv2531c | silent (Ala841) | 9867 | - | | 2865760 | A | G | 2608.77 | SNP | Rv2542 | Thr211Ala | 32 | - | | 2865882 | T | C | 2485.77 | SNP | Rv2542 | silent (Val251) | 9901 | - | | 2866551 | C | G | 40.77 | SNP | Rv2543 (lppA) | silent (Ala28) | 9867 | - | | 2866569 | C | A | 109.77 | SNP | Rv2543 (lppA) | silent (Thr34) | 9871 | - | | 2866578 | C | A | 152.77 | SNP | Rv2543 (lppA) | His37Gln | 23 | - | | 2866580 | A | G | 219.77 | SNP | Rv2543 (lppA) | Asn38Ser | 34 | - | | 2866607 | G | A | 191.77 | SNP | Rv2543 (lppA) | Gly47Asp | 6 | - | | 2867207 | C | G | 2589.77 | SNP | Rv2544 (lppB) | silent (Ala28) | 9867 | - | | 2867230 | G | A | 1985.77 | SNP | Rv2544 (lppB) | Gly36Asp | 6 | - | | 2867231 | C | T | 2024.77 | SNP | Rv2544 (lppB) | silent (Gly36) | 9935 | - | | 2867236 | A | G | 2085.77 | SNP | Rv2544 (lppB) | Asn38Ser | 34 | - | | 2867240 | C | T | 2101.77 | SNP | Rv2544 (lppB) | silent (Pro39) | 9926 | - | | 2867245 | A | C | 2202.77 | SNP | Rv2544 (lppB) | Lys41Thr | 8 | - | | 2867251 | C | G | 2162.77 | SNP | Rv2544 (lppB) | Pro43Arg | 4 | - | | 2867254 | A | G | 2050.77 | SNP | Rv2544 (lppB) | His44Arg | 10 | - | | 2867263 | G | A | 2334.77 | SNP | Rv2544 (lppB) | Gly47Asp | 6 | - | | 2877378 | G | C | 2570.77 | SNP | Rv2557 | Ala103Pro | 13 | - | | 2880702 | G | C | 2872.77 | SNP | Rv2560 | Val210Leu | 15 | - | | 2881337 | C | T | 1581.77 | SNP | intergenic |  |  | - | | 2881569 | A | G | 1343.77 | SNP | Rv2561 | Glu54Gly | 7 | - | | 2881597 | AG | A | 2069.73 | DEL | Rv2561 |  |  | - | | 2884068 | A | C | 2356.77 | SNP | Rv2564 (glnQ) | Met(s)243Leu | 3 | - | | 2886570 | A | G | 2849.77 | SNP | Rv2566 | silent (Glu66) | 9865 | - | | 2888201 | T | C | 1453.77 | SNP | Rv2566 | Leu610Pro | 2 | - | | 2889633 | T | C | 1560.77 | SNP | Rv2566 | silent (Ala1087) | 9867 | - | | 2891267 | C | T | 2421.77 | SNP | Rv2567 | silent (Gly491) | 9935 | - | | 2891728 | A | G | 1730.77 | SNP | Rv2567 | Gln645Arg | 10 | - | | 2894208 | G | A | 1545.77 | SNP | Rv2569c | silent (Ser67) | 9840 | - | | 2895016 | C | A | 1363.77 | SNP | Rv2571c | silent (Ala315) | 9867 | - | | 2895473 | A | G | 2305.77 | SNP | Rv2571c | Phe163Ser | 3 | - | | 2895848 | C | CA | 4681.73 | INS | Rv2571c |  |  | - | | 2897660 | T | C | 1478.77 | SNP | Rv2572c (aspS) | silent (Ala48) | 9867 | - | | 2898522 | T | C | 1489.77 | SNP | Rv2573 | silent (Tyr160) | 9945 | - | | 2903050 | G | A | 1915.77 | SNP | Rv2578c | Thr161Ile | 7 | - | | 2910461 | G | T | 1774.77 | SNP | Rv2584c (apt) | Ala147Glu | 10 | - | | 2910852 | C | G | 1671.77 | SNP | Rv2584c (apt) | Ala17Pro | 13 | - | | 2911293 | C | G | 1502.77 | SNP | Rv2585c | Cys462Ser | 11 | - | | 2912294 | T | G | 2179.77 | SNP | Rv2585c | silent (Ala128) | 9867 | - | | 2923391 | T | C | 1253.77 | SNP | Rv2592c (ruvB) | silent (Pro281) | 9926 | - | | 2925462 | T | A | 2180.77 | SNP | intergenic |  |  | - | | 2925962 | T | C | 2689.77 | SNP | Rv2596 (vapC40) | Cys77Arg | 1 | - | | 2926153 | T | C | 2276.77 | SNP | intergenic |  |  | - | | 2927939 | T | C | 2404.77 | SNP | intergenic |  |  | - | | 2939373 | G | C | 1632.77 | SNP | Rv2611c | Ser197Cys | 5 | - | | 2939657 | T | C | 794.77 | SNP | Rv2611c | Ile102Met(s) | 6 | - | | 2941179 | G | A | 2517.77 | SNP | Rv2613c | silent (Arg6) | 9913 | genotype | | 2943675 | G | A | 296.78 | SNP | Rv2615c (PE\_PGRS45) | silent (Gly437) | 9935 | - | | 2943711 | G | GCCGCCGTTT | 1137.73 | INS | Rv2615c (PE\_PGRS45) |  |  | - | | 2945768 | G | A | 1618.77 | SNP | Rv2616 | Ala147Thr | 22 | - | | 2948524 | T | A | 1930.77 | SNP | Rv2621c | Glu12Val(s) | 17 | - | | 2949251 | G | A | 1489.77 | SNP | Rv2622 | Val206Ile | 33 | - | | 2954439 | T | C | 2437.77 | SNP | Rv2627c | Arg104Gly | 1 | - | | 2958693 | G | A | 3010.77 | SNP | Rv2631 | silent (Ala374) | 9867 | - | | 2959256 | TATCCAATC | T | 7816.73 | DEL | intergenic |  |  | - | | 2959265 | A | T | 1918.77 | SNP | intergenic |  |  | - | | 2960592 | C | T | 273.78 | SNP | Rv2634c (PE\_PGRS46) | Gly617Asp | 6 | - | | 2964594 | G | A | 1600.77 | SNP | Rv2638 | Ala64Thr | 22 | - | | 2967741 | TG | T | 2582.73 | DEL | Rv2643 (arsC) |  |  | - | | 2974933 | A | G | 825.77 | SNP | Rv2650c | Ile101Thr | 11 | - | | 2975227 | T | G | 1661.77 | SNP | Rv2650c | Asn3Thr | 13 | - | | 2983613 | G | A | 306.77 | SNP | Rv2666 | silent (Gly181) | 9935 | - | | 2984740 | A | G | 1407.77 | SNP | Rv2668 | His3Arg | 10 | - | | 2988630 | C | G | 701.77 | SNP | Rv2672 | His317Asp | 4 | - | | 2989431 | G | A | 2517.77 | SNP | Rv2673 (aftC) | silent (Leu47) | 9947 | - | | 2994187 | T | C | 1118.77 | SNP | Rv2678c (hemE) | Leu292Leu(s) | 4 | - | | 2995548 | C | A | 1264.77 | SNP | Rv2679 (echA15) | Thr145Asn | 9 | - | | 3003115 | C | G | 2069.77 | SNP | Rv2685 (arsB1) | Ala378Gly | 21 | - | | 3005185 | G | T | 2557.77 | SNP | Rv2688c | Pro156Thr | 5 | - | | 3009692 | A | G | 2423.77 | SNP | Rv2691 (ceoB) | Thr117Ala | 32 | - | | 3009903 | C | T | 2326.77 | SNP | Rv2691 (ceoB) | Pro187Leu | 3 | - | | 3010014 | G | A | 2387.77 | SNP | Rv2691 (ceoB) | Gly224Glu | 4 | - | | 3010420 | A | G | 1626.77 | SNP | Rv2692 (ceoC) | Ile133Val | 57 | - | | 3011837 | A | G | 2189.77 | SNP | intergenic |  |  | - | | 3017465 | T | C | 1971.77 | SNP | Rv2702 (ppgK) | Ile203Thr | 11 | - | | 3017599 | A | C | 2448.77 | SNP | Rv2702 (ppgK) | Asn248His | 18 | - | | 3023684 | G | A | 2316.77 | SNP | Rv2711 (ideR) | silent (Thr40) | 9871 | - | | 3024753 | T | C | 2245.77 | SNP | Rv2712c | silent (Ala192) | 9867 | - | | 3027548 | C | T | 2256.77 | SNP | Rv2714 | Pro162Ser | 17 | - | | 3027798 | T | C | 1854.77 | SNP | Rv2714 | Val(s)245Ala | 9867 | - | | 3031168 | A | G | 1550.77 | SNP | Rv2719c | Tyr124His | 4 | - | | 3037030 | G | C | 2567.77 | SNP | Rv2724c (fadE20) | Arg88Gly | 1 | - | | 3037377 | T | C | 936.77 | SNP | intergenic |  |  | - | | 3040344 | C | T | 1389.77 | SNP | Rv2727c (miaA) | silent (Glu142) | 9865 | - | | 3041032 | G | A | 1777.77 | SNP | Rv2728c | silent (Leu144) | 9947 | - | | 3041871 | G | T | 2412.77 | SNP | Rv2729c | Ala202Glu | 10 | - | | 3043700 | C | T | 1281.77 | SNP | Rv2731 | silent (Ala225) | 9867 | - | | 3054081 | A | G | 1819.77 | SNP | Rv2741 (PE\_PGRS47) | silent (Gly56) | 9935 | - | | 3054321 | A | G | 600.77 | SNP | Rv2741 (PE\_PGRS47) | silent (Gly136) | 9935 | - | | 3054724 | A | G | 80.28 | SNP | Rv2741 (PE\_PGRS47) | Ser271Gly | 21 | - | | 3059321 | G | A | 2847.77 | SNP | Rv2747 (argA) | Val(s)20Val | 13 | - | | 3059811 | CT | C | 819.73 | DEL | intergenic |  |  | - | | 3059829 | G | GA | 833.73 | INS | intergenic |  |  | - | | 3069167 | A | G | 1829.77 | SNP | Rv2756c (hsdM) | Leu306Pro | 2 | - | | 3069566 | C | T | 3328.77 | SNP | Rv2756c (hsdM) | Gly173Asp | 6 | - | | 3072285 | A | C | 2290.77 | SNP | Rv2761c (hsdS) | Leu119Arg | 1 | - | | 3076172 | C | T | 552.77 | SNP | Rv2766c | Asp67Asn | 36 | - | | 3078178 | C | T | 2323.77 | SNP | Rv2769c (PE27) | Val(s)270Met(s) | 9867 | - | | 3079877 | A | G | 1623.77 | SNP | Rv2770c (PPE44) | Phe194Ser | 3 | - | | 3080795 | A | G | 2375.77 | SNP | Rv2771c | Leu80Pro | 2 | - | | 3086788 | T | C | 3127.77 | SNP | intergenic |  |  | - | | 3089299 | C | G | 1784.77 | SNP | Rv2782c (pepR) | Gly355Arg | 0 | - | | 3103682 | T | C | 2087.77 | SNP | Rv2794c (pptT) | Met(s)87Val(s) | 9867 | - | | 3104189 | A | G | 3253.77 | SNP | Rv2795c | silent (Cys241) | 9973 | - | | 3111280 | G | A | 1436.77 | SNP | Rv2802c | Leu182Phe | 6 | - | | 3112700 | C | T | 1492.77 | SNP | Rv2804c | Gly132Asp | 6 | - | | 3112877 | G | A | 1454.77 | SNP | Rv2804c; Rv2805 | Ala73Val; Gly4Asp | 13; 6 | - | | 3113018 | ACGAAGCCAGGGC | A | 9267.73 | DEL | Rv2804c; Rv2805 |  |  | - | | 3113872 | A | T | 1851.77 | SNP | Rv2807 | Glu72Val(s) | 17 | - | | 3114515 | CA | C | 4151.73 | DEL | Rv2807 |  |  | - | | 3115384 | G | C | 3204.83 | SNP | intergenic |  |  | - | | 3117533 | G | A | 3615.77 | SNP | Rv2812 | Arg239Gln | 9 | - | | 3118000 | A | G | 1184.77 | SNP | Rv2812 | Arg395Gly | 1 | - | | 3122775 | C | T | 126.90 | SNP | intergenic |  |  | - | | 3122780 | GT | G | 140.87 | DEL | intergenic |  |  | - | | 3122783 | AACTAGAG | A | 437.87 | DEL | intergenic |  |  | - | | 3122793 | GGTGTCGAA | G | 525.87 | DEL | intergenic |  |  | - | | 3122806 | G | T | 126.90 | SNP | intergenic |  |  | - | | 3122807 | C | G | 135.90 | SNP | intergenic |  |  | - | | 3122809 | C | G | 132.90 | SNP | intergenic |  |  | - | | 3122810 | G | A | 126.90 | SNP | intergenic |  |  | - | | 3131469 | T | TTGTCGGCGA | 9130.73 | INS | Rv2823c |  |  | - | | 3132265 | G | A | 3022.77 | SNP | Rv2824c | silent (Ser150) | 9840 | - | | 3133016 | C | G | 231.77 | SNP | Rv2825c | Arg175Thr | 2 | - | | 3133054 | C | G | 2652.77 | SNP | Rv2825c | silent (Ser162) | 9840 | - | | 3133055 | G | C | 2555.77 | SNP | Rv2825c | Ser162Trp | 1 | - | | 3133536 | T | C | 3445.77 | SNP | Rv2825c | Lys2Glu | 4 | - | | 3135272 | G | A | 2699.77 | SNP | Rv2827c | Ala71Val | 13 | - | | 3135950 | C | G | 300.78 | SNP | Rv2828c | silent (Ser128) | 9840 | - | | 3135951 | G | C | 296.78 | SNP | Rv2828c | Ser128Trp | 1 | - | | 3137058 | G | A | 1350.77 | SNP | Rv2830c (vapB22) | Ala56Val(s) | 9867 | - | | 3162805 | C | G | 238.80 | SNP | Rv2853 (PE\_PGRS48) | Arg180Gly | 1 | - | | 3165807 | G | A | 2635.77 | SNP | Rv2855 (mtr) | Val(s)201Val | 13 | - | | 3168492 | G | A | 1830.77 | SNP | intergenic |  |  | - | | 3174013 | G | A | 2289.77 | SNP | Rv2861c (mapB) | Pro2Leu | 3 | - | | 3174496 | A | G | 1761.77 | SNP | Rv2862c | Cys50Arg | 1 | - | | 3177884 | C | A | 2483.77 | SNP | Rv2866 (relG) | silent (Arg21) | 9913 | - | | 3180988 | C | A | 1247.77 | SNP | Rv2869c (rip) | Val259Phe | 0 | - | | 3183561 | G | C | 167.77 | SNP | Rv2872 (vapC43) | silent (Pro60) | 9926 | - | | 3186860 | T | G | 2213.77 | SNP | Rv2874 (dipZ) | Tyr672Asp | 0 | - | | 3189242 | A | G | 2043.77 | SNP | Rv2878c (mpt53) | silent (Ala52) | 9867 | - | | 3190145 | TC | T | 2556.73 | DEL | Rv2880c |  |  | - | | 3194241 | C | CG | 2082.73 | INS | Rv2885c |  |  | - | | 3200478 | G | A | 804.77 | SNP | Rv2891 | silent (Leu71) | 9947 | - | | 3202731 | A | C | 1527.77 | SNP | Rv2893 | silent (Gly104) | 9935 | - | | 3207373 | G | T | 1309.77 | SNP | Rv2897c | Asp190Glu | 56 | - | | 3208600 | G | A | 2905.77 | SNP | Rv2899c (fdhD) | silent (Tyr269) | 9945 | - | | 3212723 | C | A | 2409.77 | SNP | Rv2902c (rnhB) | silent (Ala78) | 9867 | - | | 3219500 | T | A | 2007.77 | SNP | Rv2912c | Lys121Met(s) | 2 | - | | 3221361 | G | C | 3246.77 | SNP | Rv2913c | Ala113Gly | 21 | - | | 3226181 | A | C | 1378.77 | SNP | Rv2916c (ffh) | silent (Arg35) | 9913 | - | | 3228143 | G | T | 1092.77 | SNP | Rv2917 | Arg594Leu | 1 | - | | 3232703 | G | A | 1863.77 | SNP | intergenic |  |  | - | | 3232759 | G | A | 2036.77 | SNP | intergenic |  |  | - | | 3236442 | G | A | 2225.77 | SNP | Rv2922c (smc) | silent (His455) | 9912 | - | | 3236497 | A | T | 1963.77 | SNP | Rv2922c (smc) | Val(s)437Glu | 10 | - | | 3244113 | T | C | 3227.04 | SNP | Rv2930 (fadD26) | silent (Pro139) | 9926 | - | | 3247298 | C | A | 1671.77 | SNP | Rv2931 (ppsA) | silent (Gly618) | 9935 | - | | 3247304 | C | G | 1346.77 | SNP | Rv2931 (ppsA) | Val620Val(s) | 18 | - | | 3247305 | AACCCT | A | 3220.76 | DEL | Rv2931 (ppsA) |  |  | - | | 3247313 | C | CACCAG | 3035.73 | INS | Rv2931 (ppsA) |  |  | - | | 3247319 | C | T | 1545.77 | SNP | Rv2931 (ppsA) | silent (Gly625) | 9935 | - | | 3247340 | G | A | 1764.77 | SNP | Rv2931 (ppsA) | Val(s)632Val | 13 | - | | 3247851 | G | A | 1536.77 | SNP | Rv2931 (ppsA) | Ala803Thr | 22 | - | | 3247853 | C | T | 1481.77 | SNP | Rv2931 (ppsA) | silent (Ala803) | 9867 | - | | 3247856 | G | C | 1543.77 | SNP | Rv2931 (ppsA) | silent (Arg804) | 9913 | - | | 3247864 | C | CTAGG | 3147.73 | INS | Rv2931 (ppsA) |  |  | - | | 3247865 | GCAAA | G | 3461.73 | DEL | Rv2931 (ppsA) |  |  | - | | 3247874 | G | A | 1502.77 | SNP | Rv2931 (ppsA) | silent (Arg810) | 9913 | - | | 3247877 | T | C | 1510.77 | SNP | Rv2931 (ppsA) | silent (Phe811) | 9946 | - | | 3247883 | T | C | 1792.77 | SNP | Rv2931 (ppsA) | silent (Ser813) | 9840 | - | | 3248074 | G | A | 1792.77 | SNP | Rv2931 (ppsA) | Arg877His | 8 | - | | 3248075 | C | T | 1839.77 | SNP | Rv2931 (ppsA) | silent (Arg877) | 9913 | - | | 3251047 | C | A | 2718.77 | SNP | Rv2931 (ppsA) | Ala1868Glu | 10 | - | | 3256494 | A | G | 2452.77 | SNP | Rv2933 (ppsC) | silent (Gly270) | 9935 | - | | 3266030 | A | G | 979.77 | SNP | Rv2934 (ppsD) | silent (Ser1261) | 9840 | - | | 3269581 | A | G | 2722.77 | SNP | Rv2935 (ppsE) | silent (Ala615) | 9867 | - | | 3270784 | A | G | 2108.77 | SNP | Rv2935 (ppsE) | silent (Gln1016) | 9876 | - | | 3273107 | C | A | 2543.77 | SNP | Rv2936 (drrA) | silent (Ala298) | 9867 | genotype | | 3275485 | G | C | 2757.77 | SNP | Rv2939 (papA5) | silent (Ser179) | 9840 | - | | 3287998 | C | T | 1915.77 | SNP | intergenic |  |  | - | | 3296371 | G | GCCGCGGC | 2994.92 | INS | Rv2947c (pks15) |  |  | - (CGGCGCCG would be genotype) | | 3296843 | A | G | 1396.77 | SNP | Rv2947c (pks15) | Val(s)333Ala | 9867 | - | | 3302589 | A | C | 3517.77 | SNP | intergenic |  |  | - | | 3306441 | G | A | 1781.77 | SNP | Rv2953 | Arg388Gln | 9 | - | | 3308606 | G | A | 3464.77 | SNP | intergenic |  |  | - | | 3312620 | C | A | 3355.77 | SNP | Rv2959c | Glu73Asp | 53 | - | | 3314412 | A | G | 2372.77 | SNP | Rv2962c | silent (Ala237) | 9867 | - | | 3315725 | CG | C | 2942.73 | DEL | Rv2963 |  |  | - | | 3316955 | A | C | 2593.77 | SNP | Rv2964 (purU) | Met(s)143Leu | 3 | - | | 3326554 | A | C | 2590.77 | SNP | Rv2971 | Asn152His | 18 | genotype | | 3331361 | ACG | A | 2085.73 | DEL | Rv2974c; Rv2975c |  |  | - | | 3335708 | G | C | 1960.77 | SNP | Rv2979c | Pro14Arg | 4 | - | | 3336561 | TAAGTGCCGATCGCAAGCGC GGCGCTTGCGCCGGGCGCGG CGGGTCGGCACCATCGGGCT A | T | 828.73 | DEL | intergenic |  |  | - | | 3336646 | T | A | 299.77 | SNP | intergenic |  |  | - | | 3336825 | T | C | 1687.77 | SNP | Rv2981c (ddlA) | Thr365Ala | 32 | - | | 3338194 | C | A | 1503.77 | SNP | Rv2982c (gpdA2) | Arg269Leu | 1 | - | | 3338603 | G | C | 1859.77 | SNP | Rv2982c (gpdA2) | Pro133Ala | 22 | - | | 3348536 | C | T | 997.77 | SNP | intergenic |  |  | - | | 3349917 | G | A | 1812.77 | SNP | Rv2992c (gltS) | His121Tyr | 4 | - | | 3352932 | C | G | 2169.77 | SNP | Rv2995c (leuB) | silent (Thr179) | 9871 | - | | 3355949 | A | C | 1538.77 | SNP | Rv2997 | Asp284Ala | 10 | - | | 3357464 | C | G | 3106.77 | SNP | intergenic |  |  | - | | 3358235 | A | T | 2481.77 | SNP | Rv2999 (lppY) | Met(s)212Leu(s) | 9867 | - | | 3363338 | A | G | 2538.77 | SNP | intergenic |  |  | - | | 3367765 | G | A | 2038.77 | SNP | Rv3009c (gatB) | silent (Gly343) | 9935 | - | | 3380804 | T | G | 2018.77 | SNP | Rv3022A (PE29) | Ile64Leu | 22 | - | | 3385218 | C | T | 1729.77 | SNP | Rv3026c | Gly287Glu | 4 | - | | 3393640 | C | T | 2110.77 | SNP | Rv3033 | silent (Asn87) | 9822 | - | | 3395654 | G | A | 1154.77 | SNP | Rv3035 | silent (Leu92) | 9947 | - | | 3401871 | A | G | 1961.77 | SNP | Rv3041c | silent (Ala16) | 9867 | - | | 3402816 | C | T | 2166.77 | SNP | Rv3042c (serB2) | Gly116Glu | 4 | - | | 3404376 | C | G | 1913.77 | SNP | Rv3043c (ctaD) | silent (Thr182) | 9871 | - | | 3413785 | C | T | 2722.77 | SNP | Rv3051c (nrdE) | Val128Ile | 33 | - | | 3415180 | ACACCTAGGGGGTGG | A | 8517.73 | DEL | intergenic |  |  | - | | 3420825 | A | G | 2417.77 | SNP | Rv3059 (cyp136) | Asp445Gly | 11 | - | | 3425854 | C | T | 1886.77 | SNP | Rv3062 (ligB) | Pro91Ser | 17 | - | | 3428897 | G | C | 1663.77 | SNP | Rv3063 (cstA) | Arg552Pro | 5 | - | | 3428917 | C | A | 1584.77 | SNP | Rv3063 (cstA) | Arg559Ser | 11 | - | | 3437007 | G | C | 1422.77 | SNP | Rv3074 | Ala77Pro | 13 | - | | 3440464 | T | G | 2134.77 | SNP | Rv3077 | silent (Arg308) | 9913 | - | | 3440468 | G | C | 2238.77 | SNP | Rv3077 | Gly310Arg | 0 | - | | 3442529 | G | A | 2068.77 | SNP | Rv3079c | silent (Leu23) | 9947 | - | | 3446128 | A | G | 2345.77 | SNP | Rv3081 | Asp30Gly | 11 | - | | 3448955 | CG | C | 1628.73 | DEL | Rv3083 |  |  | - | | 3450725 | T | C | 2208.77 | SNP | Rv3084 (lipR) | silent (Val243) | 9901 | - | | 3451405 | G | C | 2063.77 | SNP | Rv3085 | Val(s)162Val | 13 | - | | 3454263 | C | G | 2217.77 | SNP | Rv3087 | Leu447Val | 11 | - | | 3455686 | G | C | 3543.77 | SNP | Rv3088 (tgs4) | silent (Leu449) | 9947 | - | | 3456666 | A | G | 2272.77 | SNP | Rv3089 (fadD13) | silent (Ala302) | 9867 | - | | 3459081 | G | C | 2524.77 | SNP | Rv3090 | Ala291Pro | 13 | - | | 3462135 | G | C | 1808.77 | SNP | Rv3093c | Cys210Trp | 0 | - | | 3466919 | C | G | 946.77 | SNP | Rv3097c (lipY) | Gly58Ala | 21 | - | | 3467465 | C | G | 3174.77 | SNP | Rv3098c | silent (Ala66) | 9867 | - | | 3473996 | G | GA | 3017.73 | INS | intergenic |  |  | - | | 3478698 | G | A | 100.77 | SNP | Rv3109 (moaA1) | silent (Pro350) | 9926 | - | | 3480474 | G | A | 3612.77 | SNP | Rv3113 | Gly134Glu | 4 | - | | 3480789 | T | C | 4185.77 | SNP | Rv3114 | Ser11Pro | 12 | - | | 3485117 | A | C | 3352.77 | SNP | intergenic |  |  | - | | 3486977 | A | G | 3724.77 | SNP | Rv3121 (cyp141) | Lys157Glu | 4 | - | | 3488122 | G | A | 1709.77 | SNP | Rv3122 | Gly12Arg | 0 | - | | 3489665 | C | T | 3050.77 | SNP | Rv3124 (moaR1) | Pro54Ser | 17 | - | | 3499497 | G | C | 3194.83 | SNP | Rv3133c (devR) | Ala140Gly | 21 | - | | 3500243 | G | C | 2723.77 | SNP | Rv3134c | silent (Ala169) | 9867 | - | | 3503895 | C | T | 2194.77 | SNP | Rv3137 | Pro168Leu | 3 | - | | 3505027 | G | A | 572.77 | SNP | Rv3138 (pflA) | Arg278His | 8 | - | | 3506650 | A | C | 1686.77 | SNP | Rv3139 (fadE24) | Ile430Leu | 22 | - | | 3509615 | A | T | 245.78 | SNP | intergenic |  |  | - | | 3510120 | T | G | 1386.77 | SNP | Rv3144c (PPE52) | Lys400Gln | 6 | - | | 3515467 | C | T | 2274.77 | SNP | Rv3150 (nuoF) | Pro19Leu | 3 | - | | 3518167 | A | G | 953.77 | SNP | Rv3151 (nuoG) | Ile474Met(s) | 6 | - | | 3518555 | A | G | 1062.77 | SNP | Rv3151 (nuoG) | Thr604Ala | 32 | - | | 3526137 | T | G | 2082.77 | SNP | Rv3158 (nuoN) | silent (Ala116) | 9867 | - | | 3528064 | T | C | 200.77 | SNP | Rv3159c (PPE53) | Asn367Ser | 34 | - | | 3528065 | T | C | 188.77 | SNP | Rv3159c (PPE53) | Asn367Asp | 42 | - | | 3528066 | G | A | 195.77 | SNP | Rv3159c (PPE53) | silent (Asn366) | 9822 | - | | 3528072 | A | G | 249.77 | SNP | Rv3159c (PPE53) | silent (Ser364) | 9840 | - | | 3528084 | A | G | 284.77 | SNP | Rv3159c (PPE53) | silent (Gly360) | 9935 | - | | 3528087 | G | A | 283.77 | SNP | Rv3159c (PPE53) | silent (Ile359) | 9872 | - | | 3528099 | A | C | 250.77 | SNP | Rv3159c (PPE53) | silent (Gly355) | 9935 | - | | 3528102 | C | A | 275.77 | SNP | Rv3159c (PPE53) | silent (Ser354) | 9840 | - | | 3528117 | T | C | 267.77 | SNP | Rv3159c (PPE53) | silent (Leu349) | 9947 | - | | 3528119 | G | T | 296.77 | SNP | Rv3159c (PPE53) | Leu349Ile | 9 | - | | 3528120 | A | G | 332.77 | SNP | Rv3159c (PPE53) | silent (Asn348) | 9822 | - | | 3528129 | G | T | 314.77 | SNP | Rv3159c (PPE53) | silent (Gly345) | 9935 | - | | 3528140 | G | A | 386.77 | SNP | Rv3159c (PPE53) | Leu342Leu(s) | 4 | - | | 3528144 | G | A | 361.77 | SNP | Rv3159c (PPE53) | silent (Gly340) | 9935 | - | | 3528158 | A | G | 440.77 | SNP | Rv3159c (PPE53) | Leu(s)336Leu | 3 | - | | 3528159 | G | A | 513.77 | SNP | Rv3159c (PPE53) | silent (Asn335) | 9822 | - | | 3528165 | G | A | 725.77 | SNP | Rv3159c (PPE53) | silent (Gly333) | 9935 | - | | 3528192 | G | A | 602.77 | SNP | Rv3159c (PPE53) | silent (Asn324) | 9822 | - | | 3528198 | A | G | 586.77 | SNP | Rv3159c (PPE53) | silent (Asn322) | 9822 | - | | 3528971 | TCGC | T | 3791.73 | DEL | Rv3159c (PPE53) |  |  | - | | 3529067 | G | C | 1723.77 | SNP | Rv3159c (PPE53) | Arg33Gly | 1 | - | | 3530955 | C | G | 2649.77 | SNP | Rv3161c | Val62Leu | 15 | - | | 3539353 | G | A | 1698.77 | SNP | Rv3170 (aofH) | silent (Gln283) | 9876 | - | | 3556275 | A | G | 2322.77 | SNP | Rv3190c | Leu138Pro | 2 | - | | 3557253 | C | T | 3425.77 | SNP | intergenic |  |  | - | | 3562338 | G | T | 3016.77 | SNP | Rv3193c | Leu279Met(s) | 4 | - | | 3568199 | G | A | 1700.77 | SNP | Rv3197 | silent (Gln392) | 9876 | - | | 3580636 | CT | C | 2812.73 | DEL | intergenic |  |  | - | | 3581414 | A | G | 2369.77 | SNP | Rv3204 | Thr34Ala | 32 | - | | 3582694 | C | T | 3000.77 | SNP | Rv3206c (moeB1) | silent (Lys339) | 9926 | - | | 3590686 | G | GC | 3183.75 | INS | intergenic |  |  | - | | 3591063 | T | C | 1310.77 | SNP | Rv3213c | Lys144Glu | 4 | - | | 3600576 | C | T | 2201.77 | SNP | Rv3224 | silent (Cys242) | 9973 | - | | 3604821 | G | C | 771.77 | SNP | Rv3228 | silent (Ala32) | 9867 | - | | 3610391 | A | AC | 5964.73 | INS | Rv3234c (tgs3) |  |  | - | | 3611286 | GC | G | 3221.60 | DEL | intergenic |  |  | - | | 3614982 | T | C | 3044.77 | SNP | Rv3239c | silent (Leu874) | 9947 | - | | 3621423 | A | G | 1958.77 | SNP | intergenic |  |  | - | | 3622441 | A | C | 1535.77 | SNP | Rv3243c | Val217Val(s) | 18 | - | | 3625065 | T | G | 1558.77 | SNP | Rv3245c (mtrB) | Met(s)517Leu | 3 | - | | 3630122 | G | T | 1922.77 | SNP | Rv3249c | Ser89STOP | 35 | - | | 3631671 | G | GT | 5161.73 | INS | Rv3252c (alkB) |  |  | - | | 3634408 | T | C | 1304.77 | SNP | Rv3254 | Leu245Pro | 2 | - | | 3636991 | G | A | 920.77 | SNP | Rv3256c | Pro109Ser | 17 | - | | 3638093 | G | A | 1916.77 | SNP | Rv3257c (pmmA) | Ser206Leu(s) | 35 | - | | 3645524 | C | T | 2362.77 | SNP | Rv3264c (manB) | Asp152Asn | 36 | - | | 3653988 | G | A | 1314.77 | SNP | Rv3272 | Val(s)181Met(s) | 9867 | - | | 3656206 | C | A | 1741.77 | SNP | Rv3273 | Arg524Ser | 11 | - | | 3663889 | C | A | 128.77 | SNP | Rv3281 (accE5) | Asn67Lys | 25 | - | | 3668233 | G | A | 2251.77 | SNP | Rv3286c (sigF) | Ser241Leu | 1 | - | | 3670040 | C | T | 2064.77 | SNP | Rv3289c | silent (Ala124) | 9867 | - | | 3678094 | C | T | 2597.77 | SNP | Rv3296 (lhr) | silent (Ala440) | 9867 | - | | 3679764 | G | A | 626.77 | SNP | Rv3296 (lhr) | Ser997Asn | 20 | - | | 3681548 | A | C | 2416.77 | SNP | Rv3297 (nei) | silent (Arg77) | 9913 | - | | 3685510 | T | C | 1503.77 | SNP | Rv3299c (atsB) | Ser152Gly | 21 | - | | 3689523 | G | T | 1863.77 | SNP | Rv3303c (lpdA) | Cys472STOP | 3 | - | | 3690016 | A | G | 1326.77 | SNP | Rv3303c (lpdA) | Leu(s)308Ser | 28 | - | | 3693681 | A | C | 2190.77 | SNP | Rv3306c (amiB1) | silent (Ala103) | 9867 | - | | 3697585 | C | T | 1834.77 | SNP | Rv3310 (sapM) | Leu130Phe | 6 | - | | 3704596 | G | C | 2528.77 | SNP | Rv3317 (sdhD) | Val(s)54Leu | 3 | - | | 3706260 | G | C | 2013.77 | SNP | Rv3318 (sdhA) | Gly421Arg | 0 | - | | 3710382 | T | A | 58.28 | SNP | intergenic |  |  | - | | 3711910 | G | A | 332.78 | SNP | Rv3327 | Trp54STOP | 0 | - | | 3712372 | A | G | 165.84 | SNP | Rv3327 | silent (Leu208) | 9947 | - | | 3714211 | G | T | 1608.77 | SNP | Rv3328c (sigJ) | Pro41Gln | 6 | - | | 3714639 | G | A | 1944.77 | SNP | Rv3329 | Arg83His | 8 | - | | 3714757 | A | C | 1981.77 | SNP | Rv3329 | Gln122His | 20 | - | | 3715775 | C | T | 1792.77 | SNP | intergenic |  |  | - | | 3718357 | C | T | 1849.77 | SNP | Rv3331 (sugI) | Pro423Leu | 3 | - | | 3721806 | G | C | 2858.77 | SNP | Rv3335c | silent (Gly265) | 9935 | - | | 3729342 | C | T | 1107.77 | SNP | Rv3342 | Ala240Val(s) | 9867 | - | | 3729597 | G | C | 793.77 | SNP | Rv3343c (PPE54) | Leu2447Val | 11 | - | | 3730371 | C | T | 2329.77 | SNP | Rv3343c (PPE54) | Gly2189Ser | 16 | - | | 3730453 | C | T | 1918.77 | SNP | Rv3343c (PPE54) | Val(s)2161Val | 13 | - | | 3730466 | A | G | 1900.77 | SNP | Rv3343c (PPE54) | Ile2157Thr | 11 | - | | 3730675 | CCCGGTGCCAGAGTTAAAGA ACCCCACATTA | C | 3619.73 | DEL | Rv3343c (PPE54) |  |  | - | | 3732113 | C | G | 1095.77 | SNP | Rv3343c (PPE54) | Arg1608Pro | 5 | - | | 3732114 | G | T | 1046.77 | SNP | Rv3343c (PPE54) | silent (Arg1608) | 9913 | - | | 3732181 | C | T | 1068.77 | SNP | Rv3343c (PPE54) | Val(s)1585Val | 13 | - | | 3732310 | G | A | 946.77 | SNP | Rv3343c (PPE54) | silent (Asn1542) | 9822 | - | | 3732344 | G | A | 1105.77 | SNP | Rv3343c (PPE54) | Ala1531Val | 13 | - | | 3732517 | A | G | 840.77 | SNP | Rv3343c (PPE54) | silent (Ile1473) | 9872 | - | | 3732525 | A | T | 884.77 | SNP | Rv3343c (PPE54) | Phe1471Ile | 7 | - | | 3732553 | A | G | 877.77 | SNP | Rv3343c (PPE54) | silent (Ile1461) | 9872 | - | | 3732624 | A | G | 1289.77 | SNP | Rv3343c (PPE54) | Leu(s)1438Leu | 3 | - | | 3734112 | C | T | 2171.77 | SNP | Rv3343c (PPE54) | Val942Ile | 33 | - | | 3735802 | C | G | 1372.77 | SNP | Rv3343c (PPE54) | silent (Thr378) | 9871 | - | | 3736024 | A | G | 660.77 | SNP | Rv3343c (PPE54) | silent (Gly304) | 9935 | - | | 3736072 | A | G | 86.77 | SNP | Rv3343c (PPE54) | silent (Ile288) | 9872 | - | | 3736080 | A | T | 113.77 | SNP | Rv3343c (PPE54) | Phe286Ile | 7 | - | | 3736108 | A | G | 160.77 | SNP | Rv3343c (PPE54) | silent (Ile276) | 9872 | - | | 3736628 | T | G | 1278.77 | SNP | Rv3343c (PPE54) | Glu103Ala | 17 | - | | 3738364 | G | A | 537.77 | SNP | Rv3345c (PE\_PGRS50) | Arg1471Trp | 2 | - | | 3738416 | TG | T | 398.73 | DEL | Rv3345c (PE\_PGRS50) |  |  | - | | 3739496 | GACACCGCCA | G | 538.87 | DEL | Rv3345c (PE\_PGRS50) |  |  | - | | 3740181 | T | C | 717.77 | SNP | Rv3345c (PE\_PGRS50) | Asn865Ser | 34 | - | | 3741240 | C | T | 757.77 | SNP | Rv3345c (PE\_PGRS50) | Gly512Asp | 6 | - | | 3742165 | T | C | 138.90 | SNP | Rv3345c (PE\_PGRS50) | Thr204Ala | 32 | - | | 3742174 | C | A | 96.03 | SNP | Rv3345c (PE\_PGRS50) | Ala201Ser | 28 | - | | 3742183 | C | A | 66.28 | SNP | Rv3345c (PE\_PGRS50) | Ala198Ser | 28 | - | | 3746409 | A | G | 1270.77 | SNP | Rv3347c (PPE55) | Leu2259Pro | 2 | - | | 3749653 | G | A | 2963.77 | SNP | Rv3347c (PPE55) | Gln1178STOP | 8 | - | | 3749763 | G | T | 3070.77 | SNP | Rv3347c (PPE55) | Ser1141STOP | 35 | - | | 3750177 | A | T | 2042.77 | SNP | Rv3347c (PPE55) | Phe1003Tyr | 21 | - | | 3750178 | A | C | 2106.77 | SNP | Rv3347c (PPE55) | Phe1003Val | 1 | - | | 3750185 | C | G | 2031.77 | SNP | Rv3347c (PPE55) | silent (Ser1000) | 9840 | - | | 3750187 | A | T | 1958.77 | SNP | Rv3347c (PPE55) | Ser1000Thr | 32 | - | | 3750188 | C | G | 2065.77 | SNP | Rv3347c (PPE55) | Met(s)999Ile | 2 | - | | 3750193 | G | A | 1832.77 | SNP | Rv3347c (PPE55) | Leu998Phe | 6 | - | | 3750205 | C | T | 2067.77 | SNP | Rv3347c (PPE55) | Asp994Asn | 36 | - | | 3750209 | A | G | 1840.77 | SNP | Rv3347c (PPE55) | silent (Asn992) | 9822 | - | | 3750210 | T | G | 1992.77 | SNP | Rv3347c (PPE55) | Asn992Thr | 13 | - | | 3750407 | G | C | 2028.77 | SNP | Rv3347c (PPE55) | silent (Gly926) | 9935 | - | | 3750417 | A | T | 1986.77 | SNP | Rv3347c (PPE55) | Phe923Tyr | 21 | - | | 3750421 | T | C | 1965.77 | SNP | Rv3347c (PPE55) | Ser922Gly | 21 | - | | 3750584 | G | A | 999.77 | SNP | Rv3347c (PPE55) | silent (Asn867) | 9822 | - | | 3752003 | G | C | 1626.77 | SNP | Rv3347c (PPE55) | Val394Val(s) | 18 | - | | 3752006 | G | A | 1536.77 | SNP | Rv3347c (PPE55) | silent (Asn393) | 9822 | - | | 3752007 | T | C | 1626.77 | SNP | Rv3347c (PPE55) | Asn393Ser | 34 | - | | 3752008 | T | C | 1545.77 | SNP | Rv3347c (PPE55) | Asn393Asp | 42 | - | | 3752012 | C | G | 1611.77 | SNP | Rv3347c (PPE55) | silent (Pro391) | 9926 | - | | 3752207 | A | G | 1836.77 | SNP | Rv3347c (PPE55) | silent (Ile326) | 9872 | - | | 3753116 | C | T | 778.77 | SNP | Rv3347c (PPE55) | silent (Pro23) | 9926 | - | | 3753164 | T | G | 1387.77 | SNP | Rv3347c (PPE55) | silent (Pro7) | 9926 | - | | 3754169 | G | C | 173.84 | SNP | Rv3348 | Leu(s)135Phe | 1 | - | | 3758363 | T | C | 2269.77 | SNP | Rv3350c (PPE56) | Ile2914Val | 57 | - | | 3758999 | C | T | 1229.77 | SNP | Rv3350c (PPE56) | Gly2702Ser | 16 | - | | 3762496 | G | A | 1636.77 | SNP | Rv3350c (PPE56) | Thr1536Ile | 7 | - | | 3763313 | C | T | 829.77 | SNP | Rv3350c (PPE56) | Ala1264Thr | 22 | - | | 3763724 | A | G | 1686.77 | SNP | Rv3350c (PPE56) | Phe1127Leu | 13 | - | | 3765861 | G | A | 102.77 | SNP | Rv3350c (PPE56) | silent (Asn414) | 9822 | - | | 3767962 | A | G | 1098.77 | SNP | Rv3351c | Val60Ala | 18 | - | | 3771009 | T | G | 1928.77 | SNP | Rv3357 (relJ) | silent (Ser79) | 9840 | - | | 3772833 | CA | C | 3731.73 | DEL | Rv3360 |  |  | - | | 3787466 | G | C | 2715.77 | SNP | Rv3372 (otsB2) | Glu385Gln | 27 | - | | 3794867 | C | CCA | 5149.73 | INS | Rv3379c (dxs2) |  |  | - | | 3798095 | A | C | 5235.77 | SNP | Rv3383c (idsB) | Val132Gly | 5 | - | | 3799116 | A | G | 4387.77 | SNP | intergenic |  |  | - | | 3799512 | C | T | 1985.77 | SNP | Rv3384c (vapC46) | Ala42Thr | 22 | - | | 3815477 | G | T | 1448.77 | SNP | Rv3398c (idsA1) | silent (Ala210) | 9867 | - | | 3817117 | C | A | 1770.77 | SNP | Rv3399 | Ala330Glu | 10 | - | | 3820407 | A | G | 365.77 | SNP | intergenic |  |  | - | | 3820446 | G | C | 73.77 | SNP | intergenic |  |  | - | | 3820449 | T | G | 55.77 | SNP | intergenic |  |  | - | | 3820545 | A | G | 553.77 | SNP | intergenic |  |  | - | | 3821503 | A | C | 2043.77 | SNP | Rv3402c | Leu130Arg | 1 | - | | 3821845 | G | T | 2785.77 | SNP | Rv3402c | Ala16Glu | 10 | - | | 3823159 | A | T | 1995.77 | SNP | Rv3403c | silent (Val235) | 9901 | - | | 3826684 | C | T | 1563.77 | SNP | Rv3408 (vapC47) | Ser46Leu(s) | 35 | - | | 3829770 | T | C | 2063.77 | SNP | Rv3410c (guaB3) | silent (Pro47) | 9926 | - | | 3830695 | A | G | 1195.77 | SNP | Rv3411c (guaB2) | silent (Ala275) | 9867 | - | | 3835223 | C | T | 2077.77 | SNP | intergenic |  |  | - | | 3837887 | C | T | 1603.77 | SNP | Rv3419c (gcp) | Gly235Ser | 16 | - | | 3838871 | A | G | 1914.77 | SNP | Rv3420c (rimI) | silent (Ala64) | 9867 | - | | 3841652 | T | A | 163.77 | SNP | intergenic |  |  | - | | 3841654 | T | G | 309.77 | SNP | intergenic |  |  | - | | 3841662 | T | C | 529.77 | SNP | intergenic |  |  | - | | 3841663 | C | T | 406.77 | SNP | intergenic |  |  | - | | 3842228 | A | G | 42.77 | SNP | intergenic |  |  | - | | 3842452 | C | A | 42.77 | SNP | Rv3425 (PPE57) | Gln72Lys | 12 | - | | 3842620 | A | G | 3966.77 | SNP | Rv3425 (PPE57) | Thr128Ala | 32 | - | | 3842625 | A | G | 4145.77 | SNP | Rv3425 (PPE57) | silent (Pro129) | 9926 | - | | 3843001 | G | A | 530.77 | SNP | intergenic |  |  | - | | 3843024 | A | C | 596.77 | SNP | intergenic |  |  | - | | 3843025 | A | G | 517.77 | SNP | intergenic |  |  | - | | 3843032 | G | A | 507.77 | SNP | intergenic |  |  | - | | 3843354 | A | G | 234.77 | SNP | Rv3426 (PPE58) | Thr107Ala | 32 | - | | 3843356 | T | C | 277.77 | SNP | Rv3426 (PPE58) | silent (Thr107) | 9871 | - | | 3843361 | C | A | 261.77 | SNP | Rv3426 (PPE58) | Ala109Asp | 6 | - | | 3843362 | C | A | 267.77 | SNP | Rv3426 (PPE58) | silent (Ala109) | 9867 | - | | 3843363 | A | G | 278.77 | SNP | Rv3426 (PPE58) | Asn110Asp | 42 | - | | 3843407 | CG | C | 4411.73 | DEL | Rv3426 (PPE58) |  |  | - | | 3843696 | T | A | 484.77 | SNP | Rv3426 (PPE58) | Leu(s)221Met(s) | 9867 | - | | 3843704 | G | C | 512.77 | SNP | Rv3426 (PPE58) | silent (Thr223) | 9871 | - | | 3843714 | T | C | 725.77 | SNP | Rv3426 (PPE58) | Cys227Arg | 1 | - | | 3843749 | G | T | 580.77 | SNP | intergenic |  |  | - | | 3843751 | G | T | 594.77 | SNP | intergenic |  |  | - | | 3843752 | A | G | 654.77 | SNP | intergenic |  |  | - | | 3843753 | G | A | 605.77 | SNP | intergenic |  |  | - | | 3843760 | T | C | 677.77 | SNP | intergenic |  |  | - | | 3844756 | GC | G | 4484.73 | DEL | Rv3428c |  |  | - | | 3844992 | T | A | 3805.77 | SNP | Rv3428c | Ser327Cys | 5 | - | | 3845695 | C | T | 3303.77 | SNP | Rv3428c | silent (Arg92) | 9913 | - | | 3846591 | C | T | 142.77 | SNP | intergenic |  |  | - | | 3846605 | G | A | 1533.77 | SNP | intergenic |  |  | - | | 3846607 | A | C | 1655.77 | SNP | intergenic |  |  | - | | 3846622 | G | T | 1603.77 | SNP | intergenic |  |  | - | | 3846687 | A | G | 1634.77 | SNP | intergenic |  |  | - | | 3846704 | A | G | 1339.77 | SNP | intergenic |  |  | - | | 3846707 | A | C | 1332.77 | SNP | intergenic |  |  | - | | 3846716 | C | T | 227.77 | SNP | intergenic |  |  | - | | 3846727 | C | T | 211.77 | SNP | intergenic |  |  | - | | 3846728 | A | G | 203.77 | SNP | intergenic |  |  | - | | 3846741 | G | T | 305.77 | SNP | intergenic |  |  | - | | 3846743 | C | G | 388.77 | SNP | intergenic |  |  | - | | 3846764 | C | G | 616.77 | SNP | intergenic |  |  | - | | 3846774 | T | G | 480.77 | SNP | intergenic |  |  | - | | 3846777 | C | A | 533.77 | SNP | intergenic |  |  | - | | 3846779 | T | G | 587.77 | SNP | intergenic |  |  | - | | 3846840 | G | GCT | 3345.73 | INS | intergenic |  |  | - | | 3846843 | CAAA | C | 1477.87 | DEL | intergenic |  |  | - | | 3846851 | G | A | 687.77 | SNP | intergenic |  |  | - | | 3846852 | C | G | 756.77 | SNP | intergenic |  |  | - | | 3846853 | T | C | 648.77 | SNP | intergenic |  |  | - | | 3846857 | G | A | 696.77 | SNP | intergenic |  |  | - | | 3846860 | T | G | 737.77 | SNP | intergenic |  |  | - | | 3846866 | C | A | 635.78 | SNP | intergenic |  |  | - | | 3846881 | AT | A | 946.79 | DEL | intergenic |  |  | - | | 3846886 | A | T | 691.77 | SNP | intergenic |  |  | - | | 3846897 | T | G | 776.74 | SNP | intergenic |  |  | - | | 3847010 | G | C | 93.77 | SNP | intergenic |  |  | - | | 3847014 | C | G | 86.77 | SNP | intergenic |  |  | - | | 3847022 | T | C | 118.77 | SNP | intergenic |  |  | - | | 3847039 | G | A | 167.77 | SNP | intergenic |  |  | - | | 3847052 | G | A | 169.77 | SNP | intergenic |  |  | - | | 3847073 | G | C | 132.77 | SNP | intergenic |  |  | - | | 3847074 | C | G | 103.77 | SNP | intergenic |  |  | - | | 3847087 | G | A | 138.77 | SNP | intergenic |  |  | - | | 3847090 | G | C | 159.77 | SNP | intergenic |  |  | - | | 3847099 | G | A | 150.77 | SNP | intergenic |  |  | - | | 3847112 | T | A | 165.77 | SNP | intergenic |  |  | - | | 3847137 | T | C | 31.77 | SNP | intergenic |  |  | - | | 3847153 | A | C | 34.77 | SNP | intergenic |  |  | - | | 3847154 | A | G | 64.77 | SNP | intergenic |  |  | - | | 3859893 | C | T | 1899.77 | SNP | Rv3440c | silent (Glu28) | 9865 | - | | 3860216 | G | A | 1111.77 | SNP | Rv3441c (mrsA) | silent (Ala385) | 9867 | - | | 3862472 | GA | G | 2934.73 | DEL | intergenic |  |  | - | | 3864565 | T | G | 1833.77 | SNP | Rv3447c (eccC4) | Glu1225Ala | 17 | - | | 3864995 | T | C | 2446.77 | SNP | Rv3447c (eccC4) | Ser1082Gly | 21 | - | | 3869355 | T | C | 2047.77 | SNP | Rv3448 (eccD4) | Ile335Thr | 11 | - | | 3870010 | A | G | 1258.77 | SNP | Rv3449 (mycP4) | Thr87Ala | 32 | - | | 3871246 | T | C | 1043.77 | SNP | Rv3450c (eccB4) | silent (Gly417) | 9935 | - | | 3874722 | AT | A | 5604.73 | DEL | Rv3453 |  |  | - | | 3874745 | G | C | 3402.77 | SNP | intergenic |  |  | - | | 3877421 | A | G | 2883.77 | SNP | Rv3456c (rplQ) | silent (Pro4) | 9926 | - | | 3880175 | C | A | 1775.77 | SNP | intergenic |  |  | - | | 3880675 | T | TA | 6313.73 | INS | intergenic |  |  | - | | 3883683 | G | C | 441.77 | SNP | Rv3466 | Val(s)53Val | 13 | - | | 3883686 | T | G | 411.77 | SNP | Rv3466 | silent (Gly54) | 9935 | - | | 3883690 | G | A | 503.77 | SNP | Rv3466 | Ala56Thr | 22 | - | | 3883707 | T | C | 494.77 | SNP | Rv3466 | silent (Leu61) | 9947 | - | | 3883711 | G | A | 414.77 | SNP | Rv3466 | Ala63Thr | 22 | - | | 3883796 | G | T | 129.77 | SNP | Rv3466 | Arg91Leu | 1 | - | | 3883797 | G | A | 134.77 | SNP | Rv3466 | silent (Arg91) | 9913 | - | | 3883845 | T | C | 46.77 | SNP | Rv3466 | silent (Gly107) | 9935 | - | | 3883852 | C | T | 241.77 | SNP | Rv3466 | silent (Leu110) | 9947 | - | | 3883854 | A | G | 195.77 | SNP | Rv3466 | silent (Leu110) | 9947 | - | | 3884697 | C | G | 215.80 | SNP | Rv3467 | Ala245Gly | 21 | - | | 3885886 | T | C | 2659.77 | SNP | Rv3468c | Ile62Val | 57 | - | | 3892671 | A | G | 3405.77 | SNP | Rv3476c (kgtP) | silent (Val350) | 9901 | - | | 3893480 | G | A | 2364.77 | SNP | Rv3476c (kgtP) | Leu81Phe | 6 | - | | 3895727 | C | A | 2152.77 | SNP | intergenic |  |  | - | | 3896031 | C | G | 3049.77 | SNP | Rv3479 | Ala71Gly | 21 | - | | 3896340 | T | G | 1969.77 | SNP | Rv3479 | Leu174Arg | 1 | - | | 3898408 | A | G | 3001.77 | SNP | Rv3479 | silent (Ala863) | 9867 | - | | 3906462 | A | AT | 3072.73 | INS | Rv3487c (lipF) |  |  | - | | 3908062 | C | T | 1932.77 | SNP | intergenic |  |  | - | | 3909235 | G | C | 2594.77 | SNP | Rv3490 (otsA) | Val(s)334Leu | 3 | - | | 3918649 | T | C | 1359.77 | SNP | Rv3499c (mce4A) | silent (Gly184) | 9935 | - | | 3928257 | GGGCGGTACC | G | 202 | DEL | Rv3507 (PE\_PGRS53) |  |  | - | | 3928448 | C | CCT | 294.80 | INS | Rv3507 (PE\_PGRS53) |  |  | - | | 3928784 | C | CCGGCGGGGCCGGCGGTAA | 2558.73 | INS | Rv3507 (PE\_PGRS53) |  |  | - | | 3929084 | C | G | 194.77 | SNP | Rv3507 (PE\_PGRS53) | Ala839Gly | 21 | - | | 3931139 | T | C | 293.77 | SNP | Rv3508 (PE\_PGRS54) | silent (Asp45) | 9859 | - | | 3934542 | T | G | 118.03 | SNP | Rv3508 (PE\_PGRS54) | Ser1180Ala | 35 | - | | 3934699 | G | A | 163.84 | SNP | Rv3508 (PE\_PGRS54) | Ser1232Asn | 20 | - | | 3934733 | G | C | 135.82 | SNP | Rv3508 (PE\_PGRS54) | silent (Gly1243) | 9935 | - | | 3934734 | G | A | 86.78 | SNP | Rv3508 (PE\_PGRS54) | Ala1244Thr | 22 | - | | 3934878 | G | A | 209.77 | SNP | Rv3508 (PE\_PGRS54) | Asp1292Asn | 36 | - | | 3934897 | T | C | 180.77 | SNP | Rv3508 (PE\_PGRS54) | Phe1298Ser | 3 | - | | 3940802 | A | G | 386.77 | SNP | Rv3511 (PE\_PGRS55) | Asn396Asp | 42 | - | | 3941351 | ACCGGCGGCAACGCCGGCG | A | 2108.73 | DEL | Rv3511 (PE\_PGRS55) |  |  | - | | 3941497 | G | C | 56.77 | SNP | Rv3511 (PE\_PGRS55) | silent (Gly627) | 9935 | - | | 3941572 | C | CGGCCAACAA | 698.73 | INS | Rv3511 (PE\_PGRS55) |  |  | - | | 3941723 | AC | A | 185.80 | DEL | Rv3511 (PE\_PGRS55) |  |  | - | | 3941834 | G | C | 295.77 | SNP | intergenic |  |  | - | | 3941836 | C | A | 289.77 | SNP | intergenic |  |  | - | | 3942481 | C | G | 245.80 | SNP | intergenic |  |  | - | | 3942640 | T | C | 368.77 | SNP | intergenic |  |  | - | | 3943019 | C | G | 176.90 | SNP | intergenic |  |  | - | | 3943079 | C | A | 116.03 | SNP | intergenic |  |  | - | | 3943160 | C | T | 31.74 | SNP | intergenic |  |  | - | | 3945192 | C | T | 1001.77 | SNP | Rv3513c (fadD18) | Arg186His | 8 | - | | 3947395 | G | A | 88.77 | SNP | Rv3514 (PE\_PGRS57) | silent (Gly534) | 9935 | - | | 3948404 | T | G | 50.28 | SNP | Rv3514 (PE\_PGRS57) | Cys871Gly | 1 | - | | 3948414 | T | G | 62.28 | SNP | Rv3514 (PE\_PGRS57) | Val874Gly | 5 | - | | 3948417 | T | G | 63.28 | SNP | Rv3514 (PE\_PGRS57) | Val875Gly | 5 | - | | 3948541 | AGGCGGCACC | A | 691.73 | DEL | Rv3514 (PE\_PGRS57) |  |  | - | | 3948612 | ACGGCGGCAC | A | 667.73 | DEL | Rv3514 (PE\_PGRS57) |  |  | - | | 3949531 | C | G | 258.96 | SNP | Rv3514 (PE\_PGRS57) | silent (Gly1246) | 9935 | - | | 3949532 | C | G | 231.80 | SNP | Rv3514 (PE\_PGRS57) | Pro1247Ala | 22 | - | | 3949767 | C | G | 57.28 | SNP | Rv3514 (PE\_PGRS57) | Thr1325Ser | 38 | - | | 3950099 | GACGGTGGTA | G | 2011.73 | DEL | Rv3514 (PE\_PGRS57) |  |  | - | | 3952800 | G | A | 1646.77 | SNP | Rv3516 (echA19) | Gly86Asp | 6 | - | | 3954846 | G | A | 2903.77 | SNP | Rv3518c (cyp142) | Leu226Leu(s) | 4 | - | | 3958403 | A | G | 2572.77 | SNP | Rv3521 | Asn295Asp | 42 | - | | 3959418 | C | T | 1488.77 | SNP | Rv3522 (ltp4) | Thr324Ile | 7 | - | | 3964930 | C | T | 5041.77 | SNP | Rv3528c | Gly88Glu | 4 | - | | 3981983 | G | A | 1971.77 | SNP | Rv3543c (fadE29) | silent (Pro386) | 9926 | - | | 3984321 | G | A | 2490.77 | SNP | Rv3545c (cyp125) | silent (His375) | 9912 | - | | 3989504 | G | A | 2401.77 | SNP | Rv3550 (echA20) | Val118Ile | 33 | - | | 3990093 | C | T | 2122.77 | SNP | Rv3551 | silent (Val66) | 9901 | - | | 3994101 | A | G | 2342.77 | SNP | Rv3554 (fdxB) | Ile473Val | 57 | - | | 3994305 | C | T | 1816.77 | SNP | Rv3554 (fdxB) | Leu541Leu(s) | 4 | - | | 3998041 | GTT | G | 3917.73 | DEL | Rv3558 (PPE64) |  |  | - | | 4004896 | G | A | 1368.77 | SNP | Rv3563 (fadE32) | silent (Gln202) | 9876 | - | | 4005114 | G | C | 1581.77 | SNP | Rv3563 (fadE32) | Trp275Ser | 5 | - | | 4005335 | C | T | 1405.77 | SNP | Rv3564 (fadE33) | Ala30Val(s) | 9867 | - | | 4005607 | T | C | 1258.77 | SNP | Rv3564 (fadE33) | Leu(s)121Leu | 3 | - | | 4007004 | G | A | 1916.77 | SNP | Rv3565 (aspB) | Val269Ile | 33 | - | | 4007272 | G | A | 1822.77 | SNP | Rv3565 (aspB) | Arg358Gln | 9 | - | | 4008747 | A | G | 2030.77 | SNP | Rv3567c (hsaB) | Ile179Thr | 11 | - | | 4012286 | C | T | 2067.77 | SNP | intergenic |  |  | - | | 4024273 | T | C | 2266.77 | SNP | Rv3581c (ispF) | Val25Val(s) | 18 | - | | 4026899 | G | A | 1870.77 | SNP | Rv3585 (radA) | silent (Gln152) | 9876 | - | | 4028752 | A | G | 1776.77 | SNP | Rv3586 | Thr288Ala | 32 | - | | 4033711 | A | G | 2231.77 | SNP | Rv3591c | Val(s)111Ala | 9867 | - | | 4034827 | C | T | 2537.77 | SNP | Rv3593 (lpqF) | Ala159Val(s) | 9867 | - | | 4035033 | G | A | 2334.77 | SNP | Rv3593 (lpqF) | Ala228Thr | 22 | - | | 4037283 | T | G | 386.77 | SNP | Rv3595c (PE\_PGRS59) | silent (Gly256) | 9935 | - | | 4044872 | G | A | 1914.77 | SNP | Rv3602c (panC) | silent (Gly113) | 9935 | - | | 4052349 | T | G | 2090.77 | SNP | Rv3610c (ftsH) | Lys179Gln | 6 | - | | 4052966 | C | T | 1419.77 | SNP | Rv3611 | Pro6Leu | 3 | - | | 4055801 | G | A | 2214.77 | SNP | Rv3616c (espA) | Thr192Ile | 7 | - | | 4056416 | C | A | 4150.77 | SNP | intergenic |  |  | - | | 4057036 | A | G | 3115.77 | SNP | intergenic |  |  | - | | 4059186 | G | T | 1278.77 | SNP | Rv3618 | silent (Ala163) | 9867 | - | | 4059904 | A | G | 1422.77 | SNP | intergenic |  |  | - | | 4060100 | G | A | 617.77 | SNP | Rv3619c (esxV) | Leu57Leu(s) | 4 | - | | 4060201 | G | A | 472.77 | SNP | Rv3619c (esxV) | Ser23Leu(s) | 35 | - | | 4060210 | T | A | 428.77 | SNP | Rv3619c (esxV) | Gln20Leu | 6 | - | | 4060230 | G | A | 358.77 | SNP | Rv3619c (esxV) | silent (His13) | 9912 | - | | 4061892 | G | A | 2134.77 | SNP | intergenic |  |  | - | | 4065300 | G | T | 2071.77 | SNP | Rv3626c | silent (Arg202) | 9913 | - | | 4069292 | G | A | 1123.77 | SNP | Rv3630 | Ala40Thr | 22 | - | | 4080057 | GCCAGAA | G | 7697.73 | DEL | Rv3641c (fic) |  |  | - | | 4082100 | C | T | 1097.77 | SNP | Rv3644c | Val208Ile | 33 | - | | 4087479 | TCCGCATCGTCGCCGAACTA GGTCCGACTGCCCGGCTCCT CAGCGGACCCCAGCGGGA | T | 1539.73 | DEL | intergenic |  |  | - | | 4087880 | G | A | 1224.77 | SNP | Rv3647c | silent (Pro103) | 9926 | - | | 4088543 | T | TCCTTTTCTTCTGGGTGCGG TGCACCG | 7093.73 | INS | intergenic |  |  | - | | 4089058 | T | C | 1611.77 | SNP | Rv3649 | Leu93Pro | 2 | - | | 4090453 | C | G | 1379.77 | SNP | Rv3649 | Ala558Gly | 21 | - | | 4092376 | T | C | 2407.77 | SNP | Rv3651 | Ile179Thr | 11 | - | | 4094346 | T | TCGGCGCCGGCGGCGCCGG | 4250.73 | INS | Rv3653 (PE\_PGRS61) |  |  | - | | 4095001 | CG | C | 2448.73 | DEL | Rv3655c |  |  | - | | 4095295 | T | C | 2526.77 | SNP | Rv3655c | silent (Glu2) | 9865 | - | | 4096636 | C | T | 972.77 | SNP | Rv3658c | Gly102Ser | 16 | - | | 4097490 | A | AC | 1941.73 | INS | Rv3659c |  |  | - | | 4097569 | G | T | 861.77 | SNP | Rv3659c | silent (Ala142) | 9867 | - | | 4098522 | C | G | 1509.77 | SNP | Rv3660c | silent (Gly209) | 9935 | - | | 4100975 | T | C | 1135.77 | SNP | intergenic |  |  | - | | 4107074 | T | C | 2163.77 | SNP | Rv3666c (dppA) | Gln4Arg | 10 | - | | 4108386 | G | C | 1738.77 | SNP | Rv3667 (acs) | Ala199Pro | 13 | - | | 4111303 | G | C | 2492.77 | SNP | Rv3669 | Val(s)159Val | 13 | - | | 4112429 | T | C | 2661.77 | SNP | Rv3671c | Ile363Val | 57 | - | | 4115952 | C | T | 2701.77 | SNP | intergenic |  |  | - | | 4117467 | C | G | 1929.77 | SNP | Rv3677c | Asp196His | 3 | - | | 4120926 | A | G | 685.77 | SNP | Rv3680 | Asn378Asp | 42 | - | | 4120983 | A | G | 1813.77 | SNP | intergenic |  |  | - | | 4122287 | G | T | 1532.77 | SNP | Rv3682 (ponA2) | silent (Ala124) | 9867 | - | | 4124254 | C | G | 639.77 | SNP | Rv3682 (ponA2) | Pro780Arg | 4 | - | | 4132509 | G | A | 1685.77 | SNP | intergenic |  |  | - | | 4133010 | G | A | 1941.77 | SNP | Rv3691 | Ala165Thr | 22 | - | | 4135103 | C | T | 1395.77 | SNP | Rv3693 | silent (Arg126) | 9913 | - | | 4138622 | G | A | 2199.77 | SNP | Rv3696c (glpK) | silent (Arg378) | 9913 | - | | 4142192 | G | A | 1700.77 | SNP | Rv3699 | Gly50Glu | 4 | - | | 4145150 | G | C | 2424.77 | SNP | Rv3702c | Val155Val(s) | 18 | - | | 4145737 | A | G | 1642.77 | SNP | Rv3703c | silent (Tyr385) | 9945 | - | | 4148801 | C | A | 2133.77 | SNP | Rv3705c | Glu54Asp | 53 | - | | 4151855 | A | G | 1602.77 | SNP | Rv3708c (asd) | silent (Pro121) | 9926 | - | | 4156099 | C | A | 2091.77 | SNP | Rv3711c (dnaQ) | Val(s)211Leu(s) | 9867 | - | | 4156503 | C | T | 1743.77 | SNP | Rv3711c (dnaQ) | Gly76Asp | 6 | - | | 4159195 | T | C | 1918.77 | SNP | Rv3714c | silent (Pro209) | 9926 | - | | 4162339 | A | G | 3219.84 | SNP | Rv3719 | Thr12Ala | 32 | - | | 4166290 | G | A | 2754.77 | SNP | Rv3721c (dnaZX) | His148Tyr | 4 | - | | 4166441 | G | A | 1974.77 | SNP | Rv3721c (dnaZX) | silent (His97) | 9912 | - | | 4169852 | G | T | 1994.77 | SNP | Rv3724B (cut5b) | Ala83Ser | 28 | - | | 4170739 | G | C | 2974.77 | SNP | Rv3725 | Val176Leu | 15 | - | | 4170964 | G | GA | 4013.73 | INS | Rv3725 |  |  | - | | 4171479 | C | G | 1960.77 | SNP | Rv3726 | Ala20Gly | 21 | - | | 4176359 | C | T | 715.77 | SNP | Rv3728 | Pro496Leu | 3 | - | | 4179089 | C | T | 1938.77 | SNP | Rv3729 | Pro269Ser | 17 | - | | 4182695 | G | A | 2094.77 | SNP | Rv3731 (ligC) | Arg313His | 8 | - | | 4187485 | T | C | 2700.77 | SNP | Rv3736 | silent (Ala284) | 9867 | - | | 4187817 | A | G | 1882.77 | SNP | Rv3737 | Asp40Gly | 11 | - | | 4195390 | A | T | 1690.77 | SNP | intergenic |  |  | - | | 4195799 | C | A | 1827.77 | SNP | Rv3744 (nmtR) | silent (Gly120) | 9935 | - | | 4197138 | C | CT | 2611.73 | INS | intergenic |  |  | - | | 4198611 | CG | C | 3256.73 | DEL | intergenic |  |  | - | | 4199024 | C | G | 2687.77 | SNP | Rv3751 | Leu51Val | 11 | - | | 4204441 | A | G | 2247.77 | SNP | Rv3759c (proX) | silent (His311) | 9912 | - | | 4205120 | A | G | 2752.77 | SNP | Rv3759c (proX) | Leu85Pro | 2 | - | | 4210274 | A | G | 2471.77 | SNP | Rv3764c (tcrY) | Cys246Arg | 1 | - | | 4215484 | G | C | 1949.77 | SNP | Rv3770c | Pro98Ala | 22 | - | | 4217557 | A | G | 2184.77 | SNP | Rv3772 (hisC2) | Thr142Ala | 32 | - | | 4221490 | C | G | 1777.77 | SNP | Rv3776 | silent (Leu134) | 9947 | - | | 4222073 | A | G | 969.77 | SNP | Rv3776 | Met(s)329Val(s) | 9867 | - | | 4222131 | C | T | 1122.77 | SNP | Rv3776 | Ala348Val | 13 | - | | 4222533 | C | T | 1752.77 | SNP | Rv3776 | Ala482Val | 13 | - | | 4222882 | A | G | 1653.77 | SNP | Rv3777 | silent (Leu63) | 9947 | - | | 4223172 | T | C | 1325.77 | SNP | Rv3777 | Val160Ala | 18 | - | | 4226275 | C | T | 2906.77 | SNP | Rv3779 | Pro431Ser | 17 | - | | 4231859 | CGCGGTCTGTGCGCCGGGCG CACGCACCGAATTCGAGCTA TTAGCGGCCGCGGCAAGGGA CGCATTCGGCCTGGACGTCC ACCCAGCGGT | C | 1546.73 | DEL | Rv3785 |  |  | - | | 4232555 | T | C | 2680.77 | SNP | Rv3786c | Gln348Arg | 10 | - | | 4240172 | G | A | 2727.77 | SNP | Rv3793 (embC) | Val(s)104Met(s) | 9867 | - | | 4241562 | G | A | 1495.77 | SNP | Rv3793 (embC) | Arg567His | 8 | genotype | | 4242075 | G | A | 1427.77 | SNP | Rv3793 (embC) | Arg738Gln | 9 | genotype | | 4242643 | C | T | 1201.77 | SNP | Rv3793 (embC) | silent (Arg927) | 9913 | genotype | | 4247429 | A | G | 2293.77 | SNP | Rv3795 (embB) | Met(s)306Val(s) | 9867 | resistance | | 4251297 | G | C | 1640.77 | SNP | Rv3797 (fadE35) | silent (Gly71) | 9935 | - | | 4254006 | G | T | 34.74 | SNP | Rv3798 | silent (Leu338) | 9947 | - | | 4255922 | A | G | 2861.77 | SNP | Rv3799c (accD4) | silent (His9) | 9912 | - | | 4257220 | A | G | 2304.77 | SNP | Rv3800c (pks13) | silent (Arg1309) | 9913 | - | | 4258447 | G | A | 1105.77 | SNP | Rv3800c (pks13) | silent (Arg900) | 9913 | - | | 4262388 | C | T | 2143.77 | SNP | Rv3801c (fadD32) | Gly227Ser | 16 | - | | 4266647 | A | G | 2053.77 | SNP | Rv3804c (fbpA) | silent (Val4) | 9901 | - | | 4272744 | G | A | 1942.77 | SNP | Rv3809c (glf) | silent (Thr244) | 9871 | - | | 4282707 | C | G | 3759.77 | SNP | Rv3818 | Pro87Ala | 22 | - | | 4286826 | A | G | 3349.77 | SNP | Rv3822 | Lys36Glu | 4 | - | | 4287164 | A | G | 2475.77 | SNP | Rv3822 | silent (Gly148) | 9935 | - | | 4292941 | G | A | 3241.77 | SNP | Rv3824c (papA1) | silent (His78) | 9912 | - | | 4296015 | G | A | 3305.77 | SNP | Rv3825c (pks2) | silent (Asp1197) | 9859 | - | | 4301870 | T | C | 1490.77 | SNP | Rv3827c | His307Arg | 10 | - | | 4302036 | T | C | 2384.77 | SNP | Rv3827c | Thr252Ala | 32 | - | | 4303842 | A | G | 2213.77 | SNP | Rv3829c | silent (Asp389) | 9859 | - | | 4305243 | G | A | 1810.77 | SNP | Rv3830c | Pro148Leu | 3 | - | | 4306155 | C | T | 1519.77 | SNP | Rv3831 | silent (Ser133) | 9840 | - | | 4306896 | C | T | 2542.77 | SNP | Rv3833 | silent (Ala10) | 9867 | - | | 4307179 | G | A | 2420.77 | SNP | Rv3833 | Val105Ile | 33 | - | | 4313128 | C | T | 1960.77 | SNP | Rv3839 | Pro122Ser | 17 | - | | 4313156 | G | A | 2418.77 | SNP | Rv3839 | Arg131Gln | 9 | - | | 4314645 | A | G | 1826.77 | SNP | Rv3841 (bfrB) | silent (Leu156) | 9947 | - | | 4314800 | G | A | 2730.77 | SNP | Rv3842c (glpQ1) | His255Tyr | 4 | - | | 4317264 | G | A | 2447.77 | SNP | intergenic |  |  | - | | 4321515 | C | A | 2783.77 | SNP | intergenic |  |  | - | | 4322039 | AC | A | 2988.73 | DEL | Rv3847 |  |  | - | | 4326284 | A | C | 4169.77 | SNP | Rv3854c (ethA) | Leu397Arg | 1 | - | | 4328154 | G | A | 3520.77 | SNP | Rv3855 (ethR) | silent (Thr202) | 9871 | - | | 4328492 | G | A | 1714.77 | SNP | Rv3856c | Ala306Val | 13 | - | | 4329782 | G | A | 3167.77 | SNP | intergenic |  |  | - | | 4336991 | C | T | 788.77 | SNP | Rv3860 | Ala72Val | 13 | - | | 4338595 | GC | G | 6754.73 | DEL | intergenic |  |  | - | | 4338732 | G | A | 2848.77 | SNP | intergenic |  |  | - | | 4343784 | G | A | 2038.77 | SNP | Rv3868 (eccA1) | silent (Lys157) | 9926 | - | | 4345548 | G | A | 3215.74 | SNP | Rv3869 (eccB1) | Met(s)170Ile | 2 | - | | 4348820 | G | GGC | 3362.73 | INS | intergenic |  |  | - | | 4351039 | G | T | 2168.77 | SNP | Rv3872 (PE35) | Glu99STOP | 17 | - | | 4355319 | C | G | 2946.77 | SNP | Rv3877 (eccD1) | Leu105Val(s) | 4 | - | | 4356110 | G | C | 2148.77 | SNP | Rv3877 (eccD1) | silent (Leu368) | 9947 | - | | 4357597 | C | G | 1549.77 | SNP | Rv3879c (espK) | Cys729Ser | 11 | - | | 4359165 | G | C | 943.77 | SNP | Rv3879c (espK) | silent (Thr206) | 9871 | - | | 4366195 | T | C | 2260.77 | SNP | Rv3884c (eccA2) | Glu215Gly | 7 | - | | 4366272 | G | C | 1840.77 | SNP | Rv3884c (eccA2) | silent (Ala189) | 9867 | - | | 4372353 | G | C | 1300.77 | SNP | Rv3888c | silent (Arg118) | 9913 | - | | 4372540 | A | C | 2420.77 | SNP | Rv3888c | Val56Gly | 5 | - | | 4372813 | C | A | 2602.77 | SNP | Rv3889c (espG2) | Arg273Leu | 1 | - | | 4373259 | G | A | 1985.77 | SNP | Rv3889c (espG2) | silent (Ala124) | 9867 | - | | 4375452 | G | A | 1677.77 | SNP | Rv3892c (PPE69) | Leu78Leu(s) | 4 | - | | 4375628 | G | T | 1796.77 | SNP | Rv3892c (PPE69) | Thr19Lys | 11 | - | | 4379680 | C | G | 2791.77 | SNP | Rv3894c (eccC2) | Arg258Pro | 5 | - | | 4380158 | C | T | 2655.77 | SNP | Rv3894c (eccC2) | Glu99Lys | 7 | - | | 4380159 | C | A | 2633.77 | SNP | Rv3894c (eccC2) | Leu(s)98Phe | 1 | - | | 4382054 | T | C | 2210.77 | SNP | Rv3896c | silent (Ala266) | 9867 | - | | 4382275 | G | T | 1497.77 | SNP | Rv3896c | Gln193Lys | 12 | - | | 4383144 | C | CCGGGG | 3993.73 | INS | Rv3897c |  |  | - | | 4383655 | A | G | 1458.77 | SNP | Rv3898c | STOP111Gln | 3 | - | | 4384007 | C | G | 2023.77 | SNP | intergenic |  |  | - | | 4386228 | T | C | 2616.77 | SNP | Rv3900c | silent (Leu27) | 9947 | - | | 4386248 | G | C | 2465.77 | SNP | Rv3900c | Pro21Ala | 22 | - | | 4388414 | G | A | 3103.77 | SNP | Rv3903c | silent (Asp673) | 9859 | - | | 4390471 | C | A | 2374.77 | SNP | Rv3904c (esxE) | Gly80Val | 3 | - | | 4393178 | A | G | 1813.77 | SNP | intergenic |  |  | - | | 4396495 | C | A | 1247.77 | SNP | Rv3909 | silent (Gly768) | 9935 | - | | 4397736 | C | T | 2547.77 | SNP | Rv3910 | silent (Ile380) | 9872 | - | | 4400660 | AC | A | 3963.73 | DEL | Rv3911 (sigM) |  |  | - | | 4401509 | C | T | 2438.77 | SNP | Rv3912 | Arg214Trp | 2 | - | | 4407588 | T | C | 2354.77 | SNP | Rv3919c (gid) | silent (Ala205) | 9867 | genotype | | 4408087 | CG | C | 3316.73 | DEL | Rv3919c (gid) |  |  | - | | 4408923 | C | T | 2487.77 | SNP | intergenic |  |  | - | | 4409954 | G | C | 3944.77 | SNP | Rv3921c | Ala39Gly | 21 | - | |  | | export |

elog
