# Supplementary material for: Patterns of compensatory mutations in rpoA/B/C genes of multidrug resistant M. tuberculosis in Uganda
Source: PLoS One. 2025 Dec 4;20(12):e0328957. doi: 10.1371/journal.pone.0328957 (PMC12677784; doi:10.1371/journal.pone.0328957)
Supplement: S2 File — (ZIP) [file pone.0328957.s002.zip › Variants K_S11_L001_001.bam.html]

 

Calling SNPs/INDELs (computing variant list in .vcf format) from K\_S11\_L001\_001.bam

*by SAMtools/BCFtools:*

Howto

Important aspects

This takes up to one hour!!! **Please wait ...**

Variants K\_S11\_L001\_001.bam

|  |  |
| --- | --- |
| Variants |  |

|  |  |
| --- | --- |
| |  | | --- | | *by GATK* | |

|  |  |  |
| --- | --- | --- |
| |  | | --- | | K\_S11\_L001\_001.bam | | | computed 2016-10-27 using PhyResSE v1.0 (Ref. NC\_000962.3) | |

|  |  |
| --- | --- |
| 1398  variants called Export in VCF format |  |

|  |  |  |  |  |  |  |  |  |  |  |  |  |  |  |  |  |  |  |  |  |  |  |  |  |  |  |  |  |  |  |  |  |  |  |  |  |  |  |  |  |  |  |  |  |  |  |  |  |  |  |  |  |  |  |  |  |  |  |  |  |  |  |  |  |  |  |  |  |  |  |  |  |  |  |  |  |  |  |  |  |  |  |  |  |  |  |  |  |  |  |  |  |  |  |  |  |  |  |  |  |  |  |  |  |  |  |  |  |  |  |  |  |  |  |  |  |  |  |  |  |  |  |  |  |  |  |  |  |  |  |  |  |  |  |  |  |  |  |  |  |  |  |  |  |  |  |  |  |  |  |  |  |  |  |  |  |  |  |  |  |  |  |  |  |  |  |  |  |  |  |  |  |  |  |  |  |  |  |  |  |  |  |  |  |  |  |  |  |  |  |  |  |  |  |  |  |  |  |  |  |  |  |  |  |  |  |  |  |  |  |  |  |  |  |  |  |  |  |  |  |  |  |  |  |  |  |  |  |  |  |  |  |  |  |  |  |  |  |  |  |  |  |  |  |  |  |  |  |  |  |  |  |  |  |  |  |  |  |  |  |  |  |  |  |  |  |  |  |  |  |  |  |  |  |  |  |  |  |  |  |  |  |  |  |  |  |  |  |  |  |  |  |  |  |  |  |  |  |  |  |  |  |  |  |  |  |  |  |  |  |  |  |  |  |  |  |  |  |  |  |  |  |  |  |  |  |  |  |  |  |  |  |  |  |  |  |  |  |  |  |  |  |  |  |  |  |  |  |  |  |  |  |  |  |  |  |  |  |  |  |  |  |  |  |  |  |  |  |  |  |  |  |  |  |  |  |  |  |  |  |  |  |  |  |  |  |  |  |  |  |  |  |  |  |  |  |  |  |  |  |  |  |  |  |  |  |  |  |  |  |  |  |  |  |  |  |  |  |  |  |  |  |  |  |  |  |  |  |  |  |  |  |  |  |  |  |  |  |  |  |  |  |  |  |  |  |  |  |  |  |  |  |  |  |  |  |  |  |  |  |  |  |  |  |  |  |  |  |  |  |  |  |  |  |  |  |  |  |  |  |  |  |  |  |  |  |  |  |  |  |  |  |  |  |  |  |  |  |  |  |  |  |  |  |  |  |  |  |  |  |  |  |  |  |  |  |  |  |  |  |  |  |  |  |  |  |  |  |  |  |  |  |  |  |  |  |  |  |  |  |  |  |  |  |  |  |  |  |  |  |  |  |  |  |  |  |  |  |  |  |  |  |  |  |  |  |  |  |  |  |  |  |  |  |  |  |  |  |  |  |  |  |  |  |  |  |  |  |  |  |  |  |  |  |  |  |  |  |  |  |  |  |  |  |  |  |  |  |  |  |  |  |  |  |  |  |  |  |  |  |  |  |  |  |  |  |  |  |  |  |  |  |  |  |  |  |  |  |  |  |  |  |  |  |  |  |  |  |  |  |  |  |  |  |  |  |  |  |  |  |  |  |  |  |  |  |  |  |  |  |  |  |  |  |  |  |  |  |  |  |  |  |  |  |  |  |  |  |  |  |  |  |  |  |  |  |  |  |  |  |  |  |  |  |  |  |  |  |  |  |  |  |  |  |  |  |  |  |  |  |  |  |  |  |  |  |  |  |  |  |  |  |  |  |  |  |  |  |  |  |  |  |  |  |  |  |  |  |  |  |  |  |  |  |  |  |  |  |  |  |  |  |  |  |  |  |  |  |  |  |  |  |  |  |  |  |  |  |  |  |  |  |  |  |  |  |  |  |  |  |  |  |  |  |  |  |  |  |  |  |  |  |  |  |  |  |  |  |  |  |  |  |  |  |  |  |  |  |  |  |  |  |  |  |  |  |  |  |  |  |  |  |  |  |  |  |  |  |  |  |  |  |  |  |  |  |  |  |  |  |  |  |  |  |  |  |  |  |  |  |  |  |  |  |  |  |  |  |  |  |  |  |  |  |  |  |  |  |  |  |  |  |  |  |  |  |  |  |  |  |  |  |  |  |  |  |  |  |  |  |  |  |  |  |  |  |  |  |  |  |  |  |  |  |  |  |  |  |  |  |  |  |  |  |  |  |  |  |  |  |  |  |  |  |  |  |  |  |  |  |  |  |  |  |  |  |  |  |  |  |  |  |  |  |  |  |  |  |  |  |  |  |  |  |  |  |  |  |  |  |  |  |  |  |  |  |  |  |  |  |  |  |  |  |  |  |  |  |  |  |  |  |  |  |  |  |  |  |  |  |  |  |  |  |  |  |  |  |  |  |  |  |  |  |  |  |  |  |  |  |  |  |  |  |  |  |  |  |  |  |  |  |  |  |  |  |  |  |  |  |  |  |  |  |  |  |  |  |  |  |  |  |  |  |  |  |  |  |  |  |  |  |  |  |  |  |  |  |  |  |  |  |  |  |  |  |  |  |  |  |  |  |  |  |  |  |  |  |  |  |  |  |  |  |  |  |  |  |  |  |  |  |  |  |  |  |  |  |  |  |  |  |  |  |  |  |  |  |  |  |  |  |  |  |  |  |  |  |  |  |  |  |  |  |  |  |  |  |  |  |  |  |  |  |  |  |  |  |  |  |  |  |  |  |  |  |  |  |  |  |  |  |  |  |  |  |  |  |  |  |  |  |  |  |  |  |  |  |  |  |  |  |  |  |  |  |  |  |  |  |  |  |  |  |  |  |  |  |  |  |  |  |  |  |  |  |  |  |  |  |  |  |  |  |  |  |  |  |  |  |  |  |  |  |  |  |  |  |  |  |  |  |  |  |  |  |  |  |  |  |  |  |  |  |  |  |  |  |  |  |  |  |  |  |  |  |  |  |  |  |  |  |  |  |  |  |  |  |  |  |  |  |  |  |  |  |  |  |  |  |  |  |  |  |  |  |  |  |  |  |  |  |  |  |  |  |  |  |  |  |  |  |  |  |  |  |  |  |  |  |  |  |  |  |  |  |  |  |  |  |  |  |  |  |  |  |  |  |  |  |  |  |  |  |  |  |  |  |  |  |  |  |  |  |  |  |  |  |  |  |  |  |  |  |  |  |  |  |  |  |  |  |  |  |  |  |  |  |  |  |  |  |  |  |  |  |  |  |  |  |  |  |  |  |  |  |  |  |  |  |  |  |  |  |  |  |  |  |  |  |  |  |  |  |  |  |  |  |  |  |  |  |  |  |  |  |  |  |  |  |  |  |  |  |  |  |  |  |  |  |  |  |  |  |  |  |  |  |  |  |  |  |  |  |  |  |  |  |  |  |  |  |  |  |  |  |  |  |  |  |  |  |  |  |  |  |  |  |  |  |  |  |  |  |  |  |  |  |  |  |  |  |  |  |  |  |  |  |  |  |  |  |  |  |  |  |  |  |  |  |  |  |  |  |  |  |  |  |  |  |  |  |  |  |  |  |  |  |  |  |  |  |  |  |  |  |  |  |  |  |  |  |  |  |  |  |  |  |  |  |  |  |  |  |  |  |  |  |  |  |  |  |  |  |  |  |  |  |  |  |  |  |  |  |  |  |  |  |  |  |  |  |  |  |  |  |  |  |  |  |  |  |  |  |  |  |  |  |  |  |  |  |  |  |  |  |  |  |  |  |  |  |  |  |  |  |  |  |  |  |  |  |  |  |  |  |  |  |  |  |  |  |  |  |  |  |  |  |  |  |  |  |  |  |  |  |  |  |  |  |  |  |  |  |  |  |  |  |  |  |  |  |  |  |  |  |  |  |  |  |  |  |  |  |  |  |  |  |  |  |  |  |  |  |  |  |  |  |  |  |  |  |  |  |  |  |  |  |  |  |  |  |  |  |  |  |  |  |  |  |  |  |  |  |  |  |  |  |  |  |  |  |  |  |  |  |  |  |  |  |  |  |  |  |  |  |  |  |  |  |  |  |  |  |  |  |  |  |  |  |  |  |  |  |  |  |  |  |  |  |  |  |  |  |  |  |  |  |  |  |  |  |  |  |  |  |  |  |  |  |  |  |  |  |  |  |  |  |  |  |  |  |  |  |  |  |  |  |  |  |  |  |  |  |  |  |  |  |  |  |  |  |  |  |  |  |  |  |  |  |  |  |  |  |  |  |  |  |  |  |  |  |  |  |  |  |  |  |  |  |  |  |  |  |  |  |  |  |  |  |  |  |  |  |  |  |  |  |  |  |  |  |  |  |  |  |  |  |  |  |  |  |  |  |  |  |  |  |  |  |  |  |  |  |  |  |  |  |  |  |  |  |  |  |  |  |  |  |  |  |  |  |  |  |  |  |  |  |  |  |  |  |  |  |  |  |  |  |  |  |  |  |  |  |  |  |  |  |  |  |  |  |  |  |  |  |  |  |  |  |  |  |  |  |  |  |  |  |  |  |  |  |  |  |  |  |  |  |  |  |  |  |  |  |  |  |  |  |  |  |  |  |  |  |  |  |  |  |  |  |  |  |  |  |  |  |  |  |  |  |  |  |  |  |  |  |  |  |  |  |  |  |  |  |  |  |  |  |  |  |  |  |  |  |  |  |  |  |  |  |  |  |  |  |  |  |  |  |  |  |  |  |  |  |  |  |  |  |  |  |  |  |  |  |  |  |  |  |  |  |  |  |  |  |  |  |  |  |  |  |  |  |  |  |  |  |  |  |  |  |  |  |  |  |  |  |  |  |  |  |  |  |  |  |  |  |  |  |  |  |  |  |  |  |  |  |  |  |  |  |  |  |  |  |  |  |  |  |  |  |  |  |  |  |  |  |  |  |  |  |  |  |  |  |  |  |  |  |  |  |  |  |  |  |  |  |  |  |  |  |  |  |  |  |  |  |  |  |  |  |  |  |  |  |  |  |  |  |  |  |  |  |  |  |  |  |  |  |  |  |  |  |  |  |  |  |  |  |  |  |  |  |  |  |  |  |  |  |  |  |  |  |  |  |  |  |  |  |  |  |  |  |  |  |  |  |  |  |  |  |  |  |  |  |  |  |  |  |  |  |  |  |  |  |  |  |  |  |  |  |  |  |  |  |  |  |  |  |  |  |  |  |  |  |  |  |  |  |  |  |  |  |  |  |  |  |  |  |  |  |  |  |  |  |  |  |  |  |  |  |  |  |  |  |  |  |  |  |  |  |  |  |  |  |  |  |  |  |  |  |  |  |  |  |  |  |  |  |  |  |  |  |  |  |  |  |  |  |  |  |  |  |  |  |  |  |  |  |  |  |  |  |  |  |  |  |  |  |  |  |  |  |  |  |  |  |  |  |  |  |  |  |  |  |  |  |  |  |  |  |  |  |  |  |  |  |  |  |  |  |  |  |  |  |  |  |  |  |  |  |  |  |  |  |  |  |  |  |  |  |  |  |  |  |  |  |  |  |  |  |  |  |  |  |  |  |  |  |  |  |  |  |  |  |  |  |  |  |  |  |  |  |  |  |  |  |  |  |  |  |  |  |  |  |  |  |  |  |  |  |  |  |  |  |  |  |  |  |  |  |  |  |  |  |  |  |  |  |  |  |  |  |  |  |  |  |  |  |  |  |  |  |  |  |  |  |  |  |  |  |  |  |  |  |  |  |  |  |  |  |  |  |  |  |  |  |  |  |  |  |  |  |  |  |  |  |  |  |  |  |  |  |  |  |  |  |  |  |  |  |  |  |  |  |  |  |  |  |  |  |  |  |  |  |  |  |  |  |  |  |  |  |  |  |  |  |  |  |  |  |  |  |  |  |  |  |  |  |  |  |  |  |  |  |  |  |  |  |  |  |  |  |  |  |  |  |  |  |  |  |  |  |  |  |  |  |  |  |  |  |  |  |  |  |  |  |  |  |  |  |  |  |  |  |  |  |  |  |  |  |  |  |  |  |  |  |  |  |  |  |  |  |  |  |  |  |  |  |  |  |  |  |  |  |  |  |  |  |  |  |  |  |  |  |  |  |  |  |  |  |  |  |  |  |  |  |  |  |  |  |  |  |  |  |  |  |  |  |  |  |  |  |  |  |  |  |  |  |  |  |  |  |  |  |  |  |  |  |  |  |  |  |  |  |  |  |  |  |  |  |  |  |  |  |  |  |  |  |  |  |  |  |  |  |  |  |  |  |  |  |  |  |  |  |  |  |  |  |  |  |  |  |  |  |  |  |  |  |  |  |  |  |  |  |  |  |  |  |  |  |  |  |  |  |  |  |  |  |  |  |  |  |  |  |  |  |  |  |  |  |  |  |  |  |  |  |  |  |  |  |  |  |  |  |  |  |  |  |  |  |  |  |  |  |  |  |  |  |  |  |  |  |  |  |  |  |  |  |  |  |  |  |  |  |  |  |  |  |  |  |  |  |  |  |  |  |  |  |  |  |  |  |  |  |  |  |  |  |  |  |  |  |  |  |  |  |  |  |  |  |  |  |  |  |  |  |  |  |  |  |  |  |  |  |  |  |  |  |  |  |  |  |  |  |  |  |  |  |  |  |  |  |  |  |  |  |  |  |  |  |  |  |  |  |  |  |  |  |  |  |  |  |  |  |  |  |  |  |  |  |  |  |  |  |  |  |  |  |  |  |  |  |  |  |  |  |  |  |  |  |  |  |  |  |  |  |  |  |  |  |  |  |  |  |  |  |  |  |  |  |  |  |  |  |  |  |  |  |  |  |  |  |  |  |  |  |  |  |  |  |  |  |  |  |  |  |  |  |  |  |  |  |  |  |  |  |  |  |  |  |  |  |  |  |  |  |  |  |  |  |  |  |  |  |  |  |  |  |  |  |  |  |  |  |  |  |  |  |  |  |  |  |  |  |  |  |  |  |  |  |  |  |  |  |  |  |  |  |  |  |  |  |  |  |  |  |  |  |  |  |  |  |  |  |  |  |  |  |  |  |  |  |  |  |  |  |  |  |  |  |  |  |  |  |  |  |  |  |  |  |  |  |  |  |  |  |  |  |  |  |  |  |  |  |  |  |  |  |  |  |  |  |  |  |  |  |  |  |  |  |  |  |  |  |  |  |  |  |  |  |  |  |  |  |  |  |  |  |  |  |  |  |  |  |  |  |  |  |  |  |  |  |  |  |  |  |  |  |  |  |  |  |  |  |  |  |  |  |  |  |  |  |  |  |  |  |  |  |  |  |  |  |  |  |  |  |  |  |  |  |  |  |  |  |  |  |  |  |  |  |  |  |  |  |  |  |  |  |  |  |  |  |  |  |  |  |  |  |  |  |  |  |  |  |  |  |  |  |  |  |  |  |  |  |  |  |  |  |  |  |  |  |  |  |  |  |  |  |  |  |  |  |  |  |  |  |  |  |  |  |  |  |  |  |  |  |  |  |  |  |  |  |  |  |  |  |  |  |  |  |  |  |  |  |  |  |  |  |  |  |  |  |  |  |  |  |  |  |  |  |  |  |  |  |  |  |  |  |  |  |  |  |  |  |  |  |  |  |  |  |  |  |  |  |  |  |  |  |  |  |  |  |  |  |  |  |  |  |  |  |  |  |  |  |  |  |  |  |  |  |  |  |  |  |  |  |  |  |  |  |  |  |  |  |  |  |  |  |  |  |  |  |  |  |  |  |  |  |  |  |  |  |  |  |  |  |  |  |  |  |  |  |  |  |  |  |  |  |  |  |  |  |  |  |  |  |  |  |  |  |  |  |  |  |  |  |  |  |  |  |  |  |  |  |  |  |  |  |  |  |  |  |  |  |  |  |  |  |  |  |  |  |  |  |  |  |  |  |  |  |  |  |  |  |  |  |  |  |  |  |  |  |  |  |  |  |  |  |  |  |  |  |  |  |  |  |  |  |  |  |  |  |  |  |  |  |  |  |  |  |  |  |  |  |  |  |  |  |  |  |  |  |  |  |  |  |  |  |  |  |  |  |  |  |  |  |  |  |  |  |  |  |  |  |  |  |  |  |  |  |  |  |  |  |  |  |  |  |  |  |  |  |  |  |  |  |  |  |  |  |  |  |  |  |  |  |  |  |  |  |  |  |  |  |  |  |  |  |  |  |  |  |  |  |  |  |  |  |  |  |  |  |  |  |  |  |  |  |  |  |  |  |  |  |  |  |  |  |  |  |  |  |  |  |  |  |  |  |  |  |  |  |  |  |  |  |  |  |  |  |  |  |  |  |  |  |  |  |  |  |  |  |  |  |  |  |  |  |  |  |  |  |  |  |  |  |  |  |  |  |  |  |  |  |  |  |  |  |  |  |  |  |  |  |  |  |  |  |  |  |  |  |  |  |  |  |  |  |  |  |  |  |  |  |  |  |  |  |  |  |  |  |  |  |  |  |  |  |  |  |  |  |  |  |  |  |  |  |  |  |  |  |  |  |  |  |  |  |  |  |  |  |  |  |  |  |  |  |  |  |  |  |  |  |  |  |  |  |  |  |  |  |  |  |  |  |  |  |  |  |  |  |  |  |  |  |  |  |  |  |  |  |  |  |  |  |  |  |  |  |  |  |  |  |  |  |  |  |  |  |  |  |  |  |  |  |  |  |  |  |  |  |  |  |  |  |  |  |  |  |  |  |  |  |  |  |  |  |  |  |  |  |  |  |  |  |  |  |  |  |  |  |  |  |  |  |  |  |  |  |  |  |  |  |  |  |  |  |  |  |  |  |  |  |  |  |  |  |  |  |  |  |  |  |  |  |  |  |  |  |  |  |  |  |  |  |  |  |  |  |  |  |  |  |  |  |  |  |  |  |  |  |  |  |  |  |  |  |  |  |  |  |  |  |  |  |  |  |  |  |  |  |  |  |  |  |  |  |  |  |  |  |  |  |  |  |  |  |  |  |  |  |  |  |  |  |  |  |  |  |  |  |  |  |  |  |  |  |  |  |  |  |  |  |  |  |  |  |  |  |  |  |  |  |  |  |  |  |  |  |  |  |  |  |  |  |  |  |  |  |  |  |  |  |  |  |  |  |  |  |  |  |  |  |  |  |  |  |  |  |  |  |  |  |  |  |  |  |  |  |  |  |  |  |  |  |  |  |  |  |  |  |  |  |  |  |  |  |  |  |  |  |  |  |  |  |  |  |  |  |  |  |  |  |  |  |  |  |  |  |  |  |  |  |  |  |  |  |  |  |  |  |  |  |  |  |  |  |  |  |  |  |  |  |  |  |  |  |  |  |  |  |  |  |  |  |  |  |  |  |  |  |  |  |  |  |  |  |  |  |  |  |  |  |  |  |  |  |  |  |  |  |  |  |  |  |  |  |  |  |  |  |  |  |  |  |  |  |  |  |  |  |  |  |  |  |  |  |  |  |  |  |  |  |  |  |  |  |  |  |  |  |  |  |  |  |  |  |  |  |  |  |  |  |  |  |  |  |  |  |  |  |  |  |  |  |  |  |  |  |  |  |  |  |  |  |  |  |  |  |  |  |  |  |  |  |  |  |  |  |  |  |  |  |  |  |  |  |  |  |  |  |  |  |  |  |  |  |  |  |  |  |  |  |  |  |  |  |  |  |  |  |  |  |  |  |  |  |  |  |  |  |  |  |  |  |  |  |  |  |  |  |  |  |  |  |  |  |  |  |  |  |  |  |  |  |  |  |  |  |  |  |  |  |  |  |  |  |  |  |  |  |  |  |  |  |  |  |  |  |  |  |  |  |  |  |  |  |  |  |  |  |  |  |  |  |  |  |  |  |  |  |  |  |  |  |  |  |  |  |  |  |  |  |  |  |  |  |  |  |  |  |  |  |  |  |  |  |  |  |  |  |  |  |  |  |  |  |  |  |  |  |  |  |  |  |  |  |  |  |  |  |  |  |  |  |  |  |  |  |  |  |  |  |  |  |  |  |  |  |  |  |  |  |  |  |  |  |  |  |  |  |  |  |  |  |  |  |  |  |  |  |  |  |  |  |  |  |  |  |  |  |  |  |  |  |  |  |  |  |  |  |  |  |  |  |  |  |  |  |  |  |  |  |  |  |  |  |  |  |  |  |  |  |  |  |  |  |  |  |  |  |  |  |  |  |  |  |  |  |  |  |  |  |  |  |  |  |  |  |  |  |  |  |  |  |  |  |  |  |  |  |  |  |  |  |  |  |  |  |  |  |  |  |  |  |  |  |  |  |  |  |  |  |  |  |  |  |  |  |  |  |  |  |  |  |  |  |  |  |  |  |  |  |  |  |  |  |  |  |  |  |  |  |  |  |  |  |  |  |  |  |  |  |  |  |  |  |  |  |  |  |  |  |  |  |  |  |  |  |  |  |  |  |  |  |  |  |  |  |  |  |  |  |  |  |  |  |  |  |  |  |  |  |  |  |  |  |  |  |  |  |  |  |  |  |  |  |  |  |  |  |  |  |  |  |  |  |  |  |  |  |  |  |  |  |  |  |  |  |  |  |  |  |  |  |  |  |  |  |  |  |  |  |  |  |  |  |  |  |  |  |  |  |  |  |  |  |  |  |  |  |  |  |  |  |  |  |  |  |  |  |  |  |  |  |  |  |  |  |  |  |  |  |  |  |  |  |  |  |  |  |  |  |  |  |  |  |  |  |  |  |  |  |  |  |  |  |  |  |  |  |  |  |  |  |  |  |  |  |  |  |  |  |  |  |  |  |  |  |  |  |  |  |  |  |  |  |  |  |  |  |  |  |  |  |  |  |  |  |  |  |  |  |  |  |  |  |  |  |  |  |  |  |  |  |  |  |  |  |  |  |  |  |  |  |  |  |  |  |  |  |  |  |  |  |  |  |  |  |  |  |  |  |  |  |  |  |  |  |  |  |  |  |  |  |  |  |  |  |  |  |  |  |  |  |  |  |  |  |  |  |  |  |  |  |  |  |  |  |  |  |  |  |  |  |  |  |  |  |  |  |  |  |  |  |  |  |  |  |  |  |  |  |  |  |  |  |  |  |  |  |  |  |  |  |  |  |  |  |  |  |  |  |  |  |  |  |  |  |  |  |  |  |  |  |  |  |  |  |  |  |  |  |  |  |  |  |  |  |  |  |  |  |  |  |  |  |  |  |  |  |  |  |  |  |  |  |  |  |  |  |  |  |  |  |  |  |  |  |  |  |  |  |  |  |  |  |  |  |  |  |  |  |  |  |  |  |  |  |  |  |  |  |  |  |  |  |  |  |  |  |  |  |  |  |  |  |  |  |  |  |  |  |  |  |  |  |  |  |  |  |  |  |  |  |  |  |  |  |  |  |  |  |  |  |  |  |  |  |  |  |  |  |  |  |  |  |  |  |  |  |  |  |  |  |  |  |  |  |  |  |  |  |  |  |  |  |  |  |  |  |  |  |  |  |  |  |  |  |  |  |  |  |  |  |  |  |  |  |  |  |  |  |  |  |  |  |  |  |  |  |  |  |  |  |  |  |  |  |  |  |  |  |  |  |  |  |  |  |  |  |  |  |  |  |  |  |  |  |  |  |  |  |  |  |  |  |  |  |  |  |  |  |  |  |  |  |  |  |  |  |  |  |  |  |  |  |  |  |  |  |  |  |  |  |  |  |  |  |  |  |  |  |  |  |  |  |  |  |  |  |  |  |  |  |  |  |  |  |  |  |  |  |  |  |  |  |  |  |  |  |  |  |  |  |  |  |  |  |  |  |  |  |  |  |  |  |  |  |  |  |  |  |  |  |  |  |  |  |  |  |  |  |  |  |  |  |  |  |  |  |  |  |  |  |  |  |  |  |  |  |  |  |  |  |  |  |  |  |  |  |  |  |  |  |  |  |  |  |  |  |  |  |  |  |  |  |  |  |  |  |  |  |  |  |  |  |  |  |  |  |  |  |  |  |  |  |  |  |  |  |  |  |  |  |  |  |  |  |  |  |  |  |  |  |  |  |  |  |  |  |  |  |  |  |  |  |  |  |  |  |  |  |  |  |  |  |  |  |  |  |  |  |  |  |  |  |  |  |  |  |  |  |  |  |  |  |  |  |  |  |  |  |  |  |  |  |  |  |  |  |  |  |  |  |  |  |  |  |  |  |  |  |  |  |  |  |  |  |  |  |  |  |  |  |  |  |  |  |  |  |  |  |  |  |  |  |  |  |  |  |  |  |  |  |  |  |  |  |  |  |  |  |  |  |  |  |  |  |  |  |  |  |  |  |  |  |  |  |  |  |  |  |  |  |  |  |  |  |  |  |  |  |  |  |  |  |  |  |  |  |  |  |  |  |  |  |  |  |  |  |  |  |  |  |  |  |  |  |  |  |  |  |  |  |  |  |  |  |  |  |  |  |  |  |  |  |  |  |  |  |  |  |  |  |  |  |  |  |  |  |  |  |  |  |  |  |  |  |  |  |  |  |  |  |  |  |  |  |  |  |  |  |  |  |  |  |  |  |  |  |  |  |  |  |  |  |  |  |  |  |  |  |  |  |  |  |  |  |  |  |  |  |  |  |  |  |  |  |  |  |  |  |  |  |  |  |  |  |  |  |  |  |  |  |  |  |  |  |  |  |  |  |  |  |  |  |  |  |  |  |  |  |  |  |  |  |  |  |  |  |  |  |  |  |  |  |  |  |  |  |  |  |  |  |  |  |  |  |  |  |  |  |  |  |  |  |  |  |  |  |  |  |  |  |  |  |  |  |  |  |  |  |  |  |  |  |  |  |  |  |  |  |  |  |  |  |  |  |  |  |  |  |  |  |  |  |  |  |  |  |  |  |  |  |  |  |  |  |  |  |  |  |  |  |  |  |  |  |  |  |  |  |  |  |  |  |  |  |  |  |  |  |  |  |  |  |  |  |  |  |  |  |  |  |  |  |  |  |  |  |  |  |  |  |  |  |  |  |  |  |  |  |  |  |  |  |  |  |  |  |  |  |  |  |  |  |  |  |  |  |  |  |  |  |  |  |  |  |  |  |  |  |  |  |  |  |  |  |  |  |  |  |  |  |  |  |  |  |  |  |  |  |  |  |  |  |  |  |  |  |  |  |  |  |  |  |  |  |  |  |  |  |  |  |  |  |  |  |  |  |  |  |  |  |  |  |  |  |  |  |  |  |  |  |  |  |  |  |  |  |  |  |  |  |  |  |  |  |  |  |  |  |  |  |  |  |  |  |  |  |  |  |  |  |  |  |  |  |  |  |  |  |  |  |  |  |  |  |  |  |  |  |  |  |  |  |  |  |  |  |  |  |  |  |  |  |  |  |  |  |  |  |  |  |  |  |  |  |  |  |  |  |  |  |  |  |  |  |  |  |  |  |  |  |  |  |  |  |  |  |  |  |  |  |  |  |  |  |  |  |  |  |  |  |  |  |  |  |  |  |  |  |  |  |  |  |  |  |  |  |  |  |  |  |  |  |  |  |  |  |  |  |  |  |  |  |  |  |  |  |  |  |  |  |  |  |  |  |  |  |  |  |  |  |  |  |  |  |  |  |  |  |  |  |  |  |  |  |  |  |  |  |  |  |  |  |  |  |  |  |  |  |  |  |  |  |  |  |  |  |  |  |  |  |  |  |  |  |  |  |  |  |  |  |  |  |  |  |  |  |  |  |  |  |  |  |  |  |  |  |  |  |  |  |  |  |  |  |  |  |  |  |  |  |  |  |  |  |  |  |  |  |  |  |  |  |  |  |  |  |  |  |  |  |  |  |  |  |  |  |  |  |  |  |  |  |  |  |  |  |  |  |  |  |  |  |  |  |  |  |  |  |  |  |  |  |  |  |  |  |  |  |  |  |  |  |  |  |  |  |  |  |  |  |  |  |  |  |  |  |  |  |  |  |  |  |  |  |  |  |  |  |  |  |  |  |  |  |  |  |  |  |  |  |  |  |  |  |  |  |  |  |  |  |  |  |  |  |  |  |  |  |  |  |  |  |  |  |  |  |  |  |  |  |  |  |  |  |  |  |  |  |  |  |  |  |  |  |  |  |  |  |  |  |  |  |  |  |  |  |  |  |  |  |  |  |  |  |  |  |  |  |  |  |  |  |  |  |  |  |  |  |  |  |  |  |  |  |  |  |  |  |  |  |  |  |  |  |  |  |  |  |  |  |  |  |  |  |  |  |  |  |  |  |  |  |  |  |  |  |  |  |  |  |  |  |  |  |  |  |  |  |  |  |  |  |  |  |  |  |  |  |  |  |  |  |  |  |  |  |  |  |  |  |  |  |  |  |  |  |  |  |  |  |  |  |  |  |  |  |  |  |  |  |  |  |  |  |  |  |  |  |  |  |  |  |  |  |  |  |  |  |  |  |  |  |  |  |  |  |  |  |  |  |  |  |  |  |  |  |  |  |  |  |  |  |  |  |  |  |  |  |  |  |  |  |  |  |  |  |  |  |  |  |  |  |  |  |  |  |  |  |  |  |  |  |  |  |  |  |  |  |  |  |  |  |  |  |  |  |  |  |  |  |  |  |  |  |  |  |  |  |  |  |  |  |  |  |  |  |  |  |  |  |  |  |  |  |  |  |  |  |  |  |  |  |  |  |  |  |  |  |  |  |  |  |  |  |  |  |  |  |  |  |  |  |  |  |  |  |  |  |  |  |  |  |  |  |  |  |  |  |  |  |  |  |  |  |  |  |  |  |  |  |  |  |  |  |  |  |  |  |  |  |  |  |  |  |  |  |  |  |  |  |  |  |  |  |  |  |  |  |  |  |  |  |  |  |  |  |  |  |  |  |  |  |  |  |  |  |  |  |  |  |  |  |  |  |  |  |  |  |  |  |  |  |  |  |  |  |  |  |  |  |  |  |  |  |  |  |  |  |  |  |  |  |  |  |  |  |  |  |  |  |  |  |  |  |  |  |  |  |  |  |  |  |  |  |  |  |  |  |  |  |  |  |  |  |  |  |  |  |  |  |  |  |  |  |  |  |  |  |  |  |  |  |  |  |  |  |  |  |  |  |  |  |  |  |  |  |  |  |  |  |  |  |  |  |  |  |  |  |  |  |  |  |  |  |  |  |  |  |  |  |  |  |  |  |  |  |  |  |  |  |  |  |  |  |  |  |  |  |  |  |  |  |  |  |  |  |  |  |  |  |  |  |  |  |  |  |  |  |  |  |  |  |  |  |  |  |  |  |  |  |  |  |  |  |  |  |  |  |  |  |  |  |  |  |  |  |  |  |  |  |  |  |  |  |  |  |  |  |  |  |  |  |  |  |  |  |  |  |  |  |  |  |  |  |  |  |  |  |  |  |  |  |  |  |  |  |  |  |  |  |  |  |  |  |  |  |  |  |  |  |  |  |  |  |  |  |  |  |  |  |  |  |  |  |  |  |  |  |  |  |  |  |  |  |  |  |  |  |  |  |  |  |  |  |  |  |  |  |  |  |  |  |  |  |  |  |  |  |  |  |  |  |  |  |  |  |  |  |  |  |  |  |  |  |  |  |  |  |  |  |  |  |  |  |  |  |  |  |  |  |  |  |  |  |  |  |  |  |  |  |  |  |  |  |  |  |  |  |  |  |  |  |  |  |  |  |  |  |  |  |  |  |  |  |  |  |  |  |  |  |  |  |  |  |  |  |  |  |  |  |  |  |  |  |  |  |  |  |  |  |  |  |  |  |  |  |  |  |  |  |  |  |  |  |  |  |  |  |  |  |  |  |  |  |  |  |  |  |  |  |  |  |  |  |  |  |  |  |  |  |  |  |  |  |  |  |  |  |  |  |  |  |  |  |  |  |  |  |  |  |  |  |  |  |  |  |  |  |  |  |  |  |  |  |  |  |  |  |  |  |  |  |  |  |  |  |  |  |  |  |  |  |  |  |  |  |  |  |  |  |  |  |  |  |  |  |  |  |  |  |  |  |  |  |  |  |  |  |  |  |  |  |  |  |  |  |  |  |  |  |  |  |  |  |  |  |  |  |  |  |  |  |  |  |  |  |  |  |  |  |  |  |  |  |  |  |  |  |  |  |  |  |  |  |  |  |  |  |  |  |  |  |  |  |  |  |  |  |  |  |  |  |  |  |  |  |  |  |  |  |  |  |  |  |  |  |  |  |  |  |  |  |  |  |  |  |  |  |  |  |  |  |  |  |  |  |  |  |  |  |  |  |  |  |  |  |  |  |  |  |  |  |  |  |  |  |  |  |  |  |  |  |  |  |  |  |  |  |  |  |  |  |  |  |  |  |  |  |  |  |  |  |  |  |  |  |  |  |  |  |  |  |  |  |  |  |  |  |  |  |  |  |  |  |  |  |  |  |  |  |  |  |  |  |  |  |  |  |  |  |  |  |  |  |  |  |  |  |  |  |  |  |  |  |  |  |  |  |  |  |  |  |  |  |  |  |  |  |  |  |  |  |  |  |  |  |  |  |  |  |  |  |  |  |  |  |  |  |  |  |  |  |  |  |  |  |  |  |  |  |  |  |  |  |  |  |  |  |  |  |  |  |  |  |  |  |  |  |  |  |  |  |  |  |  |  |  |  |  |  |  |  |  |  |  |  |  |  |  |  |  |  |  |  |  |  |  |  |  |  |  |  |  |  |  |  |  |  |  |  |  |  |  |  |  |  |  |  |  |  |  |  |  |  |  |  |  |  |  |  |  |  |  |  |  |  |  |  |  |  |  |  |  |  |  |  |  |  |  |  |  |  |  |  |  |  |  |  |  |  |  |  |  |  |  |  |  |  |  |  |  |  |  |  |  |  |  |  |  |  |  |  |  |  |  |  |  |  |  |  |  |  |  |  |  |  |  |  |  |  |  |  |  |  |  |  |  |  |  |  |  |  |  |  |  |  |  |  |  |  |  |  |  |  |  |  |  |  |  |  |  |  |  |  |  |  |  |  |  |  |  |  |  |  |  |  |  |  |  |  |  |  |  |  |  |  |  |  |  |  |  |  |  |  |  |  |  |  |  |  |  |  |  |  |  |  |  |  |  |  |  |  |  |  |  |  |  |  |  |  |  |  |  |  |  |  |  |  |  |  |  |  |  |  |  |  |  |  |  |  |  |  |  |  |  |  |  |  |  |  |  |  |  |  |  |  |  |  |  |  |  |  |  |  |  |  |  |  |  |  |  |  |  |  |  |  |  |  |  |  |  |  |  |  |  |  |  |  |  |  |  |  |  |  |  |  |  |  |  |  |  |  |  |  |  |  |  |  |  |  |  |  |  |  |  |  |  |  |  |  |  |  |  |  |  |  |  |  |  |  |  |  |  |  |  |  |  |  |  |  |  |  |  |  |  |  |  |  |  |  |  |  |  |  |  |  |  |  |  |  |  |  |  |  |  |  |  |  |  |  |  |  |  |  |  |  |  |  |  |  |  |  |  |  |  |  |  |  |  |  |  |  |  |  |  |  |  |  |  |  |  |  |  |  |  |  |  |  |  |  |  |  |  |  |  |  |  |  |  |  |  |  |  |  |  |  |  |  |  |  |  |  |  |  |  |  |  |  |  |  |  |  |  |  |  |  |  |  |  |  |  |  |  |  |  |  |  |  |  |  |  |  |  |  |  |  |  |  |  |  |  |  |  |  |  |  |  |  |  |  |  |  |  |  |  |  |  |  |  |  |  |  |  |  |  |  |  |  |  |  |  |  |  |  |  |  |  |  |  |  |  |  |  |  |  |  |  |  |  |  |  |  |  |  |  |  |  |  |  |  |  |  |  |  |  |  |  |  |  |  |  |  |  |  |  |  |  |  |  |  |  |  |  |  |  |  |  |  |  |  |  |  |  |  |  |  |  |  |  |  |  |  |  |  |  |  |  |  |  |  |  |  |  |  |  |  |  |  |  |  |  |  |  |  |  |  |  |  |  |  |  |  |  |  |  |  |  |  |  |  |  |  |  |  |  |  |  |  |  |  |  |  |  |  |  |  |  |  |  |  |  |  |  |  |  |  |  |  |  |  |  |  |  |  |  |  |  |  |  |  |  |  |  |  |  |  |  |  |  |  |  |  |  |  |  |  |  |  |  |  |  |  |  |  |  |  |  |  |  |  |  |  |  |  |  |  |  |  |  |  |  |  |  |  |  |  |  |  |  |  |  |  |  |  |  |  |  |  |  |  |  |  |  |  |  |  |  |  |  |  |  |  |  |  |  |  |  |  |  |  |  |  |  |  |  |  |  |  |  |  |  |  |  |  |  |  |  |  |  |  |  |  |  |  |  |  |  |  |  |  |  |  |  |  |  |  |  |  |  |  |  |  |  |  |  |  |  |  |  |  |  |  |  |  |  |  |  |  |  |  |  |  |  |  |  |  |  |  |  |  |  |  |  |  |  |  |  |  |  |  |  |  |  |  |  |  |  |  |  |  |  |  |  |  |  |  |  |  |  |  |  |  |  |  |  |  |  |  |  |  |  |  |  |  |  |  |  |  |  |  |  |  |  |  |  |  |  |  |  |  |  |  |  |  |  |  |  |  |  |  |  |  |  |  |  |  |  |  |  |  |  |  |  |  |  |  |  |  |  |  |  |  |  |  |  |  |  |  |  |  |  |  |  |  |  |  |  |  |  |  |  |  |  |  |  |  |  |  |  |  |  |  |  |  |  |  |  |  |  |  |  |  |  |  |  |  |  |  |  |  |  |  |  |  |  |  |  |  |  |  |  |  |  |  |  |  |  |  |  |  |  |  |  |  |  |  |  |  |  |  |  |  |  |  |  |  |  |  |  |  |  |  |  |  |  |  |  |  |  |  |  |  |  |  |  |  |  |  |  |  |  |  |  |  |  |  |  |  |  |  |  |  |  |  |  |  |  |  |  |  |  |  |  |  |  |  |  |  |  |  |  |  |  |  |  |  |  |  |  |  |  |  |  |  |  |  |  |  |  |  |  |  |  |  |  |  |  |  |  |  |  |  |  |  |  |  |  |  |  |  |  |  |  |  |  |  |  |  |  |  |  |  |  |  |  |  |  |  |  |  |  |  |  |  |  |  |  |  |  |  |  |  |  |  |  |  |  |  |  |  |  |  |  |  |  |  |  |  |  |  |  |  |  |  |  |  |  |  |  |  |  |  |  |  |  |  |  |  |  |  |  |  |  |  |  |  |  |  |  |  |  |  |  |  |  |  |  |  |  |  |  |  |  |  |  |  |  |  |  |  |  |  |  |  |  |  |  |  |  |  |  |  |  |  |  |  |  |  |  |  |  |  |  |  |  |  |  |  |  |  |  |  |  |  |  |  |  |  |  |  |  |  |  |  |  |  |  |  |  |  |  |  |  |  |  |  |  |  |  |  |  |  |  |  |  |  |  |  |  |  |  |  |  |  |  |  |  |  |  |  |  |  |  |  |  |  |  |  |  |  |  |  |  |  |  |  |  |  |  |  |  |  |  |  |  |  |  |  |  |  |  |  |  |  |  |  |  |  |  |  |  |  |  |  |  |  |  |  |  |  |  |  |  |  |  |  |  |  |  |  |  |  |  |  |  |  |  |  |  |  |  |  |  |  |  |  |  |  |  |  |  |  |  |  |  |  |  |  |  |  |  |  |  |  |  |  |  |  |  |  |  |  |  |  |  |  |  |  |  |  |  |  |  |  |  |  |  |  |  |  |  |  |  |  |  |  |  |  |  |  |  |  |  |  |  |  |  |  |  |  |  |  |  |  |  |  |  |  |  |  |  |  |  |  |  |  |  |  |  |  |  |  |  |  |  |  |  |  |  |  |  |  |  |  |  |  |  |  |  |  |  |  |  |  |  |  |  |  |  |  |  |  |  |  |  |  |  |  |  |  |  |  |  |  |  |  |  |  |  |  |  |  |  |  |  |  |  |  |  |  |  |  |  |  |  |  |  |  |  |  |  |  |  |  |  |  |  |  |  |  |  |  |  |  |  |  |  |  |  |  |  |  |  |  |  |  |  |  |  |  |  |  |  |  |  |  |  |  |  |  |  |  |  |  |  |  |  |  |  |  |  |  |  |  |  |  |  |  |  |  |  |  |  |  |  |  |  |  |  |  |  |  |  |  |  |  |  |  |  |  |  |  |  |  |  |  |  |  |  |  |  |  |  |  |  |  |  |  |  |  |  |  |  |  |  |  |  |  |  |  |  |  |  |  |  |  |  |  |  |  |  |  |  |  |  |  |  |  |  |  |  |  |  |  |  |  |  |  |  |  |  |  |  |  |  |  |  |  |  |  |  |  |  |  |  |  |  |  |  |  |  |  |  |  |  |  |  |  |  |  |  |  |  |  |  |  |  |  |  |  |  |  |  |  |  |  |  |  |  |  |  |  |  |  |  |  |  |  |  |  |  |  |  |  |  |  |  |  |  |  |  |  |  |  |  |  |  |  |  |  |  |  |  |  |  |  |  |  |  |  |  |  |  |  |  |  |  |  |  |  |  |  |  |  |  |  |  |  |  |  |  |  |  |  |  |  |  |  |  |  |  |  |  |  |  |  |  |  |  |  |  |  |  |  |  |  |  |  |  |  |  |  |  |  |  |  |  |  |  |  |  |  |  |  |  |  |  |  |  |  |  |  |  |  |  |  |  |  |  |  |  |  |  |  |  |  |  |  |  |  |  |  |  |  |  |  |  |  |  |  |  |  |  |  |  |  |  |  |  |  |  |  |  |  |  |  |  |  |  |  |  |  |  |  |  |  |  |  |  |  |  |  |  |  |  |  |  |  |  |  |  |  |  |  |  |  |  |  |  |  |  |  |  |  |  |  |  |  |  |  |  |  |  |  |  |  |  |  |  |  |  |  |  |  |  |  |  |  |  |  |  |  |  |  |  |  |  |  |  |  |  |  |  |  |  |  |  |  |  |  |  |  |  |  |  |  |  |  |  |  |  |  |  |  |  |  |  |  |  |  |  |  |  |  |  |  |  |  |  |  |  |  |  |  |  |  |  |  |  |  |  |  |  |  |  |  |  |  |  |  |  |  |  |  |  |  |  |  |  |  |  |  |  |  |  |  |  |  |  |  |  |  |  |  |  |  |  |  |  |  |  |  |  |  |  |  |  |  |  |  |  |  |  |  |  |  |  |  |  |  |  |  |  |  |  |  |  |  |  |  |  |  |  |  |  |  |  |  |  |  |  |  |  |  |  |  |  |  |  |  |  |  |  |  |  |  |  |  |  |  |  |  |  |  |  |  |  |  |  |  |  |  |  |  |  |  |  |  |  |  |  |  |  |  |  |  |  |  |  |  |  |  |  |  |  |  |  |  |  |  |  |  |  |  |  |  |  |  |  |  |  |  |  |  |  |  |  |  |  |  |  |  |  |  |  |  |  |  |  |  |  |  |  |  |  |  |  |  |  |  |  |  |  |  |  |  |  |  |  |  |  |  |  |  |  |  |  |  |  |  |  |  |  |  |  |  |  |  |  |  |  |  |  |  |  |  |  |  |  |  |  |  |  |  |  |  |  |  |  |  |  |  |  |  |  |  |  |  |  |  |  |  |  |  |  |  |  |  |  |  |  |  |  |  |  |  |  |  |  |  |  |  |  |  |  |  |  |  |  |  |  |  |  |  |  |  |  |  |  |  |  |  |  |  |  |  |  |  |  |  |  |  |  |  |  |  |  |  |  |  |  |  |  |  |  |  |  |  |  |  |  |  |  |  |  |  |  |  |  |  |  |  |  |  |  |  |  |  |  |  |  |  |  |  |  |  |  |  |  |  |  |  |  |  |  |  |  |  |  |  |  |  |  |  |  |  |  |  |  |  |  |  |  |  |  |  |  |  |  |  |  |  |  |  |  |  |  |  |  |  |  |  |  |  |  |  |  |  |  |  |  |  |  |  |  |  |  |  |  |  |  |  |  |  |  |  |  |  |  |  |  |  |  |  |  |  |  |  |  |  |  |  |  |  |  |  |  |  |  |  |  |  |  |  |  |  |  |  |  |  |  |  |  |  |  |  |  |  |  |  |  |  |  |  |  |  |  |  |  |  |  |  |  |  |  |  |  |  |  |  |  |  |  |  |  |  |  |  |  |  |  |  |  |  |  |  |  |  |  |  |  |  |  |  |  |  |  |  |  |  |  |  |  |  |  |  |  |  |  |  |  |  |  |  |  |  |  |  |  |  |  |  |  |  |  |  |  |  |  |  |  |  |  |  |  |  |  |  |  |  |  |  |  |  |  |  |  |  |  |  |  |  |  |  |  |  |  |  |  |  |  |  |  |  |  |  |  |  |  |  |  |  |  |  |  |  |  |  |  |  |  |  |  |  |  |  |  |  |  |  |  |  |  |  |  |  |  |  |  |  |  |  |  |  |  |  |  |  |  |  |  |  |  |  |  |  |  |  |  |  |  |  |  |  |  |  |  |  |  |  |  |  |  |  |  |  |  |  |  |  |  |  |  |  |  |  |  |  |  |  |  |  |  |  |  |  |  |  |  |  |  |  |  |  |  |  |  |  |  |  |  |  |  |  |  |  |  |  |  |  |  |  |  |  |  |  |  |  |  |  |  |  |  |  |  |  |  |  |  |  |  |  |  |  |  |  |  |  |  |  |  |  |  |  |  |  |  |  |  |  |  |  |  |  |  |  |  |  |  |  |  |  |  |  |  |  |  |  |  |  |  |  |  |  |  |  |  |  |  |  |  |  |  |  |  |  |  |  |  |  |  |  |  |  |  |  |  |  |  |  |  |  |  |  |  |  |  |  |  |  |  |  |  |  |  |  |  |  |  |  |  |  |  |  |  |  |  |  |  |  |  |  |  |  |  |  |  |  |  |  |  |  |  |  |  |  |  |  |  |  |  |  |  |  |  |  |  |  |  |  |  |  |  |  |  |  |  |  |  |  |  |  |  |  |  |  |  |  |  |  |  |  |  |  |  |  |  |  |  |  |  |  |  |  |  |  |  |  |  |  |  |  |  |  |  |  |  |  |  |  |  |  |  |  |  |  |  |  |  |  |  |  |  |  |  |  |  |  |  |  |  |  |  |  |  |  |  |  |  |  |  |  |  |  |  |  |  |  |  |  |  |  |  |  |  |  |  |  |  |  |  |  |  |  |  |  |  |  |  |  |  |  |  |  |  |  |  |  |  |  |  |  |  |  |  |  |  |  |  |  |  |  |  |  |  |  |  |  |  |  |  |  |  |  |  |  |  |  |  |  |  |  |  |  |  |  |  |  |  |  |  |  |  |  |  |  |  |  |  |  |  |  |  |  |  |  |  |  |  |  |  |  |  |  |  |  |  |  |  |  |  |  |  |  |  |  |  |  |  |  |  |  |  |  |  |  |  |  |  |  |  |  |  |  |  |  |  |  |  |  |  |  |  |  |  |  |  |  |  |  |  |  |  |  |  |  |  |  |  |  |  |  |  |  |  |  |  |  |  |  |  |  |  |  |  |  |  |  |  |  |  |  |  |  |  |  |  |  |  |  |  |  |  |  |  |  |  |  |  |  |  |  |  |  |  |  |  |  |  |  |  |  |  |  |  |  |  |  |  |  |  |  |  |  |  |  |  |  |  |  |  |  |  |  |  |  |  |  |  |  |  |  |  |  |  |  |  |  |  |  |  |  |  |  |  |  |  |  |  |  |  |  |  |  |  |  |  |  |  |  |  |  |  |  |  |  |  |  |  |  |  |  |  |  |  |  |  |  |  |  |  |  |  |  |  |  |  |  |  |  |  |  |  |  |  |  |  |  |  |  |  |  |  |  |  |  |  |  |  |  |  |  |  |  |  |  |  |  |  |  |  |  |  |  |  |  |  |  |  |  |  |  |  |  |  |  |  |  |  |  |  |  |  |  |  |  |  |  |  |  |  |  |  |  |  |  |  |  |  |  |  |  |  |  |  |  |  |  |  |  |  |  |  |  |  |  |  |  |  |  |  |  |  |  |  |  |  |  |  |  |  |  |  |  |  |  |  |  |  |  |  |  |  |  |  |  |  |  |  |  |  |  |  |  |  |  |  |  |  |  |  |  |  |  |  |  |  |  |  |  |  |  |  |  |  |  |  |  |  |  |  |  |  |  |  |  |  |  |  |  |  |  |  |  |  |  |  |  |  |  |  |  |  |  |  |  |  |  |  |  |  |  |  |  |  |  |  |  |  |  |  |  |  |  |  |  |  |  |  |  |  |  |  |  |  |  |  |  |  |  |  |  |  |  |  |  |  |  |  |  |  |  |  |  |  |  |  |  |  |  |  |  |  |  |  |  |  |  |  |  |  |  |  |  |  |  |  |  |  |  |  |  |  |  |  |  |  |  |  |  |  |  |  |  |  |  |  |  |  |  |  |  |  |  |  |  |  |  |  |  |  |  |  |  |  |  |  |  |  |  |  |  |  |  |  |  |  |  |  |  |  |  |  |  |  |  |  |  |  |  |  |  |  |  |  |  |  |  |  |  |  |  |  |  |  |  |  |  |  |  |  |  |  |  |  |  |  |  |  |  |  |  |  |  |  |  |  |  |  |  |  |  |  |  |  |  |  |  |  |  |  |  |  |  |  |  |  |  |  |  |  |  |  |  |  |  |  |  |  |  |  |  |  |  |  |  |  |  |  |  |  |  |  |  |  |  |  |  |  |  |  |  |  |  |  |  |  |  |  |  |  |  |  |  |  |  |  |  |  |  |  |  |  |  |  |  |  |  |  |  |  |  |  |  |  |  |  |  |  |  |  |  |  |  |  |  |  |  |  |  |  |  |  |  |  |  |  |  |  |  |  |  |  |  |  |  |  |  |  |  |  |  |  |  |  |  |  |  |  |  |  |  |  |  |  |  |  |  |  |  |  |  |  |  |  |  |  |  |  |  |  |  |  |  |  |  |  |  |  |  |  |  |  |  |  |  |  |  |  |  |  |  |  |  |  |  |  |  |  |  |  |  |  |  |  |  |  |  |  |  |  |  |  |  |  |  |  |  |  |  |  |  |  |  |  |  |  |  |  |  |  |  |  |  |  |  |  |  |  |  |  |  |  |  |  |  |  |  |  |  |  |  |  |  |  |  |  |  |  |  |  |  |  |  |  |  |  |  |  |  |  |  |  |  |  |  |  |  |  |  |  |  |  |  |  |  |  |  |  |  |  |  |  |  |  |  |  |  |  |  |  |  |  |  |  |  |  |  |  |  |  |  |  |  |  |  |  |  |  |  |  |  |  |  |  |  |  |  |  |  |  |  |  |  |  |  |  |  |  |  |  |  |  |  |  |  |  |  |  |  |  |  |  |  |  |  |  |  |  |  |  |  |  |  |  |  |  |  |  |  |  |  |  |  |  |  |  |  |  |  |  |  |  |  |  |  |  |  |  |  |  |  |  |  |  |  |  |  |  |  |  |  |  |  |  |  |  |  |  |  |  |  |  |  |  |  |  |  |  |  |  |  |  |  |  |  |  |  |  |  |  |  |  |  |  |  |  |  |  |  |  |  |  |  |  |  |  |  |  |  |  |  |  |  |  |  |  |  |  |  |  |  |  |  |  |  |  |  |  |  |  |  |  |  |  |  |  |  |  |  |  |  |  |  |  |  |  |  |  |  |  |  |  |  |  |  |  |  |  |  |  |  |  |  |  |  |  |  |  |  |  |  |  |  |  |  |  |  |  |  |  |  |  |  |  |  |  |  |  |  |  |  |  |  |  |  |  |  |  |  |  |  |  |  |  |  |  |  |  |  |  |  |  |  |  |  |  |  |  |  |  |  |  |  |  |  |  |  |  |  |  |  |  |  |  |  |  |  |  |  |  |  |  |  |  |  |  |  |  |  |  |  |  |  |  |  |  |  |  |  |  |  |  |  |  |  |  |  |  |  |  |  |  |  |  |  |  |  |  |  |  |  |  |  |  |  |  |  |  |  |  |  |  |  |  |  |  |  |  |  |  |  |  |  |  |  |  |  |  |  |  |  |  |  |  |  |  |  |  |  |  |  |  |  |  |  |  |  |  |  |  |  |  |  |  |  |  |  |  |  |  |  |  |  |  |  |  |  |  |  |  |  |  |  |  |  |  |  |  |  |  |  |  |  |  |  |  |  |  |  |  |  |  |  |  |  |  |  |  |  |  |  |  |  |  |  |  |  |  |  |  |  |  |  |  |  |  |  |  |  |  |  |  |  |  |  |  |  |  |  |  |  |  |  |  |  |  |  |  |  |  |  |  |  |  |  |  |  |  |  |  |  |  |  |  |  |  |  |  |  |  |  |  |  |  |  |  |  |  |  |  |  |  |  |  |  |  |  |  |  |  |  |  |  |  |  |  |  |  |  |  |  |  |  |  |  |  |  |  |  |  |  |  |  |  |  |  |  |  |  |  |  |  |  |  |  |  |  |  |  |  |  |  |  |  |  |  |  |  |  |  |  |  |  |  |  |  |  |  |  |  |  |  |  |  |  |  |  |  |  |  |  |  |  |  |  |  |  |  |  |  |  |  |  |  |  |  |  |  |  |  |  |  |  |  |  |  |  |  |  |  |  |  |  |  |  |  |  |  |  |  |  |  |  |  |  |  |  |  |  |  |  |  |  |  |  |  |  |  |  |  |  |  |  |  |  |  |  |  |  |  |  |  |  |  |  |  |  |  |  |  |  |  |  |  |  |  |  |  |  |  |  |  |  |  |  |  |  |  |  |  |  |  |  |  |  |  |  |  |  |  |  |  |  |  |  |  |  |  |  |  |  |  |  |  |  |  |  |  |  |  |  |  |  |  |  |  |  |  |  |  |  |  |  |  |  |  |  |  |  |  |  |  |  |  |  |  |  |  |  |  |  |  |  |  |  |  |  |  |  |  |  |  |  |  |  |  |  |  |  |  |  |  |  |  |  |  |  |  |  |  |  |  |  |  |  |  |  |  |  |  |  |  |  |  |  |  |  |  |  |  |  |  |  |  |  |  |  |  |  |  |  |  |  |  |  |  |  |  |  |  |  |  |  |  |  |  |  |  |  |  |  |  |  |  |  |  |  |  |  |  |  |  |  |  |  |  |  |  |  |  |  |  |  |  |  |  |  |  |  |  |  |  |  |  |  |  |  |  |  |  |  |  |  |  |  |  |  |  |  |  |  |  |  |  |  |  |  |  |  |  |  |  |  |  |  |  |  |  |  |  |  |  |  |  |  |  |  |  |  |  |  |  |  |  |  |  |  |  |  |  |  |  |  |  |  |  |  |  |  |  |  |  |  |  |  |  |  |  |  |  |  |  |  |  |  |  |  |  |  |  |  |  |  |  |  |  |  |  |  |  |  |  |  |  |  |  |  |  |  |  |  |  |  |  |  |  |  |  |  |  |  |  |  |  |  |  |  |  |  |  |  |  |  |  |  |  |  |  |  |  |  |  |  |  |  |  |  |  |  |  |  |  |  |  |  |  |  |  |  |  |  |  |  |  |  |  |  |  |  |  |  |  |  |  |  |  |  |  |  |  |  |  |  |  |  |  |  |  |  |  |  |  |  |  |  |  |  |  |  |  |  |  |  |  |  |  |  |  |  |  |  |  |  |  |  |  |  |  |  |  |  |  |  |  |  |  |  |  |  |  |  |  |  |  |  |  |  |  |  |  |  |  |  |  |  |  |  |  |  |  |  |  |  |  |  |  |  |  |  |  |  |  |  |  |  |  |  |  |  |  |  |  |  |  |  |  |  |  |  |  |  |  |  |  |  |  |  |  |  |  |  |  |  |  |  |  |  |  |  |  |  |  |  |  |  |  |  |  |  |  |  |  |  |  |  |  |  |  |  |  |  |  |  |  |  |  |  |  |  |  |  |  |  |  |  |  |  |  |  |  |  |  |  |  |  |  |  |  |  |  |  |  |  |  |  |  |  |  |  |  |  |  |  |  |  |  |  |  |  |  |  |  |  |  |  |  |  |  |  |  |  |  |  |  |  |  |  |  |  |  |  |  |  |  |  |  |  |  |  |  |  |  |  |  |  |  |  |  |  |  |  |  |  |  |  |  |  |  |  |  |  |  |  |  |  |  |  |  |  |  |  |  |  |  |  |  |  |  |  |  |  |  |  |  |  |  |  |  |  |  |  |  |  |  |  |  |  |  |  |  |  |  |  |  |  |  |  |  |  |  |  |  |  |  |  |  |  |  |  |  |  |  |  |  |  |  |  |  |  |  |  |  |  |  |  |  |  |  |  |  |  |  |  |  |  |  |  |  |  |  |  |  |  |  |  |  |  |  |  |  |  |  |  |  |  |  |  |  |  |  |  |  |  |  |  |  |  |  |  |  |  |  |  |  |  |  |  |  |  |  |  |  |  |  |  |  |  |  |  |  |  |  |  |  |  |  |  |  |  |  |  |  |  |  |  |  |  |  |  |  |  |  |  |  |  |  |  |  |  |  |  |  |  |  |  |  |  |  |  |  |  |  |  |  |  |  |  |  |  |  |  |  |  |  |  |  |  |  |  |  |  |  |  |  |  |  |  |  |  |  |  |  |  |  |  |  |  |  |  |  |  |  |  |  |  |  |  |  |  |  |  |  |  |  |  |  |  |  |  |  |  |  |  |  |  |  |  |  |  |  |  |  |  |  |  |  |  |  |  |  |  |  |  |  |  |  |  |  |  |  |  |  |  |  |  |  |  |  |  |  |  |  |  |  |  |  |  |  |  |  |  |  |  |  |  |  |  |  |  |  |  |  |  |  |  |  |
| --- | --- | --- | --- | --- | --- | --- | --- | --- | --- | --- | --- | --- | --- | --- | --- | --- | --- | --- | --- | --- | --- | --- | --- | --- | --- | --- | --- | --- | --- | --- | --- | --- | --- | --- | --- | --- | --- | --- | --- | --- | --- | --- | --- | --- | --- | --- | --- | --- | --- | --- | --- | --- | --- | --- | --- | --- | --- | --- | --- | --- | --- | --- | --- | --- | --- | --- | --- | --- | --- | --- | --- | --- | --- | --- | --- | --- | --- | --- | --- | --- | --- | --- | --- | --- | --- | --- | --- | --- | --- | --- | --- | --- | --- | --- | --- | --- | --- | --- | --- | --- | --- | --- | --- | --- | --- | --- | --- | --- | --- | --- | --- | --- | --- | --- | --- | --- | --- | --- | --- | --- | --- | --- | --- | --- | --- | --- | --- | --- | --- | --- | --- | --- | --- | --- | --- | --- | --- | --- | --- | --- | --- | --- | --- | --- | --- | --- | --- | --- | --- | --- | --- | --- | --- | --- | --- | --- | --- | --- | --- | --- | --- | --- | --- | --- | --- | --- | --- | --- | --- | --- | --- | --- | --- | --- | --- | --- | --- | --- | --- | --- | --- | --- | --- | --- | --- | --- | --- | --- | --- | --- | --- | --- | --- | --- | --- | --- | --- | --- | --- | --- | --- | --- | --- | --- | --- | --- | --- | --- | --- | --- | --- | --- | --- | --- | --- | --- | --- | --- | --- | --- | --- | --- | --- | --- | --- | --- | --- | --- | --- | --- | --- | --- | --- | --- | --- | --- | --- | --- | --- | --- | --- | --- | --- | --- | --- | --- | --- | --- | --- | --- | --- | --- | --- | --- | --- | --- | --- | --- | --- | --- | --- | --- | --- | --- | --- | --- | --- | --- | --- | --- | --- | --- | --- | --- | --- | --- | --- | --- | --- | --- | --- | --- | --- | --- | --- | --- | --- | --- | --- | --- | --- | --- | --- | --- | --- | --- | --- | --- | --- | --- | --- | --- | --- | --- | --- | --- | --- | --- | --- | --- | --- | --- | --- | --- | --- | --- | --- | --- | --- | --- | --- | --- | --- | --- | --- | --- | --- | --- | --- | --- | --- | --- | --- | --- | --- | --- | --- | --- | --- | --- | --- | --- | --- | --- | --- | --- | --- | --- | --- | --- | --- | --- | --- | --- | --- | --- | --- | --- | --- | --- | --- | --- | --- | --- | --- | --- | --- | --- | --- | --- | --- | --- | --- | --- | --- | --- | --- | --- | --- | --- | --- | --- | --- | --- | --- | --- | --- | --- | --- | --- | --- | --- | --- | --- | --- | --- | --- | --- | --- | --- | --- | --- | --- | --- | --- | --- | --- | --- | --- | --- | --- | --- | --- | --- | --- | --- | --- | --- | --- | --- | --- | --- | --- | --- | --- | --- | --- | --- | --- | --- | --- | --- | --- | --- | --- | --- | --- | --- | --- | --- | --- | --- | --- | --- | --- | --- | --- | --- | --- | --- | --- | --- | --- | --- | --- | --- | --- | --- | --- | --- | --- | --- | --- | --- | --- | --- | --- | --- | --- | --- | --- | --- | --- | --- | --- | --- | --- | --- | --- | --- | --- | --- | --- | --- | --- | --- | --- | --- | --- | --- | --- | --- | --- | --- | --- | --- | --- | --- | --- | --- | --- | --- | --- | --- | --- | --- | --- | --- | --- | --- | --- | --- | --- | --- | --- | --- | --- | --- | --- | --- | --- | --- | --- | --- | --- | --- | --- | --- | --- | --- | --- | --- | --- | --- | --- | --- | --- | --- | --- | --- | --- | --- | --- | --- | --- | --- | --- | --- | --- | --- | --- | --- | --- | --- | --- | --- | --- | --- | --- | --- | --- | --- | --- | --- | --- | --- | --- | --- | --- | --- | --- | --- | --- | --- | --- | --- | --- | --- | --- | --- | --- | --- | --- | --- | --- | --- | --- | --- | --- | --- | --- | --- | --- | --- | --- | --- | --- | --- | --- | --- | --- | --- | --- | --- | --- | --- | --- | --- | --- | --- | --- | --- | --- | --- | --- | --- | --- | --- | --- | --- | --- | --- | --- | --- | --- | --- | --- | --- | --- | --- | --- | --- | --- | --- | --- | --- | --- | --- | --- | --- | --- | --- | --- | --- | --- | --- | --- | --- | --- | --- | --- | --- | --- | --- | --- | --- | --- | --- | --- | --- | --- | --- | --- | --- | --- | --- | --- | --- | --- | --- | --- | --- | --- | --- | --- | --- | --- | --- | --- | --- | --- | --- | --- | --- | --- | --- | --- | --- | --- | --- | --- | --- | --- | --- | --- | --- | --- | --- | --- | --- | --- | --- | --- | --- | --- | --- | --- | --- | --- | --- | --- | --- | --- | --- | --- | --- | --- | --- | --- | --- | --- | --- | --- | --- | --- | --- | --- | --- | --- | --- | --- | --- | --- | --- | --- | --- | --- | --- | --- | --- | --- | --- | --- | --- | --- | --- | --- | --- | --- | --- | --- | --- | --- | --- | --- | --- | --- | --- | --- | --- | --- | --- | --- | --- | --- | --- | --- | --- | --- | --- | --- | --- | --- | --- | --- | --- | --- | --- | --- | --- | --- | --- | --- | --- | --- | --- | --- | --- | --- | --- | --- | --- | --- | --- | --- | --- | --- | --- | --- | --- | --- | --- | --- | --- | --- | --- | --- | --- | --- | --- | --- | --- | --- | --- | --- | --- | --- | --- | --- | --- | --- | --- | --- | --- | --- | --- | --- | --- | --- | --- | --- | --- | --- | --- | --- | --- | --- | --- | --- | --- | --- | --- | --- | --- | --- | --- | --- | --- | --- | --- | --- | --- | --- | --- | --- | --- | --- | --- | --- | --- | --- | --- | --- | --- | --- | --- | --- | --- | --- | --- | --- | --- | --- | --- | --- | --- | --- | --- | --- | --- | --- | --- | --- | --- | --- | --- | --- | --- | --- | --- | --- | --- | --- | --- | --- | --- | --- | --- | --- | --- | --- | --- | --- | --- | --- | --- | --- | --- | --- | --- | --- | --- | --- | --- | --- | --- | --- | --- | --- | --- | --- | --- | --- | --- | --- | --- | --- | --- | --- | --- | --- | --- | --- | --- | --- | --- | --- | --- | --- | --- | --- | --- | --- | --- | --- | --- | --- | --- | --- | --- | --- | --- | --- | --- | --- | --- | --- | --- | --- | --- | --- | --- | --- | --- | --- | --- | --- | --- | --- | --- | --- | --- | --- | --- | --- | --- | --- | --- | --- | --- | --- | --- | --- | --- | --- | --- | --- | --- | --- | --- | --- | --- | --- | --- | --- | --- | --- | --- | --- | --- | --- | --- | --- | --- | --- | --- | --- | --- | --- | --- | --- | --- | --- | --- | --- | --- | --- | --- | --- | --- | --- | --- | --- | --- | --- | --- | --- | --- | --- | --- | --- | --- | --- | --- | --- | --- | --- | --- | --- | --- | --- | --- | --- | --- | --- | --- | --- | --- | --- | --- | --- | --- | --- | --- | --- | --- | --- | --- | --- | --- | --- | --- | --- | --- | --- | --- | --- | --- | --- | --- | --- | --- | --- | --- | --- | --- | --- | --- | --- | --- | --- | --- | --- | --- | --- | --- | --- | --- | --- | --- | --- | --- | --- | --- | --- | --- | --- | --- | --- | --- | --- | --- | --- | --- | --- | --- | --- | --- | --- | --- | --- | --- | --- | --- | --- | --- | --- | --- | --- | --- | --- | --- | --- | --- | --- | --- | --- | --- | --- | --- | --- | --- | --- | --- | --- | --- | --- | --- | --- | --- | --- | --- | --- | --- | --- | --- | --- | --- | --- | --- | --- | --- | --- | --- | --- | --- | --- | --- | --- | --- | --- | --- | --- | --- | --- | --- | --- | --- | --- | --- | --- | --- | --- | --- | --- | --- | --- | --- | --- | --- | --- | --- | --- | --- | --- | --- | --- | --- | --- | --- | --- | --- | --- | --- | --- | --- | --- | --- | --- | --- | --- | --- | --- | --- | --- | --- | --- | --- | --- | --- | --- | --- | --- | --- | --- | --- | --- | --- | --- | --- | --- | --- | --- | --- | --- | --- | --- | --- | --- | --- | --- | --- | --- | --- | --- | --- | --- | --- | --- | --- | --- | --- | --- | --- | --- | --- | --- | --- | --- | --- | --- | --- | --- | --- | --- | --- | --- | --- | --- | --- | --- | --- | --- | --- | --- | --- | --- | --- | --- | --- | --- | --- | --- | --- | --- | --- | --- | --- | --- | --- | --- | --- | --- | --- | --- | --- | --- | --- | --- | --- | --- | --- | --- | --- | --- | --- | --- | --- | --- | --- | --- | --- | --- | --- | --- | --- | --- | --- | --- | --- | --- | --- | --- | --- | --- | --- | --- | --- | --- | --- | --- | --- | --- | --- | --- | --- | --- | --- | --- | --- | --- | --- | --- | --- | --- | --- | --- | --- | --- | --- | --- | --- | --- | --- | --- | --- | --- | --- | --- | --- | --- | --- | --- | --- | --- | --- | --- | --- | --- | --- | --- | --- | --- | --- | --- | --- | --- | --- | --- | --- | --- | --- | --- | --- | --- | --- | --- | --- | --- | --- | --- | --- | --- | --- | --- | --- | --- | --- | --- | --- | --- | --- | --- | --- | --- | --- | --- | --- | --- | --- | --- | --- | --- | --- | --- | --- | --- | --- | --- | --- | --- | --- | --- | --- | --- | --- | --- | --- | --- | --- | --- | --- | --- | --- | --- | --- | --- | --- | --- | --- | --- | --- | --- | --- | --- | --- | --- | --- | --- | --- | --- | --- | --- | --- | --- | --- | --- | --- | --- | --- | --- | --- | --- | --- | --- | --- | --- | --- | --- | --- | --- | --- | --- | --- | --- | --- | --- | --- | --- | --- | --- | --- | --- | --- | --- | --- | --- | --- | --- | --- | --- | --- | --- | --- | --- | --- | --- | --- | --- | --- | --- | --- | --- | --- | --- | --- | --- | --- | --- | --- | --- | --- | --- | --- | --- | --- | --- | --- | --- | --- | --- | --- | --- | --- | --- | --- | --- | --- | --- | --- | --- | --- | --- | --- | --- | --- | --- | --- | --- | --- | --- | --- | --- | --- | --- | --- | --- | --- | --- | --- | --- | --- | --- | --- | --- | --- | --- | --- | --- | --- | --- | --- | --- | --- | --- | --- | --- | --- | --- | --- | --- | --- | --- | --- | --- | --- | --- | --- | --- | --- | --- | --- | --- | --- | --- | --- | --- | --- | --- | --- | --- | --- | --- | --- | --- | --- | --- | --- | --- | --- | --- | --- | --- | --- | --- | --- | --- | --- | --- | --- | --- | --- | --- | --- | --- | --- | --- | --- | --- | --- | --- | --- | --- | --- | --- | --- | --- | --- | --- | --- | --- | --- | --- | --- | --- | --- | --- | --- | --- | --- | --- | --- | --- | --- | --- | --- | --- | --- | --- | --- | --- | --- | --- | --- | --- | --- | --- | --- | --- | --- | --- | --- | --- | --- | --- | --- | --- | --- | --- | --- | --- | --- | --- | --- | --- | --- | --- | --- | --- | --- | --- | --- | --- | --- | --- | --- | --- | --- | --- | --- | --- | --- | --- | --- | --- | --- | --- | --- | --- | --- | --- | --- | --- | --- | --- | --- | --- | --- | --- | --- | --- | --- | --- | --- | --- | --- | --- | --- | --- | --- | --- | --- | --- | --- | --- | --- | --- | --- | --- | --- | --- | --- | --- | --- | --- | --- | --- | --- | --- | --- | --- | --- | --- | --- | --- | --- | --- | --- | --- | --- | --- | --- | --- | --- | --- | --- | --- | --- | --- | --- | --- | --- | --- | --- | --- | --- | --- | --- | --- | --- | --- | --- | --- | --- | --- | --- | --- | --- | --- | --- | --- | --- | --- | --- | --- | --- | --- | --- | --- | --- | --- | --- | --- | --- | --- | --- | --- | --- | --- | --- | --- | --- | --- | --- | --- | --- | --- | --- | --- | --- | --- | --- | --- | --- | --- | --- | --- | --- | --- | --- | --- | --- | --- | --- | --- | --- | --- | --- | --- | --- | --- | --- | --- | --- | --- | --- | --- | --- | --- | --- | --- | --- | --- | --- | --- | --- | --- | --- | --- | --- | --- | --- | --- | --- | --- | --- | --- | --- | --- | --- | --- | --- | --- | --- | --- | --- | --- | --- | --- | --- | --- | --- | --- | --- | --- | --- | --- | --- | --- | --- | --- | --- | --- | --- | --- | --- | --- | --- | --- | --- | --- | --- | --- | --- | --- | --- | --- | --- | --- | --- | --- | --- | --- | --- | --- | --- | --- | --- | --- | --- | --- | --- | --- | --- | --- | --- | --- | --- | --- | --- | --- | --- | --- | --- | --- | --- | --- | --- | --- | --- | --- | --- | --- | --- | --- | --- | --- | --- | --- | --- | --- | --- | --- | --- | --- | --- | --- | --- | --- | --- | --- | --- | --- | --- | --- | --- | --- | --- | --- | --- | --- | --- | --- | --- | --- | --- | --- | --- | --- | --- | --- | --- | --- | --- | --- | --- | --- | --- | --- | --- | --- | --- | --- | --- | --- | --- | --- | --- | --- | --- | --- | --- | --- | --- | --- | --- | --- | --- | --- | --- | --- | --- | --- | --- | --- | --- | --- | --- | --- | --- | --- | --- | --- | --- | --- | --- | --- | --- | --- | --- | --- | --- | --- | --- | --- | --- | --- | --- | --- | --- | --- | --- | --- | --- | --- | --- | --- | --- | --- | --- | --- | --- | --- | --- | --- | --- | --- | --- | --- | --- | --- | --- | --- | --- | --- | --- | --- | --- | --- | --- | --- | --- | --- | --- | --- | --- | --- | --- | --- | --- | --- | --- | --- | --- | --- | --- | --- | --- | --- | --- | --- | --- | --- | --- | --- | --- | --- | --- | --- | --- | --- | --- | --- | --- | --- | --- | --- | --- | --- | --- | --- | --- | --- | --- | --- | --- | --- | --- | --- | --- | --- | --- | --- | --- | --- | --- | --- | --- | --- | --- | --- | --- | --- | --- | --- | --- | --- | --- | --- | --- | --- | --- | --- | --- | --- | --- | --- | --- | --- | --- | --- | --- | --- | --- | --- | --- | --- | --- | --- | --- | --- | --- | --- | --- | --- | --- | --- | --- | --- | --- | --- | --- | --- | --- | --- | --- | --- | --- | --- | --- | --- | --- | --- | --- | --- | --- | --- | --- | --- | --- | --- | --- | --- | --- | --- | --- | --- | --- | --- | --- | --- | --- | --- | --- | --- | --- | --- | --- | --- | --- | --- | --- | --- | --- | --- | --- | --- | --- | --- | --- | --- | --- | --- | --- | --- | --- | --- | --- | --- | --- | --- | --- | --- | --- | --- | --- | --- | --- | --- | --- | --- | --- | --- | --- | --- | --- | --- | --- | --- | --- | --- | --- | --- | --- | --- | --- | --- | --- | --- | --- | --- | --- | --- | --- | --- | --- | --- | --- | --- | --- | --- | --- | --- | --- | --- | --- | --- | --- | --- | --- | --- | --- | --- | --- | --- | --- | --- | --- | --- | --- | --- | --- | --- | --- | --- | --- | --- | --- | --- | --- | --- | --- | --- | --- | --- | --- | --- | --- | --- | --- | --- | --- | --- | --- | --- | --- | --- | --- | --- | --- | --- | --- | --- | --- | --- | --- | --- | --- | --- | --- | --- | --- | --- | --- | --- | --- | --- | --- | --- | --- | --- | --- | --- | --- | --- | --- | --- | --- | --- | --- | --- | --- | --- | --- | --- | --- | --- | --- | --- | --- | --- | --- | --- | --- | --- | --- | --- | --- | --- | --- | --- | --- | --- | --- | --- | --- | --- | --- | --- | --- | --- | --- | --- | --- | --- | --- | --- | --- | --- | --- | --- | --- | --- | --- | --- | --- | --- | --- | --- | --- | --- | --- | --- | --- | --- | --- | --- | --- | --- | --- | --- | --- | --- | --- | --- | --- | --- | --- | --- | --- | --- | --- | --- | --- | --- | --- | --- | --- | --- | --- | --- | --- | --- | --- | --- | --- | --- | --- | --- | --- | --- | --- | --- | --- | --- | --- | --- | --- | --- | --- | --- | --- | --- | --- | --- | --- | --- | --- | --- | --- | --- | --- | --- | --- | --- | --- | --- | --- | --- | --- | --- | --- | --- | --- | --- | --- | --- | --- | --- | --- | --- | --- | --- | --- | --- | --- | --- | --- | --- | --- | --- | --- | --- | --- | --- | --- | --- | --- | --- | --- | --- | --- | --- | --- | --- | --- | --- | --- | --- | --- | --- | --- | --- | --- | --- | --- | --- | --- | --- | --- | --- | --- | --- | --- | --- | --- | --- | --- | --- | --- | --- | --- | --- | --- | --- | --- | --- | --- | --- | --- | --- | --- | --- | --- | --- | --- | --- | --- | --- | --- | --- | --- | --- | --- | --- | --- | --- | --- | --- | --- | --- | --- | --- | --- | --- | --- | --- | --- | --- | --- | --- | --- | --- | --- | --- | --- | --- | --- | --- | --- | --- | --- | --- | --- | --- | --- | --- | --- | --- | --- | --- | --- | --- | --- | --- | --- | --- | --- | --- | --- | --- | --- | --- | --- | --- | --- | --- | --- | --- | --- | --- | --- | --- | --- | --- | --- | --- | --- | --- | --- | --- | --- | --- | --- | --- | --- | --- | --- | --- | --- | --- | --- | --- | --- | --- | --- | --- | --- | --- | --- | --- | --- | --- | --- | --- | --- | --- | --- | --- | --- | --- | --- | --- | --- | --- | --- | --- | --- | --- | --- | --- | --- | --- | --- | --- | --- | --- | --- | --- | --- | --- | --- | --- | --- | --- | --- | --- | --- | --- | --- | --- | --- | --- | --- | --- | --- | --- | --- | --- | --- | --- | --- | --- | --- | --- | --- | --- | --- | --- | --- | --- | --- | --- | --- | --- | --- | --- | --- | --- | --- | --- | --- | --- | --- | --- | --- | --- | --- | --- | --- | --- | --- | --- | --- | --- | --- | --- | --- | --- | --- | --- | --- | --- | --- | --- | --- | --- | --- | --- | --- | --- | --- | --- | --- | --- | --- | --- | --- | --- | --- | --- | --- | --- | --- | --- | --- | --- | --- | --- | --- | --- | --- | --- | --- | --- | --- | --- | --- | --- | --- | --- | --- | --- | --- | --- | --- | --- | --- | --- | --- | --- | --- | --- | --- | --- | --- | --- | --- | --- | --- | --- | --- | --- | --- | --- | --- | --- | --- | --- | --- | --- | --- | --- | --- | --- | --- | --- | --- | --- | --- | --- | --- | --- | --- | --- | --- | --- | --- | --- | --- | --- | --- | --- | --- | --- | --- | --- | --- | --- | --- | --- | --- | --- | --- | --- | --- | --- | --- | --- | --- | --- | --- | --- | --- | --- | --- | --- | --- | --- | --- | --- | --- | --- | --- | --- | --- | --- | --- | --- | --- | --- | --- | --- | --- | --- | --- | --- | --- | --- | --- | --- | --- | --- | --- | --- | --- | --- | --- | --- | --- | --- | --- | --- | --- | --- | --- | --- | --- | --- | --- | --- | --- | --- | --- | --- | --- | --- | --- | --- | --- | --- | --- | --- | --- | --- | --- | --- | --- | --- | --- | --- | --- | --- | --- | --- | --- | --- | --- | --- | --- | --- | --- | --- | --- | --- | --- | --- | --- | --- | --- | --- | --- | --- | --- | --- | --- | --- | --- | --- | --- | --- | --- | --- | --- | --- | --- | --- | --- | --- | --- | --- | --- | --- | --- | --- | --- | --- | --- | --- | --- | --- | --- | --- | --- | --- | --- | --- | --- | --- | --- | --- | --- | --- | --- | --- | --- | --- | --- | --- | --- | --- | --- | --- | --- | --- | --- | --- | --- | --- | --- | --- | --- | --- | --- | --- | --- | --- | --- | --- | --- | --- | --- | --- | --- | --- | --- | --- | --- | --- | --- | --- | --- | --- | --- | --- | --- | --- | --- | --- | --- | --- | --- | --- | --- | --- | --- | --- | --- | --- | --- | --- | --- | --- | --- | --- | --- | --- | --- | --- | --- | --- | --- | --- | --- | --- | --- | --- | --- | --- | --- | --- | --- | --- | --- | --- | --- | --- | --- | --- | --- | --- | --- | --- | --- | --- | --- | --- | --- | --- | --- | --- | --- | --- | --- | --- | --- | --- | --- | --- | --- | --- | --- | --- | --- | --- | --- | --- | --- | --- | --- | --- | --- | --- | --- | --- | --- | --- | --- | --- | --- | --- | --- | --- | --- | --- | --- | --- | --- | --- | --- | --- | --- | --- | --- | --- | --- | --- | --- | --- | --- | --- | --- | --- | --- | --- | --- | --- | --- | --- | --- | --- | --- | --- | --- | --- | --- | --- | --- | --- | --- | --- | --- | --- | --- | --- | --- | --- | --- | --- | --- | --- | --- | --- | --- | --- | --- | --- | --- | --- | --- | --- | --- | --- | --- | --- | --- | --- | --- | --- | --- | --- | --- | --- | --- | --- | --- | --- | --- | --- | --- | --- | --- | --- | --- | --- | --- | --- | --- | --- | --- | --- | --- | --- | --- | --- | --- | --- | --- | --- | --- | --- | --- | --- | --- | --- | --- | --- | --- | --- | --- | --- | --- | --- | --- | --- | --- | --- | --- | --- | --- | --- | --- | --- | --- | --- | --- | --- | --- | --- | --- | --- | --- | --- | --- | --- | --- | --- | --- | --- | --- | --- | --- | --- | --- | --- | --- | --- | --- | --- | --- | --- | --- | --- | --- | --- | --- | --- | --- | --- | --- | --- | --- | --- | --- | --- | --- | --- | --- | --- | --- | --- | --- | --- | --- | --- | --- | --- | --- | --- | --- | --- | --- | --- | --- | --- | --- | --- | --- | --- | --- | --- | --- | --- | --- | --- | --- | --- | --- | --- | --- | --- | --- | --- | --- | --- | --- | --- | --- | --- | --- | --- | --- | --- | --- | --- | --- | --- | --- | --- | --- | --- | --- | --- | --- | --- | --- | --- | --- | --- | --- | --- | --- | --- | --- | --- | --- | --- | --- | --- | --- | --- | --- | --- | --- | --- | --- | --- | --- | --- | --- | --- | --- | --- | --- | --- | --- | --- | --- | --- | --- | --- | --- | --- | --- | --- | --- | --- | --- | --- | --- | --- | --- | --- | --- | --- | --- | --- | --- | --- | --- | --- | --- | --- | --- | --- | --- | --- | --- | --- | --- | --- | --- | --- | --- | --- | --- | --- | --- | --- | --- | --- | --- | --- | --- | --- | --- | --- | --- | --- | --- | --- | --- | --- | --- | --- | --- | --- | --- | --- | --- | --- | --- | --- | --- | --- | --- | --- | --- | --- | --- | --- | --- | --- | --- | --- | --- | --- | --- | --- | --- | --- | --- | --- | --- | --- | --- | --- | --- | --- | --- | --- | --- | --- | --- | --- | --- | --- | --- | --- | --- | --- | --- | --- | --- | --- | --- | --- | --- | --- | --- | --- | --- | --- | --- | --- | --- | --- | --- | --- | --- | --- | --- | --- | --- | --- | --- | --- | --- | --- | --- | --- | --- | --- | --- | --- | --- | --- | --- | --- | --- | --- | --- | --- | --- | --- | --- | --- | --- | --- | --- | --- | --- | --- | --- | --- | --- | --- | --- | --- | --- | --- | --- | --- | --- | --- | --- | --- | --- | --- | --- | --- | --- | --- | --- | --- | --- | --- | --- | --- | --- | --- | --- | --- | --- | --- | --- | --- | --- | --- | --- | --- | --- | --- | --- | --- | --- | --- | --- | --- | --- | --- | --- | --- | --- | --- | --- | --- | --- | --- | --- | --- | --- | --- | --- | --- | --- | --- | --- | --- | --- | --- | --- | --- | --- | --- | --- | --- | --- | --- | --- | --- | --- | --- | --- | --- | --- | --- | --- | --- | --- | --- | --- | --- | --- | --- | --- | --- | --- | --- | --- | --- | --- | --- | --- | --- | --- | --- | --- | --- | --- | --- | --- | --- | --- | --- | --- | --- | --- | --- | --- | --- | --- | --- | --- | --- | --- | --- | --- | --- | --- | --- | --- | --- | --- | --- | --- | --- | --- | --- | --- | --- | --- | --- | --- | --- | --- | --- | --- | --- | --- | --- | --- | --- | --- | --- | --- | --- | --- | --- | --- | --- | --- | --- | --- | --- | --- | --- | --- | --- | --- | --- | --- | --- | --- | --- | --- | --- | --- | --- | --- | --- | --- | --- | --- | --- | --- | --- | --- | --- | --- | --- | --- | --- | --- | --- | --- | --- | --- | --- | --- | --- | --- | --- | --- | --- | --- | --- | --- | --- | --- | --- | --- | --- | --- | --- | --- | --- | --- | --- | --- | --- | --- | --- | --- | --- | --- | --- | --- | --- | --- | --- | --- | --- | --- | --- | --- | --- | --- | --- | --- | --- | --- | --- | --- | --- | --- | --- | --- | --- | --- | --- | --- | --- | --- | --- | --- | --- | --- | --- | --- | --- | --- | --- | --- | --- | --- | --- | --- | --- | --- | --- | --- | --- | --- | --- | --- | --- | --- | --- | --- | --- | --- | --- | --- | --- | --- | --- | --- | --- | --- | --- | --- | --- | --- | --- | --- | --- | --- | --- | --- | --- | --- | --- | --- | --- | --- | --- | --- | --- | --- | --- | --- | --- | --- | --- | --- | --- | --- | --- | --- | --- | --- | --- | --- | --- | --- | --- | --- | --- | --- | --- | --- | --- | --- | --- | --- | --- | --- | --- | --- | --- | --- | --- | --- | --- | --- | --- | --- | --- | --- | --- | --- | --- | --- | --- | --- | --- | --- | --- | --- | --- | --- | --- | --- | --- | --- | --- | --- | --- | --- | --- | --- | --- | --- | --- | --- | --- | --- | --- | --- | --- | --- | --- | --- | --- | --- | --- | --- | --- | --- | --- | --- | --- | --- | --- | --- | --- | --- | --- | --- | --- | --- | --- | --- | --- | --- | --- | --- | --- | --- | --- | --- | --- | --- | --- | --- | --- | --- | --- | --- | --- | --- | --- | --- | --- | --- | --- | --- | --- | --- | --- | --- | --- | --- | --- | --- | --- | --- | --- | --- | --- | --- | --- | --- | --- | --- | --- | --- | --- | --- | --- | --- | --- | --- | --- | --- | --- | --- | --- | --- | --- | --- | --- | --- | --- | --- | --- | --- | --- | --- | --- | --- | --- | --- | --- | --- | --- | --- | --- | --- | --- | --- | --- | --- | --- | --- | --- | --- | --- | --- | --- | --- | --- | --- | --- | --- | --- | --- | --- | --- | --- | --- | --- | --- | --- | --- | --- | --- | --- | --- | --- | --- | --- | --- | --- | --- | --- | --- | --- | --- | --- | --- | --- | --- | --- | --- | --- | --- | --- | --- | --- | --- | --- | --- | --- | --- | --- | --- | --- | --- | --- | --- | --- | --- | --- | --- | --- | --- | --- | --- | --- | --- | --- | --- | --- | --- | --- | --- | --- | --- | --- | --- | --- | --- | --- | --- | --- | --- | --- | --- | --- | --- | --- | --- | --- | --- | --- | --- | --- | --- | --- | --- | --- | --- | --- | --- | --- | --- | --- | --- | --- | --- | --- | --- | --- | --- | --- | --- | --- | --- | --- | --- | --- | --- | --- | --- | --- | --- | --- | --- | --- | --- | --- | --- | --- | --- | --- | --- | --- | --- | --- | --- | --- | --- | --- | --- | --- | --- | --- | --- | --- | --- | --- | --- | --- | --- | --- | --- | --- | --- | --- | --- | --- | --- | --- | --- | --- | --- | --- | --- | --- | --- | --- | --- | --- | --- | --- | --- | --- | --- | --- | --- | --- | --- | --- | --- | --- | --- | --- | --- | --- | --- | --- | --- | --- | --- | --- | --- | --- | --- | --- | --- | --- | --- | --- | --- | --- | --- | --- | --- | --- | --- | --- | --- | --- | --- | --- | --- | --- | --- | --- | --- | --- | --- | --- | --- | --- | --- | --- | --- | --- | --- | --- | --- | --- | --- | --- | --- | --- | --- | --- | --- | --- | --- | --- | --- | --- | --- | --- | --- | --- | --- | --- | --- | --- | --- | --- | --- | --- | --- | --- | --- | --- | --- | --- | --- | --- | --- | --- | --- | --- | --- | --- | --- | --- | --- | --- | --- | --- | --- | --- | --- | --- | --- | --- | --- | --- | --- | --- | --- | --- | --- | --- | --- | --- | --- | --- | --- | --- | --- | --- | --- | --- | --- | --- | --- | --- | --- | --- | --- | --- | --- | --- | --- | --- | --- | --- | --- | --- | --- | --- | --- | --- | --- | --- | --- | --- | --- | --- | --- | --- | --- | --- | --- | --- | --- | --- | --- | --- | --- | --- | --- | --- | --- | --- | --- | --- | --- | --- | --- | --- | --- | --- | --- | --- | --- | --- | --- | --- | --- | --- | --- | --- | --- | --- | --- | --- | --- | --- | --- | --- | --- | --- | --- | --- | --- | --- | --- | --- | --- | --- | --- | --- | --- | --- | --- | --- | --- | --- | --- | --- | --- | --- | --- | --- | --- | --- | --- | --- | --- | --- | --- | --- | --- | --- | --- | --- | --- | --- | --- | --- | --- | --- | --- | --- | --- | --- | --- | --- | --- | --- | --- | --- | --- | --- | --- | --- | --- | --- | --- | --- | --- | --- | --- | --- | --- | --- | --- | --- | --- | --- | --- | --- | --- | --- | --- | --- | --- | --- | --- | --- | --- | --- | --- | --- | --- | --- | --- | --- | --- | --- | --- | --- | --- | --- | --- | --- | --- | --- | --- | --- | --- | --- | --- | --- | --- | --- | --- | --- | --- | --- | --- | --- | --- | --- | --- | --- | --- | --- | --- | --- | --- | --- | --- | --- | --- | --- | --- | --- | --- | --- | --- | --- | --- | --- | --- | --- | --- | --- | --- | --- | --- | --- | --- | --- | --- | --- | --- | --- | --- | --- | --- | --- | --- | --- | --- | --- | --- | --- | --- | --- | --- | --- | --- | --- | --- | --- | --- | --- | --- | --- | --- | --- | --- | --- | --- | --- | --- | --- | --- | --- | --- | --- | --- | --- | --- | --- | --- | --- | --- | --- | --- | --- | --- | --- | --- | --- | --- | --- | --- | --- | --- | --- | --- | --- | --- | --- | --- | --- | --- | --- | --- | --- | --- | --- | --- | --- | --- | --- | --- | --- | --- | --- | --- | --- | --- | --- | --- | --- | --- | --- | --- | --- | --- | --- | --- | --- | --- | --- | --- | --- | --- | --- | --- | --- | --- | --- | --- | --- | --- | --- | --- | --- | --- | --- | --- | --- | --- | --- | --- | --- | --- | --- | --- | --- | --- | --- | --- | --- | --- | --- | --- | --- | --- | --- | --- | --- | --- | --- | --- | --- | --- | --- | --- | --- | --- | --- | --- | --- | --- | --- | --- | --- | --- | --- | --- | --- | --- | --- | --- | --- | --- | --- | --- | --- | --- | --- | --- | --- | --- | --- | --- | --- | --- | --- | --- | --- | --- | --- | --- | --- | --- | --- | --- | --- | --- | --- | --- | --- | --- | --- | --- | --- | --- | --- | --- | --- | --- | --- | --- | --- | --- | --- | --- | --- | --- | --- | --- | --- | --- | --- | --- | --- | --- | --- | --- | --- | --- | --- | --- | --- | --- | --- | --- | --- | --- | --- | --- | --- | --- | --- | --- | --- | --- | --- | --- | --- | --- | --- | --- | --- | --- | --- | --- | --- | --- | --- | --- | --- | --- | --- | --- | --- | --- | --- | --- | --- | --- | --- | --- | --- | --- | --- | --- | --- | --- | --- | --- | --- | --- | --- | --- | --- | --- | --- | --- | --- | --- | --- | --- | --- | --- | --- | --- | --- | --- | --- | --- | --- | --- | --- | --- | --- | --- | --- | --- | --- | --- | --- | --- | --- | --- | --- | --- | --- | --- | --- | --- | --- | --- | --- | --- | --- | --- | --- | --- | --- | --- | --- | --- | --- | --- | --- | --- | --- | --- | --- | --- | --- | --- | --- | --- | --- | --- | --- | --- | --- | --- | --- | --- | --- | --- | --- | --- | --- | --- | --- | --- | --- | --- | --- | --- | --- | --- | --- | --- | --- | --- | --- | --- | --- | --- | --- | --- | --- | --- | --- | --- | --- | --- | --- | --- | --- | --- | --- | --- | --- | --- | --- | --- | --- | --- | --- | --- | --- | --- | --- | --- | --- | --- | --- | --- | --- | --- | --- | --- | --- | --- | --- | --- | --- | --- | --- | --- | --- | --- | --- | --- | --- | --- | --- | --- | --- | --- | --- | --- | --- | --- | --- | --- | --- | --- | --- | --- | --- | --- | --- | --- | --- | --- | --- | --- | --- | --- | --- | --- | --- | --- | --- | --- | --- | --- | --- | --- | --- | --- | --- | --- | --- | --- | --- | --- | --- | --- | --- | --- | --- | --- | --- | --- | --- | --- | --- | --- | --- | --- | --- | --- | --- | --- | --- | --- | --- | --- | --- | --- | --- | --- | --- | --- | --- | --- | --- | --- | --- | --- | --- | --- | --- | --- | --- | --- | --- | --- | --- | --- | --- | --- | --- | --- | --- | --- | --- | --- | --- | --- | --- | --- | --- | --- | --- | --- | --- | --- | --- | --- | --- | --- | --- | --- | --- | --- | --- | --- | --- | --- | --- | --- | --- | --- | --- | --- | --- | --- | --- | --- | --- | --- | --- | --- | --- | --- | --- | --- | --- | --- | --- | --- | --- | --- | --- | --- | --- | --- | --- | --- | --- | --- | --- | --- | --- | --- | --- | --- | --- | --- | --- | --- | --- | --- | --- | --- | --- | --- | --- | --- | --- | --- | --- | --- | --- | --- | --- | --- | --- | --- | --- | --- | --- | --- | --- | --- | --- | --- | --- | --- | --- | --- | --- | --- | --- | --- | --- | --- | --- | --- | --- | --- | --- | --- | --- | --- | --- | --- | --- | --- | --- | --- | --- | --- | --- | --- | --- | --- | --- | --- | --- | --- | --- | --- | --- | --- | --- | --- | --- | --- | --- | --- | --- | --- | --- | --- | --- | --- | --- | --- | --- | --- | --- | --- | --- | --- | --- | --- | --- | --- | --- | --- | --- | --- | --- | --- | --- | --- | --- | --- | --- | --- | --- | --- | --- | --- | --- | --- | --- | --- | --- | --- | --- | --- | --- | --- | --- | --- | --- | --- | --- | --- | --- | --- | --- | --- | --- | --- | --- | --- | --- | --- | --- | --- | --- | --- | --- | --- | --- | --- | --- | --- | --- | --- | --- | --- | --- | --- | --- | --- | --- | --- | --- | --- | --- | --- | --- | --- | --- | --- | --- | --- | --- | --- | --- | --- | --- | --- | --- | --- | --- | --- | --- | --- | --- | --- | --- | --- | --- | --- | --- | --- | --- | --- | --- | --- | --- | --- | --- | --- | --- | --- | --- | --- | --- | --- | --- | --- | --- | --- | --- | --- | --- | --- | --- | --- | --- | --- | --- | --- | --- | --- | --- | --- | --- | --- | --- | --- | --- | --- | --- | --- | --- | --- | --- | --- | --- | --- | --- | --- | --- | --- | --- | --- | --- | --- | --- | --- | --- | --- | --- | --- | --- | --- | --- | --- | --- | --- | --- | --- | --- | --- | --- | --- | --- | --- | --- | --- | --- | --- | --- | --- | --- | --- | --- | --- | --- | --- | --- | --- | --- | --- | --- | --- | --- | --- | --- | --- | --- | --- | --- | --- | --- | --- | --- | --- | --- | --- | --- | --- | --- | --- | --- | --- | --- | --- | --- | --- | --- | --- | --- | --- | --- | --- | --- | --- | --- | --- | --- | --- | --- | --- | --- | --- | --- | --- | --- | --- | --- | --- | --- | --- | --- | --- | --- | --- | --- | --- | --- | --- | --- | --- | --- | --- | --- | --- | --- | --- | --- | --- | --- | --- | --- | --- | --- | --- | --- | --- | --- | --- | --- | --- | --- | --- | --- | --- | --- | --- | --- | --- | --- | --- | --- | --- | --- | --- | --- | --- | --- | --- | --- | --- | --- | --- | --- | --- | --- | --- | --- | --- | --- | --- | --- | --- | --- | --- | --- | --- | --- | --- | --- | --- | --- | --- | --- | --- | --- | --- | --- | --- | --- | --- | --- | --- | --- | --- | --- | --- | --- | --- | --- | --- | --- | --- | --- | --- | --- | --- | --- | --- | --- | --- | --- | --- | --- | --- | --- | --- | --- | --- | --- | --- | --- | --- | --- | --- | --- | --- | --- | --- | --- | --- | --- | --- | --- | --- | --- | --- | --- | --- | --- | --- | --- | --- | --- | --- | --- | --- | --- | --- | --- | --- | --- | --- | --- | --- | --- | --- | --- | --- | --- | --- | --- | --- | --- | --- | --- | --- | --- | --- | --- | --- | --- | --- | --- | --- | --- | --- | --- | --- | --- | --- | --- | --- | --- | --- | --- | --- | --- | --- | --- | --- | --- | --- | --- | --- | --- | --- | --- | --- | --- | --- | --- | --- | --- | --- | --- | --- | --- | --- | --- | --- | --- | --- | --- | --- | --- | --- | --- | --- | --- | --- | --- | --- | --- | --- | --- | --- | --- | --- | --- | --- | --- | --- | --- | --- | --- | --- | --- | --- | --- | --- | --- | --- | --- | --- | --- | --- | --- | --- | --- | --- | --- | --- | --- | --- | --- | --- | --- | --- | --- | --- | --- | --- | --- | --- | --- | --- | --- | --- | --- | --- | --- | --- | --- | --- | --- | --- | --- | --- | --- | --- | --- | --- | --- | --- | --- | --- | --- | --- | --- | --- | --- | --- | --- | --- | --- | --- | --- | --- | --- | --- | --- | --- | --- | --- | --- | --- | --- | --- | --- | --- | --- | --- | --- | --- | --- | --- | --- | --- | --- | --- | --- | --- | --- | --- | --- | --- | --- | --- | --- | --- | --- | --- | --- | --- | --- | --- | --- | --- | --- | --- | --- | --- | --- | --- | --- | --- | --- | --- | --- | --- | --- | --- | --- | --- | --- | --- | --- | --- | --- | --- | --- | --- | --- | --- | --- | --- | --- | --- | --- | --- | --- | --- | --- | --- | --- | --- | --- | --- | --- | --- | --- | --- | --- | --- | --- | --- | --- | --- | --- | --- | --- | --- | --- | --- | --- | --- | --- | --- | --- | --- | --- | --- | --- | --- | --- | --- | --- | --- | --- | --- | --- | --- | --- | --- | --- | --- | --- | --- | --- | --- | --- | --- | --- | --- | --- | --- | --- | --- | --- | --- | --- | --- | --- | --- | --- | --- | --- | --- | --- | --- | --- | --- | --- | --- | --- | --- | --- | --- | --- | --- | --- | --- | --- | --- | --- | --- | --- | --- | --- | --- | --- | --- | --- | --- | --- | --- | --- | --- | --- | --- | --- | --- | --- | --- | --- | --- | --- | --- | --- | --- | --- | --- | --- | --- | --- | --- | --- | --- | --- | --- | --- | --- | --- | --- | --- | --- | --- | --- | --- | --- | --- | --- | --- | --- | --- | --- | --- | --- | --- | --- | --- | --- | --- | --- | --- | --- | --- | --- | --- | --- | --- | --- | --- | --- | --- | --- | --- | --- | --- | --- | --- | --- | --- | --- | --- | --- | --- | --- | --- | --- | --- | --- | --- | --- | --- | --- | --- | --- | --- | --- | --- | --- | --- | --- | --- | --- | --- | --- | --- | --- | --- | --- | --- | --- | --- | --- | --- | --- | --- | --- | --- | --- | --- | --- | --- | --- | --- | --- | --- | --- | --- | --- | --- | --- | --- | --- | --- | --- | --- | --- | --- | --- | --- | --- | --- | --- | --- | --- | --- | --- | --- | --- | --- | --- | --- | --- | --- | --- | --- | --- | --- | --- | --- | --- | --- | --- | --- | --- | --- | --- | --- | --- | --- | --- | --- | --- | --- | --- | --- | --- | --- | --- | --- | --- | --- | --- | --- | --- | --- | --- | --- | --- | --- | --- | --- | --- | --- | --- | --- | --- | --- | --- | --- | --- | --- | --- | --- | --- | --- | --- | --- | --- | --- | --- | --- | --- | --- | --- | --- | --- | --- | --- | --- | --- | --- | --- | --- | --- | --- | --- | --- | --- | --- | --- | --- | --- | --- | --- | --- | --- | --- | --- | --- | --- | --- | --- | --- | --- | --- | --- | --- | --- | --- | --- | --- | --- | --- | --- | --- | --- | --- | --- | --- | --- | --- | --- | --- | --- | --- | --- | --- | --- | --- | --- | --- | --- | --- | --- | --- | --- | --- | --- | --- | --- | --- | --- | --- | --- | --- | --- | --- | --- | --- | --- | --- | --- | --- | --- | --- | --- | --- | --- | --- | --- | --- | --- | --- | --- | --- | --- | --- | --- | --- | --- | --- | --- | --- | --- | --- | --- | --- | --- | --- | --- | --- | --- | --- | --- | --- | --- | --- | --- | --- | --- | --- | --- | --- | --- | --- | --- | --- | --- | --- | --- | --- | --- | --- | --- | --- | --- | --- | --- | --- | --- | --- | --- | --- | --- | --- | --- | --- | --- | --- | --- | --- | --- | --- | --- | --- | --- | --- | --- | --- | --- | --- | --- | --- | --- | --- | --- | --- | --- | --- | --- | --- | --- | --- | --- | --- | --- | --- | --- | --- | --- | --- | --- | --- | --- | --- | --- | --- | --- | --- | --- | --- | --- | --- | --- | --- | --- | --- | --- | --- | --- | --- | --- | --- | --- | --- | --- | --- | --- | --- | --- | --- | --- | --- | --- | --- | --- | --- | --- | --- | --- | --- | --- | --- | --- | --- | --- | --- | --- | --- | --- | --- | --- | --- | --- | --- | --- | --- | --- | --- | --- | --- | --- | --- | --- | --- | --- | --- | --- | --- | --- | --- | --- | --- | --- | --- | --- | --- | --- | --- | --- | --- | --- | --- | --- | --- | --- | --- | --- | --- | --- | --- | --- | --- | --- | --- | --- | --- | --- | --- | --- | --- | --- | --- | --- | --- | --- | --- | --- | --- | --- | --- | --- | --- | --- | --- | --- | --- | --- | --- | --- | --- | --- | --- | --- | --- | --- | --- | --- | --- | --- | --- | --- | --- | --- | --- | --- | --- | --- | --- | --- | --- | --- | --- | --- | --- | --- | --- | --- | --- | --- | --- | --- | --- | --- | --- | --- | --- | --- | --- | --- | --- | --- | --- | --- | --- | --- | --- | --- | --- | --- | --- | --- | --- | --- | --- | --- | --- | --- | --- | --- | --- | --- | --- | --- | --- | --- | --- | --- | --- | --- | --- | --- | --- | --- | --- | --- | --- | --- | --- | --- | --- | --- | --- | --- | --- | --- | --- | --- | --- | --- | --- | --- | --- | --- | --- | --- | --- | --- | --- | --- | --- | --- | --- | --- | --- | --- | --- | --- | --- | --- | --- | --- | --- | --- | --- | --- | --- | --- | --- | --- | --- | --- | --- | --- | --- | --- | --- | --- | --- | --- | --- | --- | --- | --- | --- | --- | --- | --- | --- | --- | --- | --- | --- | --- | --- | --- | --- | --- | --- | --- | --- | --- | --- | --- | --- | --- | --- | --- | --- | --- | --- | --- | --- | --- | --- | --- | --- | --- | --- | --- | --- | --- | --- | --- | --- | --- | --- | --- | --- | --- | --- | --- | --- | --- | --- | --- | --- | --- | --- | --- | --- | --- | --- | --- | --- | --- | --- | --- | --- | --- | --- | --- | --- | --- | --- | --- | --- | --- | --- | --- | --- | --- | --- | --- | --- | --- | --- | --- | --- | --- | --- | --- | --- | --- | --- | --- | --- | --- | --- | --- | --- | --- | --- | --- | --- | --- | --- | --- | --- | --- | --- | --- | --- | --- | --- | --- | --- | --- | --- | --- | --- | --- | --- | --- | --- | --- | --- | --- | --- | --- | --- | --- | --- | --- | --- | --- | --- | --- | --- | --- | --- | --- | --- | --- | --- | --- | --- | --- | --- | --- | --- | --- | --- | --- | --- | --- | --- | --- | --- | --- | --- | --- | --- | --- | --- | --- | --- | --- | --- | --- | --- | --- | --- | --- | --- | --- | --- | --- | --- | --- | --- | --- | --- | --- | --- | --- | --- | --- | --- | --- | --- | --- | --- | --- | --- | --- | --- | --- | --- | --- | --- | --- | --- | --- | --- | --- | --- | --- | --- | --- | --- | --- | --- | --- | --- | --- | --- | --- | --- | --- | --- | --- | --- | --- | --- | --- | --- | --- | --- | --- | --- | --- | --- | --- | --- | --- | --- | --- | --- | --- | --- | --- | --- | --- | --- | --- | --- | --- | --- | --- | --- | --- | --- | --- | --- | --- | --- | --- | --- | --- | --- | --- | --- | --- | --- | --- | --- | --- | --- | --- | --- | --- | --- | --- | --- | --- | --- | --- | --- | --- | --- | --- | --- | --- | --- | --- | --- | --- | --- | --- | --- | --- | --- | --- | --- | --- | --- | --- | --- | --- | --- | --- | --- | --- | --- | --- | --- | --- | --- | --- | --- | --- | --- | --- | --- | --- | --- | --- | --- | --- | --- | --- | --- | --- | --- | --- | --- | --- | --- | --- | --- | --- | --- | --- | --- | --- | --- | --- | --- | --- | --- | --- | --- | --- | --- | --- | --- | --- | --- | --- | --- | --- | --- | --- | --- | --- | --- | --- | --- | --- | --- | --- | --- | --- | --- | --- | --- | --- | --- | --- | --- | --- | --- | --- | --- | --- | --- | --- | --- | --- | --- | --- | --- | --- | --- | --- | --- | --- | --- | --- | --- | --- | --- | --- | --- | --- | --- | --- | --- | --- | --- | --- | --- | --- | --- | --- | --- | --- | --- | --- | --- | --- | --- | --- | --- | --- | --- | --- | --- | --- | --- | --- | --- | --- | --- | --- | --- | --- | --- | --- | --- | --- | --- | --- | --- | --- | --- | --- | --- | --- | --- | --- | --- | --- | --- | --- | --- | --- | --- | --- | --- | --- | --- | --- | --- | --- | --- | --- | --- | --- | --- | --- | --- | --- | --- | --- | --- | --- | --- | --- | --- | --- | --- | --- | --- | --- | --- | --- | --- | --- | --- | --- | --- | --- | --- | --- | --- | --- | --- | --- | --- | --- | --- | --- | --- | --- | --- | --- | --- | --- | --- | --- | --- | --- | --- | --- | --- | --- | --- | --- | --- | --- | --- | --- | --- | --- | --- | --- | --- | --- | --- | --- | --- | --- | --- | --- | --- | --- | --- | --- | --- | --- | --- | --- | --- | --- | --- | --- | --- | --- | --- | --- | --- | --- | --- | --- | --- | --- | --- | --- | --- | --- | --- | --- | --- | --- | --- | --- | --- | --- | --- | --- | --- | --- | --- | --- | --- | --- | --- | --- | --- | --- | --- | --- | --- | --- | --- | --- | --- | --- | --- | --- | --- | --- | --- | --- | --- | --- | --- | --- | --- | --- | --- | --- | --- | --- | --- | --- | --- | --- | --- | --- | --- | --- | --- | --- | --- | --- | --- | --- | --- | --- | --- | --- | --- | --- | --- | --- | --- | --- | --- | --- | --- | --- | --- | --- | --- | --- | --- | --- | --- | --- | --- | --- | --- | --- | --- | --- | --- | --- | --- | --- | --- | --- | --- | --- | --- | --- | --- | --- | --- | --- | --- | --- | --- | --- | --- | --- | --- | --- | --- | --- | --- | --- | --- | --- | --- | --- | --- | --- | --- | --- | --- | --- | --- | --- | --- | --- | --- | --- | --- | --- | --- | --- | --- | --- | --- | --- | --- | --- | --- | --- | --- | --- | --- | --- | --- | --- | --- | --- | --- | --- | --- | --- | --- | --- | --- | --- | --- | --- | --- | --- | --- | --- | --- | --- | --- | --- | --- | --- | --- | --- | --- | --- | --- | --- | --- | --- | --- | --- | --- | --- | --- | --- | --- | --- | --- | --- | --- | --- | --- | --- | --- | --- | --- | --- | --- | --- | --- | --- | --- | --- | --- | --- | --- | --- | --- | --- | --- | --- | --- | --- | --- | --- | --- | --- | --- | --- | --- | --- | --- | --- | --- | --- | --- | --- | --- | --- | --- | --- | --- | --- | --- | --- | --- | --- | --- | --- | --- | --- | --- | --- | --- | --- | --- | --- | --- | --- | --- | --- | --- | --- | --- | --- | --- | --- | --- | --- | --- | --- | --- | --- | --- | --- | --- | --- | --- | --- | --- | --- | --- | --- | --- | --- | --- | --- | --- | --- | --- | --- | --- | --- | --- | --- | --- | --- | --- | --- | --- | --- | --- | --- | --- | --- | --- | --- | --- | --- | --- | --- | --- | --- | --- | --- | --- | --- | --- | --- | --- | --- | --- | --- | --- | --- | --- | --- | --- | --- | --- | --- | --- | --- | --- | --- | --- | --- | --- | --- | --- | --- | --- | --- | --- | --- | --- | --- | --- | --- | --- | --- | --- | --- | --- | --- | --- | --- | --- | --- | --- | --- | --- | --- | --- | --- | --- | --- | --- | --- | --- | --- | --- | --- | --- | --- | --- | --- | --- | --- | --- | --- | --- | --- | --- | --- | --- | --- | --- | --- | --- | --- | --- | --- | --- | --- | --- | --- | --- | --- | --- | --- | --- | --- | --- | --- | --- | --- | --- | --- | --- | --- | --- | --- | --- | --- | --- | --- | --- | --- | --- | --- | --- | --- | --- | --- | --- | --- | --- | --- | --- | --- | --- | --- | --- | --- | --- | --- | --- | --- | --- | --- | --- | --- | --- | --- | --- | --- | --- | --- | --- | --- | --- | --- | --- | --- | --- | --- | --- | --- | --- | --- | --- | --- | --- | --- | --- | --- | --- | --- | --- | --- | --- | --- | --- | --- | --- | --- | --- | --- | --- | --- | --- | --- | --- | --- | --- | --- | --- | --- | --- | --- | --- | --- | --- | --- | --- | --- | --- | --- | --- | --- | --- | --- | --- | --- | --- | --- | --- | --- | --- | --- | --- | --- | --- | --- | --- | --- | --- | --- | --- | --- | --- | --- | --- | --- | --- | --- | --- | --- | --- | --- | --- | --- | --- | --- | --- | --- | --- | --- | --- | --- | --- | --- | --- | --- | --- | --- | --- | --- | --- | --- | --- | --- | --- | --- | --- | --- | --- | --- | --- | --- | --- | --- | --- | --- | --- | --- | --- | --- | --- | --- | --- | --- | --- | --- | --- | --- | --- | --- | --- | --- | --- | --- | --- | --- | --- | --- | --- | --- | --- | --- | --- | --- | --- | --- | --- | --- | --- | --- | --- | --- | --- | --- | --- | --- | --- | --- | --- | --- | --- | --- | --- | --- | --- | --- | --- | --- | --- | --- | --- | --- | --- | --- | --- | --- | --- | --- | --- | --- | --- | --- | --- | --- | --- | --- | --- | --- | --- | --- | --- | --- | --- | --- | --- | --- | --- | --- | --- | --- | --- | --- | --- | --- | --- | --- | --- | --- | --- | --- | --- | --- | --- | --- | --- | --- | --- | --- | --- | --- | --- | --- | --- | --- | --- | --- | --- | --- | --- | --- | --- | --- | --- | --- | --- | --- | --- | --- | --- | --- | --- | --- | --- | --- | --- | --- | --- | --- | --- | --- | --- | --- | --- | --- | --- | --- | --- | --- | --- | --- | --- | --- | --- | --- | --- | --- | --- | --- | --- | --- | --- | --- | --- | --- | --- | --- | --- | --- | --- | --- | --- | --- | --- | --- | --- | --- | --- | --- | --- | --- | --- | --- | --- | --- | --- | --- | --- | --- | --- | --- | --- | --- | --- | --- | --- | --- | --- | --- | --- | --- | --- | --- | --- | --- | --- | --- | --- | --- | --- | --- | --- | --- | --- | --- | --- | --- | --- | --- | --- | --- | --- | --- | --- | --- | --- | --- | --- | --- | --- | --- | --- | --- | --- | --- | --- | --- | --- | --- | --- | --- | --- | --- | --- | --- | --- | --- | --- | --- | --- | --- | --- | --- | --- | --- | --- | --- | --- | --- | --- | --- | --- | --- | --- | --- | --- | --- | --- | --- | --- | --- | --- | --- | --- | --- | --- | --- | --- | --- | --- | --- | --- | --- | --- | --- | --- | --- | --- | --- | --- | --- | --- | --- | --- | --- | --- | --- | --- | --- | --- | --- | --- | --- | --- | --- | --- | --- | --- | --- | --- | --- | --- | --- | --- | --- | --- | --- | --- | --- | --- | --- | --- | --- | --- | --- | --- | --- | --- | --- | --- | --- | --- | --- | --- | --- | --- | --- | --- | --- | --- | --- | --- | --- | --- | --- | --- | --- | --- | --- | --- | --- | --- | --- | --- | --- | --- | --- | --- | --- | --- | --- | --- | --- | --- | --- | --- | --- | --- | --- | --- | --- | --- | --- | --- | --- | --- | --- | --- | --- | --- | --- | --- | --- | --- | --- | --- | --- | --- | --- | --- | --- | --- | --- | --- | --- | --- | --- | --- | --- | --- | --- | --- | --- | --- | --- | --- | --- | --- | --- | --- | --- | --- | --- | --- | --- | --- | --- | --- | --- | --- | --- | --- | --- | --- | --- | --- | --- | --- | --- | --- | --- | --- | --- | --- | --- | --- | --- | --- | --- | --- | --- | --- | --- | --- | --- | --- | --- | --- | --- | --- | --- | --- | --- | --- | --- | --- | --- | --- | --- | --- | --- | --- | --- | --- | --- | --- | --- | --- | --- | --- | --- | --- | --- | --- | --- | --- | --- | --- | --- | --- | --- | --- | --- | --- | --- | --- | --- | --- | --- | --- | --- | --- | --- | --- | --- | --- | --- | --- | --- | --- | --- | --- | --- | --- | --- | --- | --- | --- | --- | --- | --- | --- | --- | --- | --- | --- | --- | --- | --- | --- | --- | --- | --- | --- | --- | --- | --- | --- | --- | --- | --- | --- | --- | --- | --- | --- | --- | --- | --- | --- | --- | --- | --- | --- | --- | --- | --- | --- | --- | --- | --- | --- | --- | --- | --- | --- | --- | --- | --- | --- | --- | --- | --- | --- | --- | --- | --- | --- | --- | --- | --- | --- | --- | --- | --- | --- | --- | --- | --- | --- | --- | --- | --- | --- | --- | --- | --- | --- | --- | --- | --- | --- | --- | --- | --- | --- | --- | --- | --- | --- | --- | --- | --- | --- | --- | --- | --- | --- | --- | --- | --- | --- | --- | --- | --- | --- | --- | --- | --- | --- | --- | --- | --- | --- | --- | --- | --- | --- | --- | --- | --- | --- | --- | --- | --- | --- | --- | --- | --- | --- | --- | --- | --- | --- | --- | --- | --- | --- | --- | --- | --- | --- | --- | --- | --- | --- | --- | --- | --- | --- | --- | --- | --- | --- | --- | --- | --- | --- | --- | --- | --- | --- | --- | --- | --- | --- | --- | --- | --- | --- | --- | --- | --- | --- | --- | --- | --- | --- | --- | --- | --- | --- | --- | --- | --- | --- | --- | --- | --- | --- | --- | --- | --- | --- | --- | --- | --- | --- | --- | --- | --- | --- | --- | --- | --- | --- | --- | --- | --- | --- | --- | --- | --- | --- | --- | --- | --- | --- | --- | --- | --- | --- | --- | --- | --- | --- | --- | --- | --- | --- | --- | --- | --- | --- | --- | --- | --- | --- | --- | --- | --- | --- | --- | --- | --- | --- | --- | --- | --- | --- | --- | --- | --- | --- | --- | --- | --- | --- | --- | --- | --- | --- | --- | --- | --- | --- | --- | --- | --- | --- | --- | --- | --- | --- | --- | --- | --- | --- | --- | --- | --- | --- | --- | --- | --- | --- | --- | --- | --- | --- | --- | --- | --- | --- | --- | --- | --- | --- | --- | --- | --- | --- | --- | --- | --- | --- | --- | --- | --- | --- | --- | --- | --- | --- | --- | --- | --- | --- | --- | --- | --- | --- | --- | --- | --- | --- | --- | --- | --- | --- | --- | --- | --- | --- | --- | --- | --- | --- | --- | --- | --- | --- | --- | --- | --- | --- | --- | --- | --- | --- | --- | --- | --- | --- | --- | --- | --- | --- | --- | --- | --- | --- | --- | --- | --- | --- | --- | --- | --- | --- | --- | --- | --- | --- | --- | --- | --- | --- | --- | --- | --- | --- | --- | --- | --- | --- | --- | --- | --- | --- | --- | --- | --- | --- | --- | --- | --- | --- | --- | --- | --- | --- | --- | --- | --- | --- | --- | --- | --- | --- | --- | --- | --- | --- | --- | --- | --- | --- | --- | --- | --- | --- | --- | --- | --- | --- | --- | --- | --- | --- | --- | --- | --- | --- | --- | --- | --- | --- | --- | --- | --- | --- | --- | --- | --- | --- | --- | --- | --- | --- | --- | --- | --- | --- | --- | --- | --- | --- | --- | --- | --- | --- | --- | --- | --- | --- | --- | --- | --- | --- | --- | --- | --- | --- | --- | --- | --- | --- | --- | --- | --- | --- | --- | --- | --- | --- | --- | --- | --- | --- | --- | --- | --- | --- | --- | --- | --- | --- | --- | --- | --- | --- | --- | --- | --- | --- | --- | --- | --- | --- | --- | --- | --- | --- | --- | --- | --- | --- | --- | --- | --- | --- | --- | --- | --- | --- | --- | --- | --- | --- | --- | --- | --- | --- | --- | --- | --- | --- | --- | --- | --- | --- | --- | --- | --- | --- | --- | --- | --- | --- | --- | --- | --- | --- | --- | --- | --- | --- | --- | --- | --- | --- | --- | --- | --- | --- | --- | --- | --- | --- | --- | --- | --- | --- | --- | --- | --- | --- | --- | --- | --- | --- | --- | --- | --- | --- | --- | --- | --- | --- | --- | --- | --- | --- | --- | --- | --- | --- | --- | --- | --- | --- | --- | --- | --- | --- | --- | --- | --- | --- | --- | --- | --- | --- | --- | --- | --- | --- | --- | --- | --- | --- | --- | --- | --- | --- | --- | --- | --- | --- | --- | --- | --- | --- | --- | --- | --- | --- | --- | --- | --- | --- | --- | --- | --- | --- | --- | --- | --- | --- | --- | --- | --- | --- | --- | --- | --- | --- | --- | --- | --- | --- | --- | --- | --- | --- | --- | --- | --- | --- | --- | --- | --- | --- | --- | --- | --- | --- | --- | --- | --- | --- | --- | --- | --- | --- | --- | --- | --- | --- | --- | --- | --- | --- | --- | --- | --- | --- | --- | --- | --- | --- | --- | --- | --- | --- | --- | --- | --- | --- | --- | --- | --- | --- | --- | --- | --- | --- | --- | --- | --- | --- | --- | --- | --- | --- | --- | --- | --- | --- | --- | --- | --- | --- | --- | --- | --- | --- | --- | --- | --- | --- | --- | --- | --- | --- | --- | --- | --- | --- | --- | --- | --- | --- | --- | --- | --- | --- | --- | --- | --- | --- | --- | --- | --- | --- | --- | --- | --- | --- | --- | --- | --- | --- | --- | --- | --- | --- | --- | --- | --- | --- | --- | --- | --- | --- | --- | --- | --- | --- | --- | --- | --- | --- | --- | --- | --- | --- | --- | --- | --- | --- | --- | --- | --- | --- | --- | --- | --- | --- | --- | --- | --- | --- | --- | --- | --- | --- | --- | --- | --- | --- | --- | --- | --- | --- | --- | --- | --- | --- | --- | --- | --- | --- | --- | --- | --- | --- | --- | --- | --- | --- | --- | --- | --- | --- | --- | --- | --- | --- | --- | --- | --- | --- | --- | --- | --- | --- | --- | --- | --- | --- | --- | --- | --- | --- | --- | --- | --- | --- | --- | --- | --- | --- | --- | --- | --- | --- | --- | --- | --- | --- | --- | --- | --- | --- | --- | --- | --- | --- | --- | --- | --- | --- | --- | --- | --- | --- | --- | --- | --- | --- | --- | --- | --- | --- | --- | --- | --- | --- | --- | --- | --- | --- | --- | --- | --- | --- | --- | --- | --- | --- | --- | --- | --- | --- | --- | --- | --- | --- | --- | --- | --- | --- | --- | --- | --- | --- | --- | --- | --- | --- | --- | --- | --- | --- | --- | --- | --- | --- | --- | --- | --- | --- | --- | --- | --- | --- | --- | --- | --- | --- | --- | --- | --- | --- | --- | --- | --- | --- | --- | --- | --- | --- | --- | --- | --- | --- | --- | --- | --- | --- | --- | --- | --- | --- | --- | --- | --- | --- | --- | --- | --- | --- | --- | --- | --- | --- | --- | --- | --- | --- | --- | --- | --- | --- | --- | --- | --- | --- | --- | --- | --- | --- | --- | --- | --- | --- | --- | --- | --- | --- | --- | --- | --- | --- | --- | --- | --- | --- | --- | --- | --- | --- | --- | --- | --- | --- | --- | --- | --- | --- | --- | --- | --- | --- | --- | --- | --- | --- | --- | --- | --- | --- | --- | --- | --- | --- | --- | --- | --- | --- | --- | --- | --- | --- | --- | --- | --- | --- | --- | --- | --- | --- | --- | --- | --- | --- | --- | --- | --- | --- | --- | --- | --- | --- | --- | --- | --- | --- | --- | --- | --- | --- | --- | --- | --- | --- | --- | --- | --- | --- | --- | --- | --- | --- | --- | --- | --- | --- | --- | --- | --- | --- | --- | --- | --- | --- | --- | --- | --- | --- | --- | --- | --- | --- | --- | --- | --- | --- | --- | --- | --- | --- | --- | --- | --- | --- | --- | --- | --- | --- | --- | --- | --- | --- | --- | --- | --- | --- | --- | --- | --- | --- | --- | --- | --- | --- | --- | --- | --- | --- | --- | --- | --- | --- | --- | --- | --- | --- | --- | --- | --- | --- | --- | --- | --- | --- | --- | --- | --- | --- | --- | --- | --- | --- | --- | --- | --- | --- | --- | --- | --- | --- | --- | --- | --- | --- | --- | --- | --- | --- | --- | --- | --- | --- | --- | --- | --- | --- | --- | --- | --- | --- | --- | --- | --- | --- | --- | --- | --- | --- | --- | --- | --- | --- | --- | --- | --- | --- | --- | --- | --- | --- | --- | --- | --- | --- | --- | --- | --- | --- | --- | --- | --- | --- | --- | --- | --- | --- | --- | --- | --- | --- | --- | --- | --- | --- | --- | --- | --- | --- | --- | --- | --- | --- | --- | --- | --- | --- | --- | --- | --- | --- | --- | --- | --- | --- | --- | --- | --- | --- | --- | --- | --- | --- | --- | --- | --- | --- | --- | --- | --- | --- | --- | --- | --- | --- | --- | --- | --- | --- | --- | --- | --- | --- | --- | --- | --- | --- | --- | --- | --- | --- | --- | --- | --- | --- | --- | --- | --- | --- | --- | --- | --- | --- | --- | --- | --- | --- | --- | --- | --- | --- | --- | --- | --- | --- | --- | --- | --- | --- | --- | --- | --- | --- | --- | --- | --- | --- | --- | --- | --- | --- | --- | --- | --- | --- | --- | --- | --- | --- | --- | --- | --- | --- | --- | --- | --- | --- | --- | --- | --- | --- | --- | --- | --- | --- | --- | --- | --- | --- | --- | --- | --- | --- | --- | --- | --- | --- | --- | --- | --- | --- | --- | --- | --- | --- | --- | --- | --- | --- | --- | --- | --- | --- | --- | --- | --- | --- | --- | --- | --- | --- | --- | --- | --- | --- | --- | --- | --- | --- | --- | --- | --- | --- | --- | --- | --- | --- | --- | --- | --- | --- | --- | --- | --- | --- | --- | --- | --- | --- | --- | --- | --- | --- | --- | --- | --- | --- | --- | --- | --- | --- | --- | --- | --- | --- | --- | --- | --- | --- | --- | --- | --- | --- | --- | --- | --- | --- | --- | --- | --- | --- | --- | --- | --- | --- | --- | --- | --- | --- | --- | --- | --- | --- | --- | --- | --- | --- | --- | --- | --- | --- | --- | --- | --- | --- | --- | --- | --- | --- | --- | --- | --- | --- | --- | --- | --- | --- | --- | --- | --- | --- | --- | --- | --- | --- | --- | --- | --- | --- | --- | --- | --- | --- | --- | --- | --- | --- | --- | --- | --- | --- | --- | --- | --- | --- | --- | --- | --- | --- | --- | --- | --- | --- | --- | --- | --- | --- | --- | --- | --- | --- | --- | --- | --- | --- | --- | --- | --- | --- | --- | --- | --- | --- | --- | --- | --- | --- | --- | --- | --- | --- | --- | --- | --- | --- | --- | --- | --- | --- | --- | --- | --- | --- | --- | --- | --- | --- | --- | --- | --- | --- | --- | --- | --- | --- | --- | --- | --- | --- | --- | --- | --- | --- | --- | --- | --- | --- | --- | --- | --- | --- | --- | --- | --- | --- | --- | --- | --- | --- | --- | --- | --- | --- | --- | --- | --- | --- | --- | --- | --- | --- | --- | --- | --- | --- | --- | --- | --- | --- | --- | --- | --- | --- | --- | --- | --- | --- | --- | --- | --- | --- | --- | --- | --- | --- | --- | --- | --- | --- | --- | --- | --- | --- | --- | --- | --- | --- | --- | --- | --- | --- | --- | --- | --- | --- | --- | --- | --- | --- | --- | --- | --- | --- | --- | --- | --- | --- | --- | --- | --- | --- | --- | --- | --- | --- | --- | --- | --- | --- | --- | --- | --- | --- | --- | --- | --- | --- | --- | --- | --- | --- | --- | --- | --- | --- | --- | --- | --- | --- | --- | --- | --- | --- | --- | --- | --- | --- | --- | --- | --- | --- | --- | --- | --- | --- | --- | --- | --- | --- | --- | --- | --- | --- | --- | --- | --- | --- | --- | --- | --- | --- | --- | --- | --- | --- | --- | --- | --- | --- | --- | --- | --- | --- | --- | --- | --- | --- | --- | --- | --- | --- | --- | --- | --- | --- | --- | --- | --- | --- | --- | --- | --- | --- | --- | --- | --- | --- | --- | --- | --- | --- | --- | --- | --- | --- | --- | --- | --- | --- | --- | --- | --- | --- | --- | --- | --- | --- | --- | --- | --- | --- | --- | --- | --- | --- | --- | --- | --- | --- | --- | --- | --- | --- | --- | --- | --- | --- | --- | --- | --- | --- | --- | --- | --- | --- | --- | --- | --- | --- | --- | --- | --- | --- | --- | --- | --- | --- | --- | --- | --- | --- | --- | --- | --- | --- | --- | --- | --- | --- | --- | --- | --- | --- | --- | --- | --- | --- | --- | --- | --- | --- | --- | --- | --- | --- | --- | --- | --- | --- | --- | --- | --- | --- | --- | --- | --- | --- | --- | --- | --- | --- | --- | --- | --- | --- | --- | --- | --- | --- | --- | --- | --- | --- | --- | --- | --- | --- | --- | --- | --- | --- | --- | --- | --- | --- | --- | --- | --- | --- | --- | --- | --- | --- | --- | --- | --- | --- | --- | --- | --- | --- | --- | --- | --- | --- | --- | --- | --- | --- | --- | --- | --- | --- | --- | --- | --- | --- | --- | --- | --- | --- | --- | --- | --- | --- | --- | --- | --- | --- | --- | --- | --- | --- | --- | --- | --- | --- | --- | --- | --- | --- | --- | --- | --- | --- | --- | --- | --- | --- | --- | --- | --- | --- | --- | --- | --- | --- | --- | --- | --- | --- | --- | --- | --- | --- | --- | --- | --- | --- | --- | --- | --- | --- | --- | --- | --- | --- | --- | --- | --- | --- | --- | --- | --- | --- | --- | --- | --- | --- | --- | --- | --- | --- | --- | --- | --- | --- | --- | --- | --- | --- | --- | --- | --- | --- | --- | --- | --- | --- | --- | --- | --- | --- | --- | --- | --- | --- | --- | --- | --- | --- | --- | --- | --- | --- | --- | --- | --- | --- | --- | --- | --- | --- | --- | --- | --- | --- | --- | --- | --- | --- | --- | --- | --- | --- | --- | --- | --- | --- | --- | --- | --- | --- | --- | --- | --- | --- | --- | --- | --- | --- | --- | --- | --- | --- | --- | --- | --- | --- | --- | --- | --- | --- | --- | --- | --- | --- | --- | --- | --- | --- | --- | --- | --- | --- | --- | --- | --- | --- | --- | --- | --- | --- | --- | --- | --- | --- | --- | --- | --- | --- | --- | --- | --- | --- | --- | --- | --- | --- | --- | --- | --- | --- | --- | --- | --- | --- | --- | --- | --- | --- | --- | --- | --- | --- | --- | --- | --- | --- | --- | --- | --- | --- | --- | --- | --- | --- | --- | --- | --- | --- | --- | --- | --- | --- | --- | --- | --- | --- | --- | --- | --- | --- | --- | --- | --- | --- | --- | --- | --- | --- | --- | --- | --- | --- | --- | --- | --- | --- | --- | --- | --- | --- | --- | --- | --- | --- | --- | --- | --- | --- | --- | --- | --- | --- | --- | --- | --- | --- | --- | --- | --- | --- | --- | --- | --- | --- | --- | --- | --- | --- | --- | --- | --- | --- | --- | --- | --- | --- | --- | --- | --- | --- | --- | --- | --- | --- | --- | --- | --- | --- | --- | --- | --- | --- | --- | --- | --- | --- | --- | --- | --- | --- | --- | --- | --- | --- | --- | --- | --- | --- | --- | --- | --- | --- | --- | --- | --- | --- | --- | --- | --- | --- | --- | --- | --- | --- | --- | --- | --- | --- | --- | --- | --- | --- | --- | --- | --- | --- | --- | --- | --- | --- | --- | --- | --- | --- | --- | --- | --- | --- | --- | --- | --- | --- | --- | --- | --- | --- | --- | --- | --- | --- | --- | --- | --- | --- | --- | --- | --- | --- | --- | --- | --- | --- | --- | --- | --- | --- | --- | --- | --- | --- | --- | --- | --- | --- | --- | --- | --- | --- | --- | --- | --- | --- | --- | --- | --- | --- | --- | --- | --- | --- | --- | --- | --- | --- | --- | --- | --- | --- | --- | --- | --- | --- | --- | --- | --- | --- | --- | --- | --- | --- | --- | --- | --- | --- | --- | --- | --- | --- | --- | --- | --- | --- | --- | --- | --- | --- | --- | --- | --- | --- | --- | --- | --- | --- | --- | --- | --- | --- | --- | --- | --- | --- | --- | --- | --- | --- | --- | --- | --- | --- | --- | --- | --- | --- | --- | --- | --- | --- | --- | --- | --- | --- | --- | --- | --- | --- | --- | --- | --- | --- | --- | --- | --- | --- | --- | --- | --- | --- | --- | --- | --- | --- | --- | --- | --- | --- | --- | --- | --- | --- | --- | --- | --- | --- | --- | --- | --- | --- | --- | --- | --- | --- | --- | --- | --- | --- | --- | --- | --- | --- | --- | --- | --- | --- | --- | --- | --- | --- | --- | --- | --- | --- | --- | --- | --- | --- | --- | --- | --- | --- | --- | --- | --- | --- | --- | --- | --- | --- | --- | --- | --- | --- | --- | --- | --- | --- | --- | --- | --- | --- | --- | --- | --- | --- | --- | --- | --- | --- | --- | --- | --- | --- | --- | --- | --- | --- | --- | --- | --- | --- | --- | --- | --- | --- | --- | --- | --- | --- | --- | --- | --- | --- | --- | --- | --- | --- | --- | --- | --- | --- | --- | --- | --- | --- | --- | --- | --- | --- | --- | --- | --- | --- | --- | --- | --- | --- | --- | --- | --- | --- | --- | --- | --- | --- | --- | --- | --- | --- | --- | --- | --- | --- | --- | --- | --- | --- | --- | --- | --- | --- | --- | --- | --- | --- | --- | --- | --- | --- | --- | --- | --- | --- | --- | --- | --- | --- | --- | --- | --- | --- | --- | --- | --- | --- | --- | --- | --- | --- | --- | --- | --- | --- | --- | --- | --- | --- | --- | --- | --- | --- | --- | --- | --- | --- | --- | --- | --- | --- | --- | --- | --- | --- | --- | --- | --- | --- | --- | --- | --- | --- | --- | --- | --- | --- | --- | --- | --- | --- | --- | --- | --- | --- | --- | --- | --- | --- | --- | --- | --- | --- | --- | --- | --- | --- | --- | --- | --- | --- | --- | --- | --- | --- | --- | --- | --- | --- | --- | --- | --- | --- | --- | --- | --- | --- | --- | --- | --- | --- | --- | --- | --- | --- | --- | --- | --- | --- | --- | --- | --- | --- | --- | --- | --- | --- | --- | --- | --- | --- | --- | --- | --- | --- | --- | --- | --- | --- | --- | --- | --- | --- | --- | --- | --- | --- | --- | --- | --- | --- | --- | --- | --- | --- | --- | --- | --- | --- | --- | --- | --- | --- | --- | --- | --- | --- | --- | --- | --- | --- | --- | --- | --- | --- | --- | --- | --- | --- | --- | --- | --- | --- | --- | --- | --- | --- | --- | --- | --- | --- | --- | --- | --- | --- | --- | --- | --- | --- | --- | --- | --- | --- | --- | --- | --- | --- | --- | --- | --- | --- | --- | --- | --- | --- | --- | --- | --- | --- | --- | --- | --- | --- | --- | --- | --- | --- | --- | --- | --- | --- | --- | --- | --- | --- | --- | --- | --- | --- | --- | --- | --- | --- | --- | --- | --- | --- | --- | --- | --- | --- | --- | --- | --- | --- | --- | --- | --- | --- | --- | --- | --- | --- | --- | --- | --- | --- | --- | --- | --- | --- | --- | --- | --- | --- | --- | --- | --- | --- | --- | --- | --- | --- | --- | --- | --- | --- | --- | --- | --- | --- | --- | --- | --- | --- | --- | --- | --- | --- | --- | --- | --- | --- | --- | --- | --- | --- | --- | --- | --- | --- | --- | --- | --- | --- | --- | --- | --- | --- | --- | --- | --- | --- | --- | --- | --- | --- | --- | --- | --- | --- | --- | --- | --- | --- | --- | --- | --- | --- | --- | --- | --- | --- | --- | --- | --- | --- | --- | --- | --- | --- | --- | --- | --- | --- | --- | --- | --- | --- | --- | --- | --- | --- | --- | --- | --- | --- | --- | --- | --- | --- | --- | --- | --- | --- | --- | --- | --- | --- | --- | --- | --- | --- | --- | --- | --- | --- | --- | --- | --- | --- | --- | --- | --- | --- | --- | --- | --- | --- | --- | --- | --- | --- | --- | --- | --- | --- | --- | --- | --- | --- | --- | --- | --- | --- | --- | --- | --- | --- | --- | --- | --- | --- | --- | --- | --- | --- | --- | --- | --- | --- | --- | --- | --- | --- | --- | --- | --- | --- | --- | --- | --- | --- | --- | --- | --- | --- | --- | --- | --- | --- | --- | --- | --- | --- | --- | --- | --- | --- | --- | --- | --- | --- | --- | --- | --- | --- | --- | --- | --- | --- | --- | --- | --- | --- | --- | --- | --- | --- | --- | --- | --- | --- | --- | --- | --- | --- | --- | --- | --- | --- | --- | --- | --- | --- | --- | --- | --- | --- | --- | --- | --- | --- | --- | --- | --- | --- | --- | --- | --- | --- | --- | --- | --- | --- | --- | --- | --- | --- | --- | --- | --- | --- | --- | --- | --- | --- | --- | --- | --- | --- | --- | --- | --- | --- | --- | --- | --- | --- | --- | --- | --- | --- | --- | --- | --- | --- | --- | --- | --- | --- | --- | --- | --- | --- | --- | --- | --- | --- | --- | --- | --- | --- | --- | --- | --- | --- | --- | --- | --- | --- | --- | --- | --- | --- | --- | --- | --- | --- | --- | --- | --- | --- | --- | --- | --- | --- | --- | --- | --- | --- | --- | --- | --- | --- | --- | --- | --- | --- | --- | --- | --- | --- | --- | --- | --- | --- | --- | --- | --- | --- | --- | --- | --- | --- | --- | --- | --- | --- | --- | --- | --- | --- | --- | --- | --- | --- | --- | --- | --- | --- | --- | --- | --- | --- | --- | --- | --- | --- | --- | --- | --- | --- | --- | --- | --- | --- | --- | --- | --- | --- | --- | --- | --- | --- | --- | --- | --- | --- | --- | --- | --- | --- | --- | --- | --- | --- | --- | --- | --- | --- | --- | --- | --- | --- | --- | --- | --- | --- | --- | --- | --- | --- | --- | --- | --- | --- | --- | --- | --- | --- | --- | --- | --- | --- | --- | --- | --- | --- | --- | --- | --- | --- | --- | --- | --- | --- | --- | --- | --- | --- | --- | --- | --- | --- | --- | --- | --- | --- | --- | --- | --- | --- | --- | --- | --- | --- | --- | --- | --- | --- | --- | --- | --- | --- | --- | --- | --- | --- | --- | --- | --- | --- | --- | --- | --- | --- | --- | --- | --- | --- | --- | --- | --- | --- | --- | --- | --- | --- | --- | --- | --- | --- | --- | --- | --- | --- | --- | --- | --- | --- | --- | --- | --- | --- | --- | --- | --- | --- | --- | --- | --- | --- | --- | --- | --- | --- | --- | --- | --- | --- | --- | --- | --- | --- | --- | --- | --- | --- | --- | --- | --- | --- | --- | --- | --- | --- | --- | --- | --- | --- | --- | --- | --- | --- | --- | --- | --- | --- | --- | --- | --- | --- | --- | --- | --- | --- | --- | --- | --- | --- | --- | --- | --- | --- | --- | --- | --- | --- | --- | --- | --- | --- | --- | --- | --- | --- | --- | --- | --- | --- | --- | --- | --- | --- | --- | --- | --- | --- | --- | --- | --- | --- | --- | --- | --- | --- | --- | --- | --- | --- | --- | --- | --- | --- | --- | --- | --- | --- | --- | --- | --- | --- | --- | --- | --- | --- | --- | --- | --- | --- | --- | --- | --- | --- | --- | --- | --- | --- | --- | --- | --- | --- | --- | --- | --- | --- | --- | --- | --- | --- | --- | --- | --- | --- | --- | --- | --- | --- | --- | --- | --- | --- | --- | --- | --- | --- | --- | --- | --- | --- | --- | --- | --- | --- | --- | --- | --- | --- | --- | --- | --- | --- | --- | --- | --- | --- | --- | --- | --- | --- | --- | --- | --- | --- | --- | --- | --- | --- | --- | --- | --- | --- | --- | --- | --- | --- | --- | --- | --- | --- | --- | --- | --- | --- | --- | --- | --- | --- | --- | --- | --- | --- | --- | --- | --- | --- | --- | --- | --- | --- | --- | --- | --- | --- | --- | --- | --- | --- | --- | --- | --- | --- | --- | --- | --- | --- | --- | --- | --- | --- | --- | --- | --- | --- | --- | --- | --- | --- | --- | --- | --- | --- | --- | --- | --- | --- | --- | --- | --- | --- | --- | --- | --- | --- | --- | --- | --- | --- | --- | --- | --- | --- | --- | --- | --- | --- | --- | --- | --- | --- | --- | --- | --- | --- | --- | --- | --- | --- | --- | --- | --- | --- | --- | --- | --- | --- | --- | --- | --- | --- | --- | --- | --- | --- | --- | --- | --- | --- | --- | --- | --- | --- | --- | --- | --- | --- | --- | --- | --- | --- | --- | --- | --- | --- | --- | --- | --- | --- | --- | --- | --- | --- | --- | --- | --- | --- | --- | --- | --- | --- | --- | --- | --- | --- | --- | --- | --- | --- | --- | --- | --- | --- | --- | --- | --- | --- | --- | --- | --- | --- | --- | --- | --- | --- | --- | --- | --- | --- | --- | --- | --- | --- | --- | --- | --- | --- | --- | --- | --- | --- | --- | --- | --- | --- | --- | --- | --- | --- | --- | --- | --- | --- | --- | --- | --- | --- | --- | --- | --- | --- | --- | --- | --- | --- | --- | --- | --- | --- | --- | --- | --- | --- | --- | --- | --- | --- | --- | --- | --- | --- | --- | --- | --- | --- | --- | --- | --- | --- | --- | --- | --- | --- | --- | --- | --- | --- | --- | --- | --- | --- | --- | --- | --- | --- | --- | --- | --- | --- | --- | --- | --- | --- | --- | --- | --- | --- | --- | --- | --- | --- | --- | --- | --- | --- | --- | --- | --- | --- | --- | --- | --- | --- | --- | --- | --- | --- | --- | --- | --- | --- | --- | --- | --- | --- | --- | --- | --- | --- | --- | --- | --- | --- | --- | --- | --- | --- | --- | --- | --- | --- | --- | --- | --- | --- | --- | --- | --- | --- | --- | --- | --- | --- | --- | --- | --- | --- | --- | --- | --- | --- | --- | --- | --- | --- | --- | --- | --- | --- | --- | --- | --- | --- | --- | --- | --- | --- | --- | --- | --- | --- | --- | --- | --- | --- | --- | --- | --- | --- | --- | --- | --- | --- | --- | --- | --- | --- | --- | --- | --- | --- | --- | --- | --- | --- | --- | --- | --- | --- | --- | --- | --- | --- | --- | --- | --- | --- | --- | --- | --- | --- | --- | --- | --- | --- | --- | --- | --- | --- | --- | --- | --- | --- | --- | --- | --- | --- | --- | --- | --- | --- | --- | --- | --- | --- | --- | --- | --- | --- | --- | --- | --- | --- | --- | --- | --- | --- | --- | --- | --- | --- | --- | --- | --- | --- | --- | --- | --- | --- | --- | --- | --- | --- | --- | --- | --- | --- | --- | --- | --- | --- | --- | --- | --- | --- | --- | --- | --- | --- | --- | --- | --- | --- | --- | --- | --- | --- | --- | --- | --- | --- | --- | --- | --- | --- | --- | --- | --- | --- | --- | --- | --- | --- | --- | --- | --- | --- | --- | --- | --- | --- | --- | --- | --- | --- | --- | --- | --- | --- | --- | --- | --- | --- | --- | --- | --- | --- | --- | --- | --- | --- | --- | --- | --- | --- | --- | --- | --- | --- | --- | --- | --- | --- | --- | --- | --- | --- | --- | --- | --- | --- | --- | --- | --- | --- | --- | --- | --- | --- | --- | --- | --- | --- | --- | --- | --- | --- | --- | --- | --- | --- | --- | --- | --- | --- | --- | --- | --- | --- | --- | --- | --- | --- | --- | --- | --- | --- | --- | --- | --- | --- | --- | --- | --- | --- | --- | --- | --- | --- | --- | --- | --- | --- | --- | --- | --- | --- | --- | --- | --- | --- | --- | --- | --- | --- | --- | --- | --- | --- | --- | --- | --- | --- | --- | --- | --- | --- | --- | --- | --- | --- | --- | --- | --- | --- | --- | --- | --- | --- | --- | --- | --- | --- | --- | --- | --- | --- | --- | --- | --- | --- | --- | --- | --- | --- | --- | --- | --- | --- | --- | --- | --- | --- | --- | --- | --- | --- | --- | --- | --- | --- | --- | --- | --- | --- | --- | --- | --- | --- | --- | --- | --- | --- | --- | --- | --- | --- | --- | --- | --- | --- | --- | --- | --- | --- | --- | --- | --- | --- | --- | --- | --- | --- | --- | --- | --- | --- | --- | --- | --- | --- | --- | --- | --- | --- | --- | --- | --- | --- | --- | --- | --- | --- | --- | --- | --- | --- | --- | --- | --- | --- | --- | --- | --- | --- | --- | --- | --- | --- | --- | --- | --- | --- | --- | --- | --- | --- | --- | --- | --- | --- | --- | --- | --- | --- | --- | --- | --- | --- | --- | --- | --- | --- | --- | --- | --- | --- | --- | --- | --- | --- | --- | --- | --- | --- | --- | --- | --- | --- | --- | --- | --- | --- | --- | --- | --- | --- | --- | --- | --- | --- | --- | --- | --- | --- | --- | --- | --- | --- | --- | --- | --- | --- | --- | --- | --- | --- | --- | --- | --- | --- | --- | --- | --- | --- | --- | --- | --- | --- | --- | --- | --- | --- | --- | --- | --- | --- | --- | --- | --- | --- | --- | --- | --- | --- | --- | --- | --- | --- | --- | --- | --- | --- | --- | --- | --- | --- | --- | --- | --- | --- | --- | --- | --- | --- | --- | --- | --- | --- | --- | --- | --- | --- | --- | --- | --- | --- | --- | --- | --- | --- | --- | --- | --- | --- | --- | --- | --- | --- | --- | --- | --- | --- | --- | --- | --- | --- | --- | --- | --- | --- | --- | --- | --- | --- | --- | --- | --- | --- | --- | --- | --- | --- | --- | --- | --- | --- | --- | --- | --- | --- | --- | --- | --- | --- | --- | --- | --- | --- | --- | --- | --- | --- | --- | --- | --- | --- | --- | --- | --- | --- | --- | --- | --- | --- | --- | --- | --- | --- | --- | --- | --- | --- | --- | --- | --- | --- | --- | --- | --- | --- | --- | --- | --- | --- | --- | --- | --- | --- | --- | --- | --- | --- | --- | --- | --- | --- | --- | --- | --- | --- | --- | --- | --- | --- | --- | --- | --- | --- | --- | --- | --- | --- | --- | --- | --- | --- | --- | --- | --- | --- |
| |  |  |  |  |  |  |  |  |  | | --- | --- | --- | --- | --- | --- | --- | --- | --- | | **Position** | **Reference** | **Sample** | **Quality** | **Type** | **Region** | **AA Exchange** | **PAM1** | **Known Variant** | | 840 | G | T | 45.77 | SNP | Rv0001 (dnaA) | Glu280Asp | 53 | - | | 1977 | A | G | 80.28 | SNP | intergenic |  |  | - | | 4013 | T | C | 280.78 | SNP | Rv0003 (recF) | Ile245Thr | 11 | - | | 4323 | G | T | 47.77 | SNP | Rv0003 (recF) | Gln348His | 20 | - | | 6140 | G | T | 236.84 | SNP | Rv0005 (gyrB) | Val(s)301Leu(s) | 9867 | - | | 7362 | G | C | 88.28 | SNP | Rv0006 (gyrA) | Glu21Gln | 27 | - | | 7585 | G | C | 106.28 | SNP | Rv0006 (gyrA) | Ser95Thr | 32 | genotype | | 9304 | G | A | 472.77 | SNP | Rv0006 (gyrA) | Gly668Asp | 6 | - | | 11841 | G | C | 85.28 | SNP | intergenic |  |  | - | | 11879 | A | G | 115.03 | SNP | Rv0008c | Ser145Pro | 12 | - | | 12555 | C | A | 38.77 | SNP | Rv0009 (ppiA) | Leu30Met(s) | 4 | - | | 14785 | T | C | 165.90 | SNP | Rv0012 | Cys233Arg | 1 | - | | 17608 | G | C | 94.28 | SNP | Rv0015c (pknA) | Ser385Arg | 6 | - | | 26959 | C | G | 106.28 | SNP | intergenic |  |  | - | | 33457 | C | T | 85.28 | SNP | Rv0030 | silent (His78) | 9912 | - | | 33551 | T | G | 142.03 | SNP | Rv0030 | STOP110Gly | 21 | - | | 34044 | T | C | 143.03 | SNP | intergenic |  |  | - | | 35097 | T | C | 275.80 | SNP | Rv0032 (bioF2) | Ile268Thr | 11 | - | | 37031 | C | G | 199.84 | SNP | Rv0034 | silent (Ala55) | 9867 | - | | 38821 | C | A | 31.77 | SNP | Rv0035 (fadD34) | silent (Leu521) | 9947 | - | | 40162 | C | T | 209.78 | SNP | Rv0037c | Met(s)347Ile | 2 | - | | 42744 | C | A | 37.77 | SNP | Rv0040c (mtc28) | Gly208Cys | 0 | - | | 42967 | G | C | 99.28 | SNP | Rv0040c (mtc28) | silent (Pro133) | 9926 | - | | 43958 | C | A | 48.78 | SNP | Rv0041 (leuS) | His133Asn | 21 | - | | 47036 | C | T | 170.84 | SNP | Rv0042c | Gly58Arg | 0 | - | | 47995 | C | A | 31.77 | SNP | Rv0043c | Glu36STOP | 17 | - | | 55553 | C | T | 48.74 | SNP | Rv0050 (ponA1) | Pro631Ser | 17 | - | | 62049 | A | G | 408.77 | SNP | Rv0058 (dnaB) | Arg552Gly | 1 | - | | 63771 | C | T | 163.85 | SNP | Rv0059 | Pro191Leu | 3 | - | | 63842 | C | A | 31.77 | SNP | Rv0059 | Pro215Thr | 5 | - | | 64250 | C | A | 38.77 | SNP | Rv0060 | silent (Pro114) | 9926 | - | | 69989 | G | A | 126.03 | SNP | Rv0064 | Gly457Asp | 6 | - | | 70533 | G | T | 167.90 | SNP | Rv0064 | silent (Ser638) | 9840 | - | | 70816 | A | G | 213.84 | SNP | Rv0064 | Asn733Asp | 42 | - | | 75940 | G | C | 119.03 | SNP | Rv0068 | Val(s)214Leu | 3 | - | | 79504 | TCGGTGGACC | T | 505.87 | DEL | Rv0071 |  |  | - | | 80616 | C | G | 166.90 | SNP | intergenic |  |  | - | | 88007 | T | A | 267.78 | SNP | intergenic |  |  | - | | 91747 | G | T | 48.78 | SNP | Rv0083 | Gly450Cys | 0 | - | | 92199 | T | G | 50.74 | SNP | Rv0083 | silent (Thr600) | 9871 | - | | 92388 | A | G | 94.28 | SNP | Rv0084 (hycD) | Ile21Val | 57 | - | | 92765 | C | A | 32.77 | SNP | Rv0084 (hycD) | Phe146Leu | 13 | - | | 99588 | C | A | 46.78 | SNP | intergenic |  |  | - | | 100142 | C | T | 181.96 | SNP | Rv0091 (mtn) | silent (Thr153) | 9871 | - | | 100767 | A | G | 185.90 | SNP | Rv0092 (ctpA) | Asp62Gly | 11 | - | | 101727 | G | A | 91.28 | SNP | Rv0092 (ctpA) | Gly382Glu | 4 | - | | 104712 | C | T | 101.28 | SNP | intergenic |  |  | - | | 104915 | T | C | 34.77 | SNP | Rv0095c | Thr101Ala | 32 | - | | 104941 | T | C | 62.74 | SNP | Rv0095c | Gln92Arg | 10 | - | | 104942 | G | C | 52.74 | SNP | Rv0095c | Gln92Glu | 35 | - | | 104944 | G | A | 33.74 | SNP | Rv0095c | Ala91Val | 13 | - | | 105045 | G | C | 139.03 | SNP | Rv0095c | Asp57Glu | 56 | - | | 116000 | T | G | 173.90 | SNP | Rv0101 (nrp) | Val2000Val(s) | 18 | - | | 119579 | G | T | 32.77 | SNP | Rv0102 | Trp622Cys | 0 | - | | 122109 | A | G | 116.03 | SNP | Rv0103c (ctpB) | Leu(s)22Ser | 28 | - | | 125638 | G | T | 38.77 | SNP | intergenic |  |  | - | | 125830 | G | GA | 282.75 | INS | Rv0107c (ctpI) |  |  | - | | 131174 | T | TG | 344.74 | INS | intergenic |  |  | - | | 133445 | G | A | 368.77 | SNP | Rv0110 | silent (Gly142) | 9935 | - | | 133839 | C | T | 255.78 | SNP | intergenic |  |  | - | | 133862 | G | A | 197.80 | SNP | intergenic |  |  | - | | 139784 | G | T | 37.77 | SNP | intergenic |  |  | - | | 140550 | G | T | 34.77 | SNP | Rv0116c (ldtA) | Pro158Gln | 6 | - | | 146087 | T | C | 42.74 | SNP | Rv0120c (fusA2) | Asn562Ser | 34 | - | | 150584 | T | C | 65.28 | SNP | Rv0124 (PE\_PGRS2) | Leu351Pro | 2 | - | | 154283 | T | C | 42.74 | SNP | Rv0127 (mak) | Ser18Pro | 12 | - | | 155762 | G | T | 33.77 | SNP | Rv0128 | silent (Ala32) | 9867 | - | | 156698 | C | A | 38.77 | SNP | Rv0129c (fbpC) | silent (Pro301) | 9926 | - | | 157292 | C | T | 154.90 | SNP | Rv0129c (fbpC) | silent (Glu103) | 9865 | genotype | | 158000 | G | A | 280.78 | SNP | Rv0130 (htdZ) | Ala52Thr | 22 | - | | 158717 | G | T | 37.77 | SNP | Rv0131c (fadE1) | silent (Ile314) | 9872 | - | | 162581 | G | A | 123.90 | SNP | Rv0134 (ephF) | Gly271Ser | 16 | - | | 167008 | C | A | 38.77 | SNP | Rv0140 | silent (Val33) | 9901 | - | | 168739 | C | A | 42.77 | SNP | Rv0143c | Glu482STOP | 17 | - | | 176303 | C | T | 66.03 | SNP | Rv0149 | His202Tyr | 4 | - | | 177857 | G | A | 45.74 | SNP | Rv0151c (PE1) | Leu485Leu(s) | 4 | - | | 177999 | C | T | 74.28 | SNP | Rv0151c (PE1) | silent (Pro437) | 9926 | - | | 180025 | C | T | 280.80 | SNP | Rv0152c (PE2) | Gly291Glu | 4 | - | | 183903 | G | T | 44.77 | SNP | Rv0155 (pntAa) | silent (Leu94) | 9947 | - | | 188800 | T | C | 258.80 | SNP | Rv0159c (PE3) | Thr14Ala | 32 | - | | 194681 | G | C | 81.28 | SNP | Rv0165c (mce1R) | silent (Leu45) | 9947 | - | | 196144 | C | A | 31.77 | SNP | Rv0166 (fadD5) | silent (Val384) | 9901 | - | | 196642 | C | T | 249.78 | SNP | Rv0166 (fadD5) | silent (Asn550) | 9822 | - | | 201672 | C | T | 47.74 | SNP | Rv0171 (mce1C) | silent (Ala247) | 9867 | - | | 203269 | C | T | 84.28 | SNP | Rv0172 (mce1D) | Ala265Val(s) | 9867 | - | | 206339 | T | C | 248.80 | SNP | Rv0174 (mce1F) | Leu370Pro | 2 | - | | 207226 | T | C | 95.28 | SNP | Rv0175 | Met(s)138Thr | 22 | - | | 207431 | C | A | 31.77 | SNP | Rv0175 | silent (Ala206) | 9867 | - | | 223080 | T | C | 224.84 | SNP | Rv0191 | silent (Tyr264) | 9945 | - | | 223942 | T | C | 53.74 | SNP | Rv0192 | Ser127Pro | 12 | - | | 227098 | T | C | 55.74 | SNP | Rv0194 | Met(s)74Thr | 22 | - | | 228069 | G | A | 192.80 | SNP | Rv0194 | Val(s)398Met(s) | 9867 | - | | 228168 | G | C | 403.77 | SNP | Rv0194 | Gly431Arg | 0 | - | | 231114 | C | G | 164.90 | SNP | Rv0195 | silent (Ala72) | 9867 | - | | 231657 | C | A | 47.77 | SNP | Rv0196 | Pro4Gln | 6 | - | | 234051 | G | A | 131.03 | SNP | Rv0197 | silent (Pro607) | 9926 | - | | 234477 | T | G | 240.84 | SNP | Rv0197 | Tyr749STOP | 2 | - | | 234496 | C | CGT | 764.73 | INS | Rv0197 |  |  | - | | 239026 | C | A | 34.77 | SNP | Rv0202c (mmpL11) | Arg756Leu | 1 | - | | 250591 | G | T | 52.77 | SNP | Rv0210 | Asp158Tyr | 0 | - | | 251404 | G | T | 30.77 | SNP | Rv0210 | Glu429STOP | 17 | - | | 257397 | C | A | 36.77 | SNP | Rv0214 (fadD4) | Pro445Gln | 6 | - | | 261869 | T | C | 193.84 | SNP | Rv0218 | Cys316Arg | 1 | - | | 263118 | G | T | 31.77 | SNP | Rv0220 (lipC) | Asp103Tyr | 0 | - | | 265554 | A | C | 47.74 | SNP | Rv0222 (echA1) | silent (Val16) | 9901 | - | | 265841 | C | A | 35.77 | SNP | Rv0222 (echA1) | Ala112Glu | 10 | - | | 267820 | C | A | 38.77 | SNP | intergenic |  |  | - | | 278681 | C | G | 137.90 | SNP | Rv0233 (nrdB) | His33Asp | 4 | - | | 282188 | G | A | 125.03 | SNP | Rv0235c | Arg143Cys | 1 | - | | 283614 | T | C | 133.03 | SNP | Rv0236c (aftD) | Ser1080Gly | 21 | - | | 287449 | C | A | 39.78 | SNP | Rv0237 (lpqI) | silent (Val88) | 9901 | - | | 288150 | G | T | 40.77 | SNP | Rv0237 (lpqI) | Arg322Leu | 1 | - | | 288521 | G | T | 36.77 | SNP | Rv0238 | Glu32STOP | 17 | - | | 291036 | C | A | 43.77 | SNP | Rv0242c (fabG4) | Gly332Cys | 0 | - | | 293704 | CT | C | 154.87 | DEL | intergenic |  |  | - | | 294241 | C | A | 31.77 | SNP | Rv0244c (fadE5) | Asp465Tyr | 0 | - | | 300229 | C | A | 46.78 | SNP | Rv0248c | Arg192Leu | 1 | - | | 302711 | A | G | 217.84 | SNP | intergenic |  |  | - | | 310973 | G | A | 109.03 | SNP | Rv0259c | Ala182Val(s) | 9867 | - | | 311613 | G | T | 34.74 | SNP | Rv0260c | silent (Val349) | 9901 | - | | 316129 | C | G | 51.74 | SNP | Rv0264c | Gly96Ala | 21 | - | | 317285 | G | T | 35.77 | SNP | Rv0265c | silent (Val73) | 9901 | - | | 323043 | C | A | 44.77 | SNP | Rv0268c | silent (Arg77) | 9913 | - | | 324968 | G | T | 124.03 | SNP | Rv0270 (fadD2) | Val(s)134Val | 13 | - | | 330975 | G | A | 187.84 | SNP | Rv0274; Rv0275c | Arg185Gln; silent (Ala228) | 9; 9867 | - | | 331673 | C | T | 34.74 | SNP | intergenic |  |  | - | | 333892 | G | C | 43.74 | SNP | Rv0278c (PE\_PGRS3) | Arg807Gly | 1 | - | | 334641 | G | C | 48.74 | SNP | Rv0278c (PE\_PGRS3) | Ala557Gly | 21 | - | | 335922 | C | G | 34.77 | SNP | Rv0278c (PE\_PGRS3) | Gly130Ala | 21 | - | | 335927 | A | G | 32.77 | SNP | Rv0278c (PE\_PGRS3) | silent (Asn128) | 9822 | - | | 335971 | A | G | 45.77 | SNP | Rv0278c (PE\_PGRS3) | Leu(s)114Leu | 3 | - | | 336504 | G | T | 97.77 | SNP | intergenic |  |  | - | | 336535 | T | G | 126.77 | SNP | intergenic |  |  | - | | 336537 | T | G | 77.77 | SNP | intergenic |  |  | - | | 336540 | G | T | 124.77 | SNP | intergenic |  |  | - | | 336546 | T | G | 125.77 | SNP | intergenic |  |  | - | | 336557 | C | CT | 217.73 | INS | intergenic |  |  | - | | 336590 | G | C | 57.77 | SNP | Rv0279c (PE\_PGRS4) | Ile828Met(s) | 6 | - | | 336592 | T | G | 52.77 | SNP | Rv0279c (PE\_PGRS4) | Ile828Leu | 22 | - | | 336611 | G | C | 59.77 | SNP | Rv0279c (PE\_PGRS4) | silent (Ala821) | 9867 | - | | 336620 | T | C | 54.77 | SNP | Rv0279c (PE\_PGRS4) | silent (Thr818) | 9871 | - | | 338844 | A | G | 62.77 | SNP | Rv0279c (PE\_PGRS4) | Val(s)77Ala | 9867 | - | | 338845 | C | T | 50.77 | SNP | Rv0279c (PE\_PGRS4) | Val(s)77Met(s) | 9867 | - | | 338876 | G | A | 121.77 | SNP | Rv0279c (PE\_PGRS4) | silent (Ser66) | 9840 | - | | 338903 | G | C | 139.77 | SNP | Rv0279c (PE\_PGRS4) | silent (Ala57) | 9867 | - | | 338960 | T | C | 173.77 | SNP | Rv0279c (PE\_PGRS4) | silent (Ala38) | 9867 | - | | 338963 | T | C | 195.77 | SNP | Rv0279c (PE\_PGRS4) | silent (Thr37) | 9871 | - | | 338990 | G | T | 55.77 | SNP | Rv0279c (PE\_PGRS4) | silent (Ala28) | 9867 | - | | 338997 | G | C | 84.77 | SNP | Rv0279c (PE\_PGRS4) | Ala26Gly | 21 | - | | 338998 | C | T | 47.77 | SNP | Rv0279c (PE\_PGRS4) | Ala26Thr | 22 | - | | 348115 | C | A | 31.77 | SNP | Rv0284 (eccC3) | silent (Ile827) | 9872 | - | | 356296 | C | A | 30.77 | SNP | Rv0292 (eccE3) | silent (Val139) | 9901 | - | | 356331 | C | A | 31.77 | SNP | Rv0292 (eccE3) | Ser151STOP | 35 | - | | 356528 | A | G | 173.90 | SNP | Rv0292 (eccE3) | Asn217Asp | 42 | - | | 373282 | TA | T | 216.80 | DEL | Rv0305c (PPE6) |  |  | - | | 376774 | T | C | 33.90 | SNP | Rv0307c | silent (Ala94) | 9867 | - | | 384380 | A | C | 181.90 | SNP | Rv0315 | Lys260Thr | 8 | - | | 386432 | C | G | 286.78 | SNP | Rv0318c | Gly223Ala | 21 | - | | 386710 | G | T | 31.77 | SNP | Rv0318c | silent (Val130) | 9901 | - | | 387324 | G | T | 31.78 | SNP | Rv0319 (pcp) | Glu59Asp | 53 | - | | 388311 | T | C | 356.78 | SNP | Rv0320 | Leu(s)142Leu | 3 | - | | 390828 | T | C | 61.74 | SNP | Rv0323c | Ser142Gly | 21 | - | | 392560 | G | T | 32.77 | SNP | Rv0326 | Gln96His | 20 | - | | 403980 | G | A | 43.74 | SNP | Rv0338c | Ala621Val | 13 | - | | 404326 | T | C | 212.84 | SNP | Rv0338c | Arg506Gly | 1 | - | | 405740 | G | T | 39.77 | SNP | Rv0338c | silent (Val34) | 9901 | - | | 409695 | C | A | 35.77 | SNP | Rv0341 (iniB) | Leu112Met(s) | 4 | - | | 412297 | TGCG | T | 319.80 | DEL | Rv0342 (iniA) |  |  | - | | 412302 | G | A | 111.03 | SNP | Rv0342 (iniA) | Gly489Ser | 16 | - | | 414486 | C | T | 45.79 | SNP | Rv0344c (lpqJ) | silent (Glu152) | 9865 | - | | 415483 | C | A | 32.77 | SNP | intergenic |  |  | - | | 418973 | G | T | 31.77 | SNP | Rv0349 | Asp9Tyr | 0 | - | | 420008 | A | G | 215.84 | SNP | Rv0350 (dnaK) | silent (Ala58) | 9867 | - | | 424320 | T | TC | 173.87 | INS | Rv0354c (PPE7) |  |  | - | | 427310 | TTGCCGAGGTTTGCAC | T | 1575.74 | DEL | Rv0355c (PPE8) |  |  | - | | 438716 | G | T | 61.77 | SNP | Rv0360c | silent (Pro8) | 9926 | - | | 438965 | C | A | 34.77 | SNP | Rv0361 | silent (Ile48) | 9872 | - | | 444351 | G | T | 103.03 | SNP | Rv0366c | Asn155Lys | 25 | - | | 451993 | C | A | 106.03 | SNP | Rv0374c | Gly96Val(s) | 21 | - | | 452967 | C | A | 36.77 | SNP | Rv0375c | Arg63Leu | 1 | - | | 454295 | T | C | 244.78 | SNP | Rv0376c | silent (Pro26) | 9926 | - | | 459399 | A | C | 106.28 | SNP | intergenic |  |  | - | | 467508 | C | CG | 183.87 | INS | Rv0388c (PPE9) |  |  | - | | 467516 | G | C | 99.28 | SNP | Rv0388c (PPE9) | silent (Ser162) | 9840 | - | | 467526 | C | G | 99.28 | SNP | Rv0388c (PPE9) | Gly159Ala | 21 | - | | 467546 | G | C | 97.28 | SNP | Rv0388c (PPE9) | Asp152Glu | 56 | - | | 467557 | A | C | 94.28 | SNP | Rv0388c (PPE9) | Leu(s)149Val(s) | 9867 | - | | 467564 | A | C | 62.74 | SNP | Rv0388c (PPE9) | His146Gln | 23 | - | | 467585 | G | C | 48.74 | SNP | Rv0388c (PPE9) | His139Gln | 23 | - | | 467621 | T | G | 62.74 | SNP | Rv0388c (PPE9) | silent (Gly127) | 9935 | - | | 467638 | G | T | 83.28 | SNP | Rv0388c (PPE9) | Gln122Lys | 12 | - | | 472705 | T | C | 82.28 | SNP | intergenic |  |  | - | | 475178 | T | C | 343.78 | SNP | Rv0395 | Val80Ala | 18 | - | | 478358 | C | T | 70.28 | SNP | Rv0399c (lpqK) | Glu67Lys | 7 | - | | 481099 | C | A | 31.77 | SNP | Rv0402c (mmpL1) | silent (Arg711) | 9913 | - | | 485178 | G | T | 36.77 | SNP | Rv0404 (fadD30) | Gly401Val | 3 | - | | 489935 | G | C | 215.84 | SNP | Rv0405 (pks6); Rv0406c | Arg1402Pro; silent (Thr257) | 5; 9871 | - | | 498557 | C | A | 68.28 | SNP | Rv0412c | Asp355Tyr | 0 | - | | 501641 | G | T | 50.77 | SNP | Rv0415 (thiO) | Arg165Leu | 1 | - | | 502589 | C | G | 131.03 | SNP | Rv0417 (thiG) | Ser75Cys | 5 | - | | 503354 | G | C | 119.03 | SNP | intergenic |  |  | - | | 504421 | G | T | 37.77 | SNP | Rv0418 (lpqL) | Arg309Leu | 1 | - | | 510831 | TCCGGGGGGCGCA | T | 1129.75 | DEL | Rv0425c (ctpH) |  |  | - | | 510914 | G | T | 31.77 | SNP | Rv0425c (ctpH) | silent (Arg1470) | 9913 | - | | 513257 | T | C | 53.74 | SNP | Rv0425c (ctpH) | Met(s)689Val(s) | 9867 | - | | 515241 | G | T | 43.77 | SNP | Rv0425c (ctpH) | silent (Val27) | 9901 | - | | 519273 | G | T | 33.77 | SNP | Rv0431 | silent (Ser67) | 9840 | - | | 520890 | C | A | 32.77 | SNP | Rv0433 | Phe189Leu | 13 | - | | 523676 | G | T | 44.77 | SNP | Rv0435c | silent (Ala286) | 9867 | - | | 532492 | C | A | 42.77 | SNP | Rv0443 | Gln33Lys | 12 | - | | 534691 | C | T | 112.03 | SNP | Rv0446c | Trp175STOP | 0 | - | | 541201 | A | G | 142.03 | SNP | Rv0450c (mmpL4) | silent (Leu97) | 9947 | - | | 551525 | A | C | 52.74 | SNP | Rv0459 | silent (Arg110) | 9913 | - | | 557133 | G | A | 291.78 | SNP | Rv0466 | Val226Ile | 33 | - | | 563420 | T | C | 228.84 | SNP | Rv0472c | Glu3Gly | 7 | - | | 573262 | A | G | 251.80 | SNP | Rv0484c | silent (Gly180) | 9935 | - | | 573326 | A | T | 142.90 | SNP | Rv0484c | Leu159Gln | 3 | - | | 589536 | G | A | 187.78 | SNP | Rv0499 | silent (Leu118) | 9947 | - | | 597816 | A | G | 144.03 | SNP | Rv0507 (mmpL2) | silent (Ala206) | 9867 | - | | 598475 | G | A | 129.90 | SNP | Rv0507 (mmpL2) | Arg426His | 8 | - | | 602651 | G | T | 31.77 | SNP | Rv0510 (hemC) | silent (Ala265) | 9867 | - | | 608037 | A | C | 141.03 | SNP | Rv0515 | His496Pro | 5 | - | | 608224 | G | T | 37.77 | SNP | Rv0516c | silent (Val104) | 9901 | - | | 610120 | T | G | 462.77 | SNP | intergenic |  |  | - | | 620973 | C | A | 35.77 | SNP | Rv0530 | His23Asn | 21 | - | | 623021 | G | T | 58.74 | SNP | Rv0532 (PE\_PGRS6) | Val(s)77Leu(s) | 9867 | - | | 624620 | G | T | 55.77 | SNP | Rv0533c (fabH) | silent (Thr287) | 9871 | - | | 630722 | G | C | 195.90 | SNP | Rv0538 | Arg228Pro | 5 | - | | 632330 | G | T | 43.74 | SNP | Rv0539 | silent (Arg196) | 9913 | - | | 637319 | G | A | 141.90 | SNP | Rv0545c (pitA) | Pro49Ser | 17 | - | | 648002 | T | G | 94.28 | SNP | Rv0556 | Leu15Arg | 1 | - | | 649313 | C | T | 46.77 | SNP | Rv0557 (mgtA) | Arg260Trp | 2 | - | | 650811 | C | A | 44.77 | SNP | Rv0560c | Gly232Cys | 0 | - | | 652902 | G | T | 37.77 | SNP | Rv0562 (grcC1) | Met(s)44Ile | 2 | - | | 656478 | G | T | 30.77 | SNP | Rv0565c | silent (Leu331) | 9947 | - | | 661383 | G | T | 38.77 | SNP | Rv0570 (nrdZ) | Arg30Leu | 1 | - | | 665293 | A | G | 382.77 | SNP | Rv0572c | Phe31Leu | 13 | - | | 667659 | C | T | 247.80 | SNP | Rv0574c | Asp246Asn | 36 | - | | 668161 | G | T | 38.77 | SNP | Rv0574c | silent (Thr78) | 9871 | - | | 671510 | C | A | 34.77 | SNP | Rv0577 (TB27.3) | Phe115Leu | 13 | - | | 672491 | C | G | 43.74 | SNP | Rv0578c (PE\_PGRS7) | silent (Gly1142) | 9935 | - | | 673238 | A | G | 175.90 | SNP | Rv0578c (PE\_PGRS7) | silent (His893) | 9912 | - | | 685461 | C | G | 90.28 | SNP | Rv0587 (yrbE2A) | silent (Ala111) | 9867 | - | | 685608 | T | C | 174.84 | SNP | Rv0587 (yrbE2A) | silent (Leu160) | 9947 | - | | 686972 | T | C | 239.84 | SNP | Rv0589 (mce2A) | Phe51Ser | 3 | - | | 690465 | T | G | 128.03 | SNP | Rv0591 (mce2C) | silent (Leu469) | 9947 | - | | 694271 | G | T | 40.77 | SNP | Rv0594 (mce2F) | Lys345Asn | 13 | - | | 698968 | G | A | 261.78 | SNP | Rv0601c | silent (Gly9) | 9935 | - | | 709226 | G | A | 44.74 | SNP | Rv0613c | Thr97Ile | 7 | - | | 718152 | C | A | 38.77 | SNP | Rv0626 (vapB5) | Pro43Gln | 6 | - | | 726703 | C | A | 148.03 | SNP | Rv0631c (recC) | Arg535Met(s) | 2 | - | | 729268 | G | T | 46.77 | SNP | Rv0632c (echA3) | Pro4Gln | 6 | - | | 733798 | G | C | 44.74 | SNP | Rv0638 (secE1) | Ser21Thr | 32 | - | | 736084 | G | T | 40.77 | SNP | Rv0641 (rplA) | Gly190Cys | 0 | - | | 736710 | T | C | 185.90 | SNP | Rv0642c (mmaA4) | Asn165Ser | 34 | - | | 737008 | G | C | 151.03 | SNP | Rv0642c (mmaA4) | Leu66Val(s) | 4 | - | | 738936 | C | A | 45.77 | SNP | Rv0644c (mmaA2) | Trp75Cys | 0 | - | | 741628 | C | A | 31.77 | SNP | Rv0647c | silent (Ala330) | 9867 | - | | 742516 | C | A | 32.74 | SNP | Rv0647c | silent (Pro34) | 9926 | - | | 746981 | C | G | 41.88 | SNP | Rv0649 (fabD2) | Pro207Ala | 22 | - | | 749858 | G | T | 33.77 | SNP | Rv0653c | silent (Pro24) | 9926 | - | | 753098 | G | A | 39.74 | SNP | Rv0656c (vapC6) | silent (Cys90) | 9973 | - | | 753438 | G | A | 61.74 | SNP | intergenic |  |  | - | | 761155 | C | T | 234.80 | SNP | Rv0667 (rpoB) | Ser450Leu(s) | 35 | resistance | | 762101 | C | T | 315.78 | SNP | Rv0667 (rpoB) | silent (Arg765) | 9913 | - | | 764817 | T | C | 100.28 | SNP | Rv0668 (rpoC) | Val(s)483Ala | 9867 | - | | 764995 | C | G | 62.74 | SNP | Rv0668 (rpoC) | silent (Ala542) | 9867 | genotype | | 769962 | GC | G | 246.77 | DEL | Rv0670 (end) |  |  | - | | 773431 | G | C | 267.80 | SNP | Rv0673 (echA4) | Gln103His | 20 | - | | 773809 | G | A | 61.74 | SNP | Rv0673 (echA4) | silent (Glu229) | 9865 | - | | 775639 | T | C | 90.28 | SNP | Rv0676c (mmpL5) | Ile948Val | 57 | - | | 783754 | G | T | 45.77 | SNP | Rv0684 (fusA1) | Asp424Tyr | 0 | - | | 784440 | G | T | 229.80 | SNP | Rv0684 (fusA1) | silent (Ala652) | 9867 | - | | 785436 | C | A | 44.77 | SNP | Rv0685 (tuf) | Arg206Ser | 11 | - | | 787839 | C | A | 38.77 | SNP | Rv0687 | silent (Ser247) | 9840 | - | | 790180 | A | G | 85.28 | SNP | Rv0690c | silent (Asp298) | 9859 | - | | 793688 | C | A | 35.77 | SNP | Rv0694 (lldD1) | silent (Val118) | 9901 | - | | 796509 | G | T | 79.28 | SNP | Rv0696 | Gly331Cys | 0 | - | | 798685 | G | T | 40.77 | SNP | intergenic |  |  | - | | 802261 | G | T | 32.77 | SNP | Rv0703 (rplW) | Lys43Asn | 13 | - | | 807805 | G | T | 32.77 | SNP | Rv0711 (atsA) | Asp491Tyr | 0 | - | | 810301 | G | T | 36.77 | SNP | Rv0713 | Arg119Leu | 1 | - | | 820483 | G | T | 48.74 | SNP | Rv0727c (fucA) | Ala6Asp | 6 | - | | 820668 | G | T | 32.77 | SNP | Rv0728c (serA2) | Pro270Gln | 6 | - | | 821693 | G | T | 42.77 | SNP | Rv0729 (xylB) | Asp63Tyr | 0 | - | | 837033 | A | G | 48.74 | SNP | Rv0746 (PE\_PGRS9) | Thr445Ala | 32 | - | | 839334 | A | G | 50.74 | SNP | Rv0747 (PE\_PGRS10) | Lys295Arg | 19 | - | | 839516 | A | G | 65.88 | SNP | Rv0747 (PE\_PGRS10) | Thr356Ala | 32 | - | | 839519 | C | G | 80.28 | SNP | Rv0747 (PE\_PGRS10) | Leu357Val(s) | 4 | - | | 839520 | T | C | 80.28 | SNP | Rv0747 (PE\_PGRS10) | Leu357Pro | 2 | - | | 839534 | A | C | 77.28 | SNP | Rv0747 (PE\_PGRS10) | Ile362Leu | 22 | - | | 841494 | C | G | 182.84 | SNP | Rv0749 (vapC31) | silent (Leu89) | 9947 | - | | 841495 | A | G | 180.84 | SNP | Rv0749 (vapC31) | Met(s)90Val(s) | 9867 | - | | 841605 | C | T | 191.80 | SNP | Rv0749 (vapC31) | silent (Asp126) | 9859 | - | | 841764 | G | C | 215.84 | SNP | Rv0749A | silent (Thr37) | 9871 | - | | 848632 | G | T | 59.77 | SNP | Rv0755c (PPE12) | Ser470STOP | 35 | - | | 850918 | G | T | 33.74 | SNP | Rv0756c | silent (Pro183) | 9926 | - | | 851602 | C | A | 32.77 | SNP | intergenic |  |  | - | | 852910 | C | T | 120.90 | SNP | Rv0758 (phoR) | Pro172Leu | 3 | - | | 854252 | GC | G | 244.75 | DEL | intergenic |  |  | - | | 856665 | G | T | 31.77 | SNP | Rv0763c | silent (Val5) | 9901 | - | | 857696 | A | G | 147.90 | SNP | Rv0764c (cyp51) | silent (Ala114) | 9867 | - | | 876051 | G | T | 31.77 | SNP | Rv0782 (ptrBb) | Leu(s)440Phe | 1 | - | | 882257 | T | C | 235.80 | SNP | Rv0787 | Tyr267His | 4 | - | | 885542 | G | C | 79.28 | SNP | Rv0791c | Ser100Cys | 5 | - | | 888526 | G | T | 35.77 | SNP | Rv0794c | silent (Ala37) | 9867 | - | | 888774 | G | A | 79.77 | SNP | intergenic |  |  | - | | 893733 | T | G | 142.03 | SNP | Rv0800 (pepC) | Leu139Arg | 1 | - | | 897590 | G | T | 34.77 | SNP | Rv0803 (purL) | Glu591STOP | 17 | - | | 900221 | T | C | 272.80 | SNP | Rv0806c (cpsY) | Val370Val(s) | 18 | - | | 903550 | T | C | 88.28 | SNP | Rv0808 (purF) | silent (Ala480) | 9867 | - | | 903913 | T | C | 141.03 | SNP | Rv0809 (purM) | silent (Gly63) | 9935 | - | | 906857 | A | G | 150.03 | SNP | Rv0812 | Ile145Met(s) | 6 | - | | 909280 | A | C | 225.84 | SNP | Rv0815c (cysA2) | silent (Ala13) | 9867 | - | | 913263 | G | T | 43.77 | SNP | Rv0820 (phoT) | Asp180Tyr | 0 | - | | 914430 | C | A | 32.77 | SNP | Rv0822c | Gly628Trp | 0 | - | | 918583 | G | T | 33.77 | SNP | Rv0824c (desA1) | silent (Pro56) | 9926 | - | | 919494 | G | T | 59.74 | SNP | Rv0825c | silent (Ala20) | 9867 | - | | 921813 | C | G | 194.90 | SNP | Rv0829 | Ala80Gly | 21 | - | | 923356 | C | G | 95.28 | SNP | Rv0831c | silent (Ala118) | 9867 | - | | 937811 | C | A | 41.77 | SNP | Rv0841 | silent (Thr73) | 9871 | - | | 942479 | T | C | 226.80 | SNP | intergenic |  |  | - | | 943861 | G | T | 37.77 | SNP | Rv0846c | Arg112Ser | 11 | - | | 945214 | G | A | 56.74 | SNP | Rv0848 (cysK2) | Gly93Ser | 16 | - | | 947429 | T | A | 241.18 | SNP | Rv0850 | Ser40Thr | 32 | - | | 947430 | C | A | 262.89 | SNP | Rv0850 | Ser40STOP | 35 | - | | 949535 | T | C | 48.74 | SNP | Rv0853c (pdc) | silent (Ala528) | 9867 | - | | 955524 | A | G | 229.84 | SNP | Rv0859 (fadA) | Ser150Gly | 21 | - | | 956189 | C | A | 42.77 | SNP | Rv0859 (fadA) | silent (Thr371) | 9871 | - | | 965801 | G | T | 34.77 | SNP | intergenic |  |  | - | | 971624 | G | T | 32.77 | SNP | Rv0873 (fadE10) | Glu374STOP | 17 | - | | 976896 | TTG | T | 838.73 | DEL | Rv0878c (PPE13) |  |  | - | | 977060 | T | G | 219.96 | SNP | Rv0878c (PPE13) | Thr382Pro | 4 | - | | 979704 | G | C | 364.77 | SNP | Rv0881 | Gly115Arg | 0 | - | | 980908 | C | A | 34.77 | SNP | Rv0883c | silent (Leu120) | 9947 | - | | 986463 | G | C | 51.74 | SNP | intergenic |  |  | - | | 990001 | G | C | 99.28 | SNP | Rv0890c | Pro866Ala | 22 | - | | 990533 | T | C | 142.03 | SNP | Rv0890c | silent (Thr688) | 9871 | - | | 993346 | A | C | 293.78 | SNP | Rv0891c | Val37Gly | 5 | - | | 1004729 | C | T | 126.03 | SNP | Rv0902c (prrB) | silent (Arg371) | 9913 | - | | 1007586 | C | A | 42.77 | SNP | Rv0904c (accD3) | Gly199Trp | 0 | - | | 1010204 | C | CG | 354.74 | INS | Rv0907 |  |  | - | | 1017238 | C | T | 43.77 | SNP | Rv0913c | silent (Pro496) | 9926 | - | | 1026201 | C | A | 43.77 | SNP | Rv0920c | Gly206Trp | 0 | - | | 1031736 | G | T | 36.77 | SNP | Rv0924c (mntH) | silent (Ala43) | 9867 | - | | 1035784 | C | A | 36.77 | SNP | Rv0928 (pstS3) | silent (Leu294) | 9947 | - | | 1037012 | T | C | 95.28 | SNP | Rv0930 (pstA1) | Met(s)5Thr | 22 | - | | 1037355 | T | C | 209.84 | SNP | Rv0930 (pstA1) | silent (Thr119) | 9871 | - | | 1037911 | C | T | 239.80 | SNP | Rv0930 (pstA1) | Arg305STOP | 2 | - | | 1041445 | C | T | 60.74 | SNP | Rv0933 (pstB) | Thr61Met(s) | 32 | - | | 1045396 | C | A | 37.77 | SNP | Rv0937c (mku) | Asp209Tyr | 0 | - | | 1046084 | G | T | 54.77 | SNP | intergenic |  |  | - | | 1047165 | T | C | 223.84 | SNP | Rv0938 (ligD) | Cys344Arg | 1 | - | | 1048840 | C | A | 37.77 | SNP | Rv0939 | silent (Thr143) | 9871 | - | | 1050173 | C | T | 267.78 | SNP | Rv0939 | Pro588Ser | 17 | - | | 1055049 | C | T | 192.84 | SNP | Rv0946c (pgi) | Arg546His | 8 | - | | 1057788 | T | G | 431.77 | SNP | Rv0948c | Lys59Thr | 8 | - | | 1060929 | G | T | 31.77 | SNP | Rv0950c | silent (Val242) | 9901 | - | | 1061676 | GTGC | G | 805.73 | DEL | intergenic |  |  | - | | 1062026 | G | T | 34.77 | SNP | Rv0951 (sucC) | silent (Pro21) | 9926 | - | | 1062424 | C | A | 73.77 | SNP | Rv0951 (sucC) | Ser154Tyr | 1 | - | | 1064365 | C | A | 34.77 | SNP | Rv0953c | Gly200Cys | 0 | - | | 1068151 | T | C | 197.84 | SNP | Rv0956 (purN) | silent (His197) | 9912 | - | | 1068432 | A | G | 150.03 | SNP | Rv0957 (purH) | silent (Pro76) | 9926 | - | | 1070702 | T | C | 47.74 | SNP | Rv0958 | Ser274Pro | 12 | - | | 1071797 | C | G | 178.90 | SNP | Rv0959 | silent (Gly181) | 9935 | - | | 1072601 | G | T | 35.77 | SNP | Rv0959 | silent (Leu449) | 9947 | - | | 1074558 | G | A | 228.78 | SNP | Rv0962c (lprP) | Pro186Leu | 3 | - | | 1075279 | T | C | 99.28 | SNP | intergenic |  |  | - | | 1076309 | G | T | 132.03 | SNP | Rv0964c | Pro124Thr | 5 | - | | 1077312 | A | G | 178.90 | SNP | Rv0966c | Val(s)175Ala | 9867 | - | | 1079927 | C | A | 30.29 | SNP | Rv0969 (ctpV) | silent (Thr395) | 9871 | - | | 1081681 | T | C | 182.84 | SNP | Rv0970 | silent (Val210) | 9901 | - | | 1083533 | G | A | 291.78 | SNP | Rv0972c (fadE12) | Ser73Leu(s) | 35 | - | | 1093322 | C | T | 102.03 | SNP | intergenic |  |  | - | | 1093406 | A | G | 186.90 | SNP | Rv0978c (PE\_PGRS17) | silent (Val317) | 9901 | - | | 1095364 | T | G | 191.84 | SNP | Rv0980c (PE\_PGRS18) | Asn363Thr | 13 | - | | 1095493 | G | T | 44.77 | SNP | Rv0980c (PE\_PGRS18) | Pro320Gln | 6 | - | | 1096633 | T | G | 62.74 | SNP | intergenic |  |  | - | | 1096697 | G | T | 41.77 | SNP | intergenic |  |  | - | | 1100234 | T | C | 50.74 | SNP | Rv0983 (pepD) | Leu390Pro | 2 | - | | 1103249 | C | T | 208.84 | SNP | Rv0987 | silent (Ala236) | 9867 | - | | 1106422 | T | C | 182.90 | SNP | Rv0989c (grcC2) | Ile321Val | 57 | - | | 1107434 | A | T | 135.03 | SNP | intergenic |  |  | - | | 1107917 | G | T | 90.03 | SNP | Rv0990c | His61Gln | 23 | - | | 1109935 | C | A | 33.77 | SNP | Rv0993 (galU) | Leu222Ile | 9 | - | | 1109975 | A | G | 272.80 | SNP | Rv0993 (galU) | Gln235Arg | 10 | - | | 1127648 | C | A | 138.03 | SNP | Rv1008 (tatD) | Thr187Asn | 9 | - | | 1129987 | C | T | 174.18 | SNP | Rv1010 (ksgA) | Thr279Met(s) | 32 | - | | 1130181 | T | C | 107.03 | SNP | intergenic |  |  | - | | 1130374 | G | T | 38.77 | SNP | Rv1011 (ispE) | Asp62Tyr | 0 | - | | 1130506 | G | T | 38.77 | SNP | Rv1011 (ispE) | Gly106Cys | 0 | - | | 1133836 | C | A | 36.74 | SNP | Rv1014c (pth) | Gly25Trp | 0 | - | | 1134208 | G | T | 34.77 | SNP | Rv1015c (rplY) | Leu121Met(s) | 4 | - | | 1135232 | G | C | 61.74 | SNP | Rv1016c (lpqT) | Ser78Arg | 6 | - | | 1149551 | C | T | 127.03 | SNP | Rv1028c (kdpD) | silent (Glu712) | 9865 | - | | 1150321 | TGCCCGACAGCAAG | T | 1556.74 | DEL | Rv1028c (kdpD) |  |  | - | | 1150585 | G | A | 155.90 | SNP | Rv1028c (kdpD) | Pro368Ser | 17 | - | | 1153416 | C | A | 31.77 | SNP | Rv1029 (kdpA) | Leu469Ile | 9 | - | | 1159161 | GCCCA | G | 598.74 | DEL | Rv1034c |  |  | - | | 1163134 | T | C | 42.79 | SNP | Rv1040c (PE8) | silent (Gly81) | 9935 | - | | 1163455 | G | T | 35.77 | SNP | intergenic |  |  | - | | 1164495 | C | T | 119.03 | SNP | intergenic |  |  | - | | 1165521 | T | TA | 269.77 | INS | intergenic |  |  | - | | 1168715 | C | CT | 344.74 | INS | Rv1046c |  |  | - | | 1176522 | C | A | 44.77 | SNP | intergenic |  |  | - | | 1178116 | T | C | 106.28 | SNP | Rv1056 | silent (Thr163) | 9871 | - | | 1194531 | C | G | 183.90 | SNP | Rv1070c (echA8) | Glu171Asp | 53 | - | | 1194783 | C | A | 35.77 | SNP | Rv1070c (echA8) | Trp87Cys | 0 | - | | 1198891 | G | T | 35.77 | SNP | Rv1074c (fadA3) | silent (Thr161) | 9871 | - | | 1199547 | G | A | 227.78 | SNP | Rv1075c | Pro275Leu | 3 | - | | 1200418 | A | G | 151.03 | SNP | intergenic |  |  | - | | 1201158 | C | A | 38.77 | SNP | Rv1076 (lipU) | Pro131Gln | 6 | - | | 1201289 | G | A | 270.78 | SNP | Rv1076 (lipU) | Glu175Lys | 7 | - | | 1206400 | G | A | 234.80 | SNP | Rv1081c | Pro7Ser | 17 | - | | 1218737 | G | T | 78.03 | SNP | Rv1091 (PE\_PGRS22) | Gly757Cys | 0 | - | | 1219628 | C | A | 67.77 | SNP | Rv1092c (coaA) | Gly187Trp | 0 | - | | 1220680 | T | C | 198.84 | SNP | Rv1093 (glyA1) | Val36Ala | 18 | - | | 1224367 | T | C | 142.03 | SNP | intergenic |  |  | - | | 1228344 | G | T | 38.77 | SNP | Rv1099c (glpX) | Pro114His | 3 | - | | 1231691 | G | T | 35.77 | SNP | Rv1104 | Asp131Tyr | 0 | - | | 1234336 | C | A | 41.77 | SNP | Rv1108c (xseA) | Glu375Asp | 53 | - | | 1244937 | C | A | 41.77 | SNP | Rv1121 (zwf1) | Arg411Ser | 11 | - | | 1248321 | C | A | 47.78 | SNP | Rv1125 | silent (Val80) | 9901 | - | | 1248978 | T | C | 272.80 | SNP | Rv1125 | silent (Ala299) | 9867 | - | | 1253671 | G | T | 35.77 | SNP | Rv1129c | silent (Pro288) | 9926 | - | | 1265645 | C | A | 37.77 | SNP | Rv1138c | Asp282Tyr | 0 | - | | 1268517 | G | T | 41.77 | SNP | Rv1141c (echA11) | silent (Arg165) | 9913 | - | | 1269127 | G | T | 40.77 | SNP | intergenic |  |  | - | | 1269398 | G | T | 38.77 | SNP | Rv1142c (echA10) | silent (Val187) | 9901 | - | | 1272269 | C | T | 88.28 | SNP | intergenic |  |  | - | | 1272302 | C | A | 39.77 | SNP | intergenic |  |  | - | | 1275833 | C | A | 40.77 | SNP | intergenic |  |  | - | | 1276938 | G | C | 197.84 | SNP | Rv1148c | Leu271Val | 11 | - | | 1281118 | T | C | 183.90 | SNP | Rv1154c | Thr123Ala | 32 | - | | 1281263 | G | T | 35.77 | SNP | Rv1154c | silent (Thr74) | 9871 | - | | 1281443 | C | G | 182.90 | SNP | Rv1154c; Rv1155 | Lys14Asn; Val5Val(s) | 13; 18 | - | | 1288691 | C | A | 32.77 | SNP | Rv1161 (narG) | Ser455STOP | 35 | - | | 1292102 | A | G | 219.84 | SNP | Rv1162 (narH) | silent (Pro346) | 9926 | - | | 1296241 | C | A | 34.74 | SNP | Rv1166 (lpqW) | silent (Val30) | 9901 | - | | 1296368 | G | T | 36.77 | SNP | Rv1166 (lpqW) | Asp73Tyr | 0 | - | | 1297327 | G | A | 82.28 | SNP | Rv1166 (lpqW) | Val(s)392Val | 13 | - | | 1297999 | T | G | 264.80 | SNP | Rv1166 (lpqW) | silent (Ser616) | 9840 | - | | 1299894 | G | T | 41.77 | SNP | Rv1169c (lipX) | silent (Val77) | 9901 | - | | 1303710 | G | T | 42.77 | SNP | Rv1173 (fbiC) | Leu(s)260Phe | 1 | - | | 1305593 | C | G | 99.80 | SNP | intergenic |  |  | - | | 1307598 | C | G | 40.74 | SNP | Rv1175c (fadH) | Cys210Ser | 11 | - | | 1308985 | G | T | 34.77 | SNP | intergenic |  |  | - | | 1313337 | A | AG | 402.74 | INS | intergenic |  |  | - | | 1313338 | A | C | 129.03 | SNP | intergenic |  |  | - | | 1315191 | A | C | 150.90 | SNP | Rv1180 (pks3) | STOP489Tyr | 1 | - | | 1315884 | G | A | 88.28 | SNP | Rv1181 (pks4) | silent (Ala217) | 9867 | - | | 1325105 | C | A | 31.77 | SNP | Rv1184c | silent (Thr169) | 9871 | - | | 1327806 | G | T | 37.77 | SNP | Rv1186c | silent (Pro500) | 9926 | - | | 1327890 | G | A | 194.90 | SNP | Rv1186c | silent (Asp472) | 9859 | - | | 1328668 | C | A | 35.77 | SNP | Rv1186c | Arg213Leu | 1 | - | | 1328687 | G | C | 228.80 | SNP | Rv1186c | Pro207Ala | 22 | - | | 1336354 | G | T | 30.77 | SNP | Rv1193 (fadD36) | silent (Leu187) | 9947 | - | | 1337673 | C | A | 37.77 | SNP | Rv1194c | Gly281Trp | 0 | - | | 1340500 | G | T | 61.74 | SNP | Rv1196 (PPE18) | Met(s)384Ile | 2 | - | | 1345645 | C | A | 69.77 | SNP | Rv1202 (dapE) | Ser129STOP | 35 | - | | 1354437 | A | C | 51.84 | SNP | Rv1211 | silent (Ala65) | 9867 | - | | 1357114 | C | T | 71.77 | SNP | intergenic |  |  | - | | 1360209 | T | C | 133.03 | SNP | Rv1217c | silent (Ala531) | 9867 | - | | 1362815 | G | T | 37.77 | SNP | Rv1219c | Leu183Met(s) | 4 | - | | 1365837 | C | CG | 254.75 | INS | intergenic |  |  | - | | 1366844 | G | T | 31.77 | SNP | Rv1223 (htrA) | Gly324Cys | 0 | - | | 1366951 | C | A | 31.77 | SNP | Rv1223 (htrA) | silent (Pro359) | 9926 | - | | 1373170 | G | C | 97.28 | SNP | Rv1230c | Pro343Arg | 4 | - | | 1373553 | G | A | 111.03 | SNP | Rv1230c | silent (Gly215) | 9935 | - | | 1373853 | G | T | 34.77 | SNP | Rv1230c | silent (Ala115) | 9867 | - | | 1374065 | T | C | 106.28 | SNP | Rv1230c | Ser45Gly | 21 | - | | 1375724 | A | C | 98.28 | SNP | Rv1232c | Cys149Gly | 1 | - | | 1376950 | A | AG | 230.77 | INS | intergenic |  |  | - | | 1378345 | C | A | 32.77 | SNP | Rv1235 (lpqY) | silent (Val274) | 9901 | - | | 1381851 | C | A | 32.77 | SNP | Rv1238 (sugC) | Pro390Thr | 5 | - | | 1382628 | T | C | 62.74 | SNP | Rv1239c (corA) | Lys139Glu | 4 | - | | 1389738 | G | A | 116.85 | SNP | Rv1248c | silent (Asp1105) | 9859 | - | | 1390110 | G | T | 30.77 | SNP | Rv1248c | Phe981Leu | 13 | - | | 1393626 | A | G | 106.28 | SNP | Rv1249c | silent (Leu119) | 9947 | - | | 1396922 | T | C | 257.80 | SNP | Rv1251c | silent (Thr773) | 9871 | - | | 1404169 | T | G | 115.03 | SNP | Rv1256c (cyp130) | Lys145Asn | 13 | - | | 1409769 | G | T | 42.77 | SNP | Rv1261c | silent (Thr55) | 9871 | - | | 1411210 | T | G | 51.74 | SNP | Rv1263 (amiB2) | Val260Val(s) | 18 | - | | 1413148 | C | T | 141.90 | SNP | intergenic |  |  | - | | 1414021 | C | T | 101.03 | SNP | Rv1266c (pknH) | Arg607Gln | 9 | - | | 1423519 | G | T | 39.77 | SNP | Rv1273c | His178Asn | 21 | - | | 1428506 | G | T | 112.03 | SNP | Rv1278 | Ala365Ser | 28 | - | | 1434353 | G | C | 130.03 | SNP | Rv1281c (oppD) | Leu307Val | 11 | - | | 1436936 | G | T | 37.77 | SNP | Rv1283c (oppB) | Pro61His | 3 | - | | 1440469 | C | G | 91.28 | SNP | Rv1286 (cysN) | silent (Pro521) | 9926 | - | | 1445474 | G | A | 202.84 | SNP | intergenic |  |  | - | | 1445781 | A | G | 265.80 | SNP | Rv1291c | silent (Ala18) | 9867 | - | | 1446923 | T | G | 136.03 | SNP | Rv1292 (argS) | Ile182Ser | 2 | - | | 1450005 | C | A | 32.77 | SNP | Rv1294 (thrA) | Leu211Met(s) | 4 | - | | 1452071 | C | A | 94.03 | SNP | Rv1296 (thrB) | silent (Gly25) | 9935 | - | | 1453040 | C | A | 32.77 | SNP | intergenic |  |  | - | | 1457144 | C | T | 124.90 | SNP | Rv1300 (hemK) | Arg194Cys | 1 | - | | 1459563 | G | T | 71.77 | SNP | intergenic |  |  | - | | 1463557 | G | T | 32.77 | SNP | Rv1308 (atpA) | Val(s)110Val | 13 | - | | 1466056 | G | T | 31.77 | SNP | Rv1310 (atpD) | silent (Leu72) | 9947 | - | | 1471659 | C | T | 142.03 | SNP | intergenic |  |  | - | | 1480024 | G | T | 185.80 | SNP | Rv1318c | Phe267Leu | 13 | - | | 1480945 | C | G | 155.77 | SNP | Rv1319c | silent (Thr519) | 9871 | - | | 1480948 | C | T | 102.77 | SNP | Rv1319c | silent (Glu518) | 9865 | - | | 1481185 | A | C | 480.77 | SNP | Rv1319c | Asp439Glu | 56 | - | | 1482067 | G | T | 42.77 | SNP | Rv1319c | silent (Ala145) | 9867 | - | | 1482627 | T | C | 371.77 | SNP | Rv1320c | Thr531Ala | 32 | - | | 1484708 | A | C | 311.10 | SNP | Rv1321 | Ser144Arg | 6 | - | | 1485961 | C | A | 32.77 | SNP | Rv1323 (fadA4) | Leu34Met(s) | 4 | - | | 1496243 | C | A | 192.84 | SNP | Rv1328 (glgP) | silent (Gly560) | 9935 | - | | 1496791 | A | T | 127.03 | SNP | Rv1328 (glgP) | Tyr743Phe | 28 | - | | 1499274 | C | G | 143.03 | SNP | Rv1330c (pncB1) | Gly429Ala | 21 | - | | 1503554 | C | A | 45.77 | SNP | Rv1336 (cysM) | Pro54Gln | 6 | - | | 1507533 | G | T | 32.77 | SNP | Rv1340 (rphA) | STOP260Leu | 3 | - | | 1509350 | C | A | 36.77 | SNP | Rv1345 (mbtM) | Arg24Ser | 11 | - | | 1512031 | T | G | 183.90 | SNP | Rv1347c (mbtK) | Asp192Ala | 10 | - | | 1521235 | G | T | 32.77 | SNP | Rv1354c | silent (Val214) | 9901 | - | | 1525854 | C | A | 46.77 | SNP | Rv1357c | silent (Thr121) | 9871 | - | | 1526819 | C | A | 258.80 | SNP | Rv1358 | silent (Arg70) | 9913 | - | | 1527449 | G | GT | 271.77 | INS | Rv1358 |  |  | - | | 1533241 | G | C | 106.28 | SNP | Rv1361c (PPE19) | silent (Thr131) | 9871 | - | | 1533583 | G | A | 86.28 | SNP | Rv1361c (PPE19) | silent (Tyr17) | 9945 | - | | 1536251 | G | T | 34.74 | SNP | Rv1364c | Ala465Glu | 10 | - | | 1547125 | T | C | 322.78 | SNP | Rv1374c | Thr136Ala | 32 | - | | 1549673 | C | T | 93.28 | SNP | Rv1376 | Leu176Leu(s) | 4 | - | | 1552547 | G | A | 147.03 | SNP | Rv1378c | Arg37Trp | 2 | - | | 1561739 | G | A | 131.90 | SNP | Rv1386 (PE15) | silent (Leu92) | 9947 | - | | 1563717 | C | T | 180.90 | SNP | Rv1388 (mihF) | silent (Val8) | 9901 | - | | 1570566 | C | A | 186.84 | SNP | Rv1394c (cyp132) | Arg135Leu | 1 | - | | 1573660 | T | G | 212.84 | SNP | Rv1396c (PE\_PGRS25) | Arg66Ser | 11 | - | | 1575983 | C | A | 40.77 | SNP | Rv1400c (lipI) | Glu272STOP | 17 | - | | 1586249 | C | T | 316.78 | SNP | Rv1410c | silent (Gln506) | 9876 | - | | 1588456 | A | G | 118.03 | SNP | Rv1411c (lprG) | silent (Arg9) | 9913 | - | | 1588899 | G | T | 280.78 | SNP | Rv1412 (ribC) | silent (Ala111) | 9867 | - | | 1596354 | G | T | 35.77 | SNP | Rv1421 | Gly126Cys | 0 | - | | 1602617 | C | T | 199.78 | SNP | Rv1427c (fadD12) | Val438Ile | 33 | - | | 1603660 | C | A | 42.77 | SNP | Rv1427c (fadD12) | Arg90Met(s) | 2 | - | | 1609604 | G | T | 35.77 | SNP | Rv1431 | Glu508STOP | 17 | - | | 1609840 | A | G | 144.03 | SNP | Rv1431 | silent (Pro586) | 9926 | - | | 1610899 | G | T | 36.77 | SNP | Rv1432 | Gly351Cys | 0 | - | | 1613035 | T | C | 158.90 | SNP | intergenic |  |  | - | | 1613960 | G | T | 81.28 | SNP | Rv1436 (gap) | silent (Ala218) | 9867 | - | | 1615519 | C | A | 33.77 | SNP | Rv1437 (pgk) | silent (Pro397) | 9926 | - | | 1624791 | C | G | 40.74 | SNP | Rv1446c (opcA) | Arg192Pro | 5 | - | | 1625347 | C | CG | 449.73 | INS | Rv1446c (opcA) |  |  | - | | 1629264 | G | C | 61.74 | SNP | Rv1449c (tkt) | Asp312Glu | 56 | - | | 1630148 | A | C | 444.77 | SNP | Rv1449c (tkt) | Tyr18Asp | 0 | - | | 1632349 | GC | G | 144.87 | DEL | Rv1450c (PE\_PGRS27) |  |  | - | | 1636826 | C | A | 44.28 | SNP | Rv1452c (PE\_PGRS28) | silent (Gly468) | 9935 | - | | 1636983 | C | G | 43.74 | SNP | Rv1452c (PE\_PGRS28) | Gly416Ala | 21 | - | | 1638911 | C | A | 55.77 | SNP | Rv1453 | silent (Leu177) | 9947 | - | | 1639505 | G | T | 58.77 | SNP | Rv1453 | Glu375Asp | 53 | - | | 1639594 | C | A | 210.80 | SNP | Rv1453 | Pro405Gln | 6 | - | | 1643450 | C | A | 38.77 | SNP | Rv1458c | Asp271Tyr | 0 | - | | 1645802 | T | C | 142.03 | SNP | Rv1459c | Lys113Glu | 4 | - | | 1650457 | C | A | 38.77 | SNP | Rv1462 | Ser311STOP | 35 | - | | 1654611 | C | A | 54.77 | SNP | Rv1467c (fadE15) | Glu298STOP | 17 | - | | 1657964 | C | A | 34.77 | SNP | Rv1469 (ctpD) | silent (Val334) | 9901 | - | | 1659483 | G | T | 32.77 | SNP | Rv1471 (trxB1) | silent (Pro38) | 9926 | - | | 1664913 | G | T | 32.77 | SNP | Rv1475c (acn) | silent (Ile378) | 9872 | - | | 1667901 | G | T | 38.77 | SNP | Rv1477 (ripA) | Met(s)304Ile | 2 | - | | 1673442 | G | T | 31.74 | SNP | Rv1483 (fabG1) | Val(s)1Val | 13 | - | | 1676290 | C | A | 138.90 | SNP | Rv1486c | Lys198Asn | 13 | - | | 1682202 | G | T | 40.77 | SNP | Rv1492 (mutA) | Gly16Trp | 0 | - | | 1689349 | C | T | 177.84 | SNP | Rv1498c | Arg191His | 8 | - | | 1691799 | C | T | 169.90 | SNP | Rv1500 | Thr317Ile | 7 | - | | 1692141 | A | C | 183.90 | SNP | Rv1501 | silent (Ile84) | 9872 | - | | 1692795 | G | C | 362.78 | SNP | intergenic |  |  | - | | 1693561 | A | G | 87.28 | SNP | Rv1502 | Tyr213Cys | 3 | - | | 1698911 | G | A | 46.28 | SNP | Rv1508c | silent (Gly328) | 9935 | - | | 1700898 | G | T | 31.77 | SNP | Rv1509 | silent (Leu229) | 9947 | - | | 1703808 | C | A | 32.77 | SNP | Rv1511 (gmdA) | Phe245Leu | 13 | - | | 1704704 | T | C | 263.80 | SNP | Rv1512 (epiA) | silent (Ser204) | 9840 | - | | 1706119 | T | C | 356.78 | SNP | Rv1514c | silent (Ser159) | 9840 | - | | 1706433 | C | A | 44.77 | SNP | Rv1514c | Gly55Trp | 0 | - | | 1709899 | A | C | 216.84 | SNP | Rv1518 | Asn86His | 18 | - | | 1718761 | C | T | 184.84 | SNP | Rv1524 | silent (Gly12) | 9935 | - | | 1719322 | G | A | 162.84 | SNP | Rv1524 | silent (Leu199) | 9947 | - | | 1724120 | G | A | 89.03 | SNP | Rv1527c (pks5) | silent (Asp1430) | 9859 | - | | 1724281 | G | T | 32.77 | SNP | Rv1527c (pks5) | Gln1377Lys | 12 | - | | 1728837 | A | G | 134.03 | SNP | intergenic |  |  | - | | 1732104 | G | T | 34.77 | SNP | Rv1530 (adh) | silent (Leu244) | 9947 | - | | 1734994 | C | T | 148.90 | SNP | Rv1534 | silent (Ala87) | 9867 | - | | 1736638 | C | T | 84.28 | SNP | Rv1536 (ileS) | silent (Arg40) | 9913 | - | | 1740771 | A | C | 246.80 | SNP | Rv1537 (dinX) | Thr306Pro | 4 | - | | 1744126 | C | A | 46.79 | SNP | Rv1541c (lprI) | Trp82Cys | 0 | - | | 1750023 | C | A | 34.77 | SNP | Rv1547 (dnaE1) | Pro777Gln | 6 | - | | 1752561 | T | C | 211.84 | SNP | Rv1548c (PPE21) | Asp258Gly | 11 | - | | 1753519 | G | GC | 315.74 | INS | Rv1549 (fadD11.1) |  |  | - | | 1755599 | C | T | 137.03 | SNP | Rv1551 (plsB1) | Ala52Val | 13 | - | | 1760292 | A | G | 226.84 | SNP | Rv1554 (frdC) | Met(s)40Val(s) | 9867 | - | | 1771222 | G | T | 31.77 | SNP | Rv1564c (treX) | Ser127Tyr | 1 | - | | 1771320 | G | A | 269.78 | SNP | Rv1564c (treX) | silent (Asp94) | 9859 | - | | 1773289 | C | A | 42.77 | SNP | Rv1565c | Gly181Trp | 0 | - | | 1773613 | C | A | 32.77 | SNP | Rv1565c | Gly73Trp | 0 | - | | 1775186 | C | A | 34.77 | SNP | intergenic |  |  | - | | 1777213 | C | G | 247.80 | SNP | Rv1569 (bioF1) | Ala171Gly | 21 | - | | 1778430 | T | C | 177.90 | SNP | Rv1570 (bioD) | Met(s)191Thr | 22 | - | | 1789591 | T | C | 55.74 | SNP | Rv1588c | Leu82Leu(s) | 4 | - | | 1789593 | A | G | 44.74 | SNP | Rv1588c | silent (Leu82) | 9947 | - | | 1789742 | G | C | 69.77 | SNP | Rv1588c | Thr32Ser | 38 | - | | 1789746 | A | G | 70.77 | SNP | Rv1588c | Leu(s)31Leu | 3 | - | | 1789756 | A | G | 69.77 | SNP | Rv1588c | silent (Ser27) | 9840 | - | | 1791495 | G | T | 37.77 | SNP | Rv1590 | Gln54His | 20 | - | | 1792822 | C | A | 37.77 | SNP | Rv1592c | Asp307Tyr | 0 | - | | 1793769 | C | T | 130.03 | SNP | intergenic |  |  | - | | 1798355 | G | A | 221.80 | SNP | Rv1597 | Gly21Asp | 6 | - | | 1798577 | C | A | 33.77 | SNP | Rv1597 | Ala95Glu | 10 | - | | 1802005 | C | A | 37.77 | SNP | Rv1600 (hisC1) | silent (Val370) | 9901 | - | | 1803265 | G | A | 247.80 | SNP | Rv1602 (hisH) | Ser201Asn | 20 | - | | 1804409 | C | A | 144.90 | SNP | Rv1604 (impA) | Pro124Gln | 6 | - | | 1805948 | C | T | 337.77 | SNP | Rv1606 (hisI) | Thr99Ile | 7 | - | | 1807070 | C | A | 34.77 | SNP | Rv1607 (chaA) | Pro297Gln | 6 | - | | 1812918 | G | T | 37.77 | SNP | Rv1613 (trpA) | Gly187Val(s) | 21 | - | | 1817976 | A | T | 190.84 | SNP | Rv1618 (tesB1) | His121Leu | 4 | - | | 1833968 | G | T | 42.77 | SNP | Rv1630 (rpsA) | Gly143Trp | 0 | - | | 1836286 | G | C | 108.03 | SNP | intergenic |  |  | - | | 1840076 | G | T | 33.77 | SNP | Rv1634 | silent (Leu303) | 9947 | - | | 1840204 | C | A | 37.77 | SNP | Rv1634 | Pro346Gln | 6 | - | | 1844706 | G | A | 189.84 | SNP | Rv1638 (uvrA) | silent (Glu322) | 9865 | - | | 1845120 | C | A | 35.77 | SNP | Rv1638 (uvrA) | silent (Leu460) | 9947 | - | | 1847811 | A | C | 70.18 | SNP | Rv1639c | silent (Pro216) | 9926 | - | | 1847919 | C | G | 87.28 | SNP | Rv1639c | silent (Thr180) | 9871 | - | | 1848421 | G | T | 44.77 | SNP | Rv1639c | Pro13Gln | 6 | - | | 1848634 | C | A | 37.77 | SNP | Rv1640c (lysX) | Met(s)1134Ile | 2 | - | | 1851969 | C | A | 53.77 | SNP | Rv1640c (lysX) | Asp23Tyr | 0 | - | | 1852185 | C | T | 37.74 | SNP | intergenic |  |  | - | | 1854001 | G | T | 39.77 | SNP | Rv1644 (tsnR) | Met(s)132Ile | 2 | - | | 1854300 | T | C | 170.90 | SNP | Rv1644 (tsnR) | Leu232Pro | 2 | - | | 1856777 | G | C | 166.90 | SNP | Rv1647 | Ala2Pro | 13 | - | | 1858200 | G | T | 35.77 | SNP | Rv1648 | Trp157Leu(s) | 0 | - | | 1858714 | C | A | 38.77 | SNP | intergenic |  |  | - | | 1861274 | G | A | 79.28 | SNP | Rv1650 (pheT) | Arg506His | 8 | - | | 1865577 | T | C | 210.84 | SNP | Rv1652 (argC) | Met(s)1Thr | 22 | - | | 1869146 | C | A | 31.77 | SNP | Rv1655 (argD) | Arg142Ser | 11 | - | | 1874378 | C | A | 36.77 | SNP | Rv1660 (pks10) | silent (Ile73) | 9872 | - | | 1875445 | G | T | 43.77 | SNP | Rv1661 (pks7) | Gly48Cys | 0 | - | | 1885610 | G | T | 71.77 | SNP | Rv1662 (pks8) | Gly1303Cys | 0 | - | | 1885618 | G | T | 30.77 | SNP | Rv1662 (pks8) | Met(s)1305Ile | 2 | - | | 1885772 | G | A | 132.90 | SNP | Rv1662 (pks8) | Ala1357Thr | 22 | - | | 1894300 | G | GGTCTTGCCGC | 783.77 | INS | Rv1668c |  |  | - | | 1899351 | T | C | 158.90 | SNP | Rv1674c | Glu189Gly | 7 | - | | 1901493 | T | C | 227.84 | SNP | Rv1676 | silent (Ser149) | 9840 | - | | 1907296 | G | C | 195.90 | SNP | Rv1682 | silent (Ala298) | 9867 | - | | 1908517 | C | A | 34.77 | SNP | Rv1683 | silent (Val308) | 9901 | - | | 1911307 | G | T | 34.77 | SNP | Rv1685c | silent (Leu31) | 9947 | - | | 1931179 | C | A | 140.03 | SNP | Rv1704c (cycA) | Arg93Leu | 1 | - | | 1933988 | G | A | 67.28 | SNP | intergenic |  |  | - | | 1937205 | C | A | 30.77 | SNP | Rv1708 | Phe282Leu | 13 | - | | 1941717 | G | A | 109.28 | SNP | intergenic |  |  | - | | 1943039 | C | T | 170.80 | SNP | Rv1715 (fadB3) | silent (Ile127) | 9872 | - | | 1943592 | C | T | 127.03 | SNP | Rv1716 | Pro6Leu | 3 | - | | 1944107 | A | G | 99.28 | SNP | Rv1716 | Ser178Gly | 21 | - | | 1944402 | T | C | 177.90 | SNP | Rv1716 | Val276Ala | 18 | - | | 1944642 | CT | C | 189.87 | DEL | Rv1717 |  |  | - | | 1947903 | G | T | 266.80 | SNP | Rv1722 | Val(s)15Leu(s) | 9867 | - | | 1950767 | T | C | 244.80 | SNP | Rv1724c | silent (Lys95) | 9926 | - | | 1953293 | C | A | 34.77 | SNP | Rv1727 | silent (Val8) | 9901 | - | | 1954030 | C | A | 34.77 | SNP | Rv1728c | Arg202Leu | 1 | - | | 1960284 | C | A | 212.84 | SNP | Rv1733c | Gln68His | 20 | - | | 1962936 | C | A | 37.77 | SNP | Rv1736c (narX) | Met(s)417Ile | 2 | - | | 1964165 | C | A | 38.77 | SNP | Rv1736c (narX) | Gly8Cys | 0 | - | | 1967237 | C | A | 154.84 | SNP | Rv1739c | Arg134Leu | 1 | - | | 1967429 | A | G | 164.90 | SNP | Rv1739c | Leu70Pro | 2 | - | | 1981056 | C | T | 106.28 | SNP | intergenic |  |  | - | | 1983313 | T | G | 225.84 | SNP | Rv1753c (PPE24) | Asn488Thr | 13 | - | | 2007785 | C | T | 223.80 | SNP | intergenic |  |  | - | | 2013233 | G | T | 43.77 | SNP | Rv1779c | Pro416His | 3 | - | | 2014210 | C | A | 41.77 | SNP | Rv1779c | Val(s)90Val | 13 | - | | 2019942 | A | G | 159.90 | SNP | Rv1783 (eccC5) | Gln229Arg | 10 | - | | 2022868 | T | C | 35.74 | SNP | Rv1783 (eccC5) | silent (Ser1204) | 9840 | - | | 2023569 | G | T | 76.77 | SNP | Rv1785c (cyp143) | Leu354Met(s) | 4 | - | | 2030983 | G | T | 35.77 | SNP | intergenic |  |  | - | | 2032425 | T | C | 151.03 | SNP | Rv1795 (eccD5) | silent (Gly62) | 9935 | - | | 2045310 | A | G | 61.74 | SNP | Rv1803c (PE\_PGRS32) | silent (Ile511) | 9872 | - | | 2046750 | G | A | 86.28 | SNP | Rv1803c (PE\_PGRS32) | silent (Asn31) | 9822 | - | | 2049065 | T | C | 168.90 | SNP | intergenic |  |  | - | | 2049097 | G | C | 138.03 | SNP | intergenic |  |  | - | | 2050196 | C | A | 32.77 | SNP | Rv1808 (PPE32) | silent (Ile92) | 9872 | - | | 2051746 | T | C | 274.78 | SNP | Rv1809 (PPE33) | silent (Ala155) | 9867 | - | | 2052035 | G | T | 208.84 | SNP | Rv1809 (PPE33) | Val(s)252Leu(s) | 9867 | - | | 2053682 | C | T | 137.03 | SNP | Rv1811 (mgtC) | silent (Ile80) | 9872 | genotype | | 2055271 | A | G | 124.03 | SNP | Rv1812c | Leu30Pro | 2 | - | | 2057774 | A | T | 93.28 | SNP | Rv1815 | Ile83Phe | 8 | - | | 2061000 | G | T | 40.77 | SNP | Rv1817 | Gly469Val(s) | 21 | - | | 2061023 | G | T | 38.77 | SNP | Rv1817 | Gly477STOP | 21 | - | | 2071939 | G | T | 34.77 | SNP | intergenic |  |  | - | | 2073389 | G | T | 57.74 | SNP | Rv1828 | Leu(s)103Phe | 1 | - | | 2075087 | T | C | 135.90 | SNP | Rv1830 | Tyr83His | 4 | - | | 2084526 | G | A | 57.77 | SNP | Rv1836c | silent (Pro37) | 9926 | - | | 2087308 | C | A | 38.77 | SNP | Rv1838c (vapC13) | Met(s)115Ile | 2 | - | | 2096186 | A | G | 43.74 | SNP | Rv1846c (blaI) | silent (Thr138) | 9871 | - | | 2096452 | G | T | 228.77 | SNP | Rv1846c (blaI) | Gln50Lys | 12 | - | | 2097990 | A | C | 129.03 | SNP | Rv1850 (ureC) | silent (Ala10) | 9867 | - | | 2105350 | C | A | 40.77 | SNP | Rv1857 (modA) | silent (Val122) | 9901 | - | | 2108141 | T | C | 106.28 | SNP | Rv1860 (apa) | Phe136Leu | 13 | - | | 2109523 | C | CG | 414.73 | INS | intergenic |  |  | - | | 2116903 | C | T | 234.78 | SNP | Rv1867 | silent (Gly380) | 9935 | - | | 2117796 | G | T | 41.77 | SNP | Rv1868 | silent (Arg150) | 9913 | - | | 2123169 | T | G | 127.03 | SNP | intergenic |  |  | - | | 2126366 | G | C | 97.28 | SNP | Rv1877 | Val155Leu | 15 | - | | 2132315 | C | A | 38.77 | SNP | Rv1881c (lppE) | Leu(s)5Phe | 1 | - | | 2133468 | T | TTCGCATGCCGTCACC | 1937.73 | INS | Rv1883c |  |  | - | | 2134215 | T | C | 175.90 | SNP | Rv1884c (rpfC) | His16Arg | 10 | - | | 2135870 | T | C | 137.03 | SNP | intergenic |  |  | - | | 2135900 | T | G | 61.77 | SNP | intergenic |  |  | - | | 2136166 | G | A | 139.90 | SNP | intergenic |  |  | - | | 2137521 | A | ACTCCGATCAC | 1453.73 | INS | Rv1888c |  |  | - | | 2139961 | C | A | 34.77 | SNP | Rv1891 | Pro74Gln | 6 | - | | 2140365 | A | G | 44.74 | SNP | Rv1892 | silent (Gly67) | 9935 | - | | 2143328 | G | C | 315.78 | SNP | Rv1895 | Val(s)270Leu | 3 | - | | 2146848 | G | T | 33.77 | SNP | Rv1900c (lipJ) | silent (Thr262) | 9871 | - | | 2147022 | A | C | 141.03 | SNP | Rv1900c (lipJ) | Ile204Met(s) | 6 | - | | 2155168 | C | G | 48.74 | SNP | Rv1908c (katG) | Ser315Thr | 32 | resistance | | 2156042 | C | A | 33.77 | SNP | Rv1908c (katG) | Gly24Cys | 0 | - | | 2156245 | C | A | 30.77 | SNP | Rv1909c (furA) | silent (Ser116) | 9840 | - | | 2161857 | G | A | 31.77 | SNP | Rv1916 (aceAb) | Ala98Thr | 22 | - | | 2163790 | A | C | 92.28 | SNP | Rv1917c (PPE34) | silent (Pro1174) | 9926 | - | | 2164363 | A | C | 42.94 | SNP | Rv1917c (PPE34) | silent (Thr983) | 9871 | - | | 2165286 | A | C | 146.03 | SNP | Rv1917c (PPE34) | Ser676Ala | 35 | - | | 2165554 | A | G | 91.28 | SNP | Rv1917c (PPE34) | silent (Gly586) | 9935 | - | | 2168141 | G | T | 33.77 | SNP | Rv1918c (PPE35) | Phe824Leu | 13 | - | | 2174216 | A | G | 88.28 | SNP | Rv1922 | Val50Val(s) | 18 | - | | 2177264 | C | A | 37.77 | SNP | Rv1925 (fadD31) | Arg60Ser | 11 | - | | 2187226 | C | T | 30.74 | SNP | intergenic |  |  | - | | 2190409 | C | A | 30.77 | SNP | Rv1937 | silent (Val638) | 9901 | - | | 2197148 | C | A | 268.78 | SNP | Rv1945 | Thr387Asn | 9 | - | | 2201905 | G | T | 70.77 | SNP | Rv1955 (higB) | Asp63Tyr | 0 | - | | 2202943 | C | A | 35.77 | SNP | Rv1957 | silent (Pro120) | 9926 | - | | 2206271 | C | A | 41.78 | SNP | Rv1963c (mce3R) | Glu178STOP | 17 | - | | 2207525 | C | T | 243.78 | SNP | intergenic |  |  | - | | 2207591 | T | TC | 389.74 | INS | intergenic |  |  | - | | 2208983 | C | A | 40.77 | SNP | Rv1965 (yrbE3B) | silent (Ala159) | 9867 | - | | 2210663 | G | T | 37.77 | SNP | Rv1967 (mce3B) | silent (Leu21) | 9947 | - | | 2211826 | A | G | 329.78 | SNP | Rv1968 (mce3C) | silent (Lys67) | 9926 | - | | 2213265 | G | A | 60.74 | SNP | Rv1969 (mce3D) | silent (Arg137) | 9913 | - | | 2213450 | C | A | 32.77 | SNP | Rv1969 (mce3D) | Ser199Tyr | 1 | - | | 2215928 | G | T | 33.77 | SNP | Rv1971 (mce3F) | silent (Ala224) | 9867 | - | | 2216248 | C | G | 107.03 | SNP | Rv1971 (mce3F) | Pro331Arg | 4 | - | | 2216443 | C | A | 109.03 | SNP | Rv1971 (mce3F) | Ala396Glu | 10 | - | | 2220348 | G | T | 47.77 | SNP | Rv1977 | Asp199Tyr | 0 | - | | 2220512 | T | G | 133.03 | SNP | Rv1977 | silent (Ser253) | 9840 | - | | 2223293 | T | C | 50.74 | SNP | intergenic |  |  | - | | 2228967 | A | G | 52.74 | SNP | intergenic |  |  | - | | 2231132 | G | A | 149.90 | SNP | Rv1987 | Ser36Asn | 20 | - | | 2237053 | G | A | 49.74 | SNP | Rv1992c (ctpG) | Pro85Leu | 3 | - | | 2245532 | T | C | 332.78 | SNP | Rv2000 | silent (Cys108) | 9973 | - | | 2246507 | C | A | 33.77 | SNP | Rv2000 | silent (Val433) | 9901 | - | | 2246744 | C | A | 32.77 | SNP | Rv2000 | Phe512Leu | 13 | - | | 2246884 | G | T | 31.77 | SNP | Rv2001 | Gly18Val | 3 | - | | 2251999 | A | G | 47.74 | SNP | intergenic |  |  | - | | 2255372 | G | T | 30.77 | SNP | Rv2006 (otsB1) | Arg1124Leu | 1 | - | | 2257780 | T | G | 130.03 | SNP | Rv2008c | Ile55Leu | 22 | - | | 2260525 | C | T | 67.28 | SNP | intergenic |  |  | - | | 2264782 | C | A | 221.78 | SNP | Rv2017 | Ala262Glu | 10 | - | | 2265059 | T | G | 326.78 | SNP | intergenic |  |  | - | | 2265695 | G | T | 32.77 | SNP | Rv2018 | Arg139Met(s) | 2 | - | | 2266487 | G | C | 156.77 | SNP | Rv2020c | silent (Leu78) | 9947 | - | | 2266504 | T | TA | 327.73 | INS | Rv2020c |  |  | - | | 2266508 | A | T | 134.77 | SNP | Rv2020c | Asp71Glu | 56 | - | | 2266511 | GT | G | 311.73 | DEL | Rv2020c |  |  | - | | 2266517 | T | C | 192.77 | SNP | Rv2020c | silent (Glu68) | 9865 | - | | 2266550 | G | T | 178.77 | SNP | Rv2020c | silent (Gly57) | 9935 | - | | 2266553 | C | G | 206.77 | SNP | Rv2020c | silent (Ser56) | 9840 | - | | 2266583 | C | G | 292.77 | SNP | Rv2020c | Glu46Asp | 53 | - | | 2266598 | G | C | 194.77 | SNP | Rv2020c | silent (Leu41) | 9947 | - | | 2266604 | C | G | 199.77 | SNP | Rv2020c | silent (Ser39) | 9840 | - | | 2266613 | G | GC | 394.73 | INS | Rv2020c |  |  | - | | 2266624 | G | T | 156.77 | SNP | Rv2020c | Leu33Ile | 9 | - | | 2267372 | A | G | 240.84 | SNP | Rv2022c | Val(s)118Ala | 9867 | - | | 2269153 | C | A | 45.77 | SNP | Rv2024c | Arg363Met(s) | 2 | - | | 2269780 | T | C | 62.74 | SNP | Rv2024c | Asp154Gly | 11 | - | | 2270102 | A | G | 62.74 | SNP | Rv2024c | Trp47Arg | 8 | - | | 2276244 | G | T | 74.77 | SNP | Rv2029c (pfkB) | Leu61Ile | 9 | - | | 2278442 | C | G | 99.28 | SNP | Rv2030c | silent (Arg15) | 9913 | - | | 2282787 | C | T | 337.77 | SNP | Rv2037c | Cys312Tyr | 3 | - | | 2283030 | A | G | 61.74 | SNP | Rv2037c | Ile231Thr | 11 | - | | 2284850 | G | T | 40.77 | SNP | Rv2039c | silent (Val264) | 9901 | - | | 2285251 | C | A | 305.77 | SNP | Rv2039c | Val131Phe | 0 | - | | 2287121 | A | G | 205.84 | SNP | Rv2041c | silent (Asp242) | 9859 | - | | 2289080 | C | A | 34.77 | SNP | Rv2043c (pncA) | silent (Pro54) | 9926 | - | | 2291967 | G | T | 34.77 | SNP | Rv2047c | Arg854Ser | 11 | - | | 2296042 | G | C | 129.03 | SNP | Rv2048c (pks12) | Pro3649Ala | 22 | - | | 2300237 | A | G | 255.80 | SNP | Rv2048c (pks12) | silent (Ala2250) | 9867 | - | | 2300546 | A | T | 44.74 | SNP | Rv2048c (pks12) | His2147Gln | 23 | - | | 2300552 | T | G | 51.74 | SNP | Rv2048c (pks12) | silent (Pro2145) | 9926 | - | | 2300555 | A | G | 50.74 | SNP | Rv2048c (pks12) | silent (Asp2144) | 9859 | - | | 2302033 | G | A | 105.03 | SNP | Rv2048c (pks12) | Arg1652Cys | 1 | - | | 2304218 | C | A | 31.74 | SNP | Rv2048c (pks12) | silent (Ala923) | 9867 | - | | 2312847 | G | A | 183.84 | SNP | Rv2053c (fxsA) | Ala68Val | 13 | - | | 2312970 | C | A | 36.77 | SNP | Rv2053c (fxsA) | Trp27Leu(s) | 0 | - | | 2320073 | C | A | 41.77 | SNP | Rv2062c (cobN) | silent (Ser227) | 9840 | - | | 2327492 | C | T | 57.28 | SNP | Rv2069 (sigC); Rv2070c (cobK) | silent (Leu183); silent (STOP245) | 9947; 9867 | - | | 2329533 | A | G | 62.74 | SNP | Rv2072c (cobL) | Leu205Pro | 2 | - | | 2334007 | A | G | 62.74 | SNP | Rv2077c | silent (Ala96) | 9867 | - | | 2335075 | A | G | 172.90 | SNP | Rv2078 | Glu6Gly | 7 | - | | 2335494 | A | G | 291.78 | SNP | Rv2079 | Tyr47Cys | 3 | - | | 2337204 | C | A | 42.77 | SNP | Rv2079 | Pro617Gln | 6 | - | | 2340106 | G | C | 44.74 | SNP | Rv2082 | Met(s)466Ile | 2 | - | | 2340621 | C | G | 170.90 | SNP | Rv2082 | Pro638Arg | 4 | - | | 2341636 | C | G | 50.74 | SNP | Rv2083 | Leu256Val(s) | 4 | - | | 2345037 | C | A | 210.80 | SNP | Rv2088 (pknJ) | silent (Leu209) | 9947 | - | | 2348446 | C | G | 164.90 | SNP | Rv2090 | Phe358Leu(s) | 2 | - | | 2351681 | C | A | 35.77 | SNP | Rv2092c (helY) | Gly125Val(s) | 21 | - | | 2355694 | C | A | 32.77 | SNP | Rv2097c (pafA) | Val(s)328Val | 13 | - | | 2361377 | G | T | 38.77 | SNP | Rv2101 (helZ) | Gly380Cys | 0 | - | | 2361604 | C | G | 49.74 | SNP | Rv2101 (helZ) | Val455Val(s) | 18 | - | | 2362041 | C | A | 123.03 | SNP | Rv2101 (helZ) | Pro601Gln | 6 | - | | 2367697 | G | T | 63.77 | SNP | intergenic |  |  | - | | 2368564 | TA | T | 301.75 | DEL | intergenic |  |  | - | | 2378473 | C | T | 224.78 | SNP | Rv2119 | Arg30Trp | 2 | - | | 2382085 | AGT | A | 381.75 | DEL | Rv2123 (PPE37) |  |  | - | | 2385695 | C | T | 124.03 | SNP | Rv2124c (metH) | Gly125Arg | 0 | - | | 2386389 | G | A | 227.80 | SNP | Rv2125 | Gly33Ser | 16 | - | | 2387733 | T | C | 49.74 | SNP | Rv2126c (PE\_PGRS37) | silent (Glu80) | 9865 | - | | 2396569 | G | T | 32.77 | SNP | Rv2136c | silent (Val90) | 9901 | - | | 2401883 | C | T | 38.74 | SNP | intergenic |  |  | - | | 2405025 | G | T | 30.77 | SNP | Rv2145c (wag31) | Ser125STOP | 35 | - | | 2415656 | G | C | 62.74 | SNP | Rv2155c (murD) | Arg247Gly | 1 | - | | 2416382 | G | T | 48.79 | SNP | Rv2155c (murD) | Leu5Met(s) | 4 | - | | 2419851 | G | T | 32.77 | SNP | Rv2158c (murE) | Pro253His | 3 | - | | 2424925 | A | G | 41.79 | SNP | intergenic |  |  | - | | 2426242 | C | A | 41.77 | SNP | Rv2163c (pbpB) | silent (Ser282) | 9840 | - | | 2436853 | C | A | 31.77 | SNP | Rv2174 (mptA) | silent (Val315) | 9901 | - | | 2437606 | C | A | 187.84 | SNP | Rv2175c | Arg94Leu | 1 | - | | 2438829 | C | A | 34.77 | SNP | Rv2176 (pknL) | Gln297Lys | 12 | - | | 2438919 | A | C | 183.90 | SNP | Rv2176 (pknL) | Thr327Pro | 4 | - | | 2439166 | CCATTTCGGCA | C | 481.94 | DEL | intergenic |  |  | - | | 2439182 | A | T | 102.28 | SNP | intergenic |  |  | - | | 2439204 | A | G | 104.28 | SNP | intergenic |  |  | - | | 2440926 | G | T | 122.90 | SNP | Rv2178c (aroG) | Asp265Glu | 56 | - | | 2443188 | G | A | 101.28 | SNP | Rv2180c | silent (His9) | 9912 | - | | 2452756 | C | A | 149.03 | SNP | Rv2190c | Ala173Ser | 28 | - | | 2454142 | G | T | 51.77 | SNP | Rv2191 | Val(s)108Val | 13 | - | | 2454738 | T | G | 62.74 | SNP | Rv2191 | Leu307Arg | 1 | - | | 2462871 | G | A | 100.28 | SNP | Rv2198c (mmpS3) | silent (Ala59) | 9867 | - | | 2463094 | G | A | 84.28 | SNP | intergenic |  |  | - | | 2471781 | C | A | 37.77 | SNP | Rv2207 (cobT) | Ala124Glu | 10 | - | | 2475801 | G | T | 67.77 | SNP | Rv2210c (ilvE) | Pro57His | 3 | - | | 2477971 | G | T | 35.77 | SNP | Rv2212 | Trp261Leu(s) | 0 | - | | 2499726 | G | A | 58.74 | SNP | Rv2226 | Asp299Asn | 36 | - | | 2502073 | C | T | 222.78 | SNP | Rv2228c | silent (Arg222) | 9913 | - | | 2502996 | G | T | 32.77 | SNP | Rv2229c | silent (Ile159) | 9872 | - | | 2503625 | C | T | 36.28 | SNP | Rv2230c | silent (Leu328) | 9947 | - | | 2507847 | C | T | 283.78 | SNP | Rv2235 | Pro71Ser | 17 | - | | 2509140 | G | C | 45.74 | SNP | Rv2236c (cobD) | Ser79Cys | 5 | - | | 2509722 | A | G | 213.84 | SNP | Rv2237 | silent (Pro78) | 9926 | - | | 2511353 | G | T | 32.77 | SNP | Rv2239c | silent (Ala100) | 9867 | - | | 2511379 | G | T | 63.77 | SNP | Rv2239c | Leu92Met(s) | 4 | - | | 2513483 | G | T | 38.77 | SNP | Rv2241 (aceE) | Met(s)315Ile | 2 | - | | 2521342 | T | C | 347.78 | SNP | Rv2247 (accD6) | silent (Asp200) | 9859 | - | | 2521855 | G | T | 76.77 | SNP | Rv2247 (accD6) | Met(s)371Ile | 2 | - | | 2521918 | G | T | 42.77 | SNP | Rv2247 (accD6) | Met(s)392Ile | 2 | - | | 2523205 | G | GCGC | 274.87 | INS | intergenic |  |  | - | | 2525722 | CG | C | 305.74 | DEL | Rv2250A; Rv2251 |  |  | - | | 2527676 | G | A | 139.90 | SNP | Rv2252 | Gly230Ser | 16 | - | | 2529680 | A | G | 263.78 | SNP | Rv2256c | silent (Thr65) | 9871 | - | | 2530030 | C | A | 33.77 | SNP | Rv2257c | Asp265Tyr | 0 | - | | 2531742 | A | G | 257.80 | SNP | Rv2258c | silent (Ala52) | 9867 | - | | 2540721 | C | A | 30.77 | SNP | Rv2266 (cyp124) | silent (Ala206) | 9867 | - | | 2561866 | C | A | 32.77 | SNP | Rv2289 (cdh) | silent (Pro64) | 9926 | - | | 2569199 | G | T | 37.77 | SNP | Rv2298 | Asp40Tyr | 0 | - | | 2571592 | G | T | 34.77 | SNP | Rv2299c (htpG) | silent (Ala137) | 9867 | - | | 2573756 | C | A | 83.28 | SNP | intergenic |  |  | - | | 2586127 | A | G | 233.84 | SNP | Rv2314c | silent (Gly388) | 9935 | - | | 2588184 | C | A | 157.84 | SNP | Rv2315c | silent (Arg207) | 9913 | - | | 2594475 | G | T | 39.77 | SNP | Rv2321c (rocD2) | silent (Thr75) | 9871 | - | | 2594515 | G | T | 34.77 | SNP | Rv2321c (rocD2) | Ser62STOP | 35 | - | | 2597096 | G | T | 36.77 | SNP | Rv2325c | silent (Pro254) | 9926 | - | | 2598400 | A | G | 132.03 | SNP | Rv2326c | silent (Asn516) | 9822 | - | | 2599493 | C | A | 34.77 | SNP | Rv2326c | Arg152Leu | 1 | - | | 2599821 | C | A | 218.80 | SNP | Rv2326c | Ala43Ser | 28 | - | | 2601798 | G | T | 41.77 | SNP | Rv2328 (PE23) | Gln356His | 20 | - | | 2612632 | C | A | 57.74 | SNP | Rv2337c | Gly119Val | 3 | - | | 2617632 | A | AT | 370.74 | INS | intergenic |  |  | - | | 2617633 | C | T | 173.84 | SNP | intergenic |  |  | - | | 2630795 | C | A | 32.77 | SNP | Rv2351c (plcA) | Met(s)427Ile | 2 | - | | 2637425 | C | A | 31.77 | SNP | intergenic |  |  | - | | 2640492 | C | A | 33.77 | SNP | Rv2357c (glyS) | Gln191His | 20 | - | | 2648861 | C | T | 387.77 | SNP | Rv2367c | Val18Ile | 33 | - | | 2652895 | G | T | 38.77 | SNP | Rv2373c (dnaJ2) | His365Asn | 21 | - | | 2654371 | G | A | 141.90 | SNP | Rv2374c (hrcA) | silent (Asn241) | 9822 | - | | 2656225 | A | G | 239.84 | SNP | Rv2377c (mbtH) | Val69Ala | 18 | - | | 2660319 | C | G | 295.78 | SNP | Rv2379c (mbtF) | Glu589Asp | 53 | - | | 2664299 | G | C | 176.90 | SNP | Rv2380c (mbtE) | silent (Thr939) | 9871 | - | | 2680658 | T | G | 99.28 | SNP | intergenic |  |  | - | | 2685431 | G | T | 42.77 | SNP | Rv2391 (sirA) | Val(s)251Val | 13 | - | | 2687909 | C | A | 41.77 | SNP | Rv2393 (che1) | Pro261Gln | 6 | - | | 2695378 | C | G | 417.77 | SNP | Rv2398c (cysW) | Gly141Ala | 21 | - | | 2698585 | C | T | 68.28 | SNP | Rv2402 | silent (Tyr19) | 9945 | - | | 2704884 | A | ACAGCGACCATATCGCCGAG CT | 1530.75 | INS | Rv2407 |  |  | - | | 2705163 | G | T | 35.77 | SNP | Rv2407 | Arg156Leu | 1 | - | | 2706782 | C | A | 32.77 | SNP | Rv2409c | Met(s)184Ile | 2 | - | | 2718852 | T | G | 263.80 | SNP | intergenic |  |  | - | | 2718868 | C | A | 34.77 | SNP | intergenic |  |  | - | | 2722340 | G | A | 170.18 | SNP | Rv2425c | silent (Phe323) | 9946 | - | | 2726637 | G | A | 336.77 | SNP | Rv2428 (ahpC) | Ala149Thr | 22 | - | | 2730881 | G | T | 37.77 | SNP | Rv2435c | silent (Pro623) | 9926 | - | | 2732375 | G | T | 32.77 | SNP | Rv2435c | silent (Ile125) | 9872 | - | | 2732435 | C | A | 40.77 | SNP | Rv2435c | silent (Thr105) | 9871 | - | | 2734074 | T | C | 100.03 | SNP | Rv2436 (rbsK) | Val282Ala | 18 | - | | 2734600 | G | T | 30.77 | SNP | Rv2437 | Met(s)75Ile | 2 | - | | 2736434 | C | A | 58.28 | SNP | Rv2438c (nadE) | Arg133Leu | 1 | - | | 2747068 | C | A | 40.77 | SNP | Rv2447c (folC) | Met(s)177Ile | 2 | - | | 2748388 | C | A | 30.77 | SNP | Rv2448c (valS) | Trp613Leu(s) | 0 | - | | 2752698 | C | A | 138.90 | SNP | intergenic |  |  | - | | 2760152 | A | G | 300.78 | SNP | Rv2458 (mmuM) | Tyr125Cys | 3 | - | | 2768181 | C | T | 74.03 | SNP | intergenic |  |  | - | | 2779136 | T | C | 50.74 | SNP | Rv2476c (gdh) | Ser1043Gly | 21 | - | | 2786952 | A | G | 186.84 | SNP | Rv2482c (plsB2) | Cys778Arg | 1 | - | | 2790026 | G | T | 39.74 | SNP | Rv2483c (plsC) | His333Asn | 21 | - | | 2791061 | A | G | 89.28 | SNP | Rv2484c | silent (Thr478) | 9871 | - | | 2797986 | G | T | 43.77 | SNP | Rv2488c | silent (Ser965) | 9840 | - | | 2804116 | G | A | 109.10 | SNP | Rv2490c (PE\_PGRS43) | silent (Gly707) | 9935 | - | | 2804314 | A | G | 106.28 | SNP | Rv2490c (PE\_PGRS43) | silent (Asp641) | 9859 | - | | 2804364 | C | A | 48.77 | SNP | Rv2490c (PE\_PGRS43) | Gly625Cys | 0 | - | | 2809621 | T | C | 138.03 | SNP | Rv2495c (bkdC) | Thr107Ala | 32 | - | | 2814789 | C | A | 34.77 | SNP | Rv2500c (fadE19) | silent (Ala41) | 9867 | - | | 2818837 | A | G | 182.90 | SNP | Rv2503c (scoB) | silent (Gly97) | 9935 | - | | 2821342 | C | T | 59.74 | SNP | Rv2505c (fadD35) | silent (Ala85) | 9867 | - | | 2822953 | G | T | 62.77 | SNP | Rv2507 | silent (Pro172) | 9926 | - | | 2823224 | G | T | 37.77 | SNP | Rv2507 | Gly263Trp | 0 | - | | 2827984 | G | T | 201.84 | SNP | intergenic |  |  | - | | 2828019 | T | C | 167.90 | SNP | intergenic |  |  | - | | 2830525 | C | A | 440.77 | SNP | Rv2513 | Thr122Lys | 11 | - | | 2835895 | C | A | 37.77 | SNP | Rv2519 (PE26) | silent (Thr37) | 9871 | - | | 2840351 | G | T | 34.77 | SNP | Rv2524c (fas) | Phe2994Leu | 13 | - | | 2850386 | G | T | 31.77 | SNP | Rv2525c | silent (Ile63) | 9872 | - | | 2854056 | G | T | 40.77 | SNP | Rv2529 | Gln394His | 20 | - | | 2855259 | A | G | 132.03 | SNP | Rv2531c | silent (Ala841) | 9867 | - | | 2856038 | G | A | 129.90 | SNP | Rv2531c | His582Tyr | 4 | - | | 2857014 | G | A | 138.90 | SNP | Rv2531c | silent (Thr256) | 9871 | - | | 2858011 | T | C | 200.79 | SNP | Rv2532c | Met(s)82Val(s) | 9867 | - | | 2865760 | A | G | 206.84 | SNP | Rv2542 | Thr211Ala | 32 | - | | 2865882 | T | C | 179.90 | SNP | Rv2542 | silent (Val251) | 9901 | - | | 2868659 | C | G | 86.80 | SNP | Rv2547 (vapB19) | silent (Ala18) | 9867 | - | | 2871048 | C | T | 107.03 | SNP | Rv2551c | silent (Leu49) | 9947 | - | | 2875643 | G | T | 39.77 | SNP | Rv2555c (alaS) | silent (Ile281) | 9872 | - | | 2880702 | G | C | 124.03 | SNP | Rv2560 | Val210Leu | 15 | - | | 2881455 | A | G | 132.03 | SNP | Rv2561 | Tyr16Cys | 3 | - | | 2888201 | T | C | 106.28 | SNP | Rv2566 | Leu610Pro | 2 | - | | 2889633 | T | C | 253.80 | SNP | Rv2566 | silent (Ala1087) | 9867 | - | | 2891267 | C | T | 74.28 | SNP | Rv2567 | silent (Gly491) | 9935 | - | | 2891728 | A | G | 214.84 | SNP | Rv2567 | Gln645Arg | 10 | - | | 2892808 | C | A | 40.77 | SNP | Rv2568c | Gly222Cys | 0 | - | | 2893238 | C | A | 42.74 | SNP | Rv2568c | silent (Arg78) | 9913 | - | | 2894208 | G | A | 61.74 | SNP | Rv2569c | silent (Ser67) | 9840 | - | | 2894854 | C | T | 119.90 | SNP | Rv2570 | Gln115STOP | 8 | - | | 2900469 | G | T | 42.77 | SNP | Rv2576c | silent (Pro74) | 9926 | - | | 2901874 | G | T | 41.77 | SNP | Rv2577 | silent (Ser319) | 9840 | - | | 2902158 | A | C | 144.03 | SNP | Rv2577 | Asp414Ala | 10 | - | | 2906918 | A | T | 84.28 | SNP | Rv2582 (ppiB) | silent (Leu35) | 9947 | - | | 2909083 | C | A | 35.77 | SNP | Rv2583c (relA) | Glu372Asp | 53 | - | | 2911293 | C | G | 122.03 | SNP | Rv2585c | Cys462Ser | 11 | - | | 2915025 | C | A | 31.77 | SNP | Rv2587c (secD) | Asp238Tyr | 0 | - | | 2923391 | T | C | 97.28 | SNP | Rv2592c (ruvB) | silent (Pro281) | 9926 | - | | 2927939 | T | C | 253.80 | SNP | intergenic |  |  | - | | 2939373 | G | C | 196.84 | SNP | Rv2611c | Ser197Cys | 5 | - | | 2939657 | T | C | 97.28 | SNP | Rv2611c | Ile102Met(s) | 6 | - | | 2945167 | G | T | 183.78 | SNP | intergenic |  |  | - | | 2946157 | T | C | 353.78 | SNP | Rv2617c | Asn44Ser | 34 | - | | 2948516 | C | A | 46.77 | SNP | Rv2621c | Gly15Trp | 0 | - | | 2950725 | G | T | 32.77 | SNP | Rv2624c | Pro195Thr | 5 | - | | 2954439 | T | C | 62.74 | SNP | Rv2627c | Arg104Gly | 1 | - | | 2963096 | G | T | 40.77 | SNP | Rv2636 | Trp128Cys | 0 | - | | 2968913 | T | C | 99.28 | SNP | intergenic |  |  | - | | 2971004 | G | T | 57.74 | SNP | Rv2646 | Asp152Tyr | 0 | - | | 2974933 | A | G | 143.03 | SNP | Rv2650c | Ile101Thr | 11 | - | | 2982204 | G | T | 31.77 | SNP | Rv2664 | silent (Ala36) | 9867 | - | | 2984740 | A | G | 94.28 | SNP | Rv2668 | His3Arg | 10 | - | | 3001464 | G | T | 33.74 | SNP | Rv2684 (arsA) | Gly284Val(s) | 21 | - | | 3005185 | G | T | 157.90 | SNP | Rv2688c | Pro156Thr | 5 | - | | 3006767 | A | C | 282.85 | SNP | Rv2689c | Leu99Arg | 1 | - | | 3007562 | G | T | 36.77 | SNP | Rv2690c | Arg550Ser | 11 | - | | 3009692 | A | G | 210.84 | SNP | Rv2691 (ceoB) | Thr117Ala | 32 | - | | 3017465 | T | C | 184.90 | SNP | Rv2702 (ppgK) | Ile203Thr | 11 | - | | 3024976 | C | A | 37.77 | SNP | Rv2712c | Arg118Leu | 1 | - | | 3026431 | C | A | 35.77 | SNP | Rv2713 (sthA) | His331Asn | 21 | - | | 3029610 | G | A | 107.96 | SNP | Rv2716 | Ala147Thr | 22 | - | | 3039811 | C | A | 40.77 | SNP | intergenic |  |  | - | | 3046775 | G | T | 31.77 | SNP | intergenic |  |  | - | | 3054081 | A | G | 209.84 | SNP | Rv2741 (PE\_PGRS47) | silent (Gly56) | 9935 | - | | 3066294 | G | T | 30.77 | SNP | Rv2753c (dapA) | silent (Pro277) | 9926 | - | | 3073868 | T | C | 345.77 | SNP | Rv2764c (thyA) | Thr202Ala | 32 | genotype | | 3077039 | C | A | 96.03 | SNP | Rv2768c (PPE43) | Gly347Val | 3 | - | | 3080795 | A | G | 257.80 | SNP | Rv2771c | Leu80Pro | 2 | - | | 3083977 | G | T | 289.78 | SNP | Rv2776c | Asp109Glu | 56 | - | | 3084074 | C | T | 208.84 | SNP | Rv2776c | Gly77Asp | 6 | - | | 3086788 | T | C | 127.03 | SNP | intergenic |  |  | - | | 3089679 | G | A | 49.74 | SNP | Rv2782c (pepR) | Pro228Leu | 3 | - | | 3099269 | A | C | 248.80 | SNP | Rv2790c (ltp1) | Phe301Val | 1 | - | | 3099383 | C | A | 36.77 | SNP | Rv2790c (ltp1) | Gly263Cys | 0 | - | | 3101119 | G | T | 148.03 | SNP | Rv2791c | Arg155Ser | 11 | - | | 3101964 | G | A | 183.84 | SNP | Rv2792c | Leu67Leu(s) | 4 | - | | 3103682 | T | C | 50.74 | SNP | Rv2794c (pptT) | Met(s)87Val(s) | 9867 | - | | 3104561 | G | T | 44.77 | SNP | Rv2795c | Phe117Leu | 13 | - | | 3108674 | A | C | 55.74 | SNP | Rv2800 | silent (Arg87) | 9913 | - | | 3113872 | A | T | 93.03 | SNP | Rv2807 | Glu72Val(s) | 17 | - | | 3118000 | A | G | 454.77 | SNP | Rv2812 | Arg395Gly | 1 | - | | 3131011 | C | A | 41.77 | SNP | Rv2823c | Asp255Tyr | 0 | - | | 3131469 | T | TTGTCGGCGA | 912.75 | INS | Rv2823c |  |  | - | | 3131860 | G | T | 40.77 | SNP | Rv2824c | Phe285Leu | 13 | - | | 3133536 | T | C | 405.77 | SNP | Rv2825c | Lys2Glu | 4 | - | | 3136677 | C | A | 39.77 | SNP | Rv2829c (vapC22) | Val(s)112Val | 13 | - | | 3137058 | G | A | 54.28 | SNP | Rv2830c (vapB22) | Ala56Val(s) | 9867 | - | | 3140525 | T | C | 168.90 | SNP | Rv2834c (ugpE) | Met(s)264Val(s) | 9867 | - | | 3141497 | G | T | 58.77 | SNP | Rv2835c (ugpA) | silent (Ile242) | 9872 | - | | 3144306 | C | A | 31.77 | SNP | Rv2837c | Gly114Cys | 0 | - | | 3149188 | G | T | 72.77 | SNP | Rv2841c (nusA) | silent (Arg81) | 9913 | - | | 3155168 | C | A | 32.77 | SNP | Rv2847c (cysG) | Arg235Ile | 2 | - | | 3158935 | G | C | 53.74 | SNP | Rv2850c | Arg374Gly | 1 | - | | 3160545 | G | T | 30.77 | SNP | intergenic |  |  | - | | 3165074 | T | C | 62.74 | SNP | Rv2854 | Val(s)308Ala | 9867 | - | | 3175702 | T | C | 302.78 | SNP | Rv2864c | Ile522Val | 57 | - | | 3177320 | G | T | 34.77 | SNP | intergenic |  |  | - | | 3177884 | C | A | 179.90 | SNP | Rv2866 (relG) | silent (Arg21) | 9913 | - | | 3183561 | G | C | 86.77 | SNP | Rv2872 (vapC43) | silent (Pro60) | 9926 | - | | 3186860 | T | G | 298.78 | SNP | Rv2874 (dipZ) | Tyr672Asp | 0 | - | | 3191027 | G | A | 383.77 | SNP | Rv2881c (cdsA) | Leu199Leu(s) | 4 | - | | 3197732 | G | T | 37.77 | SNP | Rv2888c (amiC) | Pro185Gln | 6 | - | | 3198853 | G | T | 34.77 | SNP | Rv2889c (tsf) | Phe85Leu | 13 | - | | 3199518 | G | T | 31.77 | SNP | Rv2890c (rpsB) | silent (Ala155) | 9867 | - | | 3207297 | G | T | 205.84 | SNP | Rv2897c | Leu216Met(s) | 4 | - | | 3214010 | G | T | 34.77 | SNP | Rv2904c (rplS) | His82Asn | 21 | - | | 3214790 | C | T | 115.90 | SNP | Rv2905 (lppW) | Leu55Leu(s) | 4 | - | | 3216838 | G | T | 35.77 | SNP | Rv2907c (rimM) | silent (Val18) | 9901 | - | | 3216994 | C | A | 42.77 | SNP | Rv2908c | Gly52Trp | 0 | - | | 3220187 | G | T | 37.77 | SNP | Rv2913c | silent (Leu504) | 9947 | - | | 3225205 | C | A | 42.77 | SNP | Rv2916c (ffh) | Gly361Cys | 0 | - | | 3226181 | A | C | 231.84 | SNP | Rv2916c (ffh) | silent (Arg35) | 9913 | - | | 3228143 | G | T | 35.74 | SNP | Rv2917 | Arg594Leu | 1 | - | | 3228359 | C | A | 38.74 | SNP | Rv2918c (glnD) | Val(s)774Val | 13 | - | | 3232815 | A | G | 188.84 | SNP | intergenic |  |  | - | | 3236230 | C | A | 188.80 | SNP | Rv2922c (smc) | Arg526Leu | 1 | - | | 3237503 | G | T | 33.77 | SNP | Rv2922c (smc) | silent (Arg102) | 9913 | - | | 3243630 | G | A | 299.78 | SNP | intergenic |  |  | - | | 3247851 | G | A | 262.78 | SNP | Rv2931 (ppsA) | Ala803Thr | 22 | - | | 3247853 | C | T | 296.78 | SNP | Rv2931 (ppsA) | silent (Ala803) | 9867 | - | | 3247856 | G | C | 304.78 | SNP | Rv2931 (ppsA) | silent (Arg804) | 9913 | - | | 3247864 | C | CTAGG | 539.73 | INS | Rv2931 (ppsA) |  |  | - | | 3247865 | GCAAA | G | 522.73 | DEL | Rv2931 (ppsA) |  |  | - | | 3247874 | G | A | 291.78 | SNP | Rv2931 (ppsA) | silent (Arg810) | 9913 | - | | 3247877 | T | C | 309.78 | SNP | Rv2931 (ppsA) | silent (Phe811) | 9946 | - | | 3247883 | T | C | 395.77 | SNP | Rv2931 (ppsA) | silent (Ser813) | 9840 | - | | 3248074 | G | A | 125.03 | SNP | Rv2931 (ppsA) | Arg877His | 8 | - | | 3248075 | C | T | 131.03 | SNP | Rv2931 (ppsA) | silent (Arg877) | 9913 | - | | 3256494 | A | G | 84.28 | SNP | Rv2933 (ppsC) | silent (Gly270) | 9935 | - | | 3260301 | A | G | 120.90 | SNP | Rv2933 (ppsC) | silent (Gly1539) | 9935 | - | | 3267743 | A | G | 49.74 | SNP | Rv2935 (ppsE) | Ile3Val | 57 | - | | 3269581 | A | G | 135.03 | SNP | Rv2935 (ppsE) | silent (Ala615) | 9867 | - | | 3270784 | A | G | 369.77 | SNP | Rv2935 (ppsE) | silent (Gln1016) | 9876 | - | | 3278675 | C | T | 202.78 | SNP | Rv2940c (mas) | silent (Leu1347) | 9947 | - | | 3279233 | C | A | 32.77 | SNP | Rv2940c (mas) | silent (Gly1161) | 9935 | - | | 3281677 | C | A | 41.77 | SNP | Rv2940c (mas) | Gly347Trp | 0 | - | | 3284995 | C | A | 40.77 | SNP | Rv2941 (fadD28) | Pro554His | 3 | - | | 3285080 | C | A | 40.77 | SNP | Rv2942 (mmpL7) | Pro4Gln | 6 | - | | 3290224 | C | A | 31.77 | SNP | Rv2943A; Rv2944 | Pro174Thr; silent (Leu145) | 5; 9947 | - | | 3292737 | T | G | 89.28 | SNP | Rv2946c (pks1) | Gln1206Pro | 8 | - | | 3296843 | A | G | 99.28 | SNP | Rv2947c (pks15) | Val(s)333Ala | 9867 | - | | 3296935 | C | A | 46.77 | SNP | Rv2947c (pks15) | silent (Leu302) | 9947 | - | | 3300495 | C | A | 39.77 | SNP | Rv2949c | Gly26Cys | 0 | - | | 3301728 | G | T | 32.77 | SNP | Rv2950c (fadD29) | Ser243STOP | 35 | - | | 3308606 | G | A | 98.03 | SNP | intergenic |  |  | - | | 3309770 | C | A | 46.77 | SNP | Rv2957 | Leu101Met(s) | 4 | - | | 3312632 | C | T | 61.74 | SNP | Rv2959c | Trp69STOP | 0 | - | | 3313931 | C | A | 44.77 | SNP | Rv2962c | Gly398Trp | 0 | - | | 3336825 | T | C | 215.84 | SNP | Rv2981c (ddlA) | Thr365Ala | 32 | - | | 3338603 | G | C | 133.03 | SNP | Rv2982c (gpdA2) | Pro133Ala | 22 | - | | 3339794 | G | T | 37.77 | SNP | intergenic |  |  | - | | 3341172 | G | T | 35.77 | SNP | Rv2984 (ppk1) | Arg440Leu | 1 | - | | 3344552 | G | T | 41.77 | SNP | Rv2987c (leuD) | silent (Pro26) | 9926 | - | | 3347142 | C | A | 37.77 | SNP | Rv2990c | Arg193Leu | 1 | - | | 3354863 | G | T | 35.77 | SNP | Rv2996c (serA1) | silent (Val69) | 9901 | - | | 3354896 | C | T | 124.81 | SNP | Rv2996c (serA1) | silent (Glu58) | 9865 | - | | 3358235 | A | T | 45.74 | SNP | Rv2999 (lppY) | Met(s)212Leu(s) | 9867 | - | | 3359789 | G | A | 266.78 | SNP | Rv3001c (ilvC) | silent (Ala266) | 9867 | - | | 3360951 | G | T | 37.77 | SNP | Rv3002c (ilvN) | silent (Leu60) | 9947 | - | | 3361562 | C | A | 32.77 | SNP | Rv3003c (ilvB1) | Trp475Cys | 0 | - | | 3363338 | A | G | 166.90 | SNP | intergenic |  |  | - | | 3363378 | C | A | 40.77 | SNP | Rv3004 (cfp6) | Leu11Met(s) | 4 | - | | 3367765 | G | A | 79.28 | SNP | Rv3009c (gatB) | silent (Gly343) | 9935 | - | | 3377271 | G | C | 43.74 | SNP | Rv3018c (PPE46) | Leu325Val(s) | 4 | - | | 3377281 | T | C | 32.74 | SNP | Rv3018c (PPE46) | silent (Gly321) | 9935 | - | | 3377293 | G | C | 33.74 | SNP | Rv3018c (PPE46) | silent (Ala317) | 9867 | - | | 3377305 | T | C | 106.28 | SNP | Rv3018c (PPE46) | Val313Val(s) | 18 | - | | 3377314 | A | C | 78.28 | SNP | Rv3018c (PPE46) | Val310Val(s) | 18 | - | | 3377320 | A | C | 95.28 | SNP | Rv3018c (PPE46) | silent (Ala308) | 9867 | - | | 3377326 | G | A | 77.28 | SNP | Rv3018c (PPE46) | silent (Ala306) | 9867 | - | | 3377347 | C | A | 118.03 | SNP | Rv3018c (PPE46) | silent (Gly299) | 9935 | - | | 3379742 | T | C | 88.28 | SNP | intergenic |  |  | - | | 3379751 | A | C | 92.28 | SNP | intergenic |  |  | - | | 3379757 | A | C | 115.03 | SNP | intergenic |  |  | - | | 3379763 | G | A | 78.03 | SNP | intergenic |  |  | - | | 3379784 | C | A | 118.03 | SNP | intergenic |  |  | - | | 3379788 | C | G | 135.03 | SNP | intergenic |  |  | - | | 3380740 | C | T | 284.78 | SNP | Rv3022A (PE29) | Gly85Asp | 6 | - | | 3381007 | T | TA | 638.73 | INS | intergenic |  |  | - | | 3402026 | C | A | 37.77 | SNP | Rv3042c (serB2) | silent (Thr379) | 9871 | - | | 3402816 | C | T | 307.78 | SNP | Rv3042c (serB2) | Gly116Glu | 4 | - | | 3403120 | C | A | 37.77 | SNP | Rv3042c (serB2) | Asp15Tyr | 0 | - | | 3403216 | AT | A | 234.80 | DEL | Rv3043c (ctaD) |  |  | - | | 3405343 | C | A | 30.77 | SNP | Rv3044 (fecB) | silent (Arg70) | 9913 | - | | 3417899 | C | A | 30.77 | SNP | Rv3057c | Arg255Leu | 1 | - | | 3418328 | T | G | 97.28 | SNP | Rv3057c | Asp112Ala | 10 | - | | 3418330 | G | A | 103.28 | SNP | Rv3057c | silent (His111) | 9912 | - | | 3420892 | C | A | 34.77 | SNP | Rv3059 (cyp136) | silent (Pro467) | 9926 | - | | 3425854 | C | T | 240.78 | SNP | Rv3062 (ligB) | Pro91Ser | 17 | - | | 3426795 | C | G | 197.84 | SNP | Rv3062 (ligB) | silent (Ser404) | 9840 | - | | 3428917 | C | A | 143.90 | SNP | Rv3063 (cstA) | Arg559Ser | 11 | - | | 3429202 | T | G | 204.84 | SNP | Rv3063 (cstA) | Tyr654Asp | 0 | - | | 3431326 | G | A | 159.90 | SNP | intergenic |  |  | - | | 3434715 | C | A | 30.77 | SNP | Rv3071 | silent (Ser84) | 9840 | - | | 3440464 | T | G | 106.28 | SNP | Rv3077 | silent (Arg308) | 9913 | - | | 3440468 | G | C | 106.28 | SNP | Rv3077 | Gly310Arg | 0 | - | | 3440522 | G | T | 39.77 | SNP | Rv3077 | Asp328Tyr | 0 | - | | 3456666 | A | G | 62.74 | SNP | Rv3089 (fadD13) | silent (Ala302) | 9867 | - | | 3460986 | G | A | 67.81 | SNP | Rv3092c | Pro250Leu | 3 | - | | 3461662 | C | A | 31.77 | SNP | Rv3092c | Asp25Tyr | 0 | - | | 3462135 | G | C | 120.03 | SNP | Rv3093c | Cys210Trp | 0 | - | | 3462683 | C | A | 31.77 | SNP | Rv3093c | Asp28Tyr | 0 | - | | 3470579 | A | G | 268.80 | SNP | Rv3101c (ftsX) | Leu(s)34Ser | 28 | - | | 3473923 | G | T | 42.77 | SNP | intergenic |  |  | - | | 3473996 | G | GA | 487.73 | INS | intergenic |  |  | - | | 3480260 | C | A | 35.77 | SNP | Rv3113 | silent (Arg63) | 9913 | - | | 3480511 | C | A | 71.77 | SNP | Rv3113 | silent (Pro146) | 9926 | - | | 3485267 | G | T | 34.77 | SNP | Rv3119 (moaE1) | Asp46Tyr | 0 | - | | 3486977 | A | G | 213.84 | SNP | Rv3121 (cyp141) | Lys157Glu | 4 | - | | 3487186 | C | A | 34.77 | SNP | Rv3121 (cyp141) | silent (Val226) | 9901 | - | | 3503231 | G | C | 186.90 | SNP | Rv3136A | Ala16Gly | 21 | - | | 3503895 | C | T | 179.84 | SNP | Rv3137 | Pro168Leu | 3 | - | | 3504154 | G | T | 30.77 | SNP | Rv3137 | silent (Leu254) | 9947 | - | | 3505027 | G | A | 76.77 | SNP | Rv3138 (pflA) | Arg278His | 8 | - | | 3507152 | G | T | 46.79 | SNP | Rv3140 (fadE23) | silent (Leu121) | 9947 | - | | 3509818 | G | T | 32.77 | SNP | Rv3143 | silent (Arg55) | 9913 | - | | 3514512 | G | C | 147.53 | SNP | Rv3148 (nuoD) | Gly392Ala | 21 | genotype | | 3517541 | G | T | 44.77 | SNP | Rv3151 (nuoG) | Asp266Tyr | 0 | - | | 3518167 | A | G | 132.03 | SNP | Rv3151 (nuoG) | Ile474Met(s) | 6 | - | | 3518555 | A | G | 62.74 | SNP | Rv3151 (nuoG) | Thr604Ala | 32 | - | | 3525088 | G | A | 240.80 | SNP | Rv3157 (nuoM) | Val(s)319Val | 13 | - | | 3525547 | G | T | 36.77 | SNP | Rv3157 (nuoM) | Met(s)472Ile | 2 | - | | 3532808 | G | T | 33.77 | SNP | Rv3163c | Arg36Ser | 11 | - | | 3546934 | G | T | 35.77 | SNP | intergenic |  |  | - | | 3547558 | G | T | 32.77 | SNP | intergenic |  |  | - | | 3548641 | T | C | 97.28 | SNP | Rv3179 | Tyr342His | 4 | - | | 3556275 | A | G | 216.84 | SNP | Rv3190c | Leu138Pro | 2 | - | | 3561155 | G | A | 177.84 | SNP | Rv3193c | Ala673Val | 13 | - | | 3564832 | G | T | 30.77 | SNP | Rv3195 | Asp157Tyr | 0 | - | | 3573486 | G | T | 40.77 | SNP | Rv3200c | Arg62Ser | 11 | - | | 3580636 | CT | C | 328.74 | DEL | intergenic |  |  | - | | 3581414 | A | G | 119.03 | SNP | Rv3204 | Thr34Ala | 32 | - | | 3584741 | G | T | 35.77 | SNP | intergenic |  |  | - | | 3590686 | G | GC | 194.87 | INS | intergenic |  |  | - | | 3591063 | T | C | 96.28 | SNP | Rv3213c | Lys144Glu | 4 | - | | 3594124 | C | T | 147.90 | SNP | Rv3217c | Ala38Thr | 22 | - | | 3595483 | A | ACTGGCAGCGTAGT | 594.87 | INS | intergenic |  |  | - | | 3604821 | G | C | 313.78 | SNP | Rv3228 | silent (Ala32) | 9867 | - | | 3607692 | C | A | 31.77 | SNP | Rv3230c | Arg188Leu | 1 | - | | 3610060 | C | A | 35.77 | SNP | Rv3233c | silent (Gly104) | 9935 | - | | 3610441 | C | T | 147.03 | SNP | Rv3234c (tgs3) | Arg250His | 8 | - | | 3612009 | C | T | 92.28 | SNP | Rv3236c | Ala370Thr | 22 | - | | 3612594 | C | A | 38.77 | SNP | Rv3236c | Asp175Tyr | 0 | - | | 3614982 | T | C | 62.74 | SNP | Rv3239c | silent (Leu874) | 9947 | - | | 3617576 | C | A | 63.77 | SNP | Rv3239c | Gly10Trp | 0 | - | | 3620611 | C | A | 38.77 | SNP | Rv3241c | STOP215Leu | 3 | - | | 3622441 | A | C | 92.28 | SNP | Rv3243c | Val217Val(s) | 18 | - | | 3648491 | G | T | 69.77 | SNP | Rv3267 | Asp203Tyr | 0 | - | | 3651699 | G | T | 40.77 | SNP | Rv3270 (ctpC) | Gly392Cys | 0 | - | | 3655324 | G | T | 36.77 | SNP | Rv3273 | Glu230STOP | 17 | - | | 3666663 | C | A | 31.77 | SNP | Rv3285 (accA3) | Gln103Lys | 12 | - | | 3669254 | C | A | 33.77 | SNP | Rv3287c (rsbW) | silent (Leu45) | 9947 | - | | 3677743 | G | T | 33.77 | SNP | Rv3296 (lhr) | silent (Ser323) | 9840 | - | | 3680644 | C | T | 87.03 | SNP | Rv3296 (lhr) | silent (Ala1290) | 9867 | - | | 3687121 | G | T | 35.77 | SNP | Rv3301c (phoY1) | Arg153Ser | 11 | - | | 3689523 | G | T | 151.84 | SNP | Rv3303c (lpdA) | Cys472STOP | 3 | - | | 3692140 | C | T | 128.10 | SNP | Rv3305c (amiA1) | silent (Leu223) | 9947 | - | | 3692757 | G | T | 67.28 | SNP | Rv3305c (amiA1) | Leu18Met(s) | 4 | - | | 3697180 | C | A | 40.77 | SNP | intergenic |  |  | - | | 3704106 | G | T | 36.77 | SNP | Rv3316 (sdhC) | Trp2Leu(s) | 0 | - | | 3704492 | C | A | 46.77 | SNP | Rv3317 (sdhD) | Pro19Gln | 6 | - | | 3711910 | G | A | 37.74 | SNP | Rv3327 | Trp54STOP | 0 | - | | 3714211 | G | T | 91.90 | SNP | Rv3328c (sigJ) | Pro41Gln | 6 | - | | 3714757 | A | C | 139.03 | SNP | Rv3329 | Gln122His | 20 | - | | 3718357 | C | T | 96.28 | SNP | Rv3331 (sugI) | Pro423Leu | 3 | - | | 3720264 | C | A | 31.77 | SNP | Rv3333c | silent (Thr173) | 9871 | - | | 3721806 | G | C | 168.90 | SNP | Rv3335c | silent (Gly265) | 9935 | - | | 3723899 | C | A | 40.77 | SNP | Rv3337 | silent (Arg82) | 9913 | - | | 3724503 | C | A | 32.77 | SNP | Rv3338 | silent (Ile200) | 9872 | - | | 3730327 | G | A | 73.28 | SNP | Rv3343c (PPE54) | silent (Phe2203) | 9946 | - | | 3730411 | G | A | 34.74 | SNP | Rv3343c (PPE54) | silent (Gly2175) | 9935 | - | | 3732706 | G | A | 40.74 | SNP | Rv3343c (PPE54) | silent (Gly1410) | 9935 | - | | 3736628 | T | G | 60.74 | SNP | Rv3343c (PPE54) | Glu103Ala | 17 | - | | 3776706 | C | T | 51.88 | SNP | Rv3365c | Ala266Thr | 22 | - | | 3794884 | G | A | 46.28 | SNP | intergenic |  |  | - | | 3798095 | A | C | 106.28 | SNP | Rv3383c (idsB) | Val132Gly | 5 | - | | 3800663 | G | A | 150.90 | SNP | Rv3386 | Arg191His | 8 | - | | 3804415 | G | T | 35.74 | SNP | Rv3389c (htdY) | Pro126Gln | 6 | - | | 3814668 | C | A | 37.77 | SNP | Rv3397c (phyA) | Asp111Tyr | 0 | - | | 3815341 | T | A | 196.22 | SNP | Rv3398c (idsA1) | Lys256STOP | 2 | - | | 3817117 | C | A | 62.28 | SNP | Rv3399 | Ala330Glu | 10 | - | | 3821785 | G | T | 42.77 | SNP | Rv3402c | Pro36His | 3 | - | | 3822349 | C | A | 34.77 | SNP | Rv3403c | Glu505Asp | 53 | - | | 3823159 | A | T | 55.28 | SNP | Rv3403c | silent (Val235) | 9901 | - | | 3825372 | G | T | 34.77 | SNP | Rv3406 | Gly15Cys | 0 | - | | 3825560 | C | G | 227.84 | SNP | Rv3406 | silent (Leu77) | 9947 | - | | 3826684 | C | T | 80.28 | SNP | Rv3408 (vapC47) | Ser46Leu(s) | 35 | - | | 3829152 | G | A | 60.03 | SNP | Rv3410c (guaB3) | silent (Leu253) | 9947 | - | | 3829770 | T | C | 88.28 | SNP | Rv3410c (guaB3) | silent (Pro47) | 9926 | - | | 3831496 | C | A | 34.77 | SNP | Rv3411c (guaB2) | silent (Leu8) | 9947 | - | | 3838871 | A | G | 52.74 | SNP | Rv3420c (rimI) | silent (Ala64) | 9867 | - | | 3847364 | C | T | 47.74 | SNP | Rv3429 (PPE59) | Ala67Val | 13 | - | | 3847367 | G | A | 38.74 | SNP | Rv3429 (PPE59) | Gly68Glu | 4 | - | | 3847378 | G | C | 49.74 | SNP | Rv3429 (PPE59) | Asp72His | 3 | - | | 3847380 | C | A | 43.74 | SNP | Rv3429 (PPE59) | Asp72Glu | 56 | - | | 3858912 | T | G | 61.74 | SNP | Rv3439c | Ile251Leu | 22 | - | | 3859376 | C | T | 48.74 | SNP | Rv3439c | Gly96Glu | 4 | - | | 3859893 | C | T | 72.28 | SNP | Rv3440c | silent (Glu28) | 9865 | - | | 3861806 | A | C | 61.74 | SNP | Rv3442c (rpsI) | Phe49Val | 1 | - | | 3862425 | G | T | 31.77 | SNP | intergenic |  |  | - | | 3862455 | G | T | 31.77 | SNP | intergenic |  |  | - | | 3862472 | GA | G | 340.74 | DEL | intergenic |  |  | - | | 3873392 | T | G | 55.74 | SNP | Rv3451 (cut3) | Leu259Arg | 1 | - | | 3874191 | T | C | 131.03 | SNP | intergenic |  |  | - | | 3877421 | A | G | 174.90 | SNP | Rv3456c (rplQ) | silent (Pro4) | 9926 | - | | 3878536 | C | A | 42.77 | SNP | intergenic |  |  | - | | 3878878 | A | G | 210.84 | SNP | Rv3458c (rpsD) | silent (Ser129) | 9840 | - | | 3881187 | G | A | 297.78 | SNP | Rv3463 | Gly94Asp | 6 | - | | 3882227 | G | T | 51.77 | SNP | Rv3464 (rmlB) | Asp131Tyr | 0 | - | | 3883626 | A | G | 97.28 | SNP | Rv3466 | silent (Pro34) | 9926 | - | | 3884906 | A | G | 221.84 | SNP | Rv3467 | Lys315Glu | 4 | - | | 3885886 | T | C | 106.28 | SNP | Rv3468c | Ile62Val | 57 | - | | 3892671 | A | G | 310.78 | SNP | Rv3476c (kgtP) | silent (Val350) | 9901 | - | | 3893989 | C | A | 34.77 | SNP | intergenic |  |  | - | | 3896340 | T | G | 183.90 | SNP | Rv3479 | Leu174Arg | 1 | - | | 3898408 | A | G | 393.77 | SNP | Rv3479 | silent (Ala863) | 9867 | - | | 3910936 | C | A | 36.77 | SNP | Rv3492c | Leu(s)4Phe | 1 | - | | 3910976 | C | A | 34.77 | SNP | Rv3493c | Asp234Tyr | 0 | - | | 3918089 | G | T | 42.77 | SNP | Rv3499c (mce4A) | Pro371Gln | 6 | - | | 3919373 | G | T | 31.77 | SNP | Rv3500c (yrbE4B) | silent (Thr230) | 9871 | - | | 3922282 | C | A | 38.77 | SNP | intergenic |  |  | - | | 3924316 | G | T | 40.77 | SNP | Rv3505 (fadE27) | Asp207Tyr | 0 | - | | 3924329 | G | T | 43.77 | SNP | Rv3505 (fadE27) | Arg211Met(s) | 2 | - | | 3930930 | G | T | 40.77 | SNP | intergenic |  |  | - | | 3934699 | G | A | 49.28 | SNP | Rv3508 (PE\_PGRS54) | Ser1232Asn | 20 | - | | 3934733 | G | C | 30.77 | SNP | Rv3508 (PE\_PGRS54) | silent (Gly1243) | 9935 | - | | 3942640 | T | C | 113.03 | SNP | intergenic |  |  | - | | 3952800 | G | A | 322.78 | SNP | Rv3516 (echA19) | Gly86Asp | 6 | - | | 3954096 | C | A | 33.77 | SNP | Rv3517 | silent (Val222) | 9901 | - | | 3957681 | T | C | 85.28 | SNP | Rv3521 | Val54Ala | 18 | - | | 3958403 | A | G | 232.84 | SNP | Rv3521 | Asn295Asp | 42 | - | | 3959418 | C | T | 160.90 | SNP | Rv3522 (ltp4) | Thr324Ile | 7 | - | | 3961452 | C | A | 34.77 | SNP | Rv3524 | Pro233His | 3 | - | | 3964463 | AG | A | 356.74 | DEL | intergenic |  |  | - | | 3977226 | G | A | 162.90 | SNP | Rv3538 | Leu(s)55Leu | 3 | genotype | | 3978500 | G | T | 32.77 | SNP | Rv3539 (PPE63) | Asp148Tyr | 0 | - | | 3979606 | C | A | 41.77 | SNP | Rv3540c (ltp2) | Gly352Trp | 0 | - | | 3983271 | T | G | 233.80 | SNP | Rv3544c (fadE28) | Ile292Leu | 22 | - | | 3991245 | G | T | 31.77 | SNP | Rv3552 | Asp159Tyr | 0 | - | | 4005607 | T | C | 160.90 | SNP | Rv3564 (fadE33) | Leu(s)121Leu | 3 | - | | 4006218 | C | A | 72.77 | SNP | Rv3565 (aspB) | Leu7Met(s) | 4 | - | | 4011800 | G | T | 38.77 | SNP | Rv3570c (hsaA) | silent (Val157) | 9901 | - | | 4024273 | T | C | 91.78 | SNP | Rv3581c (ispF) | Val25Val(s) | 18 | - | | 4025308 | G | T | 35.77 | SNP | Rv3583c | silent (Thr79) | 9871 | - | | 4026899 | G | A | 160.90 | SNP | Rv3585 (radA) | silent (Gln152) | 9876 | - | | 4030318 | C | A | 34.77 | SNP | Rv3588c (canB) | Glu59Asp | 53 | - | | 4034827 | C | T | 56.03 | SNP | Rv3593 (lpqF) | Ala159Val(s) | 9867 | - | | 4037283 | T | G | 106.28 | SNP | Rv3595c (PE\_PGRS59) | silent (Gly256) | 9935 | - | | 4038287 | G | A | 185.84 | SNP | Rv3596c (clpC1) | silent (Asn806) | 9822 | - | | 4042761 | G | A | 76.28 | SNP | Rv3598c (lysS) | silent (Asp60) | 9859 | - | | 4043365 | G | T | 336.78 | SNP | Rv3600c | silent (Ala165) | 9867 | - | | 4046003 | G | A | 63.28 | SNP | Rv3603c | Ala39Val(s) | 9867 | - | | 4051871 | C | A | 37.74 | SNP | Rv3610c (ftsH) | Arg338Leu | 1 | - | | 4052970 | G | A | 80.28 | SNP | Rv3611 | silent (Ala7) | 9867 | - | | 4055801 | G | A | 235.80 | SNP | Rv3616c (espA) | Thr192Ile | 7 | - | | 4059904 | A | G | 169.90 | SNP | intergenic |  |  | - | | 4063225 | C | A | 41.77 | SNP | Rv3623 (lpqG) | silent (Val233) | 9901 | - | | 4075957 | C | A | 132.03 | SNP | Rv3636 | Ala69Asp | 6 | - | | 4086945 | C | A | 32.77 | SNP | Rv3646c (topA) | Glu105STOP | 17 | - | | 4088346 | G | A | 253.78 | SNP | Rv3648c (cspA) | silent (Thr62) | 9871 | - | | 4093531 | G | A | 196.84 | SNP | intergenic |  |  | - | | 4097996 | C | A | 91.03 | SNP | intergenic |  |  | - | | 4100975 | T | C | 87.28 | SNP | intergenic |  |  | - | | 4101175 | C | A | 41.77 | SNP | intergenic |  |  | - | | 4108713 | G | T | 31.77 | SNP | Rv3667 (acs) | Gly308Cys | 0 | - | | 4109354 | A | C | 79.28 | SNP | Rv3667 (acs) | silent (Leu521) | 9947 | - | | 4111303 | G | C | 118.03 | SNP | Rv3669 | Val(s)159Val | 13 | - | | 4117042 | G | T | 30.77 | SNP | Rv3676 (crp) | Glu189STOP | 17 | - | | 4120926 | A | G | 65.77 | SNP | Rv3680 | Asn378Asp | 42 | - | | 4120983 | A | G | 157.77 | SNP | intergenic |  |  | - | | 4122671 | C | A | 34.77 | SNP | Rv3682 (ponA2) | silent (Val252) | 9901 | - | | 4124972 | G | T | 38.77 | SNP | Rv3683 | Asp186Tyr | 0 | - | | 4135112 | G | A | 113.03 | SNP | Rv3693 | Met(s)129Ile | 2 | - | | 4142744 | G | T | 31.77 | SNP | Rv3699 | STOP234Leu | 3 | - | | 4143321 | C | A | 42.77 | SNP | Rv3700c | Val(s)200Val | 13 | - | | 4144077 | C | A | 30.77 | SNP | Rv3701c | Leu(s)280Phe | 1 | - | | 4146330 | A | G | 201.84 | SNP | Rv3703c | Leu(s)188Leu | 3 | - | | 4148286 | G | T | 33.77 | SNP | intergenic |  |  | - | | 4155152 | G | T | 44.77 | SNP | Rv3710 (leuA) | Glu471Asp | 53 | - | | 4156099 | C | A | 77.28 | SNP | Rv3711c (dnaQ) | Val(s)211Leu(s) | 9867 | - | | 4157176 | A | G | 95.28 | SNP | Rv3712 | Thr66Ala | 32 | - | | 4162339 | A | G | 184.90 | SNP | Rv3719 | Thr12Ala | 32 | - | | 4163922 | G | T | 38.77 | SNP | Rv3720 | Asp63Tyr | 0 | - | | 4163944 | A | G | 173.90 | SNP | Rv3720 | His70Arg | 10 | - | | 4166294 | G | T | 43.77 | SNP | Rv3721c (dnaZX) | silent (Pro146) | 9926 | - | | 4166432 | C | A | 45.77 | SNP | Rv3721c (dnaZX) | Val(s)100Val | 13 | - | | 4173551 | G | T | 33.77 | SNP | Rv3727 | Gln199His | 20 | - | | 4173672 | C | A | 36.77 | SNP | Rv3727 | Leu240Ile | 9 | - | | 4174564 | T | C | 217.53 | SNP | Rv3727 | Ile537Thr | 11 | - | | 4174775 | C | A | 46.77 | SNP | intergenic |  |  | - | | 4175207 | G | T | 59.74 | SNP | Rv3728 | Gly112Val(s) | 21 | - | | 4182695 | G | A | 103.28 | SNP | Rv3731 (ligC) | Arg313His | 8 | - | | 4187485 | T | C | 223.84 | SNP | Rv3736 | silent (Ala284) | 9867 | - | | 4187817 | A | G | 53.74 | SNP | Rv3737 | Asp40Gly | 11 | - | | 4189980 | C | A | 36.77 | SNP | Rv3738c (PPE66) | Gly85Cys | 0 | - | | 4197138 | C | CT | 243.14 | INS | intergenic |  |  | - | | 4198611 | CG | C | 254.75 | DEL | intergenic |  |  | - | | 4201274 | G | T | 47.78 | SNP | Rv3754 (tyrA) | Arg285Leu | 1 | - | | 4204441 | A | G | 272.78 | SNP | Rv3759c (proX) | silent (His311) | 9912 | - | | 4210274 | A | G | 50.74 | SNP | Rv3764c (tcrY) | Cys246Arg | 1 | - | | 4210353 | C | A | 35.77 | SNP | Rv3764c (tcrY) | Val(s)219Val | 13 | - | | 4214963 | G | A | 171.90 | SNP | intergenic |  |  | - | | 4218350 | T | C | 577.77 | SNP | Rv3773c | Lys159Arg | 19 | - | | 4220174 | G | A | 472.77 | SNP | Rv3775 (lipE) | Asp164Asn | 36 | - | | 4221490 | C | G | 613.77 | SNP | Rv3776 | silent (Leu134) | 9947 | - | | 4222073 | A | G | 264.78 | SNP | Rv3776 | Met(s)329Val(s) | 9867 | - | | 4222882 | A | G | 499.77 | SNP | Rv3777 | silent (Leu63) | 9947 | - | | 4226700 | C | G | 697.77 | SNP | Rv3779 | His572Gln | 23 | - | | 4227572 | G | T | 46.77 | SNP | Rv3781 (rfbE) | Trp15Leu(s) | 0 | - | | 4233299 | G | A | 527.77 | SNP | Rv3786c | Thr100Ile | 7 | - | | 4235959 | G | T | 46.77 | SNP | Rv3790 (dprE1) | Gly61Trp | 0 | - | | 4238682 | G | T | 38.77 | SNP | Rv3792 (aftA) | Asp251Tyr | 0 | - | | 4242643 | C | T | 271.78 | SNP | Rv3793 (embC) | silent (Arg927) | 9913 | genotype | | 4249742 | C | A | 32.77 | SNP | Rv3795 (embB) | Gln1077Lys | 12 | - | | 4251097 | G | T | 36.77 | SNP | Rv3797 (fadE35) | Asp5Tyr | 0 | - | | 4252546 | C | T | 569.77 | SNP | Rv3797 (fadE35) | Arg488Trp | 2 | - | | 4255196 | G | T | 45.77 | SNP | Rv3799c (accD4) | silent (Ser251) | 9840 | - | | 4255922 | A | G | 236.80 | SNP | Rv3799c (accD4) | silent (His9) | 9912 | - | | 4257220 | A | G | 49.74 | SNP | Rv3800c (pks13) | silent (Arg1309) | 9913 | - | | 4257843 | C | A | 35.74 | SNP | Rv3800c (pks13) | Gly1102Cys | 0 | - | | 4264218 | C | A | 183.82 | SNP | Rv3802c | Val50Phe | 0 | - | | 4264219 | T | G | 231.80 | SNP | Rv3802c | silent (Gly49) | 9935 | - | | 4264410 | C | G | 218.84 | SNP | intergenic |  |  | - | | 4274251 | G | T | 45.77 | SNP | Rv3810 (pirG) | silent (Leu171) | 9947 | - | | 4288405 | G | T | 86.28 | SNP | Rv3823c (mmpL8) | Ala1042Glu | 10 | - | | 4289741 | C | A | 38.77 | SNP | Rv3823c (mmpL8) | Asp597Tyr | 0 | - | | 4293072 | G | A | 92.28 | SNP | Rv3824c (papA1) | Leu35Phe | 6 | - | | 4302036 | T | C | 320.78 | SNP | Rv3827c | Thr252Ala | 32 | - | | 4306155 | C | T | 174.90 | SNP | Rv3831 | silent (Ser133) | 9840 | - | | 4307179 | G | A | 149.90 | SNP | Rv3833 | Val105Ile | 33 | - | | 4309229 | G | T | 35.77 | SNP | Rv3835 | silent (Ala61) | 9867 | - | | 4311871 | G | A | 59.28 | SNP | Rv3838c (pheA) | His267Tyr | 4 | - | | 4315384 | T | C | 96.28 | SNP | Rv3842c (glpQ1) | Asp60Gly | 11 | - | | 4315614 | G | T | 48.77 | SNP | Rv3843c | Ala328Glu | 10 | - | | 4317407 | G | T | 34.77 | SNP | intergenic |  |  | - | | 4319652 | G | A | 294.78 | SNP | intergenic |  |  | - | | 4320050 | A | G | 99.28 | SNP | intergenic |  |  | - | | 4326811 | G | T | 70.28 | SNP | Rv3854c (ethA) | silent (Ile221) | 9872 | - | | 4326812 | A | T | 85.28 | SNP | Rv3854c (ethA) | Ile221Asn | 3 | - | | 4327689 | C | A | 39.77 | SNP | Rv3855 (ethR) | silent (Val47) | 9901 | - | | 4328019 | C | T | 60.74 | SNP | Rv3855 (ethR) | silent (Arg157) | 9913 | - | | 4336090 | A | AT | 339.75 | INS | intergenic |  |  | - | | 4337923 | G | T | 35.77 | SNP | Rv3860 | Asp383Tyr | 0 | - | | 4338208 | G | T | 42.77 | SNP | Rv3861; Rv3862c (whiB6) | Arg88Leu; Pro105Gln | 1; 6 | - | | 4338595 | GC | G | 323.75 | DEL | intergenic |  |  | - | | 4338732 | G | A | 114.90 | SNP | intergenic |  |  | - | | 4340330 | T | G | 325.78 | SNP | Rv3864 (espE) | Leu(s)21Val(s) | 9867 | - | | 4342973 | G | T | 30.77 | SNP | Rv3867 (espH) | Val(s)68Val | 13 | - | | 4345329 | C | T | 275.78 | SNP | Rv3869 (eccB1) | silent (Asn97) | 9822 | - | | 4346727 | G | T | 37.77 | SNP | Rv3870 (eccCa1) | Gly83Cys | 0 | - | | 4347130 | T | C | 129.03 | SNP | Rv3870 (eccCa1) | Leu217Pro | 2 | - | | 4347381 | G | T | 54.77 | SNP | Rv3870 (eccCa1) | Asp301Tyr | 0 | - | | 4351039 | G | T | 89.28 | SNP | Rv3872 (PE35) | Glu99STOP | 17 | - | | 4356110 | G | C | 274.80 | SNP | Rv3877 (eccD1) | silent (Leu368) | 9947 | - | | 4356516 | G | T | 43.77 | SNP | Rv3877 (eccD1) | Asp504Tyr | 0 | - | | 4357303 | C | A | 36.77 | SNP | Rv3878 (espJ) | Pro204Gln | 6 | - | | 4358304 | G | T | 35.77 | SNP | Rv3879c (espK) | silent (Ile493) | 9872 | - | | 4359195 | G | A | 118.03 | SNP | Rv3879c (espK) | silent (Gly196) | 9935 | - | | 4366113 | C | A | 46.77 | SNP | Rv3884c (eccA2) | silent (Arg242) | 9913 | - | | 4372661 | T | C | 90.28 | SNP | Rv3888c | Ile16Val | 57 | - | | 4372913 | G | A | 59.74 | SNP | Rv3889c (espG2) | Arg240Cys | 1 | - | | 4373475 | C | G | 126.03 | SNP | Rv3889c (espG2) | Val(s)52Val | 13 | - | | 4373496 | C | G | 130.03 | SNP | Rv3889c (espG2) | Val(s)45Val | 13 | - | | 4374799 | C | A | 36.77 | SNP | Rv3892c (PPE69) | silent (Ser295) | 9840 | - | | 4375628 | G | T | 60.74 | SNP | Rv3892c (PPE69) | Thr19Lys | 11 | - | | 4377447 | G | A | 192.84 | SNP | Rv3894c (eccC2) | silent (Asp1002) | 9859 | - | | 4379680 | C | G | 295.78 | SNP | Rv3894c (eccC2) | Arg258Pro | 5 | - | | 4380720 | C | A | 41.77 | SNP | Rv3895c (eccB2) | silent (Ser407) | 9840 | - | | 4382054 | T | C | 142.03 | SNP | Rv3896c | silent (Ala266) | 9867 | - | | 4382275 | G | T | 125.03 | SNP | Rv3896c | Gln193Lys | 12 | - | | 4383094 | A | G | 83.28 | SNP | Rv3897c | Cys183Arg | 1 | - | | 4391553 | C | T | 316.77 | SNP | Rv3906c | silent (Pro18) | 9926 | - | | 4395964 | C | A | 78.28 | SNP | Rv3909 | silent (Thr591) | 9871 | - | | 4400040 | C | A | 35.77 | SNP | Rv3910 | silent (Val1148) | 9901 | - | | 4400660 | AC | A | 164.87 | DEL | Rv3911 (sigM) |  |  | - | | 4403900 | A | G | 128.03 | SNP | Rv3915 | Met(s)237Val(s) | 9867 | - | | 4407980 | G | C | 253.53 | SNP | Rv3919c (gid) | Pro75Ala | 22 | - | | 4408156 | A | C | 202.31 | SNP | Rv3919c (gid) | Leu16Arg | 1 | genotype | | 4409893 | G | T | 30.77 | SNP | Rv3921c | Phe59Leu | 13 | - | |  | | export |

elog
