# Supplementary material for: Patterns of compensatory mutations in rpoA/B/C genes of multidrug resistant M. tuberculosis in Uganda
Source: PLoS One. 2025 Dec 4;20(12):e0328957. doi: 10.1371/journal.pone.0328957 (PMC12677784; doi:10.1371/journal.pone.0328957)
Supplement: S2 File — (ZIP) [file pone.0328957.s002.zip › Variants L_S12_L001_001.bam.html]

 

Calling SNPs/INDELs (computing variant list in .vcf format) from L\_S12\_L001\_001.bam

*by SAMtools/BCFtools:*

Howto

Important aspects

This takes up to one hour!!! **Please wait ...**

Variants L\_S12\_L001\_001.bam

|  |  |
| --- | --- |
| Variants |  |

|  |  |
| --- | --- |
| |  | | --- | | *by GATK* | |

|  |  |  |
| --- | --- | --- |
| |  | | --- | | L\_S12\_L001\_001.bam | | | computed 2016-10-27 using PhyResSE v1.0 (Ref. NC\_000962.3) | |

|  |  |
| --- | --- |
| 1428  variants called Export in VCF format |  |

|  |  |  |  |  |  |  |  |  |  |  |  |  |  |  |  |  |  |  |  |  |  |  |  |  |  |  |  |  |  |  |  |  |  |  |  |  |  |  |  |  |  |  |  |  |  |  |  |  |  |  |  |  |  |  |  |  |  |  |  |  |  |  |  |  |  |  |  |  |  |  |  |  |  |  |  |  |  |  |  |  |  |  |  |  |  |  |  |  |  |  |  |  |  |  |  |  |  |  |  |  |  |  |  |  |  |  |  |  |  |  |  |  |  |  |  |  |  |  |  |  |  |  |  |  |  |  |  |  |  |  |  |  |  |  |  |  |  |  |  |  |  |  |  |  |  |  |  |  |  |  |  |  |  |  |  |  |  |  |  |  |  |  |  |  |  |  |  |  |  |  |  |  |  |  |  |  |  |  |  |  |  |  |  |  |  |  |  |  |  |  |  |  |  |  |  |  |  |  |  |  |  |  |  |  |  |  |  |  |  |  |  |  |  |  |  |  |  |  |  |  |  |  |  |  |  |  |  |  |  |  |  |  |  |  |  |  |  |  |  |  |  |  |  |  |  |  |  |  |  |  |  |  |  |  |  |  |  |  |  |  |  |  |  |  |  |  |  |  |  |  |  |  |  |  |  |  |  |  |  |  |  |  |  |  |  |  |  |  |  |  |  |  |  |  |  |  |  |  |  |  |  |  |  |  |  |  |  |  |  |  |  |  |  |  |  |  |  |  |  |  |  |  |  |  |  |  |  |  |  |  |  |  |  |  |  |  |  |  |  |  |  |  |  |  |  |  |  |  |  |  |  |  |  |  |  |  |  |  |  |  |  |  |  |  |  |  |  |  |  |  |  |  |  |  |  |  |  |  |  |  |  |  |  |  |  |  |  |  |  |  |  |  |  |  |  |  |  |  |  |  |  |  |  |  |  |  |  |  |  |  |  |  |  |  |  |  |  |  |  |  |  |  |  |  |  |  |  |  |  |  |  |  |  |  |  |  |  |  |  |  |  |  |  |  |  |  |  |  |  |  |  |  |  |  |  |  |  |  |  |  |  |  |  |  |  |  |  |  |  |  |  |  |  |  |  |  |  |  |  |  |  |  |  |  |  |  |  |  |  |  |  |  |  |  |  |  |  |  |  |  |  |  |  |  |  |  |  |  |  |  |  |  |  |  |  |  |  |  |  |  |  |  |  |  |  |  |  |  |  |  |  |  |  |  |  |  |  |  |  |  |  |  |  |  |  |  |  |  |  |  |  |  |  |  |  |  |  |  |  |  |  |  |  |  |  |  |  |  |  |  |  |  |  |  |  |  |  |  |  |  |  |  |  |  |  |  |  |  |  |  |  |  |  |  |  |  |  |  |  |  |  |  |  |  |  |  |  |  |  |  |  |  |  |  |  |  |  |  |  |  |  |  |  |  |  |  |  |  |  |  |  |  |  |  |  |  |  |  |  |  |  |  |  |  |  |  |  |  |  |  |  |  |  |  |  |  |  |  |  |  |  |  |  |  |  |  |  |  |  |  |  |  |  |  |  |  |  |  |  |  |  |  |  |  |  |  |  |  |  |  |  |  |  |  |  |  |  |  |  |  |  |  |  |  |  |  |  |  |  |  |  |  |  |  |  |  |  |  |  |  |  |  |  |  |  |  |  |  |  |  |  |  |  |  |  |  |  |  |  |  |  |  |  |  |  |  |  |  |  |  |  |  |  |  |  |  |  |  |  |  |  |  |  |  |  |  |  |  |  |  |  |  |  |  |  |  |  |  |  |  |  |  |  |  |  |  |  |  |  |  |  |  |  |  |  |  |  |  |  |  |  |  |  |  |  |  |  |  |  |  |  |  |  |  |  |  |  |  |  |  |  |  |  |  |  |  |  |  |  |  |  |  |  |  |  |  |  |  |  |  |  |  |  |  |  |  |  |  |  |  |  |  |  |  |  |  |  |  |  |  |  |  |  |  |  |  |  |  |  |  |  |  |  |  |  |  |  |  |  |  |  |  |  |  |  |  |  |  |  |  |  |  |  |  |  |  |  |  |  |  |  |  |  |  |  |  |  |  |  |  |  |  |  |  |  |  |  |  |  |  |  |  |  |  |  |  |  |  |  |  |  |  |  |  |  |  |  |  |  |  |  |  |  |  |  |  |  |  |  |  |  |  |  |  |  |  |  |  |  |  |  |  |  |  |  |  |  |  |  |  |  |  |  |  |  |  |  |  |  |  |  |  |  |  |  |  |  |  |  |  |  |  |  |  |  |  |  |  |  |  |  |  |  |  |  |  |  |  |  |  |  |  |  |  |  |  |  |  |  |  |  |  |  |  |  |  |  |  |  |  |  |  |  |  |  |  |  |  |  |  |  |  |  |  |  |  |  |  |  |  |  |  |  |  |  |  |  |  |  |  |  |  |  |  |  |  |  |  |  |  |  |  |  |  |  |  |  |  |  |  |  |  |  |  |  |  |  |  |  |  |  |  |  |  |  |  |  |  |  |  |  |  |  |  |  |  |  |  |  |  |  |  |  |  |  |  |  |  |  |  |  |  |  |  |  |  |  |  |  |  |  |  |  |  |  |  |  |  |  |  |  |  |  |  |  |  |  |  |  |  |  |  |  |  |  |  |  |  |  |  |  |  |  |  |  |  |  |  |  |  |  |  |  |  |  |  |  |  |  |  |  |  |  |  |  |  |  |  |  |  |  |  |  |  |  |  |  |  |  |  |  |  |  |  |  |  |  |  |  |  |  |  |  |  |  |  |  |  |  |  |  |  |  |  |  |  |  |  |  |  |  |  |  |  |  |  |  |  |  |  |  |  |  |  |  |  |  |  |  |  |  |  |  |  |  |  |  |  |  |  |  |  |  |  |  |  |  |  |  |  |  |  |  |  |  |  |  |  |  |  |  |  |  |  |  |  |  |  |  |  |  |  |  |  |  |  |  |  |  |  |  |  |  |  |  |  |  |  |  |  |  |  |  |  |  |  |  |  |  |  |  |  |  |  |  |  |  |  |  |  |  |  |  |  |  |  |  |  |  |  |  |  |  |  |  |  |  |  |  |  |  |  |  |  |  |  |  |  |  |  |  |  |  |  |  |  |  |  |  |  |  |  |  |  |  |  |  |  |  |  |  |  |  |  |  |  |  |  |  |  |  |  |  |  |  |  |  |  |  |  |  |  |  |  |  |  |  |  |  |  |  |  |  |  |  |  |  |  |  |  |  |  |  |  |  |  |  |  |  |  |  |  |  |  |  |  |  |  |  |  |  |  |  |  |  |  |  |  |  |  |  |  |  |  |  |  |  |  |  |  |  |  |  |  |  |  |  |  |  |  |  |  |  |  |  |  |  |  |  |  |  |  |  |  |  |  |  |  |  |  |  |  |  |  |  |  |  |  |  |  |  |  |  |  |  |  |  |  |  |  |  |  |  |  |  |  |  |  |  |  |  |  |  |  |  |  |  |  |  |  |  |  |  |  |  |  |  |  |  |  |  |  |  |  |  |  |  |  |  |  |  |  |  |  |  |  |  |  |  |  |  |  |  |  |  |  |  |  |  |  |  |  |  |  |  |  |  |  |  |  |  |  |  |  |  |  |  |  |  |  |  |  |  |  |  |  |  |  |  |  |  |  |  |  |  |  |  |  |  |  |  |  |  |  |  |  |  |  |  |  |  |  |  |  |  |  |  |  |  |  |  |  |  |  |  |  |  |  |  |  |  |  |  |  |  |  |  |  |  |  |  |  |  |  |  |  |  |  |  |  |  |  |  |  |  |  |  |  |  |  |  |  |  |  |  |  |  |  |  |  |  |  |  |  |  |  |  |  |  |  |  |  |  |  |  |  |  |  |  |  |  |  |  |  |  |  |  |  |  |  |  |  |  |  |  |  |  |  |  |  |  |  |  |  |  |  |  |  |  |  |  |  |  |  |  |  |  |  |  |  |  |  |  |  |  |  |  |  |  |  |  |  |  |  |  |  |  |  |  |  |  |  |  |  |  |  |  |  |  |  |  |  |  |  |  |  |  |  |  |  |  |  |  |  |  |  |  |  |  |  |  |  |  |  |  |  |  |  |  |  |  |  |  |  |  |  |  |  |  |  |  |  |  |  |  |  |  |  |  |  |  |  |  |  |  |  |  |  |  |  |  |  |  |  |  |  |  |  |  |  |  |  |  |  |  |  |  |  |  |  |  |  |  |  |  |  |  |  |  |  |  |  |  |  |  |  |  |  |  |  |  |  |  |  |  |  |  |  |  |  |  |  |  |  |  |  |  |  |  |  |  |  |  |  |  |  |  |  |  |  |  |  |  |  |  |  |  |  |  |  |  |  |  |  |  |  |  |  |  |  |  |  |  |  |  |  |  |  |  |  |  |  |  |  |  |  |  |  |  |  |  |  |  |  |  |  |  |  |  |  |  |  |  |  |  |  |  |  |  |  |  |  |  |  |  |  |  |  |  |  |  |  |  |  |  |  |  |  |  |  |  |  |  |  |  |  |  |  |  |  |  |  |  |  |  |  |  |  |  |  |  |  |  |  |  |  |  |  |  |  |  |  |  |  |  |  |  |  |  |  |  |  |  |  |  |  |  |  |  |  |  |  |  |  |  |  |  |  |  |  |  |  |  |  |  |  |  |  |  |  |  |  |  |  |  |  |  |  |  |  |  |  |  |  |  |  |  |  |  |  |  |  |  |  |  |  |  |  |  |  |  |  |  |  |  |  |  |  |  |  |  |  |  |  |  |  |  |  |  |  |  |  |  |  |  |  |  |  |  |  |  |  |  |  |  |  |  |  |  |  |  |  |  |  |  |  |  |  |  |  |  |  |  |  |  |  |  |  |  |  |  |  |  |  |  |  |  |  |  |  |  |  |  |  |  |  |  |  |  |  |  |  |  |  |  |  |  |  |  |  |  |  |  |  |  |  |  |  |  |  |  |  |  |  |  |  |  |  |  |  |  |  |  |  |  |  |  |  |  |  |  |  |  |  |  |  |  |  |  |  |  |  |  |  |  |  |  |  |  |  |  |  |  |  |  |  |  |  |  |  |  |  |  |  |  |  |  |  |  |  |  |  |  |  |  |  |  |  |  |  |  |  |  |  |  |  |  |  |  |  |  |  |  |  |  |  |  |  |  |  |  |  |  |  |  |  |  |  |  |  |  |  |  |  |  |  |  |  |  |  |  |  |  |  |  |  |  |  |  |  |  |  |  |  |  |  |  |  |  |  |  |  |  |  |  |  |  |  |  |  |  |  |  |  |  |  |  |  |  |  |  |  |  |  |  |  |  |  |  |  |  |  |  |  |  |  |  |  |  |  |  |  |  |  |  |  |  |  |  |  |  |  |  |  |  |  |  |  |  |  |  |  |  |  |  |  |  |  |  |  |  |  |  |  |  |  |  |  |  |  |  |  |  |  |  |  |  |  |  |  |  |  |  |  |  |  |  |  |  |  |  |  |  |  |  |  |  |  |  |  |  |  |  |  |  |  |  |  |  |  |  |  |  |  |  |  |  |  |  |  |  |  |  |  |  |  |  |  |  |  |  |  |  |  |  |  |  |  |  |  |  |  |  |  |  |  |  |  |  |  |  |  |  |  |  |  |  |  |  |  |  |  |  |  |  |  |  |  |  |  |  |  |  |  |  |  |  |  |  |  |  |  |  |  |  |  |  |  |  |  |  |  |  |  |  |  |  |  |  |  |  |  |  |  |  |  |  |  |  |  |  |  |  |  |  |  |  |  |  |  |  |  |  |  |  |  |  |  |  |  |  |  |  |  |  |  |  |  |  |  |  |  |  |  |  |  |  |  |  |  |  |  |  |  |  |  |  |  |  |  |  |  |  |  |  |  |  |  |  |  |  |  |  |  |  |  |  |  |  |  |  |  |  |  |  |  |  |  |  |  |  |  |  |  |  |  |  |  |  |  |  |  |  |  |  |  |  |  |  |  |  |  |  |  |  |  |  |  |  |  |  |  |  |  |  |  |  |  |  |  |  |  |  |  |  |  |  |  |  |  |  |  |  |  |  |  |  |  |  |  |  |  |  |  |  |  |  |  |  |  |  |  |  |  |  |  |  |  |  |  |  |  |  |  |  |  |  |  |  |  |  |  |  |  |  |  |  |  |  |  |  |  |  |  |  |  |  |  |  |  |  |  |  |  |  |  |  |  |  |  |  |  |  |  |  |  |  |  |  |  |  |  |  |  |  |  |  |  |  |  |  |  |  |  |  |  |  |  |  |  |  |  |  |  |  |  |  |  |  |  |  |  |  |  |  |  |  |  |  |  |  |  |  |  |  |  |  |  |  |  |  |  |  |  |  |  |  |  |  |  |  |  |  |  |  |  |  |  |  |  |  |  |  |  |  |  |  |  |  |  |  |  |  |  |  |  |  |  |  |  |  |  |  |  |  |  |  |  |  |  |  |  |  |  |  |  |  |  |  |  |  |  |  |  |  |  |  |  |  |  |  |  |  |  |  |  |  |  |  |  |  |  |  |  |  |  |  |  |  |  |  |  |  |  |  |  |  |  |  |  |  |  |  |  |  |  |  |  |  |  |  |  |  |  |  |  |  |  |  |  |  |  |  |  |  |  |  |  |  |  |  |  |  |  |  |  |  |  |  |  |  |  |  |  |  |  |  |  |  |  |  |  |  |  |  |  |  |  |  |  |  |  |  |  |  |  |  |  |  |  |  |  |  |  |  |  |  |  |  |  |  |  |  |  |  |  |  |  |  |  |  |  |  |  |  |  |  |  |  |  |  |  |  |  |  |  |  |  |  |  |  |  |  |  |  |  |  |  |  |  |  |  |  |  |  |  |  |  |  |  |  |  |  |  |  |  |  |  |  |  |  |  |  |  |  |  |  |  |  |  |  |  |  |  |  |  |  |  |  |  |  |  |  |  |  |  |  |  |  |  |  |  |  |  |  |  |  |  |  |  |  |  |  |  |  |  |  |  |  |  |  |  |  |  |  |  |  |  |  |  |  |  |  |  |  |  |  |  |  |  |  |  |  |  |  |  |  |  |  |  |  |  |  |  |  |  |  |  |  |  |  |  |  |  |  |  |  |  |  |  |  |  |  |  |  |  |  |  |  |  |  |  |  |  |  |  |  |  |  |  |  |  |  |  |  |  |  |  |  |  |  |  |  |  |  |  |  |  |  |  |  |  |  |  |  |  |  |  |  |  |  |  |  |  |  |  |  |  |  |  |  |  |  |  |  |  |  |  |  |  |  |  |  |  |  |  |  |  |  |  |  |  |  |  |  |  |  |  |  |  |  |  |  |  |  |  |  |  |  |  |  |  |  |  |  |  |  |  |  |  |  |  |  |  |  |  |  |  |  |  |  |  |  |  |  |  |  |  |  |  |  |  |  |  |  |  |  |  |  |  |  |  |  |  |  |  |  |  |  |  |  |  |  |  |  |  |  |  |  |  |  |  |  |  |  |  |  |  |  |  |  |  |  |  |  |  |  |  |  |  |  |  |  |  |  |  |  |  |  |  |  |  |  |  |  |  |  |  |  |  |  |  |  |  |  |  |  |  |  |  |  |  |  |  |  |  |  |  |  |  |  |  |  |  |  |  |  |  |  |  |  |  |  |  |  |  |  |  |  |  |  |  |  |  |  |  |  |  |  |  |  |  |  |  |  |  |  |  |  |  |  |  |  |  |  |  |  |  |  |  |  |  |  |  |  |  |  |  |  |  |  |  |  |  |  |  |  |  |  |  |  |  |  |  |  |  |  |  |  |  |  |  |  |  |  |  |  |  |  |  |  |  |  |  |  |  |  |  |  |  |  |  |  |  |  |  |  |  |  |  |  |  |  |  |  |  |  |  |  |  |  |  |  |  |  |  |  |  |  |  |  |  |  |  |  |  |  |  |  |  |  |  |  |  |  |  |  |  |  |  |  |  |  |  |  |  |  |  |  |  |  |  |  |  |  |  |  |  |  |  |  |  |  |  |  |  |  |  |  |  |  |  |  |  |  |  |  |  |  |  |  |  |  |  |  |  |  |  |  |  |  |  |  |  |  |  |  |  |  |  |  |  |  |  |  |  |  |  |  |  |  |  |  |  |  |  |  |  |  |  |  |  |  |  |  |  |  |  |  |  |  |  |  |  |  |  |  |  |  |  |  |  |  |  |  |  |  |  |  |  |  |  |  |  |  |  |  |  |  |  |  |  |  |  |  |  |  |  |  |  |  |  |  |  |  |  |  |  |  |  |  |  |  |  |  |  |  |  |  |  |  |  |  |  |  |  |  |  |  |  |  |  |  |  |  |  |  |  |  |  |  |  |  |  |  |  |  |  |  |  |  |  |  |  |  |  |  |  |  |  |  |  |  |  |  |  |  |  |  |  |  |  |  |  |  |  |  |  |  |  |  |  |  |  |  |  |  |  |  |  |  |  |  |  |  |  |  |  |  |  |  |  |  |  |  |  |  |  |  |  |  |  |  |  |  |  |  |  |  |  |  |  |  |  |  |  |  |  |  |  |  |  |  |  |  |  |  |  |  |  |  |  |  |  |  |  |  |  |  |  |  |  |  |  |  |  |  |  |  |  |  |  |  |  |  |  |  |  |  |  |  |  |  |  |  |  |  |  |  |  |  |  |  |  |  |  |  |  |  |  |  |  |  |  |  |  |  |  |  |  |  |  |  |  |  |  |  |  |  |  |  |  |  |  |  |  |  |  |  |  |  |  |  |  |  |  |  |  |  |  |  |  |  |  |  |  |  |  |  |  |  |  |  |  |  |  |  |  |  |  |  |  |  |  |  |  |  |  |  |  |  |  |  |  |  |  |  |  |  |  |  |  |  |  |  |  |  |  |  |  |  |  |  |  |  |  |  |  |  |  |  |  |  |  |  |  |  |  |  |  |  |  |  |  |  |  |  |  |  |  |  |  |  |  |  |  |  |  |  |  |  |  |  |  |  |  |  |  |  |  |  |  |  |  |  |  |  |  |  |  |  |  |  |  |  |  |  |  |  |  |  |  |  |  |  |  |  |  |  |  |  |  |  |  |  |  |  |  |  |  |  |  |  |  |  |  |  |  |  |  |  |  |  |  |  |  |  |  |  |  |  |  |  |  |  |  |  |  |  |  |  |  |  |  |  |  |  |  |  |  |  |  |  |  |  |  |  |  |  |  |  |  |  |  |  |  |  |  |  |  |  |  |  |  |  |  |  |  |  |  |  |  |  |  |  |  |  |  |  |  |  |  |  |  |  |  |  |  |  |  |  |  |  |  |  |  |  |  |  |  |  |  |  |  |  |  |  |  |  |  |  |  |  |  |  |  |  |  |  |  |  |  |  |  |  |  |  |  |  |  |  |  |  |  |  |  |  |  |  |  |  |  |  |  |  |  |  |  |  |  |  |  |  |  |  |  |  |  |  |  |  |  |  |  |  |  |  |  |  |  |  |  |  |  |  |  |  |  |  |  |  |  |  |  |  |  |  |  |  |  |  |  |  |  |  |  |  |  |  |  |  |  |  |  |  |  |  |  |  |  |  |  |  |  |  |  |  |  |  |  |  |  |  |  |  |  |  |  |  |  |  |  |  |  |  |  |  |  |  |  |  |  |  |  |  |  |  |  |  |  |  |  |  |  |  |  |  |  |  |  |  |  |  |  |  |  |  |  |  |  |  |  |  |  |  |  |  |  |  |  |  |  |  |  |  |  |  |  |  |  |  |  |  |  |  |  |  |  |  |  |  |  |  |  |  |  |  |  |  |  |  |  |  |  |  |  |  |  |  |  |  |  |  |  |  |  |  |  |  |  |  |  |  |  |  |  |  |  |  |  |  |  |  |  |  |  |  |  |  |  |  |  |  |  |  |  |  |  |  |  |  |  |  |  |  |  |  |  |  |  |  |  |  |  |  |  |  |  |  |  |  |  |  |  |  |  |  |  |  |  |  |  |  |  |  |  |  |  |  |  |  |  |  |  |  |  |  |  |  |  |  |  |  |  |  |  |  |  |  |  |  |  |  |  |  |  |  |  |  |  |  |  |  |  |  |  |  |  |  |  |  |  |  |  |  |  |  |  |  |  |  |  |  |  |  |  |  |  |  |  |  |  |  |  |  |  |  |  |  |  |  |  |  |  |  |  |  |  |  |  |  |  |  |  |  |  |  |  |  |  |  |  |  |  |  |  |  |  |  |  |  |  |  |  |  |  |  |  |  |  |  |  |  |  |  |  |  |  |  |  |  |  |  |  |  |  |  |  |  |  |  |  |  |  |  |  |  |  |  |  |  |  |  |  |  |  |  |  |  |  |  |  |  |  |  |  |  |  |  |  |  |  |  |  |  |  |  |  |  |  |  |  |  |  |  |  |  |  |  |  |  |  |  |  |  |  |  |  |  |  |  |  |  |  |  |  |  |  |  |  |  |  |  |  |  |  |  |  |  |  |  |  |  |  |  |  |  |  |  |  |  |  |  |  |  |  |  |  |  |  |  |  |  |  |  |  |  |  |  |  |  |  |  |  |  |  |  |  |  |  |  |  |  |  |  |  |  |  |  |  |  |  |  |  |  |  |  |  |  |  |  |  |  |  |  |  |  |  |  |  |  |  |  |  |  |  |  |  |  |  |  |  |  |  |  |  |  |  |  |  |  |  |  |  |  |  |  |  |  |  |  |  |  |  |  |  |  |  |  |  |  |  |  |  |  |  |  |  |  |  |  |  |  |  |  |  |  |  |  |  |  |  |  |  |  |  |  |  |  |  |  |  |  |  |  |  |  |  |  |  |  |  |  |  |  |  |  |  |  |  |  |  |  |  |  |  |  |  |  |  |  |  |  |  |  |  |  |  |  |  |  |  |  |  |  |  |  |  |  |  |  |  |  |  |  |  |  |  |  |  |  |  |  |  |  |  |  |  |  |  |  |  |  |  |  |  |  |  |  |  |  |  |  |  |  |  |  |  |  |  |  |  |  |  |  |  |  |  |  |  |  |  |  |  |  |  |  |  |  |  |  |  |  |  |  |  |  |  |  |  |  |  |  |  |  |  |  |  |  |  |  |  |  |  |  |  |  |  |  |  |  |  |  |  |  |  |  |  |  |  |  |  |  |  |  |  |  |  |  |  |  |  |  |  |  |  |  |  |  |  |  |  |  |  |  |  |  |  |  |  |  |  |  |  |  |  |  |  |  |  |  |  |  |  |  |  |  |  |  |  |  |  |  |  |  |  |  |  |  |  |  |  |  |  |  |  |  |  |  |  |  |  |  |  |  |  |  |  |  |  |  |  |  |  |  |  |  |  |  |  |  |  |  |  |  |  |  |  |  |  |  |  |  |  |  |  |  |  |  |  |  |  |  |  |  |  |  |  |  |  |  |  |  |  |  |  |  |  |  |  |  |  |  |  |  |  |  |  |  |  |  |  |  |  |  |  |  |  |  |  |  |  |  |  |  |  |  |  |  |  |  |  |  |  |  |  |  |  |  |  |  |  |  |  |  |  |  |  |  |  |  |  |  |  |  |  |  |  |  |  |  |  |  |  |  |  |  |  |  |  |  |  |  |  |  |  |  |  |  |  |  |  |  |  |  |  |  |  |  |  |  |  |  |  |  |  |  |  |  |  |  |  |  |  |  |  |  |  |  |  |  |  |  |  |  |  |  |  |  |  |  |  |  |  |  |  |  |  |  |  |  |  |  |  |  |  |  |  |  |  |  |  |  |  |  |  |  |  |  |  |  |  |  |  |  |  |  |  |  |  |  |  |  |  |  |  |  |  |  |  |  |  |  |  |  |  |  |  |  |  |  |  |  |  |  |  |  |  |  |  |  |  |  |  |  |  |  |  |  |  |  |  |  |  |  |  |  |  |  |  |  |  |  |  |  |  |  |  |  |  |  |  |  |  |  |  |  |  |  |  |  |  |  |  |  |  |  |  |  |  |  |  |  |  |  |  |  |  |  |  |  |  |  |  |  |  |  |  |  |  |  |  |  |  |  |  |  |  |  |  |  |  |  |  |  |  |  |  |  |  |  |  |  |  |  |  |  |  |  |  |  |  |  |  |  |  |  |  |  |  |  |  |  |  |  |  |  |  |  |  |  |  |  |  |  |  |  |  |  |  |  |  |  |  |  |  |  |  |  |  |  |  |  |  |  |  |  |  |  |  |  |  |  |  |  |  |  |  |  |  |  |  |  |  |  |  |  |  |  |  |  |  |  |  |  |  |  |  |  |  |  |  |  |  |  |  |  |  |  |  |  |  |  |  |  |  |  |  |  |  |  |  |  |  |  |  |  |  |  |  |  |  |  |  |  |  |  |  |  |  |  |  |  |  |  |  |  |  |  |  |  |  |  |  |  |  |  |  |  |  |  |  |  |  |  |  |  |  |  |  |  |  |  |  |  |  |  |  |  |  |  |  |  |  |  |  |  |  |  |  |  |  |  |  |  |  |  |  |  |  |  |  |  |  |  |  |  |  |  |  |  |  |  |  |  |  |  |  |  |  |  |  |  |  |  |  |  |  |  |  |  |  |  |  |  |  |  |  |  |  |  |  |  |  |  |  |  |  |  |  |  |  |  |  |  |  |  |  |  |  |  |  |  |  |  |  |  |  |  |  |  |  |  |  |  |  |  |  |  |  |  |  |  |  |  |  |  |  |  |  |  |  |  |  |  |  |  |  |  |  |  |  |  |  |  |  |  |  |  |  |  |  |  |  |  |  |  |  |  |  |  |  |  |  |  |  |  |  |  |  |  |  |  |  |  |  |  |  |  |  |  |  |  |  |  |  |  |  |  |  |  |  |  |  |  |  |  |  |  |  |  |  |  |  |  |  |  |  |  |  |  |  |  |  |  |  |  |  |  |  |  |  |  |  |  |  |  |  |  |  |  |  |  |  |  |  |  |  |  |  |  |  |  |  |  |  |  |  |  |  |  |  |  |  |  |  |  |  |  |  |  |  |  |  |  |  |  |  |  |  |  |  |  |  |  |  |  |  |  |  |  |  |  |  |  |  |  |  |  |  |  |  |  |  |  |  |  |  |  |  |  |  |  |  |  |  |  |  |  |  |  |  |  |  |  |  |  |  |  |  |  |  |  |  |  |  |  |  |  |  |  |  |  |  |  |  |  |  |  |  |  |  |  |  |  |  |  |  |  |  |  |  |  |  |  |  |  |  |  |  |  |  |  |  |  |  |  |  |  |  |  |  |  |  |  |  |  |  |  |  |  |  |  |  |  |  |  |  |  |  |  |  |  |  |  |  |  |  |  |  |  |  |  |  |  |  |  |  |  |  |  |  |  |  |  |  |  |  |  |  |  |  |  |  |  |  |  |  |  |  |  |  |  |  |  |  |  |  |  |  |  |  |  |  |  |  |  |  |  |  |  |  |  |  |  |  |  |  |  |  |  |  |  |  |  |  |  |  |  |  |  |  |  |  |  |  |  |  |  |  |  |  |  |  |  |  |  |  |  |  |  |  |  |  |  |  |  |  |  |  |  |  |  |  |  |  |  |  |  |  |  |  |  |  |  |  |  |  |  |  |  |  |  |  |  |  |  |  |  |  |  |  |  |  |  |  |  |  |  |  |  |  |  |  |  |  |  |  |  |  |  |  |  |  |  |  |  |  |  |  |  |  |  |  |  |  |  |  |  |  |  |  |  |  |  |  |  |  |  |  |  |  |  |  |  |  |  |  |  |  |  |  |  |  |  |  |  |  |  |  |  |  |  |  |  |  |  |  |  |  |  |  |  |  |  |  |  |  |  |  |  |  |  |  |  |  |  |  |  |  |  |  |  |  |  |  |  |  |  |  |  |  |  |  |  |  |  |  |  |  |  |  |  |  |  |  |  |  |  |  |  |  |  |  |  |  |  |  |  |  |  |  |  |  |  |  |  |  |  |  |  |  |  |  |  |  |  |  |  |  |  |  |  |  |  |  |  |  |  |  |  |  |  |  |  |  |  |  |  |  |  |  |  |  |  |  |  |  |  |  |  |  |  |  |  |  |  |  |  |  |  |  |  |  |  |  |  |  |  |  |  |  |  |  |  |  |  |  |  |  |  |  |  |  |  |  |  |  |  |  |  |  |  |  |  |  |  |  |  |  |  |  |  |  |  |  |  |  |  |  |  |  |  |  |  |  |  |  |  |  |  |  |  |  |  |  |  |  |  |  |  |  |  |  |  |  |  |  |  |  |  |  |  |  |  |  |  |  |  |  |  |  |  |  |  |  |  |  |  |  |  |  |  |  |  |  |  |  |  |  |  |  |  |  |  |  |  |  |  |  |  |  |  |  |  |  |  |  |  |  |  |  |  |  |  |  |  |  |  |  |  |  |  |  |  |  |  |  |  |  |  |  |  |  |  |  |  |  |  |  |  |  |  |  |  |  |  |  |  |  |  |  |  |  |  |  |  |  |  |  |  |  |  |  |  |  |  |  |  |  |  |  |  |  |  |  |  |  |  |  |  |  |  |  |  |  |  |  |  |  |  |  |  |  |  |  |  |  |  |  |  |  |  |  |  |  |  |  |  |  |  |  |  |  |  |  |  |  |  |  |  |  |  |  |  |  |  |  |  |  |  |  |  |  |  |  |  |  |  |  |  |  |  |  |  |  |  |  |  |  |  |  |  |  |  |  |  |  |  |  |  |  |  |  |  |  |  |  |  |  |  |  |  |  |  |  |  |  |  |  |  |  |  |  |  |  |  |  |  |  |  |  |  |  |  |  |  |  |  |  |  |  |  |  |  |  |  |  |  |  |  |  |  |  |  |  |  |  |  |  |  |  |  |  |  |  |  |  |  |  |  |  |  |  |  |  |  |  |  |  |  |  |  |  |  |  |  |  |  |  |  |  |  |  |  |  |  |  |  |  |  |  |  |  |  |  |  |  |  |  |  |  |  |  |  |  |  |  |  |  |  |  |  |  |  |  |  |  |  |  |  |  |  |  |  |  |  |  |  |  |  |  |  |  |  |  |  |  |  |  |  |  |  |  |  |  |  |  |  |  |  |  |  |  |  |  |  |  |  |  |  |  |  |  |  |  |  |  |  |  |  |  |  |  |  |  |  |  |  |  |  |  |  |  |  |  |  |  |  |  |  |  |  |  |  |  |  |  |  |  |  |  |  |  |  |  |  |  |  |  |  |  |  |  |  |  |  |  |  |  |  |  |  |  |  |  |  |  |  |  |  |  |  |  |  |  |  |  |  |  |  |  |  |  |  |  |  |  |  |  |  |  |  |  |  |  |  |  |  |  |  |  |  |  |  |  |  |  |  |  |  |  |  |  |  |  |  |  |  |  |  |  |  |  |  |  |  |  |  |  |  |  |  |  |  |  |  |  |  |  |  |  |  |  |  |  |  |  |  |  |  |  |  |  |  |  |  |  |  |  |  |  |  |  |  |  |  |  |  |  |  |  |  |  |  |  |  |  |  |  |  |  |  |  |  |  |  |  |  |  |  |  |  |  |  |  |  |  |  |  |  |  |  |  |  |  |  |  |  |  |  |  |  |  |  |  |  |  |  |  |  |  |  |  |  |  |  |  |  |  |  |  |  |  |  |  |  |  |  |  |  |  |  |  |  |  |  |  |  |  |  |  |  |  |  |  |  |  |  |  |  |  |  |  |  |  |  |  |  |  |  |  |  |  |  |  |  |  |  |  |  |  |  |  |  |  |  |  |  |  |  |  |  |  |  |  |  |  |  |  |  |  |  |  |  |  |  |  |  |  |  |  |  |  |  |  |  |  |  |  |  |  |  |  |  |  |  |  |  |  |  |  |  |  |  |  |  |  |  |  |  |  |  |  |  |  |  |  |  |  |  |  |  |  |  |  |  |  |  |  |  |  |  |  |  |  |  |  |  |  |  |  |  |  |  |  |  |  |  |  |  |  |  |  |  |  |  |  |  |  |  |  |  |  |  |  |  |  |  |  |  |  |  |  |  |  |  |  |  |  |  |  |  |  |  |  |  |  |  |  |  |  |  |  |  |  |  |  |  |  |  |  |  |  |  |  |  |  |  |  |  |  |  |  |  |  |  |  |  |  |  |  |  |  |  |  |  |  |  |  |  |  |  |  |  |  |  |  |  |  |  |  |  |  |  |  |  |  |  |  |  |  |  |  |  |  |  |  |  |  |  |  |  |  |  |  |  |  |  |  |  |  |  |  |  |  |  |  |  |  |  |  |  |  |  |  |  |  |  |  |  |  |  |  |  |  |  |  |  |  |  |  |  |  |  |  |  |  |  |  |  |  |  |  |  |  |  |  |  |  |  |  |  |  |  |  |  |  |  |  |  |  |  |  |  |  |  |  |  |  |  |  |  |  |  |  |  |  |  |  |  |  |  |  |  |  |  |  |  |  |  |  |  |  |  |  |  |  |  |  |  |  |  |  |  |  |  |  |  |  |  |  |  |  |  |  |  |  |  |  |  |  |  |  |  |  |  |  |  |  |  |  |  |  |  |  |  |  |  |  |  |  |  |  |  |  |  |  |  |  |  |  |  |  |  |  |  |  |  |  |  |  |  |  |  |  |  |  |  |  |  |  |  |  |  |  |  |  |  |  |  |  |  |  |  |  |  |  |  |  |  |  |  |  |  |  |  |  |  |  |  |  |  |  |  |  |  |  |  |  |  |  |  |  |  |  |  |  |  |  |  |  |  |  |  |  |  |  |  |  |  |  |  |  |  |  |  |  |  |  |  |  |  |  |  |  |  |  |  |  |  |  |  |  |  |  |  |  |  |  |  |  |  |  |  |  |  |  |  |  |  |  |  |  |  |  |  |  |  |  |  |  |  |  |  |  |  |  |  |  |  |  |  |  |  |  |  |  |  |  |  |  |  |  |  |  |  |  |  |  |  |  |  |  |  |  |  |  |  |  |  |  |  |  |  |  |  |  |  |  |  |  |  |  |  |  |  |  |  |  |  |  |  |  |  |  |  |  |  |  |  |  |  |  |  |  |  |  |  |  |  |  |  |  |  |  |  |  |  |  |  |  |  |  |  |  |  |  |  |  |  |  |  |  |  |  |  |  |  |  |  |  |  |  |  |  |  |  |  |  |  |  |  |  |  |  |  |  |  |  |  |  |  |  |  |  |  |  |  |  |  |  |  |  |  |  |  |  |  |  |  |  |  |  |  |  |  |  |  |  |  |  |  |  |  |  |  |  |  |  |  |  |  |  |  |  |  |  |  |  |  |  |  |  |  |  |  |  |  |  |  |  |  |  |  |  |  |  |  |  |  |  |  |  |  |  |  |  |  |  |  |  |  |  |  |  |  |  |  |  |  |  |  |  |  |  |  |  |  |  |  |  |  |  |  |  |  |  |  |  |  |  |  |  |  |  |  |  |  |  |  |  |  |  |  |  |  |  |  |  |  |  |  |  |  |  |  |  |  |  |  |  |  |  |  |  |  |  |  |  |  |  |  |  |  |  |  |  |  |  |  |  |  |  |  |  |  |  |  |  |  |  |  |  |  |  |  |  |  |  |  |  |  |  |  |  |  |  |  |  |  |  |  |  |  |  |  |  |  |  |  |  |  |  |  |  |  |  |  |  |  |  |  |  |  |  |  |  |  |  |  |  |  |  |  |  |  |  |  |  |  |  |  |  |  |  |  |  |  |  |  |  |  |  |  |  |  |  |  |  |  |  |  |  |  |  |  |  |  |  |  |  |  |  |  |  |  |  |  |  |  |  |  |  |  |  |  |  |  |  |  |  |  |  |  |  |  |  |  |  |  |  |  |  |  |  |  |  |  |  |  |  |  |  |  |  |  |  |  |  |  |  |  |  |  |  |  |  |  |  |  |  |  |  |  |  |  |  |  |  |  |  |  |  |  |  |  |  |  |  |  |  |  |  |  |  |  |  |  |  |  |  |  |  |  |  |  |  |  |  |  |  |  |  |  |  |  |  |  |  |  |  |  |  |  |  |  |  |  |  |  |  |  |  |  |  |  |  |  |  |  |  |  |  |  |  |  |  |  |  |  |  |  |  |  |  |  |  |  |  |  |  |  |  |  |  |  |  |  |  |  |  |  |  |  |  |  |  |  |  |  |  |  |  |  |  |  |  |  |  |  |  |  |  |  |  |  |  |  |  |  |  |  |  |  |  |  |  |  |  |  |  |  |  |  |  |  |  |  |  |  |  |  |  |  |  |  |  |  |  |  |  |  |  |  |  |  |  |  |  |  |  |  |  |  |  |  |  |  |  |  |  |  |  |  |  |  |  |  |  |  |  |  |  |  |  |  |  |  |  |  |  |  |  |  |  |  |  |  |  |  |  |  |  |  |  |  |  |  |  |  |  |  |  |  |  |  |  |  |  |  |  |  |  |  |  |  |  |  |  |  |  |  |  |  |  |  |  |  |  |  |  |  |  |  |  |  |  |  |  |  |  |  |  |  |  |  |  |  |  |  |  |  |  |  |  |  |  |  |  |  |  |  |  |  |  |  |  |  |  |  |  |  |  |  |  |  |  |  |  |  |  |  |  |  |  |  |  |  |  |  |  |  |  |  |  |  |  |  |  |  |  |  |  |  |  |  |  |  |  |  |  |  |  |  |  |  |  |  |  |  |  |  |  |  |  |  |  |  |  |  |  |  |  |  |  |  |  |  |  |  |  |  |  |  |  |  |  |  |  |  |  |  |  |  |  |  |  |  |  |  |  |  |  |  |  |  |  |  |  |  |  |  |  |  |  |  |  |  |  |  |  |  |  |  |  |  |  |  |  |  |  |  |  |  |  |  |  |  |  |  |  |  |  |  |  |  |  |  |  |  |  |  |  |  |  |  |  |  |  |  |  |  |  |  |  |  |  |  |  |  |  |  |  |  |  |  |  |  |  |  |  |  |  |  |  |  |  |  |  |  |  |  |  |  |  |  |  |  |  |  |  |  |  |  |  |  |  |  |  |  |  |  |  |  |  |  |  |  |  |  |  |  |  |  |  |  |  |  |  |  |  |  |  |  |  |  |  |  |  |  |  |  |  |  |  |  |  |  |  |  |  |  |  |  |  |  |  |  |  |  |  |  |  |  |  |  |  |  |  |  |  |  |  |  |  |  |  |  |  |  |  |  |  |  |  |  |  |  |  |  |  |  |  |  |  |  |  |  |  |  |  |  |  |  |  |  |  |  |  |  |  |  |  |  |  |  |  |  |  |  |  |  |  |  |  |  |  |  |  |  |  |  |  |  |  |  |  |  |  |  |  |  |  |  |  |  |  |  |  |  |  |  |  |  |  |  |  |  |  |  |  |  |  |  |  |  |  |  |  |  |  |  |  |  |  |  |  |  |  |  |  |  |  |  |  |  |  |  |  |  |  |  |  |  |  |  |  |  |  |  |  |  |  |  |  |  |  |  |  |  |  |  |  |  |  |  |  |  |  |  |  |  |  |  |  |  |  |  |  |  |  |  |  |  |  |  |  |  |  |  |  |  |  |  |  |  |  |  |  |  |  |  |  |  |  |  |  |  |  |  |  |  |  |  |  |  |  |  |  |  |  |  |  |  |  |  |  |  |  |  |  |  |  |  |  |  |  |  |  |  |  |  |  |  |  |  |  |  |  |  |  |  |  |  |  |  |  |  |  |  |  |  |  |  |  |  |  |  |  |  |  |  |  |  |  |  |  |  |  |  |  |  |  |  |  |  |  |  |  |  |  |  |  |  |  |  |  |  |  |  |  |  |  |  |  |  |  |  |  |  |  |  |  |  |  |  |  |  |  |  |  |  |  |  |  |  |  |  |  |  |  |  |  |  |  |  |  |  |  |  |  |  |  |  |  |  |  |  |  |  |  |  |  |  |  |  |  |  |  |  |  |  |  |  |  |  |  |  |  |  |  |  |  |  |  |  |  |  |  |  |  |  |  |  |  |  |  |  |  |  |  |  |  |  |  |  |  |  |  |  |  |  |  |  |  |  |  |  |  |  |  |  |  |  |  |  |  |  |  |  |  |  |  |  |  |  |  |  |  |  |  |  |  |  |  |  |  |  |  |  |  |  |  |  |  |  |  |  |  |  |  |  |  |  |  |  |  |  |  |  |  |  |  |  |  |  |  |  |  |  |  |  |  |  |  |  |  |  |  |  |  |  |  |  |  |  |  |  |  |  |  |  |  |  |  |  |  |  |  |  |  |  |  |  |  |  |  |  |  |  |  |  |  |  |  |  |  |  |  |  |  |  |  |  |  |  |  |  |  |  |  |  |  |  |  |  |  |  |  |  |  |  |  |  |  |  |  |  |  |  |  |  |  |  |  |  |  |  |  |  |  |  |  |  |  |  |  |  |  |  |  |  |  |  |  |  |  |  |  |  |  |  |  |  |  |  |  |  |  |  |  |  |  |  |  |  |  |  |  |  |  |  |  |  |  |  |  |  |  |  |  |  |  |  |  |  |  |  |  |  |  |  |  |  |  |  |  |  |  |  |  |  |  |  |  |  |  |  |  |  |  |  |  |  |  |  |  |  |  |  |  |  |  |  |  |  |  |  |  |  |  |  |  |  |  |  |  |  |  |  |  |  |  |  |  |  |  |  |  |  |  |  |  |  |  |  |  |  |  |  |  |  |  |  |  |  |  |  |  |  |  |  |  |  |  |  |  |  |  |  |  |  |  |  |  |  |  |  |  |  |  |  |  |  |  |  |  |  |  |  |  |  |  |  |  |  |  |  |  |  |  |  |  |  |  |  |  |  |  |  |  |  |  |  |  |  |  |  |  |  |  |  |  |  |  |  |  |  |  |  |  |  |  |  |  |  |  |  |  |  |  |  |  |  |  |  |  |  |  |  |  |  |  |  |  |  |  |  |  |  |  |  |  |  |  |  |  |  |  |  |  |  |  |  |  |  |  |  |  |  |  |  |  |  |  |  |  |  |  |  |  |  |  |  |  |  |  |  |  |  |  |  |  |  |  |  |  |  |  |  |  |  |  |  |  |  |  |  |  |  |  |  |  |  |  |  |  |  |  |  |  |  |  |  |  |  |  |  |  |  |  |  |  |  |  |  |  |  |  |  |  |  |  |  |  |  |  |  |  |  |  |  |  |  |  |  |  |  |  |  |  |  |  |  |  |  |  |  |  |  |  |  |  |  |  |  |  |  |  |  |  |  |  |  |  |  |  |  |  |  |  |  |  |  |  |  |  |  |  |  |  |  |  |  |  |  |  |  |  |  |  |  |  |  |  |  |  |  |  |  |  |  |  |  |  |  |  |  |  |  |  |  |  |  |  |  |  |  |  |  |  |  |  |  |  |  |  |  |  |  |  |  |  |  |  |  |  |  |  |  |  |  |  |  |  |  |  |  |  |  |  |  |  |  |  |  |  |  |  |  |  |  |  |  |  |  |  |  |  |  |  |  |  |  |  |  |  |  |  |  |  |  |  |  |  |  |  |  |  |  |  |  |  |  |  |  |  |  |  |  |  |  |  |  |  |  |  |  |  |  |  |  |  |  |  |  |  |  |  |  |  |  |  |  |  |  |  |  |  |  |  |  |  |  |  |  |  |  |  |  |  |  |  |  |  |  |  |  |  |  |  |  |  |  |  |  |  |  |  |  |  |  |  |  |  |  |  |  |  |  |  |  |  |  |  |  |  |  |  |  |  |  |  |  |  |  |  |  |  |  |  |  |  |  |  |  |  |  |  |  |  |  |  |  |  |  |  |  |  |  |  |  |  |  |  |  |  |  |  |  |  |  |  |  |  |  |  |  |  |  |  |  |  |  |  |  |  |  |  |  |  |  |  |  |  |  |  |  |  |  |  |  |  |  |  |  |  |  |  |  |  |  |  |  |  |  |  |  |  |  |  |  |  |  |  |  |  |  |  |  |  |  |  |  |  |  |  |  |  |  |  |  |  |  |  |  |  |  |  |  |  |  |  |  |  |  |  |  |  |  |  |  |  |  |  |  |  |  |  |  |  |  |  |  |  |  |  |  |  |  |  |  |  |  |  |  |  |  |  |  |  |  |  |  |  |  |  |  |  |  |  |  |  |  |  |  |  |  |  |  |  |  |  |  |  |  |  |  |  |  |  |  |  |  |  |  |  |  |  |  |  |  |  |  |  |  |  |  |  |  |  |  |  |  |  |  |  |  |  |  |  |  |  |  |  |  |  |  |  |  |  |  |  |  |  |  |  |  |  |  |  |  |  |  |  |  |  |  |  |  |  |  |  |  |  |  |  |  |  |  |  |  |  |  |  |  |  |  |  |  |  |  |  |  |  |  |  |  |  |  |  |  |  |  |  |  |  |  |  |  |  |  |  |  |  |  |  |  |  |  |  |  |  |  |  |  |  |  |  |  |  |  |  |  |  |  |  |  |  |  |  |  |  |  |  |  |  |  |  |  |  |  |  |  |  |  |  |  |  |  |  |  |  |  |  |  |  |  |  |  |  |  |  |  |  |  |  |  |  |  |  |  |  |  |  |  |  |  |  |  |  |  |  |  |  |  |  |  |  |  |  |  |  |  |  |  |  |  |  |  |  |  |  |  |  |  |  |  |  |  |  |  |  |  |  |  |  |  |  |  |  |  |  |  |  |  |  |  |  |  |  |  |  |  |  |  |  |  |  |  |  |  |  |  |  |  |  |  |  |  |  |  |  |  |  |  |  |  |  |  |  |  |  |  |  |  |  |  |  |  |  |  |  |  |  |  |  |  |  |  |  |  |  |  |  |  |  |  |  |  |  |  |  |  |  |  |  |  |  |  |  |  |  |  |  |  |  |  |  |  |  |  |  |  |  |  |  |  |  |  |  |  |  |  |  |  |  |  |  |  |  |  |  |  |  |  |  |  |  |  |  |  |  |  |  |  |  |  |  |  |  |  |  |  |  |  |  |  |  |  |  |  |  |  |  |  |  |  |  |  |  |  |  |  |  |  |  |  |  |  |  |  |  |  |  |  |  |  |  |  |  |  |  |  |  |  |  |  |  |  |  |  |  |  |  |  |  |  |  |  |  |  |  |  |  |  |  |  |  |  |  |  |  |  |  |  |  |  |  |  |  |  |  |  |  |  |  |  |  |  |  |  |  |  |  |  |  |  |  |  |  |  |  |  |  |  |  |  |  |  |  |  |  |  |  |  |  |  |  |  |  |  |  |  |  |  |  |  |  |  |  |  |  |  |  |  |  |  |  |  |  |  |  |  |  |  |  |  |  |  |  |  |  |  |  |  |  |  |  |  |  |  |  |  |  |  |  |  |  |  |  |  |  |  |  |  |  |  |  |  |  |  |  |  |  |  |  |  |  |  |  |  |  |  |  |  |  |  |  |  |  |  |  |  |  |  |  |  |  |  |  |  |  |  |  |  |  |  |  |  |  |  |  |  |  |  |  |  |  |  |  |  |  |  |  |  |  |  |  |  |  |  |  |  |  |  |  |  |  |  |  |  |  |  |  |  |  |  |  |  |  |  |  |  |  |  |  |  |  |  |  |  |  |  |  |  |  |  |  |  |  |  |  |  |  |  |  |  |  |  |  |  |  |  |  |  |  |  |  |  |  |  |  |  |  |  |  |  |  |  |  |  |  |  |  |  |  |  |  |  |  |  |  |  |  |  |  |  |  |  |  |  |  |  |  |  |  |  |  |  |  |  |  |  |  |  |  |  |  |  |  |  |  |  |  |  |  |  |  |  |  |  |  |  |  |  |  |  |  |  |  |  |  |  |  |  |  |  |  |  |  |  |  |  |  |  |  |  |  |  |  |  |  |  |  |  |  |  |  |  |  |  |  |  |  |  |  |  |  |  |  |  |  |  |  |  |  |  |  |  |  |  |  |  |  |  |  |  |  |  |  |  |  |  |  |  |  |  |  |  |  |  |  |  |  |  |  |  |  |  |  |  |  |  |  |  |  |  |  |  |  |  |  |  |  |  |  |  |  |  |  |  |  |  |  |  |  |  |  |  |  |  |  |  |  |  |  |  |  |  |  |  |  |  |  |  |  |  |  |  |  |  |  |  |  |  |  |  |  |  |  |  |  |  |  |  |  |  |  |  |  |  |  |  |  |  |  |  |  |  |  |  |  |  |  |  |  |  |  |  |  |  |  |  |  |  |  |  |  |  |  |  |  |  |  |  |  |  |  |  |  |  |  |  |  |  |  |  |  |  |  |  |  |  |  |  |  |  |  |  |  |  |  |  |  |  |  |  |  |  |  |  |  |  |  |  |  |  |  |  |  |  |  |  |  |  |  |  |  |  |  |  |  |  |  |  |  |  |  |  |  |  |  |  |  |  |  |  |  |  |  |  |  |  |  |  |  |  |  |  |  |  |  |  |  |  |  |  |  |  |  |  |  |  |  |  |  |  |  |  |  |  |  |  |  |  |  |  |  |  |  |  |  |  |  |  |  |  |  |  |  |  |  |  |  |  |  |  |  |  |  |  |  |  |  |  |  |  |  |  |  |  |  |  |  |  |  |  |  |  |  |  |  |  |  |  |  |  |  |  |  |  |  |  |  |  |  |  |  |  |  |  |  |  |  |  |  |  |  |  |  |  |  |  |  |  |  |  |  |  |  |  |  |  |  |  |  |  |  |  |  |  |  |  |  |  |  |  |  |  |  |  |  |  |  |  |  |  |  |  |  |  |  |  |  |  |  |  |  |  |  |  |  |  |  |  |  |  |  |  |  |  |  |  |  |  |  |  |  |  |  |  |  |  |  |  |  |  |  |  |  |  |  |  |  |  |  |  |  |  |  |  |  |  |  |  |  |  |  |  |  |  |  |  |  |  |  |  |  |  |  |  |  |  |  |  |  |  |  |  |  |  |  |  |  |  |  |  |  |  |  |  |  |  |  |  |  |  |  |  |  |  |  |  |  |  |  |  |  |  |  |  |  |  |  |  |  |  |  |  |  |  |  |  |  |  |  |  |  |  |  |  |  |  |  |  |  |  |  |  |  |  |  |  |  |  |  |  |  |  |  |  |  |  |  |  |  |  |  |  |  |  |  |  |  |  |  |  |  |  |  |  |  |  |  |  |  |  |  |  |  |  |  |  |  |  |  |  |  |  |  |  |  |  |  |  |  |  |  |  |  |  |  |  |  |  |  |  |  |  |  |  |  |  |  |  |  |  |  |  |  |  |  |  |  |  |  |  |  |  |  |  |  |  |  |  |  |  |  |  |  |  |  |  |  |  |  |  |  |  |  |  |  |  |  |  |  |  |  |  |  |  |  |  |  |  |  |  |  |  |  |  |  |  |  |  |  |  |  |  |  |  |  |  |  |  |  |  |  |  |  |  |  |  |  |  |  |  |  |  |  |  |  |  |  |  |  |  |  |  |  |  |  |  |  |  |  |  |  |  |  |  |  |  |  |  |  |  |  |  |  |  |  |  |  |  |  |  |  |  |  |  |  |  |  |  |  |  |  |  |  |  |  |  |  |  |  |  |  |  |  |  |  |  |  |  |  |  |  |  |  |  |  |  |  |  |  |  |  |  |  |  |  |  |  |  |  |  |  |  |  |  |  |  |  |  |  |  |  |  |  |  |  |  |  |  |  |  |  |  |  |  |  |  |  |  |  |  |  |  |  |  |  |  |  |  |  |  |  |  |  |  |  |  |  |  |  |  |  |  |  |  |  |  |  |  |  |  |  |  |  |  |  |  |  |  |  |  |  |  |  |  |  |  |  |  |  |  |  |  |  |  |  |  |  |  |  |  |  |  |  |  |  |  |  |  |  |  |  |  |  |  |  |  |  |  |  |  |  |  |  |  |  |  |  |  |  |  |  |  |  |  |  |  |  |  |  |  |  |  |  |  |  |  |  |  |  |  |  |  |  |  |  |  |  |  |  |  |  |  |  |  |  |  |  |  |  |  |  |  |  |  |  |  |  |  |  |  |  |  |  |  |  |  |  |  |  |  |  |  |  |  |  |  |  |  |  |  |  |  |  |  |  |  |  |  |  |  |  |  |  |  |  |  |  |  |  |  |  |  |  |  |  |  |  |  |  |  |  |  |  |  |  |  |  |  |  |  |  |  |  |  |  |  |  |  |  |  |  |  |  |  |  |  |  |  |  |  |  |  |  |  |  |  |  |  |  |  |  |  |  |  |  |  |  |  |  |  |  |  |  |  |  |  |  |  |  |  |  |  |  |  |  |  |  |  |  |  |  |  |  |  |  |  |  |  |  |  |  |  |  |  |  |  |  |  |  |  |  |  |  |  |  |  |  |  |  |  |  |  |  |  |  |  |  |  |  |  |  |  |  |  |  |  |  |  |  |  |  |  |  |  |  |  |  |  |  |  |  |  |  |  |  |  |  |  |  |  |  |  |  |  |  |  |  |  |  |  |  |  |  |  |  |  |  |  |  |  |  |  |  |  |  |  |  |  |  |  |  |  |  |  |  |  |  |  |  |  |  |  |  |  |  |  |  |  |  |  |  |  |  |  |  |  |  |  |  |  |  |  |  |  |  |  |  |  |  |  |  |  |  |  |  |  |  |  |  |  |  |  |  |  |  |  |  |  |  |  |  |  |  |  |  |  |  |  |  |  |  |  |  |  |  |  |  |  |  |  |  |  |  |  |  |  |  |  |  |  |  |  |  |  |  |  |  |  |  |  |  |  |  |  |  |  |  |  |  |  |  |  |  |  |  |  |  |  |  |  |  |  |  |  |  |  |  |  |  |  |  |  |  |  |  |  |  |  |  |  |  |  |  |  |  |  |  |  |  |  |  |  |  |  |  |  |  |  |  |  |  |  |  |  |  |  |  |  |  |  |  |  |  |  |  |  |  |  |  |  |  |  |  |  |  |  |  |  |  |  |  |  |  |  |  |  |  |  |  |  |  |  |  |  |  |  |  |  |  |  |  |  |  |  |  |  |  |  |  |  |  |  |  |  |  |  |  |  |  |  |  |  |  |  |  |  |  |  |  |  |  |  |  |  |  |  |  |  |  |  |  |  |  |  |  |  |  |  |  |  |  |  |  |  |  |  |  |  |  |  |  |  |  |  |  |  |  |  |  |  |  |  |  |  |  |  |  |  |  |  |  |  |  |  |  |  |  |  |  |  |  |  |  |  |  |  |  |  |  |  |  |  |  |  |  |  |  |  |  |  |  |  |  |  |  |  |  |  |  |  |  |  |  |  |  |  |  |  |  |  |  |  |  |  |  |  |  |  |  |  |  |  |  |  |  |  |  |  |  |  |  |  |  |  |  |  |  |  |  |  |  |  |  |  |  |  |  |  |  |  |  |  |  |  |  |  |  |  |  |  |  |  |  |  |  |  |  |  |  |  |  |  |  |  |  |  |  |  |  |  |  |  |  |  |  |  |  |  |  |  |  |  |  |  |  |  |  |  |  |  |  |  |  |  |  |  |  |  |  |  |  |  |  |  |  |  |  |  |  |  |  |  |  |  |  |  |  |  |  |  |  |  |  |  |  |  |  |  |  |  |  |  |  |  |  |  |  |  |  |  |  |  |  |  |  |  |  |  |  |  |  |  |  |  |  |  |  |  |  |  |  |  |  |  |  |  |  |  |  |  |  |  |  |  |  |  |  |  |  |  |  |  |  |  |  |  |  |  |  |  |  |  |  |  |  |  |  |  |  |  |  |  |  |  |  |  |  |  |  |  |  |  |  |  |  |  |  |  |  |  |  |  |  |  |  |  |  |  |  |  |  |  |  |  |  |  |  |  |  |  |  |  |  |  |  |  |  |  |  |  |  |  |  |  |  |  |  |  |  |  |  |  |  |  |  |  |  |  |  |  |  |  |  |  |  |  |  |  |  |  |  |  |  |  |  |  |  |  |  |  |  |  |  |  |  |  |  |  |  |  |  |  |  |  |  |  |  |  |  |  |  |  |  |  |  |  |  |  |  |  |  |  |  |  |  |  |  |  |  |  |  |  |  |  |  |  |  |  |  |  |  |  |  |  |  |  |  |  |  |  |  |  |  |  |  |  |  |  |  |  |  |  |  |  |  |  |  |  |  |  |  |  |  |  |  |  |  |  |  |  |  |  |  |  |  |  |  |  |  |  |  |  |  |  |  |  |  |  |  |  |  |  |  |  |  |  |  |  |  |  |  |  |  |  |  |  |  |  |  |  |  |  |  |  |  |  |  |  |  |  |  |  |  |  |  |  |  |  |  |  |  |  |  |  |  |  |  |  |  |  |  |  |  |  |  |  |  |  |  |  |  |  |  |  |  |  |  |  |  |  |  |  |  |  |  |  |  |  |  |  |  |  |  |  |  |  |  |  |  |  |  |  |  |  |  |  |  |  |  |  |  |  |  |  |  |  |  |  |  |  |  |  |  |  |  |  |  |  |  |  |  |  |  |  |  |  |  |  |  |  |  |  |  |  |  |  |  |  |  |  |  |  |  |  |  |  |  |  |  |  |  |  |  |  |  |  |  |  |  |  |  |  |  |  |  |  |  |  |  |  |  |  |  |  |  |  |  |  |  |  |  |  |  |  |  |  |  |  |  |  |  |  |  |  |  |  |  |  |  |  |  |  |  |  |  |  |  |  |  |  |  |  |  |  |  |  |  |  |  |  |  |  |  |  |  |  |  |  |  |  |  |  |  |  |  |  |  |  |  |  |  |  |  |  |  |  |  |  |  |  |  |  |  |  |  |  |  |  |  |  |  |  |  |  |  |  |  |  |  |  |  |  |  |  |  |  |  |  |  |  |  |  |  |  |  |  |  |  |  |  |  |  |  |  |  |  |  |  |  |  |  |  |  |  |  |  |  |  |  |  |  |  |  |  |  |  |  |  |  |  |  |  |  |  |  |  |  |  |  |  |  |  |  |  |  |  |  |  |  |  |  |  |  |  |  |  |  |  |  |  |  |  |  |  |  |  |  |  |  |  |  |  |  |  |  |  |  |  |  |  |  |  |  |  |  |  |  |  |  |  |  |  |  |  |  |  |  |  |  |  |  |  |  |  |  |  |  |  |  |  |  |  |  |  |  |  |  |  |  |  |  |  |  |  |  |  |  |  |  |  |  |  |  |  |  |  |  |  |  |  |  |  |  |  |  |  |  |  |  |  |  |  |  |  |  |  |  |  |  |  |  |  |  |  |  |  |  |  |  |  |  |  |  |  |  |  |  |  |  |  |  |  |  |  |
| --- | --- | --- | --- | --- | --- | --- | --- | --- | --- | --- | --- | --- | --- | --- | --- | --- | --- | --- | --- | --- | --- | --- | --- | --- | --- | --- | --- | --- | --- | --- | --- | --- | --- | --- | --- | --- | --- | --- | --- | --- | --- | --- | --- | --- | --- | --- | --- | --- | --- | --- | --- | --- | --- | --- | --- | --- | --- | --- | --- | --- | --- | --- | --- | --- | --- | --- | --- | --- | --- | --- | --- | --- | --- | --- | --- | --- | --- | --- | --- | --- | --- | --- | --- | --- | --- | --- | --- | --- | --- | --- | --- | --- | --- | --- | --- | --- | --- | --- | --- | --- | --- | --- | --- | --- | --- | --- | --- | --- | --- | --- | --- | --- | --- | --- | --- | --- | --- | --- | --- | --- | --- | --- | --- | --- | --- | --- | --- | --- | --- | --- | --- | --- | --- | --- | --- | --- | --- | --- | --- | --- | --- | --- | --- | --- | --- | --- | --- | --- | --- | --- | --- | --- | --- | --- | --- | --- | --- | --- | --- | --- | --- | --- | --- | --- | --- | --- | --- | --- | --- | --- | --- | --- | --- | --- | --- | --- | --- | --- | --- | --- | --- | --- | --- | --- | --- | --- | --- | --- | --- | --- | --- | --- | --- | --- | --- | --- | --- | --- | --- | --- | --- | --- | --- | --- | --- | --- | --- | --- | --- | --- | --- | --- | --- | --- | --- | --- | --- | --- | --- | --- | --- | --- | --- | --- | --- | --- | --- | --- | --- | --- | --- | --- | --- | --- | --- | --- | --- | --- | --- | --- | --- | --- | --- | --- | --- | --- | --- | --- | --- | --- | --- | --- | --- | --- | --- | --- | --- | --- | --- | --- | --- | --- | --- | --- | --- | --- | --- | --- | --- | --- | --- | --- | --- | --- | --- | --- | --- | --- | --- | --- | --- | --- | --- | --- | --- | --- | --- | --- | --- | --- | --- | --- | --- | --- | --- | --- | --- | --- | --- | --- | --- | --- | --- | --- | --- | --- | --- | --- | --- | --- | --- | --- | --- | --- | --- | --- | --- | --- | --- | --- | --- | --- | --- | --- | --- | --- | --- | --- | --- | --- | --- | --- | --- | --- | --- | --- | --- | --- | --- | --- | --- | --- | --- | --- | --- | --- | --- | --- | --- | --- | --- | --- | --- | --- | --- | --- | --- | --- | --- | --- | --- | --- | --- | --- | --- | --- | --- | --- | --- | --- | --- | --- | --- | --- | --- | --- | --- | --- | --- | --- | --- | --- | --- | --- | --- | --- | --- | --- | --- | --- | --- | --- | --- | --- | --- | --- | --- | --- | --- | --- | --- | --- | --- | --- | --- | --- | --- | --- | --- | --- | --- | --- | --- | --- | --- | --- | --- | --- | --- | --- | --- | --- | --- | --- | --- | --- | --- | --- | --- | --- | --- | --- | --- | --- | --- | --- | --- | --- | --- | --- | --- | --- | --- | --- | --- | --- | --- | --- | --- | --- | --- | --- | --- | --- | --- | --- | --- | --- | --- | --- | --- | --- | --- | --- | --- | --- | --- | --- | --- | --- | --- | --- | --- | --- | --- | --- | --- | --- | --- | --- | --- | --- | --- | --- | --- | --- | --- | --- | --- | --- | --- | --- | --- | --- | --- | --- | --- | --- | --- | --- | --- | --- | --- | --- | --- | --- | --- | --- | --- | --- | --- | --- | --- | --- | --- | --- | --- | --- | --- | --- | --- | --- | --- | --- | --- | --- | --- | --- | --- | --- | --- | --- | --- | --- | --- | --- | --- | --- | --- | --- | --- | --- | --- | --- | --- | --- | --- | --- | --- | --- | --- | --- | --- | --- | --- | --- | --- | --- | --- | --- | --- | --- | --- | --- | --- | --- | --- | --- | --- | --- | --- | --- | --- | --- | --- | --- | --- | --- | --- | --- | --- | --- | --- | --- | --- | --- | --- | --- | --- | --- | --- | --- | --- | --- | --- | --- | --- | --- | --- | --- | --- | --- | --- | --- | --- | --- | --- | --- | --- | --- | --- | --- | --- | --- | --- | --- | --- | --- | --- | --- | --- | --- | --- | --- | --- | --- | --- | --- | --- | --- | --- | --- | --- | --- | --- | --- | --- | --- | --- | --- | --- | --- | --- | --- | --- | --- | --- | --- | --- | --- | --- | --- | --- | --- | --- | --- | --- | --- | --- | --- | --- | --- | --- | --- | --- | --- | --- | --- | --- | --- | --- | --- | --- | --- | --- | --- | --- | --- | --- | --- | --- | --- | --- | --- | --- | --- | --- | --- | --- | --- | --- | --- | --- | --- | --- | --- | --- | --- | --- | --- | --- | --- | --- | --- | --- | --- | --- | --- | --- | --- | --- | --- | --- | --- | --- | --- | --- | --- | --- | --- | --- | --- | --- | --- | --- | --- | --- | --- | --- | --- | --- | --- | --- | --- | --- | --- | --- | --- | --- | --- | --- | --- | --- | --- | --- | --- | --- | --- | --- | --- | --- | --- | --- | --- | --- | --- | --- | --- | --- | --- | --- | --- | --- | --- | --- | --- | --- | --- | --- | --- | --- | --- | --- | --- | --- | --- | --- | --- | --- | --- | --- | --- | --- | --- | --- | --- | --- | --- | --- | --- | --- | --- | --- | --- | --- | --- | --- | --- | --- | --- | --- | --- | --- | --- | --- | --- | --- | --- | --- | --- | --- | --- | --- | --- | --- | --- | --- | --- | --- | --- | --- | --- | --- | --- | --- | --- | --- | --- | --- | --- | --- | --- | --- | --- | --- | --- | --- | --- | --- | --- | --- | --- | --- | --- | --- | --- | --- | --- | --- | --- | --- | --- | --- | --- | --- | --- | --- | --- | --- | --- | --- | --- | --- | --- | --- | --- | --- | --- | --- | --- | --- | --- | --- | --- | --- | --- | --- | --- | --- | --- | --- | --- | --- | --- | --- | --- | --- | --- | --- | --- | --- | --- | --- | --- | --- | --- | --- | --- | --- | --- | --- | --- | --- | --- | --- | --- | --- | --- | --- | --- | --- | --- | --- | --- | --- | --- | --- | --- | --- | --- | --- | --- | --- | --- | --- | --- | --- | --- | --- | --- | --- | --- | --- | --- | --- | --- | --- | --- | --- | --- | --- | --- | --- | --- | --- | --- | --- | --- | --- | --- | --- | --- | --- | --- | --- | --- | --- | --- | --- | --- | --- | --- | --- | --- | --- | --- | --- | --- | --- | --- | --- | --- | --- | --- | --- | --- | --- | --- | --- | --- | --- | --- | --- | --- | --- | --- | --- | --- | --- | --- | --- | --- | --- | --- | --- | --- | --- | --- | --- | --- | --- | --- | --- | --- | --- | --- | --- | --- | --- | --- | --- | --- | --- | --- | --- | --- | --- | --- | --- | --- | --- | --- | --- | --- | --- | --- | --- | --- | --- | --- | --- | --- | --- | --- | --- | --- | --- | --- | --- | --- | --- | --- | --- | --- | --- | --- | --- | --- | --- | --- | --- | --- | --- | --- | --- | --- | --- | --- | --- | --- | --- | --- | --- | --- | --- | --- | --- | --- | --- | --- | --- | --- | --- | --- | --- | --- | --- | --- | --- | --- | --- | --- | --- | --- | --- | --- | --- | --- | --- | --- | --- | --- | --- | --- | --- | --- | --- | --- | --- | --- | --- | --- | --- | --- | --- | --- | --- | --- | --- | --- | --- | --- | --- | --- | --- | --- | --- | --- | --- | --- | --- | --- | --- | --- | --- | --- | --- | --- | --- | --- | --- | --- | --- | --- | --- | --- | --- | --- | --- | --- | --- | --- | --- | --- | --- | --- | --- | --- | --- | --- | --- | --- | --- | --- | --- | --- | --- | --- | --- | --- | --- | --- | --- | --- | --- | --- | --- | --- | --- | --- | --- | --- | --- | --- | --- | --- | --- | --- | --- | --- | --- | --- | --- | --- | --- | --- | --- | --- | --- | --- | --- | --- | --- | --- | --- | --- | --- | --- | --- | --- | --- | --- | --- | --- | --- | --- | --- | --- | --- | --- | --- | --- | --- | --- | --- | --- | --- | --- | --- | --- | --- | --- | --- | --- | --- | --- | --- | --- | --- | --- | --- | --- | --- | --- | --- | --- | --- | --- | --- | --- | --- | --- | --- | --- | --- | --- | --- | --- | --- | --- | --- | --- | --- | --- | --- | --- | --- | --- | --- | --- | --- | --- | --- | --- | --- | --- | --- | --- | --- | --- | --- | --- | --- | --- | --- | --- | --- | --- | --- | --- | --- | --- | --- | --- | --- | --- | --- | --- | --- | --- | --- | --- | --- | --- | --- | --- | --- | --- | --- | --- | --- | --- | --- | --- | --- | --- | --- | --- | --- | --- | --- | --- | --- | --- | --- | --- | --- | --- | --- | --- | --- | --- | --- | --- | --- | --- | --- | --- | --- | --- | --- | --- | --- | --- | --- | --- | --- | --- | --- | --- | --- | --- | --- | --- | --- | --- | --- | --- | --- | --- | --- | --- | --- | --- | --- | --- | --- | --- | --- | --- | --- | --- | --- | --- | --- | --- | --- | --- | --- | --- | --- | --- | --- | --- | --- | --- | --- | --- | --- | --- | --- | --- | --- | --- | --- | --- | --- | --- | --- | --- | --- | --- | --- | --- | --- | --- | --- | --- | --- | --- | --- | --- | --- | --- | --- | --- | --- | --- | --- | --- | --- | --- | --- | --- | --- | --- | --- | --- | --- | --- | --- | --- | --- | --- | --- | --- | --- | --- | --- | --- | --- | --- | --- | --- | --- | --- | --- | --- | --- | --- | --- | --- | --- | --- | --- | --- | --- | --- | --- | --- | --- | --- | --- | --- | --- | --- | --- | --- | --- | --- | --- | --- | --- | --- | --- | --- | --- | --- | --- | --- | --- | --- | --- | --- | --- | --- | --- | --- | --- | --- | --- | --- | --- | --- | --- | --- | --- | --- | --- | --- | --- | --- | --- | --- | --- | --- | --- | --- | --- | --- | --- | --- | --- | --- | --- | --- | --- | --- | --- | --- | --- | --- | --- | --- | --- | --- | --- | --- | --- | --- | --- | --- | --- | --- | --- | --- | --- | --- | --- | --- | --- | --- | --- | --- | --- | --- | --- | --- | --- | --- | --- | --- | --- | --- | --- | --- | --- | --- | --- | --- | --- | --- | --- | --- | --- | --- | --- | --- | --- | --- | --- | --- | --- | --- | --- | --- | --- | --- | --- | --- | --- | --- | --- | --- | --- | --- | --- | --- | --- | --- | --- | --- | --- | --- | --- | --- | --- | --- | --- | --- | --- | --- | --- | --- | --- | --- | --- | --- | --- | --- | --- | --- | --- | --- | --- | --- | --- | --- | --- | --- | --- | --- | --- | --- | --- | --- | --- | --- | --- | --- | --- | --- | --- | --- | --- | --- | --- | --- | --- | --- | --- | --- | --- | --- | --- | --- | --- | --- | --- | --- | --- | --- | --- | --- | --- | --- | --- | --- | --- | --- | --- | --- | --- | --- | --- | --- | --- | --- | --- | --- | --- | --- | --- | --- | --- | --- | --- | --- | --- | --- | --- | --- | --- | --- | --- | --- | --- | --- | --- | --- | --- | --- | --- | --- | --- | --- | --- | --- | --- | --- | --- | --- | --- | --- | --- | --- | --- | --- | --- | --- | --- | --- | --- | --- | --- | --- | --- | --- | --- | --- | --- | --- | --- | --- | --- | --- | --- | --- | --- | --- | --- | --- | --- | --- | --- | --- | --- | --- | --- | --- | --- | --- | --- | --- | --- | --- | --- | --- | --- | --- | --- | --- | --- | --- | --- | --- | --- | --- | --- | --- | --- | --- | --- | --- | --- | --- | --- | --- | --- | --- | --- | --- | --- | --- | --- | --- | --- | --- | --- | --- | --- | --- | --- | --- | --- | --- | --- | --- | --- | --- | --- | --- | --- | --- | --- | --- | --- | --- | --- | --- | --- | --- | --- | --- | --- | --- | --- | --- | --- | --- | --- | --- | --- | --- | --- | --- | --- | --- | --- | --- | --- | --- | --- | --- | --- | --- | --- | --- | --- | --- | --- | --- | --- | --- | --- | --- | --- | --- | --- | --- | --- | --- | --- | --- | --- | --- | --- | --- | --- | --- | --- | --- | --- | --- | --- | --- | --- | --- | --- | --- | --- | --- | --- | --- | --- | --- | --- | --- | --- | --- | --- | --- | --- | --- | --- | --- | --- | --- | --- | --- | --- | --- | --- | --- | --- | --- | --- | --- | --- | --- | --- | --- | --- | --- | --- | --- | --- | --- | --- | --- | --- | --- | --- | --- | --- | --- | --- | --- | --- | --- | --- | --- | --- | --- | --- | --- | --- | --- | --- | --- | --- | --- | --- | --- | --- | --- | --- | --- | --- | --- | --- | --- | --- | --- | --- | --- | --- | --- | --- | --- | --- | --- | --- | --- | --- | --- | --- | --- | --- | --- | --- | --- | --- | --- | --- | --- | --- | --- | --- | --- | --- | --- | --- | --- | --- | --- | --- | --- | --- | --- | --- | --- | --- | --- | --- | --- | --- | --- | --- | --- | --- | --- | --- | --- | --- | --- | --- | --- | --- | --- | --- | --- | --- | --- | --- | --- | --- | --- | --- | --- | --- | --- | --- | --- | --- | --- | --- | --- | --- | --- | --- | --- | --- | --- | --- | --- | --- | --- | --- | --- | --- | --- | --- | --- | --- | --- | --- | --- | --- | --- | --- | --- | --- | --- | --- | --- | --- | --- | --- | --- | --- | --- | --- | --- | --- | --- | --- | --- | --- | --- | --- | --- | --- | --- | --- | --- | --- | --- | --- | --- | --- | --- | --- | --- | --- | --- | --- | --- | --- | --- | --- | --- | --- | --- | --- | --- | --- | --- | --- | --- | --- | --- | --- | --- | --- | --- | --- | --- | --- | --- | --- | --- | --- | --- | --- | --- | --- | --- | --- | --- | --- | --- | --- | --- | --- | --- | --- | --- | --- | --- | --- | --- | --- | --- | --- | --- | --- | --- | --- | --- | --- | --- | --- | --- | --- | --- | --- | --- | --- | --- | --- | --- | --- | --- | --- | --- | --- | --- | --- | --- | --- | --- | --- | --- | --- | --- | --- | --- | --- | --- | --- | --- | --- | --- | --- | --- | --- | --- | --- | --- | --- | --- | --- | --- | --- | --- | --- | --- | --- | --- | --- | --- | --- | --- | --- | --- | --- | --- | --- | --- | --- | --- | --- | --- | --- | --- | --- | --- | --- | --- | --- | --- | --- | --- | --- | --- | --- | --- | --- | --- | --- | --- | --- | --- | --- | --- | --- | --- | --- | --- | --- | --- | --- | --- | --- | --- | --- | --- | --- | --- | --- | --- | --- | --- | --- | --- | --- | --- | --- | --- | --- | --- | --- | --- | --- | --- | --- | --- | --- | --- | --- | --- | --- | --- | --- | --- | --- | --- | --- | --- | --- | --- | --- | --- | --- | --- | --- | --- | --- | --- | --- | --- | --- | --- | --- | --- | --- | --- | --- | --- | --- | --- | --- | --- | --- | --- | --- | --- | --- | --- | --- | --- | --- | --- | --- | --- | --- | --- | --- | --- | --- | --- | --- | --- | --- | --- | --- | --- | --- | --- | --- | --- | --- | --- | --- | --- | --- | --- | --- | --- | --- | --- | --- | --- | --- | --- | --- | --- | --- | --- | --- | --- | --- | --- | --- | --- | --- | --- | --- | --- | --- | --- | --- | --- | --- | --- | --- | --- | --- | --- | --- | --- | --- | --- | --- | --- | --- | --- | --- | --- | --- | --- | --- | --- | --- | --- | --- | --- | --- | --- | --- | --- | --- | --- | --- | --- | --- | --- | --- | --- | --- | --- | --- | --- | --- | --- | --- | --- | --- | --- | --- | --- | --- | --- | --- | --- | --- | --- | --- | --- | --- | --- | --- | --- | --- | --- | --- | --- | --- | --- | --- | --- | --- | --- | --- | --- | --- | --- | --- | --- | --- | --- | --- | --- | --- | --- | --- | --- | --- | --- | --- | --- | --- | --- | --- | --- | --- | --- | --- | --- | --- | --- | --- | --- | --- | --- | --- | --- | --- | --- | --- | --- | --- | --- | --- | --- | --- | --- | --- | --- | --- | --- | --- | --- | --- | --- | --- | --- | --- | --- | --- | --- | --- | --- | --- | --- | --- | --- | --- | --- | --- | --- | --- | --- | --- | --- | --- | --- | --- | --- | --- | --- | --- | --- | --- | --- | --- | --- | --- | --- | --- | --- | --- | --- | --- | --- | --- | --- | --- | --- | --- | --- | --- | --- | --- | --- | --- | --- | --- | --- | --- | --- | --- | --- | --- | --- | --- | --- | --- | --- | --- | --- | --- | --- | --- | --- | --- | --- | --- | --- | --- | --- | --- | --- | --- | --- | --- | --- | --- | --- | --- | --- | --- | --- | --- | --- | --- | --- | --- | --- | --- | --- | --- | --- | --- | --- | --- | --- | --- | --- | --- | --- | --- | --- | --- | --- | --- | --- | --- | --- | --- | --- | --- | --- | --- | --- | --- | --- | --- | --- | --- | --- | --- | --- | --- | --- | --- | --- | --- | --- | --- | --- | --- | --- | --- | --- | --- | --- | --- | --- | --- | --- | --- | --- | --- | --- | --- | --- | --- | --- | --- | --- | --- | --- | --- | --- | --- | --- | --- | --- | --- | --- | --- | --- | --- | --- | --- | --- | --- | --- | --- | --- | --- | --- | --- | --- | --- | --- | --- | --- | --- | --- | --- | --- | --- | --- | --- | --- | --- | --- | --- | --- | --- | --- | --- | --- | --- | --- | --- | --- | --- | --- | --- | --- | --- | --- | --- | --- | --- | --- | --- | --- | --- | --- | --- | --- | --- | --- | --- | --- | --- | --- | --- | --- | --- | --- | --- | --- | --- | --- | --- | --- | --- | --- | --- | --- | --- | --- | --- | --- | --- | --- | --- | --- | --- | --- | --- | --- | --- | --- | --- | --- | --- | --- | --- | --- | --- | --- | --- | --- | --- | --- | --- | --- | --- | --- | --- | --- | --- | --- | --- | --- | --- | --- | --- | --- | --- | --- | --- | --- | --- | --- | --- | --- | --- | --- | --- | --- | --- | --- | --- | --- | --- | --- | --- | --- | --- | --- | --- | --- | --- | --- | --- | --- | --- | --- | --- | --- | --- | --- | --- | --- | --- | --- | --- | --- | --- | --- | --- | --- | --- | --- | --- | --- | --- | --- | --- | --- | --- | --- | --- | --- | --- | --- | --- | --- | --- | --- | --- | --- | --- | --- | --- | --- | --- | --- | --- | --- | --- | --- | --- | --- | --- | --- | --- | --- | --- | --- | --- | --- | --- | --- | --- | --- | --- | --- | --- | --- | --- | --- | --- | --- | --- | --- | --- | --- | --- | --- | --- | --- | --- | --- | --- | --- | --- | --- | --- | --- | --- | --- | --- | --- | --- | --- | --- | --- | --- | --- | --- | --- | --- | --- | --- | --- | --- | --- | --- | --- | --- | --- | --- | --- | --- | --- | --- | --- | --- | --- | --- | --- | --- | --- | --- | --- | --- | --- | --- | --- | --- | --- | --- | --- | --- | --- | --- | --- | --- | --- | --- | --- | --- | --- | --- | --- | --- | --- | --- | --- | --- | --- | --- | --- | --- | --- | --- | --- | --- | --- | --- | --- | --- | --- | --- | --- | --- | --- | --- | --- | --- | --- | --- | --- | --- | --- | --- | --- | --- | --- | --- | --- | --- | --- | --- | --- | --- | --- | --- | --- | --- | --- | --- | --- | --- | --- | --- | --- | --- | --- | --- | --- | --- | --- | --- | --- | --- | --- | --- | --- | --- | --- | --- | --- | --- | --- | --- | --- | --- | --- | --- | --- | --- | --- | --- | --- | --- | --- | --- | --- | --- | --- | --- | --- | --- | --- | --- | --- | --- | --- | --- | --- | --- | --- | --- | --- | --- | --- | --- | --- | --- | --- | --- | --- | --- | --- | --- | --- | --- | --- | --- | --- | --- | --- | --- | --- | --- | --- | --- | --- | --- | --- | --- | --- | --- | --- | --- | --- | --- | --- | --- | --- | --- | --- | --- | --- | --- | --- | --- | --- | --- | --- | --- | --- | --- | --- | --- | --- | --- | --- | --- | --- | --- | --- | --- | --- | --- | --- | --- | --- | --- | --- | --- | --- | --- | --- | --- | --- | --- | --- | --- | --- | --- | --- | --- | --- | --- | --- | --- | --- | --- | --- | --- | --- | --- | --- | --- | --- | --- | --- | --- | --- | --- | --- | --- | --- | --- | --- | --- | --- | --- | --- | --- | --- | --- | --- | --- | --- | --- | --- | --- | --- | --- | --- | --- | --- | --- | --- | --- | --- | --- | --- | --- | --- | --- | --- | --- | --- | --- | --- | --- | --- | --- | --- | --- | --- | --- | --- | --- | --- | --- | --- | --- | --- | --- | --- | --- | --- | --- | --- | --- | --- | --- | --- | --- | --- | --- | --- | --- | --- | --- | --- | --- | --- | --- | --- | --- | --- | --- | --- | --- | --- | --- | --- | --- | --- | --- | --- | --- | --- | --- | --- | --- | --- | --- | --- | --- | --- | --- | --- | --- | --- | --- | --- | --- | --- | --- | --- | --- | --- | --- | --- | --- | --- | --- | --- | --- | --- | --- | --- | --- | --- | --- | --- | --- | --- | --- | --- | --- | --- | --- | --- | --- | --- | --- | --- | --- | --- | --- | --- | --- | --- | --- | --- | --- | --- | --- | --- | --- | --- | --- | --- | --- | --- | --- | --- | --- | --- | --- | --- | --- | --- | --- | --- | --- | --- | --- | --- | --- | --- | --- | --- | --- | --- | --- | --- | --- | --- | --- | --- | --- | --- | --- | --- | --- | --- | --- | --- | --- | --- | --- | --- | --- | --- | --- | --- | --- | --- | --- | --- | --- | --- | --- | --- | --- | --- | --- | --- | --- | --- | --- | --- | --- | --- | --- | --- | --- | --- | --- | --- | --- | --- | --- | --- | --- | --- | --- | --- | --- | --- | --- | --- | --- | --- | --- | --- | --- | --- | --- | --- | --- | --- | --- | --- | --- | --- | --- | --- | --- | --- | --- | --- | --- | --- | --- | --- | --- | --- | --- | --- | --- | --- | --- | --- | --- | --- | --- | --- | --- | --- | --- | --- | --- | --- | --- | --- | --- | --- | --- | --- | --- | --- | --- | --- | --- | --- | --- | --- | --- | --- | --- | --- | --- | --- | --- | --- | --- | --- | --- | --- | --- | --- | --- | --- | --- | --- | --- | --- | --- | --- | --- | --- | --- | --- | --- | --- | --- | --- | --- | --- | --- | --- | --- | --- | --- | --- | --- | --- | --- | --- | --- | --- | --- | --- | --- | --- | --- | --- | --- | --- | --- | --- | --- | --- | --- | --- | --- | --- | --- | --- | --- | --- | --- | --- | --- | --- | --- | --- | --- | --- | --- | --- | --- | --- | --- | --- | --- | --- | --- | --- | --- | --- | --- | --- | --- | --- | --- | --- | --- | --- | --- | --- | --- | --- | --- | --- | --- | --- | --- | --- | --- | --- | --- | --- | --- | --- | --- | --- | --- | --- | --- | --- | --- | --- | --- | --- | --- | --- | --- | --- | --- | --- | --- | --- | --- | --- | --- | --- | --- | --- | --- | --- | --- | --- | --- | --- | --- | --- | --- | --- | --- | --- | --- | --- | --- | --- | --- | --- | --- | --- | --- | --- | --- | --- | --- | --- | --- | --- | --- | --- | --- | --- | --- | --- | --- | --- | --- | --- | --- | --- | --- | --- | --- | --- | --- | --- | --- | --- | --- | --- | --- | --- | --- | --- | --- | --- | --- | --- | --- | --- | --- | --- | --- | --- | --- | --- | --- | --- | --- | --- | --- | --- | --- | --- | --- | --- | --- | --- | --- | --- | --- | --- | --- | --- | --- | --- | --- | --- | --- | --- | --- | --- | --- | --- | --- | --- | --- | --- | --- | --- | --- | --- | --- | --- | --- | --- | --- | --- | --- | --- | --- | --- | --- | --- | --- | --- | --- | --- | --- | --- | --- | --- | --- | --- | --- | --- | --- | --- | --- | --- | --- | --- | --- | --- | --- | --- | --- | --- | --- | --- | --- | --- | --- | --- | --- | --- | --- | --- | --- | --- | --- | --- | --- | --- | --- | --- | --- | --- | --- | --- | --- | --- | --- | --- | --- | --- | --- | --- | --- | --- | --- | --- | --- | --- | --- | --- | --- | --- | --- | --- | --- | --- | --- | --- | --- | --- | --- | --- | --- | --- | --- | --- | --- | --- | --- | --- | --- | --- | --- | --- | --- | --- | --- | --- | --- | --- | --- | --- | --- | --- | --- | --- | --- | --- | --- | --- | --- | --- | --- | --- | --- | --- | --- | --- | --- | --- | --- | --- | --- | --- | --- | --- | --- | --- | --- | --- | --- | --- | --- | --- | --- | --- | --- | --- | --- | --- | --- | --- | --- | --- | --- | --- | --- | --- | --- | --- | --- | --- | --- | --- | --- | --- | --- | --- | --- | --- | --- | --- | --- | --- | --- | --- | --- | --- | --- | --- | --- | --- | --- | --- | --- | --- | --- | --- | --- | --- | --- | --- | --- | --- | --- | --- | --- | --- | --- | --- | --- | --- | --- | --- | --- | --- | --- | --- | --- | --- | --- | --- | --- | --- | --- | --- | --- | --- | --- | --- | --- | --- | --- | --- | --- | --- | --- | --- | --- | --- | --- | --- | --- | --- | --- | --- | --- | --- | --- | --- | --- | --- | --- | --- | --- | --- | --- | --- | --- | --- | --- | --- | --- | --- | --- | --- | --- | --- | --- | --- | --- | --- | --- | --- | --- | --- | --- | --- | --- | --- | --- | --- | --- | --- | --- | --- | --- | --- | --- | --- | --- | --- | --- | --- | --- | --- | --- | --- | --- | --- | --- | --- | --- | --- | --- | --- | --- | --- | --- | --- | --- | --- | --- | --- | --- | --- | --- | --- | --- | --- | --- | --- | --- | --- | --- | --- | --- | --- | --- | --- | --- | --- | --- | --- | --- | --- | --- | --- | --- | --- | --- | --- | --- | --- | --- | --- | --- | --- | --- | --- | --- | --- | --- | --- | --- | --- | --- | --- | --- | --- | --- | --- | --- | --- | --- | --- | --- | --- | --- | --- | --- | --- | --- | --- | --- | --- | --- | --- | --- | --- | --- | --- | --- | --- | --- | --- | --- | --- | --- | --- | --- | --- | --- | --- | --- | --- | --- | --- | --- | --- | --- | --- | --- | --- | --- | --- | --- | --- | --- | --- | --- | --- | --- | --- | --- | --- | --- | --- | --- | --- | --- | --- | --- | --- | --- | --- | --- | --- | --- | --- | --- | --- | --- | --- | --- | --- | --- | --- | --- | --- | --- | --- | --- | --- | --- | --- | --- | --- | --- | --- | --- | --- | --- | --- | --- | --- | --- | --- | --- | --- | --- | --- | --- | --- | --- | --- | --- | --- | --- | --- | --- | --- | --- | --- | --- | --- | --- | --- | --- | --- | --- | --- | --- | --- | --- | --- | --- | --- | --- | --- | --- | --- | --- | --- | --- | --- | --- | --- | --- | --- | --- | --- | --- | --- | --- | --- | --- | --- | --- | --- | --- | --- | --- | --- | --- | --- | --- | --- | --- | --- | --- | --- | --- | --- | --- | --- | --- | --- | --- | --- | --- | --- | --- | --- | --- | --- | --- | --- | --- | --- | --- | --- | --- | --- | --- | --- | --- | --- | --- | --- | --- | --- | --- | --- | --- | --- | --- | --- | --- | --- | --- | --- | --- | --- | --- | --- | --- | --- | --- | --- | --- | --- | --- | --- | --- | --- | --- | --- | --- | --- | --- | --- | --- | --- | --- | --- | --- | --- | --- | --- | --- | --- | --- | --- | --- | --- | --- | --- | --- | --- | --- | --- | --- | --- | --- | --- | --- | --- | --- | --- | --- | --- | --- | --- | --- | --- | --- | --- | --- | --- | --- | --- | --- | --- | --- | --- | --- | --- | --- | --- | --- | --- | --- | --- | --- | --- | --- | --- | --- | --- | --- | --- | --- | --- | --- | --- | --- | --- | --- | --- | --- | --- | --- | --- | --- | --- | --- | --- | --- | --- | --- | --- | --- | --- | --- | --- | --- | --- | --- | --- | --- | --- | --- | --- | --- | --- | --- | --- | --- | --- | --- | --- | --- | --- | --- | --- | --- | --- | --- | --- | --- | --- | --- | --- | --- | --- | --- | --- | --- | --- | --- | --- | --- | --- | --- | --- | --- | --- | --- | --- | --- | --- | --- | --- | --- | --- | --- | --- | --- | --- | --- | --- | --- | --- | --- | --- | --- | --- | --- | --- | --- | --- | --- | --- | --- | --- | --- | --- | --- | --- | --- | --- | --- | --- | --- | --- | --- | --- | --- | --- | --- | --- | --- | --- | --- | --- | --- | --- | --- | --- | --- | --- | --- | --- | --- | --- | --- | --- | --- | --- | --- | --- | --- | --- | --- | --- | --- | --- | --- | --- | --- | --- | --- | --- | --- | --- | --- | --- | --- | --- | --- | --- | --- | --- | --- | --- | --- | --- | --- | --- | --- | --- | --- | --- | --- | --- | --- | --- | --- | --- | --- | --- | --- | --- | --- | --- | --- | --- | --- | --- | --- | --- | --- | --- | --- | --- | --- | --- | --- | --- | --- | --- | --- | --- | --- | --- | --- | --- | --- | --- | --- | --- | --- | --- | --- | --- | --- | --- | --- | --- | --- | --- | --- | --- | --- | --- | --- | --- | --- | --- | --- | --- | --- | --- | --- | --- | --- | --- | --- | --- | --- | --- | --- | --- | --- | --- | --- | --- | --- | --- | --- | --- | --- | --- | --- | --- | --- | --- | --- | --- | --- | --- | --- | --- | --- | --- | --- | --- | --- | --- | --- | --- | --- | --- | --- | --- | --- | --- | --- | --- | --- | --- | --- | --- | --- | --- | --- | --- | --- | --- | --- | --- | --- | --- | --- | --- | --- | --- | --- | --- | --- | --- | --- | --- | --- | --- | --- | --- | --- | --- | --- | --- | --- | --- | --- | --- | --- | --- | --- | --- | --- | --- | --- | --- | --- | --- | --- | --- | --- | --- | --- | --- | --- | --- | --- | --- | --- | --- | --- | --- | --- | --- | --- | --- | --- | --- | --- | --- | --- | --- | --- | --- | --- | --- | --- | --- | --- | --- | --- | --- | --- | --- | --- | --- | --- | --- | --- | --- | --- | --- | --- | --- | --- | --- | --- | --- | --- | --- | --- | --- | --- | --- | --- | --- | --- | --- | --- | --- | --- | --- | --- | --- | --- | --- | --- | --- | --- | --- | --- | --- | --- | --- | --- | --- | --- | --- | --- | --- | --- | --- | --- | --- | --- | --- | --- | --- | --- | --- | --- | --- | --- | --- | --- | --- | --- | --- | --- | --- | --- | --- | --- | --- | --- | --- | --- | --- | --- | --- | --- | --- | --- | --- | --- | --- | --- | --- | --- | --- | --- | --- | --- | --- | --- | --- | --- | --- | --- | --- | --- | --- | --- | --- | --- | --- | --- | --- | --- | --- | --- | --- | --- | --- | --- | --- | --- | --- | --- | --- | --- | --- | --- | --- | --- | --- | --- | --- | --- | --- | --- | --- | --- | --- | --- | --- | --- | --- | --- | --- | --- | --- | --- | --- | --- | --- | --- | --- | --- | --- | --- | --- | --- | --- | --- | --- | --- | --- | --- | --- | --- | --- | --- | --- | --- | --- | --- | --- | --- | --- | --- | --- | --- | --- | --- | --- | --- | --- | --- | --- | --- | --- | --- | --- | --- | --- | --- | --- | --- | --- | --- | --- | --- | --- | --- | --- | --- | --- | --- | --- | --- | --- | --- | --- | --- | --- | --- | --- | --- | --- | --- | --- | --- | --- | --- | --- | --- | --- | --- | --- | --- | --- | --- | --- | --- | --- | --- | --- | --- | --- | --- | --- | --- | --- | --- | --- | --- | --- | --- | --- | --- | --- | --- | --- | --- | --- | --- | --- | --- | --- | --- | --- | --- | --- | --- | --- | --- | --- | --- | --- | --- | --- | --- | --- | --- | --- | --- | --- | --- | --- | --- | --- | --- | --- | --- | --- | --- | --- | --- | --- | --- | --- | --- | --- | --- | --- | --- | --- | --- | --- | --- | --- | --- | --- | --- | --- | --- | --- | --- | --- | --- | --- | --- | --- | --- | --- | --- | --- | --- | --- | --- | --- | --- | --- | --- | --- | --- | --- | --- | --- | --- | --- | --- | --- | --- | --- | --- | --- | --- | --- | --- | --- | --- | --- | --- | --- | --- | --- | --- | --- | --- | --- | --- | --- | --- | --- | --- | --- | --- | --- | --- | --- | --- | --- | --- | --- | --- | --- | --- | --- | --- | --- | --- | --- | --- | --- | --- | --- | --- | --- | --- | --- | --- | --- | --- | --- | --- | --- | --- | --- | --- | --- | --- | --- | --- | --- | --- | --- | --- | --- | --- | --- | --- | --- | --- | --- | --- | --- | --- | --- | --- | --- | --- | --- | --- | --- | --- | --- | --- | --- | --- | --- | --- | --- | --- | --- | --- | --- | --- | --- | --- | --- | --- | --- | --- | --- | --- | --- | --- | --- | --- | --- | --- | --- | --- | --- | --- | --- | --- | --- | --- | --- | --- | --- | --- | --- | --- | --- | --- | --- | --- | --- | --- | --- | --- | --- | --- | --- | --- | --- | --- | --- | --- | --- | --- | --- | --- | --- | --- | --- | --- | --- | --- | --- | --- | --- | --- | --- | --- | --- | --- | --- | --- | --- | --- | --- | --- | --- | --- | --- | --- | --- | --- | --- | --- | --- | --- | --- | --- | --- | --- | --- | --- | --- | --- | --- | --- | --- | --- | --- | --- | --- | --- | --- | --- | --- | --- | --- | --- | --- | --- | --- | --- | --- | --- | --- | --- | --- | --- | --- | --- | --- | --- | --- | --- | --- | --- | --- | --- | --- | --- | --- | --- | --- | --- | --- | --- | --- | --- | --- | --- | --- | --- | --- | --- | --- | --- | --- | --- | --- | --- | --- | --- | --- | --- | --- | --- | --- | --- | --- | --- | --- | --- | --- | --- | --- | --- | --- | --- | --- | --- | --- | --- | --- | --- | --- | --- | --- | --- | --- | --- | --- | --- | --- | --- | --- | --- | --- | --- | --- | --- | --- | --- | --- | --- | --- | --- | --- | --- | --- | --- | --- | --- | --- | --- | --- | --- | --- | --- | --- | --- | --- | --- | --- | --- | --- | --- | --- | --- | --- | --- | --- | --- | --- | --- | --- | --- | --- | --- | --- | --- | --- | --- | --- | --- | --- | --- | --- | --- | --- | --- | --- | --- | --- | --- | --- | --- | --- | --- | --- | --- | --- | --- | --- | --- | --- | --- | --- | --- | --- | --- | --- | --- | --- | --- | --- | --- | --- | --- | --- | --- | --- | --- | --- | --- | --- | --- | --- | --- | --- | --- | --- | --- | --- | --- | --- | --- | --- | --- | --- | --- | --- | --- | --- | --- | --- | --- | --- | --- | --- | --- | --- | --- | --- | --- | --- | --- | --- | --- | --- | --- | --- | --- | --- | --- | --- | --- | --- | --- | --- | --- | --- | --- | --- | --- | --- | --- | --- | --- | --- | --- | --- | --- | --- | --- | --- | --- | --- | --- | --- | --- | --- | --- | --- | --- | --- | --- | --- | --- | --- | --- | --- | --- | --- | --- | --- | --- | --- | --- | --- | --- | --- | --- | --- | --- | --- | --- | --- | --- | --- | --- | --- | --- | --- | --- | --- | --- | --- | --- | --- | --- | --- | --- | --- | --- | --- | --- | --- | --- | --- | --- | --- | --- | --- | --- | --- | --- | --- | --- | --- | --- | --- | --- | --- | --- | --- | --- | --- | --- | --- | --- | --- | --- | --- | --- | --- | --- | --- | --- | --- | --- | --- | --- | --- | --- | --- | --- | --- | --- | --- | --- | --- | --- | --- | --- | --- | --- | --- | --- | --- | --- | --- | --- | --- | --- | --- | --- | --- | --- | --- | --- | --- | --- | --- | --- | --- | --- | --- | --- | --- | --- | --- | --- | --- | --- | --- | --- | --- | --- | --- | --- | --- | --- | --- | --- | --- | --- | --- | --- | --- | --- | --- | --- | --- | --- | --- | --- | --- | --- | --- | --- | --- | --- | --- | --- | --- | --- | --- | --- | --- | --- | --- | --- | --- | --- | --- | --- | --- | --- | --- | --- | --- | --- | --- | --- | --- | --- | --- | --- | --- | --- | --- | --- | --- | --- | --- | --- | --- | --- | --- | --- | --- | --- | --- | --- | --- | --- | --- | --- | --- | --- | --- | --- | --- | --- | --- | --- | --- | --- | --- | --- | --- | --- | --- | --- | --- | --- | --- | --- | --- | --- | --- | --- | --- | --- | --- | --- | --- | --- | --- | --- | --- | --- | --- | --- | --- | --- | --- | --- | --- | --- | --- | --- | --- | --- | --- | --- | --- | --- | --- | --- | --- | --- | --- | --- | --- | --- | --- | --- | --- | --- | --- | --- | --- | --- | --- | --- | --- | --- | --- | --- | --- | --- | --- | --- | --- | --- | --- | --- | --- | --- | --- | --- | --- | --- | --- | --- | --- | --- | --- | --- | --- | --- | --- | --- | --- | --- | --- | --- | --- | --- | --- | --- | --- | --- | --- | --- | --- | --- | --- | --- | --- | --- | --- | --- | --- | --- | --- | --- | --- | --- | --- | --- | --- | --- | --- | --- | --- | --- | --- | --- | --- | --- | --- | --- | --- | --- | --- | --- | --- | --- | --- | --- | --- | --- | --- | --- | --- | --- | --- | --- | --- | --- | --- | --- | --- | --- | --- | --- | --- | --- | --- | --- | --- | --- | --- | --- | --- | --- | --- | --- | --- | --- | --- | --- | --- | --- | --- | --- | --- | --- | --- | --- | --- | --- | --- | --- | --- | --- | --- | --- | --- | --- | --- | --- | --- | --- | --- | --- | --- | --- | --- | --- | --- | --- | --- | --- | --- | --- | --- | --- | --- | --- | --- | --- | --- | --- | --- | --- | --- | --- | --- | --- | --- | --- | --- | --- | --- | --- | --- | --- | --- | --- | --- | --- | --- | --- | --- | --- | --- | --- | --- | --- | --- | --- | --- | --- | --- | --- | --- | --- | --- | --- | --- | --- | --- | --- | --- | --- | --- | --- | --- | --- | --- | --- | --- | --- | --- | --- | --- | --- | --- | --- | --- | --- | --- | --- | --- | --- | --- | --- | --- | --- | --- | --- | --- | --- | --- | --- | --- | --- | --- | --- | --- | --- | --- | --- | --- | --- | --- | --- | --- | --- | --- | --- | --- | --- | --- | --- | --- | --- | --- | --- | --- | --- | --- | --- | --- | --- | --- | --- | --- | --- | --- | --- | --- | --- | --- | --- | --- | --- | --- | --- | --- | --- | --- | --- | --- | --- | --- | --- | --- | --- | --- | --- | --- | --- | --- | --- | --- | --- | --- | --- | --- | --- | --- | --- | --- | --- | --- | --- | --- | --- | --- | --- | --- | --- | --- | --- | --- | --- | --- | --- | --- | --- | --- | --- | --- | --- | --- | --- | --- | --- | --- | --- | --- | --- | --- | --- | --- | --- | --- | --- | --- | --- | --- | --- | --- | --- | --- | --- | --- | --- | --- | --- | --- | --- | --- | --- | --- | --- | --- | --- | --- | --- | --- | --- | --- | --- | --- | --- | --- | --- | --- | --- | --- | --- | --- | --- | --- | --- | --- | --- | --- | --- | --- | --- | --- | --- | --- | --- | --- | --- | --- | --- | --- | --- | --- | --- | --- | --- | --- | --- | --- | --- | --- | --- | --- | --- | --- | --- | --- | --- | --- | --- | --- | --- | --- | --- | --- | --- | --- | --- | --- | --- | --- | --- | --- | --- | --- | --- | --- | --- | --- | --- | --- | --- | --- | --- | --- | --- | --- | --- | --- | --- | --- | --- | --- | --- | --- | --- | --- | --- | --- | --- | --- | --- | --- | --- | --- | --- | --- | --- | --- | --- | --- | --- | --- | --- | --- | --- | --- | --- | --- | --- | --- | --- | --- | --- | --- | --- | --- | --- | --- | --- | --- | --- | --- | --- | --- | --- | --- | --- | --- | --- | --- | --- | --- | --- | --- | --- | --- | --- | --- | --- | --- | --- | --- | --- | --- | --- | --- | --- | --- | --- | --- | --- | --- | --- | --- | --- | --- | --- | --- | --- | --- | --- | --- | --- | --- | --- | --- | --- | --- | --- | --- | --- | --- | --- | --- | --- | --- | --- | --- | --- | --- | --- | --- | --- | --- | --- | --- | --- | --- | --- | --- | --- | --- | --- | --- | --- | --- | --- | --- | --- | --- | --- | --- | --- | --- | --- | --- | --- | --- | --- | --- | --- | --- | --- | --- | --- | --- | --- | --- | --- | --- | --- | --- | --- | --- | --- | --- | --- | --- | --- | --- | --- | --- | --- | --- | --- | --- | --- | --- | --- | --- | --- | --- | --- | --- | --- | --- | --- | --- | --- | --- | --- | --- | --- | --- | --- | --- | --- | --- | --- | --- | --- | --- | --- | --- | --- | --- | --- | --- | --- | --- | --- | --- | --- | --- | --- | --- | --- | --- | --- | --- | --- | --- | --- | --- | --- | --- | --- | --- | --- | --- | --- | --- | --- | --- | --- | --- | --- | --- | --- | --- | --- | --- | --- | --- | --- | --- | --- | --- | --- | --- | --- | --- | --- | --- | --- | --- | --- | --- | --- | --- | --- | --- | --- | --- | --- | --- | --- | --- | --- | --- | --- | --- | --- | --- | --- | --- | --- | --- | --- | --- | --- | --- | --- | --- | --- | --- | --- | --- | --- | --- | --- | --- | --- | --- | --- | --- | --- | --- | --- | --- | --- | --- | --- | --- | --- | --- | --- | --- | --- | --- | --- | --- | --- | --- | --- | --- | --- | --- | --- | --- | --- | --- | --- | --- | --- | --- | --- | --- | --- | --- | --- | --- | --- | --- | --- | --- | --- | --- | --- | --- | --- | --- | --- | --- | --- | --- | --- | --- | --- | --- | --- | --- | --- | --- | --- | --- | --- | --- | --- | --- | --- | --- | --- | --- | --- | --- | --- | --- | --- | --- | --- | --- | --- | --- | --- | --- | --- | --- | --- | --- | --- | --- | --- | --- | --- | --- | --- | --- | --- | --- | --- | --- | --- | --- | --- | --- | --- | --- | --- | --- | --- | --- | --- | --- | --- | --- | --- | --- | --- | --- | --- | --- | --- | --- | --- | --- | --- | --- | --- | --- | --- | --- | --- | --- | --- | --- | --- | --- | --- | --- | --- | --- | --- | --- | --- | --- | --- | --- | --- | --- | --- | --- | --- | --- | --- | --- | --- | --- | --- | --- | --- | --- | --- | --- | --- | --- | --- | --- | --- | --- | --- | --- | --- | --- | --- | --- | --- | --- | --- | --- | --- | --- | --- | --- | --- | --- | --- | --- | --- | --- | --- | --- | --- | --- | --- | --- | --- | --- | --- | --- | --- | --- | --- | --- | --- | --- | --- | --- | --- | --- | --- | --- | --- | --- | --- | --- | --- | --- | --- | --- | --- | --- | --- | --- | --- | --- | --- | --- | --- | --- | --- | --- | --- | --- | --- | --- | --- | --- | --- | --- | --- | --- | --- | --- | --- | --- | --- | --- | --- | --- | --- | --- | --- | --- | --- | --- | --- | --- | --- | --- | --- | --- | --- | --- | --- | --- | --- | --- | --- | --- | --- | --- | --- | --- | --- | --- | --- | --- | --- | --- | --- | --- | --- | --- | --- | --- | --- | --- | --- | --- | --- | --- | --- | --- | --- | --- | --- | --- | --- | --- | --- | --- | --- | --- | --- | --- | --- | --- | --- | --- | --- | --- | --- | --- | --- | --- | --- | --- | --- | --- | --- | --- | --- | --- | --- | --- | --- | --- | --- | --- | --- | --- | --- | --- | --- | --- | --- | --- | --- | --- | --- | --- | --- | --- | --- | --- | --- | --- | --- | --- | --- | --- | --- | --- | --- | --- | --- | --- | --- | --- | --- | --- | --- | --- | --- | --- | --- | --- | --- | --- | --- | --- | --- | --- | --- | --- | --- | --- | --- | --- | --- | --- | --- | --- | --- | --- | --- | --- | --- | --- | --- | --- | --- | --- | --- | --- | --- | --- | --- | --- | --- | --- | --- | --- | --- | --- | --- | --- | --- | --- | --- | --- | --- | --- | --- | --- | --- | --- | --- | --- | --- | --- | --- | --- | --- | --- | --- | --- | --- | --- | --- | --- | --- | --- | --- | --- | --- | --- | --- | --- | --- | --- | --- | --- | --- | --- | --- | --- | --- | --- | --- | --- | --- | --- | --- | --- | --- | --- | --- | --- | --- | --- | --- | --- | --- | --- | --- | --- | --- | --- | --- | --- | --- | --- | --- | --- | --- | --- | --- | --- | --- | --- | --- | --- | --- | --- | --- | --- | --- | --- | --- | --- | --- | --- | --- | --- | --- | --- | --- | --- | --- | --- | --- | --- | --- | --- | --- | --- | --- | --- | --- | --- | --- | --- | --- | --- | --- | --- | --- | --- | --- | --- | --- | --- | --- | --- | --- | --- | --- | --- | --- | --- | --- | --- | --- | --- | --- | --- | --- | --- | --- | --- | --- | --- | --- | --- | --- | --- | --- | --- | --- | --- | --- | --- | --- | --- | --- | --- | --- | --- | --- | --- | --- | --- | --- | --- | --- | --- | --- | --- | --- | --- | --- | --- | --- | --- | --- | --- | --- | --- | --- | --- | --- | --- | --- | --- | --- | --- | --- | --- | --- | --- | --- | --- | --- | --- | --- | --- | --- | --- | --- | --- | --- | --- | --- | --- | --- | --- | --- | --- | --- | --- | --- | --- | --- | --- | --- | --- | --- | --- | --- | --- | --- | --- | --- | --- | --- | --- | --- | --- | --- | --- | --- | --- | --- | --- | --- | --- | --- | --- | --- | --- | --- | --- | --- | --- | --- | --- | --- | --- | --- | --- | --- | --- | --- | --- | --- | --- | --- | --- | --- | --- | --- | --- | --- | --- | --- | --- | --- | --- | --- | --- | --- | --- | --- | --- | --- | --- | --- | --- | --- | --- | --- | --- | --- | --- | --- | --- | --- | --- | --- | --- | --- | --- | --- | --- | --- | --- | --- | --- | --- | --- | --- | --- | --- | --- | --- | --- | --- | --- | --- | --- | --- | --- | --- | --- | --- | --- | --- | --- | --- | --- | --- | --- | --- | --- | --- | --- | --- | --- | --- | --- | --- | --- | --- | --- | --- | --- | --- | --- | --- | --- | --- | --- | --- | --- | --- | --- | --- | --- | --- | --- | --- | --- | --- | --- | --- | --- | --- | --- | --- | --- | --- | --- | --- | --- | --- | --- | --- | --- | --- | --- | --- | --- | --- | --- | --- | --- | --- | --- | --- | --- | --- | --- | --- | --- | --- | --- | --- | --- | --- | --- | --- | --- | --- | --- | --- | --- | --- | --- | --- | --- | --- | --- | --- | --- | --- | --- | --- | --- | --- | --- | --- | --- | --- | --- | --- | --- | --- | --- | --- | --- | --- | --- | --- | --- | --- | --- | --- | --- | --- | --- | --- | --- | --- | --- | --- | --- | --- | --- | --- | --- | --- | --- | --- | --- | --- | --- | --- | --- | --- | --- | --- | --- | --- | --- | --- | --- | --- | --- | --- | --- | --- | --- | --- | --- | --- | --- | --- | --- | --- | --- | --- | --- | --- | --- | --- | --- | --- | --- | --- | --- | --- | --- | --- | --- | --- | --- | --- | --- | --- | --- | --- | --- | --- | --- | --- | --- | --- | --- | --- | --- | --- | --- | --- | --- | --- | --- | --- | --- | --- | --- | --- | --- | --- | --- | --- | --- | --- | --- | --- | --- | --- | --- | --- | --- | --- | --- | --- | --- | --- | --- | --- | --- | --- | --- | --- | --- | --- | --- | --- | --- | --- | --- | --- | --- | --- | --- | --- | --- | --- | --- | --- | --- | --- | --- | --- | --- | --- | --- | --- | --- | --- | --- | --- | --- | --- | --- | --- | --- | --- | --- | --- | --- | --- | --- | --- | --- | --- | --- | --- | --- | --- | --- | --- | --- | --- | --- | --- | --- | --- | --- | --- | --- | --- | --- | --- | --- | --- | --- | --- | --- | --- | --- | --- | --- | --- | --- | --- | --- | --- | --- | --- | --- | --- | --- | --- | --- | --- | --- | --- | --- | --- | --- | --- | --- | --- | --- | --- | --- | --- | --- | --- | --- | --- | --- | --- | --- | --- | --- | --- | --- | --- | --- | --- | --- | --- | --- | --- | --- | --- | --- | --- | --- | --- | --- | --- | --- | --- | --- | --- | --- | --- | --- | --- | --- | --- | --- | --- | --- | --- | --- | --- | --- | --- | --- | --- | --- | --- | --- | --- | --- | --- | --- | --- | --- | --- | --- | --- | --- | --- | --- | --- | --- | --- | --- | --- | --- | --- | --- | --- | --- | --- | --- | --- | --- | --- | --- | --- | --- | --- | --- | --- | --- | --- | --- | --- | --- | --- | --- | --- | --- | --- | --- | --- | --- | --- | --- | --- | --- | --- | --- | --- | --- | --- | --- | --- | --- | --- | --- | --- | --- | --- | --- | --- | --- | --- | --- | --- | --- | --- | --- | --- | --- | --- | --- | --- | --- | --- | --- | --- | --- | --- | --- | --- | --- | --- | --- | --- | --- | --- | --- | --- | --- | --- | --- | --- | --- | --- | --- | --- | --- | --- | --- | --- | --- | --- | --- | --- | --- | --- | --- | --- | --- | --- | --- | --- | --- | --- | --- | --- | --- | --- | --- | --- | --- | --- | --- | --- | --- | --- | --- | --- | --- | --- | --- | --- | --- | --- | --- | --- | --- | --- | --- | --- | --- | --- | --- | --- | --- | --- | --- | --- | --- | --- | --- | --- | --- | --- | --- | --- | --- | --- | --- | --- | --- | --- | --- | --- | --- | --- | --- | --- | --- | --- | --- | --- | --- | --- | --- | --- | --- | --- | --- | --- | --- | --- | --- | --- | --- | --- | --- | --- | --- | --- | --- | --- | --- | --- | --- | --- | --- | --- | --- | --- | --- | --- | --- | --- | --- | --- | --- | --- | --- | --- | --- | --- | --- | --- | --- | --- | --- | --- | --- | --- | --- | --- | --- | --- | --- | --- | --- | --- | --- | --- | --- | --- | --- | --- | --- | --- | --- | --- | --- | --- | --- | --- | --- | --- | --- | --- | --- | --- | --- | --- | --- | --- | --- | --- | --- | --- | --- | --- | --- | --- | --- | --- | --- | --- | --- | --- | --- | --- | --- | --- | --- | --- | --- | --- | --- | --- | --- | --- | --- | --- | --- | --- | --- | --- | --- | --- | --- | --- | --- | --- | --- | --- | --- | --- | --- | --- | --- | --- | --- | --- | --- | --- | --- | --- | --- | --- | --- | --- | --- | --- | --- | --- | --- | --- | --- | --- | --- | --- | --- | --- | --- | --- | --- | --- | --- | --- | --- | --- | --- | --- | --- | --- | --- | --- | --- | --- | --- | --- | --- | --- | --- | --- | --- | --- | --- | --- | --- | --- | --- | --- | --- | --- | --- | --- | --- | --- | --- | --- | --- | --- | --- | --- | --- | --- | --- | --- | --- | --- | --- | --- | --- | --- | --- | --- | --- | --- | --- | --- | --- | --- | --- | --- | --- | --- | --- | --- | --- | --- | --- | --- | --- | --- | --- | --- | --- | --- | --- | --- | --- | --- | --- | --- | --- | --- | --- | --- | --- | --- | --- | --- | --- | --- | --- | --- | --- | --- | --- | --- | --- | --- | --- | --- | --- | --- | --- | --- | --- | --- | --- | --- | --- | --- | --- | --- | --- | --- | --- | --- | --- | --- | --- | --- | --- | --- | --- | --- | --- | --- | --- | --- | --- | --- | --- | --- | --- | --- | --- | --- | --- | --- | --- | --- | --- | --- | --- | --- | --- | --- | --- | --- | --- | --- | --- | --- | --- | --- | --- | --- | --- | --- | --- | --- | --- | --- | --- | --- | --- | --- | --- | --- | --- | --- | --- | --- | --- | --- | --- | --- | --- | --- | --- | --- | --- | --- | --- | --- | --- | --- | --- | --- | --- | --- | --- | --- | --- | --- | --- | --- | --- | --- | --- | --- | --- | --- | --- | --- | --- | --- | --- | --- | --- | --- | --- | --- | --- | --- | --- | --- | --- | --- | --- | --- | --- | --- | --- | --- | --- | --- | --- | --- | --- | --- | --- | --- | --- | --- | --- | --- | --- | --- | --- | --- | --- | --- | --- | --- | --- | --- | --- | --- | --- | --- | --- | --- | --- | --- | --- | --- | --- | --- | --- | --- | --- | --- | --- | --- | --- | --- | --- | --- | --- | --- | --- | --- | --- | --- | --- | --- | --- | --- | --- | --- | --- | --- | --- | --- | --- | --- | --- | --- | --- | --- | --- | --- | --- | --- | --- | --- | --- | --- | --- | --- | --- | --- | --- | --- | --- | --- | --- | --- | --- | --- | --- | --- | --- | --- | --- | --- | --- | --- | --- | --- | --- | --- | --- | --- | --- | --- | --- | --- | --- | --- | --- | --- | --- | --- | --- | --- | --- | --- | --- | --- | --- | --- | --- | --- | --- | --- | --- | --- | --- | --- | --- | --- | --- | --- | --- | --- | --- | --- | --- | --- | --- | --- | --- | --- | --- | --- | --- | --- | --- | --- | --- | --- | --- | --- | --- | --- | --- | --- | --- | --- | --- | --- | --- | --- | --- | --- | --- | --- | --- | --- | --- | --- | --- | --- | --- | --- | --- | --- | --- | --- | --- | --- | --- | --- | --- | --- | --- | --- | --- | --- | --- | --- | --- | --- | --- | --- | --- | --- | --- | --- | --- | --- | --- | --- | --- | --- | --- | --- | --- | --- | --- | --- | --- | --- | --- | --- | --- | --- | --- | --- | --- | --- | --- | --- | --- | --- | --- | --- | --- | --- | --- | --- | --- | --- | --- | --- | --- | --- | --- | --- | --- | --- | --- | --- | --- | --- | --- | --- | --- | --- | --- | --- | --- | --- | --- | --- | --- | --- | --- | --- | --- | --- | --- | --- | --- | --- | --- | --- | --- | --- | --- | --- | --- | --- | --- | --- | --- | --- | --- | --- | --- | --- | --- | --- | --- | --- | --- | --- | --- | --- | --- | --- | --- | --- | --- | --- | --- | --- | --- | --- | --- | --- | --- | --- | --- | --- | --- | --- | --- | --- | --- | --- | --- | --- | --- | --- | --- | --- | --- | --- | --- | --- | --- | --- | --- | --- | --- | --- | --- | --- | --- | --- | --- | --- | --- | --- | --- | --- | --- | --- | --- | --- | --- | --- | --- | --- | --- | --- | --- | --- | --- | --- | --- | --- | --- | --- | --- | --- | --- | --- | --- | --- | --- | --- | --- | --- | --- | --- | --- | --- | --- | --- | --- | --- | --- | --- | --- | --- | --- | --- | --- | --- | --- | --- | --- | --- | --- | --- | --- | --- | --- | --- | --- | --- | --- | --- | --- | --- | --- | --- | --- | --- | --- | --- | --- | --- | --- | --- | --- | --- | --- | --- | --- | --- | --- | --- | --- | --- | --- | --- | --- | --- | --- | --- | --- | --- | --- | --- | --- | --- | --- | --- | --- | --- | --- | --- | --- | --- | --- | --- | --- | --- | --- | --- | --- | --- | --- | --- | --- | --- | --- | --- | --- | --- | --- | --- | --- | --- | --- | --- | --- | --- | --- | --- | --- | --- | --- | --- | --- | --- | --- | --- | --- | --- | --- | --- | --- | --- | --- | --- | --- | --- | --- | --- | --- | --- | --- | --- | --- | --- | --- | --- | --- | --- | --- | --- | --- | --- | --- | --- | --- | --- | --- | --- | --- | --- | --- | --- | --- | --- | --- | --- | --- | --- | --- | --- | --- | --- | --- | --- | --- | --- | --- | --- | --- | --- | --- | --- | --- | --- | --- | --- | --- | --- | --- | --- | --- | --- | --- | --- | --- | --- | --- | --- | --- | --- | --- | --- | --- | --- | --- | --- | --- | --- | --- | --- | --- | --- | --- | --- | --- | --- | --- | --- | --- | --- | --- | --- | --- | --- | --- | --- | --- | --- | --- | --- | --- | --- | --- | --- | --- | --- | --- | --- | --- | --- | --- | --- | --- | --- | --- | --- | --- | --- | --- | --- | --- | --- | --- | --- | --- | --- | --- | --- | --- | --- | --- | --- | --- | --- | --- | --- | --- | --- | --- | --- | --- | --- | --- | --- | --- | --- | --- | --- | --- | --- | --- | --- | --- | --- | --- | --- | --- | --- | --- | --- | --- | --- | --- | --- | --- | --- | --- | --- | --- | --- | --- | --- | --- | --- | --- | --- | --- | --- | --- | --- | --- | --- | --- | --- | --- | --- | --- | --- | --- | --- | --- | --- | --- | --- | --- | --- | --- | --- | --- | --- | --- | --- | --- | --- | --- | --- | --- | --- | --- | --- | --- | --- | --- | --- | --- | --- | --- | --- | --- | --- | --- | --- | --- | --- | --- | --- | --- | --- | --- | --- | --- | --- | --- | --- | --- | --- | --- | --- | --- | --- | --- | --- | --- | --- | --- | --- | --- | --- | --- | --- | --- | --- | --- | --- | --- | --- | --- | --- | --- | --- | --- | --- | --- | --- | --- | --- | --- | --- | --- | --- | --- | --- | --- | --- | --- | --- | --- | --- | --- | --- | --- | --- | --- | --- | --- | --- | --- | --- | --- | --- | --- | --- | --- | --- | --- | --- | --- | --- | --- | --- | --- | --- | --- | --- | --- | --- | --- | --- | --- | --- | --- | --- | --- | --- | --- | --- | --- | --- | --- | --- | --- | --- | --- | --- | --- | --- | --- | --- | --- | --- | --- | --- | --- | --- | --- | --- | --- | --- | --- | --- | --- | --- | --- | --- | --- | --- | --- | --- | --- | --- | --- | --- | --- | --- | --- | --- | --- | --- | --- | --- | --- | --- | --- | --- | --- | --- | --- | --- | --- | --- | --- | --- | --- | --- | --- | --- | --- | --- | --- | --- | --- | --- | --- | --- | --- | --- | --- | --- | --- | --- | --- | --- | --- | --- | --- | --- | --- | --- | --- | --- | --- | --- | --- | --- | --- | --- | --- | --- | --- | --- | --- | --- | --- | --- | --- | --- | --- | --- | --- | --- | --- | --- | --- | --- | --- | --- | --- | --- | --- | --- | --- | --- | --- | --- | --- | --- | --- | --- | --- | --- | --- | --- | --- | --- | --- | --- | --- | --- | --- | --- | --- | --- | --- | --- | --- | --- | --- | --- | --- | --- | --- | --- | --- | --- | --- | --- | --- | --- | --- | --- | --- | --- | --- | --- | --- | --- | --- | --- | --- | --- | --- | --- | --- | --- | --- | --- | --- | --- | --- | --- | --- | --- | --- | --- | --- | --- | --- | --- | --- | --- | --- | --- | --- | --- | --- | --- | --- | --- | --- | --- | --- | --- | --- | --- | --- | --- | --- | --- | --- | --- | --- | --- | --- | --- | --- | --- | --- | --- | --- | --- | --- | --- | --- | --- | --- | --- | --- | --- | --- | --- | --- | --- | --- | --- | --- | --- | --- | --- | --- | --- | --- | --- | --- | --- | --- | --- | --- | --- | --- | --- | --- | --- | --- | --- | --- | --- | --- | --- | --- | --- | --- | --- | --- | --- | --- | --- | --- | --- | --- | --- | --- | --- | --- | --- | --- | --- | --- | --- | --- | --- | --- | --- | --- | --- | --- | --- | --- | --- | --- | --- | --- | --- | --- | --- | --- | --- | --- | --- | --- | --- | --- | --- | --- | --- | --- | --- | --- | --- | --- | --- | --- | --- | --- | --- | --- | --- | --- | --- | --- | --- | --- | --- | --- | --- | --- | --- | --- | --- | --- | --- | --- | --- | --- | --- | --- | --- | --- | --- | --- | --- | --- | --- | --- | --- | --- | --- | --- | --- | --- | --- | --- | --- | --- | --- | --- | --- | --- | --- | --- | --- | --- | --- | --- | --- | --- | --- | --- | --- | --- | --- | --- | --- | --- | --- | --- | --- | --- | --- | --- | --- | --- | --- | --- | --- | --- | --- | --- | --- | --- | --- | --- | --- | --- | --- | --- | --- | --- | --- | --- | --- | --- | --- | --- | --- | --- | --- | --- | --- | --- | --- | --- | --- | --- | --- | --- | --- | --- | --- | --- | --- | --- | --- | --- | --- | --- | --- | --- | --- | --- | --- | --- | --- | --- | --- | --- | --- | --- | --- | --- | --- | --- | --- | --- | --- | --- | --- | --- | --- | --- | --- | --- | --- | --- | --- | --- | --- | --- | --- | --- | --- | --- | --- | --- | --- | --- | --- | --- | --- | --- | --- | --- | --- | --- | --- | --- | --- | --- | --- | --- | --- | --- | --- | --- | --- | --- | --- | --- | --- | --- | --- | --- | --- | --- | --- | --- | --- | --- | --- | --- | --- | --- | --- | --- | --- | --- | --- | --- | --- | --- | --- | --- | --- | --- | --- | --- | --- | --- | --- | --- | --- | --- | --- | --- | --- | --- | --- | --- | --- | --- | --- | --- | --- | --- | --- | --- | --- | --- | --- | --- | --- | --- | --- | --- | --- | --- | --- | --- | --- | --- | --- | --- | --- | --- | --- | --- | --- | --- | --- | --- | --- | --- | --- | --- | --- | --- | --- | --- | --- | --- | --- | --- | --- | --- | --- | --- | --- | --- | --- | --- | --- | --- | --- | --- | --- | --- | --- | --- | --- | --- | --- | --- | --- | --- | --- | --- | --- | --- | --- | --- | --- | --- | --- | --- | --- | --- | --- | --- | --- | --- | --- | --- | --- | --- | --- | --- | --- | --- | --- | --- | --- | --- | --- | --- | --- | --- | --- | --- | --- | --- | --- | --- | --- | --- | --- | --- | --- | --- | --- | --- | --- | --- | --- | --- | --- | --- | --- | --- | --- | --- | --- | --- | --- | --- | --- | --- | --- | --- | --- | --- | --- | --- | --- | --- | --- | --- | --- | --- | --- | --- | --- | --- | --- | --- | --- | --- | --- | --- | --- | --- | --- | --- | --- | --- | --- | --- | --- | --- | --- | --- | --- | --- | --- | --- | --- | --- | --- | --- | --- | --- | --- | --- | --- | --- | --- | --- | --- | --- | --- | --- | --- | --- | --- | --- | --- | --- | --- | --- | --- | --- | --- | --- | --- | --- | --- | --- | --- | --- | --- | --- | --- | --- | --- | --- | --- | --- | --- | --- | --- | --- | --- | --- | --- | --- | --- | --- | --- | --- | --- | --- | --- | --- | --- | --- | --- | --- | --- | --- | --- | --- | --- | --- | --- | --- | --- | --- | --- | --- | --- | --- | --- | --- | --- | --- | --- | --- | --- | --- | --- | --- | --- | --- | --- | --- | --- | --- | --- | --- | --- | --- | --- | --- | --- | --- | --- | --- | --- | --- | --- | --- | --- | --- | --- | --- | --- | --- | --- | --- | --- | --- | --- | --- | --- | --- | --- | --- | --- | --- | --- | --- | --- | --- | --- | --- | --- | --- | --- | --- | --- | --- | --- | --- | --- | --- | --- | --- | --- | --- | --- | --- | --- | --- | --- | --- | --- | --- | --- | --- | --- | --- | --- | --- | --- | --- | --- | --- | --- | --- | --- | --- | --- | --- | --- | --- | --- | --- | --- | --- | --- | --- | --- | --- | --- | --- | --- | --- | --- | --- | --- | --- | --- | --- | --- | --- | --- | --- | --- | --- | --- | --- | --- | --- | --- | --- | --- | --- | --- | --- | --- | --- | --- | --- | --- | --- | --- | --- | --- | --- | --- | --- | --- | --- | --- | --- | --- | --- | --- | --- | --- | --- | --- | --- | --- | --- | --- | --- | --- | --- | --- | --- | --- | --- | --- | --- | --- | --- | --- | --- | --- | --- | --- | --- | --- | --- | --- | --- | --- | --- | --- | --- | --- | --- | --- | --- | --- | --- | --- | --- | --- | --- | --- | --- | --- | --- | --- | --- | --- | --- | --- | --- | --- | --- | --- | --- | --- | --- | --- | --- | --- | --- | --- | --- | --- | --- | --- | --- | --- | --- | --- | --- | --- | --- | --- | --- | --- | --- | --- | --- | --- | --- | --- | --- | --- | --- | --- | --- | --- | --- | --- | --- | --- | --- | --- | --- | --- | --- | --- | --- | --- | --- | --- | --- | --- | --- | --- | --- | --- | --- | --- | --- | --- | --- | --- | --- | --- | --- | --- | --- | --- | --- | --- | --- | --- | --- | --- | --- | --- | --- | --- | --- | --- | --- | --- | --- | --- | --- | --- | --- | --- | --- | --- | --- | --- | --- | --- | --- | --- | --- | --- | --- | --- | --- | --- | --- | --- | --- | --- | --- | --- | --- | --- | --- | --- | --- | --- | --- | --- | --- | --- | --- | --- | --- | --- | --- | --- | --- | --- | --- | --- | --- | --- | --- | --- | --- | --- | --- | --- | --- | --- | --- | --- | --- | --- | --- | --- | --- | --- | --- | --- | --- | --- | --- | --- | --- | --- | --- | --- | --- | --- | --- | --- | --- | --- | --- | --- | --- | --- | --- | --- | --- | --- | --- | --- | --- | --- | --- | --- | --- | --- | --- | --- | --- | --- | --- | --- | --- | --- | --- | --- | --- | --- | --- | --- | --- | --- | --- | --- | --- | --- | --- | --- | --- | --- | --- | --- | --- | --- | --- | --- | --- | --- | --- | --- | --- | --- | --- | --- | --- | --- | --- | --- | --- | --- | --- | --- | --- | --- | --- | --- | --- | --- | --- | --- | --- | --- | --- | --- | --- | --- | --- | --- | --- | --- | --- | --- | --- | --- | --- | --- | --- | --- | --- | --- | --- | --- | --- | --- | --- | --- | --- | --- | --- | --- | --- | --- | --- | --- | --- | --- | --- | --- | --- | --- | --- | --- | --- | --- | --- | --- | --- | --- | --- | --- | --- | --- | --- | --- | --- | --- | --- | --- | --- | --- | --- | --- | --- | --- | --- | --- | --- | --- | --- | --- | --- | --- | --- | --- | --- | --- | --- | --- | --- | --- | --- | --- | --- | --- | --- | --- | --- | --- | --- | --- | --- | --- | --- | --- | --- | --- | --- | --- | --- | --- | --- | --- | --- | --- | --- | --- | --- | --- | --- | --- | --- | --- | --- | --- | --- | --- | --- | --- | --- | --- | --- | --- | --- | --- | --- | --- | --- | --- | --- | --- | --- | --- | --- | --- | --- | --- | --- | --- | --- | --- | --- | --- | --- | --- | --- | --- | --- | --- | --- | --- | --- | --- | --- | --- | --- | --- | --- | --- | --- | --- | --- | --- | --- | --- | --- | --- | --- | --- | --- | --- | --- | --- | --- | --- | --- | --- | --- | --- | --- | --- | --- | --- | --- | --- | --- | --- | --- | --- | --- | --- | --- | --- | --- | --- | --- | --- | --- | --- | --- | --- | --- | --- | --- | --- | --- | --- | --- | --- | --- | --- | --- | --- | --- | --- | --- | --- | --- | --- | --- | --- | --- | --- | --- | --- | --- | --- | --- | --- | --- | --- | --- | --- | --- | --- | --- | --- | --- | --- | --- | --- | --- | --- | --- | --- | --- | --- | --- | --- | --- | --- | --- | --- | --- | --- | --- | --- | --- | --- | --- | --- | --- | --- | --- | --- | --- | --- | --- | --- | --- | --- | --- | --- | --- | --- | --- | --- | --- | --- | --- | --- | --- | --- | --- | --- | --- | --- | --- | --- | --- | --- | --- | --- | --- | --- | --- | --- | --- | --- | --- | --- | --- | --- | --- | --- | --- | --- | --- | --- | --- | --- | --- | --- | --- | --- | --- | --- | --- | --- | --- | --- | --- | --- | --- | --- | --- | --- | --- | --- | --- | --- | --- | --- | --- | --- | --- | --- | --- | --- | --- | --- | --- | --- | --- | --- | --- | --- | --- | --- | --- | --- | --- | --- | --- | --- | --- | --- | --- | --- | --- | --- | --- | --- | --- | --- | --- | --- | --- | --- | --- | --- | --- | --- | --- | --- | --- | --- | --- | --- | --- | --- | --- | --- | --- | --- | --- | --- | --- | --- | --- | --- | --- | --- | --- | --- | --- | --- | --- | --- | --- | --- | --- | --- | --- | --- | --- | --- | --- | --- | --- | --- | --- | --- | --- | --- | --- | --- | --- | --- | --- | --- | --- | --- | --- | --- | --- | --- | --- | --- | --- | --- | --- | --- | --- | --- | --- | --- | --- | --- | --- | --- | --- | --- | --- | --- | --- | --- | --- | --- | --- | --- | --- | --- | --- | --- | --- | --- | --- | --- | --- | --- | --- | --- | --- | --- | --- | --- | --- | --- | --- | --- | --- | --- | --- | --- | --- | --- | --- | --- | --- | --- | --- | --- | --- | --- | --- | --- | --- | --- | --- | --- | --- | --- | --- | --- | --- | --- | --- | --- | --- | --- | --- | --- | --- | --- | --- | --- | --- | --- | --- | --- | --- | --- | --- | --- | --- | --- | --- | --- | --- | --- | --- | --- | --- | --- | --- | --- | --- | --- | --- | --- | --- | --- | --- | --- | --- | --- | --- | --- | --- | --- | --- | --- | --- | --- | --- | --- | --- | --- | --- | --- | --- | --- | --- | --- | --- | --- | --- | --- | --- | --- | --- | --- | --- | --- | --- | --- | --- | --- | --- | --- | --- | --- | --- | --- | --- | --- | --- | --- | --- | --- | --- | --- | --- | --- | --- | --- | --- | --- | --- | --- | --- | --- | --- | --- | --- | --- | --- | --- | --- | --- | --- | --- | --- | --- | --- | --- | --- | --- | --- | --- | --- | --- | --- | --- | --- | --- | --- | --- | --- | --- | --- | --- | --- | --- | --- | --- | --- | --- | --- | --- | --- | --- | --- | --- | --- | --- | --- | --- | --- | --- | --- | --- | --- | --- | --- | --- | --- | --- | --- | --- | --- | --- | --- | --- | --- | --- | --- | --- | --- | --- | --- | --- | --- | --- | --- | --- | --- | --- | --- | --- | --- | --- | --- | --- | --- | --- | --- | --- | --- | --- | --- | --- | --- | --- | --- | --- | --- | --- | --- | --- | --- | --- | --- | --- | --- | --- | --- | --- | --- | --- | --- | --- | --- | --- | --- | --- | --- | --- | --- | --- | --- | --- | --- | --- | --- | --- | --- | --- | --- | --- | --- | --- | --- | --- | --- | --- | --- | --- | --- | --- | --- | --- | --- | --- | --- | --- | --- | --- | --- | --- | --- | --- | --- | --- | --- | --- | --- | --- | --- | --- | --- | --- | --- | --- | --- | --- | --- | --- | --- | --- | --- | --- | --- | --- | --- | --- | --- | --- | --- | --- | --- | --- | --- | --- | --- | --- | --- | --- | --- | --- | --- | --- | --- | --- | --- | --- | --- | --- | --- | --- | --- | --- | --- | --- | --- | --- | --- | --- | --- | --- | --- | --- | --- | --- | --- | --- | --- | --- | --- | --- | --- | --- | --- | --- | --- | --- | --- | --- | --- | --- | --- | --- | --- | --- | --- | --- | --- | --- | --- | --- | --- | --- | --- | --- | --- | --- | --- | --- | --- | --- | --- | --- | --- | --- | --- | --- | --- | --- | --- | --- | --- | --- | --- | --- | --- | --- | --- | --- | --- | --- | --- | --- | --- | --- | --- | --- | --- | --- | --- | --- | --- | --- | --- | --- | --- | --- | --- | --- | --- | --- | --- | --- | --- | --- | --- | --- | --- | --- | --- | --- | --- | --- | --- | --- | --- | --- | --- | --- | --- | --- | --- | --- | --- | --- | --- | --- | --- | --- | --- | --- | --- | --- | --- | --- | --- | --- | --- | --- | --- | --- | --- | --- | --- | --- | --- | --- | --- | --- | --- | --- | --- | --- | --- | --- | --- | --- | --- | --- | --- | --- | --- | --- | --- | --- | --- | --- | --- | --- | --- | --- | --- | --- | --- | --- | --- | --- | --- | --- | --- | --- | --- | --- | --- | --- | --- | --- | --- | --- | --- | --- | --- | --- | --- | --- | --- | --- | --- | --- | --- | --- | --- | --- | --- | --- | --- | --- | --- | --- | --- | --- | --- | --- | --- | --- | --- | --- | --- | --- | --- | --- | --- | --- | --- | --- | --- | --- | --- | --- | --- | --- | --- | --- | --- | --- | --- | --- | --- | --- | --- | --- | --- | --- | --- | --- | --- | --- | --- | --- | --- | --- | --- | --- | --- | --- | --- | --- | --- | --- | --- | --- | --- | --- | --- | --- | --- | --- | --- | --- | --- | --- | --- | --- | --- | --- | --- | --- | --- | --- | --- | --- | --- | --- | --- | --- | --- | --- | --- | --- | --- | --- | --- | --- | --- | --- | --- | --- | --- | --- | --- | --- | --- | --- | --- | --- | --- | --- | --- | --- | --- | --- | --- | --- | --- | --- | --- | --- | --- | --- | --- | --- | --- | --- | --- | --- | --- | --- | --- | --- | --- | --- | --- | --- | --- | --- | --- | --- | --- | --- | --- | --- | --- | --- | --- | --- | --- | --- | --- | --- | --- | --- | --- | --- | --- | --- | --- | --- | --- | --- | --- | --- | --- | --- | --- | --- | --- | --- | --- | --- | --- | --- | --- | --- | --- | --- | --- | --- | --- | --- | --- | --- | --- | --- | --- | --- | --- | --- | --- | --- | --- | --- | --- | --- | --- | --- | --- | --- | --- | --- | --- | --- | --- | --- | --- | --- | --- | --- | --- | --- | --- | --- | --- | --- | --- | --- | --- | --- | --- | --- | --- | --- | --- | --- | --- | --- | --- | --- | --- | --- | --- | --- | --- | --- | --- | --- | --- | --- | --- | --- | --- | --- | --- | --- | --- | --- | --- | --- | --- | --- | --- | --- | --- | --- | --- | --- | --- | --- | --- | --- | --- | --- | --- | --- | --- | --- | --- | --- | --- | --- | --- | --- | --- | --- | --- | --- | --- | --- | --- | --- | --- | --- | --- | --- | --- | --- | --- | --- | --- | --- | --- | --- | --- | --- | --- | --- | --- | --- | --- | --- | --- | --- | --- | --- | --- | --- | --- | --- | --- | --- | --- | --- | --- | --- | --- | --- | --- | --- | --- | --- | --- | --- | --- | --- | --- | --- | --- | --- | --- | --- | --- | --- | --- | --- | --- | --- | --- | --- | --- | --- | --- | --- | --- | --- | --- | --- | --- | --- | --- | --- | --- | --- | --- | --- | --- | --- | --- | --- | --- | --- | --- | --- | --- | --- | --- | --- | --- | --- | --- | --- | --- | --- | --- | --- | --- | --- | --- | --- | --- | --- | --- | --- | --- | --- | --- | --- | --- | --- | --- | --- | --- | --- | --- | --- | --- | --- | --- | --- | --- | --- | --- | --- | --- | --- | --- | --- | --- | --- | --- | --- | --- | --- | --- | --- | --- | --- | --- | --- | --- | --- | --- | --- | --- | --- | --- | --- | --- | --- | --- | --- | --- | --- | --- | --- | --- | --- | --- | --- | --- | --- | --- | --- | --- | --- | --- | --- | --- | --- | --- | --- | --- | --- | --- | --- | --- | --- | --- | --- | --- | --- | --- | --- | --- | --- | --- | --- | --- | --- | --- | --- | --- | --- | --- | --- | --- | --- | --- | --- | --- | --- | --- | --- | --- | --- | --- | --- | --- | --- | --- | --- | --- | --- | --- | --- | --- | --- | --- | --- | --- | --- | --- | --- | --- | --- | --- | --- | --- | --- | --- | --- | --- | --- | --- | --- | --- | --- | --- | --- | --- | --- | --- | --- | --- | --- | --- | --- | --- | --- | --- | --- | --- | --- | --- | --- | --- | --- | --- | --- | --- | --- | --- | --- | --- | --- | --- | --- | --- | --- | --- | --- | --- | --- | --- | --- | --- | --- | --- | --- | --- | --- | --- | --- | --- | --- | --- | --- | --- | --- | --- | --- | --- | --- | --- | --- | --- | --- | --- | --- | --- | --- | --- | --- | --- | --- | --- | --- | --- | --- | --- | --- | --- | --- | --- | --- | --- | --- | --- | --- | --- | --- | --- | --- | --- | --- | --- | --- | --- | --- | --- | --- | --- | --- | --- | --- | --- | --- | --- | --- | --- | --- | --- | --- | --- | --- | --- | --- | --- | --- | --- | --- | --- | --- | --- | --- | --- | --- | --- | --- | --- | --- | --- | --- | --- | --- | --- | --- | --- | --- | --- | --- | --- | --- | --- | --- | --- | --- | --- | --- | --- | --- | --- | --- | --- | --- | --- | --- | --- | --- | --- | --- | --- | --- | --- | --- | --- | --- | --- | --- | --- | --- | --- | --- | --- | --- | --- | --- | --- | --- | --- | --- | --- | --- | --- | --- | --- | --- | --- | --- | --- | --- | --- | --- | --- | --- | --- | --- | --- | --- | --- | --- | --- | --- | --- | --- | --- | --- | --- | --- | --- | --- | --- | --- | --- | --- | --- | --- | --- | --- | --- | --- | --- | --- | --- | --- | --- | --- | --- | --- | --- | --- | --- | --- | --- | --- | --- | --- | --- | --- | --- | --- | --- | --- | --- | --- | --- | --- | --- | --- | --- | --- | --- | --- | --- | --- | --- | --- | --- | --- | --- | --- | --- | --- | --- | --- | --- | --- | --- | --- | --- | --- | --- | --- | --- | --- | --- | --- | --- | --- | --- | --- | --- | --- | --- | --- | --- | --- | --- | --- | --- | --- | --- | --- | --- | --- | --- | --- | --- | --- | --- | --- | --- | --- | --- | --- | --- | --- | --- | --- | --- | --- | --- | --- | --- | --- | --- | --- | --- | --- | --- | --- | --- | --- | --- | --- | --- | --- | --- | --- | --- | --- | --- | --- | --- | --- | --- | --- | --- | --- | --- | --- | --- | --- | --- | --- | --- | --- | --- | --- | --- | --- | --- | --- | --- | --- | --- | --- | --- | --- | --- | --- | --- | --- | --- | --- | --- | --- | --- | --- | --- | --- | --- | --- | --- | --- | --- | --- | --- | --- | --- | --- | --- | --- | --- | --- | --- | --- | --- | --- | --- | --- | --- | --- | --- | --- | --- | --- | --- | --- | --- | --- | --- | --- | --- | --- | --- | --- | --- | --- | --- | --- | --- | --- | --- | --- | --- | --- | --- | --- | --- | --- | --- | --- | --- | --- | --- | --- | --- | --- | --- | --- | --- | --- | --- | --- | --- | --- | --- | --- | --- | --- | --- | --- | --- | --- | --- | --- | --- | --- | --- | --- | --- | --- | --- | --- | --- | --- | --- | --- | --- | --- | --- | --- | --- | --- | --- | --- | --- | --- | --- | --- | --- | --- | --- | --- | --- | --- | --- | --- | --- | --- | --- | --- | --- | --- | --- | --- | --- | --- | --- | --- | --- | --- | --- | --- | --- | --- | --- | --- | --- | --- | --- | --- | --- | --- | --- | --- | --- | --- | --- | --- | --- | --- | --- | --- | --- | --- | --- | --- | --- | --- | --- | --- | --- | --- | --- | --- | --- | --- | --- | --- | --- | --- | --- | --- | --- | --- | --- | --- | --- | --- | --- | --- | --- | --- | --- | --- | --- | --- | --- | --- | --- | --- | --- | --- | --- | --- | --- | --- | --- | --- | --- | --- | --- | --- | --- | --- | --- | --- | --- | --- | --- | --- | --- | --- | --- | --- | --- | --- | --- | --- | --- | --- | --- | --- | --- | --- | --- | --- | --- | --- | --- | --- | --- | --- | --- | --- | --- | --- | --- | --- | --- | --- | --- | --- | --- | --- | --- | --- | --- | --- | --- | --- | --- | --- | --- | --- | --- | --- | --- | --- | --- | --- | --- | --- | --- | --- | --- | --- | --- | --- | --- | --- | --- | --- | --- | --- | --- | --- | --- | --- | --- | --- | --- | --- | --- | --- | --- | --- | --- | --- | --- | --- | --- | --- | --- | --- | --- | --- | --- | --- | --- | --- | --- | --- | --- | --- | --- | --- | --- | --- | --- | --- | --- | --- | --- | --- | --- | --- | --- | --- | --- | --- | --- | --- | --- | --- | --- | --- | --- | --- | --- | --- | --- | --- | --- | --- | --- | --- | --- | --- | --- | --- | --- | --- | --- | --- | --- | --- | --- | --- | --- | --- | --- | --- | --- | --- | --- | --- | --- | --- | --- | --- | --- | --- | --- | --- | --- | --- | --- | --- | --- | --- | --- | --- | --- | --- | --- | --- | --- | --- | --- | --- | --- | --- | --- | --- | --- | --- | --- | --- | --- | --- | --- | --- | --- | --- | --- | --- | --- | --- | --- | --- | --- | --- | --- | --- | --- | --- | --- | --- | --- | --- | --- | --- | --- | --- | --- | --- | --- | --- | --- | --- | --- | --- | --- | --- | --- | --- | --- | --- | --- | --- | --- | --- | --- | --- | --- | --- | --- | --- | --- | --- | --- | --- | --- | --- | --- | --- | --- | --- | --- | --- | --- | --- | --- | --- | --- | --- | --- | --- | --- | --- | --- | --- | --- | --- | --- | --- | --- | --- | --- | --- | --- | --- | --- | --- | --- | --- | --- | --- | --- | --- | --- | --- | --- | --- | --- | --- | --- | --- | --- | --- | --- | --- | --- | --- | --- | --- | --- | --- | --- | --- | --- | --- | --- | --- | --- | --- | --- | --- | --- | --- | --- | --- | --- | --- | --- | --- | --- | --- | --- | --- | --- | --- | --- | --- | --- | --- | --- | --- | --- | --- | --- | --- | --- | --- | --- | --- | --- | --- | --- | --- | --- | --- | --- | --- | --- | --- | --- | --- | --- | --- | --- | --- | --- | --- | --- | --- | --- | --- | --- | --- | --- | --- | --- | --- | --- | --- | --- | --- | --- | --- | --- | --- | --- | --- | --- | --- | --- | --- | --- | --- | --- | --- | --- | --- | --- | --- | --- | --- | --- | --- | --- | --- | --- | --- | --- | --- | --- | --- | --- | --- | --- | --- | --- | --- | --- | --- | --- | --- | --- | --- | --- | --- | --- | --- | --- | --- | --- | --- | --- | --- | --- | --- | --- | --- | --- | --- | --- | --- | --- | --- | --- | --- | --- | --- | --- | --- | --- | --- | --- | --- | --- | --- | --- | --- | --- | --- | --- | --- | --- | --- | --- | --- | --- | --- | --- | --- | --- | --- | --- | --- | --- | --- | --- | --- | --- | --- | --- | --- | --- | --- | --- | --- | --- | --- | --- | --- | --- | --- | --- | --- | --- | --- | --- | --- | --- | --- | --- | --- | --- | --- | --- | --- | --- | --- | --- | --- | --- | --- | --- | --- | --- | --- | --- | --- | --- | --- | --- | --- | --- | --- | --- | --- | --- | --- | --- | --- | --- | --- | --- | --- | --- | --- | --- | --- | --- | --- | --- | --- | --- | --- | --- | --- | --- | --- | --- | --- | --- | --- | --- | --- | --- | --- | --- | --- | --- | --- | --- | --- | --- | --- | --- | --- | --- | --- | --- | --- | --- | --- | --- | --- | --- | --- | --- | --- | --- | --- | --- | --- | --- | --- | --- | --- | --- | --- | --- | --- | --- | --- | --- | --- | --- | --- | --- | --- | --- | --- | --- | --- | --- | --- | --- | --- | --- | --- | --- | --- | --- | --- | --- | --- | --- | --- | --- | --- | --- | --- | --- | --- | --- | --- | --- | --- | --- | --- | --- | --- | --- | --- | --- | --- | --- | --- | --- | --- | --- | --- | --- | --- | --- | --- | --- | --- | --- | --- | --- | --- | --- | --- | --- | --- | --- | --- | --- | --- | --- | --- | --- | --- | --- | --- | --- | --- | --- | --- | --- | --- | --- | --- | --- | --- | --- | --- | --- | --- | --- | --- | --- | --- | --- | --- | --- | --- | --- | --- | --- | --- | --- | --- | --- | --- | --- | --- | --- | --- | --- | --- | --- | --- | --- | --- | --- | --- | --- | --- | --- | --- | --- | --- | --- | --- | --- | --- | --- | --- | --- | --- | --- | --- | --- | --- | --- | --- | --- | --- | --- | --- | --- | --- | --- | --- | --- | --- | --- | --- | --- | --- | --- | --- | --- | --- | --- | --- | --- | --- | --- | --- | --- | --- | --- | --- | --- | --- | --- | --- | --- | --- | --- | --- | --- | --- | --- | --- | --- | --- | --- | --- | --- | --- | --- | --- | --- | --- | --- | --- | --- | --- | --- | --- | --- | --- | --- | --- | --- | --- | --- | --- | --- | --- | --- | --- | --- | --- | --- | --- | --- | --- | --- | --- | --- | --- | --- | --- | --- | --- | --- | --- | --- | --- | --- | --- | --- | --- | --- | --- | --- | --- | --- | --- | --- | --- | --- | --- | --- | --- | --- | --- | --- | --- | --- | --- | --- | --- | --- | --- | --- | --- | --- | --- | --- | --- | --- | --- | --- | --- | --- | --- | --- | --- | --- | --- | --- | --- | --- | --- | --- | --- | --- | --- | --- | --- | --- | --- | --- | --- | --- | --- | --- | --- | --- | --- | --- | --- | --- | --- | --- | --- | --- | --- | --- | --- | --- | --- | --- | --- | --- | --- | --- | --- | --- | --- | --- | --- | --- | --- | --- | --- | --- | --- | --- | --- | --- | --- | --- | --- | --- | --- | --- | --- | --- | --- |
| |  |  |  |  |  |  |  |  |  | | --- | --- | --- | --- | --- | --- | --- | --- | --- | | **Position** | **Reference** | **Sample** | **Quality** | **Type** | **Region** | **AA Exchange** | **PAM1** | **Known Variant** | | 1977 | A | G | 1605.77 | SNP | intergenic |  |  | - | | 4013 | T | C | 2720.77 | SNP | Rv0003 (recF) | Ile245Thr | 11 | - | | 7362 | G | C | 1763.77 | SNP | Rv0006 (gyrA) | Glu21Gln | 27 | - | | 7585 | G | C | 2606.77 | SNP | Rv0006 (gyrA) | Ser95Thr | 32 | genotype | | 8688 | G | T | 2064.77 | SNP | Rv0006 (gyrA) | Ala463Ser | 28 | - | | 9304 | G | A | 1727.77 | SNP | Rv0006 (gyrA) | Gly668Asp | 6 | - | | 9841 | C | T | 2561.77 | SNP | intergenic |  |  | - | | 9944 | A | C | 1524.77 | SNP | Rv0007 | Ser11Arg | 6 | - | | 11879 | A | G | 1968.77 | SNP | Rv0008c | Ser145Pro | 12 | - | | 14785 | T | C | 2931.77 | SNP | Rv0012 | Cys233Arg | 1 | - | | 15517 | C | G | 2618.77 | SNP | Rv0013 (trpG) | Arg202Gly | 1 | - | | 21795 | G | A | 192.31 | SNP | Rv0018c (pstP) | Pro463Ser | 17 | - | | 24698 | GCCGCGTTGCTCGGGGTAA | G | 3808.73 | DEL | Rv0020c (fhaA) |  |  | - | | 26959 | C | G | 1984.77 | SNP | intergenic |  |  | - | | 27492 | T | A | 1449.74 | SNP | intergenic |  |  | - | | 33231 | CG | C | 3372.73 | DEL | Rv0030 |  |  | - | | 33929 | C | T | 2976.77 | SNP | intergenic |  |  | - | | 34044 | T | C | 2988.77 | SNP | intergenic |  |  | - | | 41432 | C | T | 2629.84 | SNP | Rv0038 | silent (Ile43) | 9872 | - | | 42967 | G | C | 2209.77 | SNP | Rv0040c (mtc28) | silent (Pro133) | 9926 | - | | 43722 | C | T | 2315.77 | SNP | Rv0041 (leuS) | Pro54Leu | 3 | - | | 49857 | G | A | 2194.77 | SNP | Rv0045c | Pro28Leu | 3 | - | | 50557 | T | C | 1573.77 | SNP | Rv0046c (ino1) | Arg190Gly | 1 | - | | 51949 | A | G | 2659.77 | SNP | Rv0048c | Val250Ala | 18 | - | | 54394 | A | G | 2798.77 | SNP | Rv0050 (ponA1) | silent (Ala244) | 9867 | - | | 55553 | C | T | 1019.77 | SNP | Rv0050 (ponA1) | Pro631Ser | 17 | - | | 62049 | A | G | 1763.77 | SNP | Rv0058 (dnaB) | Arg552Gly | 1 | - | | 67121 | A | G | 2057.77 | SNP | Rv0063 | Thr67Ala | 32 | - | | 69989 | G | A | 2629.77 | SNP | Rv0064 | Gly457Asp | 6 | - | | 70816 | A | G | 1859.77 | SNP | Rv0064 | Asn733Asp | 42 | - | | 71336 | G | C | 262.80 | SNP | Rv0064 | Arg906Pro | 5 | - | | 71584 | C | CCGAGCGCTGTTCTGGCGCT AATCTGACGCTAGAATAG | 14570.73 | INS | intergenic |  |  | - | | 74956 | T | C | 2943.77 | SNP | Rv0067c | silent (Lys81) | 9926 | - | | 75940 | G | C | 2125.77 | SNP | Rv0068 | Val(s)214Leu | 3 | - | | 79504 | T | TCGGTGGACCCGGTGGACC | 5528.73 | INS | Rv0071 |  |  | - | | 80616 | C | G | 1741.77 | SNP | intergenic |  |  | - | | 82437 | GT | G | 3266.73 | DEL | Rv0073 |  |  | - | | 84830 | GC | G | 3840.73 | DEL | Rv0075 |  |  | - | | 84832 | C | T | 2242.77 | SNP | Rv0075 | silent (Ala279) | 9867 | - | | 90749 | C | G | 1648.77 | SNP | Rv0083 | Ala117Gly | 21 | - | | 92199 | T | G | 2186.77 | SNP | Rv0083 | silent (Thr600) | 9871 | - | | 103181 | A | G | 1999.77 | SNP | Rv0093c | silent (Gly161) | 9935 | - | | 116000 | T | G | 2091.77 | SNP | Rv0101 (nrp) | Val2000Val(s) | 18 | - | | 122109 | A | G | 1905.77 | SNP | Rv0103c (ctpB) | Leu(s)22Ser | 28 | - | | 125830 | G | GA | 3188.80 | INS | Rv0107c (ctpI) |  |  | - | | 130401 | G | C | 1439.77 | SNP | Rv0107c (ctpI) | silent (Gly47) | 9935 | - | | 131174 | T | TG | 2883.73 | INS | intergenic |  |  | - | | 132417 | C | G | 87.28 | SNP | Rv0109 (PE\_PGRS1) | Arg346Gly | 1 | - | | 133839 | C | T | 2228.77 | SNP | intergenic |  |  | - | | 146087 | T | C | 2892.77 | SNP | Rv0120c (fusA2) | Asn562Ser | 34 | - | | 154283 | T | C | 2479.77 | SNP | Rv0127 (mak) | Ser18Pro | 12 | - | | 161514 | C | T | 1760.77 | SNP | intergenic |  |  | - | | 161572 | A | G | 1996.77 | SNP | intergenic |  |  | - | | 169303 | CA | C | 2143.90 | DEL | Rv0143c |  |  | - | | 177857 | G | A | 1691.77 | SNP | Rv0151c (PE1) | Leu485Leu(s) | 4 | - | | 188800 | T | C | 1206.77 | SNP | Rv0159c (PE3) | Thr14Ala | 32 | - | | 193735 | C | G | 2970.77 | SNP | Rv0164 (TB18.5) | Ala37Gly | 21 | - | | 194681 | G | C | 1576.77 | SNP | Rv0165c (mce1R) | silent (Leu45) | 9947 | - | | 196642 | C | T | 2993.77 | SNP | Rv0166 (fadD5) | silent (Asn550) | 9822 | - | | 202675 | T | C | 2604.77 | SNP | Rv0172 (mce1D) | Ile67Thr | 11 | - | | 206339 | T | C | 1722.77 | SNP | Rv0174 (mce1F) | Leu370Pro | 2 | - | | 215234 | A | G | 2139.77 | SNP | Rv0184 | Asn89Ser | 34 | - | | 217306 | C | G | 2103.77 | SNP | Rv0186 (bglS) | Val346Val(s) | 18 | - | | 221845 | C | T | 3228.29 | SNP | intergenic |  |  | - | | 223942 | T | C | 710.77 | SNP | Rv0192 | Ser127Pro | 12 | - | | 225323 | T | C | 2046.77 | SNP | Rv0193c | Lys417Glu | 4 | - | | 227098 | T | C | 2610.77 | SNP | Rv0194 | Met(s)74Thr | 22 | - | | 231114 | C | G | 2168.77 | SNP | Rv0195 | silent (Ala72) | 9867 | - | | 234477 | T | G | 1884.77 | SNP | Rv0197 | Tyr749STOP | 2 | - | | 234496 | C | CGT | 4180.73 | INS | Rv0197 |  |  | - | | 240639 | C | T | 2037.77 | SNP | Rv0202c (mmpL11) | Met(s)218Ile | 2 | - | | 260116 | T | C | 2022.77 | SNP | Rv0217c (lipW) | Asp239Gly | 11 | - | | 261869 | T | C | 1744.77 | SNP | Rv0218 | Cys316Arg | 1 | - | | 265554 | A | C | 2338.77 | SNP | Rv0222 (echA1) | silent (Val16) | 9901 | - | | 278681 | C | G | 2030.77 | SNP | Rv0233 (nrdB) | His33Asp | 4 | - | | 279515 | GCGGCCTCGGCCTAGGCCTG GCGAGCAGACGCAAAATCGC CCAATTTCGTGCCGAATTGG GCGATTTTGCGTCTGC | G | 7203.94 | DEL | Rv0233 (nrdB) |  |  | - | | 283614 | T | C | 1258.77 | SNP | Rv0236c (aftD) | Ser1080Gly | 21 | - | | 285772 | A | C | 1408.77 | SNP | Rv0236c (aftD) | silent (Pro360) | 9926 | - | | 285871 | A | G | 1415.77 | SNP | Rv0236c (aftD) | silent (Val327) | 9901 | - | | 293628 | A | AC | 1888.73 | INS | intergenic |  |  | - | | 295644 | C | T | 887.77 | SNP | intergenic |  |  | - | | 304923 | A | G | 2192.77 | SNP | Rv0252 (nirB) | silent (Lys686) | 9926 | - | | 310973 | G | A | 2170.77 | SNP | Rv0259c | Ala182Val(s) | 9867 | - | | 311613 | G | T | 2071.77 | SNP | Rv0260c | silent (Val349) | 9901 | - | | 317540 | G | C | 1662.77 | SNP | Rv0266c (oplA) | silent (Ala1205) | 9867 | - | | 326122 | A | C | 2283.77 | SNP | Rv0270 (fadD2) | Asp519Ala | 10 | - | | 328464 | C | T | 2631.77 | SNP | intergenic |  |  | - | | 333883 | CGGCGCCGCG | C | 1554.73 | DEL | Rv0278c (PE\_PGRS3) |  |  | - | | 334641 | G | C | 161.77 | SNP | Rv0278c (PE\_PGRS3) | Ala557Gly | 21 | - | | 335810 | CCCGCCGGCGCCGCCGTTG | C | 1525.75 | DEL | Rv0278c (PE\_PGRS3) |  |  | - | | 336380 | A | T | 119.77 | SNP | intergenic |  |  | - | | 336400 | C | G | 164.77 | SNP | intergenic |  |  | - | | 336403 | C | G | 164.77 | SNP | intergenic |  |  | - | | 336405 | A | G | 128.77 | SNP | intergenic |  |  | - | | 336504 | G | T | 696.77 | SNP | intergenic |  |  | - | | 336535 | T | G | 569.77 | SNP | intergenic |  |  | - | | 336537 | T | G | 551.77 | SNP | intergenic |  |  | - | | 336540 | G | T | 474.77 | SNP | intergenic |  |  | - | | 336546 | T | G | 583.77 | SNP | intergenic |  |  | - | | 336557 | C | CT | 850.73 | INS | intergenic |  |  | - | | 336560 | T | C | 489.77 | SNP | Rv0279c (PE\_PGRS4) | silent (STOP838) | 9867 | - | | 336562 | A | ATGG | 1168.73 | INS | Rv0279c (PE\_PGRS4) |  |  | - | | 336590 | G | C | 514.77 | SNP | Rv0279c (PE\_PGRS4) | Ile828Met(s) | 6 | - | | 336592 | T | G | 475.77 | SNP | Rv0279c (PE\_PGRS4) | Ile828Leu | 22 | - | | 336611 | G | C | 410.77 | SNP | Rv0279c (PE\_PGRS4) | silent (Ala821) | 9867 | - | | 336617 | G | C | 365.77 | SNP | Rv0279c (PE\_PGRS4) | silent (Pro819) | 9926 | - | | 336620 | T | C | 377.77 | SNP | Rv0279c (PE\_PGRS4) | silent (Thr818) | 9871 | - | | 336691 | T | C | 48.28 | SNP | Rv0279c (PE\_PGRS4) | Ser795Gly | 21 | - | | 336707 | G | A | 64.28 | SNP | Rv0279c (PE\_PGRS4) | silent (Asp789) | 9859 | - | | 336708 | T | C | 50.28 | SNP | Rv0279c (PE\_PGRS4) | Asp789Gly | 11 | - | | 336710 | A | G | 33.74 | SNP | Rv0279c (PE\_PGRS4) | silent (Ala788) | 9867 | - | | 336728 | G | A | 169.77 | SNP | Rv0279c (PE\_PGRS4) | silent (Gly782) | 9935 | - | | 337820 | G | A | 260.22 | SNP | Rv0279c (PE\_PGRS4) | silent (Gly418) | 9935 | - | | 337959 | A | C | 82.28 | SNP | Rv0279c (PE\_PGRS4) | Ile372Ser | 2 | - | | 338020 | A | C | 86.28 | SNP | Rv0279c (PE\_PGRS4) | Cys352Gly | 1 | - | | 338100 | T | C | 394.77 | SNP | Rv0279c (PE\_PGRS4) | Asn325Ser | 34 | - | | 338453 | A | G | 113.03 | SNP | Rv0279c (PE\_PGRS4) | silent (Ala207) | 9867 | - | | 338600 | GCCGCCGTTGCCGCCGGCC | G | 955.73 | DEL | Rv0279c (PE\_PGRS4) |  |  | - | | 338682 | T | G | 201.77 | SNP | Rv0279c (PE\_PGRS4) | Asp131Ala | 10 | - | | 338683 | C | G | 176.77 | SNP | Rv0279c (PE\_PGRS4) | Asp131His | 3 | - | | 338685 | C | G | 159.77 | SNP | Rv0279c (PE\_PGRS4) | Gly130Ala | 21 | - | | 338690 | A | G | 170.77 | SNP | Rv0279c (PE\_PGRS4) | silent (Asn128) | 9822 | - | | 338692 | T | C | 157.77 | SNP | Rv0279c (PE\_PGRS4) | Asn128Asp | 42 | - | | 340372 | T | C | 653.77 | SNP | Rv0280 (PPE3) | Ser337Pro | 12 | - | | 346275 | C | G | 2341.77 | SNP | Rv0284 (eccC3) | Pro214Arg | 4 | - | | 350738 | C | T | 2520.77 | SNP | Rv0286 (PPE4) | silent (Thr268) | 9871 | - | | 353766 | T | C | 1887.77 | SNP | Rv0290 (eccD3) | silent (Ile228) | 9872 | - | | 353767 | C | T | 1752.80 | SNP | Rv0290 (eccD3) | Pro229Ser | 17 | - | | 355803 | G | T | 1460.77 | SNP | Rv0291 (mycP3) | Ala436Ser | 28 | - | | 356528 | A | G | 1726.77 | SNP | Rv0292 (eccE3) | Asn217Asp | 42 | - | | 364498 | TG | T | 2728.73 | DEL | intergenic |  |  | - | | 367824 | A | G | 2205.77 | SNP | Rv0304c (PPE5) | silent (Ser1647) | 9840 | - | | 372913 | A | C | 2144.77 | SNP | Rv0305c (PPE6) | silent (Gly933) | 9935 | - | | 373282 | TA | T | 2385.73 | DEL | Rv0305c (PPE6) |  |  | - | | 376774 | T | C | 1913.77 | SNP | Rv0307c | silent (Ala94) | 9867 | - | | 378407 | G | T | 2869.77 | SNP | Rv0309 | Gln159His | 20 | - | | 384380 | A | C | 2261.77 | SNP | Rv0315 | Lys260Thr | 8 | - | | 386432 | C | G | 2098.77 | SNP | Rv0318c | Gly223Ala | 21 | - | | 386971 | G | C | 1752.77 | SNP | Rv0318c | silent (Leu43) | 9947 | - | | 390828 | T | C | 2351.77 | SNP | Rv0323c | Ser142Gly | 21 | - | | 391853 | A | G | 2083.77 | SNP | Rv0324 | Thr168Ala | 32 | - | | 396634 | A | G | 1918.77 | SNP | Rv0331 | Glu145Gly | 7 | - | | 401678 | C | A | 1445.77 | SNP | Rv0336 | Pro496His | 3 | - | | 401693 | C | G | 1606.77 | SNP | Rv0336 | Pro501Arg | 4 | - | | 403980 | G | A | 2543.77 | SNP | Rv0338c | Ala621Val | 13 | - | | 404326 | T | C | 1825.77 | SNP | Rv0338c | Arg506Gly | 1 | - | | 407877 | T | C | 2040.89 | SNP | Rv0339c | Glu191Gly | 7 | - | | 409989 | G | A | 709.78 | SNP | Rv0341 (iniB) | Val210Ile | 33 | - | | 414486 | C | T | 1889.77 | SNP | Rv0344c (lpqJ) | silent (Glu152) | 9865 | - | | 420008 | A | G | 2764.77 | SNP | Rv0350 (dnaK) | silent (Ala58) | 9867 | - | | 423722 | C | T | 2447.77 | SNP | Rv0353 (hspR) | silent (Thr28) | 9871 | - | | 424320 | T | TC | 1949.73 | INS | Rv0354c (PPE7) |  |  | - | | 425510 | TCGCCGATGTTGGCGCTGCC CAGGTTGTAGA | T | 2721.73 | DEL | Rv0355c (PPE8) |  |  | - | | 427310 | TTGCCGAGGTTTGCAC | T | 7557.73 | DEL | Rv0355c (PPE8) |  |  | - | | 433515 | G | A | 1931.77 | SNP | Rv0355c (PPE8) | Leu389Leu(s) | 4 | - | | 447411 | G | T | 2199.77 | SNP | Rv0370c | silent (Arg211) | 9913 | - | | 452063 | T | G | 1477.77 | SNP | Rv0374c | Thr73Pro | 4 | - | | 454295 | T | C | 2847.77 | SNP | Rv0376c | silent (Pro26) | 9926 | - | | 457452 | T | G | 1382.77 | SNP | Rv0381c | silent (Thr124) | 9871 | - | | 459399 | A | C | 2118.77 | SNP | intergenic |  |  | - | | 467497 | C | CG | 1991.73 | INS | Rv0388c (PPE9) |  |  | - | | 467508 | C | CG | 2355.73 | INS | Rv0388c (PPE9) |  |  | - | | 467516 | G | C | 1372.77 | SNP | Rv0388c (PPE9) | silent (Ser162) | 9840 | - | | 467526 | C | G | 1424.77 | SNP | Rv0388c (PPE9) | Gly159Ala | 21 | - | | 467546 | G | C | 1616.77 | SNP | Rv0388c (PPE9) | Asp152Glu | 56 | - | | 467557 | A | C | 1426.77 | SNP | Rv0388c (PPE9) | Leu(s)149Val(s) | 9867 | - | | 467564 | A | C | 1585.77 | SNP | Rv0388c (PPE9) | His146Gln | 23 | - | | 467585 | G | C | 1979.77 | SNP | Rv0388c (PPE9) | His139Gln | 23 | - | | 467590 | T | C | 2045.77 | SNP | Rv0388c (PPE9) | Thr138Ala | 32 | - | | 467621 | T | G | 1841.77 | SNP | Rv0388c (PPE9) | silent (Gly127) | 9935 | - | | 467638 | G | T | 1768.77 | SNP | Rv0388c (PPE9) | Gln122Lys | 12 | - | | 475178 | T | C | 1590.77 | SNP | Rv0395 | Val80Ala | 18 | - | | 475330 | C | T | 2579.77 | SNP | Rv0395 | Leu131Leu(s) | 4 | - | | 483935 | T | G | 2507.77 | SNP | intergenic |  |  | - | | 485670 | G | A | 2635.77 | SNP | Rv0404 (fadD30) | Ser565Asn | 20 | - | | 488572 | G | T | 1683.77 | SNP | Rv0405 (pks6) | Ala948Ser | 28 | - | | 489935 | G | C | 2562.77 | SNP | Rv0405 (pks6); Rv0406c | Arg1402Pro; silent (Thr257) | 5; 9871 | - | | 498557 | C | A | 2043.77 | SNP | Rv0412c | Asp355Tyr | 0 | - | | 502589 | C | G | 2425.77 | SNP | Rv0417 (thiG) | Ser75Cys | 5 | - | | 503213 | T | C | 1922.77 | SNP | intergenic |  |  | - | | 503354 | G | C | 3077.77 | SNP | intergenic |  |  | - | | 506920 | C | T | 2202.77 | SNP | Rv0420c | Ala18Thr | 22 | - | | 510675 | G | T | 2052.77 | SNP | intergenic |  |  | - | | 513257 | T | C | 1091.77 | SNP | Rv0425c (ctpH) | Met(s)689Val(s) | 9867 | - | | 514876 | G | C | 1902.77 | SNP | Rv0425c (ctpH) | Ala149Gly | 21 | - | | 515110 | T | G | 1764.77 | SNP | Rv0425c (ctpH) | Glu71Ala | 17 | - | | 524891 | C | A | 2000.77 | SNP | Rv0436c (pssA) | Gly167Val | 3 | - | | 541201 | A | G | 2639.77 | SNP | Rv0450c (mmpL4) | silent (Leu97) | 9947 | - | | 548792 | G | T | 2197.77 | SNP | Rv0457c | silent (Arg272) | 9913 | - | | 551525 | A | C | 1583.77 | SNP | Rv0459 | silent (Arg110) | 9913 | - | | 553613 | G | T | 2172.77 | SNP | Rv0462 (lpdC) | Ala334Ser | 28 | - | | 565655 | A | G | 2313.77 | SNP | intergenic |  |  | - | | 573262 | A | G | 1768.77 | SNP | Rv0484c | silent (Gly180) | 9935 | - | | 576077 | C | T | 1606.77 | SNP | Rv0486 (mshA) | Leu244Leu(s) | 4 | genotype | | 580772 | T | A | 728.18 | SNP | intergenic |  |  | - | | 580773 | GGGGGCACCACCCGCTTGCG GGGGA | G | 7240.73 | DEL | intergenic |  |  | - | | 585891 | C | G | 2397.77 | SNP | Rv0495c | Glu142Gln | 27 | - | | 590436 | T | C | 2464.77 | SNP | Rv0500 (proC) | silent (Ala118) | 9867 | - | | 591505 | C | A | 1991.77 | SNP | Rv0500B | Arg11Ser | 11 | - | | 594138 | G | A | 2444.77 | SNP | Rv0503c (cmaA2) | silent (Ile214) | 9872 | - | | 597816 | A | G | 2150.77 | SNP | Rv0507 (mmpL2) | silent (Ala206) | 9867 | - | | 598475 | G | A | 2430.77 | SNP | Rv0507 (mmpL2) | Arg426His | 8 | - | | 610120 | T | G | 1821.77 | SNP | intergenic |  |  | - | | 611463 | G | A | 1583.77 | SNP | Rv0519c | silent (Thr204) | 9871 | - | | 624025 | C | G | 62.74 | SNP | Rv0532 (PE\_PGRS6) | Ser411Arg | 6 | - | | 625536 | A | G | 2000.77 | SNP | intergenic |  |  | - | | 630722 | G | C | 1186.77 | SNP | Rv0538 | Arg228Pro | 5 | - | | 637319 | G | A | 1692.77 | SNP | Rv0545c (pitA) | Pro49Ser | 17 | - | | 648002 | T | G | 3583.77 | SNP | Rv0556 | Leu15Arg | 1 | - | | 651149 | G | A | 2162.77 | SNP | Rv0560c | Ala119Val | 13 | - | | 657425 | C | T | 2661.77 | SNP | Rv0565c | Gly16Ser | 16 | - | | 663410 | A | C | 50.77 | SNP | intergenic |  |  | - | | 663418 | A | C | 64.77 | SNP | intergenic |  |  | - | | 663419 | G | A | 68.77 | SNP | intergenic |  |  | - | | 663420 | C | A | 52.77 | SNP | intergenic |  |  | - | | 665293 | A | G | 2673.77 | SNP | Rv0572c | Phe31Leu | 13 | - | | 669398 | T | C | 1734.77 | SNP | Rv0575c | silent (Gln116) | 9876 | - | | 671406 | A | ATC | 4793.73 | INS | Rv0577 (TB27.3) |  |  | - | | 672491 | C | G | 303.78 | SNP | Rv0578c (PE\_PGRS7) | silent (Gly1142) | 9935 | - | | 673238 | A | G | 36.74 | SNP | Rv0578c (PE\_PGRS7) | silent (His893) | 9912 | - | | 685461 | C | G | 2358.77 | SNP | Rv0587 (yrbE2A) | silent (Ala111) | 9867 | - | | 685608 | T | C | 2707.77 | SNP | Rv0587 (yrbE2A) | silent (Leu160) | 9947 | - | | 686972 | T | C | 2701.77 | SNP | Rv0589 (mce2A) | Phe51Ser | 3 | - | | 690465 | T | G | 886.77 | SNP | Rv0591 (mce2C) | silent (Leu469) | 9947 | - | | 695231 | C | T | 2536.77 | SNP | Rv0595c (vapC4); Rv0596c (vapB4) | Val(s)1Met(s); Trp85STOP | 9867; 0 | - | | 698968 | G | A | 1358.77 | SNP | Rv0601c | silent (Gly9) | 9935 | - | | 705333 | C | G | 82.77 | SNP | Rv0610c | Asp193His | 3 | - | | 712693 | A | G | 1625.77 | SNP | intergenic |  |  | - | | 716547 | T | G | 2222.77 | SNP | Rv0623 (vapB30) | Val46Val(s) | 18 | - | | 732110 | T | G | 3132.77 | SNP | Rv0635 (hadA) | Cys61Gly | 1 | - | | 738902 | C | T | 2044.77 | SNP | Rv0644c (mmaA2) | Asp87Asn | 36 | - | | 751297 | A | AC | 2788.73 | INS | Rv0654 |  |  | - | | 752141 | A | G | 2469.77 | SNP | Rv0655 (mkl) | Ile209Val | 57 | - | | 753562 | A | G | 2678.77 | SNP | Rv0657c (vapB6) | Ile19Thr | 11 | - | | 754186 | A | G | 1477.77 | SNP | Rv0658c | Leu75Pro | 2 | - | | 756216 | A | G | 2077.77 | SNP | Rv0663 (atsD) | Asp27Gly | 11 | - | | 758322 | A | G | 2169.77 | SNP | Rv0663 (atsD) | Asp729Gly | 11 | - | | 761139 | C | T | 1859.77 | SNP | Rv0667 (rpoB) | His445Tyr | 4 | resistance | | 761489 | G | A | 1627.77 | SNP | Rv0667 (rpoB) | silent (Glu561) | 9865 | - | | 767414 | G | A | 1513.77 | SNP | intergenic |  |  | - | | 775639 | T | C | 1537.77 | SNP | Rv0676c (mmpL5) | Ile948Val | 57 | - | | 781395 | T | C | 2130.77 | SNP | intergenic (Rv0682-165nt) |  |  | - | | 795991 | T | G | 2215.77 | SNP | Rv0696 | Phe158Cys | 0 | - | | 799136 | AC | A | 4034.73 | DEL | Rv0698 |  |  | - | | 800204 | T | C | 2086.77 | SNP | intergenic |  |  | - | | 811637 | C | A | 2227.77 | SNP | Rv0714 (rplN) | Pro89Thr | 5 | - | | 820483 | G | T | 1452.77 | SNP | Rv0727c (fucA) | Ala6Asp | 6 | - | | 836150 | TG | T | 151.87 | DEL | Rv0746 (PE\_PGRS9) |  |  | - | | 836538 | A | G | 106.28 | SNP | Rv0746 (PE\_PGRS9) | Asn280Asp | 42 | - | | 836658 | A | G | 56.74 | SNP | Rv0746 (PE\_PGRS9) | Thr320Ala | 32 | - | | 837033 | A | G | 462.77 | SNP | Rv0746 (PE\_PGRS9) | Thr445Ala | 32 | - | | 839269 | A | G | 112.84 | SNP | Rv0747 (PE\_PGRS10) | silent (Gly273) | 9935 | - | | 839279 | G | A | 213.77 | SNP | Rv0747 (PE\_PGRS10) | Asp277Asn | 36 | - | | 839284 | C | G | 81.77 | SNP | Rv0747 (PE\_PGRS10) | silent (Ala278) | 9867 | - | | 839291 | T | C | 37.77 | SNP | Rv0747 (PE\_PGRS10) | Phe281Leu | 13 | - | | 839295 | T | C | 31.77 | SNP | Rv0747 (PE\_PGRS10) | Phe282Ser | 3 | - | | 839309 | T | G | 225.77 | SNP | Rv0747 (PE\_PGRS10) | Ser287Ala | 35 | - | | 839334 | A | G | 94.28 | SNP | Rv0747 (PE\_PGRS10) | Lys295Arg | 19 | - | | 839348 | A | G | 80.78 | SNP | Rv0747 (PE\_PGRS10) | Ser300Gly | 21 | - | | 839515 | G | A | 344.77 | SNP | Rv0747 (PE\_PGRS10) | silent (Ala355) | 9867 | - | | 839516 | A | G | 392.77 | SNP | Rv0747 (PE\_PGRS10) | Thr356Ala | 32 | - | | 839519 | C | G | 405.77 | SNP | Rv0747 (PE\_PGRS10) | Leu357Val(s) | 4 | - | | 839520 | T | C | 385.77 | SNP | Rv0747 (PE\_PGRS10) | Leu357Pro | 2 | - | | 839534 | A | C | 379.77 | SNP | Rv0747 (PE\_PGRS10) | Ile362Leu | 22 | - | | 840272 | G | T | 35.77 | SNP | Rv0747 (PE\_PGRS10) | Ala608Ser | 28 | - | | 841764 | G | C | 2296.77 | SNP | Rv0749A | silent (Thr37) | 9871 | - | | 849486 | G | C | 1499.77 | SNP | Rv0755c (PPE12) | Asn185Lys | 25 | - | | 849983 | C | T | 1003.77 | SNP | Rv0755c (PPE12) | Ala20Thr | 22 | - | | 852910 | C | T | 1374.77 | SNP | Rv0758 (phoR) | Pro172Leu | 3 | - | | 853360 | A | G | 2048.77 | SNP | Rv0758 (phoR) | Asp322Gly | 11 | - | | 854252 | GC | G | 2406.74 | DEL | intergenic |  |  | - | | 857696 | A | G | 1826.77 | SNP | Rv0764c (cyp51) | silent (Ala114) | 9867 | - | | 857791 | A | G | 1693.77 | SNP | Rv0764c (cyp51) | Phe83Leu | 13 | - | | 863766 | G | T | 1277.77 | SNP | Rv0770 | Ala171Ser | 28 | - | | 864468 | A | G | 1907.77 | SNP | Rv0771 | Glu110Gly | 7 | - | | 874835 | C | CCG | 6109.73 | INS | Rv0781 (ptrBa); Rv0782 (ptrBb) |  |  | - | | 880562 | G | T | 2298.77 | SNP | Rv0785 | Cys408Phe | 0 | - | | 882257 | T | C | 2370.77 | SNP | Rv0787 | Tyr267His | 4 | - | | 888774 | G | A | 1288.77 | SNP | intergenic |  |  | - | | 890549 | G | A | 598.77 | SNP | Rv0797 | Trp54STOP | 0 | - | | 893636 | G | A | 1913.77 | SNP | Rv0800 (pepC) | Gly107Arg | 0 | - | | 893733 | T | G | 2351.77 | SNP | Rv0800 (pepC) | Leu139Arg | 1 | - | | 893895 | C | T | 1063.77 | SNP | Rv0800 (pepC) | Ala193Val(s) | 9867 | - | | 900221 | T | C | 2430.77 | SNP | Rv0806c (cpsY) | Val370Val(s) | 18 | - | | 903550 | T | C | 2054.77 | SNP | Rv0808 (purF) | silent (Ala480) | 9867 | - | | 903913 | T | C | 1954.77 | SNP | Rv0809 (purM) | silent (Gly63) | 9935 | - | | 906857 | A | G | 1951.77 | SNP | Rv0812 | Ile145Met(s) | 6 | - | | 919424 | C | G | 1872.77 | SNP | Rv0825c | Val44Leu | 15 | - | | 921813 | C | G | 2153.77 | SNP | Rv0829 | Ala80Gly | 21 | - | | 927110 | A | G | 100.28 | SNP | Rv0833 (PE\_PGRS13) | Ser584Gly | 21 | - | | 927385 | A | G | 93.28 | SNP | Rv0833 (PE\_PGRS13) | silent (Gly675) | 9935 | - | | 928228 | G | T | 530.77 | SNP | Rv0834c (PE\_PGRS14) | Ala753Asp | 6 | - | | 928917 | C | G | 175.78 | SNP | Rv0834c (PE\_PGRS14) | silent (Gly523) | 9935 | - | | 928918 | C | G | 149.78 | SNP | Rv0834c (PE\_PGRS14) | Gly523Ala | 21 | - | | 932881 | C | T | 1849.77 | SNP | Rv0836c | Gly18Arg | 0 | - | | 936189 | G | A | 1766.77 | SNP | Rv0839 | Ala205Thr | 22 | - | | 945214 | G | A | 2859.77 | SNP | Rv0848 (cysK2) | Gly93Ser | 16 | - | | 949535 | T | C | 1605.77 | SNP | Rv0853c (pdc) | silent (Ala528) | 9867 | - | | 954906 | C | T | 1823.77 | SNP | Rv0858c (dapC) | silent (Arg5) | 9913 | - | | 955524 | A | G | 1632.77 | SNP | Rv0859 (fadA) | Ser150Gly | 21 | - | | 956679 | C | G | 1712.77 | SNP | Rv0860 (fadB) | silent (Ala129) | 9867 | - | | 957117 | T | C | 1865.77 | SNP | Rv0860 (fadB) | silent (Asp275) | 9859 | - | | 968426 | A | AGCCGGGTTG | 1092.74 | INS | Rv0872c (PE\_PGRS15) |  |  | - | | 976897 | TG | T | 3151.73 | DEL | Rv0878c (PPE13) |  |  | - | | 979704 | G | C | 2726.77 | SNP | Rv0881 | Gly115Arg | 0 | - | | 986463 | G | C | 2750.77 | SNP | intergenic |  |  | - | | 990001 | G | C | 2065.77 | SNP | Rv0890c | Pro866Ala | 22 | - | | 993346 | A | C | 3335.77 | SNP | Rv0891c | Val37Gly | 5 | - | | 1007505 | C | T | 2334.77 | SNP | Rv0904c (accD3) | Glu226Lys | 7 | - | | 1010204 | C | CG | 4106.73 | INS | Rv0907 |  |  | - | | 1025106 | T | C | 3585.77 | SNP | Rv0919 | silent (Phe141) | 9946 | - | | 1037012 | T | C | 1421.77 | SNP | Rv0930 (pstA1) | Met(s)5Thr | 22 | - | | 1037911 | C | T | 2315.77 | SNP | Rv0930 (pstA1) | Arg305STOP | 2 | - | | 1038677 | C | T | 2101.77 | SNP | Rv0931c (pknD) | Gly413Glu | 4 | - | | 1047165 | T | C | 1509.77 | SNP | Rv0938 (ligD) | Cys344Arg | 1 | - | | 1057990 | G | A | 2221.77 | SNP | intergenic |  |  | - | | 1068151 | T | C | 2396.77 | SNP | Rv0956 (purN) | silent (His197) | 9912 | - | | 1068432 | A | G | 1894.77 | SNP | Rv0957 (purH) | silent (Pro76) | 9926 | - | | 1070702 | T | C | 1742.77 | SNP | Rv0958 | Ser274Pro | 12 | - | | 1074558 | G | A | 1797.77 | SNP | Rv0962c (lprP) | Pro186Leu | 3 | - | | 1075279 | T | C | 2437.77 | SNP | intergenic |  |  | - | | 1076309 | G | T | 2903.77 | SNP | Rv0964c | Pro124Thr | 5 | - | | 1077312 | A | G | 1500.77 | SNP | Rv0966c | Val(s)175Ala | 9867 | - | | 1079927 | C | A | 1699.77 | SNP | Rv0969 (ctpV) | silent (Thr395) | 9871 | - | | 1080191 | T | C | 1212.77 | SNP | Rv0969 (ctpV) | silent (Pro483) | 9926 | - | | 1081681 | T | C | 1556.77 | SNP | Rv0970 | silent (Val210) | 9901 | - | | 1087193 | G | C | 1906.77 | SNP | Rv0974c (accD2) | Asn51Lys | 25 | - | | 1089922 | G | T | 1389.77 | SNP | Rv0976c | Pro85His | 3 | - | | 1093406 | A | G | 1686.77 | SNP | Rv0978c (PE\_PGRS17) | silent (Val317) | 9901 | - | | 1093928 | G | A | 57.28 | SNP | Rv0978c (PE\_PGRS17) | silent (Asn143) | 9822 | - | | 1094885 | C | T | 2363.77 | SNP | intergenic |  |  | - | | 1096299 | C | G | 1486.77 | SNP | Rv0980c (PE\_PGRS18) | silent (Ala51) | 9867 | - | | 1096633 | T | G | 2878.77 | SNP | intergenic |  |  | - | | 1100234 | T | C | 1728.77 | SNP | Rv0983 (pepD) | Leu390Pro | 2 | - | | 1106422 | T | C | 4105.77 | SNP | Rv0989c (grcC2) | Ile321Val | 57 | - | | 1109975 | A | G | 2903.77 | SNP | Rv0993 (galU) | Gln235Arg | 10 | - | | 1126889 | G | C | 2354.77 | SNP | Rv1007c (metS) | Arg39Gly | 1 | - | | 1127648 | C | A | 3177.77 | SNP | Rv1008 (tatD) | Thr187Asn | 9 | - | | 1149551 | C | T | 1843.77 | SNP | Rv1028c (kdpD) | silent (Glu712) | 9865 | - | | 1150585 | G | A | 1822.77 | SNP | Rv1028c (kdpD) | Pro368Ser | 17 | - | | 1163134 | T | C | 2052.77 | SNP | Rv1040c (PE8) | silent (Gly81) | 9935 | - | | 1164571 | A | G | 1482.77 | SNP | intergenic |  |  | - | | 1165521 | T | TA | 2896.73 | INS | intergenic |  |  | - | | 1168715 | C | CT | 2864.73 | INS | Rv1046c |  |  | - | | 1170404 | C | A | 165.84 | SNP | Rv1047 | Gln328Lys | 12 | - | | 1178116 | T | C | 3921.77 | SNP | Rv1056 | silent (Thr163) | 9871 | - | | 1187117 | C | T | 1372.77 | SNP | Rv1064c (lpqV) | silent (Glu69) | 9865 | - | | 1189606 | A | G | 77.77 | SNP | Rv1067c (PE\_PGRS19) | silent (Gly273) | 9935 | - | | 1189613 | G | A | 64.77 | SNP | Rv1067c (PE\_PGRS19) | Ala271Val | 13 | - | | 1190093 | A | C | 1373.77 | SNP | Rv1067c (PE\_PGRS19) | Leu(s)111Trp | 0 | - | | 1191497 | T | A | 47.74 | SNP | Rv1068c (PE\_PGRS20) | Thr218Ser | 38 | - | | 1191741 | G | A | 288.78 | SNP | Rv1068c (PE\_PGRS20) | silent (Tyr136) | 9945 | - | | 1200418 | A | G | 2199.77 | SNP | intergenic |  |  | - | | 1200722 | T | C | 2474.77 | SNP | intergenic |  |  | - | | 1220680 | T | C | 3173.77 | SNP | Rv1093 (glyA1) | Val36Ala | 18 | - | | 1224367 | T | C | 1681.77 | SNP | intergenic |  |  | - | | 1225462 | T | C | 1683.77 | SNP | Rv1097c | Asp228Gly | 11 | - | | 1226282 | T | G | 2422.77 | SNP | Rv1098c (fum) | silent (Ala428) | 9867 | - | | 1233285 | G | A | 2237.77 | SNP | Rv1106c | silent (Tyr224) | 9945 | - | | 1237536 | C | G | 1699.77 | SNP | Rv1111c | silent (Pro219) | 9926 | - | | 1243509 | G | T | 1663.77 | SNP | intergenic |  |  | - | | 1248978 | T | C | 2085.77 | SNP | Rv1125 | silent (Ala299) | 9867 | - | | 1251199 | C | T | 2228.77 | SNP | Rv1127c (ppdK) | Gly69Glu | 4 | - | | 1252164 | T | C | 2327.77 | SNP | Rv1128c | Glu270Gly | 7 | - | | 1254624 | C | A | 2910.77 | SNP | Rv1130 (prpD) | silent (Arg24) | 9913 | - | | 1257825 | CG | C | 3867.73 | DEL | Rv1132 |  |  | - | | 1270608 | G | T | 2304.77 | SNP | Rv1143 (mcr) | Val183Phe | 0 | - | | 1275957 | T | C | 2183.77 | SNP | intergenic |  |  | - | | 1281118 | T | C | 2512.77 | SNP | Rv1154c | Thr123Ala | 32 | - | | 1292102 | A | G | 1904.77 | SNP | Rv1162 (narH) | silent (Pro346) | 9926 | - | | 1306259 | A | G | 2418.77 | SNP | Rv1175c (fadH) | silent (Ala656) | 9867 | - | | 1307598 | C | G | 1346.77 | SNP | Rv1175c (fadH) | Cys210Ser | 11 | - | | 1312147 | T | C | 1935.77 | SNP | Rv1179c | Thr385Ala | 32 | - | | 1313337 | A | AG | 2962.73 | INS | intergenic |  |  | - | | 1313338 | A | C | 1821.77 | SNP | intergenic |  |  | - | | 1315191 | A | C | 1948.77 | SNP | Rv1180 (pks3) | STOP489Tyr | 1 | - | | 1315884 | G | A | 1472.77 | SNP | Rv1181 (pks4) | silent (Ala217) | 9867 | - | | 1327890 | G | A | 1854.77 | SNP | Rv1186c | silent (Asp472) | 9859 | - | | 1328687 | G | C | 2296.77 | SNP | Rv1186c | Pro207Ala | 22 | - | | 1340578 | T | C | 55.77 | SNP | intergenic |  |  | - | | 1340688 | C | T | 489.77 | SNP | Rv1197 (esxK) | silent (His10) | 9912 | - | | 1342581 | T | C | 698.77 | SNP | Rv1199c | Thr9Ala | 32 | - | | 1344964 | C | T | 2228.77 | SNP | Rv1201c (dapD) | Arg69His | 8 | - | | 1346347 | C | T | 1138.77 | SNP | Rv1203c | Ala187Thr | 22 | - | | 1359180 | G | A | 2448.77 | SNP | Rv1215c | Arg89Trp | 2 | - | | 1360209 | T | C | 1791.77 | SNP | Rv1217c | silent (Ala531) | 9867 | - | | 1362006 | T | C | 3210.76 | SNP | Rv1218c | Gln243Arg | 10 | - | | 1365837 | C | CG | 2148.73 | INS | intergenic |  |  | - | | 1374065 | T | C | 1107.77 | SNP | Rv1230c | Ser45Gly | 21 | - | | 1375446 | C | T | 1785.77 | SNP | Rv1232c | silent (Leu241) | 9947 | - | | 1375724 | A | C | 1894.77 | SNP | Rv1232c | Cys149Gly | 1 | - | | 1382628 | T | C | 2562.77 | SNP | Rv1239c (corA) | Lys139Glu | 4 | - | | 1393626 | A | G | 1526.77 | SNP | Rv1249c | silent (Leu119) | 9947 | - | | 1395555 | C | G | 1674.77 | SNP | Rv1250 | Phe459Leu(s) | 2 | - | | 1396922 | T | C | 1877.77 | SNP | Rv1251c | silent (Thr773) | 9871 | - | | 1401599 | C | G | 1577.77 | SNP | Rv1253 (deaD) | Arg544Gly | 1 | - | | 1403052 | A | C | 2073.77 | SNP | Rv1255c | Phe112Cys | 0 | - | | 1409766 | C | T | 2394.77 | SNP | Rv1261c | silent (Thr56) | 9871 | - | | 1411210 | T | G | 2490.77 | SNP | Rv1263 (amiB2) | Val260Val(s) | 18 | - | | 1413148 | C | T | 1296.77 | SNP | intergenic |  |  | - | | 1414021 | C | T | 1409.77 | SNP | Rv1266c (pknH) | Arg607Gln | 9 | - | | 1416222 | A | G | 109.77 | SNP | Rv1267c (embR) | Phe376Leu | 13 | - | | 1416232 | A | G | 140.77 | SNP | Rv1267c (embR) | silent (Cys372) | 9973 | - | | 1416234 | A | C | 121.77 | SNP | Rv1267c (embR) | Cys372Gly | 1 | - | | 1422920 | T | C | 1701.77 | SNP | Rv1273c | silent (Thr377) | 9871 | - | | 1440469 | C | G | 2678.77 | SNP | Rv1286 (cysN) | silent (Pro521) | 9926 | - | | 1441794 | A | G | 2264.77 | SNP | Rv1288 | Val149Val(s) | 18 | - | | 1442141 | C | G | 1680.77 | SNP | Rv1288 | Thr265Arg | 1 | - | | 1443428 | G | A | 343.77 | SNP | intergenic |  |  | - | | 1445781 | A | G | 2992.77 | SNP | Rv1291c | silent (Ala18) | 9867 | - | | 1457144 | C | T | 1388.77 | SNP | Rv1300 (hemK) | Arg194Cys | 1 | - | | 1466779 | C | T | 2336.77 | SNP | Rv1310 (atpD) | silent (Ile313) | 9872 | - | | 1468208 | A | C | 1698.77 | SNP | Rv1313c | Leu433Arg | 1 | - | | 1471659 | C | T | 2851.77 | SNP | intergenic |  |  | - | | 1480233 | A | C | 40.77 | SNP | Rv1318c | Phe198Val | 1 | - | | 1480945 | C | G | 1207.77 | SNP | Rv1319c | silent (Thr519) | 9871 | - | | 1480948 | C | T | 1154.77 | SNP | Rv1319c | silent (Glu518) | 9865 | - | | 1480972 | T | C | 1070.77 | SNP | Rv1319c | silent (Glu510) | 9865 | - | | 1482627 | T | C | 2005.77 | SNP | Rv1320c | Thr531Ala | 32 | - | | 1482908 | G | A | 1910.77 | SNP | Rv1320c | Ala437Val | 13 | - | | 1484708 | A | C | 2545.77 | SNP | Rv1321 | Ser144Arg | 6 | - | | 1487796 | C | A | 1889.77 | SNP | Rv1324 | silent (Ile212) | 9872 | genotype | | 1488405 | CG | C | 145 | DEL | Rv1325c (PE\_PGRS24) |  |  | - | | 1488409 | T | TC | 130 | INS | Rv1325c (PE\_PGRS24) |  |  | - | | 1488421 | G | A | 107.77 | SNP | Rv1325c (PE\_PGRS24) | silent (Gly515) | 9935 | - | | 1488424 | G | A | 124.77 | SNP | Rv1325c (PE\_PGRS24) | silent (Ala514) | 9867 | - | | 1488433 | A | G | 130.90 | SNP | Rv1325c (PE\_PGRS24) | silent (Asp511) | 9859 | - | | 1488434 | T | G | 126.90 | SNP | Rv1325c (PE\_PGRS24) | Asp511Ala | 10 | - | | 1488435 | C | A | 122.90 | SNP | Rv1325c (PE\_PGRS24) | Asp511Tyr | 0 | - | | 1499274 | C | G | 1282.77 | SNP | Rv1330c (pncB1) | Gly429Ala | 21 | - | | 1515256 | A | C | 2478.77 | SNP | Rv1348 (irtA) | Asp737Ala | 10 | - | | 1524904 | A | G | 2095.77 | SNP | intergenic |  |  | - | | 1526819 | C | A | 2090.77 | SNP | Rv1358 | silent (Arg70) | 9913 | - | | 1533241 | G | C | 2025.77 | SNP | Rv1361c (PPE19) | silent (Thr131) | 9871 | - | | 1533583 | G | A | 1995.77 | SNP | Rv1361c (PPE19) | silent (Tyr17) | 9945 | - | | 1536251 | G | T | 2253.77 | SNP | Rv1364c | Ala465Glu | 10 | - | | 1543803 | C | T | 2662.77 | SNP | Rv1371 | Leu149Phe | 6 | - | | 1547125 | T | C | 3311.77 | SNP | Rv1374c | Thr136Ala | 32 | - | | 1549986 | A | G | 1534.77 | SNP | Rv1376 | Gln280Arg | 10 | - | | 1552547 | G | A | 1229.77 | SNP | Rv1378c | Arg37Trp | 2 | - | | 1563717 | C | T | 1761.77 | SNP | Rv1388 (mihF) | silent (Val8) | 9901 | - | | 1568018 | C | T | 1548.77 | SNP | Rv1392 (metK) | silent (Asp398) | 9859 | - | | 1570566 | C | A | 2174.77 | SNP | Rv1394c (cyp132) | Arg135Leu | 1 | - | | 1573660 | T | G | 1268.77 | SNP | Rv1396c (PE\_PGRS25) | Arg66Ser | 11 | - | | 1574120 | G | A | 2122.77 | SNP | Rv1397c (vapC10) | Arg132Trp | 2 | - | | 1588899 | G | T | 1784.77 | SNP | Rv1412 (ribC) | silent (Ala111) | 9867 | - | | 1600586 | C | T | 2092.77 | SNP | Rv1425 | Ser310Leu(s) | 35 | - | | 1600721 | C | T | 2056.77 | SNP | Rv1425 | Thr355Met(s) | 32 | - | | 1605170 | C | T | 2001.77 | SNP | Rv1429 | Ala98Val(s) | 9867 | - | | 1609840 | A | G | 1632.77 | SNP | Rv1431 | silent (Pro586) | 9926 | - | | 1612624 | T | TATCGGTACCGGTGCGCCAG GG | 6725.77 | INS | Rv1435c |  |  | - | | 1613035 | T | C | 2515.77 | SNP | intergenic |  |  | - | | 1616354 | T | G | 1206.77 | SNP | intergenic |  |  | - | | 1618610 | AGCTGCCGCCGCCGGTGCCG CCGGCGCCGCCGTCGCCGCC GGCGCCGCCGGCGCCGGCGC T | A | 2961.77 | DEL | Rv1441c (PE\_PGRS26) |  |  | - | | 1622092 | C | T | 1796.77 | SNP | intergenic |  |  | - | | 1624791 | C | G | 1598.77 | SNP | Rv1446c (opcA) | Arg192Pro | 5 | - | | 1626857 | C | T | 1417.77 | SNP | Rv1447c (zwf2) | Val36Ile | 33 | - | | 1627351 | T | C | 1657.56 | SNP | Rv1448c (tal) | Thr244Ala | 32 | - | | 1630148 | A | C | 2470.77 | SNP | Rv1449c (tkt) | Tyr18Asp | 0 | - | | 1633431 | C | A | 75.77 | SNP | Rv1450c (PE\_PGRS27) | silent (Gly399) | 9935 | - | | 1634609 | C | T | 2856.77 | SNP | Rv1450c (PE\_PGRS27) | Ala7Thr | 22 | - | | 1634610 | C | T | 2826.77 | SNP | Rv1450c (PE\_PGRS27) | Val(s)6Val | 13 | - | | 1634633 | A | T | 588.77 | SNP | intergenic |  |  | - | | 1634636 | T | A | 408.77 | SNP | intergenic |  |  | - | | 1634806 | C | T | 3002.77 | SNP | intergenic |  |  | - | | 1636826 | C | A | 50.28 | SNP | Rv1452c (PE\_PGRS28) | silent (Gly468) | 9935 | - | | 1636881 | A | G | 45.74 | SNP | Rv1452c (PE\_PGRS28) | Ile450Thr | 11 | - | | 1636980 | G | T | 167.84 | SNP | Rv1452c (PE\_PGRS28) | Pro417His | 3 | - | | 1636981 | G | T | 160.90 | SNP | Rv1452c (PE\_PGRS28) | Pro417Thr | 5 | - | | 1636983 | C | G | 166.90 | SNP | Rv1452c (PE\_PGRS28) | Gly416Ala | 21 | - | | 1636996 | G | C | 39.77 | SNP | Rv1452c (PE\_PGRS28) | Arg412Gly | 1 | - | | 1638182 | C | T | 42.74 | SNP | Rv1452c (PE\_PGRS28) | silent (Ser16) | 9840 | - | | 1638183 | G | A | 43.74 | SNP | Rv1452c (PE\_PGRS28) | Ser16Leu(s) | 35 | - | | 1638188 | C | T | 45.74 | SNP | Rv1452c (PE\_PGRS28) | silent (Ala14) | 9867 | - | | 1638191 | C | G | 49.74 | SNP | Rv1452c (PE\_PGRS28) | silent (Ala13) | 9867 | - | | 1638194 | G | C | 81.28 | SNP | Rv1452c (PE\_PGRS28) | silent (Ala12) | 9867 | - | | 1639594 | C | A | 1554.77 | SNP | Rv1453 | Pro405Gln | 6 | - | | 1645802 | T | C | 2798.77 | SNP | Rv1459c | Lys113Glu | 4 | - | | 1647444 | C | T | 2511.77 | SNP | Rv1461 | silent (His152) | 9912 | - | | 1650072 | A | G | 2045.77 | SNP | Rv1462 | Asn183Asp | 42 | - | | 1652756 | C | G | 2794.77 | SNP | Rv1464 (csd) | Phe413Leu(s) | 2 | - | | 1670814 | C | T | 1538.77 | SNP | Rv1480 | silent (Gly134) | 9935 | - | | 1676290 | C | A | 2506.77 | SNP | Rv1486c | Lys198Asn | 13 | - | | 1677603 | G | A | 2082.77 | SNP | Rv1488 | silent (Glu69) | 9865 | - | | 1689349 | C | T | 1675.77 | SNP | Rv1498c | Arg191His | 8 | - | | 1692141 | A | C | 3017.77 | SNP | Rv1501 | silent (Ile84) | 9872 | - | | 1693561 | A | G | 4161.77 | SNP | Rv1502 | Tyr213Cys | 3 | - | | 1698911 | G | A | 2227.77 | SNP | Rv1508c | silent (Gly328) | 9935 | - | | 1706119 | T | C | 1818.77 | SNP | Rv1514c | silent (Ser159) | 9840 | - | | 1709432 | C | T | 1767.77 | SNP | Rv1517 | Leu188Phe | 6 | - | | 1710354 | C | G | 2156.77 | SNP | Rv1518 | silent (Thr237) | 9871 | - | | 1713192 | A | G | 2237.77 | SNP | Rv1521 (fadD25) | Val297Val(s) | 18 | - | | 1714439 | C | T | 2204.77 | SNP | Rv1522c (mmpL12) | silent (Glu1058) | 9865 | - | | 1719823 | A | G | 2379.77 | SNP | Rv1524 | silent (Lys366) | 9926 | - | | 1720444 | T | G | 1906.77 | SNP | Rv1525 (wbbL2) | Val143Gly | 5 | - | | 1728022 | C | G | 2210.77 | SNP | Rv1527c (pks5) | Gly130Arg | 0 | - | | 1728837 | A | G | 2518.77 | SNP | intergenic |  |  | - | | 1732204 | A | G | 1637.77 | SNP | Rv1530 (adh) | Thr278Ala | 32 | - | | 1733166 | GA | G | 4439.73 | DEL | Rv1532c |  |  | - | | 1747327 | C | T | 1738.77 | SNP | Rv1546 | Leu45Leu(s) | 4 | - | | 1752561 | T | C | 776.77 | SNP | Rv1548c (PPE21) | Asp258Gly | 11 | - | | 1753519 | G | GC | 1789.73 | INS | Rv1549 (fadD11.1) |  |  | - | | 1759252 | G | T | 2250.77 | SNP | Rv1552 (frdA) | silent (Ser524) | 9840 | genotype | | 1760292 | A | G | 2679.77 | SNP | Rv1554 (frdC) | Met(s)40Val(s) | 9867 | - | | 1778430 | T | C | 1255.77 | SNP | Rv1570 (bioD) | Met(s)191Thr | 22 | - | | 1789446 | C | T | 156.84 | SNP | Rv1588c | Val131Ile | 33 | - | | 1789516 | A | G | 324.77 | SNP | Rv1588c | silent (Gly107) | 9935 | - | | 1789564 | C | T | 339.77 | SNP | Rv1588c | silent (Arg91) | 9913 | - | | 1789565 | C | A | 310.77 | SNP | Rv1588c | Arg91Leu | 1 | - | | 1789650 | C | T | 639.77 | SNP | Rv1588c | Ala63Thr | 22 | - | | 1789654 | A | G | 824.77 | SNP | Rv1588c | silent (Leu61) | 9947 | - | | 1789671 | C | T | 860.77 | SNP | Rv1588c | Ala56Thr | 22 | - | | 1789675 | A | C | 899.77 | SNP | Rv1588c | silent (Gly54) | 9935 | - | | 1789678 | C | G | 974.77 | SNP | Rv1588c | Val(s)53Val | 13 | - | | 1798355 | G | A | 1637.77 | SNP | Rv1597 | Gly21Asp | 6 | - | | 1798880 | T | C | 1693.77 | SNP | Rv1597 | Phe196Ser | 3 | - | | 1803265 | G | A | 2453.77 | SNP | Rv1602 (hisH) | Ser201Asn | 20 | - | | 1804409 | C | A | 1982.77 | SNP | Rv1604 (impA) | Pro124Gln | 6 | - | | 1808795 | A | C | 2387.77 | SNP | Rv1609 (trpE) | Asp298Ala | 10 | - | | 1817856 | A | C | 2319.77 | SNP | Rv1618 (tesB1) | Glu81Ala | 17 | - | | 1817976 | A | T | 2549.77 | SNP | Rv1618 (tesB1) | His121Leu | 4 | - | | 1836286 | G | C | 1383.77 | SNP | intergenic |  |  | - | | 1837590 | T | C | 2062.77 | SNP | Rv1633 (uvrB) | silent (Asp172) | 9859 | - | | 1838097 | A | G | 1539.77 | SNP | Rv1633 (uvrB) | silent (Ser341) | 9840 | - | | 1846766 | CCG | C | 3011.73 | DEL | Rv1638A |  |  | - | | 1847919 | C | G | 1739.77 | SNP | Rv1639c | silent (Thr180) | 9871 | - | | 1849381 | G | A | 1865.77 | SNP | Rv1640c (lysX) | silent (Ile885) | 9872 | - | | 1854300 | T | C | 1892.77 | SNP | Rv1644 (tsnR) | Leu232Pro | 2 | - | | 1856777 | G | C | 1971.77 | SNP | Rv1647 | Ala2Pro | 13 | - | | 1860528 | T | C | 1298.88 | SNP | Rv1650 (pheT) | silent (Cys257) | 9973 | - | | 1865210 | T | C | 904.77 | SNP | Rv1651c (PE\_PGRS30) | Tyr58Cys | 3 | - | | 1867614 | C | T | 1826.77 | SNP | Rv1653 (argJ) | silent (Ala328) | 9867 | - | | 1867838 | G | A | 2110.77 | SNP | Rv1653 (argJ) | Ser403Asn | 20 | - | | 1868780 | A | G | 1896.77 | SNP | Rv1655 (argD) | Met(s)20Val(s) | 9867 | - | | 1872211 | G | A | 1989.77 | SNP | Rv1658 (argG) | silent (Ala283) | 9867 | - | | 1872597 | C | T | 1052.77 | SNP | intergenic |  |  | - | | 1880850 | G | A | 2358.77 | SNP | Rv1661 (pks7) | Leu(s)1849Leu | 3 | - | | 1885772 | G | A | 1806.77 | SNP | Rv1662 (pks8) | Ala1357Thr | 22 | - | | 1891101 | CA | C | 2376.73 | DEL | intergenic |  |  | - | | 1894300 | G | GGTCTTGCCGC | 6875.73 | INS | Rv1668c |  |  | - | | 1894422 | A | G | 1900.77 | SNP | Rv1668c | silent (Asp307) | 9859 | - | | 1898633 | G | A | 2234.77 | SNP | Rv1673c | silent (Asp200) | 9859 | - | | 1901493 | T | C | 2389.77 | SNP | Rv1676 | silent (Ser149) | 9840 | - | | 1907296 | G | C | 2922.77 | SNP | Rv1682 | silent (Ala298) | 9867 | - | | 1916137 | A | G | 1338.77 | SNP | Rv1691 | silent (Leu63) | 9947 | - | | 1917972 | A | G | 1729.77 | SNP | Rv1694 (tlyA) | silent (Leu11) | 9947 | - | | 1931179 | C | A | 1989.77 | SNP | Rv1704c (cycA) | Arg93Leu | 1 | - | | 1931629 | T | G | 1639.77 | SNP | Rv1705c (PPE22) | Gln342His | 20 | - | | 1933953 | A | G | 2776.77 | SNP | intergenic |  |  | - | | 1933988 | G | A | 2811.77 | SNP | intergenic |  |  | - | | 1944107 | A | G | 2041.77 | SNP | Rv1716 | Ser178Gly | 21 | - | | 1944402 | T | C | 1848.77 | SNP | Rv1716 | Val276Ala | 18 | - | | 1945045 | G | A | 1481.77 | SNP | Rv1718 | silent (Leu79) | 9947 | - | | 1945054 | G | A | 1581.77 | SNP | Rv1718 | silent (Gly82) | 9935 | - | | 1950767 | T | C | 3015.77 | SNP | Rv1724c | silent (Lys95) | 9926 | - | | 1960284 | C | A | 1998.77 | SNP | Rv1733c | Gln68His | 20 | - | | 1967237 | C | A | 1886.77 | SNP | Rv1739c | Arg134Leu | 1 | - | | 1983057 | T | C | 261.77 | SNP | Rv1753c (PPE24) | silent (Pro573) | 9926 | - | | 1983291 | C | T | 62.77 | SNP | Rv1753c (PPE24) | silent (Pro495) | 9926 | - | | 1983313 | T | G | 946.77 | SNP | Rv1753c (PPE24) | Asn488Thr | 13 | - | | 1983981 | G | A | 1214.77 | SNP | Rv1753c (PPE24) | silent (Gly265) | 9935 | - | | 1989457 | C | T | 2297.77 | SNP | Rv1758 (cut1) | Pro139Leu | 3 | - | | 1993808 | A | T | 1641.77 | SNP | Rv1760 | Glu219Val(s) | 17 | - | | 2008217 | T | G | 1793.77 | SNP | Rv1774 | Val129Gly | 5 | - | | 2022868 | T | C | 1081.77 | SNP | Rv1783 (eccC5) | silent (Ser1204) | 9840 | - | | 2025913 | T | C | 164.56 | SNP | Rv1787 (PPE25) | Ser205Pro | 12 | - | | 2029087 | G | C | 370.77 | SNP | Rv1790 (PPE27) | Leu(s)221Phe | 1 | - | | 2029104 | G | A | 93.77 | SNP | Rv1790 (PPE27) | Arg227Gln | 9 | - | | 2045310 | A | G | 358.77 | SNP | Rv1803c (PE\_PGRS32) | silent (Ile511) | 9872 | - | | 2049065 | T | C | 1921.77 | SNP | intergenic |  |  | - | | 2049097 | G | C | 2094.77 | SNP | intergenic |  |  | - | | 2051746 | T | C | 1757.77 | SNP | Rv1809 (PPE33) | silent (Ala155) | 9867 | - | | 2052035 | G | T | 1962.77 | SNP | Rv1809 (PPE33) | Val(s)252Leu(s) | 9867 | - | | 2055271 | A | G | 2266.77 | SNP | Rv1812c | Leu30Pro | 2 | - | | 2057591 | C | T | 1307.77 | SNP | Rv1815 | Pro22Ser | 17 | - | | 2057774 | A | T | 2035.77 | SNP | Rv1815 | Ile83Phe | 8 | - | | 2059831 | T | G | 1019.74 | SNP | Rv1817 | silent (Gly79) | 9935 | - | | 2061433 | T | TCCGCCGGCG | 615.73 | INS | Rv1818c (PE\_PGRS33) |  |  | - | | 2063685 | C | T | 1401.77 | SNP | Rv1819c (bacA) | Leu(s)348Leu | 3 | - | | 2063911 | A | G | 2279.77 | SNP | Rv1819c (bacA) | Ile273Thr | 11 | - | | 2066470 | G | A | 1532.77 | SNP | Rv1821 (secA2) | Gly5Asp | 6 | - | | 2074458 | G | C | 1281.77 | SNP | intergenic |  |  | - | | 2074754 | C | T | 2018.77 | SNP | intergenic |  |  | - | | 2094911 | ACAGCGT | A | 6582.73 | DEL | Rv1844c (gnd1) |  |  | - | | 2096186 | A | G | 1844.77 | SNP | Rv1846c (blaI) | silent (Thr138) | 9871 | - | | 2100906 | C | T | 1358.77 | SNP | Rv1852 (ureG) | silent (Asp189) | 9859 | - | | 2108141 | T | C | 808.77 | SNP | Rv1860 (apa) | Phe136Leu | 13 | - | | 2109523 | C | CG | 3278.73 | INS | intergenic |  |  | - | | 2116903 | C | T | 2161.77 | SNP | Rv1867 | silent (Gly380) | 9935 | - | | 2123169 | T | G | 3185.74 | SNP | intergenic |  |  | - | | 2123182 | A | C | 3233.06 | SNP | Rv1873 | silent (Ser3) | 9840 | - | | 2128870 | A | G | 1985.77 | SNP | Rv1878 (glnA3) | silent (Leu283) | 9947 | - | | 2133468 | T | TTCGCATGCCGTCACC | 32729.73 | INS | Rv1883c |  |  | - | | 2135870 | T | C | 1593.77 | SNP | intergenic |  |  | - | | 2137521 | A | ACTCCGATCAC | 12716.73 | INS | Rv1888c |  |  | - | | 2143328 | G | C | 2503.77 | SNP | Rv1895 | Val(s)270Leu | 3 | - | | 2145740 | TC | T | 2238.73 | DEL | Rv1899c (lppD) |  |  | - | | 2147022 | A | C | 2757.77 | SNP | Rv1900c (lipJ) | Ile204Met(s) | 6 | - | | 2151148 | C | G | 2190.77 | SNP | Rv1904 | Ile65Met(s) | 6 | - | | 2153246 | T | G | 2570.77 | SNP | Rv1907c | silent (Arg213) | 9913 | - | | 2155168 | C | G | 2527.77 | SNP | Rv1908c (katG) | Ser315Thr | 32 | resistance | | 2156847 | G | A | 2026.77 | SNP | Rv1910c | silent (Thr151) | 9871 | - | | 2160998 | G | A | 1989.77 | SNP | Rv1915 (aceAa) | Gly179Asp | 6 | - | | 2161343 | G | GT | 1885.73 | INS | Rv1915 (aceAa) |  |  | - | | 2163375 | T | C | 732.77 | SNP | Rv1917c (PPE34) | Asn1313Asp | 42 | - | | 2163400 | A | G | 526.77 | SNP | Rv1917c (PPE34) | silent (Gly1304) | 9935 | - | | 2163403 | A | G | 487.77 | SNP | Rv1917c (PPE34) | silent (Pro1303) | 9926 | - | | 2163412 | A | G | 792.77 | SNP | Rv1917c (PPE34) | silent (Val1300) | 9901 | - | | 2163415 | C | A | 685.77 | SNP | Rv1917c (PPE34) | silent (Pro1299) | 9926 | - | | 2163417 | G | C | 815.77 | SNP | Rv1917c (PPE34) | Pro1299Ala | 22 | - | | 2163419 | C | T | 661.77 | SNP | Rv1917c (PPE34) | Ser1298Asn | 20 | - | | 2163421 | C | G | 675.77 | SNP | Rv1917c (PPE34) | silent (Thr1297) | 9871 | - | | 2163444 | T | C | 766.77 | SNP | Rv1917c (PPE34) | Asn1290Asp | 42 | - | | 2163790 | A | C | 1410.77 | SNP | Rv1917c (PPE34) | silent (Pro1174) | 9926 | - | | 2165286 | A | C | 1575.77 | SNP | Rv1917c (PPE34) | Ser676Ala | 35 | - | | 2165503 | T | A | 2646.77 | SNP | Rv1917c (PPE34) | silent (Ala603) | 9867 | - | | 2173033 | A | G | 1963.77 | SNP | Rv1921c (lppF) | Trp255Arg | 8 | - | | 2180817 | ATCGCCTTG | A | 10747.73 | DEL | Rv1928c |  |  | - | | 2181026 | G | C | 2438.77 | SNP | Rv1928c | silent (Pro64) | 9926 | - | | 2185342 | C | T | 1276.77 | SNP | Rv1934c (fadE17) | Asp283Asn | 36 | - | | 2186785 | C | T | 1627.77 | SNP | Rv1935c (echA13) | silent (Gln125) | 9876 | - | | 2196715 | G | C | 1906.77 | SNP | Rv1945 | Val243Leu | 15 | - | | 2196964 | A | C | 1424.77 | SNP | Rv1945 | Asn326His | 18 | - | | 2196969 | G | C | 1338.77 | SNP | Rv1945 | silent (Ala327) | 9867 | - | | 2196970 | C | A | 1272.77 | SNP | Rv1945 | His328Asn | 21 | - | | 2205283 | C | T | 2551.77 | SNP | Rv1962A (vapB35) | silent (Arg89) | 9913 | - | | 2207591 | T | TC | 5423.73 | INS | intergenic |  |  | - | | 2211826 | A | G | 1326.77 | SNP | Rv1968 (mce3C) | silent (Lys67) | 9926 | - | | 2216035 | A | G | 1419.77 | SNP | Rv1971 (mce3F) | Glu260Gly | 7 | - | | 2216443 | C | A | 1850.77 | SNP | Rv1971 (mce3F) | Ala396Glu | 10 | - | | 2220512 | T | G | 2495.77 | SNP | Rv1977 | silent (Ser253) | 9840 | - | | 2223293 | T | C | 2716.77 | SNP | intergenic |  |  | - | | 2225237 | T | C | 2186.77 | SNP | intergenic |  |  | - | | 2225238 | A | C | 2014.77 | SNP | intergenic |  |  | - | | 2226319 | C | G | 2112.77 | SNP | Rv1983 (PE\_PGRS35) | Arg26Gly | 1 | - | | 2228925 | A | C | 1743.77 | SNP | intergenic |  |  | - | | 2228967 | A | G | 1955.77 | SNP | intergenic |  |  | - | | 2237059 | G | T | 2362.77 | SNP | Rv1992c (ctpG) | Thr83Asn | 9 | - | | 2243199 | C | G | 2226.77 | SNP | Rv1998c | Glu175Gln | 27 | - | | 2244768 | G | A | 2095.77 | SNP | Rv1999c | Ala124Val(s) | 9867 | - | | 2247677 | A | C | 2611.77 | SNP | Rv2002 (fabG3) | silent (Ile6) | 9872 | - | | 2251179 | C | T | 1830.77 | SNP | Rv2005c | silent (Pro235) | 9926 | - | | 2251999 | A | G | 1949.77 | SNP | intergenic |  |  | - | | 2260151 | A | G | 903.77 | SNP | intergenic |  |  | - | | 2260154 | C | T | 879.77 | SNP | intergenic |  |  | - | | 2260171 | T | C | 1320.77 | SNP | intergenic |  |  | - | | 2260174 | C | T | 1251.77 | SNP | intergenic |  |  | - | | 2260196 | C | CA | 1797.73 | INS | intergenic |  |  | - | | 2260199 | C | T | 1308.77 | SNP | intergenic |  |  | - | | 2260212 | G | T | 870.77 | SNP | intergenic |  |  | - | | 2260214 | G | C | 1017.77 | SNP | intergenic |  |  | - | | 2260220 | C | T | 942.77 | SNP | intergenic |  |  | - | | 2260222 | C | G | 1023.77 | SNP | intergenic |  |  | - | | 2260231 | T | C | 995.77 | SNP | intergenic |  |  | - | | 2260525 | C | T | 1454.77 | SNP | intergenic |  |  | - | | 2262026 | T | G | 1247.77 | SNP | Rv2015c | silent (Ala349) | 9867 | - | | 2264782 | C | A | 2213.77 | SNP | Rv2017 | Ala262Glu | 10 | - | | 2265059 | T | G | 2367.77 | SNP | intergenic |  |  | - | | 2266487 | G | C | 1403.77 | SNP | Rv2020c | silent (Leu78) | 9947 | - | | 2266504 | T | TA | 2511.73 | INS | Rv2020c |  |  | - | | 2266508 | A | T | 1251.77 | SNP | Rv2020c | Asp71Glu | 56 | - | | 2266511 | GT | G | 2202.73 | DEL | Rv2020c |  |  | - | | 2266517 | T | C | 1376.77 | SNP | Rv2020c | silent (Glu68) | 9865 | - | | 2266550 | G | T | 1622.77 | SNP | Rv2020c | silent (Gly57) | 9935 | - | | 2266553 | C | G | 1630.77 | SNP | Rv2020c | silent (Ser56) | 9840 | - | | 2266583 | C | G | 1669.77 | SNP | Rv2020c | Glu46Asp | 53 | - | | 2266598 | G | C | 1741.77 | SNP | Rv2020c | silent (Leu41) | 9947 | - | | 2266604 | C | G | 1536.77 | SNP | Rv2020c | silent (Ser39) | 9840 | - | | 2266613 | G | GC | 2763.73 | INS | Rv2020c |  |  | - | | 2266624 | G | T | 1719.77 | SNP | Rv2020c | Leu33Ile | 9 | - | | 2267223 | G | A | 2365.77 | SNP | Rv2022c | Gln168STOP | 8 | - | | 2267533 | C | T | 3406.77 | SNP | Rv2022c | Val(s)64Val | 13 | - | | 2269780 | T | C | 1606.77 | SNP | Rv2024c | Asp154Gly | 11 | - | | 2270102 | A | G | 2004.77 | SNP | Rv2024c | Trp47Arg | 8 | - | | 2277272 | A | G | 2070.77 | SNP | Rv2030c | silent (Tyr405) | 9945 | - | | 2280734 | G | A | 1923.77 | SNP | Rv2033c | His117Tyr | 4 | - | | 2282787 | C | T | 1906.77 | SNP | Rv2037c | Cys312Tyr | 3 | - | | 2285251 | C | A | 2660.77 | SNP | Rv2039c | Val131Phe | 0 | - | | 2287121 | A | G | 2039.77 | SNP | Rv2041c | silent (Asp242) | 9859 | - | | 2296042 | G | C | 1672.77 | SNP | Rv2048c (pks12) | Pro3649Ala | 22 | - | | 2296181 | A | G | 647.77 | SNP | Rv2048c (pks12) | silent (Gly3602) | 9935 | - | | 2300237 | A | G | 712.77 | SNP | Rv2048c (pks12) | silent (Ala2250) | 9867 | - | | 2300546 | A | T | 1199.77 | SNP | Rv2048c (pks12) | His2147Gln | 23 | - | | 2300552 | T | G | 1067.77 | SNP | Rv2048c (pks12) | silent (Pro2145) | 9926 | - | | 2300555 | A | G | 1132.77 | SNP | Rv2048c (pks12) | silent (Asp2144) | 9859 | - | | 2300664 | G | A | 1311.77 | SNP | Rv2048c (pks12) | Pro2108Leu | 3 | - | | 2311099 | C | G | 1296.77 | SNP | Rv2052c | silent (Pro473) | 9926 | - | | 2316510 | G | A | 1058.88 | SNP | Rv2059; Rv2060 | Gly446Asp; Val78Ile | 6; 33 | - | | 2329533 | A | G | 1270.77 | SNP | Rv2072c (cobL) | Leu205Pro | 2 | - | | 2334007 | A | G | 2344.77 | SNP | Rv2077c | silent (Ala96) | 9867 | - | | 2335075 | A | G | 2046.77 | SNP | Rv2078 | Glu6Gly | 7 | - | | 2335494 | A | G | 1947.77 | SNP | Rv2079 | Tyr47Cys | 3 | - | | 2338275 | G | C | 328.77 | SNP | Rv2081c | silent (Thr77) | 9871 | - | | 2338457 | G | A | 955.77 | SNP | Rv2081c | Arg17Cys | 1 | - | | 2338677 | T | C | 1081.77 | SNP | intergenic |  |  | - | | 2338679 | C | T | 978.77 | SNP | intergenic |  |  | - | | 2338692 | C | G | 1191.77 | SNP | intergenic |  |  | - | | 2338701 | C | T | 903.77 | SNP | intergenic |  |  | - | | 2338702 | A | G | 988.77 | SNP | intergenic |  |  | - | | 2338704 | C | G | 974.77 | SNP | intergenic |  |  | - | | 2338707 | C | T | 930.77 | SNP | intergenic |  |  | - | | 2338716 | G | C | 1545.77 | SNP | Rv2082 | Gly3Ala | 21 | - | | 2338720 | T | C | 1584.77 | SNP | Rv2082 | silent (Asp4) | 9859 | - | | 2338768 | G | T | 1712.77 | SNP | Rv2082 | silent (Pro20) | 9926 | - | | 2338773 | G | A | 1686.77 | SNP | Rv2082 | Arg22Gln | 9 | - | | 2338810 | T | C | 1649.77 | SNP | Rv2082 | silent (Arg34) | 9913 | - | | 2338811 | A | G | 1708.77 | SNP | Rv2082 | Lys35Glu | 4 | - | | 2338866 | T | G | 2011.77 | SNP | Rv2082 | Leu53Arg | 1 | - | | 2338912 | A | C | 2075.77 | SNP | Rv2082 | silent (Arg68) | 9913 | - | | 2338961 | G | A | 4673.77 | SNP | Rv2082 | Val85Ile | 33 | - | | 2338990 | G | C | 4715.77 | SNP | Rv2082 | silent (Ala94) | 9867 | - | | 2338994 | G | A | 4716.77 | SNP | Rv2082 | Ala96Thr | 22 | - | | 2340621 | C | G | 3306.77 | SNP | Rv2082 | Pro638Arg | 4 | - | | 2341636 | C | G | 1090.77 | SNP | Rv2083 | Leu256Val(s) | 4 | - | | 2345037 | C | A | 1659.77 | SNP | Rv2088 (pknJ) | silent (Leu209) | 9947 | - | | 2348446 | C | G | 998.77 | SNP | Rv2090 | Phe358Leu(s) | 2 | - | | 2356054 | G | A | 1136.94 | SNP | Rv2097c (pafA) | silent (Val208) | 9901 | - | | 2358029 | T | TG | 1934.73 | INS | intergenic |  |  | - | | 2359051 | T | C | 1634.77 | SNP | Rv2100 | silent (Asp221) | 9859 | - | | 2359072 | G | A | 1626.77 | SNP | Rv2100 | silent (Ala228) | 9867 | - | | 2359075 | G | A | 1716.77 | SNP | Rv2100 | silent (Leu229) | 9947 | - | | 2361492 | T | G | 897.77 | SNP | Rv2101 (helZ) | Leu418Arg | 1 | - | | 2361604 | C | G | 1182.77 | SNP | Rv2101 (helZ) | Val455Val(s) | 18 | - | | 2362041 | C | A | 2299.77 | SNP | Rv2101 (helZ) | Pro601Gln | 6 | - | | 2368564 | TA | T | 4738.73 | DEL | intergenic |  |  | - | | 2369326 | C | G | 2055.77 | SNP | Rv2109c (prcA) | Arg135Pro | 5 | - | | 2372550 | G | C | 1251.77 | SNP | Rv2112c (dop) | Pro7Arg | 4 | - | | 2384873 | C | T | 1087.82 | SNP | Rv2124c (metH) | Asp399Asn | 36 | - | | 2386389 | G | A | 1684.77 | SNP | Rv2125 | Gly33Ser | 16 | - | | 2387733 | T | C | 475.77 | SNP | Rv2126c (PE\_PGRS37) | silent (Glu80) | 9865 | - | | 2393341 | C | A | 2711.77 | SNP | intergenic |  |  | - | | 2394922 | C | T | 1144.77 | SNP | Rv2134c | Val(s)106Met(s) | 9867 | - | | 2396728 | G | A | 1539.77 | SNP | Rv2136c | silent (Gly37) | 9935 | - | | 2396770 | G | C | 1767.77 | SNP | Rv2136c | silent (Ser23) | 9840 | - | | 2401825 | T | C | 880.77 | SNP | intergenic |  |  | - | | 2403871 | G | T | 983.77 | SNP | Rv2143 | Ala299Ser | 28 | - | | 2408744 | T | C | 1337.77 | SNP | Rv2150c (ftsZ) | Ile261Val | 57 | - | | 2411730 | G | C | 1776.77 | SNP | Rv2152c (murC) | silent (Ser131) | 9840 | genotype | | 2415656 | G | C | 1496.77 | SNP | Rv2155c (murD) | Arg247Gly | 1 | - | | 2420503 | A | G | 1282.77 | SNP | Rv2158c (murE) | Leu(s)36Leu | 3 | - | | 2424925 | A | G | 1318.77 | SNP | intergenic |  |  | - | | 2428471 | T | C | 2114.77 | SNP | Rv2165c | Arg319Gly | 1 | - | | 2439519 | G | A | 1032.77 | SNP | Rv2177c | silent (Arg143) | 9913 | - | | 2440926 | G | T | 2424.77 | SNP | Rv2178c (aroG) | Asp265Glu | 56 | - | | 2441970 | G | A | 1543.77 | SNP | Rv2179c | silent (Phe116) | 9946 | - | | 2446461 | G | A | 1449.96 | SNP | Rv2184c | silent (Arg162) | 9913 | - | | 2449222 | C | G | 1770.77 | SNP | Rv2187 (fadD15) | Pro355Ala | 22 | - | | 2462871 | G | A | 1718.77 | SNP | Rv2198c (mmpS3) | silent (Ala59) | 9867 | - | | 2477573 | C | G | 2118.77 | SNP | Rv2212 | silent (Leu128) | 9947 | - | | 2478266 | A | C | 1738.77 | SNP | Rv2212 | Glu359Asp | 53 | - | | 2480676 | G | A | 1507.77 | SNP | Rv2214c (ephD) | silent (Leu342) | 9947 | - | | 2482888 | G | A | 1167.77 | SNP | Rv2215 (dlaT) | silent (Ala308) | 9867 | - | | 2483263 | A | C | 2318.77 | SNP | Rv2215 (dlaT) | silent (Ser433) | 9840 | - | | 2499726 | G | A | 2352.77 | SNP | Rv2226 | Asp299Asn | 36 | - | | 2507412 | G | A | 1300.77 | SNP | Rv2234 (ptpA) | Leu(s)89Leu | 3 | - | | 2509140 | G | C | 1452.77 | SNP | Rv2236c (cobD) | Ser79Cys | 5 | - | | 2509722 | A | G | 1942.77 | SNP | Rv2237 | silent (Pro78) | 9926 | - | | 2516567 | G | C | 2315.77 | SNP | intergenic |  |  | - | | 2521342 | T | C | 1826.77 | SNP | Rv2247 (accD6) | silent (Asp200) | 9859 | - | | 2523205 | G | GCGC | 2272.73 | INS | intergenic |  |  | - | | 2523423 | C | T | 1683.77 | SNP | Rv2249c (glpD1) | Glu457Lys | 7 | - | | 2525722 | CG | C | 2157.73 | DEL | Rv2250A; Rv2251 |  |  | - | | 2525776 | C | T | 1580.77 | SNP | Rv2250A; Rv2251 | silent (Cys125); Ala71Val | 9973; 13 | - | | 2526974 | T | C | 1978.77 | SNP | Rv2251 | silent (Pro470) | 9926 | - | | 2529680 | A | G | 2032.77 | SNP | Rv2256c | silent (Thr65) | 9871 | - | | 2531742 | A | G | 2315.77 | SNP | Rv2258c | silent (Ala52) | 9867 | - | | 2532017 | G | C | 58.77 | SNP | intergenic |  |  | - | | 2534562 | GGA | G | 3024.73 | DEL | Rv2262c |  |  | - | | 2540441 | T | C | 1764.77 | SNP | Rv2266 (cyp124) | Leu113Pro | 2 | - | | 2547673 | ACGC | A | 6656.73 | DEL | Rv2275 |  |  | - | | 2559151 | C | T | 1750.77 | SNP | Rv2286c | Gly140Asp | 6 | - | | 2573756 | C | A | 2548.77 | SNP | intergenic |  |  | - | | 2578626 | A | G | 1962.77 | SNP | Rv2307c | Met(s)24Thr | 22 | - | | 2586127 | A | G | 1622.77 | SNP | Rv2314c | silent (Gly388) | 9935 | - | | 2589216 | G | C | 2896.77 | SNP | Rv2316 (uspA) | Val(s)127Leu | 3 | - | | 2595605 | C | T | 1999.77 | SNP | Rv2323c | Ser222Asn | 20 | - | | 2598400 | A | G | 2208.77 | SNP | Rv2326c | silent (Asn516) | 9822 | - | | 2607715 | T | C | 2835.77 | SNP | Rv2333c (stp) | Ile203Val | 57 | - | | 2608117 | C | A | 1826.77 | SNP | Rv2333c (stp) | Asp69Tyr | 0 | - | | 2615585 | A | G | 2646.77 | SNP | Rv2339 (mmpL9) | Tyr298Cys | 3 | - | | 2617610 | C | T | 2364.77 | SNP | intergenic |  |  | - | | 2625834 | C | A | 1374.77 | SNP | intergenic |  |  | - | | 2626244 | G | C | 131.77 | SNP | Rv2347c (esxP) | silent (Ser92) | 9840 | - | | 2626247 | G | C | 141.77 | SNP | Rv2347c (esxP) | silent (Ala91) | 9867 | - | | 2626271 | G | A | 71.77 | SNP | Rv2347c (esxP) | silent (Asn83) | 9822 | - | | 2626274 | G | C | 71.77 | SNP | Rv2347c (esxP) | silent (Ala82) | 9867 | - | | 2626280 | G | A | 60.77 | SNP | Rv2347c (esxP) | silent (Arg80) | 9913 | - | | 2626283 | A | G | 83.77 | SNP | Rv2347c (esxP) | silent (Val79) | 9901 | - | | 2626304 | G | A | 71.77 | SNP | Rv2347c (esxP) | silent (His72) | 9912 | - | | 2626490 | G | A | 1212.96 | SNP | Rv2347c (esxP) | silent (His10) | 9912 | - | | 2626513 | T | A | 1219.77 | SNP | Rv2347c (esxP) | Thr3Ser | 38 | - | | 2626514 | T | G | 1237.74 | SNP | Rv2347c (esxP) | silent (Ala2) | 9867 | - | | 2630158 | C | G | 334.77 | SNP | Rv2350c (plcB) | silent (Arg54) | 9913 | - | | 2630161 | A | G | 341.77 | SNP | Rv2350c (plcB) | silent (Asn53) | 9822 | - | | 2630173 | C | G | 110.77 | SNP | Rv2350c (plcB) | Leu(s)49Phe | 1 | - | | 2630176 | C | G | 105.77 | SNP | Rv2350c (plcB) | Leu(s)48Phe | 1 | - | | 2630182 | G | A | 90.77 | SNP | Rv2350c (plcB) | silent (Ile46) | 9872 | - | | 2630184 | T | A | 58.77 | SNP | Rv2350c (plcB) | Ile46Phe | 8 | - | | 2631226 | C | A | 2163.77 | SNP | Rv2351c (plcA) | Asp284Tyr | 0 | - | | 2631556 | C | G | 135.77 | SNP | Rv2351c (plcA) | Gly174Arg | 0 | - | | 2631565 | T | C | 263.77 | SNP | Rv2351c (plcA) | Ile171Val | 57 | - | | 2631574 | T | C | 335.77 | SNP | Rv2351c (plcA) | Thr168Ala | 32 | - | | 2631583 | G | A | 432.77 | SNP | Rv2351c (plcA) | Leu165Leu(s) | 4 | - | | 2631599 | G | A | 714.77 | SNP | Rv2351c (plcA) | silent (Ile159) | 9872 | - | | 2631620 | A | G | 660.77 | SNP | Rv2351c (plcA) | silent (Gly152) | 9935 | - | | 2631962 | T | G | 58.77 | SNP | Rv2351c (plcA) | silent (Gly38) | 9935 | - | | 2631967 | G | A | 64.77 | SNP | Rv2351c (plcA) | Pro37Ser | 17 | - | | 2631968 | A | G | 120.77 | SNP | Rv2351c (plcA) | silent (Cys36) | 9973 | - | | 2631971 | A | G | 156.77 | SNP | Rv2351c (plcA) | silent (Pro35) | 9926 | - | | 2631977 | G | C | 162.77 | SNP | Rv2351c (plcA) | silent (Ala33) | 9867 | - | | 2637541 | C | T | 2024.77 | SNP | intergenic |  |  | - | | 2642383 | C | T | 1891.77 | SNP | Rv2360c | Ala66Thr | 22 | - | | 2656225 | A | G | 2661.77 | SNP | Rv2377c (mbtH) | Val69Ala | 18 | - | | 2660319 | C | G | 1748.77 | SNP | Rv2379c (mbtF) | Glu589Asp | 53 | - | | 2678199 | C | T | 1793.77 | SNP | Rv2385 (mbtJ) | silent (Thr157) | 9871 | - | | 2680658 | T | G | 3177.77 | SNP | intergenic |  |  | - | | 2695378 | C | G | 2611.77 | SNP | Rv2398c (cysW) | Gly141Ala | 21 | - | | 2704884 | A | ACAGCGACCATATCGCCGAG CT | 1908.73 | INS | Rv2407 |  |  | - | | 2713795 | C | T | 1853.77 | SNP | intergenic |  |  | - | | 2718852 | T | G | 1951.77 | SNP | intergenic |  |  | - | | 2720895 | G | A | 302.77 | SNP | Rv2424c | His295Tyr | 4 | - | | 2720952 | T | C | 131.90 | SNP | Rv2424c | Thr276Ala | 32 | - | | 2720954 | G | A | 92.90 | SNP | Rv2424c | Thr275Ile | 7 | - | | 2721562 | C | G | 2005.77 | SNP | Rv2424c | silent (Ala72) | 9867 | - | | 2734074 | T | C | 580.77 | SNP | Rv2436 (rbsK) | Val282Ala | 18 | - | | 2737572 | C | A | 1297.77 | SNP | Rv2439c (proB) | Ala226Ser | 28 | - | | 2748087 | G | A | 1775.77 | SNP | Rv2448c (valS) | silent (Ser713) | 9840 | - | | 2751804 | C | T | 935.77 | SNP | Rv2450c (rpfE) | Arg126Gln | 9 | - | | 2752661 | TCCACACGCCGTGGTGACCT GCGCCACAACGACTTGC | T | 24572.73 | DEL | intergenic |  |  | - | | 2759741 | G | A | 2424.77 | SNP | intergenic |  |  | - | | 2760152 | A | G | 2068.77 | SNP | Rv2458 (mmuM) | Tyr125Cys | 3 | - | | 2779136 | T | C | 1528.77 | SNP | Rv2476c (gdh) | Ser1043Gly | 21 | - | | 2786952 | A | G | 2339.77 | SNP | Rv2482c (plsB2) | Cys778Arg | 1 | - | | 2796214 | G | GC | 826.73 | INS | Rv2487c (PE\_PGRS42) |  |  | - | | 2809621 | T | C | 2751.77 | SNP | Rv2495c (bkdC) | Thr107Ala | 32 | - | | 2818837 | A | G | 1664.77 | SNP | Rv2503c (scoB) | silent (Gly97) | 9935 | - | | 2819636 | C | T | 1460.77 | SNP | Rv2504c (scoA) | Gly79Arg | 0 | - | | 2821342 | C | T | 882.77 | SNP | Rv2505c (fadD35) | silent (Ala85) | 9867 | - | | 2822416 | C | G | 1727.77 | SNP | intergenic |  |  | - | | 2827984 | G | T | 1597.77 | SNP | intergenic |  |  | - | | 2828019 | T | C | 1938.77 | SNP | intergenic |  |  | - | | 2828822 | G | T | 162.84 | SNP | Rv2512c | Gln328Lys | 12 | - | | 2829779 | T | C | 824.77 | SNP | Rv2512c | Thr9Ala | 32 | - | | 2829796 | G | A | 892.77 | SNP | Rv2512c | Ser3Phe | 2 | - | | 2830525 | C | A | 2275.77 | SNP | Rv2513 | Thr122Lys | 11 | - | | 2830960 | G | A | 2432.77 | SNP | Rv2514c | Arg127Cys | 1 | - | | 2838564 | C | G | 1301.77 | SNP | Rv2522c | silent (Pro326) | 9926 | - | | 2851736 | C | G | 2287.77 | SNP | intergenic |  |  | - | | 2855259 | A | G | 1615.77 | SNP | Rv2531c | silent (Ala841) | 9867 | - | | 2865760 | A | G | 2339.77 | SNP | Rv2542 | Thr211Ala | 32 | - | | 2865882 | T | C | 2404.77 | SNP | Rv2542 | silent (Val251) | 9901 | - | | 2866503 | G | A | 79.77 | SNP | Rv2543 (lppA) | silent (Pro12) | 9926 | - | | 2866551 | C | G | 327.77 | SNP | Rv2543 (lppA) | silent (Ala28) | 9867 | - | | 2866569 | C | A | 475.77 | SNP | Rv2543 (lppA) | silent (Thr34) | 9871 | - | | 2866578 | C | A | 519.77 | SNP | Rv2543 (lppA) | His37Gln | 23 | - | | 2866580 | A | G | 547.77 | SNP | Rv2543 (lppA) | Asn38Ser | 34 | - | | 2866607 | G | A | 751.77 | SNP | Rv2543 (lppA) | Gly47Asp | 6 | - | | 2866647 | G | A | 728.77 | SNP | Rv2543 (lppA) | silent (Lys60) | 9926 | - | | 2866671 | G | A | 550.77 | SNP | Rv2543 (lppA) | silent (Glu68) | 9865 | - | | 2866677 | A | C | 525.77 | SNP | Rv2543 (lppA) | silent (Leu70) | 9947 | - | | 2866863 | C | G | 1067.77 | SNP | Rv2543 (lppA) | silent (Ala132) | 9867 | - | | 2866876 | A | G | 1169.77 | SNP | Rv2543 (lppA) | Ile137Val | 57 | - | | 2866880 | C | T | 1167.77 | SNP | Rv2543 (lppA) | Ala138Val | 13 | - | | 2866882 | G | A | 1354.77 | SNP | Rv2543 (lppA) | Ala139Thr | 22 | - | | 2867207 | C | G | 917.77 | SNP | Rv2544 (lppB) | silent (Ala28) | 9867 | - | | 2867230 | G | A | 546.77 | SNP | Rv2544 (lppB) | Gly36Asp | 6 | - | | 2867231 | C | T | 533.77 | SNP | Rv2544 (lppB) | silent (Gly36) | 9935 | - | | 2867236 | A | G | 514.77 | SNP | Rv2544 (lppB) | Asn38Ser | 34 | - | | 2867240 | C | T | 545.77 | SNP | Rv2544 (lppB) | silent (Pro39) | 9926 | - | | 2867245 | A | C | 506.77 | SNP | Rv2544 (lppB) | Lys41Thr | 8 | - | | 2867251 | C | G | 517.77 | SNP | Rv2544 (lppB) | Pro43Arg | 4 | - | | 2867254 | A | G | 447.77 | SNP | Rv2544 (lppB) | His44Arg | 10 | - | | 2867263 | G | A | 468.77 | SNP | Rv2544 (lppB) | Gly47Asp | 6 | - | | 2867298 | C | A | 261.77 | SNP | Rv2544 (lppB) | His59Asn | 21 | - | | 2867347 | A | G | 164.77 | SNP | Rv2544 (lppB) | Gln75Arg | 10 | - | | 2880702 | G | C | 2159.77 | SNP | Rv2560 | Val210Leu | 15 | - | | 2881582 | GT | G | 2225.73 | DEL | Rv2561 |  |  | - | | 2881597 | AG | A | 2314.73 | DEL | Rv2561 |  |  | - | | 2888201 | T | C | 1372.77 | SNP | Rv2566 | Leu610Pro | 2 | - | | 2889633 | T | C | 1617.77 | SNP | Rv2566 | silent (Ala1087) | 9867 | - | | 2891267 | C | T | 2642.77 | SNP | Rv2567 | silent (Gly491) | 9935 | - | | 2891700 | T | G | 1925.77 | SNP | Rv2567 | Ser636Ala | 35 | - | | 2891728 | A | G | 1735.77 | SNP | Rv2567 | Gln645Arg | 10 | - | | 2894208 | G | A | 1783.77 | SNP | Rv2569c | silent (Ser67) | 9840 | - | | 2903556 | T | C | 3033.77 | SNP | intergenic |  |  | - | | 2910461 | G | T | 1748.77 | SNP | Rv2584c (apt) | Ala147Glu | 10 | - | | 2911293 | C | G | 1970.77 | SNP | Rv2585c | Cys462Ser | 11 | - | | 2912294 | T | G | 1965.77 | SNP | Rv2585c | silent (Ala128) | 9867 | - | | 2921808 | C | G | 1656.77 | SNP | Rv2591 (PE\_PGRS44) | silent (Ala86) | 9867 | - | | 2923264 | G | A | 900.31 | SNP | Rv2592c (ruvB) | Leu324Leu(s) | 4 | - | | 2923391 | T | C | 1360.77 | SNP | Rv2592c (ruvB) | silent (Pro281) | 9926 | - | | 2927939 | T | C | 2228.77 | SNP | intergenic |  |  | - | | 2939373 | G | C | 1734.77 | SNP | Rv2611c | Ser197Cys | 5 | - | | 2939657 | T | C | 1194.77 | SNP | Rv2611c | Ile102Met(s) | 6 | - | | 2942377 | G | A | 1904.77 | SNP | Rv2614c (thrS) | silent (Gly297) | 9935 | - | | 2943878 | C | G | 244.89 | SNP | Rv2615c (PE\_PGRS45) | Gly370Arg | 0 | - | | 2954439 | T | C | 3252.77 | SNP | Rv2627c | Arg104Gly | 1 | - | | 2954571 | A | C | 2512.77 | SNP | Rv2627c | Leu60Val | 11 | - | | 2973463 | A | T | 31.74 | SNP | intergenic |  |  | - | | 2974933 | A | G | 1157.77 | SNP | Rv2650c | Ile101Thr | 11 | - | | 2983613 | G | A | 186.80 | SNP | Rv2666 | silent (Gly181) | 9935 | - | | 2984740 | A | G | 955.77 | SNP | Rv2668 | His3Arg | 10 | - | | 2988815 | C | G | 1470.77 | SNP | Rv2672 | Asn378Lys | 25 | - | | 3001083 | C | G | 3004.77 | SNP | Rv2684 (arsA) | Pro157Arg | 4 | - | | 3003084 | GC | G | 3257.73 | DEL | Rv2685 (arsB1) |  |  | - | | 3005185 | G | T | 1901.77 | SNP | Rv2688c | Pro156Thr | 5 | - | | 3009692 | A | G | 2622.77 | SNP | Rv2691 (ceoB) | Thr117Ala | 32 | - | | 3015047 | T | C | 2721.77 | SNP | intergenic |  |  | - | | 3016852 | G | A | 929.96 | SNP | intergenic |  |  | - | | 3017465 | T | C | 2966.77 | SNP | Rv2702 (ppgK) | Ile203Thr | 11 | - | | 3020457 | C | T | 1919.77 | SNP | Rv2706c | Val(s)1Met(s) | 9867 | - | | 3021962 | G | A | 2426.77 | SNP | Rv2709 | Glu42Lys | 7 | - | | 3030196 | TGGTCTGCTGTGTGTACG | T | 7959.73 | DEL | Rv2717c |  |  | - | | 3030214 | T | A | 1091.31 | SNP | Rv2717c | Thr50Ser | 38 | - | | 3037377 | T | C | 964.77 | SNP | intergenic |  |  | - | | 3041871 | G | T | 1785.77 | SNP | Rv2729c | Ala202Glu | 10 | - | | 3054081 | A | G | 2497.77 | SNP | Rv2741 (PE\_PGRS47) | silent (Gly56) | 9935 | - | | 3054321 | A | G | 476.77 | SNP | Rv2741 (PE\_PGRS47) | silent (Gly136) | 9935 | - | | 3056663 | G | A | 1440.77 | SNP | Rv2743c | silent (Cys190) | 9973 | - | | 3066280 | G | A | 2267.77 | SNP | Rv2753c (dapA) | Thr282Ile | 7 | - | | 3069167 | A | G | 1785.77 | SNP | Rv2756c (hsdM) | Leu306Pro | 2 | - | | 3078178 | C | T | 1298.77 | SNP | Rv2769c (PE27) | Val(s)270Met(s) | 9867 | - | | 3080795 | A | G | 3486.77 | SNP | Rv2771c | Leu80Pro | 2 | - | | 3086742 | A | C | 2801.77 | SNP | Rv2779c | Phe5Val | 1 | - | | 3086788 | T | C | 2571.77 | SNP | intergenic |  |  | - | | 3095662 | C | A | 1758.77 | SNP | Rv2787 | silent (Thr184) | 9871 | - | | 3097028 | G | A | 2204.77 | SNP | Rv2788 (sirR) | Ala24Thr | 22 | - | | 3098405 | G | A | 1717.77 | SNP | Rv2789c (fadE21) | silent (Asn178) | 9822 | - | | 3100202 | T | G | 2132.77 | SNP | Rv2791c | STOP460Cys | 1 | - | | 3103461 | G | A | 2391.77 | SNP | Rv2794c (pptT) | silent (Tyr160) | 9945 | - | | 3103682 | T | C | 2081.77 | SNP | Rv2794c (pptT) | Met(s)87Val(s) | 9867 | - | | 3104336 | T | C | 2791.77 | SNP | Rv2795c | silent (Glu192) | 9865 | - | | 3113872 | A | T | 1623.77 | SNP | Rv2807 | Glu72Val(s) | 17 | - | | 3118000 | A | G | 1471.77 | SNP | Rv2812 | Arg395Gly | 1 | - | | 3124014 | A | C | 2541.77 | SNP | Rv2817c | Ser324Ala | 35 | - | | 3124352 | C | A | 2459.77 | SNP | Rv2817c | Ser211Ile | 1 | - | | 3127717 | T | C | 1848.77 | SNP | Rv2820c | Thr186Ala | 32 | - | | 3131469 | T | TTGTCGGCGA | 6048.73 | INS | Rv2823c |  |  | - | | 3133536 | T | C | 2612.77 | SNP | Rv2825c | Lys2Glu | 4 | - | | 3137058 | G | A | 2153.77 | SNP | Rv2830c (vapB22) | Ala56Val(s) | 9867 | - | | 3138077 | A | G | 826.77 | SNP | intergenic |  |  | - | | 3141827 | C | G | 2813.77 | SNP | Rv2835c (ugpA) | silent (Leu132) | 9947 | - | | 3162805 | C | G | 248.78 | SNP | Rv2853 (PE\_PGRS48) | Arg180Gly | 1 | - | | 3177884 | C | A | 1602.77 | SNP | Rv2866 (relG) | silent (Arg21) | 9913 | - | | 3186588 | ACGG | A | 3111.73 | DEL | Rv2874 (dipZ) |  |  | - | | 3186664 | A | C | 1982.77 | SNP | Rv2874 (dipZ) | silent (Ala606) | 9867 | - | | 3186860 | T | G | 1793.77 | SNP | Rv2874 (dipZ) | Tyr672Asp | 0 | - | | 3190145 | TC | T | 2763.73 | DEL | Rv2880c |  |  | - | | 3192431 | T | C | 2589.77 | SNP | Rv2883c (pyrH) | Asp243Gly | 11 | - | | 3198496 | G | A | 1292.77 | SNP | Rv2889c (tsf) | silent (Ile204) | 9872 | - | | 3201711 | T | G | 587.77 | SNP | Rv2892c (PPE45) | Thr104Pro | 4 | - | | 3218122 | A | C | 2655.77 | SNP | Rv2910c | Ile50Ser | 2 | - | | 3226181 | A | C | 1573.77 | SNP | Rv2916c (ffh) | silent (Arg35) | 9913 | - | | 3228143 | G | T | 1586.77 | SNP | Rv2917 | Arg594Leu | 1 | - | | 3232703 | G | A | 258.77 | SNP | intergenic |  |  | - | | 3232759 | G | A | 2183.77 | SNP | intergenic |  |  | - | | 3232766 | T | C | 124.77 | SNP | intergenic |  |  | - | | 3232822 | T | C | 98.77 | SNP | intergenic |  |  | - | | 3233940 | G | C | 1399.77 | SNP | Rv2921c (ftsY) | Ala67Gly | 21 | - | | 3247316 | C | G | 2259.77 | SNP | Rv2931 (ppsA) | Asp624Glu | 56 | - | | 3247851 | G | A | 1295.77 | SNP | Rv2931 (ppsA) | Ala803Thr | 22 | - | | 3247853 | C | T | 1260.77 | SNP | Rv2931 (ppsA) | silent (Ala803) | 9867 | - | | 3247856 | G | C | 1305.77 | SNP | Rv2931 (ppsA) | silent (Arg804) | 9913 | - | | 3247864 | C | CTAGG | 3143.73 | INS | Rv2931 (ppsA) |  |  | - | | 3247865 | GCAAA | G | 3101.73 | DEL | Rv2931 (ppsA) |  |  | - | | 3247874 | G | A | 1316.77 | SNP | Rv2931 (ppsA) | silent (Arg810) | 9913 | - | | 3247877 | T | C | 1360.77 | SNP | Rv2931 (ppsA) | silent (Phe811) | 9946 | - | | 3247883 | T | C | 1825.77 | SNP | Rv2931 (ppsA) | silent (Ser813) | 9840 | - | | 3248074 | G | A | 1902.77 | SNP | Rv2931 (ppsA) | Arg877His | 8 | - | | 3248075 | C | T | 1987.77 | SNP | Rv2931 (ppsA) | silent (Arg877) | 9913 | - | | 3256494 | A | G | 2277.77 | SNP | Rv2933 (ppsC) | silent (Gly270) | 9935 | - | | 3261813 | C | G | 1106.77 | SNP | Rv2933 (ppsC) | Asp2043Glu | 56 | - | | 3269581 | A | G | 1991.77 | SNP | Rv2935 (ppsE) | silent (Ala615) | 9867 | - | | 3270784 | A | G | 1415.77 | SNP | Rv2935 (ppsE) | silent (Gln1016) | 9876 | - | | 3290870 | G | C | 2187.77 | SNP | Rv2945c (lppX) | silent (Ser152) | 9840 | - | | 3296843 | A | G | 874.77 | SNP | Rv2947c (pks15) | Val(s)333Ala | 9867 | - | | 3308606 | G | A | 3060.77 | SNP | intergenic |  |  | - | | 3311262 | C | G | 1681.77 | SNP | Rv2958c | Val247Leu | 15 | - | | 3315177 | G | A | 2724.77 | SNP | intergenic |  |  | - | | 3315213 | T | C | 2404.77 | SNP | intergenic |  |  | - | | 3336587 | T | A | 536.77 | SNP | intergenic |  |  | - | | 3336620 | TA | T | 193.73 | DEL | intergenic |  |  | - | | 3336646 | T | A | 135.77 | SNP | intergenic |  |  | - | | 3336705 | A | T | 71.77 | SNP | intergenic |  |  | - | | 3336825 | T | C | 1545.77 | SNP | Rv2981c (ddlA) | Thr365Ala | 32 | - | | 3338603 | G | C | 2326.77 | SNP | Rv2982c (gpdA2) | Pro133Ala | 22 | - | | 3351817 | C | T | 2370.77 | SNP | Rv2994 | silent (Ser183) | 9840 | - | | 3352932 | C | G | 1909.77 | SNP | Rv2995c (leuB) | silent (Thr179) | 9871 | - | | 3358235 | A | T | 2679.77 | SNP | Rv2999 (lppY) | Met(s)212Leu(s) | 9867 | - | | 3363338 | A | G | 1938.77 | SNP | intergenic |  |  | - | | 3365382 | C | A | 1060.77 | SNP | Rv3006 (lppZ) | Thr225Lys | 11 | - | | 3366950 | G | A | 2487.77 | SNP | Rv3008 | Asp103Asn | 36 | - | | 3367765 | G | A | 1252.77 | SNP | Rv3009c (gatB) | silent (Gly343) | 9935 | - | | 3379708 | G | C | 160.90 | SNP | intergenic |  |  | - | | 3379712 | G | C | 121.03 | SNP | intergenic |  |  | - | | 3379718 | T | C | 166.90 | SNP | intergenic |  |  | - | | 3379726 | C | A | 85.28 | SNP | intergenic |  |  | - | | 3379730 | G | C | 42.74 | SNP | intergenic |  |  | - | | 3379732 | C | T | 42.74 | SNP | intergenic |  |  | - | | 3379735 | A | C | 49.74 | SNP | intergenic |  |  | - | | 3379736 | C | A | 80.28 | SNP | intergenic |  |  | - | | 3379742 | T | C | 234.80 | SNP | intergenic |  |  | - | | 3379751 | A | C | 296.78 | SNP | intergenic |  |  | - | | 3379757 | A | C | 366.77 | SNP | intergenic |  |  | - | | 3379763 | G | A | 448.77 | SNP | intergenic |  |  | - | | 3379784 | C | A | 708.77 | SNP | intergenic |  |  | - | | 3379788 | C | G | 839.77 | SNP | intergenic |  |  | - | | 3381641 | G | T | 103.03 | SNP | Rv3023c | Gln328Lys | 12 | - | | 3383966 | G | C | 2146.77 | SNP | Rv3025c (iscS) | Val367Val(s) | 18 | - | | 3384213 | A | G | 2460.77 | SNP | Rv3025c (iscS) | Val285Ala | 18 | - | | 3391074 | G | C | 1437.77 | SNP | Rv3031 | Val385Leu | 15 | - | | 3400580 | G | A | 1897.77 | SNP | Rv3040c | Ser160Phe | 2 | - | | 3401871 | A | G | 1603.77 | SNP | Rv3041c | silent (Ala16) | 9867 | - | | 3402816 | C | T | 2089.77 | SNP | Rv3042c (serB2) | Gly116Glu | 4 | - | | 3404376 | C | G | 1629.77 | SNP | Rv3043c (ctaD) | silent (Thr182) | 9871 | - | | 3404670 | G | A | 2405.77 | SNP | Rv3043c (ctaD) | silent (Thr84) | 9871 | - | | 3406045 | G | A | 2492.77 | SNP | Rv3044 (fecB) | Ala304Thr | 22 | - | | 3408301 | GC | G | 2638.75 | DEL | Rv3047c |  |  | - | | 3412445 | G | A | 2573.77 | SNP | Rv3051c (nrdE) | silent (Ile574) | 9872 | - | | 3415180 | ACACCTAGGGGGTGG | A | 9046.73 | DEL | intergenic |  |  | - | | 3416734 | G | A | 2156.77 | SNP | Rv3056 (dinP) | silent (Leu10) | 9947 | - | | 3422104 | G | A | 2516.77 | SNP | Rv3060c | silent (Val370) | 9901 | - | | 3422687 | G | A | 2556.77 | SNP | Rv3060c | Thr176Ile | 7 | - | | 3425854 | C | T | 2168.77 | SNP | Rv3062 (ligB) | Pro91Ser | 17 | - | | 3428917 | C | A | 2110.77 | SNP | Rv3063 (cstA) | Arg559Ser | 11 | - | | 3432635 | C | A | 2269.78 | SNP | Rv3068c (pgmA) | Val(s)330Leu(s) | 9867 | - | | 3440464 | T | G | 2010.77 | SNP | Rv3077 | silent (Arg308) | 9913 | - | | 3440468 | G | C | 1999.77 | SNP | Rv3077 | Gly310Arg | 0 | - | | 3442970 | C | T | 1459.77 | SNP | Rv3080c (pknK) | Ala1007Thr | 22 | - | | 3447800 | G | T | 1823.77 | SNP | Rv3082c (virS) | Asp209Glu | 56 | - | | 3450725 | T | C | 1701.77 | SNP | Rv3084 (lipR) | silent (Val243) | 9901 | - | | 3450780 | C | G | 2057.77 | SNP | Rv3084 (lipR) | Arg262Gly | 1 | - | | 3455686 | G | C | 3310.77 | SNP | Rv3088 (tgs4) | silent (Leu449) | 9947 | - | | 3456666 | A | G | 2054.77 | SNP | Rv3089 (fadD13) | silent (Ala302) | 9867 | - | | 3462135 | G | C | 1551.77 | SNP | Rv3093c | Cys210Trp | 0 | - | | 3462145 | A | AGGCGC | 3099.73 | INS | Rv3093c |  |  | - | | 3469694 | G | T | 1910.77 | SNP | Rv3100c (smpB) | silent (Ala30) | 9867 | - | | 3473996 | G | GA | 3188.01 | INS | intergenic |  |  | - | | 3477307 | G | A | 2185.77 | SNP | Rv3108 | Arg83Gln | 9 | - | | 3479354 | G | T | 2355.77 | SNP | Rv3111 (moaC1) | Ala62Ser | 28 | - | | 3480474 | G | A | 2609.77 | SNP | Rv3113 | Gly134Glu | 4 | - | | 3482432 | C | A | 232.80 | SNP | Rv3115 | Gln328Lys | 12 | - | | 3486977 | A | G | 3011.77 | SNP | Rv3121 (cyp141) | Lys157Glu | 4 | - | | 3497369 | G | A | 1258.77 | SNP | Rv3131 | silent (Leu273) | 9947 | - | | 3502160 | A | T | 600.77 | SNP | Rv3136 (PPE51) | Thr123Ser | 38 | - | | 3502166 | T | C | 622.77 | SNP | Rv3136 (PPE51) | Phe125Leu | 13 | - | | 3502169 | T | C | 565.77 | SNP | Rv3136 (PPE51) | Phe126Leu | 13 | - | | 3502171 | C | G | 516.77 | SNP | Rv3136 (PPE51) | Phe126Leu(s) | 2 | - | | 3502183 | T | C | 576.77 | SNP | Rv3136 (PPE51) | silent (Thr130) | 9871 | - | | 3502186 | G | A | 658.77 | SNP | Rv3136 (PPE51) | silent (Ala131) | 9867 | - | | 3502195 | G | A | 759.77 | SNP | Rv3136 (PPE51) | silent (Ala134) | 9867 | - | | 3502200 | C | T | 798.77 | SNP | Rv3136 (PPE51) | Thr136Ile | 7 | - | | 3502228 | C | A | 621.77 | SNP | Rv3136 (PPE51) | silent (Ala145) | 9867 | - | | 3502231 | G | A | 608.77 | SNP | Rv3136 (PPE51) | silent (Gln146) | 9876 | - | | 3502237 | C | G | 531.77 | SNP | Rv3136 (PPE51) | silent (Ala148) | 9867 | - | | 3502252 | T | C | 306.77 | SNP | Rv3136 (PPE51) | silent (Gly153) | 9935 | - | | 3502262 | G | A | 249.77 | SNP | Rv3136 (PPE51) | Ala157Thr | 22 | - | | 3503895 | C | T | 1725.77 | SNP | Rv3137 | Pro168Leu | 3 | - | | 3505027 | G | A | 651.77 | SNP | Rv3138 (pflA) | Arg278His | 8 | - | | 3518167 | A | G | 1510.77 | SNP | Rv3151 (nuoG) | Ile474Met(s) | 6 | - | | 3518555 | A | G | 1395.77 | SNP | Rv3151 (nuoG) | Thr604Ala | 32 | - | | 3528064 | T | C | 86.77 | SNP | Rv3159c (PPE53) | Asn367Ser | 34 | - | | 3528065 | T | C | 91.77 | SNP | Rv3159c (PPE53) | Asn367Asp | 42 | - | | 3528066 | G | A | 120.77 | SNP | Rv3159c (PPE53) | silent (Asn366) | 9822 | - | | 3528072 | A | G | 203.77 | SNP | Rv3159c (PPE53) | silent (Ser364) | 9840 | - | | 3528084 | A | G | 313.77 | SNP | Rv3159c (PPE53) | silent (Gly360) | 9935 | - | | 3528087 | G | A | 331.77 | SNP | Rv3159c (PPE53) | silent (Ile359) | 9872 | - | | 3528099 | A | C | 663.77 | SNP | Rv3159c (PPE53) | silent (Gly355) | 9935 | - | | 3528102 | C | A | 671.77 | SNP | Rv3159c (PPE53) | silent (Ser354) | 9840 | - | | 3528117 | T | C | 673.77 | SNP | Rv3159c (PPE53) | silent (Leu349) | 9947 | - | | 3528119 | G | T | 691.77 | SNP | Rv3159c (PPE53) | Leu349Ile | 9 | - | | 3528120 | A | G | 693.77 | SNP | Rv3159c (PPE53) | silent (Asn348) | 9822 | - | | 3528129 | G | T | 698.77 | SNP | Rv3159c (PPE53) | silent (Gly345) | 9935 | - | | 3528140 | G | A | 784.77 | SNP | Rv3159c (PPE53) | Leu342Leu(s) | 4 | - | | 3528144 | G | A | 724.77 | SNP | Rv3159c (PPE53) | silent (Gly340) | 9935 | - | | 3528158 | A | G | 886.77 | SNP | Rv3159c (PPE53) | Leu(s)336Leu | 3 | - | | 3528159 | G | A | 796.77 | SNP | Rv3159c (PPE53) | silent (Asn335) | 9822 | - | | 3528165 | G | A | 807.77 | SNP | Rv3159c (PPE53) | silent (Gly333) | 9935 | - | | 3528192 | G | A | 869.77 | SNP | Rv3159c (PPE53) | silent (Asn324) | 9822 | - | | 3528198 | A | G | 770.77 | SNP | Rv3159c (PPE53) | silent (Asn322) | 9822 | - | | 3528971 | TCGC | T | 1990.73 | DEL | Rv3159c (PPE53) |  |  | - | | 3529067 | G | C | 1414.77 | SNP | Rv3159c (PPE53) | Arg33Gly | 1 | - | | 3529142 | G | A | 1439.77 | SNP | Rv3159c (PPE53) | Pro8Ser | 17 | - | | 3535298 | C | T | 2340.77 | SNP | Rv3166c | Val(s)18Val | 13 | - | | 3549429 | T | C | 2046.77 | SNP | Rv3180c | Gln87Arg | 10 | - | | 3554067 | A | G | 66.28 | SNP | intergenic |  |  | - | | 3556275 | A | G | 2472.77 | SNP | Rv3190c | Leu138Pro | 2 | - | | 3574504 | G | A | 1457.77 | SNP | Rv3201c | Arg845Trp | 2 | - | | 3580636 | CT | C | 4145.73 | DEL | intergenic |  |  | - | | 3581414 | A | G | 2252.77 | SNP | Rv3204 | Thr34Ala | 32 | - | | 3585577 | T | C | 1614.77 | SNP | Rv3208 | Cys192Arg | 1 | - | | 3589537 | C | T | 1199.77 | SNP | Rv3212 | silent (Pro48) | 9926 | - | | 3590686 | G | GC | 3745.73 | INS | intergenic |  |  | - | | 3591063 | T | C | 1671.77 | SNP | Rv3213c | Lys144Glu | 4 | - | | 3604821 | G | C | 890.77 | SNP | Rv3228 | silent (Ala32) | 9867 | - | | 3614982 | T | C | 2560.77 | SNP | Rv3239c | silent (Leu874) | 9947 | - | | 3620484 | G | A | 2332.77 | SNP | Rv3240c (secA1) | silent (Arg16) | 9913 | - | | 3621423 | A | G | 1792.77 | SNP | intergenic |  |  | - | | 3622441 | A | C | 1655.77 | SNP | Rv3243c | Val217Val(s) | 18 | - | | 3625065 | T | G | 1911.77 | SNP | Rv3245c (mtrB) | Met(s)517Leu | 3 | - | | 3635110 | G | A | 2234.77 | SNP | Rv3255c (manA) | silent (Ala386) | 9867 | - | | 3636355 | C | T | 1600.77 | SNP | Rv3256c | Ala321Thr | 22 | - | | 3636432 | G | T | 1775.77 | SNP | Rv3256c | Thr295Lys | 11 | - | | 3666905 | C | T | 1405.77 | SNP | Rv3285 (accA3) | silent (Thr183) | 9871 | - | | 3689523 | G | T | 1748.77 | SNP | Rv3303c (lpdA) | Cys472STOP | 3 | - | | 3694373 | C | T | 1580.77 | SNP | Rv3307 (deoD) | Ala107Val | 13 | - | | 3704596 | G | C | 2003.77 | SNP | Rv3317 (sdhD) | Val(s)54Leu | 3 | - | | 3704599 | T | C | 2067.77 | SNP | Rv3317 (sdhD) | Phe55Leu | 13 | - | | 3710382 | T | A | 54.79 | SNP | intergenic |  |  | - | | 3711910 | G | A | 590.77 | SNP | Rv3327 | Trp54STOP | 0 | - | | 3714211 | G | T | 2275.77 | SNP | Rv3328c (sigJ) | Pro41Gln | 6 | - | | 3714757 | A | C | 2494.77 | SNP | Rv3329 | Gln122His | 20 | - | | 3718357 | C | T | 1409.77 | SNP | Rv3331 (sugI) | Pro423Leu | 3 | - | | 3721140 | GC | G | 4216.73 | DEL | intergenic |  |  | - | | 3721806 | G | C | 2914.77 | SNP | Rv3335c | silent (Gly265) | 9935 | - | | 3730519 | C | G | 753.77 | SNP | Rv3343c (PPE54) | silent (Thr2139) | 9871 | - | | 3730642 | G | A | 118.03 | SNP | Rv3343c (PPE54) | silent (Asn2098) | 9822 | - | | 3732247 | C | G | 139.77 | SNP | Rv3343c (PPE54) | silent (Thr1563) | 9871 | - | | 3732258 | G | C | 604.77 | SNP | Rv3343c (PPE54) | Gln1560Glu | 35 | - | | 3732310 | G | A | 802.77 | SNP | Rv3343c (PPE54) | silent (Asn1542) | 9822 | - | | 3732469 | G | A | 460.77 | SNP | Rv3343c (PPE54) | silent (Gly1489) | 9935 | - | | 3735673 | C | G | 44.74 | SNP | Rv3343c (PPE54) | Leu(s)421Phe | 1 | - | | 3735677 | A | G | 44.74 | SNP | Rv3343c (PPE54) | Val420Ala | 18 | - | | 3735709 | A | G | 57.28 | SNP | Rv3343c (PPE54) | silent (Gly409) | 9935 | - | | 3735925 | G | A | 162.90 | SNP | Rv3343c (PPE54) | silent (Asn337) | 9822 | - | | 3735931 | G | A | 74.28 | SNP | Rv3343c (PPE54) | silent (Ser335) | 9840 | - | | 3736179 | G | A | 159.84 | SNP | Rv3343c (PPE54) | Leu253Leu(s) | 4 | - | | 3736628 | T | G | 1018.77 | SNP | Rv3343c (PPE54) | Glu103Ala | 17 | - | | 3737359 | G | A | 209.41 | SNP | intergenic |  |  | - | | 3738690 | G | C | 229.80 | SNP | Rv3345c (PE\_PGRS50) | Ala1362Gly | 21 | - | | 3738701 | GGCACCGCCC | G | 1158.73 | DEL | Rv3345c (PE\_PGRS50) |  |  | - | | 3739630 | C | T | 453.77 | SNP | Rv3345c (PE\_PGRS50) | Asp1049Asn | 36 | - | | 3740904 | A | T | 362.77 | SNP | Rv3345c (PE\_PGRS50) | Ile624Asn | 3 | - | | 3740913 | T | G | 267.78 | SNP | Rv3345c (PE\_PGRS50) | Asn621Thr | 13 | - | | 3740914 | T | C | 324.77 | SNP | Rv3345c (PE\_PGRS50) | Asn621Asp | 42 | - | | 3740915 | G | A | 330.77 | SNP | Rv3345c (PE\_PGRS50) | silent (Gly620) | 9935 | - | | 3744931 | C | A | 1822.77 | SNP | Rv3347c (PPE55) | Ala2752Ser | 28 | - | | 3744937 | C | A | 1840.77 | SNP | Rv3347c (PPE55) | Val2750Phe | 0 | - | | 3744945 | G | A | 1332.77 | SNP | Rv3347c (PPE55) | Thr2747Ile | 7 | - | | 3744946 | T | A | 1355.77 | SNP | Rv3347c (PPE55) | Thr2747Ser | 38 | - | | 3744948 | C | T | 1485.77 | SNP | Rv3347c (PPE55) | Ser2746Asn | 20 | - | | 3744953 | C | A | 1380.77 | SNP | Rv3347c (PPE55) | Val(s)2744Val | 13 | - | | 3744954 | A | G | 1441.77 | SNP | Rv3347c (PPE55) | Val(s)2744Ala | 9867 | - | | 3744955 | C | T | 1474.77 | SNP | Rv3347c (PPE55) | Val(s)2744Met(s) | 9867 | - | | 3746409 | A | G | 1307.77 | SNP | Rv3347c (PPE55) | Leu2259Pro | 2 | - | | 3746708 | G | A | 509.77 | SNP | Rv3347c (PPE55) | silent (Ala2159) | 9867 | - | | 3746710 | C | G | 508.77 | SNP | Rv3347c (PPE55) | Ala2159Pro | 13 | - | | 3746719 | G | C | 569.77 | SNP | Rv3347c (PPE55) | Leu2156Val(s) | 4 | - | | 3746723 | C | G | 494.77 | SNP | Rv3347c (PPE55) | silent (Thr2154) | 9871 | - | | 3746727 | G | T | 545.77 | SNP | Rv3347c (PPE55) | Thr2153Asn | 9 | - | | 3746733 | C | T | 461.77 | SNP | Rv3347c (PPE55) | Gly2151Asp | 6 | - | | 3746734 | C | T | 457.77 | SNP | Rv3347c (PPE55) | Gly2151Ser | 16 | - | | 3747973 | A | G | 360.77 | SNP | Rv3347c (PPE55) | Phe1738Leu | 13 | - | | 3748762 | G | C | 587.77 | SNP | Rv3347c (PPE55) | Leu1475Val(s) | 4 | - | | 3748763 | G | A | 513.41 | SNP | Rv3347c (PPE55) | silent (Gly1474) | 9935 | - | | 3748773 | C | G | 299.28 | SNP | Rv3347c (PPE55) | Gly1471Ala | 21 | - | | 3748775 | G | C | 323.56 | SNP | Rv3347c (PPE55) | Phe1470Leu(s) | 2 | - | | 3748777 | A | G | 261.78 | SNP | Rv3347c (PPE55) | Phe1470Leu | 13 | - | | 3748781 | G | A | 361.78 | SNP | Rv3347c (PPE55) | silent (Val1468) | 9901 | - | | 3748788 | T | G | 582.77 | SNP | Rv3347c (PPE55) | Asn1466Thr | 13 | - | | 3748796 | G | C | 614.77 | SNP | Rv3347c (PPE55) | silent (Ala1463) | 9867 | - | | 3748799 | G | A | 599.77 | SNP | Rv3347c (PPE55) | silent (Asn1462) | 9822 | - | | 3750305 | C | G | 60.77 | SNP | Rv3347c (PPE55) | Val(s)960Val | 13 | - | | 3750397 | C | T | 35.77 | SNP | Rv3347c (PPE55) | Gly930Ser | 16 | - | | 3752207 | A | G | 2277.77 | SNP | Rv3347c (PPE55) | silent (Ile326) | 9872 | - | | 3753116 | C | T | 560.77 | SNP | Rv3347c (PPE55) | silent (Pro23) | 9926 | - | | 3753164 | T | G | 968.77 | SNP | Rv3347c (PPE55) | silent (Pro7) | 9926 | - | | 3779671 | C | CGGCAACGGT | 860.75 | INS | Rv3367 (PE\_PGRS51) |  |  | - | | 3791589 | A | C | 3430.77 | SNP | Rv3377c | Ile255Met(s) | 6 | - | | 3793286 | C | T | 2774.77 | SNP | Rv3379c (dxs2) | Asp528Asn | 36 | - | | 3798095 | A | C | 2578.77 | SNP | Rv3383c (idsB) | Val132Gly | 5 | - | | 3799068 | G | A | 2232.77 | SNP | intergenic |  |  | - | | 3815477 | G | T | 2125.77 | SNP | Rv3398c (idsA1) | silent (Ala210) | 9867 | - | | 3817117 | C | A | 1510.77 | SNP | Rv3399 | Ala330Glu | 10 | - | | 3820407 | A | G | 413.77 | SNP | intergenic |  |  | - | | 3820429 | G | A | 121.77 | SNP | intergenic |  |  | - | | 3820446 | G | C | 95.77 | SNP | intergenic |  |  | - | | 3820449 | T | G | 167.77 | SNP | intergenic |  |  | - | | 3820464 | G | A | 410.77 | SNP | intergenic |  |  | - | | 3820545 | A | G | 372.77 | SNP | intergenic |  |  | - | | 3820562 | G | C | 122.77 | SNP | intergenic |  |  | - | | 3820565 | T | G | 160.77 | SNP | intergenic |  |  | - | | 3823159 | A | T | 1677.77 | SNP | Rv3403c | silent (Val235) | 9901 | - | | 3826684 | C | T | 1527.77 | SNP | Rv3408 (vapC47) | Ser46Leu(s) | 35 | - | | 3829770 | T | C | 1603.77 | SNP | Rv3410c (guaB3) | silent (Pro47) | 9926 | - | | 3838871 | A | G | 1863.77 | SNP | Rv3420c (rimI) | silent (Ala64) | 9867 | - | | 3841652 | T | A | 54.77 | SNP | intergenic |  |  | - | | 3841654 | T | G | 207.77 | SNP | intergenic |  |  | - | | 3841662 | T | C | 391.77 | SNP | intergenic |  |  | - | | 3841663 | C | T | 318.77 | SNP | intergenic |  |  | - | | 3842452 | C | A | 254.77 | SNP | Rv3425 (PPE57) | Gln72Lys | 12 | - | | 3842461 | T | A | 625.77 | SNP | Rv3425 (PPE57) | Ser75Thr | 32 | - | | 3842581 | A | G | 373.77 | SNP | Rv3425 (PPE57) | Arg115Gly | 1 | - | | 3842582 | G | T | 373.77 | SNP | Rv3425 (PPE57) | Arg115Met(s) | 2 | - | | 3842585 | G | A | 384.77 | SNP | Rv3425 (PPE57) | Arg116His | 8 | - | | 3842620 | A | G | 1418.77 | SNP | Rv3425 (PPE57) | Thr128Ala | 32 | - | | 3842625 | A | G | 1484.77 | SNP | Rv3425 (PPE57) | silent (Pro129) | 9926 | - | | 3843001 | G | A | 125.77 | SNP | intergenic |  |  | - | | 3843024 | A | C | 232.77 | SNP | intergenic |  |  | - | | 3843025 | A | G | 207.77 | SNP | intergenic |  |  | - | | 3843032 | G | A | 155.77 | SNP | intergenic |  |  | - | | 3843354 | A | G | 75.77 | SNP | Rv3426 (PPE58) | Thr107Ala | 32 | - | | 3843356 | T | C | 113.77 | SNP | Rv3426 (PPE58) | silent (Thr107) | 9871 | - | | 3843361 | C | A | 125.77 | SNP | Rv3426 (PPE58) | Ala109Asp | 6 | - | | 3843362 | C | A | 136.77 | SNP | Rv3426 (PPE58) | silent (Ala109) | 9867 | - | | 3843363 | A | G | 133.77 | SNP | Rv3426 (PPE58) | Asn110Asp | 42 | - | | 3843407 | CG | C | 3534.73 | DEL | Rv3426 (PPE58) |  |  | - | | 3843696 | T | A | 279.77 | SNP | Rv3426 (PPE58) | Leu(s)221Met(s) | 9867 | - | | 3843704 | G | C | 353.77 | SNP | Rv3426 (PPE58) | silent (Thr223) | 9871 | - | | 3843714 | T | C | 469.77 | SNP | Rv3426 (PPE58) | Cys227Arg | 1 | - | | 3843749 | G | T | 380.77 | SNP | intergenic |  |  | - | | 3843751 | G | T | 388.77 | SNP | intergenic |  |  | - | | 3843752 | A | G | 358.77 | SNP | intergenic |  |  | - | | 3843753 | G | A | 370.77 | SNP | intergenic |  |  | - | | 3843760 | T | C | 400.77 | SNP | intergenic |  |  | - | | 3844756 | GC | G | 3004.73 | DEL | Rv3428c |  |  | - | | 3844992 | T | A | 2345.77 | SNP | Rv3428c | Ser327Cys | 5 | - | | 3846605 | G | A | 1066.77 | SNP | intergenic |  |  | - | | 3846607 | A | C | 1156.77 | SNP | intergenic |  |  | - | | 3846622 | G | T | 1166.77 | SNP | intergenic |  |  | - | | 3846687 | A | G | 1458.77 | SNP | intergenic |  |  | - | | 3846704 | A | G | 1278.77 | SNP | intergenic |  |  | - | | 3846707 | A | C | 1363.77 | SNP | intergenic |  |  | - | | 3846716 | C | T | 245.77 | SNP | intergenic |  |  | - | | 3846727 | C | T | 143.77 | SNP | intergenic |  |  | - | | 3846728 | A | G | 271.77 | SNP | intergenic |  |  | - | | 3846741 | G | T | 231.77 | SNP | intergenic |  |  | - | | 3846743 | C | G | 248.77 | SNP | intergenic |  |  | - | | 3846764 | C | G | 1026.77 | SNP | intergenic |  |  | - | | 3846773 | T | TG | 1632.73 | INS | intergenic |  |  | - | | 3846774 | T | G | 886.77 | SNP | intergenic |  |  | - | | 3846777 | C | A | 693.77 | SNP | intergenic |  |  | - | | 3846779 | T | G | 967.77 | SNP | intergenic |  |  | - | | 3846840 | G | GCT | 3275.73 | INS | intergenic |  |  | - | | 3846843 | CAAA | C | 1960.73 | DEL | intergenic |  |  | - | | 3846851 | G | A | 977.77 | SNP | intergenic |  |  | - | | 3846852 | C | G | 822.78 | SNP | intergenic |  |  | - | | 3846853 | T | C | 973.77 | SNP | intergenic |  |  | - | | 3846857 | G | A | 927.77 | SNP | intergenic |  |  | - | | 3846860 | T | G | 851.77 | SNP | intergenic |  |  | - | | 3846866 | C | A | 878.77 | SNP | intergenic |  |  | - | | 3846881 | AT | A | 1236.74 | DEL | intergenic |  |  | - | | 3846886 | A | T | 862.77 | SNP | intergenic |  |  | - | | 3846897 | T | G | 844.84 | SNP | intergenic |  |  | - | | 3847014 | C | G | 86.77 | SNP | intergenic |  |  | - | | 3847022 | T | C | 93.77 | SNP | intergenic |  |  | - | | 3847039 | G | A | 107.77 | SNP | intergenic |  |  | - | | 3847052 | G | A | 85.77 | SNP | intergenic |  |  | - | | 3847073 | G | C | 63.77 | SNP | intergenic |  |  | - | | 3847074 | C | G | 141.77 | SNP | intergenic |  |  | - | | 3847099 | G | A | 107.77 | SNP | intergenic |  |  | - | | 3847310 | G | A | 51.77 | SNP | Rv3429 (PPE59) | Gly49Asp | 6 | - | | 3847318 | C | G | 115.77 | SNP | Rv3429 (PPE59) | Gln52Glu | 35 | - | | 3847320 | G | T | 109.77 | SNP | Rv3429 (PPE59) | Gln52His | 20 | - | | 3847351 | A | T | 491.77 | SNP | Rv3429 (PPE59) | Met(s)63Leu(s) | 9867 | - | | 3847364 | C | T | 588.77 | SNP | Rv3429 (PPE59) | Ala67Val | 13 | - | | 3847367 | G | A | 592.77 | SNP | Rv3429 (PPE59) | Gly68Glu | 4 | - | | 3847378 | G | C | 1024.77 | SNP | Rv3429 (PPE59) | Asp72His | 3 | - | | 3847380 | C | A | 1008.77 | SNP | Rv3429 (PPE59) | Asp72Glu | 56 | - | | 3847546 | A | G | 1132.77 | SNP | Rv3429 (PPE59) | Thr128Ala | 32 | - | | 3847551 | A | G | 1112.77 | SNP | Rv3429 (PPE59) | silent (Pro129) | 9926 | - | | 3847562 | G | A | 886.77 | SNP | Rv3429 (PPE59) | Gly133Glu | 4 | - | | 3847563 | A | C | 835.77 | SNP | Rv3429 (PPE59) | silent (Gly133) | 9935 | - | | 3847578 | T | C | 442.77 | SNP | Rv3429 (PPE59) | silent (Tyr138) | 9945 | - | | 3847579 | C | G | 430.77 | SNP | Rv3429 (PPE59) | Gln139Glu | 35 | - | | 3847581 | G | C | 376.77 | SNP | Rv3429 (PPE59) | Gln139His | 20 | - | | 3847595 | A | G | 234.77 | SNP | Rv3429 (PPE59) | Gln144Arg | 10 | - | | 3847596 | A | C | 212.77 | SNP | Rv3429 (PPE59) | Gln144His | 20 | - | | 3847600 | A | G | 218.77 | SNP | Rv3429 (PPE59) | Ile146Val | 57 | - | | 3847608 | C | A | 170.77 | SNP | Rv3429 (PPE59) | silent (Val148) | 9901 | - | | 3859893 | C | T | 2119.77 | SNP | Rv3440c | silent (Glu28) | 9865 | - | | 3862472 | GA | G | 2449.73 | DEL | intergenic |  |  | - | | 3864995 | T | C | 2193.77 | SNP | Rv3447c (eccC4) | Ser1082Gly | 21 | - | | 3867404 | C | A | 2186.77 | SNP | Rv3447c (eccC4) | Glu279STOP | 17 | - | | 3867973 | T | C | 2963.77 | SNP | Rv3447c (eccC4) | His89Arg | 10 | - | | 3868379 | C | T | 2007.77 | SNP | Rv3448 (eccD4) | Arg10Trp | 2 | - | | 3874722 | AT | A | 4449.73 | DEL | Rv3453 |  |  | - | | 3875022 | G | C | 2103.77 | SNP | Rv3454 | Gln67His | 20 | - | | 3875230 | A | G | 2343.77 | SNP | Rv3454 | Thr137Ala | 32 | - | | 3877421 | A | G | 2267.77 | SNP | Rv3456c (rplQ) | silent (Pro4) | 9926 | - | | 3884906 | A | G | 573.85 | SNP | Rv3467 | Lys315Glu | 4 | - | | 3885886 | T | C | 2247.77 | SNP | Rv3468c | Ile62Val | 57 | - | | 3892671 | A | G | 3947.77 | SNP | Rv3476c (kgtP) | silent (Val350) | 9901 | - | | 3895036 | T | C | 2444.77 | SNP | Rv3478 (PPE60) | Val(s)204Ala | 9867 | - | | 3895399 | C | A | 740.77 | SNP | Rv3478 (PPE60) | Pro325Gln | 6 | - | | 3895400 | A | G | 746.77 | SNP | Rv3478 (PPE60) | silent (Pro325) | 9926 | - | | 3895403 | A | C | 751.77 | SNP | Rv3478 (PPE60) | silent (Ala326) | 9867 | - | | 3896340 | T | G | 1905.77 | SNP | Rv3479 | Leu174Arg | 1 | - | | 3898408 | A | G | 2079.77 | SNP | Rv3479 | silent (Ala863) | 9867 | - | | 3910737 | G | A | 1789.77 | SNP | Rv3492c | Pro71Ser | 17 | - | | 3912327 | G | A | 1868.77 | SNP | Rv3494c (mce4F) | Pro348Leu | 3 | - | | 3929843 | C | T | 267.78 | SNP | Rv3507 (PE\_PGRS53) | Ala1092Val | 13 | - | | 3930440 | C | A | 38.74 | SNP | Rv3507 (PE\_PGRS53) | Ala1291Asp | 6 | - | | 3932675 | A | G | 203.84 | SNP | Rv3508 (PE\_PGRS54) | silent (Gln557) | 9876 | - | | 3934542 | T | G | 131.03 | SNP | Rv3508 (PE\_PGRS54) | Ser1180Ala | 35 | - | | 3934690 | C | T | 167.77 | SNP | Rv3508 (PE\_PGRS54) | Ala1229Val | 13 | - | | 3934699 | G | A | 268.18 | SNP | Rv3508 (PE\_PGRS54) | Ser1232Asn | 20 | - | | 3934733 | G | C | 470.77 | SNP | Rv3508 (PE\_PGRS54) | silent (Gly1243) | 9935 | - | | 3934734 | G | A | 470.77 | SNP | Rv3508 (PE\_PGRS54) | Ala1244Thr | 22 | - | | 3939211 | T | TGTAG | 5586.73 | INS | Rv3510c |  |  | - | | 3940073 | G | T | 179.84 | SNP | Rv3511 (PE\_PGRS55) | Ala153Ser | 28 | - | | 3940802 | A | G | 106.03 | SNP | Rv3511 (PE\_PGRS55) | Asn396Asp | 42 | - | | 3941003 | G | A | 185.56 | SNP | Rv3511 (PE\_PGRS55) | Gly463Ser | 16 | - | | 3941497 | G | C | 54.77 | SNP | Rv3511 (PE\_PGRS55) | silent (Gly627) | 9935 | - | | 3941499 | C | A | 69.53 | SNP | Rv3511 (PE\_PGRS55) | Ala628Asp | 6 | - | | 3941836 | C | A | 65.77 | SNP | intergenic |  |  | - | | 3942481 | C | G | 241.80 | SNP | intergenic |  |  | - | | 3942640 | T | C | 366.77 | SNP | intergenic |  |  | - | | 3943019 | C | G | 78.28 | SNP | intergenic |  |  | - | | 3943705 | C | A | 59.77 | SNP | intergenic |  |  | - | | 3947292 | G | A | 62.90 | SNP | Rv3514 (PE\_PGRS57) | Gly500Glu | 4 | - | | 3947395 | G | A | 77.77 | SNP | Rv3514 (PE\_PGRS57) | silent (Gly534) | 9935 | - | | 3948928 | G | C | 96.77 | SNP | Rv3514 (PE\_PGRS57) | silent (Gly1045) | 9935 | - | | 3948929 | G | C | 93.77 | SNP | Rv3514 (PE\_PGRS57) | Ala1046Pro | 13 | - | | 3952800 | G | A | 1858.77 | SNP | Rv3516 (echA19) | Gly86Asp | 6 | - | | 3958403 | A | G | 2087.77 | SNP | Rv3521 | Asn295Asp | 42 | - | | 3959418 | C | T | 1816.77 | SNP | Rv3522 (ltp4) | Thr324Ile | 7 | - | | 3966934 | G | C | 2487.77 | SNP | Rv3529c | Asn35Lys | 25 | - | | 3971125 | C | T | 1167.77 | SNP | Rv3533c (PPE62) | silent (Thr443) | 9871 | - | | 3973291 | C | T | 2562.77 | SNP | Rv3534c (hsaF) | Arg101Gln | 9 | - | | 3995746 | G | T | 2335.77 | SNP | intergenic |  |  | - | | 3999707 | G | A | 2054.77 | SNP | Rv3559c | silent (Ala243) | 9867 | - | | 4005607 | T | C | 1993.77 | SNP | Rv3564 (fadE33) | Leu(s)121Leu | 3 | - | | 4014532 | C | T | 1479.77 | SNP | Rv3573c (fadE34) | Glu561Lys | 7 | - | | 4016262 | C | G | 2781.77 | SNP | intergenic |  |  | - | | 4024273 | T | C | 1953.77 | SNP | Rv3581c (ispF) | Val25Val(s) | 18 | - | | 4026899 | G | A | 1963.77 | SNP | Rv3585 (radA) | silent (Gln152) | 9876 | - | | 4034827 | C | T | 2481.77 | SNP | Rv3593 (lpqF) | Ala159Val(s) | 9867 | - | | 4037283 | T | G | 467.53 | SNP | Rv3595c (PE\_PGRS59) | silent (Gly256) | 9935 | - | | 4055801 | G | A | 2464.77 | SNP | Rv3616c (espA) | Thr192Ile | 7 | - | | 4059904 | A | G | 1872.77 | SNP | intergenic |  |  | - | | 4060100 | G | A | 1230.77 | SNP | Rv3619c (esxV) | Leu57Leu(s) | 4 | - | | 4060201 | G | A | 454.77 | SNP | Rv3619c (esxV) | Ser23Leu(s) | 35 | - | | 4060210 | T | A | 466.77 | SNP | Rv3619c (esxV) | Gln20Leu | 6 | - | | 4060230 | G | A | 471.77 | SNP | Rv3619c (esxV) | silent (His13) | 9912 | - | | 4069292 | G | A | 1334.77 | SNP | Rv3630 | Ala40Thr | 22 | - | | 4073918 | G | A | 2063.77 | SNP | Rv3635 | silent (Leu95) | 9947 | - | | 4074899 | C | G | 2255.77 | SNP | Rv3635 | silent (Ala422) | 9867 | - | | 4078617 | T | G | 2496.77 | SNP | Rv3640c | Gln378Pro | 8 | - | | 4091503 | C | T | 2078.77 | SNP | Rv3650 (PE33) | Arg91Cys | 1 | - | | 4095001 | CG | C | 3502.73 | DEL | Rv3655c |  |  | - | | 4095320 | G | A | 2785.77 | SNP | intergenic |  |  | - | | 4100134 | T | C | 2100.77 | SNP | Rv3661 | Val163Ala | 18 | - | | 4100975 | T | C | 1755.77 | SNP | intergenic |  |  | - | | 4104717 | T | G | 2304.77 | SNP | Rv3665c (dppB) | silent (Gly247) | 9935 | - | | 4111303 | G | C | 2368.77 | SNP | Rv3669 | Val(s)159Val | 13 | - | | 4120926 | A | G | 377.77 | SNP | Rv3680 | Asn378Asp | 42 | - | | 4120983 | A | G | 1199.77 | SNP | intergenic |  |  | - | | 4129917 | C | A | 2194.77 | SNP | Rv3688c | silent (Ser147) | 9840 | - | | 4151855 | A | G | 1394.77 | SNP | Rv3708c (asd) | silent (Pro121) | 9926 | - | | 4156099 | C | A | 2137.77 | SNP | Rv3711c (dnaQ) | Val(s)211Leu(s) | 9867 | - | | 4159195 | T | C | 2012.77 | SNP | Rv3714c | silent (Pro209) | 9926 | - | | 4162339 | A | G | 3616.77 | SNP | Rv3719 | Thr12Ala | 32 | - | | 4168330 | G | A | 1952.77 | SNP | intergenic |  |  | - | | 4168445 | GCGCGGACGGGAA | G | 11247.73 | DEL | intergenic |  |  | - | | 4168771 | C | T | 1937.77 | SNP | Rv3723 | Ser79Leu | 1 | - | | 4175225 | T | C | 1395.77 | SNP | Rv3728 | Val118Ala | 18 | - | | 4182695 | G | A | 1924.77 | SNP | Rv3731 (ligC) | Arg313His | 8 | - | | 4187485 | T | C | 2554.77 | SNP | Rv3736 | silent (Ala284) | 9867 | - | | 4187817 | A | G | 1806.77 | SNP | Rv3737 | Asp40Gly | 11 | - | | 4197138 | C | CT | 2571.73 | INS | intergenic |  |  | - | | 4198611 | CG | C | 3664.73 | DEL | intergenic |  |  | - | | 4204441 | A | G | 2228.77 | SNP | Rv3759c (proX) | silent (His311) | 9912 | - | | 4205120 | A | G | 1861.77 | SNP | Rv3759c (proX) | Leu85Pro | 2 | - | | 4210274 | A | G | 1816.77 | SNP | Rv3764c (tcrY) | Cys246Arg | 1 | - | | 4212976 | G | A | 2338.77 | SNP | Rv3766 | silent (Ala228) | 9867 | - | | 4219850 | A | G | 2059.77 | SNP | Rv3775 (lipE) | Ile56Val | 57 | - | | 4221490 | C | G | 2569.77 | SNP | Rv3776 | silent (Leu134) | 9947 | - | | 4222073 | A | G | 942.77 | SNP | Rv3776 | Met(s)329Val(s) | 9867 | - | | 4222882 | A | G | 2093.77 | SNP | Rv3777 | silent (Leu63) | 9947 | - | | 4223172 | T | C | 831.77 | SNP | Rv3777 | Val160Ala | 18 | - | | 4227026 | GCGA | G | 3749.73 | DEL | Rv3780 |  |  | - | | 4230033 | T | C | 2128.77 | SNP | Rv3783 (rfbD) | Val259Ala | 18 | - | | 4242643 | C | T | 1643.77 | SNP | Rv3793 (embC) | silent (Arg927) | 9913 | genotype | | 4255922 | A | G | 2107.77 | SNP | Rv3799c (accD4) | silent (His9) | 9912 | - | | 4257220 | A | G | 2063.77 | SNP | Rv3800c (pks13) | silent (Arg1309) | 9913 | - | | 4280072 | C | A | 2163.77 | SNP | Rv3815c | silent (Ala239) | 9867 | - | | 4291449 | A | C | 1916.77 | SNP | Rv3823c (mmpL8) | silent (Gly27) | 9935 | - | | 4296015 | G | A | 2674.77 | SNP | Rv3825c (pks2) | silent (Asp1197) | 9859 | - | | 4298106 | C | A | 1379.77 | SNP | Rv3825c (pks2) | silent (Ser500) | 9840 | - | | 4302036 | T | C | 1847.77 | SNP | Rv3827c | Thr252Ala | 32 | - | | 4306155 | C | T | 2301.77 | SNP | Rv3831 | silent (Ser133) | 9840 | - | | 4307179 | G | A | 2103.77 | SNP | Rv3833 | Val105Ile | 33 | - | | 4307621 | G | T | 1802.77 | SNP | Rv3833 | Arg252Leu | 1 | - | | 4309617 | G | T | 2156.77 | SNP | Rv3835 | Ala191Ser | 28 | - | | 4310449 | G | A | 1417.77 | SNP | Rv3836 | Ala17Thr | 22 | - | | 4314645 | A | G | 1911.77 | SNP | Rv3841 (bfrB) | silent (Leu156) | 9947 | - | | 4316245 | G | A | 2051.77 | SNP | Rv3843c | Pro118Ser | 17 | - | | 4316466 | G | A | 2386.77 | SNP | Rv3843c | Thr44Ile | 7 | - | | 4323883 | G | A | 2360.77 | SNP | Rv3849 (espR) | Gly129Arg | 0 | - | | 4324042 | C | T | 1608.77 | SNP | Rv3850 | Arg10Cys | 1 | - | | 4324358 | T | C | 1471.77 | SNP | Rv3850 | Ile115Thr | 11 | - | | 4338595 | GC | G | 5466.73 | DEL | intergenic |  |  | - | | 4338732 | G | A | 2616.77 | SNP | intergenic |  |  | - | | 4341354 | A | G | 2103.77 | SNP | Rv3864 (espE) | Gln362Arg | 10 | - | | 4350748 | GA | G | 2431.73 | DEL | Rv3872 (PE35) |  |  | - | | 4351039 | G | T | 2180.77 | SNP | Rv3872 (PE35) | Glu99STOP | 17 | - | | 4355141 | A | C | 2305.77 | SNP | Rv3877 (eccD1) | Glu45Asp | 53 | - | | 4356110 | G | C | 2104.77 | SNP | Rv3877 (eccD1) | silent (Leu368) | 9947 | - | | 4357597 | C | G | 2368.77 | SNP | Rv3879c (espK) | Cys729Ser | 11 | - | | 4359135 | T | TGGGGTTCCCGGGGTGATCG GGGTTCCCGGCGTGATC | 2982.73 | INS | Rv3879c (espK) |  |  | - | | 4359165 | G | C | 702.79 | SNP | Rv3879c (espK) | silent (Thr206) | 9871 | - | | 4362110 | G | C | 1351.77 | SNP | Rv3882c (eccE1) | silent (Thr437) | 9871 | - | | 4362735 | T | C | 2953.77 | SNP | Rv3882c (eccE1) | Asp229Gly | 11 | - | | 4364826 | G | C | 2789.77 | SNP | intergenic |  |  | - | | 4366195 | T | C | 1126.77 | SNP | Rv3884c (eccA2) | Glu215Gly | 7 | - | | 4366272 | G | C | 1904.77 | SNP | Rv3884c (eccA2) | silent (Ala189) | 9867 | - | | 4375628 | G | T | 2107.77 | SNP | Rv3892c (PPE69) | Thr19Lys | 11 | - | | 4376445 | G | A | 2366.77 | SNP | Rv3894c (eccC2) | silent (Pro1336) | 9926 | - | | 4378792 | A | C | 1887.77 | SNP | Rv3894c (eccC2) | Val554Gly | 5 | - | | 4379680 | C | G | 1879.77 | SNP | Rv3894c (eccC2) | Arg258Pro | 5 | - | | 4382054 | T | C | 2077.77 | SNP | Rv3896c | silent (Ala266) | 9867 | - | | 4382275 | G | T | 1955.77 | SNP | Rv3896c | Gln193Lys | 12 | - | | 4383144 | C | CCGGGG | 3119.73 | INS | Rv3897c |  |  | - | | 4383945 | C | T | 1375.77 | SNP | Rv3898c | Gly14Glu | 4 | - | | 4386228 | T | C | 2533.77 | SNP | Rv3900c | silent (Leu27) | 9947 | - | | 4388730 | T | C | 1645.77 | SNP | Rv3903c | Gln568Arg | 10 | - | | 4390391 | C | G | 1391.77 | SNP | Rv3903c | silent (Ala14) | 9867 | - | | 4393178 | A | G | 2190.77 | SNP | intergenic |  |  | - | | 4394744 | G | C | 1754.77 | SNP | Rv3909 | Val(s)185Leu | 3 | - | | 4396994 | G | C | 2167.77 | SNP | Rv3910 | Gly133Ala | 21 | - | | 4400389 | G | T | 1632.77 | SNP | Rv3911 (sigM) | silent (Ala68) | 9867 | - | | 4400660 | AC | A | 4031.73 | DEL | Rv3911 (sigM) |  |  | - | | 4403034 | G | A | 1651.77 | SNP | Rv3914 (trxC) | silent (Lys101) | 9926 | - | | 4406116 | C | A | 2015.77 | SNP | Rv3917c (parB) | Ala126Ser | 28 | - | | 4408061 | G | T | 2550.77 | SNP | Rv3919c (gid) | His48Asn | 21 | - | | 4409602 | C | T | 2191.77 | SNP | Rv3921c | silent (Pro156) | 9926 | - | | 4411276 | T | C | 2670.77 | SNP | intergenic |  |  | - | |  | | export |

elog
