# Supplementary material for: Patterns of compensatory mutations in rpoA/B/C genes of multidrug resistant M. tuberculosis in Uganda
Source: PLoS One. 2025 Dec 4;20(12):e0328957. doi: 10.1371/journal.pone.0328957 (PMC12677784; doi:10.1371/journal.pone.0328957)
Supplement: S2 File — (ZIP) [file pone.0328957.s002.zip › Variants M_S13_L001_001.bam.html]

 

Calling SNPs/INDELs (computing variant list in .vcf format) from M\_S13\_L001\_001.bam

*by SAMtools/BCFtools:*

Howto

Important aspects

This takes up to one hour!!! **Please wait ...**

Variants M\_S13\_L001\_001.bam

|  |  |
| --- | --- |
| Variants |  |

|  |  |
| --- | --- |
| |  | | --- | | *by GATK* | |

|  |  |  |
| --- | --- | --- |
| |  | | --- | | M\_S13\_L001\_001.bam | | | computed 2016-10-27 using PhyResSE v1.0 (Ref. NC\_000962.3) | |

|  |  |
| --- | --- |
| 818  variants called Export in VCF format |  |

|  |  |  |  |  |  |  |  |  |  |  |  |  |  |  |  |  |  |  |  |  |  |  |  |  |  |  |  |  |  |  |  |  |  |  |  |  |  |  |  |  |  |  |  |  |  |  |  |  |  |  |  |  |  |  |  |  |  |  |  |  |  |  |  |  |  |  |  |  |  |  |  |  |  |  |  |  |  |  |  |  |  |  |  |  |  |  |  |  |  |  |  |  |  |  |  |  |  |  |  |  |  |  |  |  |  |  |  |  |  |  |  |  |  |  |  |  |  |  |  |  |  |  |  |  |  |  |  |  |  |  |  |  |  |  |  |  |  |  |  |  |  |  |  |  |  |  |  |  |  |  |  |  |  |  |  |  |  |  |  |  |  |  |  |  |  |  |  |  |  |  |  |  |  |  |  |  |  |  |  |  |  |  |  |  |  |  |  |  |  |  |  |  |  |  |  |  |  |  |  |  |  |  |  |  |  |  |  |  |  |  |  |  |  |  |  |  |  |  |  |  |  |  |  |  |  |  |  |  |  |  |  |  |  |  |  |  |  |  |  |  |  |  |  |  |  |  |  |  |  |  |  |  |  |  |  |  |  |  |  |  |  |  |  |  |  |  |  |  |  |  |  |  |  |  |  |  |  |  |  |  |  |  |  |  |  |  |  |  |  |  |  |  |  |  |  |  |  |  |  |  |  |  |  |  |  |  |  |  |  |  |  |  |  |  |  |  |  |  |  |  |  |  |  |  |  |  |  |  |  |  |  |  |  |  |  |  |  |  |  |  |  |  |  |  |  |  |  |  |  |  |  |  |  |  |  |  |  |  |  |  |  |  |  |  |  |  |  |  |  |  |  |  |  |  |  |  |  |  |  |  |  |  |  |  |  |  |  |  |  |  |  |  |  |  |  |  |  |  |  |  |  |  |  |  |  |  |  |  |  |  |  |  |  |  |  |  |  |  |  |  |  |  |  |  |  |  |  |  |  |  |  |  |  |  |  |  |  |  |  |  |  |  |  |  |  |  |  |  |  |  |  |  |  |  |  |  |  |  |  |  |  |  |  |  |  |  |  |  |  |  |  |  |  |  |  |  |  |  |  |  |  |  |  |  |  |  |  |  |  |  |  |  |  |  |  |  |  |  |  |  |  |  |  |  |  |  |  |  |  |  |  |  |  |  |  |  |  |  |  |  |  |  |  |  |  |  |  |  |  |  |  |  |  |  |  |  |  |  |  |  |  |  |  |  |  |  |  |  |  |  |  |  |  |  |  |  |  |  |  |  |  |  |  |  |  |  |  |  |  |  |  |  |  |  |  |  |  |  |  |  |  |  |  |  |  |  |  |  |  |  |  |  |  |  |  |  |  |  |  |  |  |  |  |  |  |  |  |  |  |  |  |  |  |  |  |  |  |  |  |  |  |  |  |  |  |  |  |  |  |  |  |  |  |  |  |  |  |  |  |  |  |  |  |  |  |  |  |  |  |  |  |  |  |  |  |  |  |  |  |  |  |  |  |  |  |  |  |  |  |  |  |  |  |  |  |  |  |  |  |  |  |  |  |  |  |  |  |  |  |  |  |  |  |  |  |  |  |  |  |  |  |  |  |  |  |  |  |  |  |  |  |  |  |  |  |  |  |  |  |  |  |  |  |  |  |  |  |  |  |  |  |  |  |  |  |  |  |  |  |  |  |  |  |  |  |  |  |  |  |  |  |  |  |  |  |  |  |  |  |  |  |  |  |  |  |  |  |  |  |  |  |  |  |  |  |  |  |  |  |  |  |  |  |  |  |  |  |  |  |  |  |  |  |  |  |  |  |  |  |  |  |  |  |  |  |  |  |  |  |  |  |  |  |  |  |  |  |  |  |  |  |  |  |  |  |  |  |  |  |  |  |  |  |  |  |  |  |  |  |  |  |  |  |  |  |  |  |  |  |  |  |  |  |  |  |  |  |  |  |  |  |  |  |  |  |  |  |  |  |  |  |  |  |  |  |  |  |  |  |  |  |  |  |  |  |  |  |  |  |  |  |  |  |  |  |  |  |  |  |  |  |  |  |  |  |  |  |  |  |  |  |  |  |  |  |  |  |  |  |  |  |  |  |  |  |  |  |  |  |  |  |  |  |  |  |  |  |  |  |  |  |  |  |  |  |  |  |  |  |  |  |  |  |  |  |  |  |  |  |  |  |  |  |  |  |  |  |  |  |  |  |  |  |  |  |  |  |  |  |  |  |  |  |  |  |  |  |  |  |  |  |  |  |  |  |  |  |  |  |  |  |  |  |  |  |  |  |  |  |  |  |  |  |  |  |  |  |  |  |  |  |  |  |  |  |  |  |  |  |  |  |  |  |  |  |  |  |  |  |  |  |  |  |  |  |  |  |  |  |  |  |  |  |  |  |  |  |  |  |  |  |  |  |  |  |  |  |  |  |  |  |  |  |  |  |  |  |  |  |  |  |  |  |  |  |  |  |  |  |  |  |  |  |  |  |  |  |  |  |  |  |  |  |  |  |  |  |  |  |  |  |  |  |  |  |  |  |  |  |  |  |  |  |  |  |  |  |  |  |  |  |  |  |  |  |  |  |  |  |  |  |  |  |  |  |  |  |  |  |  |  |  |  |  |  |  |  |  |  |  |  |  |  |  |  |  |  |  |  |  |  |  |  |  |  |  |  |  |  |  |  |  |  |  |  |  |  |  |  |  |  |  |  |  |  |  |  |  |  |  |  |  |  |  |  |  |  |  |  |  |  |  |  |  |  |  |  |  |  |  |  |  |  |  |  |  |  |  |  |  |  |  |  |  |  |  |  |  |  |  |  |  |  |  |  |  |  |  |  |  |  |  |  |  |  |  |  |  |  |  |  |  |  |  |  |  |  |  |  |  |  |  |  |  |  |  |  |  |  |  |  |  |  |  |  |  |  |  |  |  |  |  |  |  |  |  |  |  |  |  |  |  |  |  |  |  |  |  |  |  |  |  |  |  |  |  |  |  |  |  |  |  |  |  |  |  |  |  |  |  |  |  |  |  |  |  |  |  |  |  |  |  |  |  |  |  |  |  |  |  |  |  |  |  |  |  |  |  |  |  |  |  |  |  |  |  |  |  |  |  |  |  |  |  |  |  |  |  |  |  |  |  |  |  |  |  |  |  |  |  |  |  |  |  |  |  |  |  |  |  |  |  |  |  |  |  |  |  |  |  |  |  |  |  |  |  |  |  |  |  |  |  |  |  |  |  |  |  |  |  |  |  |  |  |  |  |  |  |  |  |  |  |  |  |  |  |  |  |  |  |  |  |  |  |  |  |  |  |  |  |  |  |  |  |  |  |  |  |  |  |  |  |  |  |  |  |  |  |  |  |  |  |  |  |  |  |  |  |  |  |  |  |  |  |  |  |  |  |  |  |  |  |  |  |  |  |  |  |  |  |  |  |  |  |  |  |  |  |  |  |  |  |  |  |  |  |  |  |  |  |  |  |  |  |  |  |  |  |  |  |  |  |  |  |  |  |  |  |  |  |  |  |  |  |  |  |  |  |  |  |  |  |  |  |  |  |  |  |  |  |  |  |  |  |  |  |  |  |  |  |  |  |  |  |  |  |  |  |  |  |  |  |  |  |  |  |  |  |  |  |  |  |  |  |  |  |  |  |  |  |  |  |  |  |  |  |  |  |  |  |  |  |  |  |  |  |  |  |  |  |  |  |  |  |  |  |  |  |  |  |  |  |  |  |  |  |  |  |  |  |  |  |  |  |  |  |  |  |  |  |  |  |  |  |  |  |  |  |  |  |  |  |  |  |  |  |  |  |  |  |  |  |  |  |  |  |  |  |  |  |  |  |  |  |  |  |  |  |  |  |  |  |  |  |  |  |  |  |  |  |  |  |  |  |  |  |  |  |  |  |  |  |  |  |  |  |  |  |  |  |  |  |  |  |  |  |  |  |  |  |  |  |  |  |  |  |  |  |  |  |  |  |  |  |  |  |  |  |  |  |  |  |  |  |  |  |  |  |  |  |  |  |  |  |  |  |  |  |  |  |  |  |  |  |  |  |  |  |  |  |  |  |  |  |  |  |  |  |  |  |  |  |  |  |  |  |  |  |  |  |  |  |  |  |  |  |  |  |  |  |  |  |  |  |  |  |  |  |  |  |  |  |  |  |  |  |  |  |  |  |  |  |  |  |  |  |  |  |  |  |  |  |  |  |  |  |  |  |  |  |  |  |  |  |  |  |  |  |  |  |  |  |  |  |  |  |  |  |  |  |  |  |  |  |  |  |  |  |  |  |  |  |  |  |  |  |  |  |  |  |  |  |  |  |  |  |  |  |  |  |  |  |  |  |  |  |  |  |  |  |  |  |  |  |  |  |  |  |  |  |  |  |  |  |  |  |  |  |  |  |  |  |  |  |  |  |  |  |  |  |  |  |  |  |  |  |  |  |  |  |  |  |  |  |  |  |  |  |  |  |  |  |  |  |  |  |  |  |  |  |  |  |  |  |  |  |  |  |  |  |  |  |  |  |  |  |  |  |  |  |  |  |  |  |  |  |  |  |  |  |  |  |  |  |  |  |  |  |  |  |  |  |  |  |  |  |  |  |  |  |  |  |  |  |  |  |  |  |  |  |  |  |  |  |  |  |  |  |  |  |  |  |  |  |  |  |  |  |  |  |  |  |  |  |  |  |  |  |  |  |  |  |  |  |  |  |  |  |  |  |  |  |  |  |  |  |  |  |  |  |  |  |  |  |  |  |  |  |  |  |  |  |  |  |  |  |  |  |  |  |  |  |  |  |  |  |  |  |  |  |  |  |  |  |  |  |  |  |  |  |  |  |  |  |  |  |  |  |  |  |  |  |  |  |  |  |  |  |  |  |  |  |  |  |  |  |  |  |  |  |  |  |  |  |  |  |  |  |  |  |  |  |  |  |  |  |  |  |  |  |  |  |  |  |  |  |  |  |  |  |  |  |  |  |  |  |  |  |  |  |  |  |  |  |  |  |  |  |  |  |  |  |  |  |  |  |  |  |  |  |  |  |  |  |  |  |  |  |  |  |  |  |  |  |  |  |  |  |  |  |  |  |  |  |  |  |  |  |  |  |  |  |  |  |  |  |  |  |  |  |  |  |  |  |  |  |  |  |  |  |  |  |  |  |  |  |  |  |  |  |  |  |  |  |  |  |  |  |  |  |  |  |  |  |  |  |  |  |  |  |  |  |  |  |  |  |  |  |  |  |  |  |  |  |  |  |  |  |  |  |  |  |  |  |  |  |  |  |  |  |  |  |  |  |  |  |  |  |  |  |  |  |  |  |  |  |  |  |  |  |  |  |  |  |  |  |  |  |  |  |  |  |  |  |  |  |  |  |  |  |  |  |  |  |  |  |  |  |  |  |  |  |  |  |  |  |  |  |  |  |  |  |  |  |  |  |  |  |  |  |  |  |  |  |  |  |  |  |  |  |  |  |  |  |  |  |  |  |  |  |  |  |  |  |  |  |  |  |  |  |  |  |  |  |  |  |  |  |  |  |  |  |  |  |  |  |  |  |  |  |  |  |  |  |  |  |  |  |  |  |  |  |  |  |  |  |  |  |  |  |  |  |  |  |  |  |  |  |  |  |  |  |  |  |  |  |  |  |  |  |  |  |  |  |  |  |  |  |  |  |  |  |  |  |  |  |  |  |  |  |  |  |  |  |  |  |  |  |  |  |  |  |  |  |  |  |  |  |  |  |  |  |  |  |  |  |  |  |  |  |  |  |  |  |  |  |  |  |  |  |  |  |  |  |  |  |  |  |  |  |  |  |  |  |  |  |  |  |  |  |  |  |  |  |  |  |  |  |  |  |  |  |  |  |  |  |  |  |  |  |  |  |  |  |  |  |  |  |  |  |  |  |  |  |  |  |  |  |  |  |  |  |  |  |  |  |  |  |  |  |  |  |  |  |  |  |  |  |  |  |  |  |  |  |  |  |  |  |  |  |  |  |  |  |  |  |  |  |  |  |  |  |  |  |  |  |  |  |  |  |  |  |  |  |  |  |  |  |  |  |  |  |  |  |  |  |  |  |  |  |  |  |  |  |  |  |  |  |  |  |  |  |  |  |  |  |  |  |  |  |  |  |  |  |  |  |  |  |  |  |  |  |  |  |  |  |  |  |  |  |  |  |  |  |  |  |  |  |  |  |  |  |  |  |  |  |  |  |  |  |  |  |  |  |  |  |  |  |  |  |  |  |  |  |  |  |  |  |  |  |  |  |  |  |  |  |  |  |  |  |  |  |  |  |  |  |  |  |  |  |  |  |  |  |  |  |  |  |  |  |  |  |  |  |  |  |  |  |  |  |  |  |  |  |  |  |  |  |  |  |  |  |  |  |  |  |  |  |  |  |  |  |  |  |  |  |  |  |  |  |  |  |  |  |  |  |  |  |  |  |  |  |  |  |  |  |  |  |  |  |  |  |  |  |  |  |  |  |  |  |  |  |  |  |  |  |  |  |  |  |  |  |  |  |  |  |  |  |  |  |  |  |  |  |  |  |  |  |  |  |  |  |  |  |  |  |  |  |  |  |  |  |  |  |  |  |  |  |  |  |  |  |  |  |  |  |  |  |  |  |  |  |  |  |  |  |  |  |  |  |  |  |  |  |  |  |  |  |  |  |  |  |  |  |  |  |  |  |  |  |  |  |  |  |  |  |  |  |  |  |  |  |  |  |  |  |  |  |  |  |  |  |  |  |  |  |  |  |  |  |  |  |  |  |  |  |  |  |  |  |  |  |  |  |  |  |  |  |  |  |  |  |  |  |  |  |  |  |  |  |  |  |  |  |  |  |  |  |  |  |  |  |  |  |  |  |  |  |  |  |  |  |  |  |  |  |  |  |  |  |  |  |  |  |  |  |  |  |  |  |  |  |  |  |  |  |  |  |  |  |  |  |  |  |  |  |  |  |  |  |  |  |  |  |  |  |  |  |  |  |  |  |  |  |  |  |  |  |  |  |  |  |  |  |  |  |  |  |  |  |  |  |  |  |  |  |  |  |  |  |  |  |  |  |  |  |  |  |  |  |  |  |  |  |  |  |  |  |  |  |  |  |  |  |  |  |  |  |  |  |  |  |  |  |  |  |  |  |  |  |  |  |  |  |  |  |  |  |  |  |  |  |  |  |  |  |  |  |  |  |  |  |  |  |  |  |  |  |  |  |  |  |  |  |  |  |  |  |  |  |  |  |  |  |  |  |  |  |  |  |  |  |  |  |  |  |  |  |  |  |  |  |  |  |  |  |  |  |  |  |  |  |  |  |  |  |  |  |  |  |  |  |  |  |  |  |  |  |  |  |  |  |  |  |  |  |  |  |  |  |  |  |  |  |  |  |  |  |  |  |  |  |  |  |  |  |  |  |  |  |  |  |  |  |  |  |  |  |  |  |  |  |  |  |  |  |  |  |  |  |  |  |  |  |  |  |  |  |  |  |  |  |  |  |  |  |  |  |  |  |  |  |  |  |  |  |  |  |  |  |  |  |  |  |  |  |  |  |  |  |  |  |  |  |  |  |  |  |  |  |  |  |  |  |  |  |  |  |  |  |  |  |  |  |  |  |  |  |  |  |  |  |  |  |  |  |  |  |  |  |  |  |  |  |  |  |  |  |  |  |  |  |  |  |  |  |  |  |  |  |  |  |  |  |  |  |  |  |  |  |  |  |  |  |  |  |  |  |  |  |  |  |  |  |  |  |  |  |  |  |  |  |  |  |  |  |  |  |  |  |  |  |  |  |  |  |  |  |  |  |  |  |  |  |  |  |  |  |  |  |  |  |  |  |  |  |  |  |  |  |  |  |  |  |  |  |  |  |  |  |  |  |  |  |  |  |  |  |  |  |  |  |  |  |  |  |  |  |  |  |  |  |  |  |  |  |  |  |  |  |  |  |  |  |  |  |  |  |  |  |  |  |  |  |  |  |  |  |  |  |  |  |  |  |  |  |  |  |  |  |  |  |  |  |  |  |  |  |  |  |  |  |  |  |  |  |  |  |  |  |  |  |  |  |  |  |  |  |  |  |  |  |  |  |  |  |  |  |  |  |  |  |  |  |  |  |  |  |  |  |  |  |  |  |  |  |  |  |  |  |  |  |  |  |  |  |  |  |  |  |  |  |  |  |  |  |  |  |  |  |  |  |  |  |  |  |  |  |  |  |  |  |  |  |  |  |  |  |  |  |  |  |  |  |  |  |  |  |  |  |  |  |  |  |  |  |  |  |  |  |  |  |  |  |  |  |  |  |  |  |  |  |  |  |  |  |  |  |  |  |  |  |  |  |  |  |  |  |  |  |  |  |  |  |  |  |  |  |  |  |  |  |  |  |  |  |  |  |  |  |  |  |  |  |  |  |  |  |  |  |  |  |  |  |  |  |  |  |  |  |  |  |  |  |  |  |  |  |  |  |  |  |  |  |  |  |  |  |  |  |  |  |  |  |  |  |  |  |  |  |  |  |  |  |  |  |  |  |  |  |  |  |  |  |  |  |  |  |  |  |  |  |  |  |  |  |  |  |  |  |  |  |  |  |  |  |  |  |  |  |  |  |  |  |  |  |  |  |  |  |  |  |  |  |  |  |  |  |  |  |  |  |  |  |  |  |  |  |  |  |  |  |  |  |  |  |  |  |  |  |  |  |  |  |  |  |  |  |  |  |  |  |  |  |  |  |  |  |  |  |  |  |  |  |  |  |  |  |  |  |  |  |  |  |  |  |  |  |  |  |  |  |  |  |  |  |  |  |  |  |  |  |  |  |  |  |  |  |  |  |  |  |  |  |  |  |  |  |  |  |  |  |  |  |  |  |  |  |  |  |  |  |  |  |  |  |  |  |  |  |  |  |  |  |  |  |  |  |  |  |  |  |  |  |  |  |  |  |  |  |  |  |  |  |  |  |  |  |  |  |  |  |  |  |  |  |  |  |  |  |  |  |  |  |  |  |  |  |  |  |  |  |  |  |  |  |  |  |  |  |  |  |  |  |  |  |  |  |  |  |  |  |  |  |  |  |  |  |  |  |  |  |  |  |  |  |  |  |  |  |  |  |  |  |  |  |  |  |  |  |  |  |  |  |  |  |  |  |  |  |  |  |  |  |  |  |  |  |  |  |  |  |  |  |  |  |  |  |  |  |  |  |  |  |  |  |  |  |  |  |  |  |  |  |  |  |  |  |  |  |  |  |  |  |  |  |  |  |  |  |  |  |  |  |  |  |  |  |  |  |  |  |  |  |  |  |  |  |  |  |  |  |  |  |  |  |  |  |  |  |  |  |  |  |  |  |  |  |  |  |  |  |  |  |  |  |  |  |  |  |  |  |  |  |  |  |  |  |  |  |  |  |  |  |  |  |  |  |  |  |  |  |  |  |  |  |  |  |  |  |  |  |  |  |  |  |  |  |  |  |  |  |  |  |  |  |  |  |  |  |  |  |  |  |  |  |  |  |  |  |  |  |  |  |  |  |  |  |  |  |  |  |  |  |  |  |  |  |  |  |  |  |  |  |  |  |  |  |  |  |  |  |  |  |  |  |  |  |  |  |  |  |  |  |  |  |  |  |  |  |  |  |  |  |  |  |  |  |  |  |  |  |  |  |  |  |  |  |  |  |  |  |  |  |  |  |  |  |  |  |  |  |  |  |  |  |  |  |  |  |  |  |  |  |  |  |  |  |  |  |  |  |  |  |  |  |  |  |  |  |  |  |  |  |  |  |  |  |  |  |  |  |  |  |  |  |  |  |  |  |  |  |  |  |  |  |  |  |  |  |  |  |  |  |  |  |  |  |  |  |  |  |  |  |  |  |  |  |  |  |  |  |  |  |  |  |  |  |  |  |  |  |  |  |  |  |  |  |  |  |  |  |  |  |  |  |  |  |  |  |  |  |  |  |  |  |  |  |  |  |  |  |  |  |  |  |  |  |  |  |  |  |  |  |  |  |  |  |  |  |  |  |  |  |  |  |  |  |  |  |  |  |  |  |  |  |  |  |  |  |  |  |  |  |  |  |  |  |  |  |  |  |  |  |  |  |  |  |  |  |  |  |  |  |  |  |  |  |  |  |  |  |  |  |  |  |  |  |  |  |  |  |  |  |  |  |  |  |  |  |  |  |  |  |  |  |  |  |  |  |  |  |  |  |  |  |  |  |  |  |  |  |  |  |  |  |  |  |  |  |  |  |  |  |  |  |  |  |  |  |  |  |  |  |  |  |  |  |  |  |  |  |  |  |  |  |  |  |  |  |  |  |  |  |  |  |  |  |  |  |  |  |  |  |  |  |  |  |  |  |  |  |  |  |  |  |  |  |  |  |  |  |  |  |  |  |  |  |  |  |  |  |  |  |  |  |  |  |  |  |  |  |  |  |  |  |  |  |  |  |  |  |  |  |  |  |  |  |  |  |  |  |  |  |  |  |  |  |  |  |  |  |  |  |  |  |  |  |  |  |  |  |  |  |  |  |  |  |  |  |  |  |  |  |  |  |  |  |  |  |  |  |  |  |  |  |  |  |  |  |  |  |  |  |  |  |  |  |  |  |  |  |  |  |  |  |  |  |  |  |  |  |  |  |  |  |  |  |  |  |  |  |  |  |  |  |  |  |  |  |  |  |  |  |  |  |  |  |  |  |  |  |  |  |  |  |  |  |  |  |  |  |  |  |  |  |  |  |  |  |  |  |  |  |  |  |  |  |  |  |  |  |  |  |  |  |  |  |  |  |  |  |  |  |  |  |  |  |  |  |  |  |  |  |  |  |  |  |  |  |  |  |  |  |  |  |  |  |  |  |  |  |  |  |  |  |  |  |  |  |  |  |  |  |  |  |  |  |  |  |  |  |  |  |  |  |  |  |  |  |  |  |  |  |  |  |  |  |  |  |  |  |  |  |  |  |  |  |  |  |  |  |  |  |  |  |  |  |  |  |  |  |  |  |  |  |  |  |  |  |  |  |  |  |  |  |  |  |  |  |  |  |  |  |  |  |  |  |  |  |  |  |  |  |  |  |  |  |  |  |  |  |  |  |  |  |  |  |  |  |  |  |  |  |  |  |  |  |  |  |  |  |  |  |  |  |  |  |  |  |  |  |  |  |  |  |  |  |  |  |  |  |  |  |  |  |  |  |  |  |  |  |  |  |  |  |  |  |  |  |  |  |  |  |  |  |  |  |  |  |  |  |  |  |  |  |  |  |  |  |  |  |  |  |  |  |  |  |  |  |  |  |  |  |  |  |  |  |  |  |  |  |  |  |  |  |  |  |  |  |  |  |  |  |  |  |  |  |  |  |  |  |  |  |  |  |  |  |  |  |  |  |  |  |  |  |  |  |  |  |  |  |  |  |  |  |  |  |  |  |  |  |  |  |  |  |  |  |  |  |  |  |  |  |  |  |  |  |  |  |  |  |  |  |  |  |  |  |  |  |  |  |  |  |  |  |  |  |  |  |  |  |  |  |  |  |  |  |  |  |  |  |  |  |  |  |  |  |  |  |  |  |  |  |  |  |  |  |  |  |  |  |  |  |  |  |  |  |  |  |  |  |  |  |  |  |  |  |  |  |  |  |  |  |  |  |  |  |  |  |  |  |  |  |  |  |  |  |  |  |  |  |  |  |  |  |  |  |  |  |  |  |  |  |  |  |  |  |  |  |  |  |  |  |  |  |  |  |  |  |  |  |  |  |  |  |  |  |  |  |  |  |  |  |  |  |  |  |  |  |  |  |  |  |  |  |  |  |  |  |  |  |  |  |  |  |  |  |  |  |  |  |  |  |  |  |  |  |  |  |  |  |  |  |  |  |  |  |  |  |  |  |  |  |  |  |  |  |  |  |  |  |  |  |  |  |  |  |  |  |  |  |  |  |  |  |  |  |  |  |  |  |  |  |  |  |  |  |  |  |  |  |  |  |  |  |  |  |  |  |  |  |  |  |  |  |  |  |  |  |  |  |  |  |  |  |  |  |  |  |  |  |  |  |  |  |  |  |  |  |  |  |  |  |  |  |  |  |  |  |  |  |  |  |  |  |  |  |  |  |  |  |  |  |  |  |  |  |  |  |  |  |  |  |  |  |  |  |  |  |  |  |  |  |  |  |  |  |  |  |  |  |  |  |  |  |  |  |  |  |  |  |  |  |  |  |  |  |  |  |  |  |  |  |  |  |  |  |  |  |  |  |  |  |  |  |  |  |  |  |  |  |  |  |  |  |  |  |  |  |  |  |  |  |  |  |  |  |  |  |  |  |  |  |  |  |  |  |  |  |  |  |  |  |  |  |  |  |  |  |  |  |  |  |  |  |  |  |  |  |  |  |  |  |  |  |  |  |  |  |  |  |  |  |  |  |  |  |  |  |  |  |  |  |  |  |  |  |  |  |  |  |  |  |  |  |  |  |  |  |  |  |  |  |  |  |  |  |  |  |  |  |  |  |  |  |  |  |  |  |  |  |  |  |  |  |  |  |  |  |  |  |  |  |  |  |  |  |  |  |  |  |  |  |  |  |  |  |  |  |  |  |  |  |  |  |  |  |  |  |  |  |  |  |  |  |  |  |  |  |  |  |  |  |  |  |  |  |  |  |  |  |  |  |  |  |  |  |  |  |  |  |  |  |  |  |  |  |  |  |  |  |  |  |  |  |  |  |  |  |  |  |  |  |  |  |  |  |  |  |  |  |  |  |  |  |  |  |  |  |  |  |  |  |  |  |  |  |  |  |  |  |  |  |  |  |  |  |  |  |  |  |  |  |  |  |  |  |  |  |  |  |  |  |  |  |  |  |  |  |  |  |  |  |  |  |  |  |  |  |  |  |  |  |  |  |  |  |  |  |  |  |  |  |  |  |  |  |  |  |  |  |  |  |  |  |  |  |  |  |  |  |  |  |  |  |  |  |  |  |  |  |  |  |  |  |  |  |  |  |  |  |  |  |  |  |  |  |  |  |  |  |  |  |  |  |  |  |  |  |  |  |  |  |  |  |  |  |  |  |  |  |  |  |  |  |  |  |  |  |  |  |  |  |  |  |  |  |  |  |  |  |  |  |  |  |  |  |  |  |  |  |  |  |  |  |  |  |  |  |  |  |  |  |  |  |  |  |  |  |  |  |  |  |  |  |  |  |  |  |  |  |  |  |  |  |  |  |  |  |  |  |  |  |  |  |  |  |  |  |  |  |  |  |  |  |  |  |  |  |  |  |  |  |  |  |  |  |  |  |  |  |  |  |  |  |  |  |  |  |  |  |  |  |  |  |  |  |  |  |  |  |  |  |  |  |  |  |  |  |  |  |  |  |  |  |  |  |  |  |  |  |  |  |  |  |  |  |  |  |  |  |  |  |  |  |  |  |  |  |  |  |  |  |  |  |  |  |  |  |  |  |  |  |  |  |  |  |  |  |  |  |  |  |  |  |  |  |  |  |  |  |  |  |  |  |  |  |  |  |  |  |  |  |  |  |  |  |  |  |  |  |  |  |  |  |  |  |  |  |  |  |  |  |  |  |  |  |  |  |  |  |  |  |  |  |  |  |  |  |  |  |  |  |  |  |  |  |  |  |  |  |  |  |  |  |  |  |  |  |  |  |  |  |  |  |  |  |  |  |  |  |  |  |  |  |  |  |  |  |  |  |  |  |  |  |  |  |  |  |  |  |  |  |  |  |  |  |  |  |  |  |  |  |  |  |  |  |  |  |  |  |  |  |  |  |  |  |  |  |  |  |  |  |  |  |  |  |  |  |  |  |  |  |  |  |  |  |  |  |  |  |  |  |  |  |  |  |  |  |  |  |  |  |  |  |  |  |  |  |  |  |  |  |  |  |  |  |  |  |  |  |  |  |  |  |  |  |  |  |  |  |  |  |  |  |  |  |  |  |  |  |  |  |  |  |  |  |  |  |  |  |  |  |  |  |  |  |  |  |  |  |  |  |  |  |  |  |  |  |  |  |  |  |  |  |  |  |  |  |  |  |  |  |  |  |  |  |  |  |  |  |  |  |  |  |  |  |  |  |  |  |  |  |  |  |  |  |  |  |  |  |  |  |  |  |  |  |  |  |  |  |  |  |  |  |  |  |  |  |  |  |  |  |  |  |  |  |  |  |  |  |  |  |  |  |  |  |  |  |  |  |  |  |  |  |  |  |  |  |  |  |  |  |  |  |  |  |  |  |  |  |  |  |  |  |  |  |  |  |  |  |  |  |  |  |  |  |  |  |  |  |  |  |  |  |  |  |  |  |  |  |  |  |  |  |  |  |  |  |  |  |  |  |  |  |  |  |  |  |  |  |  |  |  |  |  |  |  |  |  |  |  |  |  |  |  |  |  |  |  |  |  |  |  |  |  |  |  |  |  |  |  |  |  |  |  |  |  |  |  |  |  |  |  |  |  |  |  |  |  |  |  |  |  |  |  |  |  |  |  |  |  |  |  |  |  |  |  |  |  |  |  |  |  |  |  |  |  |  |  |  |  |  |  |  |  |  |  |  |  |  |  |  |  |  |  |  |  |  |  |  |  |  |  |  |  |  |  |  |  |  |  |  |  |  |  |  |  |  |  |  |  |  |  |  |  |  |  |  |  |  |  |  |  |  |  |  |  |  |  |  |  |  |  |  |  |  |  |  |  |  |  |  |  |  |  |  |  |  |  |  |  |  |  |  |  |  |  |  |  |  |  |  |  |  |  |  |  |  |  |  |  |  |  |  |  |  |  |  |  |  |  |  |  |  |  |  |  |  |  |  |  |  |  |  |  |  |  |  |  |  |  |  |  |  |  |  |  |  |  |  |  |  |  |  |  |  |  |  |  |  |  |  |  |  |  |  |  |  |  |  |  |  |  |  |  |  |  |  |  |  |  |  |  |  |  |  |  |  |  |  |  |  |  |  |  |  |  |  |  |  |  |  |  |  |  |  |  |  |  |  |  |  |  |  |  |  |  |  |  |  |  |  |  |  |  |  |  |  |  |  |  |  |  |  |  |  |  |  |  |  |  |  |  |  |  |  |  |  |  |  |  |  |  |  |  |  |  |  |  |  |  |  |  |  |  |  |  |  |  |  |  |  |  |  |  |  |  |  |  |  |  |  |  |  |  |  |  |  |  |  |  |  |  |  |  |  |  |  |  |  |  |  |  |  |  |  |  |  |  |  |  |  |  |  |  |  |  |  |  |  |  |  |  |  |  |  |  |  |  |  |  |  |  |  |  |  |  |  |  |  |  |  |  |  |  |  |  |  |  |  |  |  |  |  |  |  |  |  |  |  |  |  |  |  |  |  |  |  |  |  |  |  |  |  |  |  |  |  |  |  |  |  |  |  |  |  |  |  |  |  |  |  |  |  |  |  |  |  |  |  |  |  |  |  |  |  |  |  |  |  |  |  |  |  |  |  |  |  |  |  |  |  |  |  |  |  |  |  |  |  |  |  |  |  |  |  |  |  |  |  |  |  |  |  |  |  |  |  |  |  |  |  |  |  |  |  |  |  |  |  |  |  |  |  |  |  |  |  |  |  |  |  |  |  |  |  |  |  |  |  |  |  |  |  |  |  |  |  |  |  |  |  |  |  |  |  |  |  |  |  |  |  |  |  |  |  |  |  |  |  |  |  |  |  |  |  |  |  |  |  |  |  |  |  |  |  |  |  |  |  |  |  |  |  |  |  |  |  |  |  |  |  |  |  |  |  |  |  |  |  |  |  |  |  |  |  |  |  |  |  |  |  |  |  |  |  |  |  |  |  |  |  |  |  |  |  |  |  |  |  |  |  |  |  |  |  |  |  |  |  |  |  |  |  |  |  |  |  |  |  |  |  |  |  |  |  |  |  |  |  |  |  |  |  |  |  |  |  |  |  |  |  |  |  |  |  |  |  |  |  |  |  |  |  |  |  |  |  |  |  |  |  |  |  |  |  |  |  |  |  |  |  |  |  |  |  |  |  |  |  |  |  |  |  |  |  |  |  |  |  |  |  |  |  |  |  |  |  |  |  |  |  |  |  |  |  |  |  |  |  |  |  |  |  |  |  |  |  |  |  |  |  |  |  |  |  |  |  |  |  |  |  |  |  |  |  |  |  |  |  |  |  |  |  |  |  |  |  |  |  |  |  |  |  |  |  |  |  |  |  |  |  |  |  |  |  |  |  |  |  |  |  |  |  |  |  |  |  |  |  |  |  |  |  |  |  |  |  |  |  |  |  |  |  |  |  |  |  |  |  |  |  |  |  |  |  |  |  |  |  |  |  |  |  |  |  |  |  |  |  |  |  |  |  |  |  |  |  |  |  |  |  |  |  |  |  |  |  |  |  |  |  |  |  |  |  |  |  |  |  |  |  |  |  |  |  |  |  |  |  |  |  |  |  |  |  |  |  |  |  |  |  |  |  |  |  |  |  |  |  |  |  |  |  |  |  |  |  |  |  |  |  |  |  |  |  |  |  |  |  |  |  |  |  |  |  |  |  |  |  |  |  |  |  |  |  |  |  |  |  |  |  |  |  |  |  |  |  |  |  |  |  |  |  |  |  |  |  |  |  |  |  |  |  |  |  |  |  |  |  |  |  |  |  |  |  |  |  |  |  |  |  |  |  |  |  |  |  |  |  |  |  |  |  |  |  |  |  |  |  |  |  |  |  |  |  |  |  |  |  |  |  |  |  |  |  |  |  |  |  |  |
| --- | --- | --- | --- | --- | --- | --- | --- | --- | --- | --- | --- | --- | --- | --- | --- | --- | --- | --- | --- | --- | --- | --- | --- | --- | --- | --- | --- | --- | --- | --- | --- | --- | --- | --- | --- | --- | --- | --- | --- | --- | --- | --- | --- | --- | --- | --- | --- | --- | --- | --- | --- | --- | --- | --- | --- | --- | --- | --- | --- | --- | --- | --- | --- | --- | --- | --- | --- | --- | --- | --- | --- | --- | --- | --- | --- | --- | --- | --- | --- | --- | --- | --- | --- | --- | --- | --- | --- | --- | --- | --- | --- | --- | --- | --- | --- | --- | --- | --- | --- | --- | --- | --- | --- | --- | --- | --- | --- | --- | --- | --- | --- | --- | --- | --- | --- | --- | --- | --- | --- | --- | --- | --- | --- | --- | --- | --- | --- | --- | --- | --- | --- | --- | --- | --- | --- | --- | --- | --- | --- | --- | --- | --- | --- | --- | --- | --- | --- | --- | --- | --- | --- | --- | --- | --- | --- | --- | --- | --- | --- | --- | --- | --- | --- | --- | --- | --- | --- | --- | --- | --- | --- | --- | --- | --- | --- | --- | --- | --- | --- | --- | --- | --- | --- | --- | --- | --- | --- | --- | --- | --- | --- | --- | --- | --- | --- | --- | --- | --- | --- | --- | --- | --- | --- | --- | --- | --- | --- | --- | --- | --- | --- | --- | --- | --- | --- | --- | --- | --- | --- | --- | --- | --- | --- | --- | --- | --- | --- | --- | --- | --- | --- | --- | --- | --- | --- | --- | --- | --- | --- | --- | --- | --- | --- | --- | --- | --- | --- | --- | --- | --- | --- | --- | --- | --- | --- | --- | --- | --- | --- | --- | --- | --- | --- | --- | --- | --- | --- | --- | --- | --- | --- | --- | --- | --- | --- | --- | --- | --- | --- | --- | --- | --- | --- | --- | --- | --- | --- | --- | --- | --- | --- | --- | --- | --- | --- | --- | --- | --- | --- | --- | --- | --- | --- | --- | --- | --- | --- | --- | --- | --- | --- | --- | --- | --- | --- | --- | --- | --- | --- | --- | --- | --- | --- | --- | --- | --- | --- | --- | --- | --- | --- | --- | --- | --- | --- | --- | --- | --- | --- | --- | --- | --- | --- | --- | --- | --- | --- | --- | --- | --- | --- | --- | --- | --- | --- | --- | --- | --- | --- | --- | --- | --- | --- | --- | --- | --- | --- | --- | --- | --- | --- | --- | --- | --- | --- | --- | --- | --- | --- | --- | --- | --- | --- | --- | --- | --- | --- | --- | --- | --- | --- | --- | --- | --- | --- | --- | --- | --- | --- | --- | --- | --- | --- | --- | --- | --- | --- | --- | --- | --- | --- | --- | --- | --- | --- | --- | --- | --- | --- | --- | --- | --- | --- | --- | --- | --- | --- | --- | --- | --- | --- | --- | --- | --- | --- | --- | --- | --- | --- | --- | --- | --- | --- | --- | --- | --- | --- | --- | --- | --- | --- | --- | --- | --- | --- | --- | --- | --- | --- | --- | --- | --- | --- | --- | --- | --- | --- | --- | --- | --- | --- | --- | --- | --- | --- | --- | --- | --- | --- | --- | --- | --- | --- | --- | --- | --- | --- | --- | --- | --- | --- | --- | --- | --- | --- | --- | --- | --- | --- | --- | --- | --- | --- | --- | --- | --- | --- | --- | --- | --- | --- | --- | --- | --- | --- | --- | --- | --- | --- | --- | --- | --- | --- | --- | --- | --- | --- | --- | --- | --- | --- | --- | --- | --- | --- | --- | --- | --- | --- | --- | --- | --- | --- | --- | --- | --- | --- | --- | --- | --- | --- | --- | --- | --- | --- | --- | --- | --- | --- | --- | --- | --- | --- | --- | --- | --- | --- | --- | --- | --- | --- | --- | --- | --- | --- | --- | --- | --- | --- | --- | --- | --- | --- | --- | --- | --- | --- | --- | --- | --- | --- | --- | --- | --- | --- | --- | --- | --- | --- | --- | --- | --- | --- | --- | --- | --- | --- | --- | --- | --- | --- | --- | --- | --- | --- | --- | --- | --- | --- | --- | --- | --- | --- | --- | --- | --- | --- | --- | --- | --- | --- | --- | --- | --- | --- | --- | --- | --- | --- | --- | --- | --- | --- | --- | --- | --- | --- | --- | --- | --- | --- | --- | --- | --- | --- | --- | --- | --- | --- | --- | --- | --- | --- | --- | --- | --- | --- | --- | --- | --- | --- | --- | --- | --- | --- | --- | --- | --- | --- | --- | --- | --- | --- | --- | --- | --- | --- | --- | --- | --- | --- | --- | --- | --- | --- | --- | --- | --- | --- | --- | --- | --- | --- | --- | --- | --- | --- | --- | --- | --- | --- | --- | --- | --- | --- | --- | --- | --- | --- | --- | --- | --- | --- | --- | --- | --- | --- | --- | --- | --- | --- | --- | --- | --- | --- | --- | --- | --- | --- | --- | --- | --- | --- | --- | --- | --- | --- | --- | --- | --- | --- | --- | --- | --- | --- | --- | --- | --- | --- | --- | --- | --- | --- | --- | --- | --- | --- | --- | --- | --- | --- | --- | --- | --- | --- | --- | --- | --- | --- | --- | --- | --- | --- | --- | --- | --- | --- | --- | --- | --- | --- | --- | --- | --- | --- | --- | --- | --- | --- | --- | --- | --- | --- | --- | --- | --- | --- | --- | --- | --- | --- | --- | --- | --- | --- | --- | --- | --- | --- | --- | --- | --- | --- | --- | --- | --- | --- | --- | --- | --- | --- | --- | --- | --- | --- | --- | --- | --- | --- | --- | --- | --- | --- | --- | --- | --- | --- | --- | --- | --- | --- | --- | --- | --- | --- | --- | --- | --- | --- | --- | --- | --- | --- | --- | --- | --- | --- | --- | --- | --- | --- | --- | --- | --- | --- | --- | --- | --- | --- | --- | --- | --- | --- | --- | --- | --- | --- | --- | --- | --- | --- | --- | --- | --- | --- | --- | --- | --- | --- | --- | --- | --- | --- | --- | --- | --- | --- | --- | --- | --- | --- | --- | --- | --- | --- | --- | --- | --- | --- | --- | --- | --- | --- | --- | --- | --- | --- | --- | --- | --- | --- | --- | --- | --- | --- | --- | --- | --- | --- | --- | --- | --- | --- | --- | --- | --- | --- | --- | --- | --- | --- | --- | --- | --- | --- | --- | --- | --- | --- | --- | --- | --- | --- | --- | --- | --- | --- | --- | --- | --- | --- | --- | --- | --- | --- | --- | --- | --- | --- | --- | --- | --- | --- | --- | --- | --- | --- | --- | --- | --- | --- | --- | --- | --- | --- | --- | --- | --- | --- | --- | --- | --- | --- | --- | --- | --- | --- | --- | --- | --- | --- | --- | --- | --- | --- | --- | --- | --- | --- | --- | --- | --- | --- | --- | --- | --- | --- | --- | --- | --- | --- | --- | --- | --- | --- | --- | --- | --- | --- | --- | --- | --- | --- | --- | --- | --- | --- | --- | --- | --- | --- | --- | --- | --- | --- | --- | --- | --- | --- | --- | --- | --- | --- | --- | --- | --- | --- | --- | --- | --- | --- | --- | --- | --- | --- | --- | --- | --- | --- | --- | --- | --- | --- | --- | --- | --- | --- | --- | --- | --- | --- | --- | --- | --- | --- | --- | --- | --- | --- | --- | --- | --- | --- | --- | --- | --- | --- | --- | --- | --- | --- | --- | --- | --- | --- | --- | --- | --- | --- | --- | --- | --- | --- | --- | --- | --- | --- | --- | --- | --- | --- | --- | --- | --- | --- | --- | --- | --- | --- | --- | --- | --- | --- | --- | --- | --- | --- | --- | --- | --- | --- | --- | --- | --- | --- | --- | --- | --- | --- | --- | --- | --- | --- | --- | --- | --- | --- | --- | --- | --- | --- | --- | --- | --- | --- | --- | --- | --- | --- | --- | --- | --- | --- | --- | --- | --- | --- | --- | --- | --- | --- | --- | --- | --- | --- | --- | --- | --- | --- | --- | --- | --- | --- | --- | --- | --- | --- | --- | --- | --- | --- | --- | --- | --- | --- | --- | --- | --- | --- | --- | --- | --- | --- | --- | --- | --- | --- | --- | --- | --- | --- | --- | --- | --- | --- | --- | --- | --- | --- | --- | --- | --- | --- | --- | --- | --- | --- | --- | --- | --- | --- | --- | --- | --- | --- | --- | --- | --- | --- | --- | --- | --- | --- | --- | --- | --- | --- | --- | --- | --- | --- | --- | --- | --- | --- | --- | --- | --- | --- | --- | --- | --- | --- | --- | --- | --- | --- | --- | --- | --- | --- | --- | --- | --- | --- | --- | --- | --- | --- | --- | --- | --- | --- | --- | --- | --- | --- | --- | --- | --- | --- | --- | --- | --- | --- | --- | --- | --- | --- | --- | --- | --- | --- | --- | --- | --- | --- | --- | --- | --- | --- | --- | --- | --- | --- | --- | --- | --- | --- | --- | --- | --- | --- | --- | --- | --- | --- | --- | --- | --- | --- | --- | --- | --- | --- | --- | --- | --- | --- | --- | --- | --- | --- | --- | --- | --- | --- | --- | --- | --- | --- | --- | --- | --- | --- | --- | --- | --- | --- | --- | --- | --- | --- | --- | --- | --- | --- | --- | --- | --- | --- | --- | --- | --- | --- | --- | --- | --- | --- | --- | --- | --- | --- | --- | --- | --- | --- | --- | --- | --- | --- | --- | --- | --- | --- | --- | --- | --- | --- | --- | --- | --- | --- | --- | --- | --- | --- | --- | --- | --- | --- | --- | --- | --- | --- | --- | --- | --- | --- | --- | --- | --- | --- | --- | --- | --- | --- | --- | --- | --- | --- | --- | --- | --- | --- | --- | --- | --- | --- | --- | --- | --- | --- | --- | --- | --- | --- | --- | --- | --- | --- | --- | --- | --- | --- | --- | --- | --- | --- | --- | --- | --- | --- | --- | --- | --- | --- | --- | --- | --- | --- | --- | --- | --- | --- | --- | --- | --- | --- | --- | --- | --- | --- | --- | --- | --- | --- | --- | --- | --- | --- | --- | --- | --- | --- | --- | --- | --- | --- | --- | --- | --- | --- | --- | --- | --- | --- | --- | --- | --- | --- | --- | --- | --- | --- | --- | --- | --- | --- | --- | --- | --- | --- | --- | --- | --- | --- | --- | --- | --- | --- | --- | --- | --- | --- | --- | --- | --- | --- | --- | --- | --- | --- | --- | --- | --- | --- | --- | --- | --- | --- | --- | --- | --- | --- | --- | --- | --- | --- | --- | --- | --- | --- | --- | --- | --- | --- | --- | --- | --- | --- | --- | --- | --- | --- | --- | --- | --- | --- | --- | --- | --- | --- | --- | --- | --- | --- | --- | --- | --- | --- | --- | --- | --- | --- | --- | --- | --- | --- | --- | --- | --- | --- | --- | --- | --- | --- | --- | --- | --- | --- | --- | --- | --- | --- | --- | --- | --- | --- | --- | --- | --- | --- | --- | --- | --- | --- | --- | --- | --- | --- | --- | --- | --- | --- | --- | --- | --- | --- | --- | --- | --- | --- | --- | --- | --- | --- | --- | --- | --- | --- | --- | --- | --- | --- | --- | --- | --- | --- | --- | --- | --- | --- | --- | --- | --- | --- | --- | --- | --- | --- | --- | --- | --- | --- | --- | --- | --- | --- | --- | --- | --- | --- | --- | --- | --- | --- | --- | --- | --- | --- | --- | --- | --- | --- | --- | --- | --- | --- | --- | --- | --- | --- | --- | --- | --- | --- | --- | --- | --- | --- | --- | --- | --- | --- | --- | --- | --- | --- | --- | --- | --- | --- | --- | --- | --- | --- | --- | --- | --- | --- | --- | --- | --- | --- | --- | --- | --- | --- | --- | --- | --- | --- | --- | --- | --- | --- | --- | --- | --- | --- | --- | --- | --- | --- | --- | --- | --- | --- | --- | --- | --- | --- | --- | --- | --- | --- | --- | --- | --- | --- | --- | --- | --- | --- | --- | --- | --- | --- | --- | --- | --- | --- | --- | --- | --- | --- | --- | --- | --- | --- | --- | --- | --- | --- | --- | --- | --- | --- | --- | --- | --- | --- | --- | --- | --- | --- | --- | --- | --- | --- | --- | --- | --- | --- | --- | --- | --- | --- | --- | --- | --- | --- | --- | --- | --- | --- | --- | --- | --- | --- | --- | --- | --- | --- | --- | --- | --- | --- | --- | --- | --- | --- | --- | --- | --- | --- | --- | --- | --- | --- | --- | --- | --- | --- | --- | --- | --- | --- | --- | --- | --- | --- | --- | --- | --- | --- | --- | --- | --- | --- | --- | --- | --- | --- | --- | --- | --- | --- | --- | --- | --- | --- | --- | --- | --- | --- | --- | --- | --- | --- | --- | --- | --- | --- | --- | --- | --- | --- | --- | --- | --- | --- | --- | --- | --- | --- | --- | --- | --- | --- | --- | --- | --- | --- | --- | --- | --- | --- | --- | --- | --- | --- | --- | --- | --- | --- | --- | --- | --- | --- | --- | --- | --- | --- | --- | --- | --- | --- | --- | --- | --- | --- | --- | --- | --- | --- | --- | --- | --- | --- | --- | --- | --- | --- | --- | --- | --- | --- | --- | --- | --- | --- | --- | --- | --- | --- | --- | --- | --- | --- | --- | --- | --- | --- | --- | --- | --- | --- | --- | --- | --- | --- | --- | --- | --- | --- | --- | --- | --- | --- | --- | --- | --- | --- | --- | --- | --- | --- | --- | --- | --- | --- | --- | --- | --- | --- | --- | --- | --- | --- | --- | --- | --- | --- | --- | --- | --- | --- | --- | --- | --- | --- | --- | --- | --- | --- | --- | --- | --- | --- | --- | --- | --- | --- | --- | --- | --- | --- | --- | --- | --- | --- | --- | --- | --- | --- | --- | --- | --- | --- | --- | --- | --- | --- | --- | --- | --- | --- | --- | --- | --- | --- | --- | --- | --- | --- | --- | --- | --- | --- | --- | --- | --- | --- | --- | --- | --- | --- | --- | --- | --- | --- | --- | --- | --- | --- | --- | --- | --- | --- | --- | --- | --- | --- | --- | --- | --- | --- | --- | --- | --- | --- | --- | --- | --- | --- | --- | --- | --- | --- | --- | --- | --- | --- | --- | --- | --- | --- | --- | --- | --- | --- | --- | --- | --- | --- | --- | --- | --- | --- | --- | --- | --- | --- | --- | --- | --- | --- | --- | --- | --- | --- | --- | --- | --- | --- | --- | --- | --- | --- | --- | --- | --- | --- | --- | --- | --- | --- | --- | --- | --- | --- | --- | --- | --- | --- | --- | --- | --- | --- | --- | --- | --- | --- | --- | --- | --- | --- | --- | --- | --- | --- | --- | --- | --- | --- | --- | --- | --- | --- | --- | --- | --- | --- | --- | --- | --- | --- | --- | --- | --- | --- | --- | --- | --- | --- | --- | --- | --- | --- | --- | --- | --- | --- | --- | --- | --- | --- | --- | --- | --- | --- | --- | --- | --- | --- | --- | --- | --- | --- | --- | --- | --- | --- | --- | --- | --- | --- | --- | --- | --- | --- | --- | --- | --- | --- | --- | --- | --- | --- | --- | --- | --- | --- | --- | --- | --- | --- | --- | --- | --- | --- | --- | --- | --- | --- | --- | --- | --- | --- | --- | --- | --- | --- | --- | --- | --- | --- | --- | --- | --- | --- | --- | --- | --- | --- | --- | --- | --- | --- | --- | --- | --- | --- | --- | --- | --- | --- | --- | --- | --- | --- | --- | --- | --- | --- | --- | --- | --- | --- | --- | --- | --- | --- | --- | --- | --- | --- | --- | --- | --- | --- | --- | --- | --- | --- | --- | --- | --- | --- | --- | --- | --- | --- | --- | --- | --- | --- | --- | --- | --- | --- | --- | --- | --- | --- | --- | --- | --- | --- | --- | --- | --- | --- | --- | --- | --- | --- | --- | --- | --- | --- | --- | --- | --- | --- | --- | --- | --- | --- | --- | --- | --- | --- | --- | --- | --- | --- | --- | --- | --- | --- | --- | --- | --- | --- | --- | --- | --- | --- | --- | --- | --- | --- | --- | --- | --- | --- | --- | --- | --- | --- | --- | --- | --- | --- | --- | --- | --- | --- | --- | --- | --- | --- | --- | --- | --- | --- | --- | --- | --- | --- | --- | --- | --- | --- | --- | --- | --- | --- | --- | --- | --- | --- | --- | --- | --- | --- | --- | --- | --- | --- | --- | --- | --- | --- | --- | --- | --- | --- | --- | --- | --- | --- | --- | --- | --- | --- | --- | --- | --- | --- | --- | --- | --- | --- | --- | --- | --- | --- | --- | --- | --- | --- | --- | --- | --- | --- | --- | --- | --- | --- | --- | --- | --- | --- | --- | --- | --- | --- | --- | --- | --- | --- | --- | --- | --- | --- | --- | --- | --- | --- | --- | --- | --- | --- | --- | --- | --- | --- | --- | --- | --- | --- | --- | --- | --- | --- | --- | --- | --- | --- | --- | --- | --- | --- | --- | --- | --- | --- | --- | --- | --- | --- | --- | --- | --- | --- | --- | --- | --- | --- | --- | --- | --- | --- | --- | --- | --- | --- | --- | --- | --- | --- | --- | --- | --- | --- | --- | --- | --- | --- | --- | --- | --- | --- | --- | --- | --- | --- | --- | --- | --- | --- | --- | --- | --- | --- | --- | --- | --- | --- | --- | --- | --- | --- | --- | --- | --- | --- | --- | --- | --- | --- | --- | --- | --- | --- | --- | --- | --- | --- | --- | --- | --- | --- | --- | --- | --- | --- | --- | --- | --- | --- | --- | --- | --- | --- | --- | --- | --- | --- | --- | --- | --- | --- | --- | --- | --- | --- | --- | --- | --- | --- | --- | --- | --- | --- | --- | --- | --- | --- | --- | --- | --- | --- | --- | --- | --- | --- | --- | --- | --- | --- | --- | --- | --- | --- | --- | --- | --- | --- | --- | --- | --- | --- | --- | --- | --- | --- | --- | --- | --- | --- | --- | --- | --- | --- | --- | --- | --- | --- | --- | --- | --- | --- | --- | --- | --- | --- | --- | --- | --- | --- | --- | --- | --- | --- | --- | --- | --- | --- | --- | --- | --- | --- | --- | --- | --- | --- | --- | --- | --- | --- | --- | --- | --- | --- | --- | --- | --- | --- | --- | --- | --- | --- | --- | --- | --- | --- | --- | --- | --- | --- | --- | --- | --- | --- | --- | --- | --- | --- | --- | --- | --- | --- | --- | --- | --- | --- | --- | --- | --- | --- | --- | --- | --- | --- | --- | --- | --- | --- | --- | --- | --- | --- | --- | --- | --- | --- | --- | --- | --- | --- | --- | --- | --- | --- | --- | --- | --- | --- | --- | --- | --- | --- | --- | --- | --- | --- | --- | --- | --- | --- | --- | --- | --- | --- | --- | --- | --- | --- | --- | --- | --- | --- | --- | --- | --- | --- | --- | --- | --- | --- | --- | --- | --- | --- | --- | --- | --- | --- | --- | --- | --- | --- | --- | --- | --- | --- | --- | --- | --- | --- | --- | --- | --- | --- | --- | --- | --- | --- | --- | --- | --- | --- | --- | --- | --- | --- | --- | --- | --- | --- | --- | --- | --- | --- | --- | --- | --- | --- | --- | --- | --- | --- | --- | --- | --- | --- | --- | --- | --- | --- | --- | --- | --- | --- | --- | --- | --- | --- | --- | --- | --- | --- | --- | --- | --- | --- | --- | --- | --- | --- | --- | --- | --- | --- | --- | --- | --- | --- | --- | --- | --- | --- | --- | --- | --- | --- | --- | --- | --- | --- | --- | --- | --- | --- | --- | --- | --- | --- | --- | --- | --- | --- | --- | --- | --- | --- | --- | --- | --- | --- | --- | --- | --- | --- | --- | --- | --- | --- | --- | --- | --- | --- | --- | --- | --- | --- | --- | --- | --- | --- | --- | --- | --- | --- | --- | --- | --- | --- | --- | --- | --- | --- | --- | --- | --- | --- | --- | --- | --- | --- | --- | --- | --- | --- | --- | --- | --- | --- | --- | --- | --- | --- | --- | --- | --- | --- | --- | --- | --- | --- | --- | --- | --- | --- | --- | --- | --- | --- | --- | --- | --- | --- | --- | --- | --- | --- | --- | --- | --- | --- | --- | --- | --- | --- | --- | --- | --- | --- | --- | --- | --- | --- | --- | --- | --- | --- | --- | --- | --- | --- | --- | --- | --- | --- | --- | --- | --- | --- | --- | --- | --- | --- | --- | --- | --- | --- | --- | --- | --- | --- | --- | --- | --- | --- | --- | --- | --- | --- | --- | --- | --- | --- | --- | --- | --- | --- | --- | --- | --- | --- | --- | --- | --- | --- | --- | --- | --- | --- | --- | --- | --- | --- | --- | --- | --- | --- | --- | --- | --- | --- | --- | --- | --- | --- | --- | --- | --- | --- | --- | --- | --- | --- | --- | --- | --- | --- | --- | --- | --- | --- | --- | --- | --- | --- | --- | --- | --- | --- | --- | --- | --- | --- | --- | --- | --- | --- | --- | --- | --- | --- | --- | --- | --- | --- | --- | --- | --- | --- | --- | --- | --- | --- | --- | --- | --- | --- | --- | --- | --- | --- | --- | --- | --- | --- | --- | --- | --- | --- | --- | --- | --- | --- | --- | --- | --- | --- | --- | --- | --- | --- | --- | --- | --- | --- | --- | --- | --- | --- | --- | --- | --- | --- | --- | --- | --- | --- | --- | --- | --- | --- | --- | --- | --- | --- | --- | --- | --- | --- | --- | --- | --- | --- | --- | --- | --- | --- | --- | --- | --- | --- | --- | --- | --- | --- | --- | --- | --- | --- | --- | --- | --- | --- | --- | --- | --- | --- | --- | --- | --- | --- | --- | --- | --- | --- | --- | --- | --- | --- | --- | --- | --- | --- | --- | --- | --- | --- | --- | --- | --- | --- | --- | --- | --- | --- | --- | --- | --- | --- | --- | --- | --- | --- | --- | --- | --- | --- | --- | --- | --- | --- | --- | --- | --- | --- | --- | --- | --- | --- | --- | --- | --- | --- | --- | --- | --- | --- | --- | --- | --- | --- | --- | --- | --- | --- | --- | --- | --- | --- | --- | --- | --- | --- | --- | --- | --- | --- | --- | --- | --- | --- | --- | --- | --- | --- | --- | --- | --- | --- | --- | --- | --- | --- | --- | --- | --- | --- | --- | --- | --- | --- | --- | --- | --- | --- | --- | --- | --- | --- | --- | --- | --- | --- | --- | --- | --- | --- | --- | --- | --- | --- | --- | --- | --- | --- | --- | --- | --- | --- | --- | --- | --- | --- | --- | --- | --- | --- | --- | --- | --- | --- | --- | --- | --- | --- | --- | --- | --- | --- | --- | --- | --- | --- | --- | --- | --- | --- | --- | --- | --- | --- | --- | --- | --- | --- | --- | --- | --- | --- | --- | --- | --- | --- | --- | --- | --- | --- | --- | --- | --- | --- | --- | --- | --- | --- | --- | --- | --- | --- | --- | --- | --- | --- | --- | --- | --- | --- | --- | --- | --- | --- | --- | --- | --- | --- | --- | --- | --- | --- | --- | --- | --- | --- | --- | --- | --- | --- | --- | --- | --- | --- | --- | --- | --- | --- | --- | --- | --- | --- | --- | --- | --- | --- | --- | --- | --- | --- | --- | --- | --- | --- | --- | --- | --- | --- | --- | --- | --- | --- | --- | --- | --- | --- | --- | --- | --- | --- | --- | --- | --- | --- | --- | --- | --- | --- | --- | --- | --- | --- | --- | --- | --- | --- | --- | --- | --- | --- | --- | --- | --- | --- | --- | --- | --- | --- | --- | --- | --- | --- | --- | --- | --- | --- | --- | --- | --- | --- | --- | --- | --- | --- | --- | --- | --- | --- | --- | --- | --- | --- | --- | --- | --- | --- | --- | --- | --- | --- | --- | --- | --- | --- | --- | --- | --- | --- | --- | --- | --- | --- | --- | --- | --- | --- | --- | --- | --- | --- | --- | --- | --- | --- | --- | --- | --- | --- | --- | --- | --- | --- | --- | --- | --- | --- | --- | --- | --- | --- | --- | --- | --- | --- | --- | --- | --- | --- | --- | --- | --- | --- | --- | --- | --- | --- | --- | --- | --- | --- | --- | --- | --- | --- | --- | --- | --- | --- | --- | --- | --- | --- | --- | --- | --- | --- | --- | --- | --- | --- | --- | --- | --- | --- | --- | --- | --- | --- | --- | --- | --- | --- | --- | --- | --- | --- | --- | --- | --- | --- | --- | --- | --- | --- | --- | --- | --- | --- | --- | --- | --- | --- | --- | --- | --- | --- | --- | --- | --- | --- | --- | --- | --- | --- | --- | --- | --- | --- | --- | --- | --- | --- | --- | --- | --- | --- | --- | --- | --- | --- | --- | --- | --- | --- | --- | --- | --- | --- | --- | --- | --- | --- | --- | --- | --- | --- | --- | --- | --- | --- | --- | --- | --- | --- | --- | --- | --- | --- | --- | --- | --- | --- | --- | --- | --- | --- | --- | --- | --- | --- | --- | --- | --- | --- | --- | --- | --- | --- | --- | --- | --- | --- | --- | --- | --- | --- | --- | --- | --- | --- | --- | --- | --- | --- | --- | --- | --- | --- | --- | --- | --- | --- | --- | --- | --- | --- | --- | --- | --- | --- | --- | --- | --- | --- | --- | --- | --- | --- | --- | --- | --- | --- | --- | --- | --- | --- | --- | --- | --- | --- | --- | --- | --- | --- | --- | --- | --- | --- | --- | --- | --- | --- | --- | --- | --- | --- | --- | --- | --- | --- | --- | --- | --- | --- | --- | --- | --- | --- | --- | --- | --- | --- | --- | --- | --- | --- | --- | --- | --- | --- | --- | --- | --- | --- | --- | --- | --- | --- | --- | --- | --- | --- | --- | --- | --- | --- | --- | --- | --- | --- | --- | --- | --- | --- | --- | --- | --- | --- | --- | --- | --- | --- | --- | --- | --- | --- | --- | --- | --- | --- | --- | --- | --- | --- | --- | --- | --- | --- | --- | --- | --- | --- | --- | --- | --- | --- | --- | --- | --- | --- | --- | --- | --- | --- | --- | --- | --- | --- | --- | --- | --- | --- | --- | --- | --- | --- | --- | --- | --- | --- | --- | --- | --- | --- | --- | --- | --- | --- | --- | --- | --- | --- | --- | --- | --- | --- | --- | --- | --- | --- | --- | --- | --- | --- | --- | --- | --- | --- | --- | --- | --- | --- | --- | --- | --- | --- | --- | --- | --- | --- | --- | --- | --- | --- | --- | --- | --- | --- | --- | --- | --- | --- | --- | --- | --- | --- | --- | --- | --- | --- | --- | --- | --- | --- | --- | --- | --- | --- | --- | --- | --- | --- | --- | --- | --- | --- | --- | --- | --- | --- | --- | --- | --- | --- | --- | --- | --- | --- | --- | --- | --- | --- | --- | --- | --- | --- | --- | --- | --- | --- | --- | --- | --- | --- | --- | --- | --- | --- | --- | --- | --- | --- | --- | --- | --- | --- | --- | --- | --- | --- | --- | --- | --- | --- | --- | --- | --- | --- | --- | --- | --- | --- | --- | --- | --- | --- | --- | --- | --- | --- | --- | --- | --- | --- | --- | --- | --- | --- | --- | --- | --- | --- | --- | --- | --- | --- | --- | --- | --- | --- | --- | --- | --- | --- | --- | --- | --- | --- | --- | --- | --- | --- | --- | --- | --- | --- | --- | --- | --- | --- | --- | --- | --- | --- | --- | --- | --- | --- | --- | --- | --- | --- | --- | --- | --- | --- | --- | --- | --- | --- | --- | --- | --- | --- | --- | --- | --- | --- | --- | --- | --- | --- | --- | --- | --- | --- | --- | --- | --- | --- | --- | --- | --- | --- | --- | --- | --- | --- | --- | --- | --- | --- | --- | --- | --- | --- | --- | --- | --- | --- | --- | --- | --- | --- | --- | --- | --- | --- | --- | --- | --- | --- | --- | --- | --- | --- | --- | --- | --- | --- | --- | --- | --- | --- | --- | --- | --- | --- | --- | --- | --- | --- | --- | --- | --- | --- | --- | --- | --- | --- | --- | --- | --- | --- | --- | --- | --- | --- | --- | --- | --- | --- | --- | --- | --- | --- | --- | --- | --- | --- | --- | --- | --- | --- | --- | --- | --- | --- | --- | --- | --- | --- | --- | --- | --- | --- | --- | --- | --- | --- | --- | --- | --- | --- | --- | --- | --- | --- | --- | --- | --- | --- | --- | --- | --- | --- | --- | --- | --- | --- | --- | --- | --- | --- | --- | --- | --- | --- | --- | --- | --- | --- | --- | --- | --- | --- | --- | --- | --- | --- | --- | --- | --- | --- | --- | --- | --- | --- | --- | --- | --- | --- | --- | --- | --- | --- | --- | --- | --- | --- | --- | --- | --- | --- | --- | --- | --- | --- | --- | --- | --- | --- | --- | --- | --- | --- | --- | --- | --- | --- | --- | --- | --- | --- | --- | --- | --- | --- | --- | --- | --- | --- | --- | --- | --- | --- | --- | --- | --- | --- | --- | --- | --- | --- | --- | --- | --- | --- | --- | --- | --- | --- | --- | --- | --- | --- | --- | --- | --- | --- | --- | --- | --- | --- | --- | --- | --- | --- | --- | --- | --- | --- | --- | --- | --- | --- | --- | --- | --- | --- | --- | --- | --- | --- | --- | --- | --- | --- | --- | --- | --- | --- | --- | --- | --- | --- | --- | --- | --- | --- | --- | --- | --- | --- | --- | --- | --- | --- | --- | --- | --- | --- | --- | --- | --- | --- | --- | --- | --- | --- | --- | --- | --- | --- | --- | --- | --- | --- | --- | --- | --- | --- | --- | --- | --- | --- | --- | --- | --- | --- | --- | --- | --- | --- | --- | --- | --- | --- | --- | --- | --- | --- | --- | --- | --- | --- | --- | --- | --- | --- | --- | --- | --- | --- | --- | --- | --- | --- | --- | --- | --- | --- | --- | --- | --- | --- | --- | --- | --- | --- | --- | --- | --- | --- | --- | --- | --- | --- | --- | --- | --- | --- | --- | --- | --- | --- | --- | --- | --- | --- | --- | --- | --- | --- | --- | --- | --- | --- | --- | --- | --- | --- | --- | --- | --- | --- | --- | --- | --- | --- | --- | --- | --- | --- | --- | --- | --- | --- | --- | --- | --- | --- | --- | --- | --- | --- | --- | --- | --- | --- | --- | --- | --- | --- | --- | --- | --- | --- | --- | --- | --- | --- | --- | --- | --- | --- | --- | --- | --- | --- | --- | --- | --- | --- | --- | --- | --- | --- | --- | --- | --- | --- | --- | --- | --- | --- | --- | --- | --- | --- | --- | --- | --- | --- | --- | --- | --- | --- | --- | --- | --- | --- | --- | --- | --- | --- | --- | --- | --- | --- | --- | --- | --- | --- | --- | --- | --- | --- | --- | --- | --- | --- | --- | --- | --- | --- | --- | --- | --- | --- | --- | --- | --- | --- | --- | --- | --- | --- | --- | --- | --- | --- | --- | --- | --- | --- | --- | --- | --- | --- | --- | --- | --- | --- | --- | --- | --- | --- | --- | --- | --- | --- | --- | --- | --- | --- | --- | --- | --- | --- | --- | --- | --- | --- | --- | --- | --- | --- | --- | --- | --- | --- | --- | --- | --- | --- | --- | --- | --- | --- | --- | --- | --- | --- | --- | --- | --- | --- | --- | --- | --- | --- | --- | --- | --- | --- | --- | --- | --- | --- | --- | --- | --- | --- | --- | --- | --- | --- | --- | --- | --- | --- | --- | --- | --- | --- | --- | --- | --- | --- | --- | --- | --- | --- | --- | --- | --- | --- | --- | --- | --- | --- | --- | --- | --- | --- | --- | --- | --- | --- | --- | --- | --- | --- | --- | --- | --- | --- | --- | --- | --- | --- | --- | --- | --- | --- | --- | --- | --- | --- | --- | --- | --- | --- | --- | --- | --- | --- | --- | --- | --- | --- | --- | --- | --- | --- | --- | --- | --- | --- | --- | --- | --- | --- | --- | --- | --- | --- | --- | --- | --- | --- | --- | --- | --- | --- | --- | --- | --- | --- | --- | --- | --- | --- | --- | --- | --- | --- | --- | --- | --- | --- | --- | --- | --- | --- | --- | --- | --- | --- | --- | --- | --- | --- | --- | --- | --- | --- | --- | --- | --- | --- | --- | --- | --- | --- | --- | --- | --- | --- | --- | --- | --- | --- | --- | --- | --- | --- | --- | --- | --- | --- | --- | --- | --- | --- | --- | --- | --- | --- | --- | --- | --- | --- | --- | --- | --- | --- | --- | --- | --- | --- | --- | --- | --- | --- | --- | --- | --- | --- | --- | --- | --- | --- | --- | --- | --- | --- | --- | --- | --- | --- | --- | --- | --- | --- | --- | --- | --- | --- | --- | --- | --- | --- | --- | --- | --- | --- | --- | --- | --- | --- | --- | --- | --- | --- | --- | --- | --- | --- | --- | --- | --- | --- | --- | --- | --- | --- | --- | --- | --- | --- | --- | --- | --- | --- | --- | --- | --- | --- | --- | --- | --- | --- | --- | --- | --- | --- | --- | --- | --- | --- | --- | --- | --- | --- | --- | --- | --- | --- | --- | --- | --- | --- | --- | --- | --- | --- | --- | --- | --- | --- | --- | --- | --- | --- | --- | --- | --- | --- | --- | --- | --- | --- | --- | --- | --- | --- | --- | --- | --- | --- | --- | --- | --- | --- | --- | --- | --- | --- | --- | --- | --- | --- | --- | --- | --- | --- | --- | --- | --- | --- | --- | --- | --- | --- | --- | --- | --- | --- | --- | --- | --- | --- | --- | --- | --- | --- | --- | --- | --- | --- | --- | --- | --- | --- | --- | --- | --- | --- | --- | --- | --- | --- | --- | --- | --- | --- | --- | --- | --- | --- | --- | --- | --- | --- | --- | --- | --- | --- | --- | --- | --- | --- | --- | --- | --- | --- | --- | --- | --- | --- | --- | --- | --- | --- | --- | --- | --- | --- | --- | --- | --- | --- | --- | --- | --- | --- | --- | --- | --- | --- | --- | --- | --- | --- | --- | --- | --- | --- | --- | --- | --- | --- | --- | --- | --- | --- | --- | --- | --- | --- | --- | --- | --- | --- | --- | --- | --- | --- | --- | --- | --- | --- | --- | --- | --- | --- | --- | --- | --- | --- | --- | --- | --- | --- | --- | --- | --- | --- | --- | --- | --- | --- | --- | --- | --- | --- | --- | --- | --- | --- | --- | --- | --- | --- | --- | --- | --- | --- | --- | --- | --- | --- | --- | --- | --- | --- | --- | --- | --- | --- | --- | --- | --- | --- | --- | --- | --- | --- | --- | --- | --- | --- | --- | --- | --- | --- | --- | --- | --- | --- | --- | --- | --- | --- | --- | --- | --- | --- | --- | --- | --- | --- | --- | --- | --- | --- | --- | --- | --- | --- | --- | --- | --- | --- | --- | --- | --- | --- | --- | --- | --- | --- | --- | --- | --- | --- | --- | --- | --- | --- | --- | --- | --- | --- | --- | --- | --- | --- | --- | --- | --- | --- | --- | --- | --- | --- | --- | --- | --- | --- | --- | --- | --- | --- | --- | --- | --- | --- | --- | --- | --- | --- | --- | --- | --- | --- | --- | --- | --- | --- | --- | --- | --- | --- | --- | --- | --- | --- | --- | --- | --- | --- | --- | --- | --- | --- | --- | --- | --- | --- | --- | --- | --- | --- | --- | --- | --- | --- | --- | --- | --- | --- | --- | --- | --- | --- | --- | --- | --- | --- | --- | --- | --- | --- | --- | --- | --- | --- | --- | --- | --- | --- | --- | --- | --- | --- | --- | --- | --- | --- | --- | --- | --- | --- | --- | --- | --- | --- | --- | --- | --- | --- | --- | --- | --- | --- | --- | --- | --- | --- | --- | --- | --- | --- | --- | --- | --- | --- | --- | --- | --- | --- | --- | --- | --- | --- | --- | --- | --- | --- | --- | --- | --- | --- | --- | --- | --- | --- | --- | --- | --- | --- | --- | --- | --- | --- | --- | --- | --- | --- | --- | --- | --- | --- | --- | --- | --- | --- | --- | --- | --- | --- | --- | --- | --- | --- | --- | --- | --- | --- | --- | --- | --- | --- | --- | --- | --- | --- | --- | --- | --- | --- | --- | --- | --- | --- | --- | --- | --- | --- | --- | --- | --- | --- | --- | --- | --- | --- | --- | --- | --- | --- | --- | --- | --- | --- | --- | --- | --- | --- | --- | --- | --- | --- | --- | --- | --- | --- | --- | --- | --- | --- | --- | --- | --- | --- | --- | --- | --- | --- | --- | --- | --- | --- | --- | --- | --- | --- | --- | --- | --- | --- | --- | --- | --- | --- | --- | --- | --- | --- | --- | --- | --- | --- | --- | --- | --- | --- | --- | --- | --- | --- | --- | --- | --- | --- | --- | --- | --- | --- | --- | --- | --- | --- | --- | --- | --- | --- | --- | --- | --- | --- | --- | --- | --- | --- | --- | --- | --- | --- | --- | --- | --- | --- | --- | --- | --- | --- | --- | --- | --- | --- | --- | --- | --- | --- | --- | --- | --- | --- | --- | --- | --- | --- | --- | --- | --- | --- | --- | --- | --- | --- | --- | --- | --- | --- | --- | --- | --- | --- | --- | --- | --- | --- | --- | --- | --- | --- | --- | --- | --- | --- | --- | --- | --- | --- | --- | --- | --- | --- | --- | --- | --- | --- | --- | --- | --- | --- | --- | --- | --- | --- | --- | --- | --- | --- | --- | --- | --- | --- | --- | --- | --- | --- | --- | --- | --- | --- | --- | --- | --- | --- | --- | --- | --- | --- | --- | --- | --- | --- | --- | --- | --- | --- | --- | --- | --- | --- | --- | --- | --- | --- | --- | --- | --- | --- | --- | --- | --- | --- | --- | --- | --- | --- | --- | --- | --- | --- | --- | --- | --- | --- | --- | --- | --- | --- | --- | --- | --- | --- | --- | --- | --- | --- | --- | --- | --- | --- | --- | --- | --- | --- | --- | --- | --- | --- | --- | --- | --- | --- | --- | --- | --- | --- | --- | --- | --- | --- | --- | --- | --- | --- | --- | --- | --- | --- | --- | --- | --- | --- | --- | --- | --- | --- | --- | --- | --- | --- | --- | --- | --- | --- | --- | --- | --- | --- | --- | --- | --- | --- | --- | --- | --- | --- | --- | --- | --- | --- | --- | --- | --- | --- | --- | --- | --- | --- | --- | --- | --- | --- | --- | --- | --- | --- | --- | --- | --- | --- | --- | --- | --- | --- | --- | --- | --- | --- | --- | --- | --- | --- | --- | --- | --- | --- | --- | --- | --- | --- | --- | --- | --- | --- | --- | --- | --- | --- | --- | --- | --- | --- | --- | --- | --- | --- | --- | --- | --- | --- | --- | --- | --- | --- | --- | --- | --- | --- | --- | --- | --- | --- | --- | --- | --- | --- | --- | --- | --- | --- | --- | --- | --- | --- | --- | --- | --- | --- | --- | --- | --- | --- | --- | --- | --- | --- | --- | --- | --- | --- | --- | --- | --- | --- | --- | --- | --- | --- | --- | --- | --- | --- | --- | --- | --- | --- | --- | --- | --- | --- | --- | --- | --- | --- | --- | --- | --- | --- | --- | --- | --- | --- | --- | --- | --- | --- | --- | --- | --- | --- | --- | --- | --- | --- | --- | --- | --- | --- | --- | --- | --- | --- | --- | --- | --- | --- | --- | --- | --- | --- | --- | --- | --- | --- | --- | --- | --- | --- | --- | --- | --- | --- | --- | --- | --- | --- | --- | --- | --- | --- | --- | --- | --- | --- | --- | --- | --- | --- | --- | --- | --- | --- | --- | --- | --- | --- | --- | --- | --- | --- | --- | --- | --- | --- | --- | --- | --- | --- | --- | --- | --- | --- | --- | --- | --- | --- | --- | --- | --- | --- | --- | --- | --- | --- | --- | --- | --- | --- | --- | --- | --- | --- | --- | --- | --- | --- | --- | --- | --- | --- | --- | --- | --- | --- | --- | --- | --- | --- | --- | --- | --- | --- | --- | --- | --- | --- | --- | --- | --- | --- | --- | --- | --- | --- | --- | --- | --- | --- | --- | --- | --- | --- | --- | --- | --- | --- | --- | --- | --- | --- | --- | --- | --- | --- | --- | --- | --- | --- | --- | --- | --- | --- | --- | --- | --- | --- | --- | --- | --- | --- | --- | --- | --- | --- | --- | --- | --- | --- | --- | --- | --- | --- | --- | --- | --- | --- | --- | --- | --- | --- | --- | --- | --- | --- | --- | --- | --- | --- | --- | --- | --- | --- | --- | --- | --- | --- | --- | --- | --- | --- | --- | --- | --- | --- | --- | --- | --- | --- | --- | --- | --- | --- | --- | --- | --- | --- | --- | --- | --- | --- | --- | --- | --- | --- | --- | --- | --- | --- | --- | --- | --- | --- | --- | --- | --- | --- | --- | --- | --- | --- | --- | --- | --- | --- | --- | --- | --- | --- | --- | --- | --- | --- | --- | --- | --- | --- | --- | --- | --- | --- | --- | --- | --- | --- | --- | --- | --- | --- | --- | --- | --- | --- | --- | --- | --- | --- | --- | --- | --- | --- | --- | --- | --- | --- | --- | --- | --- | --- | --- | --- | --- | --- | --- | --- | --- | --- | --- | --- | --- | --- | --- | --- | --- | --- | --- | --- | --- | --- | --- | --- | --- | --- | --- | --- | --- | --- | --- | --- | --- | --- | --- | --- | --- | --- | --- | --- | --- | --- | --- | --- | --- | --- | --- | --- | --- | --- | --- | --- | --- | --- | --- | --- | --- | --- | --- | --- | --- | --- | --- | --- | --- | --- | --- | --- | --- | --- | --- | --- | --- | --- | --- | --- | --- | --- | --- | --- | --- | --- | --- | --- | --- | --- | --- | --- | --- | --- | --- | --- | --- | --- | --- | --- | --- | --- | --- | --- | --- | --- | --- | --- | --- | --- | --- | --- | --- | --- | --- | --- | --- | --- | --- | --- | --- | --- | --- | --- | --- | --- | --- | --- | --- | --- | --- | --- | --- | --- | --- | --- | --- | --- | --- | --- | --- | --- | --- | --- | --- | --- | --- | --- | --- | --- | --- | --- | --- | --- | --- | --- | --- | --- | --- | --- | --- | --- | --- | --- | --- | --- | --- | --- | --- | --- | --- | --- | --- | --- | --- | --- | --- | --- | --- | --- | --- | --- | --- | --- | --- | --- | --- | --- | --- | --- | --- | --- | --- | --- | --- | --- | --- | --- | --- | --- | --- | --- | --- | --- | --- | --- | --- | --- | --- | --- | --- | --- | --- | --- | --- | --- | --- | --- | --- | --- | --- | --- | --- | --- | --- | --- | --- | --- | --- | --- | --- | --- | --- | --- | --- | --- | --- | --- | --- | --- | --- | --- | --- | --- | --- | --- | --- | --- | --- | --- | --- | --- | --- | --- | --- | --- | --- | --- | --- | --- | --- | --- | --- | --- | --- | --- | --- | --- | --- | --- | --- | --- | --- | --- | --- | --- | --- | --- | --- | --- | --- | --- | --- | --- | --- | --- | --- | --- | --- | --- | --- | --- | --- | --- | --- | --- | --- | --- | --- | --- | --- | --- | --- | --- | --- | --- | --- | --- | --- | --- | --- | --- | --- | --- | --- | --- | --- | --- | --- | --- | --- | --- | --- | --- | --- | --- | --- | --- | --- | --- | --- | --- | --- | --- | --- | --- | --- | --- | --- | --- | --- | --- | --- | --- | --- | --- | --- | --- | --- | --- | --- | --- | --- | --- | --- | --- | --- | --- | --- | --- | --- | --- | --- | --- | --- | --- | --- | --- | --- | --- | --- | --- | --- | --- | --- | --- | --- | --- | --- | --- | --- | --- | --- | --- | --- | --- | --- | --- | --- | --- | --- | --- | --- | --- | --- | --- | --- | --- | --- | --- | --- | --- | --- | --- | --- | --- | --- | --- | --- | --- | --- | --- | --- | --- | --- | --- | --- | --- | --- | --- | --- | --- | --- | --- | --- | --- | --- | --- | --- | --- | --- | --- | --- | --- | --- | --- | --- | --- | --- | --- | --- | --- | --- | --- | --- | --- | --- | --- | --- | --- | --- | --- | --- | --- | --- | --- | --- | --- | --- | --- | --- | --- | --- | --- | --- | --- | --- | --- | --- | --- | --- | --- | --- | --- | --- | --- | --- | --- | --- | --- | --- | --- | --- | --- | --- | --- | --- | --- | --- | --- | --- | --- | --- | --- | --- | --- | --- | --- | --- | --- | --- | --- | --- | --- | --- | --- | --- | --- | --- | --- | --- | --- | --- | --- | --- | --- | --- | --- | --- | --- | --- | --- | --- | --- | --- | --- | --- | --- | --- | --- | --- | --- | --- | --- | --- | --- | --- | --- | --- | --- | --- | --- | --- | --- | --- | --- | --- | --- | --- | --- | --- | --- | --- | --- | --- | --- | --- | --- | --- | --- | --- | --- | --- | --- | --- | --- | --- | --- | --- | --- | --- | --- | --- | --- | --- | --- | --- | --- | --- | --- | --- | --- | --- | --- | --- | --- | --- | --- | --- | --- | --- | --- | --- | --- | --- | --- | --- | --- | --- | --- | --- | --- | --- | --- | --- | --- | --- | --- | --- | --- | --- | --- | --- | --- | --- | --- | --- | --- | --- | --- | --- | --- | --- | --- | --- | --- | --- | --- | --- | --- | --- | --- | --- | --- | --- | --- | --- | --- | --- | --- | --- | --- | --- | --- | --- | --- | --- | --- | --- | --- | --- | --- | --- | --- | --- | --- | --- | --- | --- | --- | --- | --- | --- | --- | --- | --- | --- | --- | --- | --- | --- | --- | --- | --- | --- | --- | --- | --- | --- | --- | --- | --- | --- | --- | --- | --- | --- | --- | --- | --- | --- | --- | --- | --- | --- | --- | --- | --- | --- | --- | --- | --- | --- | --- | --- | --- | --- | --- | --- | --- | --- | --- | --- | --- | --- | --- | --- | --- | --- | --- | --- | --- | --- | --- | --- | --- | --- | --- | --- | --- | --- | --- | --- | --- | --- | --- | --- | --- | --- | --- | --- | --- | --- | --- | --- | --- | --- | --- | --- | --- | --- | --- | --- | --- | --- | --- | --- | --- | --- | --- | --- | --- | --- | --- | --- | --- | --- | --- | --- | --- | --- | --- | --- | --- | --- | --- | --- | --- | --- | --- | --- | --- | --- | --- | --- | --- | --- | --- | --- | --- | --- | --- | --- | --- | --- | --- | --- | --- | --- | --- | --- | --- | --- | --- | --- | --- | --- | --- | --- | --- | --- | --- | --- | --- | --- | --- | --- | --- | --- | --- | --- | --- | --- | --- | --- | --- | --- | --- | --- | --- | --- | --- | --- | --- | --- | --- | --- | --- | --- | --- | --- | --- | --- | --- | --- | --- | --- | --- | --- | --- | --- | --- | --- | --- | --- | --- | --- | --- | --- | --- | --- | --- | --- | --- | --- | --- | --- | --- | --- | --- | --- | --- | --- | --- | --- | --- | --- | --- | --- | --- | --- | --- | --- | --- | --- | --- | --- | --- | --- | --- | --- | --- | --- | --- | --- | --- | --- | --- | --- | --- | --- | --- | --- | --- | --- | --- | --- | --- | --- | --- | --- | --- | --- | --- | --- | --- | --- | --- | --- | --- | --- | --- | --- | --- | --- | --- | --- | --- | --- | --- | --- | --- | --- | --- | --- | --- | --- | --- | --- | --- | --- | --- | --- | --- | --- | --- | --- | --- | --- | --- | --- | --- | --- | --- | --- | --- | --- | --- | --- | --- | --- | --- | --- | --- | --- | --- | --- | --- | --- | --- | --- | --- | --- | --- | --- | --- | --- | --- | --- | --- | --- | --- | --- | --- | --- | --- | --- | --- | --- | --- | --- | --- | --- | --- | --- | --- | --- | --- | --- | --- | --- | --- | --- | --- | --- | --- | --- | --- | --- | --- | --- | --- | --- | --- | --- | --- | --- | --- | --- | --- | --- | --- | --- | --- | --- | --- | --- | --- | --- | --- | --- | --- | --- | --- | --- | --- | --- | --- | --- | --- | --- | --- | --- | --- | --- | --- | --- | --- | --- | --- | --- | --- | --- | --- | --- | --- | --- | --- | --- | --- | --- | --- | --- | --- | --- | --- | --- | --- | --- | --- | --- | --- | --- | --- | --- | --- | --- | --- | --- | --- | --- | --- | --- | --- | --- | --- | --- | --- | --- | --- | --- | --- | --- | --- | --- | --- | --- | --- | --- | --- | --- | --- | --- | --- | --- | --- | --- | --- | --- | --- | --- | --- | --- | --- | --- | --- | --- | --- | --- | --- | --- | --- | --- | --- | --- | --- | --- | --- | --- | --- | --- | --- | --- | --- | --- | --- | --- | --- | --- | --- | --- | --- | --- | --- | --- | --- | --- | --- | --- | --- | --- | --- | --- | --- | --- | --- | --- | --- | --- | --- | --- | --- | --- | --- | --- | --- | --- | --- | --- | --- | --- | --- | --- | --- | --- | --- | --- | --- | --- | --- | --- | --- | --- | --- | --- | --- | --- | --- | --- | --- | --- | --- | --- | --- | --- | --- | --- | --- | --- | --- |
| |  |  |  |  |  |  |  |  |  | | --- | --- | --- | --- | --- | --- | --- | --- | --- | | **Position** | **Reference** | **Sample** | **Quality** | **Type** | **Region** | **AA Exchange** | **PAM1** | **Known Variant** | | 4013 | T | C | 1641.77 | SNP | Rv0003 (recF) | Ile245Thr | 11 | - | | 7362 | G | C | 1602.77 | SNP | Rv0006 (gyrA) | Glu21Gln | 27 | - | | 10741 | C | G | 2003.77 | SNP | Rv0007 | silent (Thr276) | 9871 | - | | 11879 | A | G | 1698.77 | SNP | Rv0008c | Ser145Pro | 12 | - | | 14785 | T | C | 2400.77 | SNP | Rv0012 | Cys233Arg | 1 | - | | 19442 | G | C | 1734.77 | SNP | Rv0016c (pbpA) | Pro265Ala | 22 | - | | 26959 | C | G | 1513.77 | SNP | intergenic |  |  | - | | 31869 | G | C | 1720.77 | SNP | intergenic |  |  | - | | 34044 | T | C | 1835.77 | SNP | intergenic |  |  | - | | 37031 | C | G | 1490.77 | SNP | Rv0034 | silent (Ala55) | 9867 | - | | 37725 | CCGACACCGCCACCGCCGAC TGGCCGCTATGCACCCTCGA CGACGACGCCTACGT | C | 14912.73 | DEL | Rv0035 (fadD34) |  |  | - | | 42967 | G | C | 1577.77 | SNP | Rv0040c (mtc28) | silent (Pro133) | 9926 | - | | 54171 | C | G | 1177.77 | SNP | Rv0050 (ponA1) | Ala170Gly | 21 | - | | 55553 | C | CCGT | 1613.73 | INS | Rv0050 (ponA1) |  |  | - | | 61114 | T | C | 1064.77 | SNP | Rv0058 (dnaB) | Met(s)240Thr | 22 | - | | 62049 | A | G | 1752.77 | SNP | Rv0058 (dnaB) | Arg552Gly | 1 | - | | 69989 | G | A | 1989.77 | SNP | Rv0064 | Gly457Asp | 6 | - | | 70816 | A | G | 1095.77 | SNP | Rv0064 | Asn733Asp | 42 | - | | 71336 | G | C | 337.78 | SNP | Rv0064 | Arg906Pro | 5 | - | | 71584 | C | CCGAGCGCTGTTCTGGCGCT AATCTGACGCTAGAATAG | 12173.73 | INS | intergenic |  |  | - | | 75821 | C | G | 1846.77 | SNP | Rv0068 | Ala174Gly | 21 | - | | 75940 | G | C | 1724.77 | SNP | Rv0068 | Val(s)214Leu | 3 | - | | 79504 | T | TCGGTGGACC | 2038.73 | INS | Rv0071 |  |  | - | | 104962 | G | A | 47.74 | SNP | Rv0095c | Ala85Val(s) | 9867 | - | | 105007 | C | G | 262.80 | SNP | Rv0095c | Ser70Thr | 32 | - | | 105011 | G | A | 251.80 | SNP | Rv0095c | Leu69Leu(s) | 4 | - | | 105021 | G | A | 340.78 | SNP | Rv0095c | silent (Ser65) | 9840 | - | | 105045 | G | C | 587.77 | SNP | Rv0095c | Asp57Glu | 56 | - | | 105060 | G | A | 707.77 | SNP | Rv0095c | silent (Asp52) | 9859 | - | | 105063 | G | A | 717.77 | SNP | Rv0095c | silent (Phe51) | 9946 | - | | 116000 | T | G | 1528.77 | SNP | Rv0101 (nrp) | Val2000Val(s) | 18 | - | | 122109 | A | G | 2036.77 | SNP | Rv0103c (ctpB) | Leu(s)22Ser | 28 | - | | 124862 | G | A | 1593.77 | SNP | Rv0106 | silent (Gln163) | 9876 | - | | 125830 | G | GA | 2828.73 | INS | Rv0107c (ctpI) |  |  | - | | 128093 | C | G | 791.77 | SNP | Rv0107c (ctpI) | Glu817Gln | 27 | - | | 131174 | T | TG | 2664.73 | INS | intergenic |  |  | - | | 132417 | C | G | 131.03 | SNP | Rv0109 (PE\_PGRS1) | Arg346Gly | 1 | - | | 133839 | C | T | 1842.77 | SNP | intergenic |  |  | - | | 146087 | T | C | 2159.77 | SNP | Rv0120c (fusA2) | Asn562Ser | 34 | - | | 150795 | T | C | 781.77 | SNP | Rv0124 (PE\_PGRS2) | silent (Gly421) | 9935 | - | | 150898 | C | G | 59.77 | SNP | Rv0124 (PE\_PGRS2) | Pro456Ala | 22 | - | | 154283 | T | C | 1848.77 | SNP | Rv0127 (mak) | Ser18Pro | 12 | - | | 155468 | C | A | 928.77 | SNP | Rv0127 (mak) | silent (Arg413) | 9913 | - | | 169075 | G | A | 810.77 | SNP | Rv0143c | Leu370Leu(s) | 4 | - | | 175248 | G | A | 1845.77 | SNP | Rv0148 | Arg139His | 8 | - | | 176298 | G | A | 1988.77 | SNP | Rv0149 | Arg200His | 8 | - | | 177857 | G | A | 797.77 | SNP | Rv0151c (PE1) | Leu485Leu(s) | 4 | - | | 187034 | G | C | 1962.77 | SNP | Rv0158 | Glu84Gln | 27 | - | | 188090 | C | T | 1847.77 | SNP | Rv0159c (PE3) | Val(s)250Val | 13 | - | | 188800 | T | C | 1196.77 | SNP | Rv0159c (PE3) | Thr14Ala | 32 | - | | 192586 | C | T | 1192.77 | SNP | Rv0162c (adhE1) | Ala184Thr | 22 | - | | 201896 | A | G | 902.77 | SNP | Rv0171 (mce1C) | Asp322Gly | 11 | - | | 206339 | T | C | 1277.77 | SNP | Rv0174 (mce1F) | Leu370Pro | 2 | - | | 223942 | T | C | 1126.77 | SNP | Rv0192 | Ser127Pro | 12 | - | | 225323 | T | C | 1274.77 | SNP | Rv0193c | Lys417Glu | 4 | - | | 225500 | C | T | 1519.77 | SNP | Rv0193c | Gly358Ser | 16 | - | | 227098 | T | C | 1633.77 | SNP | Rv0194 | Met(s)74Thr | 22 | - | | 231114 | C | G | 1739.77 | SNP | Rv0195 | silent (Ala72) | 9867 | - | | 231372 | G | A | 2269.77 | SNP | Rv0195 | Val(s)158Val | 13 | - | | 234477 | T | G | 867.77 | SNP | Rv0197 | Tyr749STOP | 2 | - | | 234496 | C | CGT | 1780.73 | INS | Rv0197 |  |  | - | | 261869 | T | C | 1227.77 | SNP | Rv0218 | Cys316Arg | 1 | - | | 265554 | A | C | 1463.77 | SNP | Rv0222 (echA1) | silent (Val16) | 9901 | - | | 265710 | G | A | 1309.77 | SNP | Rv0222 (echA1) | silent (Ser68) | 9840 | - | | 271713 | T | A | 870.77 | SNP | Rv0227c | Glu376Val(s) | 17 | - | | 272685 | T | C | 1493.77 | SNP | Rv0227c | Asp52Gly | 11 | - | | 275985 | T | C | 2102.77 | SNP | intergenic |  |  | - | | 278681 | C | G | 1389.77 | SNP | Rv0233 (nrdB) | His33Asp | 4 | - | | 285498 | T | C | 764.77 | SNP | Rv0236c (aftD) | Thr452Ala | 32 | - | | 285772 | A | C | 1574.77 | SNP | Rv0236c (aftD) | silent (Pro360) | 9926 | - | | 291867 | T | A | 1145.77 | SNP | Rv0242c (fabG4) | Arg55Trp | 2 | - | | 295230 | G | A | 1930.77 | SNP | Rv0244c (fadE5) | Ala135Val | 13 | - | | 304436 | T | C | 1812.77 | SNP | Rv0252 (nirB) | Leu(s)524Ser | 28 | - | | 310973 | G | A | 1275.77 | SNP | Rv0259c | Ala182Val(s) | 9867 | - | | 311613 | G | T | 1661.77 | SNP | Rv0260c | silent (Val349) | 9901 | - | | 321551 | C | A | 969.77 | SNP | Rv0267 (narU) | Ala74Asp | 6 | - | | 325502 | C | T | 1644.77 | SNP | Rv0270 (fadD2) | silent (Val312) | 9901 | - | | 327516 | CG | C | 2510.73 | DEL | Rv0271c (fadE6) |  |  | - | | 333640 | G | A | 35.77 | SNP | Rv0278c (PE\_PGRS3) | Arg891Trp | 2 | - | | 333641 | C | T | 47.77 | SNP | Rv0278c (PE\_PGRS3) | silent (Gln890) | 9876 | - | | 333892 | G | C | 428.77 | SNP | Rv0278c (PE\_PGRS3) | Arg807Gly | 1 | - | | 334641 | G | C | 177.77 | SNP | Rv0278c (PE\_PGRS3) | Ala557Gly | 21 | - | | 335810 | CCCGCCGGCGCCGCCGTTG | C | 608.87 | DEL | Rv0278c (PE\_PGRS3) |  |  | - | | 335971 | A | G | 159.77 | SNP | Rv0278c (PE\_PGRS3) | Leu(s)114Leu | 3 | - | | 336005 | G | A | 252.77 | SNP | Rv0278c (PE\_PGRS3) | silent (Ile102) | 9872 | - | | 336047 | C | G | 293.77 | SNP | Rv0278c (PE\_PGRS3) | silent (Ala88) | 9867 | - | | 336050 | A | G | 271.77 | SNP | Rv0278c (PE\_PGRS3) | silent (Tyr87) | 9945 | - | | 336053 | G | C | 298.77 | SNP | Rv0278c (PE\_PGRS3) | silent (Ala86) | 9867 | - | | 336074 | T | C | 149.77 | SNP | Rv0278c (PE\_PGRS3) | silent (Ala79) | 9867 | - | | 336081 | A | G | 90.77 | SNP | Rv0278c (PE\_PGRS3) | Val(s)77Ala | 9867 | - | | 336082 | C | T | 86.77 | SNP | Rv0278c (PE\_PGRS3) | Val(s)77Met(s) | 9867 | - | | 336380 | A | T | 172.77 | SNP | intergenic |  |  | - | | 336400 | C | G | 247.77 | SNP | intergenic |  |  | - | | 336403 | C | G | 247.77 | SNP | intergenic |  |  | - | | 336405 | A | G | 286.77 | SNP | intergenic |  |  | - | | 336504 | G | T | 414.77 | SNP | intergenic |  |  | - | | 336535 | T | G | 486.77 | SNP | intergenic |  |  | - | | 336537 | T | G | 505.77 | SNP | intergenic |  |  | - | | 336540 | G | T | 408.77 | SNP | intergenic |  |  | - | | 336546 | T | G | 502.77 | SNP | intergenic |  |  | - | | 336557 | C | CT | 869.73 | INS | intergenic |  |  | - | | 336560 | T | C | 399.77 | SNP | Rv0279c (PE\_PGRS4) | silent (STOP838) | 9867 | - | | 336562 | A | ATGG | 834.73 | INS | Rv0279c (PE\_PGRS4) |  |  | - | | 336590 | G | C | 251.77 | SNP | Rv0279c (PE\_PGRS4) | Ile828Met(s) | 6 | - | | 336592 | T | G | 327.77 | SNP | Rv0279c (PE\_PGRS4) | Ile828Leu | 22 | - | | 336611 | G | C | 260.77 | SNP | Rv0279c (PE\_PGRS4) | silent (Ala821) | 9867 | - | | 336617 | G | C | 220.77 | SNP | Rv0279c (PE\_PGRS4) | silent (Pro819) | 9926 | - | | 336620 | T | C | 195.77 | SNP | Rv0279c (PE\_PGRS4) | silent (Thr818) | 9871 | - | | 336691 | T | C | 76.28 | SNP | Rv0279c (PE\_PGRS4) | Ser795Gly | 21 | - | | 336698 | C | G | 114.03 | SNP | Rv0279c (PE\_PGRS4) | silent (Gly792) | 9935 | - | | 336701 | A | G | 74.28 | SNP | Rv0279c (PE\_PGRS4) | silent (Gly791) | 9935 | - | | 336707 | G | A | 82.03 | SNP | Rv0279c (PE\_PGRS4) | silent (Asp789) | 9859 | - | | 336708 | T | C | 61.28 | SNP | Rv0279c (PE\_PGRS4) | Asp789Gly | 11 | - | | 336710 | A | G | 96.03 | SNP | Rv0279c (PE\_PGRS4) | silent (Ala788) | 9867 | - | | 336728 | G | A | 100.77 | SNP | Rv0279c (PE\_PGRS4) | silent (Gly782) | 9935 | - | | 337820 | G | A | 467.77 | SNP | Rv0279c (PE\_PGRS4) | silent (Gly418) | 9935 | - | | 338100 | T | C | 202.84 | SNP | Rv0279c (PE\_PGRS4) | Asn325Ser | 34 | - | | 338453 | A | G | 174.90 | SNP | Rv0279c (PE\_PGRS4) | silent (Ala207) | 9867 | - | | 338768 | G | A | 118.77 | SNP | Rv0279c (PE\_PGRS4) | silent (Ile102) | 9872 | - | | 338774 | G | A | 99.77 | SNP | Rv0279c (PE\_PGRS4) | silent (Ala100) | 9867 | - | | 338775 | G | T | 84.77 | SNP | Rv0279c (PE\_PGRS4) | Ala100Asp | 6 | - | | 338777 | G | C | 85.77 | SNP | Rv0279c (PE\_PGRS4) | silent (Leu99) | 9947 | - | | 338789 | G | C | 34.77 | SNP | Rv0279c (PE\_PGRS4) | silent (Thr95) | 9871 | - | | 338790 | G | A | 52.77 | SNP | Rv0279c (PE\_PGRS4) | Thr95Ile | 7 | - | | 338791 | T | C | 105.77 | SNP | Rv0279c (PE\_PGRS4) | Thr95Ala | 32 | - | | 338792 | G | C | 66.77 | SNP | Rv0279c (PE\_PGRS4) | silent (Ala94) | 9867 | - | | 338844 | A | G | 591.77 | SNP | Rv0279c (PE\_PGRS4) | Val(s)77Ala | 9867 | - | | 338845 | C | T | 593.77 | SNP | Rv0279c (PE\_PGRS4) | Val(s)77Met(s) | 9867 | - | | 338876 | G | A | 661.77 | SNP | Rv0279c (PE\_PGRS4) | silent (Ser66) | 9840 | - | | 338903 | G | C | 690.77 | SNP | Rv0279c (PE\_PGRS4) | silent (Ala57) | 9867 | - | | 338960 | T | C | 715.77 | SNP | Rv0279c (PE\_PGRS4) | silent (Ala38) | 9867 | - | | 338963 | T | C | 700.77 | SNP | Rv0279c (PE\_PGRS4) | silent (Thr37) | 9871 | - | | 340617 | TGCG | T | 2394.73 | DEL | Rv0280 (PPE3) |  |  | - | | 346275 | C | G | 1619.77 | SNP | Rv0284 (eccC3) | Pro214Arg | 4 | - | | 346564 | G | A | 1397.77 | SNP | Rv0284 (eccC3) | silent (Ala310) | 9867 | - | | 356528 | A | G | 1355.77 | SNP | Rv0292 (eccE3) | Asn217Asp | 42 | - | | 368087 | AGCTGCCGGTGTTGAT | A | 4063.73 | DEL | Rv0304c (PPE5) |  |  | - | | 370614 | GAT | G | 1610.73 | DEL | Rv0304c (PPE5) |  |  | - | | 373282 | TA | T | 1584.73 | DEL | Rv0305c (PPE6) |  |  | - | | 377780 | C | T | 1723.77 | SNP | Rv0308 | Pro223Leu | 3 | - | | 384380 | A | C | 1468.77 | SNP | Rv0315 | Lys260Thr | 8 | - | | 386432 | C | G | 1744.77 | SNP | Rv0318c | Gly223Ala | 21 | - | | 390828 | T | C | 1945.77 | SNP | Rv0323c | Ser142Gly | 21 | - | | 394461 | G | A | 995.77 | SNP | Rv0328 | silent (Glu117) | 9865 | - | | 403980 | G | A | 2441.77 | SNP | Rv0338c | Ala621Val | 13 | - | | 409179 | G | T | 1641.77 | SNP | intergenic |  |  | - | | 413717 | G | A | 1336.77 | SNP | Rv0343 (iniC) | Val(s)321Met(s) | 9867 | - | | 414486 | C | T | 1649.77 | SNP | Rv0344c (lpqJ) | silent (Glu152) | 9865 | - | | 417153 | C | T | 1669.77 | SNP | intergenic |  |  | - | | 420008 | A | G | 1714.77 | SNP | Rv0350 (dnaK) | silent (Ala58) | 9867 | - | | 424320 | T | TC | 1921.73 | INS | Rv0354c (PPE7) |  |  | - | | 427310 | TTGCCGAGGTTTGCAC | T | 3787.73 | DEL | Rv0355c (PPE8) |  |  | - | | 434784 | G | GCA | 3045.73 | INS | intergenic |  |  | - | | 454295 | T | C | 1552.77 | SNP | Rv0376c | silent (Pro26) | 9926 | - | | 459399 | A | C | 1823.77 | SNP | intergenic |  |  | - | | 467497 | C | CG | 1138.73 | INS | Rv0388c (PPE9) |  |  | - | | 467508 | C | CG | 1195.73 | INS | Rv0388c (PPE9) |  |  | - | | 467516 | G | C | 700.77 | SNP | Rv0388c (PPE9) | silent (Ser162) | 9840 | - | | 467526 | C | G | 887.77 | SNP | Rv0388c (PPE9) | Gly159Ala | 21 | - | | 467546 | G | C | 1090.77 | SNP | Rv0388c (PPE9) | Asp152Glu | 56 | - | | 467557 | A | C | 997.77 | SNP | Rv0388c (PPE9) | Leu(s)149Val(s) | 9867 | - | | 467564 | A | C | 1083.77 | SNP | Rv0388c (PPE9) | His146Gln | 23 | - | | 467585 | G | C | 1312.77 | SNP | Rv0388c (PPE9) | His139Gln | 23 | - | | 467590 | T | C | 1308.77 | SNP | Rv0388c (PPE9) | Thr138Ala | 32 | - | | 467621 | T | G | 1375.77 | SNP | Rv0388c (PPE9) | silent (Gly127) | 9935 | - | | 467638 | G | T | 1241.77 | SNP | Rv0388c (PPE9) | Gln122Lys | 12 | - | | 472711 | T | TTTGTGGGCC | 3026.73 | INS | intergenic |  |  | - | | 475178 | T | C | 1273.77 | SNP | Rv0395 | Val80Ala | 18 | - | | 487573 | C | G | 644.77 | SNP | Rv0405 (pks6) | Arg615Gly | 1 | - | | 493699 | C | G | 2643.77 | SNP | Rv0408 (pta) | silent (Thr638) | 9871 | - | | 499224 | C | T | 1594.77 | SNP | Rv0412c | silent (Ser132) | 9840 | - | | 502589 | C | G | 1694.77 | SNP | Rv0417 (thiG) | Ser75Cys | 5 | - | | 503354 | G | C | 2130.77 | SNP | intergenic |  |  | - | | 513257 | T | C | 1551.77 | SNP | Rv0425c (ctpH) | Met(s)689Val(s) | 9867 | - | | 515412 | A | G | 1231.77 | SNP | Rv0426c | silent (Gly135) | 9935 | - | | 533539 | C | T | 953.77 | SNP | Rv0444c (rskA) | Arg84His | 8 | - | | 537663 | C | T | 1400.77 | SNP | Rv0449c | Ala296Thr | 22 | - | | 541201 | A | G | 1768.77 | SNP | Rv0450c (mmpL4) | silent (Leu97) | 9947 | - | | 573262 | A | G | 1355.77 | SNP | Rv0484c | silent (Gly180) | 9935 | - | | 580772 | T | A | 494.77 | SNP | intergenic |  |  | - | | 580773 | GGGGGCACCACCCGCTTGCG GGGGA | G | 5424.73 | DEL | intergenic |  |  | - | | 590436 | T | C | 1260.77 | SNP | Rv0500 (proC) | silent (Ala118) | 9867 | - | | 598475 | G | A | 1562.77 | SNP | Rv0507 (mmpL2) | Arg426His | 8 | - | | 610120 | T | G | 1740.77 | SNP | intergenic |  |  | - | | 623472 | A | G | 235.84 | SNP | Rv0532 (PE\_PGRS6) | Asp227Gly | 11 | - | | 623508 | C | G | 269.80 | SNP | Rv0532 (PE\_PGRS6) | Ala239Gly | 21 | - | | 632993 | G | A | 1726.77 | SNP | Rv0540 | Gly208Arg | 0 | - | | 634388 | C | T | 905.77 | SNP | Rv0541c | Arg6His | 8 | - | | 648002 | T | G | 1861.77 | SNP | Rv0556 | Leu15Arg | 1 | - | | 655163 | C | T | 1272.77 | SNP | Rv0564c (gpdA1) | Val(s)263Met(s) | 9867 | - | | 658554 | C | A | 2014.77 | SNP | Rv0567 | Tyr78STOP | 2 | - | | 666565 | C | A | 1494.77 | SNP | Rv0573c (pncB2) | Met(s)226Ile | 2 | - | | 669398 | T | C | 1704.77 | SNP | Rv0575c | silent (Gln116) | 9876 | - | | 672491 | C | G | 393.77 | SNP | Rv0578c (PE\_PGRS7) | silent (Gly1142) | 9935 | - | | 674623 | C | T | 171.90 | SNP | Rv0578c (PE\_PGRS7) | Asp432Asn | 36 | - | | 685461 | C | G | 1378.77 | SNP | Rv0587 (yrbE2A) | silent (Ala111) | 9867 | - | | 685608 | T | C | 1403.77 | SNP | Rv0587 (yrbE2A) | silent (Leu160) | 9947 | - | | 685627 | A | G | 1435.77 | SNP | Rv0587 (yrbE2A) | Ile167Val | 57 | - | | 685637 | T | C | 1372.77 | SNP | Rv0587 (yrbE2A) | Val170Ala | 18 | - | | 686972 | T | C | 1996.77 | SNP | Rv0589 (mce2A) | Phe51Ser | 3 | - | | 690167 | C | T | 1684.77 | SNP | Rv0591 (mce2C) | Pro370Leu | 3 | - | | 690465 | T | G | 792.77 | SNP | Rv0591 (mce2C) | silent (Leu469) | 9947 | - | | 694565 | T | C | 1696.77 | SNP | Rv0594 (mce2F) | silent (Pro443) | 9926 | - | | 695154 | A | C | 1391.77 | SNP | Rv0595c (vapC4) | Phe26Leu(s) | 2 | - | | 698968 | G | A | 884.77 | SNP | Rv0601c | silent (Gly9) | 9935 | - | | 707919 | C | T | 1978.77 | SNP | Rv0613c | Gly533Ser | 16 | - | | 709367 | T | C | 1133.77 | SNP | Rv0613c; Rv0614 | Glu50Gly; silent (Ile4) | 7; 9872 | - | | 716918 | G | A | 1645.77 | SNP | Rv0624 (vapC30) | silent (Thr85) | 9871 | - | | 754186 | A | G | 1335.77 | SNP | Rv0658c | Leu75Pro | 2 | - | | 758252 | T | C | 1554.77 | SNP | Rv0663 (atsD) | Tyr706His | 4 | - | | 761139 | C | G | 1463.77 | SNP | Rv0667 (rpoB) | His445Asp | 4 | resistance | | 771927 | T | G | 1495.77 | SNP | Rv0672 (fadE8) | silent (Ala148) | 9867 | - | | 772023 | C | T | 1247.77 | SNP | Rv0672 (fadE8) | silent (Thr180) | 9871 | - | | 775639 | T | C | 1518.77 | SNP | Rv0676c (mmpL5) | Ile948Val | 57 | - | | 775715 | C | T | 1979.77 | SNP | Rv0676c (mmpL5) | silent (Arg922) | 9913 | - | | 781395 | T | C | 1928.77 | SNP | intergenic (Rv0682-165nt) |  |  | - | | 782260 | G | A | 1297.77 | SNP | Rv0683 (rpsG) | silent (Arg109) | 9913 | - | | 796876 | A | G | 2332.77 | SNP | Rv0696 | Tyr453Cys | 3 | - | | 827045 | G | A | 877.77 | SNP | Rv0734 (mapA) | Ala126Thr | 22 | - | | 827770 | G | T | 1703.77 | SNP | Rv0735 (sigL) | Met(s)76Ile | 2 | - | | 836538 | A | G | 92.28 | SNP | Rv0746 (PE\_PGRS9) | Asn280Asp | 42 | - | | 836658 | A | G | 106.28 | SNP | Rv0746 (PE\_PGRS9) | Thr320Ala | 32 | - | | 837033 | A | G | 494.77 | SNP | Rv0746 (PE\_PGRS9) | Thr445Ala | 32 | - | | 839269 | A | G | 150.77 | SNP | Rv0747 (PE\_PGRS10) | silent (Gly273) | 9935 | - | | 839279 | G | A | 237.77 | SNP | Rv0747 (PE\_PGRS10) | Asp277Asn | 36 | - | | 839284 | C | G | 60.77 | SNP | Rv0747 (PE\_PGRS10) | silent (Ala278) | 9867 | - | | 839291 | T | C | 121.77 | SNP | Rv0747 (PE\_PGRS10) | Phe281Leu | 13 | - | | 839295 | T | C | 102.77 | SNP | Rv0747 (PE\_PGRS10) | Phe282Ser | 3 | - | | 839309 | T | G | 61.77 | SNP | Rv0747 (PE\_PGRS10) | Ser287Ala | 35 | - | | 839334 | A | G | 146.03 | SNP | Rv0747 (PE\_PGRS10) | Lys295Arg | 19 | - | | 839348 | A | G | 106.28 | SNP | Rv0747 (PE\_PGRS10) | Ser300Gly | 21 | - | | 839515 | G | A | 108.77 | SNP | Rv0747 (PE\_PGRS10) | silent (Ala355) | 9867 | - | | 839516 | A | G | 115.77 | SNP | Rv0747 (PE\_PGRS10) | Thr356Ala | 32 | - | | 839519 | C | G | 85.77 | SNP | Rv0747 (PE\_PGRS10) | Leu357Val(s) | 4 | - | | 839520 | T | C | 114.77 | SNP | Rv0747 (PE\_PGRS10) | Leu357Pro | 2 | - | | 839534 | A | C | 122.77 | SNP | Rv0747 (PE\_PGRS10) | Ile362Leu | 22 | - | | 840599 | G | A | 52.74 | SNP | Rv0747 (PE\_PGRS10) | Ala717Thr | 22 | - | | 841085 | C | T | 1225.77 | SNP | Rv0748 (vapB31) | Arg47Cys | 1 | - | | 846764 | T | C | 530.77 | SNP | Rv0754 (PE\_PGRS11) | silent (Phe202) | 9946 | - | | 852910 | C | T | 1448.77 | SNP | Rv0758 (phoR) | Pro172Leu | 3 | - | | 854252 | GC | G | 1672.73 | DEL | intergenic |  |  | - | | 860323 | C | T | 695.77 | SNP | Rv0767c | Asp130Asn | 36 | - | | 867442 | A | G | 1307.77 | SNP | Rv0774c | STOP304Gln | 3 | - | | 874835 | C | CCG | 5021.73 | INS | Rv0781 (ptrBa); Rv0782 (ptrBb) |  |  | - | | 882257 | T | C | 1746.77 | SNP | Rv0787 | Tyr267His | 4 | - | | 890549 | G | A | 230.77 | SNP | Rv0797 | Trp54STOP | 0 | - | | 893733 | T | G | 2043.77 | SNP | Rv0800 (pepC) | Leu139Arg | 1 | - | | 903913 | T | C | 1393.77 | SNP | Rv0809 (purM) | silent (Gly63) | 9935 | - | | 906857 | A | G | 1210.77 | SNP | Rv0812 | Ile145Met(s) | 6 | - | | 919426 | G | GT | 2234.73 | INS | Rv0825c |  |  | - | | 919559 | T | G | 1607.77 | SNP | intergenic |  |  | - | | 921813 | C | G | 1908.77 | SNP | Rv0829 | Ala80Gly | 21 | - | | 927110 | A | G | 55.74 | SNP | Rv0833 (PE\_PGRS13) | Ser584Gly | 21 | - | | 929019 | C | T | 147.90 | SNP | Rv0834c (PE\_PGRS14) | silent (Gly489) | 9935 | - | | 932093 | A | C | 1772.77 | SNP | intergenic |  |  | - | | 955524 | A | G | 1589.77 | SNP | Rv0859 (fadA) | Ser150Gly | 21 | - | | 958487 | C | T | 841.77 | SNP | intergenic |  |  | - | | 967797 | C | T | 1742.77 | SNP | intergenic |  |  | - | | 968426 | A | AGCCGGGTTG | 1331.87 | INS | Rv0872c (PE\_PGRS15) |  |  | - | | 976092 | G | A | 1561.77 | SNP | Rv0877 | silent (Glu6) | 9865 | - | | 979704 | G | C | 1875.77 | SNP | Rv0881 | Gly115Arg | 0 | - | | 986463 | G | C | 2231.77 | SNP | intergenic |  |  | - | | 990001 | G | C | 1226.77 | SNP | Rv0890c | Pro866Ala | 22 | - | | 993346 | A | C | 1445.77 | SNP | Rv0891c | Val37Gly | 5 | - | | 1010204 | C | CG | 1982.73 | INS | Rv0907 |  |  | - | | 1025106 | T | C | 1827.77 | SNP | Rv0919 | silent (Phe141) | 9946 | - | | 1031354 | C | A | 1060.77 | SNP | Rv0924c (mntH) | Val171Phe | 0 | - | | 1032504 | A | G | 2342.77 | SNP | Rv0925c | Ser44Pro | 12 | - | | 1034314 | G | T | 1423.77 | SNP | Rv0927c | silent (Thr106) | 9871 | - | | 1037911 | C | T | 1517.77 | SNP | Rv0930 (pstA1) | Arg305STOP | 2 | - | | 1040016 | C | T | 1493.77 | SNP | Rv0932c (pstS2) | Gly345Ser | 16 | - | | 1044726 | A | G | 1975.77 | SNP | Rv0936 (pstA2) | Tyr137Cys | 3 | - | | 1067775 | C | A | 1273.77 | SNP | Rv0956 (purN) | Pro72His | 3 | - | | 1068151 | T | C | 1712.77 | SNP | Rv0956 (purN) | silent (His197) | 9912 | - | | 1074558 | G | A | 1674.77 | SNP | Rv0962c (lprP) | Pro186Leu | 3 | - | | 1076309 | G | T | 1381.77 | SNP | Rv0964c | Pro124Thr | 5 | - | | 1077312 | A | G | 1450.77 | SNP | Rv0966c | Val(s)175Ala | 9867 | - | | 1079927 | C | A | 1619.77 | SNP | Rv0969 (ctpV) | silent (Thr395) | 9871 | - | | 1081681 | T | C | 1619.77 | SNP | Rv0970 | silent (Val210) | 9901 | - | | 1093406 | A | G | 998.77 | SNP | Rv0978c (PE\_PGRS17) | silent (Val317) | 9901 | - | | 1093928 | G | A | 79.28 | SNP | Rv0978c (PE\_PGRS17) | silent (Asn143) | 9822 | - | | 1094228 | T | G | 112.03 | SNP | Rv0978c (PE\_PGRS17) | Gln43His | 20 | - | | 1094692 | G | A | 1434.77 | SNP | Rv0979c | Pro58Leu | 3 | - | | 1096633 | T | G | 1379.77 | SNP | intergenic |  |  | - | | 1100234 | T | C | 1912.77 | SNP | Rv0983 (pepD) | Leu390Pro | 2 | - | | 1103124 | G | T | 1972.77 | SNP | Rv0987 | Asp195Tyr | 0 | - | | 1109975 | A | G | 1899.77 | SNP | Rv0993 (galU) | Gln235Arg | 10 | - | | 1110064 | T | G | 1550.77 | SNP | Rv0993 (galU) | Ser265Ala | 35 | - | | 1126889 | G | C | 2327.77 | SNP | Rv1007c (metS) | Arg39Gly | 1 | - | | 1127648 | C | A | 1809.77 | SNP | Rv1008 (tatD) | Thr187Asn | 9 | - | | 1133309 | T | C | 1552.77 | SNP | intergenic |  |  | - | | 1149269 | CG | C | 2735.73 | DEL | Rv1028c (kdpD) |  |  | - | | 1150585 | G | A | 1176.77 | SNP | Rv1028c (kdpD) | Pro368Ser | 17 | - | | 1163134 | T | C | 1296.77 | SNP | Rv1040c (PE8) | silent (Gly81) | 9935 | - | | 1165521 | T | TA | 2761.73 | INS | intergenic |  |  | - | | 1168715 | C | CT | 1958.73 | INS | Rv1046c |  |  | - | | 1178959 | T | G | 39.77 | SNP | intergenic |  |  | - | | 1188319 | G | T | 367.77 | SNP | Rv1066 | Asp108Tyr | 0 | - | | 1200612 | T | C | 1526.77 | SNP | intergenic |  |  | - | | 1220680 | T | C | 2048.77 | SNP | Rv1093 (glyA1) | Val36Ala | 18 | - | | 1222682 | G | A | 1138.77 | SNP | Rv1094 (desA2) | Ala242Thr | 22 | - | | 1224367 | T | C | 799.77 | SNP | intergenic |  |  | - | | 1234605 | C | T | 898.80 | SNP | Rv1108c (xseA) | Ala286Thr | 22 | - | | 1248978 | T | C | 1210.77 | SNP | Rv1125 | silent (Ala299) | 9867 | - | | 1253847 | C | T | 1273.77 | SNP | Rv1129c | Ala230Thr | 22 | - | | 1258059 | A | G | 1473.77 | SNP | Rv1132 | silent (Thr245) | 9871 | - | | 1263093 | TGTTGTTGTTGCCGAGATTC GCAATGCCCAGGTTGTTGTT GCCGAGATTCGCAATGCCCA G | T | 2414.73 | DEL | Rv1135c (PPE16) |  |  | - | | 1281118 | T | C | 1303.77 | SNP | Rv1154c | Thr123Ala | 32 | - | | 1282968 | GC | G | 1886.73 | DEL | intergenic |  |  | - | | 1313337 | A | AG | 2658.73 | INS | intergenic |  |  | - | | 1313338 | A | C | 1541.77 | SNP | intergenic |  |  | - | | 1315191 | A | C | 1747.77 | SNP | Rv1180 (pks3) | STOP489Tyr | 1 | - | | 1315884 | G | A | 1112.77 | SNP | Rv1181 (pks4) | silent (Ala217) | 9867 | - | | 1328687 | G | C | 1369.77 | SNP | Rv1186c | Pro207Ala | 22 | - | | 1331655 | G | A | 1259.77 | SNP | Rv1188 | Arg212Gln | 9 | - | | 1340500 | G | T | 1017.77 | SNP | Rv1196 (PPE18) | Met(s)384Ile | 2 | - | | 1340664 | C | A | 361.28 | SNP | Rv1197 (esxK) | silent (Ala2) | 9867 | - | | 1340665 | T | A | 355.10 | SNP | Rv1197 (esxK) | Ser3Thr | 32 | - | | 1340784 | T | C | 314.77 | SNP | Rv1197 (esxK) | silent (Gly42) | 9935 | - | | 1340980 | TTTA | T | 549.74 | DEL | intergenic |  |  | - | | 1341023 | A | G | 184.80 | SNP | Rv1198 (esxL) | silent (Gln6) | 9876 | - | | 1341044 | C | T | 248.78 | SNP | Rv1198 (esxL) | silent (His13) | 9912 | - | | 1341099 | A | G | 350.77 | SNP | Rv1198 (esxL) | Ile32Val | 57 | - | | 1341114 | A | G | 288.78 | SNP | Rv1198 (esxL) | Thr37Ala | 32 | - | | 1341120 | A | G | 313.78 | SNP | Rv1198 (esxL) | Ser39Gly | 21 | - | | 1341148 | C | T | 525.77 | SNP | Rv1198 (esxL) | Ala48Val(s) | 9867 | - | | 1341152 | C | T | 520.77 | SNP | Rv1198 (esxL) | silent (Ala49) | 9867 | - | | 1348256 | C | T | 809.77 | SNP | Rv1204c | silent (Ala123) | 9867 | - | | 1365837 | C | CG | 1782.73 | INS | intergenic |  |  | - | | 1374065 | T | C | 991.77 | SNP | Rv1230c | Ser45Gly | 21 | - | | 1375724 | A | C | 729.77 | SNP | Rv1232c | Cys149Gly | 1 | - | | 1382628 | T | C | 1215.77 | SNP | Rv1239c (corA) | Lys139Glu | 4 | - | | 1383310 | C | G | 1801.77 | SNP | Rv1240 (mdh) | Pro33Arg | 4 | - | | 1389076 | T | C | 1334.77 | SNP | Rv1247c (relB) | Thr57Ala | 32 | - | | 1396922 | T | C | 1693.77 | SNP | Rv1251c | silent (Thr773) | 9871 | - | | 1411210 | T | G | 1516.77 | SNP | Rv1263 (amiB2) | Val260Val(s) | 18 | - | | 1414021 | C | T | 1476.77 | SNP | Rv1266c (pknH) | Arg607Gln | 9 | - | | 1431552 | C | T | 1010.77 | SNP | Rv1279 | silent (Val497) | 9901 | - | | 1438156 | C | T | 1317.77 | SNP | Rv1285 (cysD) | Ala83Val(s) | 9867 | - | | 1440469 | C | G | 1525.77 | SNP | Rv1286 (cysN) | silent (Pro521) | 9926 | - | | 1457144 | C | T | 841.77 | SNP | Rv1300 (hemK) | Arg194Cys | 1 | - | | 1461430 | T | C | 1527.77 | SNP | Rv1306 (atpF) | Ile37Thr | 11 | - | | 1471659 | C | T | 1785.77 | SNP | intergenic |  |  | - | | 1473314 | A | C | 122.77 | SNP | Rvnr01 | rRNA | rRNA | - | | 1480945 | C | G | 848.77 | SNP | Rv1319c | silent (Thr519) | 9871 | - | | 1480948 | C | T | 829.77 | SNP | Rv1319c | silent (Glu518) | 9865 | - | | 1482627 | T | C | 1055.77 | SNP | Rv1320c | Thr531Ala | 32 | - | | 1484036 | G | T | 1691.77 | SNP | Rv1320c | Thr61Lys | 11 | - | | 1484708 | A | C | 1516.77 | SNP | Rv1321 | Ser144Arg | 6 | - | | 1488433 | A | G | 142.77 | SNP | Rv1325c (PE\_PGRS24) | silent (Asp511) | 9859 | - | | 1488434 | T | G | 117.77 | SNP | Rv1325c (PE\_PGRS24) | Asp511Ala | 10 | - | | 1488435 | C | A | 104.77 | SNP | Rv1325c (PE\_PGRS24) | Asp511Tyr | 0 | - | | 1490804 | C | T | 3074.77 | SNP | Rv1326c (glgB) | silent (Pro503) | 9926 | - | | 1499274 | C | G | 825.77 | SNP | Rv1330c (pncB1) | Gly429Ala | 21 | - | | 1510293 | C | T | 1710.77 | SNP | Rv1345 (mbtM) | Ala338Val(s) | 9867 | - | | 1536251 | G | T | 1615.77 | SNP | Rv1364c | Ala465Glu | 10 | - | | 1547125 | T | C | 2019.77 | SNP | Rv1374c | Thr136Ala | 32 | - | | 1552547 | G | A | 1218.77 | SNP | Rv1378c | Arg37Trp | 2 | - | | 1562298 | T | C | 2024.77 | SNP | Rv1387 (PPE20) | Leu177Pro | 2 | - | | 1568843 | A | C | 1274.77 | SNP | Rv1393c | Tyr249Asp | 0 | - | | 1575672 | CG | C | 1418.73 | DEL | Rv1399c (nlhH) |  |  | - | | 1585223 | C | A | 1680.77 | SNP | Rv1409 (ribG) | Asp10Glu | 56 | - | | 1589842 | G | T | 1168.77 | SNP | Rv1413 | Ala153Ser | 28 | - | | 1602635 | G | C | 1418.77 | SNP | Rv1427c (fadD12) | Arg432Gly | 1 | - | | 1609501 | G | C | 1168.77 | SNP | Rv1431 | silent (Leu473) | 9947 | - | | 1612624 | T | TATCGGTACCGGTGCGCCAG GG | 4787.73 | INS | Rv1435c |  |  | - | | 1613035 | T | C | 1279.77 | SNP | intergenic |  |  | - | | 1617259 | C | G | 1244.77 | SNP | Rv1439c | Arg43Thr | 2 | - | | 1634346 | G | T | 735.77 | SNP | Rv1450c (PE\_PGRS27) | Asn94Lys | 25 | - | | 1636826 | C | A | 602.77 | SNP | Rv1452c (PE\_PGRS28) | silent (Gly468) | 9935 | - | | 1636918 | C | T | 143.77 | SNP | Rv1452c (PE\_PGRS28) | Ala438Thr | 22 | - | | 1636927 | C | T | 94.77 | SNP | Rv1452c (PE\_PGRS28) | Asp435Asn | 36 | - | | 1636928 | A | G | 102.77 | SNP | Rv1452c (PE\_PGRS28) | silent (Gly434) | 9935 | - | | 1636934 | A | G | 107.77 | SNP | Rv1452c (PE\_PGRS28) | silent (His432) | 9912 | - | | 1636936 | G | T | 84.78 | SNP | Rv1452c (PE\_PGRS28) | His432Asn | 21 | - | | 1636944 | G | T | 64.77 | SNP | Rv1452c (PE\_PGRS28) | Ala429Asp | 6 | - | | 1636945 | C | G | 99.77 | SNP | Rv1452c (PE\_PGRS28) | Ala429Pro | 13 | - | | 1636946 | C | A | 82.77 | SNP | Rv1452c (PE\_PGRS28) | silent (Gly428) | 9935 | - | | 1636980 | G | T | 194.77 | SNP | Rv1452c (PE\_PGRS28) | Pro417His | 3 | - | | 1636981 | G | T | 196.77 | SNP | Rv1452c (PE\_PGRS28) | Pro417Thr | 5 | - | | 1636983 | C | G | 192.82 | SNP | Rv1452c (PE\_PGRS28) | Gly416Ala | 21 | - | | 1639594 | C | A | 1241.77 | SNP | Rv1453 | Pro405Gln | 6 | - | | 1641132 | C | T | 1114.77 | SNP | Rv1455 | silent (Val151) | 9901 | - | | 1645980 | C | T | 1545.77 | SNP | Rv1459c | silent (Leu53) | 9947 | - | | 1650072 | A | G | 791.77 | SNP | Rv1462 | Asn183Asp | 42 | - | | 1689349 | C | T | 1060.77 | SNP | Rv1498c | Arg191His | 8 | - | | 1693561 | A | G | 2621.77 | SNP | Rv1502 | Tyr213Cys | 3 | - | | 1698911 | G | A | 1981.77 | SNP | Rv1508c | silent (Gly328) | 9935 | - | | 1706119 | T | C | 1621.77 | SNP | Rv1514c | silent (Ser159) | 9840 | - | | 1728837 | A | G | 1503.77 | SNP | intergenic |  |  | - | | 1744835 | A | G | 1395.77 | SNP | Rv1542c (glbN) | Met(s)1Thr | 22 | - | | 1752561 | T | C | 859.77 | SNP | Rv1548c (PPE21) | Asp258Gly | 11 | - | | 1753519 | G | GC | 1282.73 | INS | Rv1549 (fadD11.1) |  |  | - | | 1759252 | G | T | 1776.77 | SNP | Rv1552 (frdA) | silent (Ser524) | 9840 | genotype | | 1760292 | A | G | 1588.77 | SNP | Rv1554 (frdC) | Met(s)40Val(s) | 9867 | - | | 1778430 | T | C | 1381.77 | SNP | Rv1570 (bioD) | Met(s)191Thr | 22 | - | | 1779370 | G | C | 1613.77 | SNP | Rv1573 | silent (Thr19) | 9871 | - | | 1780586 | C | CG | 2179.73 | INS | Rv1575 |  |  | - | | 1803265 | G | A | 1704.77 | SNP | Rv1602 (hisH) | Ser201Asn | 20 | - | | 1811776 | A | C | 1505.77 | SNP | Rv1612 (trpB) | Asp217Ala | 10 | - | | 1817976 | A | T | 1828.77 | SNP | Rv1618 (tesB1) | His121Leu | 4 | - | | 1824726 | T | C | 1877.77 | SNP | Rv1623c (cydA) | Asn388Asp | 42 | - | | 1830116 | G | A | 1152.77 | SNP | Rv1628c | Ala149Val | 13 | - | | 1836286 | G | C | 1110.77 | SNP | intergenic |  |  | - | | 1848223 | C | T | 1650.77 | SNP | Rv1639c | Gly79Asp | 6 | - | | 1850250 | A | C | 1458.77 | SNP | Rv1640c (lysX) | Tyr596Asp | 0 | - | | 1855645 | G | A | 1603.77 | SNP | intergenic |  |  | - | | 1856777 | G | C | 1327.77 | SNP | Rv1647 | Ala2Pro | 13 | - | | 1868432 | C | G | 53.77 | SNP | Rv1654 (argB) | silent (Ala197) | 9867 | - | | 1872003 | T | G | 1097.77 | SNP | Rv1658 (argG) | Val214Gly | 5 | - | | 1879671 | T | C | 259.77 | SNP | Rv1661 (pks7) | silent (Gly1456) | 9935 | - | | 1879885 | G | C | 872.77 | SNP | Rv1661 (pks7) | Val1528Leu | 15 | - | | 1885772 | G | A | 838.77 | SNP | Rv1662 (pks8) | Ala1357Thr | 22 | - | | 1894300 | G | GGTCTTGCCGC | 4528.73 | INS | Rv1668c |  |  | - | | 1899295 | A | G | 1405.77 | SNP | Rv1674c | Trp208Arg | 8 | - | | 1901285 | T | C | 1573.77 | SNP | Rv1676 | Phe80Ser | 3 | - | | 1901493 | T | C | 1032.77 | SNP | Rv1676 | silent (Ser149) | 9840 | - | | 1907296 | G | C | 2022.77 | SNP | Rv1682 | silent (Ala298) | 9867 | - | | 1908018 | A | C | 1316.77 | SNP | Rv1683 | His142Pro | 5 | - | | 1917972 | A | G | 1242.77 | SNP | Rv1694 (tlyA) | silent (Leu11) | 9947 | - | | 1930144 | G | GCGGTTT | 4331.73 | INS | Rv1704c (cycA) |  |  | - | | 1931179 | C | A | 861.77 | SNP | Rv1704c (cycA) | Arg93Leu | 1 | - | | 1933988 | G | A | 2086.77 | SNP | intergenic |  |  | - | | 1944372 | G | A | 1455.77 | SNP | Rv1716 | Gly266Glu | 4 | - | | 1944402 | T | C | 1222.77 | SNP | Rv1716 | Val276Ala | 18 | - | | 1950767 | T | C | 2018.77 | SNP | Rv1724c | silent (Lys95) | 9926 | - | | 1967237 | C | A | 1418.77 | SNP | Rv1739c | Arg134Leu | 1 | - | | 1969269 | T | G | 1170.77 | SNP | Rv1743 (pknE) | Leu89Arg | 1 | - | | 2008257 | G | A | 1279.77 | SNP | Rv1774 | Trp142STOP | 0 | - | | 2022868 | T | C | 1150.77 | SNP | Rv1783 (eccC5) | silent (Ser1204) | 9840 | - | | 2030848 | A | G | 49.77 | SNP | Rv1793 (esxN) | Glu52Gly | 7 | - | | 2035650 | G | T | 1031.77 | SNP | Rv1797 (eccE5) | silent (Thr56) | 9871 | - | | 2038847 | G | A | 1442.77 | SNP | intergenic |  |  | - | | 2049001 | T | G | 1204.77 | SNP | intergenic |  |  | - | | 2049065 | T | C | 1553.77 | SNP | intergenic |  |  | - | | 2051746 | T | C | 1304.77 | SNP | Rv1809 (PPE33) | silent (Ala155) | 9867 | - | | 2052035 | G | T | 1563.77 | SNP | Rv1809 (PPE33) | Val(s)252Leu(s) | 9867 | - | | 2055271 | A | G | 2068.77 | SNP | Rv1812c | Leu30Pro | 2 | - | | 2057774 | A | T | 765.77 | SNP | Rv1815 | Ile83Phe | 8 | - | | 2096186 | A | G | 1112.77 | SNP | Rv1846c (blaI) | silent (Thr138) | 9871 | - | | 2109523 | C | CG | 2963.73 | INS | intergenic |  |  | - | | 2116903 | C | T | 1435.77 | SNP | Rv1867 | silent (Gly380) | 9935 | - | | 2118131 | G | A | 1044.77 | SNP | Rv1868 | Gly262Glu | 4 | - | | 2123119 | G | T | 1578.77 | SNP | Rv1872c (lldD2) | silent (Arg11) | 9913 | - | | 2123146 | C | T | 1676.77 | SNP | Rv1872c (lldD2) | silent (Ala2) | 9867 | - | | 2123169 | T | G | 1848.77 | SNP | intergenic |  |  | - | | 2128870 | A | G | 1239.77 | SNP | Rv1878 (glnA3) | silent (Leu283) | 9947 | - | | 2135900 | T | G | 97.77 | SNP | intergenic |  |  | - | | 2143328 | G | C | 1516.77 | SNP | Rv1895 | Val(s)270Leu | 3 | - | | 2155168 | C | G | 1216.77 | SNP | Rv1908c (katG) | Ser315Thr | 32 | resistance | | 2163375 | T | C | 273.77 | SNP | Rv1917c (PPE34) | Asn1313Asp | 42 | - | | 2163412 | A | G | 131.77 | SNP | Rv1917c (PPE34) | silent (Val1300) | 9901 | - | | 2163417 | G | C | 115.77 | SNP | Rv1917c (PPE34) | Pro1299Ala | 22 | - | | 2163419 | C | T | 64.77 | SNP | Rv1917c (PPE34) | Ser1298Asn | 20 | - | | 2163790 | A | C | 817.77 | SNP | Rv1917c (PPE34) | silent (Pro1174) | 9926 | - | | 2165286 | A | C | 940.77 | SNP | Rv1917c (PPE34) | Ser676Ala | 35 | - | | 2165479 | G | A | 60.77 | SNP | Rv1917c (PPE34) | silent (Gly611) | 9935 | - | | 2176022 | C | T | 2209.77 | SNP | Rv1923 (lipD) | Gln284STOP | 8 | - | | 2176312 | C | T | 2153.77 | SNP | Rv1923 (lipD) | silent (Asp380) | 9859 | - | | 2207591 | T | TC | 3387.73 | INS | intergenic |  |  | - | | 2211826 | A | G | 976.77 | SNP | Rv1968 (mce3C) | silent (Lys67) | 9926 | - | | 2213103 | C | T | 617.77 | SNP | Rv1969 (mce3D) | silent (Tyr83) | 9945 | - | | 2216443 | C | A | 1197.77 | SNP | Rv1971 (mce3F) | Ala396Glu | 10 | - | | 2220512 | T | G | 1590.77 | SNP | Rv1977 | silent (Ser253) | 9840 | - | | 2223293 | T | C | 1752.77 | SNP | intergenic |  |  | - | | 2228967 | A | G | 1047.77 | SNP | intergenic |  |  | - | | 2251999 | A | G | 1108.77 | SNP | intergenic |  |  | - | | 2260151 | A | G | 573.77 | SNP | intergenic |  |  | - | | 2260154 | C | T | 506.77 | SNP | intergenic |  |  | - | | 2260171 | T | C | 927.77 | SNP | intergenic |  |  | - | | 2260174 | C | T | 917.77 | SNP | intergenic |  |  | - | | 2260196 | C | CA | 1284.73 | INS | intergenic |  |  | - | | 2260199 | C | T | 926.77 | SNP | intergenic |  |  | - | | 2260212 | G | T | 575.77 | SNP | intergenic |  |  | - | | 2260214 | G | C | 563.77 | SNP | intergenic |  |  | - | | 2260220 | C | T | 484.77 | SNP | intergenic |  |  | - | | 2260222 | C | G | 465.77 | SNP | intergenic |  |  | - | | 2260231 | T | C | 587.77 | SNP | intergenic |  |  | - | | 2260525 | C | T | 1440.77 | SNP | intergenic |  |  | - | | 2261540 | G | GAC | 2495.73 | INS | Rv2014 |  |  | - | | 2264782 | C | A | 1346.77 | SNP | Rv2017 | Ala262Glu | 10 | - | | 2266487 | G | C | 1436.77 | SNP | Rv2020c | silent (Leu78) | 9947 | - | | 2266504 | T | TA | 2416.73 | INS | Rv2020c |  |  | - | | 2266508 | A | T | 1041.77 | SNP | Rv2020c | Asp71Glu | 56 | - | | 2266511 | GT | G | 1849.73 | DEL | Rv2020c |  |  | - | | 2266517 | T | C | 1128.77 | SNP | Rv2020c | silent (Glu68) | 9865 | - | | 2266550 | G | T | 1172.77 | SNP | Rv2020c | silent (Gly57) | 9935 | - | | 2266553 | C | G | 1206.77 | SNP | Rv2020c | silent (Ser56) | 9840 | - | | 2266583 | C | G | 1294.77 | SNP | Rv2020c | Glu46Asp | 53 | - | | 2266598 | G | C | 1071.77 | SNP | Rv2020c | silent (Leu41) | 9947 | - | | 2266604 | C | G | 1038.77 | SNP | Rv2020c | silent (Ser39) | 9840 | - | | 2266613 | G | GC | 1798.73 | INS | Rv2020c |  |  | - | | 2266624 | G | T | 1296.77 | SNP | Rv2020c | Leu33Ile | 9 | - | | 2269780 | T | C | 770.77 | SNP | Rv2024c | Asp154Gly | 11 | - | | 2270102 | A | G | 1490.77 | SNP | Rv2024c | Trp47Arg | 8 | - | | 2271246 | C | T | 1394.77 | SNP | Rv2025c | Arg168Lys | 37 | - | | 2282787 | C | T | 1334.77 | SNP | Rv2037c | Cys312Tyr | 3 | - | | 2285251 | C | A | 1833.77 | SNP | Rv2039c | Val131Phe | 0 | - | | 2287121 | A | G | 2040.77 | SNP | Rv2041c | silent (Asp242) | 9859 | - | | 2296042 | G | C | 1410.77 | SNP | Rv2048c (pks12) | Pro3649Ala | 22 | - | | 2300237 | A | G | 600.77 | SNP | Rv2048c (pks12) | silent (Ala2250) | 9867 | - | | 2309577 | G | A | 1202.77 | SNP | Rv2051c (ppm1) | silent (Gly393) | 9935 | - | | 2319092 | A | T | 775.77 | SNP | Rv2062c (cobN) | Phe554Leu | 13 | - | | 2323385 | G | C | 88.77 | SNP | Rv2066 (cobI) | Ala71Pro | 13 | - | | 2335494 | A | G | 1189.77 | SNP | Rv2079 | Tyr47Cys | 3 | - | | 2339710 | G | C | 299.78 | SNP | Rv2082 | silent (Ala334) | 9867 | - | | 2340621 | C | G | 1257.77 | SNP | Rv2082 | Pro638Arg | 4 | - | | 2341636 | C | G | 794.77 | SNP | Rv2083 | Leu256Val(s) | 4 | - | | 2343288 | G | A | 1482.77 | SNP | Rv2085 | Ala88Thr | 22 | - | | 2357162 | TCGC | T | 311.73 | DEL | intergenic |  |  | - | | 2362041 | C | A | 1277.77 | SNP | Rv2101 (helZ) | Pro601Gln | 6 | - | | 2368564 | TA | T | 2792.73 | DEL | intergenic |  |  | - | | 2378813 | A | G | 900.77 | SNP | Rv2119 | Tyr143Cys | 3 | - | | 2386389 | G | A | 1433.77 | SNP | Rv2125 | Gly33Ser | 16 | - | | 2387733 | T | C | 216.84 | SNP | Rv2126c (PE\_PGRS37) | silent (Glu80) | 9865 | - | | 2415656 | G | C | 993.77 | SNP | Rv2155c (murD) | Arg247Gly | 1 | - | | 2419881 | T | C | 1362.77 | SNP | Rv2158c (murE) | Asn243Ser | 34 | - | | 2424925 | A | G | 837.77 | SNP | intergenic |  |  | - | | 2499726 | G | A | 1666.77 | SNP | Rv2226 | Asp299Asn | 36 | - | | 2509722 | A | G | 1859.77 | SNP | Rv2237 | silent (Pro78) | 9926 | - | | 2516216 | C | G | 1309.77 | SNP | Rv2242 | Arg305Gly | 1 | - | | 2521342 | T | C | 1060.77 | SNP | Rv2247 (accD6) | silent (Asp200) | 9859 | - | | 2523205 | G | GCGC | 3685.73 | INS | intergenic |  |  | - | | 2525722 | CG | C | 1648.73 | DEL | Rv2250A; Rv2251 |  |  | - | | 2532017 | G | C | 51.77 | SNP | intergenic |  |  | - | | 2534562 | GGA | G | 1956.73 | DEL | Rv2262c |  |  | - | | 2536892 | T | C | 37.77 | SNP | Rv2264c | Thr487Ala | 32 | - | | 2537142 | G | C | 834.77 | SNP | Rv2264c | silent (Arg403) | 9913 | - | | 2544815 | C | T | 1521.77 | SNP | Rv2270 (lppN) | Pro40Ser | 17 | - | | 2576090 | C | A | 1917.77 | SNP | Rv2305 | silent (Arg94) | 9913 | - | | 2582003 | G | A | 1216.77 | SNP | Rv2309c | Ala99Val | 13 | - | | 2586127 | A | G | 1388.77 | SNP | Rv2314c | silent (Gly388) | 9935 | - | | 2588544 | G | C | 1564.77 | SNP | Rv2315c | Val87Val(s) | 18 | - | | 2598400 | A | G | 1638.77 | SNP | Rv2326c | silent (Asn516) | 9822 | - | | 2600227 | A | G | 1769.77 | SNP | Rv2327 | Val80Val(s) | 18 | - | | 2611949 | T | C | 1565.77 | SNP | Rv2337c | Thr347Ala | 32 | - | | 2612632 | C | A | 1077.77 | SNP | Rv2337c | Gly119Val | 3 | - | | 2616990 | C | T | 2470.77 | SNP | Rv2339 (mmpL9) | silent (Thr766) | 9871 | - | | 2626004 | G | A | 1646.77 | SNP | Rv2346c (esxO) | Leu57Leu(s) | 4 | - | | 2626011 | G | A | 1492.77 | SNP | Rv2346c (esxO) | silent (Ile54) | 9872 | - | | 2626018 | T | C | 208.77 | SNP | Rv2346c (esxO) | Glu52Gly | 7 | - | | 2626056 | A | G | 84.77 | SNP | Rv2346c (esxO) | silent (Gly39) | 9935 | - | | 2626095 | C | G | 694.77 | SNP | Rv2346c (esxO) | silent (Ala26) | 9867 | - | | 2626149 | A | C | 510.77 | SNP | Rv2346c (esxO) | silent (Gly8) | 9935 | - | | 2626194 | GTAA | G | 5931.73 | DEL | intergenic |  |  | - | | 2626244 | G | C | 146.77 | SNP | Rv2347c (esxP) | silent (Ser92) | 9840 | - | | 2626247 | G | C | 79.77 | SNP | Rv2347c (esxP) | silent (Ala91) | 9867 | - | | 2626271 | G | A | 76.77 | SNP | Rv2347c (esxP) | silent (Asn83) | 9822 | - | | 2626274 | G | C | 85.77 | SNP | Rv2347c (esxP) | silent (Ala82) | 9867 | - | | 2626280 | G | A | 36.77 | SNP | Rv2347c (esxP) | silent (Arg80) | 9913 | - | | 2626283 | A | G | 94.77 | SNP | Rv2347c (esxP) | silent (Val79) | 9901 | - | | 2626288 | G | A | 37.77 | SNP | Rv2347c (esxP) | Leu78Leu(s) | 4 | - | | 2626295 | A | C | 111.77 | SNP | Rv2347c (esxP) | silent (Arg75) | 9913 | - | | 2626304 | G | A | 57.77 | SNP | Rv2347c (esxP) | silent (His72) | 9912 | - | | 2626397 | C | G | 298.77 | SNP | Rv2347c (esxP) | silent (Ser41) | 9840 | - | | 2626400 | G | A | 288.77 | SNP | Rv2347c (esxP) | silent (Ile40) | 9872 | - | | 2626600 | G | A | 92.77 | SNP | intergenic |  |  | - | | 2638997 | G | A | 783.77 | SNP | Rv2356c (PPE40) | Ser180Leu(s) | 35 | - | | 2650964 | A | C | 154.77 | SNP | Rv2370c | Ser199Ala | 35 | - | | 2656225 | A | G | 1569.77 | SNP | Rv2377c (mbtH) | Val69Ala | 18 | - | | 2718852 | T | G | 1439.77 | SNP | intergenic |  |  | - | | 2734074 | T | C | 688.77 | SNP | Rv2436 (rbsK) | Val282Ala | 18 | - | | 2751804 | C | T | 877.77 | SNP | Rv2450c (rpfE) | Arg126Gln | 9 | - | | 2752698 | C | A | 2184.77 | SNP | intergenic |  |  | - | | 2760152 | A | G | 1257.77 | SNP | Rv2458 (mmuM) | Tyr125Cys | 3 | - | | 2774944 | C | T | 915.77 | SNP | Rv2471 (aglA) | Arg461Cys | 1 | - | | 2779136 | T | C | 1275.77 | SNP | Rv2476c (gdh) | Ser1043Gly | 21 | - | | 2786952 | A | G | 1075.77 | SNP | Rv2482c (plsB2) | Cys778Arg | 1 | - | | 2794588 | A | C | 767.77 | SNP | Rv2486 (echA14) | Glu80Ala | 17 | - | | 2801672 | GC | G | 997.73 | DEL | Rv2490c (PE\_PGRS43) |  |  | - | | 2803137 | C | T | 222.88 | SNP | Rv2490c (PE\_PGRS43) | Ala1034Thr | 22 | - | | 2809621 | T | C | 1529.77 | SNP | Rv2495c (bkdC) | Thr107Ala | 32 | - | | 2818837 | A | G | 1340.77 | SNP | Rv2503c (scoB) | silent (Gly97) | 9935 | - | | 2827984 | G | T | 1557.77 | SNP | intergenic |  |  | - | | 2828019 | T | C | 1511.77 | SNP | intergenic |  |  | - | | 2828605 | T | C | 831.77 | SNP | Rv2512c | Glu400Gly | 7 | - | | 2830525 | C | A | 1478.77 | SNP | Rv2513 | Thr122Lys | 11 | - | | 2855259 | A | G | 1113.77 | SNP | Rv2531c | silent (Ala841) | 9867 | - | | 2865760 | A | G | 2304.77 | SNP | Rv2542 | Thr211Ala | 32 | - | | 2865882 | T | C | 2329.77 | SNP | Rv2542 | silent (Val251) | 9901 | - | | 2866489 | T | C | 907.77 | SNP | Rv2543 (lppA) | Ser8Pro | 12 | - | | 2866503 | G | A | 751.77 | SNP | Rv2543 (lppA) | silent (Pro12) | 9926 | - | | 2866569 | C | A | 159.90 | SNP | Rv2543 (lppA) | silent (Thr34) | 9871 | - | | 2866574 | A | G | 165.90 | SNP | Rv2543 (lppA) | Asp36Gly | 11 | - | | 2866575 | T | C | 165.90 | SNP | Rv2543 (lppA) | silent (Asp36) | 9859 | - | | 2866578 | C | A | 99.28 | SNP | Rv2543 (lppA) | His37Gln | 23 | - | | 2866584 | T | C | 165.90 | SNP | Rv2543 (lppA) | silent (Pro39) | 9926 | - | | 2866589 | C | A | 159.90 | SNP | Rv2543 (lppA) | Thr41Lys | 11 | - | | 2866595 | G | C | 165.90 | SNP | Rv2543 (lppA) | Arg43Pro | 5 | - | | 2866598 | G | A | 152.90 | SNP | Rv2543 (lppA) | Arg44His | 8 | - | | 2866642 | A | C | 51.74 | SNP | Rv2543 (lppA) | Asn59His | 18 | - | | 2866647 | G | A | 38.74 | SNP | Rv2543 (lppA) | silent (Lys60) | 9926 | - | | 2888201 | T | C | 1261.77 | SNP | Rv2566 | Leu610Pro | 2 | - | | 2889633 | T | C | 1535.77 | SNP | Rv2566 | silent (Ala1087) | 9867 | - | | 2891267 | C | T | 1293.77 | SNP | Rv2567 | silent (Gly491) | 9935 | - | | 2891728 | A | G | 1638.77 | SNP | Rv2567 | Gln645Arg | 10 | - | | 2892168 | G | A | 942.77 | SNP | Rv2567 | Glu792Lys | 7 | - | | 2894208 | G | A | 950.77 | SNP | Rv2569c | silent (Ser67) | 9840 | - | | 2894710 | G | A | 986.77 | SNP | Rv2570 | Gly67Arg | 0 | - | | 2910461 | G | T | 1098.77 | SNP | Rv2584c (apt) | Ala147Glu | 10 | - | | 2911293 | C | G | 1331.77 | SNP | Rv2585c | Cys462Ser | 11 | - | | 2912294 | T | G | 1565.77 | SNP | Rv2585c | silent (Ala128) | 9867 | - | | 2923391 | T | C | 839.77 | SNP | Rv2592c (ruvB) | silent (Pro281) | 9926 | - | | 2927939 | T | C | 1755.77 | SNP | intergenic |  |  | - | | 2939373 | G | C | 1422.77 | SNP | Rv2611c | Ser197Cys | 5 | - | | 2939657 | T | C | 983.77 | SNP | Rv2611c | Ile102Met(s) | 6 | - | | 2944833 | G | C | 109.03 | SNP | Rv2615c (PE\_PGRS45) | silent (Ala51) | 9867 | - | | 2944857 | T | G | 172.84 | SNP | Rv2615c (PE\_PGRS45) | Gln43His | 20 | - | | 2944932 | T | C | 260.78 | SNP | Rv2615c (PE\_PGRS45) | silent (Ala18) | 9867 | - | | 2951854 | CA | C | 1953.73 | DEL | Rv2625c |  |  | - | | 2954439 | T | C | 1586.77 | SNP | Rv2627c | Arg104Gly | 1 | - | | 2974933 | A | G | 785.77 | SNP | Rv2650c | Ile101Thr | 11 | - | | 2976594 | T | C | 1053.77 | SNP | Rv2653c | Lys106Glu | 4 | - | | 2984740 | A | G | 709.77 | SNP | Rv2668 | His3Arg | 10 | - | | 2995386 | G | C | 1050.77 | SNP | Rv2679 (echA15) | Arg91Pro | 5 | - | | 3005394 | C | G | 1406.77 | SNP | Rv2688c | Trp86Ser | 5 | - | | 3007400 | C | T | 1779.77 | SNP | Rv2690c | Val(s)604Met(s) | 9867 | - | | 3017465 | T | C | 1279.77 | SNP | Rv2702 (ppgK) | Ile203Thr | 11 | - | | 3018062 | G | A | 1256.77 | SNP | Rv2703 (sigA) | silent (Lys76) | 9926 | - | | 3026397 | G | T | 1805.77 | SNP | Rv2713 (sthA) | silent (Ala319) | 9867 | - | | 3054081 | A | G | 1595.77 | SNP | Rv2741 (PE\_PGRS47) | silent (Gly56) | 9935 | - | | 3054321 | A | G | 561.77 | SNP | Rv2741 (PE\_PGRS47) | silent (Gly136) | 9935 | - | | 3054724 | A | G | 49.74 | SNP | Rv2741 (PE\_PGRS47) | Ser271Gly | 21 | - | | 3071933 | T | C | 1661.77 | SNP | Rv2761c (hsdS) | silent (Lys236) | 9926 | - | | 3076853 | GGGAC | G | 3516.73 | DEL | intergenic |  |  | - | | 3080795 | A | G | 1362.77 | SNP | Rv2771c | Leu80Pro | 2 | - | | 3103682 | T | C | 1286.77 | SNP | Rv2794c (pptT) | Met(s)87Val(s) | 9867 | - | | 3111694 | A | C | 1149.77 | SNP | Rv2802c | Trp44Gly | 0 | - | | 3118000 | A | G | 656.77 | SNP | Rv2812 | Arg395Gly | 1 | - | | 3125268 | G | A | 1885.77 | SNP | Rv2818c | Leu293Phe | 6 | - | | 3137058 | G | A | 1160.77 | SNP | Rv2830c (vapB22) | Ala56Val(s) | 9867 | - | | 3137488 | A | C | 930.77 | SNP | Rv2831 (echA16) | Gln73Pro | 8 | - | | 3152278 | G | T | 644.77 | SNP | Rv2845c (proS) | silent (Arg225) | 9913 | - | | 3177884 | C | A | 1546.77 | SNP | Rv2866 (relG) | silent (Arg21) | 9913 | - | | 3183561 | G | C | 937.77 | SNP | Rv2872 (vapC43) | silent (Pro60) | 9926 | - | | 3186860 | T | G | 1347.77 | SNP | Rv2874 (dipZ) | Tyr672Asp | 0 | - | | 3206189 | CTCG | C | 2854.73 | DEL | Rv2896c |  |  | - | | 3216430 | G | A | 1222.77 | SNP | Rv2907c (rimM) | silent (Val154) | 9901 | - | | 3217383 | A | C | 1756.77 | SNP | Rv2909c (rpsP) | silent (Pro87) | 9926 | - | | 3220222 | G | A | 1728.77 | SNP | Rv2913c | Pro493Ser | 17 | - | | 3226181 | A | C | 1103.77 | SNP | Rv2916c (ffh) | silent (Arg35) | 9913 | - | | 3228143 | G | T | 993.77 | SNP | Rv2917 | Arg594Leu | 1 | - | | 3232192 | C | T | 860.77 | SNP | Rv2920c (amt) | silent (Gln105) | 9876 | - | | 3232759 | G | A | 405.77 | SNP | intergenic |  |  | - | | 3232815 | A | G | 79.77 | SNP | intergenic |  |  | - | | 3247316 | C | G | 1458.77 | SNP | Rv2931 (ppsA) | Asp624Glu | 56 | - | | 3247851 | G | A | 1009.77 | SNP | Rv2931 (ppsA) | Ala803Thr | 22 | - | | 3247853 | C | T | 1043.77 | SNP | Rv2931 (ppsA) | silent (Ala803) | 9867 | - | | 3247856 | G | C | 1078.77 | SNP | Rv2931 (ppsA) | silent (Arg804) | 9913 | - | | 3247864 | C | CTAGG | 2345.19 | INS | Rv2931 (ppsA) |  |  | - | | 3247865 | GCAAA | G | 2454.73 | DEL | Rv2931 (ppsA) |  |  | - | | 3247874 | G | A | 1026.77 | SNP | Rv2931 (ppsA) | silent (Arg810) | 9913 | - | | 3247877 | T | C | 1087.77 | SNP | Rv2931 (ppsA) | silent (Phe811) | 9946 | - | | 3247883 | T | C | 1395.77 | SNP | Rv2931 (ppsA) | silent (Ser813) | 9840 | - | | 3248074 | G | A | 1265.77 | SNP | Rv2931 (ppsA) | Arg877His | 8 | - | | 3248075 | C | T | 1282.77 | SNP | Rv2931 (ppsA) | silent (Arg877) | 9913 | - | | 3269581 | A | G | 1794.77 | SNP | Rv2935 (ppsE) | silent (Ala615) | 9867 | - | | 3270289 | C | A | 1283.77 | SNP | Rv2935 (ppsE) | silent (Arg851) | 9913 | - | | 3270784 | A | G | 1305.77 | SNP | Rv2935 (ppsE) | silent (Gln1016) | 9876 | - | | 3288360 | A | G | 1294.77 | SNP | intergenic |  |  | - | | 3289977 | C | G | 52.74 | SNP | Rv2943A; Rv2944 | silent (Gly91); Ala63Gly | 9935; 21 | - | | 3296843 | A | G | 1673.77 | SNP | Rv2947c (pks15) | Val(s)333Ala | 9867 | - | | 3308606 | G | A | 1571.77 | SNP | intergenic |  |  | - | | 3319755 | T | C | 1046.77 | SNP | Rv2967c (pca) | Ile1098Val | 57 | - | | 3336705 | A | T | 193.77 | SNP | intergenic |  |  | - | | 3338603 | G | C | 1292.77 | SNP | Rv2982c (gpdA2) | Pro133Ala | 22 | - | | 3358235 | A | T | 954.77 | SNP | Rv2999 (lppY) | Met(s)212Leu(s) | 9867 | - | | 3363338 | A | G | 1876.77 | SNP | intergenic |  |  | - | | 3367765 | G | A | 1173.77 | SNP | Rv3009c (gatB) | silent (Gly343) | 9935 | - | | 3369916 | G | C | 977.77 | SNP | intergenic |  |  | - | | 3396776 | T | C | 1759.77 | SNP | Rv3036c (TB22.2) | silent (Ala122) | 9867 | - | | 3415180 | ACACCTAGGGGGTGG | A | 5846.73 | DEL | intergenic |  |  | - | | 3425854 | C | T | 1581.77 | SNP | Rv3062 (ligB) | Pro91Ser | 17 | - | | 3428917 | C | A | 1201.77 | SNP | Rv3063 (cstA) | Arg559Ser | 11 | - | | 3440464 | T | G | 1839.77 | SNP | Rv3077 | silent (Arg308) | 9913 | - | | 3440468 | G | C | 1740.77 | SNP | Rv3077 | Gly310Arg | 0 | - | | 3453506 | G | A | 555.77 | SNP | Rv3087 | silent (Lys194) | 9926 | - | | 3456666 | A | G | 1373.77 | SNP | Rv3089 (fadD13) | silent (Ala302) | 9867 | - | | 3462135 | G | C | 1367.77 | SNP | Rv3093c | Cys210Trp | 0 | - | | 3486977 | A | G | 2458.77 | SNP | Rv3121 (cyp141) | Lys157Glu | 4 | - | | 3503895 | C | T | 1369.77 | SNP | Rv3137 | Pro168Leu | 3 | - | | 3505027 | G | A | 1704.77 | SNP | Rv3138 (pflA) | Arg278His | 8 | - | | 3518555 | A | G | 1393.77 | SNP | Rv3151 (nuoG) | Thr604Ala | 32 | - | | 3520810 | G | A | 1538.77 | SNP | Rv3153 (nuoI) | Asp102Asn | 36 | - | | 3540331 | G | T | 1346.77 | SNP | Rv3171c (hpx) | Leu139Met(s) | 4 | - | | 3555805 | G | A | 1470.77 | SNP | Rv3190c | Leu295Phe | 6 | - | | 3561265 | G | C | 1585.77 | SNP | Rv3193c | Val636Val(s) | 18 | - | | 3563205 | A | G | 1774.77 | SNP | intergenic |  |  | - | | 3565965 | GC | G | 2100.73 | DEL | Rv3196 |  |  | - | | 3569513 | C | T | 1219.77 | SNP | Rv3198c (uvrD2) | Gly567Arg | 0 | - | | 3580636 | CT | C | 3802.73 | DEL | intergenic |  |  | - | | 3581414 | A | G | 2135.77 | SNP | Rv3204 | Thr34Ala | 32 | - | | 3590686 | G | GC | 2048.73 | INS | intergenic |  |  | - | | 3591063 | T | C | 816.77 | SNP | Rv3213c | Lys144Glu | 4 | - | | 3591964 | C | G | 1791.77 | SNP | Rv3214 (gpm2) | Leu107Val(s) | 4 | - | | 3604821 | G | C | 554.77 | SNP | Rv3228 | silent (Ala32) | 9867 | - | | 3614130 | A | G | 1935.77 | SNP | Rv3238c | Leu90Pro | 2 | - | | 3622441 | A | C | 1228.77 | SNP | Rv3243c | Val217Val(s) | 18 | - | | 3689523 | G | T | 1386.77 | SNP | Rv3303c (lpdA) | Cys472STOP | 3 | - | | 3704596 | G | C | 1315.77 | SNP | Rv3317 (sdhD) | Val(s)54Leu | 3 | - | | 3714211 | G | T | 1473.77 | SNP | Rv3328c (sigJ) | Pro41Gln | 6 | - | | 3718357 | C | T | 1241.77 | SNP | Rv3331 (sugI) | Pro423Leu | 3 | - | | 3721806 | G | C | 1841.77 | SNP | Rv3335c | silent (Gly265) | 9935 | - | | 3730466 | A | G | 1066.94 | SNP | Rv3343c (PPE54) | Ile2157Thr | 11 | - | | 3730519 | C | G | 467.77 | SNP | Rv3343c (PPE54) | silent (Thr2139) | 9871 | - | | 3732113 | C | G | 629.77 | SNP | Rv3343c (PPE54) | Arg1608Pro | 5 | - | | 3732114 | G | T | 651.77 | SNP | Rv3343c (PPE54) | silent (Arg1608) | 9913 | - | | 3732517 | A | G | 357.77 | SNP | Rv3343c (PPE54) | silent (Ile1473) | 9872 | - | | 3732525 | A | T | 218.80 | SNP | Rv3343c (PPE54) | Phe1471Ile | 7 | - | | 3732553 | A | G | 359.77 | SNP | Rv3343c (PPE54) | silent (Ile1461) | 9872 | - | | 3732624 | A | G | 876.77 | SNP | Rv3343c (PPE54) | Leu(s)1438Leu | 3 | - | | 3736108 | A | G | 80.77 | SNP | Rv3343c (PPE54) | silent (Ile276) | 9872 | - | | 3736628 | T | G | 1152.77 | SNP | Rv3343c (PPE54) | Glu103Ala | 17 | - | | 3746409 | A | G | 529.77 | SNP | Rv3347c (PPE55) | Leu2259Pro | 2 | - | | 3752207 | A | G | 1331.77 | SNP | Rv3347c (PPE55) | silent (Ile326) | 9872 | - | | 3760724 | A | G | 1153.77 | SNP | Rv3350c (PPE56) | Leu(s)2127Leu | 3 | - | | 3763278 | G | A | 832.77 | SNP | Rv3350c (PPE56) | silent (Gly1275) | 9935 | - | | 3770705 | GC | G | 2471.73 | DEL | intergenic |  |  | - | | 3779671 | C | CGGCAACGGT | 1307.73 | INS | Rv3367 (PE\_PGRS51) |  |  | - | | 3817117 | C | A | 1176.77 | SNP | Rv3399 | Ala330Glu | 10 | - | | 3818092 | C | T | 1135.77 | SNP | Rv3401 | silent (Thr17) | 9871 | - | | 3820407 | A | G | 118.77 | SNP | intergenic |  |  | - | | 3820545 | A | G | 306.77 | SNP | intergenic |  |  | - | | 3823159 | A | T | 1211.77 | SNP | Rv3403c | silent (Val235) | 9901 | - | | 3826684 | C | T | 1102.77 | SNP | Rv3408 (vapC47) | Ser46Leu(s) | 35 | - | | 3834093 | G | A | 965.77 | SNP | Rv3415c | silent (Leu143) | 9947 | - | | 3836928 | G | C | 1610.77 | SNP | intergenic |  |  | - | | 3838871 | A | G | 1433.77 | SNP | Rv3420c (rimI) | silent (Ala64) | 9867 | - | | 3842309 | T | C | 46.74 | SNP | Rv3425 (PPE57) | Phe24Ser | 3 | - | | 3847215 | T | C | 1188.77 | SNP | Rv3429 (PPE59) | silent (Gly17) | 9935 | - | | 3847221 | T | C | 1128.77 | SNP | Rv3429 (PPE59) | silent (Gly19) | 9935 | - | | 3853976 | C | A | 2073.77 | SNP | Rv3435c | Trp273Leu(s) | 0 | - | | 3859893 | C | T | 1810.77 | SNP | Rv3440c | silent (Glu28) | 9865 | - | | 3861902 | G | C | 31.77 | SNP | Rv3442c (rpsI) | Pro17Ala | 22 | - | | 3862472 | GA | G | 2846.73 | DEL | intergenic |  |  | - | | 3877421 | A | G | 2247.77 | SNP | Rv3456c (rplQ) | silent (Pro4) | 9926 | - | | 3883626 | A | G | 913.77 | SNP | Rv3466 | silent (Pro34) | 9926 | - | | 3885382 | G | C | 2127.77 | SNP | Rv3468c | Arg230Gly | 1 | - | | 3892671 | A | G | 2162.77 | SNP | Rv3476c (kgtP) | silent (Val350) | 9901 | - | | 3895399 | C | A | 366.77 | SNP | Rv3478 (PPE60) | Pro325Gln | 6 | - | | 3895400 | A | G | 334.31 | SNP | Rv3478 (PPE60) | silent (Pro325) | 9926 | - | | 3895403 | A | C | 382.77 | SNP | Rv3478 (PPE60) | silent (Ala326) | 9867 | - | | 3896340 | T | G | 1102.77 | SNP | Rv3479 | Leu174Arg | 1 | - | | 3898408 | A | G | 1763.77 | SNP | Rv3479 | silent (Ala863) | 9867 | - | | 3928290 | C | T | 79.28 | SNP | Rv3507 (PE\_PGRS53) | silent (Gly574) | 9935 | - | | 3934542 | T | G | 94.77 | SNP | Rv3508 (PE\_PGRS54) | Ser1180Ala | 35 | - | | 3934699 | G | A | 46.77 | SNP | Rv3508 (PE\_PGRS54) | Ser1232Asn | 20 | - | | 3934733 | G | C | 144.90 | SNP | Rv3508 (PE\_PGRS54) | silent (Gly1243) | 9935 | - | | 3934734 | G | A | 135.90 | SNP | Rv3508 (PE\_PGRS54) | Ala1244Thr | 22 | - | | 3940802 | A | G | 129.03 | SNP | Rv3511 (PE\_PGRS55) | Asn396Asp | 42 | - | | 3943019 | C | G | 98.28 | SNP | intergenic |  |  | - | | 3947449 | C | A | 38.77 | SNP | Rv3514 (PE\_PGRS57) | silent (Gly552) | 9935 | - | | 3948928 | G | C | 39.77 | SNP | Rv3514 (PE\_PGRS57) | silent (Gly1045) | 9935 | - | | 3948929 | G | C | 35.77 | SNP | Rv3514 (PE\_PGRS57) | Ala1046Pro | 13 | - | | 3952800 | G | A | 1576.77 | SNP | Rv3516 (echA19) | Gly86Asp | 6 | - | | 3959418 | C | T | 1236.77 | SNP | Rv3522 (ltp4) | Thr324Ile | 7 | - | | 3969973 | G | A | 923.77 | SNP | Rv3532 (PPE61) | Gly211Arg | 0 | - | | 3983010 | A | G | 1335.77 | SNP | Rv3543c (fadE29) | Val(s)44Ala | 9867 | - | | 4009070 | G | A | 2192.77 | SNP | Rv3567c (hsaB) | silent (Cys71) | 9973 | - | | 4019214 | C | T | 1010.77 | SNP | intergenic |  |  | - | | 4024273 | T | C | 1807.77 | SNP | Rv3581c (ispF) | Val25Val(s) | 18 | - | | 4024782 | G | A | 1717.77 | SNP | Rv3582c (ispD) | silent (Thr86) | 9871 | - | | 4026899 | G | A | 1443.77 | SNP | Rv3585 (radA) | silent (Gln152) | 9876 | - | | 4034827 | C | T | 1372.77 | SNP | Rv3593 (lpqF) | Ala159Val(s) | 9867 | - | | 4037232 | AGTGCCACCG | A | 521.73 | DEL | Rv3595c (PE\_PGRS59) |  |  | - | | 4053050 | A | G | 419.22 | SNP | Rv3611 | Asn34Ser | 34 | - | | 4055801 | G | A | 1498.77 | SNP | Rv3616c (espA) | Thr192Ile | 7 | - | | 4059904 | A | G | 1496.77 | SNP | intergenic |  |  | - | | 4085398 | G | T | 1612.77 | SNP | Rv3646c (topA) | silent (Ala620) | 9867 | - | | 4091898 | C | T | 2976.77 | SNP | Rv3651 | Arg20Trp | 2 | - | | 4095001 | CG | C | 2257.73 | DEL | Rv3655c |  |  | - | | 4099265 | A | T | 1985.77 | SNP | intergenic |  |  | - | | 4100975 | T | C | 834.77 | SNP | intergenic |  |  | - | | 4111303 | G | C | 1961.77 | SNP | Rv3669 | Val(s)159Val | 13 | - | | 4112595 | G | A | 1334.77 | SNP | Rv3671c | silent (Ala307) | 9867 | - | | 4120926 | A | G | 303.77 | SNP | Rv3680 | Asn378Asp | 42 | - | | 4120983 | A | G | 812.77 | SNP | intergenic |  |  | - | | 4121491 | T | C | 2350.77 | SNP | Rv3681c (whiB4) | Ser22Gly | 21 | - | | 4131191 | G | A | 993.77 | SNP | Rv3689 | Ala279Thr | 22 | - | | 4156099 | C | A | 1593.77 | SNP | Rv3711c (dnaQ) | Val(s)211Leu(s) | 9867 | - | | 4162339 | A | G | 2458.77 | SNP | Rv3719 | Thr12Ala | 32 | - | | 4187485 | T | C | 1464.77 | SNP | Rv3736 | silent (Ala284) | 9867 | - | | 4187817 | A | G | 1347.77 | SNP | Rv3737 | Asp40Gly | 11 | - | | 4204441 | A | G | 1604.77 | SNP | Rv3759c (proX) | silent (His311) | 9912 | - | | 4210274 | A | G | 1112.77 | SNP | Rv3764c (tcrY) | Cys246Arg | 1 | - | | 4221490 | C | G | 1939.77 | SNP | Rv3776 | silent (Leu134) | 9947 | - | | 4222073 | A | G | 497.77 | SNP | Rv3776 | Met(s)329Val(s) | 9867 | - | | 4222882 | A | G | 1400.77 | SNP | Rv3777 | silent (Leu63) | 9947 | - | | 4230689 | G | GT | 2926.73 | INS | Rv3784 |  |  | - | | 4242643 | C | T | 976.77 | SNP | Rv3793 (embC) | silent (Arg927) | 9913 | genotype | | 4243222 | C | A | 1142.77 | SNP | intergenic (Rv3794-11nt) |  |  | - | | 4247730 | G | A | 1238.77 | SNP | Rv3795 (embB) | Gly406Asp | 6 | resistance | | 4249732 | C | G | 1600.77 | SNP | Rv3795 (embB) | silent (Ala1073) | 9867 | genotype | | 4264605 | C | A | 1336.77 | SNP | Rv3803c (fbpD) | Gln286His | 20 | - | | 4286939 | C | T | 1899.77 | SNP | Rv3822 | silent (Pro73) | 9926 | - | | 4302036 | T | C | 2121.77 | SNP | Rv3827c | Thr252Ala | 32 | - | | 4306155 | C | T | 1158.77 | SNP | Rv3831 | silent (Ser133) | 9840 | - | | 4306396 | G | A | 1575.77 | SNP | Rv3832c | Pro139Leu | 3 | - | | 4338595 | GC | G | 3176.71 | DEL | intergenic |  |  | - | | 4341794 | G | A | 1617.77 | SNP | Rv3865 (espF) | Ala77Thr | 22 | - | | 4348037 | G | A | 1568.77 | SNP | Rv3870 (eccCa1) | silent (Leu519) | 9947 | - | | 4351039 | G | T | 1333.77 | SNP | Rv3872 (PE35) | Glu99STOP | 17 | - | | 4356110 | G | C | 1461.77 | SNP | Rv3877 (eccD1) | silent (Leu368) | 9947 | - | | 4363115 | C | G | 1114.77 | SNP | Rv3882c (eccE1) | silent (Leu102) | 9947 | - | | 4364812 | A | G | 1998.77 | SNP | intergenic |  |  | - | | 4366272 | G | C | 1025.77 | SNP | Rv3884c (eccA2) | silent (Ala189) | 9867 | - | | 4375628 | G | T | 1569.77 | SNP | Rv3892c (PPE69) | Thr19Lys | 11 | - | | 4379680 | C | G | 2157.77 | SNP | Rv3894c (eccC2) | Arg258Pro | 5 | - | | 4382054 | T | C | 1490.77 | SNP | Rv3896c | silent (Ala266) | 9867 | - | | 4382275 | G | T | 1495.77 | SNP | Rv3896c | Gln193Lys | 12 | - | | 4383144 | C | CCGGGG | 4205.73 | INS | Rv3897c |  |  | - | | 4390775 | T | C | 966.77 | SNP | Rv3905c (esxF) | Tyr86Cys | 3 | - | | 4391579 | C | G | 1295.77 | SNP | Rv3906c | Gly10Arg | 0 | - | | 4399759 | C | T | 1370.77 | SNP | Rv3910 | Pro1055Ser | 17 | - | | 4400660 | AC | A | 2771.73 | DEL | Rv3911 (sigM) |  |  | - | | 4405840 | C | A | 1611.77 | SNP | Rv3917c (parB) | Ala218Ser | 28 | - | | 4407590 | C | T | 2200.77 | SNP | Rv3919c (gid) | Ala205Thr | 22 | - | | 4408119 | TC | T | 2338.73 | DEL | Rv3919c (gid) |  |  | - | |  | | export |

elog
