# Supplementary material for: Patterns of compensatory mutations in rpoA/B/C genes of multidrug resistant M. tuberculosis in Uganda
Source: PLoS One. 2025 Dec 4;20(12):e0328957. doi: 10.1371/journal.pone.0328957 (PMC12677784; doi:10.1371/journal.pone.0328957)
Supplement: S2 File — (ZIP) [file pone.0328957.s002.zip › Variants N_S14_L001_001.bam.html]

 

Calling SNPs/INDELs (computing variant list in .vcf format) from N\_S14\_L001\_001.bam

*by SAMtools/BCFtools:*

Howto

Important aspects

This takes up to one hour!!! **Please wait ...**

Variants N\_S14\_L001\_001.bam

|  |  |
| --- | --- |
| Variants |  |

|  |  |
| --- | --- |
| |  | | --- | | *by GATK* | |

|  |  |  |
| --- | --- | --- |
| |  | | --- | | N\_S14\_L001\_001.bam | | | computed 2016-10-27 using PhyResSE v1.0 (Ref. NC\_000962.3) | |

|  |  |
| --- | --- |
| 1382  variants called Export in VCF format |  |

|  |  |  |  |  |  |  |  |  |  |  |  |  |  |  |  |  |  |  |  |  |  |  |  |  |  |  |  |  |  |  |  |  |  |  |  |  |  |  |  |  |  |  |  |  |  |  |  |  |  |  |  |  |  |  |  |  |  |  |  |  |  |  |  |  |  |  |  |  |  |  |  |  |  |  |  |  |  |  |  |  |  |  |  |  |  |  |  |  |  |  |  |  |  |  |  |  |  |  |  |  |  |  |  |  |  |  |  |  |  |  |  |  |  |  |  |  |  |  |  |  |  |  |  |  |  |  |  |  |  |  |  |  |  |  |  |  |  |  |  |  |  |  |  |  |  |  |  |  |  |  |  |  |  |  |  |  |  |  |  |  |  |  |  |  |  |  |  |  |  |  |  |  |  |  |  |  |  |  |  |  |  |  |  |  |  |  |  |  |  |  |  |  |  |  |  |  |  |  |  |  |  |  |  |  |  |  |  |  |  |  |  |  |  |  |  |  |  |  |  |  |  |  |  |  |  |  |  |  |  |  |  |  |  |  |  |  |  |  |  |  |  |  |  |  |  |  |  |  |  |  |  |  |  |  |  |  |  |  |  |  |  |  |  |  |  |  |  |  |  |  |  |  |  |  |  |  |  |  |  |  |  |  |  |  |  |  |  |  |  |  |  |  |  |  |  |  |  |  |  |  |  |  |  |  |  |  |  |  |  |  |  |  |  |  |  |  |  |  |  |  |  |  |  |  |  |  |  |  |  |  |  |  |  |  |  |  |  |  |  |  |  |  |  |  |  |  |  |  |  |  |  |  |  |  |  |  |  |  |  |  |  |  |  |  |  |  |  |  |  |  |  |  |  |  |  |  |  |  |  |  |  |  |  |  |  |  |  |  |  |  |  |  |  |  |  |  |  |  |  |  |  |  |  |  |  |  |  |  |  |  |  |  |  |  |  |  |  |  |  |  |  |  |  |  |  |  |  |  |  |  |  |  |  |  |  |  |  |  |  |  |  |  |  |  |  |  |  |  |  |  |  |  |  |  |  |  |  |  |  |  |  |  |  |  |  |  |  |  |  |  |  |  |  |  |  |  |  |  |  |  |  |  |  |  |  |  |  |  |  |  |  |  |  |  |  |  |  |  |  |  |  |  |  |  |  |  |  |  |  |  |  |  |  |  |  |  |  |  |  |  |  |  |  |  |  |  |  |  |  |  |  |  |  |  |  |  |  |  |  |  |  |  |  |  |  |  |  |  |  |  |  |  |  |  |  |  |  |  |  |  |  |  |  |  |  |  |  |  |  |  |  |  |  |  |  |  |  |  |  |  |  |  |  |  |  |  |  |  |  |  |  |  |  |  |  |  |  |  |  |  |  |  |  |  |  |  |  |  |  |  |  |  |  |  |  |  |  |  |  |  |  |  |  |  |  |  |  |  |  |  |  |  |  |  |  |  |  |  |  |  |  |  |  |  |  |  |  |  |  |  |  |  |  |  |  |  |  |  |  |  |  |  |  |  |  |  |  |  |  |  |  |  |  |  |  |  |  |  |  |  |  |  |  |  |  |  |  |  |  |  |  |  |  |  |  |  |  |  |  |  |  |  |  |  |  |  |  |  |  |  |  |  |  |  |  |  |  |  |  |  |  |  |  |  |  |  |  |  |  |  |  |  |  |  |  |  |  |  |  |  |  |  |  |  |  |  |  |  |  |  |  |  |  |  |  |  |  |  |  |  |  |  |  |  |  |  |  |  |  |  |  |  |  |  |  |  |  |  |  |  |  |  |  |  |  |  |  |  |  |  |  |  |  |  |  |  |  |  |  |  |  |  |  |  |  |  |  |  |  |  |  |  |  |  |  |  |  |  |  |  |  |  |  |  |  |  |  |  |  |  |  |  |  |  |  |  |  |  |  |  |  |  |  |  |  |  |  |  |  |  |  |  |  |  |  |  |  |  |  |  |  |  |  |  |  |  |  |  |  |  |  |  |  |  |  |  |  |  |  |  |  |  |  |  |  |  |  |  |  |  |  |  |  |  |  |  |  |  |  |  |  |  |  |  |  |  |  |  |  |  |  |  |  |  |  |  |  |  |  |  |  |  |  |  |  |  |  |  |  |  |  |  |  |  |  |  |  |  |  |  |  |  |  |  |  |  |  |  |  |  |  |  |  |  |  |  |  |  |  |  |  |  |  |  |  |  |  |  |  |  |  |  |  |  |  |  |  |  |  |  |  |  |  |  |  |  |  |  |  |  |  |  |  |  |  |  |  |  |  |  |  |  |  |  |  |  |  |  |  |  |  |  |  |  |  |  |  |  |  |  |  |  |  |  |  |  |  |  |  |  |  |  |  |  |  |  |  |  |  |  |  |  |  |  |  |  |  |  |  |  |  |  |  |  |  |  |  |  |  |  |  |  |  |  |  |  |  |  |  |  |  |  |  |  |  |  |  |  |  |  |  |  |  |  |  |  |  |  |  |  |  |  |  |  |  |  |  |  |  |  |  |  |  |  |  |  |  |  |  |  |  |  |  |  |  |  |  |  |  |  |  |  |  |  |  |  |  |  |  |  |  |  |  |  |  |  |  |  |  |  |  |  |  |  |  |  |  |  |  |  |  |  |  |  |  |  |  |  |  |  |  |  |  |  |  |  |  |  |  |  |  |  |  |  |  |  |  |  |  |  |  |  |  |  |  |  |  |  |  |  |  |  |  |  |  |  |  |  |  |  |  |  |  |  |  |  |  |  |  |  |  |  |  |  |  |  |  |  |  |  |  |  |  |  |  |  |  |  |  |  |  |  |  |  |  |  |  |  |  |  |  |  |  |  |  |  |  |  |  |  |  |  |  |  |  |  |  |  |  |  |  |  |  |  |  |  |  |  |  |  |  |  |  |  |  |  |  |  |  |  |  |  |  |  |  |  |  |  |  |  |  |  |  |  |  |  |  |  |  |  |  |  |  |  |  |  |  |  |  |  |  |  |  |  |  |  |  |  |  |  |  |  |  |  |  |  |  |  |  |  |  |  |  |  |  |  |  |  |  |  |  |  |  |  |  |  |  |  |  |  |  |  |  |  |  |  |  |  |  |  |  |  |  |  |  |  |  |  |  |  |  |  |  |  |  |  |  |  |  |  |  |  |  |  |  |  |  |  |  |  |  |  |  |  |  |  |  |  |  |  |  |  |  |  |  |  |  |  |  |  |  |  |  |  |  |  |  |  |  |  |  |  |  |  |  |  |  |  |  |  |  |  |  |  |  |  |  |  |  |  |  |  |  |  |  |  |  |  |  |  |  |  |  |  |  |  |  |  |  |  |  |  |  |  |  |  |  |  |  |  |  |  |  |  |  |  |  |  |  |  |  |  |  |  |  |  |  |  |  |  |  |  |  |  |  |  |  |  |  |  |  |  |  |  |  |  |  |  |  |  |  |  |  |  |  |  |  |  |  |  |  |  |  |  |  |  |  |  |  |  |  |  |  |  |  |  |  |  |  |  |  |  |  |  |  |  |  |  |  |  |  |  |  |  |  |  |  |  |  |  |  |  |  |  |  |  |  |  |  |  |  |  |  |  |  |  |  |  |  |  |  |  |  |  |  |  |  |  |  |  |  |  |  |  |  |  |  |  |  |  |  |  |  |  |  |  |  |  |  |  |  |  |  |  |  |  |  |  |  |  |  |  |  |  |  |  |  |  |  |  |  |  |  |  |  |  |  |  |  |  |  |  |  |  |  |  |  |  |  |  |  |  |  |  |  |  |  |  |  |  |  |  |  |  |  |  |  |  |  |  |  |  |  |  |  |  |  |  |  |  |  |  |  |  |  |  |  |  |  |  |  |  |  |  |  |  |  |  |  |  |  |  |  |  |  |  |  |  |  |  |  |  |  |  |  |  |  |  |  |  |  |  |  |  |  |  |  |  |  |  |  |  |  |  |  |  |  |  |  |  |  |  |  |  |  |  |  |  |  |  |  |  |  |  |  |  |  |  |  |  |  |  |  |  |  |  |  |  |  |  |  |  |  |  |  |  |  |  |  |  |  |  |  |  |  |  |  |  |  |  |  |  |  |  |  |  |  |  |  |  |  |  |  |  |  |  |  |  |  |  |  |  |  |  |  |  |  |  |  |  |  |  |  |  |  |  |  |  |  |  |  |  |  |  |  |  |  |  |  |  |  |  |  |  |  |  |  |  |  |  |  |  |  |  |  |  |  |  |  |  |  |  |  |  |  |  |  |  |  |  |  |  |  |  |  |  |  |  |  |  |  |  |  |  |  |  |  |  |  |  |  |  |  |  |  |  |  |  |  |  |  |  |  |  |  |  |  |  |  |  |  |  |  |  |  |  |  |  |  |  |  |  |  |  |  |  |  |  |  |  |  |  |  |  |  |  |  |  |  |  |  |  |  |  |  |  |  |  |  |  |  |  |  |  |  |  |  |  |  |  |  |  |  |  |  |  |  |  |  |  |  |  |  |  |  |  |  |  |  |  |  |  |  |  |  |  |  |  |  |  |  |  |  |  |  |  |  |  |  |  |  |  |  |  |  |  |  |  |  |  |  |  |  |  |  |  |  |  |  |  |  |  |  |  |  |  |  |  |  |  |  |  |  |  |  |  |  |  |  |  |  |  |  |  |  |  |  |  |  |  |  |  |  |  |  |  |  |  |  |  |  |  |  |  |  |  |  |  |  |  |  |  |  |  |  |  |  |  |  |  |  |  |  |  |  |  |  |  |  |  |  |  |  |  |  |  |  |  |  |  |  |  |  |  |  |  |  |  |  |  |  |  |  |  |  |  |  |  |  |  |  |  |  |  |  |  |  |  |  |  |  |  |  |  |  |  |  |  |  |  |  |  |  |  |  |  |  |  |  |  |  |  |  |  |  |  |  |  |  |  |  |  |  |  |  |  |  |  |  |  |  |  |  |  |  |  |  |  |  |  |  |  |  |  |  |  |  |  |  |  |  |  |  |  |  |  |  |  |  |  |  |  |  |  |  |  |  |  |  |  |  |  |  |  |  |  |  |  |  |  |  |  |  |  |  |  |  |  |  |  |  |  |  |  |  |  |  |  |  |  |  |  |  |  |  |  |  |  |  |  |  |  |  |  |  |  |  |  |  |  |  |  |  |  |  |  |  |  |  |  |  |  |  |  |  |  |  |  |  |  |  |  |  |  |  |  |  |  |  |  |  |  |  |  |  |  |  |  |  |  |  |  |  |  |  |  |  |  |  |  |  |  |  |  |  |  |  |  |  |  |  |  |  |  |  |  |  |  |  |  |  |  |  |  |  |  |  |  |  |  |  |  |  |  |  |  |  |  |  |  |  |  |  |  |  |  |  |  |  |  |  |  |  |  |  |  |  |  |  |  |  |  |  |  |  |  |  |  |  |  |  |  |  |  |  |  |  |  |  |  |  |  |  |  |  |  |  |  |  |  |  |  |  |  |  |  |  |  |  |  |  |  |  |  |  |  |  |  |  |  |  |  |  |  |  |  |  |  |  |  |  |  |  |  |  |  |  |  |  |  |  |  |  |  |  |  |  |  |  |  |  |  |  |  |  |  |  |  |  |  |  |  |  |  |  |  |  |  |  |  |  |  |  |  |  |  |  |  |  |  |  |  |  |  |  |  |  |  |  |  |  |  |  |  |  |  |  |  |  |  |  |  |  |  |  |  |  |  |  |  |  |  |  |  |  |  |  |  |  |  |  |  |  |  |  |  |  |  |  |  |  |  |  |  |  |  |  |  |  |  |  |  |  |  |  |  |  |  |  |  |  |  |  |  |  |  |  |  |  |  |  |  |  |  |  |  |  |  |  |  |  |  |  |  |  |  |  |  |  |  |  |  |  |  |  |  |  |  |  |  |  |  |  |  |  |  |  |  |  |  |  |  |  |  |  |  |  |  |  |  |  |  |  |  |  |  |  |  |  |  |  |  |  |  |  |  |  |  |  |  |  |  |  |  |  |  |  |  |  |  |  |  |  |  |  |  |  |  |  |  |  |  |  |  |  |  |  |  |  |  |  |  |  |  |  |  |  |  |  |  |  |  |  |  |  |  |  |  |  |  |  |  |  |  |  |  |  |  |  |  |  |  |  |  |  |  |  |  |  |  |  |  |  |  |  |  |  |  |  |  |  |  |  |  |  |  |  |  |  |  |  |  |  |  |  |  |  |  |  |  |  |  |  |  |  |  |  |  |  |  |  |  |  |  |  |  |  |  |  |  |  |  |  |  |  |  |  |  |  |  |  |  |  |  |  |  |  |  |  |  |  |  |  |  |  |  |  |  |  |  |  |  |  |  |  |  |  |  |  |  |  |  |  |  |  |  |  |  |  |  |  |  |  |  |  |  |  |  |  |  |  |  |  |  |  |  |  |  |  |  |  |  |  |  |  |  |  |  |  |  |  |  |  |  |  |  |  |  |  |  |  |  |  |  |  |  |  |  |  |  |  |  |  |  |  |  |  |  |  |  |  |  |  |  |  |  |  |  |  |  |  |  |  |  |  |  |  |  |  |  |  |  |  |  |  |  |  |  |  |  |  |  |  |  |  |  |  |  |  |  |  |  |  |  |  |  |  |  |  |  |  |  |  |  |  |  |  |  |  |  |  |  |  |  |  |  |  |  |  |  |  |  |  |  |  |  |  |  |  |  |  |  |  |  |  |  |  |  |  |  |  |  |  |  |  |  |  |  |  |  |  |  |  |  |  |  |  |  |  |  |  |  |  |  |  |  |  |  |  |  |  |  |  |  |  |  |  |  |  |  |  |  |  |  |  |  |  |  |  |  |  |  |  |  |  |  |  |  |  |  |  |  |  |  |  |  |  |  |  |  |  |  |  |  |  |  |  |  |  |  |  |  |  |  |  |  |  |  |  |  |  |  |  |  |  |  |  |  |  |  |  |  |  |  |  |  |  |  |  |  |  |  |  |  |  |  |  |  |  |  |  |  |  |  |  |  |  |  |  |  |  |  |  |  |  |  |  |  |  |  |  |  |  |  |  |  |  |  |  |  |  |  |  |  |  |  |  |  |  |  |  |  |  |  |  |  |  |  |  |  |  |  |  |  |  |  |  |  |  |  |  |  |  |  |  |  |  |  |  |  |  |  |  |  |  |  |  |  |  |  |  |  |  |  |  |  |  |  |  |  |  |  |  |  |  |  |  |  |  |  |  |  |  |  |  |  |  |  |  |  |  |  |  |  |  |  |  |  |  |  |  |  |  |  |  |  |  |  |  |  |  |  |  |  |  |  |  |  |  |  |  |  |  |  |  |  |  |  |  |  |  |  |  |  |  |  |  |  |  |  |  |  |  |  |  |  |  |  |  |  |  |  |  |  |  |  |  |  |  |  |  |  |  |  |  |  |  |  |  |  |  |  |  |  |  |  |  |  |  |  |  |  |  |  |  |  |  |  |  |  |  |  |  |  |  |  |  |  |  |  |  |  |  |  |  |  |  |  |  |  |  |  |  |  |  |  |  |  |  |  |  |  |  |  |  |  |  |  |  |  |  |  |  |  |  |  |  |  |  |  |  |  |  |  |  |  |  |  |  |  |  |  |  |  |  |  |  |  |  |  |  |  |  |  |  |  |  |  |  |  |  |  |  |  |  |  |  |  |  |  |  |  |  |  |  |  |  |  |  |  |  |  |  |  |  |  |  |  |  |  |  |  |  |  |  |  |  |  |  |  |  |  |  |  |  |  |  |  |  |  |  |  |  |  |  |  |  |  |  |  |  |  |  |  |  |  |  |  |  |  |  |  |  |  |  |  |  |  |  |  |  |  |  |  |  |  |  |  |  |  |  |  |  |  |  |  |  |  |  |  |  |  |  |  |  |  |  |  |  |  |  |  |  |  |  |  |  |  |  |  |  |  |  |  |  |  |  |  |  |  |  |  |  |  |  |  |  |  |  |  |  |  |  |  |  |  |  |  |  |  |  |  |  |  |  |  |  |  |  |  |  |  |  |  |  |  |  |  |  |  |  |  |  |  |  |  |  |  |  |  |  |  |  |  |  |  |  |  |  |  |  |  |  |  |  |  |  |  |  |  |  |  |  |  |  |  |  |  |  |  |  |  |  |  |  |  |  |  |  |  |  |  |  |  |  |  |  |  |  |  |  |  |  |  |  |  |  |  |  |  |  |  |  |  |  |  |  |  |  |  |  |  |  |  |  |  |  |  |  |  |  |  |  |  |  |  |  |  |  |  |  |  |  |  |  |  |  |  |  |  |  |  |  |  |  |  |  |  |  |  |  |  |  |  |  |  |  |  |  |  |  |  |  |  |  |  |  |  |  |  |  |  |  |  |  |  |  |  |  |  |  |  |  |  |  |  |  |  |  |  |  |  |  |  |  |  |  |  |  |  |  |  |  |  |  |  |  |  |  |  |  |  |  |  |  |  |  |  |  |  |  |  |  |  |  |  |  |  |  |  |  |  |  |  |  |  |  |  |  |  |  |  |  |  |  |  |  |  |  |  |  |  |  |  |  |  |  |  |  |  |  |  |  |  |  |  |  |  |  |  |  |  |  |  |  |  |  |  |  |  |  |  |  |  |  |  |  |  |  |  |  |  |  |  |  |  |  |  |  |  |  |  |  |  |  |  |  |  |  |  |  |  |  |  |  |  |  |  |  |  |  |  |  |  |  |  |  |  |  |  |  |  |  |  |  |  |  |  |  |  |  |  |  |  |  |  |  |  |  |  |  |  |  |  |  |  |  |  |  |  |  |  |  |  |  |  |  |  |  |  |  |  |  |  |  |  |  |  |  |  |  |  |  |  |  |  |  |  |  |  |  |  |  |  |  |  |  |  |  |  |  |  |  |  |  |  |  |  |  |  |  |  |  |  |  |  |  |  |  |  |  |  |  |  |  |  |  |  |  |  |  |  |  |  |  |  |  |  |  |  |  |  |  |  |  |  |  |  |  |  |  |  |  |  |  |  |  |  |  |  |  |  |  |  |  |  |  |  |  |  |  |  |  |  |  |  |  |  |  |  |  |  |  |  |  |  |  |  |  |  |  |  |  |  |  |  |  |  |  |  |  |  |  |  |  |  |  |  |  |  |  |  |  |  |  |  |  |  |  |  |  |  |  |  |  |  |  |  |  |  |  |  |  |  |  |  |  |  |  |  |  |  |  |  |  |  |  |  |  |  |  |  |  |  |  |  |  |  |  |  |  |  |  |  |  |  |  |  |  |  |  |  |  |  |  |  |  |  |  |  |  |  |  |  |  |  |  |  |  |  |  |  |  |  |  |  |  |  |  |  |  |  |  |  |  |  |  |  |  |  |  |  |  |  |  |  |  |  |  |  |  |  |  |  |  |  |  |  |  |  |  |  |  |  |  |  |  |  |  |  |  |  |  |  |  |  |  |  |  |  |  |  |  |  |  |  |  |  |  |  |  |  |  |  |  |  |  |  |  |  |  |  |  |  |  |  |  |  |  |  |  |  |  |  |  |  |  |  |  |  |  |  |  |  |  |  |  |  |  |  |  |  |  |  |  |  |  |  |  |  |  |  |  |  |  |  |  |  |  |  |  |  |  |  |  |  |  |  |  |  |  |  |  |  |  |  |  |  |  |  |  |  |  |  |  |  |  |  |  |  |  |  |  |  |  |  |  |  |  |  |  |  |  |  |  |  |  |  |  |  |  |  |  |  |  |  |  |  |  |  |  |  |  |  |  |  |  |  |  |  |  |  |  |  |  |  |  |  |  |  |  |  |  |  |  |  |  |  |  |  |  |  |  |  |  |  |  |  |  |  |  |  |  |  |  |  |  |  |  |  |  |  |  |  |  |  |  |  |  |  |  |  |  |  |  |  |  |  |  |  |  |  |  |  |  |  |  |  |  |  |  |  |  |  |  |  |  |  |  |  |  |  |  |  |  |  |  |  |  |  |  |  |  |  |  |  |  |  |  |  |  |  |  |  |  |  |  |  |  |  |  |  |  |  |  |  |  |  |  |  |  |  |  |  |  |  |  |  |  |  |  |  |  |  |  |  |  |  |  |  |  |  |  |  |  |  |  |  |  |  |  |  |  |  |  |  |  |  |  |  |  |  |  |  |  |  |  |  |  |  |  |  |  |  |  |  |  |  |  |  |  |  |  |  |  |  |  |  |  |  |  |  |  |  |  |  |  |  |  |  |  |  |  |  |  |  |  |  |  |  |  |  |  |  |  |  |  |  |  |  |  |  |  |  |  |  |  |  |  |  |  |  |  |  |  |  |  |  |  |  |  |  |  |  |  |  |  |  |  |  |  |  |  |  |  |  |  |  |  |  |  |  |  |  |  |  |  |  |  |  |  |  |  |  |  |  |  |  |  |  |  |  |  |  |  |  |  |  |  |  |  |  |  |  |  |  |  |  |  |  |  |  |  |  |  |  |  |  |  |  |  |  |  |  |  |  |  |  |  |  |  |  |  |  |  |  |  |  |  |  |  |  |  |  |  |  |  |  |  |  |  |  |  |  |  |  |  |  |  |  |  |  |  |  |  |  |  |  |  |  |  |  |  |  |  |  |  |  |  |  |  |  |  |  |  |  |  |  |  |  |  |  |  |  |  |  |  |  |  |  |  |  |  |  |  |  |  |  |  |  |  |  |  |  |  |  |  |  |  |  |  |  |  |  |  |  |  |  |  |  |  |  |  |  |  |  |  |  |  |  |  |  |  |  |  |  |  |  |  |  |  |  |  |  |  |  |  |  |  |  |  |  |  |  |  |  |  |  |  |  |  |  |  |  |  |  |  |  |  |  |  |  |  |  |  |  |  |  |  |  |  |  |  |  |  |  |  |  |  |  |  |  |  |  |  |  |  |  |  |  |  |  |  |  |  |  |  |  |  |  |  |  |  |  |  |  |  |  |  |  |  |  |  |  |  |  |  |  |  |  |  |  |  |  |  |  |  |  |  |  |  |  |  |  |  |  |  |  |  |  |  |  |  |  |  |  |  |  |  |  |  |  |  |  |  |  |  |  |  |  |  |  |  |  |  |  |  |  |  |  |  |  |  |  |  |  |  |  |  |  |  |  |  |  |  |  |  |  |  |  |  |  |  |  |  |  |  |  |  |  |  |  |  |  |  |  |  |  |  |  |  |  |  |  |  |  |  |  |  |  |  |  |  |  |  |  |  |  |  |  |  |  |  |  |  |  |  |  |  |  |  |  |  |  |  |  |  |  |  |  |  |  |  |  |  |  |  |  |  |  |  |  |  |  |  |  |  |  |  |  |  |  |  |  |  |  |  |  |  |  |  |  |  |  |  |  |  |  |  |  |  |  |  |  |  |  |  |  |  |  |  |  |  |  |  |  |  |  |  |  |  |  |  |  |  |  |  |  |  |  |  |  |  |  |  |  |  |  |  |  |  |  |  |  |  |  |  |  |  |  |  |  |  |  |  |  |  |  |  |  |  |  |  |  |  |  |  |  |  |  |  |  |  |  |  |  |  |  |  |  |  |  |  |  |  |  |  |  |  |  |  |  |  |  |  |  |  |  |  |  |  |  |  |  |  |  |  |  |  |  |  |  |  |  |  |  |  |  |  |  |  |  |  |  |  |  |  |  |  |  |  |  |  |  |  |  |  |  |  |  |  |  |  |  |  |  |  |  |  |  |  |  |  |  |  |  |  |  |  |  |  |  |  |  |  |  |  |  |  |  |  |  |  |  |  |  |  |  |  |  |  |  |  |  |  |  |  |  |  |  |  |  |  |  |  |  |  |  |  |  |  |  |  |  |  |  |  |  |  |  |  |  |  |  |  |  |  |  |  |  |  |  |  |  |  |  |  |  |  |  |  |  |  |  |  |  |  |  |  |  |  |  |  |  |  |  |  |  |  |  |  |  |  |  |  |  |  |  |  |  |  |  |  |  |  |  |  |  |  |  |  |  |  |  |  |  |  |  |  |  |  |  |  |  |  |  |  |  |  |  |  |  |  |  |  |  |  |  |  |  |  |  |  |  |  |  |  |  |  |  |  |  |  |  |  |  |  |  |  |  |  |  |  |  |  |  |  |  |  |  |  |  |  |  |  |  |  |  |  |  |  |  |  |  |  |  |  |  |  |  |  |  |  |  |  |  |  |  |  |  |  |  |  |  |  |  |  |  |  |  |  |  |  |  |  |  |  |  |  |  |  |  |  |  |  |  |  |  |  |  |  |  |  |  |  |  |  |  |  |  |  |  |  |  |  |  |  |  |  |  |  |  |  |  |  |  |  |  |  |  |  |  |  |  |  |  |  |  |  |  |  |  |  |  |  |  |  |  |  |  |  |  |  |  |  |  |  |  |  |  |  |  |  |  |  |  |  |  |  |  |  |  |  |  |  |  |  |  |  |  |  |  |  |  |  |  |  |  |  |  |  |  |  |  |  |  |  |  |  |  |  |  |  |  |  |  |  |  |  |  |  |  |  |  |  |  |  |  |  |  |  |  |  |  |  |  |  |  |  |  |  |  |  |  |  |  |  |  |  |  |  |  |  |  |  |  |  |  |  |  |  |  |  |  |  |  |  |  |  |  |  |  |  |  |  |  |  |  |  |  |  |  |  |  |  |  |  |  |  |  |  |  |  |  |  |  |  |  |  |  |  |  |  |  |  |  |  |  |  |  |  |  |  |  |  |  |  |  |  |  |  |  |  |  |  |  |  |  |  |  |  |  |  |  |  |  |  |  |  |  |  |  |  |  |  |  |  |  |  |  |  |  |  |  |  |  |  |  |  |  |  |  |  |  |  |  |  |  |  |  |  |  |  |  |  |  |  |  |  |  |  |  |  |  |  |  |  |  |  |  |  |  |  |  |  |  |  |  |  |  |  |  |  |  |  |  |  |  |  |  |  |  |  |  |  |  |  |  |  |  |  |  |  |  |  |  |  |  |  |  |  |  |  |  |  |  |  |  |  |  |  |  |  |  |  |  |  |  |  |  |  |  |  |  |  |  |  |  |  |  |  |  |  |  |  |  |  |  |  |  |  |  |  |  |  |  |  |  |  |  |  |  |  |  |  |  |  |  |  |  |  |  |  |  |  |  |  |  |  |  |  |  |  |  |  |  |  |  |  |  |  |  |  |  |  |  |  |  |  |  |  |  |  |  |  |  |  |  |  |  |  |  |  |  |  |  |  |  |  |  |  |  |  |  |  |  |  |  |  |  |  |  |  |  |  |  |  |  |  |  |  |  |  |  |  |  |  |  |  |  |  |  |  |  |  |  |  |  |  |  |  |  |  |  |  |  |  |  |  |  |  |  |  |  |  |  |  |  |  |  |  |  |  |  |  |  |  |  |  |  |  |  |  |  |  |  |  |  |  |  |  |  |  |  |  |  |  |  |  |  |  |  |  |  |  |  |  |  |  |  |  |  |  |  |  |  |  |  |  |  |  |  |  |  |  |  |  |  |  |  |  |  |  |  |  |  |  |  |  |  |  |  |  |  |  |  |  |  |  |  |  |  |  |  |  |  |  |  |  |  |  |  |  |  |  |  |  |  |  |  |  |  |  |  |  |  |  |  |  |  |  |  |  |  |  |  |  |  |  |  |  |  |  |  |  |  |  |  |  |  |  |  |  |  |  |  |  |  |  |  |  |  |  |  |  |  |  |  |  |  |  |  |  |  |  |  |  |  |  |  |  |  |  |  |  |  |  |  |  |  |  |  |  |  |  |  |  |  |  |  |  |  |  |  |  |  |  |  |  |  |  |  |  |  |  |  |  |  |  |  |  |  |  |  |  |  |  |  |  |  |  |  |  |  |  |  |  |  |  |  |  |  |  |  |  |  |  |  |  |  |  |  |  |  |  |  |  |  |  |  |  |  |  |  |  |  |  |  |  |  |  |  |  |  |  |  |  |  |  |  |  |  |  |  |  |  |  |  |  |  |  |  |  |  |  |  |  |  |  |  |  |  |  |  |  |  |  |  |  |  |  |  |  |  |  |  |  |  |  |  |  |  |  |  |  |  |  |  |  |  |  |  |  |  |  |  |  |  |  |  |  |  |  |  |  |  |  |  |  |  |  |  |  |  |  |  |  |  |  |  |  |  |  |  |  |  |  |  |  |  |  |  |  |  |  |  |  |  |  |  |  |  |  |  |  |  |  |  |  |  |  |  |  |  |  |  |  |  |  |  |  |  |  |  |  |  |  |  |  |  |  |  |  |  |  |  |  |  |  |  |  |  |  |  |  |  |  |  |  |  |  |  |  |  |  |  |  |  |  |  |  |  |  |  |  |  |  |  |  |  |  |  |  |  |  |  |  |  |  |  |  |  |  |  |  |  |  |  |  |  |  |  |  |  |  |  |  |  |  |  |  |  |  |  |  |  |  |  |  |  |  |  |  |  |  |  |  |  |  |  |  |  |  |  |  |  |  |  |  |  |  |  |  |  |  |  |  |  |  |  |  |  |  |  |  |  |  |  |  |  |  |  |  |  |  |  |  |  |  |  |  |  |  |  |  |  |  |  |  |  |  |  |  |  |  |  |  |  |  |  |  |  |  |  |  |  |  |  |  |  |  |  |  |  |  |  |  |  |  |  |  |  |  |  |  |  |  |  |  |  |  |  |  |  |  |  |  |  |  |  |  |  |  |  |  |  |  |  |  |  |  |  |  |  |  |  |  |  |  |  |  |  |  |  |  |  |  |  |  |  |  |  |  |  |  |  |  |  |  |  |  |  |  |  |  |  |  |  |  |  |  |  |  |  |  |  |  |  |  |  |  |  |  |  |  |  |  |  |  |  |  |  |  |  |  |  |  |  |  |  |  |  |  |  |  |  |  |  |  |  |  |  |  |  |  |  |  |  |  |  |  |  |  |  |  |  |  |  |  |  |  |  |  |  |  |  |  |  |  |  |  |  |  |  |  |  |  |  |  |  |  |  |  |  |  |  |  |  |  |  |  |  |  |  |  |  |  |  |  |  |  |  |  |  |  |  |  |  |  |  |  |  |  |  |  |  |  |  |  |  |  |  |  |  |  |  |  |  |  |  |  |  |  |  |  |  |  |  |  |  |  |  |  |  |  |  |  |  |  |  |  |  |  |  |  |  |  |  |  |  |  |  |  |  |  |  |  |  |  |  |  |  |  |  |  |  |  |  |  |  |  |  |  |  |  |  |  |  |  |  |  |  |  |  |  |  |  |  |  |  |  |  |  |  |  |  |  |  |  |  |  |  |  |  |  |  |  |  |  |  |  |  |  |  |  |  |  |  |  |  |  |  |  |  |  |  |  |  |  |  |  |  |  |  |  |  |  |  |  |  |  |  |  |  |  |  |  |  |  |  |  |  |  |  |  |  |  |  |  |  |  |  |  |  |  |  |  |  |  |  |  |  |  |  |  |  |  |  |  |  |  |  |  |  |  |  |  |  |  |  |  |  |  |  |  |  |  |  |  |  |  |  |  |  |  |  |  |  |  |  |  |  |  |  |  |  |  |  |  |  |  |  |  |  |  |  |  |  |  |  |  |  |  |  |  |  |  |  |  |  |  |  |  |  |  |  |  |  |  |  |  |  |  |  |  |  |  |  |  |  |  |  |  |  |  |  |  |  |  |  |  |  |  |  |  |  |  |  |  |  |  |  |  |  |  |  |  |  |  |  |  |  |  |  |  |  |  |  |  |  |  |  |  |  |  |  |  |  |  |  |  |  |  |  |  |  |  |  |  |  |  |  |  |  |  |  |  |  |  |  |  |  |  |  |  |  |  |  |  |  |  |  |  |  |  |  |  |  |  |  |  |  |  |  |  |  |  |  |  |  |  |  |  |  |  |  |  |  |  |  |  |  |  |  |  |  |  |  |  |  |  |  |  |  |  |  |  |  |  |  |  |  |  |  |  |  |  |  |  |  |  |  |  |  |  |  |  |  |  |  |  |  |  |  |  |  |  |  |  |  |  |  |  |  |  |  |  |  |  |  |  |  |  |  |  |  |  |  |  |  |  |  |  |  |  |  |  |  |  |  |  |  |  |  |  |  |  |  |  |  |  |  |  |  |  |  |  |  |  |  |  |  |  |  |  |  |  |  |  |  |  |  |  |  |  |  |  |  |  |  |  |  |  |  |  |  |  |  |  |  |  |  |  |  |  |  |  |  |  |  |  |  |  |  |  |  |  |  |  |  |  |  |  |  |  |  |  |  |  |  |  |  |  |  |  |  |  |  |  |  |  |  |  |  |  |  |  |  |  |  |  |  |  |  |  |  |  |  |  |  |  |  |  |  |  |  |  |  |  |  |  |  |  |  |  |  |  |  |  |  |  |  |  |  |  |  |  |  |  |  |  |  |  |  |  |  |  |  |  |  |  |  |  |  |  |  |  |  |  |  |  |  |  |  |  |  |  |  |  |  |  |  |  |  |  |  |  |  |  |  |  |  |  |  |  |  |  |  |  |  |  |  |  |  |  |  |  |  |  |  |  |  |  |  |  |  |  |  |  |  |  |  |  |  |  |  |  |  |  |  |  |  |  |  |  |  |  |  |  |  |  |  |  |  |  |  |  |  |  |  |  |  |  |  |  |  |  |  |  |  |  |  |  |  |  |  |  |  |  |  |  |  |  |  |  |  |  |  |  |  |  |  |  |  |  |  |  |  |  |  |  |  |  |  |  |  |  |  |  |  |  |  |  |  |  |  |  |  |  |  |  |  |  |  |  |  |  |  |  |  |  |  |  |  |  |  |  |  |  |  |  |  |  |  |  |  |  |  |  |  |  |  |  |  |  |  |  |  |  |  |  |  |  |  |  |  |  |  |  |  |  |  |  |  |  |  |  |  |  |  |  |  |  |  |  |  |  |  |  |  |  |  |  |  |  |  |  |  |  |  |  |  |  |  |  |  |  |  |  |  |  |  |  |  |  |  |  |  |  |  |  |  |  |  |  |  |  |  |  |  |  |  |  |  |  |  |  |  |  |  |  |  |  |  |  |  |  |  |  |  |  |  |  |  |  |  |  |  |  |  |  |  |  |  |  |  |  |  |  |  |  |  |  |  |  |  |  |  |  |  |  |  |  |  |  |  |  |  |  |  |  |  |  |  |  |  |  |  |  |  |  |  |  |  |  |  |  |  |  |  |  |  |  |  |  |  |  |  |  |  |  |  |  |  |  |  |  |  |  |  |  |  |  |  |  |  |  |  |  |  |  |  |  |  |  |  |  |  |  |  |  |  |  |  |  |  |  |  |  |  |  |  |  |  |  |  |  |  |  |  |  |  |  |  |  |  |  |  |  |  |  |  |  |  |  |  |  |  |  |  |  |  |  |  |  |  |  |  |  |  |  |  |  |  |  |  |  |  |  |  |  |  |  |  |  |  |  |  |  |  |  |  |  |  |  |  |  |  |  |  |  |  |  |  |  |  |  |  |  |  |  |  |  |  |  |  |  |  |  |  |  |  |  |  |  |  |  |  |  |  |  |  |  |  |  |  |  |  |  |  |  |  |  |  |  |  |  |  |  |  |  |  |  |  |  |  |  |  |  |  |  |  |  |  |  |  |  |  |  |  |  |  |  |  |  |  |  |  |  |  |  |  |  |  |  |  |  |  |  |  |  |  |  |  |  |  |  |  |  |  |  |  |  |  |  |  |  |  |  |  |  |  |  |  |  |  |  |  |  |  |  |  |  |  |  |  |  |  |  |  |  |  |  |  |  |  |  |  |  |  |  |  |  |  |  |  |  |  |  |  |  |  |  |  |  |  |  |  |  |  |  |  |  |  |  |  |  |  |  |  |  |  |  |  |  |  |  |  |  |  |  |  |  |  |  |  |  |  |  |  |  |  |  |  |  |  |  |  |  |  |  |  |  |  |  |  |  |  |  |  |  |  |  |  |  |  |  |  |  |  |  |  |  |  |  |  |  |  |  |  |  |  |  |  |  |  |  |  |  |  |  |  |  |  |  |  |  |  |  |  |  |  |  |  |  |  |  |  |  |  |  |  |  |  |  |  |  |  |  |  |  |  |  |  |  |  |  |  |  |  |  |  |  |  |  |  |  |  |  |  |  |  |  |  |  |  |  |  |  |  |  |  |  |  |  |  |  |  |  |  |  |  |  |  |  |  |  |  |  |  |  |  |  |  |  |  |  |  |  |  |  |  |  |  |  |  |  |  |  |  |  |  |  |  |  |  |  |  |  |  |  |  |  |  |  |  |  |  |  |  |  |  |  |  |  |  |  |  |  |  |  |  |  |  |  |  |  |  |  |  |  |  |  |  |  |  |  |  |  |  |  |  |  |  |  |  |  |  |  |  |  |  |  |  |  |  |  |  |  |  |  |  |  |  |  |  |  |  |  |  |  |  |  |  |  |  |  |  |  |  |  |  |  |  |  |  |  |  |  |  |  |  |  |  |  |  |  |  |  |  |  |  |  |  |  |  |  |  |  |  |  |  |  |  |  |  |  |  |  |  |  |  |  |  |  |  |  |  |  |  |  |  |  |  |  |  |  |  |  |  |  |  |  |  |  |  |  |  |  |  |  |  |  |  |  |  |  |  |  |  |  |  |  |  |  |  |  |  |  |  |  |  |  |  |  |  |  |  |  |  |  |  |  |  |  |  |  |  |  |  |  |  |  |  |  |  |  |  |  |  |  |  |  |  |  |  |  |  |  |  |  |  |  |  |  |  |  |  |  |  |  |  |  |  |  |  |  |  |  |  |  |  |  |  |  |  |  |  |  |  |  |  |  |  |  |  |  |  |  |  |  |  |  |  |  |  |  |  |  |  |  |  |  |  |  |  |  |  |  |  |  |  |  |  |  |  |  |  |  |  |  |  |  |  |  |  |  |  |  |  |  |  |  |  |  |  |  |  |  |  |  |  |  |  |  |  |  |  |  |  |  |  |  |  |  |  |  |  |  |  |  |  |  |  |  |  |  |  |  |  |  |  |  |  |  |  |  |  |  |  |  |  |  |  |  |  |  |  |  |  |  |  |  |  |  |  |  |  |  |  |  |  |  |  |  |  |  |  |  |  |  |  |  |  |  |  |  |  |  |  |  |  |  |  |  |  |  |  |  |  |  |  |  |  |  |  |  |  |  |  |  |  |  |  |  |  |  |  |  |  |  |  |  |  |  |  |  |  |  |  |  |  |  |  |  |  |  |  |  |  |  |  |  |  |  |  |  |  |  |  |  |  |  |  |  |  |  |  |  |  |  |  |  |  |  |  |  |  |  |  |  |  |  |  |  |  |  |  |  |  |  |  |  |  |  |  |  |  |  |  |  |  |  |  |  |  |  |  |  |  |  |  |  |  |  |  |  |  |  |  |  |  |  |  |  |  |  |  |  |  |  |  |  |  |  |  |  |  |  |  |  |  |  |  |  |  |  |  |  |  |  |  |  |  |  |  |  |  |  |  |  |  |  |  |  |  |  |  |  |  |  |  |  |  |  |  |  |  |  |  |  |  |  |  |  |  |  |  |  |  |  |  |  |  |  |  |  |  |  |  |  |  |  |  |  |  |  |  |  |  |  |  |  |  |  |  |  |  |  |  |  |  |  |  |  |  |  |  |  |  |  |  |  |  |  |  |  |  |  |  |  |  |  |  |  |  |  |  |  |  |  |  |  |  |  |  |  |  |  |  |  |  |  |  |  |  |  |  |  |  |  |  |  |  |  |  |  |  |  |  |  |  |  |  |  |  |  |  |  |  |  |  |  |  |  |  |  |  |  |  |  |  |  |  |  |  |  |  |  |  |  |  |  |  |  |  |  |  |  |  |  |  |  |  |  |  |  |  |  |  |  |  |  |  |  |  |  |  |  |  |  |  |  |  |  |  |  |  |  |  |  |  |  |  |  |  |  |  |  |  |  |  |  |  |  |  |  |  |  |  |  |  |  |  |  |  |  |  |  |  |  |  |  |  |  |  |  |  |  |  |  |  |  |  |  |  |  |  |  |  |  |  |  |  |  |  |  |  |  |  |  |  |  |  |  |  |  |  |  |  |  |  |  |  |  |  |  |  |  |  |  |  |  |  |  |  |  |  |  |  |  |  |  |  |  |  |  |  |  |  |  |  |  |  |  |  |  |  |  |  |  |  |  |  |  |  |  |  |  |  |  |  |  |  |  |  |  |  |  |  |  |  |  |  |  |  |  |  |  |  |  |  |  |  |  |  |  |  |  |  |  |  |  |  |  |  |  |  |  |  |  |  |  |  |  |  |  |  |  |  |  |  |  |  |  |  |  |  |  |  |  |  |  |  |  |  |  |  |  |  |  |  |  |  |  |  |  |  |  |  |  |  |  |  |  |  |  |  |  |  |  |  |  |  |  |  |  |  |  |  |  |  |  |  |  |  |  |  |  |  |  |  |  |  |  |  |  |  |  |  |  |  |  |  |  |  |  |  |  |  |  |  |  |  |  |  |  |  |  |  |  |  |  |  |  |  |  |  |  |  |  |  |  |  |  |  |  |  |  |  |  |  |  |  |  |  |  |  |  |  |  |  |  |  |  |  |  |  |  |  |  |  |  |  |  |  |  |  |  |  |  |  |  |  |  |  |  |  |  |  |  |  |  |  |  |  |  |  |  |  |  |  |  |  |  |  |  |  |  |  |  |  |  |  |  |  |  |  |  |  |  |  |  |  |  |  |  |  |  |  |  |  |  |  |  |  |  |  |  |  |  |  |  |  |  |  |  |  |  |  |  |  |  |  |  |  |  |  |  |  |  |  |  |  |  |  |  |  |  |  |  |  |  |  |  |  |  |  |  |  |  |  |  |  |  |  |  |  |  |  |  |  |  |  |  |  |  |  |  |  |  |  |  |  |  |  |  |  |  |  |  |  |  |  |  |  |  |  |  |  |  |  |  |  |  |  |  |  |  |  |  |  |  |  |  |  |  |  |  |  |  |  |  |  |  |  |  |  |  |  |  |  |  |  |  |  |  |  |  |  |  |  |  |  |  |  |  |  |  |  |  |  |  |  |  |  |  |  |  |  |  |  |  |  |  |  |  |  |  |  |  |  |  |  |  |  |  |  |  |  |  |  |  |  |  |  |  |  |  |  |  |  |  |  |  |  |  |  |  |  |  |  |  |  |  |  |  |  |  |  |  |  |  |  |  |  |  |  |  |  |  |  |  |  |  |  |  |  |  |  |  |  |  |  |  |  |  |  |  |  |  |  |  |  |  |  |  |  |  |  |  |  |  |  |  |  |  |  |  |  |  |  |  |  |  |  |  |  |  |  |  |  |  |  |  |  |  |  |  |  |  |  |  |  |  |  |  |  |  |  |  |  |  |  |  |  |  |  |  |  |  |  |  |  |  |  |  |  |  |  |  |  |  |  |  |  |  |  |  |  |  |  |  |  |  |  |  |  |  |  |  |  |  |  |  |  |  |  |  |  |  |  |  |  |  |  |  |  |  |  |  |  |  |  |  |  |  |  |  |  |  |  |  |  |  |  |  |  |  |  |  |  |  |  |  |  |  |  |  |  |  |  |  |  |  |  |  |  |  |  |  |  |  |  |  |  |  |  |  |  |  |  |  |  |  |  |  |  |  |  |  |  |  |  |  |  |  |  |  |  |  |  |  |  |  |  |  |  |  |  |  |  |  |  |  |  |  |  |  |  |  |  |  |  |  |  |  |  |  |  |  |  |  |  |  |  |  |  |  |  |  |  |  |  |  |  |  |  |  |  |  |  |  |  |  |  |  |  |  |  |  |  |  |  |  |  |  |  |  |  |  |  |  |  |  |  |  |  |  |  |  |  |  |  |  |  |  |  |  |  |  |  |  |  |  |  |  |  |  |  |  |  |  |  |  |  |  |  |  |  |  |  |  |  |  |  |  |  |  |  |  |  |  |  |  |  |  |  |  |  |  |  |  |  |  |  |  |  |  |  |  |  |  |  |  |  |  |  |  |  |  |  |  |  |  |  |  |  |  |  |  |  |  |  |  |  |  |  |  |  |  |  |  |  |  |  |  |  |  |  |  |  |  |  |  |  |  |  |  |  |  |  |  |  |  |  |  |  |  |  |  |  |  |  |  |  |  |  |  |  |  |  |  |  |  |  |  |  |  |  |  |  |  |  |  |  |  |  |  |  |  |  |  |  |  |  |  |  |  |  |  |  |  |  |  |  |  |  |  |  |  |  |  |  |  |  |  |  |  |  |  |  |  |  |  |  |  |  |  |  |  |  |  |  |  |  |  |  |  |  |  |  |  |  |  |  |  |  |  |  |  |  |  |  |  |  |  |  |  |  |  |  |  |  |  |  |  |  |  |  |  |  |  |  |  |  |  |  |  |  |  |  |  |  |  |  |  |  |  |  |  |  |  |  |  |  |  |  |  |  |  |  |  |  |  |  |  |  |  |  |  |  |  |  |  |  |  |  |  |  |  |  |  |  |  |  |  |  |  |  |  |  |  |  |  |  |  |  |  |  |  |  |  |  |  |  |  |  |  |  |  |  |  |  |  |  |  |  |  |  |  |  |  |  |  |  |  |  |  |  |  |  |  |  |  |  |  |  |  |  |  |  |  |  |  |  |  |  |  |  |  |  |  |  |  |  |  |  |  |  |  |  |  |  |  |  |  |  |  |  |  |  |  |  |  |  |  |  |  |  |  |  |  |  |  |  |  |  |  |  |  |  |  |  |  |  |  |  |  |  |  |  |  |  |  |  |  |  |  |  |  |  |  |  |  |  |  |  |  |  |  |  |  |  |  |  |  |  |  |  |  |  |  |  |  |  |  |  |  |  |  |  |  |  |  |  |  |  |  |  |  |  |  |  |  |  |  |  |  |  |  |  |  |  |  |  |  |  |  |  |  |  |  |  |  |  |  |  |  |  |  |  |  |  |  |  |  |  |  |  |  |  |  |  |  |  |  |  |  |  |  |  |  |  |  |  |  |  |  |  |  |  |  |  |  |  |  |  |  |  |  |  |  |  |  |  |  |  |  |  |  |  |  |  |  |  |  |  |  |  |  |  |  |  |  |  |  |  |  |  |  |  |  |  |  |  |  |  |  |  |  |  |  |  |  |  |  |  |  |  |  |  |  |  |  |  |  |  |  |  |  |  |  |  |  |  |  |  |  |  |  |  |  |  |  |  |  |  |  |  |  |  |  |  |  |  |  |  |  |  |  |  |  |  |  |  |  |  |  |  |  |  |  |  |  |  |  |  |  |  |  |  |  |  |  |  |  |  |  |  |  |  |  |  |  |  |  |  |  |  |  |  |  |  |  |  |  |  |  |  |  |  |  |  |  |  |  |  |  |  |  |  |  |  |  |  |  |  |  |  |  |  |  |  |  |  |  |  |  |  |  |  |  |  |  |  |  |  |  |  |  |  |  |  |  |  |  |  |  |  |  |  |  |  |  |  |  |  |  |  |  |  |  |  |  |  |  |  |  |  |  |  |  |  |  |  |  |  |  |  |  |  |  |  |  |  |  |  |  |  |  |  |  |  |  |  |  |  |  |  |  |  |  |  |  |  |  |  |  |  |  |  |  |  |  |  |  |  |  |  |  |  |  |  |  |  |  |  |  |  |  |  |  |  |  |  |  |  |  |  |  |  |  |  |  |  |  |  |  |  |  |  |  |  |  |  |  |  |  |  |  |  |  |  |  |  |  |  |  |  |  |  |  |  |  |  |  |  |  |  |  |  |  |  |  |  |  |  |  |  |  |  |  |  |  |  |  |  |  |  |  |  |  |  |  |  |  |  |  |  |  |  |  |  |  |  |  |  |  |  |  |  |  |  |  |  |  |  |  |  |  |  |  |  |  |  |  |  |  |  |  |  |  |  |  |  |  |  |  |  |  |  |  |  |  |  |  |  |  |  |  |  |  |  |  |  |  |  |  |  |  |  |  |  |  |  |  |  |  |  |  |  |  |  |  |  |  |  |  |  |  |  |  |  |  |  |  |  |  |  |  |  |  |  |  |  |  |  |  |  |  |  |  |  |  |  |  |  |  |  |  |  |  |  |  |  |  |  |  |  |  |  |  |  |  |  |  |  |  |  |  |  |  |  |  |  |  |  |  |  |  |  |  |  |  |  |  |  |  |  |  |  |  |  |  |  |  |  |  |  |  |  |  |  |  |  |  |  |  |  |  |  |  |  |  |  |  |  |  |  |  |  |  |  |  |  |  |  |  |  |  |  |  |  |  |  |  |  |  |  |  |  |  |  |  |  |  |  |  |  |  |  |  |  |  |  |  |  |  |  |  |  |  |  |  |  |  |  |  |  |  |  |  |  |  |  |  |  |  |  |  |  |  |  |  |  |  |  |  |  |  |  |  |  |  |  |  |  |  |  |  |  |  |  |  |  |  |  |  |  |  |  |  |  |  |  |  |  |  |  |  |  |  |  |  |  |  |  |  |  |  |  |  |  |  |  |  |  |  |  |  |  |  |  |  |  |  |  |  |  |  |  |  |  |  |  |  |  |  |  |  |  |  |  |  |  |  |  |  |  |  |  |  |  |  |  |  |  |  |  |  |  |  |  |  |  |  |  |  |  |  |  |  |  |  |  |  |  |  |  |  |  |  |  |  |  |  |  |  |  |  |  |  |  |  |  |  |  |  |  |  |  |  |  |  |  |  |  |  |  |  |  |  |  |  |  |  |  |  |  |  |  |  |  |  |  |  |  |  |  |  |  |  |  |  |  |  |  |  |  |  |  |  |  |  |  |  |  |  |  |  |  |  |  |  |  |  |  |  |  |  |  |  |  |  |  |  |  |  |  |  |  |  |  |  |  |  |  |  |  |  |  |  |  |  |  |  |  |  |  |  |  |  |  |  |  |  |  |  |  |  |  |  |  |  |  |  |  |  |  |  |  |  |  |  |  |  |  |  |  |  |  |  |  |  |  |  |  |  |  |  |  |  |  |  |  |  |  |  |  |  |  |  |  |  |  |  |  |  |  |  |  |  |  |  |  |  |  |  |  |  |  |  |  |  |  |  |  |  |  |  |  |  |  |  |  |  |  |  |  |  |  |  |  |  |  |  |  |  |  |  |  |  |  |  |  |  |  |  |  |  |  |  |  |  |  |  |  |  |  |  |  |  |  |  |  |  |  |  |  |  |  |  |  |  |  |  |  |  |  |  |  |  |  |  |  |  |  |  |  |  |  |  |  |  |  |  |  |  |  |  |  |  |  |  |  |  |  |  |  |  |  |  |  |  |  |  |  |  |  |  |  |  |  |  |  |  |  |  |  |  |  |  |  |  |  |  |  |  |  |  |  |  |  |  |  |  |  |  |  |  |  |  |  |  |  |  |  |  |  |  |  |  |  |  |  |  |  |  |  |  |  |  |  |  |  |  |  |  |  |  |  |  |  |  |  |  |  |  |  |  |  |  |  |  |  |  |  |  |  |  |  |  |  |  |  |  |  |  |  |  |  |  |  |  |  |  |  |  |  |  |  |  |  |  |  |  |  |  |  |  |  |  |  |  |  |  |  |  |  |  |  |  |  |  |  |  |  |  |  |  |  |  |  |  |  |  |  |  |  |  |  |  |  |  |  |  |  |  |  |  |  |  |  |  |  |  |  |  |  |  |  |  |  |  |  |  |  |  |  |  |  |  |  |  |  |  |  |  |  |  |  |  |  |  |  |  |  |  |  |  |  |  |  |  |  |  |  |  |  |  |  |  |  |  |  |  |  |  |  |  |  |  |  |  |  |  |  |  |  |  |  |  |  |  |  |  |  |  |  |  |  |  |  |  |  |  |  |  |  |  |  |  |  |  |  |  |  |  |  |  |  |  |  |  |  |  |  |  |  |  |  |  |  |  |  |  |  |  |  |  |  |  |  |  |  |  |  |  |  |  |  |  |  |  |  |  |  |  |  |  |  |  |  |  |  |  |  |  |  |  |  |  |  |  |  |  |  |  |  |  |  |  |  |  |  |  |  |  |  |  |  |  |  |  |  |  |  |  |  |  |  |  |  |  |  |  |  |  |  |  |  |  |  |  |  |  |  |  |  |  |  |  |  |  |  |  |  |  |  |  |  |  |  |  |  |  |  |  |  |  |  |  |  |  |  |  |  |  |  |  |  |  |  |  |  |  |  |  |  |  |  |  |  |  |  |  |  |  |  |  |  |  |  |  |  |  |  |  |  |  |  |  |  |  |  |  |  |  |  |  |  |  |  |  |  |  |  |  |  |  |  |  |  |  |  |  |  |  |  |  |  |  |  |  |  |  |  |  |  |  |  |  |  |  |  |  |  |  |  |  |  |  |  |  |  |  |  |  |  |  |  |  |  |  |  |  |  |  |  |  |  |  |  |  |  |  |  |  |  |  |  |  |  |  |  |  |  |  |  |  |  |  |  |  |  |  |  |  |  |  |  |  |  |  |  |  |  |  |  |  |  |  |  |  |  |  |  |  |  |  |  |  |  |  |  |  |  |  |  |  |  |  |  |  |  |  |  |  |  |  |  |  |  |  |  |  |  |  |  |  |  |  |  |  |  |  |  |  |  |  |  |  |  |  |  |  |  |  |  |  |  |  |  |  |  |  |  |  |  |  |  |  |  |  |  |  |  |  |  |  |  |  |  |  |  |  |  |  |  |  |  |  |  |  |  |  |  |  |  |  |  |  |  |  |  |  |  |  |  |  |  |  |  |  |  |  |  |  |  |  |  |  |  |  |  |  |  |  |  |  |  |  |  |  |  |  |  |  |  |  |  |  |  |  |  |  |  |  |  |  |  |  |  |  |  |  |  |  |  |  |  |  |  |  |  |  |  |  |  |  |  |  |  |  |  |  |  |  |  |  |  |  |  |  |  |  |  |  |  |  |  |  |  |  |  |  |  |  |  |  |  |  |  |  |  |  |  |  |  |  |  |  |  |  |  |  |  |  |  |  |  |  |  |  |  |  |  |  |  |  |  |  |  |  |  |  |  |  |  |  |  |  |  |  |  |  |  |  |  |  |  |  |  |  |  |  |  |  |  |  |  |  |  |  |  |  |  |  |  |  |  |  |  |  |  |  |  |  |  |  |  |  |  |  |  |  |  |  |  |  |  |  |  |  |  |  |  |  |  |  |  |  |  |  |  |  |  |  |  |  |  |  |  |  |  |  |  |  |  |  |  |  |  |  |  |  |  |  |  |  |  |  |  |  |  |  |  |  |  |  |  |  |  |  |  |  |  |  |  |  |  |  |  |  |  |  |  |  |  |  |  |  |  |  |  |  |  |  |  |  |  |  |  |  |  |  |  |  |  |  |  |  |  |  |  |  |  |  |  |  |  |  |  |  |  |  |  |  |  |  |  |  |  |  |  |  |  |  |  |  |  |  |  |  |  |  |  |  |  |  |  |  |  |  |  |  |  |  |  |  |  |  |  |  |  |  |  |  |  |  |  |  |  |  |  |  |  |  |  |  |  |  |  |  |  |  |  |  |  |  |  |  |  |  |  |  |  |  |  |  |  |  |  |  |  |  |  |  |  |  |  |  |  |  |  |  |  |  |  |  |  |  |  |  |  |  |  |  |  |  |  |  |  |  |  |  |  |  |  |  |  |  |  |  |  |  |  |  |  |  |  |  |  |  |  |  |  |  |  |  |  |  |  |  |  |  |  |  |  |  |  |  |  |  |  |  |  |  |  |  |  |  |  |  |  |  |  |  |  |  |  |  |  |  |  |  |  |  |  |  |  |  |  |  |  |  |  |  |  |  |  |  |  |  |  |  |  |  |  |  |  |  |  |  |  |  |  |  |  |  |  |  |  |  |  |  |  |  |  |  |  |  |  |  |  |  |  |  |  |  |  |  |  |  |  |  |  |  |  |  |  |  |  |  |  |  |  |  |  |  |  |  |  |  |  |  |  |  |  |  |  |  |  |  |  |  |  |  |  |  |  |  |  |  |  |  |  |  |  |  |  |  |  |  |  |  |  |  |  |  |  |  |  |  |  |  |  |  |  |  |  |  |  |  |  |  |  |  |  |  |  |  |  |  |  |  |  |  |  |  |  |  |  |  |  |  |  |  |  |  |  |  |  |  |  |  |  |  |  |  |  |  |  |  |  |  |  |  |  |  |  |  |  |  |  |  |  |  |  |  |  |  |  |  |  |  |  |  |  |  |  |  |  |  |  |  |  |  |  |  |  |  |  |  |  |  |  |  |  |  |  |  |  |  |  |  |  |  |  |  |  |  |  |  |  |  |  |  |  |  |  |  |  |  |  |  |  |  |
| --- | --- | --- | --- | --- | --- | --- | --- | --- | --- | --- | --- | --- | --- | --- | --- | --- | --- | --- | --- | --- | --- | --- | --- | --- | --- | --- | --- | --- | --- | --- | --- | --- | --- | --- | --- | --- | --- | --- | --- | --- | --- | --- | --- | --- | --- | --- | --- | --- | --- | --- | --- | --- | --- | --- | --- | --- | --- | --- | --- | --- | --- | --- | --- | --- | --- | --- | --- | --- | --- | --- | --- | --- | --- | --- | --- | --- | --- | --- | --- | --- | --- | --- | --- | --- | --- | --- | --- | --- | --- | --- | --- | --- | --- | --- | --- | --- | --- | --- | --- | --- | --- | --- | --- | --- | --- | --- | --- | --- | --- | --- | --- | --- | --- | --- | --- | --- | --- | --- | --- | --- | --- | --- | --- | --- | --- | --- | --- | --- | --- | --- | --- | --- | --- | --- | --- | --- | --- | --- | --- | --- | --- | --- | --- | --- | --- | --- | --- | --- | --- | --- | --- | --- | --- | --- | --- | --- | --- | --- | --- | --- | --- | --- | --- | --- | --- | --- | --- | --- | --- | --- | --- | --- | --- | --- | --- | --- | --- | --- | --- | --- | --- | --- | --- | --- | --- | --- | --- | --- | --- | --- | --- | --- | --- | --- | --- | --- | --- | --- | --- | --- | --- | --- | --- | --- | --- | --- | --- | --- | --- | --- | --- | --- | --- | --- | --- | --- | --- | --- | --- | --- | --- | --- | --- | --- | --- | --- | --- | --- | --- | --- | --- | --- | --- | --- | --- | --- | --- | --- | --- | --- | --- | --- | --- | --- | --- | --- | --- | --- | --- | --- | --- | --- | --- | --- | --- | --- | --- | --- | --- | --- | --- | --- | --- | --- | --- | --- | --- | --- | --- | --- | --- | --- | --- | --- | --- | --- | --- | --- | --- | --- | --- | --- | --- | --- | --- | --- | --- | --- | --- | --- | --- | --- | --- | --- | --- | --- | --- | --- | --- | --- | --- | --- | --- | --- | --- | --- | --- | --- | --- | --- | --- | --- | --- | --- | --- | --- | --- | --- | --- | --- | --- | --- | --- | --- | --- | --- | --- | --- | --- | --- | --- | --- | --- | --- | --- | --- | --- | --- | --- | --- | --- | --- | --- | --- | --- | --- | --- | --- | --- | --- | --- | --- | --- | --- | --- | --- | --- | --- | --- | --- | --- | --- | --- | --- | --- | --- | --- | --- | --- | --- | --- | --- | --- | --- | --- | --- | --- | --- | --- | --- | --- | --- | --- | --- | --- | --- | --- | --- | --- | --- | --- | --- | --- | --- | --- | --- | --- | --- | --- | --- | --- | --- | --- | --- | --- | --- | --- | --- | --- | --- | --- | --- | --- | --- | --- | --- | --- | --- | --- | --- | --- | --- | --- | --- | --- | --- | --- | --- | --- | --- | --- | --- | --- | --- | --- | --- | --- | --- | --- | --- | --- | --- | --- | --- | --- | --- | --- | --- | --- | --- | --- | --- | --- | --- | --- | --- | --- | --- | --- | --- | --- | --- | --- | --- | --- | --- | --- | --- | --- | --- | --- | --- | --- | --- | --- | --- | --- | --- | --- | --- | --- | --- | --- | --- | --- | --- | --- | --- | --- | --- | --- | --- | --- | --- | --- | --- | --- | --- | --- | --- | --- | --- | --- | --- | --- | --- | --- | --- | --- | --- | --- | --- | --- | --- | --- | --- | --- | --- | --- | --- | --- | --- | --- | --- | --- | --- | --- | --- | --- | --- | --- | --- | --- | --- | --- | --- | --- | --- | --- | --- | --- | --- | --- | --- | --- | --- | --- | --- | --- | --- | --- | --- | --- | --- | --- | --- | --- | --- | --- | --- | --- | --- | --- | --- | --- | --- | --- | --- | --- | --- | --- | --- | --- | --- | --- | --- | --- | --- | --- | --- | --- | --- | --- | --- | --- | --- | --- | --- | --- | --- | --- | --- | --- | --- | --- | --- | --- | --- | --- | --- | --- | --- | --- | --- | --- | --- | --- | --- | --- | --- | --- | --- | --- | --- | --- | --- | --- | --- | --- | --- | --- | --- | --- | --- | --- | --- | --- | --- | --- | --- | --- | --- | --- | --- | --- | --- | --- | --- | --- | --- | --- | --- | --- | --- | --- | --- | --- | --- | --- | --- | --- | --- | --- | --- | --- | --- | --- | --- | --- | --- | --- | --- | --- | --- | --- | --- | --- | --- | --- | --- | --- | --- | --- | --- | --- | --- | --- | --- | --- | --- | --- | --- | --- | --- | --- | --- | --- | --- | --- | --- | --- | --- | --- | --- | --- | --- | --- | --- | --- | --- | --- | --- | --- | --- | --- | --- | --- | --- | --- | --- | --- | --- | --- | --- | --- | --- | --- | --- | --- | --- | --- | --- | --- | --- | --- | --- | --- | --- | --- | --- | --- | --- | --- | --- | --- | --- | --- | --- | --- | --- | --- | --- | --- | --- | --- | --- | --- | --- | --- | --- | --- | --- | --- | --- | --- | --- | --- | --- | --- | --- | --- | --- | --- | --- | --- | --- | --- | --- | --- | --- | --- | --- | --- | --- | --- | --- | --- | --- | --- | --- | --- | --- | --- | --- | --- | --- | --- | --- | --- | --- | --- | --- | --- | --- | --- | --- | --- | --- | --- | --- | --- | --- | --- | --- | --- | --- | --- | --- | --- | --- | --- | --- | --- | --- | --- | --- | --- | --- | --- | --- | --- | --- | --- | --- | --- | --- | --- | --- | --- | --- | --- | --- | --- | --- | --- | --- | --- | --- | --- | --- | --- | --- | --- | --- | --- | --- | --- | --- | --- | --- | --- | --- | --- | --- | --- | --- | --- | --- | --- | --- | --- | --- | --- | --- | --- | --- | --- | --- | --- | --- | --- | --- | --- | --- | --- | --- | --- | --- | --- | --- | --- | --- | --- | --- | --- | --- | --- | --- | --- | --- | --- | --- | --- | --- | --- | --- | --- | --- | --- | --- | --- | --- | --- | --- | --- | --- | --- | --- | --- | --- | --- | --- | --- | --- | --- | --- | --- | --- | --- | --- | --- | --- | --- | --- | --- | --- | --- | --- | --- | --- | --- | --- | --- | --- | --- | --- | --- | --- | --- | --- | --- | --- | --- | --- | --- | --- | --- | --- | --- | --- | --- | --- | --- | --- | --- | --- | --- | --- | --- | --- | --- | --- | --- | --- | --- | --- | --- | --- | --- | --- | --- | --- | --- | --- | --- | --- | --- | --- | --- | --- | --- | --- | --- | --- | --- | --- | --- | --- | --- | --- | --- | --- | --- | --- | --- | --- | --- | --- | --- | --- | --- | --- | --- | --- | --- | --- | --- | --- | --- | --- | --- | --- | --- | --- | --- | --- | --- | --- | --- | --- | --- | --- | --- | --- | --- | --- | --- | --- | --- | --- | --- | --- | --- | --- | --- | --- | --- | --- | --- | --- | --- | --- | --- | --- | --- | --- | --- | --- | --- | --- | --- | --- | --- | --- | --- | --- | --- | --- | --- | --- | --- | --- | --- | --- | --- | --- | --- | --- | --- | --- | --- | --- | --- | --- | --- | --- | --- | --- | --- | --- | --- | --- | --- | --- | --- | --- | --- | --- | --- | --- | --- | --- | --- | --- | --- | --- | --- | --- | --- | --- | --- | --- | --- | --- | --- | --- | --- | --- | --- | --- | --- | --- | --- | --- | --- | --- | --- | --- | --- | --- | --- | --- | --- | --- | --- | --- | --- | --- | --- | --- | --- | --- | --- | --- | --- | --- | --- | --- | --- | --- | --- | --- | --- | --- | --- | --- | --- | --- | --- | --- | --- | --- | --- | --- | --- | --- | --- | --- | --- | --- | --- | --- | --- | --- | --- | --- | --- | --- | --- | --- | --- | --- | --- | --- | --- | --- | --- | --- | --- | --- | --- | --- | --- | --- | --- | --- | --- | --- | --- | --- | --- | --- | --- | --- | --- | --- | --- | --- | --- | --- | --- | --- | --- | --- | --- | --- | --- | --- | --- | --- | --- | --- | --- | --- | --- | --- | --- | --- | --- | --- | --- | --- | --- | --- | --- | --- | --- | --- | --- | --- | --- | --- | --- | --- | --- | --- | --- | --- | --- | --- | --- | --- | --- | --- | --- | --- | --- | --- | --- | --- | --- | --- | --- | --- | --- | --- | --- | --- | --- | --- | --- | --- | --- | --- | --- | --- | --- | --- | --- | --- | --- | --- | --- | --- | --- | --- | --- | --- | --- | --- | --- | --- | --- | --- | --- | --- | --- | --- | --- | --- | --- | --- | --- | --- | --- | --- | --- | --- | --- | --- | --- | --- | --- | --- | --- | --- | --- | --- | --- | --- | --- | --- | --- | --- | --- | --- | --- | --- | --- | --- | --- | --- | --- | --- | --- | --- | --- | --- | --- | --- | --- | --- | --- | --- | --- | --- | --- | --- | --- | --- | --- | --- | --- | --- | --- | --- | --- | --- | --- | --- | --- | --- | --- | --- | --- | --- | --- | --- | --- | --- | --- | --- | --- | --- | --- | --- | --- | --- | --- | --- | --- | --- | --- | --- | --- | --- | --- | --- | --- | --- | --- | --- | --- | --- | --- | --- | --- | --- | --- | --- | --- | --- | --- | --- | --- | --- | --- | --- | --- | --- | --- | --- | --- | --- | --- | --- | --- | --- | --- | --- | --- | --- | --- | --- | --- | --- | --- | --- | --- | --- | --- | --- | --- | --- | --- | --- | --- | --- | --- | --- | --- | --- | --- | --- | --- | --- | --- | --- | --- | --- | --- | --- | --- | --- | --- | --- | --- | --- | --- | --- | --- | --- | --- | --- | --- | --- | --- | --- | --- | --- | --- | --- | --- | --- | --- | --- | --- | --- | --- | --- | --- | --- | --- | --- | --- | --- | --- | --- | --- | --- | --- | --- | --- | --- | --- | --- | --- | --- | --- | --- | --- | --- | --- | --- | --- | --- | --- | --- | --- | --- | --- | --- | --- | --- | --- | --- | --- | --- | --- | --- | --- | --- | --- | --- | --- | --- | --- | --- | --- | --- | --- | --- | --- | --- | --- | --- | --- | --- | --- | --- | --- | --- | --- | --- | --- | --- | --- | --- | --- | --- | --- | --- | --- | --- | --- | --- | --- | --- | --- | --- | --- | --- | --- | --- | --- | --- | --- | --- | --- | --- | --- | --- | --- | --- | --- | --- | --- | --- | --- | --- | --- | --- | --- | --- | --- | --- | --- | --- | --- | --- | --- | --- | --- | --- | --- | --- | --- | --- | --- | --- | --- | --- | --- | --- | --- | --- | --- | --- | --- | --- | --- | --- | --- | --- | --- | --- | --- | --- | --- | --- | --- | --- | --- | --- | --- | --- | --- | --- | --- | --- | --- | --- | --- | --- | --- | --- | --- | --- | --- | --- | --- | --- | --- | --- | --- | --- | --- | --- | --- | --- | --- | --- | --- | --- | --- | --- | --- | --- | --- | --- | --- | --- | --- | --- | --- | --- | --- | --- | --- | --- | --- | --- | --- | --- | --- | --- | --- | --- | --- | --- | --- | --- | --- | --- | --- | --- | --- | --- | --- | --- | --- | --- | --- | --- | --- | --- | --- | --- | --- | --- | --- | --- | --- | --- | --- | --- | --- | --- | --- | --- | --- | --- | --- | --- | --- | --- | --- | --- | --- | --- | --- | --- | --- | --- | --- | --- | --- | --- | --- | --- | --- | --- | --- | --- | --- | --- | --- | --- | --- | --- | --- | --- | --- | --- | --- | --- | --- | --- | --- | --- | --- | --- | --- | --- | --- | --- | --- | --- | --- | --- | --- | --- | --- | --- | --- | --- | --- | --- | --- | --- | --- | --- | --- | --- | --- | --- | --- | --- | --- | --- | --- | --- | --- | --- | --- | --- | --- | --- | --- | --- | --- | --- | --- | --- | --- | --- | --- | --- | --- | --- | --- | --- | --- | --- | --- | --- | --- | --- | --- | --- | --- | --- | --- | --- | --- | --- | --- | --- | --- | --- | --- | --- | --- | --- | --- | --- | --- | --- | --- | --- | --- | --- | --- | --- | --- | --- | --- | --- | --- | --- | --- | --- | --- | --- | --- | --- | --- | --- | --- | --- | --- | --- | --- | --- | --- | --- | --- | --- | --- | --- | --- | --- | --- | --- | --- | --- | --- | --- | --- | --- | --- | --- | --- | --- | --- | --- | --- | --- | --- | --- | --- | --- | --- | --- | --- | --- | --- | --- | --- | --- | --- | --- | --- | --- | --- | --- | --- | --- | --- | --- | --- | --- | --- | --- | --- | --- | --- | --- | --- | --- | --- | --- | --- | --- | --- | --- | --- | --- | --- | --- | --- | --- | --- | --- | --- | --- | --- | --- | --- | --- | --- | --- | --- | --- | --- | --- | --- | --- | --- | --- | --- | --- | --- | --- | --- | --- | --- | --- | --- | --- | --- | --- | --- | --- | --- | --- | --- | --- | --- | --- | --- | --- | --- | --- | --- | --- | --- | --- | --- | --- | --- | --- | --- | --- | --- | --- | --- | --- | --- | --- | --- | --- | --- | --- | --- | --- | --- | --- | --- | --- | --- | --- | --- | --- | --- | --- | --- | --- | --- | --- | --- | --- | --- | --- | --- | --- | --- | --- | --- | --- | --- | --- | --- | --- | --- | --- | --- | --- | --- | --- | --- | --- | --- | --- | --- | --- | --- | --- | --- | --- | --- | --- | --- | --- | --- | --- | --- | --- | --- | --- | --- | --- | --- | --- | --- | --- | --- | --- | --- | --- | --- | --- | --- | --- | --- | --- | --- | --- | --- | --- | --- | --- | --- | --- | --- | --- | --- | --- | --- | --- | --- | --- | --- | --- | --- | --- | --- | --- | --- | --- | --- | --- | --- | --- | --- | --- | --- | --- | --- | --- | --- | --- | --- | --- | --- | --- | --- | --- | --- | --- | --- | --- | --- | --- | --- | --- | --- | --- | --- | --- | --- | --- | --- | --- | --- | --- | --- | --- | --- | --- | --- | --- | --- | --- | --- | --- | --- | --- | --- | --- | --- | --- | --- | --- | --- | --- | --- | --- | --- | --- | --- | --- | --- | --- | --- | --- | --- | --- | --- | --- | --- | --- | --- | --- | --- | --- | --- | --- | --- | --- | --- | --- | --- | --- | --- | --- | --- | --- | --- | --- | --- | --- | --- | --- | --- | --- | --- | --- | --- | --- | --- | --- | --- | --- | --- | --- | --- | --- | --- | --- | --- | --- | --- | --- | --- | --- | --- | --- | --- | --- | --- | --- | --- | --- | --- | --- | --- | --- | --- | --- | --- | --- | --- | --- | --- | --- | --- | --- | --- | --- | --- | --- | --- | --- | --- | --- | --- | --- | --- | --- | --- | --- | --- | --- | --- | --- | --- | --- | --- | --- | --- | --- | --- | --- | --- | --- | --- | --- | --- | --- | --- | --- | --- | --- | --- | --- | --- | --- | --- | --- | --- | --- | --- | --- | --- | --- | --- | --- | --- | --- | --- | --- | --- | --- | --- | --- | --- | --- | --- | --- | --- | --- | --- | --- | --- | --- | --- | --- | --- | --- | --- | --- | --- | --- | --- | --- | --- | --- | --- | --- | --- | --- | --- | --- | --- | --- | --- | --- | --- | --- | --- | --- | --- | --- | --- | --- | --- | --- | --- | --- | --- | --- | --- | --- | --- | --- | --- | --- | --- | --- | --- | --- | --- | --- | --- | --- | --- | --- | --- | --- | --- | --- | --- | --- | --- | --- | --- | --- | --- | --- | --- | --- | --- | --- | --- | --- | --- | --- | --- | --- | --- | --- | --- | --- | --- | --- | --- | --- | --- | --- | --- | --- | --- | --- | --- | --- | --- | --- | --- | --- | --- | --- | --- | --- | --- | --- | --- | --- | --- | --- | --- | --- | --- | --- | --- | --- | --- | --- | --- | --- | --- | --- | --- | --- | --- | --- | --- | --- | --- | --- | --- | --- | --- | --- | --- | --- | --- | --- | --- | --- | --- | --- | --- | --- | --- | --- | --- | --- | --- | --- | --- | --- | --- | --- | --- | --- | --- | --- | --- | --- | --- | --- | --- | --- | --- | --- | --- | --- | --- | --- | --- | --- | --- | --- | --- | --- | --- | --- | --- | --- | --- | --- | --- | --- | --- | --- | --- | --- | --- | --- | --- | --- | --- | --- | --- | --- | --- | --- | --- | --- | --- | --- | --- | --- | --- | --- | --- | --- | --- | --- | --- | --- | --- | --- | --- | --- | --- | --- | --- | --- | --- | --- | --- | --- | --- | --- | --- | --- | --- | --- | --- | --- | --- | --- | --- | --- | --- | --- | --- | --- | --- | --- | --- | --- | --- | --- | --- | --- | --- | --- | --- | --- | --- | --- | --- | --- | --- | --- | --- | --- | --- | --- | --- | --- | --- | --- | --- | --- | --- | --- | --- | --- | --- | --- | --- | --- | --- | --- | --- | --- | --- | --- | --- | --- | --- | --- | --- | --- | --- | --- | --- | --- | --- | --- | --- | --- | --- | --- | --- | --- | --- | --- | --- | --- | --- | --- | --- | --- | --- | --- | --- | --- | --- | --- | --- | --- | --- | --- | --- | --- | --- | --- | --- | --- | --- | --- | --- | --- | --- | --- | --- | --- | --- | --- | --- | --- | --- | --- | --- | --- | --- | --- | --- | --- | --- | --- | --- | --- | --- | --- | --- | --- | --- | --- | --- | --- | --- | --- | --- | --- | --- | --- | --- | --- | --- | --- | --- | --- | --- | --- | --- | --- | --- | --- | --- | --- | --- | --- | --- | --- | --- | --- | --- | --- | --- | --- | --- | --- | --- | --- | --- | --- | --- | --- | --- | --- | --- | --- | --- | --- | --- | --- | --- | --- | --- | --- | --- | --- | --- | --- | --- | --- | --- | --- | --- | --- | --- | --- | --- | --- | --- | --- | --- | --- | --- | --- | --- | --- | --- | --- | --- | --- | --- | --- | --- | --- | --- | --- | --- | --- | --- | --- | --- | --- | --- | --- | --- | --- | --- | --- | --- | --- | --- | --- | --- | --- | --- | --- | --- | --- | --- | --- | --- | --- | --- | --- | --- | --- | --- | --- | --- | --- | --- | --- | --- | --- | --- | --- | --- | --- | --- | --- | --- | --- | --- | --- | --- | --- | --- | --- | --- | --- | --- | --- | --- | --- | --- | --- | --- | --- | --- | --- | --- | --- | --- | --- | --- | --- | --- | --- | --- | --- | --- | --- | --- | --- | --- | --- | --- | --- | --- | --- | --- | --- | --- | --- | --- | --- | --- | --- | --- | --- | --- | --- | --- | --- | --- | --- | --- | --- | --- | --- | --- | --- | --- | --- | --- | --- | --- | --- | --- | --- | --- | --- | --- | --- | --- | --- | --- | --- | --- | --- | --- | --- | --- | --- | --- | --- | --- | --- | --- | --- | --- | --- | --- | --- | --- | --- | --- | --- | --- | --- | --- | --- | --- | --- | --- | --- | --- | --- | --- | --- | --- | --- | --- | --- | --- | --- | --- | --- | --- | --- | --- | --- | --- | --- | --- | --- | --- | --- | --- | --- | --- | --- | --- | --- | --- | --- | --- | --- | --- | --- | --- | --- | --- | --- | --- | --- | --- | --- | --- | --- | --- | --- | --- | --- | --- | --- | --- | --- | --- | --- | --- | --- | --- | --- | --- | --- | --- | --- | --- | --- | --- | --- | --- | --- | --- | --- | --- | --- | --- | --- | --- | --- | --- | --- | --- | --- | --- | --- | --- | --- | --- | --- | --- | --- | --- | --- | --- | --- | --- | --- | --- | --- | --- | --- | --- | --- | --- | --- | --- | --- | --- | --- | --- | --- | --- | --- | --- | --- | --- | --- | --- | --- | --- | --- | --- | --- | --- | --- | --- | --- | --- | --- | --- | --- | --- | --- | --- | --- | --- | --- | --- | --- | --- | --- | --- | --- | --- | --- | --- | --- | --- | --- | --- | --- | --- | --- | --- | --- | --- | --- | --- | --- | --- | --- | --- | --- | --- | --- | --- | --- | --- | --- | --- | --- | --- | --- | --- | --- | --- | --- | --- | --- | --- | --- | --- | --- | --- | --- | --- | --- | --- | --- | --- | --- | --- | --- | --- | --- | --- | --- | --- | --- | --- | --- | --- | --- | --- | --- | --- | --- | --- | --- | --- | --- | --- | --- | --- | --- | --- | --- | --- | --- | --- | --- | --- | --- | --- | --- | --- | --- | --- | --- | --- | --- | --- | --- | --- | --- | --- | --- | --- | --- | --- | --- | --- | --- | --- | --- | --- | --- | --- | --- | --- | --- | --- | --- | --- | --- | --- | --- | --- | --- | --- | --- | --- | --- | --- | --- | --- | --- | --- | --- | --- | --- | --- | --- | --- | --- | --- | --- | --- | --- | --- | --- | --- | --- | --- | --- | --- | --- | --- | --- | --- | --- | --- | --- | --- | --- | --- | --- | --- | --- | --- | --- | --- | --- | --- | --- | --- | --- | --- | --- | --- | --- | --- | --- | --- | --- | --- | --- | --- | --- | --- | --- | --- | --- | --- | --- | --- | --- | --- | --- | --- | --- | --- | --- | --- | --- | --- | --- | --- | --- | --- | --- | --- | --- | --- | --- | --- | --- | --- | --- | --- | --- | --- | --- | --- | --- | --- | --- | --- | --- | --- | --- | --- | --- | --- | --- | --- | --- | --- | --- | --- | --- | --- | --- | --- | --- | --- | --- | --- | --- | --- | --- | --- | --- | --- | --- | --- | --- | --- | --- | --- | --- | --- | --- | --- | --- | --- | --- | --- | --- | --- | --- | --- | --- | --- | --- | --- | --- | --- | --- | --- | --- | --- | --- | --- | --- | --- | --- | --- | --- | --- | --- | --- | --- | --- | --- | --- | --- | --- | --- | --- | --- | --- | --- | --- | --- | --- | --- | --- | --- | --- | --- | --- | --- | --- | --- | --- | --- | --- | --- | --- | --- | --- | --- | --- | --- | --- | --- | --- | --- | --- | --- | --- | --- | --- | --- | --- | --- | --- | --- | --- | --- | --- | --- | --- | --- | --- | --- | --- | --- | --- | --- | --- | --- | --- | --- | --- | --- | --- | --- | --- | --- | --- | --- | --- | --- | --- | --- | --- | --- | --- | --- | --- | --- | --- | --- | --- | --- | --- | --- | --- | --- | --- | --- | --- | --- | --- | --- | --- | --- | --- | --- | --- | --- | --- | --- | --- | --- | --- | --- | --- | --- | --- | --- | --- | --- | --- | --- | --- | --- | --- | --- | --- | --- | --- | --- | --- | --- | --- | --- | --- | --- | --- | --- | --- | --- | --- | --- | --- | --- | --- | --- | --- | --- | --- | --- | --- | --- | --- | --- | --- | --- | --- | --- | --- | --- | --- | --- | --- | --- | --- | --- | --- | --- | --- | --- | --- | --- | --- | --- | --- | --- | --- | --- | --- | --- | --- | --- | --- | --- | --- | --- | --- | --- | --- | --- | --- | --- | --- | --- | --- | --- | --- | --- | --- | --- | --- | --- | --- | --- | --- | --- | --- | --- | --- | --- | --- | --- | --- | --- | --- | --- | --- | --- | --- | --- | --- | --- | --- | --- | --- | --- | --- | --- | --- | --- | --- | --- | --- | --- | --- | --- | --- | --- | --- | --- | --- | --- | --- | --- | --- | --- | --- | --- | --- | --- | --- | --- | --- | --- | --- | --- | --- | --- | --- | --- | --- | --- | --- | --- | --- | --- | --- | --- | --- | --- | --- | --- | --- | --- | --- | --- | --- | --- | --- | --- | --- | --- | --- | --- | --- | --- | --- | --- | --- | --- | --- | --- | --- | --- | --- | --- | --- | --- | --- | --- | --- | --- | --- | --- | --- | --- | --- | --- | --- | --- | --- | --- | --- | --- | --- | --- | --- | --- | --- | --- | --- | --- | --- | --- | --- | --- | --- | --- | --- | --- | --- | --- | --- | --- | --- | --- | --- | --- | --- | --- | --- | --- | --- | --- | --- | --- | --- | --- | --- | --- | --- | --- | --- | --- | --- | --- | --- | --- | --- | --- | --- | --- | --- | --- | --- | --- | --- | --- | --- | --- | --- | --- | --- | --- | --- | --- | --- | --- | --- | --- | --- | --- | --- | --- | --- | --- | --- | --- | --- | --- | --- | --- | --- | --- | --- | --- | --- | --- | --- | --- | --- | --- | --- | --- | --- | --- | --- | --- | --- | --- | --- | --- | --- | --- | --- | --- | --- | --- | --- | --- | --- | --- | --- | --- | --- | --- | --- | --- | --- | --- | --- | --- | --- | --- | --- | --- | --- | --- | --- | --- | --- | --- | --- | --- | --- | --- | --- | --- | --- | --- | --- | --- | --- | --- | --- | --- | --- | --- | --- | --- | --- | --- | --- | --- | --- | --- | --- | --- | --- | --- | --- | --- | --- | --- | --- | --- | --- | --- | --- | --- | --- | --- | --- | --- | --- | --- | --- | --- | --- | --- | --- | --- | --- | --- | --- | --- | --- | --- | --- | --- | --- | --- | --- | --- | --- | --- | --- | --- | --- | --- | --- | --- | --- | --- | --- | --- | --- | --- | --- | --- | --- | --- | --- | --- | --- | --- | --- | --- | --- | --- | --- | --- | --- | --- | --- | --- | --- | --- | --- | --- | --- | --- | --- | --- | --- | --- | --- | --- | --- | --- | --- | --- | --- | --- | --- | --- | --- | --- | --- | --- | --- | --- | --- | --- | --- | --- | --- | --- | --- | --- | --- | --- | --- | --- | --- | --- | --- | --- | --- | --- | --- | --- | --- | --- | --- | --- | --- | --- | --- | --- | --- | --- | --- | --- | --- | --- | --- | --- | --- | --- | --- | --- | --- | --- | --- | --- | --- | --- | --- | --- | --- | --- | --- | --- | --- | --- | --- | --- | --- | --- | --- | --- | --- | --- | --- | --- | --- | --- | --- | --- | --- | --- | --- | --- | --- | --- | --- | --- | --- | --- | --- | --- | --- | --- | --- | --- | --- | --- | --- | --- | --- | --- | --- | --- | --- | --- | --- | --- | --- | --- | --- | --- | --- | --- | --- | --- | --- | --- | --- | --- | --- | --- | --- | --- | --- | --- | --- | --- | --- | --- | --- | --- | --- | --- | --- | --- | --- | --- | --- | --- | --- | --- | --- | --- | --- | --- | --- | --- | --- | --- | --- | --- | --- | --- | --- | --- | --- | --- | --- | --- | --- | --- | --- | --- | --- | --- | --- | --- | --- | --- | --- | --- | --- | --- | --- | --- | --- | --- | --- | --- | --- | --- | --- | --- | --- | --- | --- | --- | --- | --- | --- | --- | --- | --- | --- | --- | --- | --- | --- | --- | --- | --- | --- | --- | --- | --- | --- | --- | --- | --- | --- | --- | --- | --- | --- | --- | --- | --- | --- | --- | --- | --- | --- | --- | --- | --- | --- | --- | --- | --- | --- | --- | --- | --- | --- | --- | --- | --- | --- | --- | --- | --- | --- | --- | --- | --- | --- | --- | --- | --- | --- | --- | --- | --- | --- | --- | --- | --- | --- | --- | --- | --- | --- | --- | --- | --- | --- | --- | --- | --- | --- | --- | --- | --- | --- | --- | --- | --- | --- | --- | --- | --- | --- | --- | --- | --- | --- | --- | --- | --- | --- | --- | --- | --- | --- | --- | --- | --- | --- | --- | --- | --- | --- | --- | --- | --- | --- | --- | --- | --- | --- | --- | --- | --- | --- | --- | --- | --- | --- | --- | --- | --- | --- | --- | --- | --- | --- | --- | --- | --- | --- | --- | --- | --- | --- | --- | --- | --- | --- | --- | --- | --- | --- | --- | --- | --- | --- | --- | --- | --- | --- | --- | --- | --- | --- | --- | --- | --- | --- | --- | --- | --- | --- | --- | --- | --- | --- | --- | --- | --- | --- | --- | --- | --- | --- | --- | --- | --- | --- | --- | --- | --- | --- | --- | --- | --- | --- | --- | --- | --- | --- | --- | --- | --- | --- | --- | --- | --- | --- | --- | --- | --- | --- | --- | --- | --- | --- | --- | --- | --- | --- | --- | --- | --- | --- | --- | --- | --- | --- | --- | --- | --- | --- | --- | --- | --- | --- | --- | --- | --- | --- | --- | --- | --- | --- | --- | --- | --- | --- | --- | --- | --- | --- | --- | --- | --- | --- | --- | --- | --- | --- | --- | --- | --- | --- | --- | --- | --- | --- | --- | --- | --- | --- | --- | --- | --- | --- | --- | --- | --- | --- | --- | --- | --- | --- | --- | --- | --- | --- | --- | --- | --- | --- | --- | --- | --- | --- | --- | --- | --- | --- | --- | --- | --- | --- | --- | --- | --- | --- | --- | --- | --- | --- | --- | --- | --- | --- | --- | --- | --- | --- | --- | --- | --- | --- | --- | --- | --- | --- | --- | --- | --- | --- | --- | --- | --- | --- | --- | --- | --- | --- | --- | --- | --- | --- | --- | --- | --- | --- | --- | --- | --- | --- | --- | --- | --- | --- | --- | --- | --- | --- | --- | --- | --- | --- | --- | --- | --- | --- | --- | --- | --- | --- | --- | --- | --- | --- | --- | --- | --- | --- | --- | --- | --- | --- | --- | --- | --- | --- | --- | --- | --- | --- | --- | --- | --- | --- | --- | --- | --- | --- | --- | --- | --- | --- | --- | --- | --- | --- | --- | --- | --- | --- | --- | --- | --- | --- | --- | --- | --- | --- | --- | --- | --- | --- | --- | --- | --- | --- | --- | --- | --- | --- | --- | --- | --- | --- | --- | --- | --- | --- | --- | --- | --- | --- | --- | --- | --- | --- | --- | --- | --- | --- | --- | --- | --- | --- | --- | --- | --- | --- | --- | --- | --- | --- | --- | --- | --- | --- | --- | --- | --- | --- | --- | --- | --- | --- | --- | --- | --- | --- | --- | --- | --- | --- | --- | --- | --- | --- | --- | --- | --- | --- | --- | --- | --- | --- | --- | --- | --- | --- | --- | --- | --- | --- | --- | --- | --- | --- | --- | --- | --- | --- | --- | --- | --- | --- | --- | --- | --- | --- | --- | --- | --- | --- | --- | --- | --- | --- | --- | --- | --- | --- | --- | --- | --- | --- | --- | --- | --- | --- | --- | --- | --- | --- | --- | --- | --- | --- | --- | --- | --- | --- | --- | --- | --- | --- | --- | --- | --- | --- | --- | --- | --- | --- | --- | --- | --- | --- | --- | --- | --- | --- | --- | --- | --- | --- | --- | --- | --- | --- | --- | --- | --- | --- | --- | --- | --- | --- | --- | --- | --- | --- | --- | --- | --- | --- | --- | --- | --- | --- | --- | --- | --- | --- | --- | --- | --- | --- | --- | --- | --- | --- | --- | --- | --- | --- | --- | --- | --- | --- | --- | --- | --- | --- | --- | --- | --- | --- | --- | --- | --- | --- | --- | --- | --- | --- | --- | --- | --- | --- | --- | --- | --- | --- | --- | --- | --- | --- | --- | --- | --- | --- | --- | --- | --- | --- | --- | --- | --- | --- | --- | --- | --- | --- | --- | --- | --- | --- | --- | --- | --- | --- | --- | --- | --- | --- | --- | --- | --- | --- | --- | --- | --- | --- | --- | --- | --- | --- | --- | --- | --- | --- | --- | --- | --- | --- | --- | --- | --- | --- | --- | --- | --- | --- | --- | --- | --- | --- | --- | --- | --- | --- | --- | --- | --- | --- | --- | --- | --- | --- | --- | --- | --- | --- | --- | --- | --- | --- | --- | --- | --- | --- | --- | --- | --- | --- | --- | --- | --- | --- | --- | --- | --- | --- | --- | --- | --- | --- | --- | --- | --- | --- | --- | --- | --- | --- | --- | --- | --- | --- | --- | --- | --- | --- | --- | --- | --- | --- | --- | --- | --- | --- | --- | --- | --- | --- | --- | --- | --- | --- | --- | --- | --- | --- | --- | --- | --- | --- | --- | --- | --- | --- | --- | --- | --- | --- | --- | --- | --- | --- | --- | --- | --- | --- | --- | --- | --- | --- | --- | --- | --- | --- | --- | --- | --- | --- | --- | --- | --- | --- | --- | --- | --- | --- | --- | --- | --- | --- | --- | --- | --- | --- | --- | --- | --- | --- | --- | --- | --- | --- | --- | --- | --- | --- | --- | --- | --- | --- | --- | --- | --- | --- | --- | --- | --- | --- | --- | --- | --- | --- | --- | --- | --- | --- | --- | --- | --- | --- | --- | --- | --- | --- | --- | --- | --- | --- | --- | --- | --- | --- | --- | --- | --- | --- | --- | --- | --- | --- | --- | --- | --- | --- | --- | --- | --- | --- | --- | --- | --- | --- | --- | --- | --- | --- | --- | --- | --- | --- | --- | --- | --- | --- | --- | --- | --- | --- | --- | --- | --- | --- | --- | --- | --- | --- | --- | --- | --- | --- | --- | --- | --- | --- | --- | --- | --- | --- | --- | --- | --- | --- | --- | --- | --- | --- | --- | --- | --- | --- | --- | --- | --- | --- | --- | --- | --- | --- | --- | --- | --- | --- | --- | --- | --- | --- | --- | --- | --- | --- | --- | --- | --- | --- | --- | --- | --- | --- | --- | --- | --- | --- | --- | --- | --- | --- | --- | --- | --- | --- | --- | --- | --- | --- | --- | --- | --- | --- | --- | --- | --- | --- | --- | --- | --- | --- | --- | --- | --- | --- | --- | --- | --- | --- | --- | --- | --- | --- | --- | --- | --- | --- | --- | --- | --- | --- | --- | --- | --- | --- | --- | --- | --- | --- | --- | --- | --- | --- | --- | --- | --- | --- | --- | --- | --- | --- | --- | --- | --- | --- | --- | --- | --- | --- | --- | --- | --- | --- | --- | --- | --- | --- | --- | --- | --- | --- | --- | --- | --- | --- | --- | --- | --- | --- | --- | --- | --- | --- | --- | --- | --- | --- | --- | --- | --- | --- | --- | --- | --- | --- | --- | --- | --- | --- | --- | --- | --- | --- | --- | --- | --- | --- | --- | --- | --- | --- | --- | --- | --- | --- | --- | --- | --- | --- | --- | --- | --- | --- | --- | --- | --- | --- | --- | --- | --- | --- | --- | --- | --- | --- | --- | --- | --- | --- | --- | --- | --- | --- | --- | --- | --- | --- | --- | --- | --- | --- | --- | --- | --- | --- | --- | --- | --- | --- | --- | --- | --- | --- | --- | --- | --- | --- | --- | --- | --- | --- | --- | --- | --- | --- | --- | --- | --- | --- | --- | --- | --- | --- | --- | --- | --- | --- | --- | --- | --- | --- | --- | --- | --- | --- | --- | --- | --- | --- | --- | --- | --- | --- | --- | --- | --- | --- | --- | --- | --- | --- | --- | --- | --- | --- | --- | --- | --- | --- | --- | --- | --- | --- | --- | --- | --- | --- | --- | --- | --- | --- | --- | --- | --- | --- | --- | --- | --- | --- | --- | --- | --- | --- | --- | --- | --- | --- | --- | --- | --- | --- | --- | --- | --- | --- | --- | --- | --- | --- | --- | --- | --- | --- | --- | --- | --- | --- | --- | --- | --- | --- | --- | --- | --- | --- | --- | --- | --- | --- | --- | --- | --- | --- | --- | --- | --- | --- | --- | --- | --- | --- | --- | --- | --- | --- | --- | --- | --- | --- | --- | --- | --- | --- | --- | --- | --- | --- | --- | --- | --- | --- | --- | --- | --- | --- | --- | --- | --- | --- | --- | --- | --- | --- | --- | --- | --- | --- | --- | --- | --- | --- | --- | --- | --- | --- | --- | --- | --- | --- | --- | --- | --- | --- | --- | --- | --- | --- | --- | --- | --- | --- | --- | --- | --- | --- | --- | --- | --- | --- | --- | --- | --- | --- | --- | --- | --- | --- | --- | --- | --- | --- | --- | --- | --- | --- | --- | --- | --- | --- | --- | --- | --- | --- | --- | --- | --- | --- | --- | --- | --- | --- | --- | --- | --- | --- | --- | --- | --- | --- | --- | --- | --- | --- | --- | --- | --- | --- | --- | --- | --- | --- | --- | --- | --- | --- | --- | --- | --- | --- | --- | --- | --- | --- | --- | --- | --- | --- | --- | --- | --- | --- | --- | --- | --- | --- | --- | --- | --- | --- | --- | --- | --- | --- | --- | --- | --- | --- | --- | --- | --- | --- | --- | --- | --- | --- | --- | --- | --- | --- | --- | --- | --- | --- | --- | --- | --- | --- | --- | --- | --- | --- | --- | --- | --- | --- | --- | --- | --- | --- | --- | --- | --- | --- | --- | --- | --- | --- | --- | --- | --- | --- | --- | --- | --- | --- | --- | --- | --- | --- | --- | --- | --- | --- | --- | --- | --- | --- | --- | --- | --- | --- | --- | --- | --- | --- | --- | --- | --- | --- | --- | --- | --- | --- | --- | --- | --- | --- | --- | --- | --- | --- | --- | --- | --- | --- | --- | --- | --- | --- | --- | --- | --- | --- | --- | --- | --- | --- | --- | --- | --- | --- | --- | --- | --- | --- | --- | --- | --- | --- | --- | --- | --- | --- | --- | --- | --- | --- | --- | --- | --- | --- | --- | --- | --- | --- | --- | --- | --- | --- | --- | --- | --- | --- | --- | --- | --- | --- | --- | --- | --- | --- | --- | --- | --- | --- | --- | --- | --- | --- | --- | --- | --- | --- | --- | --- | --- | --- | --- | --- | --- | --- | --- | --- | --- | --- | --- | --- | --- | --- | --- | --- | --- | --- | --- | --- | --- | --- | --- | --- | --- | --- | --- | --- | --- | --- | --- | --- | --- | --- | --- | --- | --- | --- | --- | --- | --- | --- | --- | --- | --- | --- | --- | --- | --- | --- | --- | --- | --- | --- | --- | --- | --- | --- | --- | --- | --- | --- | --- | --- | --- | --- | --- | --- | --- | --- | --- | --- | --- | --- | --- | --- | --- | --- | --- | --- | --- | --- | --- | --- | --- | --- | --- | --- | --- | --- | --- | --- | --- | --- | --- | --- | --- | --- | --- | --- | --- | --- | --- | --- | --- | --- | --- | --- | --- | --- | --- | --- | --- | --- | --- | --- | --- | --- | --- | --- | --- | --- | --- | --- | --- | --- | --- | --- | --- | --- | --- | --- | --- | --- | --- | --- | --- | --- | --- | --- | --- | --- | --- | --- | --- | --- | --- | --- | --- | --- | --- | --- | --- | --- | --- | --- | --- | --- | --- | --- | --- | --- | --- | --- | --- | --- | --- | --- | --- | --- | --- | --- | --- | --- | --- | --- | --- | --- | --- | --- | --- | --- | --- | --- | --- | --- | --- | --- | --- | --- | --- | --- | --- | --- | --- | --- | --- | --- | --- | --- | --- | --- | --- | --- | --- | --- | --- | --- | --- | --- | --- | --- | --- | --- | --- | --- | --- | --- | --- | --- | --- | --- | --- | --- | --- | --- | --- | --- | --- | --- | --- | --- | --- | --- | --- | --- | --- | --- | --- | --- | --- | --- | --- | --- | --- | --- | --- | --- | --- | --- | --- | --- | --- | --- | --- | --- | --- | --- | --- | --- | --- | --- | --- | --- | --- | --- | --- | --- | --- | --- | --- | --- | --- | --- | --- | --- | --- | --- | --- | --- | --- | --- | --- | --- | --- | --- | --- | --- | --- | --- | --- | --- | --- | --- | --- | --- | --- | --- | --- | --- | --- | --- | --- | --- | --- | --- | --- | --- | --- | --- | --- | --- | --- | --- | --- | --- | --- | --- | --- | --- | --- | --- | --- | --- | --- | --- | --- | --- | --- | --- | --- | --- | --- | --- | --- | --- | --- | --- | --- | --- | --- | --- | --- | --- | --- | --- | --- | --- | --- | --- | --- | --- | --- | --- | --- | --- | --- | --- | --- | --- | --- | --- | --- | --- | --- | --- | --- | --- | --- | --- | --- | --- | --- | --- | --- | --- | --- | --- | --- | --- | --- | --- | --- | --- | --- | --- | --- | --- | --- | --- | --- | --- | --- | --- | --- | --- | --- | --- | --- | --- | --- | --- | --- | --- | --- | --- | --- | --- | --- | --- | --- | --- | --- | --- | --- | --- | --- | --- | --- | --- | --- | --- | --- | --- | --- | --- | --- | --- | --- | --- | --- | --- | --- | --- | --- | --- | --- | --- | --- | --- | --- | --- | --- | --- | --- | --- | --- | --- | --- | --- | --- | --- | --- | --- | --- | --- | --- | --- | --- | --- | --- | --- | --- | --- | --- | --- | --- | --- | --- | --- | --- | --- | --- | --- | --- | --- | --- | --- | --- | --- | --- | --- | --- | --- | --- | --- | --- | --- | --- | --- | --- | --- | --- | --- | --- | --- | --- | --- | --- | --- | --- | --- | --- | --- | --- | --- | --- | --- | --- | --- | --- | --- | --- | --- | --- | --- | --- | --- | --- | --- | --- | --- | --- | --- | --- | --- | --- | --- | --- | --- | --- | --- | --- | --- | --- | --- | --- | --- | --- | --- | --- | --- | --- | --- | --- | --- | --- | --- | --- | --- | --- | --- | --- | --- | --- | --- | --- | --- | --- | --- | --- | --- | --- | --- | --- | --- | --- | --- | --- | --- | --- | --- | --- | --- | --- | --- | --- | --- | --- | --- | --- | --- | --- | --- | --- | --- | --- | --- | --- | --- | --- | --- | --- | --- | --- | --- | --- | --- | --- | --- | --- | --- | --- | --- | --- | --- | --- | --- | --- | --- | --- | --- | --- | --- | --- | --- | --- | --- | --- | --- | --- | --- | --- | --- | --- | --- | --- | --- | --- | --- | --- | --- | --- | --- | --- | --- | --- | --- | --- | --- | --- | --- | --- | --- | --- | --- | --- | --- | --- | --- | --- | --- | --- | --- | --- | --- | --- | --- | --- | --- | --- | --- | --- | --- | --- | --- | --- | --- | --- | --- | --- | --- | --- | --- | --- | --- | --- | --- | --- | --- | --- | --- | --- | --- | --- | --- | --- | --- | --- | --- | --- | --- | --- | --- | --- | --- | --- | --- | --- | --- | --- | --- | --- | --- | --- | --- | --- | --- | --- | --- | --- | --- | --- | --- | --- | --- | --- | --- | --- | --- | --- | --- | --- | --- | --- | --- | --- | --- | --- | --- | --- | --- | --- | --- | --- | --- | --- | --- | --- | --- | --- | --- | --- | --- | --- | --- | --- | --- | --- | --- | --- | --- | --- | --- | --- | --- | --- | --- | --- | --- | --- | --- | --- | --- | --- | --- | --- | --- | --- | --- | --- | --- | --- | --- | --- | --- | --- | --- | --- | --- | --- | --- | --- | --- | --- | --- | --- | --- | --- | --- | --- | --- | --- | --- | --- | --- | --- | --- | --- | --- | --- | --- | --- | --- | --- | --- | --- | --- | --- | --- | --- | --- | --- | --- | --- | --- | --- | --- | --- | --- | --- | --- | --- | --- | --- | --- | --- | --- | --- | --- | --- | --- | --- | --- | --- | --- | --- | --- | --- | --- | --- | --- | --- | --- | --- | --- | --- | --- | --- | --- | --- | --- | --- | --- | --- | --- | --- | --- | --- | --- | --- | --- | --- | --- | --- | --- | --- | --- | --- | --- | --- | --- | --- | --- | --- | --- | --- | --- | --- | --- | --- | --- | --- | --- | --- | --- | --- | --- | --- | --- | --- | --- | --- | --- | --- | --- | --- | --- | --- | --- | --- | --- | --- | --- | --- | --- | --- | --- | --- | --- | --- | --- | --- | --- | --- | --- | --- | --- | --- | --- | --- | --- | --- | --- | --- | --- | --- | --- | --- | --- | --- | --- | --- | --- | --- | --- | --- | --- | --- | --- | --- | --- | --- | --- | --- | --- | --- | --- | --- | --- | --- | --- | --- | --- | --- | --- | --- | --- | --- | --- | --- | --- | --- | --- | --- | --- | --- | --- | --- | --- | --- | --- | --- | --- | --- | --- | --- | --- | --- | --- | --- | --- | --- | --- | --- | --- | --- | --- | --- | --- | --- | --- | --- | --- | --- | --- | --- | --- | --- | --- | --- | --- | --- | --- | --- | --- | --- | --- | --- | --- | --- | --- | --- | --- | --- | --- | --- | --- | --- | --- | --- | --- | --- | --- | --- | --- | --- | --- | --- | --- | --- | --- | --- | --- | --- | --- | --- | --- | --- | --- | --- | --- | --- | --- | --- | --- | --- | --- | --- | --- | --- | --- | --- | --- | --- | --- | --- | --- | --- | --- | --- | --- | --- | --- | --- | --- | --- | --- | --- | --- | --- | --- | --- | --- | --- | --- | --- | --- | --- | --- | --- | --- | --- | --- | --- | --- | --- | --- | --- | --- | --- | --- | --- | --- | --- | --- | --- | --- | --- | --- | --- | --- | --- | --- | --- | --- | --- | --- | --- | --- | --- | --- | --- | --- | --- | --- | --- | --- | --- | --- | --- | --- | --- | --- | --- | --- | --- | --- | --- | --- | --- | --- | --- | --- | --- | --- | --- | --- | --- | --- | --- | --- | --- | --- | --- | --- | --- | --- | --- | --- | --- | --- | --- | --- | --- | --- | --- | --- | --- | --- | --- | --- | --- | --- | --- | --- | --- | --- | --- | --- | --- | --- | --- | --- | --- | --- | --- | --- | --- | --- | --- | --- | --- | --- | --- | --- | --- | --- | --- | --- | --- | --- | --- | --- | --- | --- | --- | --- | --- | --- | --- | --- | --- | --- | --- | --- | --- | --- | --- | --- | --- | --- | --- | --- | --- | --- | --- | --- | --- | --- | --- | --- | --- | --- | --- | --- | --- | --- | --- | --- | --- | --- | --- | --- | --- | --- | --- | --- | --- | --- | --- | --- | --- | --- | --- | --- | --- | --- | --- | --- | --- | --- | --- | --- | --- | --- | --- | --- | --- | --- | --- | --- | --- | --- | --- | --- | --- | --- | --- | --- | --- | --- | --- | --- | --- | --- | --- | --- | --- | --- | --- | --- | --- | --- | --- | --- | --- | --- | --- | --- | --- | --- | --- | --- | --- | --- | --- | --- | --- | --- | --- | --- | --- | --- | --- | --- | --- | --- | --- | --- | --- | --- | --- | --- | --- | --- | --- | --- | --- | --- | --- | --- | --- | --- | --- | --- | --- | --- | --- | --- | --- | --- | --- | --- | --- | --- | --- | --- | --- | --- | --- | --- | --- | --- | --- | --- | --- | --- | --- | --- | --- | --- | --- | --- | --- | --- | --- | --- | --- | --- | --- | --- | --- | --- | --- | --- | --- | --- | --- | --- | --- | --- | --- | --- | --- | --- | --- | --- | --- | --- | --- | --- | --- | --- | --- | --- | --- | --- | --- | --- | --- | --- | --- | --- | --- | --- | --- | --- | --- | --- | --- | --- | --- | --- | --- | --- | --- | --- | --- | --- | --- | --- | --- | --- | --- | --- | --- | --- | --- | --- | --- | --- | --- | --- | --- | --- | --- | --- | --- | --- | --- | --- | --- | --- | --- | --- | --- | --- | --- | --- | --- | --- | --- | --- | --- | --- | --- | --- | --- | --- | --- | --- | --- | --- | --- | --- | --- | --- | --- | --- | --- | --- | --- | --- | --- | --- | --- | --- | --- | --- | --- | --- | --- | --- | --- | --- | --- | --- | --- | --- | --- | --- | --- | --- | --- | --- | --- | --- | --- | --- | --- | --- | --- | --- | --- | --- | --- | --- | --- | --- | --- | --- | --- | --- | --- | --- | --- | --- | --- | --- | --- | --- | --- | --- | --- | --- | --- | --- | --- | --- | --- | --- | --- | --- | --- | --- | --- | --- | --- | --- | --- | --- | --- | --- | --- | --- | --- | --- | --- | --- | --- | --- | --- | --- | --- | --- | --- | --- | --- | --- | --- | --- | --- | --- | --- | --- | --- | --- | --- | --- | --- | --- | --- | --- | --- | --- | --- | --- | --- | --- | --- | --- | --- | --- | --- | --- | --- | --- | --- | --- | --- | --- | --- | --- | --- | --- | --- | --- | --- | --- | --- | --- | --- | --- | --- | --- | --- | --- | --- | --- | --- | --- | --- | --- | --- | --- | --- | --- | --- | --- | --- | --- | --- | --- | --- | --- | --- | --- | --- | --- | --- | --- | --- | --- | --- | --- | --- | --- | --- | --- | --- | --- | --- | --- | --- | --- | --- | --- | --- | --- | --- | --- | --- | --- | --- | --- | --- | --- | --- | --- | --- | --- | --- | --- | --- | --- | --- | --- | --- | --- | --- | --- | --- | --- | --- | --- | --- | --- | --- | --- | --- | --- | --- | --- | --- | --- | --- | --- | --- | --- | --- | --- | --- | --- | --- | --- | --- | --- | --- | --- | --- | --- | --- | --- | --- | --- | --- | --- | --- | --- | --- | --- | --- | --- | --- | --- | --- | --- | --- | --- | --- | --- | --- | --- | --- | --- | --- | --- | --- | --- | --- | --- | --- | --- | --- | --- | --- | --- | --- | --- | --- | --- | --- | --- | --- | --- | --- | --- | --- | --- | --- | --- | --- | --- | --- | --- | --- | --- | --- | --- | --- | --- | --- | --- | --- | --- | --- | --- | --- | --- | --- | --- | --- | --- | --- | --- | --- | --- | --- | --- | --- | --- | --- | --- | --- | --- | --- | --- | --- | --- | --- | --- | --- | --- | --- | --- | --- | --- | --- | --- | --- | --- | --- | --- | --- | --- | --- | --- | --- | --- | --- | --- | --- | --- | --- | --- | --- | --- | --- | --- | --- | --- | --- | --- | --- | --- | --- | --- | --- | --- | --- | --- | --- | --- | --- | --- | --- | --- | --- | --- | --- | --- | --- | --- | --- | --- | --- | --- | --- | --- | --- | --- | --- | --- | --- | --- | --- | --- | --- | --- | --- | --- | --- | --- | --- | --- | --- | --- | --- | --- | --- | --- | --- | --- | --- | --- | --- | --- | --- | --- | --- | --- | --- | --- | --- | --- | --- | --- | --- | --- | --- | --- | --- | --- | --- | --- | --- | --- | --- | --- | --- | --- | --- | --- | --- | --- | --- | --- | --- | --- | --- | --- | --- | --- | --- | --- | --- | --- | --- | --- | --- | --- | --- | --- | --- | --- | --- | --- | --- | --- | --- | --- | --- | --- | --- | --- | --- | --- | --- | --- | --- | --- | --- | --- | --- | --- | --- | --- | --- | --- | --- | --- | --- | --- | --- | --- | --- | --- | --- | --- | --- | --- | --- | --- | --- | --- | --- | --- | --- | --- | --- | --- | --- | --- | --- | --- | --- | --- | --- | --- | --- | --- | --- | --- | --- | --- | --- | --- | --- | --- | --- | --- | --- | --- | --- | --- | --- | --- | --- | --- | --- | --- | --- | --- | --- | --- | --- | --- | --- | --- | --- | --- | --- | --- | --- | --- | --- | --- | --- | --- | --- | --- | --- | --- | --- | --- | --- | --- | --- | --- | --- | --- | --- | --- | --- | --- | --- | --- | --- | --- | --- | --- | --- | --- | --- | --- | --- | --- | --- | --- | --- | --- | --- | --- | --- | --- | --- | --- | --- | --- | --- | --- | --- | --- | --- | --- | --- | --- | --- | --- | --- | --- | --- | --- | --- | --- | --- | --- | --- | --- | --- | --- | --- | --- | --- | --- | --- | --- | --- | --- | --- | --- | --- | --- | --- | --- | --- | --- | --- | --- | --- | --- | --- | --- | --- | --- | --- | --- | --- | --- | --- | --- | --- | --- | --- | --- | --- | --- | --- | --- | --- | --- | --- | --- | --- | --- | --- | --- | --- | --- | --- | --- | --- | --- | --- | --- | --- | --- | --- | --- | --- | --- | --- | --- | --- | --- | --- | --- | --- | --- | --- | --- | --- | --- | --- | --- | --- | --- | --- | --- | --- | --- | --- | --- | --- | --- | --- | --- | --- | --- | --- | --- | --- | --- | --- | --- | --- | --- | --- | --- | --- | --- | --- | --- | --- | --- | --- | --- | --- | --- | --- | --- | --- | --- | --- | --- | --- | --- | --- | --- | --- | --- | --- | --- | --- | --- | --- | --- | --- | --- | --- | --- | --- | --- | --- | --- | --- | --- | --- | --- | --- | --- | --- | --- | --- | --- | --- | --- | --- | --- | --- | --- | --- | --- | --- | --- | --- | --- | --- | --- | --- | --- | --- | --- | --- | --- | --- | --- | --- | --- | --- | --- | --- | --- | --- | --- | --- | --- | --- | --- | --- | --- | --- | --- | --- | --- | --- | --- | --- | --- | --- | --- | --- | --- | --- | --- | --- | --- | --- | --- | --- | --- | --- | --- | --- | --- | --- | --- | --- | --- | --- | --- | --- | --- | --- | --- | --- | --- | --- | --- | --- | --- | --- | --- | --- | --- | --- | --- | --- | --- | --- | --- | --- | --- | --- | --- | --- | --- | --- | --- | --- | --- | --- | --- | --- | --- | --- | --- | --- | --- | --- | --- | --- | --- | --- | --- | --- | --- | --- | --- | --- | --- | --- | --- | --- | --- | --- | --- | --- | --- | --- | --- | --- | --- | --- | --- | --- | --- | --- | --- | --- | --- | --- | --- | --- | --- | --- | --- | --- | --- | --- | --- | --- | --- | --- | --- | --- | --- | --- | --- | --- | --- | --- | --- | --- | --- | --- | --- | --- | --- | --- | --- | --- | --- | --- | --- | --- | --- | --- | --- | --- | --- | --- | --- | --- | --- | --- | --- | --- | --- | --- | --- | --- | --- | --- | --- | --- | --- | --- | --- | --- | --- | --- | --- | --- | --- | --- | --- | --- | --- | --- | --- | --- | --- | --- | --- | --- | --- | --- | --- | --- | --- | --- | --- | --- | --- | --- | --- | --- | --- | --- | --- | --- | --- | --- | --- | --- | --- | --- | --- | --- | --- | --- | --- | --- | --- | --- | --- | --- | --- | --- | --- | --- | --- | --- | --- | --- | --- | --- | --- | --- | --- | --- | --- | --- | --- | --- | --- | --- | --- | --- | --- | --- | --- | --- | --- | --- | --- | --- | --- | --- | --- | --- | --- | --- | --- | --- | --- | --- | --- | --- | --- | --- | --- | --- | --- | --- | --- | --- | --- | --- | --- | --- | --- | --- | --- | --- | --- | --- | --- | --- | --- | --- | --- | --- | --- | --- | --- | --- | --- | --- | --- | --- | --- | --- | --- | --- | --- | --- | --- | --- | --- | --- | --- | --- | --- | --- | --- | --- | --- | --- | --- | --- | --- | --- | --- | --- | --- | --- | --- | --- | --- | --- | --- | --- | --- | --- | --- | --- | --- | --- | --- | --- | --- | --- | --- | --- | --- | --- | --- | --- | --- | --- | --- | --- | --- | --- | --- | --- | --- | --- | --- | --- | --- | --- | --- | --- | --- | --- | --- | --- | --- | --- | --- | --- | --- | --- | --- | --- | --- | --- | --- | --- | --- | --- | --- | --- | --- | --- | --- | --- | --- | --- | --- | --- | --- | --- | --- | --- | --- | --- | --- | --- | --- | --- | --- | --- | --- | --- | --- | --- | --- | --- | --- | --- | --- | --- | --- | --- | --- | --- | --- | --- | --- | --- | --- | --- | --- | --- | --- | --- | --- | --- | --- | --- | --- | --- | --- | --- | --- | --- | --- | --- | --- | --- | --- | --- | --- | --- | --- | --- | --- | --- | --- | --- | --- | --- | --- | --- | --- | --- | --- | --- | --- | --- | --- | --- | --- | --- | --- | --- | --- | --- | --- | --- | --- | --- | --- | --- | --- | --- | --- | --- | --- | --- | --- | --- | --- | --- | --- | --- | --- | --- | --- | --- | --- | --- | --- | --- | --- | --- | --- | --- | --- | --- | --- | --- | --- | --- | --- | --- | --- | --- | --- | --- | --- | --- | --- | --- | --- | --- | --- | --- | --- | --- | --- | --- | --- | --- | --- | --- | --- | --- | --- | --- | --- | --- | --- | --- | --- | --- | --- | --- | --- | --- | --- | --- | --- | --- | --- | --- | --- | --- | --- | --- | --- | --- | --- | --- | --- | --- | --- | --- | --- | --- | --- | --- | --- | --- | --- | --- | --- | --- | --- | --- | --- | --- | --- | --- | --- | --- | --- | --- | --- | --- | --- | --- | --- | --- | --- | --- | --- | --- | --- | --- | --- | --- | --- | --- | --- | --- | --- | --- | --- | --- | --- | --- | --- | --- | --- | --- | --- | --- | --- | --- | --- | --- | --- | --- | --- | --- | --- | --- | --- | --- | --- | --- | --- | --- | --- | --- | --- | --- | --- | --- | --- | --- | --- | --- | --- | --- | --- | --- | --- | --- | --- | --- | --- | --- | --- | --- | --- | --- | --- | --- | --- | --- | --- | --- | --- | --- | --- | --- | --- | --- | --- | --- | --- | --- | --- | --- | --- | --- | --- | --- | --- | --- | --- | --- | --- | --- | --- | --- | --- | --- | --- | --- | --- | --- | --- | --- | --- | --- | --- | --- | --- | --- | --- | --- | --- | --- | --- | --- | --- | --- | --- | --- | --- | --- | --- | --- | --- | --- | --- | --- | --- | --- | --- | --- | --- | --- | --- | --- | --- | --- | --- | --- | --- | --- | --- | --- | --- | --- | --- | --- | --- | --- | --- | --- | --- | --- | --- | --- | --- | --- | --- | --- | --- | --- | --- | --- | --- | --- | --- | --- | --- | --- | --- | --- | --- | --- | --- | --- | --- | --- | --- | --- | --- | --- | --- | --- | --- | --- | --- | --- | --- | --- | --- | --- | --- | --- | --- | --- | --- | --- | --- | --- | --- | --- | --- | --- | --- | --- | --- | --- | --- | --- | --- | --- | --- | --- | --- | --- | --- | --- | --- | --- | --- | --- | --- | --- | --- | --- | --- | --- | --- | --- | --- | --- | --- | --- | --- | --- | --- | --- | --- | --- | --- | --- | --- | --- | --- | --- | --- | --- | --- | --- | --- | --- | --- | --- | --- | --- | --- | --- | --- | --- | --- | --- | --- | --- | --- | --- | --- | --- | --- | --- | --- | --- | --- | --- | --- | --- | --- | --- | --- | --- | --- | --- | --- | --- | --- | --- | --- | --- | --- | --- | --- | --- | --- | --- | --- | --- | --- | --- | --- | --- | --- | --- | --- | --- | --- | --- | --- | --- | --- | --- | --- | --- | --- | --- | --- | --- | --- | --- | --- | --- | --- | --- | --- | --- | --- | --- | --- | --- | --- | --- | --- | --- | --- | --- | --- | --- | --- | --- | --- | --- | --- | --- | --- | --- | --- | --- | --- | --- | --- | --- | --- | --- | --- | --- | --- | --- | --- | --- | --- | --- | --- | --- | --- | --- | --- | --- | --- | --- | --- | --- | --- | --- | --- | --- | --- | --- | --- | --- | --- | --- | --- | --- | --- | --- | --- | --- | --- | --- | --- | --- | --- | --- | --- | --- | --- | --- | --- | --- | --- | --- | --- | --- | --- | --- | --- | --- | --- | --- | --- | --- | --- | --- | --- | --- | --- | --- | --- | --- | --- | --- | --- | --- | --- | --- | --- | --- | --- | --- | --- | --- | --- | --- | --- | --- | --- | --- | --- | --- | --- | --- | --- | --- | --- | --- | --- | --- | --- | --- | --- | --- | --- | --- | --- | --- | --- | --- | --- | --- | --- | --- | --- | --- | --- | --- | --- | --- | --- | --- | --- | --- | --- | --- | --- | --- | --- | --- | --- | --- | --- | --- | --- | --- | --- | --- | --- | --- | --- | --- | --- | --- | --- | --- | --- | --- | --- | --- | --- | --- | --- | --- | --- | --- | --- | --- | --- | --- | --- | --- | --- | --- | --- | --- | --- | --- | --- | --- | --- | --- | --- | --- | --- | --- | --- | --- | --- | --- | --- | --- | --- | --- | --- | --- | --- | --- | --- | --- | --- | --- | --- | --- | --- | --- | --- | --- | --- | --- | --- | --- | --- | --- | --- | --- | --- | --- | --- | --- | --- | --- | --- | --- | --- | --- | --- | --- | --- | --- | --- | --- | --- | --- | --- | --- | --- | --- | --- | --- | --- | --- | --- | --- | --- | --- | --- | --- | --- | --- | --- | --- | --- | --- | --- | --- | --- | --- | --- | --- | --- | --- | --- | --- | --- | --- | --- | --- | --- | --- | --- | --- | --- | --- | --- | --- | --- | --- | --- | --- | --- | --- | --- | --- | --- | --- | --- | --- | --- | --- | --- | --- | --- | --- | --- | --- | --- | --- | --- | --- | --- | --- | --- | --- | --- | --- | --- | --- | --- | --- | --- | --- | --- | --- | --- | --- | --- | --- | --- | --- | --- | --- | --- | --- | --- | --- | --- | --- | --- | --- | --- | --- | --- | --- | --- | --- | --- | --- | --- | --- | --- | --- | --- | --- | --- | --- | --- | --- | --- | --- | --- | --- | --- | --- | --- | --- | --- | --- | --- | --- | --- | --- | --- | --- | --- | --- | --- | --- | --- | --- | --- | --- | --- | --- | --- | --- | --- | --- | --- | --- | --- | --- | --- | --- | --- | --- | --- | --- | --- | --- | --- | --- | --- | --- | --- | --- | --- | --- | --- | --- | --- | --- | --- | --- | --- | --- | --- | --- | --- | --- | --- | --- | --- | --- | --- | --- | --- | --- | --- | --- | --- | --- | --- | --- | --- | --- | --- | --- | --- | --- | --- | --- | --- | --- | --- | --- | --- | --- | --- | --- | --- | --- | --- | --- | --- | --- | --- | --- | --- | --- | --- | --- | --- | --- | --- | --- | --- | --- | --- | --- | --- | --- | --- | --- | --- | --- | --- | --- | --- | --- | --- | --- | --- | --- | --- | --- | --- | --- | --- | --- | --- | --- | --- | --- | --- | --- | --- | --- | --- | --- | --- | --- | --- | --- | --- | --- | --- | --- | --- | --- | --- | --- | --- | --- | --- | --- | --- | --- | --- | --- | --- | --- | --- | --- | --- | --- | --- | --- | --- | --- | --- | --- | --- | --- | --- | --- | --- | --- | --- | --- | --- | --- | --- | --- | --- | --- | --- | --- | --- | --- | --- | --- | --- | --- | --- | --- | --- | --- | --- | --- | --- | --- | --- | --- | --- | --- | --- | --- | --- | --- | --- | --- | --- | --- | --- | --- | --- | --- | --- | --- | --- | --- | --- | --- | --- | --- | --- | --- | --- | --- | --- | --- | --- | --- | --- | --- | --- | --- | --- | --- | --- | --- | --- | --- | --- | --- | --- | --- | --- | --- | --- | --- | --- | --- | --- | --- | --- | --- | --- | --- | --- | --- | --- | --- | --- | --- | --- | --- | --- | --- | --- | --- | --- | --- | --- | --- | --- | --- | --- | --- | --- | --- | --- | --- | --- | --- | --- | --- | --- | --- | --- | --- | --- | --- | --- | --- | --- | --- | --- | --- | --- | --- | --- | --- | --- | --- | --- | --- | --- | --- | --- | --- | --- | --- | --- | --- | --- | --- | --- | --- | --- | --- | --- | --- | --- | --- | --- | --- | --- | --- | --- | --- | --- | --- | --- | --- | --- | --- | --- | --- | --- | --- | --- | --- | --- | --- | --- | --- | --- | --- | --- | --- | --- | --- | --- | --- | --- | --- | --- | --- | --- | --- | --- | --- | --- | --- | --- | --- | --- | --- | --- | --- | --- | --- | --- | --- | --- | --- | --- | --- | --- | --- | --- | --- | --- | --- | --- | --- | --- | --- | --- | --- | --- | --- | --- | --- | --- | --- | --- | --- | --- | --- | --- | --- | --- | --- | --- | --- | --- | --- | --- | --- | --- | --- | --- | --- | --- | --- | --- | --- | --- | --- | --- | --- | --- | --- | --- | --- | --- | --- | --- | --- | --- | --- | --- | --- | --- | --- | --- | --- | --- | --- | --- | --- | --- | --- | --- | --- | --- | --- | --- | --- | --- | --- | --- | --- | --- | --- | --- | --- | --- | --- | --- | --- | --- | --- | --- | --- | --- | --- | --- | --- | --- | --- | --- | --- | --- | --- | --- | --- | --- | --- | --- | --- | --- | --- | --- | --- | --- | --- | --- | --- | --- | --- | --- | --- | --- | --- | --- | --- | --- | --- | --- | --- | --- | --- | --- | --- | --- | --- | --- | --- | --- | --- | --- | --- | --- | --- | --- | --- | --- | --- | --- | --- | --- | --- | --- | --- | --- | --- | --- | --- | --- | --- | --- | --- | --- | --- | --- | --- | --- | --- | --- | --- | --- | --- | --- | --- | --- | --- | --- | --- | --- | --- | --- | --- | --- | --- | --- | --- | --- | --- | --- | --- | --- | --- | --- | --- | --- | --- | --- | --- | --- | --- | --- | --- | --- | --- | --- | --- | --- | --- | --- | --- | --- | --- | --- | --- | --- | --- | --- | --- | --- | --- | --- | --- | --- | --- | --- | --- | --- | --- | --- | --- | --- | --- | --- | --- | --- | --- | --- | --- | --- | --- | --- | --- | --- | --- | --- | --- | --- | --- | --- | --- | --- | --- | --- | --- | --- | --- | --- | --- | --- | --- | --- | --- | --- | --- | --- | --- | --- | --- | --- | --- | --- | --- | --- | --- | --- | --- | --- | --- | --- | --- | --- | --- | --- | --- | --- | --- | --- | --- | --- | --- | --- | --- | --- | --- | --- | --- | --- | --- | --- | --- | --- | --- | --- | --- | --- | --- | --- | --- | --- | --- | --- | --- | --- | --- | --- | --- | --- | --- | --- | --- | --- | --- | --- | --- | --- | --- | --- | --- | --- | --- | --- | --- | --- | --- | --- | --- | --- | --- | --- | --- | --- | --- | --- | --- | --- | --- | --- | --- | --- | --- | --- | --- | --- | --- | --- | --- | --- | --- | --- | --- | --- | --- | --- | --- | --- | --- | --- | --- | --- | --- | --- | --- | --- | --- | --- | --- | --- | --- | --- | --- | --- | --- | --- | --- | --- | --- | --- | --- | --- | --- | --- | --- | --- | --- | --- | --- | --- | --- | --- | --- | --- | --- | --- | --- | --- | --- | --- | --- | --- | --- | --- | --- | --- | --- | --- | --- | --- | --- | --- | --- | --- | --- | --- | --- | --- | --- | --- | --- | --- | --- | --- | --- | --- | --- | --- | --- | --- | --- | --- | --- | --- | --- | --- | --- | --- | --- | --- | --- | --- | --- | --- | --- | --- | --- | --- | --- | --- | --- | --- | --- | --- | --- | --- | --- | --- | --- | --- | --- | --- | --- | --- | --- | --- | --- | --- | --- | --- | --- | --- | --- | --- | --- | --- | --- | --- | --- | --- | --- | --- | --- | --- | --- | --- | --- | --- | --- | --- | --- | --- | --- | --- | --- | --- | --- | --- | --- | --- | --- | --- | --- | --- | --- | --- | --- | --- | --- | --- | --- | --- | --- | --- | --- | --- | --- | --- | --- | --- | --- | --- | --- | --- | --- | --- | --- | --- | --- | --- | --- | --- | --- | --- | --- | --- | --- | --- | --- | --- | --- | --- | --- | --- | --- | --- | --- | --- | --- | --- | --- | --- | --- | --- | --- | --- | --- | --- | --- | --- | --- | --- | --- | --- | --- | --- | --- | --- | --- | --- | --- | --- | --- | --- | --- | --- | --- | --- | --- | --- | --- | --- | --- | --- | --- | --- | --- | --- | --- | --- | --- | --- | --- | --- | --- | --- | --- | --- | --- | --- | --- | --- | --- | --- | --- | --- | --- | --- | --- | --- | --- | --- | --- | --- | --- | --- | --- | --- | --- | --- | --- | --- | --- | --- | --- | --- | --- | --- | --- | --- | --- | --- | --- | --- | --- | --- | --- | --- | --- | --- | --- | --- | --- | --- | --- | --- | --- | --- | --- | --- | --- | --- | --- | --- | --- | --- | --- | --- | --- | --- | --- | --- | --- | --- | --- | --- | --- | --- | --- | --- | --- | --- | --- | --- | --- | --- | --- | --- | --- | --- | --- | --- | --- | --- | --- | --- | --- | --- | --- | --- | --- | --- | --- | --- | --- | --- | --- | --- | --- | --- | --- | --- | --- | --- | --- | --- | --- | --- | --- | --- | --- | --- | --- | --- | --- | --- | --- | --- | --- | --- | --- | --- | --- | --- | --- | --- | --- | --- | --- | --- | --- | --- | --- | --- | --- | --- | --- | --- | --- | --- | --- | --- | --- | --- | --- | --- | --- | --- | --- | --- | --- | --- | --- | --- | --- | --- | --- | --- | --- | --- | --- | --- | --- | --- | --- | --- | --- | --- | --- | --- | --- | --- | --- | --- | --- | --- | --- | --- | --- | --- | --- | --- | --- | --- | --- | --- | --- | --- | --- | --- | --- | --- | --- | --- | --- | --- | --- | --- | --- | --- | --- | --- | --- | --- | --- | --- | --- | --- | --- | --- | --- | --- | --- | --- | --- | --- | --- | --- | --- | --- | --- | --- | --- | --- | --- | --- | --- | --- | --- | --- | --- | --- | --- | --- | --- | --- | --- | --- | --- | --- | --- | --- | --- | --- | --- | --- | --- | --- | --- | --- | --- | --- | --- | --- | --- | --- | --- | --- | --- | --- | --- | --- | --- | --- | --- | --- | --- | --- | --- | --- | --- | --- | --- | --- | --- | --- | --- | --- | --- | --- | --- | --- | --- | --- | --- | --- | --- | --- | --- | --- | --- | --- | --- | --- | --- | --- | --- | --- | --- | --- | --- | --- | --- | --- | --- | --- | --- | --- | --- | --- | --- | --- | --- | --- | --- | --- | --- | --- | --- | --- | --- | --- | --- | --- | --- | --- | --- | --- | --- | --- | --- | --- | --- | --- | --- | --- | --- | --- | --- | --- | --- | --- | --- | --- | --- | --- | --- | --- | --- | --- | --- | --- | --- | --- | --- | --- | --- | --- | --- | --- | --- | --- | --- | --- | --- | --- | --- | --- | --- | --- | --- | --- | --- | --- | --- | --- | --- | --- | --- | --- | --- | --- | --- | --- | --- | --- | --- | --- | --- | --- | --- | --- | --- | --- | --- | --- | --- | --- | --- | --- | --- | --- | --- | --- | --- | --- | --- | --- | --- | --- | --- | --- | --- | --- | --- | --- | --- | --- | --- | --- | --- | --- | --- | --- | --- | --- | --- | --- | --- | --- | --- | --- | --- | --- | --- | --- | --- | --- | --- | --- | --- | --- | --- | --- | --- | --- | --- | --- | --- | --- | --- | --- | --- | --- | --- | --- | --- | --- | --- | --- | --- | --- | --- | --- | --- | --- | --- | --- | --- | --- | --- | --- | --- | --- | --- | --- | --- | --- | --- | --- | --- | --- | --- | --- | --- | --- | --- | --- | --- | --- | --- | --- | --- | --- | --- | --- | --- | --- | --- | --- | --- | --- | --- | --- | --- | --- | --- | --- | --- | --- | --- | --- | --- | --- | --- | --- | --- | --- | --- | --- | --- | --- | --- | --- | --- | --- | --- | --- | --- | --- | --- | --- | --- | --- | --- | --- | --- | --- | --- | --- | --- | --- | --- | --- | --- | --- | --- | --- | --- | --- | --- | --- | --- | --- | --- | --- | --- | --- | --- | --- | --- | --- | --- | --- | --- | --- | --- | --- | --- | --- | --- | --- | --- | --- | --- | --- | --- | --- | --- | --- | --- | --- | --- | --- | --- | --- | --- | --- | --- | --- | --- | --- | --- | --- | --- | --- | --- | --- | --- | --- | --- | --- | --- | --- | --- | --- | --- | --- | --- | --- | --- | --- | --- | --- | --- | --- | --- | --- | --- | --- | --- | --- | --- | --- | --- | --- | --- | --- | --- | --- | --- | --- | --- | --- | --- | --- | --- | --- | --- | --- | --- | --- | --- | --- | --- | --- | --- | --- | --- | --- | --- | --- | --- | --- | --- | --- | --- | --- | --- | --- | --- | --- | --- | --- | --- | --- | --- | --- | --- | --- | --- | --- | --- | --- | --- | --- | --- | --- | --- | --- | --- | --- | --- | --- | --- | --- | --- | --- | --- | --- | --- | --- | --- | --- | --- | --- | --- | --- | --- | --- | --- | --- | --- | --- | --- | --- | --- | --- | --- | --- | --- | --- | --- | --- | --- | --- | --- | --- | --- | --- | --- | --- | --- | --- | --- | --- | --- | --- | --- | --- | --- | --- | --- | --- | --- | --- | --- | --- | --- | --- | --- | --- | --- | --- | --- | --- | --- | --- | --- | --- | --- | --- | --- | --- | --- | --- | --- | --- | --- | --- | --- | --- | --- | --- | --- | --- | --- | --- | --- | --- | --- | --- | --- | --- | --- | --- | --- | --- | --- | --- | --- | --- | --- | --- | --- | --- | --- | --- | --- | --- | --- | --- | --- | --- | --- | --- | --- | --- | --- | --- | --- | --- | --- | --- | --- | --- | --- | --- | --- | --- | --- | --- | --- | --- | --- | --- | --- | --- | --- | --- | --- | --- | --- | --- | --- | --- | --- | --- | --- | --- | --- | --- | --- | --- | --- | --- | --- | --- | --- | --- | --- | --- | --- | --- | --- | --- | --- | --- | --- | --- | --- | --- | --- | --- | --- | --- | --- | --- | --- | --- | --- | --- | --- | --- | --- | --- | --- | --- | --- | --- | --- | --- | --- | --- | --- | --- | --- | --- | --- | --- | --- | --- | --- | --- | --- | --- | --- | --- | --- | --- | --- | --- | --- | --- | --- | --- | --- | --- | --- | --- | --- | --- | --- | --- | --- | --- | --- | --- | --- | --- | --- | --- | --- | --- | --- | --- | --- | --- | --- | --- | --- | --- | --- | --- | --- | --- | --- | --- | --- | --- | --- | --- | --- | --- | --- | --- | --- | --- | --- | --- | --- | --- | --- | --- | --- | --- | --- | --- | --- | --- | --- | --- | --- | --- | --- | --- | --- | --- | --- | --- | --- | --- | --- | --- | --- | --- | --- | --- | --- | --- | --- | --- | --- | --- | --- | --- | --- | --- | --- | --- | --- | --- | --- | --- | --- | --- | --- | --- | --- | --- | --- | --- | --- | --- | --- | --- | --- | --- | --- | --- | --- | --- | --- | --- | --- | --- | --- | --- | --- | --- | --- | --- | --- | --- | --- | --- | --- | --- | --- | --- | --- | --- | --- | --- | --- | --- | --- | --- | --- | --- | --- | --- | --- | --- | --- | --- | --- | --- | --- | --- | --- | --- | --- | --- | --- | --- | --- | --- | --- | --- | --- | --- | --- | --- | --- | --- | --- | --- | --- | --- | --- | --- | --- | --- | --- | --- | --- | --- | --- | --- | --- | --- | --- | --- | --- | --- | --- | --- | --- | --- | --- | --- | --- | --- | --- | --- | --- | --- | --- | --- | --- | --- | --- | --- | --- | --- | --- | --- | --- | --- | --- | --- | --- | --- | --- | --- | --- | --- | --- | --- | --- | --- | --- | --- | --- | --- | --- | --- | --- | --- | --- | --- | --- | --- | --- | --- | --- | --- | --- | --- | --- | --- | --- | --- | --- | --- | --- | --- | --- | --- | --- | --- | --- | --- | --- | --- | --- | --- | --- | --- | --- | --- | --- | --- | --- | --- | --- | --- | --- | --- | --- | --- | --- | --- | --- | --- | --- | --- | --- | --- | --- | --- | --- | --- | --- | --- | --- | --- | --- | --- | --- | --- | --- | --- | --- | --- | --- | --- | --- | --- | --- | --- | --- | --- | --- | --- | --- | --- | --- | --- | --- | --- | --- | --- | --- | --- | --- | --- | --- | --- | --- | --- | --- | --- | --- | --- | --- | --- | --- | --- | --- | --- | --- | --- | --- | --- | --- | --- | --- | --- | --- | --- | --- | --- | --- | --- | --- | --- | --- | --- | --- | --- | --- | --- | --- | --- | --- | --- | --- | --- | --- | --- | --- | --- | --- | --- | --- | --- | --- | --- | --- | --- | --- | --- | --- | --- | --- | --- | --- | --- | --- | --- | --- | --- | --- | --- | --- | --- | --- | --- | --- | --- | --- | --- | --- | --- | --- | --- | --- | --- | --- | --- | --- | --- | --- | --- | --- | --- | --- | --- | --- | --- | --- | --- | --- | --- | --- | --- | --- | --- | --- | --- | --- | --- | --- | --- | --- | --- | --- | --- | --- | --- | --- | --- | --- | --- | --- | --- | --- | --- | --- | --- | --- | --- | --- | --- | --- | --- | --- | --- | --- | --- | --- | --- | --- | --- | --- | --- | --- | --- | --- | --- | --- | --- | --- | --- | --- | --- | --- | --- | --- | --- | --- | --- | --- | --- | --- | --- | --- | --- | --- | --- | --- | --- | --- | --- | --- | --- | --- | --- | --- | --- | --- | --- | --- | --- | --- | --- | --- | --- | --- | --- | --- | --- | --- | --- | --- | --- | --- | --- | --- | --- | --- | --- | --- | --- | --- | --- | --- | --- | --- | --- | --- | --- | --- | --- | --- | --- | --- | --- | --- | --- | --- | --- | --- | --- | --- | --- | --- | --- | --- | --- | --- | --- | --- | --- | --- | --- | --- | --- | --- | --- | --- | --- | --- | --- | --- | --- | --- | --- | --- | --- | --- | --- | --- | --- | --- | --- | --- | --- | --- | --- | --- | --- | --- | --- | --- | --- | --- | --- | --- | --- | --- | --- | --- | --- | --- | --- | --- | --- | --- | --- | --- | --- | --- | --- | --- | --- | --- | --- | --- | --- | --- | --- | --- | --- | --- | --- | --- | --- | --- | --- | --- | --- | --- | --- | --- | --- | --- | --- | --- | --- | --- | --- | --- | --- | --- | --- | --- | --- | --- | --- | --- | --- | --- | --- | --- | --- | --- | --- | --- | --- | --- | --- | --- | --- | --- | --- | --- | --- | --- | --- | --- | --- | --- | --- | --- | --- | --- | --- | --- | --- | --- | --- | --- | --- | --- | --- | --- | --- | --- | --- | --- | --- | --- | --- | --- | --- | --- | --- | --- | --- | --- | --- | --- | --- | --- | --- | --- | --- | --- | --- | --- | --- | --- | --- | --- | --- | --- | --- | --- | --- | --- | --- | --- | --- | --- | --- | --- | --- | --- | --- | --- | --- | --- | --- | --- | --- | --- | --- | --- | --- | --- | --- | --- | --- | --- | --- | --- | --- | --- | --- | --- | --- | --- | --- | --- | --- | --- | --- | --- | --- | --- | --- | --- | --- | --- | --- | --- | --- | --- | --- | --- | --- | --- | --- | --- | --- | --- | --- | --- | --- | --- | --- | --- | --- | --- | --- | --- | --- | --- | --- | --- | --- | --- | --- | --- | --- | --- | --- | --- | --- | --- | --- | --- | --- | --- | --- | --- | --- | --- | --- | --- | --- | --- | --- | --- | --- | --- | --- | --- | --- | --- | --- | --- | --- | --- | --- | --- | --- | --- | --- | --- | --- | --- | --- | --- | --- | --- | --- | --- | --- | --- | --- | --- | --- | --- | --- | --- | --- | --- | --- | --- | --- | --- | --- | --- | --- | --- | --- | --- | --- | --- | --- | --- | --- | --- | --- | --- | --- | --- | --- | --- | --- | --- | --- | --- | --- | --- | --- | --- | --- | --- | --- | --- | --- | --- | --- | --- | --- | --- | --- | --- | --- | --- | --- | --- | --- | --- | --- | --- | --- | --- | --- | --- | --- | --- | --- | --- | --- | --- | --- | --- | --- | --- | --- | --- | --- | --- | --- | --- | --- | --- | --- | --- | --- | --- | --- | --- | --- | --- | --- | --- | --- | --- | --- | --- | --- | --- | --- | --- | --- | --- | --- | --- | --- | --- | --- | --- | --- | --- | --- | --- | --- | --- | --- | --- | --- | --- | --- | --- | --- | --- | --- | --- | --- | --- | --- | --- | --- | --- | --- | --- | --- | --- | --- | --- | --- | --- | --- | --- | --- | --- | --- | --- | --- | --- | --- | --- | --- | --- | --- | --- | --- | --- | --- | --- | --- | --- | --- | --- | --- | --- | --- | --- | --- | --- | --- | --- | --- | --- | --- | --- | --- | --- | --- | --- | --- | --- | --- | --- | --- | --- | --- | --- | --- | --- | --- | --- | --- | --- | --- | --- | --- | --- | --- | --- | --- | --- | --- | --- | --- | --- | --- | --- | --- | --- | --- | --- | --- | --- | --- | --- | --- | --- | --- | --- | --- | --- | --- | --- | --- | --- | --- | --- | --- | --- | --- | --- | --- | --- | --- | --- | --- | --- | --- | --- | --- | --- | --- | --- | --- | --- | --- | --- | --- | --- | --- | --- | --- | --- | --- | --- | --- | --- | --- | --- | --- | --- | --- | --- | --- | --- | --- | --- | --- | --- | --- | --- | --- | --- | --- | --- | --- | --- | --- | --- | --- | --- | --- | --- | --- | --- | --- | --- | --- | --- | --- | --- | --- | --- | --- | --- | --- | --- | --- | --- | --- | --- | --- | --- | --- | --- | --- | --- | --- | --- | --- | --- | --- | --- | --- | --- | --- | --- | --- | --- | --- | --- | --- | --- | --- | --- | --- | --- | --- | --- | --- | --- | --- | --- | --- | --- | --- | --- | --- | --- | --- | --- | --- | --- | --- | --- | --- | --- | --- | --- | --- | --- | --- | --- | --- | --- | --- | --- | --- | --- | --- | --- | --- | --- | --- | --- | --- | --- | --- | --- | --- | --- | --- | --- | --- | --- | --- | --- | --- | --- | --- | --- | --- | --- | --- | --- | --- | --- | --- | --- | --- | --- | --- | --- | --- | --- | --- | --- | --- | --- | --- | --- | --- | --- | --- | --- | --- | --- | --- | --- | --- | --- | --- | --- | --- | --- | --- | --- | --- | --- | --- | --- | --- | --- | --- | --- | --- | --- | --- | --- | --- | --- | --- | --- | --- | --- | --- | --- | --- | --- | --- | --- | --- | --- | --- | --- | --- | --- | --- | --- | --- | --- | --- | --- | --- | --- | --- | --- | --- | --- | --- | --- | --- | --- | --- | --- | --- | --- | --- | --- | --- | --- | --- | --- | --- | --- | --- | --- | --- | --- | --- | --- | --- | --- | --- | --- | --- | --- | --- | --- | --- | --- | --- | --- | --- | --- | --- | --- | --- | --- | --- | --- | --- | --- | --- | --- | --- | --- | --- | --- | --- | --- | --- | --- | --- | --- | --- | --- | --- | --- | --- | --- | --- | --- | --- | --- | --- | --- | --- | --- | --- | --- | --- | --- | --- | --- | --- | --- | --- | --- | --- | --- | --- | --- | --- | --- | --- | --- | --- | --- | --- | --- | --- | --- | --- | --- | --- | --- | --- | --- | --- | --- | --- | --- | --- | --- | --- | --- | --- | --- | --- | --- | --- | --- | --- | --- | --- | --- | --- | --- | --- | --- | --- | --- | --- | --- | --- | --- | --- | --- | --- | --- | --- | --- | --- | --- | --- | --- | --- | --- | --- | --- | --- | --- | --- | --- | --- | --- | --- | --- | --- | --- | --- | --- | --- | --- | --- | --- | --- | --- | --- | --- | --- | --- | --- | --- | --- | --- | --- | --- | --- | --- | --- | --- | --- | --- | --- | --- | --- | --- | --- | --- | --- | --- | --- | --- | --- | --- | --- |
| |  |  |  |  |  |  |  |  |  | | --- | --- | --- | --- | --- | --- | --- | --- | --- | | **Position** | **Reference** | **Sample** | **Quality** | **Type** | **Region** | **AA Exchange** | **PAM1** | **Known Variant** | | 1977 | A | G | 997.77 | SNP | intergenic |  |  | - | | 4013 | T | C | 1436.77 | SNP | Rv0003 (recF) | Ile245Thr | 11 | - | | 7362 | G | C | 1015.77 | SNP | Rv0006 (gyrA) | Glu21Gln | 27 | - | | 7585 | G | C | 1172.77 | SNP | Rv0006 (gyrA) | Ser95Thr | 32 | genotype | | 9304 | G | A | 983.77 | SNP | Rv0006 (gyrA) | Gly668Asp | 6 | - | | 11370 | C | T | 1413.77 | SNP | intergenic |  |  | - | | 11879 | A | G | 1019.77 | SNP | Rv0008c | Ser145Pro | 12 | - | | 14202 | G | C | 640.77 | SNP | Rv0012 | silent (Leu38) | 9947 | - | | 14785 | T | C | 1254.77 | SNP | Rv0012 | Cys233Arg | 1 | - | | 21795 | G | A | 208.84 | SNP | Rv0018c (pstP) | Pro463Ser | 17 | - | | 22453 | G | A | 880.77 | SNP | Rv0018c (pstP) | silent (Tyr243) | 9945 | - | | 24698 | GCCGCGTTGCTCGGGGTAA | G | 4209.37 | DEL | Rv0020c (fhaA) |  |  | - | | 26959 | C | G | 596.77 | SNP | intergenic |  |  | - | | 29482 | CA | C | 1826.73 | DEL | Rv0025 |  |  | - | | 30519 | C | T | 900.77 | SNP | Rv0026 | silent (Gly266) | 9935 | - | | 30688 | T | G | 701.77 | SNP | Rv0026 | Ser323Ala | 35 | - | | 30943 | C | T | 846.77 | SNP | Rv0026 | Pro408Ser | 17 | - | | 31077 | C | T | 1289.77 | SNP | intergenic |  |  | - | | 32349 | CCCGCGCGTCGGCGATGCGT CGCGTCGAGTCGGCGATG | C | 9221.73 | DEL | Rv0029 |  |  | - | | 34044 | T | C | 1151.77 | SNP | intergenic |  |  | - | | 37031 | C | G | 601.77 | SNP | Rv0034 | silent (Ala55) | 9867 | - | | 42967 | G | C | 814.77 | SNP | Rv0040c (mtc28) | silent (Pro133) | 9926 | - | | 50557 | T | C | 645.77 | SNP | Rv0046c (ino1) | Arg190Gly | 1 | - | | 51750 | C | T | 1084.77 | SNP | intergenic |  |  | - | | 51949 | A | G | 717.77 | SNP | Rv0048c | Val250Ala | 18 | - | | 51954 | T | A | 629.77 | SNP | Rv0048c | Glu248Asp | 53 | - | | 53785 | C | G | 1130.77 | SNP | Rv0050 (ponA1) | Ile41Met(s) | 6 | - | | 54394 | A | G | 824.77 | SNP | Rv0050 (ponA1) | silent (Ala244) | 9867 | - | | 55553 | C | CCGCCGT | 1601.73 | INS | Rv0050 (ponA1) |  |  | - | | 57393 | A | T | 679.77 | SNP | intergenic |  |  | - | | 62049 | A | G | 889.77 | SNP | Rv0058 (dnaB) | Arg552Gly | 1 | - | | 62657 | G | A | 1152.77 | SNP | Rv0058 (dnaB) | silent (Pro754) | 9926 | genotype | | 66249 | T | G | 764.77 | SNP | Rv0062 (celA1) | Leu233Arg | 1 | - | | 66285 | C | G | 674.77 | SNP | Rv0062 (celA1) | Ala245Gly | 21 | - | | 69871 | C | T | 843.77 | SNP | Rv0064 | Leu418Phe | 6 | - | | 69989 | G | A | 1193.77 | SNP | Rv0064 | Gly457Asp | 6 | - | | 70816 | A | G | 542.77 | SNP | Rv0064 | Asn733Asp | 42 | - | | 71336 | G | C | 155.90 | SNP | Rv0064 | Arg906Pro | 5 | - | | 71584 | C | CCGAGCGCTGTTCTGGCGCT AATCTGACGCTAGAATAG | 4850.73 | INS | intergenic |  |  | - | | 74390 | G | A | 1230.77 | SNP | Rv0066c (icd2) | Ala41Val | 13 | - | | 75264 | C | T | 1161.77 | SNP | intergenic |  |  | - | | 75940 | G | C | 1000.77 | SNP | Rv0068 | Val(s)214Leu | 3 | - | | 79504 | T | TCGGTGGACCCGGTGGACC | 1763.74 | INS | Rv0071 |  |  | - | | 80616 | C | G | 1284.77 | SNP | intergenic |  |  | - | | 83982 | A | G | 838.77 | SNP | Rv0074 | silent (STOP412) | 9867 | - | | 92199 | T | G | 615.77 | SNP | Rv0083 | silent (Thr600) | 9871 | - | | 95591 | G | T | 1418.77 | SNP | Rv0087 (hycE) | Glu60STOP | 17 | - | | 97388 | G | T | 1095.77 | SNP | Rv0088 | Trp154Cys | 0 | - | | 103836 | G | T | 353.80 | SNP | Rv0094c | Asn276Lys | 25 | - | | 104712 | C | T | 299.77 | SNP | intergenic |  |  | - | | 104838 | T | G | 806.77 | SNP | Rv0095c | Glu126Asp | 53 | - | | 104962 | G | A | 1254.77 | SNP | Rv0095c | Ala85Val(s) | 9867 | - | | 105045 | G | C | 1060.77 | SNP | Rv0095c | Asp57Glu | 56 | - | | 105060 | G | A | 908.77 | SNP | Rv0095c | silent (Asp52) | 9859 | - | | 105063 | G | A | 873.77 | SNP | Rv0095c | silent (Phe51) | 9946 | - | | 112247 | A | G | 747.77 | SNP | Rv0101 (nrp) | silent (Ala749) | 9867 | - | | 116000 | T | G | 1496.77 | SNP | Rv0101 (nrp) | Val2000Val(s) | 18 | - | | 122109 | A | G | 1122.77 | SNP | Rv0103c (ctpB) | Leu(s)22Ser | 28 | - | | 125830 | G | GA | 1400.73 | INS | Rv0107c (ctpI) |  |  | - | | 131174 | T | TG | 1491.73 | INS | intergenic |  |  | - | | 132417 | C | G | 93.28 | SNP | Rv0109 (PE\_PGRS1) | Arg346Gly | 1 | - | | 133839 | C | T | 1668.77 | SNP | intergenic |  |  | - | | 137850 | GT | G | 1520.73 | DEL | Rv0113 (gmhA) |  |  | - | | 144390 | G | A | 356.77 | SNP | Rv0119 (fadD7) | silent (Ala114) | 9867 | - | | 146087 | T | C | 1285.77 | SNP | Rv0120c (fusA2) | Asn562Ser | 34 | - | | 147985 | C | T | 1340.77 | SNP | Rv0121c | Ala120Thr | 22 | - | | 150338 | G | C | 72.77 | SNP | Rv0124 (PE\_PGRS2) | Gly269Ala | 21 | - | | 154283 | T | C | 1368.77 | SNP | Rv0127 (mak) | Ser18Pro | 12 | - | | 155293 | G | A | 1043.77 | SNP | Rv0127 (mak) | silent (Pro354) | 9926 | - | | 163573 | A | C | 834.77 | SNP | Rv0136 (cyp138) | silent (Arg70) | 9913 | - | | 174684 | C | A | 629.77 | SNP | Rv0147 | silent (Arg483) | 9913 | - | | 177596 | T | C | 902.77 | SNP | Rv0151c (PE1) | Ile572Val | 57 | - | | 177857 | G | A | 954.77 | SNP | Rv0151c (PE1) | Leu485Leu(s) | 4 | - | | 178946 | C | T | 762.77 | SNP | Rv0151c (PE1) | Ala122Thr | 22 | - | | 187738 | G | A | 1740.77 | SNP | Rv0159c (PE3) | Arg368Trp | 2 | - | | 188800 | T | C | 907.77 | SNP | Rv0159c (PE3) | Thr14Ala | 32 | - | | 194681 | G | C | 737.77 | SNP | Rv0165c (mce1R) | silent (Leu45) | 9947 | - | | 196642 | C | T | 1204.77 | SNP | Rv0166 (fadD5) | silent (Asn550) | 9822 | - | | 200332 | C | A | 1402.77 | SNP | Rv0170 (mce1B) | Phe146Leu | 13 | - | | 206339 | T | C | 537.77 | SNP | Rv0174 (mce1F) | Leu370Pro | 2 | - | | 216586 | A | G | 828.77 | SNP | Rv0186 (bglS) | silent (Gln106) | 9876 | - | | 220050 | C | T | 880.77 | SNP | Rv0189c (ilvD) | silent (Leu558) | 9947 | - | | 223942 | T | C | 350.77 | SNP | Rv0192 | Ser127Pro | 12 | - | | 225323 | T | C | 1114.77 | SNP | Rv0193c | Lys417Glu | 4 | - | | 227098 | T | C | 1040.77 | SNP | Rv0194 | Met(s)74Thr | 22 | - | | 231114 | C | G | 860.77 | SNP | Rv0195 | silent (Ala72) | 9867 | - | | 232574 | G | T | 907.77 | SNP | Rv0197 | Gly115Val | 3 | - | | 234477 | T | G | 941.77 | SNP | Rv0197 | Tyr749STOP | 2 | - | | 234496 | C | CGT | 1851.73 | INS | Rv0197 |  |  | - | | 251468 | G | T | 1058.77 | SNP | Rv0210 | Arg450Leu | 1 | - | | 261869 | T | C | 896.77 | SNP | Rv0218 | Cys316Arg | 1 | - | | 265554 | A | C | 1282.77 | SNP | Rv0222 (echA1) | silent (Val16) | 9901 | - | | 265968 | C | G | 837.77 | SNP | Rv0222 (echA1) | silent (Arg154) | 9913 | - | | 278681 | C | G | 740.77 | SNP | Rv0233 (nrdB) | His33Asp | 4 | - | | 283614 | T | C | 650.77 | SNP | Rv0236c (aftD) | Ser1080Gly | 21 | - | | 284623 | G | A | 644.77 | SNP | Rv0236c (aftD) | silent (Thr743) | 9871 | - | | 285772 | A | C | 537.77 | SNP | Rv0236c (aftD) | silent (Pro360) | 9926 | - | | 285871 | A | G | 584.77 | SNP | Rv0236c (aftD) | silent (Val327) | 9901 | - | | 293628 | A | AC | 1117.73 | INS | intergenic |  |  | - | | 302200 | C | T | 706.77 | SNP | Rv0251c (hsp) | silent (Gln151) | 9876 | - | | 304679 | G | T | 1441.77 | SNP | Rv0252 (nirB) | Gly605Val | 3 | - | | 304923 | A | G | 1263.77 | SNP | Rv0252 (nirB) | silent (Lys686) | 9926 | - | | 310973 | G | A | 739.77 | SNP | Rv0259c | Ala182Val(s) | 9867 | - | | 311613 | G | T | 853.77 | SNP | Rv0260c | silent (Val349) | 9901 | - | | 320038 | G | A | 1135.77 | SNP | Rv0266c (oplA) | Pro373Ser | 17 | - | | 328641 | G | T | 1515.77 | SNP | Rv0272c | silent (Thr356) | 9871 | - | | 333637 | A | G | 69.77 | SNP | Rv0278c (PE\_PGRS3) | Trp892Arg | 8 | - | | 333640 | G | A | 71.77 | SNP | Rv0278c (PE\_PGRS3) | Arg891Trp | 2 | - | | 333641 | C | T | 66.77 | SNP | Rv0278c (PE\_PGRS3) | silent (Gln890) | 9876 | - | | 333892 | G | C | 226.80 | SNP | Rv0278c (PE\_PGRS3) | Arg807Gly | 1 | - | | 334641 | G | C | 63.77 | SNP | Rv0278c (PE\_PGRS3) | Ala557Gly | 21 | - | | 335810 | CCCGCCGGCGCCGCCGTTG | C | 811.80 | DEL | Rv0278c (PE\_PGRS3) |  |  | - | | 336191 | C | T | 502.77 | SNP | Rv0278c (PE\_PGRS3) | Met(s)40Ile | 2 | - | | 336380 | A | T | 47.77 | SNP | intergenic |  |  | - | | 336400 | C | G | 108.77 | SNP | intergenic |  |  | - | | 336403 | C | G | 68.77 | SNP | intergenic |  |  | - | | 336405 | A | G | 129.77 | SNP | intergenic |  |  | - | | 336504 | G | T | 280.77 | SNP | intergenic |  |  | - | | 336535 | T | G | 153.77 | SNP | intergenic |  |  | - | | 336537 | T | G | 129.77 | SNP | intergenic |  |  | - | | 336540 | G | T | 106.77 | SNP | intergenic |  |  | - | | 336546 | T | G | 135.77 | SNP | intergenic |  |  | - | | 336560 | T | C | 114.77 | SNP | Rv0279c (PE\_PGRS4) | silent (STOP838) | 9867 | - | | 336590 | G | C | 100.77 | SNP | Rv0279c (PE\_PGRS4) | Ile828Met(s) | 6 | - | | 336592 | T | G | 31.77 | SNP | Rv0279c (PE\_PGRS4) | Ile828Leu | 22 | - | | 336611 | G | C | 94.77 | SNP | Rv0279c (PE\_PGRS4) | silent (Ala821) | 9867 | - | | 336617 | G | C | 74.77 | SNP | Rv0279c (PE\_PGRS4) | silent (Pro819) | 9926 | - | | 336620 | T | C | 57.77 | SNP | Rv0279c (PE\_PGRS4) | silent (Thr818) | 9871 | - | | 336691 | T | C | 34.74 | SNP | Rv0279c (PE\_PGRS4) | Ser795Gly | 21 | - | | 336698 | C | G | 37.74 | SNP | Rv0279c (PE\_PGRS4) | silent (Gly792) | 9935 | - | | 336701 | A | G | 42.74 | SNP | Rv0279c (PE\_PGRS4) | silent (Gly791) | 9935 | - | | 336708 | T | C | 45.74 | SNP | Rv0279c (PE\_PGRS4) | Asp789Gly | 11 | - | | 336710 | A | G | 42.74 | SNP | Rv0279c (PE\_PGRS4) | silent (Ala788) | 9867 | - | | 338020 | A | C | 55.74 | SNP | Rv0279c (PE\_PGRS4) | Cys352Gly | 1 | - | | 338100 | T | C | 199.84 | SNP | Rv0279c (PE\_PGRS4) | Asn325Ser | 34 | - | | 338453 | A | G | 128.03 | SNP | Rv0279c (PE\_PGRS4) | silent (Ala207) | 9867 | - | | 338618 | C | G | 165.90 | SNP | Rv0279c (PE\_PGRS4) | silent (Gly152) | 9935 | - | | 338768 | G | A | 40.77 | SNP | Rv0279c (PE\_PGRS4) | silent (Ile102) | 9872 | - | | 338774 | G | A | 47.77 | SNP | Rv0279c (PE\_PGRS4) | silent (Ala100) | 9867 | - | | 338775 | G | T | 46.77 | SNP | Rv0279c (PE\_PGRS4) | Ala100Asp | 6 | - | | 338777 | G | C | 53.77 | SNP | Rv0279c (PE\_PGRS4) | silent (Leu99) | 9947 | - | | 338789 | G | C | 327.78 | SNP | Rv0279c (PE\_PGRS4) | silent (Thr95) | 9871 | - | | 338790 | G | A | 302.78 | SNP | Rv0279c (PE\_PGRS4) | Thr95Ile | 7 | - | | 338791 | T | C | 344.77 | SNP | Rv0279c (PE\_PGRS4) | Thr95Ala | 32 | - | | 338792 | G | C | 316.78 | SNP | Rv0279c (PE\_PGRS4) | silent (Ala94) | 9867 | - | | 338810 | G | C | 138.77 | SNP | Rv0279c (PE\_PGRS4) | silent (Ala88) | 9867 | - | | 338813 | G | A | 130.77 | SNP | Rv0279c (PE\_PGRS4) | silent (Tyr87) | 9945 | - | | 338816 | C | G | 173.77 | SNP | Rv0279c (PE\_PGRS4) | silent (Ala86) | 9867 | - | | 338844 | A | G | 645.77 | SNP | Rv0279c (PE\_PGRS4) | Val(s)77Ala | 9867 | - | | 338845 | C | T | 587.77 | SNP | Rv0279c (PE\_PGRS4) | Val(s)77Met(s) | 9867 | - | | 338903 | G | C | 316.77 | SNP | Rv0279c (PE\_PGRS4) | silent (Ala57) | 9867 | - | | 340372 | T | C | 606.77 | SNP | Rv0280 (PPE3) | Ser337Pro | 12 | - | | 340953 | G | T | 893.77 | SNP | Rv0280 (PPE3) | silent (Ala530) | 9867 | - | | 346275 | C | G | 699.77 | SNP | Rv0284 (eccC3) | Pro214Arg | 4 | - | | 356528 | A | G | 679.77 | SNP | Rv0292 (eccE3) | Asn217Asp | 42 | - | | 361415 | G | A | 1061.77 | SNP | Rv0297 (PE\_PGRS5) | Ala28Thr | 22 | - | | 362667 | C | T | 292.78 | SNP | Rv0297 (PE\_PGRS5) | Ala445Val(s) | 9867 | - | | 372913 | A | C | 1392.77 | SNP | Rv0305c (PPE6) | silent (Gly933) | 9935 | - | | 373282 | TA | T | 1793.73 | DEL | Rv0305c (PPE6) |  |  | - | | 376774 | T | C | 772.77 | SNP | Rv0307c | silent (Ala94) | 9867 | - | | 382984 | G | A | 1408.77 | SNP | Rv0314c | silent (Ser186) | 9840 | - | | 384380 | A | C | 1169.77 | SNP | Rv0315 | Lys260Thr | 8 | - | | 386432 | C | G | 1362.77 | SNP | Rv0318c | Gly223Ala | 21 | - | | 388725 | C | T | 1296.77 | SNP | Rv0321 (dcd) | silent (Tyr48) | 9945 | - | | 390828 | T | C | 1118.77 | SNP | Rv0323c | Ser142Gly | 21 | - | | 391853 | A | G | 570.77 | SNP | Rv0324 | Thr168Ala | 32 | - | | 395784 | G | A | 1240.77 | SNP | Rv0330c | Leu102Phe | 6 | - | | 397275 | G | C | 545.77 | SNP | Rv0331 | Ala359Pro | 13 | - | | 403980 | G | A | 1220.77 | SNP | Rv0338c | Ala621Val | 13 | - | | 404326 | T | C | 1411.77 | SNP | Rv0338c | Arg506Gly | 1 | - | | 405750 | T | G | 1065.77 | SNP | Rv0338c | Tyr31Ser | 2 | - | | 414486 | C | T | 871.77 | SNP | Rv0344c (lpqJ) | silent (Glu152) | 9865 | - | | 420008 | A | G | 1136.77 | SNP | Rv0350 (dnaK) | silent (Ala58) | 9867 | - | | 424320 | T | TC | 1222.73 | INS | Rv0354c (PPE7) |  |  | - | | 427310 | TTGCCGAGGTTTGCAC | T | 2150.73 | DEL | Rv0355c (PPE8) |  |  | - | | 428186 | CTGCCGGTGTTGGTGCTGCC GGTGTTGTAGCTGCCCGTGT TGGCGATGCCCACGTTGGCG A | C | 6042.73 | DEL | Rv0355c (PPE8) |  |  | - | | 429171 | C | T | 891.77 | SNP | Rv0355c (PPE8) | Ala1837Thr | 22 | - | | 433539 | T | C | 1125.77 | SNP | Rv0355c (PPE8) | Met(s)381Val(s) | 9867 | - | | 454295 | T | C | 975.77 | SNP | Rv0376c | silent (Pro26) | 9926 | - | | 457452 | T | G | 459.77 | SNP | Rv0381c | silent (Thr124) | 9871 | - | | 459399 | A | C | 1282.77 | SNP | intergenic |  |  | - | | 463974 | T | C | 787.77 | SNP | Rv0386 | silent (His188) | 9912 | - | | 467497 | C | CG | 1089.73 | INS | Rv0388c (PPE9) |  |  | - | | 467508 | C | CG | 1129.73 | INS | Rv0388c (PPE9) |  |  | - | | 467516 | G | C | 629.77 | SNP | Rv0388c (PPE9) | silent (Ser162) | 9840 | - | | 467526 | C | G | 633.77 | SNP | Rv0388c (PPE9) | Gly159Ala | 21 | - | | 467546 | G | C | 823.77 | SNP | Rv0388c (PPE9) | Asp152Glu | 56 | - | | 467557 | A | C | 860.77 | SNP | Rv0388c (PPE9) | Leu(s)149Val(s) | 9867 | - | | 467564 | A | C | 822.77 | SNP | Rv0388c (PPE9) | His146Gln | 23 | - | | 467585 | G | C | 1004.77 | SNP | Rv0388c (PPE9) | His139Gln | 23 | - | | 467590 | T | C | 1023.77 | SNP | Rv0388c (PPE9) | Thr138Ala | 32 | - | | 467621 | T | G | 1219.77 | SNP | Rv0388c (PPE9) | silent (Gly127) | 9935 | - | | 467638 | G | T | 1238.77 | SNP | Rv0388c (PPE9) | Gln122Lys | 12 | - | | 475178 | T | C | 931.77 | SNP | Rv0395 | Val80Ala | 18 | - | | 476132 | C | A | 47.77 | SNP | Rv0397 | Pro106His | 3 | - | | 483935 | T | G | 1254.77 | SNP | intergenic |  |  | - | | 489073 | C | G | 886.77 | SNP | Rv0405 (pks6) | Leu1115Val | 11 | - | | 489935 | G | C | 1246.77 | SNP | Rv0405 (pks6); Rv0406c | Arg1402Pro; silent (Thr257) | 5; 9871 | - | | 498557 | C | A | 1223.77 | SNP | Rv0412c | Asp355Tyr | 0 | - | | 501615 | C | G | 959.77 | SNP | Rv0415 (thiO) | silent (Leu156) | 9947 | - | | 502589 | C | G | 1168.77 | SNP | Rv0417 (thiG) | Ser75Cys | 5 | - | | 503354 | G | C | 1683.77 | SNP | intergenic |  |  | - | | 513257 | T | C | 724.77 | SNP | Rv0425c (ctpH) | Met(s)689Val(s) | 9867 | - | | 514245 | C | T | 884.77 | SNP | Rv0425c (ctpH) | Val(s)359Val | 13 | genotype | | 514684 | C | T | 599.77 | SNP | Rv0425c (ctpH) | Arg213His | 8 | - | | 524891 | C | A | 833.77 | SNP | Rv0436c (pssA) | Gly167Val | 3 | - | | 541201 | A | G | 1078.77 | SNP | Rv0450c (mmpL4) | silent (Leu97) | 9947 | - | | 544461 | C | T | 269.78 | SNP | Rv0453 (PPE11) | Arg430Cys | 1 | - | | 546914 | G | A | 928.77 | SNP | intergenic |  |  | - | | 551525 | A | C | 892.77 | SNP | Rv0459 | silent (Arg110) | 9913 | - | | 565655 | A | G | 869.77 | SNP | intergenic |  |  | - | | 573262 | A | G | 1092.77 | SNP | Rv0484c | silent (Gly180) | 9935 | - | | 580772 | T | A | 256.78 | SNP | intergenic |  |  | - | | 580773 | GGGGGCACCACCCGCTTGCG GGGGA | G | 2683.74 | DEL | intergenic |  |  | - | | 590436 | T | C | 1409.77 | SNP | Rv0500 (proC) | silent (Ala118) | 9867 | - | | 595321 | T | G | 600.77 | SNP | intergenic |  |  | - | | 595399 | AT | A | 1064.73 | DEL | intergenic |  |  | - | | 597816 | A | G | 990.77 | SNP | Rv0507 (mmpL2) | silent (Ala206) | 9867 | - | | 598475 | G | A | 1185.77 | SNP | Rv0507 (mmpL2) | Arg426His | 8 | - | | 599165 | A | C | 1579.77 | SNP | Rv0507 (mmpL2) | Glu656Ala | 17 | - | | 610120 | T | G | 1426.77 | SNP | intergenic |  |  | - | | 623472 | A | G | 133.03 | SNP | Rv0532 (PE\_PGRS6) | Asp227Gly | 11 | - | | 623508 | C | G | 172.90 | SNP | Rv0532 (PE\_PGRS6) | Ala239Gly | 21 | - | | 628895 | G | A | 412.77 | SNP | Rv0537c | silent (Phe279) | 9946 | - | | 630722 | G | C | 390.77 | SNP | Rv0538 | Arg228Pro | 5 | - | | 631689 | A | G | 310.77 | SNP | intergenic |  |  | - | | 637319 | G | A | 968.77 | SNP | Rv0545c (pitA) | Pro49Ser | 17 | - | | 648002 | T | G | 1287.77 | SNP | Rv0556 | Leu15Arg | 1 | - | | 649436 | CCGGA | C | 1582.73 | DEL | Rv0557 (mgtA) |  |  | - | | 657269 | A | G | 673.77 | SNP | Rv0565c | Ser68Pro | 12 | - | | 663410 | A | C | 42.77 | SNP | intergenic |  |  | - | | 663419 | G | A | 63.77 | SNP | intergenic |  |  | - | | 663420 | C | A | 58.77 | SNP | intergenic |  |  | - | | 663429 | T | G | 116.77 | SNP | intergenic |  |  | - | | 665293 | A | G | 1059.77 | SNP | Rv0572c | Phe31Leu | 13 | - | | 669398 | T | C | 949.77 | SNP | Rv0575c | silent (Gln116) | 9876 | - | | 672491 | C | G | 166.90 | SNP | Rv0578c (PE\_PGRS7) | silent (Gly1142) | 9935 | - | | 673238 | A | G | 109.03 | SNP | Rv0578c (PE\_PGRS7) | silent (His893) | 9912 | - | | 685461 | C | G | 955.77 | SNP | Rv0587 (yrbE2A) | silent (Ala111) | 9867 | - | | 685608 | T | C | 1344.77 | SNP | Rv0587 (yrbE2A) | silent (Leu160) | 9947 | - | | 686655 | C | T | 839.77 | SNP | Rv0588 (yrbE2B) | Thr243Ile | 7 | - | | 686972 | T | C | 1413.77 | SNP | Rv0589 (mce2A) | Phe51Ser | 3 | - | | 690465 | T | G | 557.77 | SNP | Rv0591 (mce2C) | silent (Leu469) | 9947 | - | | 698828 | G | A | 431.77 | SNP | Rv0601c | Ala56Val(s) | 9867 | - | | 698968 | G | A | 758.77 | SNP | Rv0601c | silent (Gly9) | 9935 | - | | 712693 | A | G | 619.77 | SNP | intergenic |  |  | - | | 721410 | T | C | 767.77 | SNP | Rv0629c (recD) | Asp108Gly | 11 | - | | 726816 | G | C | 285.78 | SNP | Rv0631c (recC) | Phe497Leu(s) | 2 | - | | 728556 | GC | G | 1526.73 | DEL | intergenic |  |  | - | | 746045 | G | C | 703.77 | SNP | Rv0648 | silent (Leu1109) | 9947 | - | | 754186 | A | G | 1151.77 | SNP | Rv0658c | Leu75Pro | 2 | - | | 761109 | G | T | 1376.77 | SNP | Rv0667 (rpoB) | Asp435Tyr | 0 | resistance | | 765150 | G | A | 1497.77 | SNP | Rv0668 (rpoC) | Gly594Glu | 4 | genotype | | 767414 | G | A | 859.77 | SNP | intergenic |  |  | - | | 768579 | C | G | 854.77 | SNP | Rv0669c | Gly340Ala | 21 | - | | 775639 | T | C | 900.77 | SNP | Rv0676c (mmpL5) | Ile948Val | 57 | - | | 781395 | T | C | 1147.77 | SNP | intergenic (Rv0682-165nt) |  |  | - | | 782246 | G | A | 1171.77 | SNP | Rv0683 (rpsG) | Val105Ile | 33 | - | | 788259 | G | T | 839.77 | SNP | Rv0688 | Gly107Val | 3 | - | | 794480 | CG | C | 1223.73 | DEL | Rv0694 (lldD1) |  |  | - | | 800219 | T | C | 743.77 | SNP | intergenic |  |  | - | | 820483 | G | T | 898.77 | SNP | Rv0727c (fucA) | Ala6Asp | 6 | - | | 828176 | G | C | 913.77 | SNP | Rv0736 (rslA) | Asp13His | 3 | - | | 836454 | A | G | 56.74 | SNP | Rv0746 (PE\_PGRS9) | Thr252Ala | 32 | - | | 837033 | A | G | 163.90 | SNP | Rv0746 (PE\_PGRS9) | Thr445Ala | 32 | - | | 838822 | G | C | 37.77 | SNP | Rv0747 (PE\_PGRS10) | silent (Gly124) | 9935 | - | | 838828 | A | G | 33.77 | SNP | Rv0747 (PE\_PGRS10) | silent (Gly126) | 9935 | - | | 838832 | A | G | 36.77 | SNP | Rv0747 (PE\_PGRS10) | Asn128Asp | 42 | - | | 838839 | G | C | 48.77 | SNP | Rv0747 (PE\_PGRS10) | Gly130Ala | 21 | - | | 838849 | G | C | 42.77 | SNP | Rv0747 (PE\_PGRS10) | silent (Gly133) | 9935 | - | | 839279 | G | A | 31.77 | SNP | Rv0747 (PE\_PGRS10) | Asp277Asn | 36 | - | | 839284 | C | G | 31.77 | SNP | Rv0747 (PE\_PGRS10) | silent (Ala278) | 9867 | - | | 839309 | T | G | 69.77 | SNP | Rv0747 (PE\_PGRS10) | Ser287Ala | 35 | - | | 839334 | A | G | 217.80 | SNP | Rv0747 (PE\_PGRS10) | Lys295Arg | 19 | - | | 839348 | A | G | 184.84 | SNP | Rv0747 (PE\_PGRS10) | Ser300Gly | 21 | - | | 839496 | G | A | 89.77 | SNP | Rv0747 (PE\_PGRS10) | Gly349Asp | 6 | - | | 839515 | G | A | 98.77 | SNP | Rv0747 (PE\_PGRS10) | silent (Ala355) | 9867 | - | | 839516 | A | G | 131.77 | SNP | Rv0747 (PE\_PGRS10) | Thr356Ala | 32 | - | | 839519 | C | G | 109.77 | SNP | Rv0747 (PE\_PGRS10) | Leu357Val(s) | 4 | - | | 839520 | T | C | 102.77 | SNP | Rv0747 (PE\_PGRS10) | Leu357Pro | 2 | - | | 839534 | A | C | 96.77 | SNP | Rv0747 (PE\_PGRS10) | Ile362Leu | 22 | - | | 840235 | C | G | 79.77 | SNP | Rv0747 (PE\_PGRS10) | silent (Gly595) | 9935 | - | | 840272 | G | T | 55.77 | SNP | Rv0747 (PE\_PGRS10) | Ala608Ser | 28 | - | | 840278 | A | G | 35.77 | SNP | Rv0747 (PE\_PGRS10) | Ser610Gly | 21 | - | | 840298 | A | G | 31.77 | SNP | Rv0747 (PE\_PGRS10) | silent (Gly616) | 9935 | - | | 840301 | T | C | 32.77 | SNP | Rv0747 (PE\_PGRS10) | silent (Val617) | 9901 | - | | 840302 | T | C | 32.77 | SNP | Rv0747 (PE\_PGRS10) | Ser618Pro | 12 | - | | 840307 | A | C | 31.77 | SNP | Rv0747 (PE\_PGRS10) | silent (Gly619) | 9935 | - | | 840310 | G | C | 31.77 | SNP | Rv0747 (PE\_PGRS10) | silent (Gly620) | 9935 | - | | 840334 | C | G | 38.77 | SNP | Rv0747 (PE\_PGRS10) | silent (Ala628) | 9867 | - | | 840341 | C | T | 35.77 | SNP | Rv0747 (PE\_PGRS10) | Leu631Phe | 6 | - | | 840349 | C | G | 36.77 | SNP | Rv0747 (PE\_PGRS10) | silent (Leu633) | 9947 | - | | 840355 | C | T | 33.77 | SNP | Rv0747 (PE\_PGRS10) | silent (Ala635) | 9867 | - | | 840358 | C | G | 36.77 | SNP | Rv0747 (PE\_PGRS10) | silent (Ser636) | 9840 | - | | 840361 | C | T | 31.77 | SNP | Rv0747 (PE\_PGRS10) | silent (Gly637) | 9935 | - | | 840364 | G | C | 36.77 | SNP | Rv0747 (PE\_PGRS10) | silent (Gly638) | 9935 | - | | 840373 | C | T | 31.77 | SNP | Rv0747 (PE\_PGRS10) | silent (Gly641) | 9935 | - | | 840382 | T | C | 38.77 | SNP | Rv0747 (PE\_PGRS10) | silent (Gly644) | 9935 | - | | 840389 | C | T | 33.77 | SNP | Rv0747 (PE\_PGRS10) | Leu647Leu(s) | 4 | - | | 840396 | C | A | 31.77 | SNP | Rv0747 (PE\_PGRS10) | Ala649Asp | 6 | - | | 840399 | C | G | 38.77 | SNP | Rv0747 (PE\_PGRS10) | Ala650Gly | 21 | - | | 840405 | T | G | 36.77 | SNP | Rv0747 (PE\_PGRS10) | Val(s)652Gly | 21 | - | | 840407 | G | A | 33.77 | SNP | Rv0747 (PE\_PGRS10) | Val653Ile | 33 | - | | 840412 | C | T | 30.77 | SNP | Rv0747 (PE\_PGRS10) | silent (Gly654) | 9935 | - | | 840415 | C | G | 33.77 | SNP | Rv0747 (PE\_PGRS10) | silent (Gly655) | 9935 | - | | 840416 | A | G | 38.77 | SNP | Rv0747 (PE\_PGRS10) | Ile656Val | 57 | - | | 840430 | A | C | 31.77 | SNP | Rv0747 (PE\_PGRS10) | silent (Gly660) | 9935 | - | | 840496 | C | G | 34.90 | SNP | Rv0747 (PE\_PGRS10) | silent (Gly682) | 9935 | - | | 841764 | G | C | 1307.77 | SNP | Rv0749A | silent (Thr37) | 9871 | - | | 841924 | C | T | 1468.77 | SNP | intergenic |  |  | - | | 850591 | C | T | 2268.77 | SNP | intergenic |  |  | - | | 851133 | T | G | 518.77 | SNP | Rv0756c | Thr112Pro | 4 | - | | 852910 | C | T | 580.77 | SNP | Rv0758 (phoR) | Pro172Leu | 3 | - | | 854252 | GC | G | 1133.73 | DEL | intergenic |  |  | - | | 857696 | A | G | 1336.77 | SNP | Rv0764c (cyp51) | silent (Ala114) | 9867 | - | | 864184 | G | A | 1014.77 | SNP | Rv0771 | silent (Glu15) | 9865 | - | | 865737 | C | T | 835.77 | SNP | Rv0772 (purD) | silent (Ala384) | 9867 | - | | 868488 | G | A | 943.77 | SNP | Rv0775 | Glu28Lys | 7 | - | | 869440 | C | T | 1054.77 | SNP | Rv0776c | silent (Leu108) | 9947 | - | | 872108 | C | G | 467.77 | SNP | Rv0778 (cyp126) | silent (Ala226) | 9867 | - | | 874835 | C | CCG | 2520.73 | INS | Rv0781 (ptrBa); Rv0782 (ptrBb) |  |  | - | | 877224 | C | A | 1208.77 | SNP | Rv0783c (emrB) | Gly406Val | 3 | - | | 880562 | G | T | 933.77 | SNP | Rv0785 | Cys408Phe | 0 | - | | 880935 | C | T | 940.77 | SNP | Rv0785 | silent (Gly532) | 9935 | - | | 882257 | T | C | 1309.77 | SNP | Rv0787 | Tyr267His | 4 | - | | 888774 | G | A | 774.77 | SNP | intergenic |  |  | - | | 893733 | T | G | 708.77 | SNP | Rv0800 (pepC) | Leu139Arg | 1 | - | | 894060 | A | G | 78.77 | SNP | Rv0800 (pepC) | Asp248Gly | 11 | - | | 896356 | C | T | 996.77 | SNP | Rv0803 (purL) | silent (Thr179) | 9871 | - | | 897209 | G | C | 544.77 | SNP | Rv0803 (purL) | Glu464Gln | 27 | - | | 900221 | T | C | 1094.77 | SNP | Rv0806c (cpsY) | Val370Val(s) | 18 | - | | 902413 | C | T | 1034.77 | SNP | Rv0808 (purF) | silent (Val101) | 9901 | - | | 903550 | T | C | 942.77 | SNP | Rv0808 (purF) | silent (Ala480) | 9867 | - | | 903913 | T | C | 924.77 | SNP | Rv0809 (purM) | silent (Gly63) | 9935 | - | | 906857 | A | G | 714.77 | SNP | Rv0812 | Ile145Met(s) | 6 | - | | 918316 | T | C | 1038.77 | SNP | Rv0824c (desA1) | silent (Gln145) | 9876 | - | | 919574 | T | C | 1246.77 | SNP | intergenic |  |  | - | | 920747 | G | A | 670.77 | SNP | Rv0827c (kmtR) | silent (Val129) | 9901 | - | | 921813 | C | G | 1077.77 | SNP | Rv0829 | Ala80Gly | 21 | - | | 923065 | T | A | 1448.77 | SNP | Rv0831c | silent (Gly215) | 9935 | - | | 927385 | A | G | 126.03 | SNP | Rv0833 (PE\_PGRS13) | silent (Gly675) | 9935 | - | | 928075 | T | C | 276.78 | SNP | Rv0834c (PE\_PGRS14) | Asn804Ser | 34 | - | | 928076 | T | C | 231.80 | SNP | Rv0834c (PE\_PGRS14) | Asn804Asp | 42 | - | | 928158 | CTCGCCGCCG | C | 796.75 | DEL | Rv0834c (PE\_PGRS14) |  |  | - | | 929440 | T | G | 146.03 | SNP | Rv0834c (PE\_PGRS14) | Asp349Ala | 10 | - | | 931417 | C | T | 1242.77 | SNP | Rv0835 (lpqQ) | silent (Pro155) | 9926 | - | | 945214 | G | A | 887.77 | SNP | Rv0848 (cysK2) | Gly93Ser | 16 | - | | 949535 | T | C | 957.77 | SNP | Rv0853c (pdc) | silent (Ala528) | 9867 | - | | 950230 | C | T | 794.77 | SNP | Rv0853c (pdc) | Asp297Asn | 36 | - | | 954253 | C | G | 114.77 | SNP | Rv0858c (dapC) | Arg223Pro | 5 | - | | 955524 | A | G | 1279.77 | SNP | Rv0859 (fadA) | Ser150Gly | 21 | - | | 957117 | T | C | 473.77 | SNP | Rv0860 (fadB) | silent (Asp275) | 9859 | - | | 968426 | A | AGCCGGGTTG | 1366.73 | INS | Rv0872c (PE\_PGRS15) |  |  | - | | 969602 | C | G | 35.74 | SNP | Rv0872c (PE\_PGRS15) | Gly215Arg | 0 | - | | 969668 | C | A | 45.74 | SNP | Rv0872c (PE\_PGRS15) | Ala193Ser | 28 | - | | 976897 | T | G | 1338.77 | SNP | Rv0878c (PPE13) | Gln436Pro | 8 | - | | 978373 | T | C | 711.77 | SNP | intergenic |  |  | - | | 979704 | G | C | 948.77 | SNP | Rv0881 | Gly115Arg | 0 | - | | 979859 | T | C | 849.77 | SNP | Rv0881 | silent (Ala166) | 9867 | - | | 983485 | T | G | 548.77 | SNP | Rv0885 | Ser242Ala | 35 | - | | 986463 | G | C | 1643.77 | SNP | intergenic |  |  | - | | 990001 | G | C | 719.77 | SNP | Rv0890c | Pro866Ala | 22 | - | | 993346 | A | C | 1495.77 | SNP | Rv0891c | Val37Gly | 5 | - | | 1010204 | C | CG | 1922.73 | INS | Rv0907 |  |  | - | | 1025106 | T | C | 1652.77 | SNP | Rv0919 | silent (Phe141) | 9946 | - | | 1028944 | C | T | 572.77 | SNP | Rv0922 | silent (Tyr420) | 9945 | - | | 1037012 | T | C | 256.80 | SNP | Rv0930 (pstA1) | Met(s)5Thr | 22 | - | | 1037911 | C | T | 859.77 | SNP | Rv0930 (pstA1) | Arg305STOP | 2 | - | | 1047165 | T | C | 852.77 | SNP | Rv0938 (ligD) | Cys344Arg | 1 | - | | 1049508 | C | T | 653.77 | SNP | Rv0939 | Ala366Val(s) | 9867 | - | | 1068151 | T | C | 1538.77 | SNP | Rv0956 (purN) | silent (His197) | 9912 | - | | 1068432 | A | G | 1053.77 | SNP | Rv0957 (purH) | silent (Pro76) | 9926 | - | | 1070702 | T | C | 738.77 | SNP | Rv0958 | Ser274Pro | 12 | - | | 1074558 | G | A | 890.77 | SNP | Rv0962c (lprP) | Pro186Leu | 3 | - | | 1075279 | T | C | 1359.77 | SNP | intergenic |  |  | - | | 1076309 | G | T | 658.77 | SNP | Rv0964c | Pro124Thr | 5 | - | | 1077312 | A | G | 876.77 | SNP | Rv0966c | Val(s)175Ala | 9867 | - | | 1079927 | C | A | 640.77 | SNP | Rv0969 (ctpV) | silent (Thr395) | 9871 | - | | 1081681 | T | C | 803.77 | SNP | Rv0970 | silent (Val210) | 9901 | - | | 1087193 | G | C | 1094.77 | SNP | Rv0974c (accD2) | Asn51Lys | 25 | - | | 1090292 | C | G | 886.77 | SNP | intergenic |  |  | - | | 1092340 | C | CG | 319.73 | INS | Rv0977 (PE\_PGRS16) |  |  | - | | 1093406 | A | G | 867.77 | SNP | Rv0978c (PE\_PGRS17) | silent (Val317) | 9901 | - | | 1093928 | G | A | 65.28 | SNP | Rv0978c (PE\_PGRS17) | silent (Asn143) | 9822 | - | | 1095850 | A | T | 130.90 | SNP | Rv0980c (PE\_PGRS18) | Val201Asp | 1 | - | | 1095851 | C | T | 107.03 | SNP | Rv0980c (PE\_PGRS18) | Val201Ile | 33 | - | | 1095852 | A | C | 161.90 | SNP | Rv0980c (PE\_PGRS18) | silent (Gly200) | 9935 | - | | 1095855 | A | G | 157.90 | SNP | Rv0980c (PE\_PGRS18) | silent (Gly199) | 9935 | - | | 1096633 | T | G | 712.77 | SNP | intergenic |  |  | - | | 1100234 | T | C | 427.77 | SNP | Rv0983 (pepD) | Leu390Pro | 2 | - | | 1102788 | G | T | 1117.77 | SNP | Rv0987 | Val83Phe | 0 | - | | 1106422 | T | C | 2124.77 | SNP | Rv0989c (grcC2) | Ile321Val | 57 | - | | 1106877 | T | C | 762.77 | SNP | Rv0989c (grcC2) | Tyr169Cys | 3 | - | | 1109975 | A | G | 1132.77 | SNP | Rv0993 (galU) | Gln235Arg | 10 | - | | 1126889 | G | C | 725.77 | SNP | Rv1007c (metS) | Arg39Gly | 1 | - | | 1127648 | C | A | 895.77 | SNP | Rv1008 (tatD) | Thr187Asn | 9 | - | | 1148255 | G | A | 1112.77 | SNP | intergenic |  |  | - | | 1149551 | C | T | 1249.77 | SNP | Rv1028c (kdpD) | silent (Glu712) | 9865 | - | | 1150585 | G | A | 1013.77 | SNP | Rv1028c (kdpD) | Pro368Ser | 17 | - | | 1155067 | C | CG | 2238.73 | INS | Rv1030 (kdpB) |  |  | - | | 1159854 | C | T | 909.77 | SNP | Rv1035c | Ala70Thr | 22 | - | | 1163134 | T | C | 779.77 | SNP | Rv1040c (PE8) | silent (Gly81) | 9935 | - | | 1164336 | G | A | 1109.77 | SNP | intergenic |  |  | - | | 1164361 | G | A | 1277.77 | SNP | intergenic |  |  | - | | 1164571 | A | G | 884.77 | SNP | intergenic |  |  | - | | 1165521 | T | TA | 1311.73 | INS | intergenic |  |  | - | | 1168715 | C | CT | 1359.73 | INS | Rv1046c |  |  | - | | 1170404 | C | A | 133.90 | SNP | Rv1047 | Gln328Lys | 12 | - | | 1174515 | C | T | 836.77 | SNP | Rv1051c | silent (Ala62) | 9867 | - | | 1178116 | T | C | 1300.77 | SNP | Rv1056 | silent (Thr163) | 9871 | - | | 1189606 | A | G | 94.28 | SNP | Rv1067c (PE\_PGRS19) | silent (Gly273) | 9935 | - | | 1189613 | G | A | 82.28 | SNP | Rv1067c (PE\_PGRS19) | Ala271Val | 13 | - | | 1190093 | A | C | 456.77 | SNP | Rv1067c (PE\_PGRS19) | Leu(s)111Trp | 0 | - | | 1191741 | G | A | 176.90 | SNP | Rv1068c (PE\_PGRS20) | silent (Tyr136) | 9945 | - | | 1200418 | A | G | 1360.77 | SNP | intergenic |  |  | - | | 1220680 | T | C | 1009.77 | SNP | Rv1093 (glyA1) | Val36Ala | 18 | - | | 1224367 | T | C | 629.77 | SNP | intergenic |  |  | - | | 1231587 | C | T | 720.77 | SNP | Rv1104 | Pro96Leu | 3 | - | | 1237562 | G | C | 392.78 | SNP | Rv1111c | Arg211Gly | 1 | - | | 1244655 | G | T | 776.82 | SNP | Rv1121 (zwf1) | Ala317Ser | 28 | - | | 1248978 | T | C | 820.77 | SNP | Rv1125 | silent (Ala299) | 9867 | - | | 1251199 | C | T | 1029.77 | SNP | Rv1127c (ppdK) | Gly69Glu | 4 | - | | 1252164 | T | C | 982.77 | SNP | Rv1128c | Glu270Gly | 7 | - | | 1256806 | C | T | 1070.80 | SNP | Rv1131 (prpC) | silent (Asp225) | 9859 | - | | 1275957 | T | C | 913.77 | SNP | intergenic |  |  | - | | 1276321 | T | G | 966.77 | SNP | Rv1148c | Gln476His | 20 | - | | 1276322 | T | G | 976.77 | SNP | Rv1148c | Gln476Pro | 8 | - | | 1276360 | T | G | 1057.77 | SNP | Rv1148c | silent (Ile463) | 9872 | - | | 1276363 | T | G | 1048.77 | SNP | Rv1148c | silent (Arg462) | 9913 | - | | 1276366 | C | T | 1006.77 | SNP | Rv1148c | silent (Pro461) | 9926 | - | | 1276931 | T | G | 740.77 | SNP | Rv1148c | Asp273Ala | 10 | - | | 1277869 | G | GT | 1200.73 | INS | intergenic |  |  | - | | 1281118 | T | C | 1233.77 | SNP | Rv1154c | Thr123Ala | 32 | - | | 1292102 | A | G | 706.77 | SNP | Rv1162 (narH) | silent (Pro346) | 9926 | - | | 1292464 | C | T | 1020.77 | SNP | Rv1162 (narH) | Ala467Val(s) | 9867 | - | | 1302161 | C | G | 691.78 | SNP | Rv1172c (PE12) | Gly174Ala | 21 | - | | 1306259 | A | G | 1025.77 | SNP | Rv1175c (fadH) | silent (Ala656) | 9867 | - | | 1307598 | C | G | 740.77 | SNP | Rv1175c (fadH) | Cys210Ser | 11 | - | | 1312176 | C | T | 1364.77 | SNP | Rv1179c | Arg375Gln | 9 | - | | 1313337 | A | AG | 1976.73 | INS | intergenic |  |  | - | | 1313338 | A | C | 1210.77 | SNP | intergenic |  |  | - | | 1315191 | A | C | 731.77 | SNP | Rv1180 (pks3) | STOP489Tyr | 1 | - | | 1315884 | G | A | 793.77 | SNP | Rv1181 (pks4) | silent (Ala217) | 9867 | - | | 1327890 | G | A | 788.77 | SNP | Rv1186c | silent (Asp472) | 9859 | - | | 1328687 | G | C | 1030.77 | SNP | Rv1186c | Pro207Ala | 22 | - | | 1339432 | G | C | 349.78 | SNP | Rv1196 (PPE18) | silent (Ala28) | 9867 | - | | 1339435 | T | G | 352.78 | SNP | Rv1196 (PPE18) | silent (Ala29) | 9867 | - | | 1339436 | C | A | 335.78 | SNP | Rv1196 (PPE18) | Gln30Lys | 12 | - | | 1339511 | G | A | 43.74 | SNP | Rv1196 (PPE18) | Val(s)55Met(s) | 9867 | - | | 1339512 | T | C | 49.74 | SNP | Rv1196 (PPE18) | Val(s)55Ala | 9867 | - | | 1339516 | G | A | 46.74 | SNP | Rv1196 (PPE18) | silent (Gly56) | 9935 | - | | 1339837 | G | C | 72.81 | SNP | Rv1196 (PPE18) | silent (Thr163) | 9871 | - | | 1339839 | C | A | 181.84 | SNP | Rv1196 (PPE18) | Ala164Glu | 10 | - | | 1339841 | A | G | 181.84 | SNP | Rv1196 (PPE18) | Thr165Ala | 32 | - | | 1339861 | G | C | 40.74 | SNP | Rv1196 (PPE18) | Glu171Asp | 53 | - | | 1339868 | G | C | 32.77 | SNP | Rv1196 (PPE18) | Glu174Gln | 27 | - | | 1339869 | A | T | 30.78 | SNP | Rv1196 (PPE18) | Glu174Val(s) | 17 | - | | 1339894 | C | T | 255.31 | SNP | Rv1196 (PPE18) | silent (Leu182) | 9947 | - | | 1340667 | A | G | 846.77 | SNP | Rv1197 (esxK) | silent (Ser3) | 9840 | - | | 1341099 | A | G | 116.90 | SNP | Rv1198 (esxL) | Ile32Val | 57 | - | | 1341114 | A | G | 186.84 | SNP | Rv1198 (esxL) | Thr37Ala | 32 | - | | 1341120 | A | G | 153.82 | SNP | Rv1198 (esxL) | Ser39Gly | 21 | - | | 1341148 | C | T | 246.53 | SNP | Rv1198 (esxL) | Ala48Val(s) | 9867 | - | | 1341152 | C | T | 280.31 | SNP | Rv1198 (esxL) | silent (Ala49) | 9867 | - | | 1341624 | G | T | 57.28 | SNP | Rv1199c | Gln328Lys | 12 | - | | 1342581 | T | C | 448.77 | SNP | Rv1199c | Thr9Ala | 32 | - | | 1360209 | T | C | 866.77 | SNP | Rv1217c | silent (Ala531) | 9867 | - | | 1362006 | T | C | 1477.77 | SNP | Rv1218c | Gln243Arg | 10 | - | | 1365410 | G | A | 983.77 | SNP | Rv1222 (rseA) | Ala23Thr | 22 | - | | 1365837 | C | CG | 973.73 | INS | intergenic |  |  | - | | 1368961 | G | A | 361.77 | SNP | Rv1226c | silent (Leu445) | 9947 | - | | 1372703 | C | A | 795.77 | SNP | Rv1229c (mrp) | Val(s)83Leu(s) | 9867 | - | | 1374065 | T | C | 686.77 | SNP | Rv1230c | Ser45Gly | 21 | - | | 1375724 | A | C | 1089.77 | SNP | Rv1232c | Cys149Gly | 1 | - | | 1382628 | T | C | 814.77 | SNP | Rv1239c (corA) | Lys139Glu | 4 | - | | 1393626 | A | G | 838.77 | SNP | Rv1249c | silent (Leu119) | 9947 | - | | 1393994 | C | T | 1026.77 | SNP | intergenic |  |  | - | | 1396510 | G | A | 900.77 | SNP | Rv1251c | His911Tyr | 4 | - | | 1396922 | T | C | 766.77 | SNP | Rv1251c | silent (Thr773) | 9871 | - | | 1404257 | A | G | 30.77 | SNP | Rv1256c (cyp130) | Val116Ala | 18 | - | | 1411210 | T | G | 755.77 | SNP | Rv1263 (amiB2) | Val260Val(s) | 18 | - | | 1413148 | C | T | 997.77 | SNP | intergenic |  |  | - | | 1414021 | C | T | 946.77 | SNP | Rv1266c (pknH) | Arg607Gln | 9 | - | | 1416222 | A | G | 148.77 | SNP | Rv1267c (embR) | Phe376Leu | 13 | - | | 1416232 | A | G | 181.77 | SNP | Rv1267c (embR) | silent (Cys372) | 9973 | - | | 1416234 | A | C | 151.77 | SNP | Rv1267c (embR) | Cys372Gly | 1 | - | | 1426079 | C | T | 627.77 | SNP | intergenic |  |  | - | | 1440469 | C | G | 1581.77 | SNP | Rv1286 (cysN) | silent (Pro521) | 9926 | - | | 1444202 | C | G | 938.77 | SNP | Rv1290c | Leu(s)282Phe | 1 | - | | 1445781 | A | G | 642.77 | SNP | Rv1291c | silent (Ala18) | 9867 | - | | 1457144 | C | T | 297.74 | SNP | Rv1300 (hemK) | Arg194Cys | 1 | - | | 1461915 | C | G | 1532.77 | SNP | Rv1307 (atpH) | Leu25Val | 11 | - | | 1468208 | A | C | 828.77 | SNP | Rv1313c | Leu433Arg | 1 | - | | 1471539 | G | A | 1018.77 | SNP | Rv1315 (murA) | Val(s)407Met(s) | 9867 | - | | 1471659 | C | T | 1124.77 | SNP | intergenic |  |  | - | | 1480219 | C | T | 34.77 | SNP | Rv1318c | silent (Ala202) | 9867 | - | | 1480945 | C | G | 879.77 | SNP | Rv1319c | silent (Thr519) | 9871 | - | | 1480948 | C | T | 870.77 | SNP | Rv1319c | silent (Glu518) | 9865 | - | | 1480972 | T | C | 641.77 | SNP | Rv1319c | silent (Glu510) | 9865 | - | | 1481185 | A | C | 901.77 | SNP | Rv1319c | Asp439Glu | 56 | - | | 1482627 | T | C | 1028.77 | SNP | Rv1320c | Thr531Ala | 32 | - | | 1483894 | G | GT | 1656.73 | INS | Rv1320c |  |  | - | | 1484708 | A | C | 987.77 | SNP | Rv1321 | Ser144Arg | 6 | - | | 1487084 | C | T | 621.84 | SNP | intergenic |  |  | - | | 1488433 | A | G | 92.77 | SNP | Rv1325c (PE\_PGRS24) | silent (Asp511) | 9859 | - | | 1488434 | T | G | 94.77 | SNP | Rv1325c (PE\_PGRS24) | Asp511Ala | 10 | - | | 1488435 | C | A | 92.77 | SNP | Rv1325c (PE\_PGRS24) | Asp511Tyr | 0 | - | | 1495326 | A | G | 1362.77 | SNP | Rv1328 (glgP) | Lys255Glu | 4 | - | | 1499274 | C | G | 354.77 | SNP | Rv1330c (pncB1) | Gly429Ala | 21 | - | | 1519431 | C | T | 1075.77 | SNP | Rv1353c | Val(s)185Val | 13 | - | | 1526819 | C | A | 1328.77 | SNP | Rv1358 | silent (Arg70) | 9913 | - | | 1533004 | T | C | 33.77 | SNP | Rv1361c (PPE19) | silent (Gln210) | 9876 | - | | 1533016 | T | C | 116.77 | SNP | Rv1361c (PPE19) | silent (Gln206) | 9876 | - | | 1533059 | A | G | 316.77 | SNP | Rv1361c (PPE19) | Ile192Thr | 11 | - | | 1533060 | T | A | 311.77 | SNP | Rv1361c (PPE19) | Ile192Phe | 8 | - | | 1533077 | A | G | 364.77 | SNP | Rv1361c (PPE19) | Val186Ala | 18 | - | | 1533159 | C | T | 1223.77 | SNP | Rv1361c (PPE19) | Ala159Thr | 22 | - | | 1533162 | T | C | 1243.77 | SNP | Rv1361c (PPE19) | Thr158Ala | 32 | - | | 1533163 | G | C | 1280.77 | SNP | Rv1361c (PPE19) | silent (Ala157) | 9867 | - | | 1533208 | C | G | 1307.77 | SNP | Rv1361c (PPE19) | silent (Gly142) | 9935 | - | | 1533583 | G | A | 807.77 | SNP | Rv1361c (PPE19) | silent (Tyr17) | 9945 | - | | 1536251 | G | T | 916.77 | SNP | Rv1364c | Ala465Glu | 10 | - | | 1538659 | T | G | 1036.77 | SNP | Rv1366 | silent (Arg90) | 9913 | - | | 1539200 | G | T | 1195.77 | SNP | Rv1366; Rv1366A | Gly271STOP; silent (Gly7) | 21; 9935 | - | | 1546541 | A | C | 632.77 | SNP | Rv1373 | Gln177Pro | 8 | - | | 1547125 | T | C | 1207.77 | SNP | Rv1374c | Thr136Ala | 32 | - | | 1550020 | C | G | 932.77 | SNP | Rv1376 | His291Gln | 23 | - | | 1552547 | G | A | 589.77 | SNP | Rv1378c | Arg37Trp | 2 | - | | 1553633 | T | C | 671.77 | SNP | Rv1380 (pyrB) | silent (Gly134) | 9935 | - | | 1553801 | G | A | 887.77 | SNP | Rv1380 (pyrB) | Val(s)190Val | 13 | - | | 1563717 | C | T | 735.77 | SNP | Rv1388 (mihF) | silent (Val8) | 9901 | - | | 1564215 | C | G | 74.77 | SNP | Rv1388 (mihF) | silent (Gly174) | 9935 | - | | 1570566 | C | A | 1168.77 | SNP | Rv1394c (cyp132) | Arg135Leu | 1 | - | | 1572322 | G | A | 44.74 | SNP | Rv1396c (PE\_PGRS25) | silent (Asn512) | 9822 | - | | 1573660 | T | G | 880.77 | SNP | Rv1396c (PE\_PGRS25) | Arg66Ser | 11 | - | | 1580680 | C | T | 1552.77 | SNP | Rv1404 | silent (Ala30) | 9867 | - | | 1588899 | G | T | 1000.77 | SNP | Rv1412 (ribC) | silent (Ala111) | 9867 | - | | 1589383 | A | G | 1022.77 | SNP | intergenic |  |  | - | | 1591152 | C | T | 811.77 | SNP | Rv1415 (ribA2) | silent (Asp252) | 9859 | - | | 1605149 | C | T | 1116.77 | SNP | Rv1429 | Pro91Leu | 3 | - | | 1609840 | A | G | 1108.77 | SNP | Rv1431 | silent (Pro586) | 9926 | - | | 1612278 | G | GCGTCGA | 2912.73 | INS | Rv1434 |  |  | - | | 1612624 | T | TATCGGTACCGGTGCGCCAG GG | 3016.73 | INS | Rv1435c |  |  | - | | 1613035 | T | C | 941.77 | SNP | intergenic |  |  | - | | 1617735 | G | A | 540.77 | SNP | intergenic |  |  | - | | 1618615 | CCGCCGCCGGTGCCGCCGGC GCCGCCGTCGCCGCCGG | C | 1082.76 | DEL | Rv1441c (PE\_PGRS26) |  |  | - | | 1618999 | C | T | 165.90 | SNP | Rv1441c (PE\_PGRS26) | Gly229Asp | 6 | - | | 1624791 | C | G | 468.77 | SNP | Rv1446c (opcA) | Arg192Pro | 5 | - | | 1627351 | T | C | 920.77 | SNP | Rv1448c (tal) | Thr244Ala | 32 | - | | 1630148 | A | C | 1453.77 | SNP | Rv1449c (tkt) | Tyr18Asp | 0 | - | | 1632737 | C | T | 75.28 | SNP | Rv1450c (PE\_PGRS27) | Gly631Ser | 16 | - | | 1636826 | C | A | 145.90 | SNP | Rv1452c (PE\_PGRS28) | silent (Gly468) | 9935 | - | | 1636918 | C | T | 67.28 | SNP | Rv1452c (PE\_PGRS28) | Ala438Thr | 22 | - | | 1636927 | C | T | 78.28 | SNP | Rv1452c (PE\_PGRS28) | Asp435Asn | 36 | - | | 1636928 | A | G | 84.28 | SNP | Rv1452c (PE\_PGRS28) | silent (Gly434) | 9935 | - | | 1636934 | A | G | 48.74 | SNP | Rv1452c (PE\_PGRS28) | silent (His432) | 9912 | - | | 1636936 | G | T | 40.74 | SNP | Rv1452c (PE\_PGRS28) | His432Asn | 21 | - | | 1636944 | G | T | 40.74 | SNP | Rv1452c (PE\_PGRS28) | Ala429Asp | 6 | - | | 1636945 | C | G | 46.74 | SNP | Rv1452c (PE\_PGRS28) | Ala429Pro | 13 | - | | 1636946 | C | A | 31.74 | SNP | Rv1452c (PE\_PGRS28) | silent (Gly428) | 9935 | - | | 1636980 | G | T | 75.28 | SNP | Rv1452c (PE\_PGRS28) | Pro417His | 3 | - | | 1636981 | G | T | 86.28 | SNP | Rv1452c (PE\_PGRS28) | Pro417Thr | 5 | - | | 1636983 | C | G | 56.74 | SNP | Rv1452c (PE\_PGRS28) | Gly416Ala | 21 | - | | 1639594 | C | A | 1152.77 | SNP | Rv1453 | Pro405Gln | 6 | - | | 1643654 | G | T | 680.77 | SNP | Rv1458c | Leu203Ile | 9 | - | | 1644362 | C | T | 123.77 | SNP | intergenic |  |  | - | | 1645334 | C | T | 733.77 | SNP | Rv1459c | Val269Ile | 33 | - | | 1645802 | T | C | 1192.77 | SNP | Rv1459c | Lys113Glu | 4 | - | | 1646431 | G | C | 629.77 | SNP | Rv1460 | Glu82Asp | 53 | - | | 1650072 | A | G | 646.77 | SNP | Rv1462 | Asn183Asp | 42 | - | | 1668843 | A | G | 237.77 | SNP | Rv1478 | Asp142Gly | 11 | - | | 1669358 | C | A | 388.77 | SNP | Rv1479 (moxR1) | His26Asn | 21 | - | | 1676290 | C | A | 1038.77 | SNP | Rv1486c | Lys198Asn | 13 | - | | 1677388 | G | A | 993.77 | SNP | intergenic |  |  | - | | 1680631 | G | C | 878.77 | SNP | intergenic |  |  | - | | 1689349 | C | T | 1076.77 | SNP | Rv1498c | Arg191His | 8 | - | | 1689571 | C | G | 1314.77 | SNP | Rv1498c | Cys117Ser | 11 | - | | 1692141 | A | C | 1500.77 | SNP | Rv1501 | silent (Ile84) | 9872 | - | | 1693561 | A | G | 1854.77 | SNP | Rv1502 | Tyr213Cys | 3 | - | | 1698911 | G | A | 1089.77 | SNP | Rv1508c | silent (Gly328) | 9935 | - | | 1704554 | C | T | 931.77 | SNP | Rv1512 (epiA) | silent (Ile154) | 9872 | - | | 1706119 | T | C | 1032.77 | SNP | Rv1514c | silent (Ser159) | 9840 | - | | 1709432 | C | T | 957.77 | SNP | Rv1517 | Leu188Phe | 6 | - | | 1711645 | T | C | 1091.77 | SNP | Rv1520 | silent (Gly206) | 9935 | - | | 1711692 | G | A | 1027.77 | SNP | Rv1520 | Arg222Gln | 9 | - | | 1713192 | A | G | 1540.77 | SNP | Rv1521 (fadD25) | Val297Val(s) | 18 | - | | 1724722 | T | C | 590.77 | SNP | Rv1527c (pks5) | Thr1230Ala | 32 | - | | 1728622 | C | G | 1315.77 | SNP | intergenic |  |  | - | | 1728837 | A | G | 1145.77 | SNP | intergenic |  |  | - | | 1752561 | T | C | 501.77 | SNP | Rv1548c (PPE21) | Asp258Gly | 11 | - | | 1753519 | G | GC | 2110.73 | INS | Rv1549 (fadD11.1) |  |  | - | | 1759252 | G | T | 768.77 | SNP | Rv1552 (frdA) | silent (Ser524) | 9840 | genotype | | 1760292 | A | G | 1048.77 | SNP | Rv1554 (frdC) | Met(s)40Val(s) | 9867 | - | | 1769469 | C | A | 1273.77 | SNP | Rv1564c (treX) | silent (Ala711) | 9867 | - | | 1772742 | G | A | 750.77 | SNP | Rv1565c | Ala363Val(s) | 9867 | - | | 1778430 | T | C | 817.77 | SNP | Rv1570 (bioD) | Met(s)191Thr | 22 | - | | 1789446 | C | T | 175.56 | SNP | Rv1588c | Val131Ile | 33 | - | | 1789516 | A | G | 199.77 | SNP | Rv1588c | silent (Gly107) | 9935 | - | | 1789564 | C | T | 533.77 | SNP | Rv1588c | silent (Arg91) | 9913 | - | | 1789565 | C | A | 508.77 | SNP | Rv1588c | Arg91Leu | 1 | - | | 1789650 | C | T | 383.77 | SNP | Rv1588c | Ala63Thr | 22 | - | | 1789654 | A | G | 488.77 | SNP | Rv1588c | silent (Leu61) | 9947 | - | | 1789671 | C | T | 419.77 | SNP | Rv1588c | Ala56Thr | 22 | - | | 1789675 | A | C | 486.77 | SNP | Rv1588c | silent (Gly54) | 9935 | - | | 1789678 | C | G | 344.77 | SNP | Rv1588c | Val(s)53Val | 13 | - | | 1792777 | T | C | 973.77 | SNP | Rv1592c | Ile322Val | 57 | - | | 1792778 | T | C | 1160.77 | SNP | Rv1592c | silent (Glu321) | 9865 | - | | 1798355 | G | A | 1001.77 | SNP | Rv1597 | Gly21Asp | 6 | - | | 1803265 | G | A | 962.77 | SNP | Rv1602 (hisH) | Ser201Asn | 20 | - | | 1804409 | C | A | 902.77 | SNP | Rv1604 (impA) | Pro124Gln | 6 | - | | 1817976 | A | T | 1296.77 | SNP | Rv1618 (tesB1) | His121Leu | 4 | - | | 1833684 | G | C | 1512.77 | SNP | Rv1630 (rpsA) | Arg48Pro | 5 | - | | 1834130 | G | A | 1184.77 | SNP | Rv1630 (rpsA) | Glu197Lys | 7 | - | | 1836286 | G | C | 764.77 | SNP | intergenic |  |  | - | | 1847919 | C | G | 738.77 | SNP | Rv1639c | silent (Thr180) | 9871 | - | | 1849119 | G | C | 1148.77 | SNP | Rv1640c (lysX) | Arg973Gly | 1 | - | | 1854300 | T | C | 1033.77 | SNP | Rv1644 (tsnR) | Leu232Pro | 2 | - | | 1856777 | G | C | 1003.77 | SNP | Rv1647 | Ala2Pro | 13 | - | | 1864811 | T | C | 259.78 | SNP | Rv1651c (PE\_PGRS30) | Asn191Ser | 34 | - | | 1868578 | C | T | 788.77 | SNP | Rv1654 (argB) | Ser246Leu(s) | 35 | - | | 1872906 | G | T | 592.77 | SNP | Rv1659 (argH) | Ala90Ser | 28 | - | | 1875207 | C | T | 623.77 | SNP | Rv1660 (pks10) | Leu350Leu(s) | 4 | - | | 1879671 | T | C | 60.77 | SNP | Rv1661 (pks7) | silent (Gly1456) | 9935 | - | | 1884697 | G | A | 781.77 | SNP | Rv1662 (pks8) | silent (Gly998) | 9935 | - | | 1885772 | G | A | 814.77 | SNP | Rv1662 (pks8) | Ala1357Thr | 22 | - | | 1885840 | G | A | 767.77 | SNP | Rv1662 (pks8) | silent (Glu1379) | 9865 | - | | 1894300 | G | GGTCTTGCCGC | 3732.73 | INS | Rv1668c |  |  | - | | 1894422 | A | G | 894.77 | SNP | Rv1668c | silent (Asp307) | 9859 | - | | 1896387 | T | C | 923.77 | SNP | Rv1670 | Trp90Arg | 8 | - | | 1901493 | T | C | 803.77 | SNP | Rv1676 | silent (Ser149) | 9840 | - | | 1907296 | G | C | 1301.77 | SNP | Rv1682 | silent (Ala298) | 9867 | - | | 1916137 | A | G | 431.77 | SNP | Rv1691 | silent (Leu63) | 9947 | - | | 1917972 | A | G | 526.77 | SNP | Rv1694 (tlyA) | silent (Leu11) | 9947 | - | | 1931179 | C | A | 872.77 | SNP | Rv1704c (cycA) | Arg93Leu | 1 | - | | 1931564 | G | A | 439.77 | SNP | Rv1705c (PPE22) | Thr364Met(s) | 32 | - | | 1933988 | G | A | 1683.77 | SNP | intergenic |  |  | - | | 1940258 | G | A | 972.77 | SNP | Rv1712 (cmk) | silent (Glu220) | 9865 | - | | 1944107 | A | G | 498.77 | SNP | Rv1716 | Ser178Gly | 21 | - | | 1944402 | T | C | 784.77 | SNP | Rv1716 | Val276Ala | 18 | - | | 1950767 | T | C | 1069.77 | SNP | Rv1724c | silent (Lys95) | 9926 | - | | 1951626 | GC | G | 804.73 | DEL | Rv1725c |  |  | - | | 1952601 | C | T | 590.77 | SNP | Rv1726 | silent (His250) | 9912 | - | | 1956524 | G | A | 1350.77 | SNP | Rv1730c | Thr241Met(s) | 32 | - | | 1960284 | C | A | 849.77 | SNP | Rv1733c | Gln68His | 20 | - | | 1960391 | G | A | 1262.77 | SNP | Rv1733c | Pro33Ser | 17 | - | | 1962071 | AGG | A | 1630.73 | DEL | intergenic |  |  | - | | 1964719 | C | T | 894.77 | SNP | Rv1737c (narK2) | Val(s)218Met(s) | 9867 | - | | 1967237 | C | A | 949.77 | SNP | Rv1739c | Arg134Leu | 1 | - | | 1983135 | T | G | 59.77 | SNP | Rv1753c (PPE24) | silent (Pro547) | 9926 | - | | 1983195 | A | G | 86.28 | SNP | Rv1753c (PPE24) | silent (Gly527) | 9935 | - | | 1983198 | C | G | 110.03 | SNP | Rv1753c (PPE24) | Val(s)526Val | 13 | - | | 1983313 | T | G | 830.77 | SNP | Rv1753c (PPE24) | Asn488Thr | 13 | - | | 1992323 | GC | G | 172.80 | DEL | Rv1759c (wag22) |  |  | - | | 1993808 | A | T | 770.77 | SNP | Rv1760 | Glu219Val(s) | 17 | - | | 2007502 | G | A | 943.77 | SNP | Rv1773c | His89Tyr | 4 | - | | 2008870 | C | G | 758.77 | SNP | Rv1774 | Leu347Val | 11 | - | | 2014185 | C | T | 403.77 | SNP | Rv1779c | Ala99Thr | 22 | - | | 2020144 | G | A | 901.77 | SNP | Rv1783 (eccC5) | silent (Glu296) | 9865 | - | | 2022868 | T | C | 680.77 | SNP | Rv1783 (eccC5) | silent (Ser1204) | 9840 | - | | 2026148 | C | T | 781.77 | SNP | Rv1787 (PPE25) | Ala283Val(s) | 9867 | - | | 2030634 | G | C | 88.28 | SNP | intergenic |  |  | - | | 2030717 | G | T | 48.74 | SNP | Rv1793 (esxN) | silent (Gly8) | 9935 | - | | 2030720 | C | T | 88.28 | SNP | Rv1793 (esxN) | silent (Asp9) | 9859 | - | | 2030848 | A | G | 52.77 | SNP | Rv1793 (esxN) | Glu52Gly | 7 | - | | 2030855 | T | C | 315.77 | SNP | Rv1793 (esxN) | silent (Ile54) | 9872 | - | | 2030862 | T | C | 266.77 | SNP | Rv1793 (esxN) | Leu(s)57Leu | 3 | - | | 2030942 | G | A | 793.77 | SNP | Rv1793 (esxN) | silent (Ala83) | 9867 | - | | 2037698 | G | A | 752.77 | SNP | Rv1798 (eccA5) | silent (Thr333) | 9871 | - | | 2039901 | C | T | 870.77 | SNP | Rv1800 (PPE28) | Ala150Val | 13 | - | | 2045310 | A | G | 513.77 | SNP | Rv1803c (PE\_PGRS32) | silent (Ile511) | 9872 | - | | 2049065 | T | C | 853.77 | SNP | intergenic |  |  | - | | 2049097 | G | C | 864.77 | SNP | intergenic |  |  | - | | 2051746 | T | C | 890.77 | SNP | Rv1809 (PPE33) | silent (Ala155) | 9867 | - | | 2052035 | G | T | 1268.77 | SNP | Rv1809 (PPE33) | Val(s)252Leu(s) | 9867 | - | | 2054805 | G | A | 1047.77 | SNP | Rv1812c | silent (Pro185) | 9926 | - | | 2055271 | A | G | 1020.77 | SNP | Rv1812c | Leu30Pro | 2 | - | | 2056450 | T | C | 1005.77 | SNP | intergenic |  |  | - | | 2057141 | G | A | 1371.77 | SNP | Rv1814 (erg3) | silent (Pro207) | 9926 | - | | 2057774 | A | T | 682.77 | SNP | Rv1815 | Ile83Phe | 8 | - | | 2061433 | T | TCCGCCGGCG | 633.80 | INS | Rv1818c (PE\_PGRS33) |  |  | - | | 2065383 | C | T | 853.77 | SNP | Rv1820 (ilvG) | silent (Ala195) | 9867 | - | | 2074514 | G | C | 197.84 | SNP | intergenic |  |  | - | | 2074754 | C | T | 1238.77 | SNP | intergenic |  |  | - | | 2081449 | T | TA | 1741.73 | INS | Rv1835c |  |  | - | | 2087935 | A | G | 628.77 | SNP | intergenic |  |  | - | | 2090921 | C | T | 791.77 | SNP | Rv1842c | Gly389Ser | 16 | - | | 2094911 | ACAGCGT | A | 2481.73 | DEL | Rv1844c (gnd1) |  |  | - | | 2096186 | A | G | 649.77 | SNP | Rv1846c (blaI) | silent (Thr138) | 9871 | - | | 2108141 | T | C | 263.78 | SNP | Rv1860 (apa) | Phe136Leu | 13 | - | | 2109523 | C | CG | 1719.73 | INS | intergenic |  |  | - | | 2115210 | A | C | 718.77 | SNP | Rv1866 | Asn691His | 18 | - | | 2116903 | C | T | 914.77 | SNP | Rv1867 | silent (Gly380) | 9935 | - | | 2123169 | T | G | 1112.77 | SNP | intergenic |  |  | - | | 2128870 | A | G | 804.77 | SNP | Rv1878 (glnA3) | silent (Leu283) | 9947 | - | | 2132152 | T | C | 1798.77 | SNP | Rv1881c (lppE) | Ile60Val | 57 | - | | 2133468 | T | TTCGCATGCCGTCACC | 1213.73 | INS | Rv1883c |  |  | - | | 2134325 | C | T | 1418.77 | SNP | Rv1885c | Arg183Gln | 9 | - | | 2135870 | T | C | 786.77 | SNP | intergenic |  |  | - | | 2135900 | T | G | 401.77 | SNP | intergenic |  |  | - | | 2137521 | A | ACTCCGATCAC | 5056.73 | INS | Rv1888c |  |  | - | | 2143328 | G | C | 917.77 | SNP | Rv1895 | Val(s)270Leu | 3 | - | | 2147022 | A | C | 966.77 | SNP | Rv1900c (lipJ) | Ile204Met(s) | 6 | - | | 2152952 | A | C | 1261.77 | SNP | intergenic |  |  | - | | 2155168 | C | G | 807.77 | SNP | Rv1908c (katG) | Ser315Thr | 32 | resistance | | 2157856 | G | A | 407.77 | SNP | Rv1911c (lppC) | silent (Val44) | 9901 | - | | 2158582 | G | A | 1151.77 | SNP | Rv1912c (fadB5) | silent (Gly170) | 9935 | - | | 2160998 | G | A | 1011.77 | SNP | Rv1915 (aceAa) | Gly179Asp | 6 | - | | 2161343 | G | GT | 1080.73 | INS | Rv1915 (aceAa) |  |  | - | | 2162803 | T | C | 836.77 | SNP | intergenic |  |  | - | | 2163375 | T | C | 1416.77 | SNP | Rv1917c (PPE34) | Asn1313Asp | 42 | - | | 2163412 | A | G | 753.77 | SNP | Rv1917c (PPE34) | silent (Val1300) | 9901 | - | | 2163415 | C | A | 787.77 | SNP | Rv1917c (PPE34) | silent (Pro1299) | 9926 | - | | 2163417 | G | C | 760.77 | SNP | Rv1917c (PPE34) | Pro1299Ala | 22 | - | | 2163419 | C | T | 677.77 | SNP | Rv1917c (PPE34) | Ser1298Asn | 20 | - | | 2163421 | C | G | 607.77 | SNP | Rv1917c (PPE34) | silent (Thr1297) | 9871 | - | | 2163444 | T | C | 122.77 | SNP | Rv1917c (PPE34) | Asn1290Asp | 42 | - | | 2163790 | A | C | 776.77 | SNP | Rv1917c (PPE34) | silent (Pro1174) | 9926 | - | | 2165286 | A | C | 807.77 | SNP | Rv1917c (PPE34) | Ser676Ala | 35 | - | | 2165428 | T | A | 198.77 | SNP | Rv1917c (PPE34) | silent (Ala628) | 9867 | - | | 2165479 | G | A | 30.77 | SNP | Rv1917c (PPE34) | silent (Gly611) | 9935 | - | | 2165503 | T | A | 806.77 | SNP | Rv1917c (PPE34) | silent (Ala603) | 9867 | - | | 2175062 | C | T | 940.77 | SNP | Rv1922 | silent (Ala332) | 9867 | - | | 2180796 | CCGCCTTGGCCTTGTTTGAC CAT | C | 7067.73 | DEL | Rv1928c |  |  | - | | 2187274 | C | A | 1232.77 | SNP | intergenic |  |  | - | | 2202833 | G | A | 1263.77 | SNP | Rv1957 | Asp84Asn | 36 | - | | 2203754 | C | T | 1154.77 | SNP | Rv1959c (parE1) | Arg75Gln | 9 | - | | 2207591 | T | TC | 2967.73 | INS | intergenic |  |  | - | | 2211826 | A | G | 601.77 | SNP | Rv1968 (mce3C) | silent (Lys67) | 9926 | - | | 2216443 | C | A | 657.77 | SNP | Rv1971 (mce3F) | Ala396Glu | 10 | - | | 2220512 | T | G | 720.77 | SNP | Rv1977 | silent (Ser253) | 9840 | - | | 2223293 | T | C | 1485.77 | SNP | intergenic |  |  | - | | 2225456 | A | T | 1031.77 | SNP | Rv1982c (vapC36) | Leu126His | 1 | - | | 2225882 | G | A | 808.77 | SNP | Rv1982A (vapB36) | Arg74Cys | 1 | - | | 2228967 | A | G | 882.77 | SNP | intergenic |  |  | - | | 2233751 | G | C | 693.77 | SNP | intergenic |  |  | - | | 2239349 | G | A | 641.77 | SNP | Rv1996 | Ala116Thr | 22 | - | | 2247677 | A | C | 853.77 | SNP | Rv2002 (fabG3) | silent (Ile6) | 9872 | - | | 2248179 | A | G | 915.77 | SNP | Rv2002 (fabG3) | Ser174Gly | 21 | - | | 2251999 | A | G | 1143.77 | SNP | intergenic |  |  | - | | 2253453 | T | C | 1329.77 | SNP | Rv2006 (otsB1) | silent (Arg484) | 9913 | - | | 2260151 | A | G | 511.77 | SNP | intergenic |  |  | - | | 2260154 | C | T | 510.77 | SNP | intergenic |  |  | - | | 2260171 | T | C | 659.77 | SNP | intergenic |  |  | - | | 2260174 | C | T | 653.77 | SNP | intergenic |  |  | - | | 2260196 | C | CA | 841.73 | INS | intergenic |  |  | - | | 2260199 | C | T | 537.77 | SNP | intergenic |  |  | - | | 2260212 | G | T | 388.77 | SNP | intergenic |  |  | - | | 2260214 | G | C | 211.77 | SNP | intergenic |  |  | - | | 2260220 | C | T | 254.77 | SNP | intergenic |  |  | - | | 2260222 | C | G | 281.77 | SNP | intergenic |  |  | - | | 2260231 | T | C | 322.77 | SNP | intergenic |  |  | - | | 2260525 | C | T | 1015.77 | SNP | intergenic |  |  | - | | 2263760 | C | T | 853.77 | SNP | Rv2016 | Pro112Leu | 3 | - | | 2264782 | C | A | 1069.77 | SNP | Rv2017 | Ala262Glu | 10 | - | | 2265059 | T | G | 1332.77 | SNP | intergenic |  |  | - | | 2266487 | G | C | 514.77 | SNP | Rv2020c | silent (Leu78) | 9947 | - | | 2266508 | A | T | 218.77 | SNP | Rv2020c | Asp71Glu | 56 | - | | 2266517 | T | C | 263.77 | SNP | Rv2020c | silent (Glu68) | 9865 | - | | 2266550 | G | T | 599.77 | SNP | Rv2020c | silent (Gly57) | 9935 | - | | 2266553 | C | G | 615.77 | SNP | Rv2020c | silent (Ser56) | 9840 | - | | 2266583 | C | G | 669.77 | SNP | Rv2020c | Glu46Asp | 53 | - | | 2266598 | G | C | 549.77 | SNP | Rv2020c | silent (Leu41) | 9947 | - | | 2266604 | C | G | 554.77 | SNP | Rv2020c | silent (Ser39) | 9840 | - | | 2266613 | G | GC | 979.73 | INS | Rv2020c |  |  | - | | 2266624 | G | T | 644.77 | SNP | Rv2020c | Leu33Ile | 9 | - | | 2269780 | T | C | 546.77 | SNP | Rv2024c | Asp154Gly | 11 | - | | 2270102 | A | G | 873.77 | SNP | Rv2024c | Trp47Arg | 8 | - | | 2282787 | C | T | 573.77 | SNP | Rv2037c | Cys312Tyr | 3 | - | | 2285251 | C | A | 1779.77 | SNP | Rv2039c | Val131Phe | 0 | - | | 2287121 | A | G | 782.77 | SNP | Rv2041c | silent (Asp242) | 9859 | - | | 2295692 | C | T | 42.74 | SNP | Rv2048c (pks12) | Val(s)3765Val | 13 | - | | 2296042 | G | C | 511.77 | SNP | Rv2048c (pks12) | Pro3649Ala | 22 | - | | 2300237 | A | G | 503.77 | SNP | Rv2048c (pks12) | silent (Ala2250) | 9867 | - | | 2300546 | A | T | 992.77 | SNP | Rv2048c (pks12) | His2147Gln | 23 | - | | 2300552 | T | G | 974.77 | SNP | Rv2048c (pks12) | silent (Pro2145) | 9926 | - | | 2300555 | A | G | 957.77 | SNP | Rv2048c (pks12) | silent (Asp2144) | 9859 | - | | 2301965 | G | A | 611.82 | SNP | Rv2048c (pks12) | silent (Pro1674) | 9926 | - | | 2307701 | G | A | 964.77 | SNP | intergenic |  |  | - | | 2311099 | C | G | 693.77 | SNP | Rv2052c | silent (Pro473) | 9926 | - | | 2323385 | G | C | 70.77 | SNP | Rv2066 (cobI) | Ala71Pro | 13 | - | | 2325009 | C | T | 1643.77 | SNP | Rv2067c | Glu288Lys | 7 | - | | 2329533 | A | G | 960.77 | SNP | Rv2072c (cobL) | Leu205Pro | 2 | - | | 2330324 | G | A | 933.77 | SNP | Rv2073c | Arg214Cys | 1 | - | | 2334007 | A | G | 1053.77 | SNP | Rv2077c | silent (Ala96) | 9867 | - | | 2335075 | A | G | 1374.77 | SNP | Rv2078 | Glu6Gly | 7 | - | | 2335494 | A | G | 846.77 | SNP | Rv2079 | Tyr47Cys | 3 | - | | 2338194 | A | AC | 174.80 | INS | Rv2081c |  |  | - | | 2338768 | G | T | 668.77 | SNP | Rv2082 | silent (Pro20) | 9926 | - | | 2338773 | G | A | 701.77 | SNP | Rv2082 | Arg22Gln | 9 | - | | 2338866 | T | G | 714.77 | SNP | Rv2082 | Leu53Arg | 1 | - | | 2338912 | A | C | 865.77 | SNP | Rv2082 | silent (Arg68) | 9913 | - | | 2338961 | G | A | 950.77 | SNP | Rv2082 | Val85Ile | 33 | - | | 2338990 | G | C | 910.77 | SNP | Rv2082 | silent (Ala94) | 9867 | - | | 2338994 | G | A | 927.77 | SNP | Rv2082 | Ala96Thr | 22 | - | | 2339524 | G | A | 53.74 | SNP | Rv2082 | silent (Pro272) | 9926 | - | | 2339525 | G | T | 43.74 | SNP | Rv2082 | Gly273Cys | 0 | - | | 2339605 | A | G | 36.74 | SNP | Rv2082 | silent (Pro299) | 9926 | - | | 2340621 | C | G | 999.77 | SNP | Rv2082 | Pro638Arg | 4 | - | | 2341636 | C | G | 503.77 | SNP | Rv2083 | Leu256Val(s) | 4 | - | | 2345037 | C | A | 608.77 | SNP | Rv2088 (pknJ) | silent (Leu209) | 9947 | - | | 2348446 | C | G | 816.77 | SNP | Rv2090 | Phe358Leu(s) | 2 | - | | 2357268 | TGCC | T | 377.82 | DEL | intergenic |  |  | - | | 2357492 | T | A | 41.77 | SNP | intergenic |  |  | - | | 2358029 | T | TG | 889.73 | INS | intergenic |  |  | - | | 2361604 | C | G | 703.77 | SNP | Rv2101 (helZ) | Val455Val(s) | 18 | - | | 2362041 | C | A | 788.77 | SNP | Rv2101 (helZ) | Pro601Gln | 6 | - | | 2368564 | TA | T | 1558.73 | DEL | intergenic |  |  | - | | 2369326 | C | G | 1001.77 | SNP | Rv2109c (prcA) | Arg135Pro | 5 | - | | 2369971 | A | G | 105.77 | SNP | Rv2110c (prcB) | Tyr211His | 4 | - | | 2370533 | A | C | 1144.77 | SNP | Rv2110c (prcB) | silent (Ser23) | 9840 | - | | 2386389 | G | A | 696.77 | SNP | Rv2125 | Gly33Ser | 16 | - | | 2387733 | T | C | 250.80 | SNP | Rv2126c (PE\_PGRS37) | silent (Glu80) | 9865 | - | | 2396883 | G | A | 913.77 | SNP | intergenic |  |  | - | | 2401825 | T | C | 611.77 | SNP | intergenic |  |  | - | | 2415656 | G | C | 359.77 | SNP | Rv2155c (murD) | Arg247Gly | 1 | - | | 2416156 | G | A | 539.77 | SNP | Rv2155c (murD) | Thr80Ile | 7 | - | | 2424008 | G | T | 138.03 | SNP | Rv2162c (PE\_PGRS38) | silent (Ala277) | 9867 | - | | 2424925 | A | G | 597.77 | SNP | intergenic |  |  | - | | 2426630 | C | T | 846.77 | SNP | Rv2163c (pbpB) | Arg153His | 8 | - | | 2438955 | G | A | 527.79 | SNP | Rv2176 (pknL) | Gly339Arg | 0 | - | | 2439204 | A | G | 213.77 | SNP | intergenic |  |  | - | | 2439401 | A | G | 30.77 | SNP | Rv2177c | Tyr183His | 4 | - | | 2440926 | G | T | 1047.77 | SNP | Rv2178c (aroG) | Asp265Glu | 56 | - | | 2441590 | T | C | 442.77 | SNP | Rv2178c (aroG) | Gln44Arg | 10 | - | | 2449284 | C | T | 1043.77 | SNP | Rv2187 (fadD15) | silent (Tyr375) | 9945 | - | | 2454856 | C | T | 929.77 | SNP | Rv2191 | silent (Arg346) | 9913 | - | | 2460628 | C | A | 877.77 | SNP | Rv2196 (qcrB) | silent (Ala317) | 9867 | - | | 2462871 | G | A | 447.77 | SNP | Rv2198c (mmpS3) | silent (Ala59) | 9867 | - | | 2475207 | G | A | 833.77 | SNP | Rv2210c (ilvE) | Ser255Leu(s) | 35 | - | | 2487490 | AG | A | 1149.73 | DEL | intergenic |  |  | - | | 2499726 | G | A | 621.77 | SNP | Rv2226 | Asp299Asn | 36 | - | | 2506888 | T | G | 565.77 | SNP | Rv2232 (ptkA) | Val204Gly | 5 | - | | 2509140 | G | C | 536.77 | SNP | Rv2236c (cobD) | Ser79Cys | 5 | - | | 2509722 | A | G | 998.77 | SNP | Rv2237 | silent (Pro78) | 9926 | - | | 2512237 | T | TC | 1085.73 | INS | Rv2240c |  |  | - | | 2516567 | G | C | 1668.77 | SNP | intergenic |  |  | - | | 2521342 | T | C | 649.77 | SNP | Rv2247 (accD6) | silent (Asp200) | 9859 | - | | 2523205 | G | GCGC | 1358.73 | INS | intergenic |  |  | - | | 2523709 | G | A | 709.77 | SNP | Rv2249c (glpD1) | silent (Arg361) | 9913 | - | | 2525722 | CG | C | 938.73 | DEL | Rv2250A; Rv2251 |  |  | - | | 2526026 | G | A | 819.77 | SNP | Rv2251 | silent (Ala154) | 9867 | - | | 2526974 | T | C | 697.77 | SNP | Rv2251 | silent (Pro470) | 9926 | - | | 2527120 | C | T | 609.77 | SNP | Rv2252 | silent (Asp44) | 9859 | - | | 2529680 | A | G | 717.77 | SNP | Rv2256c | silent (Thr65) | 9871 | - | | 2531742 | A | G | 1304.77 | SNP | Rv2258c | silent (Ala52) | 9867 | - | | 2534562 | GGA | G | 1219.73 | DEL | Rv2262c |  |  | - | | 2537780 | G | A | 953.77 | SNP | Rv2264c | Arg191Trp | 2 | - | | 2540326 | T | A | 1416.77 | SNP | Rv2266 (cyp124) | Tyr75Asn | 4 | - | | 2557095 | G | T | 978.77 | SNP | Rv2284 (lipM) | silent (Leu317) | 9947 | - | | 2559686 | C | T | 1481.77 | SNP | intergenic |  |  | - | | 2573756 | C | A | 853.77 | SNP | intergenic |  |  | - | | 2574022 | C | T | 887.77 | SNP | Rv2302 | silent (Arg70) | 9913 | - | | 2577461 | C | G | 920.77 | SNP | Rv2306A | silent (Ala118) | 9867 | - | | 2578626 | A | G | 1454.77 | SNP | Rv2307c | Met(s)24Thr | 22 | - | | 2580954 | G | C | 1608.77 | SNP | Rv2308 | Arg179Pro | 5 | - | | 2582348 | G | T | 729.77 | SNP | intergenic |  |  | - | | 2585809 | G | A | 953.77 | SNP | Rv2313c | Ala33Val(s) | 9867 | - | | 2586127 | A | G | 568.77 | SNP | Rv2314c | silent (Gly388) | 9935 | - | | 2589216 | G | C | 1414.77 | SNP | Rv2316 (uspA) | Val(s)127Leu | 3 | - | | 2598400 | A | G | 1081.77 | SNP | Rv2326c | silent (Asn516) | 9822 | - | | 2603797 | G | A | 1123.77 | SNP | Rv2330c (lppP) | silent (Ile142) | 9872 | - | | 2608117 | C | A | 799.77 | SNP | Rv2333c (stp) | Asp69Tyr | 0 | - | | 2610702 | T | C | 1287.77 | SNP | intergenic |  |  | - | | 2611891 | T | A | 735.77 | SNP | Rv2337c | Asp366Val | 1 | - | | 2612632 | C | A | 589.77 | SNP | Rv2337c | Gly119Val | 3 | - | | 2617673 | G | A | 950.77 | SNP | Rv2340c (PE\_PGRS39) | silent (His412) | 9912 | - | | 2623232 | C | G | 1171.77 | SNP | Rv2344c (dgt) | Gly174Ala | 21 | - | | 2626004 | G | A | 1158.77 | SNP | Rv2346c (esxO) | Leu57Leu(s) | 4 | - | | 2626011 | G | A | 1089.77 | SNP | Rv2346c (esxO) | silent (Ile54) | 9872 | - | | 2626018 | T | C | 69.77 | SNP | Rv2346c (esxO) | Glu52Gly | 7 | - | | 2626101 | C | A | 345.77 | SNP | Rv2346c (esxO) | silent (Leu24) | 9947 | - | | 2626105 | A | G | 418.77 | SNP | Rv2346c (esxO) | Leu(s)23Ser | 28 | - | | 2626108 | C | G | 426.77 | SNP | Rv2346c (esxO) | Gly22Ala | 21 | - | | 2626110 | G | C | 298.77 | SNP | Rv2346c (esxO) | silent (Ala21) | 9867 | - | | 2626262 | C | T | 208.77 | SNP | Rv2347c (esxP) | silent (Glu86) | 9865 | - | | 2630158 | C | G | 268.77 | SNP | Rv2350c (plcB) | silent (Arg54) | 9913 | - | | 2630161 | A | G | 280.77 | SNP | Rv2350c (plcB) | silent (Asn53) | 9822 | - | | 2630173 | C | G | 125.77 | SNP | Rv2350c (plcB) | Leu(s)49Phe | 1 | - | | 2630176 | C | G | 158.77 | SNP | Rv2350c (plcB) | Leu(s)48Phe | 1 | - | | 2630182 | G | A | 107.77 | SNP | Rv2350c (plcB) | silent (Ile46) | 9872 | - | | 2630184 | T | A | 110.77 | SNP | Rv2350c (plcB) | Ile46Phe | 8 | - | | 2630188 | C | T | 87.77 | SNP | Rv2350c (plcB) | silent (Glu44) | 9865 | - | | 2630215 | A | G | 37.77 | SNP | Rv2350c (plcB) | silent (Pro35) | 9926 | - | | 2631556 | C | G | 243.77 | SNP | Rv2351c (plcA) | Gly174Arg | 0 | - | | 2631565 | T | C | 323.77 | SNP | Rv2351c (plcA) | Ile171Val | 57 | - | | 2631574 | T | C | 390.77 | SNP | Rv2351c (plcA) | Thr168Ala | 32 | - | | 2631583 | G | A | 348.77 | SNP | Rv2351c (plcA) | Leu165Leu(s) | 4 | - | | 2631599 | G | A | 483.77 | SNP | Rv2351c (plcA) | silent (Ile159) | 9872 | - | | 2631620 | A | G | 507.77 | SNP | Rv2351c (plcA) | silent (Gly152) | 9935 | - | | 2631962 | T | G | 161.77 | SNP | Rv2351c (plcA) | silent (Gly38) | 9935 | - | | 2631967 | G | A | 138.77 | SNP | Rv2351c (plcA) | Pro37Ser | 17 | - | | 2631968 | A | G | 169.77 | SNP | Rv2351c (plcA) | silent (Cys36) | 9973 | - | | 2631971 | A | G | 152.77 | SNP | Rv2351c (plcA) | silent (Pro35) | 9926 | - | | 2631977 | G | C | 181.77 | SNP | Rv2351c (plcA) | silent (Ala33) | 9867 | - | | 2633009 | GCATACCCGCCCCAGCACCC CC | G | 6001.73 | DEL | Rv2352c (PPE38) |  |  | - | | 2637541 | C | T | 1903.77 | SNP | intergenic |  |  | - | | 2638997 | G | A | 385.77 | SNP | Rv2356c (PPE40) | Ser180Leu(s) | 35 | - | | 2642383 | C | T | 800.77 | SNP | Rv2360c | Ala66Thr | 22 | - | | 2645270 | C | T | 806.77 | SNP | Rv2363 (amiA2) | silent (Gly317) | 9935 | - | | 2652174 | C | A | 596.77 | SNP | Rv2372c | Val218Phe | 0 | - | | 2655691 | T | C | 786.77 | SNP | Rv2376c (cfp2) | Gln142Arg | 10 | - | | 2656225 | A | G | 960.77 | SNP | Rv2377c (mbtH) | Val69Ala | 18 | - | | 2660091 | T | G | 918.77 | SNP | Rv2379c (mbtF) | silent (Ala665) | 9867 | - | | 2660319 | C | G | 836.77 | SNP | Rv2379c (mbtF) | Glu589Asp | 53 | - | | 2671061 | C | A | 712.77 | SNP | Rv2382c (mbtC) | silent (Ala181) | 9867 | - | | 2676498 | A | C | 626.77 | SNP | Rv2384 (mbtA) | Gln188Pro | 8 | - | | 2680658 | T | G | 1100.77 | SNP | intergenic |  |  | - | | 2695378 | C | G | 1254.77 | SNP | Rv2398c (cysW) | Gly141Ala | 21 | - | | 2700239 | G | A | 920.77 | SNP | Rv2402 | Val(s)571Met(s) | 9867 | - | | 2703915 | CGCGGGCCGCCCAGCGGCCC GCTGAGGAGCCGGGCAGTCA GCCCCGCCCGGCGACGAT | C | 2819.73 | DEL | intergenic |  |  | - | | 2704884 | A | ACAGCGACCATATCGCCGAG CT | 6294.73 | INS | Rv2407 |  |  | - | | 2713795 | C | T | 1051.77 | SNP | intergenic |  |  | - | | 2718852 | T | G | 1490.77 | SNP | intergenic |  |  | - | | 2720660 | C | CCATTTCGGCA | 3748.73 | INS | intergenic |  |  | - | | 2720665 | ACCGGTCGGAT | A | 3083.73 | DEL | intergenic |  |  | - | | 2720676 | T | A | 686.77 | SNP | intergenic |  |  | - | | 2721013 | A | G | 542.77 | SNP | Rv2424c | silent (Arg255) | 9913 | - | | 2721562 | C | G | 1367.77 | SNP | Rv2424c | silent (Ala72) | 9867 | - | | 2730752 | T | C | 1349.77 | SNP | Rv2435c | silent (Ser666) | 9840 | - | | 2734074 | T | C | 123.03 | SNP | Rv2436 (rbsK) | Val282Ala | 18 | - | | 2737572 | C | A | 397.77 | SNP | Rv2439c (proB) | Ala226Ser | 28 | - | | 2739174 | T | G | 722.77 | SNP | Rv2440c (obg) | silent (Gly171) | 9935 | - | | 2751804 | C | T | 578.77 | SNP | Rv2450c (rpfE) | Arg126Gln | 9 | - | | 2752698 | C | A | 1547.77 | SNP | intergenic |  |  | - | | 2752854 | G | A | 1449.77 | SNP | Rv2452c | silent (Asp47) | 9859 | - | | 2759082 | T | G | 1298.77 | SNP | Rv2457c (clpX) | Lys136Thr | 8 | - | | 2760152 | A | G | 836.77 | SNP | Rv2458 (mmuM) | Tyr125Cys | 3 | - | | 2760241 | G | A | 819.77 | SNP | Rv2458 (mmuM) | Glu155Lys | 7 | - | | 2760985 | G | T | 1242.77 | SNP | Rv2459 | silent (Leu44) | 9947 | - | | 2772619 | G | C | 1194.77 | SNP | Rv2469c | silent (Ala139) | 9867 | - | | 2773931 | T | C | 759.77 | SNP | Rv2471 (aglA) | Met(s)123Thr | 22 | - | | 2779136 | T | C | 746.77 | SNP | Rv2476c (gdh) | Ser1043Gly | 21 | - | | 2786952 | A | G | 994.77 | SNP | Rv2482c (plsB2) | Cys778Arg | 1 | - | | 2788745 | C | T | 1082.77 | SNP | Rv2482c (plsB2) | Arg180Gln | 9 | - | | 2808506 | G | C | 930.77 | SNP | Rv2494 (vapC38) | Arg66Pro | 5 | - | | 2809621 | T | C | 1023.77 | SNP | Rv2495c (bkdC) | Thr107Ala | 32 | - | | 2818837 | A | G | 915.77 | SNP | Rv2503c (scoB) | silent (Gly97) | 9935 | - | | 2821342 | C | T | 808.77 | SNP | Rv2505c (fadD35) | silent (Ala85) | 9867 | - | | 2827984 | G | T | 719.77 | SNP | intergenic |  |  | - | | 2828019 | T | C | 738.77 | SNP | intergenic |  |  | - | | 2828822 | G | T | 103.03 | SNP | Rv2512c | Gln328Lys | 12 | - | | 2829779 | T | C | 267.78 | SNP | Rv2512c | Thr9Ala | 32 | - | | 2830525 | C | A | 983.77 | SNP | Rv2513 | Thr122Lys | 11 | - | | 2832071 | G | C | 263.77 | SNP | Rv2515c | Ala174Gly | 21 | - | | 2841238 | C | A | 923.77 | SNP | Rv2524c (fas) | Ala2699Ser | 28 | - | | 2843168 | C | T | 866.77 | SNP | Rv2524c (fas) | silent (Glu2055) | 9865 | - | | 2855259 | A | G | 1149.77 | SNP | Rv2531c | silent (Ala841) | 9867 | - | | 2862052 | C | T | 462.77 | SNP | Rv2538c (aroB) | Ala209Thr | 22 | - | | 2865760 | A | G | 760.77 | SNP | Rv2542 | Thr211Ala | 32 | - | | 2865882 | T | C | 901.77 | SNP | Rv2542 | silent (Val251) | 9901 | - | | 2866551 | C | G | 382.77 | SNP | Rv2543 (lppA) | silent (Ala28) | 9867 | - | | 2866569 | C | A | 441.77 | SNP | Rv2543 (lppA) | silent (Thr34) | 9871 | - | | 2866578 | C | A | 577.77 | SNP | Rv2543 (lppA) | His37Gln | 23 | - | | 2866580 | A | G | 594.77 | SNP | Rv2543 (lppA) | Asn38Ser | 34 | - | | 2866607 | G | A | 626.77 | SNP | Rv2543 (lppA) | Gly47Asp | 6 | - | | 2866647 | G | A | 354.77 | SNP | Rv2543 (lppA) | silent (Lys60) | 9926 | - | | 2866671 | G | A | 327.77 | SNP | Rv2543 (lppA) | silent (Glu68) | 9865 | - | | 2866677 | A | C | 327.77 | SNP | Rv2543 (lppA) | silent (Leu70) | 9947 | - | | 2866863 | C | G | 181.77 | SNP | Rv2543 (lppA) | silent (Ala132) | 9867 | - | | 2866876 | A | G | 189.77 | SNP | Rv2543 (lppA) | Ile137Val | 57 | - | | 2866880 | C | T | 171.77 | SNP | Rv2543 (lppA) | Ala138Val | 13 | - | | 2866882 | G | A | 359.77 | SNP | Rv2543 (lppA) | Ala139Thr | 22 | - | | 2867207 | C | G | 810.77 | SNP | Rv2544 (lppB) | silent (Ala28) | 9867 | - | | 2867230 | G | A | 321.77 | SNP | Rv2544 (lppB) | Gly36Asp | 6 | - | | 2867231 | C | T | 293.77 | SNP | Rv2544 (lppB) | silent (Gly36) | 9935 | - | | 2867236 | A | G | 343.77 | SNP | Rv2544 (lppB) | Asn38Ser | 34 | - | | 2867240 | C | T | 287.77 | SNP | Rv2544 (lppB) | silent (Pro39) | 9926 | - | | 2867245 | A | C | 355.77 | SNP | Rv2544 (lppB) | Lys41Thr | 8 | - | | 2867251 | C | G | 261.77 | SNP | Rv2544 (lppB) | Pro43Arg | 4 | - | | 2867254 | A | G | 299.77 | SNP | Rv2544 (lppB) | His44Arg | 10 | - | | 2867263 | G | A | 340.77 | SNP | Rv2544 (lppB) | Gly47Asp | 6 | - | | 2867298 | C | A | 392.77 | SNP | Rv2544 (lppB) | His59Asn | 21 | - | | 2867347 | A | G | 327.77 | SNP | Rv2544 (lppB) | Gln75Arg | 10 | - | | 2871977 | C | G | 853.77 | SNP | Rv2552c (aroE) | silent (Ser13) | 9840 | - | | 2880702 | G | C | 1154.77 | SNP | Rv2560 | Val210Leu | 15 | - | | 2881597 | AG | A | 1118.73 | DEL | Rv2561 |  |  | - | | 2885734 | G | A | 702.77 | SNP | Rv2565 | Gly375Asp | 6 | - | | 2888201 | T | C | 459.77 | SNP | Rv2566 | Leu610Pro | 2 | - | | 2889633 | T | C | 676.77 | SNP | Rv2566 | silent (Ala1087) | 9867 | - | | 2891267 | C | T | 1195.77 | SNP | Rv2567 | silent (Gly491) | 9935 | - | | 2891728 | A | G | 828.77 | SNP | Rv2567 | Gln645Arg | 10 | - | | 2894208 | G | A | 1014.77 | SNP | Rv2569c | silent (Ser67) | 9840 | - | | 2906978 | C | T | 768.77 | SNP | Rv2582 (ppiB) | silent (Leu55) | 9947 | - | | 2910461 | G | T | 531.77 | SNP | Rv2584c (apt) | Ala147Glu | 10 | - | | 2911293 | C | G | 680.77 | SNP | Rv2585c | Cys462Ser | 11 | - | | 2912294 | T | G | 537.77 | SNP | Rv2585c | silent (Ala128) | 9867 | - | | 2913078 | C | G | 978.77 | SNP | Rv2586c (secF) | Val(s)312Leu | 3 | - | | 2917173 | G | A | 627.77 | SNP | Rv2589 (gabT) | Gly272Arg | 0 | - | | 2923391 | T | C | 368.77 | SNP | Rv2592c (ruvB) | silent (Pro281) | 9926 | - | | 2927939 | T | C | 1766.77 | SNP | intergenic |  |  | - | | 2935682 | C | A | 1347.77 | SNP | Rv2608 (PPE42) | silent (Arg213) | 9913 | - | | 2939373 | G | C | 794.77 | SNP | Rv2611c | Ser197Cys | 5 | - | | 2939657 | T | C | 471.77 | SNP | Rv2611c | Ile102Met(s) | 6 | - | | 2946570 | G | A | 913.77 | SNP | Rv2618 | Gly46Asp | 6 | - | | 2953367 | T | C | 902.77 | SNP | intergenic |  |  | - | | 2954439 | T | C | 683.77 | SNP | Rv2627c | Arg104Gly | 1 | - | | 2958749 | G | A | 1255.77 | SNP | Rv2631 | Gly393Asp | 6 | - | | 2961922 | C | T | 202.84 | SNP | Rv2634c (PE\_PGRS46) | Asp174Asn | 36 | - | | 2974933 | A | G | 444.77 | SNP | Rv2650c | Ile101Thr | 11 | - | | 2983613 | G | A | 82.28 | SNP | Rv2666 | silent (Gly181) | 9935 | - | | 2984740 | A | G | 705.77 | SNP | Rv2668 | His3Arg | 10 | - | | 3001395 | T | C | 1024.77 | SNP | Rv2684 (arsA) | Val(s)261Ala | 9867 | - | | 3005185 | G | T | 1127.77 | SNP | Rv2688c | Pro156Thr | 5 | - | | 3005618 | C | T | 900.77 | SNP | Rv2688c | silent (Ala11) | 9867 | - | | 3009692 | A | G | 935.77 | SNP | Rv2691 (ceoB) | Thr117Ala | 32 | - | | 3015140 | C | T | 1212.77 | SNP | intergenic |  |  | - | | 3017276 | A | G | 1340.77 | SNP | Rv2702 (ppgK) | Asn140Ser | 34 | - | | 3017465 | T | C | 1644.77 | SNP | Rv2702 (ppgK) | Ile203Thr | 11 | - | | 3037377 | T | C | 428.77 | SNP | intergenic |  |  | - | | 3041871 | G | T | 1041.77 | SNP | Rv2729c | Ala202Glu | 10 | - | | 3054081 | A | G | 914.77 | SNP | Rv2741 (PE\_PGRS47) | silent (Gly56) | 9935 | - | | 3054321 | A | G | 317.78 | SNP | Rv2741 (PE\_PGRS47) | silent (Gly136) | 9935 | - | | 3057137 | C | T | 708.77 | SNP | Rv2743c | silent (Lys32) | 9926 | - | | 3067901 | G | A | 883.77 | SNP | Rv2754c (thyX) | silent (Thr15) | 9871 | - | | 3069167 | A | G | 869.77 | SNP | Rv2756c (hsdM) | Leu306Pro | 2 | - | | 3078178 | C | T | 841.77 | SNP | Rv2769c (PE27) | Val(s)270Met(s) | 9867 | - | | 3080795 | A | G | 1140.77 | SNP | Rv2771c | Leu80Pro | 2 | - | | 3086208 | G | T | 736.77 | SNP | intergenic |  |  | - | | 3086788 | T | C | 1175.77 | SNP | intergenic |  |  | - | | 3089776 | G | A | 772.77 | SNP | Rv2782c (pepR) | Arg196Trp | 2 | - | | 3098497 | A | C | 959.77 | SNP | Rv2789c (fadE21) | Ser148Ala | 35 | - | | 3099091 | TCCG | T | 2154.73 | DEL | Rv2790c (ltp1) |  |  | - | | 3100151 | A | ACGACC | 3119.73 | INS | Rv2790c (ltp1) |  |  | - | | 3103682 | T | C | 758.77 | SNP | Rv2794c (pptT) | Met(s)87Val(s) | 9867 | - | | 3105748 | C | T | 876.77 | SNP | Rv2797c | Met(s)520Ile | 2 | - | | 3113491 | A | AG | 1088.73 | INS | intergenic |  |  | - | | 3113872 | A | T | 703.77 | SNP | Rv2807 | Glu72Val(s) | 17 | - | | 3114597 | C | A | 888.77 | SNP | Rv2807 | Leu314Met(s) | 4 | - | | 3118000 | A | G | 729.77 | SNP | Rv2812 | Arg395Gly | 1 | - | | 3119345 | C | T | 850.77 | SNP | intergenic |  |  | - | | 3122192 | ACCTC | A | 445.77 | DEL | intergenic |  |  | - | | 3122201 | A | AG | 245.77 | INS | intergenic |  |  | - | | 3122202 | C | T | 145.90 | SNP | intergenic |  |  | - | | 3122206 | CTGCAGCT | C | 613.77 | DEL | intergenic |  |  | - | | 3122217 | C | G | 168.84 | SNP | intergenic |  |  | - | | 3122221 | GT | G | 168.77 | DEL | intergenic |  |  | - | | 3122224 | C | CGGCGCGATTG | 686.77 | INS | intergenic |  |  | - | | 3122225 | A | T | 166.84 | SNP | intergenic |  |  | - | | 3122228 | C | CCG | 368.75 | INS | intergenic |  |  | - | | 3129359 | C | T | 1281.77 | SNP | Rv2823c | silent (Lys805) | 9926 | - | | 3131469 | T | TTGTCGGCGA | 3792.73 | INS | Rv2823c |  |  | - | | 3133536 | T | C | 1197.77 | SNP | Rv2825c | Lys2Glu | 4 | - | | 3135912 | G | C | 828.77 | SNP | Rv2828c | Thr141Arg | 1 | - | | 3137058 | G | A | 883.77 | SNP | Rv2830c (vapB22) | Ala56Val(s) | 9867 | - | | 3157349 | C | T | 1140.77 | SNP | Rv2848c (cobB) | Gly58Asp | 6 | - | | 3159998 | CAA | C | 2195.73 | DEL | Rv2850c |  |  | - | | 3162805 | C | G | 208.84 | SNP | Rv2853 (PE\_PGRS48) | Arg180Gly | 1 | - | | 3170663 | T | C | 608.77 | SNP | Rv2858c (aldC) | Thr21Ala | 32 | - | | 3177884 | C | A | 556.77 | SNP | Rv2866 (relG) | silent (Arg21) | 9913 | - | | 3183561 | G | C | 746.77 | SNP | Rv2872 (vapC43) | silent (Pro60) | 9926 | - | | 3186860 | T | G | 545.77 | SNP | Rv2874 (dipZ) | Tyr672Asp | 0 | - | | 3189433 | C | T | 648.77 | SNP | intergenic |  |  | - | | 3190145 | TC | T | 1351.73 | DEL | Rv2880c |  |  | - | | 3207290 | C | A | 831.77 | SNP | Rv2897c | Gly218Val | 3 | - | | 3219790 | G | A | 640.77 | SNP | Rv2912c | silent (Ile24) | 9872 | - | | 3226181 | A | C | 1174.77 | SNP | Rv2916c (ffh) | silent (Arg35) | 9913 | - | | 3227488 | A | G | 553.77 | SNP | Rv2917 | Ser376Gly | 21 | - | | 3228143 | G | T | 782.77 | SNP | Rv2917 | Arg594Leu | 1 | - | | 3231091 | C | A | 1111.77 | SNP | Rv2920c (amt) | Val(s)472Val | 13 | - | | 3232703 | G | A | 198.77 | SNP | intergenic |  |  | - | | 3232759 | G | A | 528.77 | SNP | intergenic |  |  | - | | 3247316 | C | G | 889.77 | SNP | Rv2931 (ppsA) | Asp624Glu | 56 | - | | 3247851 | G | A | 635.77 | SNP | Rv2931 (ppsA) | Ala803Thr | 22 | - | | 3247853 | C | T | 612.77 | SNP | Rv2931 (ppsA) | silent (Ala803) | 9867 | - | | 3247856 | G | C | 632.77 | SNP | Rv2931 (ppsA) | silent (Arg804) | 9913 | - | | 3247864 | C | CTAGG | 1297.73 | INS | Rv2931 (ppsA) |  |  | - | | 3247865 | GCAAA | G | 1378.73 | DEL | Rv2931 (ppsA) |  |  | - | | 3247874 | G | A | 545.77 | SNP | Rv2931 (ppsA) | silent (Arg810) | 9913 | - | | 3247877 | T | C | 634.77 | SNP | Rv2931 (ppsA) | silent (Phe811) | 9946 | - | | 3247883 | T | C | 778.77 | SNP | Rv2931 (ppsA) | silent (Ser813) | 9840 | - | | 3248074 | G | A | 867.77 | SNP | Rv2931 (ppsA) | Arg877His | 8 | - | | 3248075 | C | T | 903.77 | SNP | Rv2931 (ppsA) | silent (Arg877) | 9913 | - | | 3249411 | G | A | 634.03 | SNP | Rv2931 (ppsA) | Gly1323Ser | 16 | - | | 3251956 | C | G | 1098.77 | SNP | Rv2932 (ppsB) | silent (Arg295) | 9913 | - | | 3256494 | A | G | 793.77 | SNP | Rv2933 (ppsC) | silent (Gly270) | 9935 | - | | 3258274 | A | C | 316.41 | SNP | Rv2933 (ppsC) | Ser864Arg | 6 | - | | 3266288 | C | G | 180.90 | SNP | Rv2934 (ppsD) | silent (Ser1347) | 9840 | - | | 3269581 | A | G | 1031.77 | SNP | Rv2935 (ppsE) | silent (Ala615) | 9867 | - | | 3270784 | A | G | 1193.77 | SNP | Rv2935 (ppsE) | silent (Gln1016) | 9876 | - | | 3289923 | C | A | 662.77 | SNP | Rv2943A; Rv2944 | silent (Ala73); Pro45His | 9867; 3 | - | | 3296843 | A | G | 327.78 | SNP | Rv2947c (pks15) | Val(s)333Ala | 9867 | - | | 3303669 | C | A | 714.77 | SNP | Rv2951c | Ala194Ser | 28 | - | | 3308489 | C | T | 1294.77 | SNP | Rv2955c | silent (Pro19) | 9926 | - | | 3308606 | G | A | 1266.77 | SNP | intergenic |  |  | - | | 3336646 | T | A | 63.77 | SNP | intergenic |  |  | - | | 3336679 | TA | T | 232.73 | DEL | intergenic |  |  | - | | 3336825 | T | C | 834.77 | SNP | Rv2981c (ddlA) | Thr365Ala | 32 | - | | 3338603 | G | C | 927.77 | SNP | Rv2982c (gpdA2) | Pro133Ala | 22 | - | | 3352244 | A | G | 750.77 | SNP | Rv2994 | Thr326Ala | 32 | - | | 3352932 | C | G | 494.77 | SNP | Rv2995c (leuB) | silent (Thr179) | 9871 | - | | 3355161 | C | T | 615.77 | SNP | Rv2997 | silent (Arg21) | 9913 | - | | 3358235 | A | T | 1401.77 | SNP | Rv2999 (lppY) | Met(s)212Leu(s) | 9867 | - | | 3363338 | A | G | 926.77 | SNP | intergenic |  |  | - | | 3363866 | G | A | 1160.77 | SNP | Rv3005c | Leu223Leu(s) | 4 | - | | 3367765 | G | A | 675.77 | SNP | Rv3009c (gatB) | silent (Gly343) | 9935 | - | | 3381641 | G | T | 46.74 | SNP | Rv3023c | Gln328Lys | 12 | - | | 3401871 | A | G | 1090.77 | SNP | Rv3041c | silent (Ala16) | 9867 | - | | 3402816 | C | T | 1137.77 | SNP | Rv3042c (serB2) | Gly116Glu | 4 | - | | 3404376 | C | G | 953.77 | SNP | Rv3043c (ctaD) | silent (Thr182) | 9871 | - | | 3415180 | ACACCTAGGGGGTGG | A | 3534.73 | DEL | intergenic |  |  | - | | 3420388 | C | T | 1108.77 | SNP | Rv3059 (cyp136) | silent (Phe299) | 9946 | - | | 3425495 | T | C | 56.77 | SNP | intergenic |  |  | - | | 3425854 | C | T | 855.77 | SNP | Rv3062 (ligB) | Pro91Ser | 17 | - | | 3428374 | C | G | 1347.77 | SNP | Rv3063 (cstA) | His378Asp | 4 | - | | 3428917 | C | A | 1327.77 | SNP | Rv3063 (cstA) | Arg559Ser | 11 | - | | 3435765 | G | T | 976.77 | SNP | intergenic |  |  | - | | 3440464 | T | G | 892.77 | SNP | Rv3077 | silent (Arg308) | 9913 | - | | 3440468 | G | C | 925.77 | SNP | Rv3077 | Gly310Arg | 0 | - | | 3440895 | G | A | 910.77 | SNP | Rv3077 | Arg452His | 8 | - | | 3442631 | G | T | 826.77 | SNP | intergenic |  |  | - | | 3450725 | T | C | 774.77 | SNP | Rv3084 (lipR) | silent (Val243) | 9901 | - | | 3455220 | G | A | 512.77 | SNP | Rv3088 (tgs4) | Arg294Gln | 9 | - | | 3455686 | G | C | 1178.77 | SNP | Rv3088 (tgs4) | silent (Leu449) | 9947 | - | | 3456666 | A | G | 936.77 | SNP | Rv3089 (fadD13) | silent (Ala302) | 9867 | - | | 3458659 | G | A | 1112.77 | SNP | Rv3090 | Arg150His | 8 | - | | 3462135 | G | C | 648.77 | SNP | Rv3093c | Cys210Trp | 0 | - | | 3462145 | A | AGGCGC | 1212.06 | INS | Rv3093c |  |  | - | | 3465812 | G | A | 799.77 | SNP | Rv3097c (lipY) | Pro427Leu | 3 | - | | 3473996 | G | GA | 1422.73 | INS | intergenic |  |  | - | | 3480474 | G | A | 1326.77 | SNP | Rv3113 | Gly134Glu | 4 | - | | 3480722 | G | A | 1062.77 | SNP | Rv3113 | Ala217Thr | 22 | - | | 3482432 | C | A | 151.90 | SNP | Rv3115 | Gln328Lys | 12 | - | | 3486977 | A | G | 1583.77 | SNP | Rv3121 (cyp141) | Lys157Glu | 4 | - | | 3503895 | C | T | 827.77 | SNP | Rv3137 | Pro168Leu | 3 | - | | 3505027 | G | A | 1053.77 | SNP | Rv3138 (pflA) | Arg278His | 8 | - | | 3517548 | A | G | 1001.77 | SNP | Rv3151 (nuoG) | Glu268Gly | 7 | - | | 3518167 | A | G | 618.77 | SNP | Rv3151 (nuoG) | Ile474Met(s) | 6 | - | | 3518555 | A | G | 375.77 | SNP | Rv3151 (nuoG) | Thr604Ala | 32 | - | | 3528084 | A | G | 39.77 | SNP | Rv3159c (PPE53) | silent (Gly360) | 9935 | - | | 3528087 | G | A | 84.77 | SNP | Rv3159c (PPE53) | silent (Ile359) | 9872 | - | | 3528099 | A | C | 80.77 | SNP | Rv3159c (PPE53) | silent (Gly355) | 9935 | - | | 3528102 | C | A | 69.77 | SNP | Rv3159c (PPE53) | silent (Ser354) | 9840 | - | | 3528117 | T | C | 115.77 | SNP | Rv3159c (PPE53) | silent (Leu349) | 9947 | - | | 3528119 | G | T | 98.77 | SNP | Rv3159c (PPE53) | Leu349Ile | 9 | - | | 3528120 | A | G | 109.77 | SNP | Rv3159c (PPE53) | silent (Asn348) | 9822 | - | | 3528129 | G | T | 165.77 | SNP | Rv3159c (PPE53) | silent (Gly345) | 9935 | - | | 3528140 | G | A | 221.77 | SNP | Rv3159c (PPE53) | Leu342Leu(s) | 4 | - | | 3528144 | G | A | 211.77 | SNP | Rv3159c (PPE53) | silent (Gly340) | 9935 | - | | 3528158 | A | G | 274.77 | SNP | Rv3159c (PPE53) | Leu(s)336Leu | 3 | - | | 3528159 | G | A | 256.77 | SNP | Rv3159c (PPE53) | silent (Asn335) | 9822 | - | | 3528165 | G | A | 317.77 | SNP | Rv3159c (PPE53) | silent (Gly333) | 9935 | - | | 3528192 | G | A | 372.77 | SNP | Rv3159c (PPE53) | silent (Asn324) | 9822 | - | | 3528198 | A | G | 309.77 | SNP | Rv3159c (PPE53) | silent (Asn322) | 9822 | - | | 3529067 | G | C | 1798.77 | SNP | Rv3159c (PPE53) | Arg33Gly | 1 | - | | 3544710 | T | C | 704.77 | SNP | Rv3176c (mesT) | silent (Pro197) | 9926 | - | | 3556275 | A | G | 1340.77 | SNP | Rv3190c | Leu138Pro | 2 | - | | 3556607 | G | T | 957.77 | SNP | Rv3190c | Ser27Arg | 6 | - | | 3567600 | G | A | 1037.77 | SNP | Rv3197 | Val(s)193Met(s) | 9867 | - | | 3580636 | CT | C | 2443.73 | DEL | intergenic |  |  | - | | 3581414 | A | G | 967.77 | SNP | Rv3204 | Thr34Ala | 32 | - | | 3583295 | C | T | 875.77 | SNP | Rv3206c (moeB1) | Gly139Asp | 6 | - | | 3590686 | G | GC | 1074.73 | INS | intergenic |  |  | - | | 3591063 | T | C | 629.77 | SNP | Rv3213c | Lys144Glu | 4 | - | | 3595036 | C | T | 1183.77 | SNP | Rv3218 | Pro190Leu | 3 | - | | 3597737 | C | T | 1556.77 | SNP | Rv3221c (TB7.3) | Val(s)10Val | 13 | - | | 3603355 | G | A | 521.77 | SNP | intergenic |  |  | - | | 3604821 | G | C | 279.78 | SNP | Rv3228 | silent (Ala32) | 9867 | - | | 3614982 | T | C | 997.77 | SNP | Rv3239c | silent (Leu874) | 9947 | - | | 3621044 | T | C | 475.77 | SNP | Rv3241c | Ile71Val | 57 | - | | 3621423 | A | G | 1193.77 | SNP | intergenic |  |  | - | | 3622441 | A | C | 774.77 | SNP | Rv3243c | Val217Val(s) | 18 | - | | 3625065 | T | G | 930.77 | SNP | Rv3245c (mtrB) | Met(s)517Leu | 3 | - | | 3629928 | T | C | 1335.77 | SNP | Rv3249c | Thr154Ala | 32 | - | | 3649543 | G | A | 1032.77 | SNP | Rv3268 | Asp42Asn | 36 | - | | 3656121 | C | A | 71.77 | SNP | Rv3273 | silent (Arg495) | 9913 | - | | 3663889 | C | A | 137.77 | SNP | Rv3281 (accE5) | Asn67Lys | 25 | - | | 3678813 | T | A | 405.77 | SNP | Rv3296 (lhr) | Ile680Asn | 3 | - | | 3683037 | C | T | 822.77 | SNP | intergenic |  |  | - | | 3689523 | G | T | 594.77 | SNP | Rv3303c (lpdA) | Cys472STOP | 3 | - | | 3691061 | A | C | 142.77 | SNP | intergenic |  |  | - | | 3691063 | G | A | 99.77 | SNP | intergenic |  |  | - | | 3692357 | G | C | 727.77 | SNP | Rv3305c (amiA1) | Ala151Gly | 21 | - | | 3704596 | G | C | 1133.77 | SNP | Rv3317 (sdhD) | Val(s)54Leu | 3 | - | | 3709949 | G | A | 878.77 | SNP | Rv3324c (moaC3) | silent (Leu100) | 9947 | - | | 3714108 | G | A | 703.77 | SNP | Rv3328c (sigJ) | silent (Thr75) | 9871 | - | | 3714211 | G | T | 1168.77 | SNP | Rv3328c (sigJ) | Pro41Gln | 6 | - | | 3714757 | A | C | 1260.77 | SNP | Rv3329 | Gln122His | 20 | - | | 3717562 | C | CGGT | 1083.82 | INS | Rv3331 (sugI) |  |  | - | | 3718357 | C | T | 1175.77 | SNP | Rv3331 (sugI) | Pro423Leu | 3 | - | | 3721806 | G | C | 1332.77 | SNP | Rv3335c | silent (Gly265) | 9935 | - | | 3727746 | G | A | 670.77 | SNP | Rv3341 (metA) | Gly87Ser | 16 | - | | 3730390 | G | C | 1010.77 | SNP | Rv3343c (PPE54) | Phe2182Leu(s) | 2 | - | | 3730394 | G | A | 951.77 | SNP | Rv3343c (PPE54) | Ala2181Val | 13 | - | | 3730582 | G | A | 79.28 | SNP | Rv3343c (PPE54) | silent (Asn2118) | 9822 | - | | 3730648 | A | G | 38.74 | SNP | Rv3343c (PPE54) | silent (Ser2096) | 9840 | - | | 3735813 | C | G | 123.53 | SNP | Rv3343c (PPE54) | Glu375Gln | 27 | - | | 3736628 | T | G | 658.77 | SNP | Rv3343c (PPE54) | Glu103Ala | 17 | - | | 3738044 | G | T | 37.74 | SNP | intergenic |  |  | - | | 3744452 | A | C | 736.77 | SNP | Rv3347c (PPE55) | silent (Pro2911) | 9926 | - | | 3746409 | A | G | 669.77 | SNP | Rv3347c (PPE55) | Leu2259Pro | 2 | - | | 3747403 | C | A | 169.77 | SNP | Rv3347c (PPE55) | Gly1928Cys | 0 | - | | 3750177 | A | T | 56.77 | SNP | Rv3347c (PPE55) | Phe1003Tyr | 21 | - | | 3750178 | A | C | 64.77 | SNP | Rv3347c (PPE55) | Phe1003Val | 1 | - | | 3752207 | A | G | 746.77 | SNP | Rv3347c (PPE55) | silent (Ile326) | 9872 | - | | 3752809 | G | A | 578.77 | SNP | Rv3347c (PPE55) | Leu126Leu(s) | 4 | - | | 3752813 | G | A | 469.77 | SNP | Rv3347c (PPE55) | silent (Asn124) | 9822 | - | | 3752821 | T | C | 592.77 | SNP | Rv3347c (PPE55) | Met(s)122Val(s) | 9867 | - | | 3753116 | C | T | 213.80 | SNP | Rv3347c (PPE55) | silent (Pro23) | 9926 | - | | 3753164 | T | G | 436.77 | SNP | Rv3347c (PPE55) | silent (Pro7) | 9926 | - | | 3766852 | T | A | 53.74 | SNP | Rv3350c (PPE56) | Glu84Val(s) | 17 | - | | 3766858 | G | T | 52.74 | SNP | Rv3350c (PPE56) | Ala82Glu | 10 | - | | 3766859 | C | G | 54.74 | SNP | Rv3350c (PPE56) | Ala82Pro | 13 | - | | 3766860 | G | C | 55.74 | SNP | Rv3350c (PPE56) | silent (Ala81) | 9867 | - | | 3783058 | G | A | 799.77 | SNP | Rv3370c (dnaE2) | silent (Ser561) | 9840 | - | | 3786162 | G | T | 1142.77 | SNP | Rv3371 | Asp411Tyr | 0 | - | | 3791438 | T | C | 1931.77 | SNP | Rv3377c | Lys306Glu | 4 | - | | 3798095 | A | C | 1319.77 | SNP | Rv3383c (idsB) | Val132Gly | 5 | - | | 3798451 | C | G | 1563.77 | SNP | Rv3383c (idsB) | silent (Gly13) | 9935 | - | | 3805526 | T | G | 1013.77 | SNP | Rv3390 (lpqD) | Val(s)221Gly | 21 | - | | 3812821 | A | C | 1405.77 | SNP | Rv3396c (guaA) | Leu(s)420Val(s) | 9867 | - | | 3815477 | G | T | 586.28 | SNP | Rv3398c (idsA1) | silent (Ala210) | 9867 | - | | 3817117 | C | A | 788.77 | SNP | Rv3399 | Ala330Glu | 10 | - | | 3820545 | A | G | 66.77 | SNP | intergenic |  |  | - | | 3823159 | A | T | 1326.77 | SNP | Rv3403c | silent (Val235) | 9901 | - | | 3826684 | C | T | 706.77 | SNP | Rv3408 (vapC47) | Ser46Leu(s) | 35 | - | | 3829770 | T | C | 719.77 | SNP | Rv3410c (guaB3) | silent (Pro47) | 9926 | - | | 3838871 | A | G | 794.77 | SNP | Rv3420c (rimI) | silent (Ala64) | 9867 | - | | 3854174 | G | A | 903.77 | SNP | Rv3435c | Ala207Val(s) | 9867 | - | | 3859893 | C | T | 580.77 | SNP | Rv3440c | silent (Glu28) | 9865 | - | | 3862472 | GA | G | 1774.73 | DEL | intergenic |  |  | - | | 3863814 | C | A | 777.77 | SNP | Rv3446c | Gly240Cys | 0 | - | | 3864995 | T | C | 1295.77 | SNP | Rv3447c (eccC4) | Ser1082Gly | 21 | - | | 3866350 | C | T | 1002.77 | SNP | Rv3447c (eccC4) | Gly630Glu | 4 | - | | 3872726 | C | T | 738.77 | SNP | Rv3451 (cut3) | Pro37Leu | 3 | - | | 3873242 | G | A | 995.77 | SNP | Rv3451 (cut3) | Gly209Asp | 6 | - | | 3874722 | AT | A | 2566.73 | DEL | Rv3453 |  |  | - | | 3876910 | C | T | 786.77 | SNP | Rv3456c (rplQ) | Ala175Thr | 22 | - | | 3877421 | A | G | 774.77 | SNP | Rv3456c (rplQ) | silent (Pro4) | 9926 | - | | 3883626 | A | G | 723.77 | SNP | Rv3466 | silent (Pro34) | 9926 | - | | 3884791 | C | A | 249.78 | SNP | Rv3467 | Asn276Lys | 25 | - | | 3884906 | A | G | 880.77 | SNP | Rv3467 | Lys315Glu | 4 | - | | 3885886 | T | C | 967.77 | SNP | Rv3468c | Ile62Val | 57 | - | | 3887921 | A | G | 797.77 | SNP | Rv3470c (ilvB2) | silent (Phe294) | 9946 | - | | 3890310 | G | A | 1144.77 | SNP | Rv3473c (bpoA) | Arg142Trp | 2 | - | | 3892671 | A | G | 1822.77 | SNP | Rv3476c (kgtP) | silent (Val350) | 9901 | - | | 3895269 | G | C | 1019.77 | SNP | Rv3478 (PPE60) | Glu282Gln | 27 | - | | 3895399 | C | A | 324.77 | SNP | Rv3478 (PPE60) | Pro325Gln | 6 | - | | 3895400 | A | G | 341.77 | SNP | Rv3478 (PPE60) | silent (Pro325) | 9926 | - | | 3895403 | A | C | 331.77 | SNP | Rv3478 (PPE60) | silent (Ala326) | 9867 | - | | 3896340 | T | G | 871.77 | SNP | Rv3479 | Leu174Arg | 1 | - | | 3898408 | A | G | 721.77 | SNP | Rv3479 | silent (Ala863) | 9867 | - | | 3899124 | G | C | 1146.77 | SNP | Rv3480c | Leu427Val(s) | 4 | - | | 3899644 | G | C | 151.77 | SNP | Rv3480c | Val253Val(s) | 18 | - | | 3922380 | G | A | 1577.77 | SNP | intergenic |  |  | - | | 3925733 | T | G | 1094.77 | SNP | Rv3506 (fadD17) | Phe282Val | 1 | - | | 3927202 | G | A | 119.03 | SNP | Rv3507 (PE\_PGRS53) | Gly212Ser | 16 | - | | 3929089 | GACGGCGGCA | G | 533.87 | DEL | Rv3507 (PE\_PGRS53) |  |  | - | | 3934542 | T | G | 165.90 | SNP | Rv3508 (PE\_PGRS54) | Ser1180Ala | 35 | - | | 3934699 | G | A | 145.90 | SNP | Rv3508 (PE\_PGRS54) | Ser1232Asn | 20 | - | | 3940802 | A | G | 55.74 | SNP | Rv3511 (PE\_PGRS55) | Asn396Asp | 42 | - | | 3941497 | G | C | 35.77 | SNP | Rv3511 (PE\_PGRS55) | silent (Gly627) | 9935 | - | | 3941499 | C | A | 34.80 | SNP | Rv3511 (PE\_PGRS55) | Ala628Asp | 6 | - | | 3941568 | A | C | 45.77 | SNP | Rv3511 (PE\_PGRS55) | Asn651Thr | 13 | - | | 3941834 | G | C | 74.03 | SNP | intergenic |  |  | - | | 3941836 | C | A | 70.03 | SNP | intergenic |  |  | - | | 3942481 | C | G | 209.84 | SNP | intergenic |  |  | - | | 3942640 | T | C | 274.78 | SNP | intergenic |  |  | - | | 3943079 | C | A | 42.74 | SNP | intergenic |  |  | - | | 3952800 | G | A | 921.77 | SNP | Rv3516 (echA19) | Gly86Asp | 6 | - | | 3958403 | A | G | 986.77 | SNP | Rv3521 | Asn295Asp | 42 | - | | 3959418 | C | T | 871.77 | SNP | Rv3522 (ltp4) | Thr324Ile | 7 | - | | 3969763 | C | T | 539.77 | SNP | Rv3532 (PPE61) | Gln141STOP | 8 | - | | 3982149 | T | C | 650.77 | SNP | Rv3543c (fadE29) | Glu331Gly | 7 | - | | 3985547 | G | T | 1102.77 | SNP | intergenic |  |  | - | | 4002899 | C | T | 514.77 | SNP | Rv3561 (fadD3) | silent (Ala421) | 9867 | - | | 4003130 | G | A | 1285.77 | SNP | Rv3561 (fadD3) | Val(s)498Val | 13 | - | | 4005607 | T | C | 290.78 | SNP | Rv3564 (fadE33) | Leu(s)121Leu | 3 | - | | 4024273 | T | C | 942.77 | SNP | Rv3581c (ispF) | Val25Val(s) | 18 | - | | 4026899 | G | A | 606.77 | SNP | Rv3585 (radA) | silent (Gln152) | 9876 | - | | 4031626 | G | A | 169.84 | SNP | Rv3590c (PE\_PGRS58) | silent (Gly511) | 9935 | - | | 4034827 | C | T | 644.77 | SNP | Rv3593 (lpqF) | Ala159Val(s) | 9867 | - | | 4037283 | T | G | 346.78 | SNP | Rv3595c (PE\_PGRS59) | silent (Gly256) | 9935 | - | | 4047967 | A | C | 577.77 | SNP | Rv3605c | Leu72Arg | 1 | - | | 4051716 | C | T | 901.77 | SNP | Rv3610c (ftsH) | Val390Ile | 33 | - | | 4053050 | A | G | 402.77 | SNP | Rv3611 | Asn34Ser | 34 | - | | 4055801 | G | A | 1195.77 | SNP | Rv3616c (espA) | Thr192Ile | 7 | - | | 4056124 | G | A | 1197.77 | SNP | Rv3616c (espA) | silent (Leu84) | 9947 | - | | 4059904 | A | G | 842.77 | SNP | intergenic |  |  | - | | 4060100 | G | A | 638.77 | SNP | Rv3619c (esxV) | Leu57Leu(s) | 4 | - | | 4060201 | G | A | 445.77 | SNP | Rv3619c (esxV) | Ser23Leu(s) | 35 | - | | 4060210 | T | A | 412.77 | SNP | Rv3619c (esxV) | Gln20Leu | 6 | - | | 4060230 | G | A | 503.77 | SNP | Rv3619c (esxV) | silent (His13) | 9912 | - | | 4063682 | G | T | 936.77 | SNP | Rv3624c (hpt) | Leu75Met(s) | 4 | - | | 4069292 | G | A | 626.77 | SNP | Rv3630 | Ala40Thr | 22 | - | | 4086802 | G | A | 1185.77 | SNP | Rv3646c (topA) | silent (His152) | 9912 | - | | 4091760 | C | A | 1724.77 | SNP | intergenic |  |  | - | | 4094346 | T | TCGGCGCCGGCGGCGCCGG | 1779.74 | INS | Rv3653 (PE\_PGRS61) |  |  | - | | 4095001 | CG | C | 1397.73 | DEL | Rv3655c |  |  | - | | 4098261 | G | A | 158.90 | SNP | Rv3660c | silent (Ala296) | 9867 | - | | 4100971 | C | T | 883.77 | SNP | intergenic |  |  | - | | 4100975 | T | C | 806.77 | SNP | intergenic |  |  | - | | 4103884 | C | G | 125.77 | SNP | Rv3664c (dppC) | Ala198Pro | 13 | - | | 4111303 | G | C | 955.77 | SNP | Rv3669 | Val(s)159Val | 13 | - | | 4115012 | G | A | 726.77 | SNP | Rv3673c | Thr49Met(s) | 32 | - | | 4117167 | GGC | G | 2140.73 | DEL | intergenic |  |  | - | | 4120926 | A | G | 69.77 | SNP | Rv3680 | Asn378Asp | 42 | - | | 4120983 | A | G | 281.77 | SNP | intergenic |  |  | - | | 4121109 | A | C | 1220.77 | SNP | intergenic |  |  | - | | 4146314 | G | T | 753.77 | SNP | Rv3703c | Pro193Gln | 6 | - | | 4150647 | C | G | 748.77 | SNP | Rv3707c | Gly132Arg | 0 | - | | 4151855 | A | G | 738.77 | SNP | Rv3708c (asd) | silent (Pro121) | 9926 | - | | 4154051 | G | A | 1111.77 | SNP | Rv3710 (leuA) | silent (Arg104) | 9913 | - | | 4156099 | C | A | 944.77 | SNP | Rv3711c (dnaQ) | Val(s)211Leu(s) | 9867 | - | | 4159195 | T | C | 1048.77 | SNP | Rv3714c | silent (Pro209) | 9926 | - | | 4162339 | A | G | 1763.77 | SNP | Rv3719 | Thr12Ala | 32 | - | | 4182695 | G | A | 969.77 | SNP | Rv3731 (ligC) | Arg313His | 8 | - | | 4187485 | T | C | 1284.77 | SNP | Rv3736 | silent (Ala284) | 9867 | - | | 4187817 | A | G | 853.77 | SNP | Rv3737 | Asp40Gly | 11 | - | | 4192588 | G | A | 1088.77 | SNP | Rv3741c | Ala89Val(s) | 9867 | - | | 4197138 | C | CT | 1620.73 | INS | intergenic |  |  | - | | 4198611 | CG | C | 2032.73 | DEL | intergenic |  |  | - | | 4204441 | A | G | 1286.77 | SNP | Rv3759c (proX) | silent (His311) | 9912 | - | | 4205120 | A | G | 921.77 | SNP | Rv3759c (proX) | Leu85Pro | 2 | - | | 4205550 | G | A | 1165.77 | SNP | Rv3760 | Val(s)5Met(s) | 9867 | - | | 4207911 | G | A | 1153.77 | SNP | Rv3762c | silent (Ala322) | 9867 | - | | 4209518 | T | A | 634.77 | SNP | Rv3763 (lpqH) | Cys158Ser | 11 | - | | 4210274 | A | G | 966.77 | SNP | Rv3764c (tcrY) | Cys246Arg | 1 | - | | 4212840 | T | C | 1176.77 | SNP | Rv3766 | Val183Ala | 18 | - | | 4221490 | C | G | 821.77 | SNP | Rv3776 | silent (Leu134) | 9947 | - | | 4222073 | A | G | 233.80 | SNP | Rv3776 | Met(s)329Val(s) | 9867 | - | | 4222882 | A | G | 906.77 | SNP | Rv3777 | silent (Leu63) | 9947 | - | | 4223172 | T | C | 543.77 | SNP | Rv3777 | Val160Ala | 18 | - | | 4229087 | C | T | 1229.77 | SNP | Rv3782 (glfT1) | silent (Asn247) | 9822 | genotype | | 4231228 | G | GT | 2288.73 | INS | Rv3784 |  |  | - | | 4232863 | G | C | 610.77 | SNP | Rv3786c | Asp245Glu | 56 | - | | 4242643 | C | T | 889.77 | SNP | Rv3793 (embC) | silent (Arg927) | 9913 | genotype | | 4242803 | G | C | 935.77 | SNP | Rv3793 (embC) | Val(s)981Leu | 3 | genotype | | 4243622 | G | A | 744.77 | SNP | Rv3794 (embA) | silent (Thr130) | 9871 | - | | 4247729 | G | A | 681.77 | SNP | Rv3795 (embB) | Gly406Ser | 16 | resistance | | 4249408 | G | A | 758.77 | SNP | Rv3795 (embB) | silent (Pro965) | 9926 | - | | 4252878 | T | A | 295.74 | SNP | intergenic |  |  | - | | 4252879 | C | CCCACG | 1226.73 | INS | intergenic |  |  | - | | 4252881 | T | TTTTTG | 1214.73 | INS | intergenic |  |  | - | | 4255922 | A | G | 1393.77 | SNP | Rv3799c (accD4) | silent (His9) | 9912 | - | | 4257220 | A | G | 914.77 | SNP | Rv3800c (pks13) | silent (Arg1309) | 9913 | - | | 4269297 | G | C | 923.77 | SNP | Rv3806c (ubiA) | Ile179Met(s) | 6 | - | | 4270835 | T | C | 904.77 | SNP | Rv3808c (glfT2) | Gln482Arg | 10 | - | | 4275386 | G | C | 1462.77 | SNP | Rv3811 | Ala197Pro | 13 | - | | 4287109 | G | A | 1314.77 | SNP | Rv3822 | Ser130Asn | 20 | - | | 4292305 | C | T | 1369.77 | SNP | Rv3824c (papA1) | Met(s)290Ile | 2 | - | | 4296015 | G | A | 1163.77 | SNP | Rv3825c (pks2) | silent (Asp1197) | 9859 | - | | 4302036 | T | C | 881.77 | SNP | Rv3827c | Thr252Ala | 32 | - | | 4302161 | T | C | 1233.77 | SNP | Rv3827c | Gln210Arg | 10 | - | | 4306155 | C | T | 541.77 | SNP | Rv3831 | silent (Ser133) | 9840 | - | | 4306472 | G | T | 852.77 | SNP | Rv3832c | Leu114Met(s) | 4 | - | | 4306767 | G | A | 1346.77 | SNP | Rv3832c | silent (Gly15) | 9935 | - | | 4306929 | G | T | 802.77 | SNP | Rv3833 | silent (Arg21) | 9913 | - | | 4307179 | G | A | 810.77 | SNP | Rv3833 | Val105Ile | 33 | - | | 4314645 | A | G | 704.77 | SNP | Rv3841 (bfrB) | silent (Leu156) | 9947 | - | | 4319986 | C | T | 622.77 | SNP | intergenic |  |  | - | | 4323355 | G | C | 1425.77 | SNP | intergenic |  |  | - | | 4338595 | GC | G | 2298.73 | DEL | intergenic |  |  | - | | 4338732 | G | A | 921.77 | SNP | intergenic |  |  | - | | 4340006 | A | C | 831.77 | SNP | Rv3863 | silent (Ala386) | 9867 | - | | 4351039 | G | T | 1114.77 | SNP | Rv3872 (PE35) | Glu99STOP | 17 | - | | 4356110 | G | C | 732.77 | SNP | Rv3877 (eccD1) | silent (Leu368) | 9947 | - | | 4357123 | C | A | 934.77 | SNP | Rv3878 (espJ) | Thr144Lys | 11 | - | | 4357597 | C | G | 731.77 | SNP | Rv3879c (espK) | Cys729Ser | 11 | - | | 4359135 | TGGGGTTCCCGGGGTGATC | T | 7282.73 | DEL | Rv3879c (espK) |  |  | - | | 4359165 | G | C | 945.77 | SNP | Rv3879c (espK) | silent (Thr206) | 9871 | - | | 4359447 | A | G | 1567.77 | SNP | Rv3879c (espK) | silent (Asn112) | 9822 | - | | 4366195 | T | C | 524.77 | SNP | Rv3884c (eccA2) | Glu215Gly | 7 | - | | 4366272 | G | C | 660.77 | SNP | Rv3884c (eccA2) | silent (Ala189) | 9867 | - | | 4369672 | GC | G | 359.27 | DEL | Rv3886c (mycP2) |  |  | - | | 4372217 | G | A | 1039.77 | SNP | Rv3888c | His164Tyr | 4 | - | | 4374264 | G | C | 1369.77 | SNP | Rv3891c (esxD) | Gln37Glu | 35 | - | | 4375480 | C | T | 608.84 | SNP | Rv3892c (PPE69) | silent (Ala68) | 9867 | - | | 4375628 | G | T | 596.77 | SNP | Rv3892c (PPE69) | Thr19Lys | 11 | - | | 4377909 | G | A | 1285.77 | SNP | Rv3894c (eccC2) | silent (Tyr848) | 9945 | - | | 4379680 | C | G | 1015.77 | SNP | Rv3894c (eccC2) | Arg258Pro | 5 | - | | 4382054 | T | C | 1008.77 | SNP | Rv3896c | silent (Ala266) | 9867 | - | | 4382275 | G | T | 738.77 | SNP | Rv3896c | Gln193Lys | 12 | - | | 4383144 | C | CCGGGG | 2212.73 | INS | Rv3897c |  |  | - | | 4384417 | C | T | 767.77 | SNP | Rv3899c | silent (Leu321) | 9947 | - | | 4385111 | G | A | 306.78 | SNP | Rv3899c | Ala90Val | 13 | - | | 4386228 | T | C | 1158.77 | SNP | Rv3900c | silent (Leu27) | 9947 | - | | 4386709 | C | T | 1053.77 | SNP | Rv3901c | Ala36Thr | 22 | - | | 4387074 | G | A | 951.77 | SNP | intergenic |  |  | - | | 4393178 | A | G | 1124.77 | SNP | intergenic |  |  | - | | 4400246 | G | A | 491.77 | SNP | Rv3911 (sigM) | Asp21Asn | 36 | - | | 4400660 | AC | A | 816.73 | DEL | Rv3911 (sigM) |  |  | - | | 4407977 | C | T | 1318.77 | SNP | Rv3919c (gid) | Gly76Ser | 16 | - | |  | | export |

elog
