# Supplementary material for: Patterns of compensatory mutations in rpoA/B/C genes of multidrug resistant M. tuberculosis in Uganda
Source: PLoS One. 2025 Dec 4;20(12):e0328957. doi: 10.1371/journal.pone.0328957 (PMC12677784; doi:10.1371/journal.pone.0328957)
Supplement: S2 File — (ZIP) [file pone.0328957.s002.zip › Variants O_S15_L001_001.bam.html]

 

Calling SNPs/INDELs (computing variant list in .vcf format) from O\_S15\_L001\_001.bam

*by SAMtools/BCFtools:*

Howto

Important aspects

This takes up to one hour!!! **Please wait ...**

Variants O\_S15\_L001\_001.bam

|  |  |
| --- | --- |
| Variants |  |

|  |  |
| --- | --- |
| |  | | --- | | *by GATK* | |

|  |  |  |
| --- | --- | --- |
| |  | | --- | | O\_S15\_L001\_001.bam | | | computed 2016-10-27 using PhyResSE v1.0 (Ref. NC\_000962.3) | |

|  |  |
| --- | --- |
| 1178  variants called Export in VCF format |  |

|  |  |  |  |  |  |  |  |  |  |  |  |  |  |  |  |  |  |  |  |  |  |  |  |  |  |  |  |  |  |  |  |  |  |  |  |  |  |  |  |  |  |  |  |  |  |  |  |  |  |  |  |  |  |  |  |  |  |  |  |  |  |  |  |  |  |  |  |  |  |  |  |  |  |  |  |  |  |  |  |  |  |  |  |  |  |  |  |  |  |  |  |  |  |  |  |  |  |  |  |  |  |  |  |  |  |  |  |  |  |  |  |  |  |  |  |  |  |  |  |  |  |  |  |  |  |  |  |  |  |  |  |  |  |  |  |  |  |  |  |  |  |  |  |  |  |  |  |  |  |  |  |  |  |  |  |  |  |  |  |  |  |  |  |  |  |  |  |  |  |  |  |  |  |  |  |  |  |  |  |  |  |  |  |  |  |  |  |  |  |  |  |  |  |  |  |  |  |  |  |  |  |  |  |  |  |  |  |  |  |  |  |  |  |  |  |  |  |  |  |  |  |  |  |  |  |  |  |  |  |  |  |  |  |  |  |  |  |  |  |  |  |  |  |  |  |  |  |  |  |  |  |  |  |  |  |  |  |  |  |  |  |  |  |  |  |  |  |  |  |  |  |  |  |  |  |  |  |  |  |  |  |  |  |  |  |  |  |  |  |  |  |  |  |  |  |  |  |  |  |  |  |  |  |  |  |  |  |  |  |  |  |  |  |  |  |  |  |  |  |  |  |  |  |  |  |  |  |  |  |  |  |  |  |  |  |  |  |  |  |  |  |  |  |  |  |  |  |  |  |  |  |  |  |  |  |  |  |  |  |  |  |  |  |  |  |  |  |  |  |  |  |  |  |  |  |  |  |  |  |  |  |  |  |  |  |  |  |  |  |  |  |  |  |  |  |  |  |  |  |  |  |  |  |  |  |  |  |  |  |  |  |  |  |  |  |  |  |  |  |  |  |  |  |  |  |  |  |  |  |  |  |  |  |  |  |  |  |  |  |  |  |  |  |  |  |  |  |  |  |  |  |  |  |  |  |  |  |  |  |  |  |  |  |  |  |  |  |  |  |  |  |  |  |  |  |  |  |  |  |  |  |  |  |  |  |  |  |  |  |  |  |  |  |  |  |  |  |  |  |  |  |  |  |  |  |  |  |  |  |  |  |  |  |  |  |  |  |  |  |  |  |  |  |  |  |  |  |  |  |  |  |  |  |  |  |  |  |  |  |  |  |  |  |  |  |  |  |  |  |  |  |  |  |  |  |  |  |  |  |  |  |  |  |  |  |  |  |  |  |  |  |  |  |  |  |  |  |  |  |  |  |  |  |  |  |  |  |  |  |  |  |  |  |  |  |  |  |  |  |  |  |  |  |  |  |  |  |  |  |  |  |  |  |  |  |  |  |  |  |  |  |  |  |  |  |  |  |  |  |  |  |  |  |  |  |  |  |  |  |  |  |  |  |  |  |  |  |  |  |  |  |  |  |  |  |  |  |  |  |  |  |  |  |  |  |  |  |  |  |  |  |  |  |  |  |  |  |  |  |  |  |  |  |  |  |  |  |  |  |  |  |  |  |  |  |  |  |  |  |  |  |  |  |  |  |  |  |  |  |  |  |  |  |  |  |  |  |  |  |  |  |  |  |  |  |  |  |  |  |  |  |  |  |  |  |  |  |  |  |  |  |  |  |  |  |  |  |  |  |  |  |  |  |  |  |  |  |  |  |  |  |  |  |  |  |  |  |  |  |  |  |  |  |  |  |  |  |  |  |  |  |  |  |  |  |  |  |  |  |  |  |  |  |  |  |  |  |  |  |  |  |  |  |  |  |  |  |  |  |  |  |  |  |  |  |  |  |  |  |  |  |  |  |  |  |  |  |  |  |  |  |  |  |  |  |  |  |  |  |  |  |  |  |  |  |  |  |  |  |  |  |  |  |  |  |  |  |  |  |  |  |  |  |  |  |  |  |  |  |  |  |  |  |  |  |  |  |  |  |  |  |  |  |  |  |  |  |  |  |  |  |  |  |  |  |  |  |  |  |  |  |  |  |  |  |  |  |  |  |  |  |  |  |  |  |  |  |  |  |  |  |  |  |  |  |  |  |  |  |  |  |  |  |  |  |  |  |  |  |  |  |  |  |  |  |  |  |  |  |  |  |  |  |  |  |  |  |  |  |  |  |  |  |  |  |  |  |  |  |  |  |  |  |  |  |  |  |  |  |  |  |  |  |  |  |  |  |  |  |  |  |  |  |  |  |  |  |  |  |  |  |  |  |  |  |  |  |  |  |  |  |  |  |  |  |  |  |  |  |  |  |  |  |  |  |  |  |  |  |  |  |  |  |  |  |  |  |  |  |  |  |  |  |  |  |  |  |  |  |  |  |  |  |  |  |  |  |  |  |  |  |  |  |  |  |  |  |  |  |  |  |  |  |  |  |  |  |  |  |  |  |  |  |  |  |  |  |  |  |  |  |  |  |  |  |  |  |  |  |  |  |  |  |  |  |  |  |  |  |  |  |  |  |  |  |  |  |  |  |  |  |  |  |  |  |  |  |  |  |  |  |  |  |  |  |  |  |  |  |  |  |  |  |  |  |  |  |  |  |  |  |  |  |  |  |  |  |  |  |  |  |  |  |  |  |  |  |  |  |  |  |  |  |  |  |  |  |  |  |  |  |  |  |  |  |  |  |  |  |  |  |  |  |  |  |  |  |  |  |  |  |  |  |  |  |  |  |  |  |  |  |  |  |  |  |  |  |  |  |  |  |  |  |  |  |  |  |  |  |  |  |  |  |  |  |  |  |  |  |  |  |  |  |  |  |  |  |  |  |  |  |  |  |  |  |  |  |  |  |  |  |  |  |  |  |  |  |  |  |  |  |  |  |  |  |  |  |  |  |  |  |  |  |  |  |  |  |  |  |  |  |  |  |  |  |  |  |  |  |  |  |  |  |  |  |  |  |  |  |  |  |  |  |  |  |  |  |  |  |  |  |  |  |  |  |  |  |  |  |  |  |  |  |  |  |  |  |  |  |  |  |  |  |  |  |  |  |  |  |  |  |  |  |  |  |  |  |  |  |  |  |  |  |  |  |  |  |  |  |  |  |  |  |  |  |  |  |  |  |  |  |  |  |  |  |  |  |  |  |  |  |  |  |  |  |  |  |  |  |  |  |  |  |  |  |  |  |  |  |  |  |  |  |  |  |  |  |  |  |  |  |  |  |  |  |  |  |  |  |  |  |  |  |  |  |  |  |  |  |  |  |  |  |  |  |  |  |  |  |  |  |  |  |  |  |  |  |  |  |  |  |  |  |  |  |  |  |  |  |  |  |  |  |  |  |  |  |  |  |  |  |  |  |  |  |  |  |  |  |  |  |  |  |  |  |  |  |  |  |  |  |  |  |  |  |  |  |  |  |  |  |  |  |  |  |  |  |  |  |  |  |  |  |  |  |  |  |  |  |  |  |  |  |  |  |  |  |  |  |  |  |  |  |  |  |  |  |  |  |  |  |  |  |  |  |  |  |  |  |  |  |  |  |  |  |  |  |  |  |  |  |  |  |  |  |  |  |  |  |  |  |  |  |  |  |  |  |  |  |  |  |  |  |  |  |  |  |  |  |  |  |  |  |  |  |  |  |  |  |  |  |  |  |  |  |  |  |  |  |  |  |  |  |  |  |  |  |  |  |  |  |  |  |  |  |  |  |  |  |  |  |  |  |  |  |  |  |  |  |  |  |  |  |  |  |  |  |  |  |  |  |  |  |  |  |  |  |  |  |  |  |  |  |  |  |  |  |  |  |  |  |  |  |  |  |  |  |  |  |  |  |  |  |  |  |  |  |  |  |  |  |  |  |  |  |  |  |  |  |  |  |  |  |  |  |  |  |  |  |  |  |  |  |  |  |  |  |  |  |  |  |  |  |  |  |  |  |  |  |  |  |  |  |  |  |  |  |  |  |  |  |  |  |  |  |  |  |  |  |  |  |  |  |  |  |  |  |  |  |  |  |  |  |  |  |  |  |  |  |  |  |  |  |  |  |  |  |  |  |  |  |  |  |  |  |  |  |  |  |  |  |  |  |  |  |  |  |  |  |  |  |  |  |  |  |  |  |  |  |  |  |  |  |  |  |  |  |  |  |  |  |  |  |  |  |  |  |  |  |  |  |  |  |  |  |  |  |  |  |  |  |  |  |  |  |  |  |  |  |  |  |  |  |  |  |  |  |  |  |  |  |  |  |  |  |  |  |  |  |  |  |  |  |  |  |  |  |  |  |  |  |  |  |  |  |  |  |  |  |  |  |  |  |  |  |  |  |  |  |  |  |  |  |  |  |  |  |  |  |  |  |  |  |  |  |  |  |  |  |  |  |  |  |  |  |  |  |  |  |  |  |  |  |  |  |  |  |  |  |  |  |  |  |  |  |  |  |  |  |  |  |  |  |  |  |  |  |  |  |  |  |  |  |  |  |  |  |  |  |  |  |  |  |  |  |  |  |  |  |  |  |  |  |  |  |  |  |  |  |  |  |  |  |  |  |  |  |  |  |  |  |  |  |  |  |  |  |  |  |  |  |  |  |  |  |  |  |  |  |  |  |  |  |  |  |  |  |  |  |  |  |  |  |  |  |  |  |  |  |  |  |  |  |  |  |  |  |  |  |  |  |  |  |  |  |  |  |  |  |  |  |  |  |  |  |  |  |  |  |  |  |  |  |  |  |  |  |  |  |  |  |  |  |  |  |  |  |  |  |  |  |  |  |  |  |  |  |  |  |  |  |  |  |  |  |  |  |  |  |  |  |  |  |  |  |  |  |  |  |  |  |  |  |  |  |  |  |  |  |  |  |  |  |  |  |  |  |  |  |  |  |  |  |  |  |  |  |  |  |  |  |  |  |  |  |  |  |  |  |  |  |  |  |  |  |  |  |  |  |  |  |  |  |  |  |  |  |  |  |  |  |  |  |  |  |  |  |  |  |  |  |  |  |  |  |  |  |  |  |  |  |  |  |  |  |  |  |  |  |  |  |  |  |  |  |  |  |  |  |  |  |  |  |  |  |  |  |  |  |  |  |  |  |  |  |  |  |  |  |  |  |  |  |  |  |  |  |  |  |  |  |  |  |  |  |  |  |  |  |  |  |  |  |  |  |  |  |  |  |  |  |  |  |  |  |  |  |  |  |  |  |  |  |  |  |  |  |  |  |  |  |  |  |  |  |  |  |  |  |  |  |  |  |  |  |  |  |  |  |  |  |  |  |  |  |  |  |  |  |  |  |  |  |  |  |  |  |  |  |  |  |  |  |  |  |  |  |  |  |  |  |  |  |  |  |  |  |  |  |  |  |  |  |  |  |  |  |  |  |  |  |  |  |  |  |  |  |  |  |  |  |  |  |  |  |  |  |  |  |  |  |  |  |  |  |  |  |  |  |  |  |  |  |  |  |  |  |  |  |  |  |  |  |  |  |  |  |  |  |  |  |  |  |  |  |  |  |  |  |  |  |  |  |  |  |  |  |  |  |  |  |  |  |  |  |  |  |  |  |  |  |  |  |  |  |  |  |  |  |  |  |  |  |  |  |  |  |  |  |  |  |  |  |  |  |  |  |  |  |  |  |  |  |  |  |  |  |  |  |  |  |  |  |  |  |  |  |  |  |  |  |  |  |  |  |  |  |  |  |  |  |  |  |  |  |  |  |  |  |  |  |  |  |  |  |  |  |  |  |  |  |  |  |  |  |  |  |  |  |  |  |  |  |  |  |  |  |  |  |  |  |  |  |  |  |  |  |  |  |  |  |  |  |  |  |  |  |  |  |  |  |  |  |  |  |  |  |  |  |  |  |  |  |  |  |  |  |  |  |  |  |  |  |  |  |  |  |  |  |  |  |  |  |  |  |  |  |  |  |  |  |  |  |  |  |  |  |  |  |  |  |  |  |  |  |  |  |  |  |  |  |  |  |  |  |  |  |  |  |  |  |  |  |  |  |  |  |  |  |  |  |  |  |  |  |  |  |  |  |  |  |  |  |  |  |  |  |  |  |  |  |  |  |  |  |  |  |  |  |  |  |  |  |  |  |  |  |  |  |  |  |  |  |  |  |  |  |  |  |  |  |  |  |  |  |  |  |  |  |  |  |  |  |  |  |  |  |  |  |  |  |  |  |  |  |  |  |  |  |  |  |  |  |  |  |  |  |  |  |  |  |  |  |  |  |  |  |  |  |  |  |  |  |  |  |  |  |  |  |  |  |  |  |  |  |  |  |  |  |  |  |  |  |  |  |  |  |  |  |  |  |  |  |  |  |  |  |  |  |  |  |  |  |  |  |  |  |  |  |  |  |  |  |  |  |  |  |  |  |  |  |  |  |  |  |  |  |  |  |  |  |  |  |  |  |  |  |  |  |  |  |  |  |  |  |  |  |  |  |  |  |  |  |  |  |  |  |  |  |  |  |  |  |  |  |  |  |  |  |  |  |  |  |  |  |  |  |  |  |  |  |  |  |  |  |  |  |  |  |  |  |  |  |  |  |  |  |  |  |  |  |  |  |  |  |  |  |  |  |  |  |  |  |  |  |  |  |  |  |  |  |  |  |  |  |  |  |  |  |  |  |  |  |  |  |  |  |  |  |  |  |  |  |  |  |  |  |  |  |  |  |  |  |  |  |  |  |  |  |  |  |  |  |  |  |  |  |  |  |  |  |  |  |  |  |  |  |  |  |  |  |  |  |  |  |  |  |  |  |  |  |  |  |  |  |  |  |  |  |  |  |  |  |  |  |  |  |  |  |  |  |  |  |  |  |  |  |  |  |  |  |  |  |  |  |  |  |  |  |  |  |  |  |  |  |  |  |  |  |  |  |  |  |  |  |  |  |  |  |  |  |  |  |  |  |  |  |  |  |  |  |  |  |  |  |  |  |  |  |  |  |  |  |  |  |  |  |  |  |  |  |  |  |  |  |  |  |  |  |  |  |  |  |  |  |  |  |  |  |  |  |  |  |  |  |  |  |  |  |  |  |  |  |  |  |  |  |  |  |  |  |  |  |  |  |  |  |  |  |  |  |  |  |  |  |  |  |  |  |  |  |  |  |  |  |  |  |  |  |  |  |  |  |  |  |  |  |  |  |  |  |  |  |  |  |  |  |  |  |  |  |  |  |  |  |  |  |  |  |  |  |  |  |  |  |  |  |  |  |  |  |  |  |  |  |  |  |  |  |  |  |  |  |  |  |  |  |  |  |  |  |  |  |  |  |  |  |  |  |  |  |  |  |  |  |  |  |  |  |  |  |  |  |  |  |  |  |  |  |  |  |  |  |  |  |  |  |  |  |  |  |  |  |  |  |  |  |  |  |  |  |  |  |  |  |  |  |  |  |  |  |  |  |  |  |  |  |  |  |  |  |  |  |  |  |  |  |  |  |  |  |  |  |  |  |  |  |  |  |  |  |  |  |  |  |  |  |  |  |  |  |  |  |  |  |  |  |  |  |  |  |  |  |  |  |  |  |  |  |  |  |  |  |  |  |  |  |  |  |  |  |  |  |  |  |  |  |  |  |  |  |  |  |  |  |  |  |  |  |  |  |  |  |  |  |  |  |  |  |  |  |  |  |  |  |  |  |  |  |  |  |  |  |  |  |  |  |  |  |  |  |  |  |  |  |  |  |  |  |  |  |  |  |  |  |  |  |  |  |  |  |  |  |  |  |  |  |  |  |  |  |  |  |  |  |  |  |  |  |  |  |  |  |  |  |  |  |  |  |  |  |  |  |  |  |  |  |  |  |  |  |  |  |  |  |  |  |  |  |  |  |  |  |  |  |  |  |  |  |  |  |  |  |  |  |  |  |  |  |  |  |  |  |  |  |  |  |  |  |  |  |  |  |  |  |  |  |  |  |  |  |  |  |  |  |  |  |  |  |  |  |  |  |  |  |  |  |  |  |  |  |  |  |  |  |  |  |  |  |  |  |  |  |  |  |  |  |  |  |  |  |  |  |  |  |  |  |  |  |  |  |  |  |  |  |  |  |  |  |  |  |  |  |  |  |  |  |  |  |  |  |  |  |  |  |  |  |  |  |  |  |  |  |  |  |  |  |  |  |  |  |  |  |  |  |  |  |  |  |  |  |  |  |  |  |  |  |  |  |  |  |  |  |  |  |  |  |  |  |  |  |  |  |  |  |  |  |  |  |  |  |  |  |  |  |  |  |  |  |  |  |  |  |  |  |  |  |  |  |  |  |  |  |  |  |  |  |  |  |  |  |  |  |  |  |  |  |  |  |  |  |  |  |  |  |  |  |  |  |  |  |  |  |  |  |  |  |  |  |  |  |  |  |  |  |  |  |  |  |  |  |  |  |  |  |  |  |  |  |  |  |  |  |  |  |  |  |  |  |  |  |  |  |  |  |  |  |  |  |  |  |  |  |  |  |  |  |  |  |  |  |  |  |  |  |  |  |  |  |  |  |  |  |  |  |  |  |  |  |  |  |  |  |  |  |  |  |  |  |  |  |  |  |  |  |  |  |  |  |  |  |  |  |  |  |  |  |  |  |  |  |  |  |  |  |  |  |  |  |  |  |  |  |  |  |  |  |  |  |  |  |  |  |  |  |  |  |  |  |  |  |  |  |  |  |  |  |  |  |  |  |  |  |  |  |  |  |  |  |  |  |  |  |  |  |  |  |  |  |  |  |  |  |  |  |  |  |  |  |  |  |  |  |  |  |  |  |  |  |  |  |  |  |  |  |  |  |  |  |  |  |  |  |  |  |  |  |  |  |  |  |  |  |  |  |  |  |  |  |  |  |  |  |  |  |  |  |  |  |  |  |  |  |  |  |  |  |  |  |  |  |  |  |  |  |  |  |  |  |  |  |  |  |  |  |  |  |  |  |  |  |  |  |  |  |  |  |  |  |  |  |  |  |  |  |  |  |  |  |  |  |  |  |  |  |  |  |  |  |  |  |  |  |  |  |  |  |  |  |  |  |  |  |  |  |  |  |  |  |  |  |  |  |  |  |  |  |  |  |  |  |  |  |  |  |  |  |  |  |  |  |  |  |  |  |  |  |  |  |  |  |  |  |  |  |  |  |  |  |  |  |  |  |  |  |  |  |  |  |  |  |  |  |  |  |  |  |  |  |  |  |  |  |  |  |  |  |  |  |  |  |  |  |  |  |  |  |  |  |  |  |  |  |  |  |  |  |  |  |  |  |  |  |  |  |  |  |  |  |  |  |  |  |  |  |  |  |  |  |  |  |  |  |  |  |  |  |  |  |  |  |  |  |  |  |  |  |  |  |  |  |  |  |  |  |  |  |  |  |  |  |  |  |  |  |  |  |  |  |  |  |  |  |  |  |  |  |  |  |  |  |  |  |  |  |  |  |  |  |  |  |  |  |  |  |  |  |  |  |  |  |  |  |  |  |  |  |  |  |  |  |  |  |  |  |  |  |  |  |  |  |  |  |  |  |  |  |  |  |  |  |  |  |  |  |  |  |  |  |  |  |  |  |  |  |  |  |  |  |  |  |  |  |  |  |  |  |  |  |  |  |  |  |  |  |  |  |  |  |  |  |  |  |  |  |  |  |  |  |  |  |  |  |  |  |  |  |  |  |  |  |  |  |  |  |  |  |  |  |  |  |  |  |  |  |  |  |  |  |  |  |  |  |  |  |  |  |  |  |  |  |  |  |  |  |  |  |  |  |  |  |  |  |  |  |  |  |  |  |  |  |  |  |  |  |  |  |  |  |  |  |  |  |  |  |  |  |  |  |  |  |  |  |  |  |  |  |  |  |  |  |  |  |  |  |  |  |  |  |  |  |  |  |  |  |  |  |  |  |  |  |  |  |  |  |  |  |  |  |  |  |  |  |  |  |  |  |  |  |  |  |  |  |  |  |  |  |  |  |  |  |  |  |  |  |  |  |  |  |  |  |  |  |  |  |  |  |  |  |  |  |  |  |  |  |  |  |  |  |  |  |  |  |  |  |  |  |  |  |  |  |  |  |  |  |  |  |  |  |  |  |  |  |  |  |  |  |  |  |  |  |  |  |  |  |  |  |  |  |  |  |  |  |  |  |  |  |  |  |  |  |  |  |  |  |  |  |  |  |  |  |  |  |  |  |  |  |  |  |  |  |  |  |  |  |  |  |  |  |  |  |  |  |  |  |  |  |  |  |  |  |  |  |  |  |  |  |  |  |  |  |  |  |  |  |  |  |  |  |  |  |  |  |  |  |  |  |  |  |  |  |  |  |  |  |  |  |  |  |  |  |  |  |  |  |  |  |  |  |  |  |  |  |  |  |  |  |  |  |  |  |  |  |  |  |  |  |  |  |  |  |  |  |  |  |  |  |  |  |  |  |  |  |  |  |  |  |  |  |  |  |  |  |  |  |  |  |  |  |  |  |  |  |  |  |  |  |  |  |  |  |  |  |  |  |  |  |  |  |  |  |  |  |  |  |  |  |  |  |  |  |  |  |  |  |  |  |  |  |  |  |  |  |  |  |  |  |  |  |  |  |  |  |  |  |  |  |  |  |  |  |  |  |  |  |  |  |  |  |  |  |  |  |  |  |  |  |  |  |  |  |  |  |  |  |  |  |  |  |  |  |  |  |  |  |  |  |  |  |  |  |  |  |  |  |  |  |  |  |  |  |  |  |  |  |  |  |  |  |  |  |  |  |  |  |  |  |  |  |  |  |  |  |  |  |  |  |  |  |  |  |  |  |  |  |  |  |  |  |  |  |  |  |  |  |  |  |  |  |  |  |  |  |  |  |  |  |  |  |  |  |  |  |  |  |  |  |  |  |  |  |  |  |  |  |  |  |  |  |  |  |  |  |  |  |  |  |  |  |  |  |  |  |  |  |  |  |  |  |  |  |  |  |  |  |  |  |  |  |  |  |  |  |  |  |  |  |  |  |  |  |  |  |  |  |  |  |  |  |  |  |  |  |  |  |  |  |  |  |  |  |  |  |  |  |  |  |  |  |  |  |  |  |  |  |  |  |  |  |  |  |  |  |  |  |  |  |  |  |  |  |  |  |  |  |  |  |  |  |  |  |  |  |  |  |  |  |  |  |  |  |  |  |  |  |  |  |  |  |  |  |  |  |  |  |  |  |  |  |  |  |  |  |  |  |  |  |  |  |  |  |  |  |  |  |  |  |  |  |  |  |  |  |  |  |  |  |  |  |  |  |  |  |  |  |  |  |  |  |  |  |  |  |  |  |  |  |  |  |  |  |  |  |  |  |  |  |  |  |  |  |  |  |  |  |  |  |  |  |  |  |  |  |  |  |  |  |  |  |  |  |  |  |  |  |  |  |  |  |  |  |  |  |  |  |  |  |  |  |  |  |  |  |  |  |  |  |  |  |  |  |  |  |  |  |  |  |  |  |  |  |  |  |  |  |  |  |  |  |  |  |  |  |  |  |  |  |  |  |  |  |  |  |  |  |  |  |  |  |  |  |  |  |  |  |  |  |  |  |  |  |  |  |  |  |  |  |  |  |  |  |  |  |  |  |  |  |  |  |  |  |  |  |  |  |  |  |  |  |  |  |  |  |  |  |  |  |  |  |  |  |  |  |  |  |  |  |  |  |  |  |  |  |  |  |  |  |  |  |  |  |  |  |  |  |  |  |  |  |  |  |  |  |  |  |  |  |  |  |  |  |  |  |  |  |  |  |  |  |  |  |  |  |  |  |  |  |  |  |  |  |  |  |  |  |  |  |  |  |  |  |  |  |  |  |  |  |  |  |  |  |  |  |  |  |  |  |  |  |  |  |  |  |  |  |  |  |  |  |  |  |  |  |  |  |  |  |  |  |  |  |  |  |  |  |  |  |  |  |  |  |  |  |  |  |  |  |  |  |  |  |  |  |  |  |  |  |  |  |  |  |  |  |  |  |  |  |  |  |  |  |  |  |  |  |  |  |  |  |  |  |  |  |  |  |  |  |  |  |  |  |  |  |  |  |  |  |  |  |  |  |  |  |  |  |  |  |  |  |  |  |  |  |  |  |  |  |  |  |  |  |  |  |  |  |  |  |  |  |  |  |  |  |  |  |  |  |  |  |  |  |  |  |  |  |  |  |  |  |  |  |  |  |  |  |  |  |  |  |  |  |  |  |  |  |  |  |  |  |  |  |  |  |  |  |  |  |  |  |  |  |  |  |  |  |  |  |  |  |  |  |  |  |  |  |  |  |  |  |  |  |  |  |  |  |  |  |  |  |  |  |  |  |  |  |  |  |  |  |  |  |  |  |  |  |  |  |  |  |  |  |  |  |  |  |  |  |  |  |  |  |  |  |  |  |  |  |  |  |  |  |  |  |  |  |  |  |  |  |  |  |  |  |  |  |  |  |  |  |  |  |  |  |  |  |  |  |  |  |  |  |  |  |  |  |  |  |  |  |  |  |  |  |  |  |  |  |  |  |  |  |  |  |  |  |  |  |  |  |  |  |  |  |  |  |  |  |  |  |  |  |  |  |  |  |  |  |  |  |  |  |  |  |  |  |  |  |  |  |  |  |  |  |  |  |  |  |  |  |  |  |  |  |  |  |  |  |  |  |  |  |  |  |  |  |  |  |  |  |  |  |  |  |  |  |  |  |  |  |  |  |  |  |  |  |  |  |  |  |  |  |  |  |  |  |  |  |  |  |  |  |  |  |  |  |  |  |  |  |  |  |  |  |  |  |  |  |  |  |  |  |  |  |  |  |  |  |  |  |  |  |  |  |  |  |  |  |  |  |  |  |  |  |  |  |  |  |  |  |  |  |  |  |  |  |  |  |  |  |  |  |  |  |  |  |  |  |  |  |  |  |  |  |  |  |  |  |  |  |  |  |  |  |  |  |  |  |  |  |  |  |  |  |  |  |  |  |  |  |  |  |  |  |  |  |  |  |  |  |  |  |  |  |  |  |  |  |  |  |  |  |  |  |  |  |  |  |  |  |  |  |  |  |  |  |  |  |  |  |  |  |  |  |  |  |  |  |  |  |  |  |  |  |  |  |  |  |  |  |  |  |  |  |  |  |  |  |  |  |  |  |  |  |  |  |  |  |  |  |  |  |  |  |  |  |  |  |  |  |  |  |  |  |  |  |  |  |  |  |  |  |  |  |  |  |  |  |  |  |  |  |  |  |  |  |  |  |  |  |  |  |  |  |  |  |  |  |  |  |  |  |  |  |  |  |  |  |  |  |  |  |  |  |  |  |  |  |  |  |  |  |  |  |  |  |  |  |  |  |  |  |  |  |  |  |  |  |  |  |  |  |  |  |  |  |  |  |  |  |  |  |  |  |  |  |  |  |  |  |  |  |  |  |  |  |  |  |  |  |  |  |  |  |  |  |  |  |  |  |  |  |  |  |  |  |  |  |  |  |  |  |  |  |  |  |  |  |  |  |  |  |  |  |  |  |  |  |  |  |  |  |  |  |  |  |  |  |  |  |  |  |  |  |  |  |  |  |  |  |  |  |  |  |  |  |  |  |  |  |  |  |  |  |  |  |  |  |  |  |  |  |  |  |  |  |  |  |  |  |  |  |  |  |  |  |  |  |  |  |  |  |  |  |  |  |  |  |  |  |  |  |  |  |  |  |  |  |  |  |  |  |  |  |  |  |  |  |  |  |  |  |  |  |  |  |  |  |  |  |  |  |  |  |  |  |  |  |  |  |  |  |  |  |  |  |  |  |  |  |  |  |  |  |  |  |  |  |  |  |  |  |  |  |  |  |  |  |  |  |  |  |  |  |  |  |  |  |  |  |  |  |  |  |  |  |  |  |  |  |  |  |  |  |  |  |  |  |  |  |  |  |  |  |  |  |  |  |  |  |  |  |  |  |  |  |  |  |  |  |  |  |  |  |  |  |  |  |  |  |  |  |  |  |  |  |  |  |  |  |  |  |  |  |  |  |  |  |  |  |  |  |  |  |  |  |  |  |  |  |  |  |  |  |  |  |  |  |  |  |  |  |  |  |  |  |  |  |  |  |  |  |  |  |  |  |  |  |  |  |  |  |  |  |  |  |  |  |  |  |  |  |  |  |  |  |  |  |  |  |  |  |  |  |  |  |  |  |  |  |  |  |  |  |  |  |  |  |  |  |  |  |  |  |  |  |  |  |  |  |  |  |  |  |  |  |  |  |  |  |  |  |  |  |  |  |  |  |  |  |  |  |  |  |  |  |  |  |  |  |  |  |  |  |  |  |  |  |  |  |  |  |  |  |  |  |  |  |  |  |  |  |  |  |  |  |  |  |  |  |  |  |  |  |  |  |  |  |  |  |  |  |  |  |  |  |  |  |  |  |  |  |  |  |  |  |  |  |  |  |  |  |  |  |  |  |  |  |  |  |  |  |  |  |  |  |  |  |  |  |  |  |  |  |  |  |  |  |  |  |  |  |  |  |  |  |  |  |  |  |  |  |  |  |  |  |  |  |  |  |  |  |  |  |  |  |  |  |  |  |  |  |  |  |  |  |  |  |  |  |  |  |  |  |  |  |  |  |  |  |  |  |  |  |  |  |  |  |  |  |  |  |  |  |  |  |  |  |  |  |  |  |  |  |  |  |  |  |  |  |  |  |  |  |  |  |  |  |  |  |  |  |  |  |  |  |  |  |  |  |  |  |  |  |  |  |  |  |  |  |  |  |  |  |  |  |  |  |  |  |  |  |  |  |  |  |  |  |  |  |  |  |  |  |  |  |  |  |  |  |  |  |  |  |  |  |  |  |  |  |  |  |  |  |  |  |  |  |  |  |  |  |  |  |  |  |  |  |  |  |  |  |  |  |  |  |  |  |  |  |  |  |  |  |  |  |  |  |  |  |  |  |  |  |  |  |  |  |  |  |  |  |  |  |  |  |  |  |  |  |  |  |  |  |  |  |  |  |  |  |  |  |  |  |  |  |  |  |  |  |  |  |  |  |  |  |  |  |  |  |  |  |  |  |  |  |  |  |  |  |  |  |  |  |  |  |  |  |  |  |  |  |  |  |  |  |  |  |  |  |  |  |  |  |  |  |  |  |  |  |  |  |  |  |  |  |  |  |  |  |  |  |  |  |  |  |  |  |  |  |  |  |  |  |  |  |  |  |  |  |  |  |  |  |  |  |  |  |  |  |  |  |  |  |  |  |  |  |  |  |  |  |  |  |  |  |  |  |  |  |  |  |  |  |  |  |  |  |  |  |  |  |  |  |  |  |  |  |  |  |  |  |  |  |  |  |  |  |  |  |  |  |  |  |  |  |  |  |  |  |  |  |  |  |  |  |  |  |  |  |  |  |  |  |  |  |  |  |  |  |  |  |  |  |  |  |  |  |  |  |  |  |  |  |  |  |  |  |  |  |  |  |  |  |  |  |  |  |  |  |  |  |  |  |  |  |  |  |  |  |  |  |  |  |  |  |  |  |  |  |  |  |  |  |  |  |  |  |  |  |  |  |  |  |  |  |  |  |  |  |  |  |  |  |  |  |  |  |  |  |  |  |  |  |  |  |  |  |  |  |  |  |  |  |  |  |  |  |  |  |  |  |  |  |  |  |  |  |  |  |  |  |  |  |  |  |  |  |  |  |  |  |  |  |  |  |  |  |  |  |  |  |  |  |  |  |  |  |  |  |  |  |  |  |  |  |  |  |  |  |  |  |  |  |  |  |  |  |  |  |  |  |  |  |  |  |  |  |  |  |  |  |  |  |  |  |  |  |  |  |  |  |  |  |  |  |  |  |  |  |  |  |  |  |  |  |  |  |  |  |  |  |  |  |  |  |  |  |  |  |  |  |  |  |  |  |  |  |  |  |  |  |  |  |  |  |  |  |  |  |  |  |  |  |  |  |  |  |  |  |  |  |  |  |  |  |  |  |  |  |  |  |  |  |  |  |  |  |  |  |  |  |  |  |  |  |  |  |  |  |  |  |  |  |  |  |  |  |  |  |  |  |  |  |  |  |  |  |  |  |  |  |  |  |  |  |  |  |  |  |  |  |  |  |  |  |  |  |  |  |  |  |  |  |  |  |  |  |  |  |  |  |  |  |  |  |  |  |  |  |  |  |  |  |  |  |  |  |  |  |  |  |  |  |  |  |  |  |  |  |  |  |  |  |  |  |  |  |  |  |  |  |  |  |  |  |  |  |  |  |  |  |  |  |  |  |  |  |  |  |  |  |  |  |  |  |  |  |  |  |  |  |  |  |  |  |  |  |  |  |  |  |  |  |  |  |  |  |  |  |  |  |  |  |  |  |  |  |  |  |  |  |  |  |  |  |  |  |  |  |  |  |  |  |  |  |  |  |  |  |  |  |  |  |  |  |  |  |  |  |  |  |  |  |  |  |  |  |  |  |  |  |  |  |  |  |  |  |  |  |  |  |  |  |  |  |  |  |  |  |  |  |  |  |  |  |  |  |  |  |  |  |  |  |  |  |  |  |  |  |  |  |  |  |  |  |  |  |  |  |  |  |  |  |  |  |  |  |  |  |  |  |  |  |  |  |  |  |  |  |  |  |  |  |  |  |  |  |  |  |  |  |  |  |  |  |  |  |  |  |  |  |  |  |  |  |  |  |  |  |  |  |  |  |  |  |  |  |  |  |  |  |  |  |  |  |  |  |  |  |  |  |  |  |  |  |  |  |  |  |  |  |  |  |  |  |  |  |  |  |  |  |  |  |  |  |  |  |  |  |  |  |  |  |  |  |  |  |  |  |  |  |  |  |  |  |  |  |  |  |  |  |  |  |  |  |  |  |  |  |  |  |  |  |  |  |  |  |  |  |  |  |  |  |  |  |  |  |  |  |  |  |  |  |  |  |  |  |  |  |  |  |  |  |  |  |  |  |  |  |  |  |  |  |  |  |  |  |  |  |  |  |  |  |  |  |  |  |  |  |  |  |  |  |  |  |  |  |  |  |  |  |  |  |  |  |  |  |  |  |  |  |  |  |  |  |  |  |  |  |  |  |  |  |  |  |  |  |  |  |  |  |  |  |  |  |  |  |  |  |  |  |  |  |  |  |  |  |  |  |  |  |  |  |  |  |  |  |  |  |  |  |  |  |  |  |  |  |  |  |  |  |  |  |  |  |  |  |  |  |  |  |  |  |  |  |  |  |  |  |  |  |  |  |  |  |  |  |  |  |  |  |  |  |  |  |  |  |  |  |  |  |  |  |  |  |  |  |  |  |  |  |  |  |  |  |  |  |  |  |  |  |  |  |  |  |  |  |  |  |  |  |  |  |  |  |  |  |  |  |  |  |  |  |  |  |  |  |  |  |  |  |  |  |  |  |  |  |  |  |  |  |  |  |  |  |  |  |  |  |  |  |  |  |  |  |  |  |  |  |  |  |  |  |  |  |  |  |  |  |  |  |  |  |  |  |  |  |  |  |  |  |  |  |  |  |  |  |  |  |  |  |  |  |  |  |  |  |  |  |  |  |  |  |  |  |  |  |  |  |  |  |  |  |  |  |  |  |  |  |  |  |  |  |  |  |  |  |  |  |  |  |  |  |  |  |  |  |  |  |  |  |  |  |  |  |  |  |  |  |  |  |  |  |  |  |  |  |  |  |  |  |  |  |  |  |  |  |  |  |  |  |  |  |  |  |  |  |  |  |  |  |  |  |  |  |  |  |  |  |  |  |  |  |  |  |  |  |  |  |  |  |  |  |  |  |  |  |  |  |  |  |  |  |  |  |  |  |  |  |  |  |  |  |  |  |  |  |  |  |  |  |  |  |  |  |  |  |  |  |  |  |  |  |  |  |  |  |  |  |  |  |  |  |  |  |  |  |  |  |  |  |  |  |  |  |  |  |  |  |  |  |  |  |  |  |  |  |  |  |  |  |  |  |  |  |  |  |  |  |  |  |  |  |  |  |  |  |  |  |  |  |  |  |  |  |  |  |  |  |  |  |  |  |  |  |  |  |  |  |  |  |  |  |  |  |  |  |  |  |  |  |  |  |  |  |  |  |  |  |  |  |  |  |  |  |  |  |  |  |  |  |  |  |  |  |  |  |  |  |  |  |  |  |  |  |  |  |  |  |  |  |  |  |  |  |  |  |  |  |  |  |  |  |  |  |  |  |  |  |  |  |  |  |  |  |  |  |  |  |  |  |  |  |  |  |  |  |  |  |  |  |  |  |  |  |  |  |  |  |  |  |  |  |  |  |  |  |  |  |  |  |  |  |  |  |  |  |  |  |  |  |  |  |  |  |  |  |  |  |  |  |  |  |  |  |  |  |  |  |  |  |  |  |  |  |  |  |  |  |  |  |  |  |  |  |  |  |  |  |  |  |  |  |  |  |  |  |  |  |  |  |  |  |  |  |  |  |  |  |  |  |  |  |  |  |  |  |  |  |  |  |  |  |  |  |  |  |  |  |  |  |  |  |  |  |  |  |  |  |  |  |  |  |  |  |  |  |  |  |  |  |  |  |  |  |  |  |  |  |  |  |  |  |  |  |  |  |  |  |  |  |  |  |  |  |  |  |  |  |  |  |  |  |  |  |  |  |  |  |  |  |  |  |  |  |  |  |  |  |  |  |  |  |  |  |  |  |  |  |  |  |  |  |  |  |  |  |  |  |  |  |  |  |  |  |  |  |  |  |  |  |  |  |  |  |  |  |  |  |  |  |  |  |  |  |  |  |  |  |  |  |  |  |  |  |  |  |  |  |  |  |  |  |  |  |  |  |  |  |  |  |  |  |  |  |  |  |  |  |  |  |  |  |  |  |  |  |  |  |  |  |  |  |  |  |  |  |  |  |  |  |  |  |  |  |  |  |  |  |  |  |  |  |  |  |  |  |  |  |  |  |  |  |  |  |  |  |  |  |  |  |  |  |  |  |  |  |  |  |  |  |  |  |  |  |  |  |  |  |  |  |  |  |  |  |  |  |  |  |  |  |  |  |  |  |  |  |  |  |  |  |  |  |  |  |  |  |  |  |  |  |  |  |  |  |  |  |  |  |  |  |  |  |  |  |  |  |  |  |  |  |  |  |  |  |  |  |  |  |  |  |  |  |  |  |  |  |  |  |  |  |  |  |  |  |  |  |  |  |  |  |  |  |  |  |  |  |  |  |  |  |  |  |  |  |  |  |  |  |  |  |  |  |  |  |  |  |  |  |  |  |  |  |  |  |  |  |  |  |  |  |  |  |  |  |  |  |  |  |  |  |  |  |  |  |  |  |  |  |  |  |  |  |  |  |  |  |  |  |  |  |  |  |  |  |  |  |  |  |  |  |  |  |  |  |  |  |  |  |  |  |  |  |  |  |  |  |  |  |  |  |  |  |  |  |  |  |  |  |  |  |  |  |  |  |  |  |  |  |  |  |  |  |  |  |  |  |  |  |  |  |  |  |  |  |  |  |  |  |  |  |  |  |  |  |  |  |  |  |  |  |  |  |  |  |  |  |  |  |  |  |  |  |  |  |  |  |  |  |  |  |  |  |  |  |  |  |  |  |  |  |  |  |  |  |  |  |  |  |  |  |  |  |  |  |  |  |  |  |  |  |  |  |  |  |  |  |  |  |  |  |  |  |  |  |  |  |  |  |  |  |  |  |  |  |  |  |  |  |  |  |  |  |  |  |  |  |  |  |  |  |  |  |  |  |  |  |  |  |  |  |  |  |  |  |  |  |  |  |  |  |  |  |  |  |  |  |  |  |  |  |  |  |  |  |  |  |  |  |  |  |  |  |  |  |  |  |  |  |  |  |  |  |  |  |  |  |  |  |  |  |  |  |  |  |  |  |  |  |  |  |  |  |  |  |  |  |  |  |  |  |  |  |  |  |  |  |  |  |  |  |  |  |  |  |  |  |  |  |  |  |  |  |  |  |  |  |  |  |  |  |  |  |  |  |  |  |  |  |  |  |  |  |  |  |  |  |  |  |  |  |  |  |  |  |  |  |  |  |  |  |  |  |  |  |  |  |  |  |  |  |  |  |  |  |  |  |  |  |  |  |  |  |  |  |  |  |  |  |  |  |  |  |  |  |  |  |  |  |  |  |  |  |  |  |  |  |  |  |  |  |  |  |  |  |  |  |  |  |  |  |  |  |  |  |  |  |  |  |  |  |  |  |  |  |  |  |  |  |  |  |  |  |  |  |  |  |  |  |  |  |  |  |  |  |  |  |  |  |  |  |  |  |  |  |  |  |  |  |  |  |  |  |  |  |  |  |  |  |  |  |  |  |  |  |  |  |  |  |  |  |  |  |  |  |  |  |  |  |  |  |  |  |  |  |  |  |  |  |  |  |  |  |  |  |  |  |  |  |  |  |  |  |  |  |  |  |  |  |  |  |  |  |  |  |  |  |  |  |  |  |  |  |  |  |  |  |  |  |  |  |  |  |  |  |  |  |  |  |  |  |  |  |  |  |  |  |  |  |  |  |  |  |  |  |  |  |  |  |  |  |  |  |  |  |  |  |  |  |  |  |  |  |  |  |  |  |  |  |  |  |  |  |  |  |  |  |  |  |  |  |  |  |  |  |  |  |  |  |  |  |  |  |  |  |  |  |  |  |  |  |  |  |  |  |  |  |  |  |  |  |  |  |  |  |  |  |  |  |  |  |  |  |  |  |  |  |  |  |  |  |  |  |  |  |  |  |  |  |  |  |  |  |  |  |  |  |  |  |  |  |  |  |  |  |  |  |  |  |  |  |  |  |  |  |  |  |  |  |  |  |  |  |  |  |  |  |  |  |  |  |  |  |  |  |  |  |  |  |  |  |  |  |  |  |  |  |  |  |  |  |  |  |  |  |  |  |  |  |  |  |  |  |  |  |  |  |  |  |  |  |  |  |  |  |  |  |  |  |  |  |  |  |  |  |  |  |  |  |  |  |  |  |  |  |  |  |  |  |  |  |  |  |  |  |  |  |  |  |  |  |  |  |  |  |  |  |  |  |  |  |  |  |  |  |  |  |  |  |  |  |  |  |  |  |  |  |  |  |  |  |  |  |  |  |  |  |  |  |  |  |  |  |  |  |  |  |  |  |  |  |  |  |  |  |  |  |  |  |  |  |  |  |  |  |  |  |  |  |  |  |  |  |  |  |  |  |  |  |  |  |  |  |  |  |  |  |  |  |  |  |  |  |  |  |  |  |  |  |  |  |  |  |  |  |  |  |  |  |  |  |  |  |  |  |  |  |  |  |  |  |  |  |  |  |  |  |  |  |  |  |  |  |  |  |  |  |  |  |  |  |  |  |  |  |  |  |  |  |  |  |  |  |  |  |  |  |  |  |  |  |  |  |  |  |  |  |  |  |  |  |  |  |  |  |  |  |  |  |  |  |  |  |  |  |  |  |  |  |  |  |  |  |  |  |  |  |  |  |  |  |  |  |  |  |  |  |  |  |  |  |  |  |  |  |  |  |  |  |  |  |  |  |  |  |  |  |  |  |  |  |  |  |  |  |  |  |  |  |  |  |  |  |  |  |  |  |  |  |  |  |  |  |  |  |  |  |  |  |  |  |  |  |  |  |  |  |  |  |  |  |  |  |  |  |  |  |  |  |  |  |  |  |  |  |  |  |  |  |  |  |  |  |  |  |  |  |  |  |  |  |  |  |  |  |  |  |  |  |  |  |  |  |  |  |  |  |  |  |  |  |  |  |  |  |  |  |  |  |  |  |  |  |  |  |  |  |  |  |  |  |  |  |  |  |  |  |  |  |  |  |  |  |  |  |  |  |  |  |  |  |  |  |  |  |  |  |  |  |  |  |  |  |  |  |  |  |  |  |  |  |  |  |  |  |  |  |  |  |  |  |  |  |  |  |  |  |  |  |  |  |  |  |  |  |  |  |  |  |  |  |  |  |  |  |  |  |  |  |  |  |  |  |  |  |  |  |  |  |  |  |  |  |  |  |  |  |  |  |  |  |  |  |  |  |  |  |  |  |  |  |  |  |  |  |  |  |  |  |  |  |  |  |  |  |  |  |  |  |  |  |  |  |  |  |  |  |  |  |  |  |  |  |  |  |  |  |  |  |  |  |  |  |  |  |  |  |  |  |  |  |  |  |  |  |  |  |  |  |  |  |  |  |  |  |  |  |  |  |  |  |  |  |  |  |  |  |  |  |  |  |  |  |  |  |  |  |  |  |  |  |  |  |  |  |  |  |  |  |  |  |  |  |  |  |  |  |  |  |  |  |  |  |  |  |  |  |  |  |  |  |  |  |  |  |  |  |  |  |  |  |  |  |  |  |  |  |  |  |  |  |  |  |  |  |  |  |  |  |  |  |  |  |  |  |  |  |  |  |  |  |  |  |  |  |  |  |  |  |  |  |  |  |  |  |  |  |  |  |  |  |  |  |  |  |  |  |  |  |  |  |  |  |  |  |  |  |  |  |  |  |  |  |  |  |  |  |  |  |  |  |  |  |  |  |  |  |  |  |  |  |  |  |  |  |  |  |  |  |  |  |  |  |  |  |  |  |  |  |  |  |  |  |  |  |  |  |  |  |  |  |  |  |  |  |  |  |  |  |  |  |  |  |  |  |  |  |  |  |  |  |  |  |  |  |  |  |  |  |  |  |  |  |  |  |  |  |  |  |  |  |  |  |  |  |  |  |  |  |  |  |  |  |  |  |  |  |  |  |  |  |  |  |  |  |  |  |  |  |  |  |  |  |  |  |  |  |  |  |  |  |  |  |  |  |  |  |  |  |  |  |  |  |  |  |  |  |  |  |  |  |  |  |  |  |  |  |  |  |  |  |  |  |  |  |  |  |  |  |  |  |  |  |  |  |  |  |  |  |  |  |  |  |  |  |  |  |  |  |  |  |  |  |  |  |  |  |  |  |  |  |  |  |  |  |  |  |  |  |  |  |  |  |  |  |  |  |  |  |  |  |  |  |  |  |  |  |  |  |  |  |  |  |  |  |  |  |  |  |  |  |  |  |  |  |  |  |  |  |  |  |  |  |  |  |  |  |  |  |  |  |  |  |  |  |  |  |  |  |  |  |  |  |  |  |  |  |  |  |  |  |  |  |  |  |  |  |  |  |  |  |  |  |  |  |  |  |  |  |  |  |  |  |  |  |  |  |  |  |  |  |  |  |  |  |  |  |  |  |  |  |  |  |  |  |  |  |  |  |  |  |  |  |  |  |  |  |  |  |  |  |  |  |  |  |  |  |  |  |  |  |  |  |  |  |  |  |  |  |  |  |  |  |  |  |  |  |  |  |  |  |  |  |  |  |  |  |  |  |  |  |  |  |  |  |  |  |  |  |  |  |  |  |  |  |  |  |  |  |  |  |  |  |  |  |  |  |  |  |  |  |  |  |  |  |  |  |  |  |  |  |  |  |  |  |  |  |  |  |  |  |  |  |  |  |  |  |  |  |  |  |  |  |  |  |  |  |  |  |  |  |  |  |  |  |  |  |  |  |  |  |  |  |  |  |  |  |  |  |  |  |  |  |  |  |  |  |  |  |  |  |  |  |  |  |  |  |  |  |  |  |  |  |  |  |  |  |  |  |  |  |  |  |  |  |  |  |  |  |  |  |  |  |  |  |  |  |  |  |  |  |  |  |  |  |  |  |  |  |  |  |  |  |  |  |  |  |  |  |  |  |  |  |  |  |  |  |  |  |  |  |  |  |  |  |  |  |  |  |  |  |  |  |  |  |  |  |  |  |  |  |  |  |  |  |  |  |  |  |  |  |  |  |  |  |  |  |  |  |  |  |  |  |  |  |  |  |  |  |  |  |  |  |  |  |  |  |  |  |  |  |  |  |  |  |  |  |  |  |  |  |  |  |  |  |  |  |  |  |  |  |  |  |  |  |  |  |  |  |  |  |  |  |  |  |  |  |  |  |  |  |  |  |  |  |  |  |  |  |  |  |  |  |  |  |  |  |  |  |  |  |  |  |  |  |  |  |  |  |  |  |  |  |  |  |  |  |  |  |  |  |  |  |  |  |  |  |  |  |  |  |  |  |  |  |  |  |  |  |  |  |  |  |  |  |  |  |  |  |  |  |  |  |
| --- | --- | --- | --- | --- | --- | --- | --- | --- | --- | --- | --- | --- | --- | --- | --- | --- | --- | --- | --- | --- | --- | --- | --- | --- | --- | --- | --- | --- | --- | --- | --- | --- | --- | --- | --- | --- | --- | --- | --- | --- | --- | --- | --- | --- | --- | --- | --- | --- | --- | --- | --- | --- | --- | --- | --- | --- | --- | --- | --- | --- | --- | --- | --- | --- | --- | --- | --- | --- | --- | --- | --- | --- | --- | --- | --- | --- | --- | --- | --- | --- | --- | --- | --- | --- | --- | --- | --- | --- | --- | --- | --- | --- | --- | --- | --- | --- | --- | --- | --- | --- | --- | --- | --- | --- | --- | --- | --- | --- | --- | --- | --- | --- | --- | --- | --- | --- | --- | --- | --- | --- | --- | --- | --- | --- | --- | --- | --- | --- | --- | --- | --- | --- | --- | --- | --- | --- | --- | --- | --- | --- | --- | --- | --- | --- | --- | --- | --- | --- | --- | --- | --- | --- | --- | --- | --- | --- | --- | --- | --- | --- | --- | --- | --- | --- | --- | --- | --- | --- | --- | --- | --- | --- | --- | --- | --- | --- | --- | --- | --- | --- | --- | --- | --- | --- | --- | --- | --- | --- | --- | --- | --- | --- | --- | --- | --- | --- | --- | --- | --- | --- | --- | --- | --- | --- | --- | --- | --- | --- | --- | --- | --- | --- | --- | --- | --- | --- | --- | --- | --- | --- | --- | --- | --- | --- | --- | --- | --- | --- | --- | --- | --- | --- | --- | --- | --- | --- | --- | --- | --- | --- | --- | --- | --- | --- | --- | --- | --- | --- | --- | --- | --- | --- | --- | --- | --- | --- | --- | --- | --- | --- | --- | --- | --- | --- | --- | --- | --- | --- | --- | --- | --- | --- | --- | --- | --- | --- | --- | --- | --- | --- | --- | --- | --- | --- | --- | --- | --- | --- | --- | --- | --- | --- | --- | --- | --- | --- | --- | --- | --- | --- | --- | --- | --- | --- | --- | --- | --- | --- | --- | --- | --- | --- | --- | --- | --- | --- | --- | --- | --- | --- | --- | --- | --- | --- | --- | --- | --- | --- | --- | --- | --- | --- | --- | --- | --- | --- | --- | --- | --- | --- | --- | --- | --- | --- | --- | --- | --- | --- | --- | --- | --- | --- | --- | --- | --- | --- | --- | --- | --- | --- | --- | --- | --- | --- | --- | --- | --- | --- | --- | --- | --- | --- | --- | --- | --- | --- | --- | --- | --- | --- | --- | --- | --- | --- | --- | --- | --- | --- | --- | --- | --- | --- | --- | --- | --- | --- | --- | --- | --- | --- | --- | --- | --- | --- | --- | --- | --- | --- | --- | --- | --- | --- | --- | --- | --- | --- | --- | --- | --- | --- | --- | --- | --- | --- | --- | --- | --- | --- | --- | --- | --- | --- | --- | --- | --- | --- | --- | --- | --- | --- | --- | --- | --- | --- | --- | --- | --- | --- | --- | --- | --- | --- | --- | --- | --- | --- | --- | --- | --- | --- | --- | --- | --- | --- | --- | --- | --- | --- | --- | --- | --- | --- | --- | --- | --- | --- | --- | --- | --- | --- | --- | --- | --- | --- | --- | --- | --- | --- | --- | --- | --- | --- | --- | --- | --- | --- | --- | --- | --- | --- | --- | --- | --- | --- | --- | --- | --- | --- | --- | --- | --- | --- | --- | --- | --- | --- | --- | --- | --- | --- | --- | --- | --- | --- | --- | --- | --- | --- | --- | --- | --- | --- | --- | --- | --- | --- | --- | --- | --- | --- | --- | --- | --- | --- | --- | --- | --- | --- | --- | --- | --- | --- | --- | --- | --- | --- | --- | --- | --- | --- | --- | --- | --- | --- | --- | --- | --- | --- | --- | --- | --- | --- | --- | --- | --- | --- | --- | --- | --- | --- | --- | --- | --- | --- | --- | --- | --- | --- | --- | --- | --- | --- | --- | --- | --- | --- | --- | --- | --- | --- | --- | --- | --- | --- | --- | --- | --- | --- | --- | --- | --- | --- | --- | --- | --- | --- | --- | --- | --- | --- | --- | --- | --- | --- | --- | --- | --- | --- | --- | --- | --- | --- | --- | --- | --- | --- | --- | --- | --- | --- | --- | --- | --- | --- | --- | --- | --- | --- | --- | --- | --- | --- | --- | --- | --- | --- | --- | --- | --- | --- | --- | --- | --- | --- | --- | --- | --- | --- | --- | --- | --- | --- | --- | --- | --- | --- | --- | --- | --- | --- | --- | --- | --- | --- | --- | --- | --- | --- | --- | --- | --- | --- | --- | --- | --- | --- | --- | --- | --- | --- | --- | --- | --- | --- | --- | --- | --- | --- | --- | --- | --- | --- | --- | --- | --- | --- | --- | --- | --- | --- | --- | --- | --- | --- | --- | --- | --- | --- | --- | --- | --- | --- | --- | --- | --- | --- | --- | --- | --- | --- | --- | --- | --- | --- | --- | --- | --- | --- | --- | --- | --- | --- | --- | --- | --- | --- | --- | --- | --- | --- | --- | --- | --- | --- | --- | --- | --- | --- | --- | --- | --- | --- | --- | --- | --- | --- | --- | --- | --- | --- | --- | --- | --- | --- | --- | --- | --- | --- | --- | --- | --- | --- | --- | --- | --- | --- | --- | --- | --- | --- | --- | --- | --- | --- | --- | --- | --- | --- | --- | --- | --- | --- | --- | --- | --- | --- | --- | --- | --- | --- | --- | --- | --- | --- | --- | --- | --- | --- | --- | --- | --- | --- | --- | --- | --- | --- | --- | --- | --- | --- | --- | --- | --- | --- | --- | --- | --- | --- | --- | --- | --- | --- | --- | --- | --- | --- | --- | --- | --- | --- | --- | --- | --- | --- | --- | --- | --- | --- | --- | --- | --- | --- | --- | --- | --- | --- | --- | --- | --- | --- | --- | --- | --- | --- | --- | --- | --- | --- | --- | --- | --- | --- | --- | --- | --- | --- | --- | --- | --- | --- | --- | --- | --- | --- | --- | --- | --- | --- | --- | --- | --- | --- | --- | --- | --- | --- | --- | --- | --- | --- | --- | --- | --- | --- | --- | --- | --- | --- | --- | --- | --- | --- | --- | --- | --- | --- | --- | --- | --- | --- | --- | --- | --- | --- | --- | --- | --- | --- | --- | --- | --- | --- | --- | --- | --- | --- | --- | --- | --- | --- | --- | --- | --- | --- | --- | --- | --- | --- | --- | --- | --- | --- | --- | --- | --- | --- | --- | --- | --- | --- | --- | --- | --- | --- | --- | --- | --- | --- | --- | --- | --- | --- | --- | --- | --- | --- | --- | --- | --- | --- | --- | --- | --- | --- | --- | --- | --- | --- | --- | --- | --- | --- | --- | --- | --- | --- | --- | --- | --- | --- | --- | --- | --- | --- | --- | --- | --- | --- | --- | --- | --- | --- | --- | --- | --- | --- | --- | --- | --- | --- | --- | --- | --- | --- | --- | --- | --- | --- | --- | --- | --- | --- | --- | --- | --- | --- | --- | --- | --- | --- | --- | --- | --- | --- | --- | --- | --- | --- | --- | --- | --- | --- | --- | --- | --- | --- | --- | --- | --- | --- | --- | --- | --- | --- | --- | --- | --- | --- | --- | --- | --- | --- | --- | --- | --- | --- | --- | --- | --- | --- | --- | --- | --- | --- | --- | --- | --- | --- | --- | --- | --- | --- | --- | --- | --- | --- | --- | --- | --- | --- | --- | --- | --- | --- | --- | --- | --- | --- | --- | --- | --- | --- | --- | --- | --- | --- | --- | --- | --- | --- | --- | --- | --- | --- | --- | --- | --- | --- | --- | --- | --- | --- | --- | --- | --- | --- | --- | --- | --- | --- | --- | --- | --- | --- | --- | --- | --- | --- | --- | --- | --- | --- | --- | --- | --- | --- | --- | --- | --- | --- | --- | --- | --- | --- | --- | --- | --- | --- | --- | --- | --- | --- | --- | --- | --- | --- | --- | --- | --- | --- | --- | --- | --- | --- | --- | --- | --- | --- | --- | --- | --- | --- | --- | --- | --- | --- | --- | --- | --- | --- | --- | --- | --- | --- | --- | --- | --- | --- | --- | --- | --- | --- | --- | --- | --- | --- | --- | --- | --- | --- | --- | --- | --- | --- | --- | --- | --- | --- | --- | --- | --- | --- | --- | --- | --- | --- | --- | --- | --- | --- | --- | --- | --- | --- | --- | --- | --- | --- | --- | --- | --- | --- | --- | --- | --- | --- | --- | --- | --- | --- | --- | --- | --- | --- | --- | --- | --- | --- | --- | --- | --- | --- | --- | --- | --- | --- | --- | --- | --- | --- | --- | --- | --- | --- | --- | --- | --- | --- | --- | --- | --- | --- | --- | --- | --- | --- | --- | --- | --- | --- | --- | --- | --- | --- | --- | --- | --- | --- | --- | --- | --- | --- | --- | --- | --- | --- | --- | --- | --- | --- | --- | --- | --- | --- | --- | --- | --- | --- | --- | --- | --- | --- | --- | --- | --- | --- | --- | --- | --- | --- | --- | --- | --- | --- | --- | --- | --- | --- | --- | --- | --- | --- | --- | --- | --- | --- | --- | --- | --- | --- | --- | --- | --- | --- | --- | --- | --- | --- | --- | --- | --- | --- | --- | --- | --- | --- | --- | --- | --- | --- | --- | --- | --- | --- | --- | --- | --- | --- | --- | --- | --- | --- | --- | --- | --- | --- | --- | --- | --- | --- | --- | --- | --- | --- | --- | --- | --- | --- | --- | --- | --- | --- | --- | --- | --- | --- | --- | --- | --- | --- | --- | --- | --- | --- | --- | --- | --- | --- | --- | --- | --- | --- | --- | --- | --- | --- | --- | --- | --- | --- | --- | --- | --- | --- | --- | --- | --- | --- | --- | --- | --- | --- | --- | --- | --- | --- | --- | --- | --- | --- | --- | --- | --- | --- | --- | --- | --- | --- | --- | --- | --- | --- | --- | --- | --- | --- | --- | --- | --- | --- | --- | --- | --- | --- | --- | --- | --- | --- | --- | --- | --- | --- | --- | --- | --- | --- | --- | --- | --- | --- | --- | --- | --- | --- | --- | --- | --- | --- | --- | --- | --- | --- | --- | --- | --- | --- | --- | --- | --- | --- | --- | --- | --- | --- | --- | --- | --- | --- | --- | --- | --- | --- | --- | --- | --- | --- | --- | --- | --- | --- | --- | --- | --- | --- | --- | --- | --- | --- | --- | --- | --- | --- | --- | --- | --- | --- | --- | --- | --- | --- | --- | --- | --- | --- | --- | --- | --- | --- | --- | --- | --- | --- | --- | --- | --- | --- | --- | --- | --- | --- | --- | --- | --- | --- | --- | --- | --- | --- | --- | --- | --- | --- | --- | --- | --- | --- | --- | --- | --- | --- | --- | --- | --- | --- | --- | --- | --- | --- | --- | --- | --- | --- | --- | --- | --- | --- | --- | --- | --- | --- | --- | --- | --- | --- | --- | --- | --- | --- | --- | --- | --- | --- | --- | --- | --- | --- | --- | --- | --- | --- | --- | --- | --- | --- | --- | --- | --- | --- | --- | --- | --- | --- | --- | --- | --- | --- | --- | --- | --- | --- | --- | --- | --- | --- | --- | --- | --- | --- | --- | --- | --- | --- | --- | --- | --- | --- | --- | --- | --- | --- | --- | --- | --- | --- | --- | --- | --- | --- | --- | --- | --- | --- | --- | --- | --- | --- | --- | --- | --- | --- | --- | --- | --- | --- | --- | --- | --- | --- | --- | --- | --- | --- | --- | --- | --- | --- | --- | --- | --- | --- | --- | --- | --- | --- | --- | --- | --- | --- | --- | --- | --- | --- | --- | --- | --- | --- | --- | --- | --- | --- | --- | --- | --- | --- | --- | --- | --- | --- | --- | --- | --- | --- | --- | --- | --- | --- | --- | --- | --- | --- | --- | --- | --- | --- | --- | --- | --- | --- | --- | --- | --- | --- | --- | --- | --- | --- | --- | --- | --- | --- | --- | --- | --- | --- | --- | --- | --- | --- | --- | --- | --- | --- | --- | --- | --- | --- | --- | --- | --- | --- | --- | --- | --- | --- | --- | --- | --- | --- | --- | --- | --- | --- | --- | --- | --- | --- | --- | --- | --- | --- | --- | --- | --- | --- | --- | --- | --- | --- | --- | --- | --- | --- | --- | --- | --- | --- | --- | --- | --- | --- | --- | --- | --- | --- | --- | --- | --- | --- | --- | --- | --- | --- | --- | --- | --- | --- | --- | --- | --- | --- | --- | --- | --- | --- | --- | --- | --- | --- | --- | --- | --- | --- | --- | --- | --- | --- | --- | --- | --- | --- | --- | --- | --- | --- | --- | --- | --- | --- | --- | --- | --- | --- | --- | --- | --- | --- | --- | --- | --- | --- | --- | --- | --- | --- | --- | --- | --- | --- | --- | --- | --- | --- | --- | --- | --- | --- | --- | --- | --- | --- | --- | --- | --- | --- | --- | --- | --- | --- | --- | --- | --- | --- | --- | --- | --- | --- | --- | --- | --- | --- | --- | --- | --- | --- | --- | --- | --- | --- | --- | --- | --- | --- | --- | --- | --- | --- | --- | --- | --- | --- | --- | --- | --- | --- | --- | --- | --- | --- | --- | --- | --- | --- | --- | --- | --- | --- | --- | --- | --- | --- | --- | --- | --- | --- | --- | --- | --- | --- | --- | --- | --- | --- | --- | --- | --- | --- | --- | --- | --- | --- | --- | --- | --- | --- | --- | --- | --- | --- | --- | --- | --- | --- | --- | --- | --- | --- | --- | --- | --- | --- | --- | --- | --- | --- | --- | --- | --- | --- | --- | --- | --- | --- | --- | --- | --- | --- | --- | --- | --- | --- | --- | --- | --- | --- | --- | --- | --- | --- | --- | --- | --- | --- | --- | --- | --- | --- | --- | --- | --- | --- | --- | --- | --- | --- | --- | --- | --- | --- | --- | --- | --- | --- | --- | --- | --- | --- | --- | --- | --- | --- | --- | --- | --- | --- | --- | --- | --- | --- | --- | --- | --- | --- | --- | --- | --- | --- | --- | --- | --- | --- | --- | --- | --- | --- | --- | --- | --- | --- | --- | --- | --- | --- | --- | --- | --- | --- | --- | --- | --- | --- | --- | --- | --- | --- | --- | --- | --- | --- | --- | --- | --- | --- | --- | --- | --- | --- | --- | --- | --- | --- | --- | --- | --- | --- | --- | --- | --- | --- | --- | --- | --- | --- | --- | --- | --- | --- | --- | --- | --- | --- | --- | --- | --- | --- | --- | --- | --- | --- | --- | --- | --- | --- | --- | --- | --- | --- | --- | --- | --- | --- | --- | --- | --- | --- | --- | --- | --- | --- | --- | --- | --- | --- | --- | --- | --- | --- | --- | --- | --- | --- | --- | --- | --- | --- | --- | --- | --- | --- | --- | --- | --- | --- | --- | --- | --- | --- | --- | --- | --- | --- | --- | --- | --- | --- | --- | --- | --- | --- | --- | --- | --- | --- | --- | --- | --- | --- | --- | --- | --- | --- | --- | --- | --- | --- | --- | --- | --- | --- | --- | --- | --- | --- | --- | --- | --- | --- | --- | --- | --- | --- | --- | --- | --- | --- | --- | --- | --- | --- | --- | --- | --- | --- | --- | --- | --- | --- | --- | --- | --- | --- | --- | --- | --- | --- | --- | --- | --- | --- | --- | --- | --- | --- | --- | --- | --- | --- | --- | --- | --- | --- | --- | --- | --- | --- | --- | --- | --- | --- | --- | --- | --- | --- | --- | --- | --- | --- | --- | --- | --- | --- | --- | --- | --- | --- | --- | --- | --- | --- | --- | --- | --- | --- | --- | --- | --- | --- | --- | --- | --- | --- | --- | --- | --- | --- | --- | --- | --- | --- | --- | --- | --- | --- | --- | --- | --- | --- | --- | --- | --- | --- | --- | --- | --- | --- | --- | --- | --- | --- | --- | --- | --- | --- | --- | --- | --- | --- | --- | --- | --- | --- | --- | --- | --- | --- | --- | --- | --- | --- | --- | --- | --- | --- | --- | --- | --- | --- | --- | --- | --- | --- | --- | --- | --- | --- | --- | --- | --- | --- | --- | --- | --- | --- | --- | --- | --- | --- | --- | --- | --- | --- | --- | --- | --- | --- | --- | --- | --- | --- | --- | --- | --- | --- | --- | --- | --- | --- | --- | --- | --- | --- | --- | --- | --- | --- | --- | --- | --- | --- | --- | --- | --- | --- | --- | --- | --- | --- | --- | --- | --- | --- | --- | --- | --- | --- | --- | --- | --- | --- | --- | --- | --- | --- | --- | --- | --- | --- | --- | --- | --- | --- | --- | --- | --- | --- | --- | --- | --- | --- | --- | --- | --- | --- | --- | --- | --- | --- | --- | --- | --- | --- | --- | --- | --- | --- | --- | --- | --- | --- | --- | --- | --- | --- | --- | --- | --- | --- | --- | --- | --- | --- | --- | --- | --- | --- | --- | --- | --- | --- | --- | --- | --- | --- | --- | --- | --- | --- | --- | --- | --- | --- | --- | --- | --- | --- | --- | --- | --- | --- | --- | --- | --- | --- | --- | --- | --- | --- | --- | --- | --- | --- | --- | --- | --- | --- | --- | --- | --- | --- | --- | --- | --- | --- | --- | --- | --- | --- | --- | --- | --- | --- | --- | --- | --- | --- | --- | --- | --- | --- | --- | --- | --- | --- | --- | --- | --- | --- | --- | --- | --- | --- | --- | --- | --- | --- | --- | --- | --- | --- | --- | --- | --- | --- | --- | --- | --- | --- | --- | --- | --- | --- | --- | --- | --- | --- | --- | --- | --- | --- | --- | --- | --- | --- | --- | --- | --- | --- | --- | --- | --- | --- | --- | --- | --- | --- | --- | --- | --- | --- | --- | --- | --- | --- | --- | --- | --- | --- | --- | --- | --- | --- | --- | --- | --- | --- | --- | --- | --- | --- | --- | --- | --- | --- | --- | --- | --- | --- | --- | --- | --- | --- | --- | --- | --- | --- | --- | --- | --- | --- | --- | --- | --- | --- | --- | --- | --- | --- | --- | --- | --- | --- | --- | --- | --- | --- | --- | --- | --- | --- | --- | --- | --- | --- | --- | --- | --- | --- | --- | --- | --- | --- | --- | --- | --- | --- | --- | --- | --- | --- | --- | --- | --- | --- | --- | --- | --- | --- | --- | --- | --- | --- | --- | --- | --- | --- | --- | --- | --- | --- | --- | --- | --- | --- | --- | --- | --- | --- | --- | --- | --- | --- | --- | --- | --- | --- | --- | --- | --- | --- | --- | --- | --- | --- | --- | --- | --- | --- | --- | --- | --- | --- | --- | --- | --- | --- | --- | --- | --- | --- | --- | --- | --- | --- | --- | --- | --- | --- | --- | --- | --- | --- | --- | --- | --- | --- | --- | --- | --- | --- | --- | --- | --- | --- | --- | --- | --- | --- | --- | --- | --- | --- | --- | --- | --- | --- | --- | --- | --- | --- | --- | --- | --- | --- | --- | --- | --- | --- | --- | --- | --- | --- | --- | --- | --- | --- | --- | --- | --- | --- | --- | --- | --- | --- | --- | --- | --- | --- | --- | --- | --- | --- | --- | --- | --- | --- | --- | --- | --- | --- | --- | --- | --- | --- | --- | --- | --- | --- | --- | --- | --- | --- | --- | --- | --- | --- | --- | --- | --- | --- | --- | --- | --- | --- | --- | --- | --- | --- | --- | --- | --- | --- | --- | --- | --- | --- | --- | --- | --- | --- | --- | --- | --- | --- | --- | --- | --- | --- | --- | --- | --- | --- | --- | --- | --- | --- | --- | --- | --- | --- | --- | --- | --- | --- | --- | --- | --- | --- | --- | --- | --- | --- | --- | --- | --- | --- | --- | --- | --- | --- | --- | --- | --- | --- | --- | --- | --- | --- | --- | --- | --- | --- | --- | --- | --- | --- | --- | --- | --- | --- | --- | --- | --- | --- | --- | --- | --- | --- | --- | --- | --- | --- | --- | --- | --- | --- | --- | --- | --- | --- | --- | --- | --- | --- | --- | --- | --- | --- | --- | --- | --- | --- | --- | --- | --- | --- | --- | --- | --- | --- | --- | --- | --- | --- | --- | --- | --- | --- | --- | --- | --- | --- | --- | --- | --- | --- | --- | --- | --- | --- | --- | --- | --- | --- | --- | --- | --- | --- | --- | --- | --- | --- | --- | --- | --- | --- | --- | --- | --- | --- | --- | --- | --- | --- | --- | --- | --- | --- | --- | --- | --- | --- | --- | --- | --- | --- | --- | --- | --- | --- | --- | --- | --- | --- | --- | --- | --- | --- | --- | --- | --- | --- | --- | --- | --- | --- | --- | --- | --- | --- | --- | --- | --- | --- | --- | --- | --- | --- | --- | --- | --- | --- | --- | --- | --- | --- | --- | --- | --- | --- | --- | --- | --- | --- | --- | --- | --- | --- | --- | --- | --- | --- | --- | --- | --- | --- | --- | --- | --- | --- | --- | --- | --- | --- | --- | --- | --- | --- | --- | --- | --- | --- | --- | --- | --- | --- | --- | --- | --- | --- | --- | --- | --- | --- | --- | --- | --- | --- | --- | --- | --- | --- | --- | --- | --- | --- | --- | --- | --- | --- | --- | --- | --- | --- | --- | --- | --- | --- | --- | --- | --- | --- | --- | --- | --- | --- | --- | --- | --- | --- | --- | --- | --- | --- | --- | --- | --- | --- | --- | --- | --- | --- | --- | --- | --- | --- | --- | --- | --- | --- | --- | --- | --- | --- | --- | --- | --- | --- | --- | --- | --- | --- | --- | --- | --- | --- | --- | --- | --- | --- | --- | --- | --- | --- | --- | --- | --- | --- | --- | --- | --- | --- | --- | --- | --- | --- | --- | --- | --- | --- | --- | --- | --- | --- | --- | --- | --- | --- | --- | --- | --- | --- | --- | --- | --- | --- | --- | --- | --- | --- | --- | --- | --- | --- | --- | --- | --- | --- | --- | --- | --- | --- | --- | --- | --- | --- | --- | --- | --- | --- | --- | --- | --- | --- | --- | --- | --- | --- | --- | --- | --- | --- | --- | --- | --- | --- | --- | --- | --- | --- | --- | --- | --- | --- | --- | --- | --- | --- | --- | --- | --- | --- | --- | --- | --- | --- | --- | --- | --- | --- | --- | --- | --- | --- | --- | --- | --- | --- | --- | --- | --- | --- | --- | --- | --- | --- | --- | --- | --- | --- | --- | --- | --- | --- | --- | --- | --- | --- | --- | --- | --- | --- | --- | --- | --- | --- | --- | --- | --- | --- | --- | --- | --- | --- | --- | --- | --- | --- | --- | --- | --- | --- | --- | --- | --- | --- | --- | --- | --- | --- | --- | --- | --- | --- | --- | --- | --- | --- | --- | --- | --- | --- | --- | --- | --- | --- | --- | --- | --- | --- | --- | --- | --- | --- | --- | --- | --- | --- | --- | --- | --- | --- | --- | --- | --- | --- | --- | --- | --- | --- | --- | --- | --- | --- | --- | --- | --- | --- | --- | --- | --- | --- | --- | --- | --- | --- | --- | --- | --- | --- | --- | --- | --- | --- | --- | --- | --- | --- | --- | --- | --- | --- | --- | --- | --- | --- | --- | --- | --- | --- | --- | --- | --- | --- | --- | --- | --- | --- | --- | --- | --- | --- | --- | --- | --- | --- | --- | --- | --- | --- | --- | --- | --- | --- | --- | --- | --- | --- | --- | --- | --- | --- | --- | --- | --- | --- | --- | --- | --- | --- | --- | --- | --- | --- | --- | --- | --- | --- | --- | --- | --- | --- | --- | --- | --- | --- | --- | --- | --- | --- | --- | --- | --- | --- | --- | --- | --- | --- | --- | --- | --- | --- | --- | --- | --- | --- | --- | --- | --- | --- | --- | --- | --- | --- | --- | --- | --- | --- | --- | --- | --- | --- | --- | --- | --- | --- | --- | --- | --- | --- | --- | --- | --- | --- | --- | --- | --- | --- | --- | --- | --- | --- | --- | --- | --- | --- | --- | --- | --- | --- | --- | --- | --- | --- | --- | --- | --- | --- | --- | --- | --- | --- | --- | --- | --- | --- | --- | --- | --- | --- | --- | --- | --- | --- | --- | --- | --- | --- | --- | --- | --- | --- | --- | --- | --- | --- | --- | --- | --- | --- | --- | --- | --- | --- | --- | --- | --- | --- | --- | --- | --- | --- | --- | --- | --- | --- | --- | --- | --- | --- | --- | --- | --- | --- | --- | --- | --- | --- | --- | --- | --- | --- | --- | --- | --- | --- | --- | --- | --- | --- | --- | --- | --- | --- | --- | --- | --- | --- | --- | --- | --- | --- | --- | --- | --- | --- | --- | --- | --- | --- | --- | --- | --- | --- | --- | --- | --- | --- | --- | --- | --- | --- | --- | --- | --- | --- | --- | --- | --- | --- | --- | --- | --- | --- | --- | --- | --- | --- | --- | --- | --- | --- | --- | --- | --- | --- | --- | --- | --- | --- | --- | --- | --- | --- | --- | --- | --- | --- | --- | --- | --- | --- | --- | --- | --- | --- | --- | --- | --- | --- | --- | --- | --- | --- | --- | --- | --- | --- | --- | --- | --- | --- | --- | --- | --- | --- | --- | --- | --- | --- | --- | --- | --- | --- | --- | --- | --- | --- | --- | --- | --- | --- | --- | --- | --- | --- | --- | --- | --- | --- | --- | --- | --- | --- | --- | --- | --- | --- | --- | --- | --- | --- | --- | --- | --- | --- | --- | --- | --- | --- | --- | --- | --- | --- | --- | --- | --- | --- | --- | --- | --- | --- | --- | --- | --- | --- | --- | --- | --- | --- | --- | --- | --- | --- | --- | --- | --- | --- | --- | --- | --- | --- | --- | --- | --- | --- | --- | --- | --- | --- | --- | --- | --- | --- | --- | --- | --- | --- | --- | --- | --- | --- | --- | --- | --- | --- | --- | --- | --- | --- | --- | --- | --- | --- | --- | --- | --- | --- | --- | --- | --- | --- | --- | --- | --- | --- | --- | --- | --- | --- | --- | --- | --- | --- | --- | --- | --- | --- | --- | --- | --- | --- | --- | --- | --- | --- | --- | --- | --- | --- | --- | --- | --- | --- | --- | --- | --- | --- | --- | --- | --- | --- | --- | --- | --- | --- | --- | --- | --- | --- | --- | --- | --- | --- | --- | --- | --- | --- | --- | --- | --- | --- | --- | --- | --- | --- | --- | --- | --- | --- | --- | --- | --- | --- | --- | --- | --- | --- | --- | --- | --- | --- | --- | --- | --- | --- | --- | --- | --- | --- | --- | --- | --- | --- | --- | --- | --- | --- | --- | --- | --- | --- | --- | --- | --- | --- | --- | --- | --- | --- | --- | --- | --- | --- | --- | --- | --- | --- | --- | --- | --- | --- | --- | --- | --- | --- | --- | --- | --- | --- | --- | --- | --- | --- | --- | --- | --- | --- | --- | --- | --- | --- | --- | --- | --- | --- | --- | --- | --- | --- | --- | --- | --- | --- | --- | --- | --- | --- | --- | --- | --- | --- | --- | --- | --- | --- | --- | --- | --- | --- | --- | --- | --- | --- | --- | --- | --- | --- | --- | --- | --- | --- | --- | --- | --- | --- | --- | --- | --- | --- | --- | --- | --- | --- | --- | --- | --- | --- | --- | --- | --- | --- | --- | --- | --- | --- | --- | --- | --- | --- | --- | --- | --- | --- | --- | --- | --- | --- | --- | --- | --- | --- | --- | --- | --- | --- | --- | --- | --- | --- | --- | --- | --- | --- | --- | --- | --- | --- | --- | --- | --- | --- | --- | --- | --- | --- | --- | --- | --- | --- | --- | --- | --- | --- | --- | --- | --- | --- | --- | --- | --- | --- | --- | --- | --- | --- | --- | --- | --- | --- | --- | --- | --- | --- | --- | --- | --- | --- | --- | --- | --- | --- | --- | --- | --- | --- | --- | --- | --- | --- | --- | --- | --- | --- | --- | --- | --- | --- | --- | --- | --- | --- | --- | --- | --- | --- | --- | --- | --- | --- | --- | --- | --- | --- | --- | --- | --- | --- | --- | --- | --- | --- | --- | --- | --- | --- | --- | --- | --- | --- | --- | --- | --- | --- | --- | --- | --- | --- | --- | --- | --- | --- | --- | --- | --- | --- | --- | --- | --- | --- | --- | --- | --- | --- | --- | --- | --- | --- | --- | --- | --- | --- | --- | --- | --- | --- | --- | --- | --- | --- | --- | --- | --- | --- | --- | --- | --- | --- | --- | --- | --- | --- | --- | --- | --- | --- | --- | --- | --- | --- | --- | --- | --- | --- | --- | --- | --- | --- | --- | --- | --- | --- | --- | --- | --- | --- | --- | --- | --- | --- | --- | --- | --- | --- | --- | --- | --- | --- | --- | --- | --- | --- | --- | --- | --- | --- | --- | --- | --- | --- | --- | --- | --- | --- | --- | --- | --- | --- | --- | --- | --- | --- | --- | --- | --- | --- | --- | --- | --- | --- | --- | --- | --- | --- | --- | --- | --- | --- | --- | --- | --- | --- | --- | --- | --- | --- | --- | --- | --- | --- | --- | --- | --- | --- | --- | --- | --- | --- | --- | --- | --- | --- | --- | --- | --- | --- | --- | --- | --- | --- | --- | --- | --- | --- | --- | --- | --- | --- | --- | --- | --- | --- | --- | --- | --- | --- | --- | --- | --- | --- | --- | --- | --- | --- | --- | --- | --- | --- | --- | --- | --- | --- | --- | --- | --- | --- | --- | --- | --- | --- | --- | --- | --- | --- | --- | --- | --- | --- | --- | --- | --- | --- | --- | --- | --- | --- | --- | --- | --- | --- | --- | --- | --- | --- | --- | --- | --- | --- | --- | --- | --- | --- | --- | --- | --- | --- | --- | --- | --- | --- | --- | --- | --- | --- | --- | --- | --- | --- | --- | --- | --- | --- | --- | --- | --- | --- | --- | --- | --- | --- | --- | --- | --- | --- | --- | --- | --- | --- | --- | --- | --- | --- | --- | --- | --- | --- | --- | --- | --- | --- | --- | --- | --- | --- | --- | --- | --- | --- | --- | --- | --- | --- | --- | --- | --- | --- | --- | --- | --- | --- | --- | --- | --- | --- | --- | --- | --- | --- | --- | --- | --- | --- | --- | --- | --- | --- | --- | --- | --- | --- | --- | --- | --- | --- | --- | --- | --- | --- | --- | --- | --- | --- | --- | --- | --- | --- | --- | --- | --- | --- | --- | --- | --- | --- | --- | --- | --- | --- | --- | --- | --- | --- | --- | --- | --- | --- | --- | --- | --- | --- | --- | --- | --- | --- | --- | --- | --- | --- | --- | --- | --- | --- | --- | --- | --- | --- | --- | --- | --- | --- | --- | --- | --- | --- | --- | --- | --- | --- | --- | --- | --- | --- | --- | --- | --- | --- | --- | --- | --- | --- | --- | --- | --- | --- | --- | --- | --- | --- | --- | --- | --- | --- | --- | --- | --- | --- | --- | --- | --- | --- | --- | --- | --- | --- | --- | --- | --- | --- | --- | --- | --- | --- | --- | --- | --- | --- | --- | --- | --- | --- | --- | --- | --- | --- | --- | --- | --- | --- | --- | --- | --- | --- | --- | --- | --- | --- | --- | --- | --- | --- | --- | --- | --- | --- | --- | --- | --- | --- | --- | --- | --- | --- | --- | --- | --- | --- | --- | --- | --- | --- | --- | --- | --- | --- | --- | --- | --- | --- | --- | --- | --- | --- | --- | --- | --- | --- | --- | --- | --- | --- | --- | --- | --- | --- | --- | --- | --- | --- | --- | --- | --- | --- | --- | --- | --- | --- | --- | --- | --- | --- | --- | --- | --- | --- | --- | --- | --- | --- | --- | --- | --- | --- | --- | --- | --- | --- | --- | --- | --- | --- | --- | --- | --- | --- | --- | --- | --- | --- | --- | --- | --- | --- | --- | --- | --- | --- | --- | --- | --- | --- | --- | --- | --- | --- | --- | --- | --- | --- | --- | --- | --- | --- | --- | --- | --- | --- | --- | --- | --- | --- | --- | --- | --- | --- | --- | --- | --- | --- | --- | --- | --- | --- | --- | --- | --- | --- | --- | --- | --- | --- | --- | --- | --- | --- | --- | --- | --- | --- | --- | --- | --- | --- | --- | --- | --- | --- | --- | --- | --- | --- | --- | --- | --- | --- | --- | --- | --- | --- | --- | --- | --- | --- | --- | --- | --- | --- | --- | --- | --- | --- | --- | --- | --- | --- | --- | --- | --- | --- | --- | --- | --- | --- | --- | --- | --- | --- | --- | --- | --- | --- | --- | --- | --- | --- | --- | --- | --- | --- | --- | --- | --- | --- | --- | --- | --- | --- | --- | --- | --- | --- | --- | --- | --- | --- | --- | --- | --- | --- | --- | --- | --- | --- | --- | --- | --- | --- | --- | --- | --- | --- | --- | --- | --- | --- | --- | --- | --- | --- | --- | --- | --- | --- | --- | --- | --- | --- | --- | --- | --- | --- | --- | --- | --- | --- | --- | --- | --- | --- | --- | --- | --- | --- | --- | --- | --- | --- | --- | --- | --- | --- | --- | --- | --- | --- | --- | --- | --- | --- | --- | --- | --- | --- | --- | --- | --- | --- | --- | --- | --- | --- | --- | --- | --- | --- | --- | --- | --- | --- | --- | --- | --- | --- | --- | --- | --- | --- | --- | --- | --- | --- | --- | --- | --- | --- | --- | --- | --- | --- | --- | --- | --- | --- | --- | --- | --- | --- | --- | --- | --- | --- | --- | --- | --- | --- | --- | --- | --- | --- | --- | --- | --- | --- | --- | --- | --- | --- | --- | --- | --- | --- | --- | --- | --- | --- | --- | --- | --- | --- | --- | --- | --- | --- | --- | --- | --- | --- | --- | --- | --- | --- | --- | --- | --- | --- | --- | --- | --- | --- | --- | --- | --- | --- | --- | --- | --- | --- | --- | --- | --- | --- | --- | --- | --- | --- | --- | --- | --- | --- | --- | --- | --- | --- | --- | --- | --- | --- | --- | --- | --- | --- | --- | --- | --- | --- | --- | --- | --- | --- | --- | --- | --- | --- | --- | --- | --- | --- | --- | --- | --- | --- | --- | --- | --- | --- | --- | --- | --- | --- | --- | --- | --- | --- | --- | --- | --- | --- | --- | --- | --- | --- | --- | --- | --- | --- | --- | --- | --- | --- | --- | --- | --- | --- | --- | --- | --- | --- | --- | --- | --- | --- | --- | --- | --- | --- | --- | --- | --- | --- | --- | --- | --- | --- | --- | --- | --- | --- | --- | --- | --- | --- | --- | --- | --- | --- | --- | --- | --- | --- | --- | --- | --- | --- | --- | --- | --- | --- | --- | --- | --- | --- | --- | --- | --- | --- | --- | --- | --- | --- | --- | --- | --- | --- | --- | --- | --- | --- | --- | --- | --- | --- | --- | --- | --- | --- | --- | --- | --- | --- | --- | --- | --- | --- | --- | --- | --- | --- | --- | --- | --- | --- | --- | --- | --- | --- | --- | --- | --- | --- | --- | --- | --- | --- | --- | --- | --- | --- | --- | --- | --- | --- | --- | --- | --- | --- | --- | --- | --- | --- | --- | --- | --- | --- | --- | --- | --- | --- | --- | --- | --- | --- | --- | --- | --- | --- | --- | --- | --- | --- | --- | --- | --- | --- | --- | --- | --- | --- | --- | --- | --- | --- | --- | --- | --- | --- | --- | --- | --- | --- | --- | --- | --- | --- | --- | --- | --- | --- | --- | --- | --- | --- | --- | --- | --- | --- | --- | --- | --- | --- | --- | --- | --- | --- | --- | --- | --- | --- | --- | --- | --- | --- | --- | --- | --- | --- | --- | --- | --- | --- | --- | --- | --- | --- | --- | --- | --- | --- | --- | --- | --- | --- | --- | --- | --- | --- | --- | --- | --- | --- | --- | --- | --- | --- | --- | --- | --- | --- | --- | --- | --- | --- | --- | --- | --- | --- | --- | --- | --- | --- | --- | --- | --- | --- | --- | --- | --- | --- | --- | --- | --- | --- | --- | --- | --- | --- | --- | --- | --- | --- | --- | --- | --- | --- | --- | --- | --- | --- | --- | --- | --- | --- | --- | --- | --- | --- | --- | --- | --- | --- | --- | --- | --- | --- | --- | --- | --- | --- | --- | --- | --- | --- | --- | --- | --- | --- | --- | --- | --- | --- | --- | --- | --- | --- | --- | --- | --- | --- | --- | --- | --- | --- | --- | --- | --- | --- | --- | --- | --- | --- | --- | --- | --- | --- | --- | --- | --- | --- | --- | --- | --- | --- | --- | --- | --- | --- | --- | --- | --- | --- | --- | --- | --- | --- | --- | --- | --- | --- | --- | --- | --- | --- | --- | --- | --- | --- | --- | --- | --- | --- | --- | --- | --- | --- | --- | --- | --- | --- | --- | --- | --- | --- | --- | --- | --- | --- | --- | --- | --- | --- | --- | --- | --- | --- | --- | --- | --- | --- | --- | --- | --- | --- | --- | --- | --- | --- | --- | --- | --- | --- | --- | --- | --- | --- | --- | --- | --- | --- | --- | --- | --- | --- | --- | --- | --- | --- | --- | --- | --- | --- | --- | --- | --- | --- | --- | --- | --- | --- | --- | --- | --- | --- | --- | --- | --- | --- | --- | --- | --- | --- | --- | --- | --- | --- | --- | --- | --- | --- | --- | --- | --- | --- | --- | --- | --- | --- | --- | --- | --- | --- | --- | --- | --- | --- | --- | --- | --- | --- | --- | --- | --- | --- | --- | --- | --- | --- | --- | --- | --- | --- | --- | --- | --- | --- | --- | --- | --- | --- | --- | --- | --- | --- | --- | --- | --- | --- | --- | --- | --- | --- | --- | --- | --- | --- | --- | --- | --- | --- | --- | --- | --- | --- | --- | --- | --- | --- | --- | --- | --- | --- | --- | --- | --- | --- | --- | --- | --- | --- | --- | --- | --- | --- | --- | --- | --- | --- | --- | --- | --- | --- | --- | --- | --- | --- | --- | --- | --- | --- | --- | --- | --- | --- | --- | --- | --- | --- | --- | --- | --- | --- | --- | --- | --- | --- | --- | --- | --- | --- | --- | --- | --- | --- | --- | --- | --- | --- | --- | --- | --- | --- | --- | --- | --- | --- | --- | --- | --- | --- | --- | --- | --- | --- | --- | --- | --- | --- | --- | --- | --- | --- | --- | --- | --- | --- | --- | --- | --- | --- | --- | --- | --- | --- | --- | --- | --- | --- | --- | --- | --- | --- | --- | --- | --- | --- | --- | --- | --- | --- | --- | --- | --- | --- | --- | --- | --- | --- | --- | --- | --- | --- | --- | --- | --- | --- | --- | --- | --- | --- | --- | --- | --- | --- | --- | --- | --- | --- | --- | --- | --- | --- | --- | --- | --- | --- | --- | --- | --- | --- | --- | --- | --- | --- | --- | --- | --- | --- | --- | --- | --- | --- | --- | --- | --- | --- | --- | --- | --- | --- | --- | --- | --- | --- | --- | --- | --- | --- | --- | --- | --- | --- | --- | --- | --- | --- | --- | --- | --- | --- | --- | --- | --- | --- | --- | --- | --- | --- | --- | --- | --- | --- | --- | --- | --- | --- | --- | --- | --- | --- | --- | --- | --- | --- | --- | --- | --- | --- | --- | --- | --- | --- | --- | --- | --- | --- | --- | --- | --- | --- | --- | --- | --- | --- | --- | --- | --- | --- | --- | --- | --- | --- | --- | --- | --- | --- | --- | --- | --- | --- | --- | --- | --- | --- | --- | --- | --- | --- | --- | --- | --- | --- | --- | --- | --- | --- | --- | --- | --- | --- | --- | --- | --- | --- | --- | --- | --- | --- | --- | --- | --- | --- | --- | --- | --- | --- | --- | --- | --- | --- | --- | --- | --- | --- | --- | --- | --- | --- | --- | --- | --- | --- | --- | --- | --- | --- | --- | --- | --- | --- | --- | --- | --- | --- | --- | --- | --- | --- | --- | --- | --- | --- | --- | --- | --- | --- | --- | --- | --- | --- | --- | --- | --- | --- | --- | --- | --- | --- | --- | --- | --- | --- | --- | --- | --- | --- | --- | --- | --- | --- | --- | --- | --- | --- | --- | --- | --- | --- | --- | --- | --- | --- | --- | --- | --- | --- | --- | --- | --- | --- | --- | --- | --- | --- | --- | --- | --- | --- | --- | --- | --- | --- | --- | --- | --- | --- | --- | --- | --- | --- | --- | --- | --- | --- | --- | --- | --- | --- | --- | --- | --- | --- | --- | --- | --- | --- | --- | --- | --- | --- | --- | --- | --- | --- | --- | --- | --- | --- | --- | --- | --- | --- | --- | --- | --- | --- | --- | --- | --- | --- | --- | --- | --- | --- | --- | --- | --- | --- | --- | --- | --- | --- | --- | --- | --- | --- | --- | --- | --- | --- | --- | --- | --- | --- | --- | --- | --- | --- | --- | --- | --- | --- | --- | --- | --- | --- | --- | --- | --- | --- | --- | --- | --- | --- | --- | --- | --- | --- | --- | --- | --- | --- | --- | --- | --- | --- | --- | --- | --- | --- | --- | --- | --- | --- | --- | --- | --- | --- | --- | --- | --- | --- | --- | --- | --- | --- | --- | --- | --- | --- | --- | --- | --- | --- | --- | --- | --- | --- | --- | --- | --- | --- | --- | --- | --- | --- | --- | --- | --- | --- | --- | --- | --- | --- | --- | --- | --- | --- | --- | --- | --- | --- | --- | --- | --- | --- | --- | --- | --- | --- | --- | --- | --- | --- | --- | --- | --- | --- | --- | --- | --- | --- | --- | --- | --- | --- | --- | --- | --- | --- | --- | --- | --- | --- | --- | --- | --- | --- | --- | --- | --- | --- | --- | --- | --- | --- | --- | --- | --- | --- | --- | --- | --- | --- | --- | --- | --- | --- | --- | --- | --- | --- | --- | --- | --- | --- | --- | --- | --- | --- | --- | --- | --- | --- | --- | --- | --- | --- | --- | --- | --- | --- | --- | --- | --- | --- | --- | --- | --- | --- | --- | --- | --- | --- | --- | --- | --- | --- | --- | --- | --- | --- | --- | --- | --- | --- | --- | --- | --- | --- | --- | --- | --- | --- | --- | --- | --- | --- | --- | --- | --- | --- | --- | --- | --- | --- | --- | --- | --- | --- | --- | --- | --- | --- | --- | --- | --- | --- | --- | --- | --- | --- | --- | --- | --- | --- | --- | --- | --- | --- | --- | --- | --- | --- | --- | --- | --- | --- | --- | --- | --- | --- | --- | --- | --- | --- | --- | --- | --- | --- | --- | --- | --- | --- | --- | --- | --- | --- | --- | --- | --- | --- | --- | --- | --- | --- | --- | --- | --- | --- | --- | --- | --- | --- | --- | --- | --- | --- | --- | --- | --- | --- | --- | --- | --- | --- | --- | --- | --- | --- | --- | --- | --- | --- | --- | --- | --- | --- | --- | --- | --- | --- | --- | --- | --- | --- | --- | --- | --- | --- | --- | --- | --- | --- | --- | --- | --- | --- | --- | --- | --- | --- | --- | --- | --- | --- | --- | --- | --- | --- | --- | --- | --- | --- | --- | --- | --- | --- | --- | --- | --- | --- | --- | --- | --- | --- | --- | --- | --- | --- | --- | --- | --- | --- | --- | --- | --- | --- | --- | --- | --- | --- | --- | --- | --- | --- | --- | --- | --- | --- | --- | --- | --- | --- | --- | --- | --- | --- | --- | --- | --- | --- | --- | --- | --- | --- | --- | --- | --- | --- | --- | --- | --- | --- | --- | --- | --- | --- | --- | --- | --- | --- | --- | --- | --- | --- | --- | --- | --- | --- | --- | --- | --- | --- | --- | --- | --- | --- | --- | --- | --- | --- | --- | --- | --- | --- | --- | --- | --- | --- | --- | --- | --- | --- | --- | --- | --- | --- | --- | --- | --- | --- | --- | --- | --- | --- | --- | --- | --- | --- | --- | --- | --- | --- | --- | --- | --- | --- | --- | --- | --- | --- | --- | --- | --- | --- | --- | --- | --- | --- | --- | --- | --- | --- | --- | --- | --- | --- | --- | --- | --- | --- | --- | --- | --- | --- | --- | --- | --- | --- | --- | --- | --- | --- | --- | --- | --- | --- | --- | --- | --- | --- | --- | --- | --- | --- | --- | --- | --- | --- | --- | --- | --- | --- | --- | --- | --- | --- | --- | --- | --- | --- | --- | --- | --- | --- | --- | --- | --- | --- | --- | --- | --- | --- | --- | --- | --- | --- | --- | --- | --- | --- | --- | --- | --- | --- | --- | --- | --- | --- | --- | --- | --- | --- | --- | --- | --- | --- | --- | --- | --- | --- | --- | --- | --- | --- | --- | --- | --- | --- | --- | --- | --- | --- | --- | --- | --- | --- | --- | --- | --- | --- | --- | --- | --- | --- | --- | --- | --- | --- | --- | --- | --- | --- | --- | --- | --- | --- | --- | --- | --- | --- | --- | --- | --- | --- | --- | --- | --- | --- | --- | --- | --- | --- | --- | --- | --- | --- | --- | --- | --- | --- | --- | --- | --- | --- | --- | --- | --- | --- | --- | --- | --- | --- | --- | --- | --- | --- | --- | --- | --- | --- | --- | --- | --- | --- | --- | --- | --- | --- | --- | --- | --- | --- | --- | --- | --- | --- | --- | --- | --- | --- | --- | --- | --- | --- | --- | --- | --- | --- | --- | --- | --- | --- | --- | --- | --- | --- | --- | --- | --- | --- | --- | --- | --- | --- | --- | --- | --- | --- | --- | --- | --- | --- | --- | --- | --- | --- | --- | --- | --- | --- | --- | --- | --- | --- | --- | --- | --- | --- | --- | --- | --- | --- | --- | --- | --- | --- | --- | --- | --- | --- | --- | --- | --- | --- | --- | --- | --- | --- | --- | --- | --- | --- | --- | --- | --- | --- | --- | --- | --- | --- | --- | --- | --- | --- | --- | --- | --- | --- | --- | --- | --- | --- | --- | --- | --- | --- | --- | --- | --- | --- | --- | --- | --- | --- | --- | --- | --- | --- | --- | --- | --- | --- | --- | --- | --- | --- | --- | --- | --- | --- | --- | --- | --- | --- | --- | --- | --- | --- | --- | --- | --- | --- | --- | --- | --- | --- | --- | --- | --- | --- | --- | --- | --- | --- | --- | --- | --- | --- | --- | --- | --- | --- | --- | --- | --- | --- | --- | --- | --- | --- | --- | --- | --- | --- | --- | --- | --- | --- | --- | --- | --- | --- | --- | --- | --- | --- | --- | --- | --- | --- | --- | --- | --- | --- | --- | --- | --- | --- | --- | --- | --- | --- | --- | --- | --- | --- | --- | --- | --- | --- | --- | --- | --- | --- | --- | --- | --- | --- | --- | --- | --- | --- | --- | --- | --- | --- | --- | --- | --- | --- | --- | --- | --- | --- | --- | --- | --- | --- | --- | --- | --- | --- | --- | --- | --- | --- | --- | --- | --- | --- | --- | --- | --- | --- | --- | --- | --- | --- | --- | --- | --- | --- | --- | --- | --- | --- | --- | --- | --- | --- | --- | --- | --- | --- | --- | --- | --- | --- | --- | --- | --- | --- | --- | --- | --- | --- | --- | --- | --- | --- | --- | --- | --- | --- | --- | --- | --- | --- | --- | --- | --- | --- | --- | --- | --- | --- | --- | --- | --- | --- | --- | --- | --- | --- | --- | --- | --- | --- | --- | --- | --- | --- | --- | --- | --- | --- | --- | --- | --- | --- | --- | --- | --- | --- | --- | --- | --- | --- | --- | --- | --- | --- | --- | --- | --- | --- | --- | --- | --- | --- | --- | --- | --- | --- | --- | --- | --- | --- | --- | --- | --- | --- | --- | --- | --- | --- | --- | --- | --- | --- | --- | --- | --- | --- | --- | --- | --- | --- | --- | --- | --- | --- | --- | --- | --- | --- | --- | --- | --- | --- | --- | --- | --- | --- | --- | --- | --- | --- | --- | --- | --- | --- | --- | --- | --- | --- | --- | --- | --- | --- | --- | --- | --- | --- | --- | --- | --- | --- | --- | --- | --- | --- | --- | --- | --- | --- | --- | --- | --- | --- | --- | --- | --- | --- | --- | --- | --- | --- | --- | --- | --- | --- | --- | --- | --- | --- | --- | --- | --- | --- | --- | --- | --- | --- | --- | --- | --- | --- | --- | --- | --- | --- | --- | --- | --- | --- | --- | --- | --- | --- | --- | --- | --- | --- | --- | --- | --- | --- | --- | --- | --- | --- | --- | --- | --- | --- | --- | --- | --- | --- | --- | --- | --- | --- | --- | --- | --- | --- | --- | --- | --- | --- | --- | --- | --- | --- | --- | --- | --- | --- | --- | --- | --- | --- | --- | --- | --- | --- | --- | --- | --- | --- | --- | --- | --- | --- | --- | --- | --- | --- | --- | --- | --- | --- | --- | --- | --- | --- | --- | --- | --- | --- | --- | --- | --- | --- | --- | --- | --- | --- | --- | --- | --- | --- | --- | --- | --- | --- | --- | --- | --- | --- | --- | --- | --- | --- | --- | --- | --- | --- | --- | --- | --- | --- | --- | --- | --- | --- | --- | --- | --- | --- | --- | --- | --- | --- | --- | --- | --- | --- | --- | --- | --- | --- | --- | --- | --- | --- | --- | --- | --- | --- | --- | --- | --- | --- | --- | --- | --- | --- | --- | --- | --- | --- | --- | --- | --- | --- | --- | --- | --- | --- | --- | --- | --- | --- | --- | --- | --- | --- | --- | --- | --- | --- | --- | --- | --- | --- | --- | --- | --- | --- | --- | --- | --- | --- | --- | --- | --- | --- | --- | --- | --- | --- | --- | --- | --- | --- | --- | --- | --- | --- | --- | --- | --- | --- | --- | --- | --- | --- | --- | --- | --- | --- | --- | --- | --- | --- | --- | --- | --- | --- | --- | --- | --- | --- | --- | --- | --- | --- | --- | --- | --- | --- | --- | --- | --- | --- | --- | --- | --- | --- | --- | --- | --- | --- | --- | --- | --- | --- | --- | --- | --- | --- | --- | --- | --- | --- | --- | --- | --- | --- | --- | --- | --- | --- | --- | --- | --- | --- | --- | --- | --- | --- | --- | --- | --- | --- | --- | --- | --- | --- | --- | --- | --- | --- | --- | --- | --- | --- | --- | --- | --- | --- | --- | --- | --- | --- | --- | --- | --- | --- | --- | --- | --- | --- | --- | --- | --- | --- | --- | --- | --- | --- | --- | --- | --- | --- | --- | --- | --- | --- | --- | --- | --- | --- | --- | --- | --- | --- | --- | --- | --- | --- | --- | --- | --- | --- | --- | --- | --- | --- | --- | --- | --- | --- | --- | --- | --- | --- | --- | --- | --- | --- | --- | --- | --- | --- | --- | --- | --- | --- | --- | --- | --- | --- | --- | --- | --- | --- | --- | --- | --- | --- | --- | --- | --- | --- | --- | --- | --- | --- | --- | --- | --- | --- | --- | --- | --- | --- | --- | --- | --- | --- | --- | --- | --- | --- | --- | --- | --- | --- | --- | --- | --- | --- | --- | --- | --- | --- | --- | --- | --- | --- | --- | --- | --- | --- | --- | --- | --- | --- | --- | --- | --- | --- | --- | --- | --- | --- | --- | --- | --- | --- | --- | --- | --- | --- | --- | --- | --- | --- | --- | --- | --- | --- | --- | --- | --- | --- | --- | --- | --- | --- | --- | --- | --- | --- | --- | --- | --- | --- | --- | --- | --- | --- | --- | --- | --- | --- | --- | --- | --- | --- | --- | --- | --- | --- | --- | --- | --- | --- | --- | --- | --- | --- | --- | --- | --- | --- | --- | --- | --- | --- | --- | --- | --- | --- | --- | --- | --- | --- | --- | --- | --- | --- | --- | --- | --- | --- | --- | --- | --- | --- | --- | --- | --- | --- | --- | --- | --- | --- | --- | --- | --- | --- | --- | --- | --- | --- | --- | --- | --- | --- | --- | --- | --- | --- | --- | --- | --- | --- | --- | --- | --- | --- | --- | --- | --- | --- | --- | --- | --- | --- | --- | --- | --- | --- | --- | --- | --- | --- | --- | --- | --- | --- | --- | --- | --- | --- | --- | --- | --- | --- | --- | --- | --- | --- | --- | --- | --- | --- | --- | --- | --- | --- | --- | --- | --- | --- | --- | --- | --- | --- | --- | --- | --- | --- | --- | --- | --- | --- | --- | --- | --- | --- | --- | --- | --- | --- | --- | --- | --- | --- | --- | --- | --- | --- | --- | --- | --- | --- | --- | --- | --- | --- | --- | --- | --- | --- | --- | --- | --- | --- | --- | --- | --- | --- | --- | --- | --- | --- | --- | --- | --- | --- | --- | --- | --- | --- | --- | --- | --- | --- | --- | --- | --- | --- | --- | --- | --- | --- | --- | --- | --- | --- | --- | --- | --- | --- | --- | --- | --- | --- | --- | --- | --- | --- | --- | --- | --- | --- | --- | --- | --- | --- | --- | --- | --- | --- | --- | --- | --- | --- | --- | --- | --- | --- | --- | --- | --- | --- | --- | --- | --- | --- | --- | --- | --- | --- | --- | --- | --- | --- | --- | --- | --- | --- | --- | --- | --- | --- | --- | --- | --- | --- | --- | --- | --- | --- | --- | --- | --- | --- | --- | --- | --- | --- | --- | --- | --- | --- | --- | --- | --- | --- | --- | --- | --- | --- | --- | --- | --- | --- | --- | --- | --- | --- | --- | --- | --- | --- | --- | --- | --- | --- | --- | --- | --- | --- | --- | --- | --- | --- | --- | --- | --- | --- | --- | --- | --- | --- | --- | --- | --- | --- | --- | --- | --- | --- | --- | --- | --- | --- | --- | --- | --- | --- | --- | --- | --- | --- | --- | --- | --- | --- | --- | --- | --- | --- | --- | --- | --- | --- | --- | --- | --- | --- | --- | --- | --- | --- | --- | --- | --- | --- | --- | --- | --- | --- | --- | --- | --- | --- | --- | --- | --- | --- | --- | --- | --- | --- | --- | --- | --- | --- | --- | --- | --- | --- | --- | --- | --- | --- | --- | --- | --- | --- | --- | --- | --- | --- | --- | --- | --- | --- | --- | --- | --- | --- | --- | --- | --- | --- | --- | --- | --- | --- | --- | --- | --- | --- | --- | --- | --- | --- | --- | --- | --- | --- | --- | --- | --- | --- | --- | --- | --- | --- | --- | --- | --- | --- | --- | --- | --- | --- | --- | --- | --- | --- | --- | --- | --- | --- | --- | --- | --- | --- | --- | --- | --- | --- | --- | --- | --- | --- | --- | --- | --- | --- | --- | --- | --- | --- | --- | --- | --- | --- | --- | --- | --- | --- | --- | --- | --- | --- | --- | --- | --- | --- | --- | --- | --- | --- | --- | --- | --- | --- | --- | --- | --- | --- | --- | --- | --- | --- | --- | --- | --- | --- | --- | --- | --- | --- | --- | --- | --- | --- | --- | --- | --- | --- | --- | --- | --- | --- | --- | --- | --- | --- | --- | --- | --- | --- | --- | --- | --- | --- | --- | --- | --- | --- | --- | --- | --- | --- | --- | --- | --- | --- | --- | --- | --- | --- | --- | --- | --- | --- | --- | --- | --- | --- | --- | --- | --- | --- | --- | --- | --- | --- | --- | --- | --- | --- | --- | --- | --- | --- | --- | --- | --- | --- | --- | --- | --- | --- | --- | --- | --- | --- | --- | --- | --- | --- | --- | --- | --- | --- | --- | --- | --- | --- | --- | --- | --- | --- | --- | --- | --- | --- | --- | --- | --- | --- | --- | --- | --- | --- | --- | --- | --- | --- | --- | --- | --- | --- | --- | --- | --- | --- | --- | --- | --- | --- | --- | --- | --- | --- | --- | --- | --- | --- | --- | --- | --- | --- | --- | --- | --- | --- | --- | --- | --- | --- | --- | --- | --- | --- | --- | --- | --- | --- | --- | --- | --- | --- | --- | --- | --- | --- | --- | --- | --- | --- | --- | --- | --- | --- | --- | --- | --- | --- | --- | --- | --- | --- | --- | --- | --- | --- | --- | --- | --- | --- | --- | --- | --- | --- | --- | --- | --- | --- | --- | --- | --- | --- | --- | --- | --- | --- | --- | --- | --- | --- | --- | --- | --- | --- | --- | --- | --- | --- | --- | --- | --- | --- | --- | --- | --- | --- | --- | --- | --- | --- | --- | --- | --- | --- | --- | --- | --- | --- | --- | --- | --- | --- | --- | --- | --- | --- | --- | --- | --- | --- | --- | --- | --- | --- | --- | --- | --- | --- | --- | --- | --- | --- | --- | --- | --- | --- | --- | --- | --- | --- | --- | --- | --- | --- | --- | --- | --- | --- | --- | --- | --- | --- | --- | --- | --- | --- | --- | --- | --- | --- | --- | --- | --- | --- | --- | --- | --- | --- | --- | --- | --- | --- | --- | --- | --- | --- | --- | --- | --- | --- | --- | --- | --- | --- | --- | --- | --- | --- | --- | --- | --- | --- | --- | --- | --- | --- | --- | --- | --- | --- | --- | --- | --- | --- | --- | --- | --- | --- | --- | --- | --- | --- | --- | --- | --- | --- | --- | --- | --- | --- | --- | --- | --- | --- | --- | --- | --- | --- | --- | --- | --- | --- | --- | --- | --- | --- | --- | --- | --- | --- | --- | --- | --- | --- | --- | --- | --- | --- | --- | --- | --- | --- | --- | --- | --- | --- | --- | --- | --- | --- | --- | --- | --- | --- | --- | --- | --- | --- | --- | --- | --- | --- | --- | --- | --- | --- | --- | --- | --- | --- | --- | --- | --- | --- | --- | --- | --- | --- | --- | --- | --- | --- | --- | --- | --- | --- | --- | --- | --- | --- | --- | --- | --- | --- | --- | --- | --- | --- | --- | --- | --- | --- | --- | --- | --- | --- | --- | --- | --- | --- | --- | --- | --- | --- | --- | --- | --- | --- | --- | --- | --- | --- | --- | --- | --- | --- | --- | --- | --- | --- | --- | --- | --- | --- | --- | --- | --- | --- | --- | --- | --- | --- | --- | --- | --- | --- | --- | --- | --- | --- | --- | --- | --- | --- | --- | --- | --- | --- | --- | --- | --- | --- | --- | --- | --- | --- | --- | --- | --- | --- | --- | --- | --- | --- | --- | --- | --- | --- | --- | --- | --- | --- | --- | --- | --- | --- | --- | --- | --- | --- | --- | --- | --- | --- | --- | --- | --- | --- | --- | --- | --- | --- | --- | --- | --- | --- | --- | --- | --- | --- | --- | --- | --- | --- | --- | --- | --- | --- | --- | --- | --- | --- | --- | --- | --- | --- | --- | --- | --- | --- | --- | --- | --- | --- | --- | --- | --- | --- | --- | --- | --- | --- | --- | --- | --- | --- | --- | --- | --- | --- | --- | --- | --- | --- | --- | --- | --- | --- | --- | --- | --- | --- | --- | --- | --- | --- | --- | --- | --- | --- | --- | --- | --- | --- | --- | --- | --- | --- | --- | --- | --- | --- | --- | --- | --- | --- | --- | --- | --- | --- | --- | --- | --- | --- | --- | --- | --- | --- | --- | --- | --- | --- | --- | --- | --- | --- | --- | --- | --- | --- | --- | --- | --- | --- | --- | --- | --- | --- | --- | --- | --- | --- | --- | --- | --- | --- | --- | --- | --- | --- | --- | --- | --- | --- | --- | --- | --- | --- | --- | --- | --- | --- | --- | --- | --- | --- | --- | --- | --- | --- | --- | --- | --- | --- | --- | --- | --- | --- | --- | --- | --- | --- | --- | --- | --- | --- | --- | --- | --- | --- | --- | --- | --- | --- | --- | --- | --- | --- | --- | --- | --- | --- | --- | --- | --- | --- | --- | --- | --- | --- | --- | --- | --- | --- | --- | --- | --- | --- | --- | --- | --- | --- | --- | --- | --- | --- | --- | --- | --- | --- | --- | --- | --- | --- | --- | --- | --- | --- | --- | --- | --- | --- | --- | --- | --- | --- | --- | --- | --- | --- | --- | --- | --- | --- | --- | --- | --- | --- | --- | --- | --- | --- | --- | --- | --- | --- | --- | --- | --- | --- | --- | --- | --- | --- | --- | --- | --- | --- | --- | --- | --- | --- | --- | --- | --- | --- | --- | --- | --- | --- | --- | --- | --- | --- | --- | --- | --- | --- | --- | --- | --- | --- | --- | --- | --- | --- | --- | --- | --- | --- | --- | --- | --- | --- | --- | --- | --- | --- | --- | --- | --- | --- | --- | --- | --- | --- | --- | --- | --- | --- | --- | --- | --- | --- | --- | --- | --- | --- | --- | --- | --- | --- | --- | --- | --- | --- | --- | --- | --- | --- | --- | --- | --- | --- | --- | --- | --- | --- | --- | --- | --- | --- | --- | --- | --- | --- | --- | --- | --- | --- | --- | --- | --- | --- | --- | --- | --- | --- | --- | --- | --- | --- | --- | --- | --- | --- | --- | --- | --- | --- | --- | --- | --- | --- | --- | --- | --- | --- | --- | --- | --- | --- | --- | --- | --- | --- | --- | --- | --- | --- | --- | --- | --- | --- | --- | --- | --- | --- | --- | --- | --- | --- | --- | --- | --- | --- | --- | --- | --- | --- | --- | --- | --- | --- | --- | --- | --- | --- | --- | --- | --- | --- | --- | --- | --- | --- | --- | --- | --- | --- | --- | --- | --- | --- | --- | --- | --- | --- | --- | --- | --- | --- | --- | --- | --- | --- | --- | --- | --- | --- | --- | --- | --- | --- | --- | --- | --- | --- | --- | --- | --- | --- | --- | --- | --- | --- | --- | --- | --- | --- | --- | --- | --- | --- | --- | --- | --- | --- | --- | --- | --- | --- | --- | --- | --- | --- | --- | --- | --- | --- | --- | --- | --- | --- | --- | --- | --- | --- | --- | --- | --- | --- | --- | --- | --- | --- | --- | --- | --- | --- | --- | --- | --- | --- | --- | --- | --- | --- | --- | --- | --- | --- | --- | --- | --- | --- | --- | --- | --- | --- | --- | --- | --- | --- | --- | --- | --- | --- | --- | --- | --- | --- | --- | --- | --- | --- | --- | --- | --- | --- | --- | --- | --- | --- | --- | --- | --- | --- | --- | --- | --- | --- | --- | --- | --- | --- | --- | --- | --- | --- | --- | --- | --- | --- | --- | --- | --- | --- | --- | --- | --- | --- | --- | --- | --- | --- | --- | --- | --- | --- | --- | --- | --- | --- | --- | --- | --- | --- | --- | --- | --- | --- | --- | --- | --- | --- | --- | --- | --- | --- | --- | --- | --- | --- | --- | --- | --- | --- | --- | --- | --- | --- | --- | --- | --- | --- | --- | --- | --- | --- | --- | --- | --- | --- | --- | --- | --- | --- | --- | --- | --- | --- | --- | --- | --- | --- | --- | --- | --- | --- | --- | --- | --- | --- | --- | --- | --- | --- | --- | --- | --- | --- | --- | --- | --- | --- | --- | --- | --- | --- | --- | --- | --- | --- | --- | --- | --- | --- | --- | --- | --- | --- | --- | --- | --- | --- | --- | --- | --- | --- | --- | --- | --- | --- | --- | --- | --- | --- | --- | --- | --- | --- | --- | --- | --- | --- | --- | --- | --- | --- | --- | --- | --- | --- | --- | --- | --- | --- | --- | --- | --- | --- | --- | --- | --- | --- | --- | --- | --- | --- | --- | --- | --- | --- | --- | --- | --- | --- | --- | --- | --- | --- | --- | --- | --- | --- | --- | --- | --- | --- | --- | --- | --- | --- | --- | --- | --- | --- | --- | --- | --- | --- | --- | --- | --- | --- | --- | --- | --- | --- | --- | --- | --- | --- | --- | --- | --- | --- | --- | --- | --- | --- | --- | --- | --- | --- | --- | --- | --- | --- | --- | --- | --- | --- | --- | --- | --- | --- | --- | --- | --- | --- | --- | --- | --- | --- | --- | --- | --- | --- | --- | --- | --- | --- | --- | --- | --- | --- | --- | --- | --- | --- | --- | --- | --- | --- | --- | --- | --- | --- | --- | --- | --- | --- | --- | --- | --- | --- | --- | --- | --- | --- | --- | --- | --- | --- | --- | --- | --- | --- | --- | --- | --- | --- | --- | --- | --- | --- | --- | --- | --- | --- | --- | --- | --- | --- | --- | --- | --- | --- | --- | --- | --- | --- | --- | --- | --- | --- | --- | --- | --- | --- | --- | --- | --- | --- | --- | --- | --- | --- | --- | --- | --- | --- | --- | --- | --- | --- | --- | --- | --- | --- | --- | --- | --- | --- | --- | --- | --- | --- | --- | --- | --- | --- | --- | --- | --- | --- | --- | --- | --- | --- | --- | --- | --- | --- | --- | --- | --- | --- | --- | --- | --- | --- | --- | --- | --- | --- | --- | --- | --- | --- | --- | --- | --- | --- | --- | --- | --- | --- | --- | --- | --- | --- | --- | --- | --- | --- | --- | --- | --- | --- | --- | --- | --- | --- | --- | --- | --- | --- | --- | --- | --- | --- | --- | --- | --- | --- | --- | --- | --- | --- | --- | --- | --- | --- | --- | --- | --- | --- | --- | --- | --- | --- | --- | --- | --- | --- | --- | --- | --- | --- | --- | --- | --- | --- | --- | --- | --- | --- | --- | --- | --- | --- | --- | --- | --- | --- | --- | --- | --- | --- | --- | --- | --- | --- | --- | --- | --- | --- | --- | --- | --- | --- | --- | --- | --- | --- | --- | --- | --- | --- | --- | --- | --- | --- | --- | --- | --- | --- | --- | --- | --- | --- | --- | --- | --- | --- | --- | --- | --- | --- | --- | --- | --- | --- | --- | --- | --- | --- | --- | --- | --- | --- | --- | --- | --- | --- | --- | --- | --- | --- | --- | --- | --- | --- | --- | --- | --- | --- | --- | --- | --- | --- | --- | --- | --- | --- | --- | --- | --- | --- | --- | --- | --- | --- | --- | --- | --- | --- | --- | --- | --- | --- | --- | --- | --- | --- | --- | --- | --- | --- | --- | --- | --- | --- | --- | --- | --- | --- | --- | --- | --- | --- | --- | --- | --- | --- | --- | --- | --- | --- | --- | --- | --- | --- | --- | --- | --- | --- | --- | --- | --- | --- | --- | --- | --- | --- | --- | --- | --- | --- | --- | --- | --- | --- | --- | --- | --- | --- | --- | --- | --- | --- | --- | --- | --- | --- | --- | --- | --- | --- | --- | --- | --- | --- | --- | --- | --- | --- | --- | --- | --- | --- | --- | --- | --- | --- | --- | --- | --- | --- | --- | --- | --- | --- | --- | --- | --- | --- | --- | --- | --- | --- | --- | --- | --- | --- | --- | --- | --- | --- | --- | --- | --- | --- | --- | --- | --- | --- | --- | --- | --- | --- | --- | --- | --- | --- | --- | --- | --- | --- | --- | --- | --- | --- | --- | --- | --- | --- | --- | --- | --- | --- | --- | --- | --- | --- | --- | --- | --- | --- | --- | --- | --- | --- | --- | --- | --- | --- | --- | --- | --- | --- | --- | --- | --- | --- | --- | --- | --- | --- | --- | --- | --- | --- | --- | --- | --- | --- | --- | --- | --- | --- | --- | --- | --- | --- | --- | --- | --- | --- | --- | --- | --- | --- | --- | --- | --- | --- | --- | --- | --- | --- | --- | --- | --- | --- | --- | --- | --- | --- | --- | --- | --- | --- | --- | --- | --- | --- | --- | --- | --- | --- | --- | --- | --- | --- | --- | --- | --- | --- | --- | --- | --- | --- | --- | --- | --- | --- | --- | --- | --- | --- | --- | --- | --- | --- | --- | --- | --- | --- | --- | --- | --- | --- | --- | --- | --- | --- | --- | --- | --- | --- | --- | --- | --- | --- | --- | --- | --- | --- | --- | --- | --- | --- | --- | --- | --- | --- | --- | --- | --- | --- | --- | --- | --- | --- | --- | --- | --- | --- | --- | --- | --- | --- | --- | --- | --- | --- | --- | --- | --- | --- | --- | --- | --- | --- | --- | --- | --- | --- | --- | --- | --- | --- | --- | --- | --- | --- | --- | --- | --- | --- | --- | --- | --- | --- | --- | --- | --- | --- | --- | --- | --- | --- | --- | --- | --- | --- | --- | --- | --- | --- | --- | --- | --- | --- | --- | --- | --- | --- | --- | --- | --- | --- | --- | --- | --- | --- | --- | --- | --- | --- | --- | --- | --- | --- | --- | --- | --- | --- | --- | --- | --- | --- | --- | --- | --- | --- | --- | --- | --- | --- | --- | --- | --- | --- | --- | --- | --- | --- | --- | --- | --- | --- | --- | --- | --- | --- | --- | --- | --- | --- | --- | --- | --- | --- | --- | --- | --- | --- | --- | --- | --- | --- | --- | --- | --- | --- | --- | --- | --- | --- | --- | --- | --- | --- | --- | --- | --- | --- | --- | --- | --- | --- | --- | --- | --- | --- | --- | --- | --- | --- | --- | --- | --- | --- | --- | --- | --- | --- | --- | --- | --- | --- | --- | --- | --- | --- | --- | --- | --- | --- | --- | --- | --- | --- | --- | --- | --- | --- | --- | --- | --- | --- | --- | --- | --- | --- | --- | --- | --- | --- | --- | --- | --- | --- | --- | --- | --- | --- | --- | --- | --- | --- | --- | --- | --- | --- | --- | --- | --- | --- | --- | --- | --- | --- | --- | --- | --- | --- | --- | --- | --- | --- | --- | --- | --- | --- | --- | --- | --- | --- | --- | --- | --- | --- | --- | --- | --- | --- | --- | --- | --- | --- | --- | --- | --- | --- | --- | --- | --- | --- | --- | --- | --- | --- | --- | --- | --- | --- | --- | --- | --- | --- | --- | --- | --- | --- | --- | --- | --- | --- | --- | --- | --- | --- | --- | --- | --- | --- | --- | --- | --- | --- | --- | --- | --- | --- | --- | --- | --- | --- | --- | --- | --- | --- | --- | --- | --- | --- | --- | --- | --- | --- | --- | --- | --- | --- | --- | --- | --- | --- | --- | --- | --- | --- | --- | --- | --- | --- | --- | --- | --- | --- | --- | --- | --- | --- | --- | --- | --- | --- | --- | --- | --- | --- | --- | --- | --- | --- | --- | --- | --- | --- | --- | --- | --- | --- | --- | --- | --- | --- | --- | --- | --- | --- | --- | --- | --- | --- | --- | --- | --- | --- | --- | --- | --- | --- | --- | --- | --- | --- | --- | --- | --- | --- | --- | --- | --- | --- | --- | --- | --- | --- | --- | --- | --- | --- | --- | --- | --- | --- | --- | --- | --- | --- | --- | --- | --- | --- | --- | --- | --- | --- | --- | --- | --- | --- | --- | --- | --- | --- | --- | --- | --- | --- | --- | --- | --- | --- | --- | --- | --- | --- | --- | --- | --- | --- | --- | --- | --- | --- | --- | --- | --- | --- | --- | --- | --- | --- | --- | --- | --- | --- | --- | --- | --- | --- | --- | --- | --- | --- | --- | --- | --- | --- | --- | --- | --- | --- | --- |
| |  |  |  |  |  |  |  |  |  | | --- | --- | --- | --- | --- | --- | --- | --- | --- | | **Position** | **Reference** | **Sample** | **Quality** | **Type** | **Region** | **AA Exchange** | **PAM1** | **Known Variant** | | 1977 | A | G | 1062.77 | SNP | intergenic |  |  | - | | 4013 | T | C | 1866.77 | SNP | Rv0003 (recF) | Ile245Thr | 11 | - | | 7362 | G | C | 1996.77 | SNP | Rv0006 (gyrA) | Glu21Gln | 27 | - | | 7539 | A | G | 1949.77 | SNP | Rv0006 (gyrA) | Thr80Ala | 32 | genotype | | 7585 | G | C | 2151.77 | SNP | Rv0006 (gyrA) | Ser95Thr | 32 | genotype | | 9304 | G | A | 1249.77 | SNP | Rv0006 (gyrA) | Gly668Asp | 6 | - | | 11879 | A | G | 1437.77 | SNP | Rv0008c | Ser145Pro | 12 | - | | 13741 | A | C | 1855.77 | SNP | Rv0011c | silent (Gly85) | 9935 | - | | 14785 | T | C | 1521.77 | SNP | Rv0012 | Cys233Arg | 1 | - | | 18091 | G | A | 1431.77 | SNP | Rv0015c (pknA) | silent (Thr224) | 9871 | - | | 21795 | G | A | 304.78 | SNP | Rv0018c (pstP) | Pro463Ser | 17 | - | | 22334 | C | T | 1486.77 | SNP | Rv0018c (pstP) | Arg283His | 8 | - | | 26959 | C | G | 1006.77 | SNP | intergenic |  |  | - | | 32075 | T | C | 1845.77 | SNP | Rv0029 | Trp7Arg | 8 | - | | 34044 | T | C | 1680.77 | SNP | intergenic |  |  | - | | 34226 | A | G | 1772.77 | SNP | intergenic |  |  | - | | 37031 | C | G | 1518.77 | SNP | Rv0034 | silent (Ala55) | 9867 | - | | 42747 | C | T | 2244.77 | SNP | Rv0040c (mtc28) | Asp207Asn | 36 | - | | 42967 | G | C | 1311.77 | SNP | Rv0040c (mtc28) | silent (Pro133) | 9926 | - | | 47936 | G | A | 1853.77 | SNP | Rv0043c | silent (Leu55) | 9947 | - | | 50156 | C | A | 36.77 | SNP | Rv0046c (ino1) | silent (Arg323) | 9913 | - | | 52030 | A | C | 1889.77 | SNP | Rv0048c | Val(s)223Gly | 21 | - | | 54304 | C | T | 1281.77 | SNP | Rv0050 (ponA1) | silent (Leu214) | 9947 | - | | 55553 | C | CCGT | 2136.73 | INS | Rv0050 (ponA1) |  |  | - | | 57737 | G | A | 1337.77 | SNP | Rv0052 | Val110Ile | 33 | - | | 62049 | A | G | 1638.77 | SNP | Rv0058 (dnaB) | Arg552Gly | 1 | - | | 69989 | G | A | 1599.77 | SNP | Rv0064 | Gly457Asp | 6 | - | | 70816 | A | G | 1467.77 | SNP | Rv0064 | Asn733Asp | 42 | - | | 71336 | G | C | 507.77 | SNP | Rv0064 | Arg906Pro | 5 | - | | 71584 | C | CCGAGCGCTGTTCTGGCGCT AATCTGACGCTAGAATAG | 10549.73 | INS | intergenic |  |  | - | | 75940 | G | C | 1682.77 | SNP | Rv0068 | Val(s)214Leu | 3 | - | | 79498 | ACGGTGT | A | 1648.73 | DEL | Rv0071 |  |  | - | | 79503 | G | GGAC | 3905.73 | INS | Rv0071 |  |  | - | | 80616 | C | G | 1438.77 | SNP | intergenic |  |  | - | | 87257 | C | A | 1487.77 | SNP | Rv0078A | Arg182Leu | 1 | - | | 92199 | T | G | 1242.77 | SNP | Rv0083 | silent (Thr600) | 9871 | - | | 104941 | T | C | 106.28 | SNP | Rv0095c | Gln92Arg | 10 | - | | 104942 | G | C | 106.28 | SNP | Rv0095c | Gln92Glu | 35 | - | | 104944 | G | A | 106.28 | SNP | Rv0095c | Ala91Val | 13 | - | | 104962 | G | A | 142.03 | SNP | Rv0095c | Ala85Val(s) | 9867 | - | | 105021 | G | A | 283.53 | SNP | Rv0095c | silent (Ser65) | 9840 | - | | 105045 | G | C | 629.77 | SNP | Rv0095c | Asp57Glu | 56 | - | | 105060 | G | A | 680.77 | SNP | Rv0095c | silent (Asp52) | 9859 | - | | 105063 | G | A | 726.77 | SNP | Rv0095c | silent (Phe51) | 9946 | - | | 116000 | T | G | 1587.77 | SNP | Rv0101 (nrp) | Val2000Val(s) | 18 | - | | 122109 | A | G | 1639.77 | SNP | Rv0103c (ctpB) | Leu(s)22Ser | 28 | - | | 122794 | T | G | 886.77 | SNP | Rv0104 | Phe160Val | 1 | - | | 125830 | G | GA | 2835.73 | INS | Rv0107c (ctpI) |  |  | - | | 131174 | T | TG | 2304.73 | INS | intergenic |  |  | - | | 132417 | C | G | 139.90 | SNP | Rv0109 (PE\_PGRS1) | Arg346Gly | 1 | - | | 133839 | C | T | 2163.77 | SNP | intergenic |  |  | - | | 146087 | T | C | 1782.77 | SNP | Rv0120c (fusA2) | Asn562Ser | 34 | - | | 154283 | T | C | 1693.77 | SNP | Rv0127 (mak) | Ser18Pro | 12 | - | | 155948 | C | CA | 3173.72 | INS | Rv0128 |  |  | - | | 175830 | T | C | 1972.77 | SNP | Rv0149 | Leu44Pro | 2 | - | | 177857 | G | A | 1528.77 | SNP | Rv0151c (PE1) | Leu485Leu(s) | 4 | - | | 188800 | T | C | 1223.77 | SNP | Rv0159c (PE3) | Thr14Ala | 32 | - | | 194681 | G | C | 1332.77 | SNP | Rv0165c (mce1R) | silent (Leu45) | 9947 | - | | 196642 | C | T | 1865.77 | SNP | Rv0166 (fadD5) | silent (Asn550) | 9822 | - | | 206339 | T | C | 1088.77 | SNP | Rv0174 (mce1F) | Leu370Pro | 2 | - | | 207160 | G | A | 2983.77 | SNP | Rv0175 | Ser116Asn | 20 | - | | 215613 | G | A | 1052.77 | SNP | Rv0184 | silent (Ala215) | 9867 | - | | 218204 | C | T | 1463.77 | SNP | Rv0186 (bglS) | Arg646STOP | 2 | - | | 219120 | A | G | 1534.77 | SNP | Rv0187 | Asp139Gly | 11 | - | | 223942 | T | C | 1065.77 | SNP | Rv0192 | Ser127Pro | 12 | - | | 225323 | T | C | 2100.77 | SNP | Rv0193c | Lys417Glu | 4 | - | | 227098 | T | C | 2053.77 | SNP | Rv0194 | Met(s)74Thr | 22 | - | | 231114 | C | G | 1374.77 | SNP | Rv0195 | silent (Ala72) | 9867 | - | | 234477 | T | G | 1325.77 | SNP | Rv0197 | Tyr749STOP | 2 | - | | 234496 | C | CGT | 3690.73 | INS | Rv0197 |  |  | - | | 237709 | C | T | 1016.77 | SNP | Rv0200 | silent (Ala168) | 9867 | - | | 252473 | A | G | 1530.77 | SNP | Rv0211 (pckA) | Tyr231Cys | 3 | - | | 256640 | T | G | 1904.77 | SNP | Rv0214 (fadD4) | Ser193Ala | 35 | - | | 257982 | T | G | 1438.77 | SNP | Rv0215c (fadE3) | Asp292Ala | 10 | - | | 261809 | ACCG | A | 1471.73 | DEL | Rv0218 |  |  | - | | 261869 | T | C | 606.77 | SNP | Rv0218 | Cys316Arg | 1 | - | | 265244 | C | T | 1359.77 | SNP | Rv0221 | Ala393Val(s) | 9867 | - | | 265554 | A | C | 1790.77 | SNP | Rv0222 (echA1) | silent (Val16) | 9901 | - | | 275859 | G | T | 1672.77 | SNP | Rv0230c (php) | Asn35Lys | 25 | - | | 278681 | C | G | 1596.77 | SNP | Rv0233 (nrdB) | His33Asp | 4 | - | | 285772 | A | C | 1089.77 | SNP | Rv0236c (aftD) | silent (Pro360) | 9926 | - | | 285871 | A | G | 875.77 | SNP | Rv0236c (aftD) | silent (Val327) | 9901 | - | | 294314 | G | C | 42.77 | SNP | Rv0244c (fadE5) | silent (Ala440) | 9867 | - | | 294316 | C | A | 33.77 | SNP | Rv0244c (fadE5) | Ala440Ser | 28 | - | | 294341 | A | G | 73.77 | SNP | Rv0244c (fadE5) | silent (Tyr431) | 9945 | - | | 294352 | A | G | 51.77 | SNP | Rv0244c (fadE5) | Leu(s)428Leu | 3 | - | | 294362 | T | G | 56.77 | SNP | Rv0244c (fadE5) | silent (Gly424) | 9935 | - | | 294365 | A | G | 69.77 | SNP | Rv0244c (fadE5) | silent (Gly423) | 9935 | - | | 294374 | T | C | 48.77 | SNP | Rv0244c (fadE5) | silent (Gln420) | 9876 | - | | 295724 | A | G | 1413.77 | SNP | intergenic |  |  | - | | 310516 | C | T | 1708.77 | SNP | Rv0258c | silent (Gln78) | 9876 | - | | 310973 | G | A | 1114.77 | SNP | Rv0259c | Ala182Val(s) | 9867 | - | | 311613 | G | T | 1832.77 | SNP | Rv0260c | silent (Val349) | 9901 | - | | 312060 | T | G | 911.77 | SNP | Rv0260c | silent (Arg200) | 9913 | - | | 312944 | C | T | 1731.77 | SNP | Rv0261c (narK3) | Val409Ile | 33 | - | | 325038 | G | A | 1315.77 | SNP | Rv0270 (fadD2) | Gly158Ser | 16 | - | | 325039 | G | A | 1334.77 | SNP | Rv0270 (fadD2) | Gly158Asp | 6 | - | | 332357 | A | G | 2115.77 | SNP | Rv0276 | Ile204Val | 57 | - | | 333892 | G | C | 531.77 | SNP | Rv0278c (PE\_PGRS3) | Arg807Gly | 1 | - | | 336691 | T | C | 84.28 | SNP | Rv0279c (PE\_PGRS4) | Ser795Gly | 21 | - | | 336698 | C | G | 49.74 | SNP | Rv0279c (PE\_PGRS4) | silent (Gly792) | 9935 | - | | 336701 | A | G | 74.28 | SNP | Rv0279c (PE\_PGRS4) | silent (Gly791) | 9935 | - | | 336707 | G | A | 58.28 | SNP | Rv0279c (PE\_PGRS4) | silent (Asp789) | 9859 | - | | 336708 | T | C | 76.28 | SNP | Rv0279c (PE\_PGRS4) | Asp789Gly | 11 | - | | 336710 | A | G | 80.28 | SNP | Rv0279c (PE\_PGRS4) | silent (Ala788) | 9867 | - | | 337820 | G | A | 238.80 | SNP | Rv0279c (PE\_PGRS4) | silent (Gly418) | 9935 | - | | 338020 | A | C | 55.74 | SNP | Rv0279c (PE\_PGRS4) | Cys352Gly | 1 | - | | 338100 | T | C | 282.78 | SNP | Rv0279c (PE\_PGRS4) | Asn325Ser | 34 | - | | 338453 | A | G | 116.03 | SNP | Rv0279c (PE\_PGRS4) | silent (Ala207) | 9867 | - | | 338570 | G | C | 35.77 | SNP | Rv0279c (PE\_PGRS4) | silent (Ala168) | 9867 | - | | 338573 | G | C | 31.77 | SNP | Rv0279c (PE\_PGRS4) | silent (Gly167) | 9935 | - | | 338580 | T | C | 32.77 | SNP | Rv0279c (PE\_PGRS4) | Asn165Ser | 34 | - | | 338618 | C | G | 32.77 | SNP | Rv0279c (PE\_PGRS4) | silent (Gly152) | 9935 | - | | 340132 | G | A | 1534.77 | SNP | Rv0280 (PPE3) | Glu257Lys | 7 | - | | 346275 | C | G | 1468.77 | SNP | Rv0284 (eccC3) | Pro214Arg | 4 | - | | 356528 | A | G | 1460.77 | SNP | Rv0292 (eccE3) | Asn217Asp | 42 | - | | 373282 | TA | T | 2803.73 | DEL | Rv0305c (PPE6) |  |  | - | | 375714 | G | A | 2328.77 | SNP | intergenic |  |  | - | | 384380 | A | C | 1940.77 | SNP | Rv0315 | Lys260Thr | 8 | - | | 386432 | C | G | 1593.77 | SNP | Rv0318c | Gly223Ala | 21 | - | | 390828 | T | C | 1422.77 | SNP | Rv0323c | Ser142Gly | 21 | - | | 394850 | G | A | 1450.77 | SNP | Rv0329c | silent (Asp157) | 9859 | - | | 396180 | C | T | 1046.77 | SNP | intergenic |  |  | - | | 403980 | G | A | 1834.77 | SNP | Rv0338c | Ala621Val | 13 | - | | 404326 | T | C | 2007.77 | SNP | Rv0338c | Arg506Gly | 1 | - | | 410264 | C | T | 693.98 | SNP | Rv0341 (iniB) | silent (Ala301) | 9867 | - | | 412017 | C | G | 1956.77 | SNP | Rv0342 (iniA) | Gln394Glu | 35 | - | | 414486 | C | T | 1464.77 | SNP | Rv0344c (lpqJ) | silent (Glu152) | 9865 | - | | 420008 | A | G | 1415.77 | SNP | Rv0350 (dnaK) | silent (Ala58) | 9867 | - | | 424320 | T | TC | 2135.73 | INS | Rv0354c (PPE7) |  |  | - | | 427310 | TTGCCGAGGTTTGCAC | T | 5881.73 | DEL | Rv0355c (PPE8) |  |  | - | | 433654 | G | A | 1222.77 | SNP | Rv0355c (PPE8) | silent (Gly342) | 9935 | - | | 435708 | G | A | 1332.77 | SNP | Rv0357c (purA) | silent (Thr354) | 9871 | - | | 450150 | A | G | 37.77 | SNP | Rv0373c | Leu(s)552Leu | 3 | - | | 450160 | T | C | 36.77 | SNP | Rv0373c | silent (Thr548) | 9871 | - | | 454295 | T | C | 1394.77 | SNP | Rv0376c | silent (Pro26) | 9926 | - | | 457452 | T | G | 830.77 | SNP | Rv0381c | silent (Thr124) | 9871 | - | | 459399 | A | C | 1207.77 | SNP | intergenic |  |  | - | | 467497 | C | CG | 1761.73 | INS | Rv0388c (PPE9) |  |  | - | | 467508 | C | CG | 2020.73 | INS | Rv0388c (PPE9) |  |  | - | | 467516 | G | C | 1145.77 | SNP | Rv0388c (PPE9) | silent (Ser162) | 9840 | - | | 467526 | C | G | 1110.77 | SNP | Rv0388c (PPE9) | Gly159Ala | 21 | - | | 467546 | G | C | 970.77 | SNP | Rv0388c (PPE9) | Asp152Glu | 56 | - | | 467557 | A | C | 988.77 | SNP | Rv0388c (PPE9) | Leu(s)149Val(s) | 9867 | - | | 467564 | A | C | 1110.77 | SNP | Rv0388c (PPE9) | His146Gln | 23 | - | | 467585 | G | C | 1054.77 | SNP | Rv0388c (PPE9) | His139Gln | 23 | - | | 467590 | T | C | 1056.77 | SNP | Rv0388c (PPE9) | Thr138Ala | 32 | - | | 467621 | T | G | 1241.77 | SNP | Rv0388c (PPE9) | silent (Gly127) | 9935 | - | | 467638 | G | T | 1177.77 | SNP | Rv0388c (PPE9) | Gln122Lys | 12 | - | | 475178 | T | C | 1420.77 | SNP | Rv0395 | Val80Ala | 18 | - | | 488796 | G | A | 1325.77 | SNP | Rv0405 (pks6) | Val(s)1022Val | 13 | - | | 489935 | G | C | 1302.77 | SNP | Rv0405 (pks6); Rv0406c | Arg1402Pro; silent (Thr257) | 5; 9871 | - | | 492715 | C | T | 1452.77 | SNP | Rv0408 (pta) | silent (Gly310) | 9935 | - | | 493934 | T | C | 1476.77 | SNP | Rv0409 (ackA) | silent (Arg28) | 9913 | - | | 502589 | C | G | 1202.77 | SNP | Rv0417 (thiG) | Ser75Cys | 5 | - | | 503354 | G | C | 1565.77 | SNP | intergenic |  |  | - | | 513257 | T | C | 1167.77 | SNP | Rv0425c (ctpH) | Met(s)689Val(s) | 9867 | - | | 541201 | A | G | 1100.77 | SNP | Rv0450c (mmpL4) | silent (Leu97) | 9947 | - | | 551525 | A | C | 1310.77 | SNP | Rv0459 | silent (Arg110) | 9913 | - | | 559094 | A | G | 1232.77 | SNP | Rv0468 (fadB2) | Asp67Gly | 11 | - | | 573262 | A | G | 1188.77 | SNP | Rv0484c | silent (Gly180) | 9935 | - | | 580772 | T | A | 487.77 | SNP | intergenic |  |  | - | | 580773 | GGGGGCACCACCCGCTTGCG GGGGA | G | 5977.73 | DEL | intergenic |  |  | - | | 587974 | G | A | 997.77 | SNP | Rv0497 | Ala200Thr | 22 | - | | 590436 | T | C | 1501.77 | SNP | Rv0500 (proC) | silent (Ala118) | 9867 | - | | 591628 | T | C | 1217.77 | SNP | intergenic |  |  | - | | 595232 | C | T | 1570.77 | SNP | Rv0504c | Gly24Glu | 4 | - | | 597816 | A | G | 1231.77 | SNP | Rv0507 (mmpL2) | silent (Ala206) | 9867 | - | | 598475 | G | A | 1716.77 | SNP | Rv0507 (mmpL2) | Arg426His | 8 | - | | 610120 | T | G | 1524.77 | SNP | intergenic |  |  | - | | 623472 | A | G | 139.03 | SNP | Rv0532 (PE\_PGRS6) | Asp227Gly | 11 | - | | 623508 | C | G | 316.78 | SNP | Rv0532 (PE\_PGRS6) | Ala239Gly | 21 | - | | 628113 | C | T | 1001.77 | SNP | Rv0536 (galE3) | Ala289Val(s) | 9867 | - | | 630722 | G | C | 1308.77 | SNP | Rv0538 | Arg228Pro | 5 | - | | 637319 | G | A | 1442.77 | SNP | Rv0545c (pitA) | Pro49Ser | 17 | - | | 648002 | T | G | 2196.77 | SNP | Rv0556 | Leu15Arg | 1 | - | | 663410 | A | C | 38.77 | SNP | intergenic |  |  | - | | 663418 | A | C | 57.77 | SNP | intergenic |  |  | - | | 663420 | C | A | 58.77 | SNP | intergenic |  |  | - | | 663429 | T | G | 39.77 | SNP | intergenic |  |  | - | | 665293 | A | G | 1967.77 | SNP | Rv0572c | Phe31Leu | 13 | - | | 669398 | T | C | 1033.77 | SNP | Rv0575c | silent (Gln116) | 9876 | - | | 672491 | C | G | 355.77 | SNP | Rv0578c (PE\_PGRS7) | silent (Gly1142) | 9935 | - | | 673238 | A | G | 574.77 | SNP | Rv0578c (PE\_PGRS7) | silent (His893) | 9912 | - | | 684363 | C | T | 1879.77 | SNP | intergenic |  |  | - | | 685461 | C | G | 1497.77 | SNP | Rv0587 (yrbE2A) | silent (Ala111) | 9867 | - | | 685608 | T | C | 1775.77 | SNP | Rv0587 (yrbE2A) | silent (Leu160) | 9947 | - | | 685869 | G | A | 1531.77 | SNP | Rv0587 (yrbE2A) | silent (Leu247) | 9947 | - | | 686972 | T | C | 2097.77 | SNP | Rv0589 (mce2A) | Phe51Ser | 3 | - | | 690465 | T | G | 529.77 | SNP | Rv0591 (mce2C) | silent (Leu469) | 9947 | - | | 698968 | G | A | 1005.77 | SNP | Rv0601c | silent (Gly9) | 9935 | - | | 721498 | G | A | 1809.77 | SNP | Rv0629c (recD) | Leu79Leu(s) | 4 | - | | 728707 | G | T | 1761.77 | SNP | Rv0632c (echA3) | Ala191Asp | 6 | - | | 732265 | G | A | 1795.77 | SNP | Rv0635 (hadA) | Val(s)112Val | 13 | - | | 743399 | C | A | 1628.77 | SNP | Rv0648 | silent (Ala227) | 9867 | - | | 749181 | G | C | 131.77 | SNP | Rv0652 (rplL) | Glu111Asp | 53 | - | | 749187 | C | T | 117.77 | SNP | Rv0652 (rplL) | silent (Ala113) | 9867 | - | | 749191 | G | A | 73.77 | SNP | Rv0652 (rplL) | Glu115Lys | 7 | - | | 749203 | A | G | 41.77 | SNP | Rv0652 (rplL) | Lys119Glu | 4 | - | | 749204 | A | C | 39.77 | SNP | Rv0652 (rplL) | Lys119Thr | 8 | - | | 749208 | G | C | 66.77 | SNP | Rv0652 (rplL) | silent (Leu120) | 9947 | - | | 749213 | C | G | 31.77 | SNP | Rv0652 (rplL) | Ala122Gly | 21 | - | | 754186 | A | G | 1168.77 | SNP | Rv0658c | Leu75Pro | 2 | - | | 761155 | C | T | 1402.77 | SNP | Rv0667 (rpoB) | Ser450Leu(s) | 35 | resistance | | 763630 | G | C | 39.77 | SNP | Rv0668 (rpoC) | Val(s)87Val | 13 | - | | 763633 | T | C | 48.77 | SNP | Rv0668 (rpoC) | silent (Arg88) | 9913 | - | | 763636 | T | C | 60.77 | SNP | Rv0668 (rpoC) | silent (Arg89) | 9913 | - | | 772677 | C | A | 1187.77 | SNP | Rv0672 (fadE8) | silent (Gly398) | 9935 | - | | 775639 | T | C | 1610.77 | SNP | Rv0676c (mmpL5) | Ile948Val | 57 | - | | 781395 | T | C | 1737.77 | SNP | intergenic (Rv0682-165nt) |  |  | - | | 836658 | A | G | 106.28 | SNP | Rv0746 (PE\_PGRS9) | Thr320Ala | 32 | - | | 837033 | A | G | 387.77 | SNP | Rv0746 (PE\_PGRS9) | Thr445Ala | 32 | - | | 839279 | G | A | 76.77 | SNP | Rv0747 (PE\_PGRS10) | Asp277Asn | 36 | - | | 839284 | C | G | 73.77 | SNP | Rv0747 (PE\_PGRS10) | silent (Ala278) | 9867 | - | | 839309 | T | G | 111.77 | SNP | Rv0747 (PE\_PGRS10) | Ser287Ala | 35 | - | | 839334 | A | G | 91.03 | SNP | Rv0747 (PE\_PGRS10) | Lys295Arg | 19 | - | | 839348 | A | G | 125.03 | SNP | Rv0747 (PE\_PGRS10) | Ser300Gly | 21 | - | | 839515 | G | A | 246.77 | SNP | Rv0747 (PE\_PGRS10) | silent (Ala355) | 9867 | - | | 839516 | A | G | 251.77 | SNP | Rv0747 (PE\_PGRS10) | Thr356Ala | 32 | - | | 839519 | C | G | 284.77 | SNP | Rv0747 (PE\_PGRS10) | Leu357Val(s) | 4 | - | | 839534 | A | C | 223.77 | SNP | Rv0747 (PE\_PGRS10) | Ile362Leu | 22 | - | | 840496 | C | G | 122.03 | SNP | Rv0747 (PE\_PGRS10) | silent (Gly682) | 9935 | - | | 841764 | G | C | 1850.77 | SNP | Rv0749A | silent (Thr37) | 9871 | - | | 846256 | C | A | 1374.77 | SNP | Rv0754 (PE\_PGRS11) | Ala33Asp | 6 | - | | 852910 | C | T | 1022.77 | SNP | Rv0758 (phoR) | Pro172Leu | 3 | - | | 854252 | GCC | G | 3275.73 | DEL | intergenic |  |  | - | | 857696 | A | G | 1362.77 | SNP | Rv0764c (cyp51) | silent (Ala114) | 9867 | - | | 859649 | C | T | 1320.77 | SNP | Rv0766c (cyp123) | Gly142Ser | 16 | - | | 859769 | A | G | 1623.77 | SNP | Rv0766c (cyp123) | Ser102Pro | 12 | - | | 869679 | C | T | 1230.77 | SNP | Rv0776c | Gly29Arg | 0 | - | | 874787 | G | A | 1574.77 | SNP | Rv0781 (ptrBa); Rv0782 (ptrBb) | silent (Pro185); Arg19Gln | 9926; 9 | genotype | | 874835 | C | CCG | 3552.73 | INS | Rv0781 (ptrBa); Rv0782 (ptrBb) |  |  | - | | 876378 | T | C | 2020.77 | SNP | Rv0782 (ptrBb) | silent (Ala549) | 9867 | - | | 880562 | G | T | 1523.77 | SNP | Rv0785 | Cys408Phe | 0 | - | | 882257 | T | C | 1643.77 | SNP | Rv0787 | Tyr267His | 4 | - | | 890549 | G | A | 279.77 | SNP | Rv0797 | Trp54STOP | 0 | - | | 891011 | A | G | 57.77 | SNP | Rv0797 | silent (Leu208) | 9947 | - | | 893733 | T | G | 1326.77 | SNP | Rv0800 (pepC) | Leu139Arg | 1 | - | | 900221 | T | C | 1792.77 | SNP | Rv0806c (cpsY) | Val370Val(s) | 18 | - | | 903550 | T | C | 1155.77 | SNP | Rv0808 (purF) | silent (Ala480) | 9867 | - | | 903913 | T | C | 1765.77 | SNP | Rv0809 (purM) | silent (Gly63) | 9935 | - | | 906857 | A | G | 1442.77 | SNP | Rv0812 | Ile145Met(s) | 6 | - | | 919384 | T | C | 1467.77 | SNP | Rv0825c | Tyr57Cys | 3 | - | | 921813 | C | G | 1687.77 | SNP | Rv0829 | Ala80Gly | 21 | - | | 927110 | A | G | 55.74 | SNP | Rv0833 (PE\_PGRS13) | Ser584Gly | 21 | - | | 944941 | A | G | 1631.77 | SNP | Rv0848 (cysK2) | Arg2Gly | 1 | - | | 945214 | G | A | 1827.77 | SNP | Rv0848 (cysK2) | Gly93Ser | 16 | - | | 949535 | T | C | 1518.77 | SNP | Rv0853c (pdc) | silent (Ala528) | 9867 | - | | 955524 | A | G | 1295.77 | SNP | Rv0859 (fadA) | Ser150Gly | 21 | - | | 955983 | C | T | 1199.77 | SNP | Rv0859 (fadA) | Pro303Ser | 17 | - | | 956644 | C | T | 1240.77 | SNP | Rv0860 (fadB) | Leu118Leu(s) | 4 | - | | 964825 | C | T | 52.77 | SNP | Rv0867c (rpfA) | silent (Ala237) | 9867 | - | | 968426 | A | AGCCGGGTTG | 3333.73 | INS | Rv0872c (PE\_PGRS15) |  |  | - | | 976896 | TTG | T | 3291.73 | DEL | Rv0878c (PPE13) |  |  | - | | 979314 | C | T | 1377.77 | SNP | Rv0880 | silent (Ile127) | 9872 | - | | 979704 | G | C | 1566.77 | SNP | Rv0881 | Gly115Arg | 0 | - | | 984493 | C | T | 744.77 | SNP | Rv0886 (fprB) | Leu231Leu(s) | 4 | - | | 986463 | G | C | 2447.77 | SNP | intergenic |  |  | - | | 990001 | G | C | 2321.77 | SNP | Rv0890c | Pro866Ala | 22 | - | | 990626 | T | A | 1027.77 | SNP | Rv0890c | Leu657Phe | 6 | - | | 993346 | A | C | 2324.77 | SNP | Rv0891c | Val37Gly | 5 | - | | 996871 | G | A | 2041.77 | SNP | Rv0894 | Leu(s)116Leu | 3 | - | | 1010204 | C | CG | 2502.73 | INS | Rv0907 |  |  | - | | 1015344 | G | GGT | 3702.73 | INS | intergenic |  |  | - | | 1020044 | C | T | 1391.77 | SNP | intergenic |  |  | - | | 1025106 | T | C | 2253.77 | SNP | Rv0919 | silent (Phe141) | 9946 | - | | 1037012 | T | C | 830.77 | SNP | Rv0930 (pstA1) | Met(s)5Thr | 22 | - | | 1037911 | C | T | 1612.77 | SNP | Rv0930 (pstA1) | Arg305STOP | 2 | - | | 1042163 | CTGCTGCTAGCAGCGGCGGG CTG | C | 10241.73 | DEL | Rv0934 (pstS1) |  |  | - | | 1044905 | G | T | 2109.77 | SNP | Rv0936 (pstA2) | Ala197Ser | 28 | - | | 1047165 | T | C | 1278.77 | SNP | Rv0938 (ligD) | Cys344Arg | 1 | - | | 1056916 | T | G | 964.77 | SNP | intergenic |  |  | - | | 1068151 | T | C | 1983.77 | SNP | Rv0956 (purN) | silent (His197) | 9912 | - | | 1068432 | A | G | 1519.77 | SNP | Rv0957 (purH) | silent (Pro76) | 9926 | - | | 1070010 | C | A | 912.77 | SNP | Rv0958 | Pro43Gln | 6 | - | | 1070702 | T | C | 1677.77 | SNP | Rv0958 | Ser274Pro | 12 | - | | 1074558 | G | A | 1279.77 | SNP | Rv0962c (lprP) | Pro186Leu | 3 | - | | 1075279 | T | C | 2111.77 | SNP | intergenic |  |  | - | | 1076309 | G | T | 1776.77 | SNP | Rv0964c | Pro124Thr | 5 | - | | 1077312 | A | G | 1078.77 | SNP | Rv0966c | Val(s)175Ala | 9867 | - | | 1079927 | C | A | 1729.77 | SNP | Rv0969 (ctpV) | silent (Thr395) | 9871 | - | | 1081681 | T | C | 1499.77 | SNP | Rv0970 | silent (Val210) | 9901 | - | | 1087193 | G | C | 1511.77 | SNP | Rv0974c (accD2) | Asn51Lys | 25 | - | | 1093406 | A | G | 1102.77 | SNP | Rv0978c (PE\_PGRS17) | silent (Val317) | 9901 | - | | 1093928 | G | A | 89.28 | SNP | Rv0978c (PE\_PGRS17) | silent (Asn143) | 9822 | - | | 1096633 | T | G | 688.77 | SNP | intergenic |  |  | - | | 1100234 | T | C | 1299.77 | SNP | Rv0983 (pepD) | Leu390Pro | 2 | - | | 1106422 | T | C | 2339.77 | SNP | Rv0989c (grcC2) | Ile321Val | 57 | - | | 1109975 | A | G | 1873.77 | SNP | Rv0993 (galU) | Gln235Arg | 10 | - | | 1126889 | G | C | 1226.77 | SNP | Rv1007c (metS) | Arg39Gly | 1 | - | | 1127648 | C | A | 1405.77 | SNP | Rv1008 (tatD) | Thr187Asn | 9 | - | | 1149551 | C | T | 1051.77 | SNP | Rv1028c (kdpD) | silent (Glu712) | 9865 | - | | 1150585 | G | A | 1045.77 | SNP | Rv1028c (kdpD) | Pro368Ser | 17 | - | | 1163134 | T | C | 1642.77 | SNP | Rv1040c (PE8) | silent (Gly81) | 9935 | - | | 1165521 | T | TA | 2279.73 | INS | intergenic |  |  | - | | 1168715 | C | CT | 2179.73 | INS | Rv1046c |  |  | - | | 1169235 | CG | C | 2379.73 | DEL | intergenic |  |  | - | | 1169307 | C | T | 1503.77 | SNP | intergenic |  |  | - | | 1169447 | G | A | 147.90 | SNP | Rv1047 | Ala9Thr | 22 | - | | 1170404 | C | A | 118.03 | SNP | Rv1047 | Gln328Lys | 12 | - | | 1177446 | T | C | 1533.77 | SNP | Rvnt17 | tRNA | tRNA | - | | 1178116 | T | C | 2039.77 | SNP | Rv1056 | silent (Thr163) | 9871 | - | | 1184605 | C | A | 1777.77 | SNP | Rv1061 | Asp197Glu | 56 | - | | 1199762 | A | G | 1215.77 | SNP | Rv1075c | silent (Arg203) | 9913 | - | | 1200418 | A | G | 1943.77 | SNP | intergenic |  |  | - | | 1202492 | C | T | 1157.77 | SNP | Rv1077 (cbs) | Ala259Val | 13 | - | | 1204882 | A | G | 1825.77 | SNP | Rv1079 (metB) | Val272Val(s) | 18 | - | | 1213679 | T | TCGGCGACGGCGGCATCGG | 7324.73 | INS | Rv1087 (PE\_PGRS21) |  |  | - | | 1220680 | T | C | 1563.77 | SNP | Rv1093 (glyA1) | Val36Ala | 18 | - | | 1224367 | T | C | 845.77 | SNP | intergenic |  |  | - | | 1248978 | T | C | 1527.77 | SNP | Rv1125 | silent (Ala299) | 9867 | - | | 1281118 | T | C | 1847.77 | SNP | Rv1154c | Thr123Ala | 32 | - | | 1282014 | A | C | 1253.77 | SNP | intergenic |  |  | - | | 1292102 | A | G | 1196.77 | SNP | Rv1162 (narH) | silent (Pro346) | 9926 | - | | 1307897 | G | A | 1123.77 | SNP | Rv1175c (fadH) | silent (Tyr110) | 9945 | - | | 1313337 | A | AG | 2802.73 | INS | intergenic |  |  | - | | 1313338 | A | C | 1457.77 | SNP | intergenic |  |  | - | | 1315191 | A | C | 1506.77 | SNP | Rv1180 (pks3) | STOP489Tyr | 1 | - | | 1315884 | G | A | 1449.77 | SNP | Rv1181 (pks4) | silent (Ala217) | 9867 | - | | 1320059 | A | G | 1925.77 | SNP | Rv1182 (papA3) | Ile9Val | 57 | - | | 1327890 | G | A | 1897.77 | SNP | Rv1186c | silent (Asp472) | 9859 | - | | 1328222 | T | C | 1352.77 | SNP | Rv1186c | Asn362Asp | 42 | - | | 1328687 | G | C | 1408.77 | SNP | Rv1186c | Pro207Ala | 22 | - | | 1341102 | C | T | 1185.77 | SNP | Rv1198 (esxL) | Arg33Cys | 1 | - | | 1341103 | G | C | 1299.77 | SNP | Rv1198 (esxL) | Arg33Pro | 5 | - | | 1342460 | T | C | 83.28 | SNP | Rv1199c | Tyr49Cys | 3 | - | | 1342598 | G | A | 515.77 | SNP | Rv1199c | Ser3Phe | 2 | - | | 1351788 | G | A | 944.77 | SNP | Rv1207 (folP2) | Ala200Thr | 22 | - | | 1357977 | C | T | 633.82 | SNP | Rv1215c | Glu490Lys | 7 | - | | 1365837 | C | CG | 1667.73 | INS | intergenic |  |  | - | | 1371182 | G | A | 943.77 | SNP | Rv1228 (lpqX) | Gly88Glu | 4 | - | | 1374065 | T | C | 855.77 | SNP | Rv1230c | Ser45Gly | 21 | - | | 1375724 | A | C | 2148.77 | SNP | Rv1232c | Cys149Gly | 1 | - | | 1382628 | T | C | 2136.77 | SNP | Rv1239c (corA) | Lys139Glu | 4 | - | | 1385761 | GCGCCGTCGC | G | 643.80 | DEL | Rv1243c (PE\_PGRS23) |  |  | - | | 1393626 | A | G | 1413.77 | SNP | Rv1249c | silent (Leu119) | 9947 | - | | 1396922 | T | C | 1321.77 | SNP | Rv1251c | silent (Thr773) | 9871 | - | | 1411210 | T | G | 1171.77 | SNP | Rv1263 (amiB2) | Val260Val(s) | 18 | - | | 1413148 | C | T | 2199.77 | SNP | intergenic |  |  | - | | 1414021 | C | T | 1213.77 | SNP | Rv1266c (pknH) | Arg607Gln | 9 | - | | 1433114 | G | A | 1940.77 | SNP | Rv1280c (oppA) | silent (Gly109) | 9935 | - | | 1439332 | C | T | 1977.77 | SNP | Rv1286 (cysN) | silent (Val142) | 9901 | - | | 1440469 | C | G | 1648.77 | SNP | Rv1286 (cysN) | silent (Pro521) | 9926 | - | | 1445781 | A | G | 1709.77 | SNP | Rv1291c | silent (Ala18) | 9867 | - | | 1454640 | G | C | 35.77 | SNP | Rv1297 (rho) | silent (Ala479) | 9867 | - | | 1457144 | C | T | 1030.77 | SNP | Rv1300 (hemK) | Arg194Cys | 1 | - | | 1465155 | C | T | 2385.77 | SNP | Rv1309 (atpG) | Ala91Val | 13 | - | | 1468208 | A | C | 1410.77 | SNP | Rv1313c | Leu433Arg | 1 | - | | 1471659 | C | T | 2035.77 | SNP | intergenic |  |  | - | | 1472150 | T | A | 309.77 | SNP | Rvnr01 | rRNA | rRNA | - | | 1472172 | T | C | 262.77 | SNP | Rvnr01 | rRNA | rRNA | - | | 1472215 | A | G | 205.77 | SNP | Rvnr01 | rRNA | rRNA | - | | 1472225 | C | A | 187.77 | SNP | Rvnr01 | rRNA | rRNA | - | | 1472234 | T | C | 177.77 | SNP | Rvnr01 | rRNA | rRNA | - | | 1472286 | C | G | 100.77 | SNP | Rvnr01 | rRNA | rRNA | - | | 1472289 | T | G | 78.77 | SNP | Rvnr01 | rRNA | rRNA | - | | 1472290 | C | G | 94.77 | SNP | Rvnr01 | rRNA | rRNA | - | | 1472313 | G | A | 73.77 | SNP | Rvnr01 | rRNA | rRNA | - | | 1472315 | T | G | 84.77 | SNP | Rvnr01 | rRNA | rRNA | - | | 1472324 | G | C | 105.77 | SNP | Rvnr01 | rRNA | rRNA | - | | 1472325 | G | C | 82.77 | SNP | Rvnr01 | rRNA | rRNA | - | | 1472328 | G | C | 135.77 | SNP | Rvnr01 | rRNA | rRNA | - | | 1472362 | C | T | 2167.77 | SNP | Rvnr01 | rRNA | rRNA | resistance | | 1472382 | G | A | 172.77 | SNP | Rvnr01 | rRNA | rRNA | - | | 1472400 | C | T | 101.77 | SNP | Rvnr01 | rRNA | rRNA | - | | 1472422 | T | C | 97.77 | SNP | Rvnr01 | rRNA | rRNA | - | | 1472655 | G | T | 79.77 | SNP | Rvnr01 | rRNA | rRNA | - | | 1472661 | A | G | 77.77 | SNP | Rvnr01 | rRNA | rRNA | - | | 1472697 | T | C | 155.77 | SNP | Rvnr01 | rRNA | rRNA | - | | 1472713 | T | C | 264.77 | SNP | Rvnr01 | rRNA | rRNA | - | | 1472714 | A | G | 60.77 | SNP | Rvnr01 | rRNA | rRNA | - | | 1472791 | G | T | 47.77 | SNP | Rvnr01 | rRNA | rRNA | - | | 1472895 | C | T | 410.77 | SNP | Rvnr01 | rRNA | rRNA | - | | 1472952 | T | C | 165.77 | SNP | Rvnr01 | rRNA | rRNA | - | | 1472956 | T | C | 235.77 | SNP | Rvnr01 | rRNA | rRNA | - | | 1472957 | C | T | 212.77 | SNP | Rvnr01 | rRNA | rRNA | - | | 1472973 | A | C | 204.77 | SNP | Rvnr01 | rRNA | rRNA | - | | 1472987 | G | A | 312.77 | SNP | Rvnr01 | rRNA | rRNA | - | | 1472989 | G | A | 339.77 | SNP | Rvnr01 | rRNA | rRNA | - | | 1472990 | A | G | 397.77 | SNP | Rvnr01 | rRNA | rRNA | - | | 1473035 | G | A | 545.77 | SNP | Rvnr01 | rRNA | rRNA | - | | 1473055 | C | T | 340.77 | SNP | Rvnr01 | rRNA | rRNA | - | | 1473062 | T | G | 393.77 | SNP | Rvnr01 | rRNA | rRNA | - | | 1473065 | C | A | 297.77 | SNP | Rvnr01 | rRNA | rRNA | - | | 1473100 | G | A | 303.77 | SNP | Rvnr01 | rRNA | rRNA | - | | 1473110 | T | G | 234.77 | SNP | Rvnr01 | rRNA | rRNA | - | | 1473111 | A | G | 242.77 | SNP | Rvnr01 | rRNA | rRNA | - | | 1473121 | T | C | 176.77 | SNP | Rvnr01 | rRNA | rRNA | - | | 1473122 | T | A | 189.77 | SNP | Rvnr01 | rRNA | rRNA | - | | 1473145 | C | T | 253.77 | SNP | Rvnr01 | rRNA | rRNA | - | | 1473166 | G | A | 271.77 | SNP | Rvnr01 | rRNA | rRNA | - | | 1473173 | C | T | 221.77 | SNP | Rvnr01 | rRNA | rRNA | - | | 1473252 | T | C | 220.77 | SNP | Rvnr01 | rRNA | rRNA | - | | 1473266 | A | G | 231.77 | SNP | Rvnr01 | rRNA | rRNA | - | | 1473276 | A | G | 277.77 | SNP | Rvnr01 | rRNA | rRNA | - | | 1473283 | T | C | 195.77 | SNP | Rvnr01 | rRNA | rRNA | - | | 1473290 | C | T | 140.77 | SNP | Rvnr01 | rRNA | rRNA | - | | 1473301 | T | C | 224.77 | SNP | Rvnr01 | rRNA | rRNA | - | | 1473310 | T | C | 161.77 | SNP | Rvnr01 | rRNA | rRNA | - | | 1474218 | T | A | 42.77 | SNP | Rvnr02 | rRNA | rRNA | - | | 1474488 | G | T | 49.77 | SNP | Rvnr02 | rRNA | rRNA | - | | 1474516 | C | A | 126.77 | SNP | Rvnr02 | rRNA | rRNA | - | | 1474537 | G | A | 150.77 | SNP | Rvnr02 | rRNA | rRNA | - | | 1474584 | C | G | 221.77 | SNP | Rvnr02 | rRNA | rRNA | - | | 1474626 | T | C | 251.77 | SNP | Rvnr02 | rRNA | rRNA | - | | 1474627 | G | A | 241.77 | SNP | Rvnr02 | rRNA | rRNA | - | | 1474632 | G | T | 268.77 | SNP | Rvnr02 | rRNA | rRNA | - | | 1474636 | A | T | 258.77 | SNP | Rvnr02 | rRNA | rRNA | - | | 1474637 | C | T | 239.77 | SNP | Rvnr02 | rRNA | rRNA | - | | 1474638 | C | G | 242.77 | SNP | Rvnr02 | rRNA | rRNA | - | | 1474639 | G | C | 230.77 | SNP | Rvnr02 | rRNA | rRNA | - | | 1474651 | CT | C | 678.73 | DEL | Rvnr02 |  |  | - | | 1474673 | T | C | 304.77 | SNP | Rvnr02 | rRNA | rRNA | - | | 1474676 | T | A | 297.77 | SNP | Rvnr02 | rRNA | rRNA | - | | 1474692 | G | A | 163.77 | SNP | Rvnr02 | rRNA | rRNA | - | | 1474734 | G | T | 141.77 | SNP | Rvnr02 | rRNA | rRNA | - | | 1474740 | G | C | 108.77 | SNP | Rvnr02 | rRNA | rRNA | - | | 1474747 | C | G | 109.77 | SNP | Rvnr02 | rRNA | rRNA | - | | 1474749 | C | T | 101.77 | SNP | Rvnr02 | rRNA | rRNA | - | | 1474760 | A | G | 148.77 | SNP | Rvnr02 | rRNA | rRNA | - | | 1474779 | G | A | 117.77 | SNP | Rvnr02 | rRNA | rRNA | - | | 1474780 | C | T | 91.77 | SNP | Rvnr02 | rRNA | rRNA | - | | 1474794 | C | T | 105.77 | SNP | Rvnr02 | rRNA | rRNA | - | | 1474812 | G | A | 347.77 | SNP | Rvnr02 | rRNA | rRNA | - | | 1474823 | C | G | 344.77 | SNP | Rvnr02 | rRNA | rRNA | - | | 1474831 | A | G | 130.77 | SNP | Rvnr02 | rRNA | rRNA | - | | 1474904 | G | C | 306.77 | SNP | Rvnr02 | rRNA | rRNA | - | | 1474905 | T | C | 325.77 | SNP | Rvnr02 | rRNA | rRNA | - | | 1474913 | T | C | 232.77 | SNP | Rvnr02 | rRNA | rRNA | - | | 1475649 | A | G | 100.77 | SNP | Rvnr02 | rRNA | rRNA | - | | 1475659 | G | A | 169.77 | SNP | Rvnr02 | rRNA | rRNA | - | | 1475699 | C | T | 105.77 | SNP | Rvnr02 | rRNA | rRNA | - | | 1475884 | A | G | 480.77 | SNP | Rvnr02 | rRNA | rRNA | - | | 1475897 | T | C | 584.77 | SNP | Rvnr02 | rRNA | rRNA | - | | 1475900 | A | G | 526.77 | SNP | Rvnr02 | rRNA | rRNA | - | | 1475952 | A | G | 350.77 | SNP | Rvnr02 | rRNA | rRNA | - | | 1475970 | C | T | 87.77 | SNP | Rvnr02 | rRNA | rRNA | - | | 1475975 | C | T | 70.77 | SNP | Rvnr02 | rRNA | rRNA | - | | 1475977 | A | G | 71.77 | SNP | Rvnr02 | rRNA | rRNA | - | | 1475982 | G | A | 45.77 | SNP | Rvnr02 | rRNA | rRNA | - | | 1475988 | A | G | 139.77 | SNP | Rvnr02 | rRNA | rRNA | - | | 1476001 | T | C | 45.77 | SNP | Rvnr02 | rRNA | rRNA | - | | 1476214 | G | T | 71.77 | SNP | Rvnr02 | rRNA | rRNA | - | | 1476224 | A | G | 68.77 | SNP | Rvnr02 | rRNA | rRNA | - | | 1476245 | C | T | 88.77 | SNP | Rvnr02 | rRNA | rRNA | - | | 1476251 | T | C | 215.77 | SNP | Rvnr02 | rRNA | rRNA | - | | 1476260 | A | G | 231.77 | SNP | Rvnr02 | rRNA | rRNA | - | | 1476301 | A | T | 44.77 | SNP | Rvnr02 | rRNA | rRNA | - | | 1476309 | G | C | 160.77 | SNP | Rvnr02 | rRNA | rRNA | - | | 1476428 | C | T | 564.77 | SNP | Rvnr02 | rRNA | rRNA | - | | 1476466 | C | T | 378.77 | SNP | Rvnr02 | rRNA | rRNA | - | | 1476506 | T | C | 381.77 | SNP | Rvnr02 | rRNA | rRNA | - | | 1476517 | C | T | 251.77 | SNP | Rvnr02 | rRNA | rRNA | - | | 1476524 | C | A | 216.77 | SNP | Rvnr02 | rRNA | rRNA | - | | 1476528 | A | G | 206.77 | SNP | Rvnr02 | rRNA | rRNA | - | | 1476536 | G | A | 217.77 | SNP | Rvnr02 | rRNA | rRNA | - | | 1476538 | A | G | 212.77 | SNP | Rvnr02 | rRNA | rRNA | - | | 1476540 | C | G | 214.77 | SNP | Rvnr02 | rRNA | rRNA | - | | 1476584 | C | T | 389.77 | SNP | Rvnr02 | rRNA | rRNA | - | | 1476585 | A | G | 230.77 | SNP | Rvnr02 | rRNA | rRNA | - | | 1476594 | C | A | 235.77 | SNP | Rvnr02 | rRNA | rRNA | - | | 1476603 | G | T | 219.77 | SNP | Rvnr02 | rRNA | rRNA | - | | 1476608 | C | T | 205.77 | SNP | Rvnr02 | rRNA | rRNA | - | | 1476614 | A | T | 138.77 | SNP | Rvnr02 | rRNA | rRNA | - | | 1476616 | A | G | 118.77 | SNP | Rvnr02 | rRNA | rRNA | - | | 1476619 | C | T | 79.77 | SNP | Rvnr02 | rRNA | rRNA | - | | 1476628 | T | C | 153.77 | SNP | Rvnr02 | rRNA | rRNA | - | | 1476629 | C | A | 110.77 | SNP | Rvnr02 | rRNA | rRNA | - | | 1476630 | A | G | 79.77 | SNP | Rvnr02 | rRNA | rRNA | - | | 1480945 | C | G | 722.77 | SNP | Rv1319c | silent (Thr519) | 9871 | - | | 1480948 | C | T | 666.77 | SNP | Rv1319c | silent (Glu518) | 9865 | - | | 1480972 | T | C | 934.77 | SNP | Rv1319c | silent (Glu510) | 9865 | - | | 1481185 | A | C | 753.77 | SNP | Rv1319c | Asp439Glu | 56 | - | | 1481321 | A | G | 586.77 | SNP | Rv1319c | Val394Ala | 18 | - | | 1482627 | T | C | 1797.77 | SNP | Rv1320c | Thr531Ala | 32 | - | | 1483652 | A | G | 1687.77 | SNP | Rv1320c | Leu189Pro | 2 | - | | 1484708 | A | C | 1444.77 | SNP | Rv1321 | Ser144Arg | 6 | - | | 1488433 | A | G | 123.77 | SNP | Rv1325c (PE\_PGRS24) | silent (Asp511) | 9859 | - | | 1488434 | T | G | 97.77 | SNP | Rv1325c (PE\_PGRS24) | Asp511Ala | 10 | - | | 1488435 | C | A | 102.77 | SNP | Rv1325c (PE\_PGRS24) | Asp511Tyr | 0 | - | | 1499274 | C | G | 814.77 | SNP | Rv1330c (pncB1) | Gly429Ala | 21 | - | | 1514384 | C | T | 1676.77 | SNP | Rv1348 (irtA) | silent (Thr446) | 9871 | - | | 1526819 | C | A | 1169.77 | SNP | Rv1358 | silent (Arg70) | 9913 | - | | 1533208 | C | G | 917.77 | SNP | Rv1361c (PPE19) | silent (Gly142) | 9935 | - | | 1536251 | G | T | 1732.77 | SNP | Rv1364c | Ala465Glu | 10 | - | | 1537710 | AAC | A | 4713.73 | DEL | intergenic |  |  | - | | 1537771 | G | C | 2072.77 | SNP | intergenic |  |  | - | | 1544349 | T | G | 2128.77 | SNP | Rv1371 | Trp331Gly | 0 | - | | 1547125 | T | C | 1630.77 | SNP | Rv1374c | Thr136Ala | 32 | - | | 1552547 | G | A | 1023.77 | SNP | Rv1378c | Arg37Trp | 2 | - | | 1570566 | C | A | 1649.77 | SNP | Rv1394c (cyp132) | Arg135Leu | 1 | - | | 1572200 | A | G | 280.78 | SNP | Rv1396c (PE\_PGRS25) | Val553Ala | 18 | - | | 1588899 | G | T | 1388.77 | SNP | Rv1412 (ribC) | silent (Ala111) | 9867 | - | | 1597405 | G | A | 1037.77 | SNP | Rv1422 | silent (Pro175) | 9926 | - | | 1597696 | G | A | 1567.77 | SNP | Rv1422 | silent (Glu272) | 9865 | - | | 1609840 | A | G | 1611.77 | SNP | Rv1431 | silent (Pro586) | 9926 | - | | 1612624 | T | TATCGGTACCGGTGCGCCAG GG | 2219.73 | INS | Rv1435c |  |  | - | | 1613035 | T | C | 1857.77 | SNP | intergenic |  |  | - | | 1620135 | A | G | 1072.77 | SNP | Rv1442 (bisC) | silent (Gly115) | 9935 | - | | 1620843 | G | C | 1311.77 | SNP | Rv1442 (bisC) | silent (Gly351) | 9935 | - | | 1630148 | A | C | 1755.77 | SNP | Rv1449c (tkt) | Tyr18Asp | 0 | - | | 1636996 | G | C | 45.74 | SNP | Rv1452c (PE\_PGRS28) | Arg412Gly | 1 | - | | 1639594 | C | A | 1575.77 | SNP | Rv1453 | Pro405Gln | 6 | - | | 1650072 | A | G | 1060.77 | SNP | Rv1462 | Asn183Asp | 42 | - | | 1651142 | G | A | 1230.18 | SNP | Rv1463 | Ala143Thr | 22 | - | | 1655829 | G | GC | 289.74 | INS | Rv1468c (PE\_PGRS29) |  |  | - | | 1676290 | C | A | 1966.77 | SNP | Rv1486c | Lys198Asn | 13 | - | | 1689349 | C | T | 1243.77 | SNP | Rv1498c | Arg191His | 8 | - | | 1692141 | A | C | 1590.77 | SNP | Rv1501 | silent (Ile84) | 9872 | - | | 1693561 | A | G | 1982.77 | SNP | Rv1502 | Tyr213Cys | 3 | - | | 1693593 | T | G | 1556.77 | SNP | Rv1502 | Trp224Gly | 0 | - | | 1695518 | C | A | 2780.77 | SNP | Rv1505c | silent (Ser143) | 9840 | - | | 1696464 | C | G | 1947.77 | SNP | intergenic |  |  | - | | 1698911 | G | A | 1331.77 | SNP | Rv1508c | silent (Gly328) | 9935 | - | | 1706119 | T | C | 1643.77 | SNP | Rv1514c | silent (Ser159) | 9840 | - | | 1728837 | A | G | 1946.77 | SNP | intergenic |  |  | - | | 1736577 | A | G | 1656.77 | SNP | Rv1536 (ileS) | Glu20Gly | 7 | - | | 1751042 | C | T | 1792.77 | SNP | Rv1547 (dnaE1) | Pro1117Ser | 17 | - | | 1752561 | T | C | 921.77 | SNP | Rv1548c (PPE21) | Asp258Gly | 11 | - | | 1753519 | G | GC | 3302.73 | INS | Rv1549 (fadD11.1) |  |  | - | | 1759252 | G | T | 1484.77 | SNP | Rv1552 (frdA) | silent (Ser524) | 9840 | genotype | | 1760292 | A | G | 1794.77 | SNP | Rv1554 (frdC) | Met(s)40Val(s) | 9867 | - | | 1778430 | T | C | 1028.77 | SNP | Rv1570 (bioD) | Met(s)191Thr | 22 | - | | 1779370 | G | C | 1734.77 | SNP | Rv1573 | silent (Thr19) | 9871 | - | | 1780048 | A | G | 576.77 | SNP | Rv1574 | His40Arg | 10 | - | | 1780274 | A | C | 586.77 | SNP | Rv1575 | Lys26Gln | 6 | - | | 1780275 | A | G | 502.77 | SNP | Rv1575 | Lys26Arg | 19 | - | | 1780329 | T | G | 344.77 | SNP | Rv1575 | Leu44Arg | 1 | - | | 1780359 | C | T | 181.77 | SNP | Rv1575 | Ala54Val | 13 | - | | 1780586 | C | CG | 1910.73 | INS | Rv1575 |  |  | - | | 1781577 | G | A | 892.77 | SNP | Rv1576c | Thr163Ile | 7 | - | | 1788613 | C | T | 1357.77 | SNP | Rv1587c | Gly184Asp | 6 | - | | 1789507 | T | C | 134.77 | SNP | Rv1588c | silent (Leu110) | 9947 | - | | 1789509 | G | A | 113.77 | SNP | Rv1588c | silent (Leu110) | 9947 | - | | 1789516 | A | G | 121.77 | SNP | Rv1588c | silent (Gly107) | 9935 | - | | 1789564 | C | T | 162.77 | SNP | Rv1588c | silent (Arg91) | 9913 | - | | 1789565 | C | A | 205.77 | SNP | Rv1588c | Arg91Leu | 1 | - | | 1789650 | C | T | 286.77 | SNP | Rv1588c | Ala63Thr | 22 | - | | 1789654 | A | G | 262.77 | SNP | Rv1588c | silent (Leu61) | 9947 | - | | 1789671 | C | T | 324.77 | SNP | Rv1588c | Ala56Thr | 22 | - | | 1789675 | A | C | 515.77 | SNP | Rv1588c | silent (Gly54) | 9935 | - | | 1789678 | C | G | 338.77 | SNP | Rv1588c | Val(s)53Val | 13 | - | | 1789742 | G | C | 886.77 | SNP | Rv1588c | Thr32Ser | 38 | - | | 1789746 | A | G | 928.77 | SNP | Rv1588c | Leu(s)31Leu | 3 | - | | 1789766 | T | G | 1007.77 | SNP | Rv1588c | Asp24Ala | 10 | - | | 1798355 | G | A | 1380.77 | SNP | Rv1597 | Gly21Asp | 6 | - | | 1803265 | G | A | 2053.77 | SNP | Rv1602 (hisH) | Ser201Asn | 20 | - | | 1804409 | C | A | 1666.77 | SNP | Rv1604 (impA) | Pro124Gln | 6 | - | | 1808795 | A | C | 1924.77 | SNP | Rv1609 (trpE) | Asp298Ala | 10 | - | | 1814629 | G | A | 1142.77 | SNP | intergenic |  |  | - | | 1817976 | A | T | 1708.77 | SNP | Rv1618 (tesB1) | His121Leu | 4 | - | | 1836286 | G | C | 1629.77 | SNP | intergenic |  |  | - | | 1847919 | C | G | 1424.77 | SNP | Rv1639c | silent (Thr180) | 9871 | - | | 1854300 | T | C | 1350.77 | SNP | Rv1644 (tsnR) | Leu232Pro | 2 | - | | 1856777 | G | C | 1368.77 | SNP | Rv1647 | Ala2Pro | 13 | - | | 1864698 | C | T | 434.18 | SNP | Rv1651c (PE\_PGRS30) | Ala229Thr | 22 | - | | 1885772 | G | A | 1845.77 | SNP | Rv1662 (pks8) | Ala1357Thr | 22 | - | | 1894300 | G | GGTCTTGCCGC | 4031.73 | INS | Rv1668c |  |  | - | | 1901493 | T | C | 1510.77 | SNP | Rv1676 | silent (Ser149) | 9840 | - | | 1907296 | G | C | 1412.77 | SNP | Rv1682 | silent (Ala298) | 9867 | - | | 1917289 | C | T | 837.77 | SNP | Rv1692 | Arg198Trp | 2 | - | | 1917972 | A | G | 1190.77 | SNP | Rv1694 (tlyA) | silent (Leu11) | 9947 | - | | 1924500 | C | T | 1602.77 | SNP | Rv1699 (pyrG) | silent (Cys224) | 9973 | - | | 1931179 | C | A | 1654.77 | SNP | Rv1704c (cycA) | Arg93Leu | 1 | - | | 1933988 | G | A | 2056.77 | SNP | intergenic |  |  | - | | 1942489 | T | C | 996.77 | SNP | Rv1714 | Phe213Leu | 13 | - | | 1944402 | T | C | 1143.77 | SNP | Rv1716 | Val276Ala | 18 | - | | 1950767 | T | C | 1860.77 | SNP | Rv1724c | silent (Lys95) | 9926 | - | | 1955910 | T | G | 1873.77 | SNP | Rv1730c | silent (Arg446) | 9913 | - | | 1960284 | C | A | 1152.77 | SNP | Rv1733c | Gln68His | 20 | - | | 1967237 | C | A | 1608.77 | SNP | Rv1739c | Arg134Leu | 1 | - | | 1978166 | G | T | 1566.77 | SNP | Rv1750c (fadD1) | Gln468Lys | 12 | - | | 1982961 | GC | G | 1649.73 | DEL | Rv1753c (PPE24) |  |  | - | | 1983140 | T | C | 544.77 | SNP | Rv1753c (PPE24) | Thr546Ala | 32 | - | | 1983218 | T | C | 1044.77 | SNP | Rv1753c (PPE24) | Thr520Ala | 32 | - | | 1990942 | C | T | 671.77 | SNP | Rv1759c (wag22) | Gly546Ser | 16 | - | | 1992323 | G | GC | 742.73 | INS | Rv1759c (wag22) |  |  | - | | 1993808 | A | T | 1161.77 | SNP | Rv1760 | Glu219Val(s) | 17 | - | | 1994939 | G | A | 1435.77 | SNP | Rv1761c | Thr39Ile | 7 | - | | 1998852 | G | T | 1568.77 | SNP | intergenic |  |  | - | | 2009881 | T | C | 1543.77 | SNP | Rv1775 | Ile237Thr | 11 | - | | 2022868 | T | C | 1289.77 | SNP | Rv1783 (eccC5) | silent (Ser1204) | 9840 | - | | 2033748 | G | C | 2039.77 | SNP | Rv1795 (eccD5); Rv1796 (mycP5) | silent (Arg503); Gly7Ala | 9913; 21 | - | | 2045310 | A | G | 787.77 | SNP | Rv1803c (PE\_PGRS32) | silent (Ile511) | 9872 | - | | 2049065 | T | C | 1847.77 | SNP | intergenic |  |  | - | | 2049097 | G | C | 1913.77 | SNP | intergenic |  |  | - | | 2051746 | T | C | 1440.77 | SNP | Rv1809 (PPE33) | silent (Ala155) | 9867 | - | | 2052035 | G | T | 2336.77 | SNP | Rv1809 (PPE33) | Val(s)252Leu(s) | 9867 | - | | 2055271 | A | G | 1430.77 | SNP | Rv1812c | Leu30Pro | 2 | - | | 2057774 | A | T | 954.77 | SNP | Rv1815 | Ile83Phe | 8 | - | | 2060383 | C | T | 1569.77 | SNP | Rv1817 | silent (Asp263) | 9859 | - | | 2074570 | G | C | 176.77 | SNP | intergenic |  |  | - | | 2088619 | CCCGCCGTTG | C | 633.80 | DEL | Rv1840c (PE\_PGRS34) |  |  | - | | 2094911 | ACAGCGT | A | 4721.73 | DEL | Rv1844c (gnd1) |  |  | - | | 2096186 | A | G | 947.77 | SNP | Rv1846c (blaI) | silent (Thr138) | 9871 | - | | 2107371 | G | A | 928.77 | SNP | Rv1859 (modC) | silent (Pro266) | 9926 | - | | 2109523 | C | CG | 2610.73 | INS | intergenic |  |  | - | | 2116903 | C | T | 1223.77 | SNP | Rv1867 | silent (Gly380) | 9935 | - | | 2128870 | A | G | 1372.77 | SNP | Rv1878 (glnA3) | silent (Leu283) | 9947 | - | | 2133468 | T | TTCGCATGCCGTCACC | 1859.73 | INS | Rv1883c |  |  | - | | 2135870 | T | C | 1080.77 | SNP | intergenic |  |  | - | | 2143217 | T | C | 1094.77 | SNP | Rv1895 | Ser233Pro | 12 | - | | 2143328 | G | C | 1701.77 | SNP | Rv1895 | Val(s)270Leu | 3 | - | | 2147022 | A | C | 1966.77 | SNP | Rv1900c (lipJ) | Ile204Met(s) | 6 | - | | 2149855 | C | CA | 3101.73 | INS | Rv1902c (nanT) |  |  | - | | 2155168 | C | G | 1651.77 | SNP | Rv1908c (katG) | Ser315Thr | 32 | resistance | | 2163375 | T | C | 726.77 | SNP | Rv1917c (PPE34) | Asn1313Asp | 42 | - | | 2163412 | A | G | 1226.77 | SNP | Rv1917c (PPE34) | silent (Val1300) | 9901 | - | | 2163415 | C | A | 1034.77 | SNP | Rv1917c (PPE34) | silent (Pro1299) | 9926 | - | | 2163417 | G | C | 1026.77 | SNP | Rv1917c (PPE34) | Pro1299Ala | 22 | - | | 2163419 | C | T | 1065.77 | SNP | Rv1917c (PPE34) | Ser1298Asn | 20 | - | | 2163421 | C | G | 883.77 | SNP | Rv1917c (PPE34) | silent (Thr1297) | 9871 | - | | 2163444 | T | C | 193.77 | SNP | Rv1917c (PPE34) | Asn1290Asp | 42 | - | | 2163790 | A | C | 877.77 | SNP | Rv1917c (PPE34) | silent (Pro1174) | 9926 | - | | 2165286 | A | C | 1095.77 | SNP | Rv1917c (PPE34) | Ser676Ala | 35 | - | | 2165503 | T | A | 773.77 | SNP | Rv1917c (PPE34) | silent (Ala603) | 9867 | - | | 2165928 | G | T | 1274.77 | SNP | Rv1917c (PPE34) | Pro462Thr | 5 | - | | 2184781 | G | T | 1168.77 | SNP | Rv1933c (fadE18) | silent (Gly59) | 9935 | - | | 2186542 | G | A | 1095.77 | SNP | Rv1935c (echA13) | silent (Leu206) | 9947 | - | | 2196879 | T | A | 2472.77 | SNP | Rv1945 | silent (Leu297) | 9947 | - | | 2196882 | A | G | 2556.77 | SNP | Rv1945 | silent (Lys298) | 9926 | - | | 2196964 | A | C | 168.77 | SNP | Rv1945 | Asn326His | 18 | - | | 2196969 | G | C | 166.77 | SNP | Rv1945 | silent (Ala327) | 9867 | - | | 2196970 | C | A | 148.77 | SNP | Rv1945 | His328Asn | 21 | - | | 2198579 | GAACCA | G | 5485.73 | DEL | intergenic |  |  | - | | 2207591 | T | TC | 3311.73 | INS | intergenic |  |  | - | | 2211826 | A | G | 1288.77 | SNP | Rv1968 (mce3C) | silent (Lys67) | 9926 | - | | 2216443 | C | A | 1529.77 | SNP | Rv1971 (mce3F) | Ala396Glu | 10 | - | | 2220512 | T | G | 1900.77 | SNP | Rv1977 | silent (Ser253) | 9840 | - | | 2223293 | T | C | 1691.77 | SNP | intergenic |  |  | - | | 2228967 | A | G | 1647.77 | SNP | intergenic |  |  | - | | 2251999 | A | G | 1449.77 | SNP | intergenic |  |  | - | | 2260151 | A | G | 290.77 | SNP | intergenic |  |  | - | | 2260154 | C | T | 319.77 | SNP | intergenic |  |  | - | | 2260171 | T | C | 573.77 | SNP | intergenic |  |  | - | | 2260174 | C | T | 618.77 | SNP | intergenic |  |  | - | | 2260196 | C | CA | 982.73 | INS | intergenic |  |  | - | | 2260199 | C | T | 597.77 | SNP | intergenic |  |  | - | | 2260212 | G | T | 501.77 | SNP | intergenic |  |  | - | | 2260214 | G | C | 445.77 | SNP | intergenic |  |  | - | | 2260220 | C | T | 436.77 | SNP | intergenic |  |  | - | | 2260222 | C | G | 404.77 | SNP | intergenic |  |  | - | | 2260231 | T | C | 382.77 | SNP | intergenic |  |  | - | | 2260525 | C | T | 1296.77 | SNP | intergenic |  |  | - | | 2264782 | C | A | 1515.77 | SNP | Rv2017 | Ala262Glu | 10 | - | | 2265059 | T | G | 846.77 | SNP | intergenic |  |  | - | | 2266487 | G | C | 1267.77 | SNP | Rv2020c | silent (Leu78) | 9947 | - | | 2266504 | T | TA | 2171.73 | INS | Rv2020c |  |  | - | | 2266508 | A | T | 1079.77 | SNP | Rv2020c | Asp71Glu | 56 | - | | 2266511 | GT | G | 1815.73 | DEL | Rv2020c |  |  | - | | 2266517 | T | C | 1151.77 | SNP | Rv2020c | silent (Glu68) | 9865 | - | | 2266550 | G | T | 1339.77 | SNP | Rv2020c | silent (Gly57) | 9935 | - | | 2266553 | C | G | 1386.77 | SNP | Rv2020c | silent (Ser56) | 9840 | - | | 2266583 | C | G | 1692.77 | SNP | Rv2020c | Glu46Asp | 53 | - | | 2266598 | G | C | 1545.77 | SNP | Rv2020c | silent (Leu41) | 9947 | - | | 2266604 | C | G | 1498.77 | SNP | Rv2020c | silent (Ser39) | 9840 | - | | 2266613 | G | GC | 2504.73 | INS | Rv2020c |  |  | - | | 2266624 | G | T | 1306.77 | SNP | Rv2020c | Leu33Ile | 9 | - | | 2269780 | T | C | 1187.77 | SNP | Rv2024c | Asp154Gly | 11 | - | | 2270102 | A | G | 1425.77 | SNP | Rv2024c | Trp47Arg | 8 | - | | 2273627 | C | T | 1160.77 | SNP | Rv2027c (dosT) | silent (Gly294) | 9935 | - | | 2281665 | C | T | 2209.77 | SNP | Rv2035 | Pro18Ser | 17 | - | | 2282787 | C | T | 849.77 | SNP | Rv2037c | Cys312Tyr | 3 | - | | 2285251 | C | A | 1704.77 | SNP | Rv2039c | Val131Phe | 0 | - | | 2287121 | A | G | 1655.77 | SNP | Rv2041c | silent (Asp242) | 9859 | - | | 2289081 | G | T | 2154.77 | SNP | Rv2043c (pncA) | Pro54Gln | 6 | resistance | | 2296042 | G | C | 1029.77 | SNP | Rv2048c (pks12) | Pro3649Ala | 22 | - | | 2297287 | G | T | 1622.77 | SNP | Rv2048c (pks12) | Gln3234Lys | 12 | - | | 2300237 | A | G | 1168.77 | SNP | Rv2048c (pks12) | silent (Ala2250) | 9867 | - | | 2300546 | A | T | 1529.77 | SNP | Rv2048c (pks12) | His2147Gln | 23 | - | | 2300552 | T | G | 1401.77 | SNP | Rv2048c (pks12) | silent (Pro2145) | 9926 | - | | 2300555 | A | G | 1451.77 | SNP | Rv2048c (pks12) | silent (Asp2144) | 9859 | - | | 2310543 | G | A | 1399.77 | SNP | Rv2051c (ppm1) | silent (Gly71) | 9935 | - | | 2329533 | A | G | 1149.77 | SNP | Rv2072c (cobL) | Leu205Pro | 2 | - | | 2331061 | G | T | 1180.77 | SNP | Rv2074 | silent (Leu23) | 9947 | - | | 2334007 | A | G | 1419.77 | SNP | Rv2077c | silent (Ala96) | 9867 | - | | 2334290 | AGCATCTAAACCACCGTCAC CTGCGTCACCGCGGCCATCT CGCTC | A | 11726.73 | DEL | Rv2077c |  |  | - | | 2335494 | A | G | 1588.77 | SNP | Rv2079 | Tyr47Cys | 3 | - | | 2340621 | C | G | 1269.77 | SNP | Rv2082 | Pro638Arg | 4 | - | | 2341636 | C | G | 986.77 | SNP | Rv2083 | Leu256Val(s) | 4 | - | | 2345037 | C | A | 1104.77 | SNP | Rv2088 (pknJ) | silent (Leu209) | 9947 | - | | 2346672 | T | C | 1296.77 | SNP | Rv2089c (pepE) | Asp218Gly | 11 | - | | 2347643 | C | T | 1365.77 | SNP | Rv2090 | Arg91Cys | 1 | - | | 2352078 | C | G | 588.77 | SNP | intergenic |  |  | - | | 2355511 | G | A | 2012.77 | SNP | Rv2097c (pafA) | silent (Val389) | 9901 | - | | 2357268 | TGCC | T | 1076.73 | DEL | intergenic |  |  | - | | 2358104 | G | C | 754.77 | SNP | intergenic |  |  | - | | 2361604 | C | G | 1216.77 | SNP | Rv2101 (helZ) | Val455Val(s) | 18 | - | | 2362041 | C | A | 1205.77 | SNP | Rv2101 (helZ) | Pro601Gln | 6 | - | | 2368564 | TA | T | 3538.73 | DEL | intergenic |  |  | - | | 2369971 | A | G | 758.77 | SNP | Rv2110c (prcB) | Tyr211His | 4 | - | | 2374245 | C | T | 1693.77 | SNP | Rv2114 | Gln138STOP | 8 | - | | 2377785 | C | T | 1213.77 | SNP | Rv2118c | Val(s)176Val | 13 | - | | 2386389 | G | A | 1066.77 | SNP | Rv2125 | Gly33Ser | 16 | - | | 2387733 | T | C | 287.78 | SNP | Rv2126c (PE\_PGRS37) | silent (Glu80) | 9865 | - | | 2400467 | G | A | 1155.77 | SNP | Rv2141c | Thr419Ile | 7 | - | | 2412516 | T | G | 1228.77 | SNP | Rv2153c (murG) | Gln279Pro | 8 | - | | 2415656 | G | C | 970.77 | SNP | Rv2155c (murD) | Arg247Gly | 1 | - | | 2424925 | A | G | 880.77 | SNP | intergenic |  |  | - | | 2440953 | G | T | 1164.77 | SNP | Rv2178c (aroG) | silent (Arg256) | 9913 | - | | 2453645 | A | C | 1041.77 | SNP | intergenic |  |  | - | | 2465997 | T | G | 1406.77 | SNP | Rv2201 (asnB) | Ile334Ser | 2 | - | | 2476977 | G | A | 1381.77 | SNP | Rv2211c (gcvT) | Leu69Leu(s) | 4 | - | | 2499726 | G | A | 1414.77 | SNP | Rv2226 | Asp299Asn | 36 | - | | 2509140 | G | C | 813.77 | SNP | Rv2236c (cobD) | Ser79Cys | 5 | - | | 2509722 | A | G | 1339.77 | SNP | Rv2237 | silent (Pro78) | 9926 | - | | 2521342 | T | C | 1065.77 | SNP | Rv2247 (accD6) | silent (Asp200) | 9859 | - | | 2523205 | G | GCGC | 2174.73 | INS | intergenic |  |  | - | | 2525722 | CG | C | 2584.73 | DEL | Rv2250A; Rv2251 |  |  | - | | 2529680 | A | G | 1470.77 | SNP | Rv2256c | silent (Thr65) | 9871 | - | | 2531742 | A | G | 1405.77 | SNP | Rv2258c | silent (Ala52) | 9867 | - | | 2534562 | GGA | G | 2793.73 | DEL | Rv2262c |  |  | - | | 2551572 | A | C | 1516.77 | SNP | Rv2280 | Thr5Pro | 4 | - | | 2586127 | A | G | 1551.77 | SNP | Rv2314c | silent (Gly388) | 9935 | - | | 2589491 | C | T | 1685.77 | SNP | Rv2316 (uspA) | silent (Ile218) | 9872 | - | | 2598400 | A | G | 1080.77 | SNP | Rv2326c | silent (Asn516) | 9822 | - | | 2602456 | T | C | 1555.77 | SNP | Rv2329c (narK1) | Ile336Val | 57 | - | | 2612632 | C | A | 1572.77 | SNP | Rv2337c | Gly119Val | 3 | - | | 2626004 | G | A | 1074.77 | SNP | Rv2346c (esxO) | Leu57Leu(s) | 4 | - | | 2626149 | A | C | 1073.77 | SNP | Rv2346c (esxO) | silent (Gly8) | 9935 | - | | 2639364 | C | A | 311.77 | SNP | Rv2356c (PPE40) | Gly58Cys | 0 | - | | 2656225 | A | G | 1686.77 | SNP | Rv2377c (mbtH) | Val69Ala | 18 | - | | 2660319 | C | G | 978.77 | SNP | Rv2379c (mbtF) | Glu589Asp | 53 | - | | 2680658 | T | G | 1673.77 | SNP | intergenic |  |  | - | | 2695378 | C | G | 1817.77 | SNP | Rv2398c (cysW) | Gly141Ala | 21 | - | | 2703345 | C | A | 949.77 | SNP | Rv2405 | Thr26Lys | 11 | - | | 2704884 | A | ACAGCGACCATATCGCCGAG CT | 1717.73 | INS | Rv2407 |  |  | - | | 2713795 | C | T | 1406.77 | SNP | intergenic |  |  | - | | 2718852 | T | G | 1795.77 | SNP | intergenic |  |  | - | | 2734074 | T | C | 323.77 | SNP | Rv2436 (rbsK) | Val282Ala | 18 | - | | 2748712 | T | G | 1404.77 | SNP | Rv2448c (valS) | Asp505Ala | 10 | - | | 2751804 | C | T | 1187.77 | SNP | Rv2450c (rpfE) | Arg126Gln | 9 | - | | 2752698 | C | A | 2134.77 | SNP | intergenic |  |  | - | | 2760152 | A | G | 1146.77 | SNP | Rv2458 (mmuM) | Tyr125Cys | 3 | - | | 2779136 | T | C | 1245.77 | SNP | Rv2476c (gdh) | Ser1043Gly | 21 | - | | 2786952 | A | G | 1357.77 | SNP | Rv2482c (plsB2) | Cys778Arg | 1 | - | | 2795160 | C | T | 1748.77 | SNP | intergenic |  |  | - | | 2795713 | C | T | 363.78 | SNP | Rv2487c (PE\_PGRS42) | Gly558Asp | 6 | - | | 2807237 | G | A | 2005.77 | SNP | Rv2491 | Val(s)191Val | 13 | - | | 2809621 | T | C | 1578.77 | SNP | Rv2495c (bkdC) | Thr107Ala | 32 | - | | 2816034 | G | A | 1512.77 | SNP | Rv2501c (accA1) | Pro283Ser | 17 | - | | 2816296 | A | C | 1400.77 | SNP | Rv2501c (accA1) | Asp195Glu | 56 | - | | 2818837 | A | G | 1052.77 | SNP | Rv2503c (scoB) | silent (Gly97) | 9935 | - | | 2821077 | CGG | C | 3144.73 | DEL | Rv2505c (fadD35) |  |  | - | | 2821342 | C | T | 1215.77 | SNP | Rv2505c (fadD35) | silent (Ala85) | 9867 | - | | 2823309 | G | A | 1044.77 | SNP | Rv2508c | Arg429Trp | 2 | - | | 2827984 | G | T | 1163.77 | SNP | intergenic |  |  | - | | 2828019 | T | C | 1311.77 | SNP | intergenic |  |  | - | | 2828517 | A | G | 1163.77 | SNP | intergenic |  |  | - | | 2830525 | C | A | 1967.77 | SNP | Rv2513 | Thr122Lys | 11 | - | | 2836257 | G | A | 1256.77 | SNP | Rv2519 (PE26) | Gly158Asp | 6 | - | | 2840091 | C | T | 924.77 | SNP | intergenic |  |  | - | | 2855259 | A | G | 1399.77 | SNP | Rv2531c | silent (Ala841) | 9867 | - | | 2865760 | A | G | 1725.77 | SNP | Rv2542 | Thr211Ala | 32 | - | | 2865882 | T | C | 1472.77 | SNP | Rv2542 | silent (Val251) | 9901 | - | | 2881597 | AG | A | 2099.73 | DEL | Rv2561 |  |  | - | | 2888201 | T | C | 814.77 | SNP | Rv2566 | Leu610Pro | 2 | - | | 2889633 | T | C | 1337.77 | SNP | Rv2566 | silent (Ala1087) | 9867 | - | | 2891267 | C | T | 1339.77 | SNP | Rv2567 | silent (Gly491) | 9935 | - | | 2891728 | A | G | 1365.77 | SNP | Rv2567 | Gln645Arg | 10 | - | | 2894208 | G | A | 1246.77 | SNP | Rv2569c | silent (Ser67) | 9840 | - | | 2897375 | T | G | 1395.77 | SNP | Rv2572c (aspS) | silent (Arg143) | 9913 | - | | 2910461 | G | T | 1592.77 | SNP | Rv2584c (apt) | Ala147Glu | 10 | - | | 2911293 | C | G | 1284.77 | SNP | Rv2585c | Cys462Ser | 11 | - | | 2912294 | T | G | 1466.77 | SNP | Rv2585c | silent (Ala128) | 9867 | - | | 2922936 | TGGCGGTGAC | T | 1174.73 | DEL | Rv2591 (PE\_PGRS44) |  |  | - | | 2923391 | T | C | 1199.77 | SNP | Rv2592c (ruvB) | silent (Pro281) | 9926 | - | | 2927939 | T | C | 1514.77 | SNP | intergenic |  |  | - | | 2939373 | G | C | 1535.77 | SNP | Rv2611c | Ser197Cys | 5 | - | | 2939657 | T | C | 438.77 | SNP | Rv2611c | Ile102Met(s) | 6 | - | | 2944833 | G | C | 70.28 | SNP | Rv2615c (PE\_PGRS45) | silent (Ala51) | 9867 | - | | 2954439 | T | C | 1682.77 | SNP | Rv2627c | Arg104Gly | 1 | - | | 2971419 | G | A | 1627.77 | SNP | Rv2646 | Arg290His | 8 | - | | 2974933 | A | G | 829.77 | SNP | Rv2650c | Ile101Thr | 11 | - | | 2975900 | A | G | 927.77 | SNP | intergenic |  |  | - | | 2977033 | T | G | 445.84 | SNP | Rv2654c | Thr68Pro | 4 | - | | 2982955 | C | T | 1426.77 | SNP | Rv2665 | Pro86Leu | 3 | - | | 2983613 | G | A | 46.74 | SNP | Rv2666 | silent (Gly181) | 9935 | - | | 2984740 | A | G | 945.77 | SNP | Rv2668 | His3Arg | 10 | - | | 2996876 | A | C | 1164.77 | SNP | Rv2681 | silent (Leu46) | 9947 | - | | 3005185 | G | T | 1608.77 | SNP | Rv2688c | Pro156Thr | 5 | - | | 3006361 | CG | C | 2293.73 | DEL | Rv2689c |  |  | - | | 3009692 | A | G | 1791.77 | SNP | Rv2691 (ceoB) | Thr117Ala | 32 | - | | 3015966 | G | A | 665.77 | SNP | Rv2701c (suhB) | Ala257Val(s) | 9867 | - | | 3017465 | T | C | 2084.77 | SNP | Rv2702 (ppgK) | Ile203Thr | 11 | - | | 3019554 | G | T | 1591.77 | SNP | Rv2704 | Ala33Ser | 28 | - | | 3020515 | G | C | 1483.77 | SNP | intergenic |  |  | - | | 3021929 | C | T | 1844.77 | SNP | Rv2709 | Pro31Ser | 17 | - | | 3028658 | G | A | 1411.77 | SNP | Rv2715 | Leu(s)187Leu | 3 | - | | 3041871 | G | T | 1409.77 | SNP | Rv2729c | Ala202Glu | 10 | - | | 3054081 | A | G | 1658.77 | SNP | Rv2741 (PE\_PGRS47) | silent (Gly56) | 9935 | - | | 3054321 | A | G | 369.77 | SNP | Rv2741 (PE\_PGRS47) | silent (Gly136) | 9935 | - | | 3080795 | A | G | 1923.77 | SNP | Rv2771c | Leu80Pro | 2 | - | | 3100153 | G | GA | 3084.73 | INS | Rv2790c (ltp1) |  |  | - | | 3103682 | T | C | 1064.77 | SNP | Rv2794c (pptT) | Met(s)87Val(s) | 9867 | - | | 3118000 | A | G | 1451.77 | SNP | Rv2812 | Arg395Gly | 1 | - | | 3131469 | T | TTGTCGGCGA | 4903.73 | INS | Rv2823c |  |  | - | | 3133536 | T | C | 1763.77 | SNP | Rv2825c | Lys2Glu | 4 | - | | 3137058 | G | A | 1563.77 | SNP | Rv2830c (vapB22) | Ala56Val(s) | 9867 | - | | 3143100 | A | G | 1428.77 | SNP | Rv2836c (dinF) | Cys177Arg | 1 | - | | 3162805 | C | G | 230.80 | SNP | Rv2853 (PE\_PGRS48) | Arg180Gly | 1 | - | | 3170460 | C | T | 804.77 | SNP | Rv2858c (aldC) | Val(s)88Val | 13 | - | | 3175335 | C | T | 1292.77 | SNP | Rv2863 (vapC23) | Thr115Met(s) | 32 | - | | 3177884 | C | A | 1255.77 | SNP | Rv2866 (relG) | silent (Arg21) | 9913 | - | | 3181029 | A | C | 284.78 | SNP | Rv2869c (rip) | Val(s)245Gly | 21 | - | | 3186860 | T | G | 1440.77 | SNP | Rv2874 (dipZ) | Tyr672Asp | 0 | - | | 3190145 | TC | T | 1828.73 | DEL | Rv2880c |  |  | - | | 3226181 | A | C | 1343.77 | SNP | Rv2916c (ffh) | silent (Arg35) | 9913 | - | | 3228143 | G | T | 1567.77 | SNP | Rv2917 | Arg594Leu | 1 | - | | 3232759 | G | A | 343.77 | SNP | intergenic |  |  | - | | 3239643 | A | G | 32.77 | SNP | intergenic |  |  | - | | 3240568 | T | G | 1687.77 | SNP | Rv2926c | Thr202Pro | 4 | - | | 3247316 | C | G | 1140.77 | SNP | Rv2931 (ppsA) | Asp624Glu | 56 | - | | 3247851 | G | A | 1224.77 | SNP | Rv2931 (ppsA) | Ala803Thr | 22 | - | | 3247853 | C | T | 1183.77 | SNP | Rv2931 (ppsA) | silent (Ala803) | 9867 | - | | 3247856 | G | C | 1283.77 | SNP | Rv2931 (ppsA) | silent (Arg804) | 9913 | - | | 3247864 | C | CTAGG | 2870.73 | INS | Rv2931 (ppsA) |  |  | - | | 3247865 | GCAAA | G | 2851.73 | DEL | Rv2931 (ppsA) |  |  | - | | 3247874 | G | A | 1291.77 | SNP | Rv2931 (ppsA) | silent (Arg810) | 9913 | - | | 3247877 | T | C | 1336.77 | SNP | Rv2931 (ppsA) | silent (Phe811) | 9946 | - | | 3247883 | T | C | 1767.77 | SNP | Rv2931 (ppsA) | silent (Ser813) | 9840 | - | | 3248074 | G | A | 1316.77 | SNP | Rv2931 (ppsA) | Arg877His | 8 | - | | 3248075 | C | T | 1394.77 | SNP | Rv2931 (ppsA) | silent (Arg877) | 9913 | - | | 3256494 | A | G | 1988.77 | SNP | Rv2933 (ppsC) | silent (Gly270) | 9935 | - | | 3269581 | A | G | 1475.77 | SNP | Rv2935 (ppsE) | silent (Ala615) | 9867 | - | | 3270784 | A | G | 1791.77 | SNP | Rv2935 (ppsE) | silent (Gln1016) | 9876 | - | | 3296843 | A | G | 1069.77 | SNP | Rv2947c (pks15) | Val(s)333Ala | 9867 | - | | 3296924 | C | T | 571.77 | SNP | Rv2947c (pks15) | Arg306Gln | 9 | - | | 3300196 | G | A | 1976.77 | SNP | Rv2949c | silent (Phe125) | 9946 | - | | 3302802 | C | T | 1804.77 | SNP | intergenic |  |  | - | | 3304753 | G | T | 1640.77 | SNP | Rv2952 | Ala105Ser | 28 | - | | 3308606 | G | A | 2624.77 | SNP | intergenic |  |  | - | | 3317702 | C | T | 792.28 | SNP | intergenic |  |  | - | | 3336587 | T | A | 570.77 | SNP | intergenic |  |  | - | | 3336646 | T | A | 356.77 | SNP | intergenic |  |  | - | | 3336825 | T | C | 1127.77 | SNP | Rv2981c (ddlA) | Thr365Ala | 32 | - | | 3338603 | G | C | 1310.77 | SNP | Rv2982c (gpdA2) | Pro133Ala | 22 | - | | 3345749 | A | G | 1141.77 | SNP | Rv2988c (leuC) | silent (Asp109) | 9859 | - | | 3353548 | C | T | 1138.77 | SNP | Rv2996c (serA1) | Asp508Asn | 36 | - | | 3358235 | A | T | 1447.77 | SNP | Rv2999 (lppY) | Met(s)212Leu(s) | 9867 | - | | 3363338 | A | G | 1763.77 | SNP | intergenic |  |  | - | | 3366092 | A | C | 1298.77 | SNP | Rv3007c | Leu120Arg | 1 | - | | 3367765 | G | A | 706.77 | SNP | Rv3009c (gatB) | silent (Gly343) | 9935 | - | | 3371719 | G | A | 1688.77 | SNP | Rv3012c (gatC) | silent (Ile4) | 9872 | - | | 3379708 | G | C | 88.28 | SNP | intergenic |  |  | - | | 3379712 | G | C | 86.28 | SNP | intergenic |  |  | - | | 3379718 | T | C | 87.28 | SNP | intergenic |  |  | - | | 3379726 | C | A | 85.28 | SNP | intergenic |  |  | - | | 3379730 | G | C | 87.28 | SNP | intergenic |  |  | - | | 3379732 | C | T | 79.28 | SNP | intergenic |  |  | - | | 3379735 | A | C | 90.28 | SNP | intergenic |  |  | - | | 3379736 | C | A | 80.28 | SNP | intergenic |  |  | - | | 3379742 | T | C | 274.78 | SNP | intergenic |  |  | - | | 3379751 | A | C | 348.77 | SNP | intergenic |  |  | - | | 3379757 | A | C | 421.77 | SNP | intergenic |  |  | - | | 3379763 | G | A | 392.77 | SNP | intergenic |  |  | - | | 3379784 | C | A | 610.77 | SNP | intergenic |  |  | - | | 3379788 | C | G | 684.77 | SNP | intergenic |  |  | - | | 3381641 | G | T | 68.28 | SNP | Rv3023c | Gln328Lys | 12 | - | | 3382598 | C | T | 283.78 | SNP | Rv3023c | Ala9Thr | 22 | - | | 3382738 | G | A | 1023.77 | SNP | intergenic |  |  | - | | 3398280 | C | A | 1003.77 | SNP | Rv3037c | Arg4Leu | 1 | - | | 3402816 | C | T | 1817.77 | SNP | Rv3042c (serB2) | Gly116Glu | 4 | - | | 3405776 | A | G | 1187.77 | SNP | Rv3044 (fecB) | His214Arg | 10 | - | | 3409700 | CA | C | 2648.73 | DEL | Rv3049c |  |  | - | | 3415180 | ACACCTAGGGGGTGG | A | 6118.73 | DEL | intergenic |  |  | - | | 3417231 | T | C | 1618.77 | SNP | Rv3056 (dinP) | Met(s)176Thr | 22 | - | | 3425854 | C | T | 1710.77 | SNP | Rv3062 (ligB) | Pro91Ser | 17 | - | | 3428917 | C | A | 1675.77 | SNP | Rv3063 (cstA) | Arg559Ser | 11 | - | | 3440464 | T | G | 1345.77 | SNP | Rv3077 | silent (Arg308) | 9913 | - | | 3440468 | G | C | 1359.77 | SNP | Rv3077 | Gly310Arg | 0 | - | | 3456666 | A | G | 1446.77 | SNP | Rv3089 (fadD13) | silent (Ala302) | 9867 | - | | 3462135 | G | C | 775.77 | SNP | Rv3093c | Cys210Trp | 0 | - | | 3466426 | G | A | 1544.77 | SNP | Rv3097c (lipY) | silent (Val222) | 9901 | genotype | | 3467096 | C | T | 1663.77 | SNP | intergenic |  |  | - | | 3473996 | G | GA | 2677.73 | INS | intergenic |  |  | - | | 3477917 | C | T | 2076.77 | SNP | Rv3109 (moaA1) | Pro90Leu | 3 | - | | 3481458 | C | T | 779.77 | SNP | Rv3115 | Ser3Phe | 2 | - | | 3481475 | G | A | 555.77 | SNP | Rv3115 | Ala9Thr | 22 | - | | 3481596 | A | G | 126.03 | SNP | Rv3115 | Tyr49Cys | 3 | - | | 3482432 | C | A | 230.78 | SNP | Rv3115 | Gln328Lys | 12 | - | | 3482737 | T | C | 2349.77 | SNP | intergenic |  |  | - | | 3486977 | A | G | 2066.77 | SNP | Rv3121 (cyp141) | Lys157Glu | 4 | - | | 3490749 | C | T | 1505.77 | SNP | Rv3125c (PPE49) | Leu(s)301Leu | 3 | - | | 3503116 | G | A | 1347.77 | SNP | Rv3136A | silent (Val54) | 9901 | - | | 3503895 | C | T | 1724.77 | SNP | Rv3137 | Pro168Leu | 3 | - | | 3505027 | G | A | 84.77 | SNP | Rv3138 (pflA) | Arg278His | 8 | - | | 3510724 | G | A | 446.77 | SNP | Rv3144c (PPE52) | silent (Gly198) | 9935 | - | | 3518167 | A | G | 1247.77 | SNP | Rv3151 (nuoG) | Ile474Met(s) | 6 | - | | 3518555 | A | G | 1162.77 | SNP | Rv3151 (nuoG) | Thr604Ala | 32 | - | | 3535025 | G | A | 1189.77 | SNP | Rv3166c | silent (Ile109) | 9872 | - | | 3556275 | A | G | 1673.77 | SNP | Rv3190c | Leu138Pro | 2 | - | | 3562270 | G | C | 1395.77 | SNP | Rv3193c | silent (Ala301) | 9867 | - | | 3569029 | T | C | 1140.77 | SNP | intergenic |  |  | - | | 3580636 | CT | C | 2686.73 | DEL | intergenic |  |  | - | | 3581414 | A | G | 1855.77 | SNP | Rv3204 | Thr34Ala | 32 | - | | 3590686 | G | GC | 2234.73 | INS | intergenic |  |  | - | | 3591063 | T | C | 896.77 | SNP | Rv3213c | Lys144Glu | 4 | - | | 3594394 | G | C | 195.84 | SNP | intergenic |  |  | - | | 3594395 | G | A | 170.90 | SNP | intergenic |  |  | - | | 3594398 | T | C | 199.84 | SNP | intergenic |  |  | - | | 3594400 | A | G | 270.78 | SNP | intergenic |  |  | - | | 3596109 | C | T | 1461.77 | SNP | Rv3220c | Gly476Ser | 16 | - | | 3604821 | G | C | 587.77 | SNP | Rv3228 | silent (Ala32) | 9867 | - | | 3607613 | A | G | 1319.77 | SNP | Rv3230c | silent (Gly214) | 9935 | - | | 3614982 | T | C | 1540.77 | SNP | Rv3239c | silent (Leu874) | 9947 | - | | 3622441 | A | C | 1257.77 | SNP | Rv3243c | Val217Val(s) | 18 | - | | 3644061 | C | T | 2157.77 | SNP | Rv3263 | silent (Ile295) | 9872 | - | | 3663889 | C | A | 138.77 | SNP | Rv3281 (accE5) | Asn67Lys | 25 | - | | 3674122 | A | G | 1259.77 | SNP | Rv3293 (pcd) | Lys174Arg | 19 | - | | 3674918 | C | T | 1494.77 | SNP | Rv3293 (pcd) | silent (Asn439) | 9822 | - | | 3687908 | T | C | 1248.77 | SNP | Rv3302c (glpD2) | Tyr512Cys | 3 | - | | 3689523 | G | T | 1697.77 | SNP | Rv3303c (lpdA) | Cys472STOP | 3 | - | | 3692193 | C | T | 1206.77 | SNP | Rv3305c (amiA1) | Ala206Thr | 22 | - | | 3697152 | T | C | 1820.77 | SNP | intergenic |  |  | - | | 3699253 | C | G | 1685.77 | SNP | Rv3311 | Pro378Arg | 4 | - | | 3704596 | G | C | 2025.77 | SNP | Rv3317 (sdhD) | Val(s)54Leu | 3 | - | | 3714211 | G | T | 1736.77 | SNP | Rv3328c (sigJ) | Pro41Gln | 6 | - | | 3718357 | C | T | 1534.77 | SNP | Rv3331 (sugI) | Pro423Leu | 3 | - | | 3721806 | G | C | 1745.77 | SNP | Rv3335c | silent (Gly265) | 9935 | - | | 3730385 | C | G | 648.77 | SNP | Rv3343c (PPE54) | Arg2184Pro | 5 | - | | 3730386 | G | T | 662.77 | SNP | Rv3343c (PPE54) | silent (Arg2184) | 9913 | - | | 3730466 | A | G | 1693.77 | SNP | Rv3343c (PPE54) | Ile2157Thr | 11 | - | | 3730582 | G | A | 145.77 | SNP | Rv3343c (PPE54) | silent (Asn2118) | 9822 | - | | 3730624 | C | T | 456.77 | SNP | Rv3343c (PPE54) | silent (Ser2104) | 9840 | - | | 3730741 | G | A | 118.77 | SNP | Rv3343c (PPE54) | silent (Gly2065) | 9935 | - | | 3730896 | A | G | 71.77 | SNP | Rv3343c (PPE54) | Leu(s)2014Leu | 3 | - | | 3730978 | G | A | 118.77 | SNP | Rv3343c (PPE54) | silent (Gly1986) | 9935 | - | | 3732194 | A | G | 92.28 | SNP | Rv3343c (PPE54) | Ile1581Thr | 11 | - | | 3732517 | A | G | 239.77 | SNP | Rv3343c (PPE54) | silent (Ile1473) | 9872 | - | | 3732525 | A | T | 190.77 | SNP | Rv3343c (PPE54) | Phe1471Ile | 7 | - | | 3732553 | A | G | 120.77 | SNP | Rv3343c (PPE54) | silent (Ile1461) | 9872 | - | | 3732624 | A | G | 111.90 | SNP | Rv3343c (PPE54) | Leu(s)1438Leu | 3 | - | | 3732656 | G | C | 158.84 | SNP | Rv3343c (PPE54) | Ala1427Gly | 21 | - | | 3732658 | C | G | 137.90 | SNP | Rv3343c (PPE54) | Leu(s)1426Phe | 1 | - | | 3732660 | A | G | 129.90 | SNP | Rv3343c (PPE54) | Leu(s)1426Leu | 3 | - | | 3732664 | G | A | 132.90 | SNP | Rv3343c (PPE54) | silent (Val1424) | 9901 | - | | 3732666 | C | A | 136.90 | SNP | Rv3343c (PPE54) | Val1424Phe | 0 | - | | 3732667 | A | G | 138.90 | SNP | Rv3343c (PPE54) | silent (Asn1423) | 9822 | - | | 3732673 | A | C | 133.90 | SNP | Rv3343c (PPE54) | Phe1421Leu(s) | 2 | - | | 3732674 | A | C | 135.90 | SNP | Rv3343c (PPE54) | Phe1421Cys | 0 | - | | 3732706 | G | A | 507.77 | SNP | Rv3343c (PPE54) | silent (Gly1410) | 9935 | - | | 3735508 | G | A | 1806.77 | SNP | Rv3343c (PPE54) | silent (Leu476) | 9947 | - | | 3735907 | C | T | 624.77 | SNP | Rv3343c (PPE54) | silent (Ser343) | 9840 | - | | 3735931 | G | A | 422.77 | SNP | Rv3343c (PPE54) | silent (Ser335) | 9840 | - | | 3736072 | A | G | 87.77 | SNP | Rv3343c (PPE54) | silent (Ile288) | 9872 | - | | 3736080 | A | T | 86.77 | SNP | Rv3343c (PPE54) | Phe286Ile | 7 | - | | 3736108 | A | G | 207.77 | SNP | Rv3343c (PPE54) | silent (Ile276) | 9872 | - | | 3736628 | T | G | 1150.77 | SNP | Rv3343c (PPE54) | Glu103Ala | 17 | - | | 3737661 | TG | T | 600.73 | DEL | intergenic |  |  | - | | 3738516 | C | CTGCCGCCGCTGCCGCCGT | 1782.74 | INS | Rv3345c (PE\_PGRS50) |  |  | - | | 3739808 | GTTGCCGCCGTTGCCGCCGG CCGCGCCGCTCCCGTTGCCG GCGGTGCCGCCC | G | 2342.75 | DEL | Rv3345c (PE\_PGRS50) |  |  | - | | 3746409 | A | G | 1220.77 | SNP | Rv3347c (PPE55) | Leu2259Pro | 2 | - | | 3747403 | C | A | 1055.77 | SNP | Rv3347c (PPE55) | Gly1928Cys | 0 | - | | 3750993 | A | C | 551.77 | SNP | Rv3347c (PPE55) | Val(s)731Gly | 21 | - | | 3752207 | A | G | 1726.77 | SNP | Rv3347c (PPE55) | silent (Ile326) | 9872 | - | | 3752654 | A | T | 740.77 | SNP | Rv3347c (PPE55) | silent (Gly177) | 9935 | - | | 3752662 | A | G | 688.77 | SNP | Rv3347c (PPE55) | Leu(s)175Leu | 3 | - | | 3752761 | A | G | 285.78 | SNP | Rv3347c (PPE55) | Leu(s)142Leu | 3 | - | | 3752778 | A | G | 157.90 | SNP | Rv3347c (PPE55) | Val136Ala | 18 | - | | 3752809 | G | A | 42.74 | SNP | Rv3347c (PPE55) | Leu126Leu(s) | 4 | - | | 3752907 | G | A | 187.84 | SNP | Rv3347c (PPE55) | Ala93Val(s) | 9867 | - | | 3752909 | G | C | 219.80 | SNP | Rv3347c (PPE55) | silent (Ala92) | 9867 | - | | 3752910 | G | A | 151.90 | SNP | Rv3347c (PPE55) | Ala92Val | 13 | - | | 3752934 | A | T | 294.78 | SNP | Rv3347c (PPE55) | Val(s)84Glu | 10 | - | | 3753116 | C | T | 361.77 | SNP | Rv3347c (PPE55) | silent (Pro23) | 9926 | - | | 3753164 | T | G | 748.77 | SNP | Rv3347c (PPE55) | silent (Pro7) | 9926 | - | | 3758967 | G | A | 1287.77 | SNP | Rv3350c (PPE56) | silent (Gly2712) | 9935 | - | | 3766777 | A | G | 261.77 | SNP | Rv3350c (PPE56) | Leu109Pro | 2 | - | | 3766778 | G | C | 280.77 | SNP | Rv3350c (PPE56) | Leu109Val(s) | 4 | - | | 3766815 | G | C | 64.77 | SNP | Rv3350c (PPE56) | Val96Val(s) | 18 | - | | 3766816 | A | G | 33.77 | SNP | Rv3350c (PPE56) | Val96Ala | 18 | - | | 3766860 | G | C | 44.77 | SNP | Rv3350c (PPE56) | silent (Ala81) | 9867 | - | | 3779671 | C | CGGCAACGGT | 825.75 | INS | Rv3367 (PE\_PGRS51) |  |  | - | | 3787215 | A | C | 1403.77 | SNP | Rv3372 (otsB2) | Asp301Ala | 10 | - | | 3792796 | G | A | 1976.77 | SNP | Rv3378c | silent (Asn151) | 9822 | - | | 3798095 | A | C | 1817.77 | SNP | Rv3383c (idsB) | Val132Gly | 5 | - | | 3813185 | G | A | 1436.77 | SNP | Rv3396c (guaA) | silent (Gly298) | 9935 | - | | 3817117 | C | A | 1313.77 | SNP | Rv3399 | Ala330Glu | 10 | - | | 3819169 | G | A | 1400.77 | SNP | Rv3401 | silent (Pro376) | 9926 | - | | 3820407 | A | G | 56.77 | SNP | intergenic |  |  | - | | 3820545 | A | G | 183.77 | SNP | intergenic |  |  | - | | 3823159 | A | T | 1088.77 | SNP | Rv3403c | silent (Val235) | 9901 | - | | 3826684 | C | T | 1172.77 | SNP | Rv3408 (vapC47) | Ser46Leu(s) | 35 | - | | 3829770 | T | C | 1014.77 | SNP | Rv3410c (guaB3) | silent (Pro47) | 9926 | - | | 3838871 | A | G | 1252.77 | SNP | Rv3420c (rimI) | silent (Ala64) | 9867 | - | | 3841652 | T | A | 2009.77 | SNP | intergenic |  |  | - | | 3841654 | T | G | 2133.77 | SNP | intergenic |  |  | - | | 3841662 | T | C | 2396.77 | SNP | intergenic |  |  | - | | 3841663 | C | T | 2182.77 | SNP | intergenic |  |  | - | | 3842384 | A | G | 780.77 | SNP | Rv3425 (PPE57) | Asp49Gly | 11 | - | | 3842392 | G | C | 700.77 | SNP | Rv3425 (PPE57) | Asp52His | 3 | - | | 3842394 | T | G | 645.77 | SNP | Rv3425 (PPE57) | Asp52Glu | 56 | - | | 3842425 | T | A | 2519.77 | SNP | Rv3425 (PPE57) | Leu(s)63Met(s) | 9867 | - | | 3842438 | T | C | 237.77 | SNP | Rv3425 (PPE57) | Val67Ala | 18 | - | | 3842441 | A | G | 139.77 | SNP | Rv3425 (PPE57) | Glu68Gly | 7 | - | | 3842452 | C | G | 177.77 | SNP | Rv3425 (PPE57) | Gln72Glu | 35 | - | | 3842454 | A | C | 30.77 | SNP | Rv3425 (PPE57) | Gln72His | 20 | - | | 3842461 | T | A | 41.77 | SNP | Rv3425 (PPE57) | Ser75Thr | 32 | - | | 3842636 | A | G | 1630.77 | SNP | Rv3425 (PPE57) | Asp133Gly | 11 | - | | 3842637 | C | A | 1606.77 | SNP | Rv3425 (PPE57) | Asp133Glu | 56 | - | | 3843354 | A | G | 130.77 | SNP | Rv3426 (PPE58) | Thr107Ala | 32 | - | | 3843356 | T | C | 98.77 | SNP | Rv3426 (PPE58) | silent (Thr107) | 9871 | - | | 3843361 | C | A | 119.77 | SNP | Rv3426 (PPE58) | Ala109Asp | 6 | - | | 3843362 | C | A | 104.77 | SNP | Rv3426 (PPE58) | silent (Ala109) | 9867 | - | | 3843363 | A | G | 81.77 | SNP | Rv3426 (PPE58) | Asn110Asp | 42 | - | | 3843407 | CG | C | 2989.73 | DEL | Rv3426 (PPE58) |  |  | - | | 3843696 | T | A | 266.77 | SNP | Rv3426 (PPE58) | Leu(s)221Met(s) | 9867 | - | | 3843704 | G | C | 311.77 | SNP | Rv3426 (PPE58) | silent (Thr223) | 9871 | - | | 3843714 | T | C | 415.77 | SNP | Rv3426 (PPE58) | Cys227Arg | 1 | - | | 3843749 | G | T | 346.77 | SNP | intergenic |  |  | - | | 3843751 | G | T | 360.77 | SNP | intergenic |  |  | - | | 3843752 | A | G | 391.77 | SNP | intergenic |  |  | - | | 3843753 | G | A | 296.77 | SNP | intergenic |  |  | - | | 3843760 | T | C | 371.77 | SNP | intergenic |  |  | - | | 3844756 | GC | G | 2426.73 | DEL | Rv3428c |  |  | - | | 3844992 | T | A | 1361.77 | SNP | Rv3428c | Ser327Cys | 5 | - | | 3846591 | C | T | 77.77 | SNP | intergenic |  |  | - | | 3846605 | G | A | 785.77 | SNP | intergenic |  |  | - | | 3846607 | A | C | 809.77 | SNP | intergenic |  |  | - | | 3846622 | G | T | 864.77 | SNP | intergenic |  |  | - | | 3846687 | A | G | 967.77 | SNP | intergenic |  |  | - | | 3846704 | A | G | 890.77 | SNP | intergenic |  |  | - | | 3846707 | A | C | 855.77 | SNP | intergenic |  |  | - | | 3846716 | C | T | 130.77 | SNP | intergenic |  |  | - | | 3846727 | C | T | 79.77 | SNP | intergenic |  |  | - | | 3846728 | A | G | 132.77 | SNP | intergenic |  |  | - | | 3846741 | G | T | 49.77 | SNP | intergenic |  |  | - | | 3846743 | C | G | 157.77 | SNP | intergenic |  |  | - | | 3846764 | C | G | 558.77 | SNP | intergenic |  |  | - | | 3846773 | T | TG | 903.73 | INS | intergenic |  |  | - | | 3846774 | T | G | 406.77 | SNP | intergenic |  |  | - | | 3846777 | C | A | 327.77 | SNP | intergenic |  |  | - | | 3846779 | T | G | 404.77 | SNP | intergenic |  |  | - | | 3846843 | CAAA | C | 600.74 | DEL | intergenic |  |  | - | | 3846851 | G | A | 319.77 | SNP | intergenic |  |  | - | | 3846852 | C | G | 354.77 | SNP | intergenic |  |  | - | | 3846853 | T | C | 317.78 | SNP | intergenic |  |  | - | | 3846857 | G | A | 284.78 | SNP | intergenic |  |  | - | | 3846860 | T | G | 350.77 | SNP | intergenic |  |  | - | | 3846866 | C | A | 278.78 | SNP | intergenic |  |  | - | | 3846881 | AT | A | 375.74 | DEL | intergenic |  |  | - | | 3846886 | A | T | 279.78 | SNP | intergenic |  |  | - | | 3846897 | T | G | 296.78 | SNP | intergenic |  |  | - | | 3847014 | C | G | 48.74 | SNP | intergenic |  |  | - | | 3847022 | T | C | 115.03 | SNP | intergenic |  |  | - | | 3847039 | G | A | 143.90 | SNP | intergenic |  |  | - | | 3847052 | G | A | 105.03 | SNP | intergenic |  |  | - | | 3847073 | G | C | 74.28 | SNP | intergenic |  |  | - | | 3847074 | C | G | 70.28 | SNP | intergenic |  |  | - | | 3847087 | G | A | 103.03 | SNP | intergenic |  |  | - | | 3847090 | G | C | 118.03 | SNP | intergenic |  |  | - | | 3847099 | G | A | 107.03 | SNP | intergenic |  |  | - | | 3847112 | T | A | 98.03 | SNP | intergenic |  |  | - | | 3847130 | G | A | 143.90 | SNP | intergenic |  |  | - | | 3847137 | T | C | 77.28 | SNP | intergenic |  |  | - | | 3847153 | A | C | 75.28 | SNP | intergenic |  |  | - | | 3847154 | A | G | 99.03 | SNP | intergenic |  |  | - | | 3859576 | G | A | 867.77 | SNP | Rv3439c | silent (His29) | 9912 | - | | 3859893 | C | T | 886.77 | SNP | Rv3440c | silent (Glu28) | 9865 | - | | 3862472 | GA | G | 2242.73 | DEL | intergenic |  |  | - | | 3864995 | T | C | 1535.77 | SNP | Rv3447c (eccC4) | Ser1082Gly | 21 | - | | 3877421 | A | G | 1789.77 | SNP | Rv3456c (rplQ) | silent (Pro4) | 9926 | - | | 3879331 | G | A | 1384.77 | SNP | Rv3459c (rpsK) | Ser121Leu(s) | 35 | - | | 3879564 | G | C | 68.77 | SNP | Rv3459c (rpsK) | silent (Thr43) | 9871 | - | | 3879566 | T | A | 63.77 | SNP | Rv3459c (rpsK) | Thr43Ser | 38 | - | | 3884748 | G | A | 434.77 | SNP | Rv3467 | Gly262Asp | 6 | - | | 3885886 | T | C | 1694.77 | SNP | Rv3468c | Ile62Val | 57 | - | | 3892671 | A | G | 2080.77 | SNP | Rv3476c (kgtP) | silent (Val350) | 9901 | - | | 3895269 | G | C | 871.77 | SNP | Rv3478 (PPE60) | Glu282Gln | 27 | - | | 3895281 | T | C | 756.77 | SNP | Rv3478 (PPE60) | Trp286Arg | 8 | - | | 3895282 | G | A | 725.77 | SNP | Rv3478 (PPE60) | Trp286STOP | 0 | - | | 3896340 | T | G | 1173.77 | SNP | Rv3479 | Leu174Arg | 1 | - | | 3898408 | A | G | 1431.77 | SNP | Rv3479 | silent (Ala863) | 9867 | - | | 3920950 | C | T | 2233.77 | SNP | intergenic |  |  | - | | 3934542 | T | G | 158.77 | SNP | Rv3508 (PE\_PGRS54) | Ser1180Ala | 35 | - | | 3934699 | G | A | 293.77 | SNP | Rv3508 (PE\_PGRS54) | Ser1232Asn | 20 | - | | 3934733 | G | C | 225.77 | SNP | Rv3508 (PE\_PGRS54) | silent (Gly1243) | 9935 | - | | 3934734 | G | A | 215.77 | SNP | Rv3508 (PE\_PGRS54) | Ala1244Thr | 22 | - | | 3938461 | C | T | 1526.77 | SNP | Rv3510c | Arg266Gln | 9 | - | | 3940802 | A | G | 216.80 | SNP | Rv3511 (PE\_PGRS55) | Asn396Asp | 42 | - | | 3942640 | T | C | 595.77 | SNP | intergenic |  |  | - | | 3943019 | C | G | 230.80 | SNP | intergenic |  |  | - | | 3944582 | CGG | C | 234.87 | DEL | intergenic |  |  | - | | 3946929 | C | T | 241.80 | SNP | Rv3514 (PE\_PGRS57) | Ala379Val | 13 | - | | 3948414 | T | G | 47.74 | SNP | Rv3514 (PE\_PGRS57) | Val874Gly | 5 | - | | 3948417 | T | G | 47.74 | SNP | Rv3514 (PE\_PGRS57) | Val875Gly | 5 | - | | 3948928 | G | C | 41.77 | SNP | Rv3514 (PE\_PGRS57) | silent (Gly1045) | 9935 | - | | 3948929 | G | C | 53.77 | SNP | Rv3514 (PE\_PGRS57) | Ala1046Pro | 13 | - | | 3949000 | G | C | 48.77 | SNP | Rv3514 (PE\_PGRS57) | silent (Gly1069) | 9935 | - | | 3949001 | G | A | 40.77 | SNP | Rv3514 (PE\_PGRS57) | Ala1070Thr | 22 | - | | 3952800 | G | A | 1758.77 | SNP | Rv3516 (echA19) | Gly86Asp | 6 | - | | 3958403 | A | G | 1915.77 | SNP | Rv3521 | Asn295Asp | 42 | - | | 3959418 | C | T | 2115.77 | SNP | Rv3522 (ltp4) | Thr324Ile | 7 | - | | 3963665 | C | T | 1739.77 | SNP | Rv3527 | Leu21Leu(s) | 4 | - | | 3973919 | A | G | 56.77 | SNP | Rv3535c (hsaG) | silent (Asn194) | 9822 | - | | 4002574 | T | C | 1521.77 | SNP | Rv3561 (fadD3) | Val(s)313Ala | 9867 | - | | 4005607 | T | C | 1644.77 | SNP | Rv3564 (fadE33) | Leu(s)121Leu | 3 | - | | 4018414 | C | CA | 2745.73 | INS | Rv3576 (lppH) |  |  | - | | 4018415 | G | A | 1467.77 | SNP | Rv3576 (lppH) | Gly20Arg | 0 | - | | 4018802 | CAA | C | 2684.73 | DEL | Rv3576 (lppH) |  |  | - | | 4024273 | T | C | 1629.77 | SNP | Rv3581c (ispF) | Val25Val(s) | 18 | - | | 4026899 | G | A | 938.77 | SNP | Rv3585 (radA) | silent (Gln152) | 9876 | - | | 4034827 | C | T | 1972.77 | SNP | Rv3593 (lpqF) | Ala159Val(s) | 9867 | - | | 4052608 | G | A | 1949.77 | SNP | Rv3610c (ftsH) | silent (Phe92) | 9946 | - | | 4055801 | G | A | 2415.77 | SNP | Rv3616c (espA) | Thr192Ile | 7 | - | | 4059904 | A | G | 1836.77 | SNP | intergenic |  |  | - | | 4060100 | G | A | 717.77 | SNP | Rv3619c (esxV) | Leu57Leu(s) | 4 | - | | 4060201 | G | A | 288.78 | SNP | Rv3619c (esxV) | Ser23Leu(s) | 35 | - | | 4060210 | T | A | 348.77 | SNP | Rv3619c (esxV) | Gln20Leu | 6 | - | | 4060230 | G | A | 295.78 | SNP | Rv3619c (esxV) | silent (His13) | 9912 | - | | 4069292 | G | A | 1114.77 | SNP | Rv3630 | Ala40Thr | 22 | - | | 4076615 | A | C | 1093.77 | SNP | Rv3637 | silent (Ala44) | 9867 | - | | 4084504 | G | A | 87.77 | SNP | Rv3646c (topA) | silent (Ala918) | 9867 | - | | 4094986 | T | C | 909.77 | SNP | Rv3655c | silent (Pro105) | 9926 | - | | 4095001 | CG | C | 1590.73 | DEL | Rv3655c |  |  | - | | 4100975 | T | C | 1056.77 | SNP | intergenic |  |  | - | | 4109796 | G | A | 1920.77 | SNP | Rv3668c | Pro229Leu | 3 | - | | 4111303 | G | C | 2116.77 | SNP | Rv3669 | Val(s)159Val | 13 | - | | 4117361 | AC | A | 4944.73 | DEL | Rv3677c |  |  | - | | 4120926 | A | G | 117.77 | SNP | Rv3680 | Asn378Asp | 42 | - | | 4120983 | A | G | 751.77 | SNP | intergenic |  |  | - | | 4121032 | C | T | 211.77 | SNP | intergenic |  |  | - | | 4136497 | C | G | 1953.77 | SNP | Rv3694c | Lys206Asn | 13 | - | | 4139670 | C | T | 1472.77 | SNP | Rv3696c (glpK) | Cys29Tyr | 3 | - | | 4148669 | C | T | 1188.77 | SNP | Rv3705c | silent (Thr98) | 9871 | - | | 4155050 | G | A | 1798.77 | SNP | Rv3710 (leuA) | Val(s)437Val | 13 | - | | 4156099 | C | A | 1413.77 | SNP | Rv3711c (dnaQ) | Val(s)211Leu(s) | 9867 | - | | 4160407 | A | G | 1362.77 | SNP | Rv3715c (recR) | Leu(s)32Leu | 3 | - | | 4162073 | C | T | 1894.77 | SNP | Rv3718c | silent (Gln62) | 9876 | - | | 4162339 | A | G | 2315.77 | SNP | Rv3719 | Thr12Ala | 32 | - | | 4166052 | G | A | 1637.77 | SNP | Rv3721c (dnaZX) | Ala227Val | 13 | - | | 4182695 | G | A | 1254.77 | SNP | Rv3731 (ligC) | Arg313His | 8 | - | | 4187485 | T | C | 2114.77 | SNP | Rv3736 | silent (Ala284) | 9867 | - | | 4187817 | A | G | 1080.77 | SNP | Rv3737 | Asp40Gly | 11 | - | | 4189841 | T | C | 1625.77 | SNP | Rv3738c (PPE66) | Tyr131Cys | 3 | - | | 4198611 | CG | C | 1961.73 | DEL | intergenic |  |  | - | | 4204441 | A | G | 1451.77 | SNP | Rv3759c (proX) | silent (His311) | 9912 | - | | 4210274 | A | G | 1389.77 | SNP | Rv3764c (tcrY) | Cys246Arg | 1 | - | | 4212196 | A | T | 1939.77 | SNP | intergenic |  |  | - | | 4215467 | G | A | 1603.77 | SNP | Rv3770c | silent (Gly103) | 9935 | - | | 4221490 | C | G | 1259.77 | SNP | Rv3776 | silent (Leu134) | 9947 | - | | 4222073 | A | G | 770.77 | SNP | Rv3776 | Met(s)329Val(s) | 9867 | - | | 4222882 | A | G | 1950.77 | SNP | Rv3777 | silent (Leu63) | 9947 | - | | 4242643 | C | T | 1407.77 | SNP | Rv3793 (embC) | silent (Arg927) | 9913 | genotype | | 4247431 | G | C | 1839.77 | SNP | Rv3795 (embB) | Met(s)306Ile | 2 | resistance | | 4250742 | G | A | 2161.77 | SNP | Rv3796 | Gly289Ser | 16 | - | | 4255922 | A | G | 1549.77 | SNP | Rv3799c (accD4) | silent (His9) | 9912 | - | | 4257220 | A | G | 1411.77 | SNP | Rv3800c (pks13) | silent (Arg1309) | 9913 | - | | 4257849 | G | A | 1458.77 | SNP | Rv3800c (pks13) | Arg1100Trp | 2 | - | | 4260268 | G | C | 1812.77 | SNP | Rv3800c (pks13) | silent (Ala293) | 9867 | genotype | | 4269148 | A | G | 998.77 | SNP | Rv3806c (ubiA) | Val(s)229Ala | 9867 | - | | 4272408 | C | T | 2075.77 | SNP | Rv3809c (glf) | silent (Leu356) | 9947 | - | | 4287017 | G | A | 1977.77 | SNP | Rv3822 | silent (Ala99) | 9867 | - | | 4287195 | A | G | 1316.77 | SNP | Rv3822 | Thr159Ala | 32 | - | | 4287722 | G | T | 977.77 | SNP | Rv3822 | silent (Pro334) | 9926 | - | | 4302036 | T | C | 1782.77 | SNP | Rv3827c | Thr252Ala | 32 | - | | 4306155 | C | T | 1614.77 | SNP | Rv3831 | silent (Ser133) | 9840 | - | | 4311528 | G | A | 1239.77 | SNP | Rv3837c | silent (Ala60) | 9867 | - | | 4326283 | C | CAGGTCG | 5445.73 | INS | Rv3854c (ethA) |  |  | - | | 4328329 | G | C | 1759.77 | SNP | intergenic |  |  | - | | 4338595 | GC | G | 4749.73 | DEL | intergenic |  |  | - | | 4338732 | G | A | 2429.77 | SNP | intergenic |  |  | - | | 4351039 | G | T | 1680.77 | SNP | Rv3872 (PE35) | Glu99STOP | 17 | - | | 4356110 | G | C | 1555.77 | SNP | Rv3877 (eccD1) | silent (Leu368) | 9947 | - | | 4366272 | G | C | 1792.77 | SNP | Rv3884c (eccA2) | silent (Ala189) | 9867 | - | | 4366913 | G | A | 1373.77 | SNP | Rv3885c (eccE2) | Arg537Trp | 2 | - | | 4372074 | C | T | 2172.77 | SNP | Rv3888c | silent (Ser211) | 9840 | - | | 4375628 | G | T | 1686.77 | SNP | Rv3892c (PPE69) | Thr19Lys | 11 | - | | 4377461 | G | A | 1698.77 | SNP | Rv3894c (eccC2) | Leu998Phe | 6 | - | | 4378330 | G | A | 1718.77 | SNP | Rv3894c (eccC2) | Ala708Val | 13 | - | | 4379680 | C | G | 2057.77 | SNP | Rv3894c (eccC2) | Arg258Pro | 5 | - | | 4382054 | T | C | 1678.77 | SNP | Rv3896c | silent (Ala266) | 9867 | - | | 4382275 | G | T | 1802.77 | SNP | Rv3896c | Gln193Lys | 12 | - | | 4383144 | C | CCGGGG | 2755.73 | INS | Rv3897c |  |  | - | | 4400660 | AC | A | 1775.73 | DEL | Rv3911 (sigM) |  |  | - | | 4406749 | G | A | 1834.77 | SNP | Rv3918c (parA) | silent (Leu261) | 9947 | - | | 4409645 | G | A | 1633.77 | SNP | Rv3921c | Ser142Leu(s) | 35 | - | |  | | export |

elog
