# Supplementary material for: Patterns of compensatory mutations in rpoA/B/C genes of multidrug resistant M. tuberculosis in Uganda
Source: PLoS One. 2025 Dec 4;20(12):e0328957. doi: 10.1371/journal.pone.0328957 (PMC12677784; doi:10.1371/journal.pone.0328957)
Supplement: S2 File — (ZIP) [file pone.0328957.s002.zip › Variants Q_S17_L001_001.bam.html]

 

Calling SNPs/INDELs (computing variant list in .vcf format) from Q\_S17\_L001\_001.bam

*by SAMtools/BCFtools:*

Howto

Important aspects

This takes up to one hour!!! **Please wait ...**

Variants Q\_S17\_L001\_001.bam

|  |  |
| --- | --- |
| Variants |  |

|  |  |
| --- | --- |
| |  | | --- | | *by GATK* | |

|  |  |  |
| --- | --- | --- |
| |  | | --- | | Q\_S17\_L001\_001.bam | | | computed 2016-10-27 using PhyResSE v1.0 (Ref. NC\_000962.3) | |

|  |  |
| --- | --- |
| 1143  variants called Export in VCF format |  |

|  |  |  |  |  |  |  |  |  |  |  |  |  |  |  |  |  |  |  |  |  |  |  |  |  |  |  |  |  |  |  |  |  |  |  |  |  |  |  |  |  |  |  |  |  |  |  |  |  |  |  |  |  |  |  |  |  |  |  |  |  |  |  |  |  |  |  |  |  |  |  |  |  |  |  |  |  |  |  |  |  |  |  |  |  |  |  |  |  |  |  |  |  |  |  |  |  |  |  |  |  |  |  |  |  |  |  |  |  |  |  |  |  |  |  |  |  |  |  |  |  |  |  |  |  |  |  |  |  |  |  |  |  |  |  |  |  |  |  |  |  |  |  |  |  |  |  |  |  |  |  |  |  |  |  |  |  |  |  |  |  |  |  |  |  |  |  |  |  |  |  |  |  |  |  |  |  |  |  |  |  |  |  |  |  |  |  |  |  |  |  |  |  |  |  |  |  |  |  |  |  |  |  |  |  |  |  |  |  |  |  |  |  |  |  |  |  |  |  |  |  |  |  |  |  |  |  |  |  |  |  |  |  |  |  |  |  |  |  |  |  |  |  |  |  |  |  |  |  |  |  |  |  |  |  |  |  |  |  |  |  |  |  |  |  |  |  |  |  |  |  |  |  |  |  |  |  |  |  |  |  |  |  |  |  |  |  |  |  |  |  |  |  |  |  |  |  |  |  |  |  |  |  |  |  |  |  |  |  |  |  |  |  |  |  |  |  |  |  |  |  |  |  |  |  |  |  |  |  |  |  |  |  |  |  |  |  |  |  |  |  |  |  |  |  |  |  |  |  |  |  |  |  |  |  |  |  |  |  |  |  |  |  |  |  |  |  |  |  |  |  |  |  |  |  |  |  |  |  |  |  |  |  |  |  |  |  |  |  |  |  |  |  |  |  |  |  |  |  |  |  |  |  |  |  |  |  |  |  |  |  |  |  |  |  |  |  |  |  |  |  |  |  |  |  |  |  |  |  |  |  |  |  |  |  |  |  |  |  |  |  |  |  |  |  |  |  |  |  |  |  |  |  |  |  |  |  |  |  |  |  |  |  |  |  |  |  |  |  |  |  |  |  |  |  |  |  |  |  |  |  |  |  |  |  |  |  |  |  |  |  |  |  |  |  |  |  |  |  |  |  |  |  |  |  |  |  |  |  |  |  |  |  |  |  |  |  |  |  |  |  |  |  |  |  |  |  |  |  |  |  |  |  |  |  |  |  |  |  |  |  |  |  |  |  |  |  |  |  |  |  |  |  |  |  |  |  |  |  |  |  |  |  |  |  |  |  |  |  |  |  |  |  |  |  |  |  |  |  |  |  |  |  |  |  |  |  |  |  |  |  |  |  |  |  |  |  |  |  |  |  |  |  |  |  |  |  |  |  |  |  |  |  |  |  |  |  |  |  |  |  |  |  |  |  |  |  |  |  |  |  |  |  |  |  |  |  |  |  |  |  |  |  |  |  |  |  |  |  |  |  |  |  |  |  |  |  |  |  |  |  |  |  |  |  |  |  |  |  |  |  |  |  |  |  |  |  |  |  |  |  |  |  |  |  |  |  |  |  |  |  |  |  |  |  |  |  |  |  |  |  |  |  |  |  |  |  |  |  |  |  |  |  |  |  |  |  |  |  |  |  |  |  |  |  |  |  |  |  |  |  |  |  |  |  |  |  |  |  |  |  |  |  |  |  |  |  |  |  |  |  |  |  |  |  |  |  |  |  |  |  |  |  |  |  |  |  |  |  |  |  |  |  |  |  |  |  |  |  |  |  |  |  |  |  |  |  |  |  |  |  |  |  |  |  |  |  |  |  |  |  |  |  |  |  |  |  |  |  |  |  |  |  |  |  |  |  |  |  |  |  |  |  |  |  |  |  |  |  |  |  |  |  |  |  |  |  |  |  |  |  |  |  |  |  |  |  |  |  |  |  |  |  |  |  |  |  |  |  |  |  |  |  |  |  |  |  |  |  |  |  |  |  |  |  |  |  |  |  |  |  |  |  |  |  |  |  |  |  |  |  |  |  |  |  |  |  |  |  |  |  |  |  |  |  |  |  |  |  |  |  |  |  |  |  |  |  |  |  |  |  |  |  |  |  |  |  |  |  |  |  |  |  |  |  |  |  |  |  |  |  |  |  |  |  |  |  |  |  |  |  |  |  |  |  |  |  |  |  |  |  |  |  |  |  |  |  |  |  |  |  |  |  |  |  |  |  |  |  |  |  |  |  |  |  |  |  |  |  |  |  |  |  |  |  |  |  |  |  |  |  |  |  |  |  |  |  |  |  |  |  |  |  |  |  |  |  |  |  |  |  |  |  |  |  |  |  |  |  |  |  |  |  |  |  |  |  |  |  |  |  |  |  |  |  |  |  |  |  |  |  |  |  |  |  |  |  |  |  |  |  |  |  |  |  |  |  |  |  |  |  |  |  |  |  |  |  |  |  |  |  |  |  |  |  |  |  |  |  |  |  |  |  |  |  |  |  |  |  |  |  |  |  |  |  |  |  |  |  |  |  |  |  |  |  |  |  |  |  |  |  |  |  |  |  |  |  |  |  |  |  |  |  |  |  |  |  |  |  |  |  |  |  |  |  |  |  |  |  |  |  |  |  |  |  |  |  |  |  |  |  |  |  |  |  |  |  |  |  |  |  |  |  |  |  |  |  |  |  |  |  |  |  |  |  |  |  |  |  |  |  |  |  |  |  |  |  |  |  |  |  |  |  |  |  |  |  |  |  |  |  |  |  |  |  |  |  |  |  |  |  |  |  |  |  |  |  |  |  |  |  |  |  |  |  |  |  |  |  |  |  |  |  |  |  |  |  |  |  |  |  |  |  |  |  |  |  |  |  |  |  |  |  |  |  |  |  |  |  |  |  |  |  |  |  |  |  |  |  |  |  |  |  |  |  |  |  |  |  |  |  |  |  |  |  |  |  |  |  |  |  |  |  |  |  |  |  |  |  |  |  |  |  |  |  |  |  |  |  |  |  |  |  |  |  |  |  |  |  |  |  |  |  |  |  |  |  |  |  |  |  |  |  |  |  |  |  |  |  |  |  |  |  |  |  |  |  |  |  |  |  |  |  |  |  |  |  |  |  |  |  |  |  |  |  |  |  |  |  |  |  |  |  |  |  |  |  |  |  |  |  |  |  |  |  |  |  |  |  |  |  |  |  |  |  |  |  |  |  |  |  |  |  |  |  |  |  |  |  |  |  |  |  |  |  |  |  |  |  |  |  |  |  |  |  |  |  |  |  |  |  |  |  |  |  |  |  |  |  |  |  |  |  |  |  |  |  |  |  |  |  |  |  |  |  |  |  |  |  |  |  |  |  |  |  |  |  |  |  |  |  |  |  |  |  |  |  |  |  |  |  |  |  |  |  |  |  |  |  |  |  |  |  |  |  |  |  |  |  |  |  |  |  |  |  |  |  |  |  |  |  |  |  |  |  |  |  |  |  |  |  |  |  |  |  |  |  |  |  |  |  |  |  |  |  |  |  |  |  |  |  |  |  |  |  |  |  |  |  |  |  |  |  |  |  |  |  |  |  |  |  |  |  |  |  |  |  |  |  |  |  |  |  |  |  |  |  |  |  |  |  |  |  |  |  |  |  |  |  |  |  |  |  |  |  |  |  |  |  |  |  |  |  |  |  |  |  |  |  |  |  |  |  |  |  |  |  |  |  |  |  |  |  |  |  |  |  |  |  |  |  |  |  |  |  |  |  |  |  |  |  |  |  |  |  |  |  |  |  |  |  |  |  |  |  |  |  |  |  |  |  |  |  |  |  |  |  |  |  |  |  |  |  |  |  |  |  |  |  |  |  |  |  |  |  |  |  |  |  |  |  |  |  |  |  |  |  |  |  |  |  |  |  |  |  |  |  |  |  |  |  |  |  |  |  |  |  |  |  |  |  |  |  |  |  |  |  |  |  |  |  |  |  |  |  |  |  |  |  |  |  |  |  |  |  |  |  |  |  |  |  |  |  |  |  |  |  |  |  |  |  |  |  |  |  |  |  |  |  |  |  |  |  |  |  |  |  |  |  |  |  |  |  |  |  |  |  |  |  |  |  |  |  |  |  |  |  |  |  |  |  |  |  |  |  |  |  |  |  |  |  |  |  |  |  |  |  |  |  |  |  |  |  |  |  |  |  |  |  |  |  |  |  |  |  |  |  |  |  |  |  |  |  |  |  |  |  |  |  |  |  |  |  |  |  |  |  |  |  |  |  |  |  |  |  |  |  |  |  |  |  |  |  |  |  |  |  |  |  |  |  |  |  |  |  |  |  |  |  |  |  |  |  |  |  |  |  |  |  |  |  |  |  |  |  |  |  |  |  |  |  |  |  |  |  |  |  |  |  |  |  |  |  |  |  |  |  |  |  |  |  |  |  |  |  |  |  |  |  |  |  |  |  |  |  |  |  |  |  |  |  |  |  |  |  |  |  |  |  |  |  |  |  |  |  |  |  |  |  |  |  |  |  |  |  |  |  |  |  |  |  |  |  |  |  |  |  |  |  |  |  |  |  |  |  |  |  |  |  |  |  |  |  |  |  |  |  |  |  |  |  |  |  |  |  |  |  |  |  |  |  |  |  |  |  |  |  |  |  |  |  |  |  |  |  |  |  |  |  |  |  |  |  |  |  |  |  |  |  |  |  |  |  |  |  |  |  |  |  |  |  |  |  |  |  |  |  |  |  |  |  |  |  |  |  |  |  |  |  |  |  |  |  |  |  |  |  |  |  |  |  |  |  |  |  |  |  |  |  |  |  |  |  |  |  |  |  |  |  |  |  |  |  |  |  |  |  |  |  |  |  |  |  |  |  |  |  |  |  |  |  |  |  |  |  |  |  |  |  |  |  |  |  |  |  |  |  |  |  |  |  |  |  |  |  |  |  |  |  |  |  |  |  |  |  |  |  |  |  |  |  |  |  |  |  |  |  |  |  |  |  |  |  |  |  |  |  |  |  |  |  |  |  |  |  |  |  |  |  |  |  |  |  |  |  |  |  |  |  |  |  |  |  |  |  |  |  |  |  |  |  |  |  |  |  |  |  |  |  |  |  |  |  |  |  |  |  |  |  |  |  |  |  |  |  |  |  |  |  |  |  |  |  |  |  |  |  |  |  |  |  |  |  |  |  |  |  |  |  |  |  |  |  |  |  |  |  |  |  |  |  |  |  |  |  |  |  |  |  |  |  |  |  |  |  |  |  |  |  |  |  |  |  |  |  |  |  |  |  |  |  |  |  |  |  |  |  |  |  |  |  |  |  |  |  |  |  |  |  |  |  |  |  |  |  |  |  |  |  |  |  |  |  |  |  |  |  |  |  |  |  |  |  |  |  |  |  |  |  |  |  |  |  |  |  |  |  |  |  |  |  |  |  |  |  |  |  |  |  |  |  |  |  |  |  |  |  |  |  |  |  |  |  |  |  |  |  |  |  |  |  |  |  |  |  |  |  |  |  |  |  |  |  |  |  |  |  |  |  |  |  |  |  |  |  |  |  |  |  |  |  |  |  |  |  |  |  |  |  |  |  |  |  |  |  |  |  |  |  |  |  |  |  |  |  |  |  |  |  |  |  |  |  |  |  |  |  |  |  |  |  |  |  |  |  |  |  |  |  |  |  |  |  |  |  |  |  |  |  |  |  |  |  |  |  |  |  |  |  |  |  |  |  |  |  |  |  |  |  |  |  |  |  |  |  |  |  |  |  |  |  |  |  |  |  |  |  |  |  |  |  |  |  |  |  |  |  |  |  |  |  |  |  |  |  |  |  |  |  |  |  |  |  |  |  |  |  |  |  |  |  |  |  |  |  |  |  |  |  |  |  |  |  |  |  |  |  |  |  |  |  |  |  |  |  |  |  |  |  |  |  |  |  |  |  |  |  |  |  |  |  |  |  |  |  |  |  |  |  |  |  |  |  |  |  |  |  |  |  |  |  |  |  |  |  |  |  |  |  |  |  |  |  |  |  |  |  |  |  |  |  |  |  |  |  |  |  |  |  |  |  |  |  |  |  |  |  |  |  |  |  |  |  |  |  |  |  |  |  |  |  |  |  |  |  |  |  |  |  |  |  |  |  |  |  |  |  |  |  |  |  |  |  |  |  |  |  |  |  |  |  |  |  |  |  |  |  |  |  |  |  |  |  |  |  |  |  |  |  |  |  |  |  |  |  |  |  |  |  |  |  |  |  |  |  |  |  |  |  |  |  |  |  |  |  |  |  |  |  |  |  |  |  |  |  |  |  |  |  |  |  |  |  |  |  |  |  |  |  |  |  |  |  |  |  |  |  |  |  |  |  |  |  |  |  |  |  |  |  |  |  |  |  |  |  |  |  |  |  |  |  |  |  |  |  |  |  |  |  |  |  |  |  |  |  |  |  |  |  |  |  |  |  |  |  |  |  |  |  |  |  |  |  |  |  |  |  |  |  |  |  |  |  |  |  |  |  |  |  |  |  |  |  |  |  |  |  |  |  |  |  |  |  |  |  |  |  |  |  |  |  |  |  |  |  |  |  |  |  |  |  |  |  |  |  |  |  |  |  |  |  |  |  |  |  |  |  |  |  |  |  |  |  |  |  |  |  |  |  |  |  |  |  |  |  |  |  |  |  |  |  |  |  |  |  |  |  |  |  |  |  |  |  |  |  |  |  |  |  |  |  |  |  |  |  |  |  |  |  |  |  |  |  |  |  |  |  |  |  |  |  |  |  |  |  |  |  |  |  |  |  |  |  |  |  |  |  |  |  |  |  |  |  |  |  |  |  |  |  |  |  |  |  |  |  |  |  |  |  |  |  |  |  |  |  |  |  |  |  |  |  |  |  |  |  |  |  |  |  |  |  |  |  |  |  |  |  |  |  |  |  |  |  |  |  |  |  |  |  |  |  |  |  |  |  |  |  |  |  |  |  |  |  |  |  |  |  |  |  |  |  |  |  |  |  |  |  |  |  |  |  |  |  |  |  |  |  |  |  |  |  |  |  |  |  |  |  |  |  |  |  |  |  |  |  |  |  |  |  |  |  |  |  |  |  |  |  |  |  |  |  |  |  |  |  |  |  |  |  |  |  |  |  |  |  |  |  |  |  |  |  |  |  |  |  |  |  |  |  |  |  |  |  |  |  |  |  |  |  |  |  |  |  |  |  |  |  |  |  |  |  |  |  |  |  |  |  |  |  |  |  |  |  |  |  |  |  |  |  |  |  |  |  |  |  |  |  |  |  |  |  |  |  |  |  |  |  |  |  |  |  |  |  |  |  |  |  |  |  |  |  |  |  |  |  |  |  |  |  |  |  |  |  |  |  |  |  |  |  |  |  |  |  |  |  |  |  |  |  |  |  |  |  |  |  |  |  |  |  |  |  |  |  |  |  |  |  |  |  |  |  |  |  |  |  |  |  |  |  |  |  |  |  |  |  |  |  |  |  |  |  |  |  |  |  |  |  |  |  |  |  |  |  |  |  |  |  |  |  |  |  |  |  |  |  |  |  |  |  |  |  |  |  |  |  |  |  |  |  |  |  |  |  |  |  |  |  |  |  |  |  |  |  |  |  |  |  |  |  |  |  |  |  |  |  |  |  |  |  |  |  |  |  |  |  |  |  |  |  |  |  |  |  |  |  |  |  |  |  |  |  |  |  |  |  |  |  |  |  |  |  |  |  |  |  |  |  |  |  |  |  |  |  |  |  |  |  |  |  |  |  |  |  |  |  |  |  |  |  |  |  |  |  |  |  |  |  |  |  |  |  |  |  |  |  |  |  |  |  |  |  |  |  |  |  |  |  |  |  |  |  |  |  |  |  |  |  |  |  |  |  |  |  |  |  |  |  |  |  |  |  |  |  |  |  |  |  |  |  |  |  |  |  |  |  |  |  |  |  |  |  |  |  |  |  |  |  |  |  |  |  |  |  |  |  |  |  |  |  |  |  |  |  |  |  |  |  |  |  |  |  |  |  |  |  |  |  |  |  |  |  |  |  |  |  |  |  |  |  |  |  |  |  |  |  |  |  |  |  |  |  |  |  |  |  |  |  |  |  |  |  |  |  |  |  |  |  |  |  |  |  |  |  |  |  |  |  |  |  |  |  |  |  |  |  |  |  |  |  |  |  |  |  |  |  |  |  |  |  |  |  |  |  |  |  |  |  |  |  |  |  |  |  |  |  |  |  |  |  |  |  |  |  |  |  |  |  |  |  |  |  |  |  |  |  |  |  |  |  |  |  |  |  |  |  |  |  |  |  |  |  |  |  |  |  |  |  |  |  |  |  |  |  |  |  |  |  |  |  |  |  |  |  |  |  |  |  |  |  |  |  |  |  |  |  |  |  |  |  |  |  |  |  |  |  |  |  |  |  |  |  |  |  |  |  |  |  |  |  |  |  |  |  |  |  |  |  |  |  |  |  |  |  |  |  |  |  |  |  |  |  |  |  |  |  |  |  |  |  |  |  |  |  |  |  |  |  |  |  |  |  |  |  |  |  |  |  |  |  |  |  |  |  |  |  |  |  |  |  |  |  |  |  |  |  |  |  |  |  |  |  |  |  |  |  |  |  |  |  |  |  |  |  |  |  |  |  |  |  |  |  |  |  |  |  |  |  |  |  |  |  |  |  |  |  |  |  |  |  |  |  |  |  |  |  |  |  |  |  |  |  |  |  |  |  |  |  |  |  |  |  |  |  |  |  |  |  |  |  |  |  |  |  |  |  |  |  |  |  |  |  |  |  |  |  |  |  |  |  |  |  |  |  |  |  |  |  |  |  |  |  |  |  |  |  |  |  |  |  |  |  |  |  |  |  |  |  |  |  |  |  |  |  |  |  |  |  |  |  |  |  |  |  |  |  |  |  |  |  |  |  |  |  |  |  |  |  |  |  |  |  |  |  |  |  |  |  |  |  |  |  |  |  |  |  |  |  |  |  |  |  |  |  |  |  |  |  |  |  |  |  |  |  |  |  |  |  |  |  |  |  |  |  |  |  |  |  |  |  |  |  |  |  |  |  |  |  |  |  |  |  |  |  |  |  |  |  |  |  |  |  |  |  |  |  |  |  |  |  |  |  |  |  |  |  |  |  |  |  |  |  |  |  |  |  |  |  |  |  |  |  |  |  |  |  |  |  |  |  |  |  |  |  |  |  |  |  |  |  |  |  |  |  |  |  |  |  |  |  |  |  |  |  |  |  |  |  |  |  |  |  |  |  |  |  |  |  |  |  |  |  |  |  |  |  |  |  |  |  |  |  |  |  |  |  |  |  |  |  |  |  |  |  |  |  |  |  |  |  |  |  |  |  |  |  |  |  |  |  |  |  |  |  |  |  |  |  |  |  |  |  |  |  |  |  |  |  |  |  |  |  |  |  |  |  |  |  |  |  |  |  |  |  |  |  |  |  |  |  |  |  |  |  |  |  |  |  |  |  |  |  |  |  |  |  |  |  |  |  |  |  |  |  |  |  |  |  |  |  |  |  |  |  |  |  |  |  |  |  |  |  |  |  |  |  |  |  |  |  |  |  |  |  |  |  |  |  |  |  |  |  |  |  |  |  |  |  |  |  |  |  |  |  |  |  |  |  |  |  |  |  |  |  |  |  |  |  |  |  |  |  |  |  |  |  |  |  |  |  |  |  |  |  |  |  |  |  |  |  |  |  |  |  |  |  |  |  |  |  |  |  |  |  |  |  |  |  |  |  |  |  |  |  |  |  |  |  |  |  |  |  |  |  |  |  |  |  |  |  |  |  |  |  |  |  |  |  |  |  |  |  |  |  |  |  |  |  |  |  |  |  |  |  |  |  |  |  |  |  |  |  |  |  |  |  |  |  |  |  |  |  |  |  |  |  |  |  |  |  |  |  |  |  |  |  |  |  |  |  |  |  |  |  |  |  |  |  |  |  |  |  |  |  |  |  |  |  |  |  |  |  |  |  |  |  |  |  |  |  |  |  |  |  |  |  |  |  |  |  |  |  |  |  |  |  |  |  |  |  |  |  |  |  |  |  |  |  |  |  |  |  |  |  |  |  |  |  |  |  |  |  |  |  |  |  |  |  |  |  |  |  |  |  |  |  |  |  |  |  |  |  |  |  |  |  |  |  |  |  |  |  |  |  |  |  |  |  |  |  |  |  |  |  |  |  |  |  |  |  |  |  |  |  |  |  |  |  |  |  |  |  |  |  |  |  |  |  |  |  |  |  |  |  |  |  |  |  |  |  |  |  |  |  |  |  |  |  |  |  |  |  |  |  |  |  |  |  |  |  |  |  |  |  |  |  |  |  |  |  |  |  |  |  |  |  |  |  |  |  |  |  |  |  |  |  |  |  |  |  |  |  |  |  |  |  |  |  |  |  |  |  |  |  |  |  |  |  |  |  |  |  |  |  |  |  |  |  |  |  |  |  |  |  |  |  |  |  |  |  |  |  |  |  |  |  |  |  |  |  |  |  |  |  |  |  |  |  |  |  |  |  |  |  |  |  |  |  |  |  |  |  |  |  |  |  |  |  |  |  |  |  |  |  |  |  |  |  |  |  |  |  |  |  |  |  |  |  |  |  |  |  |  |  |  |  |  |  |  |  |  |  |  |  |  |  |  |  |  |  |  |  |  |  |  |  |  |  |  |  |  |  |  |  |  |  |  |  |  |  |  |  |  |  |  |  |  |  |  |  |  |  |  |  |  |  |  |  |  |  |  |  |  |  |  |  |  |  |  |  |  |  |  |  |  |  |  |  |  |  |  |  |  |  |  |  |  |  |  |  |  |  |  |  |  |  |  |  |  |  |  |  |  |  |  |  |  |  |  |  |  |  |  |  |  |  |  |  |  |  |  |  |  |  |  |  |  |  |  |  |  |  |  |  |  |  |  |  |  |  |  |  |  |  |  |  |  |  |  |  |  |  |  |  |  |  |  |  |  |  |  |  |  |  |  |  |  |  |  |  |  |  |  |  |  |  |  |  |  |  |  |  |  |  |  |  |  |  |  |  |  |  |  |  |  |  |  |  |  |  |  |  |  |  |  |  |  |  |  |  |  |  |  |  |  |  |  |  |  |  |  |  |  |  |  |  |  |  |  |  |  |  |  |  |  |  |  |  |  |  |  |  |  |  |  |  |  |  |  |  |  |  |  |  |  |  |  |  |  |  |  |  |  |  |  |  |  |  |  |  |  |  |  |  |  |  |  |  |  |  |  |  |  |  |  |  |  |  |  |  |  |  |  |  |  |  |  |  |  |  |  |  |  |  |  |  |  |  |  |  |  |  |  |  |  |  |  |  |  |  |  |  |  |  |  |  |  |  |  |  |  |  |  |  |  |  |  |  |  |  |  |  |  |  |  |  |  |  |  |  |  |  |  |  |  |  |  |  |  |  |  |  |  |  |  |  |  |  |  |  |  |  |  |  |  |  |  |  |  |  |  |  |  |  |  |  |  |  |  |  |  |  |  |  |  |  |  |  |  |  |  |  |  |  |  |  |  |  |  |  |  |  |  |  |  |  |  |  |  |  |  |  |  |  |  |  |  |  |  |  |  |  |  |  |  |  |  |  |  |  |  |  |  |  |  |  |  |  |  |  |  |  |  |  |  |  |  |  |  |  |  |  |  |  |  |  |  |  |  |  |  |  |  |  |  |  |  |  |  |  |  |  |  |  |  |  |  |  |  |  |  |  |  |  |  |  |  |  |  |  |  |  |  |  |  |  |  |  |  |  |  |  |  |  |  |  |  |  |  |  |  |  |  |  |  |  |  |  |  |  |  |  |  |  |  |  |  |  |  |  |  |  |  |  |  |  |  |  |  |  |  |  |  |  |  |  |  |  |  |  |  |  |  |  |  |  |  |  |  |  |  |  |  |  |  |  |  |  |  |  |  |  |  |  |  |  |  |  |  |  |  |  |  |  |  |  |  |  |  |  |  |  |  |  |  |  |  |  |  |  |  |  |  |  |  |  |  |  |  |  |  |  |  |  |  |  |  |  |  |  |  |  |  |  |  |  |  |  |  |  |  |  |  |  |  |  |  |  |  |  |  |  |  |  |  |  |  |  |  |  |  |  |  |  |  |  |  |  |  |  |  |  |  |  |  |  |  |  |  |  |  |  |  |  |  |  |  |  |  |  |  |  |  |  |  |  |  |  |  |  |  |  |  |  |  |  |  |  |  |  |  |  |  |  |  |  |  |  |  |  |  |  |  |  |  |  |  |  |  |  |  |  |  |  |  |  |  |  |  |  |  |  |  |  |  |  |  |  |  |  |  |  |  |  |  |  |  |  |  |  |  |  |  |  |  |  |  |  |  |  |  |  |  |  |  |  |  |  |  |  |  |  |  |  |  |  |  |  |  |  |  |  |  |  |  |  |  |  |  |  |  |  |  |  |  |  |  |  |  |  |  |  |  |  |  |  |  |  |  |  |  |  |  |  |  |  |  |  |  |  |  |  |  |  |  |  |  |  |  |  |  |  |  |  |  |  |  |  |  |  |  |  |  |  |  |  |  |  |  |  |  |  |  |  |  |  |  |  |  |  |  |  |  |  |  |  |  |  |  |  |  |  |  |  |  |  |  |  |  |  |  |  |  |  |  |  |  |  |  |  |  |  |  |  |  |  |  |  |  |  |  |  |  |  |  |  |  |  |  |  |  |  |  |  |  |  |  |  |  |  |  |  |  |  |  |  |  |  |  |  |  |  |  |  |  |  |  |  |  |  |  |  |  |  |  |  |  |  |  |  |  |  |  |  |  |  |  |  |  |  |  |  |  |  |  |  |  |  |  |  |  |  |  |  |  |  |  |  |  |  |  |  |  |  |  |  |  |  |  |  |  |  |  |  |  |  |  |  |  |  |  |  |  |  |  |  |  |  |  |  |  |  |  |  |  |  |  |  |  |  |  |  |  |  |  |  |  |  |  |  |  |  |  |  |  |  |  |  |  |  |  |  |  |  |  |  |  |  |  |  |  |  |  |  |  |  |  |  |  |  |  |  |  |  |  |  |  |  |  |  |  |  |  |  |  |  |  |  |  |  |  |  |  |  |  |  |  |  |  |  |  |  |  |  |  |  |  |  |  |  |  |  |  |  |  |  |  |  |  |  |  |  |  |  |  |  |  |  |  |  |  |  |  |  |  |  |  |  |  |  |  |  |  |  |  |  |  |  |  |  |  |  |  |  |  |  |  |  |  |  |  |  |  |  |  |  |  |  |  |  |  |  |  |  |  |  |  |  |  |  |  |  |  |  |  |  |  |  |  |  |  |  |  |  |  |  |  |  |  |  |  |  |  |  |  |  |  |  |  |  |  |  |  |  |  |  |  |  |  |  |  |  |  |  |  |  |  |  |  |  |  |  |  |  |  |  |  |  |  |  |  |  |  |  |  |  |  |  |  |  |  |  |  |  |  |  |  |  |  |  |  |  |  |  |  |  |  |  |  |  |  |  |  |  |  |  |  |  |  |  |  |  |  |  |  |  |  |  |  |  |  |  |  |  |  |  |  |  |  |  |  |  |  |  |  |  |  |  |  |  |  |  |  |  |  |  |  |  |  |  |  |  |  |  |  |  |  |  |  |  |  |  |  |  |  |  |  |  |  |  |  |  |  |  |  |  |  |  |  |  |  |  |  |  |  |  |  |  |  |  |  |  |  |  |  |  |  |  |  |  |  |  |  |  |  |  |  |  |  |  |  |  |  |  |  |  |  |  |  |  |  |  |  |  |  |  |  |  |  |  |  |  |  |  |  |  |  |  |  |  |  |  |  |  |  |  |  |  |  |  |  |  |  |  |  |  |  |  |  |  |  |  |  |  |  |  |  |  |  |  |  |  |  |  |  |  |  |  |  |  |  |  |  |  |  |  |  |  |  |  |  |  |  |  |  |  |  |  |  |  |  |  |  |  |  |  |  |  |  |  |  |  |  |  |  |  |  |  |  |  |  |  |  |  |  |  |  |  |  |  |  |  |  |  |  |  |  |  |  |  |  |  |  |  |  |  |  |  |  |  |  |  |  |  |  |  |  |  |  |  |  |  |  |  |  |  |  |  |  |  |  |  |  |  |  |  |  |  |  |  |  |  |  |  |  |  |  |  |  |  |  |  |  |  |  |  |  |  |  |  |  |  |  |  |  |  |  |  |  |  |  |  |  |  |  |  |  |  |  |  |  |  |  |  |  |  |  |  |  |  |  |  |  |  |  |  |  |  |  |  |  |  |  |  |  |  |  |  |  |  |  |  |  |  |  |  |  |  |  |  |  |  |  |  |  |  |  |  |  |  |  |  |  |  |  |  |  |  |  |  |  |  |  |  |  |  |  |  |  |  |  |  |  |  |  |  |  |  |  |  |  |  |  |  |  |  |  |  |  |  |  |  |  |  |  |  |  |  |  |  |  |  |  |  |  |  |  |  |  |  |  |  |  |  |  |  |  |  |  |  |  |  |  |  |  |  |  |  |  |  |  |  |  |  |  |  |  |  |  |  |  |  |  |  |  |  |  |  |  |  |  |  |  |  |  |  |  |  |  |  |  |  |  |  |  |  |  |  |  |  |  |  |  |  |  |  |  |  |  |  |  |  |  |  |  |  |  |  |  |  |  |  |  |  |  |  |  |  |  |  |  |  |  |  |  |  |  |  |  |  |  |  |  |  |  |  |  |  |  |  |  |  |  |  |  |  |  |  |  |  |  |  |  |  |  |  |  |  |  |  |  |  |  |  |  |  |  |  |  |  |  |  |  |  |  |  |  |  |  |  |  |  |  |  |  |  |  |  |  |  |  |  |  |  |  |  |  |  |  |  |  |  |  |  |  |  |  |  |  |  |  |  |  |  |  |  |  |  |  |  |  |  |  |  |  |  |  |  |  |  |  |  |  |  |  |  |  |  |  |  |  |  |  |  |  |  |  |  |  |  |  |  |  |  |  |  |  |  |  |  |  |  |  |  |  |  |  |  |  |  |  |  |  |  |  |  |  |  |  |  |  |  |  |  |  |  |  |  |  |  |  |  |  |  |  |  |  |  |  |  |  |  |  |  |  |  |  |  |  |  |  |  |  |  |  |  |  |  |  |  |  |  |  |  |  |  |  |  |  |  |  |  |  |  |  |  |  |  |  |  |  |  |  |  |  |  |  |  |  |  |  |  |  |  |  |  |  |  |  |  |  |  |  |  |  |  |  |  |  |  |  |  |  |  |  |  |  |  |  |  |  |  |  |  |  |  |  |  |  |  |  |  |  |  |  |  |  |  |  |  |  |  |  |  |  |  |  |  |  |  |  |  |  |  |  |  |  |  |  |  |  |  |  |  |  |  |  |  |  |  |  |  |  |  |  |  |  |  |  |  |  |  |  |  |  |  |  |  |  |  |  |  |  |  |  |  |  |  |  |  |  |  |  |  |  |  |  |  |  |  |  |  |  |  |  |  |  |  |  |  |  |  |  |  |  |  |  |  |  |  |  |  |  |  |  |  |  |  |  |  |  |  |  |  |  |  |  |  |  |  |  |  |  |  |  |  |  |  |  |  |  |  |  |  |  |  |  |  |  |  |  |  |  |  |  |  |  |  |  |  |  |  |  |  |  |  |  |  |  |  |  |  |  |  |  |  |  |  |  |  |  |  |  |  |  |  |  |  |  |  |  |  |  |  |  |  |  |  |  |  |  |  |  |  |  |  |  |  |  |  |  |  |  |  |  |  |  |  |  |  |  |  |  |  |  |  |  |  |  |  |  |  |  |  |  |  |  |  |  |  |  |  |  |  |  |  |  |  |  |  |  |  |  |  |  |  |  |  |  |  |  |  |  |  |  |  |  |  |  |  |  |  |  |  |  |  |  |  |  |  |  |  |  |  |  |  |  |  |  |  |  |  |  |  |  |  |  |  |  |  |  |  |  |  |  |  |  |  |  |  |  |  |  |  |  |  |  |  |  |  |  |  |  |  |  |  |  |  |  |  |  |  |  |  |  |  |  |  |  |  |  |  |  |  |  |  |  |  |  |  |  |  |  |  |  |  |  |  |  |  |  |  |  |  |  |  |  |  |  |  |  |  |  |  |  |  |  |  |  |  |  |  |  |  |  |  |  |  |  |  |  |  |  |  |  |  |  |  |  |  |  |  |  |  |  |  |  |  |  |  |  |  |  |  |  |  |  |  |  |  |  |  |  |  |  |  |  |  |  |  |  |  |  |  |  |  |  |  |  |  |  |  |  |  |  |  |  |  |  |  |  |  |  |  |  |  |  |  |  |  |  |  |  |  |  |  |  |  |  |  |  |  |  |  |  |  |  |  |  |  |  |  |  |  |  |  |  |  |  |  |  |  |  |  |  |  |  |  |  |  |  |  |  |  |  |  |  |  |  |  |  |  |  |  |  |  |  |  |  |  |  |  |  |  |  |  |  |  |  |  |  |  |  |  |  |  |  |  |  |  |  |  |  |  |  |  |  |  |  |  |  |  |  |  |  |  |  |  |  |  |  |  |  |  |  |  |  |  |  |  |  |  |  |  |  |  |  |  |  |  |  |  |  |  |  |  |  |  |  |  |  |  |  |  |  |  |  |  |  |  |  |  |  |  |  |  |  |  |  |  |  |  |  |  |  |  |  |  |  |  |  |  |  |  |  |  |  |  |  |  |  |  |  |  |  |  |  |  |  |  |  |  |  |  |  |  |  |  |  |  |  |  |  |  |  |  |  |  |  |  |  |  |  |  |  |  |  |  |  |  |  |  |  |  |  |  |  |  |  |  |  |  |  |  |  |  |  |  |  |  |  |  |  |  |  |  |  |  |  |  |  |  |  |  |  |  |  |  |  |  |  |  |  |  |  |  |  |  |  |  |  |  |  |  |  |  |  |  |  |  |  |  |  |  |  |  |  |  |  |  |  |  |  |  |  |  |  |  |  |  |  |  |  |  |  |  |  |  |  |  |  |  |  |  |  |  |  |  |  |  |  |  |  |  |  |  |  |  |  |  |  |  |  |  |  |  |  |  |  |  |  |  |  |  |  |  |  |  |  |  |  |  |  |  |  |  |  |  |  |  |  |  |  |  |  |  |  |  |  |  |  |  |  |  |  |  |  |  |  |  |  |  |  |  |  |  |  |  |  |  |  |  |  |  |  |  |  |  |  |  |  |  |  |  |  |  |  |  |  |  |  |  |  |  |  |  |  |  |  |  |  |  |  |  |  |  |  |  |  |  |  |  |  |  |  |  |  |  |  |  |  |  |  |  |  |  |  |  |  |  |  |  |  |  |  |  |  |  |  |  |  |  |  |  |  |  |  |  |  |  |  |  |  |  |  |  |  |  |  |  |  |  |  |  |  |  |  |  |  |  |  |  |  |  |  |  |  |  |  |  |  |  |  |  |  |  |  |  |  |  |  |  |  |  |  |  |  |  |  |  |  |  |  |  |  |  |  |  |  |  |  |  |  |  |  |  |  |  |  |  |  |  |  |  |  |  |  |  |  |  |  |  |  |  |  |  |  |  |  |  |  |  |  |  |  |  |  |  |  |  |  |  |  |  |  |  |  |  |  |  |  |  |  |  |  |  |  |  |  |  |  |  |  |  |  |  |  |  |  |  |  |  |  |  |  |  |  |  |  |  |  |  |  |  |  |  |  |  |  |  |  |  |  |  |  |  |  |  |  |  |  |  |  |  |  |  |  |  |  |  |  |  |  |  |  |  |  |  |  |  |  |  |  |  |  |  |  |  |  |  |  |  |  |  |  |  |  |  |  |  |  |  |  |  |  |  |  |  |  |  |  |  |  |  |  |  |  |  |  |  |  |  |  |  |  |  |  |  |  |  |  |  |  |  |  |  |  |  |  |  |  |  |  |  |  |  |  |  |  |  |  |  |  |  |  |  |  |  |  |  |  |  |  |  |  |  |  |  |  |  |  |  |  |  |  |  |  |  |  |  |  |  |  |  |  |  |  |  |  |  |  |  |  |  |  |  |  |  |  |  |  |  |  |  |  |  |  |  |  |  |  |  |  |  |  |  |  |  |  |  |  |  |  |  |  |  |  |  |  |  |  |  |  |  |  |  |  |  |  |  |  |  |  |  |  |  |  |  |  |  |  |  |  |  |  |  |  |  |  |  |  |  |  |  |  |  |  |  |  |  |  |  |  |  |  |  |  |  |  |  |  |  |  |  |  |  |  |  |  |  |  |  |  |  |  |  |  |  |  |  |  |  |  |  |  |  |  |  |  |  |  |  |  |  |  |  |  |  |  |  |  |  |  |  |  |  |  |  |  |  |  |  |  |  |  |  |  |  |  |  |  |  |  |  |  |  |  |  |  |  |  |  |  |  |  |  |  |  |  |  |  |  |  |  |  |  |  |  |  |  |  |  |  |  |  |  |  |  |  |  |  |  |  |  |  |  |  |  |  |  |  |  |  |  |  |  |  |  |  |  |  |  |  |  |  |  |  |  |  |  |  |  |  |  |  |  |  |  |  |  |  |  |  |  |  |  |  |  |  |  |  |  |  |  |  |  |  |  |  |  |  |  |  |  |  |  |  |  |  |  |  |  |  |  |  |  |  |  |  |  |  |  |  |  |  |  |  |  |  |  |  |  |  |  |  |  |  |  |  |  |  |  |  |  |  |  |  |  |  |  |  |  |  |  |  |  |  |  |  |  |  |  |  |  |  |  |  |  |  |  |  |  |  |  |  |  |  |  |  |  |  |  |  |  |  |  |  |  |  |  |  |  |  |  |  |  |  |  |  |  |  |  |  |  |  |  |  |  |  |  |  |  |  |  |  |  |  |  |  |  |  |  |  |  |  |  |  |  |  |  |  |  |  |  |  |  |  |  |  |  |  |  |  |  |  |  |  |  |  |  |  |  |  |  |  |  |  |  |  |  |  |  |  |  |  |  |  |  |  |  |  |  |  |  |  |  |  |  |  |  |  |  |  |  |  |  |  |  |  |  |  |  |  |  |  |  |  |  |  |  |  |  |  |  |  |  |  |  |  |  |  |  |  |  |  |  |  |  |  |  |  |  |  |  |  |  |  |  |  |  |  |  |  |  |  |  |  |  |  |  |  |  |  |  |  |  |  |  |  |  |  |  |  |  |  |  |  |  |  |  |  |  |  |  |  |  |  |  |  |  |  |  |  |  |  |  |  |  |  |  |  |  |  |  |  |  |  |  |  |  |  |  |  |  |  |  |  |  |  |  |  |  |  |  |  |  |  |  |  |  |  |  |  |  |  |  |  |  |  |  |  |  |  |  |  |  |  |  |  |  |  |  |  |  |  |  |  |  |  |  |  |  |  |  |  |  |  |  |  |  |  |  |  |  |  |  |  |  |  |  |  |  |  |  |  |  |  |  |  |  |  |  |  |  |  |  |  |  |  |  |  |  |  |  |  |  |  |  |  |  |  |  |  |  |  |  |  |  |  |  |  |  |  |  |  |  |  |  |  |  |  |  |  |  |  |  |  |  |  |  |  |  |  |  |  |  |  |  |  |  |  |  |  |  |  |  |  |  |  |  |  |  |  |  |  |  |  |  |  |  |  |  |  |  |  |  |  |  |  |  |  |  |  |  |  |  |  |  |  |  |  |  |  |  |  |  |  |  |  |  |  |  |  |  |  |  |  |  |  |  |  |  |  |  |  |  |  |  |  |  |  |  |  |  |  |  |  |  |  |  |  |  |  |  |  |  |  |  |  |  |  |  |  |  |  |  |  |  |  |  |  |  |  |  |  |  |  |  |  |  |  |  |  |  |  |  |  |  |  |  |  |  |  |  |  |  |  |  |  |  |  |  |  |  |  |  |  |  |  |  |  |  |  |  |  |  |  |  |  |  |  |  |  |  |  |  |  |  |  |  |  |  |  |  |  |  |  |  |  |  |  |  |  |  |  |  |  |  |  |  |  |  |  |  |  |  |  |  |  |  |  |  |  |  |  |  |  |  |  |  |  |  |  |  |  |  |  |  |  |  |  |  |  |  |  |  |  |  |  |  |  |  |  |  |  |  |  |  |  |  |  |  |  |  |  |  |  |  |  |  |  |  |  |  |  |  |  |  |  |  |  |  |  |  |  |  |  |  |  |  |  |  |  |  |  |  |  |  |  |  |  |  |  |  |  |  |  |  |  |  |  |  |  |  |  |  |  |  |  |  |  |  |  |  |  |  |  |  |  |  |  |  |  |  |  |  |  |  |  |  |  |  |  |  |  |  |  |  |  |  |  |  |  |  |  |  |  |  |  |  |  |  |  |  |  |  |  |  |  |  |  |  |  |  |  |  |  |  |  |  |  |  |  |  |  |  |  |  |  |  |  |  |  |  |  |  |  |  |  |  |  |  |  |  |  |  |  |  |  |  |  |  |  |  |  |  |  |  |  |  |  |  |  |  |  |  |  |  |  |  |  |  |  |  |  |  |  |  |  |  |  |  |  |  |  |  |  |  |  |  |  |  |  |  |  |  |  |  |  |  |  |  |  |  |  |  |  |  |  |  |  |  |  |  |  |  |  |  |  |  |  |  |  |  |  |  |  |  |  |  |  |  |  |  |  |  |  |  |  |  |  |  |  |  |  |  |  |  |  |  |  |  |  |  |  |  |  |  |  |  |  |  |  |  |  |  |  |  |  |  |  |  |  |  |  |  |  |  |  |  |  |  |  |  |  |  |  |  |  |  |  |  |  |  |  |  |  |  |  |  |  |  |  |  |  |  |  |  |  |  |  |  |  |  |  |  |  |  |  |  |  |  |  |  |  |  |  |  |  |  |  |  |  |  |  |  |  |  |  |  |  |  |  |  |  |  |  |  |  |  |  |  |  |  |  |  |  |  |  |  |  |  |  |  |  |  |  |  |  |  |  |  |  |  |  |  |  |  |  |  |  |  |  |  |  |  |  |  |  |  |  |  |  |  |  |  |  |  |  |  |  |  |  |  |  |  |  |  |  |  |  |  |  |  |  |  |  |  |  |  |  |  |  |  |  |  |  |  |  |  |  |  |  |  |  |  |  |  |  |  |  |  |  |  |  |  |  |  |  |  |  |  |  |  |  |  |  |  |  |  |  |  |  |  |  |  |  |  |  |  |  |  |  |  |  |  |  |  |  |  |  |  |  |  |  |  |  |  |  |  |  |  |  |  |  |  |  |  |  |  |  |  |  |  |  |  |  |  |  |  |  |  |  |  |  |  |  |  |  |  |  |  |  |  |  |  |  |  |  |  |  |  |  |  |  |  |  |  |  |  |  |  |  |  |  |  |  |  |  |  |  |  |  |  |  |  |  |  |  |  |  |  |  |  |  |  |  |  |  |  |  |  |  |  |  |  |  |  |  |  |  |  |  |  |  |  |  |  |  |  |  |  |  |  |  |  |  |  |  |  |  |  |  |  |  |  |  |  |  |  |  |  |  |  |  |  |  |  |  |  |  |  |  |  |  |  |  |  |  |  |  |  |  |  |  |  |  |  |  |  |  |  |  |  |  |  |  |  |  |  |  |  |  |  |  |  |  |  |  |  |  |  |  |  |  |  |  |  |  |  |  |  |  |  |  |  |  |  |  |  |  |  |  |  |  |  |  |  |  |  |  |  |  |  |  |  |  |  |  |  |  |  |  |  |  |  |  |  |  |  |  |  |  |  |  |  |  |  |  |  |  |  |  |  |  |  |  |  |  |  |  |  |  |  |  |  |  |  |  |  |  |  |  |  |  |  |  |  |  |  |  |  |  |  |  |  |  |  |  |  |  |  |  |  |  |  |  |  |  |  |  |  |  |  |  |  |  |  |  |  |  |  |  |  |  |  |  |  |  |  |  |  |  |  |  |  |  |  |  |  |  |  |  |  |  |  |  |  |  |  |  |  |  |  |  |  |  |  |  |  |  |  |  |  |  |  |  |  |  |  |  |  |  |  |  |  |  |  |  |  |  |  |  |  |  |  |  |  |  |  |  |  |  |  |  |  |  |  |  |  |  |  |  |  |  |  |  |  |  |  |  |  |  |  |  |  |  |  |  |  |  |  |  |  |  |  |  |  |  |  |  |  |  |  |  |  |  |  |  |  |  |  |  |  |  |  |  |  |  |  |  |  |  |  |  |  |  |  |  |  |  |  |  |  |  |  |  |  |  |  |  |  |  |  |  |  |  |  |  |  |  |  |  |  |  |  |  |  |  |  |  |  |  |  |  |  |  |  |  |  |  |  |  |  |  |  |  |  |  |  |  |  |  |  |  |  |  |  |  |  |  |  |  |  |  |  |  |  |  |  |  |  |  |  |  |  |  |  |  |  |  |  |  |  |  |  |  |  |  |  |  |  |  |  |  |  |  |  |  |  |  |  |  |  |  |  |  |  |  |  |  |  |  |  |  |  |  |  |  |  |  |  |  |  |  |  |  |  |  |  |  |  |  |  |  |  |  |  |  |  |  |  |  |  |  |  |  |  |  |  |  |  |  |  |  |  |  |  |  |  |  |  |  |  |  |  |  |  |  |  |  |  |  |  |  |  |  |  |  |  |  |  |  |  |  |  |  |  |  |  |  |  |  |  |  |  |  |  |  |  |  |  |  |  |  |  |  |  |  |  |  |  |  |  |  |  |  |  |  |  |  |  |  |  |  |  |  |  |  |  |  |  |  |  |  |  |  |  |  |  |  |  |  |  |  |  |  |  |  |  |  |  |  |  |  |  |  |  |  |  |  |  |  |  |  |  |  |  |  |  |  |  |  |  |  |  |  |  |  |  |  |  |  |  |  |  |  |  |  |  |  |  |  |  |  |  |  |  |  |  |  |  |  |  |  |  |  |  |  |  |  |  |  |  |  |  |  |  |  |  |  |  |  |  |  |  |  |  |  |  |  |  |  |  |  |  |  |  |  |  |  |  |  |  |  |  |  |  |  |  |  |  |  |  |  |  |  |  |  |  |  |  |  |  |  |  |  |  |  |  |  |  |  |  |  |  |  |  |  |  |  |  |  |  |  |  |  |  |  |  |  |  |  |  |  |  |  |  |  |  |  |  |  |  |  |  |  |  |  |  |  |  |  |  |  |  |  |  |  |  |  |  |  |  |  |  |  |  |  |  |  |  |  |  |  |  |  |  |  |  |  |  |  |  |  |  |  |  |  |  |  |  |  |  |  |  |  |  |  |  |  |  |  |  |  |  |  |  |  |  |  |  |  |  |  |  |  |  |  |  |  |  |  |  |  |  |  |  |  |  |  |  |  |  |  |  |  |  |  |  |  |  |  |  |  |  |  |  |  |  |  |  |  |  |  |  |  |  |  |  |  |  |  |  |  |  |  |  |  |  |  |  |  |  |  |  |  |  |  |  |
| --- | --- | --- | --- | --- | --- | --- | --- | --- | --- | --- | --- | --- | --- | --- | --- | --- | --- | --- | --- | --- | --- | --- | --- | --- | --- | --- | --- | --- | --- | --- | --- | --- | --- | --- | --- | --- | --- | --- | --- | --- | --- | --- | --- | --- | --- | --- | --- | --- | --- | --- | --- | --- | --- | --- | --- | --- | --- | --- | --- | --- | --- | --- | --- | --- | --- | --- | --- | --- | --- | --- | --- | --- | --- | --- | --- | --- | --- | --- | --- | --- | --- | --- | --- | --- | --- | --- | --- | --- | --- | --- | --- | --- | --- | --- | --- | --- | --- | --- | --- | --- | --- | --- | --- | --- | --- | --- | --- | --- | --- | --- | --- | --- | --- | --- | --- | --- | --- | --- | --- | --- | --- | --- | --- | --- | --- | --- | --- | --- | --- | --- | --- | --- | --- | --- | --- | --- | --- | --- | --- | --- | --- | --- | --- | --- | --- | --- | --- | --- | --- | --- | --- | --- | --- | --- | --- | --- | --- | --- | --- | --- | --- | --- | --- | --- | --- | --- | --- | --- | --- | --- | --- | --- | --- | --- | --- | --- | --- | --- | --- | --- | --- | --- | --- | --- | --- | --- | --- | --- | --- | --- | --- | --- | --- | --- | --- | --- | --- | --- | --- | --- | --- | --- | --- | --- | --- | --- | --- | --- | --- | --- | --- | --- | --- | --- | --- | --- | --- | --- | --- | --- | --- | --- | --- | --- | --- | --- | --- | --- | --- | --- | --- | --- | --- | --- | --- | --- | --- | --- | --- | --- | --- | --- | --- | --- | --- | --- | --- | --- | --- | --- | --- | --- | --- | --- | --- | --- | --- | --- | --- | --- | --- | --- | --- | --- | --- | --- | --- | --- | --- | --- | --- | --- | --- | --- | --- | --- | --- | --- | --- | --- | --- | --- | --- | --- | --- | --- | --- | --- | --- | --- | --- | --- | --- | --- | --- | --- | --- | --- | --- | --- | --- | --- | --- | --- | --- | --- | --- | --- | --- | --- | --- | --- | --- | --- | --- | --- | --- | --- | --- | --- | --- | --- | --- | --- | --- | --- | --- | --- | --- | --- | --- | --- | --- | --- | --- | --- | --- | --- | --- | --- | --- | --- | --- | --- | --- | --- | --- | --- | --- | --- | --- | --- | --- | --- | --- | --- | --- | --- | --- | --- | --- | --- | --- | --- | --- | --- | --- | --- | --- | --- | --- | --- | --- | --- | --- | --- | --- | --- | --- | --- | --- | --- | --- | --- | --- | --- | --- | --- | --- | --- | --- | --- | --- | --- | --- | --- | --- | --- | --- | --- | --- | --- | --- | --- | --- | --- | --- | --- | --- | --- | --- | --- | --- | --- | --- | --- | --- | --- | --- | --- | --- | --- | --- | --- | --- | --- | --- | --- | --- | --- | --- | --- | --- | --- | --- | --- | --- | --- | --- | --- | --- | --- | --- | --- | --- | --- | --- | --- | --- | --- | --- | --- | --- | --- | --- | --- | --- | --- | --- | --- | --- | --- | --- | --- | --- | --- | --- | --- | --- | --- | --- | --- | --- | --- | --- | --- | --- | --- | --- | --- | --- | --- | --- | --- | --- | --- | --- | --- | --- | --- | --- | --- | --- | --- | --- | --- | --- | --- | --- | --- | --- | --- | --- | --- | --- | --- | --- | --- | --- | --- | --- | --- | --- | --- | --- | --- | --- | --- | --- | --- | --- | --- | --- | --- | --- | --- | --- | --- | --- | --- | --- | --- | --- | --- | --- | --- | --- | --- | --- | --- | --- | --- | --- | --- | --- | --- | --- | --- | --- | --- | --- | --- | --- | --- | --- | --- | --- | --- | --- | --- | --- | --- | --- | --- | --- | --- | --- | --- | --- | --- | --- | --- | --- | --- | --- | --- | --- | --- | --- | --- | --- | --- | --- | --- | --- | --- | --- | --- | --- | --- | --- | --- | --- | --- | --- | --- | --- | --- | --- | --- | --- | --- | --- | --- | --- | --- | --- | --- | --- | --- | --- | --- | --- | --- | --- | --- | --- | --- | --- | --- | --- | --- | --- | --- | --- | --- | --- | --- | --- | --- | --- | --- | --- | --- | --- | --- | --- | --- | --- | --- | --- | --- | --- | --- | --- | --- | --- | --- | --- | --- | --- | --- | --- | --- | --- | --- | --- | --- | --- | --- | --- | --- | --- | --- | --- | --- | --- | --- | --- | --- | --- | --- | --- | --- | --- | --- | --- | --- | --- | --- | --- | --- | --- | --- | --- | --- | --- | --- | --- | --- | --- | --- | --- | --- | --- | --- | --- | --- | --- | --- | --- | --- | --- | --- | --- | --- | --- | --- | --- | --- | --- | --- | --- | --- | --- | --- | --- | --- | --- | --- | --- | --- | --- | --- | --- | --- | --- | --- | --- | --- | --- | --- | --- | --- | --- | --- | --- | --- | --- | --- | --- | --- | --- | --- | --- | --- | --- | --- | --- | --- | --- | --- | --- | --- | --- | --- | --- | --- | --- | --- | --- | --- | --- | --- | --- | --- | --- | --- | --- | --- | --- | --- | --- | --- | --- | --- | --- | --- | --- | --- | --- | --- | --- | --- | --- | --- | --- | --- | --- | --- | --- | --- | --- | --- | --- | --- | --- | --- | --- | --- | --- | --- | --- | --- | --- | --- | --- | --- | --- | --- | --- | --- | --- | --- | --- | --- | --- | --- | --- | --- | --- | --- | --- | --- | --- | --- | --- | --- | --- | --- | --- | --- | --- | --- | --- | --- | --- | --- | --- | --- | --- | --- | --- | --- | --- | --- | --- | --- | --- | --- | --- | --- | --- | --- | --- | --- | --- | --- | --- | --- | --- | --- | --- | --- | --- | --- | --- | --- | --- | --- | --- | --- | --- | --- | --- | --- | --- | --- | --- | --- | --- | --- | --- | --- | --- | --- | --- | --- | --- | --- | --- | --- | --- | --- | --- | --- | --- | --- | --- | --- | --- | --- | --- | --- | --- | --- | --- | --- | --- | --- | --- | --- | --- | --- | --- | --- | --- | --- | --- | --- | --- | --- | --- | --- | --- | --- | --- | --- | --- | --- | --- | --- | --- | --- | --- | --- | --- | --- | --- | --- | --- | --- | --- | --- | --- | --- | --- | --- | --- | --- | --- | --- | --- | --- | --- | --- | --- | --- | --- | --- | --- | --- | --- | --- | --- | --- | --- | --- | --- | --- | --- | --- | --- | --- | --- | --- | --- | --- | --- | --- | --- | --- | --- | --- | --- | --- | --- | --- | --- | --- | --- | --- | --- | --- | --- | --- | --- | --- | --- | --- | --- | --- | --- | --- | --- | --- | --- | --- | --- | --- | --- | --- | --- | --- | --- | --- | --- | --- | --- | --- | --- | --- | --- | --- | --- | --- | --- | --- | --- | --- | --- | --- | --- | --- | --- | --- | --- | --- | --- | --- | --- | --- | --- | --- | --- | --- | --- | --- | --- | --- | --- | --- | --- | --- | --- | --- | --- | --- | --- | --- | --- | --- | --- | --- | --- | --- | --- | --- | --- | --- | --- | --- | --- | --- | --- | --- | --- | --- | --- | --- | --- | --- | --- | --- | --- | --- | --- | --- | --- | --- | --- | --- | --- | --- | --- | --- | --- | --- | --- | --- | --- | --- | --- | --- | --- | --- | --- | --- | --- | --- | --- | --- | --- | --- | --- | --- | --- | --- | --- | --- | --- | --- | --- | --- | --- | --- | --- | --- | --- | --- | --- | --- | --- | --- | --- | --- | --- | --- | --- | --- | --- | --- | --- | --- | --- | --- | --- | --- | --- | --- | --- | --- | --- | --- | --- | --- | --- | --- | --- | --- | --- | --- | --- | --- | --- | --- | --- | --- | --- | --- | --- | --- | --- | --- | --- | --- | --- | --- | --- | --- | --- | --- | --- | --- | --- | --- | --- | --- | --- | --- | --- | --- | --- | --- | --- | --- | --- | --- | --- | --- | --- | --- | --- | --- | --- | --- | --- | --- | --- | --- | --- | --- | --- | --- | --- | --- | --- | --- | --- | --- | --- | --- | --- | --- | --- | --- | --- | --- | --- | --- | --- | --- | --- | --- | --- | --- | --- | --- | --- | --- | --- | --- | --- | --- | --- | --- | --- | --- | --- | --- | --- | --- | --- | --- | --- | --- | --- | --- | --- | --- | --- | --- | --- | --- | --- | --- | --- | --- | --- | --- | --- | --- | --- | --- | --- | --- | --- | --- | --- | --- | --- | --- | --- | --- | --- | --- | --- | --- | --- | --- | --- | --- | --- | --- | --- | --- | --- | --- | --- | --- | --- | --- | --- | --- | --- | --- | --- | --- | --- | --- | --- | --- | --- | --- | --- | --- | --- | --- | --- | --- | --- | --- | --- | --- | --- | --- | --- | --- | --- | --- | --- | --- | --- | --- | --- | --- | --- | --- | --- | --- | --- | --- | --- | --- | --- | --- | --- | --- | --- | --- | --- | --- | --- | --- | --- | --- | --- | --- | --- | --- | --- | --- | --- | --- | --- | --- | --- | --- | --- | --- | --- | --- | --- | --- | --- | --- | --- | --- | --- | --- | --- | --- | --- | --- | --- | --- | --- | --- | --- | --- | --- | --- | --- | --- | --- | --- | --- | --- | --- | --- | --- | --- | --- | --- | --- | --- | --- | --- | --- | --- | --- | --- | --- | --- | --- | --- | --- | --- | --- | --- | --- | --- | --- | --- | --- | --- | --- | --- | --- | --- | --- | --- | --- | --- | --- | --- | --- | --- | --- | --- | --- | --- | --- | --- | --- | --- | --- | --- | --- | --- | --- | --- | --- | --- | --- | --- | --- | --- | --- | --- | --- | --- | --- | --- | --- | --- | --- | --- | --- | --- | --- | --- | --- | --- | --- | --- | --- | --- | --- | --- | --- | --- | --- | --- | --- | --- | --- | --- | --- | --- | --- | --- | --- | --- | --- | --- | --- | --- | --- | --- | --- | --- | --- | --- | --- | --- | --- | --- | --- | --- | --- | --- | --- | --- | --- | --- | --- | --- | --- | --- | --- | --- | --- | --- | --- | --- | --- | --- | --- | --- | --- | --- | --- | --- | --- | --- | --- | --- | --- | --- | --- | --- | --- | --- | --- | --- | --- | --- | --- | --- | --- | --- | --- | --- | --- | --- | --- | --- | --- | --- | --- | --- | --- | --- | --- | --- | --- | --- | --- | --- | --- | --- | --- | --- | --- | --- | --- | --- | --- | --- | --- | --- | --- | --- | --- | --- | --- | --- | --- | --- | --- | --- | --- | --- | --- | --- | --- | --- | --- | --- | --- | --- | --- | --- | --- | --- | --- | --- | --- | --- | --- | --- | --- | --- | --- | --- | --- | --- | --- | --- | --- | --- | --- | --- | --- | --- | --- | --- | --- | --- | --- | --- | --- | --- | --- | --- | --- | --- | --- | --- | --- | --- | --- | --- | --- | --- | --- | --- | --- | --- | --- | --- | --- | --- | --- | --- | --- | --- | --- | --- | --- | --- | --- | --- | --- | --- | --- | --- | --- | --- | --- | --- | --- | --- | --- | --- | --- | --- | --- | --- | --- | --- | --- | --- | --- | --- | --- | --- | --- | --- | --- | --- | --- | --- | --- | --- | --- | --- | --- | --- | --- | --- | --- | --- | --- | --- | --- | --- | --- | --- | --- | --- | --- | --- | --- | --- | --- | --- | --- | --- | --- | --- | --- | --- | --- | --- | --- | --- | --- | --- | --- | --- | --- | --- | --- | --- | --- | --- | --- | --- | --- | --- | --- | --- | --- | --- | --- | --- | --- | --- | --- | --- | --- | --- | --- | --- | --- | --- | --- | --- | --- | --- | --- | --- | --- | --- | --- | --- | --- | --- | --- | --- | --- | --- | --- | --- | --- | --- | --- | --- | --- | --- | --- | --- | --- | --- | --- | --- | --- | --- | --- | --- | --- | --- | --- | --- | --- | --- | --- | --- | --- | --- | --- | --- | --- | --- | --- | --- | --- | --- | --- | --- | --- | --- | --- | --- | --- | --- | --- | --- | --- | --- | --- | --- | --- | --- | --- | --- | --- | --- | --- | --- | --- | --- | --- | --- | --- | --- | --- | --- | --- | --- | --- | --- | --- | --- | --- | --- | --- | --- | --- | --- | --- | --- | --- | --- | --- | --- | --- | --- | --- | --- | --- | --- | --- | --- | --- | --- | --- | --- | --- | --- | --- | --- | --- | --- | --- | --- | --- | --- | --- | --- | --- | --- | --- | --- | --- | --- | --- | --- | --- | --- | --- | --- | --- | --- | --- | --- | --- | --- | --- | --- | --- | --- | --- | --- | --- | --- | --- | --- | --- | --- | --- | --- | --- | --- | --- | --- | --- | --- | --- | --- | --- | --- | --- | --- | --- | --- | --- | --- | --- | --- | --- | --- | --- | --- | --- | --- | --- | --- | --- | --- | --- | --- | --- | --- | --- | --- | --- | --- | --- | --- | --- | --- | --- | --- | --- | --- | --- | --- | --- | --- | --- | --- | --- | --- | --- | --- | --- | --- | --- | --- | --- | --- | --- | --- | --- | --- | --- | --- | --- | --- | --- | --- | --- | --- | --- | --- | --- | --- | --- | --- | --- | --- | --- | --- | --- | --- | --- | --- | --- | --- | --- | --- | --- | --- | --- | --- | --- | --- | --- | --- | --- | --- | --- | --- | --- | --- | --- | --- | --- | --- | --- | --- | --- | --- | --- | --- | --- | --- | --- | --- | --- | --- | --- | --- | --- | --- | --- | --- | --- | --- | --- | --- | --- | --- | --- | --- | --- | --- | --- | --- | --- | --- | --- | --- | --- | --- | --- | --- | --- | --- | --- | --- | --- | --- | --- | --- | --- | --- | --- | --- | --- | --- | --- | --- | --- | --- | --- | --- | --- | --- | --- | --- | --- | --- | --- | --- | --- | --- | --- | --- | --- | --- | --- | --- | --- | --- | --- | --- | --- | --- | --- | --- | --- | --- | --- | --- | --- | --- | --- | --- | --- | --- | --- | --- | --- | --- | --- | --- | --- | --- | --- | --- | --- | --- | --- | --- | --- | --- | --- | --- | --- | --- | --- | --- | --- | --- | --- | --- | --- | --- | --- | --- | --- | --- | --- | --- | --- | --- | --- | --- | --- | --- | --- | --- | --- | --- | --- | --- | --- | --- | --- | --- | --- | --- | --- | --- | --- | --- | --- | --- | --- | --- | --- | --- | --- | --- | --- | --- | --- | --- | --- | --- | --- | --- | --- | --- | --- | --- | --- | --- | --- | --- | --- | --- | --- | --- | --- | --- | --- | --- | --- | --- | --- | --- | --- | --- | --- | --- | --- | --- | --- | --- | --- | --- | --- | --- | --- | --- | --- | --- | --- | --- | --- | --- | --- | --- | --- | --- | --- | --- | --- | --- | --- | --- | --- | --- | --- | --- | --- | --- | --- | --- | --- | --- | --- | --- | --- | --- | --- | --- | --- | --- | --- | --- | --- | --- | --- | --- | --- | --- | --- | --- | --- | --- | --- | --- | --- | --- | --- | --- | --- | --- | --- | --- | --- | --- | --- | --- | --- | --- | --- | --- | --- | --- | --- | --- | --- | --- | --- | --- | --- | --- | --- | --- | --- | --- | --- | --- | --- | --- | --- | --- | --- | --- | --- | --- | --- | --- | --- | --- | --- | --- | --- | --- | --- | --- | --- | --- | --- | --- | --- | --- | --- | --- | --- | --- | --- | --- | --- | --- | --- | --- | --- | --- | --- | --- | --- | --- | --- | --- | --- | --- | --- | --- | --- | --- | --- | --- | --- | --- | --- | --- | --- | --- | --- | --- | --- | --- | --- | --- | --- | --- | --- | --- | --- | --- | --- | --- | --- | --- | --- | --- | --- | --- | --- | --- | --- | --- | --- | --- | --- | --- | --- | --- | --- | --- | --- | --- | --- | --- | --- | --- | --- | --- | --- | --- | --- | --- | --- | --- | --- | --- | --- | --- | --- | --- | --- | --- | --- | --- | --- | --- | --- | --- | --- | --- | --- | --- | --- | --- | --- | --- | --- | --- | --- | --- | --- | --- | --- | --- | --- | --- | --- | --- | --- | --- | --- | --- | --- | --- | --- | --- | --- | --- | --- | --- | --- | --- | --- | --- | --- | --- | --- | --- | --- | --- | --- | --- | --- | --- | --- | --- | --- | --- | --- | --- | --- | --- | --- | --- | --- | --- | --- | --- | --- | --- | --- | --- | --- | --- | --- | --- | --- | --- | --- | --- | --- | --- | --- | --- | --- | --- | --- | --- | --- | --- | --- | --- | --- | --- | --- | --- | --- | --- | --- | --- | --- | --- | --- | --- | --- | --- | --- | --- | --- | --- | --- | --- | --- | --- | --- | --- | --- | --- | --- | --- | --- | --- | --- | --- | --- | --- | --- | --- | --- | --- | --- | --- | --- | --- | --- | --- | --- | --- | --- | --- | --- | --- | --- | --- | --- | --- | --- | --- | --- | --- | --- | --- | --- | --- | --- | --- | --- | --- | --- | --- | --- | --- | --- | --- | --- | --- | --- | --- | --- | --- | --- | --- | --- | --- | --- | --- | --- | --- | --- | --- | --- | --- | --- | --- | --- | --- | --- | --- | --- | --- | --- | --- | --- | --- | --- | --- | --- | --- | --- | --- | --- | --- | --- | --- | --- | --- | --- | --- | --- | --- | --- | --- | --- | --- | --- | --- | --- | --- | --- | --- | --- | --- | --- | --- | --- | --- | --- | --- | --- | --- | --- | --- | --- | --- | --- | --- | --- | --- | --- | --- | --- | --- | --- | --- | --- | --- | --- | --- | --- | --- | --- | --- | --- | --- | --- | --- | --- | --- | --- | --- | --- | --- | --- | --- | --- | --- | --- | --- | --- | --- | --- | --- | --- | --- | --- | --- | --- | --- | --- | --- | --- | --- | --- | --- | --- | --- | --- | --- | --- | --- | --- | --- | --- | --- | --- | --- | --- | --- | --- | --- | --- | --- | --- | --- | --- | --- | --- | --- | --- | --- | --- | --- | --- | --- | --- | --- | --- | --- | --- | --- | --- | --- | --- | --- | --- | --- | --- | --- | --- | --- | --- | --- | --- | --- | --- | --- | --- | --- | --- | --- | --- | --- | --- | --- | --- | --- | --- | --- | --- | --- | --- | --- | --- | --- | --- | --- | --- | --- | --- | --- | --- | --- | --- | --- | --- | --- | --- | --- | --- | --- | --- | --- | --- | --- | --- | --- | --- | --- | --- | --- | --- | --- | --- | --- | --- | --- | --- | --- | --- | --- | --- | --- | --- | --- | --- | --- | --- | --- | --- | --- | --- | --- | --- | --- | --- | --- | --- | --- | --- | --- | --- | --- | --- | --- | --- | --- | --- | --- | --- | --- | --- | --- | --- | --- | --- | --- | --- | --- | --- | --- | --- | --- | --- | --- | --- | --- | --- | --- | --- | --- | --- | --- | --- | --- | --- | --- | --- | --- | --- | --- | --- | --- | --- | --- | --- | --- | --- | --- | --- | --- | --- | --- | --- | --- | --- | --- | --- | --- | --- | --- | --- | --- | --- | --- | --- | --- | --- | --- | --- | --- | --- | --- | --- | --- | --- | --- | --- | --- | --- | --- | --- | --- | --- | --- | --- | --- | --- | --- | --- | --- | --- | --- | --- | --- | --- | --- | --- | --- | --- | --- | --- | --- | --- | --- | --- | --- | --- | --- | --- | --- | --- | --- | --- | --- | --- | --- | --- | --- | --- | --- | --- | --- | --- | --- | --- | --- | --- | --- | --- | --- | --- | --- | --- | --- | --- | --- | --- | --- | --- | --- | --- | --- | --- | --- | --- | --- | --- | --- | --- | --- | --- | --- | --- | --- | --- | --- | --- | --- | --- | --- | --- | --- | --- | --- | --- | --- | --- | --- | --- | --- | --- | --- | --- | --- | --- | --- | --- | --- | --- | --- | --- | --- | --- | --- | --- | --- | --- | --- | --- | --- | --- | --- | --- | --- | --- | --- | --- | --- | --- | --- | --- | --- | --- | --- | --- | --- | --- | --- | --- | --- | --- | --- | --- | --- | --- | --- | --- | --- | --- | --- | --- | --- | --- | --- | --- | --- | --- | --- | --- | --- | --- | --- | --- | --- | --- | --- | --- | --- | --- | --- | --- | --- | --- | --- | --- | --- | --- | --- | --- | --- | --- | --- | --- | --- | --- | --- | --- | --- | --- | --- | --- | --- | --- | --- | --- | --- | --- | --- | --- | --- | --- | --- | --- | --- | --- | --- | --- | --- | --- | --- | --- | --- | --- | --- | --- | --- | --- | --- | --- | --- | --- | --- | --- | --- | --- | --- | --- | --- | --- | --- | --- | --- | --- | --- | --- | --- | --- | --- | --- | --- | --- | --- | --- | --- | --- | --- | --- | --- | --- | --- | --- | --- | --- | --- | --- | --- | --- | --- | --- | --- | --- | --- | --- | --- | --- | --- | --- | --- | --- | --- | --- | --- | --- | --- | --- | --- | --- | --- | --- | --- | --- | --- | --- | --- | --- | --- | --- | --- | --- | --- | --- | --- | --- | --- | --- | --- | --- | --- | --- | --- | --- | --- | --- | --- | --- | --- | --- | --- | --- | --- | --- | --- | --- | --- | --- | --- | --- | --- | --- | --- | --- | --- | --- | --- | --- | --- | --- | --- | --- | --- | --- | --- | --- | --- | --- | --- | --- | --- | --- | --- | --- | --- | --- | --- | --- | --- | --- | --- | --- | --- | --- | --- | --- | --- | --- | --- | --- | --- | --- | --- | --- | --- | --- | --- | --- | --- | --- | --- | --- | --- | --- | --- | --- | --- | --- | --- | --- | --- | --- | --- | --- | --- | --- | --- | --- | --- | --- | --- | --- | --- | --- | --- | --- | --- | --- | --- | --- | --- | --- | --- | --- | --- | --- | --- | --- | --- | --- | --- | --- | --- | --- | --- | --- | --- | --- | --- | --- | --- | --- | --- | --- | --- | --- | --- | --- | --- | --- | --- | --- | --- | --- | --- | --- | --- | --- | --- | --- | --- | --- | --- | --- | --- | --- | --- | --- | --- | --- | --- | --- | --- | --- | --- | --- | --- | --- | --- | --- | --- | --- | --- | --- | --- | --- | --- | --- | --- | --- | --- | --- | --- | --- | --- | --- | --- | --- | --- | --- | --- | --- | --- | --- | --- | --- | --- | --- | --- | --- | --- | --- | --- | --- | --- | --- | --- | --- | --- | --- | --- | --- | --- | --- | --- | --- | --- | --- | --- | --- | --- | --- | --- | --- | --- | --- | --- | --- | --- | --- | --- | --- | --- | --- | --- | --- | --- | --- | --- | --- | --- | --- | --- | --- | --- | --- | --- | --- | --- | --- | --- | --- | --- | --- | --- | --- | --- | --- | --- | --- | --- | --- | --- | --- | --- | --- | --- | --- | --- | --- | --- | --- | --- | --- | --- | --- | --- | --- | --- | --- | --- | --- | --- | --- | --- | --- | --- | --- | --- | --- | --- | --- | --- | --- | --- | --- | --- | --- | --- | --- | --- | --- | --- | --- | --- | --- | --- | --- | --- | --- | --- | --- | --- | --- | --- | --- | --- | --- | --- | --- | --- | --- | --- | --- | --- | --- | --- | --- | --- | --- | --- | --- | --- | --- | --- | --- | --- | --- | --- | --- | --- | --- | --- | --- | --- | --- | --- | --- | --- | --- | --- | --- | --- | --- | --- | --- | --- | --- | --- | --- | --- | --- | --- | --- | --- | --- | --- | --- | --- | --- | --- | --- | --- | --- | --- | --- | --- | --- | --- | --- | --- | --- | --- | --- | --- | --- | --- | --- | --- | --- | --- | --- | --- | --- | --- | --- | --- | --- | --- | --- | --- | --- | --- | --- | --- | --- | --- | --- | --- | --- | --- | --- | --- | --- | --- | --- | --- | --- | --- | --- | --- | --- | --- | --- | --- | --- | --- | --- | --- | --- | --- | --- | --- | --- | --- | --- | --- | --- | --- | --- | --- | --- | --- | --- | --- | --- | --- | --- | --- | --- | --- | --- | --- | --- | --- | --- | --- | --- | --- | --- | --- | --- | --- | --- | --- | --- | --- | --- | --- | --- | --- | --- | --- | --- | --- | --- | --- | --- | --- | --- | --- | --- | --- | --- | --- | --- | --- | --- | --- | --- | --- | --- | --- | --- | --- | --- | --- | --- | --- | --- | --- | --- | --- | --- | --- | --- | --- | --- | --- | --- | --- | --- | --- | --- | --- | --- | --- | --- | --- | --- | --- | --- | --- | --- | --- | --- | --- | --- | --- | --- | --- | --- | --- | --- | --- | --- | --- | --- | --- | --- | --- | --- | --- | --- | --- | --- | --- | --- | --- | --- | --- | --- | --- | --- | --- | --- | --- | --- | --- | --- | --- | --- | --- | --- | --- | --- | --- | --- | --- | --- | --- | --- | --- | --- | --- | --- | --- | --- | --- | --- | --- | --- | --- | --- | --- | --- | --- | --- | --- | --- | --- | --- | --- | --- | --- | --- | --- | --- | --- | --- | --- | --- | --- | --- | --- | --- | --- | --- | --- | --- | --- | --- | --- | --- | --- | --- | --- | --- | --- | --- | --- | --- | --- | --- | --- | --- | --- | --- | --- | --- | --- | --- | --- | --- | --- | --- | --- | --- | --- | --- | --- | --- | --- | --- | --- | --- | --- | --- | --- | --- | --- | --- | --- | --- | --- | --- | --- | --- | --- | --- | --- | --- | --- | --- | --- | --- | --- | --- | --- | --- | --- | --- | --- | --- | --- | --- | --- | --- | --- | --- | --- | --- | --- | --- | --- | --- | --- | --- | --- | --- | --- | --- | --- | --- | --- | --- | --- | --- | --- | --- | --- | --- | --- | --- | --- | --- | --- | --- | --- | --- | --- | --- | --- | --- | --- | --- | --- | --- | --- | --- | --- | --- | --- | --- | --- | --- | --- | --- | --- | --- | --- | --- | --- | --- | --- | --- | --- | --- | --- | --- | --- | --- | --- | --- | --- | --- | --- | --- | --- | --- | --- | --- | --- | --- | --- | --- | --- | --- | --- | --- | --- | --- | --- | --- | --- | --- | --- | --- | --- | --- | --- | --- | --- | --- | --- | --- | --- | --- | --- | --- | --- | --- | --- | --- | --- | --- | --- | --- | --- | --- | --- | --- | --- | --- | --- | --- | --- | --- | --- | --- | --- | --- | --- | --- | --- | --- | --- | --- | --- | --- | --- | --- | --- | --- | --- | --- | --- | --- | --- | --- | --- | --- | --- | --- | --- | --- | --- | --- | --- | --- | --- | --- | --- | --- | --- | --- | --- | --- | --- | --- | --- | --- | --- | --- | --- | --- | --- | --- | --- | --- | --- | --- | --- | --- | --- | --- | --- | --- | --- | --- | --- | --- | --- | --- | --- | --- | --- | --- | --- | --- | --- | --- | --- | --- | --- | --- | --- | --- | --- | --- | --- | --- | --- | --- | --- | --- | --- | --- | --- | --- | --- | --- | --- | --- | --- | --- | --- | --- | --- | --- | --- | --- | --- | --- | --- | --- | --- | --- | --- | --- | --- | --- | --- | --- | --- | --- | --- | --- | --- | --- | --- | --- | --- | --- | --- | --- | --- | --- | --- | --- | --- | --- | --- | --- | --- | --- | --- | --- | --- | --- | --- | --- | --- | --- | --- | --- | --- | --- | --- | --- | --- | --- | --- | --- | --- | --- | --- | --- | --- | --- | --- | --- | --- | --- | --- | --- | --- | --- | --- | --- | --- | --- | --- | --- | --- | --- | --- | --- | --- | --- | --- | --- | --- | --- | --- | --- | --- | --- | --- | --- | --- | --- | --- | --- | --- | --- | --- | --- | --- | --- | --- | --- | --- | --- | --- | --- | --- | --- | --- | --- | --- | --- | --- | --- | --- | --- | --- | --- | --- | --- | --- | --- | --- | --- | --- | --- | --- | --- | --- | --- | --- | --- | --- | --- | --- | --- | --- | --- | --- | --- | --- | --- | --- | --- | --- | --- | --- | --- | --- | --- | --- | --- | --- | --- | --- | --- | --- | --- | --- | --- | --- | --- | --- | --- | --- | --- | --- | --- | --- | --- | --- | --- | --- | --- | --- | --- | --- | --- | --- | --- | --- | --- | --- | --- | --- | --- | --- | --- | --- | --- | --- | --- | --- | --- | --- | --- | --- | --- | --- | --- | --- | --- | --- | --- | --- | --- | --- | --- | --- | --- | --- | --- | --- | --- | --- | --- | --- | --- | --- | --- | --- | --- | --- | --- | --- | --- | --- | --- | --- | --- | --- | --- | --- | --- | --- | --- | --- | --- | --- | --- | --- | --- | --- | --- | --- | --- | --- | --- | --- | --- | --- | --- | --- | --- | --- | --- | --- | --- | --- | --- | --- | --- | --- | --- | --- | --- | --- | --- | --- | --- | --- | --- | --- | --- | --- | --- | --- | --- | --- | --- | --- | --- | --- | --- | --- | --- | --- | --- | --- | --- | --- | --- | --- | --- | --- | --- | --- | --- | --- | --- | --- | --- | --- | --- | --- | --- | --- | --- | --- | --- | --- | --- | --- | --- | --- | --- | --- | --- | --- | --- | --- | --- | --- | --- | --- | --- | --- | --- | --- | --- | --- | --- | --- | --- | --- | --- | --- | --- | --- | --- | --- | --- | --- | --- | --- | --- | --- | --- | --- | --- | --- | --- | --- | --- | --- | --- | --- | --- | --- | --- | --- | --- | --- | --- | --- | --- | --- | --- | --- | --- | --- | --- | --- | --- | --- | --- | --- | --- | --- | --- | --- | --- | --- | --- | --- | --- | --- | --- | --- | --- | --- | --- | --- | --- | --- | --- | --- | --- | --- | --- | --- | --- | --- | --- | --- | --- | --- | --- | --- | --- | --- | --- | --- | --- | --- | --- | --- | --- | --- | --- | --- | --- | --- | --- | --- | --- | --- | --- | --- | --- | --- | --- | --- | --- | --- | --- | --- | --- | --- | --- | --- | --- | --- | --- | --- | --- | --- | --- | --- | --- | --- | --- | --- | --- | --- | --- | --- | --- | --- | --- | --- | --- | --- | --- | --- | --- | --- | --- | --- | --- | --- | --- | --- | --- | --- | --- | --- | --- | --- | --- | --- | --- | --- | --- | --- | --- | --- | --- | --- | --- | --- | --- | --- | --- | --- | --- | --- | --- | --- | --- | --- | --- | --- | --- | --- | --- | --- | --- | --- | --- | --- | --- | --- | --- | --- | --- | --- | --- | --- | --- | --- | --- | --- | --- | --- | --- | --- | --- | --- | --- | --- | --- | --- | --- | --- | --- | --- | --- | --- | --- | --- | --- | --- | --- | --- | --- | --- | --- | --- | --- | --- | --- | --- | --- | --- | --- | --- | --- | --- | --- | --- | --- | --- | --- | --- | --- | --- | --- | --- | --- | --- | --- | --- | --- | --- | --- | --- | --- | --- | --- | --- | --- | --- | --- | --- | --- | --- | --- | --- | --- | --- | --- | --- | --- | --- | --- | --- | --- | --- | --- | --- | --- | --- | --- | --- | --- | --- | --- | --- | --- | --- | --- | --- | --- | --- | --- | --- | --- | --- | --- | --- | --- | --- | --- | --- | --- | --- | --- | --- | --- | --- | --- | --- | --- | --- | --- | --- | --- | --- | --- | --- | --- | --- | --- | --- | --- | --- | --- | --- | --- | --- | --- | --- | --- | --- | --- | --- | --- | --- | --- | --- | --- | --- | --- | --- | --- | --- | --- | --- | --- | --- | --- | --- | --- | --- | --- | --- | --- | --- | --- | --- | --- | --- | --- | --- | --- | --- | --- | --- | --- | --- | --- | --- | --- | --- | --- | --- | --- | --- | --- | --- | --- | --- | --- | --- | --- | --- | --- | --- | --- | --- | --- | --- | --- | --- | --- | --- | --- | --- | --- | --- | --- | --- | --- | --- | --- | --- | --- | --- | --- | --- | --- | --- | --- | --- | --- | --- | --- | --- | --- | --- | --- | --- | --- | --- | --- | --- | --- | --- | --- | --- | --- | --- | --- | --- | --- | --- | --- | --- | --- | --- | --- | --- | --- | --- | --- | --- | --- | --- | --- | --- | --- | --- | --- | --- | --- | --- | --- | --- | --- | --- | --- | --- | --- | --- | --- | --- | --- | --- | --- | --- | --- | --- | --- | --- | --- | --- | --- | --- | --- | --- | --- | --- | --- | --- | --- | --- | --- | --- | --- | --- | --- | --- | --- | --- | --- | --- | --- | --- | --- | --- | --- | --- | --- | --- | --- | --- | --- | --- | --- | --- | --- | --- | --- | --- | --- | --- | --- | --- | --- | --- | --- | --- | --- | --- | --- | --- | --- | --- | --- | --- | --- | --- | --- | --- | --- | --- | --- | --- | --- | --- | --- | --- | --- | --- | --- | --- | --- | --- | --- | --- | --- | --- | --- | --- | --- | --- | --- | --- | --- | --- | --- | --- | --- | --- | --- | --- | --- | --- | --- | --- | --- | --- | --- | --- | --- | --- | --- | --- | --- | --- | --- | --- | --- | --- | --- | --- | --- | --- | --- | --- | --- | --- | --- | --- | --- | --- | --- | --- | --- | --- | --- | --- | --- | --- | --- | --- | --- | --- | --- | --- | --- | --- | --- | --- | --- | --- | --- | --- | --- | --- | --- | --- | --- | --- | --- | --- | --- | --- | --- | --- | --- | --- | --- | --- | --- | --- | --- | --- | --- | --- | --- | --- | --- | --- | --- | --- | --- | --- | --- | --- | --- | --- | --- | --- | --- | --- | --- | --- | --- | --- | --- | --- | --- | --- | --- | --- | --- | --- | --- | --- | --- | --- | --- | --- | --- | --- | --- | --- | --- | --- | --- | --- | --- | --- | --- | --- | --- | --- | --- | --- | --- | --- | --- | --- | --- | --- | --- | --- | --- | --- | --- | --- | --- | --- | --- | --- | --- | --- | --- | --- | --- | --- | --- | --- | --- | --- | --- | --- | --- | --- | --- | --- | --- | --- | --- | --- | --- | --- | --- | --- | --- | --- | --- | --- | --- | --- | --- | --- | --- | --- | --- | --- | --- | --- | --- | --- | --- | --- | --- | --- | --- | --- | --- | --- | --- | --- | --- | --- | --- | --- | --- | --- | --- | --- | --- | --- | --- | --- | --- | --- | --- | --- | --- | --- | --- | --- | --- | --- | --- | --- | --- | --- | --- | --- | --- | --- | --- | --- | --- | --- | --- | --- | --- | --- | --- | --- | --- | --- | --- | --- | --- | --- | --- | --- | --- | --- | --- | --- | --- | --- | --- | --- | --- | --- | --- | --- | --- | --- | --- | --- | --- | --- | --- | --- | --- | --- | --- | --- | --- | --- | --- | --- | --- | --- | --- | --- | --- | --- | --- | --- | --- | --- | --- | --- | --- | --- | --- | --- | --- | --- | --- | --- | --- | --- | --- | --- | --- | --- | --- | --- | --- | --- | --- | --- | --- | --- | --- | --- | --- | --- | --- | --- | --- | --- | --- | --- | --- | --- | --- | --- | --- | --- | --- | --- | --- | --- | --- | --- | --- | --- | --- | --- | --- | --- | --- | --- | --- | --- | --- | --- | --- | --- | --- | --- | --- | --- | --- | --- | --- | --- | --- | --- | --- | --- | --- | --- | --- | --- | --- | --- | --- | --- | --- | --- | --- | --- | --- | --- | --- | --- | --- | --- | --- | --- | --- | --- | --- | --- | --- | --- | --- | --- | --- | --- | --- | --- | --- | --- | --- | --- | --- | --- | --- | --- | --- | --- | --- | --- | --- | --- | --- | --- | --- | --- | --- | --- | --- | --- | --- | --- | --- | --- | --- | --- | --- | --- | --- | --- | --- | --- | --- | --- | --- | --- | --- | --- | --- | --- | --- | --- | --- | --- | --- | --- | --- | --- | --- | --- | --- | --- | --- | --- | --- | --- | --- | --- | --- | --- | --- | --- | --- | --- | --- | --- | --- | --- | --- | --- | --- | --- | --- | --- | --- | --- | --- | --- | --- | --- | --- | --- | --- | --- | --- | --- | --- | --- | --- | --- | --- | --- | --- | --- | --- | --- | --- | --- | --- | --- | --- | --- | --- | --- | --- | --- | --- | --- | --- | --- | --- | --- | --- | --- | --- | --- | --- | --- | --- | --- | --- | --- | --- | --- | --- | --- | --- | --- | --- | --- | --- | --- | --- | --- | --- | --- | --- | --- | --- | --- | --- | --- | --- | --- | --- | --- | --- | --- | --- | --- | --- | --- | --- | --- | --- | --- | --- | --- | --- | --- | --- | --- | --- | --- | --- | --- | --- | --- | --- | --- | --- | --- | --- | --- | --- | --- | --- | --- | --- | --- | --- | --- | --- | --- | --- | --- | --- | --- | --- | --- | --- | --- | --- | --- | --- | --- | --- | --- | --- | --- | --- | --- | --- | --- | --- | --- | --- | --- | --- | --- | --- | --- | --- | --- | --- | --- | --- | --- | --- | --- | --- | --- | --- | --- | --- | --- | --- | --- | --- | --- | --- | --- | --- | --- | --- | --- | --- | --- | --- | --- | --- | --- | --- | --- | --- | --- | --- | --- | --- | --- | --- | --- | --- | --- | --- | --- | --- | --- | --- | --- | --- | --- | --- | --- | --- | --- | --- | --- | --- | --- | --- | --- | --- | --- | --- | --- | --- | --- | --- | --- | --- | --- | --- | --- | --- | --- | --- | --- | --- | --- | --- | --- | --- | --- | --- | --- | --- | --- | --- | --- | --- | --- | --- | --- | --- | --- | --- | --- | --- | --- | --- | --- | --- | --- | --- | --- | --- | --- | --- | --- | --- | --- | --- | --- | --- | --- | --- | --- | --- | --- | --- | --- | --- | --- | --- | --- | --- | --- | --- | --- | --- | --- | --- | --- | --- | --- | --- | --- | --- | --- | --- | --- | --- | --- | --- | --- | --- | --- | --- | --- | --- | --- | --- | --- | --- | --- | --- | --- | --- | --- | --- | --- | --- | --- | --- | --- | --- | --- | --- | --- | --- | --- | --- | --- | --- | --- | --- | --- | --- | --- | --- | --- | --- | --- | --- | --- | --- | --- | --- | --- | --- | --- | --- | --- | --- | --- | --- | --- | --- | --- | --- | --- | --- | --- | --- | --- | --- | --- | --- | --- | --- | --- | --- | --- | --- | --- | --- | --- | --- | --- | --- | --- | --- | --- | --- | --- | --- | --- | --- | --- | --- | --- | --- | --- | --- | --- | --- | --- | --- | --- | --- | --- | --- | --- | --- | --- | --- | --- | --- | --- | --- | --- | --- | --- | --- | --- | --- | --- | --- | --- | --- | --- | --- | --- | --- | --- | --- | --- | --- | --- | --- | --- | --- | --- | --- | --- | --- | --- | --- | --- | --- | --- | --- | --- | --- | --- | --- | --- | --- | --- | --- | --- | --- | --- | --- | --- | --- | --- | --- | --- | --- | --- | --- | --- | --- | --- | --- | --- | --- | --- | --- | --- | --- | --- | --- | --- | --- | --- | --- | --- | --- | --- | --- | --- | --- | --- | --- | --- | --- | --- | --- | --- | --- | --- | --- | --- | --- | --- | --- | --- | --- | --- | --- | --- | --- | --- | --- | --- | --- | --- | --- | --- | --- | --- | --- | --- | --- | --- | --- | --- | --- | --- | --- | --- | --- | --- | --- | --- | --- | --- | --- | --- | --- | --- | --- | --- | --- | --- | --- | --- | --- | --- | --- | --- | --- | --- | --- | --- | --- | --- | --- | --- | --- | --- | --- | --- | --- | --- | --- | --- | --- | --- | --- | --- | --- | --- | --- | --- | --- | --- | --- | --- | --- | --- | --- | --- | --- | --- | --- | --- | --- | --- | --- | --- | --- | --- | --- | --- | --- | --- | --- | --- | --- | --- | --- | --- | --- | --- | --- | --- | --- | --- | --- | --- | --- | --- | --- | --- | --- | --- | --- | --- | --- | --- | --- | --- | --- | --- | --- | --- | --- | --- | --- | --- | --- | --- | --- | --- | --- | --- | --- | --- | --- | --- | --- | --- | --- | --- | --- | --- | --- | --- | --- | --- | --- | --- | --- | --- | --- | --- | --- | --- | --- | --- | --- | --- | --- | --- | --- | --- | --- | --- | --- | --- | --- | --- | --- | --- | --- | --- | --- | --- | --- | --- | --- | --- | --- | --- | --- | --- | --- | --- | --- | --- | --- | --- | --- | --- | --- | --- | --- | --- | --- | --- | --- | --- | --- | --- | --- | --- | --- | --- | --- | --- | --- | --- | --- | --- | --- | --- | --- | --- | --- | --- | --- | --- | --- | --- | --- | --- | --- | --- | --- | --- | --- | --- | --- | --- | --- | --- | --- | --- | --- | --- | --- | --- | --- | --- | --- | --- | --- | --- | --- | --- | --- | --- | --- | --- | --- | --- | --- | --- | --- | --- | --- | --- | --- | --- | --- | --- | --- | --- | --- | --- | --- | --- | --- | --- | --- | --- | --- | --- | --- | --- | --- | --- | --- | --- | --- | --- | --- | --- | --- | --- | --- | --- | --- | --- | --- | --- | --- | --- | --- | --- | --- | --- | --- | --- | --- | --- | --- | --- | --- | --- | --- | --- | --- | --- | --- | --- | --- | --- | --- | --- | --- | --- | --- | --- | --- | --- | --- | --- | --- | --- | --- | --- | --- | --- | --- | --- | --- | --- | --- | --- | --- | --- | --- | --- | --- | --- | --- | --- | --- | --- | --- | --- | --- | --- | --- | --- | --- | --- | --- | --- | --- | --- | --- | --- | --- | --- | --- | --- | --- | --- | --- | --- | --- | --- | --- | --- | --- | --- | --- | --- | --- | --- | --- | --- | --- | --- | --- | --- | --- | --- | --- | --- | --- | --- | --- | --- | --- | --- | --- | --- | --- | --- | --- | --- | --- | --- | --- | --- | --- | --- | --- | --- | --- | --- | --- | --- | --- | --- | --- | --- | --- | --- | --- | --- | --- | --- | --- | --- | --- | --- | --- | --- | --- | --- | --- | --- | --- | --- | --- | --- | --- | --- | --- | --- | --- | --- | --- | --- | --- | --- | --- | --- | --- | --- | --- | --- | --- | --- | --- | --- | --- | --- | --- | --- | --- | --- | --- | --- | --- | --- | --- | --- | --- | --- | --- | --- | --- | --- | --- | --- | --- | --- | --- | --- | --- | --- | --- | --- | --- | --- | --- | --- | --- | --- | --- | --- | --- | --- | --- | --- | --- | --- | --- | --- | --- | --- | --- | --- | --- | --- | --- | --- | --- | --- | --- | --- | --- | --- | --- | --- | --- | --- | --- | --- | --- | --- | --- | --- | --- | --- | --- | --- | --- | --- | --- | --- | --- | --- | --- | --- | --- | --- | --- | --- | --- | --- | --- | --- | --- | --- | --- | --- | --- | --- | --- | --- | --- | --- | --- | --- | --- | --- | --- | --- | --- | --- | --- | --- | --- | --- | --- | --- | --- | --- | --- | --- | --- | --- | --- | --- | --- | --- | --- | --- | --- | --- | --- | --- | --- | --- | --- | --- | --- | --- | --- | --- | --- | --- | --- | --- | --- | --- | --- | --- | --- | --- | --- | --- | --- | --- | --- | --- | --- | --- | --- | --- | --- | --- | --- | --- | --- | --- | --- | --- | --- | --- | --- | --- | --- | --- | --- | --- | --- | --- | --- | --- | --- | --- | --- | --- | --- | --- | --- | --- | --- | --- | --- | --- | --- | --- | --- | --- | --- | --- | --- | --- | --- | --- | --- | --- | --- | --- | --- | --- | --- | --- | --- | --- | --- | --- | --- | --- | --- | --- | --- | --- | --- | --- | --- | --- | --- | --- | --- | --- | --- | --- | --- | --- | --- | --- | --- | --- | --- | --- | --- | --- | --- | --- | --- | --- | --- | --- | --- | --- | --- | --- | --- | --- | --- | --- | --- | --- | --- | --- | --- | --- | --- | --- | --- | --- | --- | --- | --- | --- | --- | --- | --- | --- | --- | --- | --- | --- | --- | --- | --- | --- | --- | --- | --- | --- | --- | --- | --- | --- | --- | --- | --- | --- | --- | --- | --- | --- | --- | --- | --- | --- | --- | --- | --- | --- | --- | --- | --- | --- | --- | --- | --- | --- | --- | --- | --- | --- | --- | --- | --- | --- | --- | --- | --- | --- | --- | --- | --- | --- | --- | --- | --- | --- | --- | --- | --- | --- | --- | --- | --- | --- | --- | --- | --- | --- | --- | --- | --- | --- | --- | --- | --- | --- | --- | --- | --- | --- | --- | --- | --- | --- | --- | --- | --- | --- | --- | --- | --- | --- | --- | --- | --- | --- | --- | --- | --- | --- | --- | --- | --- | --- | --- | --- | --- | --- | --- | --- | --- | --- | --- | --- | --- | --- | --- | --- | --- | --- | --- | --- | --- | --- | --- | --- | --- | --- | --- | --- | --- | --- | --- | --- | --- | --- | --- | --- | --- | --- | --- | --- | --- | --- | --- | --- | --- | --- | --- | --- | --- | --- | --- | --- | --- | --- | --- | --- | --- | --- | --- | --- | --- | --- | --- | --- | --- | --- | --- | --- | --- | --- | --- | --- | --- | --- | --- | --- | --- | --- | --- | --- | --- | --- | --- | --- | --- | --- | --- | --- | --- | --- | --- | --- | --- | --- | --- | --- | --- | --- | --- | --- | --- | --- | --- | --- | --- | --- | --- | --- | --- | --- | --- | --- | --- | --- | --- | --- | --- | --- | --- | --- | --- | --- | --- | --- | --- | --- | --- | --- | --- | --- | --- | --- | --- | --- | --- | --- | --- | --- | --- | --- | --- | --- | --- | --- | --- | --- | --- | --- | --- | --- | --- | --- | --- | --- | --- | --- | --- | --- | --- | --- | --- | --- | --- | --- | --- | --- | --- | --- | --- | --- | --- | --- | --- | --- | --- | --- | --- | --- | --- | --- | --- | --- | --- | --- | --- | --- | --- | --- | --- | --- | --- | --- | --- | --- | --- | --- | --- | --- | --- | --- | --- | --- | --- | --- | --- | --- | --- | --- | --- | --- | --- | --- | --- | --- | --- | --- | --- | --- | --- | --- | --- | --- | --- | --- | --- | --- | --- | --- | --- | --- | --- | --- | --- | --- | --- | --- | --- | --- | --- | --- | --- | --- | --- | --- | --- | --- | --- | --- | --- | --- | --- | --- | --- | --- | --- | --- | --- | --- | --- | --- | --- | --- | --- | --- | --- | --- | --- | --- | --- | --- | --- | --- | --- | --- | --- | --- | --- | --- | --- | --- | --- | --- | --- | --- | --- | --- | --- | --- | --- | --- | --- | --- | --- | --- | --- | --- | --- | --- | --- | --- | --- | --- | --- | --- | --- | --- | --- | --- | --- | --- | --- | --- | --- | --- | --- | --- | --- | --- | --- | --- | --- | --- | --- | --- | --- | --- | --- | --- | --- | --- | --- | --- | --- | --- | --- | --- | --- | --- | --- | --- | --- | --- | --- | --- | --- | --- | --- | --- | --- | --- | --- | --- | --- | --- | --- | --- | --- | --- | --- | --- | --- | --- | --- | --- | --- | --- | --- | --- | --- | --- | --- | --- | --- | --- | --- | --- | --- | --- | --- | --- | --- | --- | --- | --- | --- | --- | --- | --- | --- | --- | --- | --- | --- | --- | --- | --- | --- | --- | --- | --- | --- | --- | --- | --- | --- | --- | --- | --- | --- | --- | --- | --- | --- | --- | --- | --- | --- | --- | --- | --- | --- | --- | --- | --- | --- | --- | --- | --- | --- | --- | --- | --- | --- | --- | --- | --- | --- | --- | --- | --- | --- | --- | --- | --- | --- | --- | --- | --- | --- | --- | --- | --- | --- | --- | --- | --- | --- | --- | --- | --- | --- | --- | --- | --- | --- | --- | --- | --- | --- | --- | --- | --- | --- | --- | --- | --- | --- | --- | --- | --- | --- | --- | --- | --- | --- | --- | --- | --- | --- | --- | --- | --- | --- | --- | --- | --- | --- | --- | --- | --- | --- | --- | --- | --- | --- | --- | --- | --- | --- | --- | --- | --- | --- | --- | --- | --- | --- | --- | --- | --- | --- | --- | --- | --- | --- | --- | --- | --- | --- | --- | --- | --- | --- | --- | --- | --- | --- | --- | --- | --- | --- | --- | --- | --- | --- | --- | --- | --- | --- | --- | --- | --- | --- | --- | --- | --- | --- | --- | --- | --- | --- | --- | --- | --- | --- | --- | --- | --- | --- | --- | --- | --- | --- | --- | --- | --- | --- | --- | --- | --- | --- | --- | --- | --- | --- | --- | --- | --- | --- | --- | --- | --- | --- | --- | --- | --- | --- | --- | --- | --- | --- | --- | --- | --- | --- | --- | --- | --- | --- | --- | --- | --- | --- | --- | --- | --- | --- | --- | --- | --- | --- | --- | --- | --- | --- | --- | --- | --- | --- | --- | --- | --- | --- | --- | --- | --- | --- | --- | --- | --- | --- | --- | --- | --- | --- | --- | --- | --- | --- | --- | --- | --- | --- | --- | --- | --- | --- | --- | --- | --- | --- | --- | --- | --- | --- | --- | --- | --- | --- | --- | --- | --- | --- | --- | --- | --- | --- | --- | --- | --- | --- | --- | --- | --- | --- | --- | --- | --- | --- | --- | --- | --- | --- | --- | --- | --- | --- | --- | --- | --- | --- | --- | --- | --- | --- | --- | --- | --- | --- | --- | --- | --- | --- | --- | --- | --- | --- | --- | --- | --- | --- | --- | --- | --- | --- | --- | --- | --- | --- | --- | --- | --- | --- | --- | --- | --- | --- | --- | --- | --- | --- | --- | --- | --- | --- | --- | --- | --- | --- | --- | --- | --- | --- | --- | --- | --- | --- | --- | --- | --- | --- | --- | --- | --- | --- | --- | --- | --- | --- | --- | --- | --- | --- | --- | --- | --- | --- | --- | --- | --- | --- | --- | --- | --- | --- | --- | --- | --- | --- | --- | --- | --- | --- | --- | --- | --- | --- | --- | --- | --- | --- | --- | --- | --- | --- | --- | --- | --- | --- | --- | --- | --- | --- | --- | --- | --- | --- | --- | --- | --- | --- | --- | --- | --- | --- | --- | --- | --- | --- | --- | --- | --- | --- | --- | --- | --- | --- | --- | --- | --- | --- | --- | --- | --- | --- | --- | --- | --- | --- | --- | --- | --- | --- | --- | --- | --- | --- | --- | --- | --- | --- | --- | --- | --- | --- | --- | --- | --- | --- | --- | --- | --- | --- | --- | --- | --- | --- | --- | --- | --- | --- | --- | --- | --- | --- | --- | --- | --- | --- | --- | --- | --- | --- | --- | --- | --- | --- | --- | --- | --- | --- | --- | --- | --- | --- | --- | --- | --- | --- | --- | --- | --- | --- | --- | --- | --- | --- | --- | --- | --- | --- | --- | --- | --- | --- | --- | --- | --- | --- | --- | --- | --- | --- | --- | --- | --- | --- | --- | --- | --- | --- | --- | --- | --- | --- | --- | --- | --- | --- | --- | --- | --- | --- | --- | --- | --- | --- | --- | --- | --- | --- | --- | --- | --- | --- | --- | --- | --- | --- | --- | --- | --- | --- | --- | --- | --- | --- | --- | --- | --- | --- | --- | --- | --- | --- | --- | --- | --- | --- | --- | --- | --- | --- | --- | --- | --- | --- | --- | --- | --- | --- | --- | --- | --- | --- | --- | --- | --- | --- | --- | --- | --- | --- | --- | --- | --- | --- | --- | --- | --- | --- | --- | --- | --- | --- | --- | --- | --- | --- | --- | --- | --- | --- | --- | --- | --- | --- | --- | --- | --- | --- | --- | --- | --- | --- | --- | --- | --- | --- | --- | --- | --- | --- | --- | --- | --- | --- | --- | --- | --- | --- | --- | --- | --- | --- | --- | --- | --- | --- | --- | --- | --- | --- | --- | --- | --- | --- | --- | --- | --- | --- | --- | --- | --- | --- | --- | --- | --- | --- | --- | --- | --- | --- | --- | --- | --- | --- | --- | --- | --- | --- | --- | --- | --- | --- | --- | --- | --- | --- | --- | --- | --- | --- | --- | --- | --- | --- | --- | --- | --- | --- | --- | --- | --- | --- | --- | --- | --- | --- | --- | --- | --- | --- | --- | --- | --- | --- | --- | --- | --- | --- | --- | --- | --- | --- | --- | --- | --- | --- | --- | --- | --- | --- | --- | --- | --- | --- | --- | --- | --- | --- | --- | --- | --- | --- | --- | --- | --- | --- | --- | --- | --- | --- | --- | --- | --- | --- | --- | --- | --- | --- | --- | --- | --- | --- | --- | --- | --- | --- | --- | --- | --- | --- | --- | --- | --- | --- | --- | --- | --- | --- | --- | --- | --- | --- | --- | --- | --- | --- | --- | --- | --- | --- | --- | --- | --- | --- | --- | --- | --- | --- | --- | --- | --- | --- | --- | --- | --- | --- | --- | --- | --- | --- | --- | --- | --- | --- | --- | --- | --- | --- | --- | --- | --- | --- | --- | --- | --- | --- | --- | --- | --- | --- | --- | --- | --- | --- | --- | --- | --- | --- | --- | --- | --- | --- | --- | --- | --- | --- | --- | --- | --- | --- | --- | --- | --- | --- | --- | --- | --- | --- | --- | --- | --- | --- | --- | --- | --- | --- | --- | --- | --- | --- | --- | --- | --- | --- | --- | --- | --- | --- | --- | --- | --- | --- | --- | --- | --- | --- | --- | --- | --- | --- | --- | --- | --- | --- | --- | --- | --- | --- | --- | --- | --- | --- | --- | --- | --- | --- | --- | --- | --- | --- | --- | --- | --- | --- | --- | --- | --- | --- | --- | --- | --- | --- | --- | --- | --- | --- | --- | --- | --- | --- | --- | --- | --- | --- | --- | --- | --- | --- | --- | --- | --- | --- | --- | --- | --- | --- | --- | --- | --- | --- | --- | --- | --- | --- | --- | --- | --- | --- | --- | --- | --- | --- | --- | --- | --- | --- | --- | --- | --- | --- | --- | --- | --- | --- | --- | --- | --- | --- | --- | --- | --- | --- | --- | --- | --- | --- | --- | --- | --- | --- | --- | --- | --- | --- | --- | --- | --- | --- | --- | --- | --- | --- | --- | --- | --- | --- | --- | --- | --- | --- | --- | --- | --- | --- | --- | --- | --- | --- | --- | --- | --- | --- | --- | --- | --- | --- | --- | --- | --- | --- | --- | --- | --- | --- | --- | --- | --- | --- | --- | --- | --- | --- | --- | --- | --- | --- | --- | --- | --- | --- | --- | --- | --- | --- | --- | --- | --- | --- | --- | --- | --- | --- | --- | --- | --- | --- | --- | --- | --- | --- | --- | --- | --- | --- | --- | --- | --- | --- | --- | --- | --- | --- | --- | --- | --- | --- | --- | --- | --- | --- | --- | --- | --- | --- | --- | --- | --- | --- | --- | --- | --- | --- | --- | --- | --- | --- | --- | --- | --- | --- | --- | --- | --- | --- | --- | --- | --- | --- | --- | --- | --- | --- | --- | --- | --- | --- | --- | --- | --- | --- | --- | --- | --- | --- | --- | --- | --- | --- | --- | --- | --- | --- | --- | --- | --- | --- | --- | --- | --- | --- | --- | --- | --- | --- | --- | --- | --- | --- | --- | --- | --- | --- | --- | --- | --- | --- | --- | --- | --- | --- | --- | --- | --- | --- | --- | --- | --- | --- | --- | --- | --- | --- | --- | --- | --- | --- | --- | --- | --- | --- | --- | --- | --- | --- | --- | --- | --- | --- | --- | --- | --- | --- | --- | --- | --- | --- | --- | --- | --- | --- | --- | --- | --- | --- | --- | --- | --- | --- | --- | --- | --- | --- | --- | --- | --- | --- | --- | --- | --- | --- | --- | --- | --- | --- | --- | --- | --- | --- | --- | --- | --- | --- | --- | --- | --- | --- | --- | --- | --- | --- | --- | --- | --- | --- | --- | --- | --- | --- | --- | --- | --- | --- | --- | --- | --- | --- | --- | --- | --- | --- | --- | --- | --- | --- | --- | --- | --- | --- | --- | --- | --- | --- | --- | --- | --- | --- | --- | --- | --- | --- | --- | --- | --- | --- | --- | --- | --- | --- | --- | --- | --- | --- | --- | --- | --- | --- | --- | --- | --- | --- | --- | --- | --- | --- | --- | --- | --- | --- | --- | --- | --- | --- | --- | --- | --- | --- | --- | --- | --- | --- | --- | --- | --- | --- | --- | --- | --- | --- | --- | --- | --- | --- | --- | --- | --- | --- | --- | --- | --- | --- | --- | --- | --- | --- | --- | --- | --- | --- | --- | --- | --- | --- | --- | --- | --- | --- | --- | --- | --- | --- | --- | --- | --- | --- | --- | --- | --- | --- | --- | --- | --- | --- | --- | --- | --- | --- | --- | --- | --- | --- | --- | --- | --- | --- | --- | --- | --- | --- | --- | --- | --- | --- | --- | --- | --- | --- | --- | --- | --- | --- | --- | --- | --- | --- | --- | --- | --- | --- | --- | --- | --- | --- | --- | --- | --- | --- | --- | --- | --- | --- | --- | --- | --- | --- | --- | --- | --- | --- | --- | --- | --- | --- | --- | --- | --- | --- | --- | --- | --- | --- | --- | --- | --- | --- | --- | --- | --- | --- | --- | --- | --- | --- | --- | --- | --- | --- | --- | --- | --- | --- | --- | --- | --- | --- | --- | --- | --- | --- | --- | --- | --- | --- | --- | --- | --- | --- | --- | --- | --- | --- | --- | --- | --- | --- | --- | --- | --- | --- | --- | --- | --- | --- | --- | --- | --- | --- | --- | --- | --- | --- | --- | --- | --- | --- | --- | --- | --- | --- | --- | --- | --- | --- | --- | --- | --- | --- | --- | --- | --- | --- | --- | --- | --- | --- | --- | --- | --- | --- | --- | --- | --- | --- | --- | --- | --- | --- | --- | --- | --- | --- | --- | --- | --- | --- | --- | --- | --- | --- | --- | --- | --- | --- | --- | --- | --- | --- | --- | --- | --- | --- | --- | --- | --- | --- | --- | --- | --- | --- | --- | --- | --- | --- | --- | --- | --- | --- | --- | --- | --- | --- | --- | --- | --- | --- | --- | --- | --- | --- | --- | --- | --- | --- | --- | --- | --- | --- | --- | --- | --- | --- | --- | --- | --- | --- | --- | --- | --- | --- | --- | --- | --- | --- | --- | --- | --- | --- | --- | --- | --- | --- | --- | --- | --- | --- | --- | --- | --- | --- | --- | --- | --- | --- | --- | --- | --- | --- | --- | --- | --- | --- | --- | --- | --- | --- | --- | --- | --- | --- | --- | --- | --- | --- | --- | --- | --- | --- | --- | --- | --- | --- | --- | --- | --- | --- | --- | --- | --- | --- | --- | --- | --- | --- | --- | --- | --- | --- | --- | --- | --- | --- | --- | --- | --- | --- | --- | --- | --- | --- | --- | --- | --- | --- | --- | --- | --- | --- | --- | --- | --- | --- | --- | --- | --- | --- | --- | --- | --- | --- | --- | --- | --- | --- | --- | --- | --- | --- | --- | --- | --- | --- | --- | --- | --- | --- | --- | --- | --- | --- | --- | --- | --- | --- | --- | --- | --- | --- | --- | --- | --- | --- | --- | --- | --- | --- | --- | --- | --- | --- | --- | --- | --- | --- | --- | --- | --- | --- | --- | --- | --- | --- | --- | --- | --- | --- | --- | --- | --- | --- | --- | --- | --- | --- | --- | --- | --- | --- | --- | --- | --- | --- | --- | --- | --- | --- | --- | --- | --- | --- | --- | --- | --- | --- | --- | --- | --- | --- | --- | --- | --- | --- | --- | --- | --- | --- | --- | --- | --- | --- | --- | --- | --- | --- | --- | --- | --- | --- | --- | --- | --- | --- | --- | --- | --- | --- | --- | --- | --- | --- | --- | --- | --- | --- | --- | --- | --- | --- | --- | --- | --- | --- | --- | --- | --- | --- | --- | --- | --- | --- | --- | --- | --- | --- | --- | --- | --- | --- | --- | --- | --- | --- | --- | --- | --- | --- | --- | --- | --- | --- | --- | --- | --- | --- | --- | --- | --- | --- | --- | --- | --- | --- | --- | --- | --- | --- | --- | --- | --- | --- | --- | --- | --- | --- | --- | --- | --- | --- | --- | --- | --- | --- | --- | --- | --- | --- | --- | --- | --- | --- | --- | --- | --- | --- | --- | --- | --- | --- | --- | --- | --- | --- | --- | --- | --- | --- | --- | --- | --- | --- | --- | --- | --- | --- | --- | --- | --- | --- | --- | --- | --- | --- | --- | --- | --- | --- | --- | --- | --- | --- | --- | --- | --- | --- | --- | --- | --- | --- | --- | --- | --- | --- | --- | --- | --- | --- | --- | --- | --- | --- | --- | --- | --- | --- | --- | --- | --- | --- | --- | --- | --- | --- | --- | --- | --- | --- | --- | --- | --- | --- | --- | --- | --- | --- | --- | --- | --- | --- | --- | --- | --- | --- | --- | --- | --- | --- | --- | --- | --- | --- | --- | --- | --- | --- | --- | --- | --- | --- | --- | --- | --- | --- | --- | --- | --- | --- | --- | --- | --- | --- | --- | --- | --- | --- | --- | --- | --- | --- | --- | --- | --- | --- | --- | --- | --- | --- | --- | --- | --- | --- | --- | --- | --- | --- | --- | --- | --- | --- | --- | --- | --- | --- | --- | --- | --- | --- | --- | --- | --- | --- | --- | --- | --- | --- | --- | --- | --- | --- | --- | --- | --- | --- | --- | --- | --- | --- | --- | --- | --- | --- | --- | --- | --- | --- | --- | --- | --- | --- | --- | --- | --- | --- | --- | --- | --- | --- | --- | --- | --- | --- | --- | --- | --- | --- | --- | --- | --- | --- | --- | --- | --- | --- | --- | --- | --- | --- | --- | --- | --- | --- | --- | --- | --- | --- | --- | --- | --- | --- | --- | --- | --- | --- | --- | --- | --- | --- | --- | --- | --- | --- | --- | --- | --- | --- | --- | --- | --- | --- | --- | --- | --- | --- | --- | --- | --- | --- | --- | --- | --- | --- | --- | --- | --- | --- | --- | --- | --- | --- | --- | --- | --- | --- | --- | --- | --- | --- | --- | --- | --- | --- | --- | --- | --- | --- | --- | --- | --- | --- | --- | --- | --- | --- | --- | --- | --- | --- | --- | --- | --- | --- | --- | --- | --- | --- | --- | --- | --- | --- | --- | --- | --- | --- | --- | --- | --- | --- | --- | --- | --- | --- | --- | --- | --- | --- | --- | --- | --- | --- | --- | --- | --- | --- | --- | --- | --- | --- | --- | --- | --- | --- | --- | --- | --- | --- | --- | --- | --- | --- | --- | --- | --- | --- | --- | --- | --- | --- | --- | --- | --- | --- | --- | --- | --- | --- | --- | --- | --- | --- | --- | --- | --- | --- | --- | --- | --- | --- | --- | --- | --- | --- | --- | --- | --- | --- | --- | --- | --- | --- | --- | --- | --- | --- | --- | --- | --- | --- | --- | --- | --- | --- | --- | --- | --- | --- | --- | --- | --- | --- | --- | --- | --- | --- | --- | --- | --- | --- | --- | --- | --- | --- | --- | --- | --- | --- | --- | --- | --- | --- | --- | --- | --- | --- | --- | --- | --- | --- | --- | --- | --- | --- | --- | --- | --- | --- | --- | --- | --- | --- | --- | --- | --- | --- | --- | --- | --- | --- | --- | --- | --- | --- | --- | --- | --- | --- | --- | --- | --- | --- | --- | --- | --- | --- | --- | --- | --- | --- | --- | --- | --- | --- | --- | --- | --- | --- | --- | --- | --- | --- | --- | --- | --- | --- | --- | --- | --- | --- | --- | --- | --- | --- | --- | --- | --- | --- | --- | --- | --- | --- | --- | --- | --- | --- | --- | --- | --- | --- | --- | --- | --- | --- | --- | --- | --- | --- | --- | --- | --- | --- | --- | --- | --- | --- | --- | --- | --- | --- | --- | --- | --- | --- | --- | --- | --- | --- | --- | --- | --- | --- | --- | --- | --- | --- | --- | --- | --- | --- | --- | --- | --- | --- | --- | --- | --- | --- | --- | --- | --- | --- | --- | --- | --- | --- | --- | --- | --- | --- | --- | --- | --- | --- | --- | --- | --- | --- | --- | --- | --- | --- | --- | --- | --- | --- | --- | --- | --- | --- | --- | --- | --- | --- | --- | --- | --- | --- | --- | --- | --- | --- | --- | --- | --- | --- | --- | --- | --- | --- | --- | --- | --- | --- | --- | --- | --- | --- | --- | --- | --- | --- | --- | --- | --- | --- | --- | --- | --- | --- | --- | --- | --- | --- | --- | --- | --- | --- | --- | --- | --- | --- | --- | --- | --- | --- | --- | --- | --- | --- | --- | --- | --- | --- | --- | --- | --- | --- | --- | --- | --- | --- | --- | --- | --- | --- | --- | --- | --- | --- | --- | --- | --- | --- | --- | --- | --- | --- | --- | --- | --- | --- | --- | --- | --- | --- | --- | --- | --- | --- | --- | --- | --- | --- | --- | --- | --- | --- | --- | --- | --- | --- | --- | --- | --- | --- | --- | --- | --- | --- | --- | --- | --- | --- | --- | --- | --- | --- | --- | --- | --- | --- | --- | --- | --- | --- | --- | --- | --- | --- | --- | --- | --- | --- | --- | --- | --- | --- | --- | --- | --- | --- | --- | --- | --- | --- | --- | --- | --- | --- | --- | --- | --- | --- | --- | --- | --- | --- | --- | --- | --- | --- | --- | --- | --- | --- | --- | --- | --- | --- | --- | --- | --- | --- | --- | --- | --- | --- | --- | --- | --- | --- | --- | --- | --- | --- | --- | --- | --- | --- | --- | --- | --- | --- | --- | --- | --- | --- | --- | --- | --- | --- | --- | --- | --- | --- | --- | --- | --- | --- | --- | --- | --- | --- | --- | --- | --- | --- | --- | --- | --- | --- | --- | --- | --- | --- | --- | --- | --- | --- | --- | --- | --- | --- | --- | --- | --- | --- | --- | --- | --- | --- | --- | --- | --- | --- | --- | --- | --- | --- | --- | --- | --- | --- | --- | --- | --- | --- | --- | --- | --- | --- | --- | --- | --- | --- | --- | --- | --- | --- | --- | --- | --- | --- | --- | --- | --- | --- | --- | --- | --- | --- | --- | --- | --- | --- | --- | --- | --- | --- | --- | --- | --- | --- | --- | --- | --- | --- | --- | --- | --- | --- | --- | --- | --- | --- | --- | --- | --- | --- | --- | --- | --- | --- | --- | --- | --- | --- | --- | --- | --- | --- | --- | --- | --- | --- | --- | --- | --- | --- | --- | --- | --- | --- | --- | --- | --- | --- | --- | --- | --- | --- | --- | --- | --- | --- | --- | --- | --- | --- | --- | --- | --- | --- | --- | --- | --- | --- | --- | --- | --- | --- | --- | --- | --- | --- | --- | --- | --- | --- | --- | --- | --- | --- | --- | --- | --- | --- | --- | --- | --- | --- | --- | --- | --- | --- | --- | --- | --- | --- | --- | --- | --- | --- | --- | --- | --- | --- | --- | --- | --- | --- | --- | --- | --- | --- | --- | --- | --- | --- | --- | --- | --- | --- | --- | --- | --- | --- | --- | --- | --- | --- | --- | --- | --- | --- | --- | --- | --- | --- | --- | --- | --- | --- | --- | --- | --- | --- | --- | --- | --- | --- | --- | --- | --- | --- | --- | --- | --- | --- | --- | --- | --- | --- | --- | --- | --- | --- | --- | --- | --- | --- | --- | --- | --- | --- | --- | --- | --- | --- | --- | --- | --- | --- | --- | --- | --- | --- | --- | --- | --- | --- | --- | --- | --- | --- | --- | --- | --- | --- | --- | --- | --- | --- | --- | --- | --- | --- | --- | --- | --- | --- | --- | --- | --- | --- | --- | --- | --- | --- | --- | --- | --- | --- | --- | --- | --- | --- | --- | --- | --- | --- | --- | --- | --- | --- | --- | --- | --- | --- | --- | --- | --- | --- | --- | --- | --- | --- | --- | --- | --- | --- | --- | --- | --- | --- | --- | --- | --- | --- | --- | --- | --- | --- | --- | --- | --- | --- | --- | --- | --- | --- | --- | --- | --- | --- | --- | --- | --- | --- | --- | --- | --- | --- | --- | --- | --- | --- | --- | --- | --- | --- | --- | --- | --- | --- | --- | --- | --- | --- | --- | --- | --- | --- | --- | --- | --- | --- | --- | --- | --- | --- | --- | --- | --- | --- | --- | --- | --- | --- | --- | --- | --- | --- | --- | --- | --- | --- | --- | --- | --- | --- | --- | --- | --- | --- | --- | --- | --- |
| |  |  |  |  |  |  |  |  |  | | --- | --- | --- | --- | --- | --- | --- | --- | --- | | **Position** | **Reference** | **Sample** | **Quality** | **Type** | **Region** | **AA Exchange** | **PAM1** | **Known Variant** | | 1977 | A | G | 1826.77 | SNP | intergenic |  |  | - | | 4013 | T | C | 2501.77 | SNP | Rv0003 (recF) | Ile245Thr | 11 | - | | 6140 | G | T | 2188.77 | SNP | Rv0005 (gyrB) | Val(s)301Leu(s) | 9867 | - | | 7362 | G | C | 1309.77 | SNP | Rv0006 (gyrA) | Glu21Gln | 27 | - | | 7585 | G | C | 2099.77 | SNP | Rv0006 (gyrA) | Ser95Thr | 32 | genotype | | 9304 | G | A | 2044.77 | SNP | Rv0006 (gyrA) | Gly668Asp | 6 | - | | 11879 | A | G | 1376.77 | SNP | Rv0008c | Ser145Pro | 12 | - | | 14251 | G | A | 1230.77 | SNP | Rv0012 | Asp55Asn | 36 | - | | 14785 | T | C | 2021.77 | SNP | Rv0012 | Cys233Arg | 1 | - | | 17608 | G | C | 1359.77 | SNP | Rv0015c (pknA) | Ser385Arg | 6 | - | | 21795 | G | A | 89.28 | SNP | Rv0018c (pstP) | Pro463Ser | 17 | - | | 26959 | C | G | 1565.77 | SNP | intergenic |  |  | - | | 33457 | C | T | 1967.77 | SNP | Rv0030 | silent (His78) | 9912 | - | | 33551 | T | G | 1785.77 | SNP | Rv0030 | STOP110Gly | 21 | - | | 34044 | T | C | 2301.77 | SNP | intergenic |  |  | - | | 35097 | T | C | 2291.77 | SNP | Rv0032 (bioF2) | Ile268Thr | 11 | - | | 37031 | C | G | 1308.77 | SNP | Rv0034 | silent (Ala55) | 9867 | - | | 40162 | C | T | 1639.77 | SNP | Rv0037c | Met(s)347Ile | 2 | - | | 42967 | G | C | 1988.77 | SNP | Rv0040c (mtc28) | silent (Pro133) | 9926 | - | | 47036 | C | T | 2406.77 | SNP | Rv0042c | Gly58Arg | 0 | - | | 55553 | C | CCGT | 1927.73 | INS | Rv0050 (ponA1) |  |  | - | | 62049 | A | G | 1555.77 | SNP | Rv0058 (dnaB) | Arg552Gly | 1 | - | | 63771 | C | T | 2276.77 | SNP | Rv0059 | Pro191Leu | 3 | - | | 69989 | G | A | 2166.77 | SNP | Rv0064 | Gly457Asp | 6 | - | | 70533 | G | T | 1760.77 | SNP | Rv0064 | silent (Ser638) | 9840 | - | | 70816 | A | G | 1593.77 | SNP | Rv0064 | Asn733Asp | 42 | - | | 71336 | G | C | 202.84 | SNP | Rv0064 | Arg906Pro | 5 | - | | 71584 | C | CCGAGCGCTGTTCTGGCGCT AATCTGACGCTAGAATAG | 14639.73 | INS | intergenic |  |  | - | | 74059 | C | T | 2507.77 | SNP | Rv0066c (icd2) | silent (Lys151) | 9926 | - | | 75940 | G | C | 1675.77 | SNP | Rv0068 | Val(s)214Leu | 3 | - | | 79504 | TCGGTGGACC | T | 2908.73 | DEL | Rv0071 |  |  | - | | 80616 | C | G | 1627.77 | SNP | intergenic |  |  | - | | 84706 | CTTGCCGGGTCTGATGT | C | 12454.52 | DEL | Rv0075 |  |  | - | | 92199 | T | G | 1300.77 | SNP | Rv0083 | silent (Thr600) | 9871 | - | | 92388 | A | G | 1183.77 | SNP | Rv0084 (hycD) | Ile21Val | 57 | - | | 100142 | C | T | 1764.77 | SNP | Rv0091 (mtn) | silent (Thr153) | 9871 | - | | 100767 | A | G | 1543.77 | SNP | Rv0092 (ctpA) | Asp62Gly | 11 | - | | 101727 | G | A | 1491.77 | SNP | Rv0092 (ctpA) | Gly382Glu | 4 | - | | 103879 | C | T | 80.28 | SNP | Rv0094c | Gly262Asp | 6 | - | | 104712 | C | T | 761.77 | SNP | intergenic |  |  | - | | 104915 | T | C | 490.77 | SNP | Rv0095c | Thr101Ala | 32 | - | | 104919 | G | A | 485.77 | SNP | Rv0095c | silent (Leu99) | 9947 | - | | 104936 | T | C | 225.77 | SNP | Rv0095c | Thr94Ala | 32 | - | | 104940 | C | A | 139.77 | SNP | Rv0095c | Gln92His | 20 | - | | 104941 | T | C | 437.77 | SNP | Rv0095c | Gln92Arg | 10 | - | | 104942 | G | C | 433.77 | SNP | Rv0095c | Gln92Glu | 35 | - | | 104943 | G | C | 225.77 | SNP | Rv0095c | silent (Ala91) | 9867 | - | | 104944 | G | A | 427.77 | SNP | Rv0095c | Ala91Val | 13 | - | | 104962 | G | A | 1093.77 | SNP | Rv0095c | Ala85Val(s) | 9867 | - | | 105045 | G | C | 1836.77 | SNP | Rv0095c | Asp57Glu | 56 | - | | 116000 | T | G | 1869.77 | SNP | Rv0101 (nrp) | Val2000Val(s) | 18 | - | | 122109 | A | G | 1910.77 | SNP | Rv0103c (ctpB) | Leu(s)22Ser | 28 | - | | 125830 | G | GA | 2137.73 | INS | Rv0107c (ctpI) |  |  | - | | 131174 | T | TG | 3182.78 | INS | intergenic |  |  | - | | 132417 | C | G | 95.28 | SNP | Rv0109 (PE\_PGRS1) | Arg346Gly | 1 | - | | 133445 | G | A | 1872.77 | SNP | Rv0110 | silent (Gly142) | 9935 | - | | 133839 | C | T | 2035.77 | SNP | intergenic |  |  | - | | 133862 | G | A | 1818.77 | SNP | intergenic |  |  | - | | 146087 | T | C | 1980.77 | SNP | Rv0120c (fusA2) | Asn562Ser | 34 | - | | 150584 | T | C | 525.77 | SNP | Rv0124 (PE\_PGRS2) | Leu351Pro | 2 | - | | 154283 | T | C | 1926.77 | SNP | Rv0127 (mak) | Ser18Pro | 12 | - | | 157292 | C | T | 2692.77 | SNP | Rv0129c (fbpC) | silent (Glu103) | 9865 | genotype | | 158000 | G | A | 1701.77 | SNP | Rv0130 (htdZ) | Ala52Thr | 22 | - | | 162581 | G | A | 1024.77 | SNP | Rv0134 (ephF) | Gly271Ser | 16 | - | | 176303 | C | T | 2237.77 | SNP | Rv0149 | His202Tyr | 4 | - | | 177857 | G | A | 1569.77 | SNP | Rv0151c (PE1) | Leu485Leu(s) | 4 | - | | 177999 | C | T | 1760.77 | SNP | Rv0151c (PE1) | silent (Pro437) | 9926 | - | | 180025 | C | T | 2086.77 | SNP | Rv0152c (PE2) | Gly291Glu | 4 | - | | 188800 | T | C | 1437.77 | SNP | Rv0159c (PE3) | Thr14Ala | 32 | - | | 194681 | G | C | 1325.77 | SNP | Rv0165c (mce1R) | silent (Leu45) | 9947 | - | | 196642 | C | T | 2180.77 | SNP | Rv0166 (fadD5) | silent (Asn550) | 9822 | - | | 201672 | C | T | 1998.77 | SNP | Rv0171 (mce1C) | silent (Ala247) | 9867 | - | | 203269 | C | T | 2055.77 | SNP | Rv0172 (mce1D) | Ala265Val(s) | 9867 | - | | 206339 | T | C | 1507.77 | SNP | Rv0174 (mce1F) | Leu370Pro | 2 | - | | 207226 | T | C | 2008.77 | SNP | Rv0175 | Met(s)138Thr | 22 | - | | 212353 | C | T | 1133.77 | SNP | Rv0181c | Arg220His | 8 | - | | 223080 | T | C | 1989.77 | SNP | Rv0191 | silent (Tyr264) | 9945 | - | | 223942 | T | C | 756.77 | SNP | Rv0192 | Ser127Pro | 12 | - | | 225323 | T | C | 1220.77 | SNP | Rv0193c | Lys417Glu | 4 | - | | 227098 | T | C | 2051.77 | SNP | Rv0194 | Met(s)74Thr | 22 | - | | 228069 | G | A | 1984.77 | SNP | Rv0194 | Val(s)398Met(s) | 9867 | - | | 228168 | G | C | 2230.77 | SNP | Rv0194 | Gly431Arg | 0 | - | | 231114 | C | G | 1360.77 | SNP | Rv0195 | silent (Ala72) | 9867 | - | | 234051 | G | A | 2440.77 | SNP | Rv0197 | silent (Pro607) | 9926 | - | | 234477 | T | G | 1283.77 | SNP | Rv0197 | Tyr749STOP | 2 | - | | 234496 | C | CGT | 2962.73 | INS | Rv0197 |  |  | - | | 261869 | T | C | 1379.77 | SNP | Rv0218 | Cys316Arg | 1 | - | | 265554 | A | C | 2281.77 | SNP | Rv0222 (echA1) | silent (Val16) | 9901 | - | | 278681 | C | G | 2008.77 | SNP | Rv0233 (nrdB) | His33Asp | 4 | - | | 282188 | G | A | 1370.77 | SNP | Rv0235c | Arg143Cys | 1 | - | | 283614 | T | C | 1353.77 | SNP | Rv0236c (aftD) | Ser1080Gly | 21 | - | | 285772 | A | C | 1771.77 | SNP | Rv0236c (aftD) | silent (Pro360) | 9926 | - | | 285871 | A | G | 1226.77 | SNP | Rv0236c (aftD) | silent (Val327) | 9901 | - | | 293704 | CT | C | 1789.73 | DEL | intergenic |  |  | - | | 310973 | G | A | 1709.77 | SNP | Rv0259c | Ala182Val(s) | 9867 | - | | 311613 | G | T | 1900.77 | SNP | Rv0260c | silent (Val349) | 9901 | - | | 316129 | C | G | 1802.77 | SNP | Rv0264c | Gly96Ala | 21 | - | | 324968 | G | T | 1739.77 | SNP | Rv0270 (fadD2) | Val(s)134Val | 13 | - | | 330975 | G | A | 1796.77 | SNP | Rv0274; Rv0275c | Arg185Gln; silent (Ala228) | 9; 9867 | - | | 331673 | C | T | 1049.77 | SNP | intergenic |  |  | - | | 333584 | G | C | 31.77 | SNP | Rv0278c (PE\_PGRS3) | silent (Arg909) | 9913 | - | | 333637 | A | G | 166.77 | SNP | Rv0278c (PE\_PGRS3) | Trp892Arg | 8 | - | | 333640 | G | A | 184.77 | SNP | Rv0278c (PE\_PGRS3) | Arg891Trp | 2 | - | | 333641 | C | T | 172.77 | SNP | Rv0278c (PE\_PGRS3) | silent (Gln890) | 9876 | - | | 333892 | G | C | 440.77 | SNP | Rv0278c (PE\_PGRS3) | Arg807Gly | 1 | - | | 335810 | CCCGCCGGCGCCGCCGTTG | C | 2365.73 | DEL | Rv0278c (PE\_PGRS3) |  |  | - | | 335885 | T | G | 115.77 | SNP | Rv0278c (PE\_PGRS3) | silent (Gly142) | 9935 | - | | 335906 | T | C | 148.77 | SNP | Rv0278c (PE\_PGRS3) | Leu135Leu(s) | 4 | - | | 335919 | T | G | 191.77 | SNP | Rv0278c (PE\_PGRS3) | Asp131Ala | 10 | - | | 335920 | C | G | 135.77 | SNP | Rv0278c (PE\_PGRS3) | Asp131His | 3 | - | | 335922 | C | G | 169.77 | SNP | Rv0278c (PE\_PGRS3) | Gly130Ala | 21 | - | | 335927 | A | G | 165.77 | SNP | Rv0278c (PE\_PGRS3) | silent (Asn128) | 9822 | - | | 335929 | T | C | 165.77 | SNP | Rv0278c (PE\_PGRS3) | Asn128Asp | 42 | - | | 335971 | A | G | 350.77 | SNP | Rv0278c (PE\_PGRS3) | Leu(s)114Leu | 3 | - | | 336005 | G | A | 534.77 | SNP | Rv0278c (PE\_PGRS3) | silent (Ile102) | 9872 | - | | 336047 | C | G | 383.77 | SNP | Rv0278c (PE\_PGRS3) | silent (Ala88) | 9867 | - | | 336050 | A | G | 397.77 | SNP | Rv0278c (PE\_PGRS3) | silent (Tyr87) | 9945 | - | | 336053 | G | C | 395.77 | SNP | Rv0278c (PE\_PGRS3) | silent (Ala86) | 9867 | - | | 336074 | T | C | 269.77 | SNP | Rv0278c (PE\_PGRS3) | silent (Ala79) | 9867 | - | | 336081 | A | G | 150.77 | SNP | Rv0278c (PE\_PGRS3) | Val(s)77Ala | 9867 | - | | 336082 | C | T | 114.77 | SNP | Rv0278c (PE\_PGRS3) | Val(s)77Met(s) | 9867 | - | | 336380 | A | T | 92.77 | SNP | intergenic |  |  | - | | 336400 | C | G | 90.77 | SNP | intergenic |  |  | - | | 336405 | A | G | 68.77 | SNP | intergenic |  |  | - | | 336504 | G | T | 693.77 | SNP | intergenic |  |  | - | | 336535 | T | G | 390.77 | SNP | intergenic |  |  | - | | 336537 | T | G | 404.77 | SNP | intergenic |  |  | - | | 336540 | G | T | 386.77 | SNP | intergenic |  |  | - | | 336546 | T | G | 448.77 | SNP | intergenic |  |  | - | | 336557 | C | CT | 849.73 | INS | intergenic |  |  | - | | 336560 | T | C | 258.77 | SNP | Rv0279c (PE\_PGRS4) | silent (STOP838) | 9867 | - | | 336562 | A | ATGG | 1192.73 | INS | Rv0279c (PE\_PGRS4) |  |  | - | | 336590 | G | C | 439.77 | SNP | Rv0279c (PE\_PGRS4) | Ile828Met(s) | 6 | - | | 336592 | T | G | 395.77 | SNP | Rv0279c (PE\_PGRS4) | Ile828Leu | 22 | - | | 336611 | G | C | 335.77 | SNP | Rv0279c (PE\_PGRS4) | silent (Ala821) | 9867 | - | | 336617 | G | C | 140.77 | SNP | Rv0279c (PE\_PGRS4) | silent (Pro819) | 9926 | - | | 336620 | T | C | 106.77 | SNP | Rv0279c (PE\_PGRS4) | silent (Thr818) | 9871 | - | | 336680 | GGGGA | G | 564.76 | DEL | Rv0279c (PE\_PGRS4) |  |  | - | | 336687 | GAGC | G | 405.74 | DEL | Rv0279c (PE\_PGRS4) |  |  | - | | 336691 | T | C | 309.78 | SNP | Rv0279c (PE\_PGRS4) | Ser795Gly | 21 | - | | 336694 | A | ACACCGCC | 826.74 | INS | Rv0279c (PE\_PGRS4) |  |  | - | | 336698 | C | G | 308.78 | SNP | Rv0279c (PE\_PGRS4) | silent (Gly792) | 9935 | - | | 336701 | A | G | 304.78 | SNP | Rv0279c (PE\_PGRS4) | silent (Gly791) | 9935 | - | | 336707 | G | A | 243.78 | SNP | Rv0279c (PE\_PGRS4) | silent (Asp789) | 9859 | - | | 336708 | T | C | 229.80 | SNP | Rv0279c (PE\_PGRS4) | Asp789Gly | 11 | - | | 336710 | A | G | 272.78 | SNP | Rv0279c (PE\_PGRS4) | silent (Ala788) | 9867 | - | | 336728 | G | A | 37.77 | SNP | Rv0279c (PE\_PGRS4) | silent (Gly782) | 9935 | - | | 337820 | G | A | 159.85 | SNP | Rv0279c (PE\_PGRS4) | silent (Gly418) | 9935 | - | | 337959 | A | C | 94.28 | SNP | Rv0279c (PE\_PGRS4) | Ile372Ser | 2 | - | | 338100 | T | C | 381.77 | SNP | Rv0279c (PE\_PGRS4) | Asn325Ser | 34 | - | | 338453 | A | G | 118.03 | SNP | Rv0279c (PE\_PGRS4) | silent (Ala207) | 9867 | - | | 338719 | T | C | 65.77 | SNP | Rv0279c (PE\_PGRS4) | Thr119Ala | 32 | - | | 338768 | G | A | 194.77 | SNP | Rv0279c (PE\_PGRS4) | silent (Ile102) | 9872 | - | | 338774 | G | A | 166.77 | SNP | Rv0279c (PE\_PGRS4) | silent (Ala100) | 9867 | - | | 338775 | G | T | 172.77 | SNP | Rv0279c (PE\_PGRS4) | Ala100Asp | 6 | - | | 338777 | G | C | 179.77 | SNP | Rv0279c (PE\_PGRS4) | silent (Leu99) | 9947 | - | | 338789 | G | C | 175.77 | SNP | Rv0279c (PE\_PGRS4) | silent (Thr95) | 9871 | - | | 338790 | G | A | 154.77 | SNP | Rv0279c (PE\_PGRS4) | Thr95Ile | 7 | - | | 338791 | T | C | 185.77 | SNP | Rv0279c (PE\_PGRS4) | Thr95Ala | 32 | - | | 338792 | G | C | 179.77 | SNP | Rv0279c (PE\_PGRS4) | silent (Ala94) | 9867 | - | | 338844 | A | G | 512.77 | SNP | Rv0279c (PE\_PGRS4) | Val(s)77Ala | 9867 | - | | 338845 | C | T | 456.77 | SNP | Rv0279c (PE\_PGRS4) | Val(s)77Met(s) | 9867 | - | | 338876 | G | A | 582.77 | SNP | Rv0279c (PE\_PGRS4) | silent (Ser66) | 9840 | - | | 338903 | G | C | 687.77 | SNP | Rv0279c (PE\_PGRS4) | silent (Ala57) | 9867 | - | | 338960 | T | C | 655.77 | SNP | Rv0279c (PE\_PGRS4) | silent (Ala38) | 9867 | - | | 338963 | T | C | 611.77 | SNP | Rv0279c (PE\_PGRS4) | silent (Thr37) | 9871 | - | | 338984 | C | T | 694.77 | SNP | Rv0279c (PE\_PGRS4) | silent (Ala30) | 9867 | - | | 338990 | G | T | 667.77 | SNP | Rv0279c (PE\_PGRS4) | silent (Ala28) | 9867 | - | | 338997 | G | C | 686.77 | SNP | Rv0279c (PE\_PGRS4) | Ala26Gly | 21 | - | | 338998 | C | T | 691.77 | SNP | Rv0279c (PE\_PGRS4) | Ala26Thr | 22 | - | | 340372 | T | C | 627.77 | SNP | Rv0280 (PPE3) | Ser337Pro | 12 | - | | 346275 | C | G | 1681.77 | SNP | Rv0284 (eccC3) | Pro214Arg | 4 | - | | 356528 | A | G | 1321.77 | SNP | Rv0292 (eccE3) | Asn217Asp | 42 | - | | 373282 | TA | T | 2835.73 | DEL | Rv0305c (PPE6) |  |  | - | | 376774 | T | C | 1625.77 | SNP | Rv0307c | silent (Ala94) | 9867 | - | | 384380 | A | C | 1705.77 | SNP | Rv0315 | Lys260Thr | 8 | - | | 386432 | C | G | 1867.77 | SNP | Rv0318c | Gly223Ala | 21 | - | | 388311 | T | C | 1262.77 | SNP | Rv0320 | Leu(s)142Leu | 3 | - | | 390828 | T | C | 1689.77 | SNP | Rv0323c | Ser142Gly | 21 | - | | 403980 | G | A | 1891.77 | SNP | Rv0338c | Ala621Val | 13 | - | | 404326 | T | C | 2130.77 | SNP | Rv0338c | Arg506Gly | 1 | - | | 408723 | C | T | 1184.77 | SNP | Rv0340 | silent (Ile30) | 9872 | - | | 412297 | TGCG | T | 3182.78 | DEL | Rv0342 (iniA) |  |  | - | | 412302 | G | A | 1422.77 | SNP | Rv0342 (iniA) | Gly489Ser | 16 | - | | 414486 | C | T | 1954.77 | SNP | Rv0344c (lpqJ) | silent (Glu152) | 9865 | - | | 420008 | A | G | 2036.77 | SNP | Rv0350 (dnaK) | silent (Ala58) | 9867 | - | | 424320 | T | TC | 1791.73 | INS | Rv0354c (PPE7) |  |  | - | | 427310 | TTGCCGAGGTTTGCAC | T | 3841.73 | DEL | Rv0355c (PPE8) |  |  | - | | 444351 | G | T | 1848.77 | SNP | Rv0366c | Asn155Lys | 25 | - | | 451993 | C | A | 1796.77 | SNP | Rv0374c | Gly96Val(s) | 21 | - | | 454295 | T | C | 1911.77 | SNP | Rv0376c | silent (Pro26) | 9926 | - | | 457452 | T | G | 1410.77 | SNP | Rv0381c | silent (Thr124) | 9871 | - | | 459399 | A | C | 1959.77 | SNP | intergenic |  |  | - | | 467497 | C | CG | 2304.73 | INS | Rv0388c (PPE9) |  |  | - | | 467508 | C | CG | 2815.73 | INS | Rv0388c (PPE9) |  |  | - | | 467516 | G | C | 1832.77 | SNP | Rv0388c (PPE9) | silent (Ser162) | 9840 | - | | 467526 | C | G | 1924.77 | SNP | Rv0388c (PPE9) | Gly159Ala | 21 | - | | 467546 | G | C | 1994.77 | SNP | Rv0388c (PPE9) | Asp152Glu | 56 | - | | 467557 | A | C | 2098.77 | SNP | Rv0388c (PPE9) | Leu(s)149Val(s) | 9867 | - | | 467564 | A | C | 2267.77 | SNP | Rv0388c (PPE9) | His146Gln | 23 | - | | 467585 | G | C | 2298.77 | SNP | Rv0388c (PPE9) | His139Gln | 23 | - | | 467590 | T | C | 2233.77 | SNP | Rv0388c (PPE9) | Thr138Ala | 32 | - | | 467621 | T | G | 2170.77 | SNP | Rv0388c (PPE9) | silent (Gly127) | 9935 | - | | 467638 | G | T | 2118.77 | SNP | Rv0388c (PPE9) | Gln122Lys | 12 | - | | 472705 | T | C | 1840.77 | SNP | intergenic |  |  | - | | 475178 | T | C | 1390.77 | SNP | Rv0395 | Val80Ala | 18 | - | | 478358 | C | T | 1465.77 | SNP | Rv0399c (lpqK) | Glu67Lys | 7 | - | | 489935 | G | C | 2278.77 | SNP | Rv0405 (pks6); Rv0406c | Arg1402Pro; silent (Thr257) | 5; 9871 | - | | 498557 | C | A | 1570.77 | SNP | Rv0412c | Asp355Tyr | 0 | - | | 502589 | C | G | 1574.77 | SNP | Rv0417 (thiG) | Ser75Cys | 5 | - | | 503354 | G | C | 2399.77 | SNP | intergenic |  |  | - | | 510831 | TCCGGGGGGCGCA | T | 9069.73 | DEL | Rv0425c (ctpH) |  |  | - | | 513257 | T | C | 1918.77 | SNP | Rv0425c (ctpH) | Met(s)689Val(s) | 9867 | - | | 534691 | C | T | 2105.77 | SNP | Rv0446c | Trp175STOP | 0 | - | | 541201 | A | G | 2121.77 | SNP | Rv0450c (mmpL4) | silent (Leu97) | 9947 | - | | 545925 | G | C | 137.77 | SNP | Rv0456c (echA2) | Asp293Glu | 56 | - | | 551525 | A | C | 2427.77 | SNP | Rv0459 | silent (Arg110) | 9913 | - | | 557133 | G | A | 2152.77 | SNP | Rv0466 | Val226Ile | 33 | - | | 563420 | T | C | 2536.77 | SNP | Rv0472c | Glu3Gly | 7 | - | | 571943 | G | A | 1439.77 | SNP | Rv0483 (lprQ) | Val(s)78Val | 13 | - | | 573262 | A | G | 802.77 | SNP | Rv0484c | silent (Gly180) | 9935 | - | | 573326 | A | T | 719.77 | SNP | Rv0484c | Leu159Gln | 3 | - | | 580772 | T | A | 543.77 | SNP | intergenic |  |  | - | | 580773 | GGGGGCACCACCCGCTTGCG GGGGA | G | 4796.73 | DEL | intergenic |  |  | - | | 584438 | G | A | 43.74 | SNP | Rv0493c | Pro85Ser | 17 | - | | 589536 | G | A | 1728.77 | SNP | Rv0499 | silent (Leu118) | 9947 | - | | 590436 | T | C | 1516.77 | SNP | Rv0500 (proC) | silent (Ala118) | 9867 | - | | 597816 | A | G | 2067.77 | SNP | Rv0507 (mmpL2) | silent (Ala206) | 9867 | - | | 598475 | G | A | 2781.77 | SNP | Rv0507 (mmpL2) | Arg426His | 8 | - | | 608037 | A | C | 1420.77 | SNP | Rv0515 | His496Pro | 5 | - | | 610120 | T | G | 2220.77 | SNP | intergenic |  |  | - | | 623021 | G | T | 788.77 | SNP | Rv0532 (PE\_PGRS6) | Val(s)77Leu(s) | 9867 | - | | 630722 | G | C | 1458.77 | SNP | Rv0538 | Arg228Pro | 5 | - | | 632330 | G | T | 1063.77 | SNP | Rv0539 | silent (Arg196) | 9913 | - | | 637319 | G | A | 1407.77 | SNP | Rv0545c (pitA) | Pro49Ser | 17 | - | | 637922 | C | T | 1875.77 | SNP | Rv0546c | silent (Gln16) | 9876 | - | | 648002 | T | G | 2697.77 | SNP | Rv0556 | Leu15Arg | 1 | - | | 663419 | G | A | 45.77 | SNP | intergenic |  |  | - | | 664249 | C | T | 2047.77 | SNP | Rv0571c | silent (Gln190) | 9876 | - | | 665293 | A | G | 1755.77 | SNP | Rv0572c | Phe31Leu | 13 | - | | 667659 | C | T | 1695.77 | SNP | Rv0574c | Asp246Asn | 36 | - | | 669398 | T | C | 1540.77 | SNP | Rv0575c | silent (Gln116) | 9876 | - | | 673238 | A | G | 353.77 | SNP | Rv0578c (PE\_PGRS7) | silent (His893) | 9912 | - | | 679623 | T | C | 1544.77 | SNP | Rv0584 | Ile132Thr | 11 | - | | 685461 | C | G | 1899.77 | SNP | Rv0587 (yrbE2A) | silent (Ala111) | 9867 | - | | 685608 | T | C | 2409.77 | SNP | Rv0587 (yrbE2A) | silent (Leu160) | 9947 | - | | 686972 | T | C | 2094.77 | SNP | Rv0589 (mce2A) | Phe51Ser | 3 | - | | 690465 | T | G | 877.77 | SNP | Rv0591 (mce2C) | silent (Leu469) | 9947 | - | | 698968 | G | A | 1282.77 | SNP | Rv0601c | silent (Gly9) | 9935 | - | | 709226 | G | A | 1152.77 | SNP | Rv0613c | Thr97Ile | 7 | - | | 726703 | C | A | 581.77 | SNP | Rv0631c (recC) | Arg535Met(s) | 2 | - | | 733798 | G | C | 1208.77 | SNP | Rv0638 (secE1) | Ser21Thr | 32 | - | | 735068 | T | C | 1275.77 | SNP | Rv0640 (rplK) | Val(s)16Ala | 9867 | - | | 735075 | C | A | 1129.77 | SNP | Rv0640 (rplK) | silent (Gly18) | 9935 | - | | 736710 | T | C | 1623.77 | SNP | Rv0642c (mmaA4) | Asn165Ser | 34 | - | | 746981 | C | G | 897.77 | SNP | Rv0649 (fabD2) | Pro207Ala | 22 | - | | 752343 | A | G | 2206.77 | SNP | Rv0655 (mkl) | Glu276Gly | 7 | - | | 753098 | G | A | 963.77 | SNP | Rv0656c (vapC6) | silent (Cys90) | 9973 | - | | 753438 | G | A | 1483.77 | SNP | intergenic |  |  | - | | 754186 | A | G | 1219.77 | SNP | Rv0658c | Leu75Pro | 2 | - | | 761140 | A | G | 1695.77 | SNP | Rv0667 (rpoB) | His445Arg | 10 | resistance | | 762101 | C | T | 2181.77 | SNP | Rv0667 (rpoB) | silent (Arg765) | 9913 | - | | 764995 | C | G | 2239.77 | SNP | Rv0668 (rpoC) | silent (Ala542) | 9867 | genotype | | 769962 | GC | G | 2768.73 | DEL | Rv0670 (end) |  |  | - | | 773431 | G | C | 1968.77 | SNP | Rv0673 (echA4) | Gln103His | 20 | - | | 773809 | G | A | 1108.77 | SNP | Rv0673 (echA4) | silent (Glu229) | 9865 | - | | 775639 | T | C | 1513.77 | SNP | Rv0676c (mmpL5) | Ile948Val | 57 | - | | 781395 | T | C | 2265.77 | SNP | intergenic (Rv0682-165nt) |  |  | - | | 784440 | G | T | 1744.77 | SNP | Rv0684 (fusA1) | silent (Ala652) | 9867 | - | | 790180 | A | G | 1866.77 | SNP | Rv0690c | silent (Asp298) | 9859 | - | | 796509 | G | T | 1573.77 | SNP | Rv0696 | Gly331Cys | 0 | - | | 820483 | G | T | 1123.77 | SNP | Rv0727c (fucA) | Ala6Asp | 6 | - | | 836658 | A | G | 106.28 | SNP | Rv0746 (PE\_PGRS9) | Thr320Ala | 32 | - | | 837033 | A | G | 228.84 | SNP | Rv0746 (PE\_PGRS9) | Thr445Ala | 32 | - | | 839194 | A | G | 53.74 | SNP | Rv0747 (PE\_PGRS10) | silent (Thr248) | 9871 | - | | 839269 | A | G | 88.77 | SNP | Rv0747 (PE\_PGRS10) | silent (Gly273) | 9935 | - | | 839279 | G | A | 123.77 | SNP | Rv0747 (PE\_PGRS10) | Asp277Asn | 36 | - | | 839291 | T | C | 36.77 | SNP | Rv0747 (PE\_PGRS10) | Phe281Leu | 13 | - | | 839295 | T | C | 73.77 | SNP | Rv0747 (PE\_PGRS10) | Phe282Ser | 3 | - | | 839334 | A | G | 153.90 | SNP | Rv0747 (PE\_PGRS10) | Lys295Arg | 19 | - | | 839348 | A | G | 135.03 | SNP | Rv0747 (PE\_PGRS10) | Ser300Gly | 21 | - | | 839515 | G | A | 119.77 | SNP | Rv0747 (PE\_PGRS10) | silent (Ala355) | 9867 | - | | 839516 | A | G | 160.77 | SNP | Rv0747 (PE\_PGRS10) | Thr356Ala | 32 | - | | 839519 | C | G | 160.77 | SNP | Rv0747 (PE\_PGRS10) | Leu357Val(s) | 4 | - | | 839520 | T | C | 157.77 | SNP | Rv0747 (PE\_PGRS10) | Leu357Pro | 2 | - | | 839534 | A | C | 123.77 | SNP | Rv0747 (PE\_PGRS10) | Ile362Leu | 22 | - | | 840272 | G | T | 38.77 | SNP | Rv0747 (PE\_PGRS10) | Ala608Ser | 28 | - | | 841494 | C | G | 1577.77 | SNP | Rv0749 (vapC31) | silent (Leu89) | 9947 | - | | 841495 | A | G | 1487.77 | SNP | Rv0749 (vapC31) | Met(s)90Val(s) | 9867 | - | | 841605 | C | T | 1291.77 | SNP | Rv0749 (vapC31) | silent (Asp126) | 9859 | - | | 841764 | G | C | 2202.77 | SNP | Rv0749A | silent (Thr37) | 9871 | - | | 852910 | C | T | 1358.77 | SNP | Rv0758 (phoR) | Pro172Leu | 3 | - | | 854252 | GC | G | 2327.73 | DEL | intergenic |  |  | - | | 857696 | A | G | 1451.77 | SNP | Rv0764c (cyp51) | silent (Ala114) | 9867 | - | | 859010 | G | C | 1584.77 | SNP | Rv0766c (cyp123) | Leu355Val(s) | 4 | - | | 859131 | C | CA | 3559.73 | INS | Rv0766c (cyp123) |  |  | - | | 874835 | C | CCG | 5249.73 | INS | Rv0781 (ptrBa); Rv0782 (ptrBb) |  |  | - | | 880562 | G | T | 2609.77 | SNP | Rv0785 | Cys408Phe | 0 | - | | 882257 | T | C | 2118.77 | SNP | Rv0787 | Tyr267His | 4 | - | | 885542 | G | C | 1592.77 | SNP | Rv0791c | Ser100Cys | 5 | - | | 888774 | G | A | 809.77 | SNP | intergenic |  |  | - | | 893733 | T | G | 1403.77 | SNP | Rv0800 (pepC) | Leu139Arg | 1 | - | | 900221 | T | C | 1914.77 | SNP | Rv0806c (cpsY) | Val370Val(s) | 18 | - | | 903550 | T | C | 1323.77 | SNP | Rv0808 (purF) | silent (Ala480) | 9867 | - | | 903913 | T | C | 1056.77 | SNP | Rv0809 (purM) | silent (Gly63) | 9935 | - | | 906857 | A | G | 2118.77 | SNP | Rv0812 | Ile145Met(s) | 6 | - | | 909280 | A | C | 1361.77 | SNP | Rv0815c (cysA2) | silent (Ala13) | 9867 | - | | 921813 | C | G | 2002.77 | SNP | Rv0829 | Ala80Gly | 21 | - | | 927385 | A | G | 107.03 | SNP | Rv0833 (PE\_PGRS13) | silent (Gly675) | 9935 | - | | 927910 | ACGCCGTTGC | A | 4990.73 | DEL | Rv0834c (PE\_PGRS14) |  |  | - | | 942479 | T | C | 1260.77 | SNP | intergenic |  |  | - | | 945214 | G | A | 2205.77 | SNP | Rv0848 (cysK2) | Gly93Ser | 16 | - | | 947429 | T | A | 2195.77 | SNP | Rv0850 | Ser40Thr | 32 | - | | 947430 | C | A | 2191.77 | SNP | Rv0850 | Ser40STOP | 35 | - | | 949535 | T | C | 1784.77 | SNP | Rv0853c (pdc) | silent (Ala528) | 9867 | - | | 955524 | A | G | 1245.77 | SNP | Rv0859 (fadA) | Ser150Gly | 21 | - | | 968426 | A | AGCCGGGTTG | 2294.73 | INS | Rv0872c (PE\_PGRS15) |  |  | - | | 976896 | TTGG | T | 4224.73 | DEL | Rv0878c (PPE13) |  |  | - | | 977060 | T | G | 1040.77 | SNP | Rv0878c (PPE13) | Thr382Pro | 4 | - | | 979704 | G | C | 1984.77 | SNP | Rv0881 | Gly115Arg | 0 | - | | 986463 | G | C | 2333.77 | SNP | intergenic |  |  | - | | 990001 | G | C | 1903.77 | SNP | Rv0890c | Pro866Ala | 22 | - | | 990533 | T | C | 2458.77 | SNP | Rv0890c | silent (Thr688) | 9871 | - | | 993346 | A | C | 2645.77 | SNP | Rv0891c | Val37Gly | 5 | - | | 1004729 | C | T | 1153.77 | SNP | Rv0902c (prrB) | silent (Arg371) | 9913 | - | | 1010204 | C | CG | 3271.73 | INS | Rv0907 |  |  | - | | 1025106 | T | C | 2413.77 | SNP | Rv0919 | silent (Phe141) | 9946 | - | | 1035729 | T | C | 1776.77 | SNP | Rv0928 (pstS3) | Val276Ala | 18 | - | | 1037012 | T | C | 1267.77 | SNP | Rv0930 (pstA1) | Met(s)5Thr | 22 | - | | 1037355 | T | C | 1940.77 | SNP | Rv0930 (pstA1) | silent (Thr119) | 9871 | - | | 1037911 | C | T | 2017.77 | SNP | Rv0930 (pstA1) | Arg305STOP | 2 | - | | 1040794 | A | C | 1630.77 | SNP | Rv0932c (pstS2) | silent (Gly85) | 9935 | - | | 1041445 | C | T | 1570.77 | SNP | Rv0933 (pstB) | Thr61Met(s) | 32 | - | | 1047165 | T | C | 1456.77 | SNP | Rv0938 (ligD) | Cys344Arg | 1 | - | | 1050173 | C | T | 1853.77 | SNP | Rv0939 | Pro588Ser | 17 | - | | 1055049 | C | T | 1657.77 | SNP | Rv0946c (pgi) | Arg546His | 8 | - | | 1057788 | T | G | 1741.77 | SNP | Rv0948c | Lys59Thr | 8 | - | | 1061676 | GTGC | G | 3924.73 | DEL | intergenic |  |  | - | | 1068151 | T | C | 1662.77 | SNP | Rv0956 (purN) | silent (His197) | 9912 | - | | 1068432 | A | G | 1980.77 | SNP | Rv0957 (purH) | silent (Pro76) | 9926 | - | | 1070702 | T | C | 1594.77 | SNP | Rv0958 | Ser274Pro | 12 | - | | 1071797 | C | G | 1767.77 | SNP | Rv0959 | silent (Gly181) | 9935 | - | | 1074558 | G | A | 1947.77 | SNP | Rv0962c (lprP) | Pro186Leu | 3 | - | | 1075279 | T | C | 1754.77 | SNP | intergenic |  |  | - | | 1076309 | G | T | 1448.77 | SNP | Rv0964c | Pro124Thr | 5 | - | | 1077312 | A | G | 2181.77 | SNP | Rv0966c | Val(s)175Ala | 9867 | - | | 1079927 | C | A | 1591.77 | SNP | Rv0969 (ctpV) | silent (Thr395) | 9871 | - | | 1081681 | T | C | 1701.77 | SNP | Rv0970 | silent (Val210) | 9901 | - | | 1083533 | G | A | 1598.77 | SNP | Rv0972c (fadE12) | Ser73Leu(s) | 35 | - | | 1087193 | G | C | 2060.77 | SNP | Rv0974c (accD2) | Asn51Lys | 25 | - | | 1093322 | C | T | 1260.77 | SNP | intergenic |  |  | - | | 1093406 | A | G | 1292.77 | SNP | Rv0978c (PE\_PGRS17) | silent (Val317) | 9901 | - | | 1093886 | C | A | 97.28 | SNP | Rv0978c (PE\_PGRS17) | silent (Gly157) | 9935 | - | | 1093928 | G | A | 91.28 | SNP | Rv0978c (PE\_PGRS17) | silent (Asn143) | 9822 | - | | 1095364 | T | G | 1926.77 | SNP | Rv0980c (PE\_PGRS18) | Asn363Thr | 13 | - | | 1096508 | C | G | 258.78 | SNP | intergenic |  |  | - | | 1096510 | T | C | 307.78 | SNP | intergenic |  |  | - | | 1096633 | T | G | 1036.77 | SNP | intergenic |  |  | - | | 1100234 | T | C | 2061.77 | SNP | Rv0983 (pepD) | Leu390Pro | 2 | - | | 1103249 | C | T | 2557.77 | SNP | Rv0987 | silent (Ala236) | 9867 | - | | 1106422 | T | C | 2624.77 | SNP | Rv0989c (grcC2) | Ile321Val | 57 | - | | 1107434 | A | T | 1691.77 | SNP | intergenic |  |  | - | | 1107917 | G | T | 1481.77 | SNP | Rv0990c | His61Gln | 23 | - | | 1109975 | A | G | 1858.77 | SNP | Rv0993 (galU) | Gln235Arg | 10 | - | | 1126889 | G | C | 1844.77 | SNP | Rv1007c (metS) | Arg39Gly | 1 | - | | 1127648 | C | A | 2256.77 | SNP | Rv1008 (tatD) | Thr187Asn | 9 | - | | 1129987 | C | T | 1158.77 | SNP | Rv1010 (ksgA) | Thr279Met(s) | 32 | - | | 1130181 | T | C | 1438.77 | SNP | intergenic |  |  | - | | 1132368 | C | T | 1232.77 | SNP | Rv1013 (pks16) | silent (Thr248) | 9871 | genotype | | 1142266 | A | C | 598.77 | SNP | Rv1020 (mfd) | silent (Leu1100) | 9947 | - | | 1149551 | C | T | 2055.77 | SNP | Rv1028c (kdpD) | silent (Glu712) | 9865 | - | | 1150321 | TGCCCGACAGCAAG | T | 6659.73 | DEL | Rv1028c (kdpD) |  |  | - | | 1150585 | G | A | 1013.77 | SNP | Rv1028c (kdpD) | Pro368Ser | 17 | - | | 1159161 | GCCCA | G | 3183.75 | DEL | Rv1034c |  |  | - | | 1163134 | T | C | 1530.77 | SNP | Rv1040c (PE8) | silent (Gly81) | 9935 | - | | 1164495 | C | T | 1616.77 | SNP | intergenic |  |  | - | | 1165521 | T | TA | 2787.73 | INS | intergenic |  |  | - | | 1168715 | C | CT | 3402.73 | INS | Rv1046c |  |  | - | | 1170404 | C | A | 96.03 | SNP | Rv1047 | Gln328Lys | 12 | - | | 1178116 | T | C | 2631.77 | SNP | Rv1056 | silent (Thr163) | 9871 | - | | 1190093 | A | C | 771.77 | SNP | Rv1067c (PE\_PGRS19) | Leu(s)111Trp | 0 | - | | 1190981 | CGCCGCCGGT | C | 674.77 | DEL | Rv1068c (PE\_PGRS20) |  |  | - | | 1199547 | G | A | 2284.77 | SNP | Rv1075c | Pro275Leu | 3 | - | | 1200418 | A | G | 1687.77 | SNP | intergenic |  |  | - | | 1201289 | G | A | 1705.77 | SNP | Rv1076 (lipU) | Glu175Lys | 7 | - | | 1206400 | G | A | 1739.77 | SNP | Rv1081c | Pro7Ser | 17 | - | | 1212076 | G | A | 316.96 | SNP | Rv1087 (PE\_PGRS21) | Gly173Ser | 16 | - | | 1218737 | G | T | 300.78 | SNP | Rv1091 (PE\_PGRS22) | Gly757Cys | 0 | - | | 1220680 | T | C | 2335.77 | SNP | Rv1093 (glyA1) | Val36Ala | 18 | - | | 1224367 | T | C | 1211.77 | SNP | intergenic |  |  | - | | 1248978 | T | C | 2055.77 | SNP | Rv1125 | silent (Ala299) | 9867 | - | | 1272269 | C | T | 1797.77 | SNP | intergenic |  |  | - | | 1276938 | G | C | 1673.77 | SNP | Rv1148c | Leu271Val | 11 | - | | 1277869 | G | GT | 3727.73 | INS | intergenic |  |  | - | | 1281118 | T | C | 1806.77 | SNP | Rv1154c | Thr123Ala | 32 | - | | 1281443 | C | G | 1038.77 | SNP | Rv1154c; Rv1155 | Lys14Asn; Val5Val(s) | 13; 18 | - | | 1283492 | G | A | 496.77 | SNP | Rv1157c | Pro227Leu | 3 | - | | 1292102 | A | G | 1766.77 | SNP | Rv1162 (narH) | silent (Pro346) | 9926 | - | | 1297327 | G | A | 1071.77 | SNP | Rv1166 (lpqW) | Val(s)392Val | 13 | - | | 1297999 | T | G | 1341.77 | SNP | Rv1166 (lpqW) | silent (Ser616) | 9840 | - | | 1307598 | C | G | 1586.77 | SNP | Rv1175c (fadH) | Cys210Ser | 11 | - | | 1313337 | A | AG | 2593.73 | INS | intergenic |  |  | - | | 1313338 | A | C | 1479.77 | SNP | intergenic |  |  | - | | 1315191 | A | C | 1925.77 | SNP | Rv1180 (pks3) | STOP489Tyr | 1 | - | | 1315884 | G | A | 1681.77 | SNP | Rv1181 (pks4) | silent (Ala217) | 9867 | - | | 1327890 | G | A | 2285.77 | SNP | Rv1186c | silent (Asp472) | 9859 | - | | 1328687 | G | C | 1646.77 | SNP | Rv1186c | Pro207Ala | 22 | - | | 1341624 | G | T | 68.28 | SNP | Rv1199c | Gln328Lys | 12 | - | | 1354437 | A | C | 1716.77 | SNP | Rv1211 | silent (Ala65) | 9867 | - | | 1357114 | C | T | 645.77 | SNP | intergenic |  |  | - | | 1360209 | T | C | 1606.77 | SNP | Rv1217c | silent (Ala531) | 9867 | - | | 1365837 | C | CG | 2056.73 | INS | intergenic |  |  | - | | 1373170 | G | C | 723.77 | SNP | Rv1230c | Pro343Arg | 4 | - | | 1373553 | G | A | 1060.77 | SNP | Rv1230c | silent (Gly215) | 9935 | - | | 1374065 | T | C | 1053.77 | SNP | Rv1230c | Ser45Gly | 21 | - | | 1375724 | A | C | 1754.77 | SNP | Rv1232c | Cys149Gly | 1 | - | | 1376950 | A | AG | 1618.73 | INS | intergenic |  |  | - | | 1382628 | T | C | 2223.77 | SNP | Rv1239c (corA) | Lys139Glu | 4 | - | | 1389738 | G | A | 2459.77 | SNP | Rv1248c | silent (Asp1105) | 9859 | - | | 1393626 | A | G | 1523.77 | SNP | Rv1249c | silent (Leu119) | 9947 | - | | 1396922 | T | C | 1930.77 | SNP | Rv1251c | silent (Thr773) | 9871 | - | | 1404169 | T | G | 1500.77 | SNP | Rv1256c (cyp130) | Lys145Asn | 13 | - | | 1411210 | T | G | 1229.77 | SNP | Rv1263 (amiB2) | Val260Val(s) | 18 | - | | 1413148 | C | T | 1947.77 | SNP | intergenic |  |  | - | | 1414021 | C | T | 1594.77 | SNP | Rv1266c (pknH) | Arg607Gln | 9 | - | | 1428506 | G | T | 1226.77 | SNP | Rv1278 | Ala365Ser | 28 | - | | 1434353 | G | C | 1217.77 | SNP | Rv1281c (oppD) | Leu307Val | 11 | - | | 1440469 | C | G | 2438.77 | SNP | Rv1286 (cysN) | silent (Pro521) | 9926 | - | | 1441533 | C | G | 1406.77 | SNP | Rv1288 | Asp62Glu | 56 | - | | 1445474 | G | A | 1385.77 | SNP | intergenic |  |  | - | | 1445781 | A | G | 1468.77 | SNP | Rv1291c | silent (Ala18) | 9867 | - | | 1446923 | T | G | 1606.77 | SNP | Rv1292 (argS) | Ile182Ser | 2 | - | | 1452071 | C | A | 1157.77 | SNP | Rv1296 (thrB) | silent (Gly25) | 9935 | - | | 1457144 | C | T | 932.77 | SNP | Rv1300 (hemK) | Arg194Cys | 1 | - | | 1468208 | A | C | 1396.77 | SNP | Rv1313c | Leu433Arg | 1 | - | | 1471659 | C | T | 2555.77 | SNP | intergenic |  |  | - | | 1480024 | G | T | 1642.77 | SNP | Rv1318c | Phe267Leu | 13 | - | | 1480945 | C | G | 998.77 | SNP | Rv1319c | silent (Thr519) | 9871 | - | | 1480948 | C | T | 1016.77 | SNP | Rv1319c | silent (Glu518) | 9865 | - | | 1481185 | A | C | 2392.77 | SNP | Rv1319c | Asp439Glu | 56 | - | | 1482627 | T | C | 1697.77 | SNP | Rv1320c | Thr531Ala | 32 | - | | 1484708 | A | C | 1659.77 | SNP | Rv1321 | Ser144Arg | 6 | - | | 1488433 | A | G | 141.77 | SNP | Rv1325c (PE\_PGRS24) | silent (Asp511) | 9859 | - | | 1488434 | T | G | 141.77 | SNP | Rv1325c (PE\_PGRS24) | Asp511Ala | 10 | - | | 1488435 | C | A | 130.77 | SNP | Rv1325c (PE\_PGRS24) | Asp511Tyr | 0 | - | | 1496791 | A | T | 1308.77 | SNP | Rv1328 (glgP) | Tyr743Phe | 28 | - | | 1499274 | C | G | 1191.77 | SNP | Rv1330c (pncB1) | Gly429Ala | 21 | - | | 1512031 | T | G | 1343.77 | SNP | Rv1347c (mbtK) | Asp192Ala | 10 | - | | 1526819 | C | A | 2048.77 | SNP | Rv1358 | silent (Arg70) | 9913 | - | | 1527449 | G | GT | 3722.73 | INS | Rv1358 |  |  | - | | 1533241 | G | C | 1466.77 | SNP | Rv1361c (PPE19) | silent (Thr131) | 9871 | - | | 1533583 | G | A | 1875.77 | SNP | Rv1361c (PPE19) | silent (Tyr17) | 9945 | - | | 1536251 | G | T | 2069.77 | SNP | Rv1364c | Ala465Glu | 10 | - | | 1547125 | T | C | 1609.77 | SNP | Rv1374c | Thr136Ala | 32 | - | | 1549673 | C | T | 1574.77 | SNP | Rv1376 | Leu176Leu(s) | 4 | - | | 1552547 | G | A | 602.77 | SNP | Rv1378c | Arg37Trp | 2 | - | | 1561739 | G | A | 830.77 | SNP | Rv1386 (PE15) | silent (Leu92) | 9947 | - | | 1563717 | C | T | 1684.77 | SNP | Rv1388 (mihF) | silent (Val8) | 9901 | - | | 1564215 | C | G | 78.77 | SNP | Rv1388 (mihF) | silent (Gly174) | 9935 | - | | 1570566 | C | A | 1979.77 | SNP | Rv1394c (cyp132) | Arg135Leu | 1 | - | | 1573660 | T | G | 1001.77 | SNP | Rv1396c (PE\_PGRS25) | Arg66Ser | 11 | - | | 1586249 | C | T | 2796.77 | SNP | Rv1410c | silent (Gln506) | 9876 | - | | 1588456 | A | G | 1574.77 | SNP | Rv1411c (lprG) | silent (Arg9) | 9913 | - | | 1588899 | G | T | 1391.77 | SNP | Rv1412 (ribC) | silent (Ala111) | 9867 | - | | 1602617 | C | T | 1904.77 | SNP | Rv1427c (fadD12) | Val438Ile | 33 | - | | 1609840 | A | G | 1718.77 | SNP | Rv1431 | silent (Pro586) | 9926 | - | | 1612624 | T | TATCGGTACCGGTGCGCCAG GG | 7550.25 | INS | Rv1435c |  |  | - | | 1613035 | T | C | 1996.77 | SNP | intergenic |  |  | - | | 1613960 | G | T | 1649.77 | SNP | Rv1436 (gap) | silent (Ala218) | 9867 | - | | 1618624 | GTGCCGCCGGCGCCGCCGTC GCCGCCGGCGCCGCCGGCGC CGGCGCTGCTGCCGCCGA | G | 2439.73 | DEL | Rv1441c (PE\_PGRS26) |  |  | - | | 1624791 | C | G | 1189.77 | SNP | Rv1446c (opcA) | Arg192Pro | 5 | - | | 1625347 | C | CG | 3150.73 | INS | Rv1446c (opcA) |  |  | - | | 1630148 | A | C | 1827.77 | SNP | Rv1449c (tkt) | Tyr18Asp | 0 | - | | 1634580 | T | C | 105.77 | SNP | Rv1450c (PE\_PGRS27) | Leu16Leu(s) | 4 | - | | 1634581 | A | G | 96.77 | SNP | Rv1450c (PE\_PGRS27) | Leu16Ser | 1 | - | | 1634586 | T | C | 69.77 | SNP | Rv1450c (PE\_PGRS27) | silent (Ala14) | 9867 | - | | 1634589 | G | C | 52.77 | SNP | Rv1450c (PE\_PGRS27) | silent (Ala13) | 9867 | - | | 1634592 | C | G | 64.77 | SNP | Rv1450c (PE\_PGRS27) | silent (Ala12) | 9867 | - | | 1636826 | C | A | 225.78 | SNP | Rv1452c (PE\_PGRS28) | silent (Gly468) | 9935 | - | | 1636918 | C | T | 157.88 | SNP | Rv1452c (PE\_PGRS28) | Ala438Thr | 22 | - | | 1636927 | C | T | 88.77 | SNP | Rv1452c (PE\_PGRS28) | Asp435Asn | 36 | - | | 1636928 | A | G | 112.79 | SNP | Rv1452c (PE\_PGRS28) | silent (Gly434) | 9935 | - | | 1636934 | A | G | 111.82 | SNP | Rv1452c (PE\_PGRS28) | silent (His432) | 9912 | - | | 1636936 | G | T | 63.78 | SNP | Rv1452c (PE\_PGRS28) | His432Asn | 21 | - | | 1636944 | G | T | 49.78 | SNP | Rv1452c (PE\_PGRS28) | Ala429Asp | 6 | - | | 1636945 | C | G | 70.81 | SNP | Rv1452c (PE\_PGRS28) | Ala429Pro | 13 | - | | 1636946 | C | A | 82.79 | SNP | Rv1452c (PE\_PGRS28) | silent (Gly428) | 9935 | - | | 1636980 | G | T | 76.90 | SNP | Rv1452c (PE\_PGRS28) | Pro417His | 3 | - | | 1636981 | G | T | 107.03 | SNP | Rv1452c (PE\_PGRS28) | Pro417Thr | 5 | - | | 1636983 | C | G | 125.03 | SNP | Rv1452c (PE\_PGRS28) | Gly416Ala | 21 | - | | 1638020 | T | C | 31.74 | SNP | Rv1452c (PE\_PGRS28) | silent (Ala70) | 9867 | - | | 1638125 | T | C | 173.84 | SNP | Rv1452c (PE\_PGRS28) | silent (Ser35) | 9840 | - | | 1638140 | G | C | 188.84 | SNP | Rv1452c (PE\_PGRS28) | Ser30Arg | 6 | - | | 1638141 | C | G | 160.90 | SNP | Rv1452c (PE\_PGRS28) | Ser30Thr | 32 | - | | 1638143 | G | A | 132.90 | SNP | Rv1452c (PE\_PGRS28) | silent (Asn29) | 9822 | - | | 1638149 | T | C | 121.03 | SNP | Rv1452c (PE\_PGRS28) | Val27Val(s) | 18 | - | | 1638150 | A | G | 143.90 | SNP | Rv1452c (PE\_PGRS28) | Val27Ala | 18 | - | | 1638152 | A | G | 121.03 | SNP | Rv1452c (PE\_PGRS28) | silent (Gly26) | 9935 | - | | 1639594 | C | A | 1802.77 | SNP | Rv1453 | Pro405Gln | 6 | - | | 1645802 | T | C | 2232.77 | SNP | Rv1459c | Lys113Glu | 4 | - | | 1650072 | A | G | 1781.77 | SNP | Rv1462 | Asn183Asp | 42 | - | | 1673432 | T | C | 2180.77 | SNP | intergenic (Rv1483-8nt) |  |  | resistance | | 1676290 | C | A | 2208.77 | SNP | Rv1486c | Lys198Asn | 13 | - | | 1689349 | C | T | 2231.77 | SNP | Rv1498c | Arg191His | 8 | - | | 1691799 | C | T | 2883.77 | SNP | Rv1500 | Thr317Ile | 7 | - | | 1692141 | A | C | 2837.77 | SNP | Rv1501 | silent (Ile84) | 9872 | - | | 1692795 | G | C | 2326.77 | SNP | intergenic |  |  | - | | 1693561 | A | G | 3525.77 | SNP | Rv1502 | Tyr213Cys | 3 | - | | 1698911 | G | A | 2603.77 | SNP | Rv1508c | silent (Gly328) | 9935 | - | | 1704704 | T | C | 2007.77 | SNP | Rv1512 (epiA) | silent (Ser204) | 9840 | - | | 1706119 | T | C | 1941.77 | SNP | Rv1514c | silent (Ser159) | 9840 | - | | 1709899 | A | C | 1621.77 | SNP | Rv1518 | Asn86His | 18 | - | | 1718761 | C | T | 1460.77 | SNP | Rv1524 | silent (Gly12) | 9935 | - | | 1719322 | G | A | 863.77 | SNP | Rv1524 | silent (Leu199) | 9947 | - | | 1724120 | G | A | 1279.77 | SNP | Rv1527c (pks5) | silent (Asp1430) | 9859 | - | | 1728837 | A | G | 2164.77 | SNP | intergenic |  |  | - | | 1734994 | C | T | 1651.77 | SNP | Rv1534 | silent (Ala87) | 9867 | - | | 1736638 | C | T | 2247.77 | SNP | Rv1536 (ileS) | silent (Arg40) | 9913 | - | | 1740771 | A | C | 1601.77 | SNP | Rv1537 (dinX) | Thr306Pro | 4 | - | | 1752561 | T | C | 967.77 | SNP | Rv1548c (PPE21) | Asp258Gly | 11 | - | | 1753519 | G | GC | 1588.73 | INS | Rv1549 (fadD11.1) |  |  | - | | 1755599 | C | T | 1555.77 | SNP | Rv1551 (plsB1) | Ala52Val | 13 | - | | 1759252 | G | T | 1761.77 | SNP | Rv1552 (frdA) | silent (Ser524) | 9840 | genotype | | 1760292 | A | G | 2211.77 | SNP | Rv1554 (frdC) | Met(s)40Val(s) | 9867 | - | | 1769099 | C | A | 1739.77 | SNP | Rv1563c (treY) | Asp112Tyr | 0 | - | | 1771320 | G | A | 1772.77 | SNP | Rv1564c (treX) | silent (Asp94) | 9859 | - | | 1774036 | TTGGACGGGCGCTTGC | T | 5644.73 | DEL | Rv1566c |  |  | - | | 1777213 | C | G | 1441.77 | SNP | Rv1569 (bioF1) | Ala171Gly | 21 | - | | 1778430 | T | C | 718.77 | SNP | Rv1570 (bioD) | Met(s)191Thr | 22 | - | | 1789446 | C | T | 161.84 | SNP | Rv1588c | Val131Ile | 33 | - | | 1789516 | A | G | 586.77 | SNP | Rv1588c | silent (Gly107) | 9935 | - | | 1789564 | C | T | 664.77 | SNP | Rv1588c | silent (Arg91) | 9913 | - | | 1789565 | C | A | 549.77 | SNP | Rv1588c | Arg91Leu | 1 | - | | 1789650 | C | T | 549.77 | SNP | Rv1588c | Ala63Thr | 22 | - | | 1789654 | A | G | 623.77 | SNP | Rv1588c | silent (Leu61) | 9947 | - | | 1789671 | C | T | 671.77 | SNP | Rv1588c | Ala56Thr | 22 | - | | 1789675 | A | C | 624.77 | SNP | Rv1588c | silent (Gly54) | 9935 | - | | 1789678 | C | G | 615.77 | SNP | Rv1588c | Val(s)53Val | 13 | - | | 1789742 | G | C | 134.77 | SNP | Rv1588c | Thr32Ser | 38 | - | | 1789746 | A | G | 131.77 | SNP | Rv1588c | Leu(s)31Leu | 3 | - | | 1789756 | A | G | 75.77 | SNP | Rv1588c | silent (Ser27) | 9840 | - | | 1793769 | C | T | 1467.77 | SNP | intergenic |  |  | - | | 1798355 | G | A | 1077.77 | SNP | Rv1597 | Gly21Asp | 6 | - | | 1803265 | G | A | 2145.77 | SNP | Rv1602 (hisH) | Ser201Asn | 20 | - | | 1804409 | C | A | 1384.77 | SNP | Rv1604 (impA) | Pro124Gln | 6 | - | | 1805948 | C | T | 1832.77 | SNP | Rv1606 (hisI) | Thr99Ile | 7 | - | | 1817976 | A | T | 1697.77 | SNP | Rv1618 (tesB1) | His121Leu | 4 | - | | 1836286 | G | C | 1557.77 | SNP | intergenic |  |  | - | | 1847811 | A | C | 608.77 | SNP | Rv1639c | silent (Pro216) | 9926 | - | | 1847919 | C | G | 1529.77 | SNP | Rv1639c | silent (Thr180) | 9871 | - | | 1852185 | C | T | 1773.77 | SNP | intergenic |  |  | - | | 1854300 | T | C | 1292.77 | SNP | Rv1644 (tsnR) | Leu232Pro | 2 | - | | 1856777 | G | C | 1846.77 | SNP | Rv1647 | Ala2Pro | 13 | - | | 1861274 | G | A | 1582.77 | SNP | Rv1650 (pheT) | Arg506His | 8 | - | | 1865577 | T | C | 970.77 | SNP | Rv1652 (argC) | Met(s)1Thr | 22 | - | | 1879671 | T | C | 32.77 | SNP | Rv1661 (pks7) | silent (Gly1456) | 9935 | - | | 1885772 | G | A | 1217.77 | SNP | Rv1662 (pks8) | Ala1357Thr | 22 | - | | 1894300 | G | GGTCTTGCCGC | 4734.73 | INS | Rv1668c |  |  | - | | 1899351 | T | C | 2269.77 | SNP | Rv1674c | Glu189Gly | 7 | - | | 1901493 | T | C | 1259.77 | SNP | Rv1676 | silent (Ser149) | 9840 | - | | 1907296 | G | C | 2071.77 | SNP | Rv1682 | silent (Ala298) | 9867 | - | | 1917972 | A | G | 1241.77 | SNP | Rv1694 (tlyA) | silent (Leu11) | 9947 | - | | 1924712 | T | G | 2643.77 | SNP | Rv1699 (pyrG) | Val295Gly | 5 | - | | 1931179 | C | A | 1548.77 | SNP | Rv1704c (cycA) | Arg93Leu | 1 | - | | 1933988 | G | A | 3247.77 | SNP | intergenic |  |  | - | | 1941717 | G | A | 1617.77 | SNP | intergenic |  |  | - | | 1943039 | C | T | 1122.77 | SNP | Rv1715 (fadB3) | silent (Ile127) | 9872 | - | | 1943592 | C | T | 2139.77 | SNP | Rv1716 | Pro6Leu | 3 | - | | 1944107 | A | G | 1493.77 | SNP | Rv1716 | Ser178Gly | 21 | - | | 1944402 | T | C | 1868.77 | SNP | Rv1716 | Val276Ala | 18 | - | | 1944642 | CT | C | 2797.73 | DEL | Rv1717 |  |  | - | | 1947903 | G | T | 953.77 | SNP | Rv1722 | Val(s)15Leu(s) | 9867 | - | | 1950767 | T | C | 1555.77 | SNP | Rv1724c | silent (Lys95) | 9926 | - | | 1960284 | C | A | 1274.77 | SNP | Rv1733c | Gln68His | 20 | - | | 1967237 | C | A | 2033.77 | SNP | Rv1739c | Arg134Leu | 1 | - | | 1967429 | A | G | 1742.77 | SNP | Rv1739c | Leu70Pro | 2 | - | | 1981056 | C | T | 1354.77 | SNP | intergenic |  |  | - | | 1983057 | T | C | 208.80 | SNP | Rv1753c (PPE24) | silent (Pro573) | 9926 | - | | 1983079 | T | G | 115.03 | SNP | Rv1753c (PPE24) | Asn566Thr | 13 | - | | 1983083 | A | G | 45.74 | SNP | Rv1753c (PPE24) | Leu(s)565Leu | 3 | - | | 1983135 | T | G | 88.28 | SNP | Rv1753c (PPE24) | silent (Pro547) | 9926 | - | | 1983195 | A | G | 255.80 | SNP | Rv1753c (PPE24) | silent (Gly527) | 9935 | - | | 1983198 | C | G | 252.80 | SNP | Rv1753c (PPE24) | Val(s)526Val | 13 | - | | 1983313 | T | G | 951.77 | SNP | Rv1753c (PPE24) | Asn488Thr | 13 | - | | 2007785 | C | T | 1626.77 | SNP | intergenic |  |  | - | | 2013998 | C | T | 1407.77 | SNP | Rv1779c | Arg161His | 8 | - | | 2019942 | A | G | 1430.77 | SNP | Rv1783 (eccC5) | Gln229Arg | 10 | - | | 2022868 | T | C | 1290.77 | SNP | Rv1783 (eccC5) | silent (Ser1204) | 9840 | - | | 2032425 | T | C | 1674.77 | SNP | Rv1795 (eccD5) | silent (Gly62) | 9935 | - | | 2045310 | A | G | 221.84 | SNP | Rv1803c (PE\_PGRS32) | silent (Ile511) | 9872 | - | | 2046750 | G | A | 1706.77 | SNP | Rv1803c (PE\_PGRS32) | silent (Asn31) | 9822 | - | | 2049065 | T | C | 2014.77 | SNP | intergenic |  |  | - | | 2049097 | G | C | 1739.77 | SNP | intergenic |  |  | - | | 2051746 | T | C | 1198.77 | SNP | Rv1809 (PPE33) | silent (Ala155) | 9867 | - | | 2052035 | G | T | 1679.77 | SNP | Rv1809 (PPE33) | Val(s)252Leu(s) | 9867 | - | | 2053411 | G | C | 2094.77 | SNP | intergenic |  |  | - | | 2053682 | C | T | 1508.77 | SNP | Rv1811 (mgtC) | silent (Ile80) | 9872 | genotype | | 2055271 | A | G | 2004.77 | SNP | Rv1812c | Leu30Pro | 2 | - | | 2057774 | A | T | 1000.77 | SNP | Rv1815 | Ile83Phe | 8 | - | | 2074509 | C | G | 718.77 | SNP | intergenic |  |  | - | | 2074565 | C | G | 628.77 | SNP | intergenic |  |  | - | | 2074570 | G | C | 97.77 | SNP | intergenic |  |  | - | | 2084526 | G | A | 1356.77 | SNP | Rv1836c | silent (Pro37) | 9926 | - | | 2094911 | ACAGCGT | A | 4062.73 | DEL | Rv1844c (gnd1) |  |  | - | | 2096186 | A | G | 1463.77 | SNP | Rv1846c (blaI) | silent (Thr138) | 9871 | - | | 2097990 | A | C | 1499.77 | SNP | Rv1850 (ureC) | silent (Ala10) | 9867 | - | | 2108141 | T | C | 849.77 | SNP | Rv1860 (apa) | Phe136Leu | 13 | - | | 2109523 | C | CG | 2293.73 | INS | intergenic |  |  | - | | 2116903 | C | T | 1904.77 | SNP | Rv1867 | silent (Gly380) | 9935 | - | | 2123169 | T | G | 1911.77 | SNP | intergenic |  |  | - | | 2126366 | G | C | 1896.77 | SNP | Rv1877 | Val155Leu | 15 | - | | 2128870 | A | G | 1884.77 | SNP | Rv1878 (glnA3) | silent (Leu283) | 9947 | - | | 2133468 | T | TTCGCATGCCGTCACC | 32729.73 | INS | Rv1883c |  |  | - | | 2134215 | T | C | 1537.77 | SNP | Rv1884c (rpfC) | His16Arg | 10 | - | | 2135870 | T | C | 1351.77 | SNP | intergenic |  |  | - | | 2136166 | G | A | 1979.77 | SNP | intergenic |  |  | - | | 2137521 | A | ACTCCGATCAC | 10646.73 | INS | Rv1888c |  |  | - | | 2140365 | A | G | 1589.77 | SNP | Rv1892 | silent (Gly67) | 9935 | - | | 2143328 | G | C | 1814.77 | SNP | Rv1895 | Val(s)270Leu | 3 | - | | 2147022 | A | C | 2314.77 | SNP | Rv1900c (lipJ) | Ile204Met(s) | 6 | - | | 2155168 | C | G | 1870.77 | SNP | Rv1908c (katG) | Ser315Thr | 32 | resistance | | 2163375 | T | C | 628.77 | SNP | Rv1917c (PPE34) | Asn1313Asp | 42 | - | | 2163412 | A | G | 815.77 | SNP | Rv1917c (PPE34) | silent (Val1300) | 9901 | - | | 2163415 | C | A | 657.77 | SNP | Rv1917c (PPE34) | silent (Pro1299) | 9926 | - | | 2163417 | G | C | 793.77 | SNP | Rv1917c (PPE34) | Pro1299Ala | 22 | - | | 2163419 | C | T | 619.77 | SNP | Rv1917c (PPE34) | Ser1298Asn | 20 | - | | 2163421 | C | G | 501.77 | SNP | Rv1917c (PPE34) | silent (Thr1297) | 9871 | - | | 2163444 | T | C | 624.77 | SNP | Rv1917c (PPE34) | Asn1290Asp | 42 | - | | 2163790 | A | C | 1697.77 | SNP | Rv1917c (PPE34) | silent (Pro1174) | 9926 | - | | 2164363 | A | C | 1674.77 | SNP | Rv1917c (PPE34) | silent (Thr983) | 9871 | - | | 2165286 | A | C | 939.77 | SNP | Rv1917c (PPE34) | Ser676Ala | 35 | - | | 2165554 | A | G | 1936.77 | SNP | Rv1917c (PPE34) | silent (Gly586) | 9935 | - | | 2174216 | A | G | 1254.77 | SNP | Rv1922 | Val50Val(s) | 18 | - | | 2197148 | C | A | 2065.77 | SNP | Rv1945 | Thr387Asn | 9 | - | | 2207525 | C | T | 1983.77 | SNP | intergenic |  |  | - | | 2207591 | T | TC | 3738.73 | INS | intergenic |  |  | - | | 2211826 | A | G | 958.77 | SNP | Rv1968 (mce3C) | silent (Lys67) | 9926 | - | | 2213265 | G | A | 717.77 | SNP | Rv1969 (mce3D) | silent (Arg137) | 9913 | - | | 2216248 | C | G | 1891.77 | SNP | Rv1971 (mce3F) | Pro331Arg | 4 | - | | 2216443 | C | A | 1697.77 | SNP | Rv1971 (mce3F) | Ala396Glu | 10 | - | | 2220512 | T | G | 2162.77 | SNP | Rv1977 | silent (Ser253) | 9840 | - | | 2223293 | T | C | 1584.77 | SNP | intergenic |  |  | - | | 2228967 | A | G | 1396.77 | SNP | intergenic |  |  | - | | 2231132 | G | A | 1718.77 | SNP | Rv1987 | Ser36Asn | 20 | - | | 2237053 | G | A | 1719.77 | SNP | Rv1992c (ctpG) | Pro85Leu | 3 | - | | 2245532 | T | C | 2418.77 | SNP | Rv2000 | silent (Cys108) | 9973 | - | | 2251999 | A | G | 1602.77 | SNP | intergenic |  |  | - | | 2256545 | G | A | 1438.77 | SNP | intergenic |  |  | - | | 2257780 | T | G | 1880.77 | SNP | Rv2008c | Ile55Leu | 22 | - | | 2260525 | C | T | 1121.77 | SNP | intergenic |  |  | - | | 2264782 | C | A | 1360.77 | SNP | Rv2017 | Ala262Glu | 10 | - | | 2265059 | T | G | 2345.77 | SNP | intergenic |  |  | - | | 2266487 | G | C | 1559.77 | SNP | Rv2020c | silent (Leu78) | 9947 | - | | 2266504 | T | TA | 2320.73 | INS | Rv2020c |  |  | - | | 2266508 | A | T | 1252.77 | SNP | Rv2020c | Asp71Glu | 56 | - | | 2266511 | GT | G | 2244.73 | DEL | Rv2020c |  |  | - | | 2266517 | T | C | 1275.77 | SNP | Rv2020c | silent (Glu68) | 9865 | - | | 2266550 | G | T | 1584.77 | SNP | Rv2020c | silent (Gly57) | 9935 | - | | 2266553 | C | G | 1646.77 | SNP | Rv2020c | silent (Ser56) | 9840 | - | | 2266583 | C | G | 1533.77 | SNP | Rv2020c | Glu46Asp | 53 | - | | 2266598 | G | C | 1636.77 | SNP | Rv2020c | silent (Leu41) | 9947 | - | | 2266604 | C | G | 1504.77 | SNP | Rv2020c | silent (Ser39) | 9840 | - | | 2266613 | G | GC | 2380.73 | INS | Rv2020c |  |  | - | | 2266624 | G | T | 1551.77 | SNP | Rv2020c | Leu33Ile | 9 | - | | 2267372 | A | G | 2195.77 | SNP | Rv2022c | Val(s)118Ala | 9867 | - | | 2269780 | T | C | 1422.77 | SNP | Rv2024c | Asp154Gly | 11 | - | | 2270102 | A | G | 2018.77 | SNP | Rv2024c | Trp47Arg | 8 | - | | 2278442 | C | G | 1159.77 | SNP | Rv2030c | silent (Arg15) | 9913 | - | | 2282787 | C | T | 1165.77 | SNP | Rv2037c | Cys312Tyr | 3 | - | | 2283030 | A | G | 2269.77 | SNP | Rv2037c | Ile231Thr | 11 | - | | 2285251 | C | A | 2252.77 | SNP | Rv2039c | Val131Phe | 0 | - | | 2287121 | A | G | 1307.77 | SNP | Rv2041c | silent (Asp242) | 9859 | - | | 2288716 | G | GCA | 3536.73 | INS | Rv2043c (pncA) |  |  | - | | 2296042 | G | C | 1370.77 | SNP | Rv2048c (pks12) | Pro3649Ala | 22 | - | | 2300237 | A | G | 787.77 | SNP | Rv2048c (pks12) | silent (Ala2250) | 9867 | - | | 2300546 | A | T | 925.77 | SNP | Rv2048c (pks12) | His2147Gln | 23 | - | | 2300552 | T | G | 955.77 | SNP | Rv2048c (pks12) | silent (Pro2145) | 9926 | - | | 2300555 | A | G | 981.77 | SNP | Rv2048c (pks12) | silent (Asp2144) | 9859 | - | | 2302033 | G | A | 1035.77 | SNP | Rv2048c (pks12) | Arg1652Cys | 1 | - | | 2312847 | G | A | 1784.77 | SNP | Rv2053c (fxsA) | Ala68Val | 13 | - | | 2327492 | C | T | 1199.77 | SNP | Rv2069 (sigC); Rv2070c (cobK) | silent (Leu183); silent (STOP245) | 9947; 9867 | - | | 2329533 | A | G | 1485.77 | SNP | Rv2072c (cobL) | Leu205Pro | 2 | - | | 2334007 | A | G | 2163.77 | SNP | Rv2077c | silent (Ala96) | 9867 | - | | 2335075 | A | G | 2031.77 | SNP | Rv2078 | Glu6Gly | 7 | - | | 2335494 | A | G | 1483.77 | SNP | Rv2079 | Tyr47Cys | 3 | - | | 2340106 | G | C | 495.56 | SNP | Rv2082 | Met(s)466Ile | 2 | - | | 2340621 | C | G | 1567.77 | SNP | Rv2082 | Pro638Arg | 4 | - | | 2341636 | C | G | 907.77 | SNP | Rv2083 | Leu256Val(s) | 4 | - | | 2345037 | C | A | 1385.77 | SNP | Rv2088 (pknJ) | silent (Leu209) | 9947 | - | | 2348446 | C | G | 1332.77 | SNP | Rv2090 | Phe358Leu(s) | 2 | - | | 2357268 | TGCC | T | 381.75 | DEL | intergenic |  |  | - | | 2357492 | T | A | 44.77 | SNP | intergenic |  |  | - | | 2361604 | C | G | 1578.77 | SNP | Rv2101 (helZ) | Val455Val(s) | 18 | - | | 2362041 | C | A | 2004.77 | SNP | Rv2101 (helZ) | Pro601Gln | 6 | - | | 2368564 | TA | T | 3689.73 | DEL | intergenic |  |  | - | | 2372550 | G | C | 103.77 | SNP | Rv2112c (dop) | Pro7Arg | 4 | - | | 2378473 | C | T | 1020.03 | SNP | Rv2119 | Arg30Trp | 2 | - | | 2382085 | AGT | A | 3237.73 | DEL | Rv2123 (PPE37) |  |  | - | | 2385695 | C | T | 1017.77 | SNP | Rv2124c (metH) | Gly125Arg | 0 | - | | 2386389 | G | A | 1186.77 | SNP | Rv2125 | Gly33Ser | 16 | - | | 2387733 | T | C | 263.80 | SNP | Rv2126c (PE\_PGRS37) | silent (Glu80) | 9865 | - | | 2401825 | TGGCTCCTCCTCACCCCGTT ACCCGGGGCGCATCGTCGCC GAGCTCGATTTGATTGCCC | T | 8690.73 | DEL | intergenic |  |  | - | | 2415656 | G | C | 1153.77 | SNP | Rv2155c (murD) | Arg247Gly | 1 | - | | 2420535 | C | A | 1518.77 | SNP | Rv2158c (murE) | Gly25Val | 3 | - | | 2424925 | A | G | 1417.77 | SNP | intergenic |  |  | - | | 2427435 | C | A | 35.94 | SNP | Rv2164c | Val(s)268Val | 13 | - | | 2437606 | C | A | 2103.77 | SNP | Rv2175c | Arg94Leu | 1 | - | | 2438919 | A | C | 1161.77 | SNP | Rv2176 (pknL) | Thr327Pro | 4 | - | | 2439166 | CCATTTCGGCA | C | 3188.80 | DEL | intergenic |  |  | - | | 2439181 | A | ACCGGTCGGAT | 3156.73 | INS | intergenic |  |  | - | | 2439204 | A | G | 829.77 | SNP | intergenic |  |  | - | | 2439458 | C | T | 72.28 | SNP | Rv2177c | Ala164Thr | 22 | - | | 2440926 | G | T | 1797.77 | SNP | Rv2178c (aroG) | Asp265Glu | 56 | - | | 2443188 | G | A | 2738.77 | SNP | Rv2180c | silent (His9) | 9912 | - | | 2452756 | C | A | 1628.77 | SNP | Rv2190c | Ala173Ser | 28 | - | | 2454738 | T | G | 1690.77 | SNP | Rv2191 | Leu307Arg | 1 | - | | 2462871 | G | A | 1556.77 | SNP | Rv2198c (mmpS3) | silent (Ala59) | 9867 | - | | 2463094 | G | A | 1463.77 | SNP | intergenic |  |  | - | | 2484255 | T | G | 1614.77 | SNP | Rv2216 | silent (Ala210) | 9867 | - | | 2484327 | C | T | 1656.77 | SNP | Rv2216 | silent (Thr234) | 9871 | - | | 2499726 | G | A | 1889.77 | SNP | Rv2226 | Asp299Asn | 36 | - | | 2502073 | C | T | 1101.77 | SNP | Rv2228c | silent (Arg222) | 9913 | - | | 2503625 | C | T | 1294.77 | SNP | Rv2230c | silent (Leu328) | 9947 | - | | 2507847 | C | T | 1739.77 | SNP | Rv2235 | Pro71Ser | 17 | - | | 2509140 | G | C | 1265.77 | SNP | Rv2236c (cobD) | Ser79Cys | 5 | - | | 2509722 | A | G | 1735.77 | SNP | Rv2237 | silent (Pro78) | 9926 | - | | 2521342 | T | C | 1379.77 | SNP | Rv2247 (accD6) | silent (Asp200) | 9859 | - | | 2523205 | G | GCGC | 2612.73 | INS | intergenic |  |  | - | | 2525722 | CG | C | 1836.73 | DEL | Rv2250A; Rv2251 |  |  | - | | 2527676 | G | A | 2030.77 | SNP | Rv2252 | Gly230Ser | 16 | - | | 2529680 | A | G | 1996.77 | SNP | Rv2256c | silent (Thr65) | 9871 | - | | 2531742 | A | G | 2430.77 | SNP | Rv2258c | silent (Ala52) | 9867 | - | | 2534562 | GGA | G | 1811.73 | DEL | Rv2262c |  |  | - | | 2536625 | C | CG | 204.80 | INS | Rv2264c |  |  | - | | 2536660 | A | C | 41.77 | SNP | Rv2264c | Ile564Ser | 2 | - | | 2536892 | T | C | 64.77 | SNP | Rv2264c | Thr487Ala | 32 | - | | 2573756 | C | A | 1859.77 | SNP | intergenic |  |  | - | | 2586127 | A | G | 1612.77 | SNP | Rv2314c | silent (Gly388) | 9935 | - | | 2588184 | C | A | 1697.77 | SNP | Rv2315c | silent (Arg207) | 9913 | - | | 2589098 | A | G | 2432.77 | SNP | Rv2316 (uspA) | silent (Gly87) | 9935 | - | | 2598400 | A | G | 1998.77 | SNP | Rv2326c | silent (Asn516) | 9822 | - | | 2599821 | C | A | 2144.77 | SNP | Rv2326c | Ala43Ser | 28 | - | | 2612632 | C | A | 1510.77 | SNP | Rv2337c | Gly119Val | 3 | - | | 2617632 | A | AT | 3250.73 | INS | intergenic |  |  | - | | 2617633 | C | T | 1692.77 | SNP | intergenic |  |  | - | | 2621058 | G | A | 1122.77 | SNP | Rv2343c (dnaG) | silent (Pro465) | 9926 | - | | 2626274 | G | C | 61.77 | SNP | Rv2347c (esxP) | silent (Ala82) | 9867 | - | | 2626280 | G | A | 67.77 | SNP | Rv2347c (esxP) | silent (Arg80) | 9913 | - | | 2626295 | A | C | 41.77 | SNP | Rv2347c (esxP) | silent (Arg75) | 9913 | - | | 2648861 | C | T | 2258.77 | SNP | Rv2367c | Val18Ile | 33 | - | | 2654371 | G | A | 2499.77 | SNP | Rv2374c (hrcA) | silent (Asn241) | 9822 | - | | 2656225 | A | G | 1704.77 | SNP | Rv2377c (mbtH) | Val69Ala | 18 | - | | 2660319 | C | G | 1872.77 | SNP | Rv2379c (mbtF) | Glu589Asp | 53 | - | | 2664299 | G | C | 1179.77 | SNP | Rv2380c (mbtE) | silent (Thr939) | 9871 | - | | 2680658 | T | G | 1684.77 | SNP | intergenic |  |  | - | | 2695378 | C | G | 1878.77 | SNP | Rv2398c (cysW) | Gly141Ala | 21 | - | | 2698585 | C | T | 1966.77 | SNP | Rv2402 | silent (Tyr19) | 9945 | - | | 2704884 | A | ACAGCGACCATATCGCCGAG CT | 1638.73 | INS | Rv2407 |  |  | - | | 2713795 | C | T | 1606.77 | SNP | intergenic |  |  | - | | 2717710 | C | T | 1419.77 | SNP | Rv2419c (gpgP) | Leu(s)30Leu | 3 | - | | 2718852 | T | G | 1772.77 | SNP | intergenic |  |  | - | | 2720895 | G | A | 213.77 | SNP | Rv2424c | His295Tyr | 4 | - | | 2721013 | A | G | 186.77 | SNP | Rv2424c | silent (Arg255) | 9913 | - | | 2722340 | G | A | 1391.77 | SNP | Rv2425c | silent (Phe323) | 9946 | - | | 2734074 | T | C | 574.77 | SNP | Rv2436 (rbsK) | Val282Ala | 18 | - | | 2736434 | C | A | 2585.77 | SNP | Rv2438c (nadE) | Arg133Leu | 1 | - | | 2745889 | C | T | 1984.77 | SNP | Rv2446c | Ala84Thr | 22 | - | | 2751804 | C | T | 1224.77 | SNP | Rv2450c (rpfE) | Arg126Gln | 9 | - | | 2752698 | C | A | 2985.77 | SNP | intergenic |  |  | - | | 2760152 | A | G | 1575.77 | SNP | Rv2458 (mmuM) | Tyr125Cys | 3 | - | | 2765882 | G | A | 1662.77 | SNP | Rv2463 (lipP) | silent (Thr76) | 9871 | - | | 2768181 | C | T | 764.77 | SNP | intergenic |  |  | - | | 2771561 | C | T | 1969.77 | SNP | Rv2467 (pepN) | Ala859Val | 13 | - | | 2779136 | T | C | 1321.77 | SNP | Rv2476c (gdh) | Ser1043Gly | 21 | - | | 2786952 | A | G | 1971.77 | SNP | Rv2482c (plsB2) | Cys778Arg | 1 | - | | 2791061 | A | G | 1452.77 | SNP | Rv2484c | silent (Thr478) | 9871 | - | | 2809621 | T | C | 1489.77 | SNP | Rv2495c (bkdC) | Thr107Ala | 32 | - | | 2818837 | A | G | 1520.77 | SNP | Rv2503c (scoB) | silent (Gly97) | 9935 | - | | 2821342 | C | T | 817.77 | SNP | Rv2505c (fadD35) | silent (Ala85) | 9867 | - | | 2827984 | G | T | 1870.77 | SNP | intergenic |  |  | - | | 2828019 | T | C | 1989.77 | SNP | intergenic |  |  | - | | 2828822 | G | T | 268.78 | SNP | Rv2512c | Gln328Lys | 12 | - | | 2829779 | T | C | 654.77 | SNP | Rv2512c | Thr9Ala | 32 | - | | 2830525 | C | A | 2062.77 | SNP | Rv2513 | Thr122Lys | 11 | - | | 2855259 | A | G | 1429.77 | SNP | Rv2531c | silent (Ala841) | 9867 | - | | 2856038 | G | A | 1701.77 | SNP | Rv2531c | His582Tyr | 4 | - | | 2857014 | G | A | 1753.77 | SNP | Rv2531c | silent (Thr256) | 9871 | - | | 2858011 | T | C | 1911.77 | SNP | Rv2532c | Met(s)82Val(s) | 9867 | - | | 2865760 | A | G | 2132.77 | SNP | Rv2542 | Thr211Ala | 32 | - | | 2865882 | T | C | 2050.77 | SNP | Rv2542 | silent (Val251) | 9901 | - | | 2868659 | C | G | 1995.77 | SNP | Rv2547 (vapB19) | silent (Ala18) | 9867 | - | | 2871048 | C | T | 1182.77 | SNP | Rv2551c | silent (Leu49) | 9947 | - | | 2873093 | G | GCCA | 2002.73 | INS | Rv2553c |  |  | - | | 2880702 | G | C | 2123.77 | SNP | Rv2560 | Val210Leu | 15 | - | | 2881455 | A | G | 1465.77 | SNP | Rv2561 | Tyr16Cys | 3 | - | | 2881597 | AG | A | 2135.73 | DEL | Rv2561 |  |  | - | | 2888201 | T | C | 1167.77 | SNP | Rv2566 | Leu610Pro | 2 | - | | 2889633 | T | C | 1556.77 | SNP | Rv2566 | silent (Ala1087) | 9867 | - | | 2891267 | C | T | 1970.77 | SNP | Rv2567 | silent (Gly491) | 9935 | - | | 2891728 | A | G | 1588.77 | SNP | Rv2567 | Gln645Arg | 10 | - | | 2893238 | C | A | 1560.77 | SNP | Rv2568c | silent (Arg78) | 9913 | - | | 2894208 | G | A | 1729.77 | SNP | Rv2569c | silent (Ser67) | 9840 | - | | 2894854 | C | T | 1215.77 | SNP | Rv2570 | Gln115STOP | 8 | - | | 2902158 | A | C | 2042.77 | SNP | Rv2577 | Asp414Ala | 10 | - | | 2906918 | A | T | 1278.77 | SNP | Rv2582 (ppiB) | silent (Leu35) | 9947 | - | | 2910461 | G | T | 1540.77 | SNP | Rv2584c (apt) | Ala147Glu | 10 | - | | 2911293 | C | G | 1076.77 | SNP | Rv2585c | Cys462Ser | 11 | - | | 2912294 | T | G | 2014.77 | SNP | Rv2585c | silent (Ala128) | 9867 | - | | 2923391 | T | C | 1405.77 | SNP | Rv2592c (ruvB) | silent (Pro281) | 9926 | - | | 2927939 | T | C | 2315.77 | SNP | intergenic |  |  | - | | 2939373 | G | C | 1480.77 | SNP | Rv2611c | Ser197Cys | 5 | - | | 2939657 | T | C | 779.77 | SNP | Rv2611c | Ile102Met(s) | 6 | - | | 2945004 | C | G | 353.77 | SNP | intergenic |  |  | - | | 2945167 | G | T | 1400.77 | SNP | intergenic |  |  | - | | 2946157 | T | C | 2059.77 | SNP | Rv2617c | Asn44Ser | 34 | - | | 2954439 | T | C | 2073.77 | SNP | Rv2627c | Arg104Gly | 1 | - | | 2968913 | T | C | 1833.77 | SNP | intergenic |  |  | - | | 2974933 | A | G | 950.77 | SNP | Rv2650c | Ile101Thr | 11 | - | | 2983095 | A | G | 578.77 | SNP | Rv2666 | Thr9Ala | 32 | - | | 2984740 | A | G | 1135.77 | SNP | Rv2668 | His3Arg | 10 | - | | 3005185 | G | T | 1503.77 | SNP | Rv2688c | Pro156Thr | 5 | - | | 3006767 | A | C | 1327.77 | SNP | Rv2689c | Leu99Arg | 1 | - | | 3009692 | A | G | 2064.77 | SNP | Rv2691 (ceoB) | Thr117Ala | 32 | - | | 3017465 | T | C | 2624.77 | SNP | Rv2702 (ppgK) | Ile203Thr | 11 | - | | 3028101 | A | AC | 2033.73 | INS | Rv2715 |  |  | - | | 3029610 | G | A | 1361.77 | SNP | Rv2716 | Ala147Thr | 22 | - | | 3041871 | G | T | 1558.77 | SNP | Rv2729c | Ala202Glu | 10 | - | | 3054081 | A | G | 1717.77 | SNP | Rv2741 (PE\_PGRS47) | silent (Gly56) | 9935 | - | | 3054321 | A | G | 418.77 | SNP | Rv2741 (PE\_PGRS47) | silent (Gly136) | 9935 | - | | 3073868 | T | C | 1482.77 | SNP | Rv2764c (thyA) | Thr202Ala | 32 | genotype | | 3077039 | C | A | 1569.77 | SNP | Rv2768c (PPE43) | Gly347Val | 3 | - | | 3080795 | A | G | 2830.77 | SNP | Rv2771c | Leu80Pro | 2 | - | | 3083977 | G | T | 1453.77 | SNP | Rv2776c | Asp109Glu | 56 | - | | 3084074 | C | T | 1291.77 | SNP | Rv2776c | Gly77Asp | 6 | - | | 3086788 | T | C | 2170.77 | SNP | intergenic |  |  | - | | 3089679 | G | A | 954.77 | SNP | Rv2782c (pepR) | Pro228Leu | 3 | - | | 3099269 | A | C | 2042.77 | SNP | Rv2790c (ltp1) | Phe301Val | 1 | - | | 3101119 | G | T | 847.77 | SNP | Rv2791c | Arg155Ser | 11 | - | | 3103682 | T | C | 1534.77 | SNP | Rv2794c (pptT) | Met(s)87Val(s) | 9867 | - | | 3108055 | C | T | 1221.77 | SNP | Rv2799 | silent (Ala96) | 9867 | - | | 3108674 | A | C | 1508.77 | SNP | Rv2800 | silent (Arg87) | 9913 | - | | 3113872 | A | T | 1102.77 | SNP | Rv2807 | Glu72Val(s) | 17 | - | | 3118000 | A | G | 1166.77 | SNP | Rv2812 | Arg395Gly | 1 | - | | 3131469 | T | TTGTCGGCGA | 8465.73 | INS | Rv2823c |  |  | - | | 3133536 | T | C | 2331.77 | SNP | Rv2825c | Lys2Glu | 4 | - | | 3137058 | G | A | 1114.77 | SNP | Rv2830c (vapB22) | Ala56Val(s) | 9867 | - | | 3140525 | T | C | 1108.77 | SNP | Rv2834c (ugpE) | Met(s)264Val(s) | 9867 | - | | 3147154 | A | G | 59.77 | SNP | Rv2839c (infB) | silent (Ala240) | 9867 | - | | 3158935 | G | C | 939.77 | SNP | Rv2850c | Arg374Gly | 1 | - | | 3162805 | C | G | 131.03 | SNP | Rv2853 (PE\_PGRS48) | Arg180Gly | 1 | - | | 3165074 | T | C | 1748.77 | SNP | Rv2854 | Val(s)308Ala | 9867 | - | | 3175702 | T | C | 1344.77 | SNP | Rv2864c | Ile522Val | 57 | - | | 3177884 | C | A | 1819.77 | SNP | Rv2866 (relG) | silent (Arg21) | 9913 | - | | 3178445 | C | G | 1348.77 | SNP | intergenic |  |  | - | | 3183561 | G | C | 444.77 | SNP | Rv2872 (vapC43) | silent (Pro60) | 9926 | - | | 3186860 | T | G | 1368.77 | SNP | Rv2874 (dipZ) | Tyr672Asp | 0 | - | | 3190145 | TC | T | 2393.73 | DEL | Rv2880c |  |  | - | | 3191027 | G | A | 2115.77 | SNP | Rv2881c (cdsA) | Leu199Leu(s) | 4 | - | | 3207297 | G | T | 1151.77 | SNP | Rv2897c | Leu216Met(s) | 4 | - | | 3214790 | C | T | 1313.77 | SNP | Rv2905 (lppW) | Leu55Leu(s) | 4 | - | | 3226181 | A | C | 1586.77 | SNP | Rv2916c (ffh) | silent (Arg35) | 9913 | - | | 3228143 | G | T | 1592.77 | SNP | Rv2917 | Arg594Leu | 1 | - | | 3232718 | G | C | 648.77 | SNP | intergenic |  |  | - | | 3232815 | A | G | 2915.77 | SNP | intergenic |  |  | - | | 3236230 | C | A | 1682.77 | SNP | Rv2922c (smc) | Arg526Leu | 1 | - | | 3243630 | G | A | 2525.77 | SNP | intergenic |  |  | - | | 3247316 | C | G | 1457.77 | SNP | Rv2931 (ppsA) | Asp624Glu | 56 | - | | 3247851 | G | A | 1447.77 | SNP | Rv2931 (ppsA) | Ala803Thr | 22 | - | | 3247853 | C | T | 1384.77 | SNP | Rv2931 (ppsA) | silent (Ala803) | 9867 | - | | 3247856 | G | C | 1376.77 | SNP | Rv2931 (ppsA) | silent (Arg804) | 9913 | - | | 3247864 | C | CTAGG | 3186.76 | INS | Rv2931 (ppsA) |  |  | - | | 3247865 | GCAAA | G | 3358.73 | DEL | Rv2931 (ppsA) |  |  | - | | 3247874 | G | A | 1376.77 | SNP | Rv2931 (ppsA) | silent (Arg810) | 9913 | - | | 3247877 | T | C | 1499.77 | SNP | Rv2931 (ppsA) | silent (Phe811) | 9946 | - | | 3247883 | T | C | 1813.77 | SNP | Rv2931 (ppsA) | silent (Ser813) | 9840 | - | | 3248074 | G | A | 1410.77 | SNP | Rv2931 (ppsA) | Arg877His | 8 | - | | 3248075 | C | T | 1502.77 | SNP | Rv2931 (ppsA) | silent (Arg877) | 9913 | - | | 3256494 | A | G | 1888.77 | SNP | Rv2933 (ppsC) | silent (Gly270) | 9935 | - | | 3260301 | A | G | 1585.77 | SNP | Rv2933 (ppsC) | silent (Gly1539) | 9935 | - | | 3267743 | A | G | 1912.77 | SNP | Rv2935 (ppsE) | Ile3Val | 57 | - | | 3269581 | A | G | 2251.77 | SNP | Rv2935 (ppsE) | silent (Ala615) | 9867 | - | | 3270784 | A | G | 1556.77 | SNP | Rv2935 (ppsE) | silent (Gln1016) | 9876 | - | | 3278675 | C | T | 2537.77 | SNP | Rv2940c (mas) | silent (Leu1347) | 9947 | - | | 3292737 | T | G | 1112.77 | SNP | Rv2946c (pks1) | Gln1206Pro | 8 | - | | 3296843 | A | G | 711.77 | SNP | Rv2947c (pks15) | Val(s)333Ala | 9867 | - | | 3308606 | G | A | 2312.77 | SNP | intergenic |  |  | - | | 3312632 | C | T | 1993.77 | SNP | Rv2959c | Trp69STOP | 0 | - | | 3336587 | T | A | 606.31 | SNP | intergenic |  |  | - | | 3336646 | T | A | 540.96 | SNP | intergenic |  |  | - | | 3336825 | T | C | 1260.77 | SNP | Rv2981c (ddlA) | Thr365Ala | 32 | - | | 3338603 | G | C | 1263.77 | SNP | Rv2982c (gpdA2) | Pro133Ala | 22 | - | | 3343384 | TTGGCGGGCGCCTTGGTCGC CGCCTTCC | T | 4916.73 | DEL | Rv2986c (hupB) |  |  | - | | 3354896 | C | T | 921.77 | SNP | Rv2996c (serA1) | silent (Glu58) | 9865 | - | | 3358235 | A | T | 1945.77 | SNP | Rv2999 (lppY) | Met(s)212Leu(s) | 9867 | - | | 3363338 | A | G | 1892.77 | SNP | intergenic |  |  | - | | 3367765 | G | A | 1598.77 | SNP | Rv3009c (gatB) | silent (Gly343) | 9935 | - | | 3377271 | G | C | 44.74 | SNP | Rv3018c (PPE46) | Leu325Val(s) | 4 | - | | 3377275 | G | C | 42.74 | SNP | Rv3018c (PPE46) | silent (Ala323) | 9867 | - | | 3377289 | C | A | 44.74 | SNP | Rv3018c (PPE46) | Val(s)319Leu(s) | 9867 | - | | 3377293 | G | C | 45.74 | SNP | Rv3018c (PPE46) | silent (Ala317) | 9867 | - | | 3377298 | A | C | 47.74 | SNP | Rv3018c (PPE46) | Leu(s)316Val(s) | 9867 | - | | 3377305 | T | C | 352.77 | SNP | Rv3018c (PPE46) | Val313Val(s) | 18 | - | | 3377314 | A | C | 532.77 | SNP | Rv3018c (PPE46) | Val310Val(s) | 18 | - | | 3377320 | A | C | 577.77 | SNP | Rv3018c (PPE46) | silent (Ala308) | 9867 | - | | 3377326 | G | A | 601.77 | SNP | Rv3018c (PPE46) | silent (Ala306) | 9867 | - | | 3377347 | C | A | 728.77 | SNP | Rv3018c (PPE46) | silent (Gly299) | 9935 | - | | 3379708 | G | C | 50.74 | SNP | intergenic |  |  | - | | 3379712 | G | C | 49.74 | SNP | intergenic |  |  | - | | 3379718 | T | C | 48.74 | SNP | intergenic |  |  | - | | 3379726 | C | A | 48.74 | SNP | intergenic |  |  | - | | 3379730 | G | C | 49.74 | SNP | intergenic |  |  | - | | 3379732 | C | T | 43.74 | SNP | intergenic |  |  | - | | 3379735 | A | C | 47.74 | SNP | intergenic |  |  | - | | 3379736 | C | A | 44.74 | SNP | intergenic |  |  | - | | 3379742 | T | C | 379.77 | SNP | intergenic |  |  | - | | 3379751 | A | C | 484.77 | SNP | intergenic |  |  | - | | 3379757 | A | C | 518.77 | SNP | intergenic |  |  | - | | 3379763 | G | A | 497.77 | SNP | intergenic |  |  | - | | 3379784 | C | A | 631.77 | SNP | intergenic |  |  | - | | 3379788 | C | G | 797.77 | SNP | intergenic |  |  | - | | 3380740 | C | T | 916.77 | SNP | Rv3022A (PE29) | Gly85Asp | 6 | - | | 3402816 | C | T | 1720.77 | SNP | Rv3042c (serB2) | Gly116Glu | 4 | - | | 3403216 | AT | A | 3097.73 | DEL | Rv3043c (ctaD) |  |  | - | | 3415180 | ACACCTAGGGGGTGG | A | 6171.73 | DEL | intergenic |  |  | - | | 3418328 | T | G | 2219.77 | SNP | Rv3057c | Asp112Ala | 10 | - | | 3418330 | G | A | 2125.77 | SNP | Rv3057c | silent (His111) | 9912 | - | | 3425854 | C | T | 1621.77 | SNP | Rv3062 (ligB) | Pro91Ser | 17 | - | | 3426795 | C | G | 1742.77 | SNP | Rv3062 (ligB) | silent (Ser404) | 9840 | - | | 3428917 | C | A | 1710.77 | SNP | Rv3063 (cstA) | Arg559Ser | 11 | - | | 3429202 | T | G | 1837.77 | SNP | Rv3063 (cstA) | Tyr654Asp | 0 | - | | 3440464 | T | G | 1760.77 | SNP | Rv3077 | silent (Arg308) | 9913 | - | | 3440468 | G | C | 1718.77 | SNP | Rv3077 | Gly310Arg | 0 | - | | 3456666 | A | G | 1867.77 | SNP | Rv3089 (fadD13) | silent (Ala302) | 9867 | - | | 3460986 | G | A | 1076.77 | SNP | Rv3092c | Pro250Leu | 3 | - | | 3462135 | G | C | 1276.77 | SNP | Rv3093c | Cys210Trp | 0 | - | | 3470579 | A | G | 2043.77 | SNP | Rv3101c (ftsX) | Leu(s)34Ser | 28 | - | | 3473996 | G | GA | 2492.73 | INS | intergenic |  |  | - | | 3482432 | C | A | 234.78 | SNP | Rv3115 | Gln328Lys | 12 | - | | 3484012 | T | G | 2258.77 | SNP | Rv3117 (cysA3) | silent (Ala13) | 9867 | - | | 3486977 | A | G | 2745.77 | SNP | Rv3121 (cyp141) | Lys157Glu | 4 | - | | 3496770 | T | C | 2158.77 | SNP | Rv3131 | Leu(s)74Leu | 3 | - | | 3503231 | G | C | 1916.77 | SNP | Rv3136A | Ala16Gly | 21 | - | | 3503895 | C | T | 1208.77 | SNP | Rv3137 | Pro168Leu | 3 | - | | 3504930 | C | T | 1086.77 | SNP | Rv3138 (pflA) | Leu246Leu(s) | 4 | - | | 3505027 | G | A | 1329.77 | SNP | Rv3138 (pflA) | Arg278His | 8 | - | | 3514512 | G | C | 1471.77 | SNP | Rv3148 (nuoD) | Gly392Ala | 21 | genotype | | 3518167 | A | G | 1657.77 | SNP | Rv3151 (nuoG) | Ile474Met(s) | 6 | - | | 3518555 | A | G | 943.77 | SNP | Rv3151 (nuoG) | Thr604Ala | 32 | - | | 3538308 | G | A | 1435.77 | SNP | Rv3169 | silent (Ala357) | 9867 | - | | 3548641 | T | C | 2325.77 | SNP | Rv3179 | Tyr342His | 4 | - | | 3556275 | A | G | 2000.77 | SNP | Rv3190c | Leu138Pro | 2 | - | | 3561155 | G | A | 2732.77 | SNP | Rv3193c | Ala673Val | 13 | - | | 3580636 | CT | C | 3988.73 | DEL | intergenic |  |  | - | | 3581414 | A | G | 1430.77 | SNP | Rv3204 | Thr34Ala | 32 | - | | 3590686 | G | GC | 2071.73 | INS | intergenic |  |  | - | | 3591063 | T | C | 690.77 | SNP | Rv3213c | Lys144Glu | 4 | - | | 3594124 | C | T | 1862.77 | SNP | Rv3217c | Ala38Thr | 22 | - | | 3595483 | A | ACTGGCAGCGTAGT | 9468.73 | INS | intergenic |  |  | - | | 3604821 | G | C | 824.77 | SNP | Rv3228 | silent (Ala32) | 9867 | - | | 3610441 | C | T | 1753.77 | SNP | Rv3234c (tgs3) | Arg250His | 8 | - | | 3612009 | C | T | 989.77 | SNP | Rv3236c | Ala370Thr | 22 | - | | 3614982 | T | C | 1753.77 | SNP | Rv3239c | silent (Leu874) | 9947 | - | | 3622441 | A | C | 1114.77 | SNP | Rv3243c | Val217Val(s) | 18 | - | | 3625065 | T | G | 1136.77 | SNP | Rv3245c (mtrB) | Met(s)517Leu | 3 | - | | 3676206 | A | G | 1579.77 | SNP | Rv3295 | silent (Ala47) | 9867 | - | | 3680644 | C | T | 629.77 | SNP | Rv3296 (lhr) | silent (Ala1290) | 9867 | - | | 3689523 | G | T | 1956.77 | SNP | Rv3303c (lpdA) | Cys472STOP | 3 | - | | 3692757 | G | T | 1689.77 | SNP | Rv3305c (amiA1) | Leu18Met(s) | 4 | - | | 3704596 | G | C | 1384.77 | SNP | Rv3317 (sdhD) | Val(s)54Leu | 3 | - | | 3711910 | G | A | 906.77 | SNP | Rv3327 | Trp54STOP | 0 | - | | 3714211 | G | T | 2449.77 | SNP | Rv3328c (sigJ) | Pro41Gln | 6 | - | | 3714757 | A | C | 1895.77 | SNP | Rv3329 | Gln122His | 20 | - | | 3718357 | C | T | 1673.77 | SNP | Rv3331 (sugI) | Pro423Leu | 3 | - | | 3721806 | G | C | 2234.77 | SNP | Rv3335c | silent (Gly265) | 9935 | - | | 3730327 | G | A | 1836.77 | SNP | Rv3343c (PPE54) | silent (Phe2203) | 9946 | - | | 3730411 | G | A | 806.77 | SNP | Rv3343c (PPE54) | silent (Gly2175) | 9935 | - | | 3730741 | G | A | 2643.77 | SNP | Rv3343c (PPE54) | silent (Gly2065) | 9935 | - | | 3730978 | G | A | 221.77 | SNP | Rv3343c (PPE54) | silent (Gly1986) | 9935 | - | | 3732370 | A | G | 390.77 | SNP | Rv3343c (PPE54) | silent (Asn1522) | 9822 | - | | 3732469 | G | A | 76.28 | SNP | Rv3343c (PPE54) | silent (Gly1489) | 9935 | - | | 3732706 | G | A | 190.84 | SNP | Rv3343c (PPE54) | silent (Gly1410) | 9935 | - | | 3735931 | G | A | 255.96 | SNP | Rv3343c (PPE54) | silent (Ser335) | 9840 | - | | 3735967 | G | A | 115.22 | SNP | Rv3343c (PPE54) | silent (Ser323) | 9840 | - | | 3736179 | G | A | 134.90 | SNP | Rv3343c (PPE54) | Leu253Leu(s) | 4 | - | | 3736628 | T | G | 1087.77 | SNP | Rv3343c (PPE54) | Glu103Ala | 17 | - | | 3738535 | G | T | 157.90 | SNP | Rv3345c (PE\_PGRS50) | His1414Asn | 21 | - | | 3739913 | G | A | 744.77 | SNP | Rv3345c (PE\_PGRS50) | silent (Thr954) | 9871 | - | | 3747403 | C | A | 45.77 | SNP | Rv3347c (PPE55) | Gly1928Cys | 0 | - | | 3769550 | G | A | 138.90 | SNP | Rv3355c | silent (Tyr86) | 9945 | - | | 3776706 | C | T | 2668.77 | SNP | Rv3365c | Ala266Thr | 22 | - | | 3779671 | C | CGGCAACGGT | 516.73 | INS | Rv3367 (PE\_PGRS51) |  |  | - | | 3794884 | G | A | 1723.77 | SNP | intergenic |  |  | - | | 3798095 | A | C | 2279.77 | SNP | Rv3383c (idsB) | Val132Gly | 5 | - | | 3800663 | G | A | 1757.77 | SNP | Rv3386 | Arg191His | 8 | - | | 3817117 | C | A | 1910.77 | SNP | Rv3399 | Ala330Glu | 10 | - | | 3820407 | A | G | 93.77 | SNP | intergenic |  |  | - | | 3820545 | A | G | 148.77 | SNP | intergenic |  |  | - | | 3823159 | A | T | 1705.77 | SNP | Rv3403c | silent (Val235) | 9901 | - | | 3825560 | C | G | 1469.77 | SNP | Rv3406 | silent (Leu77) | 9947 | - | | 3826430 | A | G | 2609.77 | SNP | Rv3407 (vapB47) | Glu60Gly | 7 | - | | 3826684 | C | T | 1274.77 | SNP | Rv3408 (vapC47) | Ser46Leu(s) | 35 | - | | 3829152 | G | A | 981.77 | SNP | Rv3410c (guaB3) | silent (Leu253) | 9947 | - | | 3829770 | T | C | 1268.77 | SNP | Rv3410c (guaB3) | silent (Pro47) | 9926 | - | | 3838871 | A | G | 1761.77 | SNP | Rv3420c (rimI) | silent (Ala64) | 9867 | - | | 3847364 | C | T | 1833.77 | SNP | Rv3429 (PPE59) | Ala67Val | 13 | - | | 3847367 | G | A | 1787.77 | SNP | Rv3429 (PPE59) | Gly68Glu | 4 | - | | 3847378 | G | C | 1865.77 | SNP | Rv3429 (PPE59) | Asp72His | 3 | - | | 3847380 | C | A | 1757.77 | SNP | Rv3429 (PPE59) | Asp72Glu | 56 | - | | 3858822 | C | T | 907.77 | SNP | Rv3439c | Gly281Ser | 16 | - | | 3859376 | C | T | 993.77 | SNP | Rv3439c | Gly96Glu | 4 | - | | 3859893 | C | T | 1288.77 | SNP | Rv3440c | silent (Glu28) | 9865 | - | | 3861806 | A | C | 1522.77 | SNP | Rv3442c (rpsI) | Phe49Val | 1 | - | | 3862472 | GA | G | 2518.73 | DEL | intergenic |  |  | - | | 3864995 | T | C | 1872.77 | SNP | Rv3447c (eccC4) | Ser1082Gly | 21 | - | | 3873392 | T | G | 1579.77 | SNP | Rv3451 (cut3) | Leu259Arg | 1 | - | | 3874191 | T | C | 1387.77 | SNP | intergenic |  |  | - | | 3877421 | A | G | 1893.77 | SNP | Rv3456c (rplQ) | silent (Pro4) | 9926 | - | | 3878878 | A | G | 1737.77 | SNP | Rv3458c (rpsD) | silent (Ser129) | 9840 | - | | 3881187 | G | A | 2749.77 | SNP | Rv3463 | Gly94Asp | 6 | - | | 3883626 | A | G | 1217.77 | SNP | Rv3466 | silent (Pro34) | 9926 | - | | 3883796 | G | T | 178.84 | SNP | Rv3466 | Arg91Leu | 1 | - | | 3883797 | G | A | 144.90 | SNP | Rv3466 | silent (Arg91) | 9913 | - | | 3883845 | T | C | 165.90 | SNP | Rv3466 | silent (Gly107) | 9935 | - | | 3884906 | A | G | 1384.77 | SNP | Rv3467 | Lys315Glu | 4 | - | | 3885886 | T | C | 1500.77 | SNP | Rv3468c | Ile62Val | 57 | - | | 3892671 | A | G | 3257.77 | SNP | Rv3476c (kgtP) | silent (Val350) | 9901 | - | | 3896340 | T | G | 1365.77 | SNP | Rv3479 | Leu174Arg | 1 | - | | 3898408 | A | G | 1913.77 | SNP | Rv3479 | silent (Ala863) | 9867 | - | | 3921518 | G | A | 38.77 | SNP | Rv3502c | Pro175Ser | 17 | - | | 3934542 | T | G | 51.74 | SNP | Rv3508 (PE\_PGRS54) | Ser1180Ala | 35 | - | | 3934699 | G | A | 348.77 | SNP | Rv3508 (PE\_PGRS54) | Ser1232Asn | 20 | - | | 3934733 | G | C | 212.77 | SNP | Rv3508 (PE\_PGRS54) | silent (Gly1243) | 9935 | - | | 3934734 | G | A | 203.77 | SNP | Rv3508 (PE\_PGRS54) | Ala1244Thr | 22 | - | | 3940802 | A | G | 155.90 | SNP | Rv3511 (PE\_PGRS55) | Asn396Asp | 42 | - | | 3942481 | C | G | 314.78 | SNP | intergenic |  |  | - | | 3942640 | T | C | 419.77 | SNP | intergenic |  |  | - | | 3947357 | G | T | 32.77 | SNP | Rv3514 (PE\_PGRS57) | Ala522Ser | 28 | - | | 3952800 | G | A | 1720.77 | SNP | Rv3516 (echA19) | Gly86Asp | 6 | - | | 3958403 | A | G | 2479.77 | SNP | Rv3521 | Asn295Asp | 42 | - | | 3959418 | C | T | 1986.77 | SNP | Rv3522 (ltp4) | Thr324Ile | 7 | - | | 3964463 | AG | A | 3644.73 | DEL | intergenic |  |  | - | | 3977226 | G | A | 2142.77 | SNP | Rv3538 | Leu(s)55Leu | 3 | genotype | | 3983271 | T | G | 2201.77 | SNP | Rv3544c (fadE28) | Ile292Leu | 22 | - | | 3998059 | AGGC | A | 2740.73 | DEL | Rv3558 (PPE64) |  |  | - | | 4005607 | T | C | 1644.77 | SNP | Rv3564 (fadE33) | Leu(s)121Leu | 3 | - | | 4024273 | T | C | 1358.77 | SNP | Rv3581c (ispF) | Val25Val(s) | 18 | - | | 4026899 | G | A | 1599.77 | SNP | Rv3585 (radA) | silent (Gln152) | 9876 | - | | 4032732 | ACAG | A | 230.87 | DEL | Rv3590c (PE\_PGRS58) |  |  | - | | 4032739 | C | G | 95.28 | SNP | Rv3590c (PE\_PGRS58) | silent (Gly140) | 9935 | - | | 4032740 | C | G | 126.03 | SNP | Rv3590c (PE\_PGRS58) | Gly140Ala | 21 | - | | 4034827 | C | T | 1639.77 | SNP | Rv3593 (lpqF) | Ala159Val(s) | 9867 | - | | 4037283 | T | G | 195.90 | SNP | Rv3595c (PE\_PGRS59) | silent (Gly256) | 9935 | - | | 4038287 | G | A | 1668.77 | SNP | Rv3596c (clpC1) | silent (Asn806) | 9822 | - | | 4042761 | G | A | 2115.77 | SNP | Rv3598c (lysS) | silent (Asp60) | 9859 | - | | 4043365 | G | T | 1249.77 | SNP | Rv3600c | silent (Ala165) | 9867 | - | | 4052970 | G | A | 1378.77 | SNP | Rv3611 | silent (Ala7) | 9867 | - | | 4053050 | A | G | 711.77 | SNP | Rv3611 | Asn34Ser | 34 | - | | 4053161 | A | G | 102.03 | SNP | Rv3611 | Asn71Ser | 34 | - | | 4055801 | G | A | 1909.77 | SNP | Rv3616c (espA) | Thr192Ile | 7 | - | | 4059904 | A | G | 1469.77 | SNP | intergenic |  |  | - | | 4069292 | G | A | 1305.77 | SNP | Rv3630 | Ala40Thr | 22 | - | | 4075957 | C | A | 1799.77 | SNP | Rv3636 | Ala69Asp | 6 | - | | 4088346 | G | A | 1616.77 | SNP | Rv3648c (cspA) | silent (Thr62) | 9871 | - | | 4093531 | G | A | 1599.77 | SNP | intergenic |  |  | - | | 4093879 | TG | T | 692.74 | DEL | Rv3652 (PE\_PGRS60) |  |  | - | | 4095001 | CG | C | 3182.78 | DEL | Rv3655c |  |  | - | | 4097996 | C | A | 824.77 | SNP | intergenic |  |  | - | | 4100975 | T | C | 1668.77 | SNP | intergenic |  |  | - | | 4109354 | A | C | 1941.77 | SNP | Rv3667 (acs) | silent (Leu521) | 9947 | - | | 4111303 | G | C | 2018.77 | SNP | Rv3669 | Val(s)159Val | 13 | - | | 4120926 | A | G | 339.77 | SNP | Rv3680 | Asn378Asp | 42 | - | | 4120983 | A | G | 1029.77 | SNP | intergenic |  |  | - | | 4135112 | G | A | 1531.77 | SNP | Rv3693 | Met(s)129Ile | 2 | - | | 4146330 | A | G | 1319.77 | SNP | Rv3703c | Leu(s)188Leu | 3 | - | | 4156099 | C | A | 2016.77 | SNP | Rv3711c (dnaQ) | Val(s)211Leu(s) | 9867 | - | | 4157176 | A | G | 1381.77 | SNP | Rv3712 | Thr66Ala | 32 | - | | 4157553 | C | T | 1828.77 | SNP | Rv3712 | silent (Asn191) | 9822 | - | | 4162339 | A | G | 2924.77 | SNP | Rv3719 | Thr12Ala | 32 | - | | 4163944 | A | G | 1730.77 | SNP | Rv3720 | His70Arg | 10 | - | | 4174564 | T | C | 1048.77 | SNP | Rv3727 | Ile537Thr | 11 | - | | 4182695 | G | A | 2074.77 | SNP | Rv3731 (ligC) | Arg313His | 8 | - | | 4187485 | T | C | 1107.77 | SNP | Rv3736 | silent (Ala284) | 9867 | - | | 4187817 | A | G | 980.77 | SNP | Rv3737 | Asp40Gly | 11 | - | | 4197138 | C | CT | 2233.73 | INS | intergenic |  |  | - | | 4198611 | CG | C | 2310.73 | DEL | intergenic |  |  | - | | 4204441 | A | G | 1422.77 | SNP | Rv3759c (proX) | silent (His311) | 9912 | - | | 4210274 | A | G | 1536.77 | SNP | Rv3764c (tcrY) | Cys246Arg | 1 | - | | 4214963 | G | A | 2062.77 | SNP | intergenic |  |  | - | | 4218350 | T | C | 1675.77 | SNP | Rv3773c | Lys159Arg | 19 | - | | 4220174 | G | A | 2155.77 | SNP | Rv3775 (lipE) | Asp164Asn | 36 | - | | 4221490 | C | G | 1740.77 | SNP | Rv3776 | silent (Leu134) | 9947 | - | | 4222073 | A | G | 637.77 | SNP | Rv3776 | Met(s)329Val(s) | 9867 | - | | 4222882 | A | G | 1708.77 | SNP | Rv3777 | silent (Leu63) | 9947 | - | | 4226700 | C | G | 2487.77 | SNP | Rv3779 | His572Gln | 23 | - | | 4230016 | G | A | 1613.77 | SNP | Rv3783 (rfbD) | Val(s)253Val | 13 | - | | 4233299 | G | A | 1770.77 | SNP | Rv3786c | Thr100Ile | 7 | - | | 4242643 | C | T | 1452.77 | SNP | Rv3793 (embC) | silent (Arg927) | 9913 | genotype | | 4247730 | G | C | 1836.77 | SNP | Rv3795 (embB) | Gly406Ala | 21 | resistance | | 4252546 | C | T | 1497.77 | SNP | Rv3797 (fadE35) | Arg488Trp | 2 | - | | 4255922 | A | G | 2155.77 | SNP | Rv3799c (accD4) | silent (His9) | 9912 | - | | 4257220 | A | G | 1427.77 | SNP | Rv3800c (pks13) | silent (Arg1309) | 9913 | - | | 4264218 | C | A | 948.77 | SNP | Rv3802c | Val50Phe | 0 | - | | 4264219 | T | G | 904.77 | SNP | Rv3802c | silent (Gly49) | 9935 | - | | 4264410 | C | G | 1338.77 | SNP | intergenic |  |  | - | | 4272750 | A | C | 2010.77 | SNP | Rv3809c (glf) | Val242Val(s) | 18 | - | | 4288405 | G | T | 2617.77 | SNP | Rv3823c (mmpL8) | Ala1042Glu | 10 | - | | 4293072 | G | A | 1961.77 | SNP | Rv3824c (papA1) | Leu35Phe | 6 | - | | 4302036 | T | C | 1392.77 | SNP | Rv3827c | Thr252Ala | 32 | - | | 4306155 | C | T | 2116.77 | SNP | Rv3831 | silent (Ser133) | 9840 | - | | 4307179 | G | A | 1736.77 | SNP | Rv3833 | Val105Ile | 33 | - | | 4311871 | G | A | 1514.77 | SNP | Rv3838c (pheA) | His267Tyr | 4 | - | | 4315384 | T | C | 1808.77 | SNP | Rv3842c (glpQ1) | Asp60Gly | 11 | - | | 4319652 | G | A | 1937.77 | SNP | intergenic |  |  | - | | 4320050 | A | G | 1329.77 | SNP | intergenic |  |  | - | | 4328019 | C | T | 1552.77 | SNP | Rv3855 (ethR) | silent (Arg157) | 9913 | - | | 4336090 | A | AT | 4490.73 | INS | intergenic |  |  | - | | 4338595 | GC | G | 3740.73 | DEL | intergenic |  |  | - | | 4338732 | G | A | 2041.77 | SNP | intergenic |  |  | - | | 4340330 | T | G | 1923.77 | SNP | Rv3864 (espE) | Leu(s)21Val(s) | 9867 | - | | 4345329 | C | T | 1693.77 | SNP | Rv3869 (eccB1) | silent (Asn97) | 9822 | - | | 4350400 | T | A | 1706.77 | SNP | Rv3871 (eccCb1) | Phe525Tyr | 21 | - | | 4351039 | G | T | 1925.77 | SNP | Rv3872 (PE35) | Glu99STOP | 17 | - | | 4356110 | G | C | 1827.77 | SNP | Rv3877 (eccD1) | silent (Leu368) | 9947 | - | | 4359195 | G | A | 548.77 | SNP | Rv3879c (espK) | silent (Gly196) | 9935 | - | | 4366272 | G | C | 2037.77 | SNP | Rv3884c (eccA2) | silent (Ala189) | 9867 | - | | 4372661 | T | C | 2197.77 | SNP | Rv3888c | Ile16Val | 57 | - | | 4372913 | G | A | 2218.77 | SNP | Rv3889c (espG2) | Arg240Cys | 1 | - | | 4373475 | C | G | 2451.77 | SNP | Rv3889c (espG2) | Val(s)52Val | 13 | - | | 4373496 | C | G | 2510.77 | SNP | Rv3889c (espG2) | Val(s)45Val | 13 | - | | 4375628 | G | T | 1773.77 | SNP | Rv3892c (PPE69) | Thr19Lys | 11 | - | | 4377447 | G | A | 1379.77 | SNP | Rv3894c (eccC2) | silent (Asp1002) | 9859 | - | | 4379680 | C | G | 1848.77 | SNP | Rv3894c (eccC2) | Arg258Pro | 5 | - | | 4381944 | C | T | 2091.77 | SNP | Rv3896c | silent (STOP303) | 9867 | - | | 4382054 | T | C | 1988.77 | SNP | Rv3896c | silent (Ala266) | 9867 | - | | 4382275 | G | T | 2170.77 | SNP | Rv3896c | Gln193Lys | 12 | - | | 4383094 | A | G | 1370.77 | SNP | Rv3897c | Cys183Arg | 1 | - | | 4383144 | C | CCGGGG | 4637.73 | INS | Rv3897c |  |  | - | | 4391553 | C | T | 1208.77 | SNP | Rv3906c | silent (Pro18) | 9926 | - | | 4395964 | C | A | 1261.77 | SNP | Rv3909 | silent (Thr591) | 9871 | - | | 4400660 | AC | A | 3142.73 | DEL | Rv3911 (sigM) |  |  | - | | 4403900 | A | G | 2113.77 | SNP | Rv3915 | Met(s)237Val(s) | 9867 | - | | 4407973 | A | G | 3143.77 | SNP | Rv3919c (gid) | Val(s)77Ala | 9867 | - | | 4408156 | A | C | 2234.77 | SNP | Rv3919c (gid) | Leu16Arg | 1 | genotype | |  | | export |

elog
