# Supplementary material for: Patterns of compensatory mutations in rpoA/B/C genes of multidrug resistant M. tuberculosis in Uganda
Source: PLoS One. 2025 Dec 4;20(12):e0328957. doi: 10.1371/journal.pone.0328957 (PMC12677784; doi:10.1371/journal.pone.0328957)
Supplement: S2 File — (ZIP) [file pone.0328957.s002.zip › Variants R_S18_L001_001.bam.html]

 

Calling SNPs/INDELs (computing variant list in .vcf format) from R\_S18\_L001\_001.bam

*by SAMtools/BCFtools:*

Howto

Important aspects

This takes up to one hour!!! **Please wait ...**

Variants R\_S18\_L001\_001.bam

|  |  |
| --- | --- |
| Variants |  |

|  |  |
| --- | --- |
| |  | | --- | | *by GATK* | |

|  |  |  |
| --- | --- | --- |
| |  | | --- | | R\_S18\_L001\_001.bam | | | computed 2016-10-27 using PhyResSE v1.0 (Ref. NC\_000962.3) | |

|  |  |
| --- | --- |
| 1024  variants called Export in VCF format |  |

|  |  |  |  |  |  |  |  |  |  |  |  |  |  |  |  |  |  |  |  |  |  |  |  |  |  |  |  |  |  |  |  |  |  |  |  |  |  |  |  |  |  |  |  |  |  |  |  |  |  |  |  |  |  |  |  |  |  |  |  |  |  |  |  |  |  |  |  |  |  |  |  |  |  |  |  |  |  |  |  |  |  |  |  |  |  |  |  |  |  |  |  |  |  |  |  |  |  |  |  |  |  |  |  |  |  |  |  |  |  |  |  |  |  |  |  |  |  |  |  |  |  |  |  |  |  |  |  |  |  |  |  |  |  |  |  |  |  |  |  |  |  |  |  |  |  |  |  |  |  |  |  |  |  |  |  |  |  |  |  |  |  |  |  |  |  |  |  |  |  |  |  |  |  |  |  |  |  |  |  |  |  |  |  |  |  |  |  |  |  |  |  |  |  |  |  |  |  |  |  |  |  |  |  |  |  |  |  |  |  |  |  |  |  |  |  |  |  |  |  |  |  |  |  |  |  |  |  |  |  |  |  |  |  |  |  |  |  |  |  |  |  |  |  |  |  |  |  |  |  |  |  |  |  |  |  |  |  |  |  |  |  |  |  |  |  |  |  |  |  |  |  |  |  |  |  |  |  |  |  |  |  |  |  |  |  |  |  |  |  |  |  |  |  |  |  |  |  |  |  |  |  |  |  |  |  |  |  |  |  |  |  |  |  |  |  |  |  |  |  |  |  |  |  |  |  |  |  |  |  |  |  |  |  |  |  |  |  |  |  |  |  |  |  |  |  |  |  |  |  |  |  |  |  |  |  |  |  |  |  |  |  |  |  |  |  |  |  |  |  |  |  |  |  |  |  |  |  |  |  |  |  |  |  |  |  |  |  |  |  |  |  |  |  |  |  |  |  |  |  |  |  |  |  |  |  |  |  |  |  |  |  |  |  |  |  |  |  |  |  |  |  |  |  |  |  |  |  |  |  |  |  |  |  |  |  |  |  |  |  |  |  |  |  |  |  |  |  |  |  |  |  |  |  |  |  |  |  |  |  |  |  |  |  |  |  |  |  |  |  |  |  |  |  |  |  |  |  |  |  |  |  |  |  |  |  |  |  |  |  |  |  |  |  |  |  |  |  |  |  |  |  |  |  |  |  |  |  |  |  |  |  |  |  |  |  |  |  |  |  |  |  |  |  |  |  |  |  |  |  |  |  |  |  |  |  |  |  |  |  |  |  |  |  |  |  |  |  |  |  |  |  |  |  |  |  |  |  |  |  |  |  |  |  |  |  |  |  |  |  |  |  |  |  |  |  |  |  |  |  |  |  |  |  |  |  |  |  |  |  |  |  |  |  |  |  |  |  |  |  |  |  |  |  |  |  |  |  |  |  |  |  |  |  |  |  |  |  |  |  |  |  |  |  |  |  |  |  |  |  |  |  |  |  |  |  |  |  |  |  |  |  |  |  |  |  |  |  |  |  |  |  |  |  |  |  |  |  |  |  |  |  |  |  |  |  |  |  |  |  |  |  |  |  |  |  |  |  |  |  |  |  |  |  |  |  |  |  |  |  |  |  |  |  |  |  |  |  |  |  |  |  |  |  |  |  |  |  |  |  |  |  |  |  |  |  |  |  |  |  |  |  |  |  |  |  |  |  |  |  |  |  |  |  |  |  |  |  |  |  |  |  |  |  |  |  |  |  |  |  |  |  |  |  |  |  |  |  |  |  |  |  |  |  |  |  |  |  |  |  |  |  |  |  |  |  |  |  |  |  |  |  |  |  |  |  |  |  |  |  |  |  |  |  |  |  |  |  |  |  |  |  |  |  |  |  |  |  |  |  |  |  |  |  |  |  |  |  |  |  |  |  |  |  |  |  |  |  |  |  |  |  |  |  |  |  |  |  |  |  |  |  |  |  |  |  |  |  |  |  |  |  |  |  |  |  |  |  |  |  |  |  |  |  |  |  |  |  |  |  |  |  |  |  |  |  |  |  |  |  |  |  |  |  |  |  |  |  |  |  |  |  |  |  |  |  |  |  |  |  |  |  |  |  |  |  |  |  |  |  |  |  |  |  |  |  |  |  |  |  |  |  |  |  |  |  |  |  |  |  |  |  |  |  |  |  |  |  |  |  |  |  |  |  |  |  |  |  |  |  |  |  |  |  |  |  |  |  |  |  |  |  |  |  |  |  |  |  |  |  |  |  |  |  |  |  |  |  |  |  |  |  |  |  |  |  |  |  |  |  |  |  |  |  |  |  |  |  |  |  |  |  |  |  |  |  |  |  |  |  |  |  |  |  |  |  |  |  |  |  |  |  |  |  |  |  |  |  |  |  |  |  |  |  |  |  |  |  |  |  |  |  |  |  |  |  |  |  |  |  |  |  |  |  |  |  |  |  |  |  |  |  |  |  |  |  |  |  |  |  |  |  |  |  |  |  |  |  |  |  |  |  |  |  |  |  |  |  |  |  |  |  |  |  |  |  |  |  |  |  |  |  |  |  |  |  |  |  |  |  |  |  |  |  |  |  |  |  |  |  |  |  |  |  |  |  |  |  |  |  |  |  |  |  |  |  |  |  |  |  |  |  |  |  |  |  |  |  |  |  |  |  |  |  |  |  |  |  |  |  |  |  |  |  |  |  |  |  |  |  |  |  |  |  |  |  |  |  |  |  |  |  |  |  |  |  |  |  |  |  |  |  |  |  |  |  |  |  |  |  |  |  |  |  |  |  |  |  |  |  |  |  |  |  |  |  |  |  |  |  |  |  |  |  |  |  |  |  |  |  |  |  |  |  |  |  |  |  |  |  |  |  |  |  |  |  |  |  |  |  |  |  |  |  |  |  |  |  |  |  |  |  |  |  |  |  |  |  |  |  |  |  |  |  |  |  |  |  |  |  |  |  |  |  |  |  |  |  |  |  |  |  |  |  |  |  |  |  |  |  |  |  |  |  |  |  |  |  |  |  |  |  |  |  |  |  |  |  |  |  |  |  |  |  |  |  |  |  |  |  |  |  |  |  |  |  |  |  |  |  |  |  |  |  |  |  |  |  |  |  |  |  |  |  |  |  |  |  |  |  |  |  |  |  |  |  |  |  |  |  |  |  |  |  |  |  |  |  |  |  |  |  |  |  |  |  |  |  |  |  |  |  |  |  |  |  |  |  |  |  |  |  |  |  |  |  |  |  |  |  |  |  |  |  |  |  |  |  |  |  |  |  |  |  |  |  |  |  |  |  |  |  |  |  |  |  |  |  |  |  |  |  |  |  |  |  |  |  |  |  |  |  |  |  |  |  |  |  |  |  |  |  |  |  |  |  |  |  |  |  |  |  |  |  |  |  |  |  |  |  |  |  |  |  |  |  |  |  |  |  |  |  |  |  |  |  |  |  |  |  |  |  |  |  |  |  |  |  |  |  |  |  |  |  |  |  |  |  |  |  |  |  |  |  |  |  |  |  |  |  |  |  |  |  |  |  |  |  |  |  |  |  |  |  |  |  |  |  |  |  |  |  |  |  |  |  |  |  |  |  |  |  |  |  |  |  |  |  |  |  |  |  |  |  |  |  |  |  |  |  |  |  |  |  |  |  |  |  |  |  |  |  |  |  |  |  |  |  |  |  |  |  |  |  |  |  |  |  |  |  |  |  |  |  |  |  |  |  |  |  |  |  |  |  |  |  |  |  |  |  |  |  |  |  |  |  |  |  |  |  |  |  |  |  |  |  |  |  |  |  |  |  |  |  |  |  |  |  |  |  |  |  |  |  |  |  |  |  |  |  |  |  |  |  |  |  |  |  |  |  |  |  |  |  |  |  |  |  |  |  |  |  |  |  |  |  |  |  |  |  |  |  |  |  |  |  |  |  |  |  |  |  |  |  |  |  |  |  |  |  |  |  |  |  |  |  |  |  |  |  |  |  |  |  |  |  |  |  |  |  |  |  |  |  |  |  |  |  |  |  |  |  |  |  |  |  |  |  |  |  |  |  |  |  |  |  |  |  |  |  |  |  |  |  |  |  |  |  |  |  |  |  |  |  |  |  |  |  |  |  |  |  |  |  |  |  |  |  |  |  |  |  |  |  |  |  |  |  |  |  |  |  |  |  |  |  |  |  |  |  |  |  |  |  |  |  |  |  |  |  |  |  |  |  |  |  |  |  |  |  |  |  |  |  |  |  |  |  |  |  |  |  |  |  |  |  |  |  |  |  |  |  |  |  |  |  |  |  |  |  |  |  |  |  |  |  |  |  |  |  |  |  |  |  |  |  |  |  |  |  |  |  |  |  |  |  |  |  |  |  |  |  |  |  |  |  |  |  |  |  |  |  |  |  |  |  |  |  |  |  |  |  |  |  |  |  |  |  |  |  |  |  |  |  |  |  |  |  |  |  |  |  |  |  |  |  |  |  |  |  |  |  |  |  |  |  |  |  |  |  |  |  |  |  |  |  |  |  |  |  |  |  |  |  |  |  |  |  |  |  |  |  |  |  |  |  |  |  |  |  |  |  |  |  |  |  |  |  |  |  |  |  |  |  |  |  |  |  |  |  |  |  |  |  |  |  |  |  |  |  |  |  |  |  |  |  |  |  |  |  |  |  |  |  |  |  |  |  |  |  |  |  |  |  |  |  |  |  |  |  |  |  |  |  |  |  |  |  |  |  |  |  |  |  |  |  |  |  |  |  |  |  |  |  |  |  |  |  |  |  |  |  |  |  |  |  |  |  |  |  |  |  |  |  |  |  |  |  |  |  |  |  |  |  |  |  |  |  |  |  |  |  |  |  |  |  |  |  |  |  |  |  |  |  |  |  |  |  |  |  |  |  |  |  |  |  |  |  |  |  |  |  |  |  |  |  |  |  |  |  |  |  |  |  |  |  |  |  |  |  |  |  |  |  |  |  |  |  |  |  |  |  |  |  |  |  |  |  |  |  |  |  |  |  |  |  |  |  |  |  |  |  |  |  |  |  |  |  |  |  |  |  |  |  |  |  |  |  |  |  |  |  |  |  |  |  |  |  |  |  |  |  |  |  |  |  |  |  |  |  |  |  |  |  |  |  |  |  |  |  |  |  |  |  |  |  |  |  |  |  |  |  |  |  |  |  |  |  |  |  |  |  |  |  |  |  |  |  |  |  |  |  |  |  |  |  |  |  |  |  |  |  |  |  |  |  |  |  |  |  |  |  |  |  |  |  |  |  |  |  |  |  |  |  |  |  |  |  |  |  |  |  |  |  |  |  |  |  |  |  |  |  |  |  |  |  |  |  |  |  |  |  |  |  |  |  |  |  |  |  |  |  |  |  |  |  |  |  |  |  |  |  |  |  |  |  |  |  |  |  |  |  |  |  |  |  |  |  |  |  |  |  |  |  |  |  |  |  |  |  |  |  |  |  |  |  |  |  |  |  |  |  |  |  |  |  |  |  |  |  |  |  |  |  |  |  |  |  |  |  |  |  |  |  |  |  |  |  |  |  |  |  |  |  |  |  |  |  |  |  |  |  |  |  |  |  |  |  |  |  |  |  |  |  |  |  |  |  |  |  |  |  |  |  |  |  |  |  |  |  |  |  |  |  |  |  |  |  |  |  |  |  |  |  |  |  |  |  |  |  |  |  |  |  |  |  |  |  |  |  |  |  |  |  |  |  |  |  |  |  |  |  |  |  |  |  |  |  |  |  |  |  |  |  |  |  |  |  |  |  |  |  |  |  |  |  |  |  |  |  |  |  |  |  |  |  |  |  |  |  |  |  |  |  |  |  |  |  |  |  |  |  |  |  |  |  |  |  |  |  |  |  |  |  |  |  |  |  |  |  |  |  |  |  |  |  |  |  |  |  |  |  |  |  |  |  |  |  |  |  |  |  |  |  |  |  |  |  |  |  |  |  |  |  |  |  |  |  |  |  |  |  |  |  |  |  |  |  |  |  |  |  |  |  |  |  |  |  |  |  |  |  |  |  |  |  |  |  |  |  |  |  |  |  |  |  |  |  |  |  |  |  |  |  |  |  |  |  |  |  |  |  |  |  |  |  |  |  |  |  |  |  |  |  |  |  |  |  |  |  |  |  |  |  |  |  |  |  |  |  |  |  |  |  |  |  |  |  |  |  |  |  |  |  |  |  |  |  |  |  |  |  |  |  |  |  |  |  |  |  |  |  |  |  |  |  |  |  |  |  |  |  |  |  |  |  |  |  |  |  |  |  |  |  |  |  |  |  |  |  |  |  |  |  |  |  |  |  |  |  |  |  |  |  |  |  |  |  |  |  |  |  |  |  |  |  |  |  |  |  |  |  |  |  |  |  |  |  |  |  |  |  |  |  |  |  |  |  |  |  |  |  |  |  |  |  |  |  |  |  |  |  |  |  |  |  |  |  |  |  |  |  |  |  |  |  |  |  |  |  |  |  |  |  |  |  |  |  |  |  |  |  |  |  |  |  |  |  |  |  |  |  |  |  |  |  |  |  |  |  |  |  |  |  |  |  |  |  |  |  |  |  |  |  |  |  |  |  |  |  |  |  |  |  |  |  |  |  |  |  |  |  |  |  |  |  |  |  |  |  |  |  |  |  |  |  |  |  |  |  |  |  |  |  |  |  |  |  |  |  |  |  |  |  |  |  |  |  |  |  |  |  |  |  |  |  |  |  |  |  |  |  |  |  |  |  |  |  |  |  |  |  |  |  |  |  |  |  |  |  |  |  |  |  |  |  |  |  |  |  |  |  |  |  |  |  |  |  |  |  |  |  |  |  |  |  |  |  |  |  |  |  |  |  |  |  |  |  |  |  |  |  |  |  |  |  |  |  |  |  |  |  |  |  |  |  |  |  |  |  |  |  |  |  |  |  |  |  |  |  |  |  |  |  |  |  |  |  |  |  |  |  |  |  |  |  |  |  |  |  |  |  |  |  |  |  |  |  |  |  |  |  |  |  |  |  |  |  |  |  |  |  |  |  |  |  |  |  |  |  |  |  |  |  |  |  |  |  |  |  |  |  |  |  |  |  |  |  |  |  |  |  |  |  |  |  |  |  |  |  |  |  |  |  |  |  |  |  |  |  |  |  |  |  |  |  |  |  |  |  |  |  |  |  |  |  |  |  |  |  |  |  |  |  |  |  |  |  |  |  |  |  |  |  |  |  |  |  |  |  |  |  |  |  |  |  |  |  |  |  |  |  |  |  |  |  |  |  |  |  |  |  |  |  |  |  |  |  |  |  |  |  |  |  |  |  |  |  |  |  |  |  |  |  |  |  |  |  |  |  |  |  |  |  |  |  |  |  |  |  |  |  |  |  |  |  |  |  |  |  |  |  |  |  |  |  |  |  |  |  |  |  |  |  |  |  |  |  |  |  |  |  |  |  |  |  |  |  |  |  |  |  |  |  |  |  |  |  |  |  |  |  |  |  |  |  |  |  |  |  |  |  |  |  |  |  |  |  |  |  |  |  |  |  |  |  |  |  |  |  |  |  |  |  |  |  |  |  |  |  |  |  |  |  |  |  |  |  |  |  |  |  |  |  |  |  |  |  |  |  |  |  |  |  |  |  |  |  |  |  |  |  |  |  |  |  |  |  |  |  |  |  |  |  |  |  |  |  |  |  |  |  |  |  |  |  |  |  |  |  |  |  |  |  |  |  |  |  |  |  |  |  |  |  |  |  |  |  |  |  |  |  |  |  |  |  |  |  |  |  |  |  |  |  |  |  |  |  |  |  |  |  |  |  |  |  |  |  |  |  |  |  |  |  |  |  |  |  |  |  |  |  |  |  |  |  |  |  |  |  |  |  |  |  |  |  |  |  |  |  |  |  |  |  |  |  |  |  |  |  |  |  |  |  |  |  |  |  |  |  |  |  |  |  |  |  |  |  |  |  |  |  |  |  |  |  |  |  |  |  |  |  |  |  |  |  |  |  |  |  |  |  |  |  |  |  |  |  |  |  |  |  |  |  |  |  |  |  |  |  |  |  |  |  |  |  |  |  |  |  |  |  |  |  |  |  |  |  |  |  |  |  |  |  |  |  |  |  |  |  |  |  |  |  |  |  |  |  |  |  |  |  |  |  |  |  |  |  |  |  |  |  |  |  |  |  |  |  |  |  |  |  |  |  |  |  |  |  |  |  |  |  |  |  |  |  |  |  |  |  |  |  |  |  |  |  |  |  |  |  |  |  |  |  |  |  |  |  |  |  |  |  |  |  |  |  |  |  |  |  |  |  |  |  |  |  |  |  |  |  |  |  |  |  |  |  |  |  |  |  |  |  |  |  |  |  |  |  |  |  |  |  |  |  |  |  |  |  |  |  |  |  |  |  |  |  |  |  |  |  |  |  |  |  |  |  |  |  |  |  |  |  |  |  |  |  |  |  |  |  |  |  |  |  |  |  |  |  |  |  |  |  |  |  |  |  |  |  |  |  |  |  |  |  |  |  |  |  |  |  |  |  |  |  |  |  |  |  |  |  |  |  |  |  |  |  |  |  |  |  |  |  |  |  |  |  |  |  |  |  |  |  |  |  |  |  |  |  |  |  |  |  |  |  |  |  |  |  |  |  |  |  |  |  |  |  |  |  |  |  |  |  |  |  |  |  |  |  |  |  |  |  |  |  |  |  |  |  |  |  |  |  |  |  |  |  |  |  |  |  |  |  |  |  |  |  |  |  |  |  |  |  |  |  |  |  |  |  |  |  |  |  |  |  |  |  |  |  |  |  |  |  |  |  |  |  |  |  |  |  |  |  |  |  |  |  |  |  |  |  |  |  |  |  |  |  |  |  |  |  |  |  |  |  |  |  |  |  |  |  |  |  |  |  |  |  |  |  |  |  |  |  |  |  |  |  |  |  |  |  |  |  |  |  |  |  |  |  |  |  |  |  |  |  |  |  |  |  |  |  |  |  |  |  |  |  |  |  |  |  |  |  |  |  |  |  |  |  |  |  |  |  |  |  |  |  |  |  |  |  |  |  |  |  |  |  |  |  |  |  |  |  |  |  |  |  |  |  |  |  |  |  |  |  |  |  |  |  |  |  |  |  |  |  |  |  |  |  |  |  |  |  |  |  |  |  |  |  |  |  |  |  |  |  |  |  |  |  |  |  |  |  |  |  |  |  |  |  |  |  |  |  |  |  |  |  |  |  |  |  |  |  |  |  |  |  |  |  |  |  |  |  |  |  |  |  |  |  |  |  |  |  |  |  |  |  |  |  |  |  |  |  |  |  |  |  |  |  |  |  |  |  |  |  |  |  |  |  |  |  |  |  |  |  |  |  |  |  |  |  |  |  |  |  |  |  |  |  |  |  |  |  |  |  |  |  |  |  |  |  |  |  |  |  |  |  |  |  |  |  |  |  |  |  |  |  |  |  |  |  |  |  |  |  |  |  |  |  |  |  |  |  |  |  |  |  |  |  |  |  |  |  |  |  |  |  |  |  |  |  |  |  |  |  |  |  |  |  |  |  |  |  |  |  |  |  |  |  |  |  |  |  |  |  |  |  |  |  |  |  |  |  |  |  |  |  |  |  |  |  |  |  |  |  |  |  |  |  |  |  |  |  |  |  |  |  |  |  |  |  |  |  |  |  |  |  |  |  |  |  |  |  |  |  |  |  |  |  |  |  |  |  |  |  |  |  |  |  |  |  |  |  |  |  |  |  |  |  |  |  |  |  |  |  |  |  |  |  |  |  |  |  |  |  |  |  |  |  |  |  |  |  |  |  |  |  |  |  |  |  |  |  |  |  |  |  |  |  |  |  |  |  |  |  |  |  |  |  |  |  |  |  |  |  |  |  |  |  |  |  |  |  |  |  |  |  |  |  |  |  |  |  |  |  |  |  |  |  |  |  |  |  |  |  |  |  |  |  |  |  |  |  |  |  |  |  |  |  |  |  |  |  |  |  |  |  |  |  |  |  |  |  |  |  |  |  |  |  |  |  |  |  |  |  |  |  |  |  |  |  |  |  |  |  |  |  |  |  |  |  |  |  |  |  |  |  |  |  |  |  |  |  |  |  |  |  |  |  |  |  |  |  |  |  |  |  |  |  |  |  |  |  |  |  |  |  |  |  |  |  |  |  |  |  |  |  |  |  |  |  |  |  |  |  |  |  |  |  |  |  |  |  |  |  |  |  |  |  |  |  |  |  |  |  |  |  |  |  |  |  |  |  |  |  |  |  |  |  |  |  |  |  |  |  |  |  |  |  |  |  |  |  |  |  |  |  |  |  |  |  |  |  |  |  |  |  |  |  |  |  |  |  |  |  |  |  |  |  |  |  |  |  |  |  |  |  |  |  |  |  |  |  |  |  |  |  |  |  |  |  |  |  |  |  |  |  |  |  |  |  |  |  |  |  |  |  |  |  |  |  |  |  |  |  |  |  |  |  |  |  |  |  |  |  |  |  |  |  |  |  |  |  |  |  |  |  |  |  |  |  |  |  |  |  |  |  |  |  |  |  |  |  |  |  |  |  |  |  |  |  |  |  |  |  |  |  |  |  |  |  |  |  |  |  |  |  |  |  |  |  |  |  |  |  |  |  |  |  |  |  |  |  |  |  |  |  |  |  |  |  |  |  |  |  |  |  |  |  |  |  |  |  |  |  |  |  |  |  |  |  |  |  |  |  |  |  |  |  |  |  |  |  |  |  |  |  |  |  |  |  |  |  |  |  |  |  |  |  |  |  |  |  |  |  |  |  |  |  |  |  |  |  |  |  |  |  |  |  |  |  |  |  |  |  |  |  |  |  |  |  |  |  |  |  |  |  |  |  |  |  |  |  |  |  |  |  |  |  |  |  |  |  |  |  |  |  |  |  |  |  |  |  |  |  |  |  |  |  |  |  |  |  |  |  |  |  |  |  |  |  |  |  |  |  |  |  |  |  |  |  |  |  |  |  |  |  |  |  |  |  |  |  |  |  |  |  |  |  |  |  |  |  |  |  |  |  |  |  |  |  |  |  |  |  |  |  |  |  |  |  |  |  |  |  |  |  |  |  |  |  |  |  |  |  |  |  |  |  |  |  |  |  |  |  |  |  |  |  |  |  |  |  |  |  |  |  |  |  |  |  |  |  |  |  |  |  |  |  |  |  |  |  |  |  |  |  |  |  |  |  |  |  |  |  |  |  |  |  |  |  |  |  |  |  |  |  |  |  |  |  |  |  |  |  |  |  |  |  |  |  |  |  |  |  |  |  |  |  |  |  |  |  |  |  |  |  |  |  |  |  |  |  |  |  |  |  |  |  |  |  |  |  |  |  |  |  |  |  |  |  |  |  |  |  |  |  |  |  |  |  |  |  |  |  |  |  |  |  |  |  |  |  |  |  |  |  |  |  |  |  |  |  |  |  |  |  |  |  |  |  |  |  |  |  |  |  |  |  |  |  |  |  |  |  |  |  |  |  |  |  |  |  |  |  |  |  |  |  |  |  |  |  |  |  |  |  |  |  |  |  |  |  |  |  |  |  |  |  |  |  |  |  |  |  |  |  |  |  |  |  |  |  |  |  |  |  |  |  |  |  |  |  |  |  |  |  |  |  |  |  |  |  |  |  |  |  |  |  |  |  |  |  |  |  |  |  |  |  |  |  |  |  |  |  |  |  |  |  |  |  |  |  |  |  |  |  |  |  |  |  |  |  |  |  |  |  |  |  |  |  |  |  |  |  |  |  |  |  |  |  |  |  |  |  |  |  |  |  |  |  |  |  |  |  |  |  |  |  |  |  |  |  |  |  |  |  |  |  |  |  |  |  |  |  |  |  |  |  |  |  |  |  |  |  |  |  |  |  |  |  |  |  |  |  |  |  |  |  |  |  |  |  |  |  |  |  |  |  |  |  |  |  |  |  |  |  |  |  |  |  |  |  |  |  |  |  |  |  |  |  |  |  |  |  |  |  |  |  |  |  |  |  |  |  |  |  |  |  |  |  |  |  |  |  |  |  |  |  |  |  |  |  |  |  |  |  |  |  |  |  |  |  |  |  |  |  |  |  |  |  |  |  |  |  |  |  |  |  |  |  |  |  |  |  |  |  |  |  |  |  |  |  |  |  |  |  |  |  |  |  |  |  |  |  |  |  |  |  |  |  |  |  |  |  |  |  |  |  |  |  |  |  |  |  |  |  |  |  |  |  |  |  |  |  |  |  |  |  |  |  |  |  |  |  |  |  |  |  |  |  |  |  |  |  |  |  |  |  |  |  |  |  |  |  |  |  |  |  |  |  |  |  |  |  |  |  |  |  |  |  |  |  |  |  |  |  |  |  |  |  |  |  |  |  |  |  |  |  |  |  |  |  |  |  |  |  |  |  |  |  |  |  |  |  |  |  |  |  |  |  |  |  |  |  |  |  |  |  |  |  |  |  |  |  |  |  |  |  |  |  |  |  |  |  |  |  |  |  |  |  |  |  |  |  |  |  |  |  |  |  |  |  |  |  |  |  |  |  |  |  |  |  |  |  |  |  |  |  |  |  |  |  |  |  |  |  |  |  |  |  |  |  |  |  |  |  |  |  |  |  |  |  |  |  |  |  |  |  |  |  |  |  |  |  |  |  |  |  |  |  |  |  |  |  |  |  |  |  |  |  |  |  |  |  |  |  |  |  |  |  |  |  |  |  |  |  |  |  |  |  |  |  |  |  |  |  |  |  |  |  |  |  |  |  |  |  |  |  |  |  |  |  |  |  |  |  |  |  |  |  |  |  |  |  |  |  |  |  |  |  |  |  |  |  |  |  |  |  |  |  |  |  |  |  |  |  |  |  |  |  |  |  |  |  |  |  |  |  |  |  |  |  |  |  |  |  |  |  |  |  |  |  |  |  |  |  |  |  |  |  |  |  |  |  |  |  |  |  |  |  |  |  |  |  |  |  |  |  |  |  |  |  |  |  |  |  |  |  |  |  |  |  |  |  |  |  |  |  |  |  |  |  |  |  |  |  |  |  |  |  |  |  |  |  |  |  |  |  |  |  |  |  |  |  |  |  |  |  |  |  |  |  |  |  |  |  |  |  |  |  |  |  |  |  |  |  |  |  |  |  |  |  |  |  |  |  |  |  |  |  |  |  |  |  |  |  |  |  |  |  |  |  |  |  |  |  |  |  |  |  |  |  |  |  |  |  |  |  |  |  |  |  |  |  |  |  |  |  |  |  |  |  |  |  |  |  |  |  |  |  |  |  |  |  |  |  |  |  |  |  |  |  |  |  |  |  |  |  |  |  |  |  |  |  |  |  |  |  |  |  |  |  |  |  |  |  |  |  |  |  |  |  |  |  |  |  |  |  |  |  |  |  |  |  |  |  |  |  |  |  |  |  |  |  |  |  |  |  |  |  |  |  |  |  |  |  |  |  |  |  |  |  |  |  |  |  |  |  |  |  |  |  |  |  |  |  |  |  |  |  |  |  |  |  |  |  |  |  |  |  |  |  |  |  |  |  |  |  |  |  |  |  |  |  |  |  |  |  |  |  |  |  |  |  |  |  |  |  |  |  |  |  |  |  |  |  |  |  |  |  |  |  |  |  |  |  |  |  |  |  |  |  |  |  |  |  |  |  |  |  |  |  |  |  |  |  |  |  |  |  |  |  |  |  |  |  |  |  |  |  |  |  |  |  |  |  |  |  |  |  |  |  |  |  |  |  |  |  |  |  |  |  |  |  |  |  |  |  |  |  |  |  |  |  |  |  |  |  |  |  |  |  |  |  |  |  |  |  |  |  |  |  |  |  |  |  |  |  |  |  |  |  |  |  |  |  |  |  |  |  |  |  |  |  |  |  |  |  |  |  |  |  |  |  |  |  |  |  |  |  |  |  |  |  |  |  |  |  |  |  |  |  |  |  |  |  |  |  |  |  |  |  |  |  |  |  |  |  |  |  |  |  |  |  |  |  |  |  |  |  |  |  |  |  |  |  |  |  |  |  |  |  |  |  |  |  |  |  |  |  |  |  |  |  |  |  |  |  |  |  |  |  |  |  |  |  |  |  |  |  |  |  |  |  |  |  |  |  |  |  |  |  |  |  |  |  |  |  |  |  |  |  |  |  |  |  |  |  |  |  |  |  |  |  |  |  |  |  |  |  |  |  |  |  |  |  |  |  |  |  |  |  |  |  |  |  |  |  |  |  |  |  |  |  |  |  |  |  |  |  |  |  |  |  |  |  |  |  |  |  |  |  |  |  |  |  |  |  |  |  |  |  |  |  |  |  |  |  |  |  |  |  |  |  |  |  |  |  |  |  |  |  |  |  |  |  |  |  |  |  |  |  |  |  |  |  |  |  |  |  |  |  |  |  |  |  |  |  |  |  |  |  |  |  |  |  |  |  |  |  |  |  |  |  |  |  |  |  |  |  |  |  |  |  |  |  |  |  |  |  |  |  |  |  |  |  |  |  |  |  |  |  |  |  |  |  |  |  |  |  |  |  |  |  |  |  |  |  |  |  |  |  |  |  |  |  |  |  |  |  |  |  |  |  |  |  |  |  |  |  |  |  |  |  |  |  |  |  |  |  |  |  |  |  |  |  |  |  |  |  |  |  |  |  |  |  |  |  |  |  |  |  |  |  |  |  |  |  |  |  |  |  |  |  |  |  |  |  |  |  |  |  |  |  |  |  |  |  |  |  |  |  |  |  |  |  |  |  |  |  |  |  |  |  |  |  |  |  |  |  |  |  |  |  |  |  |  |  |  |  |  |  |  |  |  |  |  |  |  |  |  |  |  |  |  |  |  |  |  |  |  |  |  |  |  |  |  |  |  |  |  |  |  |  |  |  |  |  |  |  |  |  |  |  |  |  |  |  |  |  |  |  |  |  |  |  |  |  |  |  |  |  |  |  |  |  |  |  |  |  |  |  |  |  |  |  |  |  |  |  |  |  |  |  |  |  |  |  |  |  |  |  |  |  |  |  |  |  |  |  |  |  |  |  |  |  |  |  |  |  |  |  |  |  |  |  |  |  |  |  |  |  |  |  |  |  |  |  |  |  |  |  |  |  |  |  |  |  |  |  |  |  |  |  |  |  |  |  |  |  |  |  |  |  |  |  |  |  |  |  |  |  |  |  |  |  |  |  |  |  |  |  |  |  |  |  |  |  |  |  |  |  |  |  |  |  |  |  |  |  |  |  |  |  |  |  |  |  |  |  |  |  |  |  |  |  |  |  |  |  |  |  |  |  |  |  |  |  |  |  |  |  |  |  |  |  |  |  |  |  |  |  |  |  |  |  |  |  |  |  |  |  |  |  |  |  |  |  |  |  |  |  |  |  |  |  |  |  |  |  |  |  |  |  |  |  |  |  |  |  |  |  |  |  |  |  |  |  |  |  |  |  |  |  |  |  |  |  |  |  |  |  |  |  |  |  |  |  |  |  |  |  |  |  |  |  |  |  |  |  |  |  |  |  |  |  |  |  |  |  |  |  |  |  |  |  |  |  |  |  |  |  |  |  |  |  |  |  |  |  |  |  |  |  |  |  |  |  |  |  |  |  |  |  |  |  |  |  |  |  |  |  |  |  |  |  |  |  |  |  |  |  |  |  |  |  |  |  |  |  |  |  |  |  |  |  |  |  |  |  |  |  |  |  |  |  |  |  |  |  |  |  |  |  |  |  |  |  |  |  |  |  |  |  |  |  |  |  |  |  |  |  |  |  |  |  |  |  |  |  |  |  |  |  |  |  |  |  |  |  |  |  |  |  |  |  |  |  |  |  |  |  |  |  |  |  |  |  |  |  |  |  |  |  |  |  |  |  |  |  |  |  |  |  |  |  |  |  |  |  |  |  |  |  |  |  |  |  |  |  |  |  |  |  |  |  |  |  |  |  |  |  |  |  |  |  |  |  |  |  |  |  |  |  |  |  |  |  |  |  |  |  |  |  |  |  |  |  |  |  |  |  |  |  |  |  |  |  |  |  |  |  |  |  |  |  |  |  |  |  |  |  |  |  |  |  |  |  |  |  |  |  |  |  |  |  |  |  |  |  |  |  |  |  |  |  |  |  |  |  |  |  |  |  |  |  |  |  |  |  |  |  |  |  |  |  |  |  |  |  |  |  |  |  |  |  |  |  |  |  |  |  |  |  |  |  |  |  |  |  |  |  |  |  |  |  |  |  |  |  |  |  |  |  |  |  |  |  |  |  |  |  |  |  |  |  |  |  |  |  |  |  |  |  |  |  |  |  |  |  |  |  |  |  |  |  |  |  |  |  |  |  |  |  |  |  |  |  |  |  |  |  |  |  |  |  |  |  |  |  |  |  |  |  |  |  |  |  |  |  |  |  |  |  |  |  |  |  |  |  |  |  |  |  |  |  |  |  |  |  |  |  |  |  |  |  |  |  |  |  |  |  |  |  |  |  |  |  |  |  |  |  |  |  |  |  |  |  |  |  |  |  |  |  |  |  |  |  |  |  |  |  |  |  |  |  |  |  |  |  |  |  |  |  |  |  |  |  |  |  |  |  |  |  |  |  |  |  |  |  |  |  |  |  |  |  |  |  |  |  |  |  |  |  |  |  |  |  |  |  |  |  |  |  |  |  |  |  |  |  |  |  |  |  |  |  |  |  |  |  |  |  |  |  |  |  |  |  |  |  |  |  |  |  |  |  |  |  |  |  |  |  |  |  |  |  |  |  |  |  |  |  |  |  |  |  |  |  |  |  |  |  |  |  |  |  |  |  |  |  |  |  |  |  |  |  |  |  |  |  |  |  |  |  |  |  |  |  |  |  |  |  |  |  |  |  |  |  |  |  |  |  |  |  |  |  |  |  |  |  |  |  |  |  |  |  |  |  |  |  |  |  |  |  |  |  |  |  |  |  |  |  |  |  |  |  |  |  |  |  |  |  |  |  |  |  |  |  |  |  |  |  |  |  |  |  |  |  |  |  |  |  |  |  |  |  |  |  |  |  |  |  |  |  |  |  |  |  |  |  |  |  |  |  |  |  |  |  |  |  |  |  |  |  |  |  |  |  |  |  |  |  |  |  |  |  |  |  |  |  |  |  |  |  |  |  |  |  |  |  |  |  |  |  |  |  |  |  |  |  |  |  |  |  |  |  |  |  |  |  |  |  |  |  |  |  |  |  |  |  |  |  |  |  |  |  |  |  |  |  |  |  |  |  |  |  |  |  |  |  |  |  |  |  |  |  |  |  |  |  |  |  |  |  |  |  |  |  |  |  |  |  |  |  |  |  |  |  |  |  |  |  |  |  |  |  |  |  |  |  |  |  |  |  |  |  |  |  |  |  |  |  |  |  |  |  |  |  |  |  |  |  |  |  |  |  |  |  |  |  |  |  |  |  |  |  |  |  |  |  |  |  |  |  |  |  |  |  |  |  |  |  |  |  |  |  |  |  |  |  |  |  |  |  |  |  |  |  |  |  |  |  |  |  |  |  |  |  |  |  |  |  |  |  |  |  |  |  |  |  |  |  |  |  |  |  |  |  |  |  |  |  |  |  |  |  |  |  |  |  |  |  |  |  |  |  |  |  |  |  |  |  |  |  |  |  |  |  |  |  |  |  |  |  |  |  |  |  |  |  |  |  |  |  |  |  |  |  |  |  |  |  |  |  |  |  |  |  |  |  |  |  |  |  |  |  |  |  |  |  |  |  |  |  |  |  |  |  |  |  |  |  |  |  |  |  |  |  |  |  |  |  |  |  |  |  |  |  |  |  |  |  |  |  |  |  |  |  |  |  |  |  |  |  |  |  |  |  |  |  |  |  |  |  |  |  |  |  |  |  |  |  |  |  |  |  |  |  |  |  |  |  |  |  |  |  |  |  |  |  |  |  |  |  |  |  |  |  |  |  |  |  |  |  |  |  |  |  |  |  |  |  |  |  |  |  |  |  |  |  |  |  |  |  |  |  |  |  |  |  |  |  |  |  |  |  |  |  |  |  |  |  |  |  |  |  |  |  |  |  |  |  |  |  |  |  |  |  |  |  |  |  |  |  |  |  |  |  |  |  |  |  |  |  |  |  |  |  |  |  |  |  |  |  |  |  |  |  |  |  |  |  |  |  |  |  |  |  |  |  |  |  |  |  |  |  |  |  |  |  |  |  |  |  |  |  |  |  |  |  |  |  |  |  |  |  |  |  |  |  |  |  |  |  |  |  |  |  |  |  |  |  |  |  |  |  |  |  |  |  |  |  |  |  |  |  |  |  |  |  |  |  |  |  |  |  |  |  |  |  |  |  |  |  |  |  |  |  |  |  |  |  |  |  |  |  |  |  |  |  |  |  |  |  |  |  |  |  |  |  |  |  |  |  |  |  |  |  |  |  |  |  |  |  |  |  |  |  |  |  |  |  |  |  |  |  |  |  |  |  |  |  |  |  |  |  |  |  |  |  |  |  |  |  |  |  |  |  |  |  |  |  |  |  |  |  |  |  |  |  |  |  |  |  |  |  |  |  |  |  |  |  |  |  |  |  |  |  |  |  |  |  |  |  |  |  |  |  |  |  |  |  |  |  |  |  |  |  |  |  |  |  |  |  |  |  |  |  |  |  |  |  |  |  |  |  |  |  |  |  |  |  |  |  |  |  |  |  |  |  |  |  |  |  |  |  |  |  |  |  |  |  |  |  |  |  |  |  |  |  |  |  |  |  |  |  |  |  |  |  |  |  |  |  |  |  |  |  |  |  |  |  |  |  |  |  |  |  |  |  |  |  |  |  |  |  |  |  |  |  |  |  |  |  |  |  |  |  |  |  |  |  |  |  |  |  |  |  |  |  |  |  |  |  |  |  |  |  |  |  |  |  |  |  |  |  |  |  |  |  |  |  |  |  |  |  |  |  |  |  |  |  |  |  |  |  |  |  |  |  |  |  |  |  |  |  |  |  |  |  |  |  |  |  |  |  |  |  |  |  |  |  |  |  |  |  |  |  |  |  |  |  |  |  |  |  |  |  |  |  |  |  |  |  |  |  |  |  |  |  |  |  |  |  |  |  |  |  |  |  |  |  |  |  |  |  |  |  |  |  |  |  |  |  |  |  |  |  |  |  |  |  |  |  |  |  |  |  |  |  |  |  |  |  |  |  |  |  |  |  |  |  |  |  |  |  |  |  |  |  |  |  |  |  |  |  |  |  |  |  |  |  |  |  |  |  |  |  |  |  |  |  |  |  |  |  |  |  |  |  |  |  |  |  |  |  |  |  |  |  |  |  |  |  |  |  |  |  |  |  |  |  |  |  |  |  |  |  |  |  |  |  |  |  |  |  |  |  |  |  |  |  |  |  |  |  |  |  |  |  |  |  |  |  |  |  |  |  |  |  |  |  |  |  |  |  |  |  |  |  |  |  |  |  |  |  |  |  |  |  |  |  |  |  |  |  |  |  |  |  |  |  |  |  |  |  |  |  |  |  |  |  |  |  |  |  |  |  |  |  |  |  |  |  |  |  |  |  |  |  |  |  |  |  |  |  |  |  |  |  |  |  |  |  |  |  |  |  |  |  |  |  |  |  |  |  |  |  |  |  |  |  |  |  |  |  |  |  |  |  |  |  |  |  |  |  |  |  |  |  |  |  |  |  |  |  |  |  |  |  |  |  |  |  |  |  |  |  |  |  |  |  |  |  |  |  |  |  |  |  |  |  |  |  |  |  |  |  |  |  |  |  |  |  |  |  |  |  |  |  |  |  |  |  |  |  |  |  |  |  |  |  |  |  |  |  |  |  |  |  |  |  |  |  |  |  |  |  |  |  |  |  |  |  |  |  |  |  |  |  |  |  |  |  |  |  |  |  |  |  |  |  |  |  |  |  |  |  |  |  |  |  |  |  |  |  |  |  |  |  |  |  |  |  |  |  |  |  |  |  |  |  |  |  |  |  |  |  |  |  |  |  |  |  |  |  |  |  |  |  |  |  |  |  |  |  |  |  |  |  |  |  |  |  |  |  |  |  |  |  |  |  |  |  |  |  |  |  |  |  |  |  |  |  |  |  |  |  |  |  |  |  |  |  |  |  |  |  |  |  |  |  |  |  |  |  |  |  |  |  |  |  |  |  |  |  |  |  |  |  |  |  |  |  |  |  |  |  |  |  |  |  |  |  |  |  |  |  |  |  |  |  |  |  |  |  |  |  |  |  |  |  |  |  |  |  |  |  |  |  |  |  |  |  |  |  |  |  |  |  |  |  |  |  |  |  |  |  |  |  |  |  |  |  |  |  |  |  |  |  |  |  |  |  |  |  |  |  |  |  |  |  |  |  |  |  |  |  |  |  |  |  |  |  |  |  |  |  |  |  |  |  |  |  |  |  |  |  |  |  |  |  |  |  |  |  |  |  |  |  |  |  |  |  |  |  |  |  |  |  |  |  |  |  |  |  |  |  |  |  |  |  |  |  |  |  |  |  |  |  |  |  |  |  |  |  |  |  |  |  |  |  |  |  |  |  |  |  |  |  |  |  |  |  |  |  |  |  |  |  |  |  |  |  |  |  |  |  |  |  |  |  |  |  |  |  |  |  |  |  |  |  |  |  |  |  |  |  |  |  |  |  |  |  |  |  |  |  |  |  |  |  |  |  |  |  |  |  |  |  |  |  |  |  |  |  |  |  |  |  |  |  |  |  |  |  |  |  |  |  |  |  |  |  |  |  |  |  |  |  |  |  |  |  |  |  |  |  |  |  |  |  |  |  |  |  |  |  |  |  |  |  |  |  |  |  |  |  |  |  |  |  |  |  |  |  |  |  |  |  |  |  |  |  |  |  |  |  |  |  |  |  |  |  |  |  |  |  |  |  |  |  |  |  |  |  |  |  |  |  |  |  |  |  |  |  |  |  |  |  |  |  |  |  |  |  |  |  |  |  |  |  |  |  |  |  |  |  |  |  |  |  |  |  |  |  |  |  |  |  |  |  |  |  |  |  |  |  |
| --- | --- | --- | --- | --- | --- | --- | --- | --- | --- | --- | --- | --- | --- | --- | --- | --- | --- | --- | --- | --- | --- | --- | --- | --- | --- | --- | --- | --- | --- | --- | --- | --- | --- | --- | --- | --- | --- | --- | --- | --- | --- | --- | --- | --- | --- | --- | --- | --- | --- | --- | --- | --- | --- | --- | --- | --- | --- | --- | --- | --- | --- | --- | --- | --- | --- | --- | --- | --- | --- | --- | --- | --- | --- | --- | --- | --- | --- | --- | --- | --- | --- | --- | --- | --- | --- | --- | --- | --- | --- | --- | --- | --- | --- | --- | --- | --- | --- | --- | --- | --- | --- | --- | --- | --- | --- | --- | --- | --- | --- | --- | --- | --- | --- | --- | --- | --- | --- | --- | --- | --- | --- | --- | --- | --- | --- | --- | --- | --- | --- | --- | --- | --- | --- | --- | --- | --- | --- | --- | --- | --- | --- | --- | --- | --- | --- | --- | --- | --- | --- | --- | --- | --- | --- | --- | --- | --- | --- | --- | --- | --- | --- | --- | --- | --- | --- | --- | --- | --- | --- | --- | --- | --- | --- | --- | --- | --- | --- | --- | --- | --- | --- | --- | --- | --- | --- | --- | --- | --- | --- | --- | --- | --- | --- | --- | --- | --- | --- | --- | --- | --- | --- | --- | --- | --- | --- | --- | --- | --- | --- | --- | --- | --- | --- | --- | --- | --- | --- | --- | --- | --- | --- | --- | --- | --- | --- | --- | --- | --- | --- | --- | --- | --- | --- | --- | --- | --- | --- | --- | --- | --- | --- | --- | --- | --- | --- | --- | --- | --- | --- | --- | --- | --- | --- | --- | --- | --- | --- | --- | --- | --- | --- | --- | --- | --- | --- | --- | --- | --- | --- | --- | --- | --- | --- | --- | --- | --- | --- | --- | --- | --- | --- | --- | --- | --- | --- | --- | --- | --- | --- | --- | --- | --- | --- | --- | --- | --- | --- | --- | --- | --- | --- | --- | --- | --- | --- | --- | --- | --- | --- | --- | --- | --- | --- | --- | --- | --- | --- | --- | --- | --- | --- | --- | --- | --- | --- | --- | --- | --- | --- | --- | --- | --- | --- | --- | --- | --- | --- | --- | --- | --- | --- | --- | --- | --- | --- | --- | --- | --- | --- | --- | --- | --- | --- | --- | --- | --- | --- | --- | --- | --- | --- | --- | --- | --- | --- | --- | --- | --- | --- | --- | --- | --- | --- | --- | --- | --- | --- | --- | --- | --- | --- | --- | --- | --- | --- | --- | --- | --- | --- | --- | --- | --- | --- | --- | --- | --- | --- | --- | --- | --- | --- | --- | --- | --- | --- | --- | --- | --- | --- | --- | --- | --- | --- | --- | --- | --- | --- | --- | --- | --- | --- | --- | --- | --- | --- | --- | --- | --- | --- | --- | --- | --- | --- | --- | --- | --- | --- | --- | --- | --- | --- | --- | --- | --- | --- | --- | --- | --- | --- | --- | --- | --- | --- | --- | --- | --- | --- | --- | --- | --- | --- | --- | --- | --- | --- | --- | --- | --- | --- | --- | --- | --- | --- | --- | --- | --- | --- | --- | --- | --- | --- | --- | --- | --- | --- | --- | --- | --- | --- | --- | --- | --- | --- | --- | --- | --- | --- | --- | --- | --- | --- | --- | --- | --- | --- | --- | --- | --- | --- | --- | --- | --- | --- | --- | --- | --- | --- | --- | --- | --- | --- | --- | --- | --- | --- | --- | --- | --- | --- | --- | --- | --- | --- | --- | --- | --- | --- | --- | --- | --- | --- | --- | --- | --- | --- | --- | --- | --- | --- | --- | --- | --- | --- | --- | --- | --- | --- | --- | --- | --- | --- | --- | --- | --- | --- | --- | --- | --- | --- | --- | --- | --- | --- | --- | --- | --- | --- | --- | --- | --- | --- | --- | --- | --- | --- | --- | --- | --- | --- | --- | --- | --- | --- | --- | --- | --- | --- | --- | --- | --- | --- | --- | --- | --- | --- | --- | --- | --- | --- | --- | --- | --- | --- | --- | --- | --- | --- | --- | --- | --- | --- | --- | --- | --- | --- | --- | --- | --- | --- | --- | --- | --- | --- | --- | --- | --- | --- | --- | --- | --- | --- | --- | --- | --- | --- | --- | --- | --- | --- | --- | --- | --- | --- | --- | --- | --- | --- | --- | --- | --- | --- | --- | --- | --- | --- | --- | --- | --- | --- | --- | --- | --- | --- | --- | --- | --- | --- | --- | --- | --- | --- | --- | --- | --- | --- | --- | --- | --- | --- | --- | --- | --- | --- | --- | --- | --- | --- | --- | --- | --- | --- | --- | --- | --- | --- | --- | --- | --- | --- | --- | --- | --- | --- | --- | --- | --- | --- | --- | --- | --- | --- | --- | --- | --- | --- | --- | --- | --- | --- | --- | --- | --- | --- | --- | --- | --- | --- | --- | --- | --- | --- | --- | --- | --- | --- | --- | --- | --- | --- | --- | --- | --- | --- | --- | --- | --- | --- | --- | --- | --- | --- | --- | --- | --- | --- | --- | --- | --- | --- | --- | --- | --- | --- | --- | --- | --- | --- | --- | --- | --- | --- | --- | --- | --- | --- | --- | --- | --- | --- | --- | --- | --- | --- | --- | --- | --- | --- | --- | --- | --- | --- | --- | --- | --- | --- | --- | --- | --- | --- | --- | --- | --- | --- | --- | --- | --- | --- | --- | --- | --- | --- | --- | --- | --- | --- | --- | --- | --- | --- | --- | --- | --- | --- | --- | --- | --- | --- | --- | --- | --- | --- | --- | --- | --- | --- | --- | --- | --- | --- | --- | --- | --- | --- | --- | --- | --- | --- | --- | --- | --- | --- | --- | --- | --- | --- | --- | --- | --- | --- | --- | --- | --- | --- | --- | --- | --- | --- | --- | --- | --- | --- | --- | --- | --- | --- | --- | --- | --- | --- | --- | --- | --- | --- | --- | --- | --- | --- | --- | --- | --- | --- | --- | --- | --- | --- | --- | --- | --- | --- | --- | --- | --- | --- | --- | --- | --- | --- | --- | --- | --- | --- | --- | --- | --- | --- | --- | --- | --- | --- | --- | --- | --- | --- | --- | --- | --- | --- | --- | --- | --- | --- | --- | --- | --- | --- | --- | --- | --- | --- | --- | --- | --- | --- | --- | --- | --- | --- | --- | --- | --- | --- | --- | --- | --- | --- | --- | --- | --- | --- | --- | --- | --- | --- | --- | --- | --- | --- | --- | --- | --- | --- | --- | --- | --- | --- | --- | --- | --- | --- | --- | --- | --- | --- | --- | --- | --- | --- | --- | --- | --- | --- | --- | --- | --- | --- | --- | --- | --- | --- | --- | --- | --- | --- | --- | --- | --- | --- | --- | --- | --- | --- | --- | --- | --- | --- | --- | --- | --- | --- | --- | --- | --- | --- | --- | --- | --- | --- | --- | --- | --- | --- | --- | --- | --- | --- | --- | --- | --- | --- | --- | --- | --- | --- | --- | --- | --- | --- | --- | --- | --- | --- | --- | --- | --- | --- | --- | --- | --- | --- | --- | --- | --- | --- | --- | --- | --- | --- | --- | --- | --- | --- | --- | --- | --- | --- | --- | --- | --- | --- | --- | --- | --- | --- | --- | --- | --- | --- | --- | --- | --- | --- | --- | --- | --- | --- | --- | --- | --- | --- | --- | --- | --- | --- | --- | --- | --- | --- | --- | --- | --- | --- | --- | --- | --- | --- | --- | --- | --- | --- | --- | --- | --- | --- | --- | --- | --- | --- | --- | --- | --- | --- | --- | --- | --- | --- | --- | --- | --- | --- | --- | --- | --- | --- | --- | --- | --- | --- | --- | --- | --- | --- | --- | --- | --- | --- | --- | --- | --- | --- | --- | --- | --- | --- | --- | --- | --- | --- | --- | --- | --- | --- | --- | --- | --- | --- | --- | --- | --- | --- | --- | --- | --- | --- | --- | --- | --- | --- | --- | --- | --- | --- | --- | --- | --- | --- | --- | --- | --- | --- | --- | --- | --- | --- | --- | --- | --- | --- | --- | --- | --- | --- | --- | --- | --- | --- | --- | --- | --- | --- | --- | --- | --- | --- | --- | --- | --- | --- | --- | --- | --- | --- | --- | --- | --- | --- | --- | --- | --- | --- | --- | --- | --- | --- | --- | --- | --- | --- | --- | --- | --- | --- | --- | --- | --- | --- | --- | --- | --- | --- | --- | --- | --- | --- | --- | --- | --- | --- | --- | --- | --- | --- | --- | --- | --- | --- | --- | --- | --- | --- | --- | --- | --- | --- | --- | --- | --- | --- | --- | --- | --- | --- | --- | --- | --- | --- | --- | --- | --- | --- | --- | --- | --- | --- | --- | --- | --- | --- | --- | --- | --- | --- | --- | --- | --- | --- | --- | --- | --- | --- | --- | --- | --- | --- | --- | --- | --- | --- | --- | --- | --- | --- | --- | --- | --- | --- | --- | --- | --- | --- | --- | --- | --- | --- | --- | --- | --- | --- | --- | --- | --- | --- | --- | --- | --- | --- | --- | --- | --- | --- | --- | --- | --- | --- | --- | --- | --- | --- | --- | --- | --- | --- | --- | --- | --- | --- | --- | --- | --- | --- | --- | --- | --- | --- | --- | --- | --- | --- | --- | --- | --- | --- | --- | --- | --- | --- | --- | --- | --- | --- | --- | --- | --- | --- | --- | --- | --- | --- | --- | --- | --- | --- | --- | --- | --- | --- | --- | --- | --- | --- | --- | --- | --- | --- | --- | --- | --- | --- | --- | --- | --- | --- | --- | --- | --- | --- | --- | --- | --- | --- | --- | --- | --- | --- | --- | --- | --- | --- | --- | --- | --- | --- | --- | --- | --- | --- | --- | --- | --- | --- | --- | --- | --- | --- | --- | --- | --- | --- | --- | --- | --- | --- | --- | --- | --- | --- | --- | --- | --- | --- | --- | --- | --- | --- | --- | --- | --- | --- | --- | --- | --- | --- | --- | --- | --- | --- | --- | --- | --- | --- | --- | --- | --- | --- | --- | --- | --- | --- | --- | --- | --- | --- | --- | --- | --- | --- | --- | --- | --- | --- | --- | --- | --- | --- | --- | --- | --- | --- | --- | --- | --- | --- | --- | --- | --- | --- | --- | --- | --- | --- | --- | --- | --- | --- | --- | --- | --- | --- | --- | --- | --- | --- | --- | --- | --- | --- | --- | --- | --- | --- | --- | --- | --- | --- | --- | --- | --- | --- | --- | --- | --- | --- | --- | --- | --- | --- | --- | --- | --- | --- | --- | --- | --- | --- | --- | --- | --- | --- | --- | --- | --- | --- | --- | --- | --- | --- | --- | --- | --- | --- | --- | --- | --- | --- | --- | --- | --- | --- | --- | --- | --- | --- | --- | --- | --- | --- | --- | --- | --- | --- | --- | --- | --- | --- | --- | --- | --- | --- | --- | --- | --- | --- | --- | --- | --- | --- | --- | --- | --- | --- | --- | --- | --- | --- | --- | --- | --- | --- | --- | --- | --- | --- | --- | --- | --- | --- | --- | --- | --- | --- | --- | --- | --- | --- | --- | --- | --- | --- | --- | --- | --- | --- | --- | --- | --- | --- | --- | --- | --- | --- | --- | --- | --- | --- | --- | --- | --- | --- | --- | --- | --- | --- | --- | --- | --- | --- | --- | --- | --- | --- | --- | --- | --- | --- | --- | --- | --- | --- | --- | --- | --- | --- | --- | --- | --- | --- | --- | --- | --- | --- | --- | --- | --- | --- | --- | --- | --- | --- | --- | --- | --- | --- | --- | --- | --- | --- | --- | --- | --- | --- | --- | --- | --- | --- | --- | --- | --- | --- | --- | --- | --- | --- | --- | --- | --- | --- | --- | --- | --- | --- | --- | --- | --- | --- | --- | --- | --- | --- | --- | --- | --- | --- | --- | --- | --- | --- | --- | --- | --- | --- | --- | --- | --- | --- | --- | --- | --- | --- | --- | --- | --- | --- | --- | --- | --- | --- | --- | --- | --- | --- | --- | --- | --- | --- | --- | --- | --- | --- | --- | --- | --- | --- | --- | --- | --- | --- | --- | --- | --- | --- | --- | --- | --- | --- | --- | --- | --- | --- | --- | --- | --- | --- | --- | --- | --- | --- | --- | --- | --- | --- | --- | --- | --- | --- | --- | --- | --- | --- | --- | --- | --- | --- | --- | --- | --- | --- | --- | --- | --- | --- | --- | --- | --- | --- | --- | --- | --- | --- | --- | --- | --- | --- | --- | --- | --- | --- | --- | --- | --- | --- | --- | --- | --- | --- | --- | --- | --- | --- | --- | --- | --- | --- | --- | --- | --- | --- | --- | --- | --- | --- | --- | --- | --- | --- | --- | --- | --- | --- | --- | --- | --- | --- | --- | --- | --- | --- | --- | --- | --- | --- | --- | --- | --- | --- | --- | --- | --- | --- | --- | --- | --- | --- | --- | --- | --- | --- | --- | --- | --- | --- | --- | --- | --- | --- | --- | --- | --- | --- | --- | --- | --- | --- | --- | --- | --- | --- | --- | --- | --- | --- | --- | --- | --- | --- | --- | --- | --- | --- | --- | --- | --- | --- | --- | --- | --- | --- | --- | --- | --- | --- | --- | --- | --- | --- | --- | --- | --- | --- | --- | --- | --- | --- | --- | --- | --- | --- | --- | --- | --- | --- | --- | --- | --- | --- | --- | --- | --- | --- | --- | --- | --- | --- | --- | --- | --- | --- | --- | --- | --- | --- | --- | --- | --- | --- | --- | --- | --- | --- | --- | --- | --- | --- | --- | --- | --- | --- | --- | --- | --- | --- | --- | --- | --- | --- | --- | --- | --- | --- | --- | --- | --- | --- | --- | --- | --- | --- | --- | --- | --- | --- | --- | --- | --- | --- | --- | --- | --- | --- | --- | --- | --- | --- | --- | --- | --- | --- | --- | --- | --- | --- | --- | --- | --- | --- | --- | --- | --- | --- | --- | --- | --- | --- | --- | --- | --- | --- | --- | --- | --- | --- | --- | --- | --- | --- | --- | --- | --- | --- | --- | --- | --- | --- | --- | --- | --- | --- | --- | --- | --- | --- | --- | --- | --- | --- | --- | --- | --- | --- | --- | --- | --- | --- | --- | --- | --- | --- | --- | --- | --- | --- | --- | --- | --- | --- | --- | --- | --- | --- | --- | --- | --- | --- | --- | --- | --- | --- | --- | --- | --- | --- | --- | --- | --- | --- | --- | --- | --- | --- | --- | --- | --- | --- | --- | --- | --- | --- | --- | --- | --- | --- | --- | --- | --- | --- | --- | --- | --- | --- | --- | --- | --- | --- | --- | --- | --- | --- | --- | --- | --- | --- | --- | --- | --- | --- | --- | --- | --- | --- | --- | --- | --- | --- | --- | --- | --- | --- | --- | --- | --- | --- | --- | --- | --- | --- | --- | --- | --- | --- | --- | --- | --- | --- | --- | --- | --- | --- | --- | --- | --- | --- | --- | --- | --- | --- | --- | --- | --- | --- | --- | --- | --- | --- | --- | --- | --- | --- | --- | --- | --- | --- | --- | --- | --- | --- | --- | --- | --- | --- | --- | --- | --- | --- | --- | --- | --- | --- | --- | --- | --- | --- | --- | --- | --- | --- | --- | --- | --- | --- | --- | --- | --- | --- | --- | --- | --- | --- | --- | --- | --- | --- | --- | --- | --- | --- | --- | --- | --- | --- | --- | --- | --- | --- | --- | --- | --- | --- | --- | --- | --- | --- | --- | --- | --- | --- | --- | --- | --- | --- | --- | --- | --- | --- | --- | --- | --- | --- | --- | --- | --- | --- | --- | --- | --- | --- | --- | --- | --- | --- | --- | --- | --- | --- | --- | --- | --- | --- | --- | --- | --- | --- | --- | --- | --- | --- | --- | --- | --- | --- | --- | --- | --- | --- | --- | --- | --- | --- | --- | --- | --- | --- | --- | --- | --- | --- | --- | --- | --- | --- | --- | --- | --- | --- | --- | --- | --- | --- | --- | --- | --- | --- | --- | --- | --- | --- | --- | --- | --- | --- | --- | --- | --- | --- | --- | --- | --- | --- | --- | --- | --- | --- | --- | --- | --- | --- | --- | --- | --- | --- | --- | --- | --- | --- | --- | --- | --- | --- | --- | --- | --- | --- | --- | --- | --- | --- | --- | --- | --- | --- | --- | --- | --- | --- | --- | --- | --- | --- | --- | --- | --- | --- | --- | --- | --- | --- | --- | --- | --- | --- | --- | --- | --- | --- | --- | --- | --- | --- | --- | --- | --- | --- | --- | --- | --- | --- | --- | --- | --- | --- | --- | --- | --- | --- | --- | --- | --- | --- | --- | --- | --- | --- | --- | --- | --- | --- | --- | --- | --- | --- | --- | --- | --- | --- | --- | --- | --- | --- | --- | --- | --- | --- | --- | --- | --- | --- | --- | --- | --- | --- | --- | --- | --- | --- | --- | --- | --- | --- | --- | --- | --- | --- | --- | --- | --- | --- | --- | --- | --- | --- | --- | --- | --- | --- | --- | --- | --- | --- | --- | --- | --- | --- | --- | --- | --- | --- | --- | --- | --- | --- | --- | --- | --- | --- | --- | --- | --- | --- | --- | --- | --- | --- | --- | --- | --- | --- | --- | --- | --- | --- | --- | --- | --- | --- | --- | --- | --- | --- | --- | --- | --- | --- | --- | --- | --- | --- | --- | --- | --- | --- | --- | --- | --- | --- | --- | --- | --- | --- | --- | --- | --- | --- | --- | --- | --- | --- | --- | --- | --- | --- | --- | --- | --- | --- | --- | --- | --- | --- | --- | --- | --- | --- | --- | --- | --- | --- | --- | --- | --- | --- | --- | --- | --- | --- | --- | --- | --- | --- | --- | --- | --- | --- | --- | --- | --- | --- | --- | --- | --- | --- | --- | --- | --- | --- | --- | --- | --- | --- | --- | --- | --- | --- | --- | --- | --- | --- | --- | --- | --- | --- | --- | --- | --- | --- | --- | --- | --- | --- | --- | --- | --- | --- | --- | --- | --- | --- | --- | --- | --- | --- | --- | --- | --- | --- | --- | --- | --- | --- | --- | --- | --- | --- | --- | --- | --- | --- | --- | --- | --- | --- | --- | --- | --- | --- | --- | --- | --- | --- | --- | --- | --- | --- | --- | --- | --- | --- | --- | --- | --- | --- | --- | --- | --- | --- | --- | --- | --- | --- | --- | --- | --- | --- | --- | --- | --- | --- | --- | --- | --- | --- | --- | --- | --- | --- | --- | --- | --- | --- | --- | --- | --- | --- | --- | --- | --- | --- | --- | --- | --- | --- | --- | --- | --- | --- | --- | --- | --- | --- | --- | --- | --- | --- | --- | --- | --- | --- | --- | --- | --- | --- | --- | --- | --- | --- | --- | --- | --- | --- | --- | --- | --- | --- | --- | --- | --- | --- | --- | --- | --- | --- | --- | --- | --- | --- | --- | --- | --- | --- | --- | --- | --- | --- | --- | --- | --- | --- | --- | --- | --- | --- | --- | --- | --- | --- | --- | --- | --- | --- | --- | --- | --- | --- | --- | --- | --- | --- | --- | --- | --- | --- | --- | --- | --- | --- | --- | --- | --- | --- | --- | --- | --- | --- | --- | --- | --- | --- | --- | --- | --- | --- | --- | --- | --- | --- | --- | --- | --- | --- | --- | --- | --- | --- | --- | --- | --- | --- | --- | --- | --- | --- | --- | --- | --- | --- | --- | --- | --- | --- | --- | --- | --- | --- | --- | --- | --- | --- | --- | --- | --- | --- | --- | --- | --- | --- | --- | --- | --- | --- | --- | --- | --- | --- | --- | --- | --- | --- | --- | --- | --- | --- | --- | --- | --- | --- | --- | --- | --- | --- | --- | --- | --- | --- | --- | --- | --- | --- | --- | --- | --- | --- | --- | --- | --- | --- | --- | --- | --- | --- | --- | --- | --- | --- | --- | --- | --- | --- | --- | --- | --- | --- | --- | --- | --- | --- | --- | --- | --- | --- | --- | --- | --- | --- | --- | --- | --- | --- | --- | --- | --- | --- | --- | --- | --- | --- | --- | --- | --- | --- | --- | --- | --- | --- | --- | --- | --- | --- | --- | --- | --- | --- | --- | --- | --- | --- | --- | --- | --- | --- | --- | --- | --- | --- | --- | --- | --- | --- | --- | --- | --- | --- | --- | --- | --- | --- | --- | --- | --- | --- | --- | --- | --- | --- | --- | --- | --- | --- | --- | --- | --- | --- | --- | --- | --- | --- | --- | --- | --- | --- | --- | --- | --- | --- | --- | --- | --- | --- | --- | --- | --- | --- | --- | --- | --- | --- | --- | --- | --- | --- | --- | --- | --- | --- | --- | --- | --- | --- | --- | --- | --- | --- | --- | --- | --- | --- | --- | --- | --- | --- | --- | --- | --- | --- | --- | --- | --- | --- | --- | --- | --- | --- | --- | --- | --- | --- | --- | --- | --- | --- | --- | --- | --- | --- | --- | --- | --- | --- | --- | --- | --- | --- | --- | --- | --- | --- | --- | --- | --- | --- | --- | --- | --- | --- | --- | --- | --- | --- | --- | --- | --- | --- | --- | --- | --- | --- | --- | --- | --- | --- | --- | --- | --- | --- | --- | --- | --- | --- | --- | --- | --- | --- | --- | --- | --- | --- | --- | --- | --- | --- | --- | --- | --- | --- | --- | --- | --- | --- | --- | --- | --- | --- | --- | --- | --- | --- | --- | --- | --- | --- | --- | --- | --- | --- | --- | --- | --- | --- | --- | --- | --- | --- | --- | --- | --- | --- | --- | --- | --- | --- | --- | --- | --- | --- | --- | --- | --- | --- | --- | --- | --- | --- | --- | --- | --- | --- | --- | --- | --- | --- | --- | --- | --- | --- | --- | --- | --- | --- | --- | --- | --- | --- | --- | --- | --- | --- | --- | --- | --- | --- | --- | --- | --- | --- | --- | --- | --- | --- | --- | --- | --- | --- | --- | --- | --- | --- | --- | --- | --- | --- | --- | --- | --- | --- | --- | --- | --- | --- | --- | --- | --- | --- | --- | --- | --- | --- | --- | --- | --- | --- | --- | --- | --- | --- | --- | --- | --- | --- | --- | --- | --- | --- | --- | --- | --- | --- | --- | --- | --- | --- | --- | --- | --- | --- | --- | --- | --- | --- | --- | --- | --- | --- | --- | --- | --- | --- | --- | --- | --- | --- | --- | --- | --- | --- | --- | --- | --- | --- | --- | --- | --- | --- | --- | --- | --- | --- | --- | --- | --- | --- | --- | --- | --- | --- | --- | --- | --- | --- | --- | --- | --- | --- | --- | --- | --- | --- | --- | --- | --- | --- | --- | --- | --- | --- | --- | --- | --- | --- | --- | --- | --- | --- | --- | --- | --- | --- | --- | --- | --- | --- | --- | --- | --- | --- | --- | --- | --- | --- | --- | --- | --- | --- | --- | --- | --- | --- | --- | --- | --- | --- | --- | --- | --- | --- | --- | --- | --- | --- | --- | --- | --- | --- | --- | --- | --- | --- | --- | --- | --- | --- | --- | --- | --- | --- | --- | --- | --- | --- | --- | --- | --- | --- | --- | --- | --- | --- | --- | --- | --- | --- | --- | --- | --- | --- | --- | --- | --- | --- | --- | --- | --- | --- | --- | --- | --- | --- | --- | --- | --- | --- | --- | --- | --- | --- | --- | --- | --- | --- | --- | --- | --- | --- | --- | --- | --- | --- | --- | --- | --- | --- | --- | --- | --- | --- | --- | --- | --- | --- | --- | --- | --- | --- | --- | --- | --- | --- | --- | --- | --- | --- | --- | --- | --- | --- | --- | --- | --- | --- | --- | --- | --- | --- | --- | --- | --- | --- | --- | --- | --- | --- | --- | --- | --- | --- | --- | --- | --- | --- | --- | --- | --- | --- | --- | --- | --- | --- | --- | --- | --- | --- | --- | --- | --- | --- | --- | --- | --- | --- | --- | --- | --- | --- | --- | --- | --- | --- | --- | --- | --- | --- | --- | --- | --- | --- | --- | --- | --- | --- | --- | --- | --- | --- | --- | --- | --- | --- | --- | --- | --- | --- | --- | --- | --- | --- | --- | --- | --- | --- | --- | --- | --- | --- | --- | --- | --- | --- | --- | --- | --- | --- | --- | --- | --- | --- | --- | --- | --- | --- | --- | --- | --- | --- | --- | --- | --- | --- | --- | --- | --- | --- | --- | --- | --- | --- | --- | --- | --- | --- | --- | --- | --- | --- | --- | --- | --- | --- | --- | --- | --- | --- | --- | --- | --- | --- | --- | --- | --- | --- | --- | --- | --- | --- | --- | --- | --- | --- | --- | --- | --- | --- | --- | --- | --- | --- | --- | --- | --- | --- | --- | --- | --- | --- | --- | --- | --- | --- | --- | --- | --- | --- | --- | --- | --- | --- | --- | --- | --- | --- | --- | --- | --- | --- | --- | --- | --- | --- | --- | --- | --- | --- | --- | --- | --- | --- | --- | --- | --- | --- | --- | --- | --- | --- | --- | --- | --- | --- | --- | --- | --- | --- | --- | --- | --- | --- | --- | --- | --- | --- | --- | --- | --- | --- | --- | --- | --- | --- | --- | --- | --- | --- | --- | --- | --- | --- | --- | --- | --- | --- | --- | --- | --- | --- | --- | --- | --- | --- | --- | --- | --- | --- | --- | --- | --- | --- | --- | --- | --- | --- | --- | --- | --- | --- | --- | --- | --- | --- | --- | --- | --- | --- | --- | --- | --- | --- | --- | --- | --- | --- | --- | --- | --- | --- | --- | --- | --- | --- | --- | --- | --- | --- | --- | --- | --- | --- | --- | --- | --- | --- | --- | --- | --- | --- | --- | --- | --- | --- | --- | --- | --- | --- | --- | --- | --- | --- | --- | --- | --- | --- | --- | --- | --- | --- | --- | --- | --- | --- | --- | --- | --- | --- | --- | --- | --- | --- | --- | --- | --- | --- | --- | --- | --- | --- | --- | --- | --- | --- | --- | --- | --- | --- | --- | --- | --- | --- | --- | --- | --- | --- | --- | --- | --- | --- | --- | --- | --- | --- | --- | --- | --- | --- | --- | --- | --- | --- | --- | --- | --- | --- | --- | --- | --- | --- | --- | --- | --- | --- | --- | --- | --- | --- | --- | --- | --- | --- | --- | --- | --- | --- | --- | --- | --- | --- | --- | --- | --- | --- | --- | --- | --- | --- | --- | --- | --- | --- | --- | --- | --- | --- | --- | --- | --- | --- | --- | --- | --- | --- | --- | --- | --- | --- | --- | --- | --- | --- | --- | --- | --- | --- | --- | --- | --- | --- | --- | --- | --- | --- | --- | --- | --- | --- | --- | --- | --- | --- | --- | --- | --- | --- | --- | --- | --- | --- | --- | --- | --- | --- | --- | --- | --- | --- | --- | --- | --- | --- | --- | --- | --- | --- | --- | --- | --- | --- | --- | --- | --- | --- | --- | --- | --- | --- | --- | --- | --- | --- | --- | --- | --- | --- | --- | --- | --- | --- | --- | --- | --- | --- | --- | --- | --- | --- | --- | --- | --- | --- | --- | --- | --- | --- | --- | --- | --- | --- | --- | --- | --- | --- | --- | --- | --- | --- | --- | --- | --- | --- | --- | --- | --- | --- | --- | --- | --- | --- | --- | --- | --- | --- | --- | --- | --- | --- | --- | --- | --- | --- | --- | --- | --- | --- | --- | --- | --- | --- | --- | --- | --- | --- | --- | --- | --- | --- | --- | --- | --- | --- | --- | --- | --- | --- | --- | --- | --- | --- | --- | --- | --- | --- | --- | --- | --- | --- | --- | --- | --- | --- | --- | --- | --- | --- | --- | --- | --- | --- | --- | --- | --- | --- | --- | --- | --- | --- | --- | --- | --- | --- | --- | --- | --- | --- | --- | --- | --- | --- | --- | --- | --- | --- | --- | --- | --- | --- | --- | --- | --- | --- | --- | --- | --- | --- | --- | --- | --- | --- | --- | --- | --- | --- | --- | --- | --- | --- | --- | --- | --- | --- | --- | --- | --- | --- | --- | --- | --- | --- | --- | --- | --- | --- | --- | --- | --- | --- | --- | --- | --- | --- | --- | --- | --- | --- | --- | --- | --- | --- | --- | --- | --- | --- | --- | --- | --- | --- | --- | --- | --- | --- | --- | --- | --- | --- | --- | --- | --- | --- | --- | --- | --- | --- | --- | --- | --- | --- | --- | --- | --- | --- | --- | --- | --- | --- | --- | --- | --- | --- | --- | --- | --- | --- | --- | --- | --- | --- | --- | --- | --- | --- | --- | --- | --- | --- | --- | --- | --- | --- | --- | --- | --- | --- | --- | --- | --- | --- | --- | --- | --- | --- | --- | --- | --- | --- | --- | --- | --- | --- | --- | --- | --- | --- | --- | --- | --- | --- | --- | --- | --- | --- | --- | --- | --- | --- | --- | --- | --- | --- | --- | --- | --- | --- | --- | --- | --- | --- | --- | --- | --- | --- | --- | --- | --- | --- | --- | --- | --- | --- | --- | --- | --- | --- | --- | --- | --- | --- | --- | --- | --- | --- | --- | --- | --- | --- | --- | --- | --- | --- | --- | --- | --- | --- | --- | --- | --- | --- | --- | --- | --- | --- | --- | --- | --- | --- | --- | --- | --- | --- | --- | --- | --- | --- | --- | --- | --- | --- | --- | --- | --- | --- | --- | --- | --- | --- | --- | --- | --- | --- | --- | --- | --- | --- | --- | --- | --- | --- | --- | --- | --- | --- | --- | --- | --- | --- | --- | --- | --- | --- | --- | --- | --- | --- | --- | --- | --- | --- | --- | --- | --- | --- | --- | --- | --- | --- | --- | --- | --- | --- | --- | --- | --- | --- | --- | --- | --- | --- | --- | --- | --- | --- | --- | --- | --- | --- | --- | --- | --- | --- | --- | --- | --- | --- | --- | --- | --- | --- | --- | --- | --- | --- | --- | --- | --- | --- | --- | --- | --- | --- | --- | --- | --- | --- | --- | --- | --- | --- | --- | --- | --- | --- | --- | --- | --- | --- | --- | --- | --- | --- | --- | --- | --- | --- | --- | --- | --- | --- | --- | --- | --- | --- | --- | --- | --- | --- | --- | --- | --- | --- | --- | --- | --- | --- | --- | --- | --- | --- | --- | --- | --- | --- | --- | --- | --- | --- | --- | --- | --- | --- | --- | --- | --- | --- | --- | --- | --- | --- | --- | --- | --- | --- | --- | --- | --- | --- | --- | --- | --- | --- | --- | --- | --- | --- | --- | --- | --- | --- | --- | --- | --- | --- | --- | --- | --- | --- | --- | --- | --- | --- | --- | --- | --- | --- | --- | --- | --- | --- | --- | --- | --- | --- | --- | --- | --- | --- | --- | --- | --- | --- | --- | --- | --- | --- | --- | --- | --- | --- | --- | --- | --- | --- | --- | --- | --- | --- | --- | --- | --- | --- | --- | --- | --- | --- | --- | --- | --- | --- | --- | --- | --- | --- | --- | --- | --- | --- | --- | --- | --- | --- | --- | --- | --- | --- | --- | --- | --- | --- | --- | --- | --- | --- | --- | --- | --- | --- | --- | --- | --- | --- | --- | --- | --- | --- | --- | --- | --- | --- | --- | --- | --- | --- | --- | --- | --- | --- | --- | --- | --- | --- | --- | --- | --- | --- | --- | --- | --- | --- | --- | --- | --- | --- | --- | --- | --- | --- | --- | --- | --- | --- | --- | --- | --- | --- | --- | --- | --- | --- | --- | --- | --- | --- | --- | --- | --- | --- | --- | --- | --- | --- | --- | --- | --- | --- | --- | --- | --- | --- | --- | --- | --- | --- | --- | --- | --- | --- | --- | --- | --- | --- | --- | --- | --- | --- | --- | --- | --- | --- | --- | --- | --- | --- | --- | --- | --- | --- | --- | --- | --- | --- | --- | --- | --- | --- | --- | --- | --- | --- | --- | --- | --- | --- | --- | --- | --- | --- | --- | --- | --- | --- | --- | --- | --- | --- | --- | --- | --- | --- | --- | --- | --- | --- | --- | --- | --- | --- | --- | --- | --- | --- | --- | --- | --- | --- | --- | --- | --- | --- | --- | --- | --- | --- | --- | --- | --- | --- | --- | --- | --- | --- | --- | --- | --- | --- | --- | --- | --- | --- | --- | --- | --- | --- | --- | --- | --- | --- | --- | --- | --- | --- | --- | --- | --- | --- | --- | --- | --- | --- | --- | --- | --- | --- | --- | --- | --- | --- | --- | --- | --- | --- | --- | --- | --- | --- | --- | --- | --- | --- | --- | --- | --- | --- | --- | --- | --- | --- | --- | --- | --- | --- | --- | --- | --- | --- | --- | --- | --- | --- | --- | --- | --- | --- | --- | --- | --- | --- | --- | --- | --- | --- | --- | --- | --- | --- | --- | --- | --- | --- | --- | --- | --- | --- | --- | --- | --- | --- | --- | --- | --- | --- | --- | --- | --- | --- | --- | --- | --- | --- | --- | --- | --- | --- | --- | --- | --- | --- | --- | --- | --- | --- | --- | --- | --- | --- | --- | --- | --- | --- | --- | --- | --- | --- | --- | --- | --- | --- | --- | --- | --- | --- | --- | --- | --- | --- | --- | --- | --- | --- | --- | --- | --- | --- | --- | --- | --- | --- | --- | --- | --- | --- | --- | --- | --- | --- | --- | --- | --- | --- | --- | --- | --- | --- | --- | --- | --- | --- | --- | --- | --- | --- | --- | --- | --- | --- | --- | --- | --- | --- | --- | --- | --- | --- | --- | --- | --- | --- | --- | --- | --- | --- | --- | --- | --- | --- | --- | --- | --- | --- | --- | --- | --- | --- | --- | --- | --- | --- | --- | --- | --- | --- | --- | --- | --- | --- | --- | --- | --- | --- | --- | --- | --- | --- | --- | --- | --- | --- | --- | --- | --- | --- | --- | --- | --- | --- | --- | --- | --- | --- | --- | --- | --- | --- | --- | --- | --- | --- | --- | --- | --- | --- | --- | --- | --- | --- | --- | --- | --- | --- | --- | --- | --- | --- | --- | --- | --- | --- | --- | --- | --- | --- | --- | --- | --- | --- | --- | --- | --- | --- | --- | --- | --- | --- | --- | --- | --- | --- | --- | --- | --- | --- | --- | --- | --- | --- | --- | --- | --- | --- | --- | --- | --- | --- | --- | --- | --- | --- | --- | --- | --- | --- | --- | --- | --- | --- | --- | --- | --- | --- | --- | --- | --- | --- | --- | --- | --- | --- | --- | --- | --- | --- | --- | --- | --- | --- | --- | --- | --- | --- | --- | --- | --- | --- | --- | --- | --- | --- | --- | --- | --- | --- | --- | --- | --- | --- | --- | --- | --- | --- | --- | --- | --- | --- | --- | --- | --- | --- | --- | --- | --- | --- | --- | --- | --- | --- | --- | --- | --- | --- | --- | --- | --- | --- | --- | --- | --- | --- | --- | --- | --- | --- | --- | --- | --- | --- | --- | --- | --- | --- | --- | --- | --- | --- | --- | --- | --- | --- | --- | --- | --- | --- | --- | --- | --- | --- | --- | --- | --- | --- | --- | --- | --- | --- | --- | --- | --- | --- | --- | --- | --- | --- | --- | --- | --- | --- | --- | --- | --- | --- | --- | --- | --- | --- | --- | --- | --- | --- | --- | --- | --- | --- | --- | --- | --- | --- | --- | --- | --- | --- | --- | --- | --- | --- | --- | --- | --- | --- | --- | --- | --- | --- | --- | --- | --- | --- | --- | --- | --- | --- | --- | --- | --- | --- | --- | --- | --- | --- | --- | --- | --- | --- | --- | --- | --- | --- | --- | --- | --- | --- | --- | --- | --- | --- | --- | --- | --- | --- | --- | --- | --- | --- | --- | --- | --- | --- | --- | --- | --- | --- | --- | --- | --- | --- | --- | --- | --- | --- | --- | --- | --- | --- | --- | --- | --- | --- | --- | --- | --- | --- | --- | --- | --- | --- | --- | --- | --- | --- | --- | --- | --- | --- | --- | --- | --- | --- | --- | --- | --- | --- | --- | --- | --- | --- | --- | --- | --- | --- | --- | --- | --- | --- | --- | --- | --- | --- | --- | --- | --- | --- | --- | --- | --- | --- | --- | --- | --- | --- | --- | --- | --- | --- | --- | --- | --- | --- | --- | --- | --- | --- | --- | --- | --- | --- | --- | --- | --- | --- | --- | --- | --- | --- | --- | --- | --- | --- | --- | --- | --- | --- | --- | --- | --- | --- | --- | --- | --- | --- | --- | --- | --- | --- | --- | --- | --- | --- | --- | --- | --- | --- | --- | --- | --- | --- | --- | --- | --- | --- | --- | --- | --- | --- | --- | --- | --- | --- | --- | --- | --- | --- | --- | --- | --- | --- | --- | --- | --- | --- | --- | --- | --- | --- | --- | --- | --- | --- | --- | --- | --- | --- | --- | --- | --- | --- | --- | --- | --- | --- | --- | --- | --- | --- | --- | --- | --- | --- | --- | --- | --- | --- | --- | --- | --- | --- | --- | --- | --- | --- | --- | --- | --- | --- | --- | --- | --- | --- | --- | --- | --- | --- | --- | --- | --- | --- | --- | --- | --- | --- | --- | --- | --- | --- | --- | --- | --- | --- | --- | --- | --- | --- | --- | --- | --- | --- | --- | --- | --- | --- | --- | --- | --- | --- | --- | --- | --- | --- | --- | --- | --- | --- | --- | --- | --- | --- | --- | --- | --- | --- | --- | --- | --- | --- | --- | --- | --- | --- | --- | --- | --- | --- | --- | --- | --- | --- | --- | --- | --- | --- | --- | --- | --- | --- | --- | --- | --- | --- | --- | --- | --- | --- | --- | --- | --- | --- | --- | --- | --- | --- | --- | --- | --- | --- | --- | --- | --- | --- | --- | --- | --- | --- | --- | --- | --- | --- | --- | --- | --- | --- | --- | --- | --- | --- | --- | --- | --- | --- | --- | --- | --- | --- | --- | --- | --- | --- | --- | --- | --- | --- | --- | --- | --- | --- | --- | --- | --- | --- | --- | --- | --- | --- | --- | --- | --- | --- | --- | --- | --- | --- | --- | --- | --- | --- | --- | --- | --- | --- | --- | --- | --- | --- | --- | --- | --- | --- | --- | --- | --- | --- | --- | --- | --- | --- | --- | --- | --- | --- | --- | --- | --- | --- | --- | --- | --- | --- | --- | --- | --- | --- | --- | --- | --- | --- | --- | --- | --- | --- | --- | --- | --- | --- | --- | --- | --- | --- | --- | --- | --- | --- | --- | --- | --- | --- | --- | --- | --- | --- | --- | --- | --- | --- | --- | --- | --- | --- | --- | --- | --- | --- | --- | --- | --- | --- | --- | --- | --- | --- | --- | --- | --- | --- | --- | --- | --- | --- | --- | --- | --- | --- | --- | --- | --- | --- | --- | --- | --- | --- | --- | --- | --- | --- | --- | --- | --- | --- | --- | --- | --- | --- | --- | --- | --- | --- | --- | --- | --- | --- | --- | --- | --- | --- | --- | --- | --- | --- | --- | --- | --- | --- | --- | --- | --- | --- | --- | --- | --- | --- | --- | --- | --- | --- | --- | --- | --- | --- | --- | --- | --- | --- | --- | --- | --- | --- | --- | --- | --- | --- | --- | --- | --- | --- | --- | --- | --- | --- | --- | --- | --- | --- | --- | --- | --- | --- | --- | --- | --- | --- | --- | --- | --- | --- | --- | --- | --- | --- | --- | --- | --- | --- | --- | --- | --- | --- | --- | --- | --- | --- | --- | --- | --- | --- | --- | --- | --- | --- | --- | --- | --- | --- | --- | --- | --- | --- | --- | --- | --- | --- | --- | --- | --- | --- | --- | --- | --- | --- | --- | --- | --- | --- | --- | --- | --- | --- | --- | --- | --- | --- | --- | --- | --- | --- | --- | --- | --- | --- | --- | --- | --- | --- | --- | --- | --- | --- | --- | --- | --- | --- | --- | --- | --- | --- | --- | --- | --- | --- | --- | --- | --- | --- | --- | --- | --- | --- | --- | --- | --- | --- | --- | --- | --- | --- | --- | --- | --- | --- | --- | --- | --- | --- | --- | --- | --- | --- | --- | --- | --- | --- | --- | --- | --- | --- | --- | --- | --- | --- | --- | --- | --- | --- | --- | --- | --- | --- | --- | --- | --- | --- | --- | --- | --- | --- | --- | --- | --- | --- | --- | --- | --- | --- | --- | --- | --- | --- | --- | --- | --- | --- | --- | --- | --- | --- | --- | --- | --- | --- | --- | --- | --- | --- | --- | --- | --- | --- | --- | --- | --- | --- | --- | --- | --- | --- | --- | --- | --- | --- | --- | --- | --- | --- | --- | --- | --- | --- | --- | --- | --- | --- | --- | --- | --- | --- | --- | --- | --- | --- | --- | --- | --- | --- | --- | --- | --- | --- | --- | --- | --- | --- | --- | --- | --- | --- | --- | --- | --- | --- | --- | --- | --- | --- | --- | --- | --- | --- | --- | --- | --- | --- | --- | --- | --- | --- | --- | --- | --- | --- | --- | --- | --- | --- | --- | --- | --- | --- | --- | --- | --- | --- | --- | --- | --- | --- | --- | --- | --- | --- | --- | --- | --- | --- | --- | --- | --- | --- | --- | --- | --- | --- | --- | --- | --- | --- | --- | --- | --- | --- | --- | --- | --- | --- | --- | --- | --- | --- | --- | --- | --- | --- | --- | --- | --- | --- | --- | --- | --- | --- | --- | --- | --- | --- | --- | --- | --- | --- | --- | --- | --- | --- | --- | --- | --- | --- | --- | --- | --- | --- | --- | --- | --- | --- | --- | --- | --- | --- | --- | --- | --- | --- | --- | --- | --- | --- | --- | --- | --- | --- | --- | --- | --- | --- | --- | --- | --- | --- | --- | --- | --- | --- | --- | --- | --- | --- | --- | --- | --- | --- | --- | --- | --- | --- | --- | --- | --- | --- | --- | --- | --- | --- | --- | --- | --- | --- | --- | --- | --- | --- | --- | --- | --- | --- | --- | --- | --- | --- | --- | --- | --- | --- | --- | --- | --- | --- | --- | --- | --- | --- | --- | --- | --- | --- | --- | --- | --- | --- | --- | --- | --- | --- | --- | --- | --- | --- | --- | --- | --- | --- | --- | --- | --- | --- | --- | --- | --- | --- | --- | --- | --- | --- | --- | --- | --- | --- | --- | --- | --- | --- | --- | --- | --- | --- | --- | --- | --- | --- | --- | --- | --- | --- | --- | --- | --- | --- | --- | --- | --- | --- | --- | --- | --- | --- | --- | --- | --- | --- | --- | --- | --- | --- | --- | --- | --- | --- | --- | --- | --- | --- | --- | --- | --- | --- | --- | --- | --- | --- | --- | --- | --- | --- | --- | --- | --- | --- | --- | --- | --- | --- | --- | --- | --- | --- | --- | --- | --- | --- | --- | --- | --- | --- | --- | --- | --- | --- | --- | --- | --- | --- | --- | --- | --- | --- | --- | --- | --- | --- | --- | --- | --- | --- | --- | --- | --- | --- | --- | --- | --- | --- | --- | --- | --- | --- | --- | --- | --- | --- | --- | --- | --- | --- | --- | --- | --- | --- | --- | --- | --- | --- | --- | --- | --- | --- | --- | --- | --- | --- | --- | --- | --- | --- | --- | --- | --- | --- | --- | --- | --- | --- | --- | --- | --- | --- | --- | --- | --- | --- | --- | --- | --- | --- | --- | --- | --- | --- | --- | --- | --- | --- | --- | --- | --- | --- | --- | --- | --- | --- | --- | --- | --- | --- | --- | --- | --- | --- | --- | --- | --- | --- | --- | --- | --- | --- | --- | --- | --- | --- | --- | --- | --- | --- | --- | --- | --- | --- | --- | --- | --- | --- | --- | --- | --- | --- | --- | --- | --- | --- | --- | --- | --- | --- | --- | --- | --- | --- | --- | --- | --- | --- | --- | --- | --- | --- | --- | --- | --- | --- | --- | --- | --- | --- | --- | --- | --- | --- | --- | --- | --- | --- | --- | --- | --- | --- | --- | --- | --- | --- | --- | --- | --- | --- | --- | --- | --- | --- | --- | --- | --- | --- | --- | --- | --- | --- | --- | --- | --- | --- | --- | --- | --- | --- | --- | --- | --- | --- | --- | --- | --- | --- | --- | --- | --- | --- | --- | --- | --- | --- | --- | --- | --- | --- | --- | --- | --- | --- | --- | --- | --- | --- | --- | --- | --- | --- | --- | --- | --- | --- | --- | --- | --- | --- | --- | --- | --- | --- | --- | --- | --- | --- | --- | --- | --- | --- | --- | --- | --- | --- | --- | --- | --- | --- | --- | --- | --- | --- | --- | --- | --- | --- | --- | --- | --- | --- | --- | --- | --- | --- | --- | --- | --- | --- | --- | --- | --- | --- | --- | --- | --- | --- | --- | --- | --- | --- | --- | --- | --- | --- | --- | --- | --- | --- | --- | --- | --- | --- | --- | --- | --- | --- | --- | --- | --- | --- | --- | --- | --- | --- | --- | --- | --- | --- | --- | --- | --- | --- | --- | --- | --- | --- | --- | --- | --- | --- | --- | --- | --- | --- | --- | --- | --- | --- | --- | --- | --- | --- | --- | --- | --- | --- | --- | --- | --- | --- | --- | --- | --- | --- | --- | --- | --- | --- | --- | --- | --- | --- | --- | --- | --- | --- | --- | --- | --- | --- | --- | --- | --- | --- | --- | --- | --- | --- | --- | --- | --- | --- | --- | --- | --- | --- | --- | --- | --- | --- | --- | --- | --- | --- | --- | --- | --- | --- | --- | --- | --- | --- | --- | --- | --- | --- | --- | --- | --- | --- | --- | --- | --- | --- | --- | --- | --- | --- | --- | --- | --- | --- | --- | --- | --- | --- | --- | --- | --- | --- | --- | --- | --- | --- | --- | --- | --- | --- | --- | --- | --- | --- | --- | --- | --- | --- | --- | --- | --- | --- | --- | --- | --- | --- | --- | --- | --- | --- | --- | --- | --- | --- | --- | --- | --- | --- | --- | --- | --- | --- | --- | --- | --- | --- | --- | --- | --- | --- | --- | --- | --- | --- | --- | --- | --- | --- | --- | --- | --- | --- | --- | --- | --- | --- | --- | --- | --- | --- | --- | --- | --- | --- | --- | --- | --- | --- | --- | --- | --- | --- | --- | --- | --- | --- | --- | --- | --- | --- | --- | --- | --- | --- | --- | --- | --- | --- | --- | --- | --- | --- | --- | --- | --- | --- | --- | --- | --- | --- | --- | --- | --- | --- | --- | --- | --- | --- | --- | --- | --- | --- | --- | --- | --- | --- | --- | --- | --- | --- | --- | --- | --- | --- | --- | --- | --- | --- | --- | --- | --- | --- | --- | --- | --- | --- | --- | --- | --- | --- | --- | --- | --- | --- | --- | --- | --- | --- | --- | --- | --- | --- | --- | --- | --- | --- | --- | --- | --- | --- | --- | --- | --- | --- | --- | --- | --- | --- | --- | --- | --- | --- | --- | --- | --- | --- | --- | --- | --- | --- | --- | --- | --- | --- | --- | --- | --- | --- | --- | --- | --- | --- | --- | --- | --- | --- | --- | --- | --- | --- | --- | --- | --- | --- | --- | --- | --- | --- | --- | --- | --- | --- | --- | --- | --- | --- | --- | --- | --- | --- | --- | --- | --- | --- | --- | --- | --- | --- | --- | --- | --- | --- | --- | --- | --- | --- | --- | --- | --- | --- | --- | --- | --- | --- | --- | --- | --- | --- | --- | --- | --- | --- | --- | --- | --- | --- | --- | --- | --- | --- | --- | --- | --- | --- | --- | --- | --- | --- | --- | --- | --- | --- | --- | --- | --- | --- | --- | --- | --- | --- | --- | --- | --- | --- | --- | --- | --- | --- | --- | --- | --- | --- | --- | --- | --- | --- | --- | --- | --- | --- | --- | --- | --- | --- | --- | --- | --- | --- | --- | --- | --- | --- | --- | --- | --- | --- | --- | --- | --- | --- | --- | --- | --- | --- | --- | --- | --- | --- | --- | --- | --- | --- | --- | --- | --- | --- | --- | --- | --- | --- | --- | --- | --- | --- | --- | --- | --- | --- | --- | --- | --- | --- | --- | --- | --- | --- | --- | --- | --- | --- | --- | --- | --- | --- | --- | --- | --- | --- | --- | --- | --- | --- | --- | --- | --- | --- | --- | --- | --- | --- | --- | --- | --- | --- | --- | --- | --- | --- | --- | --- | --- | --- | --- | --- | --- | --- | --- | --- | --- | --- | --- | --- | --- | --- | --- | --- | --- | --- | --- | --- | --- | --- | --- | --- | --- | --- | --- | --- | --- | --- | --- | --- | --- | --- | --- | --- | --- | --- | --- | --- | --- | --- | --- | --- | --- | --- | --- | --- | --- | --- | --- | --- | --- | --- | --- | --- | --- | --- | --- | --- | --- | --- | --- | --- | --- | --- | --- | --- | --- | --- | --- | --- | --- | --- | --- | --- | --- | --- | --- | --- | --- | --- | --- | --- | --- | --- | --- | --- | --- | --- | --- | --- | --- | --- | --- | --- | --- | --- | --- | --- | --- | --- | --- | --- | --- | --- | --- | --- | --- | --- | --- | --- | --- | --- | --- | --- | --- | --- | --- | --- | --- | --- | --- | --- | --- | --- | --- | --- | --- | --- | --- | --- | --- | --- | --- | --- | --- | --- | --- | --- | --- | --- | --- | --- | --- | --- | --- | --- | --- | --- | --- | --- | --- | --- | --- | --- | --- | --- | --- | --- | --- | --- | --- | --- | --- | --- | --- | --- | --- | --- | --- | --- | --- | --- | --- | --- | --- | --- | --- | --- | --- | --- | --- | --- | --- | --- | --- | --- | --- | --- | --- | --- | --- | --- | --- | --- | --- | --- | --- | --- | --- | --- | --- | --- | --- | --- | --- | --- | --- | --- | --- | --- | --- | --- | --- | --- | --- | --- | --- | --- | --- | --- | --- | --- | --- | --- | --- | --- | --- | --- | --- | --- | --- | --- | --- | --- | --- | --- | --- | --- | --- | --- | --- | --- | --- | --- | --- | --- | --- | --- | --- | --- | --- | --- | --- | --- | --- | --- | --- | --- | --- | --- | --- | --- | --- | --- | --- | --- | --- | --- | --- | --- | --- | --- | --- | --- | --- | --- | --- | --- | --- | --- | --- | --- | --- | --- | --- | --- | --- | --- | --- | --- | --- | --- | --- | --- | --- | --- | --- | --- | --- | --- | --- | --- | --- | --- | --- | --- | --- | --- | --- | --- | --- | --- | --- | --- | --- | --- | --- | --- | --- | --- | --- | --- | --- | --- | --- | --- | --- | --- | --- | --- | --- | --- | --- | --- | --- | --- | --- | --- | --- | --- | --- | --- | --- | --- | --- | --- | --- | --- | --- | --- | --- | --- | --- | --- | --- | --- | --- | --- | --- | --- | --- | --- | --- | --- | --- | --- | --- | --- | --- | --- | --- | --- | --- | --- | --- | --- | --- | --- | --- | --- | --- | --- | --- | --- | --- | --- | --- | --- | --- | --- | --- | --- | --- | --- | --- | --- | --- | --- | --- | --- | --- | --- | --- | --- | --- | --- | --- | --- | --- | --- | --- | --- | --- | --- | --- | --- | --- | --- | --- | --- | --- | --- | --- | --- | --- | --- | --- | --- | --- | --- | --- | --- | --- | --- | --- | --- | --- | --- | --- | --- | --- | --- | --- | --- | --- | --- | --- | --- | --- | --- | --- | --- | --- | --- | --- | --- | --- | --- | --- | --- | --- | --- | --- | --- | --- | --- | --- | --- | --- | --- | --- | --- | --- | --- | --- | --- | --- | --- | --- | --- | --- | --- | --- | --- | --- | --- | --- | --- | --- | --- | --- | --- | --- | --- | --- | --- | --- | --- | --- | --- | --- | --- | --- | --- | --- | --- | --- | --- | --- | --- | --- | --- | --- | --- | --- | --- | --- | --- | --- | --- | --- | --- | --- | --- | --- | --- | --- | --- | --- | --- | --- | --- | --- | --- | --- | --- | --- | --- | --- | --- | --- | --- | --- | --- | --- | --- | --- | --- | --- | --- | --- | --- | --- | --- | --- | --- | --- | --- | --- | --- | --- | --- | --- | --- | --- | --- | --- | --- | --- | --- | --- | --- | --- | --- | --- | --- | --- | --- | --- | --- | --- | --- | --- | --- | --- | --- | --- | --- | --- | --- | --- | --- | --- | --- | --- | --- | --- | --- | --- | --- | --- | --- | --- | --- | --- | --- | --- | --- | --- | --- | --- | --- | --- | --- | --- | --- | --- | --- | --- | --- | --- | --- | --- | --- | --- | --- | --- | --- | --- | --- | --- | --- | --- | --- | --- | --- | --- | --- | --- | --- | --- | --- | --- | --- | --- | --- | --- | --- | --- | --- | --- | --- | --- | --- | --- | --- | --- | --- | --- | --- | --- | --- | --- | --- | --- | --- | --- | --- | --- | --- | --- | --- | --- | --- | --- | --- | --- | --- | --- | --- | --- | --- | --- | --- | --- | --- | --- | --- | --- | --- | --- | --- | --- | --- | --- | --- | --- | --- | --- | --- | --- | --- | --- | --- | --- | --- | --- | --- | --- | --- | --- | --- | --- | --- | --- | --- | --- | --- | --- | --- | --- | --- | --- | --- | --- | --- | --- | --- | --- | --- | --- | --- | --- | --- | --- | --- | --- | --- | --- | --- | --- | --- | --- | --- | --- | --- | --- | --- | --- | --- | --- | --- | --- | --- | --- | --- | --- | --- | --- | --- | --- | --- | --- | --- | --- | --- | --- | --- | --- | --- | --- | --- | --- | --- | --- | --- | --- | --- | --- | --- | --- | --- | --- | --- | --- | --- | --- | --- | --- | --- | --- | --- | --- | --- | --- | --- | --- | --- | --- | --- | --- | --- | --- | --- | --- | --- | --- | --- | --- | --- | --- | --- | --- | --- | --- | --- | --- | --- | --- | --- | --- | --- | --- | --- | --- | --- | --- | --- | --- | --- | --- | --- | --- | --- | --- | --- | --- | --- | --- | --- | --- | --- | --- | --- | --- | --- | --- | --- | --- | --- | --- | --- | --- | --- | --- | --- | --- | --- | --- | --- | --- | --- | --- | --- | --- | --- | --- | --- | --- | --- | --- | --- | --- | --- | --- | --- | --- | --- | --- | --- | --- | --- | --- | --- | --- | --- | --- | --- | --- | --- | --- | --- | --- | --- | --- | --- | --- | --- | --- | --- | --- | --- | --- | --- | --- | --- | --- | --- | --- | --- | --- | --- | --- | --- | --- | --- | --- | --- | --- | --- | --- | --- | --- | --- | --- | --- | --- | --- | --- | --- | --- | --- | --- | --- | --- | --- | --- | --- | --- | --- | --- | --- | --- | --- | --- | --- | --- | --- | --- | --- | --- | --- | --- | --- | --- | --- | --- | --- | --- | --- | --- | --- | --- | --- | --- | --- | --- | --- | --- | --- | --- | --- | --- | --- | --- | --- | --- | --- | --- | --- | --- | --- | --- | --- | --- | --- | --- | --- | --- | --- | --- | --- | --- | --- | --- | --- | --- | --- | --- | --- | --- | --- | --- | --- | --- | --- | --- | --- | --- | --- | --- | --- | --- | --- | --- | --- | --- | --- | --- | --- | --- | --- | --- | --- | --- | --- | --- | --- | --- | --- | --- | --- | --- | --- | --- | --- | --- | --- | --- | --- | --- | --- | --- | --- | --- | --- | --- | --- | --- | --- | --- | --- | --- | --- | --- | --- | --- | --- | --- | --- | --- | --- | --- | --- | --- | --- | --- | --- | --- | --- | --- | --- | --- | --- | --- | --- | --- | --- | --- | --- | --- | --- | --- | --- | --- | --- | --- | --- | --- | --- | --- | --- | --- | --- | --- | --- | --- | --- | --- | --- | --- | --- | --- | --- | --- | --- | --- | --- | --- | --- | --- | --- | --- | --- | --- | --- | --- | --- | --- | --- | --- | --- | --- | --- | --- | --- | --- | --- | --- | --- | --- | --- | --- | --- | --- | --- | --- | --- | --- | --- | --- | --- | --- | --- | --- | --- | --- | --- | --- | --- | --- | --- | --- | --- | --- | --- | --- | --- | --- | --- | --- | --- | --- | --- | --- | --- | --- | --- | --- | --- | --- | --- | --- | --- | --- | --- | --- | --- | --- | --- | --- | --- | --- | --- | --- | --- | --- | --- | --- | --- | --- | --- | --- | --- | --- | --- | --- | --- | --- | --- | --- | --- | --- | --- | --- | --- | --- | --- | --- | --- | --- | --- | --- | --- | --- | --- | --- | --- | --- | --- | --- | --- | --- | --- | --- | --- | --- | --- | --- | --- | --- | --- | --- | --- | --- | --- | --- | --- | --- | --- | --- | --- | --- | --- | --- | --- | --- | --- | --- | --- | --- | --- | --- | --- | --- | --- | --- | --- | --- | --- | --- | --- | --- | --- | --- | --- | --- | --- | --- | --- | --- | --- | --- | --- | --- | --- | --- | --- | --- | --- | --- | --- | --- | --- | --- | --- | --- | --- | --- | --- | --- | --- | --- | --- | --- | --- | --- | --- | --- | --- | --- | --- | --- | --- | --- | --- | --- | --- | --- | --- | --- | --- | --- | --- | --- | --- | --- | --- | --- | --- | --- | --- | --- | --- | --- | --- | --- | --- | --- | --- | --- | --- | --- | --- | --- | --- | --- | --- | --- | --- | --- | --- | --- | --- | --- | --- | --- | --- | --- | --- | --- | --- | --- | --- | --- | --- | --- | --- | --- | --- | --- | --- | --- | --- | --- | --- | --- | --- | --- | --- | --- | --- | --- | --- | --- | --- | --- | --- | --- | --- | --- | --- | --- | --- | --- | --- | --- | --- | --- | --- | --- | --- | --- | --- | --- | --- | --- | --- | --- | --- | --- | --- | --- | --- | --- | --- | --- | --- | --- | --- | --- | --- | --- | --- | --- | --- | --- | --- | --- | --- | --- | --- | --- | --- | --- | --- | --- | --- | --- | --- | --- | --- | --- | --- | --- | --- | --- | --- | --- | --- | --- | --- | --- | --- | --- | --- | --- | --- | --- | --- | --- | --- | --- | --- | --- | --- | --- | --- | --- | --- | --- | --- | --- | --- | --- | --- | --- | --- | --- | --- | --- | --- | --- | --- | --- | --- | --- | --- | --- | --- | --- | --- | --- | --- | --- | --- | --- | --- | --- | --- | --- | --- | --- | --- | --- | --- | --- | --- | --- | --- | --- | --- | --- | --- | --- | --- | --- | --- | --- | --- | --- | --- | --- | --- | --- | --- | --- | --- | --- | --- | --- | --- | --- | --- | --- | --- | --- | --- | --- | --- | --- | --- | --- | --- | --- | --- | --- | --- | --- | --- | --- | --- | --- | --- | --- | --- | --- | --- | --- | --- | --- | --- | --- | --- | --- | --- | --- | --- | --- | --- | --- | --- | --- | --- | --- | --- | --- | --- | --- | --- | --- | --- | --- | --- | --- | --- | --- | --- | --- | --- | --- | --- | --- | --- | --- | --- | --- | --- | --- | --- | --- | --- | --- | --- | --- | --- | --- | --- | --- | --- | --- | --- | --- | --- | --- | --- | --- | --- | --- | --- | --- | --- | --- | --- | --- | --- | --- | --- | --- | --- | --- | --- | --- | --- | --- | --- | --- | --- | --- | --- | --- | --- | --- | --- | --- | --- | --- | --- | --- | --- | --- | --- | --- | --- | --- | --- | --- | --- | --- | --- | --- | --- | --- | --- | --- | --- | --- | --- | --- | --- | --- | --- | --- | --- | --- | --- | --- | --- | --- | --- | --- | --- | --- | --- | --- | --- | --- | --- | --- | --- | --- | --- | --- | --- | --- | --- | --- | --- | --- | --- | --- | --- | --- | --- | --- | --- | --- | --- | --- | --- | --- | --- | --- | --- | --- | --- | --- | --- | --- | --- | --- | --- | --- | --- | --- | --- | --- | --- | --- | --- | --- | --- | --- | --- | --- | --- | --- | --- | --- | --- | --- | --- | --- | --- | --- | --- | --- | --- | --- | --- | --- | --- | --- | --- | --- | --- | --- | --- | --- | --- | --- | --- | --- | --- | --- | --- | --- | --- | --- | --- | --- | --- | --- | --- | --- | --- | --- | --- | --- | --- | --- | --- | --- | --- | --- | --- | --- | --- | --- | --- | --- | --- | --- | --- | --- | --- | --- | --- | --- | --- | --- | --- | --- | --- | --- | --- | --- | --- | --- | --- | --- | --- | --- | --- | --- | --- | --- | --- | --- | --- | --- | --- | --- | --- | --- | --- | --- | --- | --- | --- | --- | --- | --- | --- | --- | --- | --- | --- | --- | --- | --- | --- | --- | --- | --- | --- | --- | --- | --- | --- | --- | --- | --- | --- | --- | --- | --- | --- | --- | --- | --- | --- | --- | --- | --- | --- | --- | --- | --- | --- | --- | --- | --- | --- | --- | --- | --- | --- | --- | --- | --- | --- | --- | --- | --- | --- | --- | --- | --- | --- | --- | --- | --- | --- | --- | --- | --- | --- | --- | --- | --- | --- | --- | --- | --- | --- | --- | --- | --- | --- | --- | --- | --- | --- | --- | --- | --- | --- | --- | --- | --- | --- | --- | --- | --- | --- | --- | --- | --- | --- | --- | --- | --- | --- | --- | --- | --- | --- | --- | --- | --- | --- | --- | --- | --- | --- | --- | --- | --- | --- | --- | --- | --- | --- | --- | --- | --- | --- | --- | --- | --- | --- | --- | --- | --- | --- | --- | --- | --- | --- | --- | --- | --- | --- | --- | --- | --- | --- | --- | --- | --- | --- | --- | --- |
| |  |  |  |  |  |  |  |  |  | | --- | --- | --- | --- | --- | --- | --- | --- | --- | | **Position** | **Reference** | **Sample** | **Quality** | **Type** | **Region** | **AA Exchange** | **PAM1** | **Known Variant** | | 1977 | A | G | 1110.77 | SNP | intergenic |  |  | - | | 4013 | T | C | 1613.77 | SNP | Rv0003 (recF) | Ile245Thr | 11 | - | | 7362 | G | C | 1353.77 | SNP | Rv0006 (gyrA) | Glu21Gln | 27 | - | | 7539 | A | G | 1140.77 | SNP | Rv0006 (gyrA) | Thr80Ala | 32 | genotype | | 7585 | G | C | 1380.77 | SNP | Rv0006 (gyrA) | Ser95Thr | 32 | genotype | | 9304 | G | A | 1357.77 | SNP | Rv0006 (gyrA) | Gly668Asp | 6 | - | | 11879 | A | G | 1436.77 | SNP | Rv0008c | Ser145Pro | 12 | - | | 14773 | G | T | 1404.77 | SNP | Rv0012 | Gly229Cys | 0 | - | | 14785 | T | C | 1414.77 | SNP | Rv0012 | Cys233Arg | 1 | - | | 18091 | G | A | 705.77 | SNP | Rv0015c (pknA) | silent (Thr224) | 9871 | - | | 21795 | G | A | 183.84 | SNP | Rv0018c (pstP) | Pro463Ser | 17 | - | | 21846 | C | T | 161.90 | SNP | Rv0018c (pstP) | Glu446Lys | 7 | - | | 22334 | C | T | 875.77 | SNP | Rv0018c (pstP) | Arg283His | 8 | - | | 26959 | C | G | 1195.77 | SNP | intergenic |  |  | - | | 31282 | T | G | 1416.77 | SNP | Rv0027 | Ser32Ala | 35 | - | | 32075 | T | C | 823.77 | SNP | Rv0029 | Trp7Arg | 8 | - | | 34044 | T | C | 1610.77 | SNP | intergenic |  |  | - | | 34226 | A | G | 1920.77 | SNP | intergenic |  |  | - | | 37031 | C | G | 825.77 | SNP | Rv0034 | silent (Ala55) | 9867 | - | | 42747 | C | T | 1379.77 | SNP | Rv0040c (mtc28) | Asp207Asn | 36 | - | | 42967 | G | C | 1198.77 | SNP | Rv0040c (mtc28) | silent (Pro133) | 9926 | - | | 47904 | T | C | 268.77 | SNP | Rv0043c | His66Arg | 10 | - | | 54304 | C | T | 1190.77 | SNP | Rv0050 (ponA1) | silent (Leu214) | 9947 | - | | 55553 | C | CCGT | 1173.19 | INS | Rv0050 (ponA1) |  |  | - | | 60064 | G | A | 1723.77 | SNP | Rv0057 | Gly57Ser | 16 | - | | 62049 | A | G | 922.77 | SNP | Rv0058 (dnaB) | Arg552Gly | 1 | - | | 69989 | G | A | 1292.77 | SNP | Rv0064 | Gly457Asp | 6 | - | | 70816 | A | G | 1262.77 | SNP | Rv0064 | Asn733Asp | 42 | - | | 71336 | G | C | 300.78 | SNP | Rv0064 | Arg906Pro | 5 | - | | 71584 | C | CCGAGCGCTGTTCTGGCGCT AATCTGACGCTAGAATAG | 12891.73 | INS | intergenic |  |  | - | | 75940 | G | C | 571.77 | SNP | Rv0068 | Val(s)214Leu | 3 | - | | 79498 | ACGGTGT | A | 4712.73 | DEL | Rv0071 |  |  | - | | 80616 | C | G | 1757.77 | SNP | intergenic |  |  | - | | 82840 | T | C | 1263.77 | SNP | Rv0074 | silent (Arg31) | 9913 | - | | 87257 | C | A | 1223.77 | SNP | Rv0078A | Arg182Leu | 1 | - | | 92199 | T | G | 1187.77 | SNP | Rv0083 | silent (Thr600) | 9871 | - | | 96115 | G | A | 424.90 | SNP | Rv0087 (hycE) | silent (Arg234) | 9913 | - | | 103879 | C | T | 297.78 | SNP | Rv0094c | Gly262Asp | 6 | - | | 103962 | C | T | 138.84 | SNP | Rv0094c | silent (Pro234) | 9926 | - | | 104712 | C | T | 683.77 | SNP | intergenic |  |  | - | | 104962 | G | A | 1440.77 | SNP | Rv0095c | Ala85Val(s) | 9867 | - | | 105021 | G | A | 1051.77 | SNP | Rv0095c | silent (Ser65) | 9840 | - | | 105031 | T | G | 902.77 | SNP | Rv0095c | Asp62Ala | 10 | - | | 105045 | G | C | 1074.77 | SNP | Rv0095c | Asp57Glu | 56 | - | | 105060 | G | A | 969.77 | SNP | Rv0095c | silent (Asp52) | 9859 | - | | 105063 | G | A | 1019.77 | SNP | Rv0095c | silent (Phe51) | 9946 | - | | 116000 | T | G | 1260.77 | SNP | Rv0101 (nrp) | Val2000Val(s) | 18 | - | | 122109 | A | G | 1761.77 | SNP | Rv0103c (ctpB) | Leu(s)22Ser | 28 | - | | 122794 | T | G | 1051.77 | SNP | Rv0104 | Phe160Val | 1 | - | | 125830 | G | GA | 2039.73 | INS | Rv0107c (ctpI) |  |  | - | | 131174 | T | TG | 1925.73 | INS | intergenic |  |  | - | | 132417 | C | G | 83.28 | SNP | Rv0109 (PE\_PGRS1) | Arg346Gly | 1 | - | | 133839 | C | T | 1663.77 | SNP | intergenic |  |  | - | | 146087 | T | C | 1261.77 | SNP | Rv0120c (fusA2) | Asn562Ser | 34 | - | | 153261 | G | A | 1685.77 | SNP | Rv0126 (treS) | Arg313His | 8 | - | | 154283 | T | C | 1569.77 | SNP | Rv0127 (mak) | Ser18Pro | 12 | - | | 155948 | C | CA | 2155.73 | INS | Rv0128 |  |  | - | | 177857 | G | A | 1049.77 | SNP | Rv0151c (PE1) | Leu485Leu(s) | 4 | - | | 188800 | T | C | 1168.77 | SNP | Rv0159c (PE3) | Thr14Ala | 32 | - | | 194681 | G | C | 785.77 | SNP | Rv0165c (mce1R) | silent (Leu45) | 9947 | - | | 196642 | C | T | 1004.77 | SNP | Rv0166 (fadD5) | silent (Asn550) | 9822 | - | | 206339 | T | C | 1408.77 | SNP | Rv0174 (mce1F) | Leu370Pro | 2 | - | | 207160 | G | A | 1467.77 | SNP | Rv0175 | Ser116Asn | 20 | - | | 215613 | G | A | 420.77 | SNP | Rv0184 | silent (Ala215) | 9867 | - | | 218204 | C | T | 947.77 | SNP | Rv0186 (bglS) | Arg646STOP | 2 | - | | 219120 | A | G | 1199.77 | SNP | Rv0187 | Asp139Gly | 11 | - | | 223942 | T | C | 451.77 | SNP | Rv0192 | Ser127Pro | 12 | - | | 225323 | T | C | 1530.77 | SNP | Rv0193c | Lys417Glu | 4 | - | | 227098 | T | C | 908.77 | SNP | Rv0194 | Met(s)74Thr | 22 | - | | 231114 | C | G | 1752.77 | SNP | Rv0195 | silent (Ala72) | 9867 | - | | 234477 | T | G | 1179.77 | SNP | Rv0197 | Tyr749STOP | 2 | - | | 234496 | C | CGT | 2112.73 | INS | Rv0197 |  |  | - | | 237709 | C | T | 920.77 | SNP | Rv0200 | silent (Ala168) | 9867 | - | | 256640 | T | G | 1917.77 | SNP | Rv0214 (fadD4) | Ser193Ala | 35 | - | | 257982 | T | G | 748.77 | SNP | Rv0215c (fadE3) | Asp292Ala | 10 | - | | 261869 | T | C | 793.77 | SNP | Rv0218 | Cys316Arg | 1 | - | | 265244 | C | T | 1315.77 | SNP | Rv0221 | Ala393Val(s) | 9867 | - | | 265554 | A | C | 1185.77 | SNP | Rv0222 (echA1) | silent (Val16) | 9901 | - | | 273724 | C | A | 1453.77 | SNP | Rv0228 | Pro224Thr | 5 | - | | 275859 | G | T | 1476.77 | SNP | Rv0230c (php) | Asn35Lys | 25 | - | | 278681 | C | G | 1226.77 | SNP | Rv0233 (nrdB) | His33Asp | 4 | - | | 285772 | A | C | 853.77 | SNP | Rv0236c (aftD) | silent (Pro360) | 9926 | - | | 285871 | A | G | 466.77 | SNP | Rv0236c (aftD) | silent (Val327) | 9901 | - | | 288412 | C | T | 1405.77 | SNP | intergenic |  |  | - | | 295724 | A | G | 1270.77 | SNP | intergenic |  |  | - | | 310973 | G | A | 1190.77 | SNP | Rv0259c | Ala182Val(s) | 9867 | - | | 311613 | G | T | 1436.77 | SNP | Rv0260c | silent (Val349) | 9901 | - | | 312060 | T | G | 1058.77 | SNP | Rv0260c | silent (Arg200) | 9913 | - | | 312944 | C | T | 2342.77 | SNP | Rv0261c (narK3) | Val409Ile | 33 | - | | 325038 | G | A | 1170.77 | SNP | Rv0270 (fadD2) | Gly158Ser | 16 | - | | 325039 | G | A | 1206.77 | SNP | Rv0270 (fadD2) | Gly158Asp | 6 | - | | 332357 | A | G | 1369.77 | SNP | Rv0276 | Ile204Val | 57 | - | | 333892 | G | C | 306.78 | SNP | Rv0278c (PE\_PGRS3) | Arg807Gly | 1 | - | | 335642 | A | G | 49.74 | SNP | Rv0278c (PE\_PGRS3) | silent (Ala223) | 9867 | - | | 335643 | G | T | 43.74 | SNP | Rv0278c (PE\_PGRS3) | Ala223Asp | 6 | - | | 335651 | G | C | 51.74 | SNP | Rv0278c (PE\_PGRS3) | silent (Ala220) | 9867 | - | | 335657 | G | A | 46.74 | SNP | Rv0278c (PE\_PGRS3) | silent (Gly218) | 9935 | - | | 336691 | T | C | 87.28 | SNP | Rv0279c (PE\_PGRS4) | Ser795Gly | 21 | - | | 336698 | C | G | 50.74 | SNP | Rv0279c (PE\_PGRS4) | silent (Gly792) | 9935 | - | | 336701 | A | G | 82.28 | SNP | Rv0279c (PE\_PGRS4) | silent (Gly791) | 9935 | - | | 336707 | G | A | 68.28 | SNP | Rv0279c (PE\_PGRS4) | silent (Asp789) | 9859 | - | | 336708 | T | C | 89.28 | SNP | Rv0279c (PE\_PGRS4) | Asp789Gly | 11 | - | | 336710 | A | G | 81.28 | SNP | Rv0279c (PE\_PGRS4) | silent (Ala788) | 9867 | - | | 337820 | G | A | 59.28 | SNP | Rv0279c (PE\_PGRS4) | silent (Gly418) | 9935 | - | | 338100 | T | C | 83.28 | SNP | Rv0279c (PE\_PGRS4) | Asn325Ser | 34 | - | | 338453 | A | G | 198.84 | SNP | Rv0279c (PE\_PGRS4) | silent (Ala207) | 9867 | - | | 340132 | G | A | 1286.77 | SNP | Rv0280 (PPE3) | Glu257Lys | 7 | - | | 346275 | C | G | 1046.77 | SNP | Rv0284 (eccC3) | Pro214Arg | 4 | - | | 356528 | A | G | 902.77 | SNP | Rv0292 (eccE3) | Asn217Asp | 42 | - | | 373282 | TA | T | 1579.73 | DEL | Rv0305c (PPE6) |  |  | - | | 375504 | C | T | 844.77 | SNP | Rv0305c (PPE6) | Ala70Thr | 22 | - | | 383665 | G | T | 97.77 | SNP | Rv0315 | Ala22Ser | 28 | - | | 384380 | A | C | 1292.77 | SNP | Rv0315 | Lys260Thr | 8 | - | | 386432 | C | G | 1341.77 | SNP | Rv0318c | Gly223Ala | 21 | - | | 390828 | T | C | 1177.77 | SNP | Rv0323c | Ser142Gly | 21 | - | | 396180 | C | T | 1146.77 | SNP | intergenic |  |  | - | | 403980 | G | A | 983.77 | SNP | Rv0338c | Ala621Val | 13 | - | | 404326 | T | C | 1453.77 | SNP | Rv0338c | Arg506Gly | 1 | - | | 410264 | C | T | 368.77 | SNP | Rv0341 (iniB) | silent (Ala301) | 9867 | - | | 412017 | C | G | 1130.77 | SNP | Rv0342 (iniA) | Gln394Glu | 35 | - | | 414486 | C | T | 1287.77 | SNP | Rv0344c (lpqJ) | silent (Glu152) | 9865 | - | | 420008 | A | G | 884.77 | SNP | Rv0350 (dnaK) | silent (Ala58) | 9867 | - | | 424320 | T | TC | 1772.73 | INS | Rv0354c (PPE7) |  |  | - | | 425293 | CCCGATGCCGATGTTTCCGT TTCCGGTGTTGCCGAAGCCG ATGTTGCCGGTGCCGGTATT G | C | 2564.76 | DEL | Rv0355c (PPE8) |  |  | - | | 427310 | TTGCCGAGGTTTGCAC | T | 3122.73 | DEL | Rv0355c (PPE8) |  |  | - | | 433654 | G | A | 829.77 | SNP | Rv0355c (PPE8) | silent (Gly342) | 9935 | - | | 435708 | G | A | 1078.77 | SNP | Rv0357c (purA) | silent (Thr354) | 9871 | - | | 437022 | G | A | 698.77 | SNP | Rv0358 | Ala55Thr | 22 | - | | 446479 | G | A | 1210.77 | SNP | Rv0368c | Ala16Val(s) | 9867 | - | | 454295 | T | C | 1318.77 | SNP | Rv0376c | silent (Pro26) | 9926 | - | | 457452 | T | G | 561.77 | SNP | Rv0381c | silent (Thr124) | 9871 | - | | 459399 | A | C | 1159.77 | SNP | intergenic |  |  | - | | 460864 | C | T | 1243.77 | SNP | Rv0384c (clpB) | Arg380Gln | 9 | - | | 467497 | C | CG | 1023.73 | INS | Rv0388c (PPE9) |  |  | - | | 467508 | C | CG | 1077.73 | INS | Rv0388c (PPE9) |  |  | - | | 467516 | G | C | 701.77 | SNP | Rv0388c (PPE9) | silent (Ser162) | 9840 | - | | 467526 | C | G | 671.77 | SNP | Rv0388c (PPE9) | Gly159Ala | 21 | - | | 467546 | G | C | 605.77 | SNP | Rv0388c (PPE9) | Asp152Glu | 56 | - | | 467557 | A | C | 573.77 | SNP | Rv0388c (PPE9) | Leu(s)149Val(s) | 9867 | - | | 467564 | A | C | 584.77 | SNP | Rv0388c (PPE9) | His146Gln | 23 | - | | 467585 | G | C | 747.77 | SNP | Rv0388c (PPE9) | His139Gln | 23 | - | | 467590 | T | C | 716.77 | SNP | Rv0388c (PPE9) | Thr138Ala | 32 | - | | 467621 | T | G | 784.77 | SNP | Rv0388c (PPE9) | silent (Gly127) | 9935 | - | | 467638 | G | T | 782.77 | SNP | Rv0388c (PPE9) | Gln122Lys | 12 | - | | 475178 | T | C | 975.77 | SNP | Rv0395 | Val80Ala | 18 | - | | 485098 | T | G | 2357.77 | SNP | Rv0404 (fadD30) | silent (Arg374) | 9913 | - | | 488796 | G | A | 923.77 | SNP | Rv0405 (pks6) | Val(s)1022Val | 13 | - | | 489935 | G | C | 1486.77 | SNP | Rv0405 (pks6); Rv0406c | Arg1402Pro; silent (Thr257) | 5; 9871 | - | | 493934 | T | C | 1313.77 | SNP | Rv0409 (ackA) | silent (Arg28) | 9913 | - | | 502589 | C | G | 1700.77 | SNP | Rv0417 (thiG) | Ser75Cys | 5 | - | | 503354 | G | C | 1888.77 | SNP | intergenic |  |  | - | | 506567 | C | T | 1319.77 | SNP | Rv0419 (lpqM); Rv0420c | silent (Ala494); silent (Arg135) | 9867; 9913 | - | | 513257 | T | C | 791.77 | SNP | Rv0425c (ctpH) | Met(s)689Val(s) | 9867 | - | | 541201 | A | G | 1610.77 | SNP | Rv0450c (mmpL4) | silent (Leu97) | 9947 | - | | 545087 | C | A | 1492.77 | SNP | Rv0454 | silent (Arg85) | 9913 | - | | 551525 | A | C | 1178.77 | SNP | Rv0459 | silent (Arg110) | 9913 | - | | 573262 | A | G | 544.77 | SNP | Rv0484c | silent (Gly180) | 9935 | - | | 580772 | T | A | 312.77 | SNP | intergenic |  |  | - | | 580773 | GGGGGCACCACCCGCTTGCG GGGGA | G | 3724.73 | DEL | intergenic |  |  | - | | 587974 | G | A | 1180.77 | SNP | Rv0497 | Ala200Thr | 22 | - | | 590436 | T | C | 941.77 | SNP | Rv0500 (proC) | silent (Ala118) | 9867 | - | | 591628 | T | C | 884.77 | SNP | intergenic |  |  | - | | 595232 | C | T | 846.77 | SNP | Rv0504c | Gly24Glu | 4 | - | | 595432 | C | T | 274.77 | SNP | intergenic |  |  | - | | 597816 | A | G | 1208.77 | SNP | Rv0507 (mmpL2) | silent (Ala206) | 9867 | - | | 598475 | G | A | 2308.77 | SNP | Rv0507 (mmpL2) | Arg426His | 8 | - | | 610120 | T | G | 888.77 | SNP | intergenic |  |  | - | | 628113 | C | T | 1088.77 | SNP | Rv0536 (galE3) | Ala289Val(s) | 9867 | - | | 630722 | G | C | 984.77 | SNP | Rv0538 | Arg228Pro | 5 | - | | 632850 | C | T | 1073.77 | SNP | Rv0540 | Ala160Val | 13 | - | | 637319 | G | A | 672.77 | SNP | Rv0545c (pitA) | Pro49Ser | 17 | - | | 639917 | C | A | 441.77 | SNP | Rv0548c (menB) | Ala14Ser | 28 | - | | 648002 | T | G | 1269.77 | SNP | Rv0556 | Leu15Arg | 1 | - | | 663410 | A | C | 70.77 | SNP | intergenic |  |  | - | | 663418 | A | C | 57.77 | SNP | intergenic |  |  | - | | 663419 | G | A | 76.77 | SNP | intergenic |  |  | - | | 663420 | C | A | 68.77 | SNP | intergenic |  |  | - | | 663429 | T | G | 184.77 | SNP | intergenic |  |  | - | | 665293 | A | G | 1941.77 | SNP | Rv0572c | Phe31Leu | 13 | - | | 669398 | T | C | 851.77 | SNP | Rv0575c | silent (Gln116) | 9876 | - | | 672491 | C | G | 285.78 | SNP | Rv0578c (PE\_PGRS7) | silent (Gly1142) | 9935 | - | | 673238 | A | G | 350.77 | SNP | Rv0578c (PE\_PGRS7) | silent (His893) | 9912 | - | | 684363 | C | T | 1233.77 | SNP | intergenic |  |  | - | | 685461 | C | G | 1042.77 | SNP | Rv0587 (yrbE2A) | silent (Ala111) | 9867 | - | | 685608 | T | C | 1344.77 | SNP | Rv0587 (yrbE2A) | silent (Leu160) | 9947 | - | | 685869 | G | A | 1436.77 | SNP | Rv0587 (yrbE2A) | silent (Leu247) | 9947 | - | | 686972 | T | C | 1433.77 | SNP | Rv0589 (mce2A) | Phe51Ser | 3 | - | | 690465 | T | G | 552.77 | SNP | Rv0591 (mce2C) | silent (Leu469) | 9947 | - | | 698968 | G | A | 904.77 | SNP | Rv0601c | silent (Gly9) | 9935 | - | | 721498 | G | A | 1130.77 | SNP | Rv0629c (recD) | Leu79Leu(s) | 4 | - | | 732265 | G | A | 2214.77 | SNP | Rv0635 (hadA) | Val(s)112Val | 13 | - | | 754186 | A | G | 1010.77 | SNP | Rv0658c | Leu75Pro | 2 | - | | 775639 | T | C | 1039.77 | SNP | Rv0676c (mmpL5) | Ile948Val | 57 | - | | 781395 | T | C | 1625.77 | SNP | intergenic (Rv0682-165nt) |  |  | - | | 834270 | C | T | 228.77 | SNP | Rv0743c | silent (Ala58) | 9867 | - | | 836538 | A | G | 52.74 | SNP | Rv0746 (PE\_PGRS9) | Asn280Asp | 42 | - | | 836658 | A | G | 37.74 | SNP | Rv0746 (PE\_PGRS9) | Thr320Ala | 32 | - | | 837033 | A | G | 106.28 | SNP | Rv0746 (PE\_PGRS9) | Thr445Ala | 32 | - | | 839194 | A | G | 30.74 | SNP | Rv0747 (PE\_PGRS10) | silent (Thr248) | 9871 | - | | 839334 | A | G | 106.28 | SNP | Rv0747 (PE\_PGRS10) | Lys295Arg | 19 | - | | 839515 | G | A | 85.77 | SNP | Rv0747 (PE\_PGRS10) | silent (Ala355) | 9867 | - | | 839516 | A | G | 84.77 | SNP | Rv0747 (PE\_PGRS10) | Thr356Ala | 32 | - | | 839519 | C | G | 86.77 | SNP | Rv0747 (PE\_PGRS10) | Leu357Val(s) | 4 | - | | 839520 | T | C | 92.77 | SNP | Rv0747 (PE\_PGRS10) | Leu357Pro | 2 | - | | 839534 | A | C | 86.77 | SNP | Rv0747 (PE\_PGRS10) | Ile362Leu | 22 | - | | 839545 | C | CGG | 234.77 | INS | Rv0747 (PE\_PGRS10) |  |  | - | | 841764 | G | C | 1385.77 | SNP | Rv0749A | silent (Thr37) | 9871 | - | | 846256 | C | A | 1061.77 | SNP | Rv0754 (PE\_PGRS11) | Ala33Asp | 6 | - | | 852910 | C | T | 767.77 | SNP | Rv0758 (phoR) | Pro172Leu | 3 | - | | 854252 | GCC | G | 978.73 | DEL | intergenic |  |  | - | | 857696 | A | G | 1302.77 | SNP | Rv0764c (cyp51) | silent (Ala114) | 9867 | - | | 859649 | C | T | 1166.77 | SNP | Rv0766c (cyp123) | Gly142Ser | 16 | - | | 859769 | A | G | 1507.77 | SNP | Rv0766c (cyp123) | Ser102Pro | 12 | - | | 869679 | C | T | 1176.77 | SNP | Rv0776c | Gly29Arg | 0 | - | | 874787 | G | A | 1522.77 | SNP | Rv0781 (ptrBa); Rv0782 (ptrBb) | silent (Pro185); Arg19Gln | 9926; 9 | genotype | | 874835 | C | CCG | 3209.74 | INS | Rv0781 (ptrBa); Rv0782 (ptrBb) |  |  | - | | 876378 | T | C | 1128.77 | SNP | Rv0782 (ptrBb) | silent (Ala549) | 9867 | - | | 880562 | G | T | 1113.77 | SNP | Rv0785 | Cys408Phe | 0 | - | | 882257 | T | C | 1296.77 | SNP | Rv0787 | Tyr267His | 4 | - | | 893733 | T | G | 1062.77 | SNP | Rv0800 (pepC) | Leu139Arg | 1 | - | | 900221 | T | C | 1207.77 | SNP | Rv0806c (cpsY) | Val370Val(s) | 18 | - | | 903550 | T | C | 1467.77 | SNP | Rv0808 (purF) | silent (Ala480) | 9867 | - | | 903913 | T | C | 1122.77 | SNP | Rv0809 (purM) | silent (Gly63) | 9935 | - | | 906857 | A | G | 1102.77 | SNP | Rv0812 | Ile145Met(s) | 6 | - | | 919384 | T | C | 1708.77 | SNP | Rv0825c | Tyr57Cys | 3 | - | | 921813 | C | G | 1923.77 | SNP | Rv0829 | Ala80Gly | 21 | - | | 944941 | A | G | 1159.77 | SNP | Rv0848 (cysK2) | Arg2Gly | 1 | - | | 945214 | G | A | 1507.77 | SNP | Rv0848 (cysK2) | Gly93Ser | 16 | - | | 949535 | T | C | 979.77 | SNP | Rv0853c (pdc) | silent (Ala528) | 9867 | - | | 950119 | C | T | 631.77 | SNP | Rv0853c (pdc) | Ala334Thr | 22 | - | | 954253 | C | G | 45.77 | SNP | Rv0858c (dapC) | Arg223Pro | 5 | - | | 955524 | A | G | 1386.77 | SNP | Rv0859 (fadA) | Ser150Gly | 21 | - | | 955983 | C | T | 673.77 | SNP | Rv0859 (fadA) | Pro303Ser | 17 | - | | 956644 | C | T | 980.77 | SNP | Rv0860 (fadB) | Leu118Leu(s) | 4 | - | | 964825 | C | T | 54.77 | SNP | Rv0867c (rpfA) | silent (Ala237) | 9867 | - | | 968426 | A | AGCCGGGTTG | 1336.73 | INS | Rv0872c (PE\_PGRS15) |  |  | - | | 976896 | TTG | T | 3524.73 | DEL | Rv0878c (PPE13) |  |  | - | | 979314 | C | T | 995.77 | SNP | Rv0880 | silent (Ile127) | 9872 | - | | 979704 | G | C | 1122.77 | SNP | Rv0881 | Gly115Arg | 0 | - | | 984493 | C | T | 1040.77 | SNP | Rv0886 (fprB) | Leu231Leu(s) | 4 | - | | 986463 | G | C | 1917.77 | SNP | intergenic |  |  | - | | 990001 | G | C | 1287.77 | SNP | Rv0890c | Pro866Ala | 22 | - | | 990626 | T | A | 1032.77 | SNP | Rv0890c | Leu657Phe | 6 | - | | 993346 | A | C | 2070.77 | SNP | Rv0891c | Val37Gly | 5 | - | | 996871 | G | A | 1145.77 | SNP | Rv0894 | Leu(s)116Leu | 3 | - | | 1002282 | G | GC | 1319.73 | INS | Rv0897c |  |  | - | | 1010204 | C | CG | 2217.73 | INS | Rv0907 |  |  | - | | 1015344 | G | GGT | 2031.73 | INS | intergenic |  |  | - | | 1020044 | C | T | 1073.77 | SNP | intergenic |  |  | - | | 1025106 | T | C | 1629.77 | SNP | Rv0919 | silent (Phe141) | 9946 | - | | 1037012 | T | C | 628.77 | SNP | Rv0930 (pstA1) | Met(s)5Thr | 22 | - | | 1037911 | C | T | 1763.77 | SNP | Rv0930 (pstA1) | Arg305STOP | 2 | - | | 1044905 | G | T | 1246.77 | SNP | Rv0936 (pstA2) | Ala197Ser | 28 | - | | 1047165 | T | C | 1064.77 | SNP | Rv0938 (ligD) | Cys344Arg | 1 | - | | 1056916 | T | G | 896.77 | SNP | intergenic |  |  | - | | 1068151 | T | C | 1536.77 | SNP | Rv0956 (purN) | silent (His197) | 9912 | - | | 1068432 | A | G | 1488.77 | SNP | Rv0957 (purH) | silent (Pro76) | 9926 | - | | 1070702 | T | C | 819.77 | SNP | Rv0958 | Ser274Pro | 12 | - | | 1074558 | G | A | 1248.77 | SNP | Rv0962c (lprP) | Pro186Leu | 3 | - | | 1075279 | T | C | 1615.77 | SNP | intergenic |  |  | - | | 1076309 | G | T | 1494.77 | SNP | Rv0964c | Pro124Thr | 5 | - | | 1077312 | A | G | 656.77 | SNP | Rv0966c | Val(s)175Ala | 9867 | - | | 1079927 | C | A | 827.77 | SNP | Rv0969 (ctpV) | silent (Thr395) | 9871 | - | | 1081681 | T | C | 1022.77 | SNP | Rv0970 | silent (Val210) | 9901 | - | | 1087193 | G | C | 1197.77 | SNP | Rv0974c (accD2) | Asn51Lys | 25 | - | | 1093406 | A | G | 569.77 | SNP | Rv0978c (PE\_PGRS17) | silent (Val317) | 9901 | - | | 1093928 | G | A | 56.28 | SNP | Rv0978c (PE\_PGRS17) | silent (Asn143) | 9822 | - | | 1096470 | G | C | 584.77 | SNP | intergenic |  |  | - | | 1096633 | T | G | 1052.77 | SNP | intergenic |  |  | - | | 1100234 | T | C | 1193.77 | SNP | Rv0983 (pepD) | Leu390Pro | 2 | - | | 1106422 | T | C | 2168.77 | SNP | Rv0989c (grcC2) | Ile321Val | 57 | - | | 1109975 | A | G | 995.77 | SNP | Rv0993 (galU) | Gln235Arg | 10 | - | | 1126889 | G | C | 1280.77 | SNP | Rv1007c (metS) | Arg39Gly | 1 | - | | 1127648 | C | A | 1760.77 | SNP | Rv1008 (tatD) | Thr187Asn | 9 | - | | 1137517 | C | T | 1312.77 | SNP | Rv1018c (glmU) | Ala182Thr | 22 | - | | 1149551 | C | T | 969.77 | SNP | Rv1028c (kdpD) | silent (Glu712) | 9865 | - | | 1150585 | G | A | 699.77 | SNP | Rv1028c (kdpD) | Pro368Ser | 17 | - | | 1163134 | T | C | 1314.77 | SNP | Rv1040c (PE8) | silent (Gly81) | 9935 | - | | 1165521 | T | TA | 1884.73 | INS | intergenic |  |  | - | | 1168715 | C | CT | 2107.73 | INS | Rv1046c |  |  | - | | 1169235 | CG | C | 3013.73 | DEL | intergenic |  |  | - | | 1169307 | C | T | 1538.77 | SNP | intergenic |  |  | - | | 1170404 | C | A | 107.03 | SNP | Rv1047 | Gln328Lys | 12 | - | | 1177446 | T | C | 1529.77 | SNP | Rvnt17 | tRNA | tRNA | - | | 1178116 | T | C | 1981.77 | SNP | Rv1056 | silent (Thr163) | 9871 | - | | 1184605 | C | A | 1199.77 | SNP | Rv1061 | Asp197Glu | 56 | - | | 1199762 | A | G | 1360.77 | SNP | Rv1075c | silent (Arg203) | 9913 | - | | 1200418 | A | G | 1532.77 | SNP | intergenic |  |  | - | | 1204882 | A | G | 1513.77 | SNP | Rv1079 (metB) | Val272Val(s) | 18 | - | | 1208969 | T | C | 484.77 | SNP | Rv1084 | Val445Ala | 18 | - | | 1220680 | T | C | 1399.77 | SNP | Rv1093 (glyA1) | Val36Ala | 18 | - | | 1224367 | T | C | 1097.77 | SNP | intergenic |  |  | - | | 1248978 | T | C | 1143.77 | SNP | Rv1125 | silent (Ala299) | 9867 | - | | 1276588 | C | G | 1018.77 | SNP | Rv1148c | silent (Ala387) | 9867 | - | | 1281118 | T | C | 1360.77 | SNP | Rv1154c | Thr123Ala | 32 | - | | 1292102 | A | G | 1326.77 | SNP | Rv1162 (narH) | silent (Pro346) | 9926 | - | | 1313337 | A | AG | 1609.73 | INS | intergenic |  |  | - | | 1313338 | A | C | 1063.77 | SNP | intergenic |  |  | - | | 1315191 | A | C | 1146.77 | SNP | Rv1180 (pks3) | STOP489Tyr | 1 | - | | 1315884 | G | A | 785.77 | SNP | Rv1181 (pks4) | silent (Ala217) | 9867 | - | | 1327890 | G | A | 1212.77 | SNP | Rv1186c | silent (Asp472) | 9859 | - | | 1328222 | T | C | 1209.77 | SNP | Rv1186c | Asn362Asp | 42 | - | | 1328687 | G | C | 802.77 | SNP | Rv1186c | Pro207Ala | 22 | - | | 1341102 | C | T | 1033.77 | SNP | Rv1198 (esxL) | Arg33Cys | 1 | - | | 1341103 | G | C | 1155.77 | SNP | Rv1198 (esxL) | Arg33Pro | 5 | - | | 1341624 | G | T | 152.90 | SNP | Rv1199c | Gln328Lys | 12 | - | | 1357977 | C | T | 615.77 | SNP | Rv1215c | Glu490Lys | 7 | - | | 1365837 | C | CG | 1407.73 | INS | intergenic |  |  | - | | 1371182 | G | A | 939.77 | SNP | Rv1228 (lpqX) | Gly88Glu | 4 | - | | 1374065 | T | C | 755.77 | SNP | Rv1230c | Ser45Gly | 21 | - | | 1375724 | A | C | 1360.77 | SNP | Rv1232c | Cys149Gly | 1 | - | | 1382628 | T | C | 1491.77 | SNP | Rv1239c (corA) | Lys139Glu | 4 | - | | 1393626 | A | G | 644.77 | SNP | Rv1249c | silent (Leu119) | 9947 | - | | 1396922 | T | C | 1485.77 | SNP | Rv1251c | silent (Thr773) | 9871 | - | | 1411210 | T | G | 1078.77 | SNP | Rv1263 (amiB2) | Val260Val(s) | 18 | - | | 1413148 | C | T | 1471.77 | SNP | intergenic |  |  | - | | 1414021 | C | T | 981.77 | SNP | Rv1266c (pknH) | Arg607Gln | 9 | - | | 1433114 | G | A | 1628.77 | SNP | Rv1280c (oppA) | silent (Gly109) | 9935 | - | | 1439332 | C | T | 1128.77 | SNP | Rv1286 (cysN) | silent (Val142) | 9901 | - | | 1440469 | C | G | 2161.77 | SNP | Rv1286 (cysN) | silent (Pro521) | 9926 | - | | 1445781 | A | G | 1203.77 | SNP | Rv1291c | silent (Ala18) | 9867 | - | | 1457144 | C | T | 711.77 | SNP | Rv1300 (hemK) | Arg194Cys | 1 | - | | 1465155 | C | T | 1562.77 | SNP | Rv1309 (atpG) | Ala91Val | 13 | - | | 1468208 | A | C | 775.77 | SNP | Rv1313c | Leu433Arg | 1 | - | | 1468380 | T | C | 325.78 | SNP | Rv1313c | Lys376Glu | 4 | - | | 1471659 | C | T | 1736.77 | SNP | intergenic |  |  | - | | 1475855 | A | G | 1538.77 | SNP | Rvnr02 | rRNA | rRNA | - | | 1480174 | C | G | 112.77 | SNP | Rv1318c | silent (Leu217) | 9947 | - | | 1480176 | G | T | 77.77 | SNP | Rv1318c | Leu217Met(s) | 4 | - | | 1480186 | C | T | 41.77 | SNP | Rv1318c | silent (Ala213) | 9867 | - | | 1480189 | C | G | 81.77 | SNP | Rv1318c | Leu(s)212Phe | 1 | - | | 1480197 | G | C | 51.77 | SNP | Rv1318c | Arg210Gly | 1 | - | | 1480198 | T | C | 30.77 | SNP | Rv1318c | silent (Pro209) | 9926 | - | | 1480205 | G | A | 36.77 | SNP | Rv1318c | Pro207Leu | 3 | - | | 1480219 | C | T | 78.77 | SNP | Rv1318c | silent (Ala202) | 9867 | - | | 1480945 | C | G | 948.77 | SNP | Rv1319c | silent (Thr519) | 9871 | - | | 1480948 | C | T | 912.77 | SNP | Rv1319c | silent (Glu518) | 9865 | - | | 1480972 | T | C | 845.77 | SNP | Rv1319c | silent (Glu510) | 9865 | - | | 1481185 | A | C | 550.77 | SNP | Rv1319c | Asp439Glu | 56 | - | | 1481321 | A | G | 591.77 | SNP | Rv1319c | Val394Ala | 18 | - | | 1481468 | G | T | 920.77 | SNP | Rv1319c | Ala345Asp | 6 | - | | 1482627 | T | C | 1417.77 | SNP | Rv1320c | Thr531Ala | 32 | - | | 1483652 | A | G | 1443.77 | SNP | Rv1320c | Leu189Pro | 2 | - | | 1484708 | A | C | 1156.77 | SNP | Rv1321 | Ser144Arg | 6 | - | | 1488433 | A | G | 41.77 | SNP | Rv1325c (PE\_PGRS24) | silent (Asp511) | 9859 | - | | 1488435 | C | A | 32.77 | SNP | Rv1325c (PE\_PGRS24) | Asp511Tyr | 0 | - | | 1499274 | C | G | 978.77 | SNP | Rv1330c (pncB1) | Gly429Ala | 21 | - | | 1526819 | C | A | 2041.77 | SNP | Rv1358 | silent (Arg70) | 9913 | - | | 1532827 | A | T | 195.90 | SNP | Rv1361c (PPE19) | Phe269Leu | 13 | - | | 1536251 | G | T | 1045.77 | SNP | Rv1364c | Ala465Glu | 10 | - | | 1537710 | AAC | A | 3514.73 | DEL | intergenic |  |  | - | | 1537771 | G | C | 1530.77 | SNP | intergenic |  |  | - | | 1544349 | T | G | 1764.77 | SNP | Rv1371 | Trp331Gly | 0 | - | | 1547125 | T | C | 1212.77 | SNP | Rv1374c | Thr136Ala | 32 | - | | 1552547 | G | A | 1055.77 | SNP | Rv1378c | Arg37Trp | 2 | - | | 1564215 | C | G | 40.77 | SNP | Rv1388 (mihF) | silent (Gly174) | 9935 | - | | 1570566 | C | A | 1426.77 | SNP | Rv1394c (cyp132) | Arg135Leu | 1 | - | | 1572200 | A | G | 171.84 | SNP | Rv1396c (PE\_PGRS25) | Val553Ala | 18 | - | | 1588899 | G | T | 1048.77 | SNP | Rv1412 (ribC) | silent (Ala111) | 9867 | - | | 1597405 | G | A | 455.77 | SNP | Rv1422 | silent (Pro175) | 9926 | - | | 1597696 | G | A | 915.77 | SNP | Rv1422 | silent (Glu272) | 9865 | - | | 1604691 | A | G | 1801.77 | SNP | Rv1428c | silent (Pro23) | 9926 | - | | 1609840 | A | G | 828.77 | SNP | Rv1431 | silent (Pro586) | 9926 | - | | 1612624 | T | TATCGGTACCGGTGCGCCAG GG | 2570.73 | INS | Rv1435c |  |  | - | | 1613035 | T | C | 1936.77 | SNP | intergenic |  |  | - | | 1618616 | C | T | 52.77 | SNP | Rv1441c (PE\_PGRS26) | Gly357Ser | 16 | - | | 1620135 | A | G | 860.77 | SNP | Rv1442 (bisC) | silent (Gly115) | 9935 | - | | 1623955 | C | T | 1095.77 | SNP | Rv1445c (devB) | Arg168His | 8 | - | | 1630148 | A | C | 1648.77 | SNP | Rv1449c (tkt) | Tyr18Asp | 0 | - | | 1633344 | A | G | 157.90 | SNP | Rv1450c (PE\_PGRS27) | silent (Asn428) | 9822 | - | | 1634633 | A | T | 896.77 | SNP | intergenic |  |  | - | | 1634636 | T | A | 932.77 | SNP | intergenic |  |  | - | | 1636991 | T | C | 47.74 | SNP | Rv1452c (PE\_PGRS28) | silent (Gly413) | 9935 | - | | 1636996 | G | C | 84.28 | SNP | Rv1452c (PE\_PGRS28) | Arg412Gly | 1 | - | | 1637006 | G | A | 83.28 | SNP | Rv1452c (PE\_PGRS28) | silent (Val408) | 9901 | - | | 1637009 | G | A | 83.28 | SNP | Rv1452c (PE\_PGRS28) | silent (Gly407) | 9935 | - | | 1637012 | A | G | 86.28 | SNP | Rv1452c (PE\_PGRS28) | silent (Gly406) | 9935 | - | | 1637015 | A | G | 51.74 | SNP | Rv1452c (PE\_PGRS28) | silent (Ala405) | 9867 | - | | 1637018 | G | C | 85.28 | SNP | Rv1452c (PE\_PGRS28) | silent (Gly404) | 9935 | - | | 1638182 | C | T | 37.74 | SNP | Rv1452c (PE\_PGRS28) | silent (Ser16) | 9840 | - | | 1638183 | G | A | 36.74 | SNP | Rv1452c (PE\_PGRS28) | Ser16Leu(s) | 35 | - | | 1638188 | C | T | 37.74 | SNP | Rv1452c (PE\_PGRS28) | silent (Ala14) | 9867 | - | | 1638191 | C | G | 40.74 | SNP | Rv1452c (PE\_PGRS28) | silent (Ala13) | 9867 | - | | 1638194 | G | C | 41.74 | SNP | Rv1452c (PE\_PGRS28) | silent (Ala12) | 9867 | - | | 1638211 | T | C | 146.90 | SNP | Rv1452c (PE\_PGRS28) | Thr7Ala | 32 | - | | 1638212 | T | C | 152.90 | SNP | Rv1452c (PE\_PGRS28) | Val6Val(s) | 18 | - | | 1639594 | C | A | 1015.77 | SNP | Rv1453 | Pro405Gln | 6 | - | | 1644362 | C | T | 131.77 | SNP | intergenic |  |  | - | | 1650072 | A | G | 1253.77 | SNP | Rv1462 | Asn183Asp | 42 | - | | 1655829 | G | GC | 287.73 | INS | Rv1468c (PE\_PGRS29) |  |  | - | | 1676290 | C | A | 1648.77 | SNP | Rv1486c | Lys198Asn | 13 | - | | 1689349 | C | T | 1508.77 | SNP | Rv1498c | Arg191His | 8 | - | | 1692141 | A | C | 2333.77 | SNP | Rv1501 | silent (Ile84) | 9872 | - | | 1693561 | A | G | 2687.77 | SNP | Rv1502 | Tyr213Cys | 3 | - | | 1695518 | C | A | 2399.77 | SNP | Rv1505c | silent (Ser143) | 9840 | - | | 1696464 | C | G | 1314.77 | SNP | intergenic |  |  | - | | 1698911 | G | A | 1529.77 | SNP | Rv1508c | silent (Gly328) | 9935 | - | | 1706119 | T | C | 1105.77 | SNP | Rv1514c | silent (Ser159) | 9840 | - | | 1728837 | A | G | 1514.77 | SNP | intergenic |  |  | - | | 1736577 | A | G | 1422.77 | SNP | Rv1536 (ileS) | Glu20Gly | 7 | - | | 1751042 | C | T | 1198.77 | SNP | Rv1547 (dnaE1) | Pro1117Ser | 17 | - | | 1752561 | T | C | 902.77 | SNP | Rv1548c (PPE21) | Asp258Gly | 11 | - | | 1753519 | G | GC | 1805.73 | INS | Rv1549 (fadD11.1) |  |  | - | | 1759252 | G | T | 1291.77 | SNP | Rv1552 (frdA) | silent (Ser524) | 9840 | genotype | | 1760292 | A | G | 1363.77 | SNP | Rv1554 (frdC) | Met(s)40Val(s) | 9867 | - | | 1778430 | T | C | 640.77 | SNP | Rv1570 (bioD) | Met(s)191Thr | 22 | - | | 1779370 | G | C | 1541.77 | SNP | Rv1573 | silent (Thr19) | 9871 | - | | 1780048 | A | G | 595.77 | SNP | Rv1574 | His40Arg | 10 | - | | 1780274 | A | C | 197.77 | SNP | Rv1575 | Lys26Gln | 6 | - | | 1780275 | A | G | 169.77 | SNP | Rv1575 | Lys26Arg | 19 | - | | 1780586 | C | CG | 1402.73 | INS | Rv1575 |  |  | - | | 1781577 | G | A | 660.85 | SNP | Rv1576c | Thr163Ile | 7 | - | | 1788086 | C | CT | 1078.73 | INS | Rv1586c |  |  | - | | 1788613 | C | T | 858.77 | SNP | Rv1587c | Gly184Asp | 6 | - | | 1789446 | C | T | 94.03 | SNP | Rv1588c | Val131Ile | 33 | - | | 1789516 | A | G | 215.80 | SNP | Rv1588c | silent (Gly107) | 9935 | - | | 1789564 | C | T | 157.84 | SNP | Rv1588c | silent (Arg91) | 9913 | - | | 1789565 | C | A | 161.84 | SNP | Rv1588c | Arg91Leu | 1 | - | | 1789650 | C | T | 443.77 | SNP | Rv1588c | Ala63Thr | 22 | - | | 1789654 | A | G | 521.77 | SNP | Rv1588c | silent (Leu61) | 9947 | - | | 1789671 | C | T | 611.77 | SNP | Rv1588c | Ala56Thr | 22 | - | | 1789675 | A | C | 676.77 | SNP | Rv1588c | silent (Gly54) | 9935 | - | | 1789678 | C | G | 610.77 | SNP | Rv1588c | Val(s)53Val | 13 | - | | 1789766 | T | G | 996.77 | SNP | Rv1588c | Asp24Ala | 10 | - | | 1798355 | G | A | 1335.77 | SNP | Rv1597 | Gly21Asp | 6 | - | | 1803265 | G | A | 1741.77 | SNP | Rv1602 (hisH) | Ser201Asn | 20 | - | | 1804409 | C | A | 1152.77 | SNP | Rv1604 (impA) | Pro124Gln | 6 | - | | 1808795 | A | C | 1625.77 | SNP | Rv1609 (trpE) | Asp298Ala | 10 | - | | 1814629 | G | A | 806.77 | SNP | intergenic |  |  | - | | 1817976 | A | T | 1758.77 | SNP | Rv1618 (tesB1) | His121Leu | 4 | - | | 1836286 | G | C | 960.77 | SNP | intergenic |  |  | - | | 1847919 | C | G | 553.77 | SNP | Rv1639c | silent (Thr180) | 9871 | - | | 1854300 | T | C | 595.77 | SNP | Rv1644 (tsnR) | Leu232Pro | 2 | - | | 1856777 | G | C | 1094.77 | SNP | Rv1647 | Ala2Pro | 13 | - | | 1864698 | C | T | 347.77 | SNP | Rv1651c (PE\_PGRS30) | Ala229Thr | 22 | - | | 1885772 | G | A | 824.77 | SNP | Rv1662 (pks8) | Ala1357Thr | 22 | - | | 1894300 | G | GGTCTTGCCGC | 2905.73 | INS | Rv1668c |  |  | - | | 1901493 | T | C | 1194.77 | SNP | Rv1676 | silent (Ser149) | 9840 | - | | 1907296 | G | C | 1535.77 | SNP | Rv1682 | silent (Ala298) | 9867 | - | | 1917289 | C | T | 437.77 | SNP | Rv1692 | Arg198Trp | 2 | - | | 1917972 | A | G | 841.77 | SNP | Rv1694 (tlyA) | silent (Leu11) | 9947 | - | | 1931179 | C | A | 1286.77 | SNP | Rv1704c (cycA) | Arg93Leu | 1 | - | | 1933988 | G | A | 1942.77 | SNP | intergenic |  |  | - | | 1942489 | T | C | 605.77 | SNP | Rv1714 | Phe213Leu | 13 | - | | 1944402 | T | C | 939.77 | SNP | Rv1716 | Val276Ala | 18 | - | | 1950767 | T | C | 2225.77 | SNP | Rv1724c | silent (Lys95) | 9926 | - | | 1955910 | T | G | 1486.77 | SNP | Rv1730c | silent (Arg446) | 9913 | - | | 1960284 | C | A | 1442.77 | SNP | Rv1733c | Gln68His | 20 | - | | 1967237 | C | A | 1146.77 | SNP | Rv1739c | Arg134Leu | 1 | - | | 1978166 | G | T | 963.77 | SNP | Rv1750c (fadD1) | Gln468Lys | 12 | - | | 1982961 | GC | G | 1333.50 | DEL | Rv1753c (PPE24) |  |  | - | | 1987394 | C | T | 1598.77 | SNP | intergenic |  |  | - | | 1989042 | A | T | 69.28 | SNP | Rv1758 (cut1) | Met(s)1Leu(s) | 9867 | - | | 1989043 | T | C | 89.28 | SNP | Rv1758 (cut1) | Met(s)1Thr | 22 | - | | 1989044 | G | A | 83.28 | SNP | Rv1758 (cut1) | Met(s)1Ile | 2 | - | | 1990942 | C | T | 186.90 | SNP | Rv1759c (wag22) | Gly546Ser | 16 | - | | 1992323 | G | GCCCC,GCCC | 489.19 | MUL | Rv1759c (wag22) |  |  | - | | 1993808 | A | T | 1218.77 | SNP | Rv1760 | Glu219Val(s) | 17 | - | | 1994939 | G | A | 1495.77 | SNP | Rv1761c | Thr39Ile | 7 | - | | 2009881 | T | C | 848.77 | SNP | Rv1775 | Ile237Thr | 11 | - | | 2015696 | C | T | 887.77 | SNP | Rv1781c (malQ) | Arg594Gln | 9 | - | | 2022868 | T | C | 838.77 | SNP | Rv1783 (eccC5) | silent (Ser1204) | 9840 | - | | 2033748 | G | C | 1721.77 | SNP | Rv1795 (eccD5); Rv1796 (mycP5) | silent (Arg503); Gly7Ala | 9913; 21 | - | | 2038672 | T | C | 182.90 | SNP | intergenic |  |  | - | | 2045310 | A | G | 556.77 | SNP | Rv1803c (PE\_PGRS32) | silent (Ile511) | 9872 | - | | 2049065 | T | C | 1010.77 | SNP | intergenic |  |  | - | | 2049097 | G | C | 990.77 | SNP | intergenic |  |  | - | | 2051117 | C | T | 868.77 | SNP | Rv1808 (PPE32) | silent (Ser399) | 9840 | - | | 2051746 | T | C | 758.77 | SNP | Rv1809 (PPE33) | silent (Ala155) | 9867 | - | | 2052035 | G | T | 1348.77 | SNP | Rv1809 (PPE33) | Val(s)252Leu(s) | 9867 | - | | 2055271 | A | G | 1228.77 | SNP | Rv1812c | Leu30Pro | 2 | - | | 2057774 | A | T | 1159.77 | SNP | Rv1815 | Ile83Phe | 8 | - | | 2074570 | G | C | 54.77 | SNP | intergenic |  |  | - | | 2088619 | CCCGCCGTTG | C | 499.73 | DEL | Rv1840c (PE\_PGRS34) |  |  | - | | 2094911 | ACAGCGT | A | 3551.73 | DEL | Rv1844c (gnd1) |  |  | - | | 2096186 | A | G | 687.77 | SNP | Rv1846c (blaI) | silent (Thr138) | 9871 | - | | 2103768 | C | A | 1271.77 | SNP | Rv1855c | Ala114Ser | 28 | - | | 2109523 | C | CG | 1478.73 | INS | intergenic |  |  | - | | 2116903 | C | T | 1525.77 | SNP | Rv1867 | silent (Gly380) | 9935 | - | | 2128870 | A | G | 2251.77 | SNP | Rv1878 (glnA3) | silent (Leu283) | 9947 | - | | 2130552 | C | T | 1251.77 | SNP | Rv1880c (cyp140) | Val436Ile | 33 | - | | 2133468 | T | TTCGCATGCCGTCACC | 2918.73 | INS | Rv1883c |  |  | - | | 2135870 | T | C | 1133.77 | SNP | intergenic |  |  | - | | 2143217 | T | C | 1181.77 | SNP | Rv1895 | Ser233Pro | 12 | - | | 2143328 | G | C | 1455.77 | SNP | Rv1895 | Val(s)270Leu | 3 | - | | 2147022 | A | C | 1660.77 | SNP | Rv1900c (lipJ) | Ile204Met(s) | 6 | - | | 2149855 | C | CA | 2460.73 | INS | Rv1902c (nanT) |  |  | - | | 2154339 | T | G | 1300.77 | SNP | Rv1908c (katG) | silent (Ala591) | 9867 | - | | 2163375 | T | C | 626.77 | SNP | Rv1917c (PPE34) | Asn1313Asp | 42 | - | | 2163412 | A | G | 1059.77 | SNP | Rv1917c (PPE34) | silent (Val1300) | 9901 | - | | 2163415 | C | A | 1006.77 | SNP | Rv1917c (PPE34) | silent (Pro1299) | 9926 | - | | 2163417 | G | C | 1142.77 | SNP | Rv1917c (PPE34) | Pro1299Ala | 22 | - | | 2163419 | C | T | 1054.77 | SNP | Rv1917c (PPE34) | Ser1298Asn | 20 | - | | 2163421 | C | G | 978.77 | SNP | Rv1917c (PPE34) | silent (Thr1297) | 9871 | - | | 2163444 | T | C | 84.77 | SNP | Rv1917c (PPE34) | Asn1290Asp | 42 | - | | 2163790 | A | C | 952.77 | SNP | Rv1917c (PPE34) | silent (Pro1174) | 9926 | - | | 2165286 | A | C | 1071.77 | SNP | Rv1917c (PPE34) | Ser676Ala | 35 | - | | 2165503 | T | A | 1065.77 | SNP | Rv1917c (PPE34) | silent (Ala603) | 9867 | - | | 2165928 | G | T | 1270.77 | SNP | Rv1917c (PPE34) | Pro462Thr | 5 | - | | 2184781 | G | T | 1162.77 | SNP | Rv1933c (fadE18) | silent (Gly59) | 9935 | - | | 2196879 | T | A | 1904.77 | SNP | Rv1945 | silent (Leu297) | 9947 | - | | 2196882 | A | G | 1981.77 | SNP | Rv1945 | silent (Lys298) | 9926 | - | | 2196964 | A | C | 453.77 | SNP | Rv1945 | Asn326His | 18 | - | | 2196969 | G | C | 427.77 | SNP | Rv1945 | silent (Ala327) | 9867 | - | | 2196970 | C | A | 355.77 | SNP | Rv1945 | His328Asn | 21 | - | | 2198579 | GAACCA | G | 5311.73 | DEL | intergenic |  |  | - | | 2207591 | T | TC | 2343.73 | INS | intergenic |  |  | - | | 2211826 | A | G | 1067.77 | SNP | Rv1968 (mce3C) | silent (Lys67) | 9926 | - | | 2213306 | G | T | 901.77 | SNP | Rv1969 (mce3D) | Arg151Leu | 1 | - | | 2216443 | C | A | 1097.77 | SNP | Rv1971 (mce3F) | Ala396Glu | 10 | - | | 2220512 | T | G | 1261.77 | SNP | Rv1977 | silent (Ser253) | 9840 | - | | 2223293 | T | C | 1273.77 | SNP | intergenic |  |  | - | | 2228967 | A | G | 1481.77 | SNP | intergenic |  |  | - | | 2233073 | G | A | 1404.77 | SNP | Rv1989c | Ala76Val(s) | 9867 | - | | 2234247 | CG | C | 2652.73 | DEL | intergenic |  |  | - | | 2251999 | A | G | 1635.77 | SNP | intergenic |  |  | - | | 2260151 | A | G | 679.77 | SNP | intergenic |  |  | - | | 2260154 | C | T | 569.77 | SNP | intergenic |  |  | - | | 2260171 | T | C | 737.77 | SNP | intergenic |  |  | - | | 2260174 | C | T | 861.77 | SNP | intergenic |  |  | - | | 2260196 | C | CA | 1117.73 | INS | intergenic |  |  | - | | 2260199 | C | T | 692.77 | SNP | intergenic |  |  | - | | 2260212 | G | T | 656.77 | SNP | intergenic |  |  | - | | 2260214 | G | C | 730.77 | SNP | intergenic |  |  | - | | 2260220 | C | T | 684.77 | SNP | intergenic |  |  | - | | 2260222 | C | G | 721.77 | SNP | intergenic |  |  | - | | 2260231 | T | C | 737.77 | SNP | intergenic |  |  | - | | 2260525 | C | T | 860.77 | SNP | intergenic |  |  | - | | 2264782 | C | A | 1211.77 | SNP | Rv2017 | Ala262Glu | 10 | - | | 2265059 | T | G | 757.77 | SNP | intergenic |  |  | - | | 2266487 | G | C | 917.77 | SNP | Rv2020c | silent (Leu78) | 9947 | - | | 2266504 | T | TA | 1355.73 | INS | Rv2020c |  |  | - | | 2266508 | A | T | 615.77 | SNP | Rv2020c | Asp71Glu | 56 | - | | 2266511 | GT | G | 1039.73 | DEL | Rv2020c |  |  | - | | 2266517 | T | C | 517.77 | SNP | Rv2020c | silent (Glu68) | 9865 | - | | 2266550 | G | T | 869.77 | SNP | Rv2020c | silent (Gly57) | 9935 | - | | 2266553 | C | G | 791.77 | SNP | Rv2020c | silent (Ser56) | 9840 | - | | 2266583 | C | G | 1112.77 | SNP | Rv2020c | Glu46Asp | 53 | - | | 2266598 | G | C | 1092.77 | SNP | Rv2020c | silent (Leu41) | 9947 | - | | 2266604 | C | G | 1073.77 | SNP | Rv2020c | silent (Ser39) | 9840 | - | | 2266613 | G | GC | 1623.73 | INS | Rv2020c |  |  | - | | 2266624 | G | T | 946.77 | SNP | Rv2020c | Leu33Ile | 9 | - | | 2269780 | T | C | 781.77 | SNP | Rv2024c | Asp154Gly | 11 | - | | 2270102 | A | G | 1089.77 | SNP | Rv2024c | Trp47Arg | 8 | - | | 2273627 | C | T | 772.77 | SNP | Rv2027c (dosT) | silent (Gly294) | 9935 | - | | 2282787 | C | T | 1201.77 | SNP | Rv2037c | Cys312Tyr | 3 | - | | 2284819 | C | G | 1038.77 | SNP | Rv2039c | Val(s)275Leu | 3 | - | | 2285251 | C | A | 1959.77 | SNP | Rv2039c | Val131Phe | 0 | - | | 2285906 | G | A | 2071.77 | SNP | Rv2040c | Leu209Leu(s) | 4 | - | | 2287121 | A | G | 1821.77 | SNP | Rv2041c | silent (Asp242) | 9859 | - | | 2296042 | G | C | 867.77 | SNP | Rv2048c (pks12) | Pro3649Ala | 22 | - | | 2297287 | G | T | 1187.77 | SNP | Rv2048c (pks12) | Gln3234Lys | 12 | - | | 2300237 | A | G | 740.77 | SNP | Rv2048c (pks12) | silent (Ala2250) | 9867 | - | | 2300546 | A | T | 1137.77 | SNP | Rv2048c (pks12) | His2147Gln | 23 | - | | 2300552 | T | G | 1091.77 | SNP | Rv2048c (pks12) | silent (Pro2145) | 9926 | - | | 2300555 | A | G | 1120.77 | SNP | Rv2048c (pks12) | silent (Asp2144) | 9859 | - | | 2310543 | G | A | 896.77 | SNP | Rv2051c (ppm1) | silent (Gly71) | 9935 | - | | 2329533 | A | G | 907.77 | SNP | Rv2072c (cobL) | Leu205Pro | 2 | - | | 2331061 | G | T | 1093.77 | SNP | Rv2074 | silent (Leu23) | 9947 | - | | 2334007 | A | G | 1502.77 | SNP | Rv2077c | silent (Ala96) | 9867 | - | | 2334290 | AGCATCTAAACCACCGTCAC CTGCGTCACCGCGGCCATCT CGCTC | A | 11707.73 | DEL | Rv2077c |  |  | - | | 2335494 | A | G | 869.77 | SNP | Rv2079 | Tyr47Cys | 3 | - | | 2340621 | C | G | 1174.77 | SNP | Rv2082 | Pro638Arg | 4 | - | | 2341636 | C | G | 602.77 | SNP | Rv2083 | Leu256Val(s) | 4 | - | | 2345037 | C | A | 831.77 | SNP | Rv2088 (pknJ) | silent (Leu209) | 9947 | - | | 2346672 | T | C | 1194.77 | SNP | Rv2089c (pepE) | Asp218Gly | 11 | - | | 2355511 | G | A | 1572.77 | SNP | Rv2097c (pafA) | silent (Val389) | 9901 | - | | 2357492 | T | A | 32.74 | SNP | intergenic |  |  | - | | 2358104 | G | C | 951.77 | SNP | intergenic |  |  | - | | 2361604 | C | G | 981.77 | SNP | Rv2101 (helZ) | Val455Val(s) | 18 | - | | 2362041 | C | A | 695.79 | SNP | Rv2101 (helZ) | Pro601Gln | 6 | - | | 2368564 | TA | T | 2431.73 | DEL | intergenic |  |  | - | | 2369971 | A | G | 771.77 | SNP | Rv2110c (prcB) | Tyr211His | 4 | - | | 2373353 | G | A | 1451.77 | SNP | Rv2113 | Gly242Ser | 16 | - | | 2374245 | C | T | 1771.77 | SNP | Rv2114 | Gln138STOP | 8 | - | | 2377785 | C | T | 1254.77 | SNP | Rv2118c | Val(s)176Val | 13 | - | | 2386389 | G | A | 876.77 | SNP | Rv2125 | Gly33Ser | 16 | - | | 2387733 | T | C | 87.28 | SNP | Rv2126c (PE\_PGRS37) | silent (Glu80) | 9865 | - | | 2400467 | G | A | 1265.77 | SNP | Rv2141c | Thr419Ile | 7 | - | | 2415656 | G | C | 655.77 | SNP | Rv2155c (murD) | Arg247Gly | 1 | - | | 2424925 | A | G | 929.77 | SNP | intergenic |  |  | - | | 2439204 | A | G | 842.77 | SNP | intergenic |  |  | - | | 2439519 | G | A | 569.77 | SNP | Rv2177c | silent (Arg143) | 9913 | - | | 2440953 | G | T | 923.77 | SNP | Rv2178c (aroG) | silent (Arg256) | 9913 | - | | 2453645 | A | C | 1572.77 | SNP | intergenic |  |  | - | | 2465997 | T | G | 1332.77 | SNP | Rv2201 (asnB) | Ile334Ser | 2 | - | | 2478845 | G | A | 858.77 | SNP | Rv2213 (pepB) | Val(s)170Met(s) | 9867 | - | | 2499726 | G | A | 1150.77 | SNP | Rv2226 | Asp299Asn | 36 | - | | 2509140 | G | C | 746.77 | SNP | Rv2236c (cobD) | Ser79Cys | 5 | - | | 2509722 | A | G | 771.77 | SNP | Rv2237 | silent (Pro78) | 9926 | - | | 2521342 | T | C | 930.77 | SNP | Rv2247 (accD6) | silent (Asp200) | 9859 | - | | 2523205 | G | GCGC | 1696.73 | INS | intergenic |  |  | - | | 2524418 | G | A | 1033.77 | SNP | Rv2249c (glpD1) | Ala125Val(s) | 9867 | - | | 2525722 | CG | C | 1290.73 | DEL | Rv2250A; Rv2251 |  |  | - | | 2529680 | A | G | 1195.77 | SNP | Rv2256c | silent (Thr65) | 9871 | - | | 2531742 | A | G | 1558.77 | SNP | Rv2258c | silent (Ala52) | 9867 | - | | 2532123 | G | C | 594.77 | SNP | intergenic |  |  | - | | 2534562 | GGA | G | 2312.73 | DEL | Rv2262c |  |  | - | | 2551572 | A | C | 1026.77 | SNP | Rv2280 | Thr5Pro | 4 | - | | 2586127 | A | G | 1041.77 | SNP | Rv2314c | silent (Gly388) | 9935 | - | | 2589491 | C | T | 1305.77 | SNP | Rv2316 (uspA) | silent (Ile218) | 9872 | - | | 2598400 | A | G | 653.77 | SNP | Rv2326c | silent (Asn516) | 9822 | - | | 2602456 | T | C | 1332.77 | SNP | Rv2329c (narK1) | Ile336Val | 57 | - | | 2612632 | C | A | 1030.77 | SNP | Rv2337c | Gly119Val | 3 | - | | 2626004 | G | A | 1224.77 | SNP | Rv2346c (esxO) | Leu57Leu(s) | 4 | - | | 2626149 | A | C | 1345.77 | SNP | Rv2346c (esxO) | silent (Gly8) | 9935 | - | | 2630158 | C | G | 428.77 | SNP | Rv2350c (plcB) | silent (Arg54) | 9913 | - | | 2630161 | A | G | 406.77 | SNP | Rv2350c (plcB) | silent (Asn53) | 9822 | - | | 2630173 | C | G | 204.77 | SNP | Rv2350c (plcB) | Leu(s)49Phe | 1 | - | | 2630176 | C | G | 176.77 | SNP | Rv2350c (plcB) | Leu(s)48Phe | 1 | - | | 2630182 | G | A | 182.77 | SNP | Rv2350c (plcB) | silent (Ile46) | 9872 | - | | 2630184 | T | A | 177.77 | SNP | Rv2350c (plcB) | Ile46Phe | 8 | - | | 2630188 | C | T | 251.77 | SNP | Rv2350c (plcB) | silent (Glu44) | 9865 | - | | 2630206 | T | G | 137.77 | SNP | Rv2350c (plcB) | silent (Gly38) | 9935 | - | | 2630211 | G | A | 64.77 | SNP | Rv2350c (plcB) | Pro37Ser | 17 | - | | 2630215 | A | G | 64.77 | SNP | Rv2350c (plcB) | silent (Pro35) | 9926 | - | | 2631556 | C | G | 173.77 | SNP | Rv2351c (plcA) | Gly174Arg | 0 | - | | 2631565 | T | C | 258.77 | SNP | Rv2351c (plcA) | Ile171Val | 57 | - | | 2631574 | T | C | 319.77 | SNP | Rv2351c (plcA) | Thr168Ala | 32 | - | | 2631583 | G | A | 338.77 | SNP | Rv2351c (plcA) | Leu165Leu(s) | 4 | - | | 2631599 | G | A | 308.77 | SNP | Rv2351c (plcA) | silent (Ile159) | 9872 | - | | 2631620 | A | G | 352.77 | SNP | Rv2351c (plcA) | silent (Gly152) | 9935 | - | | 2631899 | A | G | 71.77 | SNP | Rv2351c (plcA) | silent (Tyr59) | 9945 | - | | 2631914 | C | G | 45.77 | SNP | Rv2351c (plcA) | silent (Arg54) | 9913 | - | | 2631932 | C | G | 40.77 | SNP | Rv2351c (plcA) | Leu(s)48Phe | 1 | - | | 2631944 | C | T | 83.77 | SNP | Rv2351c (plcA) | silent (Glu44) | 9865 | - | | 2631962 | T | G | 271.77 | SNP | Rv2351c (plcA) | silent (Gly38) | 9935 | - | | 2631967 | G | A | 314.77 | SNP | Rv2351c (plcA) | Pro37Ser | 17 | - | | 2631968 | A | G | 261.77 | SNP | Rv2351c (plcA) | silent (Cys36) | 9973 | - | | 2631971 | A | G | 286.77 | SNP | Rv2351c (plcA) | silent (Pro35) | 9926 | - | | 2631977 | G | C | 422.77 | SNP | Rv2351c (plcA) | silent (Ala33) | 9867 | - | | 2656225 | A | G | 1371.77 | SNP | Rv2377c (mbtH) | Val69Ala | 18 | - | | 2660319 | C | G | 1159.77 | SNP | Rv2379c (mbtF) | Glu589Asp | 53 | - | | 2680658 | T | G | 1344.77 | SNP | intergenic |  |  | - | | 2695378 | C | G | 1497.77 | SNP | Rv2398c (cysW) | Gly141Ala | 21 | - | | 2704884 | A | ACAGCGACCATATCGCCGAG CT | 32729.73 | INS | Rv2407 |  |  | - | | 2713795 | C | T | 1012.77 | SNP | intergenic |  |  | - | | 2717758 | C | T | 856.77 | SNP | Rv2419c (gpgP) | silent (Thr14) | 9871 | - | | 2718852 | T | G | 1177.77 | SNP | intergenic |  |  | - | | 2734074 | T | C | 352.77 | SNP | Rv2436 (rbsK) | Val282Ala | 18 | - | | 2748712 | T | G | 1153.77 | SNP | Rv2448c (valS) | Asp505Ala | 10 | - | | 2751804 | C | T | 453.77 | SNP | Rv2450c (rpfE) | Arg126Gln | 9 | - | | 2752698 | C | A | 2102.77 | SNP | intergenic |  |  | - | | 2760152 | A | G | 1058.77 | SNP | Rv2458 (mmuM) | Tyr125Cys | 3 | - | | 2779136 | T | C | 758.77 | SNP | Rv2476c (gdh) | Ser1043Gly | 21 | - | | 2786952 | A | G | 828.77 | SNP | Rv2482c (plsB2) | Cys778Arg | 1 | - | | 2795160 | C | T | 697.77 | SNP | intergenic |  |  | - | | 2795713 | C | T | 148.90 | SNP | Rv2487c (PE\_PGRS42) | Gly558Asp | 6 | - | | 2807237 | G | A | 1842.77 | SNP | Rv2491 | Val(s)191Val | 13 | - | | 2809621 | T | C | 992.77 | SNP | Rv2495c (bkdC) | Thr107Ala | 32 | - | | 2813883 | G | T | 1333.77 | SNP | Rv2500c (fadE19) | Asp343Glu | 56 | - | | 2816034 | G | A | 1275.77 | SNP | Rv2501c (accA1) | Pro283Ser | 17 | - | | 2816296 | A | C | 1221.77 | SNP | Rv2501c (accA1) | Asp195Glu | 56 | - | | 2818837 | A | G | 1628.77 | SNP | Rv2503c (scoB) | silent (Gly97) | 9935 | - | | 2821077 | CGG | C | 3863.73 | DEL | Rv2505c (fadD35) |  |  | - | | 2821342 | C | T | 1021.77 | SNP | Rv2505c (fadD35) | silent (Ala85) | 9867 | - | | 2823309 | G | A | 1167.77 | SNP | Rv2508c | Arg429Trp | 2 | - | | 2827984 | G | T | 1141.77 | SNP | intergenic |  |  | - | | 2828019 | T | C | 1446.77 | SNP | intergenic |  |  | - | | 2828517 | A | G | 1111.77 | SNP | intergenic |  |  | - | | 2830525 | C | A | 1771.77 | SNP | Rv2513 | Thr122Lys | 11 | - | | 2836257 | G | A | 1093.77 | SNP | Rv2519 (PE26) | Gly158Asp | 6 | - | | 2840091 | C | T | 458.77 | SNP | intergenic |  |  | - | | 2855259 | A | G | 1209.77 | SNP | Rv2531c | silent (Ala841) | 9867 | - | | 2865760 | A | G | 1414.77 | SNP | Rv2542 | Thr211Ala | 32 | - | | 2865882 | T | C | 1155.77 | SNP | Rv2542 | silent (Val251) | 9901 | - | | 2881597 | AG | A | 1568.73 | DEL | Rv2561 |  |  | - | | 2888201 | T | C | 371.77 | SNP | Rv2566 | Leu610Pro | 2 | - | | 2889633 | T | C | 855.77 | SNP | Rv2566 | silent (Ala1087) | 9867 | - | | 2891267 | C | T | 972.77 | SNP | Rv2567 | silent (Gly491) | 9935 | - | | 2891728 | A | G | 692.77 | SNP | Rv2567 | Gln645Arg | 10 | - | | 2894208 | G | A | 909.77 | SNP | Rv2569c | silent (Ser67) | 9840 | - | | 2897375 | T | G | 381.77 | SNP | Rv2572c (aspS) | silent (Arg143) | 9913 | - | | 2910461 | G | T | 747.77 | SNP | Rv2584c (apt) | Ala147Glu | 10 | - | | 2911293 | C | G | 921.77 | SNP | Rv2585c | Cys462Ser | 11 | - | | 2912294 | T | G | 1516.77 | SNP | Rv2585c | silent (Ala128) | 9867 | - | | 2922936 | TGGCGGTGAC | T | 880.75 | DEL | Rv2591 (PE\_PGRS44) |  |  | - | | 2923391 | T | C | 701.77 | SNP | Rv2592c (ruvB) | silent (Pro281) | 9926 | - | | 2927939 | T | C | 1616.77 | SNP | intergenic |  |  | - | | 2939373 | G | C | 1054.77 | SNP | Rv2611c | Ser197Cys | 5 | - | | 2939657 | T | C | 559.77 | SNP | Rv2611c | Ile102Met(s) | 6 | - | | 2944932 | T | C | 32.74 | SNP | Rv2615c (PE\_PGRS45) | silent (Ala18) | 9867 | - | | 2945042 | G | C | 192.84 | SNP | intergenic |  |  | - | | 2945044 | C | T | 228.80 | SNP | intergenic |  |  | - | | 2954439 | T | C | 1479.77 | SNP | Rv2627c | Arg104Gly | 1 | - | | 2971419 | G | A | 1600.77 | SNP | Rv2646 | Arg290His | 8 | - | | 2974933 | A | G | 559.77 | SNP | Rv2650c | Ile101Thr | 11 | - | | 2975900 | A | G | 851.77 | SNP | intergenic |  |  | - | | 2977033 | T | G | 425.56 | SNP | Rv2654c | Thr68Pro | 4 | - | | 2982955 | C | T | 1392.77 | SNP | Rv2665 | Pro86Leu | 3 | - | | 2983613 | G | A | 90.77 | SNP | Rv2666 | silent (Gly181) | 9935 | - | | 2984740 | A | G | 652.77 | SNP | Rv2668 | His3Arg | 10 | - | | 2986821 | C | T | 836.77 | SNP | Rv2670c | Arg7His | 8 | - | | 2996876 | A | C | 1175.77 | SNP | Rv2681 | silent (Leu46) | 9947 | - | | 3005185 | G | T | 1281.77 | SNP | Rv2688c | Pro156Thr | 5 | - | | 3006361 | CG | C | 1908.73 | DEL | Rv2689c |  |  | - | | 3009692 | A | G | 1247.77 | SNP | Rv2691 (ceoB) | Thr117Ala | 32 | - | | 3015966 | G | A | 639.77 | SNP | Rv2701c (suhB) | Ala257Val(s) | 9867 | - | | 3017465 | T | C | 1966.77 | SNP | Rv2702 (ppgK) | Ile203Thr | 11 | - | | 3019554 | G | T | 1405.77 | SNP | Rv2704 | Ala33Ser | 28 | - | | 3020515 | G | C | 1398.77 | SNP | intergenic |  |  | - | | 3021929 | C | T | 1944.77 | SNP | Rv2709 | Pro31Ser | 17 | - | | 3028658 | G | A | 975.77 | SNP | Rv2715 | Leu(s)187Leu | 3 | - | | 3041871 | G | T | 1039.77 | SNP | Rv2729c | Ala202Glu | 10 | - | | 3053777 | G | T | 1796.77 | SNP | intergenic |  |  | - | | 3054081 | A | G | 1284.77 | SNP | Rv2741 (PE\_PGRS47) | silent (Gly56) | 9935 | - | | 3054321 | A | G | 267.78 | SNP | Rv2741 (PE\_PGRS47) | silent (Gly136) | 9935 | - | | 3056080 | G | A | 1063.77 | SNP | Rv2742c | Pro90Leu | 3 | - | | 3065279 | T | C | 1263.77 | SNP | Rv2752c | Thr305Ala | 32 | - | | 3069451 | C | T | 1488.77 | SNP | Rv2756c (hsdM) | silent (Lys211) | 9926 | - | | 3070855 | G | T | 1680.77 | SNP | intergenic |  |  | - | | 3080795 | A | G | 1846.77 | SNP | Rv2771c | Leu80Pro | 2 | - | | 3100153 | G | GA | 2101.73 | INS | Rv2790c (ltp1) |  |  | - | | 3103682 | T | C | 1527.77 | SNP | Rv2794c (pptT) | Met(s)87Val(s) | 9867 | - | | 3118000 | A | G | 857.77 | SNP | Rv2812 | Arg395Gly | 1 | - | | 3131469 | T | TTGTCGGCGA | 3311.73 | INS | Rv2823c |  |  | - | | 3133536 | T | C | 1799.77 | SNP | Rv2825c | Lys2Glu | 4 | - | | 3135912 | G | C | 664.77 | SNP | Rv2828c | Thr141Arg | 1 | - | | 3136335 | G | A | 607.77 | SNP | Rv2828A | Arg89Trp | 2 | - | | 3137058 | G | A | 1391.77 | SNP | Rv2830c (vapB22) | Ala56Val(s) | 9867 | - | | 3143100 | A | G | 622.77 | SNP | Rv2836c (dinF) | Cys177Arg | 1 | - | | 3162805 | C | G | 206.84 | SNP | Rv2853 (PE\_PGRS48) | Arg180Gly | 1 | - | | 3170460 | C | T | 604.77 | SNP | Rv2858c (aldC) | Val(s)88Val | 13 | - | | 3175335 | C | T | 1005.77 | SNP | Rv2863 (vapC23) | Thr115Met(s) | 32 | - | | 3177884 | C | A | 1234.77 | SNP | Rv2866 (relG) | silent (Arg21) | 9913 | - | | 3181029 | A | C | 205.84 | SNP | Rv2869c (rip) | Val(s)245Gly | 21 | - | | 3183561 | G | C | 320.77 | SNP | Rv2872 (vapC43) | silent (Pro60) | 9926 | - | | 3186860 | T | G | 1233.77 | SNP | Rv2874 (dipZ) | Tyr672Asp | 0 | - | | 3189481 | CGCGGCCAGCTGTCCGCAGC GCTGGCTGATTCGCCTGCGC TCCAGCTACCCGCCTACG | C | 648.73 | DEL | intergenic |  |  | - | | 3190145 | TC | T | 1255.73 | DEL | Rv2880c |  |  | - | | 3226181 | A | C | 1219.77 | SNP | Rv2916c (ffh) | silent (Arg35) | 9913 | - | | 3228143 | G | T | 844.77 | SNP | Rv2917 | Arg594Leu | 1 | - | | 3232703 | G | A | 623.77 | SNP | intergenic |  |  | - | | 3240568 | T | G | 879.77 | SNP | Rv2926c | Thr202Pro | 4 | - | | 3247316 | C | G | 691.77 | SNP | Rv2931 (ppsA) | Asp624Glu | 56 | - | | 3247851 | G | A | 722.77 | SNP | Rv2931 (ppsA) | Ala803Thr | 22 | - | | 3247853 | C | T | 701.77 | SNP | Rv2931 (ppsA) | silent (Ala803) | 9867 | - | | 3247856 | G | C | 712.77 | SNP | Rv2931 (ppsA) | silent (Arg804) | 9913 | - | | 3247864 | C | CTAGG | 1535.06 | INS | Rv2931 (ppsA) |  |  | - | | 3247865 | GCAAA | G | 1728.73 | DEL | Rv2931 (ppsA) |  |  | - | | 3247874 | G | A | 678.77 | SNP | Rv2931 (ppsA) | silent (Arg810) | 9913 | - | | 3247877 | T | C | 715.77 | SNP | Rv2931 (ppsA) | silent (Phe811) | 9946 | - | | 3247883 | T | C | 1074.77 | SNP | Rv2931 (ppsA) | silent (Ser813) | 9840 | - | | 3248074 | G | A | 866.77 | SNP | Rv2931 (ppsA) | Arg877His | 8 | - | | 3248075 | C | T | 923.77 | SNP | Rv2931 (ppsA) | silent (Arg877) | 9913 | - | | 3256494 | A | G | 1181.77 | SNP | Rv2933 (ppsC) | silent (Gly270) | 9935 | - | | 3269581 | A | G | 1113.77 | SNP | Rv2935 (ppsE) | silent (Ala615) | 9867 | - | | 3270784 | A | G | 953.77 | SNP | Rv2935 (ppsE) | silent (Gln1016) | 9876 | - | | 3294332 | G | A | 941.77 | SNP | Rv2946c (pks1) | silent (Gly674) | 9935 | - | | 3296843 | A | G | 628.77 | SNP | Rv2947c (pks15) | Val(s)333Ala | 9867 | - | | 3300196 | G | A | 1615.77 | SNP | Rv2949c | silent (Phe125) | 9946 | - | | 3304753 | G | T | 1062.77 | SNP | Rv2952 | Ala105Ser | 28 | - | | 3308606 | G | A | 2000.77 | SNP | intergenic |  |  | - | | 3313000 | TG | T | 1871.73 | DEL | Rv2960c |  |  | - | | 3317702 | C | T | 1016.77 | SNP | intergenic |  |  | - | | 3319327 | C | G | 1018.77 | SNP | Rv2966c | Val(s)47Val | 13 | - | | 3336587 | T | A | 443.77 | SNP | intergenic |  |  | - | | 3336646 | T | A | 233.78 | SNP | intergenic |  |  | - | | 3336825 | T | C | 720.77 | SNP | Rv2981c (ddlA) | Thr365Ala | 32 | - | | 3338603 | G | C | 827.77 | SNP | Rv2982c (gpdA2) | Pro133Ala | 22 | - | | 3345749 | A | G | 1056.77 | SNP | Rv2988c (leuC) | silent (Asp109) | 9859 | - | | 3353548 | C | T | 861.77 | SNP | Rv2996c (serA1) | Asp508Asn | 36 | - | | 3358235 | A | T | 1601.77 | SNP | Rv2999 (lppY) | Met(s)212Leu(s) | 9867 | - | | 3363338 | A | G | 1428.77 | SNP | intergenic |  |  | - | | 3366092 | A | C | 1120.77 | SNP | Rv3007c | Leu120Arg | 1 | - | | 3367765 | G | A | 1479.77 | SNP | Rv3009c (gatB) | silent (Gly343) | 9935 | - | | 3371719 | G | A | 1428.77 | SNP | Rv3012c (gatC) | silent (Ile4) | 9872 | - | | 3373744 | C | T | 2272.77 | SNP | Rv3014c (ligA) | Glu293Lys | 7 | - | | 3379708 | G | C | 345.84 | SNP | intergenic |  |  | - | | 3379712 | G | C | 401.77 | SNP | intergenic |  |  | - | | 3379718 | T | C | 375.77 | SNP | intergenic |  |  | - | | 3379726 | C | A | 283.78 | SNP | intergenic |  |  | - | | 3379730 | G | C | 303.78 | SNP | intergenic |  |  | - | | 3379732 | C | T | 265.78 | SNP | intergenic |  |  | - | | 3379735 | A | C | 344.77 | SNP | intergenic |  |  | - | | 3379736 | C | A | 341.77 | SNP | intergenic |  |  | - | | 3379742 | T | C | 624.77 | SNP | intergenic |  |  | - | | 3379751 | A | C | 777.77 | SNP | intergenic |  |  | - | | 3379757 | A | C | 848.77 | SNP | intergenic |  |  | - | | 3379763 | G | A | 760.77 | SNP | intergenic |  |  | - | | 3379784 | C | A | 922.77 | SNP | intergenic |  |  | - | | 3379788 | C | G | 1170.77 | SNP | intergenic |  |  | - | | 3380439 | G | GC | 220.80 | INS | intergenic |  |  | - | | 3381641 | G | T | 127.90 | SNP | Rv3023c | Gln328Lys | 12 | - | | 3382477 | T | C | 154.90 | SNP | Rv3023c | Tyr49Cys | 3 | - | | 3382598 | C | T | 432.77 | SNP | Rv3023c | Ala9Thr | 22 | - | | 3382615 | G | A | 613.77 | SNP | Rv3023c | Ser3Phe | 2 | - | | 3402816 | C | T | 3054.77 | SNP | Rv3042c (serB2) | Gly116Glu | 4 | - | | 3405776 | A | G | 1809.77 | SNP | Rv3044 (fecB) | His214Arg | 10 | - | | 3409700 | CA | C | 3692.73 | DEL | Rv3049c |  |  | - | | 3415180 | ACACCTAGGGGGTGG | A | 7735.73 | DEL | intergenic |  |  | - | | 3417231 | T | C | 2633.77 | SNP | Rv3056 (dinP) | Met(s)176Thr | 22 | - | | 3425854 | C | T | 2273.77 | SNP | Rv3062 (ligB) | Pro91Ser | 17 | - | | 3428917 | C | A | 2129.77 | SNP | Rv3063 (cstA) | Arg559Ser | 11 | - | | 3435199 | T | C | 1830.77 | SNP | Rv3071 | Trp246Arg | 8 | - | | 3440464 | T | G | 2022.77 | SNP | Rv3077 | silent (Arg308) | 9913 | - | | 3440468 | G | C | 2147.77 | SNP | Rv3077 | Gly310Arg | 0 | - | | 3456666 | A | G | 2139.77 | SNP | Rv3089 (fadD13) | silent (Ala302) | 9867 | - | | 3462135 | G | C | 1573.77 | SNP | Rv3093c | Cys210Trp | 0 | - | | 3466426 | G | A | 2771.77 | SNP | Rv3097c (lipY) | silent (Val222) | 9901 | genotype | | 3467096 | C | T | 1567.77 | SNP | intergenic |  |  | - | | 3473996 | G | GA | 3616.73 | INS | intergenic |  |  | - | | 3477917 | C | T | 2835.77 | SNP | Rv3109 (moaA1) | Pro90Leu | 3 | - | | 3481475 | G | A | 1089.77 | SNP | Rv3115 | Ala9Thr | 22 | - | | 3482737 | T | C | 2957.77 | SNP | intergenic |  |  | - | | 3486977 | A | G | 3228.29 | SNP | Rv3121 (cyp141) | Lys157Glu | 4 | - | | 3490749 | C | T | 1596.77 | SNP | Rv3125c (PPE49) | Leu(s)301Leu | 3 | - | | 3503895 | C | T | 1975.77 | SNP | Rv3137 | Pro168Leu | 3 | - | | 3505027 | G | A | 202.77 | SNP | Rv3138 (pflA) | Arg278His | 8 | - | | 3518167 | A | G | 1051.77 | SNP | Rv3151 (nuoG) | Ile474Met(s) | 6 | - | | 3518555 | A | G | 949.77 | SNP | Rv3151 (nuoG) | Thr604Ala | 32 | - | | 3556275 | A | G | 2152.77 | SNP | Rv3190c | Leu138Pro | 2 | - | | 3562270 | G | C | 2210.77 | SNP | Rv3193c | silent (Ala301) | 9867 | - | | 3569029 | T | C | 1466.77 | SNP | intergenic |  |  | - | | 3580636 | CT | C | 2693.73 | DEL | intergenic |  |  | - | | 3581414 | A | G | 1735.77 | SNP | Rv3204 | Thr34Ala | 32 | - | | 3590686 | G | GC | 2412.73 | INS | intergenic |  |  | - | | 3591063 | T | C | 1566.77 | SNP | Rv3213c | Lys144Glu | 4 | - | | 3594394 | G | C | 421.77 | SNP | intergenic |  |  | - | | 3594395 | G | A | 355.77 | SNP | intergenic |  |  | - | | 3594398 | T | C | 472.77 | SNP | intergenic |  |  | - | | 3594400 | A | G | 536.77 | SNP | intergenic |  |  | - | | 3604821 | G | C | 412.77 | SNP | Rv3228 | silent (Ala32) | 9867 | - | | 3605038 | A | G | 931.77 | SNP | Rv3228 | Thr105Ala | 32 | - | | 3607613 | A | G | 1884.77 | SNP | Rv3230c | silent (Gly214) | 9935 | - | | 3614982 | T | C | 2921.77 | SNP | Rv3239c | silent (Leu874) | 9947 | - | | 3622441 | A | C | 1872.77 | SNP | Rv3243c | Val217Val(s) | 18 | - | | 3643222 | G | A | 2730.77 | SNP | Rv3263 | Val16Ile | 33 | - | | 3644061 | C | T | 2758.77 | SNP | Rv3263 | silent (Ile295) | 9872 | - | | 3663889 | C | A | 103.77 | SNP | Rv3281 (accE5) | Asn67Lys | 25 | - | | 3674918 | C | T | 1315.77 | SNP | Rv3293 (pcd) | silent (Asn439) | 9822 | - | | 3687908 | T | C | 1694.77 | SNP | Rv3302c (glpD2) | Tyr512Cys | 3 | - | | 3689523 | G | T | 777.77 | SNP | Rv3303c (lpdA) | Cys472STOP | 3 | - | | 3691061 | A | C | 128.77 | SNP | intergenic |  |  | - | | 3691063 | G | A | 140.77 | SNP | intergenic |  |  | - | | 3692193 | C | T | 1458.77 | SNP | Rv3305c (amiA1) | Ala206Thr | 22 | - | | 3695799 | T | C | 234.77 | SNP | Rv3308 (pmmB) | silent (Gly312) | 9935 | - | | 3697152 | T | C | 2405.77 | SNP | intergenic |  |  | - | | 3699253 | C | G | 2002.77 | SNP | Rv3311 | Pro378Arg | 4 | - | | 3704596 | G | C | 1915.77 | SNP | Rv3317 (sdhD) | Val(s)54Leu | 3 | - | | 3711910 | G | A | 389.77 | SNP | Rv3327 | Trp54STOP | 0 | - | | 3714211 | G | T | 1485.77 | SNP | Rv3328c (sigJ) | Pro41Gln | 6 | - | | 3718357 | C | T | 2114.77 | SNP | Rv3331 (sugI) | Pro423Leu | 3 | - | | 3721806 | G | C | 2043.77 | SNP | Rv3335c | silent (Gly265) | 9935 | - | | 3730385 | C | G | 809.77 | SNP | Rv3343c (PPE54) | Arg2184Pro | 5 | - | | 3730386 | G | T | 788.77 | SNP | Rv3343c (PPE54) | silent (Arg2184) | 9913 | - | | 3730466 | A | G | 1893.77 | SNP | Rv3343c (PPE54) | Ile2157Thr | 11 | - | | 3730582 | G | A | 126.77 | SNP | Rv3343c (PPE54) | silent (Asn2118) | 9822 | - | | 3730624 | C | T | 573.77 | SNP | Rv3343c (PPE54) | silent (Ser2104) | 9840 | - | | 3730642 | G | A | 74.77 | SNP | Rv3343c (PPE54) | silent (Asn2098) | 9822 | - | | 3730741 | G | A | 332.77 | SNP | Rv3343c (PPE54) | silent (Gly2065) | 9935 | - | | 3730825 | A | G | 47.77 | SNP | Rv3343c (PPE54) | silent (Ile2037) | 9872 | - | | 3730896 | A | G | 149.77 | SNP | Rv3343c (PPE54) | Leu(s)2014Leu | 3 | - | | 3730978 | G | A | 161.77 | SNP | Rv3343c (PPE54) | silent (Gly1986) | 9935 | - | | 3731461 | C | G | 44.74 | SNP | Rv3343c (PPE54) | silent (Thr1825) | 9871 | - | | 3732194 | A | G | 181.84 | SNP | Rv3343c (PPE54) | Ile1581Thr | 11 | - | | 3732517 | A | G | 292.77 | SNP | Rv3343c (PPE54) | silent (Ile1473) | 9872 | - | | 3732525 | A | T | 219.77 | SNP | Rv3343c (PPE54) | Phe1471Ile | 7 | - | | 3732553 | A | G | 135.77 | SNP | Rv3343c (PPE54) | silent (Ile1461) | 9872 | - | | 3732706 | G | A | 410.77 | SNP | Rv3343c (PPE54) | silent (Gly1410) | 9935 | - | | 3735508 | G | A | 2344.77 | SNP | Rv3343c (PPE54) | silent (Leu476) | 9947 | - | | 3735907 | C | T | 820.77 | SNP | Rv3343c (PPE54) | silent (Ser343) | 9840 | - | | 3735931 | G | A | 761.77 | SNP | Rv3343c (PPE54) | silent (Ser335) | 9840 | - | | 3735967 | G | A | 670.77 | SNP | Rv3343c (PPE54) | silent (Ser323) | 9840 | - | | 3736628 | T | G | 1326.77 | SNP | Rv3343c (PPE54) | Glu103Ala | 17 | - | | 3737661 | TG | T | 254.77 | DEL | intergenic |  |  | - | | 3738027 | T | G | 62.74 | SNP | intergenic |  |  | - | | 3738131 | C | G | 50.74 | SNP | intergenic |  |  | - | | 3738516 | C | CTGCCGCCGCTGCCGCCGT | 2233.74 | INS | Rv3345c (PE\_PGRS50) |  |  | - | | 3739808 | GTTGCCGCCGTTGCCGCCGG CCGCGCCGCTCCCGTTGCCG GCGGTGCCGCCC | G | 3291.73 | DEL | Rv3345c (PE\_PGRS50) |  |  | - | | 3746409 | A | G | 1417.77 | SNP | Rv3347c (PPE55) | Leu2259Pro | 2 | - | | 3747403 | C | A | 1136.77 | SNP | Rv3347c (PPE55) | Gly1928Cys | 0 | - | | 3752207 | A | G | 2130.77 | SNP | Rv3347c (PPE55) | silent (Ile326) | 9872 | - | | 3752654 | A | T | 1149.77 | SNP | Rv3347c (PPE55) | silent (Gly177) | 9935 | - | | 3752662 | A | G | 1054.77 | SNP | Rv3347c (PPE55) | Leu(s)175Leu | 3 | - | | 3752761 | A | G | 425.77 | SNP | Rv3347c (PPE55) | Leu(s)142Leu | 3 | - | | 3752778 | A | G | 308.78 | SNP | Rv3347c (PPE55) | Val136Ala | 18 | - | | 3752791 | CTG | C | 352.77 | DEL | Rv3347c (PPE55) |  |  | - | | 3752797 | C | CGT | 322.77 | INS | Rv3347c (PPE55) |  |  | - | | 3752809 | G | A | 110.03 | SNP | Rv3347c (PPE55) | Leu126Leu(s) | 4 | - | | 3752813 | G | A | 48.74 | SNP | Rv3347c (PPE55) | silent (Asn124) | 9822 | - | | 3752821 | T | C | 43.74 | SNP | Rv3347c (PPE55) | Met(s)122Val(s) | 9867 | - | | 3752907 | G | A | 76.03 | SNP | Rv3347c (PPE55) | Ala93Val(s) | 9867 | - | | 3752909 | G | C | 92.28 | SNP | Rv3347c (PPE55) | silent (Ala92) | 9867 | - | | 3752910 | G | A | 126.90 | SNP | Rv3347c (PPE55) | Ala92Val | 13 | - | | 3752934 | A | T | 128.90 | SNP | Rv3347c (PPE55) | Val(s)84Glu | 10 | - | | 3753116 | C | T | 611.77 | SNP | Rv3347c (PPE55) | silent (Pro23) | 9926 | - | | 3753164 | T | G | 1311.77 | SNP | Rv3347c (PPE55) | silent (Pro7) | 9926 | - | | 3766777 | A | G | 323.77 | SNP | Rv3350c (PPE56) | Leu109Pro | 2 | - | | 3766778 | G | C | 402.77 | SNP | Rv3350c (PPE56) | Leu109Val(s) | 4 | - | | 3766815 | G | C | 247.77 | SNP | Rv3350c (PPE56) | Val96Val(s) | 18 | - | | 3766816 | A | G | 209.77 | SNP | Rv3350c (PPE56) | Val96Ala | 18 | - | | 3766819 | C | G | 218.77 | SNP | Rv3350c (PPE56) | Gly95Ala | 21 | - | | 3766858 | G | T | 81.77 | SNP | Rv3350c (PPE56) | Ala82Glu | 10 | - | | 3766859 | C | G | 93.77 | SNP | Rv3350c (PPE56) | Ala82Pro | 13 | - | | 3766860 | G | C | 124.77 | SNP | Rv3350c (PPE56) | silent (Ala81) | 9867 | - | | 3779671 | C | CGGCAACGGT | 697.82 | INS | Rv3367 (PE\_PGRS51) |  |  | - | | 3792796 | G | A | 3861.77 | SNP | Rv3378c | silent (Asn151) | 9822 | - | | 3798095 | A | C | 3026.77 | SNP | Rv3383c (idsB) | Val132Gly | 5 | - | | 3803867 | AAATCGCCCAATTTCGTGCC GAATTGGGCGATTTTGCGTC TGCTCGGCGCAG | A | 1740.80 | DEL | intergenic |  |  | - | | 3817117 | C | A | 1782.77 | SNP | Rv3399 | Ala330Glu | 10 | - | | 3819169 | G | A | 1835.77 | SNP | Rv3401 | silent (Pro376) | 9926 | - | | 3820545 | A | G | 1438.77 | SNP | intergenic |  |  | - | | 3823159 | A | T | 2174.77 | SNP | Rv3403c | silent (Val235) | 9901 | - | | 3826684 | C | T | 1532.77 | SNP | Rv3408 (vapC47) | Ser46Leu(s) | 35 | - | | 3829770 | T | C | 1946.77 | SNP | Rv3410c (guaB3) | silent (Pro47) | 9926 | - | | 3838871 | A | G | 1673.77 | SNP | Rv3420c (rimI) | silent (Ala64) | 9867 | - | | 3840262 | G | A | 2375.77 | SNP | Rv3423c (alr) | His387Tyr | 4 | - | | 3842384 | A | G | 1566.77 | SNP | Rv3425 (PPE57) | Asp49Gly | 11 | - | | 3842392 | G | C | 1580.77 | SNP | Rv3425 (PPE57) | Asp52His | 3 | - | | 3842394 | T | G | 1613.77 | SNP | Rv3425 (PPE57) | Asp52Glu | 56 | - | | 3842425 | T | A | 1049.77 | SNP | Rv3425 (PPE57) | Leu(s)63Met(s) | 9867 | - | | 3842438 | T | C | 836.77 | SNP | Rv3425 (PPE57) | Val67Ala | 18 | - | | 3842441 | A | G | 829.77 | SNP | Rv3425 (PPE57) | Glu68Gly | 7 | - | | 3842452 | C | G | 571.77 | SNP | Rv3425 (PPE57) | Gln72Glu | 35 | - | | 3842454 | A | C | 547.77 | SNP | Rv3425 (PPE57) | Gln72His | 20 | - | | 3842461 | T | A | 557.77 | SNP | Rv3425 (PPE57) | Ser75Thr | 32 | - | | 3859576 | G | A | 1139.77 | SNP | Rv3439c | silent (His29) | 9912 | - | | 3859893 | C | T | 1286.77 | SNP | Rv3440c | silent (Glu28) | 9865 | - | | 3862472 | GA | G | 2798.73 | DEL | intergenic |  |  | - | | 3864995 | T | C | 2241.77 | SNP | Rv3447c (eccC4) | Ser1082Gly | 21 | - | | 3877421 | A | G | 2340.77 | SNP | Rv3456c (rplQ) | silent (Pro4) | 9926 | - | | 3879331 | G | A | 1946.77 | SNP | Rv3459c (rpsK) | Ser121Leu(s) | 35 | - | | 3884748 | G | A | 546.77 | SNP | Rv3467 | Gly262Asp | 6 | - | | 3885886 | T | C | 2008.77 | SNP | Rv3468c | Ile62Val | 57 | - | | 3892671 | A | G | 3383.77 | SNP | Rv3476c (kgtP) | silent (Val350) | 9901 | - | | 3895269 | G | C | 1030.77 | SNP | Rv3478 (PPE60) | Glu282Gln | 27 | - | | 3895281 | T | C | 1188.77 | SNP | Rv3478 (PPE60) | Trp286Arg | 8 | - | | 3895282 | G | A | 1130.77 | SNP | Rv3478 (PPE60) | Trp286STOP | 0 | - | | 3896340 | T | G | 2128.77 | SNP | Rv3479 | Leu174Arg | 1 | - | | 3898408 | A | G | 1947.77 | SNP | Rv3479 | silent (Ala863) | 9867 | - | | 3920950 | C | T | 2182.77 | SNP | intergenic |  |  | - | | 3934542 | T | G | 119.77 | SNP | Rv3508 (PE\_PGRS54) | Ser1180Ala | 35 | - | | 3934699 | G | A | 97.94 | SNP | Rv3508 (PE\_PGRS54) | Ser1232Asn | 20 | - | | 3934733 | G | C | 43.78 | SNP | Rv3508 (PE\_PGRS54) | silent (Gly1243) | 9935 | - | | 3934734 | G | A | 55.77 | SNP | Rv3508 (PE\_PGRS54) | Ala1244Thr | 22 | - | | 3940802 | A | G | 139.90 | SNP | Rv3511 (PE\_PGRS55) | Asn396Asp | 42 | - | | 3942640 | T | C | 310.78 | SNP | intergenic |  |  | - | | 3944104 | A | C | 41.77 | SNP | intergenic |  |  | - | | 3944582 | CGG | C | 883.27 | DEL | intergenic |  |  | - | | 3946929 | C | T | 118.03 | SNP | Rv3514 (PE\_PGRS57) | Ala379Val | 13 | - | | 3952800 | G | A | 2575.77 | SNP | Rv3516 (echA19) | Gly86Asp | 6 | - | | 3956678 | T | C | 2008.77 | SNP | Rv3520c | Thr231Ala | 32 | - | | 3958403 | A | G | 2321.77 | SNP | Rv3521 | Asn295Asp | 42 | - | | 3959418 | C | T | 2350.77 | SNP | Rv3522 (ltp4) | Thr324Ile | 7 | - | | 3963665 | C | T | 2586.77 | SNP | Rv3527 | Leu21Leu(s) | 4 | - | | 4005607 | T | C | 1359.77 | SNP | Rv3564 (fadE33) | Leu(s)121Leu | 3 | - | | 4018414 | C | CA | 4200.73 | INS | Rv3576 (lppH) |  |  | - | | 4018415 | G | A | 2293.77 | SNP | Rv3576 (lppH) | Gly20Arg | 0 | - | | 4018802 | CAA | C | 4489.73 | DEL | Rv3576 (lppH) |  |  | - | | 4024273 | T | C | 2094.77 | SNP | Rv3581c (ispF) | Val25Val(s) | 18 | - | | 4026899 | G | A | 1610.77 | SNP | Rv3585 (radA) | silent (Gln152) | 9876 | - | | 4034827 | C | T | 2414.77 | SNP | Rv3593 (lpqF) | Ala159Val(s) | 9867 | - | | 4052608 | G | A | 2226.77 | SNP | Rv3610c (ftsH) | silent (Phe92) | 9946 | - | | 4055801 | G | A | 2966.77 | SNP | Rv3616c (espA) | Thr192Ile | 7 | - | | 4059904 | A | G | 1914.77 | SNP | intergenic |  |  | - | | 4060100 | G | A | 1404.77 | SNP | Rv3619c (esxV) | Leu57Leu(s) | 4 | - | | 4060201 | G | A | 584.77 | SNP | Rv3619c (esxV) | Ser23Leu(s) | 35 | - | | 4060210 | T | A | 543.77 | SNP | Rv3619c (esxV) | Gln20Leu | 6 | - | | 4060230 | G | A | 552.77 | SNP | Rv3619c (esxV) | silent (His13) | 9912 | - | | 4069292 | G | A | 1537.77 | SNP | Rv3630 | Ala40Thr | 22 | - | | 4076615 | A | C | 1757.77 | SNP | Rv3637 | silent (Ala44) | 9867 | - | | 4094986 | T | C | 2188.77 | SNP | Rv3655c | silent (Pro105) | 9926 | - | | 4095001 | CG | C | 3714.73 | DEL | Rv3655c |  |  | - | | 4100975 | T | C | 1461.77 | SNP | intergenic |  |  | - | | 4109796 | G | A | 2247.77 | SNP | Rv3668c | Pro229Leu | 3 | - | | 4111303 | G | C | 1967.77 | SNP | Rv3669 | Val(s)159Val | 13 | - | | 4117361 | AC | A | 3291.73 | DEL | Rv3677c |  |  | - | | 4120926 | A | G | 405.77 | SNP | Rv3680 | Asn378Asp | 42 | - | | 4120983 | A | G | 985.77 | SNP | intergenic |  |  | - | | 4121032 | C | T | 533.77 | SNP | intergenic |  |  | - | | 4126440 | G | A | 1718.77 | SNP | Rv3684 | silent (Ala334) | 9867 | - | | 4136497 | C | G | 1969.77 | SNP | Rv3694c | Lys206Asn | 13 | - | | 4139670 | C | T | 2099.77 | SNP | Rv3696c (glpK) | Cys29Tyr | 3 | - | | 4148669 | C | T | 2230.77 | SNP | Rv3705c | silent (Thr98) | 9871 | - | | 4155050 | G | A | 2798.77 | SNP | Rv3710 (leuA) | Val(s)437Val | 13 | - | | 4156099 | C | A | 1497.77 | SNP | Rv3711c (dnaQ) | Val(s)211Leu(s) | 9867 | - | | 4160407 | A | G | 2419.77 | SNP | Rv3715c (recR) | Leu(s)32Leu | 3 | - | | 4162073 | C | T | 1574.77 | SNP | Rv3718c | silent (Gln62) | 9876 | - | | 4162339 | A | G | 3113.77 | SNP | Rv3719 | Thr12Ala | 32 | - | | 4182695 | G | A | 1194.77 | SNP | Rv3731 (ligC) | Arg313His | 8 | - | | 4187485 | T | C | 1304.77 | SNP | Rv3736 | silent (Ala284) | 9867 | - | | 4187817 | A | G | 1262.77 | SNP | Rv3737 | Asp40Gly | 11 | - | | 4189841 | T | C | 1091.77 | SNP | Rv3738c (PPE66) | Tyr131Cys | 3 | - | | 4192547 | C | A | 1179.77 | SNP | Rv3741c | Gly103STOP | 21 | - | | 4198611 | CG | C | 2072.73 | DEL | intergenic |  |  | - | | 4204441 | A | G | 1090.77 | SNP | Rv3759c (proX) | silent (His311) | 9912 | - | | 4210274 | A | G | 1195.77 | SNP | Rv3764c (tcrY) | Cys246Arg | 1 | - | | 4212196 | A | T | 1737.77 | SNP | intergenic |  |  | - | | 4215467 | G | A | 929.77 | SNP | Rv3770c | silent (Gly103) | 9935 | - | | 4215784 | C | T | 1546.77 | SNP | intergenic |  |  | - | | 4221490 | C | G | 1046.77 | SNP | Rv3776 | silent (Leu134) | 9947 | - | | 4222073 | A | G | 168.90 | SNP | Rv3776 | Met(s)329Val(s) | 9867 | - | | 4222882 | A | G | 1274.77 | SNP | Rv3777 | silent (Leu63) | 9947 | - | | 4242643 | C | T | 464.77 | SNP | Rv3793 (embC) | silent (Arg927) | 9913 | genotype | | 4245092 | C | T | 1173.77 | SNP | Rv3794 (embA) | silent (Gly620) | 9935 | - | | 4250742 | G | A | 2260.77 | SNP | Rv3796 | Gly289Ser | 16 | - | | 4255922 | A | G | 1989.77 | SNP | Rv3799c (accD4) | silent (His9) | 9912 | - | | 4257220 | A | G | 914.77 | SNP | Rv3800c (pks13) | silent (Arg1309) | 9913 | - | | 4257849 | G | A | 890.77 | SNP | Rv3800c (pks13) | Arg1100Trp | 2 | - | | 4260268 | G | C | 1242.77 | SNP | Rv3800c (pks13) | silent (Ala293) | 9867 | genotype | | 4279825 | AGCC | A | 2884.73 | DEL | Rv3814c |  |  | - | | 4287017 | G | A | 2180.77 | SNP | Rv3822 | silent (Ala99) | 9867 | - | | 4287195 | A | G | 1393.77 | SNP | Rv3822 | Thr159Ala | 32 | - | | 4302036 | T | C | 696.77 | SNP | Rv3827c | Thr252Ala | 32 | - | | 4306155 | C | T | 1134.77 | SNP | Rv3831 | silent (Ser133) | 9840 | - | | 4311528 | G | A | 839.77 | SNP | Rv3837c | silent (Ala60) | 9867 | - | | 4328329 | G | C | 1129.77 | SNP | intergenic |  |  | - | | 4335830 | G | A | 1173.77 | SNP | Rv3859c (gltB) | silent (Gly84) | 9935 | - | | 4338595 | GC | G | 4064.73 | DEL | intergenic |  |  | - | | 4338732 | G | A | 2026.77 | SNP | intergenic |  |  | - | | 4346852 | G | A | 1018.77 | SNP | Rv3870 (eccCa1) | Val(s)124Val | 13 | - | | 4351039 | G | T | 1147.77 | SNP | Rv3872 (PE35) | Glu99STOP | 17 | - | | 4356110 | G | C | 1210.77 | SNP | Rv3877 (eccD1) | silent (Leu368) | 9947 | - | | 4366272 | G | C | 1047.77 | SNP | Rv3884c (eccA2) | silent (Ala189) | 9867 | - | | 4375628 | G | T | 866.77 | SNP | Rv3892c (PPE69) | Thr19Lys | 11 | - | | 4377461 | G | A | 1146.77 | SNP | Rv3894c (eccC2) | Leu998Phe | 6 | - | | 4379680 | C | G | 1671.77 | SNP | Rv3894c (eccC2) | Arg258Pro | 5 | - | | 4382054 | T | C | 1173.77 | SNP | Rv3896c | silent (Ala266) | 9867 | - | | 4382275 | G | T | 1093.77 | SNP | Rv3896c | Gln193Lys | 12 | - | | 4383144 | C | CCGGGG | 3195.79 | INS | Rv3897c |  |  | - | | 4400660 | AC | A | 1857.73 | DEL | Rv3911 (sigM) |  |  | - | | 4406749 | G | A | 1802.77 | SNP | Rv3918c (parA) | silent (Leu261) | 9947 | - | | 4409645 | G | A | 2046.77 | SNP | Rv3921c | Ser142Leu(s) | 35 | - | |  | | export |

elog
