# Supplementary material for: Patterns of compensatory mutations in rpoA/B/C genes of multidrug resistant M. tuberculosis in Uganda
Source: PLoS One. 2025 Dec 4;20(12):e0328957. doi: 10.1371/journal.pone.0328957 (PMC12677784; doi:10.1371/journal.pone.0328957)
Supplement: S2 File — (ZIP) [file pone.0328957.s002.zip › Variants T_S20_L001_001.bam.html]

 

Calling SNPs/INDELs (computing variant list in .vcf format) from T\_S20\_L001\_001.bam

*by SAMtools/BCFtools:*

Howto

Important aspects

This takes up to one hour!!! **Please wait ...**

Variants T\_S20\_L001\_001.bam

|  |  |
| --- | --- |
| Variants |  |

|  |  |
| --- | --- |
| |  | | --- | | *by GATK* | |

|  |  |  |
| --- | --- | --- |
| |  | | --- | | T\_S20\_L001\_001.bam | | | computed 2016-10-27 using PhyResSE v1.0 (Ref. NC\_000962.3) | |

|  |  |
| --- | --- |
| 1176  variants called Export in VCF format |  |

|  |  |  |  |  |  |  |  |  |  |  |  |  |  |  |  |  |  |  |  |  |  |  |  |  |  |  |  |  |  |  |  |  |  |  |  |  |  |  |  |  |  |  |  |  |  |  |  |  |  |  |  |  |  |  |  |  |  |  |  |  |  |  |  |  |  |  |  |  |  |  |  |  |  |  |  |  |  |  |  |  |  |  |  |  |  |  |  |  |  |  |  |  |  |  |  |  |  |  |  |  |  |  |  |  |  |  |  |  |  |  |  |  |  |  |  |  |  |  |  |  |  |  |  |  |  |  |  |  |  |  |  |  |  |  |  |  |  |  |  |  |  |  |  |  |  |  |  |  |  |  |  |  |  |  |  |  |  |  |  |  |  |  |  |  |  |  |  |  |  |  |  |  |  |  |  |  |  |  |  |  |  |  |  |  |  |  |  |  |  |  |  |  |  |  |  |  |  |  |  |  |  |  |  |  |  |  |  |  |  |  |  |  |  |  |  |  |  |  |  |  |  |  |  |  |  |  |  |  |  |  |  |  |  |  |  |  |  |  |  |  |  |  |  |  |  |  |  |  |  |  |  |  |  |  |  |  |  |  |  |  |  |  |  |  |  |  |  |  |  |  |  |  |  |  |  |  |  |  |  |  |  |  |  |  |  |  |  |  |  |  |  |  |  |  |  |  |  |  |  |  |  |  |  |  |  |  |  |  |  |  |  |  |  |  |  |  |  |  |  |  |  |  |  |  |  |  |  |  |  |  |  |  |  |  |  |  |  |  |  |  |  |  |  |  |  |  |  |  |  |  |  |  |  |  |  |  |  |  |  |  |  |  |  |  |  |  |  |  |  |  |  |  |  |  |  |  |  |  |  |  |  |  |  |  |  |  |  |  |  |  |  |  |  |  |  |  |  |  |  |  |  |  |  |  |  |  |  |  |  |  |  |  |  |  |  |  |  |  |  |  |  |  |  |  |  |  |  |  |  |  |  |  |  |  |  |  |  |  |  |  |  |  |  |  |  |  |  |  |  |  |  |  |  |  |  |  |  |  |  |  |  |  |  |  |  |  |  |  |  |  |  |  |  |  |  |  |  |  |  |  |  |  |  |  |  |  |  |  |  |  |  |  |  |  |  |  |  |  |  |  |  |  |  |  |  |  |  |  |  |  |  |  |  |  |  |  |  |  |  |  |  |  |  |  |  |  |  |  |  |  |  |  |  |  |  |  |  |  |  |  |  |  |  |  |  |  |  |  |  |  |  |  |  |  |  |  |  |  |  |  |  |  |  |  |  |  |  |  |  |  |  |  |  |  |  |  |  |  |  |  |  |  |  |  |  |  |  |  |  |  |  |  |  |  |  |  |  |  |  |  |  |  |  |  |  |  |  |  |  |  |  |  |  |  |  |  |  |  |  |  |  |  |  |  |  |  |  |  |  |  |  |  |  |  |  |  |  |  |  |  |  |  |  |  |  |  |  |  |  |  |  |  |  |  |  |  |  |  |  |  |  |  |  |  |  |  |  |  |  |  |  |  |  |  |  |  |  |  |  |  |  |  |  |  |  |  |  |  |  |  |  |  |  |  |  |  |  |  |  |  |  |  |  |  |  |  |  |  |  |  |  |  |  |  |  |  |  |  |  |  |  |  |  |  |  |  |  |  |  |  |  |  |  |  |  |  |  |  |  |  |  |  |  |  |  |  |  |  |  |  |  |  |  |  |  |  |  |  |  |  |  |  |  |  |  |  |  |  |  |  |  |  |  |  |  |  |  |  |  |  |  |  |  |  |  |  |  |  |  |  |  |  |  |  |  |  |  |  |  |  |  |  |  |  |  |  |  |  |  |  |  |  |  |  |  |  |  |  |  |  |  |  |  |  |  |  |  |  |  |  |  |  |  |  |  |  |  |  |  |  |  |  |  |  |  |  |  |  |  |  |  |  |  |  |  |  |  |  |  |  |  |  |  |  |  |  |  |  |  |  |  |  |  |  |  |  |  |  |  |  |  |  |  |  |  |  |  |  |  |  |  |  |  |  |  |  |  |  |  |  |  |  |  |  |  |  |  |  |  |  |  |  |  |  |  |  |  |  |  |  |  |  |  |  |  |  |  |  |  |  |  |  |  |  |  |  |  |  |  |  |  |  |  |  |  |  |  |  |  |  |  |  |  |  |  |  |  |  |  |  |  |  |  |  |  |  |  |  |  |  |  |  |  |  |  |  |  |  |  |  |  |  |  |  |  |  |  |  |  |  |  |  |  |  |  |  |  |  |  |  |  |  |  |  |  |  |  |  |  |  |  |  |  |  |  |  |  |  |  |  |  |  |  |  |  |  |  |  |  |  |  |  |  |  |  |  |  |  |  |  |  |  |  |  |  |  |  |  |  |  |  |  |  |  |  |  |  |  |  |  |  |  |  |  |  |  |  |  |  |  |  |  |  |  |  |  |  |  |  |  |  |  |  |  |  |  |  |  |  |  |  |  |  |  |  |  |  |  |  |  |  |  |  |  |  |  |  |  |  |  |  |  |  |  |  |  |  |  |  |  |  |  |  |  |  |  |  |  |  |  |  |  |  |  |  |  |  |  |  |  |  |  |  |  |  |  |  |  |  |  |  |  |  |  |  |  |  |  |  |  |  |  |  |  |  |  |  |  |  |  |  |  |  |  |  |  |  |  |  |  |  |  |  |  |  |  |  |  |  |  |  |  |  |  |  |  |  |  |  |  |  |  |  |  |  |  |  |  |  |  |  |  |  |  |  |  |  |  |  |  |  |  |  |  |  |  |  |  |  |  |  |  |  |  |  |  |  |  |  |  |  |  |  |  |  |  |  |  |  |  |  |  |  |  |  |  |  |  |  |  |  |  |  |  |  |  |  |  |  |  |  |  |  |  |  |  |  |  |  |  |  |  |  |  |  |  |  |  |  |  |  |  |  |  |  |  |  |  |  |  |  |  |  |  |  |  |  |  |  |  |  |  |  |  |  |  |  |  |  |  |  |  |  |  |  |  |  |  |  |  |  |  |  |  |  |  |  |  |  |  |  |  |  |  |  |  |  |  |  |  |  |  |  |  |  |  |  |  |  |  |  |  |  |  |  |  |  |  |  |  |  |  |  |  |  |  |  |  |  |  |  |  |  |  |  |  |  |  |  |  |  |  |  |  |  |  |  |  |  |  |  |  |  |  |  |  |  |  |  |  |  |  |  |  |  |  |  |  |  |  |  |  |  |  |  |  |  |  |  |  |  |  |  |  |  |  |  |  |  |  |  |  |  |  |  |  |  |  |  |  |  |  |  |  |  |  |  |  |  |  |  |  |  |  |  |  |  |  |  |  |  |  |  |  |  |  |  |  |  |  |  |  |  |  |  |  |  |  |  |  |  |  |  |  |  |  |  |  |  |  |  |  |  |  |  |  |  |  |  |  |  |  |  |  |  |  |  |  |  |  |  |  |  |  |  |  |  |  |  |  |  |  |  |  |  |  |  |  |  |  |  |  |  |  |  |  |  |  |  |  |  |  |  |  |  |  |  |  |  |  |  |  |  |  |  |  |  |  |  |  |  |  |  |  |  |  |  |  |  |  |  |  |  |  |  |  |  |  |  |  |  |  |  |  |  |  |  |  |  |  |  |  |  |  |  |  |  |  |  |  |  |  |  |  |  |  |  |  |  |  |  |  |  |  |  |  |  |  |  |  |  |  |  |  |  |  |  |  |  |  |  |  |  |  |  |  |  |  |  |  |  |  |  |  |  |  |  |  |  |  |  |  |  |  |  |  |  |  |  |  |  |  |  |  |  |  |  |  |  |  |  |  |  |  |  |  |  |  |  |  |  |  |  |  |  |  |  |  |  |  |  |  |  |  |  |  |  |  |  |  |  |  |  |  |  |  |  |  |  |  |  |  |  |  |  |  |  |  |  |  |  |  |  |  |  |  |  |  |  |  |  |  |  |  |  |  |  |  |  |  |  |  |  |  |  |  |  |  |  |  |  |  |  |  |  |  |  |  |  |  |  |  |  |  |  |  |  |  |  |  |  |  |  |  |  |  |  |  |  |  |  |  |  |  |  |  |  |  |  |  |  |  |  |  |  |  |  |  |  |  |  |  |  |  |  |  |  |  |  |  |  |  |  |  |  |  |  |  |  |  |  |  |  |  |  |  |  |  |  |  |  |  |  |  |  |  |  |  |  |  |  |  |  |  |  |  |  |  |  |  |  |  |  |  |  |  |  |  |  |  |  |  |  |  |  |  |  |  |  |  |  |  |  |  |  |  |  |  |  |  |  |  |  |  |  |  |  |  |  |  |  |  |  |  |  |  |  |  |  |  |  |  |  |  |  |  |  |  |  |  |  |  |  |  |  |  |  |  |  |  |  |  |  |  |  |  |  |  |  |  |  |  |  |  |  |  |  |  |  |  |  |  |  |  |  |  |  |  |  |  |  |  |  |  |  |  |  |  |  |  |  |  |  |  |  |  |  |  |  |  |  |  |  |  |  |  |  |  |  |  |  |  |  |  |  |  |  |  |  |  |  |  |  |  |  |  |  |  |  |  |  |  |  |  |  |  |  |  |  |  |  |  |  |  |  |  |  |  |  |  |  |  |  |  |  |  |  |  |  |  |  |  |  |  |  |  |  |  |  |  |  |  |  |  |  |  |  |  |  |  |  |  |  |  |  |  |  |  |  |  |  |  |  |  |  |  |  |  |  |  |  |  |  |  |  |  |  |  |  |  |  |  |  |  |  |  |  |  |  |  |  |  |  |  |  |  |  |  |  |  |  |  |  |  |  |  |  |  |  |  |  |  |  |  |  |  |  |  |  |  |  |  |  |  |  |  |  |  |  |  |  |  |  |  |  |  |  |  |  |  |  |  |  |  |  |  |  |  |  |  |  |  |  |  |  |  |  |  |  |  |  |  |  |  |  |  |  |  |  |  |  |  |  |  |  |  |  |  |  |  |  |  |  |  |  |  |  |  |  |  |  |  |  |  |  |  |  |  |  |  |  |  |  |  |  |  |  |  |  |  |  |  |  |  |  |  |  |  |  |  |  |  |  |  |  |  |  |  |  |  |  |  |  |  |  |  |  |  |  |  |  |  |  |  |  |  |  |  |  |  |  |  |  |  |  |  |  |  |  |  |  |  |  |  |  |  |  |  |  |  |  |  |  |  |  |  |  |  |  |  |  |  |  |  |  |  |  |  |  |  |  |  |  |  |  |  |  |  |  |  |  |  |  |  |  |  |  |  |  |  |  |  |  |  |  |  |  |  |  |  |  |  |  |  |  |  |  |  |  |  |  |  |  |  |  |  |  |  |  |  |  |  |  |  |  |  |  |  |  |  |  |  |  |  |  |  |  |  |  |  |  |  |  |  |  |  |  |  |  |  |  |  |  |  |  |  |  |  |  |  |  |  |  |  |  |  |  |  |  |  |  |  |  |  |  |  |  |  |  |  |  |  |  |  |  |  |  |  |  |  |  |  |  |  |  |  |  |  |  |  |  |  |  |  |  |  |  |  |  |  |  |  |  |  |  |  |  |  |  |  |  |  |  |  |  |  |  |  |  |  |  |  |  |  |  |  |  |  |  |  |  |  |  |  |  |  |  |  |  |  |  |  |  |  |  |  |  |  |  |  |  |  |  |  |  |  |  |  |  |  |  |  |  |  |  |  |  |  |  |  |  |  |  |  |  |  |  |  |  |  |  |  |  |  |  |  |  |  |  |  |  |  |  |  |  |  |  |  |  |  |  |  |  |  |  |  |  |  |  |  |  |  |  |  |  |  |  |  |  |  |  |  |  |  |  |  |  |  |  |  |  |  |  |  |  |  |  |  |  |  |  |  |  |  |  |  |  |  |  |  |  |  |  |  |  |  |  |  |  |  |  |  |  |  |  |  |  |  |  |  |  |  |  |  |  |  |  |  |  |  |  |  |  |  |  |  |  |  |  |  |  |  |  |  |  |  |  |  |  |  |  |  |  |  |  |  |  |  |  |  |  |  |  |  |  |  |  |  |  |  |  |  |  |  |  |  |  |  |  |  |  |  |  |  |  |  |  |  |  |  |  |  |  |  |  |  |  |  |  |  |  |  |  |  |  |  |  |  |  |  |  |  |  |  |  |  |  |  |  |  |  |  |  |  |  |  |  |  |  |  |  |  |  |  |  |  |  |  |  |  |  |  |  |  |  |  |  |  |  |  |  |  |  |  |  |  |  |  |  |  |  |  |  |  |  |  |  |  |  |  |  |  |  |  |  |  |  |  |  |  |  |  |  |  |  |  |  |  |  |  |  |  |  |  |  |  |  |  |  |  |  |  |  |  |  |  |  |  |  |  |  |  |  |  |  |  |  |  |  |  |  |  |  |  |  |  |  |  |  |  |  |  |  |  |  |  |  |  |  |  |  |  |  |  |  |  |  |  |  |  |  |  |  |  |  |  |  |  |  |  |  |  |  |  |  |  |  |  |  |  |  |  |  |  |  |  |  |  |  |  |  |  |  |  |  |  |  |  |  |  |  |  |  |  |  |  |  |  |  |  |  |  |  |  |  |  |  |  |  |  |  |  |  |  |  |  |  |  |  |  |  |  |  |  |  |  |  |  |  |  |  |  |  |  |  |  |  |  |  |  |  |  |  |  |  |  |  |  |  |  |  |  |  |  |  |  |  |  |  |  |  |  |  |  |  |  |  |  |  |  |  |  |  |  |  |  |  |  |  |  |  |  |  |  |  |  |  |  |  |  |  |  |  |  |  |  |  |  |  |  |  |  |  |  |  |  |  |  |  |  |  |  |  |  |  |  |  |  |  |  |  |  |  |  |  |  |  |  |  |  |  |  |  |  |  |  |  |  |  |  |  |  |  |  |  |  |  |  |  |  |  |  |  |  |  |  |  |  |  |  |  |  |  |  |  |  |  |  |  |  |  |  |  |  |  |  |  |  |  |  |  |  |  |  |  |  |  |  |  |  |  |  |  |  |  |  |  |  |  |  |  |  |  |  |  |  |  |  |  |  |  |  |  |  |  |  |  |  |  |  |  |  |  |  |  |  |  |  |  |  |  |  |  |  |  |  |  |  |  |  |  |  |  |  |  |  |  |  |  |  |  |  |  |  |  |  |  |  |  |  |  |  |  |  |  |  |  |  |  |  |  |  |  |  |  |  |  |  |  |  |  |  |  |  |  |  |  |  |  |  |  |  |  |  |  |  |  |  |  |  |  |  |  |  |  |  |  |  |  |  |  |  |  |  |  |  |  |  |  |  |  |  |  |  |  |  |  |  |  |  |  |  |  |  |  |  |  |  |  |  |  |  |  |  |  |  |  |  |  |  |  |  |  |  |  |  |  |  |  |  |  |  |  |  |  |  |  |  |  |  |  |  |  |  |  |  |  |  |  |  |  |  |  |  |  |  |  |  |  |  |  |  |  |  |  |  |  |  |  |  |  |  |  |  |  |  |  |  |  |  |  |  |  |  |  |  |  |  |  |  |  |  |  |  |  |  |  |  |  |  |  |  |  |  |  |  |  |  |  |  |  |  |  |  |  |  |  |  |  |  |  |  |  |  |  |  |  |  |  |  |  |  |  |  |  |  |  |  |  |  |  |  |  |  |  |  |  |  |  |  |  |  |  |  |  |  |  |  |  |  |  |  |  |  |  |  |  |  |  |  |  |  |  |  |  |  |  |  |  |  |  |  |  |  |  |  |  |  |  |  |  |  |  |  |  |  |  |  |  |  |  |  |  |  |  |  |  |  |  |  |  |  |  |  |  |  |  |  |  |  |  |  |  |  |  |  |  |  |  |  |  |  |  |  |  |  |  |  |  |  |  |  |  |  |  |  |  |  |  |  |  |  |  |  |  |  |  |  |  |  |  |  |  |  |  |  |  |  |  |  |  |  |  |  |  |  |  |  |  |  |  |  |  |  |  |  |  |  |  |  |  |  |  |  |  |  |  |  |  |  |  |  |  |  |  |  |  |  |  |  |  |  |  |  |  |  |  |  |  |  |  |  |  |  |  |  |  |  |  |  |  |  |  |  |  |  |  |  |  |  |  |  |  |  |  |  |  |  |  |  |  |  |  |  |  |  |  |  |  |  |  |  |  |  |  |  |  |  |  |  |  |  |  |  |  |  |  |  |  |  |  |  |  |  |  |  |  |  |  |  |  |  |  |  |  |  |  |  |  |  |  |  |  |  |  |  |  |  |  |  |  |  |  |  |  |  |  |  |  |  |  |  |  |  |  |  |  |  |  |  |  |  |  |  |  |  |  |  |  |  |  |  |  |  |  |  |  |  |  |  |  |  |  |  |  |  |  |  |  |  |  |  |  |  |  |  |  |  |  |  |  |  |  |  |  |  |  |  |  |  |  |  |  |  |  |  |  |  |  |  |  |  |  |  |  |  |  |  |  |  |  |  |  |  |  |  |  |  |  |  |  |  |  |  |  |  |  |  |  |  |  |  |  |  |  |  |  |  |  |  |  |  |  |  |  |  |  |  |  |  |  |  |  |  |  |  |  |  |  |  |  |  |  |  |  |  |  |  |  |  |  |  |  |  |  |  |  |  |  |  |  |  |  |  |  |  |  |  |  |  |  |  |  |  |  |  |  |  |  |  |  |  |  |  |  |  |  |  |  |  |  |  |  |  |  |  |  |  |  |  |  |  |  |  |  |  |  |  |  |  |  |  |  |  |  |  |  |  |  |  |  |  |  |  |  |  |  |  |  |  |  |  |  |  |  |  |  |  |  |  |  |  |  |  |  |  |  |  |  |  |  |  |  |  |  |  |  |  |  |  |  |  |  |  |  |  |  |  |  |  |  |  |  |  |  |  |  |  |  |  |  |  |  |  |  |  |  |  |  |  |  |  |  |  |  |  |  |  |  |  |  |  |  |  |  |  |  |  |  |  |  |  |  |  |  |  |  |  |  |  |  |  |  |  |  |  |  |  |  |  |  |  |  |  |  |  |  |  |  |  |  |  |  |  |  |  |  |  |  |  |  |  |  |  |  |  |  |  |  |  |  |  |  |  |  |  |  |  |  |  |  |  |  |  |  |  |  |  |  |  |  |  |  |  |  |  |  |  |  |  |  |  |  |  |  |  |  |  |  |  |  |  |  |  |  |  |  |  |  |  |  |  |  |  |  |  |  |  |  |  |  |  |  |  |  |  |  |  |  |  |  |  |  |  |  |  |  |  |  |  |  |  |  |  |  |  |  |  |  |  |  |  |  |  |  |  |  |  |  |  |  |  |  |  |  |  |  |  |  |  |  |  |  |  |  |  |  |  |  |  |  |  |  |  |  |  |  |  |  |  |  |  |  |  |  |  |  |  |  |  |  |  |  |  |  |  |  |  |  |  |  |  |  |  |  |  |  |  |  |  |  |  |  |  |  |  |  |  |  |  |  |  |  |  |  |  |  |  |  |  |  |  |  |  |  |  |  |  |  |  |  |  |  |  |  |  |  |  |  |  |  |  |  |  |  |  |  |  |  |  |  |  |  |  |  |  |  |  |  |  |  |  |  |  |  |  |  |  |  |  |  |  |  |  |  |  |  |  |  |  |  |  |  |  |  |  |  |  |  |  |  |  |  |  |  |  |  |  |  |  |  |  |  |  |  |  |  |  |  |  |  |  |  |  |  |  |  |  |  |  |  |  |  |  |  |  |  |  |  |  |  |  |  |  |  |  |  |  |  |  |  |  |  |  |  |  |  |  |  |  |  |  |  |  |  |  |  |  |  |  |  |  |  |  |  |  |  |  |  |  |  |  |  |  |  |  |  |  |  |  |  |  |  |  |  |  |  |  |  |  |  |  |  |  |  |  |  |  |  |  |  |  |  |  |  |  |  |  |  |  |  |  |  |  |  |  |  |  |  |  |  |  |  |  |  |  |  |  |  |  |  |  |  |  |  |  |  |  |  |  |  |  |  |  |  |  |  |  |  |  |  |  |  |  |  |  |  |  |  |  |  |  |  |  |  |  |  |  |  |  |  |  |  |  |  |  |  |  |  |  |  |  |  |  |  |  |  |  |  |  |  |  |  |  |  |  |  |  |  |  |  |  |  |  |  |  |  |  |  |  |  |  |  |  |  |  |  |  |  |  |  |  |  |  |  |  |  |  |  |  |  |  |  |  |  |  |  |  |  |  |  |  |  |  |  |  |  |  |  |  |  |  |  |  |  |  |  |  |  |  |  |  |  |  |  |  |  |  |  |  |  |  |  |  |  |  |  |  |  |  |  |  |  |  |  |  |  |  |  |  |  |  |  |  |  |  |  |  |  |  |  |  |  |  |  |  |  |  |  |  |  |  |  |  |  |  |  |  |  |  |  |  |  |  |  |  |  |  |  |  |  |  |  |  |  |  |  |  |  |  |  |  |  |  |  |  |  |  |  |  |  |  |  |  |  |  |  |  |  |  |  |  |  |  |  |  |  |  |  |  |  |  |  |  |  |  |  |  |  |  |  |  |  |  |  |  |  |  |  |  |  |  |  |  |  |  |  |  |  |  |  |  |  |  |  |  |  |  |  |  |  |  |  |  |  |  |  |  |  |  |  |  |  |  |  |  |  |  |  |  |  |  |  |  |  |  |  |  |  |  |  |  |  |  |  |  |  |  |  |  |  |  |  |  |  |  |  |  |  |  |  |  |  |  |  |  |  |  |  |  |  |  |  |  |  |  |  |  |  |  |  |  |  |  |  |  |  |  |  |  |  |  |  |  |  |  |  |  |  |  |  |  |  |  |  |  |  |  |  |  |  |  |  |  |  |  |  |  |  |  |  |  |  |  |  |  |  |  |  |  |  |  |  |  |  |  |  |  |  |  |  |  |  |  |  |  |  |  |  |  |  |  |  |  |  |  |  |  |  |  |  |  |  |  |  |  |  |  |  |  |  |  |  |  |  |  |  |  |  |  |  |  |  |  |  |  |  |  |  |  |  |  |  |  |  |  |  |  |  |  |  |  |  |  |  |  |  |  |  |  |  |  |  |  |  |  |  |  |  |  |  |  |  |  |  |  |  |  |  |  |  |  |  |  |  |  |  |  |  |  |  |  |  |  |  |  |  |  |  |  |  |  |  |  |  |  |  |  |  |  |  |  |  |  |  |  |  |  |  |  |  |  |  |  |  |  |  |  |  |  |  |  |  |  |  |  |  |  |  |  |  |  |  |  |  |  |  |  |  |  |  |  |  |  |  |  |  |  |  |  |  |  |  |  |  |  |  |  |  |  |  |  |  |  |  |  |  |  |  |  |  |  |  |  |  |  |  |  |  |  |  |  |  |  |  |  |  |  |  |  |  |  |  |  |  |  |  |  |  |  |  |  |  |  |  |  |  |  |  |  |  |  |  |  |  |  |  |  |  |  |  |  |  |  |  |  |  |  |  |  |  |  |  |  |  |  |  |  |  |  |  |  |  |  |  |  |  |  |  |  |  |  |  |  |  |  |  |  |  |  |  |  |  |  |  |  |  |  |  |  |  |  |  |  |  |  |  |  |  |  |  |  |  |  |  |  |  |  |  |  |  |  |  |  |  |  |  |  |  |  |  |  |  |  |  |  |  |  |  |  |  |  |  |  |  |  |  |  |  |  |  |  |  |  |  |  |  |  |  |  |  |  |  |  |  |  |  |  |  |  |  |  |  |  |  |  |  |  |  |  |  |  |  |  |  |  |  |  |  |  |  |  |  |  |  |  |  |  |  |  |  |  |  |  |  |  |  |  |  |  |  |  |  |  |  |  |  |  |  |  |  |  |  |  |  |  |  |  |  |  |  |  |  |  |  |  |  |  |  |  |  |  |  |  |  |  |  |  |  |  |  |  |  |  |  |  |  |  |  |  |  |  |  |  |  |  |  |  |  |  |  |  |  |  |  |  |  |  |  |  |  |  |  |  |  |  |  |  |  |  |  |  |  |  |  |  |  |  |  |  |  |  |  |  |  |  |  |  |  |  |  |  |  |  |  |  |  |  |  |  |  |  |  |  |  |  |  |  |  |  |  |  |  |  |  |  |  |  |  |  |  |  |  |  |  |  |  |  |  |  |  |  |  |  |  |  |  |  |  |  |  |  |  |  |  |  |  |  |  |  |  |  |  |  |  |  |  |  |  |  |  |  |  |  |  |  |  |  |  |  |  |  |  |  |  |  |  |  |  |  |  |  |  |  |  |  |  |  |  |  |  |  |  |  |  |  |  |  |  |  |  |  |  |  |  |  |  |  |  |  |  |  |  |  |  |  |  |  |  |  |  |  |  |  |  |  |  |  |  |  |  |  |  |  |  |  |  |  |  |  |  |  |  |  |  |  |  |  |  |  |  |  |  |  |  |  |  |  |  |  |  |  |  |  |  |  |  |  |  |  |  |  |  |  |  |  |  |  |  |  |  |  |  |  |  |  |  |  |  |  |  |  |  |  |  |  |  |  |  |  |  |  |  |  |  |  |  |  |  |  |  |  |  |  |  |  |  |  |  |  |  |  |  |  |  |  |  |  |  |  |  |  |  |  |  |  |  |  |  |  |  |  |  |  |  |  |  |  |  |  |  |  |  |  |  |  |  |  |  |  |  |  |  |  |  |  |  |  |  |  |  |  |  |  |  |  |  |  |  |  |  |  |  |  |  |  |  |  |  |  |  |  |  |  |  |  |  |  |  |  |  |  |  |  |  |  |  |  |  |  |  |  |  |  |  |  |  |  |  |  |  |  |  |  |  |  |  |  |  |  |  |  |  |  |  |  |  |  |  |  |  |  |  |  |  |  |  |  |  |  |  |  |  |  |  |  |  |  |  |  |  |  |  |  |  |  |  |  |  |  |  |  |  |  |  |  |  |  |  |  |  |  |  |  |  |  |  |  |  |  |  |  |  |  |  |  |  |  |  |  |  |  |  |  |  |  |  |  |  |  |  |  |  |  |  |  |  |  |  |  |  |  |  |  |  |  |  |  |  |  |  |  |  |  |  |  |  |  |  |  |  |  |  |  |  |  |  |  |  |  |  |  |  |  |  |  |  |  |  |  |  |  |  |  |  |  |  |  |  |  |  |  |  |  |  |  |  |  |  |  |  |  |  |  |  |  |  |  |  |  |  |  |  |  |  |  |  |  |  |  |  |  |  |  |  |  |  |  |  |  |  |  |  |  |  |  |  |  |  |  |  |  |  |  |  |  |  |  |  |  |  |  |  |  |  |  |  |  |  |  |  |  |  |  |  |  |  |  |  |  |  |  |  |  |  |  |  |  |  |  |  |  |  |  |  |  |  |  |  |  |  |  |  |  |  |  |  |  |  |  |  |  |  |  |  |  |  |  |  |  |  |  |  |  |  |  |  |  |  |  |  |  |  |  |  |  |  |  |  |  |  |  |  |  |  |  |  |  |  |  |  |  |  |  |  |  |  |  |  |  |  |  |  |  |  |  |  |  |  |  |  |  |  |  |  |  |  |  |  |  |  |  |  |  |  |  |  |  |  |  |  |  |  |  |  |  |  |  |  |  |  |  |  |  |  |  |  |  |  |  |  |  |  |  |  |  |  |  |  |  |  |  |  |  |  |  |  |  |  |  |  |  |  |  |  |  |  |  |  |  |  |  |  |  |  |  |  |  |  |  |  |  |  |  |  |  |  |  |  |  |  |  |  |  |  |  |  |  |  |  |  |  |  |  |  |  |  |  |  |  |  |  |  |  |  |  |  |  |  |  |  |  |  |  |  |  |  |  |  |  |  |  |  |  |  |  |  |  |  |  |  |  |  |  |  |  |  |  |  |  |  |  |  |  |  |  |  |  |  |  |  |  |  |  |  |  |  |  |  |  |  |  |  |  |  |  |  |  |  |  |  |  |  |  |  |  |  |  |  |  |  |  |  |  |  |  |  |  |  |  |  |  |  |  |  |  |  |  |  |  |  |  |  |  |  |  |  |  |  |  |  |  |  |  |  |  |  |  |  |  |  |  |  |  |  |  |  |  |  |  |  |  |  |  |  |  |  |  |  |  |  |  |  |  |  |  |  |  |  |  |  |  |  |  |  |  |  |  |  |  |  |  |  |  |  |  |  |  |  |  |  |  |  |  |  |  |  |  |  |  |  |  |  |  |  |  |  |  |  |  |  |  |  |  |  |  |  |  |  |  |  |  |  |  |  |  |  |  |  |  |  |  |  |  |  |  |  |  |  |  |  |  |  |  |  |  |  |  |  |  |  |  |  |  |  |  |  |  |  |  |  |  |  |  |  |  |  |  |  |  |  |  |  |  |  |  |  |  |  |  |  |  |  |  |  |  |  |  |  |  |  |  |  |  |  |  |  |  |  |  |  |  |  |  |  |  |  |  |  |  |  |  |  |  |  |  |  |  |  |  |  |  |  |  |  |  |  |  |  |  |  |  |  |  |  |  |  |  |  |  |  |  |  |  |  |  |  |  |  |  |  |  |  |  |  |  |  |  |  |  |  |  |  |  |  |  |  |  |  |  |  |  |  |  |  |  |  |  |  |  |  |  |  |  |  |  |  |  |  |  |  |  |  |  |  |  |  |  |  |  |  |  |  |  |  |  |  |  |  |  |  |  |  |  |  |  |  |  |  |  |  |  |  |  |  |  |  |  |  |  |  |  |  |  |  |  |  |  |  |  |  |  |  |  |  |  |  |  |  |  |  |  |  |  |  |  |  |  |  |  |  |  |  |  |  |  |  |  |  |  |  |  |  |  |  |  |  |  |  |  |  |  |  |  |  |  |  |  |  |  |  |  |  |  |  |  |  |  |  |  |  |  |  |  |  |  |  |  |  |  |  |  |  |  |  |  |  |  |  |  |  |  |  |  |  |  |  |  |  |  |  |  |  |  |  |  |  |  |  |  |  |  |  |  |  |  |  |  |  |  |  |  |  |  |  |  |  |  |  |  |  |  |  |  |  |  |  |  |  |  |  |  |  |  |  |  |  |  |  |  |  |  |  |  |  |  |  |  |  |  |  |  |  |  |  |  |  |  |  |  |  |  |  |  |  |  |  |  |  |  |  |  |  |  |  |  |  |  |  |  |  |  |  |  |  |  |  |  |  |  |  |  |  |  |  |  |  |  |  |  |  |  |  |  |  |  |  |  |  |  |  |  |  |  |  |  |  |  |  |  |  |  |  |  |  |  |  |  |  |  |  |  |  |  |  |  |  |  |  |  |  |  |  |  |  |  |  |  |  |  |  |  |  |  |  |  |  |  |  |  |  |  |  |  |  |  |  |  |  |  |  |  |  |  |  |  |  |  |  |  |  |  |  |  |  |  |  |  |  |  |  |  |  |  |  |  |  |  |  |  |  |  |  |  |  |  |  |  |  |  |  |  |  |  |  |  |  |  |  |  |  |  |  |  |  |  |  |  |  |  |  |  |  |  |  |  |  |  |  |  |  |  |  |  |  |  |  |  |  |  |  |  |  |  |  |  |  |  |  |  |  |  |  |  |  |  |  |  |  |  |  |  |  |  |  |  |  |  |  |  |  |  |  |  |  |  |  |  |  |  |  |  |  |  |  |  |  |  |  |  |  |  |  |  |  |  |  |  |  |  |  |  |  |  |  |  |  |  |  |  |  |  |  |  |  |  |  |  |  |  |  |  |  |  |  |  |  |  |  |  |  |  |  |  |  |  |  |  |  |  |  |  |  |  |  |  |  |  |  |  |  |  |  |  |  |  |  |  |  |  |  |  |  |  |  |  |  |  |  |  |  |  |  |  |  |  |  |  |  |  |  |  |  |  |  |  |  |  |  |  |  |  |  |  |  |  |  |  |  |  |  |  |  |  |  |  |  |  |  |  |  |  |  |  |  |  |  |  |  |  |  |  |  |  |  |  |  |  |  |  |  |  |  |  |  |  |  |  |  |  |  |  |  |  |  |  |  |  |  |  |  |  |  |  |  |  |  |  |  |  |  |  |  |  |  |  |  |  |  |  |  |  |  |  |  |  |  |  |  |  |  |  |  |  |  |  |  |  |  |  |  |  |  |  |  |  |  |  |  |  |  |  |  |  |  |  |  |  |  |  |  |  |  |  |  |  |  |  |  |  |  |  |  |  |  |  |  |  |  |  |  |  |  |  |  |  |  |  |  |  |  |  |  |  |  |  |  |  |  |  |  |  |  |  |  |  |  |  |  |  |  |  |  |  |  |  |  |  |  |  |  |  |  |  |  |  |  |  |  |  |  |  |  |  |  |  |  |  |  |  |  |  |  |  |  |  |  |  |  |  |  |  |  |  |  |  |  |  |  |  |  |  |  |  |  |  |  |  |  |  |  |  |  |  |  |  |  |  |  |  |  |  |  |  |  |  |  |  |  |  |  |  |  |  |  |  |  |  |  |  |  |  |  |  |  |  |  |  |  |  |  |  |  |  |  |  |  |  |  |  |  |  |  |  |  |  |  |  |  |  |  |  |  |  |  |  |  |  |  |  |  |  |  |  |  |  |  |  |  |  |  |  |  |  |  |  |  |  |  |  |  |  |  |  |  |  |  |  |  |  |  |  |  |  |  |  |  |  |  |  |  |  |  |  |  |  |  |  |  |  |  |  |  |  |  |  |  |  |  |  |  |  |  |  |  |  |  |  |  |  |  |  |  |  |  |  |  |  |  |  |  |  |  |  |  |  |  |  |  |  |  |  |  |  |  |  |  |  |  |  |  |  |  |  |  |  |  |  |  |  |  |  |  |  |  |  |  |  |  |  |  |  |  |  |  |  |  |  |  |  |  |  |  |  |  |  |  |  |  |  |  |  |  |  |  |  |  |  |  |  |  |  |  |  |  |  |  |  |  |  |  |  |  |  |  |  |  |  |  |  |  |  |  |  |  |  |  |  |  |  |  |  |  |  |  |  |  |  |  |  |  |  |  |  |  |  |  |  |  |  |  |  |  |  |  |  |  |  |  |  |  |  |  |  |  |  |  |  |  |  |  |  |  |  |  |  |  |  |  |  |  |  |  |  |  |  |  |  |  |  |  |  |  |  |  |  |  |  |  |  |  |  |  |  |  |  |  |  |  |  |  |  |  |  |  |  |  |  |  |  |  |  |  |  |  |  |  |  |  |  |  |  |  |  |  |  |  |  |  |  |  |  |  |  |  |  |  |  |  |  |  |  |  |  |  |  |  |  |  |  |  |  |  |  |  |  |  |  |  |  |  |  |  |  |  |  |  |  |  |  |  |  |  |  |  |  |  |  |  |  |  |  |  |  |  |  |  |  |  |  |  |  |  |  |  |  |  |  |  |  |  |  |  |  |  |  |  |  |  |  |  |  |  |  |  |  |  |  |  |  |  |  |  |  |  |  |  |  |  |  |  |  |  |  |  |  |  |  |  |  |  |  |  |  |  |  |  |  |  |  |  |  |  |  |  |  |  |  |  |  |  |  |  |  |  |  |  |  |  |  |  |  |  |  |  |  |  |  |  |  |  |  |  |  |  |  |  |  |  |  |  |  |  |  |  |  |  |  |  |  |  |  |  |  |  |  |  |  |  |  |  |  |  |  |  |  |  |  |  |  |  |  |  |  |  |  |  |  |  |  |  |  |  |  |  |  |  |  |  |  |  |  |  |  |  |  |  |  |  |  |  |  |  |  |  |  |  |  |  |  |  |  |  |  |  |  |  |  |  |  |  |  |  |  |  |  |  |  |  |  |  |  |  |  |  |  |  |  |  |  |  |  |  |  |  |  |  |  |  |  |  |  |  |  |  |  |  |  |  |  |  |  |  |  |  |  |  |  |  |  |  |  |  |  |  |  |  |  |  |  |  |  |  |  |  |  |  |  |  |  |  |  |  |  |  |  |  |  |  |  |  |  |  |  |  |  |  |  |  |  |  |  |  |  |  |  |  |  |  |  |  |  |  |  |  |  |  |  |  |  |  |  |  |  |  |  |  |  |  |  |  |  |  |  |  |  |  |  |  |  |  |  |  |  |  |  |  |  |  |  |  |  |  |  |  |  |  |  |  |  |  |  |  |  |  |  |  |  |  |  |  |  |  |  |  |  |  |  |  |  |  |  |  |  |  |  |  |  |  |  |  |  |  |  |  |  |  |  |  |  |  |  |  |  |  |  |  |  |  |  |  |  |  |  |  |  |  |  |  |  |  |  |  |  |  |  |  |  |  |  |  |  |  |  |  |  |  |  |  |  |  |  |  |  |  |  |  |  |  |  |  |  |  |  |  |  |  |  |  |  |  |  |  |  |  |  |  |  |  |  |  |  |  |  |  |  |  |  |  |  |  |  |  |  |  |  |  |  |  |  |  |  |  |  |  |  |  |  |  |  |  |  |  |  |  |  |  |  |  |  |  |  |  |  |  |  |  |  |  |  |  |  |  |  |  |  |  |  |  |  |  |  |  |  |  |  |  |  |  |  |  |  |  |  |  |  |  |  |  |  |  |  |  |  |  |  |  |  |  |  |  |  |  |  |  |  |  |  |  |  |  |  |  |  |  |  |  |  |  |  |  |  |  |  |  |  |  |  |  |  |  |  |  |  |  |  |  |  |  |  |  |  |  |  |  |  |  |  |  |  |  |  |  |  |  |  |  |  |  |  |  |  |  |  |  |  |  |  |  |  |  |  |  |  |  |  |  |  |  |  |  |  |  |  |  |  |  |  |  |  |  |  |  |  |  |  |  |  |  |  |  |  |  |  |  |  |  |  |  |  |  |  |  |  |  |  |  |  |  |  |  |  |  |  |  |  |  |  |  |  |  |  |  |  |  |  |  |  |  |  |  |  |  |  |  |  |  |  |  |  |  |  |  |  |  |  |  |  |  |  |  |  |  |  |  |  |  |  |  |  |  |  |  |  |  |  |  |  |  |  |  |  |  |  |  |  |  |  |  |  |  |  |  |  |  |  |  |  |  |  |  |  |  |  |  |  |  |  |  |  |  |  |  |  |  |  |  |  |  |  |  |  |  |  |  |  |  |  |  |  |  |  |  |  |  |  |  |  |  |  |  |  |  |  |  |  |  |  |  |  |  |  |  |  |  |  |  |  |  |  |  |  |  |  |  |  |  |  |  |  |  |  |  |  |  |  |  |  |  |  |  |  |  |  |  |  |  |  |  |  |  |  |  |  |  |  |  |  |  |  |  |  |  |  |  |  |  |  |  |  |  |  |  |  |  |  |  |  |  |  |  |  |  |  |  |  |  |  |  |  |  |  |  |  |  |  |  |  |  |  |  |  |  |  |  |  |  |  |  |  |  |  |  |  |  |  |  |  |  |  |  |  |  |  |  |  |  |  |  |  |  |  |  |  |  |  |  |  |  |  |  |  |  |  |  |  |  |  |  |  |  |  |  |  |  |  |  |  |  |  |  |  |  |  |  |  |  |  |  |  |  |  |  |  |  |  |  |  |  |  |  |  |  |  |  |  |  |  |  |  |  |  |  |  |  |  |  |  |  |  |  |  |  |  |  |  |  |  |  |  |  |  |  |  |  |  |  |  |  |  |  |  |  |  |  |  |  |  |  |  |  |  |  |  |  |  |  |  |  |  |  |  |  |  |  |  |  |  |  |  |  |  |  |  |  |  |  |  |  |  |  |  |  |  |  |  |  |  |  |  |  |  |  |  |  |  |  |  |  |  |  |  |  |  |  |  |  |  |  |  |  |  |  |  |  |  |  |  |  |  |  |  |  |  |  |  |  |  |  |  |  |  |  |  |  |  |  |  |  |  |  |  |  |  |  |  |  |  |  |  |  |  |  |  |  |  |  |  |  |  |  |  |  |  |  |  |  |  |  |  |  |  |  |  |  |  |  |  |  |  |  |  |  |  |  |  |  |  |  |  |  |  |  |  |  |  |  |  |  |  |  |  |  |  |  |  |  |  |  |  |  |  |  |  |  |  |  |  |  |  |  |  |  |  |  |  |  |  |  |  |  |  |  |  |  |  |  |  |  |  |  |  |  |  |  |  |  |  |  |  |  |  |  |  |  |  |  |  |  |  |  |  |  |  |  |  |  |  |  |  |  |  |  |  |  |  |  |  |  |  |  |  |  |  |  |  |  |  |  |  |  |  |  |  |  |  |  |  |  |  |  |  |  |  |  |  |  |  |  |  |  |  |  |  |  |  |  |  |  |  |  |  |  |  |  |  |  |  |  |  |  |  |  |  |  |  |  |  |  |  |  |  |  |  |  |  |  |  |  |  |  |  |  |  |  |  |  |  |  |  |  |  |  |  |  |  |  |  |  |  |  |  |  |  |  |  |  |  |  |  |  |  |  |  |  |  |  |  |  |  |  |  |  |  |  |  |  |  |  |  |  |  |  |  |  |  |  |  |  |  |  |  |  |  |  |  |  |  |  |  |  |  |  |  |  |  |  |  |  |  |  |  |  |  |  |  |  |  |  |  |  |  |  |  |  |  |  |  |  |  |  |  |  |  |  |  |  |  |  |  |  |  |  |  |  |  |  |  |  |  |  |  |  |  |  |  |  |  |  |  |  |  |  |  |  |  |  |  |  |  |  |  |  |  |  |  |  |  |  |  |  |  |  |  |  |  |  |  |  |  |  |  |  |  |  |  |  |  |  |  |  |  |  |  |  |  |  |  |  |  |  |  |  |  |  |  |  |  |  |  |  |  |  |  |  |  |  |  |  |  |  |  |  |  |  |  |  |  |  |  |  |  |  |  |  |  |  |  |  |  |  |  |  |  |  |  |  |  |  |  |  |  |  |  |  |  |  |  |  |  |  |  |  |  |  |  |  |  |  |  |  |  |  |  |  |  |  |  |  |  |  |  |  |  |  |  |  |  |  |  |  |  |  |  |  |  |  |  |  |  |  |  |  |  |  |  |  |  |  |  |  |  |  |  |  |  |  |  |  |  |  |  |  |  |  |  |  |  |  |  |  |  |  |  |  |  |  |  |  |  |  |  |  |  |  |  |  |  |  |  |  |  |  |  |  |  |  |  |  |  |  |  |  |  |  |  |  |  |  |  |  |  |  |  |  |  |  |  |  |  |  |  |  |  |  |  |  |  |  |  |  |  |  |  |  |  |  |  |  |  |  |  |  |  |  |  |  |  |  |  |  |  |  |  |  |  |  |  |  |  |  |  |  |  |  |  |  |  |  |  |  |  |  |  |  |  |  |  |  |  |  |  |  |  |  |  |  |  |  |  |  |  |  |  |  |  |  |  |  |  |  |  |  |  |  |  |  |  |  |  |  |  |  |  |  |  |  |  |  |  |  |  |  |  |  |  |  |  |  |  |  |  |  |  |  |  |  |  |  |  |  |  |  |  |  |  |  |  |  |  |  |  |  |  |  |  |  |  |  |  |  |  |  |  |  |  |  |  |  |  |  |  |  |  |  |  |  |  |  |  |  |  |  |  |  |  |  |  |  |  |  |  |  |  |  |  |  |  |  |  |  |  |  |  |  |  |  |  |  |  |  |  |  |  |  |  |  |  |  |  |  |  |  |  |  |  |  |  |  |  |  |  |  |  |  |  |  |  |  |  |  |  |  |  |  |  |  |  |  |  |  |  |  |  |  |  |  |  |  |  |  |  |  |  |  |  |  |  |  |  |  |  |  |  |  |  |  |  |  |  |  |  |  |  |  |  |  |  |  |  |  |  |  |  |  |  |  |  |  |  |  |  |  |  |  |  |  |  |  |  |  |  |  |  |  |  |  |  |  |  |  |  |  |  |  |  |  |  |  |  |  |  |  |  |  |  |  |  |  |  |  |  |  |  |  |  |  |  |  |  |  |  |  |  |  |  |  |  |  |  |  |  |  |  |  |  |  |  |  |  |  |  |  |  |  |  |  |  |  |  |  |  |  |  |  |  |  |  |  |  |  |  |  |  |  |  |  |  |  |  |  |  |  |  |  |  |  |  |  |  |  |  |  |  |  |  |  |  |  |  |  |  |  |  |  |  |  |  |  |  |  |  |  |  |  |  |  |  |  |  |  |  |  |  |  |  |  |  |  |  |  |  |  |  |  |  |  |  |  |  |  |  |  |  |  |  |  |  |  |  |  |  |  |  |  |  |  |  |  |  |  |  |  |  |  |  |  |  |  |  |  |  |  |  |  |  |  |  |  |  |  |  |  |  |  |  |  |  |  |  |  |  |  |  |  |  |  |  |  |  |  |  |  |  |  |  |  |  |  |  |  |  |  |  |  |  |  |  |  |  |  |  |  |  |  |  |  |  |  |  |  |  |  |  |  |  |  |  |  |  |  |  |  |  |  |  |  |  |  |  |  |  |  |  |  |  |  |  |  |  |  |  |  |  |  |  |  |  |  |  |  |  |  |  |  |  |  |  |  |  |  |  |  |  |  |  |  |  |  |  |  |  |  |  |  |  |  |  |  |  |  |  |  |  |  |  |  |  |  |  |  |  |  |  |  |  |  |  |  |  |  |  |  |  |  |  |  |  |  |  |  |  |  |  |  |  |  |  |  |  |  |  |  |  |  |  |  |  |  |  |  |  |  |  |  |  |  |  |  |  |  |  |  |  |  |  |  |  |  |  |  |  |  |  |  |  |  |  |  |  |  |  |  |  |  |  |  |  |  |  |  |  |  |  |  |  |  |  |  |  |  |  |  |  |  |  |  |  |  |  |  |  |  |  |  |  |  |  |  |  |  |  |  |  |  |  |  |  |  |  |  |  |  |  |  |  |  |  |  |  |  |  |  |  |  |  |  |  |  |  |  |  |  |  |  |  |  |  |  |  |  |  |  |  |  |  |  |  |  |  |  |  |  |  |  |  |  |  |  |  |  |  |  |  |  |  |  |  |  |  |  |  |  |  |  |  |  |  |  |  |  |  |  |  |  |  |  |  |  |  |  |  |  |  |  |  |  |  |  |  |  |  |  |  |  |  |  |  |  |  |  |  |  |  |  |  |  |  |  |  |  |  |  |  |  |  |  |  |  |  |  |  |  |  |  |  |  |  |  |  |  |  |  |  |  |  |  |  |  |  |  |  |  |  |  |  |  |  |  |  |  |  |  |  |  |  |  |  |  |  |  |  |  |  |  |  |  |  |  |  |  |  |  |  |  |  |  |  |  |  |  |  |  |  |  |  |  |  |  |  |  |  |  |  |  |  |  |  |  |  |  |  |  |  |  |  |  |  |  |  |  |  |  |  |  |  |  |  |  |  |  |  |  |  |  |  |  |  |  |  |  |  |  |  |  |  |  |  |  |  |  |  |  |  |  |  |  |  |  |  |  |  |  |  |  |  |  |  |  |  |  |  |  |  |  |  |  |  |  |  |  |  |  |  |  |  |  |  |  |  |  |  |  |  |  |  |  |  |  |  |  |  |  |  |  |  |  |  |  |  |  |  |  |  |  |  |  |  |  |  |  |  |  |  |  |  |  |  |  |  |  |  |  |  |  |  |  |  |  |  |  |  |  |  |  |  |  |  |  |  |  |  |  |  |  |  |  |  |  |  |  |  |  |  |  |  |  |  |  |  |  |  |  |  |  |  |  |  |  |  |  |  |  |  |  |  |  |  |  |  |  |  |  |  |  |  |  |  |  |  |  |  |  |  |  |  |  |  |  |  |  |  |  |  |  |  |  |  |  |  |  |  |  |  |  |  |  |  |  |  |  |  |  |  |  |  |  |  |  |  |  |  |  |  |  |  |  |  |  |  |  |  |  |  |  |  |  |  |  |  |  |  |  |  |  |  |  |  |  |  |  |  |  |  |  |  |  |  |  |  |  |  |  |  |  |  |  |  |  |  |  |  |  |  |  |  |  |  |  |  |  |  |  |  |  |  |  |  |  |  |  |  |  |  |  |  |  |  |  |  |  |  |  |  |  |  |  |  |  |  |  |  |  |  |  |  |  |  |  |  |  |  |  |  |  |  |  |  |  |  |  |  |  |  |  |  |  |  |  |  |  |  |  |  |  |  |  |  |  |  |  |  |  |  |  |  |  |  |  |  |  |  |  |  |  |  |  |  |  |  |  |  |  |  |  |
| --- | --- | --- | --- | --- | --- | --- | --- | --- | --- | --- | --- | --- | --- | --- | --- | --- | --- | --- | --- | --- | --- | --- | --- | --- | --- | --- | --- | --- | --- | --- | --- | --- | --- | --- | --- | --- | --- | --- | --- | --- | --- | --- | --- | --- | --- | --- | --- | --- | --- | --- | --- | --- | --- | --- | --- | --- | --- | --- | --- | --- | --- | --- | --- | --- | --- | --- | --- | --- | --- | --- | --- | --- | --- | --- | --- | --- | --- | --- | --- | --- | --- | --- | --- | --- | --- | --- | --- | --- | --- | --- | --- | --- | --- | --- | --- | --- | --- | --- | --- | --- | --- | --- | --- | --- | --- | --- | --- | --- | --- | --- | --- | --- | --- | --- | --- | --- | --- | --- | --- | --- | --- | --- | --- | --- | --- | --- | --- | --- | --- | --- | --- | --- | --- | --- | --- | --- | --- | --- | --- | --- | --- | --- | --- | --- | --- | --- | --- | --- | --- | --- | --- | --- | --- | --- | --- | --- | --- | --- | --- | --- | --- | --- | --- | --- | --- | --- | --- | --- | --- | --- | --- | --- | --- | --- | --- | --- | --- | --- | --- | --- | --- | --- | --- | --- | --- | --- | --- | --- | --- | --- | --- | --- | --- | --- | --- | --- | --- | --- | --- | --- | --- | --- | --- | --- | --- | --- | --- | --- | --- | --- | --- | --- | --- | --- | --- | --- | --- | --- | --- | --- | --- | --- | --- | --- | --- | --- | --- | --- | --- | --- | --- | --- | --- | --- | --- | --- | --- | --- | --- | --- | --- | --- | --- | --- | --- | --- | --- | --- | --- | --- | --- | --- | --- | --- | --- | --- | --- | --- | --- | --- | --- | --- | --- | --- | --- | --- | --- | --- | --- | --- | --- | --- | --- | --- | --- | --- | --- | --- | --- | --- | --- | --- | --- | --- | --- | --- | --- | --- | --- | --- | --- | --- | --- | --- | --- | --- | --- | --- | --- | --- | --- | --- | --- | --- | --- | --- | --- | --- | --- | --- | --- | --- | --- | --- | --- | --- | --- | --- | --- | --- | --- | --- | --- | --- | --- | --- | --- | --- | --- | --- | --- | --- | --- | --- | --- | --- | --- | --- | --- | --- | --- | --- | --- | --- | --- | --- | --- | --- | --- | --- | --- | --- | --- | --- | --- | --- | --- | --- | --- | --- | --- | --- | --- | --- | --- | --- | --- | --- | --- | --- | --- | --- | --- | --- | --- | --- | --- | --- | --- | --- | --- | --- | --- | --- | --- | --- | --- | --- | --- | --- | --- | --- | --- | --- | --- | --- | --- | --- | --- | --- | --- | --- | --- | --- | --- | --- | --- | --- | --- | --- | --- | --- | --- | --- | --- | --- | --- | --- | --- | --- | --- | --- | --- | --- | --- | --- | --- | --- | --- | --- | --- | --- | --- | --- | --- | --- | --- | --- | --- | --- | --- | --- | --- | --- | --- | --- | --- | --- | --- | --- | --- | --- | --- | --- | --- | --- | --- | --- | --- | --- | --- | --- | --- | --- | --- | --- | --- | --- | --- | --- | --- | --- | --- | --- | --- | --- | --- | --- | --- | --- | --- | --- | --- | --- | --- | --- | --- | --- | --- | --- | --- | --- | --- | --- | --- | --- | --- | --- | --- | --- | --- | --- | --- | --- | --- | --- | --- | --- | --- | --- | --- | --- | --- | --- | --- | --- | --- | --- | --- | --- | --- | --- | --- | --- | --- | --- | --- | --- | --- | --- | --- | --- | --- | --- | --- | --- | --- | --- | --- | --- | --- | --- | --- | --- | --- | --- | --- | --- | --- | --- | --- | --- | --- | --- | --- | --- | --- | --- | --- | --- | --- | --- | --- | --- | --- | --- | --- | --- | --- | --- | --- | --- | --- | --- | --- | --- | --- | --- | --- | --- | --- | --- | --- | --- | --- | --- | --- | --- | --- | --- | --- | --- | --- | --- | --- | --- | --- | --- | --- | --- | --- | --- | --- | --- | --- | --- | --- | --- | --- | --- | --- | --- | --- | --- | --- | --- | --- | --- | --- | --- | --- | --- | --- | --- | --- | --- | --- | --- | --- | --- | --- | --- | --- | --- | --- | --- | --- | --- | --- | --- | --- | --- | --- | --- | --- | --- | --- | --- | --- | --- | --- | --- | --- | --- | --- | --- | --- | --- | --- | --- | --- | --- | --- | --- | --- | --- | --- | --- | --- | --- | --- | --- | --- | --- | --- | --- | --- | --- | --- | --- | --- | --- | --- | --- | --- | --- | --- | --- | --- | --- | --- | --- | --- | --- | --- | --- | --- | --- | --- | --- | --- | --- | --- | --- | --- | --- | --- | --- | --- | --- | --- | --- | --- | --- | --- | --- | --- | --- | --- | --- | --- | --- | --- | --- | --- | --- | --- | --- | --- | --- | --- | --- | --- | --- | --- | --- | --- | --- | --- | --- | --- | --- | --- | --- | --- | --- | --- | --- | --- | --- | --- | --- | --- | --- | --- | --- | --- | --- | --- | --- | --- | --- | --- | --- | --- | --- | --- | --- | --- | --- | --- | --- | --- | --- | --- | --- | --- | --- | --- | --- | --- | --- | --- | --- | --- | --- | --- | --- | --- | --- | --- | --- | --- | --- | --- | --- | --- | --- | --- | --- | --- | --- | --- | --- | --- | --- | --- | --- | --- | --- | --- | --- | --- | --- | --- | --- | --- | --- | --- | --- | --- | --- | --- | --- | --- | --- | --- | --- | --- | --- | --- | --- | --- | --- | --- | --- | --- | --- | --- | --- | --- | --- | --- | --- | --- | --- | --- | --- | --- | --- | --- | --- | --- | --- | --- | --- | --- | --- | --- | --- | --- | --- | --- | --- | --- | --- | --- | --- | --- | --- | --- | --- | --- | --- | --- | --- | --- | --- | --- | --- | --- | --- | --- | --- | --- | --- | --- | --- | --- | --- | --- | --- | --- | --- | --- | --- | --- | --- | --- | --- | --- | --- | --- | --- | --- | --- | --- | --- | --- | --- | --- | --- | --- | --- | --- | --- | --- | --- | --- | --- | --- | --- | --- | --- | --- | --- | --- | --- | --- | --- | --- | --- | --- | --- | --- | --- | --- | --- | --- | --- | --- | --- | --- | --- | --- | --- | --- | --- | --- | --- | --- | --- | --- | --- | --- | --- | --- | --- | --- | --- | --- | --- | --- | --- | --- | --- | --- | --- | --- | --- | --- | --- | --- | --- | --- | --- | --- | --- | --- | --- | --- | --- | --- | --- | --- | --- | --- | --- | --- | --- | --- | --- | --- | --- | --- | --- | --- | --- | --- | --- | --- | --- | --- | --- | --- | --- | --- | --- | --- | --- | --- | --- | --- | --- | --- | --- | --- | --- | --- | --- | --- | --- | --- | --- | --- | --- | --- | --- | --- | --- | --- | --- | --- | --- | --- | --- | --- | --- | --- | --- | --- | --- | --- | --- | --- | --- | --- | --- | --- | --- | --- | --- | --- | --- | --- | --- | --- | --- | --- | --- | --- | --- | --- | --- | --- | --- | --- | --- | --- | --- | --- | --- | --- | --- | --- | --- | --- | --- | --- | --- | --- | --- | --- | --- | --- | --- | --- | --- | --- | --- | --- | --- | --- | --- | --- | --- | --- | --- | --- | --- | --- | --- | --- | --- | --- | --- | --- | --- | --- | --- | --- | --- | --- | --- | --- | --- | --- | --- | --- | --- | --- | --- | --- | --- | --- | --- | --- | --- | --- | --- | --- | --- | --- | --- | --- | --- | --- | --- | --- | --- | --- | --- | --- | --- | --- | --- | --- | --- | --- | --- | --- | --- | --- | --- | --- | --- | --- | --- | --- | --- | --- | --- | --- | --- | --- | --- | --- | --- | --- | --- | --- | --- | --- | --- | --- | --- | --- | --- | --- | --- | --- | --- | --- | --- | --- | --- | --- | --- | --- | --- | --- | --- | --- | --- | --- | --- | --- | --- | --- | --- | --- | --- | --- | --- | --- | --- | --- | --- | --- | --- | --- | --- | --- | --- | --- | --- | --- | --- | --- | --- | --- | --- | --- | --- | --- | --- | --- | --- | --- | --- | --- | --- | --- | --- | --- | --- | --- | --- | --- | --- | --- | --- | --- | --- | --- | --- | --- | --- | --- | --- | --- | --- | --- | --- | --- | --- | --- | --- | --- | --- | --- | --- | --- | --- | --- | --- | --- | --- | --- | --- | --- | --- | --- | --- | --- | --- | --- | --- | --- | --- | --- | --- | --- | --- | --- | --- | --- | --- | --- | --- | --- | --- | --- | --- | --- | --- | --- | --- | --- | --- | --- | --- | --- | --- | --- | --- | --- | --- | --- | --- | --- | --- | --- | --- | --- | --- | --- | --- | --- | --- | --- | --- | --- | --- | --- | --- | --- | --- | --- | --- | --- | --- | --- | --- | --- | --- | --- | --- | --- | --- | --- | --- | --- | --- | --- | --- | --- | --- | --- | --- | --- | --- | --- | --- | --- | --- | --- | --- | --- | --- | --- | --- | --- | --- | --- | --- | --- | --- | --- | --- | --- | --- | --- | --- | --- | --- | --- | --- | --- | --- | --- | --- | --- | --- | --- | --- | --- | --- | --- | --- | --- | --- | --- | --- | --- | --- | --- | --- | --- | --- | --- | --- | --- | --- | --- | --- | --- | --- | --- | --- | --- | --- | --- | --- | --- | --- | --- | --- | --- | --- | --- | --- | --- | --- | --- | --- | --- | --- | --- | --- | --- | --- | --- | --- | --- | --- | --- | --- | --- | --- | --- | --- | --- | --- | --- | --- | --- | --- | --- | --- | --- | --- | --- | --- | --- | --- | --- | --- | --- | --- | --- | --- | --- | --- | --- | --- | --- | --- | --- | --- | --- | --- | --- | --- | --- | --- | --- | --- | --- | --- | --- | --- | --- | --- | --- | --- | --- | --- | --- | --- | --- | --- | --- | --- | --- | --- | --- | --- | --- | --- | --- | --- | --- | --- | --- | --- | --- | --- | --- | --- | --- | --- | --- | --- | --- | --- | --- | --- | --- | --- | --- | --- | --- | --- | --- | --- | --- | --- | --- | --- | --- | --- | --- | --- | --- | --- | --- | --- | --- | --- | --- | --- | --- | --- | --- | --- | --- | --- | --- | --- | --- | --- | --- | --- | --- | --- | --- | --- | --- | --- | --- | --- | --- | --- | --- | --- | --- | --- | --- | --- | --- | --- | --- | --- | --- | --- | --- | --- | --- | --- | --- | --- | --- | --- | --- | --- | --- | --- | --- | --- | --- | --- | --- | --- | --- | --- | --- | --- | --- | --- | --- | --- | --- | --- | --- | --- | --- | --- | --- | --- | --- | --- | --- | --- | --- | --- | --- | --- | --- | --- | --- | --- | --- | --- | --- | --- | --- | --- | --- | --- | --- | --- | --- | --- | --- | --- | --- | --- | --- | --- | --- | --- | --- | --- | --- | --- | --- | --- | --- | --- | --- | --- | --- | --- | --- | --- | --- | --- | --- | --- | --- | --- | --- | --- | --- | --- | --- | --- | --- | --- | --- | --- | --- | --- | --- | --- | --- | --- | --- | --- | --- | --- | --- | --- | --- | --- | --- | --- | --- | --- | --- | --- | --- | --- | --- | --- | --- | --- | --- | --- | --- | --- | --- | --- | --- | --- | --- | --- | --- | --- | --- | --- | --- | --- | --- | --- | --- | --- | --- | --- | --- | --- | --- | --- | --- | --- | --- | --- | --- | --- | --- | --- | --- | --- | --- | --- | --- | --- | --- | --- | --- | --- | --- | --- | --- | --- | --- | --- | --- | --- | --- | --- | --- | --- | --- | --- | --- | --- | --- | --- | --- | --- | --- | --- | --- | --- | --- | --- | --- | --- | --- | --- | --- | --- | --- | --- | --- | --- | --- | --- | --- | --- | --- | --- | --- | --- | --- | --- | --- | --- | --- | --- | --- | --- | --- | --- | --- | --- | --- | --- | --- | --- | --- | --- | --- | --- | --- | --- | --- | --- | --- | --- | --- | --- | --- | --- | --- | --- | --- | --- | --- | --- | --- | --- | --- | --- | --- | --- | --- | --- | --- | --- | --- | --- | --- | --- | --- | --- | --- | --- | --- | --- | --- | --- | --- | --- | --- | --- | --- | --- | --- | --- | --- | --- | --- | --- | --- | --- | --- | --- | --- | --- | --- | --- | --- | --- | --- | --- | --- | --- | --- | --- | --- | --- | --- | --- | --- | --- | --- | --- | --- | --- | --- | --- | --- | --- | --- | --- | --- | --- | --- | --- | --- | --- | --- | --- | --- | --- | --- | --- | --- | --- | --- | --- | --- | --- | --- | --- | --- | --- | --- | --- | --- | --- | --- | --- | --- | --- | --- | --- | --- | --- | --- | --- | --- | --- | --- | --- | --- | --- | --- | --- | --- | --- | --- | --- | --- | --- | --- | --- | --- | --- | --- | --- | --- | --- | --- | --- | --- | --- | --- | --- | --- | --- | --- | --- | --- | --- | --- | --- | --- | --- | --- | --- | --- | --- | --- | --- | --- | --- | --- | --- | --- | --- | --- | --- | --- | --- | --- | --- | --- | --- | --- | --- | --- | --- | --- | --- | --- | --- | --- | --- | --- | --- | --- | --- | --- | --- | --- | --- | --- | --- | --- | --- | --- | --- | --- | --- | --- | --- | --- | --- | --- | --- | --- | --- | --- | --- | --- | --- | --- | --- | --- | --- | --- | --- | --- | --- | --- | --- | --- | --- | --- | --- | --- | --- | --- | --- | --- | --- | --- | --- | --- | --- | --- | --- | --- | --- | --- | --- | --- | --- | --- | --- | --- | --- | --- | --- | --- | --- | --- | --- | --- | --- | --- | --- | --- | --- | --- | --- | --- | --- | --- | --- | --- | --- | --- | --- | --- | --- | --- | --- | --- | --- | --- | --- | --- | --- | --- | --- | --- | --- | --- | --- | --- | --- | --- | --- | --- | --- | --- | --- | --- | --- | --- | --- | --- | --- | --- | --- | --- | --- | --- | --- | --- | --- | --- | --- | --- | --- | --- | --- | --- | --- | --- | --- | --- | --- | --- | --- | --- | --- | --- | --- | --- | --- | --- | --- | --- | --- | --- | --- | --- | --- | --- | --- | --- | --- | --- | --- | --- | --- | --- | --- | --- | --- | --- | --- | --- | --- | --- | --- | --- | --- | --- | --- | --- | --- | --- | --- | --- | --- | --- | --- | --- | --- | --- | --- | --- | --- | --- | --- | --- | --- | --- | --- | --- | --- | --- | --- | --- | --- | --- | --- | --- | --- | --- | --- | --- | --- | --- | --- | --- | --- | --- | --- | --- | --- | --- | --- | --- | --- | --- | --- | --- | --- | --- | --- | --- | --- | --- | --- | --- | --- | --- | --- | --- | --- | --- | --- | --- | --- | --- | --- | --- | --- | --- | --- | --- | --- | --- | --- | --- | --- | --- | --- | --- | --- | --- | --- | --- | --- | --- | --- | --- | --- | --- | --- | --- | --- | --- | --- | --- | --- | --- | --- | --- | --- | --- | --- | --- | --- | --- | --- | --- | --- | --- | --- | --- | --- | --- | --- | --- | --- | --- | --- | --- | --- | --- | --- | --- | --- | --- | --- | --- | --- | --- | --- | --- | --- | --- | --- | --- | --- | --- | --- | --- | --- | --- | --- | --- | --- | --- | --- | --- | --- | --- | --- | --- | --- | --- | --- | --- | --- | --- | --- | --- | --- | --- | --- | --- | --- | --- | --- | --- | --- | --- | --- | --- | --- | --- | --- | --- | --- | --- | --- | --- | --- | --- | --- | --- | --- | --- | --- | --- | --- | --- | --- | --- | --- | --- | --- | --- | --- | --- | --- | --- | --- | --- | --- | --- | --- | --- | --- | --- | --- | --- | --- | --- | --- | --- | --- | --- | --- | --- | --- | --- | --- | --- | --- | --- | --- | --- | --- | --- | --- | --- | --- | --- | --- | --- | --- | --- | --- | --- | --- | --- | --- | --- | --- | --- | --- | --- | --- | --- | --- | --- | --- | --- | --- | --- | --- | --- | --- | --- | --- | --- | --- | --- | --- | --- | --- | --- | --- | --- | --- | --- | --- | --- | --- | --- | --- | --- | --- | --- | --- | --- | --- | --- | --- | --- | --- | --- | --- | --- | --- | --- | --- | --- | --- | --- | --- | --- | --- | --- | --- | --- | --- | --- | --- | --- | --- | --- | --- | --- | --- | --- | --- | --- | --- | --- | --- | --- | --- | --- | --- | --- | --- | --- | --- | --- | --- | --- | --- | --- | --- | --- | --- | --- | --- | --- | --- | --- | --- | --- | --- | --- | --- | --- | --- | --- | --- | --- | --- | --- | --- | --- | --- | --- | --- | --- | --- | --- | --- | --- | --- | --- | --- | --- | --- | --- | --- | --- | --- | --- | --- | --- | --- | --- | --- | --- | --- | --- | --- | --- | --- | --- | --- | --- | --- | --- | --- | --- | --- | --- | --- | --- | --- | --- | --- | --- | --- | --- | --- | --- | --- | --- | --- | --- | --- | --- | --- | --- | --- | --- | --- | --- | --- | --- | --- | --- | --- | --- | --- | --- | --- | --- | --- | --- | --- | --- | --- | --- | --- | --- | --- | --- | --- | --- | --- | --- | --- | --- | --- | --- | --- | --- | --- | --- | --- | --- | --- | --- | --- | --- | --- | --- | --- | --- | --- | --- | --- | --- | --- | --- | --- | --- | --- | --- | --- | --- | --- | --- | --- | --- | --- | --- | --- | --- | --- | --- | --- | --- | --- | --- | --- | --- | --- | --- | --- | --- | --- | --- | --- | --- | --- | --- | --- | --- | --- | --- | --- | --- | --- | --- | --- | --- | --- | --- | --- | --- | --- | --- | --- | --- | --- | --- | --- | --- | --- | --- | --- | --- | --- | --- | --- | --- | --- | --- | --- | --- | --- | --- | --- | --- | --- | --- | --- | --- | --- | --- | --- | --- | --- | --- | --- | --- | --- | --- | --- | --- | --- | --- | --- | --- | --- | --- | --- | --- | --- | --- | --- | --- | --- | --- | --- | --- | --- | --- | --- | --- | --- | --- | --- | --- | --- | --- | --- | --- | --- | --- | --- | --- | --- | --- | --- | --- | --- | --- | --- | --- | --- | --- | --- | --- | --- | --- | --- | --- | --- | --- | --- | --- | --- | --- | --- | --- | --- | --- | --- | --- | --- | --- | --- | --- | --- | --- | --- | --- | --- | --- | --- | --- | --- | --- | --- | --- | --- | --- | --- | --- | --- | --- | --- | --- | --- | --- | --- | --- | --- | --- | --- | --- | --- | --- | --- | --- | --- | --- | --- | --- | --- | --- | --- | --- | --- | --- | --- | --- | --- | --- | --- | --- | --- | --- | --- | --- | --- | --- | --- | --- | --- | --- | --- | --- | --- | --- | --- | --- | --- | --- | --- | --- | --- | --- | --- | --- | --- | --- | --- | --- | --- | --- | --- | --- | --- | --- | --- | --- | --- | --- | --- | --- | --- | --- | --- | --- | --- | --- | --- | --- | --- | --- | --- | --- | --- | --- | --- | --- | --- | --- | --- | --- | --- | --- | --- | --- | --- | --- | --- | --- | --- | --- | --- | --- | --- | --- | --- | --- | --- | --- | --- | --- | --- | --- | --- | --- | --- | --- | --- | --- | --- | --- | --- | --- | --- | --- | --- | --- | --- | --- | --- | --- | --- | --- | --- | --- | --- | --- | --- | --- | --- | --- | --- | --- | --- | --- | --- | --- | --- | --- | --- | --- | --- | --- | --- | --- | --- | --- | --- | --- | --- | --- | --- | --- | --- | --- | --- | --- | --- | --- | --- | --- | --- | --- | --- | --- | --- | --- | --- | --- | --- | --- | --- | --- | --- | --- | --- | --- | --- | --- | --- | --- | --- | --- | --- | --- | --- | --- | --- | --- | --- | --- | --- | --- | --- | --- | --- | --- | --- | --- | --- | --- | --- | --- | --- | --- | --- | --- | --- | --- | --- | --- | --- | --- | --- | --- | --- | --- | --- | --- | --- | --- | --- | --- | --- | --- | --- | --- | --- | --- | --- | --- | --- | --- | --- | --- | --- | --- | --- | --- | --- | --- | --- | --- | --- | --- | --- | --- | --- | --- | --- | --- | --- | --- | --- | --- | --- | --- | --- | --- | --- | --- | --- | --- | --- | --- | --- | --- | --- | --- | --- | --- | --- | --- | --- | --- | --- | --- | --- | --- | --- | --- | --- | --- | --- | --- | --- | --- | --- | --- | --- | --- | --- | --- | --- | --- | --- | --- | --- | --- | --- | --- | --- | --- | --- | --- | --- | --- | --- | --- | --- | --- | --- | --- | --- | --- | --- | --- | --- | --- | --- | --- | --- | --- | --- | --- | --- | --- | --- | --- | --- | --- | --- | --- | --- | --- | --- | --- | --- | --- | --- | --- | --- | --- | --- | --- | --- | --- | --- | --- | --- | --- | --- | --- | --- | --- | --- | --- | --- | --- | --- | --- | --- | --- | --- | --- | --- | --- | --- | --- | --- | --- | --- | --- | --- | --- | --- | --- | --- | --- | --- | --- | --- | --- | --- | --- | --- | --- | --- | --- | --- | --- | --- | --- | --- | --- | --- | --- | --- | --- | --- | --- | --- | --- | --- | --- | --- | --- | --- | --- | --- | --- | --- | --- | --- | --- | --- | --- | --- | --- | --- | --- | --- | --- | --- | --- | --- | --- | --- | --- | --- | --- | --- | --- | --- | --- | --- | --- | --- | --- | --- | --- | --- | --- | --- | --- | --- | --- | --- | --- | --- | --- | --- | --- | --- | --- | --- | --- | --- | --- | --- | --- | --- | --- | --- | --- | --- | --- | --- | --- | --- | --- | --- | --- | --- | --- | --- | --- | --- | --- | --- | --- | --- | --- | --- | --- | --- | --- | --- | --- | --- | --- | --- | --- | --- | --- | --- | --- | --- | --- | --- | --- | --- | --- | --- | --- | --- | --- | --- | --- | --- | --- | --- | --- | --- | --- | --- | --- | --- | --- | --- | --- | --- | --- | --- | --- | --- | --- | --- | --- | --- | --- | --- | --- | --- | --- | --- | --- | --- | --- | --- | --- | --- | --- | --- | --- | --- | --- | --- | --- | --- | --- | --- | --- | --- | --- | --- | --- | --- | --- | --- | --- | --- | --- | --- | --- | --- | --- | --- | --- | --- | --- | --- | --- | --- | --- | --- | --- | --- | --- | --- | --- | --- | --- | --- | --- | --- | --- | --- | --- | --- | --- | --- | --- | --- | --- | --- | --- | --- | --- | --- | --- | --- | --- | --- | --- | --- | --- | --- | --- | --- | --- | --- | --- | --- | --- | --- | --- | --- | --- | --- | --- | --- | --- | --- | --- | --- | --- | --- | --- | --- | --- | --- | --- | --- | --- | --- | --- | --- | --- | --- | --- | --- | --- | --- | --- | --- | --- | --- | --- | --- | --- | --- | --- | --- | --- | --- | --- | --- | --- | --- | --- | --- | --- | --- | --- | --- | --- | --- | --- | --- | --- | --- | --- | --- | --- | --- | --- | --- | --- | --- | --- | --- | --- | --- | --- | --- | --- | --- | --- | --- | --- | --- | --- | --- | --- | --- | --- | --- | --- | --- | --- | --- | --- | --- | --- | --- | --- | --- | --- | --- | --- | --- | --- | --- | --- | --- | --- | --- | --- | --- | --- | --- | --- | --- | --- | --- | --- | --- | --- | --- | --- | --- | --- | --- | --- | --- | --- | --- | --- | --- | --- | --- | --- | --- | --- | --- | --- | --- | --- | --- | --- | --- | --- | --- | --- | --- | --- | --- | --- | --- | --- | --- | --- | --- | --- | --- | --- | --- | --- | --- | --- | --- | --- | --- | --- | --- | --- | --- | --- | --- | --- | --- | --- | --- | --- | --- | --- | --- | --- | --- | --- | --- | --- | --- | --- | --- | --- | --- | --- | --- | --- | --- | --- | --- | --- | --- | --- | --- | --- | --- | --- | --- | --- | --- | --- | --- | --- | --- | --- | --- | --- | --- | --- | --- | --- | --- | --- | --- | --- | --- | --- | --- | --- | --- | --- | --- | --- | --- | --- | --- | --- | --- | --- | --- | --- | --- | --- | --- | --- | --- | --- | --- | --- | --- | --- | --- | --- | --- | --- | --- | --- | --- | --- | --- | --- | --- | --- | --- | --- | --- | --- | --- | --- | --- | --- | --- | --- | --- | --- | --- | --- | --- | --- | --- | --- | --- | --- | --- | --- | --- | --- | --- | --- | --- | --- | --- | --- | --- | --- | --- | --- | --- | --- | --- | --- | --- | --- | --- | --- | --- | --- | --- | --- | --- | --- | --- | --- | --- | --- | --- | --- | --- | --- | --- | --- | --- | --- | --- | --- | --- | --- | --- | --- | --- | --- | --- | --- | --- | --- | --- | --- | --- | --- | --- | --- | --- | --- | --- | --- | --- | --- | --- | --- | --- | --- | --- | --- | --- | --- | --- | --- | --- | --- | --- | --- | --- | --- | --- | --- | --- | --- | --- | --- | --- | --- | --- | --- | --- | --- | --- | --- | --- | --- | --- | --- | --- | --- | --- | --- | --- | --- | --- | --- | --- | --- | --- | --- | --- | --- | --- | --- | --- | --- | --- | --- | --- | --- | --- | --- | --- | --- | --- | --- | --- | --- | --- | --- | --- | --- | --- | --- | --- | --- | --- | --- | --- | --- | --- | --- | --- | --- | --- | --- | --- | --- | --- | --- | --- | --- | --- | --- | --- | --- | --- | --- | --- | --- | --- | --- | --- | --- | --- | --- | --- | --- | --- | --- | --- | --- | --- | --- | --- | --- | --- | --- | --- | --- | --- | --- | --- | --- | --- | --- | --- | --- | --- | --- | --- | --- | --- | --- | --- | --- | --- | --- | --- | --- | --- | --- | --- | --- | --- | --- | --- | --- | --- | --- | --- | --- | --- | --- | --- | --- | --- | --- | --- | --- | --- | --- | --- | --- | --- | --- | --- | --- | --- | --- | --- | --- | --- | --- | --- | --- | --- | --- | --- | --- | --- | --- | --- | --- | --- | --- | --- | --- | --- | --- | --- | --- | --- | --- | --- | --- | --- | --- | --- | --- | --- | --- | --- | --- | --- | --- | --- | --- | --- | --- | --- | --- | --- | --- | --- | --- | --- | --- | --- | --- | --- | --- | --- | --- | --- | --- | --- | --- | --- | --- | --- | --- | --- | --- | --- | --- | --- | --- | --- | --- | --- | --- | --- | --- | --- | --- | --- | --- | --- | --- | --- | --- | --- | --- | --- | --- | --- | --- | --- | --- | --- | --- | --- | --- | --- | --- | --- | --- | --- | --- | --- | --- | --- | --- | --- | --- | --- | --- | --- | --- | --- | --- | --- | --- | --- | --- | --- | --- | --- | --- | --- | --- | --- | --- | --- | --- | --- | --- | --- | --- | --- | --- | --- | --- | --- | --- | --- | --- | --- | --- | --- | --- | --- | --- | --- | --- | --- | --- | --- | --- | --- | --- | --- | --- | --- | --- | --- | --- | --- | --- | --- | --- | --- | --- | --- | --- | --- | --- | --- | --- | --- | --- | --- | --- | --- | --- | --- | --- | --- | --- | --- | --- | --- | --- | --- | --- | --- | --- | --- | --- | --- | --- | --- | --- | --- | --- | --- | --- | --- | --- | --- | --- | --- | --- | --- | --- | --- | --- | --- | --- | --- | --- | --- | --- | --- | --- | --- | --- | --- | --- | --- | --- | --- | --- | --- | --- | --- | --- | --- | --- | --- | --- | --- | --- | --- | --- | --- | --- | --- | --- | --- | --- | --- | --- | --- | --- | --- | --- | --- | --- | --- | --- | --- | --- | --- | --- | --- | --- | --- | --- | --- | --- | --- | --- | --- | --- | --- | --- | --- | --- | --- | --- | --- | --- | --- | --- | --- | --- | --- | --- | --- | --- | --- | --- | --- | --- | --- | --- | --- | --- | --- | --- | --- | --- | --- | --- | --- | --- | --- | --- | --- | --- | --- | --- | --- | --- | --- | --- | --- | --- | --- | --- | --- | --- | --- | --- | --- | --- | --- | --- | --- | --- | --- | --- | --- | --- | --- | --- | --- | --- | --- | --- | --- | --- | --- | --- | --- | --- | --- | --- | --- | --- | --- | --- | --- | --- | --- | --- | --- | --- | --- | --- | --- | --- | --- | --- | --- | --- | --- | --- | --- | --- | --- | --- | --- | --- | --- | --- | --- | --- | --- | --- | --- | --- | --- | --- | --- | --- | --- | --- | --- | --- | --- | --- | --- | --- | --- | --- | --- | --- | --- | --- | --- | --- | --- | --- | --- | --- | --- | --- | --- | --- | --- | --- | --- | --- | --- | --- | --- | --- | --- | --- | --- | --- | --- | --- | --- | --- | --- | --- | --- | --- | --- | --- | --- | --- | --- | --- | --- | --- | --- | --- | --- | --- | --- | --- | --- | --- | --- | --- | --- | --- | --- | --- | --- | --- | --- | --- | --- | --- | --- | --- | --- | --- | --- | --- | --- | --- | --- | --- | --- | --- | --- | --- | --- | --- | --- | --- | --- | --- | --- | --- | --- | --- | --- | --- | --- | --- | --- | --- | --- | --- | --- | --- | --- | --- | --- | --- | --- | --- | --- | --- | --- | --- | --- | --- | --- | --- | --- | --- | --- | --- | --- | --- | --- | --- | --- | --- | --- | --- | --- | --- | --- | --- | --- | --- | --- | --- | --- | --- | --- | --- | --- | --- | --- | --- | --- | --- | --- | --- | --- | --- | --- | --- | --- | --- | --- | --- | --- | --- | --- | --- | --- | --- | --- | --- | --- | --- | --- | --- | --- | --- | --- | --- | --- | --- | --- | --- | --- | --- | --- | --- | --- | --- | --- | --- | --- | --- | --- | --- | --- | --- | --- | --- | --- | --- | --- | --- | --- | --- | --- | --- | --- | --- | --- | --- | --- | --- | --- | --- | --- | --- | --- | --- | --- | --- | --- | --- | --- | --- | --- | --- | --- | --- | --- | --- | --- | --- | --- | --- | --- | --- | --- | --- | --- | --- | --- | --- | --- | --- | --- | --- | --- | --- | --- | --- | --- | --- | --- | --- | --- | --- | --- | --- | --- | --- | --- | --- | --- | --- | --- | --- | --- | --- | --- | --- | --- | --- | --- | --- | --- | --- | --- | --- | --- | --- | --- | --- | --- | --- | --- | --- | --- | --- | --- | --- | --- | --- | --- | --- | --- | --- | --- | --- | --- | --- | --- | --- | --- | --- | --- | --- | --- | --- | --- | --- | --- | --- | --- | --- | --- | --- | --- | --- | --- | --- | --- | --- | --- | --- | --- | --- | --- | --- | --- | --- | --- | --- | --- | --- | --- | --- | --- | --- | --- | --- | --- | --- | --- | --- | --- | --- | --- | --- | --- | --- | --- | --- | --- | --- | --- | --- | --- | --- | --- | --- | --- | --- | --- | --- | --- | --- | --- | --- | --- | --- | --- | --- | --- | --- | --- | --- | --- | --- | --- | --- | --- | --- | --- | --- | --- | --- | --- | --- | --- | --- | --- | --- | --- | --- | --- | --- | --- | --- | --- | --- | --- | --- | --- | --- | --- | --- | --- | --- | --- | --- | --- | --- | --- | --- | --- | --- | --- | --- | --- | --- | --- | --- | --- | --- | --- | --- | --- | --- | --- | --- | --- | --- | --- | --- | --- | --- | --- | --- | --- | --- | --- | --- | --- | --- | --- | --- | --- | --- | --- | --- | --- | --- | --- | --- | --- | --- | --- | --- | --- | --- | --- | --- | --- | --- | --- | --- | --- | --- | --- | --- | --- | --- | --- | --- | --- | --- | --- | --- | --- | --- | --- | --- | --- | --- | --- | --- | --- | --- | --- | --- | --- | --- | --- | --- | --- | --- | --- | --- | --- | --- | --- | --- | --- | --- | --- | --- | --- | --- | --- | --- | --- | --- | --- | --- | --- | --- | --- | --- | --- | --- | --- | --- | --- | --- | --- | --- | --- | --- | --- | --- | --- | --- | --- | --- | --- | --- | --- | --- | --- | --- | --- | --- | --- | --- | --- | --- | --- | --- | --- | --- | --- | --- | --- | --- | --- | --- | --- | --- | --- | --- | --- | --- | --- | --- | --- | --- | --- | --- | --- | --- | --- | --- | --- | --- | --- | --- | --- | --- | --- | --- | --- | --- | --- | --- | --- | --- | --- | --- | --- | --- | --- | --- | --- | --- | --- | --- | --- | --- | --- | --- | --- | --- | --- | --- | --- | --- | --- | --- | --- | --- | --- | --- | --- | --- | --- | --- | --- | --- | --- | --- | --- | --- | --- | --- | --- | --- | --- | --- | --- | --- | --- | --- | --- | --- | --- | --- | --- | --- | --- | --- | --- | --- | --- | --- | --- | --- | --- | --- | --- | --- | --- | --- | --- | --- | --- | --- | --- | --- | --- | --- | --- | --- | --- | --- | --- | --- | --- | --- | --- | --- | --- | --- | --- | --- | --- | --- | --- | --- | --- | --- | --- | --- | --- | --- | --- | --- | --- | --- | --- | --- | --- | --- | --- | --- | --- | --- | --- | --- | --- | --- | --- | --- | --- | --- | --- | --- | --- | --- | --- | --- | --- | --- | --- | --- | --- | --- | --- | --- | --- | --- | --- | --- | --- | --- | --- | --- | --- | --- | --- | --- | --- | --- | --- | --- | --- | --- | --- | --- | --- | --- | --- | --- | --- | --- | --- | --- | --- | --- | --- | --- | --- | --- | --- | --- | --- | --- | --- | --- | --- | --- | --- | --- | --- | --- | --- | --- | --- | --- | --- | --- | --- | --- | --- | --- | --- | --- | --- | --- | --- | --- | --- | --- | --- | --- | --- | --- | --- | --- | --- | --- | --- | --- | --- | --- | --- | --- | --- | --- | --- | --- | --- | --- | --- | --- | --- | --- | --- | --- | --- | --- | --- | --- | --- | --- | --- | --- | --- | --- | --- | --- | --- | --- | --- | --- | --- | --- | --- | --- | --- | --- | --- | --- | --- | --- | --- | --- | --- | --- | --- | --- | --- | --- | --- | --- | --- | --- | --- | --- | --- | --- | --- | --- | --- | --- | --- | --- | --- | --- | --- | --- | --- | --- | --- | --- | --- | --- | --- | --- | --- | --- | --- | --- | --- | --- | --- | --- | --- | --- | --- | --- | --- | --- | --- | --- | --- | --- | --- | --- | --- | --- | --- | --- | --- | --- | --- | --- | --- | --- | --- | --- | --- | --- | --- | --- | --- | --- | --- | --- | --- | --- | --- | --- | --- | --- | --- | --- | --- | --- | --- | --- | --- | --- | --- | --- | --- | --- | --- | --- | --- | --- | --- | --- | --- | --- | --- | --- | --- | --- | --- | --- | --- | --- | --- | --- | --- | --- | --- | --- | --- | --- | --- | --- | --- | --- | --- | --- | --- | --- | --- | --- | --- | --- | --- | --- | --- | --- | --- | --- | --- | --- | --- | --- | --- | --- | --- | --- | --- | --- | --- | --- | --- | --- | --- | --- | --- | --- | --- | --- | --- | --- | --- | --- | --- | --- | --- | --- | --- | --- | --- | --- | --- | --- | --- | --- | --- | --- | --- | --- | --- | --- | --- | --- | --- | --- | --- | --- | --- | --- | --- | --- | --- | --- | --- | --- | --- | --- | --- | --- | --- | --- | --- | --- | --- | --- | --- | --- | --- | --- | --- | --- | --- | --- | --- | --- | --- | --- | --- | --- | --- | --- | --- | --- | --- | --- | --- | --- | --- | --- | --- | --- | --- | --- | --- | --- | --- | --- | --- | --- | --- | --- | --- | --- | --- | --- | --- | --- | --- | --- | --- | --- | --- | --- | --- | --- | --- | --- | --- | --- | --- | --- | --- | --- | --- | --- | --- | --- | --- | --- | --- | --- | --- | --- | --- | --- | --- | --- | --- | --- | --- | --- | --- | --- | --- | --- | --- | --- | --- | --- | --- | --- | --- | --- | --- | --- | --- | --- | --- | --- | --- | --- | --- | --- | --- | --- | --- | --- | --- | --- | --- | --- | --- | --- | --- | --- | --- | --- | --- | --- | --- | --- | --- | --- | --- | --- | --- | --- | --- | --- | --- | --- | --- | --- | --- | --- | --- | --- | --- | --- | --- | --- | --- | --- | --- | --- | --- | --- | --- | --- | --- | --- | --- | --- | --- | --- | --- | --- | --- | --- | --- | --- | --- | --- | --- | --- | --- | --- | --- | --- | --- | --- | --- | --- | --- | --- | --- | --- | --- | --- | --- | --- | --- | --- | --- | --- | --- | --- | --- | --- | --- | --- | --- | --- | --- | --- | --- | --- | --- | --- | --- | --- | --- | --- | --- | --- | --- | --- | --- | --- | --- | --- | --- | --- | --- | --- | --- | --- | --- | --- | --- | --- | --- | --- | --- | --- | --- | --- | --- | --- | --- | --- | --- | --- | --- | --- | --- | --- | --- | --- | --- | --- | --- | --- | --- | --- | --- | --- | --- | --- | --- | --- | --- | --- | --- | --- | --- | --- | --- | --- | --- | --- | --- | --- | --- | --- | --- | --- | --- | --- | --- | --- | --- | --- | --- | --- | --- | --- | --- | --- | --- | --- | --- | --- | --- | --- | --- | --- | --- | --- | --- | --- | --- | --- | --- | --- | --- | --- | --- | --- | --- | --- | --- | --- | --- | --- | --- | --- | --- | --- | --- | --- | --- | --- | --- | --- | --- | --- | --- | --- | --- | --- | --- | --- | --- | --- | --- | --- | --- | --- | --- | --- | --- | --- | --- | --- | --- | --- | --- | --- | --- | --- | --- | --- | --- | --- | --- | --- | --- | --- | --- | --- | --- | --- | --- | --- | --- | --- | --- | --- | --- | --- | --- | --- | --- | --- | --- | --- | --- | --- | --- | --- | --- | --- | --- | --- | --- | --- | --- | --- | --- | --- | --- | --- | --- | --- | --- | --- | --- | --- | --- | --- | --- | --- | --- | --- | --- | --- | --- | --- | --- | --- | --- | --- | --- | --- | --- | --- | --- | --- | --- | --- | --- | --- | --- | --- | --- | --- | --- | --- | --- | --- | --- | --- | --- | --- | --- | --- | --- | --- | --- | --- | --- | --- | --- | --- | --- | --- | --- | --- | --- | --- | --- | --- | --- | --- | --- | --- | --- | --- | --- | --- | --- | --- | --- | --- | --- | --- | --- | --- | --- | --- | --- | --- | --- | --- | --- | --- | --- | --- | --- | --- | --- | --- | --- | --- | --- | --- | --- | --- | --- | --- | --- | --- | --- | --- | --- | --- | --- | --- | --- | --- | --- | --- | --- | --- | --- | --- | --- | --- | --- | --- | --- | --- | --- | --- | --- | --- | --- | --- | --- | --- | --- | --- | --- | --- | --- | --- | --- | --- | --- | --- | --- | --- | --- | --- | --- | --- | --- | --- | --- | --- | --- | --- | --- | --- | --- | --- | --- | --- | --- | --- | --- | --- | --- | --- | --- | --- | --- | --- | --- | --- | --- | --- | --- | --- | --- | --- | --- | --- | --- | --- | --- | --- | --- | --- | --- | --- | --- | --- | --- | --- | --- | --- | --- | --- | --- | --- | --- | --- | --- | --- | --- | --- | --- | --- | --- | --- | --- | --- | --- | --- | --- | --- | --- | --- | --- | --- | --- | --- | --- | --- | --- | --- | --- | --- | --- | --- | --- | --- | --- | --- | --- | --- | --- | --- | --- | --- | --- | --- | --- | --- | --- | --- | --- | --- | --- | --- | --- | --- | --- | --- | --- | --- | --- | --- | --- | --- | --- | --- | --- | --- | --- | --- | --- | --- | --- | --- | --- | --- | --- | --- | --- | --- | --- | --- | --- | --- | --- | --- | --- | --- | --- | --- | --- | --- | --- | --- | --- | --- | --- | --- | --- | --- | --- | --- | --- | --- | --- | --- | --- | --- | --- | --- | --- | --- | --- | --- | --- | --- | --- | --- | --- | --- | --- | --- | --- | --- | --- | --- | --- | --- | --- | --- | --- | --- | --- | --- | --- | --- | --- | --- | --- | --- | --- | --- | --- | --- | --- | --- | --- | --- | --- | --- | --- | --- | --- | --- | --- | --- | --- | --- | --- | --- | --- | --- | --- | --- | --- | --- | --- | --- | --- | --- | --- | --- | --- | --- | --- | --- | --- | --- | --- | --- | --- | --- | --- | --- | --- | --- | --- | --- | --- | --- | --- | --- | --- | --- | --- | --- | --- | --- | --- | --- | --- | --- | --- | --- | --- | --- | --- | --- | --- | --- | --- | --- | --- | --- | --- | --- | --- | --- | --- | --- | --- | --- | --- | --- | --- | --- | --- | --- | --- | --- | --- | --- | --- | --- | --- | --- | --- | --- | --- | --- | --- | --- | --- | --- | --- | --- | --- | --- | --- | --- | --- | --- | --- | --- | --- | --- | --- | --- | --- | --- | --- | --- | --- | --- | --- | --- | --- | --- | --- | --- | --- | --- | --- | --- | --- | --- | --- | --- | --- | --- | --- | --- | --- | --- | --- | --- | --- | --- | --- | --- | --- | --- | --- | --- | --- | --- | --- | --- | --- | --- | --- | --- | --- | --- | --- | --- | --- | --- | --- | --- | --- | --- | --- | --- | --- | --- | --- | --- | --- | --- | --- | --- | --- | --- | --- | --- | --- | --- | --- | --- | --- | --- | --- | --- | --- | --- | --- | --- | --- | --- | --- | --- | --- | --- | --- | --- | --- | --- | --- | --- | --- | --- | --- | --- | --- | --- | --- | --- | --- | --- | --- | --- | --- | --- | --- | --- | --- | --- | --- | --- | --- | --- | --- | --- | --- | --- | --- | --- | --- | --- | --- | --- | --- | --- | --- | --- | --- | --- | --- | --- | --- | --- | --- | --- | --- | --- | --- | --- | --- | --- | --- | --- | --- | --- | --- | --- | --- | --- | --- | --- | --- | --- | --- | --- | --- | --- | --- | --- | --- | --- | --- | --- | --- | --- | --- | --- | --- | --- | --- | --- | --- | --- | --- | --- | --- | --- | --- | --- | --- | --- | --- | --- | --- | --- | --- | --- | --- | --- | --- | --- | --- | --- | --- | --- | --- | --- | --- | --- | --- | --- | --- | --- | --- | --- | --- | --- | --- | --- | --- | --- | --- | --- | --- | --- | --- | --- | --- | --- | --- | --- | --- | --- | --- | --- | --- | --- | --- | --- | --- | --- | --- | --- | --- | --- | --- | --- | --- | --- | --- | --- | --- | --- | --- | --- | --- | --- | --- | --- | --- | --- | --- | --- | --- | --- | --- | --- | --- | --- | --- | --- | --- | --- | --- | --- | --- | --- | --- | --- | --- | --- | --- | --- | --- | --- | --- | --- | --- | --- | --- | --- | --- | --- | --- | --- | --- | --- | --- | --- | --- | --- | --- | --- | --- | --- | --- | --- | --- | --- | --- | --- | --- | --- | --- | --- | --- | --- | --- | --- | --- | --- | --- | --- | --- | --- | --- | --- | --- | --- | --- | --- | --- | --- | --- | --- | --- | --- | --- | --- | --- | --- | --- | --- | --- | --- | --- | --- | --- | --- | --- | --- | --- | --- | --- | --- | --- | --- | --- | --- | --- | --- | --- | --- | --- | --- | --- | --- | --- | --- | --- | --- | --- | --- | --- | --- | --- | --- | --- | --- | --- | --- | --- | --- | --- | --- | --- | --- | --- | --- | --- | --- | --- | --- | --- | --- | --- | --- | --- | --- | --- | --- | --- | --- | --- | --- | --- | --- | --- | --- | --- | --- | --- | --- | --- | --- | --- | --- | --- | --- | --- | --- | --- | --- | --- | --- | --- | --- | --- | --- | --- | --- | --- | --- | --- | --- | --- | --- | --- | --- | --- | --- | --- | --- | --- | --- | --- | --- | --- | --- | --- | --- | --- | --- | --- | --- | --- | --- | --- | --- | --- | --- | --- | --- | --- | --- | --- | --- | --- | --- | --- | --- | --- | --- | --- | --- | --- | --- | --- | --- | --- | --- | --- | --- | --- | --- | --- | --- | --- | --- | --- | --- | --- | --- | --- | --- | --- | --- | --- | --- | --- | --- | --- | --- | --- | --- | --- | --- | --- | --- | --- | --- | --- | --- | --- | --- | --- | --- | --- | --- | --- | --- | --- | --- | --- | --- | --- | --- | --- | --- | --- | --- | --- | --- | --- | --- | --- | --- | --- | --- | --- | --- | --- | --- | --- | --- | --- | --- | --- | --- | --- | --- | --- | --- | --- | --- | --- | --- | --- | --- | --- | --- | --- | --- | --- | --- | --- | --- | --- | --- | --- | --- | --- | --- | --- | --- | --- | --- | --- | --- | --- | --- | --- | --- | --- | --- | --- | --- | --- | --- | --- | --- | --- | --- | --- | --- | --- | --- | --- | --- | --- | --- | --- | --- | --- | --- | --- | --- | --- | --- | --- | --- | --- | --- | --- | --- | --- | --- | --- | --- | --- | --- | --- | --- | --- | --- | --- | --- | --- | --- | --- | --- | --- | --- | --- | --- | --- | --- | --- | --- | --- | --- | --- | --- | --- | --- | --- | --- | --- | --- | --- | --- | --- | --- | --- | --- | --- | --- | --- | --- | --- | --- | --- | --- | --- | --- | --- | --- | --- | --- | --- | --- | --- | --- | --- | --- | --- | --- | --- | --- | --- | --- | --- | --- | --- | --- | --- | --- | --- | --- | --- | --- | --- | --- | --- | --- | --- | --- | --- | --- | --- | --- | --- | --- | --- | --- | --- | --- | --- | --- | --- | --- | --- | --- | --- | --- | --- | --- | --- | --- | --- | --- | --- | --- | --- | --- | --- | --- | --- | --- | --- | --- | --- | --- | --- | --- | --- | --- | --- | --- | --- | --- | --- | --- | --- | --- | --- | --- | --- | --- | --- | --- | --- | --- | --- | --- | --- | --- | --- | --- | --- | --- | --- | --- | --- | --- | --- | --- | --- | --- | --- | --- | --- | --- | --- | --- | --- | --- | --- | --- | --- | --- | --- | --- | --- | --- | --- | --- | --- | --- | --- | --- | --- | --- | --- | --- | --- | --- | --- | --- | --- | --- | --- | --- | --- | --- | --- | --- | --- | --- | --- | --- | --- | --- | --- | --- | --- | --- | --- | --- | --- | --- | --- | --- | --- | --- | --- | --- | --- | --- | --- | --- | --- | --- | --- | --- | --- | --- | --- | --- | --- | --- | --- | --- | --- | --- | --- | --- | --- | --- | --- | --- | --- | --- | --- | --- | --- | --- | --- | --- | --- | --- | --- | --- | --- | --- | --- | --- | --- | --- | --- | --- | --- | --- | --- | --- | --- | --- | --- | --- | --- | --- | --- | --- | --- | --- | --- | --- | --- | --- | --- | --- | --- | --- | --- | --- | --- | --- | --- | --- | --- | --- | --- | --- | --- | --- | --- | --- | --- | --- | --- | --- | --- | --- | --- | --- | --- | --- | --- | --- | --- | --- | --- | --- | --- | --- | --- | --- | --- | --- | --- | --- | --- | --- | --- | --- | --- | --- | --- | --- | --- | --- | --- | --- | --- | --- | --- | --- | --- | --- | --- | --- | --- | --- | --- | --- | --- | --- | --- | --- | --- | --- | --- | --- | --- | --- | --- | --- | --- | --- | --- | --- | --- | --- | --- | --- | --- | --- | --- | --- | --- | --- | --- | --- | --- | --- | --- | --- | --- | --- | --- | --- | --- | --- | --- | --- | --- | --- | --- | --- | --- | --- | --- | --- | --- | --- | --- | --- | --- | --- | --- | --- | --- | --- | --- | --- | --- | --- | --- | --- | --- | --- | --- | --- | --- | --- | --- | --- | --- | --- | --- | --- | --- | --- | --- | --- | --- | --- | --- | --- | --- | --- | --- | --- | --- | --- | --- | --- | --- | --- | --- | --- | --- | --- | --- | --- | --- | --- | --- | --- | --- | --- | --- | --- | --- | --- | --- | --- | --- | --- | --- | --- | --- | --- | --- | --- | --- | --- | --- | --- | --- | --- | --- | --- | --- | --- | --- | --- | --- | --- | --- | --- | --- | --- | --- | --- | --- | --- | --- | --- | --- | --- | --- | --- | --- | --- | --- | --- | --- | --- | --- | --- | --- | --- | --- | --- | --- | --- | --- | --- | --- | --- | --- | --- | --- | --- | --- | --- | --- | --- | --- | --- | --- | --- | --- | --- | --- | --- | --- | --- | --- | --- | --- | --- | --- | --- | --- | --- | --- | --- | --- | --- | --- | --- | --- | --- | --- | --- | --- | --- | --- | --- | --- | --- | --- | --- | --- | --- | --- | --- | --- | --- | --- | --- | --- | --- | --- | --- | --- | --- | --- | --- | --- | --- | --- | --- | --- | --- | --- | --- | --- | --- | --- | --- | --- | --- | --- | --- | --- | --- | --- | --- | --- | --- | --- | --- | --- | --- | --- | --- | --- | --- | --- | --- | --- | --- | --- | --- | --- | --- | --- | --- | --- | --- | --- | --- | --- | --- | --- | --- | --- | --- | --- | --- | --- | --- | --- | --- | --- | --- | --- | --- | --- | --- | --- | --- | --- | --- | --- | --- | --- | --- | --- | --- | --- | --- | --- | --- | --- | --- | --- | --- | --- | --- | --- | --- | --- | --- | --- | --- | --- | --- | --- | --- | --- | --- | --- | --- | --- | --- | --- | --- | --- | --- | --- | --- | --- | --- | --- | --- | --- | --- | --- | --- | --- | --- | --- | --- | --- | --- | --- | --- | --- | --- | --- | --- | --- | --- | --- | --- | --- | --- | --- | --- | --- | --- | --- | --- | --- | --- | --- | --- | --- | --- | --- | --- | --- | --- | --- | --- | --- | --- | --- | --- | --- | --- | --- | --- | --- | --- | --- | --- | --- | --- | --- | --- | --- | --- | --- | --- | --- | --- | --- | --- | --- | --- | --- | --- | --- | --- | --- | --- | --- | --- | --- | --- | --- | --- | --- | --- | --- | --- | --- | --- | --- | --- | --- | --- | --- | --- | --- | --- | --- | --- | --- | --- | --- | --- | --- | --- | --- | --- | --- | --- | --- | --- | --- | --- | --- | --- | --- | --- | --- | --- | --- | --- | --- | --- | --- | --- | --- | --- | --- | --- | --- | --- | --- | --- | --- | --- | --- | --- | --- | --- | --- | --- | --- | --- | --- | --- | --- | --- | --- | --- | --- | --- | --- | --- | --- | --- | --- | --- | --- | --- | --- | --- | --- | --- | --- | --- | --- | --- | --- | --- | --- | --- | --- | --- | --- | --- | --- | --- | --- | --- | --- | --- | --- | --- | --- | --- | --- | --- | --- | --- | --- | --- | --- | --- | --- | --- | --- | --- | --- | --- | --- | --- | --- | --- | --- | --- | --- | --- | --- | --- | --- | --- | --- | --- | --- | --- | --- | --- | --- | --- | --- | --- | --- | --- | --- | --- | --- | --- | --- | --- | --- | --- | --- | --- | --- | --- | --- | --- | --- | --- | --- | --- | --- | --- | --- | --- | --- | --- | --- | --- | --- | --- | --- | --- | --- | --- | --- | --- | --- | --- | --- | --- | --- | --- | --- | --- | --- | --- | --- | --- | --- | --- | --- | --- | --- | --- | --- | --- | --- | --- | --- | --- | --- | --- | --- | --- | --- | --- | --- | --- | --- | --- | --- | --- | --- | --- | --- | --- | --- | --- | --- | --- | --- | --- | --- | --- | --- | --- | --- | --- | --- | --- | --- | --- | --- | --- | --- | --- | --- | --- | --- | --- | --- | --- | --- | --- | --- | --- | --- | --- | --- | --- | --- | --- | --- | --- | --- | --- | --- | --- | --- | --- | --- | --- | --- | --- | --- | --- | --- | --- | --- | --- | --- | --- | --- | --- | --- | --- | --- | --- | --- | --- | --- | --- | --- | --- | --- | --- | --- | --- | --- | --- | --- | --- | --- | --- | --- | --- | --- | --- | --- | --- | --- | --- | --- | --- | --- | --- | --- | --- | --- | --- | --- | --- | --- | --- | --- | --- | --- | --- | --- | --- | --- | --- | --- | --- | --- | --- | --- | --- | --- | --- | --- | --- | --- | --- | --- | --- | --- | --- | --- | --- | --- | --- | --- | --- | --- | --- | --- | --- | --- | --- | --- | --- | --- | --- | --- | --- | --- | --- | --- | --- | --- | --- | --- | --- | --- | --- | --- | --- | --- | --- | --- | --- | --- | --- | --- | --- | --- | --- | --- | --- | --- | --- | --- | --- | --- | --- | --- | --- | --- | --- | --- | --- | --- | --- | --- | --- | --- | --- | --- | --- | --- | --- | --- | --- | --- | --- | --- | --- | --- | --- | --- | --- | --- | --- | --- | --- | --- | --- | --- | --- | --- | --- | --- | --- | --- | --- | --- | --- | --- | --- | --- | --- | --- | --- | --- | --- | --- | --- | --- | --- | --- | --- | --- | --- | --- | --- | --- | --- | --- | --- | --- | --- | --- | --- | --- | --- | --- | --- | --- | --- | --- | --- | --- | --- | --- | --- | --- | --- | --- | --- | --- | --- | --- | --- | --- | --- | --- | --- | --- | --- | --- | --- | --- | --- | --- | --- | --- | --- | --- | --- | --- | --- | --- | --- | --- | --- | --- | --- | --- | --- | --- | --- | --- | --- | --- | --- | --- | --- | --- | --- | --- | --- | --- | --- | --- | --- | --- | --- | --- | --- | --- | --- | --- | --- | --- | --- | --- | --- | --- | --- | --- | --- | --- | --- | --- | --- | --- | --- | --- | --- | --- | --- | --- | --- | --- | --- | --- | --- | --- | --- | --- | --- | --- | --- | --- | --- | --- | --- | --- | --- | --- | --- | --- | --- | --- | --- | --- | --- | --- | --- | --- | --- | --- | --- | --- | --- | --- | --- | --- | --- | --- | --- | --- | --- | --- | --- | --- | --- | --- | --- | --- | --- | --- | --- | --- | --- | --- | --- | --- | --- | --- | --- | --- | --- | --- | --- | --- | --- | --- | --- | --- | --- | --- | --- | --- | --- | --- | --- | --- | --- | --- | --- | --- | --- | --- | --- | --- | --- | --- | --- | --- | --- | --- | --- | --- | --- | --- | --- | --- | --- | --- | --- | --- | --- | --- | --- | --- | --- | --- | --- | --- | --- | --- | --- | --- | --- | --- | --- | --- | --- | --- | --- | --- | --- | --- | --- | --- | --- | --- | --- | --- | --- | --- | --- | --- | --- | --- | --- | --- | --- | --- | --- | --- | --- | --- | --- | --- | --- | --- | --- | --- | --- | --- | --- | --- | --- | --- | --- | --- | --- | --- | --- | --- | --- | --- | --- | --- | --- | --- | --- | --- | --- | --- | --- | --- | --- | --- | --- | --- | --- | --- | --- | --- | --- | --- | --- | --- | --- | --- | --- | --- | --- | --- | --- | --- | --- | --- | --- | --- | --- | --- | --- | --- | --- | --- | --- | --- | --- | --- | --- | --- | --- | --- | --- | --- | --- | --- | --- | --- | --- | --- | --- | --- | --- | --- | --- | --- | --- | --- | --- | --- | --- | --- | --- | --- | --- | --- | --- | --- | --- | --- | --- | --- | --- | --- | --- | --- | --- | --- | --- | --- | --- | --- | --- | --- | --- | --- | --- | --- | --- | --- | --- | --- | --- | --- | --- | --- | --- | --- | --- | --- | --- | --- | --- | --- | --- | --- | --- | --- | --- | --- | --- | --- | --- | --- | --- | --- | --- | --- | --- | --- | --- | --- | --- | --- | --- | --- | --- | --- | --- | --- | --- | --- | --- | --- | --- | --- | --- | --- | --- | --- | --- | --- | --- | --- | --- | --- | --- | --- | --- | --- | --- | --- | --- | --- | --- | --- | --- | --- | --- | --- | --- | --- | --- | --- | --- | --- | --- | --- | --- | --- | --- | --- | --- | --- | --- | --- | --- | --- | --- | --- | --- | --- | --- | --- | --- | --- | --- | --- | --- | --- | --- | --- | --- | --- | --- | --- | --- | --- | --- | --- | --- | --- | --- | --- | --- | --- | --- | --- | --- | --- | --- | --- | --- | --- | --- | --- | --- | --- | --- | --- | --- | --- | --- | --- | --- | --- | --- | --- | --- | --- | --- | --- | --- | --- | --- | --- | --- | --- | --- | --- | --- | --- | --- | --- | --- | --- | --- | --- | --- | --- | --- | --- | --- | --- | --- | --- | --- | --- | --- | --- | --- | --- | --- | --- | --- | --- | --- | --- | --- | --- | --- | --- | --- | --- | --- | --- | --- | --- | --- | --- | --- | --- | --- | --- | --- | --- | --- | --- | --- | --- | --- | --- | --- | --- | --- | --- | --- | --- | --- | --- | --- | --- | --- | --- | --- | --- | --- | --- | --- | --- | --- | --- | --- | --- | --- | --- | --- | --- | --- | --- | --- | --- | --- | --- | --- | --- | --- | --- | --- | --- | --- | --- | --- | --- | --- | --- | --- | --- | --- | --- | --- | --- | --- | --- | --- | --- | --- | --- | --- | --- | --- | --- | --- | --- | --- | --- | --- | --- | --- | --- | --- | --- | --- | --- | --- | --- | --- | --- | --- | --- | --- | --- | --- | --- | --- | --- | --- | --- | --- | --- | --- | --- | --- | --- | --- | --- | --- | --- | --- | --- | --- | --- | --- | --- | --- | --- | --- | --- | --- | --- | --- | --- | --- | --- | --- | --- | --- | --- | --- | --- | --- | --- | --- | --- | --- | --- | --- | --- | --- | --- | --- | --- | --- | --- | --- | --- | --- | --- | --- | --- | --- | --- | --- | --- | --- | --- | --- | --- | --- | --- | --- | --- | --- | --- | --- | --- | --- | --- | --- | --- | --- | --- | --- | --- | --- | --- | --- | --- | --- | --- | --- | --- | --- | --- | --- | --- | --- | --- | --- | --- | --- | --- | --- | --- | --- | --- | --- | --- | --- | --- | --- | --- | --- | --- | --- | --- | --- | --- | --- | --- | --- | --- | --- | --- | --- | --- | --- | --- | --- | --- | --- | --- | --- | --- | --- | --- | --- | --- | --- | --- | --- | --- | --- | --- | --- | --- | --- | --- | --- | --- | --- | --- | --- | --- | --- | --- | --- | --- | --- | --- | --- | --- | --- | --- | --- | --- | --- | --- | --- | --- | --- | --- | --- | --- | --- | --- | --- | --- | --- | --- | --- | --- | --- | --- | --- | --- | --- | --- | --- | --- | --- | --- | --- | --- | --- | --- | --- | --- | --- | --- | --- | --- | --- | --- | --- | --- | --- | --- | --- | --- | --- | --- | --- | --- | --- | --- | --- | --- | --- | --- | --- | --- | --- | --- | --- | --- | --- | --- | --- | --- | --- | --- | --- | --- | --- | --- | --- | --- | --- | --- | --- | --- | --- | --- | --- | --- | --- | --- | --- | --- | --- | --- | --- | --- | --- | --- | --- | --- | --- | --- | --- | --- | --- | --- | --- | --- | --- | --- | --- | --- | --- | --- | --- | --- | --- | --- | --- | --- | --- | --- | --- | --- | --- | --- | --- | --- | --- | --- | --- | --- | --- | --- | --- | --- | --- | --- | --- | --- | --- | --- | --- | --- | --- | --- | --- | --- | --- | --- | --- | --- | --- | --- | --- | --- | --- | --- | --- | --- | --- | --- | --- | --- | --- | --- | --- | --- | --- | --- | --- | --- | --- | --- | --- | --- | --- | --- | --- | --- | --- | --- | --- | --- | --- | --- | --- | --- | --- | --- | --- | --- | --- | --- | --- | --- | --- | --- | --- | --- | --- | --- | --- | --- | --- | --- | --- | --- | --- | --- | --- | --- | --- | --- | --- | --- | --- | --- | --- | --- | --- | --- | --- | --- | --- | --- | --- | --- | --- | --- | --- | --- | --- | --- | --- | --- | --- | --- | --- | --- | --- | --- | --- | --- | --- | --- | --- | --- | --- | --- | --- | --- | --- | --- | --- | --- | --- | --- | --- | --- | --- | --- | --- | --- | --- | --- | --- | --- | --- | --- | --- | --- | --- | --- | --- | --- | --- | --- | --- | --- | --- | --- | --- | --- | --- | --- | --- | --- | --- | --- | --- | --- | --- | --- | --- | --- | --- | --- | --- | --- | --- | --- | --- | --- | --- | --- | --- | --- | --- | --- | --- | --- | --- | --- | --- | --- | --- | --- | --- | --- | --- | --- | --- | --- | --- | --- | --- | --- | --- | --- | --- | --- | --- | --- | --- | --- | --- | --- | --- | --- | --- | --- | --- | --- | --- | --- | --- | --- | --- | --- | --- | --- | --- | --- | --- | --- | --- | --- | --- | --- | --- | --- | --- | --- | --- | --- | --- | --- | --- | --- | --- | --- | --- | --- | --- | --- | --- | --- | --- | --- | --- | --- | --- | --- | --- | --- | --- | --- | --- | --- | --- | --- | --- | --- | --- | --- | --- | --- | --- | --- | --- | --- | --- | --- | --- | --- | --- | --- | --- | --- | --- | --- | --- | --- | --- | --- | --- | --- | --- | --- | --- | --- | --- | --- | --- | --- | --- | --- | --- | --- | --- | --- | --- | --- | --- | --- | --- | --- | --- | --- | --- | --- | --- | --- | --- | --- | --- | --- | --- | --- | --- | --- | --- | --- | --- | --- | --- | --- | --- | --- | --- | --- | --- | --- | --- | --- | --- | --- | --- | --- | --- | --- | --- | --- | --- | --- | --- | --- | --- | --- | --- | --- | --- | --- | --- | --- | --- | --- | --- | --- | --- | --- | --- | --- | --- | --- | --- | --- | --- | --- | --- | --- | --- | --- | --- | --- | --- | --- | --- | --- | --- | --- | --- | --- | --- | --- | --- | --- | --- | --- | --- | --- | --- | --- | --- | --- | --- | --- | --- | --- | --- | --- | --- | --- | --- | --- | --- | --- | --- | --- | --- | --- | --- | --- | --- | --- | --- | --- | --- | --- | --- | --- | --- | --- | --- | --- | --- | --- | --- | --- | --- | --- | --- | --- | --- | --- | --- | --- | --- | --- | --- | --- | --- | --- | --- | --- | --- | --- | --- | --- | --- | --- | --- | --- | --- | --- | --- | --- | --- | --- | --- | --- | --- | --- | --- | --- | --- | --- | --- | --- | --- | --- | --- | --- | --- | --- | --- | --- | --- | --- | --- | --- | --- | --- | --- | --- | --- | --- | --- | --- | --- | --- | --- | --- | --- | --- | --- | --- | --- | --- | --- | --- | --- | --- | --- | --- | --- | --- | --- | --- | --- | --- | --- | --- | --- | --- | --- | --- | --- | --- | --- | --- | --- | --- | --- | --- | --- | --- | --- | --- | --- | --- | --- | --- | --- | --- | --- | --- | --- | --- | --- | --- | --- | --- | --- | --- | --- | --- | --- | --- | --- | --- | --- | --- | --- | --- | --- | --- | --- | --- | --- | --- | --- | --- | --- | --- | --- | --- | --- | --- | --- | --- | --- | --- | --- | --- | --- | --- | --- | --- | --- | --- | --- | --- | --- | --- | --- | --- | --- | --- | --- | --- | --- | --- | --- | --- | --- | --- | --- | --- | --- | --- | --- | --- | --- | --- | --- | --- | --- | --- | --- | --- | --- | --- | --- | --- | --- | --- | --- | --- | --- | --- | --- | --- | --- | --- | --- | --- | --- | --- | --- | --- | --- | --- | --- | --- | --- | --- | --- | --- | --- | --- | --- | --- | --- | --- | --- | --- | --- | --- | --- | --- | --- | --- | --- | --- | --- | --- | --- | --- | --- | --- | --- | --- | --- | --- | --- | --- | --- | --- | --- | --- | --- | --- | --- | --- | --- | --- | --- | --- | --- | --- | --- | --- | --- | --- | --- | --- | --- | --- | --- | --- | --- | --- | --- | --- | --- | --- | --- | --- | --- | --- | --- | --- | --- | --- | --- | --- | --- | --- | --- | --- | --- | --- | --- | --- | --- | --- | --- | --- | --- | --- | --- | --- | --- | --- | --- | --- | --- | --- | --- | --- | --- | --- | --- | --- | --- | --- | --- | --- | --- | --- | --- | --- | --- | --- | --- | --- | --- | --- | --- | --- | --- | --- | --- | --- | --- | --- | --- | --- | --- | --- | --- | --- | --- | --- | --- | --- | --- | --- | --- | --- | --- | --- | --- | --- | --- | --- | --- | --- | --- | --- | --- | --- | --- | --- | --- | --- | --- | --- | --- | --- | --- | --- | --- | --- | --- | --- | --- | --- | --- | --- | --- | --- | --- | --- | --- | --- | --- | --- | --- | --- | --- | --- | --- | --- | --- | --- | --- | --- | --- | --- | --- | --- | --- | --- | --- | --- | --- | --- | --- | --- | --- | --- | --- | --- | --- | --- | --- | --- | --- | --- | --- | --- | --- | --- | --- | --- | --- | --- | --- | --- | --- | --- | --- | --- | --- | --- | --- | --- | --- | --- | --- | --- | --- | --- | --- | --- | --- | --- | --- | --- | --- | --- | --- | --- | --- | --- | --- | --- | --- | --- | --- | --- | --- | --- | --- | --- | --- | --- | --- | --- | --- | --- | --- | --- | --- | --- | --- | --- | --- | --- | --- | --- | --- | --- | --- | --- | --- | --- | --- | --- | --- | --- | --- | --- | --- | --- | --- | --- | --- | --- | --- | --- | --- | --- | --- | --- | --- | --- | --- | --- | --- | --- | --- | --- | --- | --- | --- | --- | --- | --- | --- | --- | --- | --- | --- | --- | --- | --- | --- | --- | --- | --- | --- | --- | --- | --- | --- | --- | --- | --- | --- | --- | --- | --- | --- | --- | --- | --- | --- | --- | --- | --- | --- | --- | --- | --- | --- | --- | --- | --- | --- | --- | --- | --- | --- | --- | --- | --- | --- | --- | --- | --- | --- | --- | --- | --- | --- | --- | --- | --- | --- | --- | --- | --- | --- | --- | --- | --- | --- | --- | --- | --- | --- | --- | --- | --- | --- | --- | --- | --- | --- | --- | --- | --- | --- | --- | --- | --- | --- | --- | --- | --- | --- | --- | --- | --- | --- | --- | --- | --- | --- | --- | --- | --- | --- | --- | --- | --- | --- | --- | --- | --- | --- | --- | --- | --- | --- | --- | --- | --- | --- | --- | --- | --- | --- | --- | --- | --- | --- | --- | --- | --- | --- | --- | --- | --- | --- | --- | --- | --- | --- | --- | --- | --- | --- | --- | --- | --- | --- | --- | --- | --- | --- | --- | --- | --- | --- | --- | --- | --- | --- | --- | --- | --- | --- | --- | --- | --- | --- | --- | --- | --- | --- | --- | --- | --- | --- | --- | --- | --- | --- | --- | --- | --- | --- | --- | --- | --- | --- | --- | --- | --- | --- | --- | --- | --- | --- | --- | --- | --- | --- | --- | --- | --- | --- | --- | --- | --- | --- | --- | --- | --- | --- | --- | --- | --- | --- | --- | --- | --- | --- | --- | --- | --- | --- | --- | --- | --- | --- | --- | --- | --- | --- | --- | --- | --- | --- | --- | --- | --- | --- | --- | --- | --- | --- | --- | --- | --- | --- | --- | --- | --- | --- | --- | --- | --- | --- | --- | --- | --- | --- | --- | --- | --- | --- | --- | --- | --- | --- | --- | --- | --- | --- | --- | --- | --- | --- | --- | --- | --- | --- | --- | --- | --- | --- | --- | --- | --- | --- | --- | --- | --- | --- | --- | --- | --- | --- | --- | --- | --- | --- | --- | --- | --- | --- | --- | --- | --- | --- | --- | --- | --- | --- | --- | --- | --- | --- | --- | --- | --- | --- | --- | --- | --- | --- | --- | --- | --- | --- | --- | --- | --- | --- | --- | --- | --- | --- | --- | --- | --- | --- | --- | --- | --- | --- | --- | --- | --- | --- | --- | --- | --- | --- | --- | --- | --- | --- | --- | --- | --- | --- | --- | --- | --- | --- | --- | --- | --- | --- | --- | --- | --- | --- | --- | --- | --- | --- | --- | --- | --- | --- | --- | --- | --- | --- | --- | --- | --- | --- | --- | --- | --- | --- | --- | --- | --- | --- | --- | --- | --- | --- | --- | --- | --- | --- | --- | --- | --- | --- | --- | --- | --- | --- | --- | --- | --- | --- | --- | --- | --- | --- | --- | --- | --- | --- | --- | --- | --- | --- | --- | --- | --- | --- | --- | --- | --- | --- | --- | --- | --- | --- | --- | --- | --- | --- | --- | --- | --- | --- | --- | --- | --- | --- | --- | --- | --- | --- | --- | --- | --- | --- | --- | --- | --- | --- | --- | --- | --- | --- | --- | --- | --- | --- | --- | --- | --- | --- | --- | --- | --- | --- | --- | --- | --- | --- | --- | --- | --- | --- | --- | --- | --- | --- | --- | --- | --- | --- | --- | --- | --- | --- | --- | --- | --- | --- | --- | --- | --- | --- | --- | --- | --- | --- | --- | --- | --- | --- | --- | --- | --- | --- | --- | --- | --- | --- | --- | --- | --- | --- | --- | --- | --- | --- | --- | --- | --- | --- | --- | --- | --- | --- | --- | --- | --- | --- | --- | --- | --- | --- | --- | --- | --- | --- | --- | --- | --- | --- | --- | --- | --- | --- | --- | --- | --- | --- | --- | --- | --- | --- | --- | --- | --- | --- | --- | --- | --- | --- | --- | --- | --- | --- | --- | --- | --- | --- | --- | --- | --- | --- | --- | --- | --- | --- | --- | --- | --- | --- | --- | --- | --- | --- | --- | --- | --- | --- | --- | --- | --- | --- | --- | --- | --- | --- | --- | --- | --- | --- | --- | --- | --- | --- | --- | --- | --- | --- | --- | --- | --- | --- | --- | --- | --- | --- | --- | --- | --- | --- | --- | --- | --- | --- | --- | --- | --- | --- | --- | --- | --- | --- | --- | --- | --- | --- | --- | --- | --- | --- | --- | --- | --- | --- | --- | --- | --- | --- | --- | --- | --- | --- | --- | --- | --- | --- | --- | --- | --- | --- | --- | --- | --- | --- | --- | --- | --- | --- | --- | --- | --- | --- | --- | --- | --- | --- | --- | --- | --- | --- | --- | --- | --- | --- | --- | --- | --- | --- | --- | --- | --- | --- | --- | --- | --- | --- | --- | --- | --- | --- | --- | --- | --- | --- | --- | --- | --- | --- | --- | --- | --- | --- | --- | --- | --- | --- | --- | --- | --- | --- | --- | --- | --- | --- | --- | --- | --- | --- | --- | --- | --- | --- | --- | --- | --- | --- | --- | --- | --- | --- | --- | --- | --- | --- | --- | --- | --- | --- | --- | --- | --- | --- | --- | --- | --- | --- |
| |  |  |  |  |  |  |  |  |  | | --- | --- | --- | --- | --- | --- | --- | --- | --- | | **Position** | **Reference** | **Sample** | **Quality** | **Type** | **Region** | **AA Exchange** | **PAM1** | **Known Variant** | | 1977 | A | G | 547.77 | SNP | intergenic |  |  | - | | 4013 | T | C | 1252.77 | SNP | Rv0003 (recF) | Ile245Thr | 11 | - | | 7219 | C | G | 32.77 | SNP | Rv0005 (gyrB) | silent (Arg660) | 9913 | - | | 7225 | T | C | 33.77 | SNP | Rv0005 (gyrB) | silent (Phe662) | 9946 | - | | 7362 | G | C | 694.77 | SNP | Rv0006 (gyrA) | Glu21Gln | 27 | - | | 7394 | T | C | 36.77 | SNP | Rv0006 (gyrA) | silent (Tyr31) | 9945 | - | | 7421 | G | C | 34.77 | SNP | Rv0006 (gyrA) | silent (Ala40) | 9867 | - | | 7427 | G | C | 38.77 | SNP | Rv0006 (gyrA) | silent (Pro42) | 9926 | - | | 7442 | G | C | 45.77 | SNP | Rv0006 (gyrA) | silent (Gly47) | 9935 | - | | 7451 | C | G | 31.77 | SNP | Rv0006 (gyrA) | silent (Pro50) | 9926 | - | | 7539 | A | G | 626.77 | SNP | Rv0006 (gyrA) | Thr80Ala | 32 | genotype | | 7585 | G | C | 947.77 | SNP | Rv0006 (gyrA) | Ser95Thr | 32 | genotype | | 7607 | C | G | 93.77 | SNP | Rv0006 (gyrA) | silent (Pro102) | 9926 | - | | 7614 | C | A | 96.77 | SNP | Rv0006 (gyrA) | Leu105Met(s) | 4 | - | | 7629 | G | A | 80.77 | SNP | Rv0006 (gyrA) | Val(s)110Met(s) | 9867 | - | | 7631 | G | C | 99.77 | SNP | Rv0006 (gyrA) | Val(s)110Val | 13 | - | | 9304 | G | A | 862.77 | SNP | Rv0006 (gyrA) | Gly668Asp | 6 | - | | 11879 | A | G | 494.77 | SNP | Rv0008c | Ser145Pro | 12 | - | | 13741 | A | C | 1411.77 | SNP | Rv0011c | silent (Gly85) | 9935 | - | | 14785 | T | C | 972.77 | SNP | Rv0012 | Cys233Arg | 1 | - | | 18091 | G | A | 273.78 | SNP | Rv0015c (pknA) | silent (Thr224) | 9871 | - | | 22334 | C | T | 691.77 | SNP | Rv0018c (pstP) | Arg283His | 8 | - | | 26959 | C | G | 746.77 | SNP | intergenic |  |  | - | | 32075 | T | C | 930.77 | SNP | Rv0029 | Trp7Arg | 8 | - | | 34044 | T | C | 789.77 | SNP | intergenic |  |  | - | | 34226 | A | G | 949.77 | SNP | intergenic |  |  | - | | 37031 | C | G | 508.77 | SNP | Rv0034 | silent (Ala55) | 9867 | - | | 42747 | C | T | 622.77 | SNP | Rv0040c (mtc28) | Asp207Asn | 36 | - | | 42967 | G | C | 804.77 | SNP | Rv0040c (mtc28) | silent (Pro133) | 9926 | - | | 47936 | G | A | 847.77 | SNP | Rv0043c | silent (Leu55) | 9947 | - | | 50393 | C | G | 36.77 | SNP | Rv0046c (ino1) | silent (Arg244) | 9913 | - | | 50399 | A | G | 41.77 | SNP | Rv0046c (ino1) | silent (Arg242) | 9913 | - | | 50402 | T | C | 45.77 | SNP | Rv0046c (ino1) | silent (Glu241) | 9865 | - | | 54304 | C | T | 1125.77 | SNP | Rv0050 (ponA1) | silent (Leu214) | 9947 | - | | 55553 | C | CCGT | 529.74 | INS | Rv0050 (ponA1) |  |  | - | | 57737 | G | A | 996.77 | SNP | Rv0052 | Val110Ile | 33 | - | | 62049 | A | G | 686.77 | SNP | Rv0058 (dnaB) | Arg552Gly | 1 | - | | 69989 | G | A | 1132.77 | SNP | Rv0064 | Gly457Asp | 6 | - | | 70816 | A | G | 957.77 | SNP | Rv0064 | Asn733Asp | 42 | - | | 71336 | G | C | 295.78 | SNP | Rv0064 | Arg906Pro | 5 | - | | 71584 | C | CCGAGCGCTGTTCTGGCGCT AATCTGACGCTAGAATAG | 8455.73 | INS | intergenic |  |  | - | | 75940 | G | C | 768.77 | SNP | Rv0068 | Val(s)214Leu | 3 | - | | 79503 | G | GGAC | 1944.73 | INS | Rv0071 |  |  | - | | 80616 | C | G | 524.77 | SNP | intergenic |  |  | - | | 87257 | C | A | 702.77 | SNP | Rv0078A | Arg182Leu | 1 | - | | 92199 | T | G | 370.77 | SNP | Rv0083 | silent (Thr600) | 9871 | - | | 104962 | G | A | 96.28 | SNP | Rv0095c | Ala85Val(s) | 9867 | - | | 105021 | G | A | 103.03 | SNP | Rv0095c | silent (Ser65) | 9840 | - | | 105045 | G | C | 254.80 | SNP | Rv0095c | Asp57Glu | 56 | - | | 105060 | G | A | 277.78 | SNP | Rv0095c | silent (Asp52) | 9859 | - | | 105063 | G | A | 286.78 | SNP | Rv0095c | silent (Phe51) | 9946 | - | | 116000 | T | G | 606.77 | SNP | Rv0101 (nrp) | Val2000Val(s) | 18 | - | | 122109 | A | G | 1090.77 | SNP | Rv0103c (ctpB) | Leu(s)22Ser | 28 | - | | 122794 | T | G | 499.77 | SNP | Rv0104 | Phe160Val | 1 | - | | 125830 | G | GA | 1382.73 | INS | Rv0107c (ctpI) |  |  | - | | 131174 | T | TG | 1110.73 | INS | intergenic |  |  | - | | 133839 | C | T | 1041.77 | SNP | intergenic |  |  | - | | 146087 | T | C | 794.77 | SNP | Rv0120c (fusA2) | Asn562Ser | 34 | - | | 154283 | T | C | 1195.77 | SNP | Rv0127 (mak) | Ser18Pro | 12 | - | | 155948 | C | CA | 1552.73 | INS | Rv0128 |  |  | - | | 175830 | T | C | 945.77 | SNP | Rv0149 | Leu44Pro | 2 | - | | 177857 | G | A | 915.77 | SNP | Rv0151c (PE1) | Leu485Leu(s) | 4 | - | | 188800 | T | C | 581.77 | SNP | Rv0159c (PE3) | Thr14Ala | 32 | - | | 194681 | G | C | 521.77 | SNP | Rv0165c (mce1R) | silent (Leu45) | 9947 | - | | 196642 | C | T | 969.77 | SNP | Rv0166 (fadD5) | silent (Asn550) | 9822 | - | | 206339 | T | C | 853.77 | SNP | Rv0174 (mce1F) | Leu370Pro | 2 | - | | 207160 | G | A | 1271.77 | SNP | Rv0175 | Ser116Asn | 20 | - | | 215613 | G | A | 424.77 | SNP | Rv0184 | silent (Ala215) | 9867 | - | | 218204 | C | T | 451.77 | SNP | Rv0186 (bglS) | Arg646STOP | 2 | - | | 219120 | A | G | 825.77 | SNP | Rv0187 | Asp139Gly | 11 | - | | 223942 | T | C | 378.77 | SNP | Rv0192 | Ser127Pro | 12 | - | | 225323 | T | C | 661.77 | SNP | Rv0193c | Lys417Glu | 4 | - | | 227098 | T | C | 1066.77 | SNP | Rv0194 | Met(s)74Thr | 22 | - | | 231114 | C | G | 800.77 | SNP | Rv0195 | silent (Ala72) | 9867 | - | | 234477 | T | G | 914.77 | SNP | Rv0197 | Tyr749STOP | 2 | - | | 234496 | C | CGT | 1833.73 | INS | Rv0197 |  |  | - | | 237709 | C | T | 669.77 | SNP | Rv0200 | silent (Ala168) | 9867 | - | | 252473 | A | G | 1028.77 | SNP | Rv0211 (pckA) | Tyr231Cys | 3 | - | | 256640 | T | G | 1046.77 | SNP | Rv0214 (fadD4) | Ser193Ala | 35 | - | | 257982 | T | G | 596.77 | SNP | Rv0215c (fadE3) | Asp292Ala | 10 | - | | 261809 | ACCG | A | 720.70 | DEL | Rv0218 |  |  | - | | 261869 | T | C | 313.78 | SNP | Rv0218 | Cys316Arg | 1 | - | | 265244 | C | T | 888.77 | SNP | Rv0221 | Ala393Val(s) | 9867 | - | | 265554 | A | C | 862.77 | SNP | Rv0222 (echA1) | silent (Val16) | 9901 | - | | 275859 | G | T | 981.77 | SNP | Rv0230c (php) | Asn35Lys | 25 | - | | 278681 | C | G | 597.77 | SNP | Rv0233 (nrdB) | His33Asp | 4 | - | | 285772 | A | C | 492.77 | SNP | Rv0236c (aftD) | silent (Pro360) | 9926 | - | | 285871 | A | G | 257.78 | SNP | Rv0236c (aftD) | silent (Val327) | 9901 | - | | 295724 | A | G | 1081.77 | SNP | intergenic |  |  | - | | 310516 | C | T | 526.77 | SNP | Rv0258c | silent (Gln78) | 9876 | - | | 310973 | G | A | 805.77 | SNP | Rv0259c | Ala182Val(s) | 9867 | - | | 311613 | G | T | 1004.77 | SNP | Rv0260c | silent (Val349) | 9901 | - | | 312060 | T | G | 1001.77 | SNP | Rv0260c | silent (Arg200) | 9913 | - | | 312944 | C | T | 937.77 | SNP | Rv0261c (narK3) | Val409Ile | 33 | - | | 325038 | G | A | 917.77 | SNP | Rv0270 (fadD2) | Gly158Ser | 16 | - | | 325039 | G | A | 956.77 | SNP | Rv0270 (fadD2) | Gly158Asp | 6 | - | | 332357 | A | G | 684.77 | SNP | Rv0276 | Ile204Val | 57 | - | | 333892 | G | C | 51.28 | SNP | Rv0278c (PE\_PGRS3) | Arg807Gly | 1 | - | | 334988 | A | G | 42.74 | SNP | Rv0278c (PE\_PGRS3) | silent (Gly441) | 9935 | - | | 335722 | G | A | 42.74 | SNP | Rv0278c (PE\_PGRS3) | Leu197Leu(s) | 4 | - | | 335725 | T | C | 42.74 | SNP | Rv0278c (PE\_PGRS3) | Met(s)196Val(s) | 9867 | - | | 335732 | G | C | 42.74 | SNP | Rv0278c (PE\_PGRS3) | silent (Gly193) | 9935 | - | | 335749 | A | C | 43.74 | SNP | Rv0278c (PE\_PGRS3) | Ser188Ala | 35 | - | | 335762 | A | G | 41.74 | SNP | Rv0278c (PE\_PGRS3) | silent (Ser183) | 9840 | - | | 335766 | C | G | 39.74 | SNP | Rv0278c (PE\_PGRS3) | Ser182Thr | 32 | - | | 335789 | G | C | 33.74 | SNP | Rv0278c (PE\_PGRS3) | silent (Ala174) | 9867 | - | | 335799 | T | C | 41.74 | SNP | Rv0278c (PE\_PGRS3) | Asn171Ser | 34 | - | | 336691 | T | C | 73.28 | SNP | Rv0279c (PE\_PGRS4) | Ser795Gly | 21 | - | | 336698 | C | G | 72.28 | SNP | Rv0279c (PE\_PGRS4) | silent (Gly792) | 9935 | - | | 336701 | A | G | 82.28 | SNP | Rv0279c (PE\_PGRS4) | silent (Gly791) | 9935 | - | | 336707 | G | A | 65.28 | SNP | Rv0279c (PE\_PGRS4) | silent (Asp789) | 9859 | - | | 336708 | T | C | 79.28 | SNP | Rv0279c (PE\_PGRS4) | Asp789Gly | 11 | - | | 336710 | A | G | 84.28 | SNP | Rv0279c (PE\_PGRS4) | silent (Ala788) | 9867 | - | | 337959 | A | C | 71.28 | SNP | Rv0279c (PE\_PGRS4) | Ile372Ser | 2 | - | | 338020 | A | C | 99.28 | SNP | Rv0279c (PE\_PGRS4) | Cys352Gly | 1 | - | | 338100 | T | C | 42.74 | SNP | Rv0279c (PE\_PGRS4) | Asn325Ser | 34 | - | | 338453 | A | G | 43.74 | SNP | Rv0279c (PE\_PGRS4) | silent (Ala207) | 9867 | - | | 340132 | G | A | 556.77 | SNP | Rv0280 (PPE3) | Glu257Lys | 7 | - | | 346275 | C | G | 771.77 | SNP | Rv0284 (eccC3) | Pro214Arg | 4 | - | | 356528 | A | G | 529.77 | SNP | Rv0292 (eccE3) | Asn217Asp | 42 | - | | 373282 | TA | T | 1023.73 | DEL | Rv0305c (PPE6) |  |  | - | | 375714 | G | A | 1167.77 | SNP | intergenic |  |  | - | | 384380 | A | C | 1070.77 | SNP | Rv0315 | Lys260Thr | 8 | - | | 386432 | C | G | 827.77 | SNP | Rv0318c | Gly223Ala | 21 | - | | 390828 | T | C | 690.77 | SNP | Rv0323c | Ser142Gly | 21 | - | | 394850 | G | A | 699.77 | SNP | Rv0329c | silent (Asp157) | 9859 | - | | 396180 | C | T | 859.77 | SNP | intergenic |  |  | - | | 403980 | G | A | 1148.77 | SNP | Rv0338c | Ala621Val | 13 | - | | 404326 | T | C | 884.77 | SNP | Rv0338c | Arg506Gly | 1 | - | | 410264 | C | T | 116.77 | SNP | Rv0341 (iniB) | silent (Ala301) | 9867 | - | | 412017 | C | G | 1011.77 | SNP | Rv0342 (iniA) | Gln394Glu | 35 | - | | 414486 | C | T | 920.77 | SNP | Rv0344c (lpqJ) | silent (Glu152) | 9865 | - | | 420008 | A | G | 794.77 | SNP | Rv0350 (dnaK) | silent (Ala58) | 9867 | - | | 420985 | G | A | 33.77 | SNP | Rv0350 (dnaK) | Arg384Lys | 37 | - | | 421022 | G | C | 33.77 | SNP | Rv0350 (dnaK) | silent (Arg396) | 9913 | - | | 421030 | C | T | 32.77 | SNP | Rv0350 (dnaK) | Thr399Ile | 7 | - | | 421031 | T | C | 68.77 | SNP | Rv0350 (dnaK) | silent (Thr399) | 9871 | - | | 421037 | C | G | 58.77 | SNP | Rv0350 (dnaK) | silent (Thr401) | 9871 | - | | 421040 | C | G | 33.77 | SNP | Rv0350 (dnaK) | silent (Thr402) | 9871 | - | | 421055 | A | G | 45.77 | SNP | Rv0350 (dnaK) | silent (Gln407) | 9876 | - | | 421061 | G | C | 51.77 | SNP | Rv0350 (dnaK) | silent (Ser409) | 9840 | - | | 424320 | T | TC | 867.73 | INS | Rv0354c (PPE7) |  |  | - | | 425293 | CCCGATGCCGATGTTTCCGT TTCCGGTGTTGCCGAAGCCG ATGTTGCCGGTGCCGGTATT G | C | 1161.87 | DEL | Rv0355c (PPE8) |  |  | - | | 427310 | TTGCCGAGGTTTGCAC | T | 2823.73 | DEL | Rv0355c (PPE8) |  |  | - | | 433654 | G | A | 522.77 | SNP | Rv0355c (PPE8) | silent (Gly342) | 9935 | - | | 435708 | G | A | 563.77 | SNP | Rv0357c (purA) | silent (Thr354) | 9871 | - | | 454295 | T | C | 910.77 | SNP | Rv0376c | silent (Pro26) | 9926 | - | | 457452 | T | G | 345.84 | SNP | Rv0381c | silent (Thr124) | 9871 | - | | 459399 | A | C | 740.77 | SNP | intergenic |  |  | - | | 467497 | C | CG | 474.73 | INS | Rv0388c (PPE9) |  |  | - | | 467508 | C | CG | 527.73 | INS | Rv0388c (PPE9) |  |  | - | | 467516 | G | C | 318.78 | SNP | Rv0388c (PPE9) | silent (Ser162) | 9840 | - | | 467526 | C | G | 298.78 | SNP | Rv0388c (PPE9) | Gly159Ala | 21 | - | | 467546 | G | C | 439.77 | SNP | Rv0388c (PPE9) | Asp152Glu | 56 | - | | 467557 | A | C | 490.77 | SNP | Rv0388c (PPE9) | Leu(s)149Val(s) | 9867 | - | | 467564 | A | C | 489.77 | SNP | Rv0388c (PPE9) | His146Gln | 23 | - | | 467585 | G | C | 676.77 | SNP | Rv0388c (PPE9) | His139Gln | 23 | - | | 467590 | T | C | 582.77 | SNP | Rv0388c (PPE9) | Thr138Ala | 32 | - | | 467621 | T | G | 687.77 | SNP | Rv0388c (PPE9) | silent (Gly127) | 9935 | - | | 467638 | G | T | 749.77 | SNP | Rv0388c (PPE9) | Gln122Lys | 12 | - | | 475178 | T | C | 659.77 | SNP | Rv0395 | Val80Ala | 18 | - | | 488796 | G | A | 521.77 | SNP | Rv0405 (pks6) | Val(s)1022Val | 13 | - | | 489935 | G | C | 804.77 | SNP | Rv0405 (pks6); Rv0406c | Arg1402Pro; silent (Thr257) | 5; 9871 | - | | 492715 | C | T | 653.77 | SNP | Rv0408 (pta) | silent (Gly310) | 9935 | - | | 493934 | T | C | 772.77 | SNP | Rv0409 (ackA) | silent (Arg28) | 9913 | - | | 502589 | C | G | 914.77 | SNP | Rv0417 (thiG) | Ser75Cys | 5 | - | | 503354 | G | C | 1113.77 | SNP | intergenic |  |  | - | | 513257 | T | C | 828.77 | SNP | Rv0425c (ctpH) | Met(s)689Val(s) | 9867 | - | | 541201 | A | G | 720.77 | SNP | Rv0450c (mmpL4) | silent (Leu97) | 9947 | - | | 551525 | A | C | 796.77 | SNP | Rv0459 | silent (Arg110) | 9913 | - | | 559094 | A | G | 497.77 | SNP | Rv0468 (fadB2) | Asp67Gly | 11 | - | | 573262 | A | G | 502.77 | SNP | Rv0484c | silent (Gly180) | 9935 | - | | 580772 | T | A | 292.78 | SNP | intergenic |  |  | - | | 580773 | GGGGGCACCACCCGCTTGCG GGGGA | G | 3230.05 | DEL | intergenic |  |  | - | | 587974 | G | A | 478.77 | SNP | Rv0497 | Ala200Thr | 22 | - | | 590436 | T | C | 347.77 | SNP | Rv0500 (proC) | silent (Ala118) | 9867 | - | | 591628 | T | C | 692.77 | SNP | intergenic |  |  | - | | 595232 | C | T | 829.77 | SNP | Rv0504c | Gly24Glu | 4 | - | | 597816 | A | G | 569.77 | SNP | Rv0507 (mmpL2) | silent (Ala206) | 9867 | - | | 598475 | G | A | 867.77 | SNP | Rv0507 (mmpL2) | Arg426His | 8 | - | | 610120 | T | G | 909.77 | SNP | intergenic |  |  | - | | 628113 | C | T | 595.77 | SNP | Rv0536 (galE3) | Ala289Val(s) | 9867 | - | | 630722 | G | C | 494.77 | SNP | Rv0538 | Arg228Pro | 5 | - | | 637319 | G | A | 909.77 | SNP | Rv0545c (pitA) | Pro49Ser | 17 | - | | 639351 | A | C | 41.77 | SNP | Rv0548c (menB) | silent (Arg202) | 9913 | - | | 648002 | T | G | 1065.77 | SNP | Rv0556 | Leu15Arg | 1 | - | | 663410 | A | C | 75.77 | SNP | intergenic |  |  | - | | 663418 | A | C | 97.77 | SNP | intergenic |  |  | - | | 663419 | G | A | 117.77 | SNP | intergenic |  |  | - | | 663420 | C | A | 89.77 | SNP | intergenic |  |  | - | | 663429 | T | G | 114.77 | SNP | intergenic |  |  | - | | 665293 | A | G | 982.77 | SNP | Rv0572c | Phe31Leu | 13 | - | | 669398 | T | C | 758.77 | SNP | Rv0575c | silent (Gln116) | 9876 | - | | 672491 | C | G | 51.74 | SNP | Rv0578c (PE\_PGRS7) | silent (Gly1142) | 9935 | - | | 672770 | A | G | 32.74 | SNP | Rv0578c (PE\_PGRS7) | silent (Gly1049) | 9935 | - | | 673238 | A | G | 125.03 | SNP | Rv0578c (PE\_PGRS7) | silent (His893) | 9912 | - | | 684363 | C | T | 1020.77 | SNP | intergenic |  |  | - | | 685461 | C | G | 536.77 | SNP | Rv0587 (yrbE2A) | silent (Ala111) | 9867 | - | | 685608 | T | C | 639.77 | SNP | Rv0587 (yrbE2A) | silent (Leu160) | 9947 | - | | 685869 | G | A | 1013.77 | SNP | Rv0587 (yrbE2A) | silent (Leu247) | 9947 | - | | 686972 | T | C | 1137.77 | SNP | Rv0589 (mce2A) | Phe51Ser | 3 | - | | 690465 | T | G | 392.77 | SNP | Rv0591 (mce2C) | silent (Leu469) | 9947 | - | | 698968 | G | A | 632.77 | SNP | Rv0601c | silent (Gly9) | 9935 | - | | 721498 | G | A | 1041.77 | SNP | Rv0629c (recD) | Leu79Leu(s) | 4 | - | | 728707 | G | T | 936.77 | SNP | Rv0632c (echA3) | Ala191Asp | 6 | - | | 732265 | G | A | 742.77 | SNP | Rv0635 (hadA) | Val(s)112Val | 13 | - | | 743399 | C | A | 917.77 | SNP | Rv0648 | silent (Ala227) | 9867 | - | | 754186 | A | G | 535.77 | SNP | Rv0658c | Leu75Pro | 2 | - | | 760982 | G | C | 57.77 | SNP | Rv0667 (rpoB) | silent (Arg392) | 9913 | - | | 760991 | G | C | 62.77 | SNP | Rv0667 (rpoB) | silent (Arg395) | 9913 | - | | 761003 | C | G | 53.77 | SNP | Rv0667 (rpoB) | silent (Thr399) | 9871 | - | | 761015 | G | C | 39.77 | SNP | Rv0667 (rpoB) | Val(s)403Val | 13 | - | | 761027 | A | G | 47.77 | SNP | Rv0667 (rpoB) | silent (Thr407) | 9871 | - | | 761036 | G | C | 35.77 | SNP | Rv0667 (rpoB) | silent (Thr410) | 9871 | - | | 761037 | T | C | 39.77 | SNP | Rv0667 (rpoB) | Leu(s)411Leu | 3 | - | | 761057 | G | C | 49.77 | SNP | Rv0667 (rpoB) | Val(s)417Val | 13 | - | | 761064 | G | T | 34.77 | SNP | Rv0667 (rpoB) | Ala420Ser | 28 | - | | 761066 | G | C | 70.77 | SNP | Rv0667 (rpoB) | silent (Ala420) | 9867 | - | | 761155 | C | T | 1046.77 | SNP | Rv0667 (rpoB) | Ser450Leu(s) | 35 | resistance | | 763528 | G | T | 30.77 | SNP | Rv0668 (rpoC) | silent (Gly53) | 9935 | - | | 763531 | G | C | 36.77 | SNP | Rv0668 (rpoC) | silent (Pro54) | 9926 | - | | 763558 | C | T | 47.77 | SNP | Rv0668 (rpoC) | silent (Gly63) | 9935 | - | | 763960 | T | G | 32.77 | SNP | Rv0668 (rpoC) | Val197Val(s) | 18 | - | | 772677 | C | A | 465.77 | SNP | Rv0672 (fadE8) | silent (Gly398) | 9935 | - | | 775639 | T | C | 633.77 | SNP | Rv0676c (mmpL5) | Ile948Val | 57 | - | | 781395 | T | C | 1135.77 | SNP | intergenic (Rv0682-165nt) |  |  | - | | 782655 | A | G | 41.77 | SNP | Rv0684 (fusA1) | silent (Glu57) | 9865 | - | | 782661 | A | G | 37.77 | SNP | Rv0684 (fusA1) | silent (Glu59) | 9865 | - | | 785783 | G | T | 51.77 | SNP | Rv0685 (tuf) | silent (Arg321) | 9913 | - | | 785813 | G | C | 63.77 | SNP | Rv0685 (tuf) | silent (Pro331) | 9926 | - | | 805271 | C | G | 40.77 | SNP | Rv0708 (rplP) | Ile54Met(s) | 6 | - | | 805295 | C | A | 53.77 | SNP | Rv0708 (rplP) | silent (Gly62) | 9935 | - | | 837033 | A | G | 145.03 | SNP | Rv0746 (PE\_PGRS9) | Thr445Ala | 32 | - | | 839334 | A | G | 90.28 | SNP | Rv0747 (PE\_PGRS10) | Lys295Arg | 19 | - | | 839348 | A | G | 63.28 | SNP | Rv0747 (PE\_PGRS10) | Ser300Gly | 21 | - | | 839515 | G | A | 108.03 | SNP | Rv0747 (PE\_PGRS10) | silent (Ala355) | 9867 | - | | 839516 | A | G | 116.03 | SNP | Rv0747 (PE\_PGRS10) | Thr356Ala | 32 | - | | 839519 | C | G | 118.03 | SNP | Rv0747 (PE\_PGRS10) | Leu357Val(s) | 4 | - | | 839520 | T | C | 117.03 | SNP | Rv0747 (PE\_PGRS10) | Leu357Pro | 2 | - | | 839534 | A | C | 115.03 | SNP | Rv0747 (PE\_PGRS10) | Ile362Leu | 22 | - | | 840496 | C | G | 54.74 | SNP | Rv0747 (PE\_PGRS10) | silent (Gly682) | 9935 | - | | 841764 | G | C | 784.77 | SNP | Rv0749A | silent (Thr37) | 9871 | - | | 846256 | C | A | 458.77 | SNP | Rv0754 (PE\_PGRS11) | Ala33Asp | 6 | - | | 852910 | C | T | 424.77 | SNP | Rv0758 (phoR) | Pro172Leu | 3 | - | | 854252 | GCC | G | 2079.73 | DEL | intergenic |  |  | - | | 857696 | A | G | 1042.77 | SNP | Rv0764c (cyp51) | silent (Ala114) | 9867 | - | | 859649 | C | T | 866.77 | SNP | Rv0766c (cyp123) | Gly142Ser | 16 | - | | 859769 | A | G | 972.77 | SNP | Rv0766c (cyp123) | Ser102Pro | 12 | - | | 869679 | C | T | 800.77 | SNP | Rv0776c | Gly29Arg | 0 | - | | 874787 | G | A | 1033.77 | SNP | Rv0781 (ptrBa); Rv0782 (ptrBb) | silent (Pro185); Arg19Gln | 9926; 9 | genotype | | 874835 | C | CCG | 2245.73 | INS | Rv0781 (ptrBa); Rv0782 (ptrBb) |  |  | - | | 876378 | T | C | 565.77 | SNP | Rv0782 (ptrBb) | silent (Ala549) | 9867 | - | | 880562 | G | T | 855.77 | SNP | Rv0785 | Cys408Phe | 0 | - | | 882257 | T | C | 1275.77 | SNP | Rv0787 | Tyr267His | 4 | - | | 890549 | G | A | 216.77 | SNP | Rv0797 | Trp54STOP | 0 | - | | 893733 | T | G | 821.77 | SNP | Rv0800 (pepC) | Leu139Arg | 1 | - | | 900221 | T | C | 1001.77 | SNP | Rv0806c (cpsY) | Val370Val(s) | 18 | - | | 903550 | T | C | 712.77 | SNP | Rv0808 (purF) | silent (Ala480) | 9867 | - | | 903913 | T | C | 826.77 | SNP | Rv0809 (purM) | silent (Gly63) | 9935 | - | | 906857 | A | G | 838.77 | SNP | Rv0812 | Ile145Met(s) | 6 | - | | 919384 | T | C | 854.77 | SNP | Rv0825c | Tyr57Cys | 3 | - | | 921813 | C | G | 711.77 | SNP | Rv0829 | Ala80Gly | 21 | - | | 944941 | A | G | 1047.77 | SNP | Rv0848 (cysK2) | Arg2Gly | 1 | - | | 945214 | G | A | 779.77 | SNP | Rv0848 (cysK2) | Gly93Ser | 16 | - | | 949535 | T | C | 868.77 | SNP | Rv0853c (pdc) | silent (Ala528) | 9867 | - | | 955524 | A | G | 521.77 | SNP | Rv0859 (fadA) | Ser150Gly | 21 | - | | 955983 | C | T | 435.77 | SNP | Rv0859 (fadA) | Pro303Ser | 17 | - | | 956644 | C | T | 647.77 | SNP | Rv0860 (fadB) | Leu118Leu(s) | 4 | - | | 968426 | A | AGCCGGGTTG | 1324.73 | INS | Rv0872c (PE\_PGRS15) |  |  | - | | 976896 | TTG | T | 1699.73 | DEL | Rv0878c (PPE13) |  |  | - | | 979314 | C | T | 504.77 | SNP | Rv0880 | silent (Ile127) | 9872 | - | | 979704 | G | C | 844.77 | SNP | Rv0881 | Gly115Arg | 0 | - | | 984493 | C | T | 601.77 | SNP | Rv0886 (fprB) | Leu231Leu(s) | 4 | - | | 986463 | G | C | 1018.77 | SNP | intergenic |  |  | - | | 990001 | G | C | 596.77 | SNP | Rv0890c | Pro866Ala | 22 | - | | 990626 | T | A | 662.77 | SNP | Rv0890c | Leu657Phe | 6 | - | | 993346 | A | C | 1156.77 | SNP | Rv0891c | Val37Gly | 5 | - | | 996871 | G | A | 1414.77 | SNP | Rv0894 | Leu(s)116Leu | 3 | - | | 1010204 | C | CG | 1852.73 | INS | Rv0907 |  |  | - | | 1015344 | G | GGT | 990.73 | INS | intergenic |  |  | - | | 1020044 | C | T | 739.77 | SNP | intergenic |  |  | - | | 1025106 | T | C | 1214.77 | SNP | Rv0919 | silent (Phe141) | 9946 | - | | 1037012 | T | C | 392.77 | SNP | Rv0930 (pstA1) | Met(s)5Thr | 22 | - | | 1037911 | C | T | 902.77 | SNP | Rv0930 (pstA1) | Arg305STOP | 2 | - | | 1042163 | CTGCTGCTAGCAGCGGCGGG CTG | C | 4472.73 | DEL | Rv0934 (pstS1) |  |  | - | | 1044905 | G | T | 766.77 | SNP | Rv0936 (pstA2) | Ala197Ser | 28 | - | | 1047165 | T | C | 562.77 | SNP | Rv0938 (ligD) | Cys344Arg | 1 | - | | 1056916 | T | G | 691.77 | SNP | intergenic |  |  | - | | 1068151 | T | C | 1163.77 | SNP | Rv0956 (purN) | silent (His197) | 9912 | - | | 1068432 | A | G | 687.77 | SNP | Rv0957 (purH) | silent (Pro76) | 9926 | - | | 1070010 | C | A | 723.77 | SNP | Rv0958 | Pro43Gln | 6 | - | | 1070702 | T | C | 399.77 | SNP | Rv0958 | Ser274Pro | 12 | - | | 1074558 | G | A | 784.77 | SNP | Rv0962c (lprP) | Pro186Leu | 3 | - | | 1075279 | T | C | 1216.77 | SNP | intergenic |  |  | - | | 1076309 | G | T | 839.77 | SNP | Rv0964c | Pro124Thr | 5 | - | | 1077312 | A | G | 828.77 | SNP | Rv0966c | Val(s)175Ala | 9867 | - | | 1079927 | C | A | 634.77 | SNP | Rv0969 (ctpV) | silent (Thr395) | 9871 | - | | 1081681 | T | C | 850.77 | SNP | Rv0970 | silent (Val210) | 9901 | - | | 1087193 | G | C | 1024.77 | SNP | Rv0974c (accD2) | Asn51Lys | 25 | - | | 1093406 | A | G | 680.77 | SNP | Rv0978c (PE\_PGRS17) | silent (Val317) | 9901 | - | | 1093928 | G | A | 224.78 | SNP | Rv0978c (PE\_PGRS17) | silent (Asn143) | 9822 | - | | 1096633 | T | G | 909.77 | SNP | intergenic |  |  | - | | 1100234 | T | C | 756.77 | SNP | Rv0983 (pepD) | Leu390Pro | 2 | - | | 1106422 | T | C | 1091.77 | SNP | Rv0989c (grcC2) | Ile321Val | 57 | - | | 1109975 | A | G | 959.77 | SNP | Rv0993 (galU) | Gln235Arg | 10 | - | | 1126889 | G | C | 768.77 | SNP | Rv1007c (metS) | Arg39Gly | 1 | - | | 1127648 | C | A | 929.77 | SNP | Rv1008 (tatD) | Thr187Asn | 9 | - | | 1149551 | C | T | 868.77 | SNP | Rv1028c (kdpD) | silent (Glu712) | 9865 | - | | 1150585 | G | A | 548.77 | SNP | Rv1028c (kdpD) | Pro368Ser | 17 | - | | 1163134 | T | C | 1013.77 | SNP | Rv1040c (PE8) | silent (Gly81) | 9935 | - | | 1165521 | T | TA | 1662.73 | INS | intergenic |  |  | - | | 1168715 | C | CT | 1980.73 | INS | Rv1046c |  |  | - | | 1169235 | CG | C | 1897.73 | DEL | intergenic |  |  | - | | 1169307 | C | T | 796.77 | SNP | intergenic |  |  | - | | 1169447 | G | A | 163.90 | SNP | Rv1047 | Ala9Thr | 22 | - | | 1170404 | C | A | 152.84 | SNP | Rv1047 | Gln328Lys | 12 | - | | 1177446 | T | C | 745.77 | SNP | Rvnt17 | tRNA | tRNA | - | | 1178116 | T | C | 1479.77 | SNP | Rv1056 | silent (Thr163) | 9871 | - | | 1184605 | C | A | 827.77 | SNP | Rv1061 | Asp197Glu | 56 | - | | 1199762 | A | G | 456.77 | SNP | Rv1075c | silent (Arg203) | 9913 | - | | 1200418 | A | G | 755.77 | SNP | intergenic |  |  | - | | 1202492 | C | T | 605.82 | SNP | Rv1077 (cbs) | Ala259Val | 13 | - | | 1204882 | A | G | 1152.77 | SNP | Rv1079 (metB) | Val272Val(s) | 18 | - | | 1213679 | T | TCGGCGACGGCGGCATCGG | 1939.74 | INS | Rv1087 (PE\_PGRS21) |  |  | - | | 1220680 | T | C | 911.77 | SNP | Rv1093 (glyA1) | Val36Ala | 18 | - | | 1222919 | C | G | 31.77 | SNP | intergenic |  |  | - | | 1224367 | T | C | 684.77 | SNP | intergenic |  |  | - | | 1248978 | T | C | 1103.77 | SNP | Rv1125 | silent (Ala299) | 9867 | - | | 1276794 | T | C | 40.74 | SNP | Rv1148c | Ile319Val | 57 | - | | 1281118 | T | C | 1091.77 | SNP | Rv1154c | Thr123Ala | 32 | - | | 1282014 | A | C | 435.77 | SNP | intergenic |  |  | - | | 1292102 | A | G | 556.77 | SNP | Rv1162 (narH) | silent (Pro346) | 9926 | - | | 1307897 | G | A | 838.77 | SNP | Rv1175c (fadH) | silent (Tyr110) | 9945 | - | | 1313337 | A | AG | 909.73 | INS | intergenic |  |  | - | | 1313338 | A | C | 577.77 | SNP | intergenic |  |  | - | | 1315191 | A | C | 446.77 | SNP | Rv1180 (pks3) | STOP489Tyr | 1 | - | | 1315884 | G | A | 653.77 | SNP | Rv1181 (pks4) | silent (Ala217) | 9867 | - | | 1320059 | A | G | 1182.77 | SNP | Rv1182 (papA3) | Ile9Val | 57 | - | | 1327890 | G | A | 953.77 | SNP | Rv1186c | silent (Asp472) | 9859 | - | | 1328222 | T | C | 633.77 | SNP | Rv1186c | Asn362Asp | 42 | - | | 1328687 | G | C | 835.77 | SNP | Rv1186c | Pro207Ala | 22 | - | | 1341102 | C | T | 813.77 | SNP | Rv1198 (esxL) | Arg33Cys | 1 | - | | 1341103 | G | C | 880.77 | SNP | Rv1198 (esxL) | Arg33Pro | 5 | - | | 1342598 | G | A | 365.77 | SNP | Rv1199c | Ser3Phe | 2 | - | | 1351788 | G | A | 756.77 | SNP | Rv1207 (folP2) | Ala200Thr | 22 | - | | 1357977 | C | T | 382.31 | SNP | Rv1215c | Glu490Lys | 7 | - | | 1365837 | C | CG | 1102.73 | INS | intergenic |  |  | - | | 1371182 | G | A | 614.77 | SNP | Rv1228 (lpqX) | Gly88Glu | 4 | - | | 1374065 | T | C | 698.77 | SNP | Rv1230c | Ser45Gly | 21 | - | | 1375724 | A | C | 891.77 | SNP | Rv1232c | Cys149Gly | 1 | - | | 1382628 | T | C | 746.77 | SNP | Rv1239c (corA) | Lys139Glu | 4 | - | | 1385761 | GCGCCGTCGC | G | 640.80 | DEL | Rv1243c (PE\_PGRS23) |  |  | - | | 1393626 | A | G | 94.28 | SNP | Rv1249c | silent (Leu119) | 9947 | - | | 1396922 | T | C | 924.77 | SNP | Rv1251c | silent (Thr773) | 9871 | - | | 1411210 | T | G | 1023.77 | SNP | Rv1263 (amiB2) | Val260Val(s) | 18 | - | | 1413148 | C | T | 929.77 | SNP | intergenic |  |  | - | | 1414021 | C | T | 682.77 | SNP | Rv1266c (pknH) | Arg607Gln | 9 | - | | 1433114 | G | A | 670.77 | SNP | Rv1280c (oppA) | silent (Gly109) | 9935 | - | | 1439332 | C | T | 819.77 | SNP | Rv1286 (cysN) | silent (Val142) | 9901 | - | | 1440469 | C | G | 982.77 | SNP | Rv1286 (cysN) | silent (Pro521) | 9926 | - | | 1445781 | A | G | 593.77 | SNP | Rv1291c | silent (Ala18) | 9867 | - | | 1457144 | C | T | 396.77 | SNP | Rv1300 (hemK) | Arg194Cys | 1 | - | | 1465155 | C | T | 1031.77 | SNP | Rv1309 (atpG) | Ala91Val | 13 | - | | 1468208 | A | C | 727.77 | SNP | Rv1313c | Leu433Arg | 1 | - | | 1471659 | C | T | 1196.77 | SNP | intergenic |  |  | - | | 1472106 | G | A | 32.77 | SNP | Rvnr01 | rRNA | rRNA | - | | 1472150 | T | A | 184.77 | SNP | Rvnr01 | rRNA | rRNA | - | | 1472172 | T | C | 213.77 | SNP | Rvnr01 | rRNA | rRNA | - | | 1472215 | A | G | 186.77 | SNP | Rvnr01 | rRNA | rRNA | - | | 1472225 | C | A | 117.77 | SNP | Rvnr01 | rRNA | rRNA | - | | 1472234 | T | C | 133.77 | SNP | Rvnr01 | rRNA | rRNA | - | | 1472362 | C | T | 1316.77 | SNP | Rvnr01 | rRNA | rRNA | resistance | | 1472382 | G | A | 41.77 | SNP | Rvnr01 | rRNA | rRNA | - | | 1472530 | G | A | 184.77 | SNP | Rvnr01 | rRNA | rRNA | - | | 1472582 | G | A | 147.77 | SNP | Rvnr01 | rRNA | rRNA | - | | 1472583 | T | C | 132.77 | SNP | Rvnr01 | rRNA | rRNA | - | | 1472655 | G | T | 148.77 | SNP | Rvnr01 | rRNA | rRNA | - | | 1472660 | T | C | 73.77 | SNP | Rvnr01 | rRNA | rRNA | - | | 1472661 | A | G | 181.77 | SNP | Rvnr01 | rRNA | rRNA | - | | 1472697 | T | C | 211.77 | SNP | Rvnr01 | rRNA | rRNA | - | | 1472713 | T | C | 211.77 | SNP | Rvnr01 | rRNA | rRNA | - | | 1472895 | C | T | 156.77 | SNP | Rvnr01 | rRNA | rRNA | - | | 1472987 | G | A | 32.77 | SNP | Rvnr01 | rRNA | rRNA | - | | 1472990 | A | G | 46.77 | SNP | Rvnr01 | rRNA | rRNA | - | | 1473035 | G | A | 276.77 | SNP | Rvnr01 | rRNA | rRNA | - | | 1473055 | C | T | 108.77 | SNP | Rvnr01 | rRNA | rRNA | - | | 1473062 | T | G | 123.77 | SNP | Rvnr01 | rRNA | rRNA | - | | 1473065 | C | A | 119.77 | SNP | Rvnr01 | rRNA | rRNA | - | | 1473100 | G | A | 109.77 | SNP | Rvnr01 | rRNA | rRNA | - | | 1473110 | T | G | 52.77 | SNP | Rvnr01 | rRNA | rRNA | - | | 1473111 | A | G | 47.77 | SNP | Rvnr01 | rRNA | rRNA | - | | 1473121 | T | C | 48.77 | SNP | Rvnr01 | rRNA | rRNA | - | | 1473122 | T | A | 100.77 | SNP | Rvnr01 | rRNA | rRNA | - | | 1473145 | C | T | 42.77 | SNP | Rvnr01 | rRNA | rRNA | - | | 1473252 | T | C | 30.77 | SNP | Rvnr01 | rRNA | rRNA | - | | 1473844 | C | T | 109.77 | SNP | Rvnr02 | rRNA | rRNA | - | | 1473870 | G | A | 39.77 | SNP | Rvnr02 | rRNA | rRNA | - | | 1473871 | T | C | 64.77 | SNP | Rvnr02 | rRNA | rRNA | - | | 1473876 | G | A | 44.77 | SNP | Rvnr02 | rRNA | rRNA | - | | 1473887 | T | C | 63.77 | SNP | Rvnr02 | rRNA | rRNA | - | | 1473899 | A | G | 36.77 | SNP | Rvnr02 | rRNA | rRNA | - | | 1474348 | C | T | 73.77 | SNP | Rvnr02 | rRNA | rRNA | - | | 1474353 | A | G | 46.77 | SNP | Rvnr02 | rRNA | rRNA | - | | 1474356 | T | C | 53.77 | SNP | Rvnr02 | rRNA | rRNA | - | | 1474359 | C | G | 84.77 | SNP | Rvnr02 | rRNA | rRNA | - | | 1474362 | A | G | 56.77 | SNP | Rvnr02 | rRNA | rRNA | - | | 1474376 | T | G | 61.77 | SNP | Rvnr02 | rRNA | rRNA | - | | 1474465 | G | C | 50.77 | SNP | Rvnr02 | rRNA | rRNA | - | | 1474812 | G | A | 88.77 | SNP | Rvnr02 | rRNA | rRNA | - | | 1474823 | C | G | 74.77 | SNP | Rvnr02 | rRNA | rRNA | - | | 1474904 | G | C | 70.77 | SNP | Rvnr02 | rRNA | rRNA | - | | 1474905 | T | C | 65.77 | SNP | Rvnr02 | rRNA | rRNA | - | | 1475066 | C | G | 50.77 | SNP | Rvnr02 | rRNA | rRNA | - | | 1475067 | A | G | 41.77 | SNP | Rvnr02 | rRNA | rRNA | - | | 1475079 | T | C | 79.77 | SNP | Rvnr02 | rRNA | rRNA | - | | 1475080 | G | C | 61.77 | SNP | Rvnr02 | rRNA | rRNA | - | | 1475088 | A | G | 71.77 | SNP | Rvnr02 | rRNA | rRNA | - | | 1475114 | C | T | 65.77 | SNP | Rvnr02 | rRNA | rRNA | - | | 1475116 | G | A | 57.77 | SNP | Rvnr02 | rRNA | rRNA | - | | 1475897 | T | C | 190.77 | SNP | Rvnr02 | rRNA | rRNA | - | | 1475900 | A | G | 142.77 | SNP | Rvnr02 | rRNA | rRNA | - | | 1475952 | A | G | 188.77 | SNP | Rvnr02 | rRNA | rRNA | - | | 1475970 | C | T | 121.77 | SNP | Rvnr02 | rRNA | rRNA | - | | 1475975 | C | T | 119.77 | SNP | Rvnr02 | rRNA | rRNA | - | | 1475977 | A | G | 107.77 | SNP | Rvnr02 | rRNA | rRNA | - | | 1475982 | G | A | 113.77 | SNP | Rvnr02 | rRNA | rRNA | - | | 1475988 | A | G | 179.77 | SNP | Rvnr02 | rRNA | rRNA | - | | 1475997 | A | T | 135.77 | SNP | Rvnr02 | rRNA | rRNA | - | | 1476001 | T | C | 136.77 | SNP | Rvnr02 | rRNA | rRNA | - | | 1476200 | A | T | 123.77 | SNP | Rvnr02 | rRNA | rRNA | - | | 1476201 | C | T | 107.77 | SNP | Rvnr02 | rRNA | rRNA | - | | 1476214 | G | T | 134.77 | SNP | Rvnr02 | rRNA | rRNA | - | | 1476245 | C | T | 45.77 | SNP | Rvnr02 | rRNA | rRNA | - | | 1476251 | T | C | 46.77 | SNP | Rvnr02 | rRNA | rRNA | - | | 1476428 | C | T | 250.77 | SNP | Rvnr02 | rRNA | rRNA | - | | 1476466 | C | T | 74.77 | SNP | Rvnr02 | rRNA | rRNA | - | | 1476517 | C | T | 65.77 | SNP | Rvnr02 | rRNA | rRNA | - | | 1476538 | A | G | 30.77 | SNP | Rvnr02 | rRNA | rRNA | - | | 1476540 | C | G | 35.77 | SNP | Rvnr02 | rRNA | rRNA | - | | 1476584 | C | T | 45.77 | SNP | Rvnr02 | rRNA | rRNA | - | | 1480174 | C | G | 54.77 | SNP | Rv1318c | silent (Leu217) | 9947 | - | | 1480176 | G | T | 44.77 | SNP | Rv1318c | Leu217Met(s) | 4 | - | | 1480233 | A | C | 50.77 | SNP | Rv1318c | Phe198Val | 1 | - | | 1480945 | C | G | 360.77 | SNP | Rv1319c | silent (Thr519) | 9871 | - | | 1480948 | C | T | 401.77 | SNP | Rv1319c | silent (Glu518) | 9865 | - | | 1480972 | T | C | 598.77 | SNP | Rv1319c | silent (Glu510) | 9865 | - | | 1481185 | A | C | 385.77 | SNP | Rv1319c | Asp439Glu | 56 | - | | 1481321 | A | G | 557.77 | SNP | Rv1319c | Val394Ala | 18 | - | | 1482627 | T | C | 1089.77 | SNP | Rv1320c | Thr531Ala | 32 | - | | 1483652 | A | G | 833.77 | SNP | Rv1320c | Leu189Pro | 2 | - | | 1484708 | A | C | 721.77 | SNP | Rv1321 | Ser144Arg | 6 | - | | 1499274 | C | G | 608.77 | SNP | Rv1330c (pncB1) | Gly429Ala | 21 | - | | 1514384 | C | T | 801.77 | SNP | Rv1348 (irtA) | silent (Thr446) | 9871 | - | | 1526819 | C | A | 918.77 | SNP | Rv1358 | silent (Arg70) | 9913 | - | | 1532827 | A | T | 46.77 | SNP | Rv1361c (PPE19) | Phe269Leu | 13 | - | | 1533208 | C | G | 497.90 | SNP | Rv1361c (PPE19) | silent (Gly142) | 9935 | - | | 1536251 | G | T | 1110.77 | SNP | Rv1364c | Ala465Glu | 10 | - | | 1537710 | AAC | A | 2614.73 | DEL | intergenic |  |  | - | | 1537771 | G | C | 1052.77 | SNP | intergenic |  |  | - | | 1544349 | T | G | 895.77 | SNP | Rv1371 | Trp331Gly | 0 | - | | 1547125 | T | C | 1112.77 | SNP | Rv1374c | Thr136Ala | 32 | - | | 1552547 | G | A | 563.77 | SNP | Rv1378c | Arg37Trp | 2 | - | | 1570566 | C | A | 1120.77 | SNP | Rv1394c (cyp132) | Arg135Leu | 1 | - | | 1572200 | A | G | 54.74 | SNP | Rv1396c (PE\_PGRS25) | Val553Ala | 18 | - | | 1588899 | G | T | 528.77 | SNP | Rv1412 (ribC) | silent (Ala111) | 9867 | - | | 1597405 | G | A | 245.78 | SNP | Rv1422 | silent (Pro175) | 9926 | - | | 1597696 | G | A | 376.77 | SNP | Rv1422 | silent (Glu272) | 9865 | - | | 1609840 | A | G | 653.77 | SNP | Rv1431 | silent (Pro586) | 9926 | - | | 1612624 | T | TATCGGTACCGGTGCGCCAG GG | 2907.73 | INS | Rv1435c |  |  | - | | 1613035 | T | C | 939.77 | SNP | intergenic |  |  | - | | 1620135 | A | G | 537.77 | SNP | Rv1442 (bisC) | silent (Gly115) | 9935 | - | | 1620843 | G | C | 450.77 | SNP | Rv1442 (bisC) | silent (Gly351) | 9935 | - | | 1630148 | A | C | 1054.77 | SNP | Rv1449c (tkt) | Tyr18Asp | 0 | - | | 1636991 | T | C | 79.28 | SNP | Rv1452c (PE\_PGRS28) | silent (Gly413) | 9935 | - | | 1636996 | G | C | 118.03 | SNP | Rv1452c (PE\_PGRS28) | Arg412Gly | 1 | - | | 1637006 | G | A | 85.03 | SNP | Rv1452c (PE\_PGRS28) | silent (Val408) | 9901 | - | | 1637009 | G | A | 101.03 | SNP | Rv1452c (PE\_PGRS28) | silent (Gly407) | 9935 | - | | 1637012 | A | G | 85.28 | SNP | Rv1452c (PE\_PGRS28) | silent (Gly406) | 9935 | - | | 1637015 | A | G | 87.28 | SNP | Rv1452c (PE\_PGRS28) | silent (Ala405) | 9867 | - | | 1637018 | G | C | 112.03 | SNP | Rv1452c (PE\_PGRS28) | silent (Gly404) | 9935 | - | | 1639594 | C | A | 958.77 | SNP | Rv1453 | Pro405Gln | 6 | - | | 1650072 | A | G | 541.77 | SNP | Rv1462 | Asn183Asp | 42 | - | | 1651142 | G | A | 491.77 | SNP | Rv1463 | Ala143Thr | 22 | - | | 1676290 | C | A | 900.77 | SNP | Rv1486c | Lys198Asn | 13 | - | | 1689349 | C | T | 510.77 | SNP | Rv1498c | Arg191His | 8 | - | | 1692141 | A | C | 1492.77 | SNP | Rv1501 | silent (Ile84) | 9872 | - | | 1693561 | A | G | 1005.77 | SNP | Rv1502 | Tyr213Cys | 3 | - | | 1693593 | T | G | 916.77 | SNP | Rv1502 | Trp224Gly | 0 | - | | 1695518 | C | A | 1112.77 | SNP | Rv1505c | silent (Ser143) | 9840 | - | | 1696464 | C | G | 750.77 | SNP | intergenic |  |  | - | | 1698911 | G | A | 819.77 | SNP | Rv1508c | silent (Gly328) | 9935 | - | | 1706119 | T | C | 883.77 | SNP | Rv1514c | silent (Ser159) | 9840 | - | | 1728837 | A | G | 1198.77 | SNP | intergenic |  |  | - | | 1736577 | A | G | 770.77 | SNP | Rv1536 (ileS) | Glu20Gly | 7 | - | | 1751042 | C | T | 873.77 | SNP | Rv1547 (dnaE1) | Pro1117Ser | 17 | - | | 1752561 | T | C | 467.77 | SNP | Rv1548c (PPE21) | Asp258Gly | 11 | - | | 1753519 | G | GC | 534.73 | INS | Rv1549 (fadD11.1) |  |  | - | | 1759252 | G | T | 1080.77 | SNP | Rv1552 (frdA) | silent (Ser524) | 9840 | genotype | | 1760292 | A | G | 1176.77 | SNP | Rv1554 (frdC) | Met(s)40Val(s) | 9867 | - | | 1778430 | T | C | 377.77 | SNP | Rv1570 (bioD) | Met(s)191Thr | 22 | - | | 1779370 | G | C | 842.77 | SNP | Rv1573 | silent (Thr19) | 9871 | - | | 1780048 | A | G | 250.77 | SNP | Rv1574 | His40Arg | 10 | - | | 1780274 | A | C | 101.77 | SNP | Rv1575 | Lys26Gln | 6 | - | | 1780275 | A | G | 127.77 | SNP | Rv1575 | Lys26Arg | 19 | - | | 1780586 | C | CG | 820.73 | INS | Rv1575 |  |  | - | | 1781577 | G | A | 381.77 | SNP | Rv1576c | Thr163Ile | 7 | - | | 1788613 | C | T | 523.77 | SNP | Rv1587c | Gly184Asp | 6 | - | | 1789507 | T | C | 71.77 | SNP | Rv1588c | silent (Leu110) | 9947 | - | | 1789509 | G | A | 76.77 | SNP | Rv1588c | silent (Leu110) | 9947 | - | | 1789516 | A | G | 206.77 | SNP | Rv1588c | silent (Gly107) | 9935 | - | | 1789564 | C | T | 110.77 | SNP | Rv1588c | silent (Arg91) | 9913 | - | | 1789565 | C | A | 194.77 | SNP | Rv1588c | Arg91Leu | 1 | - | | 1789650 | C | T | 342.77 | SNP | Rv1588c | Ala63Thr | 22 | - | | 1789654 | A | G | 381.77 | SNP | Rv1588c | silent (Leu61) | 9947 | - | | 1789671 | C | T | 430.77 | SNP | Rv1588c | Ala56Thr | 22 | - | | 1789675 | A | C | 354.77 | SNP | Rv1588c | silent (Gly54) | 9935 | - | | 1789678 | C | G | 338.77 | SNP | Rv1588c | Val(s)53Val | 13 | - | | 1789742 | G | C | 375.77 | SNP | Rv1588c | Thr32Ser | 38 | - | | 1789746 | A | G | 399.77 | SNP | Rv1588c | Leu(s)31Leu | 3 | - | | 1789766 | T | G | 702.77 | SNP | Rv1588c | Asp24Ala | 10 | - | | 1798355 | G | A | 641.77 | SNP | Rv1597 | Gly21Asp | 6 | - | | 1803265 | G | A | 1190.77 | SNP | Rv1602 (hisH) | Ser201Asn | 20 | - | | 1804409 | C | A | 641.77 | SNP | Rv1604 (impA) | Pro124Gln | 6 | - | | 1808795 | A | C | 776.77 | SNP | Rv1609 (trpE) | Asp298Ala | 10 | - | | 1814629 | G | A | 447.77 | SNP | intergenic |  |  | - | | 1817976 | A | T | 1070.77 | SNP | Rv1618 (tesB1) | His121Leu | 4 | - | | 1836286 | G | C | 770.77 | SNP | intergenic |  |  | - | | 1847919 | C | G | 857.77 | SNP | Rv1639c | silent (Thr180) | 9871 | - | | 1854300 | T | C | 575.77 | SNP | Rv1644 (tsnR) | Leu232Pro | 2 | - | | 1856777 | G | C | 946.77 | SNP | Rv1647 | Ala2Pro | 13 | - | | 1864698 | C | T | 261.78 | SNP | Rv1651c (PE\_PGRS30) | Ala229Thr | 22 | - | | 1885772 | G | A | 837.77 | SNP | Rv1662 (pks8) | Ala1357Thr | 22 | - | | 1894300 | G | GGTCTTGCCGC | 1717.73 | INS | Rv1668c |  |  | - | | 1901493 | T | C | 544.77 | SNP | Rv1676 | silent (Ser149) | 9840 | - | | 1907296 | G | C | 836.77 | SNP | Rv1682 | silent (Ala298) | 9867 | - | | 1917289 | C | T | 216.80 | SNP | Rv1692 | Arg198Trp | 2 | - | | 1917972 | A | G | 674.77 | SNP | Rv1694 (tlyA) | silent (Leu11) | 9947 | - | | 1924500 | C | T | 754.77 | SNP | Rv1699 (pyrG) | silent (Cys224) | 9973 | - | | 1931179 | C | A | 1118.77 | SNP | Rv1704c (cycA) | Arg93Leu | 1 | - | | 1933988 | G | A | 1116.77 | SNP | intergenic |  |  | - | | 1942489 | T | C | 539.77 | SNP | Rv1714 | Phe213Leu | 13 | - | | 1944402 | T | C | 642.77 | SNP | Rv1716 | Val276Ala | 18 | - | | 1950767 | T | C | 1141.77 | SNP | Rv1724c | silent (Lys95) | 9926 | - | | 1955910 | T | G | 652.77 | SNP | Rv1730c | silent (Arg446) | 9913 | - | | 1960284 | C | A | 803.77 | SNP | Rv1733c | Gln68His | 20 | - | | 1967237 | C | A | 547.77 | SNP | Rv1739c | Arg134Leu | 1 | - | | 1978166 | G | T | 1015.77 | SNP | Rv1750c (fadD1) | Gln468Lys | 12 | - | | 1982961 | GC | G | 551.73 | DEL | Rv1753c (PPE24) |  |  | - | | 1983140 | T | C | 34.10 | SNP | Rv1753c (PPE24) | Thr546Ala | 32 | - | | 1983218 | T | C | 306.98 | SNP | Rv1753c (PPE24) | Thr520Ala | 32 | - | | 1989042 | A | T | 99.03 | SNP | Rv1758 (cut1) | Met(s)1Leu(s) | 9867 | - | | 1989043 | T | C | 120.03 | SNP | Rv1758 (cut1) | Met(s)1Thr | 22 | - | | 1989044 | G | A | 114.03 | SNP | Rv1758 (cut1) | Met(s)1Ile | 2 | - | | 1990942 | C | T | 141.03 | SNP | Rv1759c (wag22) | Gly546Ser | 16 | - | | 1992323 | G | GC | 554.75 | INS | Rv1759c (wag22) |  |  | - | | 1993808 | A | T | 819.77 | SNP | Rv1760 | Glu219Val(s) | 17 | - | | 1994939 | G | A | 1355.77 | SNP | Rv1761c | Thr39Ile | 7 | - | | 1998852 | G | T | 838.77 | SNP | intergenic |  |  | - | | 2009881 | T | C | 596.77 | SNP | Rv1775 | Ile237Thr | 11 | - | | 2022868 | T | C | 569.77 | SNP | Rv1783 (eccC5) | silent (Ser1204) | 9840 | - | | 2033748 | G | C | 891.77 | SNP | Rv1795 (eccD5); Rv1796 (mycP5) | silent (Arg503); Gly7Ala | 9913; 21 | - | | 2045310 | A | G | 139.53 | SNP | Rv1803c (PE\_PGRS32) | silent (Ile511) | 9872 | - | | 2049065 | T | C | 614.77 | SNP | intergenic |  |  | - | | 2049097 | G | C | 656.77 | SNP | intergenic |  |  | - | | 2051746 | T | C | 703.77 | SNP | Rv1809 (PPE33) | silent (Ala155) | 9867 | - | | 2052035 | G | T | 847.77 | SNP | Rv1809 (PPE33) | Val(s)252Leu(s) | 9867 | - | | 2055271 | A | G | 587.77 | SNP | Rv1812c | Leu30Pro | 2 | - | | 2057774 | A | T | 570.77 | SNP | Rv1815 | Ile83Phe | 8 | - | | 2060383 | C | T | 597.77 | SNP | Rv1817 | silent (Asp263) | 9859 | - | | 2074570 | G | C | 61.77 | SNP | intergenic |  |  | - | | 2094911 | ACAGCGT | A | 2872.73 | DEL | Rv1844c (gnd1) |  |  | - | | 2096186 | A | G | 576.77 | SNP | Rv1846c (blaI) | silent (Thr138) | 9871 | - | | 2107371 | G | A | 649.77 | SNP | Rv1859 (modC) | silent (Pro266) | 9926 | - | | 2109523 | C | CG | 1699.73 | INS | intergenic |  |  | - | | 2116903 | C | T | 663.77 | SNP | Rv1867 | silent (Gly380) | 9935 | - | | 2128870 | A | G | 565.77 | SNP | Rv1878 (glnA3) | silent (Leu283) | 9947 | - | | 2133468 | T | TTCGCATGCCGTCACC | 1831.73 | INS | Rv1883c |  |  | - | | 2135870 | T | C | 581.77 | SNP | intergenic |  |  | - | | 2143217 | T | C | 690.77 | SNP | Rv1895 | Ser233Pro | 12 | - | | 2143328 | G | C | 1015.77 | SNP | Rv1895 | Val(s)270Leu | 3 | - | | 2147022 | A | C | 1237.77 | SNP | Rv1900c (lipJ) | Ile204Met(s) | 6 | - | | 2149855 | C | CA | 1387.73 | INS | Rv1902c (nanT) |  |  | - | | 2155168 | C | G | 935.77 | SNP | Rv1908c (katG) | Ser315Thr | 32 | resistance | | 2163375 | T | C | 486.77 | SNP | Rv1917c (PPE34) | Asn1313Asp | 42 | - | | 2163412 | A | G | 326.77 | SNP | Rv1917c (PPE34) | silent (Val1300) | 9901 | - | | 2163415 | C | A | 400.77 | SNP | Rv1917c (PPE34) | silent (Pro1299) | 9926 | - | | 2163417 | G | C | 305.77 | SNP | Rv1917c (PPE34) | Pro1299Ala | 22 | - | | 2163419 | C | T | 308.77 | SNP | Rv1917c (PPE34) | Ser1298Asn | 20 | - | | 2163421 | C | G | 305.77 | SNP | Rv1917c (PPE34) | silent (Thr1297) | 9871 | - | | 2163790 | A | C | 662.77 | SNP | Rv1917c (PPE34) | silent (Pro1174) | 9926 | - | | 2165286 | A | C | 657.77 | SNP | Rv1917c (PPE34) | Ser676Ala | 35 | - | | 2165503 | T | A | 531.77 | SNP | Rv1917c (PPE34) | silent (Ala603) | 9867 | - | | 2165928 | G | T | 726.77 | SNP | Rv1917c (PPE34) | Pro462Thr | 5 | - | | 2184781 | G | T | 614.77 | SNP | Rv1933c (fadE18) | silent (Gly59) | 9935 | - | | 2186542 | G | A | 649.77 | SNP | Rv1935c (echA13) | silent (Leu206) | 9947 | - | | 2196879 | T | A | 1533.77 | SNP | Rv1945 | silent (Leu297) | 9947 | - | | 2196882 | A | G | 1624.77 | SNP | Rv1945 | silent (Lys298) | 9926 | - | | 2196964 | A | C | 137.77 | SNP | Rv1945 | Asn326His | 18 | - | | 2196969 | G | C | 123.77 | SNP | Rv1945 | silent (Ala327) | 9867 | - | | 2196970 | C | A | 115.77 | SNP | Rv1945 | His328Asn | 21 | - | | 2198579 | GAACCA | G | 2309.73 | DEL | intergenic |  |  | - | | 2207591 | T | TC | 1999.73 | INS | intergenic |  |  | - | | 2211826 | A | G | 461.77 | SNP | Rv1968 (mce3C) | silent (Lys67) | 9926 | - | | 2216443 | C | A | 652.77 | SNP | Rv1971 (mce3F) | Ala396Glu | 10 | - | | 2220512 | T | G | 1335.77 | SNP | Rv1977 | silent (Ser253) | 9840 | - | | 2223293 | T | C | 891.77 | SNP | intergenic |  |  | - | | 2228967 | A | G | 812.77 | SNP | intergenic |  |  | - | | 2251999 | A | G | 888.77 | SNP | intergenic |  |  | - | | 2260151 | A | G | 529.77 | SNP | intergenic |  |  | - | | 2260154 | C | T | 464.77 | SNP | intergenic |  |  | - | | 2260171 | T | C | 595.77 | SNP | intergenic |  |  | - | | 2260174 | C | T | 585.77 | SNP | intergenic |  |  | - | | 2260196 | C | CA | 951.73 | INS | intergenic |  |  | - | | 2260199 | C | T | 629.77 | SNP | intergenic |  |  | - | | 2260212 | G | T | 515.77 | SNP | intergenic |  |  | - | | 2260214 | G | C | 472.77 | SNP | intergenic |  |  | - | | 2260220 | C | T | 433.77 | SNP | intergenic |  |  | - | | 2260222 | C | G | 450.77 | SNP | intergenic |  |  | - | | 2260231 | T | C | 430.77 | SNP | intergenic |  |  | - | | 2260525 | C | T | 581.77 | SNP | intergenic |  |  | - | | 2264782 | C | A | 668.41 | SNP | Rv2017 | Ala262Glu | 10 | - | | 2265059 | T | G | 647.77 | SNP | intergenic |  |  | - | | 2266487 | G | C | 472.77 | SNP | Rv2020c | silent (Leu78) | 9947 | - | | 2266504 | T | TA | 627.73 | INS | Rv2020c |  |  | - | | 2266508 | A | T | 312.77 | SNP | Rv2020c | Asp71Glu | 56 | - | | 2266511 | GT | G | 553.73 | DEL | Rv2020c |  |  | - | | 2266517 | T | C | 369.77 | SNP | Rv2020c | silent (Glu68) | 9865 | - | | 2266550 | G | T | 506.77 | SNP | Rv2020c | silent (Gly57) | 9935 | - | | 2266553 | C | G | 557.77 | SNP | Rv2020c | silent (Ser56) | 9840 | - | | 2266583 | C | G | 618.77 | SNP | Rv2020c | Glu46Asp | 53 | - | | 2266598 | G | C | 673.77 | SNP | Rv2020c | silent (Leu41) | 9947 | - | | 2266604 | C | G | 651.77 | SNP | Rv2020c | silent (Ser39) | 9840 | - | | 2266613 | G | GC | 1155.73 | INS | Rv2020c |  |  | - | | 2266624 | G | T | 868.77 | SNP | Rv2020c | Leu33Ile | 9 | - | | 2269780 | T | C | 366.77 | SNP | Rv2024c | Asp154Gly | 11 | - | | 2270102 | A | G | 683.77 | SNP | Rv2024c | Trp47Arg | 8 | - | | 2273627 | C | T | 699.77 | SNP | Rv2027c (dosT) | silent (Gly294) | 9935 | - | | 2281665 | C | T | 1372.77 | SNP | Rv2035 | Pro18Ser | 17 | - | | 2282787 | C | T | 381.77 | SNP | Rv2037c | Cys312Tyr | 3 | - | | 2285251 | C | A | 727.77 | SNP | Rv2039c | Val131Phe | 0 | - | | 2287121 | A | G | 806.77 | SNP | Rv2041c | silent (Asp242) | 9859 | - | | 2289081 | G | T | 788.77 | SNP | Rv2043c (pncA) | Pro54Gln | 6 | resistance | | 2296042 | G | C | 563.77 | SNP | Rv2048c (pks12) | Pro3649Ala | 22 | - | | 2297287 | G | T | 323.77 | SNP | Rv2048c (pks12) | Gln3234Lys | 12 | - | | 2300237 | A | G | 438.77 | SNP | Rv2048c (pks12) | silent (Ala2250) | 9867 | - | | 2300546 | A | T | 373.77 | SNP | Rv2048c (pks12) | His2147Gln | 23 | - | | 2300552 | T | G | 382.77 | SNP | Rv2048c (pks12) | silent (Pro2145) | 9926 | - | | 2300555 | A | G | 390.77 | SNP | Rv2048c (pks12) | silent (Asp2144) | 9859 | - | | 2310543 | G | A | 492.77 | SNP | Rv2051c (ppm1) | silent (Gly71) | 9935 | - | | 2329533 | A | G | 448.77 | SNP | Rv2072c (cobL) | Leu205Pro | 2 | - | | 2331061 | G | T | 386.77 | SNP | Rv2074 | silent (Leu23) | 9947 | - | | 2334007 | A | G | 1111.77 | SNP | Rv2077c | silent (Ala96) | 9867 | - | | 2334290 | AGCATCTAAACCACCGTCAC CTGCGTCACCGCGGCCATCT CGCTC | A | 6552.73 | DEL | Rv2077c |  |  | - | | 2335494 | A | G | 681.77 | SNP | Rv2079 | Tyr47Cys | 3 | - | | 2340621 | C | G | 621.77 | SNP | Rv2082 | Pro638Arg | 4 | - | | 2341636 | C | G | 304.78 | SNP | Rv2083 | Leu256Val(s) | 4 | - | | 2345037 | C | A | 663.77 | SNP | Rv2088 (pknJ) | silent (Leu209) | 9947 | - | | 2346672 | T | C | 710.77 | SNP | Rv2089c (pepE) | Asp218Gly | 11 | - | | 2347643 | C | T | 855.77 | SNP | Rv2090 | Arg91Cys | 1 | - | | 2352078 | C | G | 183.84 | SNP | intergenic |  |  | - | | 2355511 | G | A | 511.10 | SNP | Rv2097c (pafA) | silent (Val389) | 9901 | - | | 2358104 | G | C | 268.80 | SNP | intergenic |  |  | - | | 2361604 | C | G | 728.77 | SNP | Rv2101 (helZ) | Val455Val(s) | 18 | - | | 2362041 | C | A | 753.77 | SNP | Rv2101 (helZ) | Pro601Gln | 6 | - | | 2368564 | TA | T | 1645.73 | DEL | intergenic |  |  | - | | 2369971 | A | G | 342.31 | SNP | Rv2110c (prcB) | Tyr211His | 4 | - | | 2374245 | C | T | 1004.77 | SNP | Rv2114 | Gln138STOP | 8 | - | | 2377785 | C | T | 1016.77 | SNP | Rv2118c | Val(s)176Val | 13 | - | | 2386389 | G | A | 598.77 | SNP | Rv2125 | Gly33Ser | 16 | - | | 2387733 | T | C | 50.74 | SNP | Rv2126c (PE\_PGRS37) | silent (Glu80) | 9865 | - | | 2400467 | G | A | 936.77 | SNP | Rv2141c | Thr419Ile | 7 | - | | 2412516 | T | G | 356.77 | SNP | Rv2153c (murG) | Gln279Pro | 8 | - | | 2415656 | G | C | 468.77 | SNP | Rv2155c (murD) | Arg247Gly | 1 | - | | 2424925 | A | G | 699.77 | SNP | intergenic |  |  | - | | 2440953 | G | T | 1117.77 | SNP | Rv2178c (aroG) | silent (Arg256) | 9913 | - | | 2453645 | A | C | 536.79 | SNP | intergenic |  |  | - | | 2465997 | T | G | 453.77 | SNP | Rv2201 (asnB) | Ile334Ser | 2 | - | | 2476977 | G | A | 638.77 | SNP | Rv2211c (gcvT) | Leu69Leu(s) | 4 | - | | 2499726 | G | A | 707.77 | SNP | Rv2226 | Asp299Asn | 36 | - | | 2509140 | G | C | 624.77 | SNP | Rv2236c (cobD) | Ser79Cys | 5 | - | | 2509722 | A | G | 578.77 | SNP | Rv2237 | silent (Pro78) | 9926 | - | | 2521342 | T | C | 943.77 | SNP | Rv2247 (accD6) | silent (Asp200) | 9859 | - | | 2523205 | G | GCGC | 1368.73 | INS | intergenic |  |  | - | | 2525722 | CG | C | 1263.73 | DEL | Rv2250A; Rv2251 |  |  | - | | 2529680 | A | G | 865.77 | SNP | Rv2256c | silent (Thr65) | 9871 | - | | 2531742 | A | G | 670.77 | SNP | Rv2258c | silent (Ala52) | 9867 | - | | 2532017 | G | C | 78.77 | SNP | intergenic |  |  | - | | 2534562 | GGA | G | 1316.73 | DEL | Rv2262c |  |  | - | | 2551572 | A | C | 788.77 | SNP | Rv2280 | Thr5Pro | 4 | - | | 2586127 | A | G | 651.77 | SNP | Rv2314c | silent (Gly388) | 9935 | - | | 2589491 | C | T | 878.77 | SNP | Rv2316 (uspA) | silent (Ile218) | 9872 | - | | 2598400 | A | G | 813.77 | SNP | Rv2326c | silent (Asn516) | 9822 | - | | 2602456 | T | C | 959.77 | SNP | Rv2329c (narK1) | Ile336Val | 57 | - | | 2612632 | C | A | 606.77 | SNP | Rv2337c | Gly119Val | 3 | - | | 2626004 | G | A | 794.77 | SNP | Rv2346c (esxO) | Leu57Leu(s) | 4 | - | | 2626149 | A | C | 712.77 | SNP | Rv2346c (esxO) | silent (Gly8) | 9935 | - | | 2639364 | C | A | 77.77 | SNP | Rv2356c (PPE40) | Gly58Cys | 0 | - | | 2656225 | A | G | 1106.77 | SNP | Rv2377c (mbtH) | Val69Ala | 18 | - | | 2660319 | C | G | 550.77 | SNP | Rv2379c (mbtF) | Glu589Asp | 53 | - | | 2680658 | T | G | 1179.77 | SNP | intergenic |  |  | - | | 2695378 | C | G | 962.77 | SNP | Rv2398c (cysW) | Gly141Ala | 21 | - | | 2703345 | C | A | 715.77 | SNP | Rv2405 | Thr26Lys | 11 | - | | 2704884 | A | ACAGCGACCATATCGCCGAG CT | 32729.73 | INS | Rv2407 |  |  | - | | 2713795 | C | T | 880.77 | SNP | intergenic |  |  | - | | 2718852 | T | G | 1518.77 | SNP | intergenic |  |  | - | | 2734074 | T | C | 97.03 | SNP | Rv2436 (rbsK) | Val282Ala | 18 | - | | 2748712 | T | G | 810.77 | SNP | Rv2448c (valS) | Asp505Ala | 10 | - | | 2751804 | C | T | 227.80 | SNP | Rv2450c (rpfE) | Arg126Gln | 9 | - | | 2752698 | C | A | 1012.77 | SNP | intergenic |  |  | - | | 2760152 | A | G | 527.77 | SNP | Rv2458 (mmuM) | Tyr125Cys | 3 | - | | 2769765 | C | G | 34.77 | SNP | Rv2467 (pepN) | silent (Pro260) | 9926 | - | | 2769768 | A | G | 35.77 | SNP | Rv2467 (pepN) | silent (Glu261) | 9865 | - | | 2769780 | C | G | 33.77 | SNP | Rv2467 (pepN) | silent (Gly265) | 9935 | - | | 2769783 | A | G | 72.77 | SNP | Rv2467 (pepN) | silent (Ala266) | 9867 | - | | 2769789 | A | G | 53.77 | SNP | Rv2467 (pepN) | silent (Glu268) | 9865 | - | | 2779136 | T | C | 308.78 | SNP | Rv2476c (gdh) | Ser1043Gly | 21 | - | | 2786952 | A | G | 1145.77 | SNP | Rv2482c (plsB2) | Cys778Arg | 1 | - | | 2795160 | C | T | 734.77 | SNP | intergenic |  |  | - | | 2807237 | G | A | 884.77 | SNP | Rv2491 | Val(s)191Val | 13 | - | | 2809621 | T | C | 687.77 | SNP | Rv2495c (bkdC) | Thr107Ala | 32 | - | | 2816034 | G | A | 442.77 | SNP | Rv2501c (accA1) | Pro283Ser | 17 | - | | 2816296 | A | C | 662.77 | SNP | Rv2501c (accA1) | Asp195Glu | 56 | - | | 2818837 | A | G | 520.77 | SNP | Rv2503c (scoB) | silent (Gly97) | 9935 | - | | 2820654 | C | G | 48.77 | SNP | Rv2505c (fadD35) | Ala315Pro | 13 | - | | 2820667 | A | G | 42.77 | SNP | Rv2505c (fadD35) | silent (Ile310) | 9872 | - | | 2821077 | CGG | C | 1544.73 | DEL | Rv2505c (fadD35) |  |  | - | | 2821342 | C | T | 562.77 | SNP | Rv2505c (fadD35) | silent (Ala85) | 9867 | - | | 2823309 | G | A | 916.77 | SNP | Rv2508c | Arg429Trp | 2 | - | | 2827984 | G | T | 916.77 | SNP | intergenic |  |  | - | | 2828019 | T | C | 975.77 | SNP | intergenic |  |  | - | | 2828517 | A | G | 1210.77 | SNP | intergenic |  |  | - | | 2830525 | C | A | 931.77 | SNP | Rv2513 | Thr122Lys | 11 | - | | 2836257 | G | A | 744.77 | SNP | Rv2519 (PE26) | Gly158Asp | 6 | - | | 2840091 | C | T | 221.80 | SNP | intergenic |  |  | - | | 2855259 | A | G | 825.77 | SNP | Rv2531c | silent (Ala841) | 9867 | - | | 2865760 | A | G | 1150.77 | SNP | Rv2542 | Thr211Ala | 32 | - | | 2865882 | T | C | 921.77 | SNP | Rv2542 | silent (Val251) | 9901 | - | | 2881597 | AG | A | 1116.73 | DEL | Rv2561 |  |  | - | | 2888201 | T | C | 399.77 | SNP | Rv2566 | Leu610Pro | 2 | - | | 2889633 | T | C | 679.77 | SNP | Rv2566 | silent (Ala1087) | 9867 | - | | 2891267 | C | T | 959.77 | SNP | Rv2567 | silent (Gly491) | 9935 | - | | 2891728 | A | G | 566.77 | SNP | Rv2567 | Gln645Arg | 10 | - | | 2894208 | G | A | 719.77 | SNP | Rv2569c | silent (Ser67) | 9840 | - | | 2897375 | T | G | 353.77 | SNP | Rv2572c (aspS) | silent (Arg143) | 9913 | - | | 2910461 | G | T | 773.77 | SNP | Rv2584c (apt) | Ala147Glu | 10 | - | | 2911293 | C | G | 837.77 | SNP | Rv2585c | Cys462Ser | 11 | - | | 2912294 | T | G | 920.77 | SNP | Rv2585c | silent (Ala128) | 9867 | - | | 2922936 | TGGCGGTGAC | T | 763.77 | DEL | Rv2591 (PE\_PGRS44) |  |  | - | | 2923391 | T | C | 416.77 | SNP | Rv2592c (ruvB) | silent (Pro281) | 9926 | - | | 2927939 | T | C | 708.77 | SNP | intergenic |  |  | - | | 2939373 | G | C | 804.77 | SNP | Rv2611c | Ser197Cys | 5 | - | | 2939657 | T | C | 419.77 | SNP | Rv2611c | Ile102Met(s) | 6 | - | | 2944833 | G | C | 73.28 | SNP | Rv2615c (PE\_PGRS45) | silent (Ala51) | 9867 | - | | 2954439 | T | C | 698.77 | SNP | Rv2627c | Arg104Gly | 1 | - | | 2971419 | G | A | 1062.77 | SNP | Rv2646 | Arg290His | 8 | - | | 2974933 | A | G | 357.77 | SNP | Rv2650c | Ile101Thr | 11 | - | | 2975900 | A | G | 302.78 | SNP | intergenic |  |  | - | | 2977033 | T | G | 237.78 | SNP | Rv2654c | Thr68Pro | 4 | - | | 2982955 | C | T | 582.77 | SNP | Rv2665 | Pro86Leu | 3 | - | | 2983613 | G | A | 90.03 | SNP | Rv2666 | silent (Gly181) | 9935 | - | | 2984740 | A | G | 370.77 | SNP | Rv2668 | His3Arg | 10 | - | | 2996876 | A | C | 627.77 | SNP | Rv2681 | silent (Leu46) | 9947 | - | | 3005185 | G | T | 732.77 | SNP | Rv2688c | Pro156Thr | 5 | - | | 3006361 | CG | C | 1485.73 | DEL | Rv2689c |  |  | - | | 3009692 | A | G | 899.77 | SNP | Rv2691 (ceoB) | Thr117Ala | 32 | - | | 3015966 | G | A | 408.77 | SNP | Rv2701c (suhB) | Ala257Val(s) | 9867 | - | | 3017465 | T | C | 1411.77 | SNP | Rv2702 (ppgK) | Ile203Thr | 11 | - | | 3019385 | G | C | 33.77 | SNP | Rv2703 (sigA) | silent (Pro517) | 9926 | - | | 3019554 | G | T | 529.77 | SNP | Rv2704 | Ala33Ser | 28 | - | | 3020515 | G | C | 882.77 | SNP | intergenic |  |  | - | | 3021929 | C | T | 794.77 | SNP | Rv2709 | Pro31Ser | 17 | - | | 3028658 | G | A | 903.77 | SNP | Rv2715 | Leu(s)187Leu | 3 | - | | 3041871 | G | T | 836.77 | SNP | Rv2729c | Ala202Glu | 10 | - | | 3054081 | A | G | 1074.77 | SNP | Rv2741 (PE\_PGRS47) | silent (Gly56) | 9935 | - | | 3054321 | A | G | 727.77 | SNP | Rv2741 (PE\_PGRS47) | silent (Gly136) | 9935 | - | | 3080795 | A | G | 1686.77 | SNP | Rv2771c | Leu80Pro | 2 | - | | 3092280 | G | C | 102.77 | SNP | Rv2783c (gpsI) | silent (Leu106) | 9947 | - | | 3092283 | C | G | 108.77 | SNP | Rv2783c (gpsI) | silent (Arg105) | 9913 | - | | 3092292 | C | G | 108.77 | SNP | Rv2783c (gpsI) | silent (Leu102) | 9947 | - | | 3092304 | G | C | 49.77 | SNP | Rv2783c (gpsI) | silent (Thr98) | 9871 | - | | 3092310 | G | C | 52.77 | SNP | Rv2783c (gpsI) | silent (Pro96) | 9926 | - | | 3092313 | T | C | 57.77 | SNP | Rv2783c (gpsI) | silent (Arg95) | 9913 | - | | 3092340 | G | C | 49.77 | SNP | Rv2783c (gpsI) | silent (Pro86) | 9926 | - | | 3092349 | G | C | 31.77 | SNP | Rv2783c (gpsI) | silent (Gly83) | 9935 | - | | 3092355 | C | G | 42.77 | SNP | Rv2783c (gpsI) | silent (Ala81) | 9867 | - | | 3092358 | A | G | 53.77 | SNP | Rv2783c (gpsI) | silent (Tyr80) | 9945 | - | | 3092364 | G | C | 32.77 | SNP | Rv2783c (gpsI) | silent (Arg78) | 9913 | - | | 3100153 | G | GA | 1477.73 | INS | Rv2790c (ltp1) |  |  | - | | 3103682 | T | C | 323.78 | SNP | Rv2794c (pptT) | Met(s)87Val(s) | 9867 | - | | 3118000 | A | G | 642.77 | SNP | Rv2812 | Arg395Gly | 1 | - | | 3131469 | T | TTGTCGGCGA | 3341.73 | INS | Rv2823c |  |  | - | | 3133536 | T | C | 1277.77 | SNP | Rv2825c | Lys2Glu | 4 | - | | 3137058 | G | A | 940.77 | SNP | Rv2830c (vapB22) | Ala56Val(s) | 9867 | - | | 3143100 | A | G | 326.78 | SNP | Rv2836c (dinF) | Cys177Arg | 1 | - | | 3162805 | C | G | 85.28 | SNP | Rv2853 (PE\_PGRS48) | Arg180Gly | 1 | - | | 3170460 | C | T | 717.77 | SNP | Rv2858c (aldC) | Val(s)88Val | 13 | - | | 3175335 | C | T | 763.77 | SNP | Rv2863 (vapC23) | Thr115Met(s) | 32 | - | | 3177884 | C | A | 656.77 | SNP | Rv2866 (relG) | silent (Arg21) | 9913 | - | | 3179866 | A | C | 30.77 | SNP | Rv2868c (gcpE) | silent (Pro222) | 9926 | - | | 3179881 | G | C | 68.77 | SNP | Rv2868c (gcpE) | Val217Val(s) | 18 | - | | 3179884 | A | G | 75.77 | SNP | Rv2868c (gcpE) | silent (Gly216) | 9935 | - | | 3179896 | T | C | 73.77 | SNP | Rv2868c (gcpE) | silent (Pro212) | 9926 | - | | 3179909 | C | T | 88.77 | SNP | Rv2868c (gcpE) | Arg208Gln | 9 | - | | 3179911 | T | C | 81.77 | SNP | Rv2868c (gcpE) | silent (Ala207) | 9867 | - | | 3179914 | A | G | 55.77 | SNP | Rv2868c (gcpE) | silent (Ala206) | 9867 | - | | 3179917 | A | G | 68.77 | SNP | Rv2868c (gcpE) | silent (Leu205) | 9947 | - | | 3179923 | C | T | 95.77 | SNP | Rv2868c (gcpE) | silent (Glu203) | 9865 | - | | 3179933 | G | C | 80.77 | SNP | Rv2868c (gcpE) | Ala200Gly | 21 | - | | 3179934 | C | G | 94.77 | SNP | Rv2868c (gcpE) | Ala200Pro | 13 | - | | 3179941 | C | G | 112.77 | SNP | Rv2868c (gcpE) | Val(s)197Val | 13 | - | | 3179944 | C | G | 93.77 | SNP | Rv2868c (gcpE) | Val(s)196Val | 13 | - | | 3181029 | A | C | 321.78 | SNP | Rv2869c (rip) | Val(s)245Gly | 21 | - | | 3183561 | G | C | 72.77 | SNP | Rv2872 (vapC43) | silent (Pro60) | 9926 | - | | 3186860 | T | G | 800.77 | SNP | Rv2874 (dipZ) | Tyr672Asp | 0 | - | | 3189523 | C | T | 615.77 | SNP | intergenic |  |  | - | | 3190145 | TC | T | 864.73 | DEL | Rv2880c |  |  | - | | 3226181 | A | C | 346.78 | SNP | Rv2916c (ffh) | silent (Arg35) | 9913 | - | | 3228143 | G | T | 299.78 | SNP | Rv2917 | Arg594Leu | 1 | - | | 3232759 | G | A | 176.77 | SNP | intergenic |  |  | - | | 3240568 | T | G | 649.77 | SNP | Rv2926c | Thr202Pro | 4 | - | | 3247316 | C | G | 803.77 | SNP | Rv2931 (ppsA) | Asp624Glu | 56 | - | | 3247851 | G | A | 362.77 | SNP | Rv2931 (ppsA) | Ala803Thr | 22 | - | | 3247853 | C | T | 366.77 | SNP | Rv2931 (ppsA) | silent (Ala803) | 9867 | - | | 3247856 | G | C | 374.77 | SNP | Rv2931 (ppsA) | silent (Arg804) | 9913 | - | | 3247864 | C | CTAGG | 886.75 | INS | Rv2931 (ppsA) |  |  | - | | 3247865 | GCAAA | G | 807.73 | DEL | Rv2931 (ppsA) |  |  | - | | 3247874 | G | A | 285.78 | SNP | Rv2931 (ppsA) | silent (Arg810) | 9913 | - | | 3247877 | T | C | 330.78 | SNP | Rv2931 (ppsA) | silent (Phe811) | 9946 | - | | 3247883 | T | C | 412.77 | SNP | Rv2931 (ppsA) | silent (Ser813) | 9840 | - | | 3248074 | G | A | 244.80 | SNP | Rv2931 (ppsA) | Arg877His | 8 | - | | 3248075 | C | T | 227.80 | SNP | Rv2931 (ppsA) | silent (Arg877) | 9913 | - | | 3256494 | A | G | 511.77 | SNP | Rv2933 (ppsC) | silent (Gly270) | 9935 | - | | 3269581 | A | G | 791.77 | SNP | Rv2935 (ppsE) | silent (Ala615) | 9867 | - | | 3270784 | A | G | 610.77 | SNP | Rv2935 (ppsE) | silent (Gln1016) | 9876 | - | | 3296843 | A | G | 616.77 | SNP | Rv2947c (pks15) | Val(s)333Ala | 9867 | - | | 3296924 | C | T | 375.77 | SNP | Rv2947c (pks15) | Arg306Gln | 9 | - | | 3300196 | G | A | 1427.77 | SNP | Rv2949c | silent (Phe125) | 9946 | - | | 3302802 | C | T | 468.77 | SNP | intergenic |  |  | - | | 3304753 | G | T | 856.77 | SNP | Rv2952 | Ala105Ser | 28 | - | | 3308606 | G | A | 939.77 | SNP | intergenic |  |  | - | | 3317702 | C | T | 865.77 | SNP | intergenic |  |  | - | | 3336587 | T | A | 172.80 | SNP | intergenic |  |  | - | | 3336646 | T | A | 48.94 | SNP | intergenic |  |  | - | | 3336825 | T | C | 651.77 | SNP | Rv2981c (ddlA) | Thr365Ala | 32 | - | | 3338603 | G | C | 554.77 | SNP | Rv2982c (gpdA2) | Pro133Ala | 22 | - | | 3345749 | A | G | 787.77 | SNP | Rv2988c (leuC) | silent (Asp109) | 9859 | - | | 3353548 | C | T | 683.77 | SNP | Rv2996c (serA1) | Asp508Asn | 36 | - | | 3358235 | A | T | 914.77 | SNP | Rv2999 (lppY) | Met(s)212Leu(s) | 9867 | - | | 3363338 | A | G | 629.77 | SNP | intergenic |  |  | - | | 3366092 | A | C | 669.77 | SNP | Rv3007c | Leu120Arg | 1 | - | | 3367765 | G | A | 693.77 | SNP | Rv3009c (gatB) | silent (Gly343) | 9935 | - | | 3371719 | G | A | 841.77 | SNP | Rv3012c (gatC) | silent (Ile4) | 9872 | - | | 3379708 | G | C | 236.80 | SNP | intergenic |  |  | - | | 3379712 | G | C | 195.84 | SNP | intergenic |  |  | - | | 3379718 | T | C | 230.80 | SNP | intergenic |  |  | - | | 3379726 | C | A | 230.80 | SNP | intergenic |  |  | - | | 3379730 | G | C | 212.80 | SNP | intergenic |  |  | - | | 3379732 | C | T | 182.84 | SNP | intergenic |  |  | - | | 3379735 | A | C | 230.80 | SNP | intergenic |  |  | - | | 3379736 | C | A | 203.80 | SNP | intergenic |  |  | - | | 3379742 | T | C | 416.77 | SNP | intergenic |  |  | - | | 3379751 | A | C | 404.82 | SNP | intergenic |  |  | - | | 3379757 | A | C | 510.77 | SNP | intergenic |  |  | - | | 3379763 | G | A | 532.77 | SNP | intergenic |  |  | - | | 3379784 | C | A | 599.77 | SNP | intergenic |  |  | - | | 3379788 | C | G | 715.77 | SNP | intergenic |  |  | - | | 3382598 | C | T | 252.80 | SNP | Rv3023c | Ala9Thr | 22 | - | | 3382738 | G | A | 647.77 | SNP | intergenic |  |  | - | | 3398280 | C | A | 498.77 | SNP | Rv3037c | Arg4Leu | 1 | - | | 3402816 | C | T | 913.77 | SNP | Rv3042c (serB2) | Gly116Glu | 4 | - | | 3405776 | A | G | 890.77 | SNP | Rv3044 (fecB) | His214Arg | 10 | - | | 3409700 | CA | C | 1877.73 | DEL | Rv3049c |  |  | - | | 3415180 | ACACCTAGGGGGTGG | A | 2587.73 | DEL | intergenic |  |  | - | | 3417231 | T | C | 946.77 | SNP | Rv3056 (dinP) | Met(s)176Thr | 22 | - | | 3425854 | C | T | 625.77 | SNP | Rv3062 (ligB) | Pro91Ser | 17 | - | | 3428917 | C | A | 1077.77 | SNP | Rv3063 (cstA) | Arg559Ser | 11 | - | | 3440464 | T | G | 686.77 | SNP | Rv3077 | silent (Arg308) | 9913 | - | | 3440468 | G | C | 689.77 | SNP | Rv3077 | Gly310Arg | 0 | - | | 3456666 | A | G | 653.77 | SNP | Rv3089 (fadD13) | silent (Ala302) | 9867 | - | | 3462135 | G | C | 489.77 | SNP | Rv3093c | Cys210Trp | 0 | - | | 3466426 | G | A | 813.78 | SNP | Rv3097c (lipY) | silent (Val222) | 9901 | genotype | | 3467096 | C | T | 800.77 | SNP | intergenic |  |  | - | | 3473996 | G | GA | 1178.73 | INS | intergenic |  |  | - | | 3477917 | C | T | 1051.77 | SNP | Rv3109 (moaA1) | Pro90Leu | 3 | - | | 3481458 | C | T | 369.77 | SNP | Rv3115 | Ser3Phe | 2 | - | | 3481475 | G | A | 292.78 | SNP | Rv3115 | Ala9Thr | 22 | - | | 3481596 | A | G | 49.74 | SNP | Rv3115 | Tyr49Cys | 3 | - | | 3482432 | C | A | 46.74 | SNP | Rv3115 | Gln328Lys | 12 | - | | 3482737 | T | C | 848.77 | SNP | intergenic |  |  | - | | 3486977 | A | G | 1534.77 | SNP | Rv3121 (cyp141) | Lys157Glu | 4 | - | | 3490749 | C | T | 523.77 | SNP | Rv3125c (PPE49) | Leu(s)301Leu | 3 | - | | 3503116 | G | A | 719.77 | SNP | Rv3136A | silent (Val54) | 9901 | - | | 3503895 | C | T | 602.77 | SNP | Rv3137 | Pro168Leu | 3 | - | | 3505027 | G | A | 744.77 | SNP | Rv3138 (pflA) | Arg278His | 8 | - | | 3510724 | G | A | 165.84 | SNP | Rv3144c (PPE52) | silent (Gly198) | 9935 | - | | 3518167 | A | G | 376.77 | SNP | Rv3151 (nuoG) | Ile474Met(s) | 6 | - | | 3518555 | A | G | 563.77 | SNP | Rv3151 (nuoG) | Thr604Ala | 32 | - | | 3523550 | A | C | 36.77 | SNP | Rv3156 (nuoL) | silent (Arg439) | 9913 | - | | 3535025 | G | A | 631.77 | SNP | Rv3166c | silent (Ile109) | 9872 | - | | 3556275 | A | G | 962.77 | SNP | Rv3190c | Leu138Pro | 2 | - | | 3562270 | G | C | 880.77 | SNP | Rv3193c | silent (Ala301) | 9867 | - | | 3569029 | T | C | 765.77 | SNP | intergenic |  |  | - | | 3580636 | CT | C | 1857.73 | DEL | intergenic |  |  | - | | 3581414 | A | G | 1293.77 | SNP | Rv3204 | Thr34Ala | 32 | - | | 3590686 | G | GC | 1214.73 | INS | intergenic |  |  | - | | 3591063 | T | C | 535.77 | SNP | Rv3213c | Lys144Glu | 4 | - | | 3594394 | G | C | 159.90 | SNP | intergenic |  |  | - | | 3594395 | G | A | 168.84 | SNP | intergenic |  |  | - | | 3594398 | T | C | 174.84 | SNP | intergenic |  |  | - | | 3594400 | A | G | 130.03 | SNP | intergenic |  |  | - | | 3596109 | C | T | 1111.77 | SNP | Rv3220c | Gly476Ser | 16 | - | | 3604821 | G | C | 317.78 | SNP | Rv3228 | silent (Ala32) | 9867 | - | | 3607613 | A | G | 790.77 | SNP | Rv3230c | silent (Gly214) | 9935 | - | | 3614982 | T | C | 1147.77 | SNP | Rv3239c | silent (Leu874) | 9947 | - | | 3622441 | A | C | 871.77 | SNP | Rv3243c | Val217Val(s) | 18 | - | | 3644061 | C | T | 1321.77 | SNP | Rv3263 | silent (Ile295) | 9872 | - | | 3663298 | C | A | 78.77 | SNP | Rv3280 (accD5) | Leu413Ile | 9 | - | | 3663311 | G | C | 39.77 | SNP | Rv3280 (accD5) | Gly417Ala | 21 | - | | 3663321 | C | G | 37.77 | SNP | Rv3280 (accD5) | silent (Thr420) | 9871 | - | | 3663324 | G | C | 71.77 | SNP | Rv3280 (accD5) | Val(s)421Val | 13 | - | | 3663327 | A | C | 72.77 | SNP | Rv3280 (accD5) | silent (Pro422) | 9926 | - | | 3663331 | A | G | 98.77 | SNP | Rv3280 (accD5) | Ile424Val | 57 | - | | 3674122 | A | G | 660.77 | SNP | Rv3293 (pcd) | Lys174Arg | 19 | - | | 3674918 | C | T | 976.77 | SNP | Rv3293 (pcd) | silent (Asn439) | 9822 | - | | 3687908 | T | C | 472.77 | SNP | Rv3302c (glpD2) | Tyr512Cys | 3 | - | | 3689523 | G | T | 567.77 | SNP | Rv3303c (lpdA) | Cys472STOP | 3 | - | | 3692193 | C | T | 720.77 | SNP | Rv3305c (amiA1) | Ala206Thr | 22 | - | | 3697152 | T | C | 1371.77 | SNP | intergenic |  |  | - | | 3699253 | C | G | 402.77 | SNP | Rv3311 | Pro378Arg | 4 | - | | 3704596 | G | C | 949.77 | SNP | Rv3317 (sdhD) | Val(s)54Leu | 3 | - | | 3714211 | G | T | 1056.77 | SNP | Rv3328c (sigJ) | Pro41Gln | 6 | - | | 3718357 | C | T | 643.77 | SNP | Rv3331 (sugI) | Pro423Leu | 3 | - | | 3721806 | G | C | 1062.77 | SNP | Rv3335c | silent (Gly265) | 9935 | - | | 3730385 | C | G | 321.77 | SNP | Rv3343c (PPE54) | Arg2184Pro | 5 | - | | 3730386 | G | T | 334.77 | SNP | Rv3343c (PPE54) | silent (Arg2184) | 9913 | - | | 3730466 | A | G | 1220.77 | SNP | Rv3343c (PPE54) | Ile2157Thr | 11 | - | | 3730624 | C | T | 153.77 | SNP | Rv3343c (PPE54) | silent (Ser2104) | 9840 | - | | 3730642 | G | A | 97.77 | SNP | Rv3343c (PPE54) | silent (Asn2098) | 9822 | - | | 3730741 | G | A | 123.77 | SNP | Rv3343c (PPE54) | silent (Gly2065) | 9935 | - | | 3730789 | A | G | 45.77 | SNP | Rv3343c (PPE54) | silent (Ile2049) | 9872 | - | | 3730797 | A | T | 75.77 | SNP | Rv3343c (PPE54) | Phe2047Ile | 7 | - | | 3730825 | A | G | 120.77 | SNP | Rv3343c (PPE54) | silent (Ile2037) | 9872 | - | | 3730896 | A | G | 283.77 | SNP | Rv3343c (PPE54) | Leu(s)2014Leu | 3 | - | | 3730978 | G | A | 223.77 | SNP | Rv3343c (PPE54) | silent (Gly1986) | 9935 | - | | 3732194 | A | G | 161.90 | SNP | Rv3343c (PPE54) | Ile1581Thr | 11 | - | | 3732517 | A | G | 149.77 | SNP | Rv3343c (PPE54) | silent (Ile1473) | 9872 | - | | 3732525 | A | T | 98.77 | SNP | Rv3343c (PPE54) | Phe1471Ile | 7 | - | | 3732553 | A | G | 120.77 | SNP | Rv3343c (PPE54) | silent (Ile1461) | 9872 | - | | 3732624 | A | G | 64.85 | SNP | Rv3343c (PPE54) | Leu(s)1438Leu | 3 | - | | 3732658 | C | G | 47.74 | SNP | Rv3343c (PPE54) | Leu(s)1426Phe | 1 | - | | 3732660 | A | G | 48.74 | SNP | Rv3343c (PPE54) | Leu(s)1426Leu | 3 | - | | 3732664 | G | A | 43.74 | SNP | Rv3343c (PPE54) | silent (Val1424) | 9901 | - | | 3732666 | C | A | 35.74 | SNP | Rv3343c (PPE54) | Val1424Phe | 0 | - | | 3732673 | A | C | 31.74 | SNP | Rv3343c (PPE54) | Phe1421Leu(s) | 2 | - | | 3732674 | A | C | 45.74 | SNP | Rv3343c (PPE54) | Phe1421Cys | 0 | - | | 3732706 | G | A | 319.77 | SNP | Rv3343c (PPE54) | silent (Gly1410) | 9935 | - | | 3735508 | G | A | 1475.77 | SNP | Rv3343c (PPE54) | silent (Leu476) | 9947 | - | | 3735907 | C | T | 218.03 | SNP | Rv3343c (PPE54) | silent (Ser343) | 9840 | - | | 3735931 | G | A | 142.90 | SNP | Rv3343c (PPE54) | silent (Ser335) | 9840 | - | | 3735967 | G | A | 96.28 | SNP | Rv3343c (PPE54) | silent (Ser323) | 9840 | - | | 3736072 | A | G | 89.77 | SNP | Rv3343c (PPE54) | silent (Ile288) | 9872 | - | | 3736080 | A | T | 139.77 | SNP | Rv3343c (PPE54) | Phe286Ile | 7 | - | | 3736108 | A | G | 187.77 | SNP | Rv3343c (PPE54) | silent (Ile276) | 9872 | - | | 3736628 | T | G | 694.77 | SNP | Rv3343c (PPE54) | Glu103Ala | 17 | - | | 3746409 | A | G | 418.77 | SNP | Rv3347c (PPE55) | Leu2259Pro | 2 | - | | 3747403 | C | A | 319.77 | SNP | Rv3347c (PPE55) | Gly1928Cys | 0 | - | | 3750993 | A | C | 218.80 | SNP | Rv3347c (PPE55) | Val(s)731Gly | 21 | - | | 3752207 | A | G | 879.77 | SNP | Rv3347c (PPE55) | silent (Ile326) | 9872 | - | | 3752654 | A | T | 388.77 | SNP | Rv3347c (PPE55) | silent (Gly177) | 9935 | - | | 3752662 | A | G | 320.78 | SNP | Rv3347c (PPE55) | Leu(s)175Leu | 3 | - | | 3752761 | A | G | 251.78 | SNP | Rv3347c (PPE55) | Leu(s)142Leu | 3 | - | | 3752778 | A | G | 155.90 | SNP | Rv3347c (PPE55) | Val136Ala | 18 | - | | 3752791 | CTG | C | 265.80 | DEL | Rv3347c (PPE55) |  |  | - | | 3752907 | G | A | 61.74 | SNP | Rv3347c (PPE55) | Ala93Val(s) | 9867 | - | | 3752909 | G | C | 113.03 | SNP | Rv3347c (PPE55) | silent (Ala92) | 9867 | - | | 3752910 | G | A | 72.28 | SNP | Rv3347c (PPE55) | Ala92Val | 13 | - | | 3752934 | A | T | 201.84 | SNP | Rv3347c (PPE55) | Val(s)84Glu | 10 | - | | 3753116 | C | T | 397.77 | SNP | Rv3347c (PPE55) | silent (Pro23) | 9926 | - | | 3753164 | T | G | 617.77 | SNP | Rv3347c (PPE55) | silent (Pro7) | 9926 | - | | 3758967 | G | A | 576.77 | SNP | Rv3350c (PPE56) | silent (Gly2712) | 9935 | - | | 3766777 | A | G | 33.77 | SNP | Rv3350c (PPE56) | Leu109Pro | 2 | - | | 3766778 | G | C | 30.77 | SNP | Rv3350c (PPE56) | Leu109Val(s) | 4 | - | | 3787215 | A | C | 768.77 | SNP | Rv3372 (otsB2) | Asp301Ala | 10 | - | | 3792796 | G | A | 1409.77 | SNP | Rv3378c | silent (Asn151) | 9822 | - | | 3798095 | A | C | 1173.77 | SNP | Rv3383c (idsB) | Val132Gly | 5 | - | | 3803867 | AAATCGCCCAATTTCGTGCC GAATTGGGCGATTTTGCGTC TGCTCGGCGCAG | A | 2717.80 | DEL | intergenic |  |  | - | | 3813185 | G | A | 737.77 | SNP | Rv3396c (guaA) | silent (Gly298) | 9935 | - | | 3817117 | C | A | 425.77 | SNP | Rv3399 | Ala330Glu | 10 | - | | 3819169 | G | A | 707.77 | SNP | Rv3401 | silent (Pro376) | 9926 | - | | 3820407 | A | G | 48.77 | SNP | intergenic |  |  | - | | 3820545 | A | G | 48.77 | SNP | intergenic |  |  | - | | 3823159 | A | T | 739.77 | SNP | Rv3403c | silent (Val235) | 9901 | - | | 3826684 | C | T | 819.77 | SNP | Rv3408 (vapC47) | Ser46Leu(s) | 35 | - | | 3829770 | T | C | 509.77 | SNP | Rv3410c (guaB3) | silent (Pro47) | 9926 | - | | 3838871 | A | G | 454.77 | SNP | Rv3420c (rimI) | silent (Ala64) | 9867 | - | | 3841652 | T | A | 1274.77 | SNP | intergenic |  |  | - | | 3841654 | T | G | 1434.77 | SNP | intergenic |  |  | - | | 3841662 | T | C | 1497.77 | SNP | intergenic |  |  | - | | 3841663 | C | T | 1390.77 | SNP | intergenic |  |  | - | | 3842384 | A | G | 391.77 | SNP | Rv3425 (PPE57) | Asp49Gly | 11 | - | | 3842392 | G | C | 337.77 | SNP | Rv3425 (PPE57) | Asp52His | 3 | - | | 3842394 | T | G | 390.77 | SNP | Rv3425 (PPE57) | Asp52Glu | 56 | - | | 3842425 | T | A | 2302.77 | SNP | Rv3425 (PPE57) | Leu(s)63Met(s) | 9867 | - | | 3842438 | T | C | 45.77 | SNP | Rv3425 (PPE57) | Val67Ala | 18 | - | | 3842452 | C | G | 109.77 | SNP | Rv3425 (PPE57) | Gln72Glu | 35 | - | | 3842636 | A | G | 1238.77 | SNP | Rv3425 (PPE57) | Asp133Gly | 11 | - | | 3842637 | C | A | 1107.77 | SNP | Rv3425 (PPE57) | Asp133Glu | 56 | - | | 3843354 | A | G | 340.77 | SNP | Rv3426 (PPE58) | Thr107Ala | 32 | - | | 3843356 | T | C | 336.77 | SNP | Rv3426 (PPE58) | silent (Thr107) | 9871 | - | | 3843361 | C | A | 291.77 | SNP | Rv3426 (PPE58) | Ala109Asp | 6 | - | | 3843362 | C | A | 296.77 | SNP | Rv3426 (PPE58) | silent (Ala109) | 9867 | - | | 3843363 | A | G | 308.77 | SNP | Rv3426 (PPE58) | Asn110Asp | 42 | - | | 3843407 | CG | C | 1073.73 | DEL | Rv3426 (PPE58) |  |  | - | | 3843531 | A | G | 35.77 | SNP | Rv3426 (PPE58) | Met(s)166Val(s) | 9867 | - | | 3843536 | G | A | 51.77 | SNP | Rv3426 (PPE58) | silent (Ala167) | 9867 | - | | 3843696 | T | A | 200.77 | SNP | Rv3426 (PPE58) | Leu(s)221Met(s) | 9867 | - | | 3843704 | G | C | 261.77 | SNP | Rv3426 (PPE58) | silent (Thr223) | 9871 | - | | 3843714 | T | C | 248.77 | SNP | Rv3426 (PPE58) | Cys227Arg | 1 | - | | 3843749 | G | T | 213.77 | SNP | intergenic |  |  | - | | 3843751 | G | T | 251.77 | SNP | intergenic |  |  | - | | 3843752 | A | G | 262.77 | SNP | intergenic |  |  | - | | 3843753 | G | A | 273.77 | SNP | intergenic |  |  | - | | 3843760 | T | C | 365.77 | SNP | intergenic |  |  | - | | 3844756 | GC | G | 1588.73 | DEL | Rv3428c |  |  | - | | 3844992 | T | A | 918.77 | SNP | Rv3428c | Ser327Cys | 5 | - | | 3846605 | G | A | 606.77 | SNP | intergenic |  |  | - | | 3846607 | A | C | 679.77 | SNP | intergenic |  |  | - | | 3846622 | G | T | 731.77 | SNP | intergenic |  |  | - | | 3846687 | A | G | 822.77 | SNP | intergenic |  |  | - | | 3846704 | A | G | 665.77 | SNP | intergenic |  |  | - | | 3846707 | A | C | 630.77 | SNP | intergenic |  |  | - | | 3846716 | C | T | 143.77 | SNP | intergenic |  |  | - | | 3846727 | C | T | 148.77 | SNP | intergenic |  |  | - | | 3846728 | A | G | 149.77 | SNP | intergenic |  |  | - | | 3846741 | G | T | 143.77 | SNP | intergenic |  |  | - | | 3846743 | C | G | 130.77 | SNP | intergenic |  |  | - | | 3846764 | C | G | 477.77 | SNP | intergenic |  |  | - | | 3846773 | T | TG | 819.73 | INS | intergenic |  |  | - | | 3846774 | T | G | 396.77 | SNP | intergenic |  |  | - | | 3846777 | C | A | 343.77 | SNP | intergenic |  |  | - | | 3846779 | T | G | 404.77 | SNP | intergenic |  |  | - | | 3846840 | G | GCT | 1487.73 | INS | intergenic |  |  | - | | 3846843 | CAAA | C | 900.73 | DEL | intergenic |  |  | - | | 3846851 | G | A | 429.77 | SNP | intergenic |  |  | - | | 3846852 | C | G | 475.77 | SNP | intergenic |  |  | - | | 3846853 | T | C | 466.77 | SNP | intergenic |  |  | - | | 3846857 | G | A | 441.77 | SNP | intergenic |  |  | - | | 3846860 | T | G | 434.77 | SNP | intergenic |  |  | - | | 3846866 | C | A | 443.77 | SNP | intergenic |  |  | - | | 3846881 | AT | A | 572.73 | DEL | intergenic |  |  | - | | 3846886 | A | T | 449.77 | SNP | intergenic |  |  | - | | 3846897 | T | G | 429.77 | SNP | intergenic |  |  | - | | 3847010 | G | C | 108.03 | SNP | intergenic |  |  | - | | 3847014 | C | G | 149.90 | SNP | intergenic |  |  | - | | 3847022 | T | C | 118.03 | SNP | intergenic |  |  | - | | 3847039 | G | A | 186.84 | SNP | intergenic |  |  | - | | 3847052 | G | A | 141.90 | SNP | intergenic |  |  | - | | 3847073 | G | C | 157.90 | SNP | intergenic |  |  | - | | 3847074 | C | G | 156.90 | SNP | intergenic |  |  | - | | 3847087 | G | A | 147.90 | SNP | intergenic |  |  | - | | 3847090 | G | C | 154.90 | SNP | intergenic |  |  | - | | 3847099 | G | A | 144.90 | SNP | intergenic |  |  | - | | 3847112 | T | A | 79.28 | SNP | intergenic |  |  | - | | 3847130 | G | A | 110.03 | SNP | intergenic |  |  | - | | 3847137 | T | C | 113.03 | SNP | intergenic |  |  | - | | 3847153 | A | C | 151.90 | SNP | intergenic |  |  | - | | 3847154 | A | G | 154.90 | SNP | intergenic |  |  | - | | 3859576 | G | A | 421.77 | SNP | Rv3439c | silent (His29) | 9912 | - | | 3859893 | C | T | 647.77 | SNP | Rv3440c | silent (Glu28) | 9865 | - | | 3862472 | GA | G | 1012.73 | DEL | intergenic |  |  | - | | 3864995 | T | C | 702.77 | SNP | Rv3447c (eccC4) | Ser1082Gly | 21 | - | | 3877421 | A | G | 946.77 | SNP | Rv3456c (rplQ) | silent (Pro4) | 9926 | - | | 3879331 | G | A | 659.77 | SNP | Rv3459c (rpsK) | Ser121Leu(s) | 35 | - | | 3884748 | G | A | 99.03 | SNP | Rv3467 | Gly262Asp | 6 | - | | 3885886 | T | C | 608.77 | SNP | Rv3468c | Ile62Val | 57 | - | | 3892671 | A | G | 1319.77 | SNP | Rv3476c (kgtP) | silent (Val350) | 9901 | - | | 3895269 | G | C | 342.77 | SNP | Rv3478 (PPE60) | Glu282Gln | 27 | - | | 3895281 | T | C | 311.78 | SNP | Rv3478 (PPE60) | Trp286Arg | 8 | - | | 3895282 | G | A | 310.78 | SNP | Rv3478 (PPE60) | Trp286STOP | 0 | - | | 3896340 | T | G | 471.77 | SNP | Rv3479 | Leu174Arg | 1 | - | | 3898408 | A | G | 741.77 | SNP | Rv3479 | silent (Ala863) | 9867 | - | | 3920950 | C | T | 1192.77 | SNP | intergenic |  |  | - | | 3934542 | T | G | 65.77 | SNP | Rv3508 (PE\_PGRS54) | Ser1180Ala | 35 | - | | 3934733 | G | C | 105.77 | SNP | Rv3508 (PE\_PGRS54) | silent (Gly1243) | 9935 | - | | 3934734 | G | A | 77.77 | SNP | Rv3508 (PE\_PGRS54) | Ala1244Thr | 22 | - | | 3938461 | C | T | 693.77 | SNP | Rv3510c | Arg266Gln | 9 | - | | 3940802 | A | G | 99.28 | SNP | Rv3511 (PE\_PGRS55) | Asn396Asp | 42 | - | | 3942640 | T | C | 93.28 | SNP | intergenic |  |  | - | | 3948928 | G | C | 33.77 | SNP | Rv3514 (PE\_PGRS57) | silent (Gly1045) | 9935 | - | | 3948929 | G | C | 34.77 | SNP | Rv3514 (PE\_PGRS57) | Ala1046Pro | 13 | - | | 3952800 | G | A | 749.77 | SNP | Rv3516 (echA19) | Gly86Asp | 6 | - | | 3958403 | A | G | 1093.77 | SNP | Rv3521 | Asn295Asp | 42 | - | | 3959418 | C | T | 812.77 | SNP | Rv3522 (ltp4) | Thr324Ile | 7 | - | | 3963665 | C | T | 808.77 | SNP | Rv3527 | Leu21Leu(s) | 4 | - | | 4002574 | T | C | 533.77 | SNP | Rv3561 (fadD3) | Val(s)313Ala | 9867 | - | | 4005607 | T | C | 807.77 | SNP | Rv3564 (fadE33) | Leu(s)121Leu | 3 | - | | 4018414 | C | CA | 1356.73 | INS | Rv3576 (lppH) |  |  | - | | 4018415 | G | A | 782.77 | SNP | Rv3576 (lppH) | Gly20Arg | 0 | - | | 4018802 | CAA | C | 1773.73 | DEL | Rv3576 (lppH) |  |  | - | | 4024273 | T | C | 861.77 | SNP | Rv3581c (ispF) | Val25Val(s) | 18 | - | | 4026899 | G | A | 927.77 | SNP | Rv3585 (radA) | silent (Gln152) | 9876 | - | | 4034827 | C | T | 1169.77 | SNP | Rv3593 (lpqF) | Ala159Val(s) | 9867 | - | | 4052608 | G | A | 693.77 | SNP | Rv3610c (ftsH) | silent (Phe92) | 9946 | - | | 4055801 | G | A | 1124.77 | SNP | Rv3616c (espA) | Thr192Ile | 7 | - | | 4059904 | A | G | 466.77 | SNP | intergenic |  |  | - | | 4060100 | G | A | 590.77 | SNP | Rv3619c (esxV) | Leu57Leu(s) | 4 | - | | 4060201 | G | A | 281.78 | SNP | Rv3619c (esxV) | Ser23Leu(s) | 35 | - | | 4060210 | T | A | 212.78 | SNP | Rv3619c (esxV) | Gln20Leu | 6 | - | | 4060230 | G | A | 181.84 | SNP | Rv3619c (esxV) | silent (His13) | 9912 | - | | 4069292 | G | A | 892.77 | SNP | Rv3630 | Ala40Thr | 22 | - | | 4076615 | A | C | 998.77 | SNP | Rv3637 | silent (Ala44) | 9867 | - | | 4094986 | T | C | 465.28 | SNP | Rv3655c | silent (Pro105) | 9926 | - | | 4095001 | CG | C | 897.73 | DEL | Rv3655c |  |  | - | | 4100975 | T | C | 845.77 | SNP | intergenic |  |  | - | | 4109796 | G | A | 1097.77 | SNP | Rv3668c | Pro229Leu | 3 | - | | 4111303 | G | C | 792.77 | SNP | Rv3669 | Val(s)159Val | 13 | - | | 4117361 | AC | A | 1822.73 | DEL | Rv3677c |  |  | - | | 4120926 | A | G | 88.77 | SNP | Rv3680 | Asn378Asp | 42 | - | | 4120983 | A | G | 444.77 | SNP | intergenic |  |  | - | | 4121032 | C | T | 128.77 | SNP | intergenic |  |  | - | | 4136497 | C | G | 789.77 | SNP | Rv3694c | Lys206Asn | 13 | - | | 4139670 | C | T | 920.77 | SNP | Rv3696c (glpK) | Cys29Tyr | 3 | - | | 4148669 | C | T | 691.77 | SNP | Rv3705c | silent (Thr98) | 9871 | - | | 4155050 | G | A | 685.28 | SNP | Rv3710 (leuA) | Val(s)437Val | 13 | - | | 4156099 | C | A | 784.77 | SNP | Rv3711c (dnaQ) | Val(s)211Leu(s) | 9867 | - | | 4160407 | A | G | 1017.77 | SNP | Rv3715c (recR) | Leu(s)32Leu | 3 | - | | 4162073 | C | T | 691.77 | SNP | Rv3718c | silent (Gln62) | 9876 | - | | 4162339 | A | G | 1123.77 | SNP | Rv3719 | Thr12Ala | 32 | - | | 4166052 | G | A | 667.77 | SNP | Rv3721c (dnaZX) | Ala227Val | 13 | - | | 4182695 | G | A | 1022.77 | SNP | Rv3731 (ligC) | Arg313His | 8 | - | | 4187485 | T | C | 854.77 | SNP | Rv3736 | silent (Ala284) | 9867 | - | | 4187817 | A | G | 710.77 | SNP | Rv3737 | Asp40Gly | 11 | - | | 4189841 | T | C | 572.77 | SNP | Rv3738c (PPE66) | Tyr131Cys | 3 | - | | 4198611 | CG | C | 1524.73 | DEL | intergenic |  |  | - | | 4204441 | A | G | 787.77 | SNP | Rv3759c (proX) | silent (His311) | 9912 | - | | 4210274 | A | G | 675.77 | SNP | Rv3764c (tcrY) | Cys246Arg | 1 | - | | 4212196 | A | T | 1027.77 | SNP | intergenic |  |  | - | | 4215467 | G | A | 501.77 | SNP | Rv3770c | silent (Gly103) | 9935 | - | | 4216942 | C | G | 43.77 | SNP | intergenic |  |  | - | | 4221490 | C | G | 787.77 | SNP | Rv3776 | silent (Leu134) | 9947 | - | | 4222073 | A | G | 401.77 | SNP | Rv3776 | Met(s)329Val(s) | 9867 | - | | 4222882 | A | G | 743.77 | SNP | Rv3777 | silent (Leu63) | 9947 | - | | 4242643 | C | T | 523.77 | SNP | Rv3793 (embC) | silent (Arg927) | 9913 | genotype | | 4247431 | G | C | 969.77 | SNP | Rv3795 (embB) | Met(s)306Ile | 2 | resistance | | 4250742 | G | A | 1090.77 | SNP | Rv3796 | Gly289Ser | 16 | - | | 4255922 | A | G | 911.77 | SNP | Rv3799c (accD4) | silent (His9) | 9912 | - | | 4257220 | A | G | 528.77 | SNP | Rv3800c (pks13) | silent (Arg1309) | 9913 | - | | 4257849 | G | A | 659.90 | SNP | Rv3800c (pks13) | Arg1100Trp | 2 | - | | 4260268 | G | C | 749.77 | SNP | Rv3800c (pks13) | silent (Ala293) | 9867 | genotype | | 4269148 | A | G | 654.77 | SNP | Rv3806c (ubiA) | Val(s)229Ala | 9867 | - | | 4272408 | C | T | 1279.77 | SNP | Rv3809c (glf) | silent (Leu356) | 9947 | - | | 4287017 | G | A | 1390.77 | SNP | Rv3822 | silent (Ala99) | 9867 | - | | 4287195 | A | G | 1334.77 | SNP | Rv3822 | Thr159Ala | 32 | - | | 4287722 | G | T | 682.77 | SNP | Rv3822 | silent (Pro334) | 9926 | - | | 4302036 | T | C | 686.77 | SNP | Rv3827c | Thr252Ala | 32 | - | | 4306155 | C | T | 773.77 | SNP | Rv3831 | silent (Ser133) | 9840 | - | | 4311528 | G | A | 422.77 | SNP | Rv3837c | silent (Ala60) | 9867 | - | | 4326283 | C | CAGGTCG | 4713.73 | INS | Rv3854c (ethA) |  |  | - | | 4328329 | G | C | 972.77 | SNP | intergenic |  |  | - | | 4338595 | GC | G | 2126.73 | DEL | intergenic |  |  | - | | 4338732 | G | A | 1316.77 | SNP | intergenic |  |  | - | | 4351039 | G | T | 1029.77 | SNP | Rv3872 (PE35) | Glu99STOP | 17 | - | | 4356110 | G | C | 653.77 | SNP | Rv3877 (eccD1) | silent (Leu368) | 9947 | - | | 4366272 | G | C | 513.77 | SNP | Rv3884c (eccA2) | silent (Ala189) | 9867 | - | | 4366913 | G | A | 736.77 | SNP | Rv3885c (eccE2) | Arg537Trp | 2 | - | | 4372074 | C | T | 793.77 | SNP | Rv3888c | silent (Ser211) | 9840 | - | | 4375628 | G | T | 432.77 | SNP | Rv3892c (PPE69) | Thr19Lys | 11 | - | | 4377461 | G | A | 860.77 | SNP | Rv3894c (eccC2) | Leu998Phe | 6 | - | | 4378330 | G | A | 922.77 | SNP | Rv3894c (eccC2) | Ala708Val | 13 | - | | 4379680 | C | G | 567.77 | SNP | Rv3894c (eccC2) | Arg258Pro | 5 | - | | 4382054 | T | C | 866.77 | SNP | Rv3896c | silent (Ala266) | 9867 | - | | 4382275 | G | T | 1050.77 | SNP | Rv3896c | Gln193Lys | 12 | - | | 4383144 | C | CCGGGG | 1335.73 | INS | Rv3897c |  |  | - | | 4400660 | AC | A | 808.73 | DEL | Rv3911 (sigM) |  |  | - | | 4406749 | G | A | 946.77 | SNP | Rv3918c (parA) | silent (Leu261) | 9947 | - | | 4409645 | G | A | 659.79 | SNP | Rv3921c | Ser142Leu(s) | 35 | - | |  | | export |

elog
