# Supplementary material for: Patterns of compensatory mutations in rpoA/B/C genes of multidrug resistant M. tuberculosis in Uganda
Source: PLoS One. 2025 Dec 4;20(12):e0328957. doi: 10.1371/journal.pone.0328957 (PMC12677784; doi:10.1371/journal.pone.0328957)
Supplement: S2 File — (ZIP) [file pone.0328957.s002.zip › Variants U_S21_L001_001.bam.html]

 

Calling SNPs/INDELs (computing variant list in .vcf format) from U\_S21\_L001\_001.bam

*by SAMtools/BCFtools:*

Howto

Important aspects

This takes up to one hour!!! **Please wait ...**

Variants U\_S21\_L001\_001.bam

|  |  |
| --- | --- |
| Variants |  |

|  |  |
| --- | --- |
| |  | | --- | | *by GATK* | |

|  |  |  |
| --- | --- | --- |
| |  | | --- | | U\_S21\_L001\_001.bam | | | computed 2016-10-27 using PhyResSE v1.0 (Ref. NC\_000962.3) | |

|  |  |
| --- | --- |
| 1196  variants called Export in VCF format |  |

|  |  |  |  |  |  |  |  |  |  |  |  |  |  |  |  |  |  |  |  |  |  |  |  |  |  |  |  |  |  |  |  |  |  |  |  |  |  |  |  |  |  |  |  |  |  |  |  |  |  |  |  |  |  |  |  |  |  |  |  |  |  |  |  |  |  |  |  |  |  |  |  |  |  |  |  |  |  |  |  |  |  |  |  |  |  |  |  |  |  |  |  |  |  |  |  |  |  |  |  |  |  |  |  |  |  |  |  |  |  |  |  |  |  |  |  |  |  |  |  |  |  |  |  |  |  |  |  |  |  |  |  |  |  |  |  |  |  |  |  |  |  |  |  |  |  |  |  |  |  |  |  |  |  |  |  |  |  |  |  |  |  |  |  |  |  |  |  |  |  |  |  |  |  |  |  |  |  |  |  |  |  |  |  |  |  |  |  |  |  |  |  |  |  |  |  |  |  |  |  |  |  |  |  |  |  |  |  |  |  |  |  |  |  |  |  |  |  |  |  |  |  |  |  |  |  |  |  |  |  |  |  |  |  |  |  |  |  |  |  |  |  |  |  |  |  |  |  |  |  |  |  |  |  |  |  |  |  |  |  |  |  |  |  |  |  |  |  |  |  |  |  |  |  |  |  |  |  |  |  |  |  |  |  |  |  |  |  |  |  |  |  |  |  |  |  |  |  |  |  |  |  |  |  |  |  |  |  |  |  |  |  |  |  |  |  |  |  |  |  |  |  |  |  |  |  |  |  |  |  |  |  |  |  |  |  |  |  |  |  |  |  |  |  |  |  |  |  |  |  |  |  |  |  |  |  |  |  |  |  |  |  |  |  |  |  |  |  |  |  |  |  |  |  |  |  |  |  |  |  |  |  |  |  |  |  |  |  |  |  |  |  |  |  |  |  |  |  |  |  |  |  |  |  |  |  |  |  |  |  |  |  |  |  |  |  |  |  |  |  |  |  |  |  |  |  |  |  |  |  |  |  |  |  |  |  |  |  |  |  |  |  |  |  |  |  |  |  |  |  |  |  |  |  |  |  |  |  |  |  |  |  |  |  |  |  |  |  |  |  |  |  |  |  |  |  |  |  |  |  |  |  |  |  |  |  |  |  |  |  |  |  |  |  |  |  |  |  |  |  |  |  |  |  |  |  |  |  |  |  |  |  |  |  |  |  |  |  |  |  |  |  |  |  |  |  |  |  |  |  |  |  |  |  |  |  |  |  |  |  |  |  |  |  |  |  |  |  |  |  |  |  |  |  |  |  |  |  |  |  |  |  |  |  |  |  |  |  |  |  |  |  |  |  |  |  |  |  |  |  |  |  |  |  |  |  |  |  |  |  |  |  |  |  |  |  |  |  |  |  |  |  |  |  |  |  |  |  |  |  |  |  |  |  |  |  |  |  |  |  |  |  |  |  |  |  |  |  |  |  |  |  |  |  |  |  |  |  |  |  |  |  |  |  |  |  |  |  |  |  |  |  |  |  |  |  |  |  |  |  |  |  |  |  |  |  |  |  |  |  |  |  |  |  |  |  |  |  |  |  |  |  |  |  |  |  |  |  |  |  |  |  |  |  |  |  |  |  |  |  |  |  |  |  |  |  |  |  |  |  |  |  |  |  |  |  |  |  |  |  |  |  |  |  |  |  |  |  |  |  |  |  |  |  |  |  |  |  |  |  |  |  |  |  |  |  |  |  |  |  |  |  |  |  |  |  |  |  |  |  |  |  |  |  |  |  |  |  |  |  |  |  |  |  |  |  |  |  |  |  |  |  |  |  |  |  |  |  |  |  |  |  |  |  |  |  |  |  |  |  |  |  |  |  |  |  |  |  |  |  |  |  |  |  |  |  |  |  |  |  |  |  |  |  |  |  |  |  |  |  |  |  |  |  |  |  |  |  |  |  |  |  |  |  |  |  |  |  |  |  |  |  |  |  |  |  |  |  |  |  |  |  |  |  |  |  |  |  |  |  |  |  |  |  |  |  |  |  |  |  |  |  |  |  |  |  |  |  |  |  |  |  |  |  |  |  |  |  |  |  |  |  |  |  |  |  |  |  |  |  |  |  |  |  |  |  |  |  |  |  |  |  |  |  |  |  |  |  |  |  |  |  |  |  |  |  |  |  |  |  |  |  |  |  |  |  |  |  |  |  |  |  |  |  |  |  |  |  |  |  |  |  |  |  |  |  |  |  |  |  |  |  |  |  |  |  |  |  |  |  |  |  |  |  |  |  |  |  |  |  |  |  |  |  |  |  |  |  |  |  |  |  |  |  |  |  |  |  |  |  |  |  |  |  |  |  |  |  |  |  |  |  |  |  |  |  |  |  |  |  |  |  |  |  |  |  |  |  |  |  |  |  |  |  |  |  |  |  |  |  |  |  |  |  |  |  |  |  |  |  |  |  |  |  |  |  |  |  |  |  |  |  |  |  |  |  |  |  |  |  |  |  |  |  |  |  |  |  |  |  |  |  |  |  |  |  |  |  |  |  |  |  |  |  |  |  |  |  |  |  |  |  |  |  |  |  |  |  |  |  |  |  |  |  |  |  |  |  |  |  |  |  |  |  |  |  |  |  |  |  |  |  |  |  |  |  |  |  |  |  |  |  |  |  |  |  |  |  |  |  |  |  |  |  |  |  |  |  |  |  |  |  |  |  |  |  |  |  |  |  |  |  |  |  |  |  |  |  |  |  |  |  |  |  |  |  |  |  |  |  |  |  |  |  |  |  |  |  |  |  |  |  |  |  |  |  |  |  |  |  |  |  |  |  |  |  |  |  |  |  |  |  |  |  |  |  |  |  |  |  |  |  |  |  |  |  |  |  |  |  |  |  |  |  |  |  |  |  |  |  |  |  |  |  |  |  |  |  |  |  |  |  |  |  |  |  |  |  |  |  |  |  |  |  |  |  |  |  |  |  |  |  |  |  |  |  |  |  |  |  |  |  |  |  |  |  |  |  |  |  |  |  |  |  |  |  |  |  |  |  |  |  |  |  |  |  |  |  |  |  |  |  |  |  |  |  |  |  |  |  |  |  |  |  |  |  |  |  |  |  |  |  |  |  |  |  |  |  |  |  |  |  |  |  |  |  |  |  |  |  |  |  |  |  |  |  |  |  |  |  |  |  |  |  |  |  |  |  |  |  |  |  |  |  |  |  |  |  |  |  |  |  |  |  |  |  |  |  |  |  |  |  |  |  |  |  |  |  |  |  |  |  |  |  |  |  |  |  |  |  |  |  |  |  |  |  |  |  |  |  |  |  |  |  |  |  |  |  |  |  |  |  |  |  |  |  |  |  |  |  |  |  |  |  |  |  |  |  |  |  |  |  |  |  |  |  |  |  |  |  |  |  |  |  |  |  |  |  |  |  |  |  |  |  |  |  |  |  |  |  |  |  |  |  |  |  |  |  |  |  |  |  |  |  |  |  |  |  |  |  |  |  |  |  |  |  |  |  |  |  |  |  |  |  |  |  |  |  |  |  |  |  |  |  |  |  |  |  |  |  |  |  |  |  |  |  |  |  |  |  |  |  |  |  |  |  |  |  |  |  |  |  |  |  |  |  |  |  |  |  |  |  |  |  |  |  |  |  |  |  |  |  |  |  |  |  |  |  |  |  |  |  |  |  |  |  |  |  |  |  |  |  |  |  |  |  |  |  |  |  |  |  |  |  |  |  |  |  |  |  |  |  |  |  |  |  |  |  |  |  |  |  |  |  |  |  |  |  |  |  |  |  |  |  |  |  |  |  |  |  |  |  |  |  |  |  |  |  |  |  |  |  |  |  |  |  |  |  |  |  |  |  |  |  |  |  |  |  |  |  |  |  |  |  |  |  |  |  |  |  |  |  |  |  |  |  |  |  |  |  |  |  |  |  |  |  |  |  |  |  |  |  |  |  |  |  |  |  |  |  |  |  |  |  |  |  |  |  |  |  |  |  |  |  |  |  |  |  |  |  |  |  |  |  |  |  |  |  |  |  |  |  |  |  |  |  |  |  |  |  |  |  |  |  |  |  |  |  |  |  |  |  |  |  |  |  |  |  |  |  |  |  |  |  |  |  |  |  |  |  |  |  |  |  |  |  |  |  |  |  |  |  |  |  |  |  |  |  |  |  |  |  |  |  |  |  |  |  |  |  |  |  |  |  |  |  |  |  |  |  |  |  |  |  |  |  |  |  |  |  |  |  |  |  |  |  |  |  |  |  |  |  |  |  |  |  |  |  |  |  |  |  |  |  |  |  |  |  |  |  |  |  |  |  |  |  |  |  |  |  |  |  |  |  |  |  |  |  |  |  |  |  |  |  |  |  |  |  |  |  |  |  |  |  |  |  |  |  |  |  |  |  |  |  |  |  |  |  |  |  |  |  |  |  |  |  |  |  |  |  |  |  |  |  |  |  |  |  |  |  |  |  |  |  |  |  |  |  |  |  |  |  |  |  |  |  |  |  |  |  |  |  |  |  |  |  |  |  |  |  |  |  |  |  |  |  |  |  |  |  |  |  |  |  |  |  |  |  |  |  |  |  |  |  |  |  |  |  |  |  |  |  |  |  |  |  |  |  |  |  |  |  |  |  |  |  |  |  |  |  |  |  |  |  |  |  |  |  |  |  |  |  |  |  |  |  |  |  |  |  |  |  |  |  |  |  |  |  |  |  |  |  |  |  |  |  |  |  |  |  |  |  |  |  |  |  |  |  |  |  |  |  |  |  |  |  |  |  |  |  |  |  |  |  |  |  |  |  |  |  |  |  |  |  |  |  |  |  |  |  |  |  |  |  |  |  |  |  |  |  |  |  |  |  |  |  |  |  |  |  |  |  |  |  |  |  |  |  |  |  |  |  |  |  |  |  |  |  |  |  |  |  |  |  |  |  |  |  |  |  |  |  |  |  |  |  |  |  |  |  |  |  |  |  |  |  |  |  |  |  |  |  |  |  |  |  |  |  |  |  |  |  |  |  |  |  |  |  |  |  |  |  |  |  |  |  |  |  |  |  |  |  |  |  |  |  |  |  |  |  |  |  |  |  |  |  |  |  |  |  |  |  |  |  |  |  |  |  |  |  |  |  |  |  |  |  |  |  |  |  |  |  |  |  |  |  |  |  |  |  |  |  |  |  |  |  |  |  |  |  |  |  |  |  |  |  |  |  |  |  |  |  |  |  |  |  |  |  |  |  |  |  |  |  |  |  |  |  |  |  |  |  |  |  |  |  |  |  |  |  |  |  |  |  |  |  |  |  |  |  |  |  |  |  |  |  |  |  |  |  |  |  |  |  |  |  |  |  |  |  |  |  |  |  |  |  |  |  |  |  |  |  |  |  |  |  |  |  |  |  |  |  |  |  |  |  |  |  |  |  |  |  |  |  |  |  |  |  |  |  |  |  |  |  |  |  |  |  |  |  |  |  |  |  |  |  |  |  |  |  |  |  |  |  |  |  |  |  |  |  |  |  |  |  |  |  |  |  |  |  |  |  |  |  |  |  |  |  |  |  |  |  |  |  |  |  |  |  |  |  |  |  |  |  |  |  |  |  |  |  |  |  |  |  |  |  |  |  |  |  |  |  |  |  |  |  |  |  |  |  |  |  |  |  |  |  |  |  |  |  |  |  |  |  |  |  |  |  |  |  |  |  |  |  |  |  |  |  |  |  |  |  |  |  |  |  |  |  |  |  |  |  |  |  |  |  |  |  |  |  |  |  |  |  |  |  |  |  |  |  |  |  |  |  |  |  |  |  |  |  |  |  |  |  |  |  |  |  |  |  |  |  |  |  |  |  |  |  |  |  |  |  |  |  |  |  |  |  |  |  |  |  |  |  |  |  |  |  |  |  |  |  |  |  |  |  |  |  |  |  |  |  |  |  |  |  |  |  |  |  |  |  |  |  |  |  |  |  |  |  |  |  |  |  |  |  |  |  |  |  |  |  |  |  |  |  |  |  |  |  |  |  |  |  |  |  |  |  |  |  |  |  |  |  |  |  |  |  |  |  |  |  |  |  |  |  |  |  |  |  |  |  |  |  |  |  |  |  |  |  |  |  |  |  |  |  |  |  |  |  |  |  |  |  |  |  |  |  |  |  |  |  |  |  |  |  |  |  |  |  |  |  |  |  |  |  |  |  |  |  |  |  |  |  |  |  |  |  |  |  |  |  |  |  |  |  |  |  |  |  |  |  |  |  |  |  |  |  |  |  |  |  |  |  |  |  |  |  |  |  |  |  |  |  |  |  |  |  |  |  |  |  |  |  |  |  |  |  |  |  |  |  |  |  |  |  |  |  |  |  |  |  |  |  |  |  |  |  |  |  |  |  |  |  |  |  |  |  |  |  |  |  |  |  |  |  |  |  |  |  |  |  |  |  |  |  |  |  |  |  |  |  |  |  |  |  |  |  |  |  |  |  |  |  |  |  |  |  |  |  |  |  |  |  |  |  |  |  |  |  |  |  |  |  |  |  |  |  |  |  |  |  |  |  |  |  |  |  |  |  |  |  |  |  |  |  |  |  |  |  |  |  |  |  |  |  |  |  |  |  |  |  |  |  |  |  |  |  |  |  |  |  |  |  |  |  |  |  |  |  |  |  |  |  |  |  |  |  |  |  |  |  |  |  |  |  |  |  |  |  |  |  |  |  |  |  |  |  |  |  |  |  |  |  |  |  |  |  |  |  |  |  |  |  |  |  |  |  |  |  |  |  |  |  |  |  |  |  |  |  |  |  |  |  |  |  |  |  |  |  |  |  |  |  |  |  |  |  |  |  |  |  |  |  |  |  |  |  |  |  |  |  |  |  |  |  |  |  |  |  |  |  |  |  |  |  |  |  |  |  |  |  |  |  |  |  |  |  |  |  |  |  |  |  |  |  |  |  |  |  |  |  |  |  |  |  |  |  |  |  |  |  |  |  |  |  |  |  |  |  |  |  |  |  |  |  |  |  |  |  |  |  |  |  |  |  |  |  |  |  |  |  |  |  |  |  |  |  |  |  |  |  |  |  |  |  |  |  |  |  |  |  |  |  |  |  |  |  |  |  |  |  |  |  |  |  |  |  |  |  |  |  |  |  |  |  |  |  |  |  |  |  |  |  |  |  |  |  |  |  |  |  |  |  |  |  |  |  |  |  |  |  |  |  |  |  |  |  |  |  |  |  |  |  |  |  |  |  |  |  |  |  |  |  |  |  |  |  |  |  |  |  |  |  |  |  |  |  |  |  |  |  |  |  |  |  |  |  |  |  |  |  |  |  |  |  |  |  |  |  |  |  |  |  |  |  |  |  |  |  |  |  |  |  |  |  |  |  |  |  |  |  |  |  |  |  |  |  |  |  |  |  |  |  |  |  |  |  |  |  |  |  |  |  |  |  |  |  |  |  |  |  |  |  |  |  |  |  |  |  |  |  |  |  |  |  |  |  |  |  |  |  |  |  |  |  |  |  |  |  |  |  |  |  |  |  |  |  |  |  |  |  |  |  |  |  |  |  |  |  |  |  |  |  |  |  |  |  |  |  |  |  |  |  |  |  |  |  |  |  |  |  |  |  |  |  |  |  |  |  |  |  |  |  |  |  |  |  |  |  |  |  |  |  |  |  |  |  |  |  |  |  |  |  |  |  |  |  |  |  |  |  |  |  |  |  |  |  |  |  |  |  |  |  |  |  |  |  |  |  |  |  |  |  |  |  |  |  |  |  |  |  |  |  |  |  |  |  |  |  |  |  |  |  |  |  |  |  |  |  |  |  |  |  |  |  |  |  |  |  |  |  |  |  |  |  |  |  |  |  |  |  |  |  |  |  |  |  |  |  |  |  |  |  |  |  |  |  |  |  |  |  |  |  |  |  |  |  |  |  |  |  |  |  |  |  |  |  |  |  |  |  |  |  |  |  |  |  |  |  |  |  |  |  |  |  |  |  |  |  |  |  |  |  |  |  |  |  |  |  |  |  |  |  |  |  |  |  |  |  |  |  |  |  |  |  |  |  |  |  |  |  |  |  |  |  |  |  |  |  |  |  |  |  |  |  |  |  |  |  |  |  |  |  |  |  |  |  |  |  |  |  |  |  |  |  |  |  |  |  |  |  |  |  |  |  |  |  |  |  |  |  |  |  |  |  |  |  |  |  |  |  |  |  |  |  |  |  |  |  |  |  |  |  |  |  |  |  |  |  |  |  |  |  |  |  |  |  |  |  |  |  |  |  |  |  |  |  |  |  |  |  |  |  |  |  |  |  |  |  |  |  |  |  |  |  |  |  |  |  |  |  |  |  |  |  |  |  |  |  |  |  |  |  |  |  |  |  |  |  |  |  |  |  |  |  |  |  |  |  |  |  |  |  |  |  |  |  |  |  |  |  |  |  |  |  |  |  |  |  |  |  |  |  |  |  |  |  |  |  |  |  |  |  |  |  |  |  |  |  |  |  |  |  |  |  |  |  |  |  |  |  |  |  |  |  |  |  |  |  |  |  |  |  |  |  |  |  |  |  |  |  |  |  |  |  |  |  |  |  |  |  |  |  |  |  |  |  |  |  |  |  |  |  |  |  |  |  |  |  |  |  |  |  |  |  |  |  |  |  |  |  |  |  |  |  |  |  |  |  |  |  |  |  |  |  |  |  |  |  |  |  |  |  |  |  |  |  |  |  |  |  |  |  |  |  |  |  |  |  |  |  |  |  |  |  |  |  |  |  |  |  |  |  |  |  |  |  |  |  |  |  |  |  |  |  |  |  |  |  |  |  |  |  |  |  |  |  |  |  |  |  |  |  |  |  |  |  |  |  |  |  |  |  |  |  |  |  |  |  |  |  |  |  |  |  |  |  |  |  |  |  |  |  |  |  |  |  |  |  |  |  |  |  |  |  |  |  |  |  |  |  |  |  |  |  |  |  |  |  |  |  |  |  |  |  |  |  |  |  |  |  |  |  |  |  |  |  |  |  |  |  |  |  |  |  |  |  |  |  |  |  |  |  |  |  |  |  |  |  |  |  |  |  |  |  |  |  |  |  |  |  |  |  |  |  |  |  |  |  |  |  |  |  |  |  |  |  |  |  |  |  |  |  |  |  |  |  |  |  |  |  |  |  |  |  |  |  |  |  |  |  |  |  |  |  |  |  |  |  |  |  |  |  |  |  |  |  |  |  |  |  |  |  |  |  |  |  |  |  |  |  |  |  |  |  |  |  |  |  |  |  |  |  |  |  |  |  |  |  |  |  |  |  |  |  |  |  |  |  |  |  |  |  |  |  |  |  |  |  |  |  |  |  |  |  |  |  |  |  |  |  |  |  |  |  |  |  |  |  |  |  |  |  |  |  |  |  |  |  |  |  |  |  |  |  |  |  |  |  |  |  |  |  |  |  |  |  |  |  |  |  |  |  |  |  |  |  |  |  |  |  |  |  |  |  |  |  |  |  |  |  |  |  |  |  |  |  |  |  |  |  |  |  |  |  |  |  |  |  |  |  |  |  |  |  |  |  |  |  |  |  |  |  |  |  |  |  |  |  |  |  |  |  |  |  |  |  |  |  |  |  |  |  |  |  |  |  |  |  |  |  |  |  |  |  |  |  |  |  |  |  |  |  |  |  |  |  |  |  |  |  |  |  |  |  |  |  |  |  |  |  |  |  |  |  |  |  |  |  |  |  |  |  |  |  |  |  |  |  |  |  |  |  |  |  |  |  |  |  |  |  |  |  |  |  |  |  |  |  |  |  |  |  |  |  |  |  |  |  |  |  |  |  |  |  |  |  |  |  |  |  |  |  |  |  |  |  |  |  |  |  |  |  |  |  |  |  |  |  |  |  |  |  |  |  |  |  |  |  |  |  |  |  |  |  |  |  |  |  |  |  |  |  |  |  |  |  |  |  |  |  |  |  |  |  |  |  |  |  |  |  |  |  |  |  |  |  |  |  |  |  |  |  |  |  |  |  |  |  |  |  |  |  |  |  |  |  |  |  |  |  |  |  |  |  |  |  |  |  |  |  |  |  |  |  |  |  |  |  |  |  |  |  |  |  |  |  |  |  |  |  |  |  |  |  |  |  |  |  |  |  |  |  |  |  |  |  |  |  |  |  |  |  |  |  |  |  |  |  |  |  |  |  |  |  |  |  |  |  |  |  |  |  |  |  |  |  |  |  |  |  |  |  |  |  |  |  |  |  |  |  |  |  |  |  |  |  |  |  |  |  |  |  |  |  |  |  |  |  |  |  |  |  |  |  |  |  |  |  |  |  |  |  |  |  |  |  |  |  |  |  |  |  |  |  |  |  |  |  |  |  |  |  |  |  |  |  |  |  |  |  |  |  |  |  |  |  |  |  |  |  |  |  |  |  |  |  |  |  |  |  |  |  |  |  |  |  |  |  |  |  |  |  |  |  |  |  |  |  |  |  |  |  |  |  |  |  |  |  |  |  |  |  |  |  |  |  |  |  |  |  |  |  |  |  |  |  |  |  |  |  |  |  |  |  |  |  |  |  |  |  |  |  |  |  |  |  |  |  |  |  |  |  |  |  |  |  |  |  |  |  |  |  |  |  |  |  |  |  |  |  |  |  |  |  |  |  |  |  |  |  |  |  |  |  |  |  |  |  |  |  |  |  |  |  |  |  |  |  |  |  |  |  |  |  |  |  |  |  |  |  |  |  |  |  |  |  |  |  |  |  |  |  |  |  |  |  |  |  |  |  |  |  |  |  |  |  |  |  |  |  |  |  |  |  |  |  |  |  |  |  |  |  |  |  |  |  |  |  |  |  |  |  |  |  |  |  |  |  |  |  |  |  |  |  |  |  |  |  |  |  |  |  |  |  |  |  |  |  |  |  |  |  |  |  |  |  |  |  |  |  |  |  |  |  |  |  |  |  |  |  |  |  |  |  |  |  |  |  |  |  |  |  |  |  |  |  |  |  |  |  |  |  |  |  |  |  |  |  |  |  |  |  |  |  |  |  |  |  |  |  |  |  |  |  |  |  |  |  |  |  |  |  |  |  |  |  |  |  |  |  |  |  |  |  |  |  |  |  |  |  |  |  |  |  |  |  |  |  |  |  |  |  |  |  |  |  |  |  |  |  |  |  |  |  |  |  |  |  |  |  |  |  |  |  |  |  |  |  |  |  |  |  |  |  |  |  |  |  |  |  |  |  |  |  |  |  |  |  |  |  |  |  |  |  |  |  |  |  |  |  |  |  |  |  |  |  |  |  |  |  |  |  |  |  |  |  |  |  |  |  |  |  |  |  |  |  |  |  |  |  |  |  |  |  |  |  |  |  |  |  |  |  |  |  |  |  |  |  |  |  |  |  |  |  |  |  |  |  |  |  |  |  |  |  |  |  |  |  |  |  |  |  |  |  |  |  |  |  |  |  |  |  |  |  |  |  |  |  |  |  |  |  |  |  |  |  |  |  |  |  |  |  |  |  |  |  |  |  |  |  |  |  |  |  |  |  |  |  |  |  |  |  |  |  |  |  |  |  |  |  |  |  |  |  |  |  |  |  |  |  |  |  |  |  |  |  |  |  |  |  |  |  |  |  |  |  |  |  |  |  |  |  |  |  |  |  |  |  |  |  |  |  |  |  |  |  |  |  |  |  |  |  |  |  |  |  |  |  |  |  |  |  |  |  |  |  |  |  |  |  |  |  |  |  |  |  |  |  |  |  |  |  |  |  |  |  |  |  |  |  |  |  |  |  |  |  |  |  |  |  |  |  |  |  |  |  |  |  |  |  |  |  |  |  |  |  |  |  |  |  |  |  |  |  |  |  |  |  |  |  |  |  |  |  |  |  |  |  |  |  |  |  |  |  |  |  |  |  |  |  |  |  |  |  |  |  |  |  |  |  |  |  |  |  |  |  |  |  |  |  |  |  |  |  |  |  |  |  |  |  |  |  |  |  |  |  |  |  |  |  |  |  |  |  |  |  |  |  |  |  |  |  |  |  |  |  |  |  |  |  |  |  |  |  |  |  |  |  |  |  |  |  |  |  |  |  |  |  |  |  |  |  |  |  |  |  |  |  |  |  |  |  |  |  |  |  |  |  |  |  |  |  |  |  |  |  |  |  |  |  |  |  |  |  |  |  |  |  |  |  |  |  |  |  |  |  |  |  |  |  |  |  |  |  |  |  |  |  |  |  |  |  |  |  |  |  |  |  |  |  |  |  |  |  |  |  |  |  |  |  |  |  |  |  |  |  |  |  |  |  |  |  |  |  |  |  |  |  |  |  |  |  |  |  |  |  |  |  |  |  |  |  |  |  |  |  |  |  |  |  |  |  |  |  |  |  |  |  |  |  |  |  |  |  |  |  |  |  |  |  |  |  |  |  |  |  |  |  |  |  |  |  |  |  |  |  |  |  |  |  |  |  |  |  |  |  |  |  |  |  |  |  |  |  |  |  |  |  |  |  |  |  |  |  |  |  |  |  |  |  |  |  |  |  |  |  |  |  |  |  |  |  |  |  |  |  |  |  |  |  |  |  |  |  |  |  |  |  |  |  |  |  |  |  |  |  |  |  |  |  |  |  |  |  |  |  |  |  |  |  |  |  |  |  |  |  |  |  |  |  |  |  |  |  |  |  |  |  |  |  |  |  |  |  |  |  |  |  |  |  |  |  |  |  |  |  |  |  |  |  |  |  |  |  |  |  |  |  |  |  |  |  |  |  |  |  |  |  |  |  |  |  |  |  |  |  |  |  |  |  |  |  |  |  |  |  |  |  |  |  |  |  |  |  |  |  |  |  |  |  |  |  |  |  |  |  |  |  |  |  |  |  |  |  |  |  |  |  |  |  |  |  |  |  |  |  |  |  |  |  |  |  |  |  |  |  |  |  |  |  |  |  |  |  |  |  |  |  |  |  |  |  |  |  |  |  |  |  |  |  |  |  |  |  |  |  |  |  |  |  |  |  |  |  |  |  |  |  |  |  |  |  |  |  |  |  |  |  |  |  |  |  |  |  |  |  |  |  |  |  |  |  |  |  |  |  |  |  |  |  |  |  |  |  |  |  |  |  |  |  |  |  |  |  |  |  |  |  |  |  |  |  |  |  |  |  |  |  |  |  |  |  |  |  |  |  |  |  |  |  |  |  |  |  |  |  |  |  |  |  |  |  |  |  |  |  |  |  |  |  |  |  |  |  |  |  |  |  |  |  |  |  |  |  |  |  |  |  |  |  |  |  |  |  |  |  |  |  |  |  |  |  |  |  |  |  |  |  |  |  |  |  |  |  |  |  |  |  |  |  |  |  |  |  |  |  |  |  |  |  |  |  |  |  |  |  |  |  |  |  |  |  |  |  |  |  |  |  |  |  |  |  |  |  |  |  |  |  |  |  |  |  |  |  |  |  |  |  |  |  |  |  |  |  |  |  |  |  |  |  |  |  |  |  |  |  |  |  |  |  |  |  |  |  |  |  |  |  |  |  |  |  |  |  |  |  |  |  |  |  |  |  |  |  |  |  |  |  |  |  |  |  |  |  |  |  |  |  |  |  |  |  |  |  |  |  |  |  |  |  |  |  |  |  |  |  |  |  |  |  |  |  |  |  |  |  |  |  |  |  |  |  |  |  |  |  |  |  |  |  |  |  |  |  |  |  |  |  |  |  |  |  |  |  |  |  |  |  |  |  |  |  |  |  |  |  |  |  |  |  |  |  |  |  |  |  |  |  |  |  |  |  |  |  |  |  |  |  |  |  |  |  |  |  |  |  |  |  |  |  |  |  |  |  |  |  |  |  |  |  |  |  |  |  |  |  |  |  |  |  |  |  |  |  |  |  |  |  |  |  |  |  |  |  |  |  |  |  |  |  |  |  |  |  |  |  |  |  |  |  |  |  |  |  |  |  |  |  |  |  |  |  |  |  |  |  |  |  |  |  |  |  |  |  |  |  |  |  |  |  |  |  |  |  |  |  |  |  |  |  |  |  |  |  |  |  |  |  |  |  |  |  |  |  |  |  |  |  |  |  |  |  |  |  |  |  |  |  |  |  |  |  |  |  |  |  |  |  |  |  |  |  |  |  |  |  |  |  |  |  |  |  |  |  |  |  |  |  |  |  |  |  |  |  |  |  |  |  |  |  |  |  |  |  |  |  |  |  |  |  |  |  |  |  |  |  |  |  |  |  |  |  |  |  |  |  |  |  |  |  |  |  |  |  |  |  |  |  |  |  |  |  |  |  |  |  |  |  |  |  |  |  |  |  |  |  |  |  |  |  |  |  |  |  |  |  |  |  |  |  |  |  |  |  |  |  |  |  |  |  |  |  |  |  |  |  |  |  |  |  |  |  |  |  |  |  |  |  |  |  |  |  |  |  |  |  |  |  |  |  |  |  |  |  |  |  |  |  |  |  |  |  |  |  |  |  |  |  |  |  |  |  |  |  |  |  |  |  |  |  |  |  |  |  |  |  |  |  |  |  |  |  |  |  |  |  |  |  |  |  |  |  |  |  |  |  |  |  |  |  |  |  |  |  |  |  |  |  |  |  |  |  |  |  |  |  |  |  |  |  |  |  |  |  |  |  |  |  |  |  |  |  |  |  |  |  |  |  |  |  |  |  |  |  |  |  |  |  |  |  |  |  |  |  |  |  |  |  |  |  |  |  |  |  |  |  |  |  |  |  |  |  |  |  |  |  |  |  |  |  |  |  |  |  |  |  |  |  |  |  |  |  |  |  |  |  |  |  |  |  |  |  |  |  |  |  |  |  |  |  |  |  |  |  |  |  |  |  |  |  |  |  |  |  |  |  |  |  |  |  |  |  |  |  |  |  |  |  |  |  |  |  |  |  |  |  |  |  |  |  |  |  |  |  |  |  |  |  |  |  |  |  |  |  |  |  |  |  |  |  |  |  |  |  |  |  |  |  |  |  |  |  |  |  |  |  |  |  |  |  |  |  |  |  |  |  |  |  |  |  |  |  |  |  |  |  |  |  |  |  |  |  |  |  |  |  |  |  |  |  |  |  |  |  |  |  |  |  |  |  |  |  |  |  |  |  |  |  |  |  |  |  |  |  |  |  |  |  |  |  |  |  |  |  |  |  |  |  |  |  |  |  |  |  |  |  |  |  |  |  |  |  |  |  |  |  |  |  |  |  |  |  |  |  |  |  |  |  |  |  |  |  |  |  |  |  |  |  |  |  |  |  |  |  |  |  |  |  |  |  |  |  |  |  |  |  |  |  |  |  |  |  |  |  |  |  |  |  |  |  |  |  |  |  |  |  |  |  |  |  |  |  |  |  |  |  |  |  |  |  |  |  |  |  |  |  |  |  |  |  |  |  |  |  |  |  |  |  |  |  |  |  |  |  |  |  |  |  |  |  |  |  |  |  |  |  |  |  |  |  |  |  |  |  |  |  |  |  |  |  |  |  |  |  |  |  |  |  |  |  |  |  |  |  |  |  |  |  |  |  |  |  |  |  |  |  |  |  |  |  |  |  |  |  |  |  |  |  |  |  |  |  |  |  |  |  |  |  |  |  |  |  |  |  |  |  |  |  |  |  |  |  |  |  |  |  |  |  |  |  |  |  |  |  |  |  |  |  |  |  |  |  |  |  |  |  |  |  |  |  |  |  |  |  |  |  |  |  |  |  |  |  |  |  |  |  |  |  |  |  |  |  |  |  |  |  |  |  |  |  |  |  |  |  |  |  |  |  |  |  |  |  |  |  |  |  |  |  |  |  |  |  |  |  |  |  |  |  |  |  |  |  |  |  |  |  |  |  |  |  |  |  |  |  |  |  |  |  |  |  |  |  |  |  |  |  |  |  |  |  |  |  |  |  |  |  |  |  |  |  |  |  |  |  |  |  |  |  |  |  |  |  |  |  |  |  |  |  |  |  |  |  |  |  |  |  |  |  |  |  |  |  |  |  |  |  |  |  |  |  |  |  |  |  |  |  |  |  |  |  |  |  |  |  |  |  |  |  |  |  |  |  |  |  |  |  |  |  |  |  |  |  |  |  |  |  |  |  |  |  |  |  |  |  |  |  |  |  |  |  |  |  |  |  |  |  |  |  |  |  |  |  |  |  |  |  |  |  |  |  |  |  |  |  |  |  |  |  |  |  |  |  |  |  |  |  |  |  |  |  |  |  |  |  |  |  |  |  |  |  |  |  |  |  |  |  |  |  |  |  |  |  |  |  |  |  |  |  |  |  |  |  |  |  |  |  |  |  |  |  |  |  |  |  |  |  |  |  |  |  |  |  |  |  |  |  |  |  |  |  |  |  |  |  |  |  |  |  |  |  |  |  |  |  |  |  |  |  |  |  |  |  |  |  |  |  |  |  |  |  |  |  |  |  |  |  |  |  |  |  |  |  |  |  |  |  |  |  |  |  |  |  |  |  |  |  |  |  |  |  |  |  |  |  |  |  |  |  |  |  |  |  |  |  |  |  |  |  |  |  |  |  |  |  |  |  |  |  |  |  |  |  |  |  |  |  |  |  |  |  |  |  |  |  |  |  |  |  |  |  |  |  |  |  |  |  |  |  |  |  |  |  |  |  |  |  |  |  |  |  |  |  |  |  |  |  |  |  |  |  |  |  |  |  |  |  |  |  |  |  |  |  |  |  |  |  |  |  |  |  |  |  |  |  |  |  |  |  |  |  |  |  |  |  |  |  |  |  |  |  |  |  |  |  |  |  |  |  |  |  |  |  |  |  |  |  |  |  |  |  |  |  |  |  |  |  |  |  |  |  |  |  |  |  |  |  |  |  |  |  |  |  |  |  |  |  |  |  |  |  |  |  |  |  |  |  |  |  |  |  |  |  |  |  |  |  |  |  |  |  |  |  |  |  |  |  |  |  |  |  |  |  |  |  |  |  |  |  |  |  |  |  |  |  |  |  |  |  |  |  |  |  |  |  |  |  |  |  |  |  |  |  |  |  |  |  |  |  |  |  |  |  |  |  |  |  |  |  |  |  |  |  |  |  |  |  |  |  |  |  |  |  |  |  |  |  |  |  |  |  |  |  |  |  |  |  |  |  |  |  |  |  |  |  |  |  |  |  |  |  |  |  |  |  |  |  |  |  |  |  |  |  |  |  |  |  |  |  |  |  |  |  |  |  |  |  |  |  |  |  |  |  |  |  |  |  |  |  |  |  |  |  |  |  |  |  |  |  |  |  |  |  |  |  |  |  |  |  |  |  |  |  |  |  |  |  |  |  |  |  |  |  |  |  |  |  |  |  |  |  |  |  |  |  |  |  |  |  |  |  |  |  |  |  |  |  |  |  |  |  |  |  |  |  |  |  |  |  |  |  |  |  |  |  |  |  |  |  |  |  |  |  |  |  |  |  |  |  |  |  |  |  |  |  |  |  |  |  |  |  |  |  |  |  |  |  |  |  |  |  |  |  |  |  |  |  |  |  |  |  |  |  |  |  |  |  |  |  |  |  |  |  |  |  |  |  |  |  |  |  |  |  |  |  |  |  |  |  |  |  |  |  |  |  |  |  |  |  |  |  |  |  |  |  |  |  |  |  |  |  |  |  |  |  |  |  |  |  |  |  |  |  |  |  |  |  |  |  |  |  |  |  |  |  |  |  |  |  |  |  |  |  |  |  |  |  |  |  |  |  |  |  |  |  |  |  |  |  |  |  |  |  |  |  |  |  |  |  |  |  |  |  |  |  |  |  |  |  |  |  |  |  |  |  |  |  |  |  |  |  |  |  |  |  |  |  |  |  |  |  |  |  |  |  |  |  |  |  |  |  |  |  |  |  |  |  |  |  |  |  |  |  |  |  |  |  |  |  |  |  |  |  |  |  |  |  |  |  |  |  |  |  |  |  |  |  |  |  |  |  |  |  |  |  |  |  |  |  |  |  |  |  |  |  |  |  |  |  |  |  |  |  |  |  |  |  |  |  |  |  |  |  |  |  |  |  |  |  |  |  |  |  |  |  |  |  |  |  |  |  |  |  |  |  |  |  |  |  |  |  |  |  |  |  |  |  |  |  |  |  |  |  |  |  |  |  |  |  |  |  |  |  |  |  |  |  |  |  |  |  |  |  |  |  |  |  |  |  |  |  |  |  |  |  |  |  |  |  |  |  |  |  |  |  |  |  |  |  |  |  |  |  |  |  |  |  |  |  |  |  |  |  |  |  |  |  |  |  |  |  |  |  |  |  |  |  |  |  |  |  |  |  |  |  |  |  |  |  |  |  |  |  |  |  |  |  |  |  |  |  |  |  |  |  |  |  |  |  |  |  |  |  |  |  |  |  |  |  |  |  |  |  |  |  |  |  |  |  |  |  |  |  |  |  |  |  |  |  |  |  |  |  |  |  |  |  |  |  |  |  |  |  |  |  |  |  |  |  |  |  |  |  |  |  |  |  |  |  |  |  |  |  |  |  |  |  |  |  |  |  |  |  |  |  |  |  |  |  |  |  |  |  |  |  |  |  |  |  |  |  |  |  |  |  |  |  |  |  |  |  |  |  |  |  |  |  |  |  |  |  |  |  |  |  |  |  |  |  |  |  |  |  |  |  |  |  |  |  |  |  |  |  |  |  |  |  |  |  |  |  |  |  |  |  |  |  |  |  |  |  |  |  |  |  |  |  |  |  |  |  |  |  |  |  |  |  |  |  |  |  |  |  |  |  |  |  |  |  |  |  |  |  |  |  |  |  |  |  |  |  |  |  |  |  |  |  |  |  |  |  |  |  |  |  |  |  |  |  |  |  |  |  |  |  |  |  |  |  |  |  |  |  |  |  |  |  |  |  |  |  |  |  |  |  |  |  |  |  |  |  |  |  |  |  |  |  |  |  |  |  |  |  |  |  |  |  |  |  |  |  |  |  |  |  |  |  |  |  |  |  |  |  |  |  |  |  |  |  |  |  |  |  |  |  |  |  |  |  |  |  |  |  |  |  |  |  |  |  |  |  |  |  |  |  |  |  |  |  |  |  |  |  |  |  |  |  |  |  |  |  |  |  |  |  |  |  |  |  |  |  |  |  |  |  |  |  |  |  |  |  |  |  |  |  |  |  |  |  |  |  |  |  |  |  |  |  |  |  |  |  |  |  |  |  |  |  |  |  |  |  |  |  |  |  |  |  |  |  |  |  |  |  |  |  |  |  |  |  |  |  |  |  |  |  |  |  |  |  |  |  |  |  |  |  |  |  |  |  |  |  |  |  |  |  |  |  |  |  |  |  |  |  |  |  |  |  |  |  |  |  |  |  |  |  |  |  |  |  |  |  |  |  |  |  |  |  |  |  |  |  |  |  |  |  |  |  |  |  |  |  |  |  |  |  |  |  |  |  |  |  |  |  |  |  |  |  |  |  |  |  |  |  |  |  |  |  |  |  |  |  |  |  |  |  |  |  |  |  |  |  |  |  |  |  |  |  |  |  |  |  |  |  |  |  |  |  |  |  |  |  |  |  |  |  |  |  |  |  |  |  |  |  |  |  |  |  |  |  |  |  |  |  |  |  |  |  |  |  |  |  |  |  |  |  |  |  |  |  |  |  |  |  |  |  |  |  |  |  |  |  |  |  |  |  |  |  |  |  |  |  |  |  |  |  |  |  |  |  |  |  |  |  |  |  |  |  |  |  |  |  |  |  |  |  |  |  |  |  |  |  |  |  |  |  |  |  |  |  |  |  |  |  |  |  |  |  |  |  |  |  |  |  |  |  |  |  |  |  |  |  |  |  |  |  |  |  |  |  |  |  |  |  |  |  |  |  |  |  |  |  |  |  |  |  |  |  |  |  |  |  |  |  |  |  |  |  |  |  |  |  |  |  |  |  |  |  |  |  |  |  |  |  |  |  |  |  |  |  |  |  |  |  |  |  |  |  |  |  |  |  |  |  |  |  |  |  |  |  |  |  |  |  |  |  |  |  |  |  |  |  |  |  |  |  |  |  |  |  |  |  |  |  |  |  |  |  |  |  |  |  |  |  |  |  |  |  |  |  |  |  |  |  |  |  |  |  |  |  |  |  |  |  |  |  |  |  |  |  |  |  |  |  |  |  |  |  |  |  |  |  |  |  |  |  |  |  |  |  |  |  |  |  |  |  |  |  |  |  |  |  |  |  |  |  |  |  |  |  |  |  |  |  |  |  |  |  |  |  |  |  |  |  |  |  |  |  |  |  |  |  |  |  |  |  |  |  |  |  |  |  |  |  |  |  |  |  |  |  |  |  |  |  |  |  |  |  |  |  |  |  |  |  |  |  |  |  |  |  |  |  |  |  |  |  |  |  |  |  |  |  |  |  |  |  |  |  |  |  |  |  |  |  |  |  |  |  |  |  |  |  |  |  |  |  |  |  |  |  |  |  |  |  |  |  |  |  |  |  |  |  |  |  |  |  |  |  |  |  |  |  |  |  |  |  |  |  |  |  |  |  |  |  |  |  |  |  |  |  |  |  |  |  |  |  |  |  |  |  |  |  |  |  |  |  |  |  |  |  |  |  |  |  |  |  |  |  |  |  |  |  |  |  |  |  |  |  |  |  |  |  |  |  |  |  |  |  |  |  |  |  |  |  |  |  |  |  |  |  |  |  |  |  |  |  |  |  |  |  |  |  |  |  |  |  |  |  |  |  |  |  |  |  |  |  |  |  |  |  |  |  |  |  |  |  |  |  |  |  |  |  |  |  |  |  |  |  |  |  |  |  |  |  |  |  |  |  |  |  |  |  |  |  |  |  |  |  |  |  |  |  |  |  |  |  |  |  |  |  |  |  |  |  |  |  |  |  |  |  |  |  |  |  |  |  |  |  |  |  |  |  |  |  |  |  |  |  |  |  |  |  |  |  |  |  |  |  |  |  |  |  |  |  |  |  |  |  |  |  |  |  |  |  |  |  |  |  |  |  |  |  |  |  |  |  |  |  |  |  |  |  |  |  |  |  |  |  |  |  |  |  |  |  |  |  |  |  |  |  |  |  |  |  |  |  |  |  |  |  |  |  |  |  |  |  |  |  |  |  |  |  |  |  |  |  |  |  |  |  |  |  |  |  |  |  |  |  |  |  |  |  |  |  |  |  |  |  |  |  |  |  |  |  |  |  |  |  |  |  |  |  |  |  |  |  |  |  |  |  |  |  |  |  |  |  |  |  |  |  |  |  |  |  |  |  |  |  |  |  |  |  |  |  |  |  |  |  |  |  |  |  |  |  |  |  |  |  |  |  |  |  |  |  |  |  |  |  |  |  |  |  |  |  |  |  |  |  |  |  |  |  |  |  |  |  |  |  |  |  |  |  |  |  |  |  |  |  |  |  |  |  |  |  |  |  |  |  |  |  |  |  |  |  |  |  |  |  |  |  |  |  |  |  |  |  |  |  |  |  |  |  |  |  |  |  |  |  |  |  |  |  |  |  |  |  |  |  |  |  |  |  |  |  |  |  |  |  |  |  |  |  |  |  |  |  |  |  |  |  |  |  |  |  |  |  |  |  |  |  |  |  |  |  |  |  |  |  |  |  |  |  |  |  |  |  |  |  |  |  |  |  |  |  |  |  |  |  |  |  |  |  |  |  |  |  |  |  |  |  |  |  |  |  |  |  |  |  |  |  |  |  |  |  |  |  |  |  |  |  |  |  |  |  |  |  |  |  |  |  |  |  |  |  |  |  |  |  |  |  |  |  |  |  |  |  |  |  |  |  |  |  |  |  |  |  |  |  |  |  |  |  |  |  |  |  |  |  |  |  |  |  |  |  |  |  |  |  |  |  |  |  |  |  |  |  |  |  |  |  |  |  |  |  |  |  |  |  |  |  |  |  |  |  |  |  |  |  |  |  |  |  |  |  |  |  |  |  |  |  |  |  |  |  |  |  |  |  |  |  |  |  |  |  |  |  |  |  |  |  |  |  |  |  |  |  |  |  |  |  |  |  |  |  |  |  |  |  |  |  |  |  |  |  |  |  |  |  |  |  |  |  |  |  |  |  |  |  |  |  |  |  |  |  |  |  |  |  |  |  |  |  |  |  |  |  |  |  |  |  |  |  |  |  |  |  |  |  |  |  |  |  |  |  |  |  |  |  |  |  |  |  |  |  |  |  |  |  |  |  |  |  |  |  |  |  |  |  |  |  |  |  |  |  |  |  |  |  |  |  |  |  |  |  |  |  |  |  |  |  |  |  |  |  |  |  |  |  |  |  |  |  |  |  |  |  |  |  |  |  |  |  |  |  |  |  |  |  |  |  |  |  |  |  |  |  |  |  |  |  |  |  |  |  |  |  |  |  |  |  |  |  |  |  |  |  |  |  |  |  |  |  |  |  |  |  |  |  |  |  |  |  |  |  |  |  |  |  |  |  |  |  |  |  |  |  |  |  |  |  |  |  |  |  |  |  |  |  |  |  |  |  |  |  |  |  |  |  |  |  |  |  |  |  |  |  |  |  |  |  |  |  |  |  |  |  |  |  |  |  |  |  |  |  |  |  |  |  |  |  |  |  |  |  |  |  |  |  |  |  |  |  |  |  |  |  |  |  |  |  |  |  |  |  |  |  |  |  |  |  |  |  |  |  |  |  |  |  |  |  |  |  |  |  |  |  |  |  |  |  |  |  |  |  |  |  |  |  |  |  |  |  |  |  |  |  |  |  |  |  |  |  |  |  |  |  |  |  |  |  |  |  |  |  |  |  |  |  |  |  |  |  |  |  |  |  |  |  |  |  |  |  |  |  |  |  |  |  |  |  |  |  |  |  |  |  |  |  |  |  |  |  |  |  |  |  |  |  |  |  |  |  |  |  |  |  |  |  |  |  |  |  |  |  |  |  |  |  |  |  |  |  |  |  |  |  |  |  |  |  |  |  |  |  |  |  |  |  |  |  |  |  |  |  |  |  |  |  |  |  |  |  |  |  |  |  |  |  |  |  |  |  |  |  |  |  |  |  |  |  |  |  |  |  |  |  |  |  |  |  |  |  |  |  |  |  |  |  |  |  |  |  |  |  |  |  |  |  |  |  |  |  |  |  |  |  |  |  |  |  |  |  |  |  |  |  |  |  |  |  |  |  |  |  |  |  |  |  |  |  |  |  |  |  |  |  |  |  |  |  |  |  |  |  |  |  |  |  |  |  |  |  |  |  |  |  |  |  |  |  |  |  |  |  |  |  |  |  |  |  |  |  |  |  |  |  |  |  |  |  |  |  |  |  |  |  |  |  |  |  |  |  |  |  |  |  |  |  |  |  |  |  |  |  |  |  |  |  |  |  |  |  |  |  |  |  |  |  |  |  |  |  |  |  |  |  |  |  |  |  |  |  |  |  |  |  |  |  |  |  |  |  |  |  |  |  |  |  |  |  |  |  |  |  |  |  |  |  |  |  |  |  |  |  |  |  |  |  |  |  |  |  |  |  |  |  |  |  |  |  |  |  |  |  |  |  |  |  |  |  |  |  |  |  |  |  |  |  |  |  |  |  |  |  |  |  |  |  |  |  |  |  |  |  |  |  |  |  |  |  |  |  |  |  |  |  |  |  |  |  |  |  |  |  |  |  |  |  |  |  |  |  |  |  |  |  |  |  |  |  |  |  |  |  |  |  |  |  |  |  |  |  |  |  |  |  |  |  |  |  |  |  |  |  |  |  |  |  |  |  |  |  |  |  |  |  |  |  |  |  |  |  |  |  |  |  |  |  |  |  |  |  |  |  |  |  |  |  |  |  |  |  |  |  |  |  |  |  |  |  |  |  |  |  |  |  |  |  |  |  |  |  |  |  |  |  |  |  |  |  |  |  |  |  |  |  |  |  |  |  |  |  |  |  |  |  |  |  |  |  |  |  |  |  |  |  |  |  |  |  |  |  |  |  |  |  |  |  |  |  |  |  |  |  |  |  |  |  |  |  |  |  |  |  |  |  |  |  |  |  |  |  |  |  |  |  |  |  |  |  |  |  |  |  |  |  |  |  |  |  |  |  |  |  |  |  |  |  |  |  |  |  |  |  |  |  |  |  |  |  |  |  |  |  |  |  |  |  |  |  |  |  |  |  |  |  |  |  |  |  |  |  |  |  |  |  |  |  |  |  |  |  |  |  |  |  |  |  |  |  |  |  |  |  |  |  |  |  |  |  |  |  |  |  |  |  |  |  |  |  |  |  |  |  |  |  |  |  |  |  |  |  |  |  |  |  |  |  |  |  |  |  |  |  |  |  |  |  |  |  |  |  |  |  |  |  |  |  |  |  |  |  |  |  |  |  |  |  |  |  |  |  |  |  |  |  |  |  |  |  |  |  |  |  |  |  |  |  |  |  |  |  |  |  |  |  |  |  |  |  |  |  |  |  |  |  |  |  |  |  |  |  |  |  |  |  |  |  |  |  |  |  |  |  |  |  |  |  |  |  |  |  |  |  |  |  |  |  |  |  |  |  |  |  |  |  |  |  |  |  |  |  |  |  |  |  |
| --- | --- | --- | --- | --- | --- | --- | --- | --- | --- | --- | --- | --- | --- | --- | --- | --- | --- | --- | --- | --- | --- | --- | --- | --- | --- | --- | --- | --- | --- | --- | --- | --- | --- | --- | --- | --- | --- | --- | --- | --- | --- | --- | --- | --- | --- | --- | --- | --- | --- | --- | --- | --- | --- | --- | --- | --- | --- | --- | --- | --- | --- | --- | --- | --- | --- | --- | --- | --- | --- | --- | --- | --- | --- | --- | --- | --- | --- | --- | --- | --- | --- | --- | --- | --- | --- | --- | --- | --- | --- | --- | --- | --- | --- | --- | --- | --- | --- | --- | --- | --- | --- | --- | --- | --- | --- | --- | --- | --- | --- | --- | --- | --- | --- | --- | --- | --- | --- | --- | --- | --- | --- | --- | --- | --- | --- | --- | --- | --- | --- | --- | --- | --- | --- | --- | --- | --- | --- | --- | --- | --- | --- | --- | --- | --- | --- | --- | --- | --- | --- | --- | --- | --- | --- | --- | --- | --- | --- | --- | --- | --- | --- | --- | --- | --- | --- | --- | --- | --- | --- | --- | --- | --- | --- | --- | --- | --- | --- | --- | --- | --- | --- | --- | --- | --- | --- | --- | --- | --- | --- | --- | --- | --- | --- | --- | --- | --- | --- | --- | --- | --- | --- | --- | --- | --- | --- | --- | --- | --- | --- | --- | --- | --- | --- | --- | --- | --- | --- | --- | --- | --- | --- | --- | --- | --- | --- | --- | --- | --- | --- | --- | --- | --- | --- | --- | --- | --- | --- | --- | --- | --- | --- | --- | --- | --- | --- | --- | --- | --- | --- | --- | --- | --- | --- | --- | --- | --- | --- | --- | --- | --- | --- | --- | --- | --- | --- | --- | --- | --- | --- | --- | --- | --- | --- | --- | --- | --- | --- | --- | --- | --- | --- | --- | --- | --- | --- | --- | --- | --- | --- | --- | --- | --- | --- | --- | --- | --- | --- | --- | --- | --- | --- | --- | --- | --- | --- | --- | --- | --- | --- | --- | --- | --- | --- | --- | --- | --- | --- | --- | --- | --- | --- | --- | --- | --- | --- | --- | --- | --- | --- | --- | --- | --- | --- | --- | --- | --- | --- | --- | --- | --- | --- | --- | --- | --- | --- | --- | --- | --- | --- | --- | --- | --- | --- | --- | --- | --- | --- | --- | --- | --- | --- | --- | --- | --- | --- | --- | --- | --- | --- | --- | --- | --- | --- | --- | --- | --- | --- | --- | --- | --- | --- | --- | --- | --- | --- | --- | --- | --- | --- | --- | --- | --- | --- | --- | --- | --- | --- | --- | --- | --- | --- | --- | --- | --- | --- | --- | --- | --- | --- | --- | --- | --- | --- | --- | --- | --- | --- | --- | --- | --- | --- | --- | --- | --- | --- | --- | --- | --- | --- | --- | --- | --- | --- | --- | --- | --- | --- | --- | --- | --- | --- | --- | --- | --- | --- | --- | --- | --- | --- | --- | --- | --- | --- | --- | --- | --- | --- | --- | --- | --- | --- | --- | --- | --- | --- | --- | --- | --- | --- | --- | --- | --- | --- | --- | --- | --- | --- | --- | --- | --- | --- | --- | --- | --- | --- | --- | --- | --- | --- | --- | --- | --- | --- | --- | --- | --- | --- | --- | --- | --- | --- | --- | --- | --- | --- | --- | --- | --- | --- | --- | --- | --- | --- | --- | --- | --- | --- | --- | --- | --- | --- | --- | --- | --- | --- | --- | --- | --- | --- | --- | --- | --- | --- | --- | --- | --- | --- | --- | --- | --- | --- | --- | --- | --- | --- | --- | --- | --- | --- | --- | --- | --- | --- | --- | --- | --- | --- | --- | --- | --- | --- | --- | --- | --- | --- | --- | --- | --- | --- | --- | --- | --- | --- | --- | --- | --- | --- | --- | --- | --- | --- | --- | --- | --- | --- | --- | --- | --- | --- | --- | --- | --- | --- | --- | --- | --- | --- | --- | --- | --- | --- | --- | --- | --- | --- | --- | --- | --- | --- | --- | --- | --- | --- | --- | --- | --- | --- | --- | --- | --- | --- | --- | --- | --- | --- | --- | --- | --- | --- | --- | --- | --- | --- | --- | --- | --- | --- | --- | --- | --- | --- | --- | --- | --- | --- | --- | --- | --- | --- | --- | --- | --- | --- | --- | --- | --- | --- | --- | --- | --- | --- | --- | --- | --- | --- | --- | --- | --- | --- | --- | --- | --- | --- | --- | --- | --- | --- | --- | --- | --- | --- | --- | --- | --- | --- | --- | --- | --- | --- | --- | --- | --- | --- | --- | --- | --- | --- | --- | --- | --- | --- | --- | --- | --- | --- | --- | --- | --- | --- | --- | --- | --- | --- | --- | --- | --- | --- | --- | --- | --- | --- | --- | --- | --- | --- | --- | --- | --- | --- | --- | --- | --- | --- | --- | --- | --- | --- | --- | --- | --- | --- | --- | --- | --- | --- | --- | --- | --- | --- | --- | --- | --- | --- | --- | --- | --- | --- | --- | --- | --- | --- | --- | --- | --- | --- | --- | --- | --- | --- | --- | --- | --- | --- | --- | --- | --- | --- | --- | --- | --- | --- | --- | --- | --- | --- | --- | --- | --- | --- | --- | --- | --- | --- | --- | --- | --- | --- | --- | --- | --- | --- | --- | --- | --- | --- | --- | --- | --- | --- | --- | --- | --- | --- | --- | --- | --- | --- | --- | --- | --- | --- | --- | --- | --- | --- | --- | --- | --- | --- | --- | --- | --- | --- | --- | --- | --- | --- | --- | --- | --- | --- | --- | --- | --- | --- | --- | --- | --- | --- | --- | --- | --- | --- | --- | --- | --- | --- | --- | --- | --- | --- | --- | --- | --- | --- | --- | --- | --- | --- | --- | --- | --- | --- | --- | --- | --- | --- | --- | --- | --- | --- | --- | --- | --- | --- | --- | --- | --- | --- | --- | --- | --- | --- | --- | --- | --- | --- | --- | --- | --- | --- | --- | --- | --- | --- | --- | --- | --- | --- | --- | --- | --- | --- | --- | --- | --- | --- | --- | --- | --- | --- | --- | --- | --- | --- | --- | --- | --- | --- | --- | --- | --- | --- | --- | --- | --- | --- | --- | --- | --- | --- | --- | --- | --- | --- | --- | --- | --- | --- | --- | --- | --- | --- | --- | --- | --- | --- | --- | --- | --- | --- | --- | --- | --- | --- | --- | --- | --- | --- | --- | --- | --- | --- | --- | --- | --- | --- | --- | --- | --- | --- | --- | --- | --- | --- | --- | --- | --- | --- | --- | --- | --- | --- | --- | --- | --- | --- | --- | --- | --- | --- | --- | --- | --- | --- | --- | --- | --- | --- | --- | --- | --- | --- | --- | --- | --- | --- | --- | --- | --- | --- | --- | --- | --- | --- | --- | --- | --- | --- | --- | --- | --- | --- | --- | --- | --- | --- | --- | --- | --- | --- | --- | --- | --- | --- | --- | --- | --- | --- | --- | --- | --- | --- | --- | --- | --- | --- | --- | --- | --- | --- | --- | --- | --- | --- | --- | --- | --- | --- | --- | --- | --- | --- | --- | --- | --- | --- | --- | --- | --- | --- | --- | --- | --- | --- | --- | --- | --- | --- | --- | --- | --- | --- | --- | --- | --- | --- | --- | --- | --- | --- | --- | --- | --- | --- | --- | --- | --- | --- | --- | --- | --- | --- | --- | --- | --- | --- | --- | --- | --- | --- | --- | --- | --- | --- | --- | --- | --- | --- | --- | --- | --- | --- | --- | --- | --- | --- | --- | --- | --- | --- | --- | --- | --- | --- | --- | --- | --- | --- | --- | --- | --- | --- | --- | --- | --- | --- | --- | --- | --- | --- | --- | --- | --- | --- | --- | --- | --- | --- | --- | --- | --- | --- | --- | --- | --- | --- | --- | --- | --- | --- | --- | --- | --- | --- | --- | --- | --- | --- | --- | --- | --- | --- | --- | --- | --- | --- | --- | --- | --- | --- | --- | --- | --- | --- | --- | --- | --- | --- | --- | --- | --- | --- | --- | --- | --- | --- | --- | --- | --- | --- | --- | --- | --- | --- | --- | --- | --- | --- | --- | --- | --- | --- | --- | --- | --- | --- | --- | --- | --- | --- | --- | --- | --- | --- | --- | --- | --- | --- | --- | --- | --- | --- | --- | --- | --- | --- | --- | --- | --- | --- | --- | --- | --- | --- | --- | --- | --- | --- | --- | --- | --- | --- | --- | --- | --- | --- | --- | --- | --- | --- | --- | --- | --- | --- | --- | --- | --- | --- | --- | --- | --- | --- | --- | --- | --- | --- | --- | --- | --- | --- | --- | --- | --- | --- | --- | --- | --- | --- | --- | --- | --- | --- | --- | --- | --- | --- | --- | --- | --- | --- | --- | --- | --- | --- | --- | --- | --- | --- | --- | --- | --- | --- | --- | --- | --- | --- | --- | --- | --- | --- | --- | --- | --- | --- | --- | --- | --- | --- | --- | --- | --- | --- | --- | --- | --- | --- | --- | --- | --- | --- | --- | --- | --- | --- | --- | --- | --- | --- | --- | --- | --- | --- | --- | --- | --- | --- | --- | --- | --- | --- | --- | --- | --- | --- | --- | --- | --- | --- | --- | --- | --- | --- | --- | --- | --- | --- | --- | --- | --- | --- | --- | --- | --- | --- | --- | --- | --- | --- | --- | --- | --- | --- | --- | --- | --- | --- | --- | --- | --- | --- | --- | --- | --- | --- | --- | --- | --- | --- | --- | --- | --- | --- | --- | --- | --- | --- | --- | --- | --- | --- | --- | --- | --- | --- | --- | --- | --- | --- | --- | --- | --- | --- | --- | --- | --- | --- | --- | --- | --- | --- | --- | --- | --- | --- | --- | --- | --- | --- | --- | --- | --- | --- | --- | --- | --- | --- | --- | --- | --- | --- | --- | --- | --- | --- | --- | --- | --- | --- | --- | --- | --- | --- | --- | --- | --- | --- | --- | --- | --- | --- | --- | --- | --- | --- | --- | --- | --- | --- | --- | --- | --- | --- | --- | --- | --- | --- | --- | --- | --- | --- | --- | --- | --- | --- | --- | --- | --- | --- | --- | --- | --- | --- | --- | --- | --- | --- | --- | --- | --- | --- | --- | --- | --- | --- | --- | --- | --- | --- | --- | --- | --- | --- | --- | --- | --- | --- | --- | --- | --- | --- | --- | --- | --- | --- | --- | --- | --- | --- | --- | --- | --- | --- | --- | --- | --- | --- | --- | --- | --- | --- | --- | --- | --- | --- | --- | --- | --- | --- | --- | --- | --- | --- | --- | --- | --- | --- | --- | --- | --- | --- | --- | --- | --- | --- | --- | --- | --- | --- | --- | --- | --- | --- | --- | --- | --- | --- | --- | --- | --- | --- | --- | --- | --- | --- | --- | --- | --- | --- | --- | --- | --- | --- | --- | --- | --- | --- | --- | --- | --- | --- | --- | --- | --- | --- | --- | --- | --- | --- | --- | --- | --- | --- | --- | --- | --- | --- | --- | --- | --- | --- | --- | --- | --- | --- | --- | --- | --- | --- | --- | --- | --- | --- | --- | --- | --- | --- | --- | --- | --- | --- | --- | --- | --- | --- | --- | --- | --- | --- | --- | --- | --- | --- | --- | --- | --- | --- | --- | --- | --- | --- | --- | --- | --- | --- | --- | --- | --- | --- | --- | --- | --- | --- | --- | --- | --- | --- | --- | --- | --- | --- | --- | --- | --- | --- | --- | --- | --- | --- | --- | --- | --- | --- | --- | --- | --- | --- | --- | --- | --- | --- | --- | --- | --- | --- | --- | --- | --- | --- | --- | --- | --- | --- | --- | --- | --- | --- | --- | --- | --- | --- | --- | --- | --- | --- | --- | --- | --- | --- | --- | --- | --- | --- | --- | --- | --- | --- | --- | --- | --- | --- | --- | --- | --- | --- | --- | --- | --- | --- | --- | --- | --- | --- | --- | --- | --- | --- | --- | --- | --- | --- | --- | --- | --- | --- | --- | --- | --- | --- | --- | --- | --- | --- | --- | --- | --- | --- | --- | --- | --- | --- | --- | --- | --- | --- | --- | --- | --- | --- | --- | --- | --- | --- | --- | --- | --- | --- | --- | --- | --- | --- | --- | --- | --- | --- | --- | --- | --- | --- | --- | --- | --- | --- | --- | --- | --- | --- | --- | --- | --- | --- | --- | --- | --- | --- | --- | --- | --- | --- | --- | --- | --- | --- | --- | --- | --- | --- | --- | --- | --- | --- | --- | --- | --- | --- | --- | --- | --- | --- | --- | --- | --- | --- | --- | --- | --- | --- | --- | --- | --- | --- | --- | --- | --- | --- | --- | --- | --- | --- | --- | --- | --- | --- | --- | --- | --- | --- | --- | --- | --- | --- | --- | --- | --- | --- | --- | --- | --- | --- | --- | --- | --- | --- | --- | --- | --- | --- | --- | --- | --- | --- | --- | --- | --- | --- | --- | --- | --- | --- | --- | --- | --- | --- | --- | --- | --- | --- | --- | --- | --- | --- | --- | --- | --- | --- | --- | --- | --- | --- | --- | --- | --- | --- | --- | --- | --- | --- | --- | --- | --- | --- | --- | --- | --- | --- | --- | --- | --- | --- | --- | --- | --- | --- | --- | --- | --- | --- | --- | --- | --- | --- | --- | --- | --- | --- | --- | --- | --- | --- | --- | --- | --- | --- | --- | --- | --- | --- | --- | --- | --- | --- | --- | --- | --- | --- | --- | --- | --- | --- | --- | --- | --- | --- | --- | --- | --- | --- | --- | --- | --- | --- | --- | --- | --- | --- | --- | --- | --- | --- | --- | --- | --- | --- | --- | --- | --- | --- | --- | --- | --- | --- | --- | --- | --- | --- | --- | --- | --- | --- | --- | --- | --- | --- | --- | --- | --- | --- | --- | --- | --- | --- | --- | --- | --- | --- | --- | --- | --- | --- | --- | --- | --- | --- | --- | --- | --- | --- | --- | --- | --- | --- | --- | --- | --- | --- | --- | --- | --- | --- | --- | --- | --- | --- | --- | --- | --- | --- | --- | --- | --- | --- | --- | --- | --- | --- | --- | --- | --- | --- | --- | --- | --- | --- | --- | --- | --- | --- | --- | --- | --- | --- | --- | --- | --- | --- | --- | --- | --- | --- | --- | --- | --- | --- | --- | --- | --- | --- | --- | --- | --- | --- | --- | --- | --- | --- | --- | --- | --- | --- | --- | --- | --- | --- | --- | --- | --- | --- | --- | --- | --- | --- | --- | --- | --- | --- | --- | --- | --- | --- | --- | --- | --- | --- | --- | --- | --- | --- | --- | --- | --- | --- | --- | --- | --- | --- | --- | --- | --- | --- | --- | --- | --- | --- | --- | --- | --- | --- | --- | --- | --- | --- | --- | --- | --- | --- | --- | --- | --- | --- | --- | --- | --- | --- | --- | --- | --- | --- | --- | --- | --- | --- | --- | --- | --- | --- | --- | --- | --- | --- | --- | --- | --- | --- | --- | --- | --- | --- | --- | --- | --- | --- | --- | --- | --- | --- | --- | --- | --- | --- | --- | --- | --- | --- | --- | --- | --- | --- | --- | --- | --- | --- | --- | --- | --- | --- | --- | --- | --- | --- | --- | --- | --- | --- | --- | --- | --- | --- | --- | --- | --- | --- | --- | --- | --- | --- | --- | --- | --- | --- | --- | --- | --- | --- | --- | --- | --- | --- | --- | --- | --- | --- | --- | --- | --- | --- | --- | --- | --- | --- | --- | --- | --- | --- | --- | --- | --- | --- | --- | --- | --- | --- | --- | --- | --- | --- | --- | --- | --- | --- | --- | --- | --- | --- | --- | --- | --- | --- | --- | --- | --- | --- | --- | --- | --- | --- | --- | --- | --- | --- | --- | --- | --- | --- | --- | --- | --- | --- | --- | --- | --- | --- | --- | --- | --- | --- | --- | --- | --- | --- | --- | --- | --- | --- | --- | --- | --- | --- | --- | --- | --- | --- | --- | --- | --- | --- | --- | --- | --- | --- | --- | --- | --- | --- | --- | --- | --- | --- | --- | --- | --- | --- | --- | --- | --- | --- | --- | --- | --- | --- | --- | --- | --- | --- | --- | --- | --- | --- | --- | --- | --- | --- | --- | --- | --- | --- | --- | --- | --- | --- | --- | --- | --- | --- | --- | --- | --- | --- | --- | --- | --- | --- | --- | --- | --- | --- | --- | --- | --- | --- | --- | --- | --- | --- | --- | --- | --- | --- | --- | --- | --- | --- | --- | --- | --- | --- | --- | --- | --- | --- | --- | --- | --- | --- | --- | --- | --- | --- | --- | --- | --- | --- | --- | --- | --- | --- | --- | --- | --- | --- | --- | --- | --- | --- | --- | --- | --- | --- | --- | --- | --- | --- | --- | --- | --- | --- | --- | --- | --- | --- | --- | --- | --- | --- | --- | --- | --- | --- | --- | --- | --- | --- | --- | --- | --- | --- | --- | --- | --- | --- | --- | --- | --- | --- | --- | --- | --- | --- | --- | --- | --- | --- | --- | --- | --- | --- | --- | --- | --- | --- | --- | --- | --- | --- | --- | --- | --- | --- | --- | --- | --- | --- | --- | --- | --- | --- | --- | --- | --- | --- | --- | --- | --- | --- | --- | --- | --- | --- | --- | --- | --- | --- | --- | --- | --- | --- | --- | --- | --- | --- | --- | --- | --- | --- | --- | --- | --- | --- | --- | --- | --- | --- | --- | --- | --- | --- | --- | --- | --- | --- | --- | --- | --- | --- | --- | --- | --- | --- | --- | --- | --- | --- | --- | --- | --- | --- | --- | --- | --- | --- | --- | --- | --- | --- | --- | --- | --- | --- | --- | --- | --- | --- | --- | --- | --- | --- | --- | --- | --- | --- | --- | --- | --- | --- | --- | --- | --- | --- | --- | --- | --- | --- | --- | --- | --- | --- | --- | --- | --- | --- | --- | --- | --- | --- | --- | --- | --- | --- | --- | --- | --- | --- | --- | --- | --- | --- | --- | --- | --- | --- | --- | --- | --- | --- | --- | --- | --- | --- | --- | --- | --- | --- | --- | --- | --- | --- | --- | --- | --- | --- | --- | --- | --- | --- | --- | --- | --- | --- | --- | --- | --- | --- | --- | --- | --- | --- | --- | --- | --- | --- | --- | --- | --- | --- | --- | --- | --- | --- | --- | --- | --- | --- | --- | --- | --- | --- | --- | --- | --- | --- | --- | --- | --- | --- | --- | --- | --- | --- | --- | --- | --- | --- | --- | --- | --- | --- | --- | --- | --- | --- | --- | --- | --- | --- | --- | --- | --- | --- | --- | --- | --- | --- | --- | --- | --- | --- | --- | --- | --- | --- | --- | --- | --- | --- | --- | --- | --- | --- | --- | --- | --- | --- | --- | --- | --- | --- | --- | --- | --- | --- | --- | --- | --- | --- | --- | --- | --- | --- | --- | --- | --- | --- | --- | --- | --- | --- | --- | --- | --- | --- | --- | --- | --- | --- | --- | --- | --- | --- | --- | --- | --- | --- | --- | --- | --- | --- | --- | --- | --- | --- | --- | --- | --- | --- | --- | --- | --- | --- | --- | --- | --- | --- | --- | --- | --- | --- | --- | --- | --- | --- | --- | --- | --- | --- | --- | --- | --- | --- | --- | --- | --- | --- | --- | --- | --- | --- | --- | --- | --- | --- | --- | --- | --- | --- | --- | --- | --- | --- | --- | --- | --- | --- | --- | --- | --- | --- | --- | --- | --- | --- | --- | --- | --- | --- | --- | --- | --- | --- | --- | --- | --- | --- | --- | --- | --- | --- | --- | --- | --- | --- | --- | --- | --- | --- | --- | --- | --- | --- | --- | --- | --- | --- | --- | --- | --- | --- | --- | --- | --- | --- | --- | --- | --- | --- | --- | --- | --- | --- | --- | --- | --- | --- | --- | --- | --- | --- | --- | --- | --- | --- | --- | --- | --- | --- | --- | --- | --- | --- | --- | --- | --- | --- | --- | --- | --- | --- | --- | --- | --- | --- | --- | --- | --- | --- | --- | --- | --- | --- | --- | --- | --- | --- | --- | --- | --- | --- | --- | --- | --- | --- | --- | --- | --- | --- | --- | --- | --- | --- | --- | --- | --- | --- | --- | --- | --- | --- | --- | --- | --- | --- | --- | --- | --- | --- | --- | --- | --- | --- | --- | --- | --- | --- | --- | --- | --- | --- | --- | --- | --- | --- | --- | --- | --- | --- | --- | --- | --- | --- | --- | --- | --- | --- | --- | --- | --- | --- | --- | --- | --- | --- | --- | --- | --- | --- | --- | --- | --- | --- | --- | --- | --- | --- | --- | --- | --- | --- | --- | --- | --- | --- | --- | --- | --- | --- | --- | --- | --- | --- | --- | --- | --- | --- | --- | --- | --- | --- | --- | --- | --- | --- | --- | --- | --- | --- | --- | --- | --- | --- | --- | --- | --- | --- | --- | --- | --- | --- | --- | --- | --- | --- | --- | --- | --- | --- | --- | --- | --- | --- | --- | --- | --- | --- | --- | --- | --- | --- | --- | --- | --- | --- | --- | --- | --- | --- | --- | --- | --- | --- | --- | --- | --- | --- | --- | --- | --- | --- | --- | --- | --- | --- | --- | --- | --- | --- | --- | --- | --- | --- | --- | --- | --- | --- | --- | --- | --- | --- | --- | --- | --- | --- | --- | --- | --- | --- | --- | --- | --- | --- | --- | --- | --- | --- | --- | --- | --- | --- | --- | --- | --- | --- | --- | --- | --- | --- | --- | --- | --- | --- | --- | --- | --- | --- | --- | --- | --- | --- | --- | --- | --- | --- | --- | --- | --- | --- | --- | --- | --- | --- | --- | --- | --- | --- | --- | --- | --- | --- | --- | --- | --- | --- | --- | --- | --- | --- | --- | --- | --- | --- | --- | --- | --- | --- | --- | --- | --- | --- | --- | --- | --- | --- | --- | --- | --- | --- | --- | --- | --- | --- | --- | --- | --- | --- | --- | --- | --- | --- | --- | --- | --- | --- | --- | --- | --- | --- | --- | --- | --- | --- | --- | --- | --- | --- | --- | --- | --- | --- | --- | --- | --- | --- | --- | --- | --- | --- | --- | --- | --- | --- | --- | --- | --- | --- | --- | --- | --- | --- | --- | --- | --- | --- | --- | --- | --- | --- | --- | --- | --- | --- | --- | --- | --- | --- | --- | --- | --- | --- | --- | --- | --- | --- | --- | --- | --- | --- | --- | --- | --- | --- | --- | --- | --- | --- | --- | --- | --- | --- | --- | --- | --- | --- | --- | --- | --- | --- | --- | --- | --- | --- | --- | --- | --- | --- | --- | --- | --- | --- | --- | --- | --- | --- | --- | --- | --- | --- | --- | --- | --- | --- | --- | --- | --- | --- | --- | --- | --- | --- | --- | --- | --- | --- | --- | --- | --- | --- | --- | --- | --- | --- | --- | --- | --- | --- | --- | --- | --- | --- | --- | --- | --- | --- | --- | --- | --- | --- | --- | --- | --- | --- | --- | --- | --- | --- | --- | --- | --- | --- | --- | --- | --- | --- | --- | --- | --- | --- | --- | --- | --- | --- | --- | --- | --- | --- | --- | --- | --- | --- | --- | --- | --- | --- | --- | --- | --- | --- | --- | --- | --- | --- | --- | --- | --- | --- | --- | --- | --- | --- | --- | --- | --- | --- | --- | --- | --- | --- | --- | --- | --- | --- | --- | --- | --- | --- | --- | --- | --- | --- | --- | --- | --- | --- | --- | --- | --- | --- | --- | --- | --- | --- | --- | --- | --- | --- | --- | --- | --- | --- | --- | --- | --- | --- | --- | --- | --- | --- | --- | --- | --- | --- | --- | --- | --- | --- | --- | --- | --- | --- | --- | --- | --- | --- | --- | --- | --- | --- | --- | --- | --- | --- | --- | --- | --- | --- | --- | --- | --- | --- | --- | --- | --- | --- | --- | --- | --- | --- | --- | --- | --- | --- | --- | --- | --- | --- | --- | --- | --- | --- | --- | --- | --- | --- | --- | --- | --- | --- | --- | --- | --- | --- | --- | --- | --- | --- | --- | --- | --- | --- | --- | --- | --- | --- | --- | --- | --- | --- | --- | --- | --- | --- | --- | --- | --- | --- | --- | --- | --- | --- | --- | --- | --- | --- | --- | --- | --- | --- | --- | --- | --- | --- | --- | --- | --- | --- | --- | --- | --- | --- | --- | --- | --- | --- | --- | --- | --- | --- | --- | --- | --- | --- | --- | --- | --- | --- | --- | --- | --- | --- | --- | --- | --- | --- | --- | --- | --- | --- | --- | --- | --- | --- | --- | --- | --- | --- | --- | --- | --- | --- | --- | --- | --- | --- | --- | --- | --- | --- | --- | --- | --- | --- | --- | --- | --- | --- | --- | --- | --- | --- | --- | --- | --- | --- | --- | --- | --- | --- | --- | --- | --- | --- | --- | --- | --- | --- | --- | --- | --- | --- | --- | --- | --- | --- | --- | --- | --- | --- | --- | --- | --- | --- | --- | --- | --- | --- | --- | --- | --- | --- | --- | --- | --- | --- | --- | --- | --- | --- | --- | --- | --- | --- | --- | --- | --- | --- | --- | --- | --- | --- | --- | --- | --- | --- | --- | --- | --- | --- | --- | --- | --- | --- | --- | --- | --- | --- | --- | --- | --- | --- | --- | --- | --- | --- | --- | --- | --- | --- | --- | --- | --- | --- | --- | --- | --- | --- | --- | --- | --- | --- | --- | --- | --- | --- | --- | --- | --- | --- | --- | --- | --- | --- | --- | --- | --- | --- | --- | --- | --- | --- | --- | --- | --- | --- | --- | --- | --- | --- | --- | --- | --- | --- | --- | --- | --- | --- | --- | --- | --- | --- | --- | --- | --- | --- | --- | --- | --- | --- | --- | --- | --- | --- | --- | --- | --- | --- | --- | --- | --- | --- | --- | --- | --- | --- | --- | --- | --- | --- | --- | --- | --- | --- | --- | --- | --- | --- | --- | --- | --- | --- | --- | --- | --- | --- | --- | --- | --- | --- | --- | --- | --- | --- | --- | --- | --- | --- | --- | --- | --- | --- | --- | --- | --- | --- | --- | --- | --- | --- | --- | --- | --- | --- | --- | --- | --- | --- | --- | --- | --- | --- | --- | --- | --- | --- | --- | --- | --- | --- | --- | --- | --- | --- | --- | --- | --- | --- | --- | --- | --- | --- | --- | --- | --- | --- | --- | --- | --- | --- | --- | --- | --- | --- | --- | --- | --- | --- | --- | --- | --- | --- | --- | --- | --- | --- | --- | --- | --- | --- | --- | --- | --- | --- | --- | --- | --- | --- | --- | --- | --- | --- | --- | --- | --- | --- | --- | --- | --- | --- | --- | --- | --- | --- | --- | --- | --- | --- | --- | --- | --- | --- | --- | --- | --- | --- | --- | --- | --- | --- | --- | --- | --- | --- | --- | --- | --- | --- | --- | --- | --- | --- | --- | --- | --- | --- | --- | --- | --- | --- | --- | --- | --- | --- | --- | --- | --- | --- | --- | --- | --- | --- | --- | --- | --- | --- | --- | --- | --- | --- | --- | --- | --- | --- | --- | --- | --- | --- | --- | --- | --- | --- | --- | --- | --- | --- | --- | --- | --- | --- | --- | --- | --- | --- | --- | --- | --- | --- | --- | --- | --- | --- | --- | --- | --- | --- | --- | --- | --- | --- | --- | --- | --- | --- | --- | --- | --- | --- | --- | --- | --- | --- | --- | --- | --- | --- | --- | --- | --- | --- | --- | --- | --- | --- | --- | --- | --- | --- | --- | --- | --- | --- | --- | --- | --- | --- | --- | --- | --- | --- | --- | --- | --- | --- | --- | --- | --- | --- | --- | --- | --- | --- | --- | --- | --- | --- | --- | --- | --- | --- | --- | --- | --- | --- | --- | --- | --- | --- | --- | --- | --- | --- | --- | --- | --- | --- | --- | --- | --- | --- | --- | --- | --- | --- | --- | --- | --- | --- | --- | --- | --- | --- | --- | --- | --- | --- | --- | --- | --- | --- | --- | --- | --- | --- | --- | --- | --- | --- | --- | --- | --- | --- | --- | --- | --- | --- | --- | --- | --- | --- | --- | --- | --- | --- | --- | --- | --- | --- | --- | --- | --- | --- | --- | --- | --- | --- | --- | --- | --- | --- | --- | --- | --- | --- | --- | --- | --- | --- | --- | --- | --- | --- | --- | --- | --- | --- | --- | --- | --- | --- | --- | --- | --- | --- | --- | --- | --- | --- | --- | --- | --- | --- | --- | --- | --- | --- | --- | --- | --- | --- | --- | --- | --- | --- | --- | --- | --- | --- | --- | --- | --- | --- | --- | --- | --- | --- | --- | --- | --- | --- | --- | --- | --- | --- | --- | --- | --- | --- | --- | --- | --- | --- | --- | --- | --- | --- | --- | --- | --- | --- | --- | --- | --- | --- | --- | --- | --- | --- | --- | --- | --- | --- | --- | --- | --- | --- | --- | --- | --- | --- | --- | --- | --- | --- | --- | --- | --- | --- | --- | --- | --- | --- | --- | --- | --- | --- | --- | --- | --- | --- | --- | --- | --- | --- | --- | --- | --- | --- | --- | --- | --- | --- | --- | --- | --- | --- | --- | --- | --- | --- | --- | --- | --- | --- | --- | --- | --- | --- | --- | --- | --- | --- | --- | --- | --- | --- | --- | --- | --- | --- | --- | --- | --- | --- | --- | --- | --- | --- | --- | --- | --- | --- | --- | --- | --- | --- | --- | --- | --- | --- | --- | --- | --- | --- | --- | --- | --- | --- | --- | --- | --- | --- | --- | --- | --- | --- | --- | --- | --- | --- | --- | --- | --- | --- | --- | --- | --- | --- | --- | --- | --- | --- | --- | --- | --- | --- | --- | --- | --- | --- | --- | --- | --- | --- | --- | --- | --- | --- | --- | --- | --- | --- | --- | --- | --- | --- | --- | --- | --- | --- | --- | --- | --- | --- | --- | --- | --- | --- | --- | --- | --- | --- | --- | --- | --- | --- | --- | --- | --- | --- | --- | --- | --- | --- | --- | --- | --- | --- | --- | --- | --- | --- | --- | --- | --- | --- | --- | --- | --- | --- | --- | --- | --- | --- | --- | --- | --- | --- | --- | --- | --- | --- | --- | --- | --- | --- | --- | --- | --- | --- | --- | --- | --- | --- | --- | --- | --- | --- | --- | --- | --- | --- | --- | --- | --- | --- | --- | --- | --- | --- | --- | --- | --- | --- | --- | --- | --- | --- | --- | --- | --- | --- | --- | --- | --- | --- | --- | --- | --- | --- | --- | --- | --- | --- | --- | --- | --- | --- | --- | --- | --- | --- | --- | --- | --- | --- | --- | --- | --- | --- | --- | --- | --- | --- | --- | --- | --- | --- | --- | --- | --- | --- | --- | --- | --- | --- | --- | --- | --- | --- | --- | --- | --- | --- | --- | --- | --- | --- | --- | --- | --- | --- | --- | --- | --- | --- | --- | --- | --- | --- | --- | --- | --- | --- | --- | --- | --- | --- | --- | --- | --- | --- | --- | --- | --- | --- | --- | --- | --- | --- | --- | --- | --- | --- | --- | --- | --- | --- | --- | --- | --- | --- | --- | --- | --- | --- | --- | --- | --- | --- | --- | --- | --- | --- | --- | --- | --- | --- | --- | --- | --- | --- | --- | --- | --- | --- | --- | --- | --- | --- | --- | --- | --- | --- | --- | --- | --- | --- | --- | --- | --- | --- | --- | --- | --- | --- | --- | --- | --- | --- | --- | --- | --- | --- | --- | --- | --- | --- | --- | --- | --- | --- | --- | --- | --- | --- | --- | --- | --- | --- | --- | --- | --- | --- | --- | --- | --- | --- | --- | --- | --- | --- | --- | --- | --- | --- | --- | --- | --- | --- | --- | --- | --- | --- | --- | --- | --- | --- | --- | --- | --- | --- | --- | --- | --- | --- | --- | --- | --- | --- | --- | --- | --- | --- | --- | --- | --- | --- | --- | --- | --- | --- | --- | --- | --- | --- | --- | --- | --- | --- | --- | --- | --- | --- | --- | --- | --- | --- | --- | --- | --- | --- | --- | --- | --- | --- | --- | --- | --- | --- | --- | --- | --- | --- | --- | --- | --- | --- | --- | --- | --- | --- | --- | --- | --- | --- | --- | --- | --- | --- | --- | --- | --- | --- | --- | --- | --- | --- | --- | --- | --- | --- | --- | --- | --- | --- | --- | --- | --- | --- | --- | --- | --- | --- | --- | --- | --- | --- | --- | --- | --- | --- | --- | --- | --- | --- | --- | --- | --- | --- | --- | --- | --- | --- | --- | --- | --- | --- | --- | --- | --- | --- | --- | --- | --- | --- | --- | --- | --- | --- | --- | --- | --- | --- | --- | --- | --- | --- | --- | --- | --- | --- | --- | --- | --- | --- | --- | --- | --- | --- | --- | --- | --- | --- | --- | --- | --- | --- | --- | --- | --- | --- | --- | --- | --- | --- | --- | --- | --- | --- | --- | --- | --- | --- | --- | --- | --- | --- | --- | --- | --- | --- | --- | --- | --- | --- | --- | --- | --- | --- | --- | --- | --- | --- | --- | --- | --- | --- | --- | --- | --- | --- | --- | --- | --- | --- | --- | --- | --- | --- | --- | --- | --- | --- | --- | --- | --- | --- | --- | --- | --- | --- | --- | --- | --- | --- | --- | --- | --- | --- | --- | --- | --- | --- | --- | --- | --- | --- | --- | --- | --- | --- | --- | --- | --- | --- | --- | --- | --- | --- | --- | --- | --- | --- | --- | --- | --- | --- | --- | --- | --- | --- | --- | --- | --- | --- | --- | --- | --- | --- | --- | --- | --- | --- | --- | --- | --- | --- | --- | --- | --- | --- | --- | --- | --- | --- | --- | --- | --- | --- | --- | --- | --- | --- | --- | --- | --- | --- | --- | --- | --- | --- | --- | --- | --- | --- | --- | --- | --- | --- | --- | --- | --- | --- | --- | --- | --- | --- | --- | --- | --- | --- | --- | --- | --- | --- | --- | --- | --- | --- | --- | --- | --- | --- | --- | --- | --- | --- | --- | --- | --- | --- | --- | --- | --- | --- | --- | --- | --- | --- | --- | --- | --- | --- | --- | --- | --- | --- | --- | --- | --- | --- | --- | --- | --- | --- | --- | --- | --- | --- | --- | --- | --- | --- | --- | --- | --- | --- | --- | --- | --- | --- | --- | --- | --- | --- | --- | --- | --- | --- | --- | --- | --- | --- | --- | --- | --- | --- | --- | --- | --- | --- | --- | --- | --- | --- | --- | --- | --- | --- | --- | --- | --- | --- | --- | --- | --- | --- | --- | --- | --- | --- | --- | --- | --- | --- | --- | --- | --- | --- | --- | --- | --- | --- | --- | --- | --- | --- | --- | --- | --- | --- | --- | --- | --- | --- | --- | --- | --- | --- | --- | --- | --- | --- | --- | --- | --- | --- | --- | --- | --- | --- | --- | --- | --- | --- | --- | --- | --- | --- | --- | --- | --- | --- | --- | --- | --- | --- | --- | --- | --- | --- | --- | --- | --- | --- | --- | --- | --- | --- | --- | --- | --- | --- | --- | --- | --- | --- | --- | --- | --- | --- | --- | --- | --- | --- | --- | --- | --- | --- | --- | --- | --- | --- | --- | --- | --- | --- | --- | --- | --- | --- | --- | --- | --- | --- | --- | --- | --- | --- | --- | --- | --- | --- | --- | --- | --- | --- | --- | --- | --- | --- | --- | --- | --- | --- | --- | --- | --- | --- | --- | --- | --- | --- | --- | --- | --- | --- | --- | --- | --- | --- | --- | --- | --- | --- | --- | --- | --- | --- | --- | --- | --- | --- | --- | --- | --- | --- | --- | --- | --- | --- | --- | --- | --- | --- | --- | --- | --- | --- | --- | --- | --- | --- | --- | --- | --- | --- | --- | --- | --- | --- | --- | --- | --- | --- | --- | --- | --- | --- | --- | --- | --- | --- | --- | --- | --- | --- | --- | --- | --- | --- | --- | --- | --- | --- | --- | --- | --- | --- | --- | --- | --- | --- | --- | --- | --- | --- | --- | --- | --- | --- | --- | --- | --- | --- | --- | --- | --- | --- | --- | --- | --- | --- | --- | --- | --- | --- | --- | --- | --- | --- | --- | --- | --- | --- | --- | --- | --- | --- | --- | --- | --- | --- | --- | --- | --- | --- | --- | --- | --- | --- | --- | --- | --- | --- | --- | --- | --- | --- | --- | --- | --- | --- | --- | --- | --- | --- | --- | --- | --- | --- | --- | --- | --- | --- | --- | --- | --- | --- | --- | --- | --- | --- | --- | --- | --- | --- | --- | --- | --- | --- | --- | --- | --- | --- | --- | --- | --- | --- | --- | --- | --- | --- | --- | --- | --- | --- | --- | --- | --- | --- | --- | --- | --- | --- | --- | --- | --- | --- | --- | --- | --- | --- | --- | --- | --- | --- | --- | --- | --- | --- | --- | --- | --- | --- | --- | --- | --- | --- | --- | --- | --- | --- | --- | --- | --- | --- | --- | --- | --- | --- | --- | --- | --- | --- | --- | --- | --- | --- | --- | --- | --- | --- | --- | --- | --- | --- | --- | --- | --- | --- | --- | --- | --- | --- | --- | --- | --- | --- | --- | --- | --- | --- | --- | --- | --- | --- | --- | --- | --- | --- | --- | --- | --- | --- | --- | --- | --- | --- | --- | --- | --- | --- | --- | --- | --- | --- | --- | --- | --- | --- | --- | --- | --- | --- | --- | --- | --- | --- | --- | --- | --- | --- | --- | --- | --- | --- | --- | --- | --- | --- | --- | --- | --- | --- | --- | --- | --- | --- | --- | --- | --- | --- | --- | --- | --- | --- | --- | --- | --- | --- | --- | --- | --- | --- | --- | --- | --- | --- | --- | --- | --- | --- | --- | --- | --- | --- | --- | --- | --- | --- | --- | --- | --- | --- | --- | --- | --- | --- | --- | --- | --- | --- | --- | --- | --- | --- | --- | --- | --- | --- | --- | --- | --- | --- | --- | --- | --- | --- | --- | --- | --- | --- | --- | --- | --- | --- | --- | --- | --- | --- | --- | --- | --- | --- | --- | --- | --- | --- | --- | --- | --- | --- | --- | --- | --- | --- | --- | --- | --- | --- | --- | --- | --- | --- | --- | --- | --- | --- | --- | --- | --- | --- | --- | --- | --- | --- | --- | --- | --- | --- | --- | --- | --- | --- | --- | --- | --- | --- | --- | --- | --- | --- | --- | --- | --- | --- | --- | --- | --- | --- | --- | --- | --- | --- | --- | --- | --- | --- | --- | --- | --- | --- | --- | --- | --- | --- | --- | --- | --- | --- | --- | --- | --- | --- | --- | --- | --- | --- | --- | --- | --- | --- | --- | --- | --- | --- | --- | --- | --- | --- | --- | --- | --- | --- | --- | --- | --- | --- | --- | --- | --- | --- | --- | --- | --- | --- | --- | --- | --- | --- | --- | --- | --- | --- | --- | --- | --- | --- | --- | --- | --- | --- | --- | --- | --- | --- | --- | --- | --- | --- | --- | --- | --- | --- | --- | --- | --- | --- | --- | --- | --- | --- | --- | --- | --- | --- | --- | --- | --- | --- | --- | --- | --- | --- | --- | --- | --- | --- | --- | --- | --- | --- | --- | --- | --- | --- | --- | --- | --- | --- | --- | --- | --- | --- | --- | --- | --- | --- | --- | --- | --- | --- | --- | --- | --- | --- | --- | --- | --- | --- | --- | --- | --- | --- | --- | --- | --- | --- | --- | --- | --- | --- | --- | --- | --- | --- | --- | --- | --- | --- | --- | --- | --- | --- | --- | --- | --- | --- | --- | --- | --- | --- | --- | --- | --- | --- | --- | --- | --- | --- | --- | --- | --- | --- | --- | --- | --- | --- | --- | --- | --- | --- | --- | --- | --- | --- | --- | --- | --- | --- | --- | --- | --- | --- | --- | --- | --- | --- | --- | --- | --- | --- | --- | --- | --- | --- | --- | --- | --- | --- | --- | --- | --- | --- | --- | --- | --- | --- | --- | --- | --- | --- | --- | --- | --- | --- | --- | --- | --- | --- | --- | --- | --- | --- | --- | --- | --- | --- | --- | --- | --- | --- | --- | --- | --- | --- | --- | --- | --- | --- | --- | --- | --- | --- | --- | --- | --- | --- | --- | --- | --- | --- | --- | --- | --- | --- | --- | --- | --- | --- | --- | --- | --- | --- | --- | --- | --- | --- | --- | --- | --- | --- | --- | --- | --- | --- | --- | --- | --- | --- | --- | --- | --- | --- | --- | --- | --- | --- | --- | --- | --- | --- | --- | --- | --- | --- | --- | --- | --- | --- | --- | --- | --- | --- | --- | --- | --- | --- | --- | --- | --- | --- | --- | --- | --- | --- | --- | --- | --- | --- | --- | --- | --- | --- | --- | --- | --- | --- | --- | --- | --- | --- | --- | --- | --- | --- | --- | --- | --- | --- | --- | --- | --- | --- | --- | --- | --- | --- | --- | --- | --- | --- | --- | --- | --- | --- | --- | --- | --- | --- | --- | --- | --- | --- | --- | --- | --- | --- | --- | --- | --- | --- | --- | --- | --- | --- | --- | --- | --- | --- | --- | --- | --- | --- | --- | --- | --- | --- | --- | --- | --- | --- | --- | --- | --- | --- | --- | --- | --- | --- | --- | --- | --- | --- | --- | --- | --- | --- | --- | --- | --- | --- | --- | --- | --- | --- | --- | --- | --- | --- | --- | --- | --- | --- | --- | --- | --- | --- | --- | --- | --- | --- | --- | --- | --- | --- | --- | --- | --- | --- | --- | --- | --- | --- | --- | --- | --- | --- | --- | --- | --- | --- | --- | --- | --- | --- | --- | --- | --- | --- | --- | --- | --- | --- | --- | --- | --- | --- | --- | --- | --- | --- | --- | --- | --- | --- | --- | --- | --- | --- | --- | --- | --- | --- | --- | --- | --- | --- | --- | --- | --- | --- | --- | --- | --- | --- | --- | --- | --- | --- | --- | --- | --- | --- | --- | --- | --- | --- | --- | --- | --- | --- | --- | --- | --- | --- | --- | --- | --- | --- | --- | --- | --- | --- | --- | --- | --- | --- | --- | --- | --- | --- | --- | --- | --- | --- | --- | --- | --- | --- | --- | --- | --- | --- | --- | --- | --- | --- | --- | --- | --- | --- | --- | --- | --- | --- | --- | --- | --- | --- | --- | --- | --- | --- | --- | --- | --- | --- | --- | --- | --- | --- | --- | --- | --- | --- | --- | --- | --- | --- | --- | --- | --- | --- | --- | --- | --- | --- | --- | --- | --- | --- | --- | --- | --- | --- | --- | --- | --- | --- | --- | --- | --- | --- | --- | --- | --- | --- | --- | --- | --- | --- | --- | --- | --- | --- | --- | --- | --- | --- | --- | --- | --- | --- | --- | --- | --- | --- | --- | --- | --- | --- | --- | --- | --- | --- | --- | --- | --- | --- | --- | --- | --- | --- | --- | --- | --- | --- | --- | --- | --- | --- | --- | --- | --- | --- | --- | --- | --- | --- | --- | --- | --- | --- | --- | --- | --- | --- | --- | --- | --- | --- | --- | --- | --- | --- | --- | --- | --- | --- | --- | --- | --- | --- | --- | --- | --- | --- | --- | --- | --- | --- | --- | --- | --- | --- | --- | --- | --- | --- | --- | --- | --- | --- | --- | --- | --- | --- | --- | --- | --- | --- | --- | --- | --- | --- | --- | --- | --- | --- | --- | --- | --- | --- | --- | --- | --- | --- | --- | --- | --- | --- | --- | --- | --- | --- | --- | --- | --- | --- | --- | --- | --- | --- | --- | --- | --- | --- | --- | --- | --- | --- | --- | --- | --- | --- | --- | --- | --- | --- | --- | --- | --- | --- | --- | --- | --- | --- | --- | --- | --- | --- | --- | --- | --- | --- | --- | --- | --- | --- | --- | --- | --- | --- | --- | --- | --- | --- | --- | --- | --- | --- | --- | --- | --- | --- | --- | --- | --- | --- | --- | --- | --- | --- | --- | --- | --- | --- | --- | --- | --- | --- | --- | --- | --- | --- | --- | --- | --- | --- | --- | --- | --- | --- | --- | --- | --- | --- | --- | --- | --- | --- | --- | --- | --- | --- | --- | --- | --- | --- | --- | --- | --- | --- | --- | --- | --- | --- | --- | --- | --- | --- | --- | --- | --- | --- | --- | --- | --- | --- | --- | --- | --- | --- | --- | --- | --- | --- | --- | --- | --- | --- | --- | --- | --- | --- | --- | --- | --- | --- | --- | --- | --- | --- | --- | --- | --- | --- | --- | --- | --- | --- | --- | --- | --- | --- | --- | --- | --- | --- | --- | --- | --- | --- | --- | --- | --- | --- | --- | --- | --- | --- | --- | --- | --- | --- | --- | --- | --- | --- | --- | --- | --- | --- | --- | --- | --- | --- | --- | --- | --- | --- | --- | --- | --- | --- | --- | --- | --- | --- | --- | --- | --- | --- | --- | --- | --- | --- | --- | --- | --- | --- | --- | --- | --- | --- | --- | --- | --- | --- | --- | --- | --- | --- | --- | --- | --- | --- | --- | --- | --- | --- | --- | --- | --- | --- | --- | --- | --- | --- | --- | --- | --- | --- | --- | --- | --- | --- | --- | --- | --- | --- | --- | --- | --- | --- | --- | --- | --- | --- | --- | --- | --- | --- | --- | --- | --- | --- | --- | --- | --- | --- | --- | --- | --- | --- | --- | --- | --- | --- | --- | --- | --- | --- | --- | --- | --- | --- | --- | --- | --- | --- | --- | --- | --- | --- | --- | --- | --- | --- | --- | --- | --- | --- | --- | --- | --- | --- | --- | --- | --- | --- | --- | --- | --- | --- | --- | --- | --- | --- | --- | --- | --- | --- | --- | --- | --- | --- | --- | --- | --- | --- | --- | --- | --- | --- | --- | --- | --- | --- | --- | --- | --- | --- | --- | --- | --- | --- | --- | --- | --- | --- | --- | --- | --- | --- | --- | --- | --- | --- | --- | --- | --- | --- | --- | --- | --- | --- | --- | --- | --- | --- | --- | --- | --- | --- | --- | --- | --- | --- | --- | --- | --- | --- | --- | --- | --- | --- | --- | --- | --- | --- | --- | --- | --- | --- | --- | --- | --- | --- | --- | --- | --- | --- | --- | --- | --- | --- | --- | --- | --- | --- | --- | --- | --- | --- | --- | --- | --- | --- | --- | --- | --- | --- | --- | --- | --- | --- | --- | --- | --- | --- | --- | --- | --- | --- | --- | --- | --- | --- | --- | --- | --- | --- | --- | --- | --- | --- | --- | --- | --- | --- | --- | --- | --- | --- | --- | --- | --- | --- | --- | --- | --- | --- | --- | --- | --- | --- | --- | --- | --- | --- | --- | --- | --- | --- | --- | --- | --- | --- | --- | --- | --- | --- | --- | --- | --- | --- | --- | --- | --- | --- | --- | --- | --- | --- | --- | --- | --- | --- | --- | --- | --- | --- | --- | --- | --- | --- | --- | --- | --- | --- | --- | --- | --- | --- | --- | --- | --- | --- | --- | --- | --- | --- | --- | --- | --- | --- | --- | --- | --- | --- | --- | --- | --- | --- | --- | --- | --- | --- | --- | --- | --- | --- | --- | --- | --- | --- | --- | --- | --- | --- | --- | --- | --- | --- | --- | --- | --- | --- | --- | --- | --- | --- | --- | --- | --- | --- | --- | --- | --- | --- | --- | --- | --- | --- | --- | --- | --- | --- | --- | --- | --- | --- | --- | --- | --- | --- | --- | --- | --- | --- | --- | --- | --- | --- | --- | --- | --- | --- | --- | --- | --- | --- | --- | --- | --- | --- | --- | --- | --- | --- | --- | --- | --- | --- | --- | --- | --- | --- | --- | --- | --- | --- | --- | --- | --- | --- | --- | --- | --- | --- | --- | --- | --- | --- | --- | --- | --- | --- | --- | --- | --- | --- | --- | --- | --- | --- | --- | --- | --- | --- | --- | --- | --- | --- | --- | --- | --- | --- | --- | --- | --- | --- | --- | --- | --- | --- | --- | --- | --- | --- | --- | --- | --- | --- | --- | --- | --- | --- | --- | --- | --- | --- | --- | --- | --- | --- | --- | --- | --- | --- | --- | --- | --- | --- | --- | --- | --- | --- | --- | --- | --- | --- | --- | --- | --- | --- | --- | --- | --- | --- | --- | --- | --- | --- | --- | --- | --- | --- | --- | --- | --- | --- | --- | --- | --- | --- | --- | --- | --- | --- | --- | --- | --- | --- | --- | --- | --- | --- | --- | --- | --- | --- | --- | --- | --- | --- | --- | --- | --- | --- | --- | --- | --- | --- | --- | --- | --- | --- | --- | --- | --- | --- | --- | --- | --- | --- | --- | --- | --- | --- | --- | --- | --- | --- | --- | --- | --- | --- | --- | --- | --- | --- | --- | --- | --- | --- | --- | --- | --- | --- | --- | --- | --- | --- | --- | --- | --- | --- | --- | --- | --- | --- | --- | --- | --- | --- | --- | --- | --- | --- | --- | --- | --- | --- | --- | --- | --- | --- | --- | --- | --- | --- | --- | --- | --- | --- | --- | --- | --- | --- | --- | --- | --- | --- | --- | --- | --- | --- | --- | --- | --- | --- | --- | --- | --- | --- | --- | --- | --- | --- | --- | --- | --- | --- | --- | --- | --- | --- | --- | --- | --- | --- | --- | --- | --- | --- | --- | --- | --- | --- | --- | --- | --- | --- | --- | --- | --- | --- | --- | --- | --- | --- | --- | --- | --- | --- | --- | --- | --- | --- | --- | --- | --- | --- | --- | --- | --- | --- | --- | --- | --- | --- | --- | --- | --- | --- | --- | --- | --- | --- | --- | --- | --- | --- | --- | --- | --- | --- | --- | --- | --- | --- | --- | --- | --- | --- | --- | --- | --- | --- | --- | --- | --- | --- | --- | --- | --- | --- | --- | --- | --- | --- | --- | --- | --- | --- | --- | --- | --- | --- | --- | --- | --- | --- | --- | --- | --- | --- | --- | --- | --- | --- | --- | --- | --- | --- | --- | --- | --- | --- | --- | --- | --- | --- | --- | --- | --- | --- | --- | --- | --- | --- | --- | --- | --- | --- | --- | --- | --- | --- | --- | --- | --- | --- | --- | --- | --- | --- | --- | --- | --- | --- | --- | --- | --- | --- | --- | --- | --- | --- | --- | --- | --- | --- | --- | --- | --- | --- | --- | --- | --- | --- | --- | --- | --- | --- | --- | --- | --- | --- | --- | --- | --- | --- | --- | --- | --- | --- | --- | --- | --- | --- | --- | --- | --- | --- | --- | --- | --- | --- | --- | --- | --- | --- | --- | --- | --- | --- | --- | --- | --- | --- | --- | --- | --- | --- | --- | --- | --- | --- | --- | --- | --- | --- | --- | --- | --- | --- | --- | --- | --- | --- | --- | --- | --- | --- | --- | --- | --- | --- | --- | --- | --- | --- | --- | --- | --- | --- | --- | --- | --- | --- | --- | --- | --- | --- | --- | --- | --- | --- | --- | --- | --- | --- | --- | --- | --- | --- | --- | --- | --- | --- | --- | --- | --- | --- | --- | --- | --- | --- | --- | --- | --- | --- | --- | --- | --- | --- | --- | --- | --- | --- | --- | --- | --- | --- | --- | --- | --- | --- | --- | --- | --- | --- | --- | --- | --- | --- | --- | --- | --- | --- | --- | --- | --- | --- | --- | --- | --- | --- | --- | --- | --- | --- | --- | --- | --- | --- | --- | --- | --- | --- | --- | --- | --- | --- | --- | --- | --- | --- | --- | --- | --- | --- | --- | --- | --- | --- | --- | --- | --- | --- | --- | --- | --- | --- | --- | --- | --- | --- | --- | --- | --- | --- | --- | --- | --- | --- | --- | --- | --- | --- | --- | --- | --- | --- | --- | --- | --- | --- | --- | --- | --- | --- | --- | --- | --- | --- | --- | --- | --- | --- | --- | --- | --- | --- | --- | --- | --- | --- | --- | --- | --- | --- | --- | --- | --- | --- | --- | --- | --- | --- | --- | --- | --- | --- | --- | --- | --- | --- | --- | --- | --- | --- | --- | --- | --- | --- | --- | --- | --- | --- | --- | --- | --- | --- | --- | --- | --- | --- | --- | --- | --- | --- | --- | --- | --- | --- | --- | --- | --- | --- | --- | --- | --- | --- | --- | --- | --- | --- | --- | --- | --- | --- | --- | --- | --- | --- | --- | --- | --- | --- | --- | --- | --- | --- | --- | --- | --- | --- | --- | --- | --- | --- | --- | --- | --- | --- | --- | --- | --- | --- | --- | --- | --- | --- | --- | --- | --- | --- | --- | --- | --- | --- | --- | --- | --- | --- | --- | --- | --- | --- | --- | --- | --- | --- | --- | --- | --- | --- | --- | --- | --- | --- | --- | --- | --- | --- | --- | --- | --- | --- | --- | --- | --- | --- | --- | --- | --- | --- | --- | --- | --- | --- | --- | --- | --- | --- | --- | --- | --- | --- | --- | --- | --- | --- | --- | --- | --- | --- | --- | --- | --- | --- | --- | --- | --- | --- | --- | --- | --- | --- | --- | --- | --- | --- | --- | --- | --- | --- | --- | --- | --- | --- | --- | --- | --- | --- | --- | --- | --- | --- | --- | --- | --- | --- | --- | --- | --- | --- | --- | --- | --- | --- | --- | --- | --- | --- | --- | --- | --- | --- | --- | --- | --- | --- | --- | --- | --- | --- | --- | --- | --- | --- | --- | --- | --- | --- | --- | --- | --- | --- | --- | --- | --- | --- | --- | --- | --- | --- | --- | --- | --- | --- | --- | --- | --- | --- | --- | --- | --- | --- | --- | --- | --- | --- | --- | --- | --- | --- | --- | --- | --- | --- | --- | --- | --- | --- | --- | --- | --- | --- | --- | --- | --- | --- | --- | --- | --- | --- | --- | --- | --- | --- | --- | --- | --- | --- | --- | --- | --- | --- | --- | --- | --- | --- | --- | --- | --- | --- | --- | --- | --- | --- | --- | --- | --- | --- | --- | --- | --- | --- | --- | --- | --- | --- | --- | --- | --- | --- | --- | --- | --- | --- | --- | --- | --- | --- | --- | --- | --- | --- | --- | --- | --- | --- | --- | --- | --- | --- | --- | --- | --- | --- | --- | --- | --- | --- | --- | --- | --- | --- | --- | --- | --- | --- | --- | --- | --- | --- | --- | --- | --- | --- | --- | --- | --- | --- | --- | --- | --- | --- | --- | --- | --- | --- | --- | --- | --- | --- | --- | --- | --- | --- | --- | --- | --- | --- | --- | --- | --- | --- | --- | --- | --- | --- | --- | --- | --- | --- | --- | --- | --- | --- | --- | --- | --- | --- | --- | --- | --- | --- | --- | --- | --- | --- | --- | --- | --- | --- | --- | --- | --- | --- | --- | --- | --- | --- | --- | --- | --- | --- | --- | --- | --- | --- | --- | --- | --- | --- | --- | --- | --- | --- | --- | --- | --- | --- | --- | --- | --- | --- | --- | --- | --- | --- | --- | --- | --- | --- | --- | --- | --- | --- | --- | --- | --- | --- | --- | --- | --- | --- | --- | --- | --- | --- | --- | --- | --- | --- | --- | --- | --- | --- | --- | --- | --- | --- | --- | --- | --- | --- | --- | --- | --- | --- | --- | --- | --- | --- | --- | --- | --- | --- | --- | --- | --- | --- | --- | --- | --- | --- | --- | --- | --- | --- | --- | --- | --- | --- | --- | --- | --- | --- | --- | --- | --- | --- | --- | --- | --- | --- | --- | --- | --- | --- | --- | --- | --- | --- | --- | --- | --- | --- | --- | --- | --- | --- | --- | --- | --- | --- | --- | --- | --- | --- | --- | --- | --- | --- | --- | --- | --- | --- | --- | --- | --- | --- | --- | --- | --- | --- | --- | --- | --- | --- | --- | --- | --- | --- | --- | --- | --- | --- | --- | --- | --- | --- | --- | --- | --- | --- | --- | --- | --- | --- | --- | --- | --- | --- | --- | --- | --- | --- | --- | --- | --- | --- | --- | --- | --- | --- | --- | --- | --- | --- | --- | --- | --- | --- | --- | --- | --- | --- | --- | --- | --- | --- | --- | --- | --- | --- | --- | --- | --- | --- | --- | --- | --- | --- | --- | --- | --- | --- | --- | --- | --- | --- | --- | --- | --- | --- | --- | --- | --- | --- | --- | --- | --- | --- | --- | --- | --- | --- | --- | --- | --- | --- | --- | --- | --- | --- | --- | --- | --- | --- | --- | --- | --- | --- | --- | --- | --- | --- | --- | --- | --- | --- | --- | --- | --- | --- | --- | --- | --- | --- | --- | --- | --- | --- | --- | --- | --- | --- | --- | --- | --- | --- | --- | --- | --- | --- | --- | --- | --- | --- | --- | --- | --- | --- | --- | --- | --- | --- | --- | --- | --- | --- | --- | --- | --- | --- | --- | --- | --- | --- | --- | --- | --- | --- | --- | --- | --- | --- | --- | --- | --- | --- | --- | --- | --- | --- | --- | --- | --- | --- | --- | --- | --- | --- | --- | --- | --- | --- | --- | --- | --- | --- | --- | --- | --- | --- | --- | --- | --- | --- | --- | --- | --- | --- | --- | --- | --- | --- | --- | --- | --- | --- | --- | --- | --- | --- | --- | --- | --- | --- | --- | --- | --- | --- | --- | --- | --- | --- | --- | --- | --- | --- | --- | --- | --- | --- | --- | --- | --- | --- | --- | --- | --- | --- | --- | --- | --- | --- | --- | --- | --- | --- | --- | --- | --- | --- | --- | --- | --- | --- | --- | --- | --- | --- | --- | --- | --- | --- | --- | --- | --- | --- | --- | --- | --- | --- | --- | --- | --- | --- | --- | --- | --- | --- | --- | --- | --- | --- | --- | --- | --- | --- | --- | --- | --- | --- | --- | --- | --- | --- | --- | --- | --- | --- | --- | --- | --- | --- | --- | --- | --- | --- | --- | --- | --- | --- | --- | --- | --- | --- | --- | --- | --- | --- | --- | --- | --- | --- | --- | --- | --- | --- | --- | --- | --- | --- | --- | --- | --- | --- | --- | --- | --- | --- | --- | --- | --- | --- | --- | --- | --- | --- | --- | --- | --- | --- | --- | --- | --- | --- | --- | --- | --- | --- | --- | --- | --- | --- | --- | --- | --- | --- | --- | --- | --- | --- | --- | --- | --- | --- | --- | --- | --- | --- | --- | --- | --- | --- | --- | --- | --- | --- | --- | --- | --- | --- | --- | --- | --- | --- | --- | --- | --- | --- | --- | --- | --- | --- | --- | --- | --- | --- | --- | --- | --- | --- | --- | --- | --- | --- | --- | --- | --- | --- | --- | --- | --- | --- | --- | --- | --- | --- | --- | --- | --- | --- | --- | --- | --- | --- | --- | --- | --- | --- | --- | --- | --- | --- | --- | --- | --- | --- | --- | --- | --- | --- | --- | --- | --- | --- | --- | --- | --- | --- | --- | --- | --- | --- | --- | --- | --- | --- | --- | --- | --- | --- | --- | --- | --- | --- | --- | --- | --- | --- | --- | --- | --- | --- | --- | --- | --- | --- | --- | --- | --- | --- | --- | --- | --- | --- | --- | --- | --- | --- | --- | --- | --- | --- | --- | --- | --- | --- | --- | --- | --- | --- | --- | --- | --- | --- | --- | --- | --- | --- | --- | --- | --- | --- | --- | --- | --- | --- | --- | --- | --- | --- | --- | --- | --- | --- | --- | --- | --- | --- | --- | --- | --- | --- | --- | --- | --- | --- | --- | --- | --- | --- | --- | --- | --- | --- | --- | --- | --- | --- | --- | --- | --- | --- | --- | --- | --- | --- | --- | --- | --- | --- | --- | --- | --- | --- | --- | --- | --- | --- | --- | --- | --- | --- | --- | --- | --- | --- | --- | --- | --- | --- | --- | --- | --- | --- | --- | --- | --- | --- | --- | --- | --- | --- | --- | --- | --- | --- | --- | --- | --- | --- | --- | --- | --- | --- | --- | --- | --- | --- | --- | --- | --- | --- | --- | --- | --- | --- | --- | --- | --- | --- | --- | --- | --- | --- | --- | --- | --- | --- | --- | --- | --- | --- | --- | --- | --- | --- | --- | --- | --- | --- | --- | --- | --- | --- | --- | --- | --- | --- | --- | --- | --- | --- | --- | --- | --- | --- | --- | --- | --- | --- | --- | --- | --- | --- | --- | --- | --- | --- | --- | --- | --- | --- | --- | --- | --- | --- | --- | --- | --- | --- | --- | --- | --- | --- | --- | --- | --- | --- | --- | --- | --- | --- | --- | --- | --- | --- | --- | --- | --- | --- | --- | --- | --- | --- | --- | --- | --- | --- | --- | --- | --- | --- | --- | --- | --- | --- | --- | --- | --- | --- | --- | --- | --- | --- | --- | --- | --- | --- | --- | --- | --- | --- | --- | --- | --- | --- | --- | --- | --- | --- | --- | --- | --- | --- | --- | --- | --- | --- | --- | --- | --- | --- | --- | --- | --- | --- | --- | --- | --- | --- | --- | --- | --- | --- | --- | --- | --- | --- | --- | --- | --- | --- | --- | --- | --- | --- | --- | --- | --- | --- | --- | --- | --- | --- | --- | --- | --- | --- | --- | --- | --- | --- | --- | --- | --- | --- | --- | --- | --- | --- | --- | --- | --- | --- | --- | --- | --- | --- | --- | --- | --- | --- | --- | --- | --- | --- | --- | --- | --- | --- | --- | --- | --- | --- | --- | --- | --- | --- | --- | --- | --- | --- | --- | --- | --- | --- | --- | --- | --- | --- | --- | --- | --- | --- | --- | --- | --- | --- | --- | --- | --- | --- | --- | --- | --- | --- | --- | --- | --- | --- | --- | --- | --- | --- | --- | --- | --- | --- | --- | --- | --- | --- | --- | --- | --- | --- | --- | --- | --- | --- | --- | --- | --- | --- | --- | --- | --- | --- | --- | --- | --- | --- | --- | --- | --- | --- | --- | --- | --- | --- | --- | --- | --- | --- | --- | --- | --- | --- | --- | --- | --- | --- | --- | --- | --- | --- | --- | --- | --- | --- | --- | --- | --- | --- | --- | --- | --- | --- | --- | --- | --- | --- | --- | --- | --- | --- | --- | --- | --- | --- | --- | --- | --- | --- | --- | --- | --- | --- | --- | --- | --- | --- | --- | --- | --- | --- | --- | --- | --- | --- | --- | --- | --- | --- | --- | --- | --- | --- | --- | --- | --- | --- | --- | --- | --- | --- | --- | --- | --- | --- | --- | --- | --- | --- | --- | --- | --- | --- | --- | --- | --- | --- | --- | --- | --- | --- | --- | --- | --- | --- | --- | --- | --- | --- | --- | --- | --- | --- | --- | --- | --- | --- | --- | --- | --- | --- | --- | --- | --- | --- | --- | --- | --- | --- | --- | --- | --- | --- | --- | --- | --- | --- | --- | --- | --- | --- | --- | --- | --- | --- | --- | --- | --- | --- | --- | --- | --- | --- | --- | --- | --- | --- | --- | --- | --- | --- | --- | --- | --- | --- | --- | --- | --- | --- | --- | --- | --- | --- | --- | --- | --- | --- | --- | --- | --- | --- | --- | --- | --- | --- | --- | --- | --- | --- | --- | --- | --- | --- | --- | --- | --- | --- | --- | --- | --- | --- | --- | --- | --- | --- | --- | --- | --- | --- | --- | --- | --- | --- | --- | --- | --- | --- | --- | --- | --- | --- | --- | --- | --- | --- | --- | --- | --- | --- | --- | --- | --- | --- | --- | --- | --- | --- | --- | --- | --- | --- | --- | --- | --- | --- | --- | --- | --- | --- | --- | --- | --- | --- | --- | --- | --- | --- | --- | --- | --- | --- | --- | --- | --- | --- | --- | --- | --- | --- | --- | --- | --- | --- | --- | --- | --- | --- | --- | --- | --- | --- | --- | --- | --- | --- | --- | --- | --- | --- | --- | --- | --- | --- | --- | --- | --- | --- | --- | --- | --- | --- | --- | --- | --- | --- | --- | --- | --- | --- | --- | --- | --- | --- | --- | --- | --- | --- | --- | --- | --- | --- | --- | --- | --- | --- | --- | --- | --- | --- | --- | --- | --- | --- | --- | --- | --- | --- | --- | --- | --- | --- | --- | --- | --- | --- | --- | --- | --- | --- | --- | --- | --- | --- | --- | --- | --- | --- | --- | --- | --- | --- | --- | --- | --- | --- | --- | --- | --- | --- | --- | --- | --- | --- | --- | --- | --- | --- | --- | --- | --- | --- | --- | --- | --- | --- | --- | --- | --- | --- | --- | --- | --- | --- | --- | --- | --- | --- | --- | --- | --- | --- | --- | --- | --- | --- | --- | --- | --- | --- | --- | --- | --- | --- | --- | --- | --- | --- | --- | --- | --- | --- | --- | --- | --- | --- | --- | --- | --- | --- | --- | --- | --- | --- | --- | --- | --- | --- | --- | --- | --- | --- | --- | --- | --- | --- | --- | --- | --- | --- | --- | --- | --- | --- | --- | --- | --- | --- | --- | --- | --- | --- | --- | --- | --- | --- | --- | --- | --- | --- | --- | --- | --- | --- | --- | --- | --- | --- | --- | --- | --- | --- | --- | --- | --- | --- | --- | --- | --- | --- | --- | --- | --- | --- | --- | --- | --- | --- | --- | --- | --- | --- | --- | --- | --- | --- | --- | --- | --- | --- | --- | --- | --- | --- | --- | --- | --- | --- | --- | --- | --- | --- | --- | --- | --- | --- | --- | --- | --- | --- | --- | --- | --- | --- | --- | --- | --- | --- | --- | --- | --- | --- | --- | --- | --- | --- | --- | --- | --- | --- | --- | --- | --- | --- | --- | --- | --- | --- | --- | --- | --- | --- | --- | --- | --- | --- | --- | --- | --- | --- | --- | --- | --- | --- | --- | --- | --- | --- | --- | --- | --- | --- | --- | --- | --- | --- | --- | --- | --- | --- | --- | --- | --- | --- | --- | --- | --- | --- | --- | --- | --- | --- | --- | --- | --- | --- | --- | --- | --- | --- | --- | --- | --- | --- | --- | --- | --- | --- | --- | --- | --- | --- | --- | --- | --- | --- | --- | --- | --- | --- | --- | --- | --- | --- | --- | --- | --- | --- | --- | --- | --- | --- | --- | --- | --- | --- | --- | --- | --- | --- | --- | --- | --- | --- | --- | --- | --- | --- | --- | --- | --- | --- | --- | --- | --- | --- | --- | --- | --- | --- | --- | --- | --- | --- | --- | --- | --- | --- | --- | --- | --- | --- | --- | --- | --- | --- | --- | --- | --- | --- | --- | --- | --- | --- | --- | --- | --- | --- | --- | --- | --- | --- | --- | --- | --- | --- | --- | --- | --- | --- | --- | --- | --- | --- | --- | --- | --- | --- | --- | --- | --- | --- | --- | --- | --- | --- | --- | --- | --- | --- | --- | --- | --- | --- | --- | --- | --- | --- | --- | --- | --- | --- | --- | --- | --- | --- | --- | --- | --- | --- | --- | --- | --- | --- | --- | --- | --- | --- | --- | --- | --- | --- | --- | --- | --- | --- | --- | --- | --- | --- | --- | --- | --- | --- | --- | --- | --- | --- | --- | --- | --- | --- | --- | --- | --- | --- | --- | --- | --- | --- | --- | --- | --- | --- | --- | --- | --- | --- | --- | --- | --- | --- | --- | --- | --- | --- | --- | --- | --- | --- | --- | --- | --- | --- | --- | --- | --- | --- | --- | --- | --- | --- | --- | --- | --- | --- | --- | --- | --- | --- | --- | --- | --- | --- | --- | --- | --- | --- | --- | --- | --- | --- | --- | --- | --- | --- | --- | --- | --- | --- | --- | --- | --- | --- | --- | --- | --- | --- | --- | --- | --- | --- | --- | --- | --- | --- | --- | --- | --- | --- | --- | --- | --- | --- | --- | --- | --- | --- | --- | --- | --- | --- | --- | --- | --- | --- | --- | --- | --- | --- | --- | --- | --- | --- | --- | --- | --- | --- | --- | --- | --- | --- | --- | --- | --- | --- | --- | --- | --- | --- | --- | --- | --- | --- | --- | --- | --- | --- | --- | --- | --- | --- | --- | --- | --- | --- | --- | --- | --- | --- | --- | --- | --- | --- | --- | --- | --- | --- | --- | --- | --- | --- | --- | --- | --- | --- | --- | --- | --- | --- | --- | --- | --- | --- | --- | --- | --- | --- | --- | --- | --- | --- | --- | --- | --- | --- | --- | --- | --- | --- | --- | --- | --- | --- | --- | --- | --- | --- | --- | --- | --- | --- | --- | --- | --- | --- | --- | --- | --- | --- | --- | --- | --- | --- | --- | --- | --- | --- | --- | --- | --- | --- | --- | --- | --- | --- | --- | --- | --- | --- | --- | --- | --- | --- | --- | --- | --- | --- | --- | --- | --- | --- | --- | --- | --- | --- | --- | --- | --- | --- | --- | --- | --- | --- | --- | --- | --- | --- | --- | --- | --- | --- | --- | --- | --- | --- | --- | --- | --- | --- | --- | --- | --- | --- | --- | --- | --- | --- | --- | --- | --- | --- | --- | --- | --- | --- | --- | --- | --- | --- | --- | --- | --- | --- | --- | --- | --- | --- | --- | --- | --- | --- | --- | --- | --- | --- | --- | --- | --- | --- | --- | --- | --- | --- | --- | --- | --- | --- | --- | --- | --- | --- | --- | --- | --- | --- | --- | --- | --- | --- | --- | --- | --- | --- | --- | --- | --- | --- | --- | --- | --- | --- | --- | --- | --- | --- | --- | --- | --- | --- | --- | --- | --- | --- | --- | --- | --- | --- | --- | --- | --- | --- | --- | --- | --- | --- | --- | --- | --- | --- | --- | --- | --- | --- | --- | --- | --- | --- | --- | --- | --- | --- | --- | --- | --- | --- | --- | --- | --- | --- | --- | --- | --- | --- | --- | --- | --- | --- | --- | --- | --- | --- | --- | --- | --- | --- | --- | --- | --- | --- | --- | --- | --- | --- | --- | --- | --- | --- | --- | --- | --- | --- | --- | --- | --- | --- | --- | --- | --- | --- | --- | --- | --- | --- | --- | --- | --- | --- | --- | --- | --- | --- | --- | --- | --- | --- | --- | --- | --- | --- | --- | --- | --- | --- | --- | --- | --- | --- | --- | --- | --- | --- | --- | --- | --- | --- | --- | --- | --- | --- | --- | --- | --- | --- | --- | --- | --- | --- | --- | --- | --- | --- | --- | --- | --- | --- | --- | --- | --- | --- | --- | --- | --- | --- | --- | --- | --- | --- | --- | --- | --- | --- | --- | --- | --- | --- | --- | --- | --- | --- | --- | --- | --- | --- | --- | --- | --- | --- | --- | --- | --- | --- | --- | --- | --- | --- | --- | --- | --- | --- | --- | --- | --- | --- | --- | --- | --- | --- | --- | --- | --- | --- | --- | --- | --- | --- | --- | --- | --- | --- | --- | --- | --- | --- | --- | --- | --- | --- | --- | --- | --- | --- | --- | --- | --- | --- | --- | --- | --- | --- | --- | --- | --- | --- | --- | --- | --- | --- | --- | --- | --- | --- | --- | --- | --- | --- | --- | --- | --- | --- | --- | --- | --- | --- | --- | --- | --- | --- | --- | --- | --- | --- | --- | --- | --- | --- | --- | --- | --- | --- | --- | --- | --- | --- | --- | --- | --- | --- | --- | --- | --- | --- | --- | --- | --- | --- | --- | --- | --- | --- | --- | --- | --- | --- | --- | --- | --- | --- | --- | --- | --- | --- | --- | --- | --- | --- | --- | --- | --- | --- | --- | --- | --- | --- | --- | --- | --- | --- | --- | --- | --- | --- | --- | --- | --- | --- | --- | --- | --- | --- | --- | --- | --- | --- | --- | --- | --- | --- | --- | --- | --- | --- | --- | --- | --- | --- | --- | --- | --- | --- | --- | --- | --- | --- | --- | --- | --- | --- | --- | --- | --- | --- | --- | --- | --- | --- | --- | --- | --- | --- | --- | --- |
| |  |  |  |  |  |  |  |  |  | | --- | --- | --- | --- | --- | --- | --- | --- | --- | | **Position** | **Reference** | **Sample** | **Quality** | **Type** | **Region** | **AA Exchange** | **PAM1** | **Known Variant** | | 845 | T | C | 2288.77 | SNP | Rv0001 (dnaA) | Ile282Thr | 11 | - | | 1977 | A | G | 1269.77 | SNP | intergenic |  |  | - | | 4013 | T | C | 1940.77 | SNP | Rv0003 (recF) | Ile245Thr | 11 | - | | 7362 | G | C | 1534.77 | SNP | Rv0006 (gyrA) | Glu21Gln | 27 | - | | 7585 | G | C | 1575.77 | SNP | Rv0006 (gyrA) | Ser95Thr | 32 | genotype | | 8040 | G | A | 1539.77 | SNP | Rv0006 (gyrA) | Gly247Ser | 16 | genotype | | 9304 | G | A | 1593.77 | SNP | Rv0006 (gyrA) | Gly668Asp | 6 | - | | 11879 | A | G | 1690.77 | SNP | Rv0008c | Ser145Pro | 12 | - | | 14251 | G | A | 1178.77 | SNP | Rv0012 | Asp55Asn | 36 | - | | 14785 | T | C | 2109.77 | SNP | Rv0012 | Cys233Arg | 1 | - | | 17608 | G | C | 1508.77 | SNP | Rv0015c (pknA) | Ser385Arg | 6 | - | | 21795 | G | A | 157.90 | SNP | Rv0018c (pstP) | Pro463Ser | 17 | - | | 26959 | C | G | 1801.77 | SNP | intergenic |  |  | - | | 27463 | C | G | 1804.77 | SNP | intergenic |  |  | - | | 33457 | C | T | 1745.77 | SNP | Rv0030 | silent (His78) | 9912 | - | | 33551 | T | G | 2015.77 | SNP | Rv0030 | STOP110Gly | 21 | - | | 34044 | T | C | 1555.77 | SNP | intergenic |  |  | - | | 36470 | G | GC | 1715.73 | INS | Rv0032 (bioF2) |  |  | - | | 37031 | C | G | 1119.77 | SNP | Rv0034 | silent (Ala55) | 9867 | - | | 37763 | G | C | 1630.77 | SNP | Rv0035 (fadD34) | Asp169His | 3 | - | | 42967 | G | C | 1494.77 | SNP | Rv0040c (mtc28) | silent (Pro133) | 9926 | - | | 55553 | C | T | 765.77 | SNP | Rv0050 (ponA1) | Pro631Ser | 17 | - | | 62049 | A | G | 1761.77 | SNP | Rv0058 (dnaB) | Arg552Gly | 1 | - | | 63771 | C | T | 2357.77 | SNP | Rv0059 | Pro191Leu | 3 | - | | 69989 | G | A | 1275.77 | SNP | Rv0064 | Gly457Asp | 6 | - | | 70816 | A | G | 1132.77 | SNP | Rv0064 | Asn733Asp | 42 | - | | 71336 | G | C | 491.77 | SNP | Rv0064 | Arg906Pro | 5 | - | | 71584 | C | CCGAGCGCTGTTCTGGCGCT AATCTGACGCTAGAATAG | 13633.73 | INS | intergenic |  |  | - | | 74059 | C | T | 2131.77 | SNP | Rv0066c (icd2) | silent (Lys151) | 9926 | - | | 75940 | G | C | 995.77 | SNP | Rv0068 | Val(s)214Leu | 3 | - | | 79504 | TCGGTGGACC | T | 2612.73 | DEL | Rv0071 |  |  | - | | 80616 | C | G | 1382.77 | SNP | intergenic |  |  | - | | 92199 | T | G | 1732.77 | SNP | Rv0083 | silent (Thr600) | 9871 | - | | 98428 | A | G | 1706.77 | SNP | intergenic |  |  | - | | 104915 | T | C | 604.77 | SNP | Rv0095c | Thr101Ala | 32 | - | | 104919 | G | A | 625.77 | SNP | Rv0095c | silent (Leu99) | 9947 | - | | 104936 | T | C | 280.77 | SNP | Rv0095c | Thr94Ala | 32 | - | | 104940 | C | A | 241.77 | SNP | Rv0095c | Gln92His | 20 | - | | 104941 | T | C | 529.77 | SNP | Rv0095c | Gln92Arg | 10 | - | | 104942 | G | C | 502.77 | SNP | Rv0095c | Gln92Glu | 35 | - | | 104943 | G | C | 254.77 | SNP | Rv0095c | silent (Ala91) | 9867 | - | | 104944 | G | A | 448.77 | SNP | Rv0095c | Ala91Val | 13 | - | | 104962 | G | A | 771.77 | SNP | Rv0095c | Ala85Val(s) | 9867 | - | | 105045 | G | C | 1256.77 | SNP | Rv0095c | Asp57Glu | 56 | - | | 116000 | T | G | 1728.77 | SNP | Rv0101 (nrp) | Val2000Val(s) | 18 | - | | 122109 | A | G | 1875.77 | SNP | Rv0103c (ctpB) | Leu(s)22Ser | 28 | - | | 122528 | G | T | 1797.77 | SNP | Rv0104 | Arg71Leu | 1 | - | | 125830 | G | GA | 2237.73 | INS | Rv0107c (ctpI) |  |  | - | | 127974 | G | A | 708.77 | SNP | Rv0107c (ctpI) | silent (Ala856) | 9867 | - | | 130227 | C | T | 1480.77 | SNP | Rv0107c (ctpI) | Met(s)105Ile | 2 | - | | 131174 | T | TG | 2042.73 | INS | intergenic |  |  | - | | 132417 | C | G | 221.80 | SNP | Rv0109 (PE\_PGRS1) | Arg346Gly | 1 | - | | 133839 | C | T | 2462.77 | SNP | intergenic |  |  | - | | 133862 | G | A | 2549.77 | SNP | intergenic |  |  | - | | 146087 | T | C | 1622.77 | SNP | Rv0120c (fusA2) | Asn562Ser | 34 | - | | 149323 | C | T | 1477.77 | SNP | intergenic |  |  | - | | 149426 | G | A | 931.77 | SNP | intergenic |  |  | - | | 150123 | T | TG | 227.80 | INS | Rv0124 (PE\_PGRS2) |  |  | - | | 154283 | T | C | 1696.77 | SNP | Rv0127 (mak) | Ser18Pro | 12 | - | | 157292 | C | T | 1871.77 | SNP | Rv0129c (fbpC) | silent (Glu103) | 9865 | genotype | | 159726 | TGCG | T | 4083.73 | DEL | Rv0132c (fgd2) |  |  | - | | 160544 | T | G | 1675.77 | SNP | Rv0132c (fgd2) | Gln80Pro | 8 | - | | 162581 | G | A | 1666.77 | SNP | Rv0134 (ephF) | Gly271Ser | 16 | - | | 169562 | G | T | 1583.77 | SNP | Rv0143c | silent (Ala207) | 9867 | - | | 177857 | G | A | 1433.77 | SNP | Rv0151c (PE1) | Leu485Leu(s) | 4 | - | | 178941 | G | T | 1287.77 | SNP | Rv0151c (PE1) | Asn123Lys | 25 | - | | 180025 | C | T | 2139.77 | SNP | Rv0152c (PE2) | Gly291Glu | 4 | - | | 188800 | T | C | 1548.77 | SNP | Rv0159c (PE3) | Thr14Ala | 32 | - | | 194681 | G | C | 1223.77 | SNP | Rv0165c (mce1R) | silent (Leu45) | 9947 | - | | 196522 | C | T | 851.77 | SNP | Rv0166 (fadD5) | silent (Asp510) | 9859 | - | | 196642 | C | T | 927.77 | SNP | Rv0166 (fadD5) | silent (Asn550) | 9822 | - | | 200154 | T | C | 2285.77 | SNP | Rv0170 (mce1B) | Val(s)87Ala | 9867 | - | | 203269 | C | T | 1657.77 | SNP | Rv0172 (mce1D) | Ala265Val(s) | 9867 | - | | 206339 | T | C | 1387.77 | SNP | Rv0174 (mce1F) | Leu370Pro | 2 | - | | 207226 | T | C | 2021.77 | SNP | Rv0175 | Met(s)138Thr | 22 | - | | 212353 | C | T | 1028.77 | SNP | Rv0181c | Arg220His | 8 | - | | 223942 | T | C | 461.77 | SNP | Rv0192 | Ser127Pro | 12 | - | | 225323 | T | C | 1370.77 | SNP | Rv0193c | Lys417Glu | 4 | - | | 227098 | T | C | 1827.77 | SNP | Rv0194 | Met(s)74Thr | 22 | - | | 231114 | C | G | 1361.77 | SNP | Rv0195 | silent (Ala72) | 9867 | - | | 234477 | T | G | 1222.77 | SNP | Rv0197 | Tyr749STOP | 2 | - | | 234496 | C | CGT | 2577.73 | INS | Rv0197 |  |  | - | | 261869 | T | C | 1491.77 | SNP | Rv0218 | Cys316Arg | 1 | - | | 264656 | G | A | 2114.77 | SNP | Rv0221 | Arg197Gln | 9 | - | | 265554 | A | C | 1504.77 | SNP | Rv0222 (echA1) | silent (Val16) | 9901 | - | | 278681 | C | G | 1334.77 | SNP | Rv0233 (nrdB) | His33Asp | 4 | - | | 283614 | T | C | 1220.77 | SNP | Rv0236c (aftD) | Ser1080Gly | 21 | - | | 285772 | A | C | 1587.77 | SNP | Rv0236c (aftD) | silent (Pro360) | 9926 | - | | 285871 | A | G | 1423.77 | SNP | Rv0236c (aftD) | silent (Val327) | 9901 | - | | 293704 | CT | C | 2139.73 | DEL | intergenic |  |  | - | | 295724 | A | G | 1133.77 | SNP | intergenic |  |  | - | | 310973 | G | A | 877.77 | SNP | Rv0259c | Ala182Val(s) | 9867 | - | | 311613 | G | T | 1249.77 | SNP | Rv0260c | silent (Val349) | 9901 | - | | 316129 | C | G | 1434.77 | SNP | Rv0264c | Gly96Ala | 21 | - | | 316139 | C | T | 1459.77 | SNP | Rv0264c | Val93Ile | 33 | - | | 327897 | C | T | 1608.77 | SNP | Rv0271c (fadE6) | Val189Ile | 33 | - | | 333637 | A | G | 100.77 | SNP | Rv0278c (PE\_PGRS3) | Trp892Arg | 8 | - | | 333640 | G | A | 73.77 | SNP | Rv0278c (PE\_PGRS3) | Arg891Trp | 2 | - | | 333641 | C | T | 53.77 | SNP | Rv0278c (PE\_PGRS3) | silent (Gln890) | 9876 | - | | 333892 | G | C | 324.77 | SNP | Rv0278c (PE\_PGRS3) | Arg807Gly | 1 | - | | 334641 | G | C | 53.77 | SNP | Rv0278c (PE\_PGRS3) | Ala557Gly | 21 | - | | 335810 | CCCGCCGGCGCCGCCGTTG | C | 1379.75 | DEL | Rv0278c (PE\_PGRS3) |  |  | - | | 335885 | T | G | 84.77 | SNP | Rv0278c (PE\_PGRS3) | silent (Gly142) | 9935 | - | | 335906 | T | C | 111.77 | SNP | Rv0278c (PE\_PGRS3) | Leu135Leu(s) | 4 | - | | 335919 | T | G | 100.77 | SNP | Rv0278c (PE\_PGRS3) | Asp131Ala | 10 | - | | 335920 | C | G | 81.77 | SNP | Rv0278c (PE\_PGRS3) | Asp131His | 3 | - | | 335922 | C | G | 57.77 | SNP | Rv0278c (PE\_PGRS3) | Gly130Ala | 21 | - | | 335927 | A | G | 113.77 | SNP | Rv0278c (PE\_PGRS3) | silent (Asn128) | 9822 | - | | 335929 | T | C | 104.77 | SNP | Rv0278c (PE\_PGRS3) | Asn128Asp | 42 | - | | 335956 | C | T | 156.77 | SNP | Rv0278c (PE\_PGRS3) | Ala119Thr | 22 | - | | 335971 | A | G | 209.77 | SNP | Rv0278c (PE\_PGRS3) | Leu(s)114Leu | 3 | - | | 336005 | G | A | 194.77 | SNP | Rv0278c (PE\_PGRS3) | silent (Ile102) | 9872 | - | | 336047 | C | G | 154.77 | SNP | Rv0278c (PE\_PGRS3) | silent (Ala88) | 9867 | - | | 336050 | A | G | 150.77 | SNP | Rv0278c (PE\_PGRS3) | silent (Tyr87) | 9945 | - | | 336053 | G | C | 103.77 | SNP | Rv0278c (PE\_PGRS3) | silent (Ala86) | 9867 | - | | 336074 | T | C | 75.77 | SNP | Rv0278c (PE\_PGRS3) | silent (Ala79) | 9867 | - | | 336081 | A | G | 60.77 | SNP | Rv0278c (PE\_PGRS3) | Val(s)77Ala | 9867 | - | | 336082 | C | T | 50.77 | SNP | Rv0278c (PE\_PGRS3) | Val(s)77Met(s) | 9867 | - | | 336380 | A | T | 147.77 | SNP | intergenic |  |  | - | | 336400 | C | G | 73.77 | SNP | intergenic |  |  | - | | 336403 | C | G | 127.77 | SNP | intergenic |  |  | - | | 336405 | A | G | 99.77 | SNP | intergenic |  |  | - | | 336504 | G | T | 470.77 | SNP | intergenic |  |  | - | | 336535 | T | G | 411.77 | SNP | intergenic |  |  | - | | 336537 | T | G | 382.77 | SNP | intergenic |  |  | - | | 336540 | G | T | 295.77 | SNP | intergenic |  |  | - | | 336546 | T | G | 327.77 | SNP | intergenic |  |  | - | | 336557 | C | CT | 551.73 | INS | intergenic |  |  | - | | 336560 | T | C | 211.77 | SNP | Rv0279c (PE\_PGRS4) | silent (STOP838) | 9867 | - | | 336562 | A | ATGG | 713.73 | INS | Rv0279c (PE\_PGRS4) |  |  | - | | 336590 | G | C | 357.77 | SNP | Rv0279c (PE\_PGRS4) | Ile828Met(s) | 6 | - | | 336592 | T | G | 332.77 | SNP | Rv0279c (PE\_PGRS4) | Ile828Leu | 22 | - | | 336611 | G | C | 309.77 | SNP | Rv0279c (PE\_PGRS4) | silent (Ala821) | 9867 | - | | 336617 | G | C | 209.77 | SNP | Rv0279c (PE\_PGRS4) | silent (Pro819) | 9926 | - | | 336620 | T | C | 178.77 | SNP | Rv0279c (PE\_PGRS4) | silent (Thr818) | 9871 | - | | 336691 | T | C | 51.74 | SNP | Rv0279c (PE\_PGRS4) | Ser795Gly | 21 | - | | 336698 | C | G | 49.74 | SNP | Rv0279c (PE\_PGRS4) | silent (Gly792) | 9935 | - | | 336701 | A | G | 49.74 | SNP | Rv0279c (PE\_PGRS4) | silent (Gly791) | 9935 | - | | 336707 | G | A | 41.74 | SNP | Rv0279c (PE\_PGRS4) | silent (Asp789) | 9859 | - | | 336708 | T | C | 50.74 | SNP | Rv0279c (PE\_PGRS4) | Asp789Gly | 11 | - | | 336710 | A | G | 37.74 | SNP | Rv0279c (PE\_PGRS4) | silent (Ala788) | 9867 | - | | 336728 | G | A | 188.77 | SNP | Rv0279c (PE\_PGRS4) | silent (Gly782) | 9935 | - | | 337820 | G | A | 46.74 | SNP | Rv0279c (PE\_PGRS4) | silent (Gly418) | 9935 | - | | 338100 | T | C | 313.78 | SNP | Rv0279c (PE\_PGRS4) | Asn325Ser | 34 | - | | 338453 | A | G | 106.28 | SNP | Rv0279c (PE\_PGRS4) | silent (Ala207) | 9867 | - | | 338768 | G | A | 90.77 | SNP | Rv0279c (PE\_PGRS4) | silent (Ile102) | 9872 | - | | 338774 | G | A | 100.77 | SNP | Rv0279c (PE\_PGRS4) | silent (Ala100) | 9867 | - | | 338775 | G | T | 131.77 | SNP | Rv0279c (PE\_PGRS4) | Ala100Asp | 6 | - | | 338777 | G | C | 109.77 | SNP | Rv0279c (PE\_PGRS4) | silent (Leu99) | 9947 | - | | 338789 | G | C | 111.77 | SNP | Rv0279c (PE\_PGRS4) | silent (Thr95) | 9871 | - | | 338790 | G | A | 98.77 | SNP | Rv0279c (PE\_PGRS4) | Thr95Ile | 7 | - | | 338791 | T | C | 108.77 | SNP | Rv0279c (PE\_PGRS4) | Thr95Ala | 32 | - | | 338792 | G | C | 105.77 | SNP | Rv0279c (PE\_PGRS4) | silent (Ala94) | 9867 | - | | 338844 | A | G | 502.77 | SNP | Rv0279c (PE\_PGRS4) | Val(s)77Ala | 9867 | - | | 338845 | C | T | 507.77 | SNP | Rv0279c (PE\_PGRS4) | Val(s)77Met(s) | 9867 | - | | 338876 | G | A | 618.77 | SNP | Rv0279c (PE\_PGRS4) | silent (Ser66) | 9840 | - | | 338903 | G | C | 719.77 | SNP | Rv0279c (PE\_PGRS4) | silent (Ala57) | 9867 | - | | 338960 | T | C | 728.77 | SNP | Rv0279c (PE\_PGRS4) | silent (Ala38) | 9867 | - | | 338963 | T | C | 658.77 | SNP | Rv0279c (PE\_PGRS4) | silent (Thr37) | 9871 | - | | 338984 | C | T | 520.77 | SNP | Rv0279c (PE\_PGRS4) | silent (Ala30) | 9867 | - | | 338990 | G | T | 486.77 | SNP | Rv0279c (PE\_PGRS4) | silent (Ala28) | 9867 | - | | 338997 | G | C | 479.77 | SNP | Rv0279c (PE\_PGRS4) | Ala26Gly | 21 | - | | 338998 | C | T | 470.77 | SNP | Rv0279c (PE\_PGRS4) | Ala26Thr | 22 | - | | 340372 | T | C | 453.77 | SNP | Rv0280 (PPE3) | Ser337Pro | 12 | - | | 345828 | T | C | 1334.77 | SNP | Rv0284 (eccC3) | Val(s)65Ala | 9867 | - | | 346275 | C | G | 1923.77 | SNP | Rv0284 (eccC3) | Pro214Arg | 4 | - | | 356528 | A | G | 1152.77 | SNP | Rv0292 (eccE3) | Asn217Asp | 42 | - | | 361597 | C | T | 767.77 | SNP | Rv0297 (PE\_PGRS5) | silent (Ala88) | 9867 | - | | 362426 | G | C | 204.84 | SNP | Rv0297 (PE\_PGRS5) | Gly365Arg | 0 | - | | 368087 | AGCTGCCGGTGTTGAT | A | 6029.73 | DEL | Rv0304c (PPE5) |  |  | - | | 373282 | TA | T | 3139.73 | DEL | Rv0305c (PPE6) |  |  | - | | 376774 | T | C | 1496.77 | SNP | Rv0307c | silent (Ala94) | 9867 | - | | 378831 | G | A | 1580.77 | SNP | Rv0310c | silent (Ile106) | 9872 | - | | 384380 | A | C | 1741.77 | SNP | Rv0315 | Lys260Thr | 8 | - | | 386432 | C | G | 2261.77 | SNP | Rv0318c | Gly223Ala | 21 | - | | 387353 | G | A | 1427.77 | SNP | Rv0319 (pcp) | Gly69Asp | 6 | - | | 390828 | T | C | 1435.77 | SNP | Rv0323c | Ser142Gly | 21 | - | | 396601 | C | T | 1848.77 | SNP | Rv0331 | Ala134Val(s) | 9867 | - | | 403364 | G | A | 1191.77 | SNP | Rv0338c | silent (Pro826) | 9926 | genotype | | 403920 | C | T | 1761.77 | SNP | Rv0338c | Arg641His | 8 | - | | 403980 | G | A | 1806.77 | SNP | Rv0338c | Ala621Val | 13 | - | | 404326 | T | C | 1577.77 | SNP | Rv0338c | Arg506Gly | 1 | - | | 408723 | C | T | 1174.77 | SNP | Rv0340 | silent (Ile30) | 9872 | - | | 414486 | C | T | 2099.77 | SNP | Rv0344c (lpqJ) | silent (Glu152) | 9865 | - | | 420008 | A | G | 1742.77 | SNP | Rv0350 (dnaK) | silent (Ala58) | 9867 | - | | 422833 | T | C | 635.77 | SNP | Rv0352 (dnaJ1) | Leu(s)128Leu | 3 | - | | 424320 | T | TC | 1664.73 | INS | Rv0354c (PPE7) |  |  | - | | 426022 | G | C | 1665.77 | SNP | Rv0355c (PPE8) | Asn2886Lys | 25 | - | | 427310 | TTGCCGAGGTTTGCAC | T | 3566.73 | DEL | Rv0355c (PPE8) |  |  | - | | 444351 | G | T | 1392.77 | SNP | Rv0366c | Asn155Lys | 25 | - | | 457452 | T | G | 1436.77 | SNP | Rv0381c | silent (Thr124) | 9871 | - | | 459399 | A | C | 1462.77 | SNP | intergenic |  |  | - | | 463338 | G | A | 1532.77 | SNP | intergenic |  |  | - | | 467497 | C | CG | 1102.73 | INS | Rv0388c (PPE9) |  |  | - | | 467508 | C | CG | 1144.73 | INS | Rv0388c (PPE9) |  |  | - | | 467516 | G | C | 666.77 | SNP | Rv0388c (PPE9) | silent (Ser162) | 9840 | - | | 467526 | C | G | 689.77 | SNP | Rv0388c (PPE9) | Gly159Ala | 21 | - | | 467546 | G | C | 894.77 | SNP | Rv0388c (PPE9) | Asp152Glu | 56 | - | | 467557 | A | C | 999.77 | SNP | Rv0388c (PPE9) | Leu(s)149Val(s) | 9867 | - | | 467564 | A | C | 1124.77 | SNP | Rv0388c (PPE9) | His146Gln | 23 | - | | 467585 | G | C | 1250.77 | SNP | Rv0388c (PPE9) | His139Gln | 23 | - | | 467590 | T | C | 1203.77 | SNP | Rv0388c (PPE9) | Thr138Ala | 32 | - | | 467621 | T | G | 1252.77 | SNP | Rv0388c (PPE9) | silent (Gly127) | 9935 | - | | 467638 | G | T | 1409.77 | SNP | Rv0388c (PPE9) | Gln122Lys | 12 | - | | 472705 | T | C | 1295.77 | SNP | intergenic |  |  | - | | 475178 | T | C | 1421.77 | SNP | Rv0395 | Val80Ala | 18 | - | | 479632 | G | T | 1013.77 | SNP | Rv0400c (fadE7) | Thr41Asn | 9 | - | | 483745 | A | C | 1402.77 | SNP | intergenic |  |  | - | | 489935 | G | C | 1801.77 | SNP | Rv0405 (pks6); Rv0406c | Arg1402Pro; silent (Thr257) | 5; 9871 | - | | 498557 | C | A | 1854.77 | SNP | Rv0412c | Asp355Tyr | 0 | - | | 501535 | G | T | 1640.77 | SNP | Rv0415 (thiO) | Val130Phe | 0 | - | | 502589 | C | G | 1536.77 | SNP | Rv0417 (thiG) | Ser75Cys | 5 | - | | 503354 | G | C | 2605.77 | SNP | intergenic |  |  | - | | 507364 | C | G | 1474.77 | SNP | Rv0421c | Arg133Pro | 5 | - | | 513257 | T | C | 1614.77 | SNP | Rv0425c (ctpH) | Met(s)689Val(s) | 9867 | - | | 531628 | C | G | 772.77 | SNP | Rv0442c (PPE10) | Gly196Ala | 21 | - | | 534691 | C | T | 1652.77 | SNP | Rv0446c | Trp175STOP | 0 | - | | 535695 | C | T | 1408.77 | SNP | Rv0447c (ufaA1) | silent (Gly271) | 9935 | - | | 541201 | A | G | 1912.77 | SNP | Rv0450c (mmpL4) | silent (Leu97) | 9947 | - | | 545925 | G | C | 42.77 | SNP | Rv0456c (echA2) | Asp293Glu | 56 | - | | 551525 | A | C | 1292.77 | SNP | Rv0459 | silent (Arg110) | 9913 | - | | 558501 | C | T | 1909.77 | SNP | Rv0467 (icl1) | silent (Asp325) | 9859 | - | | 563420 | T | C | 2075.77 | SNP | Rv0472c | Glu3Gly | 7 | - | | 573262 | A | G | 1682.77 | SNP | Rv0484c | silent (Gly180) | 9935 | - | | 580772 | T | A | 353.77 | SNP | intergenic |  |  | - | | 580773 | GGGGGCACCACCCGCTTGCG GGGGA | G | 3240.73 | DEL | intergenic |  |  | - | | 584438 | G | A | 273.78 | SNP | Rv0493c | Pro85Ser | 17 | - | | 589536 | G | A | 1580.77 | SNP | Rv0499 | silent (Leu118) | 9947 | - | | 590436 | T | C | 1526.77 | SNP | Rv0500 (proC) | silent (Ala118) | 9867 | - | | 595287 | C | G | 1116.77 | SNP | Rv0504c | Glu6Gln | 27 | - | | 597816 | A | G | 1697.77 | SNP | Rv0507 (mmpL2) | silent (Ala206) | 9867 | - | | 598475 | G | A | 2217.77 | SNP | Rv0507 (mmpL2) | Arg426His | 8 | - | | 610120 | T | G | 1548.77 | SNP | intergenic |  |  | - | | 623508 | C | G | 62.74 | SNP | Rv0532 (PE\_PGRS6) | Ala239Gly | 21 | - | | 630722 | G | C | 1191.77 | SNP | Rv0538 | Arg228Pro | 5 | - | | 637319 | G | A | 1401.77 | SNP | Rv0545c (pitA) | Pro49Ser | 17 | - | | 637922 | C | T | 1362.77 | SNP | Rv0546c | silent (Gln16) | 9876 | - | | 642131 | C | T | 1125.77 | SNP | Rv0551c (fadD8) | silent (Lys227) | 9926 | - | | 648002 | T | G | 1787.77 | SNP | Rv0556 | Leu15Arg | 1 | - | | 663410 | A | C | 32.77 | SNP | intergenic |  |  | - | | 663429 | T | G | 61.77 | SNP | intergenic |  |  | - | | 664249 | C | T | 1486.77 | SNP | Rv0571c | silent (Gln190) | 9876 | - | | 665293 | A | G | 2004.77 | SNP | Rv0572c | Phe31Leu | 13 | - | | 669398 | T | C | 1293.77 | SNP | Rv0575c | silent (Gln116) | 9876 | - | | 672491 | C | G | 354.77 | SNP | Rv0578c (PE\_PGRS7) | silent (Gly1142) | 9935 | - | | 672512 | ACCGGTGCCG | A | 1183.73 | DEL | Rv0578c (PE\_PGRS7) |  |  | - | | 673238 | A | G | 361.77 | SNP | Rv0578c (PE\_PGRS7) | silent (His893) | 9912 | - | | 675673 | C | T | 693.77 | SNP | Rv0578c (PE\_PGRS7) | Ala82Thr | 22 | - | | 675797 | G | A | 398.77 | SNP | Rv0578c (PE\_PGRS7) | silent (Pro40) | 9926 | - | | 685461 | C | G | 1764.77 | SNP | Rv0587 (yrbE2A) | silent (Ala111) | 9867 | - | | 685608 | T | C | 1719.77 | SNP | Rv0587 (yrbE2A) | silent (Leu160) | 9947 | - | | 686972 | T | C | 2183.77 | SNP | Rv0589 (mce2A) | Phe51Ser | 3 | - | | 690465 | T | G | 864.77 | SNP | Rv0591 (mce2C) | silent (Leu469) | 9947 | - | | 698968 | G | A | 1379.77 | SNP | Rv0601c | silent (Gly9) | 9935 | - | | 709226 | G | A | 1005.77 | SNP | Rv0613c | Thr97Ile | 7 | - | | 726703 | C | A | 613.77 | SNP | Rv0631c (recC) | Arg535Met(s) | 2 | - | | 736710 | T | C | 1566.77 | SNP | Rv0642c (mmaA4) | Asn165Ser | 34 | - | | 738112 | G | C | 2430.77 | SNP | Rv0643c (mmaA3) | Ser13Cys | 5 | - | | 752343 | A | G | 1423.77 | SNP | Rv0655 (mkl) | Glu276Gly | 7 | - | | 752802 | A | C | 1266.77 | SNP | intergenic |  |  | - | | 754186 | A | G | 1707.77 | SNP | Rv0658c | Leu75Pro | 2 | - | | 761032 | A | G | 1141.77 | SNP | Rv0667 (rpoB) | Gln409Arg | 10 | - | | 761155 | C | T | 1227.77 | SNP | Rv0667 (rpoB) | Ser450Leu(s) | 35 | resistance | | 764995 | C | G | 1721.77 | SNP | Rv0668 (rpoC) | silent (Ala542) | 9867 | genotype | | 765837 | T | C | 1520.77 | SNP | Rv0668 (rpoC) | Leu823Pro | 2 | - | | 769663 | T | G | 1408.77 | SNP | intergenic |  |  | - | | 769962 | GC | G | 1944.73 | DEL | Rv0670 (end) |  |  | - | | 773809 | G | A | 610.77 | SNP | Rv0673 (echA4) | silent (Glu229) | 9865 | - | | 775419 | C | T | 1172.77 | SNP | Rv0675 (echA5) | Gln213STOP | 8 | - | | 775639 | T | C | 834.77 | SNP | Rv0676c (mmpL5) | Ile948Val | 57 | - | | 781395 | T | C | 1863.77 | SNP | intergenic (Rv0682-165nt) |  |  | - | | 781687 | A | G | 2062.77 | SNP | Rv0682 (rpsL) | Lys43Arg | 19 | resistance | | 788987 | G | A | 1600.77 | SNP | Rv0688 | Asp350Asn | 36 | - | | 794579 | T | G | 1838.77 | SNP | intergenic |  |  | - | | 796509 | G | T | 1655.77 | SNP | Rv0696 | Gly331Cys | 0 | - | | 801906 | G | C | 1539.77 | SNP | Rv0702 (rplD) | Ala149Pro | 13 | - | | 807480 | C | T | 1382.77 | SNP | Rv0711 (atsA) | silent (Asp382) | 9859 | - | | 812808 | G | A | 2832.77 | SNP | Rv0717 (rpsN1) | Trp61STOP | 0 | - | | 820483 | G | T | 829.77 | SNP | Rv0727c (fucA) | Ala6Asp | 6 | - | | 826091 | T | G | 1774.77 | SNP | Rv0732 (secY) | Leu431Arg | 1 | - | | 836272 | A | G | 62.74 | SNP | Rv0746 (PE\_PGRS9) | Glu191Gly | 7 | - | | 836291 | A | G | 56.74 | SNP | Rv0746 (PE\_PGRS9) | silent (Gly197) | 9935 | - | | 836538 | A | G | 101.28 | SNP | Rv0746 (PE\_PGRS9) | Asn280Asp | 42 | - | | 836658 | A | G | 62.74 | SNP | Rv0746 (PE\_PGRS9) | Thr320Ala | 32 | - | | 837033 | A | G | 392.77 | SNP | Rv0746 (PE\_PGRS9) | Thr445Ala | 32 | - | | 839269 | A | G | 147.80 | SNP | Rv0747 (PE\_PGRS10) | silent (Gly273) | 9935 | - | | 839279 | G | A | 201.90 | SNP | Rv0747 (PE\_PGRS10) | Asp277Asn | 36 | - | | 839291 | T | C | 194.77 | SNP | Rv0747 (PE\_PGRS10) | Phe281Leu | 13 | - | | 839295 | T | C | 194.77 | SNP | Rv0747 (PE\_PGRS10) | Phe282Ser | 3 | - | | 839334 | A | G | 62.74 | SNP | Rv0747 (PE\_PGRS10) | Lys295Arg | 19 | - | | 839348 | A | G | 62.74 | SNP | Rv0747 (PE\_PGRS10) | Ser300Gly | 21 | - | | 839471 | T | C | 91.77 | SNP | Rv0747 (PE\_PGRS10) | Leu(s)341Leu | 3 | - | | 839472 | T | A | 85.77 | SNP | Rv0747 (PE\_PGRS10) | Leu(s)341STOP | 9867 | - | | 839515 | G | A | 188.77 | SNP | Rv0747 (PE\_PGRS10) | silent (Ala355) | 9867 | - | | 839516 | A | G | 261.77 | SNP | Rv0747 (PE\_PGRS10) | Thr356Ala | 32 | - | | 839519 | C | G | 235.77 | SNP | Rv0747 (PE\_PGRS10) | Leu357Val(s) | 4 | - | | 839520 | T | C | 231.77 | SNP | Rv0747 (PE\_PGRS10) | Leu357Pro | 2 | - | | 839534 | A | C | 259.77 | SNP | Rv0747 (PE\_PGRS10) | Ile362Leu | 22 | - | | 839949 | C | G | 85.28 | SNP | Rv0747 (PE\_PGRS10) | Ala500Gly | 21 | - | | 840235 | C | G | 138.90 | SNP | Rv0747 (PE\_PGRS10) | silent (Gly595) | 9935 | - | | 840241 | C | T | 68.77 | SNP | Rv0747 (PE\_PGRS10) | silent (Gly597) | 9935 | - | | 840272 | G | T | 70.77 | SNP | Rv0747 (PE\_PGRS10) | Ala608Ser | 28 | - | | 841764 | G | C | 1872.77 | SNP | Rv0749A | silent (Thr37) | 9871 | - | | 852910 | C | T | 1476.77 | SNP | Rv0758 (phoR) | Pro172Leu | 3 | - | | 854252 | GC | G | 1495.73 | DEL | intergenic |  |  | - | | 857696 | A | G | 1972.77 | SNP | Rv0764c (cyp51) | silent (Ala114) | 9867 | - | | 859131 | C | CA | 2843.73 | INS | Rv0766c (cyp123) |  |  | - | | 859901 | G | A | 2253.77 | SNP | Rv0766c (cyp123) | Arg58Trp | 2 | - | | 862987 | C | T | 2140.77 | SNP | Rv0769 | silent (Arg192) | 9913 | - | | 863805 | T | C | 1519.77 | SNP | Rv0770 | Phe184Leu | 13 | - | | 874835 | C | CCG | 5003.73 | INS | Rv0781 (ptrBa); Rv0782 (ptrBb) |  |  | - | | 880562 | G | T | 1770.77 | SNP | Rv0785 | Cys408Phe | 0 | - | | 882257 | T | C | 1400.77 | SNP | Rv0787 | Tyr267His | 4 | - | | 885542 | G | C | 1388.77 | SNP | Rv0791c | Ser100Cys | 5 | - | | 888774 | G | A | 1080.77 | SNP | intergenic |  |  | - | | 893441 | C | T | 1349.77 | SNP | Rv0800 (pepC) | Arg42Cys | 1 | - | | 893733 | T | G | 1490.77 | SNP | Rv0800 (pepC) | Leu139Arg | 1 | - | | 894060 | A | G | 83.77 | SNP | Rv0800 (pepC) | Asp248Gly | 11 | - | | 900221 | T | C | 1163.77 | SNP | Rv0806c (cpsY) | Val370Val(s) | 18 | - | | 903550 | T | C | 1856.77 | SNP | Rv0808 (purF) | silent (Ala480) | 9867 | - | | 903913 | T | C | 1380.77 | SNP | Rv0809 (purM) | silent (Gly63) | 9935 | - | | 906857 | A | G | 1445.77 | SNP | Rv0812 | Ile145Met(s) | 6 | - | | 919393 | G | C | 1544.77 | SNP | Rv0825c | Thr54Arg | 1 | - | | 921813 | C | G | 1369.77 | SNP | Rv0829 | Ala80Gly | 21 | - | | 927385 | A | G | 85.28 | SNP | Rv0833 (PE\_PGRS13) | silent (Gly675) | 9935 | - | | 942479 | T | C | 1406.77 | SNP | intergenic |  |  | - | | 945214 | G | A | 1767.77 | SNP | Rv0848 (cysK2) | Gly93Ser | 16 | - | | 947429 | T | A | 1880.77 | SNP | Rv0850 | Ser40Thr | 32 | - | | 947430 | C | A | 1860.77 | SNP | Rv0850 | Ser40STOP | 35 | - | | 949535 | T | C | 1952.77 | SNP | Rv0853c (pdc) | silent (Ala528) | 9867 | - | | 954457 | G | A | 1507.77 | SNP | Rv0858c (dapC) | Ala155Val(s) | 9867 | - | | 955011 | A | G | 1429.77 | SNP | intergenic |  |  | - | | 955524 | A | G | 1502.77 | SNP | Rv0859 (fadA) | Ser150Gly | 21 | - | | 968140 | G | A | 1628.77 | SNP | Rv0871 (cspB) | silent (Glu81) | 9865 | - | | 968426 | A | AGCCGGGTTG | 2647.80 | INS | Rv0872c (PE\_PGRS15) |  |  | - | | 976897 | T | TGGG | 432.74 | INS | Rv0878c (PPE13) |  |  | - | | 979704 | G | C | 1199.77 | SNP | Rv0881 | Gly115Arg | 0 | - | | 986427 | A | C | 2399.77 | SNP | intergenic |  |  | - | | 986463 | G | C | 2346.77 | SNP | intergenic |  |  | - | | 990001 | G | C | 1562.77 | SNP | Rv0890c | Pro866Ala | 22 | - | | 991515 | C | T | 1619.77 | SNP | Rv0890c | Gly361Asp | 6 | - | | 993346 | A | C | 2050.77 | SNP | Rv0891c | Val37Gly | 5 | - | | 1010204 | C | CG | 2135.73 | INS | Rv0907 |  |  | - | | 1025106 | T | C | 2420.77 | SNP | Rv0919 | silent (Phe141) | 9946 | - | | 1037012 | T | C | 766.77 | SNP | Rv0930 (pstA1) | Met(s)5Thr | 22 | - | | 1037911 | C | T | 1338.77 | SNP | Rv0930 (pstA1) | Arg305STOP | 2 | - | | 1040050 | C | T | 1938.77 | SNP | Rv0932c (pstS2) | silent (Ala333) | 9867 | - | | 1040703 | C | T | 1312.77 | SNP | Rv0932c (pstS2) | Ala116Thr | 22 | - | | 1047165 | T | C | 962.77 | SNP | Rv0938 (ligD) | Cys344Arg | 1 | - | | 1054362 | G | A | 1663.77 | SNP | Rv0945 | Arg39His | 8 | - | | 1057788 | T | G | 1300.77 | SNP | Rv0948c | Lys59Thr | 8 | - | | 1061676 | GTGC | G | 4342.73 | DEL | intergenic |  |  | - | | 1068151 | T | C | 1917.77 | SNP | Rv0956 (purN) | silent (His197) | 9912 | - | | 1068432 | A | G | 1057.77 | SNP | Rv0957 (purH) | silent (Pro76) | 9926 | - | | 1070702 | T | C | 1147.77 | SNP | Rv0958 | Ser274Pro | 12 | - | | 1073033 | T | G | 1616.77 | SNP | Rv0959 | silent (Gly593) | 9935 | - | | 1074558 | G | A | 1236.77 | SNP | Rv0962c (lprP) | Pro186Leu | 3 | - | | 1075279 | T | C | 2036.77 | SNP | intergenic |  |  | - | | 1076309 | G | T | 1851.77 | SNP | Rv0964c | Pro124Thr | 5 | - | | 1077312 | A | G | 1667.77 | SNP | Rv0966c | Val(s)175Ala | 9867 | - | | 1079927 | C | A | 1198.77 | SNP | Rv0969 (ctpV) | silent (Thr395) | 9871 | - | | 1081681 | T | C | 1234.77 | SNP | Rv0970 | silent (Val210) | 9901 | - | | 1087193 | G | C | 1572.77 | SNP | Rv0974c (accD2) | Asn51Lys | 25 | - | | 1087279 | T | C | 1359.77 | SNP | Rv0974c (accD2) | Lys23Glu | 4 | - | | 1093322 | C | T | 870.77 | SNP | intergenic |  |  | - | | 1093406 | A | G | 1118.77 | SNP | Rv0978c (PE\_PGRS17) | silent (Val317) | 9901 | - | | 1096508 | C | G | 112.03 | SNP | intergenic |  |  | - | | 1096510 | T | C | 118.03 | SNP | intergenic |  |  | - | | 1096633 | T | G | 758.77 | SNP | intergenic |  |  | - | | 1100234 | T | C | 1398.77 | SNP | Rv0983 (pepD) | Leu390Pro | 2 | - | | 1103249 | C | T | 1468.77 | SNP | Rv0987 | silent (Ala236) | 9867 | - | | 1106422 | T | C | 2930.77 | SNP | Rv0989c (grcC2) | Ile321Val | 57 | - | | 1107434 | A | T | 2116.77 | SNP | intergenic |  |  | - | | 1107917 | G | T | 1363.77 | SNP | Rv0990c | His61Gln | 23 | - | | 1109975 | A | G | 1561.77 | SNP | Rv0993 (galU) | Gln235Arg | 10 | - | | 1126889 | G | C | 1809.77 | SNP | Rv1007c (metS) | Arg39Gly | 1 | - | | 1127648 | C | A | 2230.77 | SNP | Rv1008 (tatD) | Thr187Asn | 9 | - | | 1142266 | A | C | 1001.77 | SNP | Rv1020 (mfd) | silent (Leu1100) | 9947 | - | | 1144664 | G | A | 1293.77 | SNP | Rv1023 (eno) | Arg34Gln | 9 | - | | 1149551 | C | T | 1825.77 | SNP | Rv1028c (kdpD) | silent (Glu712) | 9865 | - | | 1150585 | G | A | 1365.77 | SNP | Rv1028c (kdpD) | Pro368Ser | 17 | - | | 1162274 | C | T | 1401.77 | SNP | Rv1039c (PPE15) | Ala67Thr | 22 | - | | 1163134 | T | C | 1544.77 | SNP | Rv1040c (PE8) | silent (Gly81) | 9935 | - | | 1164571 | A | G | 1882.77 | SNP | intergenic |  |  | - | | 1164809 | T | G | 598.77 | SNP | Rv1041c | Gln209His | 20 | - | | 1165521 | T | TA | 2762.73 | INS | intergenic |  |  | - | | 1168715 | C | CT | 2695.73 | INS | Rv1046c |  |  | - | | 1170404 | C | A | 188.84 | SNP | Rv1047 | Gln328Lys | 12 | - | | 1177994 | T | C | 2195.77 | SNP | Rv1056 | Tyr123His | 4 | - | | 1178116 | T | C | 2042.77 | SNP | Rv1056 | silent (Thr163) | 9871 | - | | 1189650 | C | A | 163.84 | SNP | Rv1067c (PE\_PGRS19) | Gly259Cys | 0 | - | | 1189921 | C | T | 32.74 | SNP | Rv1067c (PE\_PGRS19) | silent (Gly168) | 9935 | - | | 1190093 | A | C | 971.77 | SNP | Rv1067c (PE\_PGRS19) | Leu(s)111Trp | 0 | - | | 1190154 | C | T | 1232.77 | SNP | Rv1067c (PE\_PGRS19) | Glu91Lys | 7 | - | | 1199547 | G | A | 1325.77 | SNP | Rv1075c | Pro275Leu | 3 | - | | 1200418 | A | G | 1522.77 | SNP | intergenic |  |  | - | | 1212076 | G | A | 225.80 | SNP | Rv1087 (PE\_PGRS21) | Gly173Ser | 16 | - | | 1212326 | C | T | 73.28 | SNP | Rv1087 (PE\_PGRS21) | Ala256Val | 13 | - | | 1212616 | TCCGGTGGCGACGGCGGCGC CGGCGGGGCCGGTGGCG | T | 3819.74 | DEL | Rv1087 (PE\_PGRS21) |  |  | - | | 1217264 | A | G | 212.84 | SNP | Rv1091 (PE\_PGRS22) | Asn266Asp | 42 | - | | 1218301 | G | C | 304.78 | SNP | Rv1091 (PE\_PGRS22) | silent (Leu611) | 9947 | - | | 1218737 | G | T | 413.77 | SNP | Rv1091 (PE\_PGRS22) | Gly757Cys | 0 | - | | 1220680 | T | C | 1737.77 | SNP | Rv1093 (glyA1) | Val36Ala | 18 | - | | 1224367 | T | C | 1240.77 | SNP | intergenic |  |  | - | | 1232609 | A | G | 1552.77 | SNP | Rv1105 | Lys100Arg | 19 | - | | 1248978 | T | C | 1753.77 | SNP | Rv1125 | silent (Ala299) | 9867 | - | | 1254147 | C | G | 1246.77 | SNP | Rv1129c | Ala130Pro | 13 | - | | 1266158 | T | C | 49.77 | SNP | Rv1138c | Thr111Ala | 32 | - | | 1272010 | C | T | 1248.77 | SNP | intergenic |  |  | - | | 1277869 | G | GT | 2119.73 | INS | intergenic |  |  | - | | 1278583 | A | C | 284.78 | SNP | intergenic |  |  | - | | 1281118 | T | C | 2056.77 | SNP | Rv1154c | Thr123Ala | 32 | - | | 1285102 | G | T | 1870.77 | SNP | Rv1159 (pimE) | silent (Pro37) | 9926 | - | | 1292102 | A | G | 1195.77 | SNP | Rv1162 (narH) | silent (Pro346) | 9926 | - | | 1307598 | C | G | 1294.77 | SNP | Rv1175c (fadH) | Cys210Ser | 11 | - | | 1313337 | A | AG | 1882.73 | INS | intergenic |  |  | - | | 1313338 | A | C | 940.77 | SNP | intergenic |  |  | - | | 1315191 | A | C | 1316.77 | SNP | Rv1180 (pks3) | STOP489Tyr | 1 | - | | 1315884 | G | A | 1551.77 | SNP | Rv1181 (pks4) | silent (Ala217) | 9867 | - | | 1324724 | C | A | 1778.77 | SNP | Rv1184c | Met(s)296Ile | 2 | - | | 1327890 | G | A | 1739.77 | SNP | Rv1186c | silent (Asp472) | 9859 | - | | 1328687 | G | C | 1594.77 | SNP | Rv1186c | Pro207Ala | 22 | - | | 1339399 | C | T | 1261.77 | SNP | Rv1196 (PPE18) | silent (Tyr17) | 9945 | - | | 1339432 | G | C | 837.77 | SNP | Rv1196 (PPE18) | silent (Ala28) | 9867 | - | | 1339435 | T | G | 880.77 | SNP | Rv1196 (PPE18) | silent (Ala29) | 9867 | - | | 1339436 | C | A | 836.77 | SNP | Rv1196 (PPE18) | Gln30Lys | 12 | - | | 1341102 | C | T | 953.77 | SNP | Rv1198 (esxL) | Arg33Cys | 1 | - | | 1341103 | G | C | 1088.77 | SNP | Rv1198 (esxL) | Arg33Pro | 5 | - | | 1341624 | G | T | 46.74 | SNP | Rv1199c | Gln328Lys | 12 | - | | 1349001 | A | G | 1469.77 | SNP | Rv1205 | Met(s)95Val(s) | 9867 | - | | 1354437 | A | C | 1368.77 | SNP | Rv1211 | silent (Ala65) | 9867 | - | | 1360209 | T | C | 1652.77 | SNP | Rv1217c | silent (Ala531) | 9867 | - | | 1360652 | G | A | 2370.77 | SNP | Rv1217c | His384Tyr | 4 | - | | 1365837 | C | CG | 1707.73 | INS | intergenic |  |  | - | | 1368769 | A | C | 1400.77 | SNP | intergenic |  |  | - | | 1372887 | C | T | 875.77 | SNP | Rv1229c (mrp) | silent (Ala21) | 9867 | - | | 1374065 | T | C | 1054.77 | SNP | Rv1230c | Ser45Gly | 21 | - | | 1375724 | A | C | 1533.77 | SNP | Rv1232c | Cys149Gly | 1 | - | | 1382628 | T | C | 1555.77 | SNP | Rv1239c (corA) | Lys139Glu | 4 | - | | 1389738 | G | A | 2464.77 | SNP | Rv1248c | silent (Asp1105) | 9859 | - | | 1390527 | C | G | 1149.77 | SNP | Rv1248c | silent (Ser842) | 9840 | - | | 1390528 | G | T | 1063.77 | SNP | Rv1248c | Ser842STOP | 35 | - | | 1393626 | A | G | 1477.77 | SNP | Rv1249c | silent (Leu119) | 9947 | - | | 1396922 | T | C | 1336.77 | SNP | Rv1251c | silent (Thr773) | 9871 | - | | 1404169 | T | G | 1267.77 | SNP | Rv1256c (cyp130) | Lys145Asn | 13 | - | | 1411210 | T | G | 1747.77 | SNP | Rv1263 (amiB2) | Val260Val(s) | 18 | - | | 1413148 | C | T | 1608.77 | SNP | intergenic |  |  | - | | 1414021 | C | T | 1267.77 | SNP | Rv1266c (pknH) | Arg607Gln | 9 | - | | 1416222 | A | G | 50.77 | SNP | Rv1267c (embR) | Phe376Leu | 13 | - | | 1416232 | A | G | 51.77 | SNP | Rv1267c (embR) | silent (Cys372) | 9973 | - | | 1416234 | A | C | 53.77 | SNP | Rv1267c (embR) | Cys372Gly | 1 | - | | 1428506 | G | T | 1534.77 | SNP | Rv1278 | Ala365Ser | 28 | - | | 1431679 | A | G | 959.77 | SNP | Rv1280c (oppA) | Phe588Leu | 13 | - | | 1440469 | C | G | 1828.77 | SNP | Rv1286 (cysN) | silent (Pro521) | 9926 | - | | 1445474 | G | A | 1341.77 | SNP | intergenic |  |  | - | | 1445781 | A | G | 1588.77 | SNP | Rv1291c | silent (Ala18) | 9867 | - | | 1446923 | T | G | 1225.77 | SNP | Rv1292 (argS) | Ile182Ser | 2 | - | | 1452071 | C | A | 1112.77 | SNP | Rv1296 (thrB) | silent (Gly25) | 9935 | - | | 1457144 | C | T | 1387.77 | SNP | Rv1300 (hemK) | Arg194Cys | 1 | - | | 1471659 | C | T | 1944.77 | SNP | intergenic |  |  | - | | 1480024 | G | T | 1778.77 | SNP | Rv1318c | Phe267Leu | 13 | - | | 1480972 | T | C | 1457.77 | SNP | Rv1319c | silent (Glu510) | 9865 | - | | 1481185 | A | C | 910.77 | SNP | Rv1319c | Asp439Glu | 56 | - | | 1482627 | T | C | 1978.77 | SNP | Rv1320c | Thr531Ala | 32 | - | | 1484708 | A | C | 1678.77 | SNP | Rv1321 | Ser144Arg | 6 | - | | 1488433 | A | G | 84.77 | SNP | Rv1325c (PE\_PGRS24) | silent (Asp511) | 9859 | - | | 1488434 | T | G | 61.77 | SNP | Rv1325c (PE\_PGRS24) | Asp511Ala | 10 | - | | 1488435 | C | A | 89.77 | SNP | Rv1325c (PE\_PGRS24) | Asp511Tyr | 0 | - | | 1499274 | C | G | 898.77 | SNP | Rv1330c (pncB1) | Gly429Ala | 21 | - | | 1512031 | T | G | 1097.77 | SNP | Rv1347c (mbtK) | Asp192Ala | 10 | - | | 1512461 | G | T | 477.77 | SNP | Rv1347c (mbtK) | Gln49Lys | 12 | - | | 1519847 | C | G | 1373.77 | SNP | Rv1353c | Gly47Arg | 0 | - | | 1526819 | C | A | 1373.77 | SNP | Rv1358 | silent (Arg70) | 9913 | - | | 1527449 | G | GT | 3316.73 | INS | Rv1358 |  |  | - | | 1532743 | C | G | 661.77 | SNP | Rv1361c (PPE19) | silent (Ser297) | 9840 | - | | 1532744 | G | C | 617.77 | SNP | Rv1361c (PPE19) | Ser297Trp | 1 | - | | 1532827 | A | T | 1088.77 | SNP | Rv1361c (PPE19) | Phe269Leu | 13 | - | | 1533241 | G | C | 1819.77 | SNP | Rv1361c (PPE19) | silent (Thr131) | 9871 | - | | 1533583 | G | A | 1052.77 | SNP | Rv1361c (PPE19) | silent (Tyr17) | 9945 | - | | 1535141 | G | C | 2273.77 | SNP | Rv1363c | Asp84Glu | 56 | - | | 1536251 | G | T | 1770.77 | SNP | Rv1364c | Ala465Glu | 10 | - | | 1546530 | G | A | 1468.77 | SNP | Rv1373 | silent (Glu173) | 9865 | - | | 1547125 | T | C | 1645.77 | SNP | Rv1374c | Thr136Ala | 32 | - | | 1552547 | G | A | 1283.77 | SNP | Rv1378c | Arg37Trp | 2 | - | | 1553568 | G | A | 1317.77 | SNP | Rv1380 (pyrB) | Ala113Thr | 22 | - | | 1563717 | C | T | 1901.77 | SNP | Rv1388 (mihF) | silent (Val8) | 9901 | - | | 1564215 | C | G | 43.77 | SNP | Rv1388 (mihF) | silent (Gly174) | 9935 | - | | 1570566 | C | A | 1276.77 | SNP | Rv1394c (cyp132) | Arg135Leu | 1 | - | | 1572680 | GC | G | 541.73 | DEL | Rv1396c (PE\_PGRS25) |  |  | - | | 1573483 | C | G | 30.77 | SNP | Rv1396c (PE\_PGRS25) | silent (Gly125) | 9935 | - | | 1573497 | C | G | 33.77 | SNP | Rv1396c (PE\_PGRS25) | Asp121His | 3 | - | | 1573506 | G | C | 37.77 | SNP | Rv1396c (PE\_PGRS25) | His118Asp | 4 | - | | 1573507 | G | A | 36.77 | SNP | Rv1396c (PE\_PGRS25) | silent (Gly117) | 9935 | - | | 1573517 | G | T | 33.77 | SNP | Rv1396c (PE\_PGRS25) | Pro114Gln | 6 | - | | 1573518 | G | T | 39.77 | SNP | Rv1396c (PE\_PGRS25) | Pro114Thr | 5 | - | | 1573527 | G | C | 36.77 | SNP | Rv1396c (PE\_PGRS25) | Leu111Val(s) | 4 | - | | 1573660 | T | G | 1129.77 | SNP | Rv1396c (PE\_PGRS25) | Arg66Ser | 11 | - | | 1574776 | T | C | 2355.77 | SNP | intergenic |  |  | - | | 1586249 | C | T | 1463.77 | SNP | Rv1410c | silent (Gln506) | 9876 | - | | 1588899 | G | T | 1602.77 | SNP | Rv1412 (ribC) | silent (Ala111) | 9867 | - | | 1609840 | A | G | 1314.77 | SNP | Rv1431 | silent (Pro586) | 9926 | - | | 1612624 | T | TATCGGTACCGGTGCGCCAG GG | 6205.73 | INS | Rv1435c |  |  | - | | 1613035 | T | C | 987.77 | SNP | intergenic |  |  | - | | 1613960 | G | T | 1745.77 | SNP | Rv1436 (gap) | silent (Ala218) | 9867 | - | | 1618624 | GTGCCGCCGGCGCCGCCGTC GCCGCCGGCGCCGCCGGCGC CGGCGCTGCTGCCGCCGA | G | 2191.74 | DEL | Rv1441c (PE\_PGRS26) |  |  | - | | 1624791 | C | G | 1200.77 | SNP | Rv1446c (opcA) | Arg192Pro | 5 | - | | 1630148 | A | C | 1534.77 | SNP | Rv1449c (tkt) | Tyr18Asp | 0 | - | | 1634580 | T | C | 84.77 | SNP | Rv1450c (PE\_PGRS27) | Leu16Leu(s) | 4 | - | | 1634581 | A | G | 108.77 | SNP | Rv1450c (PE\_PGRS27) | Leu16Ser | 1 | - | | 1634586 | T | C | 133.77 | SNP | Rv1450c (PE\_PGRS27) | silent (Ala14) | 9867 | - | | 1634589 | G | C | 78.77 | SNP | Rv1450c (PE\_PGRS27) | silent (Ala13) | 9867 | - | | 1634592 | C | G | 71.77 | SNP | Rv1450c (PE\_PGRS27) | silent (Ala12) | 9867 | - | | 1636826 | C | A | 300.31 | SNP | Rv1452c (PE\_PGRS28) | silent (Gly468) | 9935 | - | | 1636918 | C | T | 251.77 | SNP | Rv1452c (PE\_PGRS28) | Ala438Thr | 22 | - | | 1636980 | G | T | 162.77 | SNP | Rv1452c (PE\_PGRS28) | Pro417His | 3 | - | | 1636981 | G | T | 153.77 | SNP | Rv1452c (PE\_PGRS28) | Pro417Thr | 5 | - | | 1636983 | C | G | 177.77 | SNP | Rv1452c (PE\_PGRS28) | Gly416Ala | 21 | - | | 1636991 | T | C | 85.77 | SNP | Rv1452c (PE\_PGRS28) | silent (Gly413) | 9935 | - | | 1636996 | G | C | 95.77 | SNP | Rv1452c (PE\_PGRS28) | Arg412Gly | 1 | - | | 1638020 | T | C | 30.74 | SNP | Rv1452c (PE\_PGRS28) | silent (Ala70) | 9867 | - | | 1638125 | T | C | 377.77 | SNP | Rv1452c (PE\_PGRS28) | silent (Ser35) | 9840 | - | | 1638140 | G | C | 368.77 | SNP | Rv1452c (PE\_PGRS28) | Ser30Arg | 6 | - | | 1638141 | C | G | 374.77 | SNP | Rv1452c (PE\_PGRS28) | Ser30Thr | 32 | - | | 1638142 | T | C | 343.77 | SNP | Rv1452c (PE\_PGRS28) | Ser30Gly | 21 | - | | 1638143 | G | A | 319.77 | SNP | Rv1452c (PE\_PGRS28) | silent (Asn29) | 9822 | - | | 1638149 | T | C | 360.77 | SNP | Rv1452c (PE\_PGRS28) | Val27Val(s) | 18 | - | | 1638150 | A | G | 368.77 | SNP | Rv1452c (PE\_PGRS28) | Val27Ala | 18 | - | | 1638152 | A | G | 357.77 | SNP | Rv1452c (PE\_PGRS28) | silent (Gly26) | 9935 | - | | 1638975 | T | G | 47.77 | SNP | Rv1453 | Ser199Ala | 35 | - | | 1639594 | C | A | 1637.77 | SNP | Rv1453 | Pro405Gln | 6 | - | | 1645802 | T | C | 2380.77 | SNP | Rv1459c | Lys113Glu | 4 | - | | 1650072 | A | G | 957.77 | SNP | Rv1462 | Asn183Asp | 42 | - | | 1651306 | C | T | 1450.77 | SNP | Rv1463 | silent (Ala197) | 9867 | - | | 1676128 | C | T | 1273.77 | SNP | Rv1486c | silent (Ser252) | 9840 | - | | 1676290 | C | A | 1648.77 | SNP | Rv1486c | Lys198Asn | 13 | - | | 1680398 | G | A | 2056.77 | SNP | Rv1490 | silent (Ala359) | 9867 | - | | 1686372 | T | A | 1739.77 | SNP | Rv1494 (mazE4) | silent (Ile34) | 9872 | - | | 1689349 | C | T | 1378.77 | SNP | Rv1498c | Arg191His | 8 | - | | 1691888 | G | A | 1838.77 | SNP | intergenic |  |  | - | | 1692141 | A | C | 2255.77 | SNP | Rv1501 | silent (Ile84) | 9872 | - | | 1692795 | G | C | 1016.77 | SNP | intergenic |  |  | - | | 1693561 | A | G | 2781.77 | SNP | Rv1502 | Tyr213Cys | 3 | - | | 1698911 | G | A | 1733.77 | SNP | Rv1508c | silent (Gly328) | 9935 | - | | 1706119 | T | C | 2390.77 | SNP | Rv1514c | silent (Ser159) | 9840 | - | | 1709828 | C | T | 1482.77 | SNP | Rv1518 | Ala62Val(s) | 9867 | - | | 1718761 | C | T | 1558.77 | SNP | Rv1524 | silent (Gly12) | 9935 | - | | 1728837 | A | G | 1291.77 | SNP | intergenic |  |  | - | | 1732239 | C | T | 1580.77 | SNP | Rv1530 (adh) | silent (Ser289) | 9840 | - | | 1734994 | C | T | 1537.77 | SNP | Rv1534 | silent (Ala87) | 9867 | - | | 1740771 | A | C | 985.77 | SNP | Rv1537 (dinX) | Thr306Pro | 4 | - | | 1752561 | T | C | 838.77 | SNP | Rv1548c (PPE21) | Asp258Gly | 11 | - | | 1753519 | G | GC | 1675.73 | INS | Rv1549 (fadD11.1) |  |  | - | | 1755519 | G | T | 1350.77 | SNP | Rv1551 (plsB1) | Met(s)25Ile | 2 | - | | 1759252 | G | T | 1080.77 | SNP | Rv1552 (frdA) | silent (Ser524) | 9840 | genotype | | 1760292 | A | G | 1318.77 | SNP | Rv1554 (frdC) | Met(s)40Val(s) | 9867 | - | | 1762615 | C | T | 1967.77 | SNP | Rv1557 (mmpL6) | Ala291Val | 13 | - | | 1775312 | A | C | 1605.77 | SNP | intergenic |  |  | - | | 1777182 | G | A | 1435.77 | SNP | Rv1569 (bioF1) | Ala161Thr | 22 | - | | 1777213 | C | G | 1315.77 | SNP | Rv1569 (bioF1) | Ala171Gly | 21 | - | | 1778430 | T | C | 884.77 | SNP | Rv1570 (bioD) | Met(s)191Thr | 22 | - | | 1779243 | T | G | 730.77 | SNP | Rv1572c | His19Pro | 5 | - | | 1789516 | A | G | 195.84 | SNP | Rv1588c | silent (Gly107) | 9935 | - | | 1789564 | C | T | 303.77 | SNP | Rv1588c | silent (Arg91) | 9913 | - | | 1789565 | C | A | 267.31 | SNP | Rv1588c | Arg91Leu | 1 | - | | 1789591 | T | C | 54.77 | SNP | Rv1588c | Leu82Leu(s) | 4 | - | | 1789593 | A | G | 79.77 | SNP | Rv1588c | silent (Leu82) | 9947 | - | | 1789650 | C | T | 593.77 | SNP | Rv1588c | Ala63Thr | 22 | - | | 1789654 | A | G | 767.77 | SNP | Rv1588c | silent (Leu61) | 9947 | - | | 1789671 | C | T | 792.77 | SNP | Rv1588c | Ala56Thr | 22 | - | | 1789675 | A | C | 806.77 | SNP | Rv1588c | silent (Gly54) | 9935 | - | | 1789678 | C | G | 791.77 | SNP | Rv1588c | Val(s)53Val | 13 | - | | 1789742 | G | C | 214.77 | SNP | Rv1588c | Thr32Ser | 38 | - | | 1789746 | A | G | 234.77 | SNP | Rv1588c | Leu(s)31Leu | 3 | - | | 1789756 | A | G | 60.77 | SNP | Rv1588c | silent (Ser27) | 9840 | - | | 1793769 | C | T | 1550.77 | SNP | intergenic |  |  | - | | 1797027 | C | T | 1173.77 | SNP | Rv1595 (nadB) | Thr408Ile | 7 | - | | 1798355 | G | A | 1603.77 | SNP | Rv1597 | Gly21Asp | 6 | - | | 1803265 | G | A | 1628.77 | SNP | Rv1602 (hisH) | Ser201Asn | 20 | - | | 1804409 | C | A | 1734.77 | SNP | Rv1604 (impA) | Pro124Gln | 6 | - | | 1805948 | C | T | 1583.77 | SNP | Rv1606 (hisI) | Thr99Ile | 7 | - | | 1815604 | G | A | 1434.77 | SNP | Rv1615 | Ala118Thr | 22 | - | | 1817976 | A | T | 1686.77 | SNP | Rv1618 (tesB1) | His121Leu | 4 | - | | 1819591 | C | T | 1393.77 | SNP | Rv1619 | silent (Pro339) | 9926 | - | | 1836286 | G | C | 1209.77 | SNP | intergenic |  |  | - | | 1837760 | A | G | 34.77 | SNP | Rv1633 (uvrB) | His229Arg | 10 | - | | 1847919 | C | G | 1673.77 | SNP | Rv1639c | silent (Thr180) | 9871 | - | | 1854300 | T | C | 1209.77 | SNP | Rv1644 (tsnR) | Leu232Pro | 2 | - | | 1856777 | G | C | 1709.77 | SNP | Rv1647 | Ala2Pro | 13 | - | | 1879671 | T | C | 78.77 | SNP | Rv1661 (pks7) | silent (Gly1456) | 9935 | - | | 1882807 | G | A | 1152.77 | SNP | Rv1662 (pks8) | silent (Gly368) | 9935 | - | | 1885772 | G | A | 1158.77 | SNP | Rv1662 (pks8) | Ala1357Thr | 22 | - | | 1885802 | C | T | 1034.77 | SNP | Rv1662 (pks8) | Pro1367Ser | 17 | - | | 1894300 | G | GGTCTTGCCGC | 6484.73 | INS | Rv1668c |  |  | - | | 1895174 | C | T | 1668.77 | SNP | Rv1668c | Asp57Asn | 36 | - | | 1899351 | T | C | 1542.77 | SNP | Rv1674c | Glu189Gly | 7 | - | | 1901493 | T | C | 1163.77 | SNP | Rv1676 | silent (Ser149) | 9840 | - | | 1907296 | G | C | 2486.77 | SNP | Rv1682 | silent (Ala298) | 9867 | - | | 1917972 | A | G | 1438.77 | SNP | Rv1694 (tlyA) | silent (Leu11) | 9947 | - | | 1931179 | C | A | 1362.77 | SNP | Rv1704c (cycA) | Arg93Leu | 1 | - | | 1933988 | G | A | 2448.77 | SNP | intergenic |  |  | - | | 1943039 | C | T | 992.77 | SNP | Rv1715 (fadB3) | silent (Ile127) | 9872 | - | | 1943592 | C | T | 1546.77 | SNP | Rv1716 | Pro6Leu | 3 | - | | 1944107 | A | G | 1129.77 | SNP | Rv1716 | Ser178Gly | 21 | - | | 1944402 | T | C | 1428.77 | SNP | Rv1716 | Val276Ala | 18 | - | | 1944642 | CT | C | 1841.73 | DEL | Rv1717 |  |  | - | | 1947903 | G | T | 1292.77 | SNP | Rv1722 | Val(s)15Leu(s) | 9867 | - | | 1950767 | T | C | 1767.77 | SNP | Rv1724c | silent (Lys95) | 9926 | - | | 1960284 | C | A | 1285.77 | SNP | Rv1733c | Gln68His | 20 | - | | 1967237 | C | A | 1376.77 | SNP | Rv1739c | Arg134Leu | 1 | - | | 1981056 | C | T | 1440.77 | SNP | intergenic |  |  | - | | 1982901 | G | T | 816.77 | SNP | Rv1753c (PPE24) | silent (Pro625) | 9926 | - | | 1983057 | T | C | 33.04 | SNP | Rv1753c (PPE24) | silent (Pro573) | 9926 | - | | 1983195 | A | G | 314.78 | SNP | Rv1753c (PPE24) | silent (Gly527) | 9935 | - | | 1983198 | C | G | 321.78 | SNP | Rv1753c (PPE24) | Val(s)526Val | 13 | - | | 1983313 | T | G | 1274.77 | SNP | Rv1753c (PPE24) | Asn488Thr | 13 | - | | 1989057 | AGAGAGGACTTCATCGATGC GCT | A | 8078.73 | DEL | Rv1758 (cut1) |  |  | - | | 1990771 | C | T | 79.03 | SNP | Rv1759c (wag22) | Ala603Thr | 22 | - | | 1990919 | G | C | 471.77 | SNP | Rv1759c (wag22) | Phe553Leu(s) | 2 | - | | 1993808 | A | T | 1558.77 | SNP | Rv1760 | Glu219Val(s) | 17 | - | | 2007545 | T | G | 967.77 | SNP | Rv1773c | silent (Ala74) | 9867 | - | | 2022868 | T | C | 1059.77 | SNP | Rv1783 (eccC5) | silent (Ser1204) | 9840 | - | | 2026008 | C | G | 75.28 | SNP | Rv1787 (PPE25) | Phe236Leu(s) | 2 | - | | 2026025 | A | G | 95.03 | SNP | Rv1787 (PPE25) | Gln242Arg | 10 | - | | 2026029 | C | T | 116.03 | SNP | Rv1787 (PPE25) | silent (Phe243) | 9946 | - | | 2026030 | T | C | 112.03 | SNP | Rv1787 (PPE25) | Phe244Leu | 13 | - | | 2026032 | C | T | 115.03 | SNP | Rv1787 (PPE25) | silent (Phe244) | 9946 | - | | 2026033 | G | A | 86.03 | SNP | Rv1787 (PPE25) | Ala245Thr | 22 | - | | 2026110 | G | A | 661.77 | SNP | Rv1787 (PPE25) | silent (Pro270) | 9926 | - | | 2037716 | T | C | 1900.77 | SNP | Rv1798 (eccA5) | silent (Arg339) | 9913 | - | | 2043557 | A | G | 1011.77 | SNP | Rv1802 (PPE30) | silent (Gly58) | 9935 | - | | 2045310 | A | G | 442.77 | SNP | Rv1803c (PE\_PGRS32) | silent (Ile511) | 9872 | - | | 2049065 | T | C | 1333.77 | SNP | intergenic |  |  | - | | 2049097 | G | C | 1262.77 | SNP | intergenic |  |  | - | | 2051746 | T | C | 1605.77 | SNP | Rv1809 (PPE33) | silent (Ala155) | 9867 | - | | 2052035 | G | T | 1568.77 | SNP | Rv1809 (PPE33) | Val(s)252Leu(s) | 9867 | - | | 2053411 | G | C | 1493.77 | SNP | intergenic |  |  | - | | 2053682 | C | T | 1484.77 | SNP | Rv1811 (mgtC) | silent (Ile80) | 9872 | genotype | | 2055271 | A | G | 1207.77 | SNP | Rv1812c | Leu30Pro | 2 | - | | 2057774 | A | T | 1372.77 | SNP | Rv1815 | Ile83Phe | 8 | - | | 2062947 | G | A | 847.77 | SNP | Rv1819c (bacA) | silent (Arg594) | 9913 | - | | 2070413 | G | C | 1472.77 | SNP | Rv1823 | Asp238His | 3 | - | | 2071576 | C | T | 1807.77 | SNP | Rv1825 | Pro181Ser | 17 | - | | 2074509 | C | G | 354.77 | SNP | intergenic |  |  | - | | 2074565 | C | G | 641.77 | SNP | intergenic |  |  | - | | 2077253 | G | A | 1855.77 | SNP | Rv1832 (gcvB) | silent (Thr459) | 9871 | - | | 2093107 | T | C | 1074.77 | SNP | Rv1843c (guaB1) | Thr198Ala | 32 | - | | 2094911 | ACAGCGT | A | 4426.73 | DEL | Rv1844c (gnd1) |  |  | - | | 2096186 | A | G | 1253.77 | SNP | Rv1846c (blaI) | silent (Thr138) | 9871 | - | | 2097990 | A | C | 1781.77 | SNP | Rv1850 (ureC) | silent (Ala10) | 9867 | - | | 2108141 | T | C | 707.77 | SNP | Rv1860 (apa) | Phe136Leu | 13 | - | | 2108838 | C | T | 1399.77 | SNP | intergenic |  |  | - | | 2109149 | C | T | 1804.77 | SNP | intergenic |  |  | - | | 2109523 | C | CG | 2485.73 | INS | intergenic |  |  | - | | 2116903 | C | T | 1382.77 | SNP | Rv1867 | silent (Gly380) | 9935 | - | | 2122395 | C | T | 1561.77 | SNP | Rv1872c (lldD2) | Val(s)253Met(s) | 9867 | - | | 2123169 | T | G | 1929.77 | SNP | intergenic |  |  | - | | 2128870 | A | G | 1417.77 | SNP | Rv1878 (glnA3) | silent (Leu283) | 9947 | - | | 2128908 | C | T | 1345.77 | SNP | Rv1878 (glnA3) | Ser296Phe | 2 | - | | 2133468 | T | TTCGCATGCCGTCACC | 1965.73 | INS | Rv1883c |  |  | - | | 2134215 | T | C | 1081.77 | SNP | Rv1884c (rpfC) | His16Arg | 10 | - | | 2135870 | T | C | 1608.77 | SNP | intergenic |  |  | - | | 2135900 | T | G | 215.77 | SNP | intergenic |  |  | - | | 2137521 | A | ACTCCGATCAC | 9731.73 | INS | Rv1888c |  |  | - | | 2143328 | G | C | 1345.77 | SNP | Rv1895 | Val(s)270Leu | 3 | - | | 2144617 | G | A | 1685.77 | SNP | Rv1897c | Pro89Leu | 3 | - | | 2147022 | A | C | 1757.77 | SNP | Rv1900c (lipJ) | Ile204Met(s) | 6 | - | | 2155168 | C | G | 1725.77 | SNP | Rv1908c (katG) | Ser315Thr | 32 | resistance | | 2163375 | T | C | 820.77 | SNP | Rv1917c (PPE34) | Asn1313Asp | 42 | - | | 2163412 | A | G | 407.77 | SNP | Rv1917c (PPE34) | silent (Val1300) | 9901 | - | | 2163415 | C | A | 408.77 | SNP | Rv1917c (PPE34) | silent (Pro1299) | 9926 | - | | 2163417 | G | C | 543.77 | SNP | Rv1917c (PPE34) | Pro1299Ala | 22 | - | | 2163419 | C | T | 212.77 | SNP | Rv1917c (PPE34) | Ser1298Asn | 20 | - | | 2163421 | C | G | 304.77 | SNP | Rv1917c (PPE34) | silent (Thr1297) | 9871 | - | | 2163444 | T | C | 297.77 | SNP | Rv1917c (PPE34) | Asn1290Asp | 42 | - | | 2163790 | A | C | 982.77 | SNP | Rv1917c (PPE34) | silent (Pro1174) | 9926 | - | | 2165286 | A | C | 1083.77 | SNP | Rv1917c (PPE34) | Ser676Ala | 35 | - | | 2165479 | GCCCACATCAATACCCAACG GGATTGCCGGAAGTGAGTAG CCATCCGGGAACACCGTAAA CGGGCCTAACCCTCCA | G | 2386.73 | DEL | Rv1917c (PPE34) |  |  | - | | 2171966 | C | G | 1371.77 | SNP | Rv1920 | Thr115Ser | 38 | - | | 2174216 | A | G | 1450.77 | SNP | Rv1922 | Val50Val(s) | 18 | - | | 2180374 | T | G | 2186.77 | SNP | Rv1927 | silent (Thr234) | 9871 | - | | 2207525 | C | T | 1603.77 | SNP | intergenic |  |  | - | | 2207591 | T | TC | 3428.73 | INS | intergenic |  |  | - | | 2211826 | A | G | 1376.77 | SNP | Rv1968 (mce3C) | silent (Lys67) | 9926 | - | | 2216248 | C | G | 1555.77 | SNP | Rv1971 (mce3F) | Pro331Arg | 4 | - | | 2216443 | C | A | 1620.77 | SNP | Rv1971 (mce3F) | Ala396Glu | 10 | - | | 2220512 | T | G | 2022.77 | SNP | Rv1977 | silent (Ser253) | 9840 | - | | 2223293 | T | C | 1657.77 | SNP | intergenic |  |  | - | | 2226837 | C | CG | 51.73 | INS | Rv1983 (PE\_PGRS35) |  |  | - | | 2228967 | A | G | 1212.77 | SNP | intergenic |  |  | - | | 2231132 | G | A | 1443.77 | SNP | Rv1987 | Ser36Asn | 20 | - | | 2236315 | A | G | 966.77 | SNP | Rv1992c (ctpG) | Phe331Ser | 3 | - | | 2245532 | T | C | 1959.77 | SNP | Rv2000 | silent (Cys108) | 9973 | - | | 2251999 | A | G | 1475.77 | SNP | intergenic |  |  | - | | 2260525 | C | T | 1637.77 | SNP | intergenic |  |  | - | | 2264782 | C | A | 1697.77 | SNP | Rv2017 | Ala262Glu | 10 | - | | 2265059 | T | G | 1136.77 | SNP | intergenic |  |  | - | | 2266487 | G | C | 1108.77 | SNP | Rv2020c | silent (Leu78) | 9947 | - | | 2266504 | T | TA | 1560.73 | INS | Rv2020c |  |  | - | | 2266508 | A | T | 817.77 | SNP | Rv2020c | Asp71Glu | 56 | - | | 2266511 | GT | G | 1418.73 | DEL | Rv2020c |  |  | - | | 2266517 | T | C | 865.77 | SNP | Rv2020c | silent (Glu68) | 9865 | - | | 2266550 | G | T | 1353.77 | SNP | Rv2020c | silent (Gly57) | 9935 | - | | 2266553 | C | G | 1472.77 | SNP | Rv2020c | silent (Ser56) | 9840 | - | | 2266583 | C | G | 1451.77 | SNP | Rv2020c | Glu46Asp | 53 | - | | 2266598 | G | C | 1406.77 | SNP | Rv2020c | silent (Leu41) | 9947 | - | | 2266604 | C | G | 1498.77 | SNP | Rv2020c | silent (Ser39) | 9840 | - | | 2266613 | G | GC | 2489.73 | INS | Rv2020c |  |  | - | | 2266624 | G | T | 1407.77 | SNP | Rv2020c | Leu33Ile | 9 | - | | 2267372 | A | G | 2523.77 | SNP | Rv2022c | Val(s)118Ala | 9867 | - | | 2269780 | T | C | 1039.77 | SNP | Rv2024c | Asp154Gly | 11 | - | | 2270102 | A | G | 1956.77 | SNP | Rv2024c | Trp47Arg | 8 | - | | 2282787 | C | T | 969.77 | SNP | Rv2037c | Cys312Tyr | 3 | - | | 2283030 | A | G | 1422.77 | SNP | Rv2037c | Ile231Thr | 11 | - | | 2285251 | C | A | 2128.77 | SNP | Rv2039c | Val131Phe | 0 | - | | 2287121 | A | G | 1399.77 | SNP | Rv2041c | silent (Asp242) | 9859 | - | | 2288065 | T | C | 1307.77 | SNP | Rv2042c | Asp206Gly | 11 | - | | 2288731 | C | T | 1490.77 | SNP | Rv2043c (pncA) | Ala171Thr | 22 | - | | 2295950 | G | A | 2625.77 | SNP | Rv2048c (pks12) | silent (Asp3679) | 9859 | - | | 2296042 | G | C | 2862.77 | SNP | Rv2048c (pks12) | Pro3649Ala | 22 | - | | 2300237 | A | G | 1114.77 | SNP | Rv2048c (pks12) | silent (Ala2250) | 9867 | - | | 2300546 | A | T | 1341.77 | SNP | Rv2048c (pks12) | His2147Gln | 23 | - | | 2300552 | T | G | 2462.77 | SNP | Rv2048c (pks12) | silent (Pro2145) | 9926 | - | | 2300555 | A | G | 2449.77 | SNP | Rv2048c (pks12) | silent (Asp2144) | 9859 | - | | 2301782 | T | C | 75.28 | SNP | Rv2048c (pks12) | Val1735Val(s) | 18 | - | | 2306306 | A | G | 897.77 | SNP | Rv2048c (pks12) | silent (Ala227) | 9867 | - | | 2306453 | G | C | 42.74 | SNP | Rv2048c (pks12) | silent (Thr178) | 9871 | - | | 2307681 | GT | G | 1714.73 | DEL | intergenic |  |  | - | | 2315669 | G | A | 1005.77 | SNP | Rv2059 | Val166Ile | 33 | - | | 2323220 | G | A | 1314.77 | SNP | Rv2066 (cobI) | Asp16Asn | 36 | - | | 2329533 | A | G | 1599.77 | SNP | Rv2072c (cobL) | Leu205Pro | 2 | - | | 2334007 | A | G | 1632.77 | SNP | Rv2077c | silent (Ala96) | 9867 | - | | 2335075 | A | G | 2189.77 | SNP | Rv2078 | Glu6Gly | 7 | - | | 2335494 | A | G | 1506.77 | SNP | Rv2079 | Tyr47Cys | 3 | - | | 2340621 | C | G | 2514.77 | SNP | Rv2082 | Pro638Arg | 4 | - | | 2341636 | C | G | 1176.77 | SNP | Rv2083 | Leu256Val(s) | 4 | - | | 2345037 | C | A | 1627.77 | SNP | Rv2088 (pknJ) | silent (Leu209) | 9947 | - | | 2347442 | G | C | 872.77 | SNP | Rv2090 | Asp24His | 3 | - | | 2348446 | C | G | 1055.77 | SNP | Rv2090 | Phe358Leu(s) | 2 | - | | 2361604 | C | G | 894.77 | SNP | Rv2101 (helZ) | Val455Val(s) | 18 | - | | 2362041 | C | A | 1136.77 | SNP | Rv2101 (helZ) | Pro601Gln | 6 | - | | 2368564 | TA | T | 3555.73 | DEL | intergenic |  |  | - | | 2372550 | G | C | 78.77 | SNP | Rv2112c (dop) | Pro7Arg | 4 | - | | 2382085 | AGT | A | 3885.73 | DEL | Rv2123 (PPE37) |  |  | - | | 2386389 | G | A | 1269.77 | SNP | Rv2125 | Gly33Ser | 16 | - | | 2387733 | T | C | 218.84 | SNP | Rv2126c (PE\_PGRS37) | silent (Glu80) | 9865 | - | | 2401825 | TGGCTCCTCCTCACCCCGTT ACCCGGGGCGCATCGTCGCC GAGCTCGATTTGATTGCCC | T | 3494.73 | DEL | intergenic |  |  | - | | 2415656 | G | C | 873.77 | SNP | Rv2155c (murD) | Arg247Gly | 1 | - | | 2424925 | A | G | 1088.77 | SNP | intergenic |  |  | - | | 2437971 | G | A | 1452.77 | SNP | Rv2176 (pknL) | Glu11Lys | 7 | - | | 2439401 | A | G | 177.84 | SNP | Rv2177c | Tyr183His | 4 | - | | 2439460 | A | G | 63.28 | SNP | Rv2177c | Ile163Thr | 11 | - | | 2440926 | G | T | 1325.77 | SNP | Rv2178c (aroG) | Asp265Glu | 56 | - | | 2456029 | A | C | 1632.77 | SNP | Rv2192c (trpD) | Ser239Ala | 35 | - | | 2462871 | G | A | 1135.77 | SNP | Rv2198c (mmpS3) | silent (Ala59) | 9867 | - | | 2463094 | G | A | 1257.77 | SNP | intergenic |  |  | - | | 2475999 | TA | T | 2574.73 | DEL | intergenic |  |  | - | | 2484255 | T | G | 1374.77 | SNP | Rv2216 | silent (Ala210) | 9867 | - | | 2499726 | G | A | 1771.77 | SNP | Rv2226 | Asp299Asn | 36 | - | | 2502073 | C | T | 742.77 | SNP | Rv2228c | silent (Arg222) | 9913 | - | | 2503625 | C | T | 1399.77 | SNP | Rv2230c | silent (Leu328) | 9947 | - | | 2509140 | G | C | 1195.77 | SNP | Rv2236c (cobD) | Ser79Cys | 5 | - | | 2509722 | A | G | 1502.77 | SNP | Rv2237 | silent (Pro78) | 9926 | - | | 2518919 | G | A | 1622.77 | SNP | Rv2245 (kasA) | Gly269Ser | 16 | genotype | | 2521342 | T | C | 1517.77 | SNP | Rv2247 (accD6) | silent (Asp200) | 9859 | - | | 2523205 | G | GCGC | 1449.73 | INS | intergenic |  |  | - | | 2525722 | CG | C | 2368.73 | DEL | Rv2250A; Rv2251 |  |  | - | | 2529680 | A | G | 1558.77 | SNP | Rv2256c | silent (Thr65) | 9871 | - | | 2531742 | A | G | 1681.77 | SNP | Rv2258c | silent (Ala52) | 9867 | - | | 2534562 | GGA | G | 2287.73 | DEL | Rv2262c |  |  | - | | 2536892 | T | C | 40.77 | SNP | Rv2264c | Thr487Ala | 32 | - | | 2537154 | G | A | 729.77 | SNP | Rv2264c | silent (Tyr399) | 9945 | - | | 2541410 | C | T | 1169.77 | SNP | intergenic |  |  | - | | 2573756 | C | A | 1639.77 | SNP | intergenic |  |  | - | | 2586127 | A | G | 1308.77 | SNP | Rv2314c | silent (Gly388) | 9935 | - | | 2598400 | A | G | 1467.77 | SNP | Rv2326c | silent (Asn516) | 9822 | - | | 2612632 | C | A | 771.77 | SNP | Rv2337c | Gly119Val | 3 | - | | 2615269 | G | T | 1429.77 | SNP | Rv2339 (mmpL9) | Ala193Ser | 28 | - | | 2621058 | G | A | 870.77 | SNP | Rv2343c (dnaG) | silent (Pro465) | 9926 | - | | 2630158 | C | G | 560.77 | SNP | Rv2350c (plcB) | silent (Arg54) | 9913 | - | | 2630161 | A | G | 559.77 | SNP | Rv2350c (plcB) | silent (Asn53) | 9822 | - | | 2630173 | C | G | 329.77 | SNP | Rv2350c (plcB) | Leu(s)49Phe | 1 | - | | 2630176 | C | G | 300.77 | SNP | Rv2350c (plcB) | Leu(s)48Phe | 1 | - | | 2630182 | G | A | 284.77 | SNP | Rv2350c (plcB) | silent (Ile46) | 9872 | - | | 2630184 | T | A | 330.77 | SNP | Rv2350c (plcB) | Ile46Phe | 8 | - | | 2630188 | C | T | 279.77 | SNP | Rv2350c (plcB) | silent (Glu44) | 9865 | - | | 2630206 | T | G | 126.77 | SNP | Rv2350c (plcB) | silent (Gly38) | 9935 | - | | 2630211 | G | A | 212.77 | SNP | Rv2350c (plcB) | Pro37Ser | 17 | - | | 2630215 | A | G | 178.77 | SNP | Rv2350c (plcB) | silent (Pro35) | 9926 | - | | 2630224 | T | G | 85.77 | SNP | Rv2350c (plcB) | silent (Gly32) | 9935 | - | | 2630233 | T | C | 77.77 | SNP | Rv2350c (plcB) | silent (Lys29) | 9926 | - | | 2631556 | C | G | 182.77 | SNP | Rv2351c (plcA) | Gly174Arg | 0 | - | | 2631565 | T | C | 288.77 | SNP | Rv2351c (plcA) | Ile171Val | 57 | - | | 2631574 | T | C | 355.77 | SNP | Rv2351c (plcA) | Thr168Ala | 32 | - | | 2631583 | G | A | 445.77 | SNP | Rv2351c (plcA) | Leu165Leu(s) | 4 | - | | 2631599 | G | A | 612.77 | SNP | Rv2351c (plcA) | silent (Ile159) | 9872 | - | | 2631620 | A | G | 488.77 | SNP | Rv2351c (plcA) | silent (Gly152) | 9935 | - | | 2631962 | T | G | 154.77 | SNP | Rv2351c (plcA) | silent (Gly38) | 9935 | - | | 2631967 | G | A | 204.77 | SNP | Rv2351c (plcA) | Pro37Ser | 17 | - | | 2631968 | A | G | 265.77 | SNP | Rv2351c (plcA) | silent (Cys36) | 9973 | - | | 2631971 | A | G | 251.77 | SNP | Rv2351c (plcA) | silent (Pro35) | 9926 | - | | 2631977 | G | C | 163.77 | SNP | Rv2351c (plcA) | silent (Ala33) | 9867 | - | | 2637223 | G | C | 1619.77 | SNP | intergenic |  |  | - | | 2638997 | G | A | 550.77 | SNP | Rv2356c (PPE40) | Ser180Leu(s) | 35 | - | | 2642375 | T | C | 1673.77 | SNP | Rv2360c | silent (Gln68) | 9876 | - | | 2654371 | G | A | 2306.77 | SNP | Rv2374c (hrcA) | silent (Asn241) | 9822 | - | | 2656225 | A | G | 1988.77 | SNP | Rv2377c (mbtH) | Val69Ala | 18 | - | | 2660319 | C | G | 1349.77 | SNP | Rv2379c (mbtF) | Glu589Asp | 53 | - | | 2680658 | T | G | 1738.77 | SNP | intergenic |  |  | - | | 2695378 | C | G | 1921.77 | SNP | Rv2398c (cysW) | Gly141Ala | 21 | - | | 2704884 | A | ACAGCGACCATATCGCCGAG CT | 32729.73 | INS | Rv2407 |  |  | - | | 2713076 | G | GCAC | 2661.73 | INS | Rv2415c |  |  | - | | 2713795 | C | T | 1114.77 | SNP | intergenic |  |  | - | | 2718852 | T | G | 2078.77 | SNP | intergenic |  |  | - | | 2721013 | A | G | 719.77 | SNP | Rv2424c | silent (Arg255) | 9913 | - | | 2733218 | C | T | 1802.77 | SNP | intergenic |  |  | - | | 2734074 | T | C | 716.77 | SNP | Rv2436 (rbsK) | Val282Ala | 18 | - | | 2736434 | C | A | 2068.77 | SNP | Rv2438c (nadE) | Arg133Leu | 1 | - | | 2745889 | C | T | 2125.77 | SNP | Rv2446c | Ala84Thr | 22 | - | | 2751804 | C | T | 948.77 | SNP | Rv2450c (rpfE) | Arg126Gln | 9 | - | | 2752698 | C | A | 2535.77 | SNP | intergenic |  |  | - | | 2754917 | C | A | 1390.77 | SNP | Rv2455c | Lys596Asn | 13 | - | | 2760152 | A | G | 1570.77 | SNP | Rv2458 (mmuM) | Tyr125Cys | 3 | - | | 2768309 | G | T | 1034.77 | SNP | Rv2466c | His192Gln | 23 | - | | 2775042 | G | A | 1058.77 | SNP | Rv2471 (aglA) | silent (Ala493) | 9867 | - | | 2779136 | T | C | 1083.77 | SNP | Rv2476c (gdh) | Ser1043Gly | 21 | - | | 2786952 | A | G | 1899.77 | SNP | Rv2482c (plsB2) | Cys778Arg | 1 | - | | 2809621 | T | C | 1351.77 | SNP | Rv2495c (bkdC) | Thr107Ala | 32 | - | | 2814961 | G | A | 1693.77 | SNP | Rv2501c (accA1) | silent (Gly640) | 9935 | - | | 2818837 | A | G | 1524.77 | SNP | Rv2503c (scoB) | silent (Gly97) | 9935 | - | | 2821342 | C | T | 975.77 | SNP | Rv2505c (fadD35) | silent (Ala85) | 9867 | - | | 2827984 | G | T | 1367.77 | SNP | intergenic |  |  | - | | 2828019 | T | C | 1358.77 | SNP | intergenic |  |  | - | | 2828822 | G | T | 155.90 | SNP | Rv2512c | Gln328Lys | 12 | - | | 2829779 | T | C | 520.77 | SNP | Rv2512c | Thr9Ala | 32 | - | | 2830525 | C | A | 1560.77 | SNP | Rv2513 | Thr122Lys | 11 | - | | 2832071 | G | C | 97.77 | SNP | Rv2515c | Ala174Gly | 21 | - | | 2855259 | A | G | 1488.77 | SNP | Rv2531c | silent (Ala841) | 9867 | - | | 2861351 | T | C | 1138.77 | SNP | Rv2537c (aroD) | Thr81Ala | 32 | - | | 2865760 | A | G | 1732.77 | SNP | Rv2542 | Thr211Ala | 32 | - | | 2865882 | T | C | 1429.77 | SNP | Rv2542 | silent (Val251) | 9901 | - | | 2871048 | C | T | 825.77 | SNP | Rv2551c | silent (Leu49) | 9947 | - | | 2880702 | G | C | 1971.77 | SNP | Rv2560 | Val210Leu | 15 | - | | 2881455 | A | G | 1080.77 | SNP | Rv2561 | Tyr16Cys | 3 | - | | 2881597 | AG | A | 1513.73 | DEL | Rv2561 |  |  | - | | 2888201 | T | C | 1185.77 | SNP | Rv2566 | Leu610Pro | 2 | - | | 2889633 | T | C | 1336.77 | SNP | Rv2566 | silent (Ala1087) | 9867 | - | | 2891267 | C | T | 2054.77 | SNP | Rv2567 | silent (Gly491) | 9935 | - | | 2891728 | A | G | 1673.77 | SNP | Rv2567 | Gln645Arg | 10 | - | | 2893238 | C | A | 1081.77 | SNP | Rv2568c | silent (Arg78) | 9913 | - | | 2894208 | G | A | 1442.77 | SNP | Rv2569c | silent (Ser67) | 9840 | - | | 2910461 | G | T | 1523.77 | SNP | Rv2584c (apt) | Ala147Glu | 10 | - | | 2911293 | C | G | 1413.77 | SNP | Rv2585c | Cys462Ser | 11 | - | | 2912294 | T | G | 1982.77 | SNP | Rv2585c | silent (Ala128) | 9867 | - | | 2923391 | T | C | 841.77 | SNP | Rv2592c (ruvB) | silent (Pro281) | 9926 | - | | 2927939 | T | C | 1943.77 | SNP | intergenic |  |  | - | | 2934747 | G | C | 1682.77 | SNP | Rv2607 (pdxH) | Val(s)184Leu | 3 | - | | 2939373 | G | C | 1112.77 | SNP | Rv2611c | Ser197Cys | 5 | - | | 2939657 | T | C | 629.77 | SNP | Rv2611c | Ile102Met(s) | 6 | - | | 2945004 | C | G | 476.77 | SNP | intergenic |  |  | - | | 2945134 | A | G | 1118.77 | SNP | intergenic |  |  | - | | 2945167 | G | T | 1137.77 | SNP | intergenic |  |  | - | | 2954439 | T | C | 1654.77 | SNP | Rv2627c | Arg104Gly | 1 | - | | 2960231 | G | T | 125.81 | SNP | Rv2634c (PE\_PGRS46) | silent (Gly737) | 9935 | - | | 2960371 | T | C | 61.77 | SNP | Rv2634c (PE\_PGRS46) | Thr691Ala | 32 | - | | 2960374 | T | G | 45.77 | SNP | Rv2634c (PE\_PGRS46) | Thr690Pro | 4 | - | | 2960375 | C | A | 59.77 | SNP | Rv2634c (PE\_PGRS46) | silent (Gly689) | 9935 | - | | 2960378 | G | A | 42.77 | SNP | Rv2634c (PE\_PGRS46) | silent (Gly688) | 9935 | - | | 2962758 | G | A | 1201.77 | SNP | Rv2636 | Ala16Thr | 22 | - | | 2966445 | C | T | 1309.77 | SNP | intergenic |  |  | - | | 2967789 | C | A | 1338.77 | SNP | Rv2643 (arsC) | Arg294Ser | 11 | - | | 2968913 | T | C | 1259.77 | SNP | intergenic |  |  | - | | 2974933 | A | G | 863.77 | SNP | Rv2650c | Ile101Thr | 11 | - | | 2980911 | G | A | 2060.77 | SNP | intergenic |  |  | - | | 2984740 | A | G | 1210.77 | SNP | Rv2668 | His3Arg | 10 | - | | 2987641 | C | A | 1206.77 | SNP | intergenic |  |  | - | | 3004091 | G | A | 1408.77 | SNP | Rv2687c | Arg220Trp | 2 | - | | 3004942 | G | A | 635.77 | SNP | Rv2688c | Arg237Cys | 1 | - | | 3005185 | G | T | 2086.77 | SNP | Rv2688c | Pro156Thr | 5 | - | | 3009692 | A | G | 1216.77 | SNP | Rv2691 (ceoB) | Thr117Ala | 32 | - | | 3017465 | T | C | 1846.77 | SNP | Rv2702 (ppgK) | Ile203Thr | 11 | - | | 3027582 | A | G | 1765.77 | SNP | Rv2714 | Glu173Gly | 7 | - | | 3029610 | G | A | 1008.77 | SNP | Rv2716 | Ala147Thr | 22 | - | | 3033577 | G | T | 1050.77 | SNP | Rv2721c | Pro348His | 3 | - | | 3041871 | G | T | 1060.77 | SNP | Rv2729c | Ala202Glu | 10 | - | | 3047124 | G | C | 1243.77 | SNP | Rv2734 | Val(s)102Leu | 3 | - | | 3054081 | A | G | 1249.77 | SNP | Rv2741 (PE\_PGRS47) | silent (Gly56) | 9935 | - | | 3054321 | A | G | 552.77 | SNP | Rv2741 (PE\_PGRS47) | silent (Gly136) | 9935 | - | | 3054724 | A | G | 46.74 | SNP | Rv2741 (PE\_PGRS47) | Ser271Gly | 21 | - | | 3061615 | T | C | 846.77 | SNP | Rv2748c (ftsK) | Met(s)298Val(s) | 9867 | - | | 3064323 | G | A | 1347.77 | SNP | Rv2751 | Arg229His | 8 | - | | 3073868 | T | C | 1362.77 | SNP | Rv2764c (thyA) | Thr202Ala | 32 | genotype | | 3077039 | C | A | 1069.77 | SNP | Rv2768c (PPE43) | Gly347Val | 3 | - | | 3080795 | A | G | 2321.77 | SNP | Rv2771c | Leu80Pro | 2 | - | | 3086788 | T | C | 1885.77 | SNP | intergenic |  |  | - | | 3095936 | C | G | 1428.77 | SNP | Rv2787 | Pro276Ala | 22 | - | | 3098835 | A | G | 1657.77 | SNP | Rv2789c (fadE21) | Leu35Pro | 2 | - | | 3101119 | G | T | 799.77 | SNP | Rv2791c | Arg155Ser | 11 | - | | 3103682 | T | C | 1219.77 | SNP | Rv2794c (pptT) | Met(s)87Val(s) | 9867 | - | | 3108055 | C | T | 1579.77 | SNP | Rv2799 | silent (Ala96) | 9867 | - | | 3108674 | A | C | 1637.77 | SNP | Rv2800 | silent (Arg87) | 9913 | - | | 3111689 | C | A | 883.77 | SNP | Rv2802c | silent (Arg45) | 9913 | - | | 3113872 | A | T | 1146.77 | SNP | Rv2807 | Glu72Val(s) | 17 | - | | 3118000 | A | G | 1108.77 | SNP | Rv2812 | Arg395Gly | 1 | - | | 3131469 | T | TTGTCGGCGA | 4698.73 | INS | Rv2823c |  |  | - | | 3133016 | C | G | 613.77 | SNP | Rv2825c | Arg175Thr | 2 | - | | 3133536 | T | C | 1542.77 | SNP | Rv2825c | Lys2Glu | 4 | - | | 3137058 | G | A | 1484.77 | SNP | Rv2830c (vapB22) | Ala56Val(s) | 9867 | - | | 3145347 | A | G | 2315.77 | SNP | Rv2839c (infB) | Leu(s)843Leu | 3 | - | | 3147170 | C | T | 291.78 | SNP | Rv2839c (infB) | Gly235Asp | 6 | - | | 3162805 | C | G | 314.78 | SNP | Rv2853 (PE\_PGRS48) | Arg180Gly | 1 | - | | 3165074 | T | C | 1367.77 | SNP | Rv2854 | Val(s)308Ala | 9867 | - | | 3175702 | T | C | 1126.77 | SNP | Rv2864c | Ile522Val | 57 | - | | 3177884 | C | A | 1434.77 | SNP | Rv2866 (relG) | silent (Arg21) | 9913 | - | | 3178445 | C | G | 1328.77 | SNP | intergenic |  |  | - | | 3183561 | G | C | 272.77 | SNP | Rv2872 (vapC43) | silent (Pro60) | 9926 | - | | 3186860 | T | G | 1114.77 | SNP | Rv2874 (dipZ) | Tyr672Asp | 0 | - | | 3188340 | G | C | 1520.77 | SNP | Rv2877c | Leu178Val | 11 | - | | 3190145 | TC | T | 1581.73 | DEL | Rv2880c |  |  | - | | 3191027 | G | A | 2126.77 | SNP | Rv2881c (cdsA) | Leu199Leu(s) | 4 | - | | 3202890 | C | T | 1183.77 | SNP | Rv2893 | silent (His157) | 9912 | - | | 3218343 | G | A | 1713.77 | SNP | Rv2911 (dacB2) | Arg2Gln | 9 | - | | 3226181 | A | C | 1097.77 | SNP | Rv2916c (ffh) | silent (Arg35) | 9913 | - | | 3226628 | C | T | 1257.77 | SNP | Rv2917 | Thr89Ile | 7 | - | | 3228143 | G | T | 1159.77 | SNP | Rv2917 | Arg594Leu | 1 | - | | 3232815 | A | G | 1212.77 | SNP | intergenic |  |  | - | | 3236230 | C | A | 782.77 | SNP | Rv2922c (smc) | Arg526Leu | 1 | - | | 3243630 | G | A | 2169.77 | SNP | intergenic |  |  | - | | 3247316 | C | G | 1401.77 | SNP | Rv2931 (ppsA) | Asp624Glu | 56 | - | | 3247403 | G | A | 1545.77 | SNP | Rv2931 (ppsA) | silent (Glu653) | 9865 | - | | 3247851 | G | A | 709.77 | SNP | Rv2931 (ppsA) | Ala803Thr | 22 | - | | 3247853 | C | T | 758.77 | SNP | Rv2931 (ppsA) | silent (Ala803) | 9867 | - | | 3247856 | G | C | 789.77 | SNP | Rv2931 (ppsA) | silent (Arg804) | 9913 | - | | 3247864 | C | CTAGG | 1846.73 | INS | Rv2931 (ppsA) |  |  | - | | 3247865 | GCAAA | G | 1892.73 | DEL | Rv2931 (ppsA) |  |  | - | | 3247874 | G | A | 729.77 | SNP | Rv2931 (ppsA) | silent (Arg810) | 9913 | - | | 3247877 | T | C | 792.77 | SNP | Rv2931 (ppsA) | silent (Phe811) | 9946 | - | | 3247883 | T | C | 1183.77 | SNP | Rv2931 (ppsA) | silent (Ser813) | 9840 | - | | 3248074 | G | A | 1039.77 | SNP | Rv2931 (ppsA) | Arg877His | 8 | - | | 3248075 | C | T | 1058.77 | SNP | Rv2931 (ppsA) | silent (Arg877) | 9913 | - | | 3256494 | A | G | 1325.77 | SNP | Rv2933 (ppsC) | silent (Gly270) | 9935 | - | | 3260301 | A | G | 934.77 | SNP | Rv2933 (ppsC) | silent (Gly1539) | 9935 | - | | 3261380 | A | G | 1119.77 | SNP | Rv2933 (ppsC) | Asn1899Ser | 34 | - | | 3266654 | T | C | 1259.77 | SNP | Rv2934 (ppsD) | silent (Gly1469) | 9935 | - | | 3269581 | A | G | 2026.77 | SNP | Rv2935 (ppsE) | silent (Ala615) | 9867 | - | | 3270784 | A | G | 1373.77 | SNP | Rv2935 (ppsE) | silent (Gln1016) | 9876 | - | | 3282688 | C | A | 1671.77 | SNP | Rv2940c (mas) | Val(s)10Leu(s) | 9867 | - | | 3290580 | CG | C | 2031.73 | DEL | intergenic |  |  | - | | 3296843 | A | G | 1075.77 | SNP | Rv2947c (pks15) | Val(s)333Ala | 9867 | - | | 3308606 | G | A | 1898.77 | SNP | intergenic |  |  | - | | 3314629 | C | T | 1894.77 | SNP | Rv2962c | Trp165STOP | 0 | - | | 3336587 | T | A | 534.77 | SNP | intergenic |  |  | - | | 3336646 | T | A | 103.77 | SNP | intergenic |  |  | - | | 3336825 | T | C | 1100.77 | SNP | Rv2981c (ddlA) | Thr365Ala | 32 | - | | 3338603 | G | C | 1388.77 | SNP | Rv2982c (gpdA2) | Pro133Ala | 22 | - | | 3351926 | T | C | 1274.77 | SNP | Rv2994 | Ser220Pro | 12 | - | | 3354896 | C | T | 715.77 | SNP | Rv2996c (serA1) | silent (Glu58) | 9865 | - | | 3358235 | A | T | 1944.77 | SNP | Rv2999 (lppY) | Met(s)212Leu(s) | 9867 | - | | 3363338 | A | G | 1965.77 | SNP | intergenic |  |  | - | | 3367765 | G | A | 1463.77 | SNP | Rv3009c (gatB) | silent (Gly343) | 9935 | - | | 3379708 | G | C | 125.03 | SNP | intergenic |  |  | - | | 3379712 | G | C | 129.03 | SNP | intergenic |  |  | - | | 3379718 | T | C | 129.03 | SNP | intergenic |  |  | - | | 3379726 | C | A | 112.03 | SNP | intergenic |  |  | - | | 3379730 | G | C | 124.03 | SNP | intergenic |  |  | - | | 3379732 | C | T | 111.03 | SNP | intergenic |  |  | - | | 3379735 | A | C | 125.03 | SNP | intergenic |  |  | - | | 3379736 | C | A | 101.03 | SNP | intergenic |  |  | - | | 3379742 | T | C | 275.78 | SNP | intergenic |  |  | - | | 3379751 | A | C | 274.78 | SNP | intergenic |  |  | - | | 3379757 | A | C | 316.78 | SNP | intergenic |  |  | - | | 3379763 | G | A | 288.78 | SNP | intergenic |  |  | - | | 3379784 | C | A | 347.77 | SNP | intergenic |  |  | - | | 3379788 | C | G | 473.77 | SNP | intergenic |  |  | - | | 3381641 | G | T | 176.84 | SNP | Rv3023c | Gln328Lys | 12 | - | | 3402816 | C | T | 1281.77 | SNP | Rv3042c (serB2) | Gly116Glu | 4 | - | | 3415180 | ACACCTAGGGGGTGG | A | 4292.79 | DEL | intergenic |  |  | - | | 3418328 | T | G | 1603.77 | SNP | Rv3057c | Asp112Ala | 10 | - | | 3418330 | G | A | 1580.77 | SNP | Rv3057c | silent (His111) | 9912 | - | | 3423184 | C | T | 1421.77 | SNP | Rv3060c | silent (Thr10) | 9871 | - | | 3424350 | G | A | 1313.77 | SNP | Rv3061c (fadE22) | Gln360STOP | 8 | - | | 3425854 | C | T | 1595.77 | SNP | Rv3062 (ligB) | Pro91Ser | 17 | - | | 3426795 | C | G | 1966.77 | SNP | Rv3062 (ligB) | silent (Ser404) | 9840 | - | | 3428917 | C | A | 1109.77 | SNP | Rv3063 (cstA) | Arg559Ser | 11 | - | | 3429202 | T | G | 2129.77 | SNP | Rv3063 (cstA) | Tyr654Asp | 0 | - | | 3440464 | T | G | 1834.77 | SNP | Rv3077 | silent (Arg308) | 9913 | - | | 3440468 | G | C | 1972.77 | SNP | Rv3077 | Gly310Arg | 0 | - | | 3445547 | G | C | 1789.77 | SNP | Rv3080c (pknK) | Arg148Gly | 1 | - | | 3454986 | C | A | 1565.77 | SNP | Rv3088 (tgs4) | Ala216Glu | 10 | - | | 3456666 | A | G | 1729.77 | SNP | Rv3089 (fadD13) | silent (Ala302) | 9867 | - | | 3460986 | G | A | 1254.77 | SNP | Rv3092c | Pro250Leu | 3 | - | | 3462135 | G | C | 1017.77 | SNP | Rv3093c | Cys210Trp | 0 | - | | 3466567 | T | C | 1220.77 | SNP | Rv3097c (lipY) | silent (Ala175) | 9867 | - | | 3473996 | G | GA | 3765.73 | INS | intergenic |  |  | - | | 3482432 | C | A | 246.78 | SNP | Rv3115 | Gln328Lys | 12 | - | | 3486660 | A | G | 2182.77 | SNP | Rv3121 (cyp141) | Asp51Gly | 11 | - | | 3486977 | A | G | 1986.77 | SNP | Rv3121 (cyp141) | Lys157Glu | 4 | - | | 3489851 | G | T | 2803.77 | SNP | Rv3124 (moaR1) | Ala116Ser | 28 | - | | 3503895 | C | T | 2032.77 | SNP | Rv3137 | Pro168Leu | 3 | - | | 3505027 | G | A | 1442.77 | SNP | Rv3138 (pflA) | Arg278His | 8 | - | | 3518167 | A | G | 1021.77 | SNP | Rv3151 (nuoG) | Ile474Met(s) | 6 | - | | 3518555 | A | G | 1121.77 | SNP | Rv3151 (nuoG) | Thr604Ala | 32 | - | | 3542262 | T | G | 1314.77 | SNP | Rv3174 | Leu42Arg | 1 | - | | 3548641 | T | C | 1468.77 | SNP | Rv3179 | Tyr342His | 4 | - | | 3554747 | G | T | 1492.77 | SNP | Rv3189 | Ala36Ser | 28 | - | | 3556275 | A | G | 1741.77 | SNP | Rv3190c | Leu138Pro | 2 | - | | 3564400 | G | A | 1202.77 | SNP | Rv3195 | Gly13Ser | 16 | - | | 3568578 | G | A | 1464.77 | SNP | Rv3197A (whiB7) | silent (Ala34) | 9867 | - | | 3580636 | CT | C | 3513.73 | DEL | intergenic |  |  | - | | 3581414 | A | G | 1632.77 | SNP | Rv3204 | Thr34Ala | 32 | - | | 3590686 | G | GC | 2220.73 | INS | intergenic |  |  | - | | 3591063 | T | C | 958.77 | SNP | Rv3213c | Lys144Glu | 4 | - | | 3594124 | C | T | 1760.77 | SNP | Rv3217c | Ala38Thr | 22 | - | | 3595483 | A | ACTGGCAGCGTAGT | 9744.73 | INS | intergenic |  |  | - | | 3604821 | G | C | 639.77 | SNP | Rv3228 | silent (Ala32) | 9867 | - | | 3610441 | C | T | 1827.77 | SNP | Rv3234c (tgs3) | Arg250His | 8 | - | | 3614982 | T | C | 1752.77 | SNP | Rv3239c | silent (Leu874) | 9947 | - | | 3622441 | A | C | 1101.77 | SNP | Rv3243c | Val217Val(s) | 18 | - | | 3625065 | T | G | 1177.77 | SNP | Rv3245c (mtrB) | Met(s)517Leu | 3 | - | | 3643392 | C | T | 1247.77 | SNP | Rv3263 | silent (Gly72) | 9935 | - | | 3643630 | G | A | 1310.77 | SNP | Rv3263 | Gly152Ser | 16 | - | | 3663889 | C | A | 33.77 | SNP | Rv3281 (accE5) | Asn67Lys | 25 | - | | 3670319 | A | AGCGGCCACAACAGCAAACC GAATC | 4874.73 | INS | Rv3289c |  |  | - | | 3689523 | G | T | 1263.77 | SNP | Rv3303c (lpdA) | Cys472STOP | 3 | - | | 3704596 | G | C | 1816.77 | SNP | Rv3317 (sdhD) | Val(s)54Leu | 3 | - | | 3711910 | G | A | 660.77 | SNP | Rv3327 | Trp54STOP | 0 | - | | 3714211 | G | T | 1042.77 | SNP | Rv3328c (sigJ) | Pro41Gln | 6 | - | | 3714757 | A | C | 1387.77 | SNP | Rv3329 | Gln122His | 20 | - | | 3718357 | C | T | 1784.77 | SNP | Rv3331 (sugI) | Pro423Leu | 3 | - | | 3721806 | G | C | 1532.77 | SNP | Rv3335c | silent (Gly265) | 9935 | - | | 3730327 | G | A | 1389.77 | SNP | Rv3343c (PPE54) | silent (Phe2203) | 9946 | - | | 3730411 | G | A | 361.77 | SNP | Rv3343c (PPE54) | silent (Gly2175) | 9935 | - | | 3730466 | A | G | 463.77 | SNP | Rv3343c (PPE54) | Ile2157Thr | 11 | - | | 3730519 | C | G | 609.77 | SNP | Rv3343c (PPE54) | silent (Thr2139) | 9871 | - | | 3730789 | A | G | 87.77 | SNP | Rv3343c (PPE54) | silent (Ile2049) | 9872 | - | | 3730797 | A | T | 45.77 | SNP | Rv3343c (PPE54) | Phe2047Ile | 7 | - | | 3730825 | A | G | 71.77 | SNP | Rv3343c (PPE54) | silent (Ile2037) | 9872 | - | | 3730896 | A | G | 148.77 | SNP | Rv3343c (PPE54) | Leu(s)2014Leu | 3 | - | | 3732089 | C | T | 43.77 | SNP | Rv3343c (PPE54) | Gly1616Asp | 6 | - | | 3732113 | C | G | 834.77 | SNP | Rv3343c (PPE54) | Arg1608Pro | 5 | - | | 3732114 | G | T | 844.77 | SNP | Rv3343c (PPE54) | silent (Arg1608) | 9913 | - | | 3732194 | A | G | 93.77 | SNP | Rv3343c (PPE54) | Ile1581Thr | 11 | - | | 3732247 | C | G | 455.77 | SNP | Rv3343c (PPE54) | silent (Thr1563) | 9871 | - | | 3735967 | G | A | 323.78 | SNP | Rv3343c (PPE54) | silent (Ser323) | 9840 | - | | 3736072 | A | G | 63.77 | SNP | Rv3343c (PPE54) | silent (Ile288) | 9872 | - | | 3736080 | A | T | 33.77 | SNP | Rv3343c (PPE54) | Phe286Ile | 7 | - | | 3736628 | T | G | 1230.77 | SNP | Rv3343c (PPE54) | Glu103Ala | 17 | - | | 3739913 | G | A | 667.77 | SNP | Rv3345c (PE\_PGRS50) | silent (Thr954) | 9871 | - | | 3741267 | GCCTTGCCGCCGTCACCGC | G | 2469.73 | DEL | Rv3345c (PE\_PGRS50) |  |  | - | | 3746409 | A | G | 699.77 | SNP | Rv3347c (PPE55) | Leu2259Pro | 2 | - | | 3752207 | A | G | 1632.77 | SNP | Rv3347c (PPE55) | silent (Ile326) | 9872 | - | | 3753116 | C | T | 590.90 | SNP | Rv3347c (PPE55) | silent (Pro23) | 9926 | - | | 3753164 | T | G | 987.77 | SNP | Rv3347c (PPE55) | silent (Pro7) | 9926 | - | | 3759051 | G | A | 1119.77 | SNP | Rv3350c (PPE56) | silent (Ser2684) | 9840 | - | | 3776706 | C | T | 1501.77 | SNP | Rv3365c | Ala266Thr | 22 | - | | 3779671 | C | CGGCAACGGT | 740.77 | INS | Rv3367 (PE\_PGRS51) |  |  | - | | 3794884 | G | A | 1710.77 | SNP | intergenic |  |  | - | | 3798095 | A | C | 2607.77 | SNP | Rv3383c (idsB) | Val132Gly | 5 | - | | 3805637 | C | T | 1547.77 | SNP | Rv3391 (acrA1) | Thr6Ile | 7 | - | | 3811327 | C | T | 1399.77 | SNP | Rv3395c | Val(s)104Met(s) | 9867 | - | | 3817117 | C | A | 1415.77 | SNP | Rv3399 | Ala330Glu | 10 | - | | 3818098 | C | T | 1682.77 | SNP | Rv3401 | silent (Leu19) | 9947 | - | | 3820407 | A | G | 142.77 | SNP | intergenic |  |  | - | | 3820444 | C | CCA | 1141.73 | INS | intergenic |  |  | - | | 3820446 | G | C | 444.77 | SNP | intergenic |  |  | - | | 3820449 | T | G | 466.77 | SNP | intergenic |  |  | - | | 3820545 | A | G | 211.77 | SNP | intergenic |  |  | - | | 3823159 | A | T | 946.77 | SNP | Rv3403c | silent (Val235) | 9901 | - | | 3825560 | C | G | 1612.77 | SNP | Rv3406 | silent (Leu77) | 9947 | - | | 3826684 | C | T | 1061.77 | SNP | Rv3408 (vapC47) | Ser46Leu(s) | 35 | - | | 3829770 | T | C | 974.77 | SNP | Rv3410c (guaB3) | silent (Pro47) | 9926 | - | | 3838871 | A | G | 1971.77 | SNP | Rv3420c (rimI) | silent (Ala64) | 9867 | - | | 3847684 | G | A | 203.84 | SNP | Rv3429 (PPE59); Rv3430c | Gly174Arg; silent (Pro374) | 0; 9926 | - | | 3850197 | G | A | 1401.77 | SNP | intergenic |  |  | - | | 3859376 | C | T | 1416.77 | SNP | Rv3439c | Gly96Glu | 4 | - | | 3859893 | C | T | 1855.77 | SNP | Rv3440c | silent (Glu28) | 9865 | - | | 3862472 | GA | G | 2405.73 | DEL | intergenic |  |  | - | | 3864995 | T | C | 1166.77 | SNP | Rv3447c (eccC4) | Ser1082Gly | 21 | - | | 3873392 | T | G | 1354.77 | SNP | Rv3451 (cut3) | Leu259Arg | 1 | - | | 3877421 | A | G | 1337.77 | SNP | Rv3456c (rplQ) | silent (Pro4) | 9926 | - | | 3884906 | A | G | 1512.77 | SNP | Rv3467 | Lys315Glu | 4 | - | | 3885886 | T | C | 1761.77 | SNP | Rv3468c | Ile62Val | 57 | - | | 3892133 | A | G | 35.74 | SNP | intergenic |  |  | - | | 3892671 | A | G | 3114.77 | SNP | Rv3476c (kgtP) | silent (Val350) | 9901 | - | | 3894032 | T | A | 1427.77 | SNP | intergenic |  |  | - | | 3894476 | C | T | 142.77 | SNP | Rv3478 (PPE60) | silent (Tyr17) | 9945 | - | | 3895691 | C | G | 1655.77 | SNP | intergenic |  |  | - | | 3896340 | T | G | 1511.77 | SNP | Rv3479 | Leu174Arg | 1 | - | | 3898408 | A | G | 1645.77 | SNP | Rv3479 | silent (Ala863) | 9867 | - | | 3903530 | G | C | 1894.77 | SNP | Rv3484 (cpsA) | silent (Ala151) | 9867 | - | | 3930896 | G | A | 1651.77 | SNP | intergenic |  |  | - | | 3934542 | T | G | 391.77 | SNP | Rv3508 (PE\_PGRS54) | Ser1180Ala | 35 | - | | 3934699 | G | A | 113.03 | SNP | Rv3508 (PE\_PGRS54) | Ser1232Asn | 20 | - | | 3936761 | A | G | 701.78 | SNP | intergenic |  |  | - | | 3940802 | A | G | 31.74 | SNP | Rv3511 (PE\_PGRS55) | Asn396Asp | 42 | - | | 3941586 | A | C | 127.03 | SNP | Rv3511 (PE\_PGRS55) | Asn657Thr | 13 | - | | 3942481 | C | G | 225.80 | SNP | intergenic |  |  | - | | 3942640 | T | C | 350.77 | SNP | intergenic |  |  | - | | 3943019 | C | G | 86.28 | SNP | intergenic |  |  | - | | 3952800 | G | A | 1311.77 | SNP | Rv3516 (echA19) | Gly86Asp | 6 | - | | 3958403 | A | G | 1591.77 | SNP | Rv3521 | Asn295Asp | 42 | - | | 3959418 | C | T | 1378.77 | SNP | Rv3522 (ltp4) | Thr324Ile | 7 | - | | 3973954 | C | T | 1062.77 | SNP | Rv3535c (hsaG) | Gly183Arg | 0 | - | | 3982763 | G | A | 1417.77 | SNP | Rv3543c (fadE29) | silent (Thr126) | 9871 | - | | 3983271 | T | G | 1856.77 | SNP | Rv3544c (fadE28) | Ile292Leu | 22 | - | | 3998059 | AGGC | A | 2810.73 | DEL | Rv3558 (PPE64) |  |  | - | | 4005607 | T | C | 1423.77 | SNP | Rv3564 (fadE33) | Leu(s)121Leu | 3 | - | | 4024273 | T | C | 1924.77 | SNP | Rv3581c (ispF) | Val25Val(s) | 18 | - | | 4026899 | G | A | 1480.77 | SNP | Rv3585 (radA) | silent (Gln152) | 9876 | - | | 4030543 | A | C | 2381.77 | SNP | Rv3589 (mutY) | silent (Ser17) | 9840 | - | | 4034827 | C | T | 1527.77 | SNP | Rv3593 (lpqF) | Ala159Val(s) | 9867 | - | | 4037283 | T | G | 225.84 | SNP | Rv3595c (PE\_PGRS59) | silent (Gly256) | 9935 | - | | 4038287 | G | A | 1720.77 | SNP | Rv3596c (clpC1) | silent (Asn806) | 9822 | - | | 4053050 | A | G | 531.77 | SNP | Rv3611 | Asn34Ser | 34 | - | | 4053161 | A | G | 81.28 | SNP | Rv3611 | Asn71Ser | 34 | - | | 4055801 | G | A | 1612.77 | SNP | Rv3616c (espA) | Thr192Ile | 7 | - | | 4059904 | A | G | 1707.77 | SNP | intergenic |  |  | - | | 4060100 | G | A | 801.77 | SNP | Rv3619c (esxV) | Leu57Leu(s) | 4 | - | | 4060201 | G | A | 562.77 | SNP | Rv3619c (esxV) | Ser23Leu(s) | 35 | - | | 4060210 | T | A | 605.77 | SNP | Rv3619c (esxV) | Gln20Leu | 6 | - | | 4060230 | G | A | 653.77 | SNP | Rv3619c (esxV) | silent (His13) | 9912 | - | | 4060334 | T | C | 1018.77 | SNP | Rv3620c (esxW) | silent (Glu86) | 9865 | - | | 4062017 | A | C | 1553.77 | SNP | Rv3622c (PE32) | Leu61Arg | 1 | - | | 4069292 | G | A | 1381.77 | SNP | Rv3630 | Ala40Thr | 22 | - | | 4075957 | C | A | 1320.77 | SNP | Rv3636 | Ala69Asp | 6 | - | | 4088346 | G | A | 1866.77 | SNP | Rv3648c (cspA) | silent (Thr62) | 9871 | - | | 4093879 | TG | T | 947.73 | DEL | Rv3652 (PE\_PGRS60) |  |  | - | | 4095001 | CG | C | 2856.73 | DEL | Rv3655c |  |  | - | | 4100975 | T | C | 1412.77 | SNP | intergenic |  |  | - | | 4111303 | G | C | 1900.77 | SNP | Rv3669 | Val(s)159Val | 13 | - | | 4120926 | A | G | 192.77 | SNP | Rv3680 | Asn378Asp | 42 | - | | 4120983 | A | G | 796.77 | SNP | intergenic |  |  | - | | 4131144 | C | T | 1388.77 | SNP | Rv3689 | Ala263Val(s) | 9867 | - | | 4135112 | G | A | 1590.77 | SNP | Rv3693 | Met(s)129Ile | 2 | - | | 4136581 | C | T | 2029.77 | SNP | Rv3694c | Val(s)178Val | 13 | - | | 4139183 | A | AC | 1609.73 | INS | Rv3696c (glpK) |  |  | - | | 4150648 | G | A | 1609.77 | SNP | Rv3707c | silent (Gly131) | 9935 | - | | 4156099 | C | A | 1371.77 | SNP | Rv3711c (dnaQ) | Val(s)211Leu(s) | 9867 | - | | 4162339 | A | G | 2366.77 | SNP | Rv3719 | Thr12Ala | 32 | - | | 4163944 | A | G | 2032.77 | SNP | Rv3720 | His70Arg | 10 | - | | 4171846 | G | T | 1179.77 | SNP | Rv3726 | Glu142Asp | 53 | - | | 4174564 | T | C | 1121.77 | SNP | Rv3727 | Ile537Thr | 11 | - | | 4182695 | G | A | 1723.77 | SNP | Rv3731 (ligC) | Arg313His | 8 | - | | 4187485 | T | C | 1748.77 | SNP | Rv3736 | silent (Ala284) | 9867 | - | | 4187817 | A | G | 1154.77 | SNP | Rv3737 | Asp40Gly | 11 | - | | 4197138 | C | CT | 1886.73 | INS | intergenic |  |  | - | | 4198611 | CG | C | 2669.73 | DEL | intergenic |  |  | - | | 4204441 | A | G | 2389.77 | SNP | Rv3759c (proX) | silent (His311) | 9912 | - | | 4210274 | A | G | 1307.77 | SNP | Rv3764c (tcrY) | Cys246Arg | 1 | - | | 4220174 | G | A | 2271.77 | SNP | Rv3775 (lipE) | Asp164Asn | 36 | - | | 4221490 | C | G | 1310.77 | SNP | Rv3776 | silent (Leu134) | 9947 | - | | 4222073 | A | G | 436.77 | SNP | Rv3776 | Met(s)329Val(s) | 9867 | - | | 4222882 | A | G | 1635.77 | SNP | Rv3777 | silent (Leu63) | 9947 | - | | 4233299 | G | A | 1577.77 | SNP | Rv3786c | Thr100Ile | 7 | - | | 4242182 | G | T | 1499.77 | SNP | Rv3793 (embC) | Ala774Ser | 28 | - | | 4242643 | C | T | 969.77 | SNP | Rv3793 (embC) | silent (Arg927) | 9913 | genotype | | 4247429 | A | G | 1652.77 | SNP | Rv3795 (embB) | Met(s)306Val(s) | 9867 | resistance | | 4255922 | A | G | 1456.77 | SNP | Rv3799c (accD4) | silent (His9) | 9912 | - | | 4257220 | A | G | 1524.77 | SNP | Rv3800c (pks13) | silent (Arg1309) | 9913 | - | | 4263305 | C | T | 1186.77 | SNP | intergenic |  |  | - | | 4264218 | C | A | 1148.77 | SNP | Rv3802c | Val50Phe | 0 | - | | 4264219 | T | G | 1143.77 | SNP | Rv3802c | silent (Gly49) | 9935 | - | | 4287011 | C | T | 2007.77 | SNP | Rv3822 | silent (Ile97) | 9872 | - | | 4291418 | G | C | 2238.77 | SNP | Rv3823c (mmpL8) | Leu38Val | 11 | - | | 4293072 | G | A | 1913.77 | SNP | Rv3824c (papA1) | Leu35Phe | 6 | - | | 4299480 | G | A | 1229.77 | SNP | Rv3825c (pks2) | silent (Ser42) | 9840 | - | | 4302036 | T | C | 1560.77 | SNP | Rv3827c | Thr252Ala | 32 | - | | 4306155 | C | T | 1609.77 | SNP | Rv3831 | silent (Ser133) | 9840 | - | | 4307179 | G | A | 1310.77 | SNP | Rv3833 | Val105Ile | 33 | - | | 4315384 | T | C | 1643.77 | SNP | Rv3842c (glpQ1) | Asp60Gly | 11 | - | | 4316046 | A | G | 1802.77 | SNP | Rv3843c | Val184Ala | 18 | - | | 4320050 | A | G | 688.77 | SNP | intergenic |  |  | - | | 4333136 | C | T | 1444.77 | SNP | Rv3859c (gltB) | silent (Lys982) | 9926 | - | | 4336090 | A | AT | 3581.73 | INS | intergenic |  |  | - | | 4338595 | GC | G | 4619.73 | DEL | intergenic |  |  | - | | 4338732 | G | A | 2390.77 | SNP | intergenic |  |  | - | | 4340330 | T | G | 1402.77 | SNP | Rv3864 (espE) | Leu(s)21Val(s) | 9867 | - | | 4347853 | T | C | 1786.77 | SNP | Rv3870 (eccCa1) | Val458Ala | 18 | - | | 4351039 | G | T | 1404.77 | SNP | Rv3872 (PE35) | Glu99STOP | 17 | - | | 4356110 | G | C | 1469.77 | SNP | Rv3877 (eccD1) | silent (Leu368) | 9947 | - | | 4361788 | G | A | 1089.77 | SNP | Rv3881c (espB) | silent (Ala46) | 9867 | - | | 4363069 | T | C | 1741.77 | SNP | Rv3882c (eccE1) | Ile118Val | 57 | - | | 4365757 | G | T | 1657.77 | SNP | Rv3884c (eccA2) | Pro361Gln | 6 | - | | 4366272 | G | C | 1616.77 | SNP | Rv3884c (eccA2) | silent (Ala189) | 9867 | - | | 4373496 | C | G | 1545.77 | SNP | Rv3889c (espG2) | Val(s)45Val | 13 | - | | 4375628 | G | T | 1754.77 | SNP | Rv3892c (PPE69) | Thr19Lys | 11 | - | | 4376098 | G | A | 1530.77 | SNP | intergenic |  |  | - | | 4379680 | C | G | 1735.77 | SNP | Rv3894c (eccC2) | Arg258Pro | 5 | - | | 4381974 | G | A | 1755.77 | SNP | Rv3896c | Ala293Val | 13 | - | | 4382054 | T | C | 1751.77 | SNP | Rv3896c | silent (Ala266) | 9867 | - | | 4382275 | G | T | 1322.77 | SNP | Rv3896c | Gln193Lys | 12 | - | | 4383144 | C | CCGGGG | 3527.73 | INS | Rv3897c |  |  | - | | 4385530 | G | C | 1547.77 | SNP | Rv3900c | Pro260Arg | 4 | - | | 4390753 | C | G | 1121.77 | SNP | Rv3905c (esxF) | silent (Ser93) | 9840 | - | | 4391553 | C | T | 1076.77 | SNP | Rv3906c | silent (Pro18) | 9926 | - | | 4400660 | AC | A | 2677.73 | DEL | Rv3911 (sigM) |  |  | - | | 4408156 | A | C | 1688.77 | SNP | Rv3919c (gid) | Leu16Arg | 1 | genotype | |  | | export |

elog
